# Analysis of Heparin Samples by Attenuated Total Reflectance Fourier-Transform Infrared Spectroscopy in the Solid State

**DOI:** 10.1021/acscentsci.2c01176

**Published:** 2023-02-14

**Authors:** Anthony J. Devlin, Courtney J. Mycroft-West, Jeremy E. Turnbull, Marcelo Andrade de Lima, Marco Guerrini, Edwin A. Yates, Mark A. Skidmore

**Affiliations:** † Centre for Glycoscience Research and Training, Keele University, Huxley Building, Keele, Staffordshire ST5 5BG, United Kingdom; ‡ The Rosalind Franklin Institute, Harwell Science and Innovation Campus, Oxfordshire OX11 0QG, United Kingdom; § Department of Biochemistry and Systems Biology, Institute of Structural, Molecular and Integrative Biology, University of Liverpool, Crown Street, Liverpool L69 7ZB, United Kingdom; ∥ Istituto di Ricerche Chimiche e Biochimiche ‘G. Ronzoni’, Via G. Colombo 81, Milan 20133, Italy

## Abstract

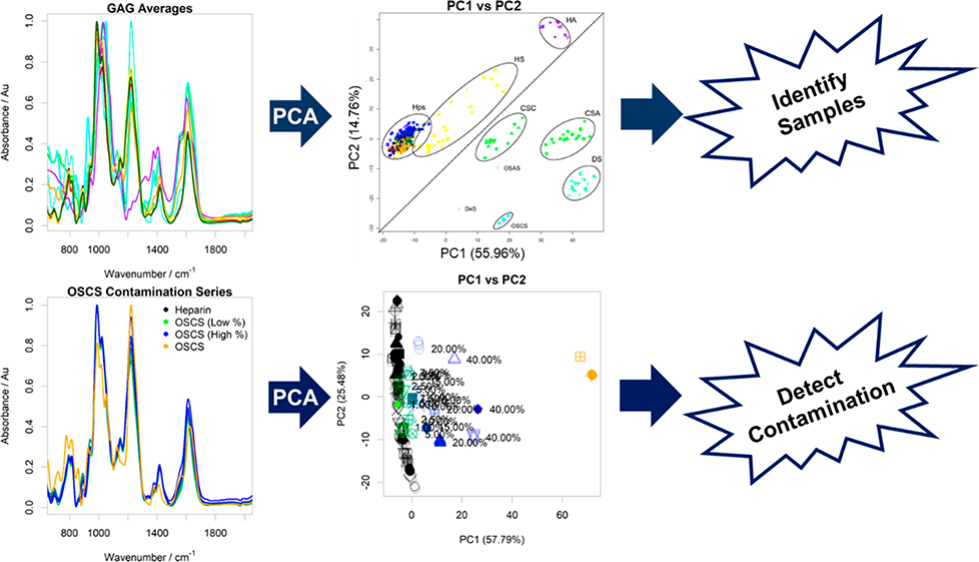

Heparin is a polydisperse, heterogeneous polysaccharide of the glycosaminoglycan (GAG) class that has found widespread clinical use as a potent anticoagulant and is classified as an essential medicine by the World Health Organization. The importance of rigorous monitoring and quality control of pharmaceutical heparin was highlighted in 2008, when the existing regulatory procedures failed to identify a life-threatening adulteration of pharmaceutical heparin with oversulfated chondroitin sulfate (OSCS). The subsequent contamination crisis resulted in the exploration of alternative approaches for which the use of multidimensional nuclear magnetic resonance (NMR) spectroscopy techniques and multivariate analysis emerged as the gold standard. This procedure is, however, technically demanding and requires access to expensive equipment. An alternative approach, utilizing attenuated total reflectance-Fourier transform infrared spectroscopy (ATR-FTIR) combined with multivariate analysis, has been developed. The method described enables the differentiation of diverse GAG samples, the classification of samples of distinct species provenance, and the detection of both established heparin contaminants and alien polysaccharides. This methodology has sensitivity comparable to that of NMR and can facilitate the rapid, cost-effective monitoring and analysis of pharmaceutical heparin. It is therefore suitable for future deployment throughout the supply chain.

## Abstract 2

ATR-FTIR spectroscopy combined with multivariate analysis enables the facile recognition of heparins, the identification of species provenance, and the detection of contaminant polysaccharides.

## Introduction

Unlike the majority of pharmaceutical agents, the widely used clinical anticoagulant heparin lacks a unique, defined structure. Heparin is a polydisperse, glycosaminoglycan (GAG) polysaccharide and natural product derived from animal tissue (primarily the intestines of pigs). Heparin possesses an inherently varied structure, but the underlying repeating unit consists of alternating 1,4-linked uronate (either β-d-glucoronate^1,2^ or, more commonly in heparin, its C-5 epimer, α-l-iduronate) and d-glucosamine. These disaccharide repeats are substituted with a variety of *N*-sulfate and *N*-acetyl groups within the glucosamine residues and *O*-sulfates within both the glucosamine and the uronate residues.

These biosynthetic modifications, which are carried out to varied extents within the chain, provide considerable sequence diversity both within heparin chains from the same heparin sample, but also between samples from different animal sources,^3^ although the composition of heparin from distinct animal sources conforms to broad ranges.^4^ The heterogeneity of pharmaceutical heparin is further increased by the practice of amalgamating materials obtained from many individual animals before processing.^5^ The need to develop sensitive methods with which to analyze heparin was highlighted by the global contamination of pharmaceutical heparin in 2007–8,^6−8^ where an unnatural, chemically modified GAG polysaccharide (over sulfated chondroitin sulfate, OSCS) was introduced as the predominant contaminant into the supply chain, resulting in at least 150 deaths and 350 other adverse events in the U.S. alone.^9^

Since the contamination events of 2007–8, there have been numerous attempts to characterize,^10^ and provide methods for, the detection of OSCS.^8,11^ However, far fewer approaches have been reported that address the more demanding challenge of detecting any potential polysaccharide contaminant. Moreover, improving the ability to differentiate between different species and/or distinct tissue sources is of particular relevance given the proposed reintroduction of bovine-sourced heparin into the U.S. market.

Analysis of the complex heterogeneous mixture that comprises pharmaceutical heparin requires heparin to be considered not as an individual, homogeneous single molecular entity typical of the majority of pharmaceutical agents (such as insulin), but rather as a collection of subtly distinct substances. Accordingly, a variety of distinct polysaccharides can be grouped in terms of their similarity under examination using a suitable analytical technique (initially, NMR spectroscopy), which is capable of distinguishing the subtle structural variations between samples using nonparametric approaches (e.g., principal component analysis, PCA). This technique also enables approved pharmaceutical heparin samples (which also demonstrate structural variability) to be grouped, according to the levels of similarity between their ^1^H NMR spectra, to form a library of accepted, bona fide heparins^12^ from which a decision can be made concerning the provenance of the test sample.^13^ This approach has also been extended to provide increased resolution by the authors using two-dimensional, heteronuclear (^1^H–^13^C) NMR.^14,15^ These strategies, nevertheless, require access to high-field NMR spectrometers and skilled technical assistance to meet the high standards required to ensure reproducibility, which imposes a significant financial cost on the production and regulation.

Many alternative and complementary spectroscopic methods have been explored previously in the quest for analysis techniques of sufficient sensitivity and have included solution-based Fourier transform infrared (FTIR) and Raman spectroscopies (reviewed in refs (4) and (11)). FTIR spectroscopy of dried films has also previously been deployed to distinguish between different GAGs.^16^

Infrared (IR) spectroscopy exploits the ability of covalent chemical bonds to absorb energy in the IR range (mid-infrared with wavelengths (∼2.5 to 50) × 10^–6^ m), resulting in excitation or vibration analogous to two masses (*m*_1_ and *m*_2_) joined by a spring, with force constant, *k*. The differential absorbance of IR light of different frequencies (measured for historical reasons in wavenumbers (cm^–1^) and which also provides convenient numbers that are proportional to energy) was recorded. In practical terms, rather than stepping progressively through the IR frequency range and measuring absorbance, almost all instruments deliver light containing wavelengths throughout the IR range over a short time span employing an interferometer, the resulting interferogram then being deconvoluted by Fourier transform (FT) to provide a spectrum consisting of wavenumbers on the *x*-axis against absorbance on the *y*-axis. To a first approximation, the frequency (ν) of the absorbed light depends on the strength of the covalent bond, represented by a force constant, *k*, and the reduced mass (μ) of the atoms involved, where μ = (*m*_1_·*m*_2_)/(*m*_1_ + *m*_2_) of the two attached atoms, of masses *m*_1_ and *m*_2_:
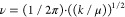
The position of an absorbance band of particular chemical groups, relevant examples being carbonyl groups (C=O), amide (NH·C=O), or sulfate groups (O–SO_3_^–^), also tends to appear at characteristic positions within the spectrum. The IR spectra of even small molecules are considerably more complicated than might be supposed, however, for several reasons. Vibrations consist not only of the main vibrational mode (principal or normal mode) but also overtones, analogous to the complex sound of a single plucked string of a musical instrument. Further, in addition to the principal mode and overtones, the vibrations of molecules consist of a variety of coupled movements, including stretching, wiggling, wagging, etc., each of which contributes to the spectrum, where the number of these vibrational modes in a molecule comprising *N* atoms is ∼3*N*. Third, in complex molecules, parts of the molecule can interact with each other, through hydrogen bonding, or with associated cations and with solvent (if present), further modifying the spectrum through subtle line broadening, small changes to the position of the signals, or the appearance of additional signals. All of these complex signals combine to generate characteristic spectral features for a particular chemical, which can provide a fingerprint and bestows considerable analytical power to FTIR for identification and differentiation purposes.

In the case of polyatomic molecules such as polymers, including polysaccharides, the spectra are the result of thousands of signals superimposed and involve very complex coupled motions. Despite this complexity, FTIR spectra of heparin are sensitive to variations in composition involving different proportions and positions of sulfation, the relative proportions of uronic acid isomers (d-GlcA and l-IdoA), and the level of *N*-acetylation.

Here, the authors utilize attenuated total reflectance-Fourier transform infrared spectroscopy (ATR-FTIR), which enables the rapid, facile analysis of heparin samples, in both the solid state (dry or powdered) and solution, via the detection of chemical signatures. When deploying FTIR for the analysis of large molecules, the total number of fundamental vibrational modes is high (∼3*N*, for *N* atoms), and the number of infrared spectral bands and their complex superposition are further increased by the presence of overtones, which correspond to harmonics of the fundamental vibrational modes. Additional nuance is provided by the internal hydrogen bonds present between constituent groups and the resulting conformational differences, which, because they change the dipoles of interacting groups, can also subtly alter the frequency of their vibrational modes. The combined effect of these factors results in the presentation of complex, but characteristic, FTIR spectra for biological macromolecules, which are very sensitive to changes in their chemical composition. The methodology presented here exploits these properties for the classification of pharmaceutical heparin samples, the detection of contaminants within pharmaceutical heparin preparations, and the determination of species origin.

The unique advantage of the dual solid/solution-state analysis of ATR-FTIR-based methodologies is that they are equally applicable for monitoring the process intermediates that arise at every step of heparin production, as they are to the quality control of the active pharmaceutical ingredient (API) and have the additional advantage of requiring minimal sample preparation. Additionally, the method avoids sample preparation in D_2_O, imposed by ^1^H NMR, thereby eliminating an additional source of variation. The highly portable nature of ATR-FTIR instrumentation, its relatively low cost, combined with relatively undemanding operation as compared to NMR, affords significant advantages while retaining comparable levels of sensitivity. Sample preparation and data collection are both straightforward and highly amenable to future adaptation to a portable format, which will be convenient for both heparin producers and regulators alike.

## Results

### Principal Component Analysis of the ATR-FTIR Spectra of Glycosaminoglycans

**Figure 1:**
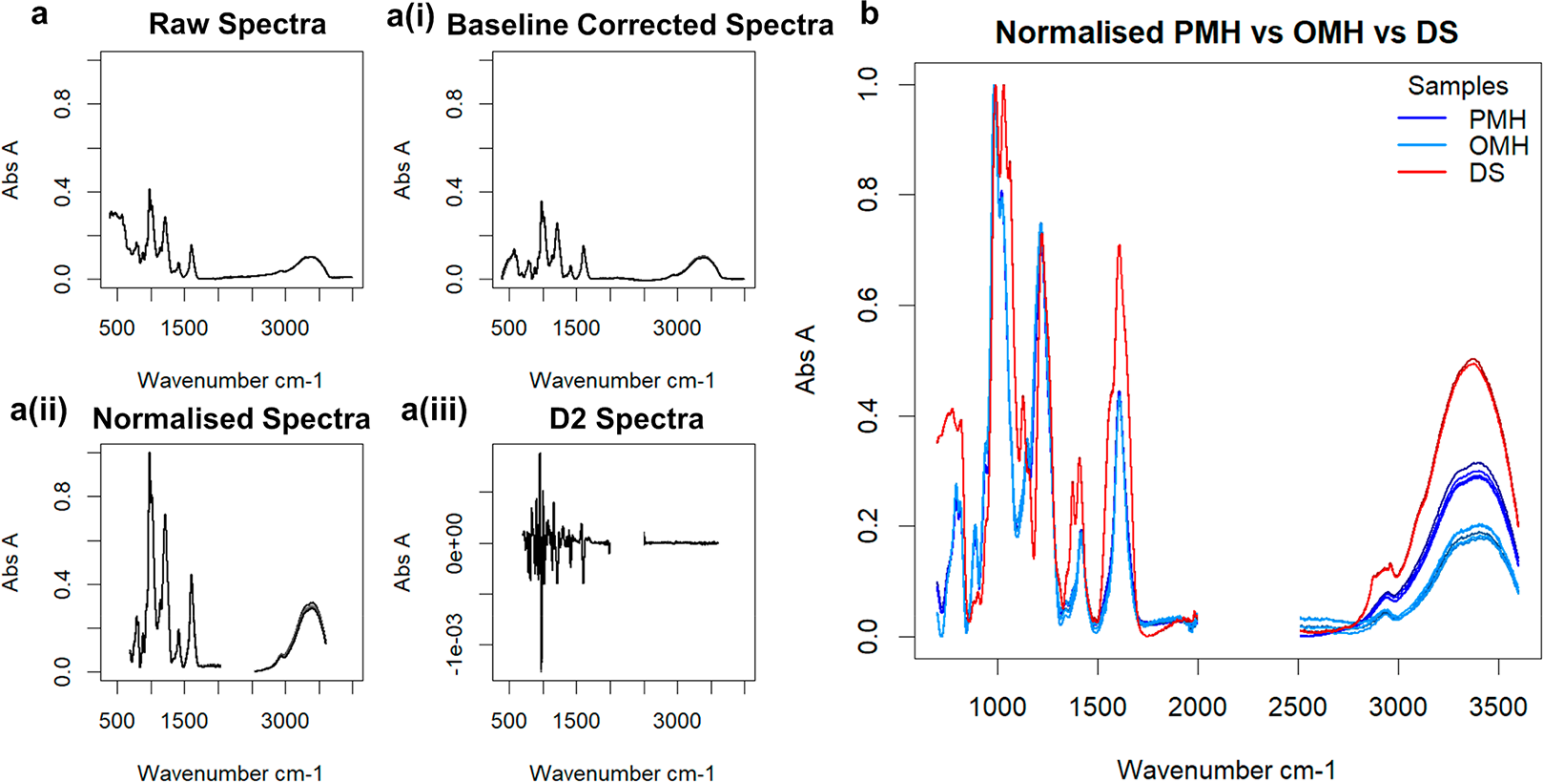
(a) Steps in the ATR-FTIR spectral preparation following recording on a dry sample of pharmaceutical heparin. (a) (i) The raw spectrum of a randomly selected heparin sample. (ii) Baseline-corrected (seventh-order polynomial) spectrum. (iii) Spectrum following normalization and removal of variable regions <700, >3600, and between 2000 and 2500 cm^–1^. (iii) The second derivative of the resultant spectrum. (b) Normalized ATR-FTIR spectra of samples of PMH, OMH, and DS (700–3600 cm^–1^) showing similarity between PMH and OMH spectra in the regions 700–2000 cm^–1^, differing significantly only in the highly variable O–H stretch region 3000–3500 cm^–1^. In contrast, DS is distinct from either PMH or OMH in the region 700–2000 cm^–1^, corresponding to structural variations.

To ascertain the viability of ATR-FTIR as a means of differentiating between heparin samples, a library of distinct GAG polysaccharides was assembled, which encompassed the structural heterogeneity of GAGs and included interspecies and interbatch variation from the same species (Figure 1). This library contained 176 heparins (Hps) comprising 69 porcine mucosal heparin samples (PMHs), 55 bovine mucosal heparins (BMHs), 33 ovine mucosal heparins (OMHs), 19 bovine lung heparins (BLHs), 31 heparan sulfates (HS), and other GAG polysaccharides (29 chondroitin sulfates (13 CS-As and 16 CS-Cs), 21 dermatan sulfates (DS), and 10 hyaluronic acids (HAs)). These additional GAGs are frequent contaminants of heparin samples, as well as being potential sources of deliberate contamination, and contain subtly different constituents (e.g., *N*-acetyl d-galactosamine and different proportions of d-GlcA and l-IdoA), linkage positions, as well as sulfate substitution patterns. The library was augmented with eight semisynthetically modified polysaccharides, comprising six oversulfated chondroitin sulfate samples (OSCSs), one oversulfated agarose sulfate sample (OSAS), and one dextran sulfate sample (DeS), to provide a sufficient breadth of structure for comparisons to be made.^12^

**Figure 2:**
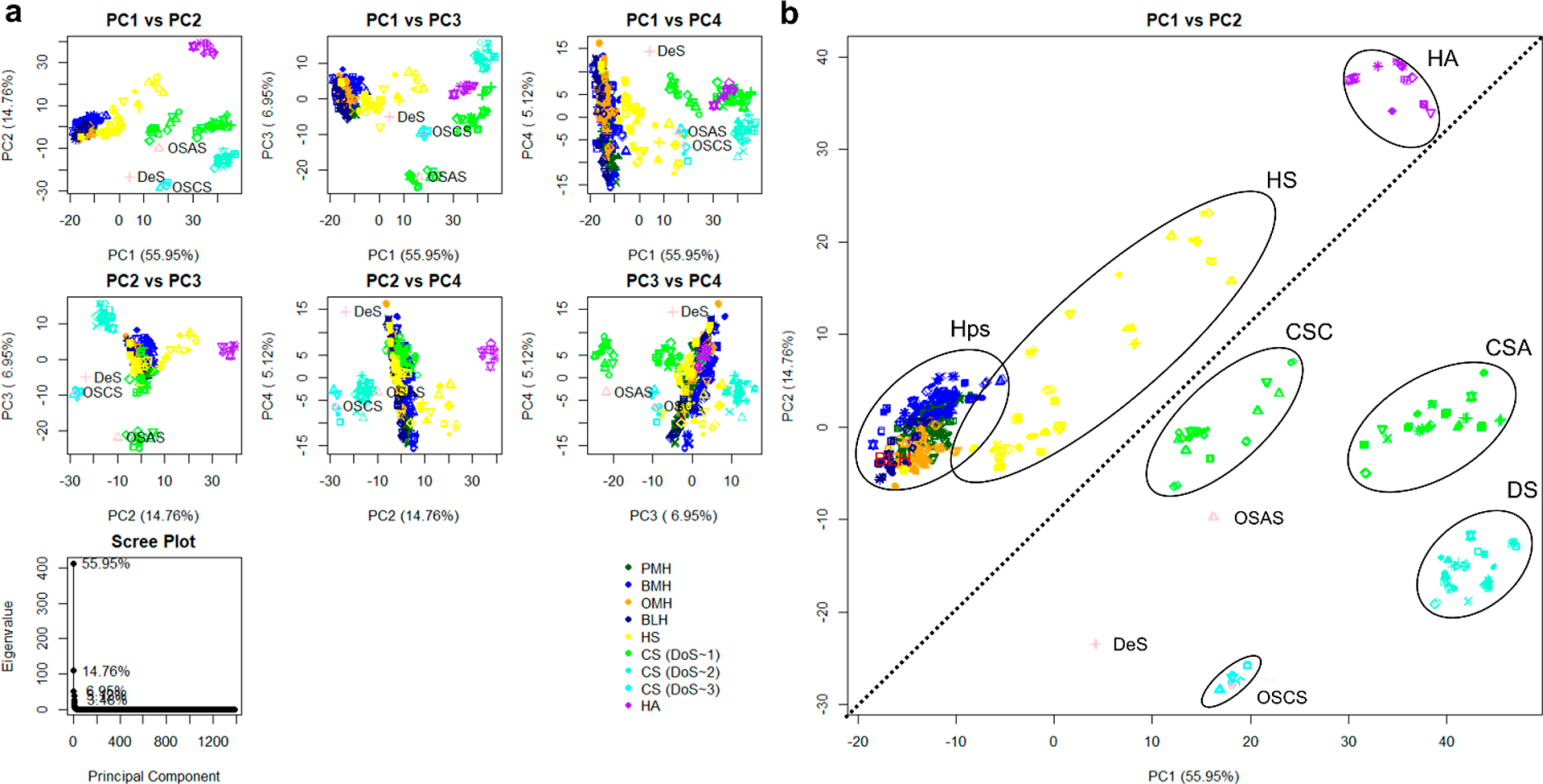
(a) PCA scores and scree plot(s) for different GAG families. At least two components are needed to separate the samples into family groups, indicating that earlier components appear to separate by gross structure, while later components separate by unique constituents. (b) Score plot for PC1 versus PC2. Glycosaminoglycan families are highlighted to show groupings, and the plot is split in half diagonally to distinguish different glycosidic bond types (β 1–4 upper left vs β 1–3/4 lower right). In all plots, 176 heparins, comprising 69 PMH (dark green), 55 BMH (blue), 33 OMH (orange), 19 BLH (dark blue), 31 HS (orange), 50 chondroitins (including 13 CS-A samples, 16 CS-C samples, and 21 DS (green for monosulfated chondroitin samples and teal for disulfated chondroitin samples)), 11 HA (purple), and 6 OSCS (including the OSCS selected for the contamination study later (aquamarine)) samples, as well as an OSAS and DeS sample, also used in the contamination study are compared. All five repeats of each sample are shown, and the polysaccharides selected for the contamination study are highlighted in lilac. The ellipses are for illustrative purposes only.

The normalized (smoothed and processed; see Materials and Methods) spectra were analyzed by PCA, and score plots of principal components 1–4 (PC1–PC4), responsible for ∼85% of the variation, were able to easily distinguish between distinct GAGs (Figure 2). The resolution between GAG samples containing either α-d- or β-d-glucosamine (GlcN) can be observed through PCs 1 and 2 (Figure 2), where HA, HS, and Hp (containing GlcN) locate toward the upper left of the plot, in contrast to CS samples (containing galactosamine; GalN), which associate at the bottom right. Within these groups, the sulfation type is distinguishable across PC1. In the region correlating with GAGs containing GlcN, three regions can be observed: the HA group, which lacks sulfation (purple), a variably sulfated HS group (yellow; HS consists of a combination of 6-*O*-sulfated, 2-*O*-sulfated, and *N*-sulfated residues, the levels of which correlate with the location of a sample within this group), and a Hp group (green, blue, and orange; containing the same sulfation positions as HS, albeit at higher amounts, with the addition of less frequent 3-*O*-sulfation). Across PC1, the ratio of *N*-sulfate (NS) to *N*-acetylation (NAc) is discernible, with samples lacking *N*-sulfation (i.e., HA and CS) locating to the right, while NS-containing GAGs (Hp and some HS samples) locate to the left. The discrimination of sulfation type is clear in the region containing galactosaminoglycan samples. Here, 6-*O*-sulfated DS and CS-C are present on the far right, while 4-*O*-sulfated CS-A presents to the left. Oversulfated chondroitin sulfate (OSCS) locates near the 4-*O*-sulfated group, while DS (containing 2-*O*-sulfated IdoA) locates further right and is distinguishable from CS-A. Oversulfated chondroitin sulfate, which also possesses 6-*O*- and 2-*O*-sulfation, locates to the left of the densest region of the CS-A cluster. It is important to note that CS polysaccharides present within the library (and indeed present naturally) are not exclusively pure CS-A or CS-C polymers, but contain variable amounts of 6-*O*-sulfation or 4-*O*-sulfation, respectively.^17,18^

The second principal component appears to accord with the degree of sulfation (DoS), orthogonal to the GlcN and GalN regions. Again, unsulfated HA resides at the top, with sulfated samples descending toward Hp, which possesses an average DoS of 2.3. Chondroitin sulfate A and CS-C, with DoS values of ∼1, locate at the top of the GalN region, moving down toward DS in the middle, which has a DoS of ∼2, and finishing at OSCS with a DoS in excess of 3. It is possible that PC3 could distinguish GAGs in terms of the detailed geometries of their sulfate and carboxylate groups, which both provide relatively large signals in FTIR spectra and are known to vary according to their disposition around the pyranose rings.^19^

### The Higher-Numbered Principal Components Reveal Increasingly Subtle Differences and Can Differentiate Synthetically Modified Polysaccharides

**Figure 3:**
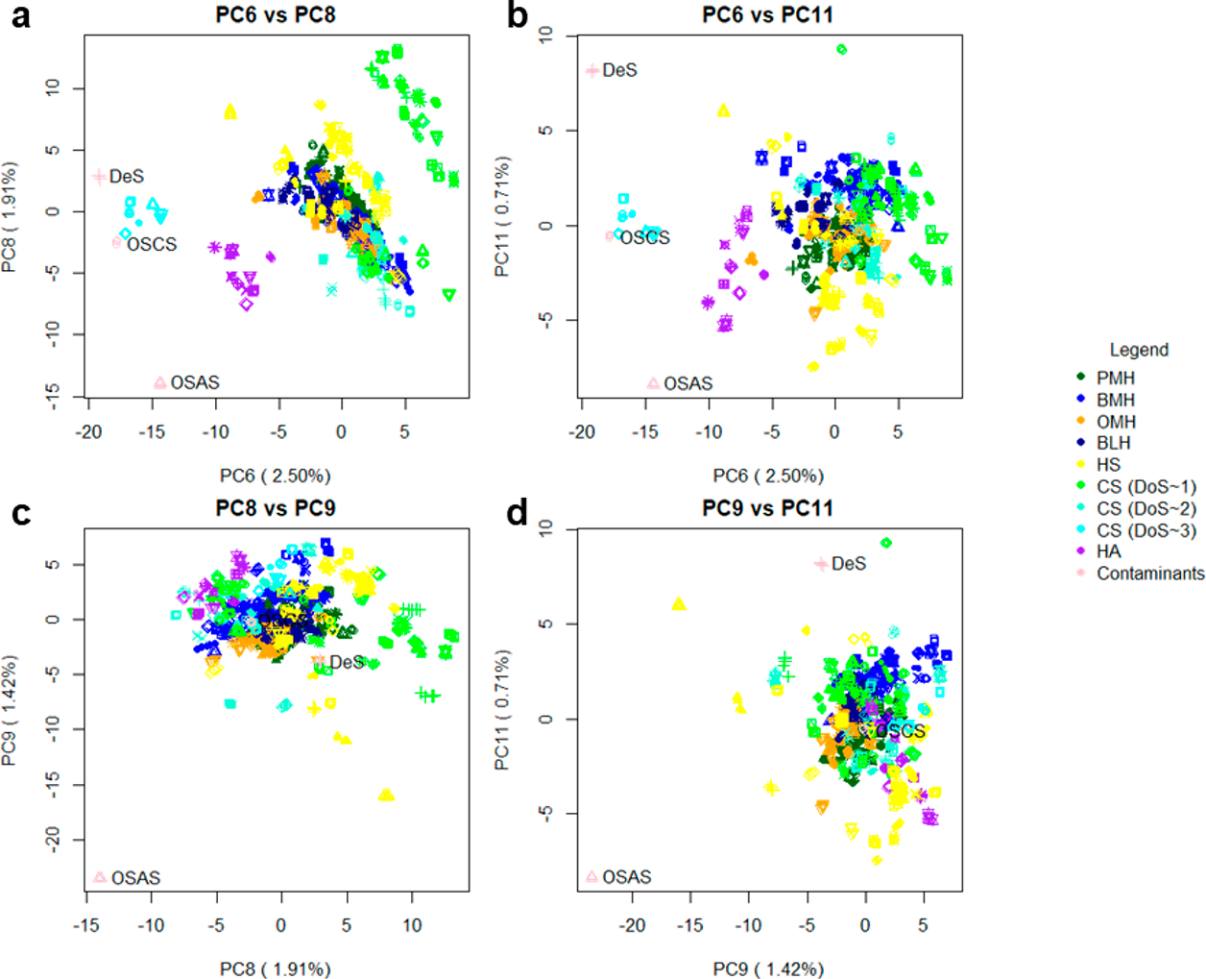
Score plots for lower PCs following the analysis of FTIR-ATR spectra of different GAG species and other polysaccharides. As the PCs cover progressively less variance, subtler changes become more apparent, amplifying the distinctness of relative outliers, such as OSCS, OSAS, and DeS. All five repeats of each spectrum are shown: (a) score plot of PC6 vs PC8, (b) score plot of PC6 vs PC11, (c) score plot of PC8 vs PC9, and (d) score plot of PC9 vs PC11.

The library also contains two additional, non-GAG carbohydrates, oversulfated agarose sulfate (OSAS) and dextran sulfate (DeS). Agarose is a d-galactose-containing polysaccharide, in which alternating d-galactose residues possess an oxygen bridge between C1 and C3, and this locates in the GalN region of the PC plot (Figure 3). Dextran sulfate, a sulfated glucose polymer, with a DoS of ∼2, consisting primarily of β(1–6) glycosidic bonds (not commonly found in GAGs), falls within the GalN-containing region, comprising groups containing ∼2 sulfates (near the DS region). It may have been hypothesized that DeS would fall within the GlcN region, as confirmed by PC2. Dextran sulfate, however, also possesses a small proportion of β(1–3) linkages, explaining the appearance in close proximity to the area containing GalN (β(1–3) or β(1–4)), albeit slightly separated from this region. The relative contribution of the OSAS and DeS samples toward the entire library is very low (0.7% of the spectra), so their contribution to the overall variation is also relatively low; hence, higher-numbered components that encompass relatively low levels of variation within the library are required to discern them from the GAG library. The unique structural features of OSAS and DeS are differentiated by PCs 6, 8, 9, and 11. PCs 8 and 9 separate OSAS entirely from the remaining polysaccharides, potentially based on their 1–3 glycosidic linkages, while PC 11 separates DeS away from the rest of the polysaccharides, presumably as a result of their β(1–6) bonds. Of particular interest is the observation that PC 6 separates all artificially sulfated (OSCS, OSAS, and DeS) polysaccharides away from all naturally occurring GAG polysaccharides.

### Detection of Known Contaminants in Porcine Mucosal Heparin

**Figure 4:**
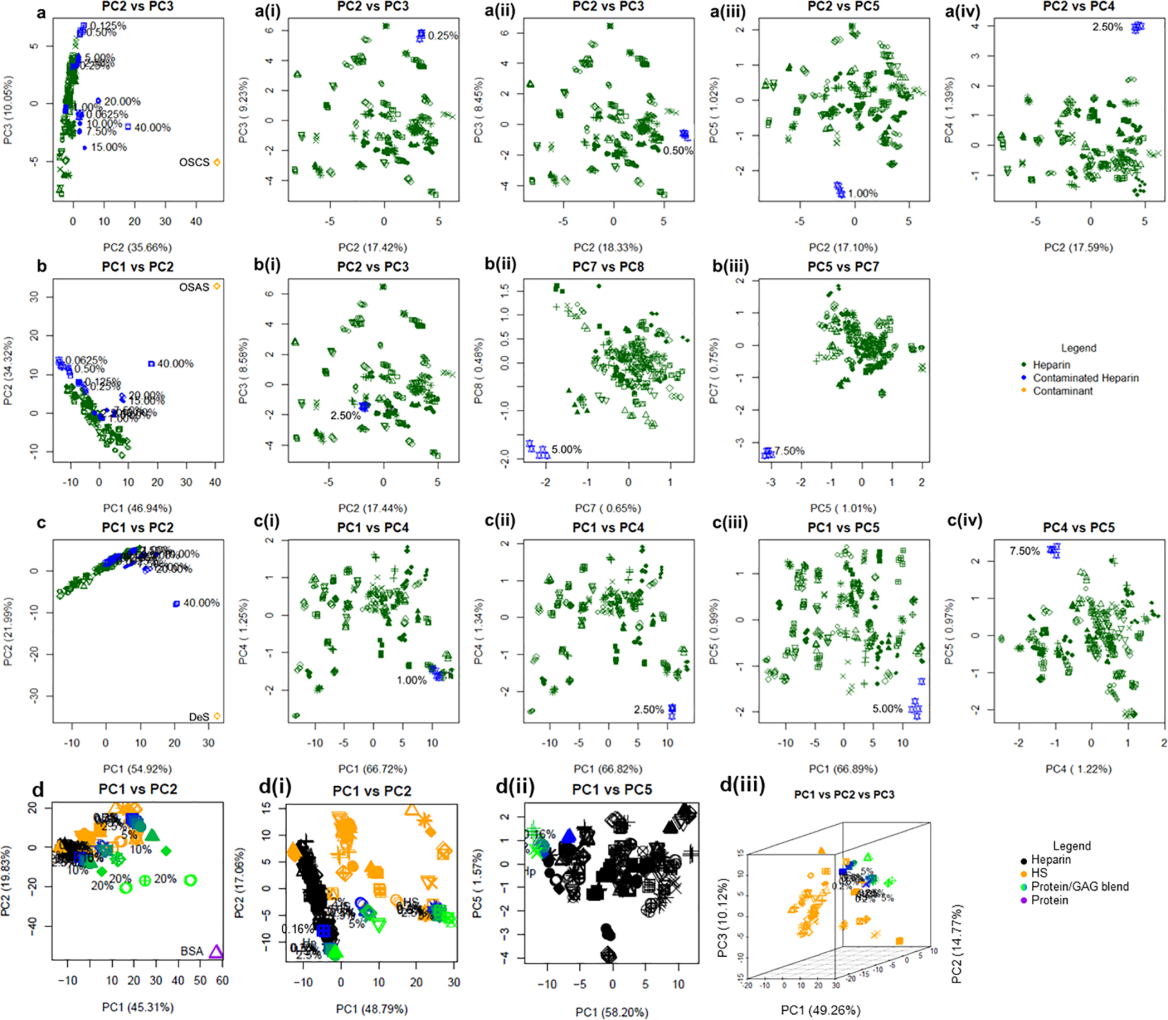
PCA score plots of FTIR-ATR spectra of the library of 69 bona fide PMH samples. These were analyzed in the presence of a randomly selected heparin contaminated deliberately with varying levels of carbohydrate contaminants OSCS (a), OSAS (b), and DeS (c), and the protein contaminant BSA (d) (confirmed earlier to contain no other contaminants, and mixed w/w% with 40%, 20%, 15%, 10%, 7.5%, 5%, 2.5%, 1%, 0.5%, 0.25%, 0.13%, and 0.06% carbohydrate contaminant or 20%, 10%, 5%, 2.5%, 1.25%, 0.63%, 0.31%, and 0.16% protein contaminant). For protein contamination, HS was also utilized and prepared in a similar manner. Differentiation down to 0.5%, 5.0%, 2.5%, and 0.31% w/w contamination was achievable, respectively. All five repeats for each sample are shown. (a) Score plot PC2 vs PC3 of heparin vs the entire contamination series. (a) (i) Score plot of PC2 vs PC3 of heparin vs 0.25% OSCS. (a) (ii) Score plot of PC2 vs PC3 of heparin vs 0.5% OSCS contamination. (a) (iii) Score plot of PC2 vs PC5 of heparin vs 1.0% OSCS contaminated heparin. (a) (iv) PC2 vs PC4 of heparin vs 2.5% OSCS contaminated heparin. (b) Score plot PC1 vs PC2 of heparin vs the entire contamination series. (b) (i) Score plot of PC2 vs PC3 of heparin vs 2.5% OSCS. (b) (ii) Score plot of PC7 vs PC8 of heparin vs 5.0% OSCS contamination. (b) (iii) Score plot of PC5 vs PC7 of heparin vs 7.5% OSAS contamination. (c) Score plot of PC1 vs PC2 of heparin vs the entire contamination series. (c) (i) Score plot of PC1 vs PC4 of heparin vs 1.0% DeS contamination. (c) (ii) Score plot of PC1 vs PC4 of heparin vs 2.5% DeS contamination. (c) (iii) Score plot of PC1 vs PC5 of heparin vs 5.0% DeS contamination. (c) (iv) Score plot of PC4 vs PC5 of heparin vs 7.5% DeS contamination. (d) Score plot of PC1 vs PC2 heparin and HS vs the three-contamination series. (d) (i) Score plot of PC1 vs PC2 heparin and HS vs the contamination series between 0.16% and 5%. (d) (ii) Score plot of PC1 vs PC5 and the heparin library vs the contamination series between 0.16% and 5%. (d) (iii) Score plot of PC1 vs PC2 vs PC3 and the HS library vs the contamination series between 0.16% and 5%.

After establishing ATR-FTIR as a tool for distinguishing polysaccharides on the basis of their structural features, we also investigated other potential practical uses. First, the detection of heparin contaminated by the addition of either a chemically modified GAG or a synthetically derivatized, non-GAG polysaccharide species was investigated. Randomly selected PMH and OSCS samples were contaminated with each other, on a defined weight-for-weight basis. The ATR-FTIR spectra of the resulting contaminated mixtures were obtained before PCA analysis as previously described (see Materials and Methods) and are presented in Figure 4a. In general, as the percentage of OSCS content increases within the PMH sample, the location within the PC score plot moves away progressively from the heparin library. Discriminatory power is only lost at the lower range of contamination (≤2.5% w/w), where the contaminated samples become spread out across the heparin library (Figure 4a (i–iv)). The fifth principal component is required to distinguish 1% w/w OSCS contamination. Below 1% w/w contamination, the contaminated samples appear on the boundary of the heparin region and, by 0.25% w/w contamination, are indistinguishable from other outlying heparins.

In a previous study employing ^1^H NMR spectroscopy,^13^ PCA was able to distinguish 2.5% w/w OSCS contamination. Oversulfated chondroitin sulfate exhibits distinct, well-defined, and characteristic chemical shifts in ^1^H NMR arising from the *N*-acetyl group, making the detection via multivariate analysis relatively straightforward. To present a stronger challenge, and to surmount the possibility of any future contamination by non-GAG polysaccharides whose ^1^H NMR chemical shifts may reside entirely within (and overlap those of) native GAGs, an *N*-acetyl free, chemically modified polysaccharide, oversulfated agarose sulfate (OSAS), was employed. The heparin sample used for OSCS contamination above was contaminated with OSAS in a comparable manner. The results (Figure 4b) were broadly similar to those for OSCS contamination; the entire contamination series extended progressively from the cluster representing the pure heparin samples, and, as for OSCS, the lower percentage contaminants merged with heparin. Using PCs 5 and 7, and 7 and 8, 7.5% w/w and 5% w/w contamination were separated strongly from the cluster of heparin samples, but by 2.5% w/w contamination, no separation was evident. Another carbohydrate, dextran sulfate (DeS), consisting of a glucopyranose polysaccharide backbone, possessing a variable sulfation pattern and a sulfation level comparable to those of heparin, was utilized in a manner akin to OSCS and OSAS (Figure 4c). The results were similar to these contaminations, the entire contamination series extending progressively from the heparin cluster. Using PCs 1 and 4, separation at the 5% w/w and 2.5% w/w levels was achieved, while 1% w/w contamination appears on the edge of the heparin cluster.

Aside from contamination with other carbohydrates, other biomolecular macromolecules, such as proteins, may also be present in carbohydrate preparations. This is of particular interest for the study of GAGs extracted from novel sources and for the analysis of routine preparations of GAGs such as HS. To explore the effects that contaminating protein absorption bands have on GAG IR spectra, a randomly selected heparin and two randomly selected HSs were blended with bovine serum albumin (BSA) at a defined weight-for-weight basis. Trends similar to those observed for other carbohydrate contaminants were observed with BSA (Figure 4d), albeit to a lower level. Samples containing 0.31% w/w BSA were observed moving to the edge of the heparin cluster, while samples containing 0.16% w/w BSA were observed within the heparin cluster (Figure 4d (ii)). Similar trends were observed with HS, where samples containing 0.31% w/w BSA protein were observed beyond the scores of other HSs (Figure 4d (iii)).

### Separation of Heparins Bound to Different Cations

The high anionic charge of heparin affords it the ability to coordinate with various cations.^20,21^ Distinct, bound cations are thought to homogenize bond angles and ring conformations, which permeate heparin^4^ and hence can influence the activities of the heparin product.^22^ Medicinally, the Na^+^ and Ca^2+^ cation forms of heparin are used, both of which possess unique monographs in the U.S., and hence observation of each would be of use. Subtle differences between sodium and calcium heparins have been noted previously,^23^ and FTIR has already been used in conjunction with circular dichroism spectroscopy to characterize the binding site of Cu^2+^ ions in heparin.^24^

**Figure 5:**
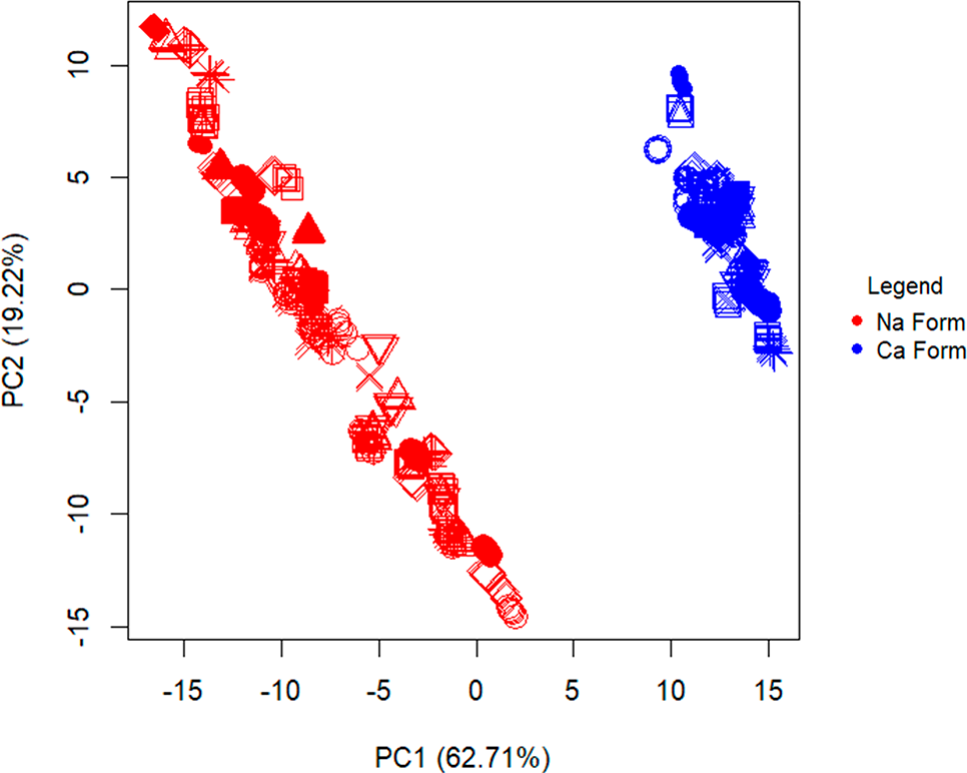
PCA score plots of FTIR-ATR spectra of sodium vs calcium heparins. Samples of commercial heparins bound to different cation forms. All five repeats of 69 sodium heparins (red) and 36 calcium heparins (blue) were compared using PCA, displaying clear separation between the two.

The ATR-FTIR spectra were recorded for 69 sodium heparins and 36 calcium heparins from commercial sources. Spectra were subsequently subjected to PCA, the results of which are plotted in Figure 5. Two clear and distinct clusters were found in PCs 1 and 2, both of which correlate with sodium or calcium heparin.

### Separation of Heparin Derived from Distinct Animal Species

In territories where the use of porcine-derived products is sensitive for religious or cultural reasons, and throughout much of South America, bovine heparin is also used as an API. Heparin from bovine sources is also in the process of being reintroduced into the North American market.^25^ Recently, heparin from distinct tissue types or nonapproved animal species has entered the market in contravention of the relevant monograph. There is significant concern that the potential exists for contamination, and it is therefore important to be able to distinguish heparin from different animal sources and tissue types. It has been established that heparin from distinct animal sources exhibits different structural features^1,3^ such as distinct sulfate patterns and molecular weights.^1,3,4^ Previously, Cabassi et al.^26^ demonstrated the sensitivity of IR spectroscopy to sulfate moieties contained within GAGs, and this is further demonstrated in Figure 2B where chondroitin sulfates of differing subtypes (A and C; defined by either 4- or 6-*O*-sulfation, respectively) are found in unique clusters. Dermatan sulfate, which generally contains a higher degree of sulfation when compared to CS, also forms a unique cluster. It is therefore proposed that distinct sulfation levels and patterning found in heparins, extracted from different animal and tissue sources, will produce unique spectra. To evaluate this, the same 176 heparins as used above, derived from different species and tissues, were subjected to PCA following ATR-FTIR spectral acquisition.

**Figure 6:**
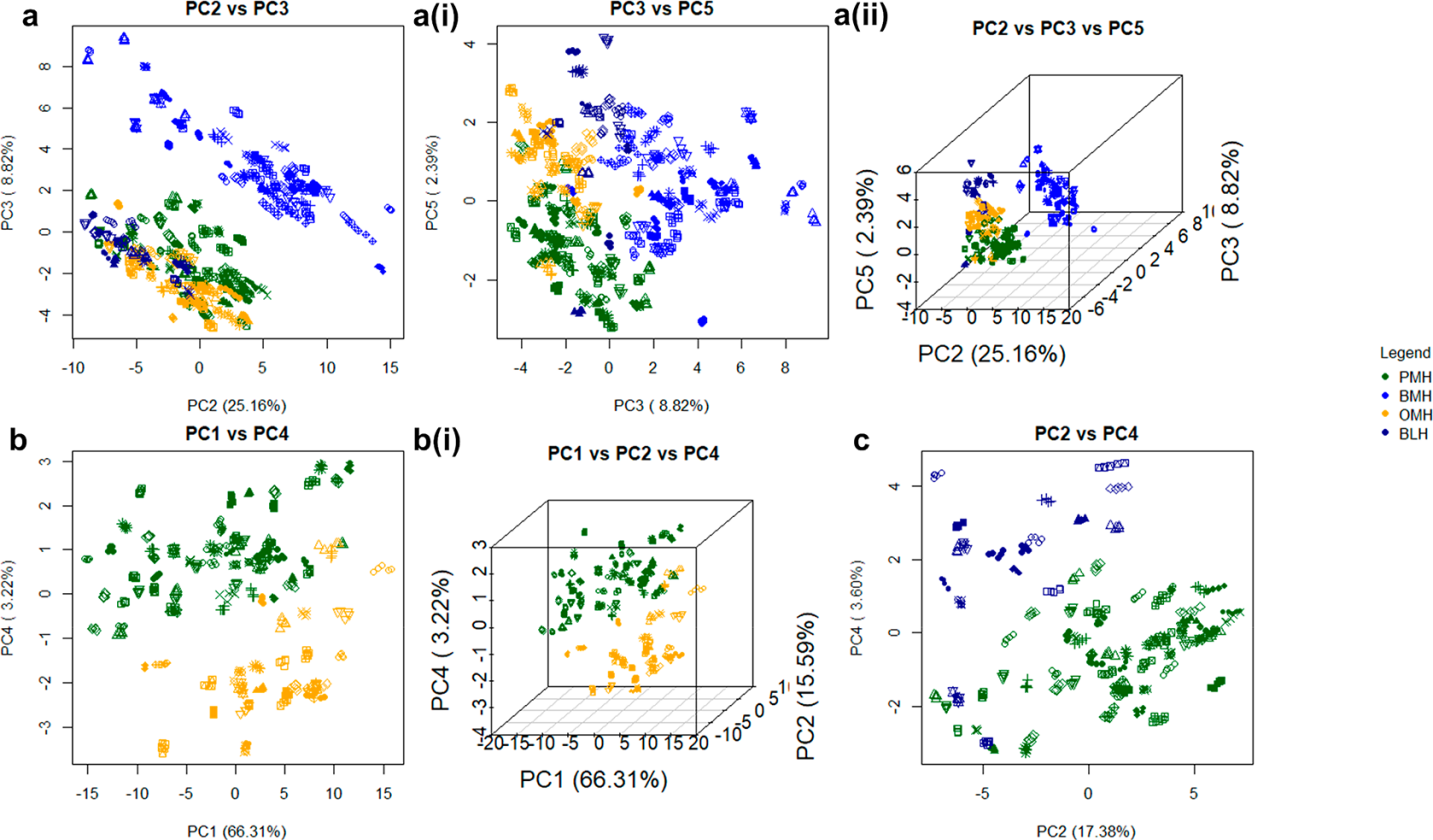
PCA score plots following the analysis of ATR-FTIR spectra of heparin samples from different animal species. Samples of heparin from each animal species can be separated into their respective clusters. All five repeats of 69 PMH (dark green), 55 BMH (blue), 33 OMH (orange), and 19 BLH (dark blue) spectra were compared using PCA. (a) All four species compared against each other. (a) Score plot of PC2 vs PC3. (a) (i) Score plot of PC3 vs PC5. (a) (ii) 3D score plot of PC2 vs PC3 vs PC5. (b) Spectra of samples of OMH and PMH are compared. Score plot of PC1 vs PC4. (b) (i) 3D score plot of PC1 vs PC2 vs PC4, respectively, and for BLH vs PMH. (c) Score plot of PC2 vs PC4 of PCA of spectra of BLH and PMH samples.

Bovine mucosal heparin separates strongly from the other heparin tissues sources employing PCs 2 and 3, however, OMH, PMH, and BLH are separated to a lesser extent (Figure 6). Across PC 5, all heparin species form one large cluster; however, four nodes within this cluster are evident, each populated with samples from a single heparin species, with BLH at the top, BMH to the right, PMH at the bottom left, and OMH at the top left. Porcine mucosal heparin, OMH, and BLH do not completely resolve, but do form distinct nodules. To further examine these samples, in Figure 6, OMH with PMH and OMH with BLH were compared, respectively. Again, the two species formed nodes at opposing ends of their combined data clusters, with a degree of crossover between the two.

### Detection of Porcine Heparin Contaminated with Bovine Mucosal Heparin

**Figure 7:**
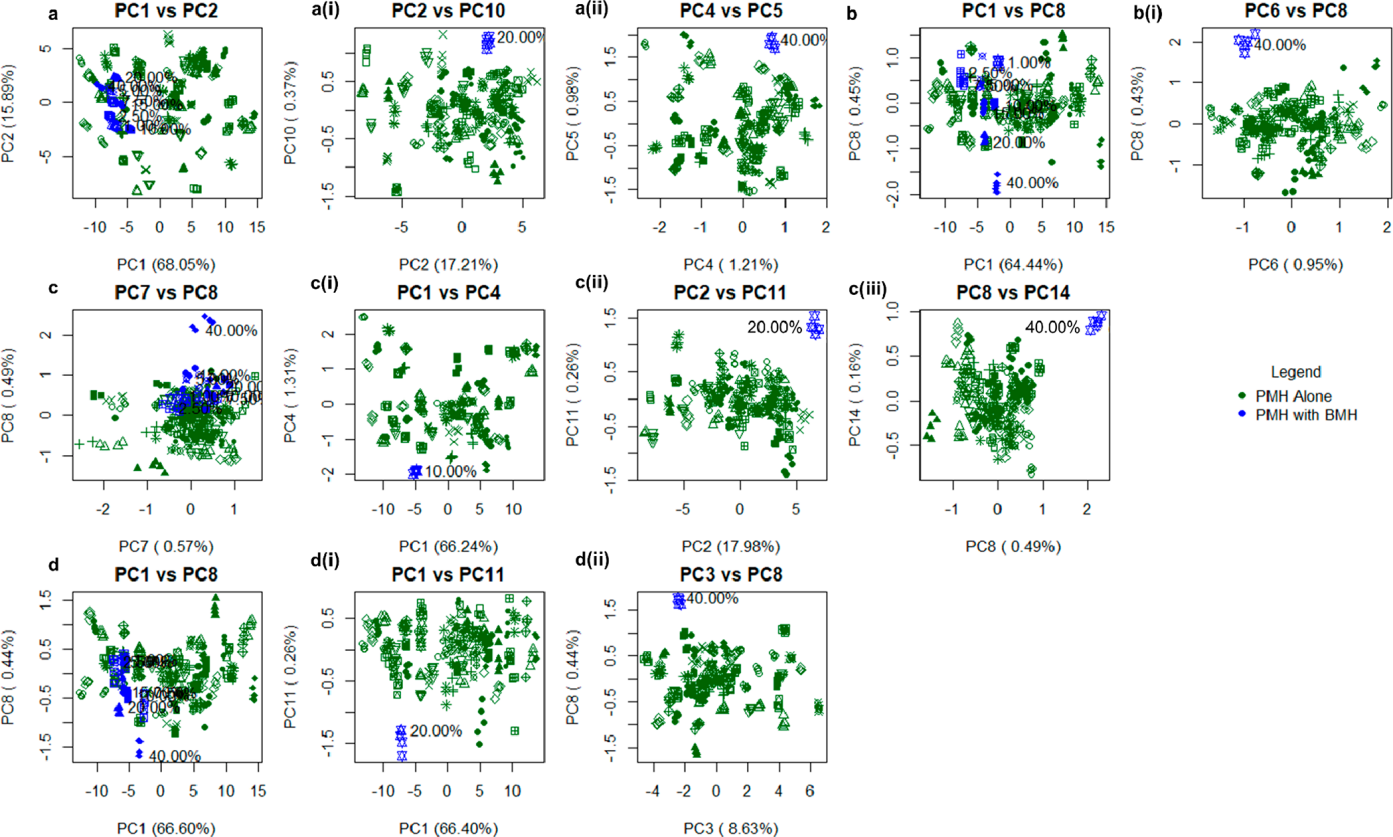
PCA score plots of FTIR-ATR spectra of the library of 69 bona fide heparin samples. These were analyzed in the presence of randomly selected PMH samples that had been contaminated deliberately with varying levels of a randomly selected BMH (confirmed to contain no other contaminants and mixed (w/w) with 40%, 20%, 15%, 10%, 7.5%, 5%, 2.5%, and 1% BMH). Four contamination series (a:d) were created using the same contaminated PMH as before, among three other randomly selected PMH samples with four randomly selected BMH samples. Series a:d correspond to Figure 2a–d. For each, the first score plot shown represents the entire contamination series against the entire PMH library. 40% contamination is detected in all cases, while 20% is detectable in series (a), (b), and (d), and 10% is detectable in series (a). (a) (i) Score plot of PC2 vs PC10 of PMH(a) vs 20.0% BMH(a) contamination. (a) (ii) Score plot of PC4 vs PC5 of PMH(a) vs 40.0% BMH(a) contamination. (b) (i) Score plot of PC6 vs PC8 of PMH(b) vs 40.0% BMH(b) contamination. (c) (i) Score plot of PC1 vs PC4 of PMH(c) vs 10.0% BMH(c) contamination. (c) (ii) Score plot of PC2 vs PC11 of PMH(c) vs 20.0% BMH(c) contamination. (c) (iii) Score plot of PC8 vs PC14 of PMH(c) vs 40.0% BMH(c) contamination. (d) (i) Score plot of PC1 vs PC11 of PMH(d) vs 20.0% BMH(d) contamination. (d) (ii) Score plot of PC3 vs PC8 of PMH(d) vs 40.0% BMH(d) contamination.

Finally, a PMH and BMH contamination series was formulated; the PMH selected for the contamination series above was employed, along with another PMH and two BMH samples selected randomly from the library. These were contaminated with each other, to form four PMH:BMH contamination series. Given that the structures of heparins from different sources are, in broad terms, similar, the comparison of any single interspecies contamination series will be sensitive to the level of structural similarity between the parental materials. For example, if a bovine-like porcine heparin is contaminated with a bovine heparin, the differences between the two will be less apparent; hence, four contamination series were employed to mitigate against this potential sampling problem. When all four contamination series were plotted against the PMH library, the contamination series emerged from the library in a linear fashion, according to the level of contamination, or clustered tightly toward one edge of the library, the 40% w/w contaminated sample being resolved from the main heparin cluster in all cases. When singularly contaminated samples were plotted against the heparin library, the 40% w/w contaminated sample could be detected in all samples, while the 20% w/w contaminated sample was separated in all series except series b (Figure 7b), and as low as 10% w/w could be resolved in series (a) (Figure 7a). High-numbered components could be employed in some cases to distinguish species; in series (a), these were PCs 5 and 10 covering 0.99% and 0.37% of the variance, respectively, and allowing separation of 20% w/w and 40% w/w contamination, respectively; in series (b), PCs 6 and 8, covering 0.95% and 0.43%, respectively, allowed separation of 40% w/w contamination; in series (c), PCs 4, 11, and 14 at 1.31%, 0.26%, and 0.16% variance enabled separation of 10%, 20%, and 40% w/w contamination, respectively; and in series (d), PCs 8 and 11, covering 0.44% and 0.26% of the variance, separated 20% and 40% w/w contamination, respectively. Such small variations are expected and arise from the high structural similarity of these molecules (Figure 7).

## Discussion

Attenuated total reflectance Fourier-transform infrared spectroscopy (ATR-FTIR) coupled with PCA was able to differentiate all GAG types in a manner similar to that of solution-based FTIR,^16^ but with significantly simpler sample preparation. Other polysaccharides were also distinguished from these, and, furthermore, the separations and groupings could be assigned on the basis of their underlying structures.

The results presented in Figure 4 demonstrate that the differentiation of samples containing even low levels of known contaminants using the ATR-FTIR approach is feasible; 0.5%, 5.0%, and 2.5% w/w of OSCS, OSAS, and DeS, respectively, were readily distinguishable by visual inspection from a preassembled library of bona fide heparins. These results are comparable to those achieved using NMR spectroscopy, which is more technically demanding and expensive than ATR-FTIR, especially since the advent of economically competitive, portable laboratory FTIR instruments. The ability to discern heparin and HS from a protein contamination was also demonstrated at a level of 0.31% w/w; however, routine techniques exist that can reliably and economically quantify protein contamination within GAGs, and these have been incorporated into pharmacopeias worldwide (e.g., Ph. Eur. 11.2 and USP-NF 2023, Issue 1). It is also clear that there is a significant level of structural complexity inherent within ATR-FTIR spectra that remains to be fully exploited and for which the earlier band assignments made previously^27−32^ for GAG FTIR cannot account. For example, the differentiation of *N*-acetyl GlcN and *N*-acetyl GalN residues, as well as information concerning the glycosidic linkage geometry that is both subtle and complex, will all require detailed complementary studies to fully decipher, and this forms the subject of ongoing investigations.

The purification of GAGs free from contaminant biomolecules is routine and undemanding for both ex vivo and in vitro biological sources, with well-established methodologies available within the scientific literature^16,33−41^ for both large- and small-scale purifications. Subsequent sample preparation for ATR-FTIR is facile and quick, and can employ solid material, avoiding dissolution and obviating any need for skilled NMR technical assistance, making the current approach both economical and accessible. The proposed method is highly sensitive and can detect structural variation between different GAG types, as well as between GAGs and other non-GAG polysaccharides. The approach can even discriminate between different cation forms of the same heparin type. It is sufficiently sensitive to distinguish between samples of heparin derived from distinct species (e.g., PMH vs BMH) and from distinct tissues within the same species (e.g., BLH vs BMH). This is of particular relevance in the context of the reintroduction of bovine-derived heparin into the U.S. market and also the need to remain vigilant to the potential addition of heparin from other, nonapproved species (e.g., ovine) into existing heparin, which would be extremely difficult to detect by current FDA methods. The present approach offers a means of readily controlling purity, origin, and process that augments the requirements of current regulatory bodies. Furthermore, it should appeal to manufacturers as a means of guaranteeing provenance and of providing confidence in their manufacturing processes at a fraction of the time and expense of NMR.

## Materials and Methods

### Materials

The details of all polysaccharide manufacturers and suppliers can be found in Tables S1–S7. All other chemicals and reagents were procured from Fisher Scientific, UK.

### Sample Preparation

Prior to the acquisition of spectra, sample preparation was performed as follows: 10 mg of dry sample was solubilized with 1 mL of ultrapure water. The solution was frozen at −80 °C and lyophilized overnight. The same preparation technique was employed for all samples to minimize variation. Care was also taken to use the same type of tubes, to minimize variation arising from different drying rates.

### Attenuated Total Reflectance Fourier-Transform Infrared Spectroscopy

Samples were recorded using a Bruker Alpha I spectrometer (Bruker, UK) in the region of 4000–400 cm^–1^, 32 scans at a resolution of 2 cm^–1^ (approximately 70 s acquisition time), *n* = 5. A background spectrum was obtained prior to recording the spectrum of each sample, using the same settings as for sample acquisition. 1–10 mg of each dried sample was placed on the crystal stage, ensuring that the entirety of the crystal was covered. A sufficient amount of sample was employed to ensure that at least 5 μm thickness was obtained, as this is the extent to which the evanescent ATR wave penetrates. The instrument stage was cleaned with water and acetone, and dried between acquisitions. Spectra were acquired using OPUS software (Bruker, UK) and exported using a CSV format.

### Fourier-Transform Infrared Spectra Processing

All data processing and subsequent analyses were performed using an Asus Vivobook Pro (M580VD-EB76) equipped with an Intel core i7- 7700HQ. Spectra were imported into R studio v1.1.463 before preliminary smoothing, employing a Savitzky–Golay algorithm (*signal* package, sgolayfilter), with a 21 neighbor, second-degree polynomial smooth. An example of the processing can be seen in Figure 1.

### Baseline Correction

Background spectra were collected prior to each sample acquisition. To further reduce the effects of environmental perturbations, each individual smoothed spectrum received a baseline correction using a seventh-order polynomial. Initially, the spectra were divided into six equally spaced regions (buckets), with the minimum absorbance value for each of these buckets, and their relevant wavenumber (*x*-axis) values were calculated. The start and end values for the spectrum were added to these values, and from the resultant 8 *x*–*y* pairs, the coefficients for a seventh-order polynomial were calculated using the *base* R *lm* function. The baseline was calculated utilizing the calculated coefficients and the original *x*-axis, before subtraction from the smoothed spectrum.

### Preparation for Principal Component Analysis

To remove the effects of inconsistent sample loading before the recording of spectra, the corrected spectra were normalized (0–1) using the equation:
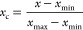
where *x* is the value to be corrected, *x*_c_ is the resultant corrected value, *x*_max_ is the maximum *x* value for the spectrum, and *x*_min_ is the minimum *x* value for the spectrum. The normalized spectra had variable regions deleted; these occur due to fluctuating CO_2_ and H_2_O levels in the environment (<700, between 2000 and 2500, and >3600 cm^–1^). The second derivative was taken using the Savitzky–Golay algorithm with 41 neighbors and a second-order polynomial. The preliminary smooth is not always required, but if this step is omitted, more neighbors are required for optimum output during this later step. It was observed that less aggressive smoothing at the beginning of the process removed anomalous baseline corrections entirely.

### Principal Component Analysis

The normalized and corrected matrix of intensities was subject to PCA using singular value decomposition with the *baseprcomp* function in R. During this process, the matrix was mean-centered, but not scaled in any other manner. Through comparison of the scree and loading plots, suitable PC scores were chosen to plot against each other as *x*–*y* scatter graphs. Low PCs were employed, assuming that the five individual repeats across this component formed compact groups.

### Defining Optimum Smoothing and Correction Parameters

To ascertain the optimum smoothing parameters, different degrees of smoothing (in terms of both neighbors and polynomial) were applied to the spectra, and the resultant PC scores were compared. If spectra were not smoothed sufficiently, their five individual repeats spread across the score plots, that is, they were dissimilar, while if the samples are oversmoothed, all samples from diverse polysaccharide types overlap, yielding no meaningful separation. The plots with the least smoothing and the most repeated sample grouping were taken forward, and these comprised 21 neighbors using a second-order polynomial for the preliminary smooth, and 41 neighbors and a second-order polynomial for the predifferentiation smooth. The optimum baseline polynomial was also defined in a similar manner, using distinct polynomials in the range of second- to ninth-order. For an *n*th-order polynomial, the spectra were divided into *n* – 1 buckets, and the same script was run, as above. Second- and third-order polynomials generated poor baselines, often resulting in early or late baseline anomalies, in which alien peaks were introduced as a consequence of their effective rigidity; for fourth-order polynomials and higher, the baselines are sufficient. A seventh-order polynomial was chosen because it yielded the fewest unusable corrections, that is, samples whose baseline becomes more curved.

### Preparation of Spectral Libraries

Prior to sample comparison using PCA, individual sample libraries were created for each polysaccharide class (PMH, OMH, BMH, BLH, CS-A, CS-C, DS, HA, HS, and OSCS). Each polysaccharide library was compared to itself with PCA, and through the use of the first 10 principal components, any outstanding samples (i.e., any that appeared distinct from the main data-cluster of the library) were removed from the spectral library. The aforementioned anomalies were due to the particularly unusual nature of the samples, abnormal bands and/or correlations between band intensities. Unique samples (OSAS and DeS) were also introduced; however, these contained far less heterogeneity, and hence a library was not required.

### Polysaccharide Sample Comparison

The various libraries were subsequently compared against each other in a series of PCA score plots, starting with all of the libraries compared against each other, including two additional sulfated polysaccharides (DeS and OSAS). To test the practical application of this method, three contamination series were created through the random selection of a PMH sample from the PMH library, and subsequent contamination on a weight-by-weight basis to create a series of contaminated samples at the levels of 0.0625, 0.125, 0.25, 0.50, 1.0, 2.5, 5.0, 7.5, 10.0, 15.0, 20.0, and 40.0% w/w. Each series contained the same PMH sample, but a different contaminant, OSCS, OSAS, or DeS. During this study, all contaminated samples were compared against the PMH library using PCA, followed by successive PCA for each individual contaminated species against the PMH library, where samples contaminated at increasing levels were compared progressively against all PMHs.

A randomly selected PMH and two randomly selected HSs were chosen to create a protein:GAG contamination series. The GAGs were contaminated to the levels of 20, 10, 5, 2.5, 1.25, 0.625, 0.3125, and 0.15625% w/w with bovine serum albumin (fraction V). The contaminated heparin was compared to the heparin library, and the contaminated HS was compared to the HS library. Samples contaminated to the level of 5% w/w and lower were also compared to their respective libraries.

All of the Hp species libraries, PMH, OMH, BMH, and BLH, were compared to each other using PCA and, following the analysis due to their clustering as opposed to separation, PMH with OMH, and PMH with BLH, underwent further comparison using PCA. A series of four PMH/BMH contamination series were then created. The comparison of PMH and BMH was selected, as these two have the highest degree of separation and represent the most likely cause of heparin contamination. The heparin used for previous contamination studies was utilized, along with three other, randomly selected PMH samples. Four BMH samples were selected at random and matched with the four PMH samples. The PMH samples were contaminated with BMH at the levels of 1.0, 2.5, 5.0, 7.5, 10.0, 15.0, 20.0, and 40.0% w/w. All contaminated samples were compared against the PMH library using PCA, followed by successive PCAs for each individual contaminated species against the PMH library, where samples contaminated at increasing levels were compared progressively against all PMHs.

## Supporting Information Available

Supplier details for carbohydrate library components, raw and processed (baseline corrected, smoothed, and normalized) ATR-FTIR spectra, second-order differential ATR-FTIR spectra, and PCA outputs for all experiments conducted (PDF)

## Abbreviations

ATR: attenuated total reflectance
BLH: bovine lung heparin
BMH: bovine mucosal heparin
BSE: bovine spongiform encephalopathy
CS-A: chondroitin sulfate-A
CS-C: chondroitin sulfate-C
DeS: dextran sulfate
DoS: degree of sulfation
DS: dermatan sulfate
FTIR: Fourier transform infrared
GAG: glycosaminoglycan
GalN: d-galactosamine
GlcN: d-glucosamine
HA: hyaluronic acid
Hp: heparin
HS: heparan sulfate
LMWH: low molecular weight heparin
OSAS: oversulfated agarose sulfate
OSCS: oversulfated chondroitin sulfate
OMH: ovine mucosal heparin
NMR: nuclear magnetic resonance
PC: principal component
PCA: principal component analysis
PMH: porcine mucosal heparin
SVD: singular value decomposition
