# Supplementary material for: Analysis of Heparin Samples by Attenuated Total Reflectance Fourier-Transform Infrared Spectroscopy in the Solid State
Source: ACS Cent Sci. 2023 Feb 14;9(3):381–92. doi: 10.1021/acscentsci.2c01176 (PMC10037494; doi:10.1021/acscentsci.2c01176)
Supplement: Supplementary file 1 — oc2c01176_si_001.pdf [file oc2c01176_si_001.pdf]

Supplementary Data for "Analysis of heparin samples by attenuated total reflectance Fourier-transform infrared (ATR-FTIR) spectroscopy in the solid-state."

Anthony Devlin1,3, Courtney J. Mycroft-West1,2, Marcelo Andrade de Lima1, Marco Guerrini3, Edwin A. Yates3,1 and Mark A. Skidmore* 1,3

1) Centre for Glycoscience Research, Keele University, Huxley Building, Keele, Staffordshire, ST5 5BG, United Kingdom. **2)** The Rosalind Franklin Institute, Harwell Science and Innovation Campus, Oxfordshire, OX11 0QG, United Kingdom. **3)** Department of Biochemistry and Systems Biology, Institute of Structural, Molecular and Integrative Biology, University of Liverpool, Crown Street, Liverpool, L69 7ZB, United Kingdom. **4)** Istituto di Ricerche Chimiche e Biochimiche 'G. Ronzoni', Via G. Colombo 81, 20133, Milan, Italy. *** Corresponding author:** m.a.skidmore@keele.ac.uk. Tel: +44 (0)1782 733945

All raw IR spectra and the spectra thereof for each preparative step are included here. For each PCA performed in the manuscript, a matrix plot for PCs 1:9, scree plot, loading plots for PCs 1:4, and score plots for PCs 1:5 as well as a page for any relevant preparation information are included here.

Graph Title Key –

"Raw": Raw data post 21neighbour, 2nd polynomial Savitsky Golay smooth.

"Normalised (0-1)": Raw spectra, following a custom 7th order polynomial baseline correction and subsequent y-axis scaling between 0 and 1.

"D2": second derivative of Normalised spectra, following a 41neighbour, 2nd order polynomial Savitsky Golay smooth.

Contents:

Page S2-S6: Tables of used samples

Page S7-S23: Raw spectra of PMH, OMH, BMH, BLH and HS.

Page S24-S40: Normalised spectra of PMH, OMH, BMH, BLH and HS.

Page S41-S57: D2 spectra of PMH, OMH, BMH, BLH and HS.

Page S58-S62: Raw spectra of CS, DS, OSCS and HA.

Page S63-S67: Normalised spectra of CS, DS, OSCS and HA.

Page S68-S72: D2 spectra of CS, DS, OSCS and HA.

*Page S73-S75:*Raw spectra of OSCS, OSAS and Des Selected for contamination, and subsequent contaminated samples.

Page S76-S78: Normalised spectra of OSCS, OSAS and Des Selected for contamination, and subsequent contaminated samples.

Page S79-S81: D2 spectra of OSCS, OSAS and Des Selected for contamination, and subsequent contaminated samples.

Page S82-S84: Raw spectra of PMH contaminated with BMH, series a:d

Page S85-S87: Normalised spectra of PMH contaminated with BMH, series a:d

Page S88-S90: D2 spectra of PMH contaminated with BMH, series a:d

Page S91-S109: PCA of Heps vs GAGs ("Everything")

Page S110-S349: PCA of OSCS contamination series, All vs PMH and each contaminated sample.

Page S350-S589: PCA of OSAS contamination series, All vs PMH and each contaminated sample.

Page S590-S879: PCA of DeS contamination series, All vs PMH and each contaminated sample.

Page S880-S935: PCA of PMH vs BMH vs OMG vs BLH, PMH vs OMH and PMH vs BLH

Page S936-S1528: PCA of PMH contaminated with BMH, All vs PMH and each contaminated sample, series a:d

Table 1: Table of PMH's used and their source(s)

Sample Name	Supplier	Batch No.
Hep 3	Acros	A0286020
Hep 4	Applichem	1X007824
Hep A	Applichem	30009511
Hep C	Applichem	40011435
Hep D	Calbiochem	375093
Hep E	Celsius	PH-89915
Hep F	Bioberica	G3060
Hep H	Leo Pharmaceuticals	DD6985
Hep J	Sigma	H3393
Hep K	Sigma	SLBQ1361V
Hep L	Sigma	SLBP2731V
Hep M	Sigma	SLBN9145V
Hep N	Sigma	SLBP6873V
Hep O	Alfa Aesar	10198820
Hep P	Acros	A0382416
Hep Q	Sigma	SLBQ1351V
Hep R	Sigma	SLBP2731V
Hep S	Applichem	6C015265
Carsyn Hep	Carbosynth	YH093541501
Hstd	EU Pharma Ref Std	Y001282
RStd	Ronzoni Institute	G9850
P1	Ronzoni Institute	G12010
P2	Ronzoni Institute	G12007
P3	Ronzoni Institute	G12006
P4	Ronzoni Institute	A5363
P5	Ronzoni Institute	
P6	Ronzoni Institute	A5675
P7	Ronzoni Institute	G7768
P8	Ronzoni Institute	G12009
P9	Ronzoni Institute	G12012
P10	Ronzoni Institute	G12013
P11	Ronzoni Institute	A353PSA
P12	Ronzoni Institute	A3201QA
P13	Ronzoni Institute	A3288QA
P14	Ronzoni Institute	A5229
P15	Ronzoni Institute	A5595
P16	Ronzoni Institute	A5605
P17	Ronzoni Institute	G7765
P19	Ronzoni Institute	G10921
P20	Ronzoni Institute	G10922
P21	Ronzoni Institute	G11653
P23	Ronzoni Institute	G11657
P24	Ronzoni Institute	G12011
P25	Ronzoni Institute	A3100A
P26	Ronzoni Institute	A5668
P27	Ronzoni Institute	G13456
P28	Ronzoni Institute	G11429
P29	Ronzoni Institute	G11444
P30	Ronzoni Institute	G1143
P31	Ronzoni Institute	A3787
P32	Ronzoni Institute	A3934
P33	Ronzoni Institute	A4077
P34	Ronzoni Institute	A4427
P35	Ronzoni Institute	A4464
P36	Ronzoni Institute	A5302
P37	Ronzoni Institute	A5306
P38	Ronzoni Institute	A5319
P39	Ronzoni Institute	A5444
P40	Ronzoni Institute	A5468
P41	Ronzoni Institute	A5569
P42	Ronzoni Institute	A5613
P43	Ronzoni Institute	A5615
P44	Ronzoni Institute	A01106023
P45	Ronzoni Institute	G11627
P47	Ronzoni Institute	G11629
P48	Ronzoni Institute	G11661
P49	Ronzoni Institute	G13100
P50	Ronzoni Institute	G13425
P51	Ronzoni Institute	G13426
P52	Ronzoni Institute	G13427
P53	Ronzoni Institute	G6202
P54	Ronzoni Institute	G6203

Table 2: Table of BMH's used and their source(s)

Sample Name	Supplier	Batch No.
B1	Ronzoni Institute	G12019
B2	Ronzoni Institute	G12018
B3	Ronzoni Institute	G12015
B4	Ronzoni Institute	G12028
B5	Ronzoni Institute	G12022
B6	Ronzoni Institute	G7850
B7	Ronzoni Institute	G7847
B9	Ronzoni Institute	G7773
B10	Ronzoni Institute	G7848
B11	Ronzoni Institute	G7844
B12	Ronzoni Institute	G7841
B13	Ronzoni Institute	G7846
B14	Ronzoni Institute	G10803
B15	Ronzoni Institute	G7845
B16	Ronzoni Institute	G12014
B18	Ronzoni Institute	G12017
B19	Ronzoni Institute	G12020
B20	Ronzoni Institute	G12021
B21	Ronzoni Institute	G12662
B22	Ronzoni Institute	G12667
B23	Ronzoni Institute	G12668
B24	Ronzoni Institute	G12669
B26	Ronzoni Institute	G12804
B27	Ronzoni Institute	G13420
B28	Ronzoni Institute	G13421
B29	Ronzoni Institute	G13422
B30	Ronzoni Institute	G13423
B31	Ronzoni Institute	G12657
B32	Ronzoni Institute	G12658
B33	Ronzoni Institute	G12659
B34	Ronzoni Institute	G12661
B35	Ronzoni Institute	G12664
B36	Ronzoni Institute	G12665
B37	Ronzoni Institute	G12666
B38	Ronzoni Institute	G13420
B39	Ronzoni Institute	G13421
B40	Ronzoni Institute	G13422
B41	Ronzoni Institute	G13423
B42	Ronzoni Institute	G12660
B43	Ronzoni Institute	G12663
B44	Ronzoni Institute	G1467
B45	Ronzoni Institute	G1468
B46	Ronzoni Institute	G1469
B47	Ronzoni Institute	G1470
B48	Ronzoni Institute	G1471
B49	Ronzoni Institute	G1472
B50	Ronzoni Institute	G1473
B51	Ronzoni Institute	G1474
B52	Ronzoni Institute	G1475
B53	Ronzoni Institute	G1476
B60	Ronzoni Institute	G12802
B61	Ronzoni Institute	G12803
B62	Ronzoni Institute	G13421
B63	Ronzoni Institute	G13422
B64	Ronzoni Institute	G13504
B65	Ronzoni Institute	G13505
B66	Ronzoni Institute	G13506

Table 3: Table of OMH's used and their source(s)

Sample Name	Supplier	Batch No.
O1	Ronzoni Institute	G12028
O2	Ronzoni Institute	G12026
O3	Ronzoni Institute	G12025
O4	Ronzoni Institute	G12024
O5	Ronzoni Institute	G12029
O6	Ronzoni Institute	G8117
O7	Ronzoni Institute	G11448
O8	Ronzoni Institute	G12077
O10	Ronzoni Institute	G1371
O11	Ronzoni Institute	G1372
O12	Ronzoni Institute	G1373
O13	Ronzoni Institute	G12205
O14	Ronzoni Institute	G12206
O15	Ronzoni Institute	G12207
O16	Ronzoni Institute	G12288
O17	Ronzoni Institute	G12301
O18	Ronzoni Institute	G12302
O19	Ronzoni Institute	G13096
O20	Ronzoni Institute	G13097
O21	Ronzoni Institute	G13098
O24	Ronzoni Institute	G13689
O25	Ronzoni Institute	G13690
O26	Ronzoni Institute	G13691
O27	Gifted by Ed Yates	NA
O28	Ronzoni Institute	G11447
O29	Ronzoni Institute	G11449
O30	Ronzoni Institute	G11480
O31	Ronzoni Institute	G11482
O32	Ronzoni Institute	G11489
O33	Ronzoni Institute	G11936
O34	Ronzoni Institute	G11937
O36	Ronzoni Institute	G10786
O38	Ronzoni Institute	GUK

Table 4: Table of BLH's used and their source(s)

Sample Name	Supplier	Batch No.
BLH1	Ronzoni Institute	G4141
BLH2	Ronzoni Institute	G46
BLH3	Ronzoni Institute	G65
BLH4	Ronzoni Institute	G121
BLH5	Ronzoni Institute	G11099
BLH6	Ronzoni Institute	G11100
BLH7	Ronzoni Institute	G11101
BLH8	Ronzoni Institute	G11388
BLH9	Ronzoni Institute	G11389
BLH10	Ronzoni Institute	G11463
BLH11	Ronzoni Institute	G12271
BLH12	Ronzoni Institute	G12272
BLH13	Ronzoni Institute	G12275
BLH14	Ronzoni Institute	G17273
BLH15	Ronzoni Institute	G17274
BLH18	Ronzoni Institute	G11757
BLH19	Ronzoni Institute	G11758
BLH20	Ronzoni Institute	G11759
BLH21	Ronzoni Institute	G10787

Table 5: Table of HAs used and their source(s)

Sample Name	Supplier	Batch No.
HAstd	Ronzoni Institute	G9980
HA1	Ronzoni Institute	G13998
HA2	Ronzoni Institute	G1039
HA3	Ronzoni Institute	G1093
HA4	Ronzoni Institute	G1824
HA5	Ronzoni Institute	G5733
HA6	Ronzoni Institute	G28
HA11	Ronzoni Institute	G5673
HA12	Ronzoni Institute	G5674
HA14	Ronzoni Institute	G45

Table 6: Table of HSs used and their source(s)

Sample Name	Supplier	Batch No.
PHS	Ronzoni Institute	G116
OHS	Ronzoni Institute	G13014
OHS	Ronzoni Institute	G13015
HS1	Ronzoni Institute	G108
HS2	Ronzoni Institute	G120
HS3	Ronzoni Institute	G1568
HS4	Ronzoni Institute	G41
HS5	Ronzoni Institute	G42
HS8	Ronzoni Institute	G101
HS9	Ronzoni Institute	G107
HS12	Ronzoni Institute	G1807
HS14	Ronzoni Institute	HHS-S
HS16	Ronzoni Institute	G34
HS17	Ronzoni Institute	G67
HS18	Ronzoni Institute	G104
HS21	Ronzoni Institute	G5021
HS22	Ronzoni Institute	LOTUKa
HS23	Ronzoni Institute	G1567
HS24	Ronzoni Institute	G7861
HS25	Ronzoni Institute	O258
HS26	Ronzoni Institute	OBGR
HS27	Ronzoni Institute	G5547
HS28	Ronzoni Institute	HS141
HS29	Ronzoni Institute	HS9335
HS30	Ronzoni Institute	HS137
HS31	Ronzoni Institute	HS138
HS32	Ronzoni Institute	HS139 - 140
HS33	Ronzoni Institute	HS149
HS34	Ronzoni Institute	HS934
HS35	Ronzoni Institute	HS20594
HS36	Ronzoni Institute	HS271192

Table 7: Table of Chondroitins used and their source(s)

Sample Name	Supplier	Batch No.
CSA1	Carbosynth	YC152881701
CSA2	Ronzoni Institute	G920
CSA3	Ronzoni Institute	G1125B
CSA5	Ronzoni Institute	G2426
CSA7	Ronzoni Institute	G167
CSA8	Ronzoni Institute	G3432
CSA9	Ronzoni Institute	G4090
DSstd	Celsus	10596
DS3	Ronzoni Institute	G1021
DS6	Ronzoni Institute	G33
DS8	Ronzoni Institute	G593
DS9	Ronzoni Institute	G5026
DS10	Ronzoni Institute	G6987
DS11	Ronzoni Institute	G904D51
DS12	Ronzoni Institute	DSB2
DS13	Ronzoni Institute	G904
DS14	Ronzoni Institute	G904/1
DS16	Ronzoni Institute	G904D44
DS17	Ronzoni Institute	G904D47
DS18	Ronzoni Institute	G904D54
DS20	Ronzoni Institute	G1084
DS21	Ronzoni Institute	G6322
DS22	Ronzoni Institute	GC180
DS23	Ronzoni Institute	GC185
DS24	Ronzoni Institute	GC181
DS25	Ronzoni Institute	GC182
DS26	Ronzoni Institute	A1908
DS27	Ronzoni Institute	DS52
CSC2	Ronzoni Institute	G1223
CSC3	Ronzoni Institute	G56
CSC4	Ronzoni Institute	G113
CSC6	Ronzoni Institute	MPS2
CSC7	Ronzoni Institute	MPS3
CS1	Ronzoni Institute	DFSTDO
CS2	Ronzoni Institute	MPSTDN
CS4	Ronzoni Institute	G267
CS5	Ronzoni Institute	G1381
CS6	Ronzoni Institute	G1673A
CS7	Ronzoni Institute	G7222
CS8	Ronzoni Institute	G11251I
CS9	Ronzoni Institute	P6098
CS11	Ronzoni Institute	G9175
CS12	Ronzoni Institute	G9176

Sample Name	***Supplier***	***Batch No.***
CS13	Ronzoni Institute	G9178
CS15	Ronzoni Institute	G8501
CS16	Ronzoni Institute	G8585
CS17	Ronzoni Institute	G8783
CS18	Ronzoni Institute	G9131
CS19	Ronzoni Institute	G9132
CS20	Ronzoni Institute	G9133
CS21	Ronzoni Institute	G91039
CS22	Ronzoni Institute	G6866
OSCS	FDA	H0M191
OSCS1a	Ronzoni Institute	G62439 (1)
OSCS1b	Ronzoni Institute	G62439 (2)
OSCS1c	Ronzoni Institute	G62439 (3)
OSCS2	Ronzoni Institute	G6243b1
OSCS3	Ronzoni Institute	G6296
OSCS4	Ronzoni Institute	G6469
OSAS	*Made in house*	NA
DeS	Fluka	1053244-42507314

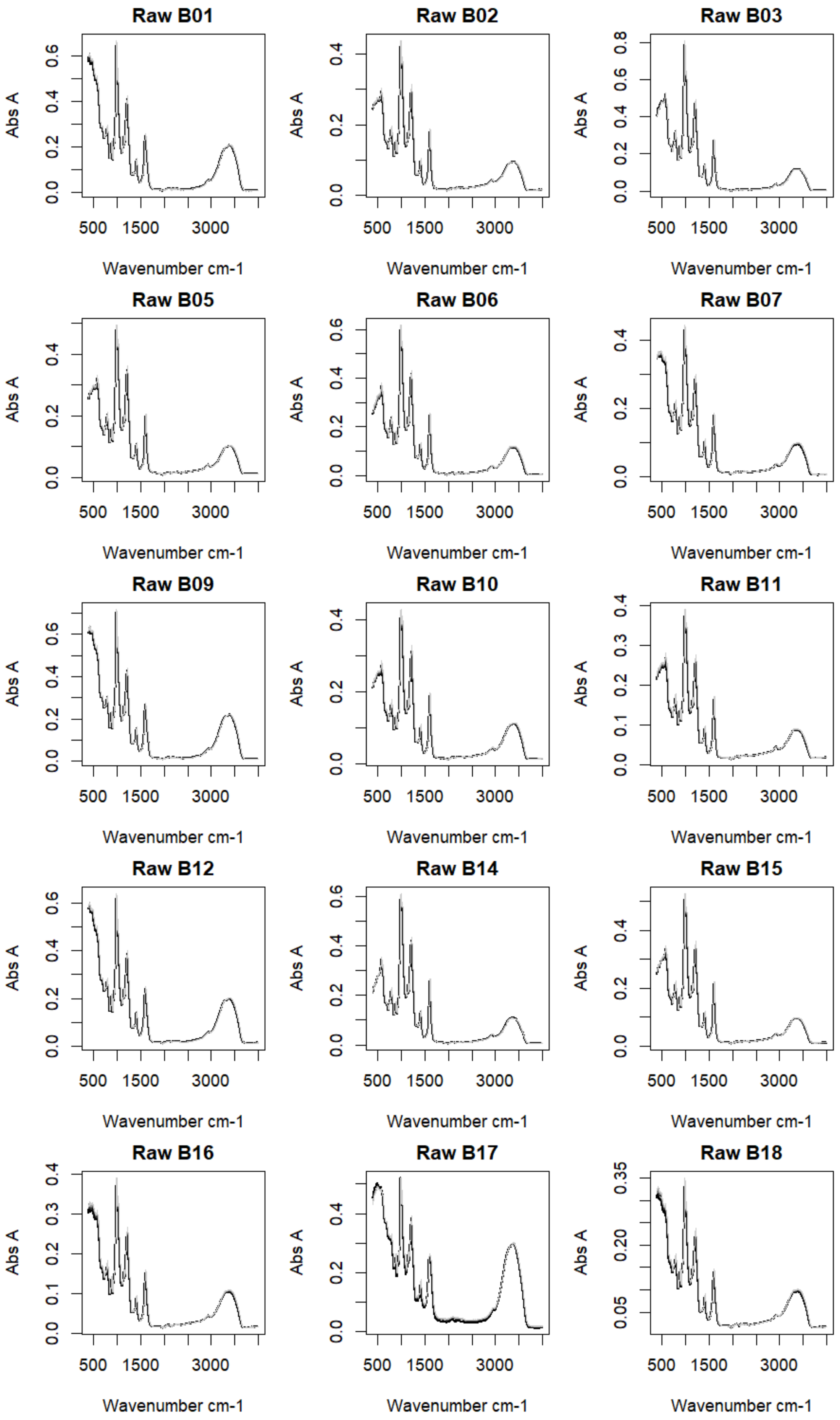
Raw B01
Raw B02
Raw B03
Raw B05
Raw B06
Raw B07
Raw B09
Raw B10
Raw B11
Raw B12
Raw B14
Raw B15
Raw B16
Raw B17
Raw B18
Abs A
Wavenumber cm-1
500
1500
3000

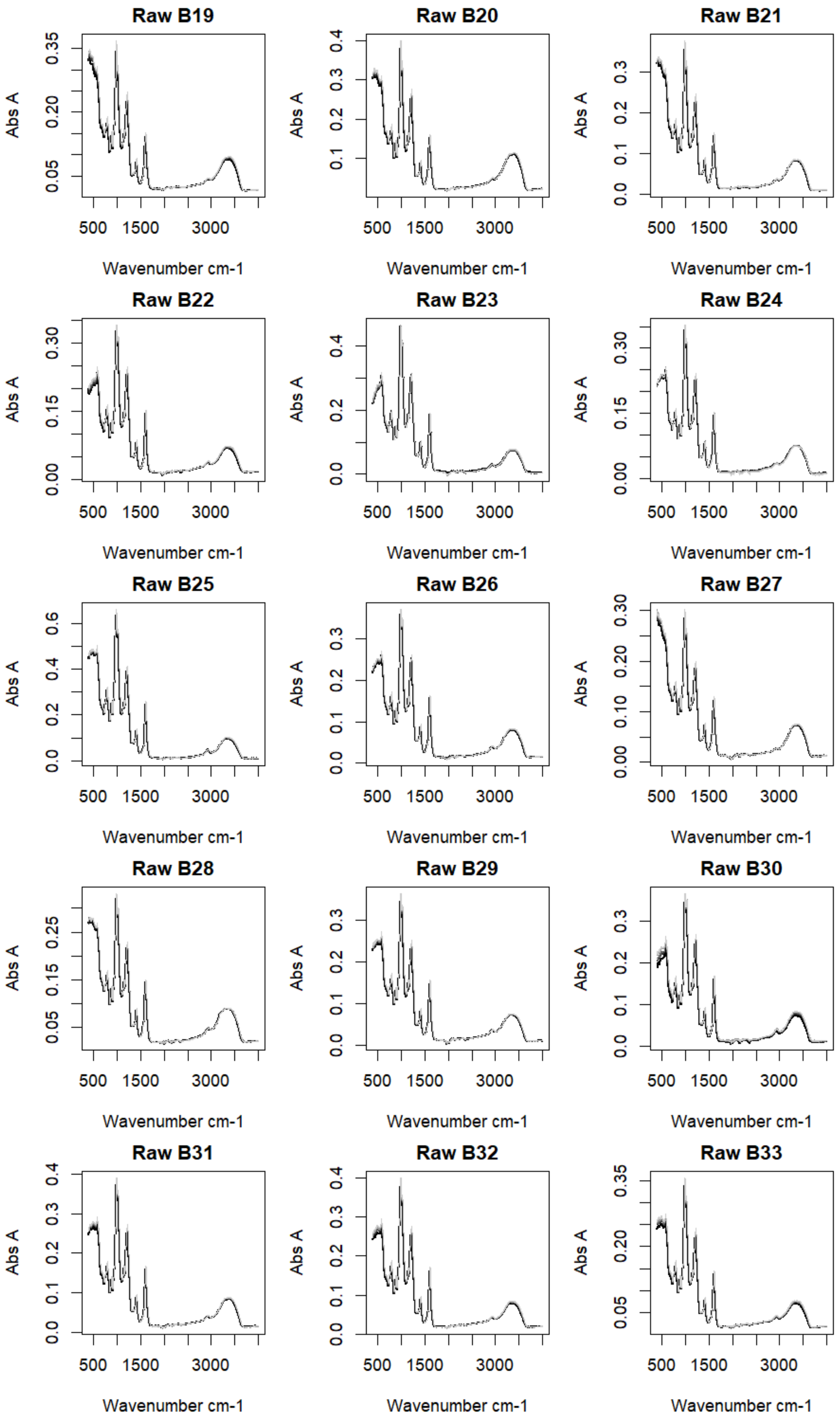
Raw B19
Abs A
0.05
0.20
0.35
500
1500
3000
Wavenumber cm-1
Raw B20
Abs A
0.1
0.2
0.3
0.4
500
1500
3000
Wavenumber cm-1
Raw B21
Abs A
0.0
0.1
0.2
0.3
500
1500
3000
Wavenumber cm-1
Raw B22
Abs A
0.00
0.15
0.30
500
1500
3000
Wavenumber cm-1
Raw B23
Abs A
0.0
0.2
0.4
500
1500
3000
Wavenumber cm-1
Raw B24
Abs A
0.00
0.15
0.30
500
1500
3000
Wavenumber cm-1
Raw B25
Abs A
0.0
0.2
0.4
0.6
500
1500
3000
Wavenumber cm-1
Raw B26
Abs A
0.0
0.1
0.2
0.3
500
1500
3000
Wavenumber cm-1
Raw B27
Abs A
0.00
0.10
0.20
0.30
500
1500
3000
Wavenumber cm-1
Raw B28
Abs A
0.05
0.15
0.25
500
1500
3000
Wavenumber cm-1
Raw B29
Abs A
0.0
0.1
0.2
0.3
500
1500
3000
Wavenumber cm-1
Raw B30
Abs A
0.0
0.1
0.2
0.3
500
1500
3000
Wavenumber cm-1
Raw B31
Abs A
0.0
0.1
0.2
0.3
0.4
500
1500
3000
Wavenumber cm-1
Raw B32
Abs A
0.0
0.1
0.2
0.3
0.4
500
1500
3000
Wavenumber cm-1
Raw B33
Abs A
0.05
0.20
0.35
500
1500
3000
Wavenumber cm-1

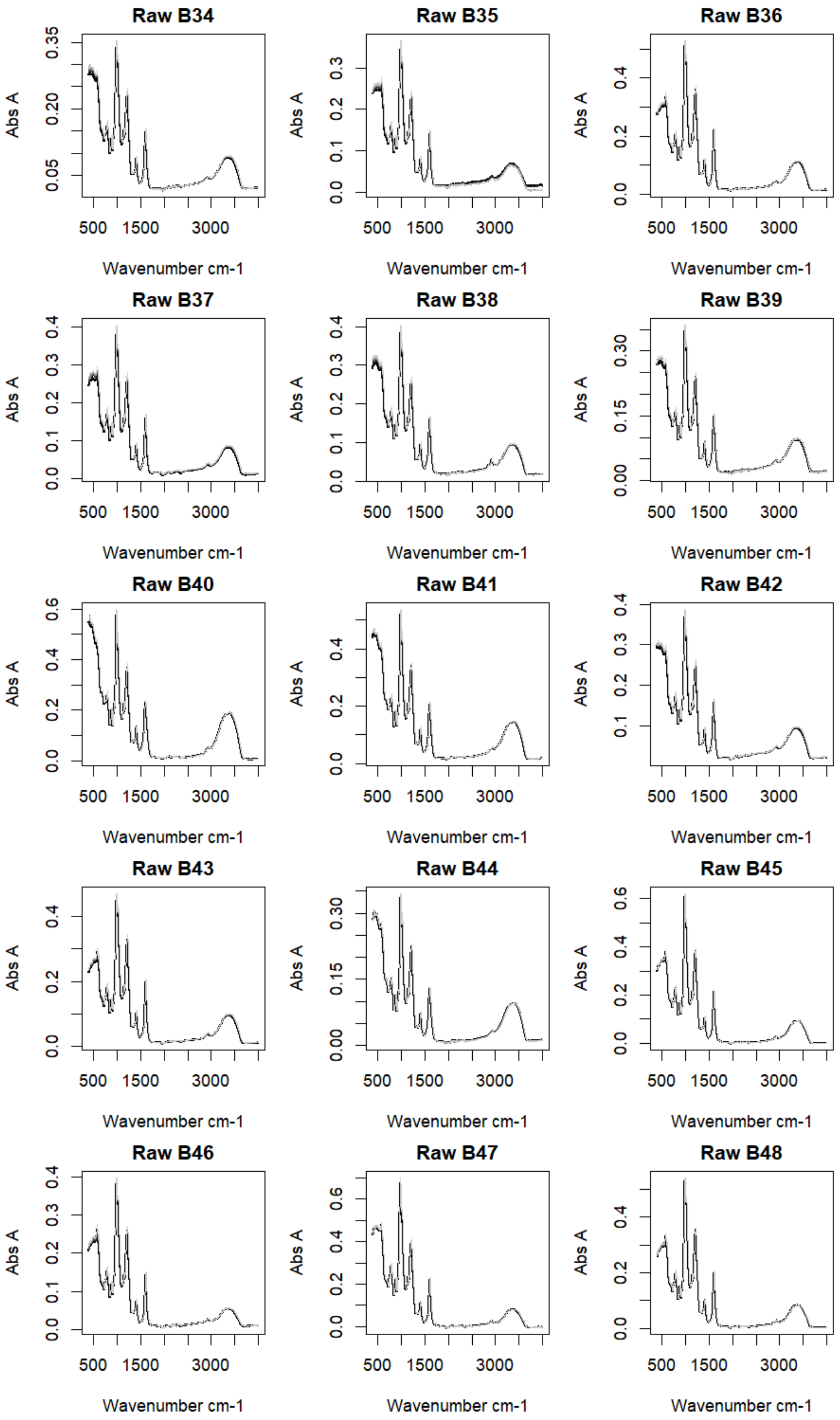
Raw B34
Raw B35
Raw B36
Raw B37
Raw B38
Raw B39
Raw B40
Raw B41
Raw B42
Raw B43
Raw B44
Raw B45
Raw B46
Raw B47
Raw B48
Abs A
Wavenumber cm-1
500 1500 3000

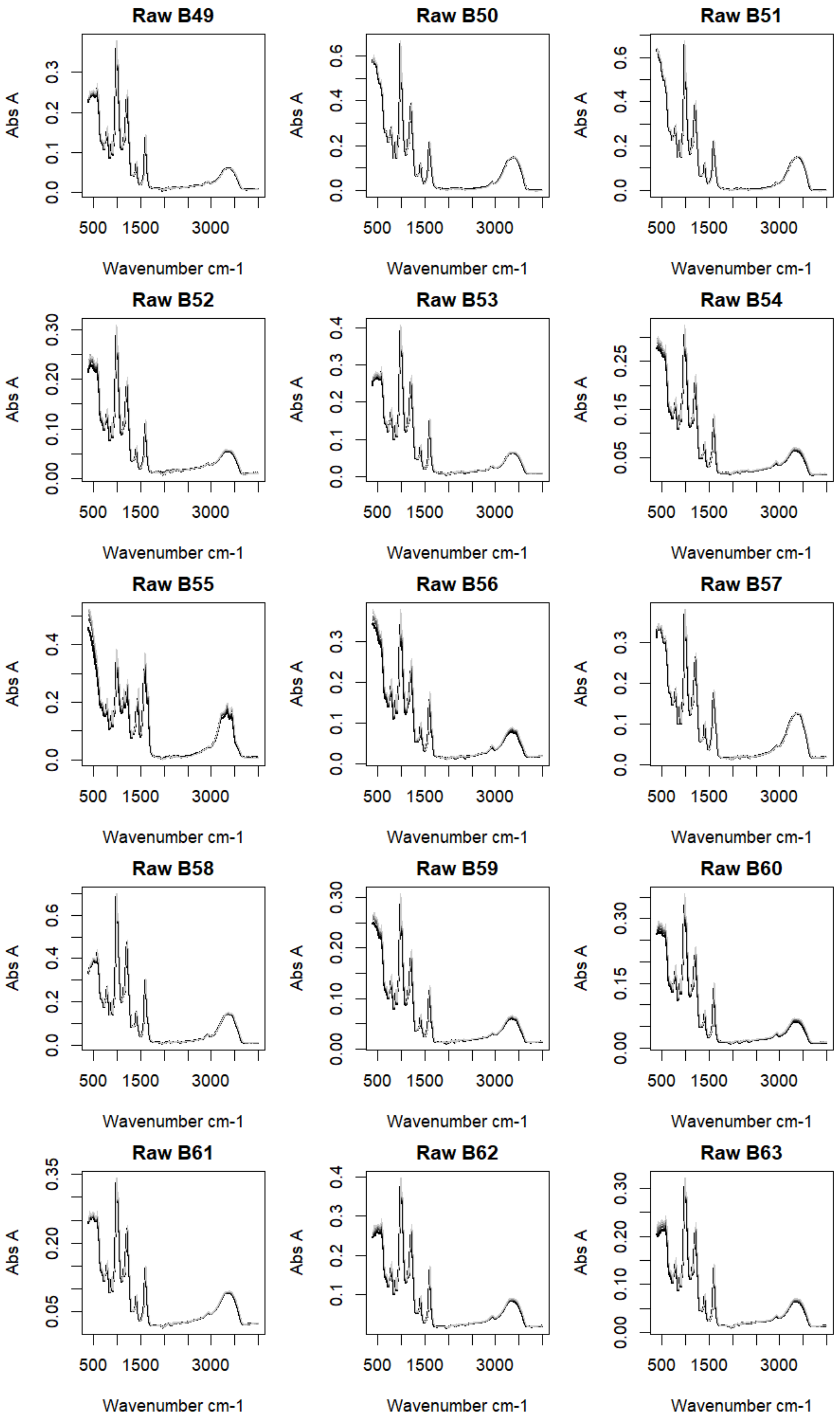

Raw B49
Raw B50
Raw B51
Raw B52
Raw B53
Raw B54
Raw B55
Raw B56
Raw B57
Raw B58
Raw B59
Raw B60
Raw B61
Raw B62
Raw B63
Abs A
Wavenumber cm-1
500
1500
3000

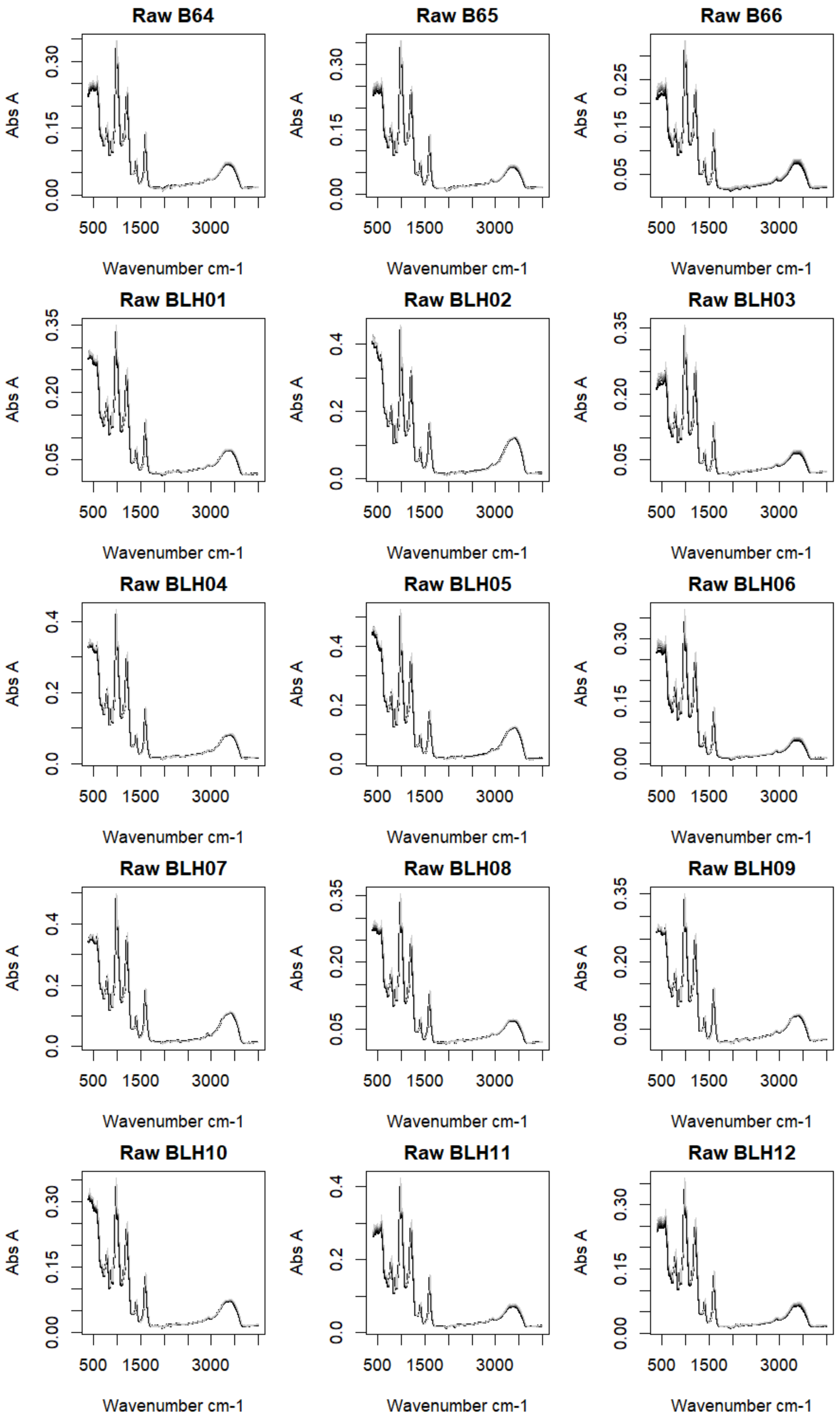
Raw B64
Raw B65
Raw B66
Raw BLH01
Raw BLH02
Raw BLH03
Raw BLH04
Raw BLH05
Raw BLH06
Raw BLH07
Raw BLH08
Raw BLH09
Raw BLH10
Raw BLH11
Raw BLH12
Abs A
Wavenumber cm-1
500 1500 3000

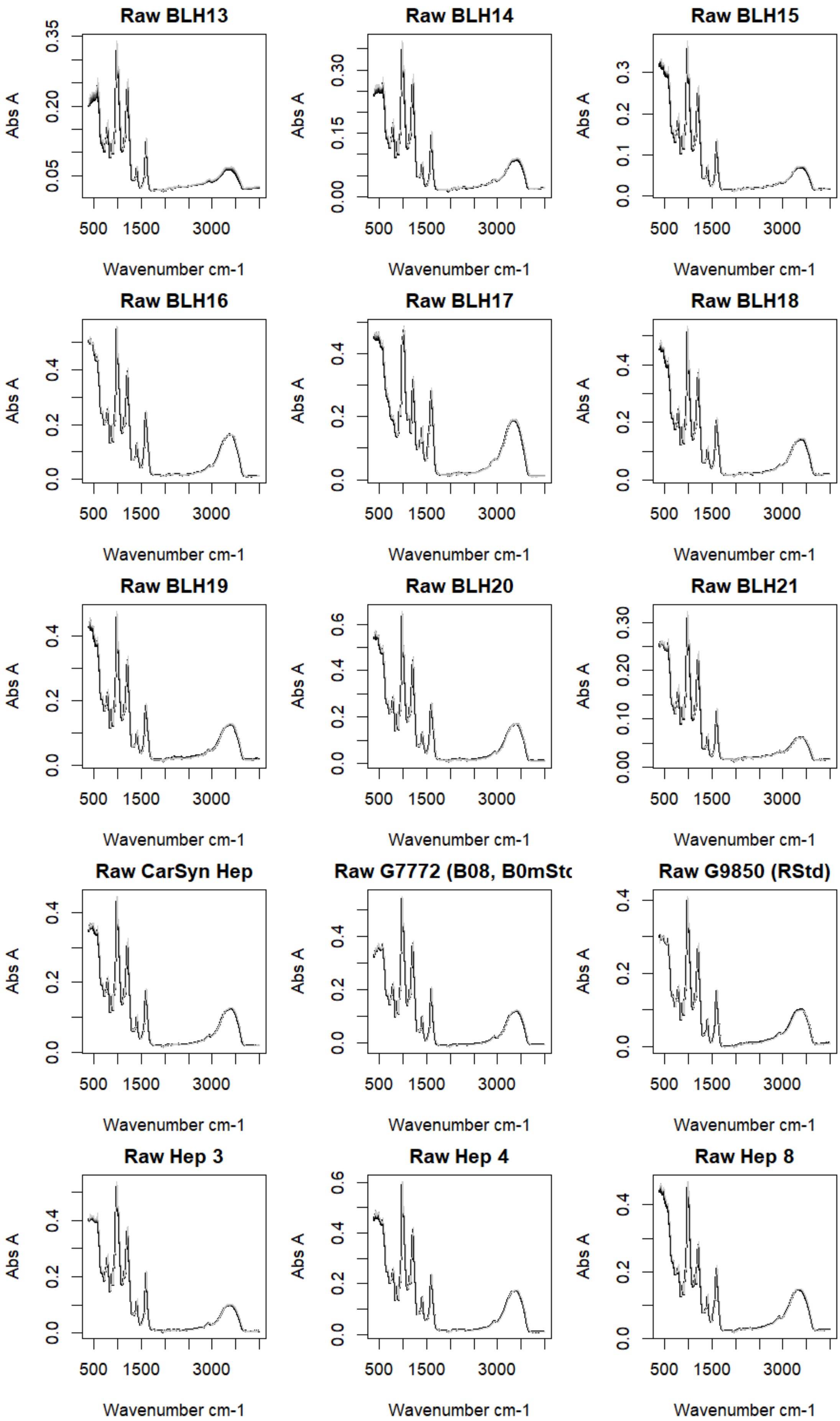

Raw BLH13
Raw BLH14
Raw BLH15
Raw BLH16
Raw BLH17
Raw BLH18
Raw BLH19
Raw BLH20
Raw BLH21
Raw CarSyn Hep
Raw G7772 (B08, B0mStc
Raw G9850 (RStd)
Raw Hep 3
Raw Hep 4
Raw Hep 8
Abs A
Wavenumber cm-1
500
1500
3000

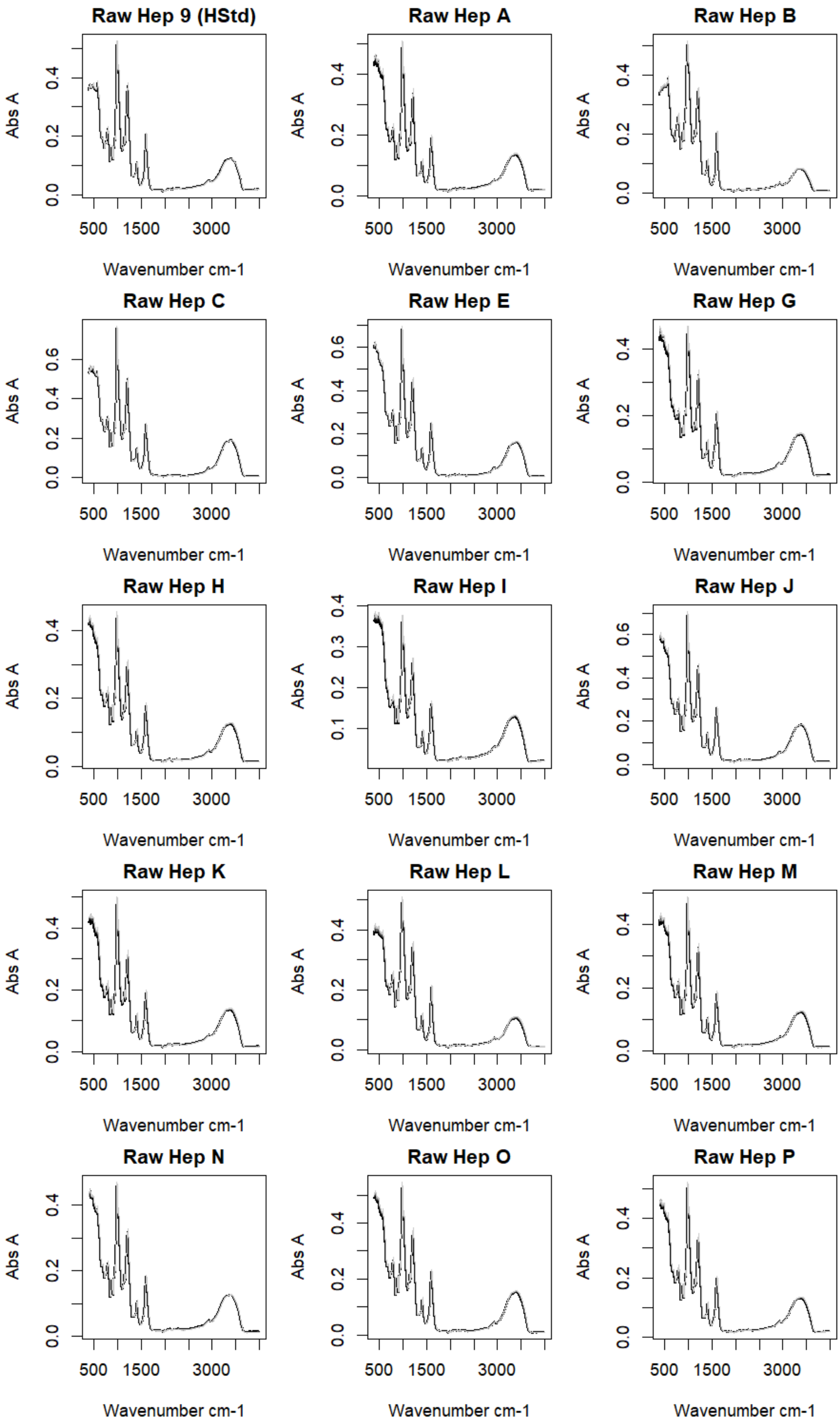
Raw Hep 9 (HStd)
Raw Hep A
Raw Hep B
Raw Hep C
Raw Hep E
Raw Hep G
Raw Hep H
Raw Hep I
Raw Hep J
Raw Hep K
Raw Hep L
Raw Hep M
Raw Hep N
Raw Hep O
Raw Hep P
Abs A
Wavenumber cm-1
500
1500
3000

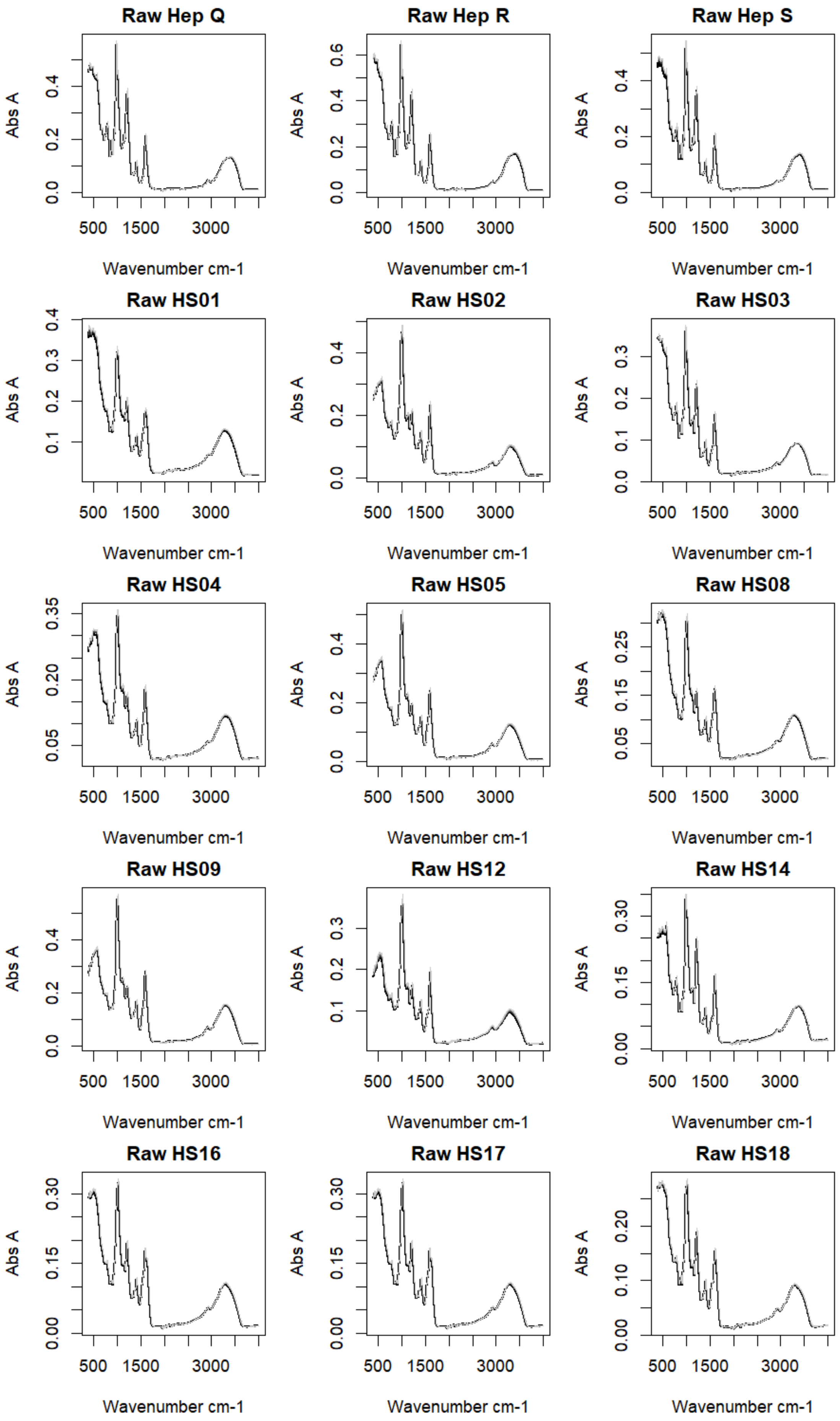
Raw Hep Q
Raw Hep R
Raw Hep S
Raw HS01
Raw HS02
Raw HS03
Raw HS04
Raw HS05
Raw HS08
Raw HS09
Raw HS12
Raw HS14
Raw HS16
Raw HS17
Raw HS18
Abs A
Wavenumber cm-1
500 1500 3000

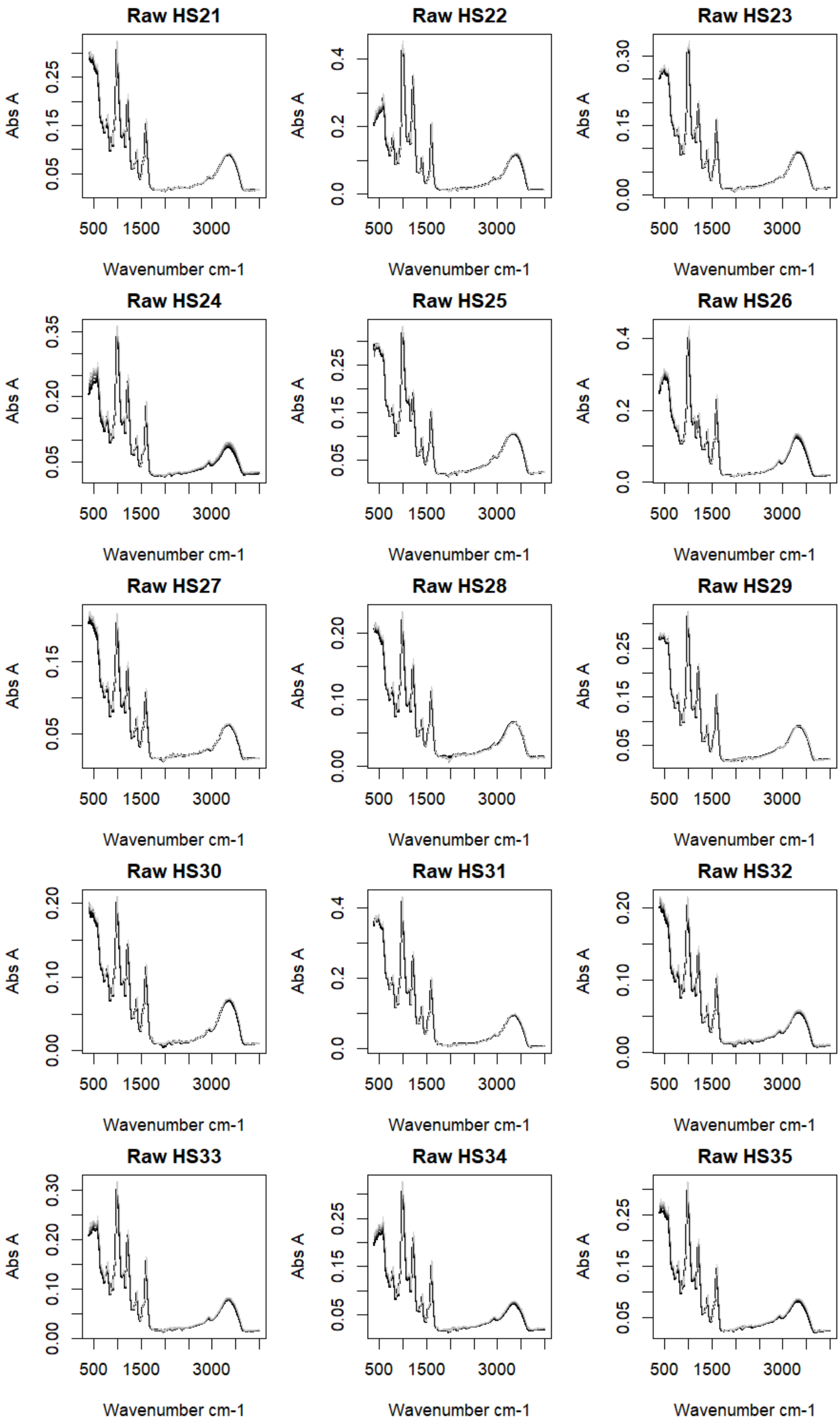
Raw HS21
Abs A
0.05 0.15 0.25
500 1500 3000
Wavenumber cm-1
Raw HS22
Abs A
0.0 0.2 0.4
500 1500 3000
Wavenumber cm-1
Raw HS23
Abs A
0.00 0.15 0.30
500 1500 3000
Wavenumber cm-1
Raw HS24
Abs A
0.05 0.20 0.35
500 1500 3000
Wavenumber cm-1
Raw HS25
Abs A
0.05 0.15 0.25
500 1500 3000
Wavenumber cm-1
Raw HS26
Abs A
0.0 0.2 0.4
500 1500 3000
Wavenumber cm-1
Raw HS27
Abs A
0.05 0.15
500 1500 3000
Wavenumber cm-1
Raw HS28
Abs A
0.00 0.10 0.20
500 1500 3000
Wavenumber cm-1
Raw HS29
Abs A
0.05 0.15 0.25
500 1500 3000
Wavenumber cm-1
Raw HS30
Abs A
0.00 0.10 0.20
500 1500 3000
Wavenumber cm-1
Raw HS31
Abs A
0.0 0.2 0.4
500 1500 3000
Wavenumber cm-1
Raw HS32
Abs A
0.00 0.10 0.20
500 1500 3000
Wavenumber cm-1
Raw HS33
Abs A
0.00 0.10 0.20 0.30
500 1500 3000
Wavenumber cm-1
Raw HS34
Abs A
0.05 0.15 0.25
500 1500 3000
Wavenumber cm-1
Raw HS35
Abs A
0.05 0.15 0.25
500 1500 3000
Wavenumber cm-1

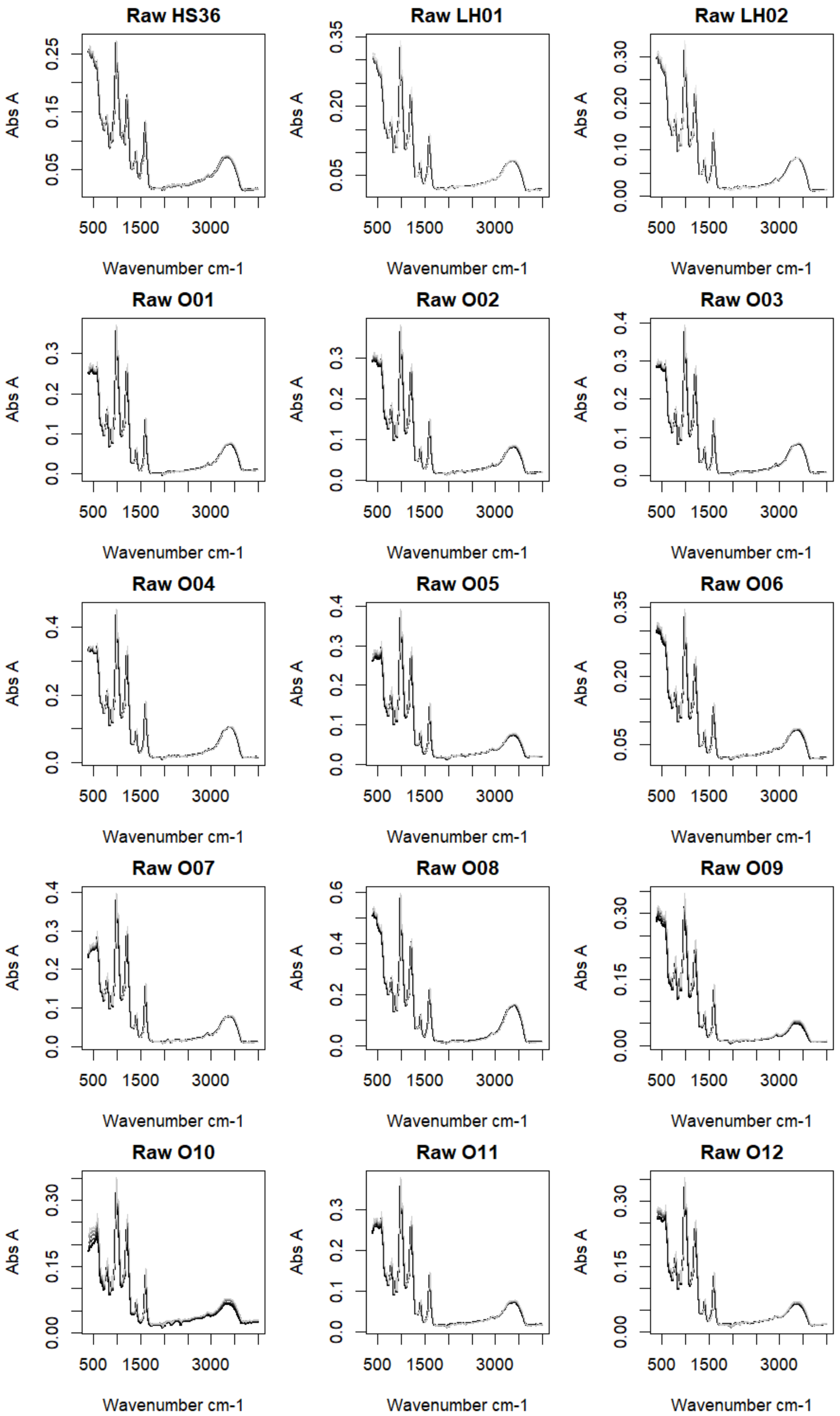
Raw HS36
Raw LH01
Raw LH02
Raw O01
Raw O02
Raw O03
Raw O04
Raw O05
Raw O06
Raw O07
Raw O08
Raw O09
Raw O10
Raw O11
Raw O12
Abs A
Wavenumber cm-1
500 1500 3000

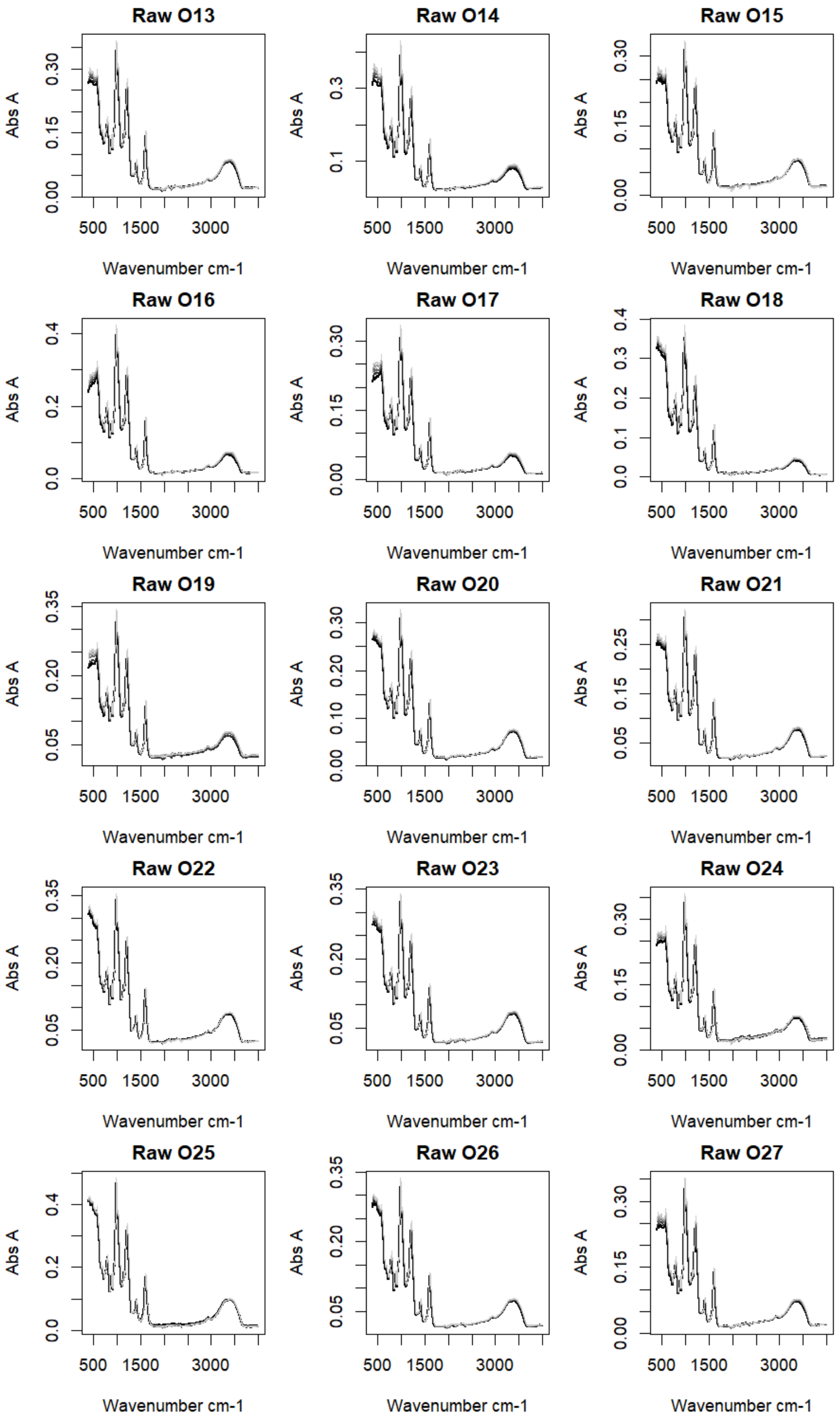
Raw O13
Raw O14
Raw O15
Raw O16
Raw O17
Raw O18
Raw O19
Raw O20
Raw O21
Raw O22
Raw O23
Raw O24
Raw O25
Raw O26
Raw O27
Abs A
Wavenumber cm-1
500
1500
3000

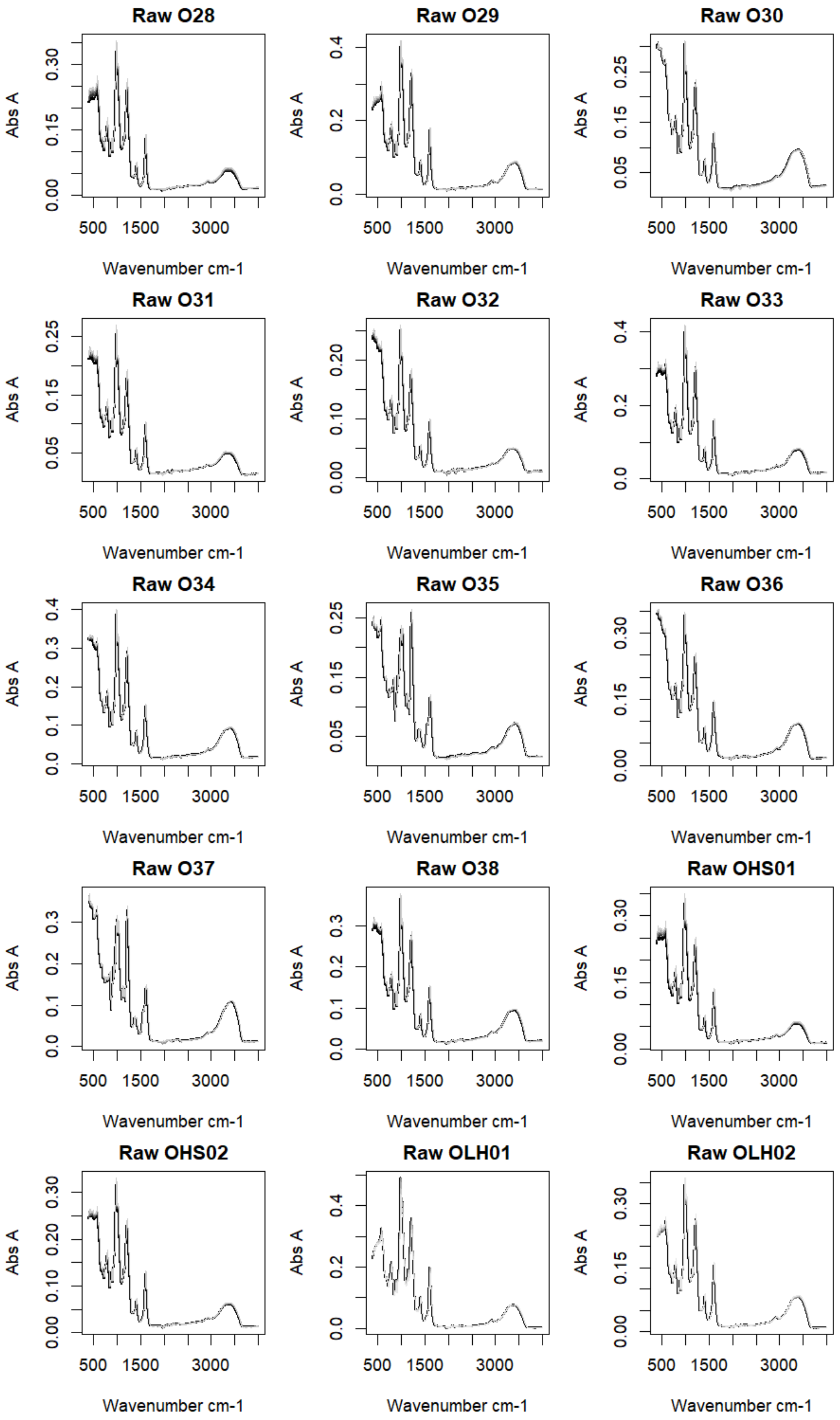

Raw O28
Raw O29
Raw O30
Raw O31
Raw O32
Raw O33
Raw O34
Raw O35
Raw O36
Raw O37
Raw O38
Raw OHS01
Raw OHS02
Raw OLH01
Raw OLH02
Abs A
Wavenumber cm-1
500
1500
3000

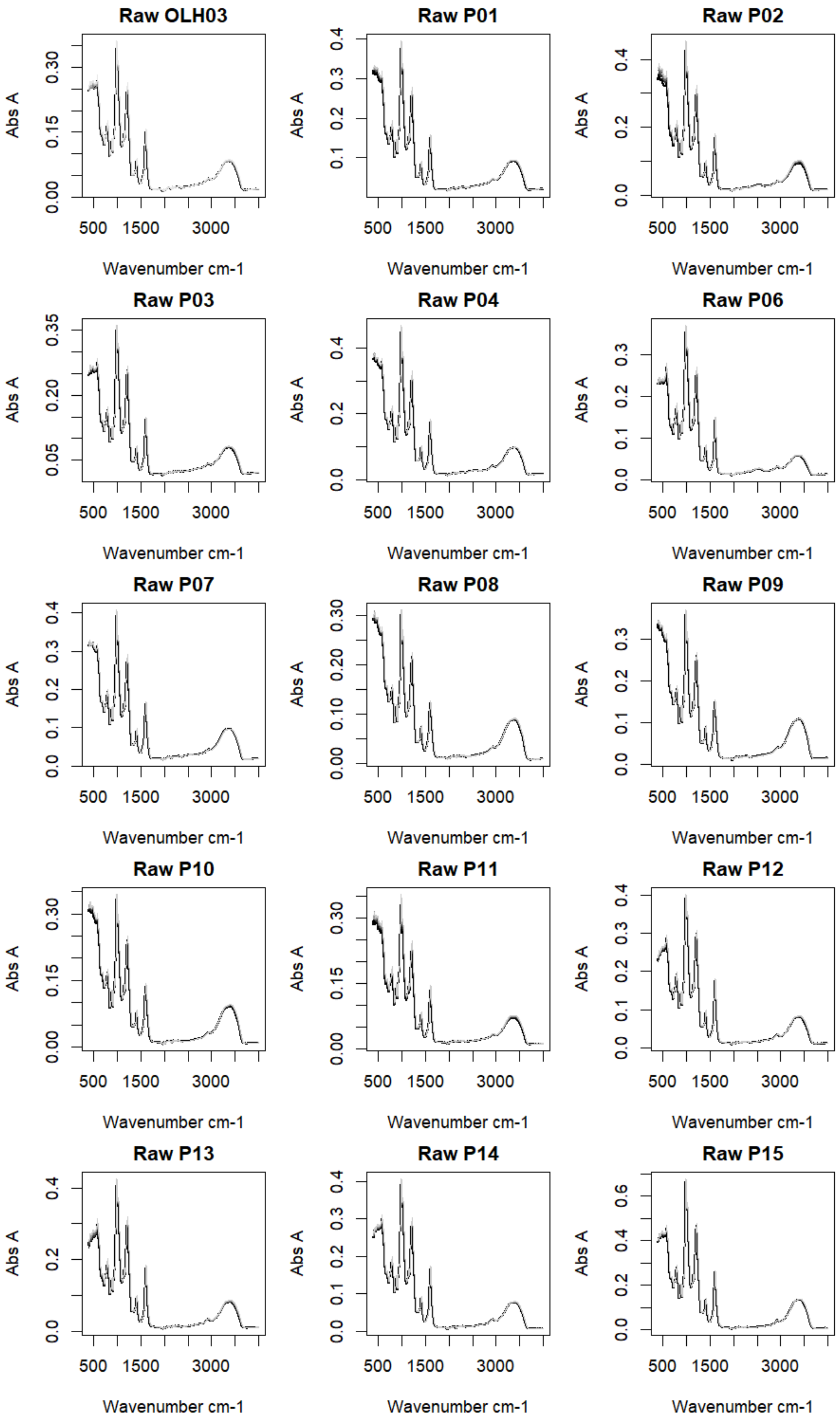

Raw OLH03
Raw P01
Raw P02
Raw P03
Raw P04
Raw P06
Raw P07
Raw P08
Raw P09
Raw P10
Raw P11
Raw P12
Raw P13
Raw P14
Raw P15
Abs A
Wavenumber cm-1
500
1500
3000

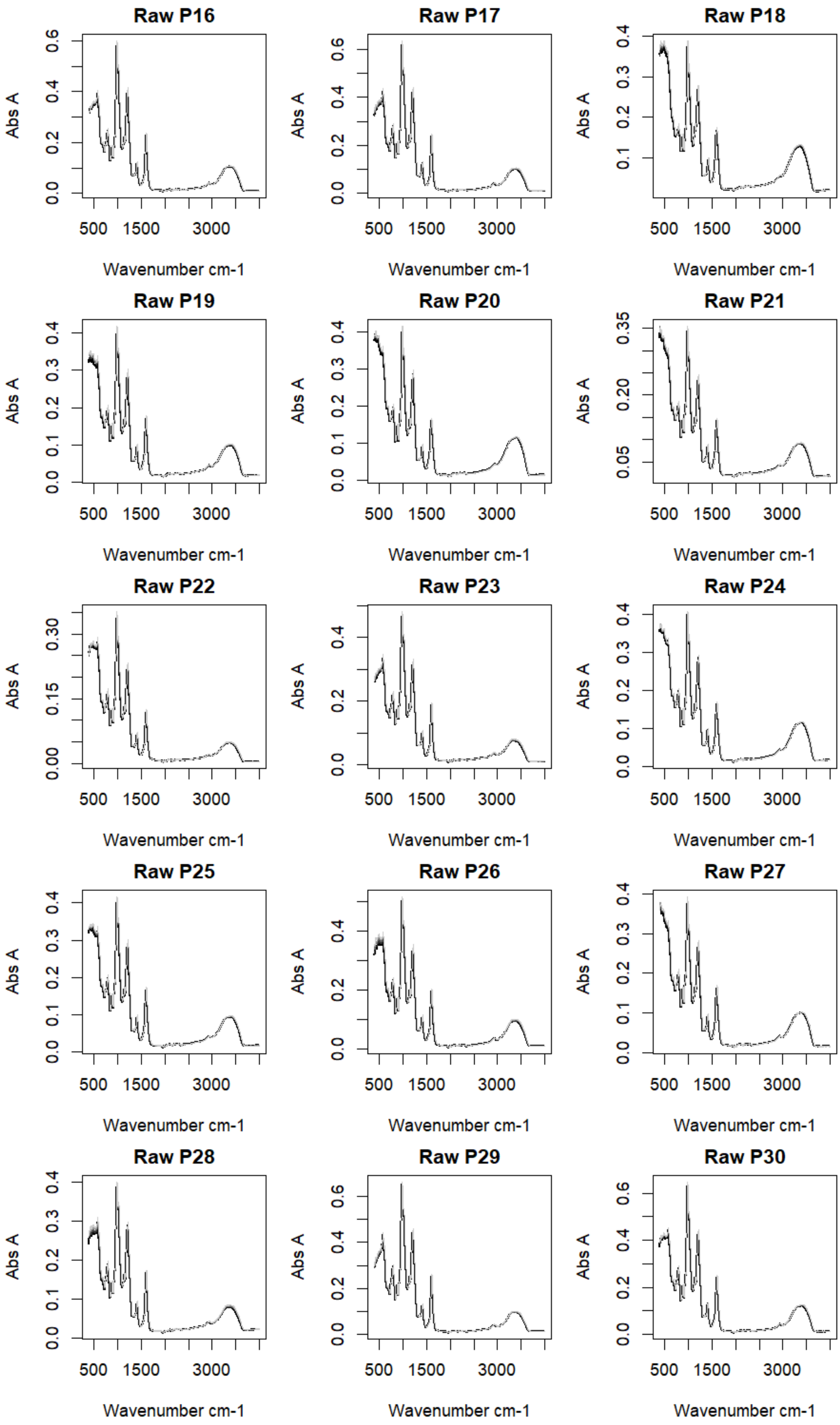
Raw P16
Raw P17
Raw P18
Raw P19
Raw P20
Raw P21
Raw P22
Raw P23
Raw P24
Raw P25
Raw P26
Raw P27
Raw P28
Raw P29
Raw P30
Abs A
Wavenumber cm-1
500
1500
3000

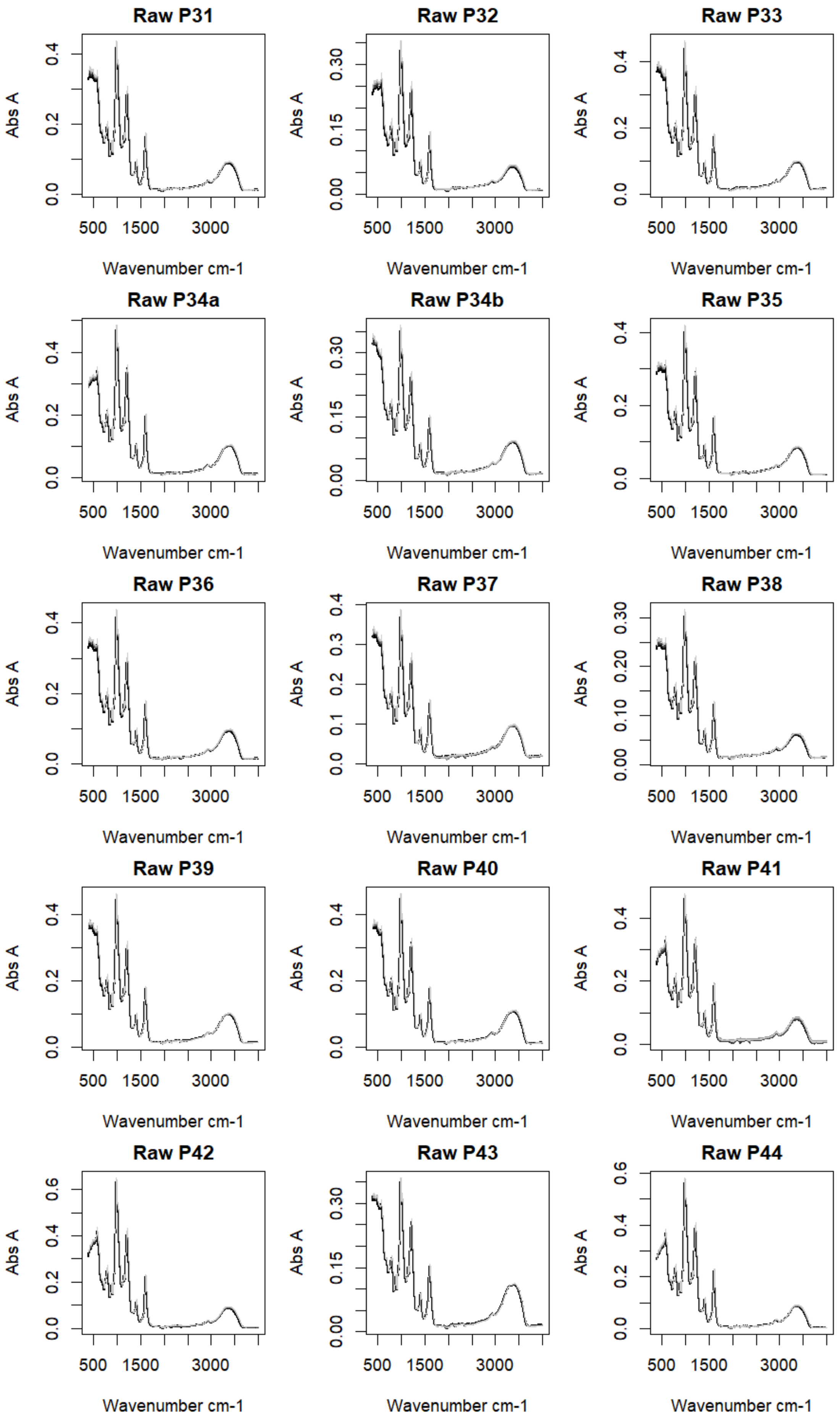
Raw P31
Raw P32
Raw P33
Raw P34a
Raw P34b
Raw P35
Raw P36
Raw P37
Raw P38
Raw P39
Raw P40
Raw P41
Raw P42
Raw P43
Raw P44
Abs A
Wavenumber cm-1
500
1500
3000

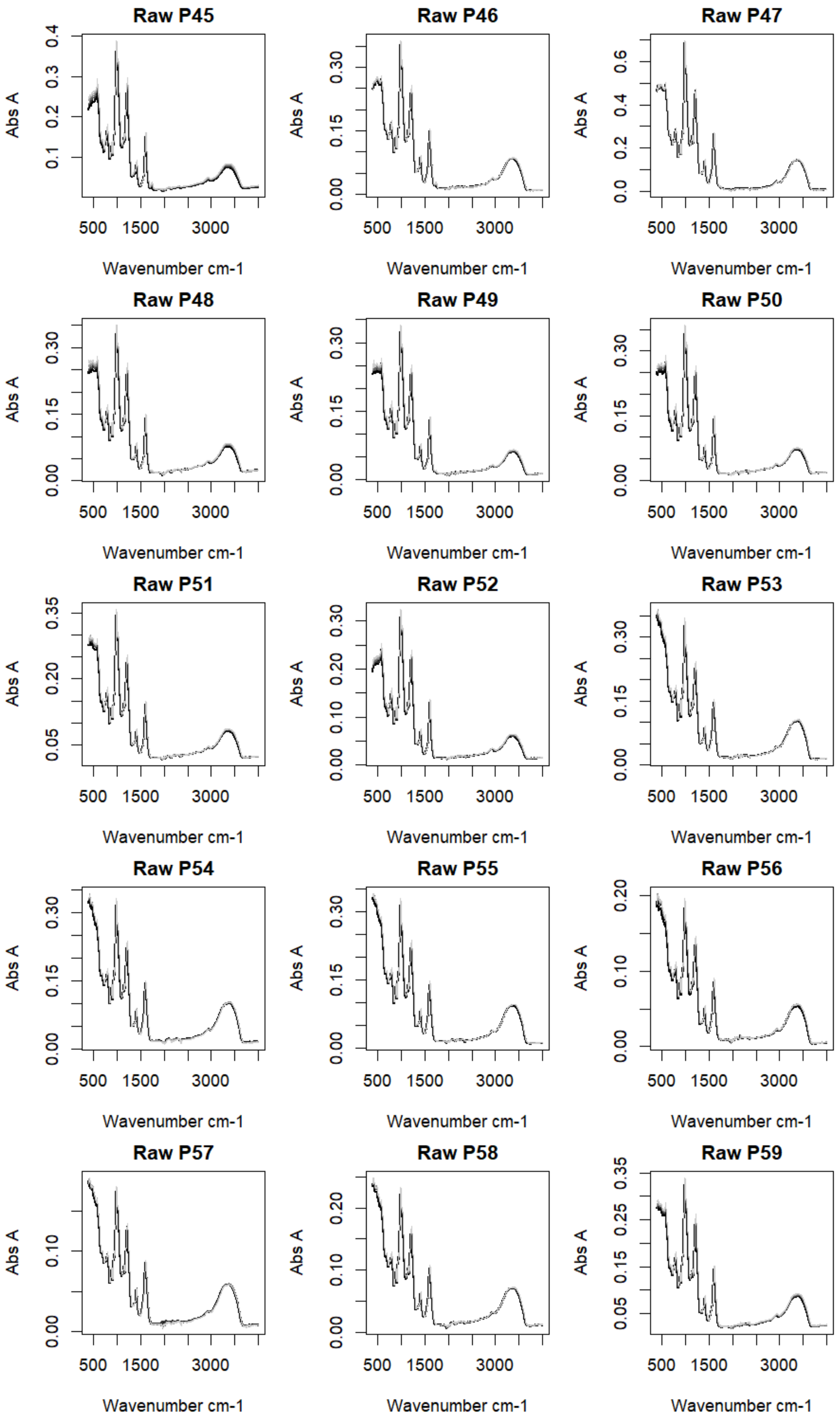
Raw P45
Raw P46
Raw P47
Raw P48
Raw P49
Raw P50
Raw P51
Raw P52
Raw P53
Raw P54
Raw P55
Raw P56
Raw P57
Raw P58
Raw P59
Abs A
Wavenumber cm-1

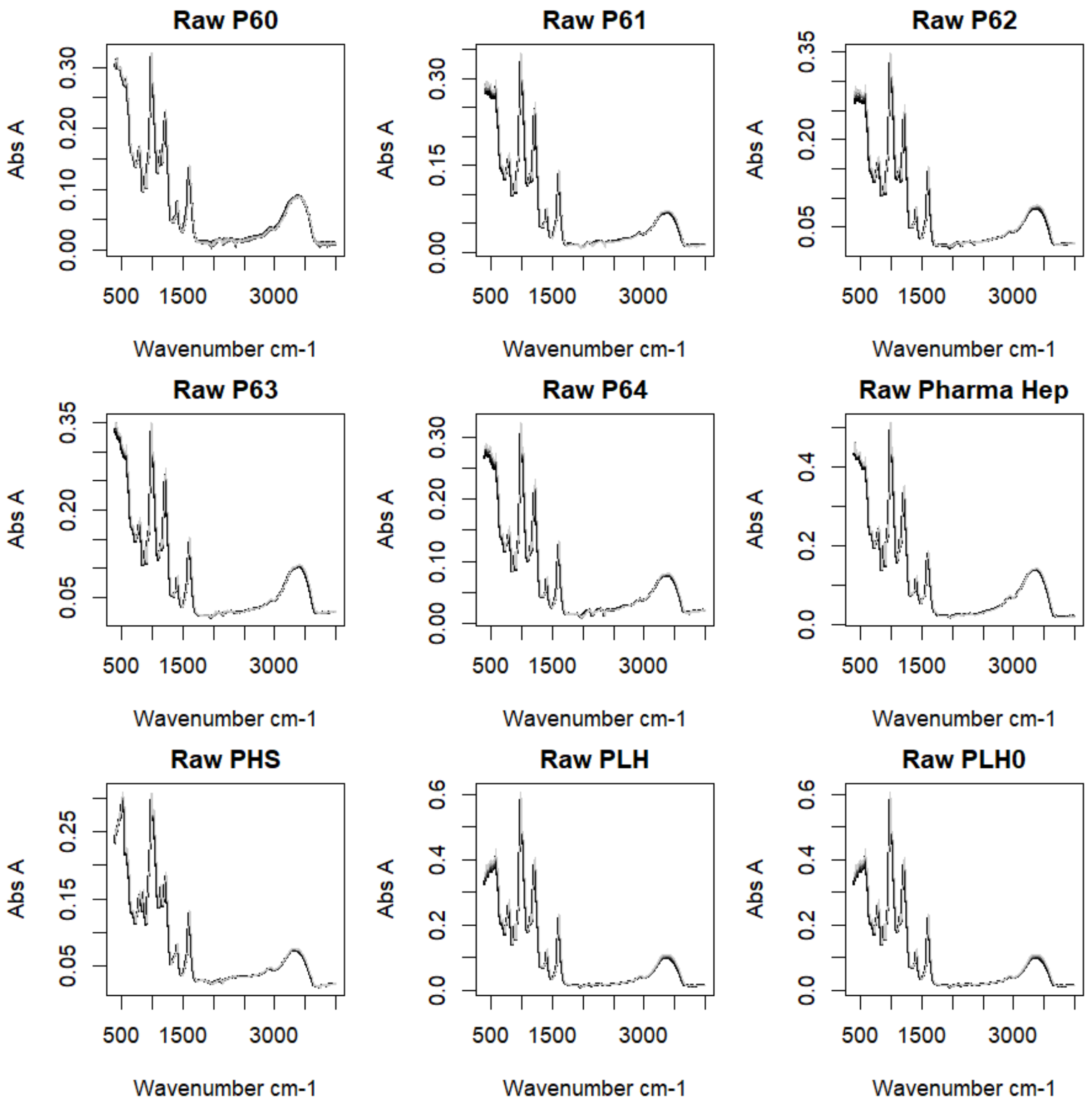
Raw P60
Raw P61
Raw P62
Raw P63
Raw P64
Raw Pharma Hep
Raw PHS
Raw PLH
Raw PLH0
Abs A
Wavenumber cm-1
500
1500
3000

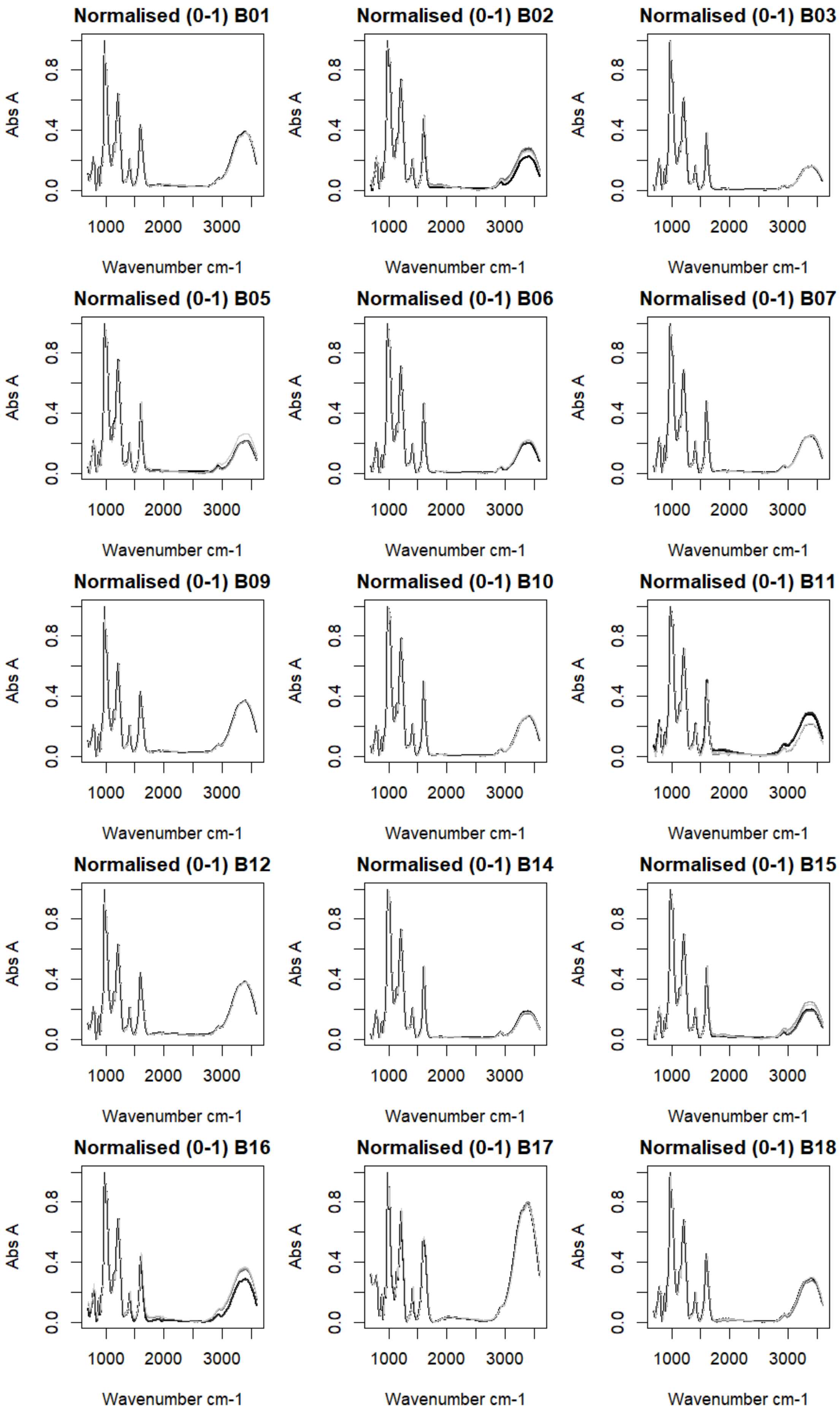
Normalised (0-1) B01
Normalised (0-1) B02
Normalised (0-1) B03
Normalised (0-1) B05
Normalised (0-1) B06
Normalised (0-1) B07
Normalised (0-1) B09
Normalised (0-1) B10
Normalised (0-1) B11
Normalised (0-1) B12
Normalised (0-1) B14
Normalised (0-1) B15
Normalised (0-1) B16
Normalised (0-1) B17
Normalised (0-1) B18
Abs A
0.0
0.4
0.8
1000
2000
3000
Wavenumber cm-1

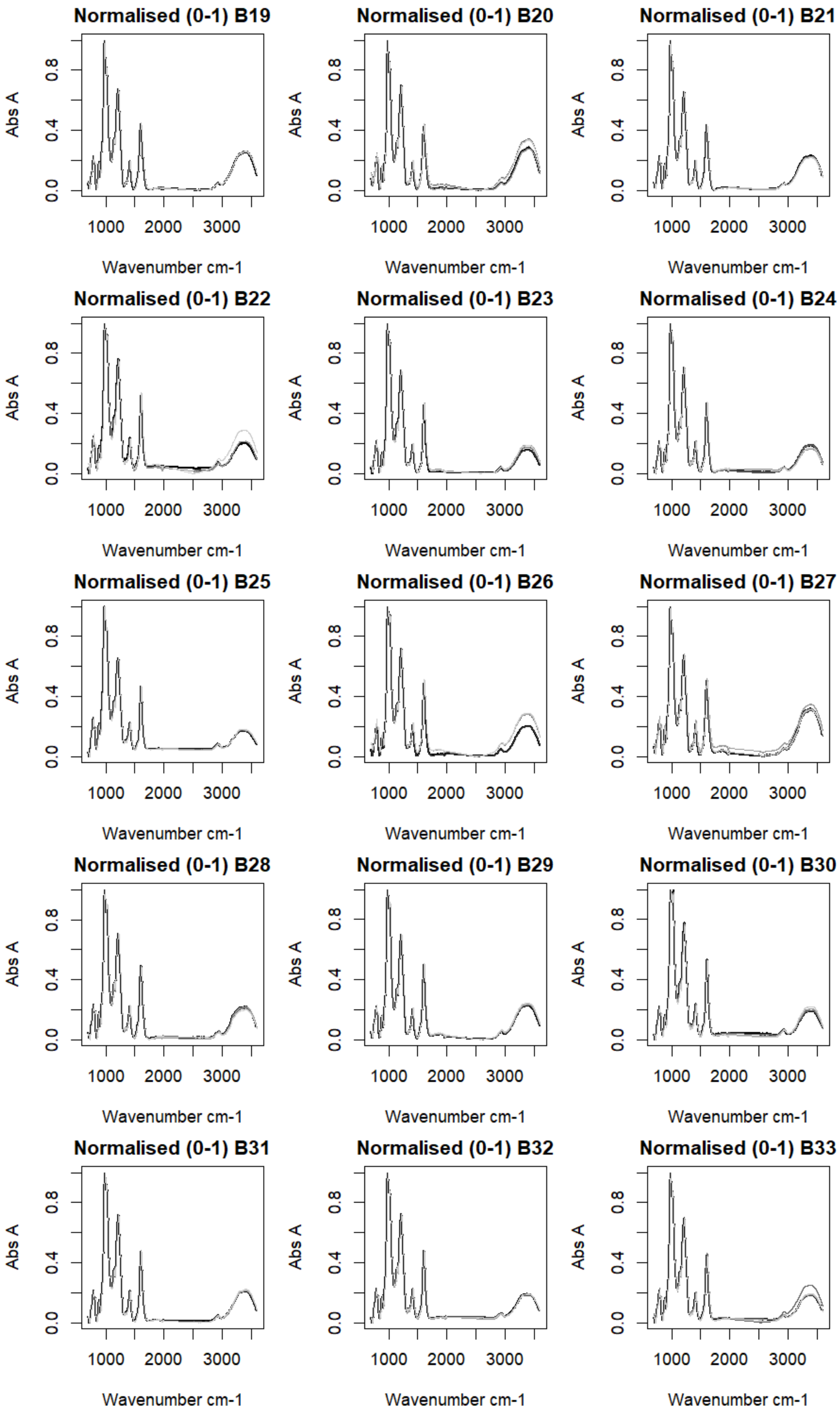
Normalised (0-1) B19
Normalised (0-1) B20
Normalised (0-1) B21
Normalised (0-1) B22
Normalised (0-1) B23
Normalised (0-1) B24
Normalised (0-1) B25
Normalised (0-1) B26
Normalised (0-1) B27
Normalised (0-1) B28
Normalised (0-1) B29
Normalised (0-1) B30
Normalised (0-1) B31
Normalised (0-1) B32
Normalised (0-1) B33
Abs A
0.0
0.4
0.8
1000
2000
3000
Wavenumber cm-1

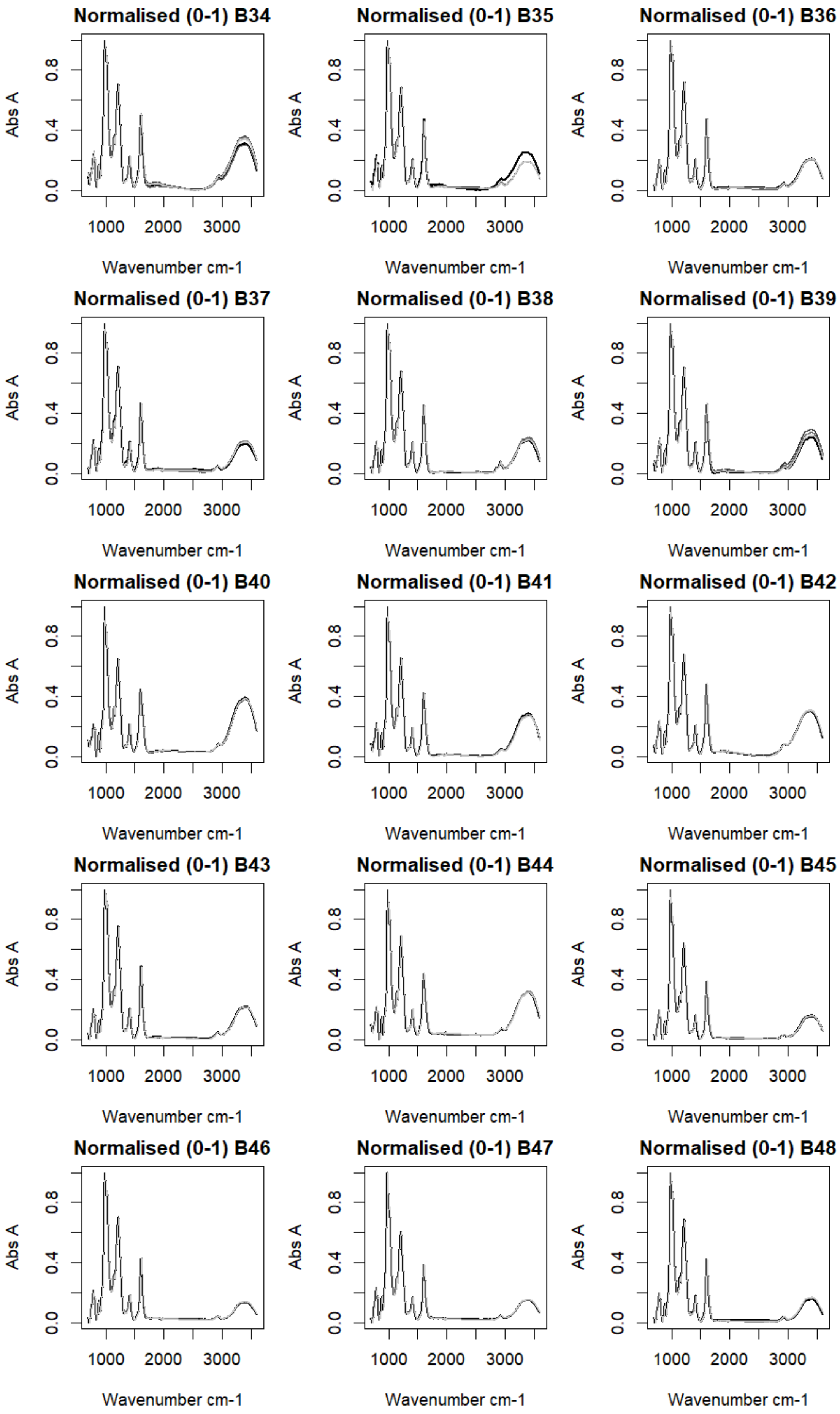
Normalised (0-1) B34
Normalised (0-1) B35
Normalised (0-1) B36
Normalised (0-1) B37
Normalised (0-1) B38
Normalised (0-1) B39
Normalised (0-1) B40
Normalised (0-1) B41
Normalised (0-1) B42
Normalised (0-1) B43
Normalised (0-1) B44
Normalised (0-1) B45
Normalised (0-1) B46
Normalised (0-1) B47
Normalised (0-1) B48
Abs A
0.0
0.4
0.8
1000
2000
3000
Wavenumber cm-1

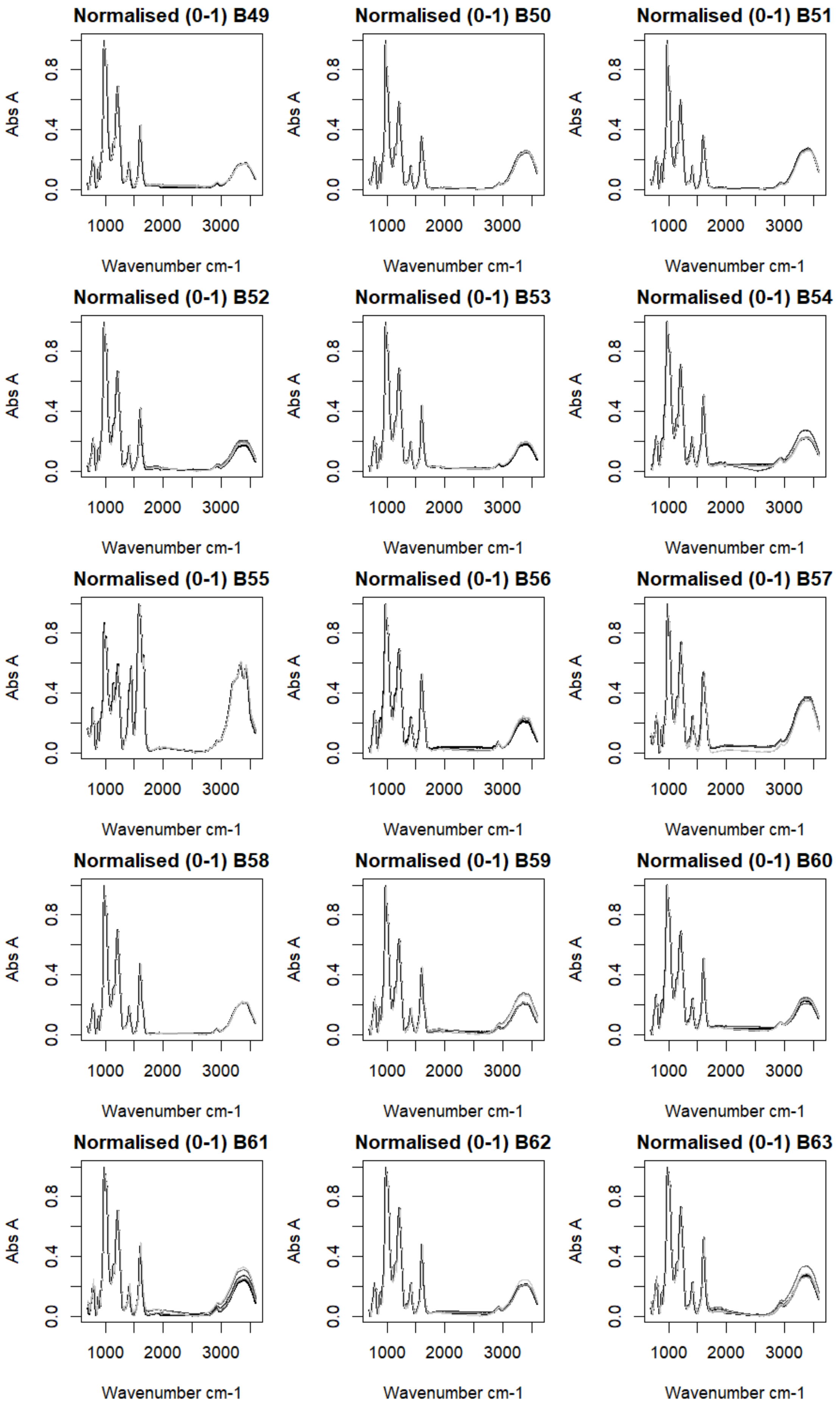
Normalised (0-1) B49
Normalised (0-1) B50
Normalised (0-1) B51
Normalised (0-1) B52
Normalised (0-1) B53
Normalised (0-1) B54
Normalised (0-1) B55
Normalised (0-1) B56
Normalised (0-1) B57
Normalised (0-1) B58
Normalised (0-1) B59
Normalised (0-1) B60
Normalised (0-1) B61
Normalised (0-1) B62
Normalised (0-1) B63
Abs A
0.0
0.4
0.8
1000
2000
3000
Wavenumber cm-1

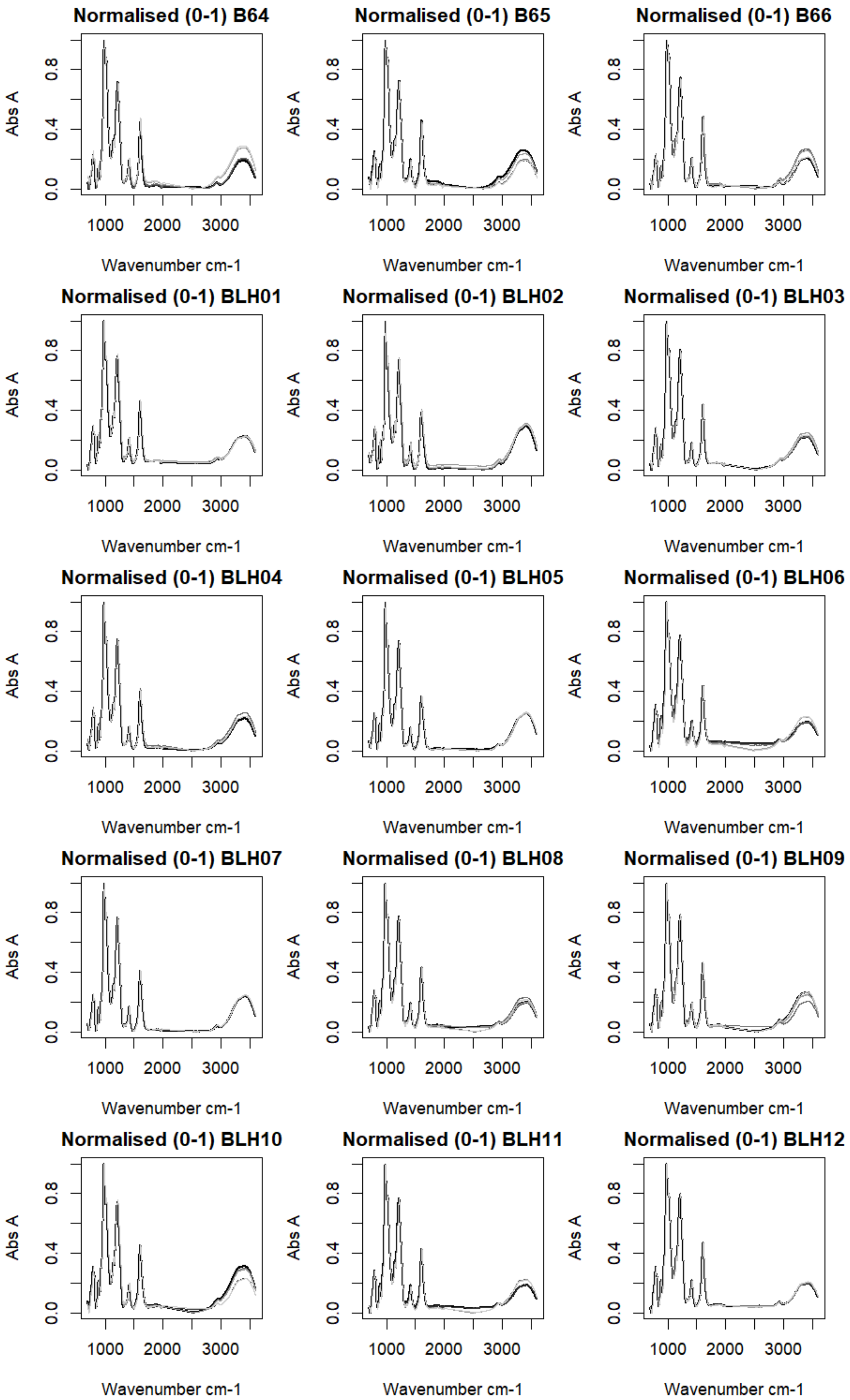
Normalised (0-1) B64
Normalised (0-1) B65
Normalised (0-1) B66
Normalised (0-1) BLH01
Normalised (0-1) BLH02
Normalised (0-1) BLH03
Normalised (0-1) BLH04
Normalised (0-1) BLH05
Normalised (0-1) BLH06
Normalised (0-1) BLH07
Normalised (0-1) BLH08
Normalised (0-1) BLH09
Normalised (0-1) BLH10
Normalised (0-1) BLH11
Normalised (0-1) BLH12
Abs A
0.0
0.4
0.8
1000
2000
3000
Wavenumber cm-1

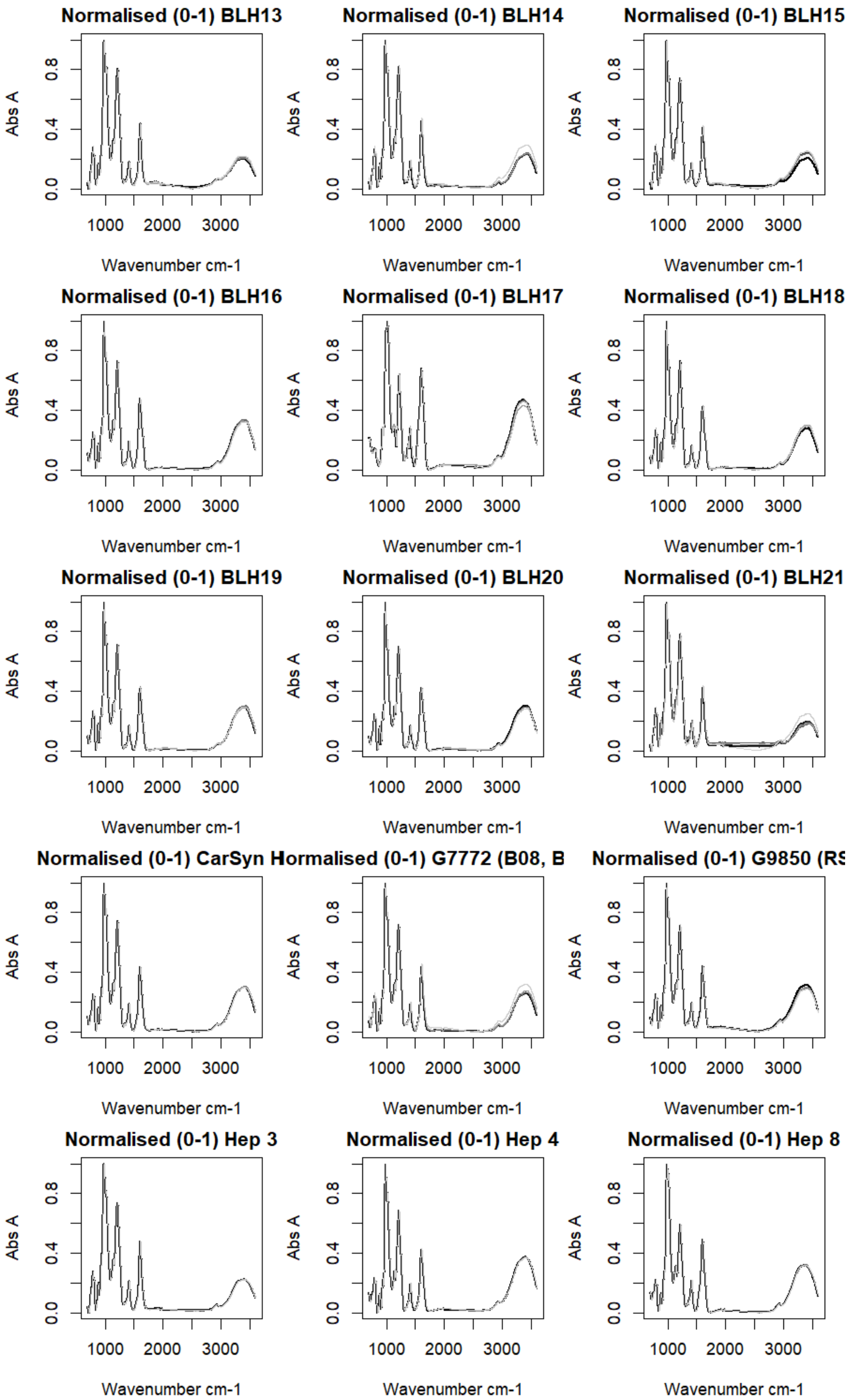

Normalised (0-1) BLH13
Normalised (0-1) BLH14
Normalised (0-1) BLH15
Normalised (0-1) BLH16
Normalised (0-1) BLH17
Normalised (0-1) BLH18
Normalised (0-1) BLH19
Normalised (0-1) BLH20
Normalised (0-1) BLH21
Normalised (0-1) CarSyn
Normalised (0-1) G7772 (B08, B
Normalised (0-1) G9850 (R
Normalised (0-1) Hep 3
Normalised (0-1) Hep 4
Normalised (0-1) Hep 8
Abs A
0.0
0.4
0.8
1000
2000
3000
Wavenumber cm-1

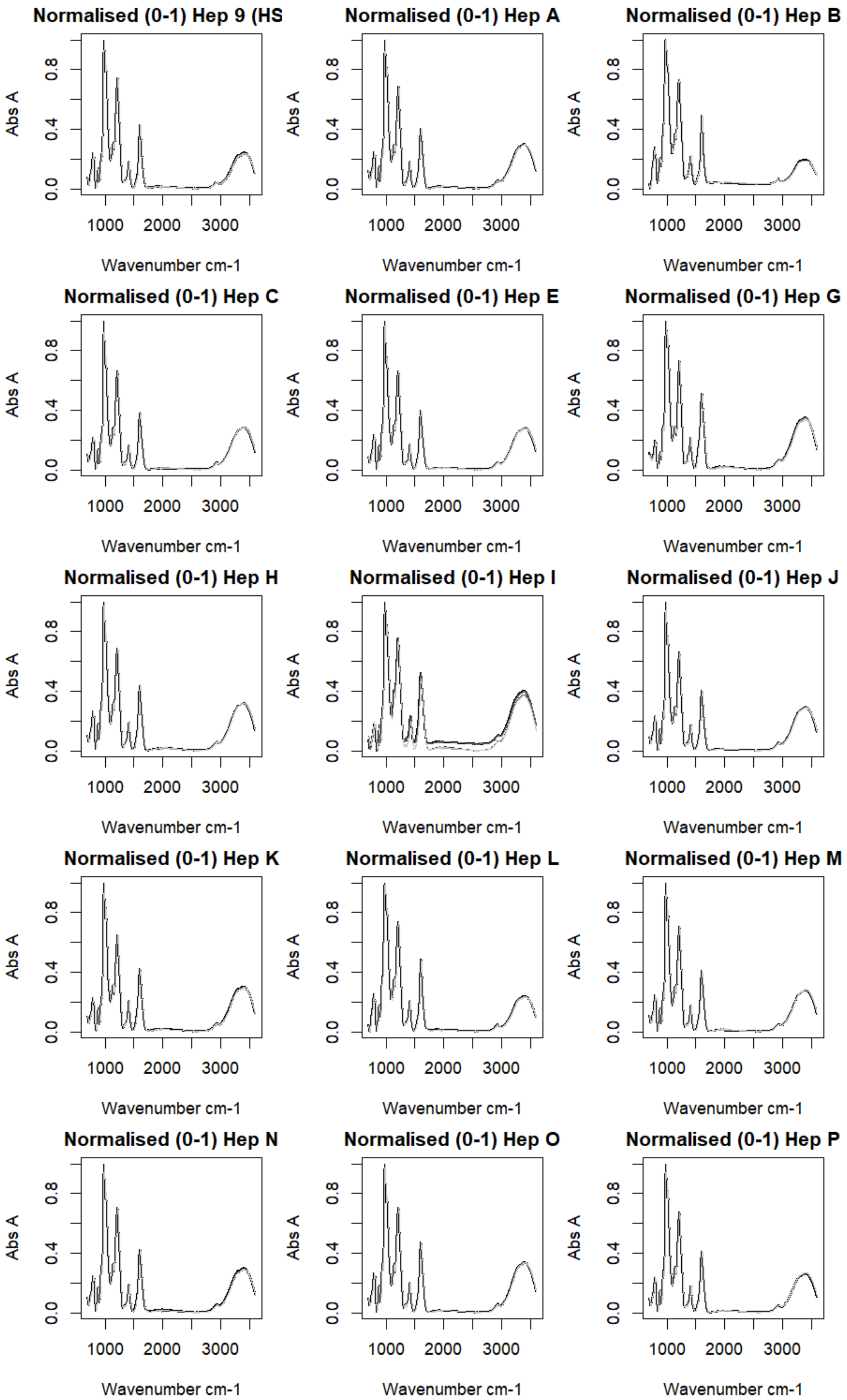
Normalised (0-1) Hep 9 (HS
Normalised (0-1) Hep A
Normalised (0-1) Hep B
Normalised (0-1) Hep C
Normalised (0-1) Hep E
Normalised (0-1) Hep G
Normalised (0-1) Hep H
Normalised (0-1) Hep I
Normalised (0-1) Hep J
Normalised (0-1) Hep K
Normalised (0-1) Hep L
Normalised (0-1) Hep M
Normalised (0-1) Hep N
Normalised (0-1) Hep O
Normalised (0-1) Hep P
Abs A
0.0
0.4
0.8
1000
2000
3000
Wavenumber cm-1

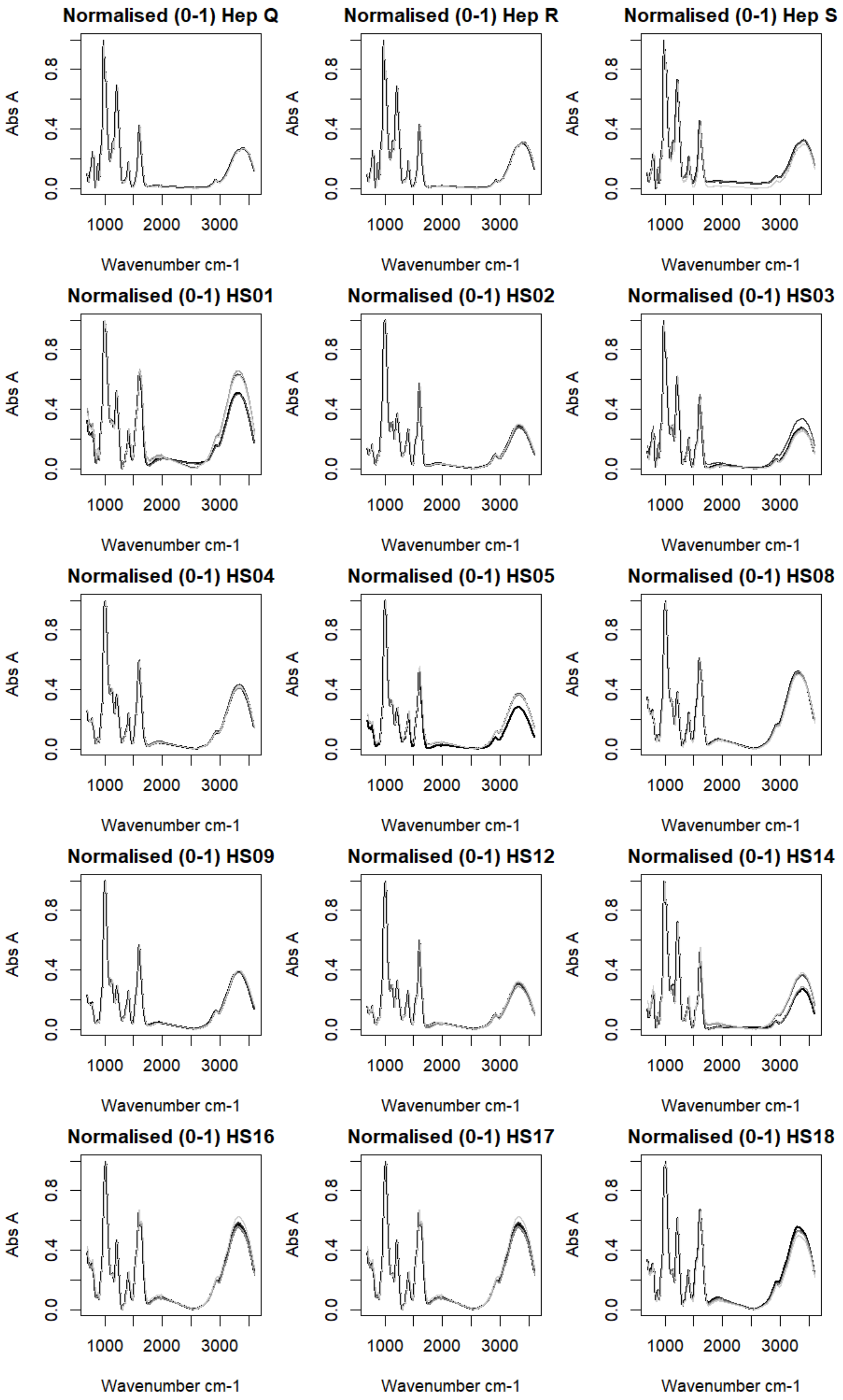
Normalised (0-1) Hep Q
Normalised (0-1) Hep R
Normalised (0-1) Hep S
Normalised (0-1) HS01
Normalised (0-1) HS02
Normalised (0-1) HS03
Normalised (0-1) HS04
Normalised (0-1) HS05
Normalised (0-1) HS08
Normalised (0-1) HS09
Normalised (0-1) HS12
Normalised (0-1) HS14
Normalised (0-1) HS16
Normalised (0-1) HS17
Normalised (0-1) HS18
Abs A
0.0
0.4
0.8
1000
2000
3000
Wavenumber cm-1

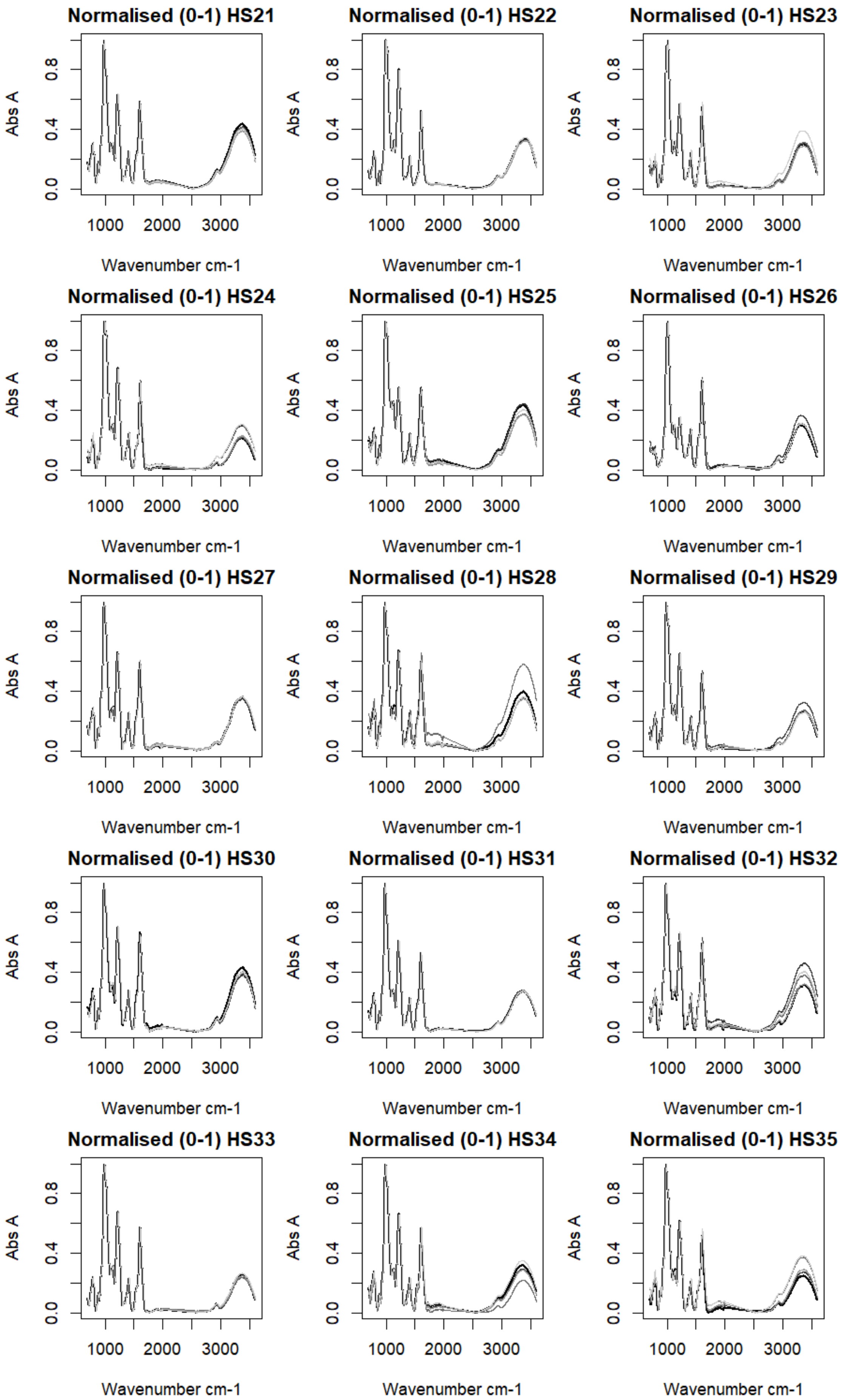
Normalised (0-1) HS21
Normalised (0-1) HS22
Normalised (0-1) HS23
Normalised (0-1) HS24
Normalised (0-1) HS25
Normalised (0-1) HS26
Normalised (0-1) HS27
Normalised (0-1) HS28
Normalised (0-1) HS29
Normalised (0-1) HS30
Normalised (0-1) HS31
Normalised (0-1) HS32
Normalised (0-1) HS33
Normalised (0-1) HS34
Normalised (0-1) HS35
Abs A
0.0
0.4
0.8
1000
2000
3000
Wavenumber cm-1

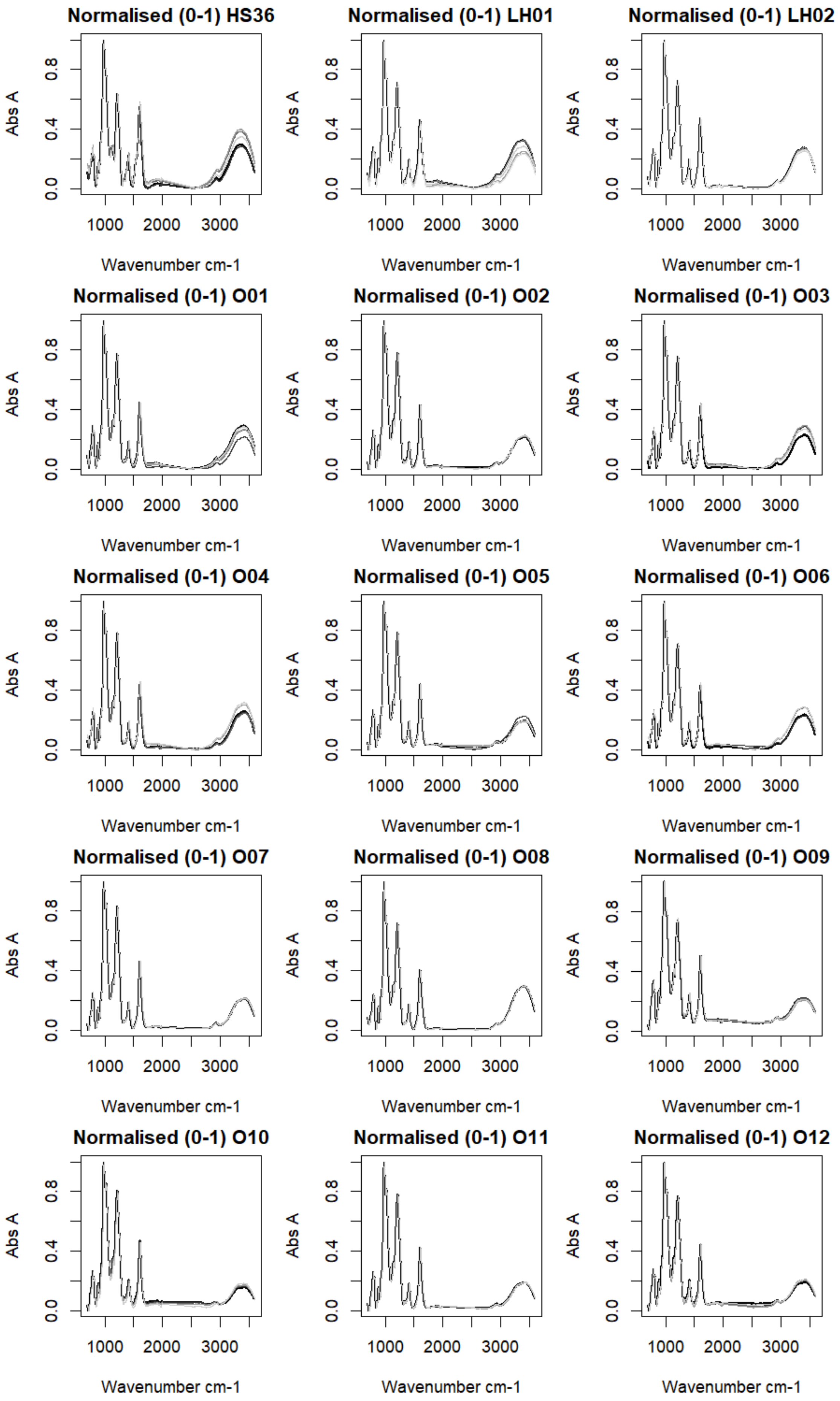
Normalised (0-1) HS36
Normalised (0-1) LH01
Normalised (0-1) LH02
Normalised (0-1) O01
Normalised (0-1) O02
Normalised (0-1) O03
Normalised (0-1) O04
Normalised (0-1) O05
Normalised (0-1) O06
Normalised (0-1) O07
Normalised (0-1) O08
Normalised (0-1) O09
Normalised (0-1) O10
Normalised (0-1) O11
Normalised (0-1) O12
Abs A
0.0
0.4
0.8
1000
2000
3000
Wavenumber cm-1

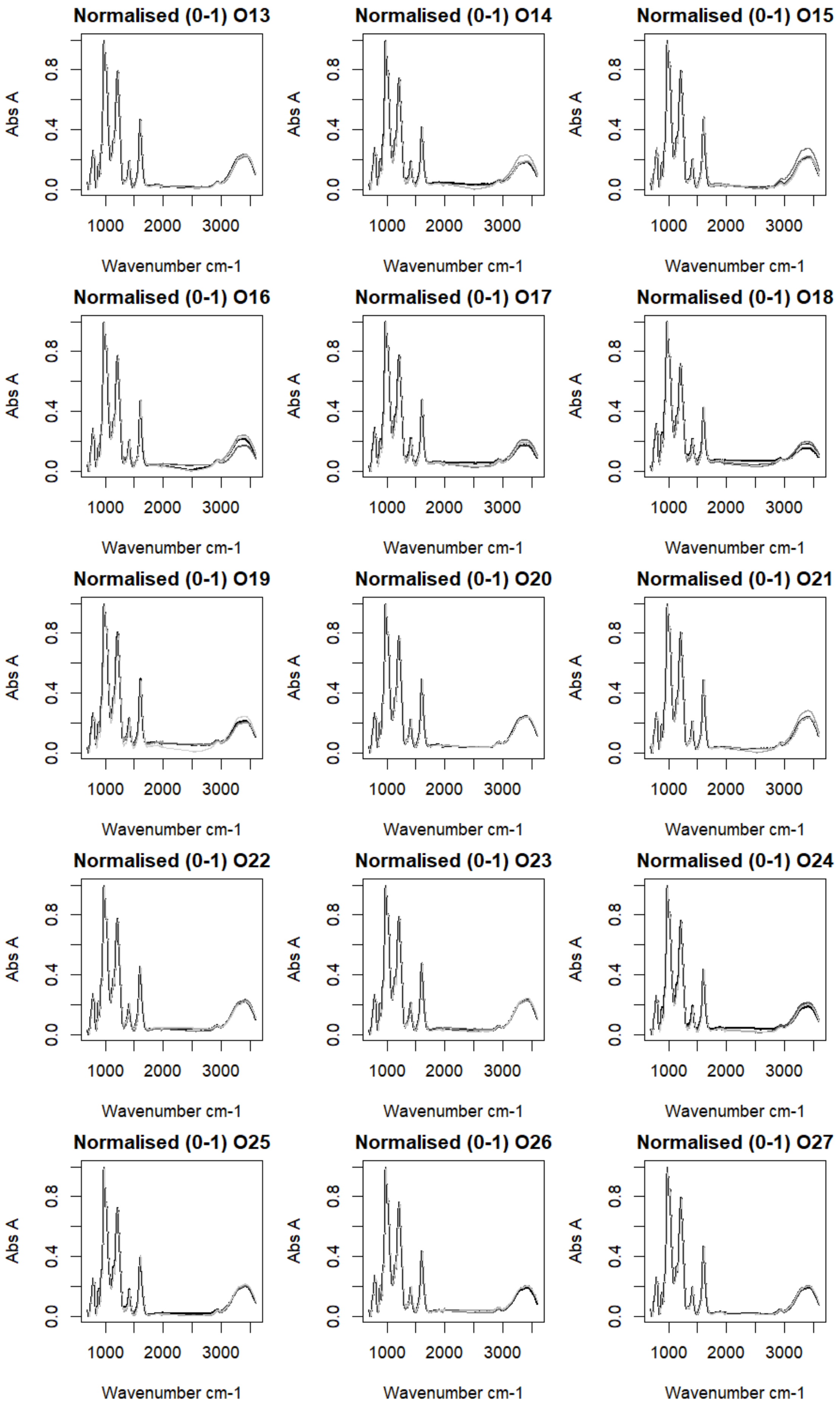
Normalised (0-1) O13
Normalised (0-1) O14
Normalised (0-1) O15
Normalised (0-1) O16
Normalised (0-1) O17
Normalised (0-1) O18
Normalised (0-1) O19
Normalised (0-1) O20
Normalised (0-1) O21
Normalised (0-1) O22
Normalised (0-1) O23
Normalised (0-1) O24
Normalised (0-1) O25
Normalised (0-1) O26
Normalised (0-1) O27
Abs A
0.0
0.4
0.8
1000
2000
3000
Wavenumber cm-1

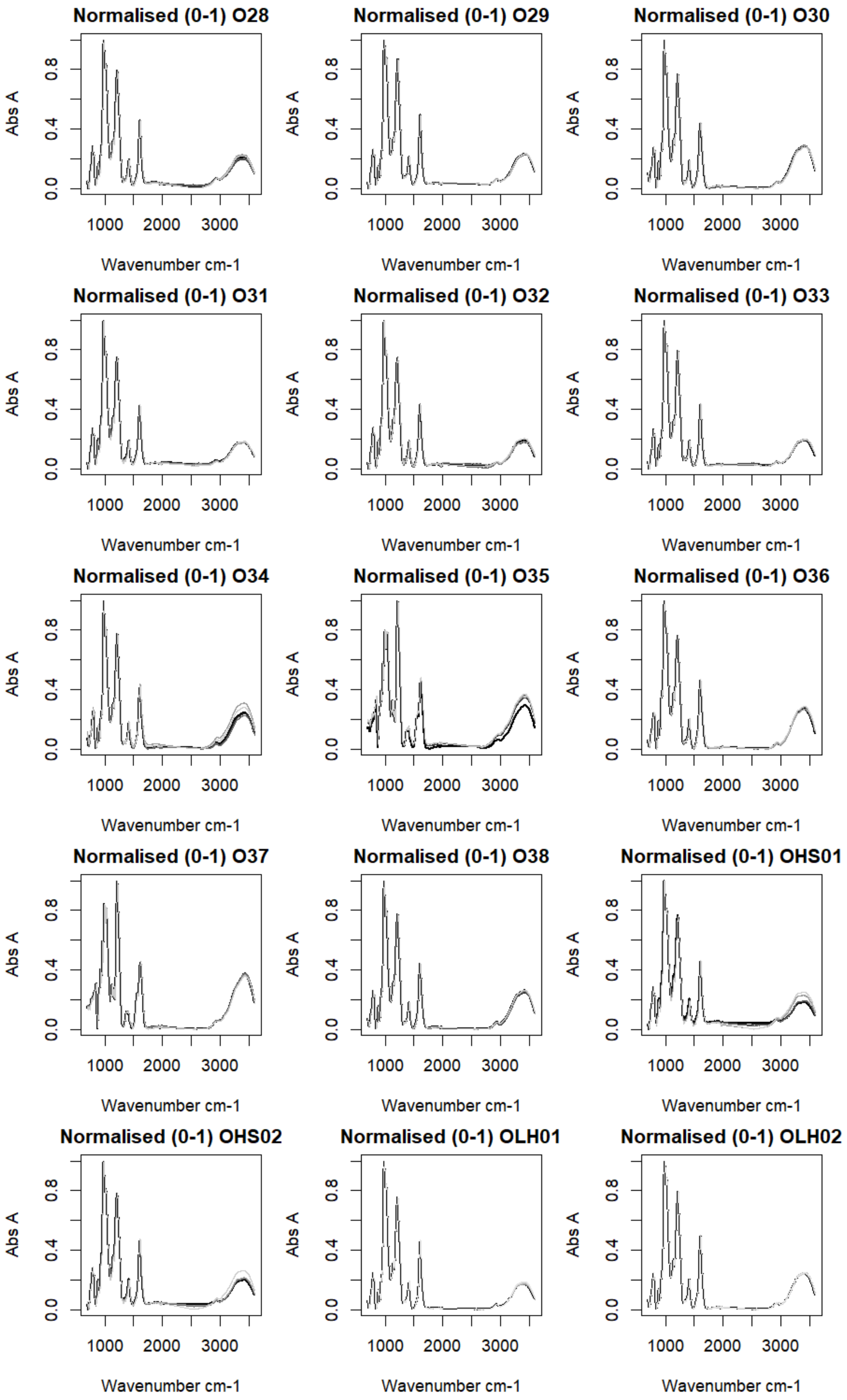
Normalised (0-1) O28
Normalised (0-1) O29
Normalised (0-1) O30
Normalised (0-1) O31
Normalised (0-1) O32
Normalised (0-1) O33
Normalised (0-1) O34
Normalised (0-1) O35
Normalised (0-1) O36
Normalised (0-1) O37
Normalised (0-1) O38
Normalised (0-1) OHS01
Normalised (0-1) OHS02
Normalised (0-1) OLH01
Normalised (0-1) OLH02
Abs A
0.0
0.4
0.8
1000
2000
3000
Wavenumber cm-1

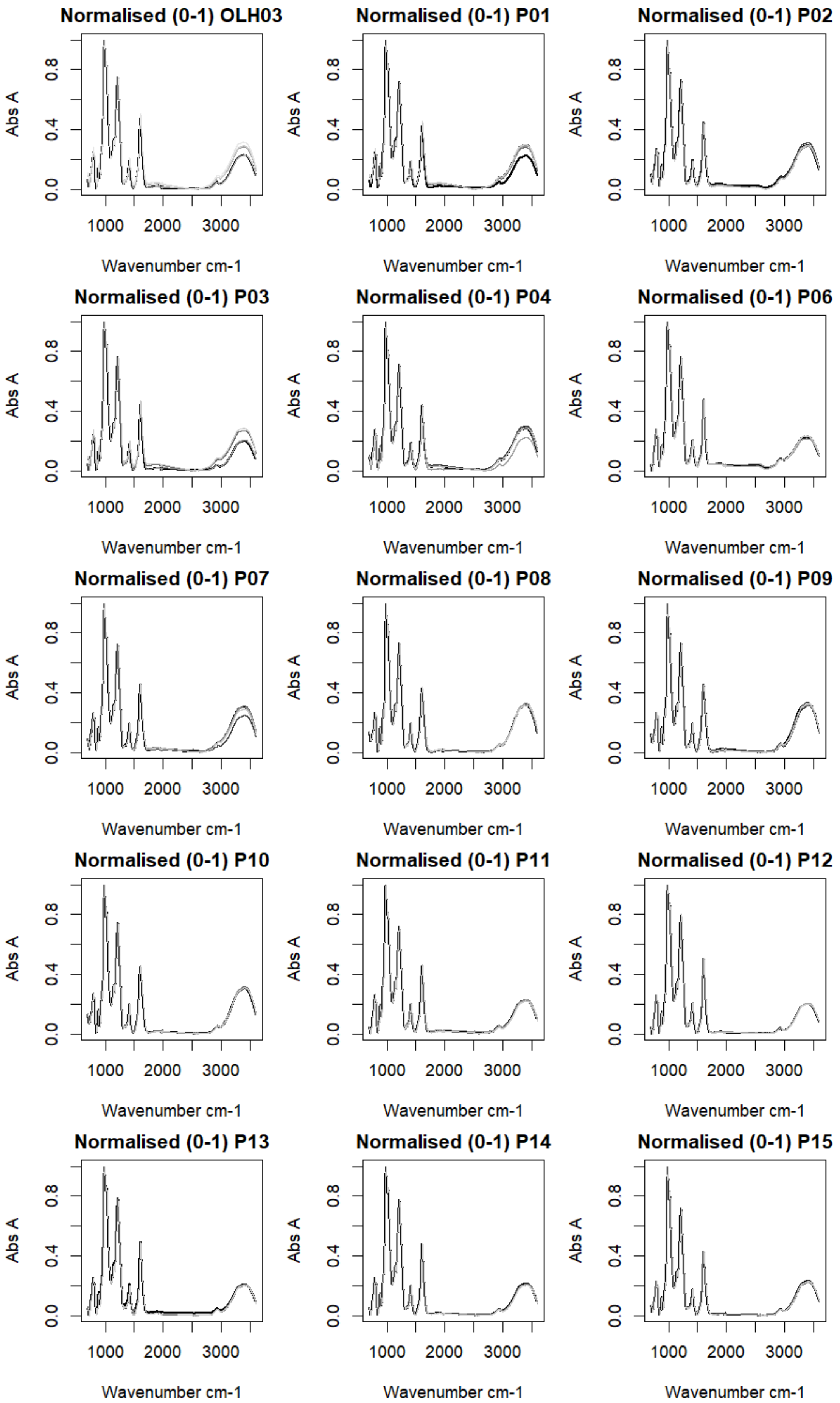
Normalised (0-1) OLH03
Normalised (0-1) P01
Normalised (0-1) P02
Normalised (0-1) P03
Normalised (0-1) P04
Normalised (0-1) P06
Normalised (0-1) P07
Normalised (0-1) P08
Normalised (0-1) P09
Normalised (0-1) P10
Normalised (0-1) P11
Normalised (0-1) P12
Normalised (0-1) P13
Normalised (0-1) P14
Normalised (0-1) P15
Abs A
0.0
0.4
0.8
1000
2000
3000
Wavenumber cm-1

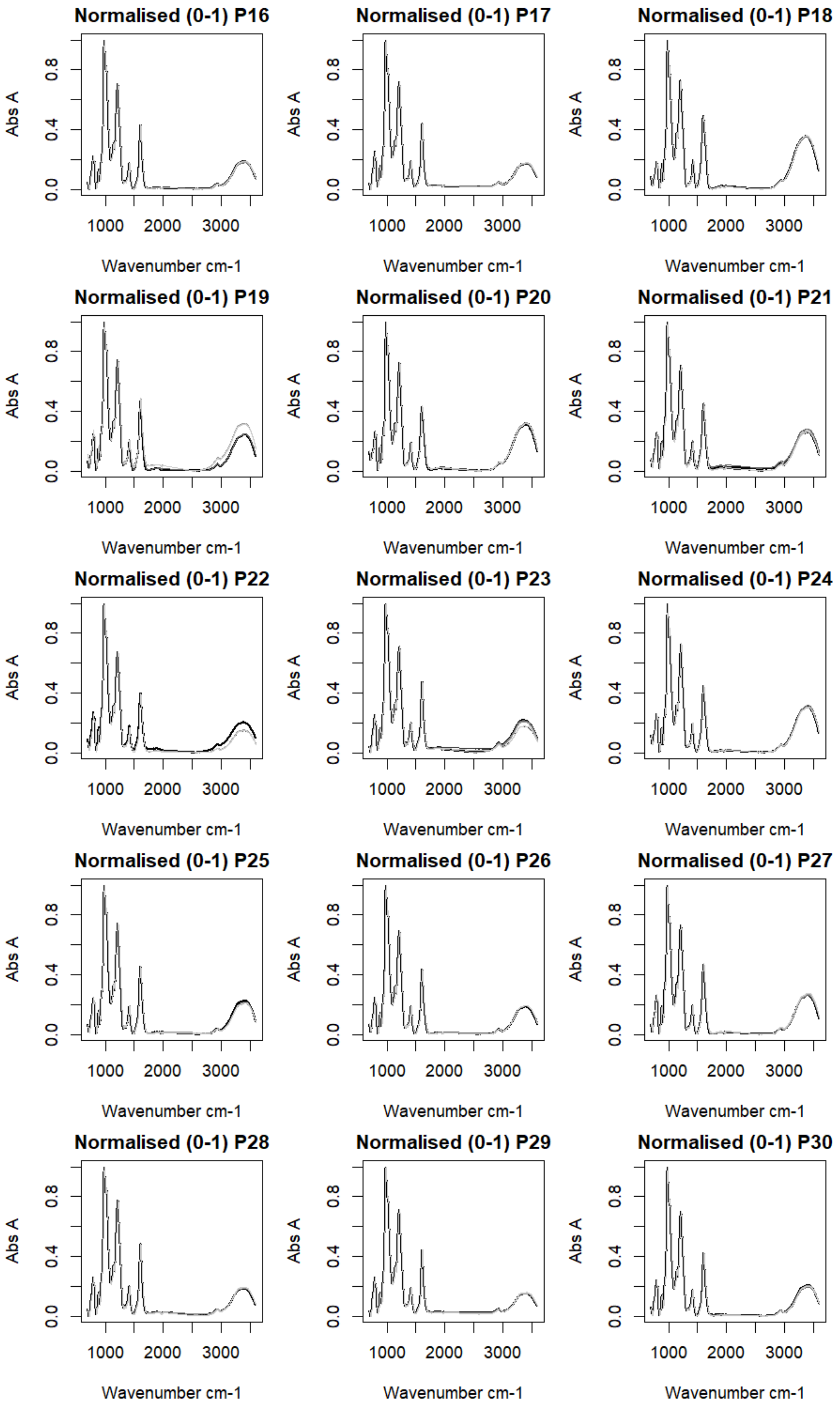
Normalised (0-1) P16
Normalised (0-1) P17
Normalised (0-1) P18
Normalised (0-1) P19
Normalised (0-1) P20
Normalised (0-1) P21
Normalised (0-1) P22
Normalised (0-1) P23
Normalised (0-1) P24
Normalised (0-1) P25
Normalised (0-1) P26
Normalised (0-1) P27
Normalised (0-1) P28
Normalised (0-1) P29
Normalised (0-1) P30
Abs A
0.0
0.4
0.8
1000
2000
3000
Wavenumber cm-1

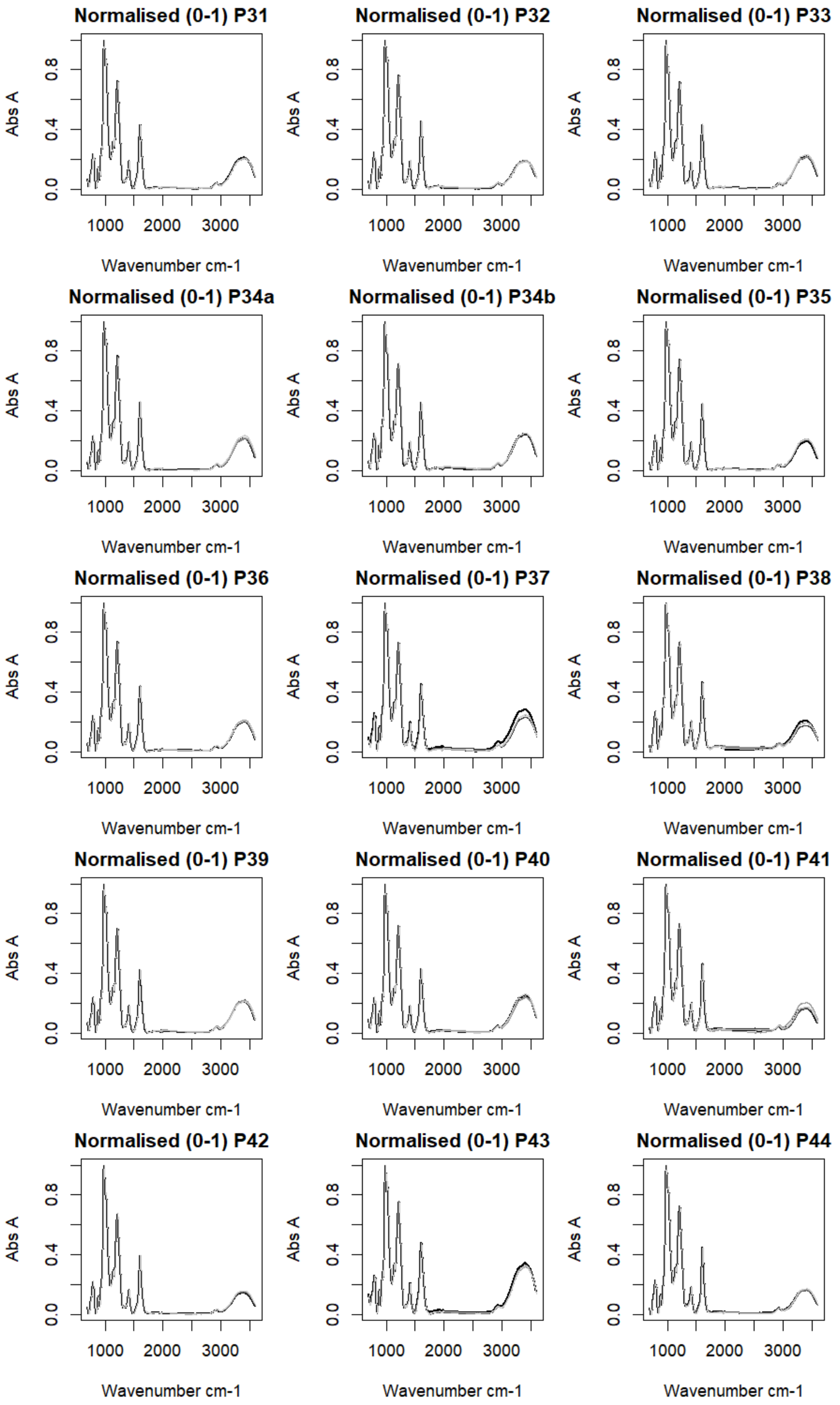
Normalised (0-1) P31
Normalised (0-1) P32
Normalised (0-1) P33
Normalised (0-1) P34a
Normalised (0-1) P34b
Normalised (0-1) P35
Normalised (0-1) P36
Normalised (0-1) P37
Normalised (0-1) P38
Normalised (0-1) P39
Normalised (0-1) P40
Normalised (0-1) P41
Normalised (0-1) P42
Normalised (0-1) P43
Normalised (0-1) P44
Abs A
0.0
0.4
0.8
1000
2000
3000
Wavenumber cm-1

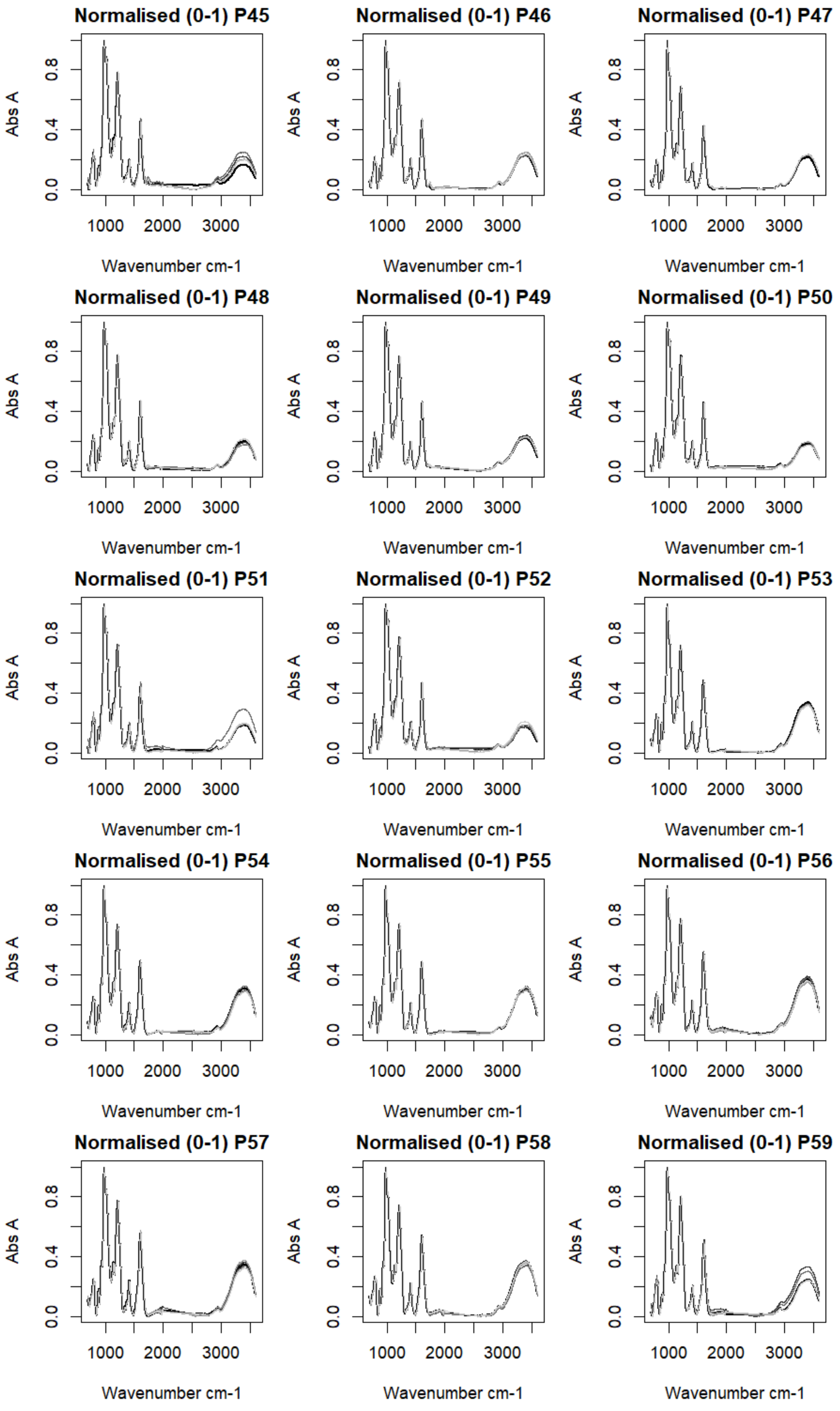
Normalised (0-1) P45
Normalised (0-1) P46
Normalised (0-1) P47
Normalised (0-1) P48
Normalised (0-1) P49
Normalised (0-1) P50
Normalised (0-1) P51
Normalised (0-1) P52
Normalised (0-1) P53
Normalised (0-1) P54
Normalised (0-1) P55
Normalised (0-1) P56
Normalised (0-1) P57
Normalised (0-1) P58
Normalised (0-1) P59
Abs A
0.0
0.4
0.8
1000
2000
3000
Wavenumber cm-1

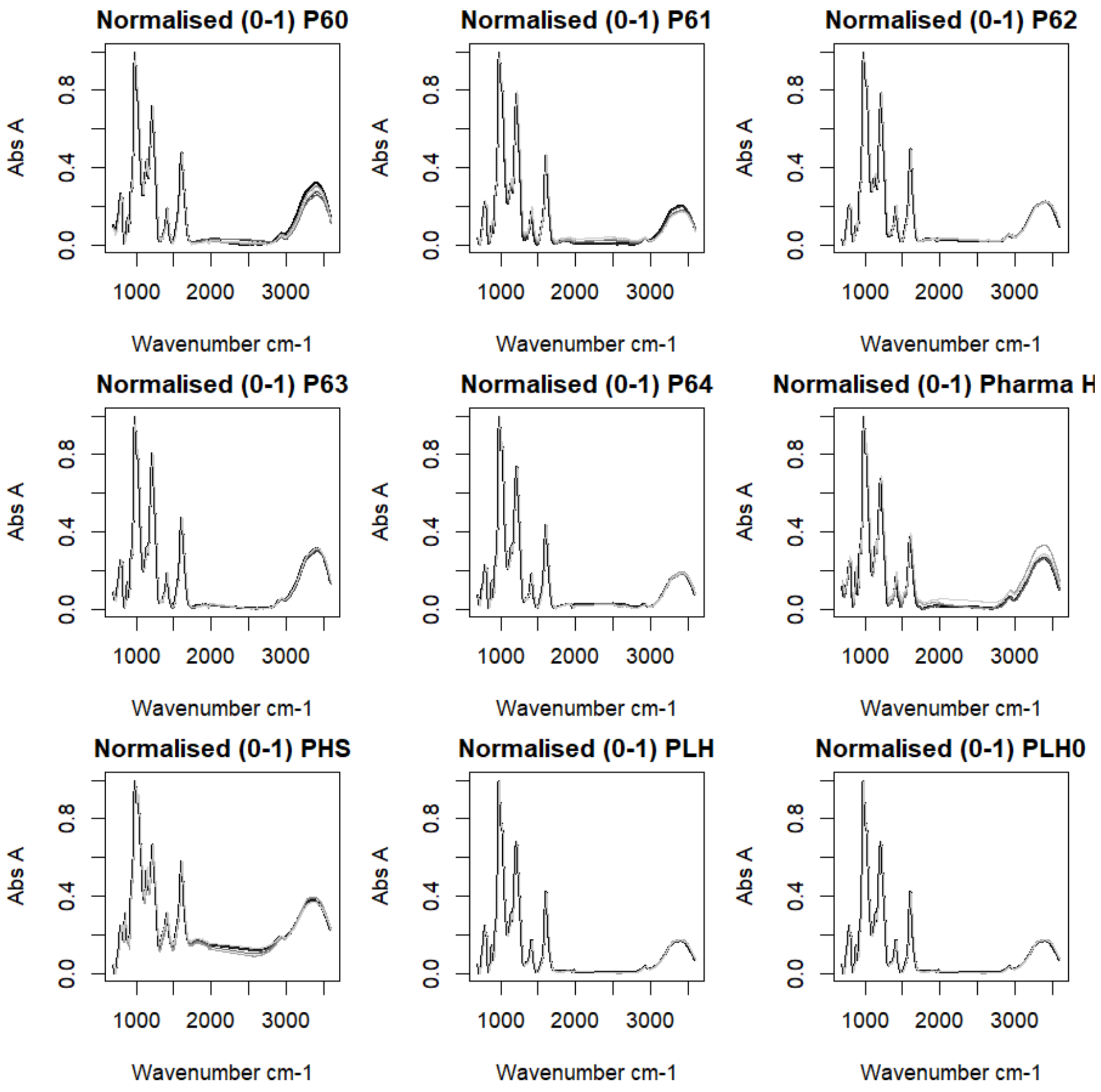
Normalised (0-1) P60
Normalised (0-1) P61
Normalised (0-1) P62
Normalised (0-1) P63
Normalised (0-1) P64
Normalised (0-1) Pharma H
Normalised (0-1) PHS
Normalised (0-1) PLH
Normalised (0-1) PLH0
Abs A
0.0
0.4
0.8
1000
2000
3000
Wavenumber cm-1

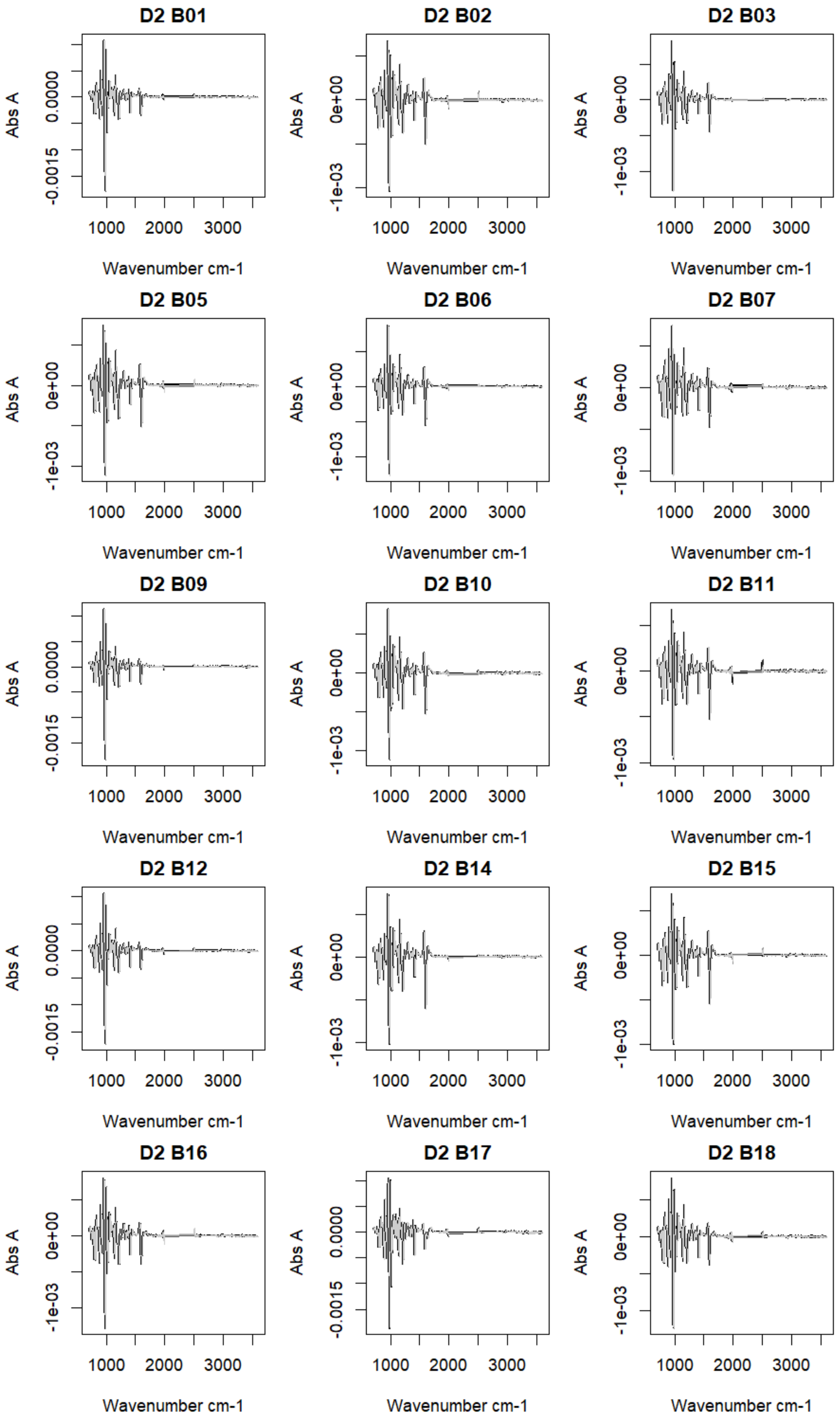
D2 B01
D2 B02
D2 B03
D2 B05
D2 B06
D2 B07
D2 B09
D2 B10
D2 B11
D2 B12
D2 B14
D2 B15
D2 B16
D2 B17
D2 B18
Abs A
Wavenumber cm-1
1000
2000
3000
0.0000
-0.0015
0e+00
-1e-03

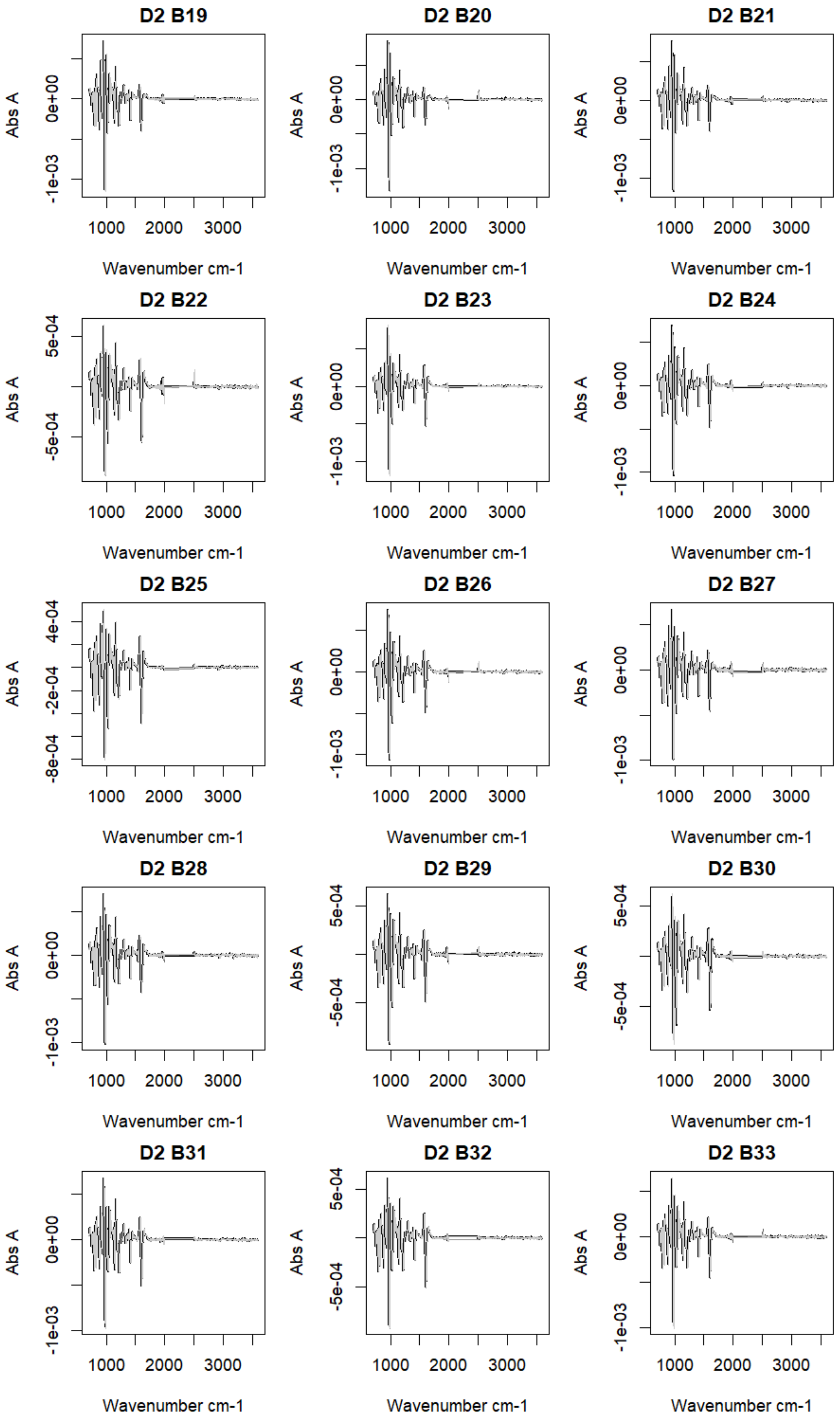
D2 B19
D2 B20
D2 B21
D2 B22
D2 B23
D2 B24
D2 B25
D2 B26
D2 B27
D2 B28
D2 B29
D2 B30
D2 B31
D2 B32
D2 B33
Abs A
Wavenumber cm-1
1000 2000 3000
0e+00
-1e-03
5e-04
-5e-04
4e-04
-2e-04
-8e-04

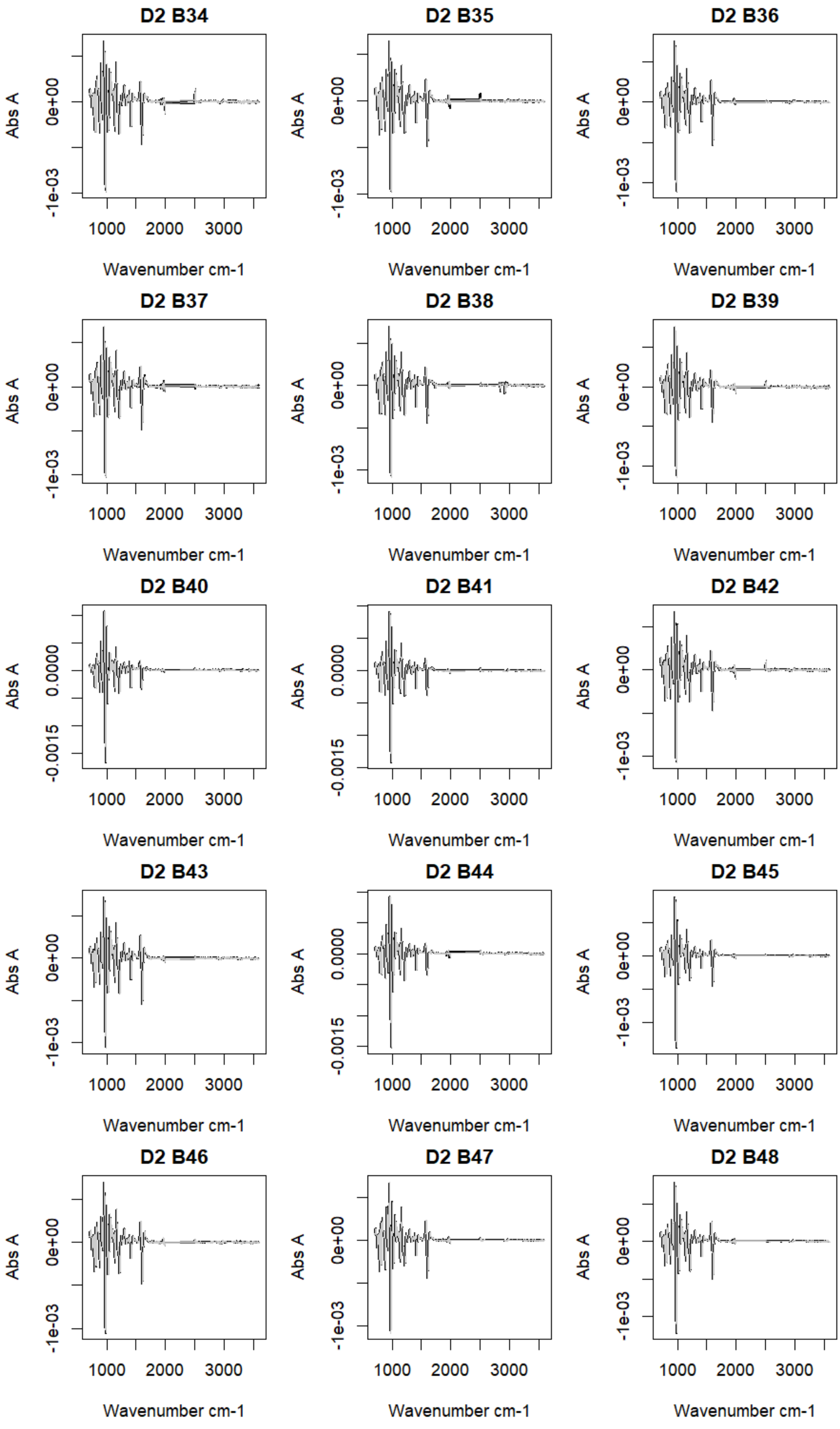
D2 B34
D2 B35
D2 B36
D2 B37
D2 B38
D2 B39
D2 B40
D2 B41
D2 B42
D2 B43
D2 B44
D2 B45
D2 B46
D2 B47
D2 B48
Abs A
0e+00
-1e-03
0.0000
-0.0015
1000
2000
3000
Wavenumber cm-1

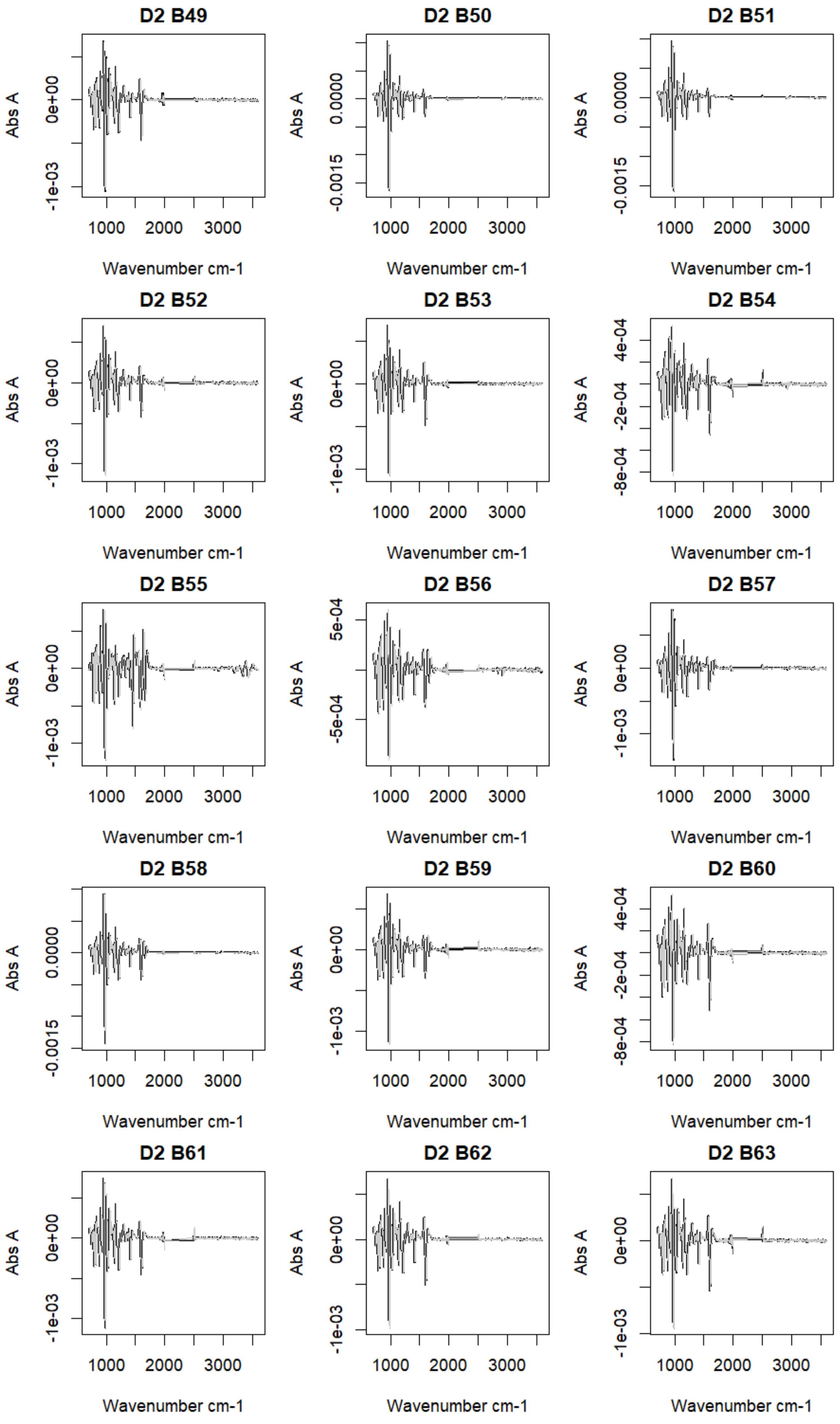
D2 B49
D2 B50
D2 B51
D2 B52
D2 B53
D2 B54
D2 B55
D2 B56
D2 B57
D2 B58
D2 B59
D2 B60
D2 B61
D2 B62
D2 B63
Abs A
Wavenumber cm-1
1000
2000
3000
0e+00
-1e-03
0.0000
-0.0015
4e-04
-2e-04
-8e-04
5e-04
-5e-04

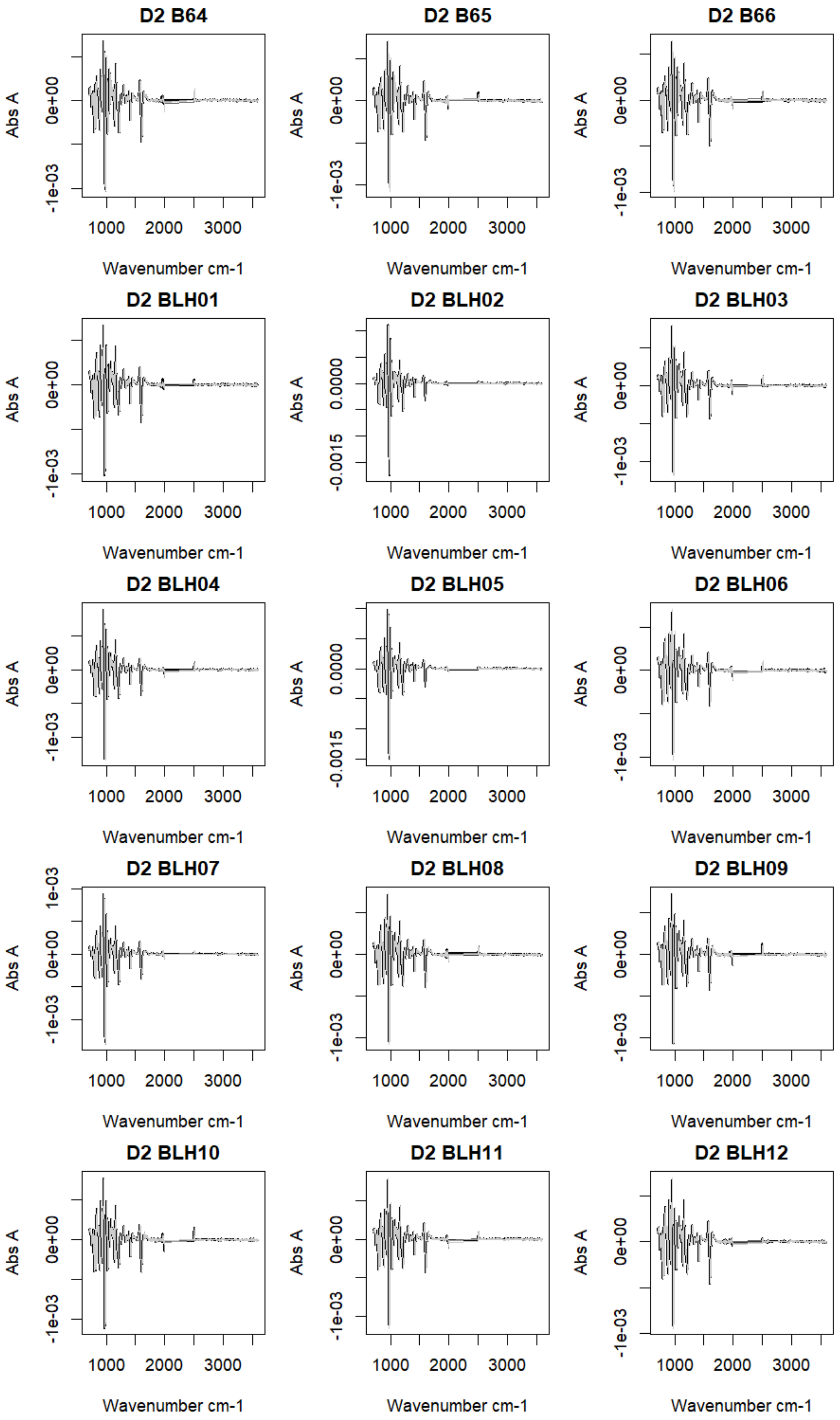
D2 B64
D2 B65
D2 B66
D2 BLH01
D2 BLH02
D2 BLH03
D2 BLH04
D2 BLH05
D2 BLH06
D2 BLH07
D2 BLH08
D2 BLH09
D2 BLH10
D2 BLH11
D2 BLH12
Abs A
0e+00
-1e-03
1e-03
0.0000
-0.0015
1000
2000
3000
Wavenumber cm-1

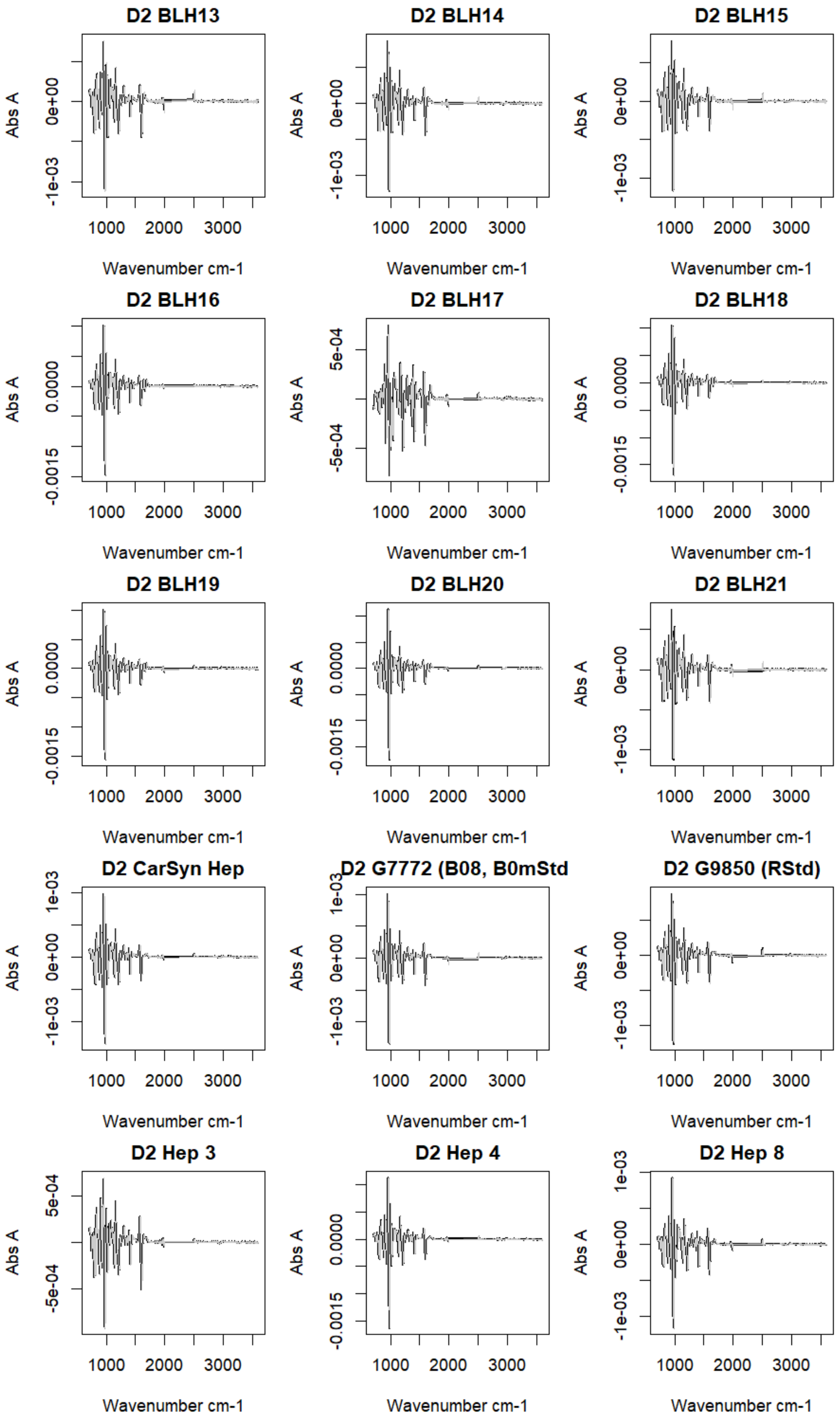
D2 BLH13
D2 BLH14
D2 BLH15
D2 BLH16
D2 BLH17
D2 BLH18
D2 BLH19
D2 BLH20
D2 BLH21
D2 CarSyn Hep
D2 G7772 (B08, B0mStd
D2 G9850 (RStd)
D2 Hep 3
D2 Hep 4
D2 Hep 8
Abs A
Wavenumber cm-1
1000
2000
3000
-1e-03
0e+00
1e-03
-0.0015
0.0000
-5e-04
5e-04

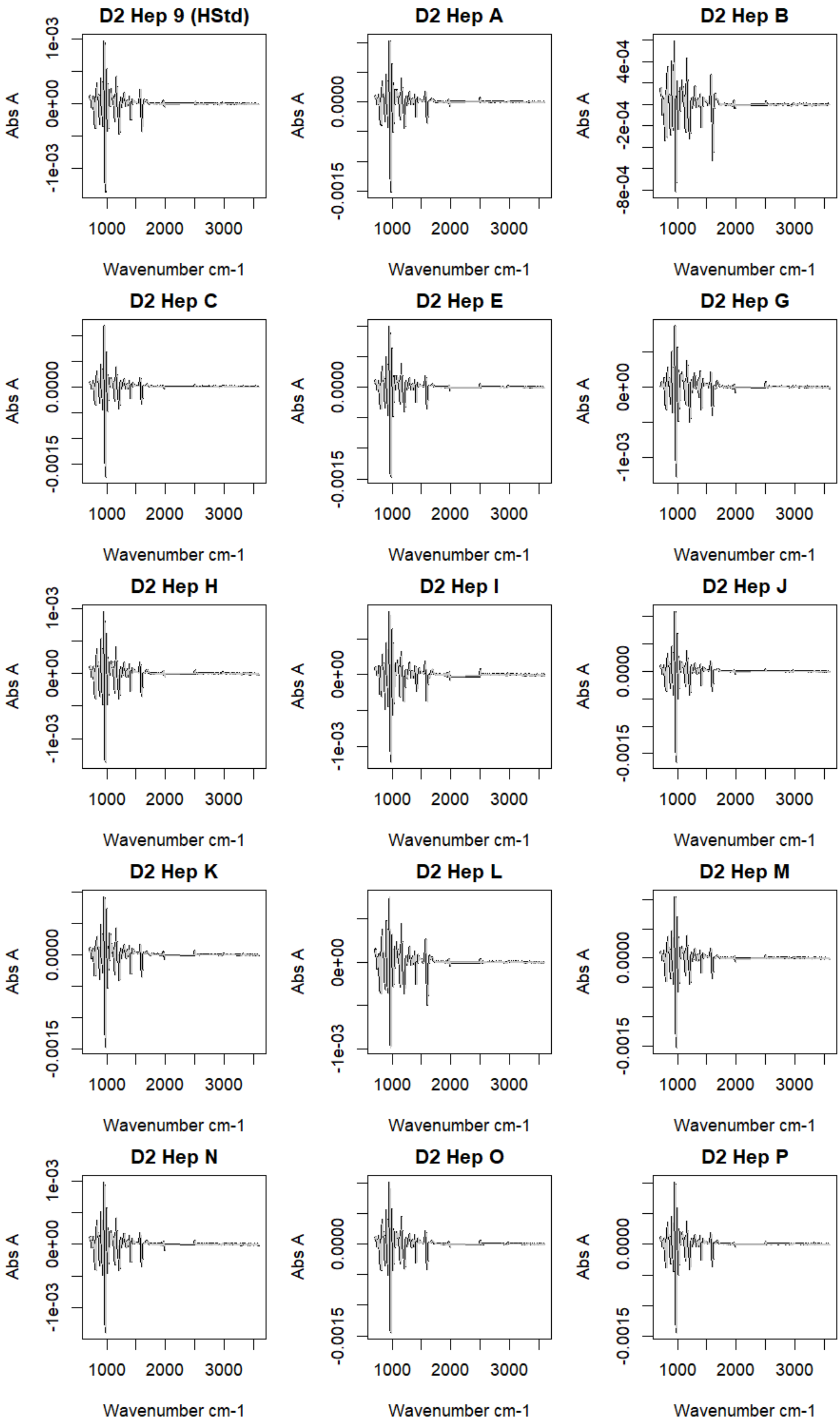
D2 Hep 9 (HStd)
D2 Hep A
D2 Hep B
D2 Hep C
D2 Hep E
D2 Hep G
D2 Hep H
D2 Hep I
D2 Hep J
D2 Hep K
D2 Hep L
D2 Hep M
D2 Hep N
D2 Hep O
D2 Hep P
Abs A
Wavenumber cm-1
1000
2000
3000
1e-03
0e+00
-1e-03
0.0000
-0.0015
4e-04
-2e-04
-8e-04

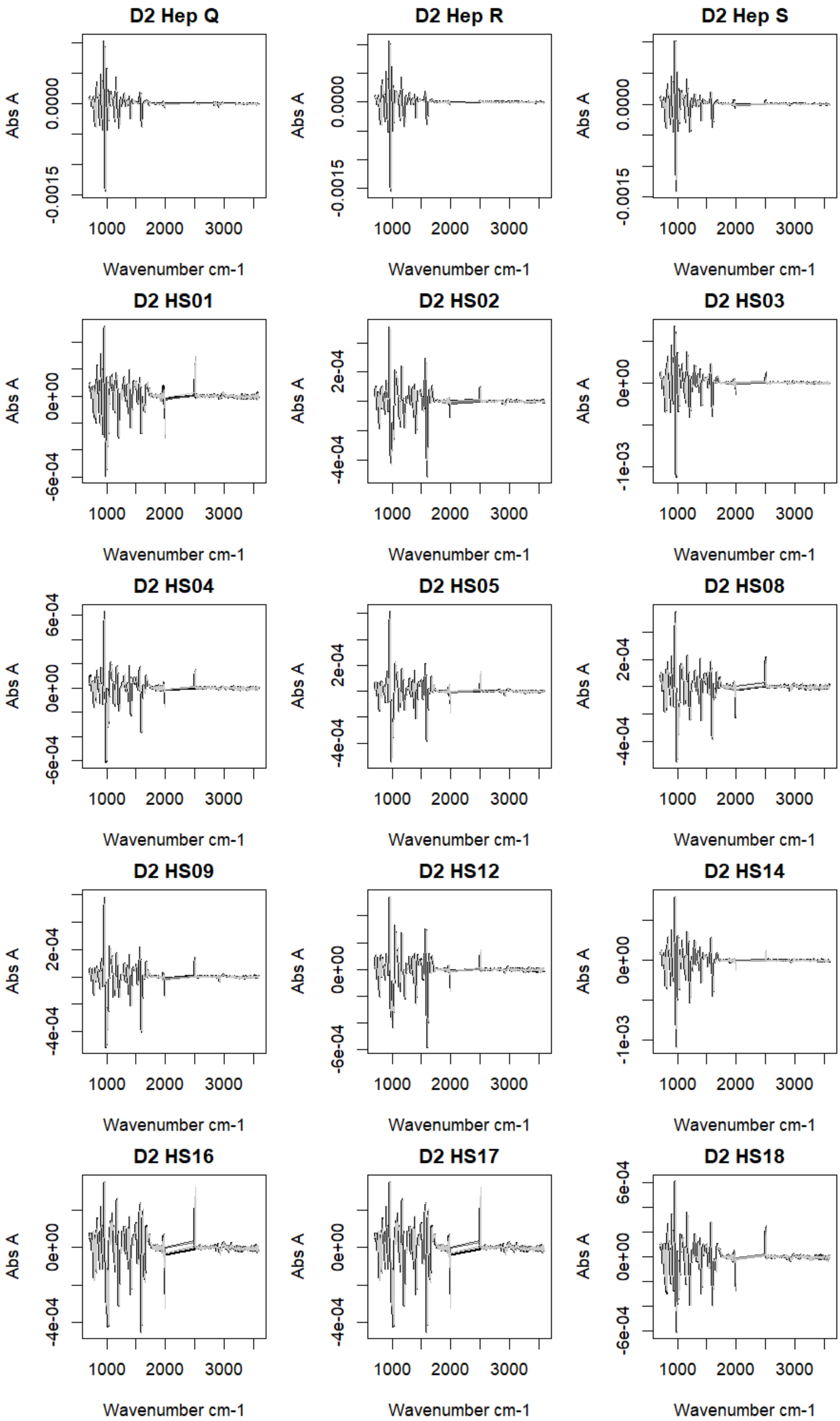
D2 Hep Q
Abs A
0.0000
-0.0015
1000 2000 3000
Wavenumber cm-1
D2 Hep R
Abs A
0.0000
-0.0015
1000 2000 3000
Wavenumber cm-1
D2 Hep S
Abs A
0.0000
-0.0015
1000 2000 3000
Wavenumber cm-1
D2 HS01
Abs A
0e+00
-6e-04
1000 2000 3000
Wavenumber cm-1
D2 HS02
Abs A
2e-04
-4e-04
1000 2000 3000
Wavenumber cm-1
D2 HS03
Abs A
0e+00
-1e-03
1000 2000 3000
Wavenumber cm-1
D2 HS04
Abs A
6e-04
0e+00
-6e-04
1000 2000 3000
Wavenumber cm-1
D2 HS05
Abs A
2e-04
-4e-04
1000 2000 3000
Wavenumber cm-1
D2 HS08
Abs A
2e-04
-4e-04
1000 2000 3000
Wavenumber cm-1
D2 HS09
Abs A
2e-04
-4e-04
1000 2000 3000
Wavenumber cm-1
D2 HS12
Abs A
0e+00
-6e-04
1000 2000 3000
Wavenumber cm-1
D2 HS14
Abs A
0e+00
-1e-03
1000 2000 3000
Wavenumber cm-1
D2 HS16
Abs A
0e+00
-4e-04
1000 2000 3000
Wavenumber cm-1
D2 HS17
Abs A
0e+00
-4e-04
1000 2000 3000
Wavenumber cm-1
D2 HS18
Abs A
6e-04
0e+00
-6e-04
1000 2000 3000
Wavenumber cm-1

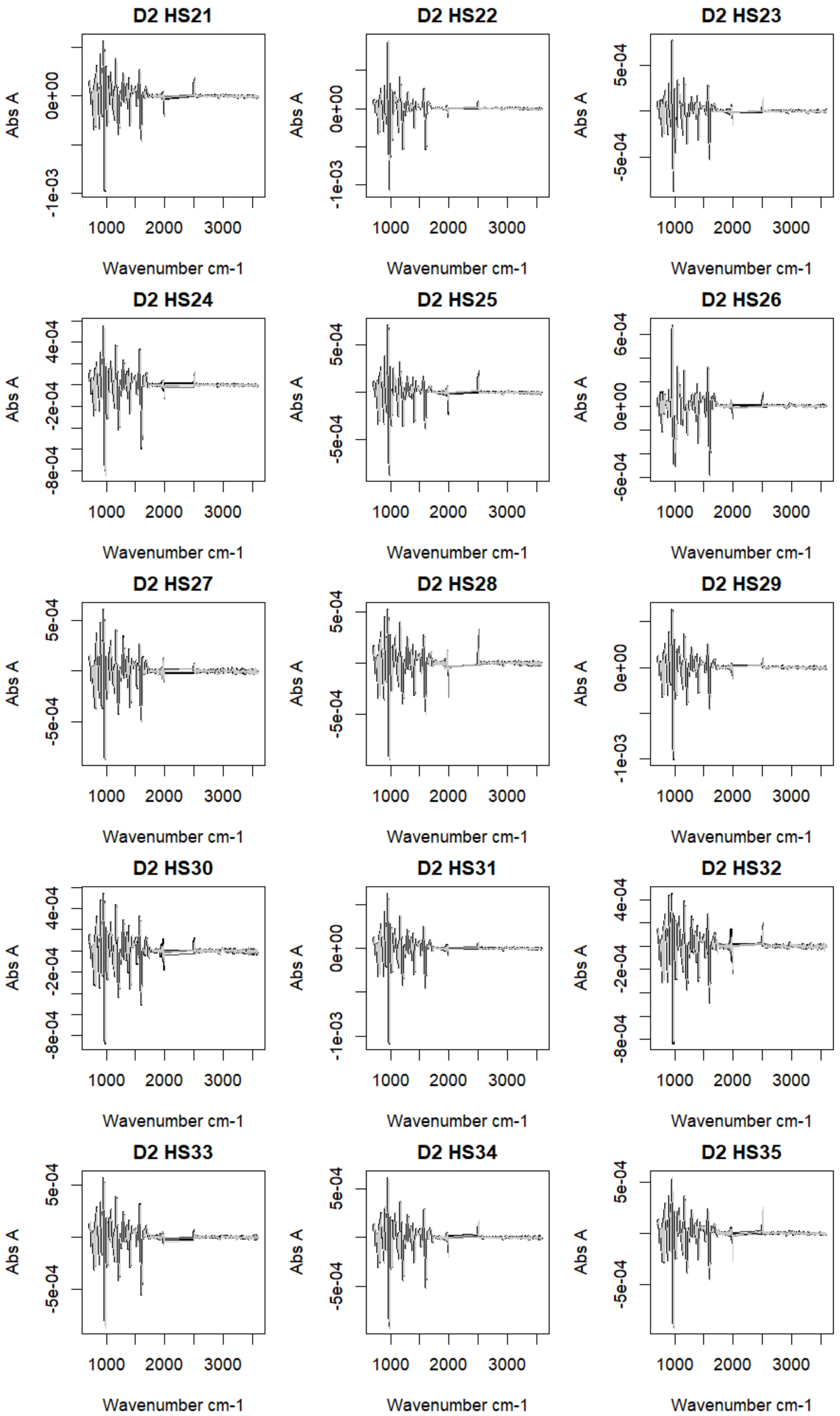
D2 HS21
D2 HS22
D2 HS23
D2 HS24
D2 HS25
D2 HS26
D2 HS27
D2 HS28
D2 HS29
D2 HS30
D2 HS31
D2 HS32
D2 HS33
D2 HS34
D2 HS35
Abs A
Wavenumber cm-1
1000 2000 3000
0e+00
-1e-03
5e-04
-5e-04
4e-04
-2e-04
-8e-04
6e-04
-6e-04

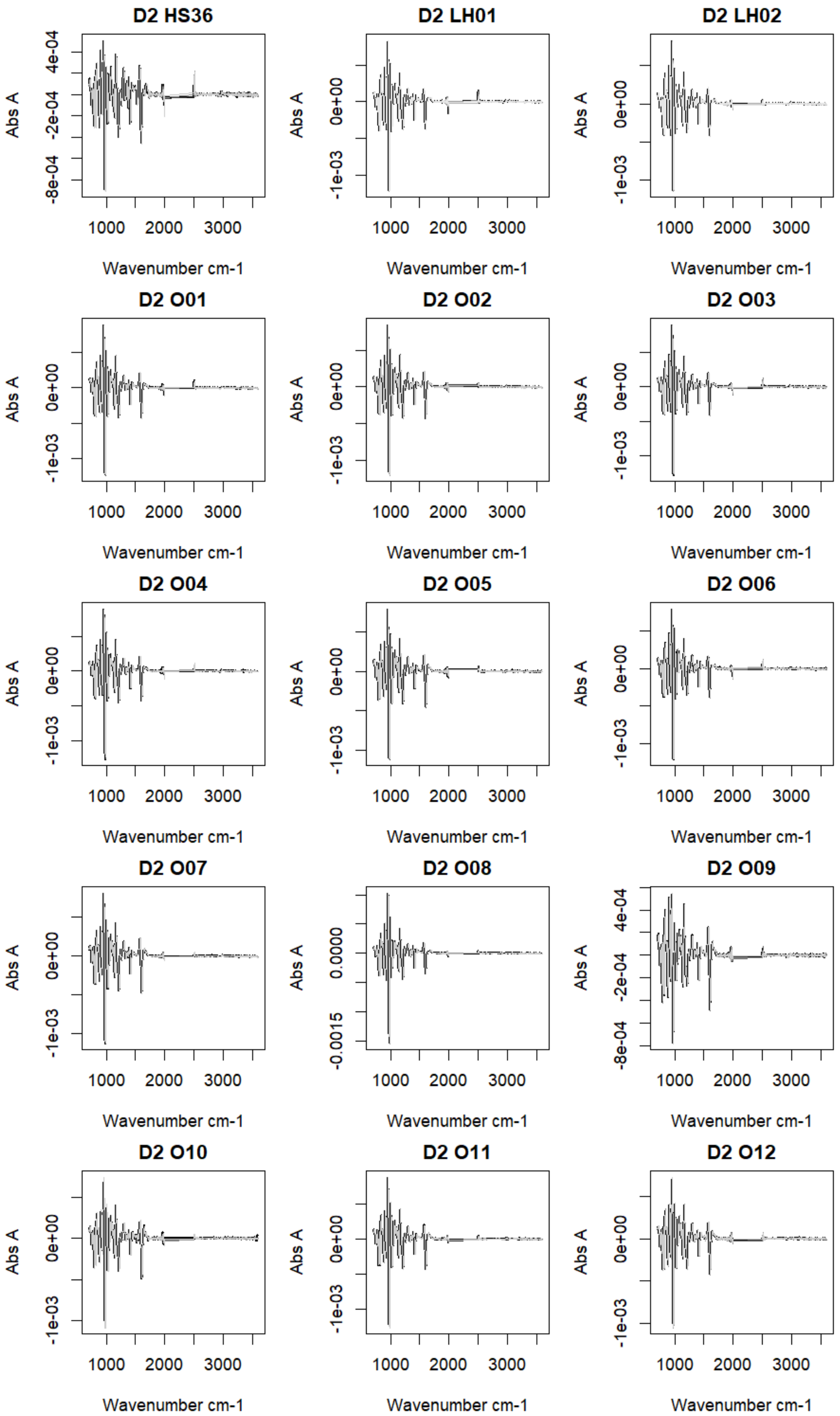
D2 HS36
D2 LH01
D2 LH02
D2 O01
D2 O02
D2 O03
D2 O04
D2 O05
D2 O06
D2 O07
D2 O08
D2 O09
D2 O10
D2 O11
D2 O12
Abs A
Wavenumber cm-1
1000
2000
3000
4e-04
-2e-04
-8e-04
0e+00
-1e-03
0.0000
-0.0015

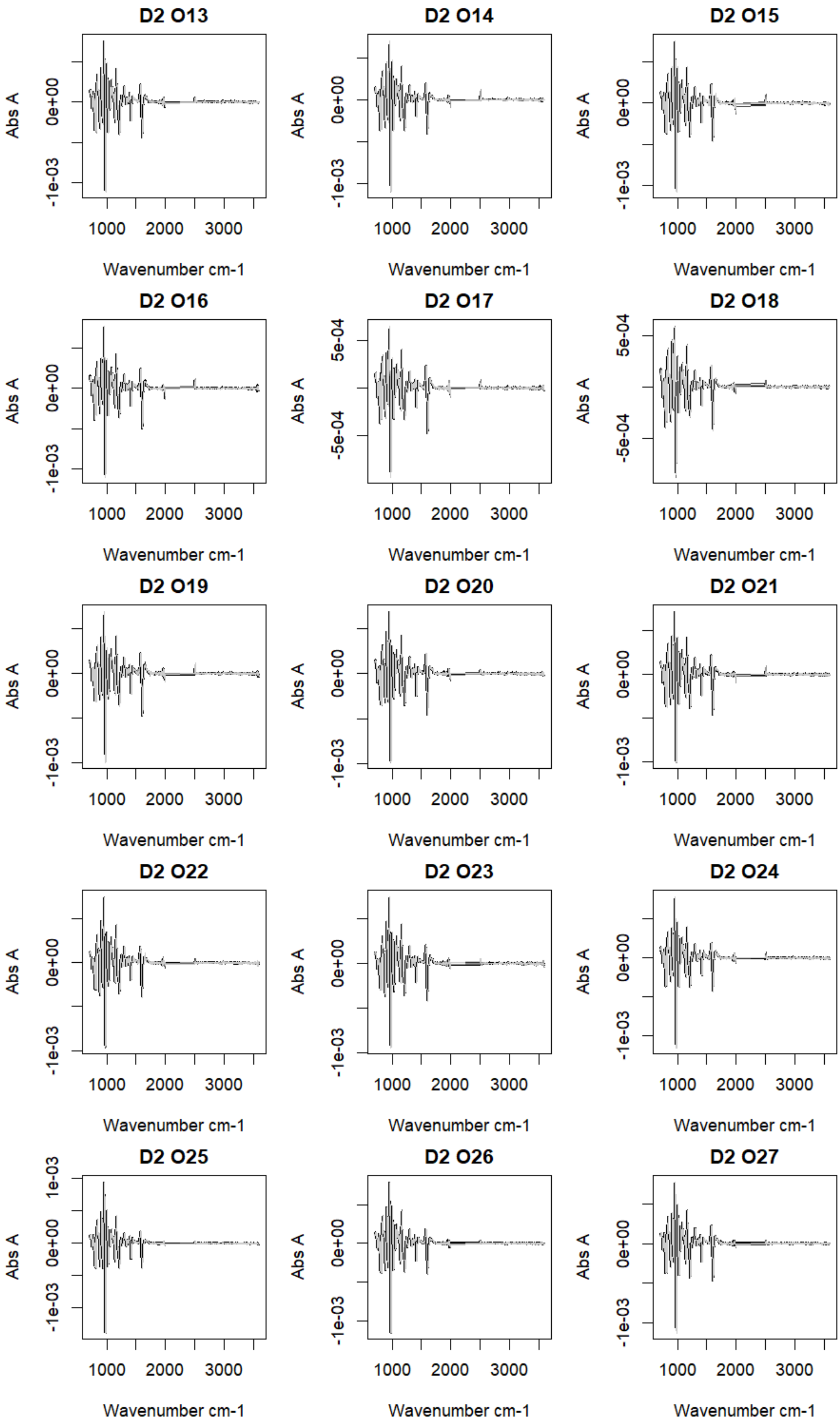
D2 O13
D2 O14
D2 O15
D2 O16
D2 O17
D2 O18
D2 O19
D2 O20
D2 O21
D2 O22
D2 O23
D2 O24
D2 O25
D2 O26
D2 O27
Abs A
Wavenumber cm-1
1000
2000
3000
0e+00
-1e-03
1e-03
5e-04
-5e-04

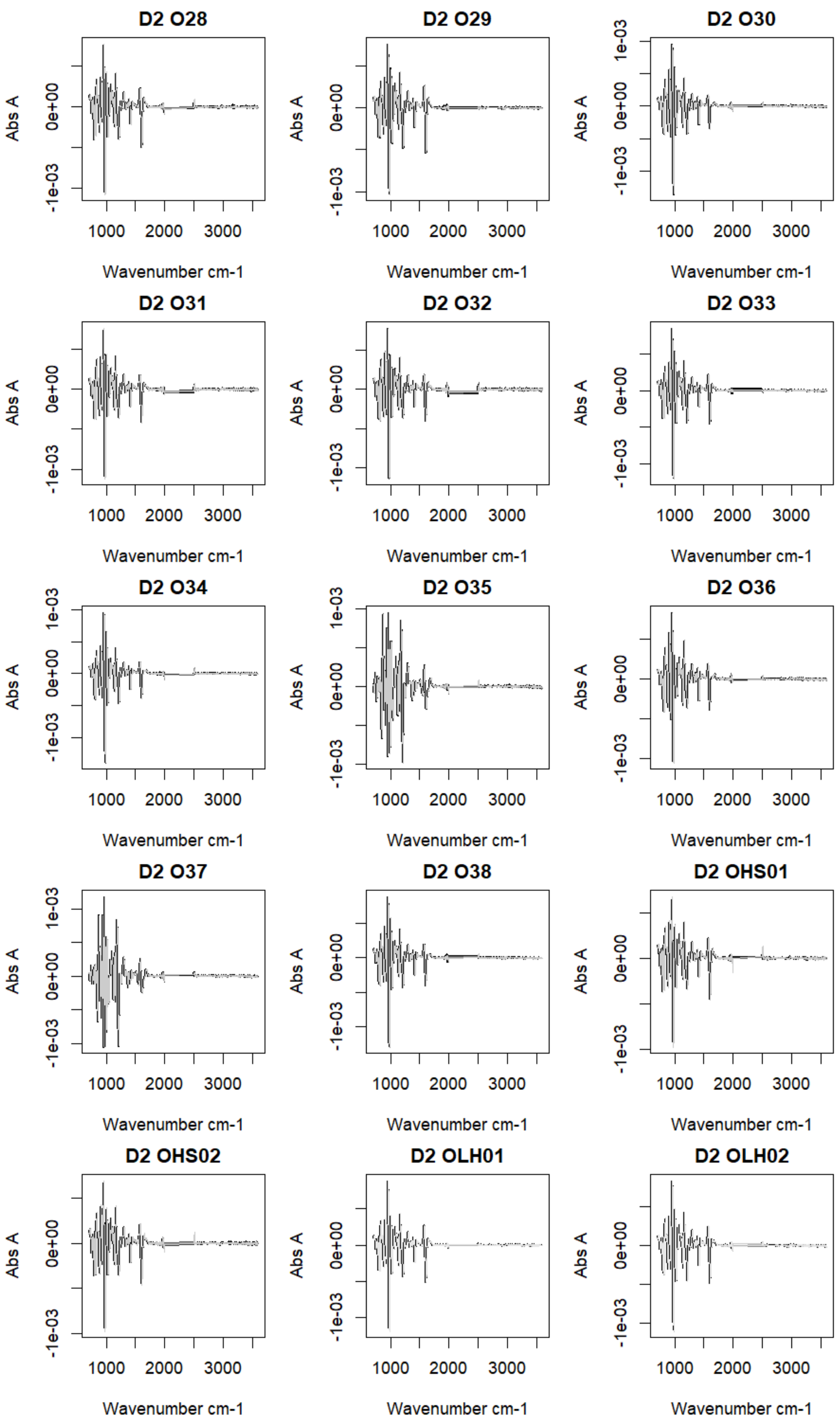
D2 O28
D2 O29
D2 O30
D2 O31
D2 O32
D2 O33
D2 O34
D2 O35
D2 O36
D2 O37
D2 O38
D2 OHS01
D2 OHS02
D2 OLH01
D2 OLH02
Abs A
1e-03
0e+00
-1e-03
1000
2000
3000
Wavenumber cm-1

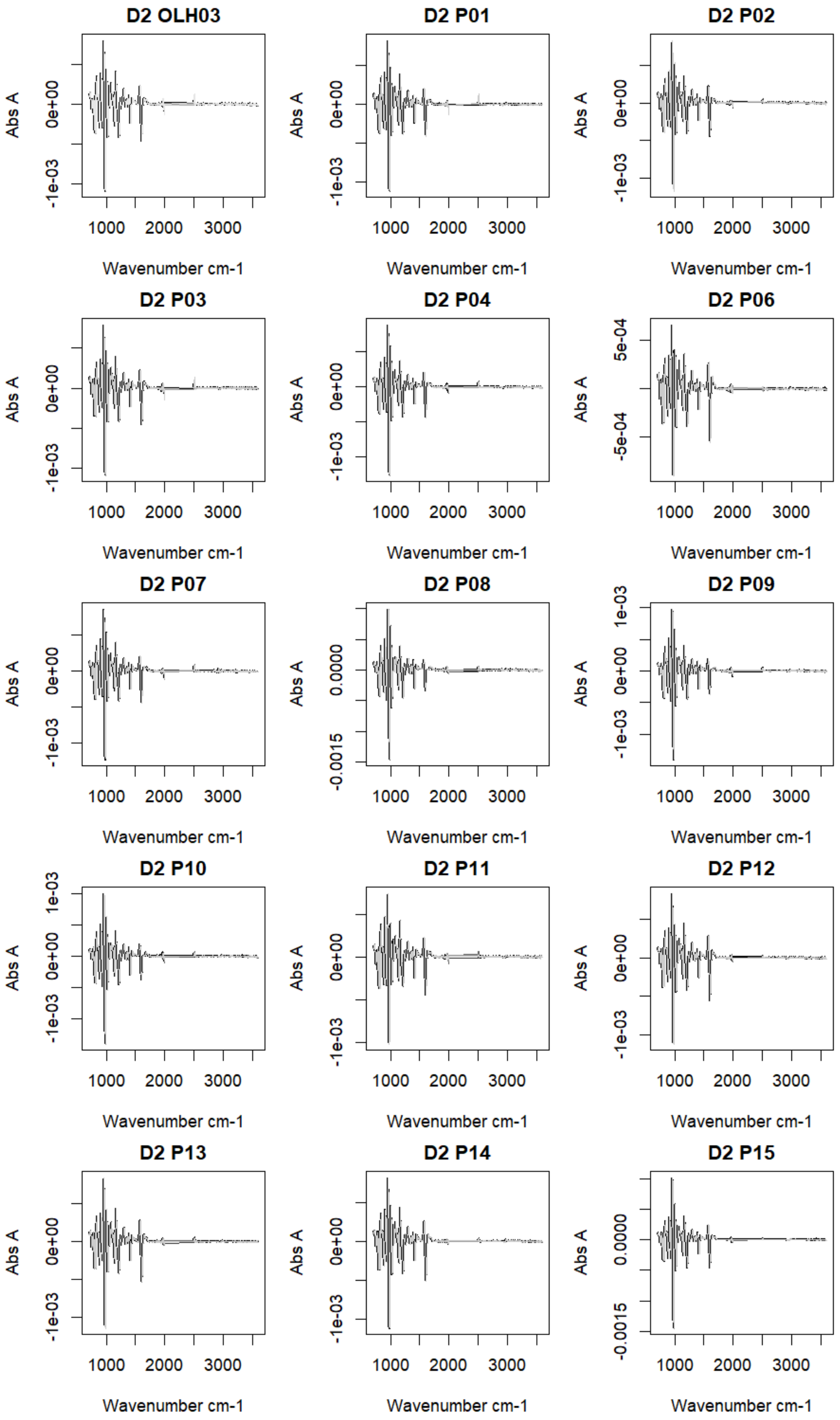
D2 OLH03
D2 P01
D2 P02
D2 P03
D2 P04
D2 P06
D2 P07
D2 P08
D2 P09
D2 P10
D2 P11
D2 P12
D2 P13
D2 P14
D2 P15
Abs A
Wavenumber cm-1
1000 2000 3000
0e+00
-1e-03
1e-03
5e-04
-5e-04
0.0000
-0.0015

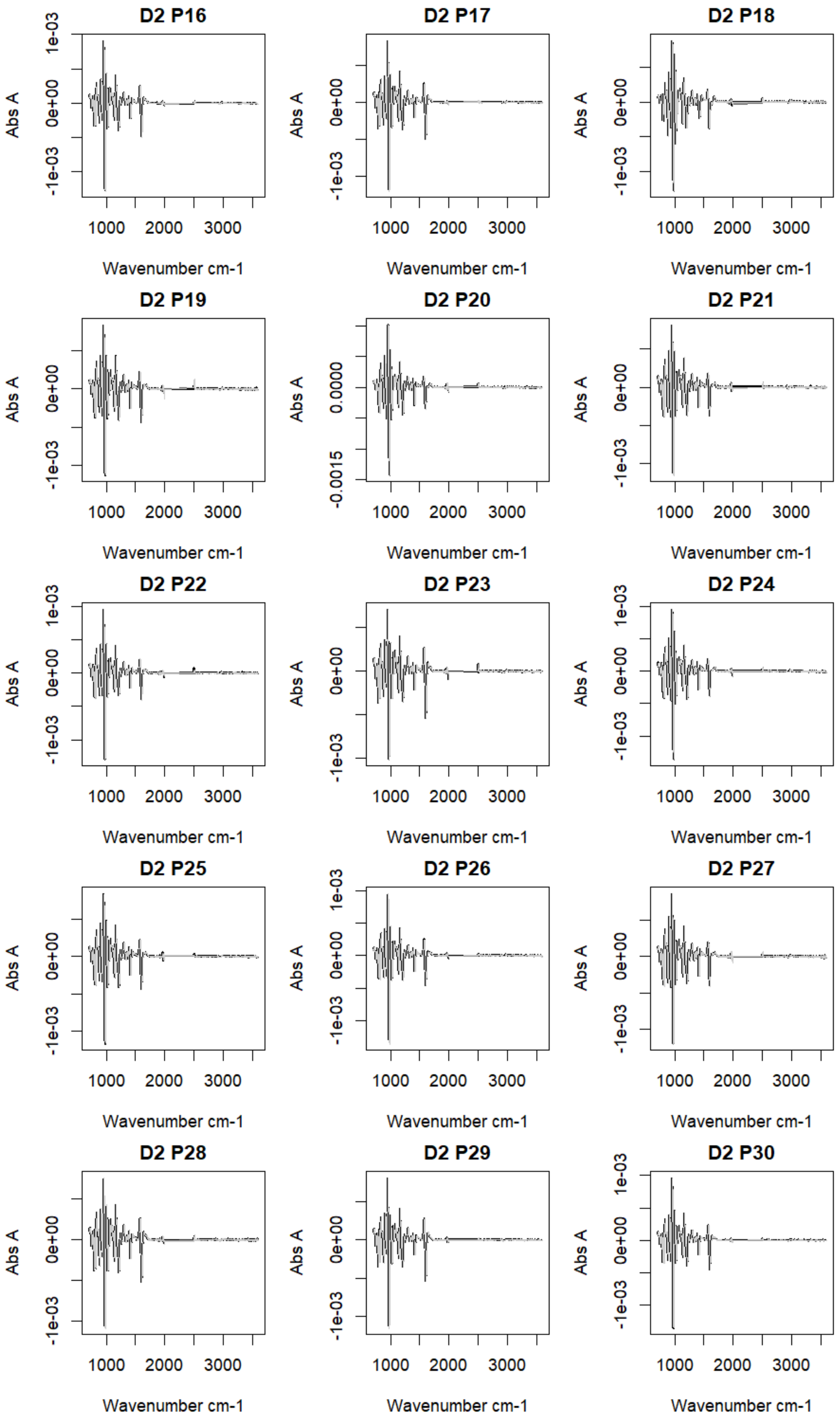
D2 P16
D2 P17
D2 P18
D2 P19
D2 P20
D2 P21
D2 P22
D2 P23
D2 P24
D2 P25
D2 P26
D2 P27
D2 P28
D2 P29
D2 P30
Abs A
1e-03
0e+00
-1e-03
0.0000
-0.0015
1000
2000
3000
Wavenumber cm-1

D2 P31

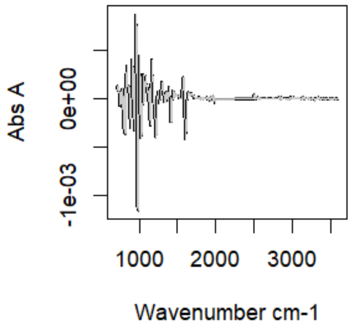

D2 P32

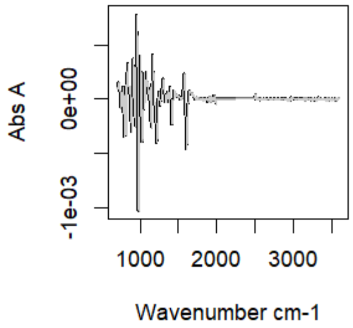

D2 P33

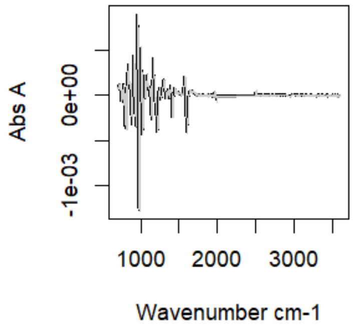

D2 P34a

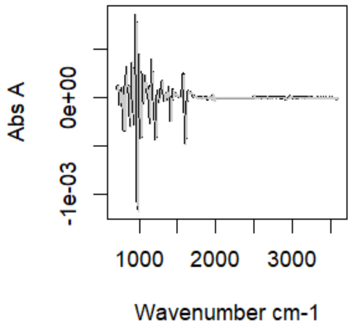

D2 P34b

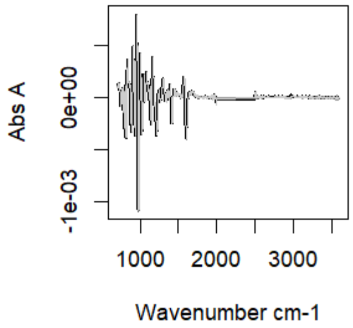

D2 P35

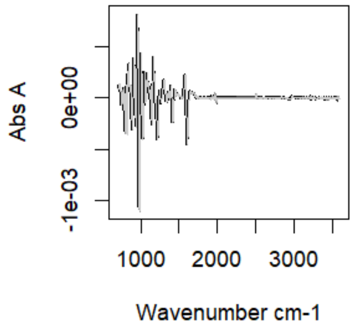

D2 P36

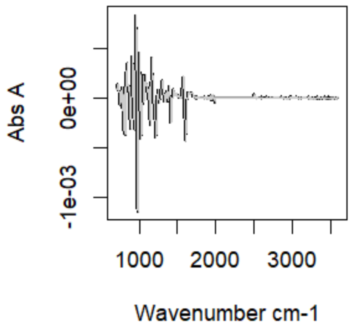

D2 P37

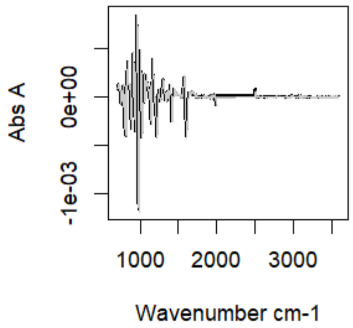

D2 P38

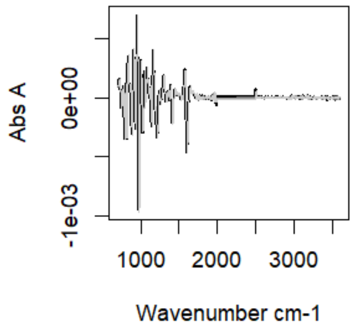

D2 P39

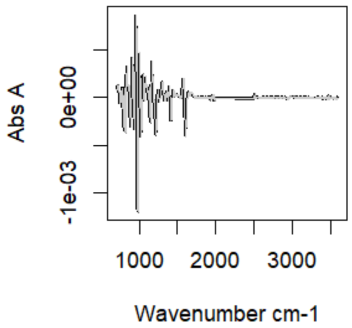

D2 P40

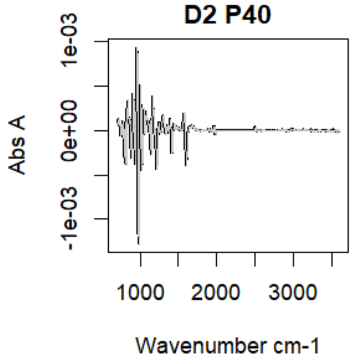

D2 P41

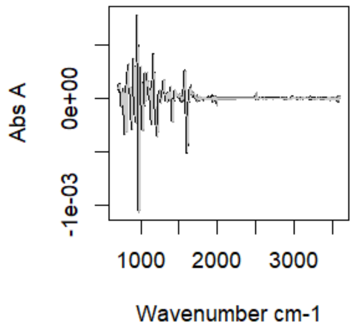

D2 P42

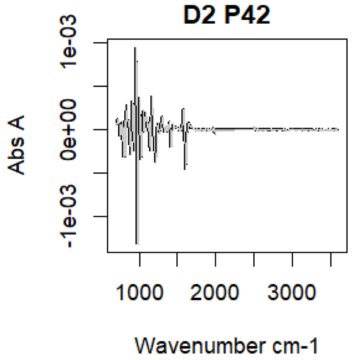

D2 P43

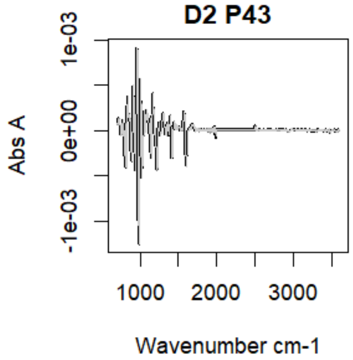

D2 P44

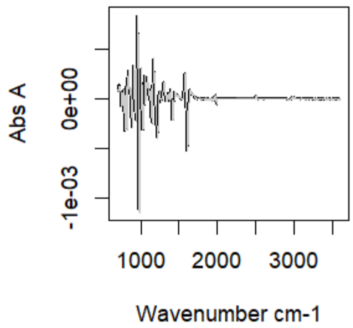

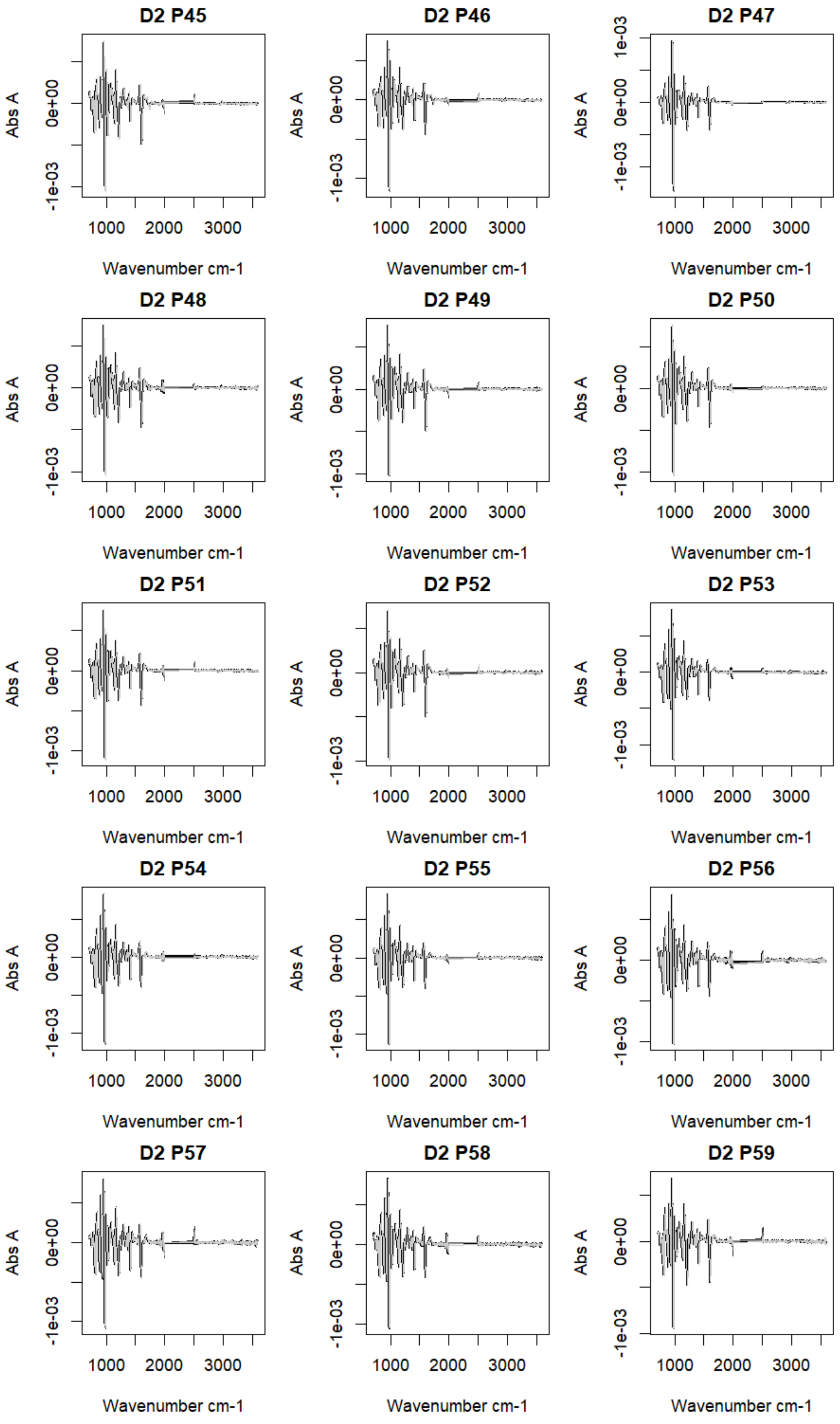
D2 P45
D2 P46
D2 P47
D2 P48
D2 P49
D2 P50
D2 P51
D2 P52
D2 P53
D2 P54
D2 P55
D2 P56
D2 P57
D2 P58
D2 P59
Abs A
-1e-03
0e+00
1e-03
1000
2000
3000
Wavenumber cm-1

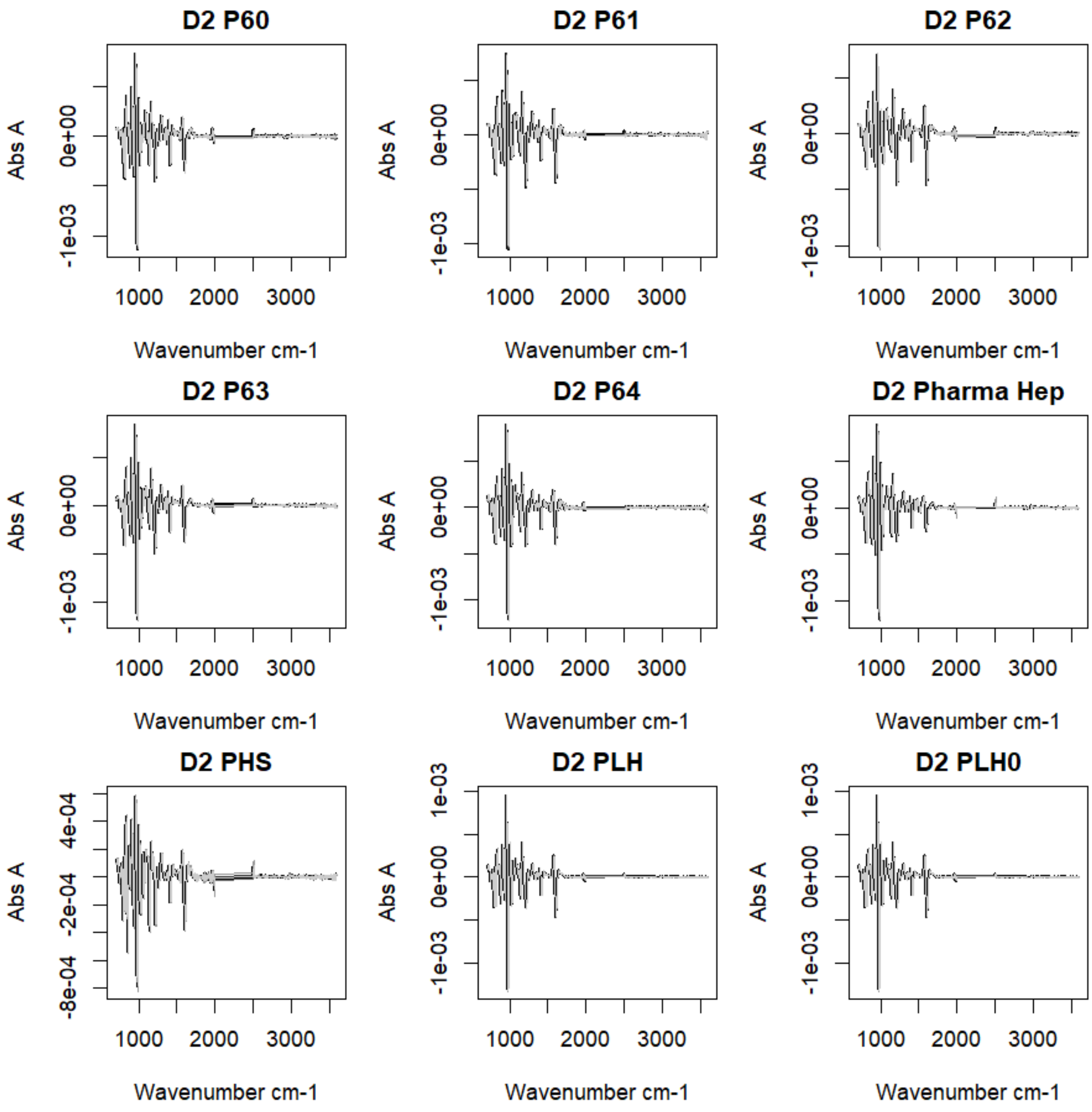
D2 P60
D2 P61
D2 P62
D2 P63
D2 P64
D2 Pharma Hep
D2 PHS
D2 PLH
D2 PLH0
Abs A
0e+00
-1e-03
1e-03
4e-04
-2e-04
-8e-04
1000
2000
3000
Wavenumber cm-1

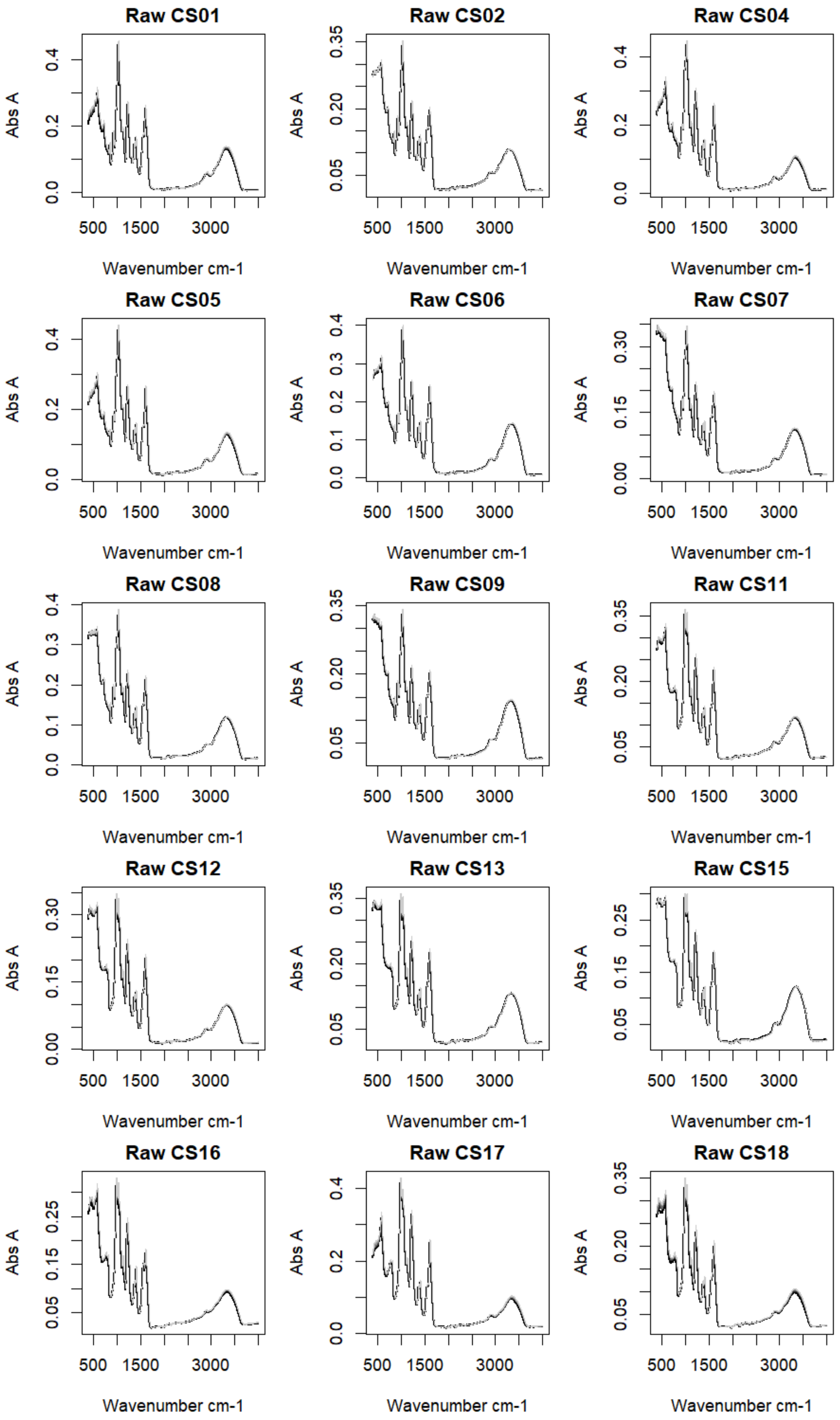
Raw CS01
Abs A
Wavenumber cm-1
Raw CS02
Abs A
Wavenumber cm-1
Raw CS04
Abs A
Wavenumber cm-1
Raw CS05
Abs A
Wavenumber cm-1
Raw CS06
Abs A
Wavenumber cm-1
Raw CS07
Abs A
Wavenumber cm-1
Raw CS08
Abs A
Wavenumber cm-1
Raw CS09
Abs A
Wavenumber cm-1
Raw CS11
Abs A
Wavenumber cm-1
Raw CS12
Abs A
Wavenumber cm-1
Raw CS13
Abs A
Wavenumber cm-1
Raw CS15
Abs A
Wavenumber cm-1
Raw CS16
Abs A
Wavenumber cm-1
Raw CS17
Abs A
Wavenumber cm-1
Raw CS18
Abs A
Wavenumber cm-1

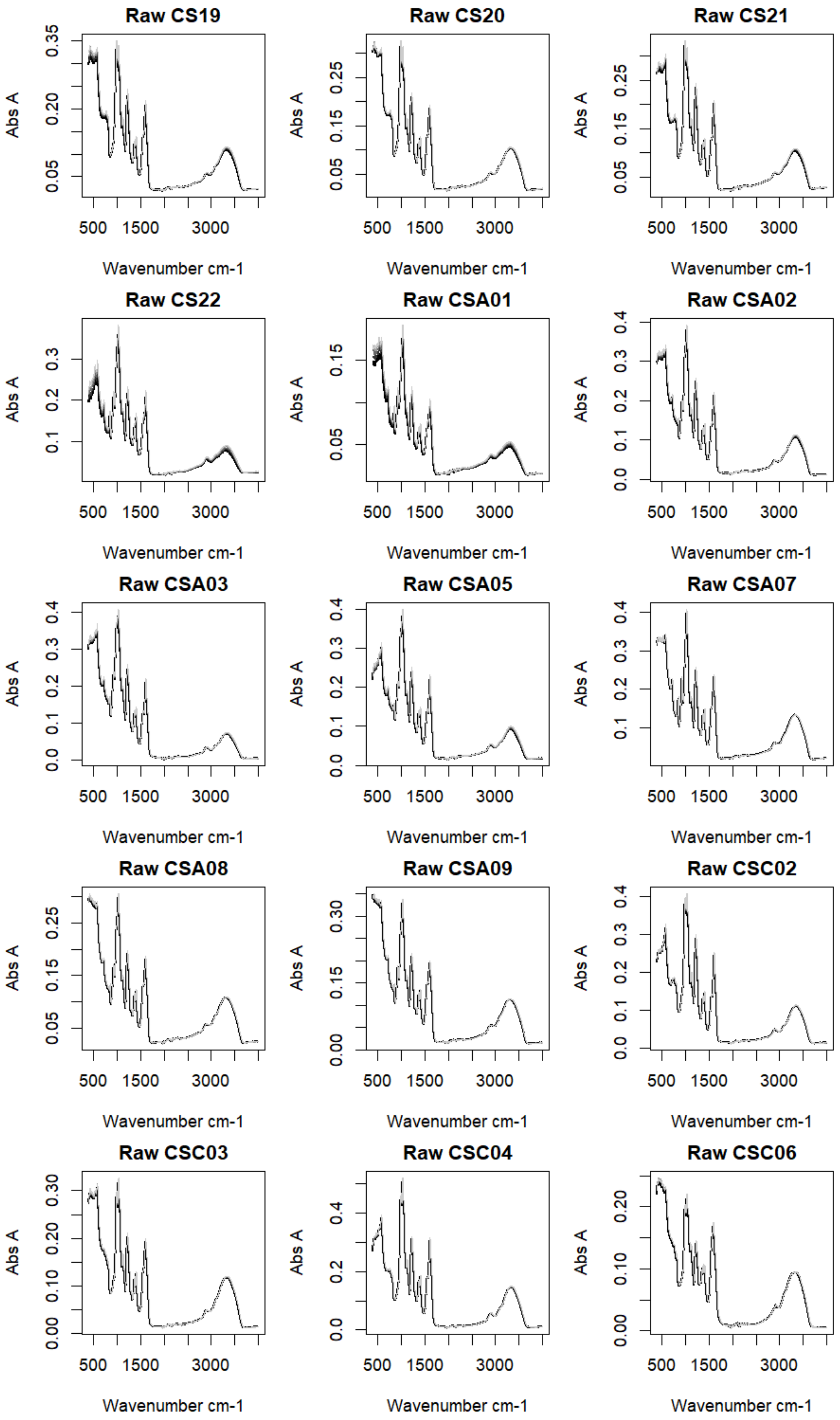
Raw CS19
Raw CS20
Raw CS21
Raw CS22
Raw CSA01
Raw CSA02
Raw CSA03
Raw CSA05
Raw CSA07
Raw CSA08
Raw CSA09
Raw CSC02
Raw CSC03
Raw CSC04
Raw CSC06
Abs A
Wavenumber cm-1
500
1500
3000

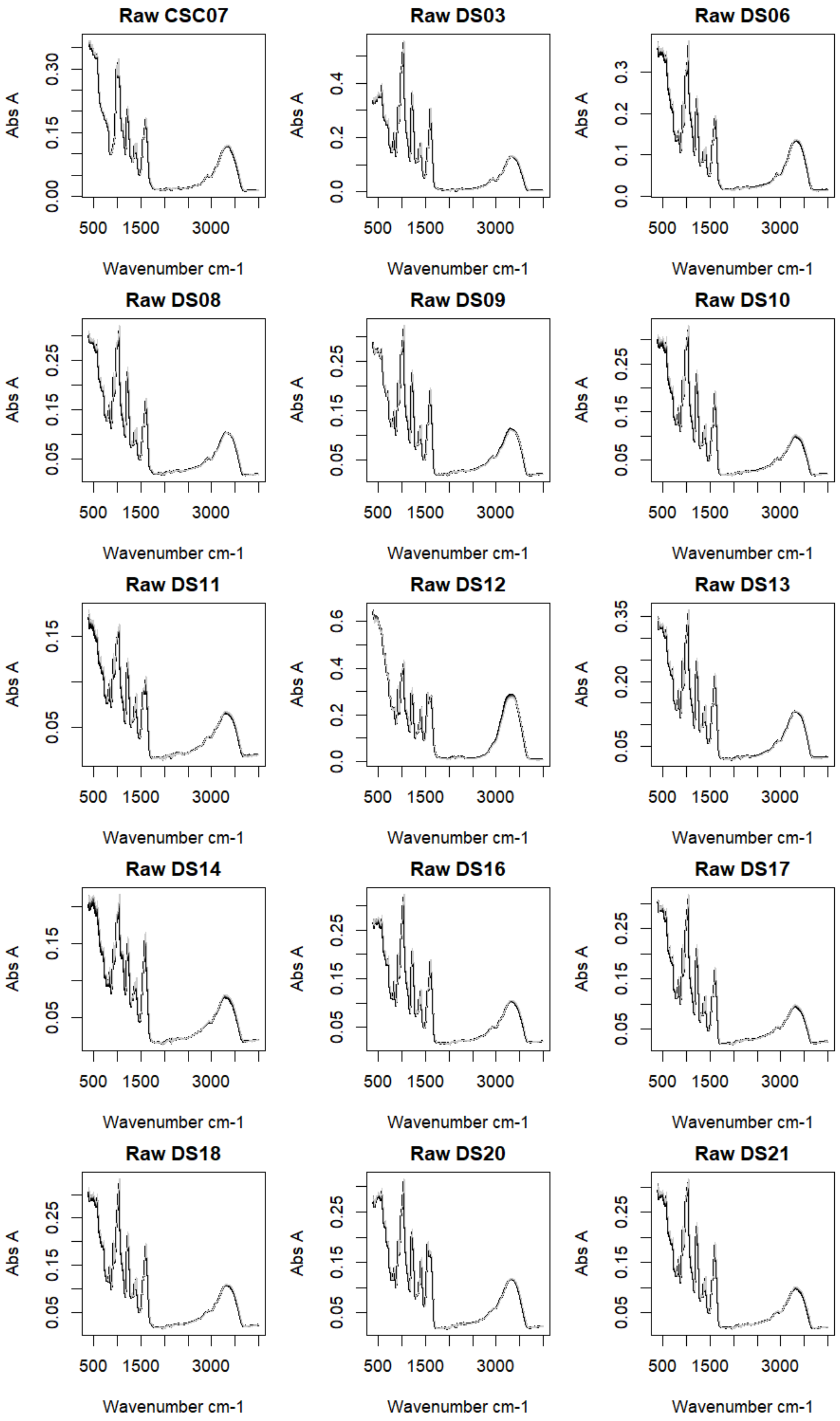
Raw CSC07
Raw DS03
Raw DS06
Raw DS08
Raw DS09
Raw DS10
Raw DS11
Raw DS12
Raw DS13
Raw DS14
Raw DS16
Raw DS17
Raw DS18
Raw DS20
Raw DS21
Abs A
Wavenumber cm-1

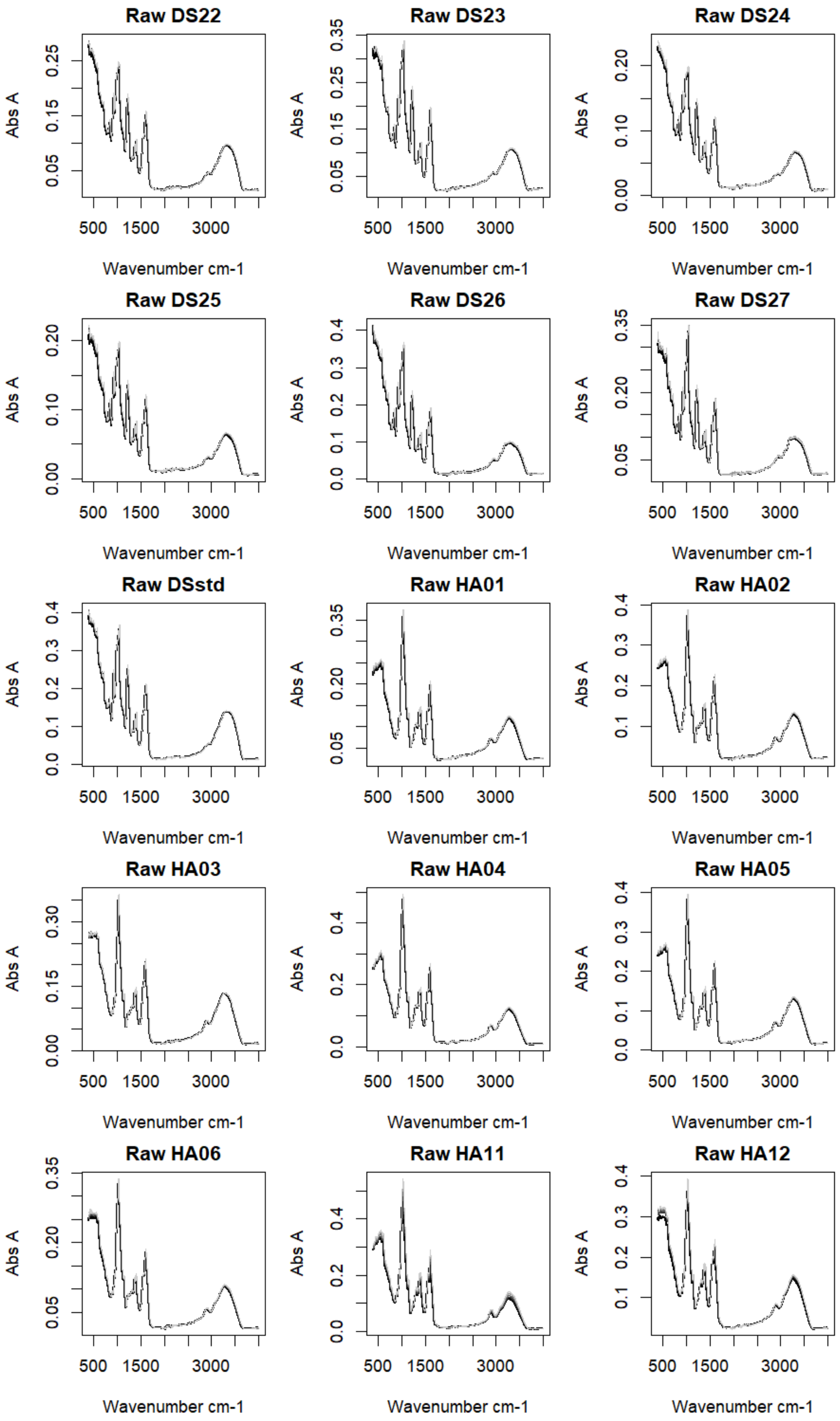
Raw DS22
Raw DS23
Raw DS24
Raw DS25
Raw DS26
Raw DS27
Raw DSstd
Raw HA01
Raw HA02
Raw HA03
Raw HA04
Raw HA05
Raw HA06
Raw HA11
Raw HA12
Abs A
Wavenumber cm-1

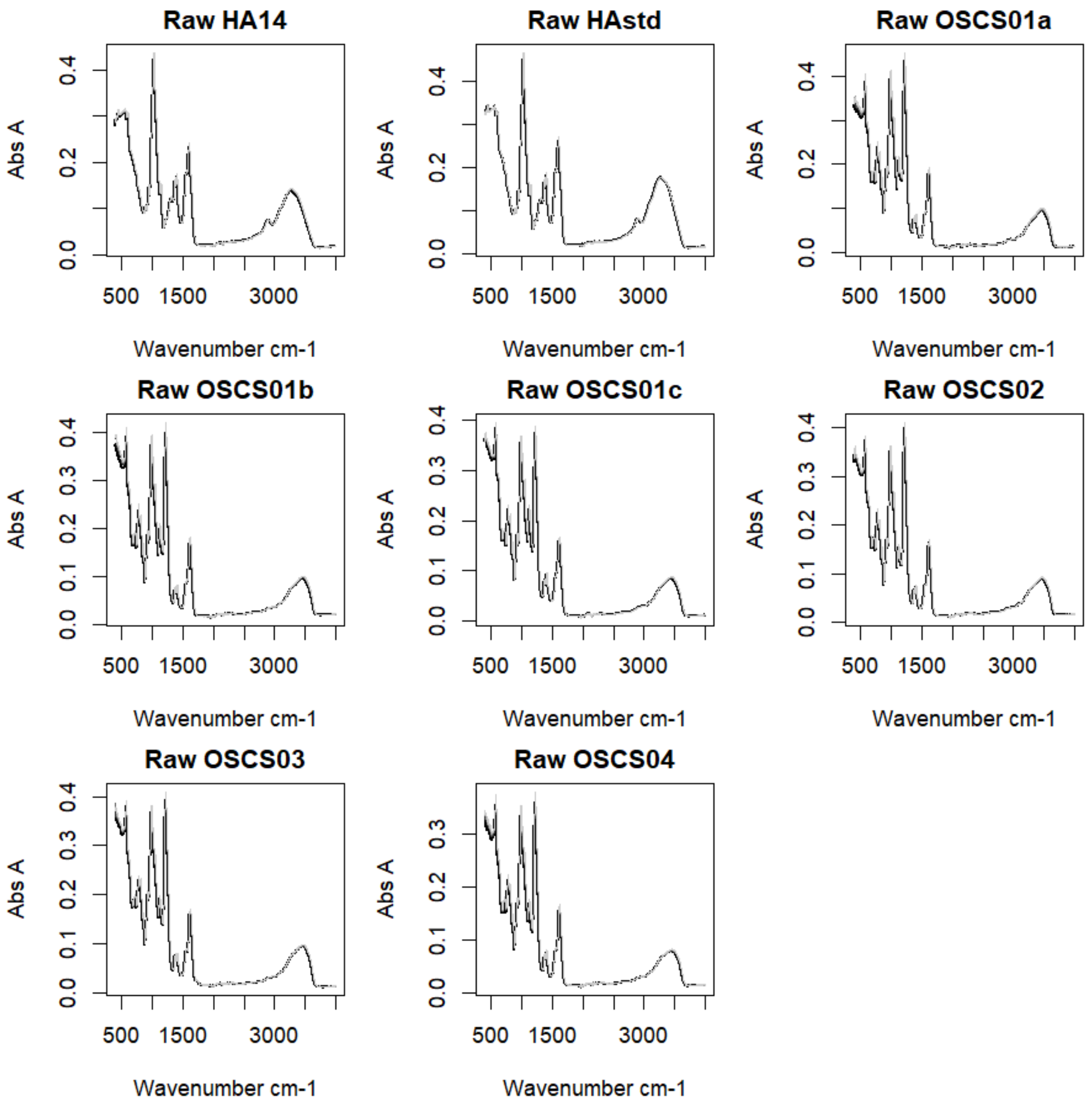
Raw HA14
Raw HAstd
Raw OSCS01a
Raw OSCS01b
Raw OSCS01c
Raw OSCS02
Raw OSCS03
Raw OSCS04
Abs A
0.0
0.1
0.2
0.3
0.4
500
1500
3000
Wavenumber cm-1

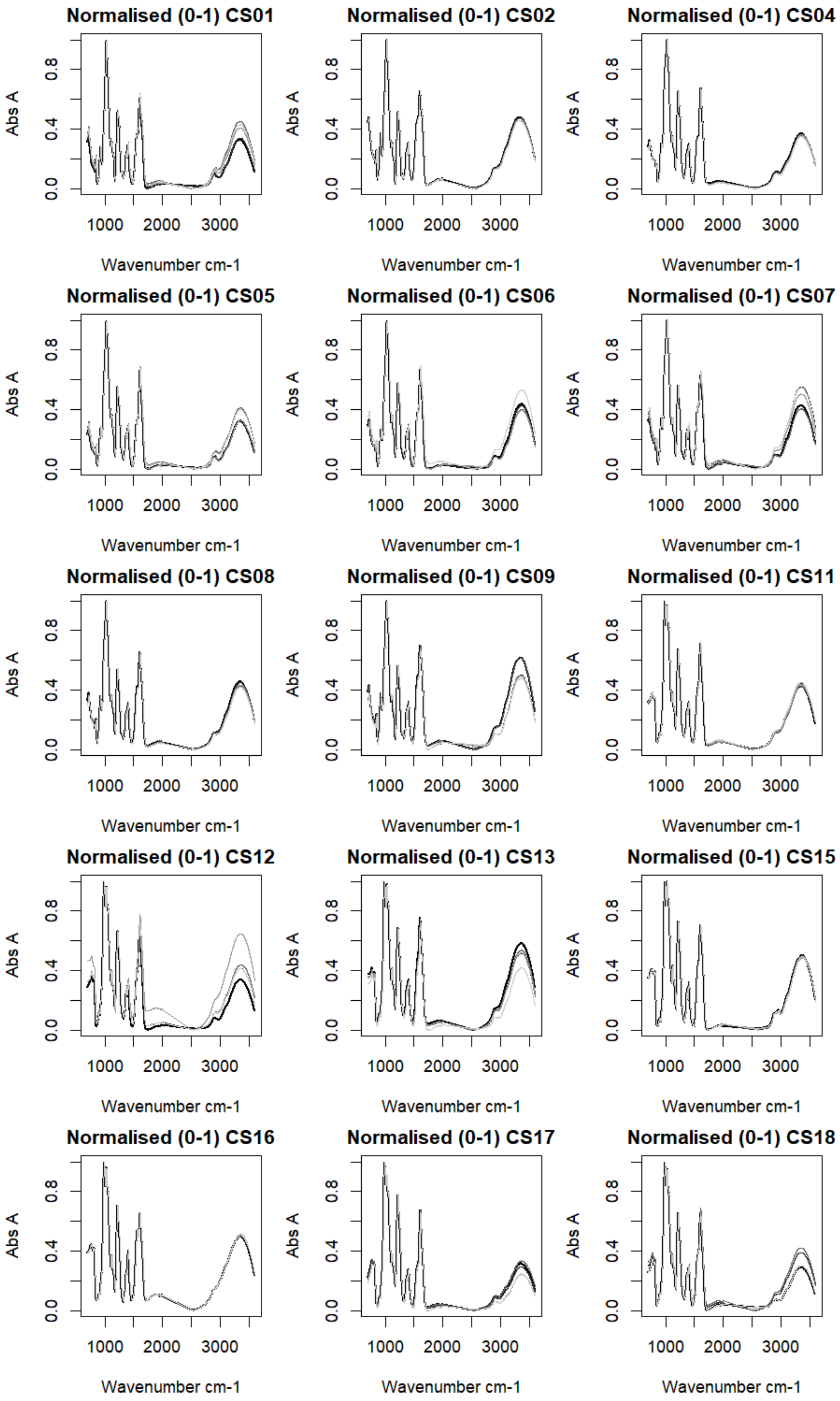

Normalised (0-1) CS01
Normalised (0-1) CS02
Normalised (0-1) CS04
Normalised (0-1) CS05
Normalised (0-1) CS06
Normalised (0-1) CS07
Normalised (0-1) CS08
Normalised (0-1) CS09
Normalised (0-1) CS11
Normalised (0-1) CS12
Normalised (0-1) CS13
Normalised (0-1) CS15
Normalised (0-1) CS16
Normalised (0-1) CS17
Normalised (0-1) CS18
Abs A
0.0
0.4
0.8
1000
2000
3000
Wavenumber cm-1

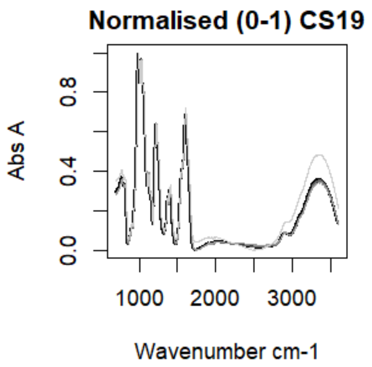
Normalised (0-1) CS19
Abs A
0.0 0.4 0.8
1000 2000 3000
Wavenumber cm-1

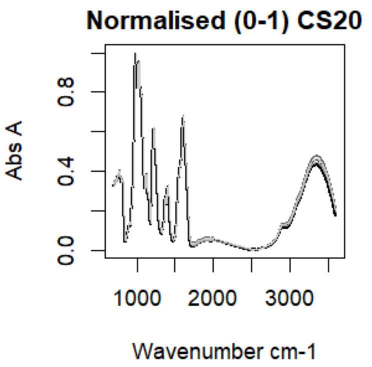
Normalised (0-1) CS20
Abs A
0.0 0.4 0.8
1000 2000 3000
Wavenumber cm-1

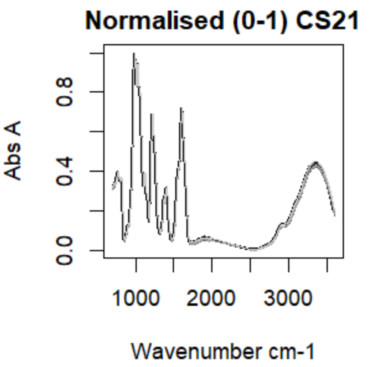
Normalised (0-1) CS21
Abs A
0.0 0.4 0.8
1000 2000 3000
Wavenumber cm-1

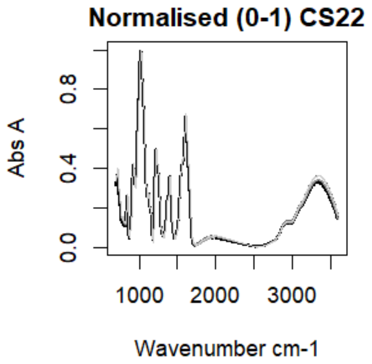
Normalised (0-1) CS22
Abs A
0.0 0.4 0.8
1000 2000 3000
Wavenumber cm-1

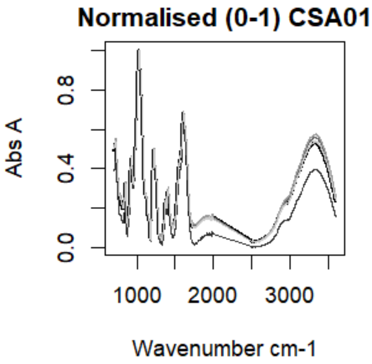
Normalised (0-1) CSA01
Abs A
0.0 0.4 0.8
1000 2000 3000
Wavenumber cm-1

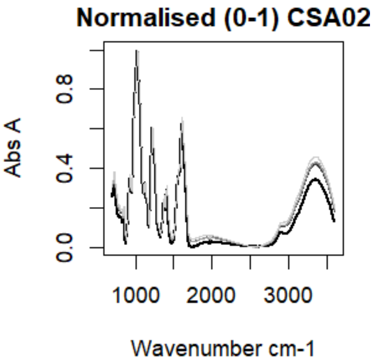
Normalised (0-1) CSA02
Abs A
0.0 0.4 0.8
1000 2000 3000
Wavenumber cm-1

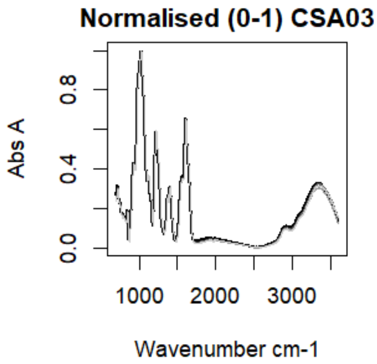
Normalised (0-1) CSA03
Abs A
0.0 0.4 0.8
1000 2000 3000
Wavenumber cm-1

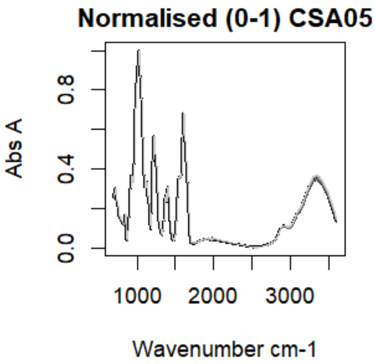
Normalised (0-1) CSA05
Abs A
0.0 0.4 0.8
1000 2000 3000
Wavenumber cm-1

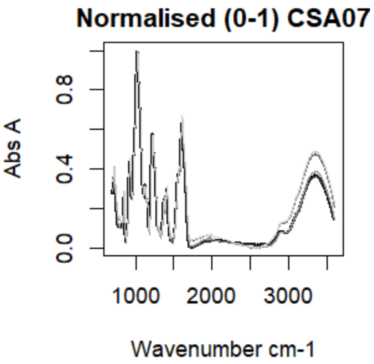
Normalised (0-1) CSA07
Abs A
0.0 0.4 0.8
1000 2000 3000
Wavenumber cm-1

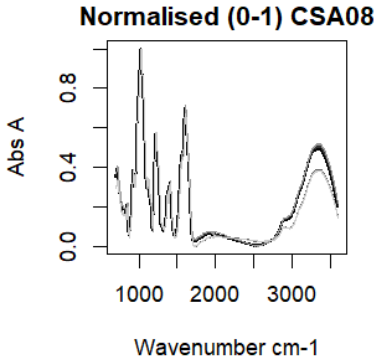
Normalised (0-1) CSA08
Abs A
0.0 0.4 0.8
1000 2000 3000
Wavenumber cm-1

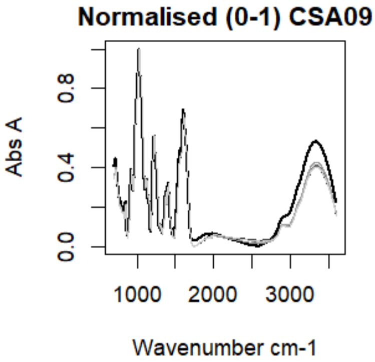
Normalised (0-1) CSA09
Abs A
0.0 0.4 0.8
1000 2000 3000
Wavenumber cm-1

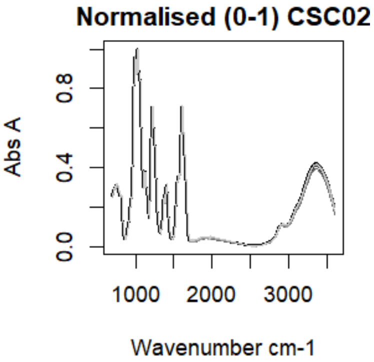
Normalised (0-1) CSC02
Abs A
0.0 0.4 0.8
1000 2000 3000
Wavenumber cm-1

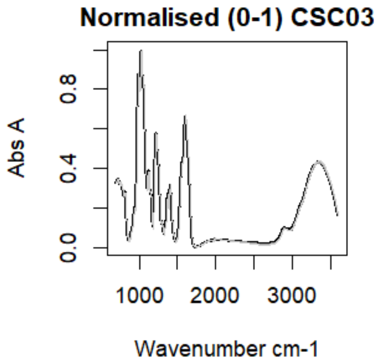
Normalised (0-1) CSC03
Abs A
0.0 0.4 0.8
1000 2000 3000
Wavenumber cm-1

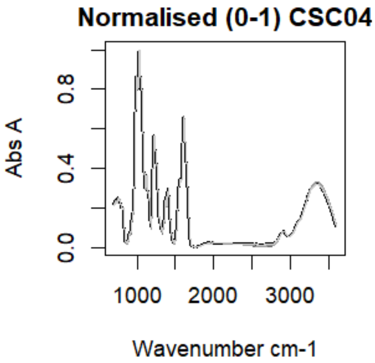
Normalised (0-1) CSC04
Abs A
0.0 0.4 0.8
1000 2000 3000
Wavenumber cm-1

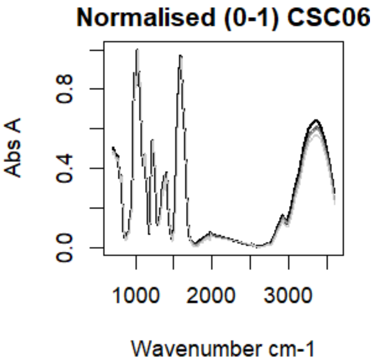
Normalised (0-1) CSC06
Abs A
0.0 0.4 0.8
1000 2000 3000
Wavenumber cm-1

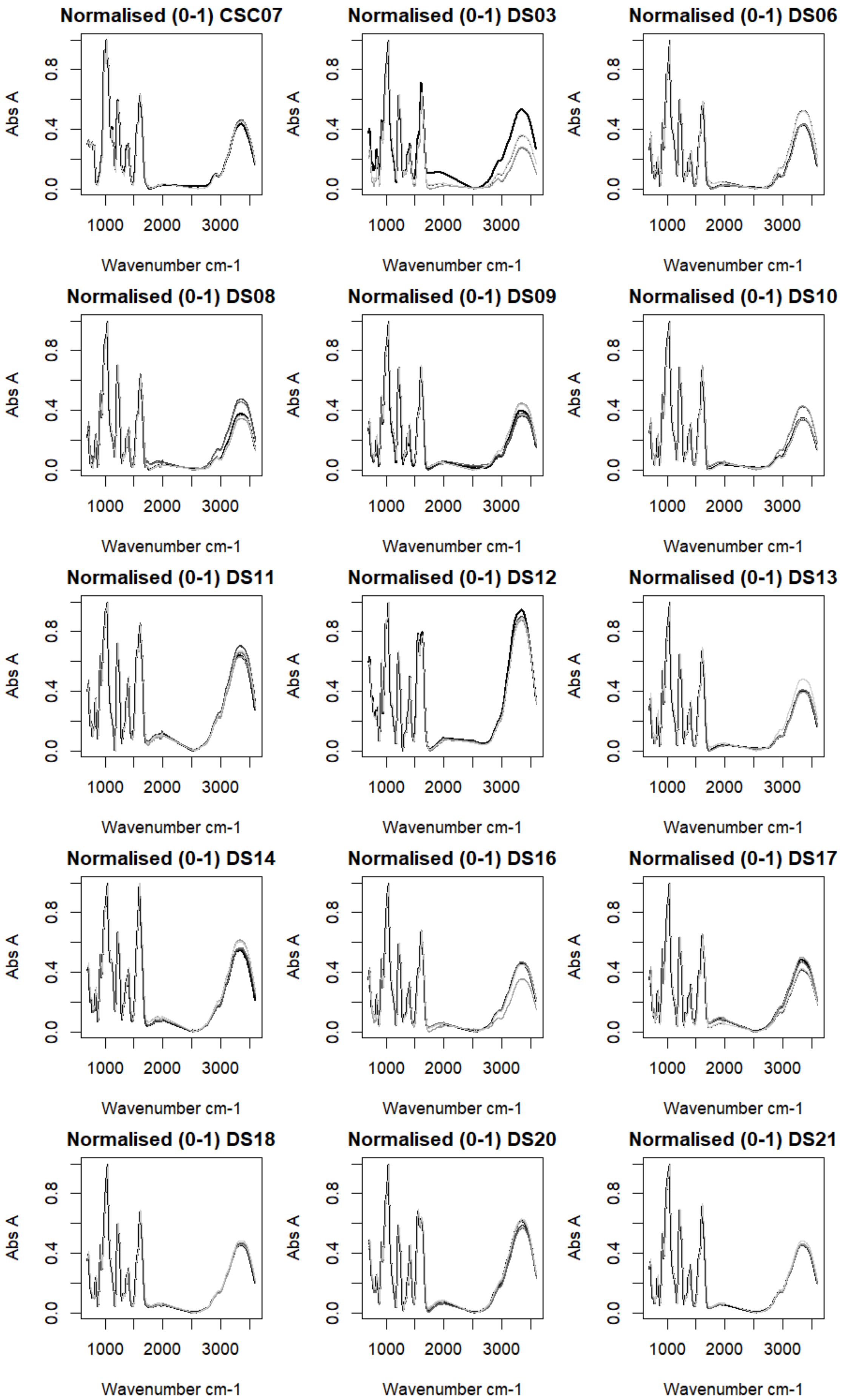
Normalised (0-1) CSC07
Normalised (0-1) DS03
Normalised (0-1) DS06
Normalised (0-1) DS08
Normalised (0-1) DS09
Normalised (0-1) DS10
Normalised (0-1) DS11
Normalised (0-1) DS12
Normalised (0-1) DS13
Normalised (0-1) DS14
Normalised (0-1) DS16
Normalised (0-1) DS17
Normalised (0-1) DS18
Normalised (0-1) DS20
Normalised (0-1) DS21
Abs A
0.0
0.4
0.8
1000
2000
3000
Wavenumber cm-1

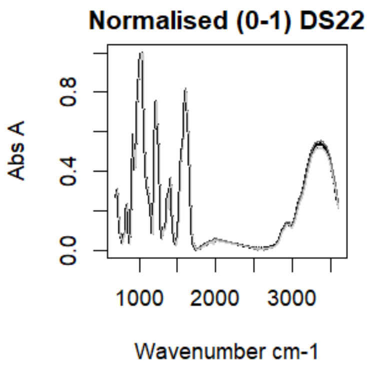
Normalised (0-1) DS22
Abs A
0.0
0.4
0.8
1000
2000
3000
Wavenumber cm-1

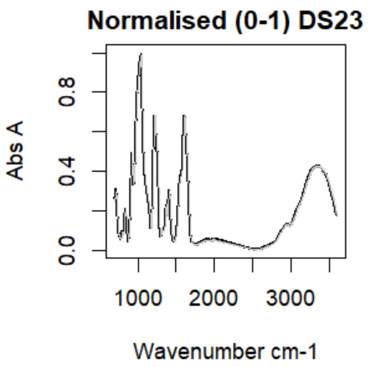
Normalised (0-1) DS23
Abs A
0.0
0.4
0.8
1000
2000
3000
Wavenumber cm-1

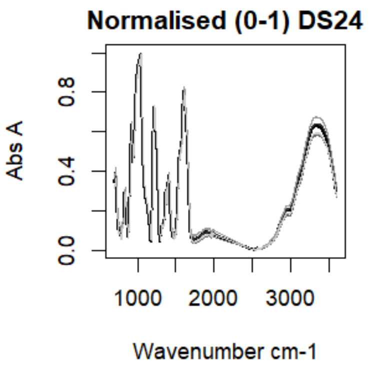
Normalised (0-1) DS24
Abs A
0.0
0.4
0.8
1000
2000
3000
Wavenumber cm-1

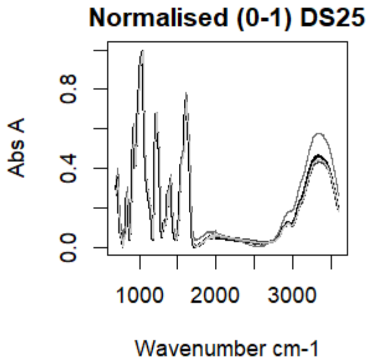
Normalised (0-1) DS25
Abs A
0.0
0.4
0.8
1000
2000
3000
Wavenumber cm-1

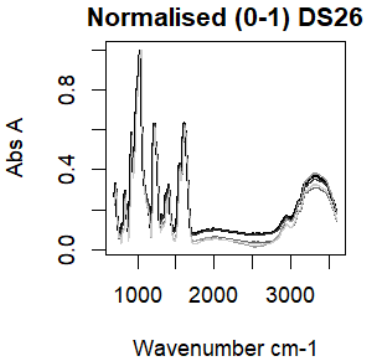
Normalised (0-1) DS26
Abs A
0.0
0.4
0.8
1000
2000
3000
Wavenumber cm-1

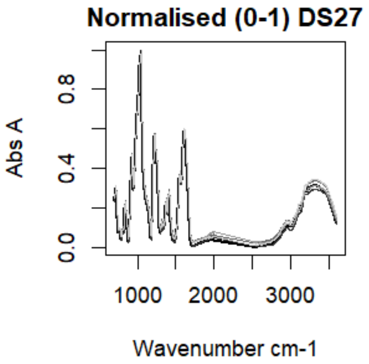
Normalised (0-1) DS27
Abs A
0.0
0.4
0.8
1000
2000
3000
Wavenumber cm-1

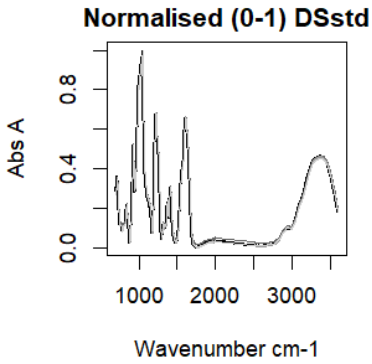
Normalised (0-1) DSstd
Abs A
0.0
0.4
0.8
1000
2000
3000
Wavenumber cm-1

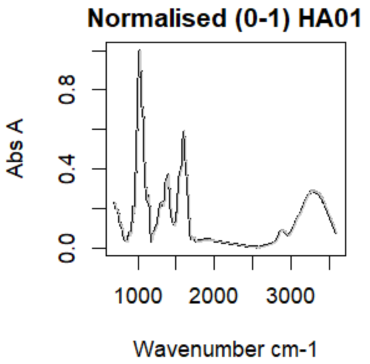
Normalised (0-1) HA01
Abs A
0.0
0.4
0.8
1000
2000
3000
Wavenumber cm-1

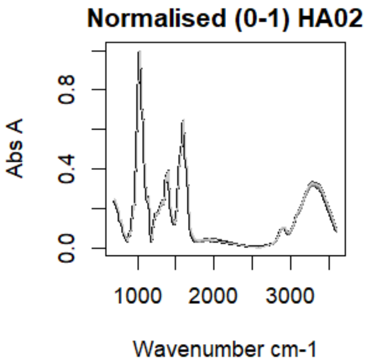
Normalised (0-1) HA02
Abs A
0.0
0.4
0.8
1000
2000
3000
Wavenumber cm-1

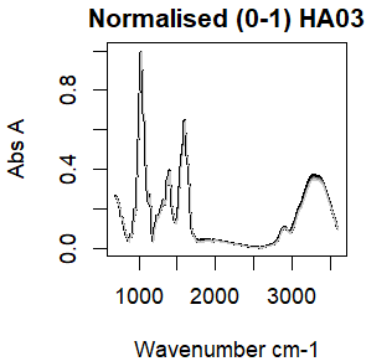
Normalised (0-1) HA03
Abs A
0.0
0.4
0.8
1000
2000
3000
Wavenumber cm-1

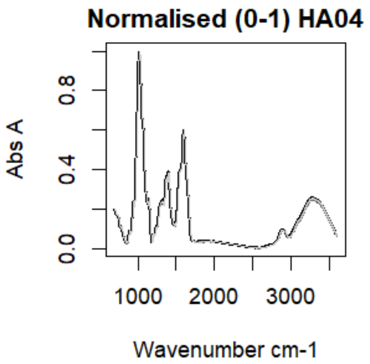
Normalised (0-1) HA04
Abs A
0.0
0.4
0.8
1000
2000
3000
Wavenumber cm-1

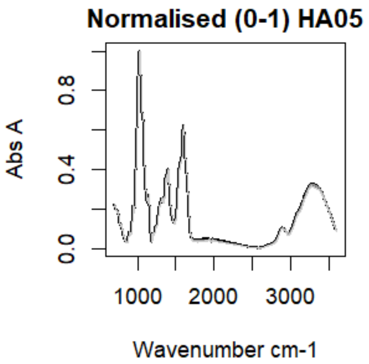
Normalised (0-1) HA05
Abs A
0.0
0.4
0.8
1000
2000
3000
Wavenumber cm-1

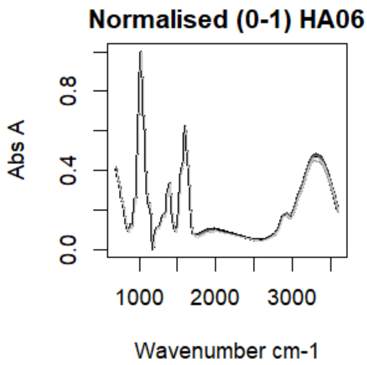
Normalised (0-1) HA06
Abs A
0.0
0.4
0.8
1000
2000
3000
Wavenumber cm-1

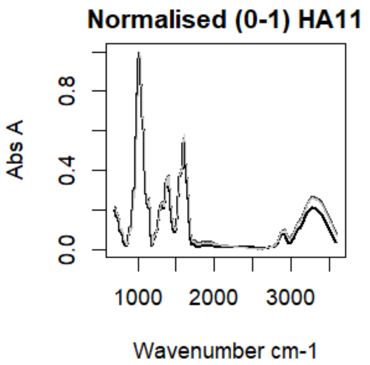
Normalised (0-1) HA11
Abs A
0.0
0.4
0.8
1000
2000
3000
Wavenumber cm-1

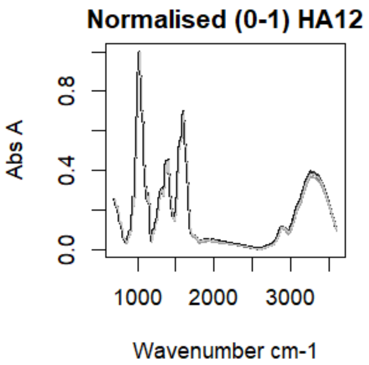
Normalised (0-1) HA12
Abs A
0.0
0.4
0.8
1000
2000
3000
Wavenumber cm-1

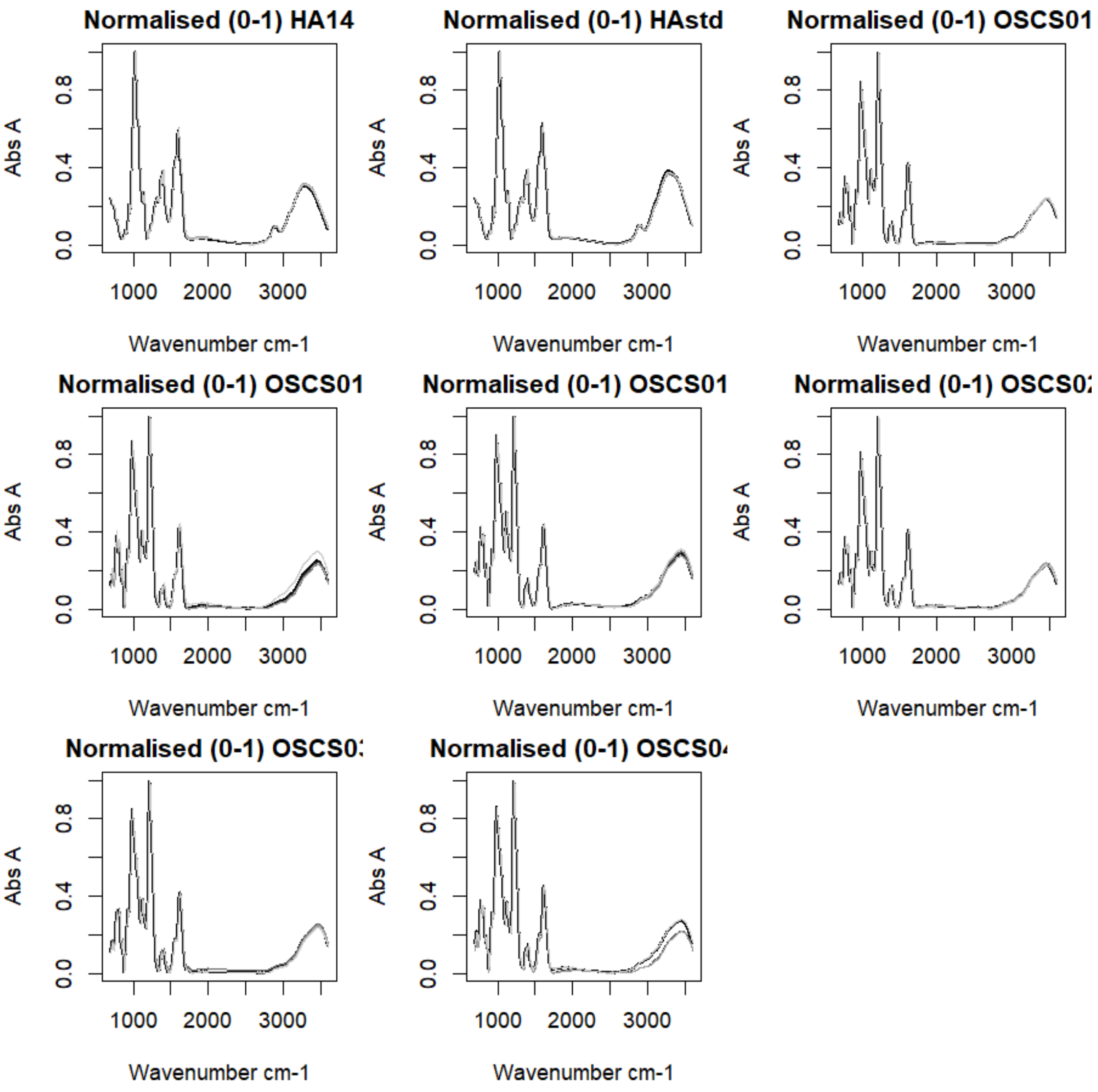
Normalised (0-1) HA14
Normalised (0-1) HAstd
Normalised (0-1) OSCS01
Normalised (0-1) OSCS01
Normalised (0-1) OSCS01
Normalised (0-1) OSCS0
Normalised (0-1) OSCS0
Normalised (0-1) OSCS0
Abs A
Wavenumber cm-1
0.0
0.4
0.8
1000
2000
3000

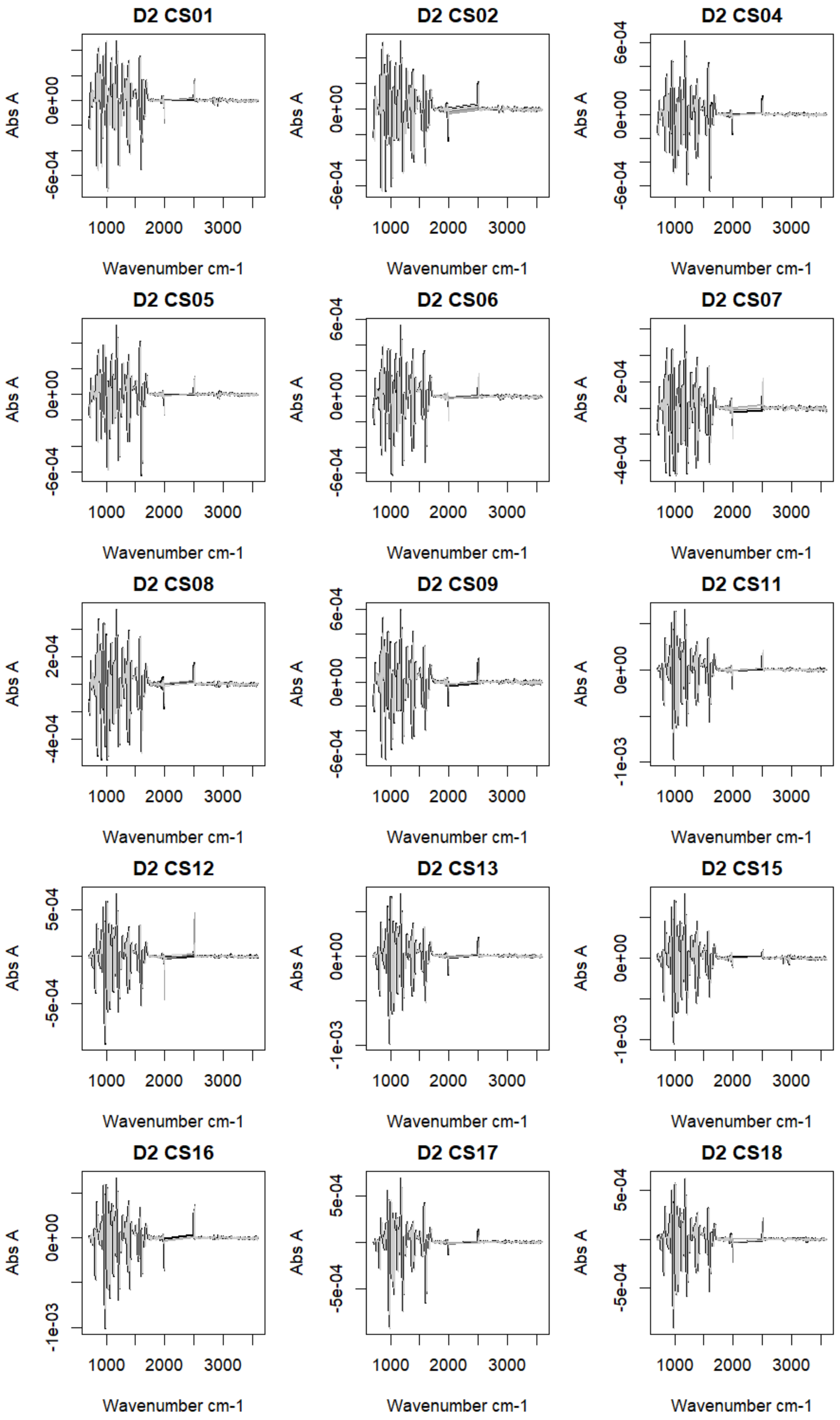
D2 CS01
D2 CS02
D2 CS04
D2 CS05
D2 CS06
D2 CS07
D2 CS08
D2 CS09
D2 CS11
D2 CS12
D2 CS13
D2 CS15
D2 CS16
D2 CS17
D2 CS18
Abs A
Wavenumber cm-1
1000
2000
3000
-6e-04
0e+00
6e-04
2e-04
-4e-04
-1e-03
5e-04
-5e-04

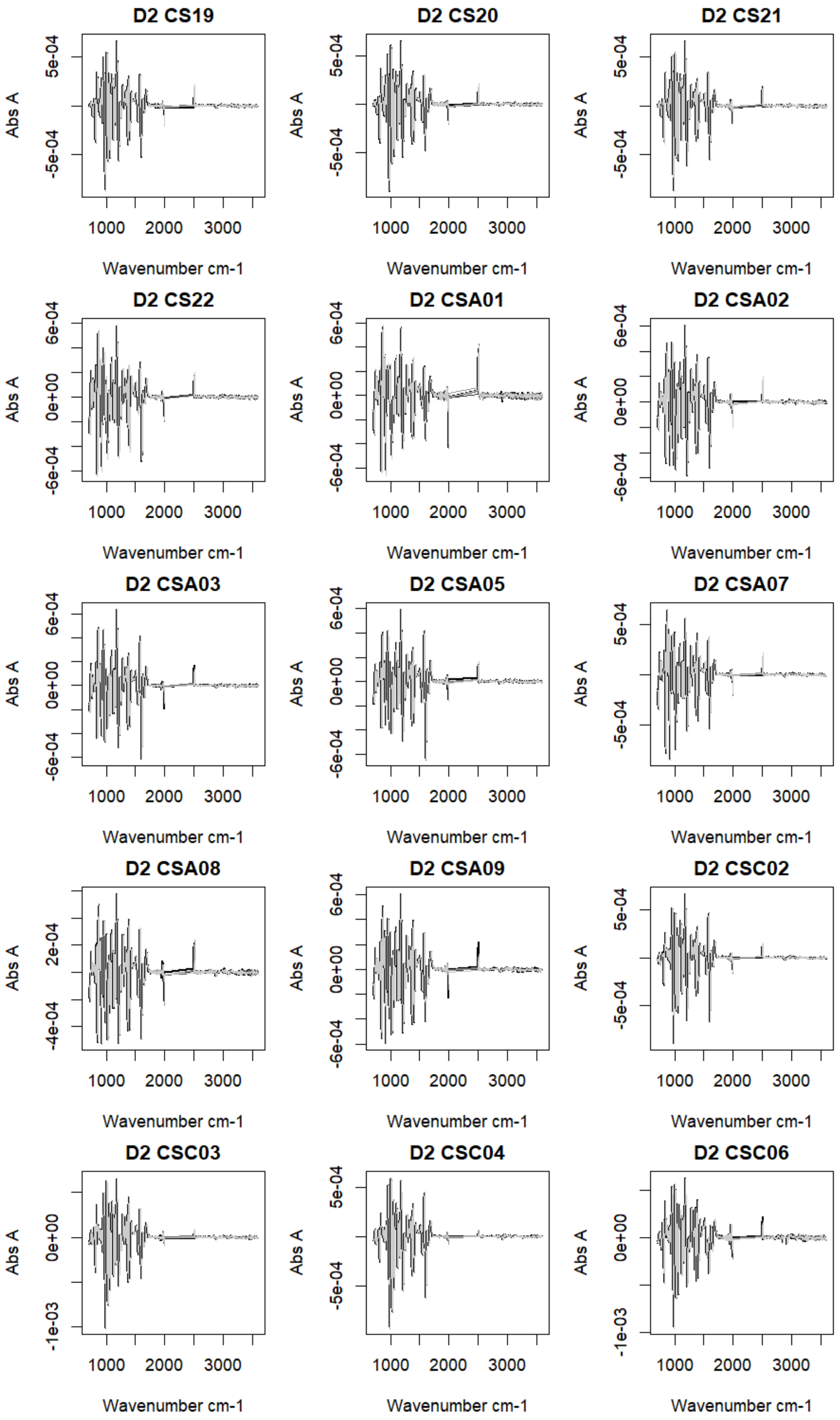
D2 CS19
D2 CS20
D2 CS21
D2 CS22
D2 CSA01
D2 CSA02
D2 CSA03
D2 CSA05
D2 CSA07
D2 CSA08
D2 CSA09
D2 CSC02
D2 CSC03
D2 CSC04
D2 CSC06
Abs A
Wavenumber cm-1
1000
2000
3000
5e-04
-5e-04
6e-04
0e+00
-6e-04
2e-04
-4e-04
-1e-03

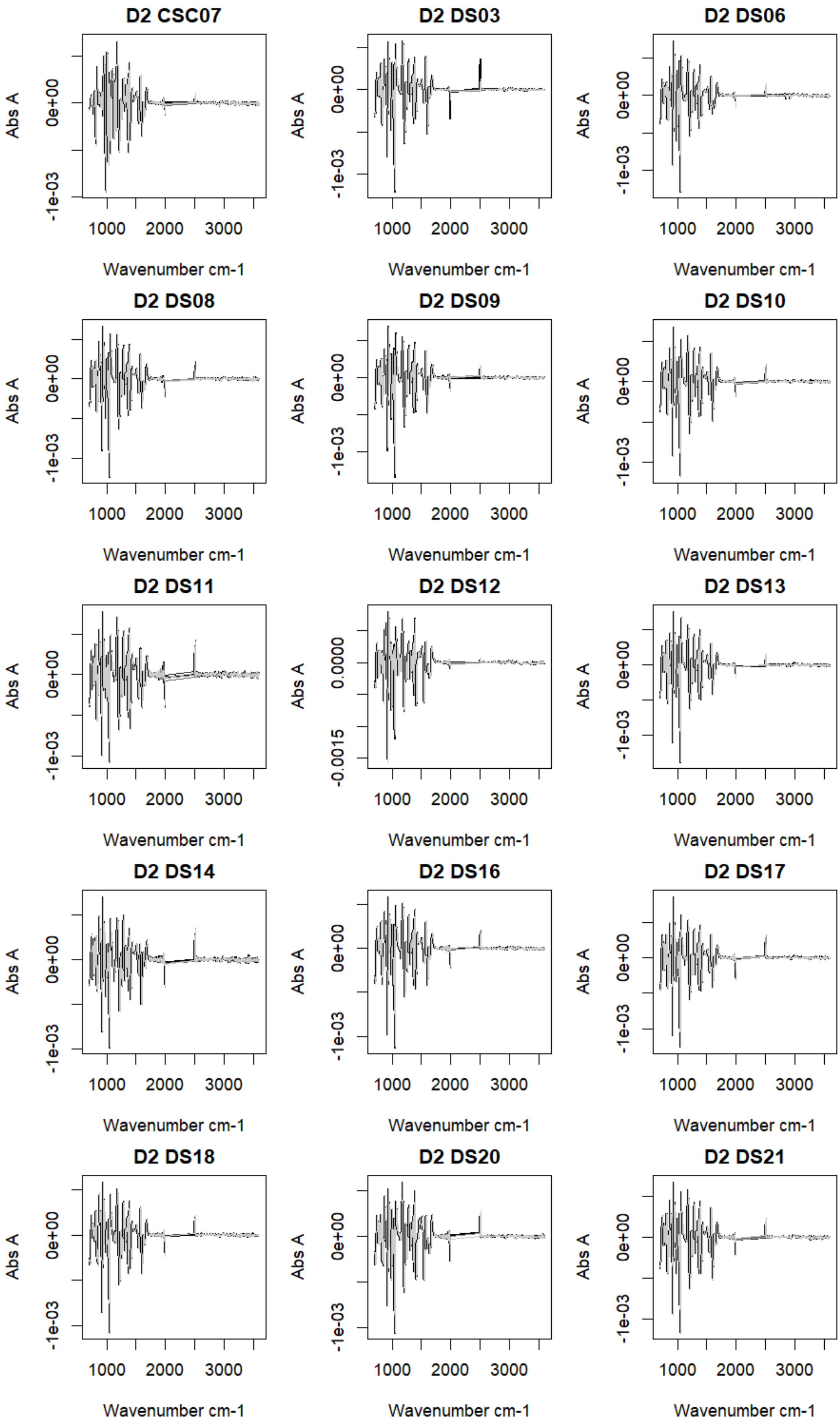
D2 CSC07
D2 DS03
D2 DS06
D2 DS08
D2 DS09
D2 DS10
D2 DS11
D2 DS12
D2 DS13
D2 DS14
D2 DS16
D2 DS17
D2 DS18
D2 DS20
D2 DS21
Abs A
0e+00
-1e-03
0.0000
-0.0015
1000
2000
3000
Wavenumber cm-1

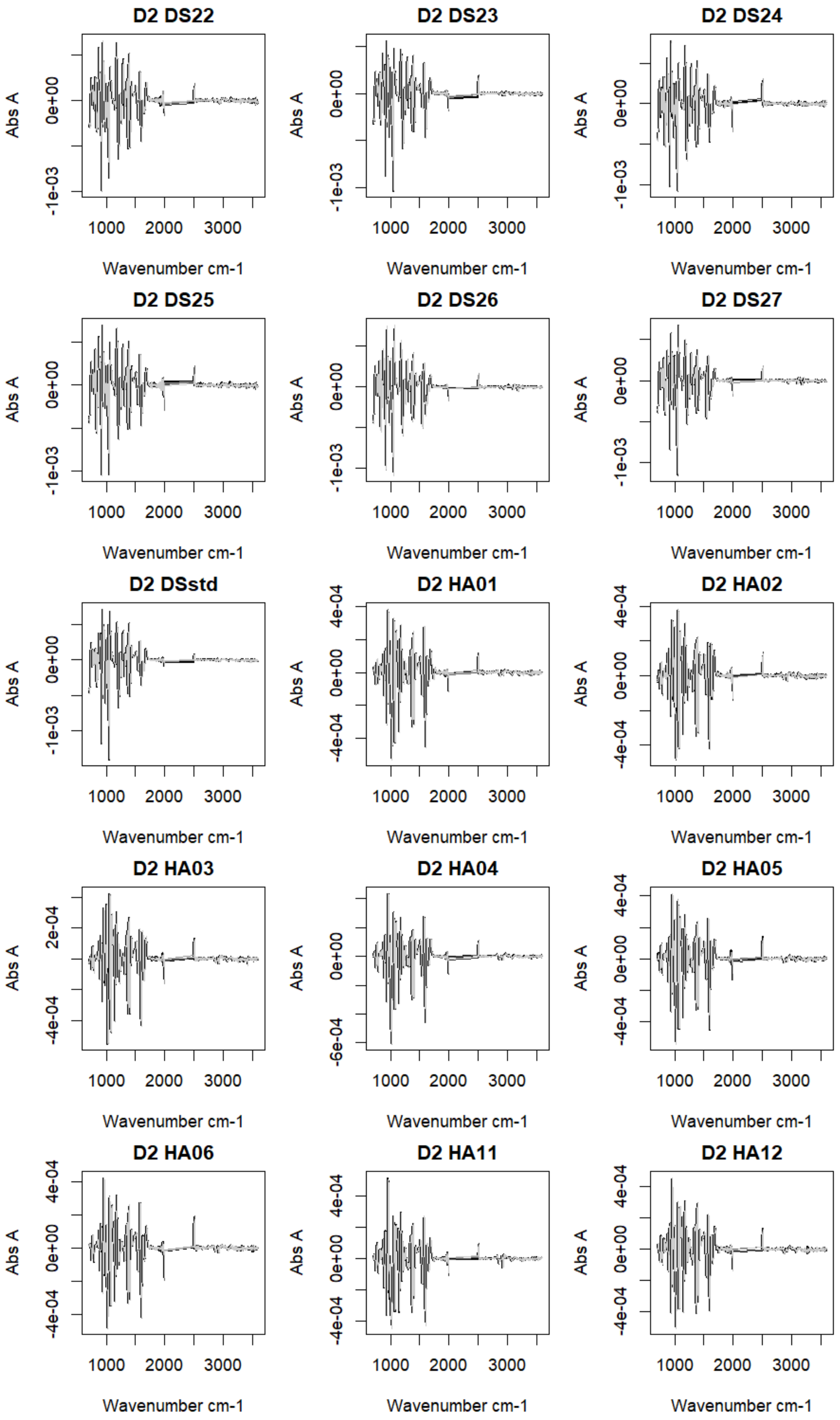
D2 DS22
D2 DS23
D2 DS24
D2 DS25
D2 DS26
D2 DS27
D2 DSstd
D2 HA01
D2 HA02
D2 HA03
D2 HA04
D2 HA05
D2 HA06
D2 HA11
D2 HA12
Abs A
Wavenumber cm-1

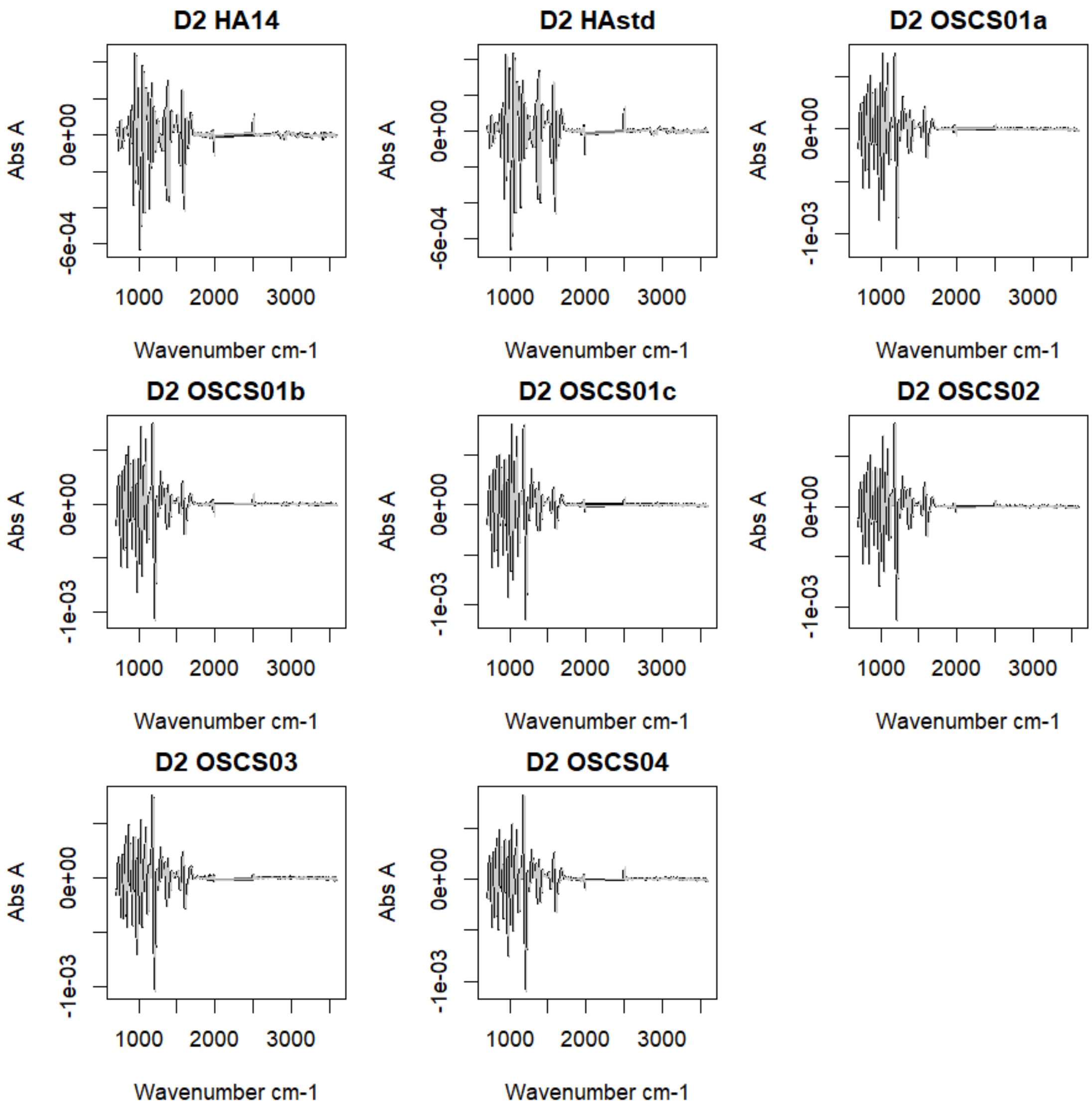
D2 HA14
D2 HAstd
D2 OSCS01a
D2 OSCS01b
D2 OSCS01c
D2 OSCS02
D2 OSCS03
D2 OSCS04
Abs A
0e+00
-6e-04
-1e-03
1000
2000
3000
Wavenumber cm-1

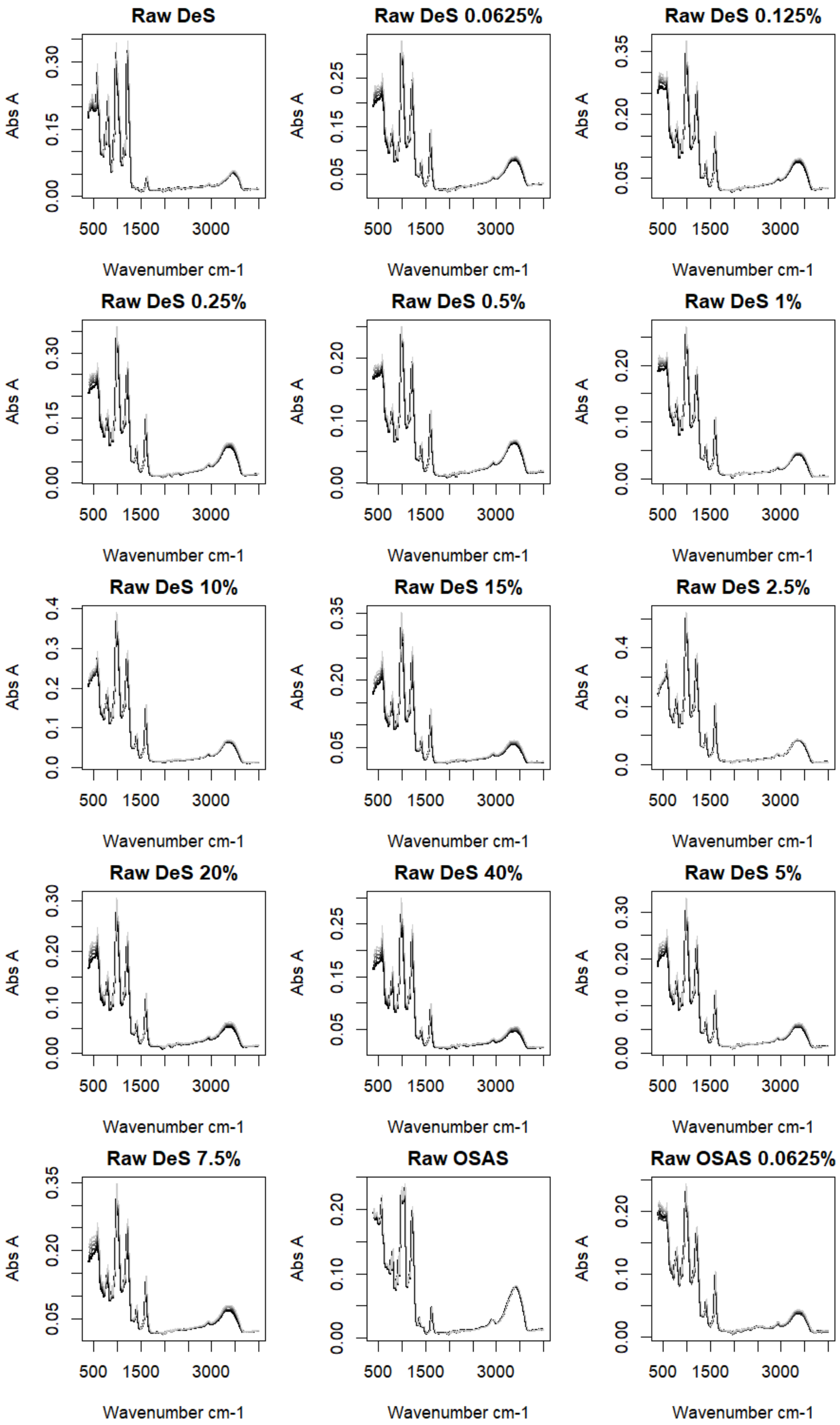
Raw DeS
Raw DeS 0.0625%
Raw DeS 0.125%
Raw DeS 0.25%
Raw DeS 0.5%
Raw DeS 1%
Raw DeS 10%
Raw DeS 15%
Raw DeS 2.5%
Raw DeS 20%
Raw DeS 40%
Raw DeS 5%
Raw DeS 7.5%
Raw OSAS
Raw OSAS 0.0625%
Abs A
Wavenumber cm-1
500
1500
3000

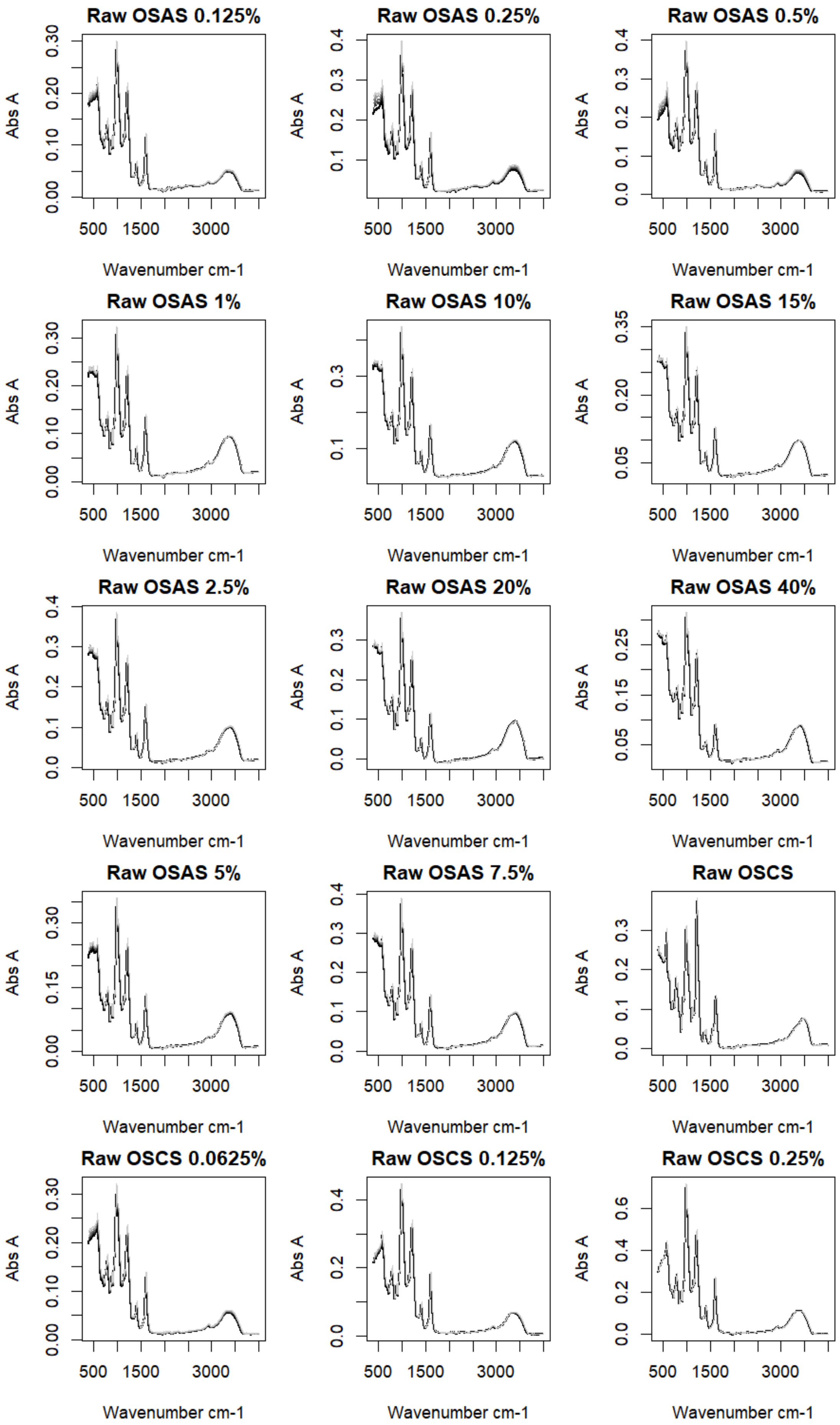

Raw OSAS 0.125%
Abs A
0.00 0.10 0.20 0.30
500 1500 3000
Wavenumber cm-1
Raw OSAS 0.25%
Abs A
0.1 0.2 0.3 0.4
500 1500 3000
Wavenumber cm-1
Raw OSAS 0.5%
Abs A
0.0 0.1 0.2 0.3 0.4
500 1500 3000
Wavenumber cm-1
Raw OSAS 1%
Abs A
0.00 0.10 0.20 0.30
500 1500 3000
Wavenumber cm-1
Raw OSAS 10%
Abs A
0.1 0.3
500 1500 3000
Wavenumber cm-1
Raw OSAS 15%
Abs A
0.05 0.20 0.35
500 1500 3000
Wavenumber cm-1
Raw OSAS 2.5%
Abs A
0.0 0.1 0.2 0.3 0.4
500 1500 3000
Wavenumber cm-1
Raw OSAS 20%
Abs A
0.0 0.1 0.2 0.3
500 1500 3000
Wavenumber cm-1
Raw OSAS 40%
Abs A
0.05 0.15 0.25
500 1500 3000
Wavenumber cm-1
Raw OSAS 5%
Abs A
0.00 0.15 0.30
500 1500 3000
Wavenumber cm-1
Raw OSAS 7.5%
Abs A
0.0 0.1 0.2 0.3 0.4
500 1500 3000
Wavenumber cm-1
Raw OSCS
Abs A
0.0 0.1 0.2 0.3
500 1500 3000
Wavenumber cm-1
Raw OSCS 0.0625%
Abs A
0.00 0.10 0.20 0.30
500 1500 3000
Wavenumber cm-1
Raw OSCS 0.125%
Abs A
0.0 0.2 0.4
500 1500 3000
Wavenumber cm-1
Raw OSCS 0.25%
Abs A
0.0 0.2 0.4 0.6
500 1500 3000
Wavenumber cm-1

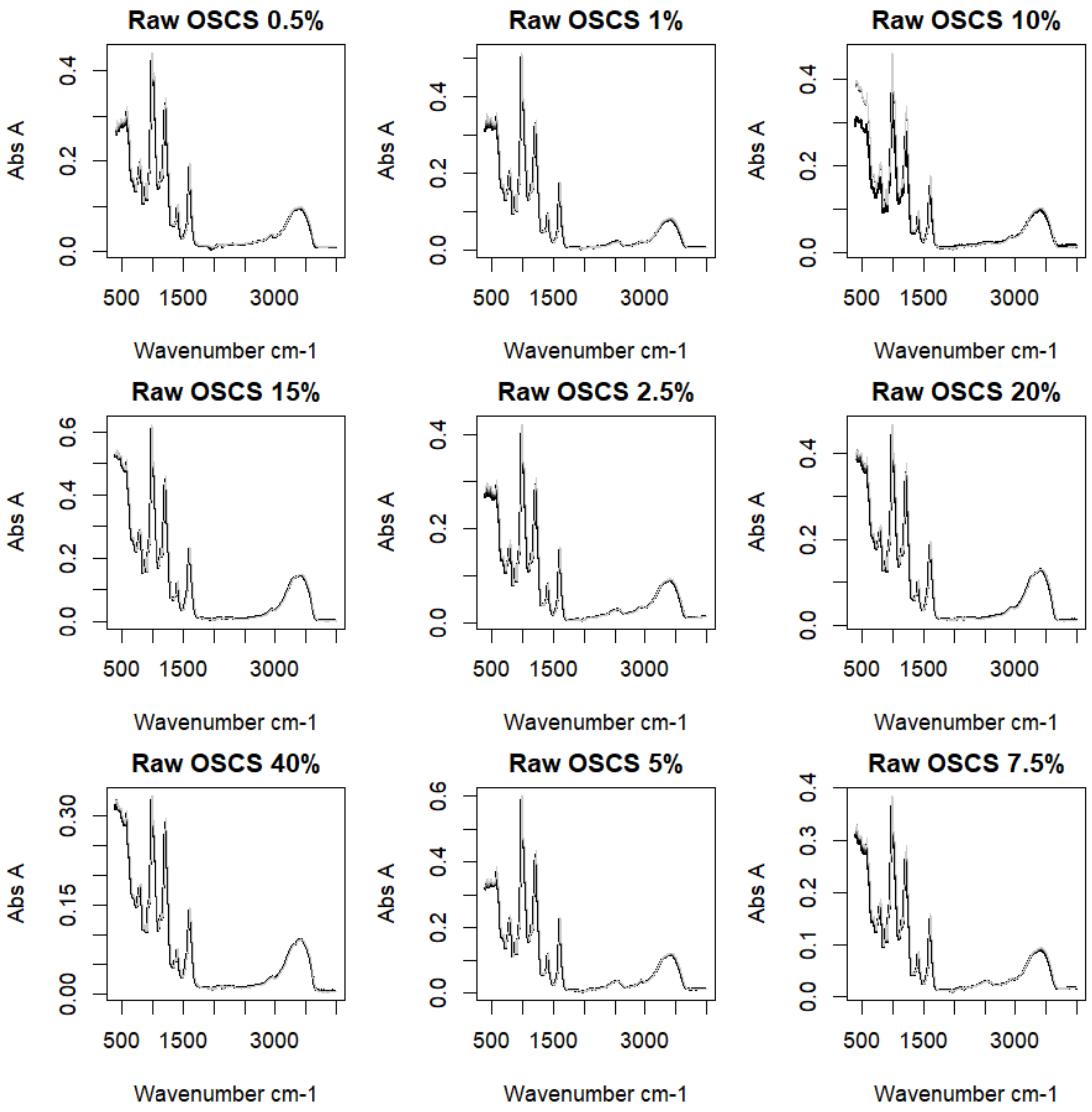
Raw OSCS 0.5%
Raw OSCS 1%
Raw OSCS 10%
Raw OSCS 15%
Raw OSCS 2.5%
Raw OSCS 20%
Raw OSCS 40%
Raw OSCS 5%
Raw OSCS 7.5%
Abs A
Wavenumber cm-1
500 1500 3000
0.0 0.2 0.4
0.0 0.2 0.4 0.6
0.00 0.15 0.30
0.0 0.1 0.2 0.3 0.4

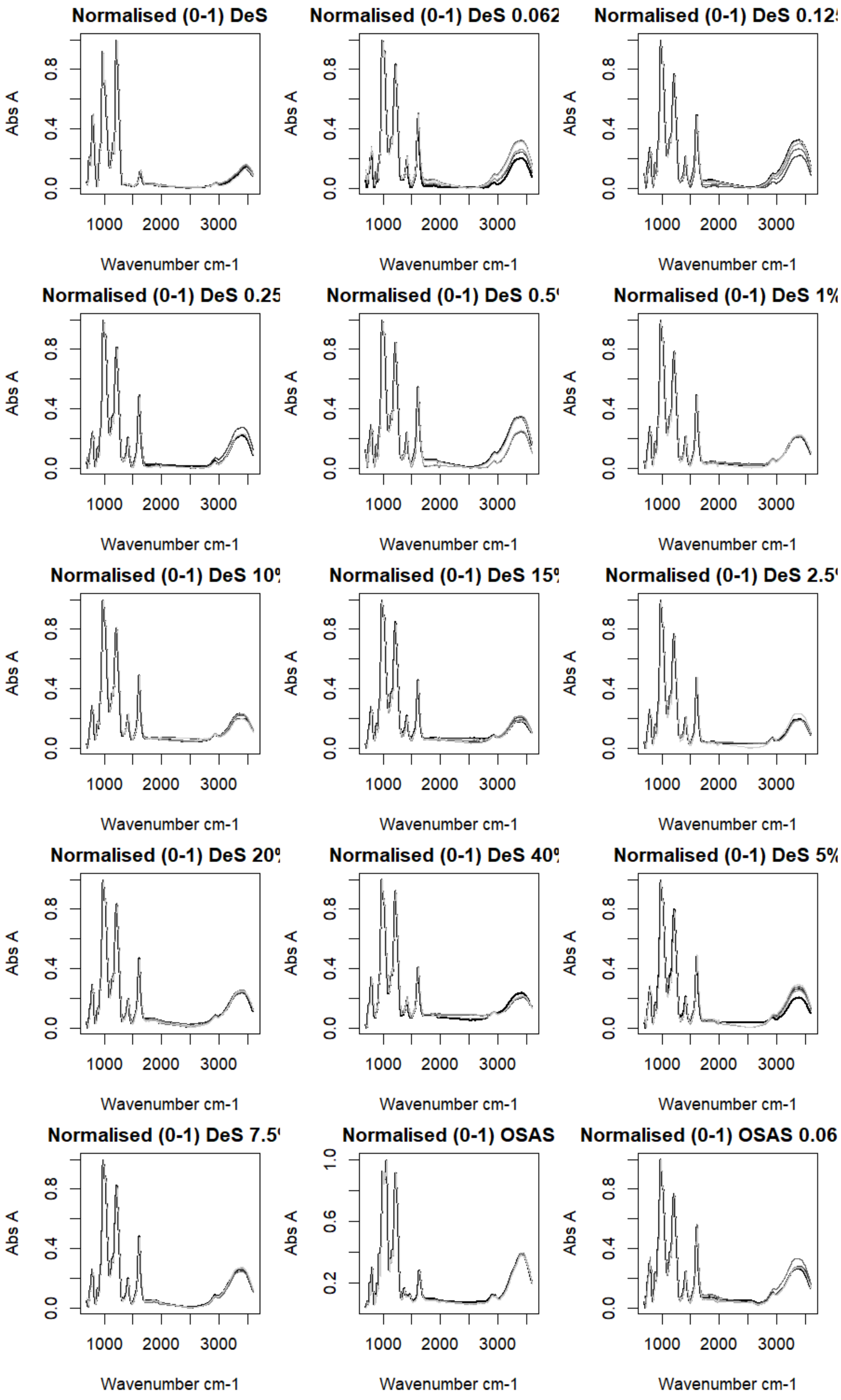
Normalised (0-1) DeS
Normalised (0-1) DeS 0.062
Normalised (0-1) DeS 0.12
Normalised (0-1) DeS 0.25
Normalised (0-1) DeS 0.5
Normalised (0-1) DeS 1%
Normalised (0-1) DeS 10
Normalised (0-1) DeS 15
Normalised (0-1) DeS 2.5
Normalised (0-1) DeS 20
Normalised (0-1) DeS 40
Normalised (0-1) DeS 5%
Normalised (0-1) DeS 7.5
Normalised (0-1) OSAS
Normalised (0-1) OSAS 0.06
Abs A
0.0
0.2
0.4
0.6
0.8
1.0
1000
2000
3000
Wavenumber cm-1

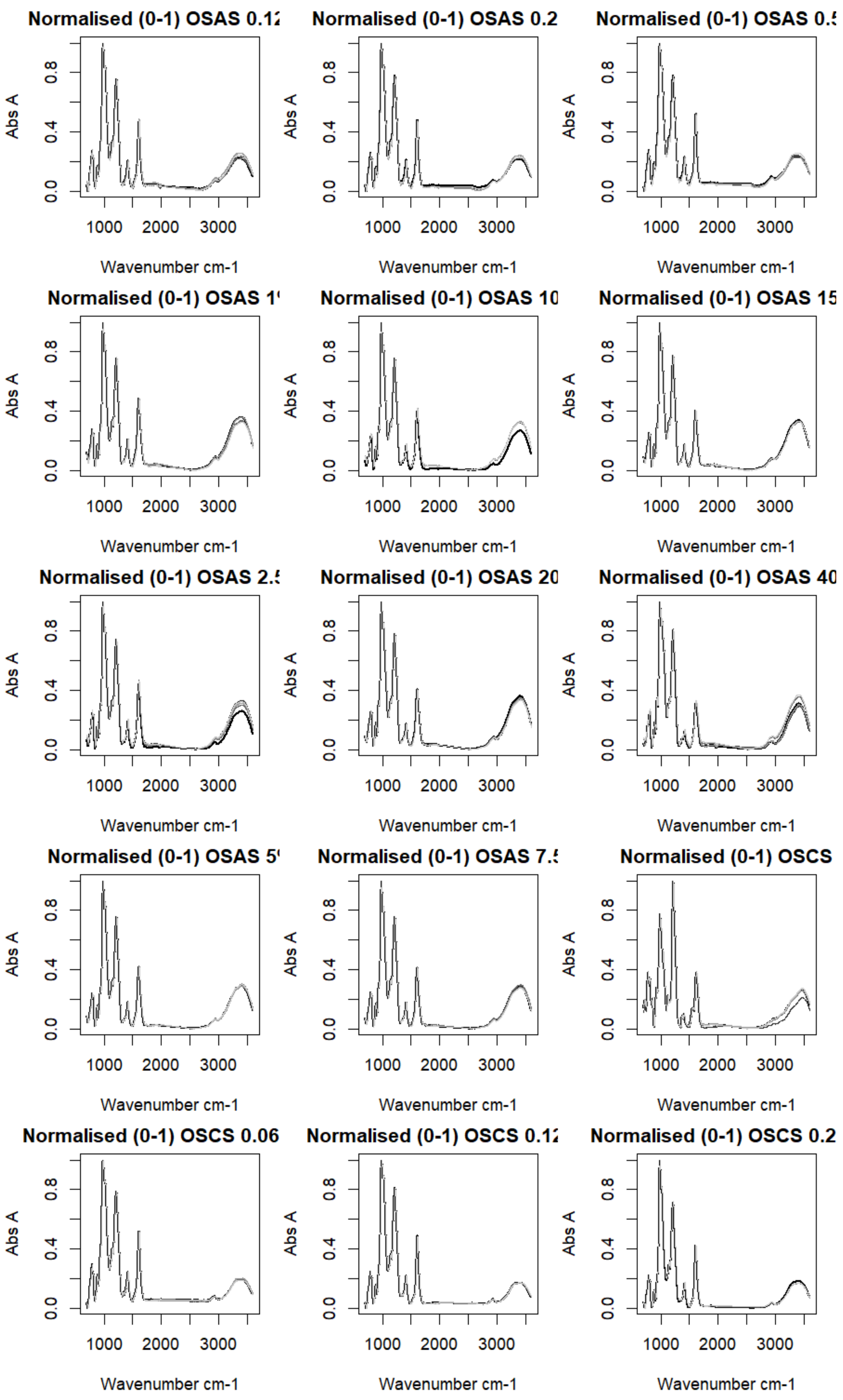
Normalised (0-1) OSAS 0.1
Normalised (0-1) OSAS 0.2
Normalised (0-1) OSAS 0.
Normalised (0-1) OSAS 1
Normalised (0-1) OSAS 10
Normalised (0-1) OSAS 15
Normalised (0-1) OSAS 2.
Normalised (0-1) OSAS 20
Normalised (0-1) OSAS 40
Normalised (0-1) OSAS 5
Normalised (0-1) OSAS 7.
Normalised (0-1) OSCS
Normalised (0-1) OSCS 0.06
Normalised (0-1) OSCS 0.1
Normalised (0-1) OSCS 0.2
Abs A
0.0
0.4
0.8
1000
2000
3000
Wavenumber cm-1

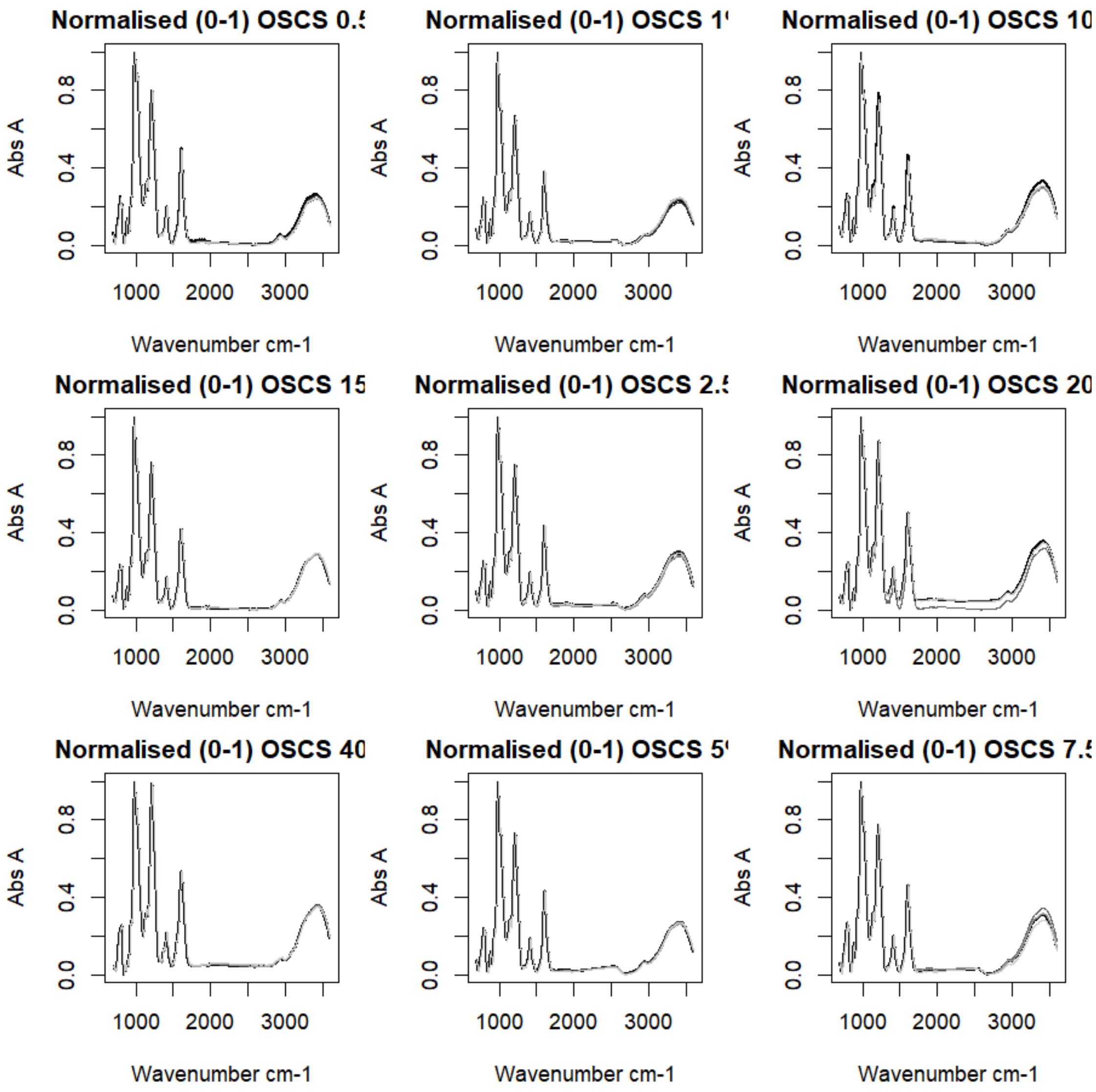
Normalised (0-1) OSCS 0.
Normalised (0-1) OSCS 1
Normalised (0-1) OSCS 10
Normalised (0-1) OSCS 15
Normalised (0-1) OSCS 2.
Normalised (0-1) OSCS 20
Normalised (0-1) OSCS 40
Normalised (0-1) OSCS 5
Normalised (0-1) OSCS 7.
Abs A
0.0
0.4
0.8
1000
2000
3000
Wavenumber cm-1

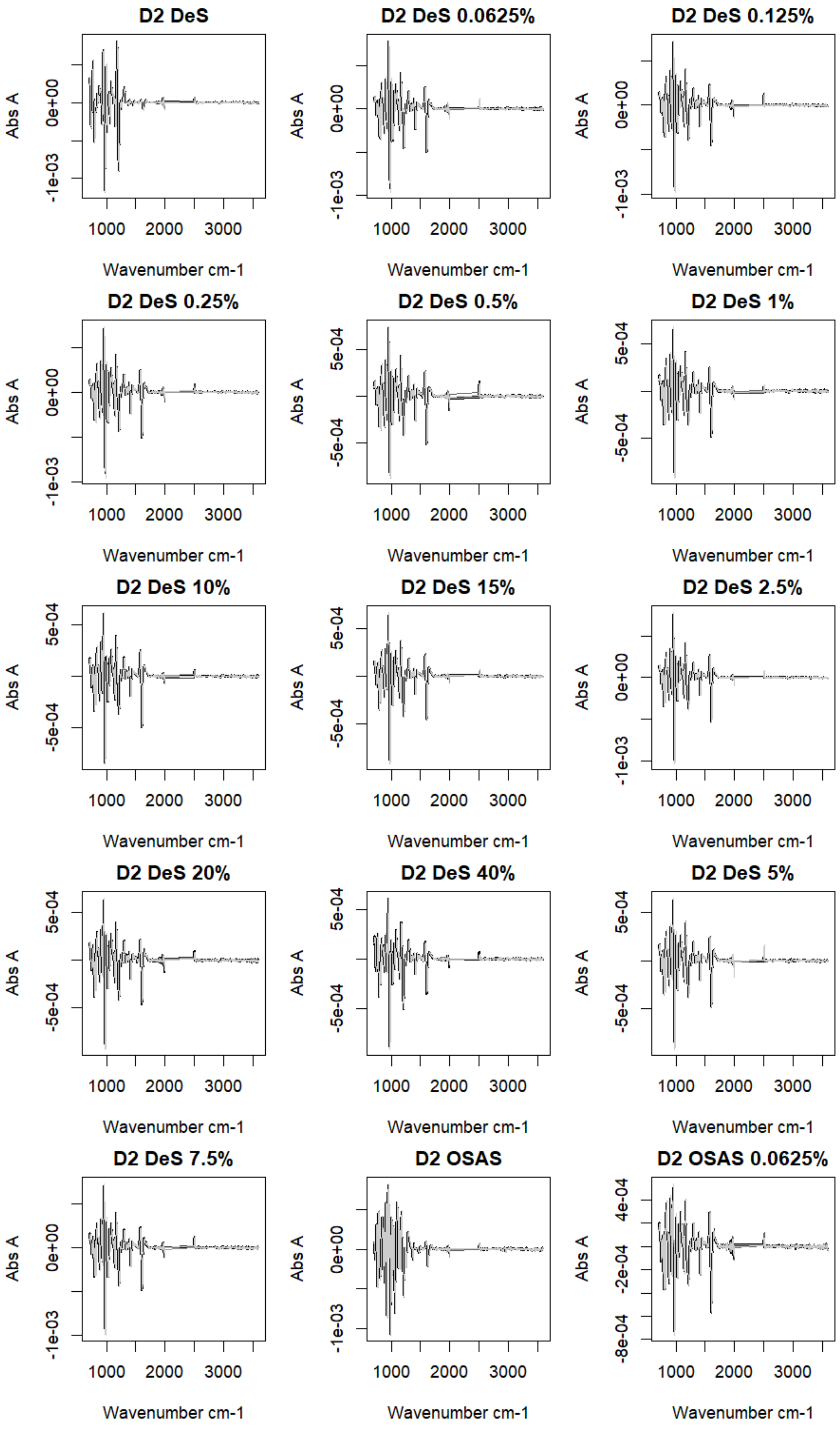
D2 DeS
D2 DeS 0.0625%
D2 DeS 0.125%
D2 DeS 0.25%
D2 DeS 0.5%
D2 DeS 1%
D2 DeS 10%
D2 DeS 15%
D2 DeS 2.5%
D2 DeS 20%
D2 DeS 40%
D2 DeS 5%
D2 DeS 7.5%
D2 OSAS
D2 OSAS 0.0625%
Abs A
Wavenumber cm-1
1000 2000 3000

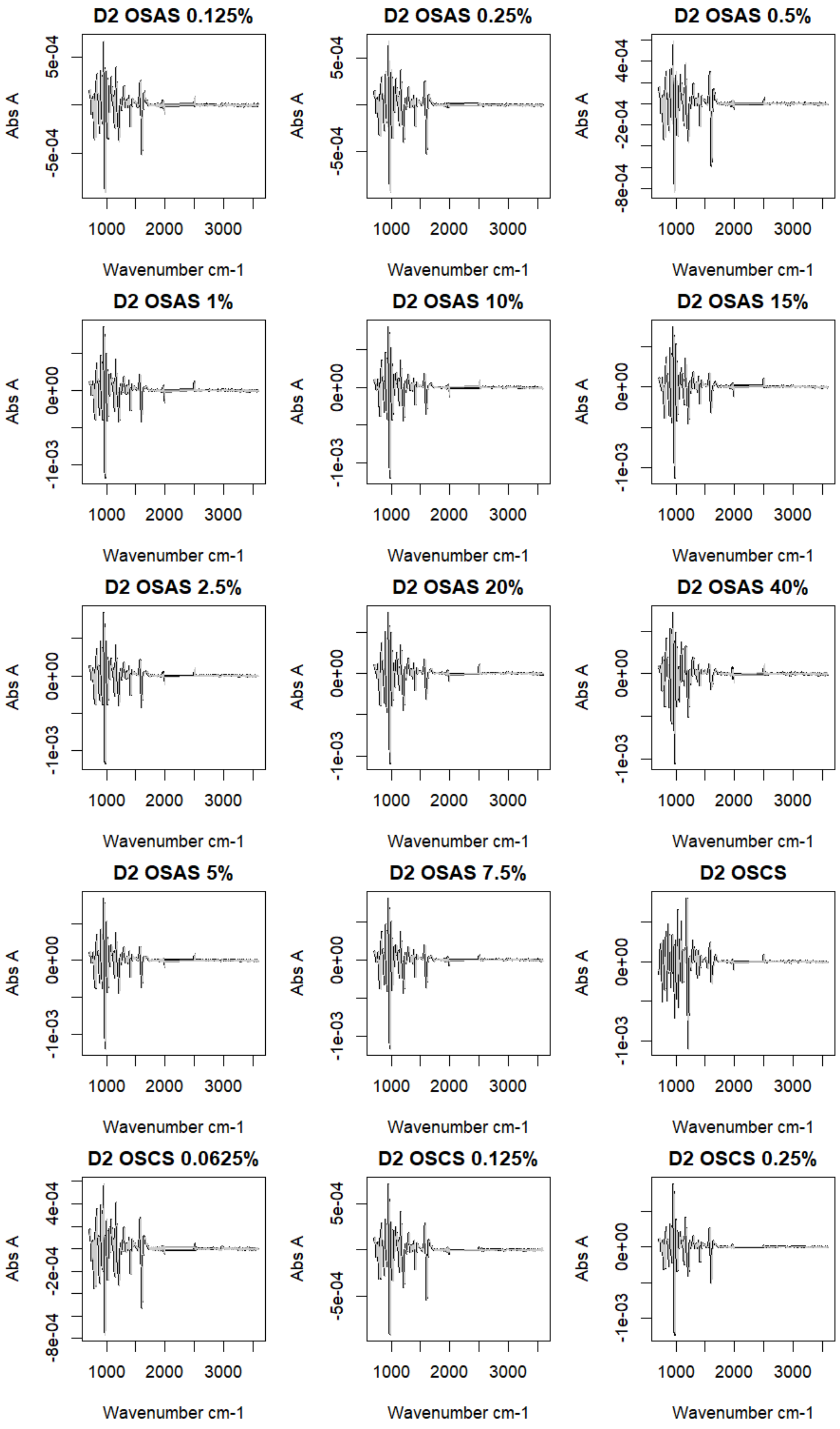
D2 OSAS 0.125%
Abs A
5e-04
-5e-04
1000
2000
3000
Wavenumber cm-1
D2 OSAS 0.25%
Abs A
5e-04
-5e-04
1000
2000
3000
Wavenumber cm-1
D2 OSAS 0.5%
Abs A
4e-04
-2e-04
-8e-04
1000
2000
3000
Wavenumber cm-1
D2 OSAS 1%
Abs A
0e+00
-1e-03
1000
2000
3000
Wavenumber cm-1
D2 OSAS 10%
Abs A
0e+00
-1e-03
1000
2000
3000
Wavenumber cm-1
D2 OSAS 15%
Abs A
0e+00
-1e-03
1000
2000
3000
Wavenumber cm-1
D2 OSAS 2.5%
Abs A
0e+00
-1e-03
1000
2000
3000
Wavenumber cm-1
D2 OSAS 20%
Abs A
0e+00
-1e-03
1000
2000
3000
Wavenumber cm-1
D2 OSAS 40%
Abs A
0e+00
-1e-03
1000
2000
3000
Wavenumber cm-1
D2 OSAS 5%
Abs A
0e+00
-1e-03
1000
2000
3000
Wavenumber cm-1
D2 OSAS 7.5%
Abs A
0e+00
-1e-03
1000
2000
3000
Wavenumber cm-1
D2 OSCS
Abs A
0e+00
-1e-03
1000
2000
3000
Wavenumber cm-1
D2 OSCS 0.0625%
Abs A
4e-04
-2e-04
-8e-04
1000
2000
3000
Wavenumber cm-1
D2 OSCS 0.125%
Abs A
5e-04
-5e-04
1000
2000
3000
Wavenumber cm-1
D2 OSCS 0.25%
Abs A
0e+00
-1e-03
1000
2000
3000
Wavenumber cm-1

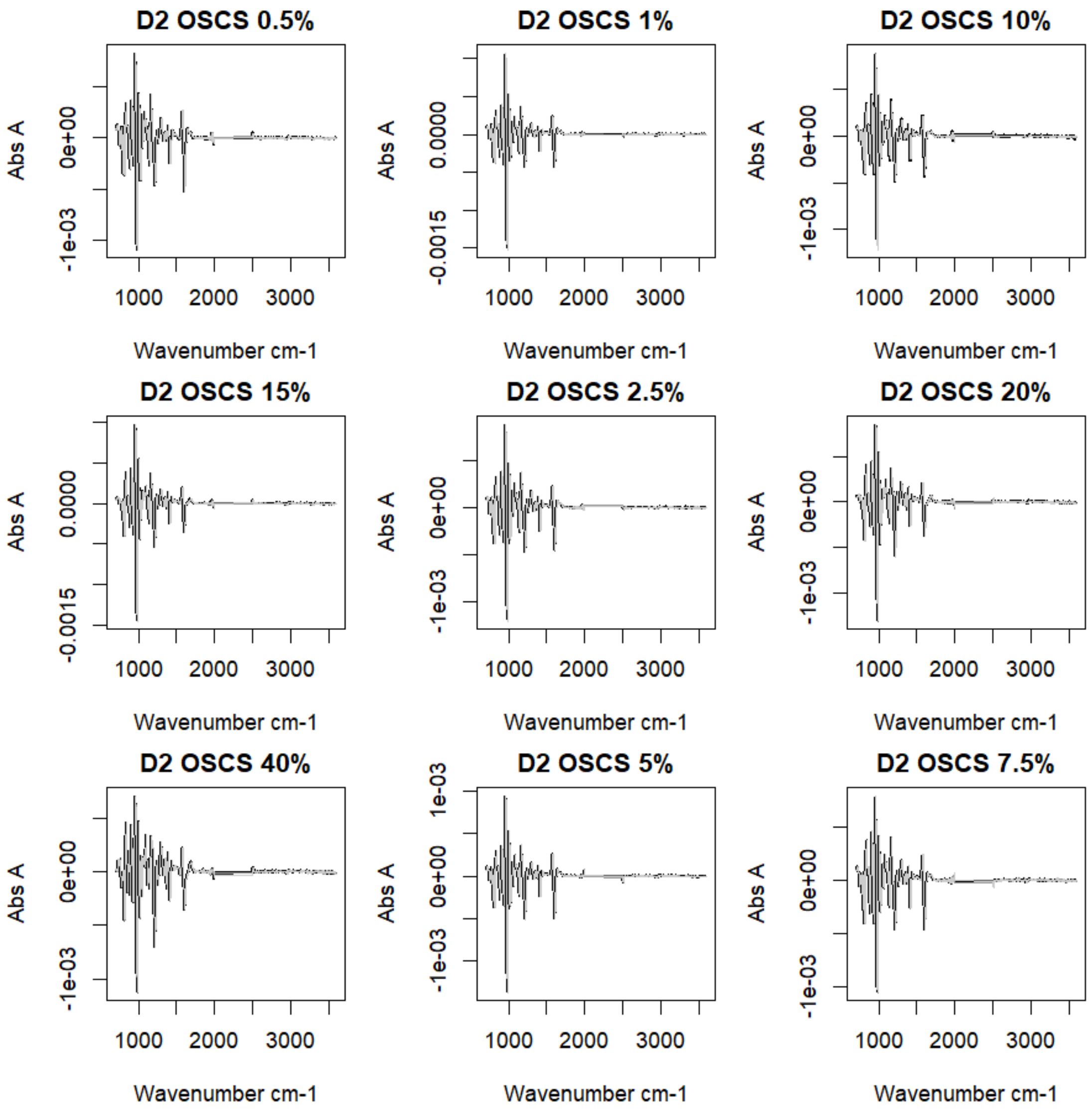
D2 OSCS 0.5%
Abs A
-1e-03
0e+00
1000
2000
3000
Wavenumber cm-1
D2 OSCS 1%
Abs A
-0.0015
0.0000
1000
2000
3000
Wavenumber cm-1
D2 OSCS 10%
Abs A
-1e-03
0e+00
1000
2000
3000
Wavenumber cm-1
D2 OSCS 15%
Abs A
-0.0015
0.0000
1000
2000
3000
Wavenumber cm-1
D2 OSCS 2.5%
Abs A
-1e-03
0e+00
1000
2000
3000
Wavenumber cm-1
D2 OSCS 20%
Abs A
-1e-03
0e+00
1000
2000
3000
Wavenumber cm-1
D2 OSCS 40%
Abs A
-1e-03
0e+00
1000
2000
3000
Wavenumber cm-1
D2 OSCS 5%
Abs A
-1e-03
0e+00
1e-03
1000
2000
3000
Wavenumber cm-1
D2 OSCS 7.5%
Abs A
-1e-03
0e+00
1000
2000
3000
Wavenumber cm-1

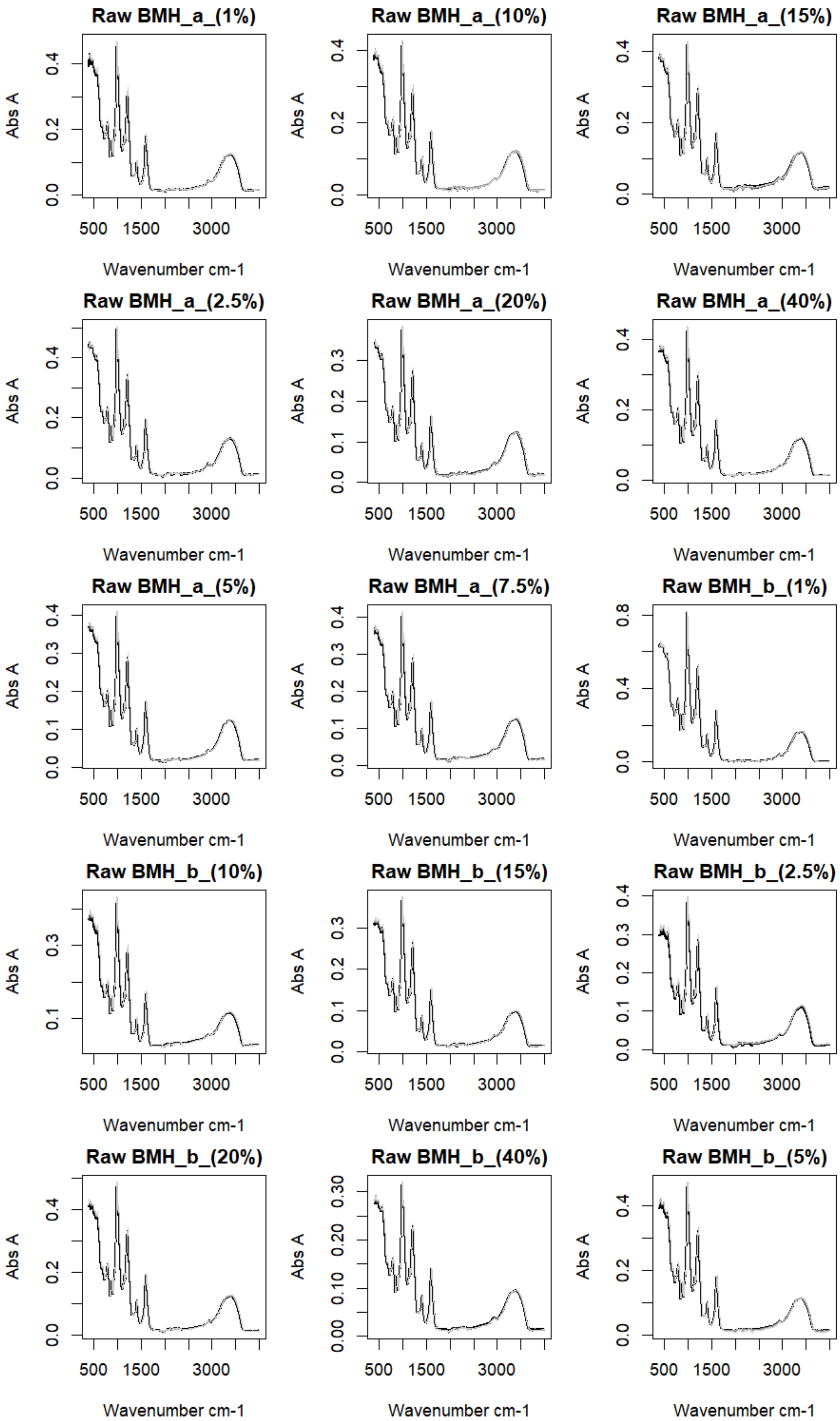
Raw BMH_a_(1%)
Raw BMH_a_(10%)
Raw BMH_a_(15%)
Raw BMH_a_(2.5%)
Raw BMH_a_(20%)
Raw BMH_a_(40%)
Raw BMH_a_(5%)
Raw BMH_a_(7.5%)
Raw BMH_b_(1%)
Raw BMH_b_(10%)
Raw BMH_b_(15%)
Raw BMH_b_(2.5%)
Raw BMH_b_(20%)
Raw BMH_b_(40%)
Raw BMH_b_(5%)
Abs A
Wavenumber cm-1
500
1500
3000

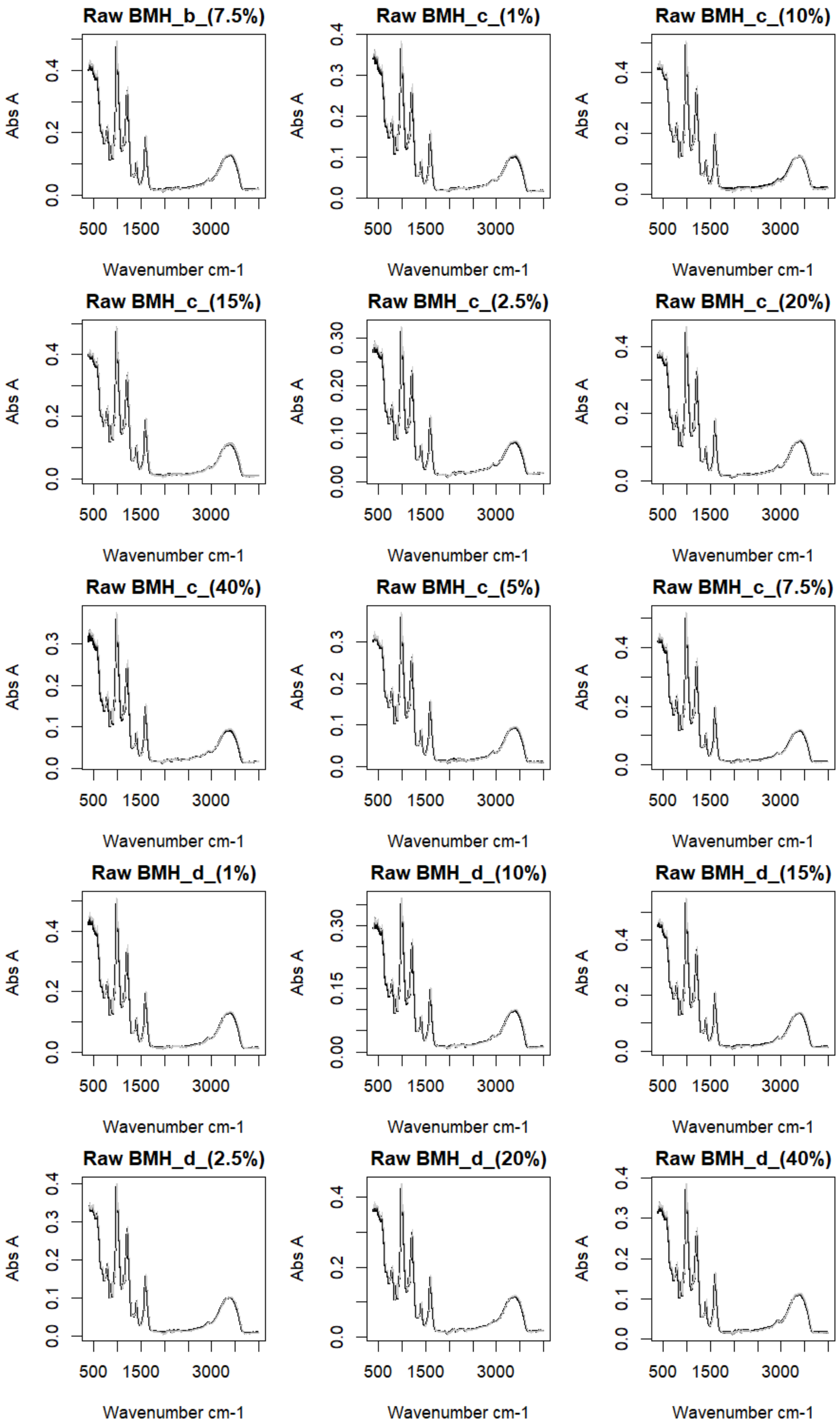
Raw BMH_b_(7.5%)
Raw BMH_c_(1%)
Raw BMH_c_(10%)
Raw BMH_c_(15%)
Raw BMH_c_(2.5%)
Raw BMH_c_(20%)
Raw BMH_c_(40%)
Raw BMH_c_(5%)
Raw BMH_c_(7.5%)
Raw BMH_d_(1%)
Raw BMH_d_(10%)
Raw BMH_d_(15%)
Raw BMH_d_(2.5%)
Raw BMH_d_(20%)
Raw BMH_d_(40%)
Abs A
Wavenumber cm-1
500
1500
3000

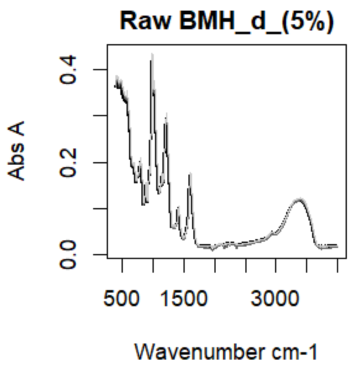

Raw BMH_d_(5%)
Abs A
0.0
0.2
0.4
500
1500
3000
Wavenumber cm-1

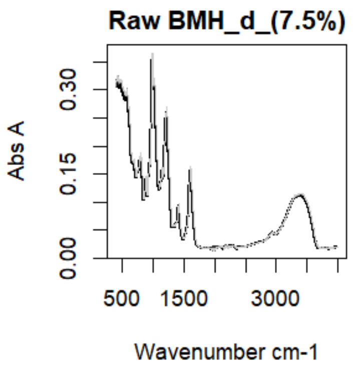

Raw BMH_d_(7.5%)
Abs A
0.00
0.15
0.30
500
1500
3000
Wavenumber cm-1

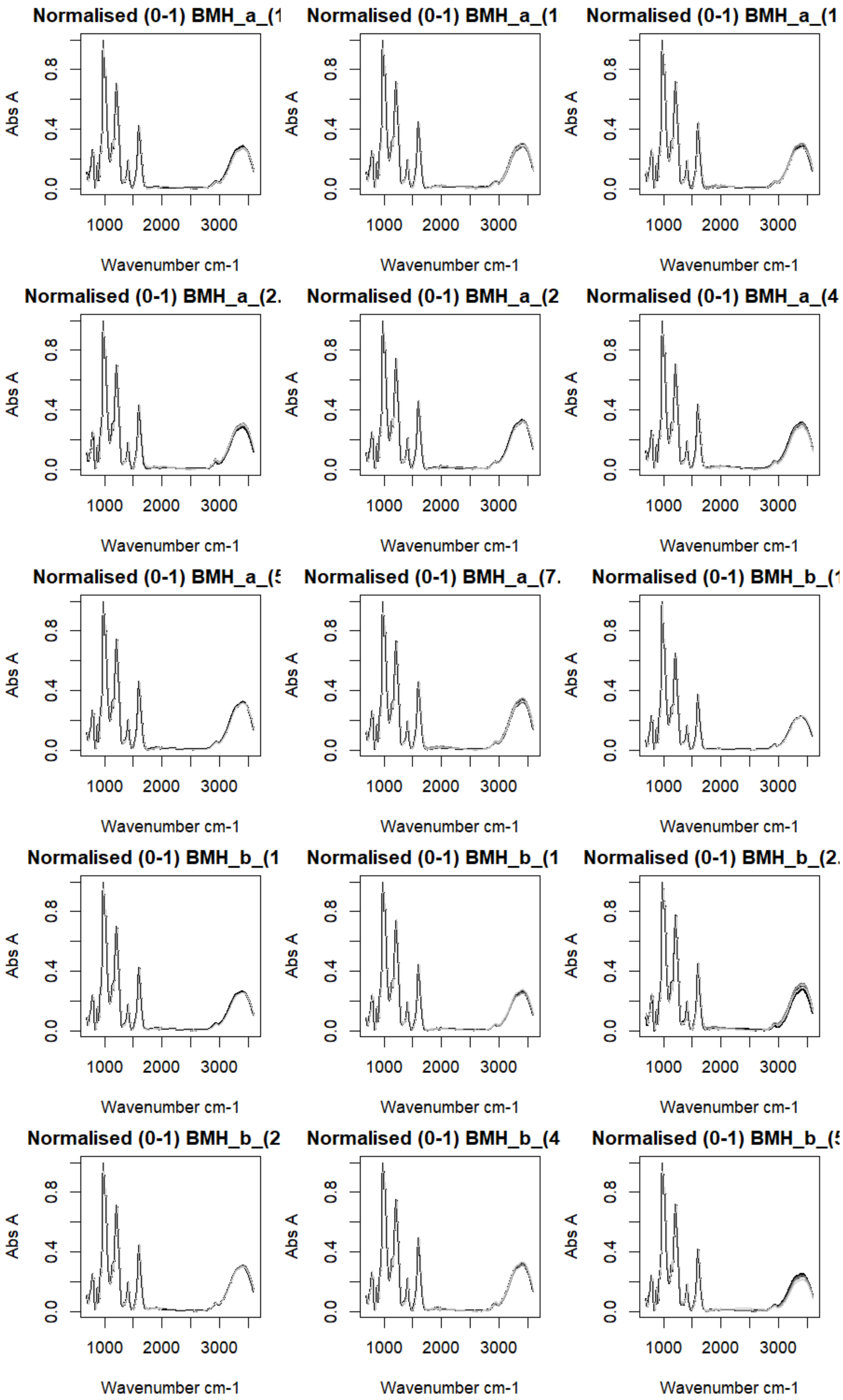

Normalised (0-1) BMH_a_(1
Normalised (0-1) BMH_a_(1
Normalised (0-1) BMH_a_(1
Normalised (0-1) BMH_a_(2.
Normalised (0-1) BMH_a_(2
Normalised (0-1) BMH_a_(4
Normalised (0-1) BMH_a_(5
Normalised (0-1) BMH_a_(7.
Normalised (0-1) BMH_b_(1
Normalised (0-1) BMH_b_(1
Normalised (0-1) BMH_b_(1
Normalised (0-1) BMH_b_(2.
Normalised (0-1) BMH_b_(2
Normalised (0-1) BMH_b_(4
Normalised (0-1) BMH_b_(5
Abs A
0.0
0.4
0.8
1000
2000
3000
Wavenumber cm-1

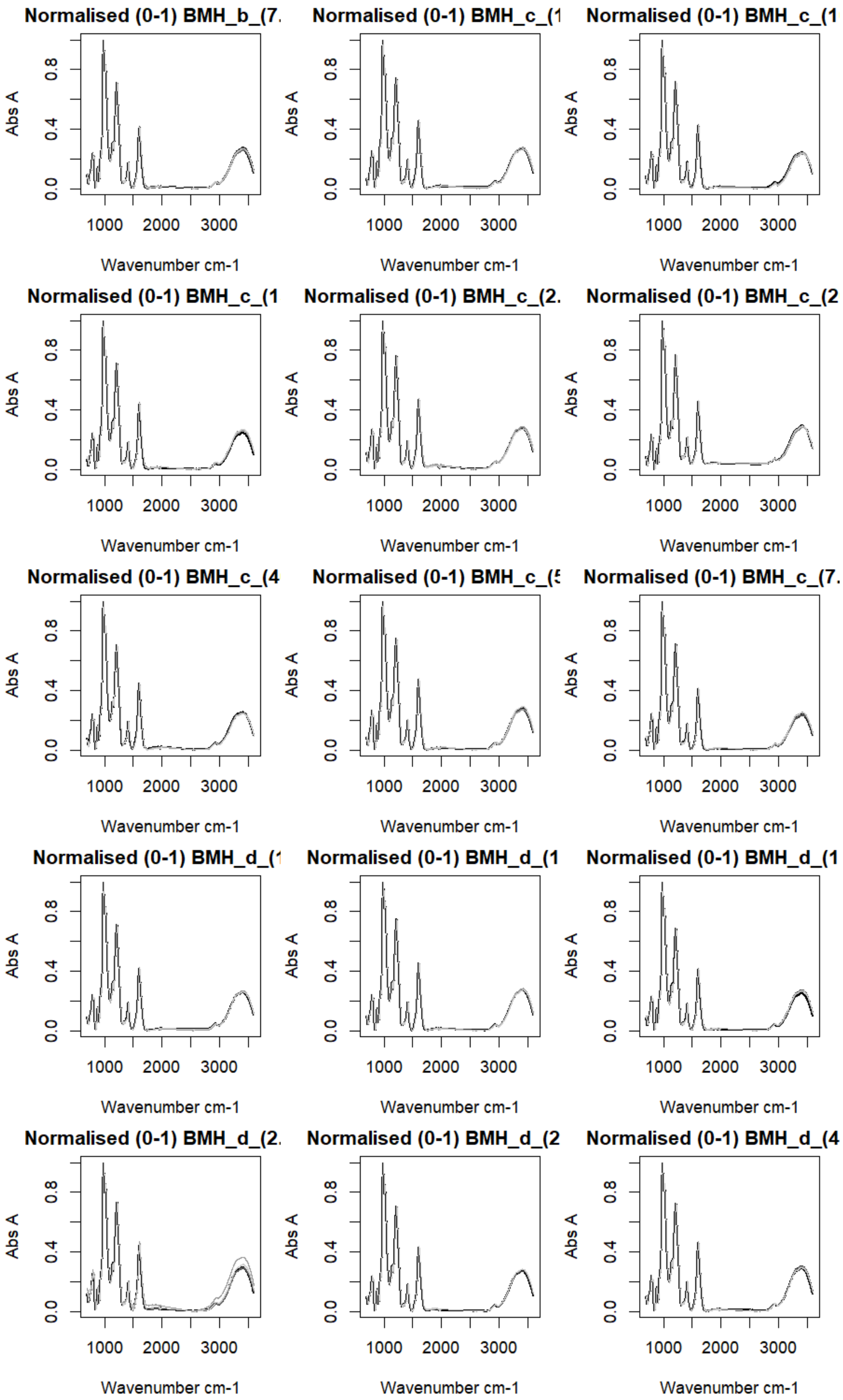
Normalised (0-1) BMH_b_(7.
Normalised (0-1) BMH_c_(1
Normalised (0-1) BMH_c_(1
Normalised (0-1) BMH_c_(1
Normalised (0-1) BMH_c_(2.
Normalised (0-1) BMH_c_(2
Normalised (0-1) BMH_c_(4
Normalised (0-1) BMH_c_(5
Normalised (0-1) BMH_c_(7.
Normalised (0-1) BMH_d_(1
Normalised (0-1) BMH_d_(1
Normalised (0-1) BMH_d_(1
Normalised (0-1) BMH_d_(2.
Normalised (0-1) BMH_d_(2
Normalised (0-1) BMH_d_(4
Abs A
0.0
0.4
0.8
1000
2000
3000
Wavenumber cm-1

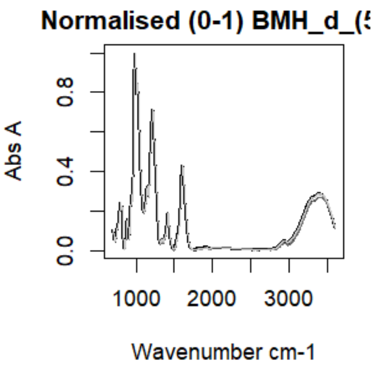
Normalised (0-1) BMH_d_(5
Abs A
0.0
0.4
0.8
1000
2000
3000
Wavenumber cm-1

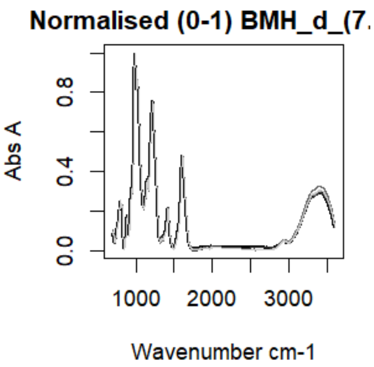
Normalised (0-1) BMH_d_(7.
Abs A
0.0
0.4
0.8
1000
2000
3000
Wavenumber cm-1

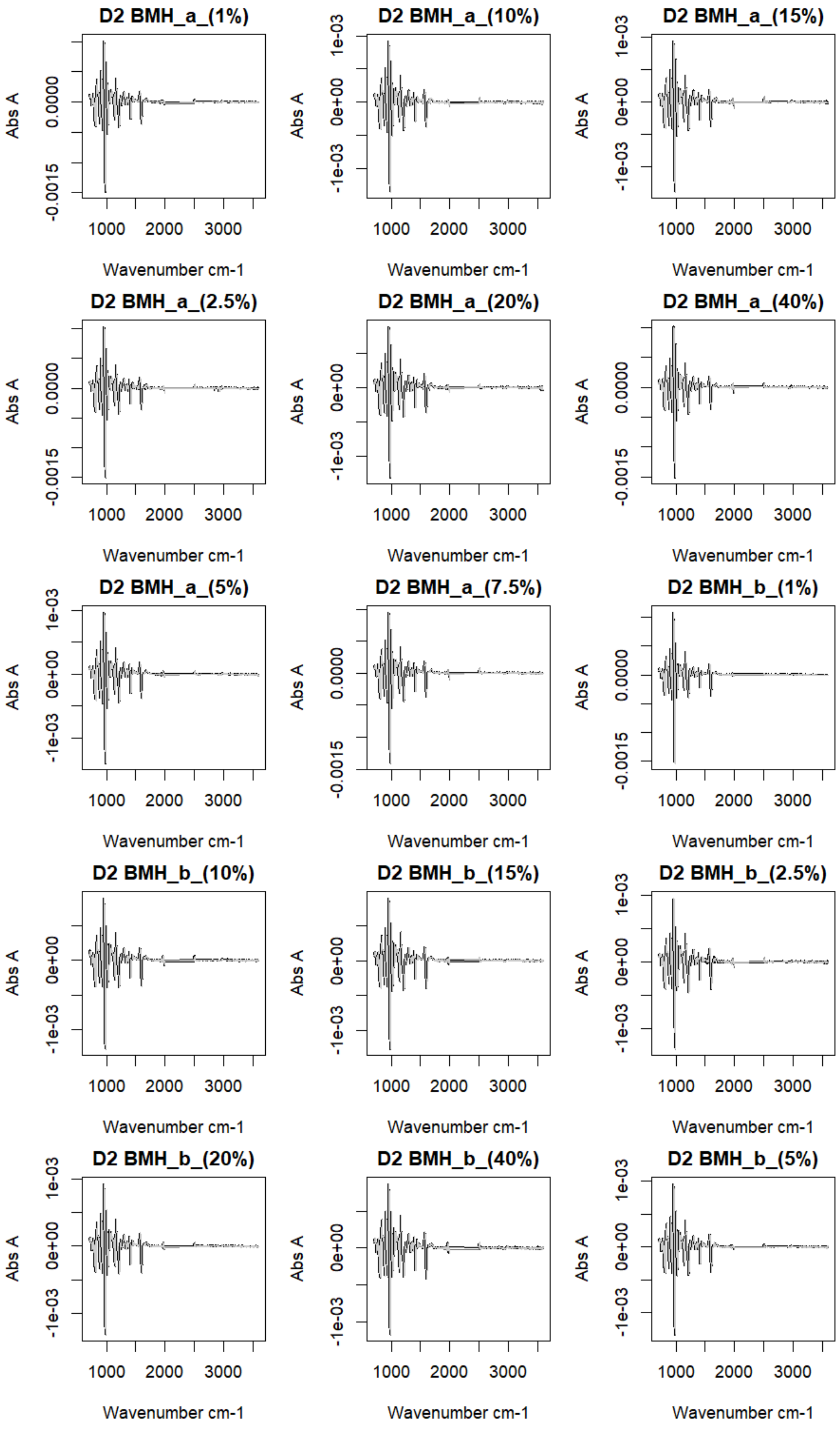
D2 BMH_a_(1%)
D2 BMH_a_(10%)
D2 BMH_a_(15%)
D2 BMH_a_(2.5%)
D2 BMH_a_(20%)
D2 BMH_a_(40%)
D2 BMH_a_(5%)
D2 BMH_a_(7.5%)
D2 BMH_b_(1%)
D2 BMH_b_(10%)
D2 BMH_b_(15%)
D2 BMH_b_(2.5%)
D2 BMH_b_(20%)
D2 BMH_b_(40%)
D2 BMH_b_(5%)
Abs A
Wavenumber cm-1
1000
2000
3000
-0.0015
0.0000
-1e-03
0e+00
1e-03

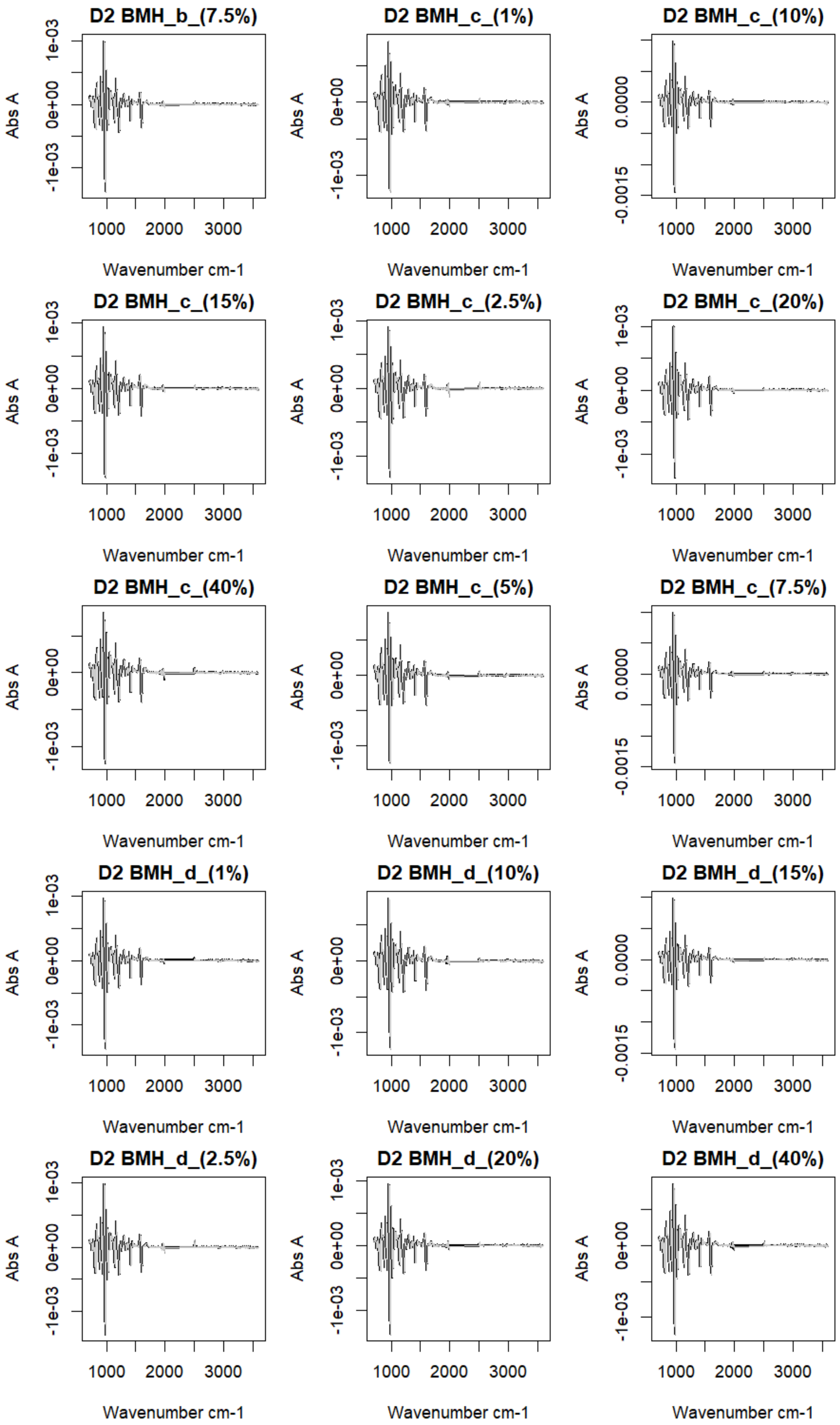
D2 BMH_b_(7.5%)
D2 BMH_c_(1%)
D2 BMH_c_(10%)
D2 BMH_c_(15%)
D2 BMH_c_(2.5%)
D2 BMH_c_(20%)
D2 BMH_c_(40%)
D2 BMH_c_(5%)
D2 BMH_c_(7.5%)
D2 BMH_d_(1%)
D2 BMH_d_(10%)
D2 BMH_d_(15%)
D2 BMH_d_(2.5%)
D2 BMH_d_(20%)
D2 BMH_d_(40%)
Abs A
Wavenumber cm-1
1000
2000
3000
1e-03
0e+00
-1e-03
0.0000
-0.0015

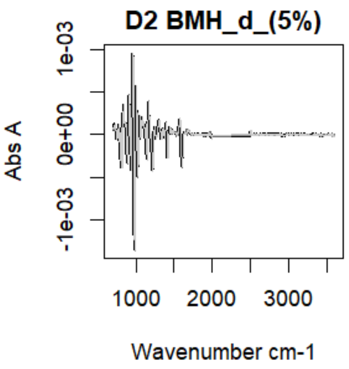
D2 BMH_d_(5%)
Abs A
1e-03
0e+00
-1e-03
1000
2000
3000
Wavenumber cm-1

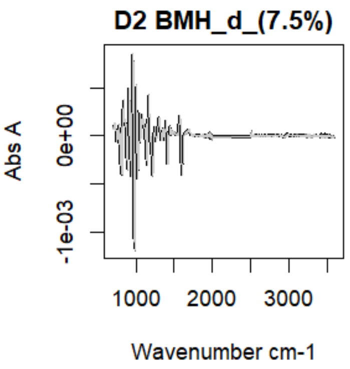
D2 BMH_d_(7.5%)
Abs A
0e+00
-1e-03
1000
2000
3000
Wavenumber cm-1

EVerything New Code D2 7th

Anthony Devlin, Keele University

PCA of PCA of Everything

Scaled Matrix PCAed

7th order polynomial baseline correction performed

Raw spectra smoothed to 21 Neighbours, 2nd order PN, Savtisky Golay

Normalisation between 0 and 100

D2 spectra smoothed to 41 Neighbours, 2nd order PN, Savtisky Golay

Regions Analysed: 3601−2501 and 2001−700cm−1

Samples Analysed: 24 BLH's 55 BMH's 31 C1's 21 C2's 6 C3's 3 Contam's
10 GAG's 31 HS's 33 OMH's 1 PLH's 69 PMH's

Keele University AD Sun Dec 09 12:39:02 2018

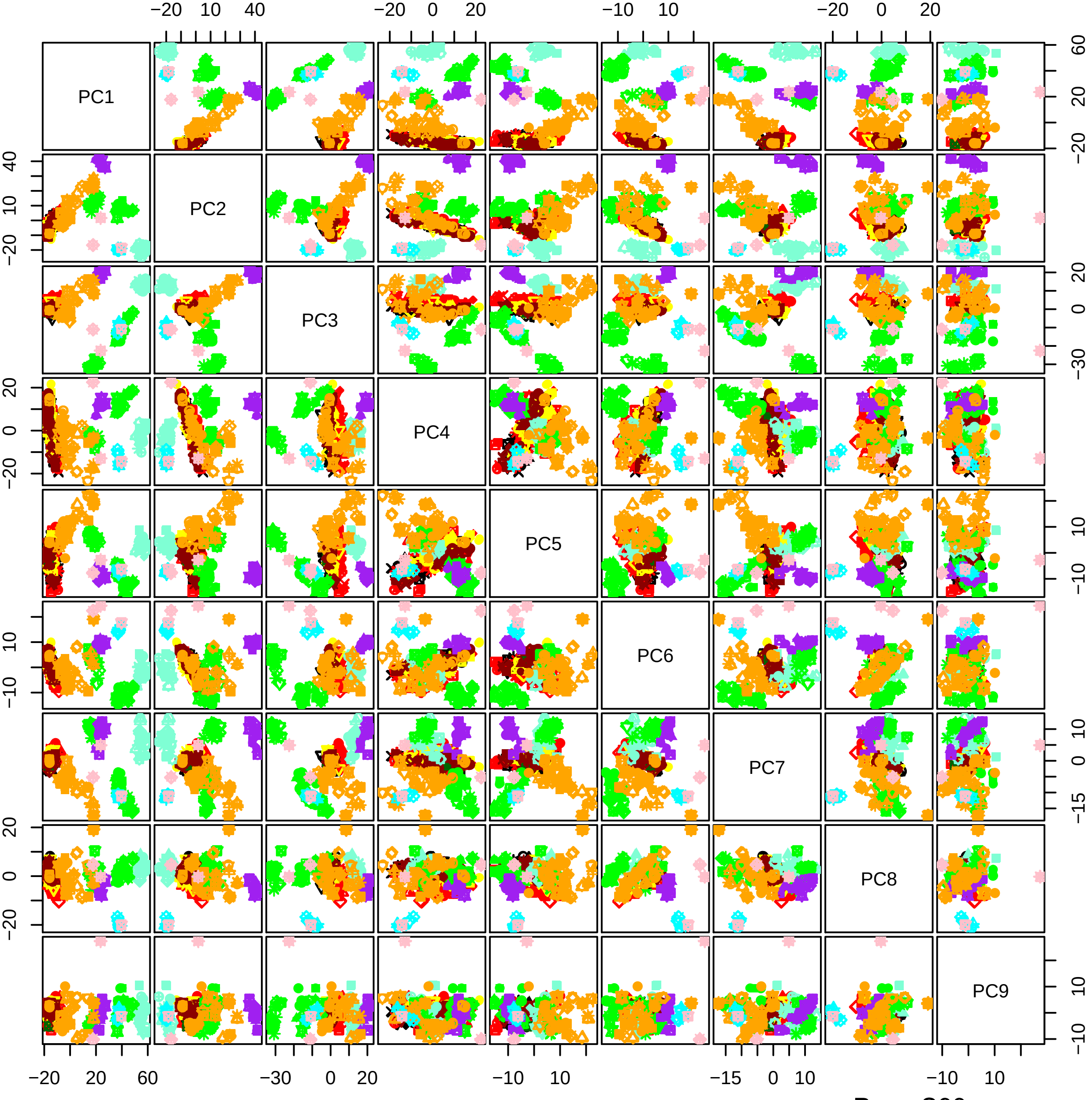
PC1
PC2
PC3
PC4
PC5
PC6
PC7
PC8
PC9

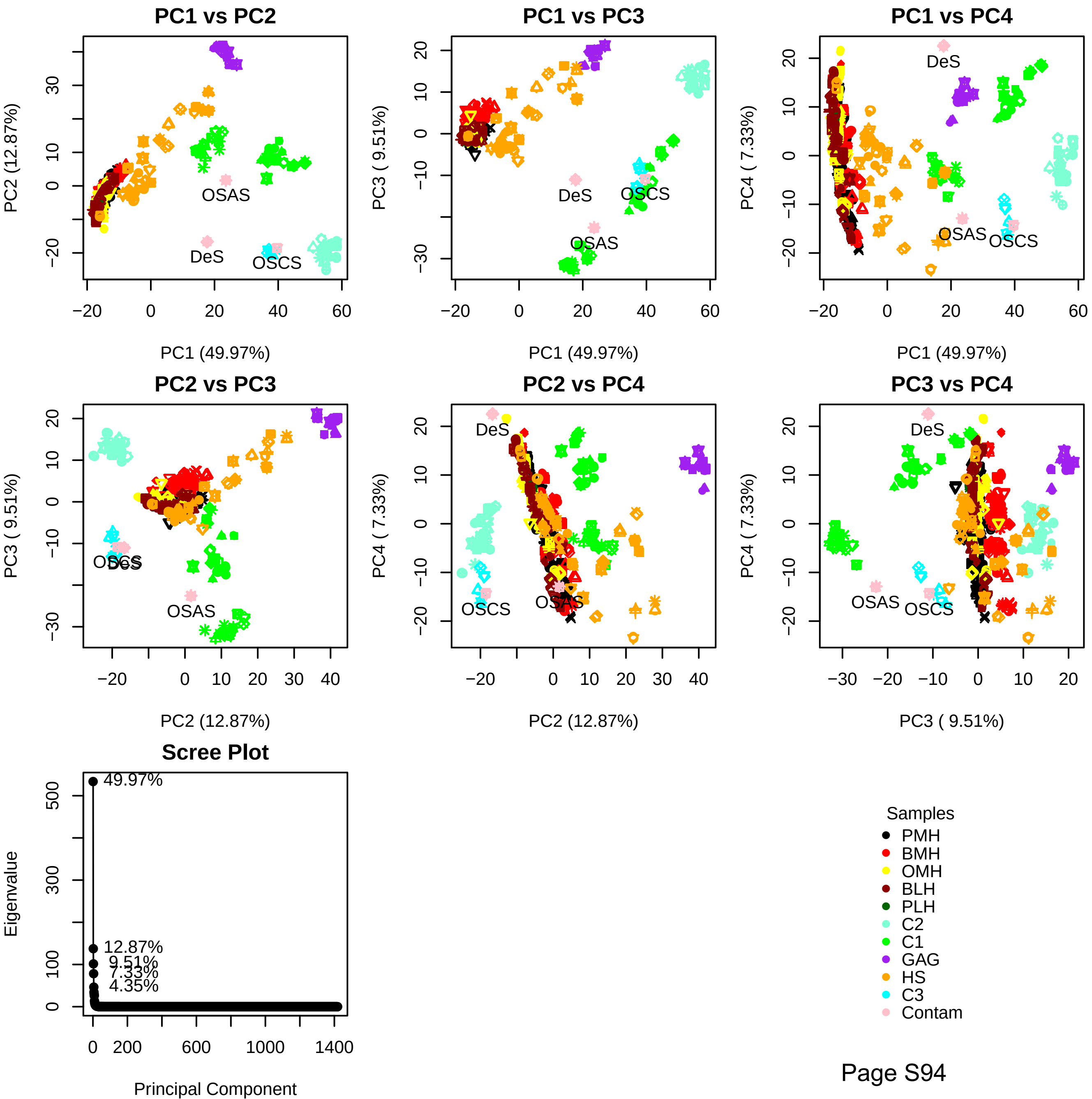
PC1 vs PC2
PC1 vs PC3
PC1 vs PC4
PC2 vs PC3
PC2 vs PC4
PC3 vs PC4
Scree Plot
PC1 (49.97%)
PC2 (12.87%)
PC3 (9.51%)
PC4 (7.33%)
DeS
OSAS
OSCS
Eigenvalue
Principal Component
49.97%
12.87%
9.51%
7.33%
4.35%
Samples
PMH
BMH
OMH
BLH
PLH
C2
C1
GAG
HS
C3
Contam

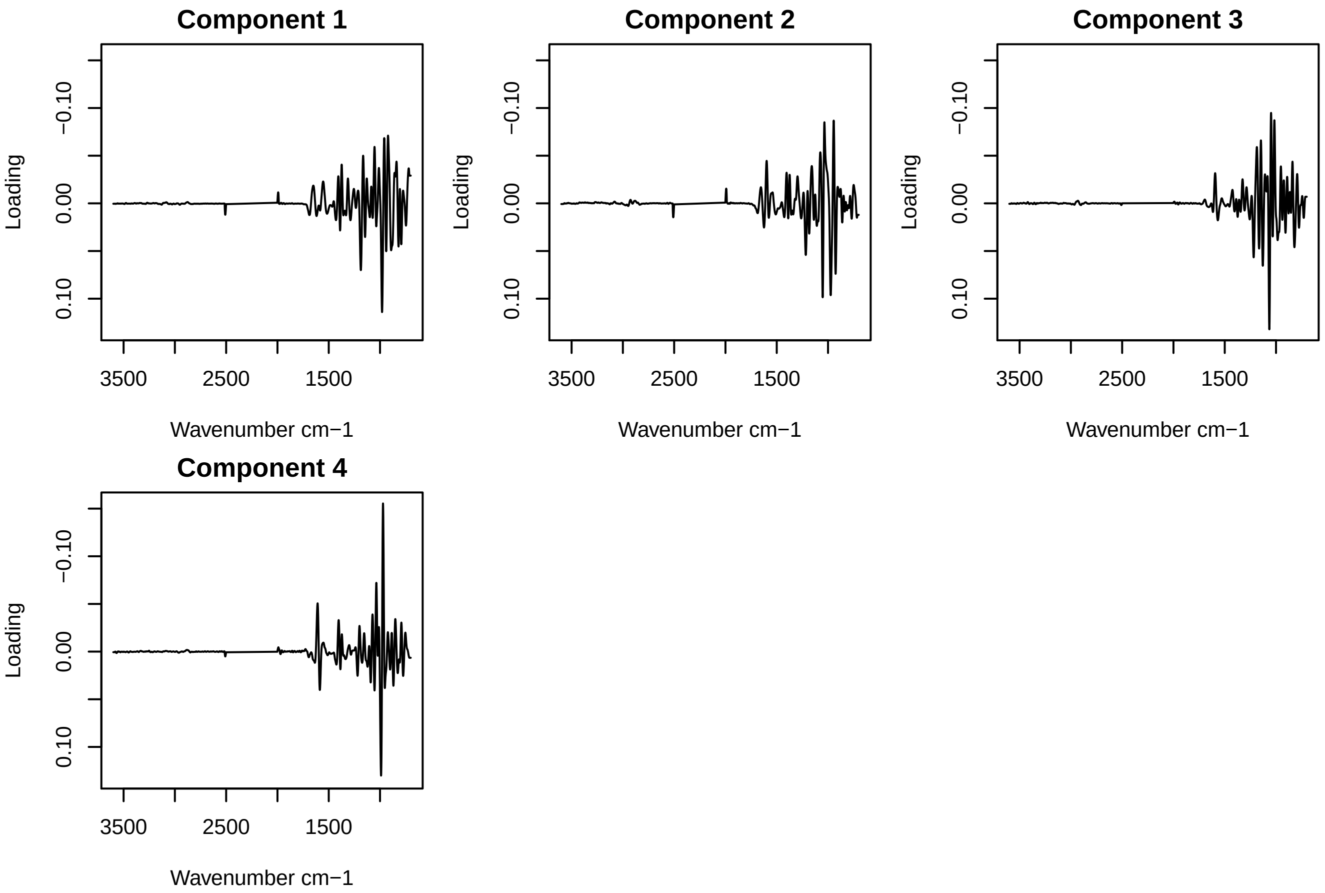
Component 1
Component 2
Component 3
Component 4
Loading
−0.10
0.00
0.10
3500
2500
1500
Wavenumber cm−1

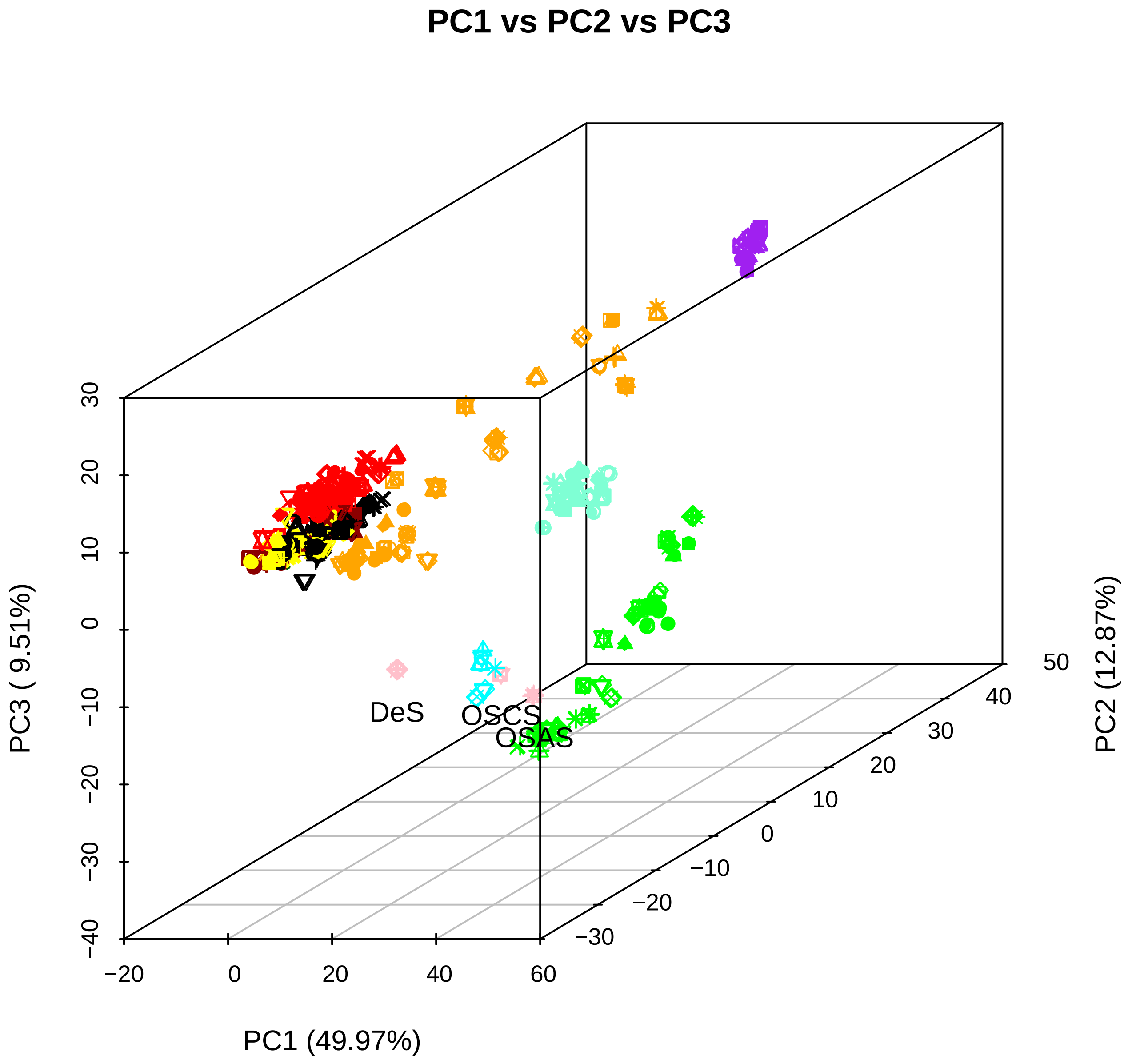
PC1 vs PC2 vs PC3
PC1 (49.97%)
PC2 (12.87%)
PC3 (9.51%)
−20
0
20
40
60
−30
−20
−10
0
10
20
30
40
50
−40
−30
−20
−10
0
10
20
30
DeS
OSCS
OSAS

PC2 vs PC3 vs PC4

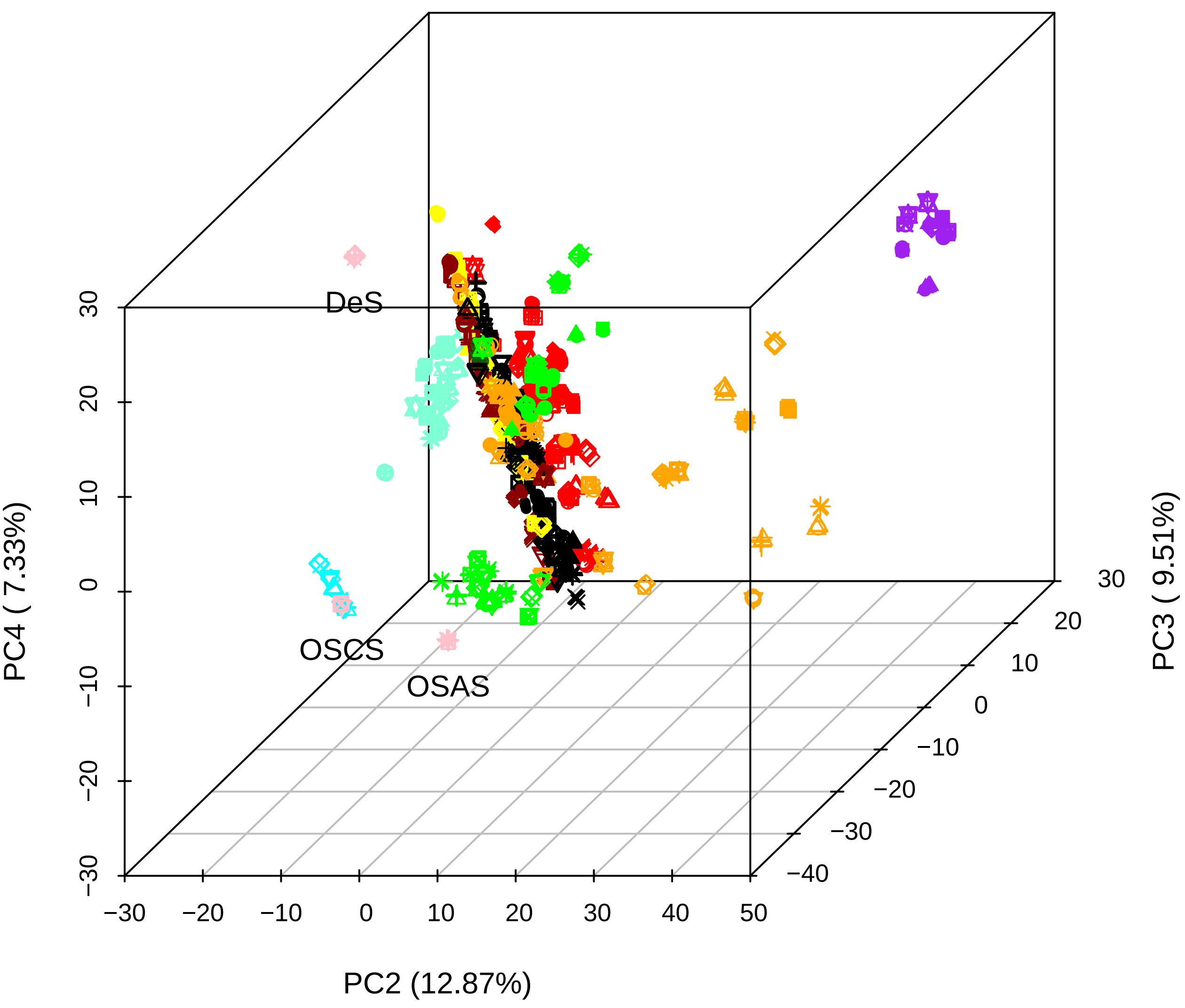

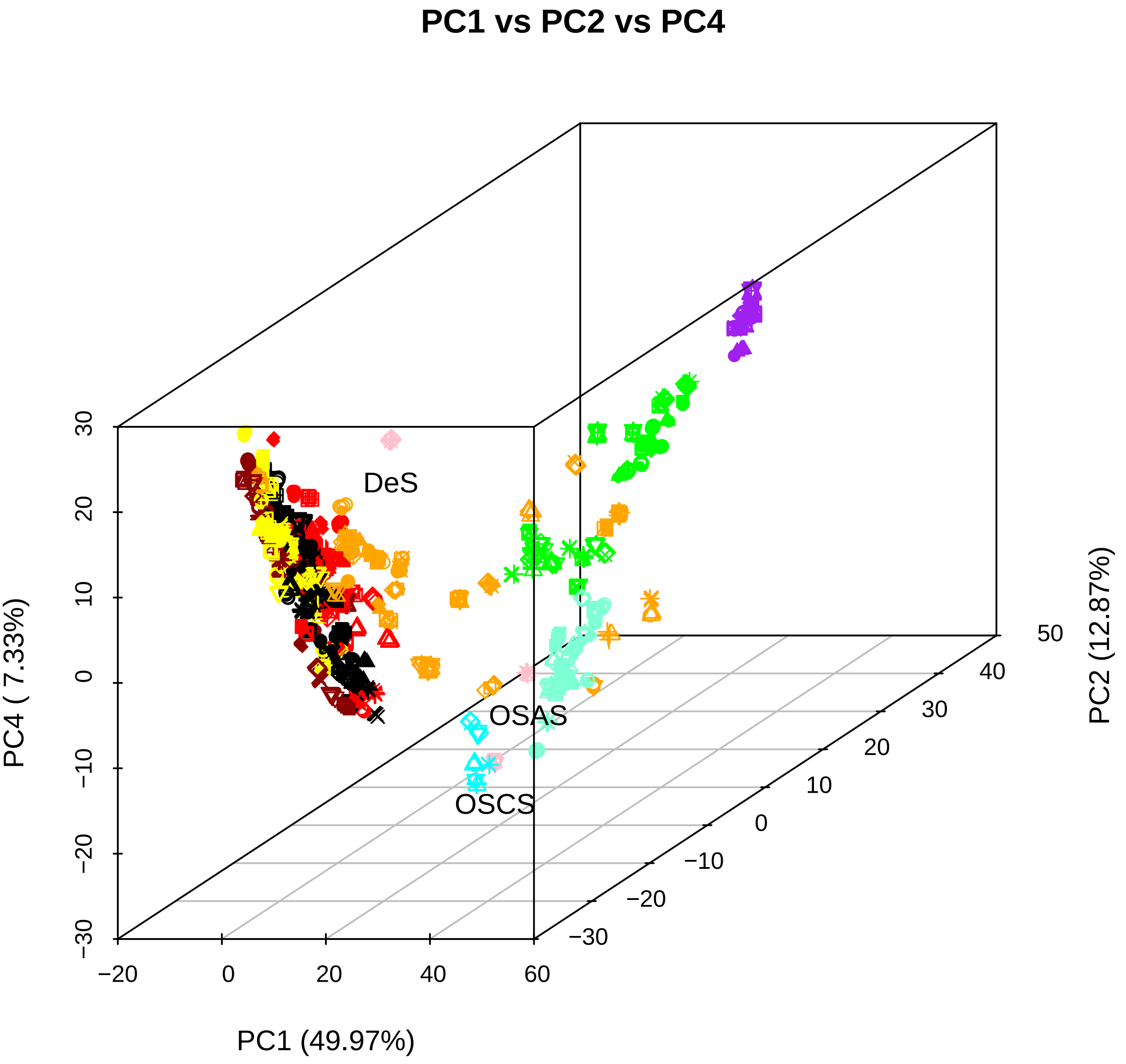

PC1 vs PC2 vs PC4
DeS
OSAS
OSCS
PC4 (7.33%)
PC1 (49.97%)
PC2 (12.87%)
30
20
10
0
−10
−20
−30
−20
0
20
40
60
50
40
30
20
10
0
−10
−20
−30

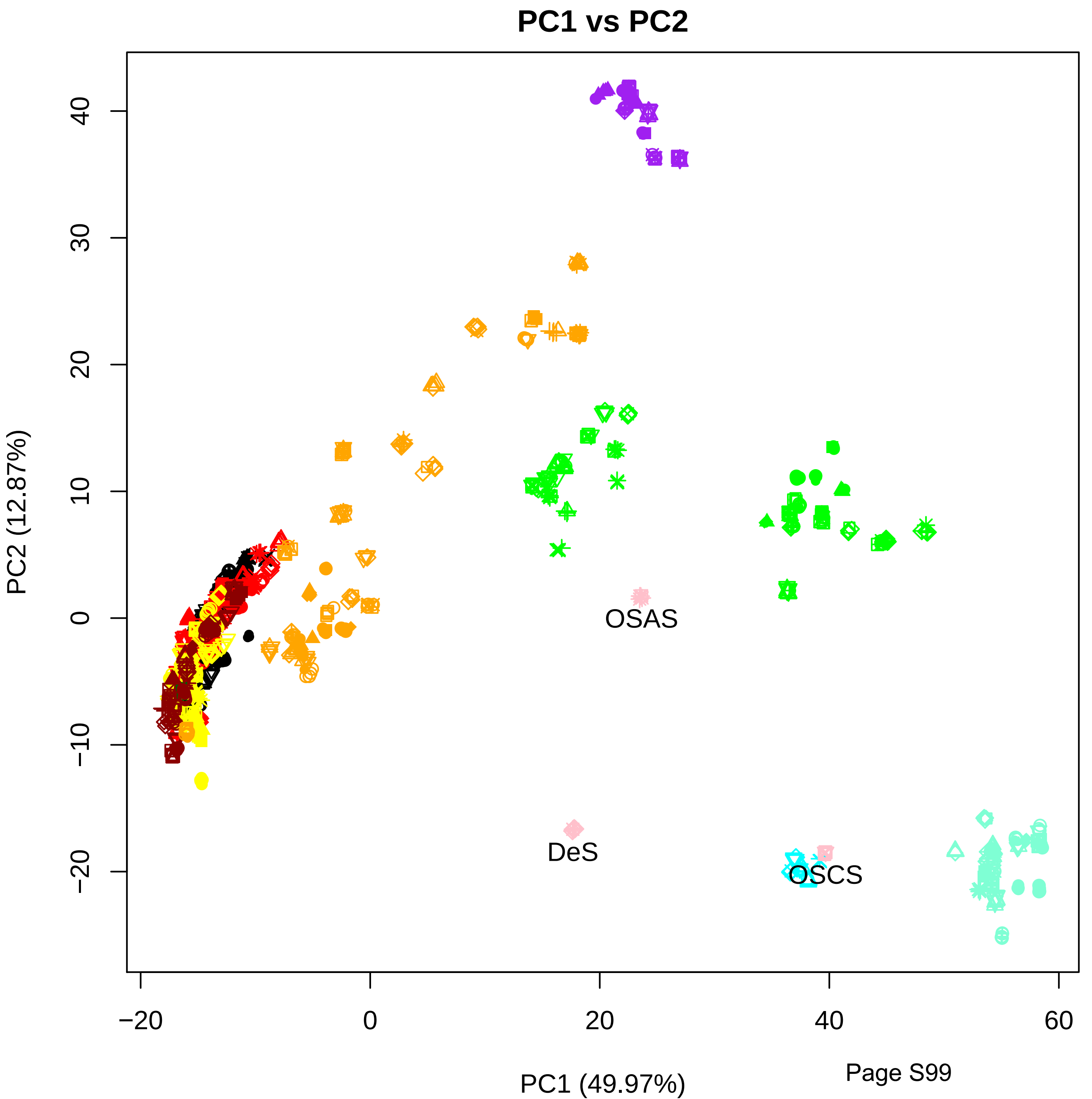
PC1 vs PC2
PC2 (12.87%)
PC1 (49.97%)
40
30
20
10
0
−10
−20
−20
0
20
40
60
OSAS
DeS
OSCS

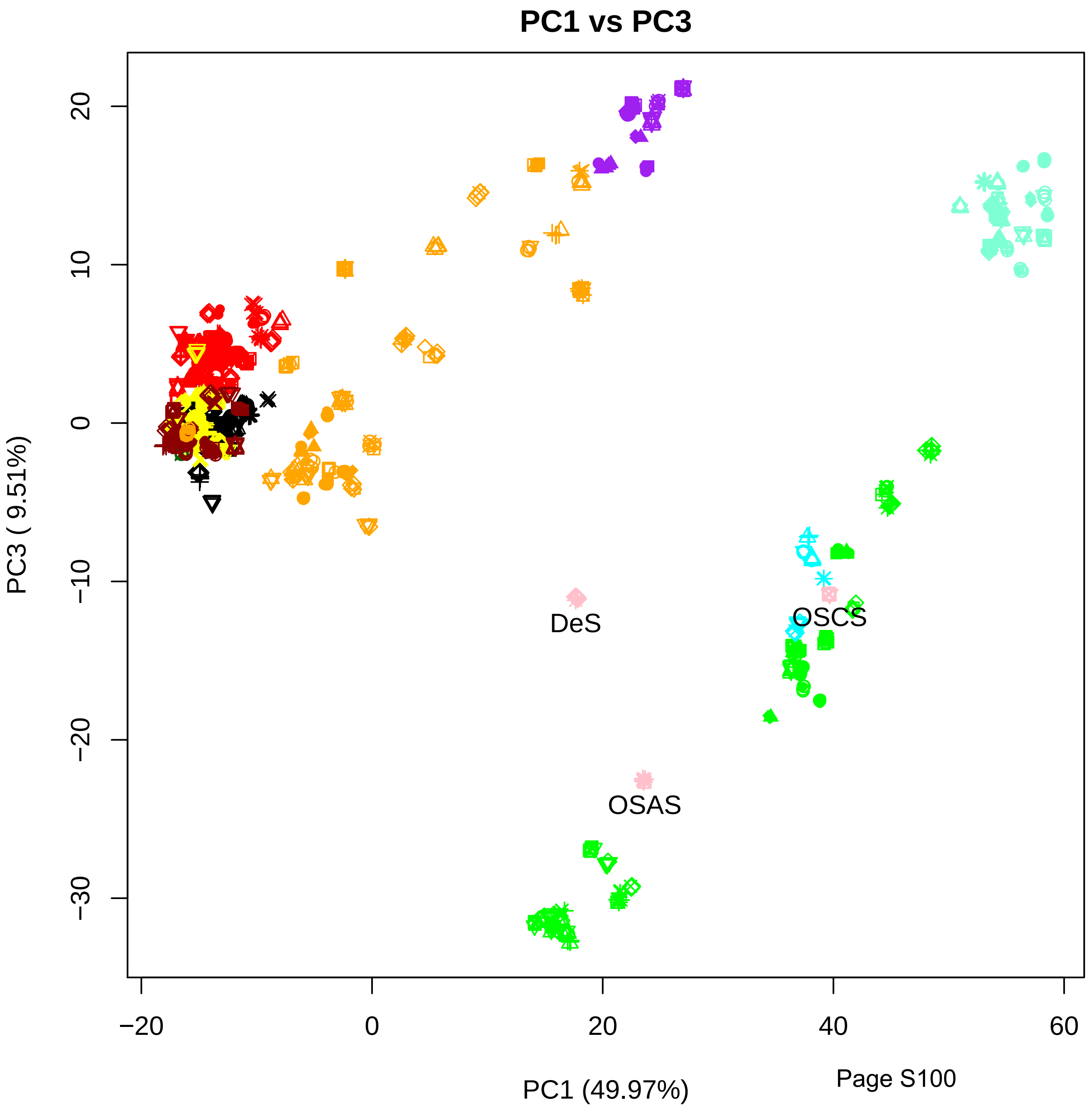
PC1 vs PC3
PC3 (9.51%)
PC1 (49.97%)
20
10
0
−10
−20
−30
−20
0
20
40
60
DeS
OSCS
OSAS

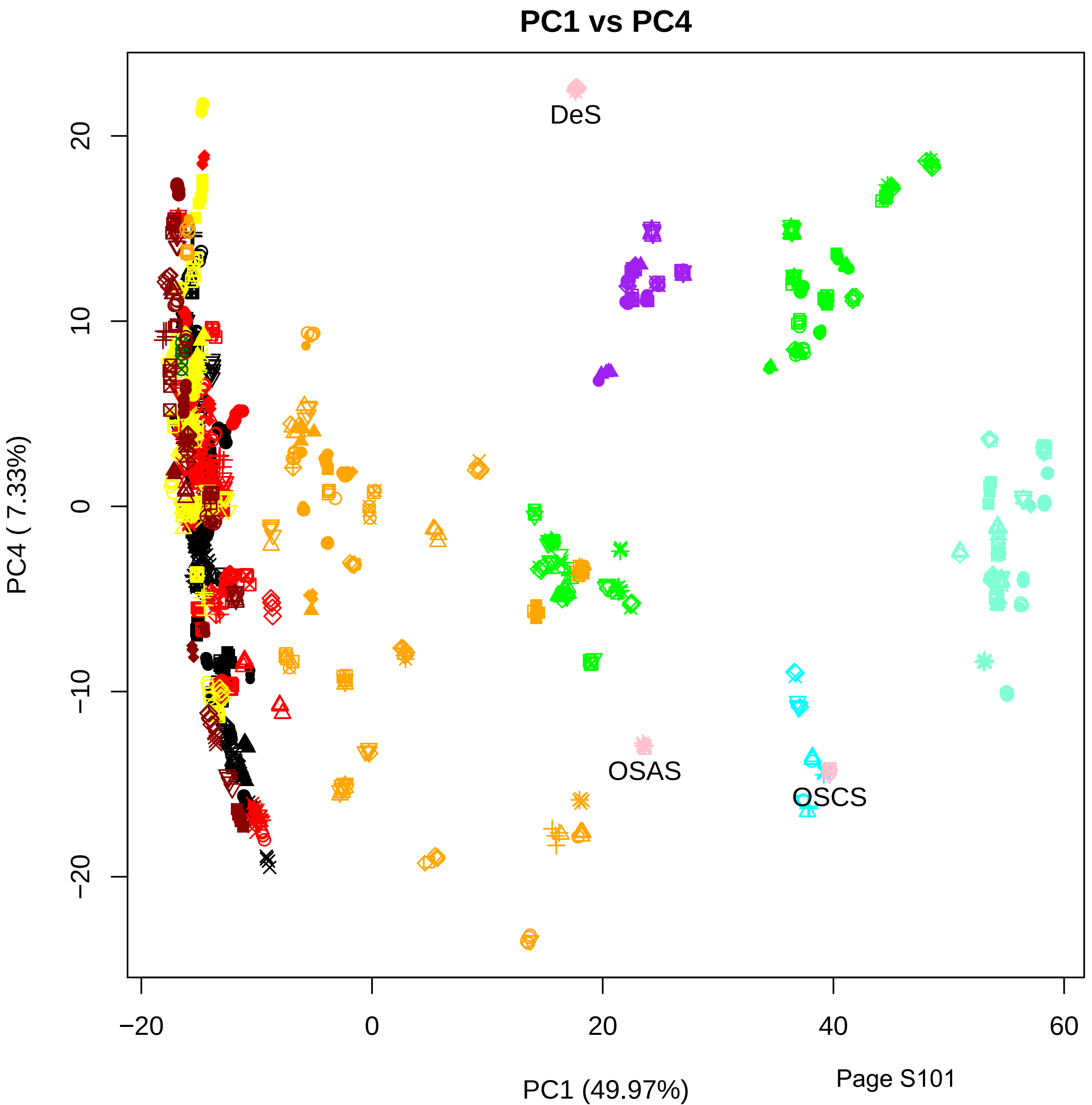
PC1 vs PC4
DeS
OSAS
OSCS
PC4 (7.33%)
PC1 (49.97%)
20
10
0
−10
−20
−20
0
20
40
60

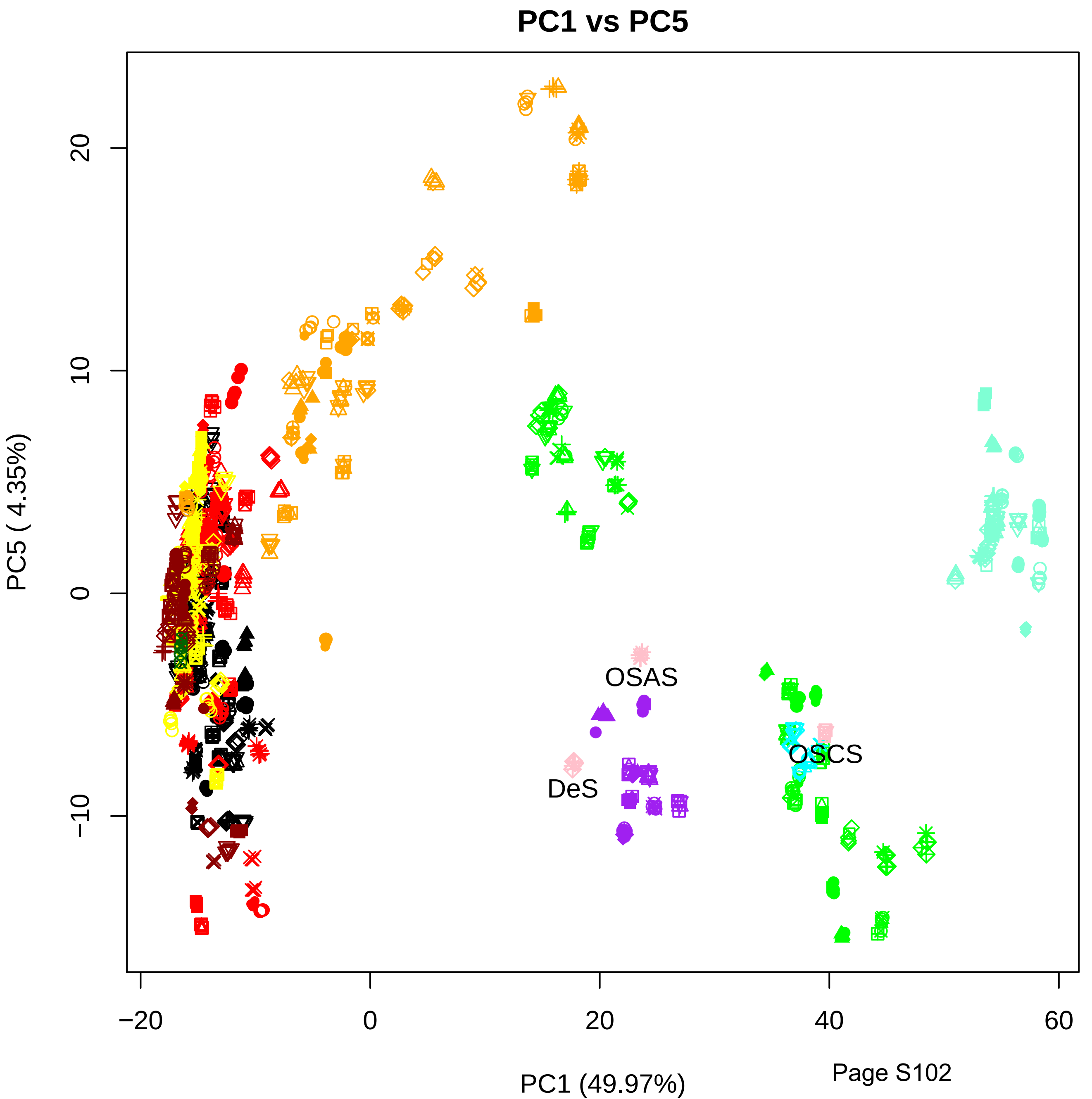
PC1 vs PC5
PC5 (4.35%)
PC1 (49.97%)
20
10
0
−10
−20
0
20
40
60
OSAS
DeS
OSCS

PC2 vs PC3

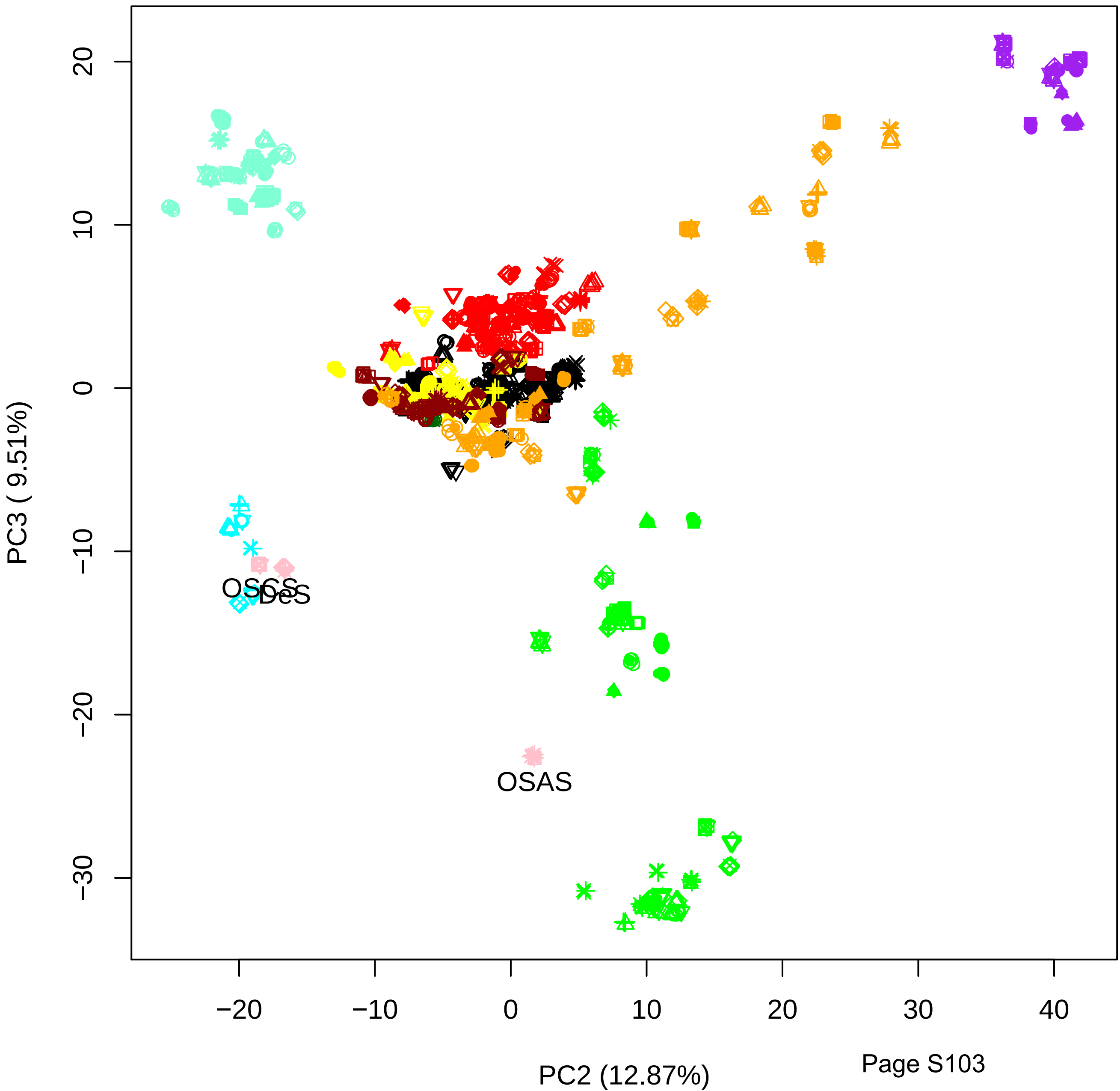

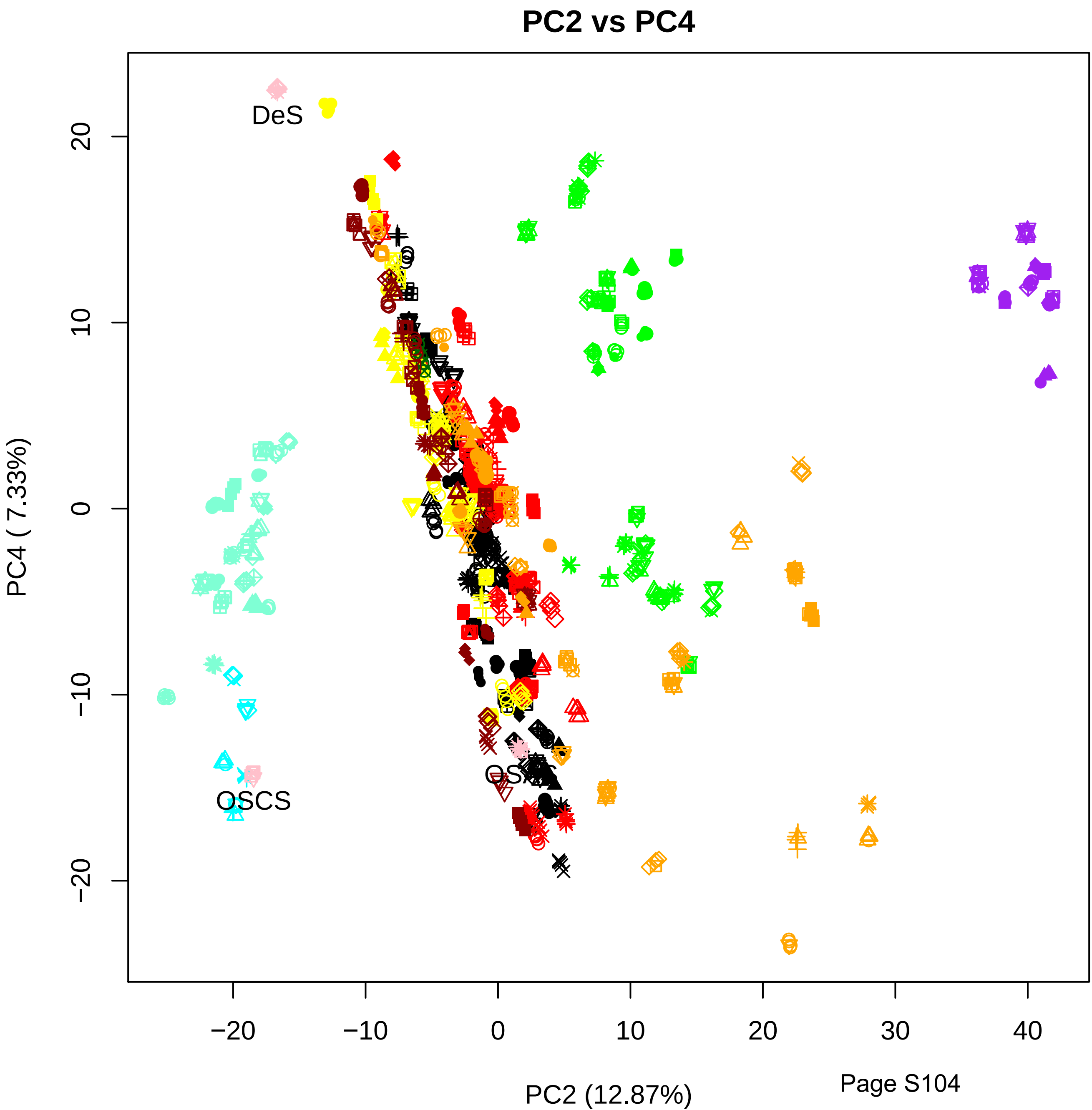

PC2 vs PC4
DeS
OSCS
OSCS
PC4 (7.33%)
PC2 (12.87%)
20
10
0
−10
−20
−20
−10
0
10
20
30
40

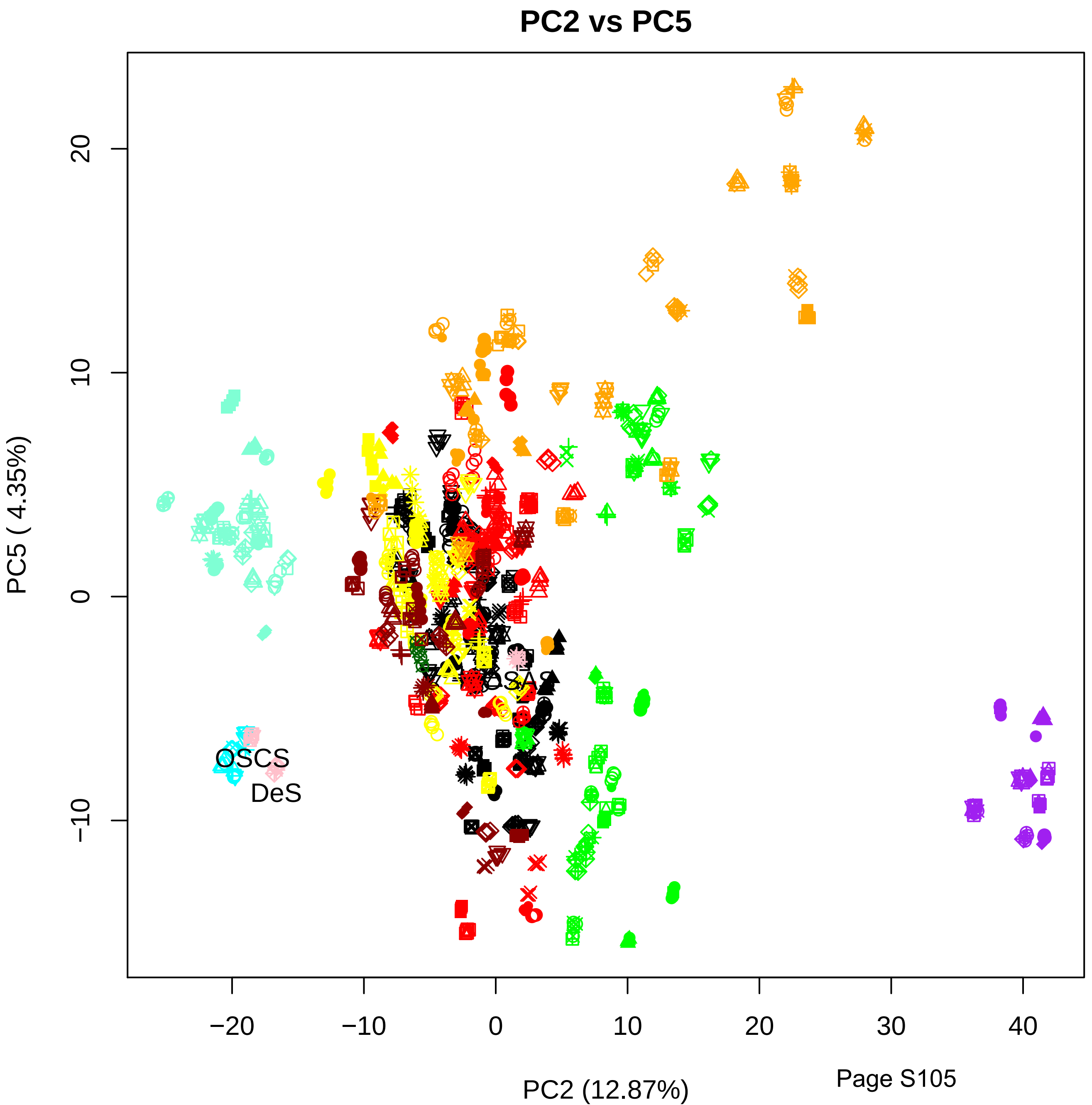

PC2 vs PC5
PC5 (4.35%)
PC2 (12.87%)
20
10
0
−10
−20
−10
0
10
20
30
40
SAS
OSCS
DeS

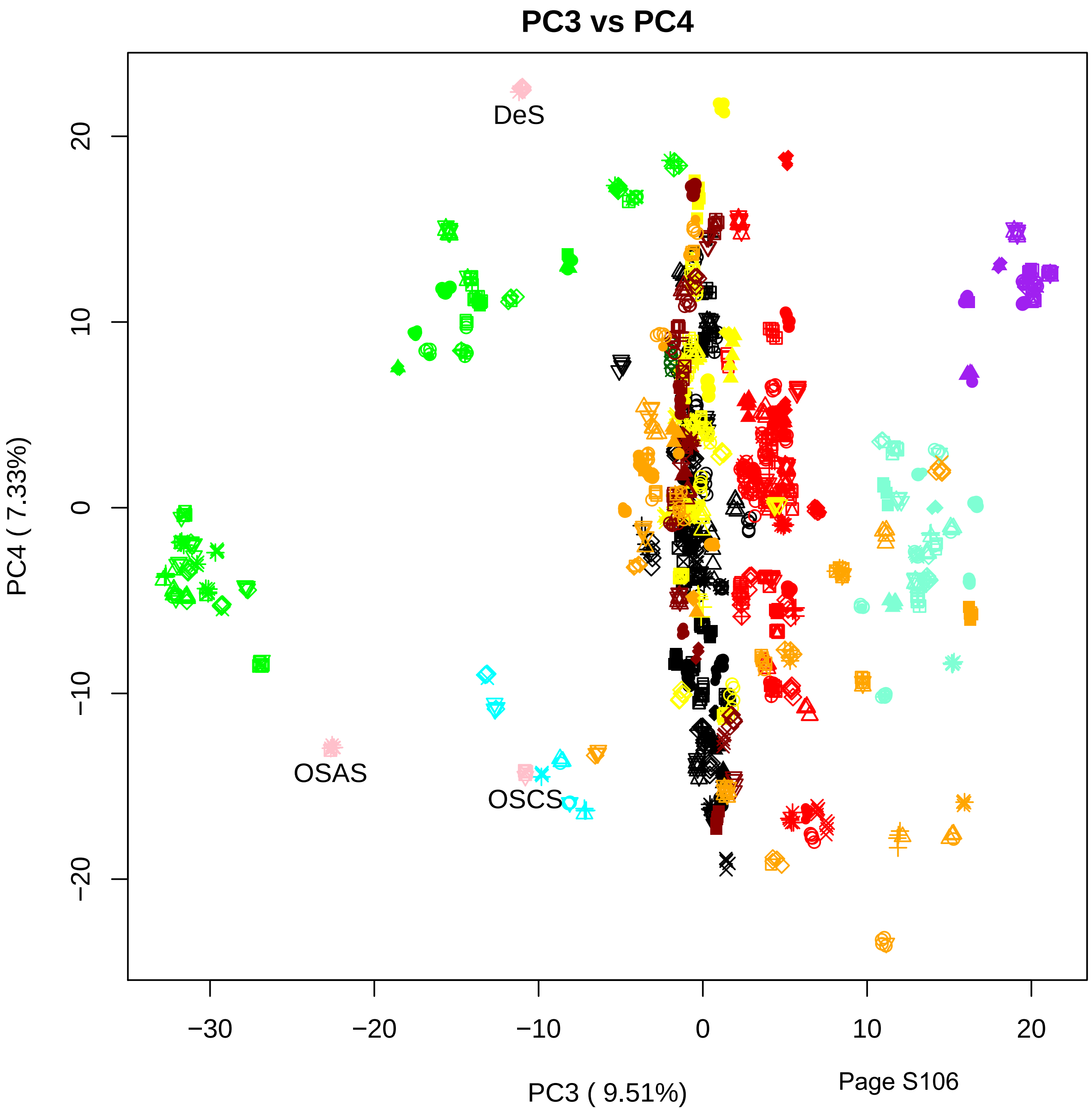
PC3 vs PC4
DeS
OSAS
OSCS
PC4 (7.33%)
PC3 (9.51%)
20
10
0
−10
−20
−30
−20
−10
0
10
20

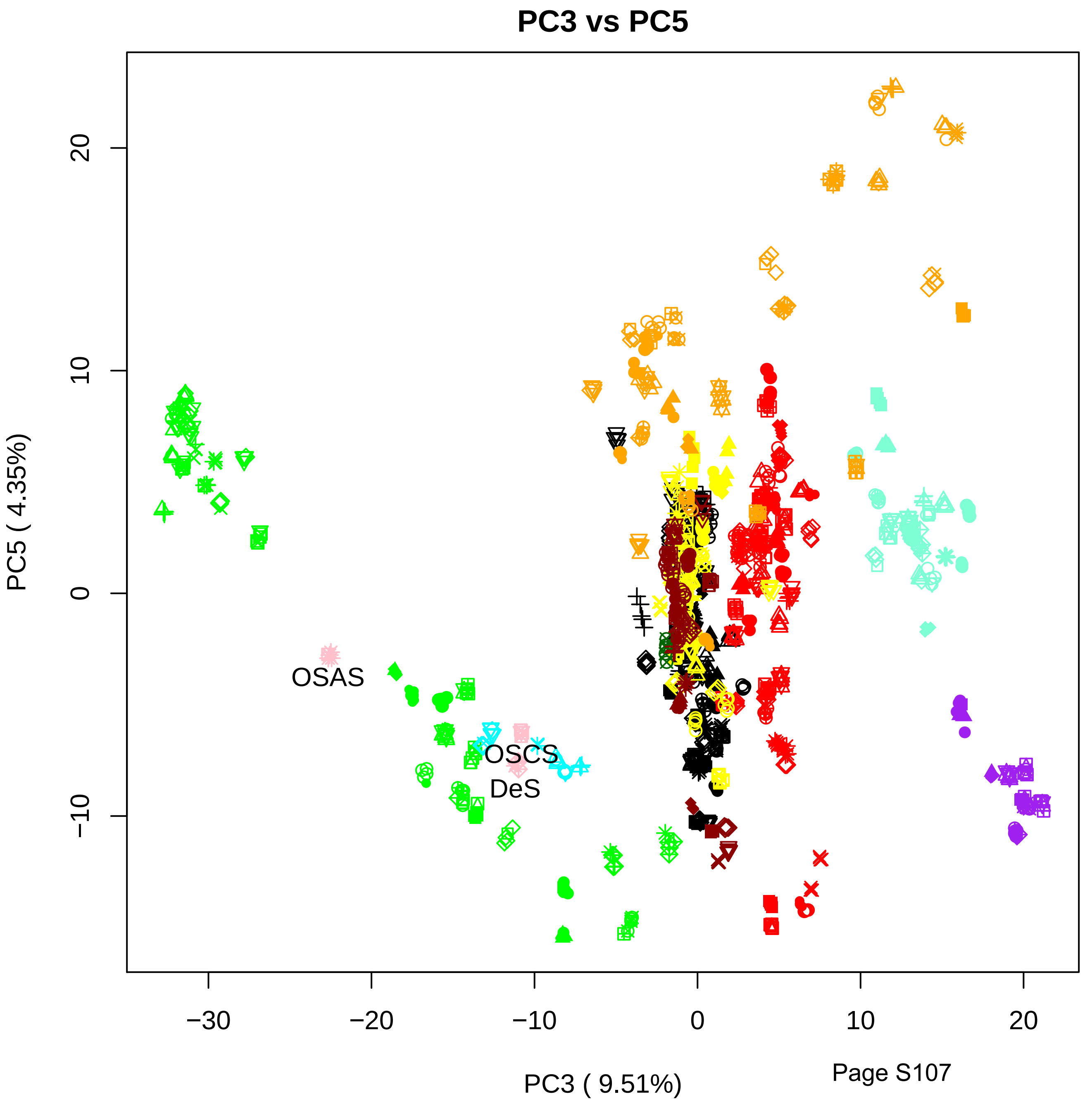
PC3 vs PC5
PC5 (4.35%)
PC3 (9.51%)
20
10
0
−10
−30
−20
−10
0
10
20
OSAS
OSCS
DeS

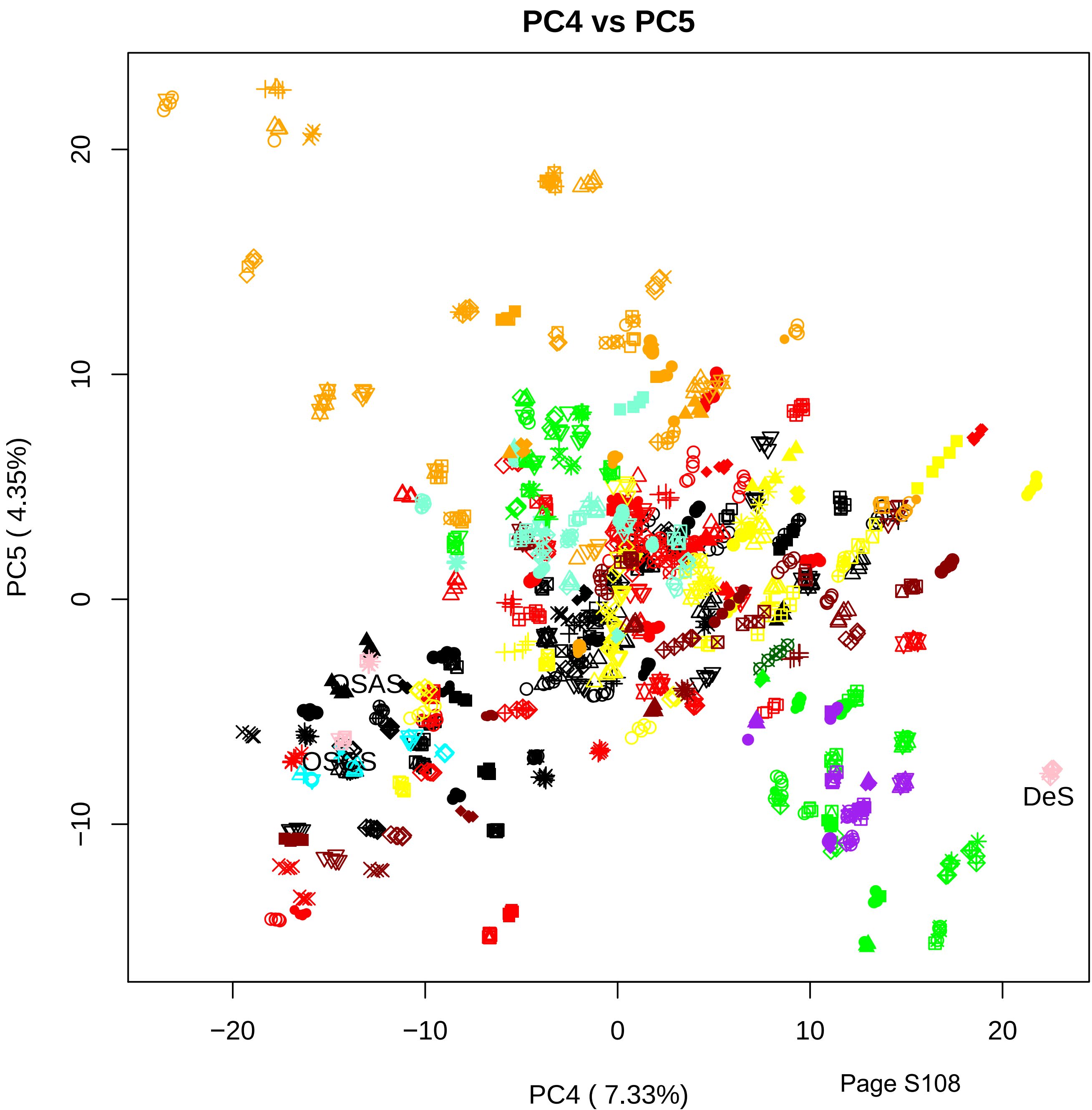
PC4 vs PC5
PC5 (4.35%)
PC4 (7.33%)
20
10
0
−10
−20
−10
0
10
20
OSAS
OSAS
DeS

OSCS Contam 7th

Anthony Devlin, Keele University

PCA of All OSCS vs PMH

Scaled Matrix PCAed

7th order polynomial baseline correction performed

Raw spectra smoothed to 21 Neighbours, 2nd order PN, Savtisky Golay

Normalisation between 0 and 100

D2 spectra smoothed to 41 Neighbours, 2nd order PN, Savtisky Golay

Regions Analysed: 3601−2501 and 2001−700cm−1

Samples Analysed: 12 Contam's 1 OSCS's 45 PMH's

Keele University AD   Sun Dec 09 16:50:25 2018

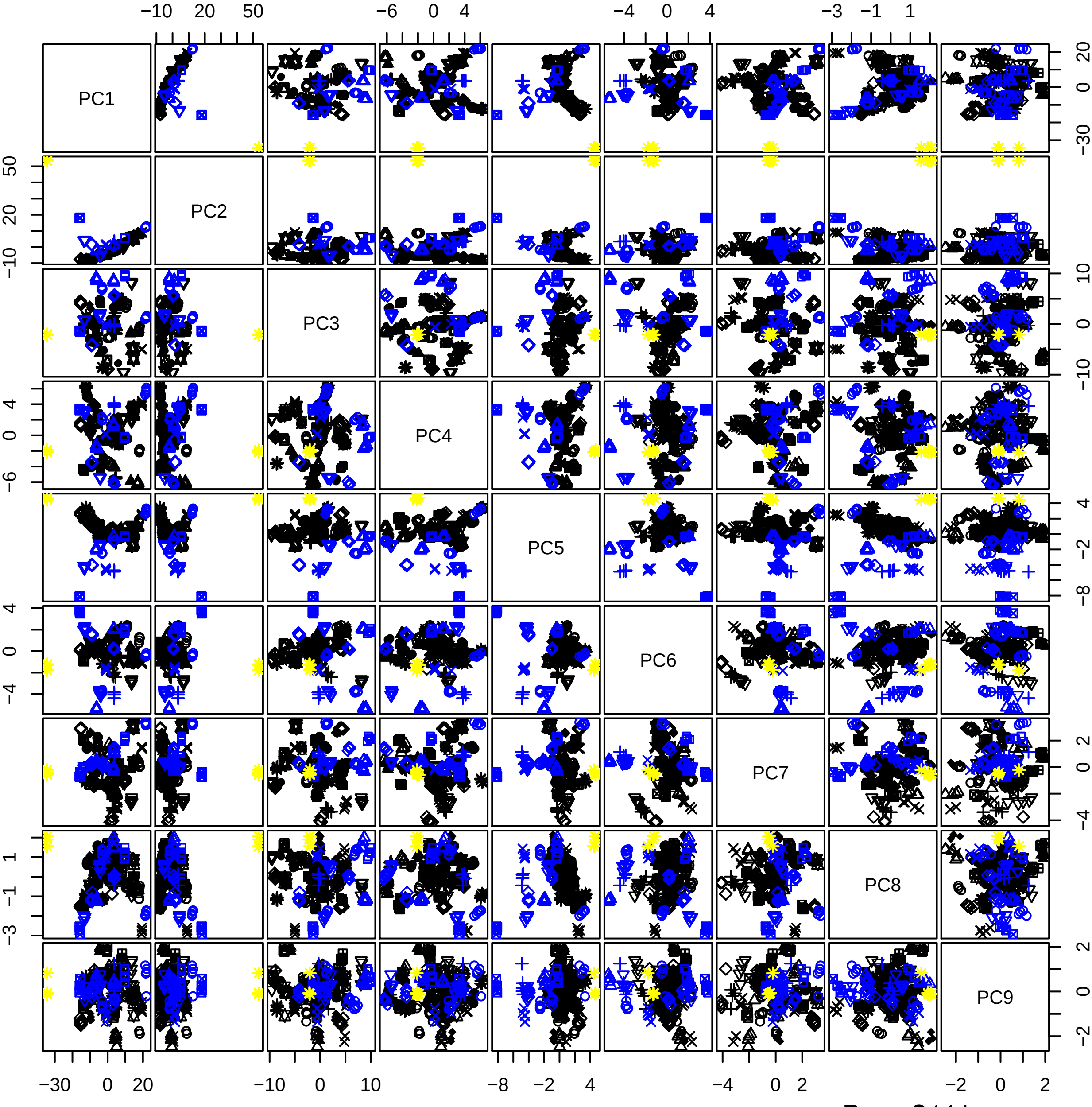
PC1
PC2
PC3
PC4
PC5
PC6
PC7
PC8
PC9
−10 20 50
−6 0 4
−4 0 4
−3 −1 1
20 0 −30
50 20 −10
10 0 −10
4 0 −6
4 −2 −8
4 0 −4
2 0 −4
1 −1 −3
2 0 −2
−30 0 20
−10 0 10
−8 −2 4
−4 0 2
−2 0 2

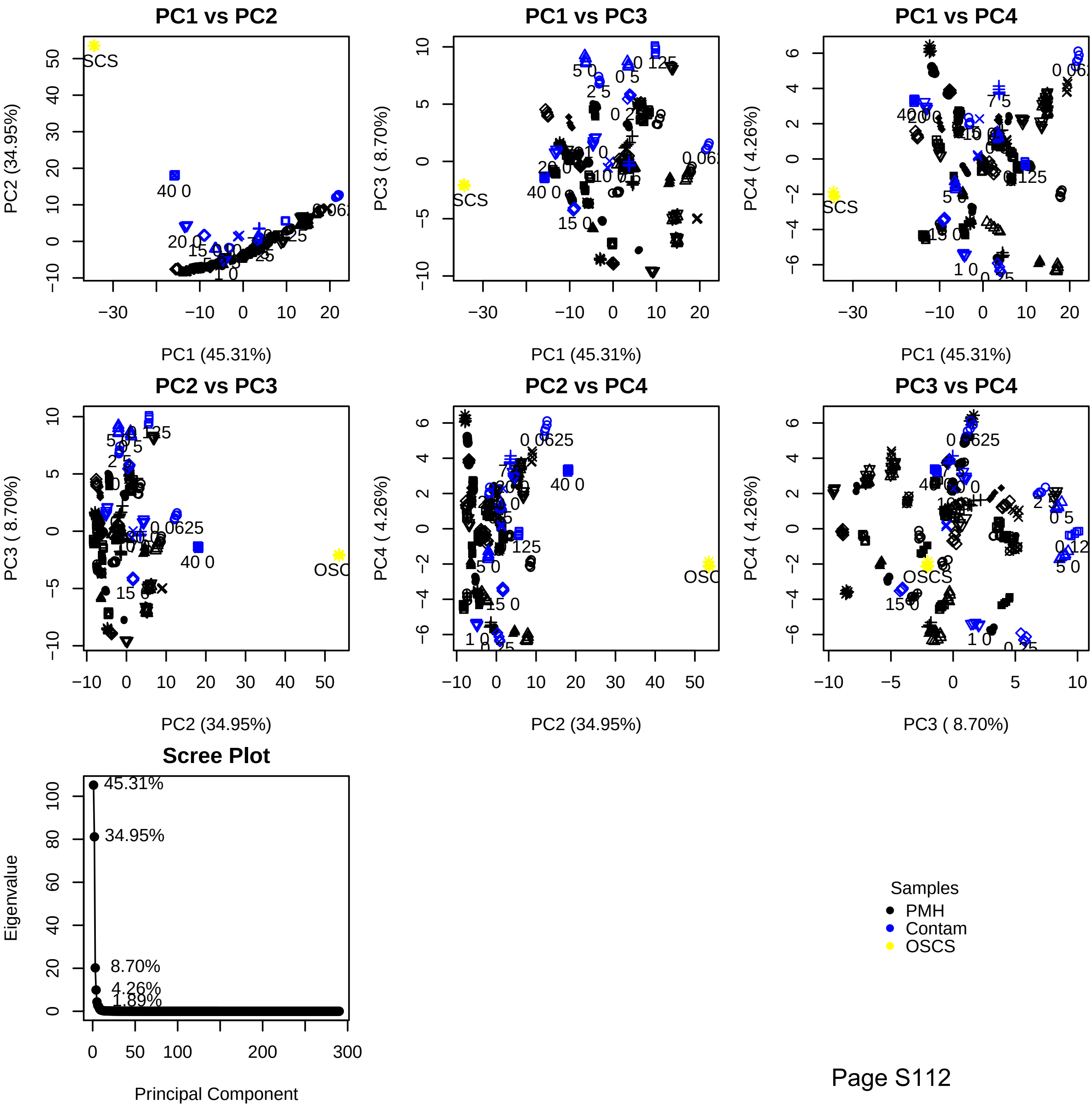

PC1 vs PC2
PC1 vs PC3
PC1 vs PC4
PC2 vs PC3
PC2 vs PC4
PC3 vs PC4
Scree Plot
PC1 (45.31%)
PC2 (34.95%)
PC3 (8.70%)
PC4 (4.26%)
Eigenvalue
Principal Component
45.31%
34.95%
8.70%
4.26%
1.89%
Samples
PMH
Contam
OSCS

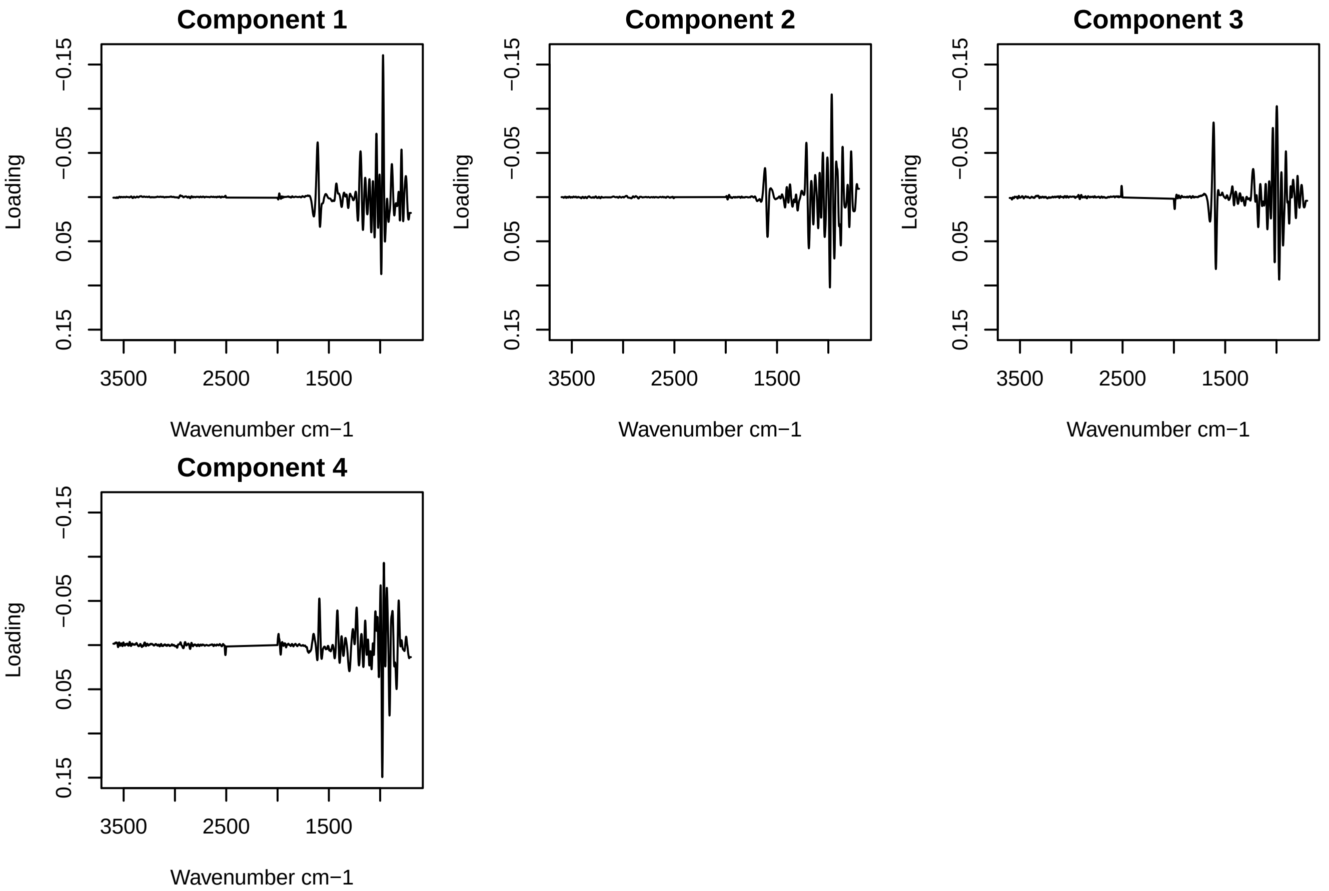

Component 1
Component 2
Component 3
Component 4
Loading
−0.15
−0.05
0.05
0.15
3500
2500
1500
Wavenumber cm−1

PC1 vs PC2 vs PC3

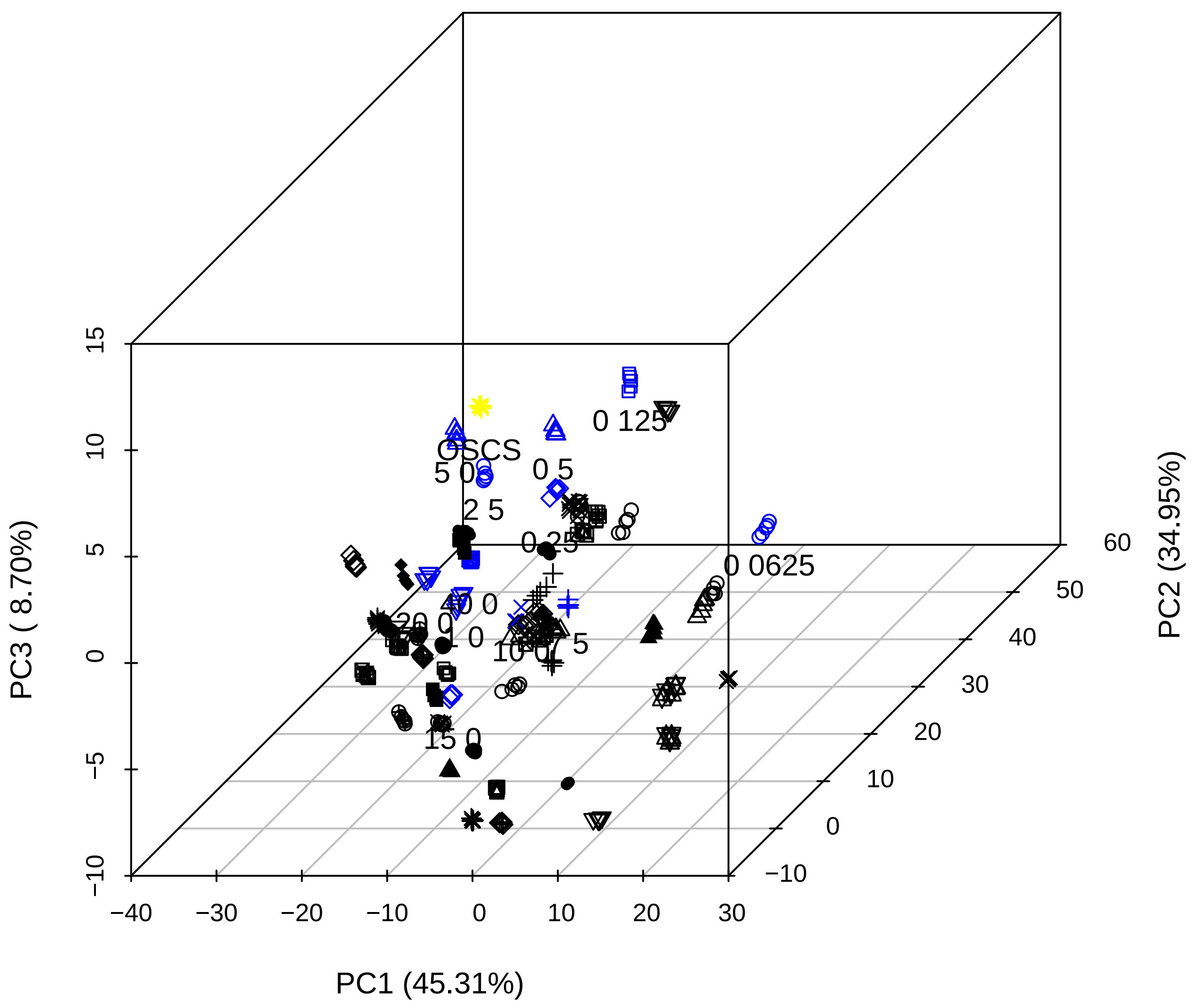

PC2 vs PC3 vs PC4

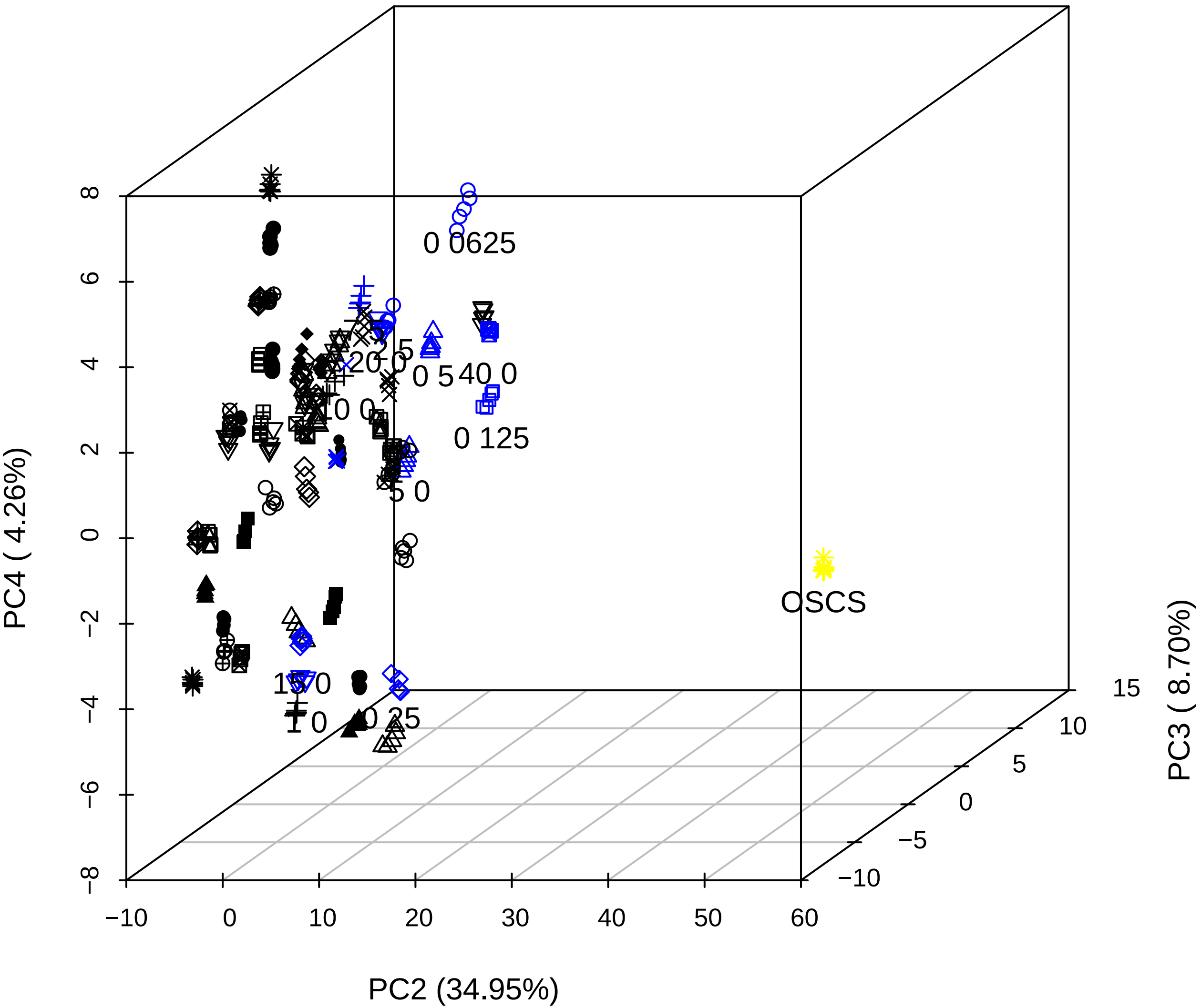

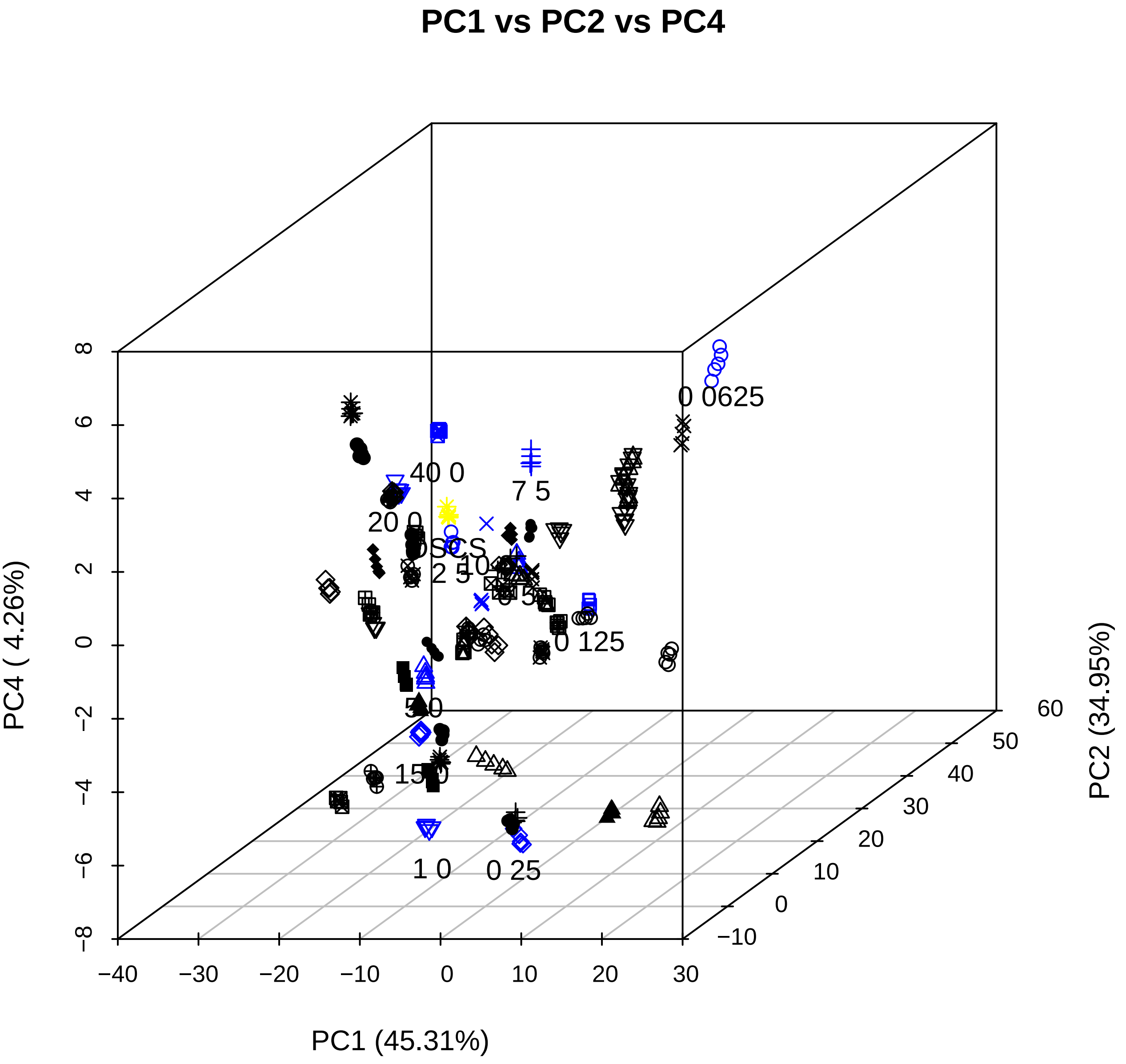
PC1 vs PC2 vs PC4
PC1 (45.31%)
PC2 (34.95%)
PC4 (4.26%)
−40
−30
−20
−10
0
10
20
30
−10
0
10
20
30
40
50
60
−8
−6
−4
−2
0
2
4
6
8
0 0625
40 0
7 5
20 0
DSCS
2 5
0 125
5 0
15 0
1 0
0 25

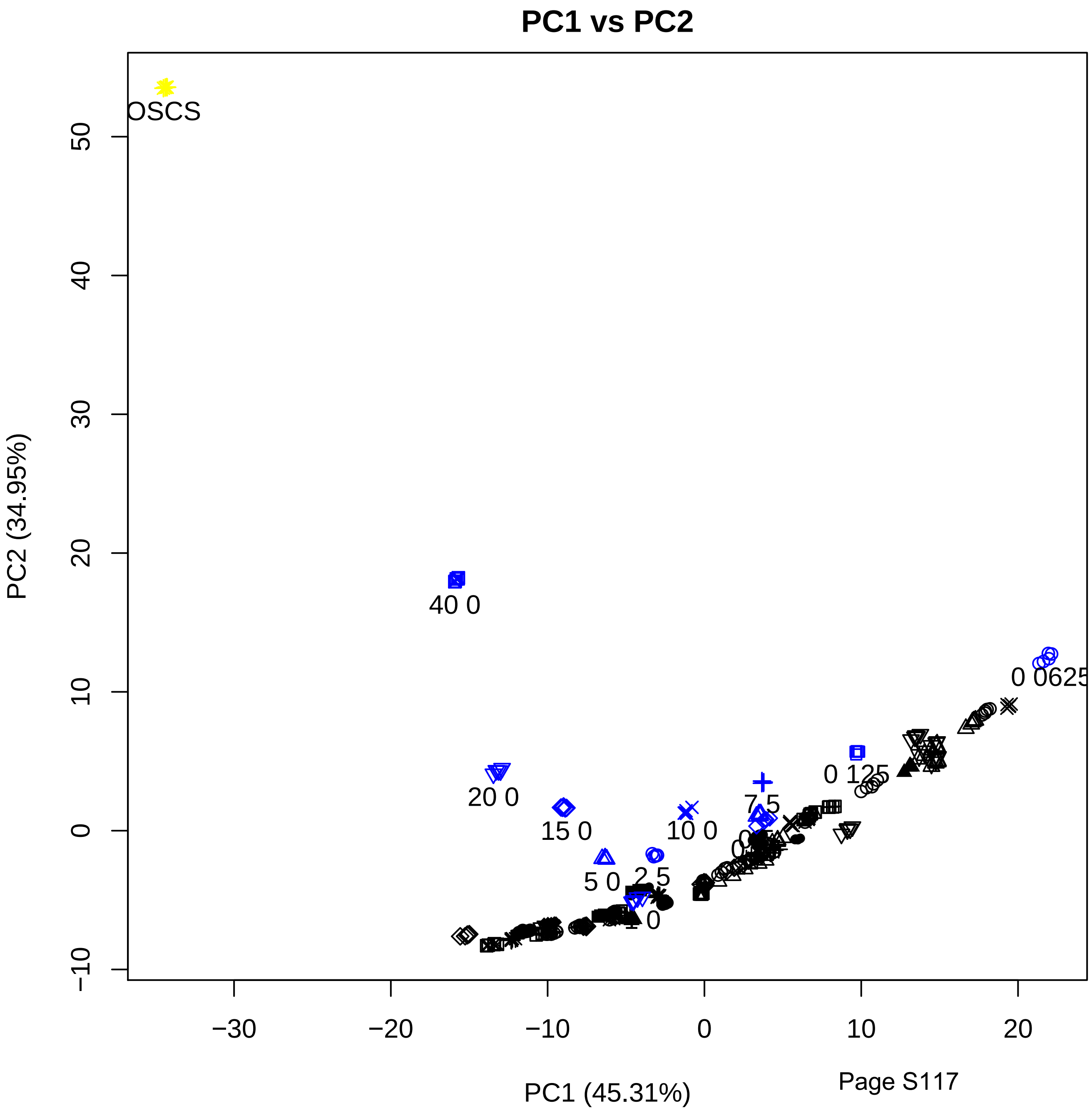
PC1 vs PC2
OSCS
40 0
20 0
15 0
10 0
5 0
2 5
1 0
7 5
0 125
0 0625
PC2 (34.95%)
PC1 (45.31%)
50
40
30
20
10
0
−10
−30
−20
−10
0
10
20

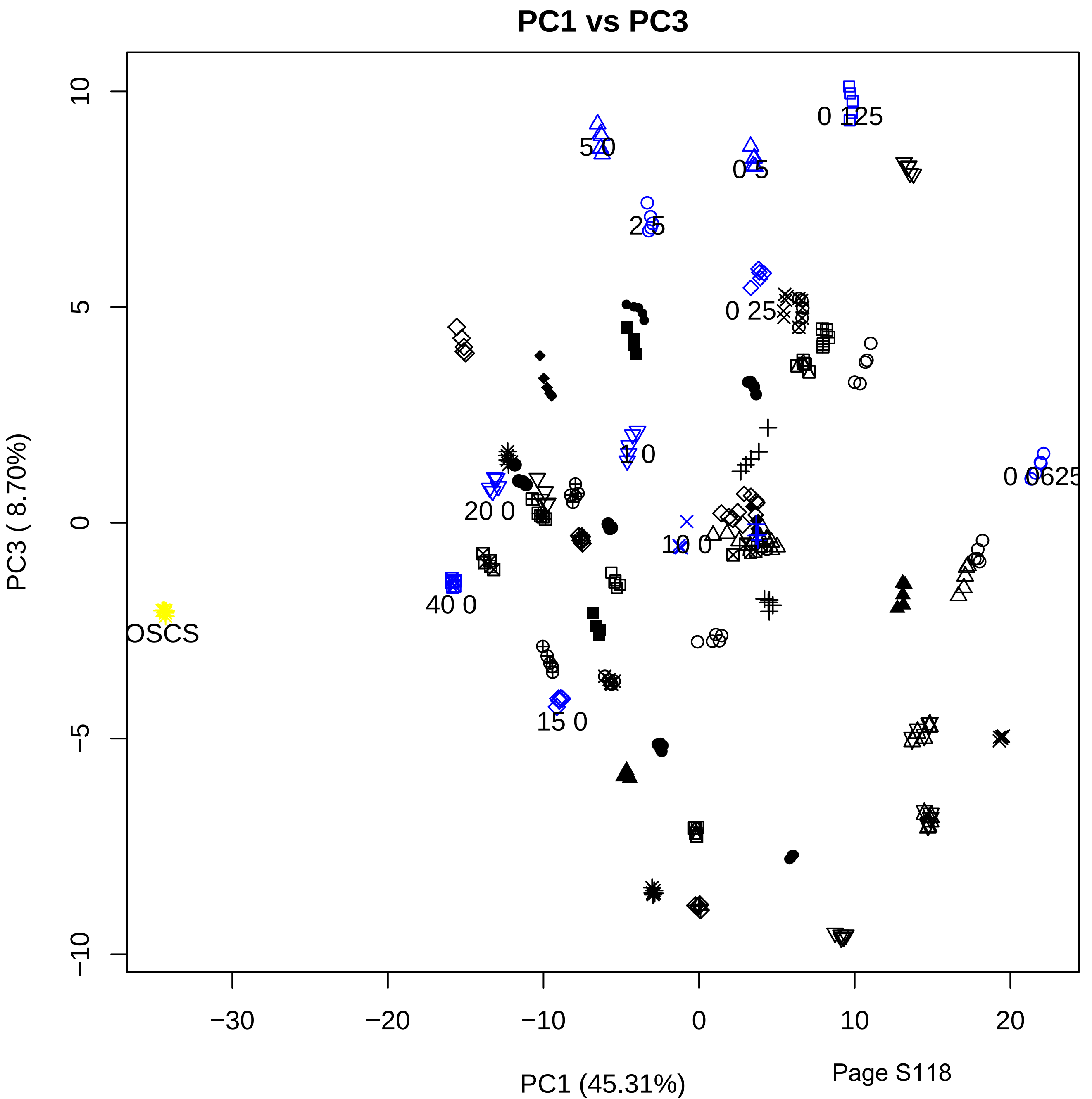

PC1 vs PC3
PC3 (8.70%)
PC1 (45.31%)
10
5
0
−5
−10
−30
−20
−10
0
10
20
0 125
5 0
0 5
2 5
0 25
1 0
0 0625
20 0
10 0
40 0
OSCS
15 0

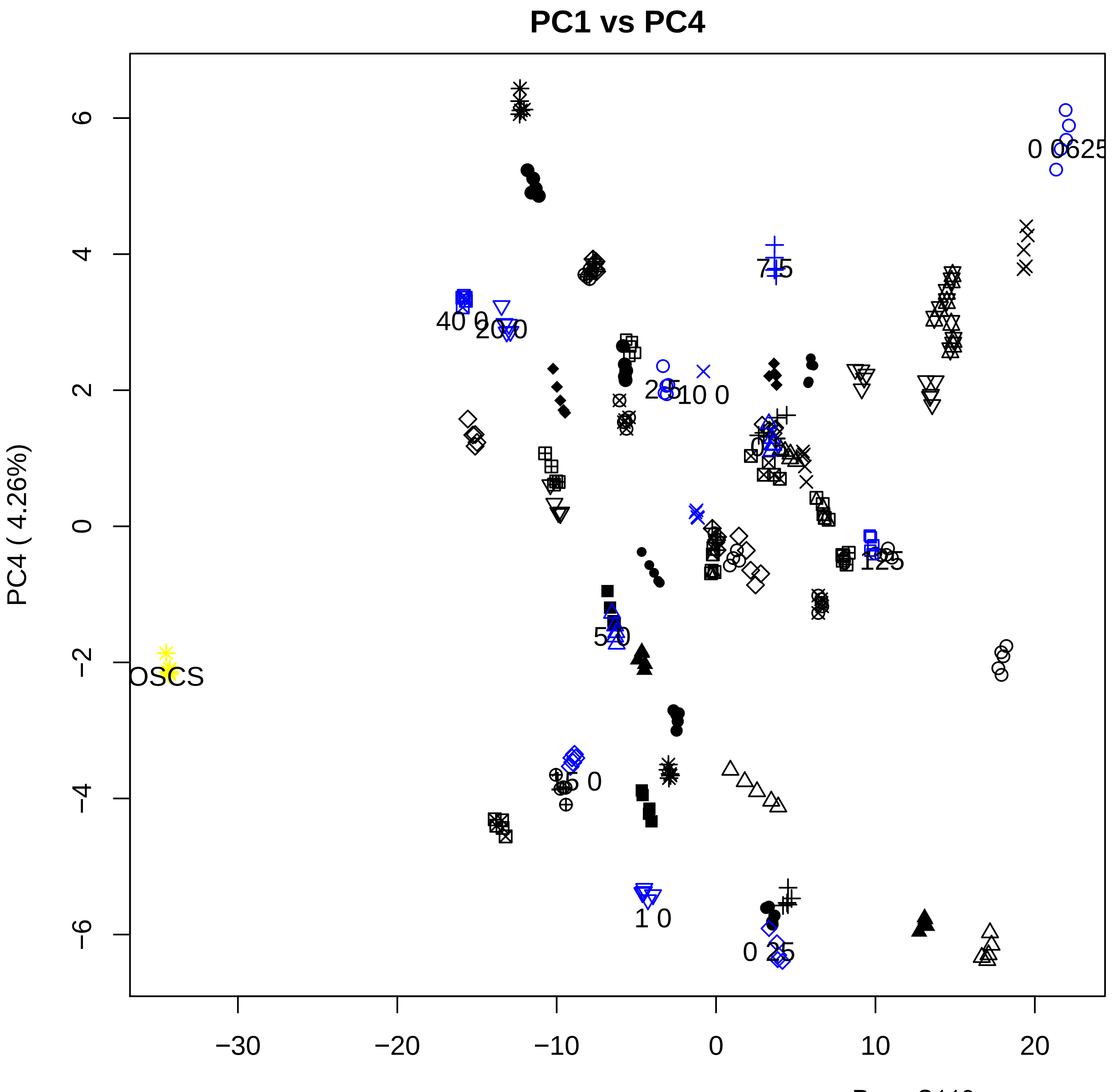
PC1 vs PC4
PC4 (4.26%)
PC1 (45.31%)
6
4
2
0
−2
−4
−6
−30
−20
−10
0
10
20
0 0625
7 5
40 0
20 0
2 5
10 0
1 25
5 0
OSCS
15 0
1 0
0 25

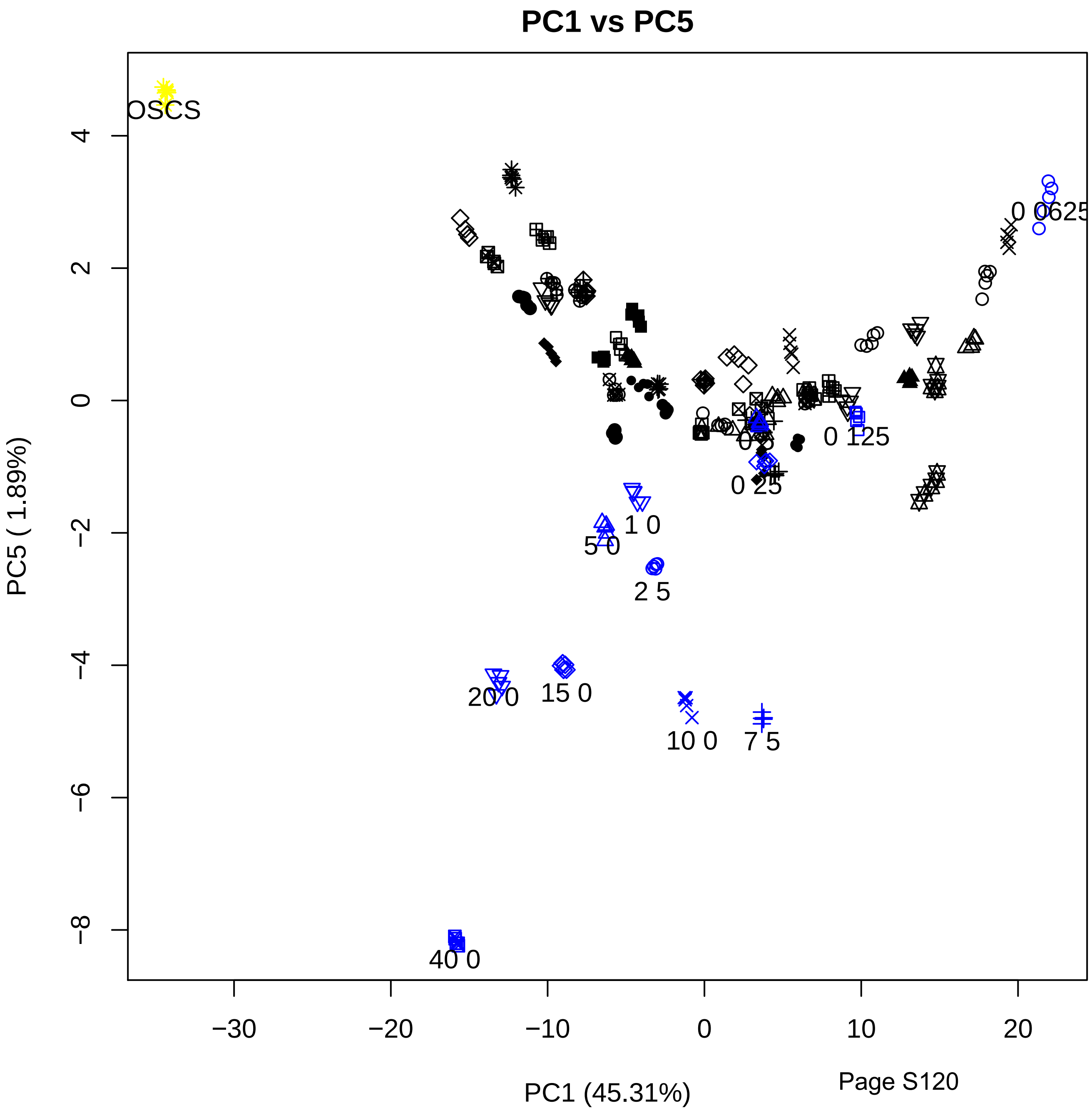
PC1 vs PC5
OSCS
0 0625
0 125
0 5
0 25
1 0
5 0
2 5
20 0
15 0
10 0
7 5
40 0
PC5 (1.89%)
PC1 (45.31%)
4
2
0
−2
−4
−6
−8
−30
−20
−10
0
10
20

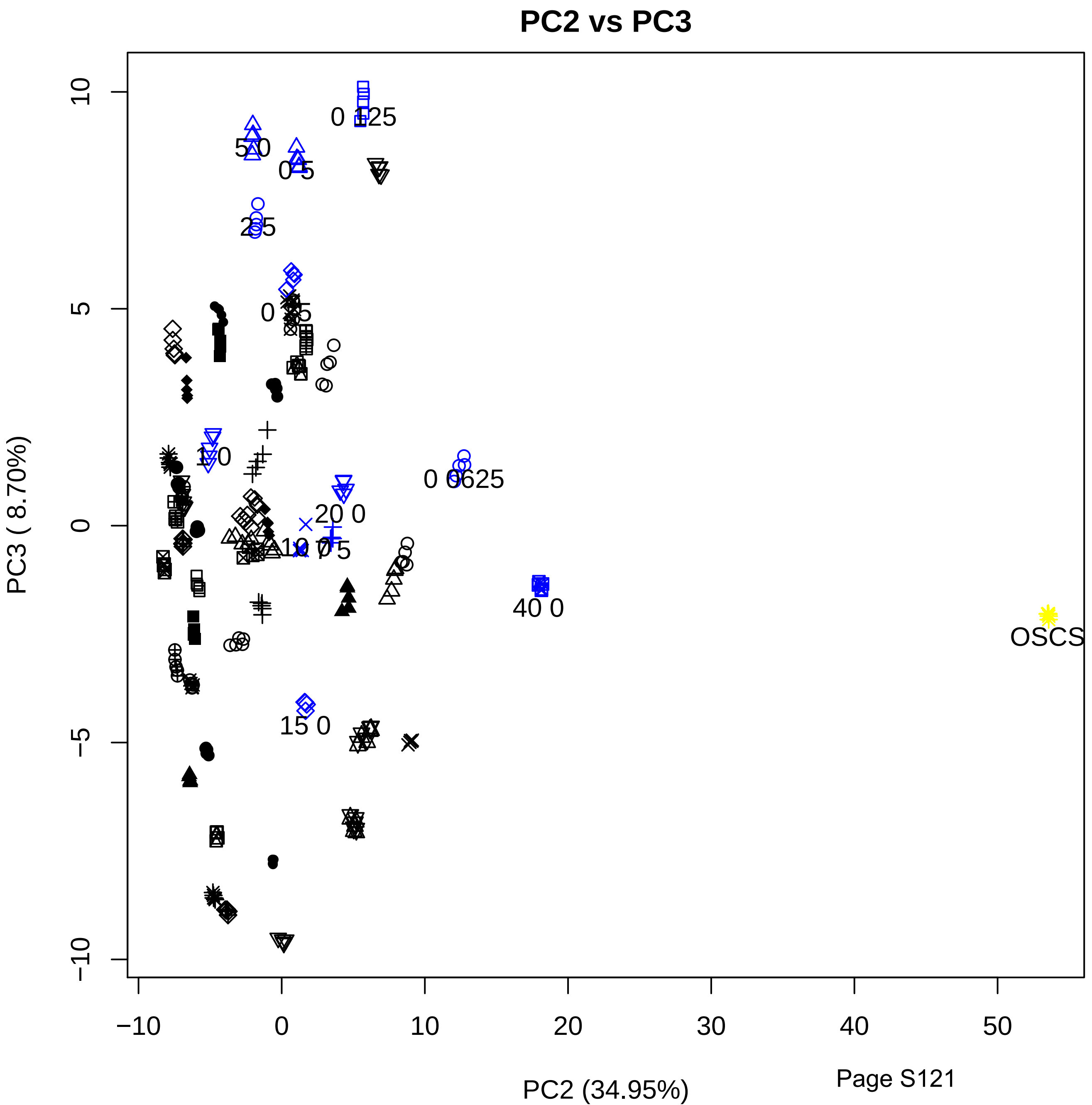
PC2 vs PC3
PC3 (8.70%)
PC2 (34.95%)
10
5
0
−5
−10
−10
0
10
20
30
40
50
0 125
5 0
0 5
2 5
1 0
0 0625
20 0
40 0
15 0
OSCS

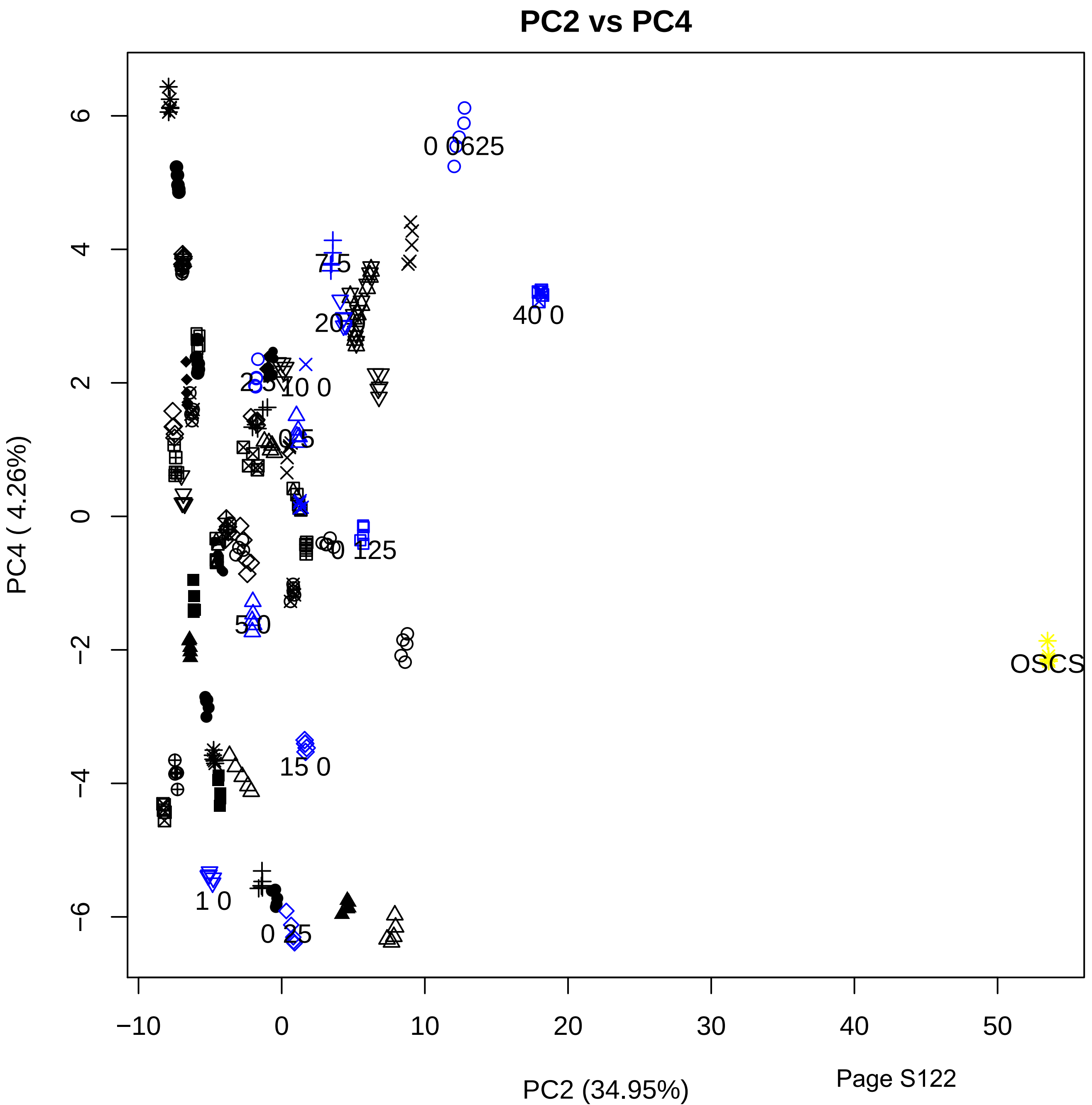
PC2 vs PC4
PC4 (4.26%)
PC2 (34.95%)
6
4
2
0
−2
−4
−6
−10
0
10
20
30
40
50
0 0625
7 5
40 0
10 0
0 125
5 0
15 0
1 0
0 25
OSCS

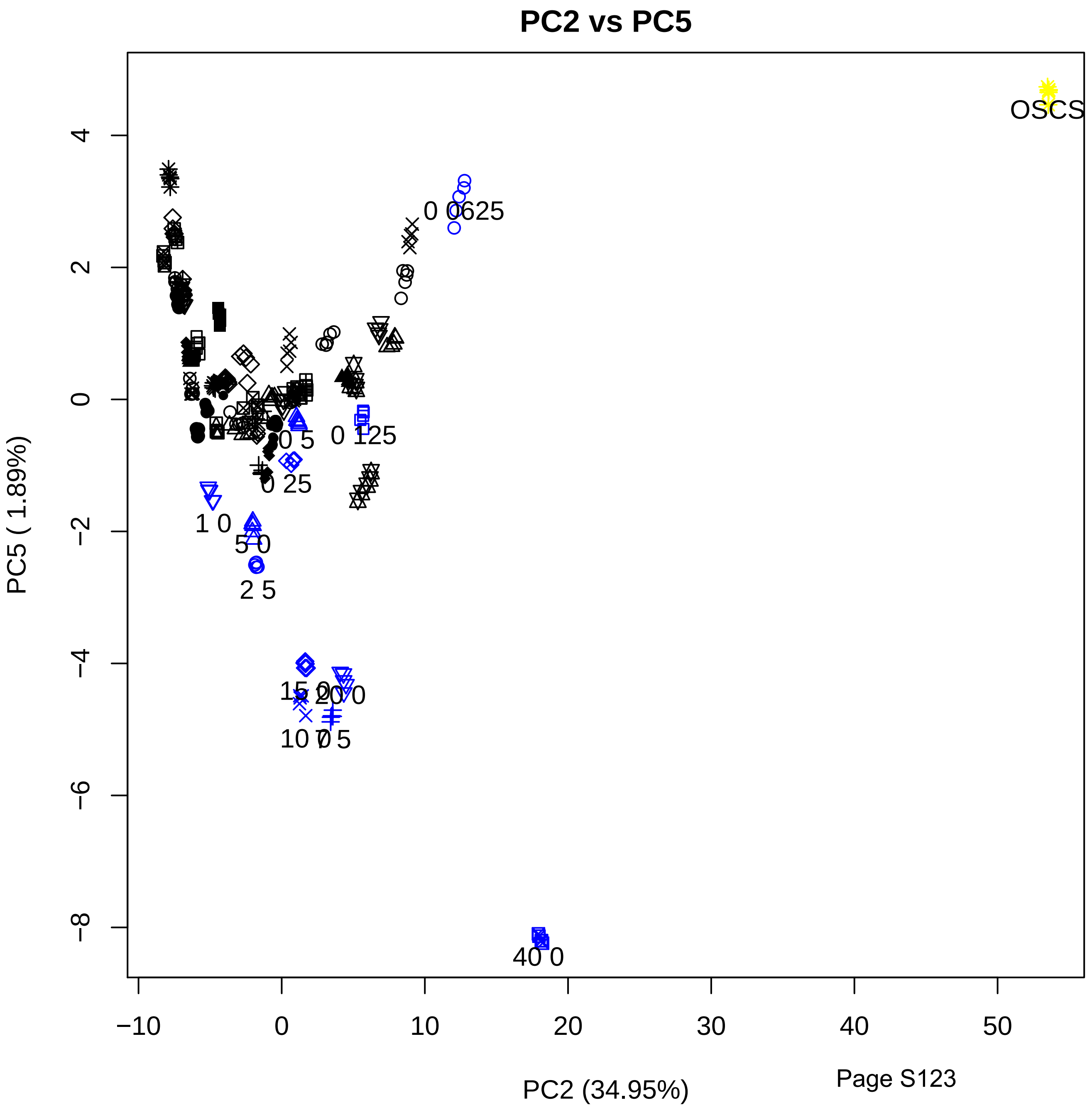
PC2 vs PC5
PC5 (1.89%)
PC2 (34.95%)
4
2
0
−2
−4
−6
−8
−10
0
10
20
30
40
50
OSCS
0 0625
0 125
0 5
0 25
1 0
5 0
2 5
15 0
20 0
10 0
7 5
40 0

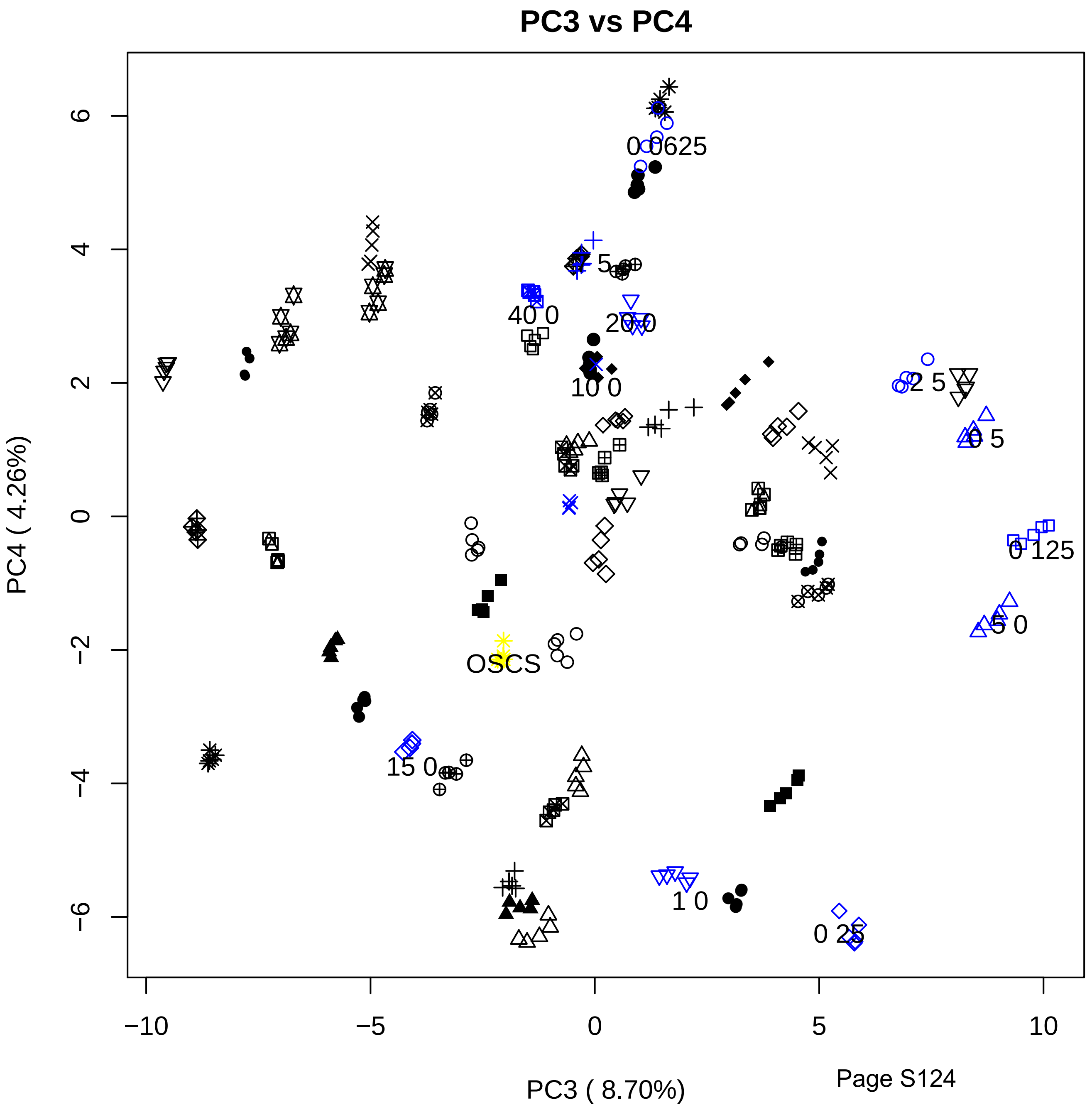
PC3 vs PC4
PC4 (4.26%)
PC3 (8.70%)
0 0625
5
40 0
20 0
10 0
2 5
0 5
0 125
5 0
OSCS
15 0
1 0
0 25

PC3 vs PC5

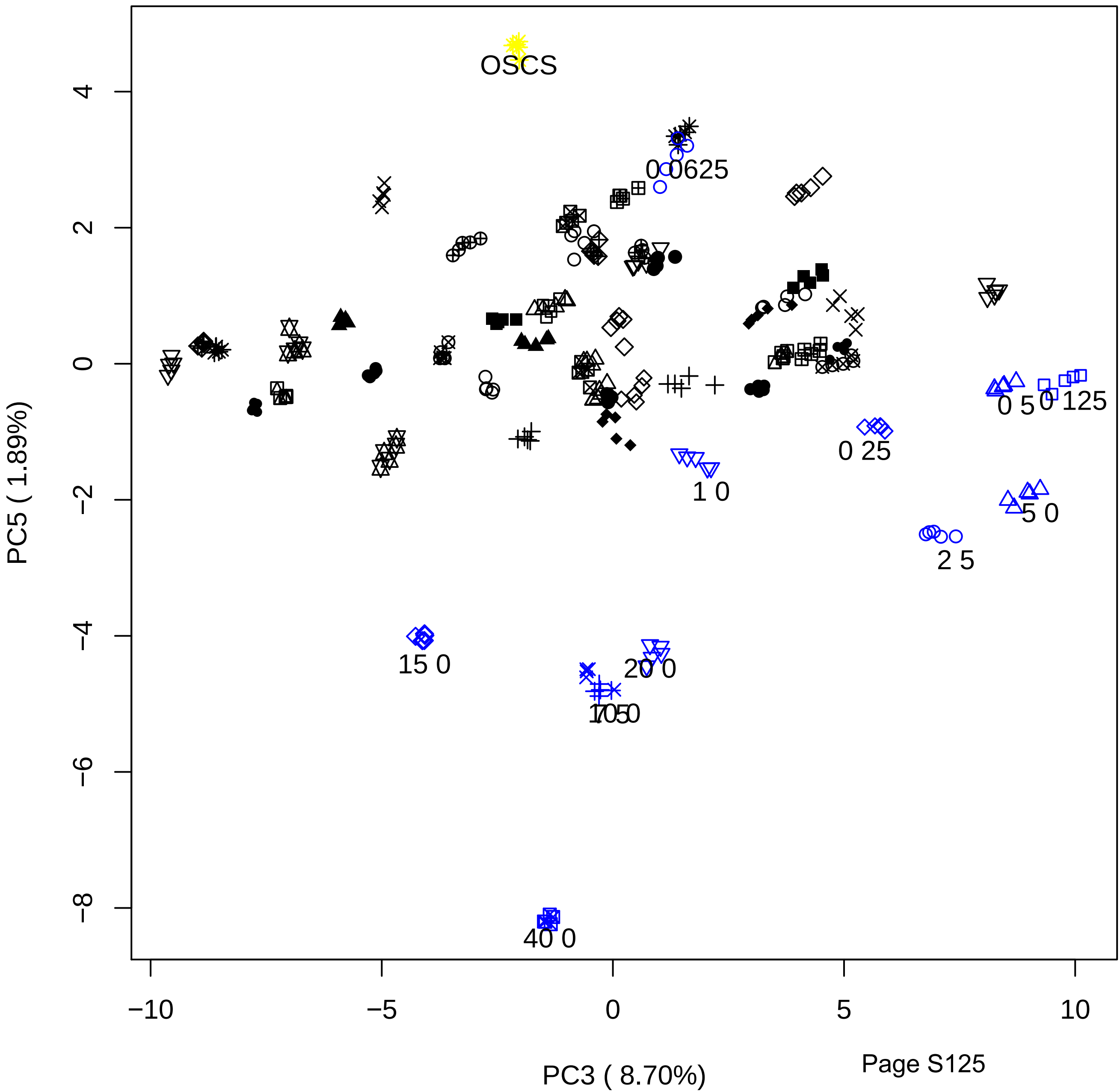
OSCS
0 0625
0 25
0 5
0 125
1 0
5 0
2 5
15 0
20 0
10
5 0
40 0
4
2
0
−2
−4
−6
−8
PC5 (1.89%)
−10
−5
0
5
10
PC3 (8.70%)

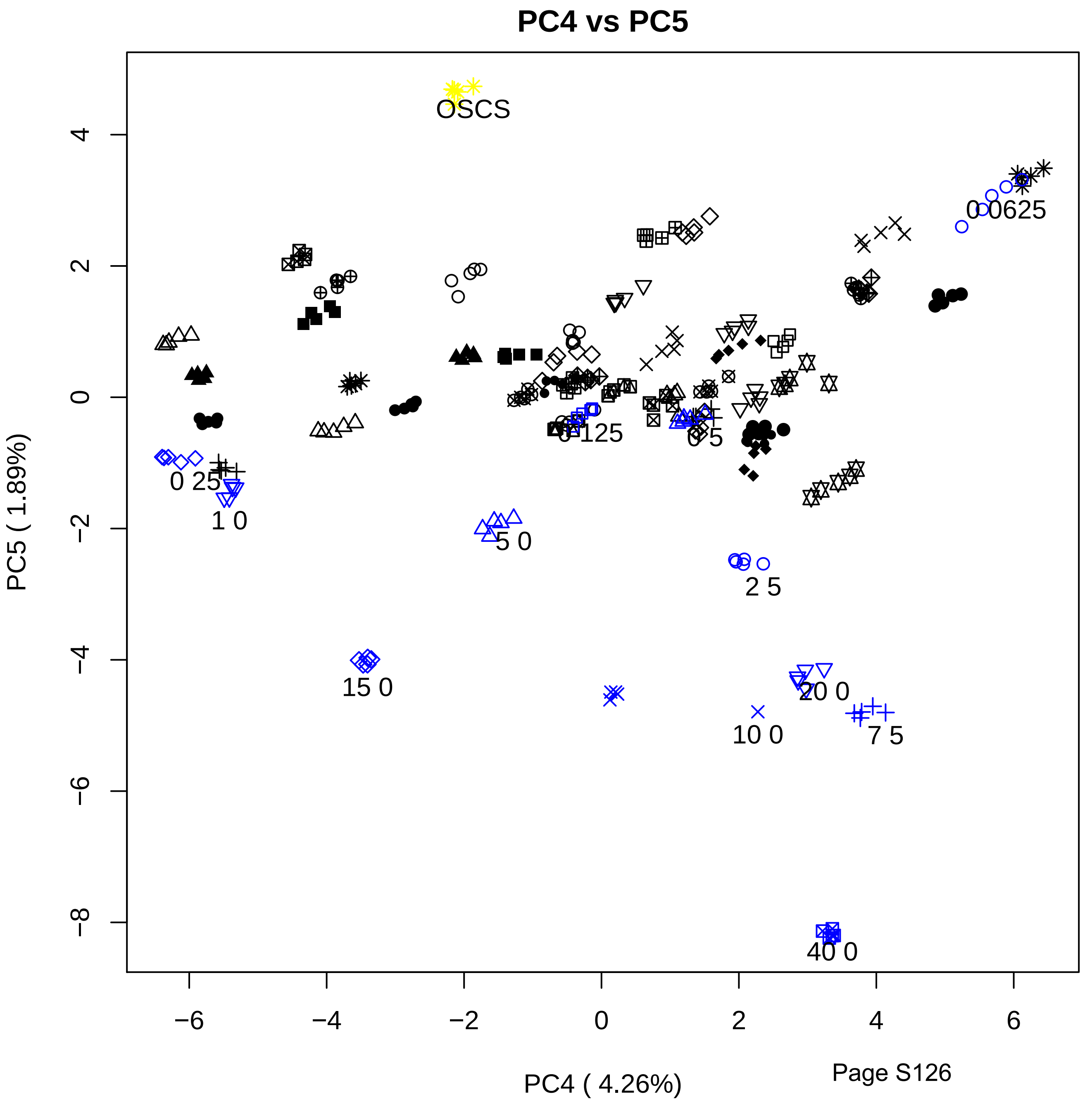
PC4 vs PC5
OSCS
0 0625
0 125
0 5
0 25
1 0
5 0
2 5
15 0
20 0
10 0
7 5
40 0
4
2
0
−2
−4
−6
−8
−6
−4
−2
0
2
4
6
PC5 (1.89%)
PC4 (4.26%)

PCA of 0.06% OSCS vs PMH

Scaled Matrix PCAed

7th order polynomial baseline correction performed

Raw spectra smoothed to 21 Neighbours, 2nd order PN, Savtisky Golay

Normalisation between 0 and 100

D2 spectra smoothed to 41 Neighbours, 2nd order PN, Savtisky Golay

Regions Analysed: 3601−2501 and 2001−700cm−1

Samples Analysed: 1 Contam's 0 OSCS's 45 PMH's

Keele University AD   Sun Dec 09 16:50:32 2018

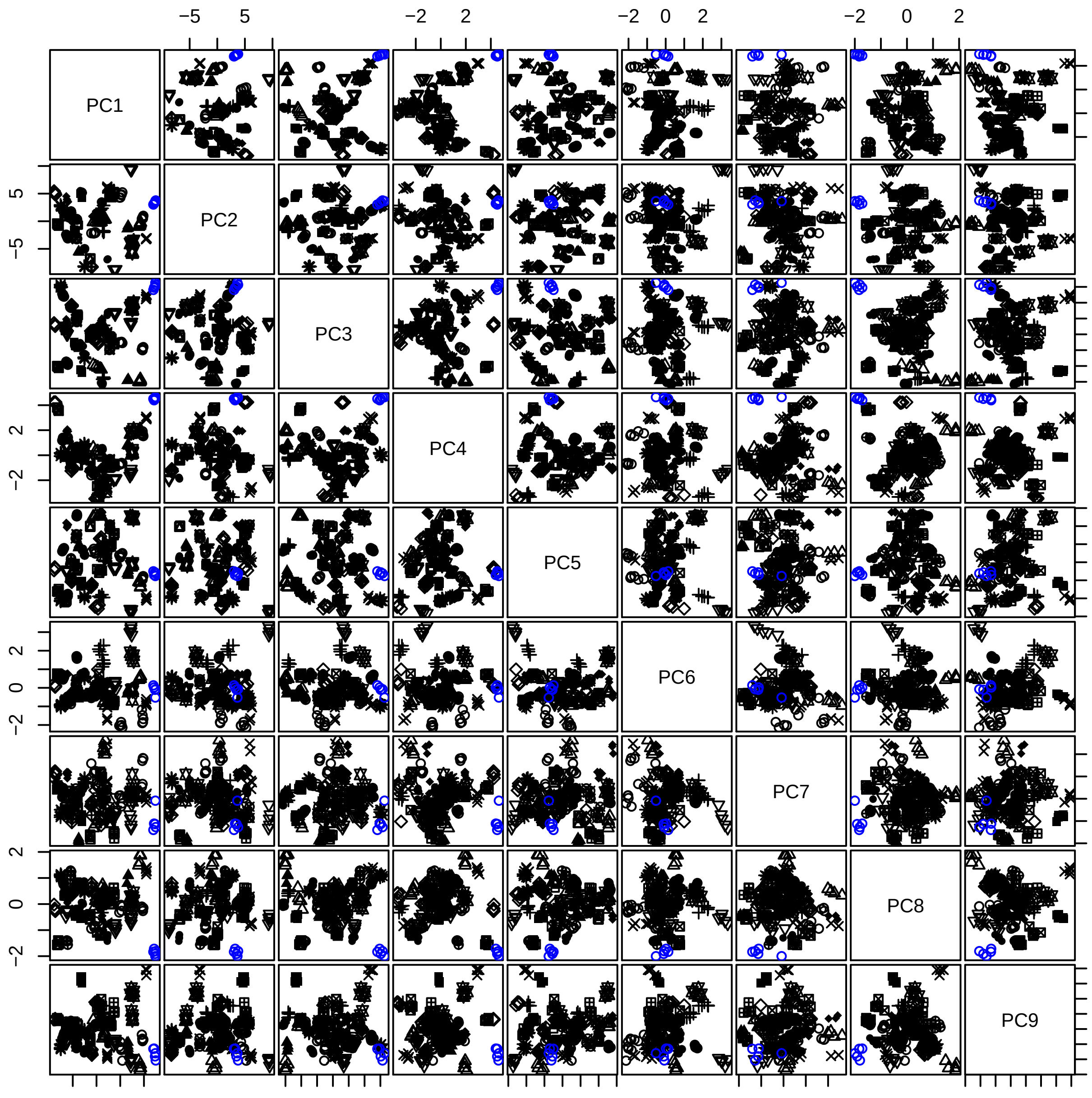
PC1
PC2
PC3
PC4
PC5
PC6
PC7
PC8
PC9

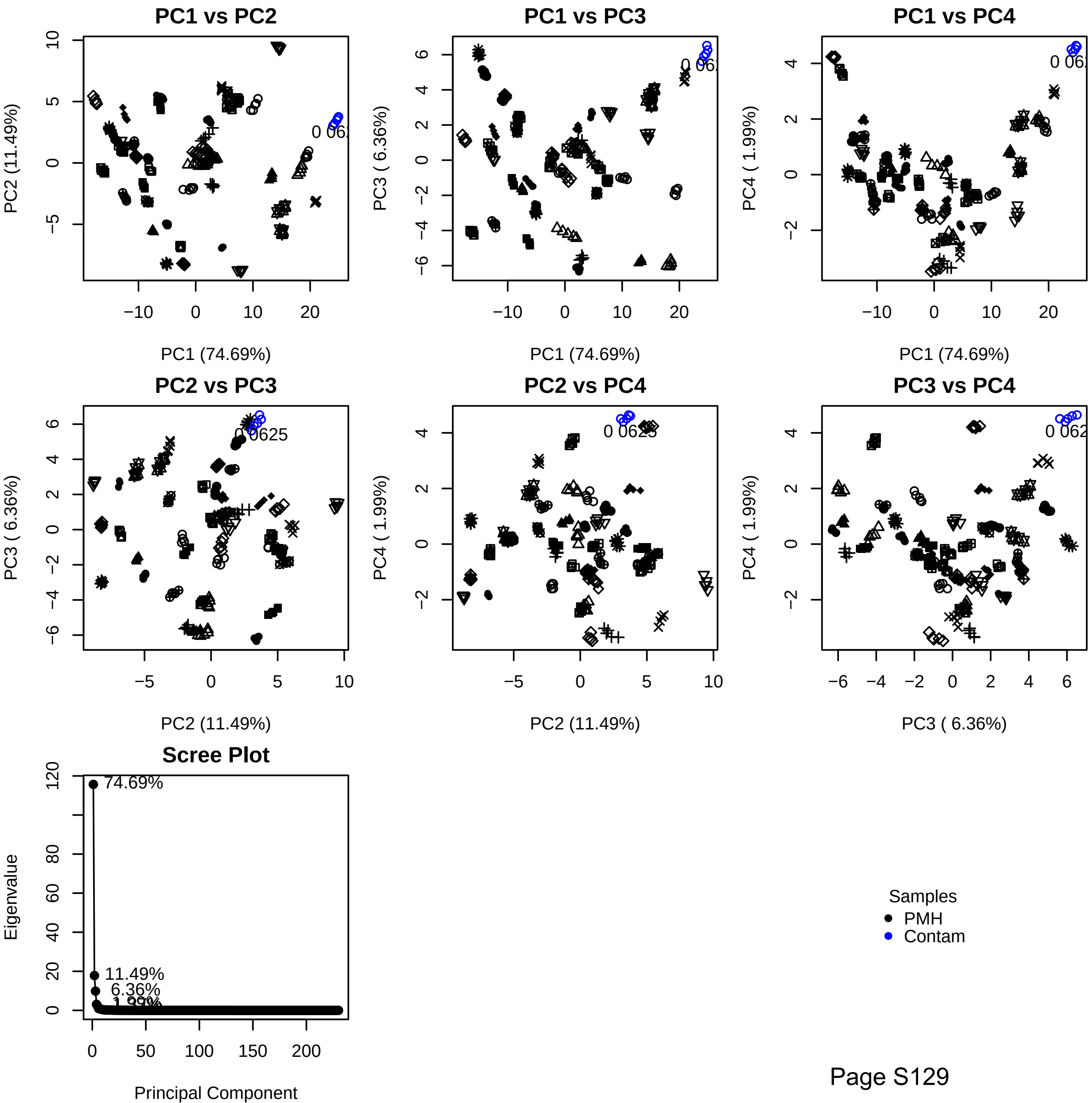
PC1 vs PC2
PC1 vs PC3
PC1 vs PC4
PC2 vs PC3
PC2 vs PC4
PC3 vs PC4
Scree Plot
PC1 (74.69%)
PC2 (11.49%)
PC3 (6.36%)
PC4 (1.99%)
Eigenvalue
Principal Component
74.69%
11.49%
6.36%
0 0625
Samples
PMH
Contam

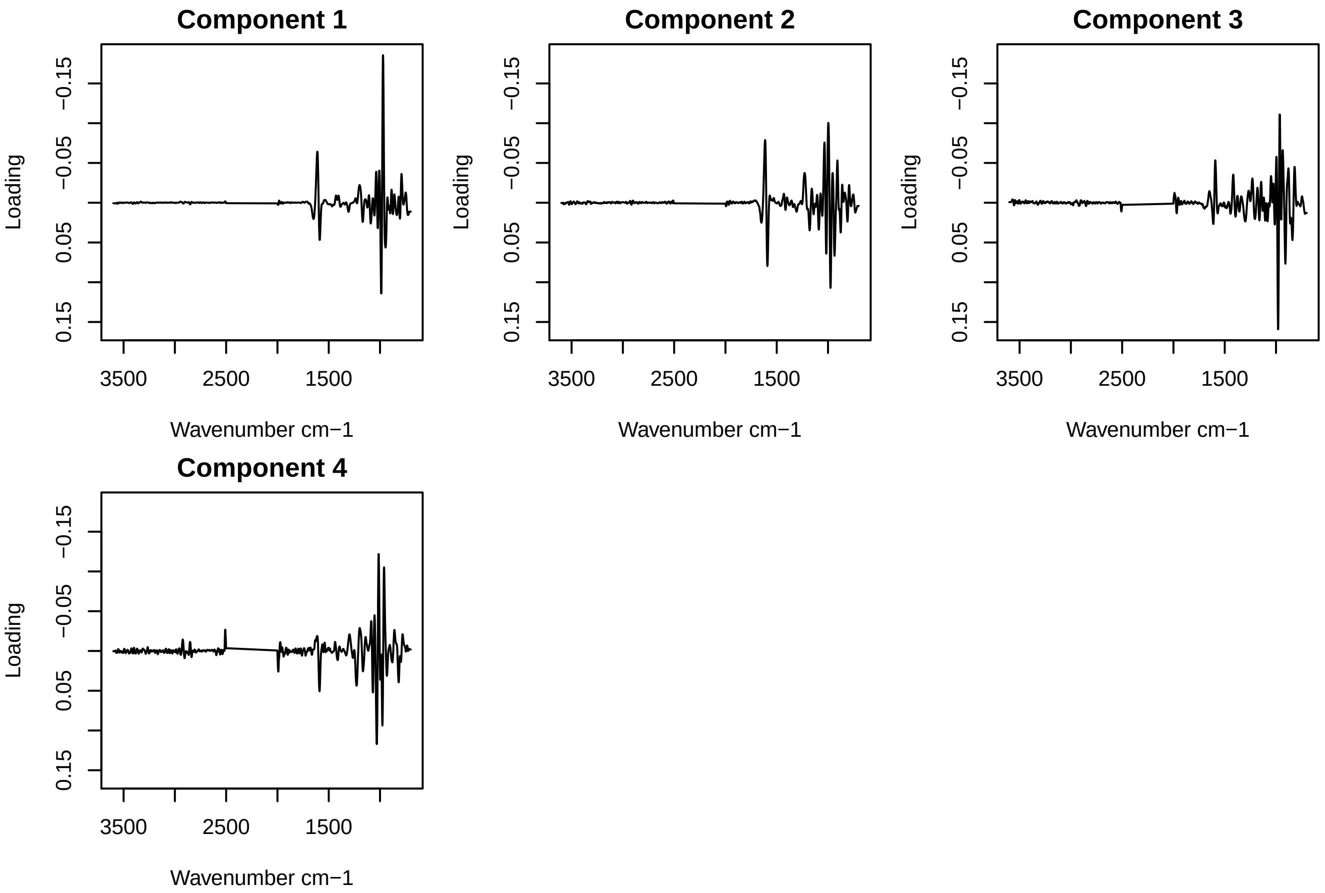

Component 1
Component 2
Component 3
Component 4
Loading
Wavenumber cm−1
−0.15
−0.05
0.05
0.15
3500
2500
1500

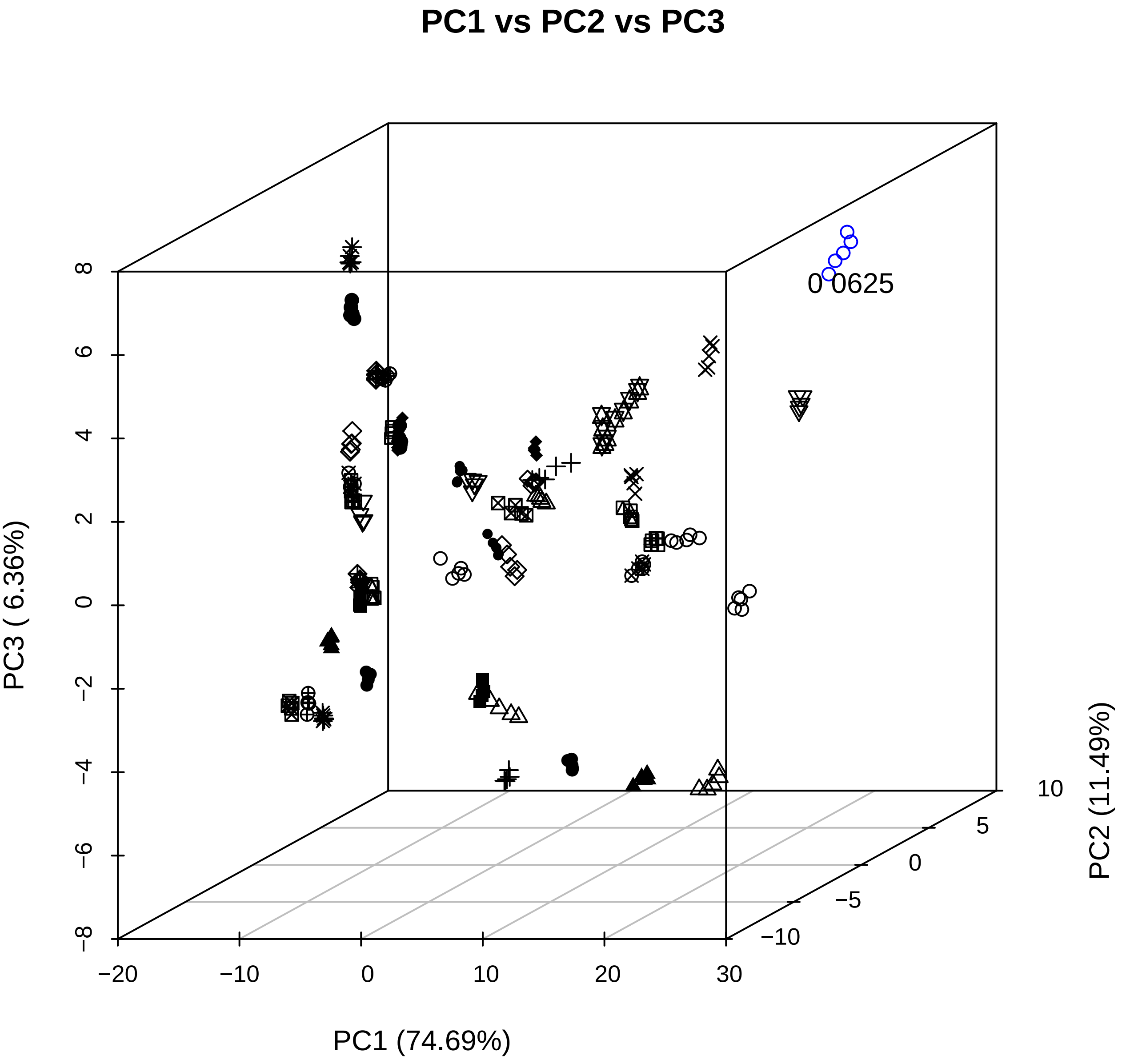

PC1 vs PC2 vs PC3
0 0625
PC1 (74.69%)
PC2 (11.49%)
PC3 (6.36%)
−20
−10
0
10
20
30
−10
−5
0
5
10
−8
−6
−4
−2
0
2
4
6
8

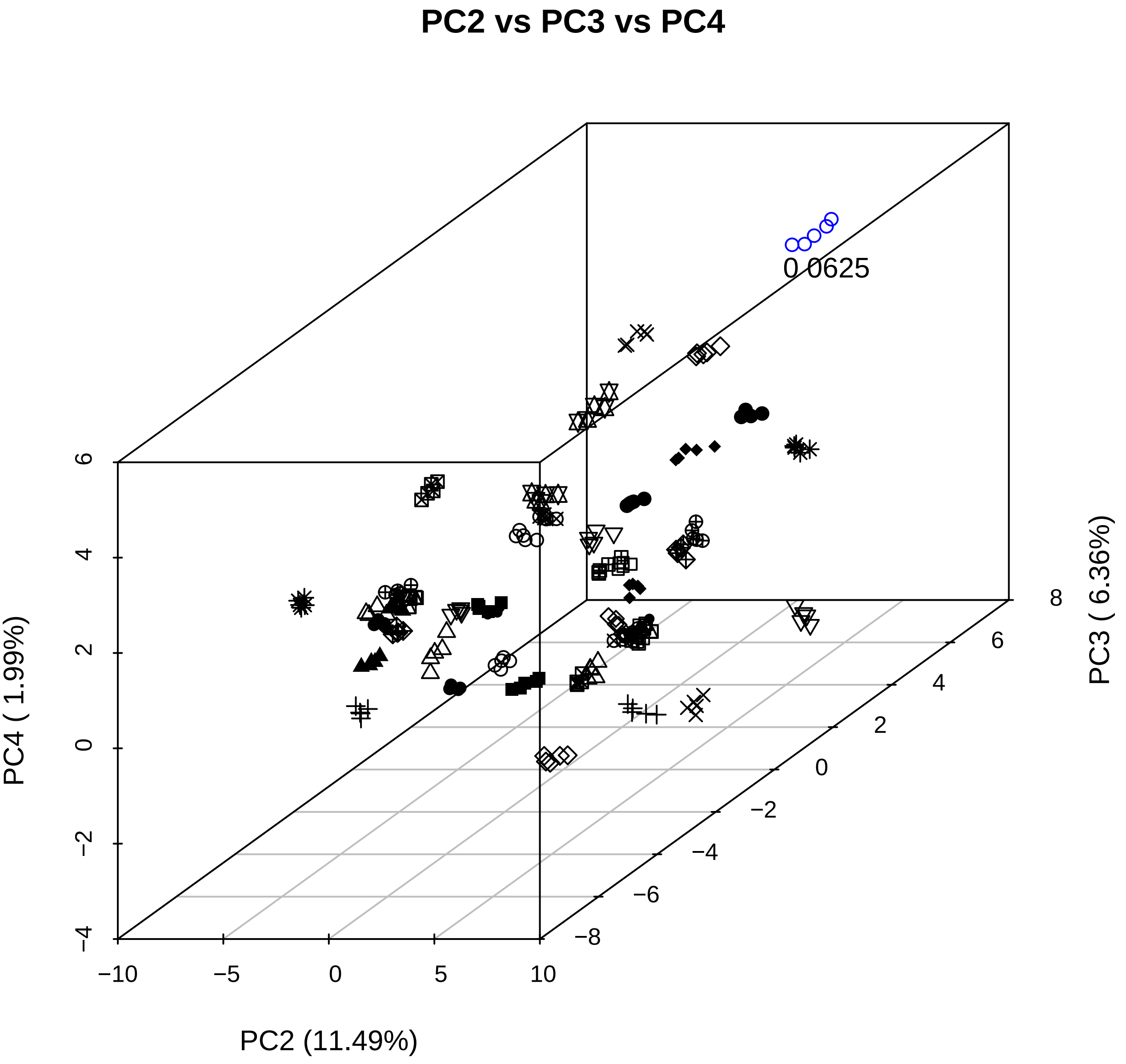
PC2 vs PC3 vs PC4
0.0625
PC2 (11.49%)
PC3 (6.36%)
PC4 (1.99%)
−10
−5
0
5
10
−8
−6
−4
−2
0
2
4
6
8
−4
−2
0
2
4
6

PC1 vs PC2 vs PC4

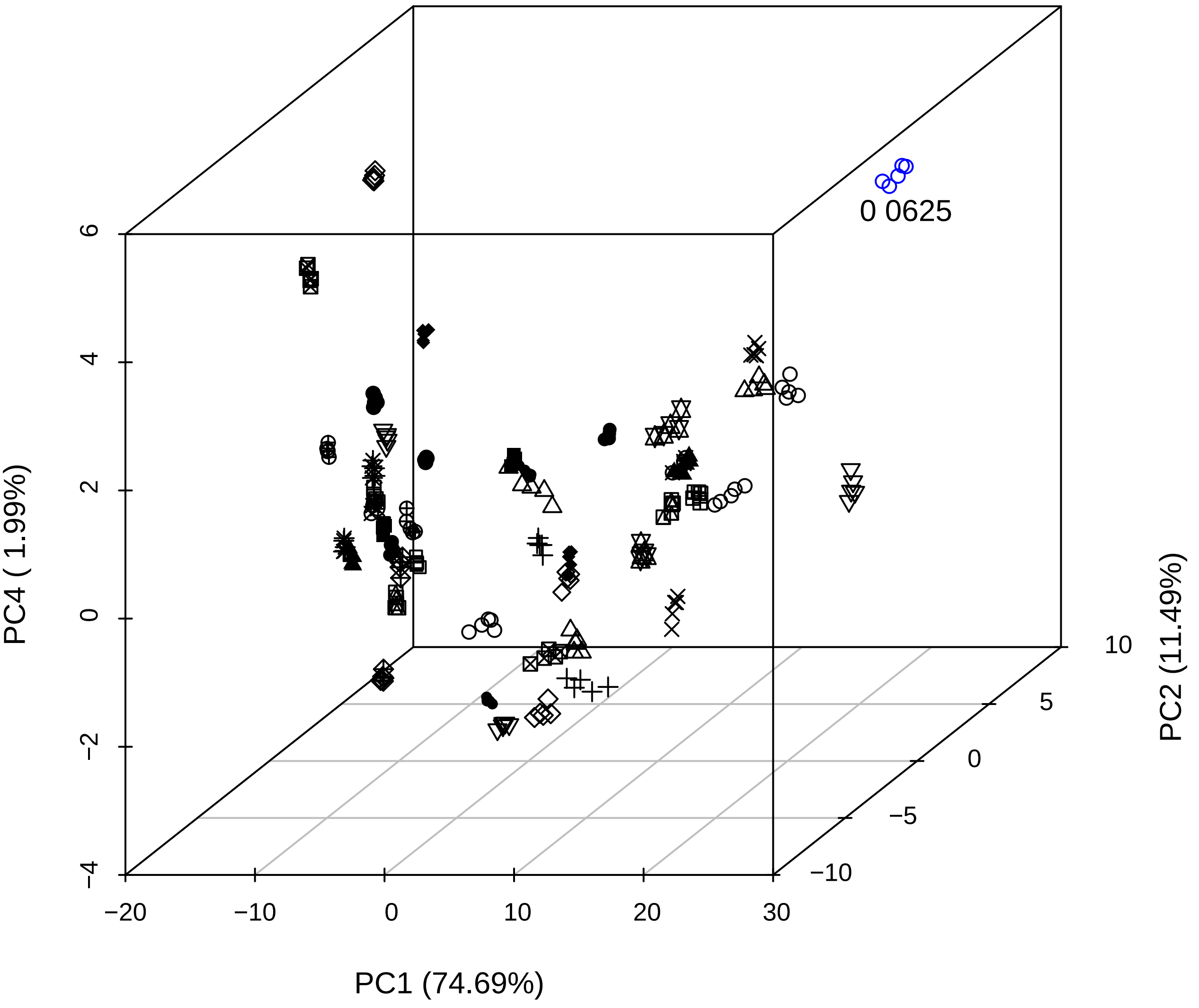

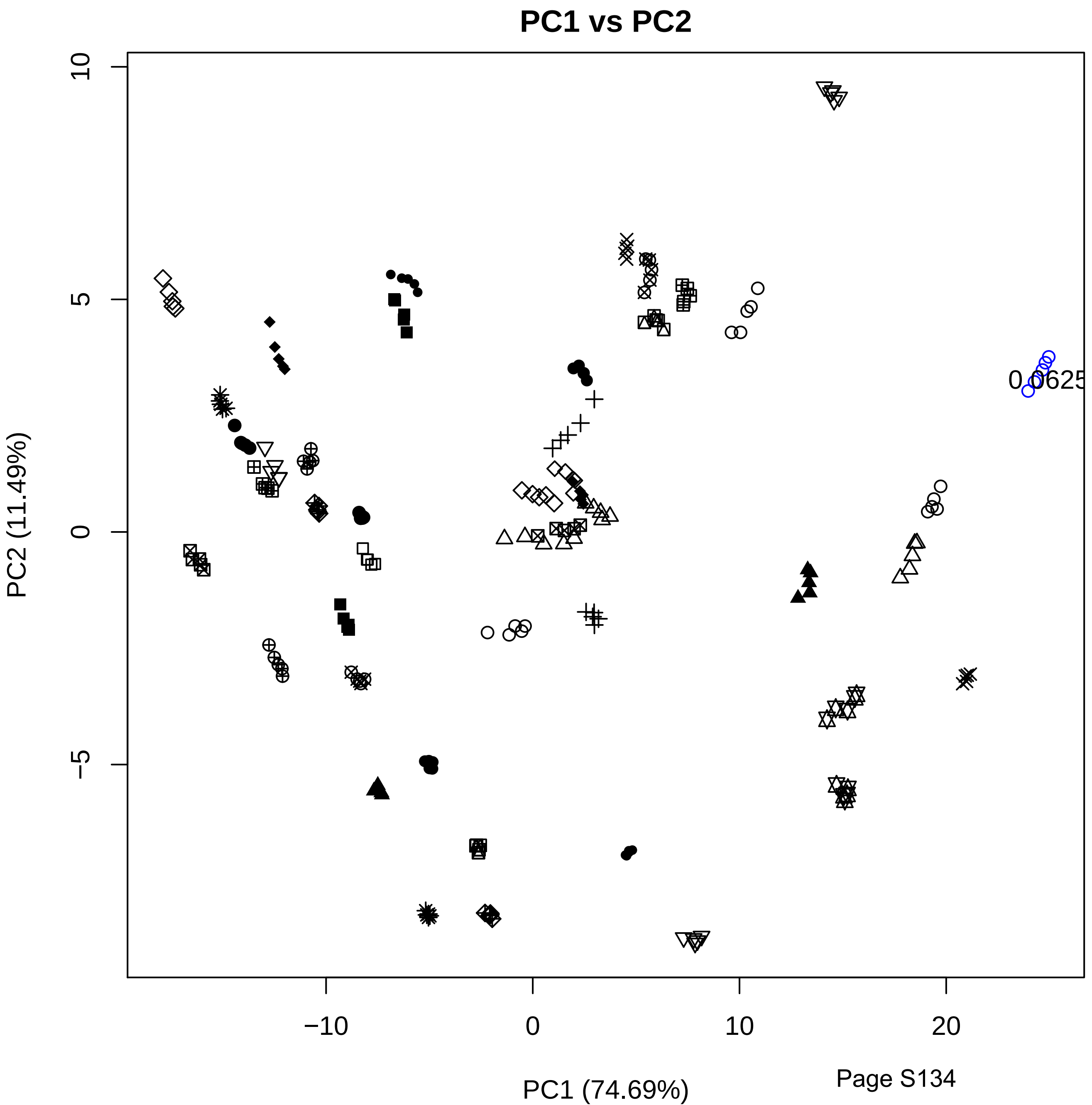
PC1 vs PC2
10
5
0
−5
PC2 (11.49%)
−10
0
10
20
PC1 (74.69%)
0.0625

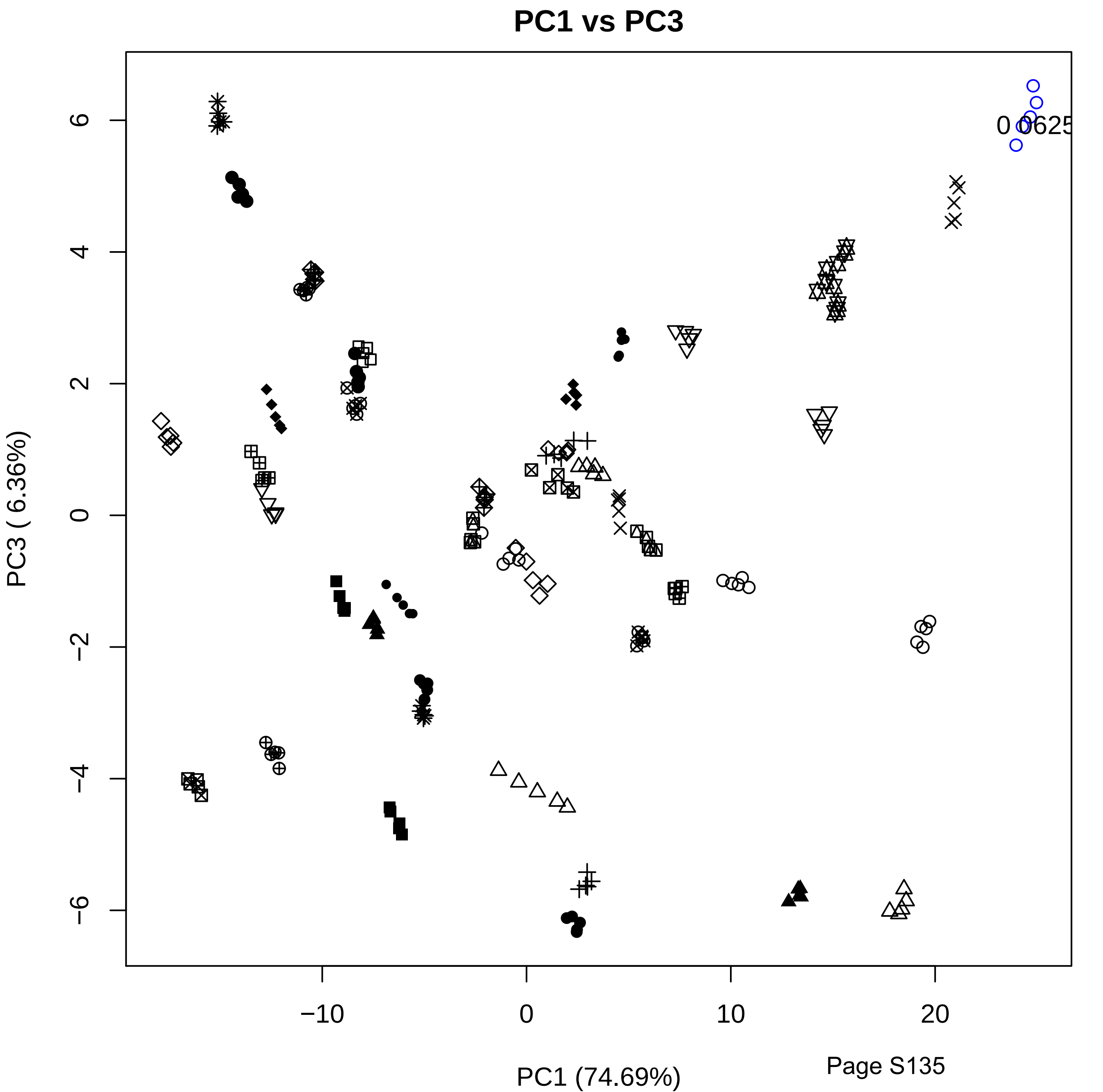
PC1 vs PC3
PC3 (6.36%)
PC1 (74.69%)
0.0625
6
4
2
0
−2
−4
−6
−10
0
10
20

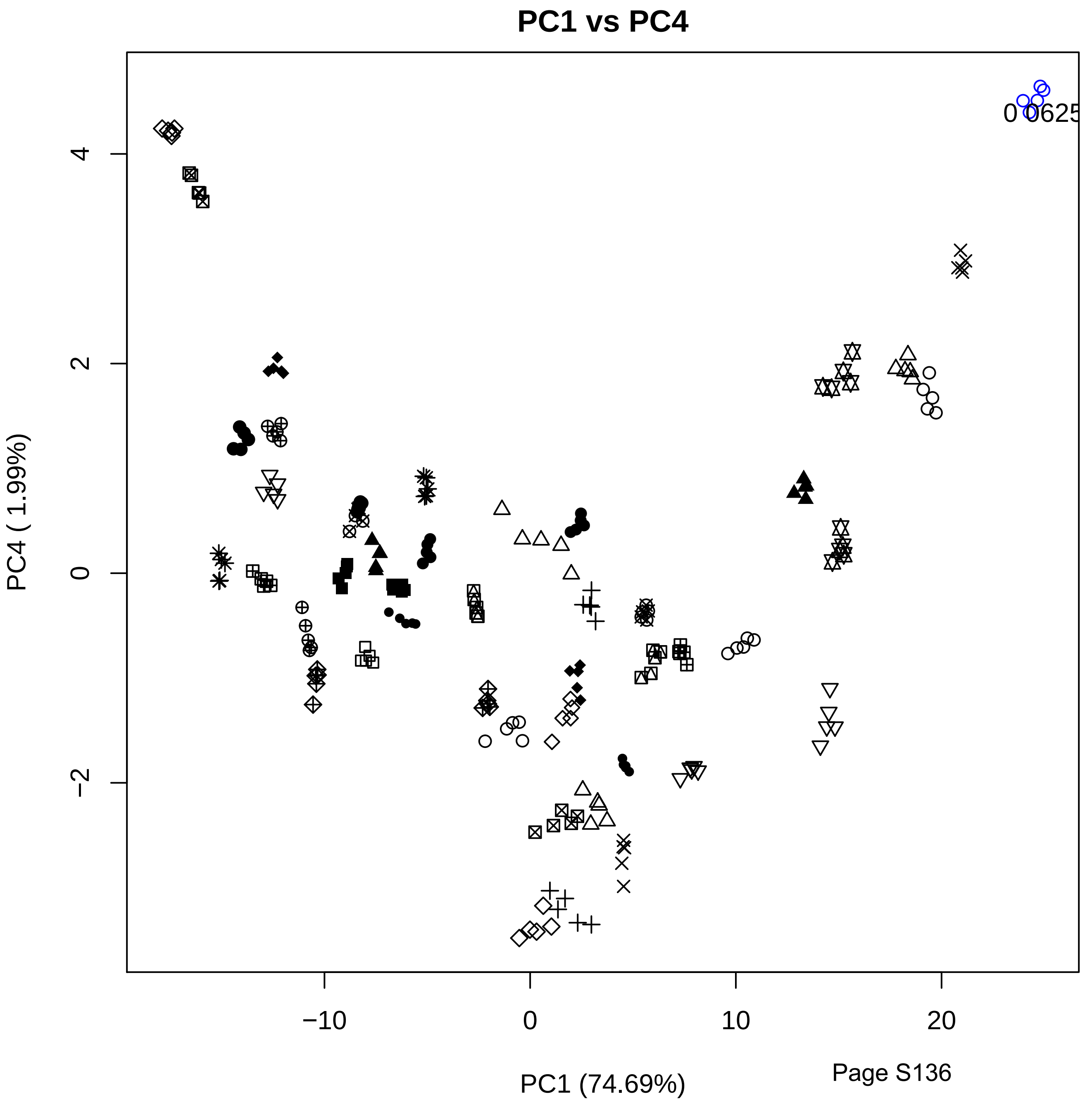
PC1 vs PC4
0 0625
4
2
0
−2
PC4 (1.99%)
−10
0
10
20
PC1 (74.69%)

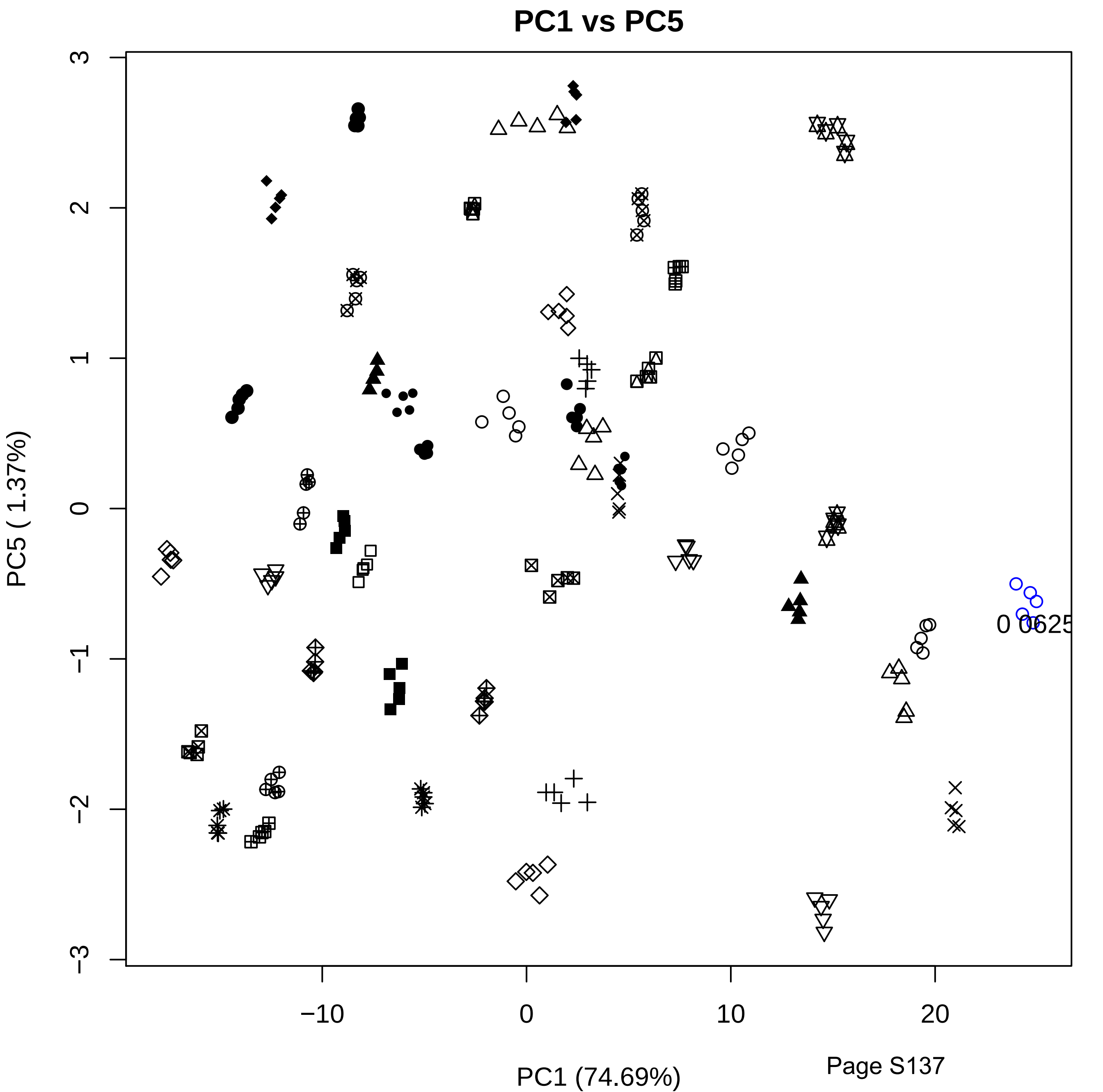

PC1 vs PC5
PC5 (1.37%)
PC1 (74.69%)
3
2
1
0
−1
−2
−3
−10
0
10
20
0 0625

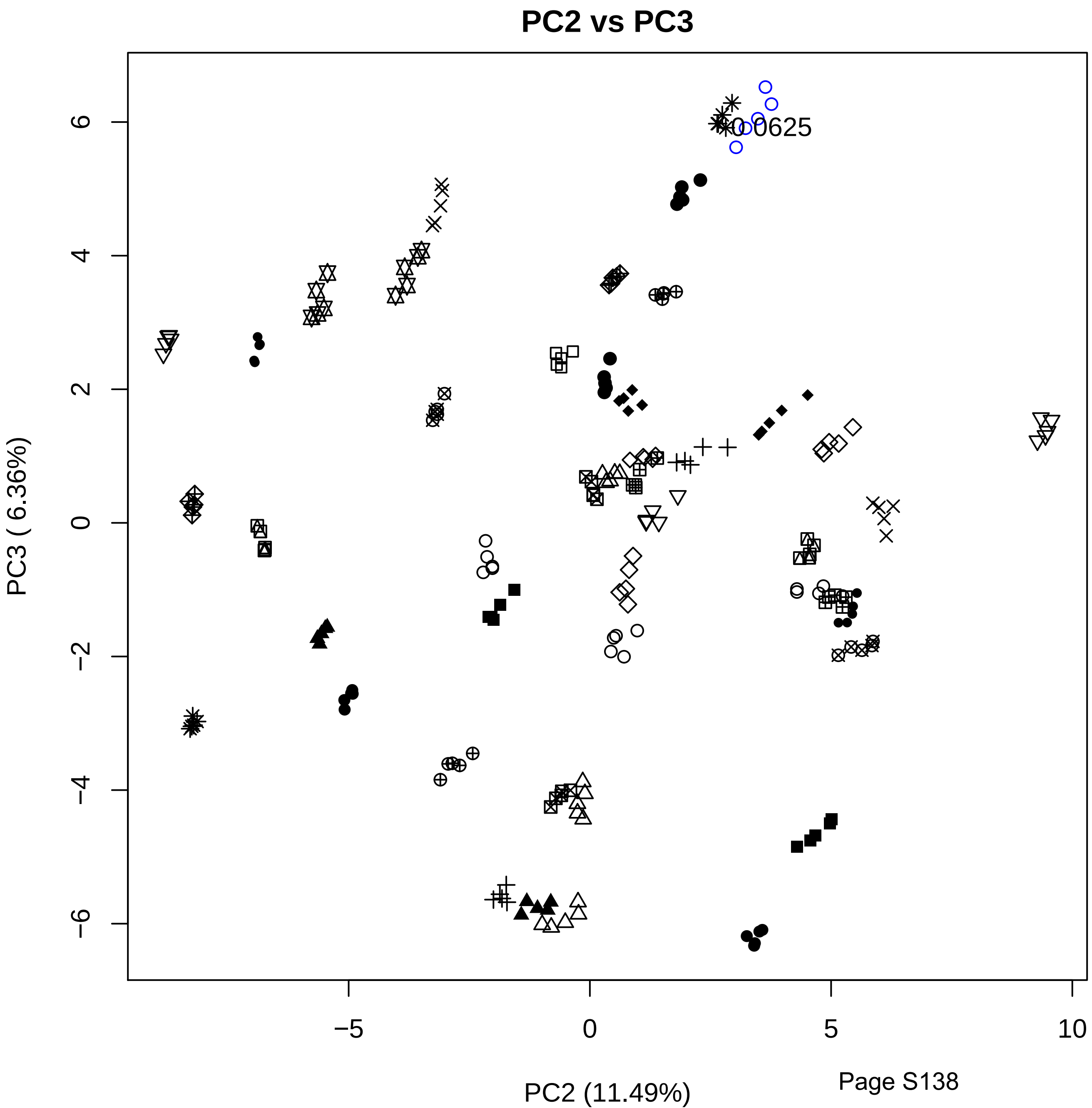
PC2 vs PC3
PC3 (6.36%)
PC2 (11.49%)
6
4
2
0
−2
−4
−6
−5
0
5
10
0625

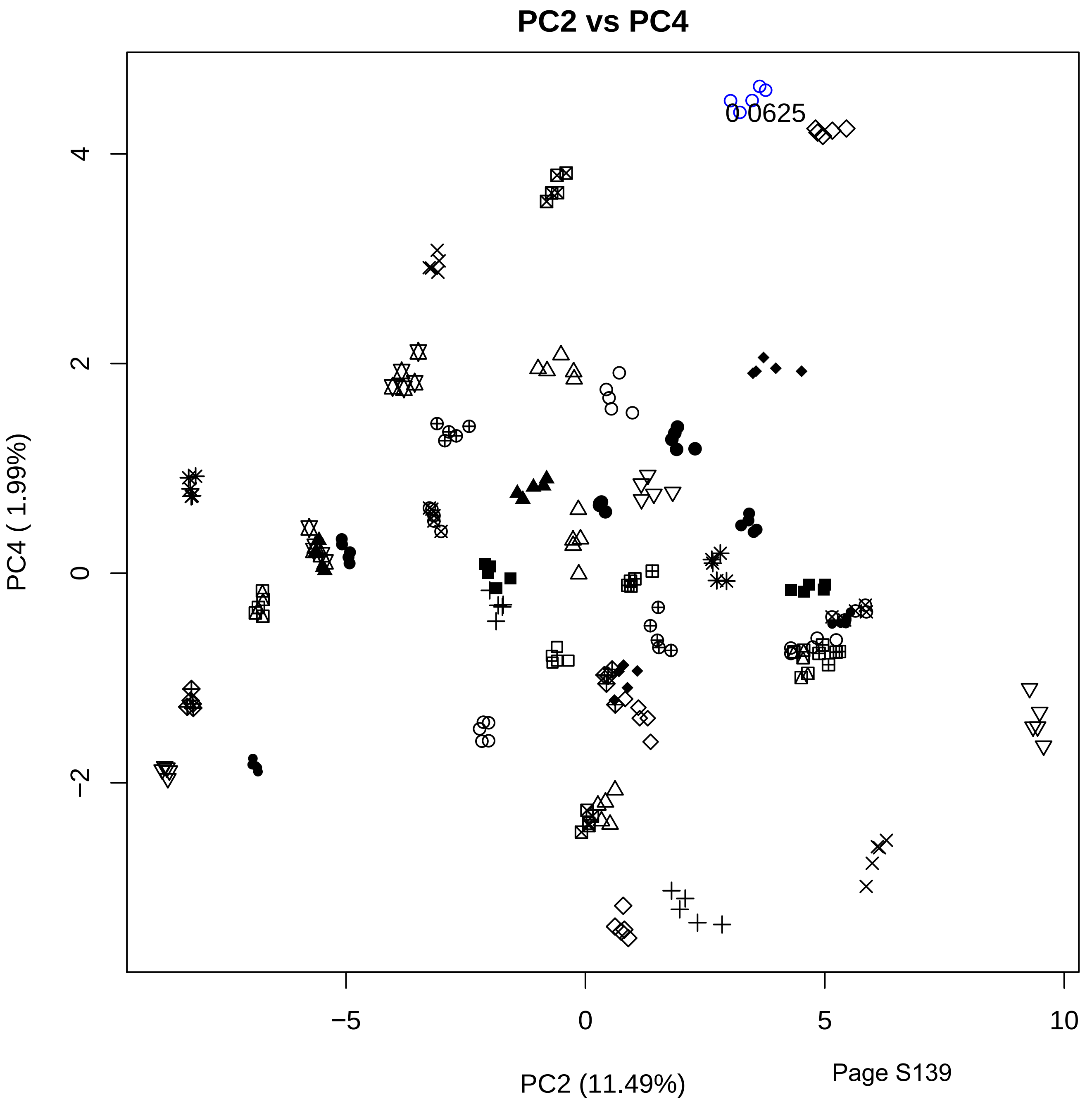
PC2 vs PC4
00625
4
2
0
−2
PC4 (1.99%)
−5
0
5
10
PC2 (11.49%)

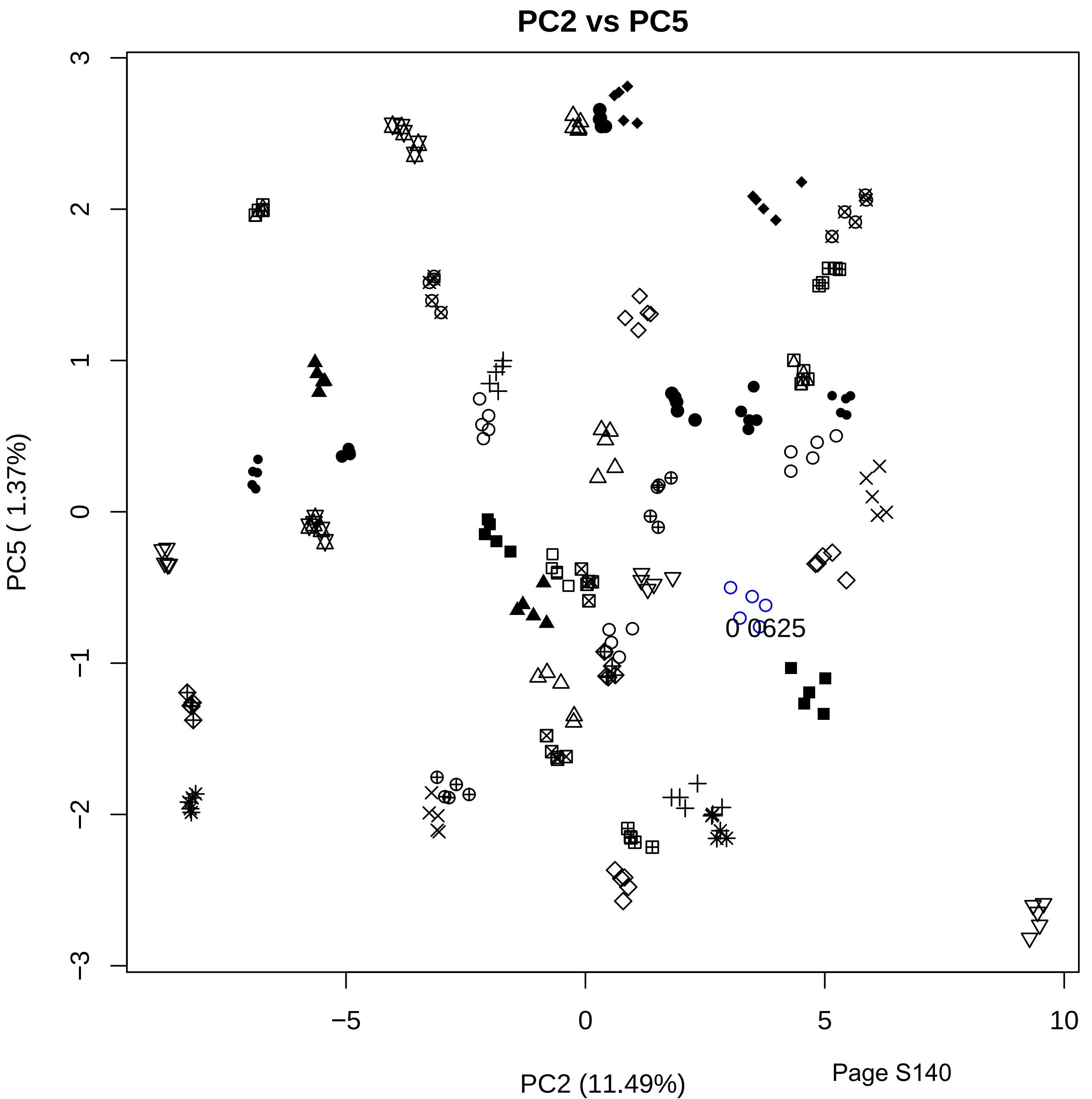
PC2 vs PC5
3
2
1
0
−1
−2
−3
PC5 (1.37%)
−5
0
5
10
PC2 (11.49%)
0.0625

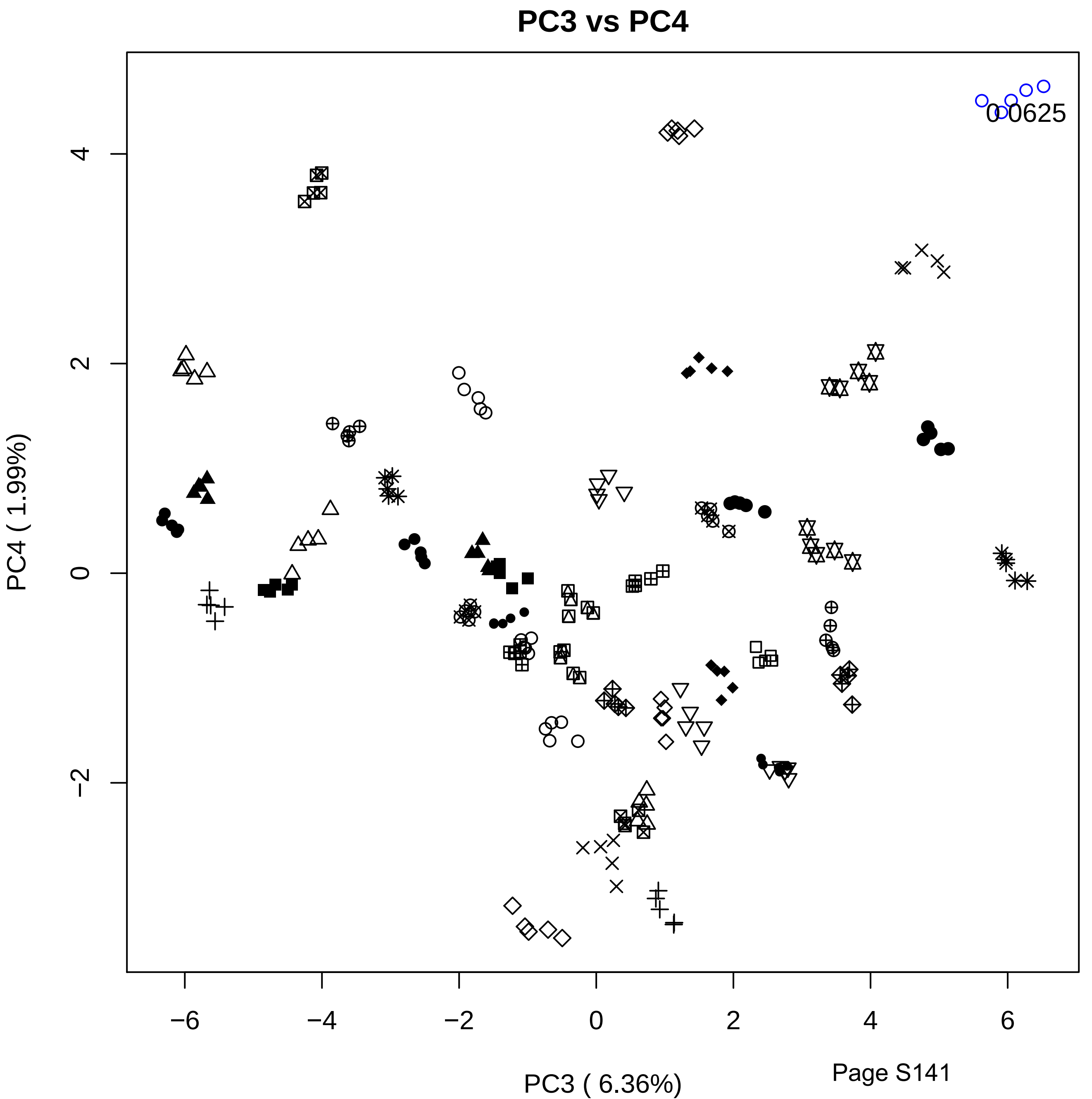
PC3 vs PC4
0.0625
PC4 (1.99%)
PC3 (6.36%)
−6
−4
−2
0
2
4
6
−2
0
2
4

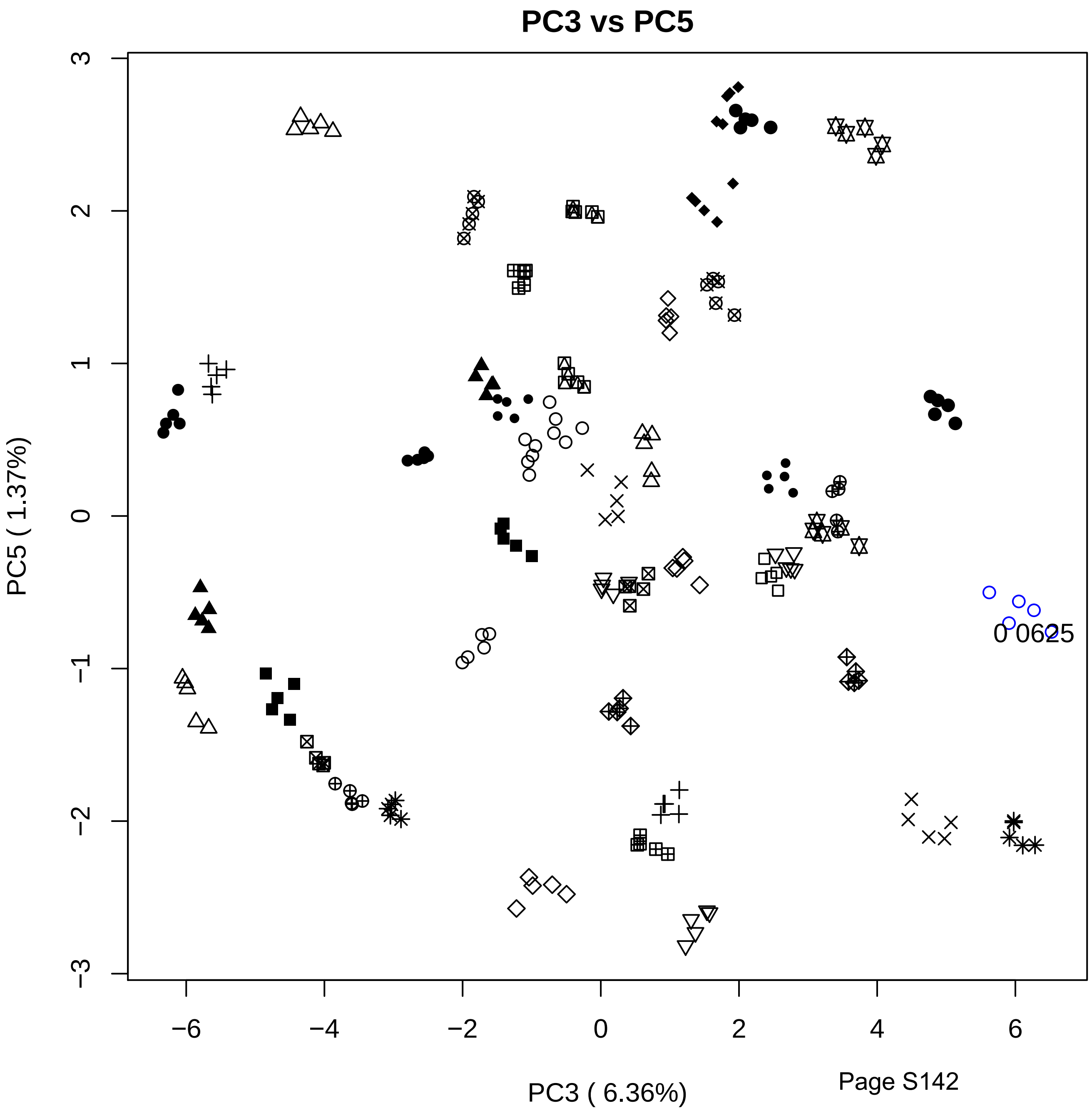
PC3 vs PC5
PC5 (1.37%)
PC3 (6.36%)
3
2
1
0
−1
−2
−3
−6
−4
−2
0
2
4
6
0 0625

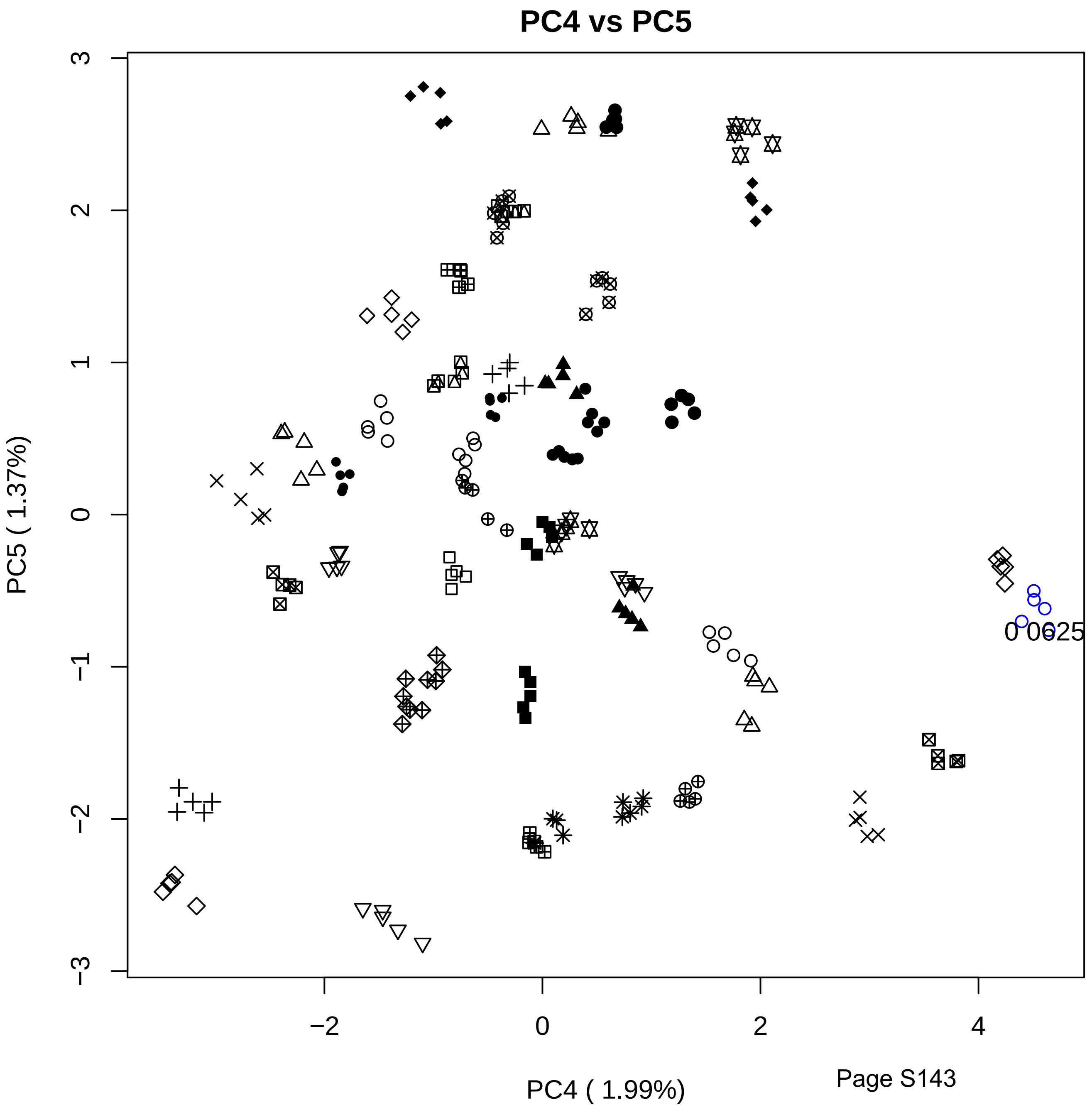
PC4 vs PC5
PC5 (1.37%)
PC4 (1.99%)
3
2
1
0
−1
−2
−3
−2
0
2
4
0 0625

PCA of 0.12% OSCS vs PMH

Scaled Matrix PCAed

7th order polynomial baseline correction performed

Raw spectra smoothed to 21 Neighbours, 2nd order PN, Savtisky Golay

Normalisation between 0 and 100

D2 spectra smoothed to 41 Neighbours, 2nd order PN, Savtisky Golay

Regions Analysed: 3601−2501 and 2001−700cm−1

Samples Analysed: 1 Contam's 0 OSCS's 45 PMH's

Keele University AD	Sun Dec 09 16:50:37 2018

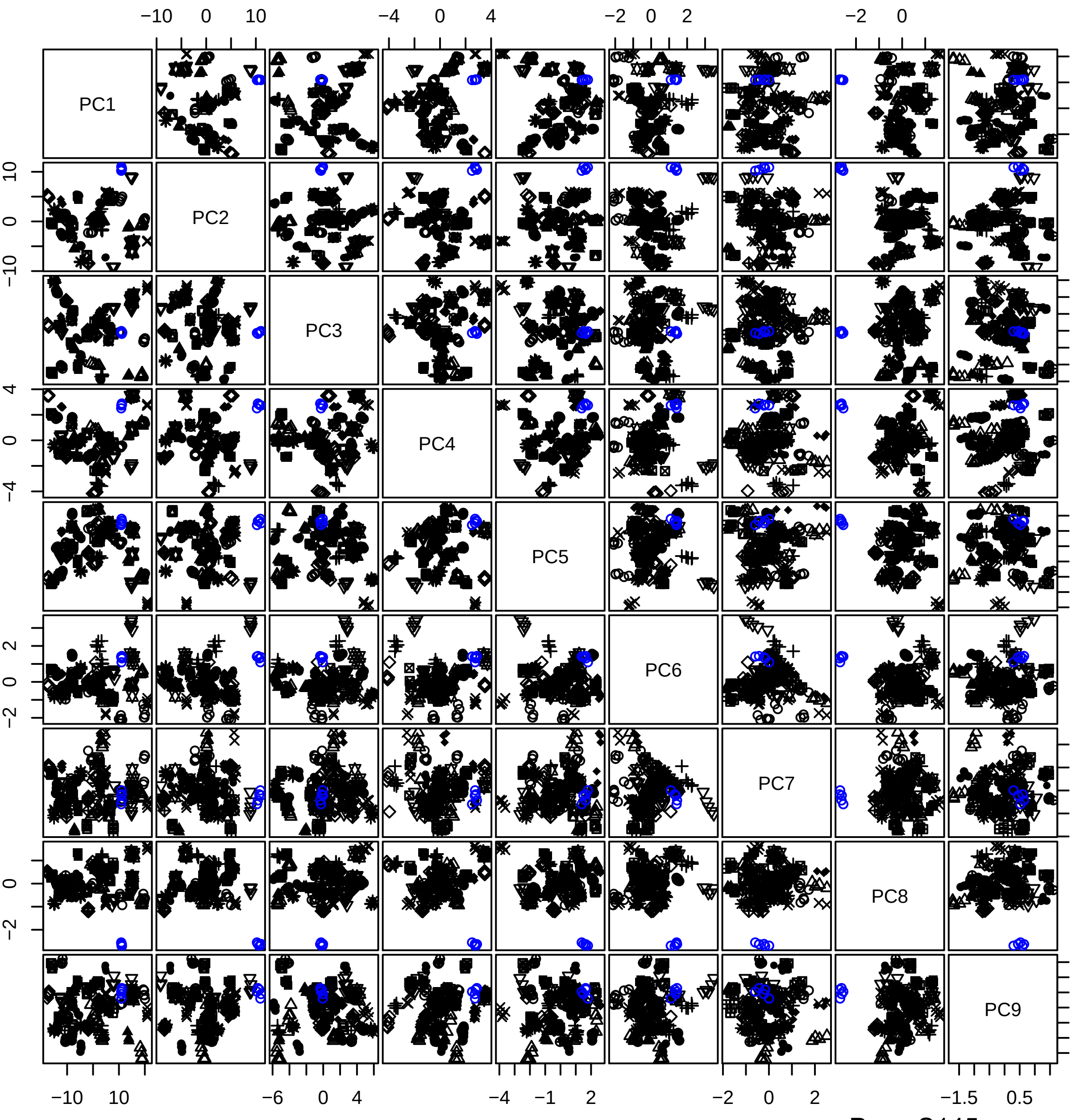
PC1
PC2
PC3
PC4
PC5
PC6
PC7
PC8
PC9

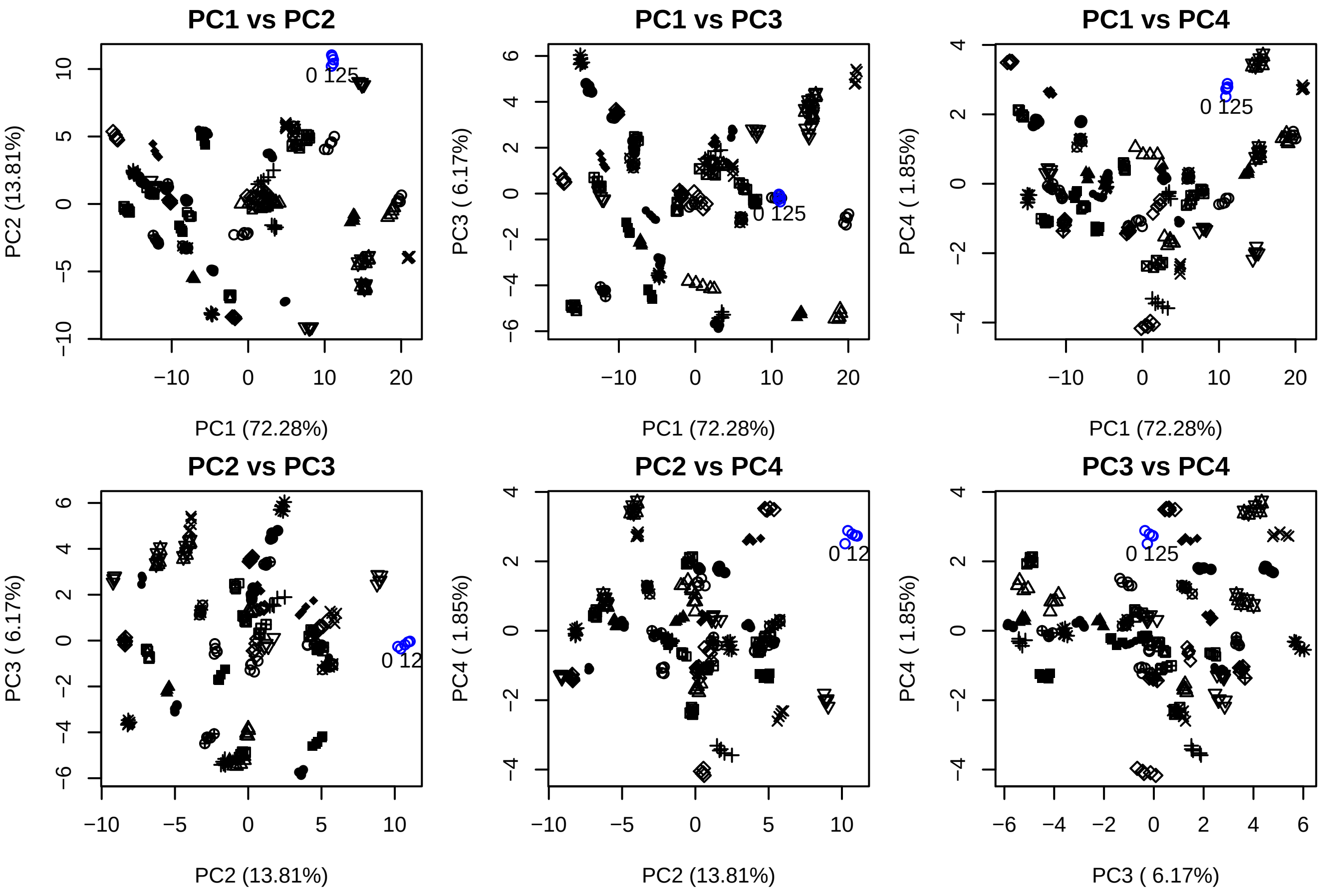
PC1 vs PC2
PC1 vs PC3
PC1 vs PC4
PC2 vs PC3
PC2 vs PC4
PC3 vs PC4
PC1 (72.28%)
PC2 (13.81%)
PC3 (6.17%)
PC4 (1.85%)
0 125

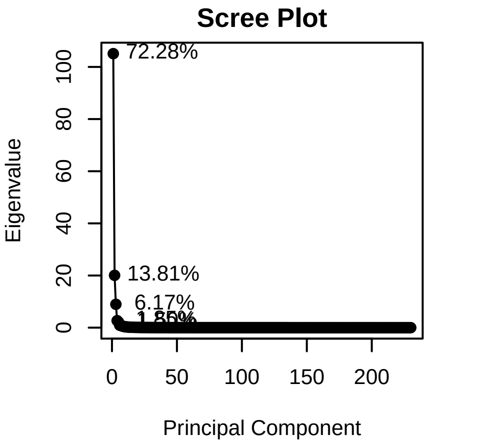
Scree Plot
Eigenvalue
Principal Component
72.28%
13.81%
6.17%

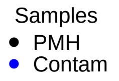
Samples
PMH
Contam

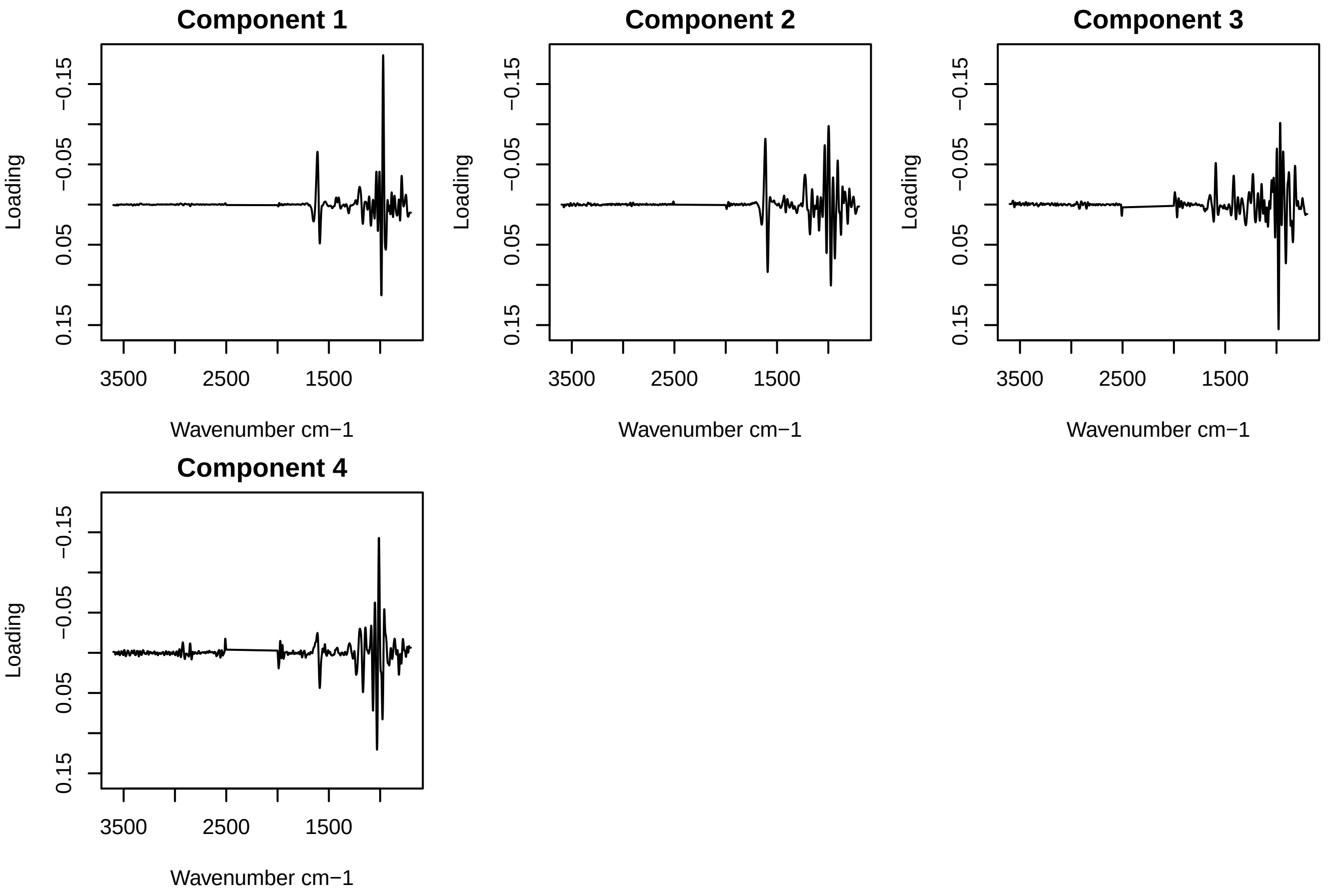
Component 1
Component 2
Component 3
Component 4
Loading
−0.15
−0.05
0.05
0.15
3500
2500
1500
Wavenumber cm−1

PC1 vs PC2 vs PC3

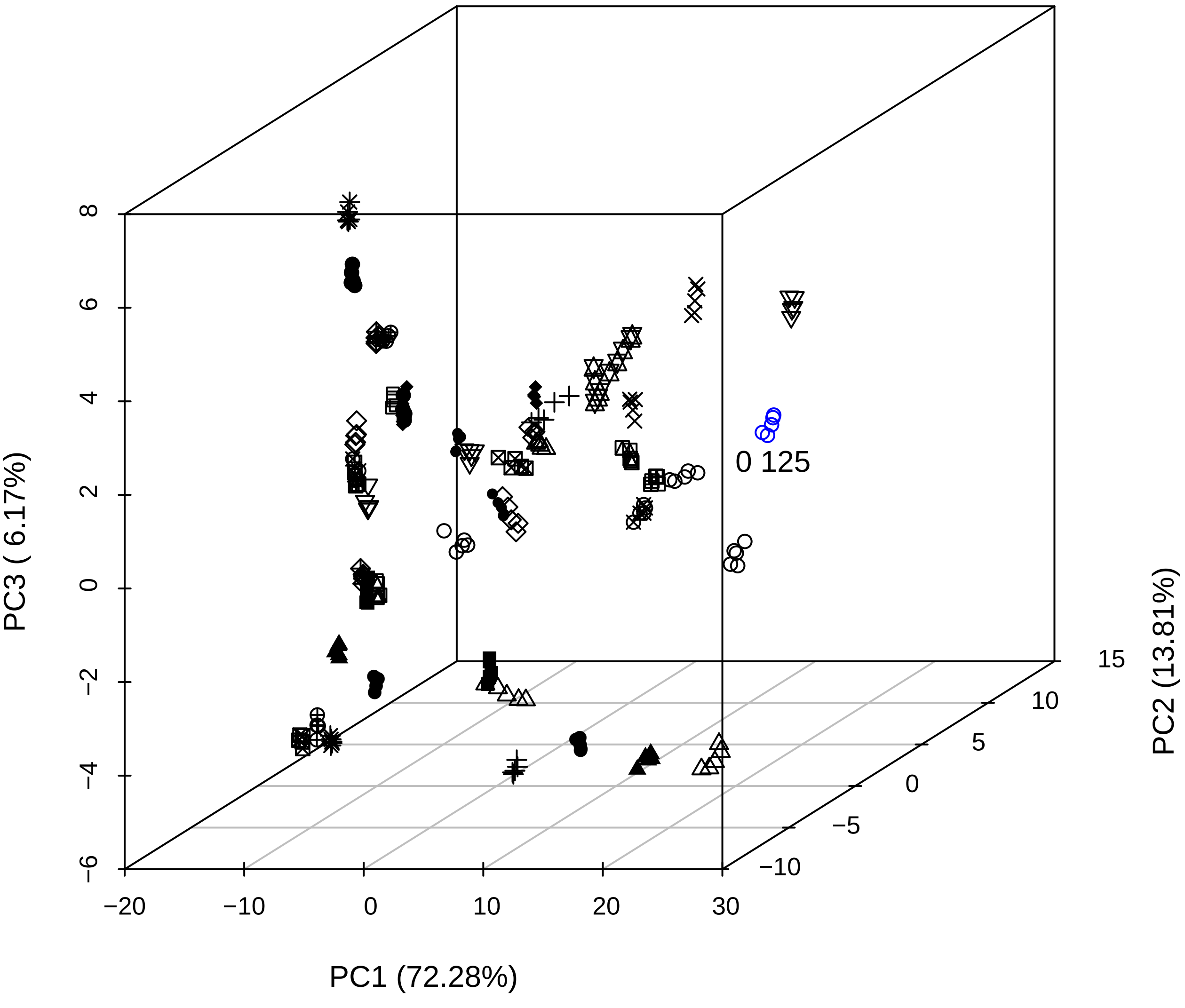

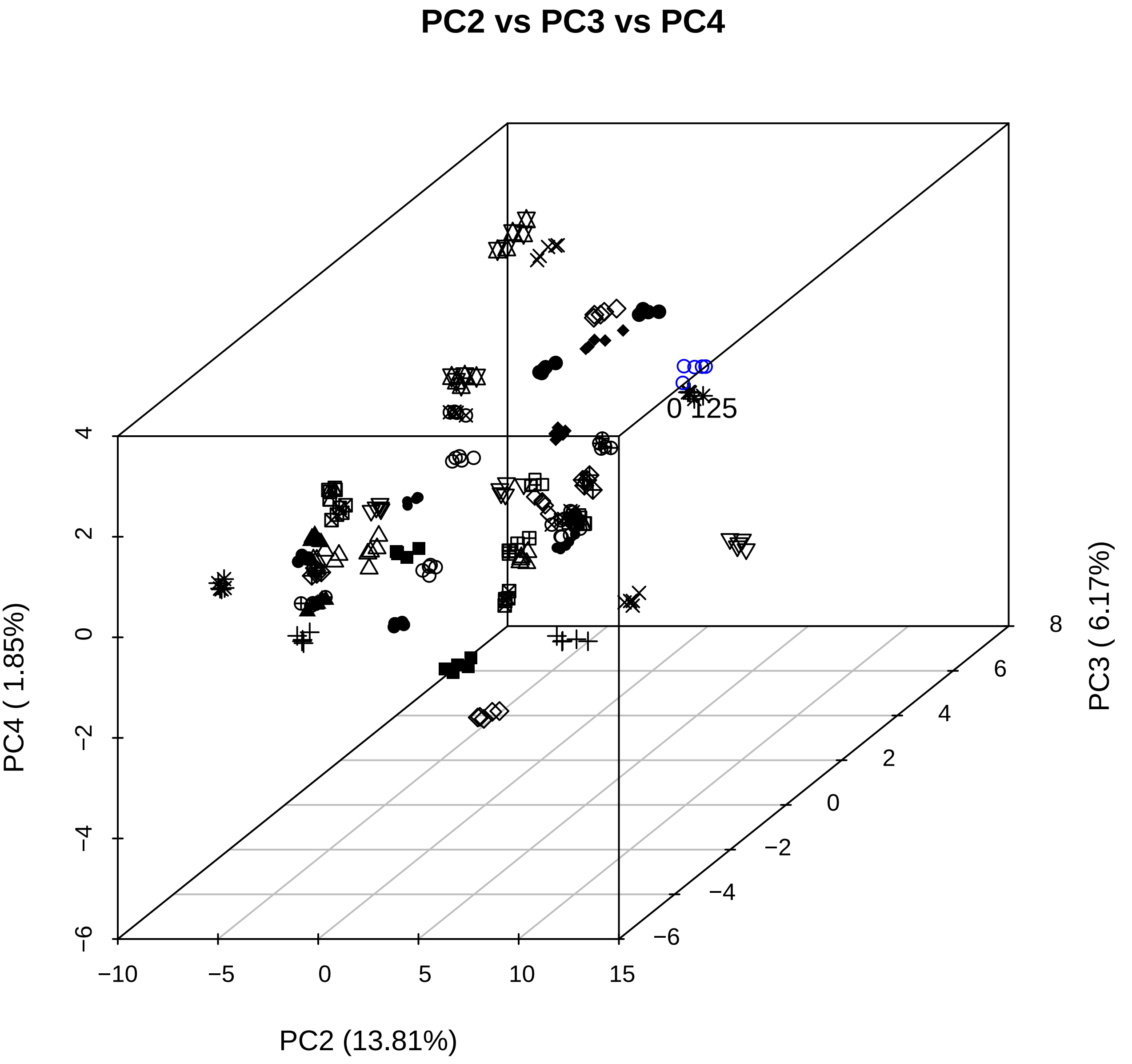
PC2 vs PC3 vs PC4
0 125
PC4 (1.85%)
PC2 (13.81%)
PC3 (6.17%)
−10
−5
0
5
10
15
−6
−4
−2
0
2
4
6
8

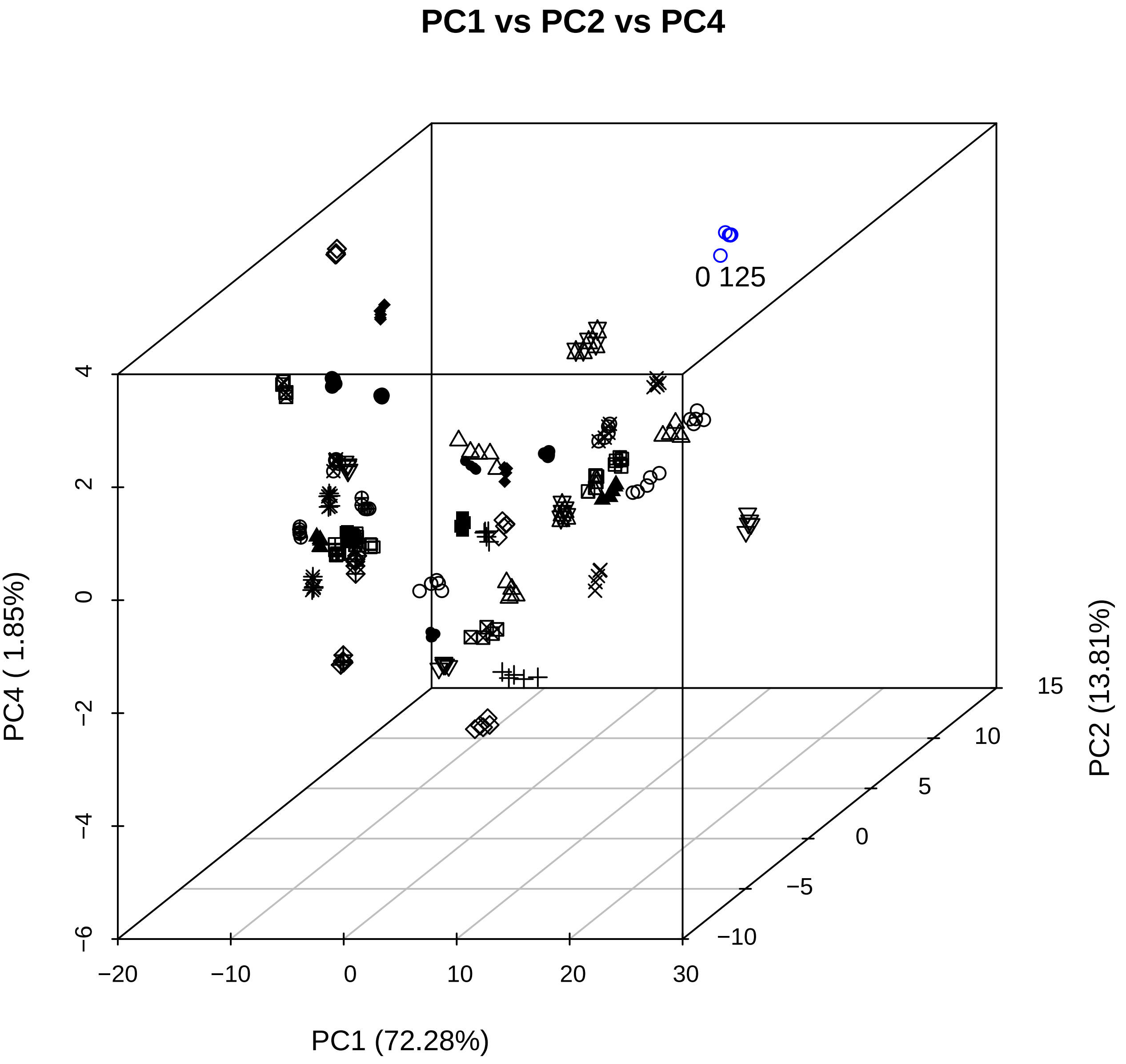

PC1 vs PC2 vs PC4
0 125
4
2
0
−2
−4
−6
PC4 (1.85%)
−20
−10
0
10
20
30
PC1 (72.28%)
−10
−5
0
5
10
15
PC2 (13.81%)

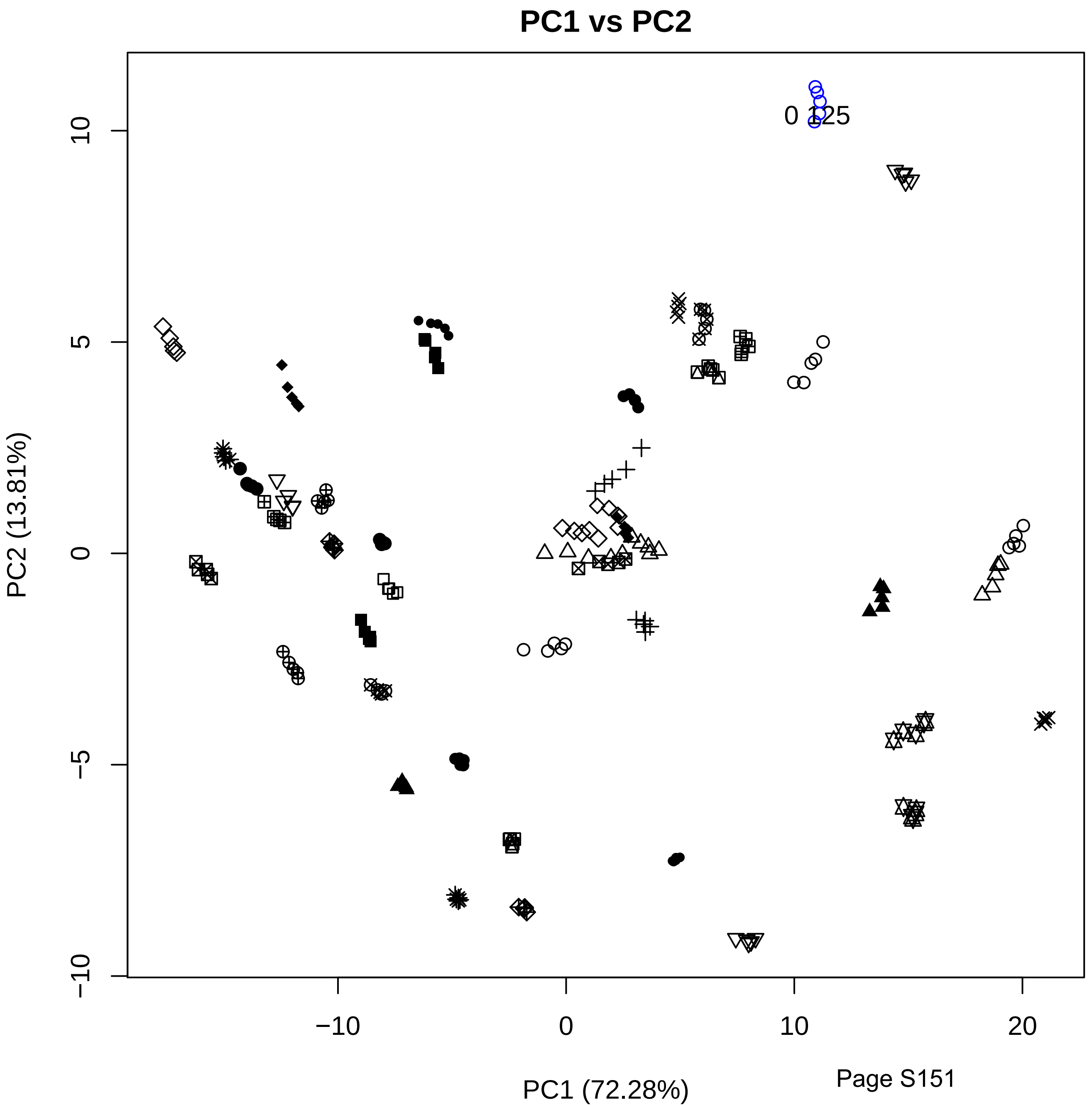
PC1 vs PC2
0 125
10
5
0
−5
−10
PC2 (13.81%)
−10
0
10
20
PC1 (72.28%)

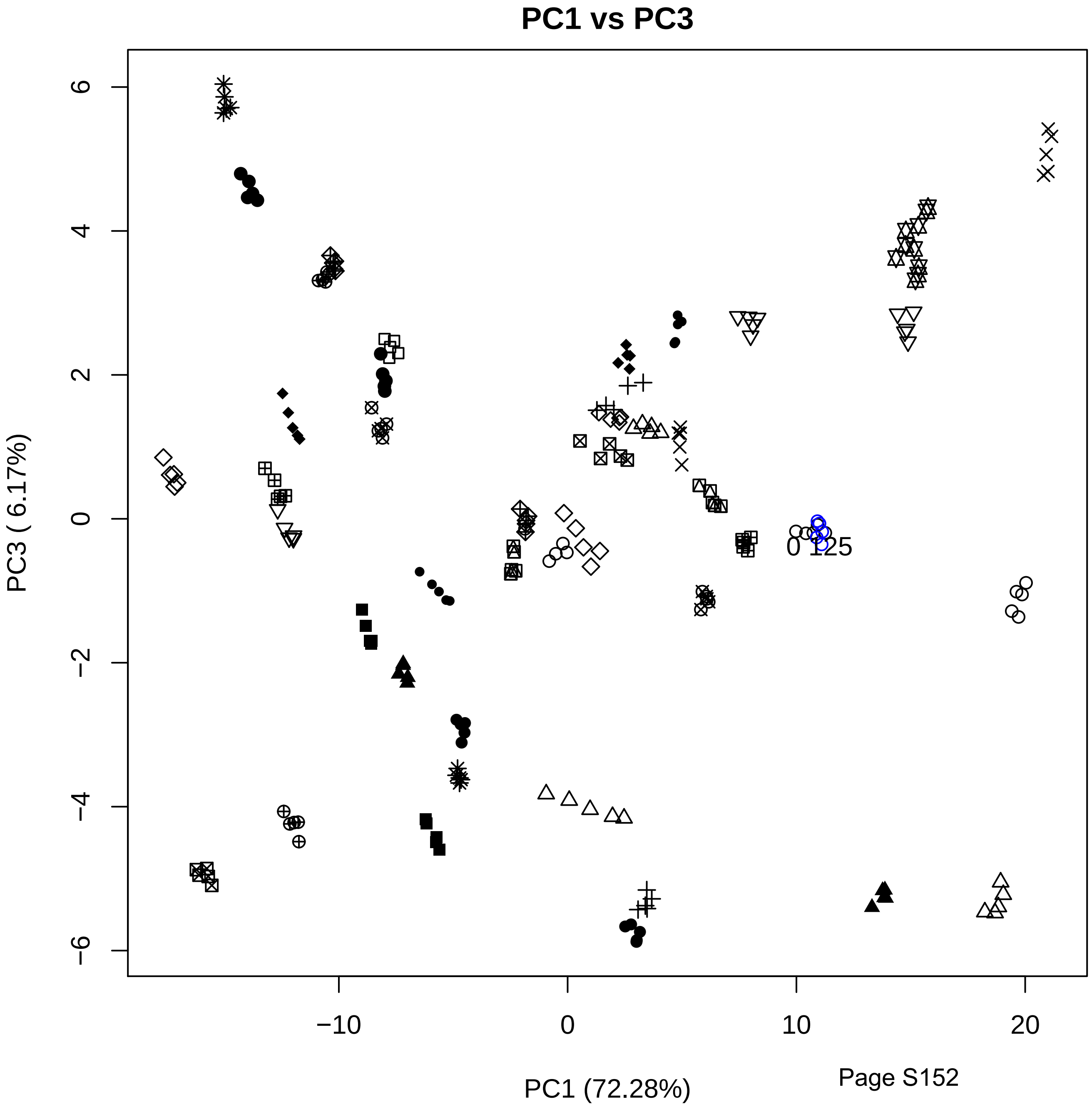
PC1 vs PC3
PC3 (6.17%)
6
4
2
0
−2
−4
−6
−10
0
10
20
PC1 (72.28%)
0 125

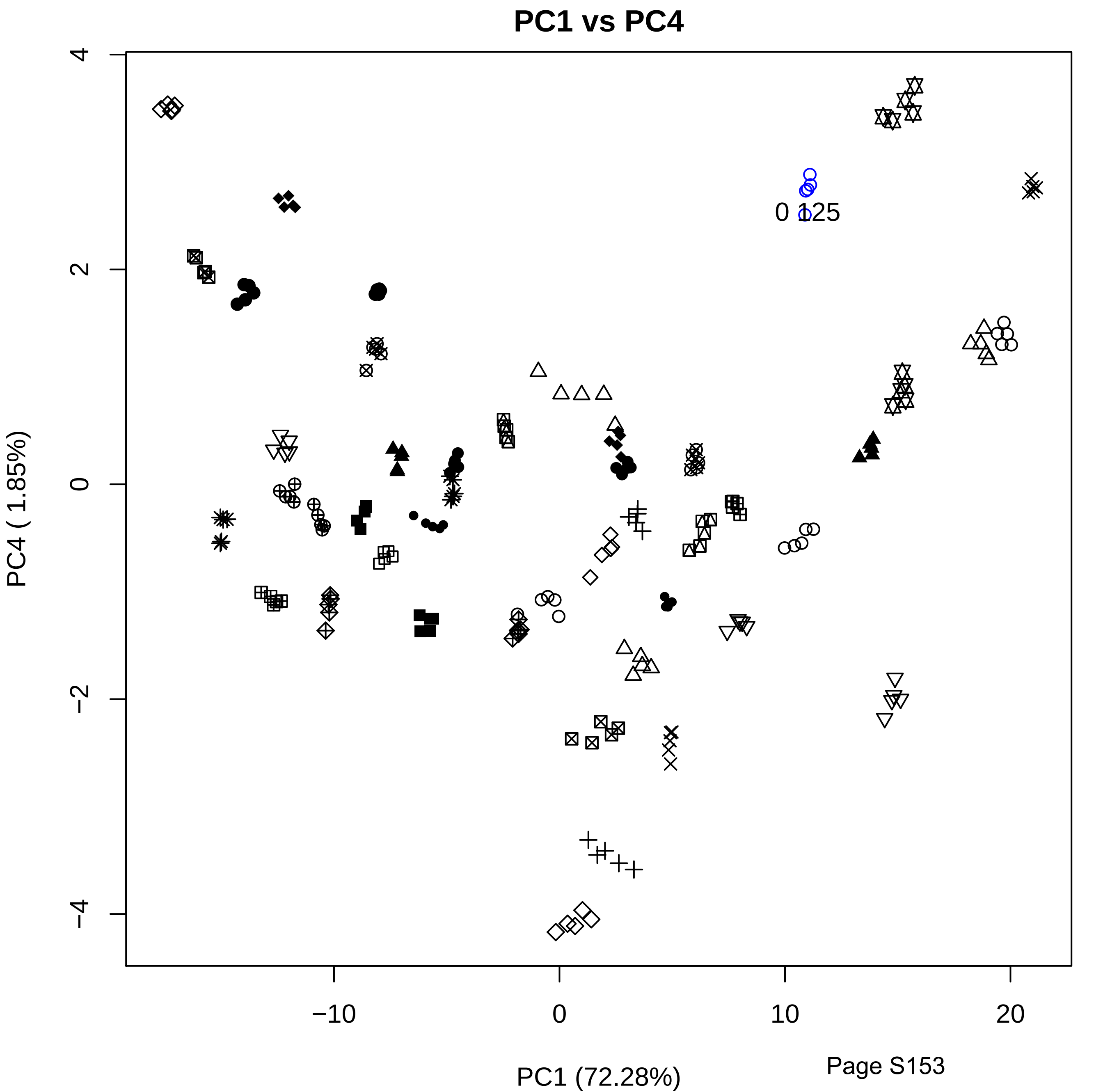

PC1 vs PC4
PC4 (1.85%)
PC1 (72.28%)
0 125
4
2
0
−2
−4
−10
0
10
20

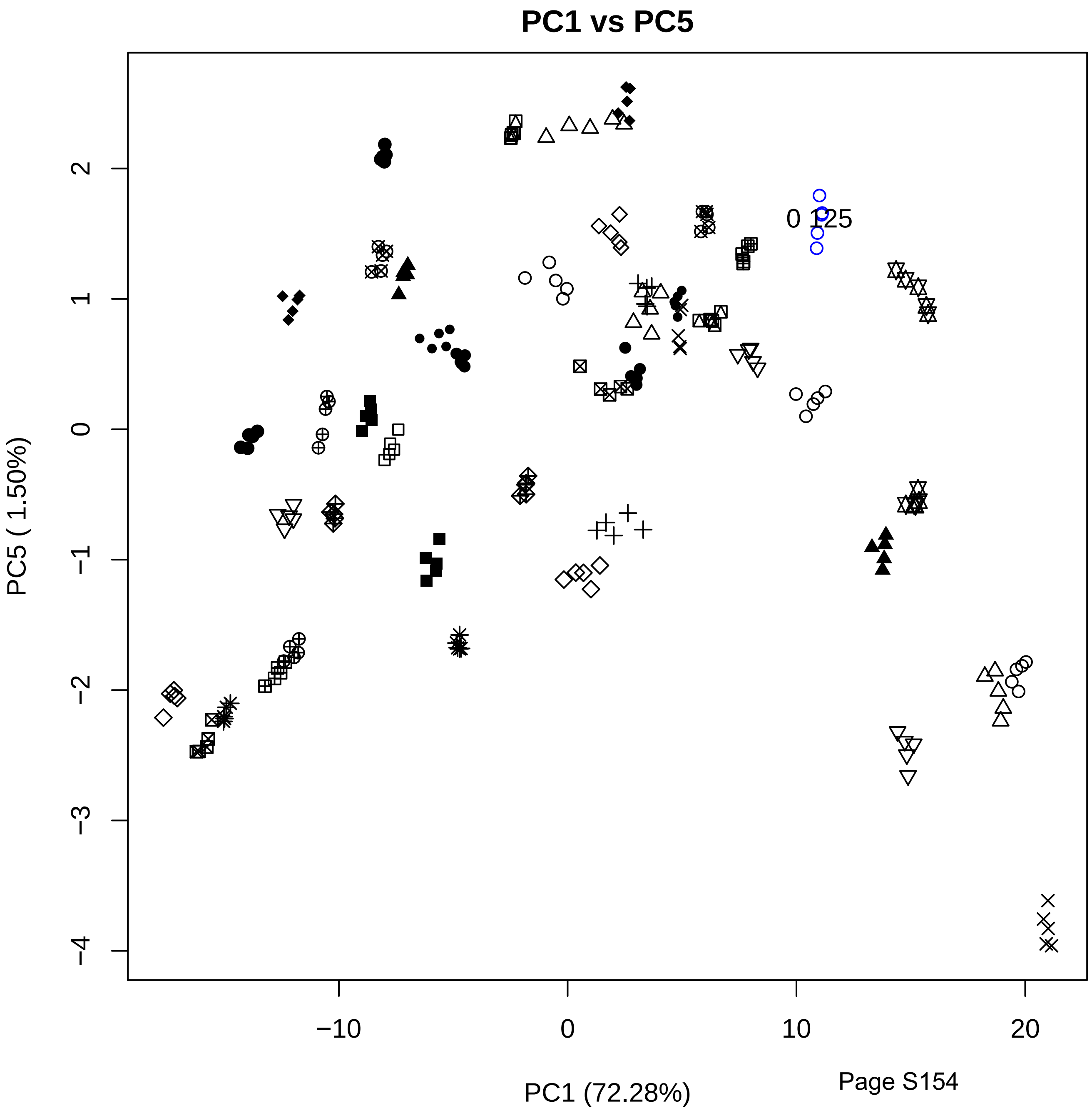
PC1 vs PC5
0 125
PC5 (1.50%)
PC1 (72.28%)
2
1
0
−1
−2
−3
−4
−10
0
10
20

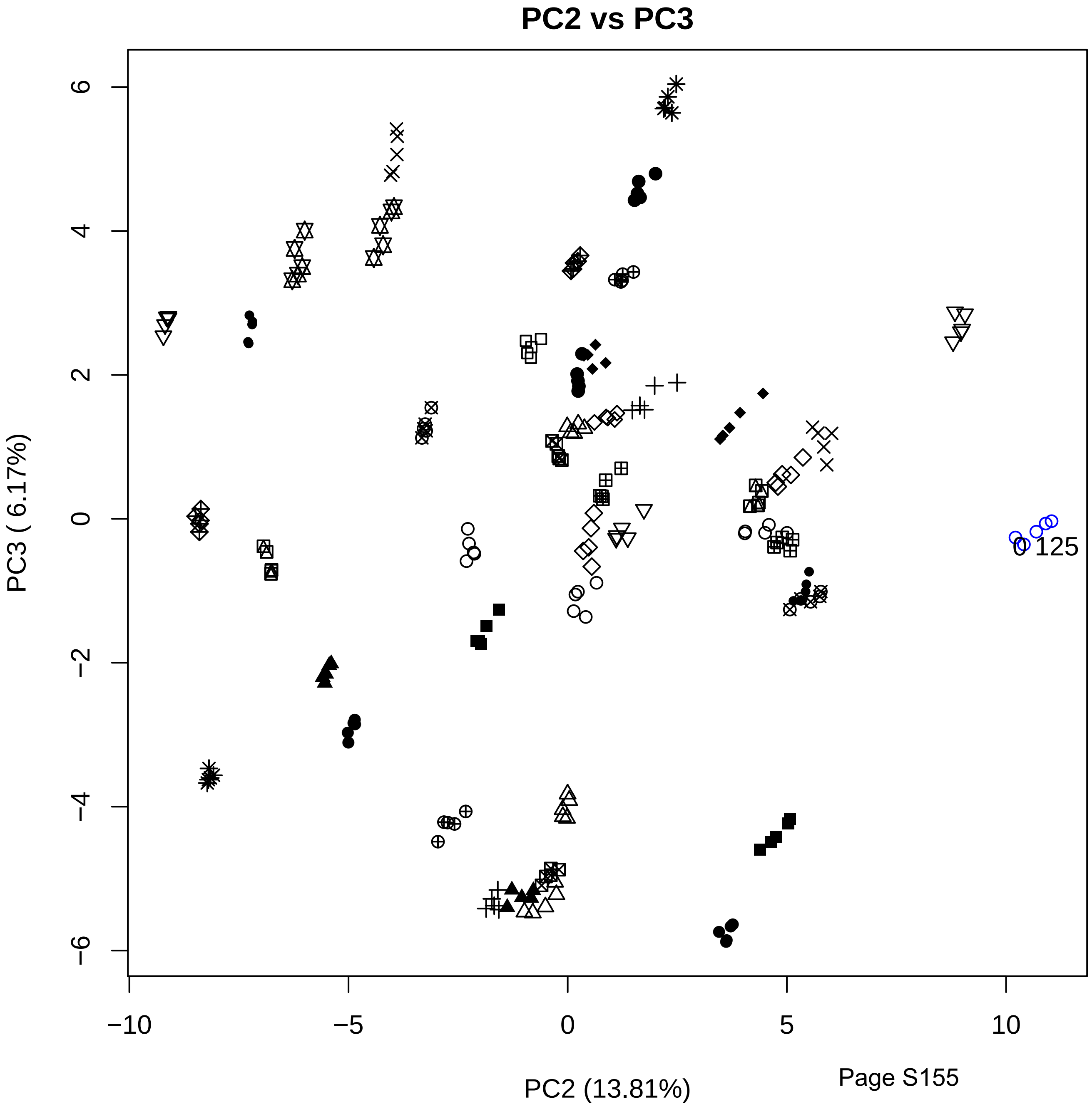
PC2 vs PC3
PC3 (6.17%)
PC2 (13.81%)
6
4
2
0
−2
−4
−6
−10
−5
0
5
10
0 125

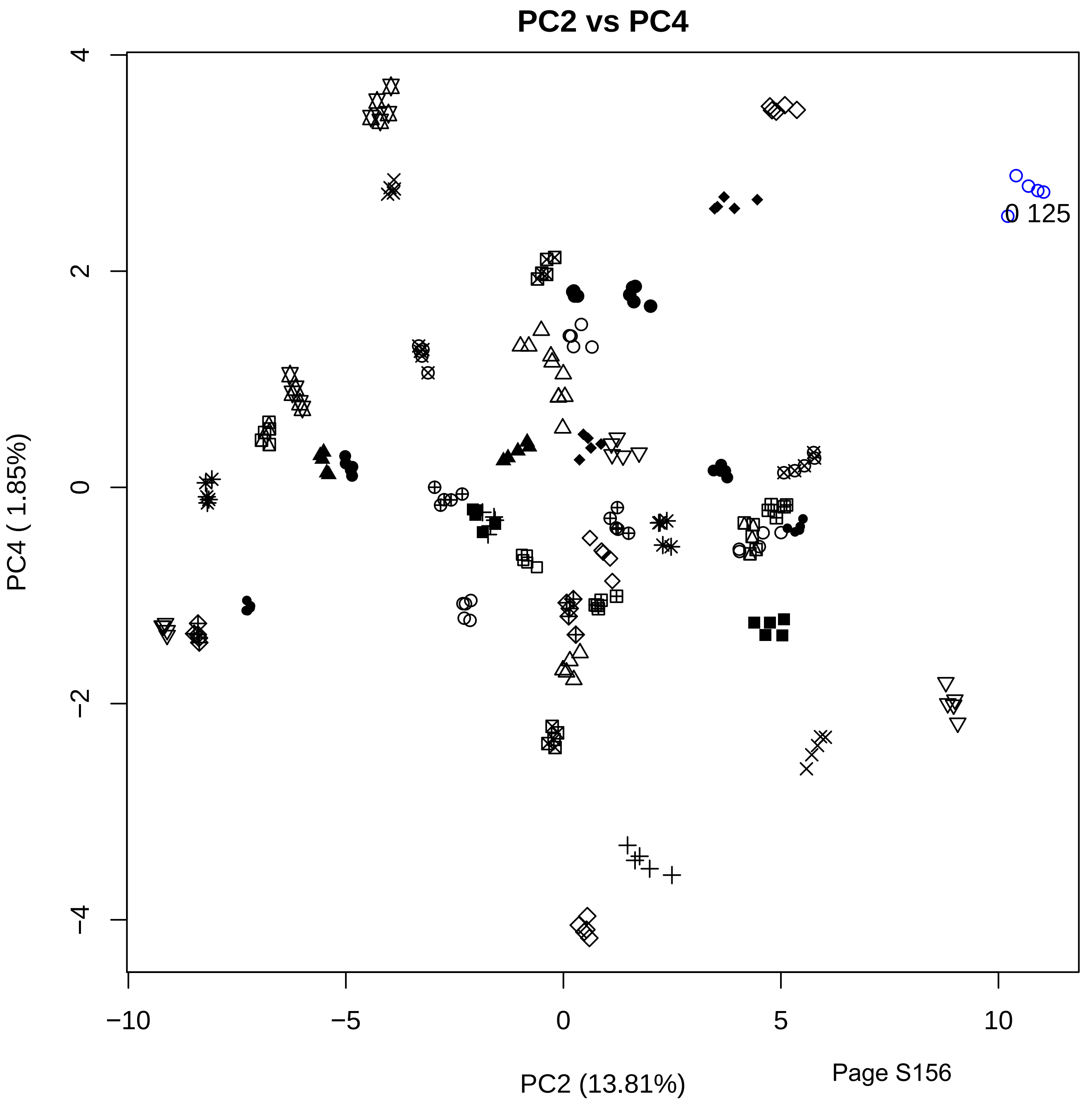

PC2 vs PC4
0 125
4
2
0
−2
−4
PC4 (1.85%)
−10
−5
0
5
10
PC2 (13.81%)

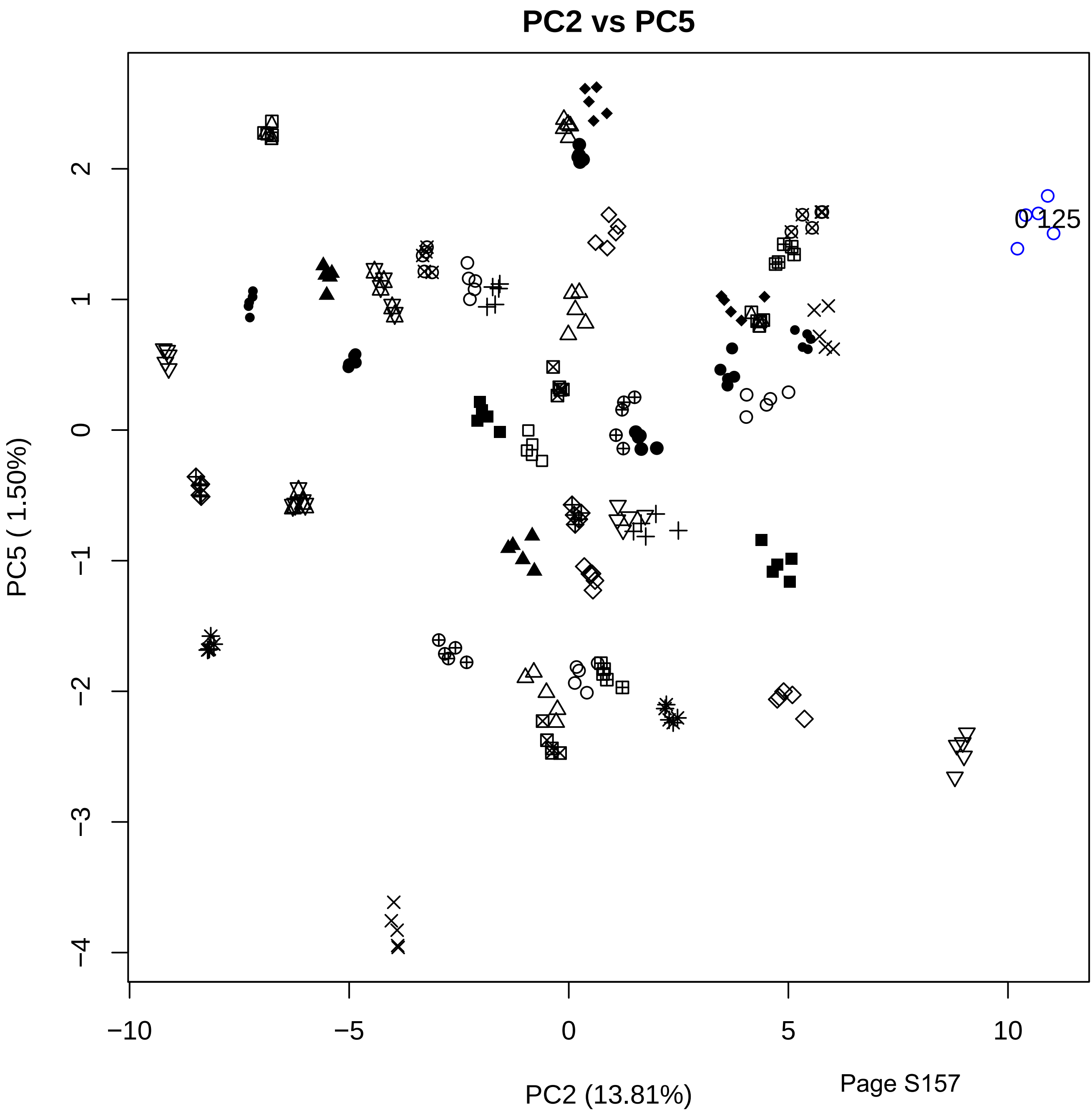
PC2 vs PC5
0 125
PC5 (1.50%)
PC2 (13.81%)
−10
−5
0
5
10
2
1
0
−1
−2
−3
−4

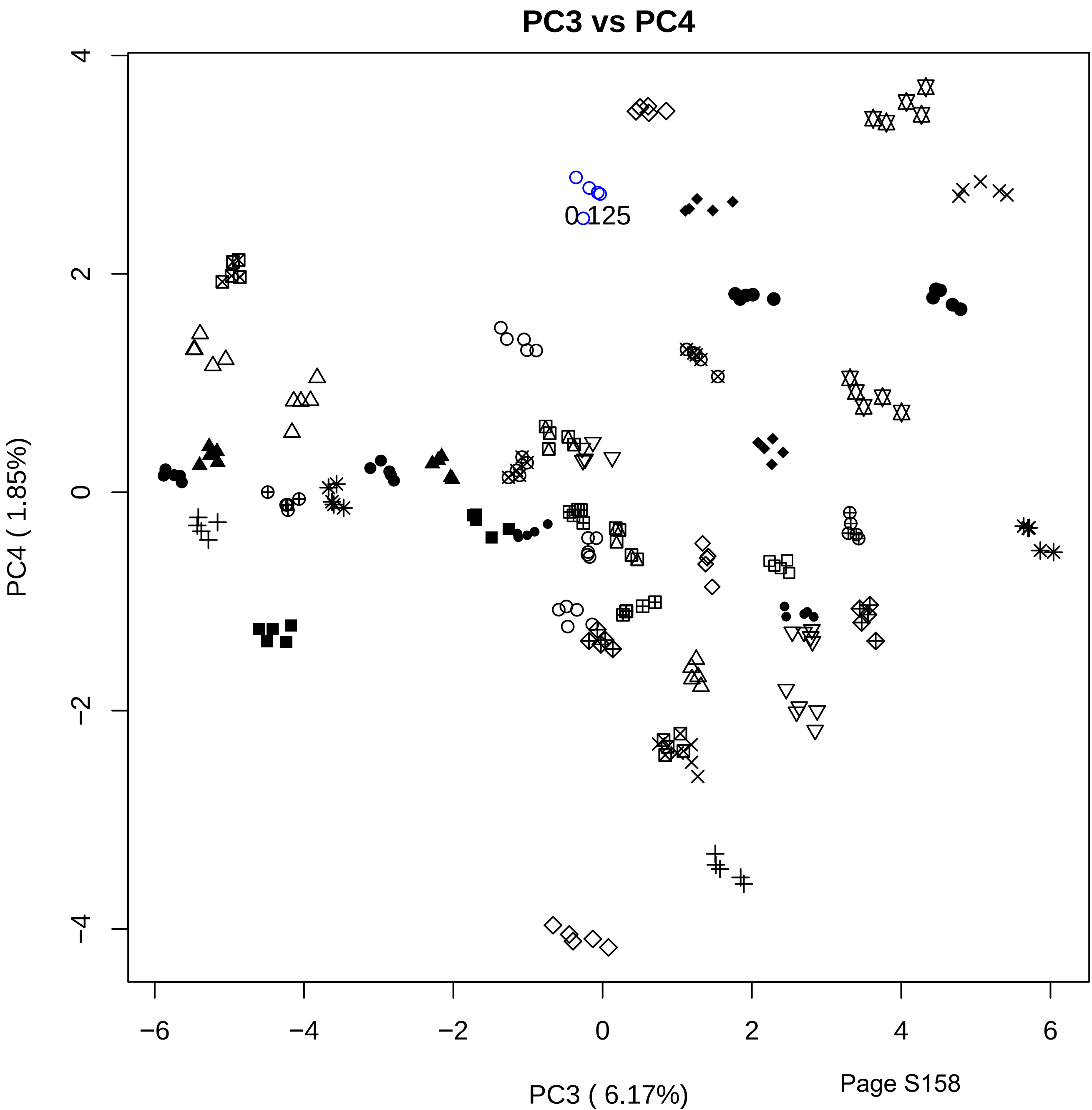

PC3 vs PC4
0.125
PC4 (1.85%)
PC3 (6.17%)
−6
−4
−2
0
2
4
6

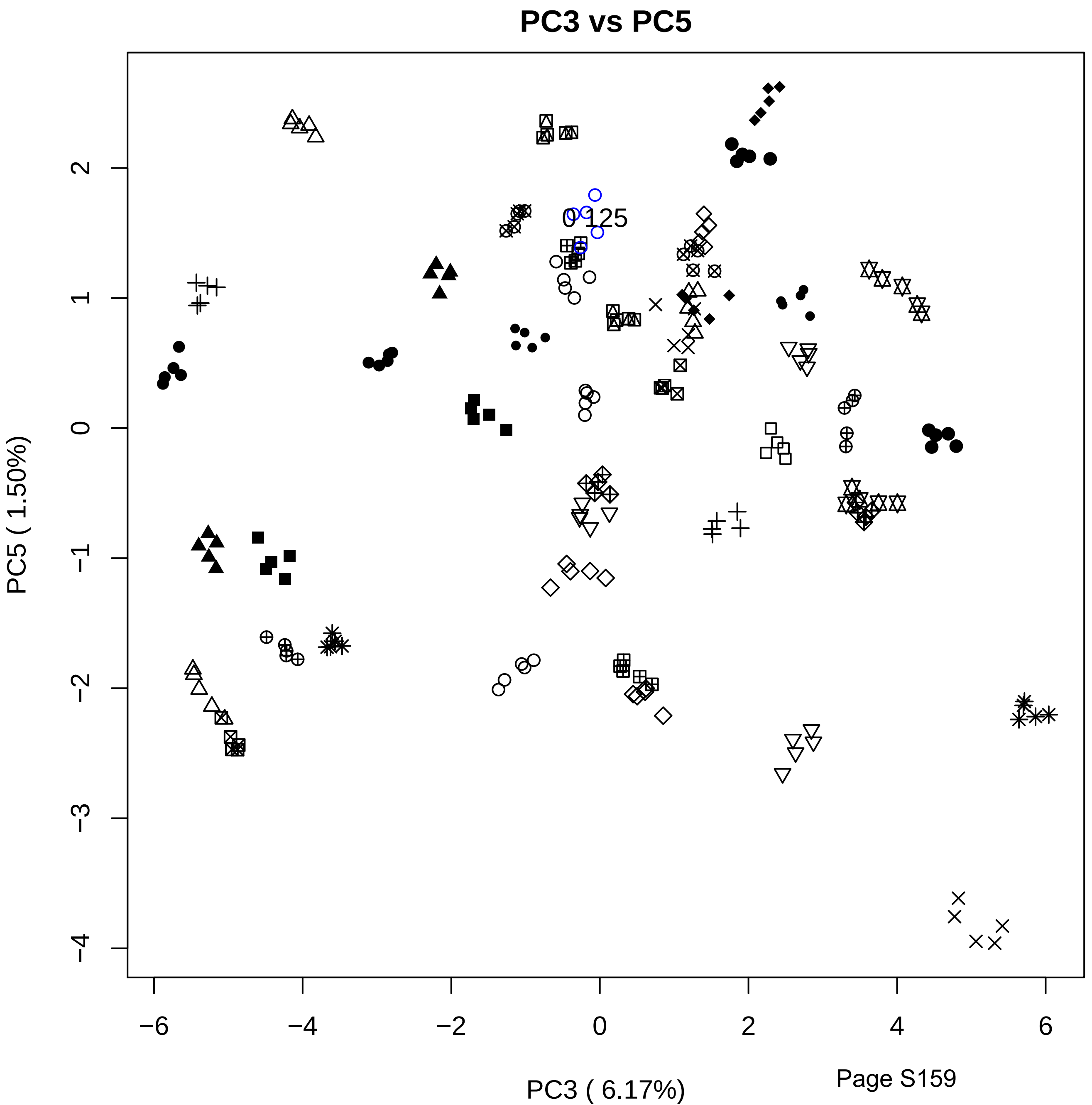
PC3 vs PC5
0 125
PC5 (1.50%)
PC3 (6.17%)
−6
−4
−2
0
2
4
6
2
1
0
−1
−2
−3
−4

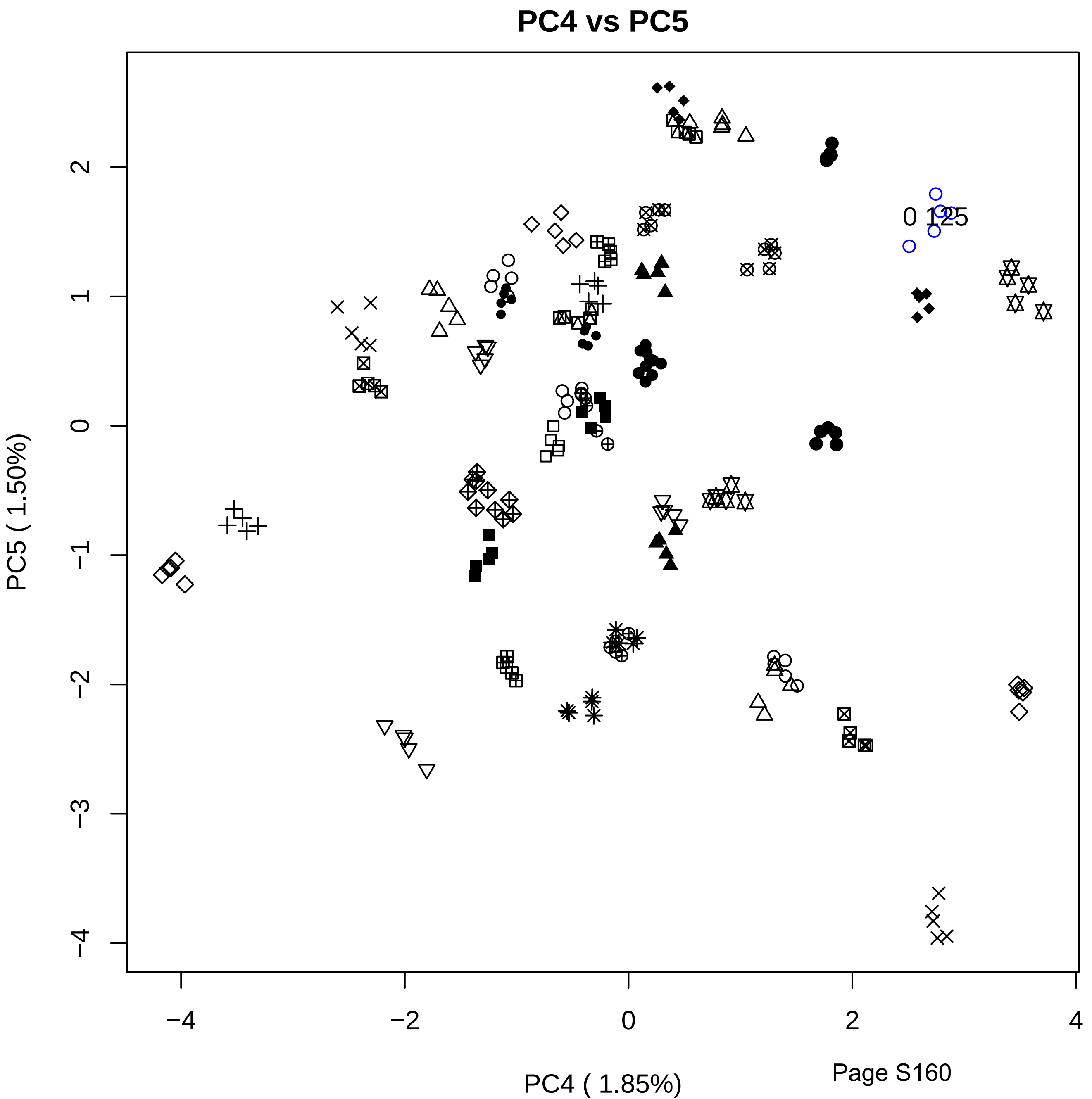
PC4 vs PC5
0 125
PC5 (1.50%)
PC4 (1.85%)
2
1
0
−1
−2
−3
−4
−4
−2
0
2
4

PCA of 0.25% OSCS vs PMH

Scaled Matrix PCAed

7th order polynomial baseline correction performed

Raw spectra smoothed to 21 Neighbours, 2nd order PN, Savtisky Golay

Normalisation between 0 and 100

D2 spectra smoothed to 41 Neighbours, 2nd order PN, Savtisky Golay

Regions Analysed: 3601−2501 and 2001−700cm−1

Samples Analysed: 1 Contam's 0 OSCS's 45 PMH's

Keele University AD   Sun Dec 09 16:50:42 2018

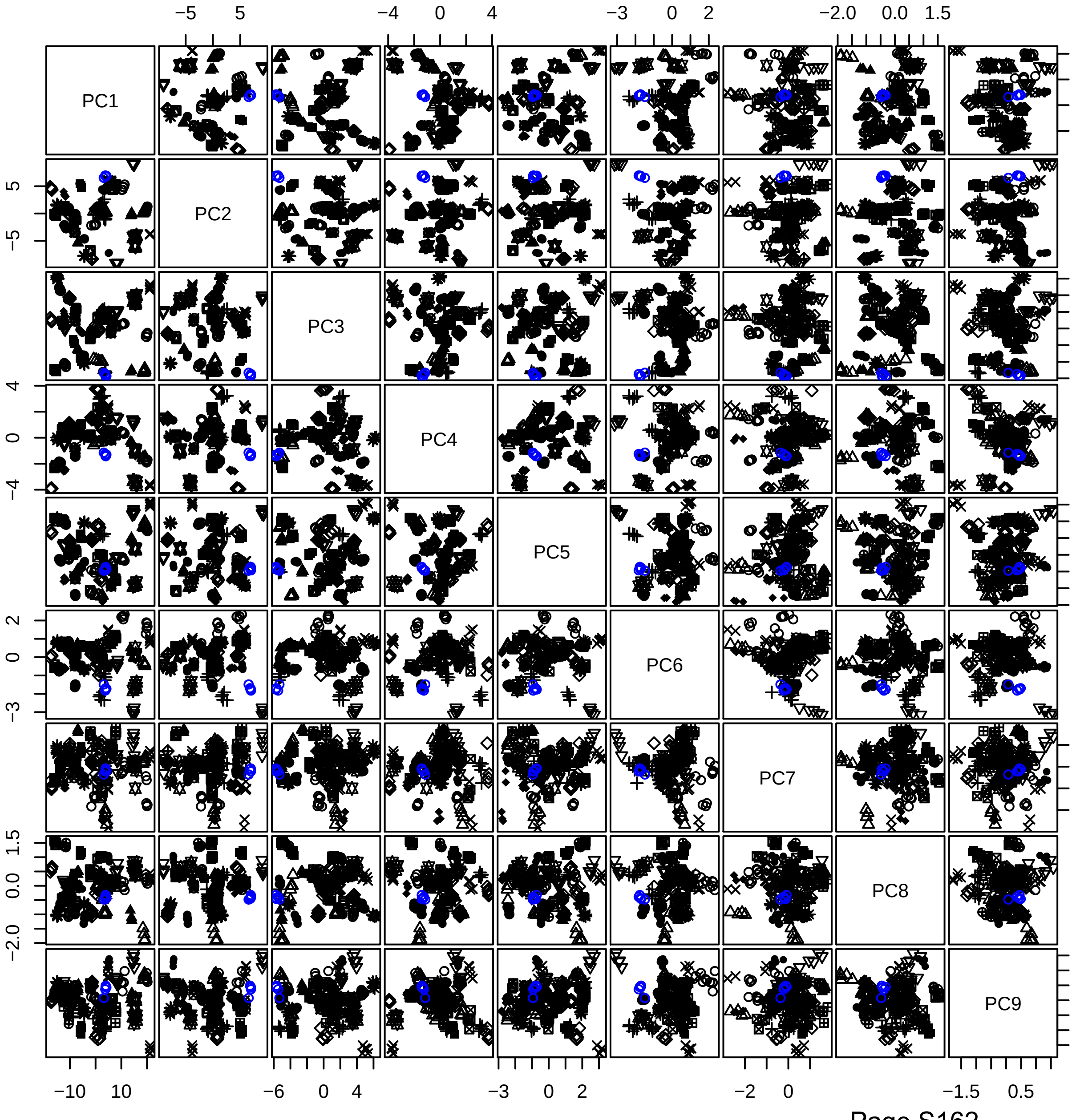

PC1
PC2
PC3
PC4
PC5
PC6
PC7
PC8
PC9

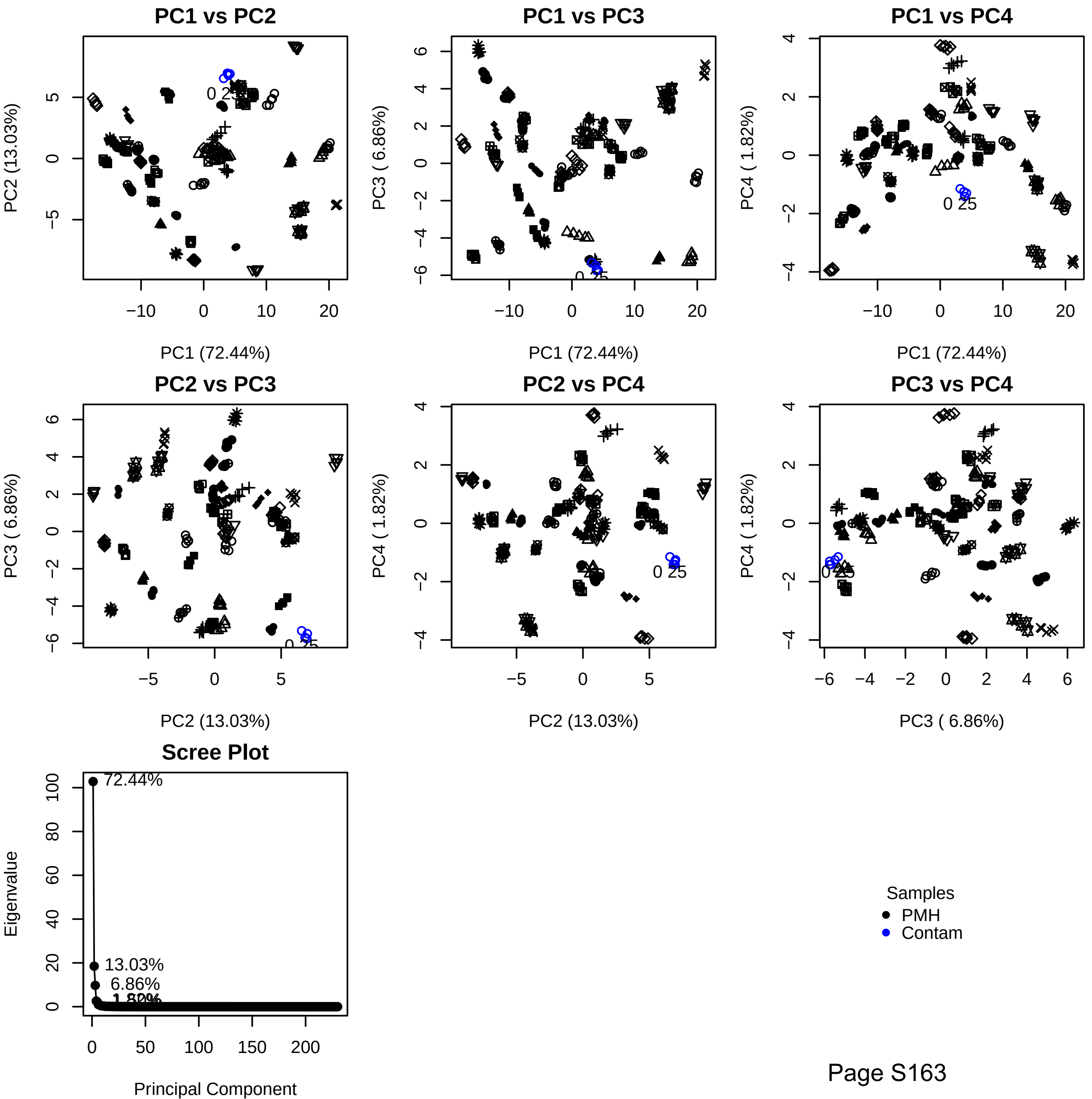
PC1 vs PC2
PC1 vs PC3
PC1 vs PC4
PC2 vs PC3
PC2 vs PC4
PC3 vs PC4
PC1 (72.44%)
PC2 (13.03%)
PC3 (6.86%)
PC4 (1.82%)
Scree Plot
Eigenvalue
Principal Component
72.44%
13.03%
6.86%
Samples
PMH
Contam

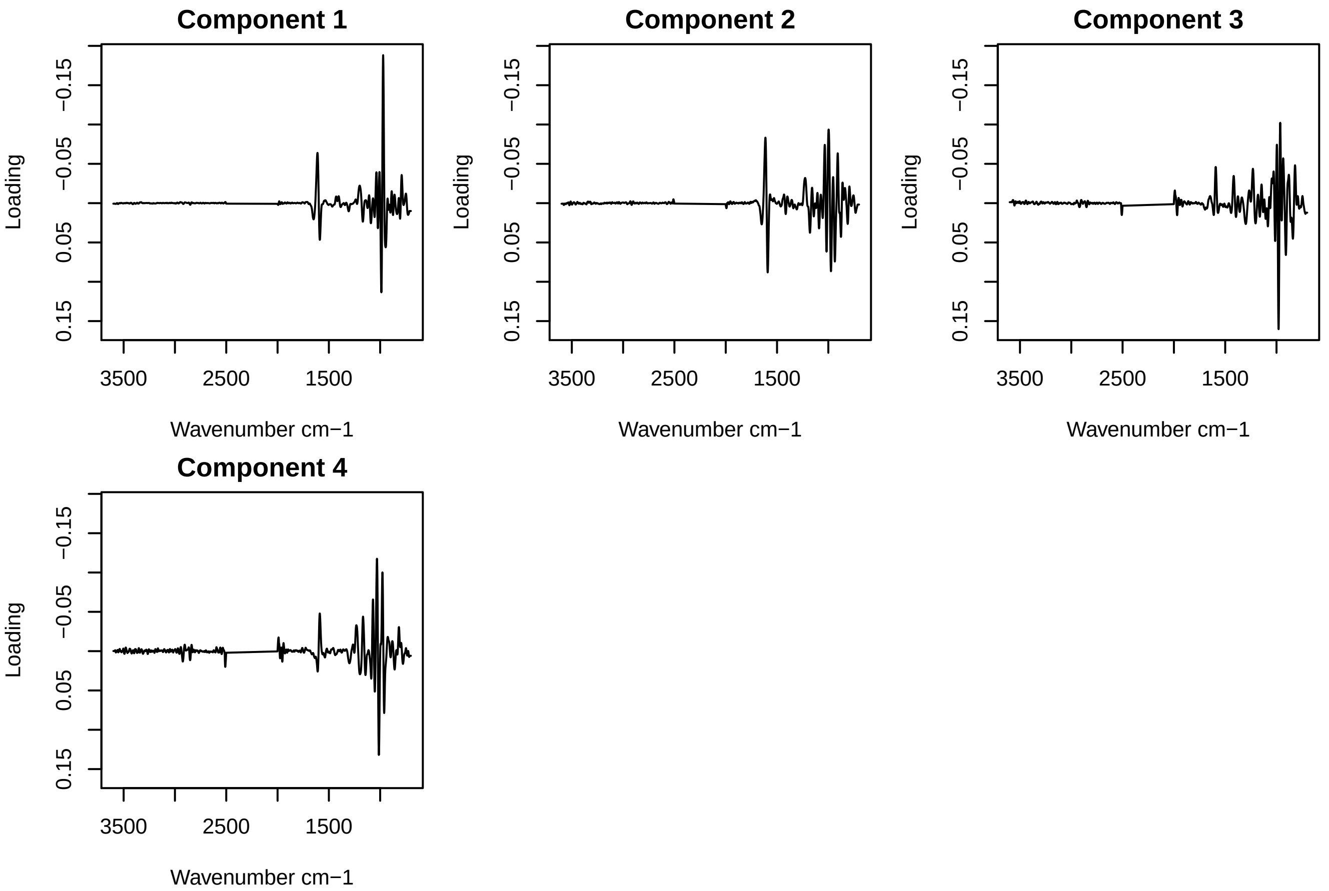
Component 1
Component 2
Component 3
Component 4
Loading
−0.15
−0.05
0.05
0.15
3500
2500
1500
Wavenumber cm−1

PC1 vs PC2 vs PC3

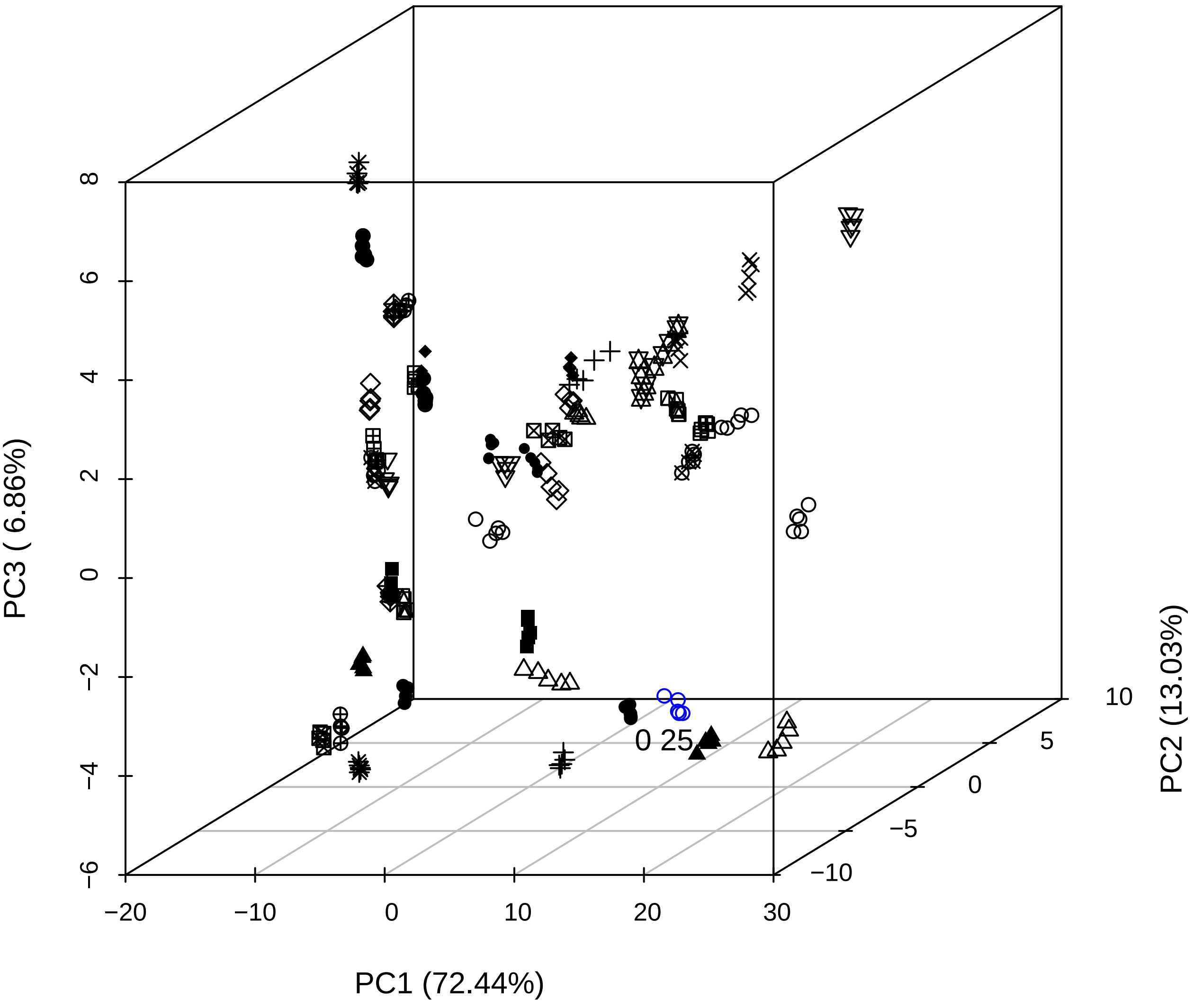

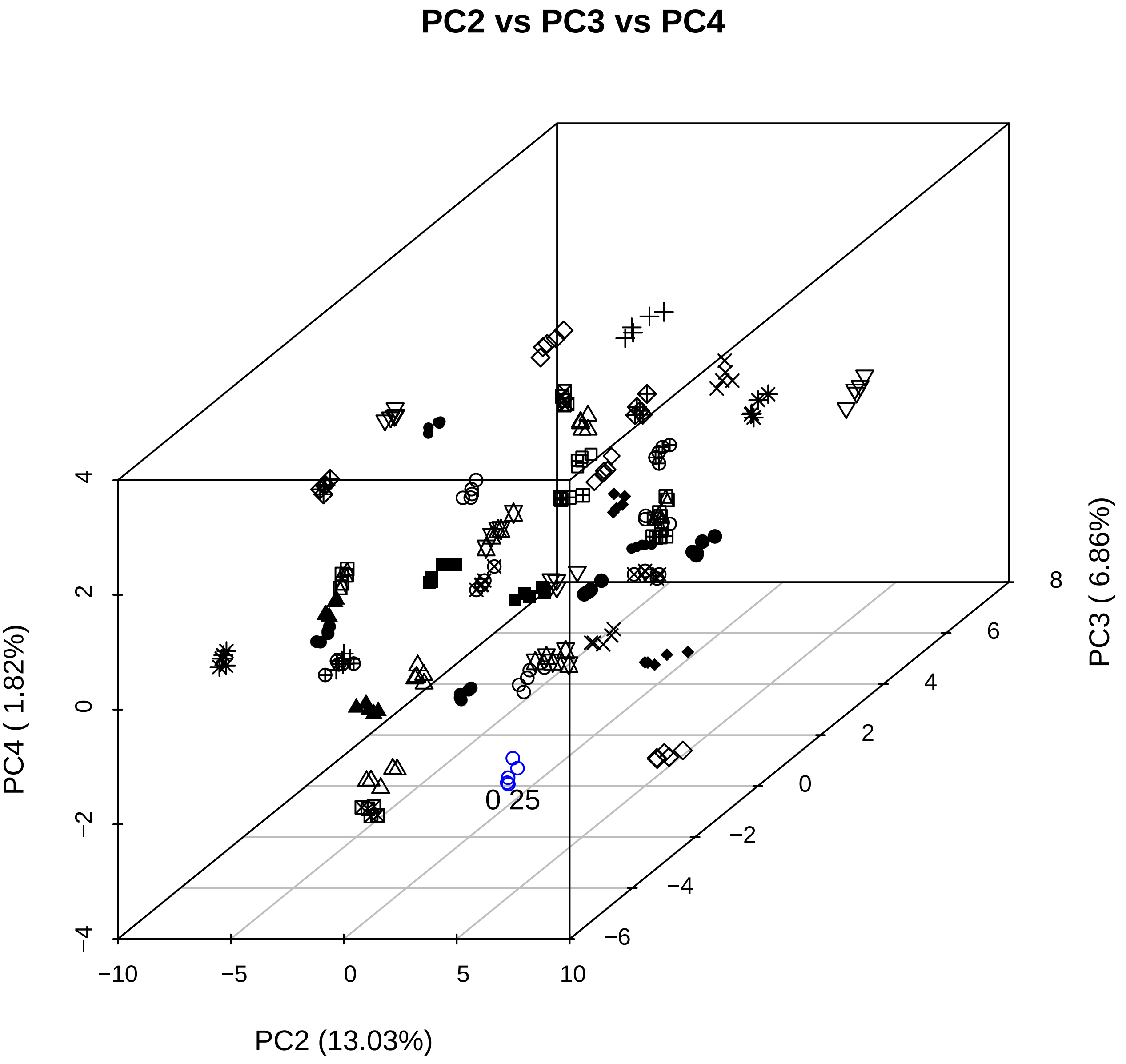
PC2 vs PC3 vs PC4
PC2 (13.03%)
PC4 (1.82%)
PC3 (6.86%)
−10
−5
0
5
10
−4
−2
0
2
4
−6
−4
−2
0
2
4
6
8
0 25

PC1 vs PC2 vs PC4

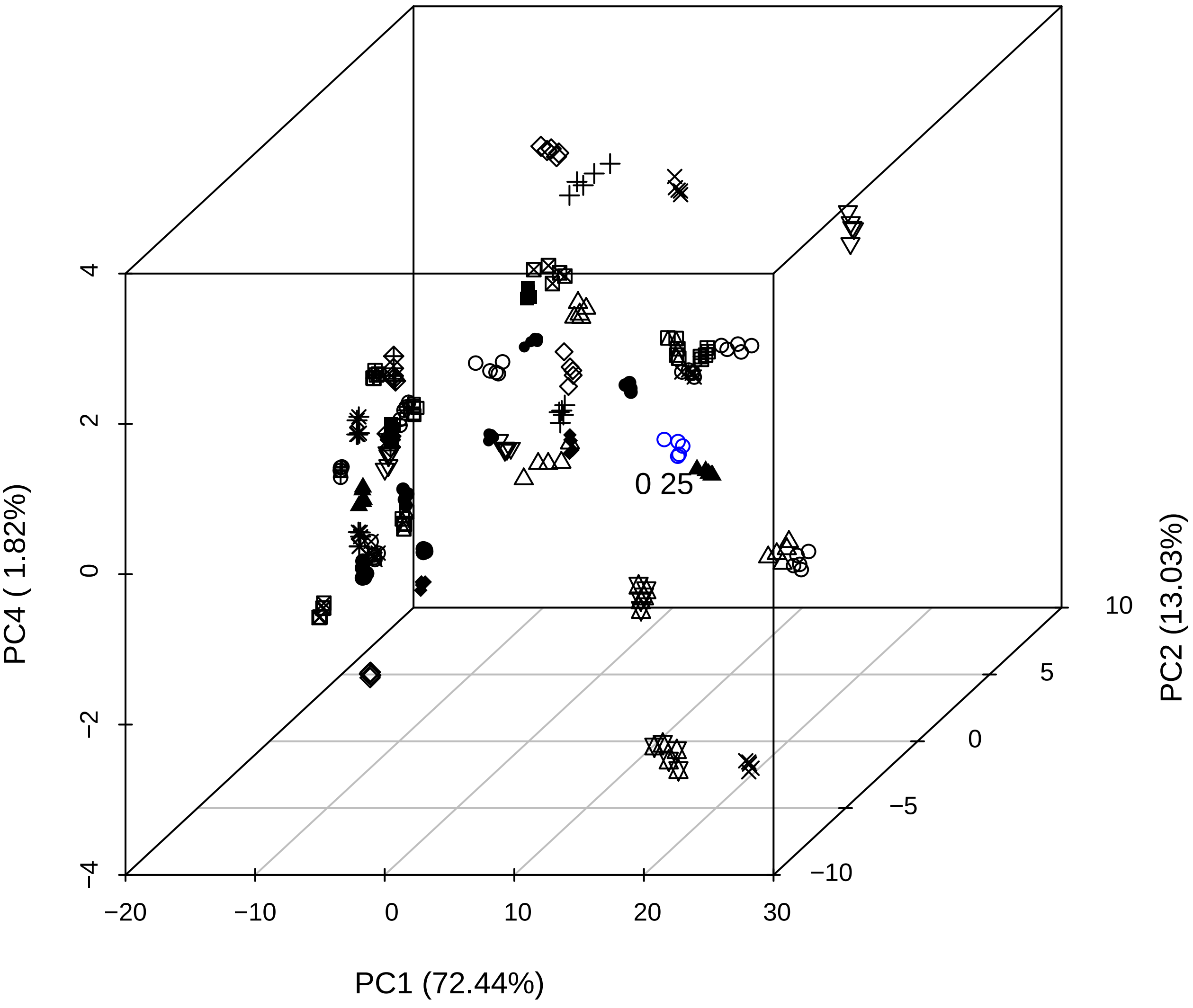

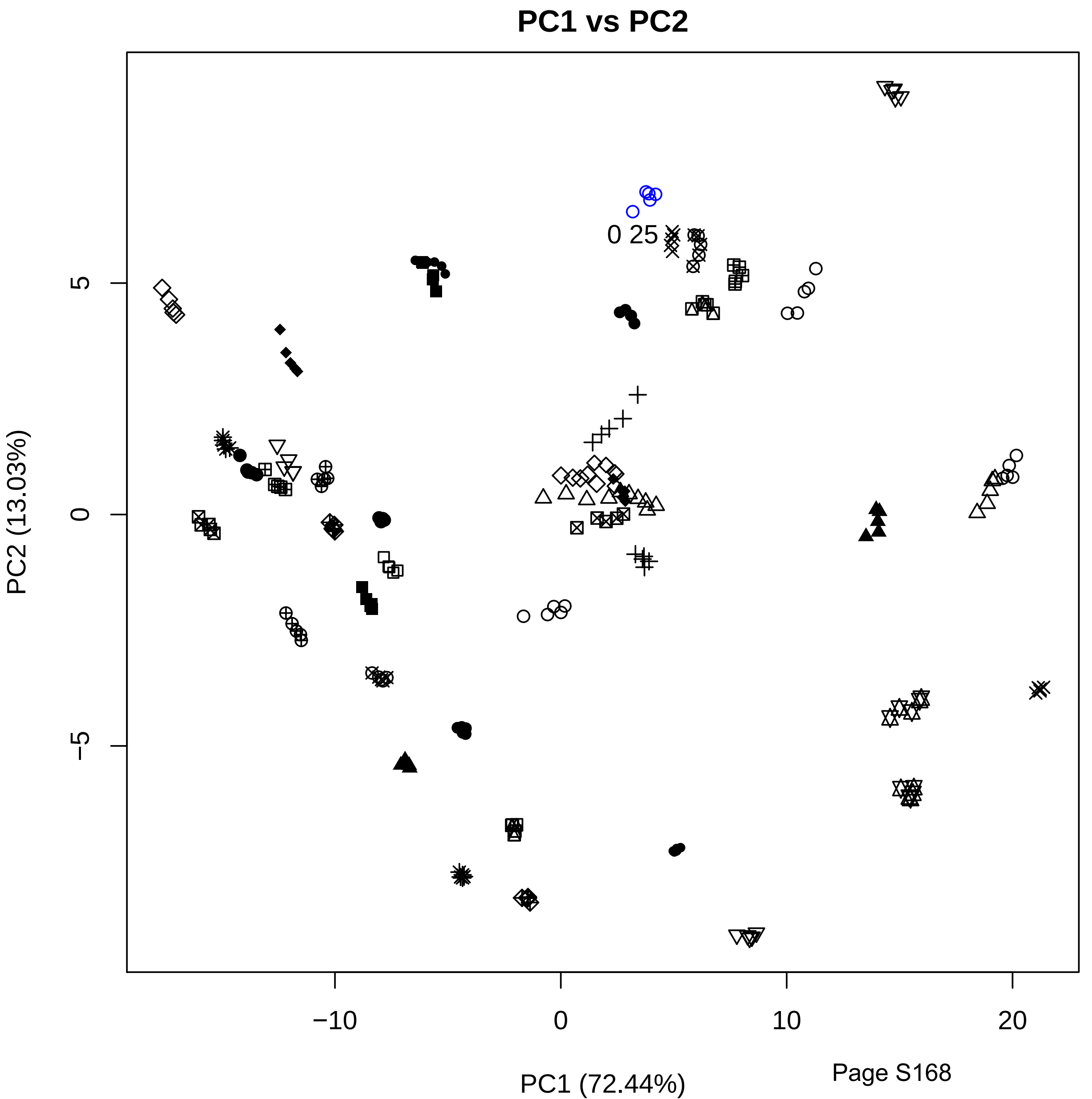
PC1 vs PC2
PC2 (13.03%)
PC1 (72.44%)
5
0
−5
−10
0
10
20
0 25

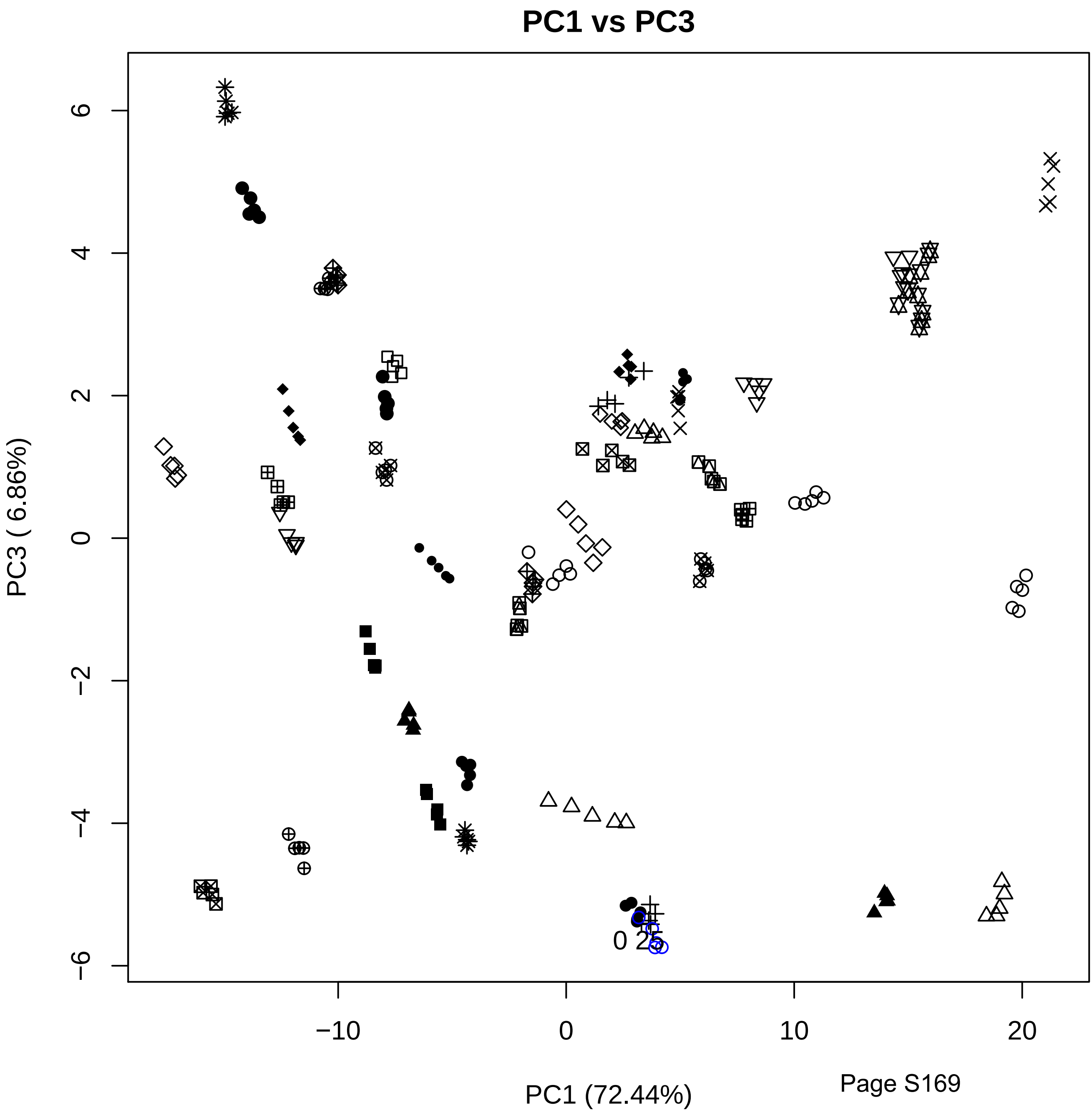
PC1 vs PC3
PC3 (6.86%)
PC1 (72.44%)
6
4
2
0
−2
−4
−6
−10
0
10
20
0 25

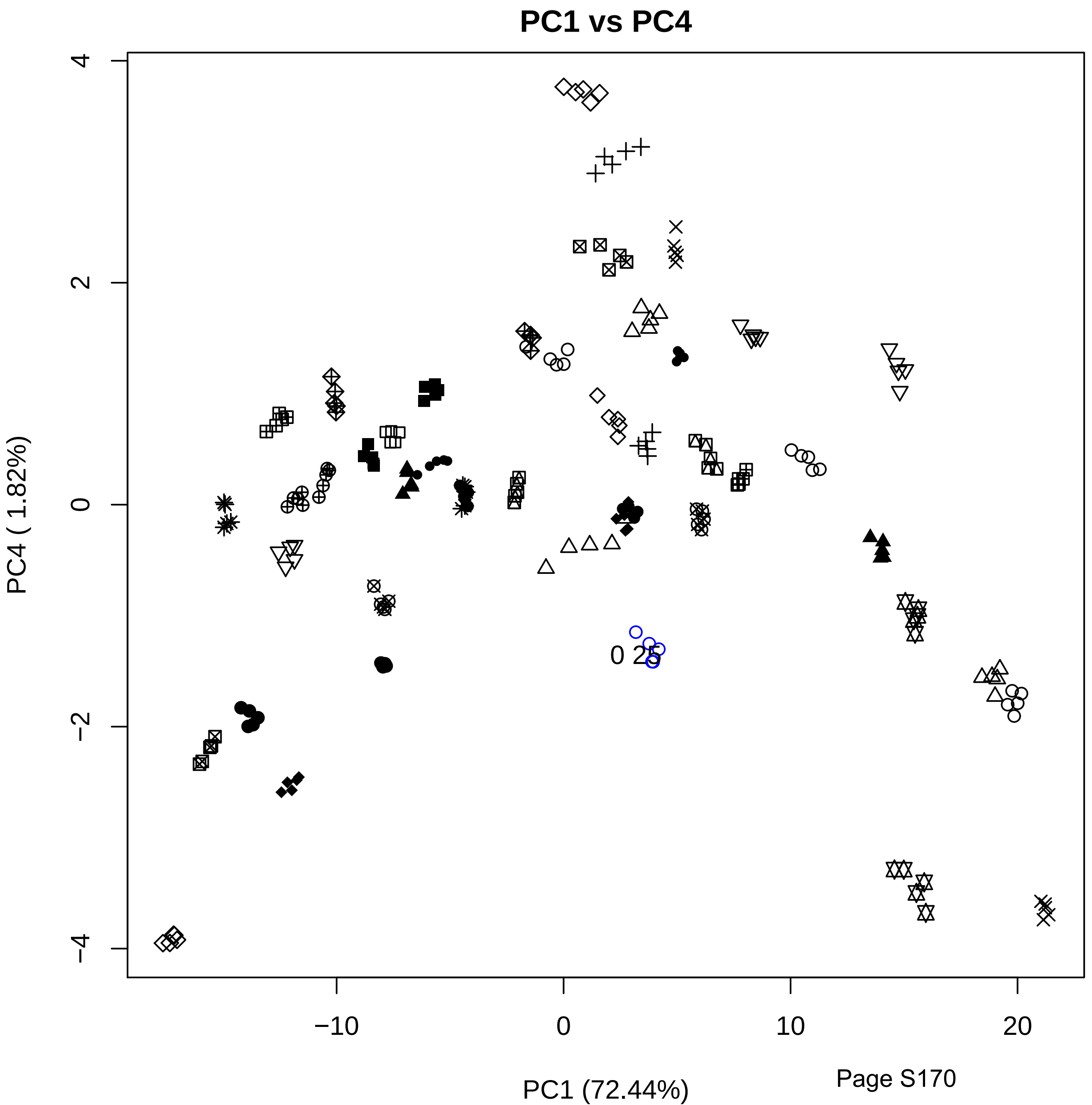
PC1 vs PC4
PC4 (1.82%)
PC1 (72.44%)
4
2
0
−2
−4
−10
0
10
20
0 25

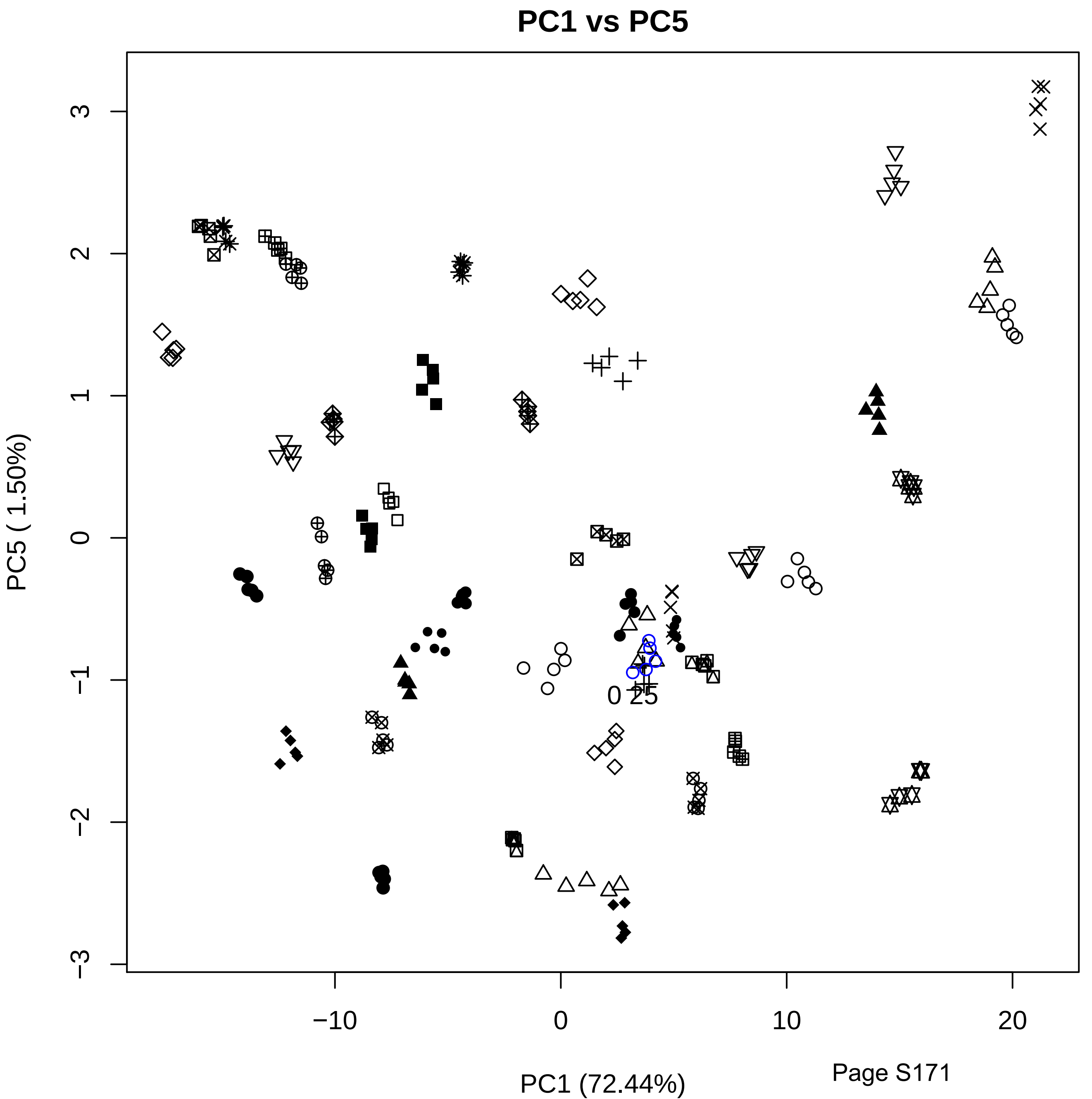

PC1 vs PC5
PC5 (1.50%)
PC1 (72.44%)
3
2
1
0
−1
−2
−3
−10
0
10
20
0 25

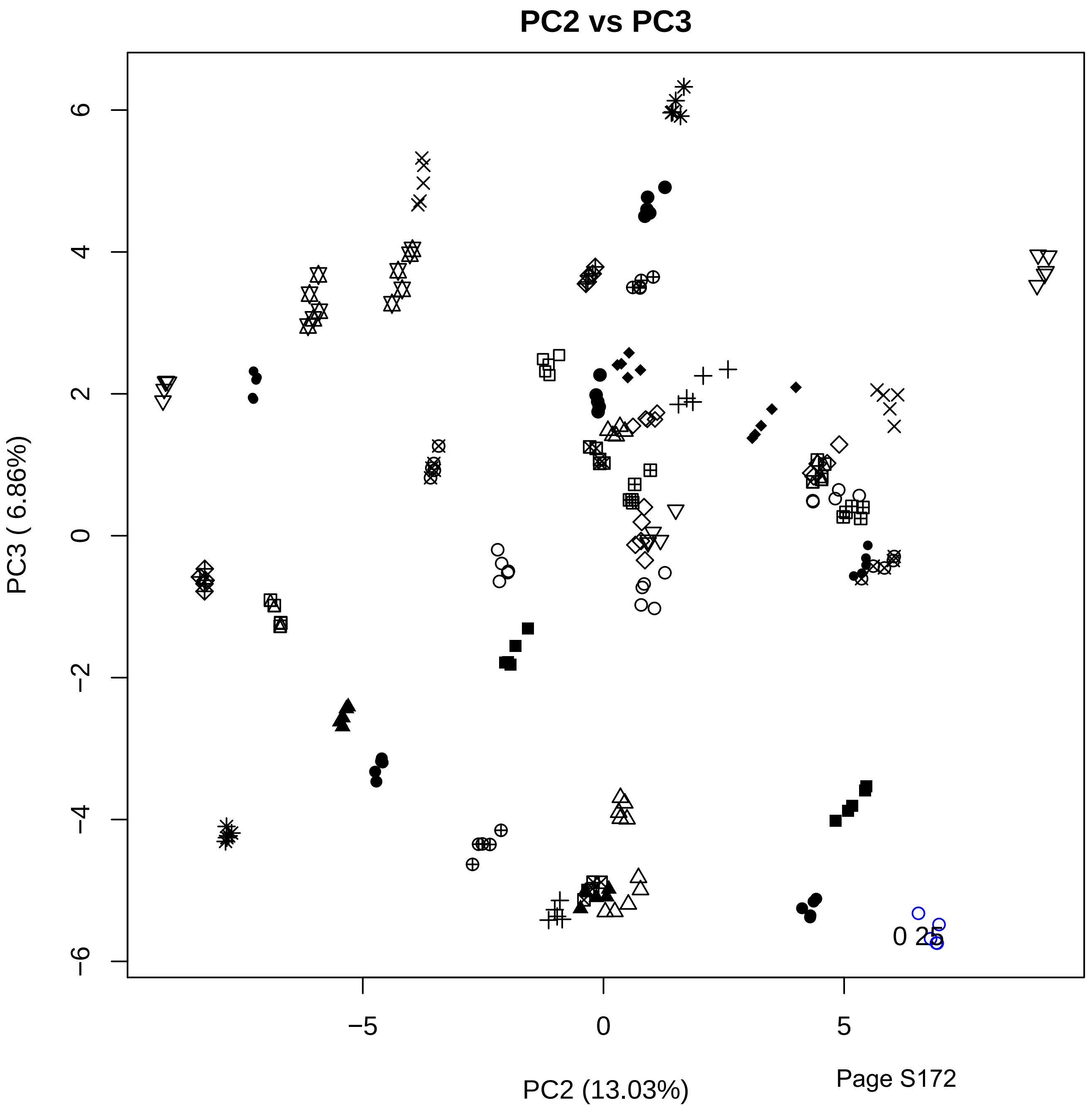
PC2 vs PC3
PC3 (6.86%)
PC2 (13.03%)
6
4
2
0
−2
−4
−6
−5
0
5
0 25

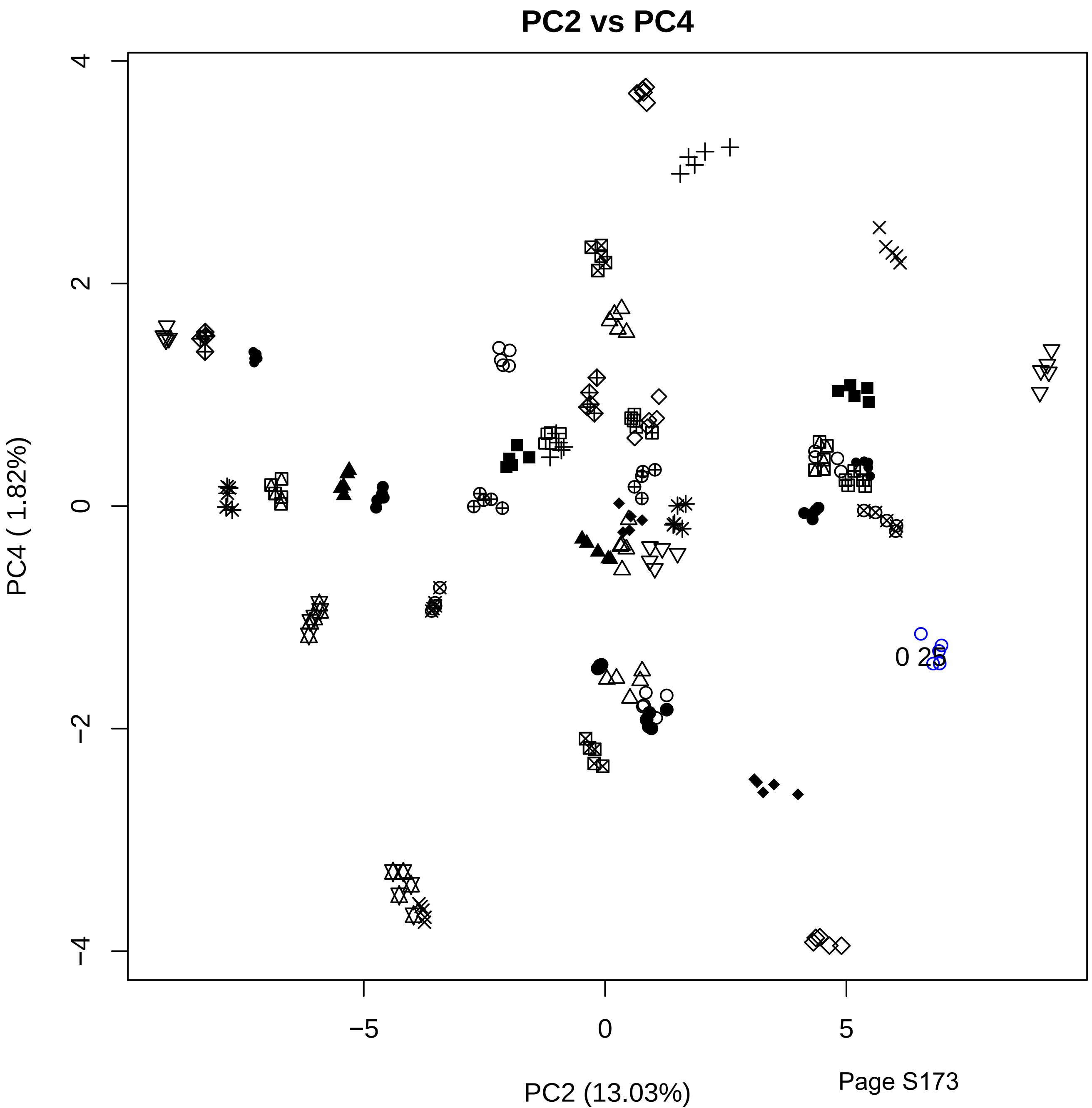
PC2 vs PC4
PC4 (1.82%)
PC2 (13.03%)
4
2
0
−2
−4
−5
0
5
0 25

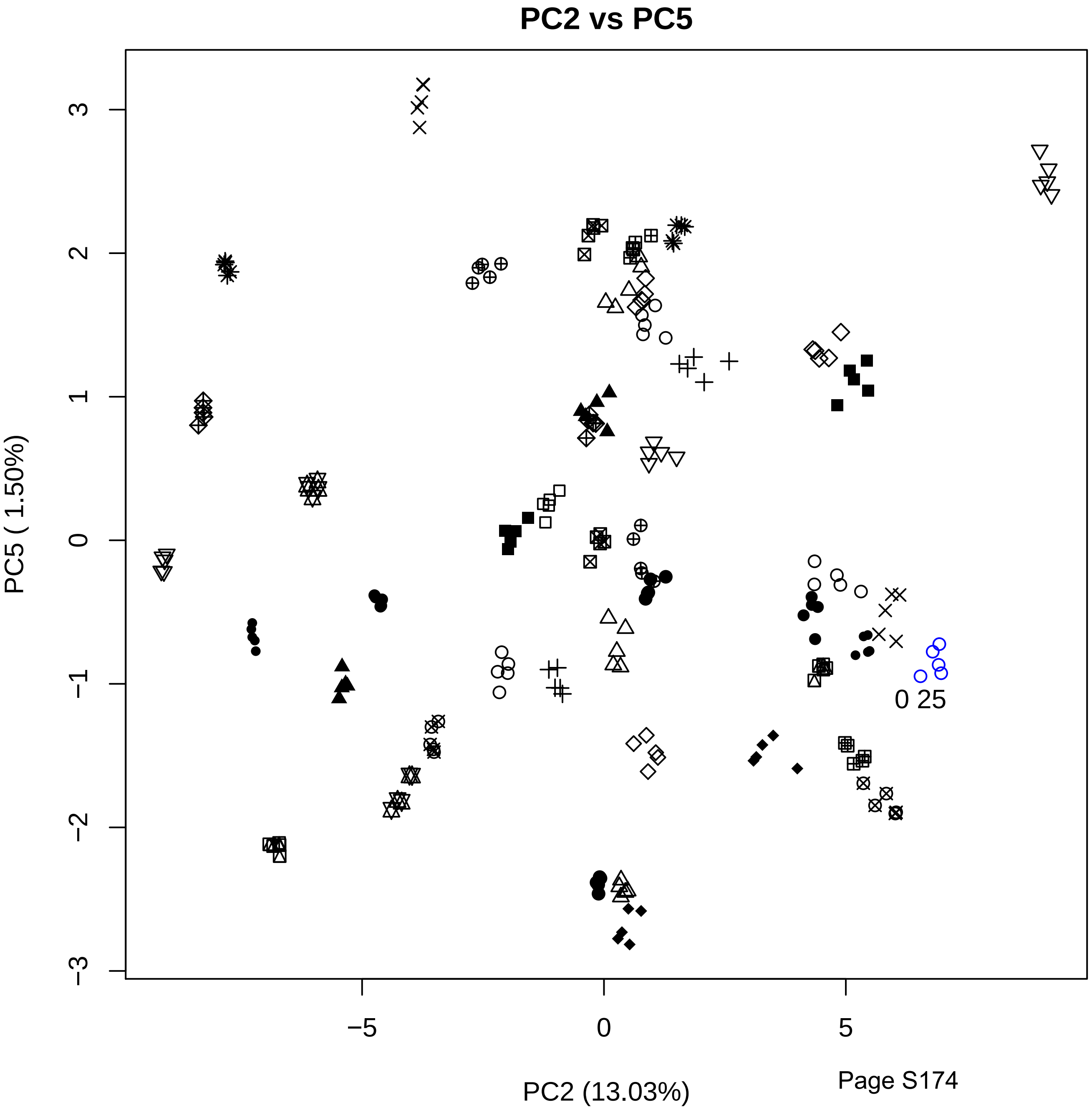
PC2 vs PC5
PC5 (1.50%)
PC2 (13.03%)
3
2
1
0
−1
−2
−3
−5
0
5
0 25

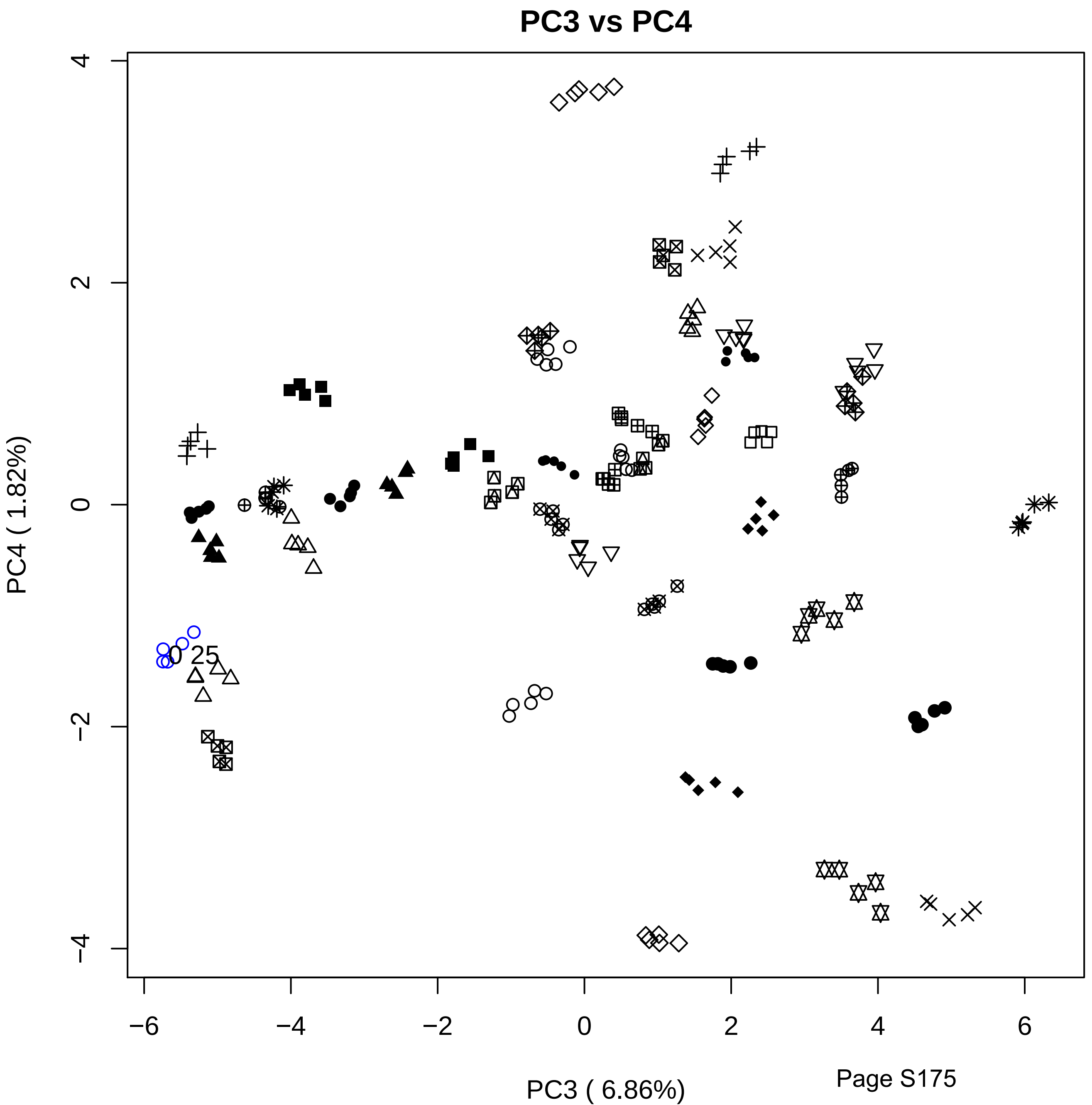
PC3 vs PC4
PC4 (1.82%)
PC3 (6.86%)
4
2
0
−2
−4
−6
−4
−2
0
2
4
6
0 25

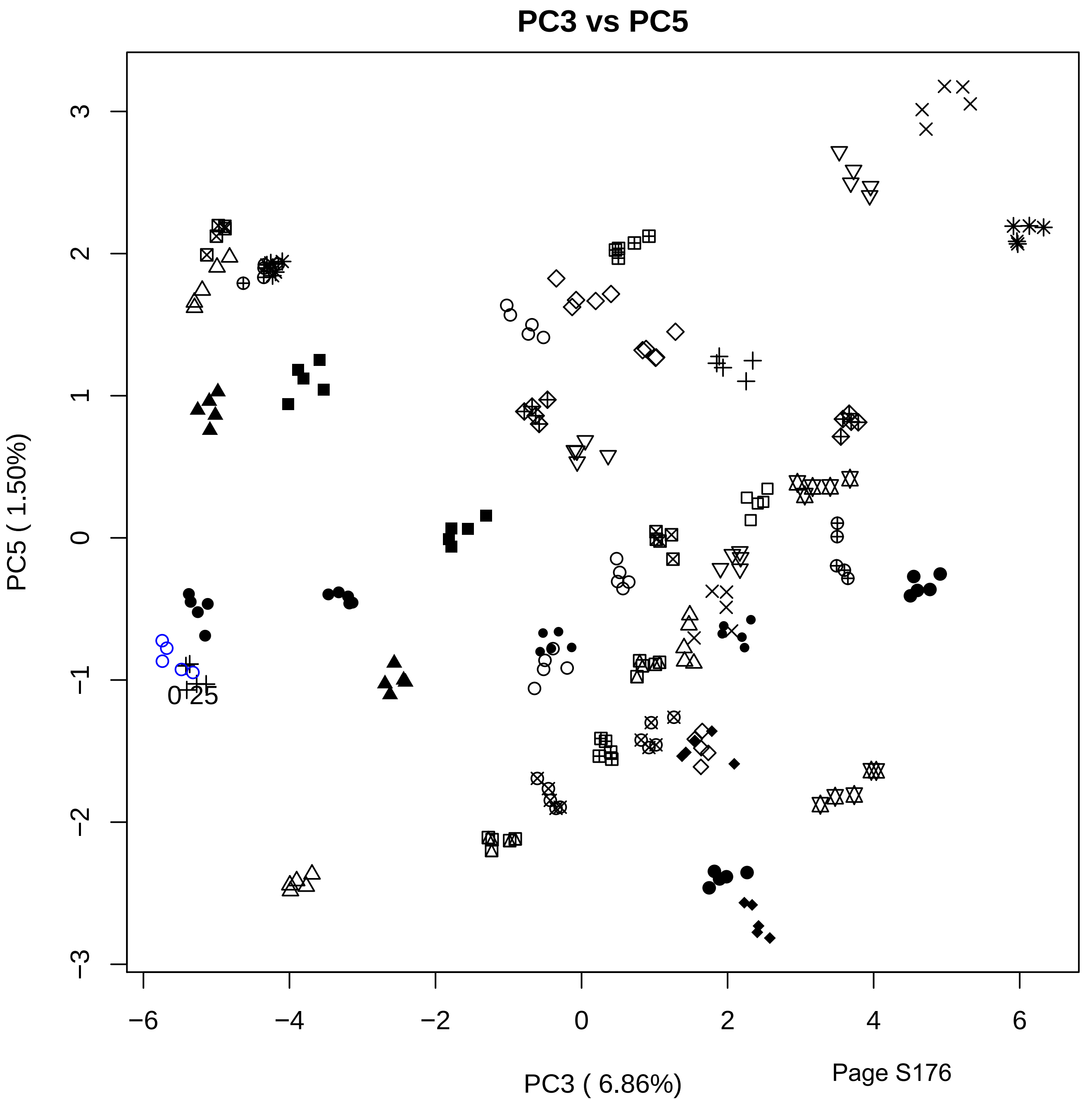

PC3 vs PC5
PC5 (1.50%)
PC3 (6.86%)
0.25
−6
−4
−2
0
2
4
6
−3
−2
−1
0
1
2
3

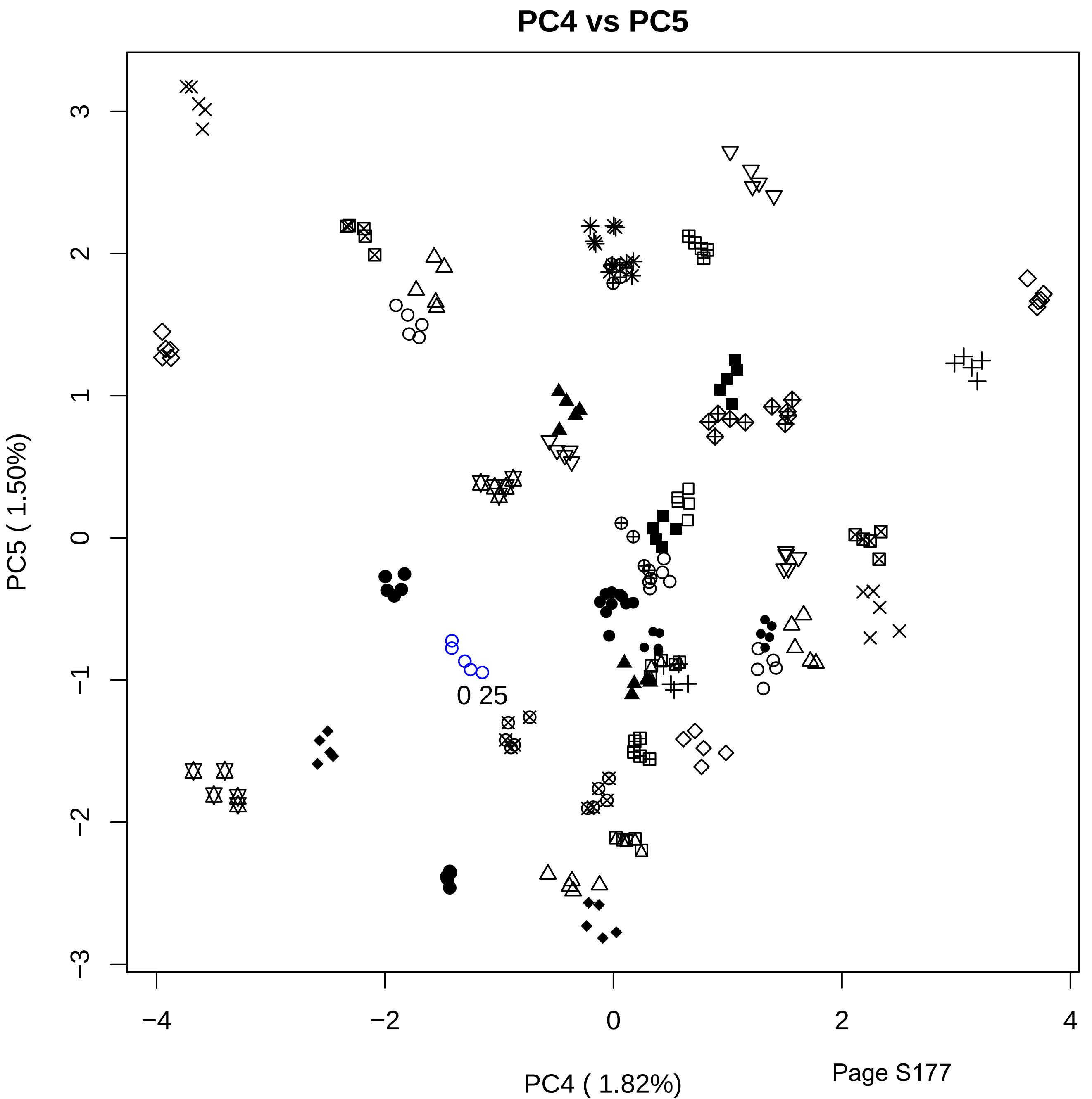
PC4 vs PC5
PC5 (1.50%)
PC4 (1.82%)
3
2
1
0
−1
−2
−3
−4
−2
0
2
4
0 25

PCA of 0.5% OSCS vs PMH

Scaled Matrix PCAed

7th order polynomial baseline correction performed

Raw spectra smoothed to 21 Neighbours, 2nd order PN, Savtisky Golay

Normalisation between 0 and 100

D2 spectra smoothed to 41 Neighbours, 2nd order PN, Savtisky Golay

Regions Analysed: 3601−2501 and 2001−700cm−1

Samples Analysed: 1 Contam's 0 OSCS's 45 PMH's

Keele University AD

Sun Dec 09 16:50:48 2018

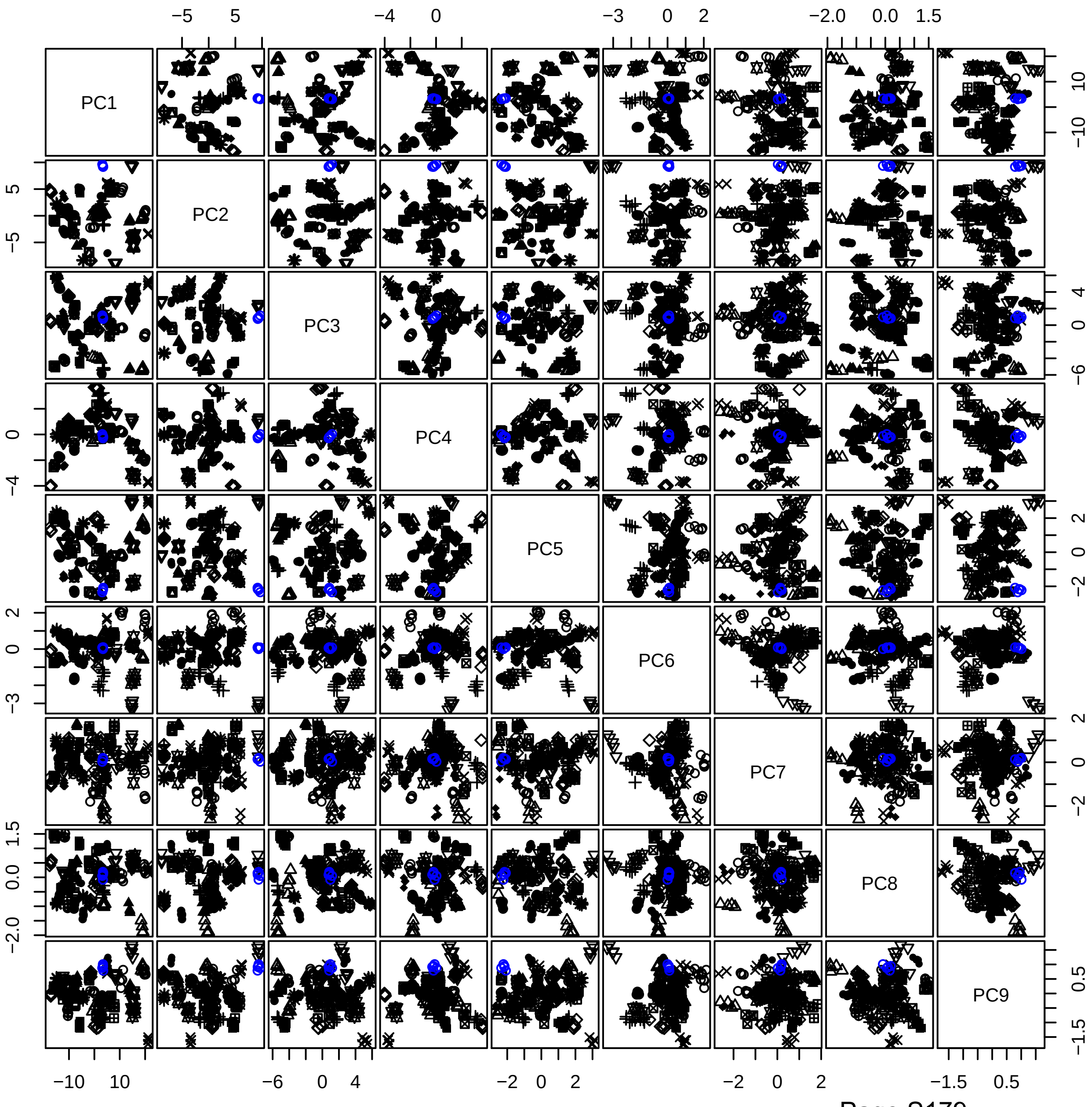
PC1
PC2
PC3
PC4
PC5
PC6
PC7
PC8
PC9
−5
5
−4
0
−3
0
2
−2.0
0.0
1.5
10
−10
5
−5
4
0
−6
0
−4
2
0
−2
2
0
−3
2
0
−2
1.5
0.0
−2.0
0.5
−1.5
−10
10
−6
0
4
−2
0
2
−2
0
2
−1.5
0.5

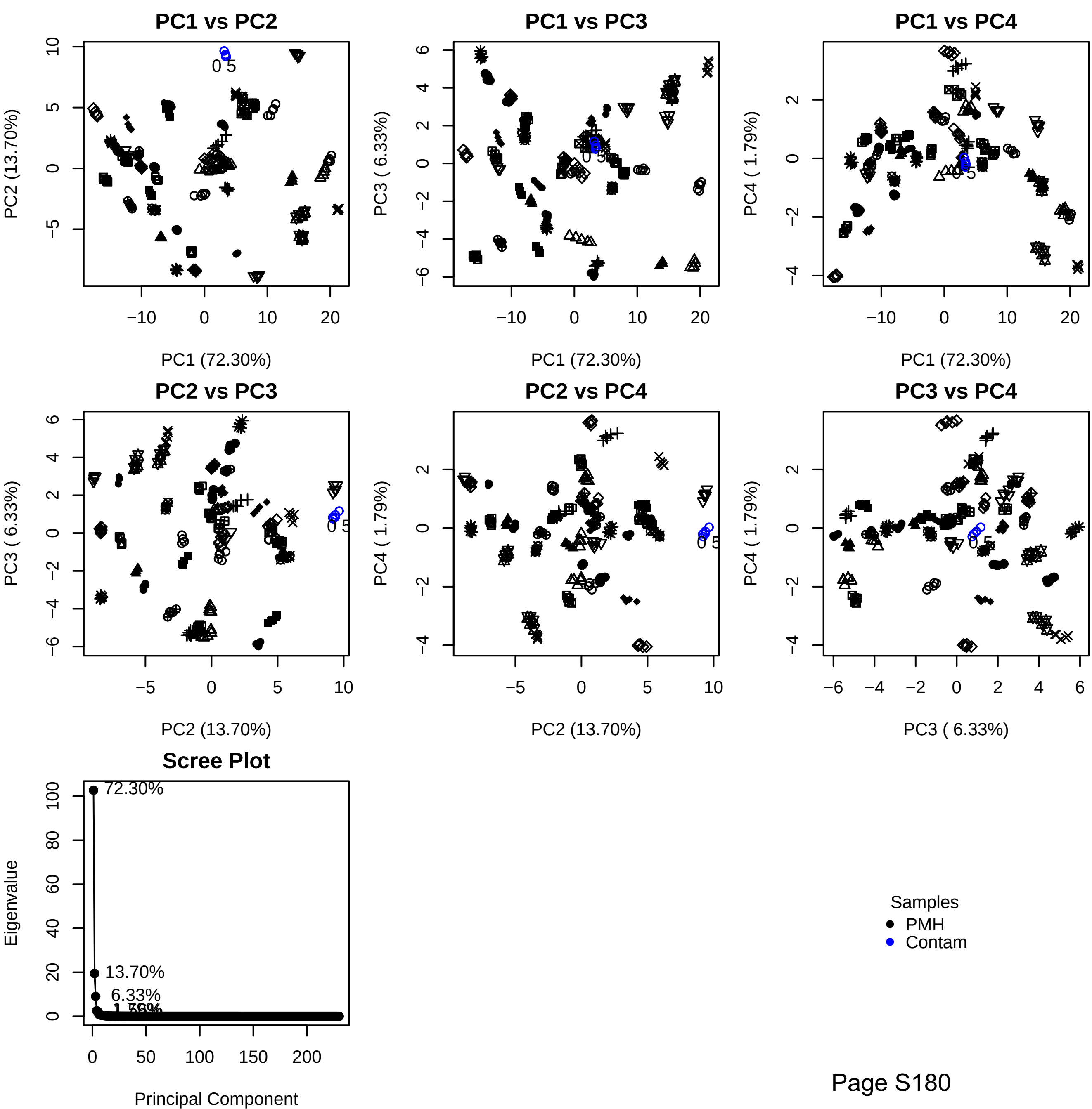
PC1 vs PC2
PC1 vs PC3
PC1 vs PC4
PC2 vs PC3
PC2 vs PC4
PC3 vs PC4
Scree Plot
PC1 (72.30%)
PC2 (13.70%)
PC3 (6.33%)
PC4 (1.79%)
Eigenvalue
Principal Component
72.30%
13.70%
6.33%
Samples
PMH
Contam

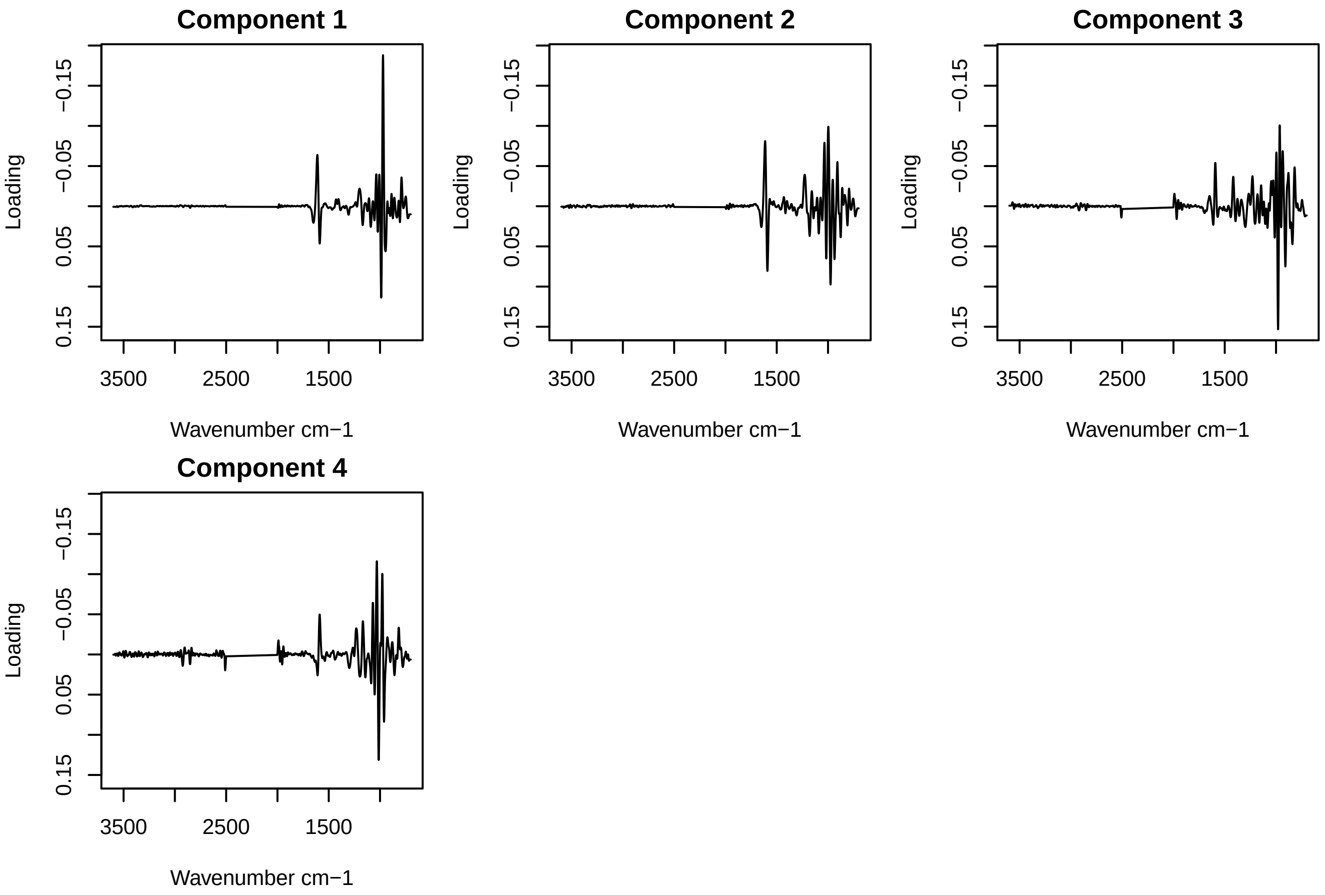

Component 1
Component 2
Component 3
Component 4
Loading
−0.15
−0.05
0.05
0.15
3500
2500
1500
Wavenumber cm−1

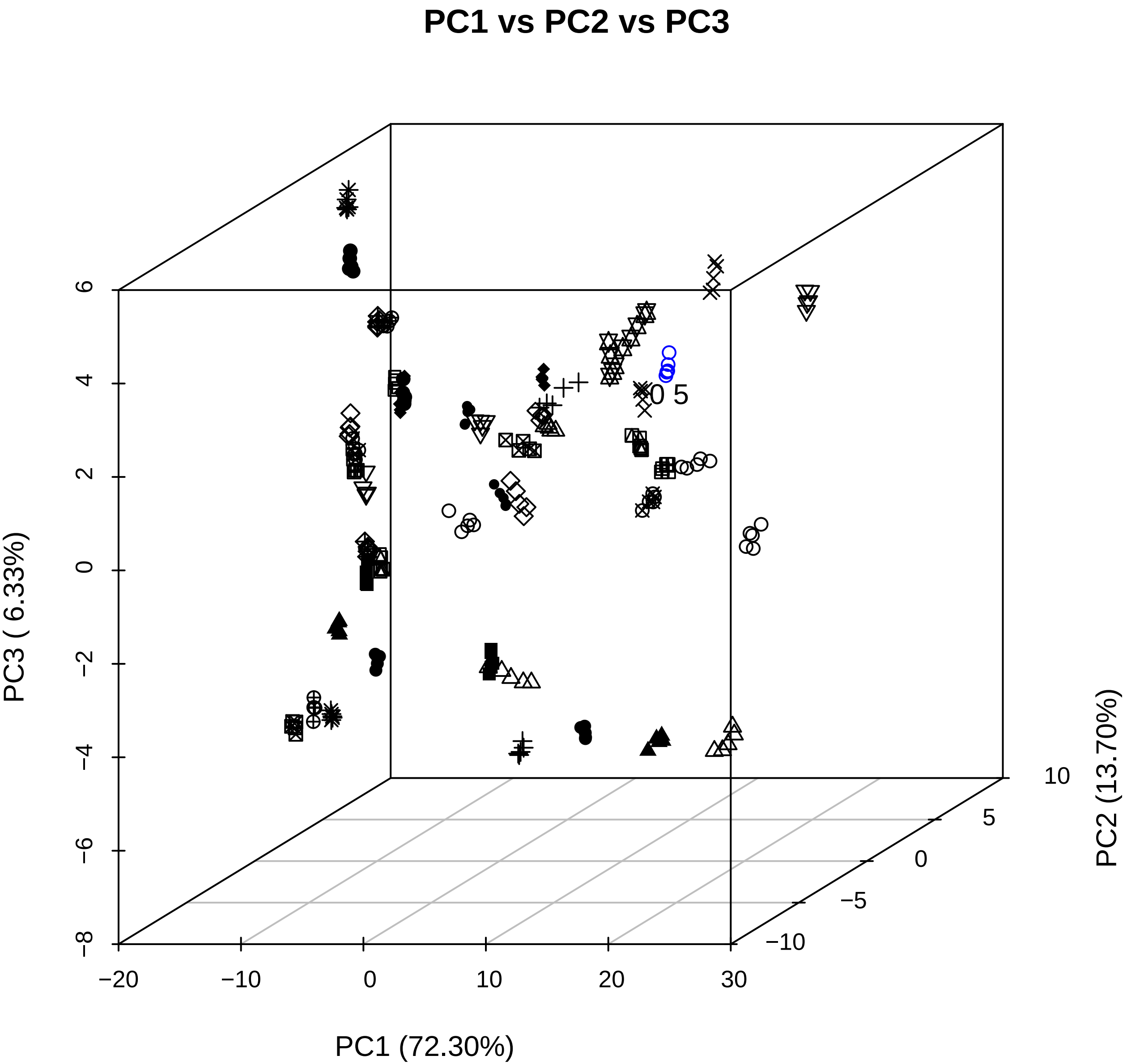
PC1 vs PC2 vs PC3
PC1 (72.30%)
PC2 (13.70%)
PC3 (6.33%)
−20
−10
0
10
20
30
−10
−5
0
5
10
−8
−6
−4
−2
0
2
4
6
0 5

PC2 vs PC3 vs PC4

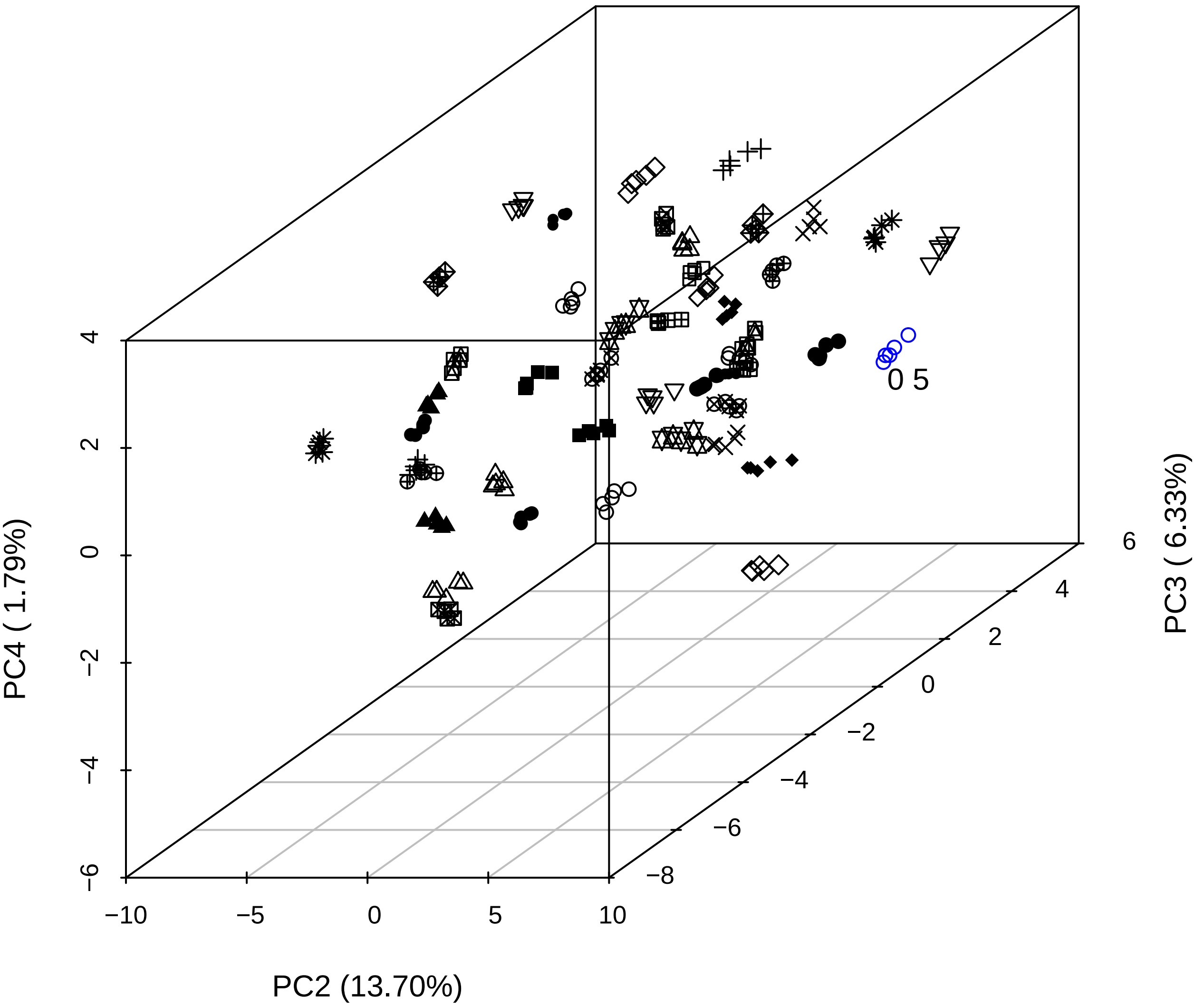

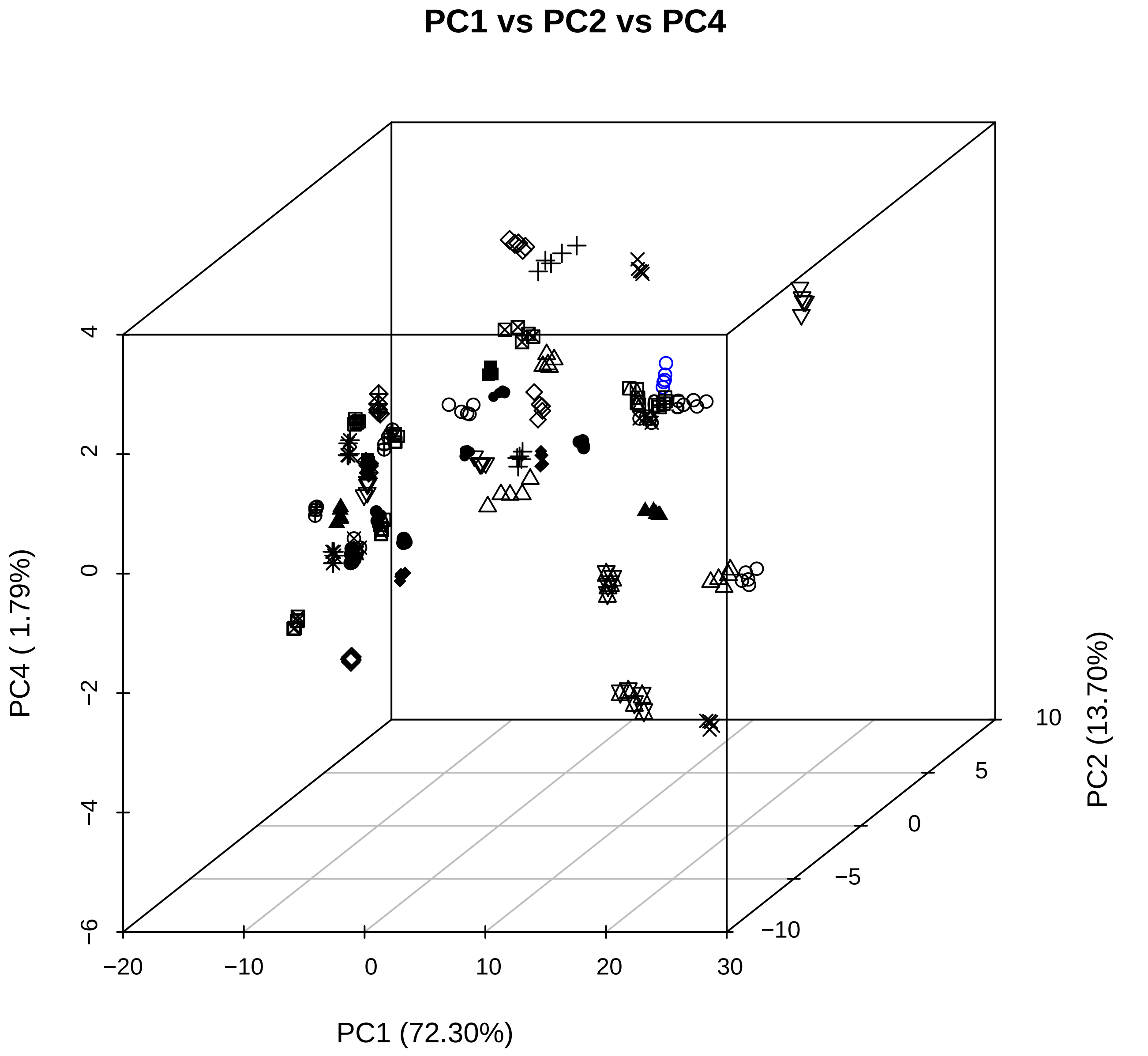
PC1 vs PC2 vs PC4
PC1 (72.30%)
PC2 (13.70%)
PC4 (1.79%)
−20
−10
0
10
20
30
−10
−5
0
5
10
−6
−4
−2
0
2
4

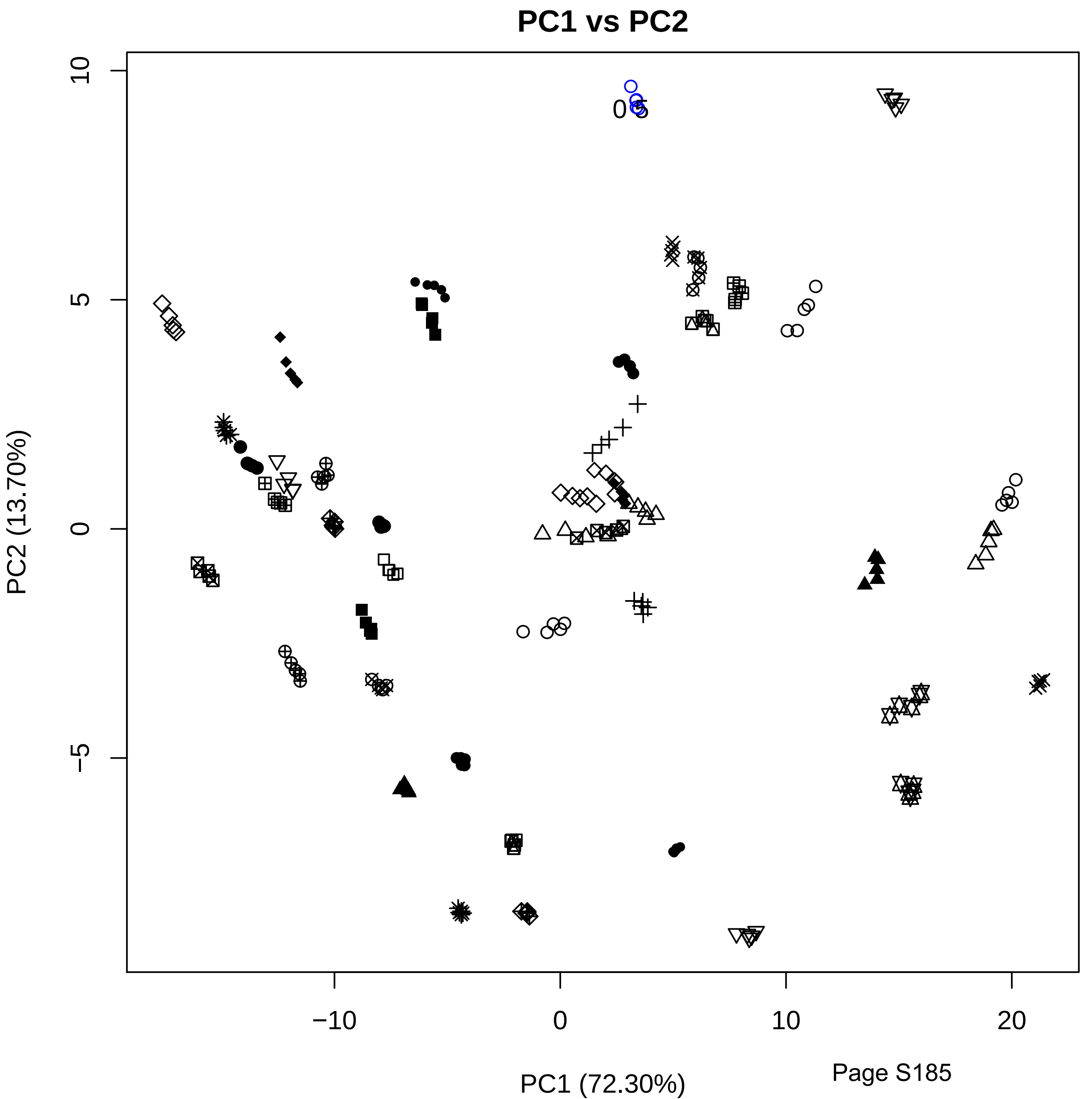
PC1 vs PC2
PC2 (13.70%)
PC1 (72.30%)
10
5
0
−5
−10
0
10
20
0 5

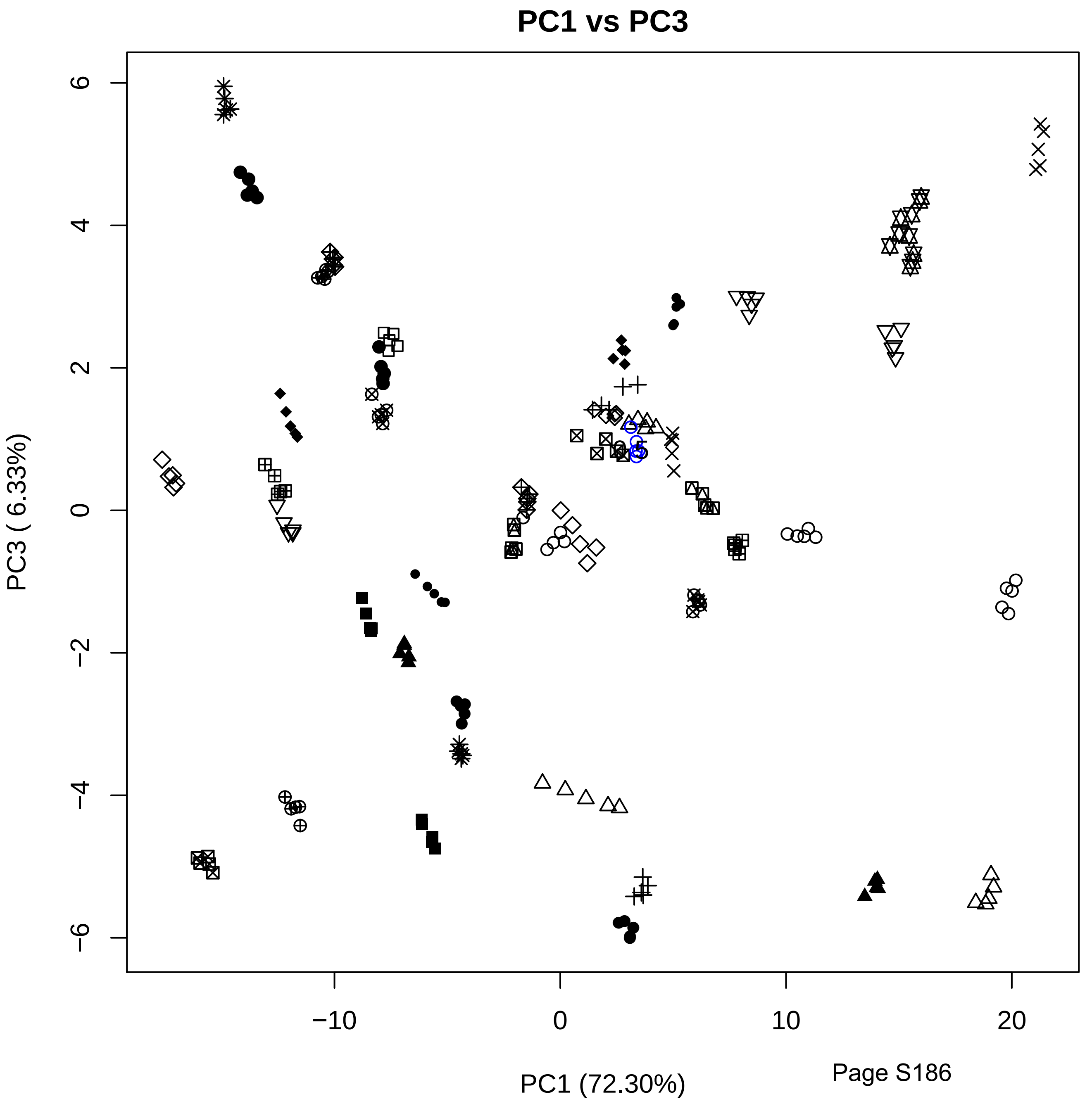
PC1 vs PC3
PC3 (6.33%)
PC1 (72.30%)
6
4
2
0
−2
−4
−6
−10
0
10
20

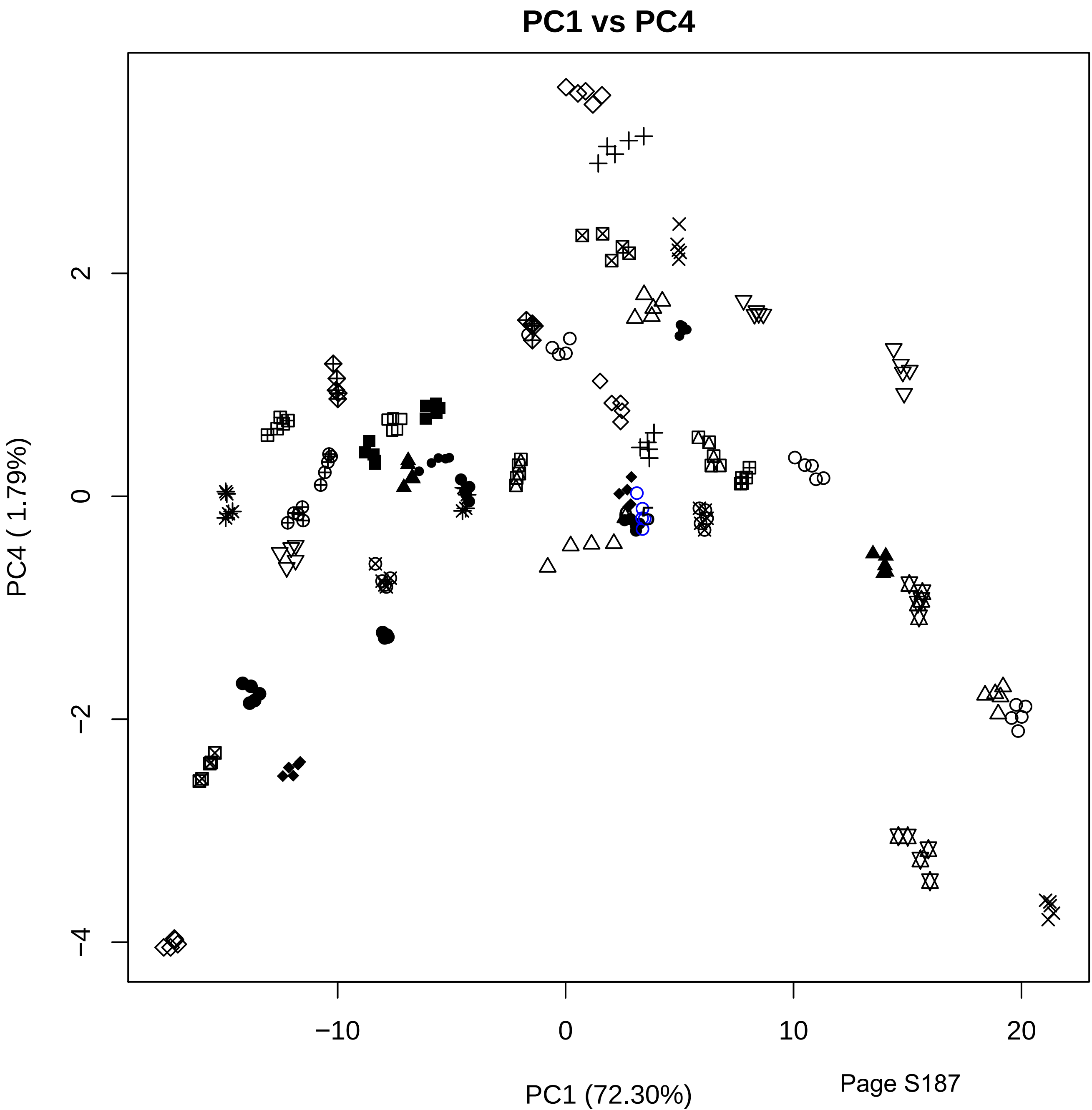
PC1 vs PC4
PC4 (1.79%)
PC1 (72.30%)
2
0
−2
−4
−10
0
10
20

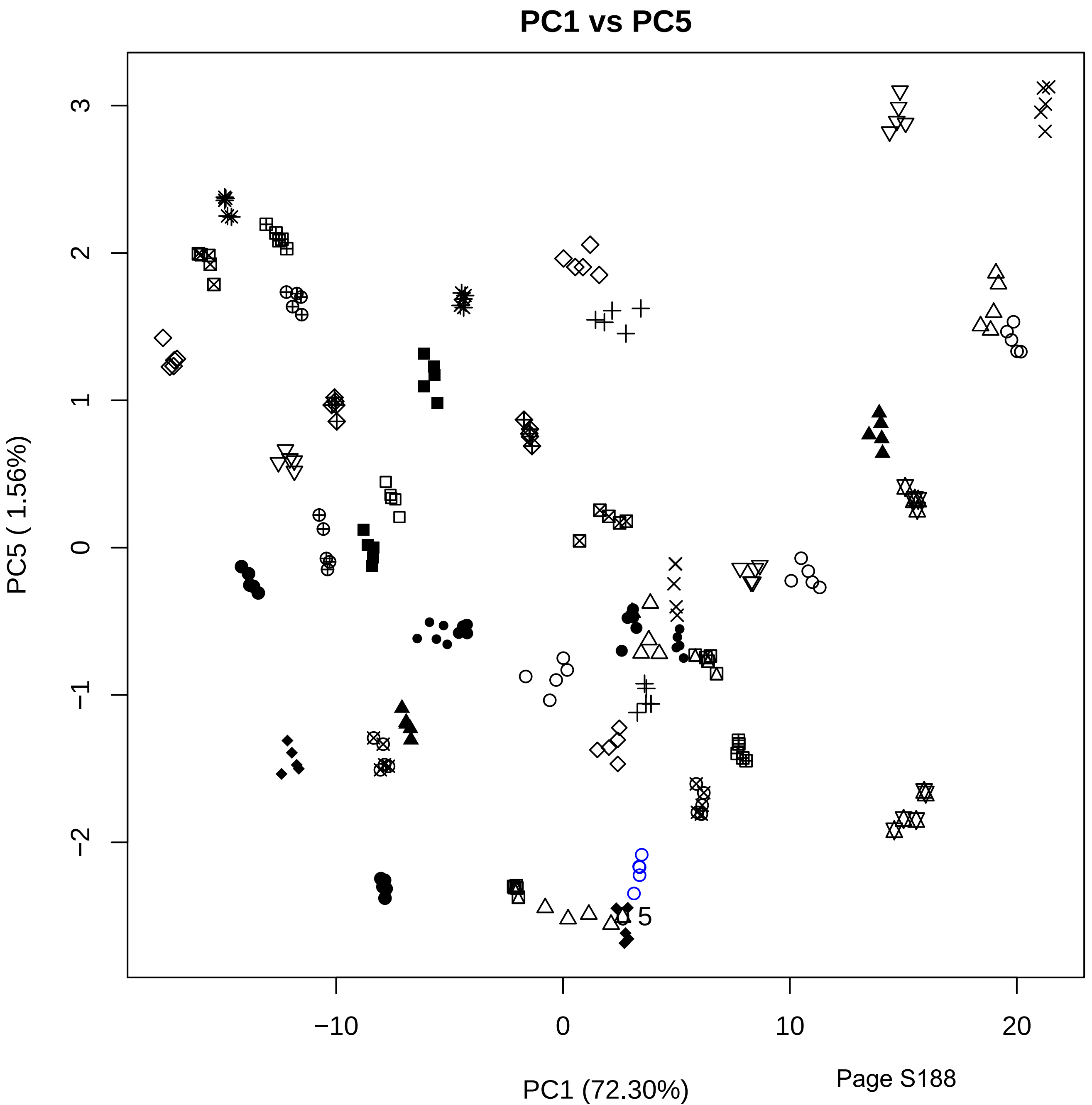
PC1 vs PC5
PC5 (1.56%)
PC1 (72.30%)
3
2
1
0
−1
−2
−10
0
10
20
5

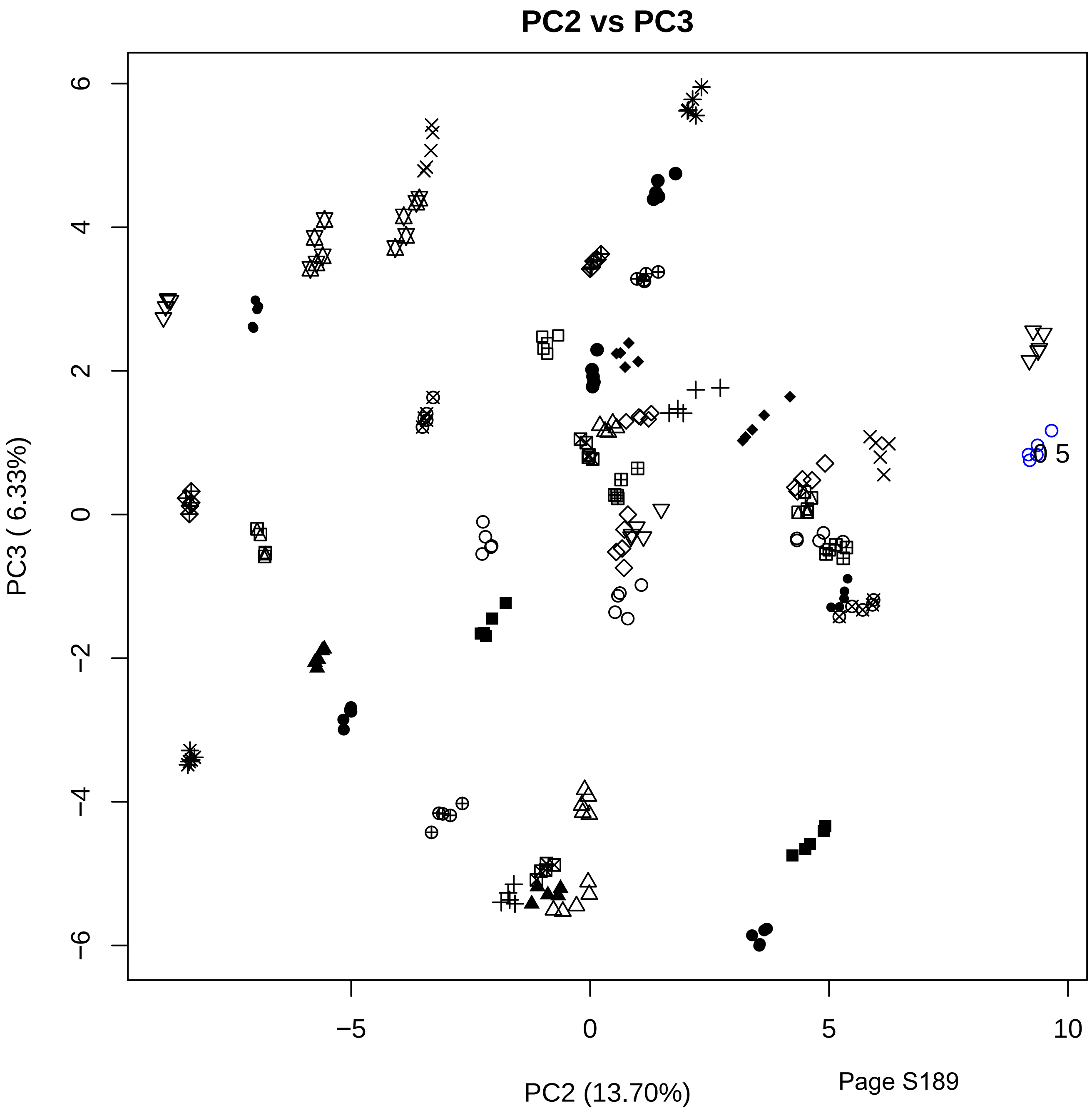

PC2 vs PC3
PC3 (6.33%)
PC2 (13.70%)
6
4
2
0
−2
−4
−6
−5
0
5
10
0 5

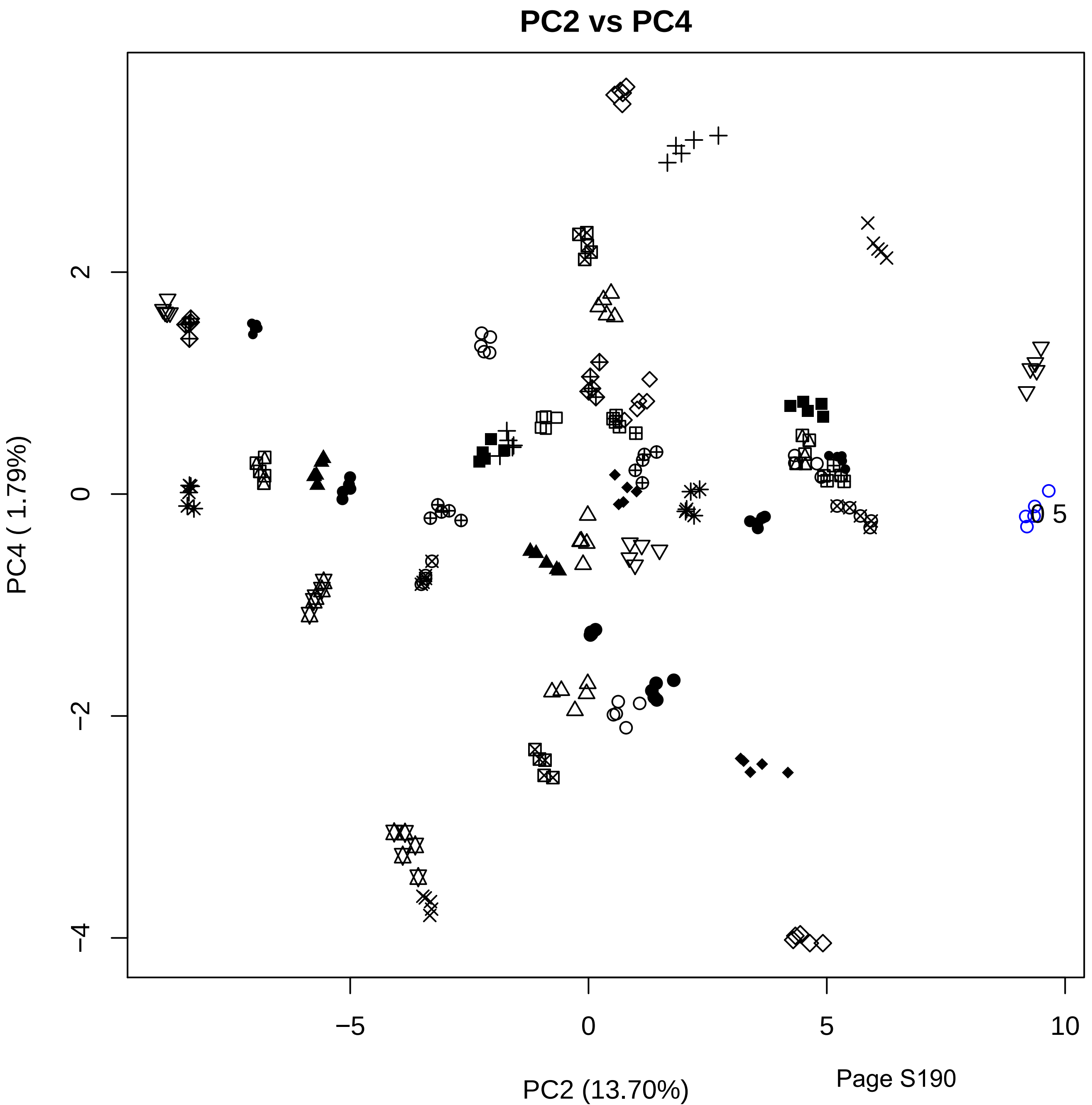
PC2 vs PC4
PC4 (1.79%)
PC2 (13.70%)
2
0
−2
−4
−5
0
5
10
0 5

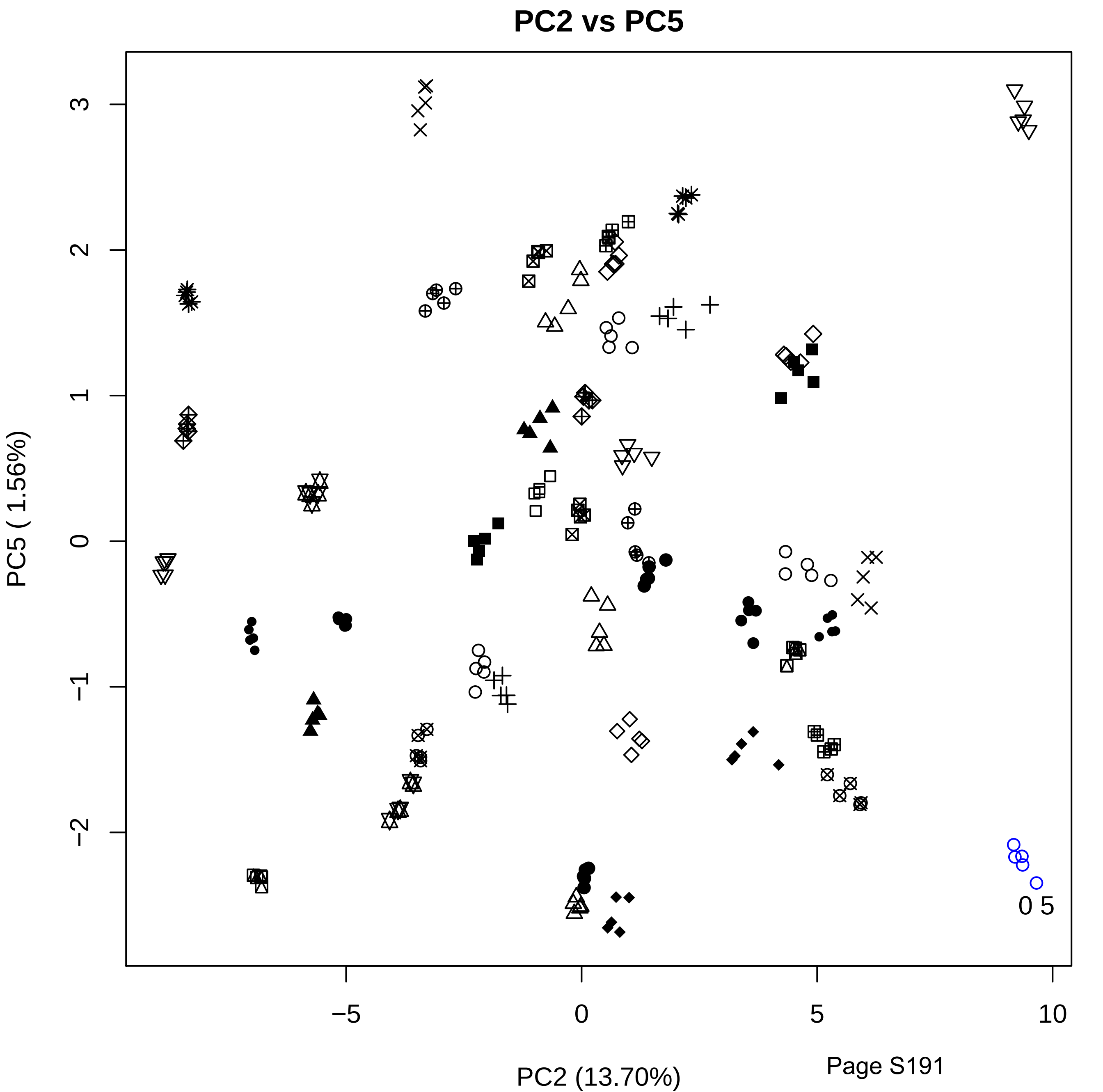
PC2 vs PC5
PC5 (1.56%)
PC2 (13.70%)
3
2
1
0
−1
−2
−5
0
5
10
0 5

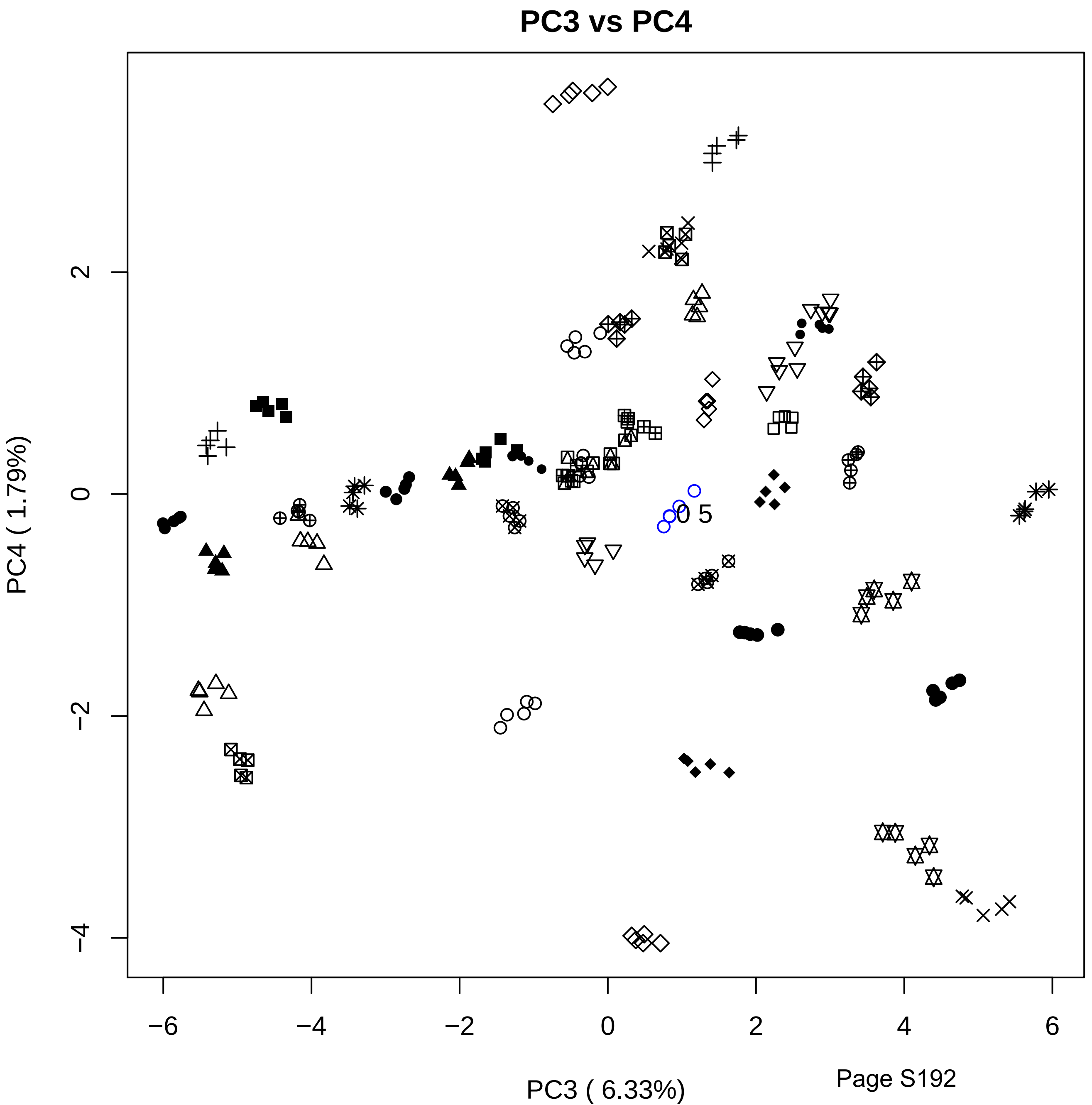

PC3 vs PC4
PC4 (1.79%)
PC3 (6.33%)
2
0
−2
−4
−6
−4
−2
0
2
4
6
0 5

PC3 vs PC5

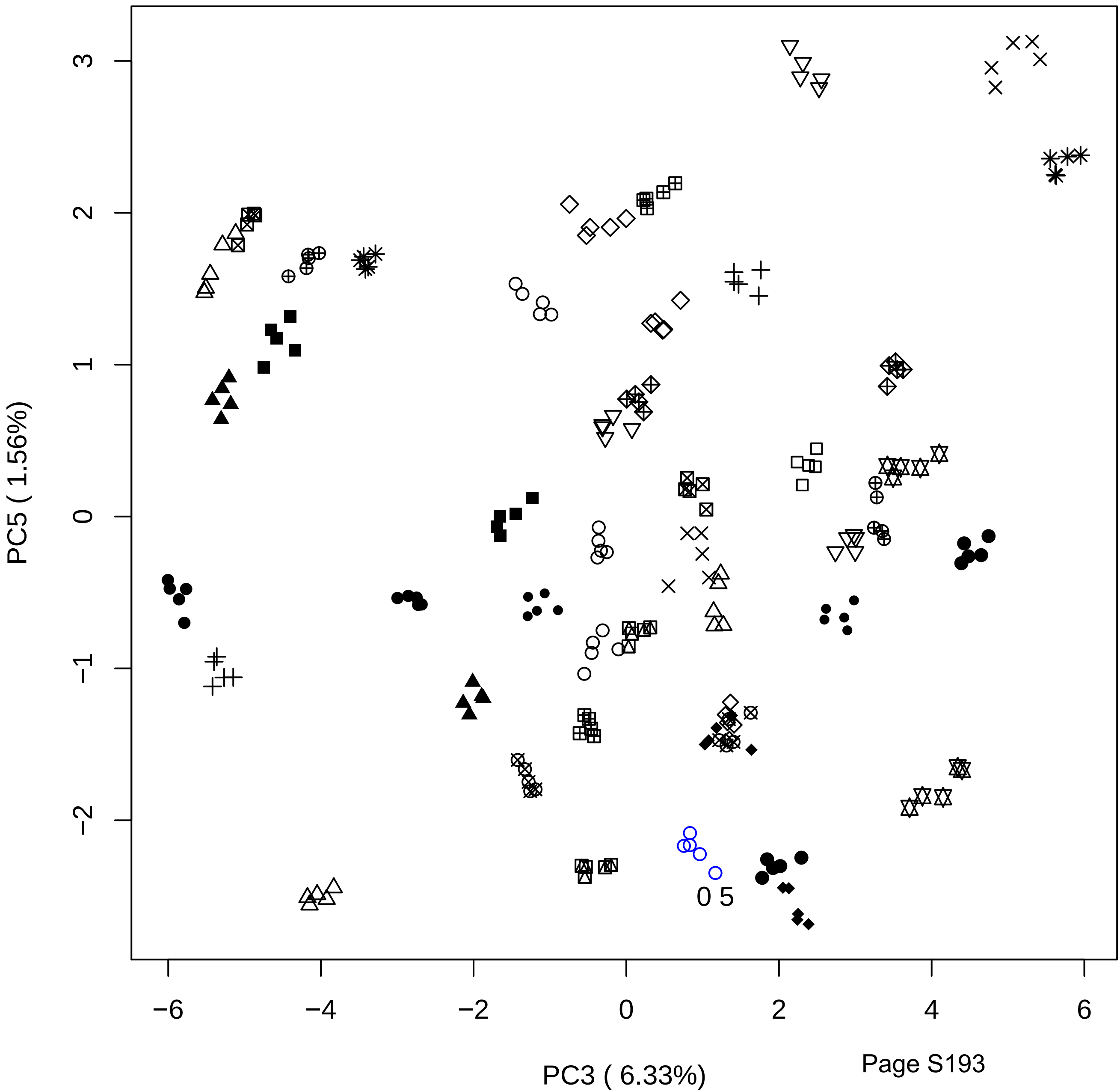

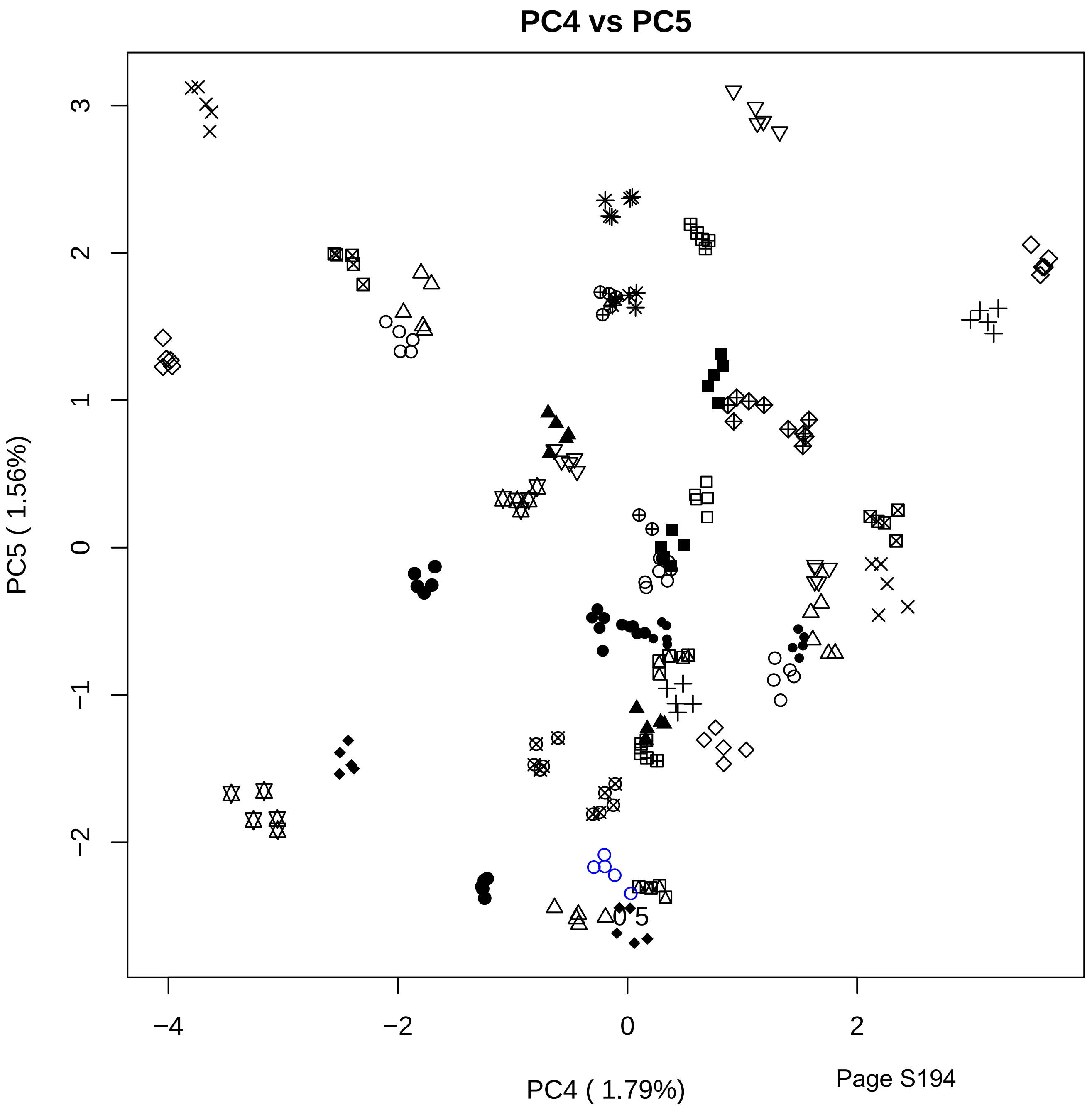

PC4 vs PC5
PC5 (1.56%)
PC4 (1.79%)
3
2
1
0
−1
−2
−4
−2
0
2
0 5

PCA of 1% OSCS vs PMH

Scaled Matrix PCAed

7th order polynomial baseline correction performed

Raw spectra smoothed to 21 Neighbours, 2nd order PN, Savtisky Golay

Normalisation between 0 and 100

D2 spectra smoothed to 41 Neighbours, 2nd order PN, Savtisky Golay

Regions Analysed: 3601−2501 and 2001−700cm−1

Samples Analysed: 1 Contam's 0 OSCS's 45 PMH's

Keele University AD Sun Dec 09 16:50:53 2018

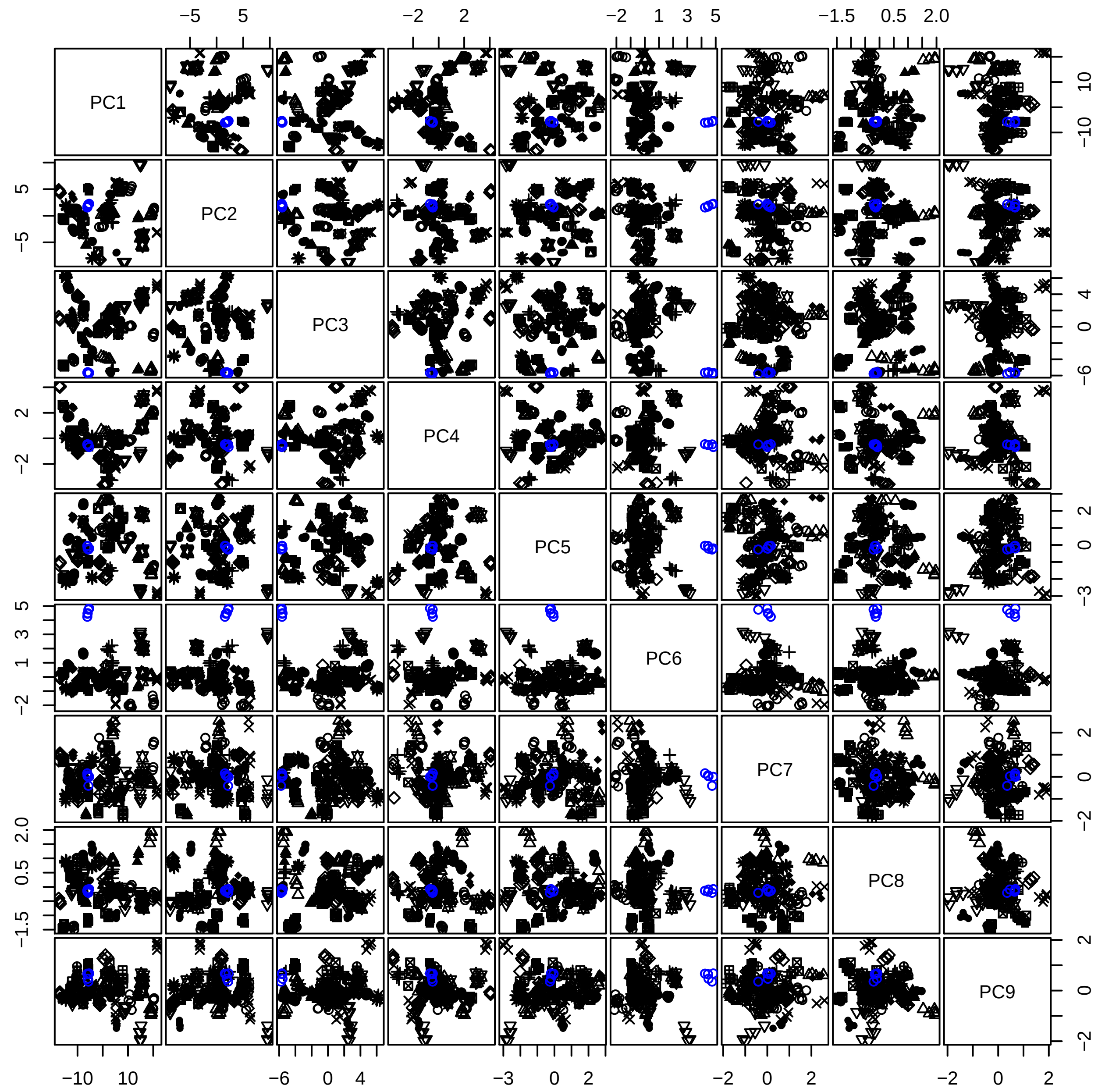
PC1
PC2
PC3
PC4
PC5
PC6
PC7
PC8
PC9

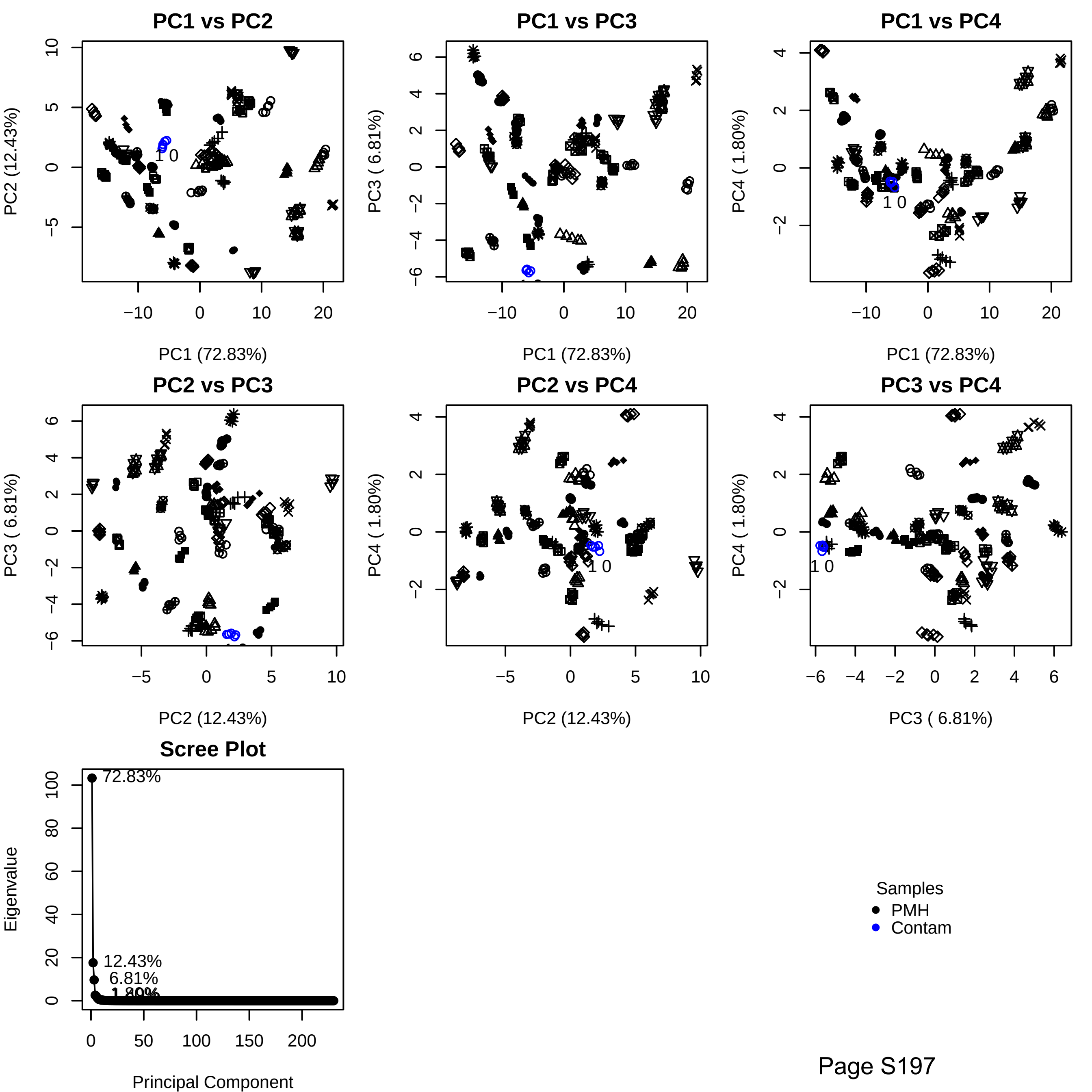
PC1 vs PC2
PC1 vs PC3
PC1 vs PC4
PC2 vs PC3
PC2 vs PC4
PC3 vs PC4
Scree Plot
PC1 (72.83%)
PC2 (12.43%)
PC3 (6.81%)
PC4 (1.80%)
Eigenvalue
Principal Component
72.83%
12.43%
6.81%
Samples
PMH
Contam

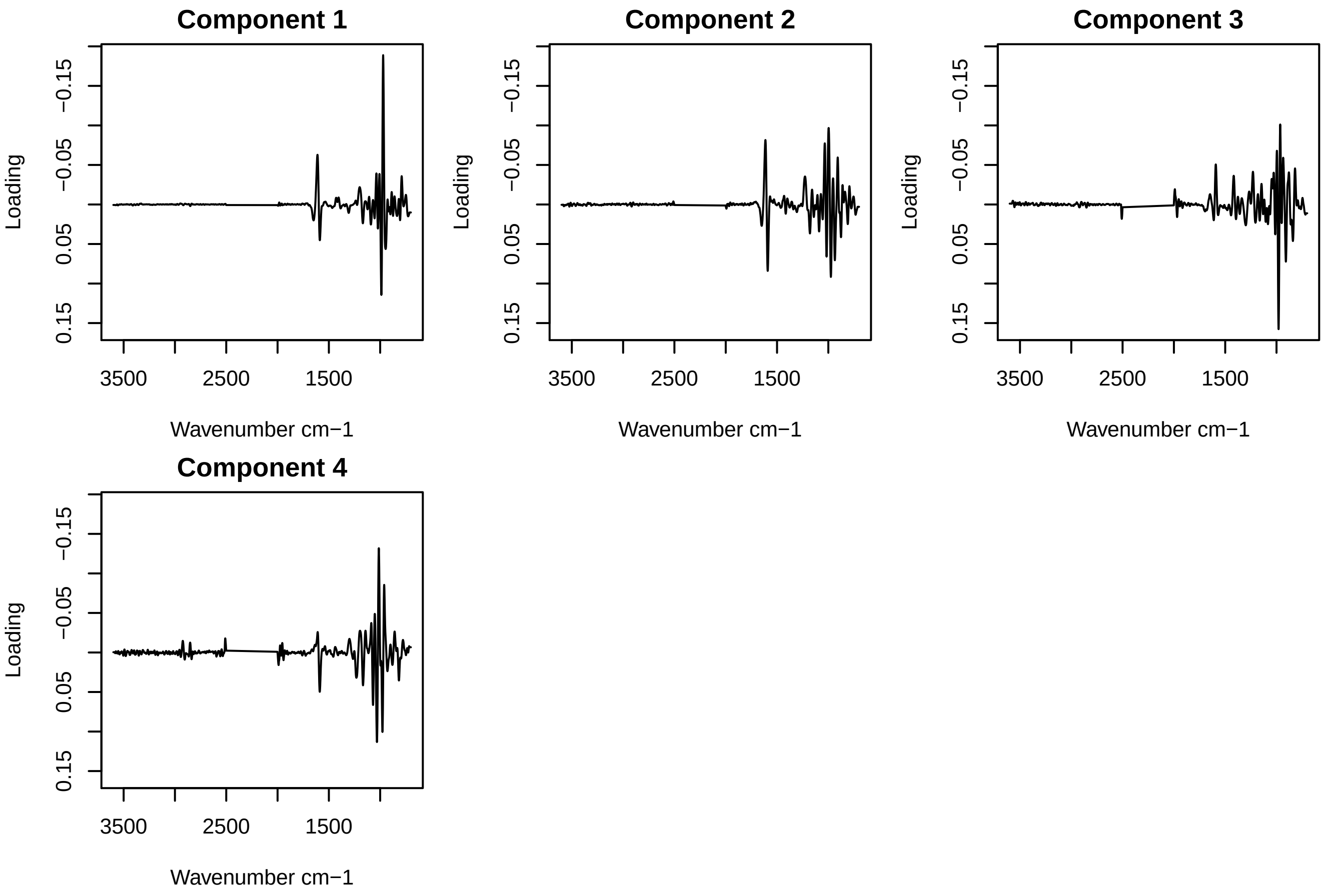
Component 1
Component 2
Component 3
Component 4
Loading
−0.15
−0.05
0.05
0.15
3500
2500
1500
Wavenumber cm−1

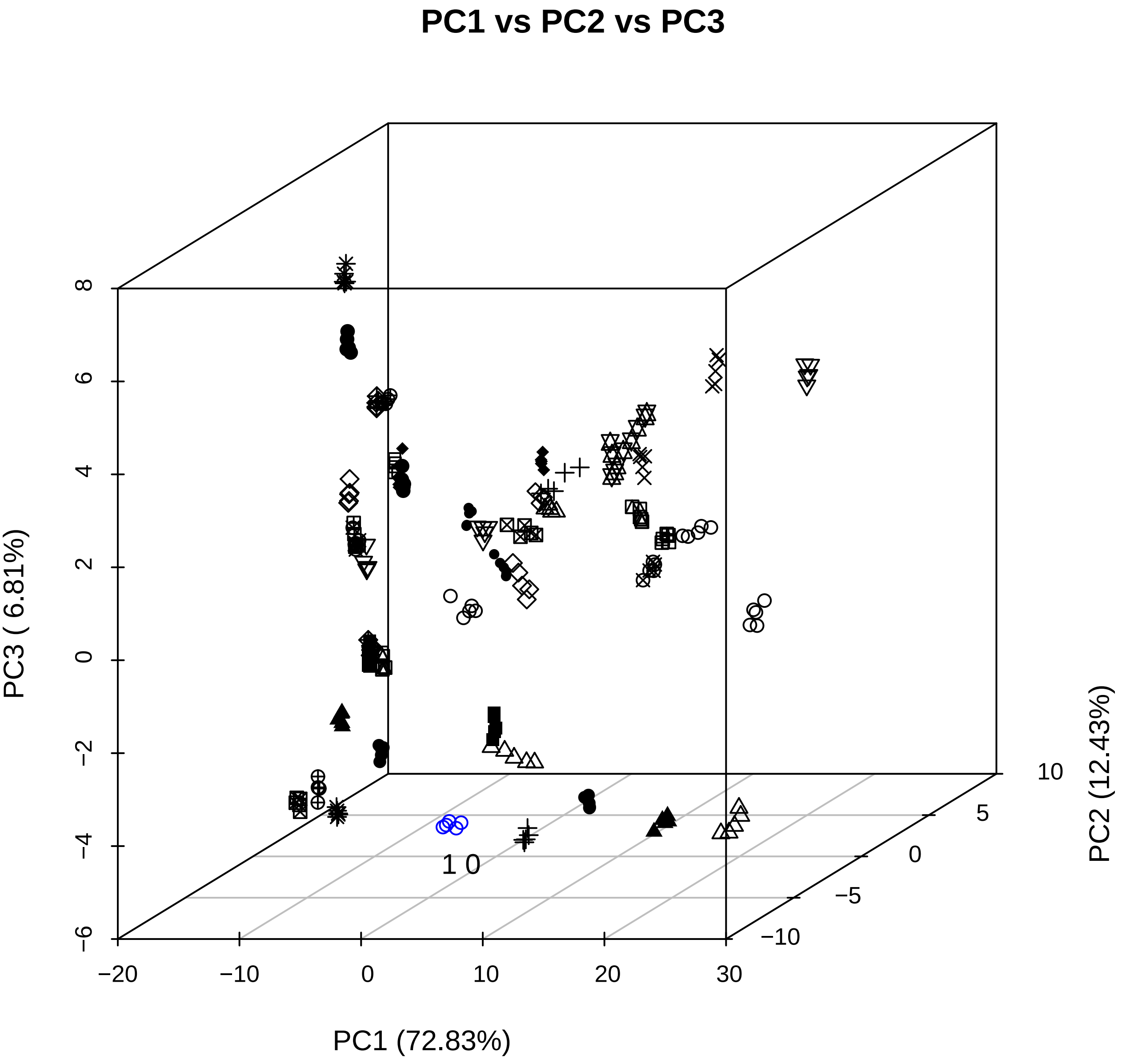
PC1 vs PC2 vs PC3
PC3 (6.81%)
8
6
4
2
0
−2
−4
−6
−20
−10
0
10
20
30
PC1 (72.83%)
PC2 (12.43%)
10
5
0
−5
−10
1 0

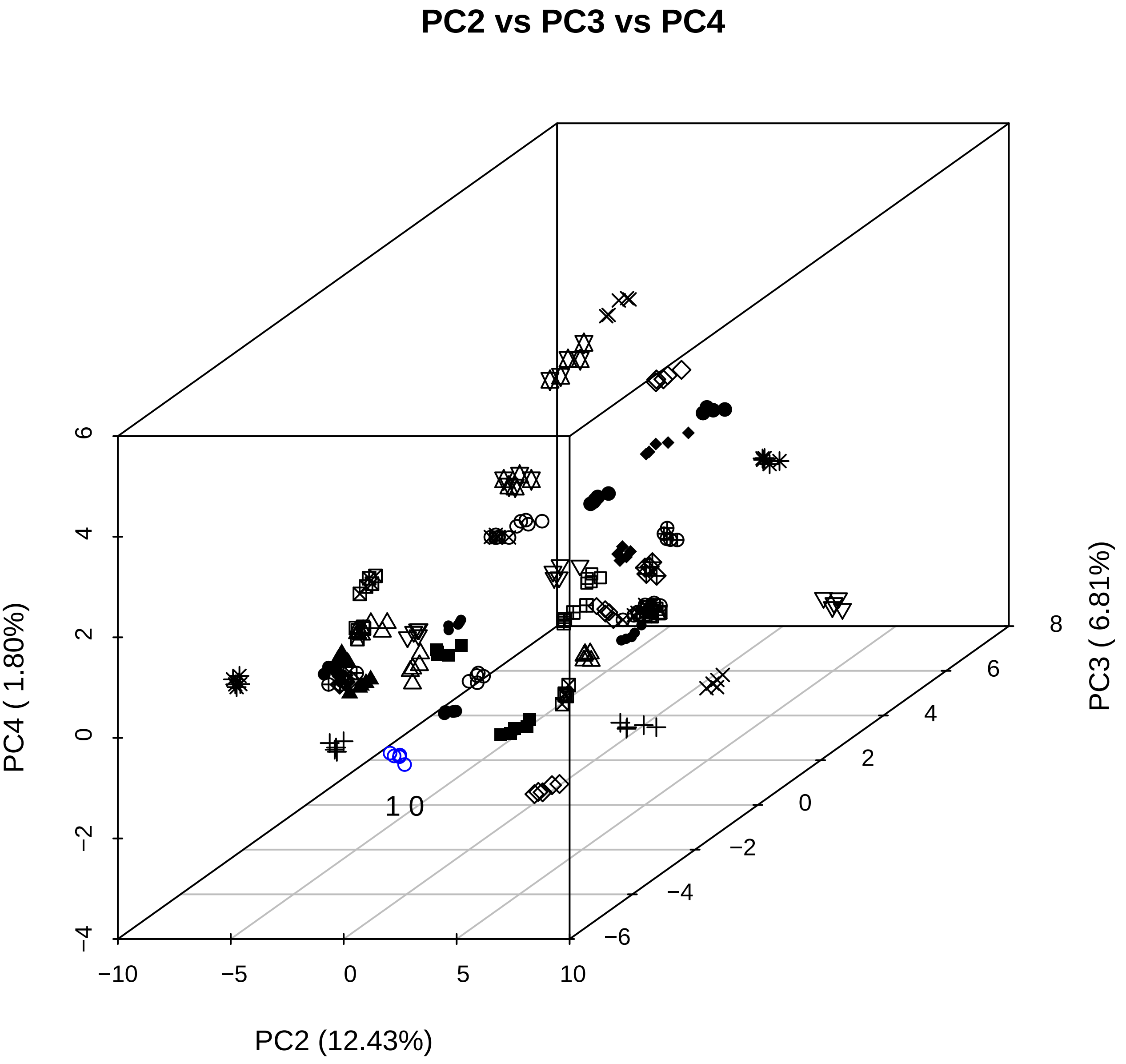

PC2 vs PC3 vs PC4
PC4 (1.80%)
PC3 (6.81%)
PC2 (12.43%)
1 0
−10
−5
0
5
10
−4
−2
0
2
4
6
−6
−4
−2
0
2
4
6
8

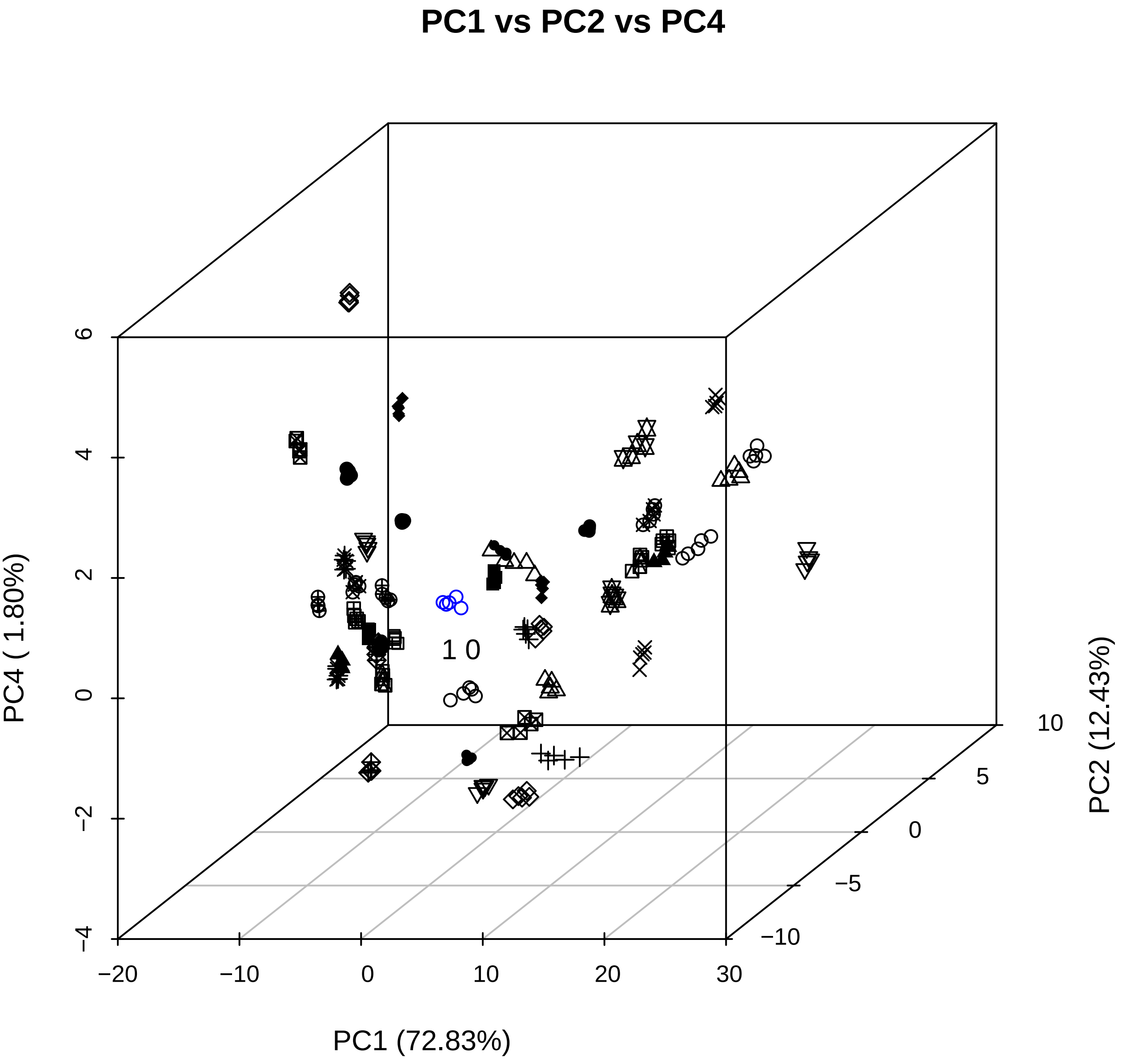

PC1 vs PC2 vs PC4
PC4 (1.80%)
PC1 (72.83%)
PC2 (12.43%)
1 0
−20
−10
0
10
20
30
−4
−2
0
2
4
6
−10
−5
0
5
10

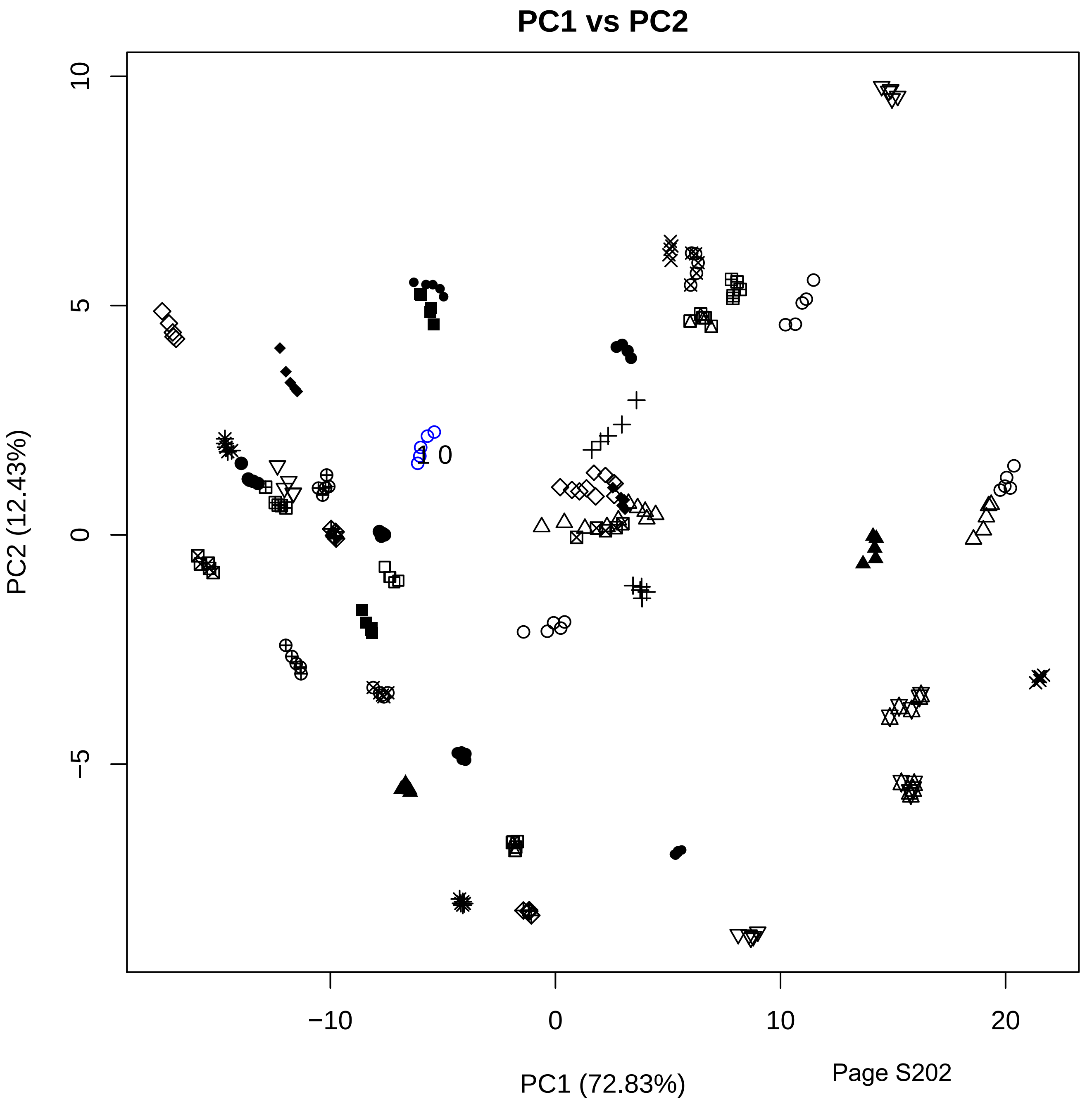
PC1 vs PC2
PC2 (12.43%)
PC1 (72.83%)
10
5
0
−5
−10
0
10
20
1 0

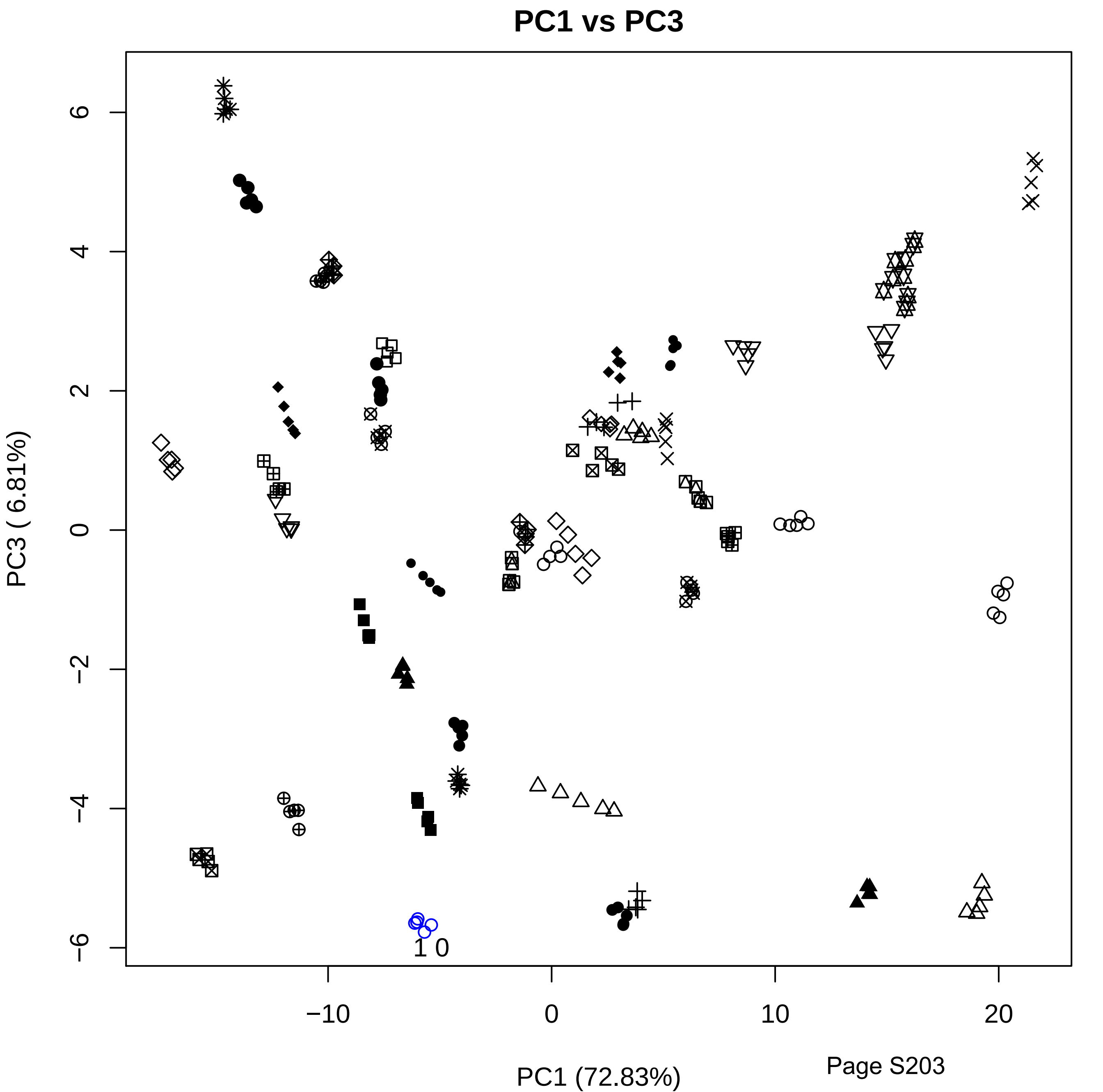
PC1 vs PC3
PC3 (6.81%)
PC1 (72.83%)
6
4
2
0
−2
−4
−6
−10
0
10
20
1 0

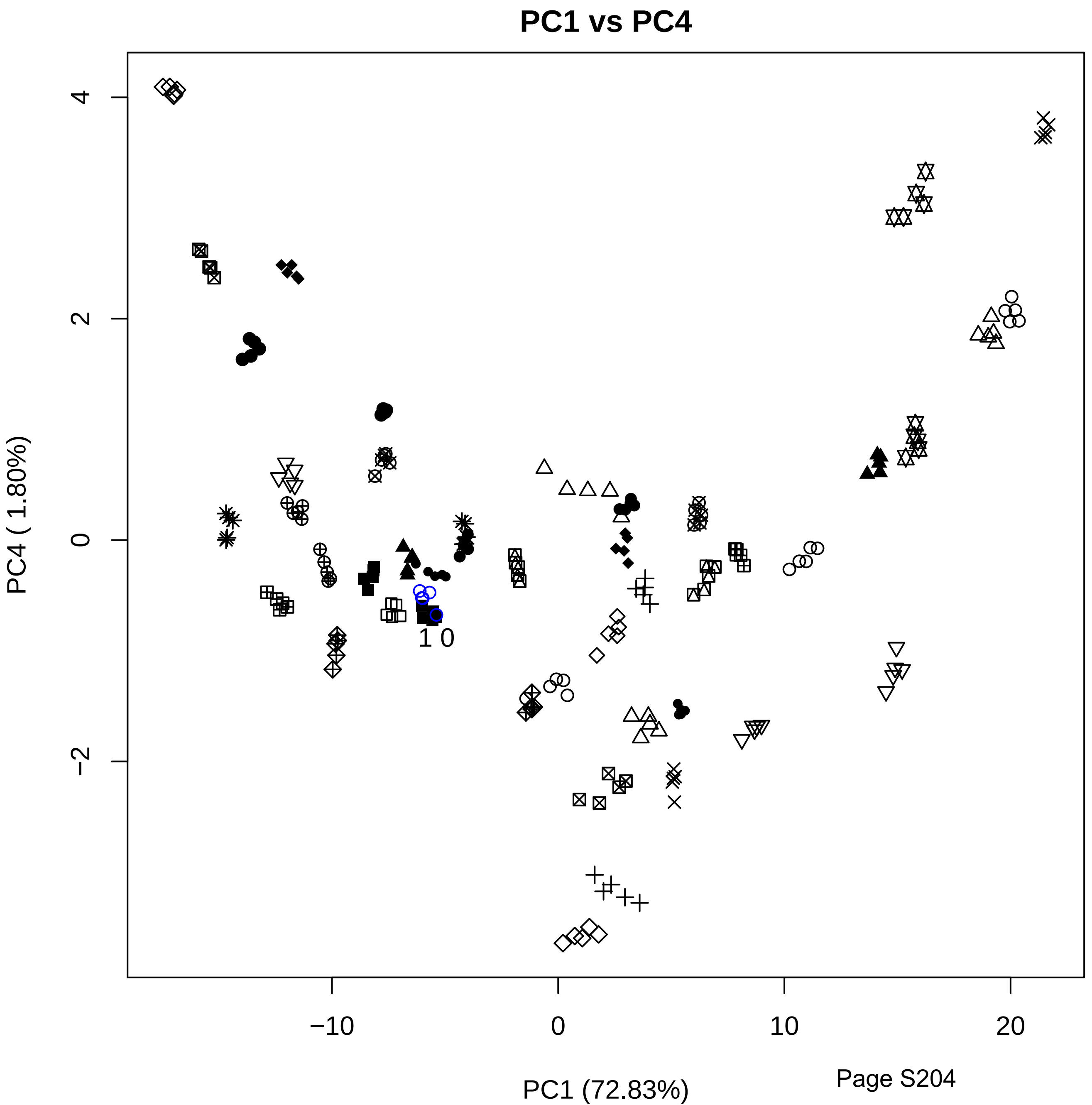
PC1 vs PC4
PC4 (1.80%)
PC1 (72.83%)
4
2
0
−2
−10
0
10
20
1 0

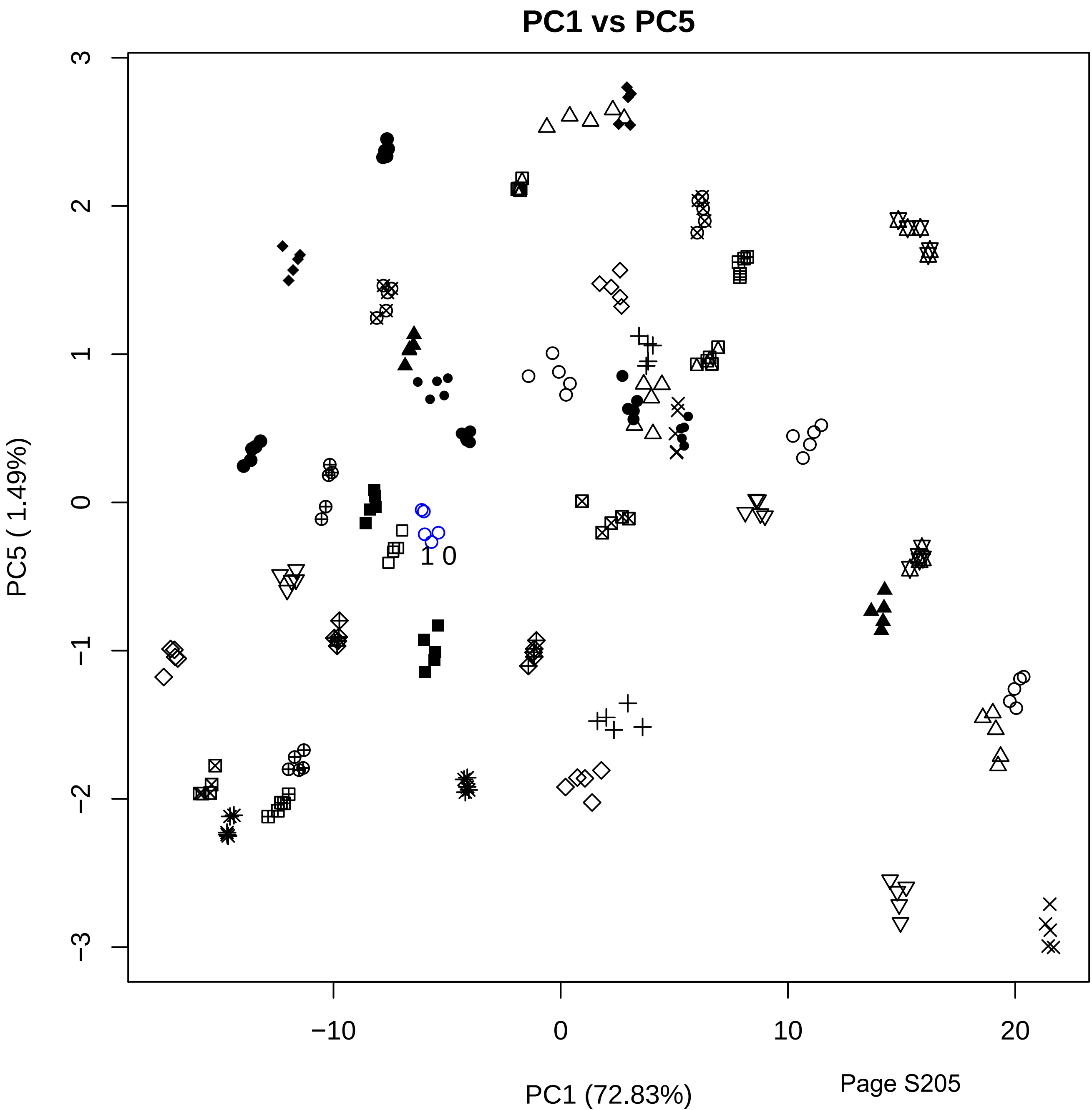

PC1 vs PC5
PC5 (1.49%)
PC1 (72.83%)
3
2
1
0
−1
−2
−3
−10
0
10
20
1 0

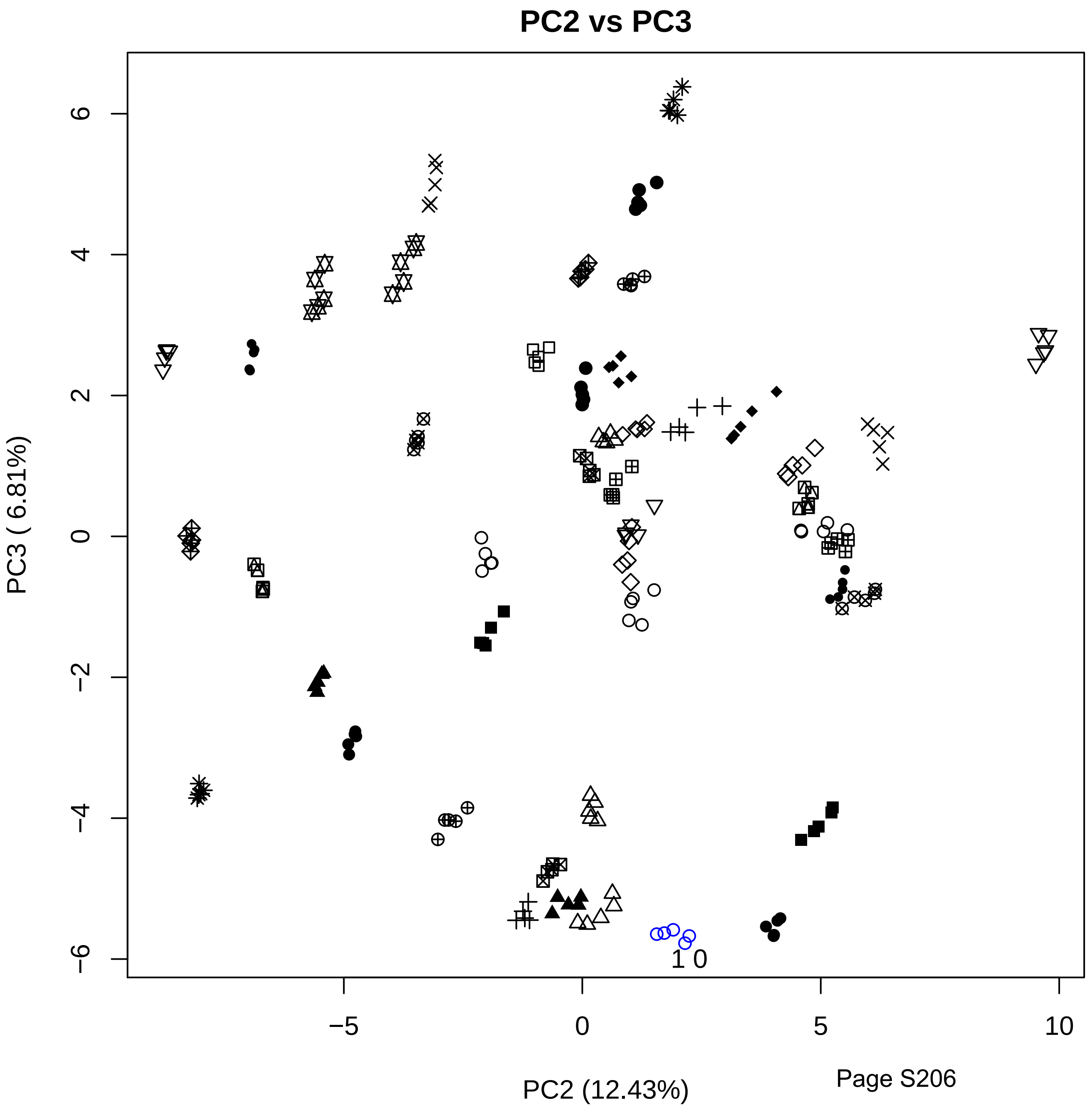
PC2 vs PC3
PC3 (6.81%)
PC2 (12.43%)
6
4
2
0
−2
−4
−6
−5
0
5
10
1 0

PC2 vs PC4

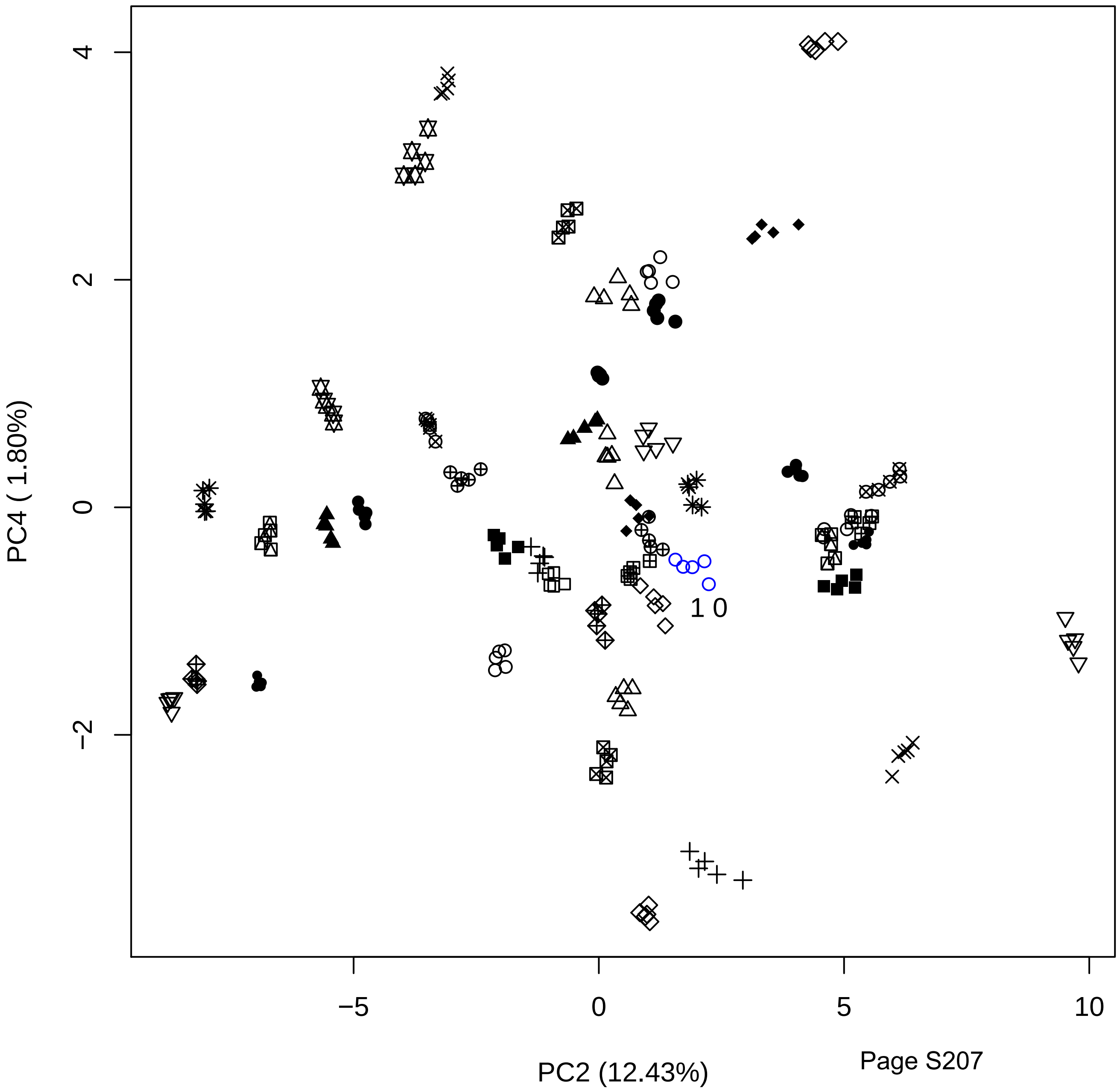

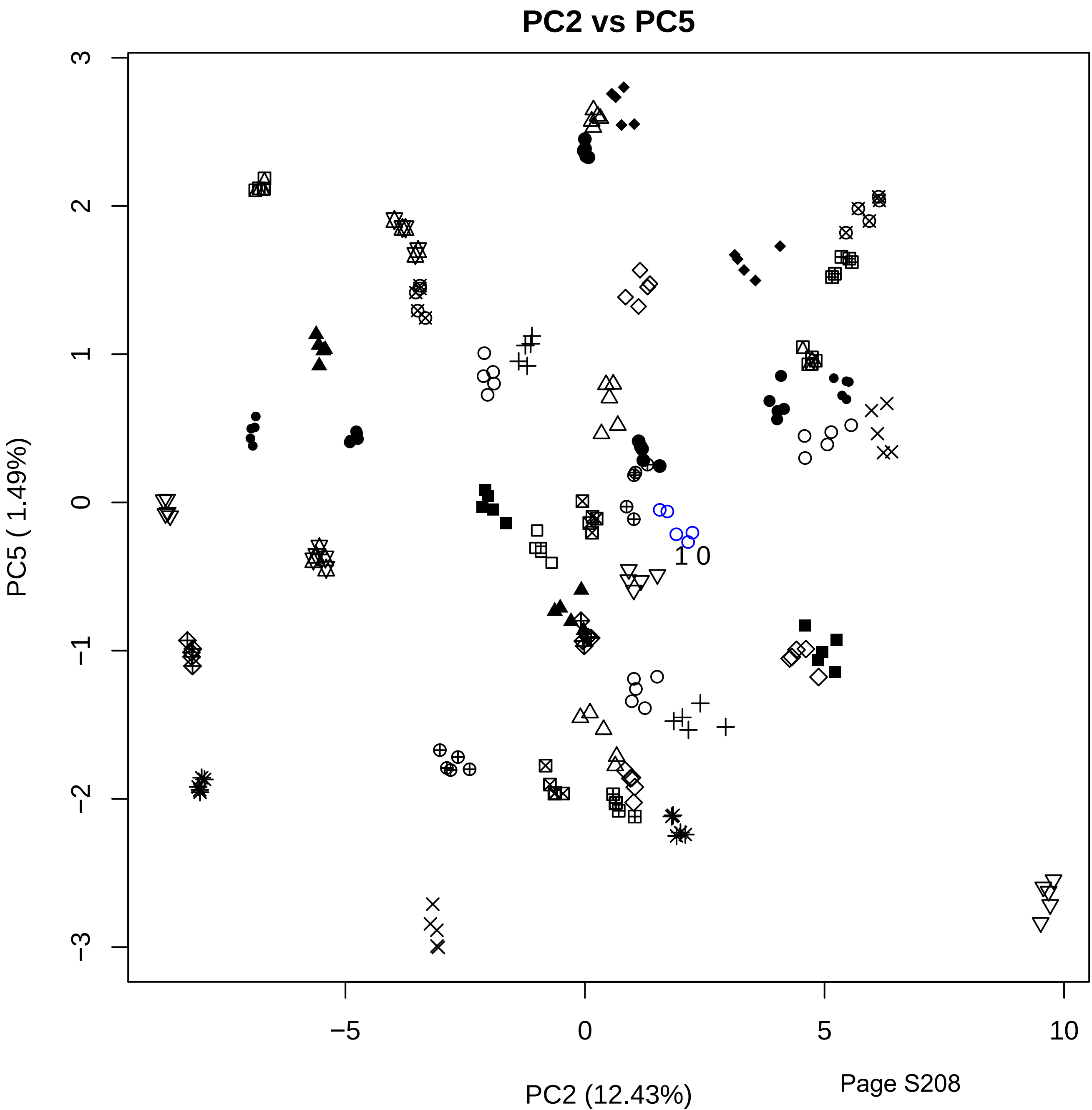

PC2 vs PC5
PC5 (1.49%)
PC2 (12.43%)
3
2
1
0
−1
−2
−3
−5
0
5
10
1 0

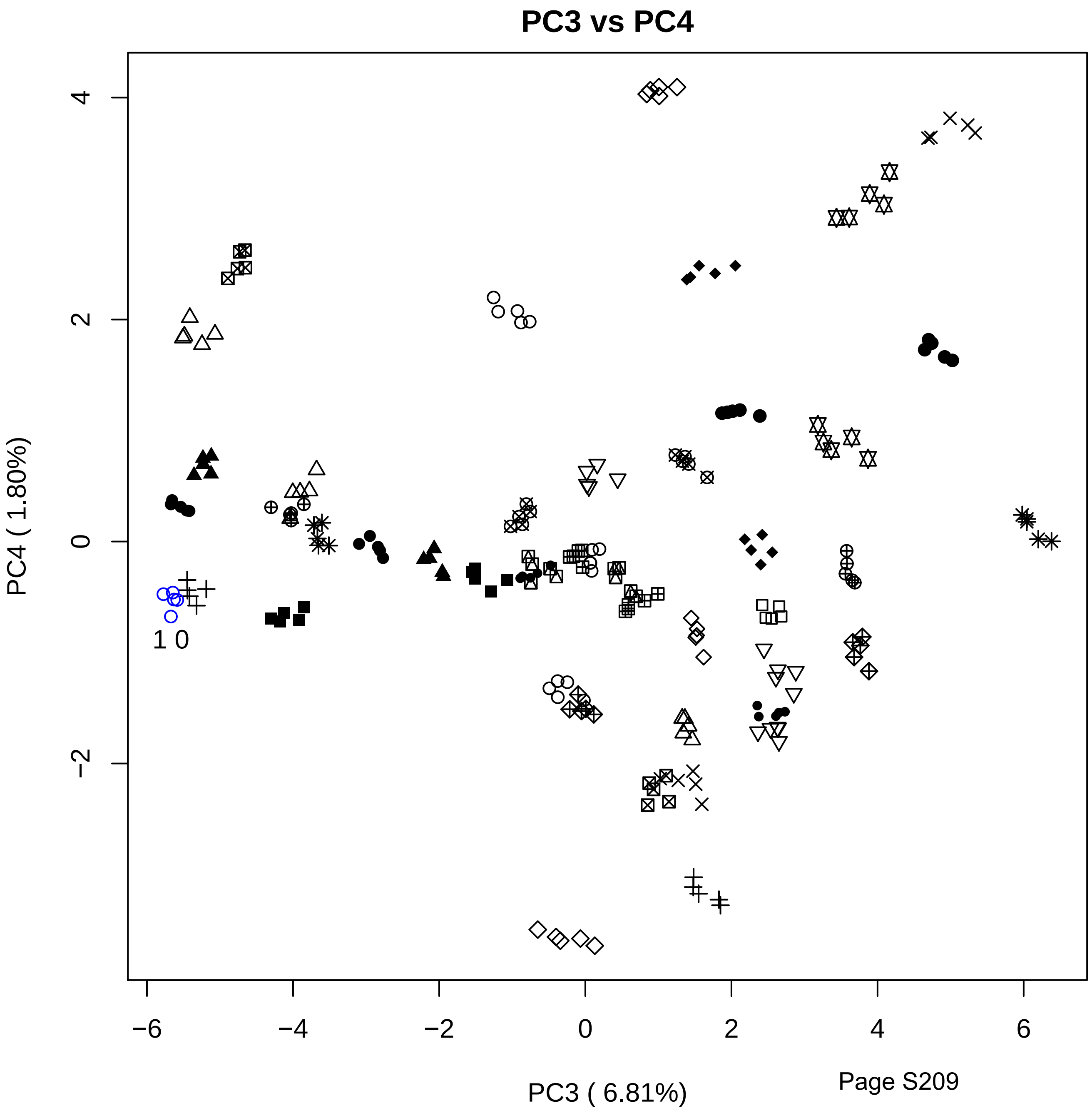

PC3 vs PC4
4
2
0
−2
PC4 (1.80%)
1 0
−6
−4
−2
0
2
4
6
PC3 (6.81%)

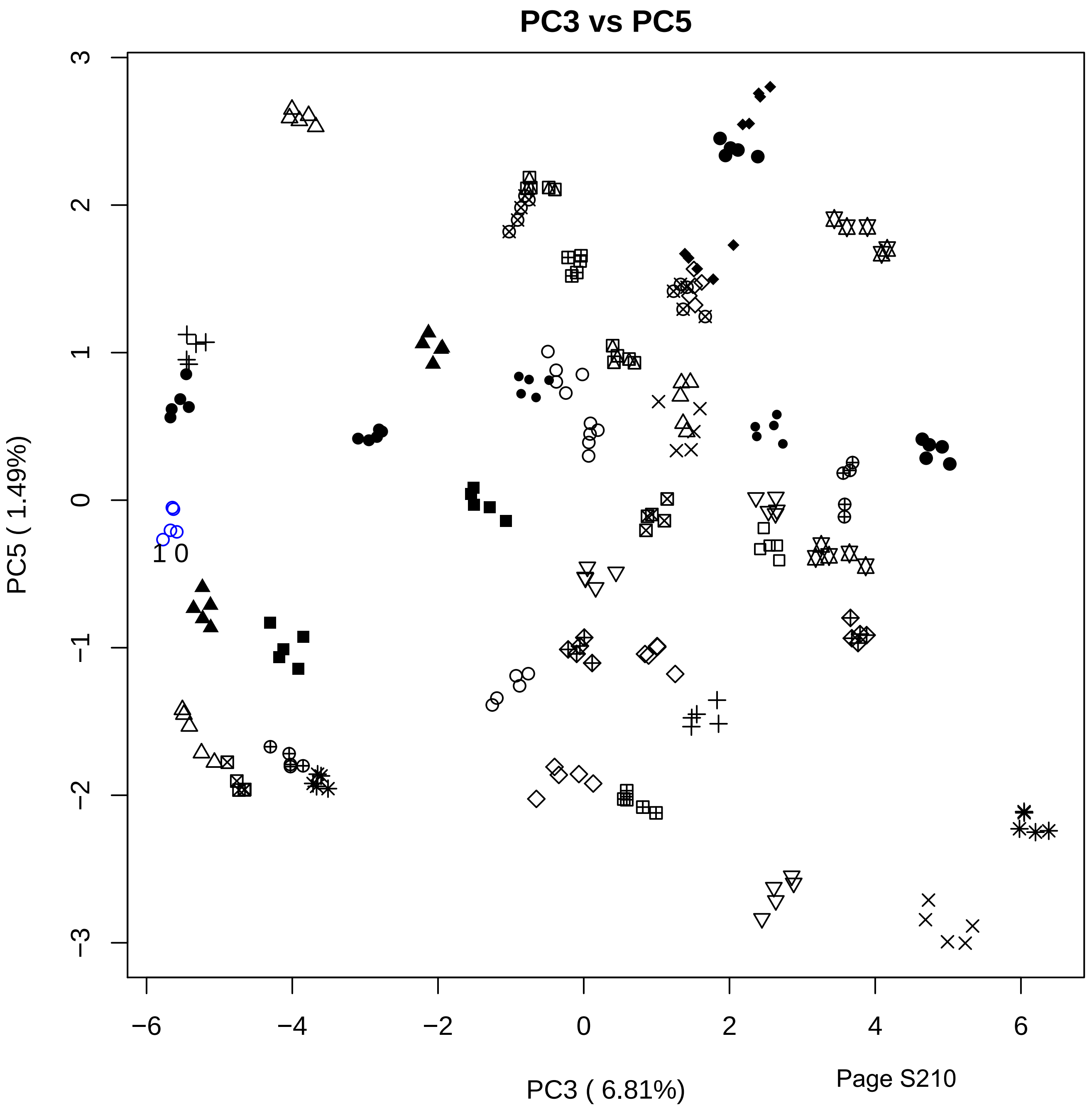
PC3 vs PC5
PC5 (1.49%)
PC3 (6.81%)
3
2
1
0
−1
−2
−3
−6
−4
−2
0
2
4
6
1 0

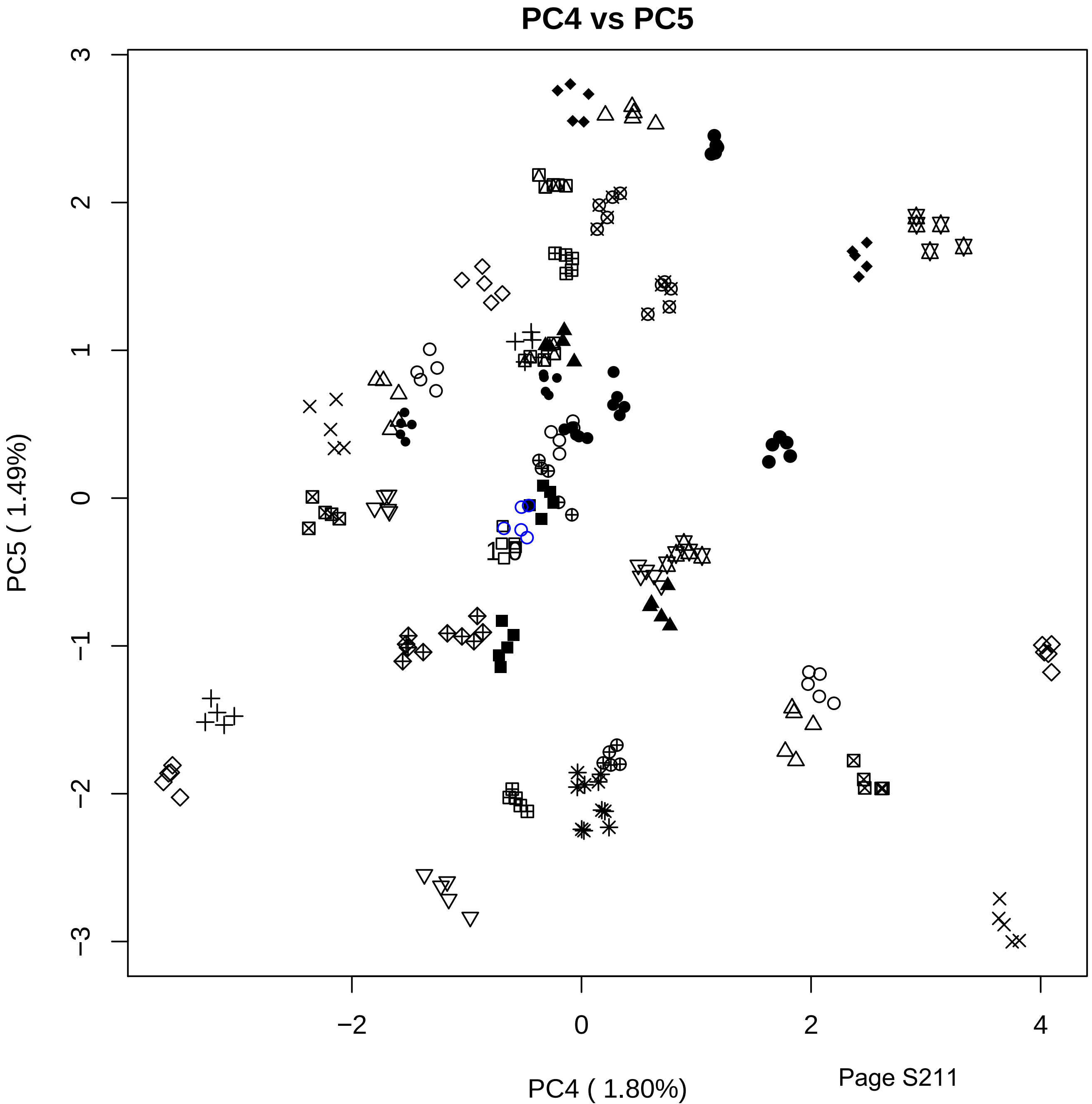
PC4 vs PC5
PC5 (1.49%)
PC4 (1.80%)
3
2
1
0
−1
−2
−3
−2
0
2
4

PCA of 2.5% OSCS vs PMH

Scaled Matrix PCAed

7th order polynomial baseline correction performed

Raw spectra smoothed to 21 Neighbours, 2nd order PN, Savtisky Golay

Normalisation between 0 and 100

D2 spectra smoothed to 41 Neighbours, 2nd order PN, Savtisky Golay

Regions Analysed: 3601−2501 and 2001−700cm−1

Samples Analysed: 1 Contam's 0 OSCS's 45 PMH's

Keele University AD   Sun Dec 09 16:50:58 2018

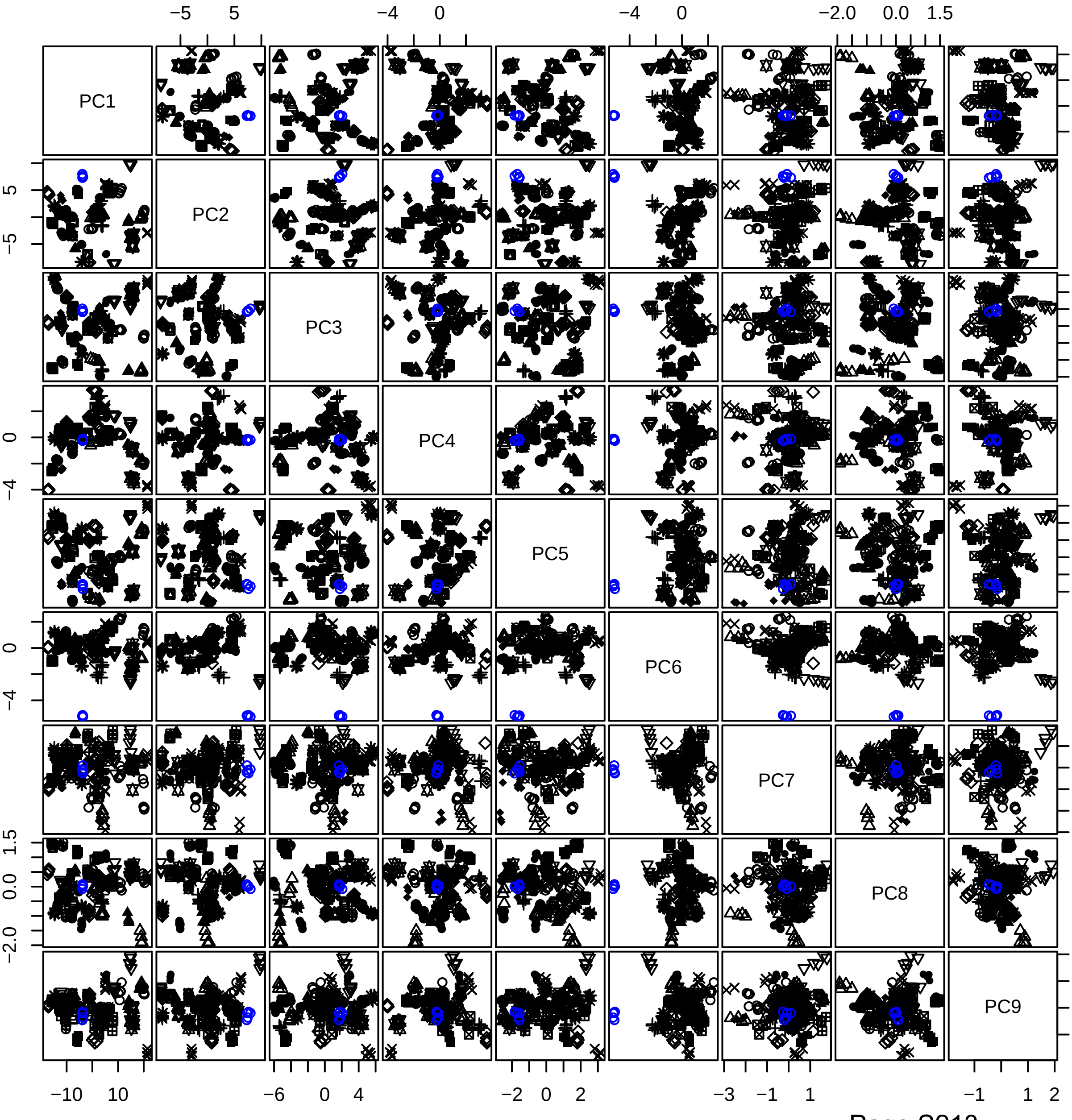
PC1
PC2
PC3
PC4
PC5
PC6
PC7
PC8
PC9

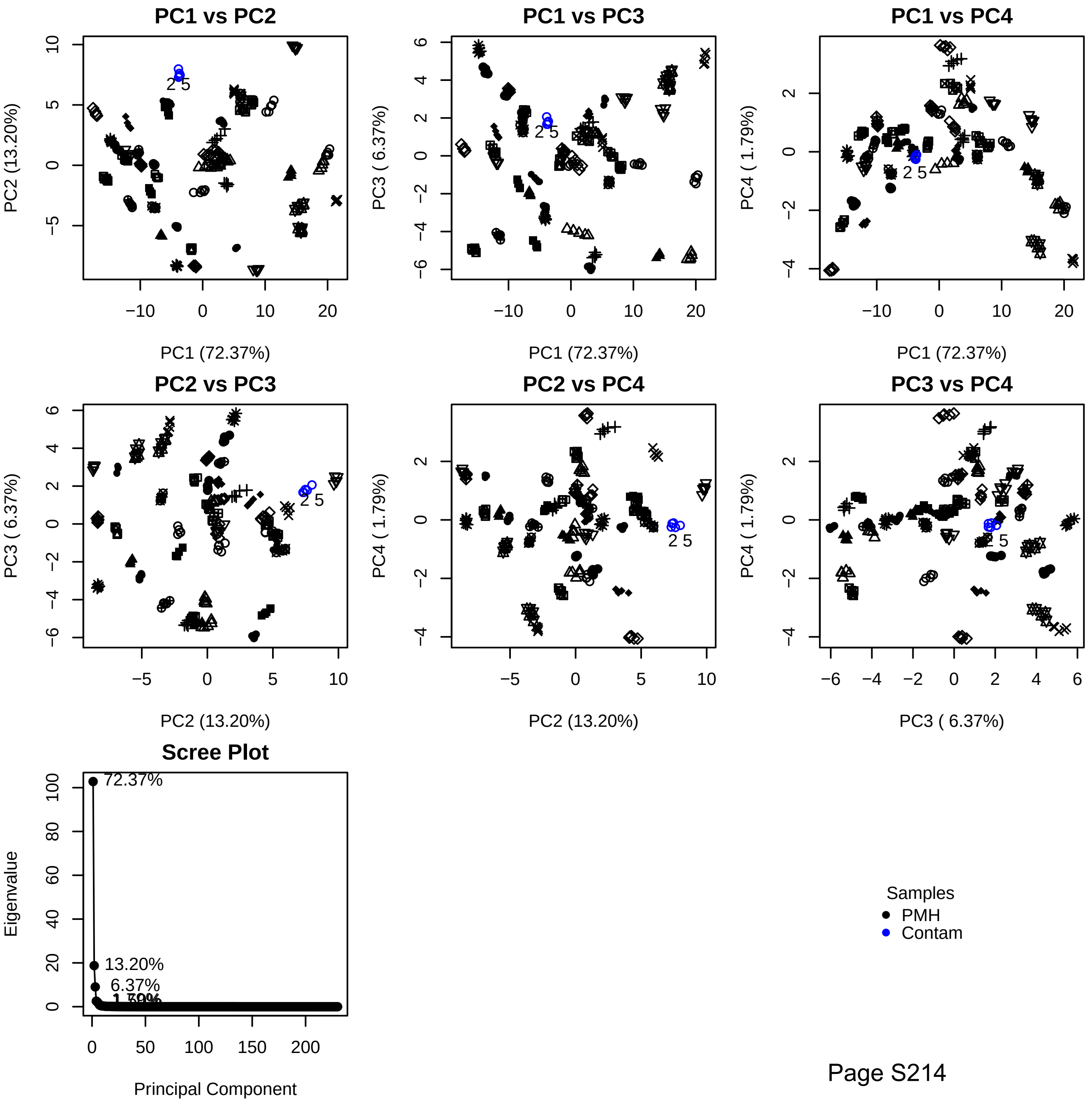
PC1 vs PC2
PC1 vs PC3
PC1 vs PC4
PC2 vs PC3
PC2 vs PC4
PC3 vs PC4
Scree Plot
PC1 (72.37%)
PC2 (13.20%)
PC3 (6.37%)
PC4 (1.79%)
2 5
Eigenvalue
Principal Component
72.37%
13.20%
6.37%
Samples
PMH
Contam

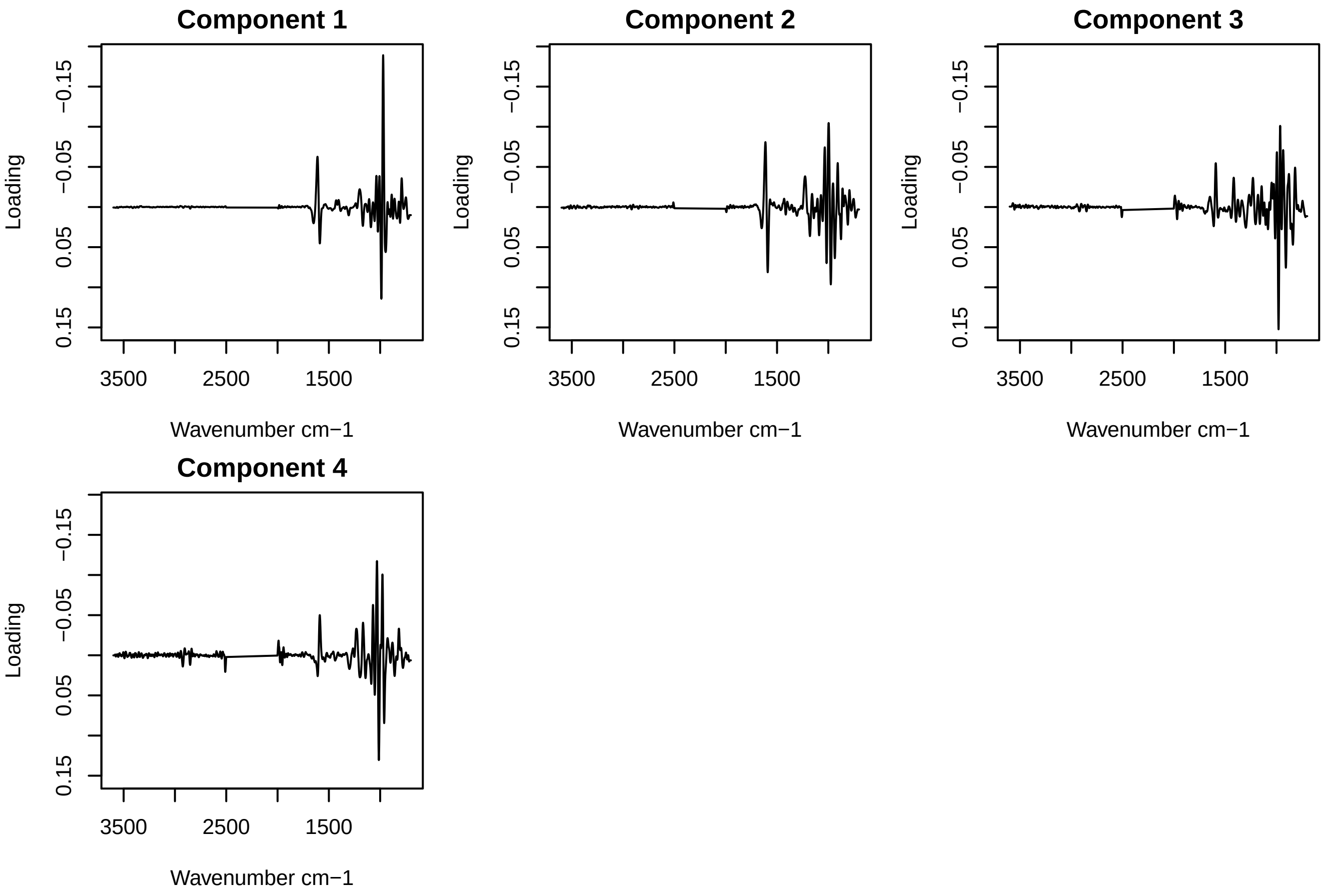
Component 1
Loading
−0.15
−0.05
0.05
0.15
3500
2500
1500
Wavenumber cm−1
Component 2
Loading
−0.15
−0.05
0.05
0.15
3500
2500
1500
Wavenumber cm−1
Component 3
Loading
−0.15
−0.05
0.05
0.15
3500
2500
1500
Wavenumber cm−1
Component 4
Loading
−0.15
−0.05
0.05
0.15
3500
2500
1500
Wavenumber cm−1

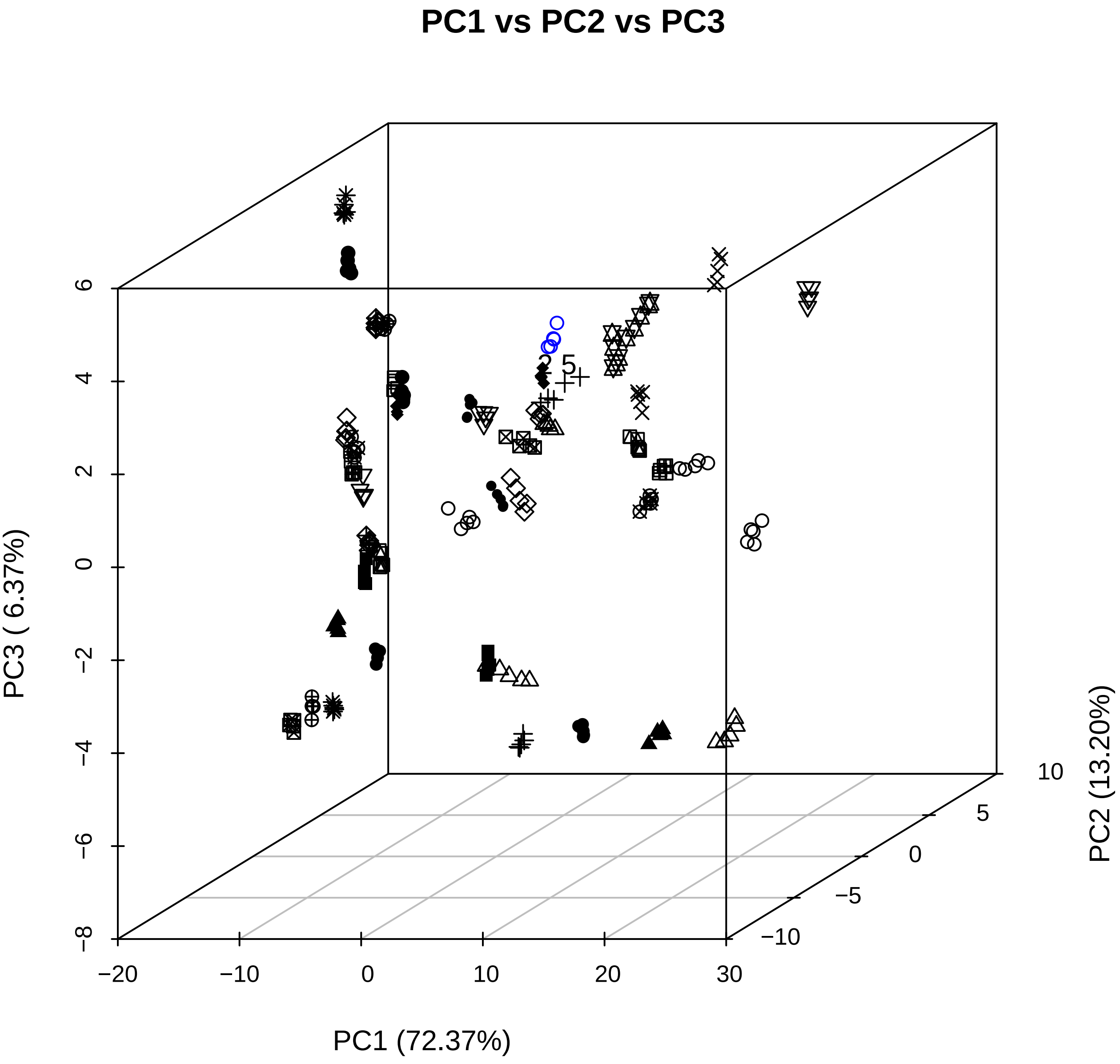
PC1 vs PC2 vs PC3
PC1 (72.37%)
PC2 (13.20%)
PC3 (6.37%)
−20
−10
0
10
20
30
−10
−5
0
5
10
−8
−6
−4
−2
0
2
4
6
2 5

PC2 vs PC3 vs PC4

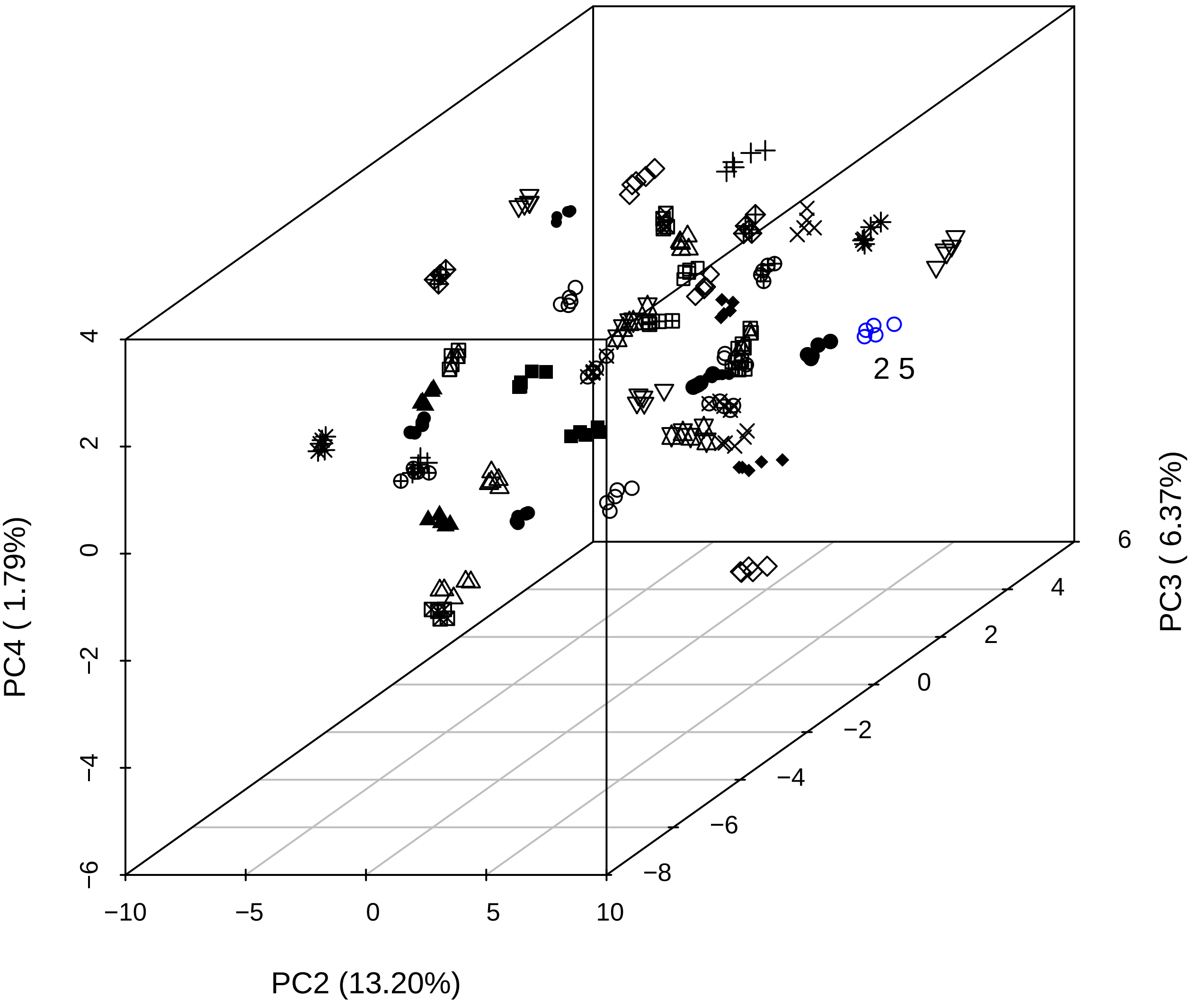

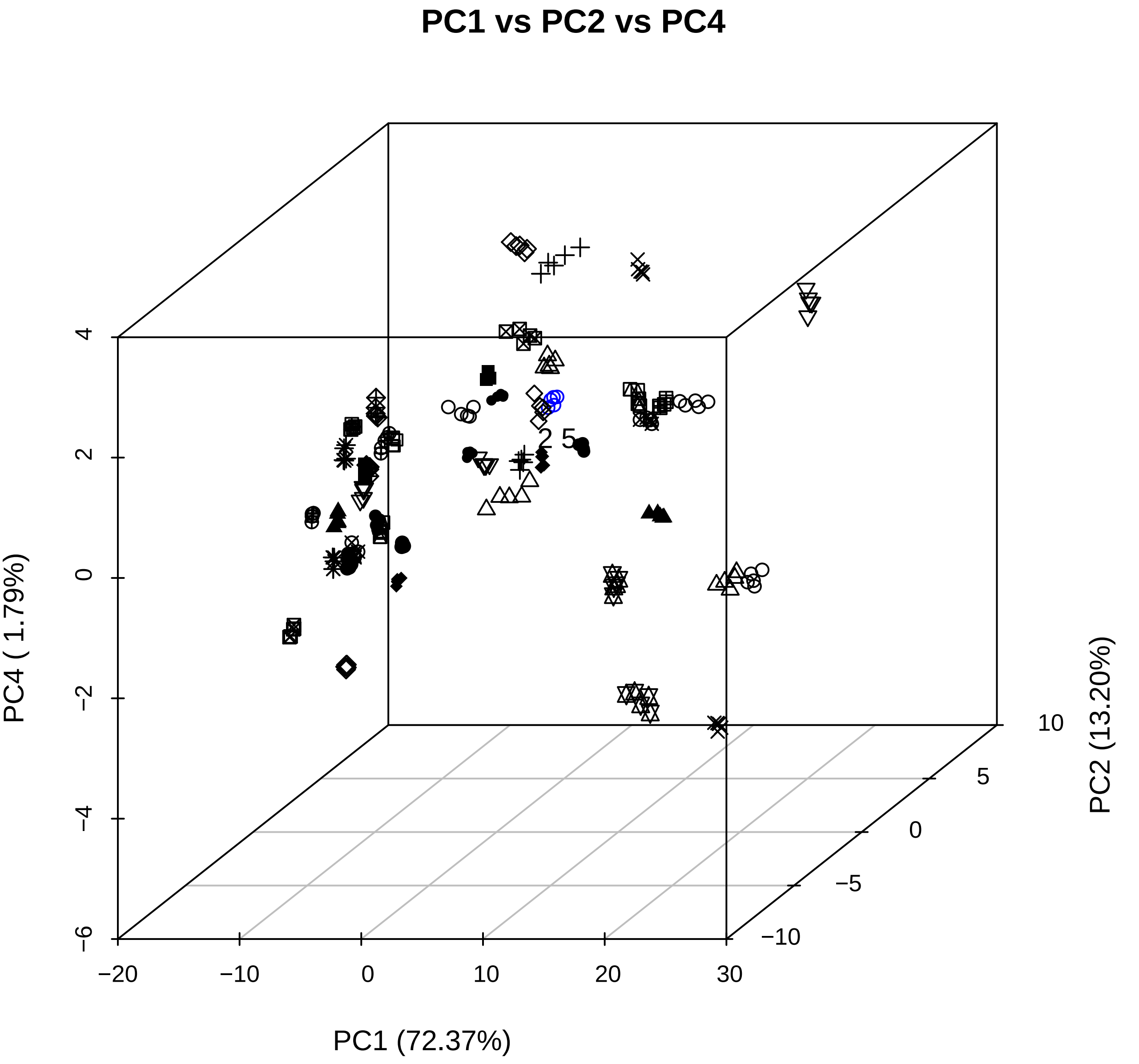

PC1 vs PC2 vs PC4
PC4 (1.79%)
PC1 (72.37%)
PC2 (13.20%)
−6
−4
−2
0
2
4
−20
−10
0
10
20
30
−10
−5
0
5
10
2
5

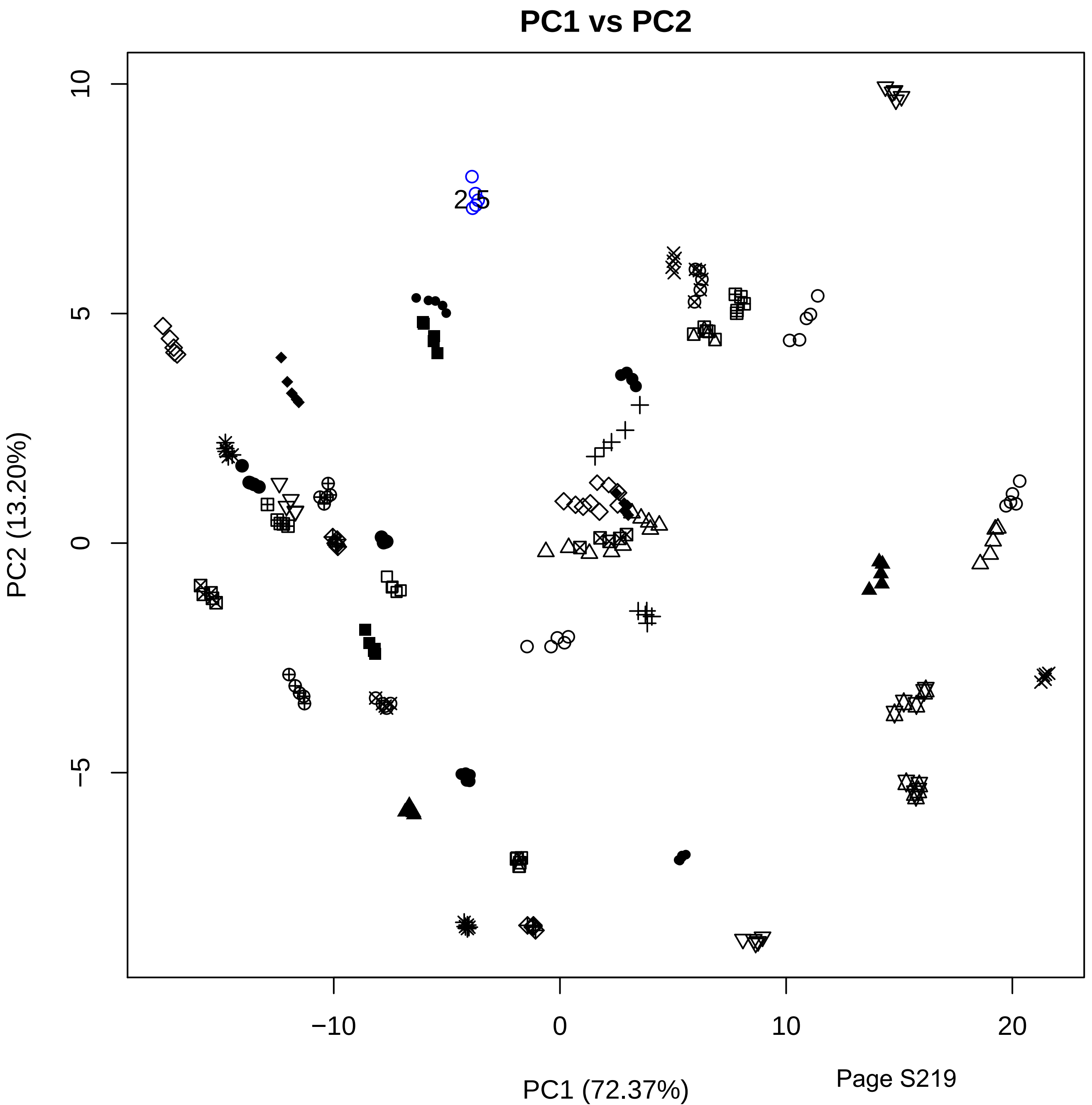
PC1 vs PC2
PC2 (13.20%)
PC1 (72.37%)
10
5
0
−5
−10
0
10
20
25

PC1 vs PC3

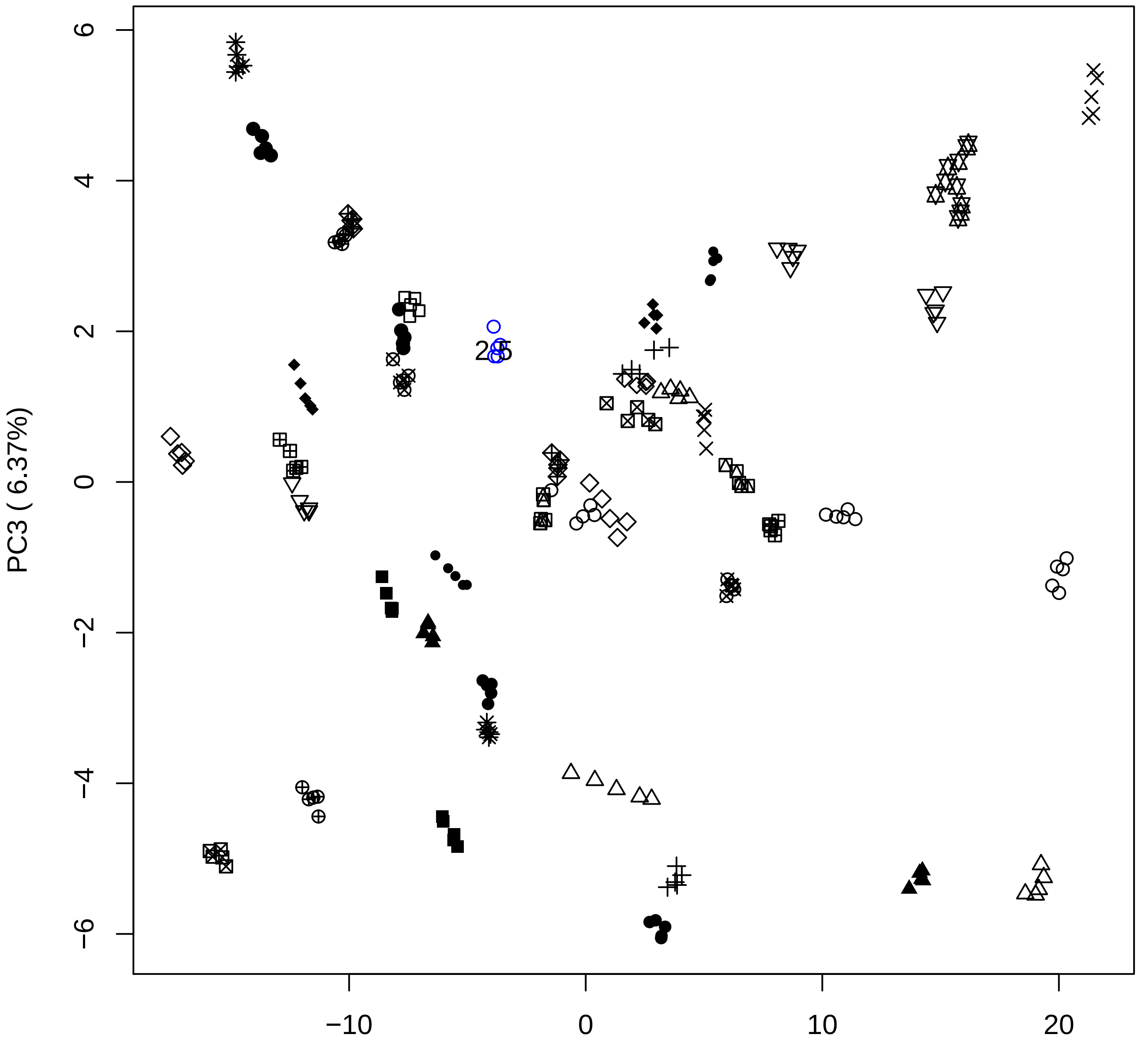

PC1 (72.37%)

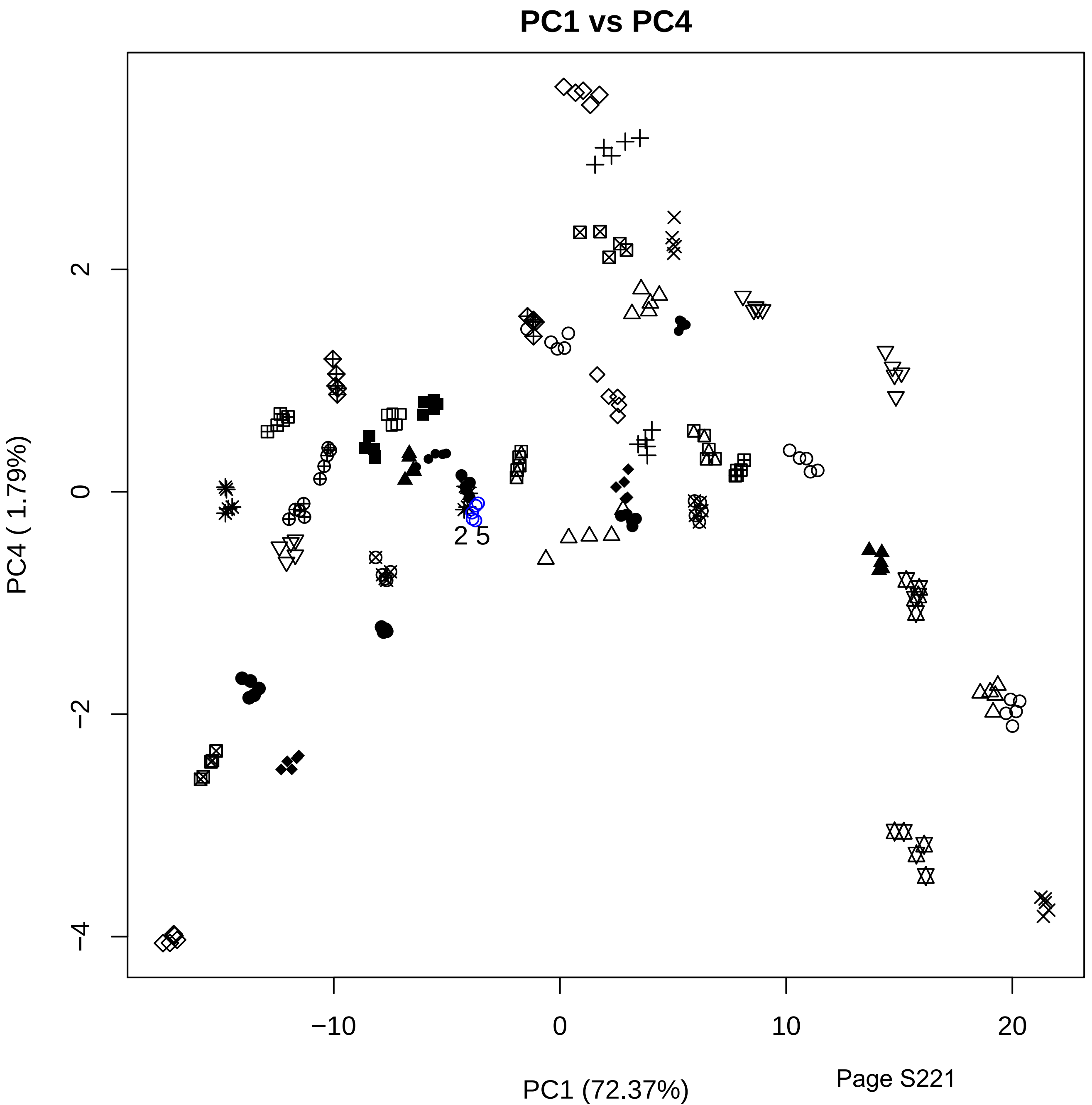
PC1 vs PC4
2
0
−2
−4
PC4 (1.79%)
−10
0
10
20
PC1 (72.37%)
2 5

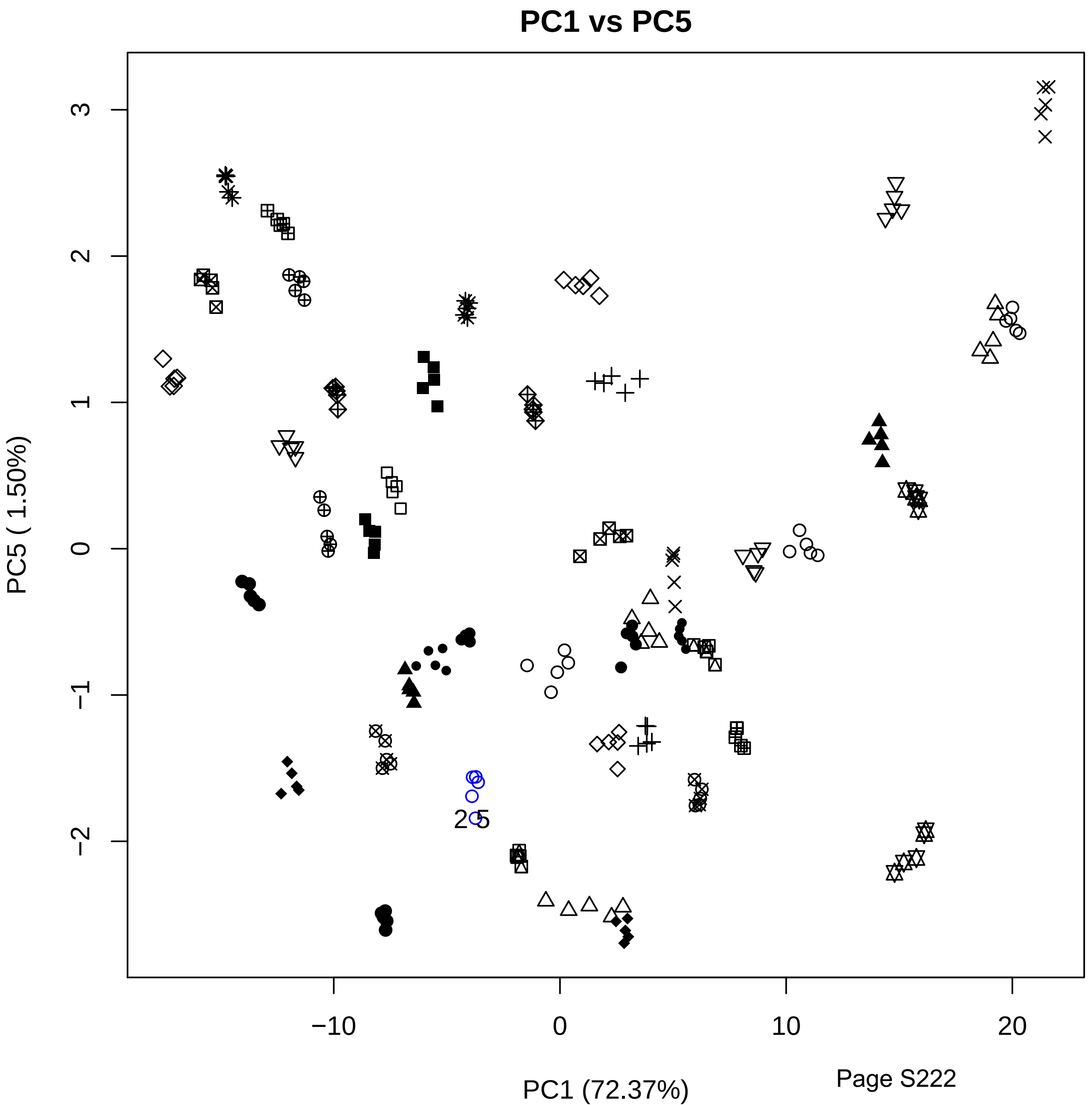

PC1 vs PC5
PC5 (1.50%)
PC1 (72.37%)
3
2
1
0
−1
−2
−10
0
10
20
25

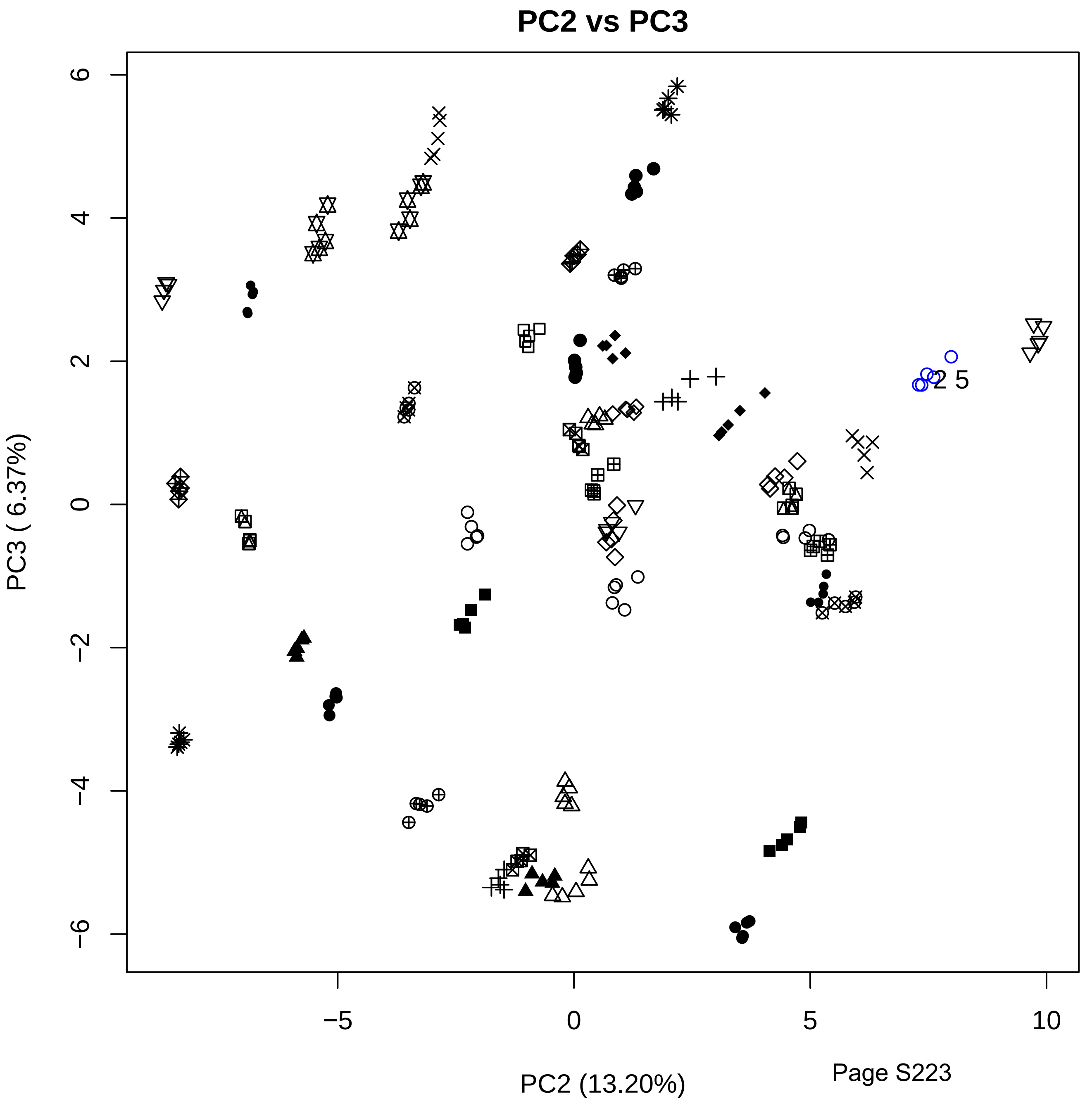
PC2 vs PC3
PC3 (6.37%)
PC2 (13.20%)
6
4
2
0
−2
−4
−6
−5
0
5
10
2 5

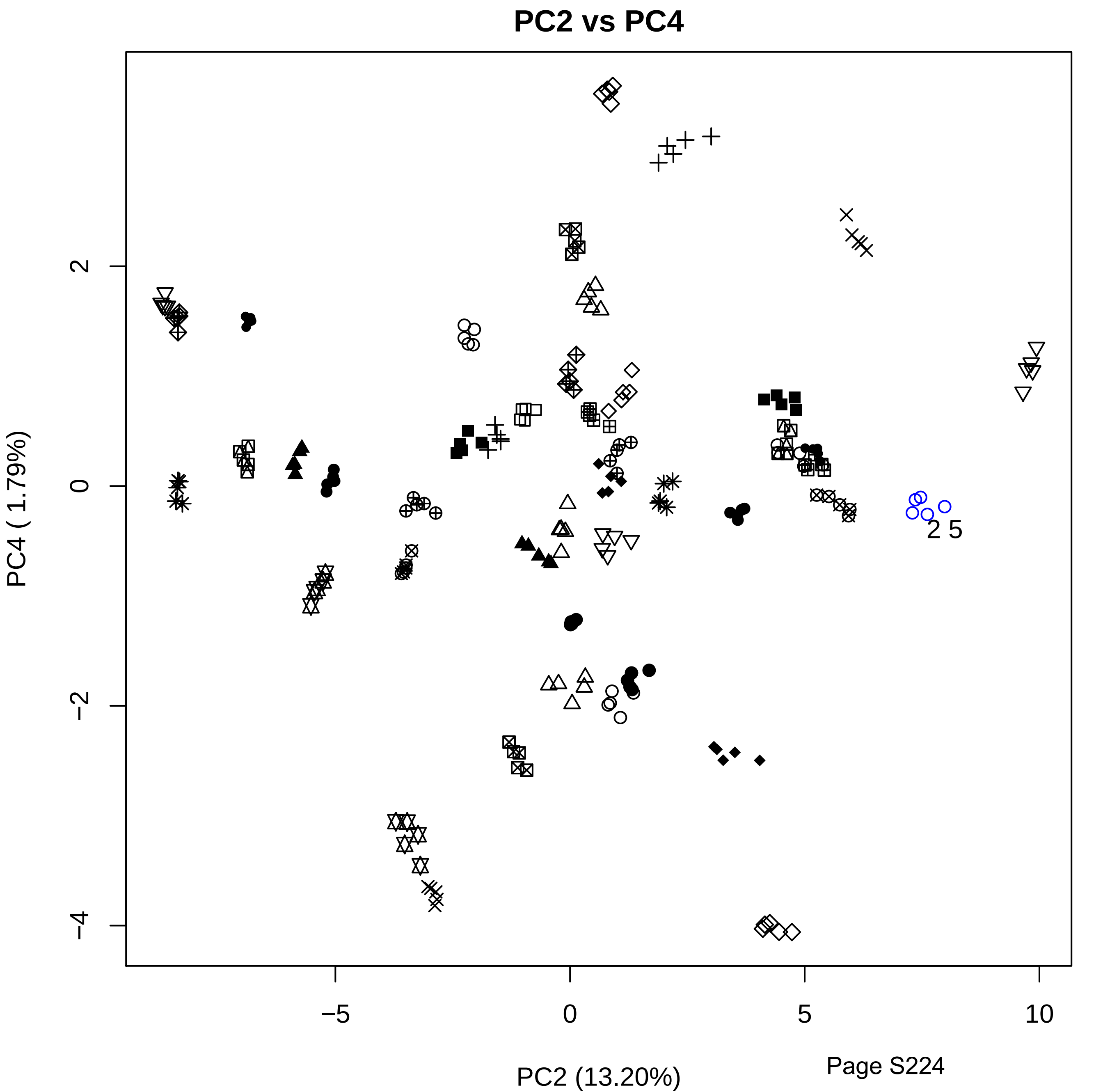
PC2 vs PC4
PC4 (1.79%)
PC2 (13.20%)
2
0
−2
−4
−5
0
5
10
2 5

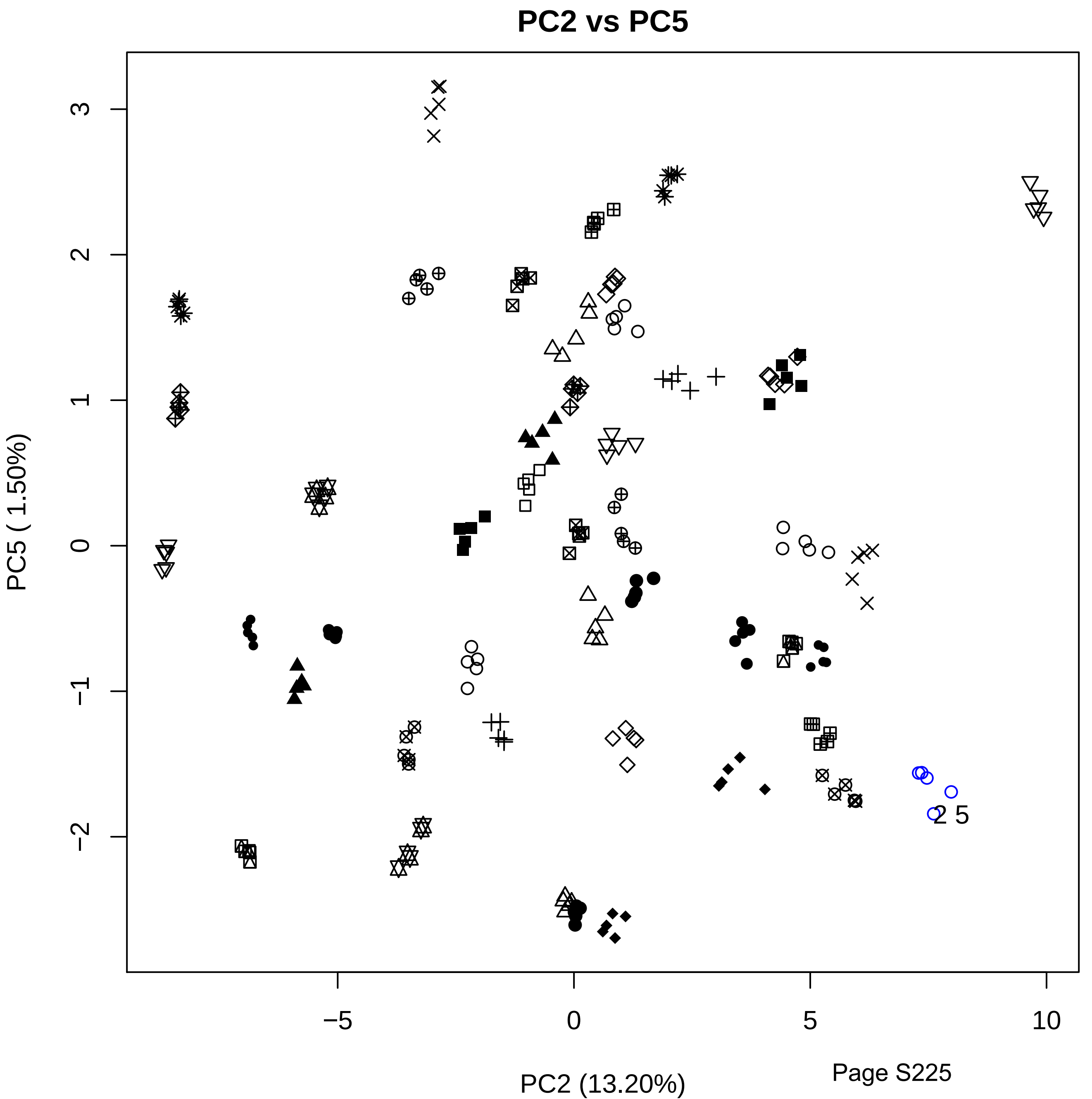
PC2 vs PC5
PC5 (1.50%)
PC2 (13.20%)
3
2
1
0
−1
−2
−5
0
5
10
2 5

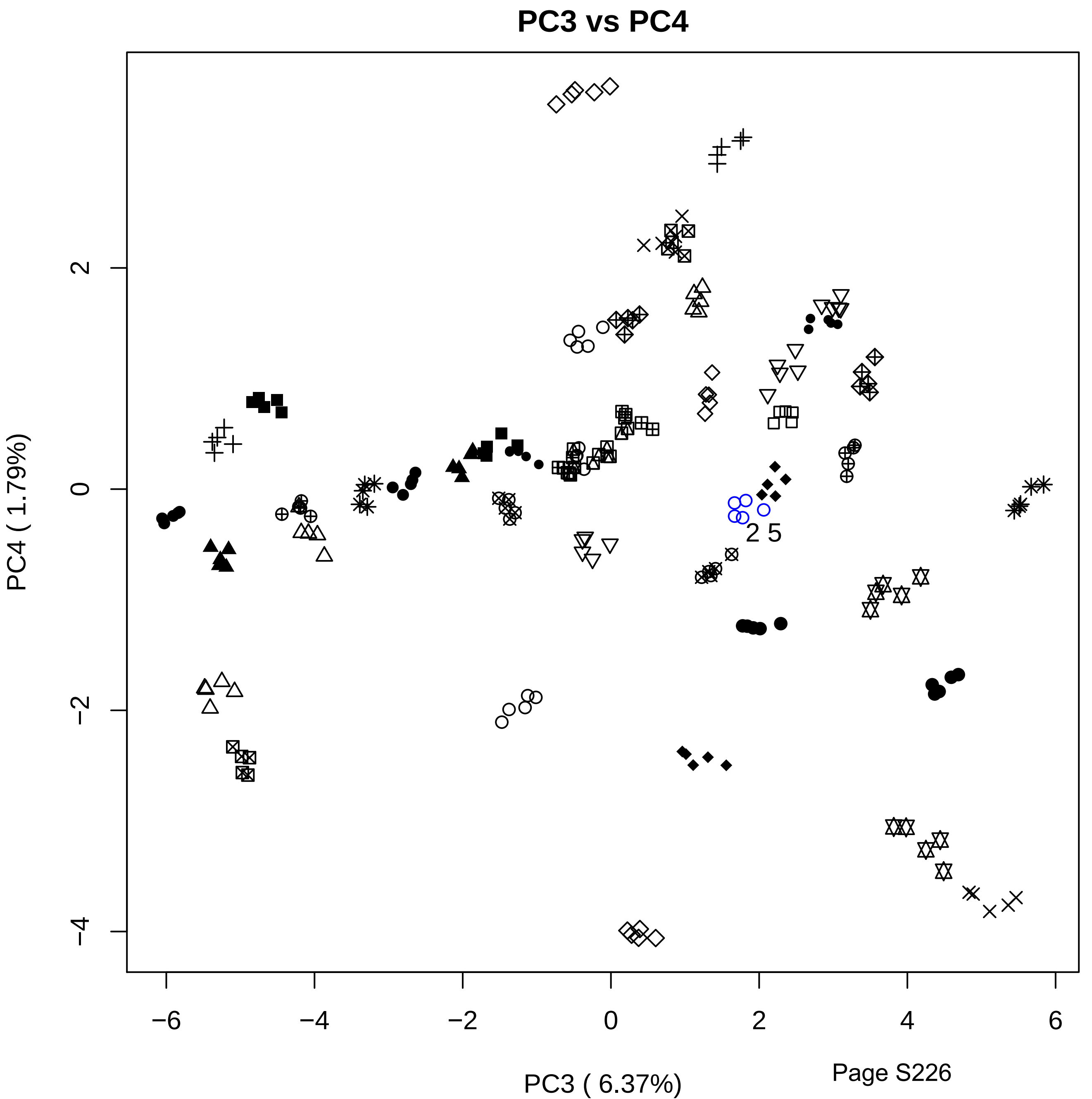

PC3 vs PC4
PC4 (1.79%)
PC3 (6.37%)
2
0
−2
−4
−6
−4
−2
0
2
4
6
2 5

PC3 vs PC5

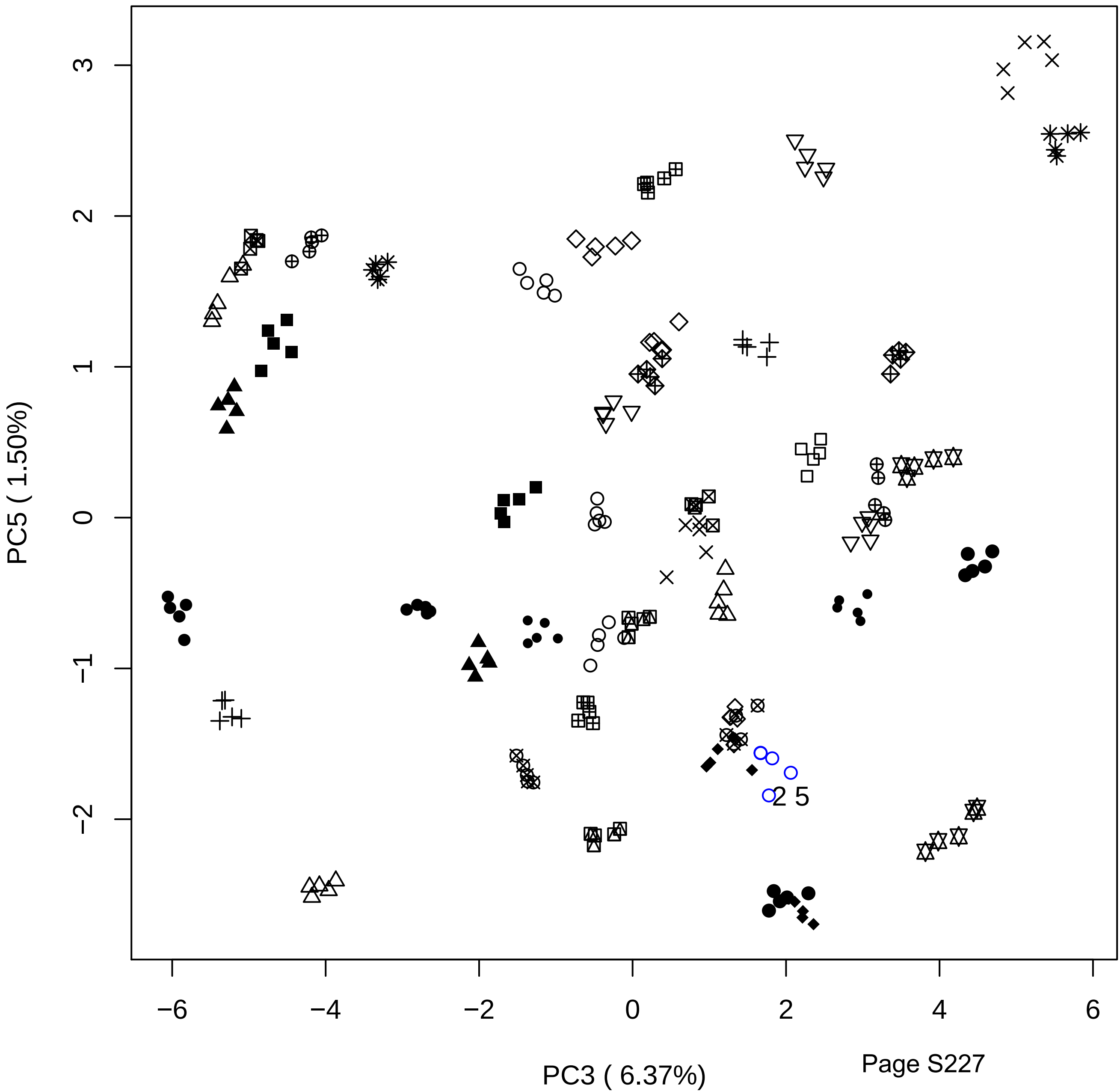

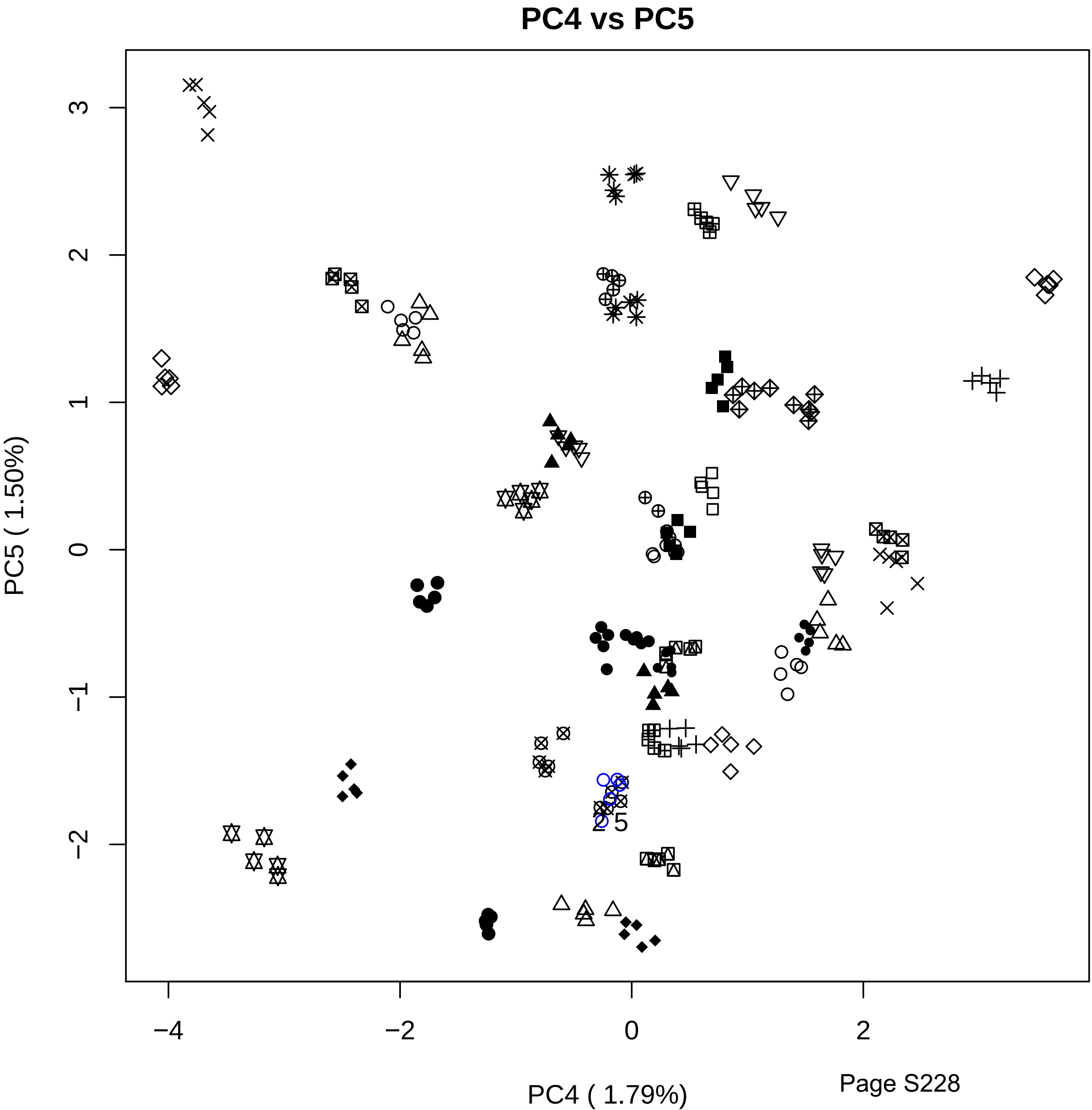
PC4 vs PC5
PC5 (1.50%)
PC4 (1.79%)
−4
−2
0
2
3
2
1
0
−1
−2

PCA of 5% OSCS vs PMH

Scaled Matrix PCAed

7th order polynomial baseline correction performed

Raw spectra smoothed to 21 Neighbours, 2nd order PN, Savtisky Golay

Normalisation between 0 and 100

D2 spectra smoothed to 41 Neighbours, 2nd order PN, Savtisky Golay

Regions Analysed: 3601−2501 and 2001−700cm−1

Samples Analysed: 1 Contam's 0 OSCS's 45 PMH's

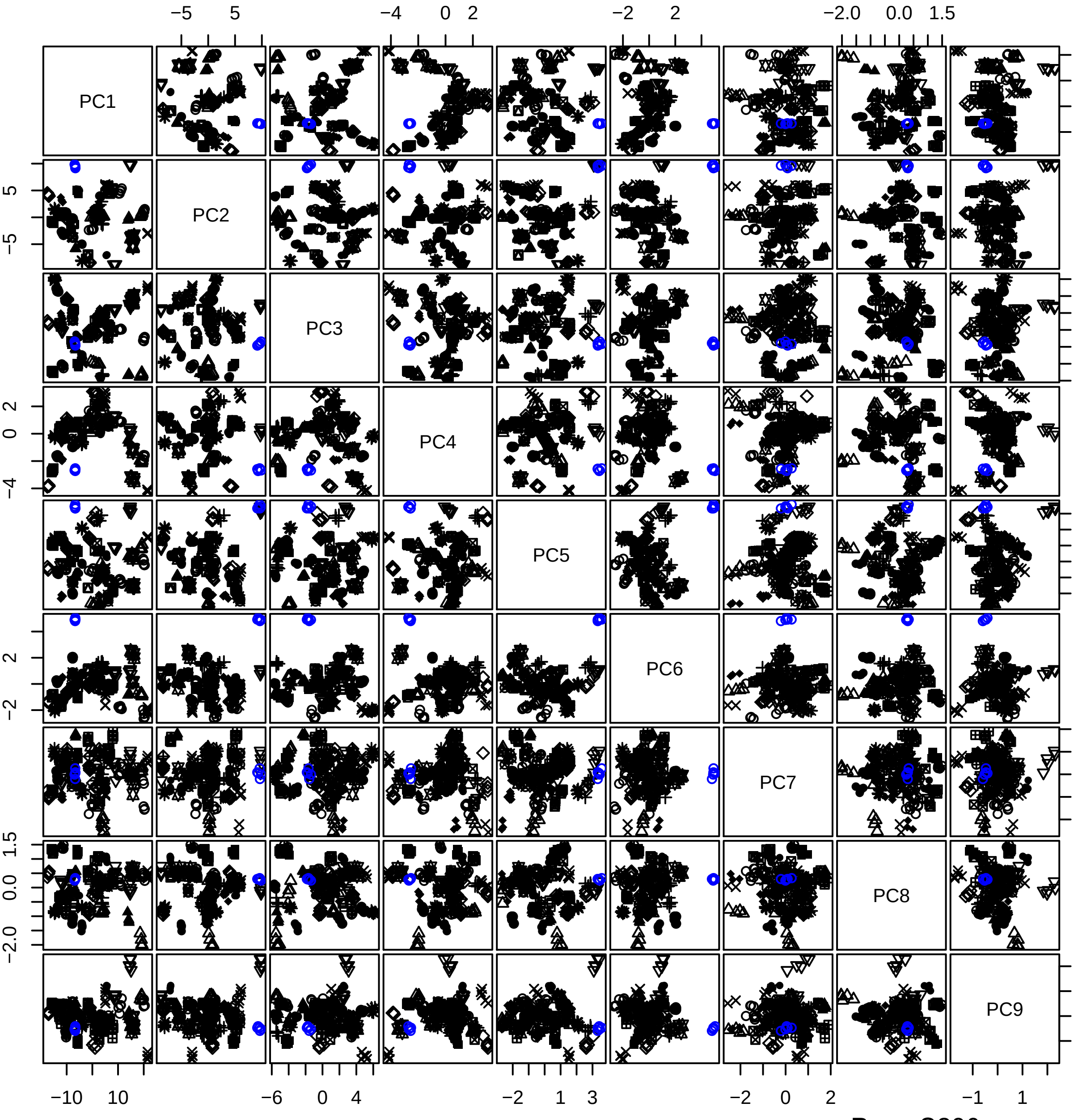
PC1
PC2
PC3
PC4
PC5
PC6
PC7
PC8
PC9
−5
5
−4
0
2
−2
2
−2.0
0.0
1.5
10
−10
−6
4
−10
10
−6
0
4
−2
1
3
−2
0
2
−1
1

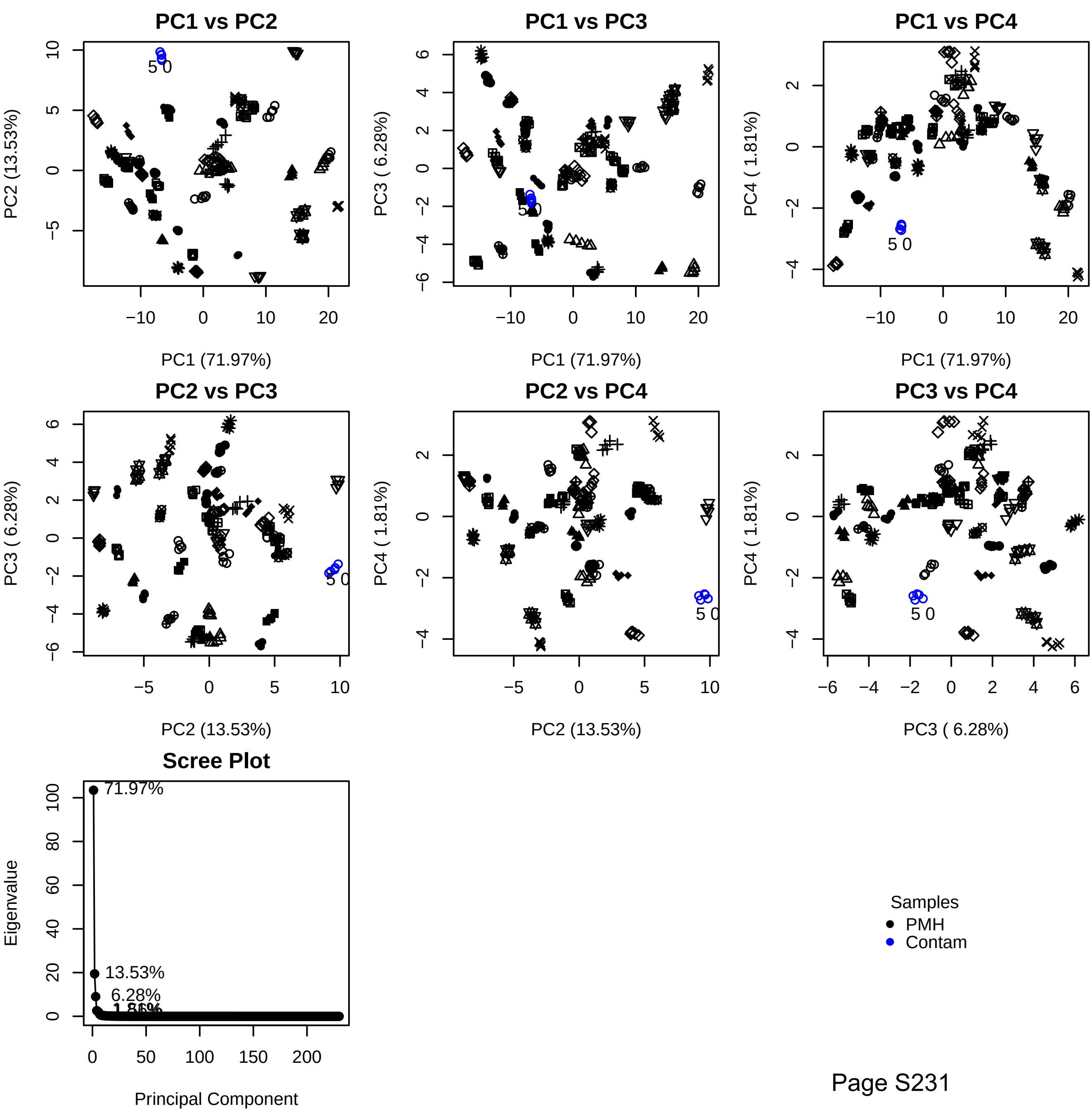
PC1 vs PC2
PC1 vs PC3
PC1 vs PC4
PC2 vs PC3
PC2 vs PC4
PC3 vs PC4
Scree Plot
PC1 (71.97%)
PC2 (13.53%)
PC3 (6.28%)
PC4 (1.81%)
Eigenvalue
Principal Component
71.97%
13.53%
6.28%
Samples
PMH
Contam

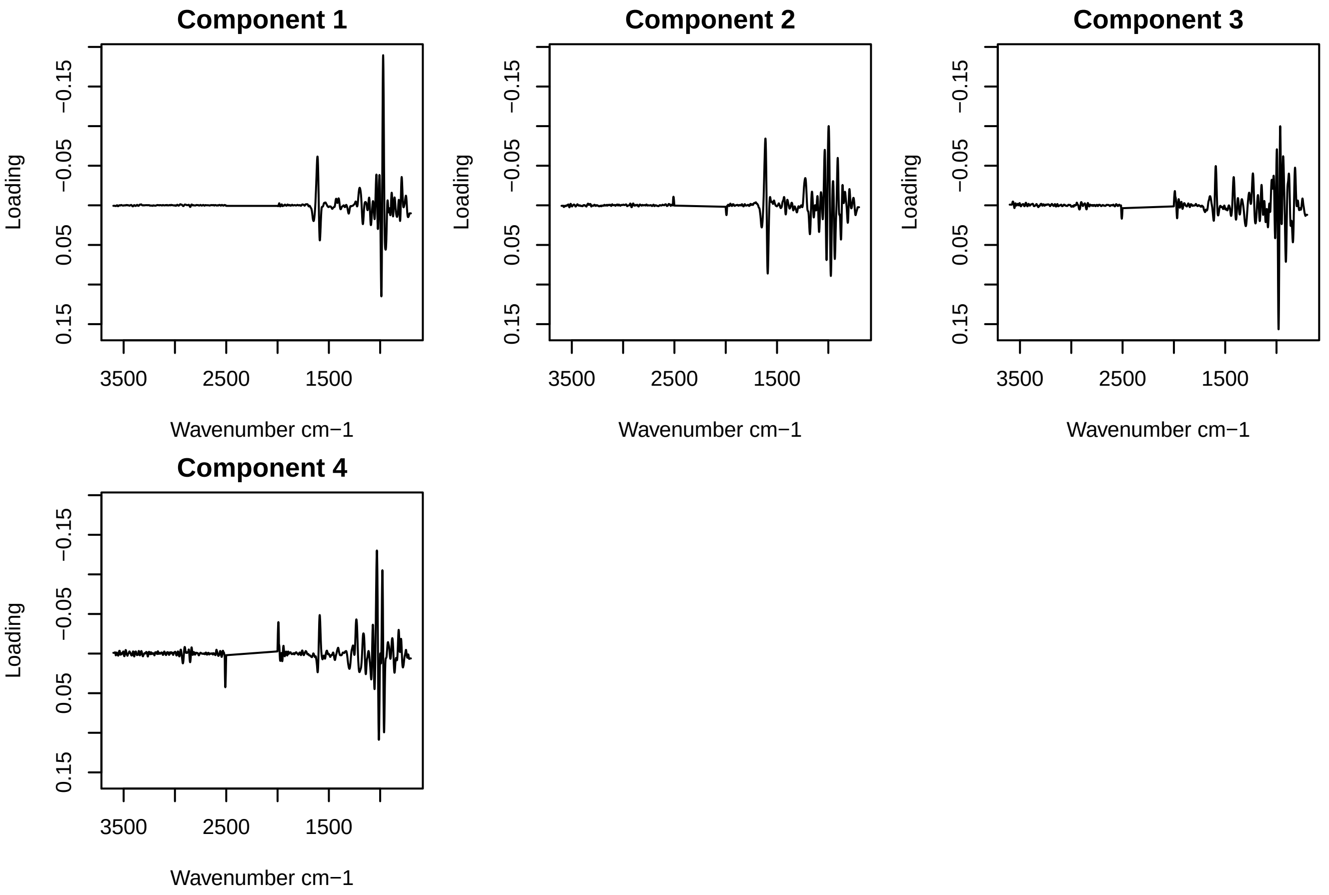
Component 1
Component 2
Component 3
Component 4
Loading
−0.15
−0.05
0.05
0.15
3500
2500
1500
Wavenumber cm−1

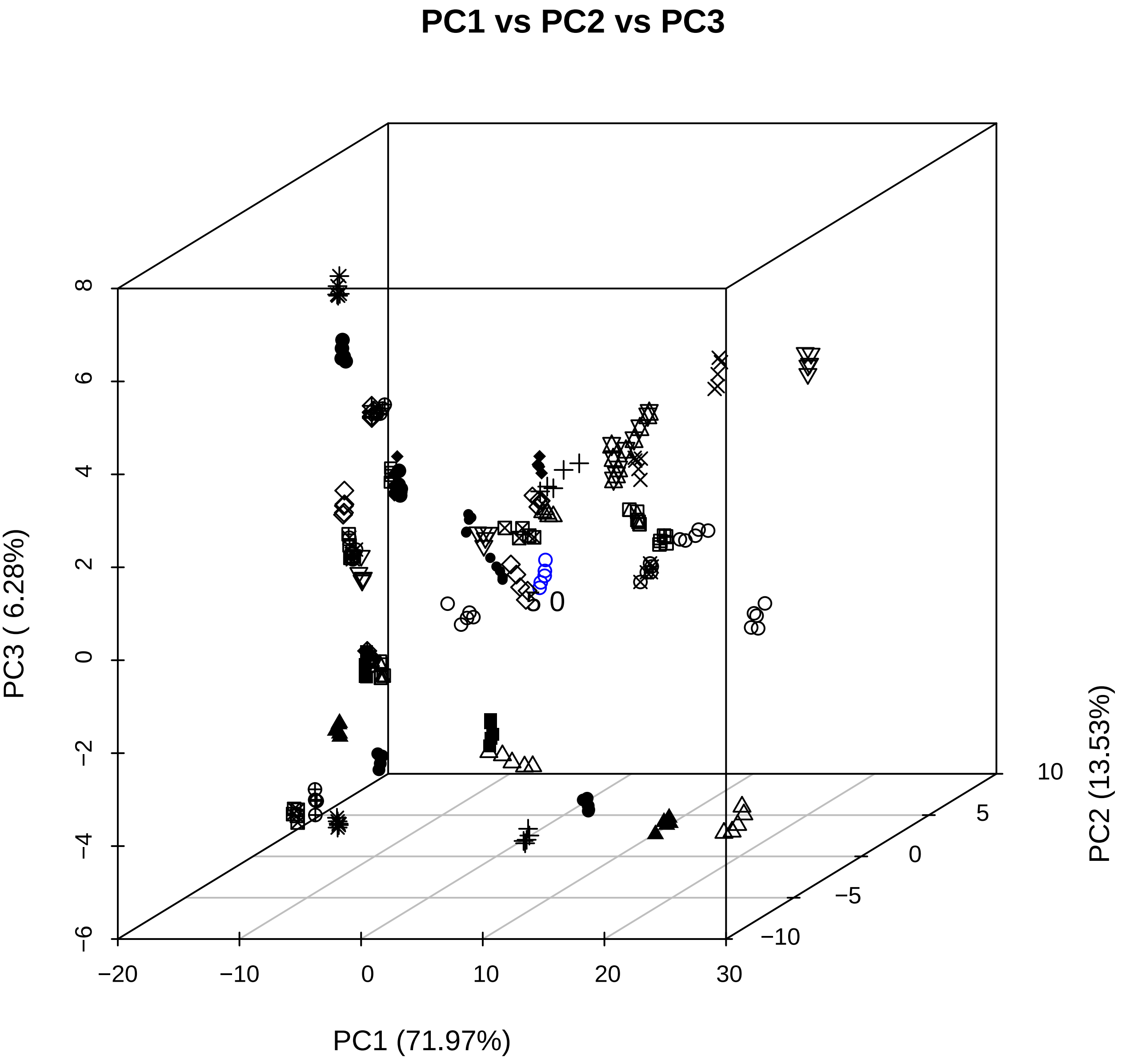

PC1 vs PC2 vs PC3
PC1 (71.97%)
PC2 (13.53%)
PC3 (6.28%)
−20
−10
0
10
20
30
−10
−5
0
5
10
−6
−4
−2
0
2
4
6
8

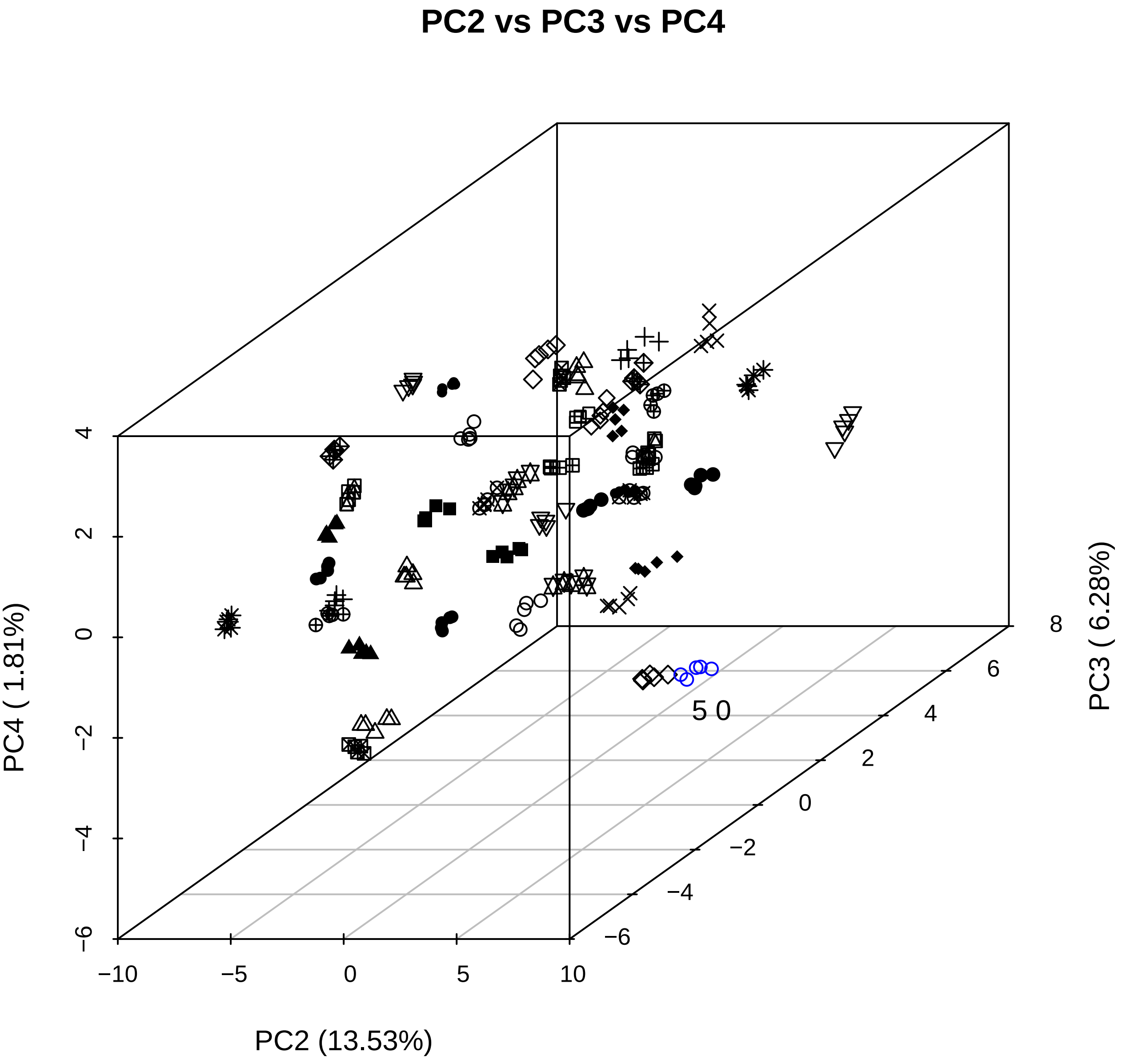
PC2 vs PC3 vs PC4
PC4 (1.81%)
PC2 (13.53%)
PC3 (6.28%)
−10
−5
0
5
10
−6
−4
−2
0
2
4
6
8
5 0

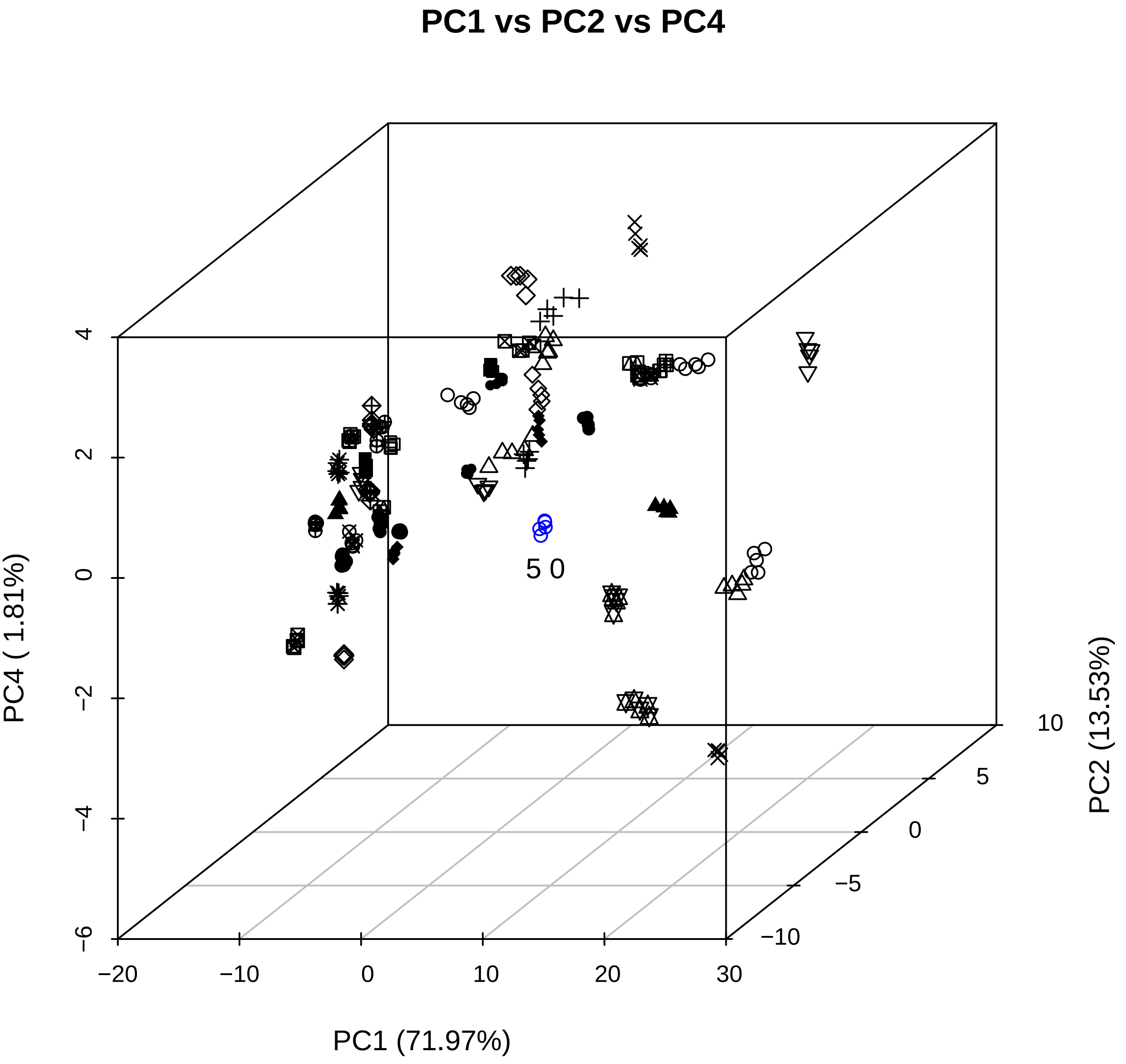

PC1 vs PC2 vs PC4
PC4 (1.81%)
PC1 (71.97%)
PC2 (13.53%)
−20
−10
0
10
20
30
−6
−4
−2
0
2
4
−10
−5
0
5
10
5 0

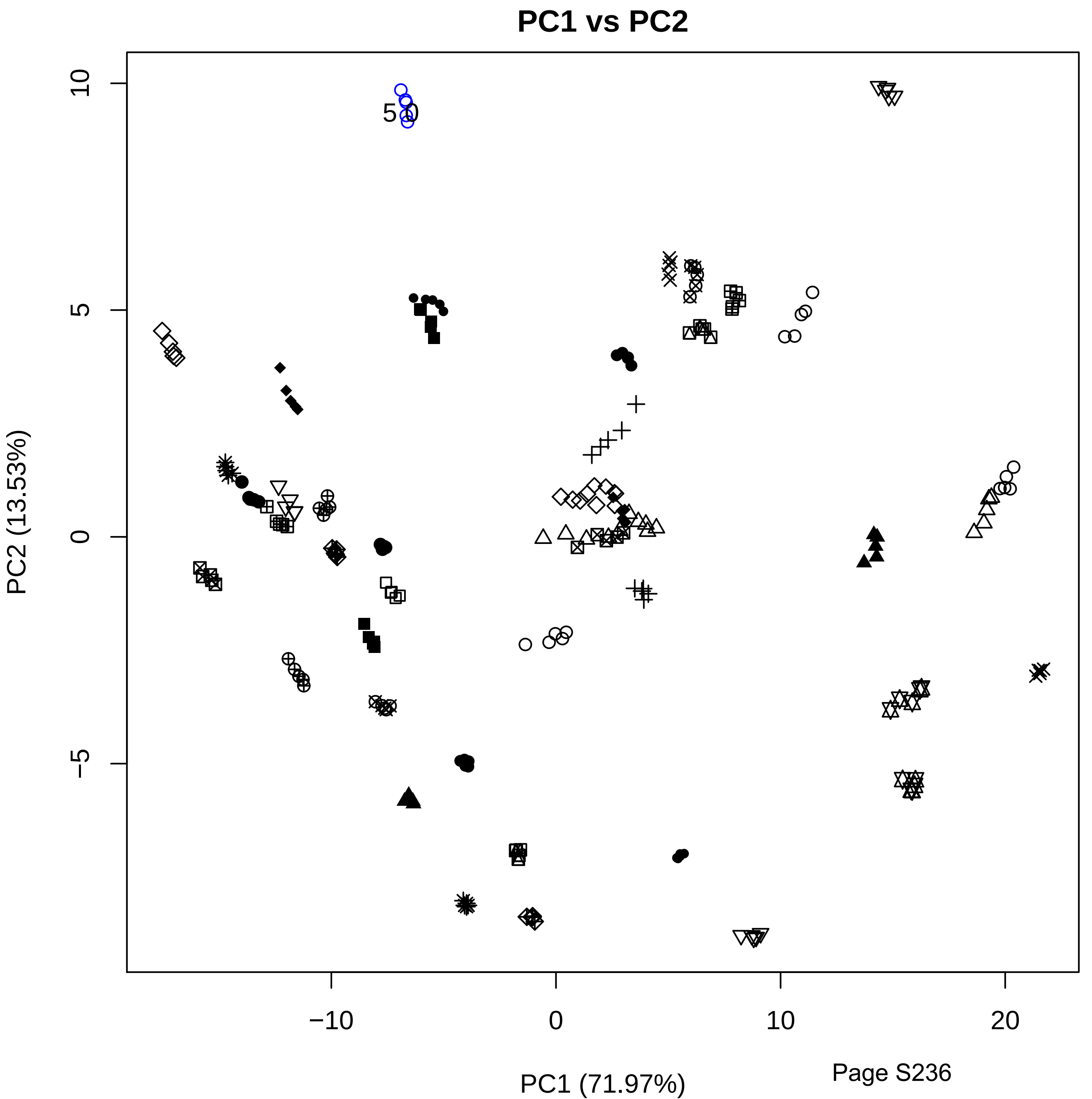
PC1 vs PC2
510
PC2 (13.53%)
PC1 (71.97%)
10
5
0
−5
−10
0
10
20

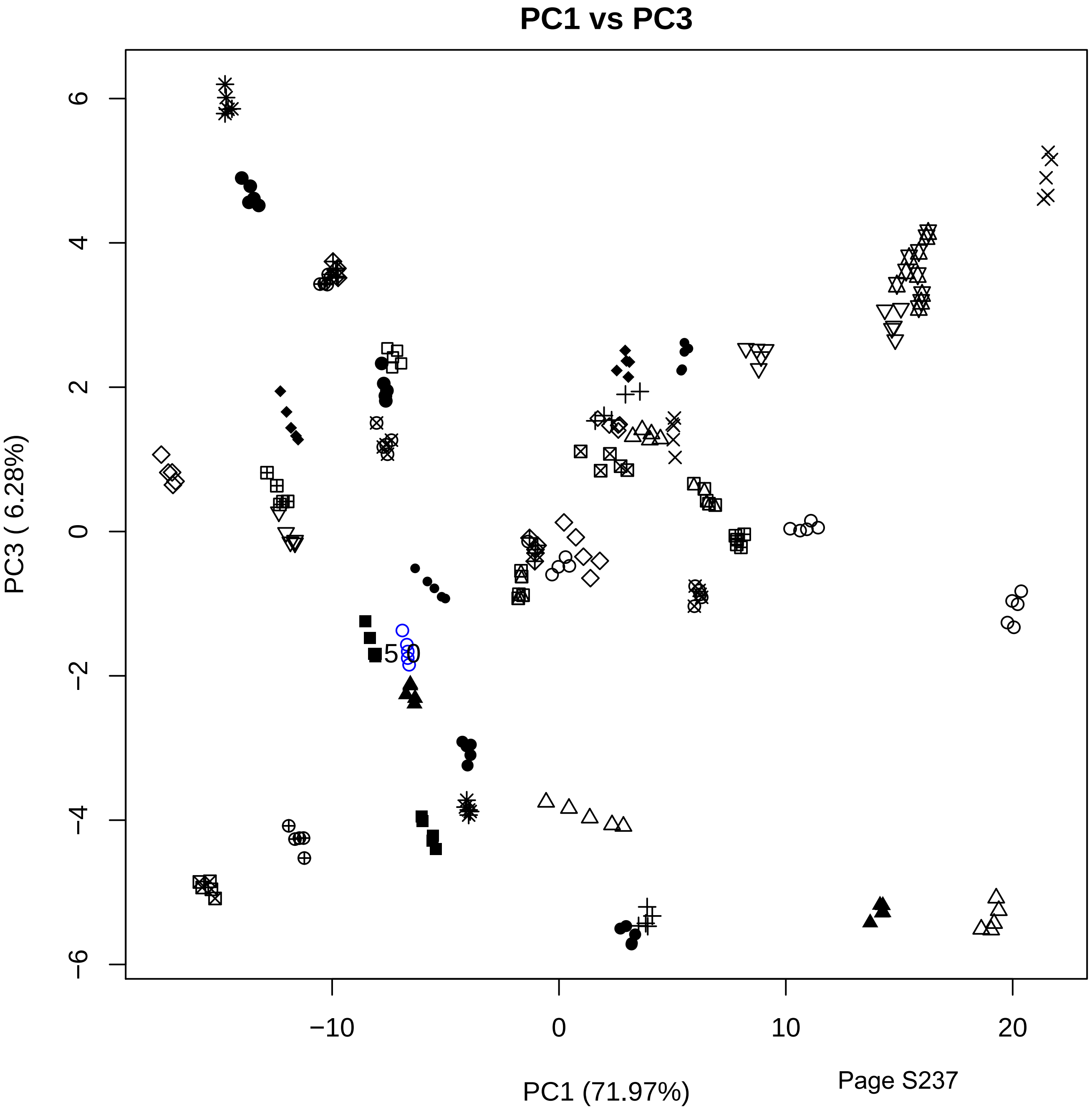
PC1 vs PC3
PC3 (6.28%)
PC1 (71.97%)
−6
−4
−2
0
2
4
6
−10
0
10
20

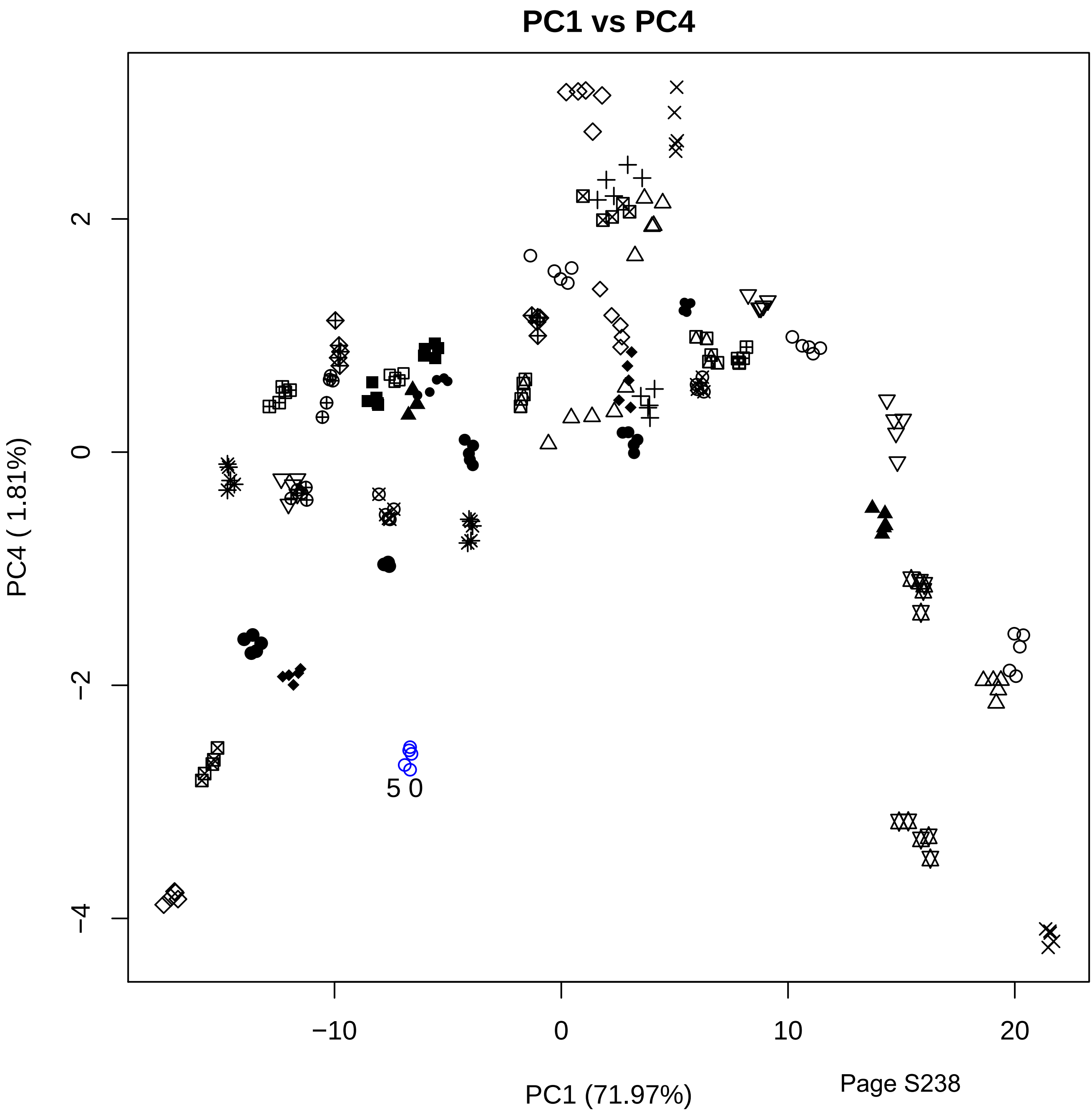
PC1 vs PC4
PC4 (1.81%)
PC1 (71.97%)
2
0
−2
−4
−10
0
10
20
5 0

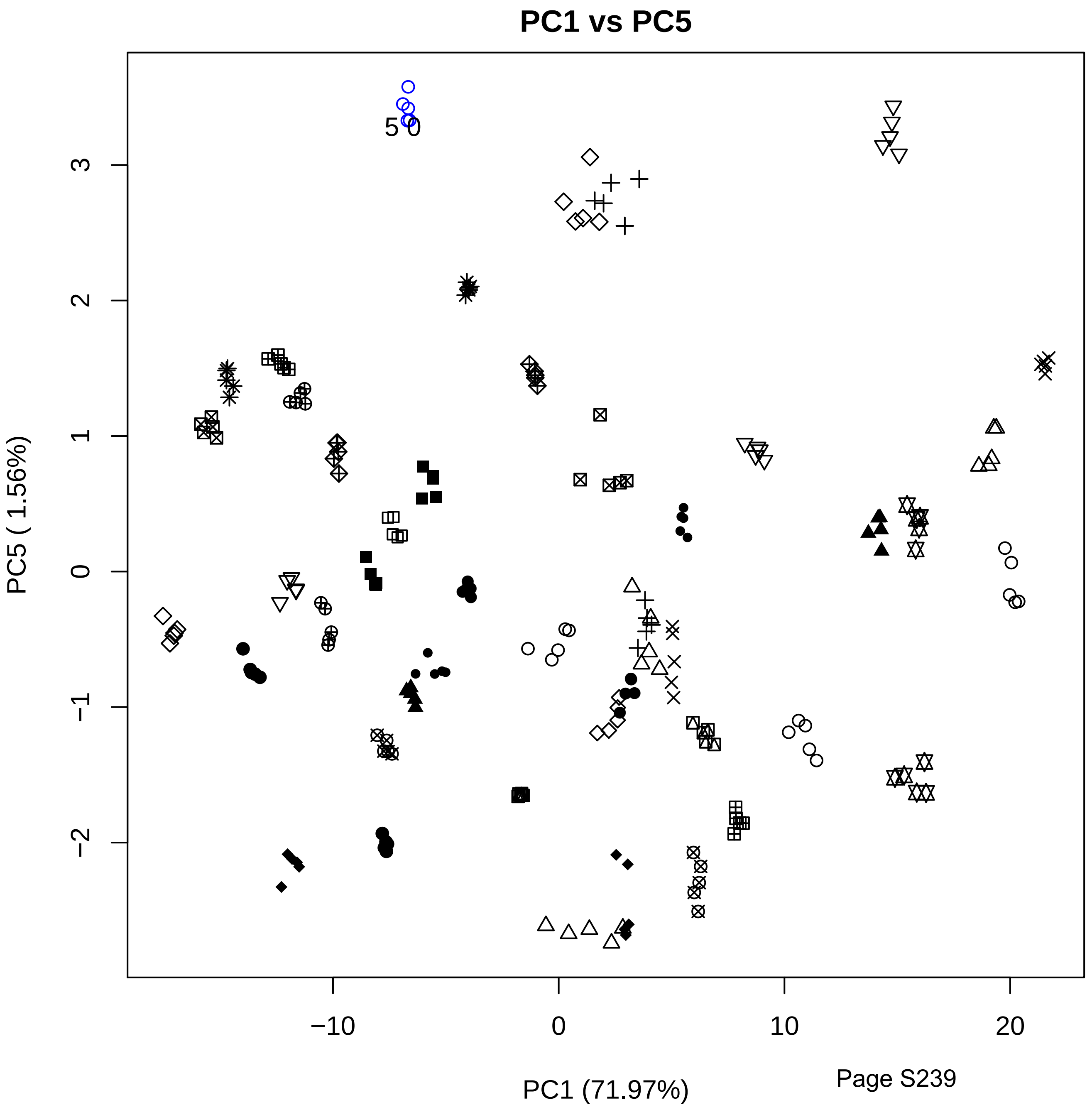
PC1 vs PC5
50
PC5 (1.56%)
3
2
1
0
−1
−2
−10
0
10
20
PC1 (71.97%)

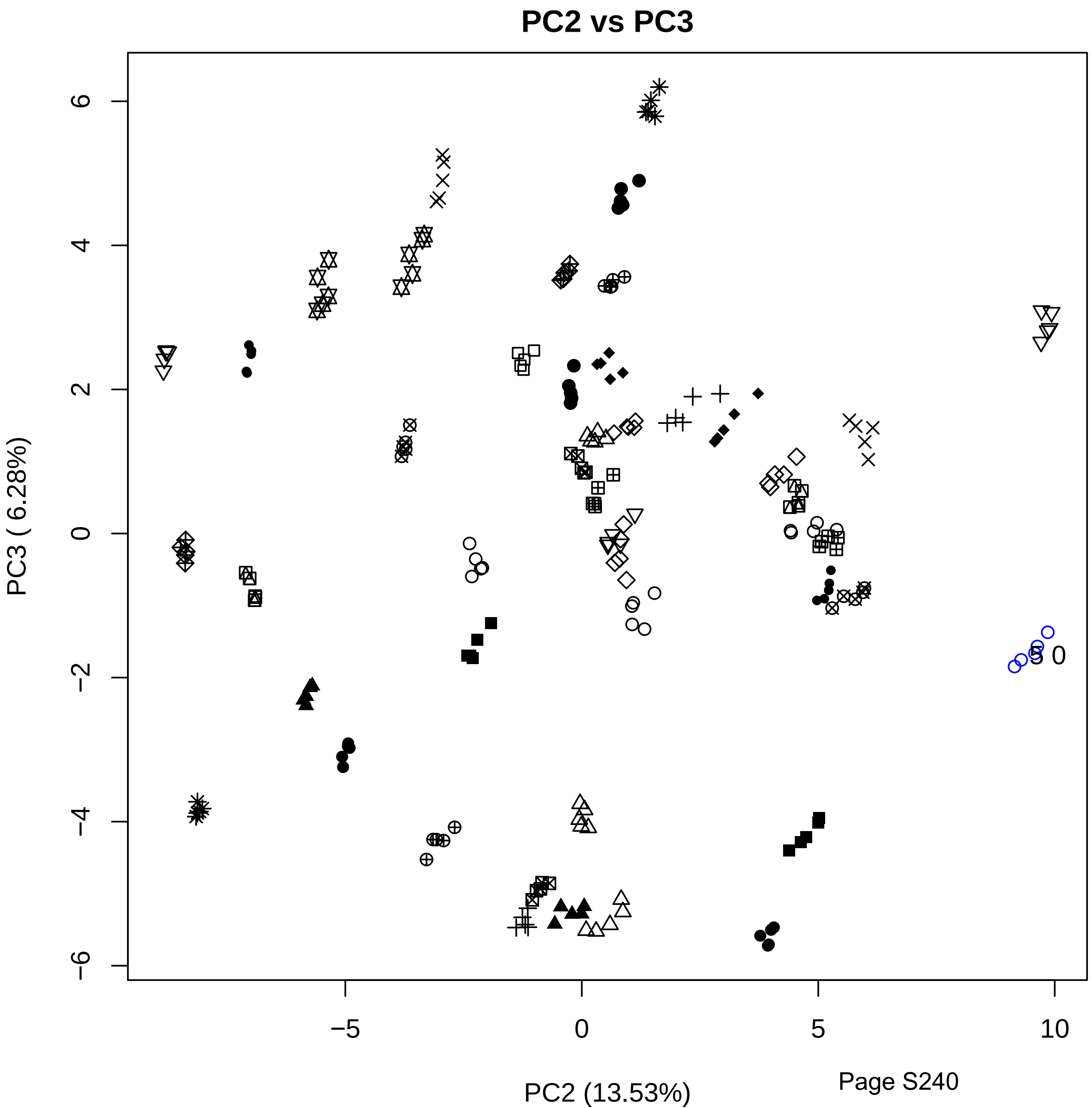
PC2 vs PC3
PC3 (6.28%)
PC2 (13.53%)
6
4
2
0
−2
−4
−6
−5
0
5
10
5 0

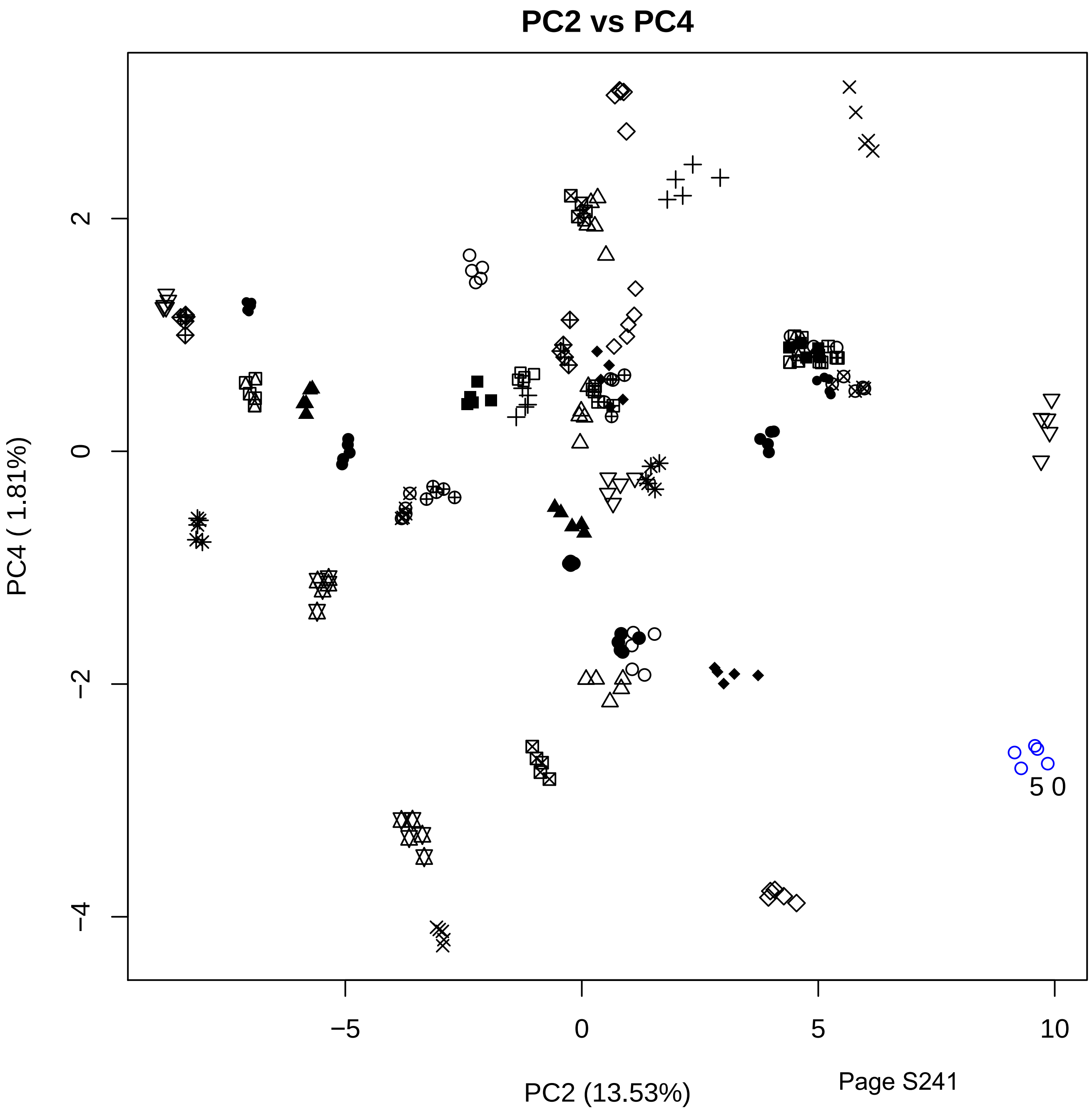
PC2 vs PC4
PC4 (1.81%)
PC2 (13.53%)
2
0
−2
−4
−5
0
5
10
5 0

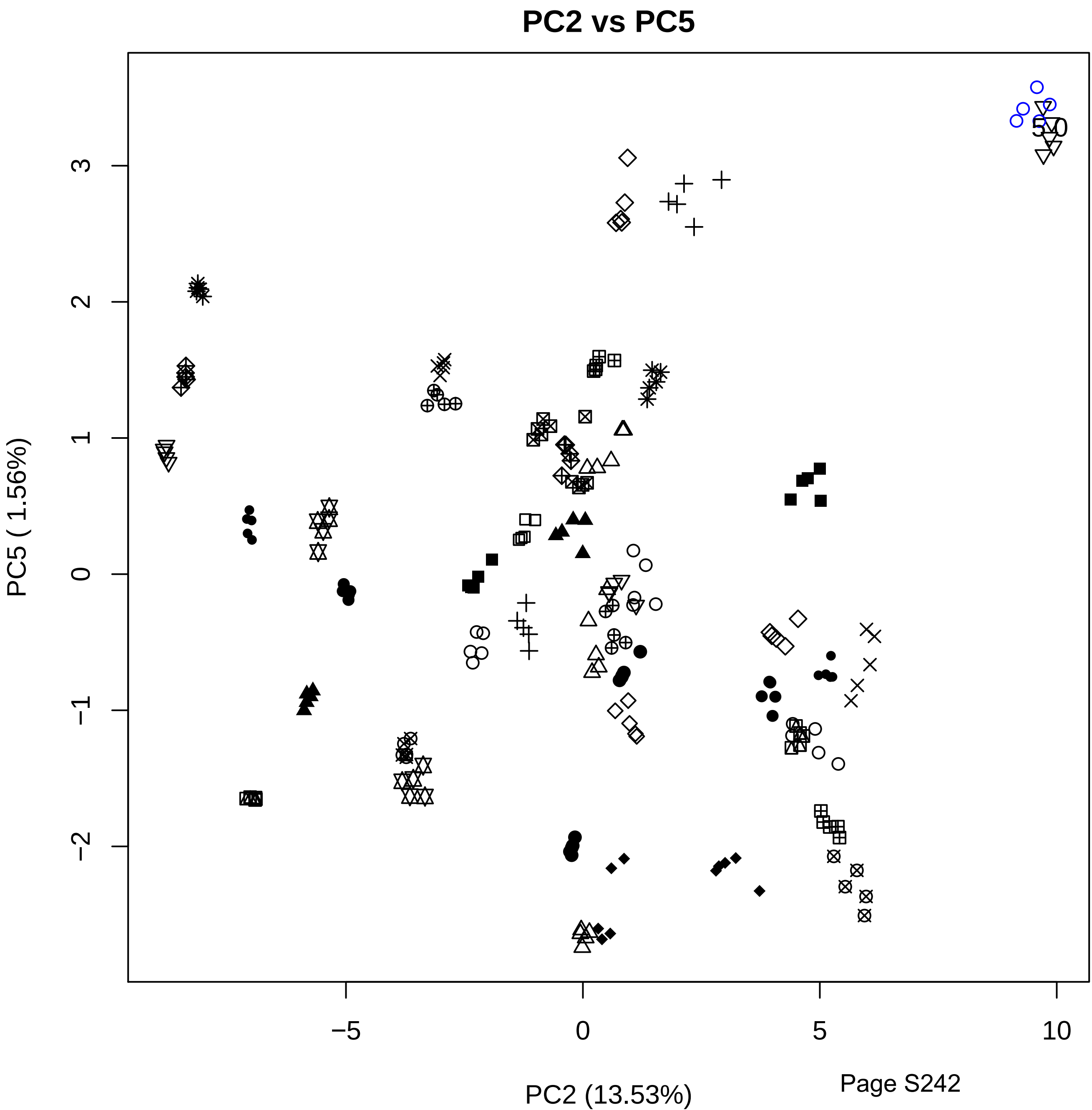
PC2 vs PC5
PC5 (1.56%)
PC2 (13.53%)
−5
0
5
10
−2
−1
0
1
2
3
50

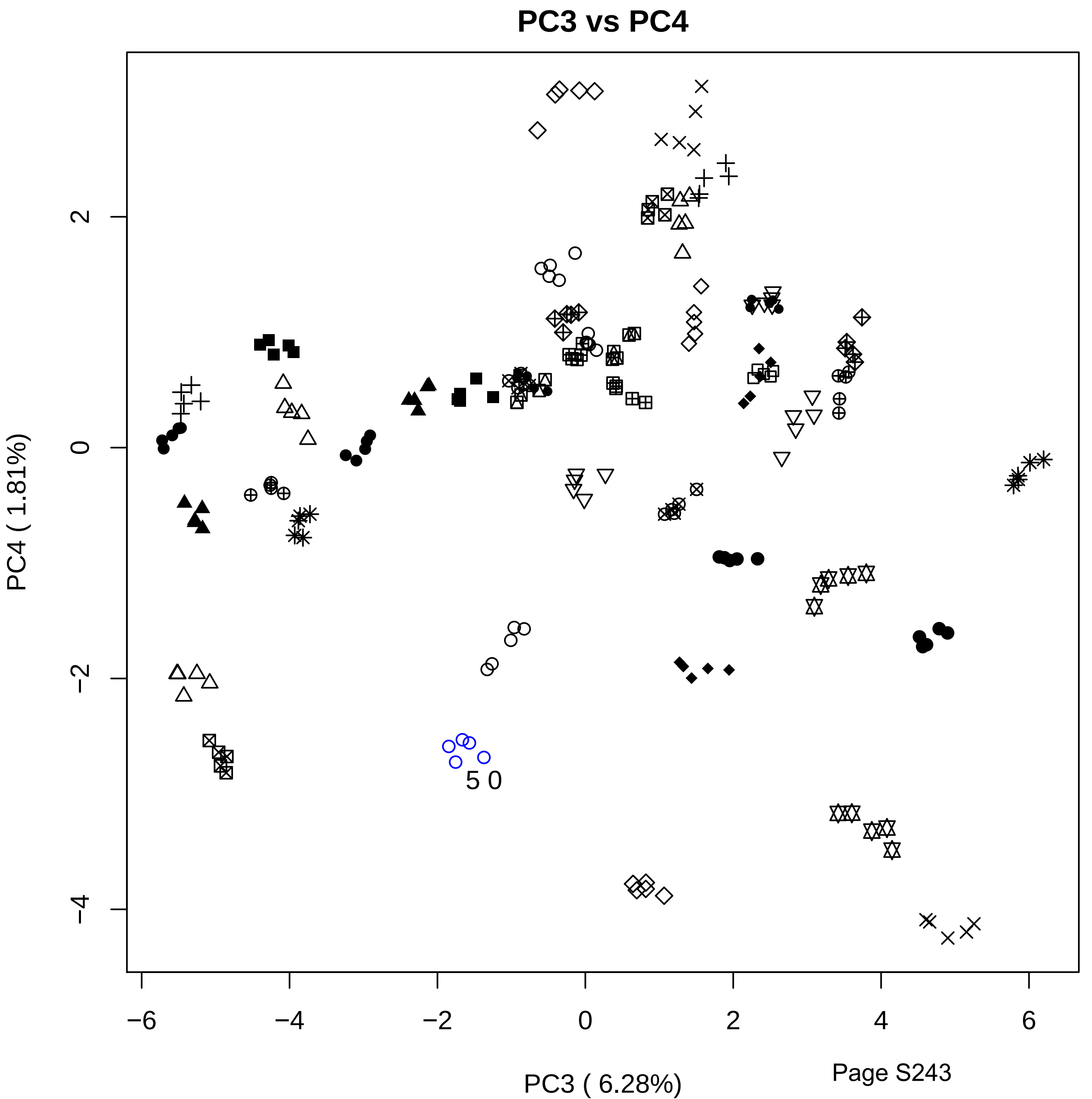
PC3 vs PC4
PC4 (1.81%)
PC3 (6.28%)
50
−6
−4
−2
0
2
4
6

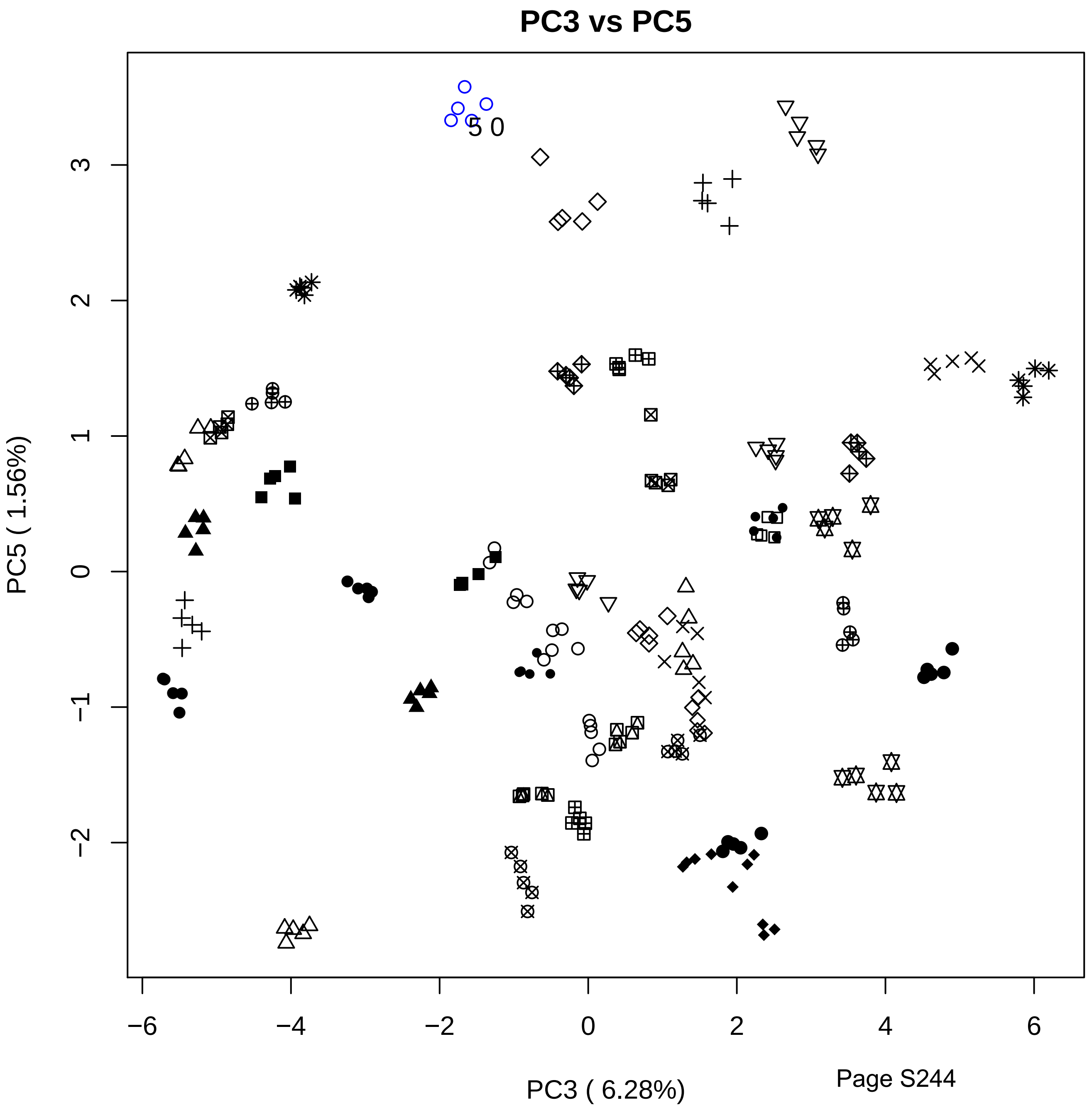

PC3 vs PC5
5 0
PC5 (1.56%)
PC3 (6.28%)
−6
−4
−2
0
2
4
6
−2
−1
0
1
2
3

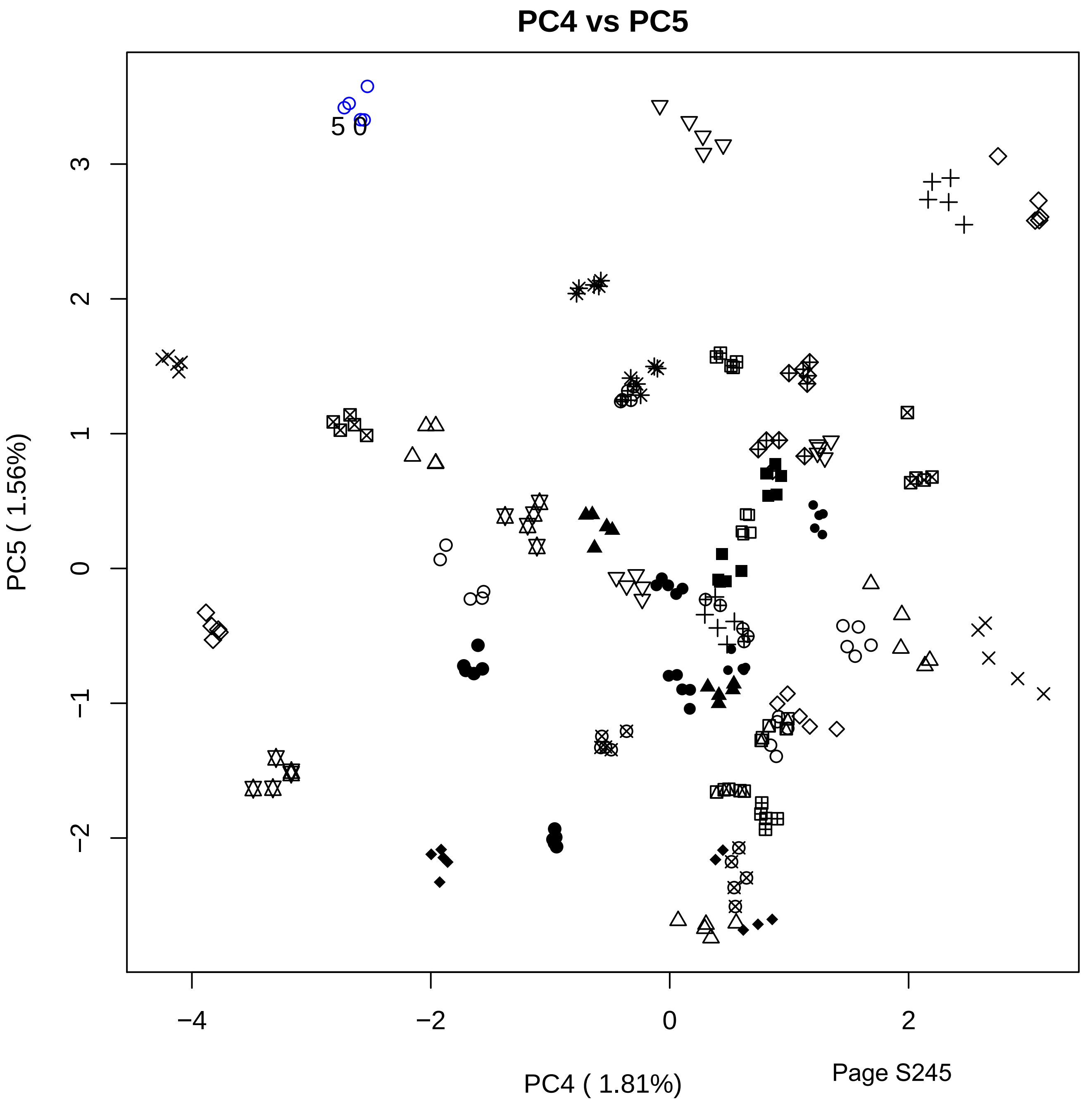
PC4 vs PC5
5 0
3
2
1
0
−1
−2
PC5 (1.56%)
−4
−2
0
2
PC4 (1.81%)

PCA of 7.5% OSCS vs PMH

Scaled Matrix PCAed

7th order polynomial baseline correction performed

Raw spectra smoothed to 21 Neighbours, 2nd order PN, Savtisky Golay

Normalisation between 0 and 100

D2 spectra smoothed to 41 Neighbours, 2nd order PN, Savtisky Golay

Regions Analysed: 3601−2501 and 2001−700cm−1

Samples Analysed: 1 Contam's 0 OSCS's 45 PMH's

Keele University AD   Sun Dec 09 16:51:09 2018

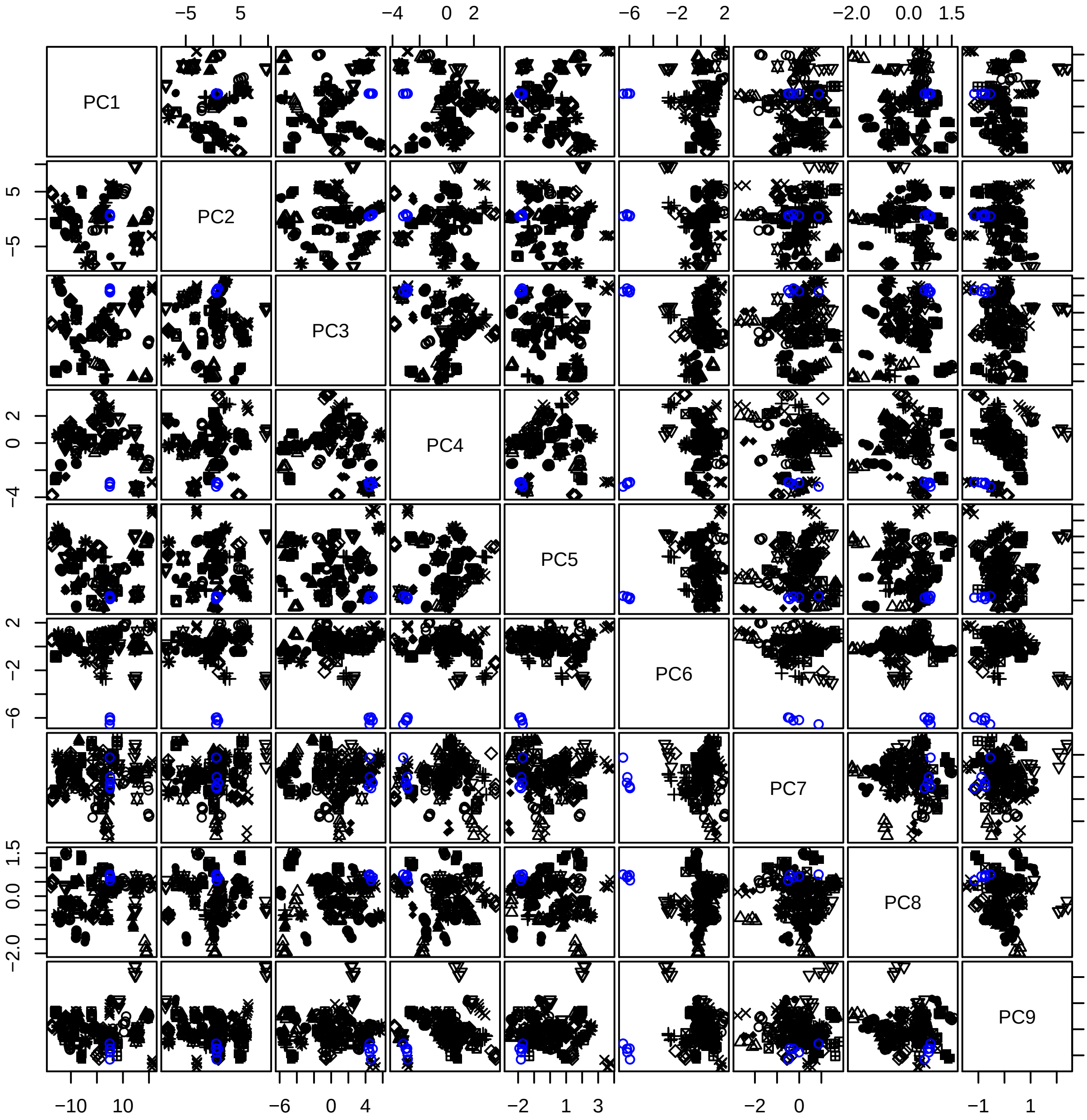
PC1
PC2
PC3
PC4
PC5
PC6
PC7
PC8
PC9

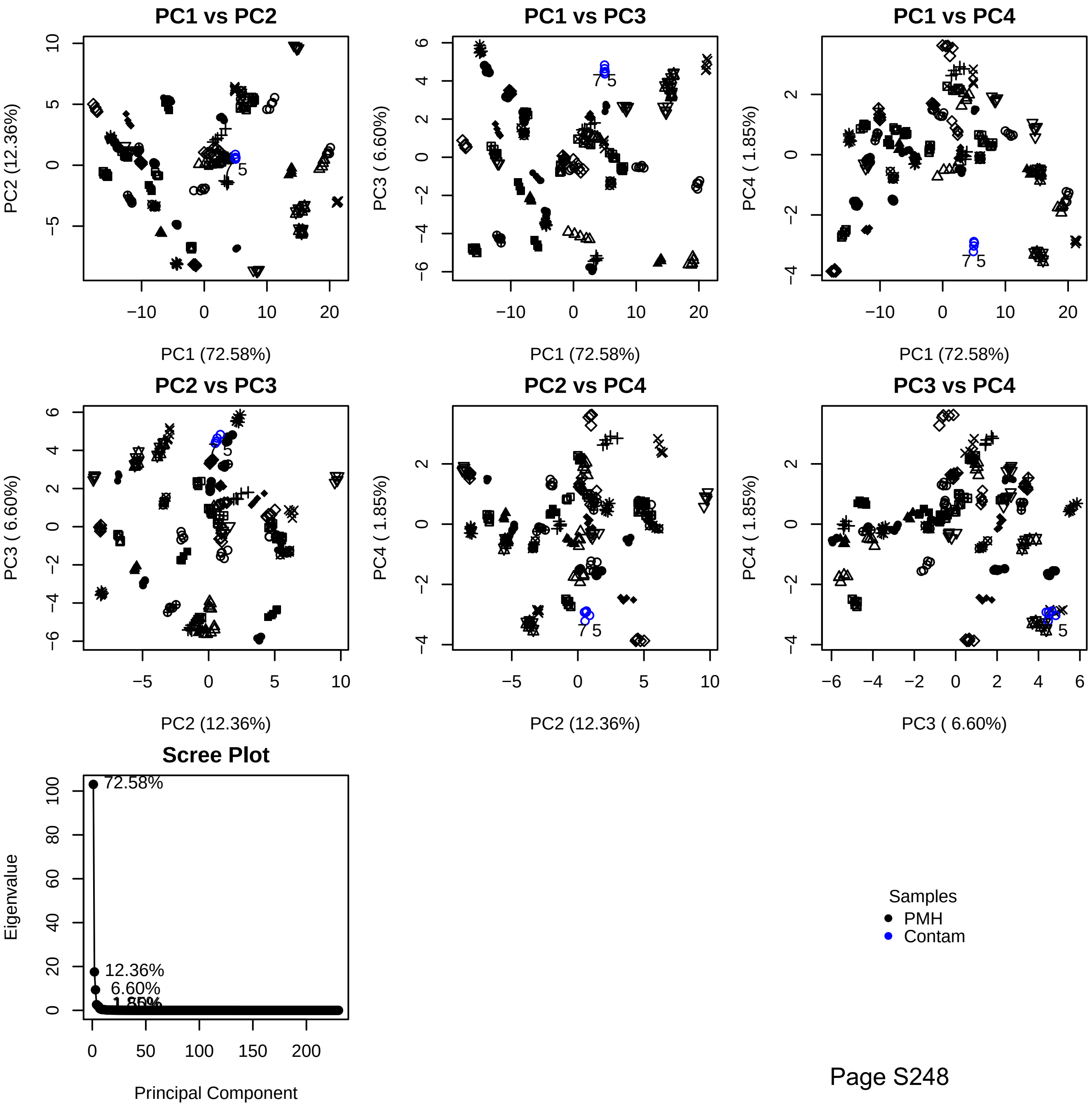
PC1 vs PC2
PC1 vs PC3
PC1 vs PC4
PC1 (72.58%)
PC2 (12.36%)
PC3 (6.60%)
PC4 (1.85%)
PC2 vs PC3
PC2 vs PC4
PC3 vs PC4
Scree Plot
72.58%
12.36%
6.60%
Eigenvalue
Principal Component
Samples
PMH
Contam

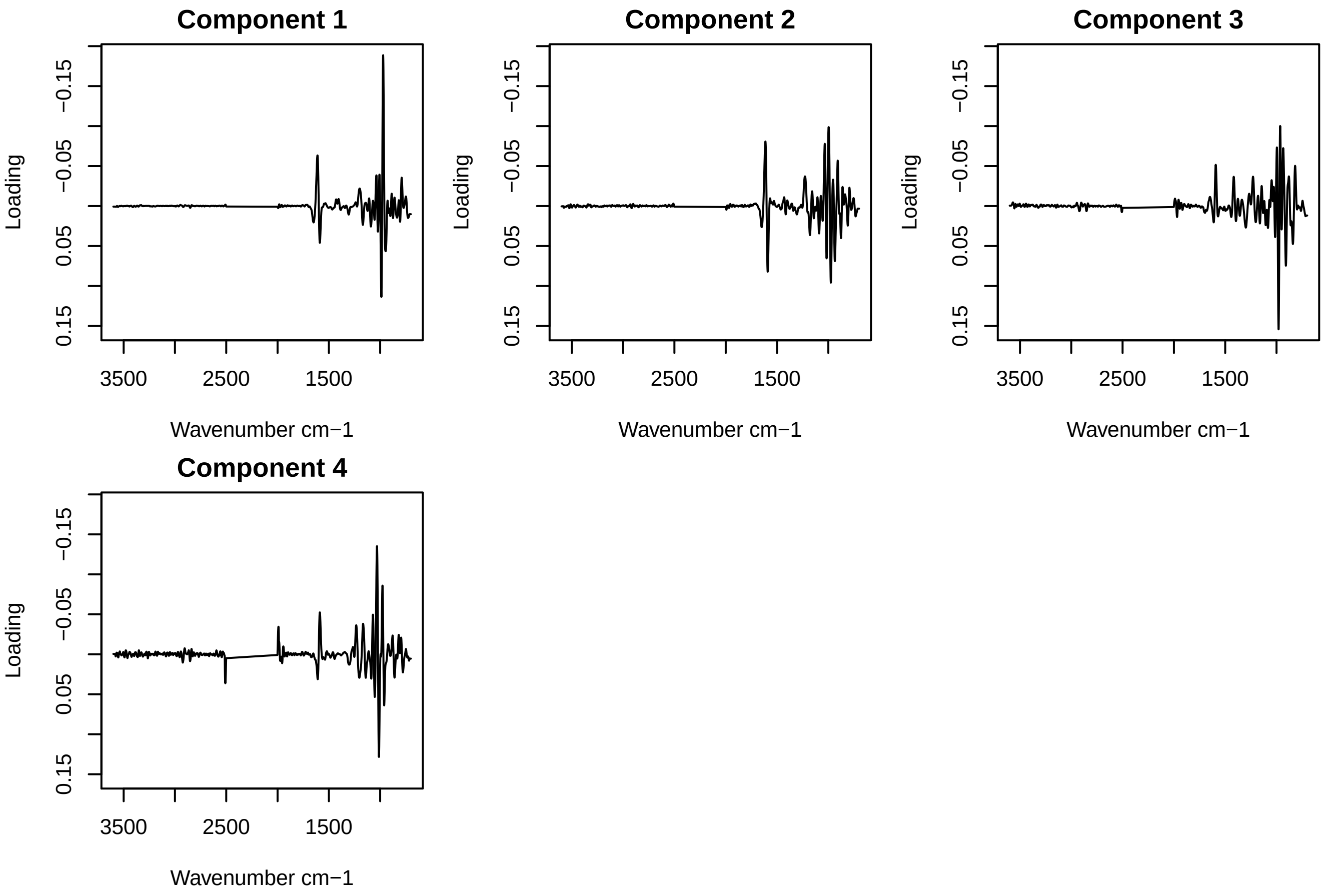
Component 1
Component 2
Component 3
Component 4
Loading
−0.15
−0.05
0.05
0.15
3500
2500
1500
Wavenumber cm−1

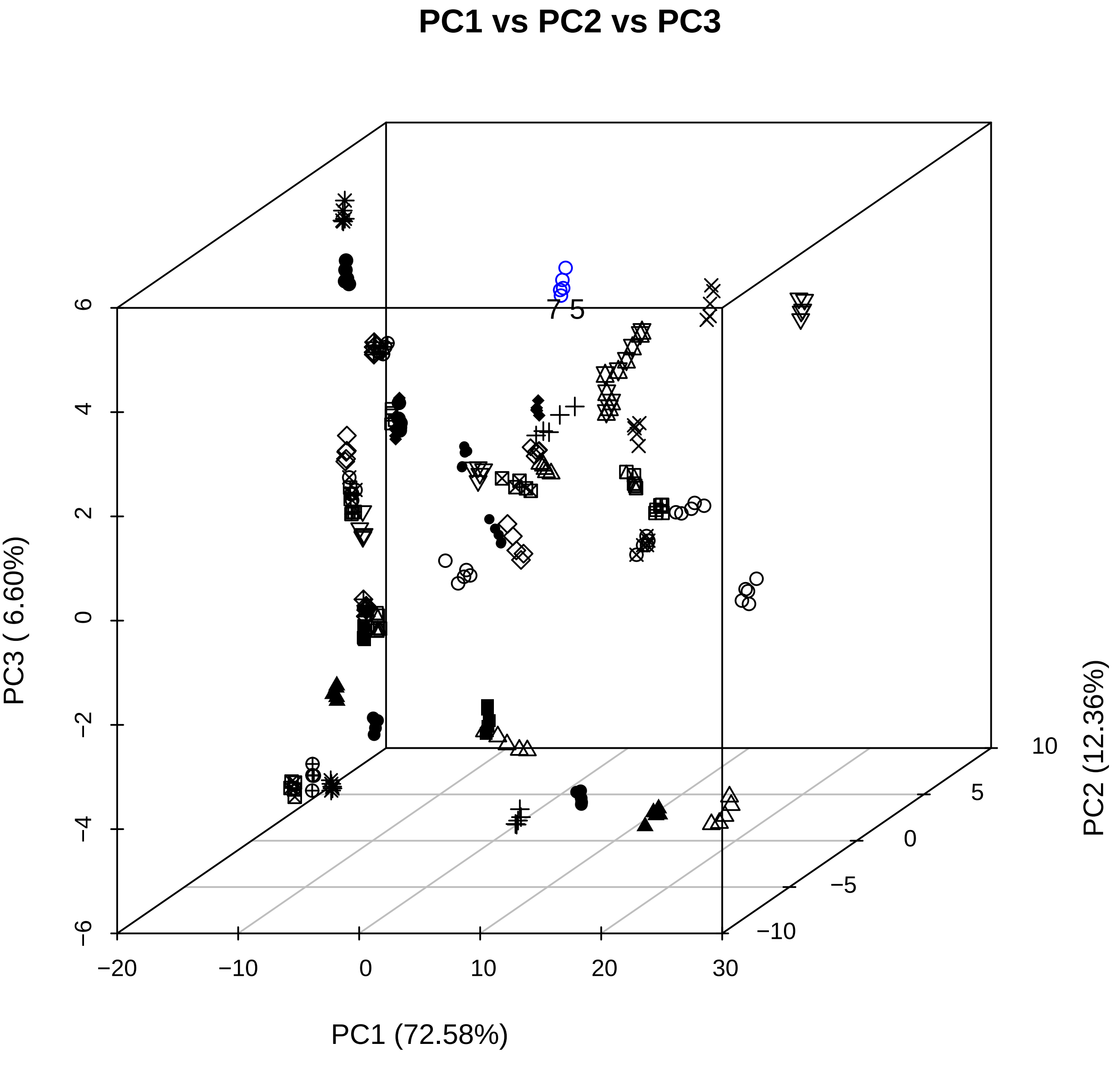
PC1 vs PC2 vs PC3
75
PC1 (72.58%)
PC2 (12.36%)
PC3 (6.60%)
−20
−10
0
10
20
30
−10
−5
0
5
10
−6
−4
−2
0
2
4
6

PC2 vs PC3 vs PC4

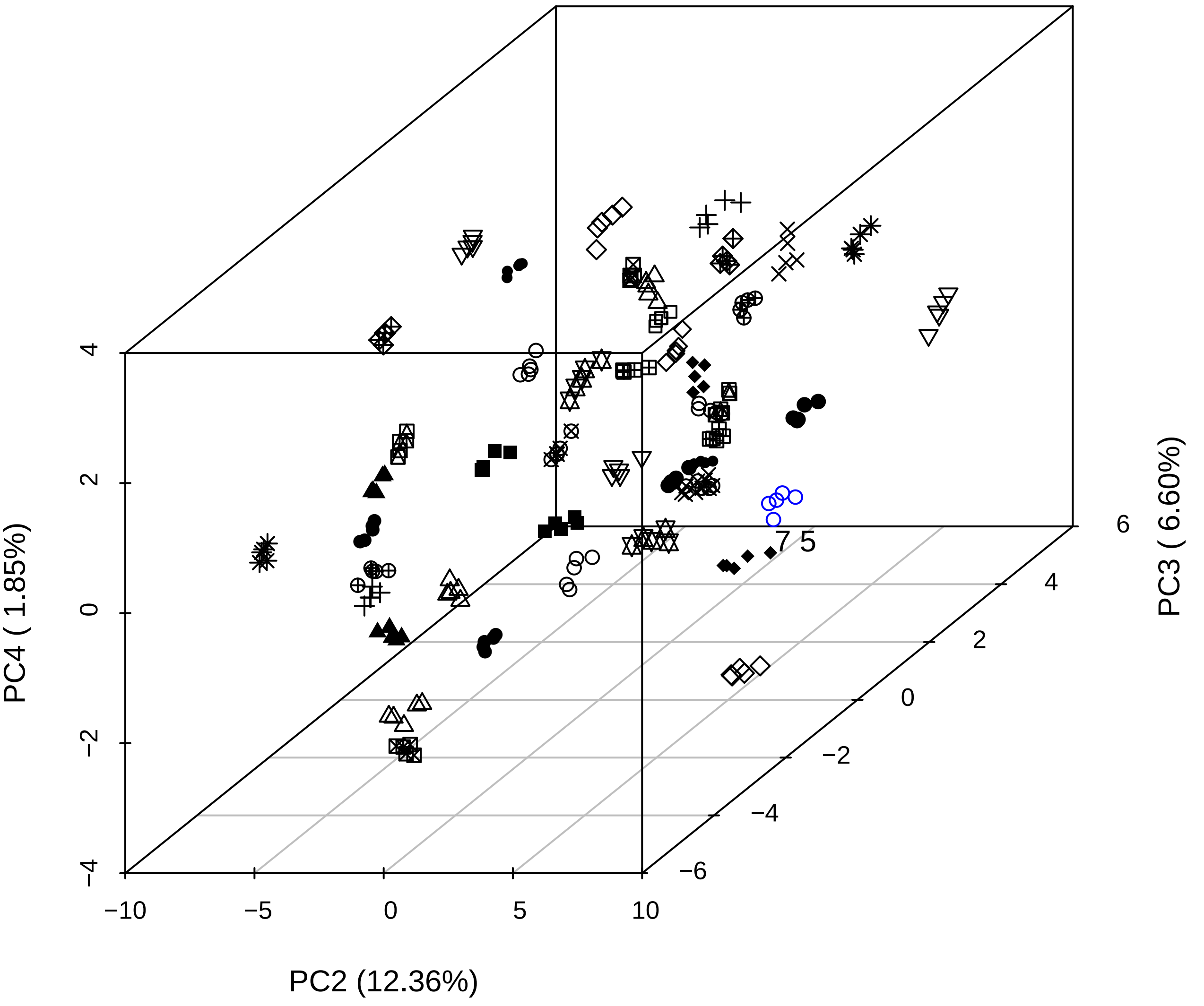

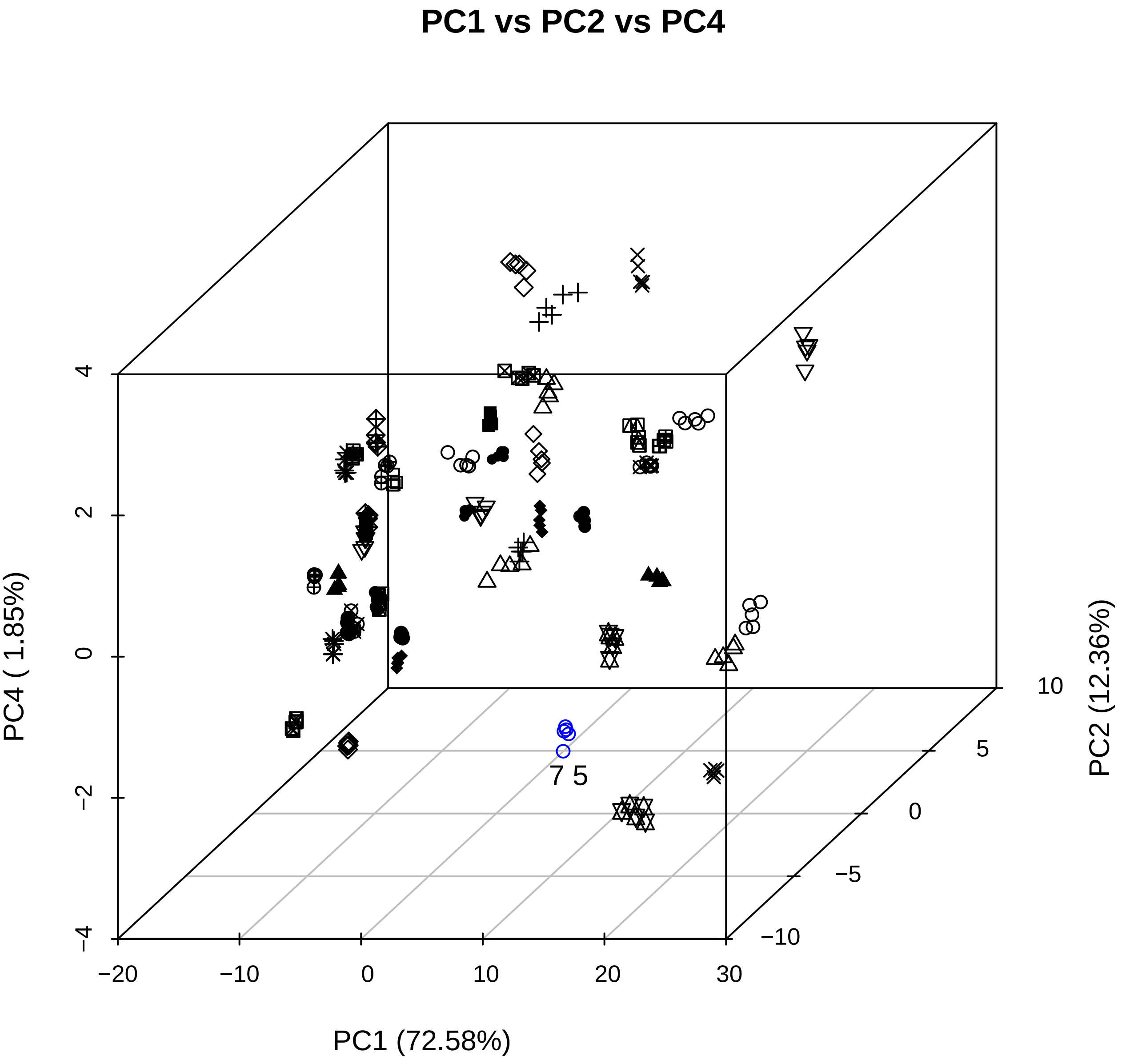

PC1 vs PC2 vs PC4
4
2
0
−2
−4
PC4 (1.85%)
−20
−10
0
10
20
30
PC1 (72.58%)
10
5
0
−5
−10
PC2 (12.36%)
7 5

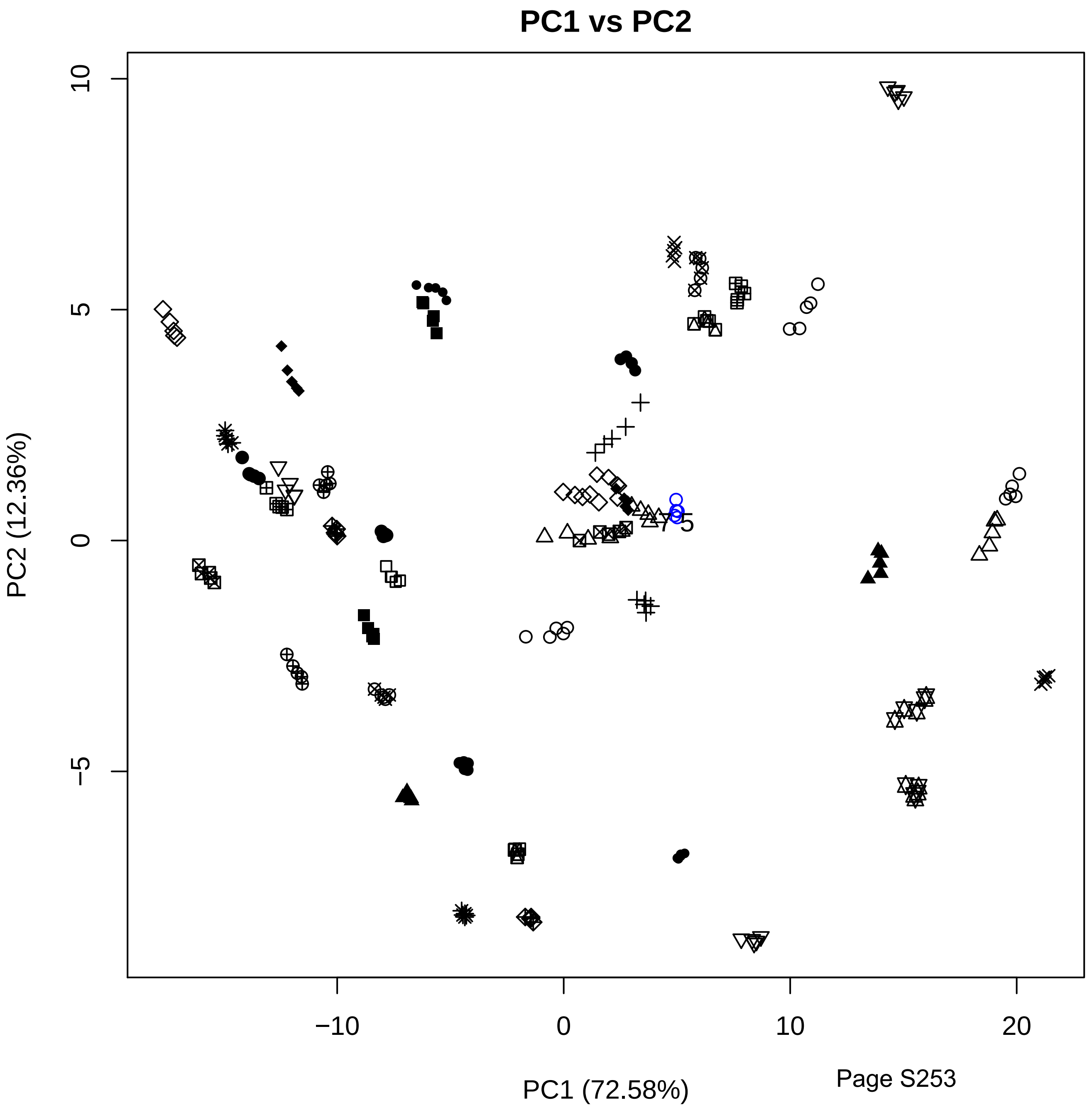
PC1 vs PC2
PC2 (12.36%)
PC1 (72.58%)
10
5
0
−5
−10
0
10
20
7
5

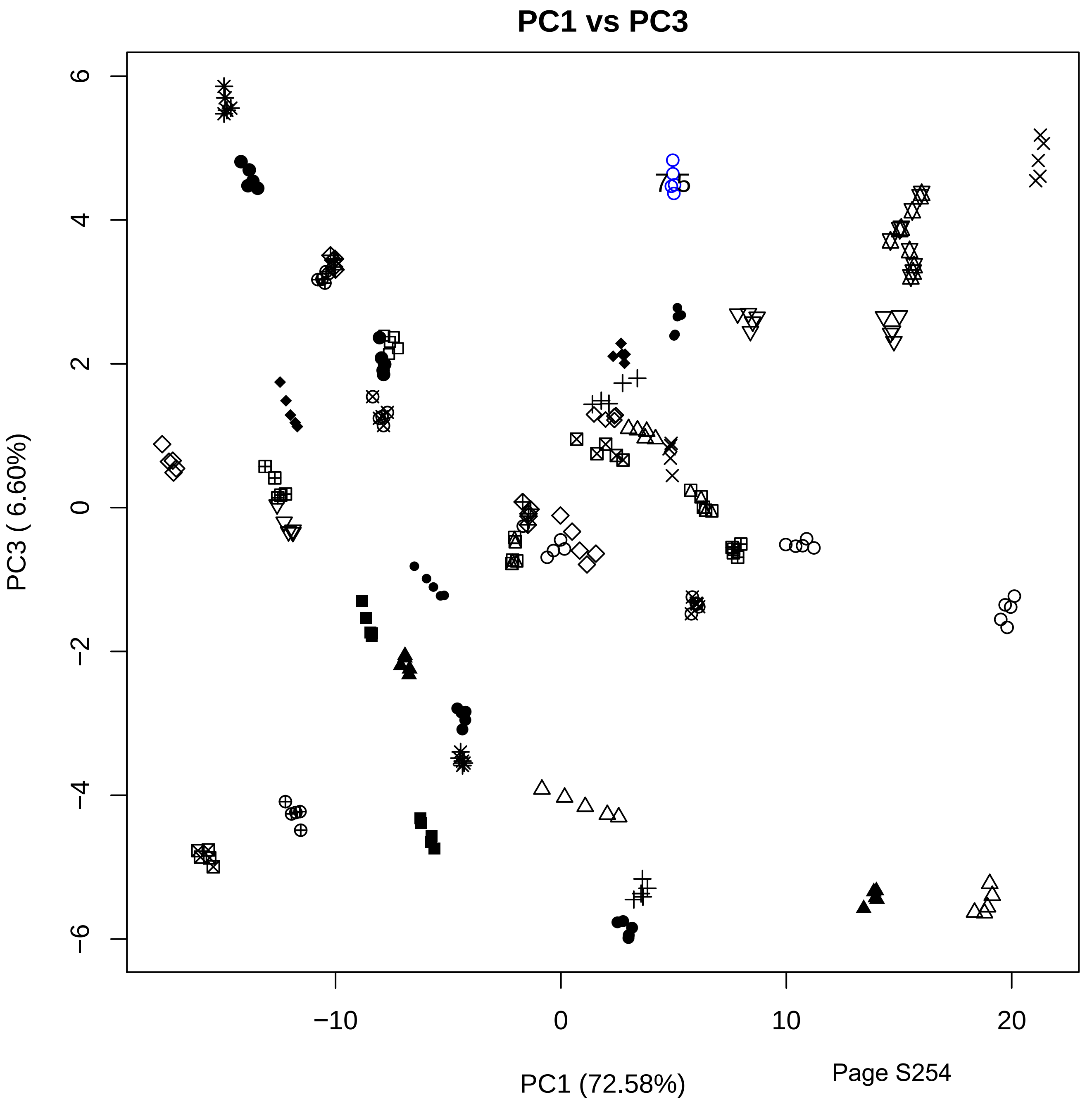

PC1 vs PC3
PC3 (6.60%)
PC1 (72.58%)
6
4
2
0
−2
−4
−6
−10
0
10
20
7.5

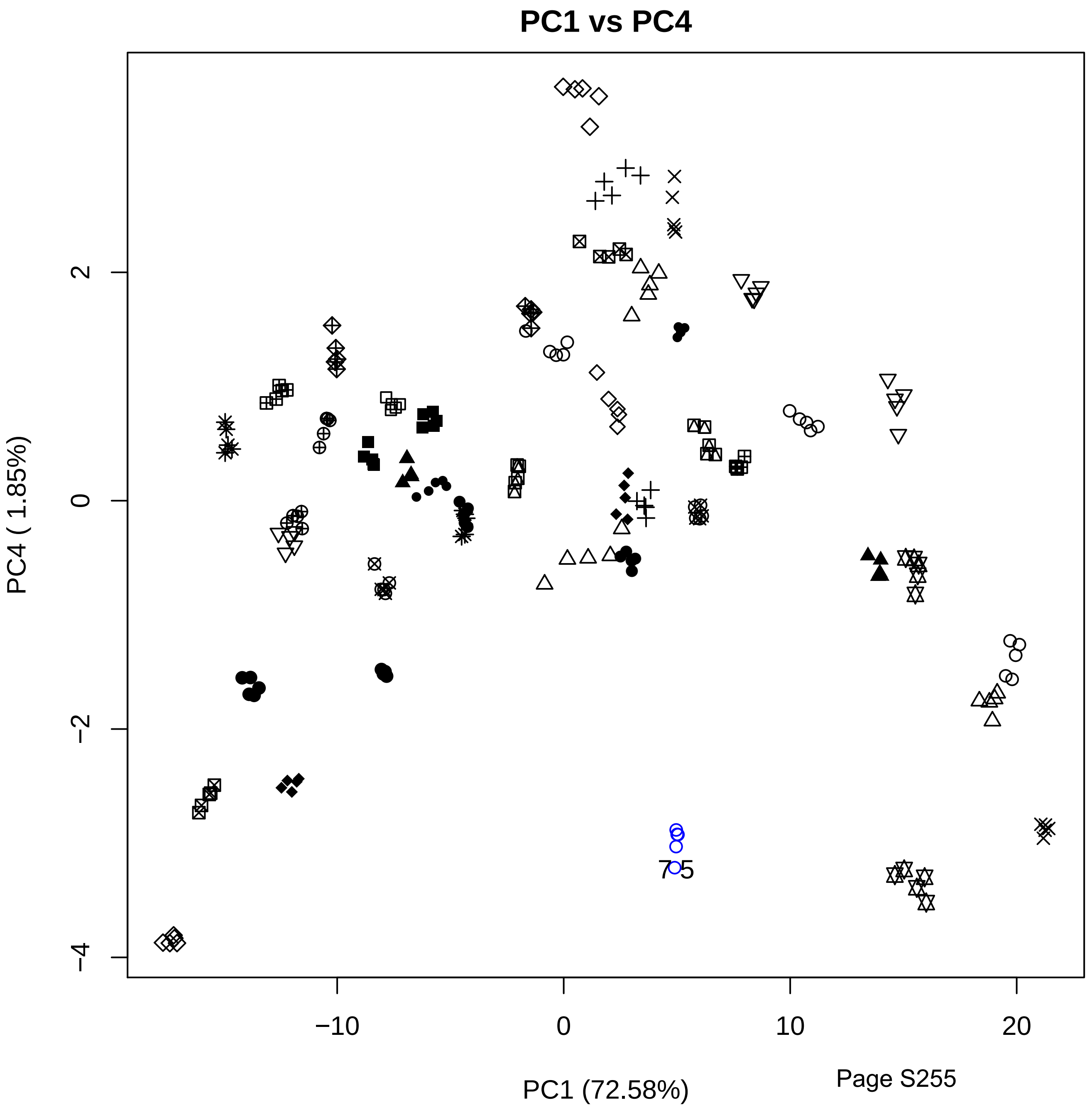
PC1 vs PC4
PC4 (1.85%)
PC1 (72.58%)
2
0
−2
−4
−10
0
10
20
75

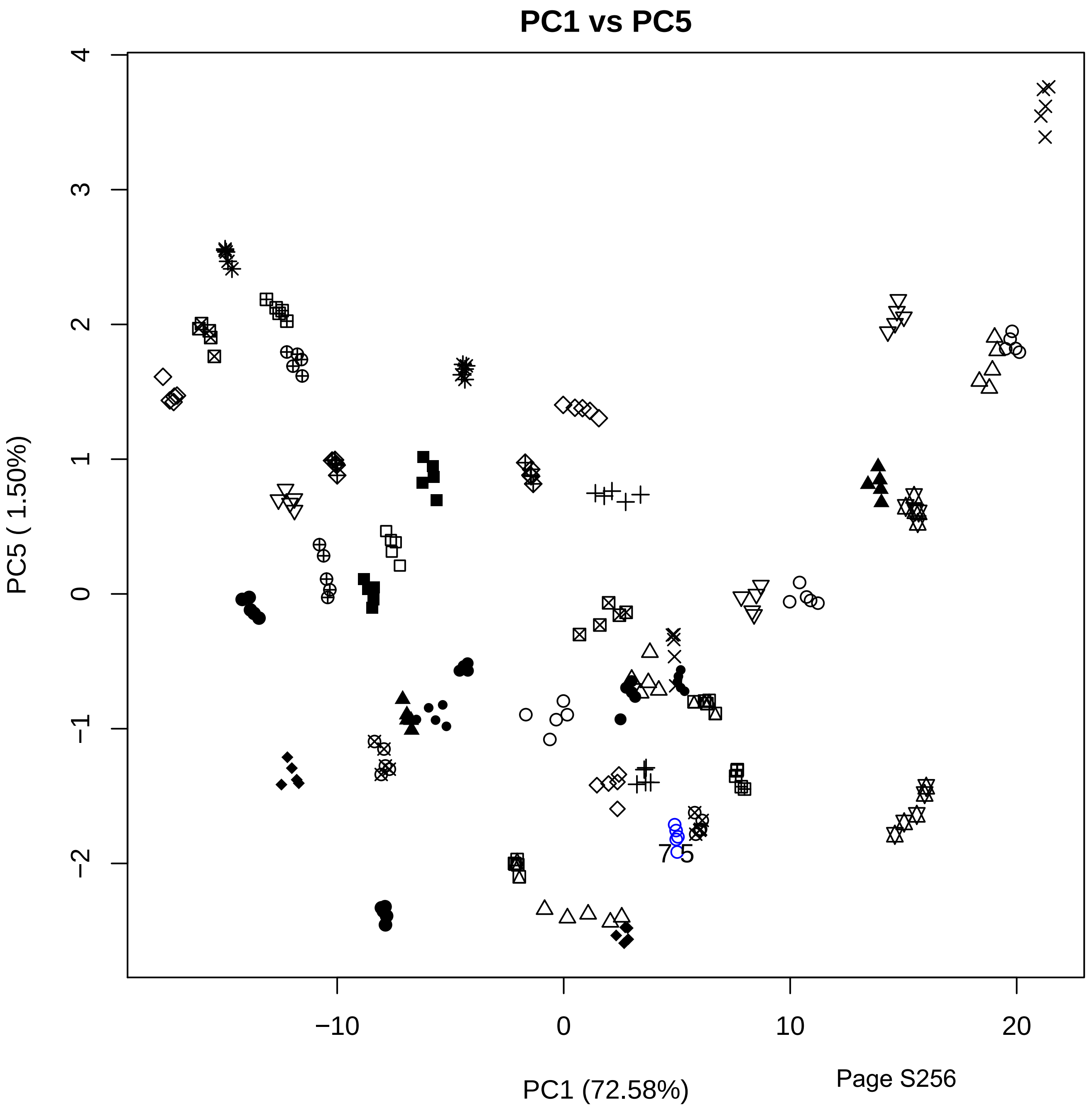
PC1 vs PC5
PC5 (1.50%)
PC1 (72.58%)
4
3
2
1
0
−1
−2
−10
0
10
20
75

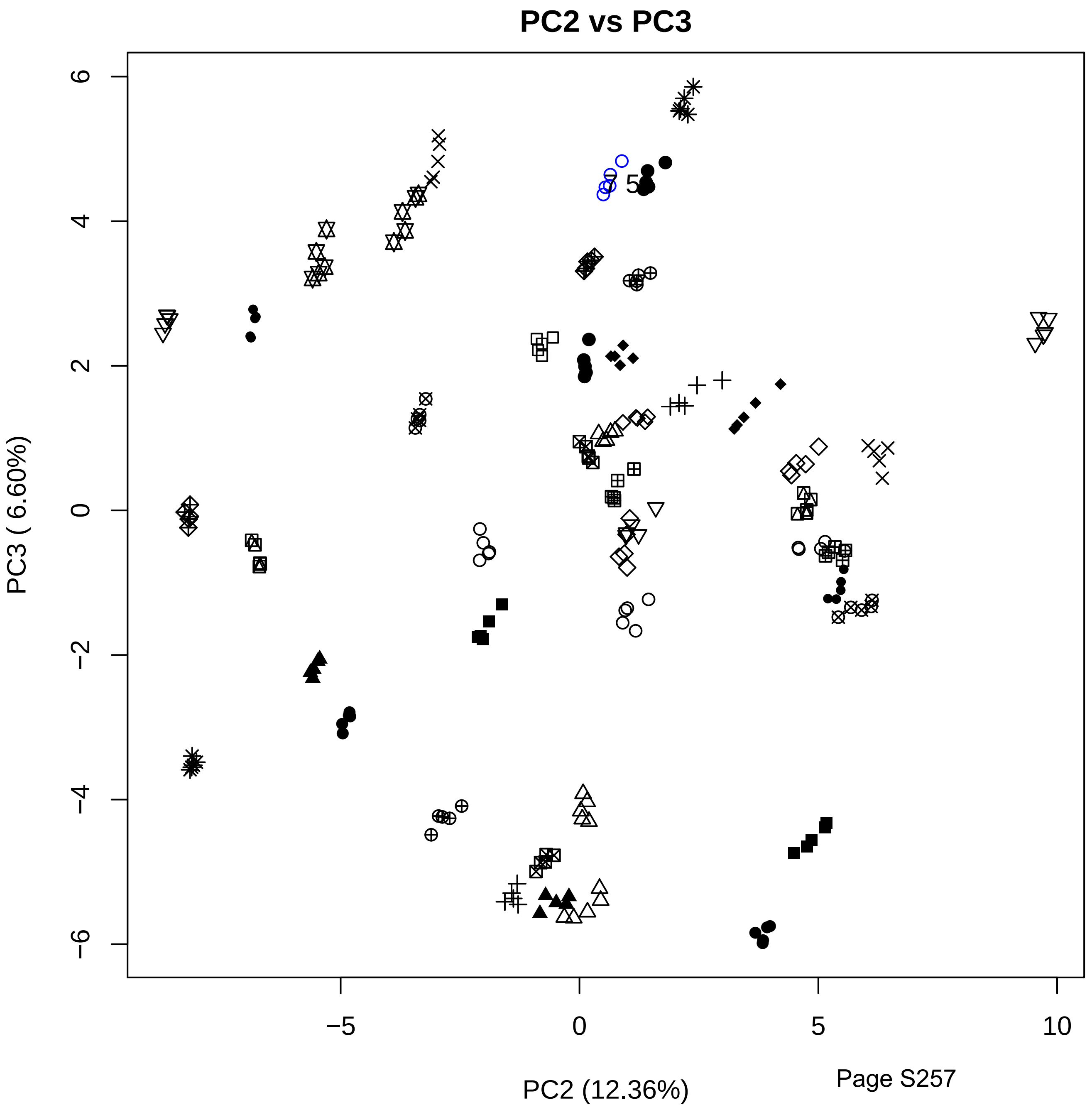
PC2 vs PC3
PC3 (6.60%)
PC2 (12.36%)
6
4
2
0
−2
−4
−6
−5
0
5
10
5

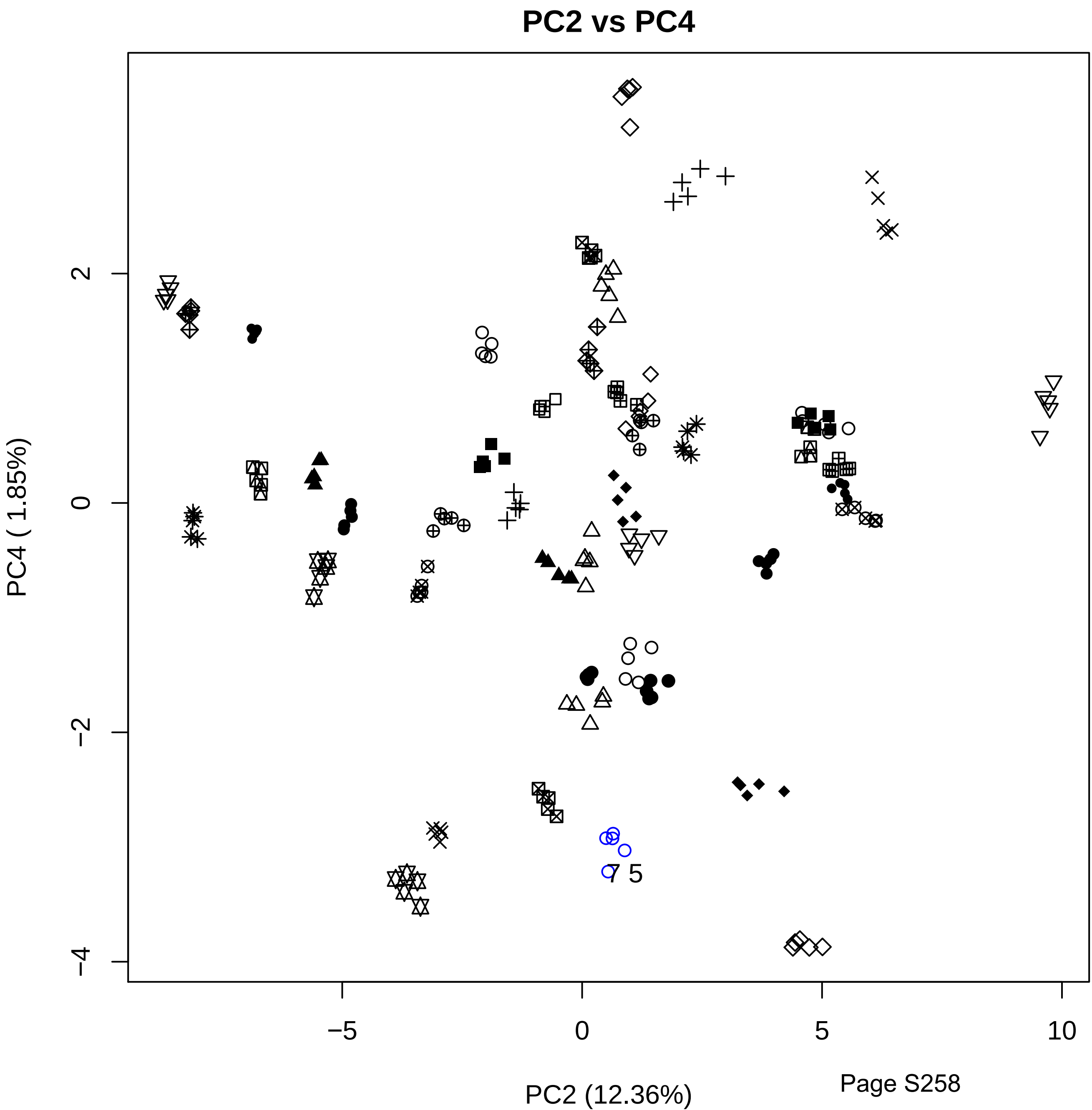
PC2 vs PC4
PC4 (1.85%)
PC2 (12.36%)
2
0
−2
−4
−5
0
5
10
7 5

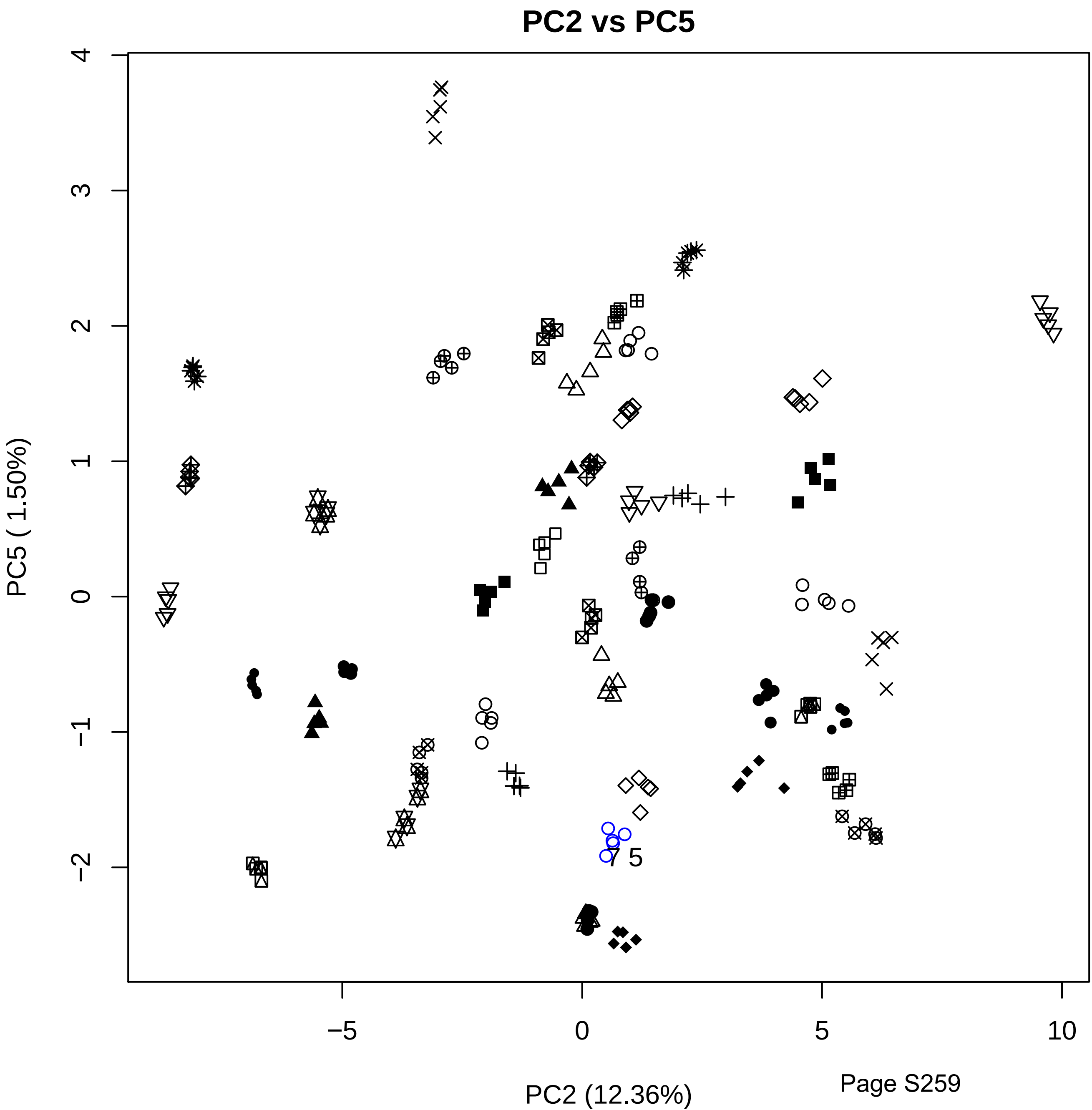
PC2 vs PC5
PC5 (1.50%)
PC2 (12.36%)
4
3
2
1
0
−1
−2
−5
0
5
10
7 5

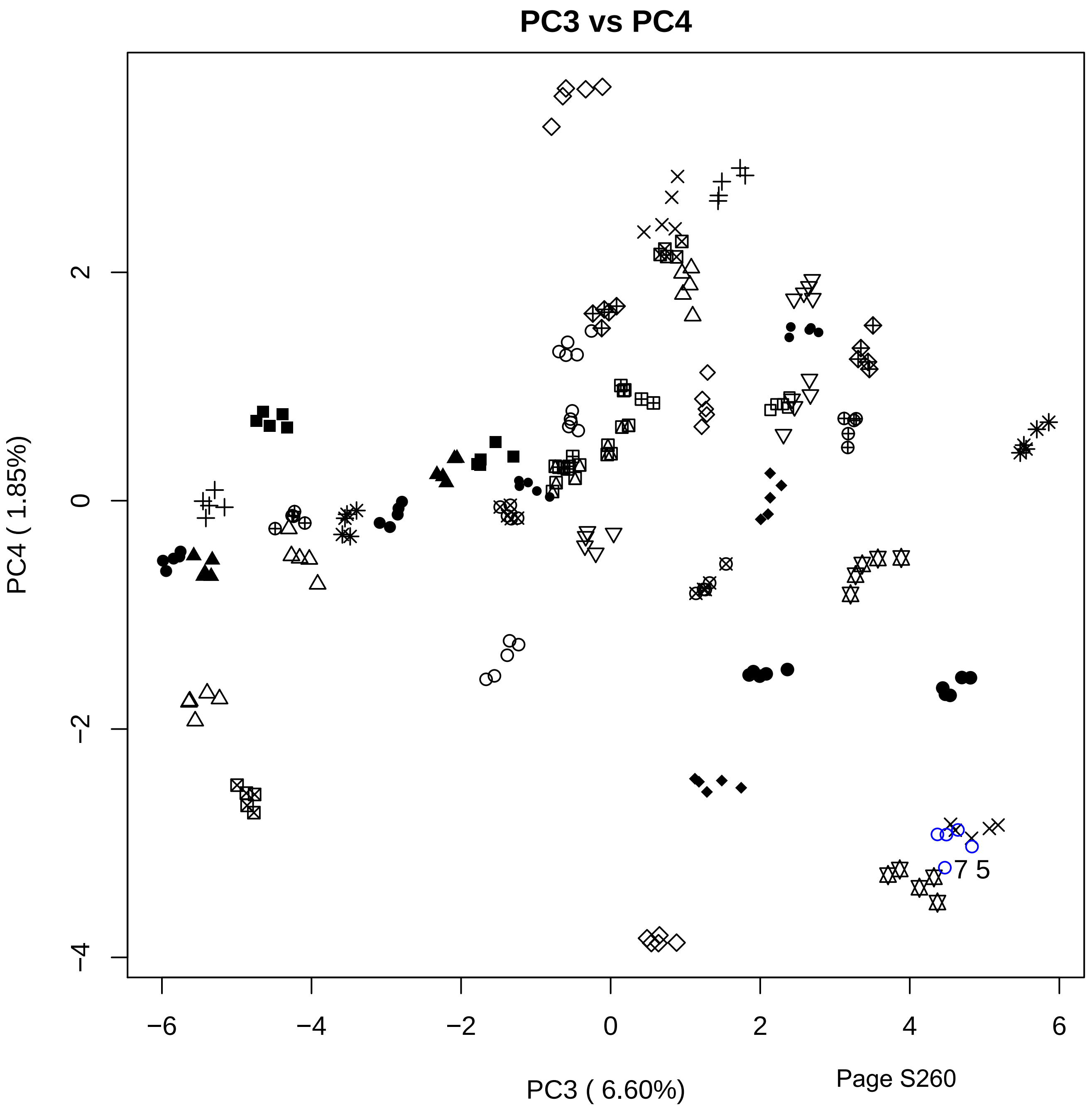

PC3 vs PC4
PC4 (1.85%)
PC3 (6.60%)
2
0
−2
−4
−6
−4
−2
0
2
4
6
7 5

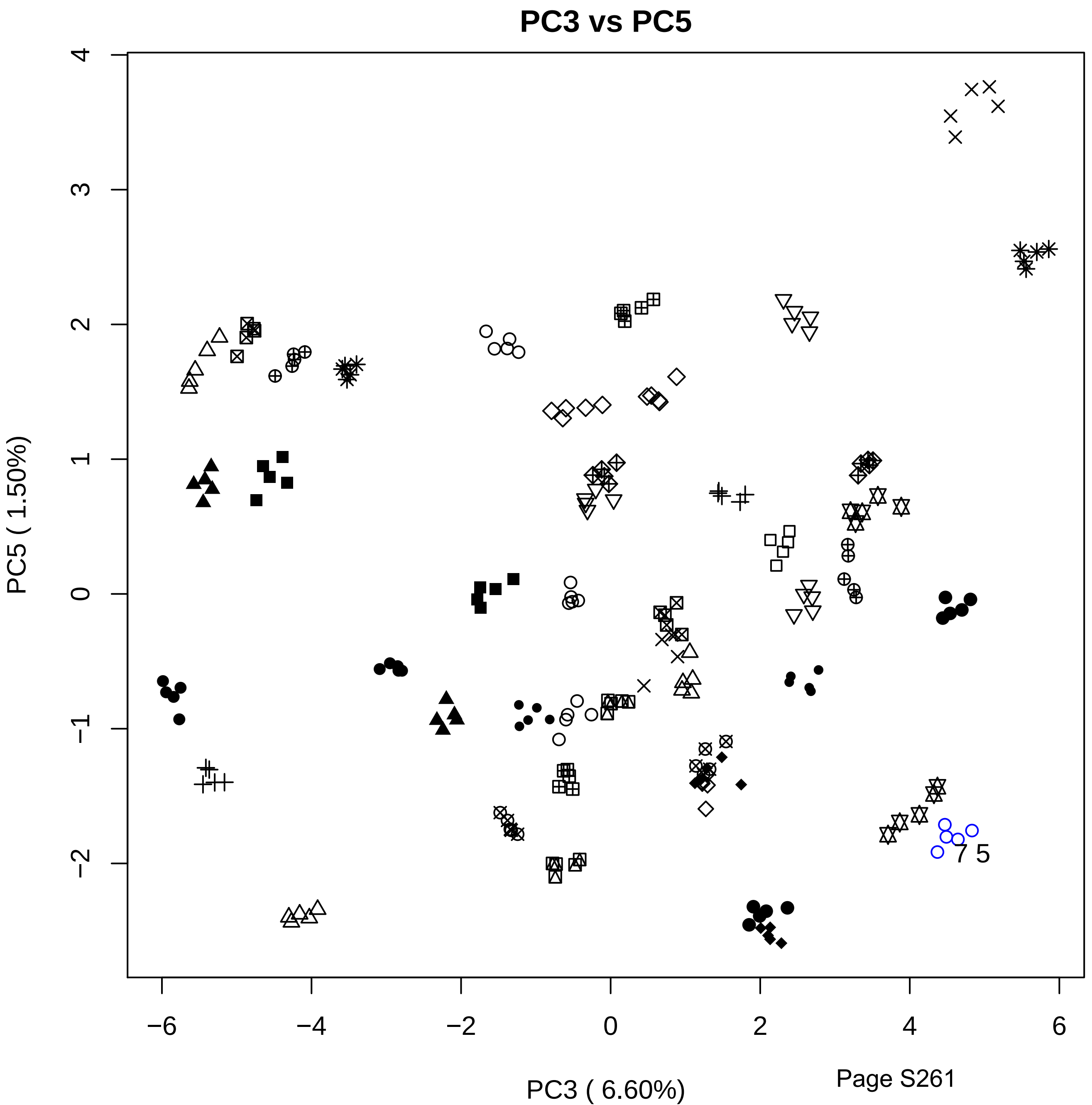

PC3 vs PC5
PC5 (1.50%)
PC3 (6.60%)
4
3
2
1
0
−1
−2
−6
−4
−2
0
2
4
6
7 5

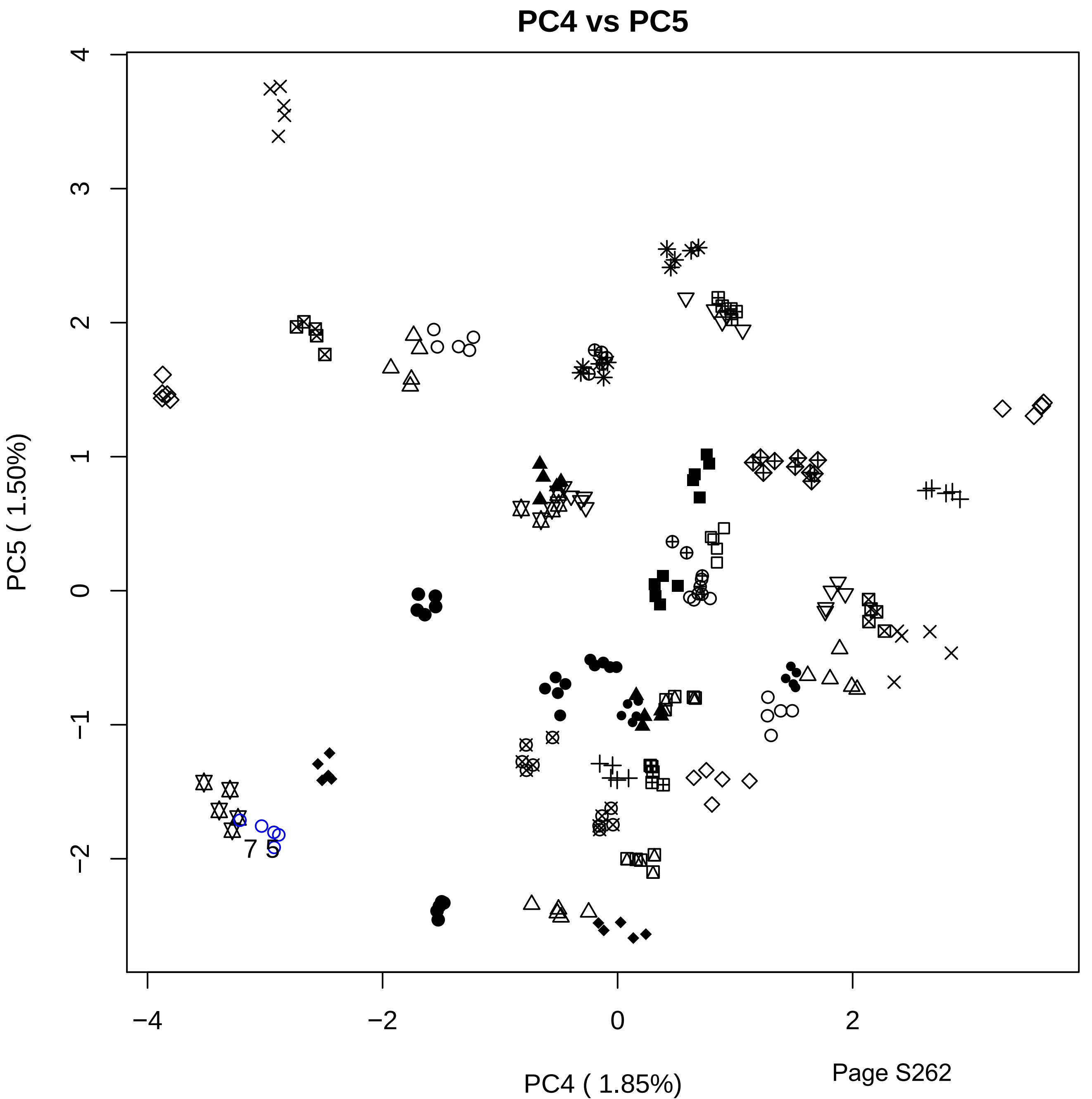
PC4 vs PC5
PC5 (1.50%)
PC4 (1.85%)
−4
−2
0
2
4
3
2
1
0
−1
−2
7 5

PCA of 10% OSCS vs PMH

Scaled Matrix PCAed

7th order polynomial baseline correction performed

Raw spectra smoothed to 21 Neighbours, 2nd order PN, Savtisky Golay

Normalisation between 0 and 100

D2 spectra smoothed to 41 Neighbours, 2nd order PN, Savtisky Golay

Regions Analysed: 3601−2501 and 2001−700cm−1

Samples Analysed: 1 Contam's 0 OSCS's 45 PMH's

Keele University AD　　　　Sun Dec 09 16:51:14 2018

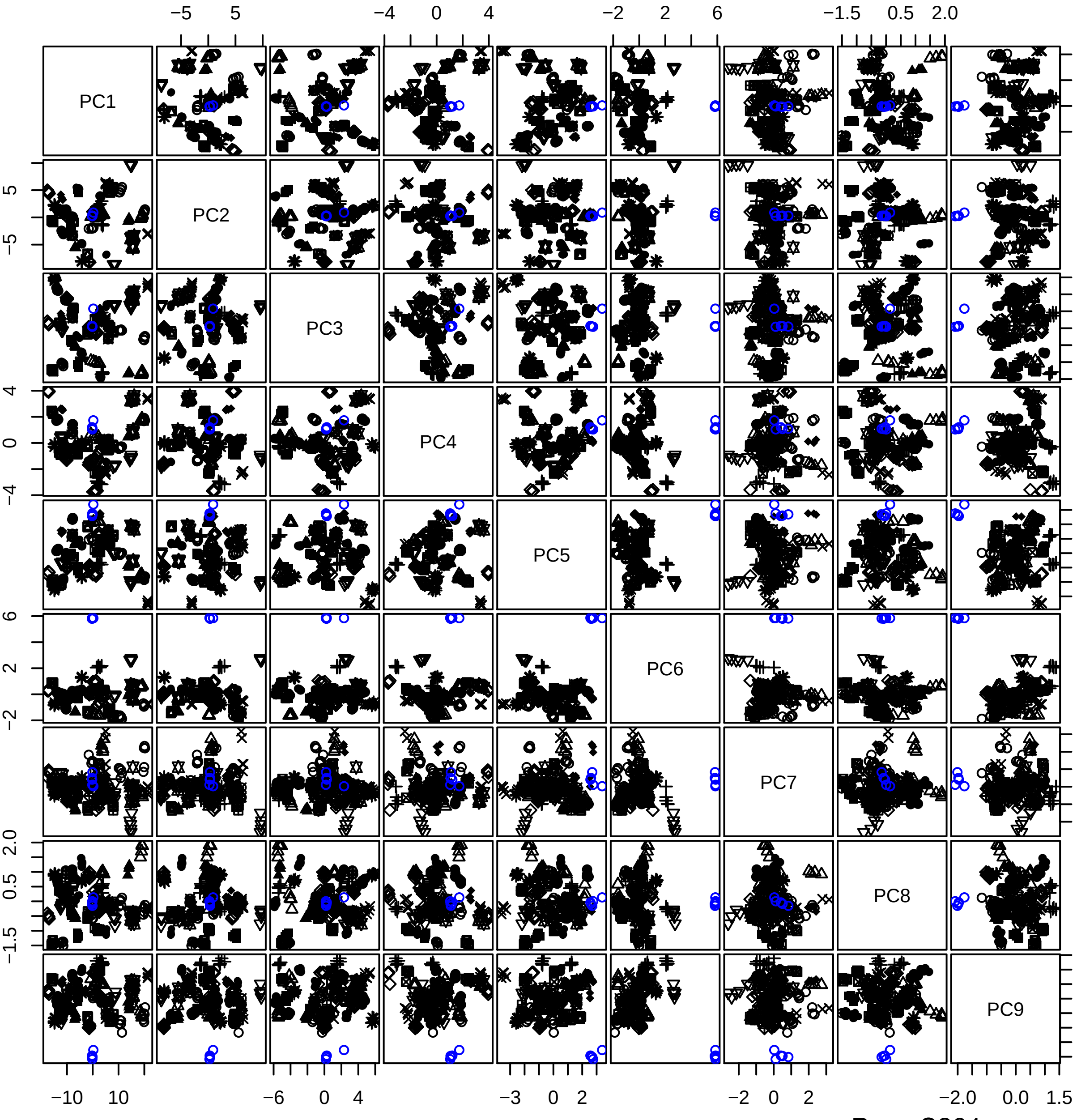
PC1
PC2
PC3
PC4
PC5
PC6
PC7
PC8
PC9

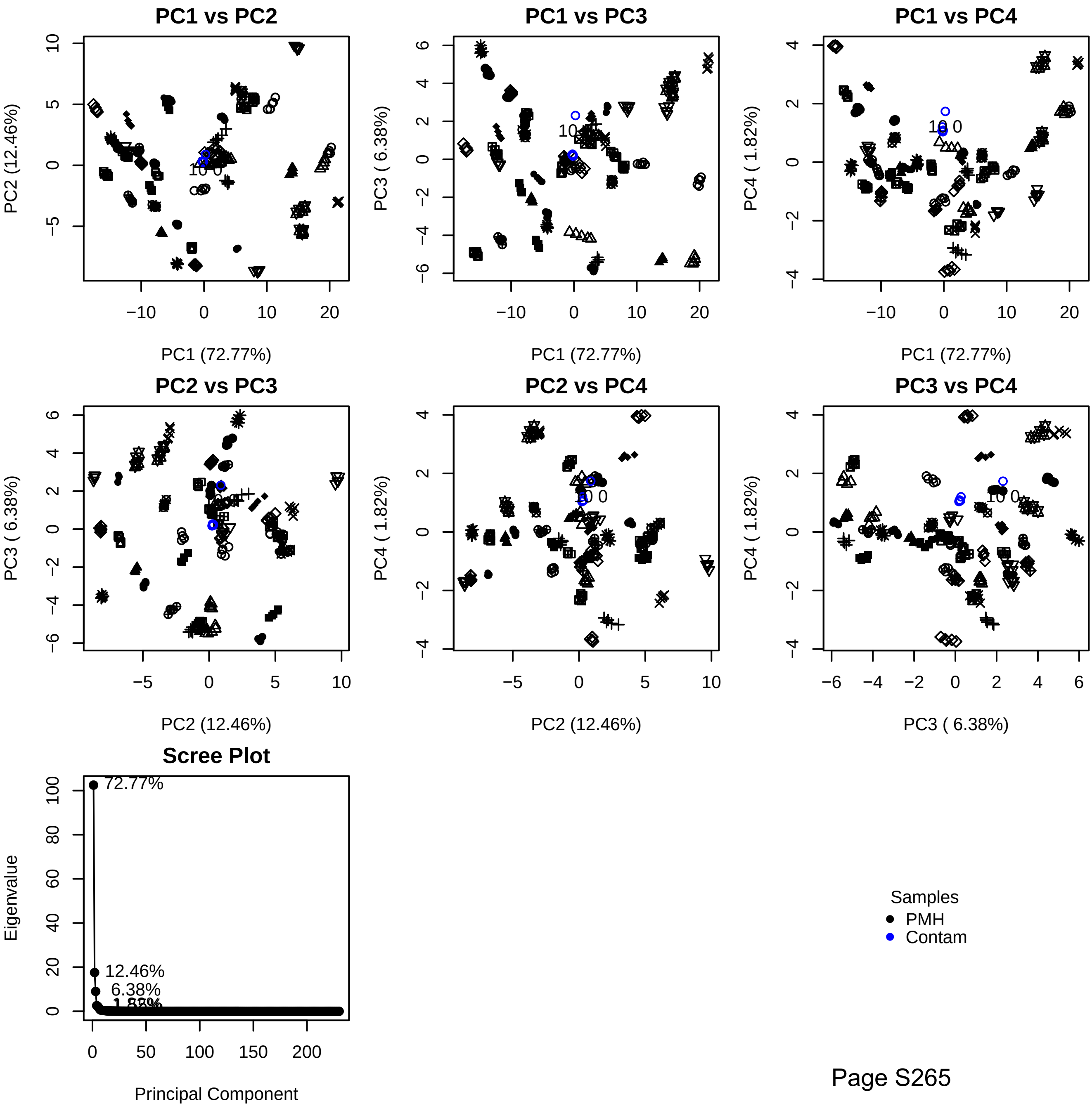

PC1 vs PC2
PC1 vs PC3
PC1 vs PC4
PC2 vs PC3
PC2 vs PC4
PC3 vs PC4
Scree Plot
PC1 (72.77%)
PC2 (12.46%)
PC3 (6.38%)
PC4 (1.82%)
Eigenvalue
Principal Component
72.77%
12.46%
6.38%
Samples
PMH
Contam

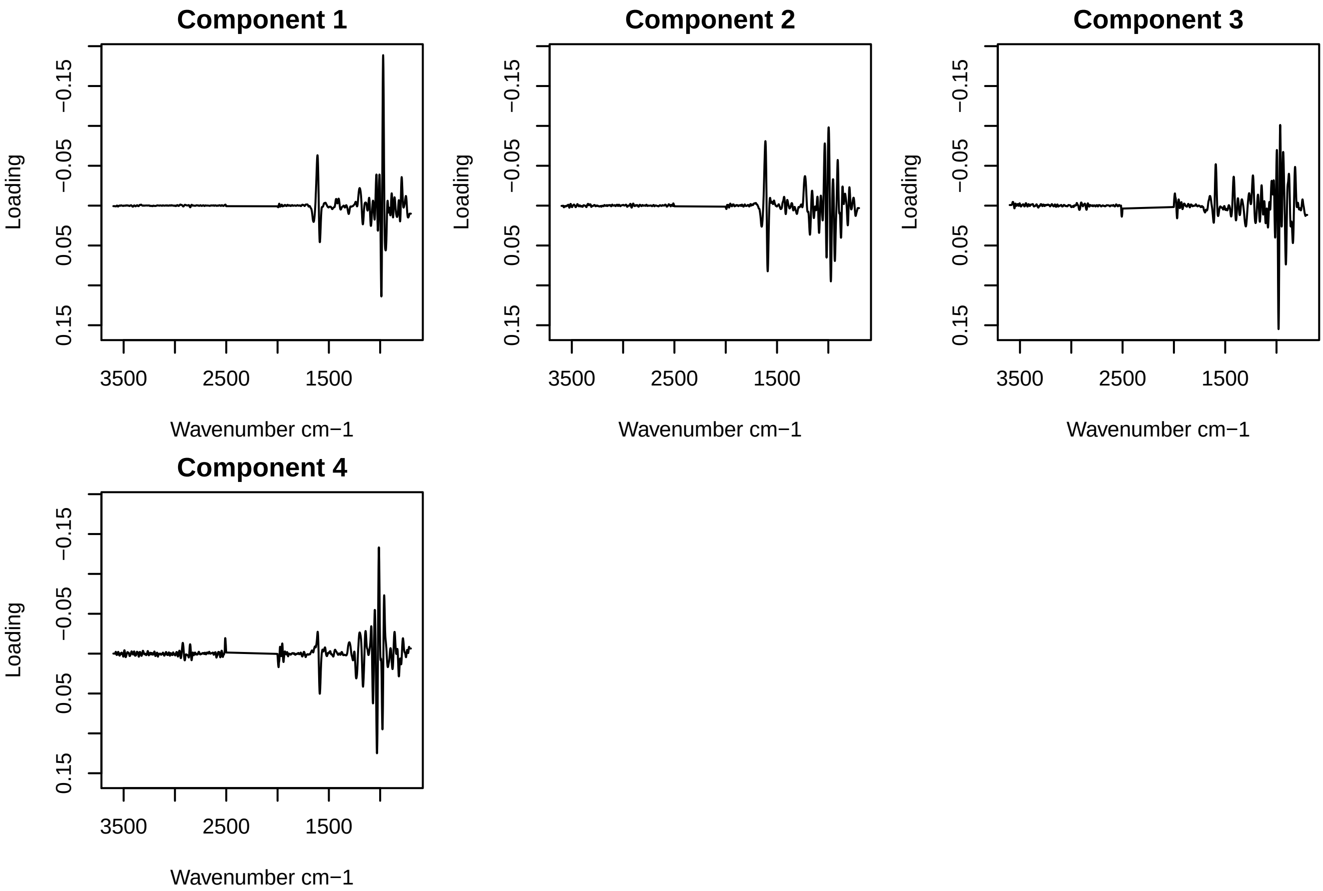

Component 1
Component 2
Component 3
Component 4
Loading
−0.15
−0.05
0.05
0.15
3500
2500
1500
Wavenumber cm−1

PC1 vs PC2 vs PC3

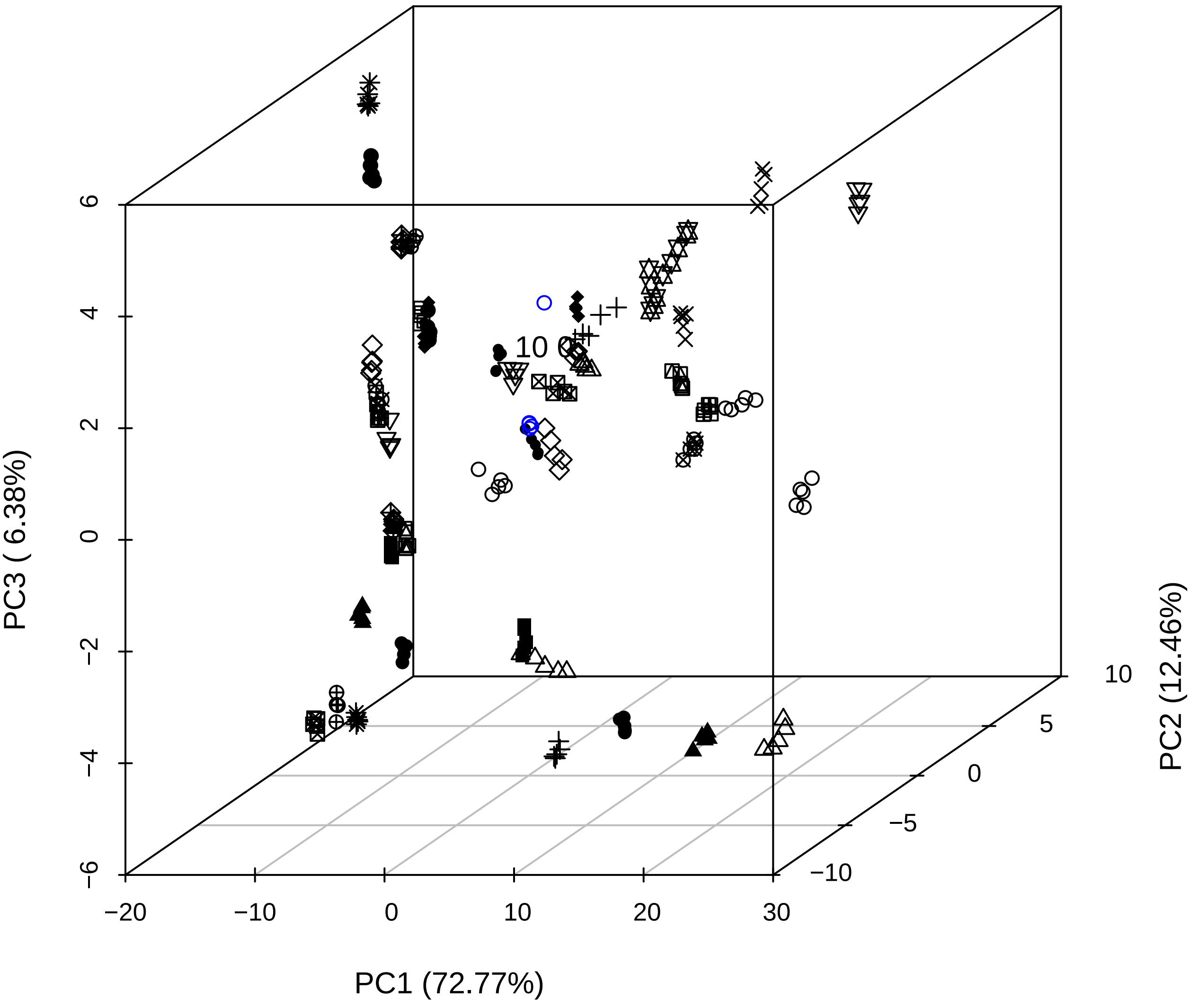

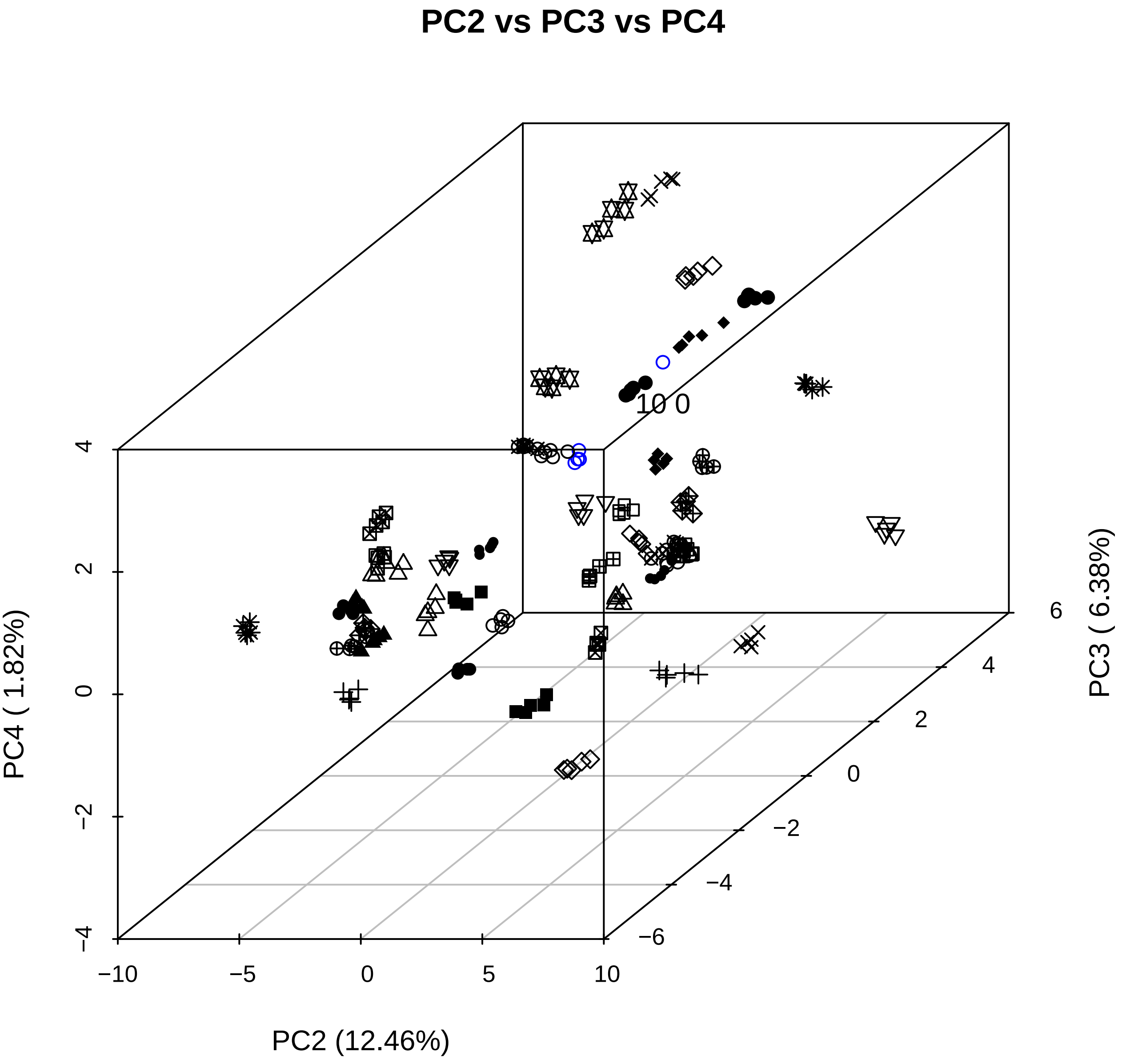
PC2 vs PC3 vs PC4
PC4 (1.82%)
PC3 (6.38%)
PC2 (12.46%)
10 0
4
2
0
−2
−4
−10
−5
0
5
10
6
4
2
0
−2
−4
−6

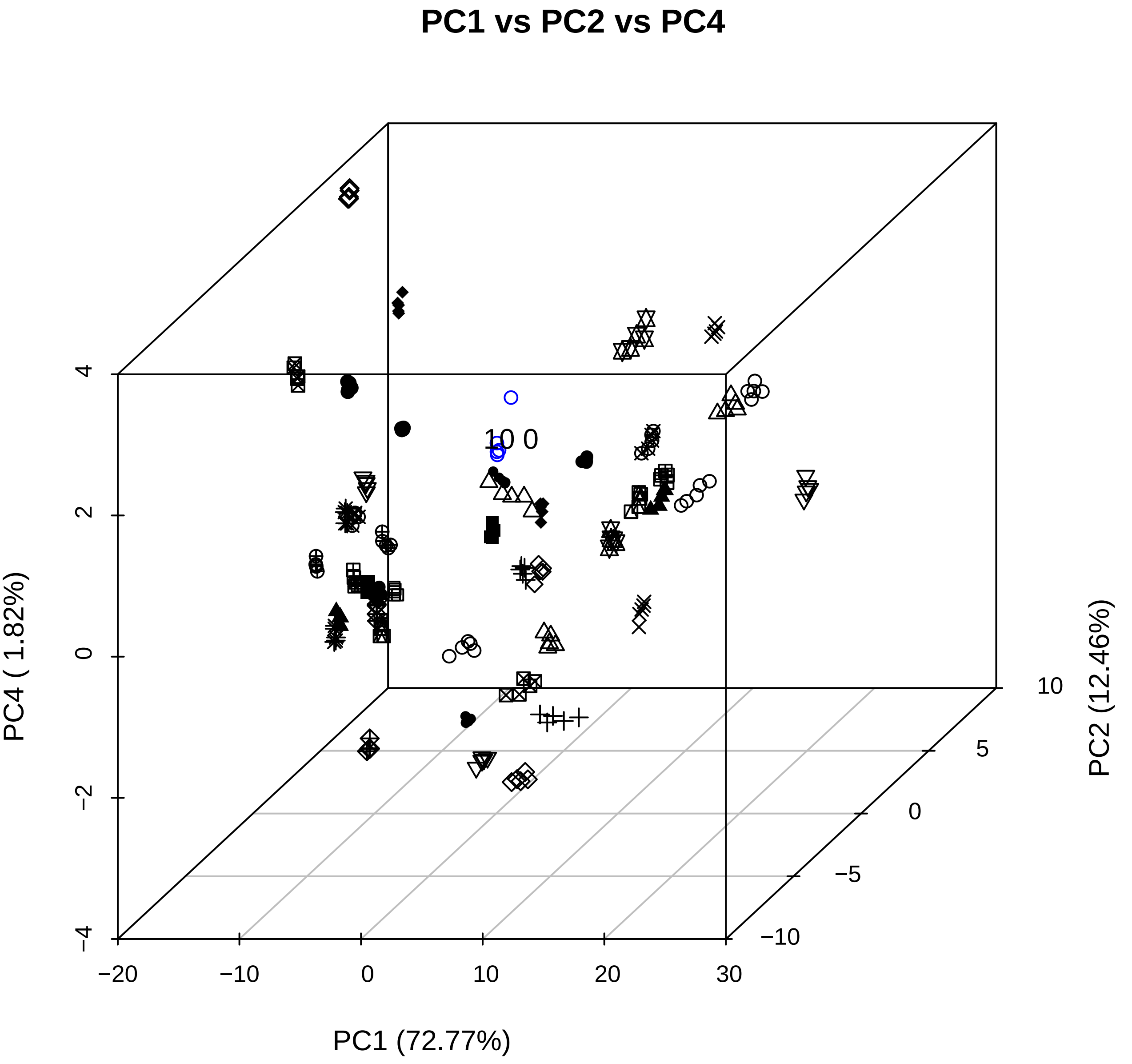
PC1 vs PC2 vs PC4
PC1 (72.77%)
PC2 (12.46%)
PC4 (1.82%)
−20
−10
0
10
20
30
−10
−5
0
5
10
−4
−2
0
2
4
10 0

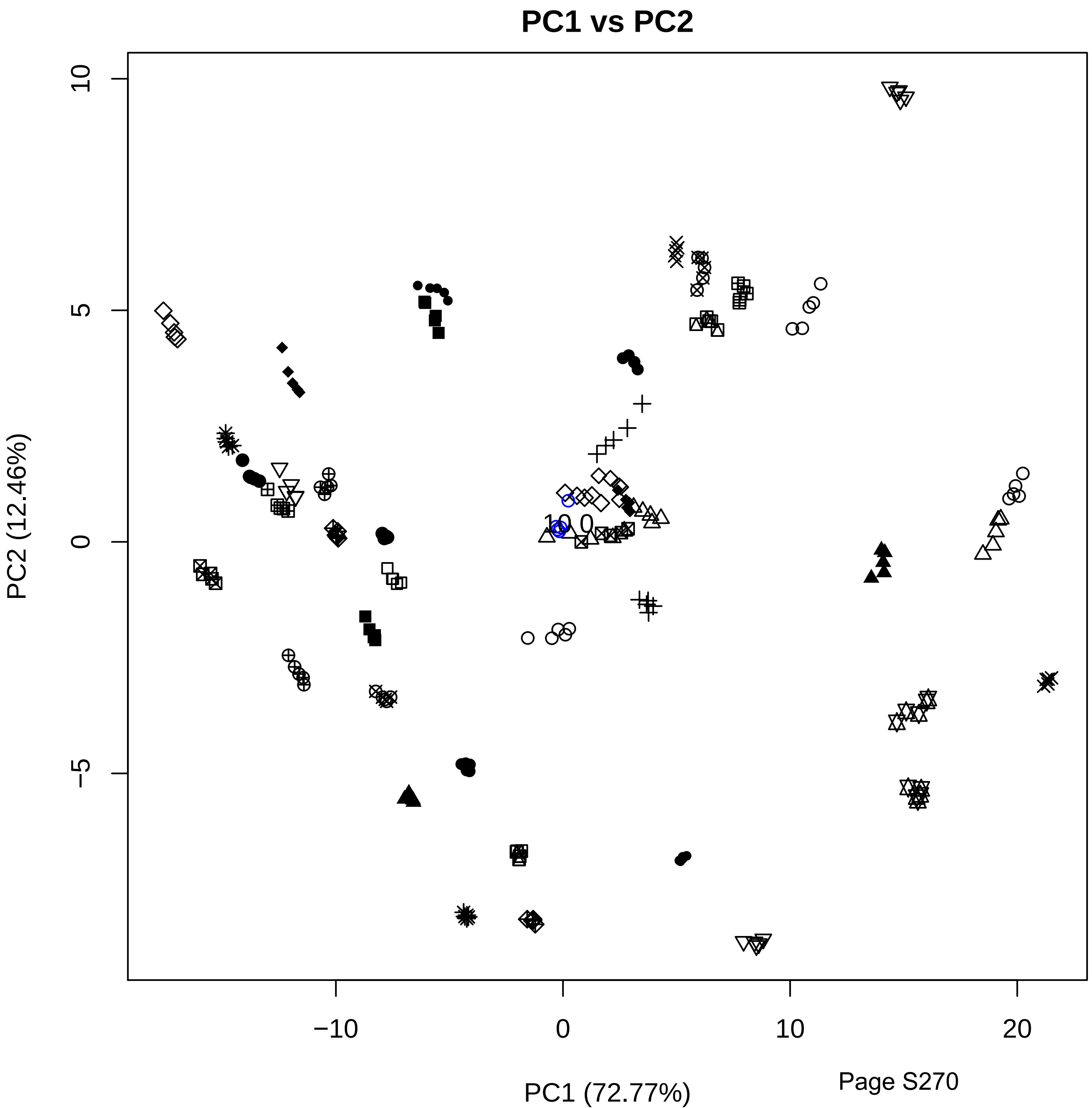
PC1 vs PC2
PC2 (12.46%)
PC1 (72.77%)
10
5
0
−5
−10
0
10
20
10 0

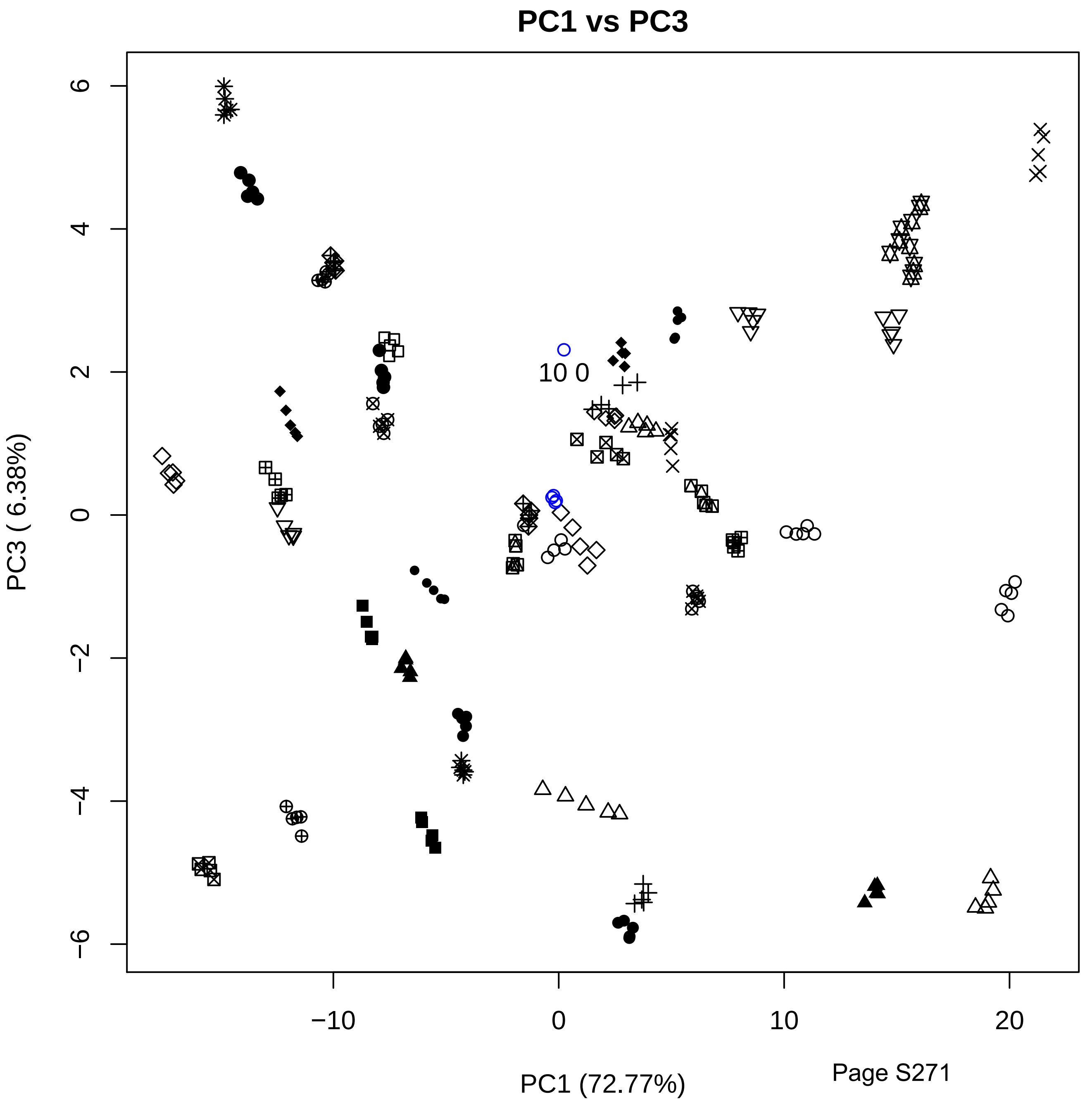

PC1 vs PC3
PC3 (6.38%)
PC1 (72.77%)
6
4
2
0
−2
−4
−6
−10
0
10
20
10 0

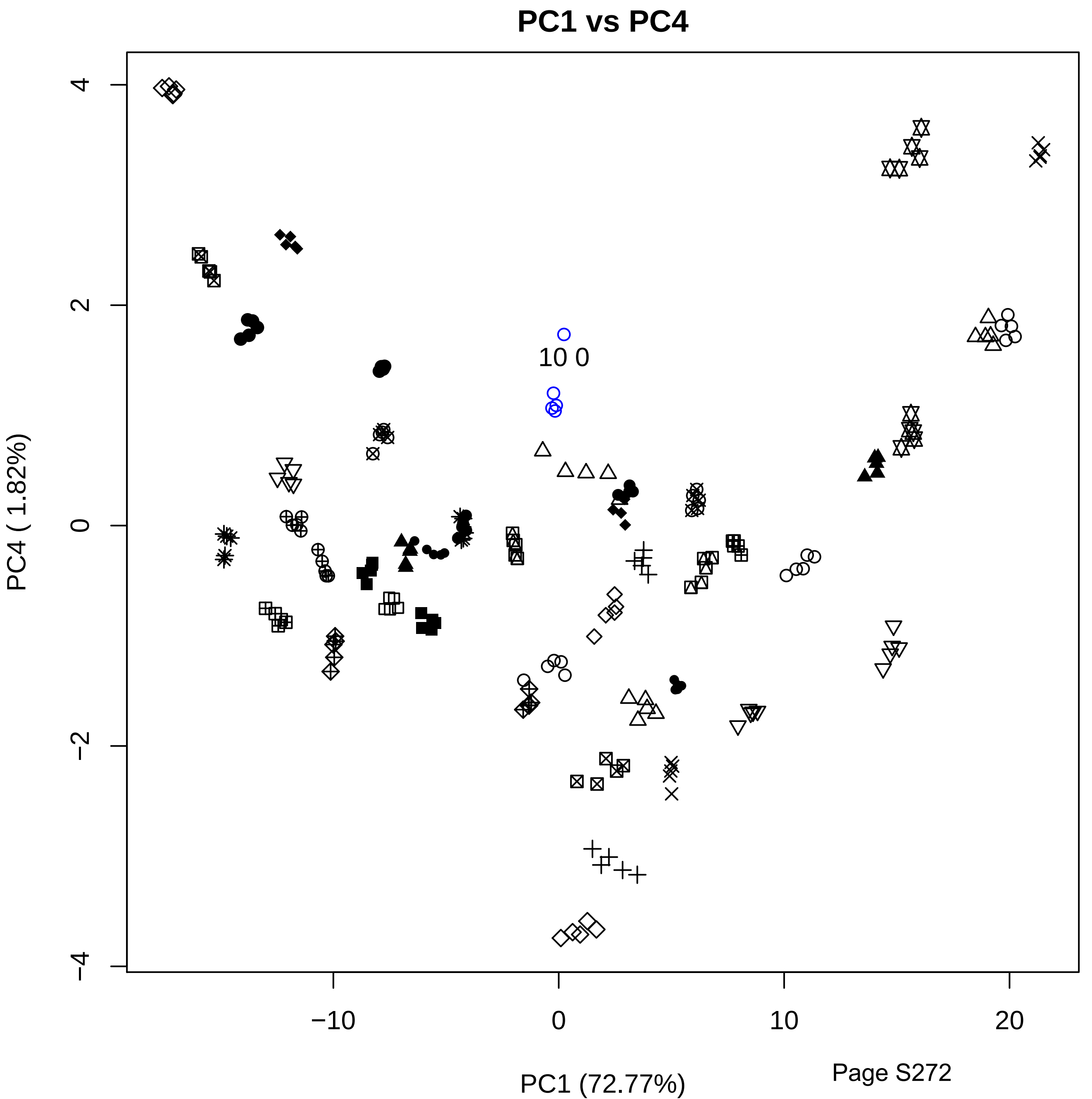
PC1 vs PC4
PC4 (1.82%)
PC1 (72.77%)
4
2
0
−2
−4
−10
0
10
20
10 0

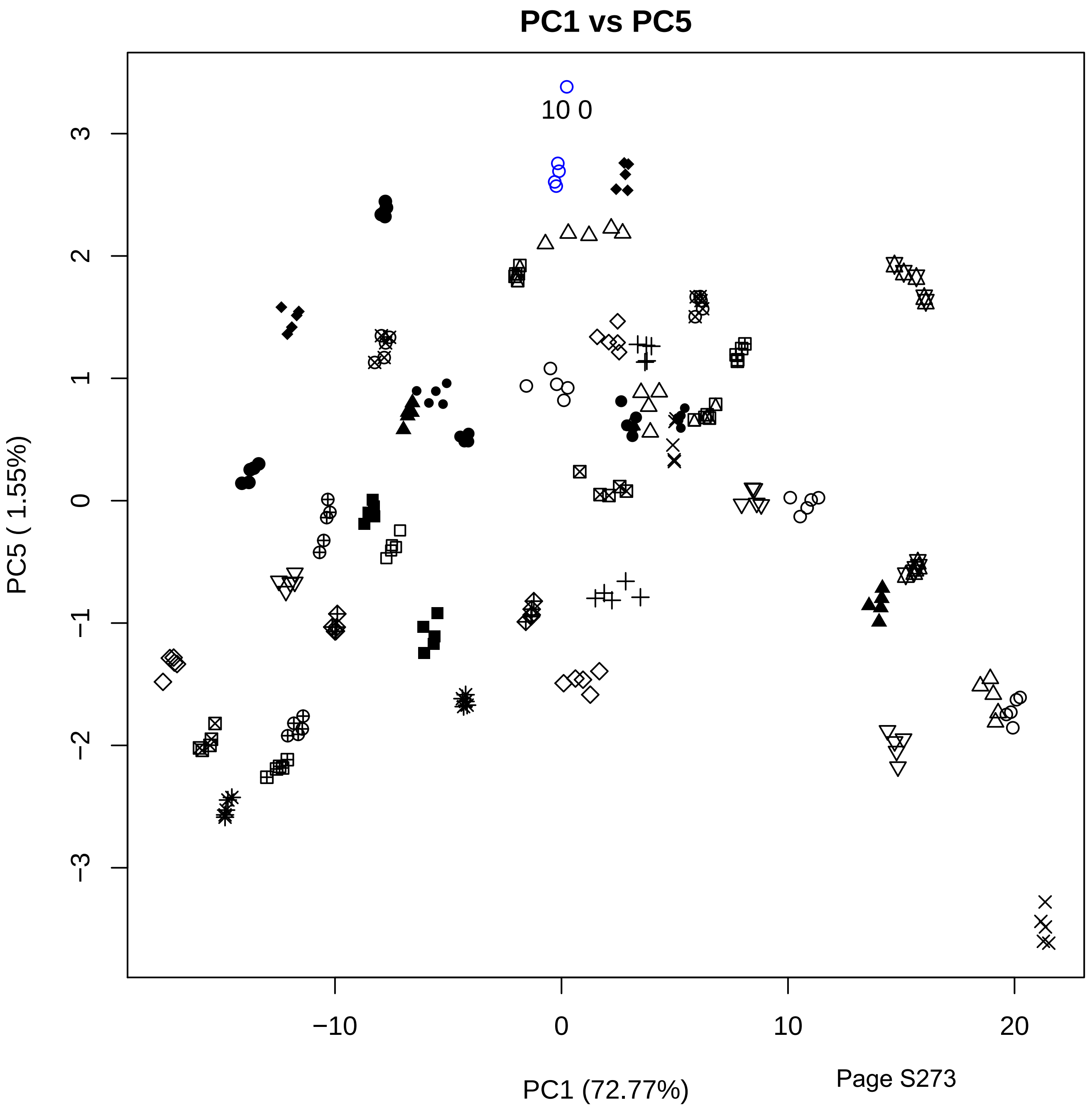
PC1 vs PC5
10 0
3
2
1
0
−1
−2
−3
PC5 (1.55%)
−10
0
10
20
PC1 (72.77%)

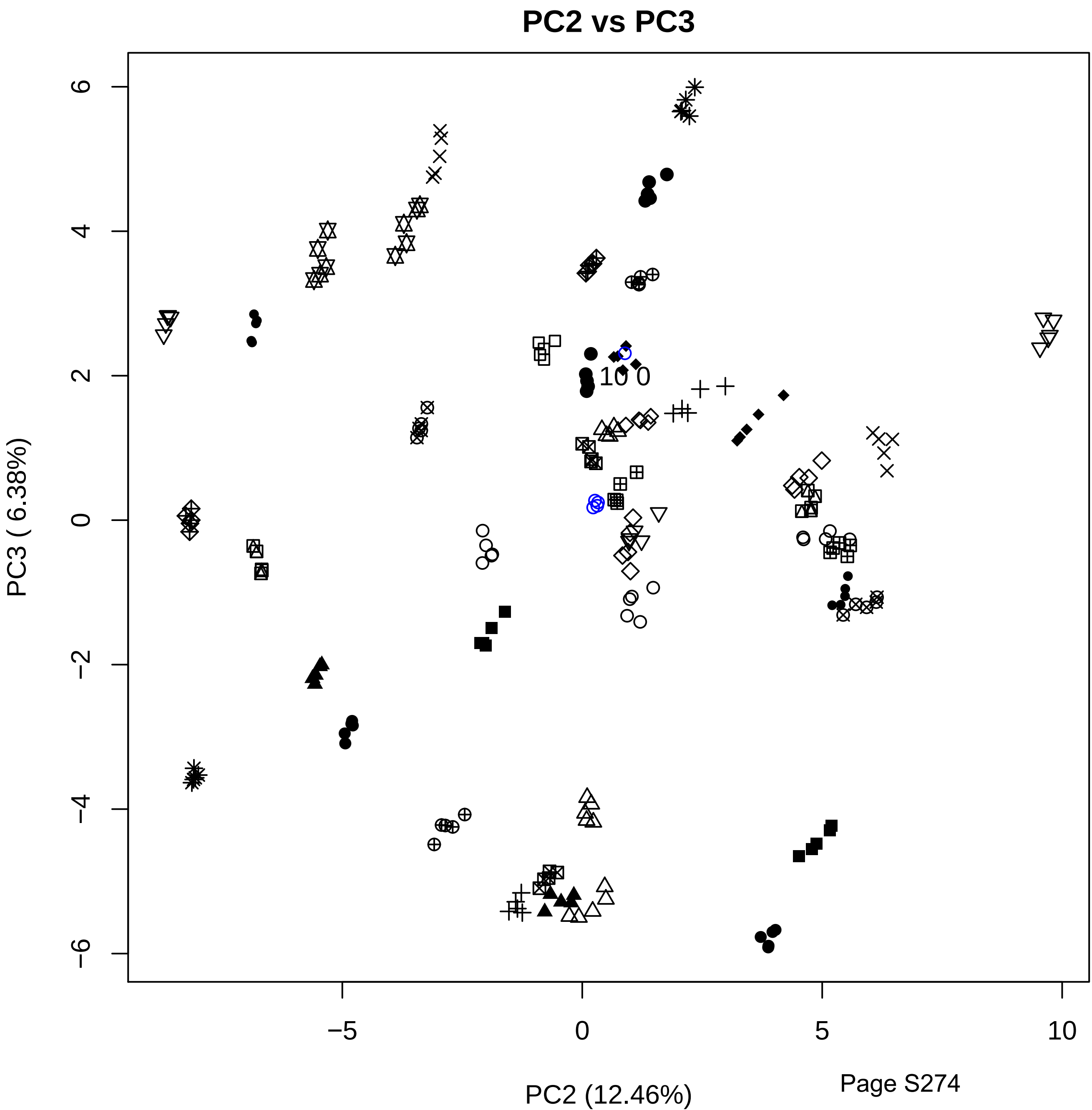
PC2 vs PC3
PC3 (6.38%)
PC2 (12.46%)
6
4
2
0
−2
−4
−6
−5
0
5
10
10 0

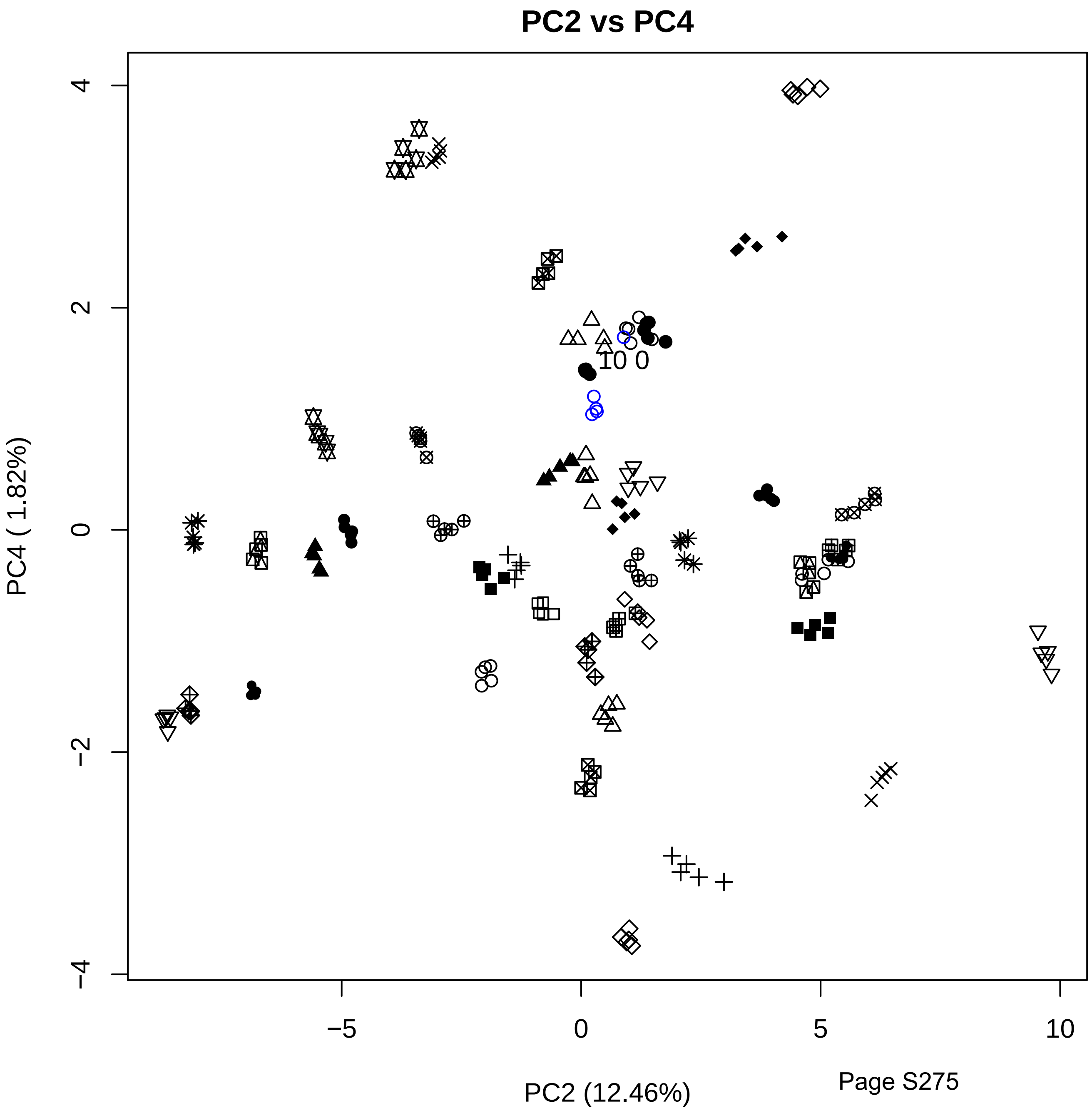
PC2 vs PC4
PC4 (1.82%)
PC2 (12.46%)
4
2
0
−2
−4
−5
0
5
10
10 0

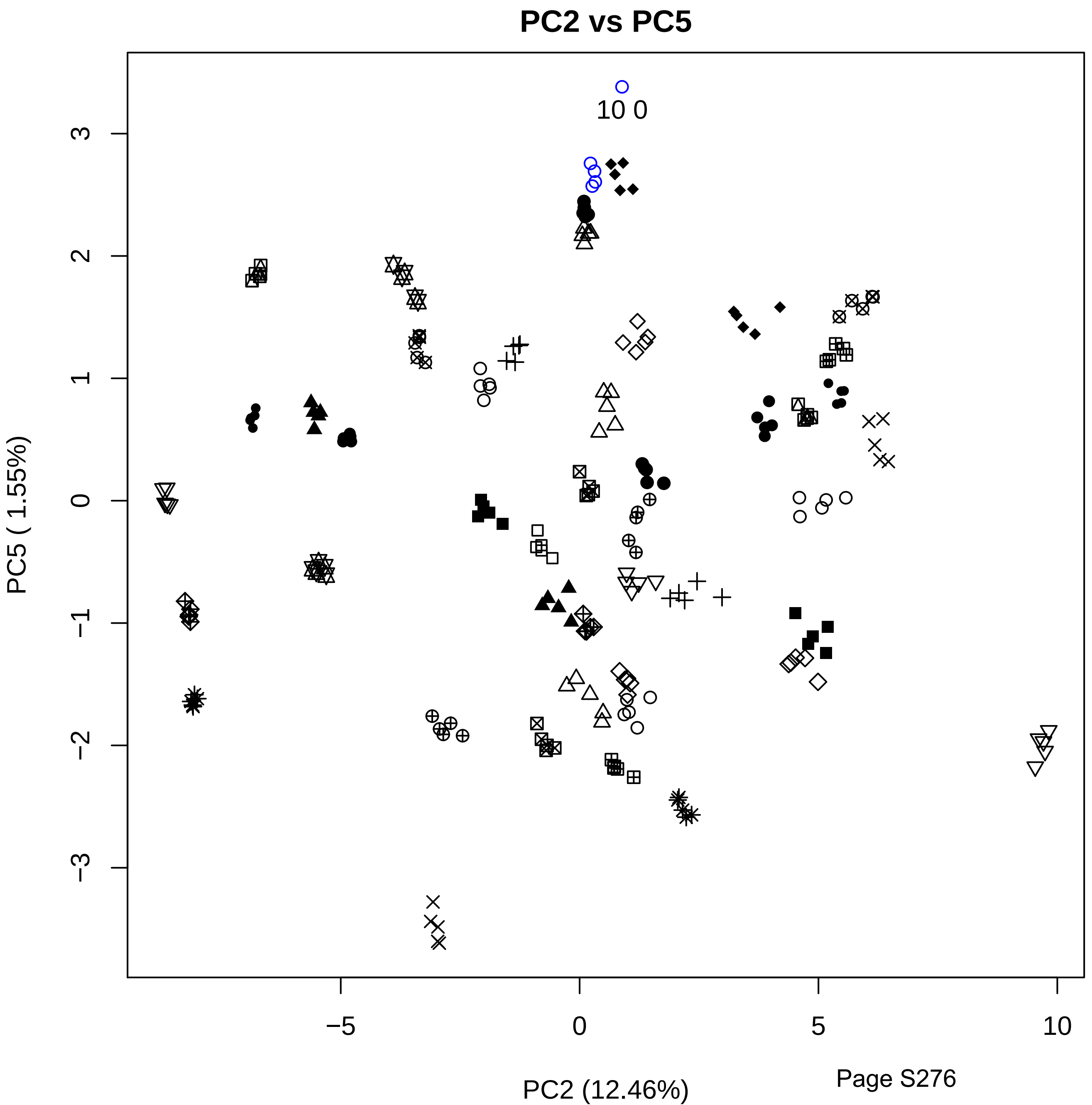
PC2 vs PC5
10 0
PC5 (1.55%)
PC2 (12.46%)
3
2
1
0
−1
−2
−3
−5
0
5
10

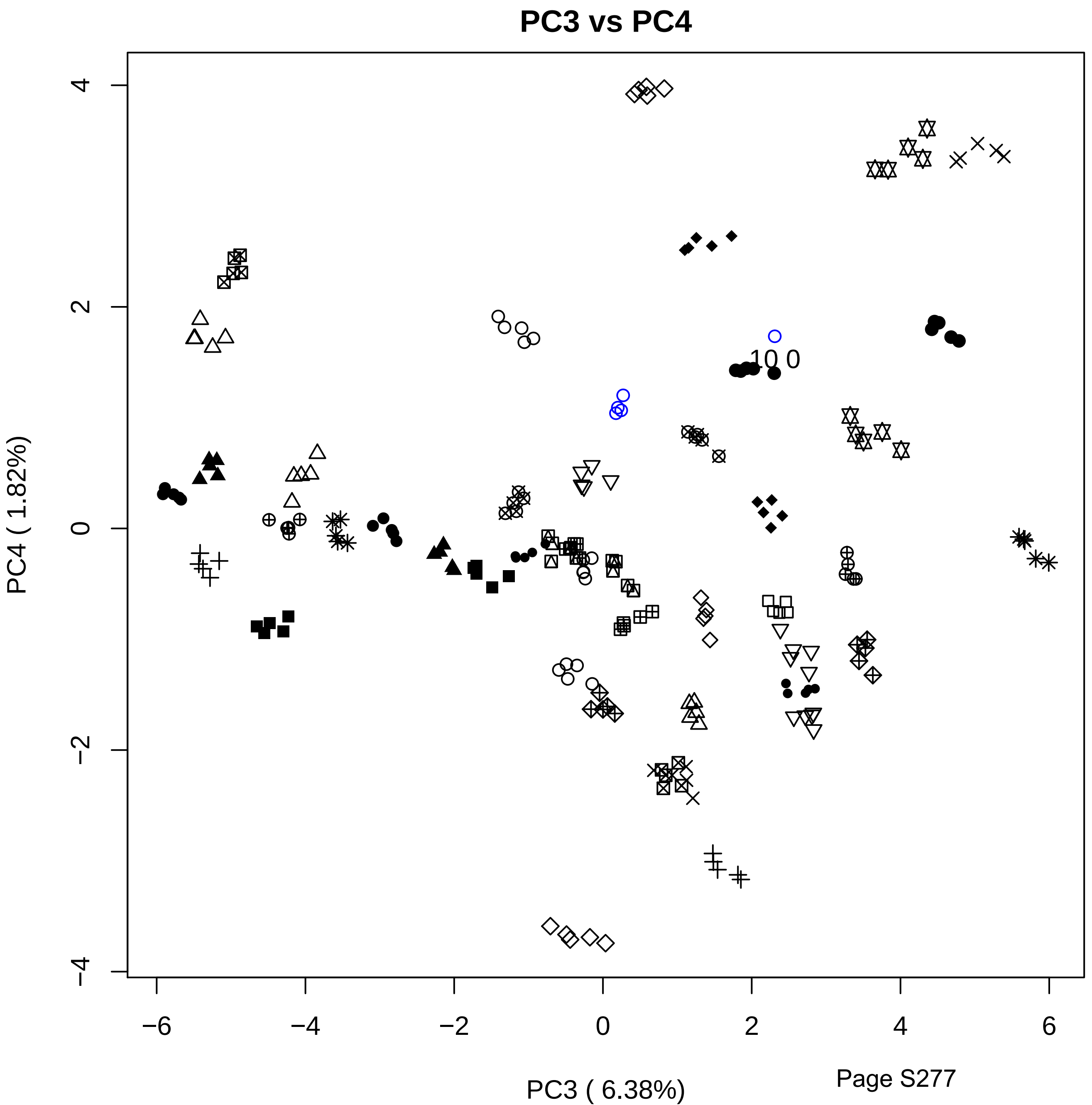

PC3 vs PC4
PC4 (1.82%)
PC3 (6.38%)
4
2
0
−2
−4
−6
−4
−2
0
2
4
6
10 0

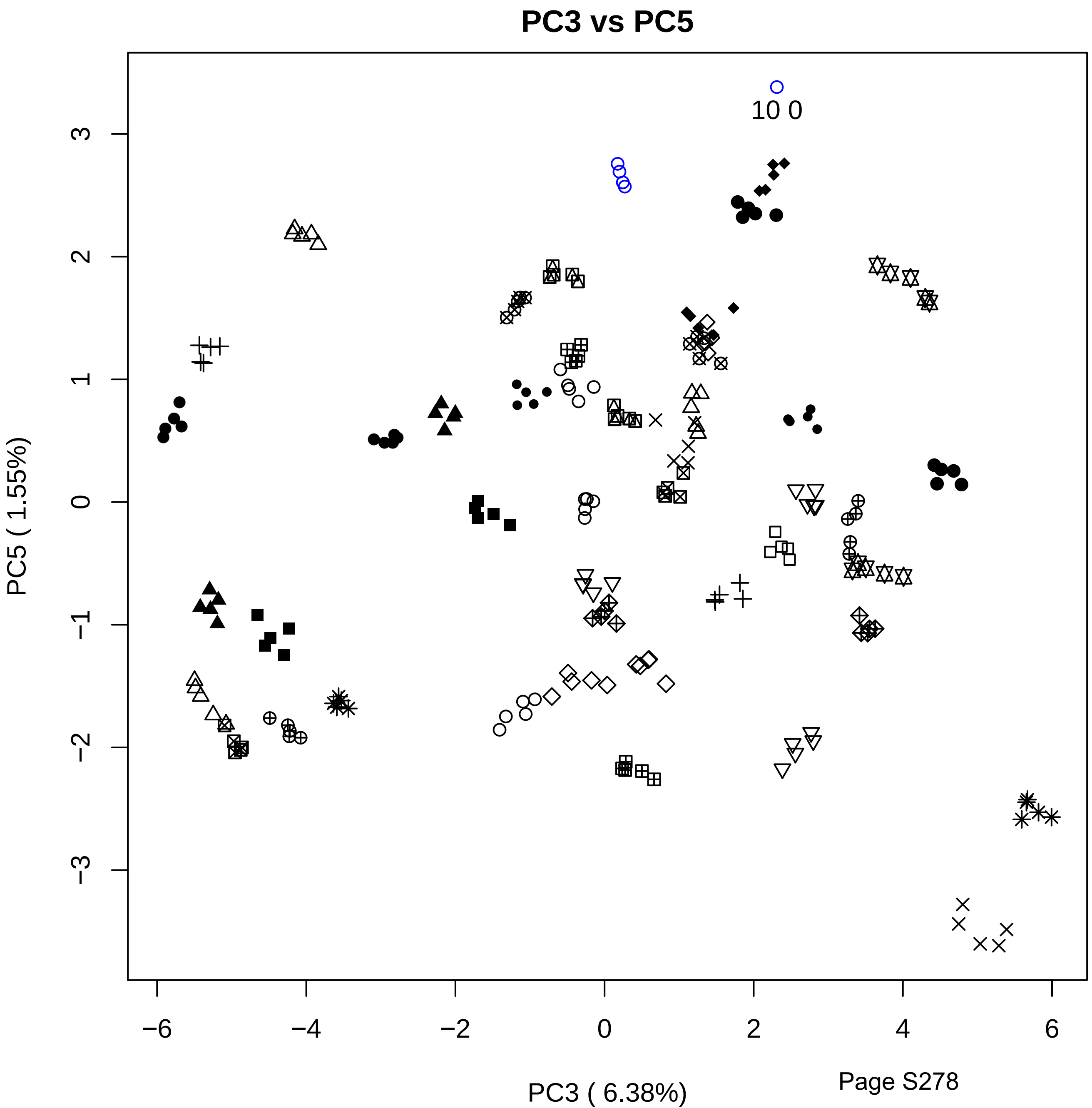

PC3 vs PC5
10 0
PC5 (1.55%)
PC3 (6.38%)
3
2
1
0
−1
−2
−3
−6
−4
−2
0
2
4
6

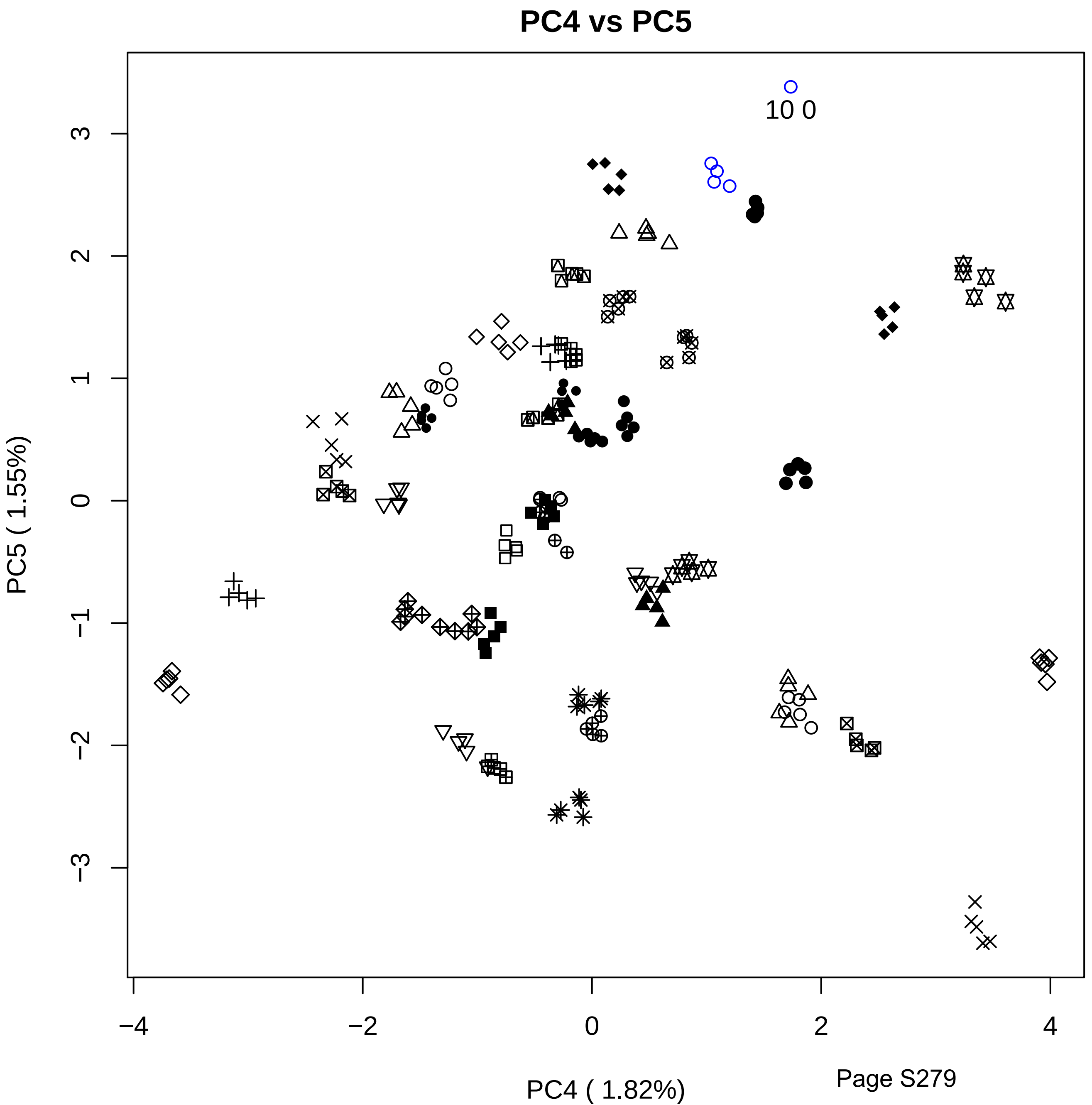

PC4 vs PC5
10 0
3
2
1
0
−1
−2
−3
PC5 (1.55%)
−4
−2
0
2
4
PC4 (1.82%)

PCA of 15% OSCS vs PMH

Scaled Matrix PCAed

7th order polynomial baseline correction performed

Raw spectra smoothed to 21 Neighbours, 2nd order PN, Savtisky Golay

Normalisation between 0 and 100

D2 spectra smoothed to 41 Neighbours, 2nd order PN, Savtisky Golay

Regions Analysed: 3601−2501 and 2001−700cm−1

Samples Analysed: 1 Contam's 0 OSCS's 45 PMH's

Keele University AD Sun Dec 09 16:51:20 2018

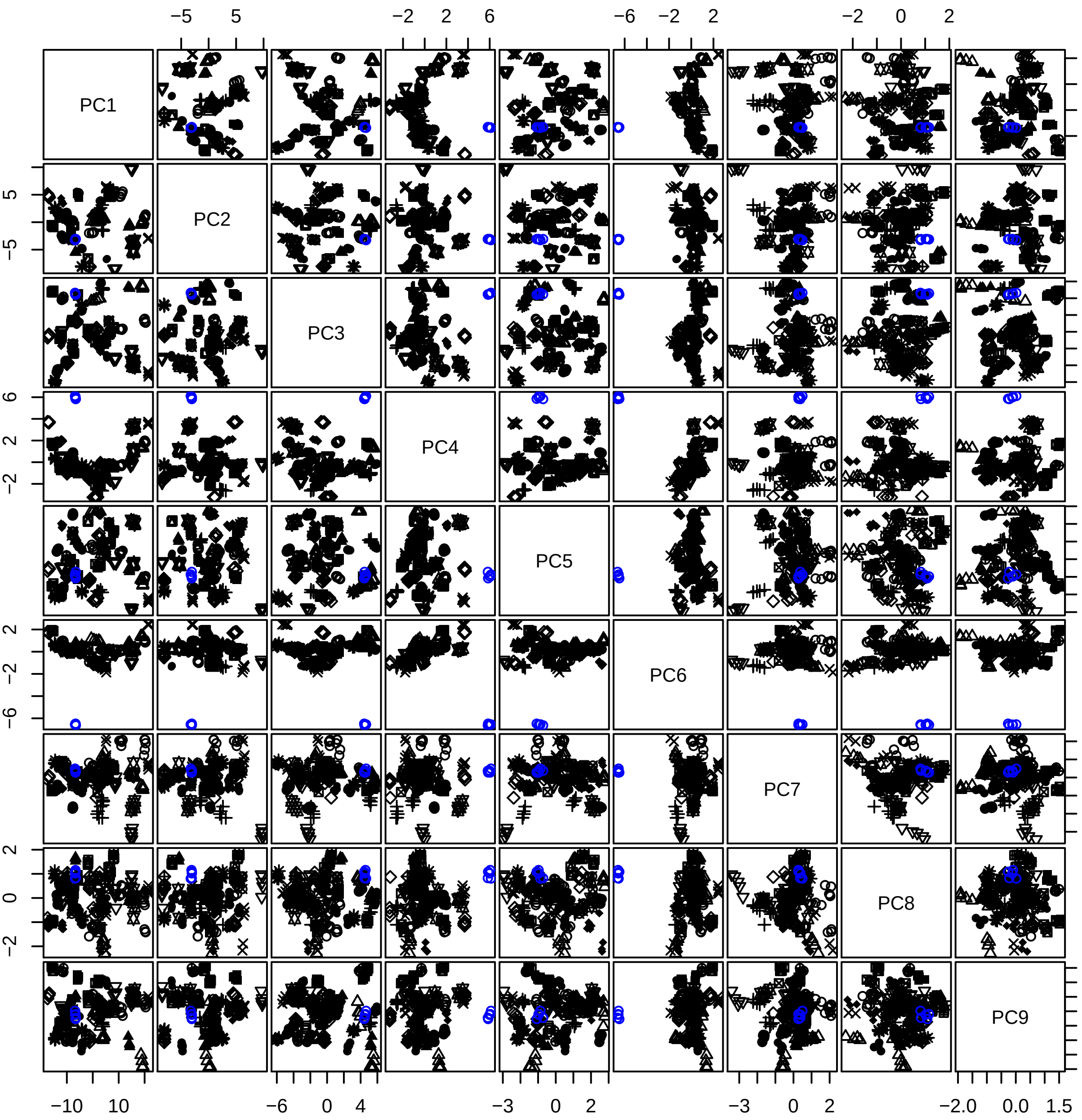
PC1
PC2
PC3
PC4
PC5
PC6
PC7
PC8
PC9

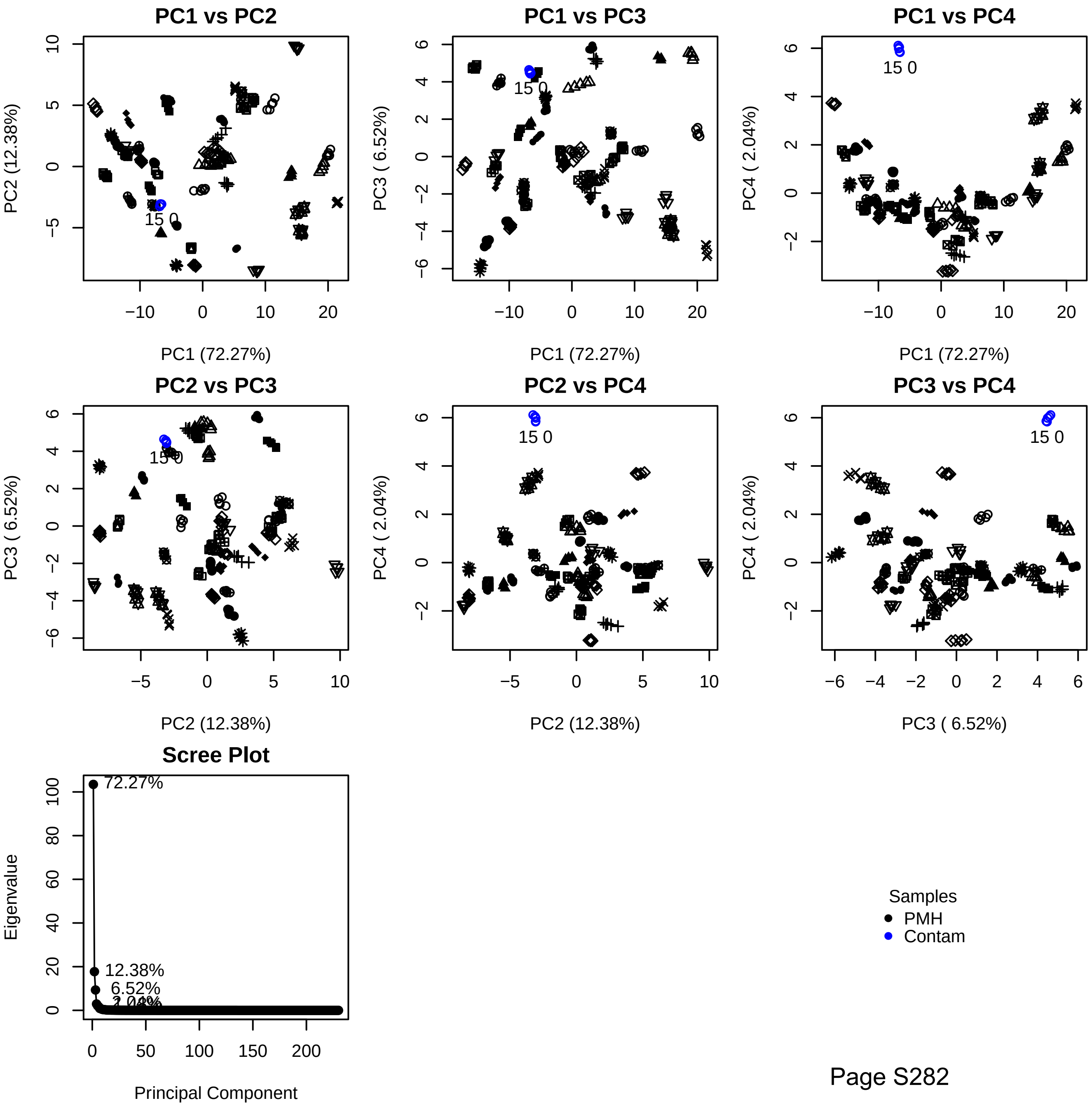
PC1 vs PC2
PC2 (12.38%)
PC1 (72.27%)
15 0
PC1 vs PC3
PC3 (6.52%)
PC1 (72.27%)
15 0
PC1 vs PC4
PC4 (2.04%)
PC1 (72.27%)
15 0
PC2 vs PC3
PC3 (6.52%)
PC2 (12.38%)
15 0
PC2 vs PC4
PC4 (2.04%)
PC2 (12.38%)
15 0
PC3 vs PC4
PC4 (2.04%)
PC3 (6.52%)
15 0
Scree Plot
Eigenvalue
Principal Component
72.27%
12.38%
6.52%
Samples
PMH
Contam

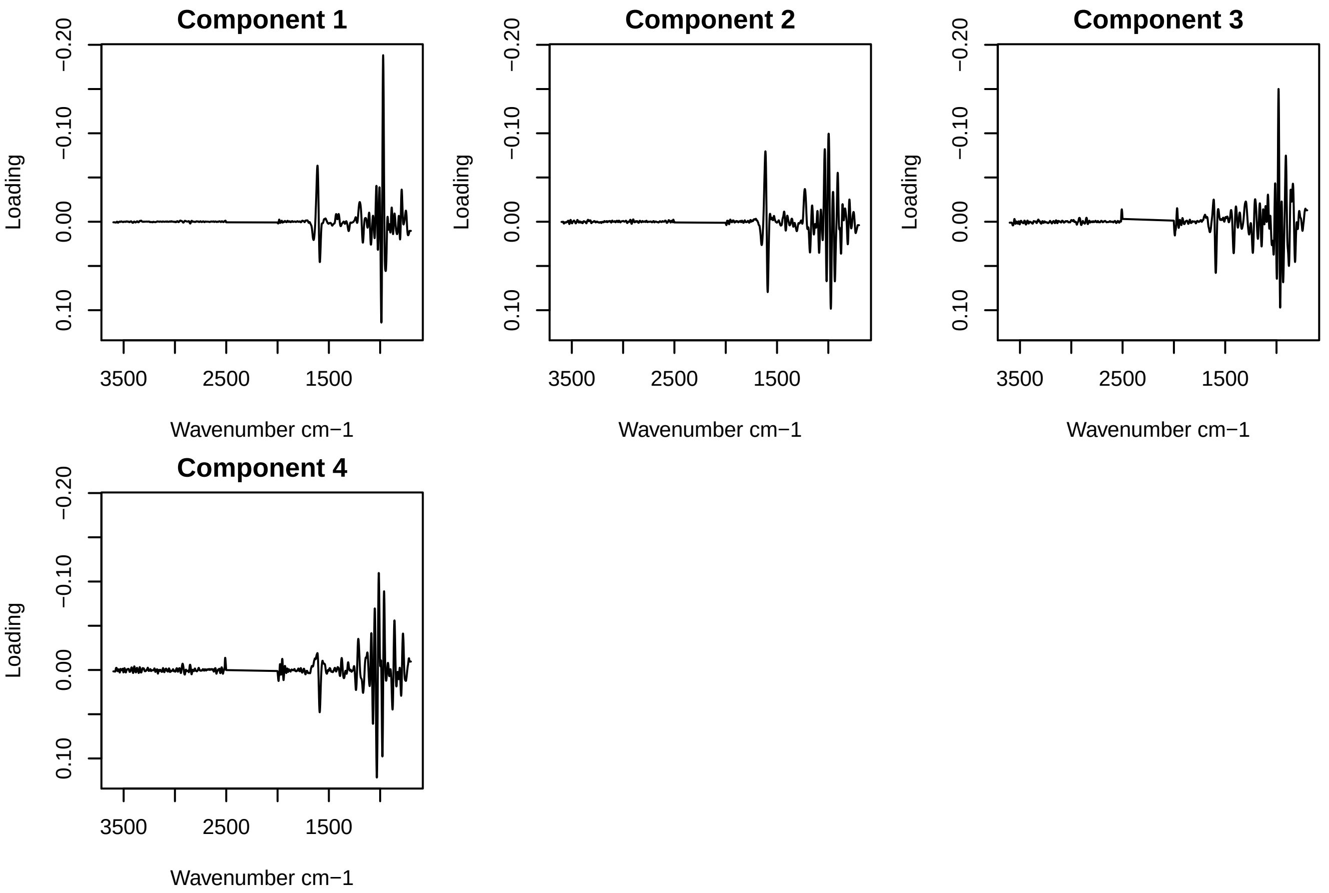

Component 1
Component 2
Component 3
Component 4
Loading
−0.20
−0.10
0.00
0.10
3500
2500
1500
Wavenumber cm−1

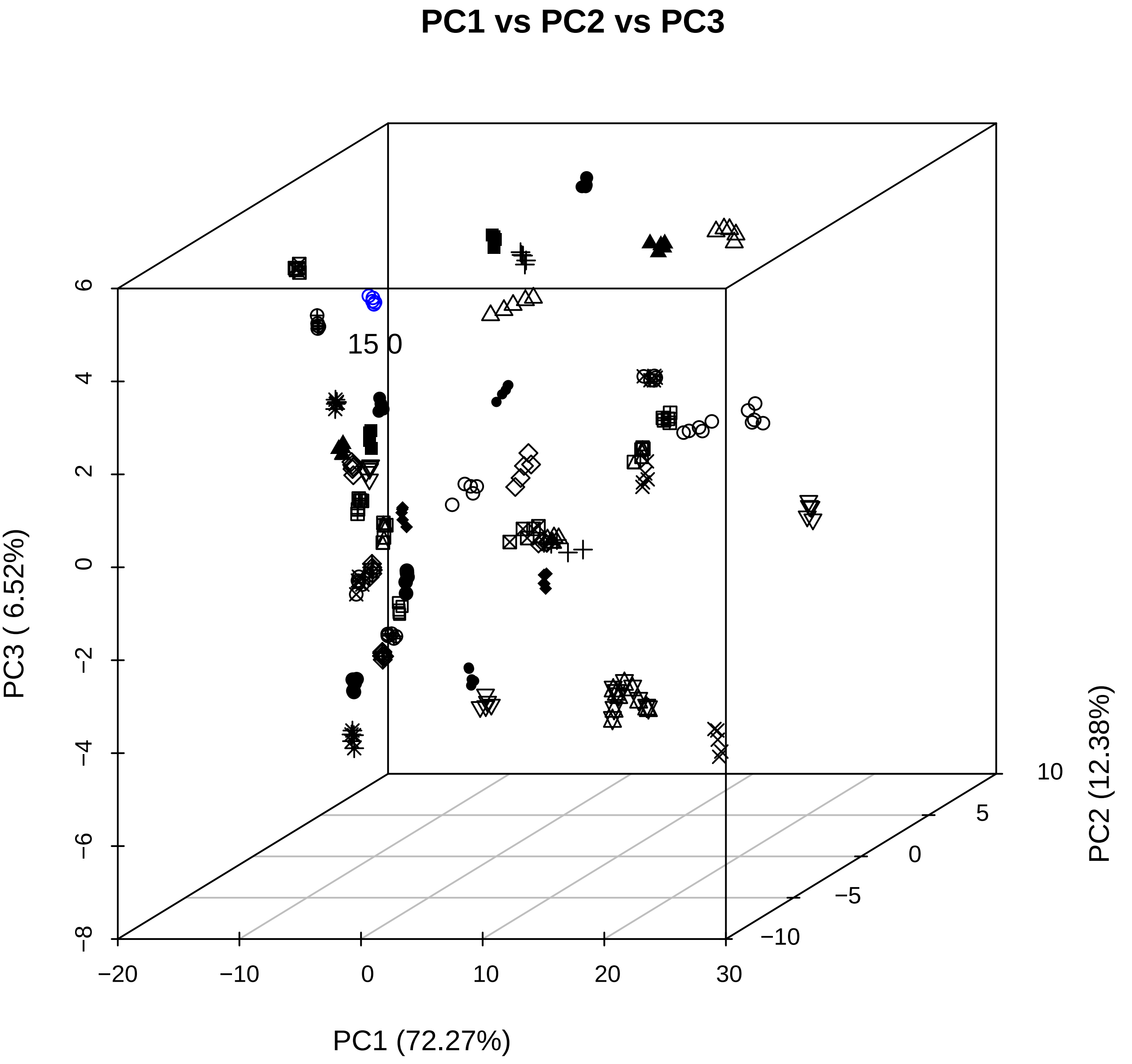
PC1 vs PC2 vs PC3
15 0
PC1 (72.27%)
PC2 (12.38%)
PC3 (6.52%)
−20
−10
0
10
20
30
−10
−5
0
5
10
−8
−6
−4
−2
0
2
4
6

PC2 vs PC3 vs PC4

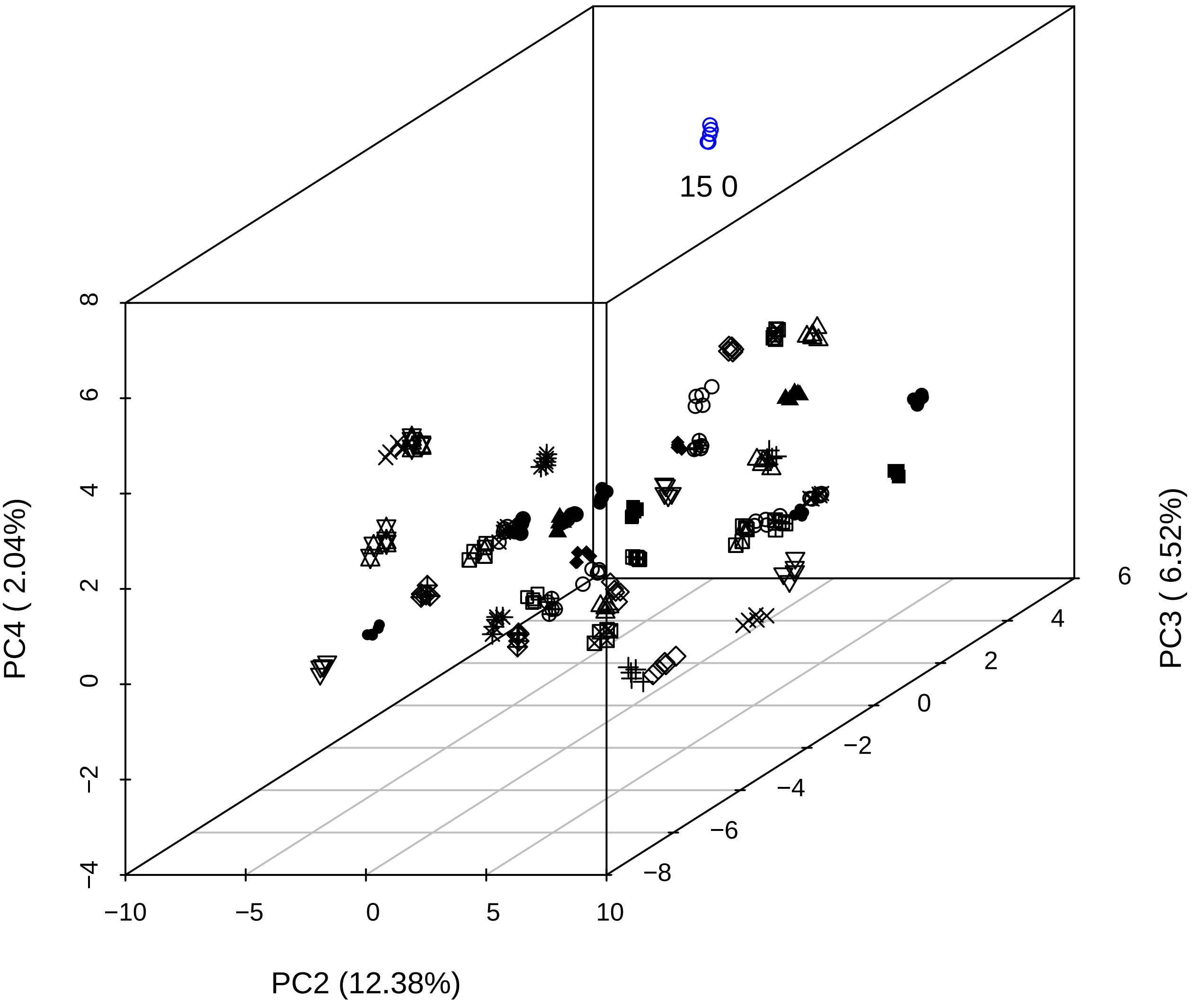

PC1 vs PC2 vs PC4

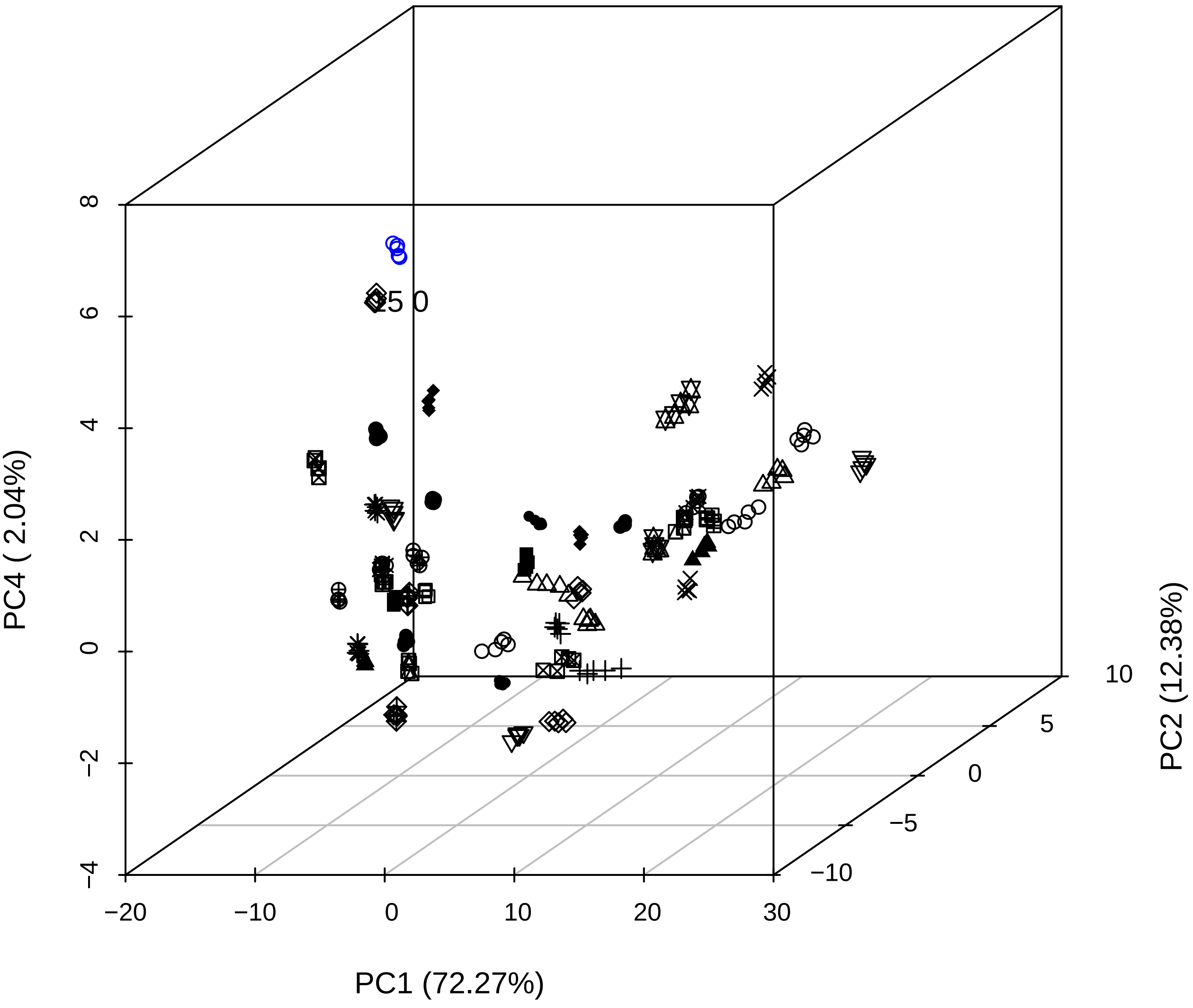

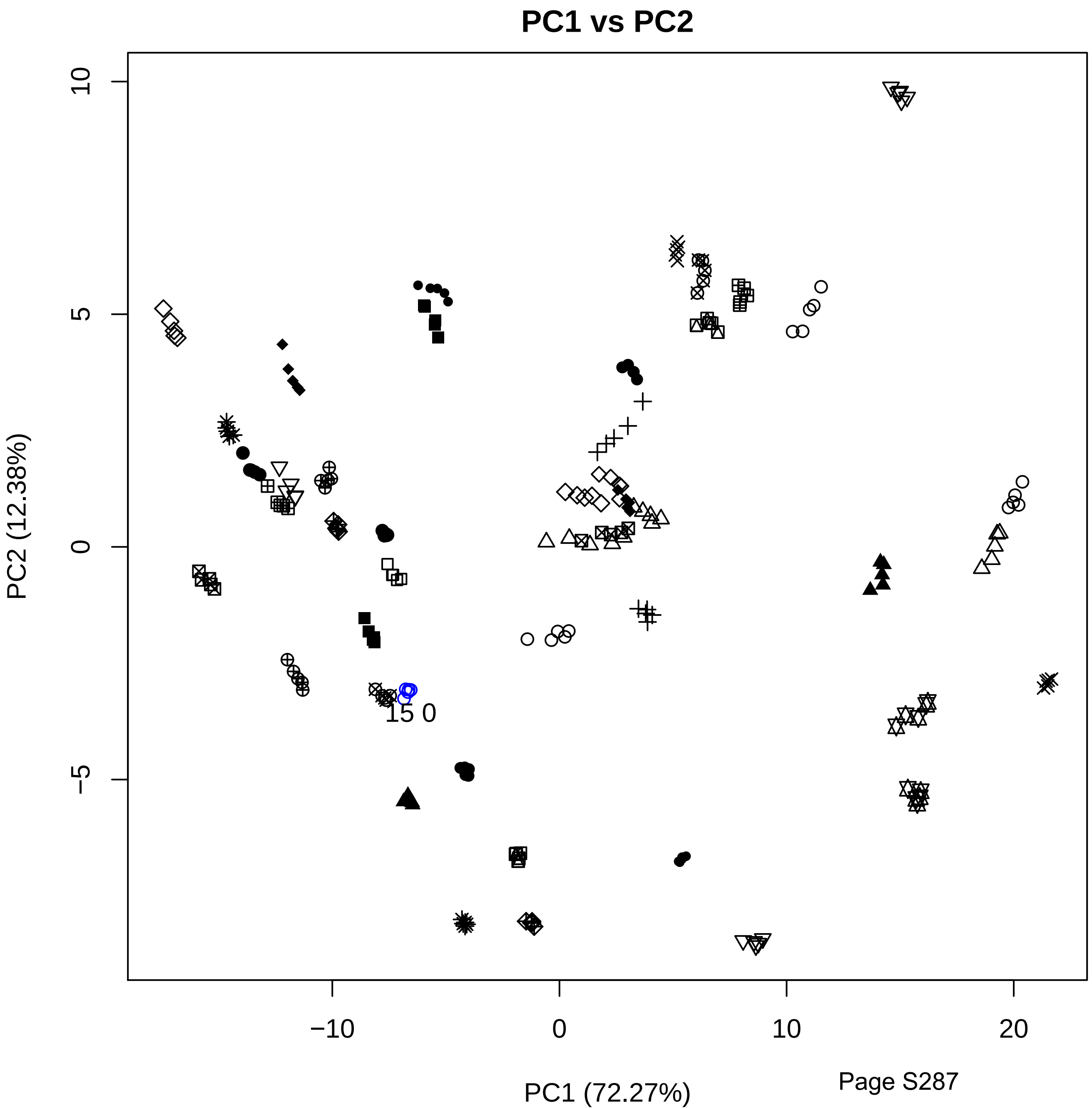
PC1 vs PC2
PC2 (12.38%)
PC1 (72.27%)
10
5
0
−5
−10
0
10
20
15 0

PC1 vs PC3

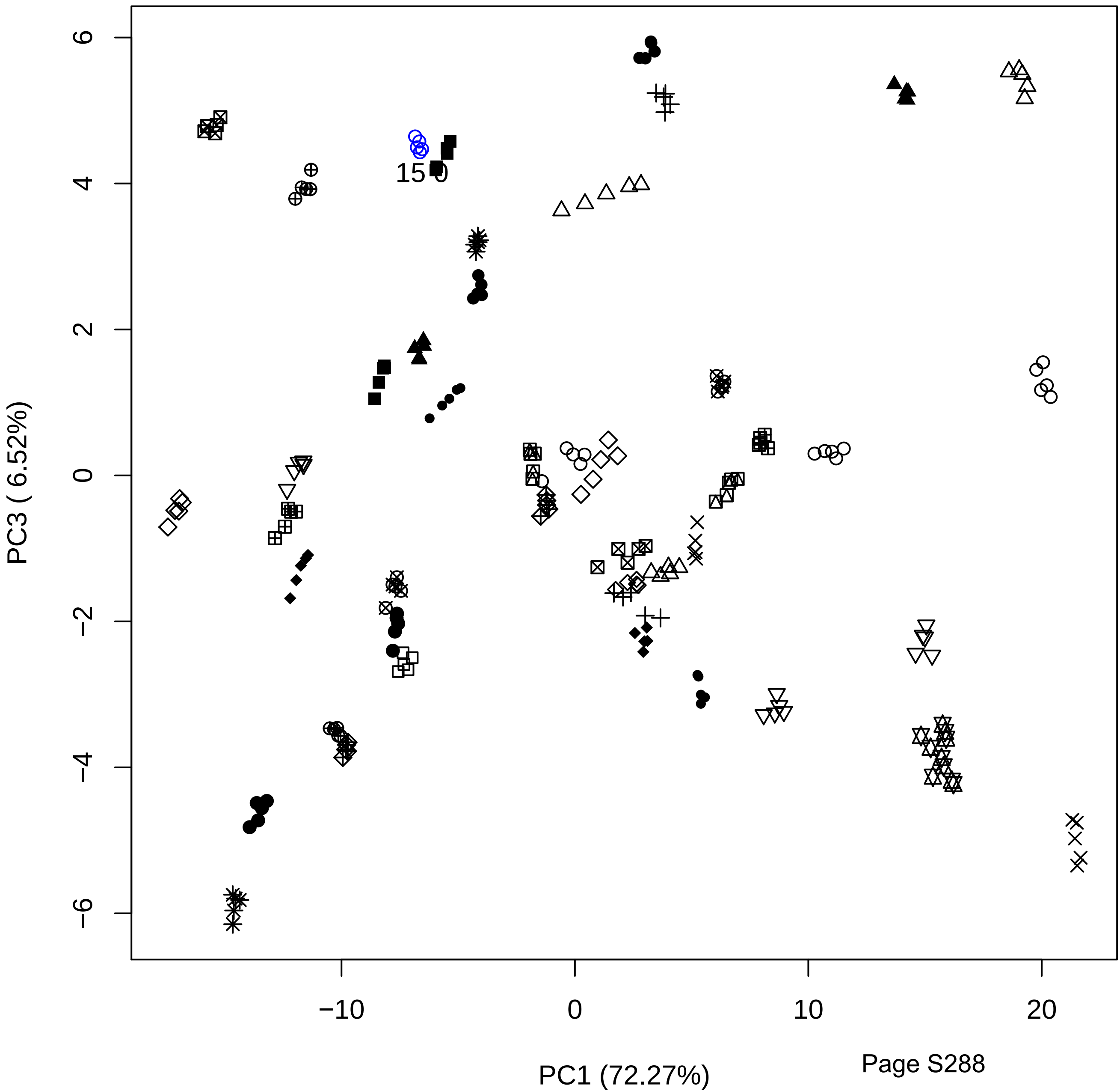

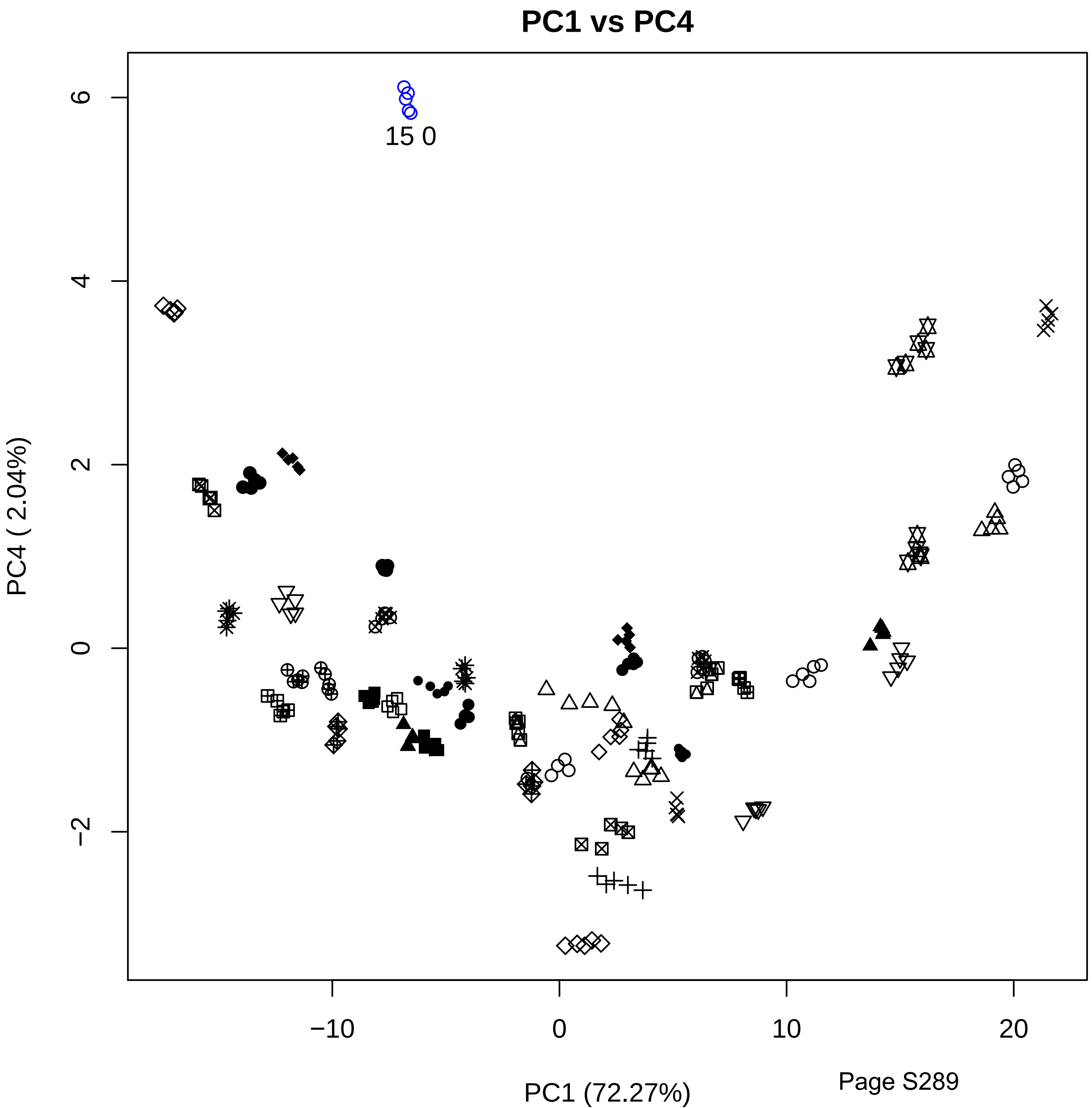
PC1 vs PC4
PC4 (2.04%)
PC1 (72.27%)
15 0
6
4
2
0
−2
−10
0
10
20

PC1 vs PC5

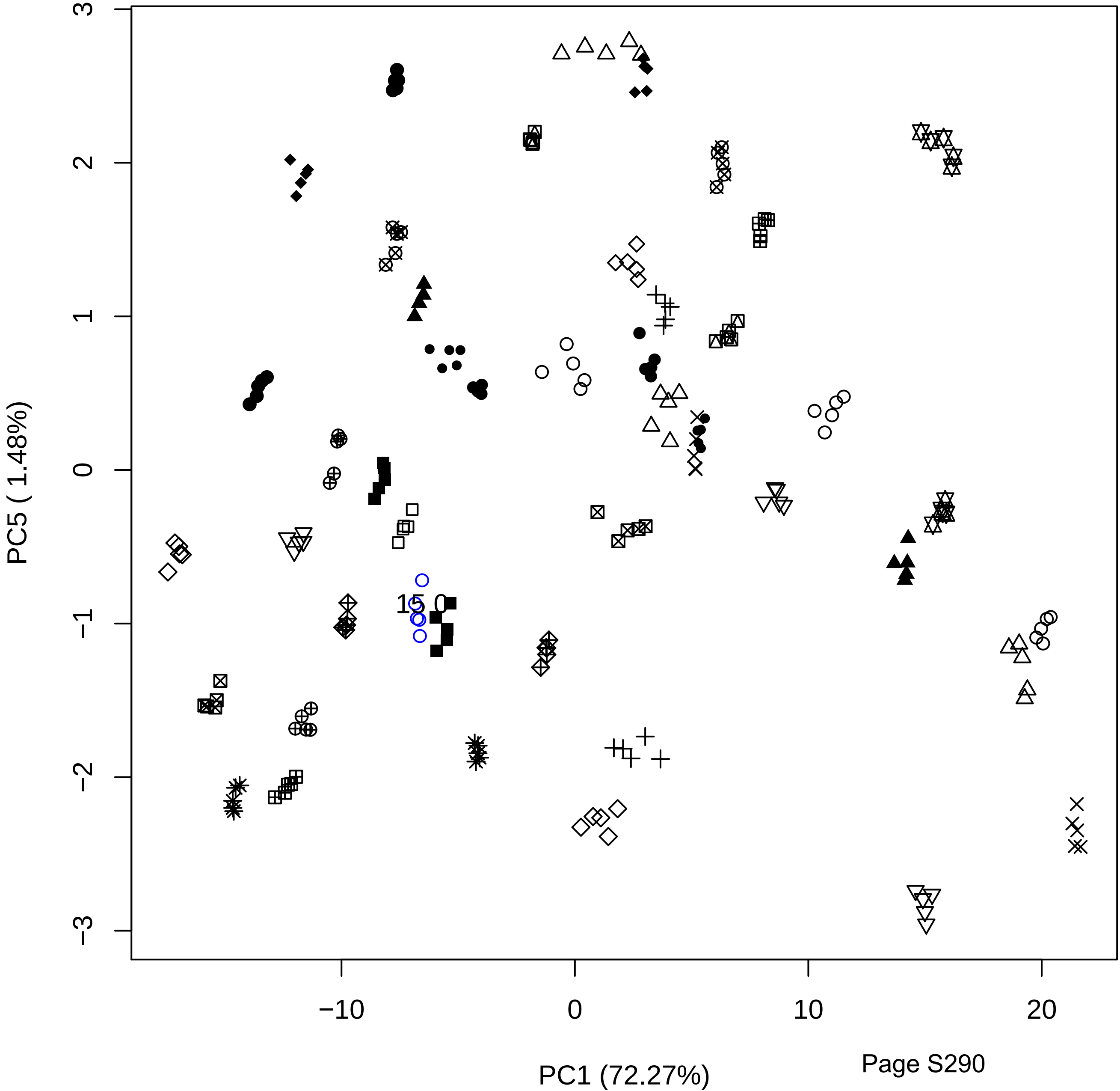

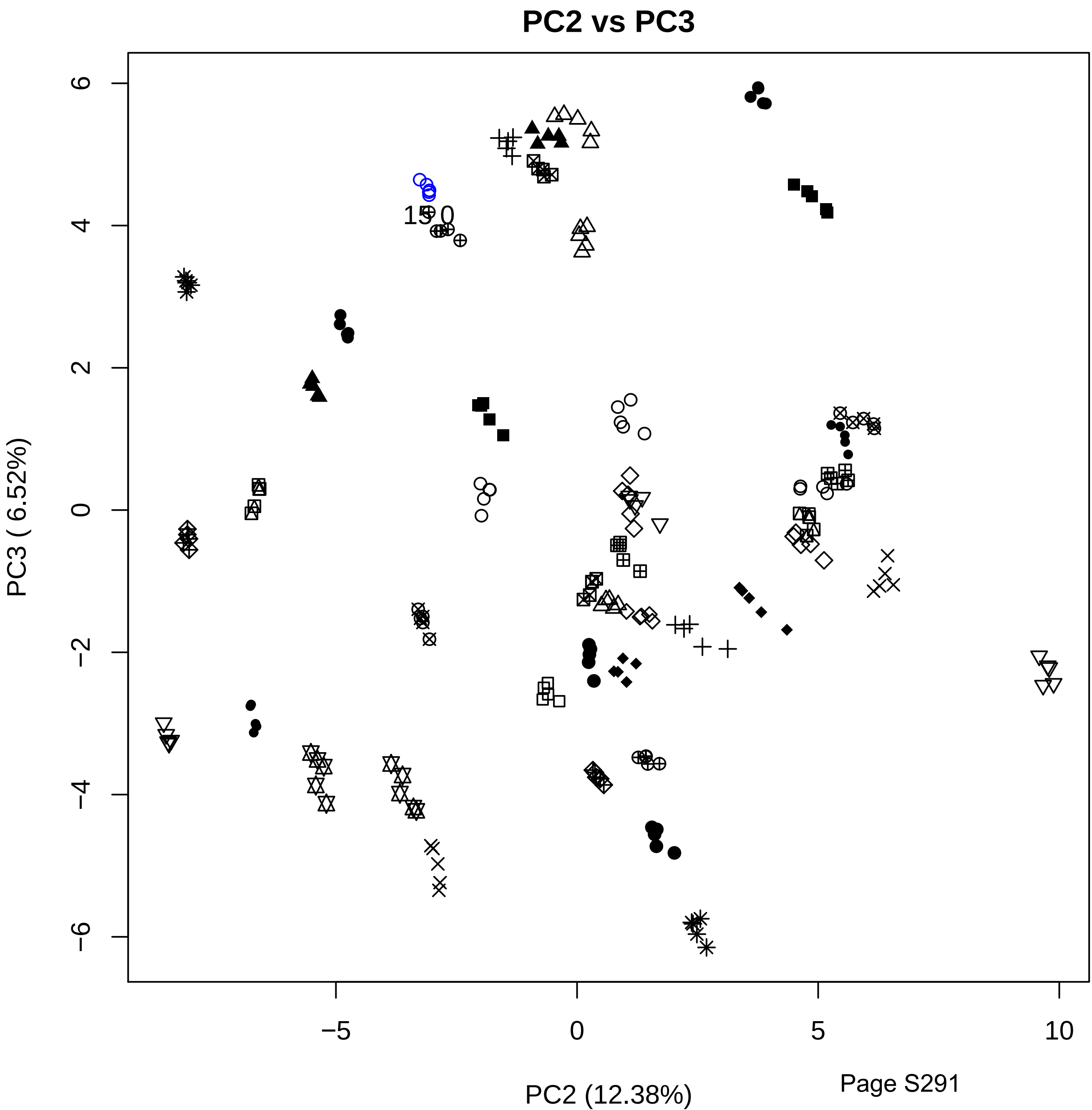
PC2 vs PC3
PC3 (6.52%)
PC2 (12.38%)
6
4
2
0
−2
−4
−6
−5
0
5
10
15 0

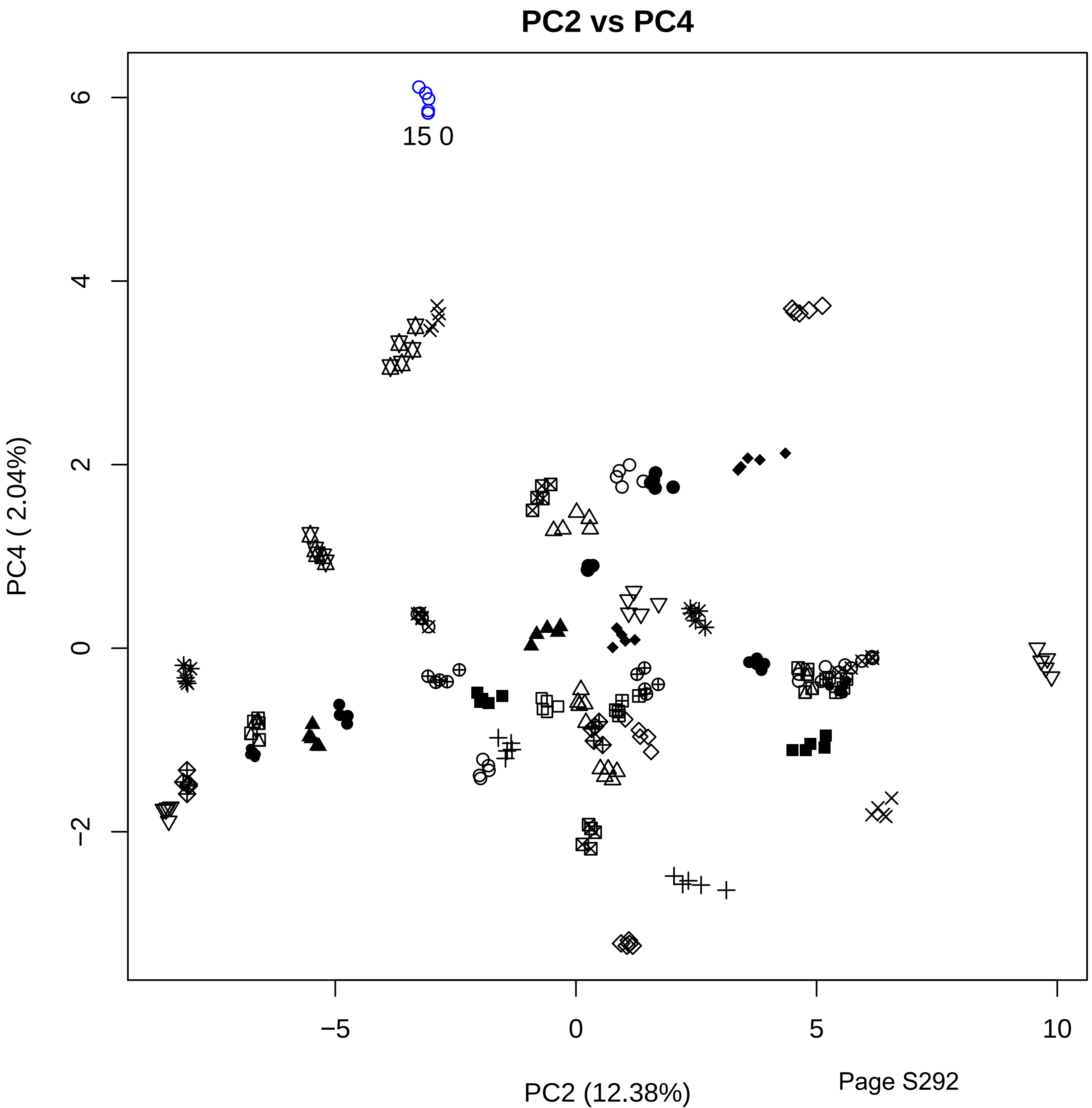

PC2 vs PC4
15 0
PC4 (2.04%)
PC2 (12.38%)
6
4
2
0
−2
−5
0
5
10

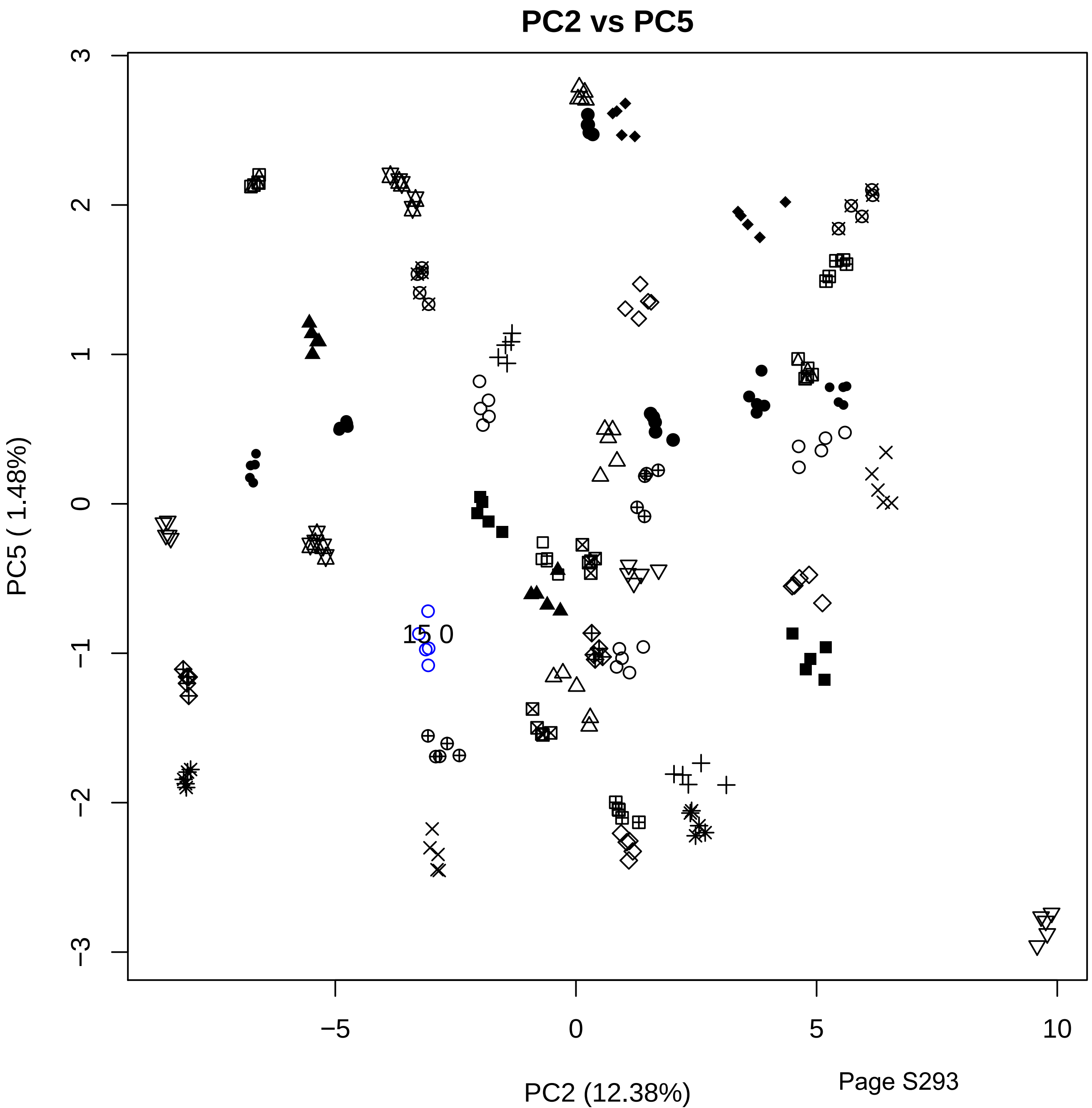
PC2 vs PC5
PC5 (1.48%)
PC2 (12.38%)
3
2
1
0
−1
−2
−3
−5
0
5
10
15 0

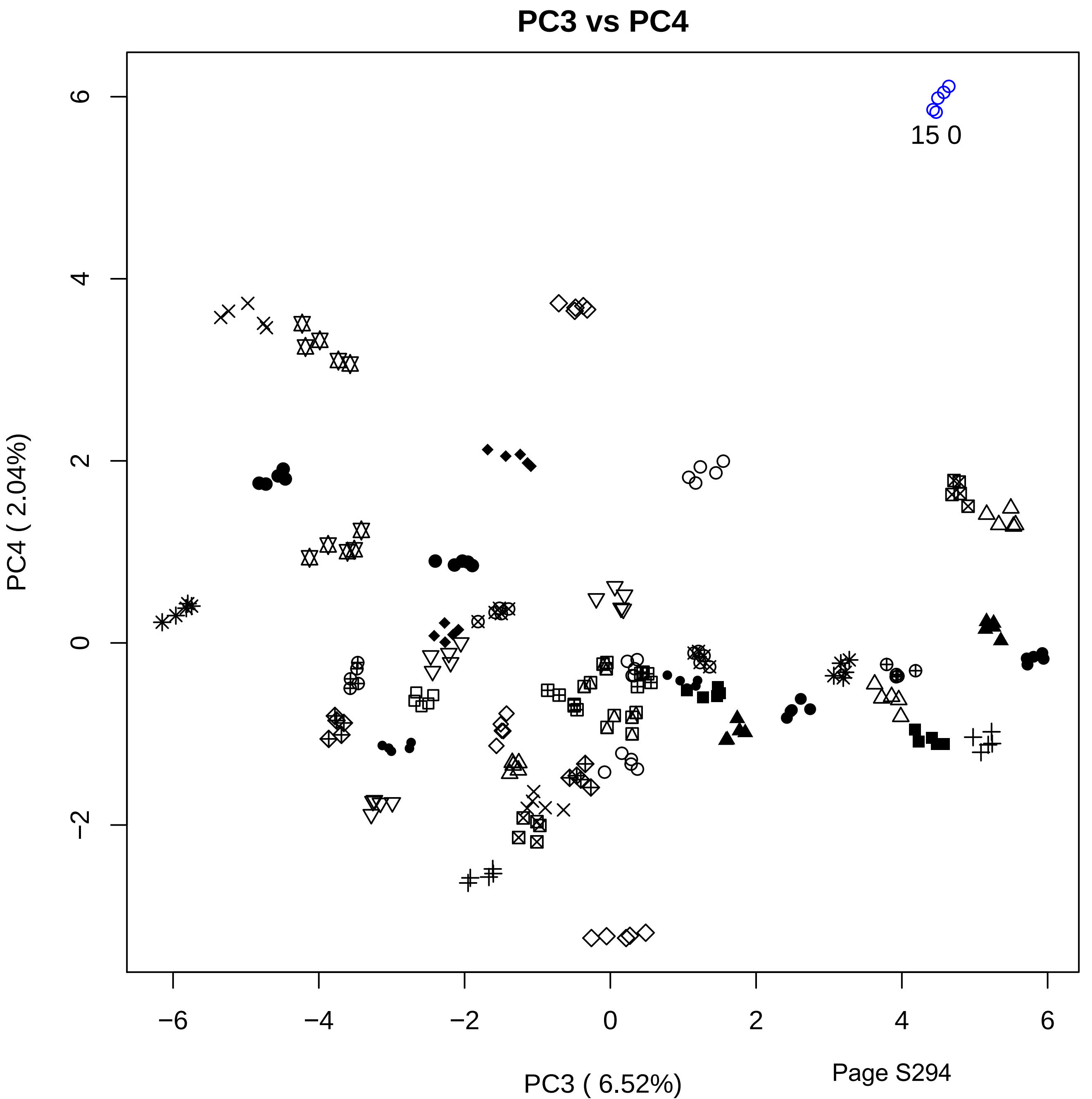
PC3 vs PC4
15 0
PC4 (2.04%)
PC3 (6.52%)
−6
−4
−2
0
2
4
6
−2
0
2
4
6

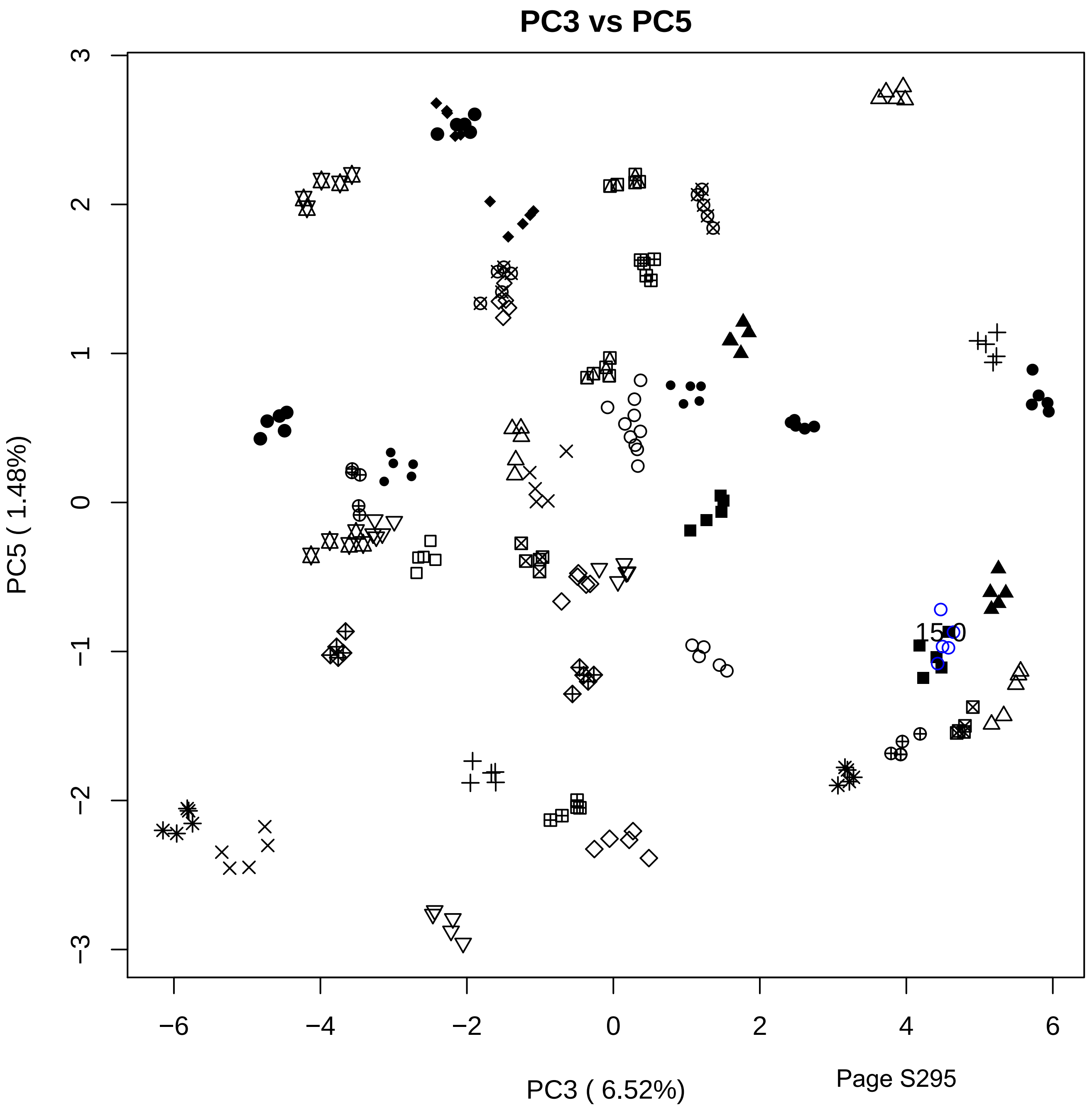
PC3 vs PC5
3
2
1
0
−1
−2
−3
PC5 (1.48%)
−6
−4
−2
0
2
4
6
PC3 (6.52%)
150

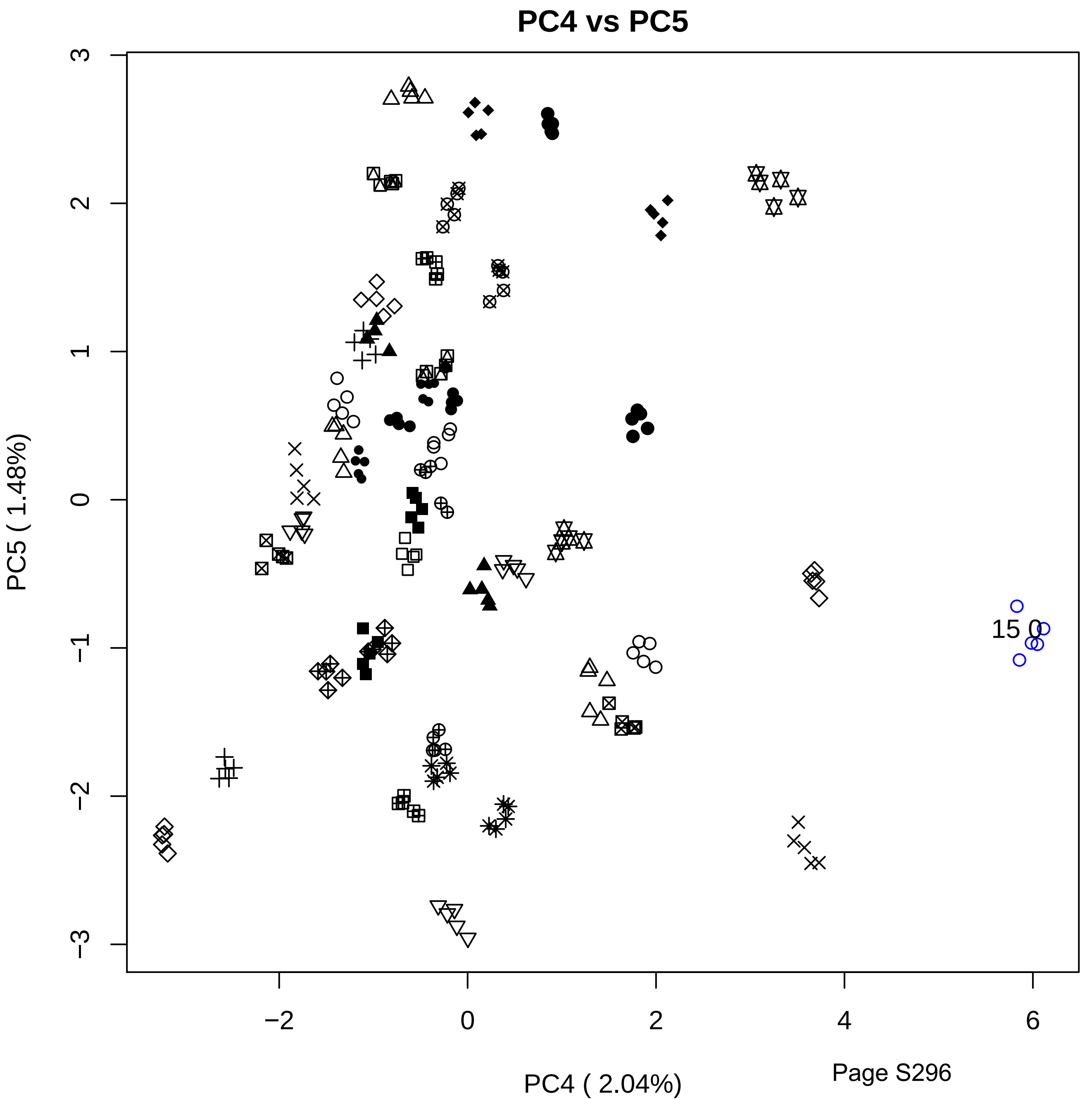
PC4 vs PC5
PC5 (1.48%)
PC4 (2.04%)
15 0
3
2
1
0
−1
−2
−3
−2
0
2
4
6

PCA of 20% OSCS vs PMH

Scaled Matrix PCAed

7th order polynomial baseline correction performed

Raw spectra smoothed to 21 Neighbours, 2nd order PN, Savtisky Golay

Normalisation between 0 and 100

D2 spectra smoothed to 41 Neighbours, 2nd order PN, Savtisky Golay

Regions Analysed: 3601−2501 and 2001−700cm−1

Samples Analysed: 1 Contam's 0 OSCS's 45 PMH's

Keele University AD   Sun Dec 09 16:51:25 2018

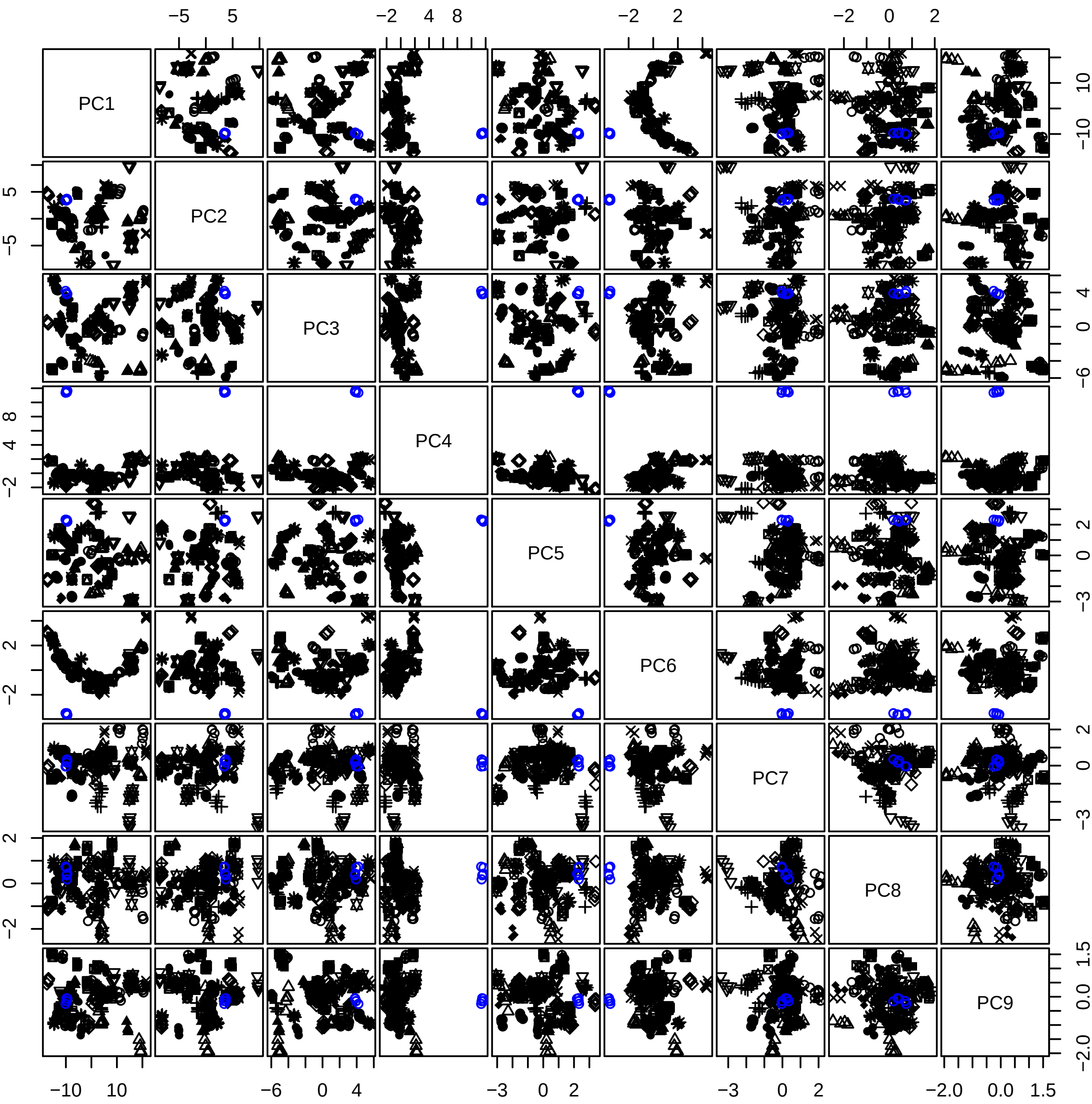
PC1
PC2
PC3
PC4
PC5
PC6
PC7
PC8
PC9
−5
5
−2
4
8
−2
2
−2
0
2
10
−10
5
−5
4
0
−6
8
4
−2
2
0
−3
2
−2
2
0
−3
2
0
−2
1.5
0.0
−2.0
−10
10
−6
0
4
−3
0
2
−3
0
2
−2.0
0.0
1.5

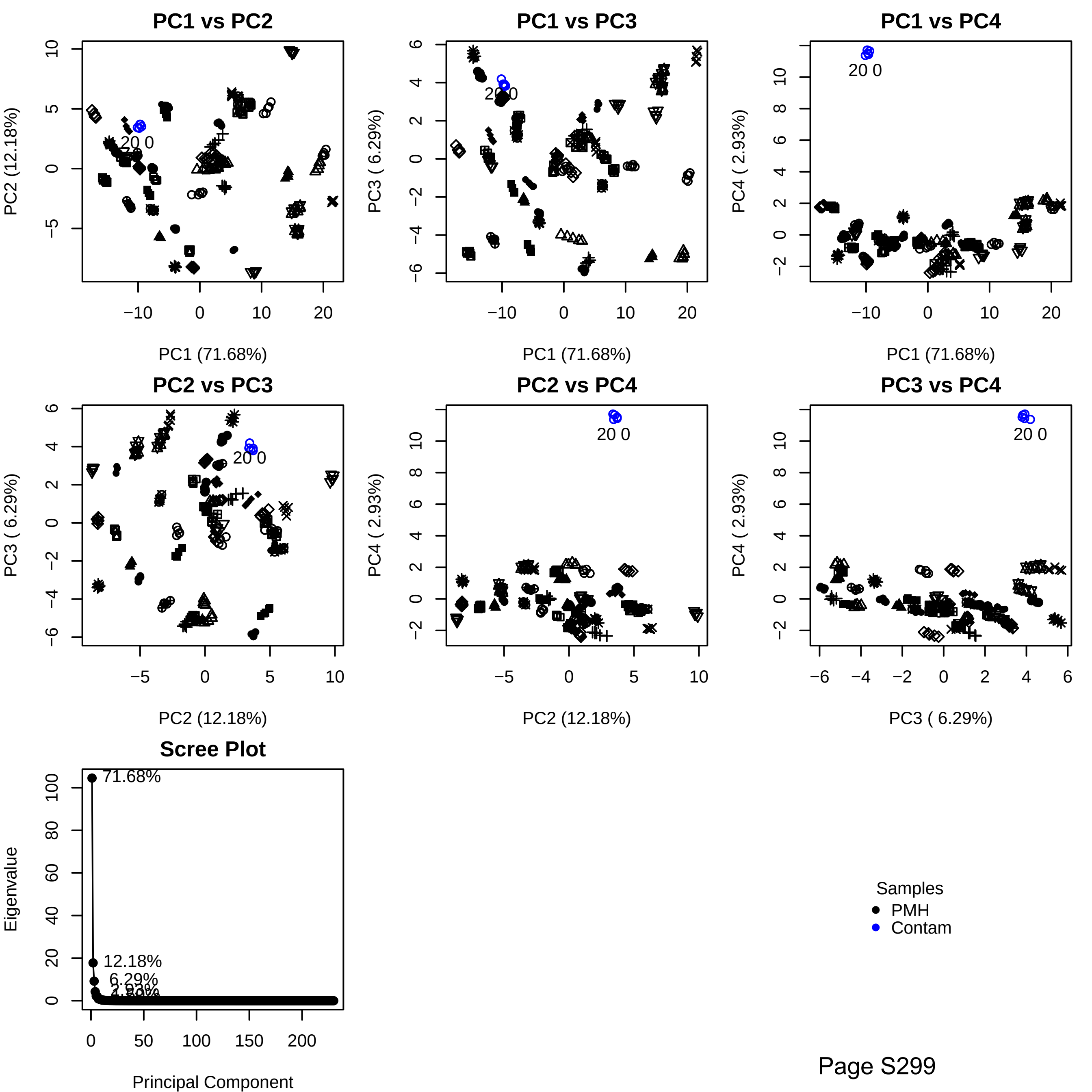
PC1 vs PC2
PC1 vs PC3
PC1 vs PC4
PC2 vs PC3
PC2 vs PC4
PC3 vs PC4
Scree Plot
PC1 (71.68%)
PC2 (12.18%)
PC3 (6.29%)
PC4 (2.93%)
20 0
Eigenvalue
Principal Component
71.68%
12.18%
6.29%
Samples
PMH
Contam

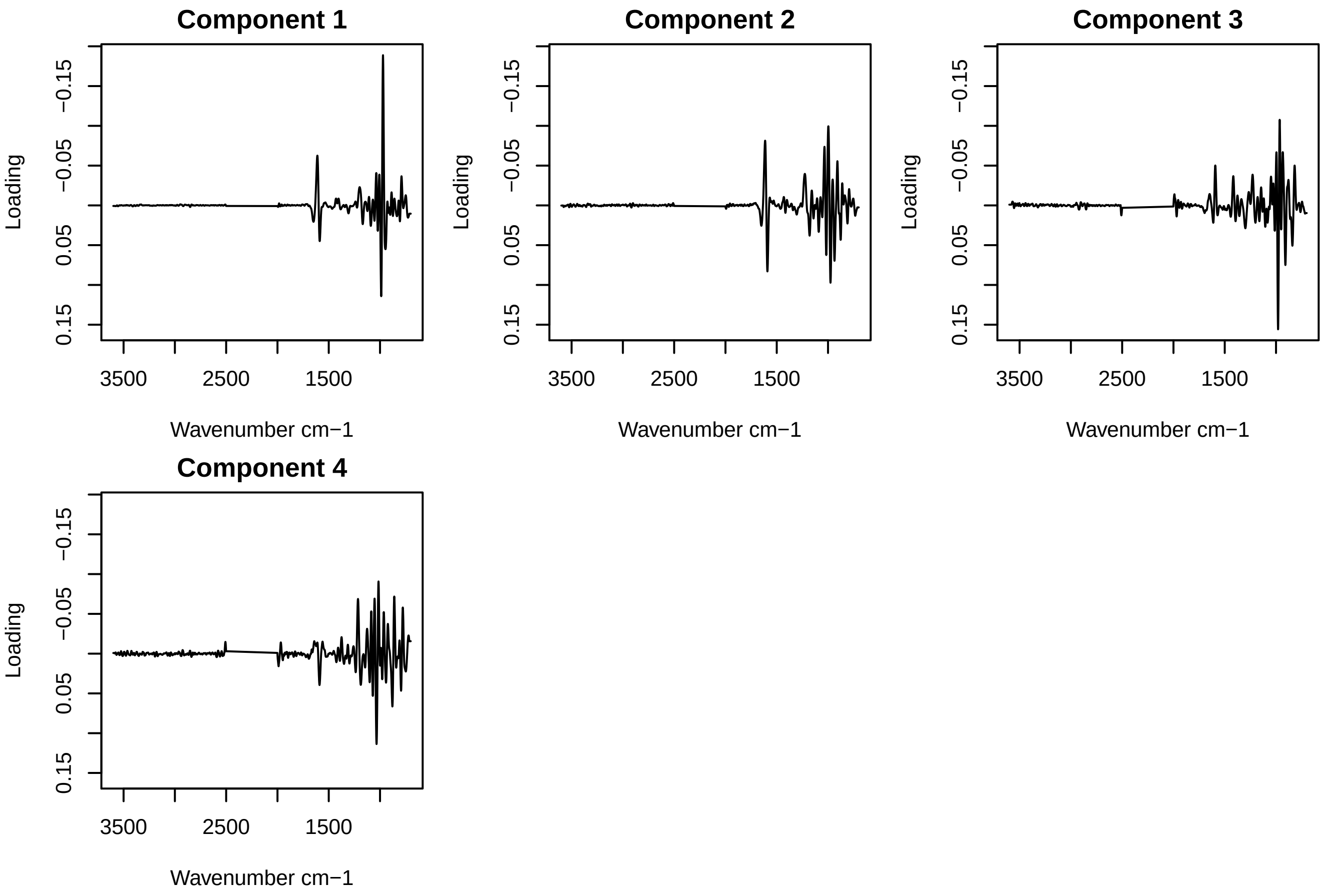
Component 1
Loading
−0.15
−0.05
0.05
0.15
3500
2500
1500
Wavenumber cm−1
Component 2
Loading
−0.15
−0.05
0.05
0.15
3500
2500
1500
Wavenumber cm−1
Component 3
Loading
−0.15
−0.05
0.05
0.15
3500
2500
1500
Wavenumber cm−1
Component 4
Loading
−0.15
−0.05
0.05
0.15
3500
2500
1500
Wavenumber cm−1

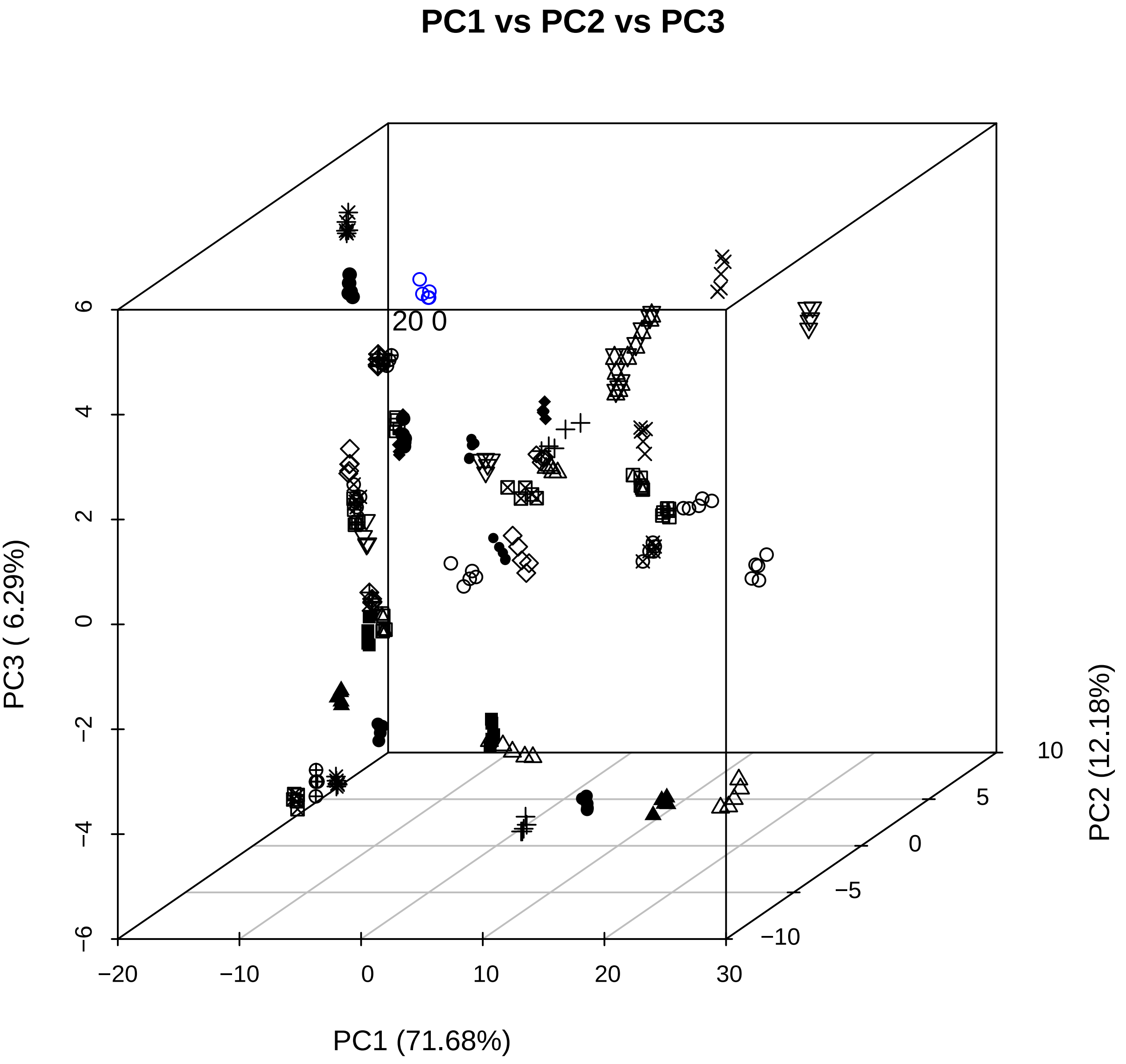

PC1 vs PC2 vs PC3
20 0
PC3 (6.29%)
PC1 (71.68%)
PC2 (12.18%)
6
4
2
0
−2
−4
−6
−20
−10
0
10
20
30
10
5
0
−5
−10

PC2 vs PC3 vs PC4

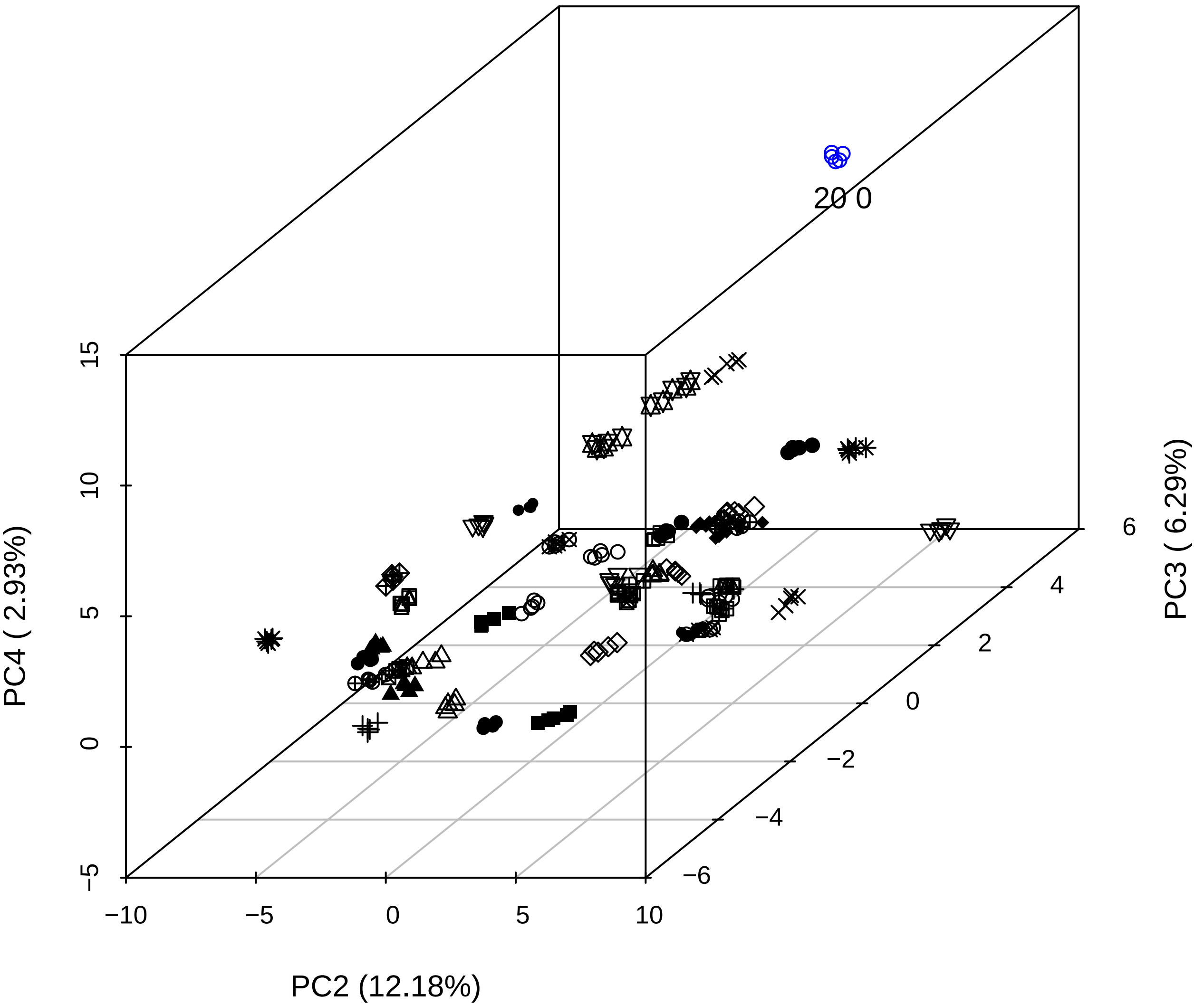

PC1 vs PC2 vs PC4

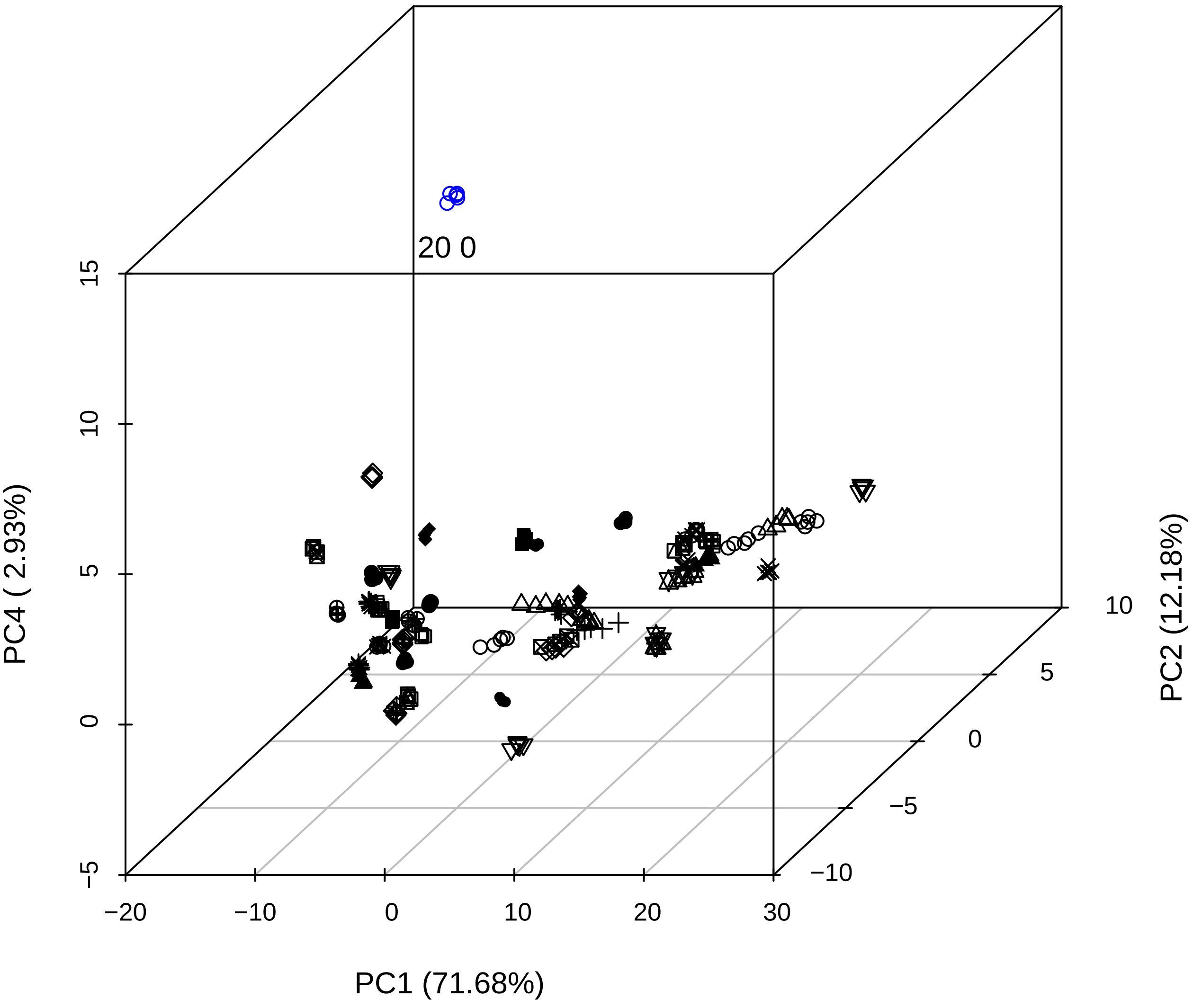

PC1 vs PC2

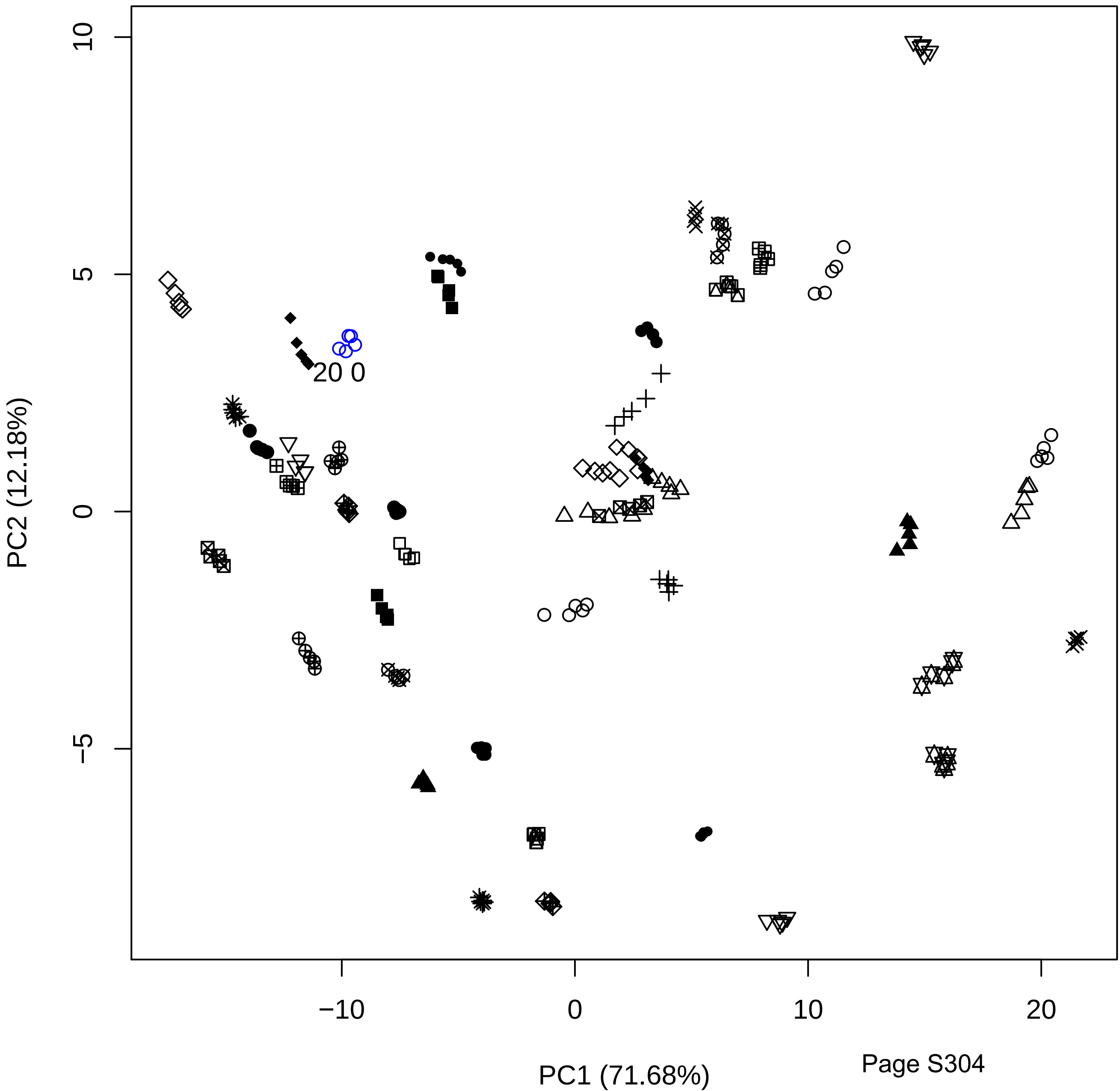

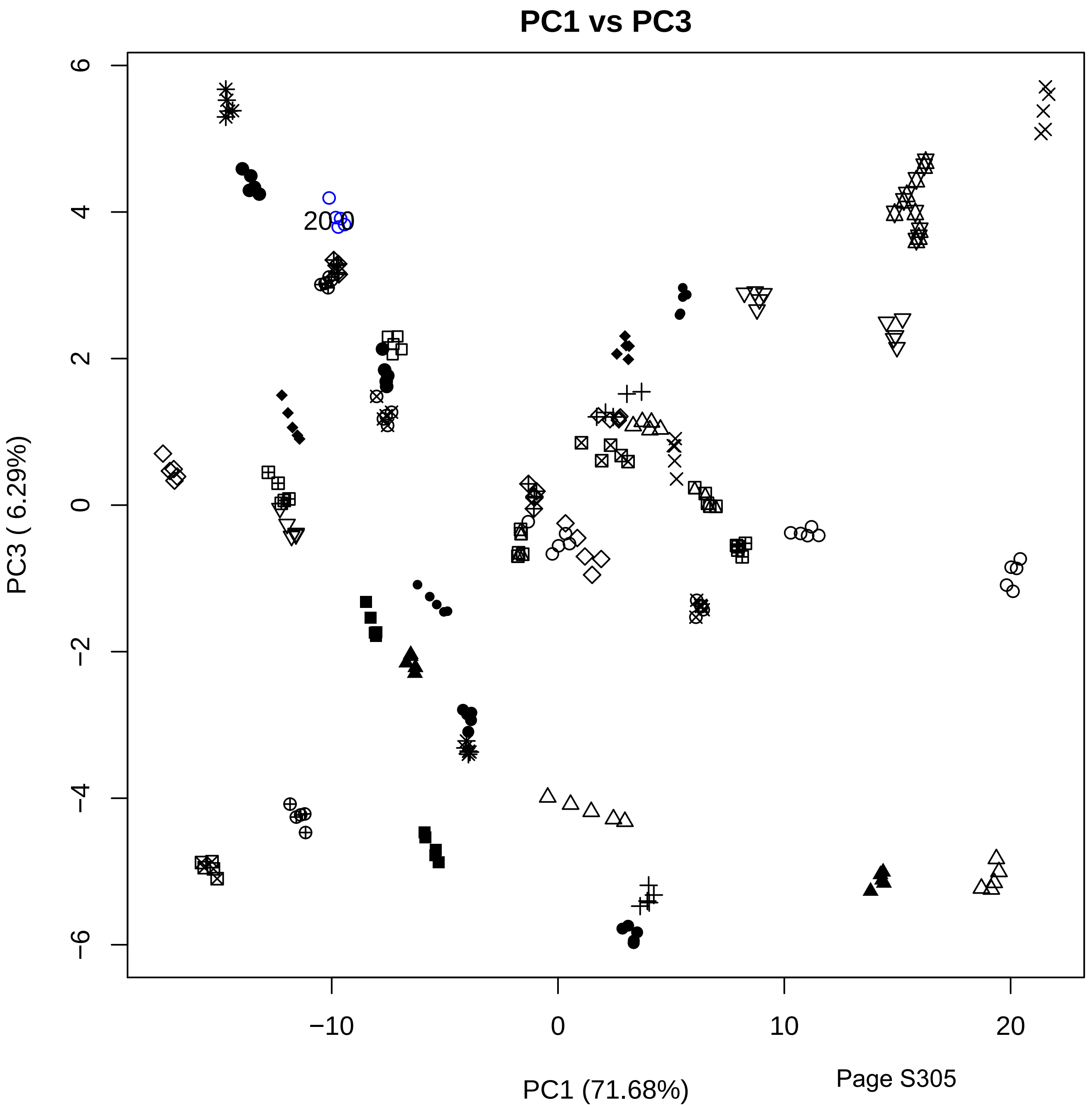
PC1 vs PC3
PC3 (6.29%)
PC1 (71.68%)
6
4
2
0
−2
−4
−6
−10
0
10
20
2010

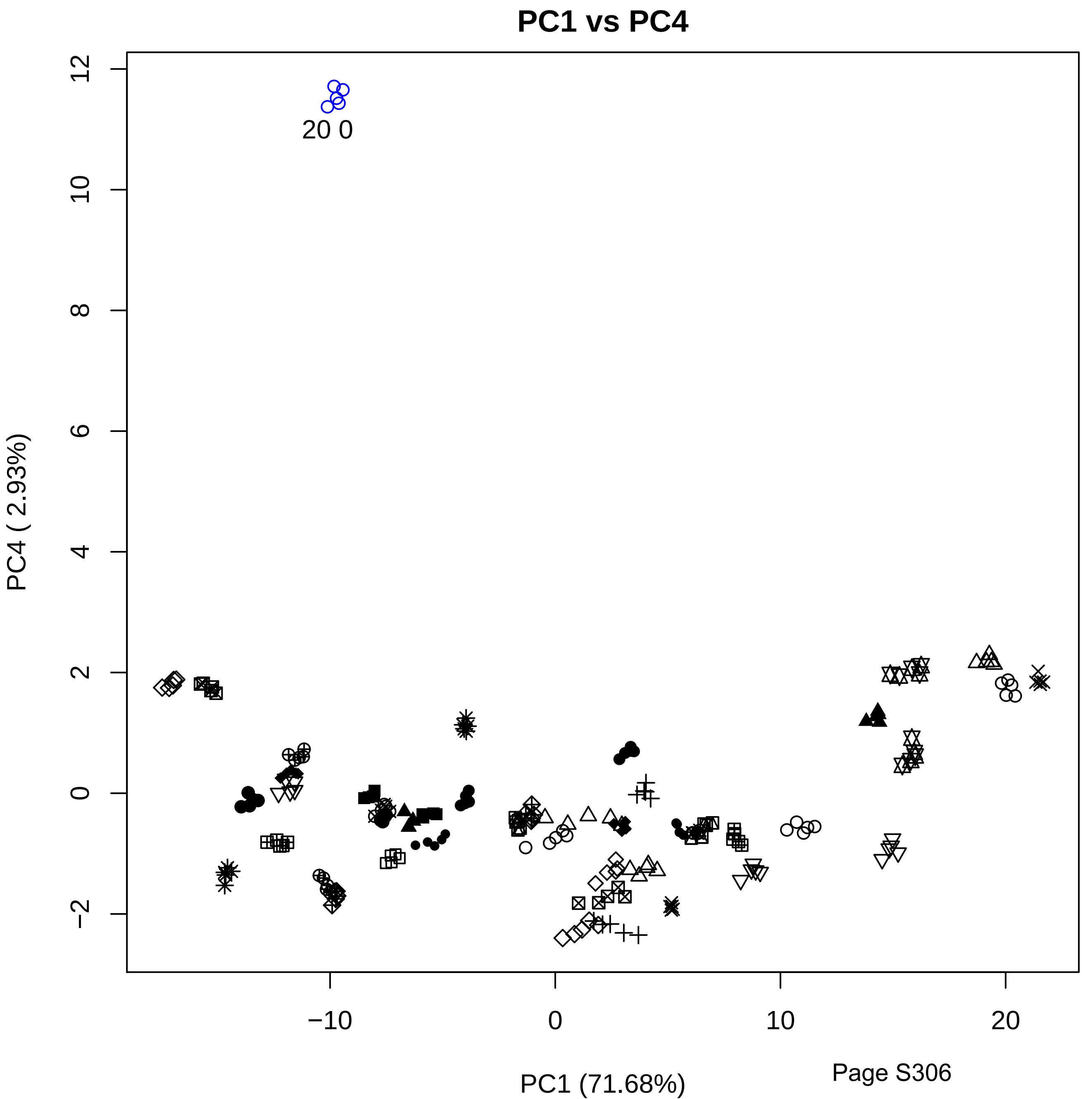

PC1 vs PC4
20 0
PC4 (2.93%)
PC1 (71.68%)
−10
0
10
20
−2
0
2
4
6
8
10
12

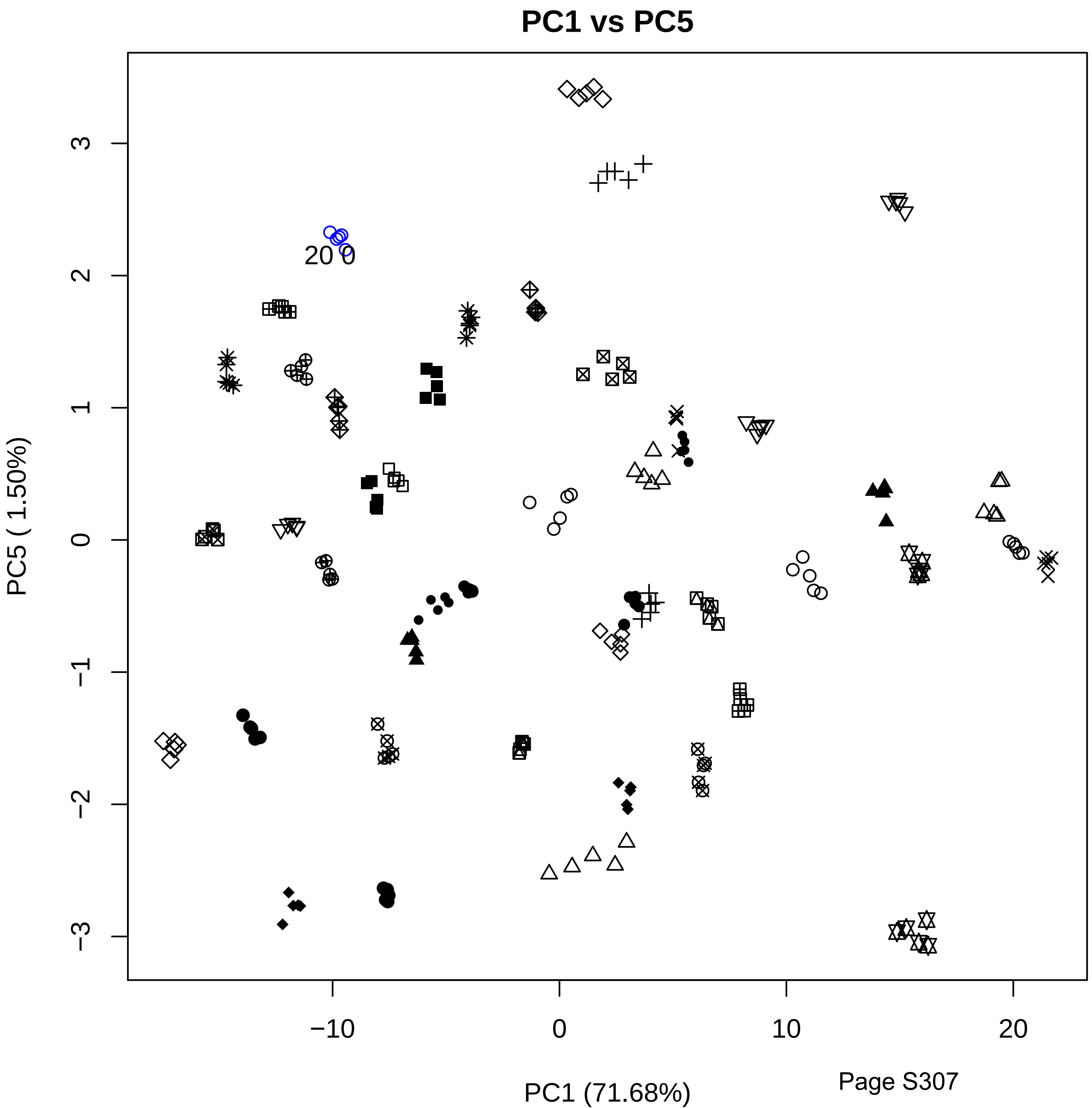
PC1 vs PC5
20 0
PC5 (1.50%)
3
2
1
0
−1
−2
−3
−10
0
10
20
PC1 (71.68%)

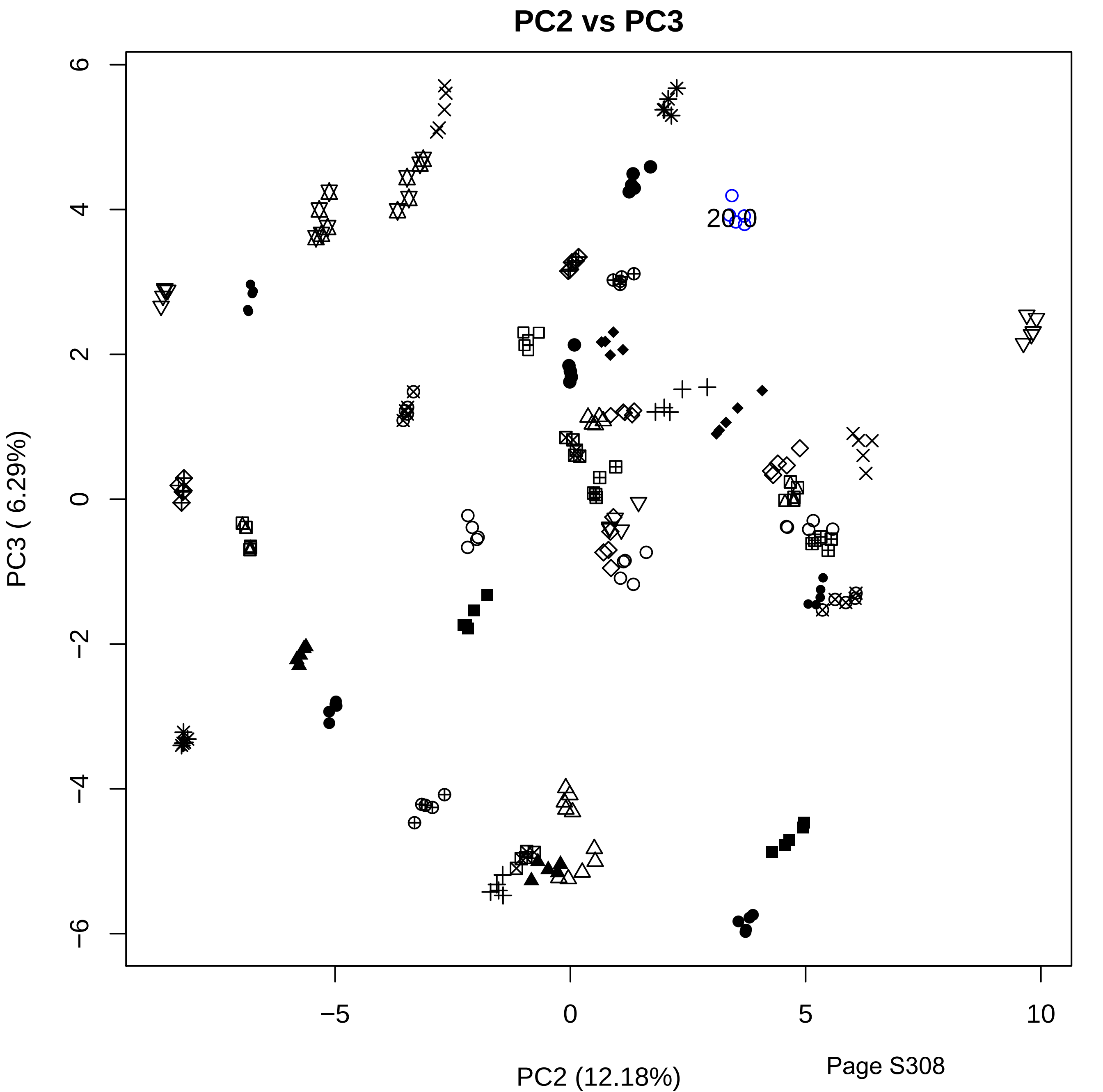

PC2 vs PC3
PC3 (6.29%)
PC2 (12.18%)
6
4
2
0
−2
−4
−6
−5
0
5
10
2010

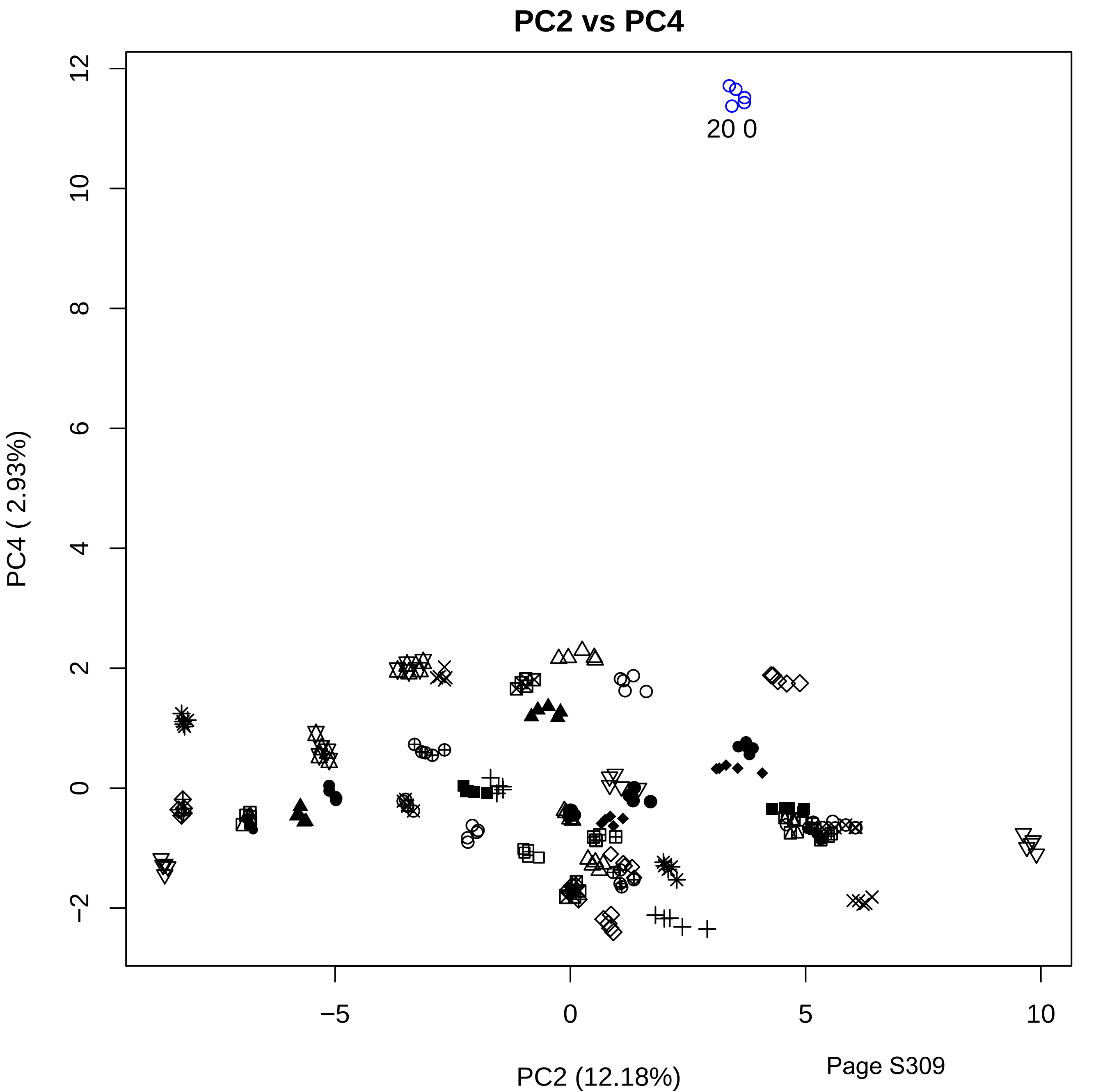
PC2 vs PC4
20 0
PC4 (2.93%)
PC2 (12.18%)
12
10
8
6
4
2
0
−2
−5
0
5
10

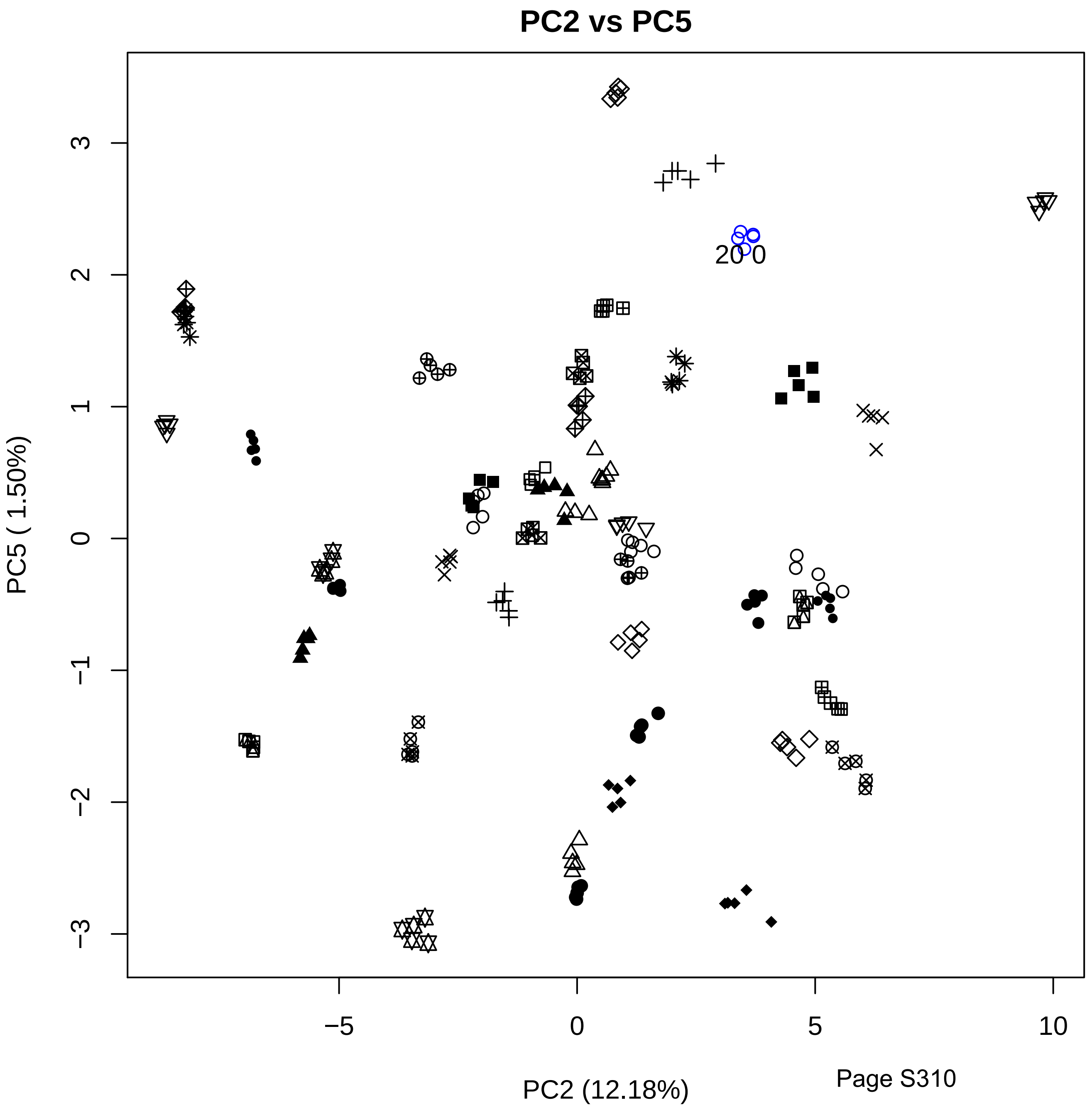

PC2 vs PC5
PC5 (1.50%)
PC2 (12.18%)
3
2
1
0
−1
−2
−3
−5
0
5
10
20 0

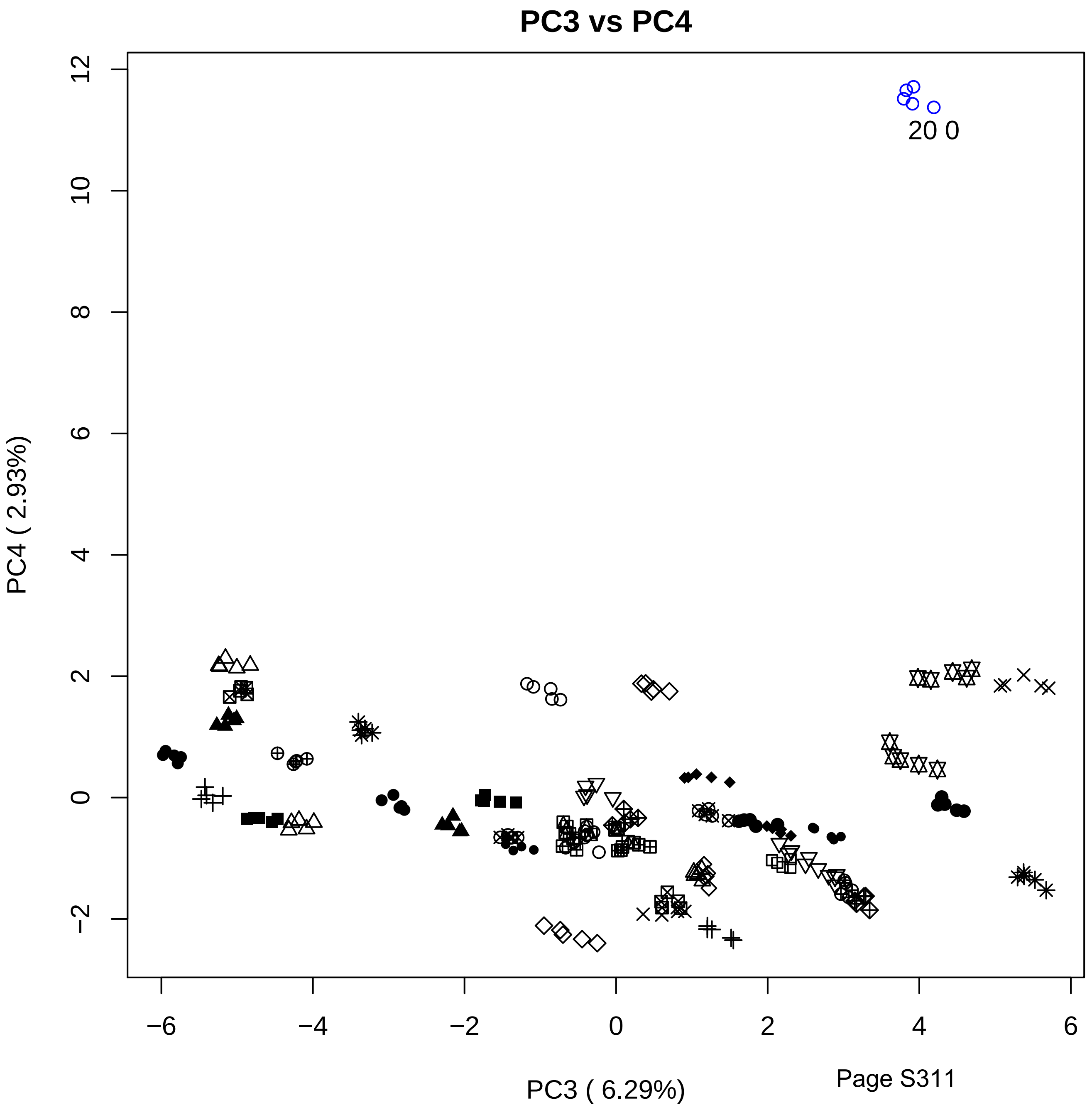

PC3 vs PC4
20 0
PC4 (2.93%)
PC3 (6.29%)
12
10
8
6
4
2
0
−2
−6
−4
−2
0
2
4
6

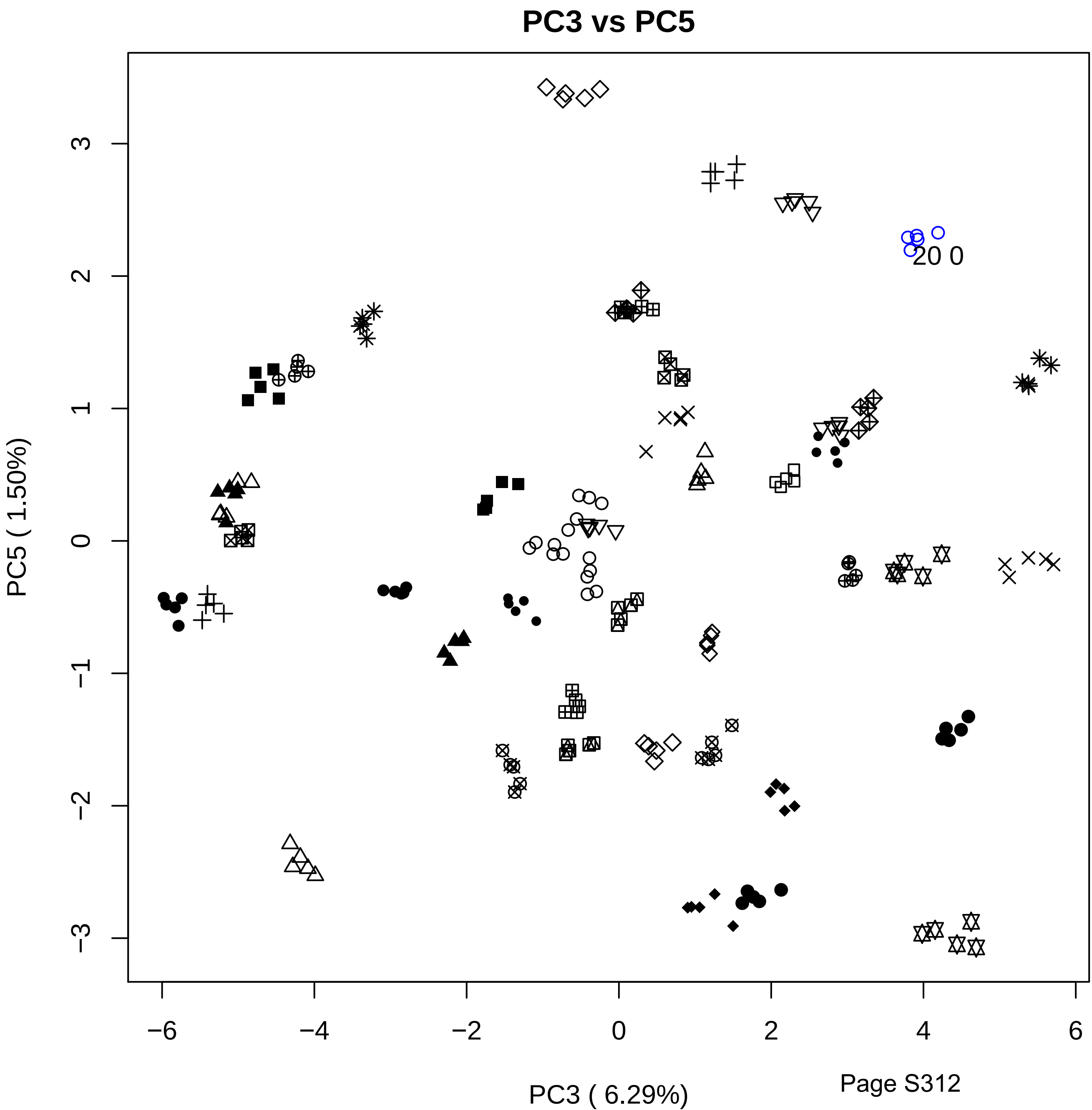
PC3 vs PC5
20 0
PC5 (1.50%)
PC3 (6.29%)
3
2
1
0
−1
−2
−3
−6
−4
−2
0
2
4
6

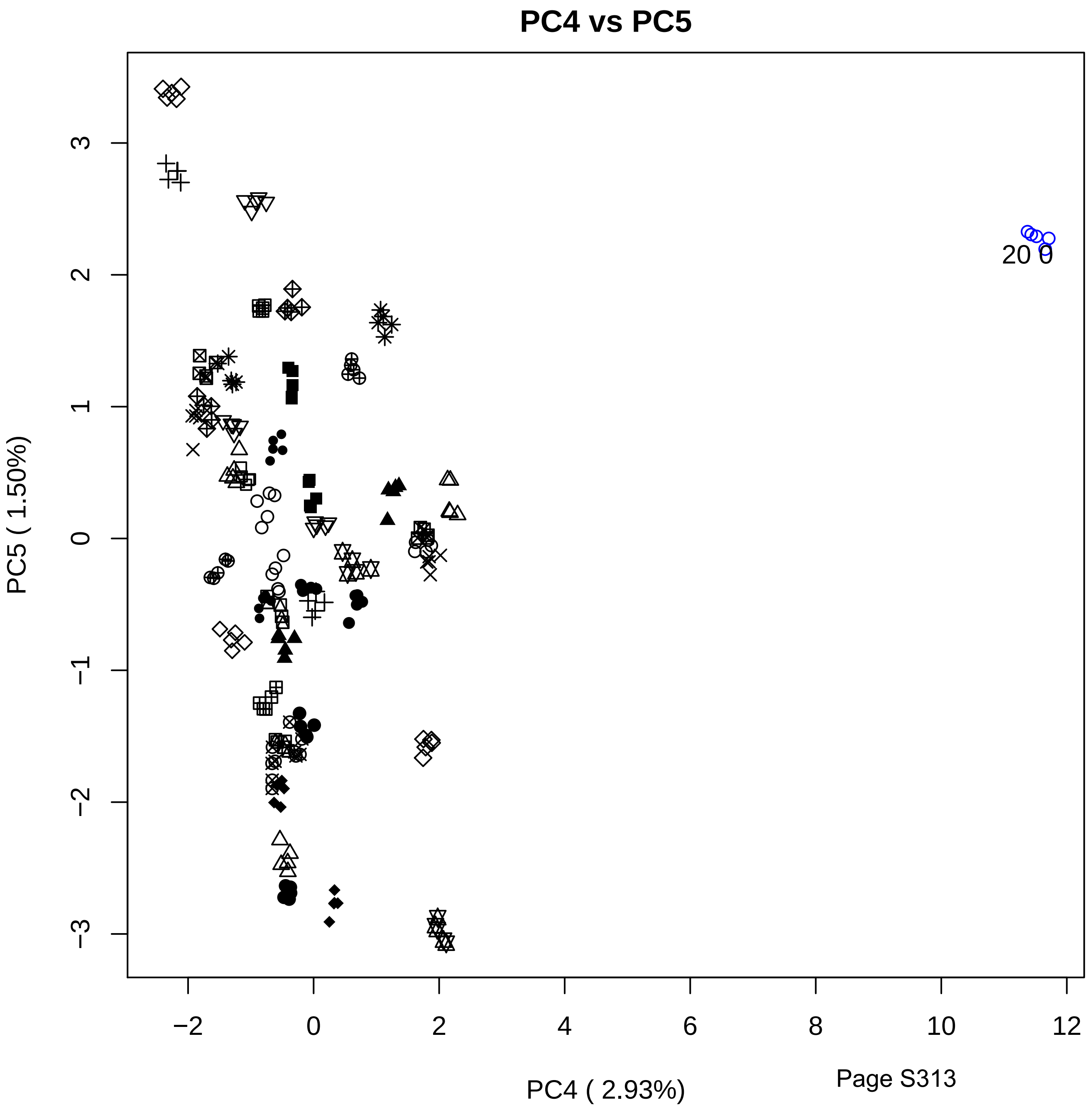
PC4 vs PC5
PC5 (1.50%)
PC4 (2.93%)
3
2
1
0
−1
−2
−3
−2
0
2
4
6
8
10
12
20 0

PCA of 40% OSCS vs PMH

Scaled Matrix PCAed

7th order polynomial baseline correction performed

Raw spectra smoothed to 21 Neighbours, 2nd order PN, Savtisky Golay

Normalisation between 0 and 100

D2 spectra smoothed to 41 Neighbours, 2nd order PN, Savtisky Golay

Regions Analysed: 3601−2501 and 2001−700cm−1

Samples Analysed: 1 Contam's 0 OSCS's 45 PMH's

Keele University AD Sun Dec 09 16:51:30 2018

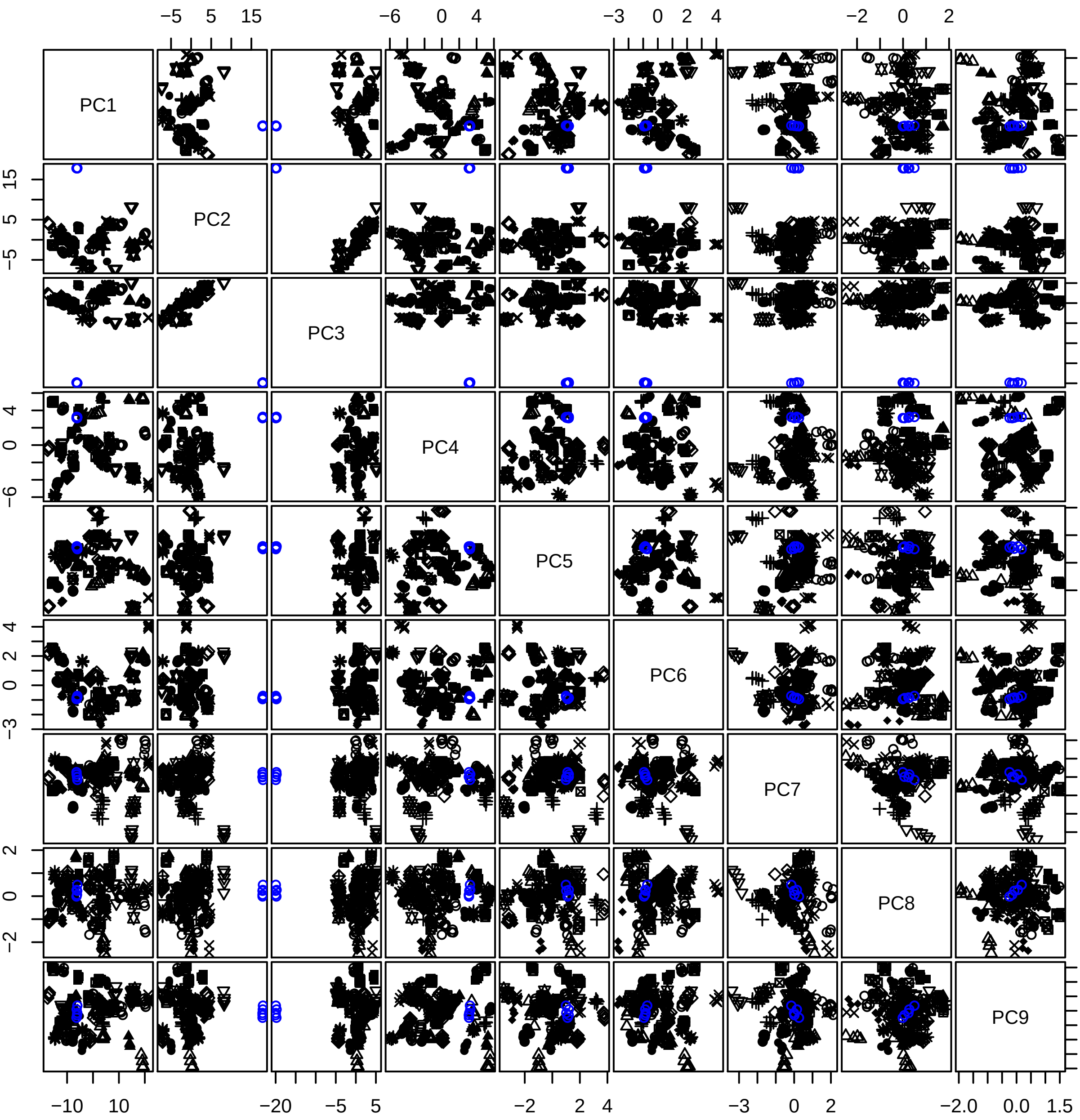
PC1
PC2
PC3
PC4
PC5
PC6
PC7
PC8
PC9

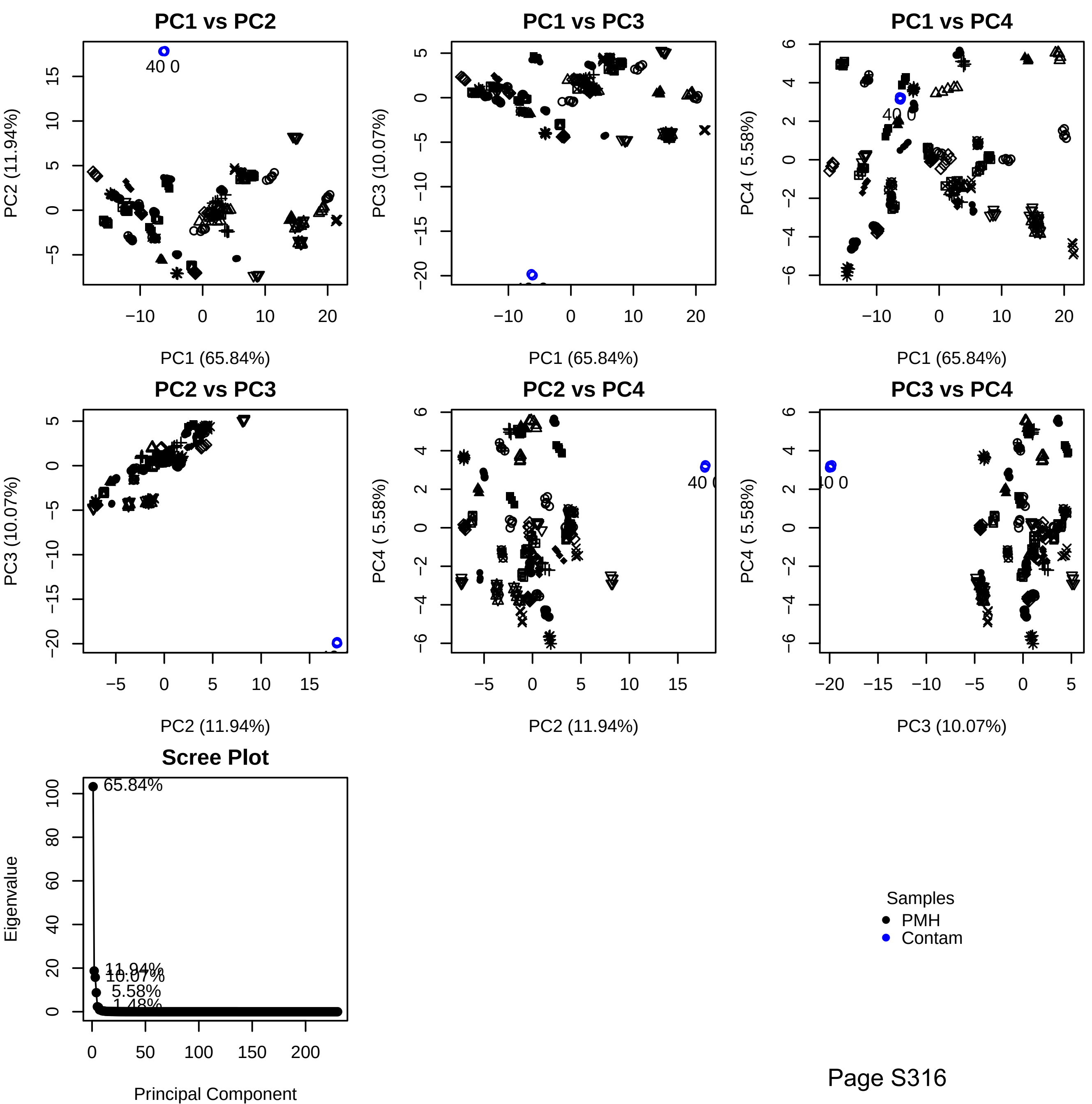
PC1 vs PC2
PC1 vs PC3
PC1 vs PC4
PC2 vs PC3
PC2 vs PC4
PC3 vs PC4
Scree Plot
PC1 (65.84%)
PC2 (11.94%)
PC3 (10.07%)
PC4 (5.58%)
40 0
Eigenvalue
Principal Component
65.84%
11.94%
10.07%
5.58%
1.48%
Samples
PMH
Contam

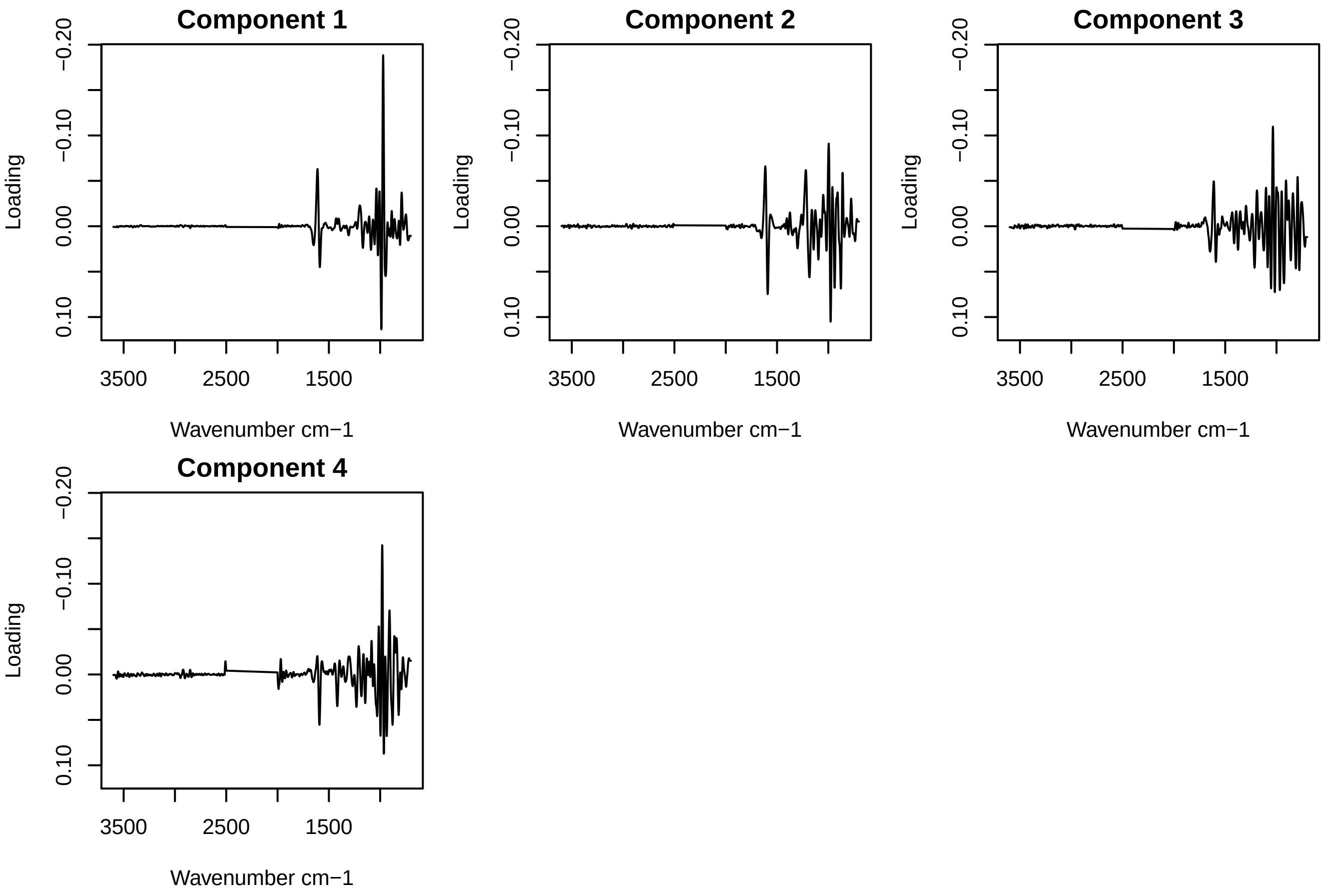
Component 1
Component 2
Component 3
Component 4
Loading
−0.20
−0.10
0.00
0.10
3500
2500
1500
Wavenumber cm−1

PC1 vs PC2 vs PC3

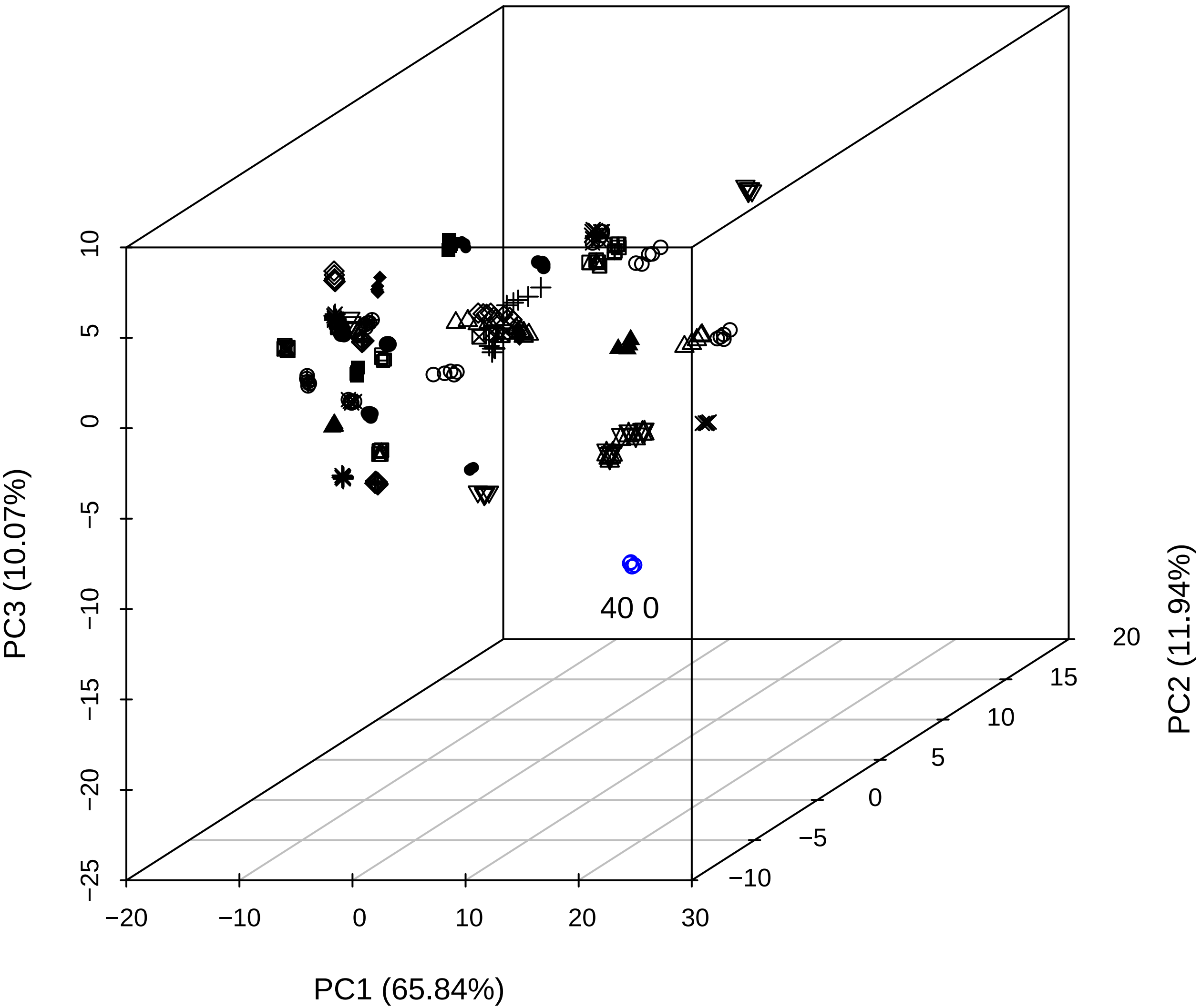

PC2 vs PC3 vs PC4

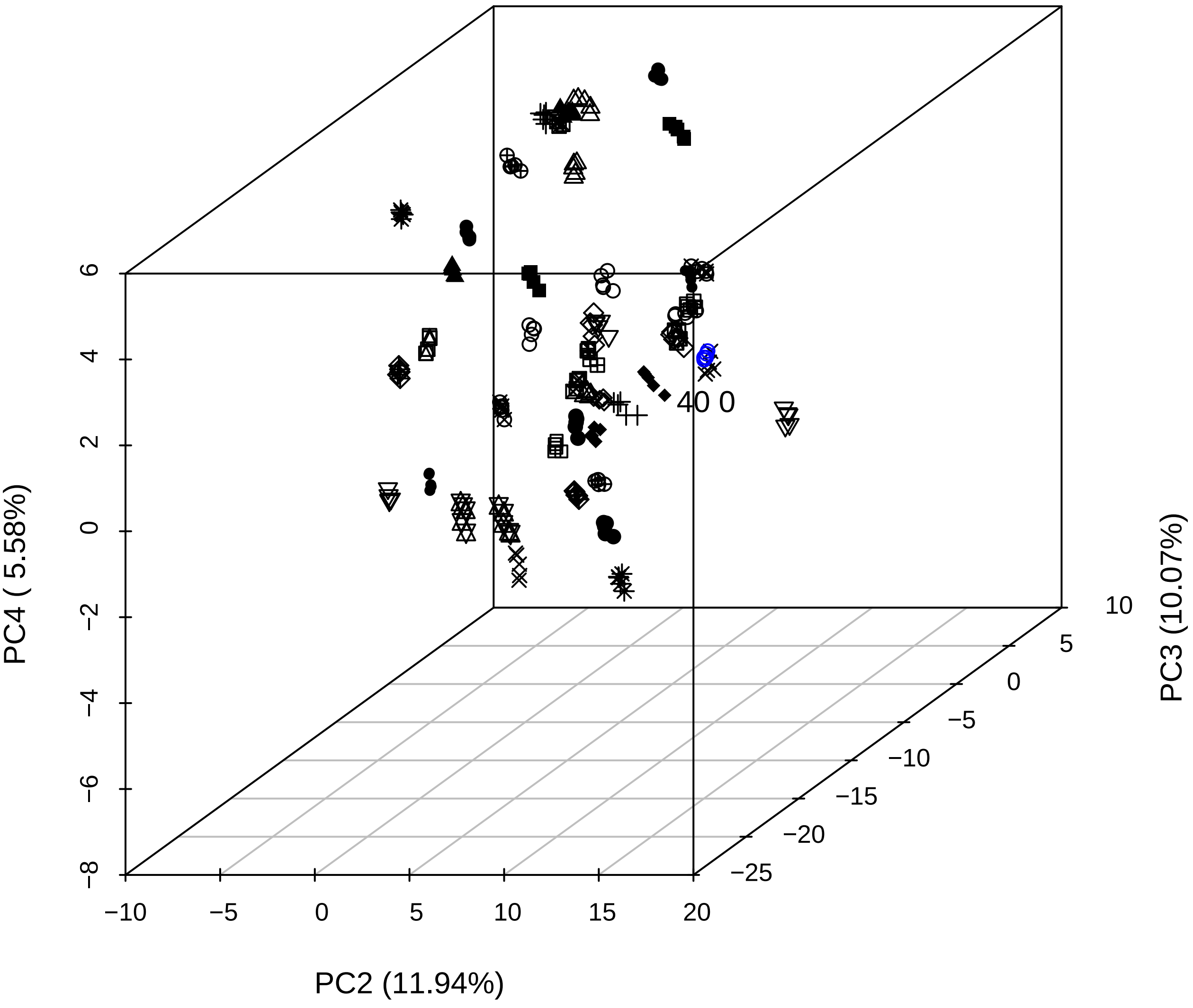

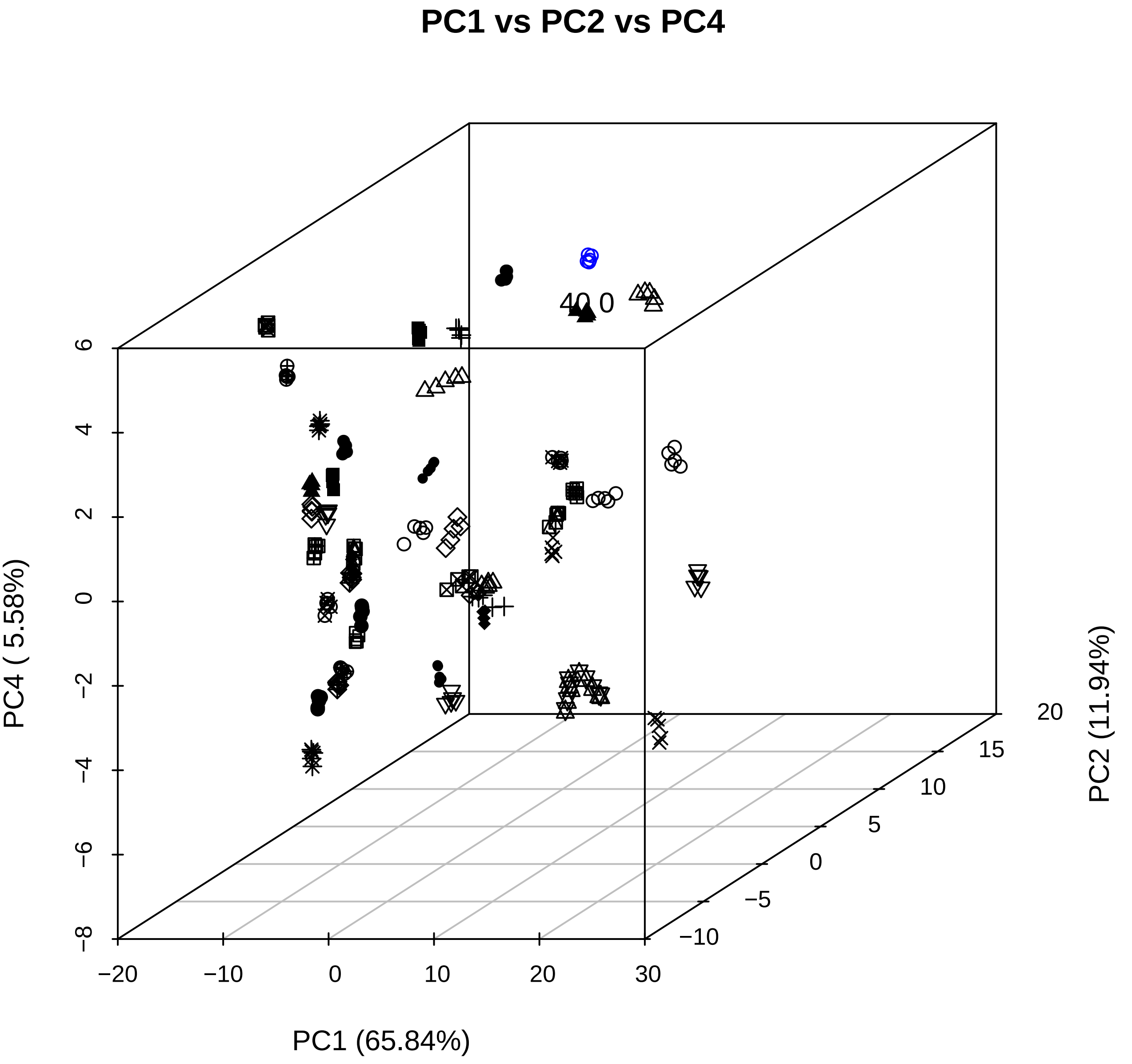
PC1 vs PC2 vs PC4
PC1 (65.84%)
PC2 (11.94%)
PC4 (5.58%)
−20
−10
0
10
20
30
−10
−5
0
5
10
15
20
−8
−6
−4
−2
0
2
4
6
40
0

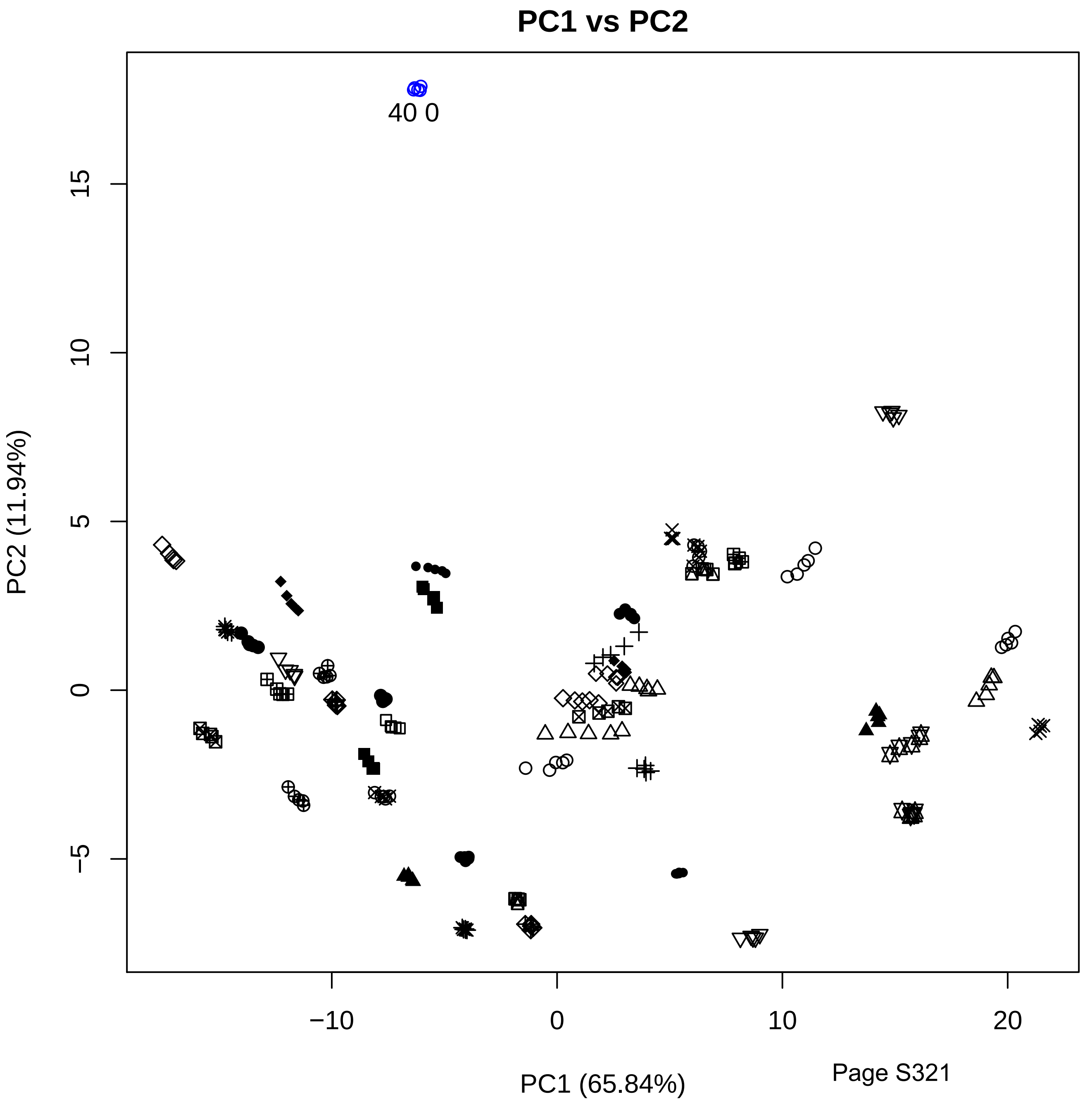
PC1 vs PC2
40 0
PC2 (11.94%)
PC1 (65.84%)
−10
0
10
20
−5
0
5
10
15

PC1 vs PC3

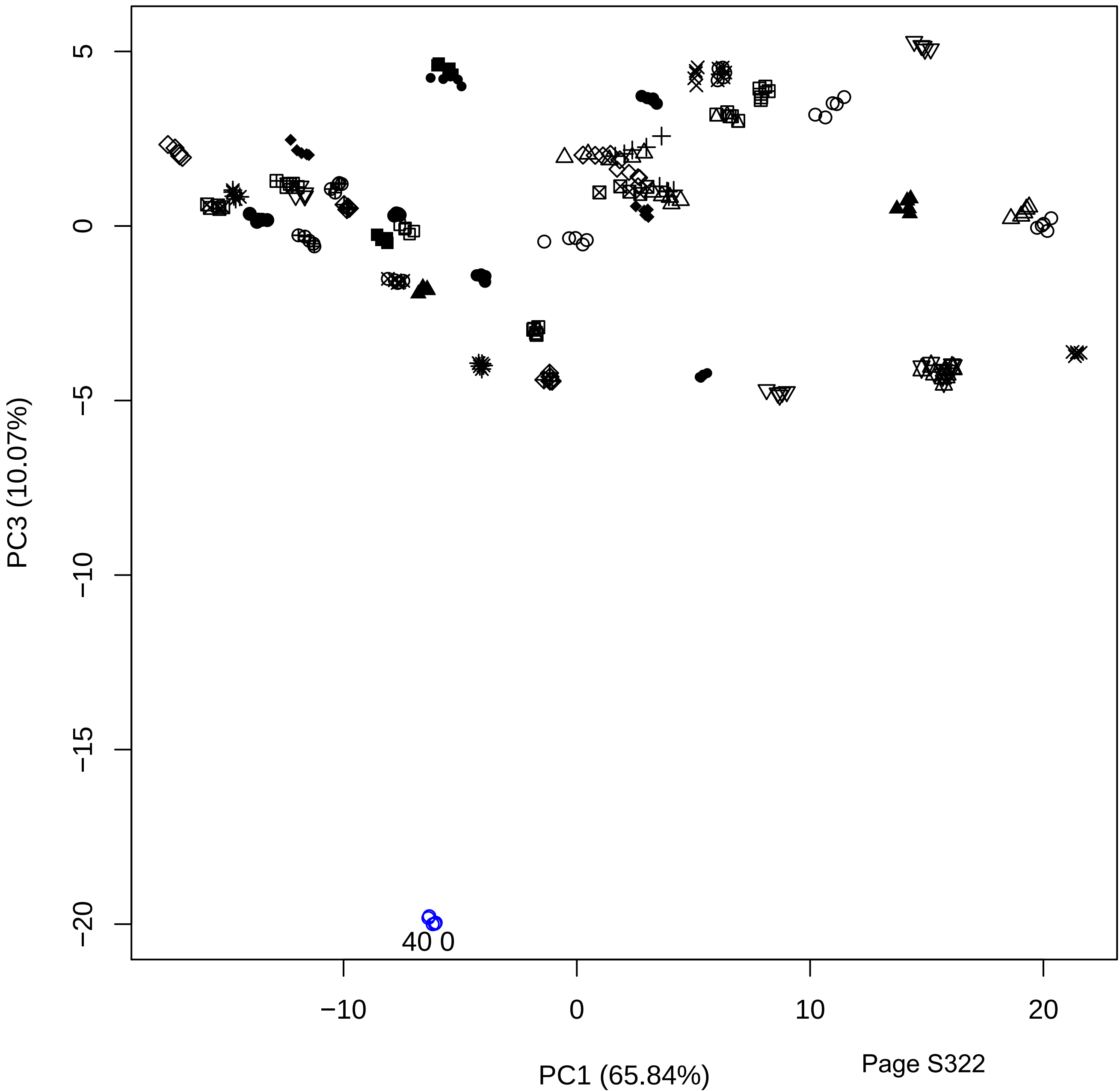

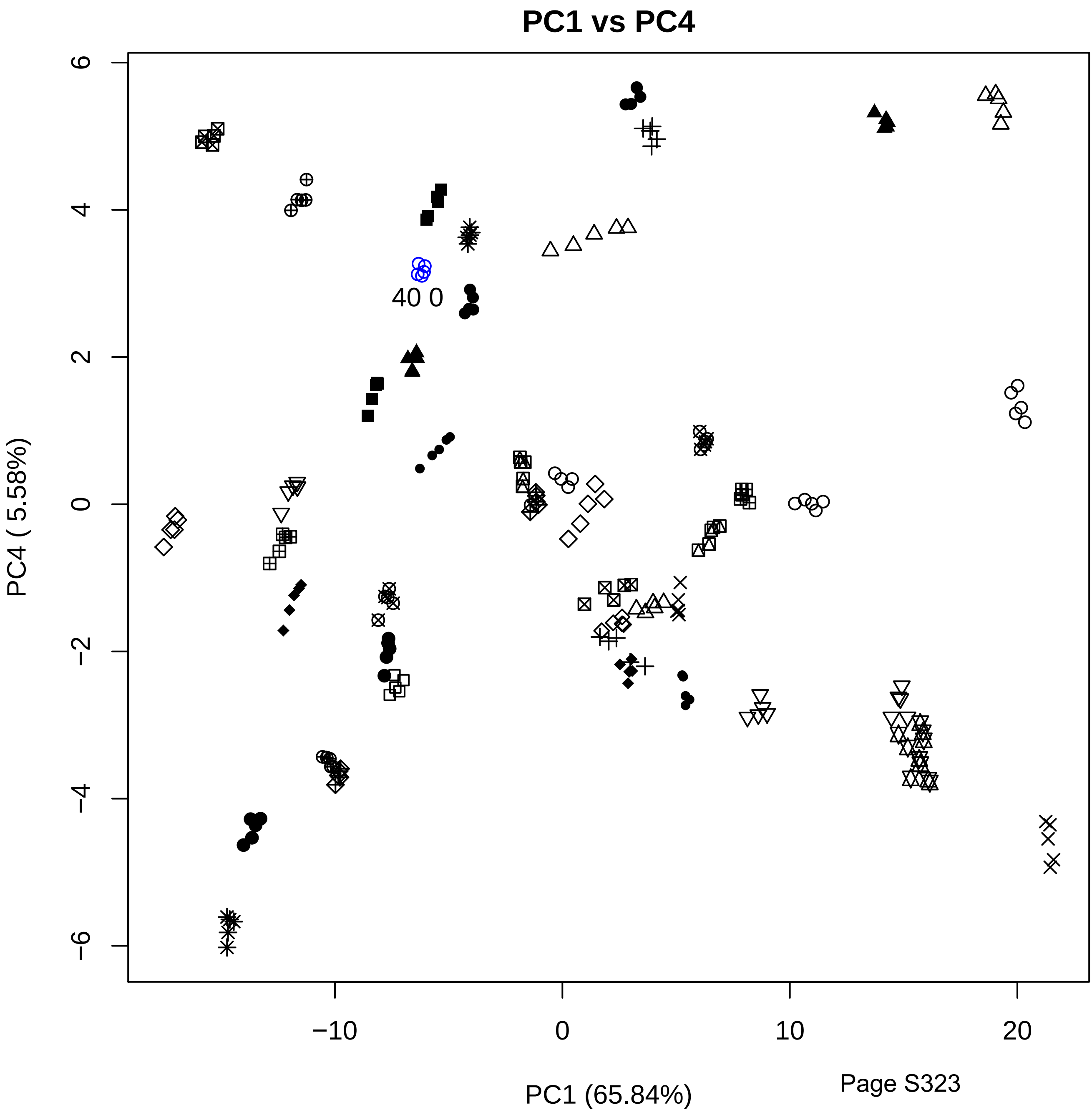
PC1 vs PC4
PC4 (5.58%)
PC1 (65.84%)
6
4
2
0
−2
−4
−6
−10
0
10
20
40 0

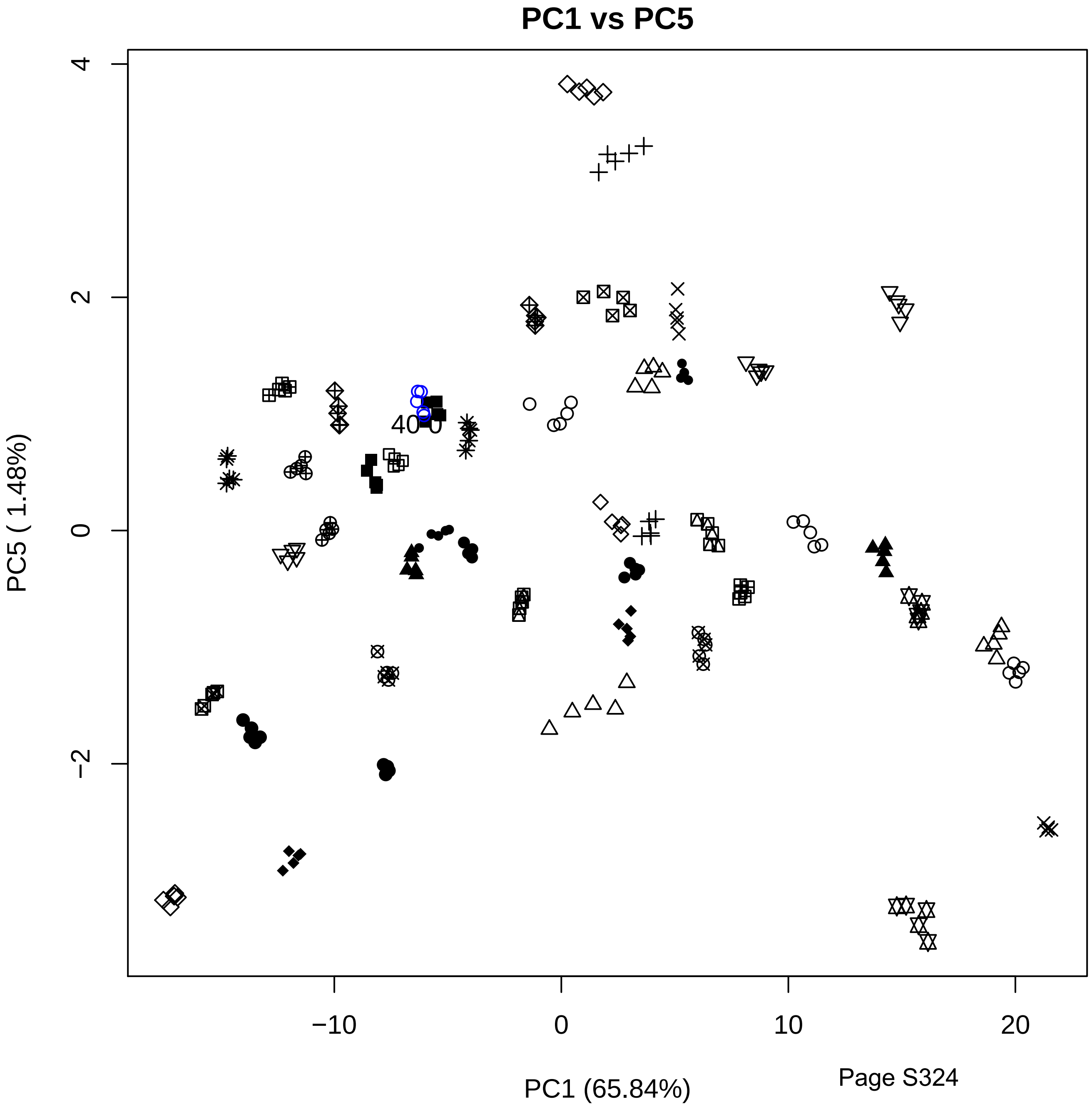
PC1 vs PC5
PC5 (1.48%)
PC1 (65.84%)
4
2
0
−2
−10
0
10
20

PC2 vs PC3

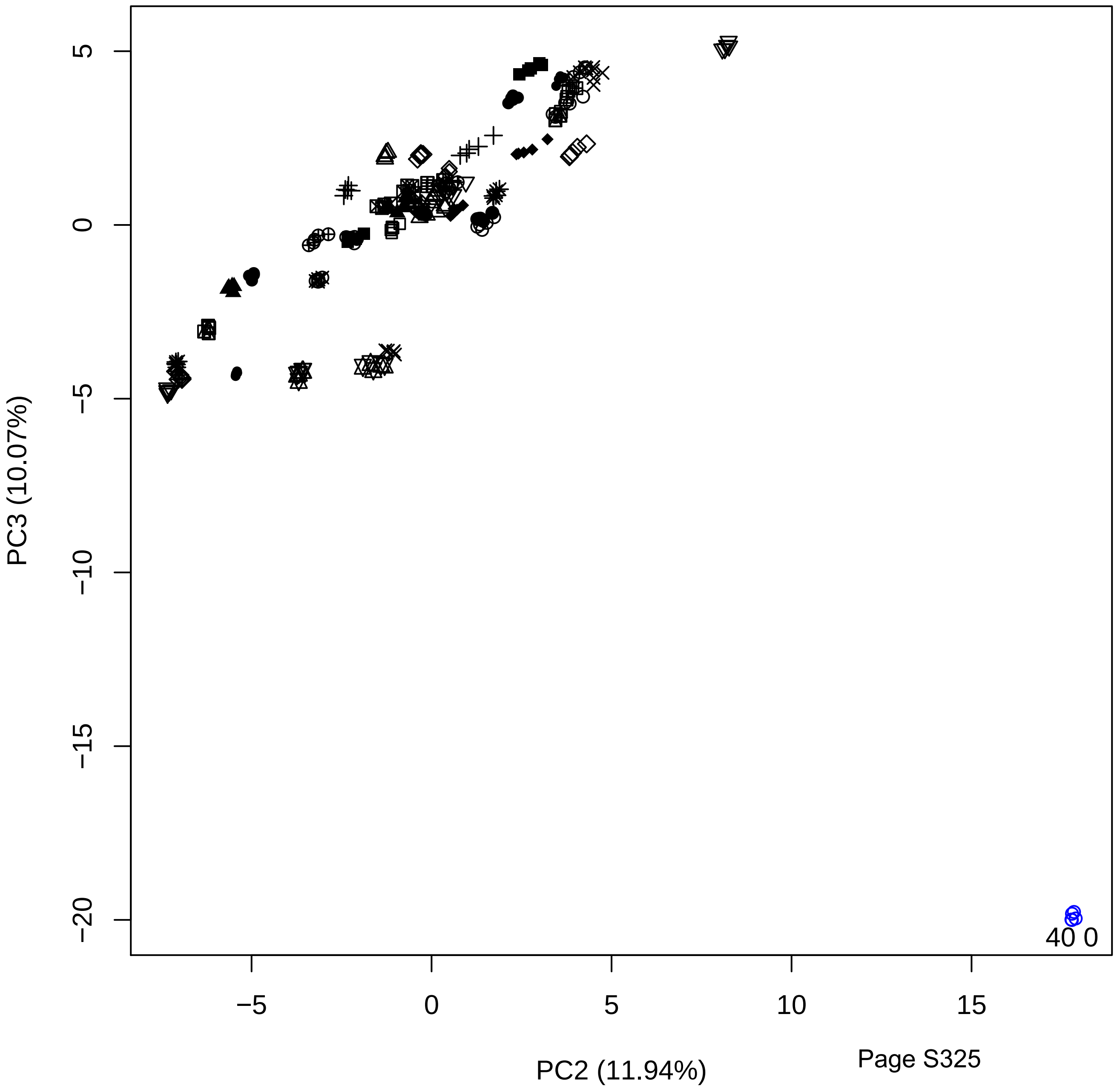

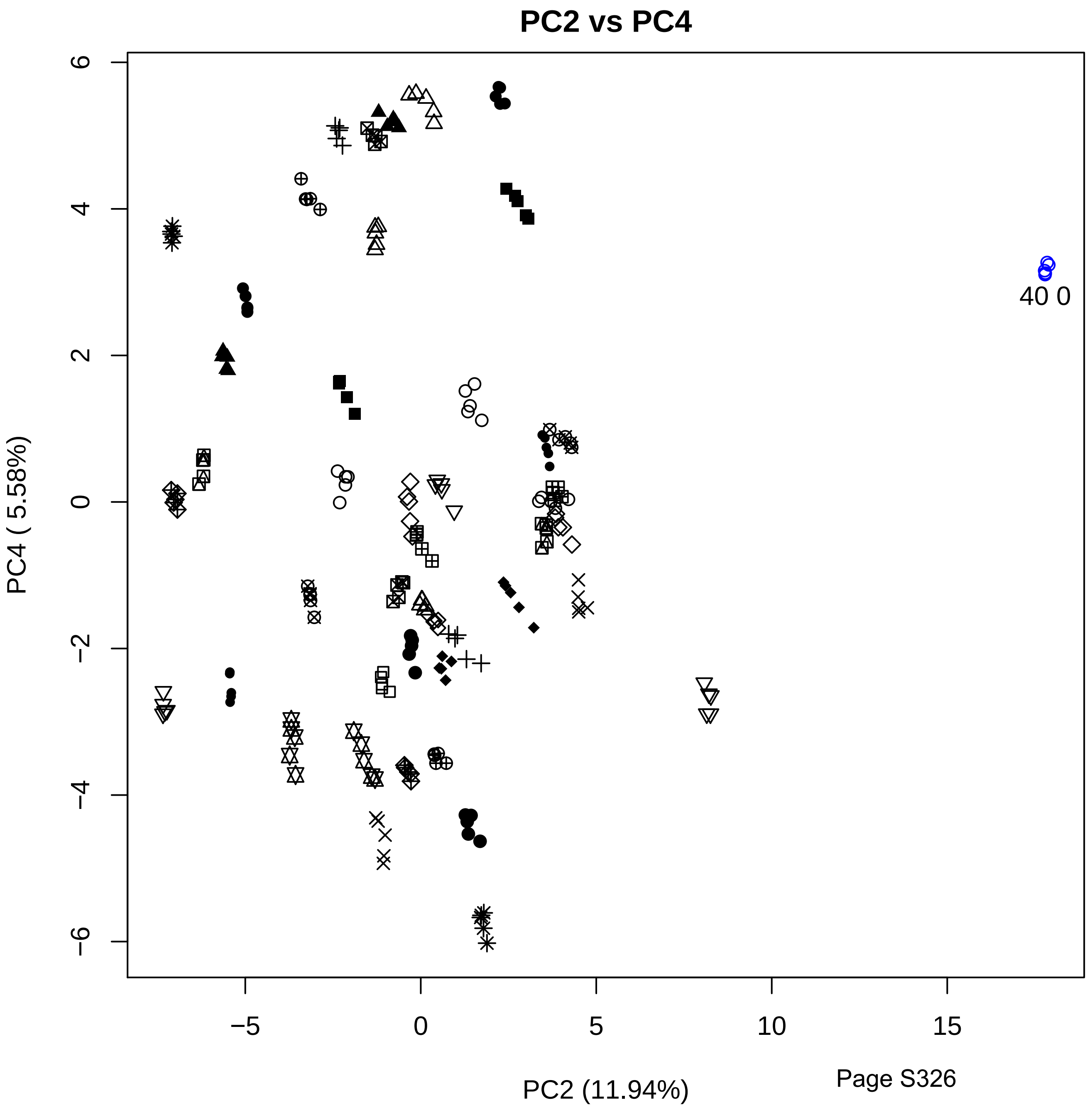

PC2 vs PC4
PC4 (5.58%)
PC2 (11.94%)
6
4
2
0
−2
−4
−6
−5
0
5
10
15
40 0

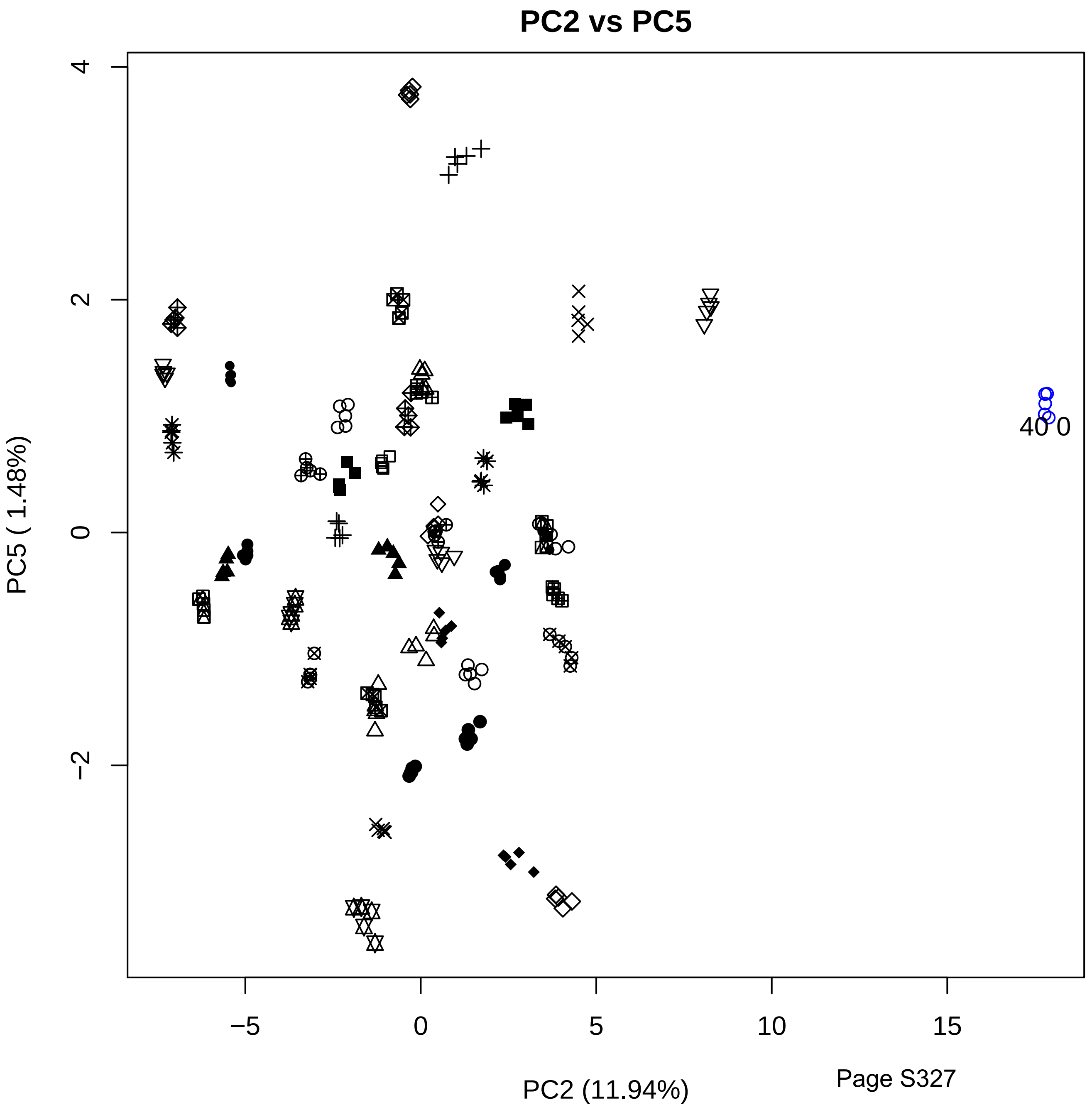
PC2 vs PC5
PC5 (1.48%)
PC2 (11.94%)
4
2
0
−2
−5
0
5
10
15
40 0

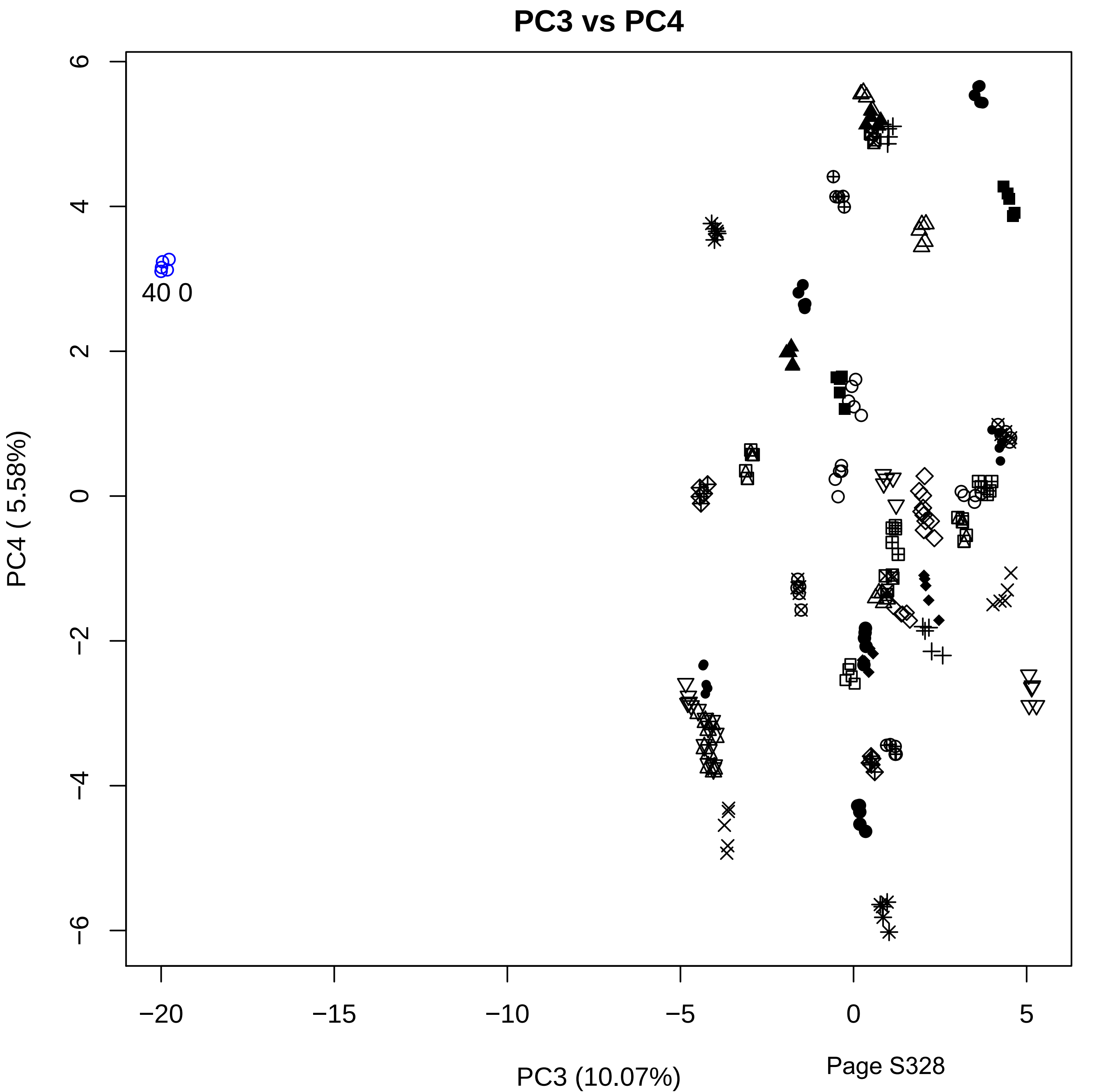
PC3 vs PC4
PC4 (5.58%)
PC3 (10.07%)
6
4
2
0
−2
−4
−6
−20
−15
−10
−5
0
5
40 0

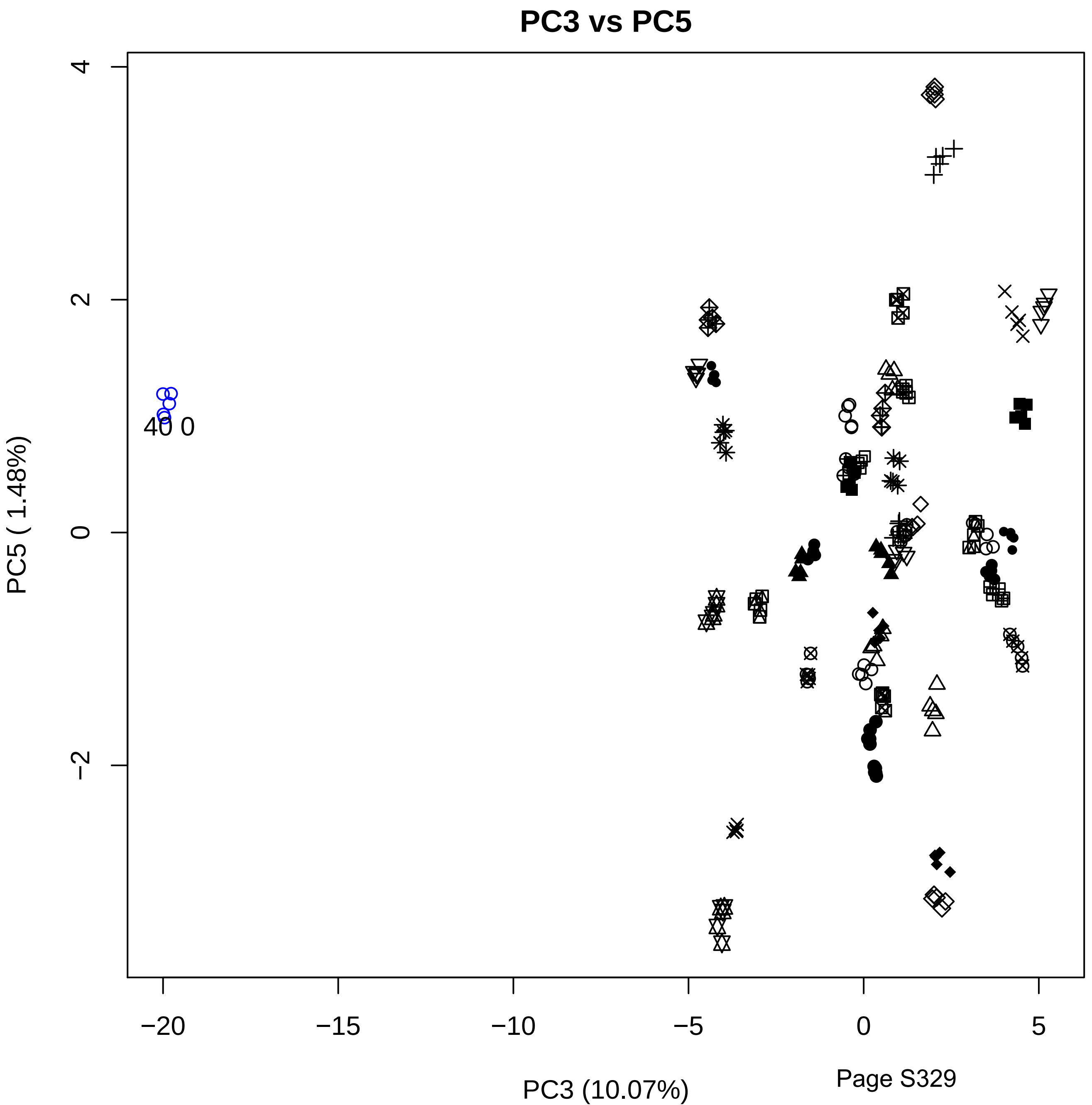

PC3 vs PC5
4
2
0
−2
PC5 (1.48%)
40 0
−20
−15
−10
−5
0
5
PC3 (10.07%)

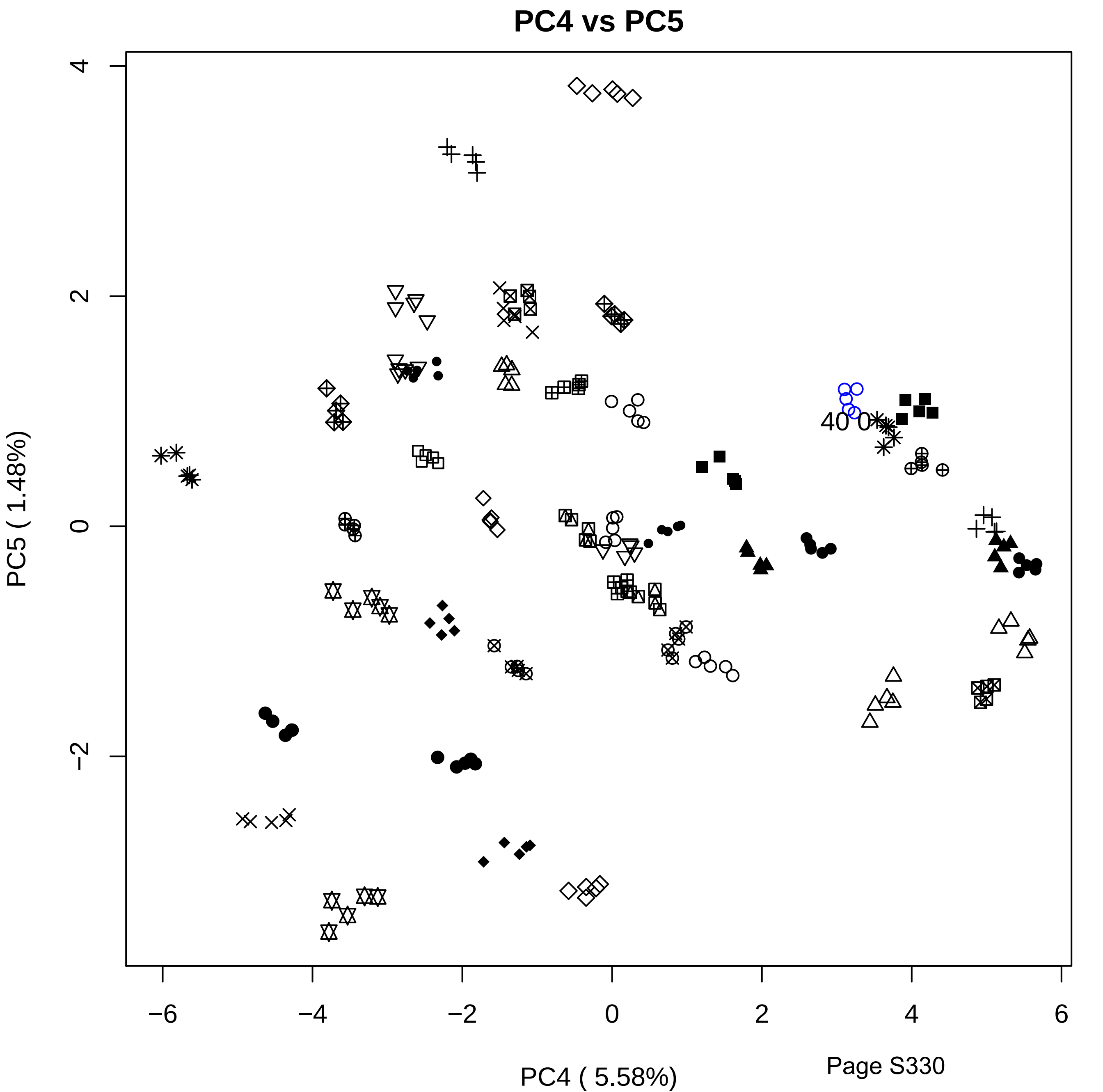
PC4 vs PC5
PC5 (1.48%)
PC4 (5.58%)
4
2
0
−2
−6
−4
−2
0
2
4
6
40 0

PCA of 100% OSCS vs PMH

Scaled Matrix PCAed

7th order polynomial baseline correction performed

Raw spectra smoothed to 21 Neighbours, 2nd order PN, Savtisky Golay

Normalisation between 0 and 100

D2 spectra smoothed to 41 Neighbours, 2nd order PN, Savtisky Golay

Regions Analysed: 3601−2501 and 2001−700cm−1

Samples Analysed: 0 Contam's 1 OSCS's 45 PMH's

Keele University AD Sun Dec 09 16:51:35 2018

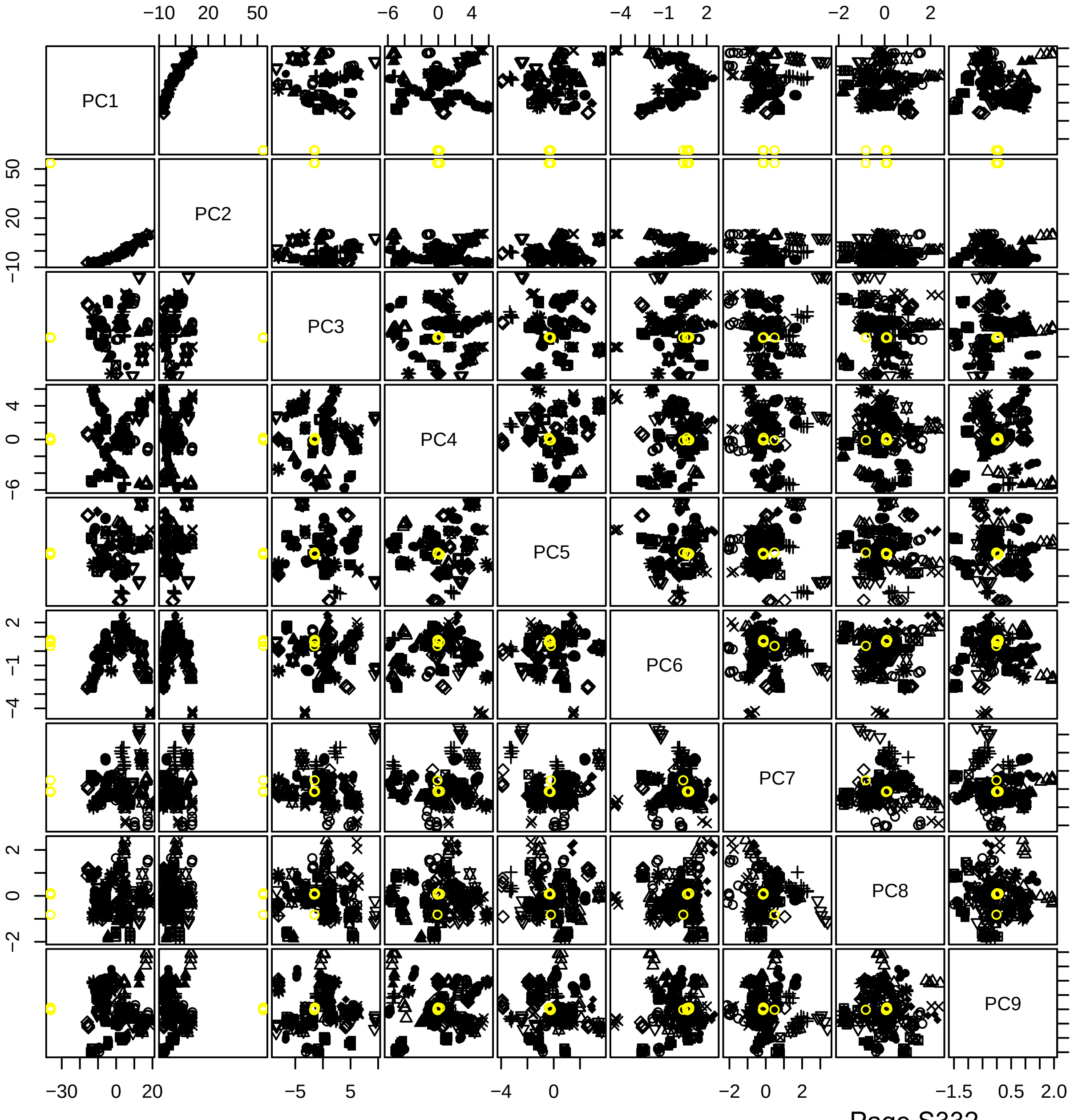
PC1
PC2
PC3
PC4
PC5
PC6
PC7
PC8
PC9
−10 20 50
−6 0 4
−4 −1 2
−2 0 2
20 0 −30
5 −5
0 −4
2 −1 −4
2 0 −2
2.0 0.5 −1.5
−30 0 20
−5 5
−4 0
−2 0 2
−1.5 0.5 2.0

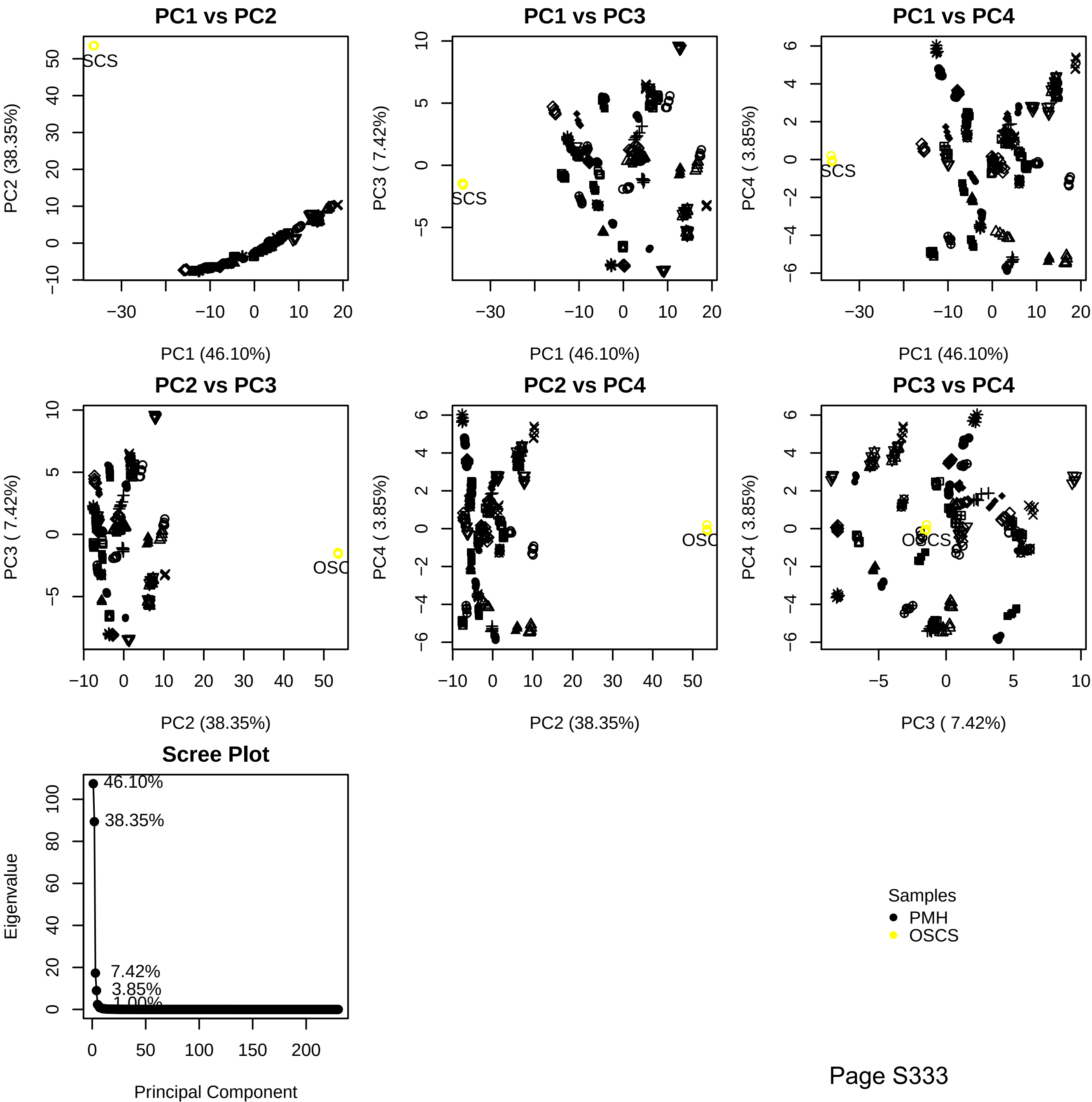
PC1 vs PC2
PC1 vs PC3
PC1 vs PC4
PC2 vs PC3
PC2 vs PC4
PC3 vs PC4
Scree Plot
PC1 (46.10%)
PC2 (38.35%)
PC3 (7.42%)
PC4 (3.85%)
SCS
OSC
OSCS
Eigenvalue
Principal Component
46.10%
38.35%
7.42%
3.85%
1.00%
Samples
PMH
OSCS

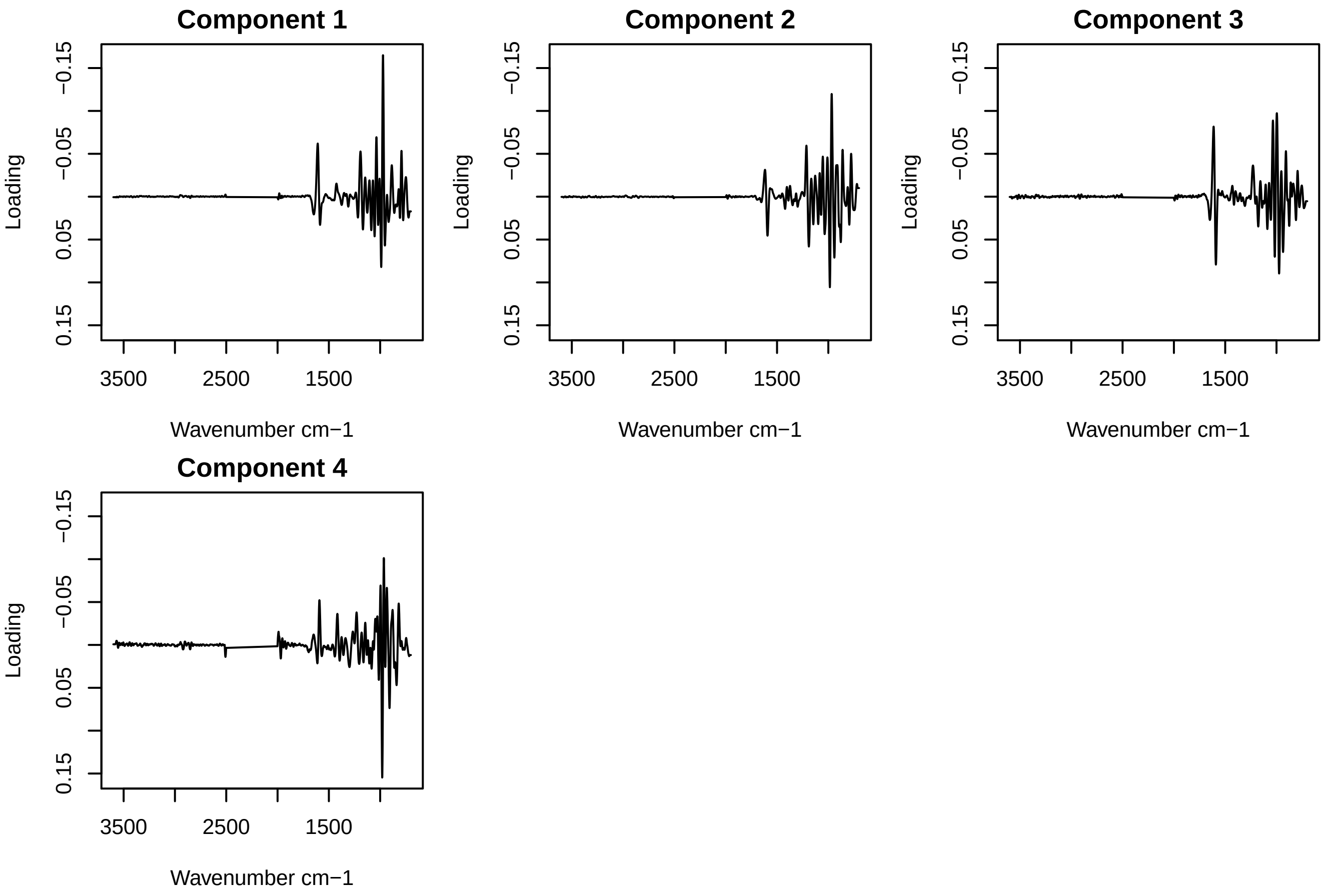
Component 1
Component 2
Component 3
Component 4
Loading
−0.15
−0.05
0.05
0.15
3500
2500
1500
Wavenumber cm−1

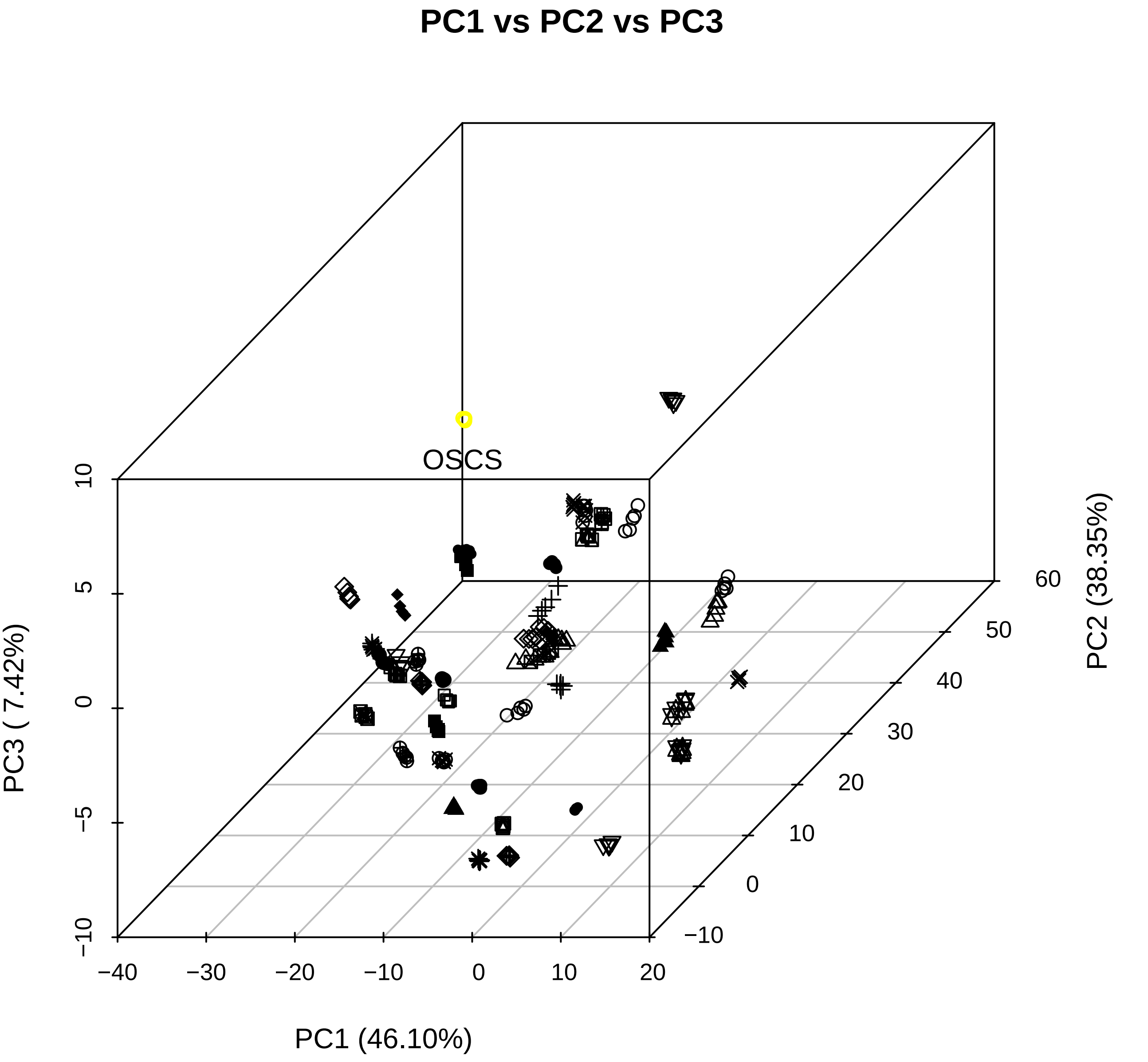
PC1 vs PC2 vs PC3
OSCS
PC1 (46.10%)
PC2 (38.35%)
PC3 (7.42%)
−40
−30
−20
−10
0
10
20
−10
0
10
20
30
40
50
60
−10
−5
0
5
10

PC2 vs PC3 vs PC4

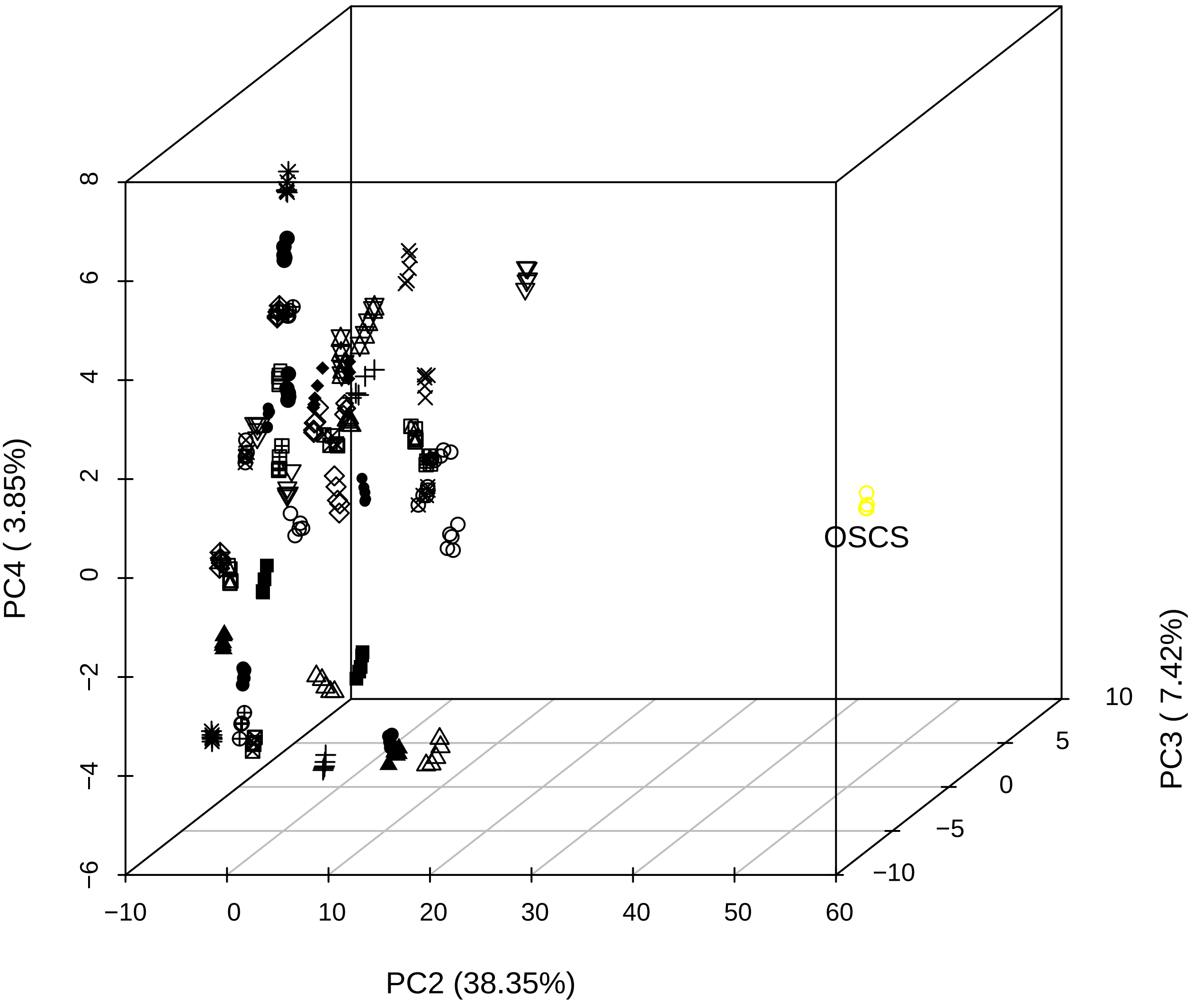

PC1 vs PC2 vs PC4

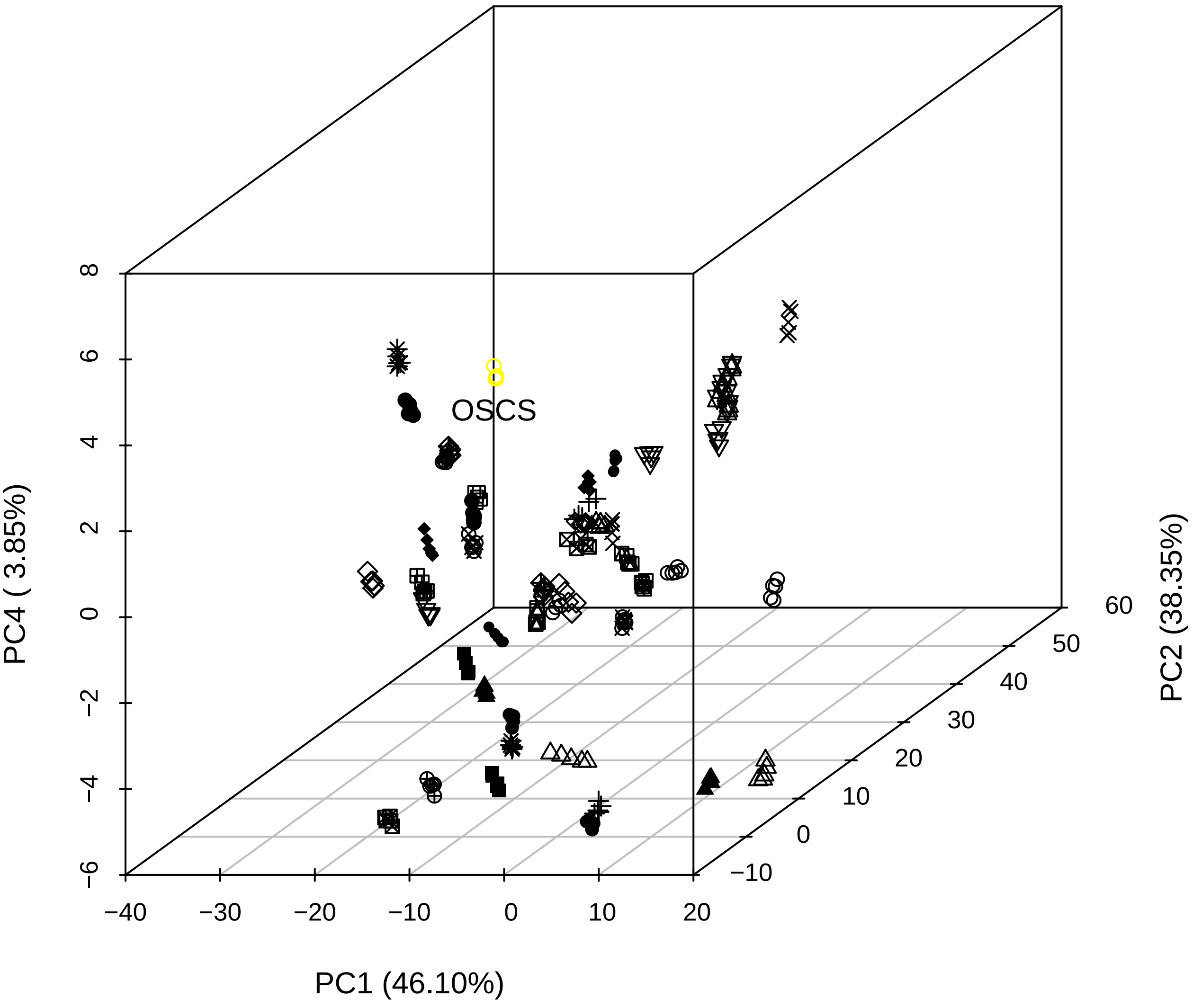

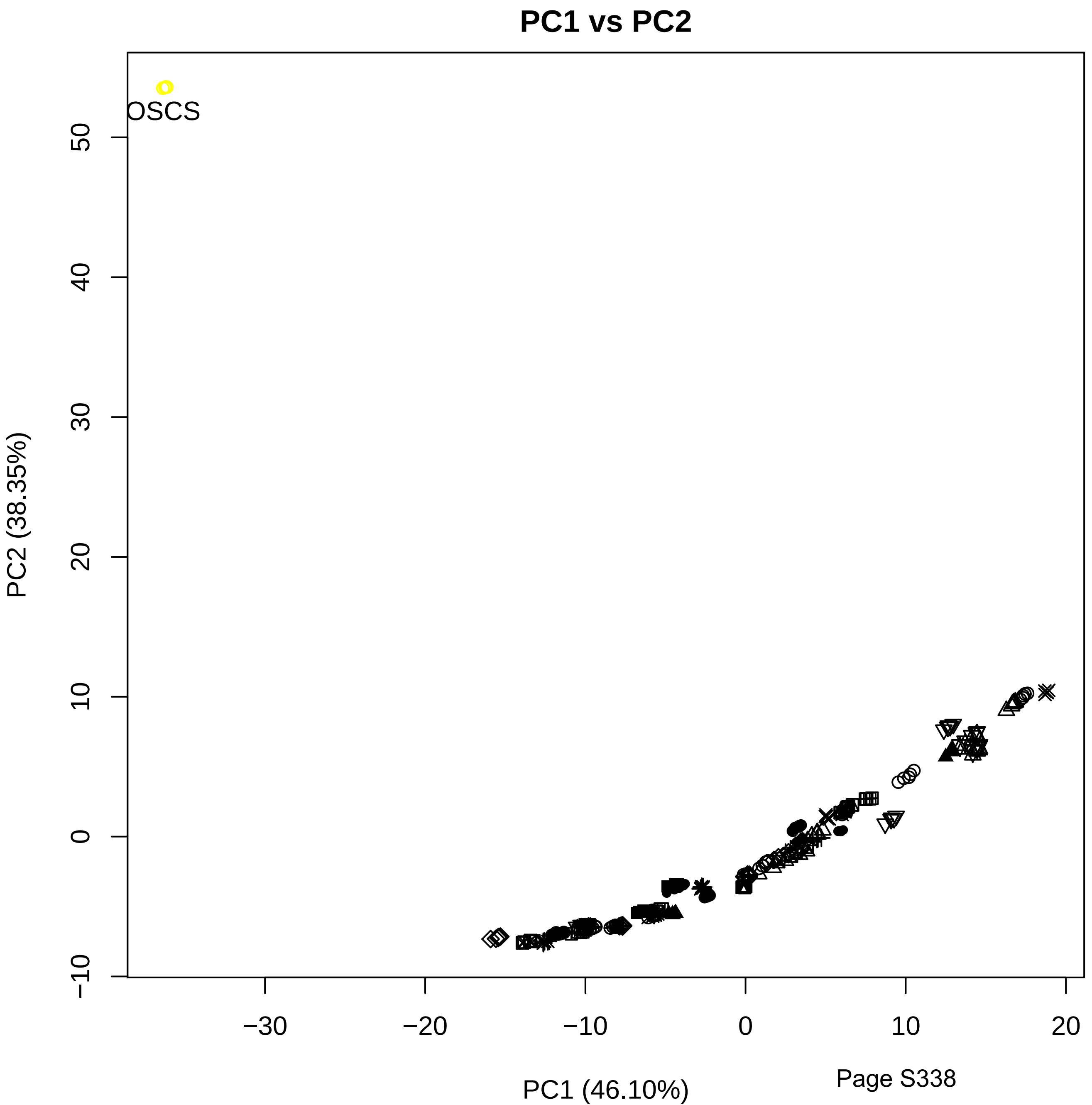
PC1 vs PC2
OSCS
PC2 (38.35%)
PC1 (46.10%)
50
40
30
20
10
0
−10
−30
−20
−10
0
10
20

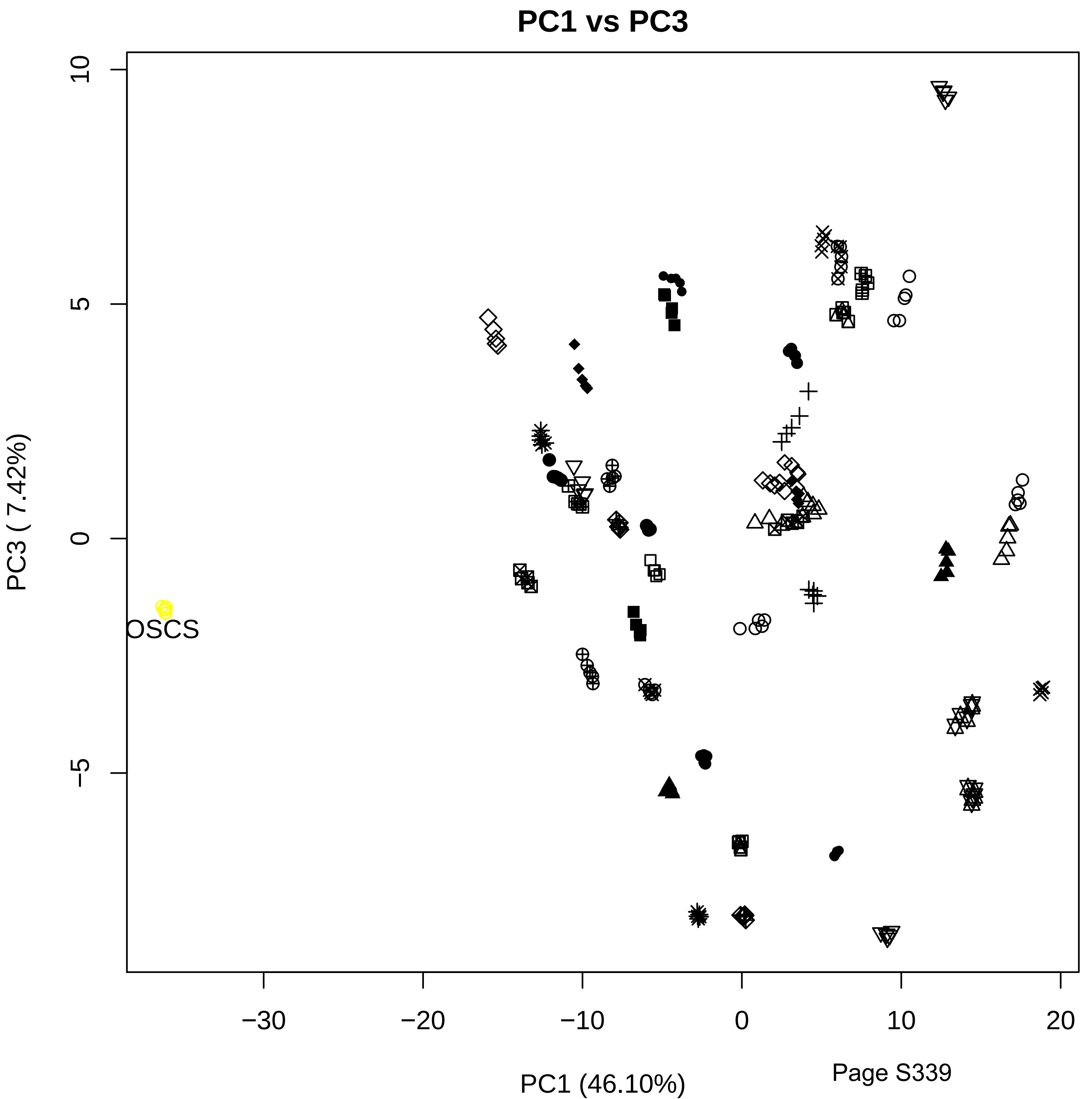

PC1 vs PC3
PC3 (7.42%)
PC1 (46.10%)
OSCS
10
5
0
−5
−30
−20
−10
0
10
20

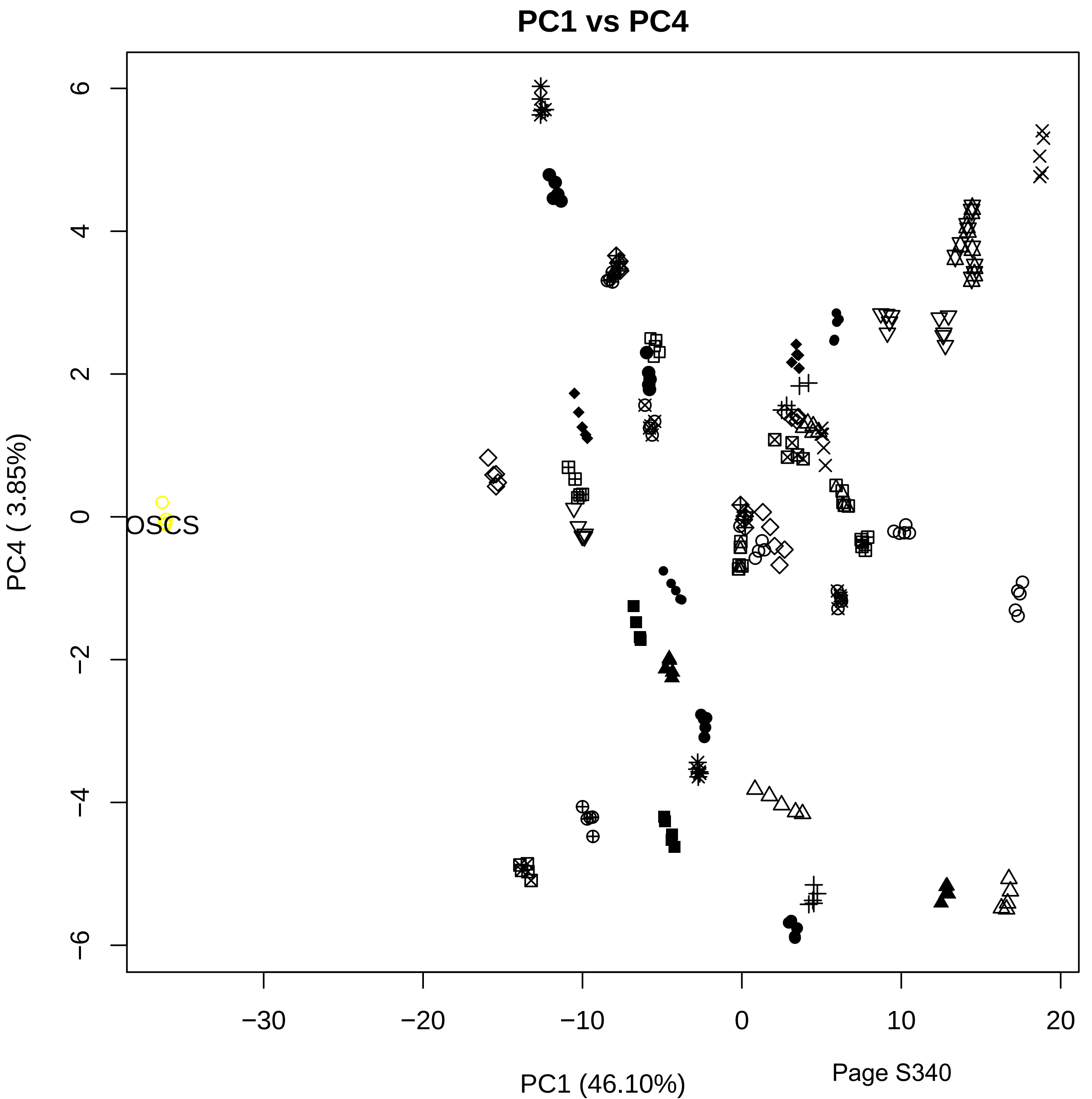
PC1 vs PC4
PC4 (3.85%)
PC1 (46.10%)
OSCS
6
4
2
0
−2
−4
−6
−30
−20
−10
0
10
20

PC1 vs PC5

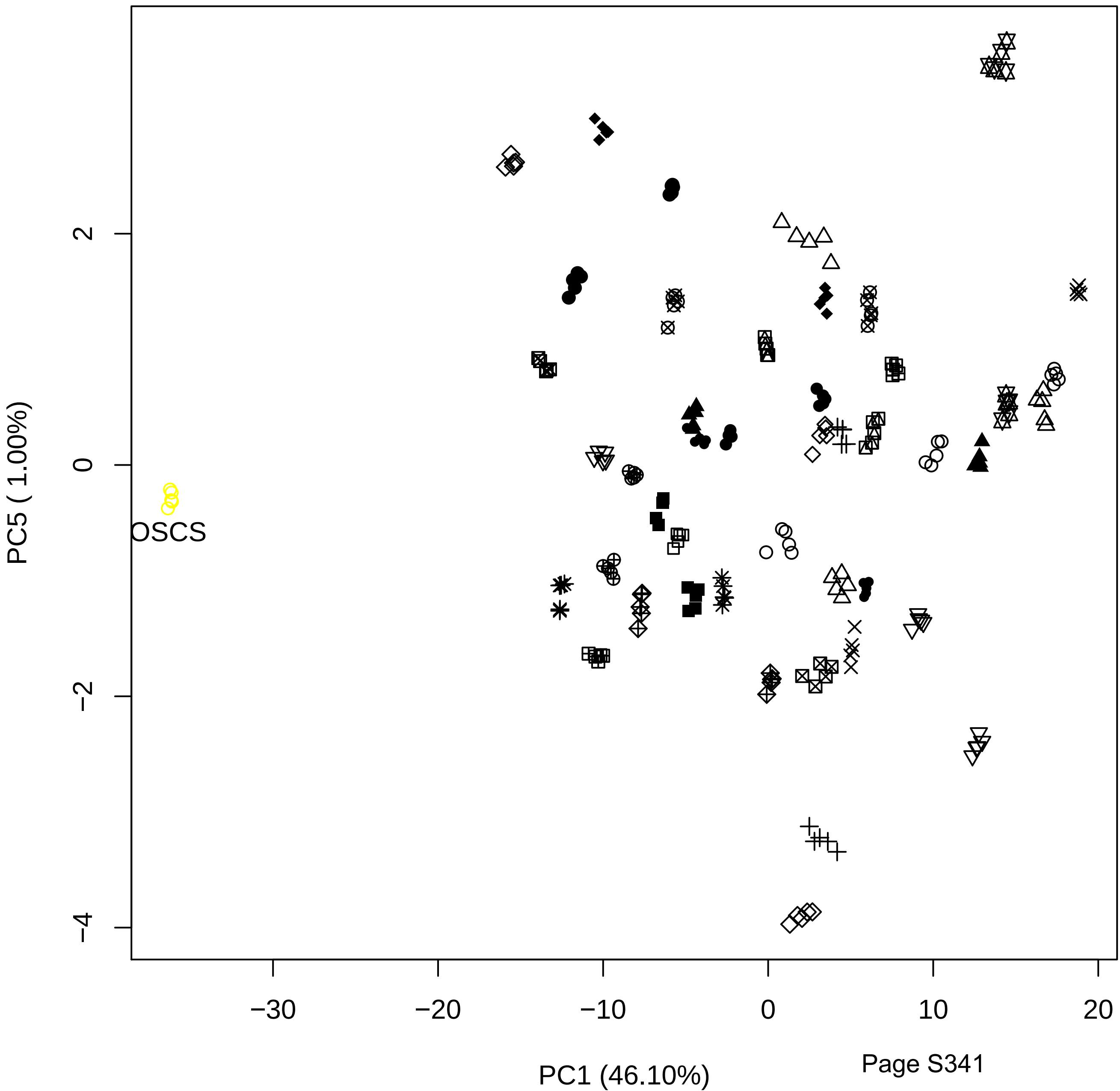

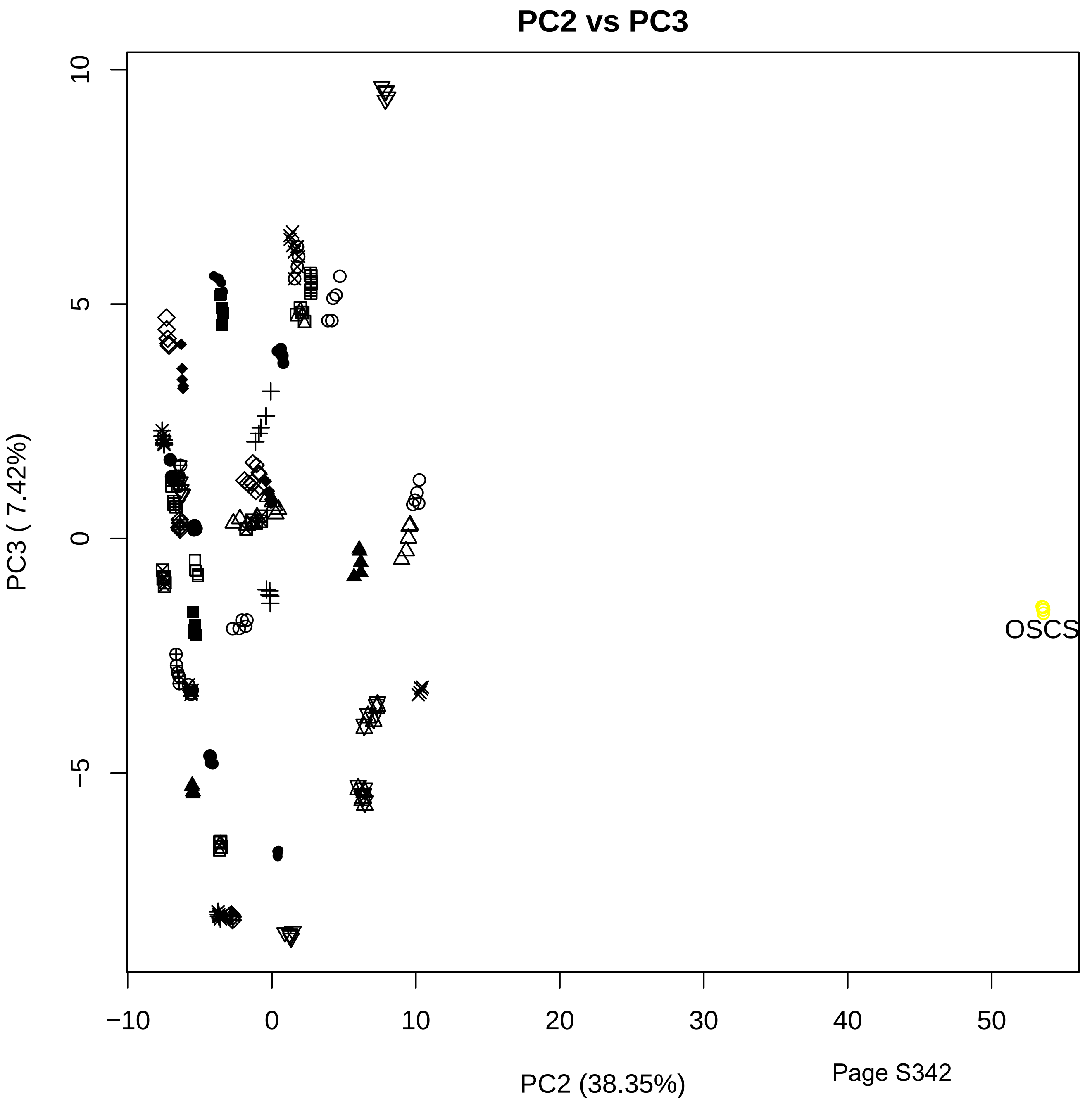
PC2 vs PC3
PC3 (7.42%)
PC2 (38.35%)
10
5
0
−5
−10
0
10
20
30
40
50
OSCS

PC2 vs PC4

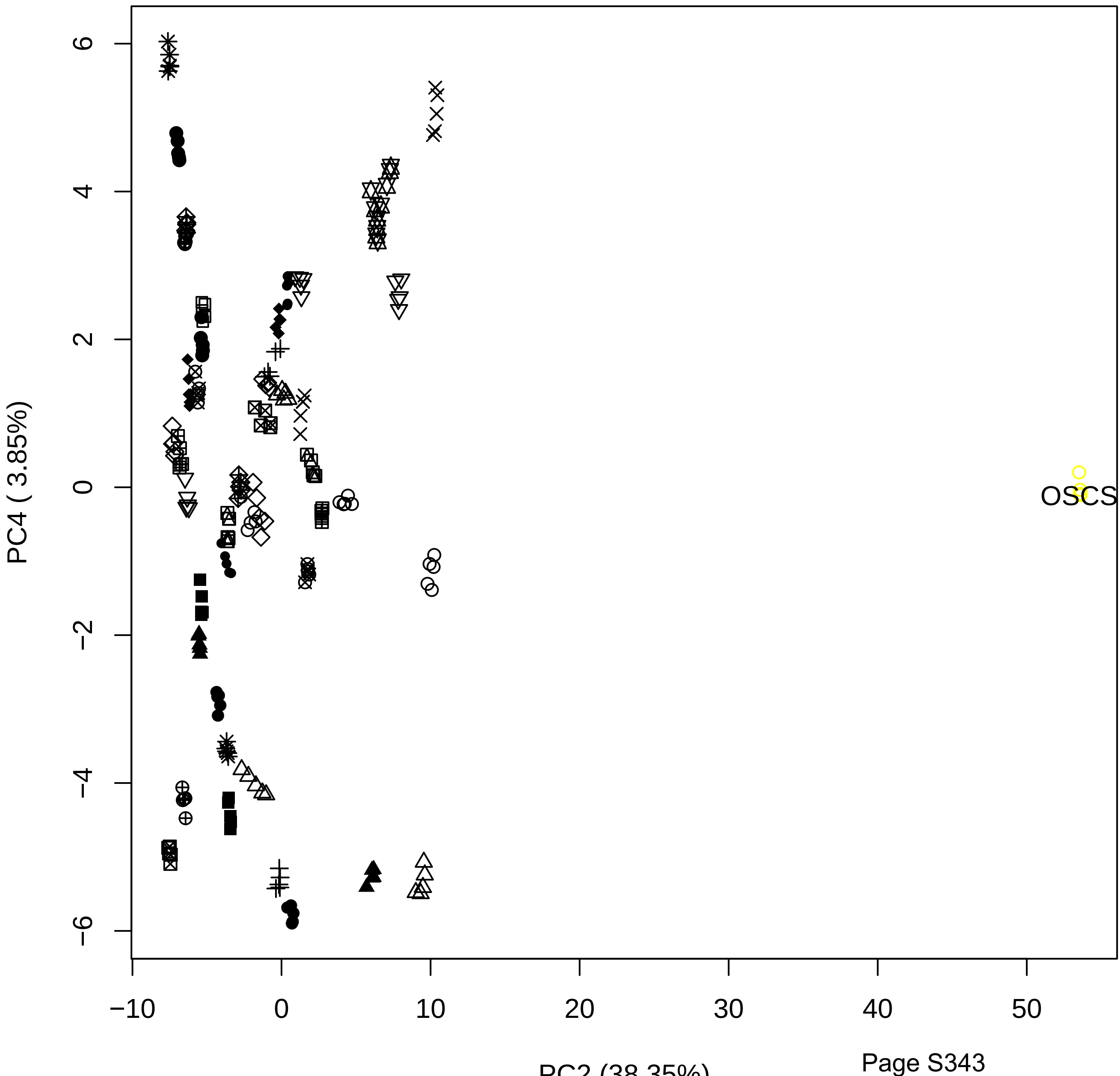

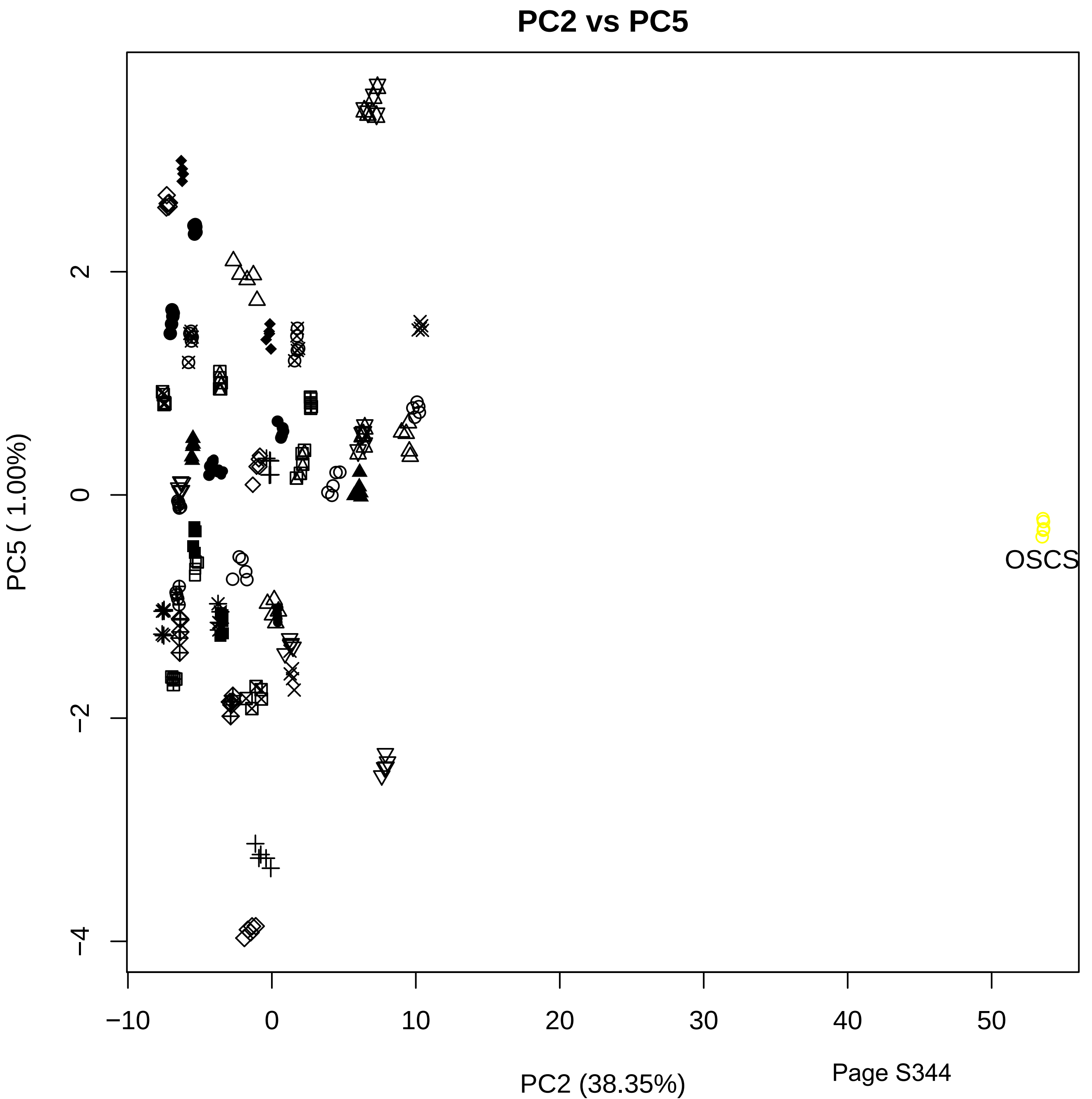
PC2 vs PC5
PC5 (1.00%)
PC2 (38.35%)
2
0
−2
−4
−10
0
10
20
30
40
50
OSCS

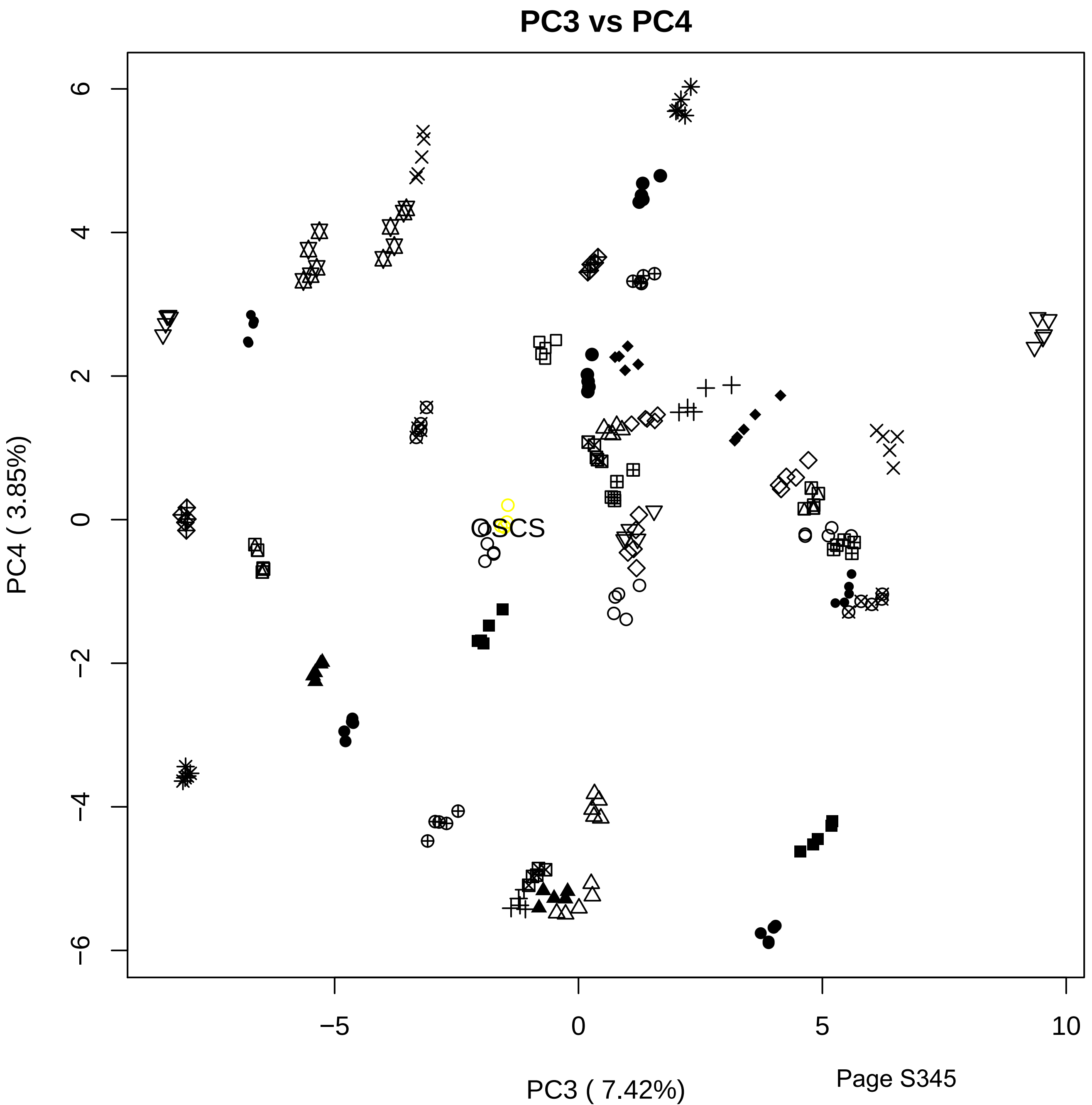
PC3 vs PC4
PC4 (3.85%)
PC3 (7.42%)
OSCS
6
4
2
0
−2
−4
−6
−5
0
5
10

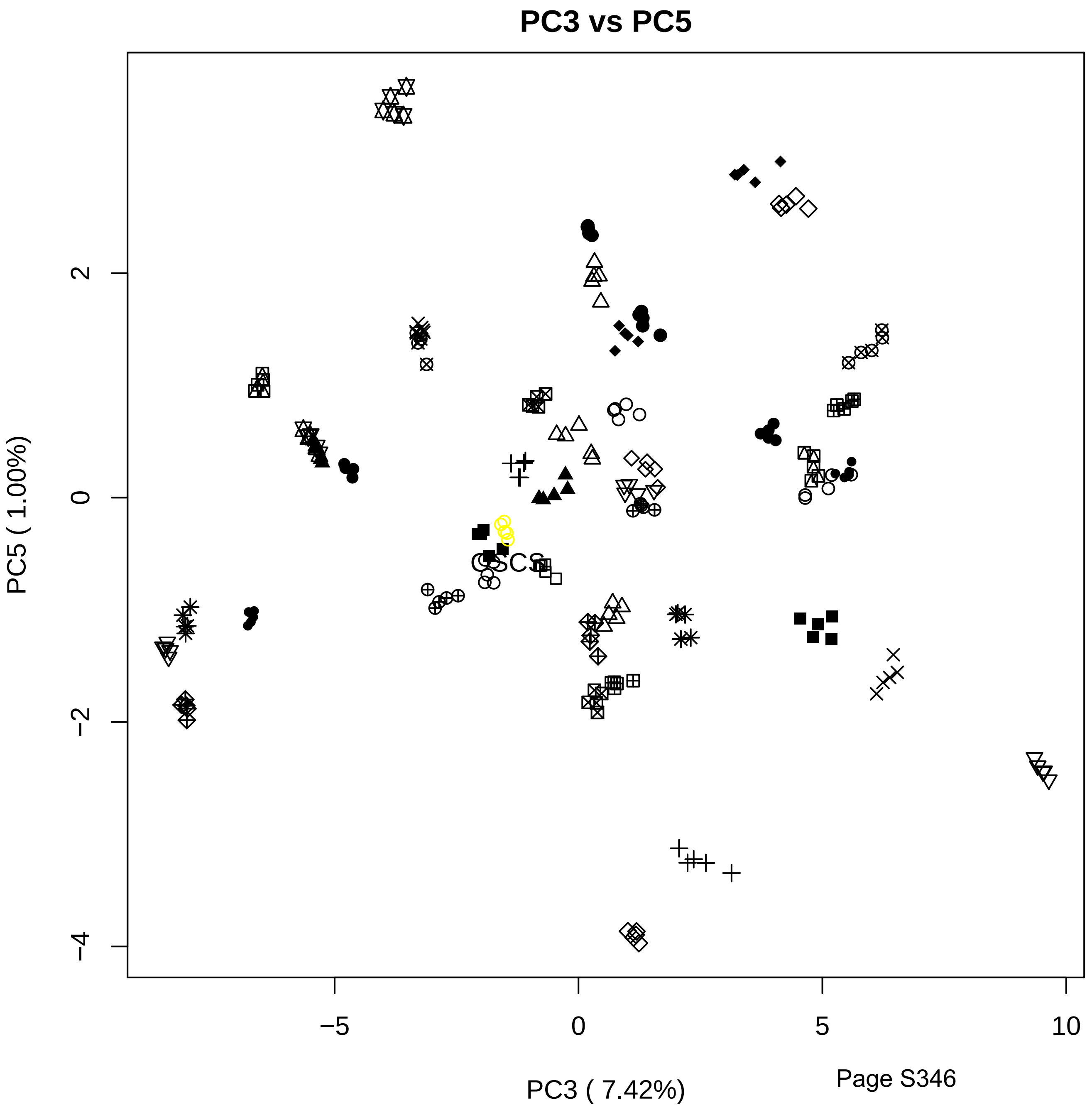
PC3 vs PC5
PC5 (1.00%)
PC3 (7.42%)
CSCS
2
0
−2
−4
−5
0
5
10

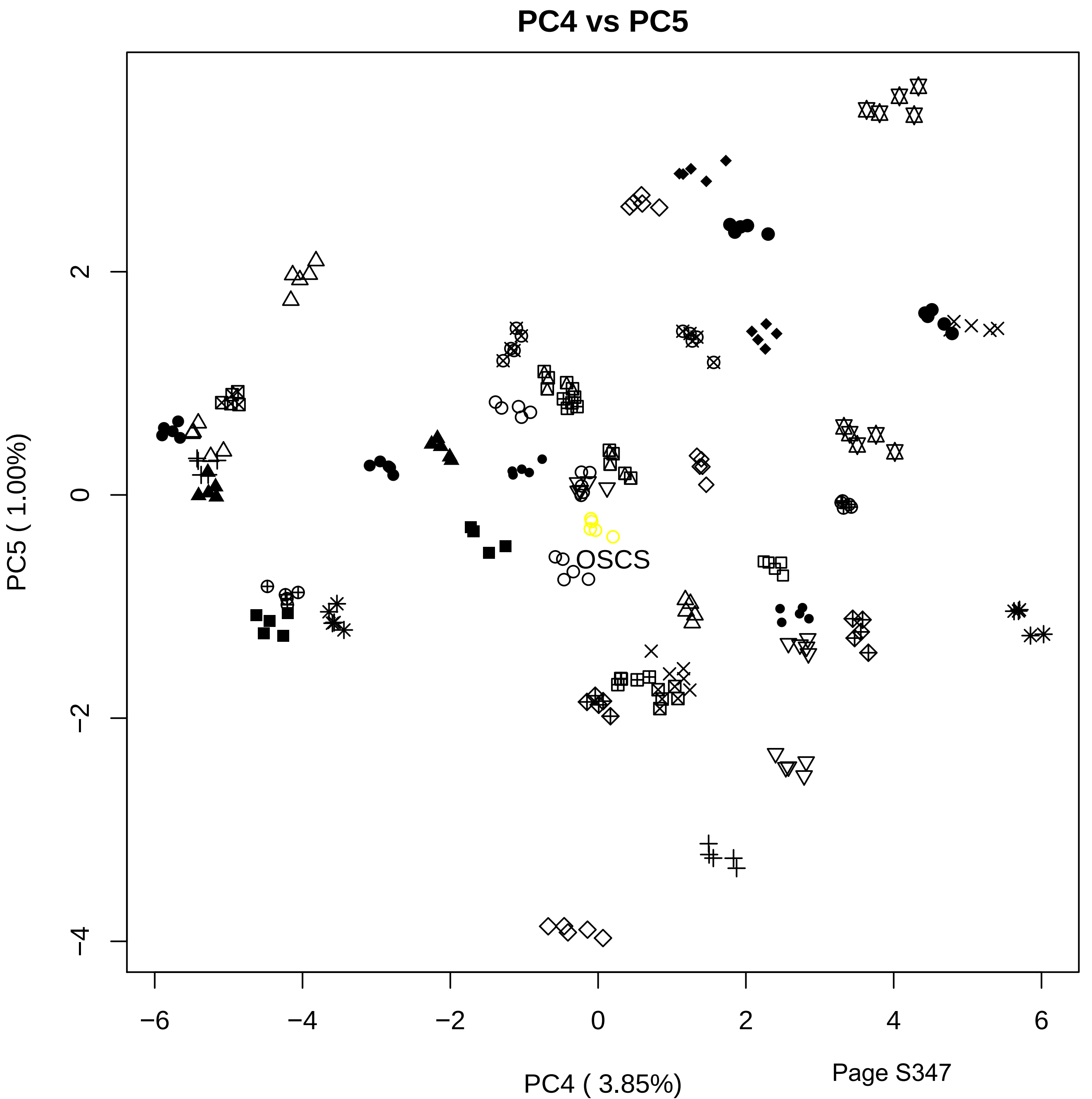
PC4 vs PC5
PC5 (1.00%)
PC4 (3.85%)
OSCS
2
0
−2
−4
−6
−4
−2
0
2
4
6

OSAS Contam 7th

Anthony Devlin, Keele University

PCA of All OSAS vs PMH

Scaled Matrix PCAed

7th order polynomial baseline correction performed

Raw spectra smoothed to 21 Neighbours, 2nd order PN, Savtisky Golay

Normalisation between 0 and 100

D2 spectra smoothed to 41 Neighbours, 2nd order PN, Savtisky Golay

Regions Analysed: 3601−2501 and 2001−700cm−1

Samples Analysed: 12 Contam's 1 OSAS's 45 PMH's

Keele University AD   Sun Dec 09 16:28:29 2018

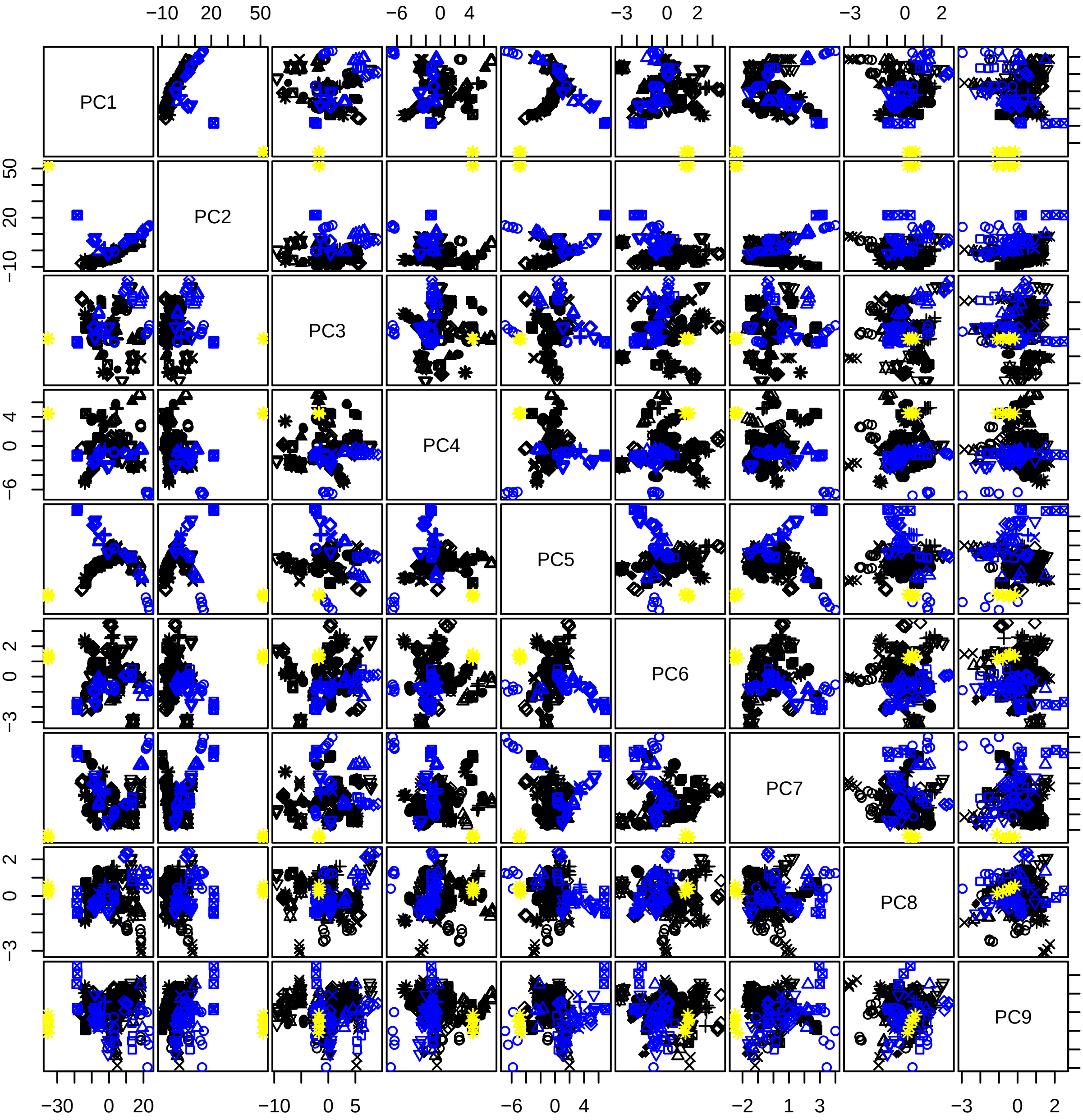
PC1
PC2
PC3
PC4
PC5
PC6
PC7
PC8
PC9
−10 20 50
−6 0 4
−3 0 2
−3 0 2
20 0 −30
50 20 −10
5 0 −10
4 0 −6
4 0 −6
2 0 −3
3 1 −2
2 0 −3
2 0 −3
−30 0 20
−10 0 5
−6 0 4
−2 1 3
−3 0 2

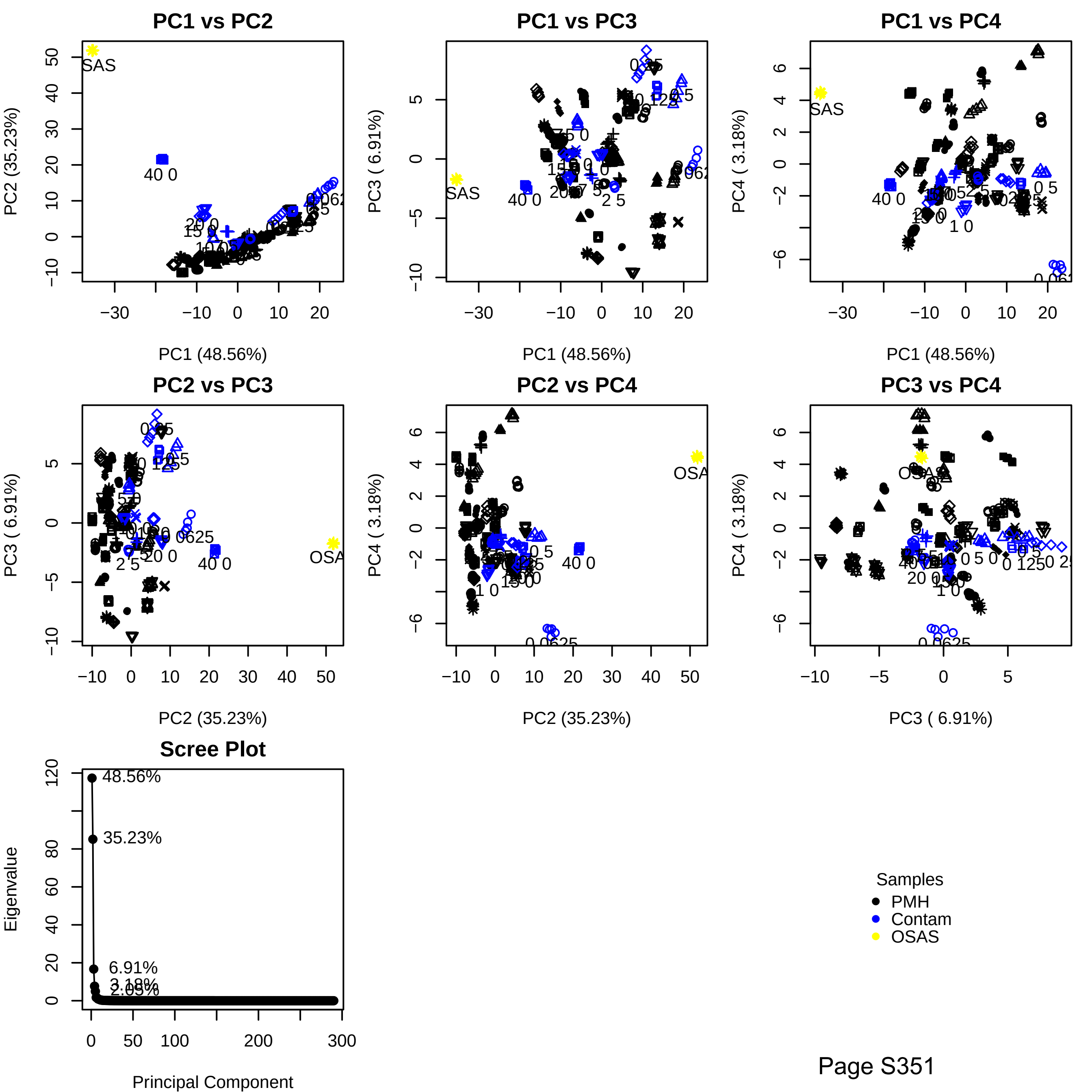
PC1 vs PC2
PC1 vs PC3
PC1 vs PC4
PC2 vs PC3
PC2 vs PC4
PC3 vs PC4
Scree Plot
PC1 (48.56%)
PC2 (35.23%)
PC3 (6.91%)
PC4 (3.18%)
Eigenvalue
Principal Component
48.56%
35.23%
6.91%
3.18%
2.05%
Samples
PMH
Contam
OSAS

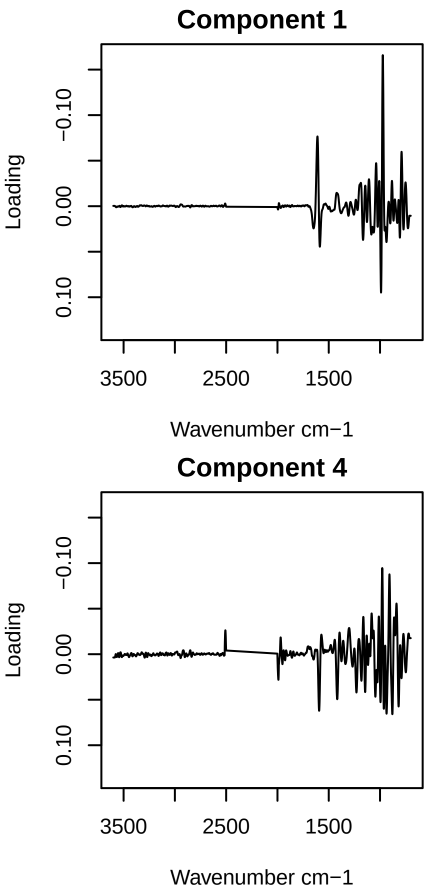

Component 1
Component 2
Component 3
Component 4
Loading
−0.10
0.00
0.10
3500
2500
1500
Wavenumber cm−1

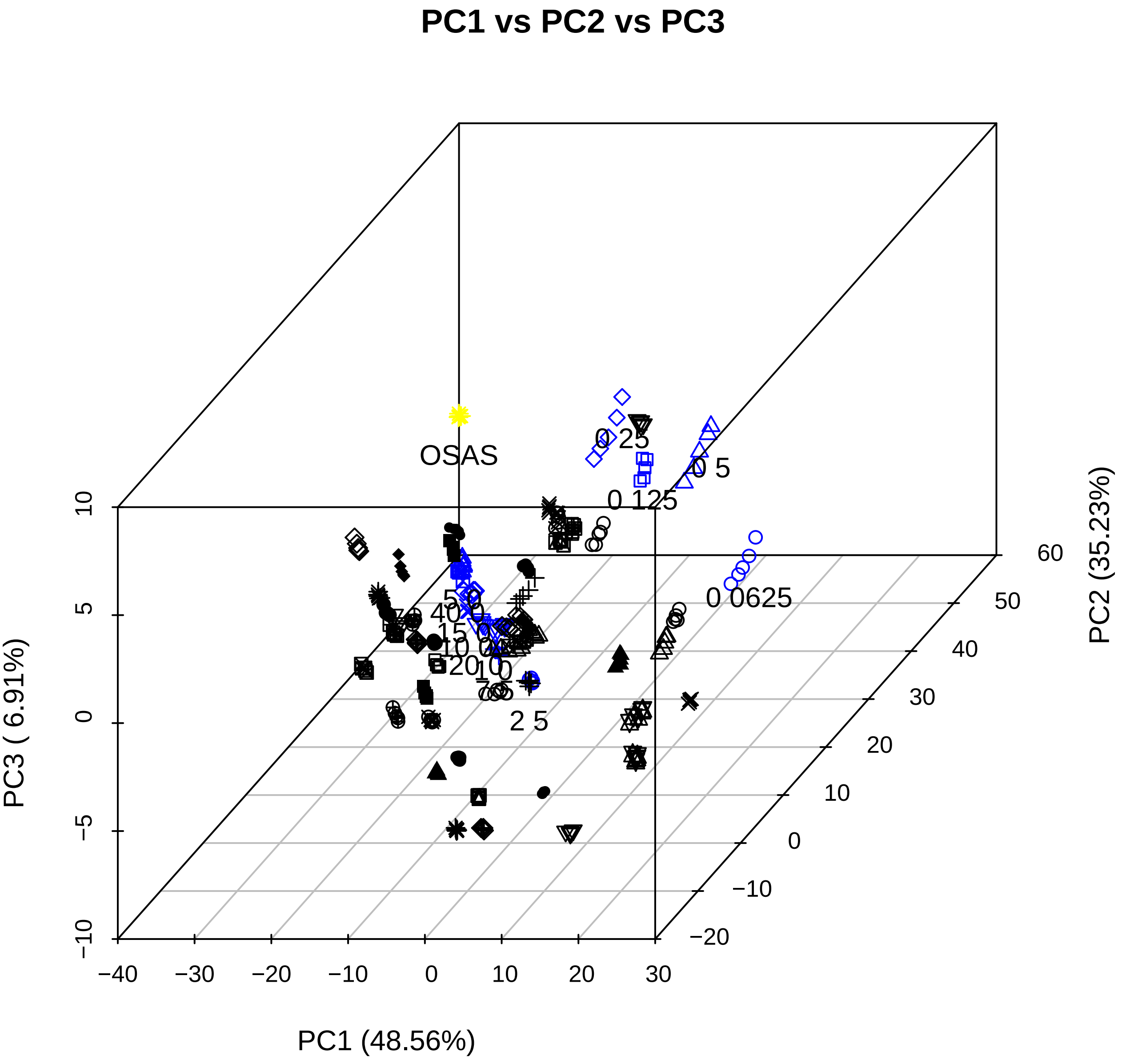
PC1 vs PC2 vs PC3
OSAS
0 25
0 5
0 125
0 0625
2 5
PC1 (48.56%)
PC2 (35.23%)
PC3 (6.91%)
−40 −30 −20 −10 0 10 20 30
−20 −10 0 10 20 30 40 50 60
−10 −5 0 5 10

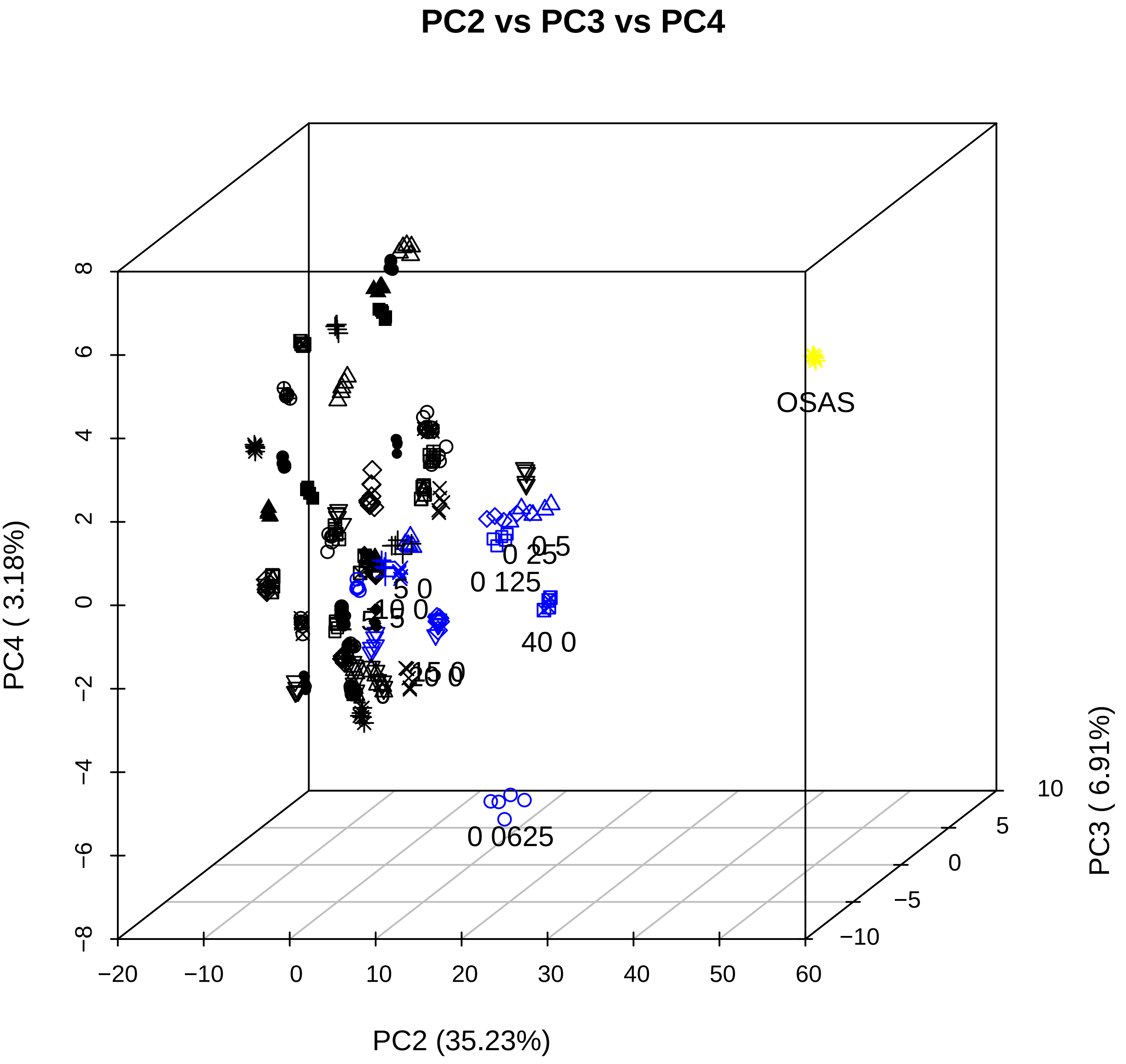

PC2 vs PC3 vs PC4
OSAS
0 5
0 25
0 125
5 0
10 0
40 0
15 0
20 0
0 0625
PC4 (3.18%)
PC2 (35.23%)
PC3 (6.91%)

PC1 vs PC2 vs PC4

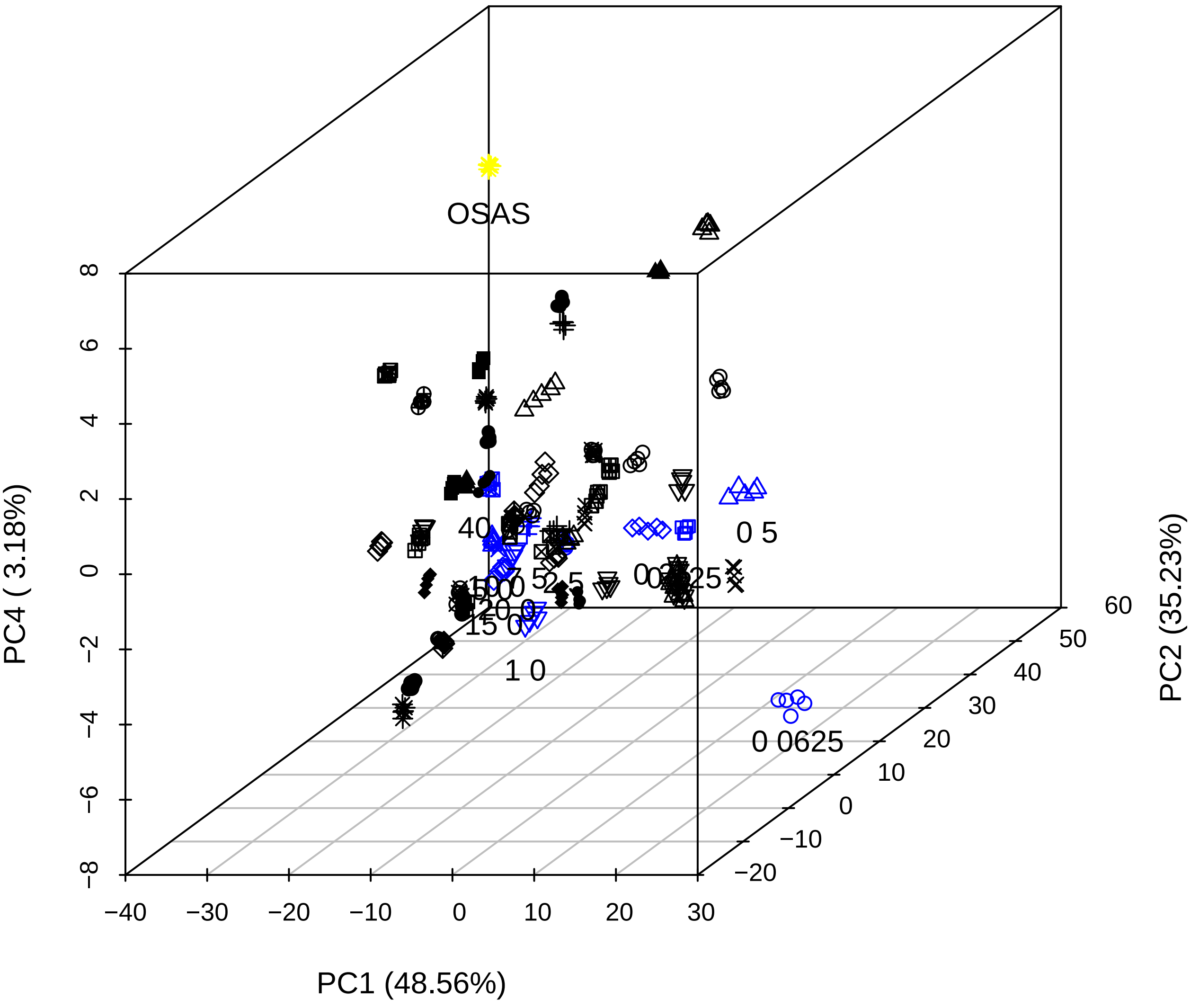

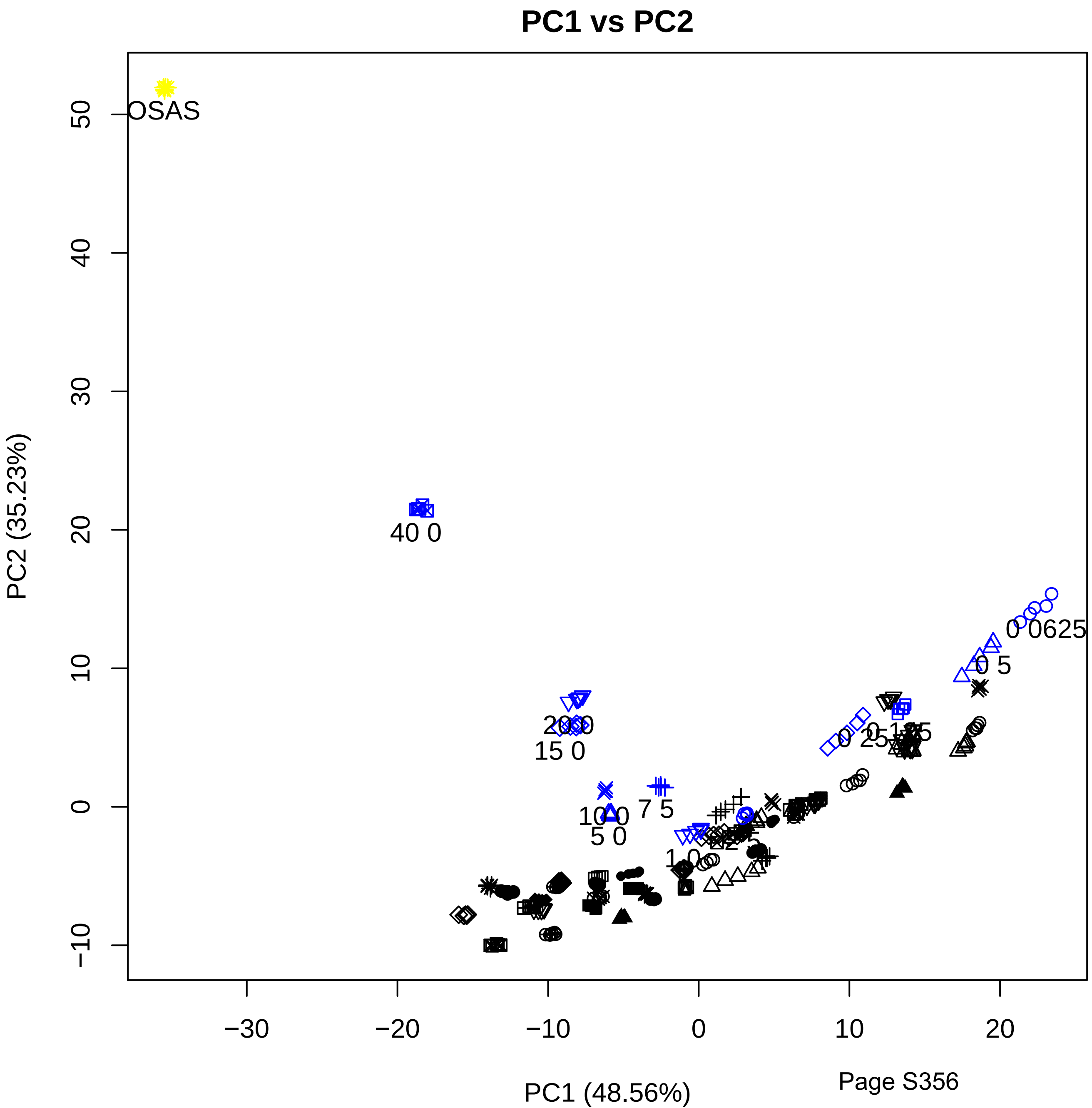

PC1 vs PC2
PC2 (35.23%)
PC1 (48.56%)
−30
−20
−10
0
10
20
50
40
30
20
10
0
−10
OSAS
40 0
20 0
15 0
10 0
5 0
7 5
1 0
0 0625
0 5
0 25

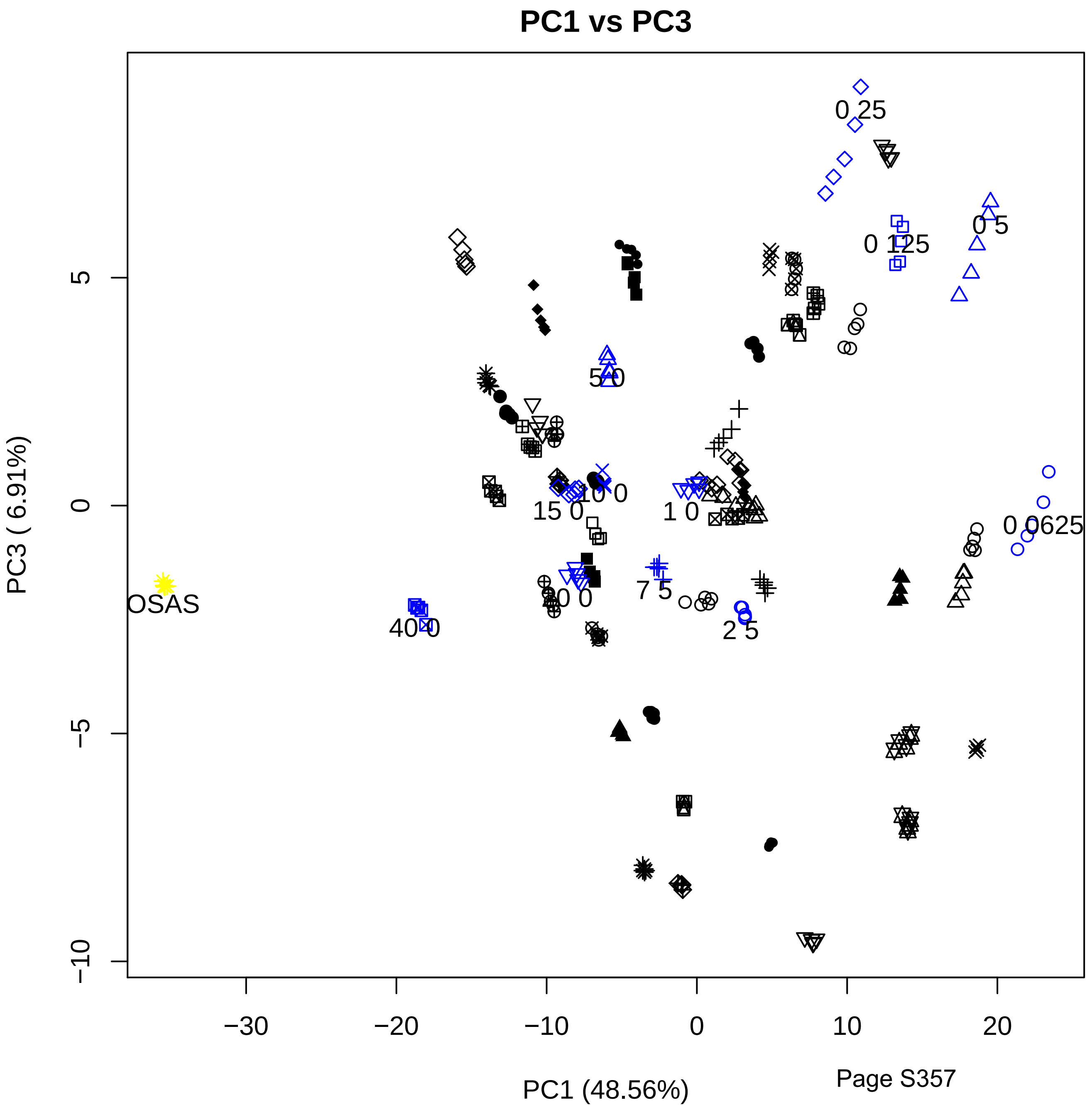
PC1 vs PC3
PC3 (6.91%)
PC1 (48.56%)
0 25
0 5
0 125
5 0
10 0
15 0
1 0
0 0625
OSAS
40 0
20 0
7 5
2 5
5
0
−5
−10
−30
−20
−10
0
10
20

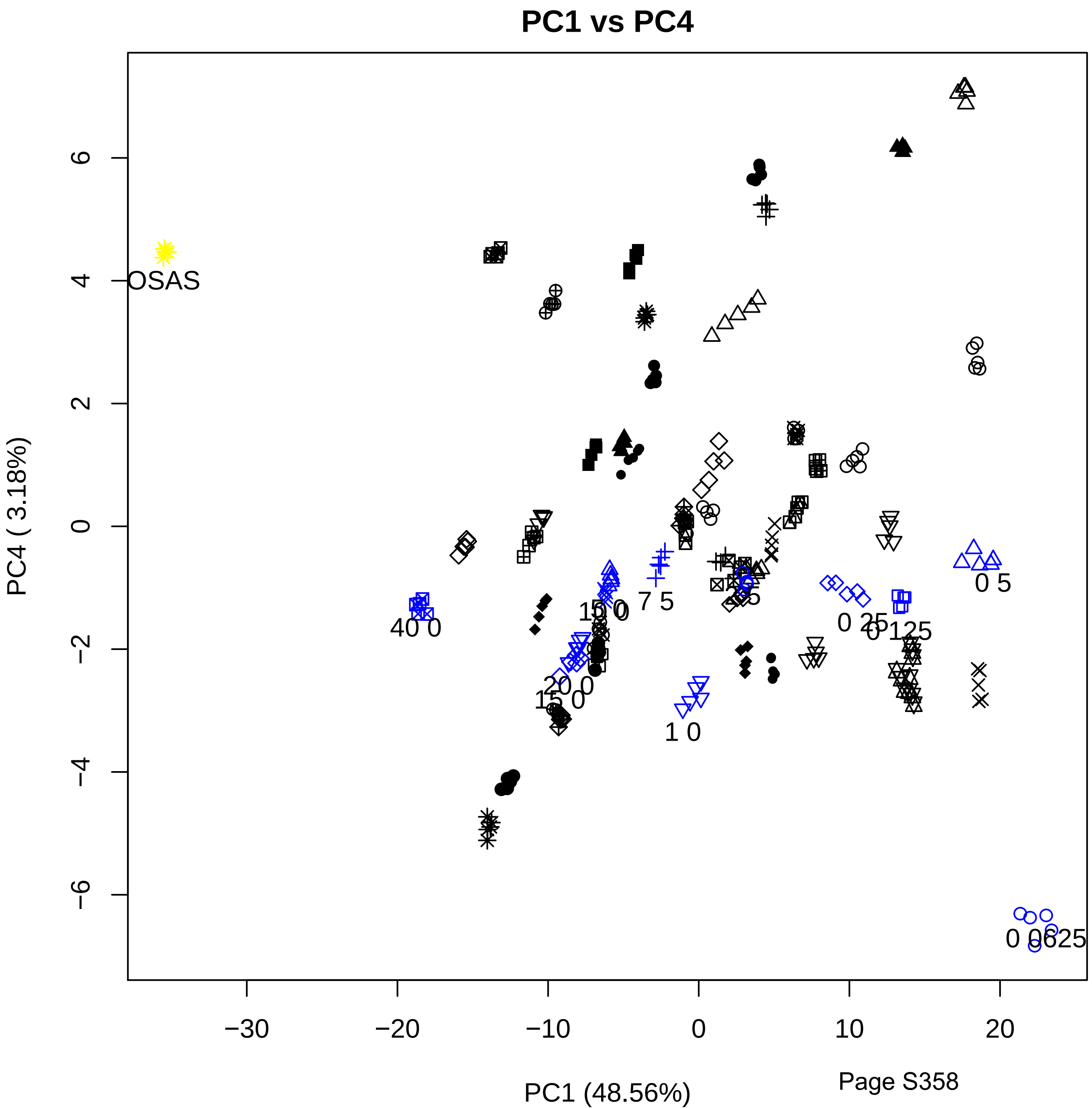
PC1 vs PC4
PC4 (3.18%)
PC1 (48.56%)
6
4
2
0
−2
−4
−6
−30
−20
−10
0
10
20
OSAS
40 0
7 5
20 0
1 0
0 25
0 5
0 0625

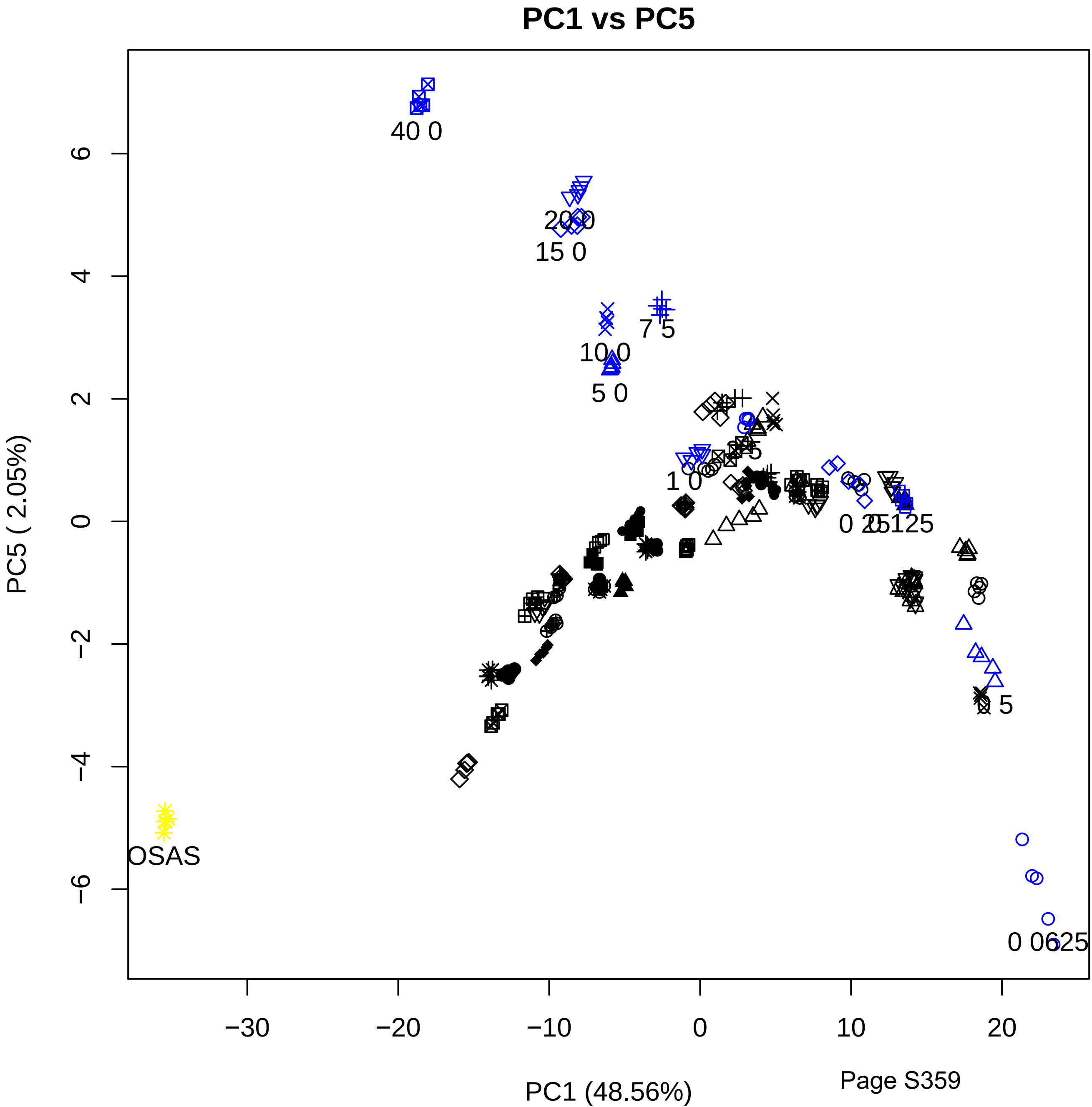
PC1 vs PC5
PC5 (2.05%)
PC1 (48.56%)
6
4
2
0
−2
−4
−6
−30
−20
−10
0
10
20
40 0
20 0
15 0
7 5
10 0
5 0
1 0
0 25
0 125
0 5
0 0625
OSAS

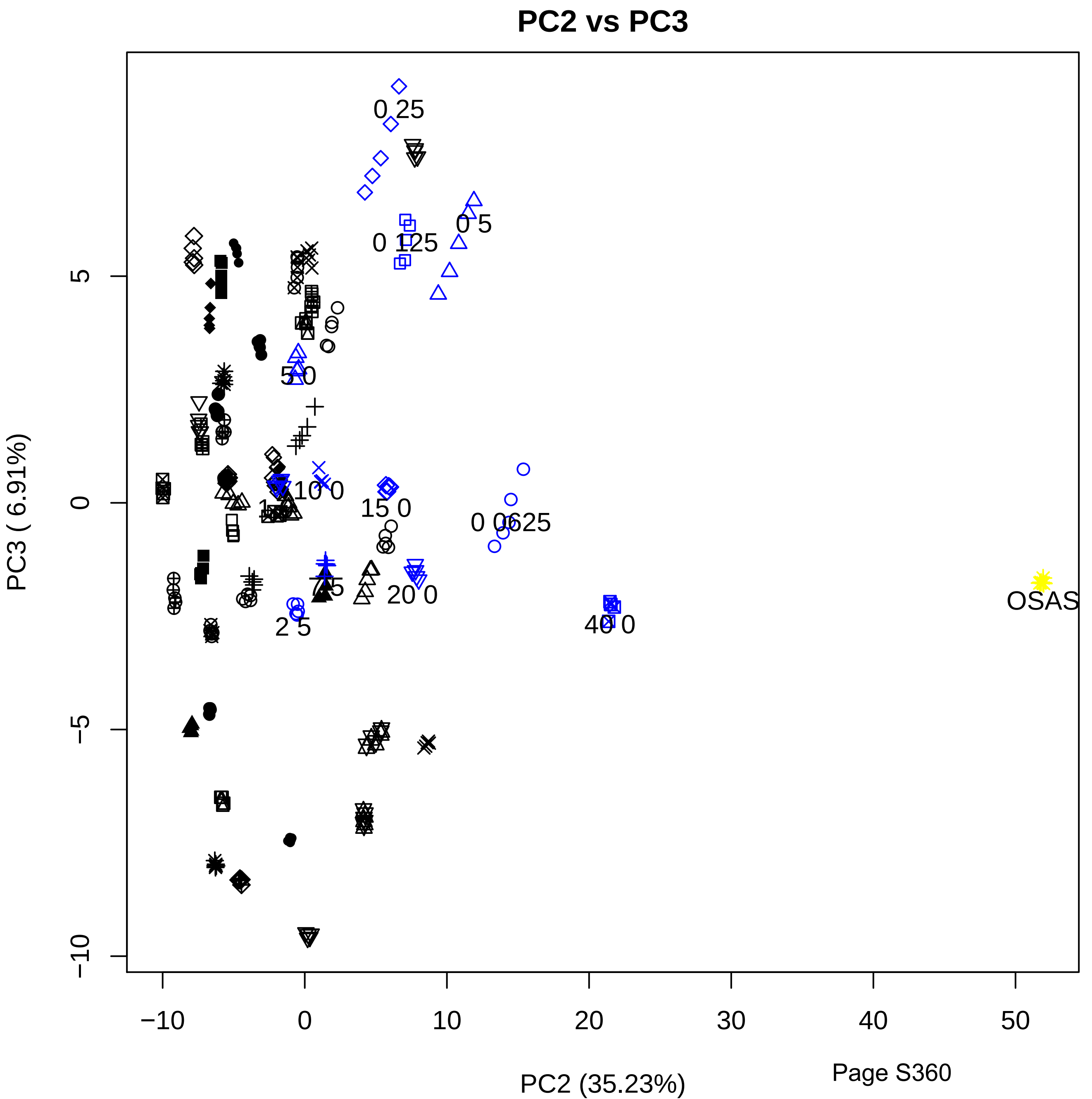
PC2 vs PC3
0 25
0 5
0 125
5 0
10 0
15 0
0 0625
20 0
2 5
40 0
OSAS
PC3 (6.91%)
PC2 (35.23%)
5
0
−5
−10
−10
0
10
20
30
40
50

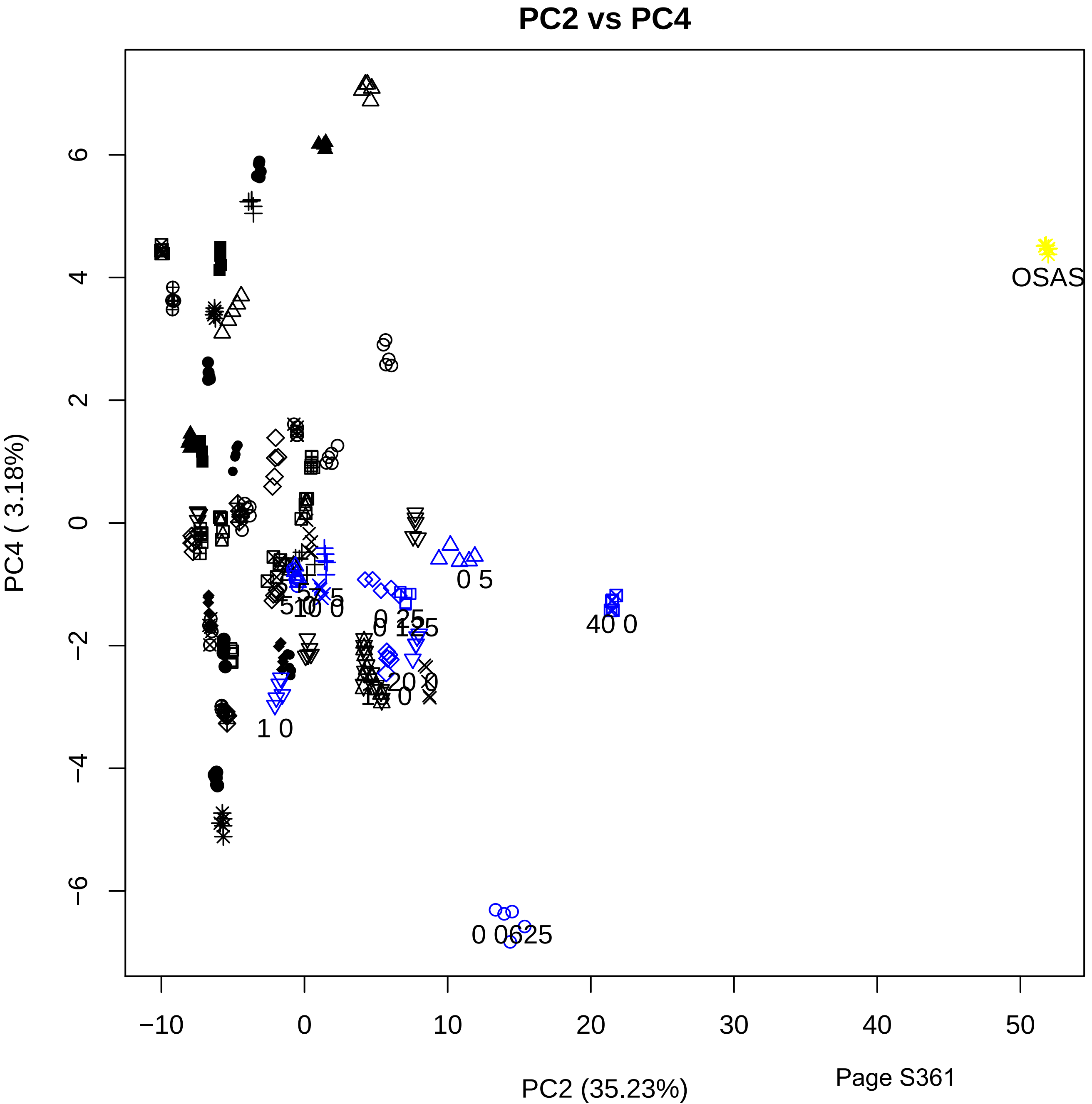

PC2 vs PC4
PC4 (3.18%)
PC2 (35.23%)
OSAS
0 5
40 0
0 0625
1 0
−10
0
10
20
30
40
50
6
4
2
0
−2
−4
−6

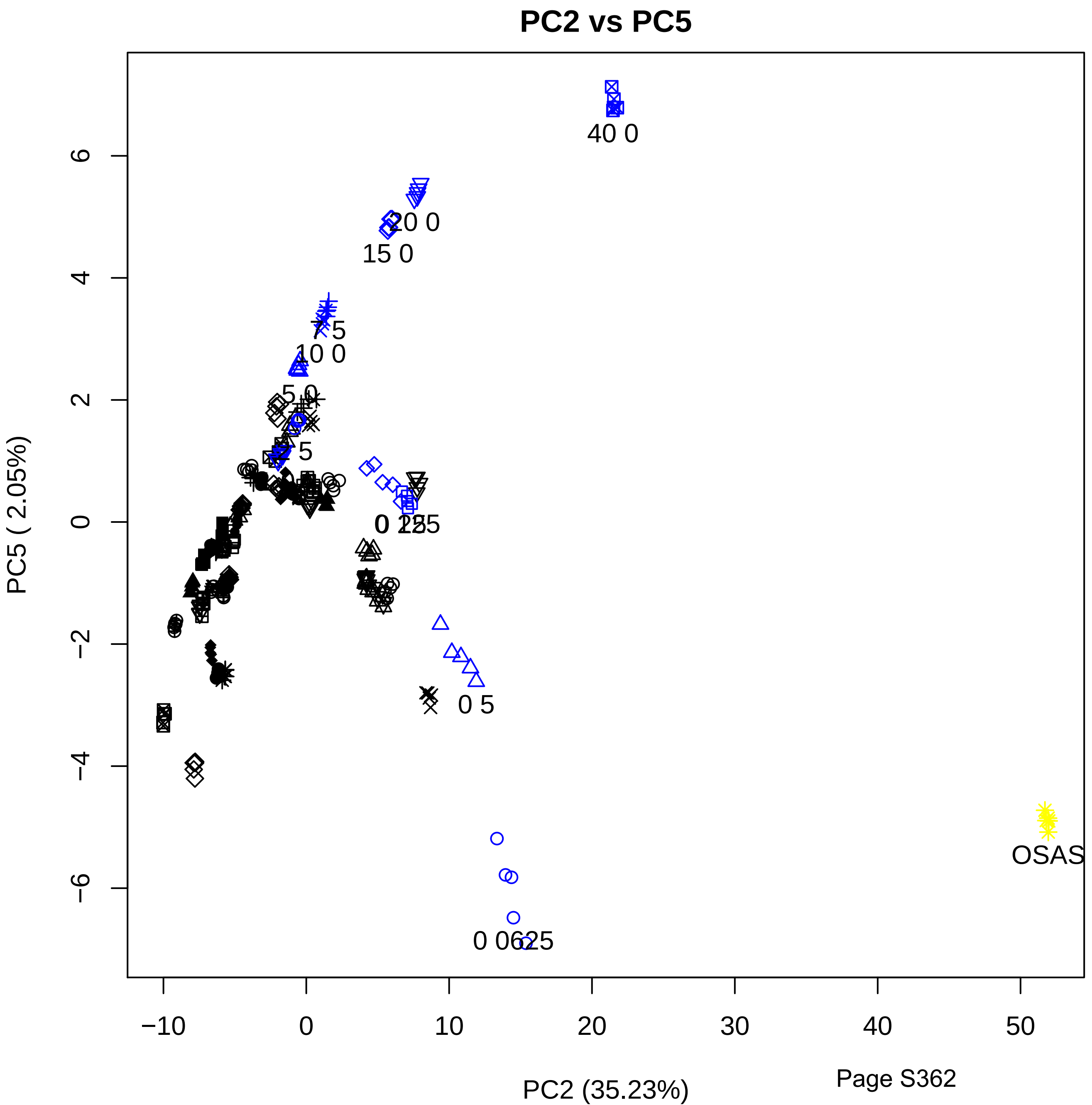
PC2 vs PC5
PC5 (2.05%)
PC2 (35.23%)
40 0
20 0
15 0
7 5
10 0
5 0
5
0 125
0 5
0 0625
OSAS
−10
0
10
20
30
40
50
6
4
2
0
−2
−4
−6

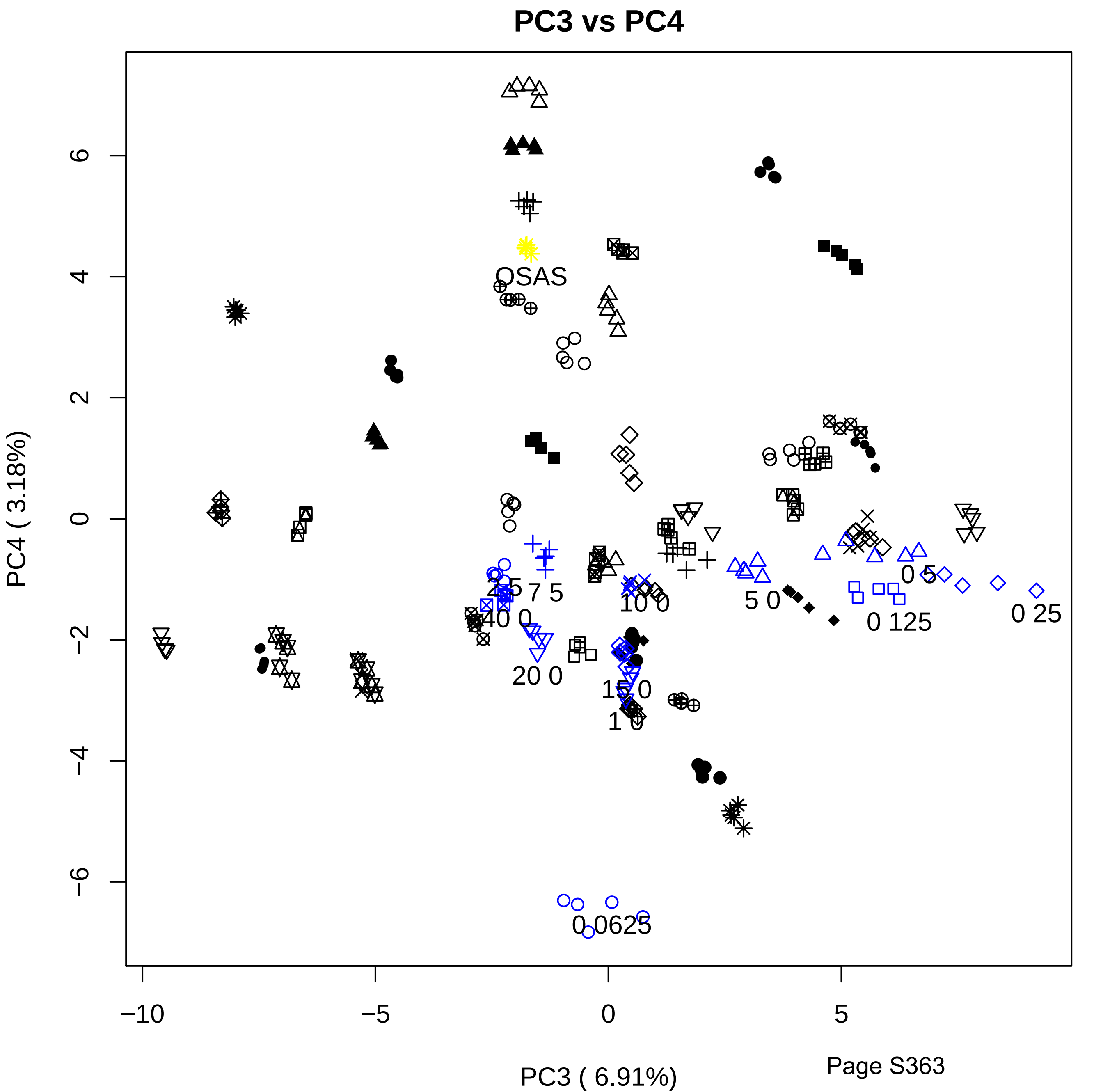
PC3 vs PC4
PC4 (3.18%)
PC3 (6.91%)
6
4
2
0
−2
−4
−6
−10
−5
0
5
OSAS
2 5
7 5
40 0
10 0
5 0
0 5
0 125
0 25
20 0
15 0
1 0
0 0625

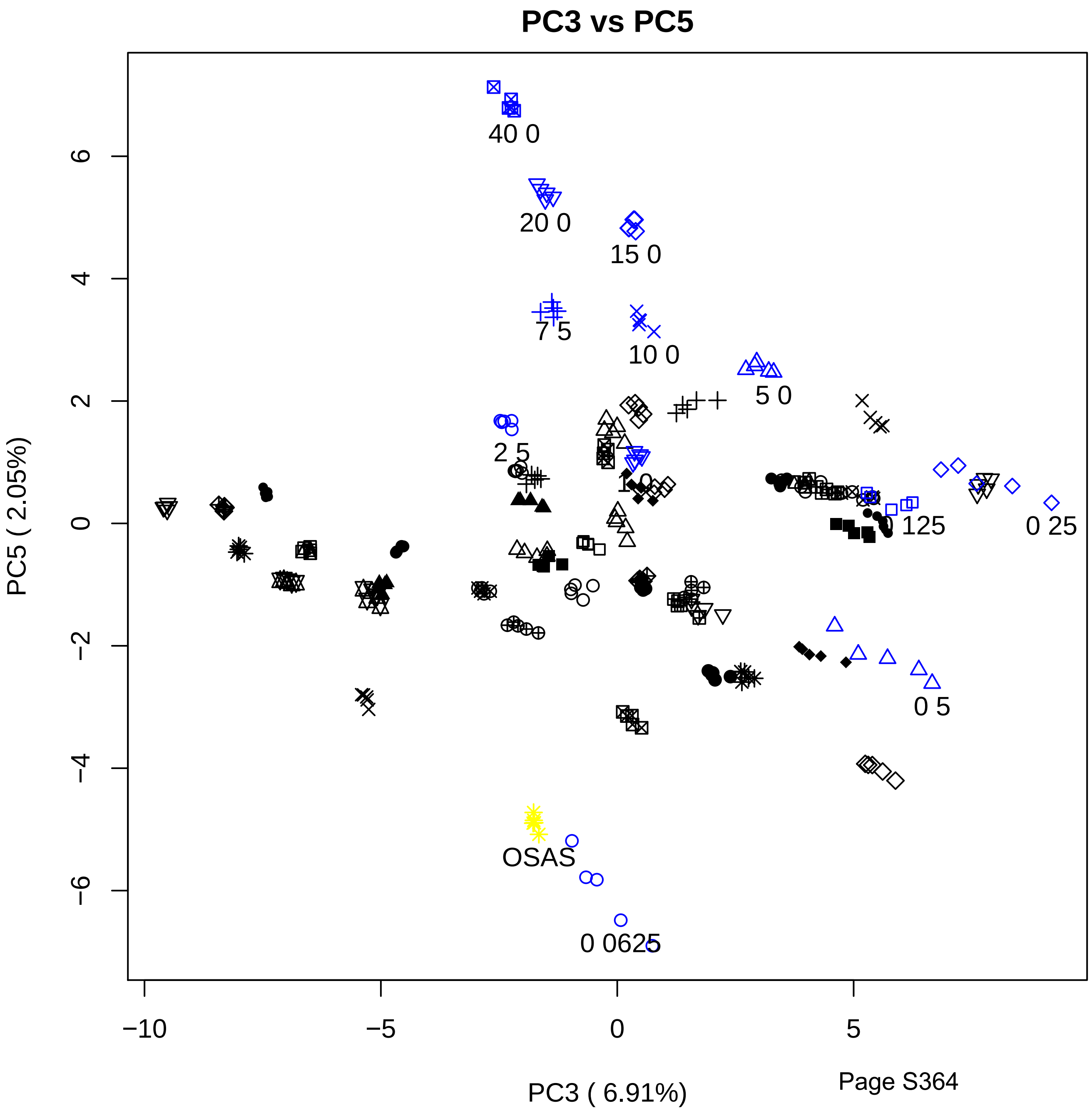

PC3 vs PC5
40 0
20 0
15 0
7 5
10 0
5 0
2 5
1 0
0 125
0 25
0 5
OSAS
0 0625
PC5 (2.05%)
PC3 (6.91%)
−10
−5
0
5
6
4
2
0
−2
−4
−6

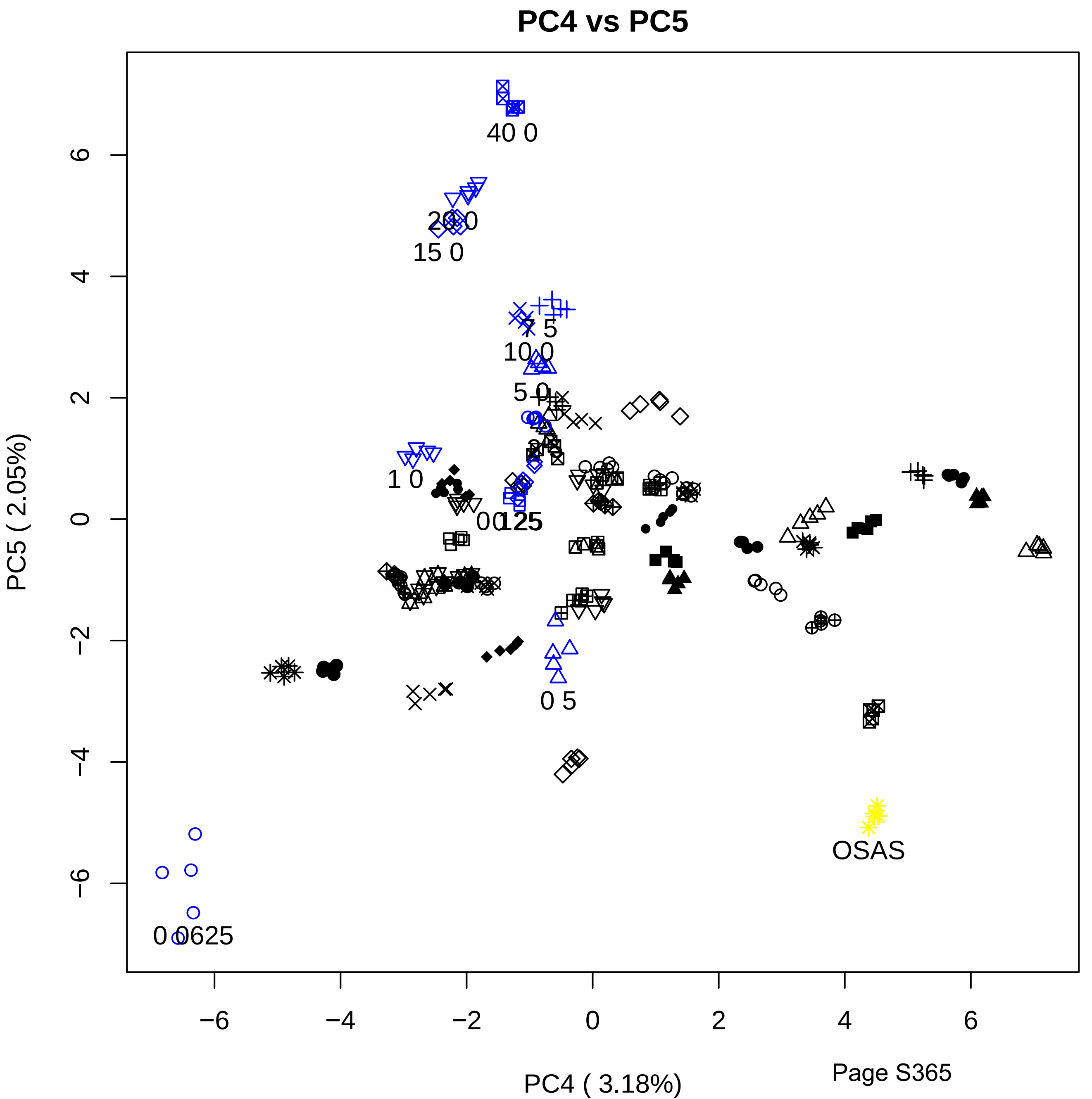
PC4 vs PC5
40 0
20 0
15 0
7 5
10 0
5 0
1 0
0 0125
0 5
OSAS
0 0625
PC5 (2.05%)
PC4 (3.18%)
6
4
2
0
−2
−4
−6
−6
−4
−2
0
2
4
6

PCA of 0.06% OSAS vs PMH

Scaled Matrix PCAed

7th order polynomial baseline correction performed

Raw spectra smoothed to 21 Neighbours, 2nd order PN, Savtisky Golay

Normalisation between 0 and 100

D2 spectra smoothed to 41 Neighbours, 2nd order PN, Savtisky Golay

Regions Analysed: 3601−2501 and 2001−700cm−1

Samples Analysed: 1 Contam's 0 OSAS's 45 PMH's

Keele University AD   Sun Dec 09 16:28:36 2018

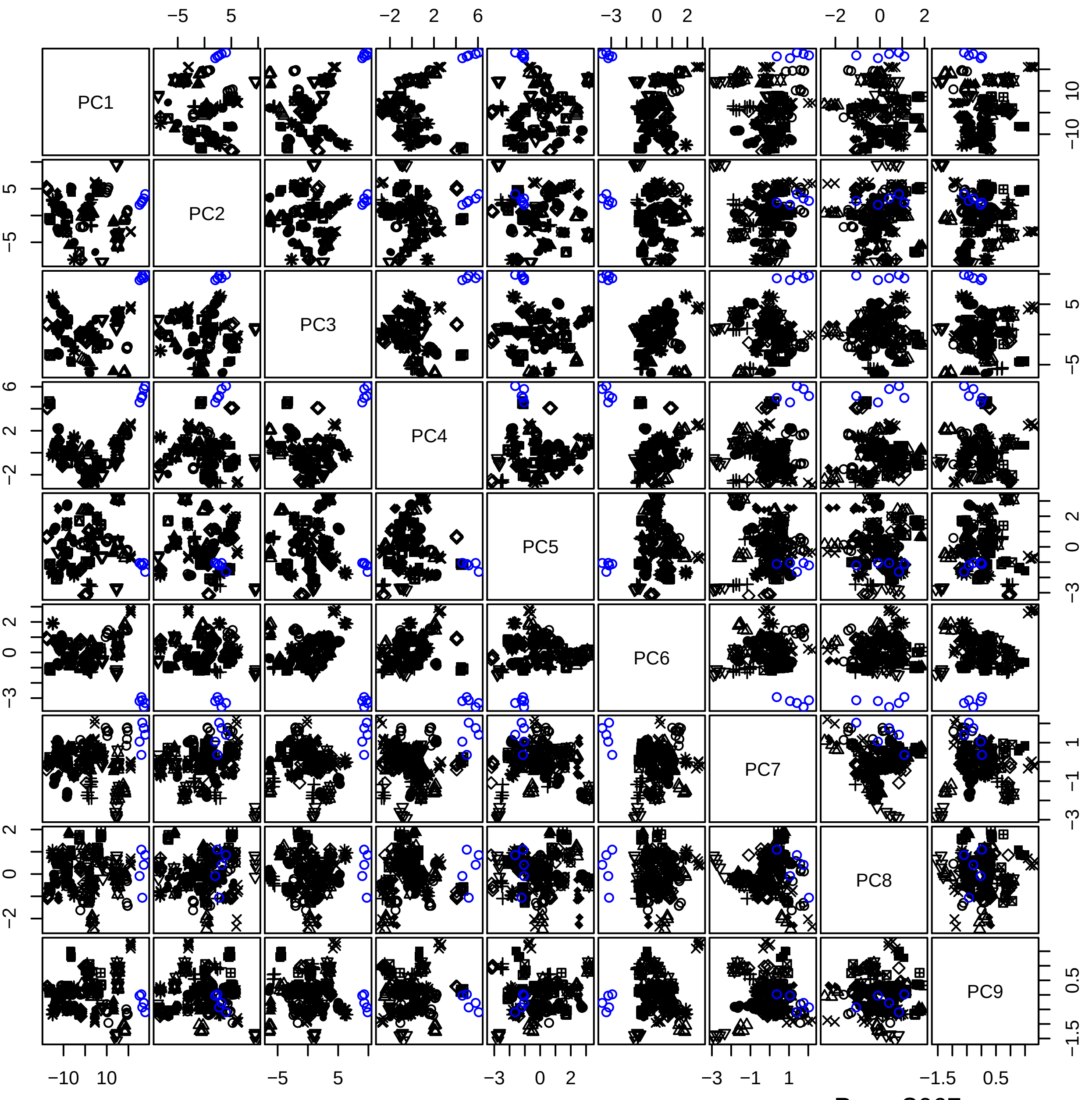
PC1
PC2
PC3
PC4
PC5
PC6
PC7
PC8
PC9

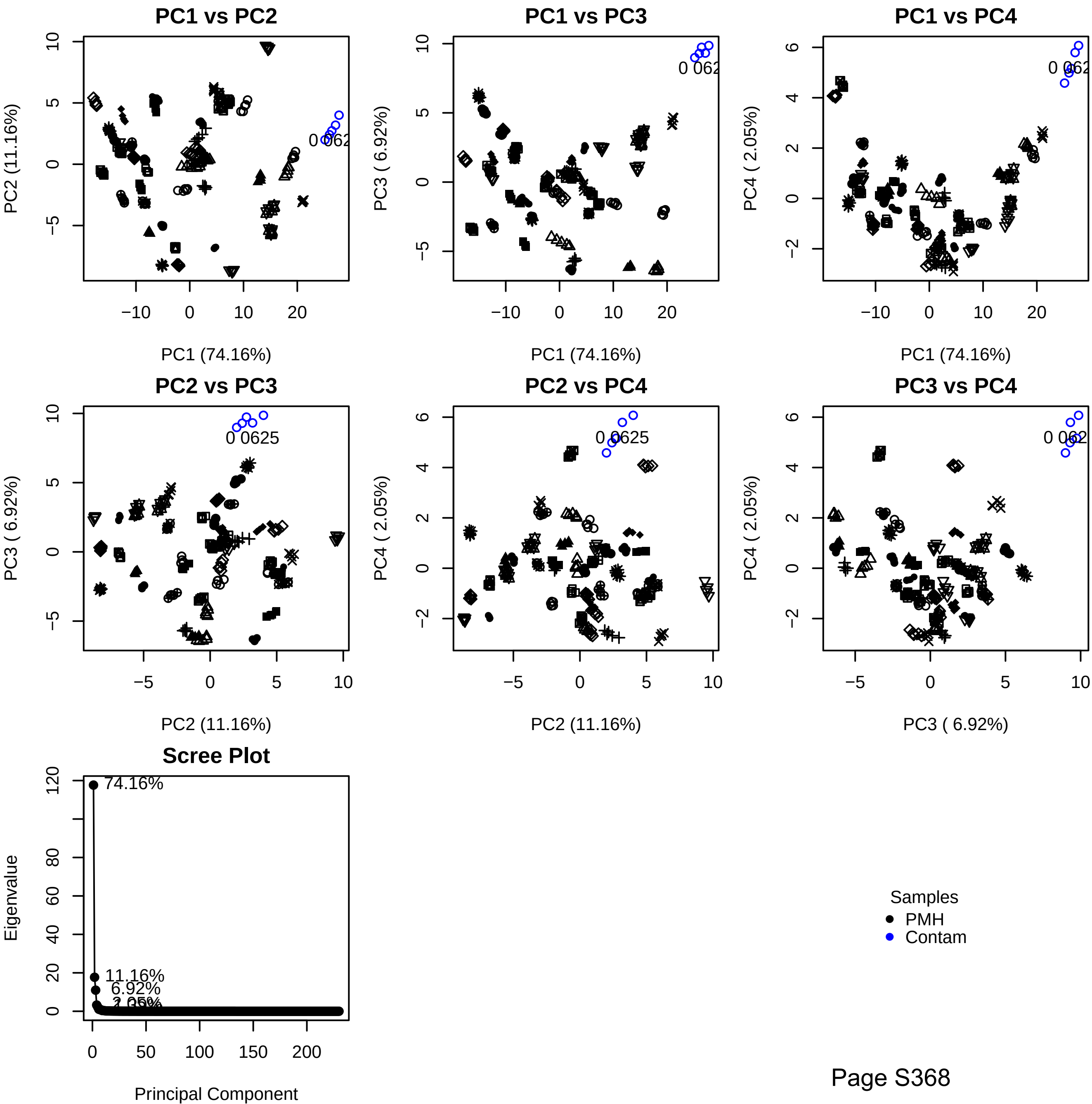
PC1 vs PC2
PC2 (11.16%)
PC1 (74.16%)
0 062
PC1 vs PC3
PC3 (6.92%)
PC1 (74.16%)
0 062
PC1 vs PC4
PC4 (2.05%)
PC1 (74.16%)
0 062
PC2 vs PC3
PC3 (6.92%)
PC2 (11.16%)
0 0625
PC2 vs PC4
PC4 (2.05%)
PC2 (11.16%)
0 0625
PC3 vs PC4
PC4 (2.05%)
PC3 (6.92%)
0 062
Scree Plot
Eigenvalue
Principal Component
74.16%
11.16%
6.92%
Samples
PMH
Contam

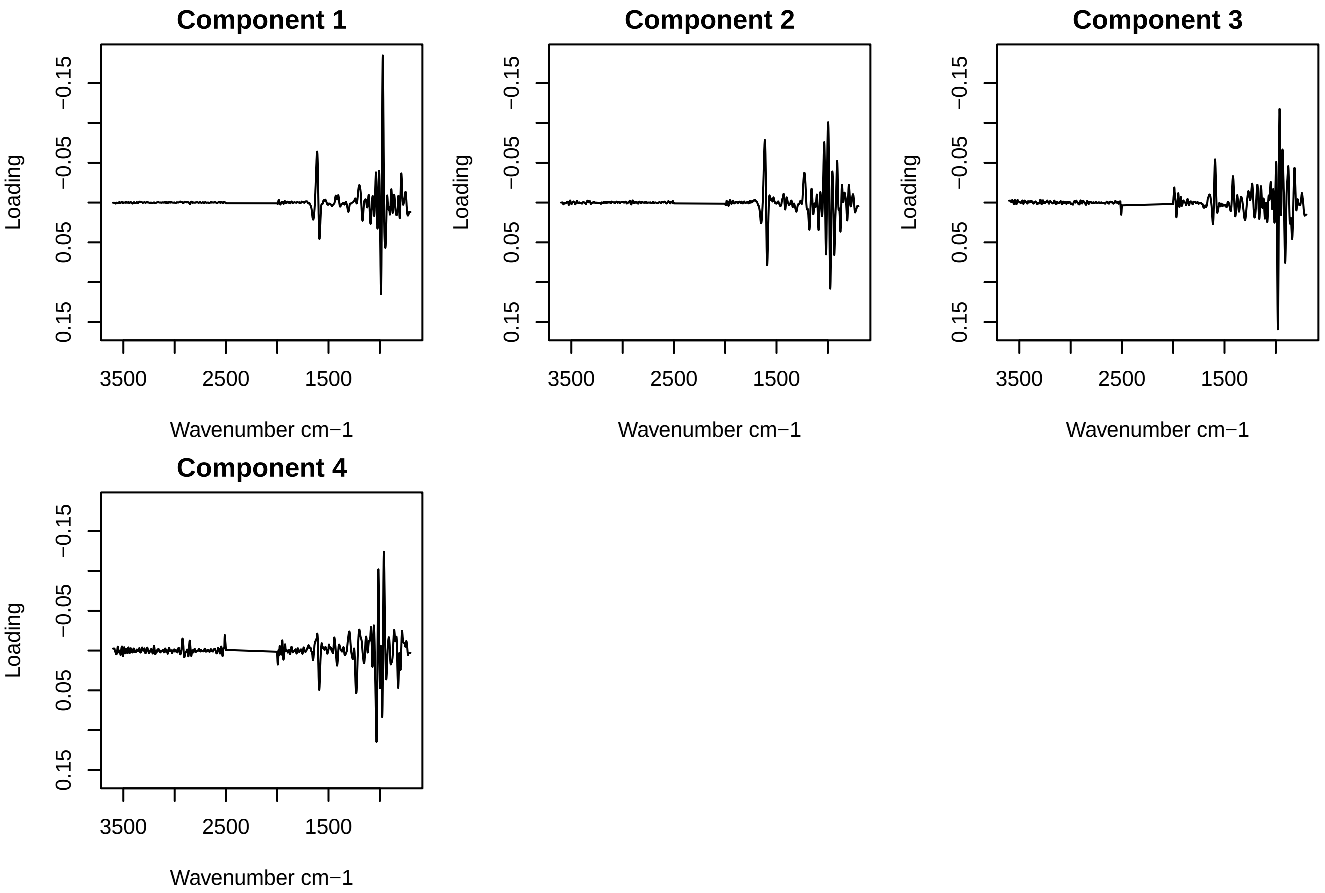
Component 1
Component 2
Component 3
Component 4
Loading
−0.15
−0.05
0.05
0.15
3500
2500
1500
Wavenumber cm−1

PC1 vs PC2 vs PC3

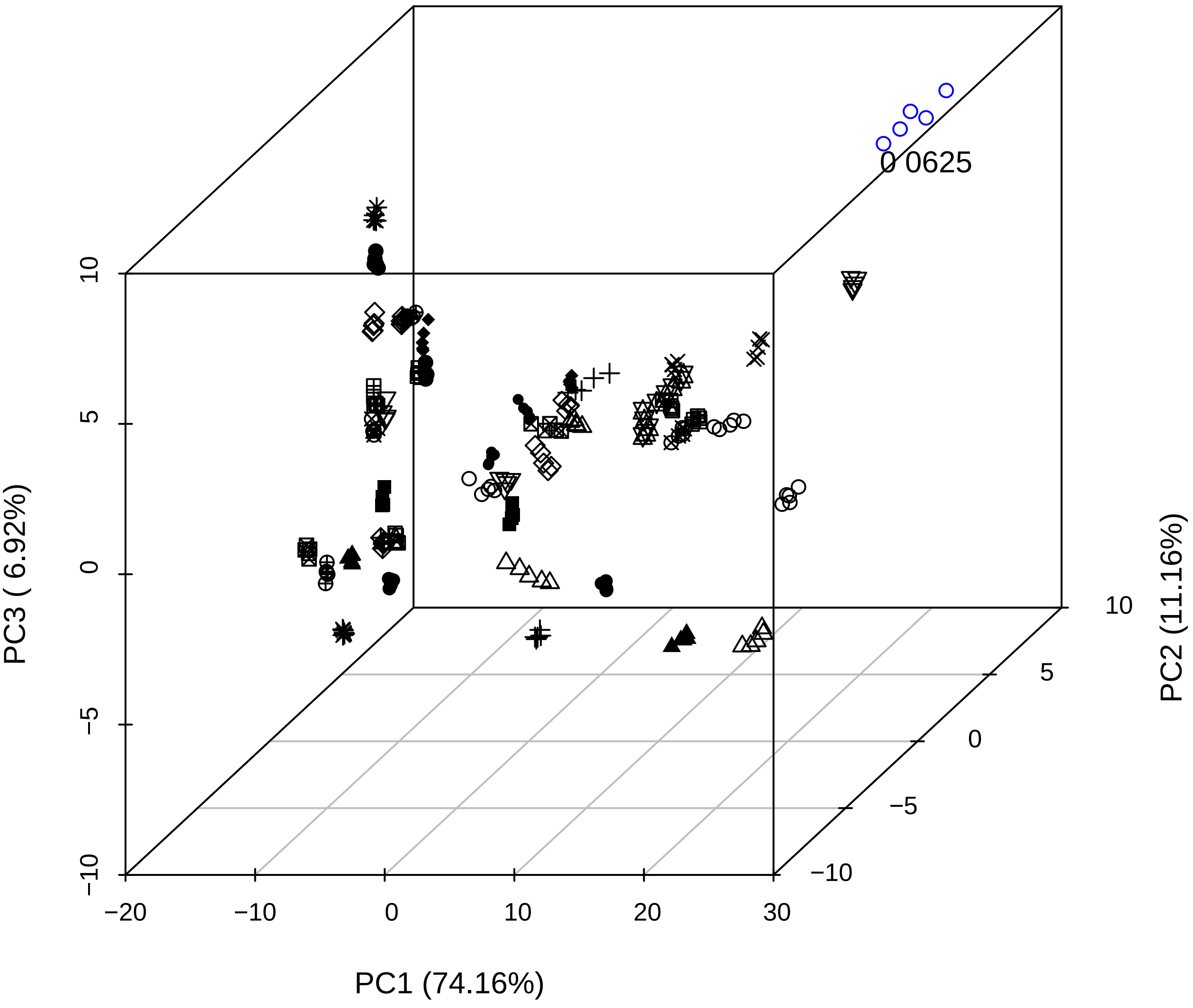

PC2 vs PC3 vs PC4

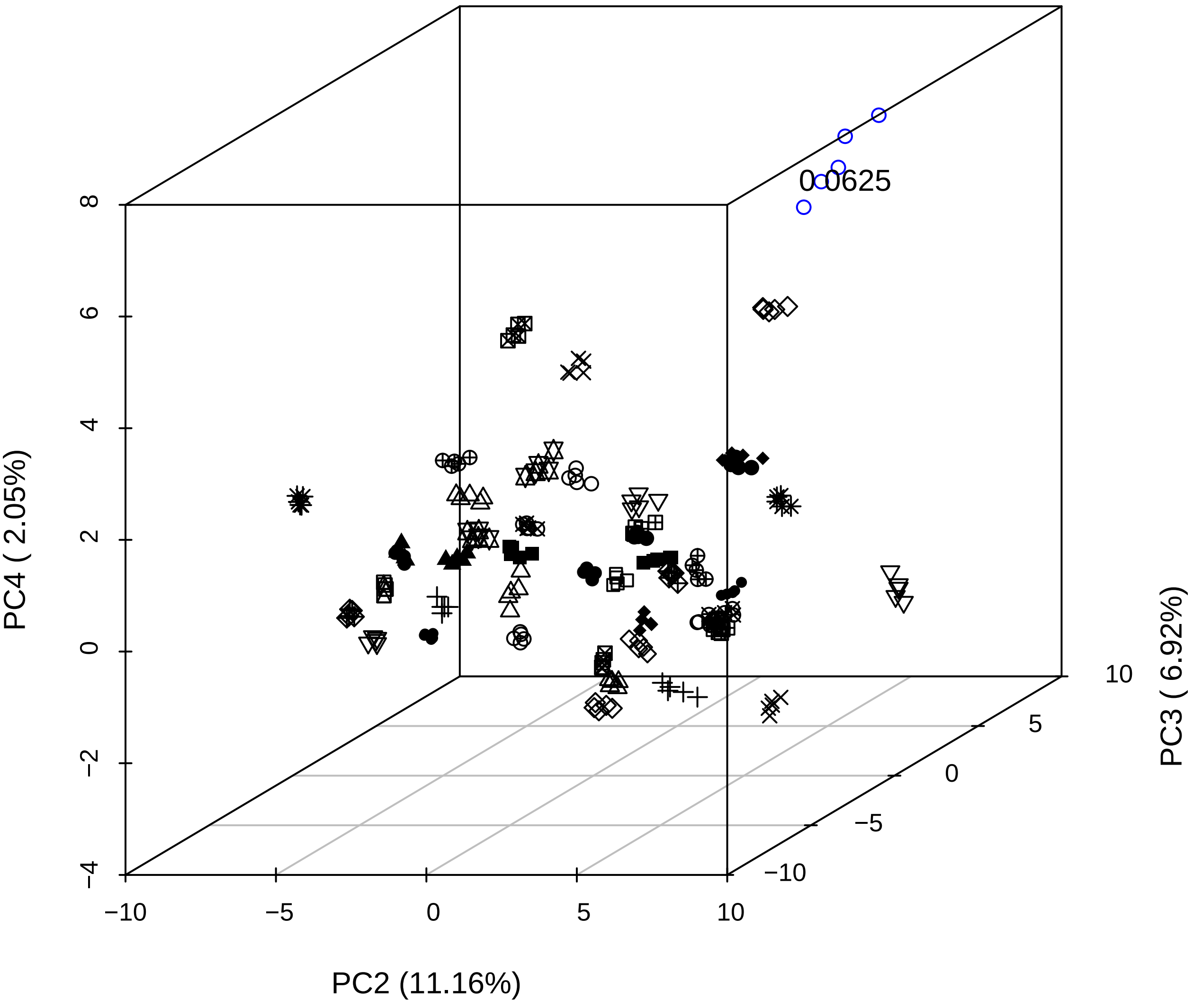

PC1 vs PC2 vs PC4

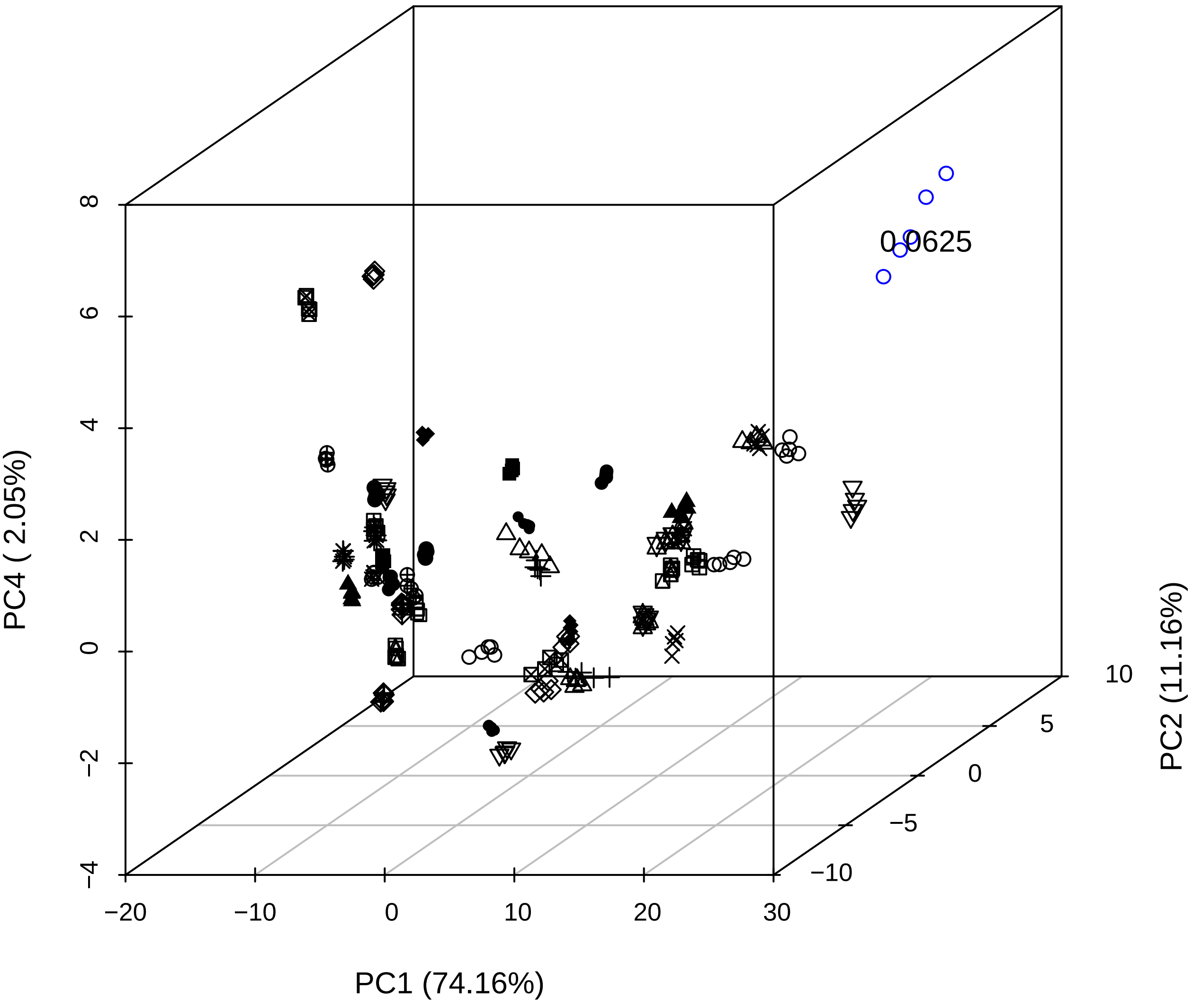

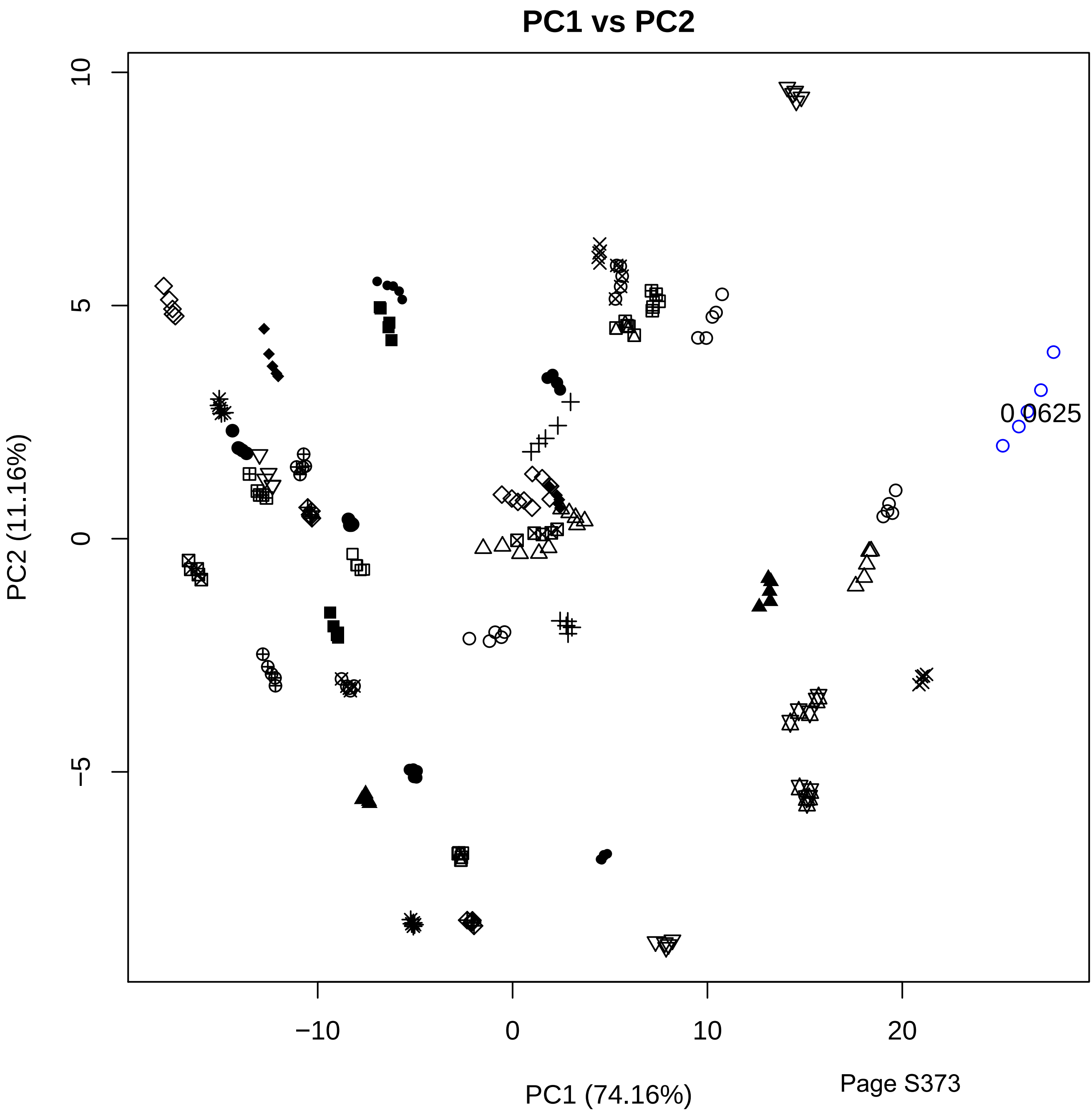
PC1 vs PC2
PC2 (11.16%)
PC1 (74.16%)
10
5
0
−5
−10
0
10
20
0.0625

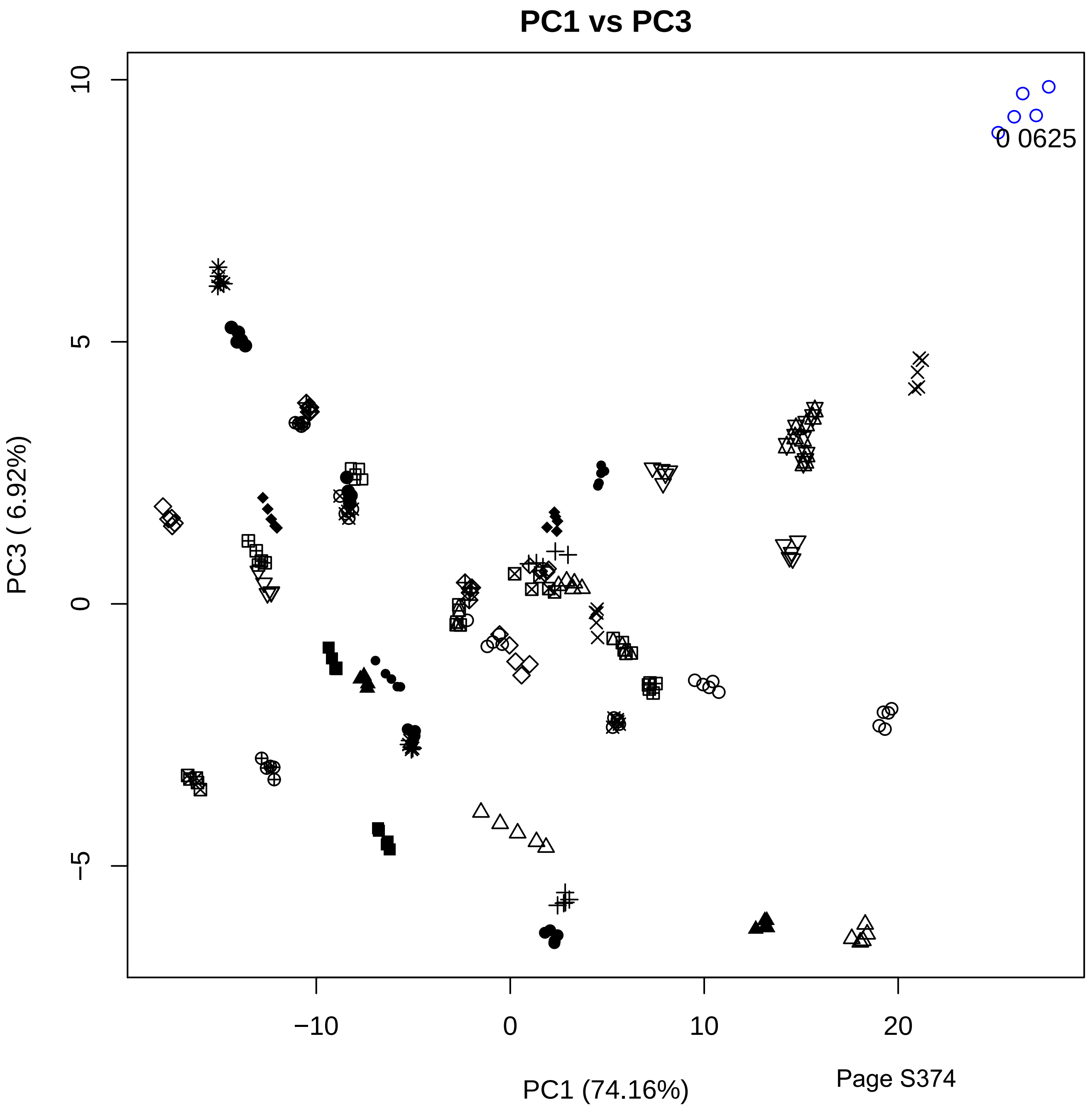
PC1 vs PC3
0 0625
10
5
0
−5
−10
0
10
20
PC3 (6.92%)
PC1 (74.16%)

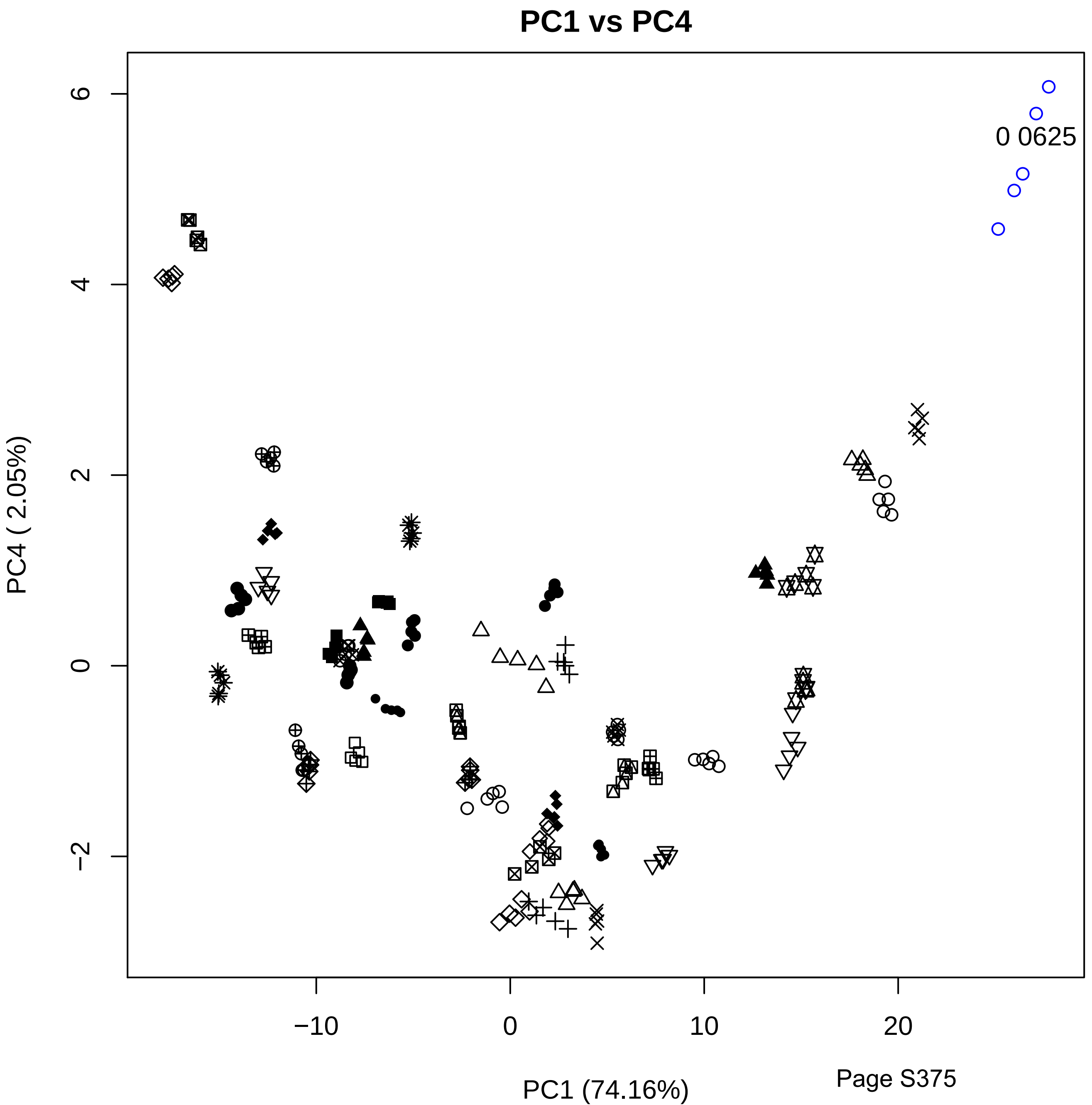
PC1 vs PC4
0 0625
PC4 (2.05%)
PC1 (74.16%)
6
4
2
0
−2
−10
0
10
20

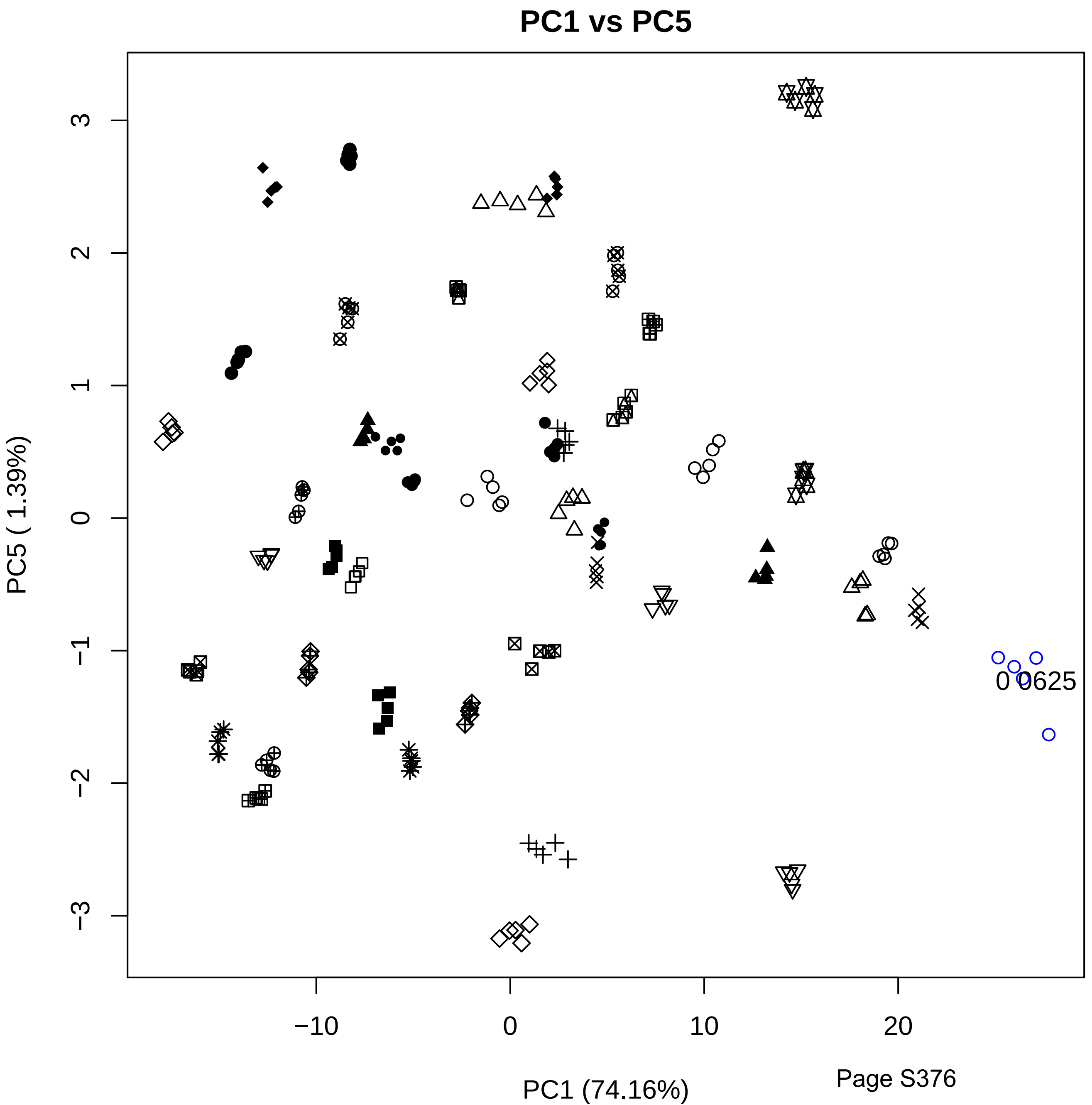

PC1 vs PC5
PC5 (1.39%)
PC1 (74.16%)
3
2
1
0
−1
−2
−3
−10
0
10
20
0 0625

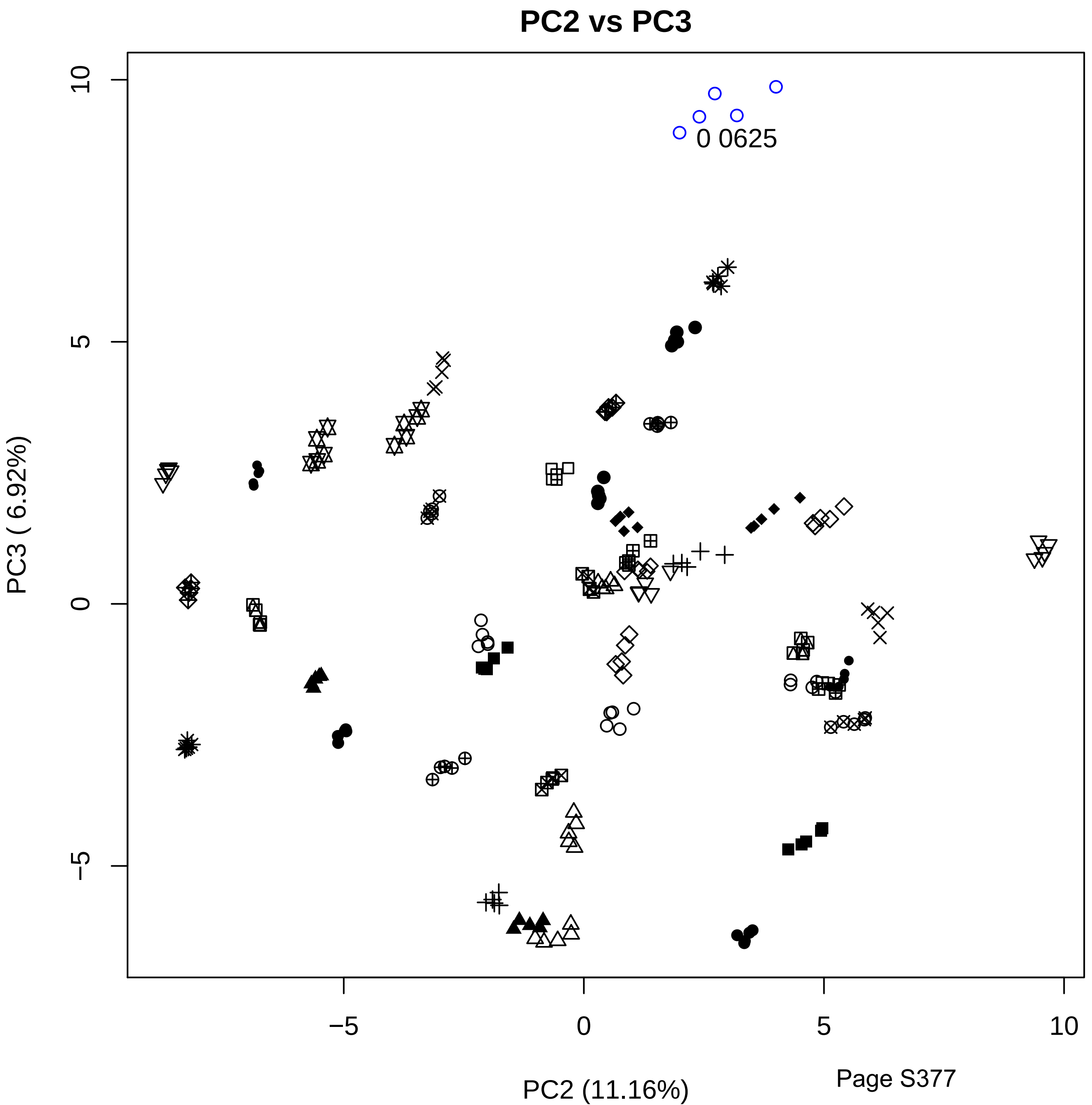
PC2 vs PC3
PC3 (6.92%)
PC2 (11.16%)
10
5
0
−5
−5
0
5
10
0 0625

PC2 vs PC4

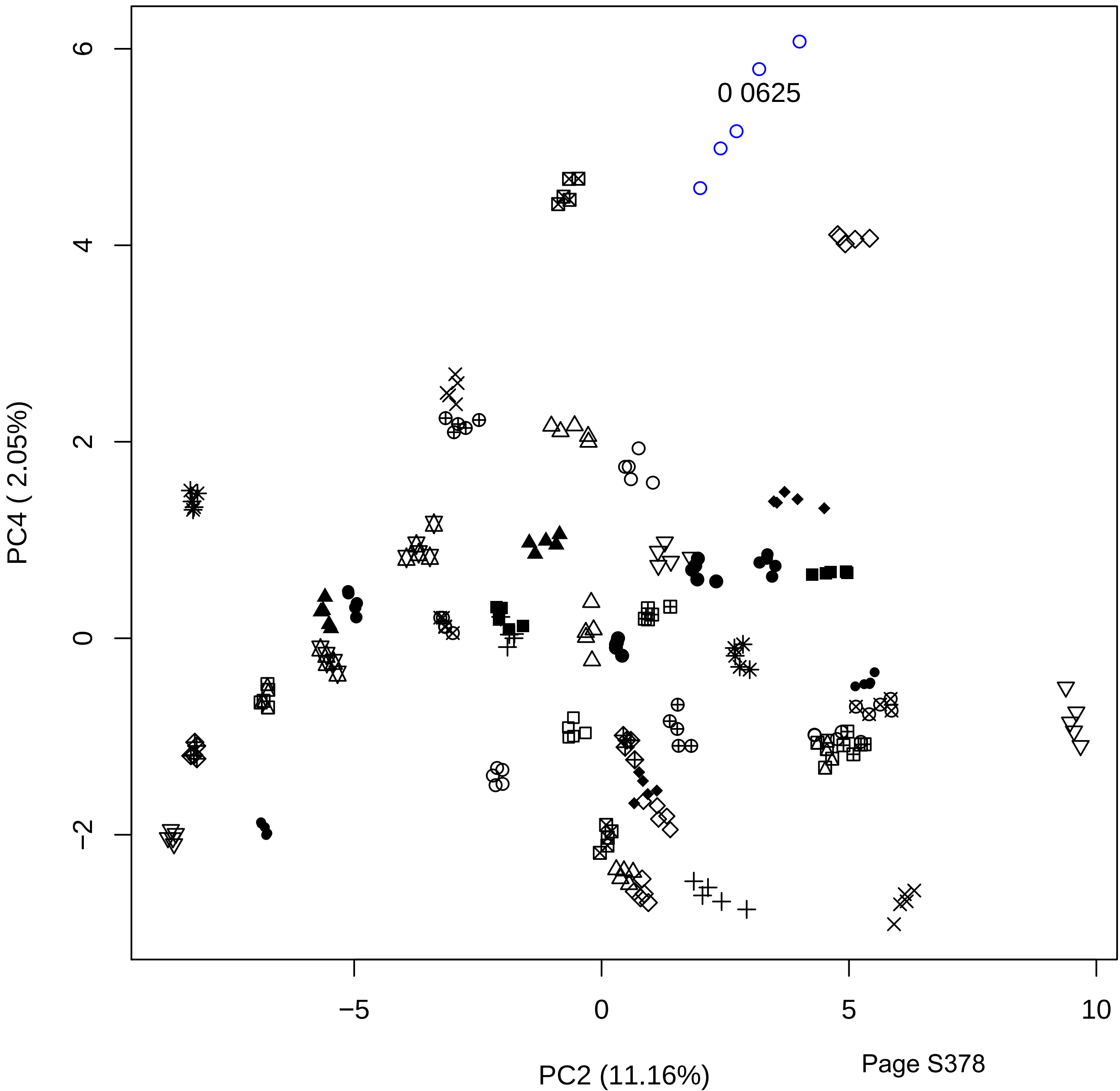

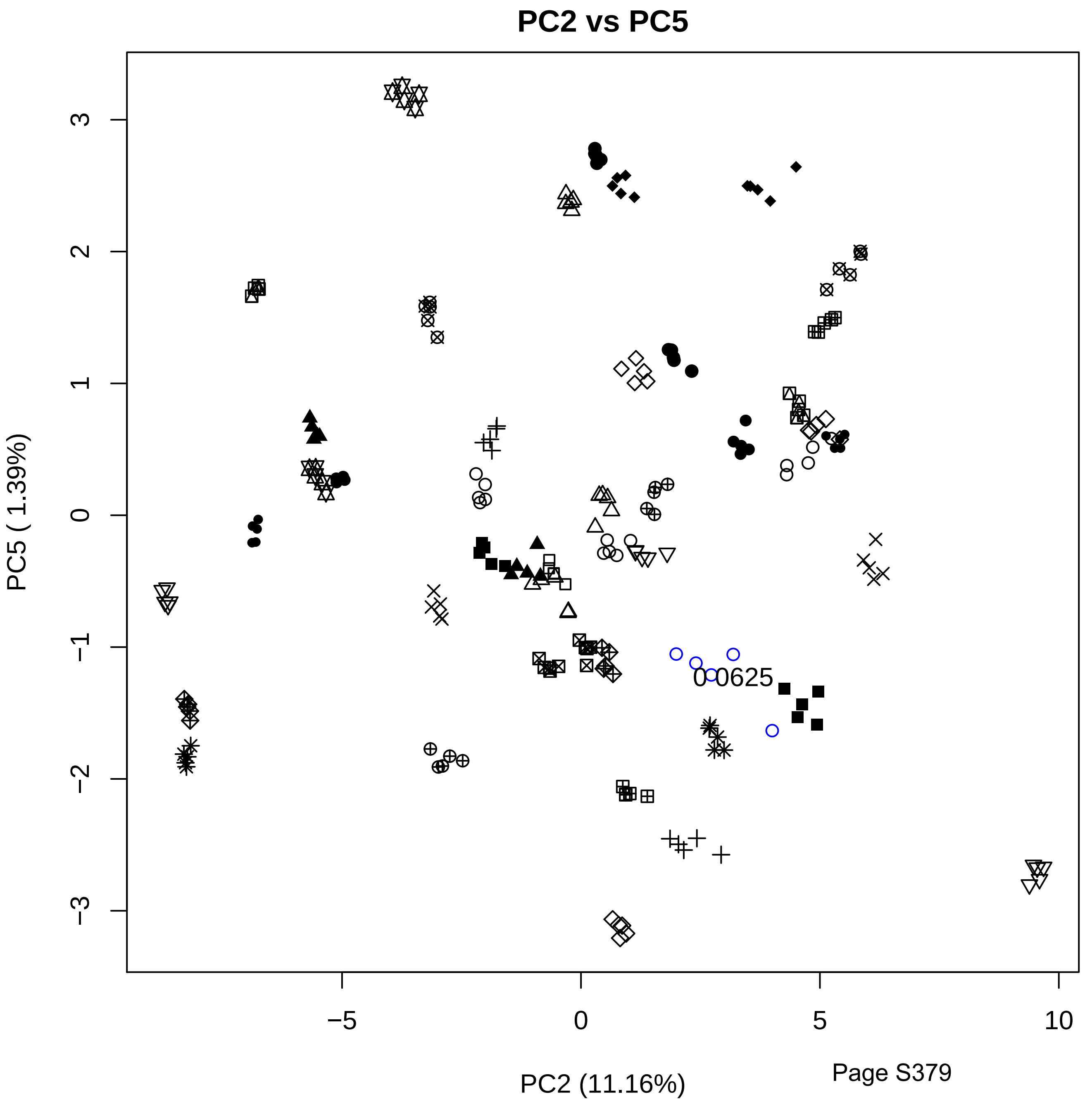
PC2 vs PC5
PC5 (1.39%)
PC2 (11.16%)
3
2
1
0
−1
−2
−3
−5
0
5
10
0.0625

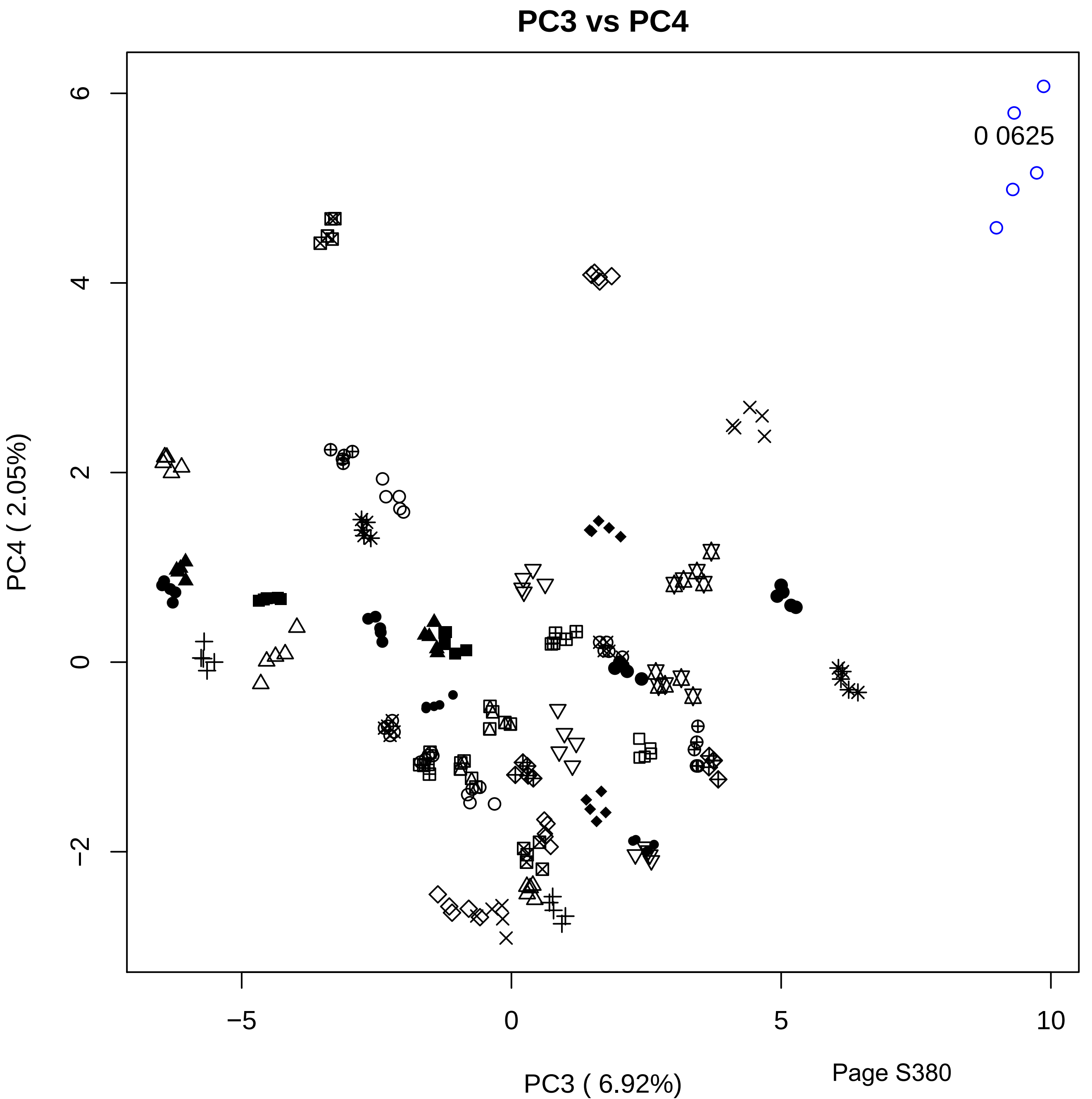

PC3 vs PC4
0 0625
PC4 (2.05%)
PC3 (6.92%)
−5
0
5
10
−2
0
2
4
6

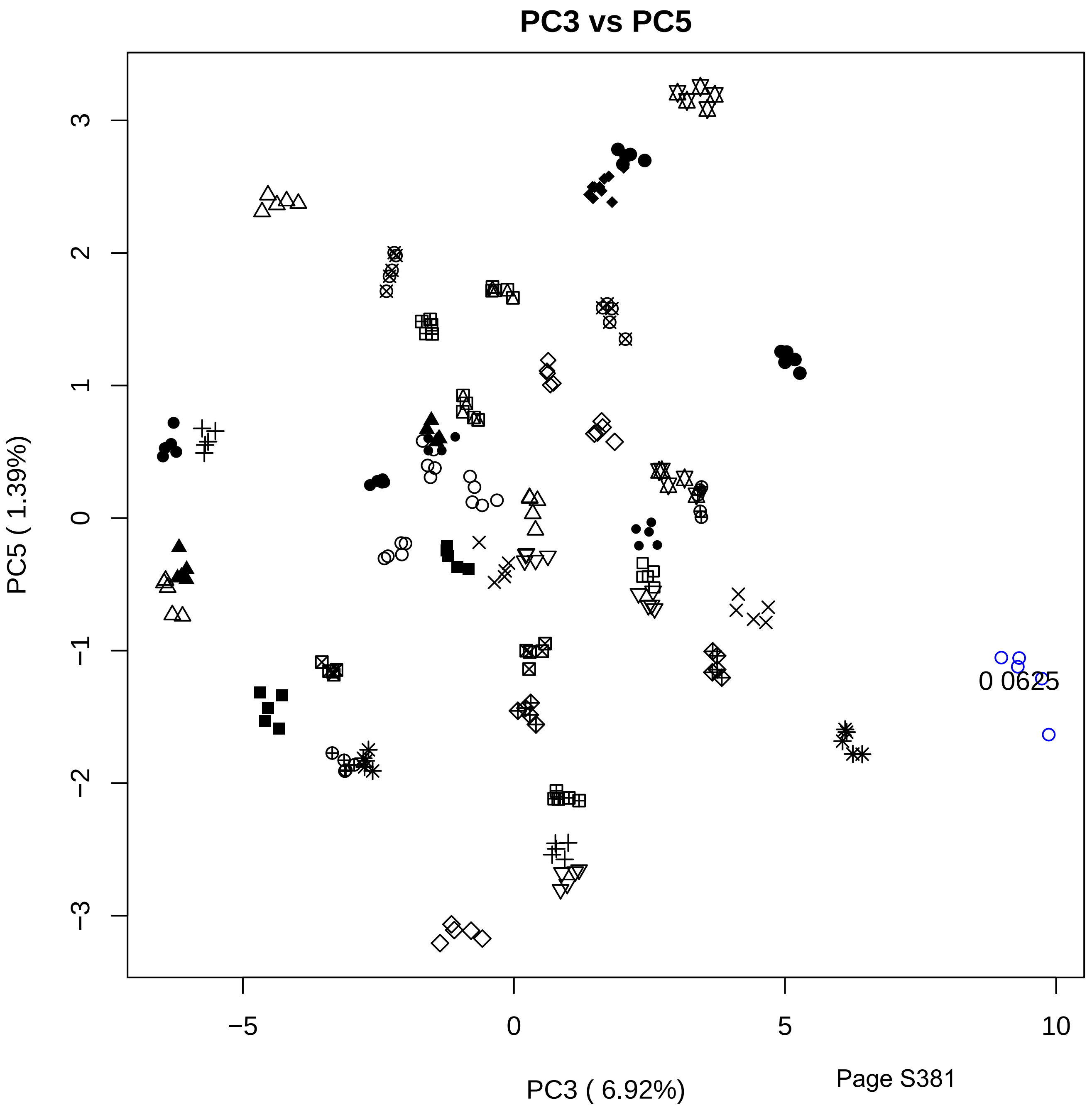
PC3 vs PC5
PC5 (1.39%)
PC3 (6.92%)
3
2
1
0
−1
−2
−3
−5
0
5
10
0 0625

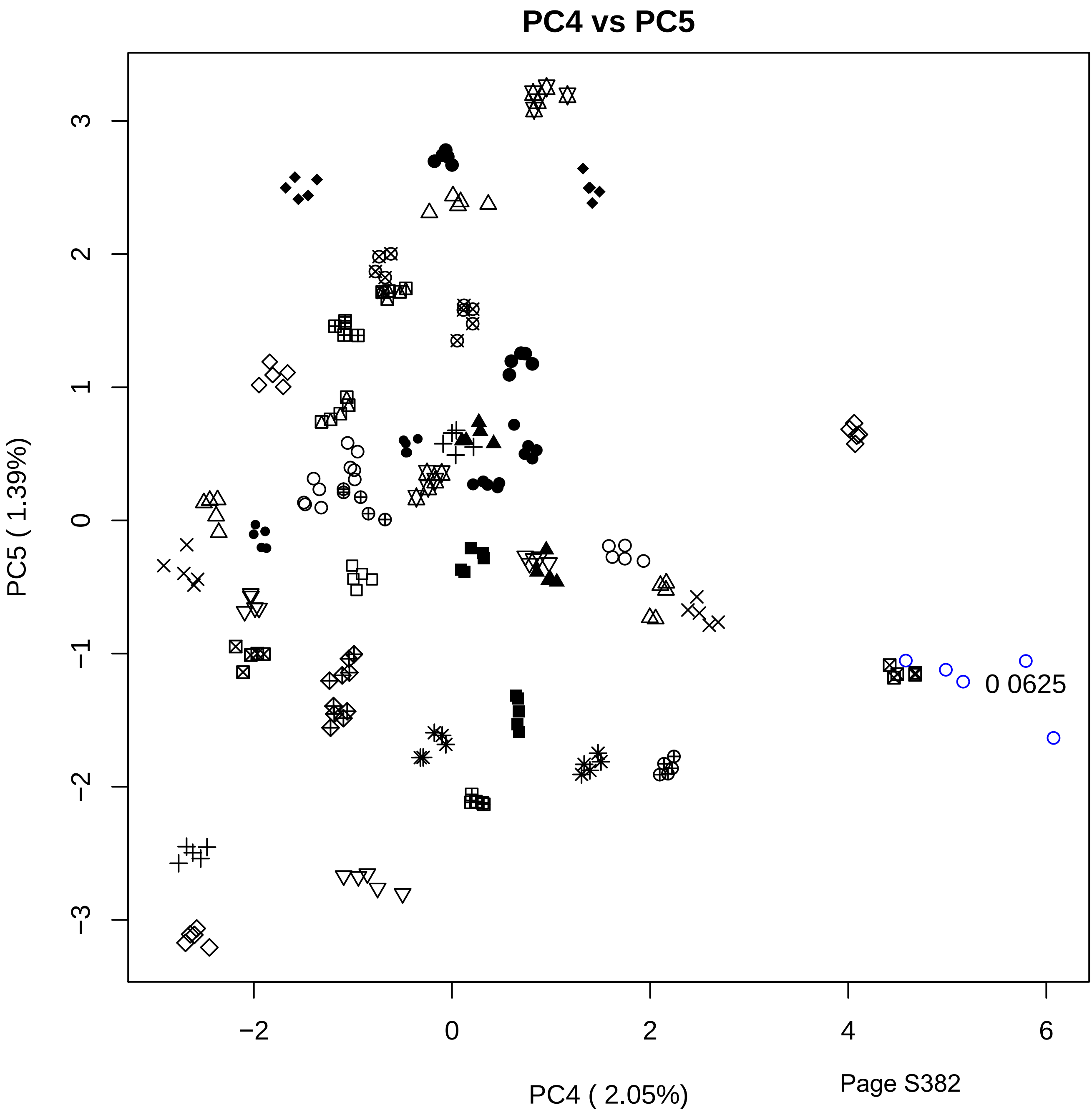

PC4 vs PC5
PC5 (1.39%)
PC4 (2.05%)
3
2
1
0
−1
−2
−3
−2
0
2
4
6
0 0625

PCA of 0.12% OSAS vs PMH

Scaled Matrix PCAed

7th order polynomial baseline correction performed

Raw spectra smoothed to 21 Neighbours, 2nd order PN, Savtisky Golay

Normalisation between 0 and 100

D2 spectra smoothed to 41 Neighbours, 2nd order PN, Savtisky Golay

Regions Analysed: 3601−2501 and 2001−700cm−1

Samples Analysed: 1 Contam's 0 OSAS's 45 PMH's

Keele University AD　　　Sun Dec 09 16:28:41 2018

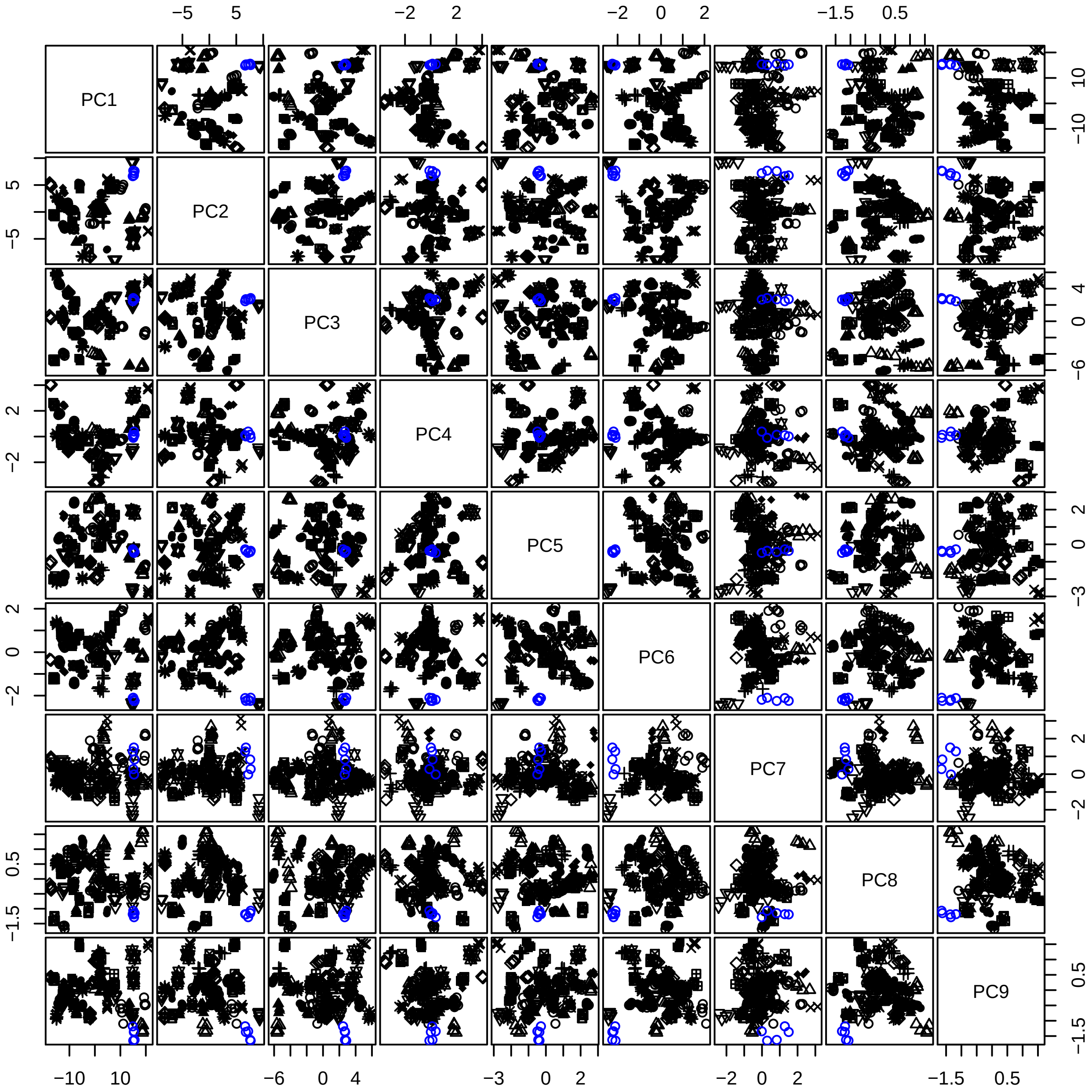
PC1
PC2
PC3
PC4
PC5
PC6
PC7
PC8
PC9

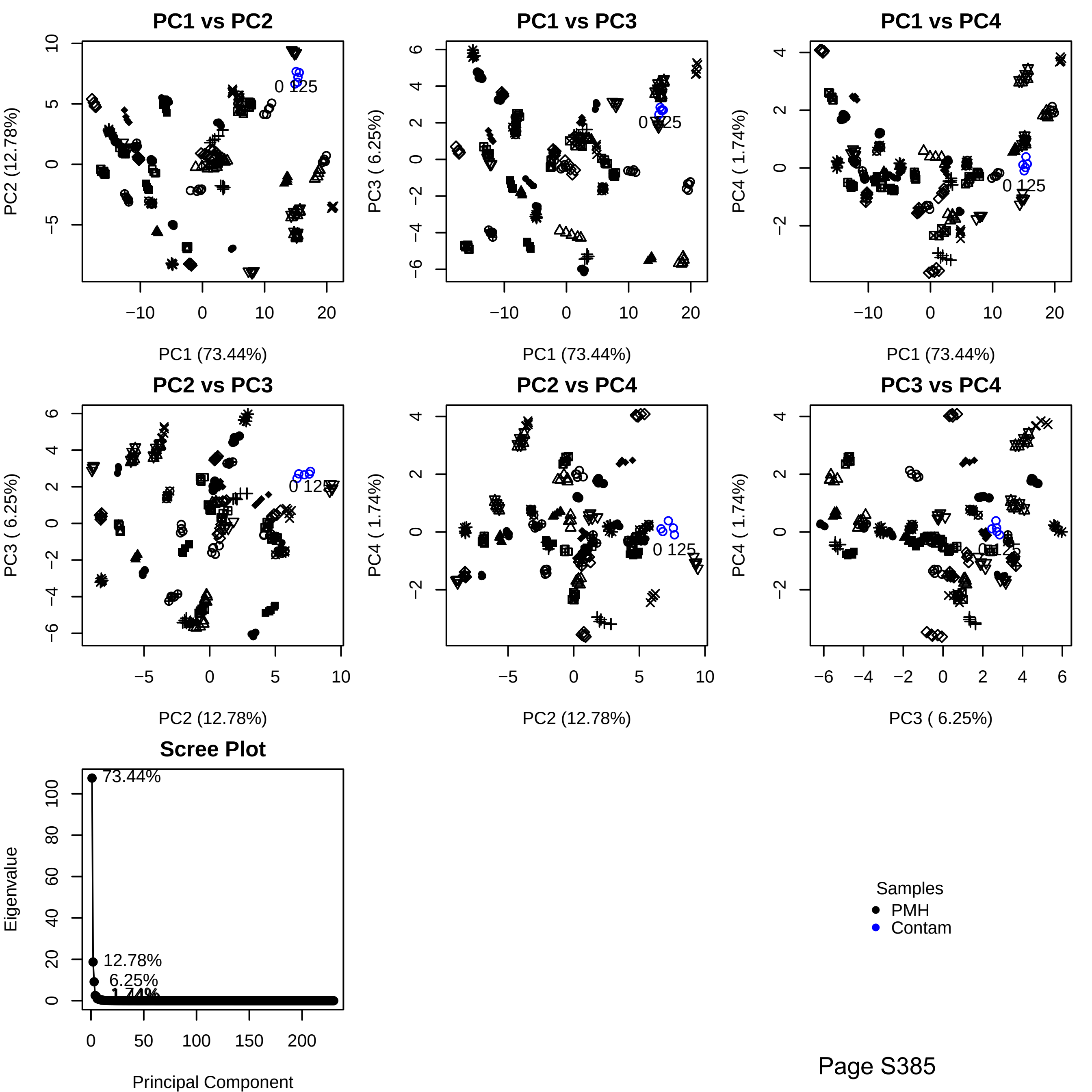
PC1 vs PC2
PC1 vs PC3
PC1 vs PC4
PC2 vs PC3
PC2 vs PC4
PC3 vs PC4
Scree Plot
PC1 (73.44%)
PC2 (12.78%)
PC3 (6.25%)
PC4 (1.74%)
Eigenvalue
Principal Component
73.44%
12.78%
6.25%
Samples
PMH
Contam

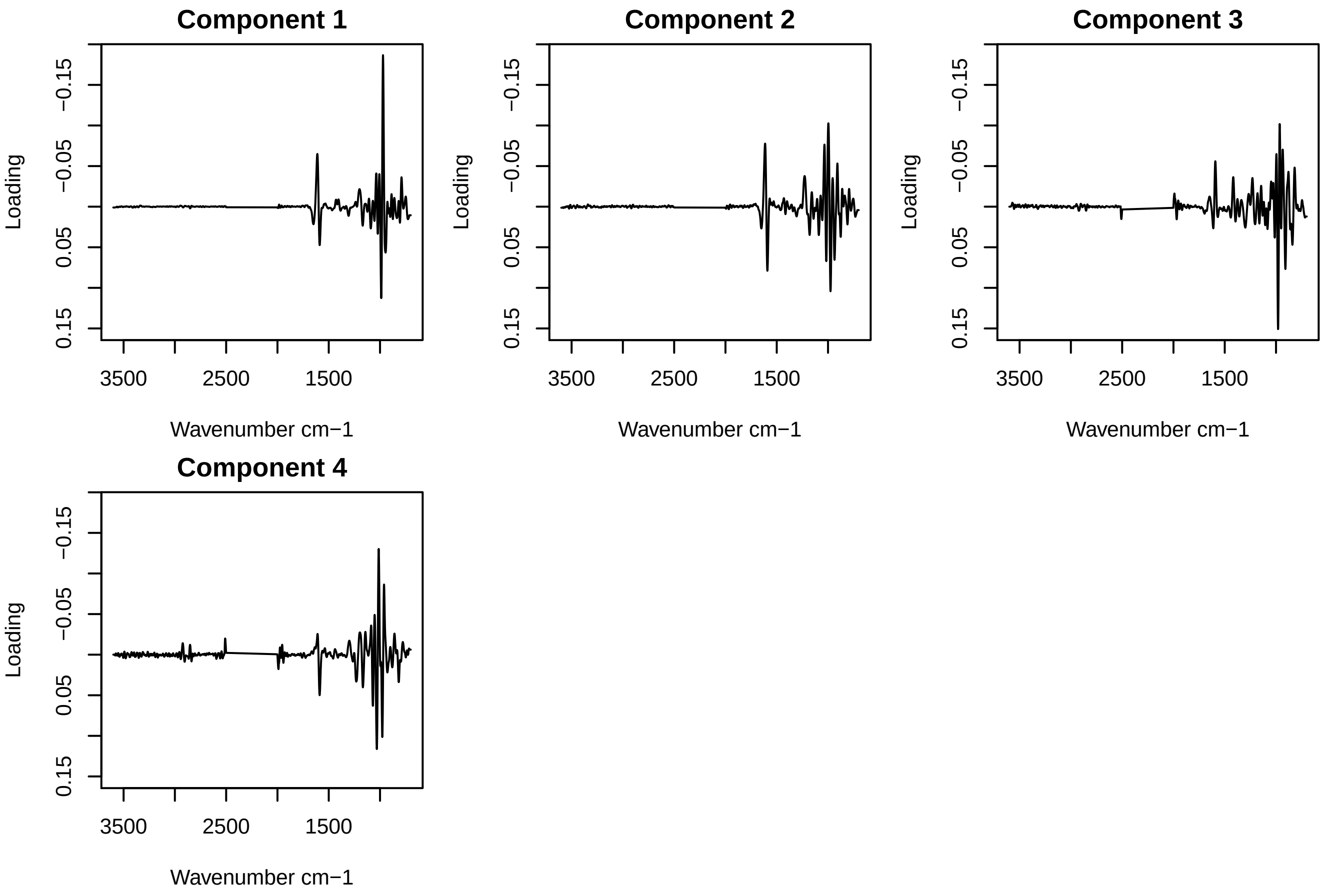
Component 1
Component 2
Component 3
Component 4
Loading
−0.15
−0.05
0.05
0.15
3500
2500
1500
Wavenumber cm−1

PC1 vs PC2 vs PC3

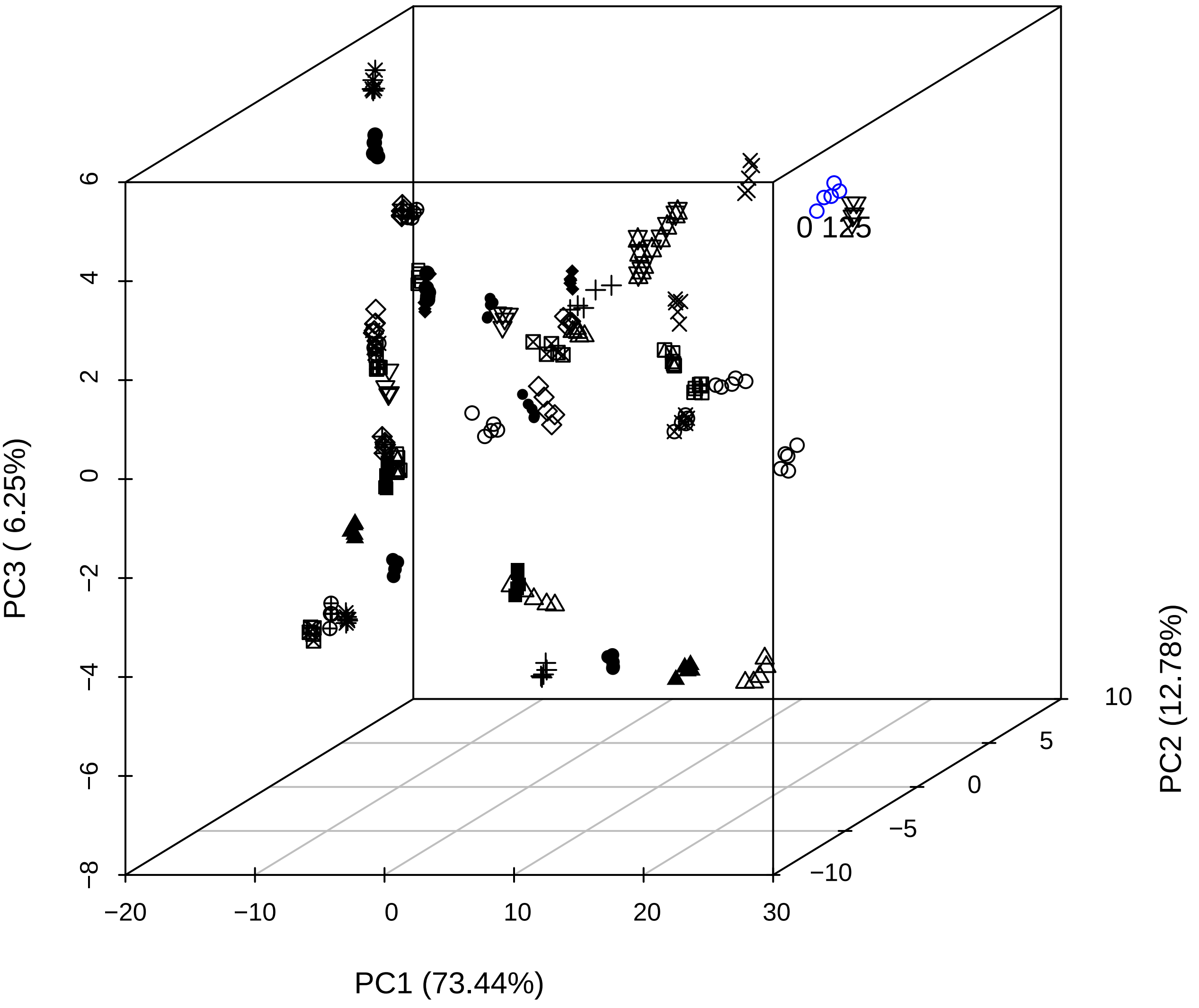

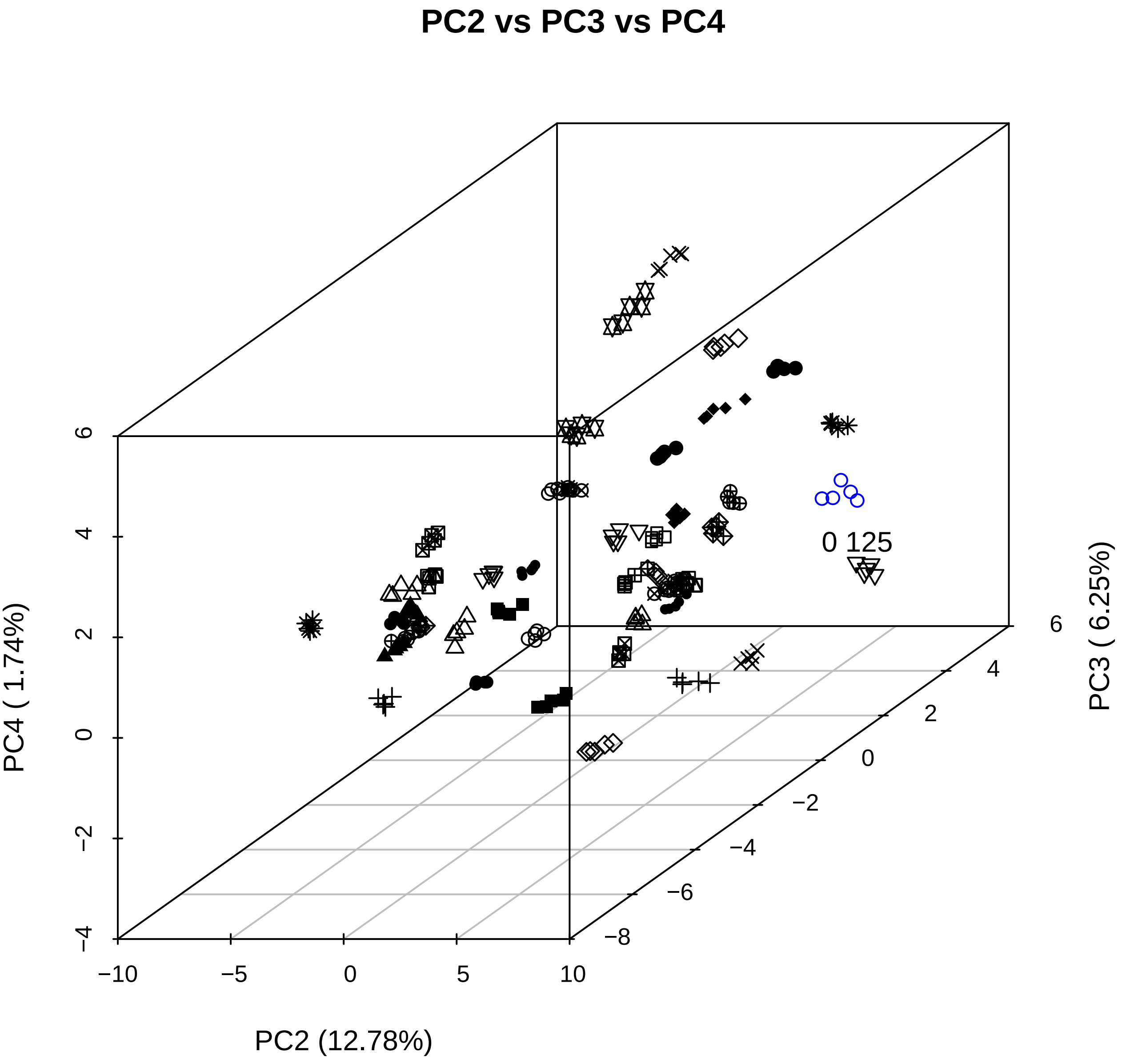
PC2 vs PC3 vs PC4
0 125
PC2 (12.78%)
PC3 (6.25%)
PC4 (1.74%)
−10
−5
0
5
10
−8
−6
−4
−2
0
2
4
6
−4
−2
0
2
4
6

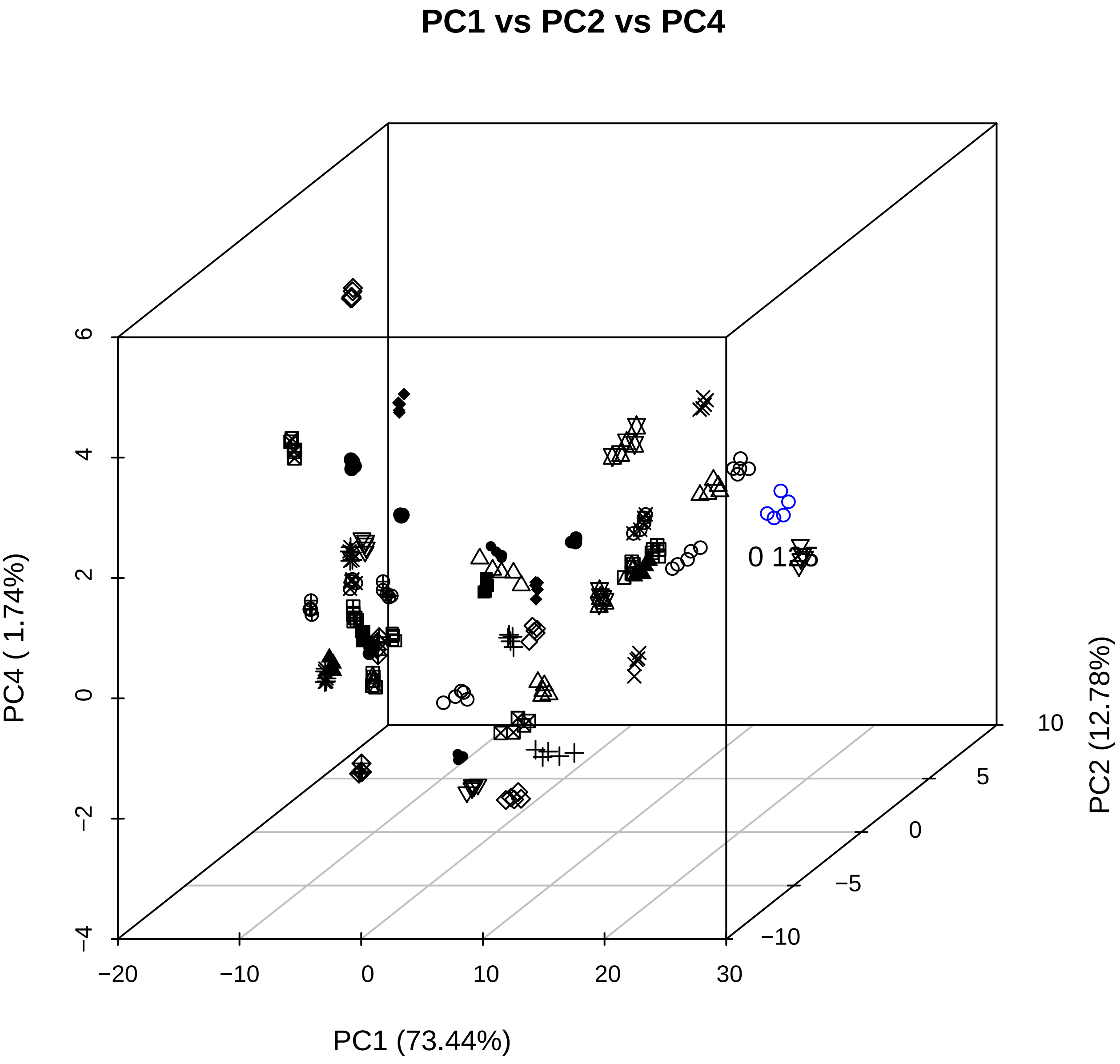
PC1 vs PC2 vs PC4
PC4 (1.74%)
PC1 (73.44%)
PC2 (12.78%)
0 125
−4
−2
0
2
4
6
−20
−10
0
10
20
30
−10
−5
0
5
10

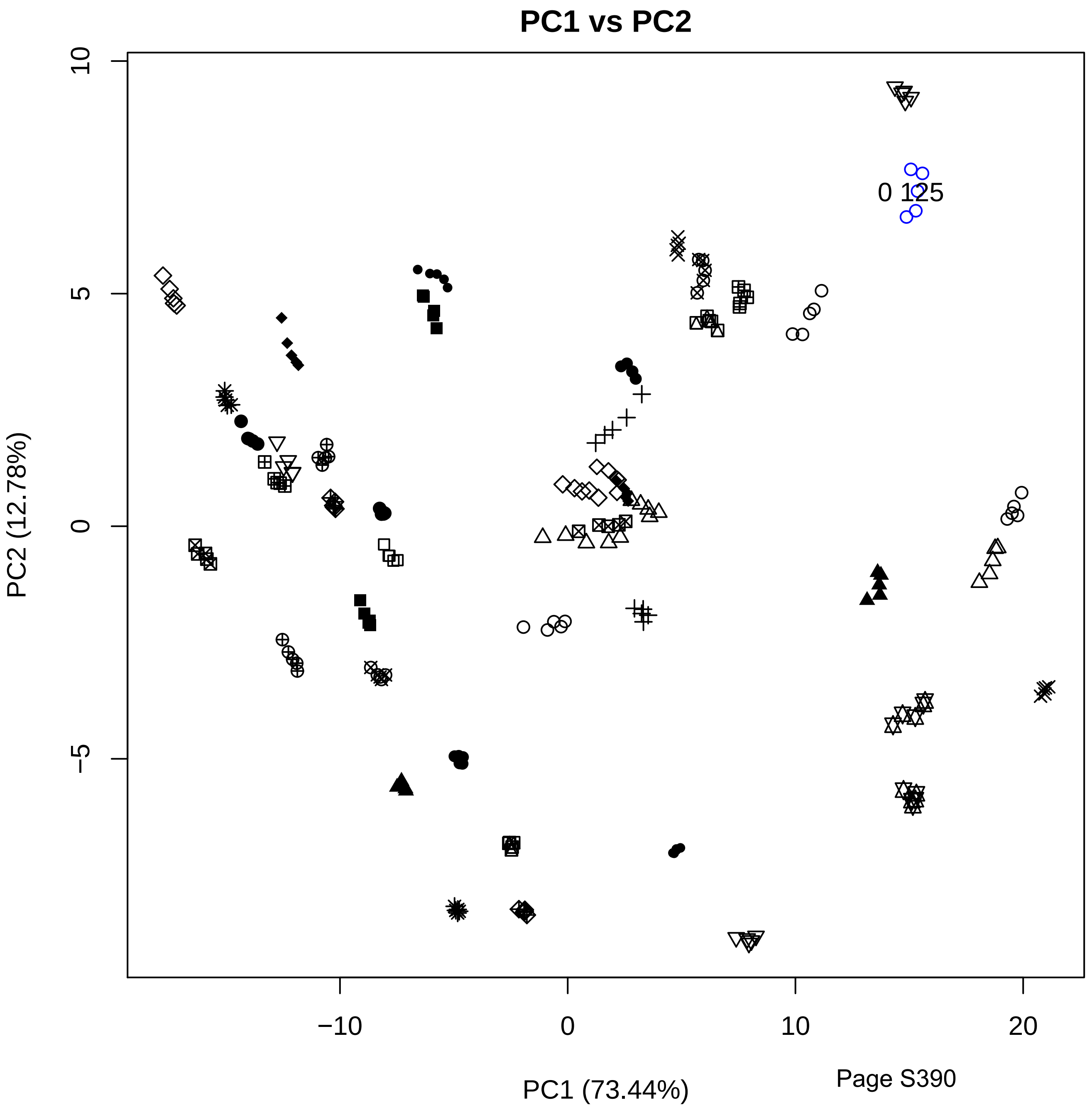

PC1 vs PC2
PC2 (12.78%)
PC1 (73.44%)
10
5
0
−5
−10
0
10
20
0 125

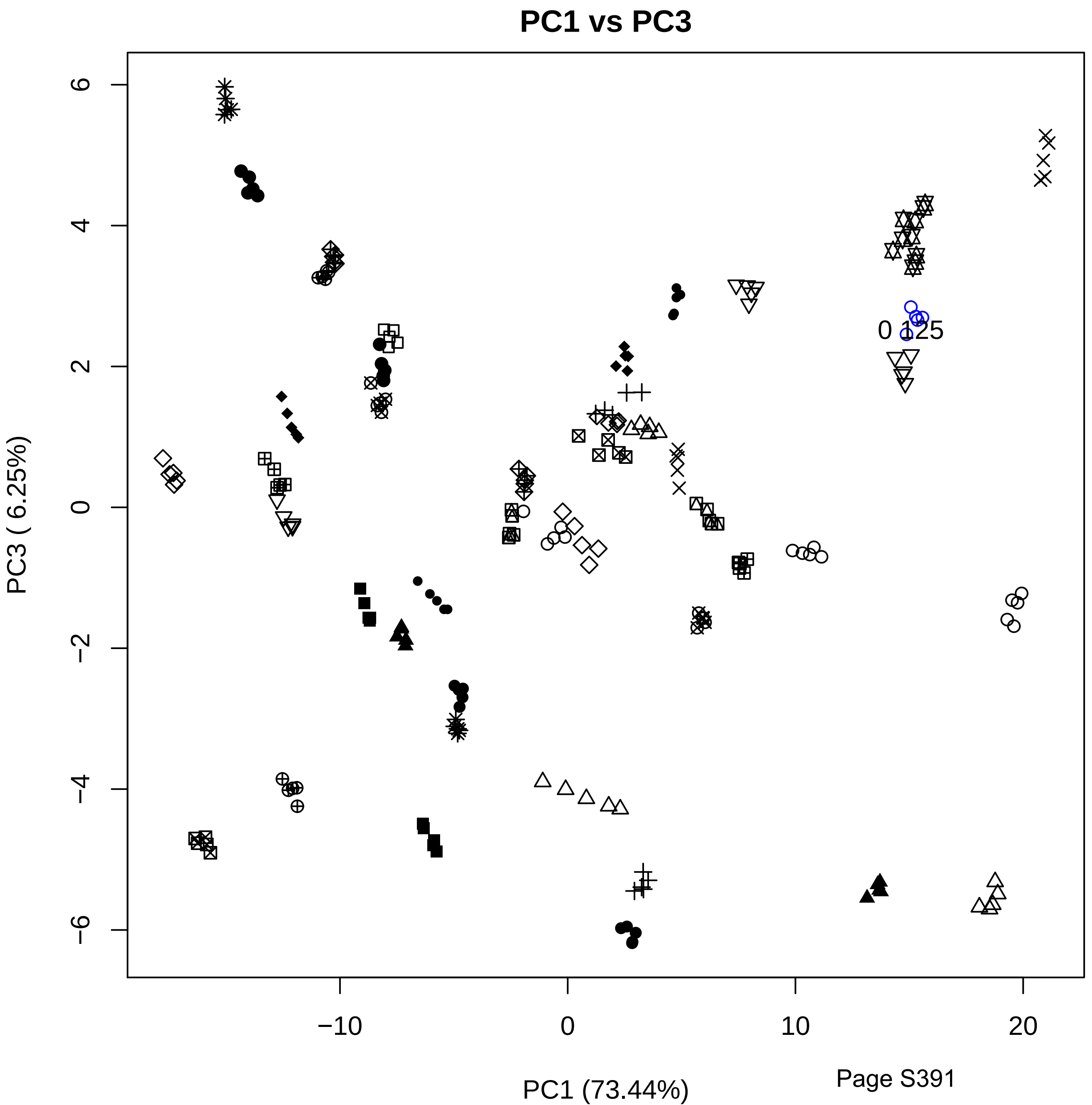
PC1 vs PC3
PC3 (6.25%)
PC1 (73.44%)
6
4
2
0
−2
−4
−6
−10
0
10
20
0 125

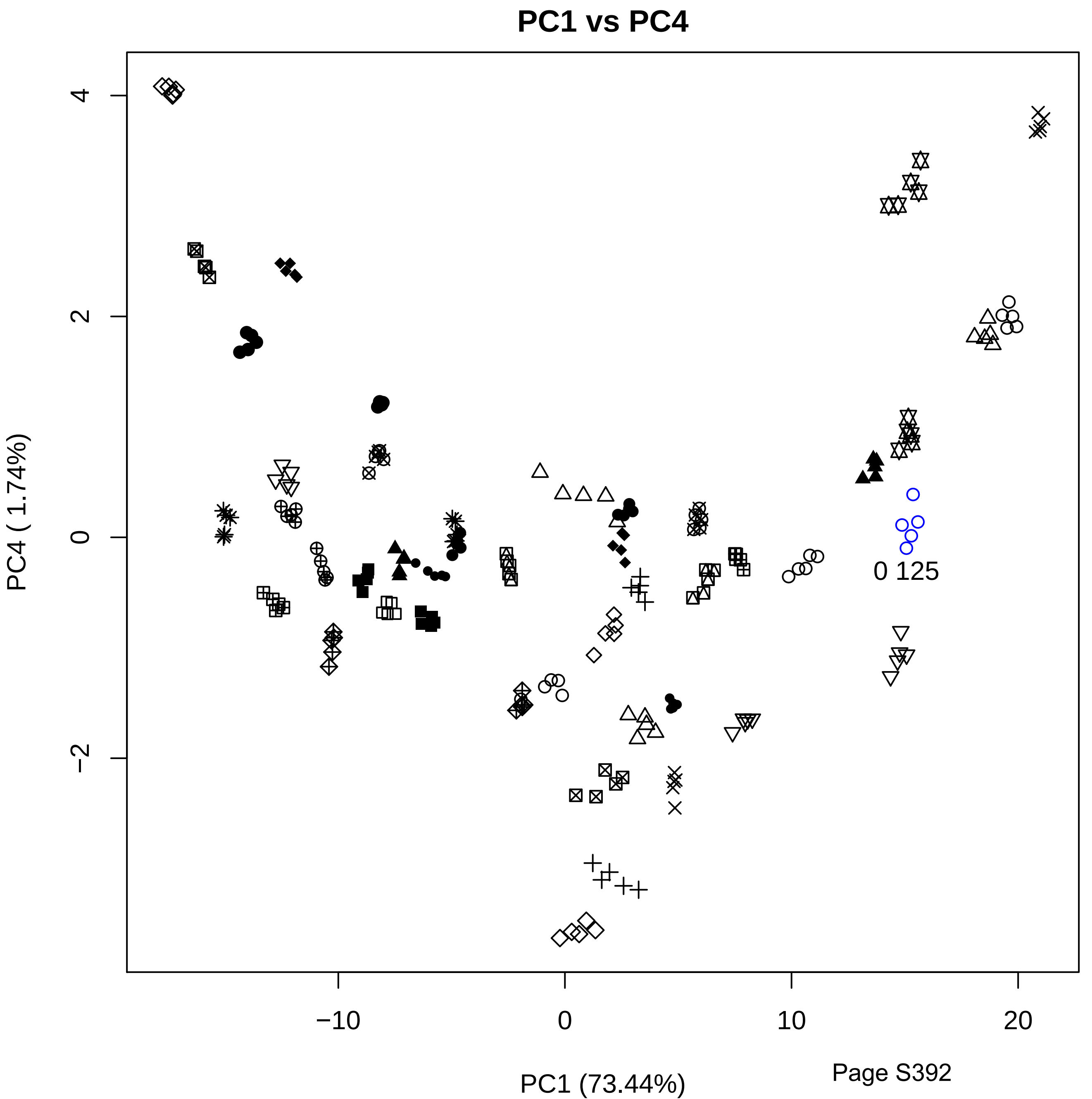
PC1 vs PC4
PC4 (1.74%)
PC1 (73.44%)
4
2
0
−2
−10
0
10
20
0 125

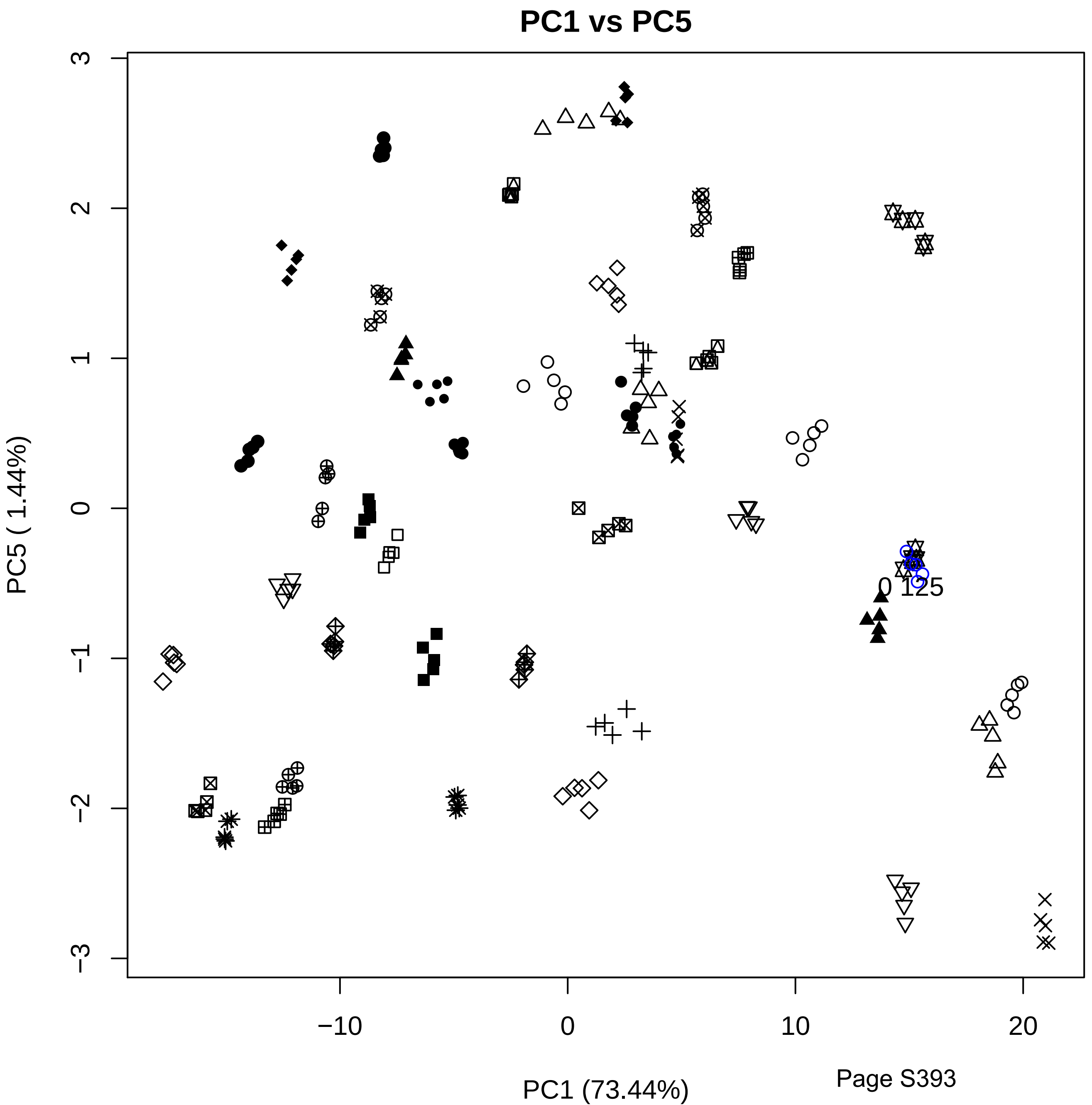
PC1 vs PC5
PC5 (1.44%)
PC1 (73.44%)
3
2
1
0
−1
−2
−3
−10
0
10
20
0 125

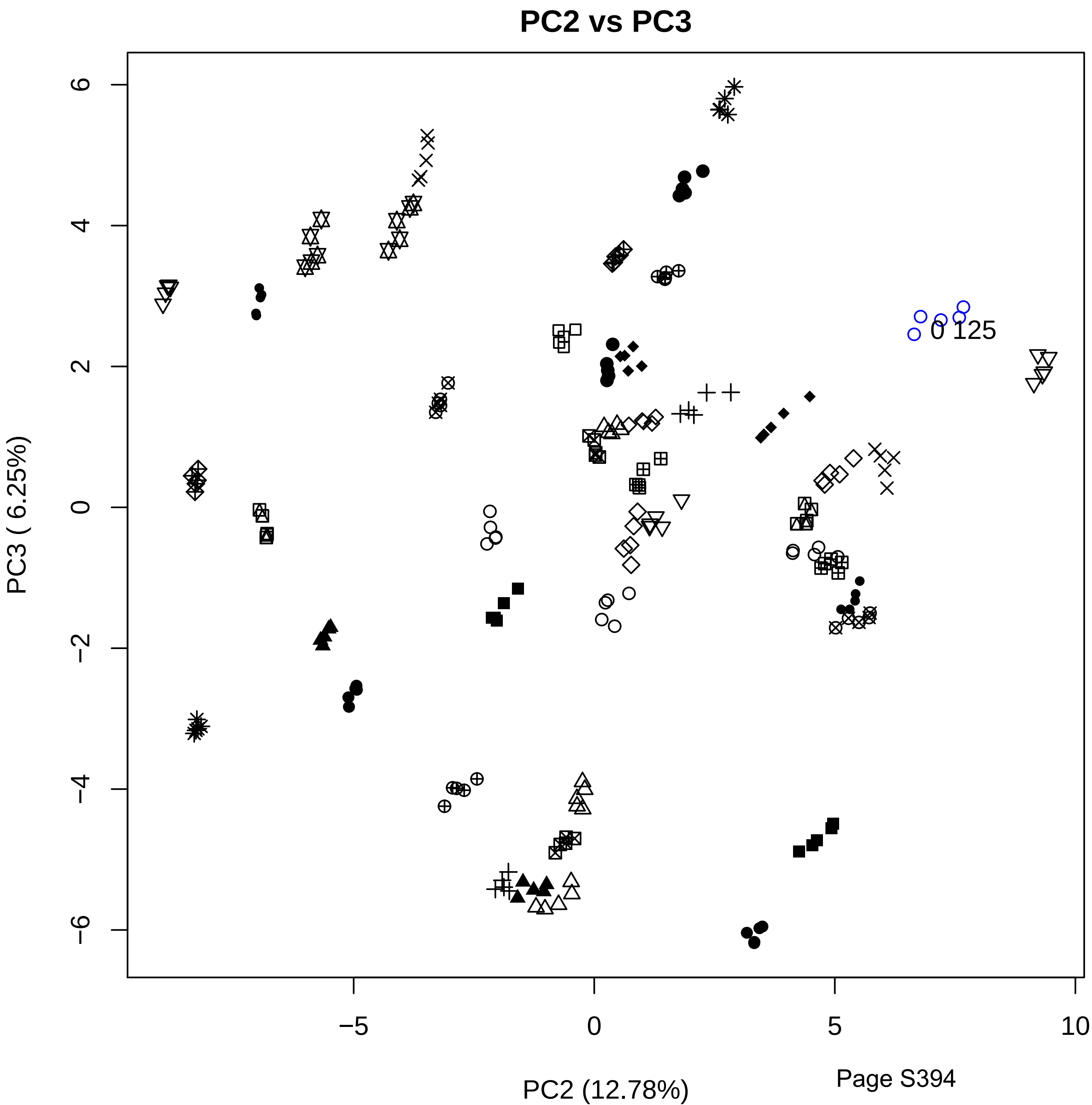
PC2 vs PC3
0 125
PC2 (12.78%)
PC3 (6.25%)
−5
0
5
10
6
4
2
0
−2
−4
−6

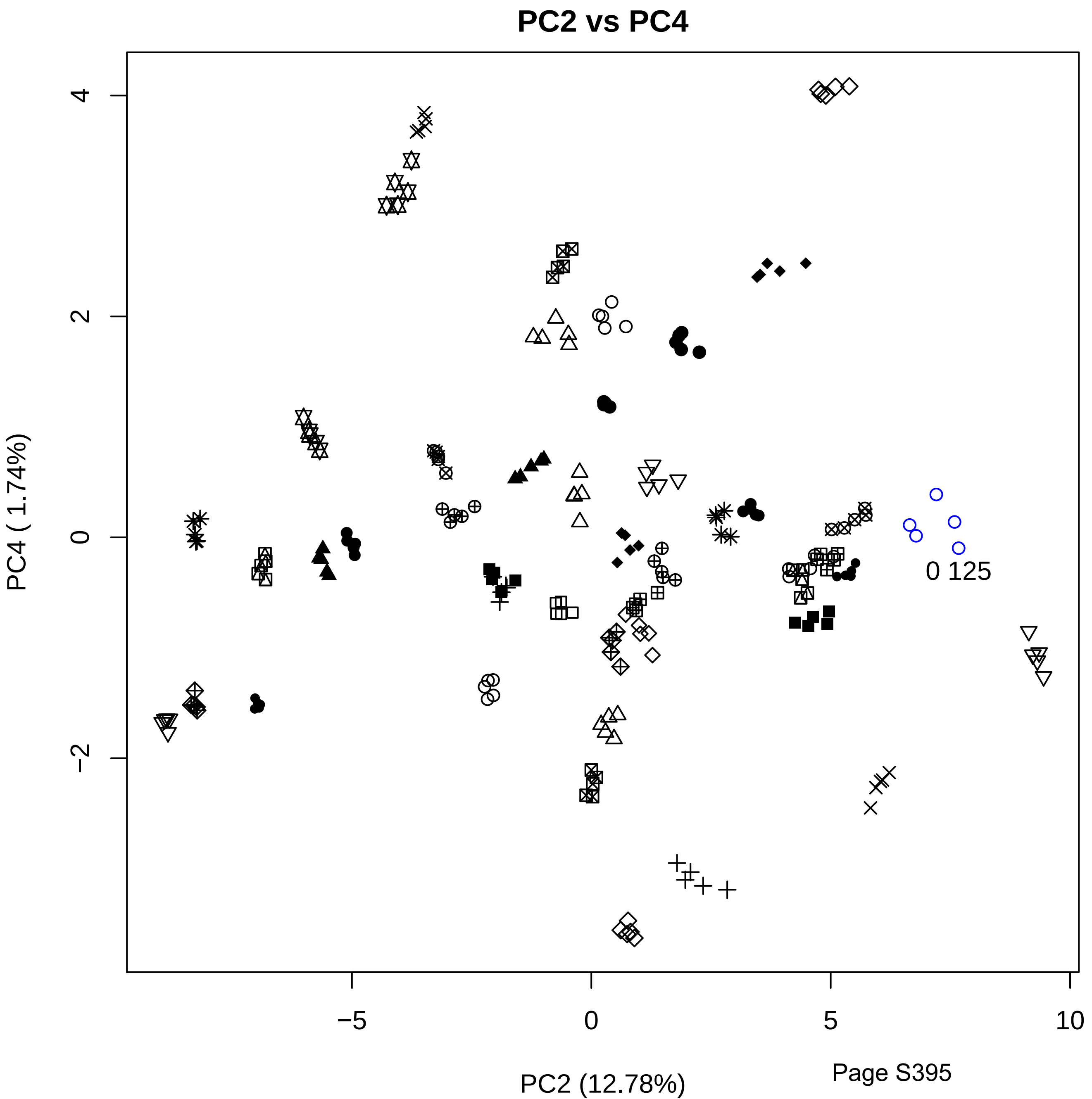

PC2 vs PC4
4
2
0
−2
PC4 (1.74%)
−5
0
5
10
PC2 (12.78%)
0 125

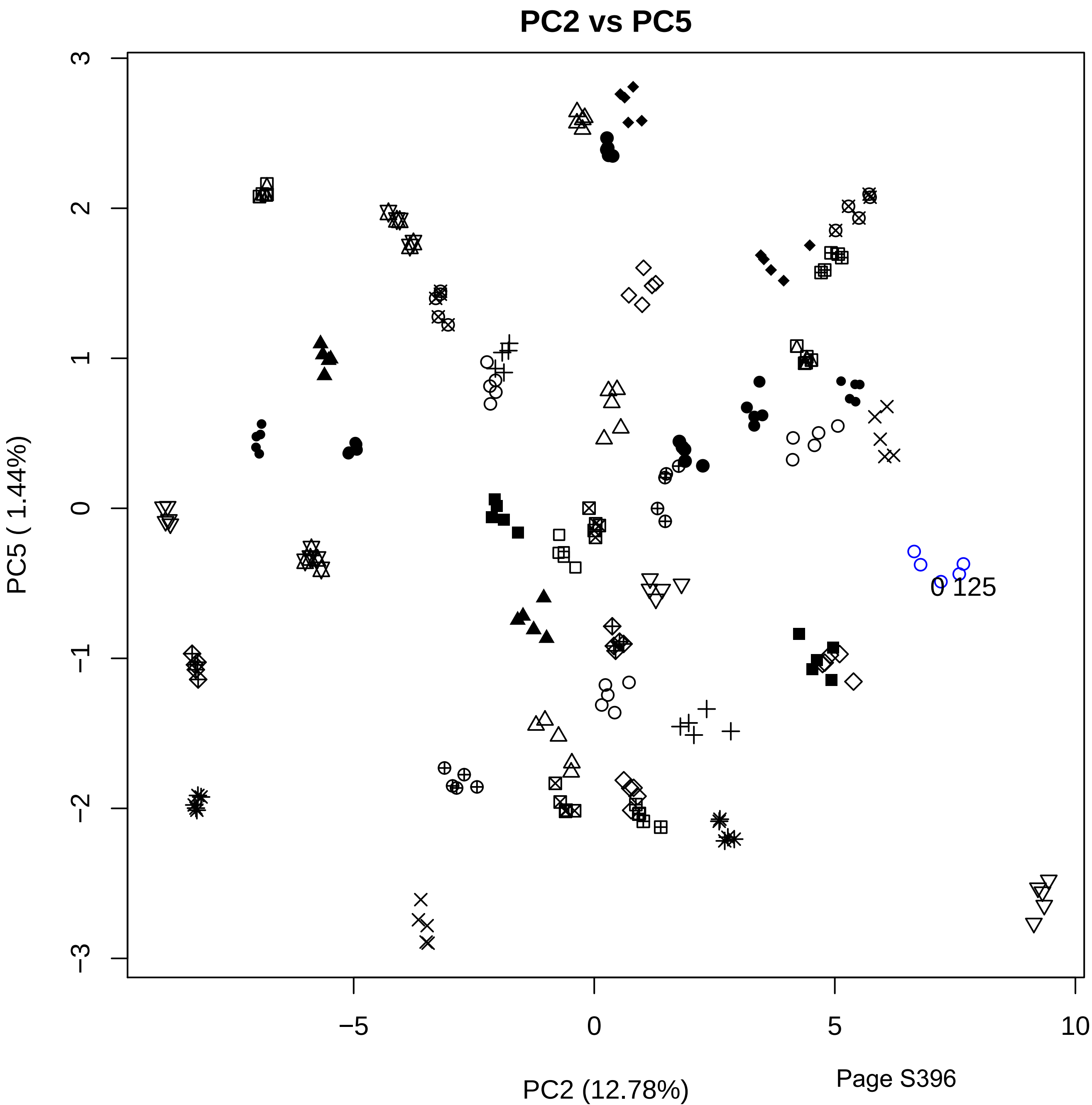
PC2 vs PC5
PC5 (1.44%)
PC2 (12.78%)
3
2
1
0
−1
−2
−3
−5
0
5
10
0 125

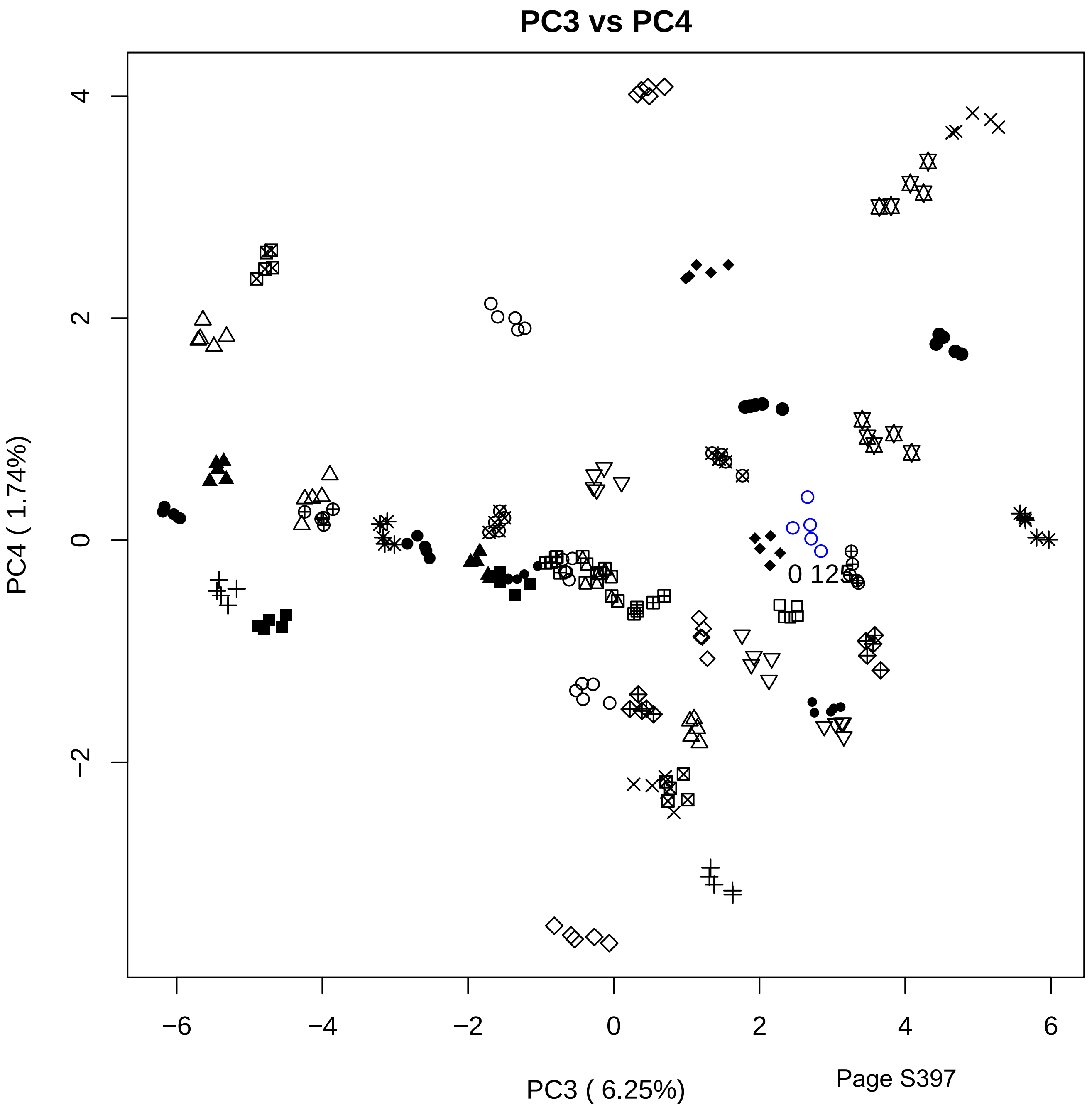
PC3 vs PC4
PC4 (1.74%)
PC3 (6.25%)
4
2
0
−2
−6
−4
−2
0
2
4
6
0 125

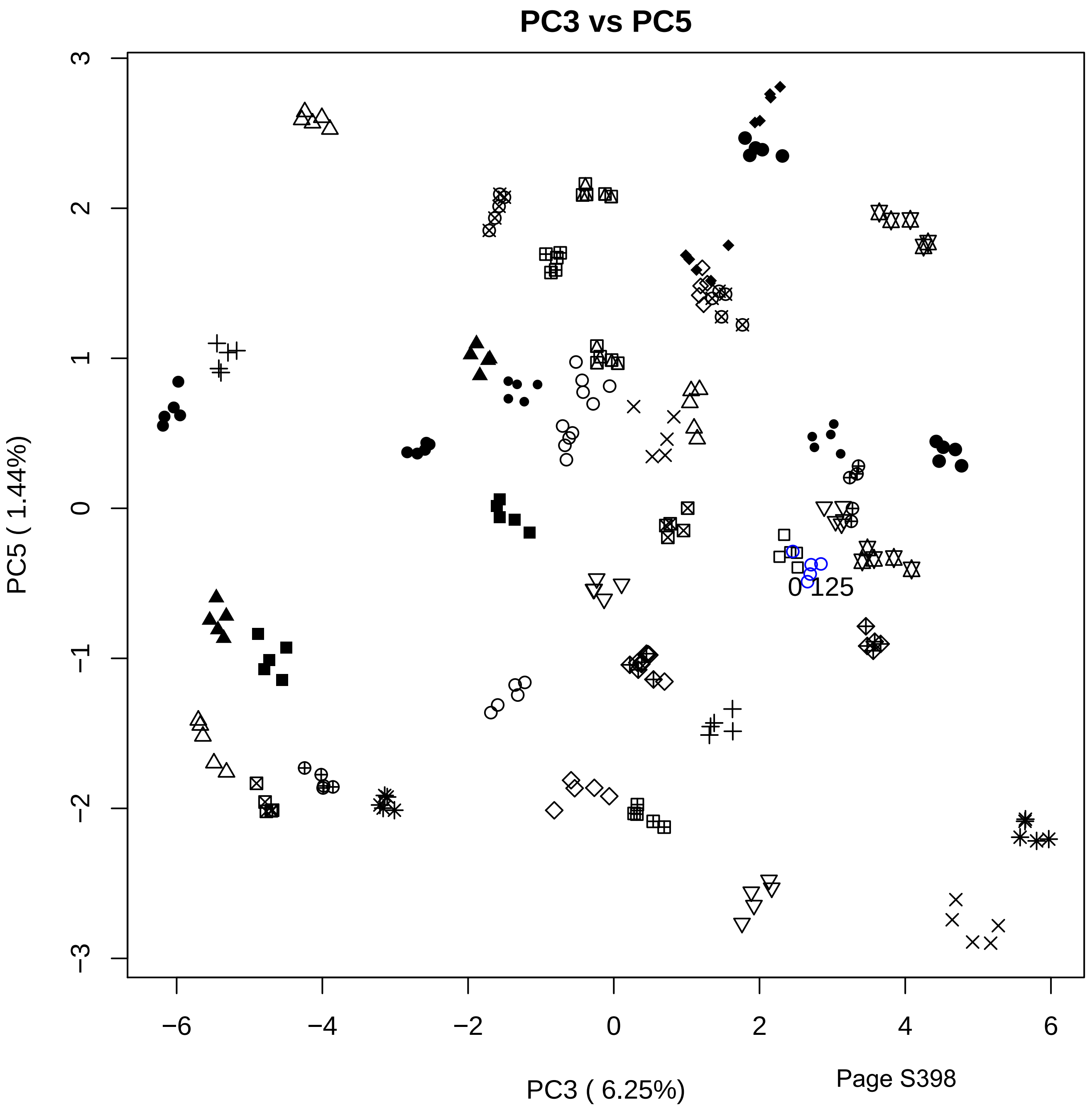
PC3 vs PC5
PC5 (1.44%)
PC3 (6.25%)
3
2
1
0
−1
−2
−3
−6
−4
−2
0
2
4
6
0 125

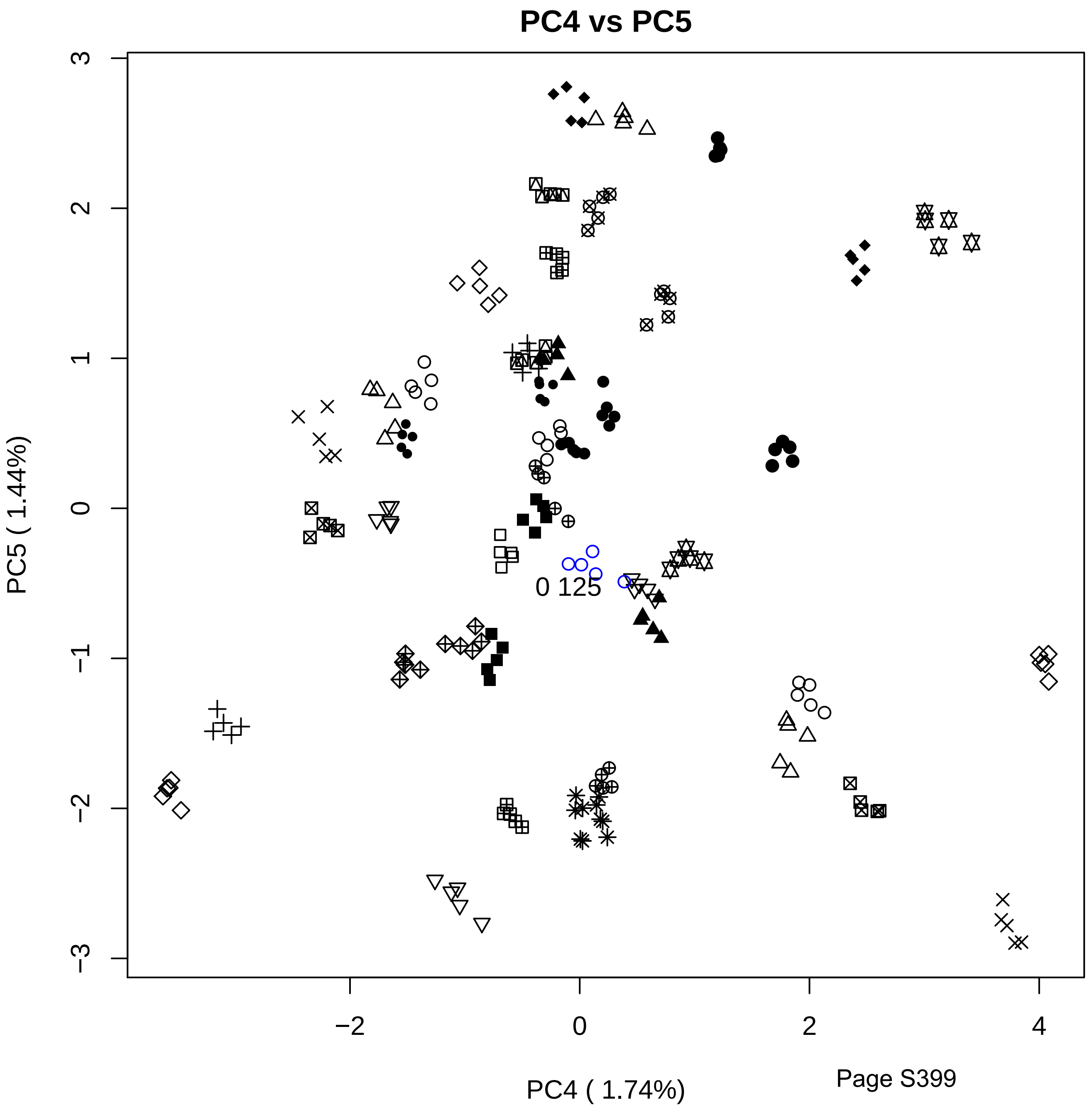

PC4 vs PC5
0 125
3
2
1
0
−1
−2
−3
−2
0
2
4
PC5 (1.44%)
PC4 (1.74%)

PCA of 0.25% OSAS vs PMH

Scaled Matrix PCAed

7th order polynomial baseline correction performed

Raw spectra smoothed to 21 Neighbours, 2nd order PN, Savtisky Golay

Normalisation between 0 and 100

D2 spectra smoothed to 41 Neighbours, 2nd order PN, Savtisky Golay

Regions Analysed: 3601−2501 and 2001−700cm−1

Samples Analysed: 1 Contam's 0 OSAS's 45 PMH's

Keele University AD   Sun Dec 09 16:28:47 2018

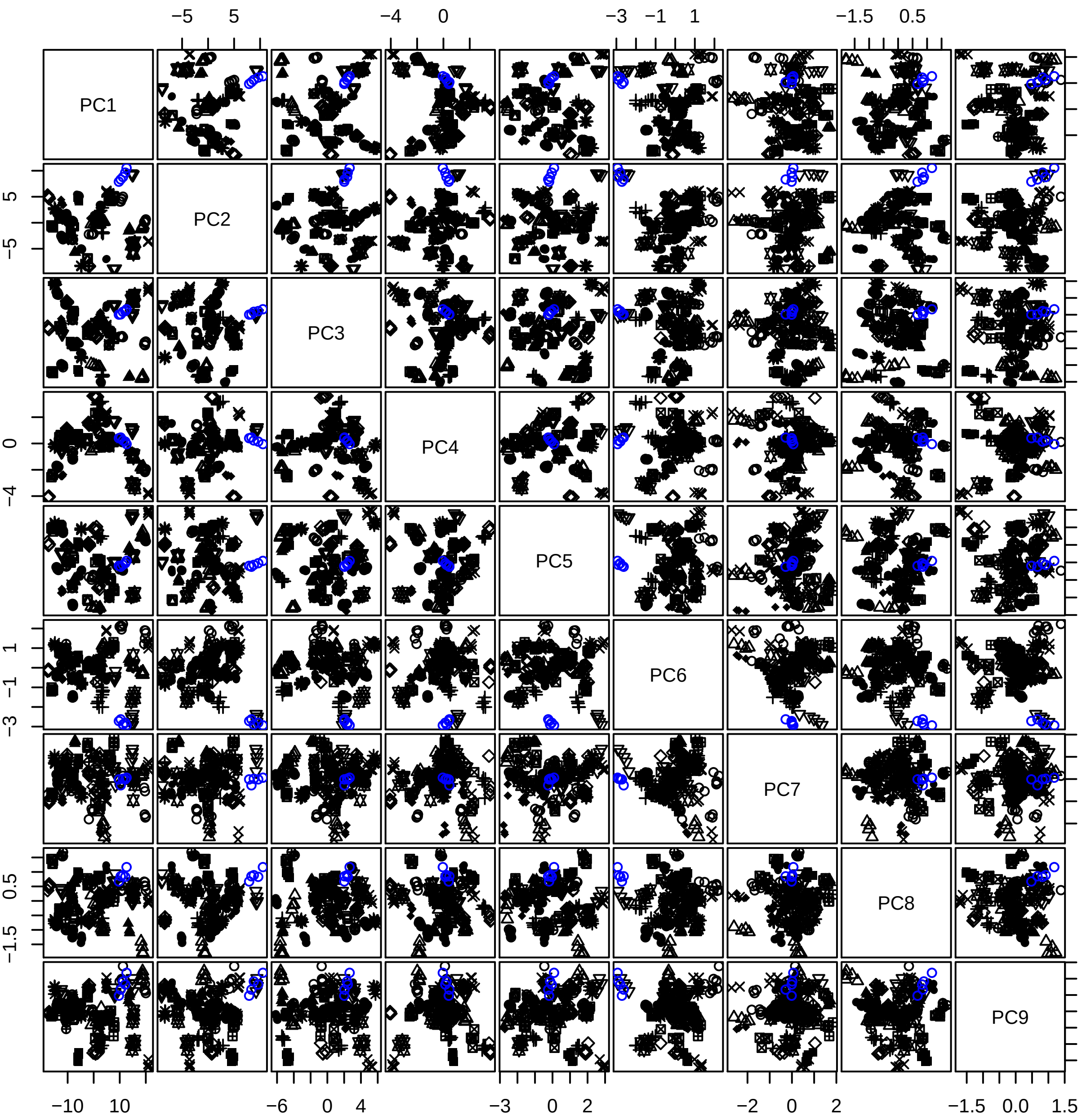

PC1
PC2
PC3
PC4
PC5
PC6
PC7
PC8
PC9

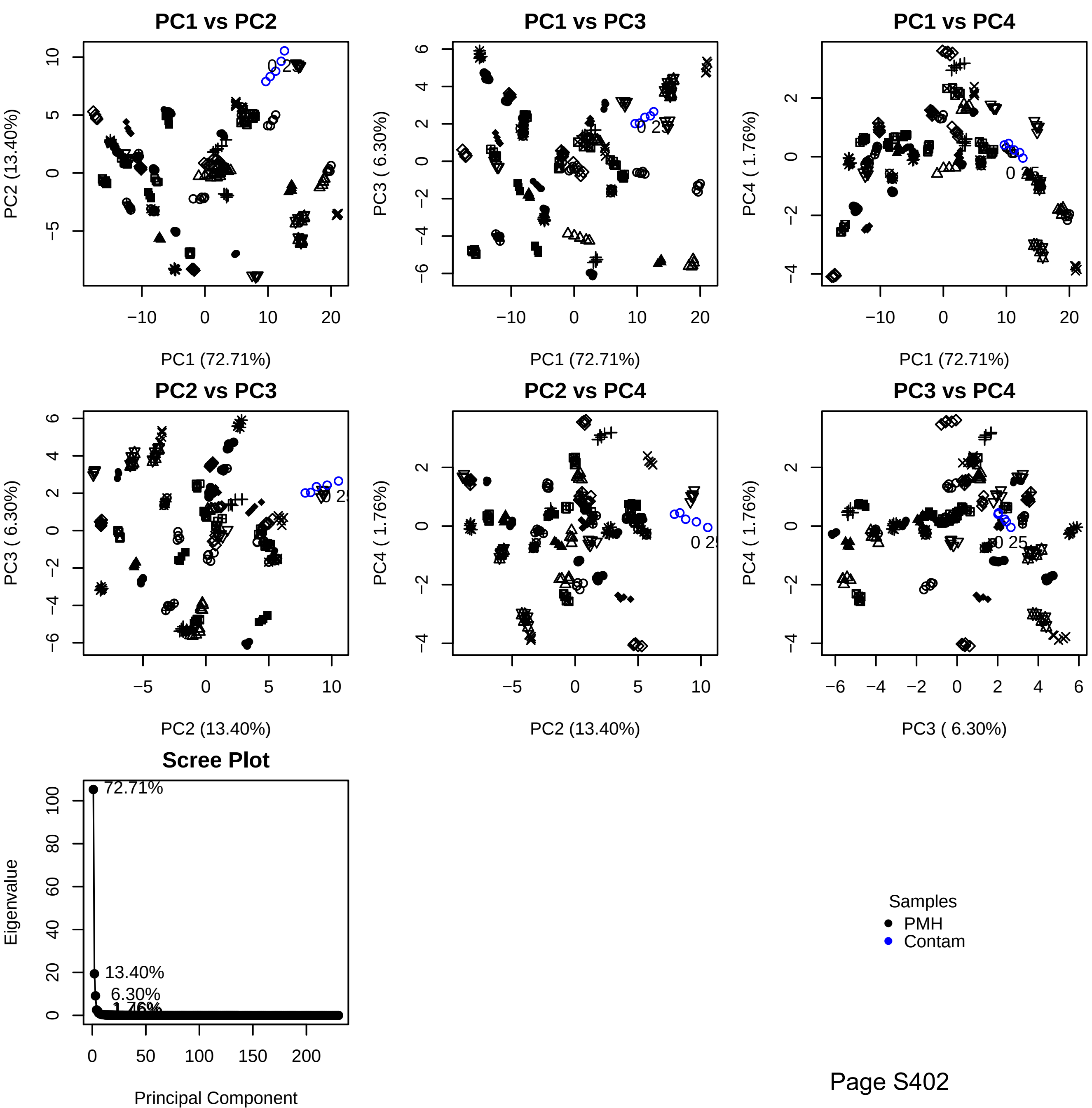

PC1 vs PC2
PC1 vs PC3
PC1 vs PC4
PC2 vs PC3
PC2 vs PC4
PC3 vs PC4
Scree Plot
PC1 (72.71%)
PC2 (13.40%)
PC3 (6.30%)
PC4 (1.76%)
Eigenvalue
Principal Component
72.71%
13.40%
6.30%
Samples
PMH
Contam

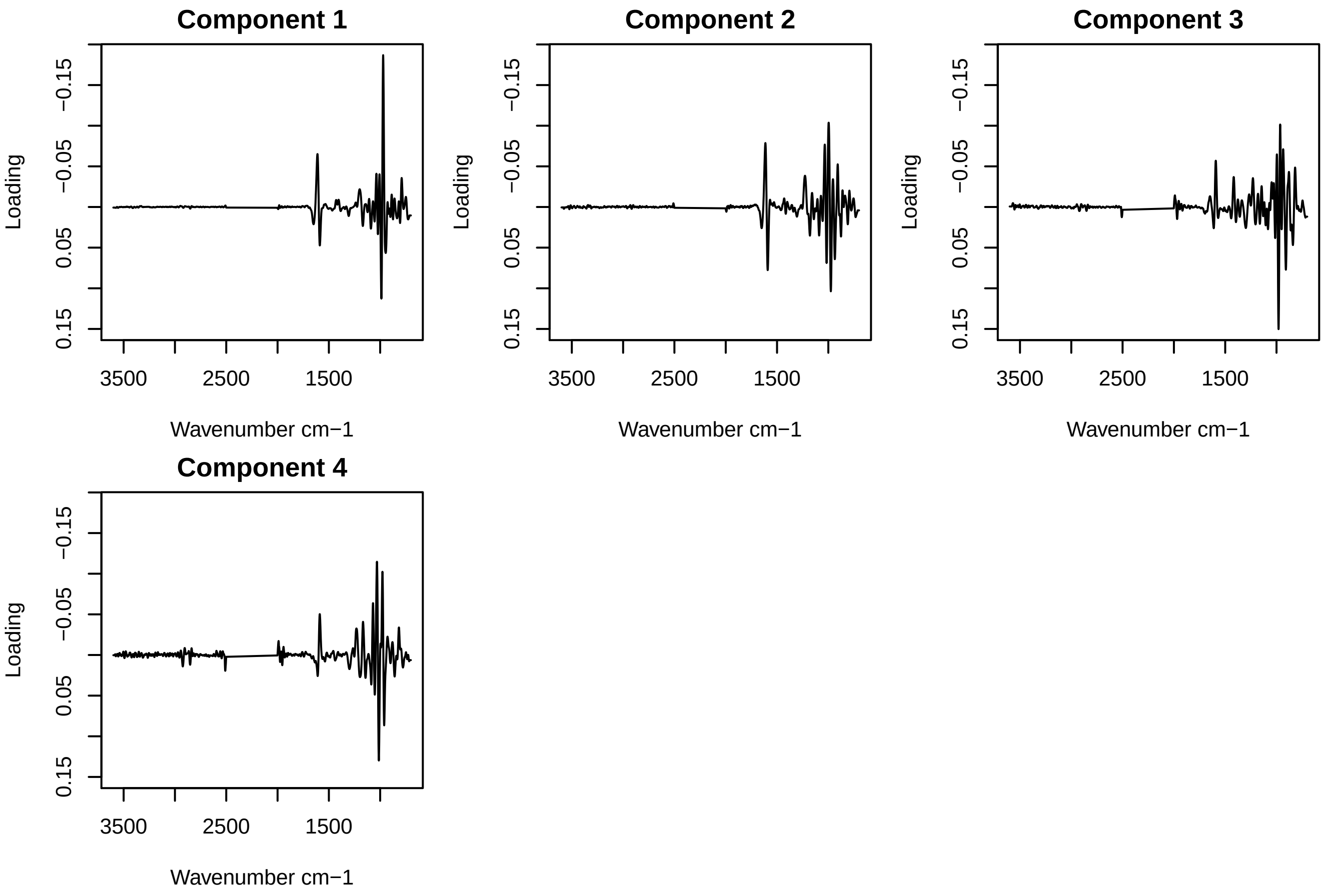

Component 1
Component 2
Component 3
Component 4
Loading
−0.15
−0.05
0.05
0.15
3500
2500
1500
Wavenumber cm−1

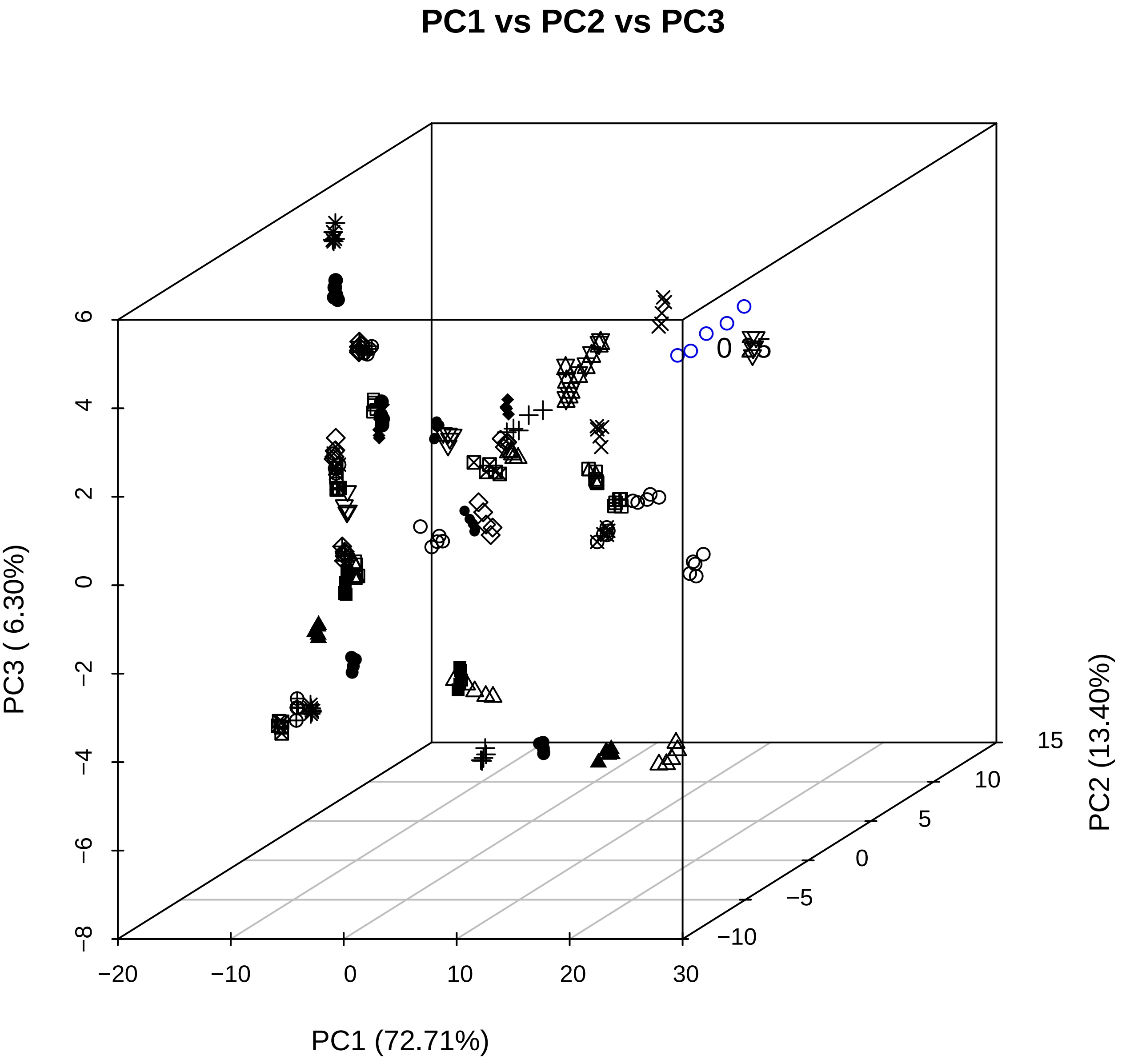
PC1 vs PC2 vs PC3
PC1 (72.71%)
PC2 (13.40%)
PC3 (6.30%)
−20
−10
0
10
20
30
−10
−5
0
5
10
15
−8
−6
−4
−2
0
2
4
6
0 25

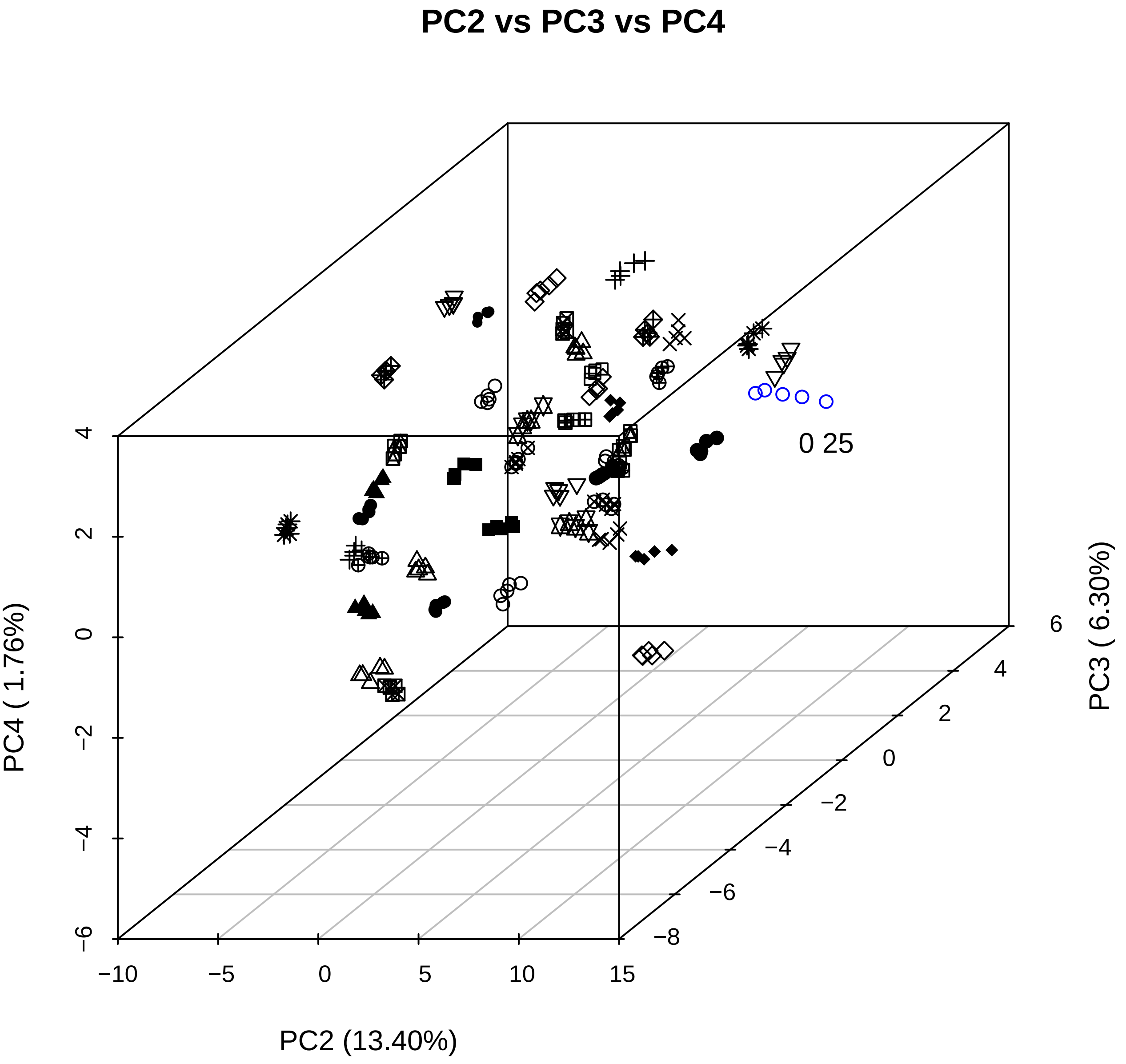
PC2 vs PC3 vs PC4
0 25
PC2 (13.40%)
PC3 (6.30%)
PC4 (1.76%)
−10
−5
0
5
10
15
−8
−6
−4
−2
0
2
4
6
−6
−4
−2
0
2
4

PC1 vs PC2 vs PC4

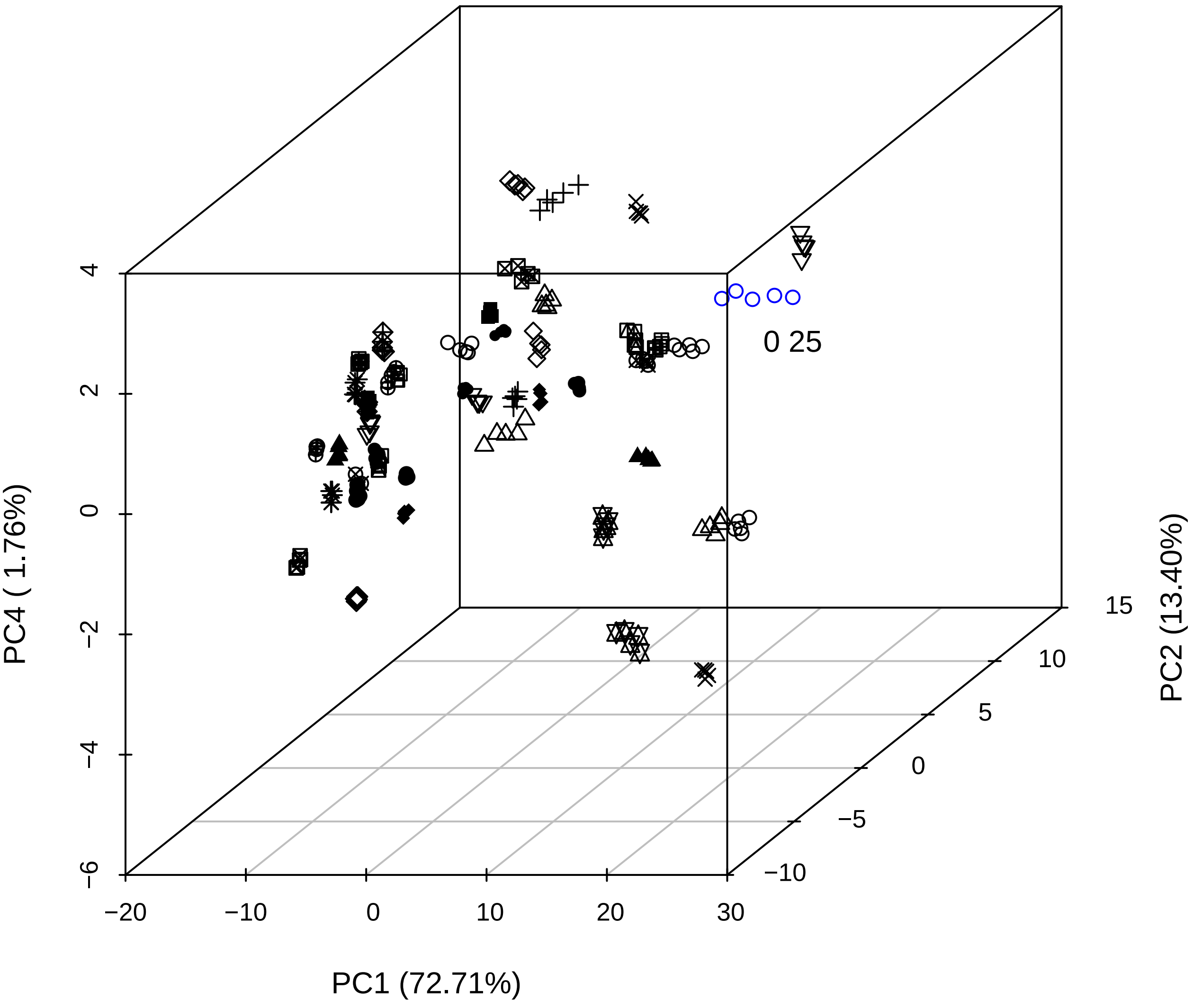

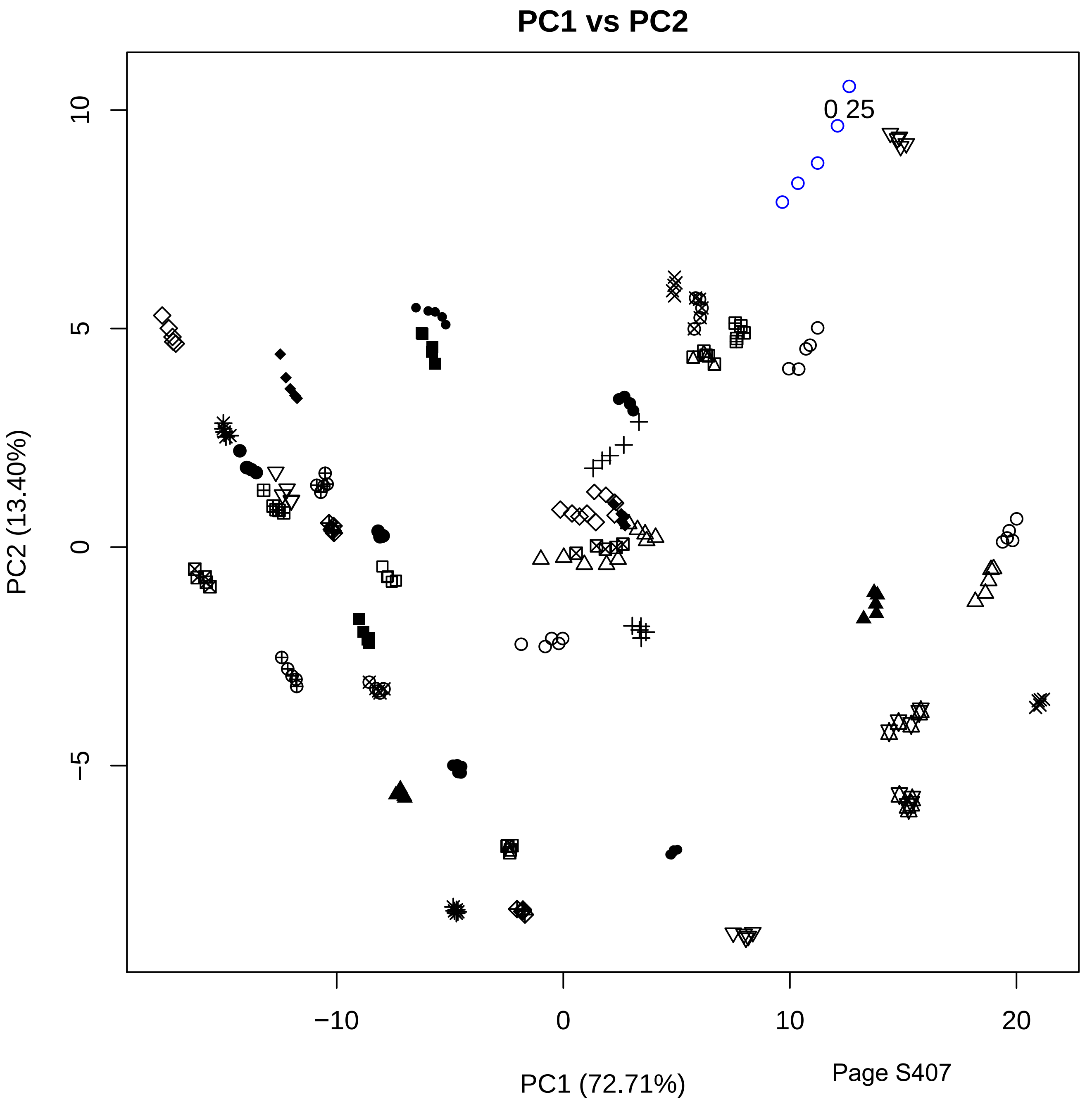

PC1 vs PC2
0 25
10
5
0
−5
−10
0
10
20
PC2 (13.40%)
PC1 (72.71%)

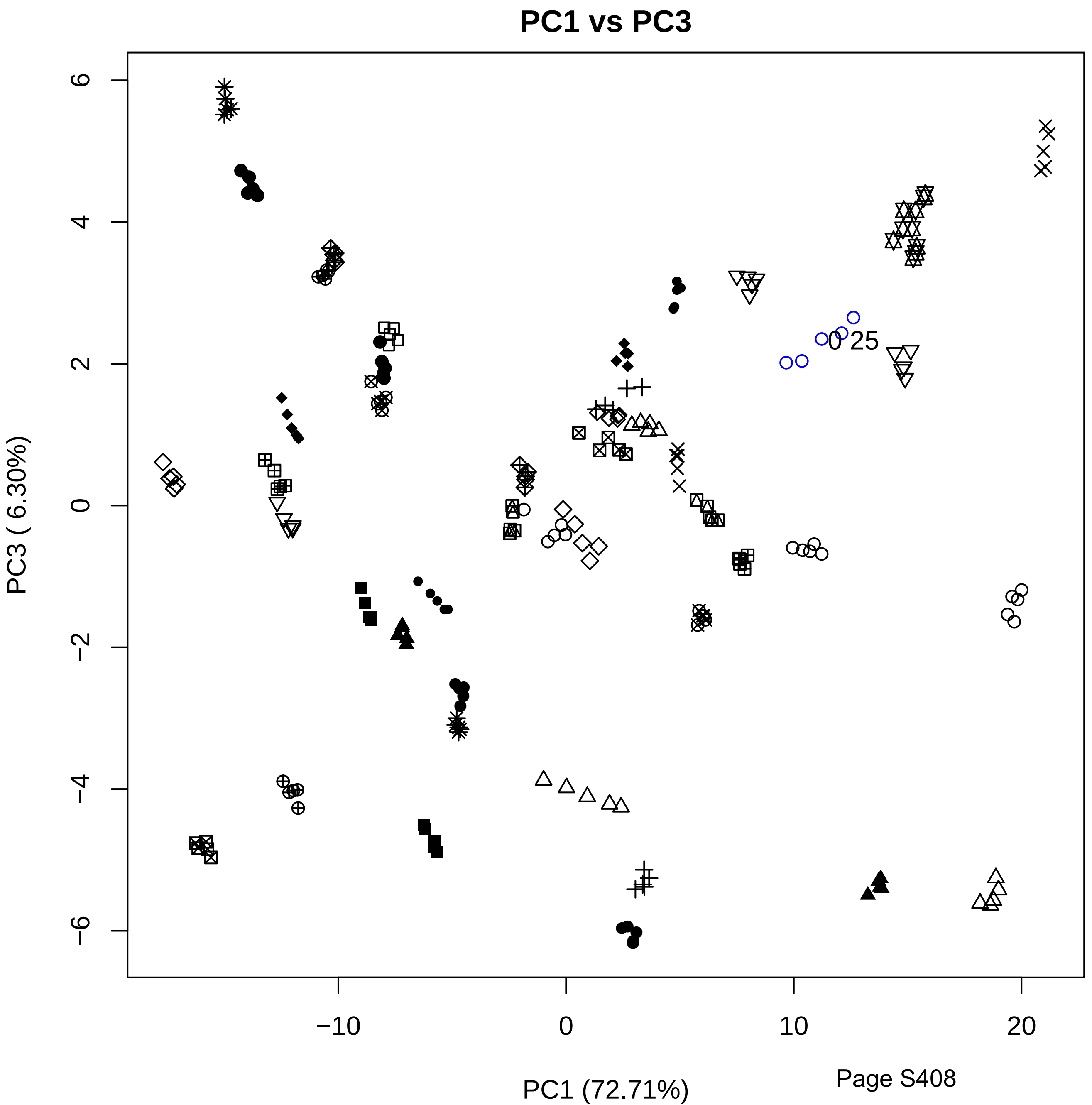

PC1 vs PC3
PC3 (6.30%)
PC1 (72.71%)
6
4
2
0
−2
−4
−6
−10
0
10
20
0 25

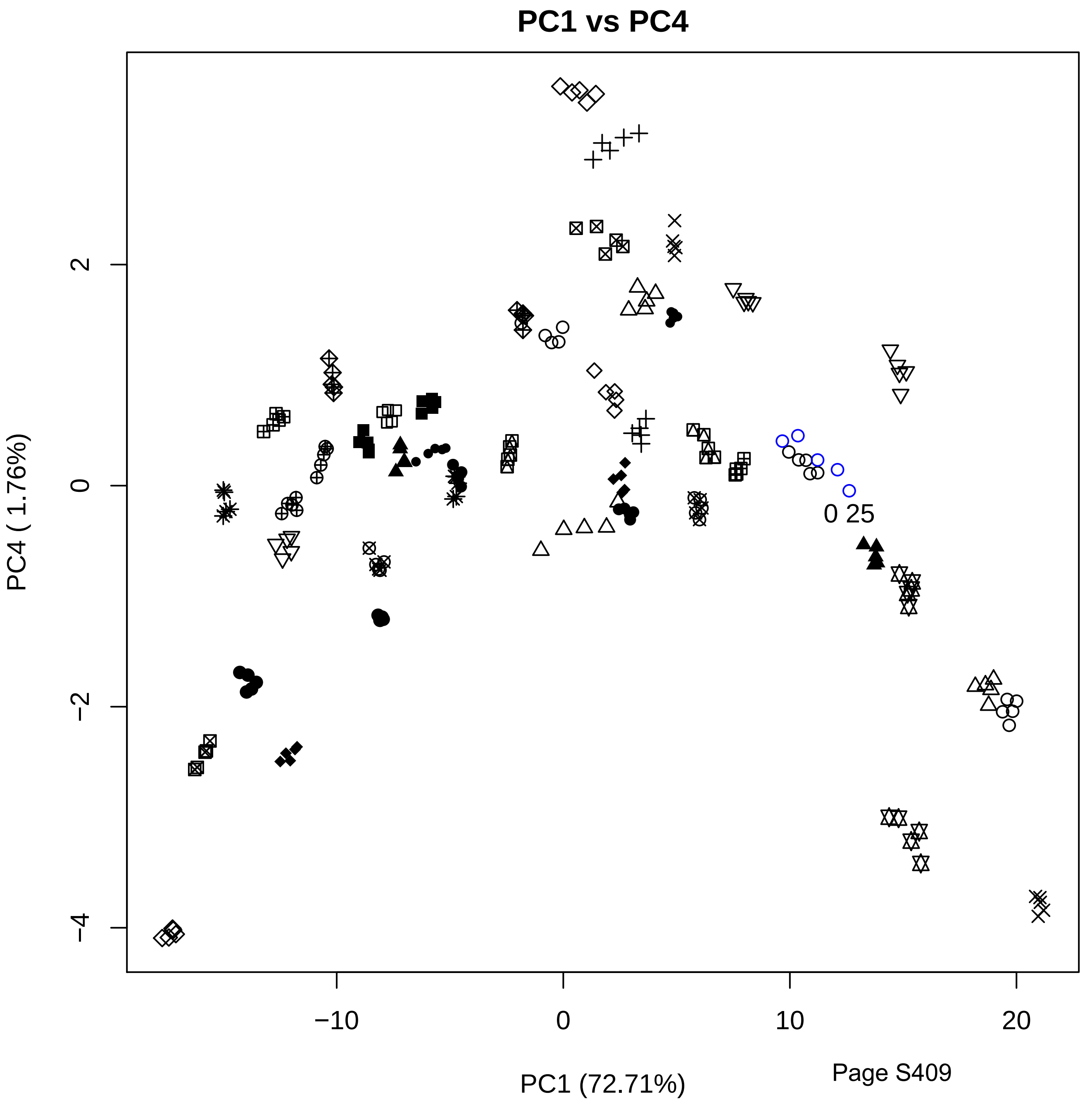
PC1 vs PC4
PC4 (1.76%)
PC1 (72.71%)
2
0
−2
−4
−10
0
10
20
0 25

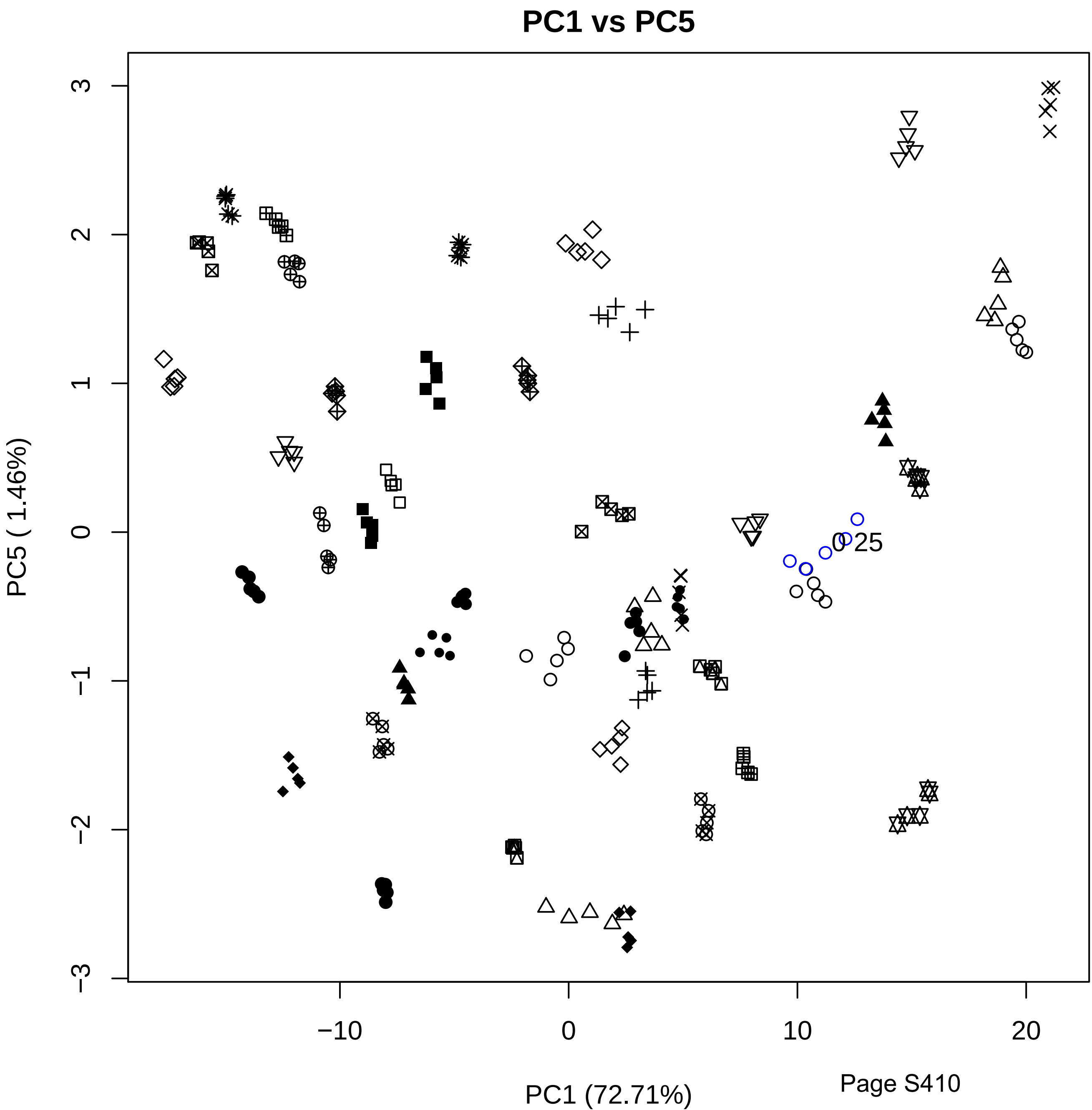
PC1 vs PC5
PC5 (1.46%)
PC1 (72.71%)
3
2
1
0
−1
−2
−3
−10
0
10
20
025

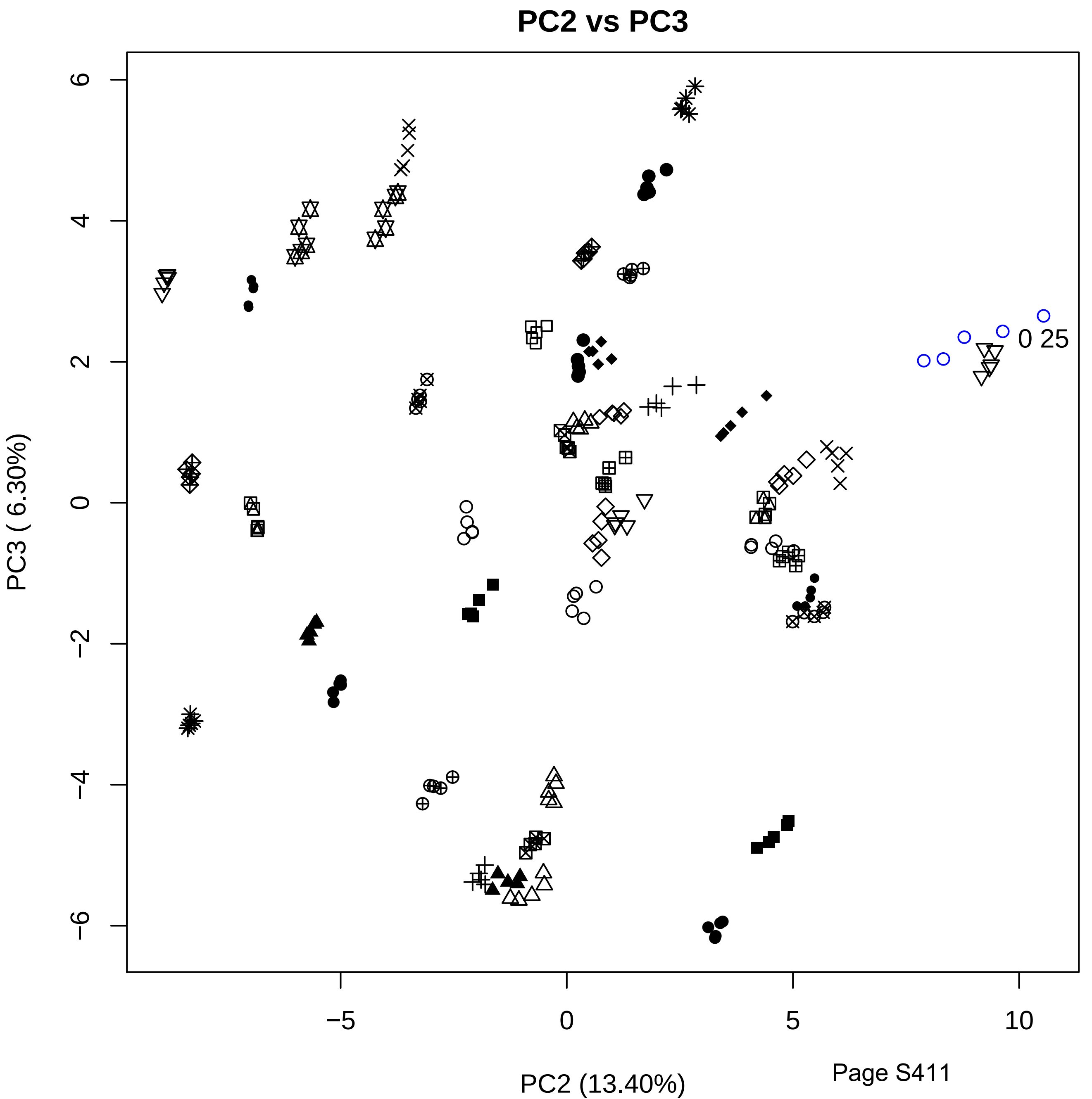
PC2 vs PC3
PC3 (6.30%)
PC2 (13.40%)
6
4
2
0
−2
−4
−6
−5
0
5
10
0 25

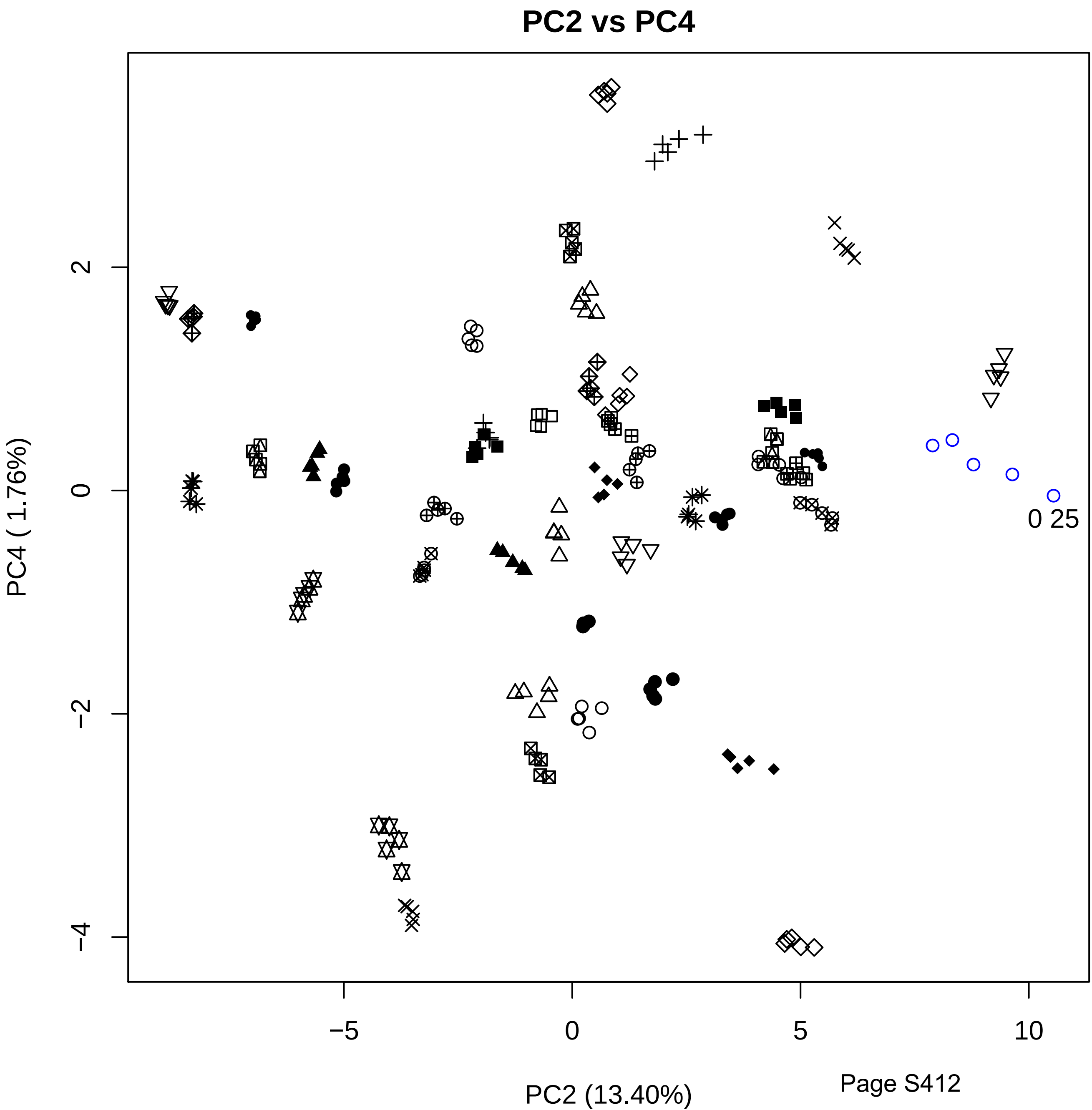
PC2 vs PC4
PC4 (1.76%)
PC2 (13.40%)
2
0
−2
−4
−5
0
5
10
0 25

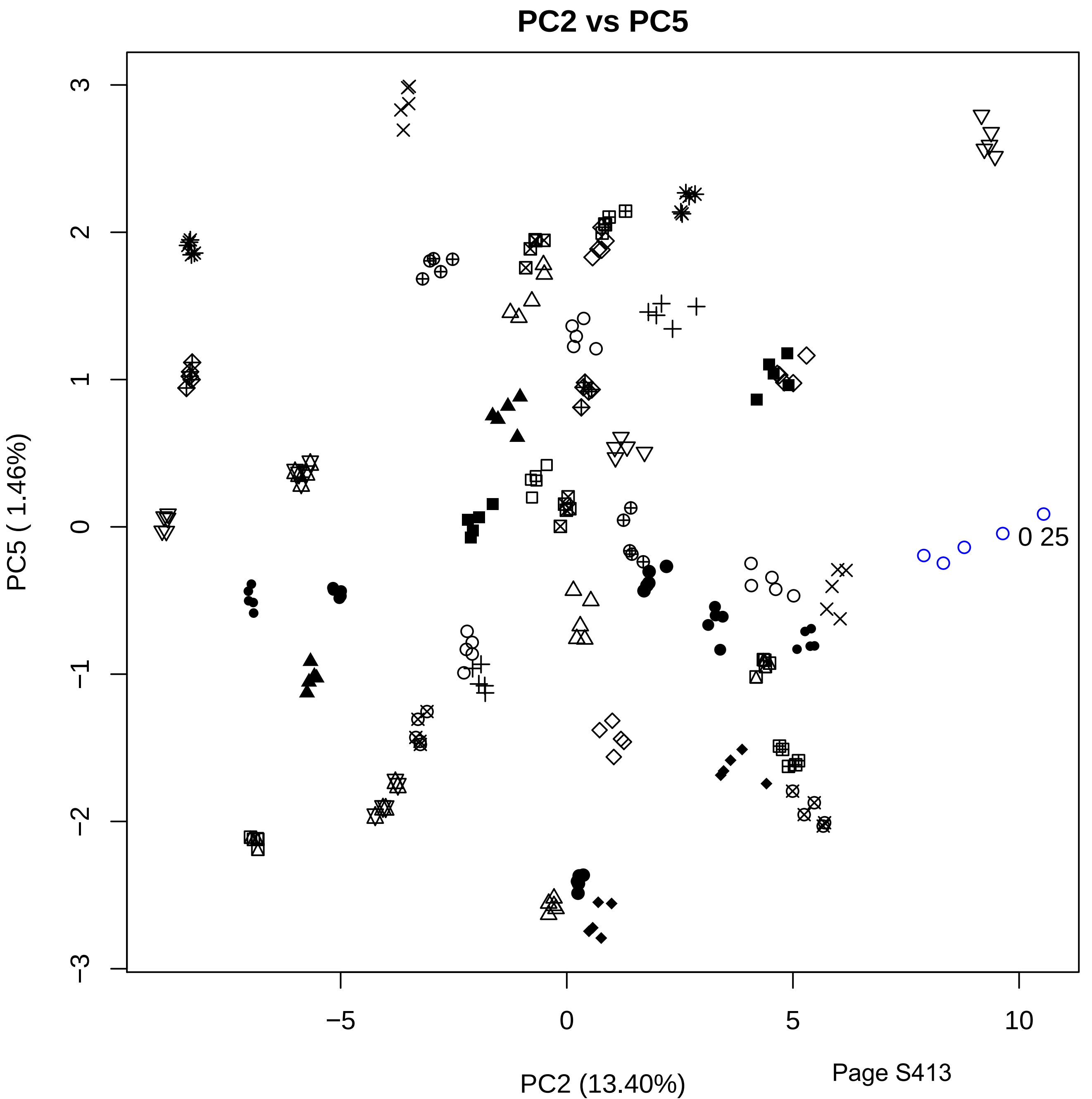

PC2 vs PC5
PC5 (1.46%)
PC2 (13.40%)
0 25
−3
−2
−1
0
1
2
3
−5
0
5
10

PC3 vs PC4

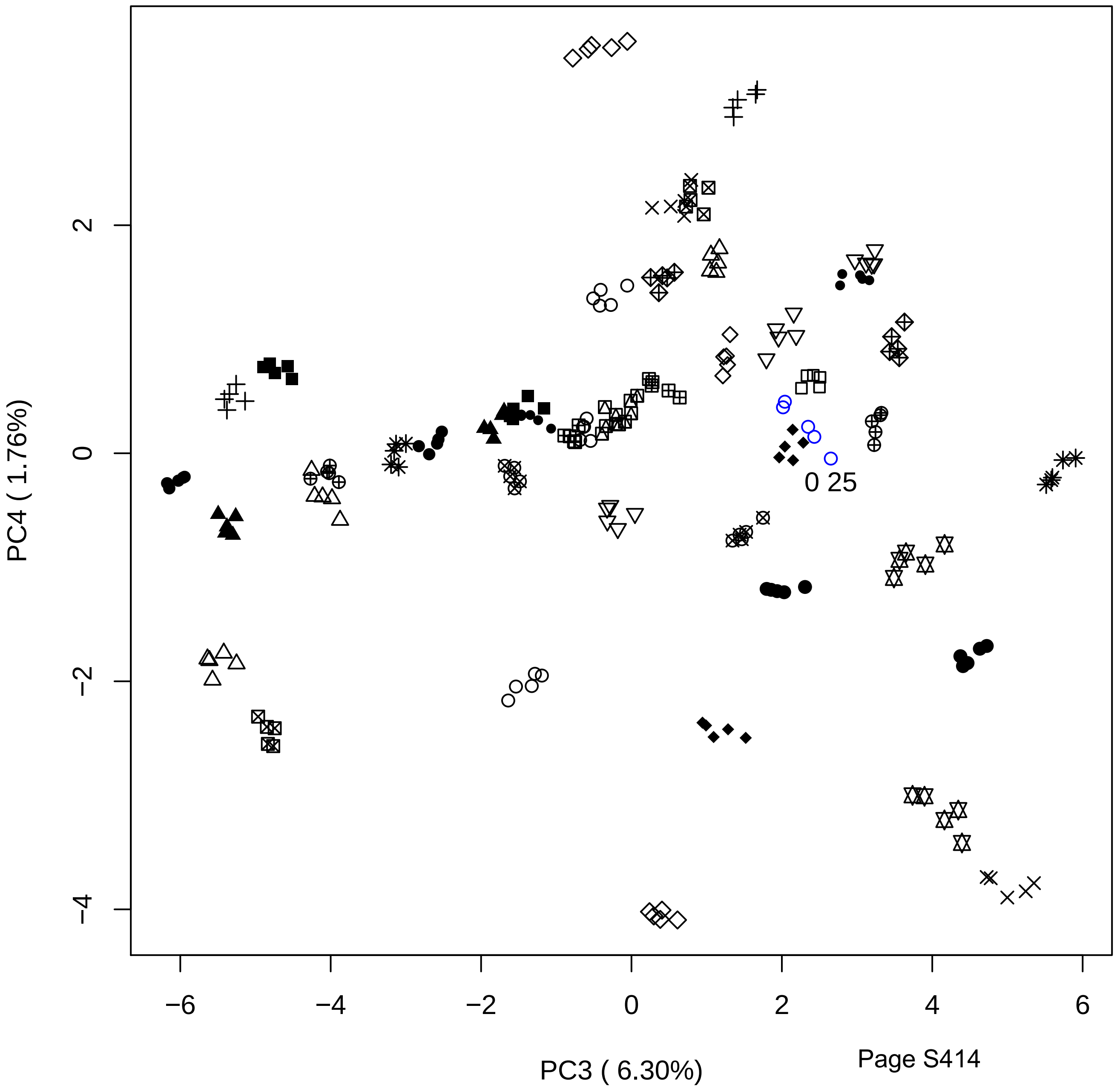

PC3 vs PC5

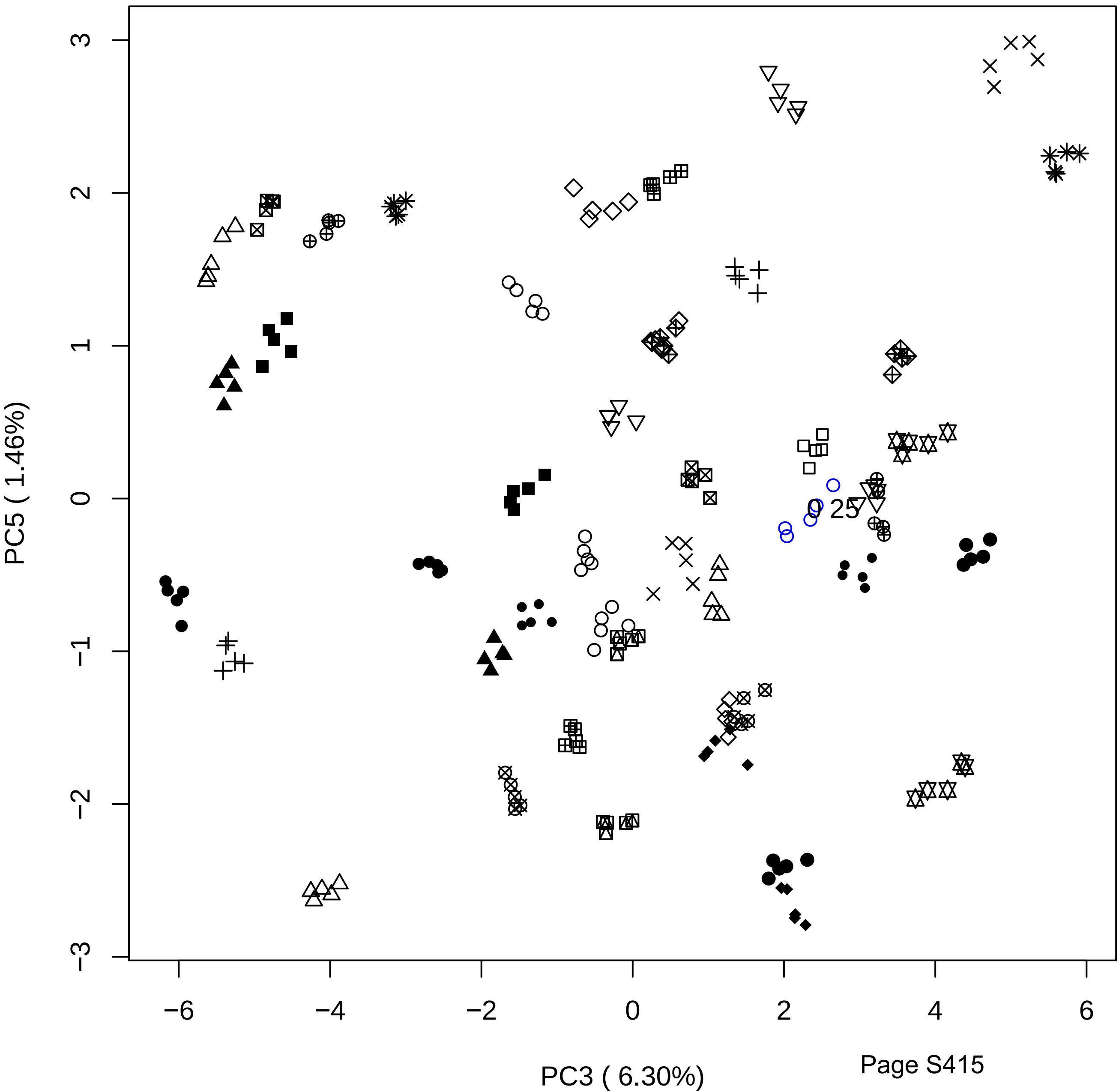

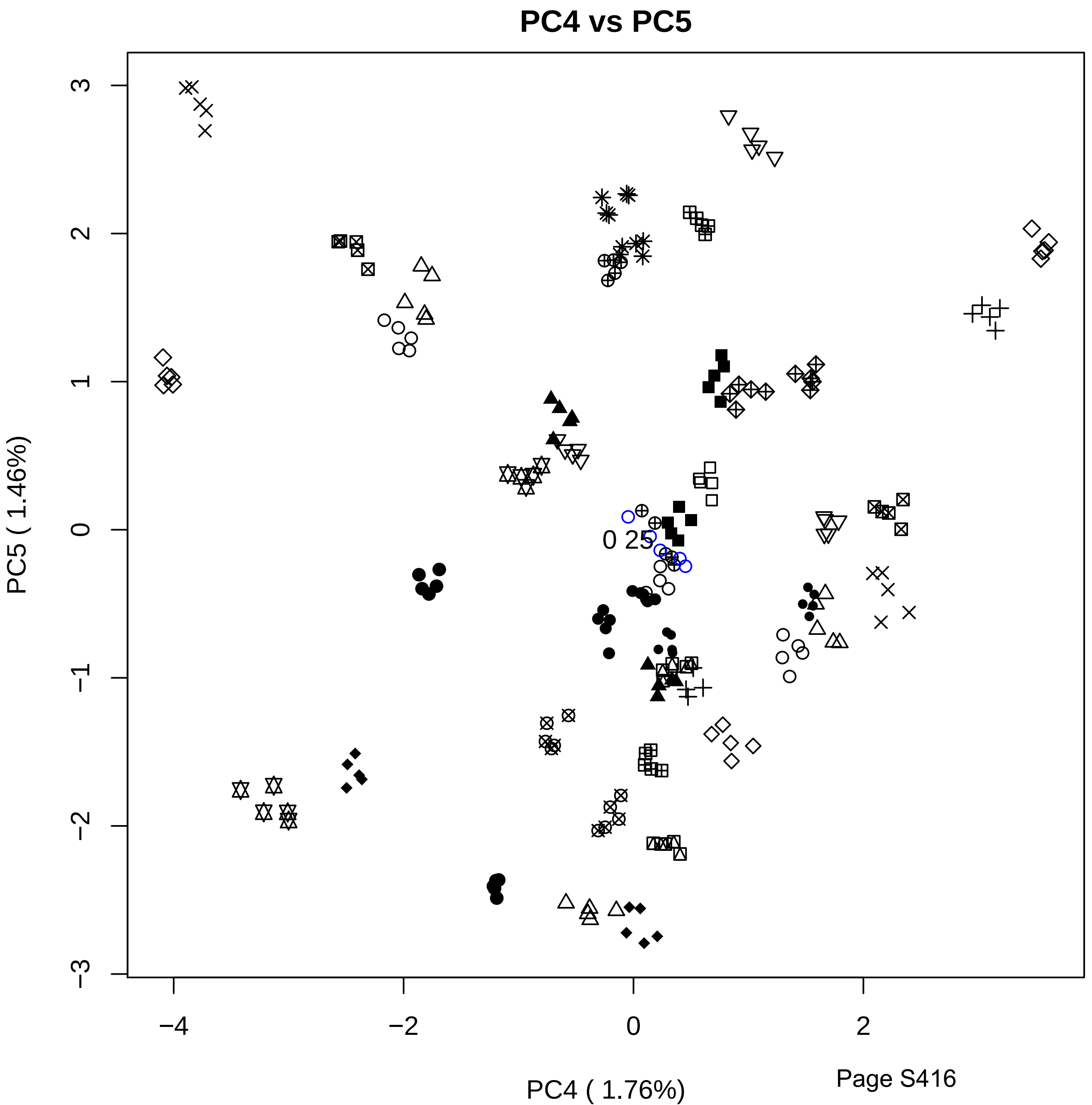

PC4 vs PC5
PC5 (1.46%)
PC4 (1.76%)
3
2
1
0
−1
−2
−3
−4
−2
0
2
0 25

PCA of 0.5% OSAS vs PMH

Scaled Matrix PCAed

7th order polynomial baseline correction performed

Raw spectra smoothed to 21 Neighbours, 2nd order PN, Savtisky Golay

Normalisation between 0 and 100

D2 spectra smoothed to 41 Neighbours, 2nd order PN, Savtisky Golay

Regions Analysed: 3601−2501 and 2001−700cm−1

Samples Analysed: 1 Contam's 0 OSAS's 45 PMH's

Keele University AD

Sun Dec 09 16:28:52 2018

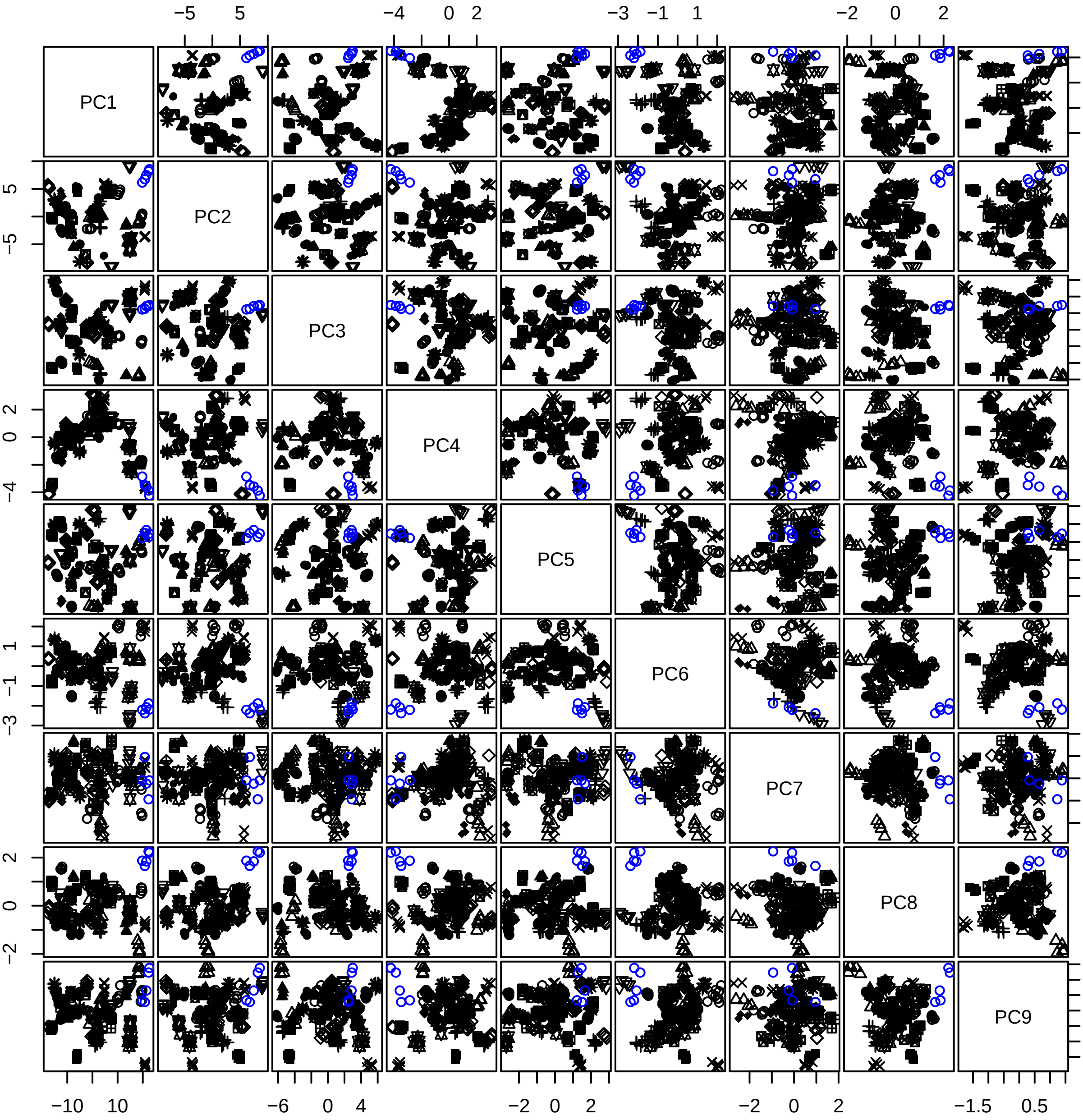
PC1
PC2
PC3
PC4
PC5
PC6
PC7
PC8
PC9

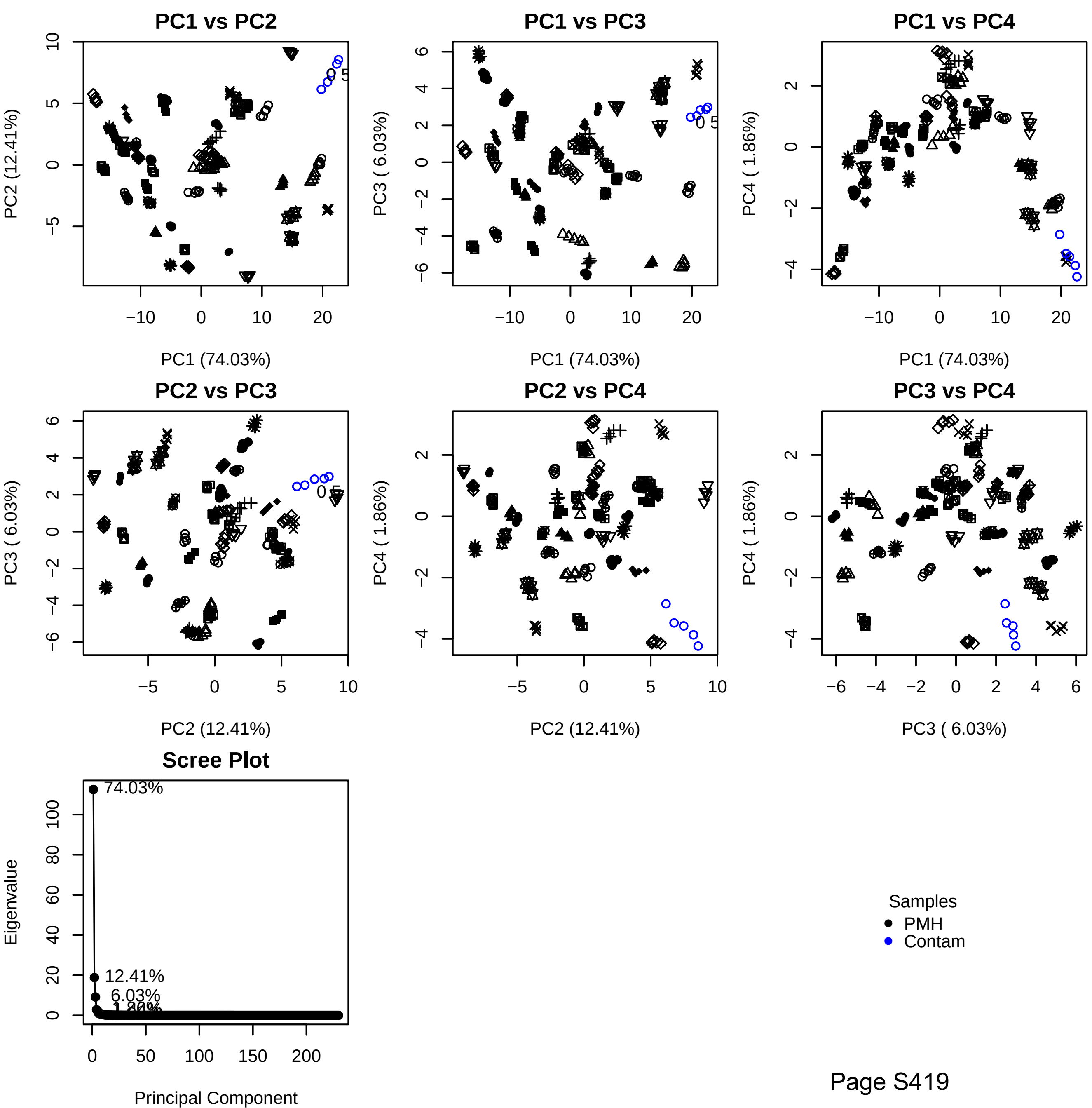
PC1 vs PC2
PC1 (74.03%)
PC2 (12.41%)
PC1 vs PC3
PC3 (6.03%)
PC1 vs PC4
PC4 (1.86%)
PC2 vs PC3
PC2 vs PC4
PC3 vs PC4
Scree Plot
Eigenvalue
Principal Component
74.03%
12.41%
6.03%
Samples
PMH
Contam

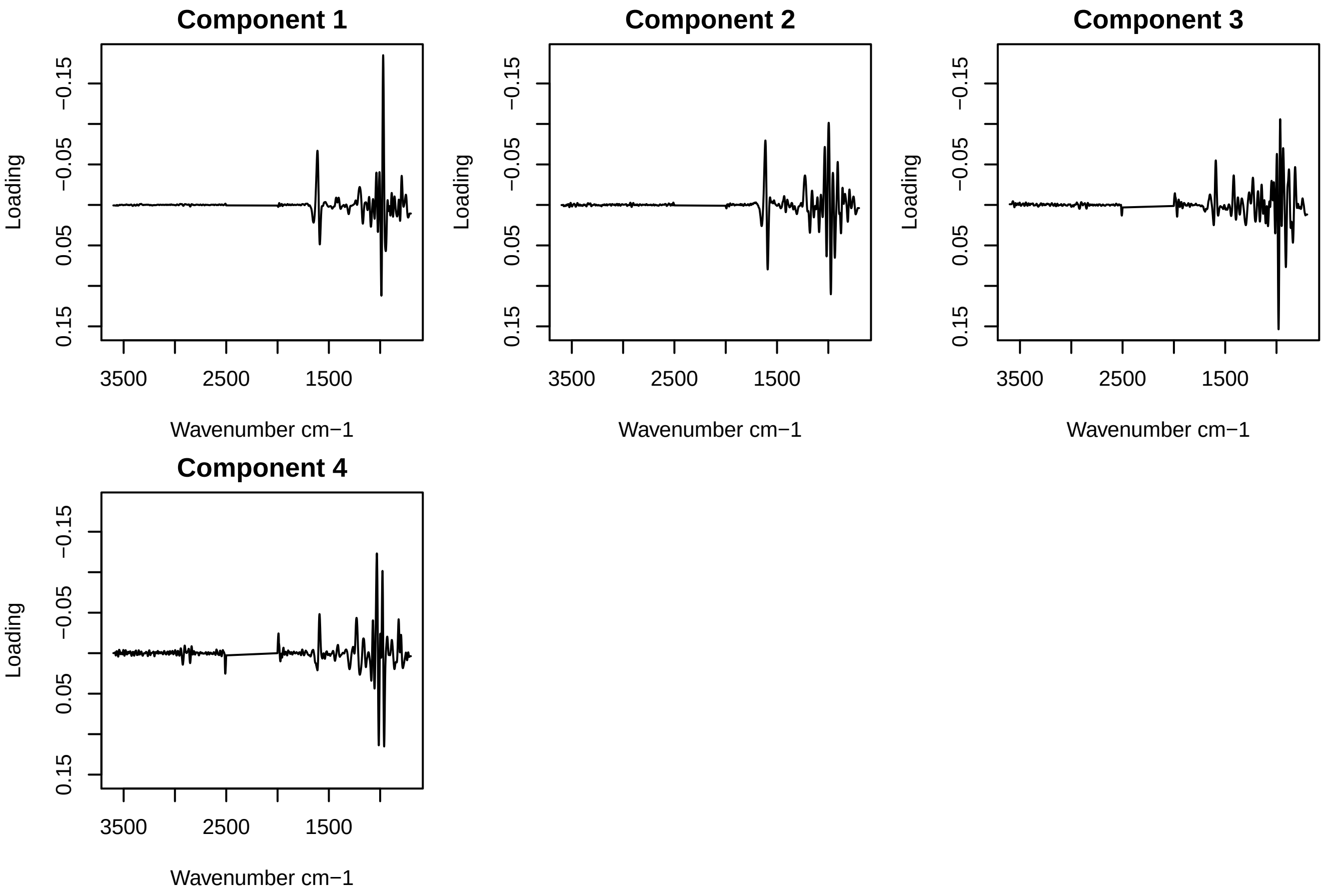

Component 1
Component 2
Component 3
Component 4
Loading
−0.15
−0.05
0.05
0.15
3500
2500
1500
Wavenumber cm−1

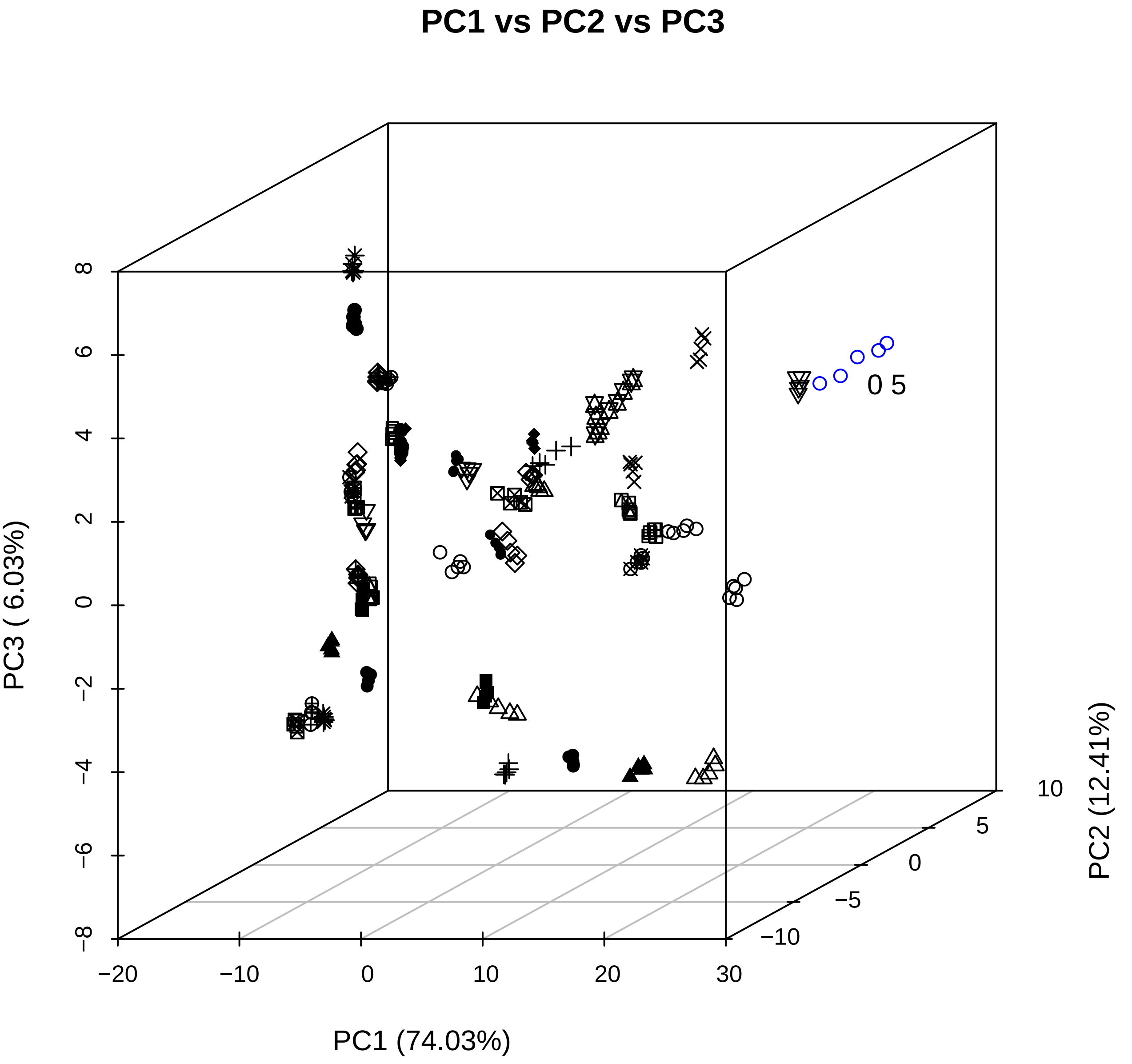

PC1 vs PC2 vs PC3
PC3 (6.03%)
8
6
4
2
0
−2
−4
−6
−8
−20
−10
0
10
20
30
PC1 (74.03%)
10
5
0
−5
−10
PC2 (12.41%)
0 5

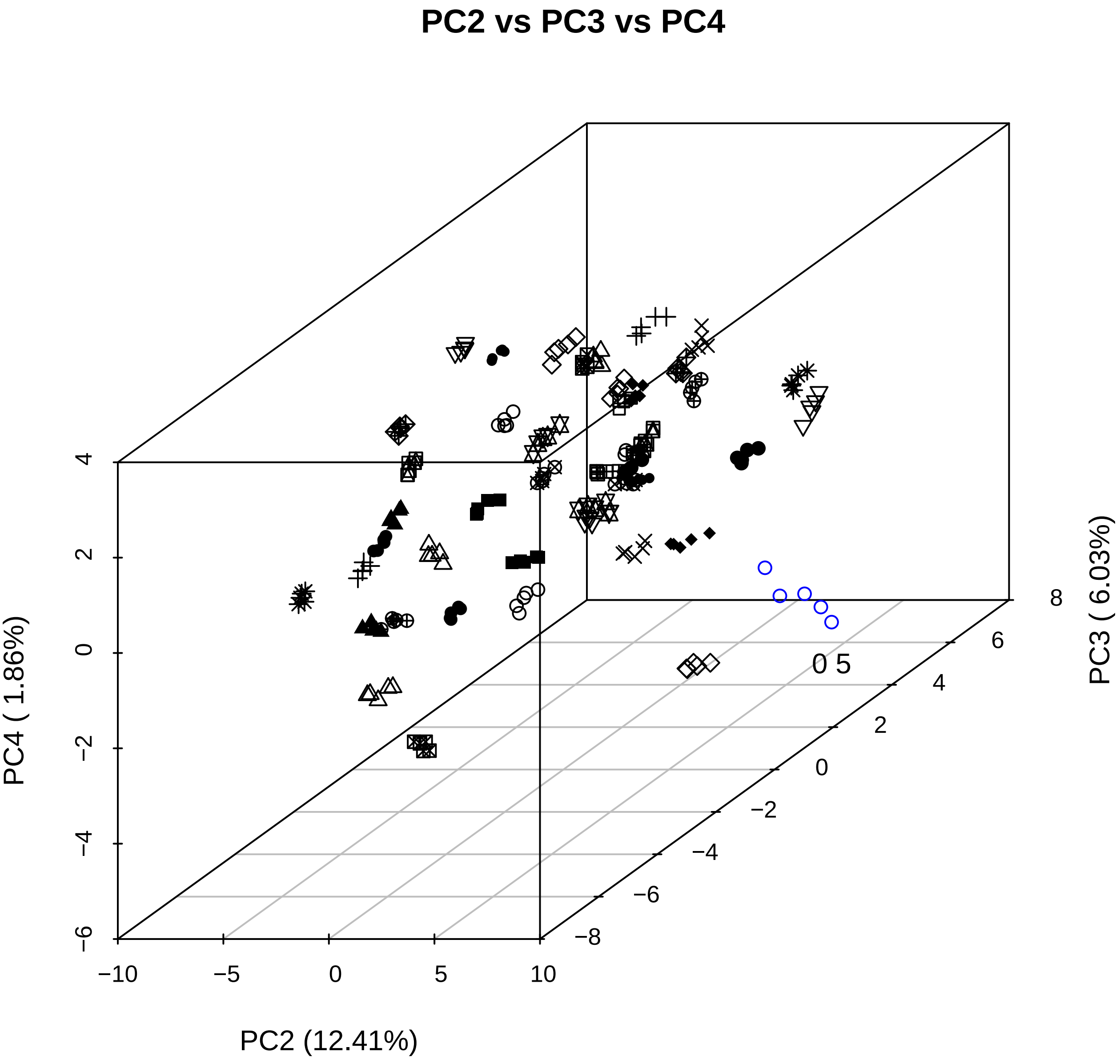
PC2 vs PC3 vs PC4
PC2 (12.41%)
PC4 (1.86%)
PC3 (6.03%)
−10
−5
0
5
10
−6
−4
−2
0
2
4
−8
−6
−4
−2
0
2
4
6
8
0 5

PC1 vs PC2 vs PC4

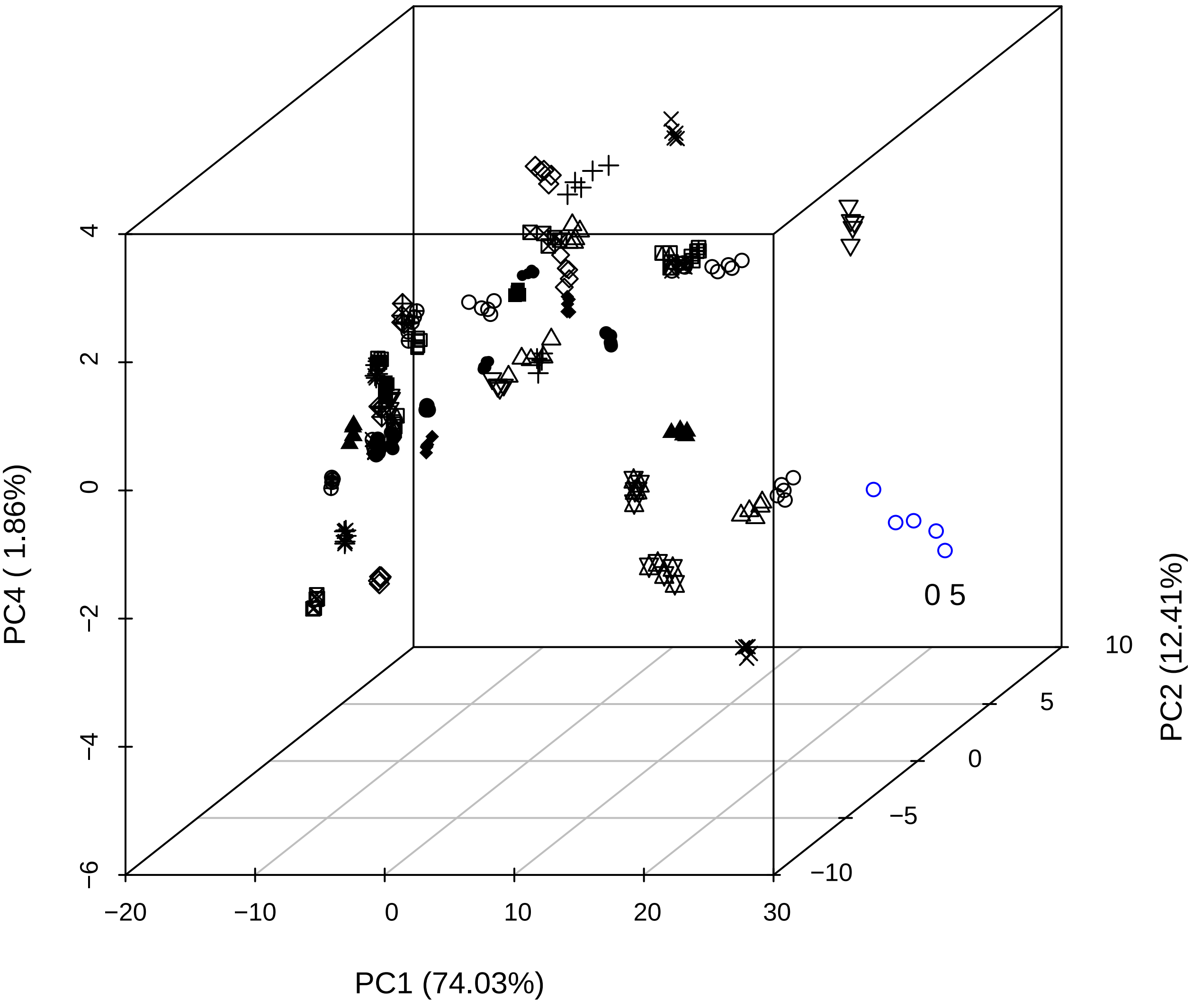

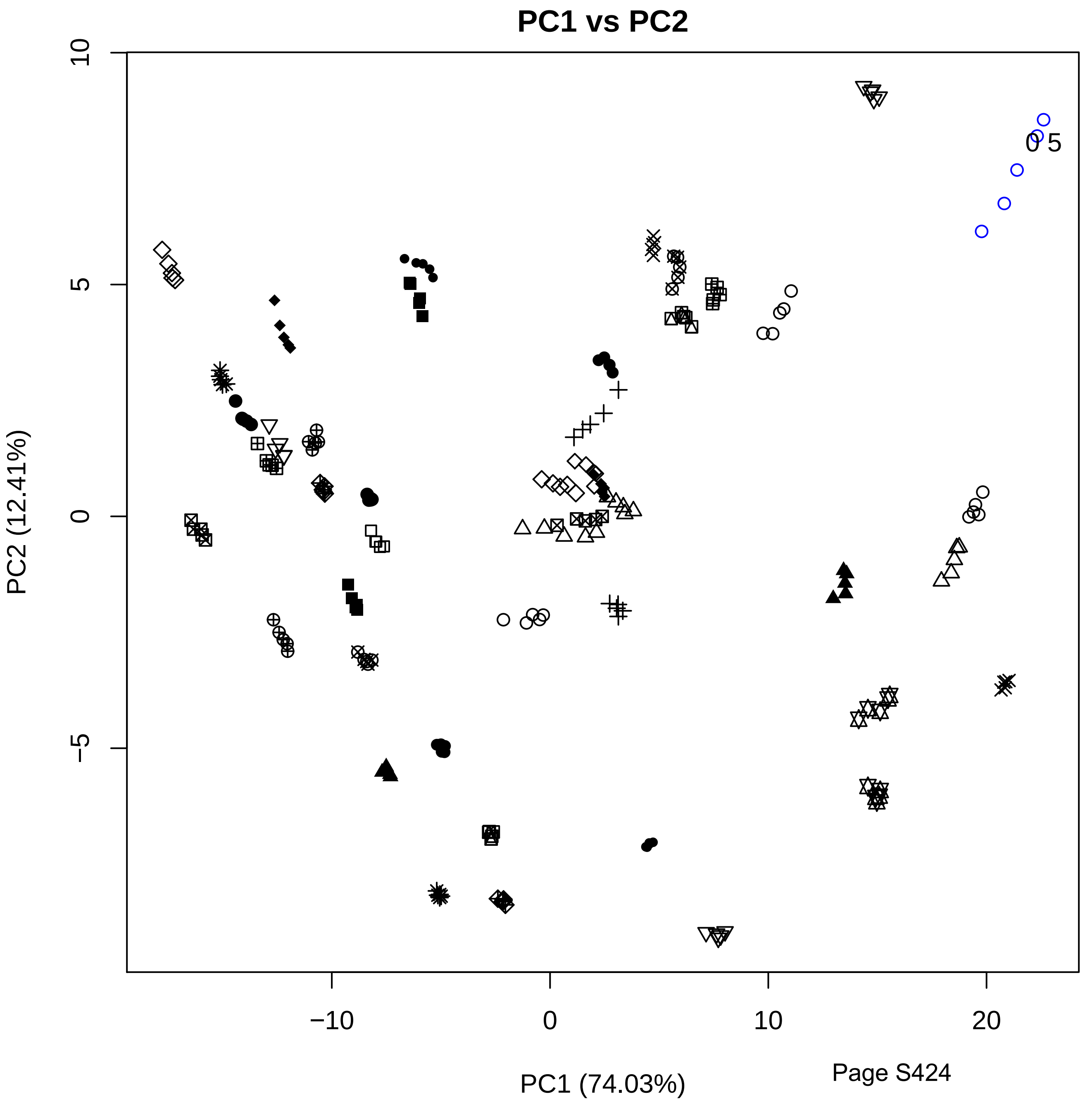
PC1 vs PC2
PC2 (12.41%)
PC1 (74.03%)
10
5
0
−5
−10
0
10
20
0 5

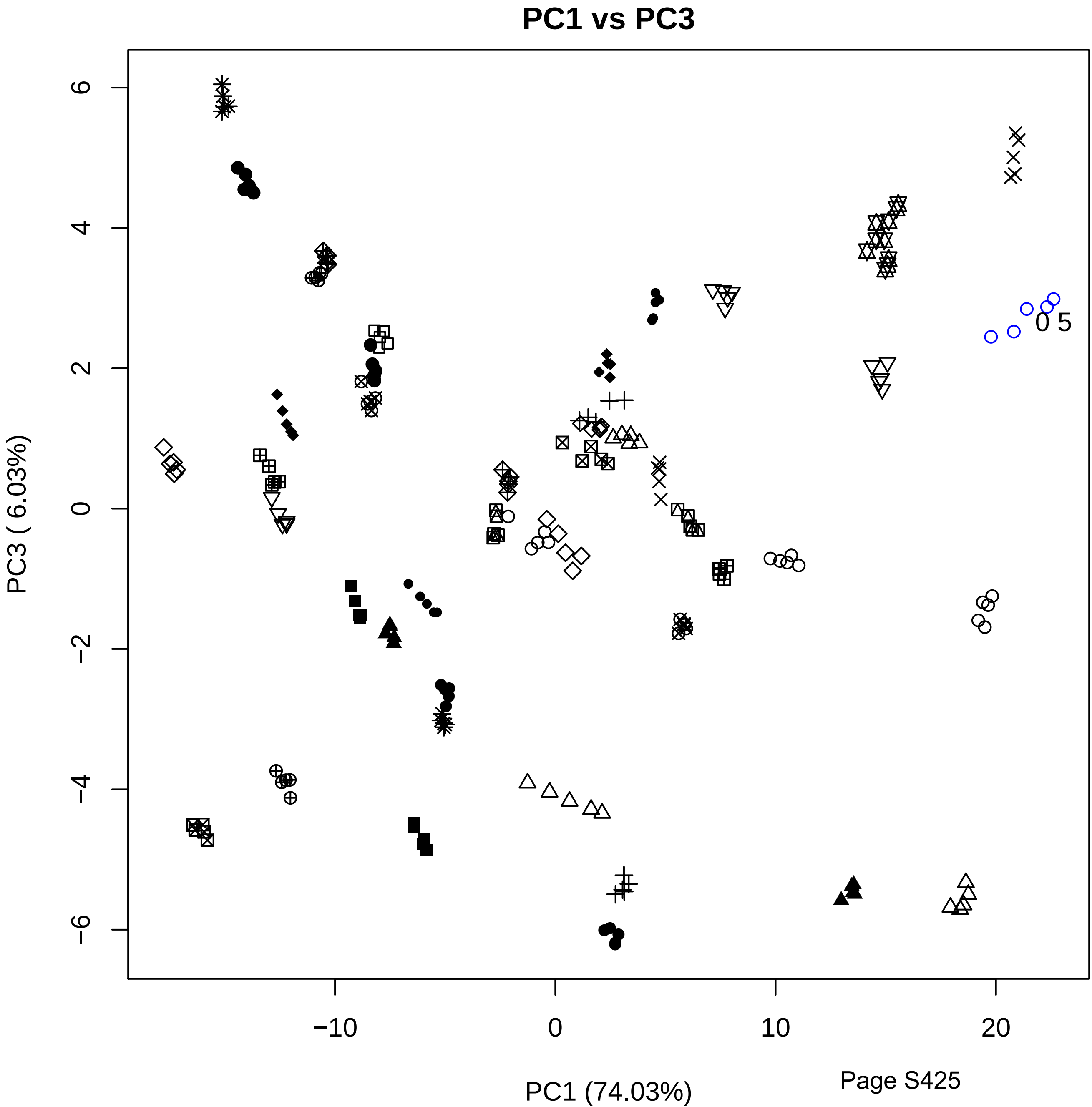
PC1 vs PC3
PC3 (6.03%)
PC1 (74.03%)
6
4
2
0
−2
−4
−6
−10
0
10
20
0 5

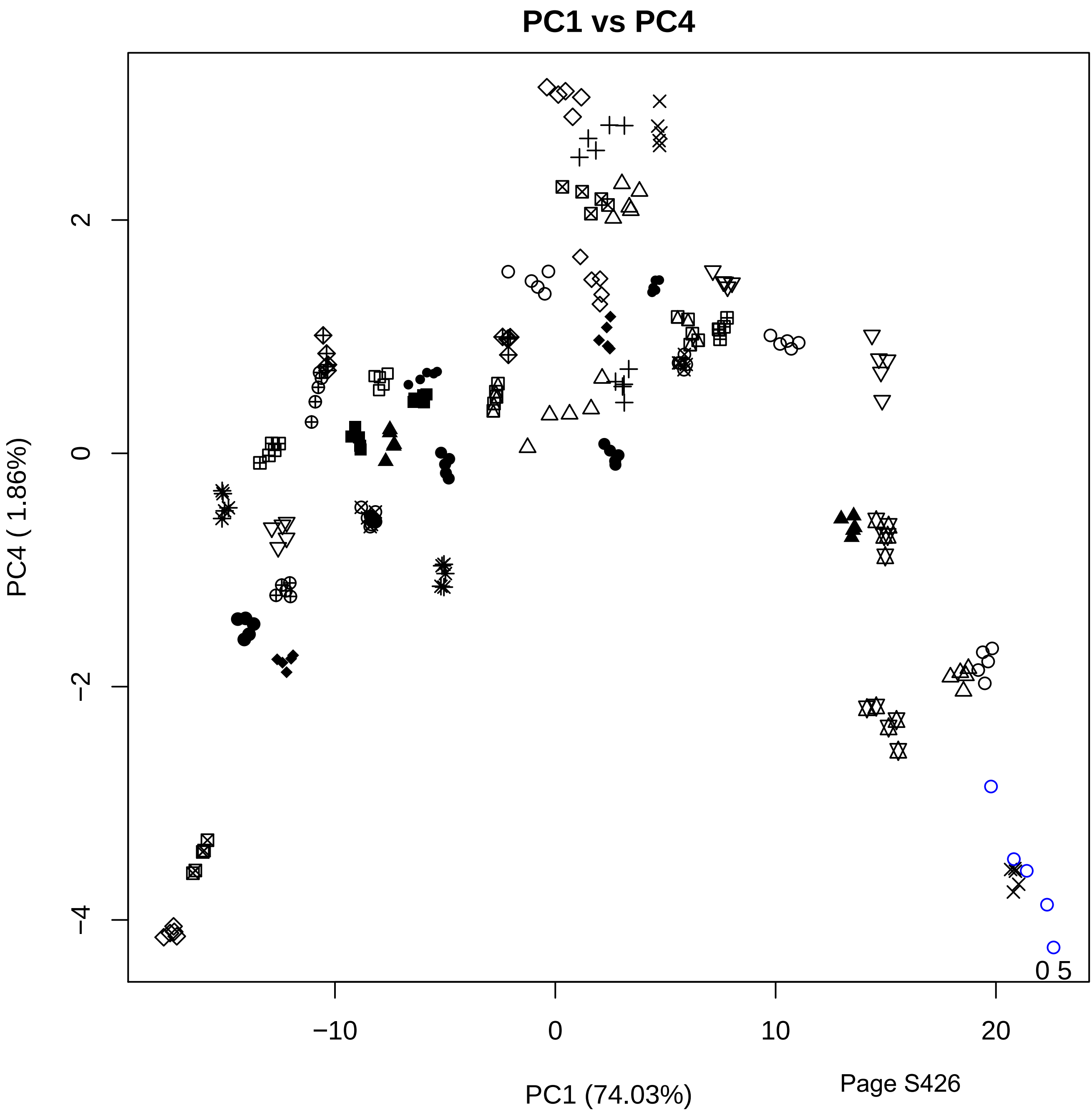
PC1 vs PC4
PC4 (1.86%)
PC1 (74.03%)
2
0
−2
−4
−10
0
10
20
0 5

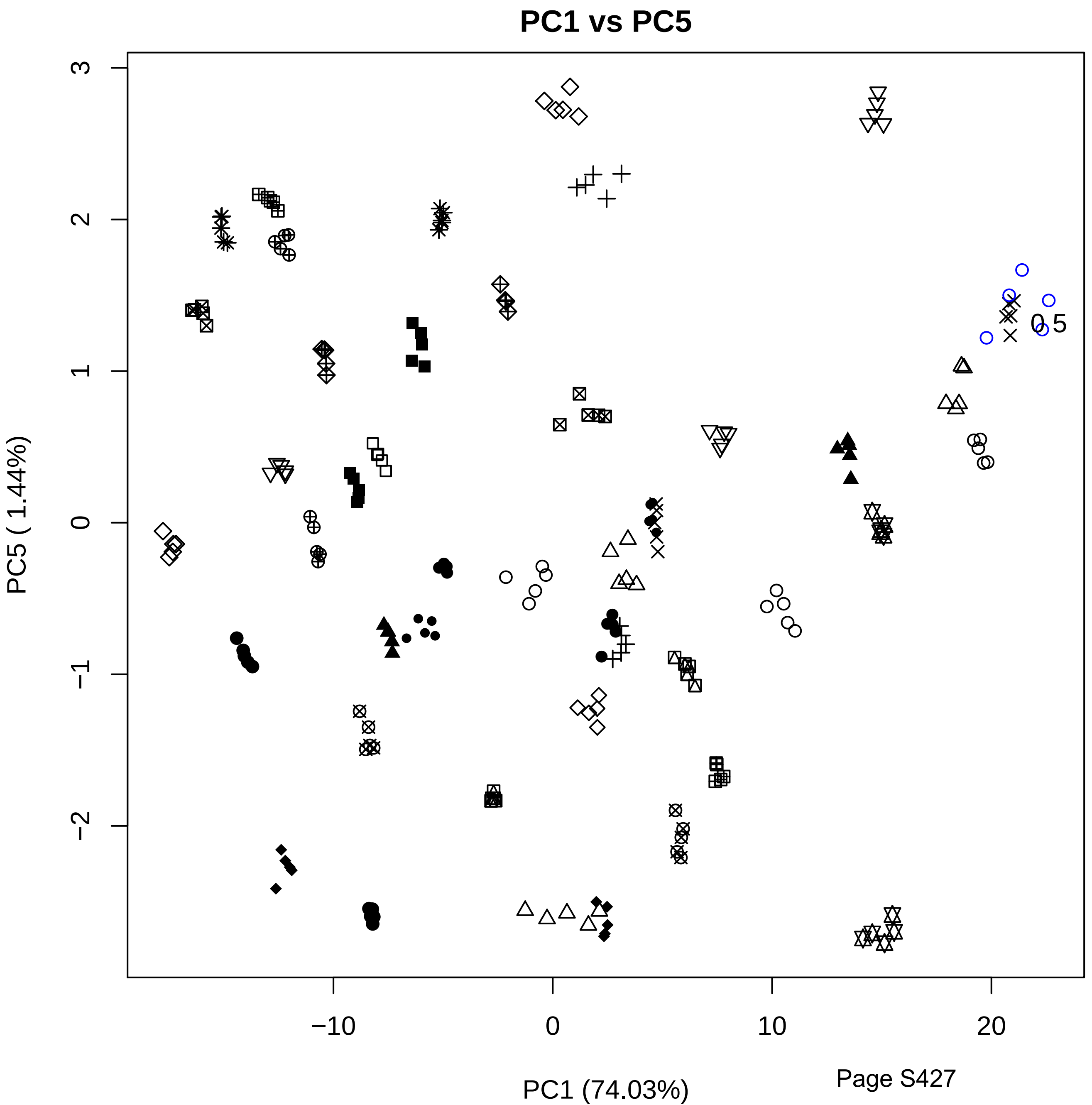

PC1 vs PC5
PC5 (1.44%)
PC1 (74.03%)
0.5

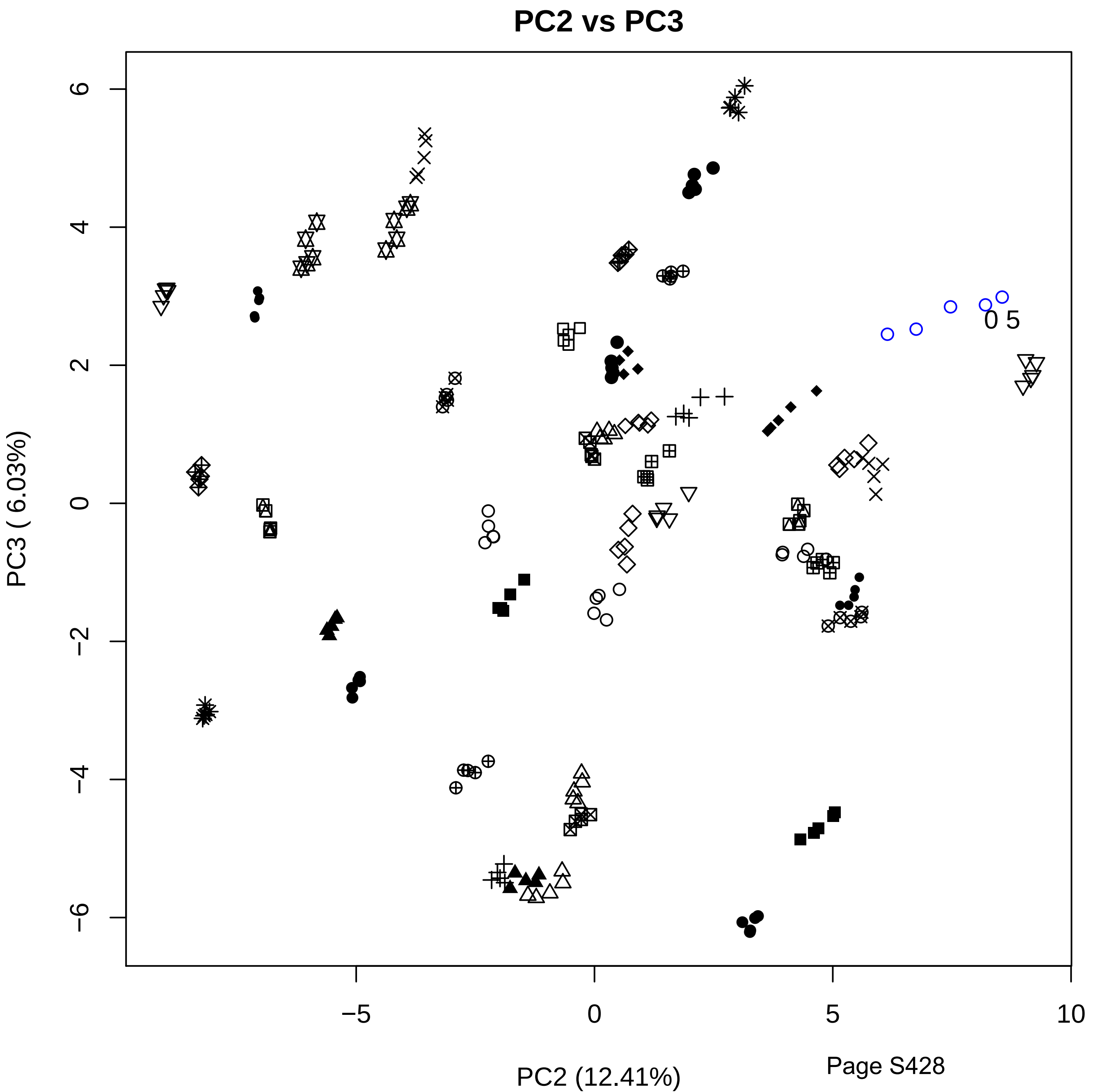
PC2 vs PC3
PC3 (6.03%)
PC2 (12.41%)
6
4
2
0
−2
−4
−6
−5
0
5
10
0 5

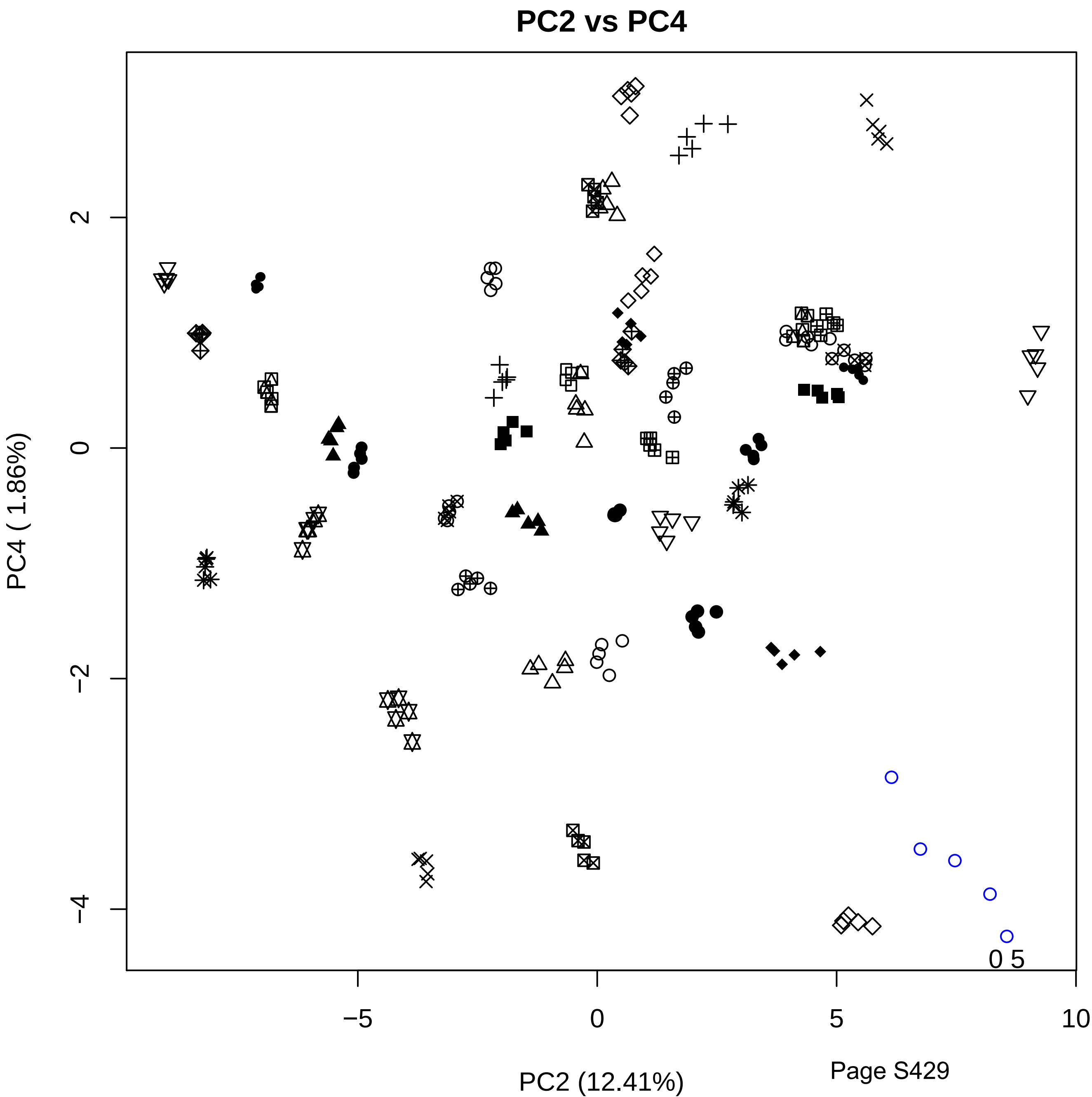
PC2 vs PC4
PC4 (1.86%)
PC2 (12.41%)
2
0
−2
−4
−5
0
5
10
0 5

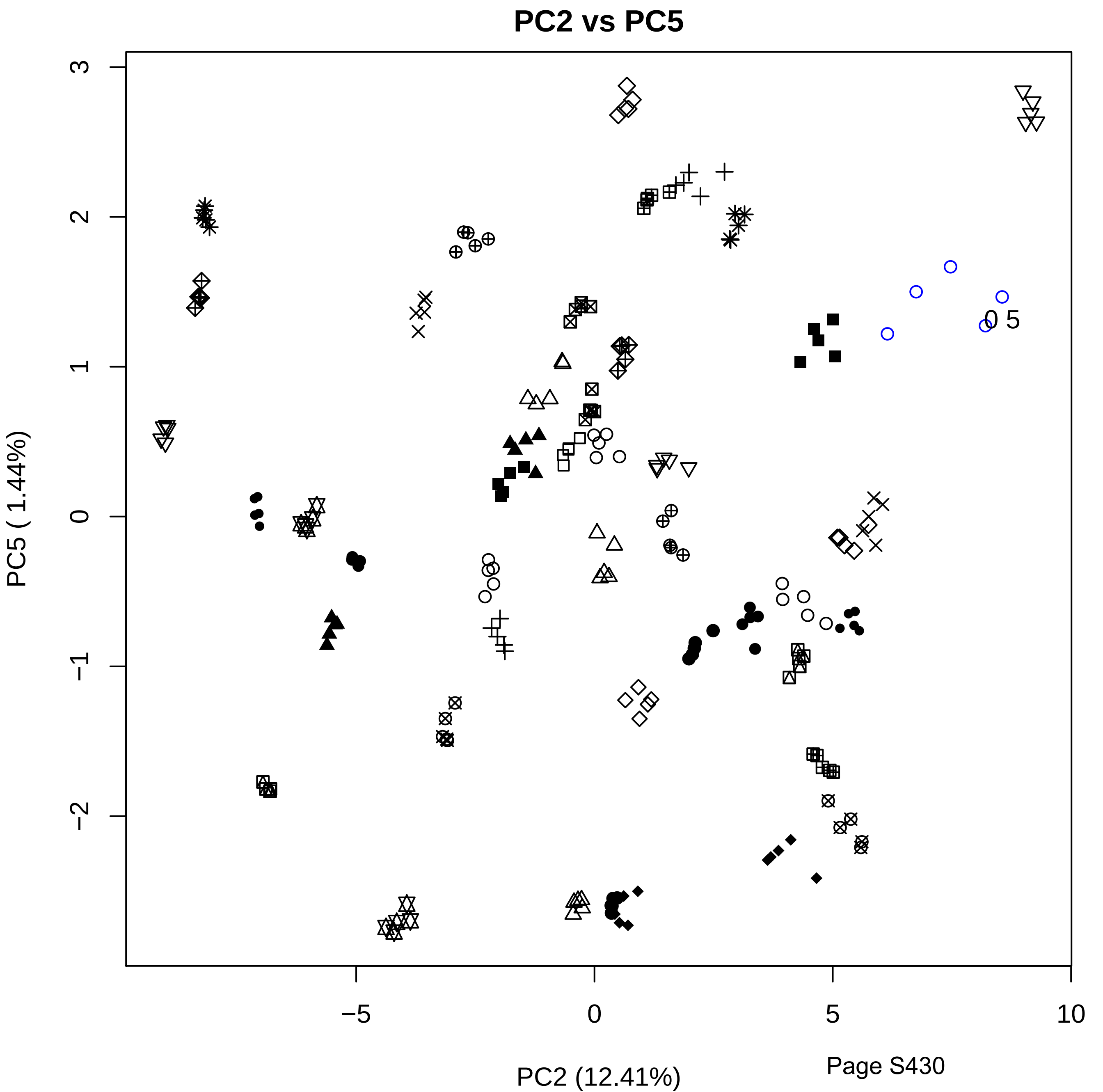

PC2 vs PC5
PC5 (1.44%)
PC2 (12.41%)
3
2
1
0
−1
−2
−5
0
5
10
0 5

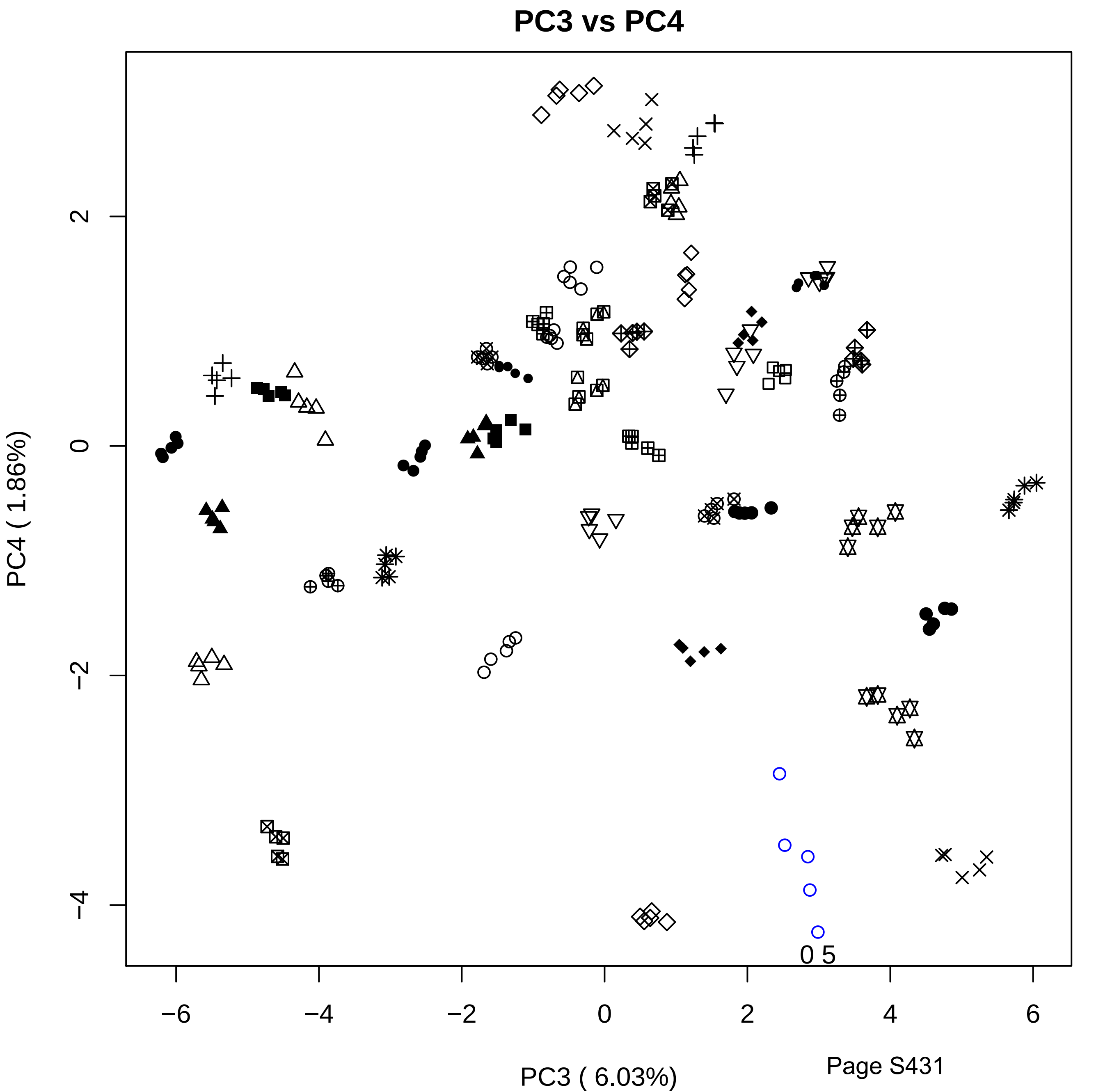
PC3 vs PC4
PC4 (1.86%)
PC3 (6.03%)
2
0
−2
−4
−6
−4
−2
0
2
4
6
0 5

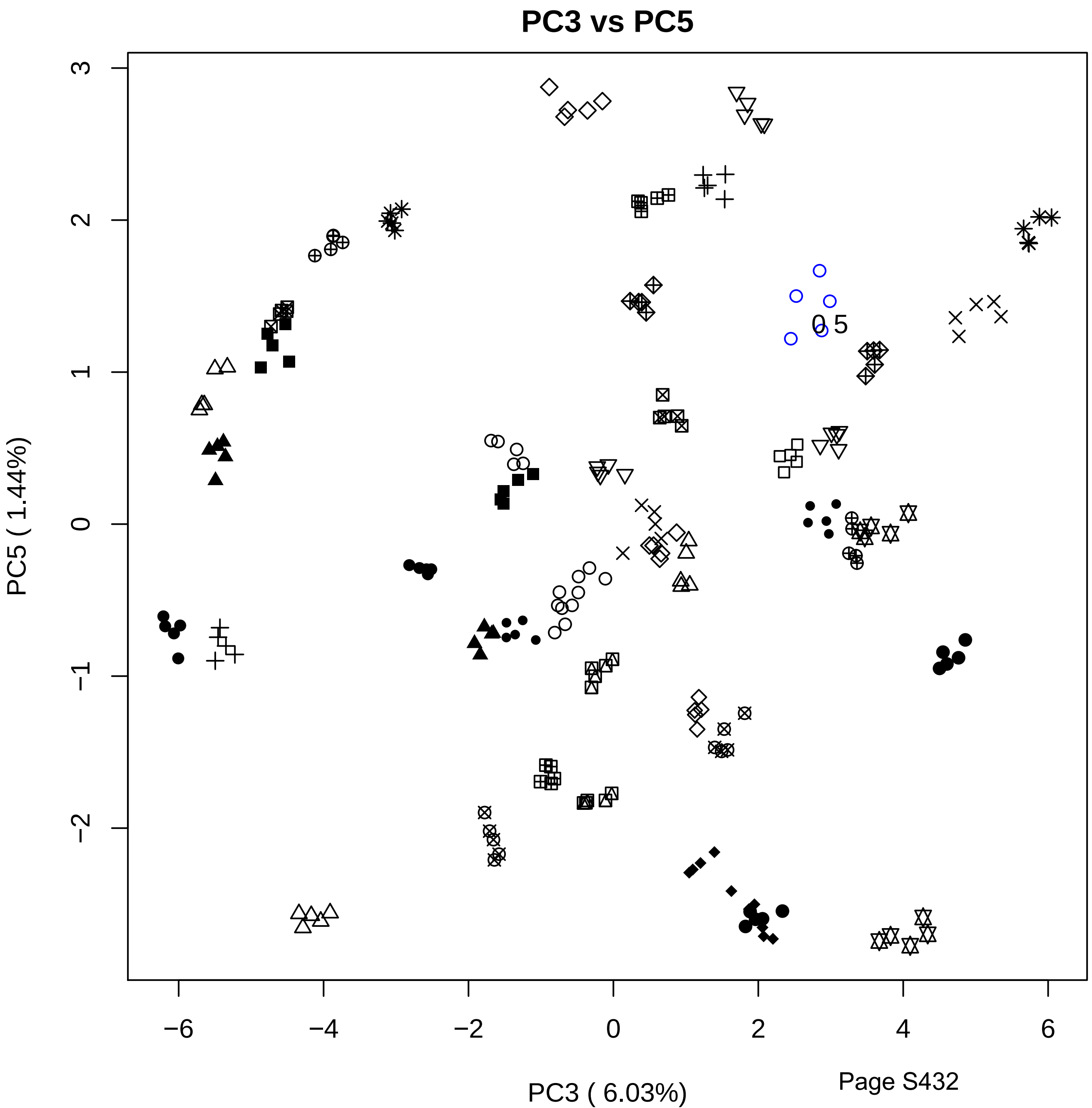

PC3 vs PC5
PC5 (1.44%)
PC3 (6.03%)
3
2
1
0
−1
−2
−6
−4
−2
0
2
4
6
0 5

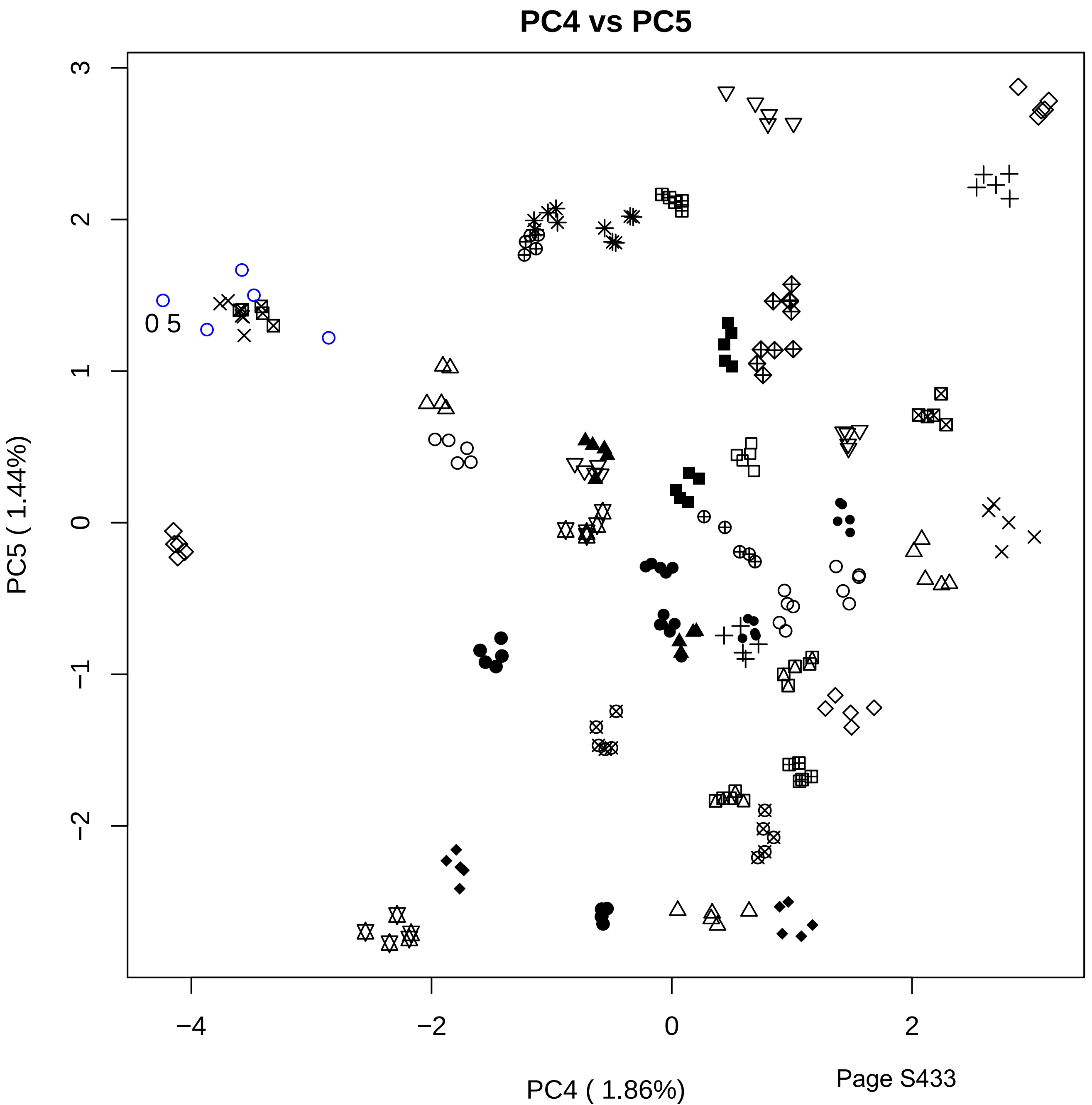
PC4 vs PC5
PC5 (1.44%)
PC4 (1.86%)
0 5
3
2
1
0
−1
−2
−4
−2
0
2

PCA of 1% OSAS vs PMH

Scaled Matrix PCAed

7th order polynomial baseline correction performed

Raw spectra smoothed to 21 Neighbours, 2nd order PN, Savtisky Golay

Normalisation between 0 and 100

D2 spectra smoothed to 41 Neighbours, 2nd order PN, Savtisky Golay

Regions Analysed: 3601−2501 and 2001−700cm−1

Samples Analysed: 1 Contam's 0 OSAS's 45 PMH's

Keele University AD Sun Dec 09 16:28:57 2018

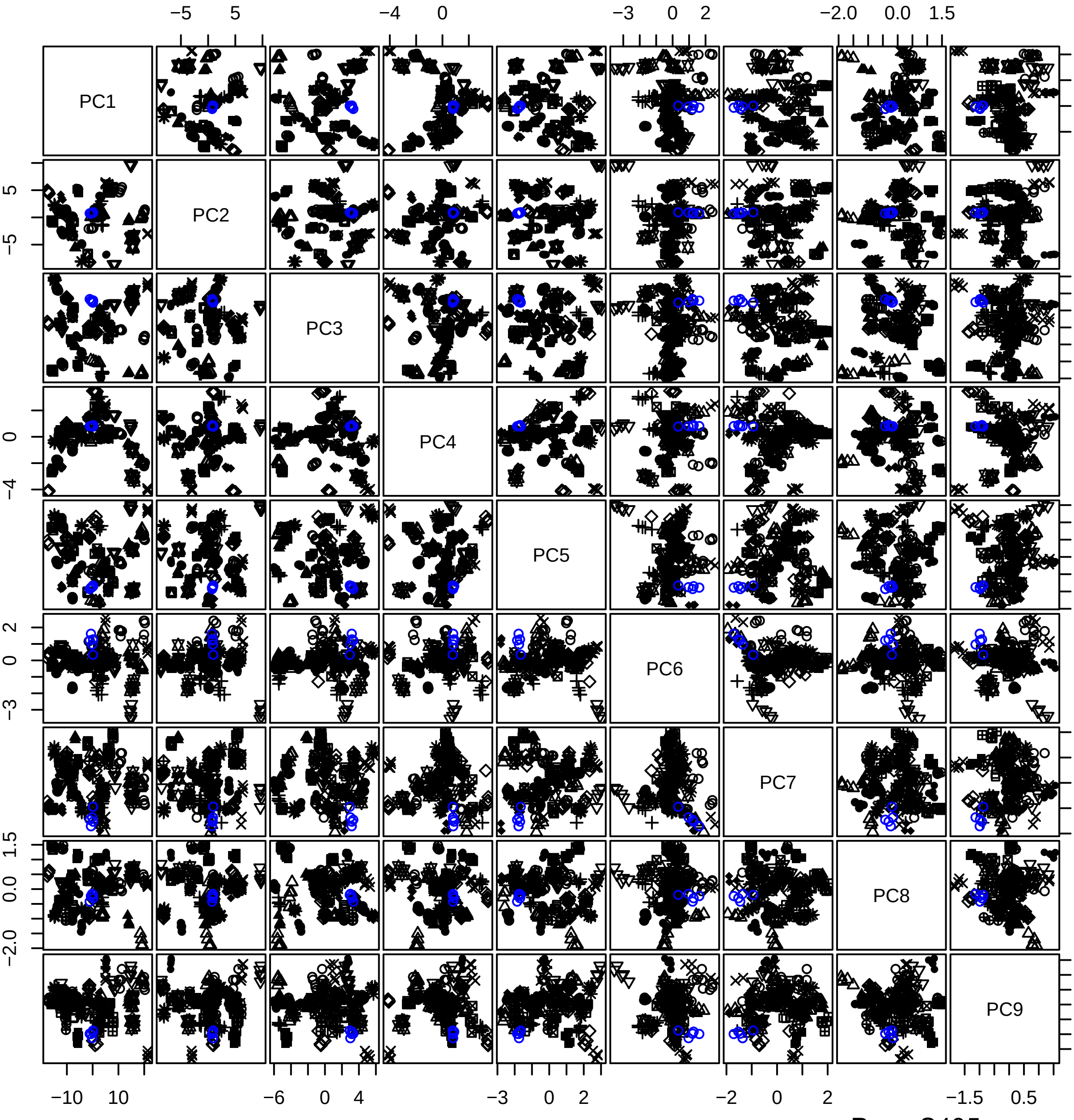
PC1
PC2
PC3
PC4
PC5
PC6
PC7
PC8
PC9

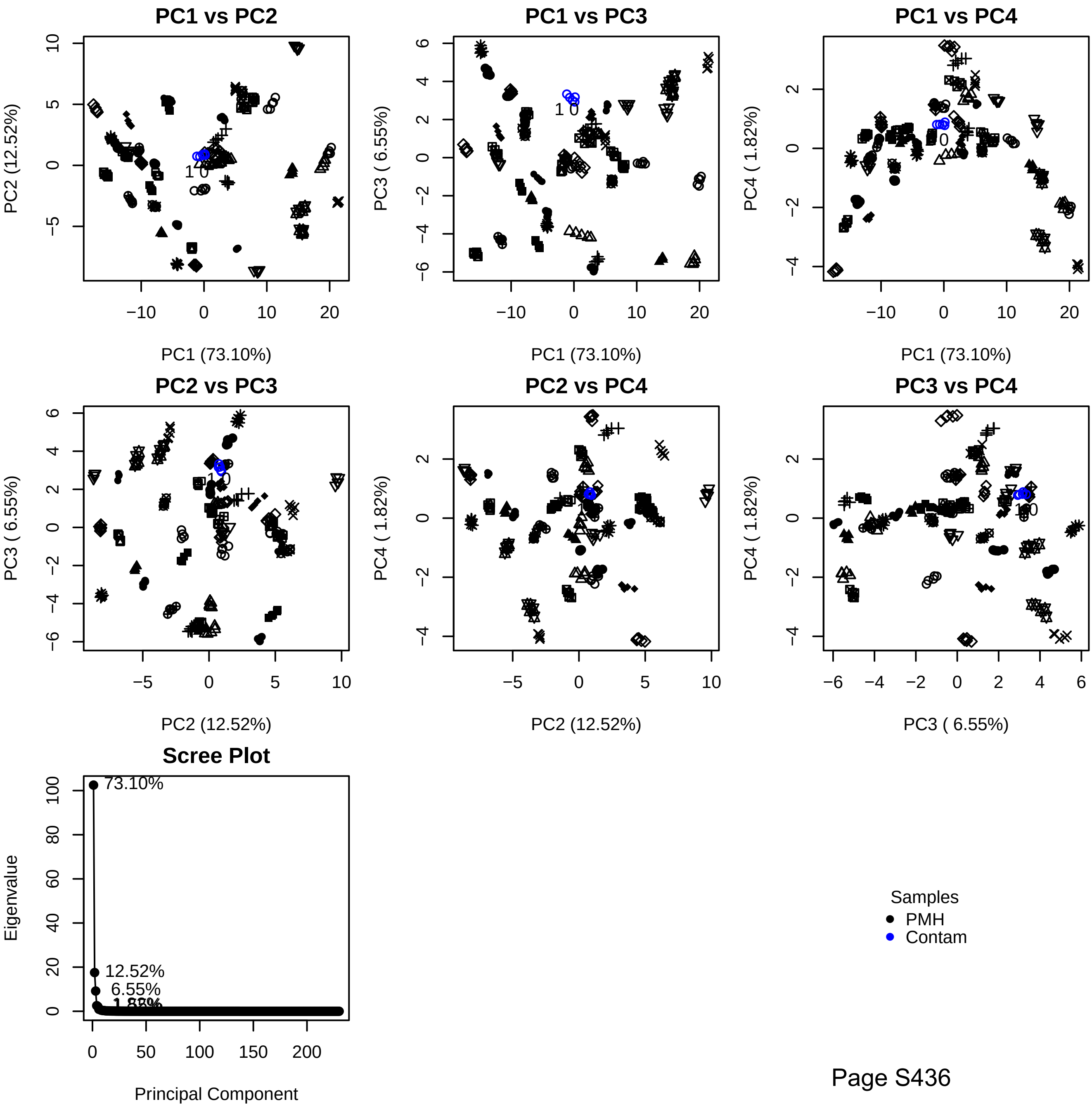
PC1 vs PC2
PC1 vs PC3
PC1 vs PC4
PC2 vs PC3
PC2 vs PC4
PC3 vs PC4
Scree Plot
PC1 (73.10%)
PC2 (12.52%)
PC3 (6.55%)
PC4 (1.82%)
Eigenvalue
Principal Component
73.10%
12.52%
6.55%
Samples
PMH
Contam

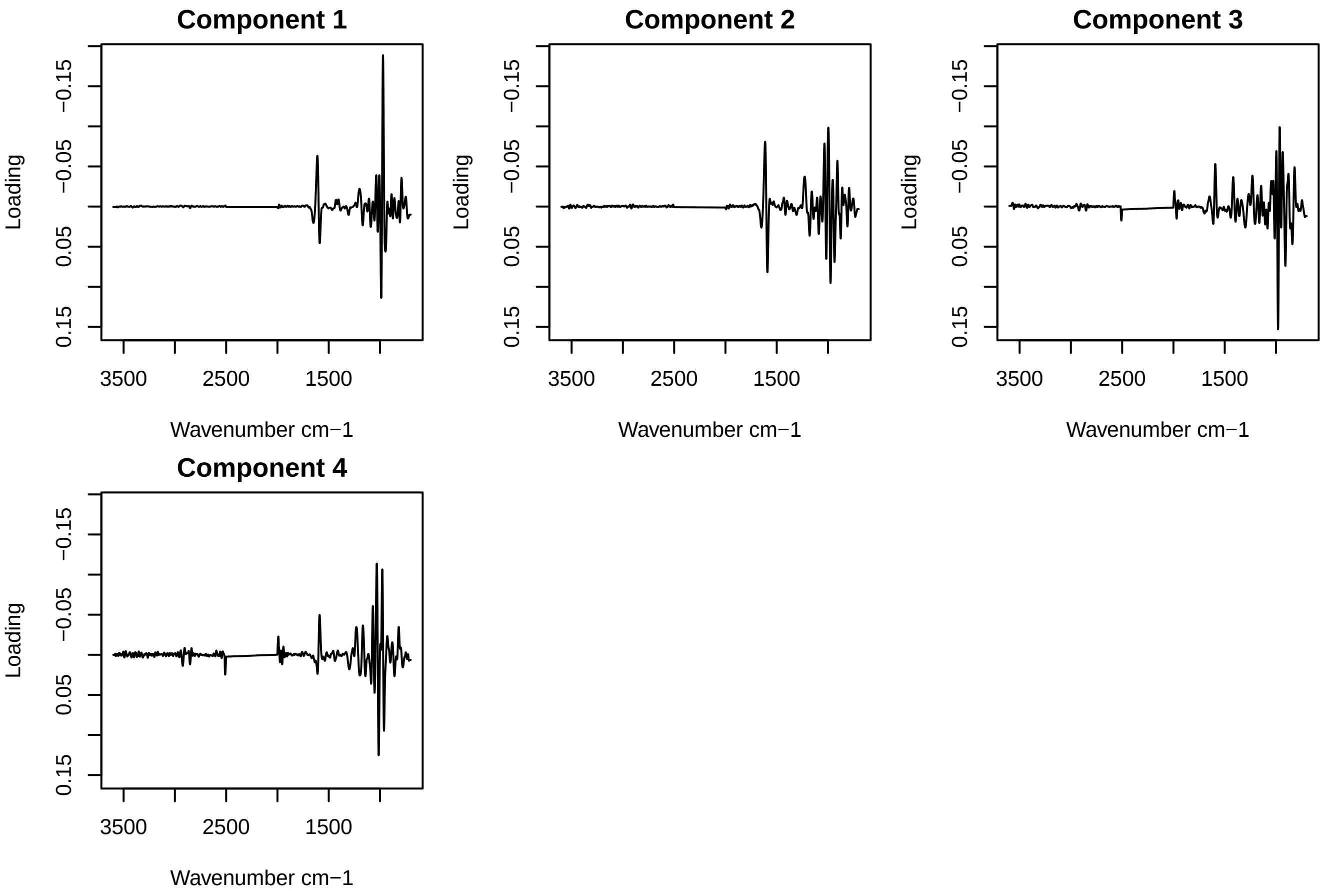

Component 1
Component 2
Component 3
Component 4
Loading
−0.15
−0.05
0.05
0.15
3500
2500
1500
Wavenumber cm−1

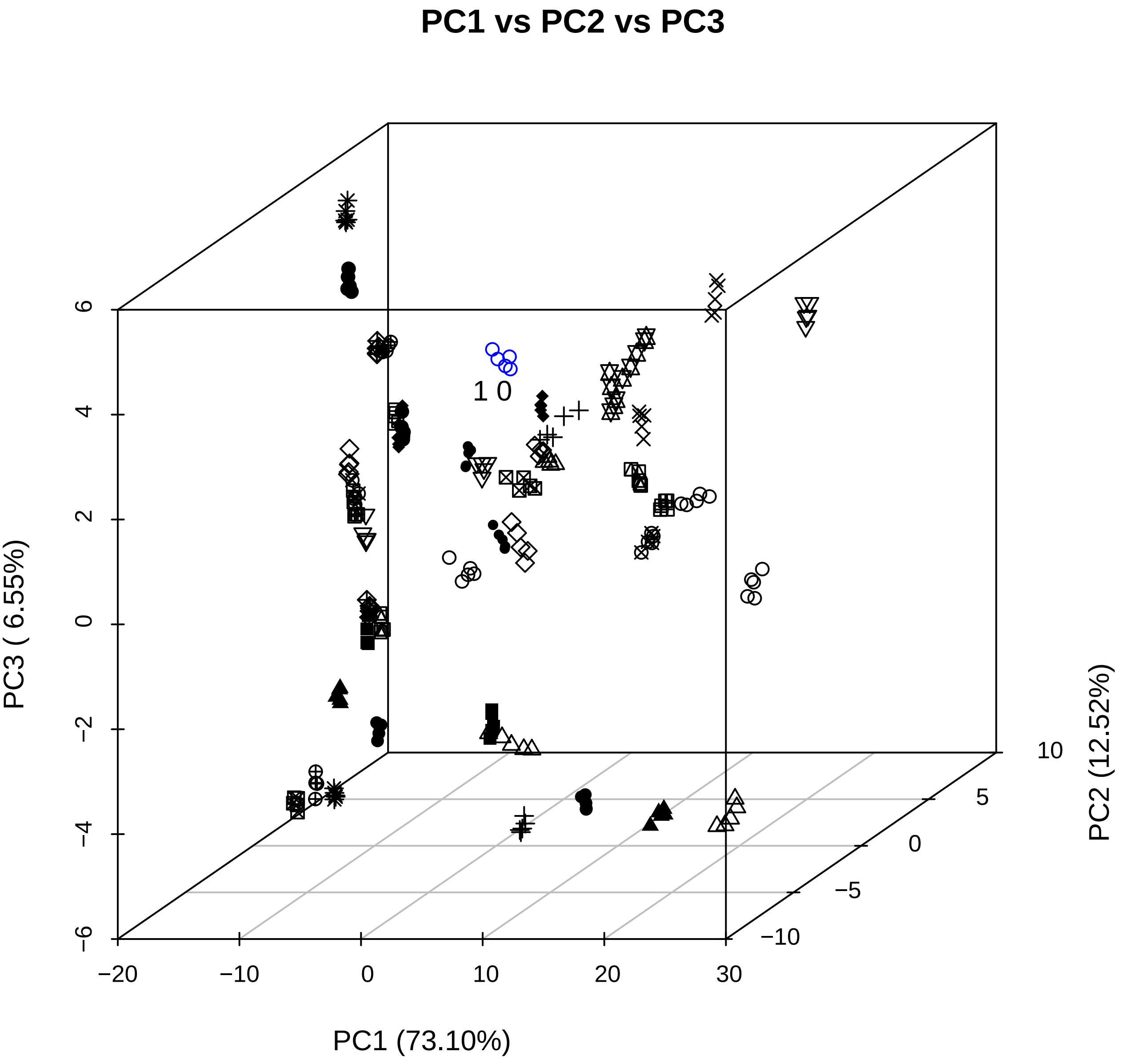

PC1 vs PC2 vs PC3
PC1 (73.10%)
PC2 (12.52%)
PC3 (6.55%)
−20
−10
0
10
20
30
−10
−5
0
5
10
−6
−4
−2
0
2
4
6
1 0

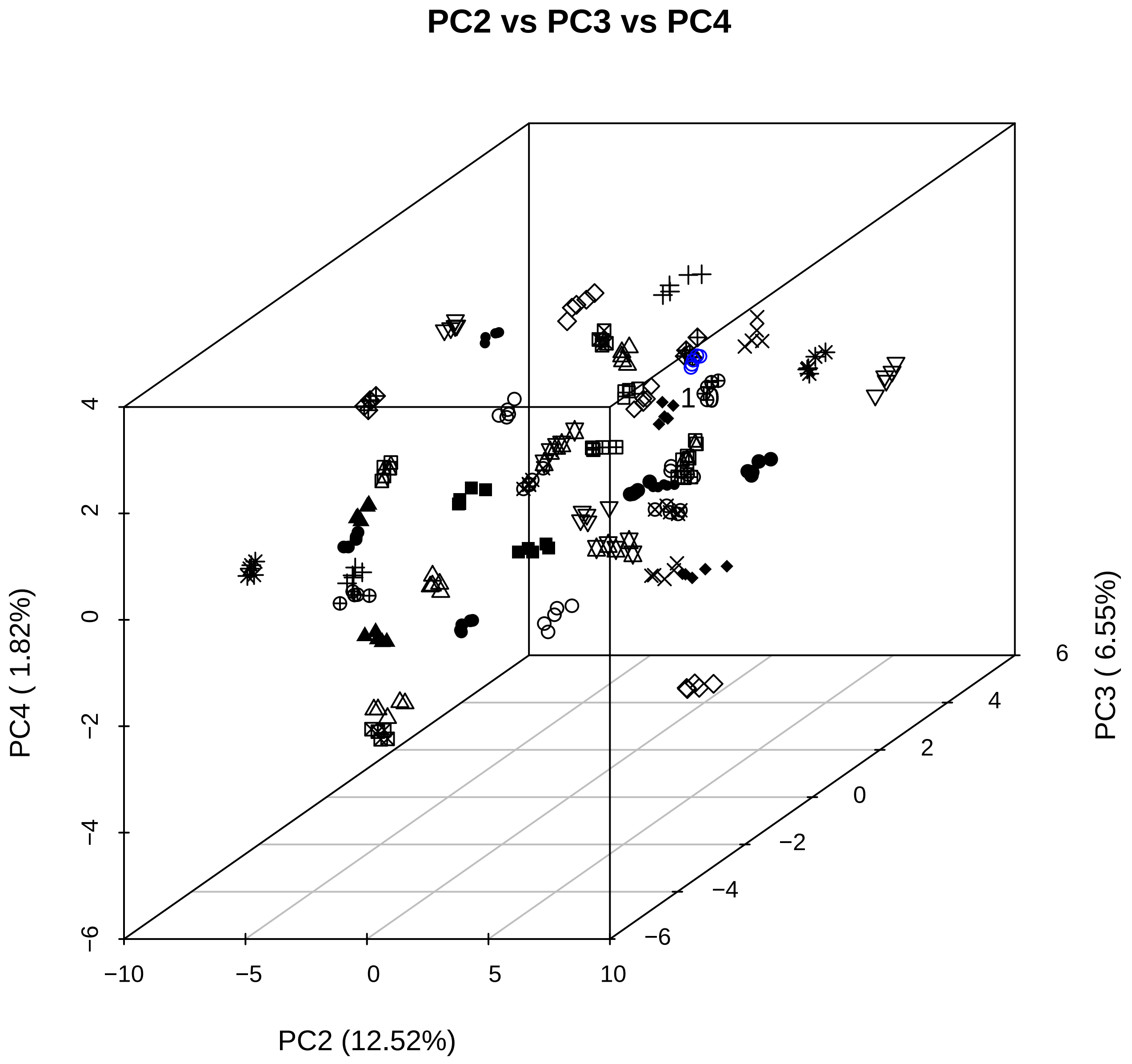
PC2 vs PC3 vs PC4
PC4 (1.82%)
PC2 (12.52%)
PC3 (6.55%)
−10
−5
0
5
10
−6
−4
−2
0
2
4
6
16

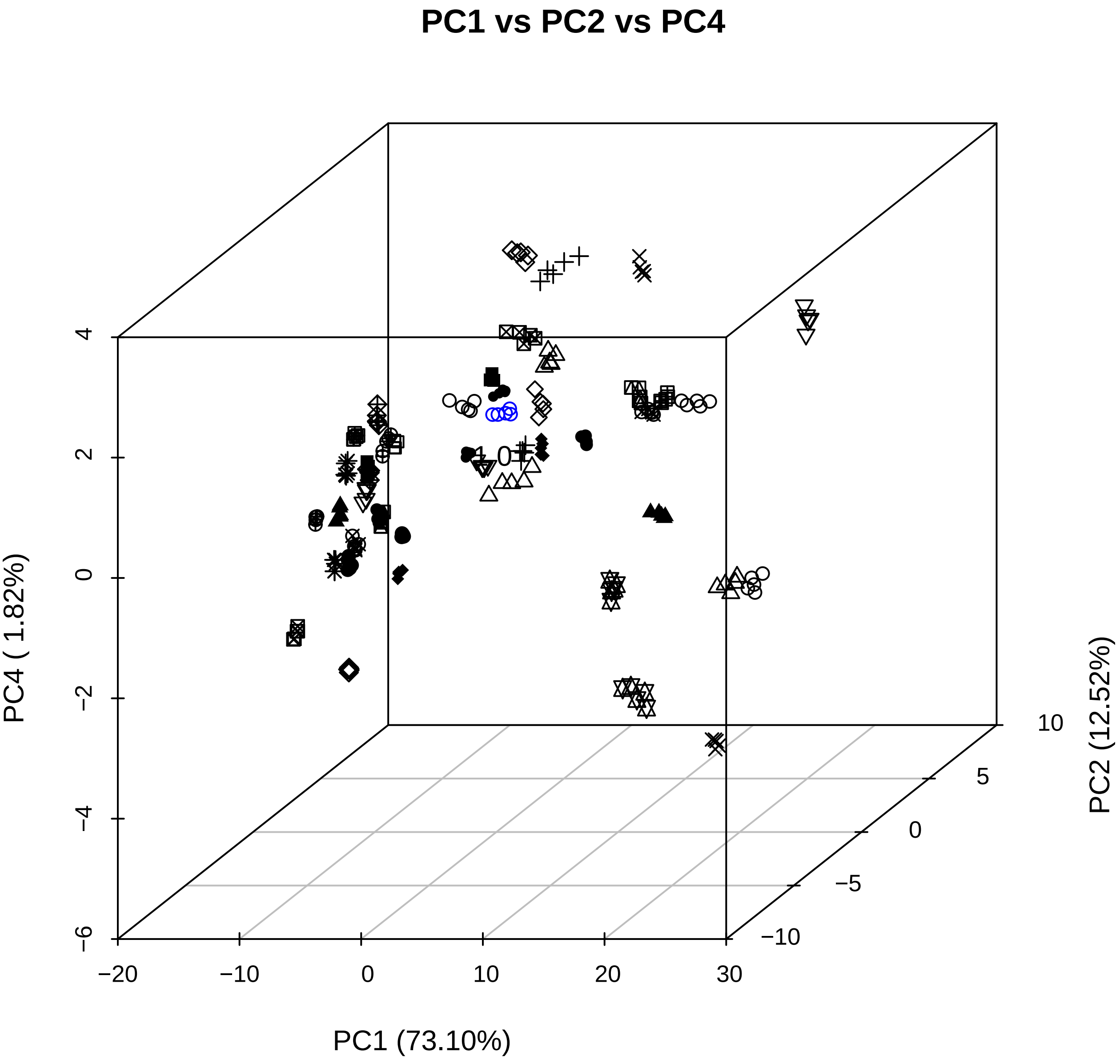
PC1 vs PC2 vs PC4
PC4 (1.82%)
4
2
0
−2
−4
−6
−20
−10
0
10
20
30
PC1 (73.10%)
PC2 (12.52%)
10
5
0
−5
−10

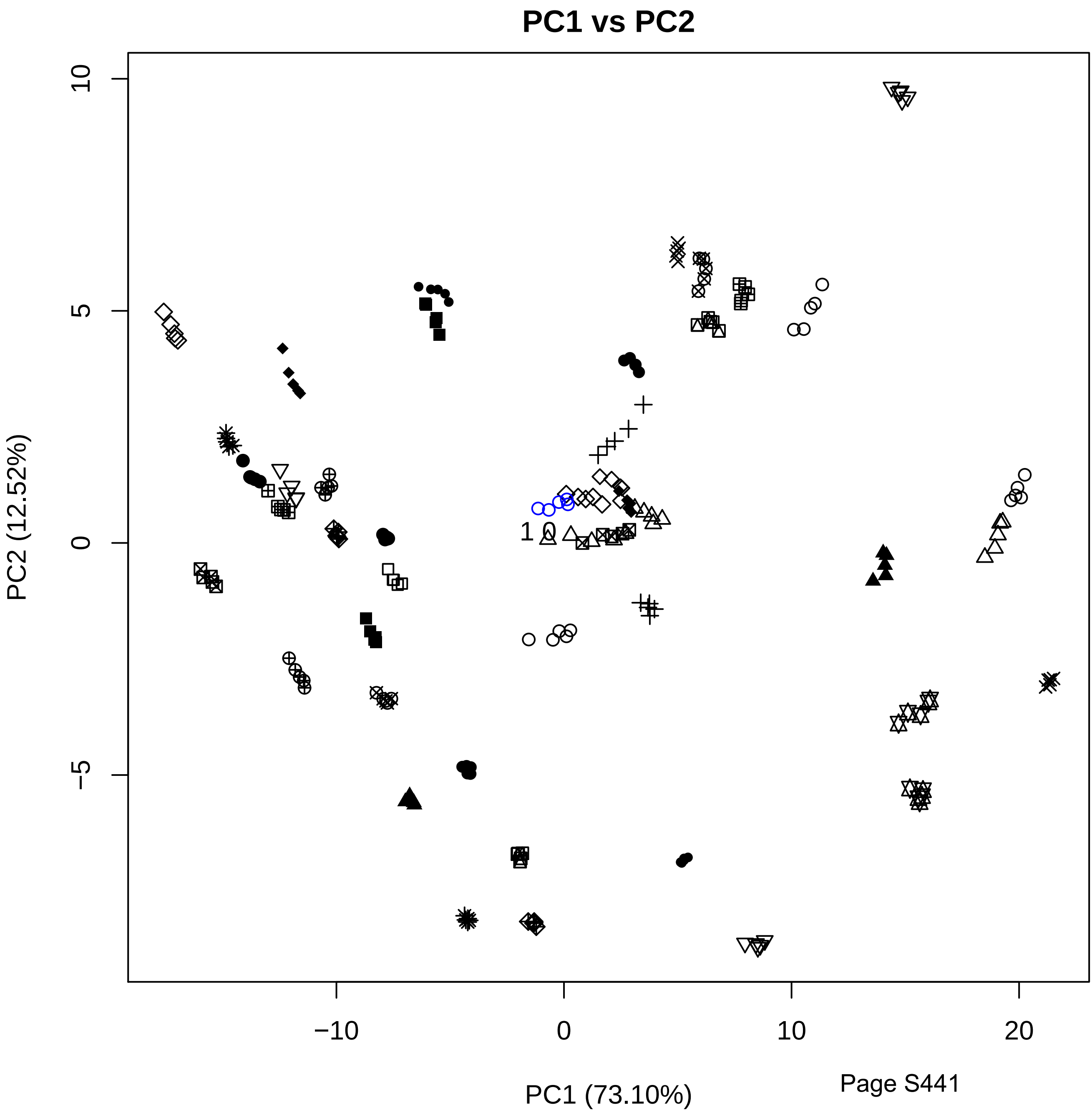
PC1 vs PC2
10
5
0
−5
PC2 (12.52%)
10
−10
0
10
20
PC1 (73.10%)

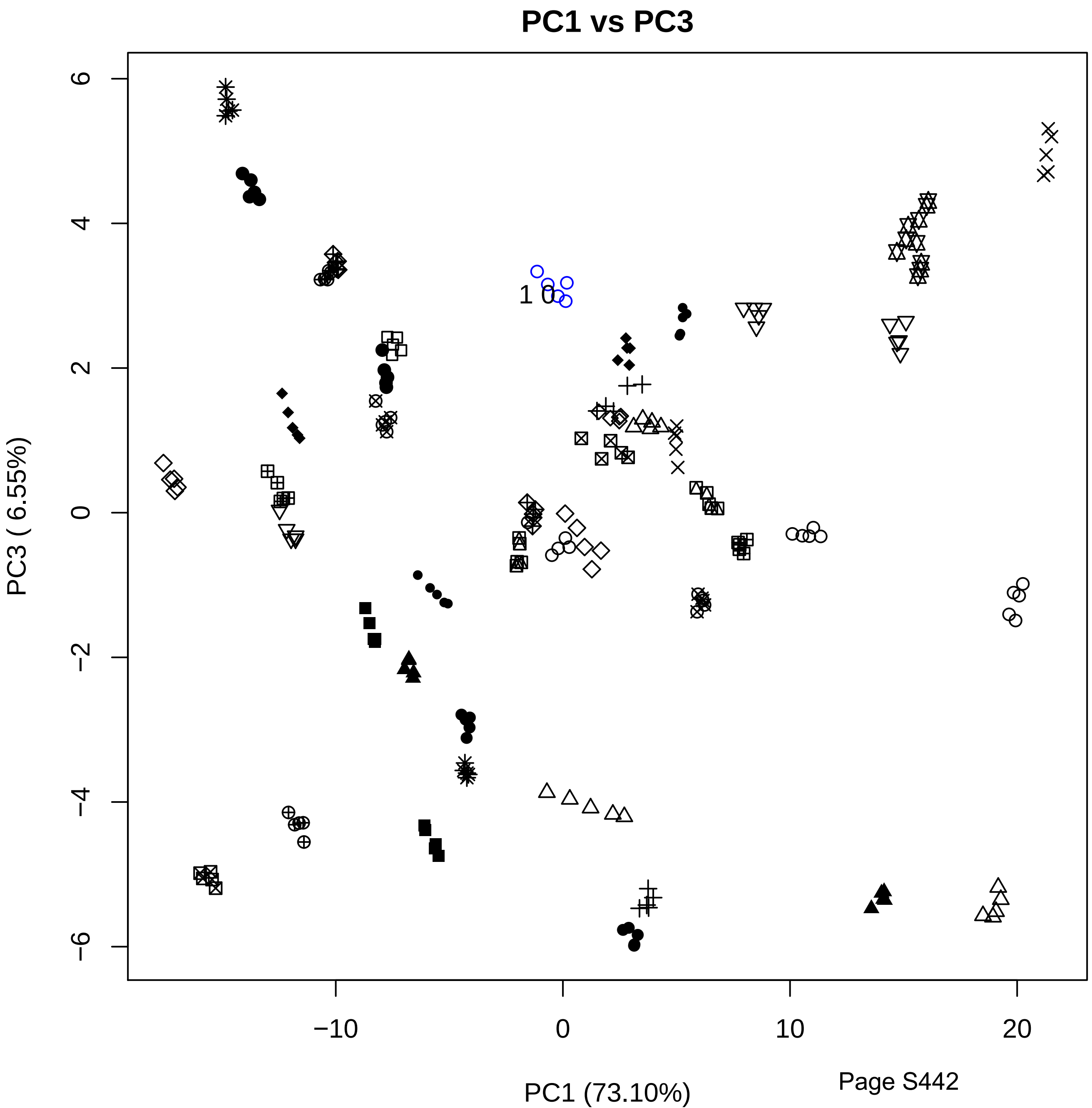
PC1 vs PC3
PC3 (6.55%)
PC1 (73.10%)
6
4
2
0
−2
−4
−6
−10
0
10
20
1 0

PC1 vs PC4

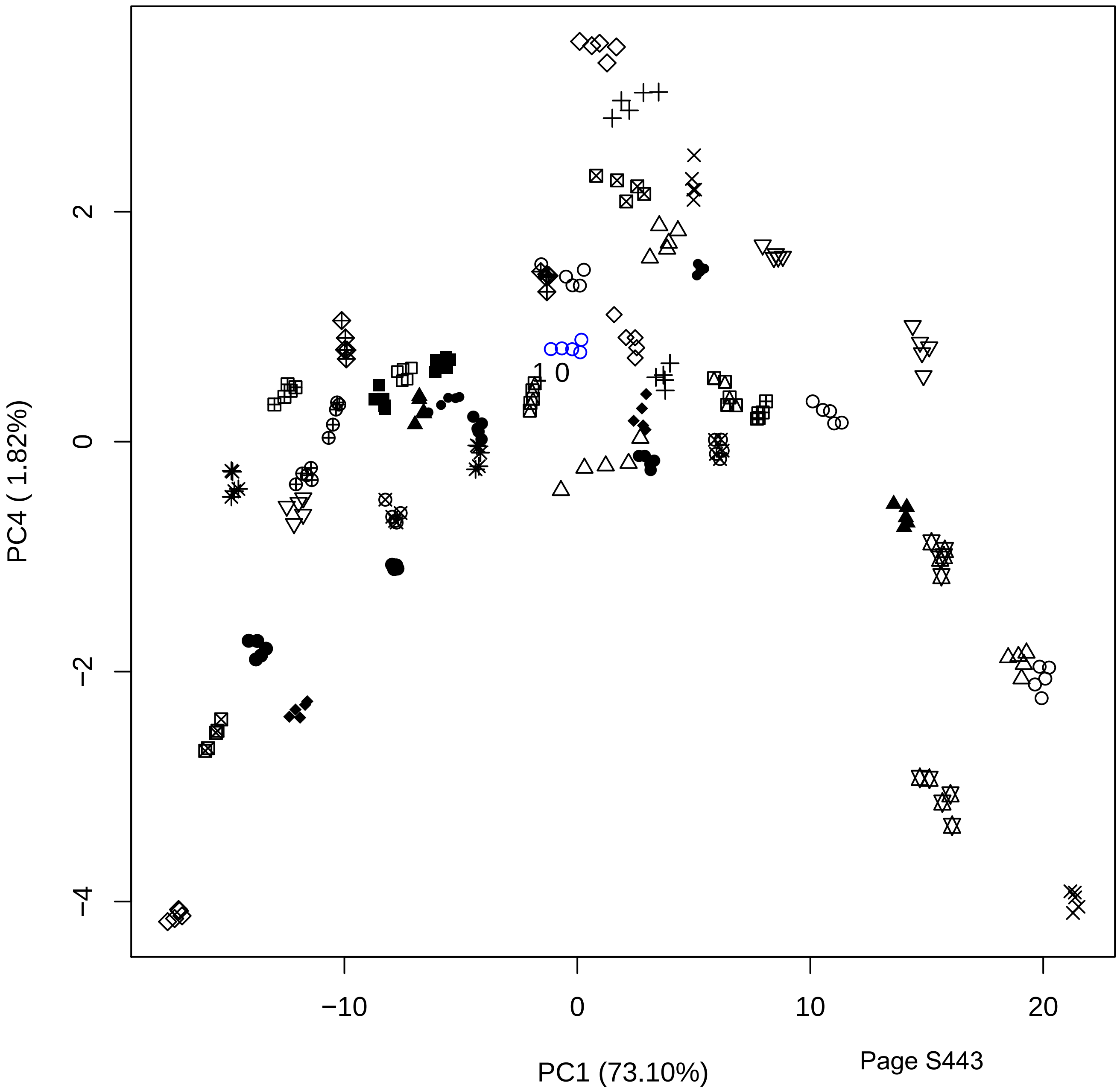

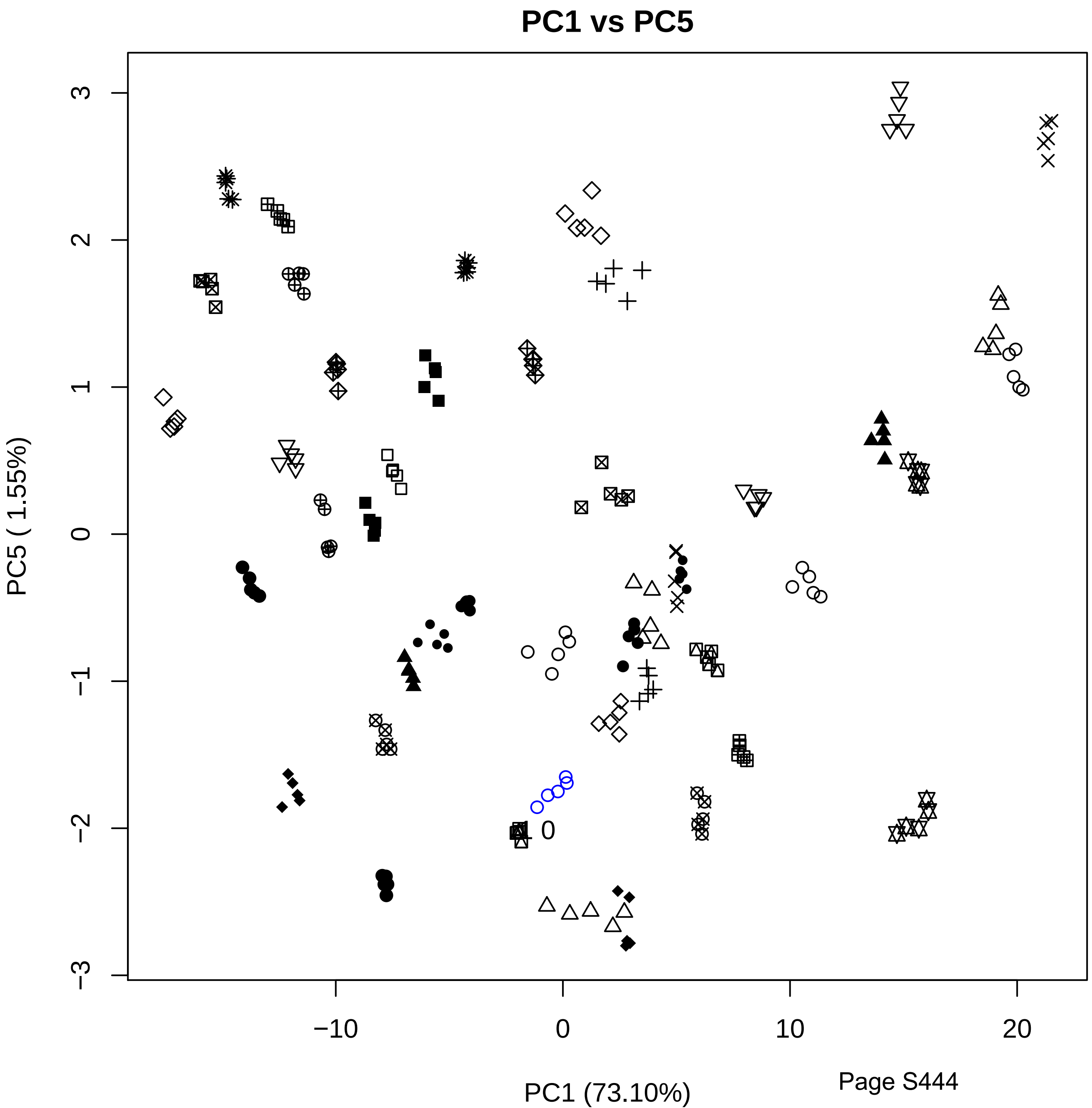

PC1 vs PC5
PC5 (1.55%)
PC1 (73.10%)
3
2
1
0
−1
−2
−3
−10
0
10
20

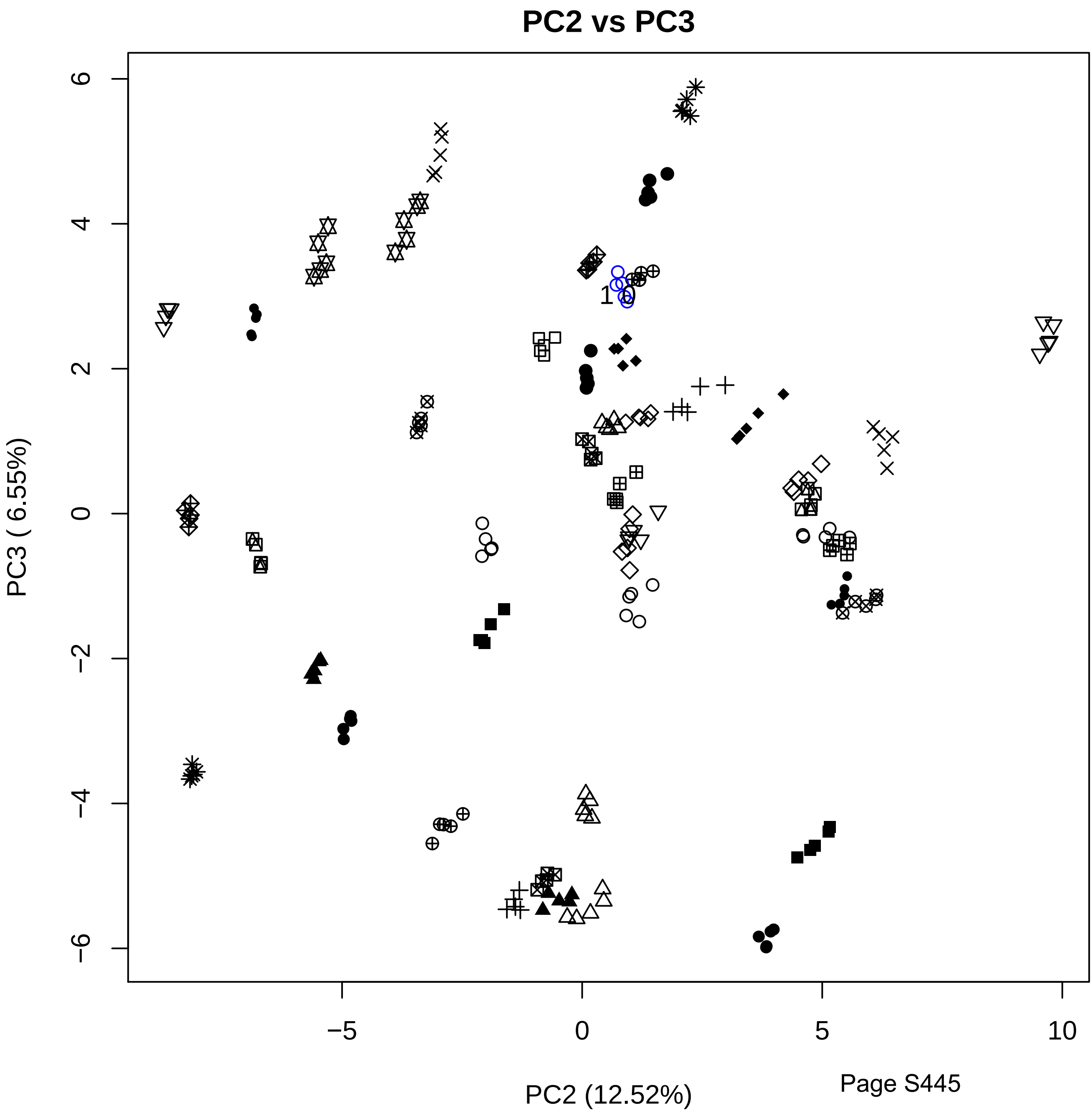
PC2 vs PC3
PC3 (6.55%)
PC2 (12.52%)
6
4
2
0
−2
−4
−6
−5
0
5
10
1 0

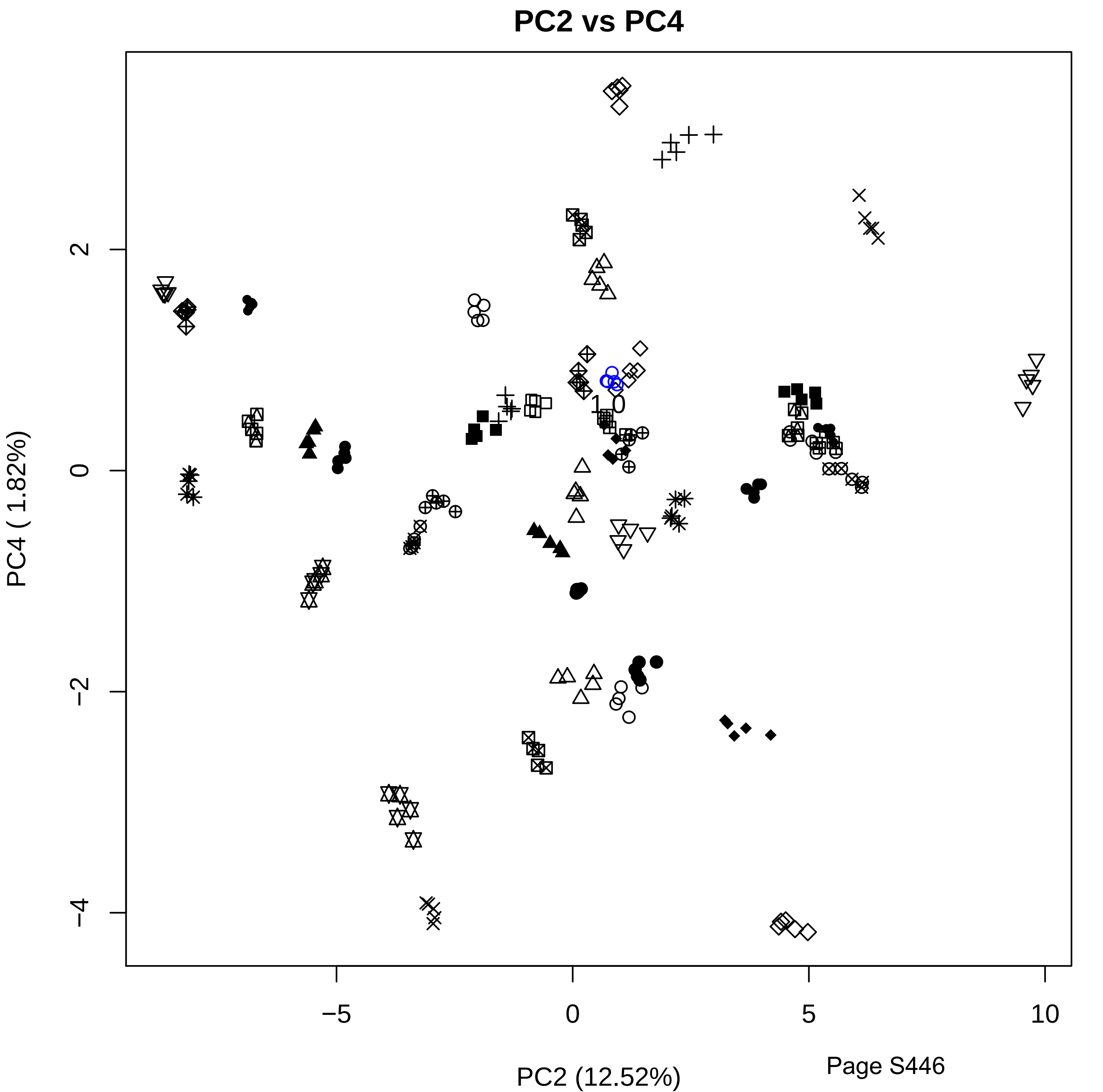
PC2 vs PC4
PC4 (1.82%)
PC2 (12.52%)
2
0
−2
−4
−5
0
5
10
10

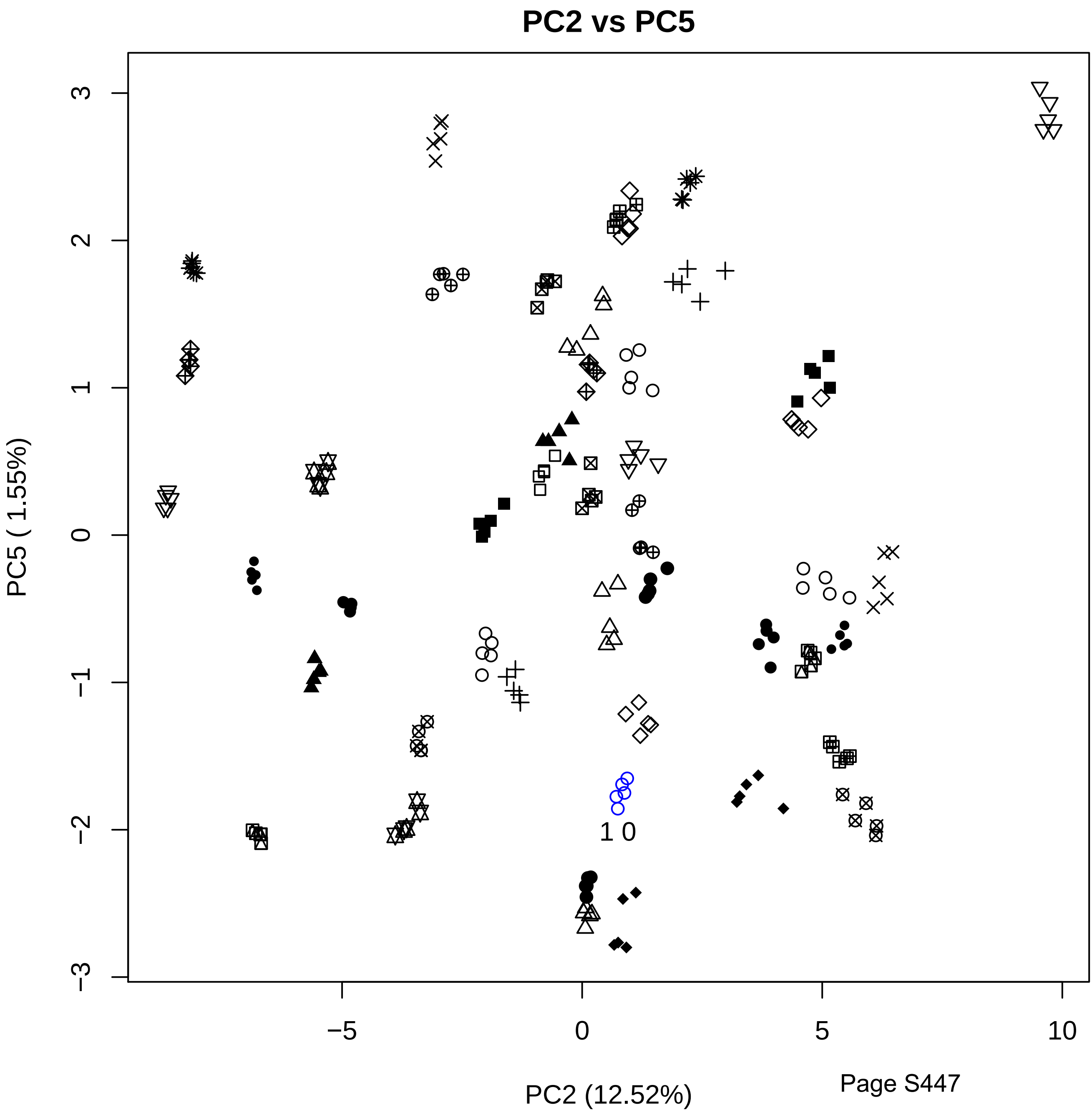
PC2 vs PC5
PC5 (1.55%)
PC2 (12.52%)
3
2
1
0
−1
−2
−3
−5
0
5
10
1 0

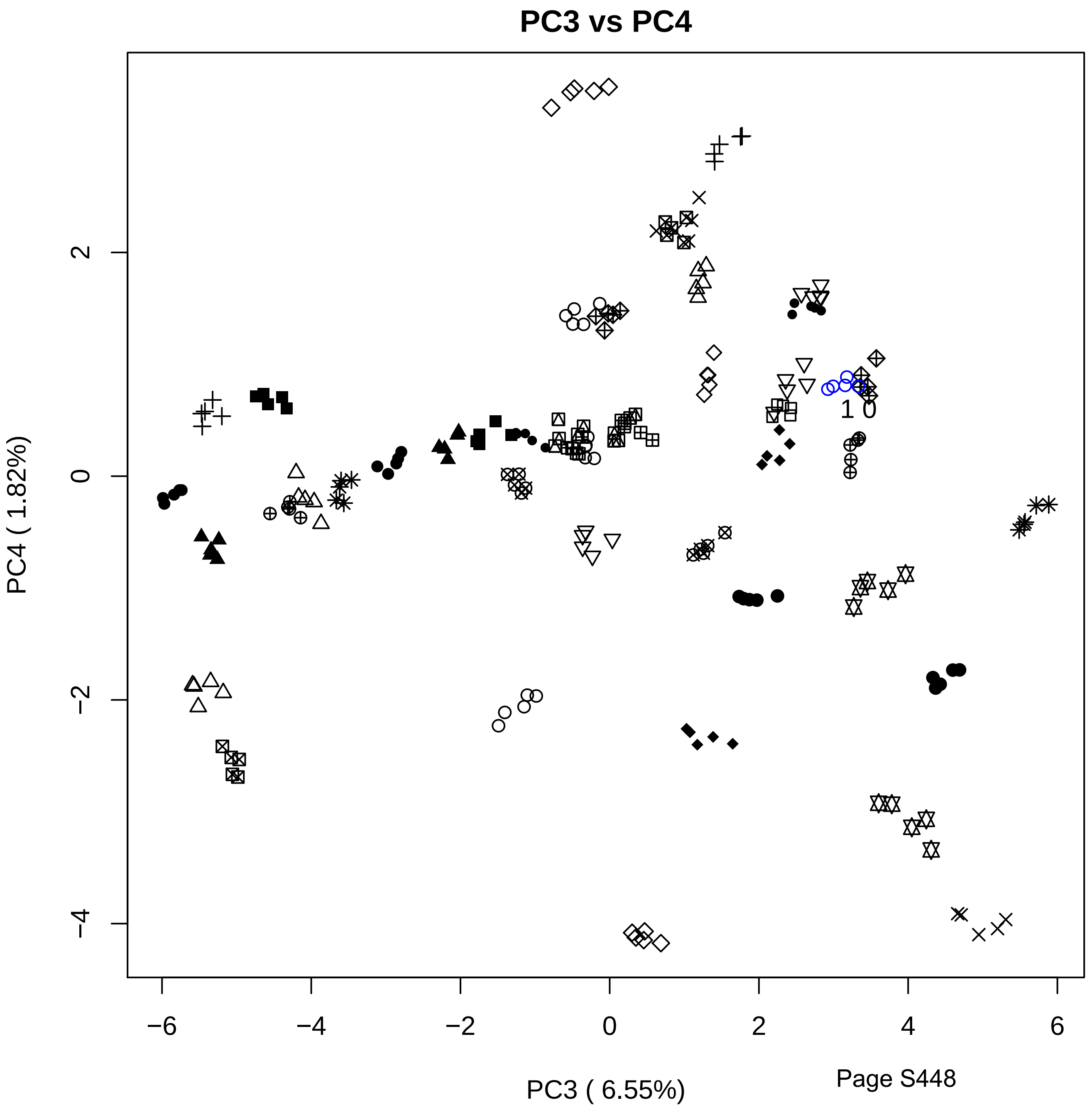
PC3 vs PC4
PC4 (1.82%)
PC3 (6.55%)
-6
-4
-2
0
2
4
6
-4
-2
0
2
1 0

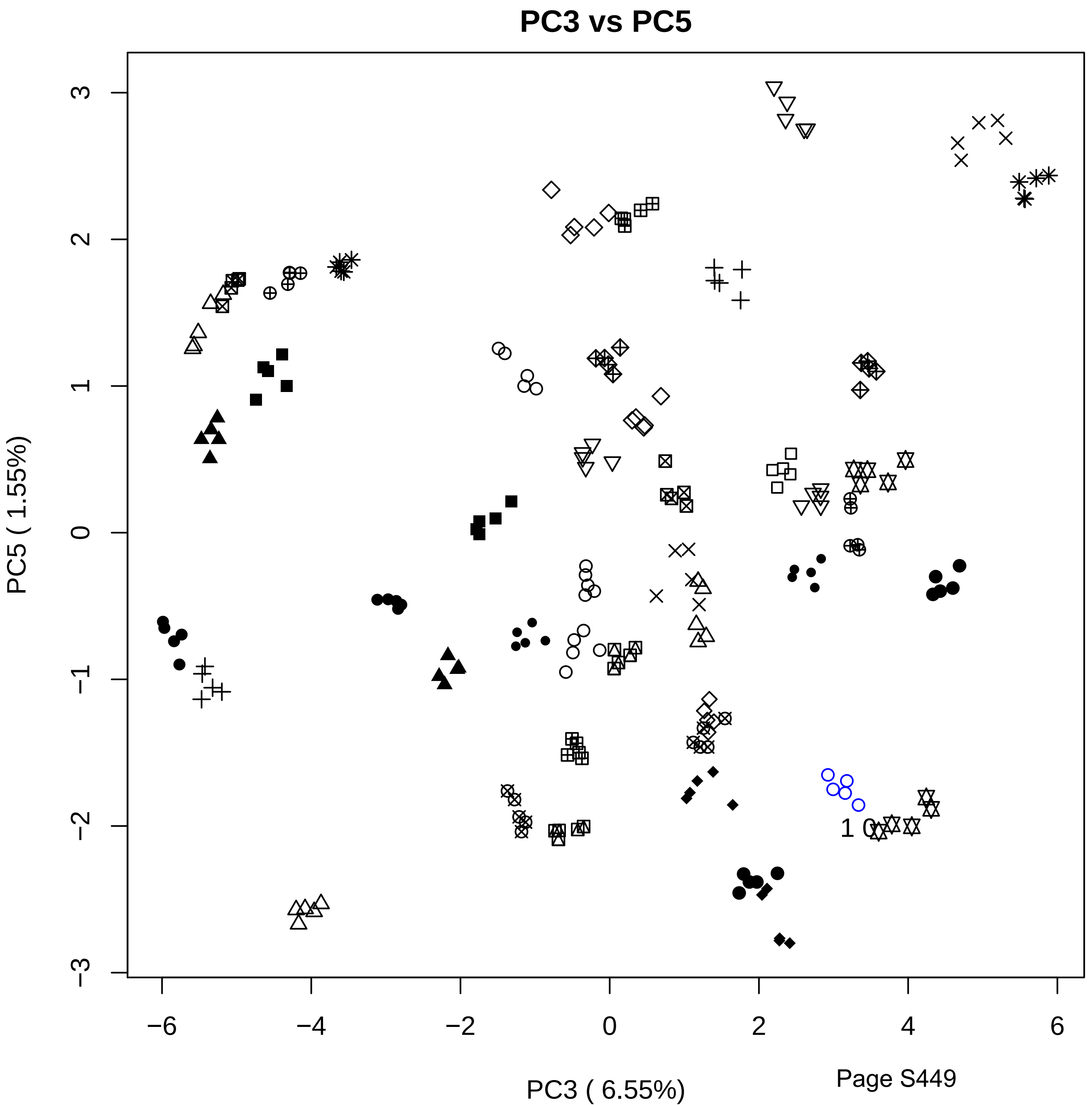
PC3 vs PC5
PC5 (1.55%)
PC3 (6.55%)
3
2
1
0
−1
−2
−3
−6
−4
−2
0
2
4
6
1 0

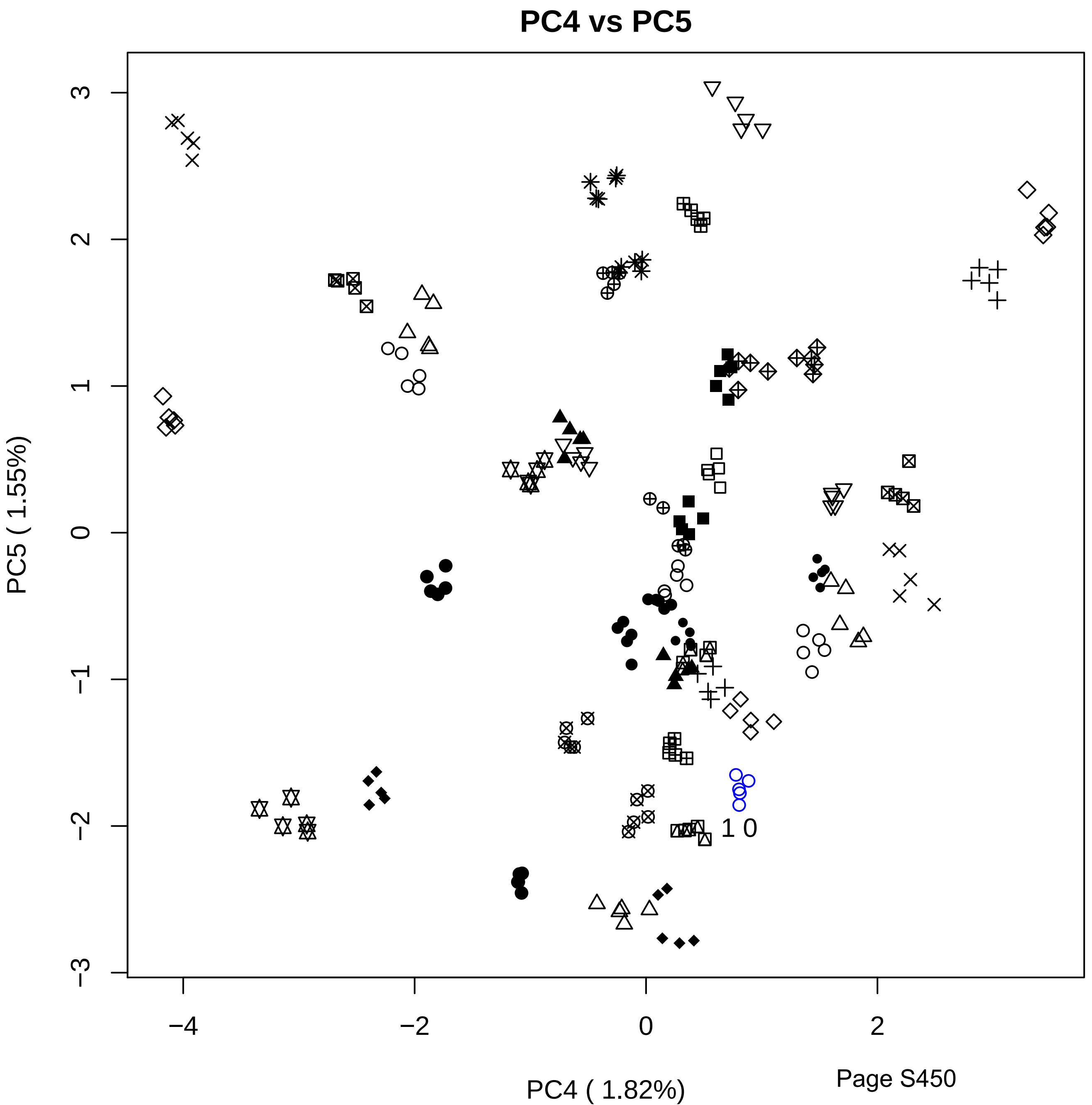
PC4 vs PC5
PC5 (1.55%)
PC4 (1.82%)
−4
−2
0
2
−3
−2
−1
0
1
2
3
1 0

PCA of 2.5% OSAS vs PMH

Scaled Matrix PCAed

7th order polynomial baseline correction performed

Raw spectra smoothed to 21 Neighbours, 2nd order PN, Savtisky Golay

Normalisation between 0 and 100

D2 spectra smoothed to 41 Neighbours, 2nd order PN, Savtisky Golay

Regions Analysed: 3601−2501 and 2001−700cm−1

Samples Analysed: 1 Contam's 0 OSAS's 45 PMH's

Keele University AD   Sun Dec 09 16:29:02 2018

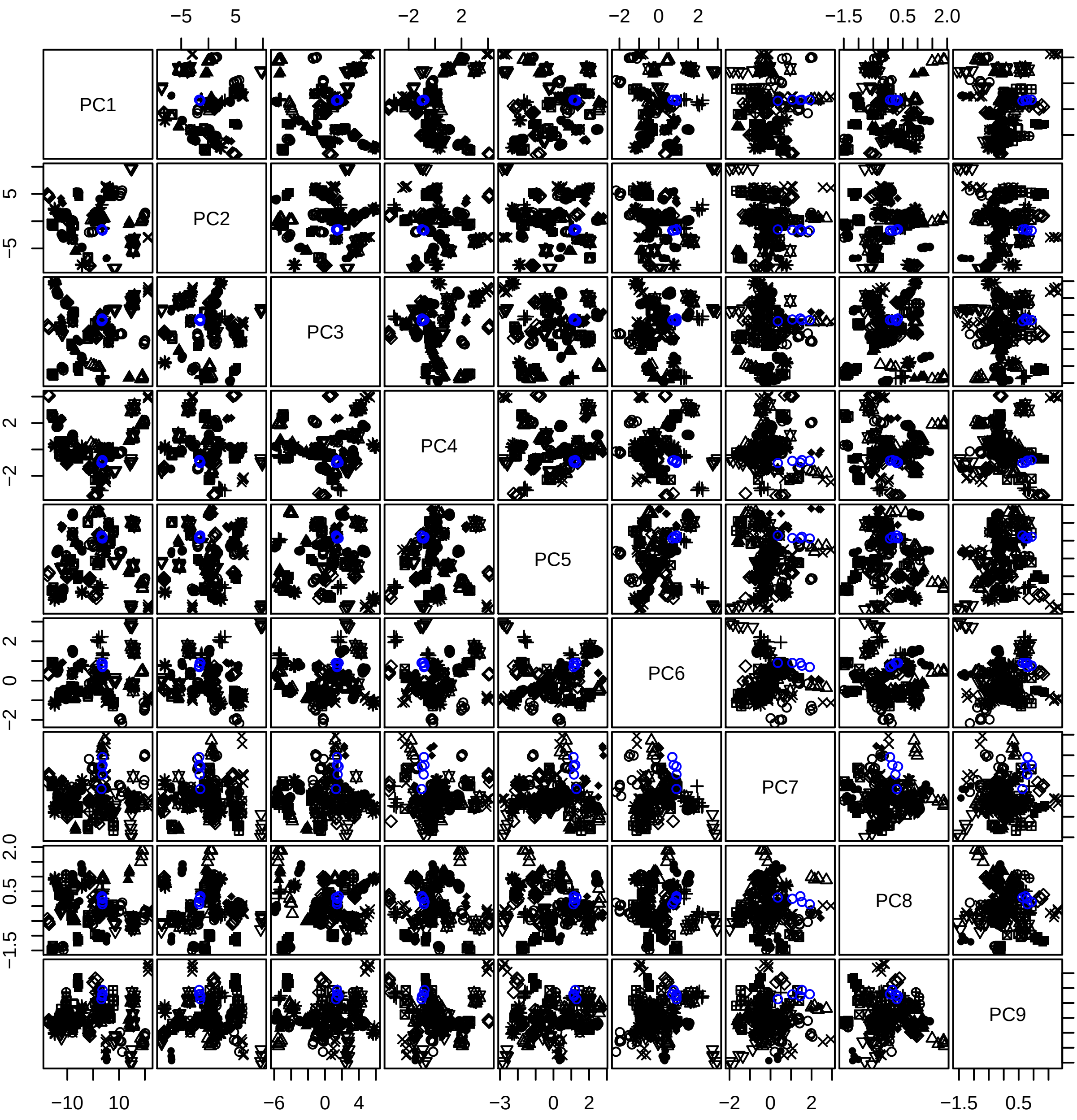
PC1
PC2
PC3
PC4
PC5
PC6
PC7
PC8
PC9
−5 5
−2 2
−2 0 2
−1.5 0.5 2.0
10
−10
5
−5
4
0
−6
2
−2
2
0
−3
2
0
−2
2
0
−2
2.0
0.5
−1.5
0.5
−1.5
−10 10
−6 0 4
−3 0 2
−2 0 2
−1.5 0.5

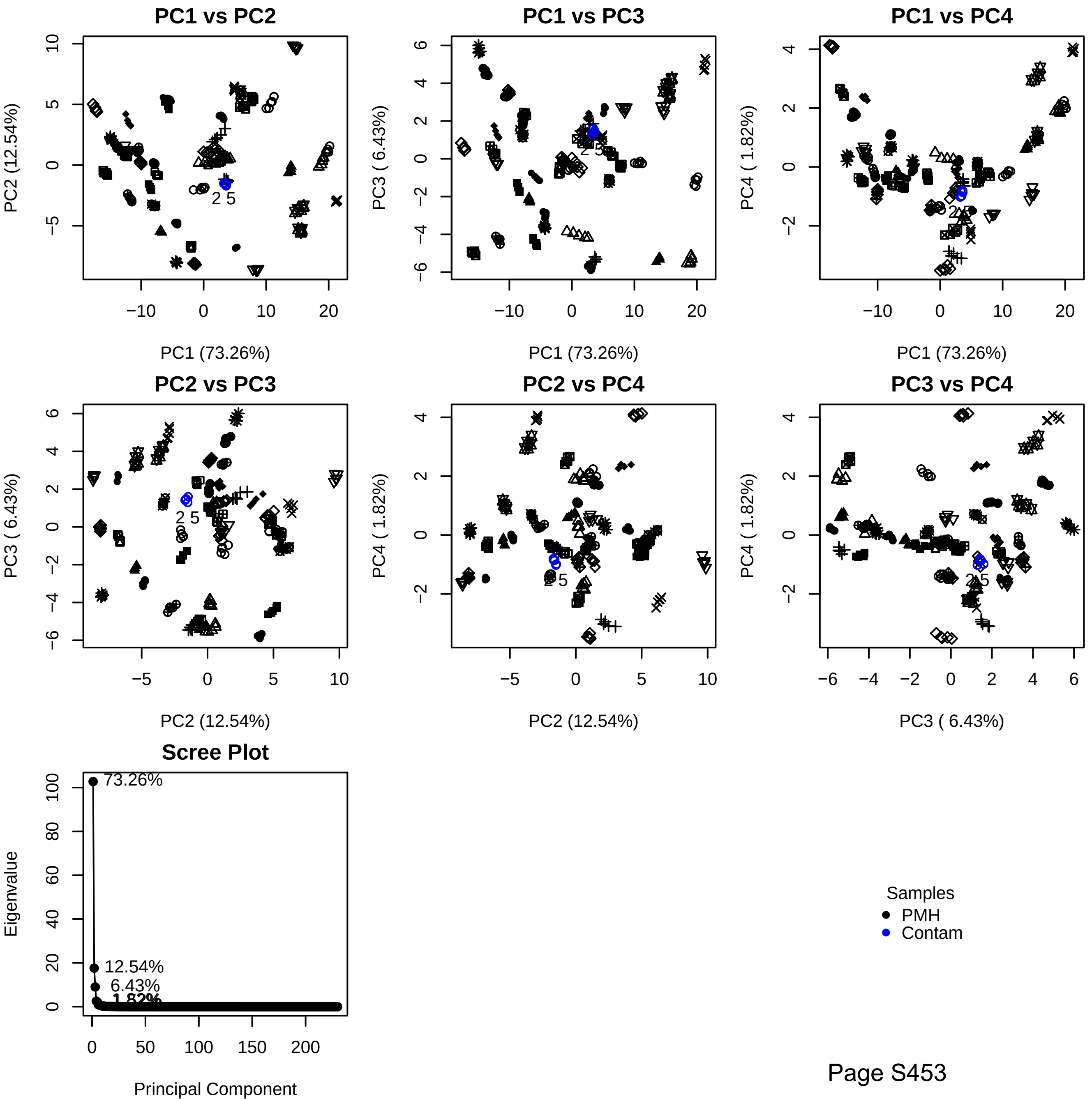
PC1 vs PC2
PC1 vs PC3
PC1 vs PC4
PC2 vs PC3
PC2 vs PC4
PC3 vs PC4
Scree Plot
PC1 (73.26%)
PC2 (12.54%)
PC3 (6.43%)
PC4 (1.82%)
Eigenvalue
Principal Component
73.26%
12.54%
6.43%
1.82%
2 5
Samples
PMH
Contam

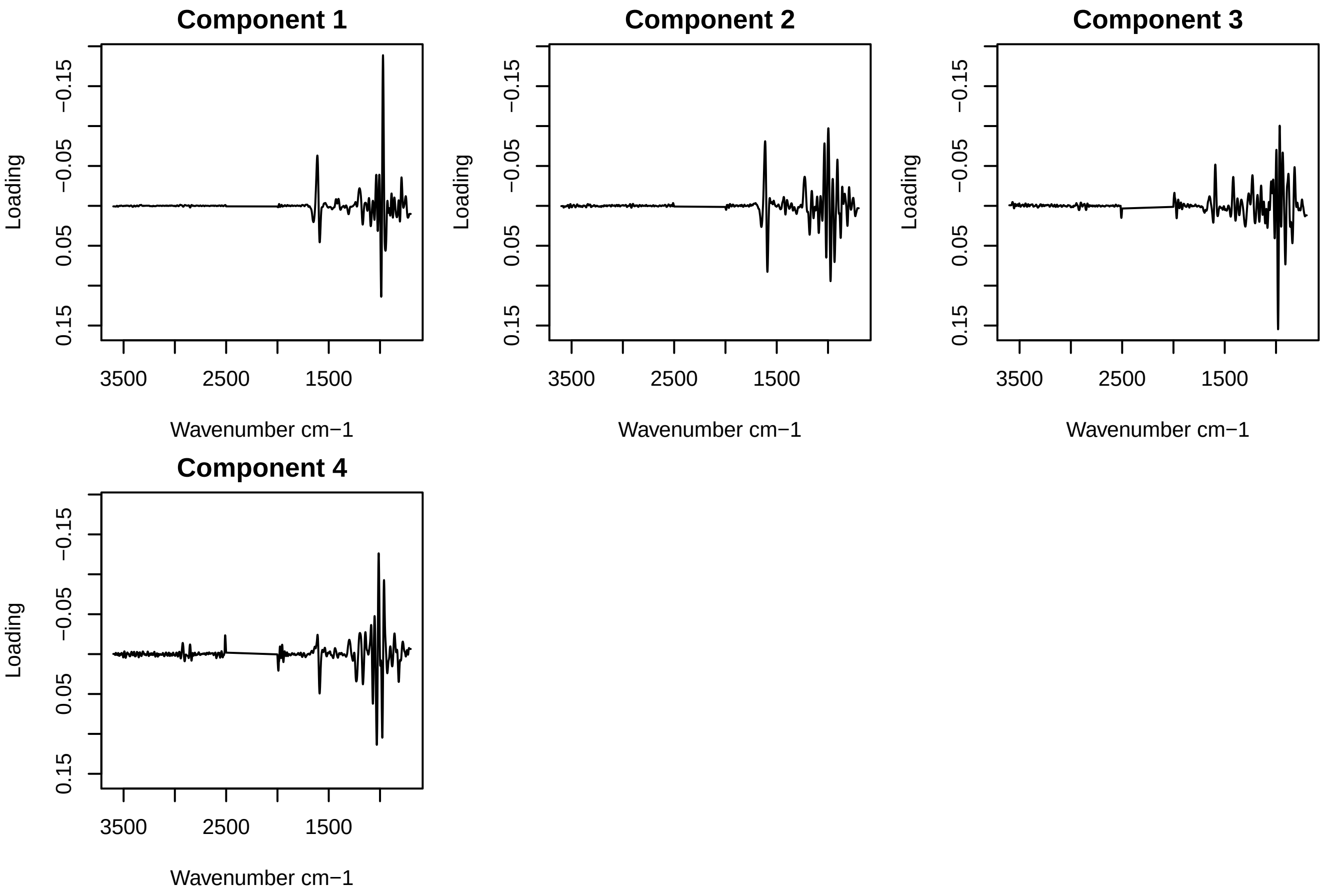
Component 1
Component 2
Component 3
Component 4
Loading
−0.15
−0.05
0.05
0.15
3500
2500
1500
Wavenumber cm−1

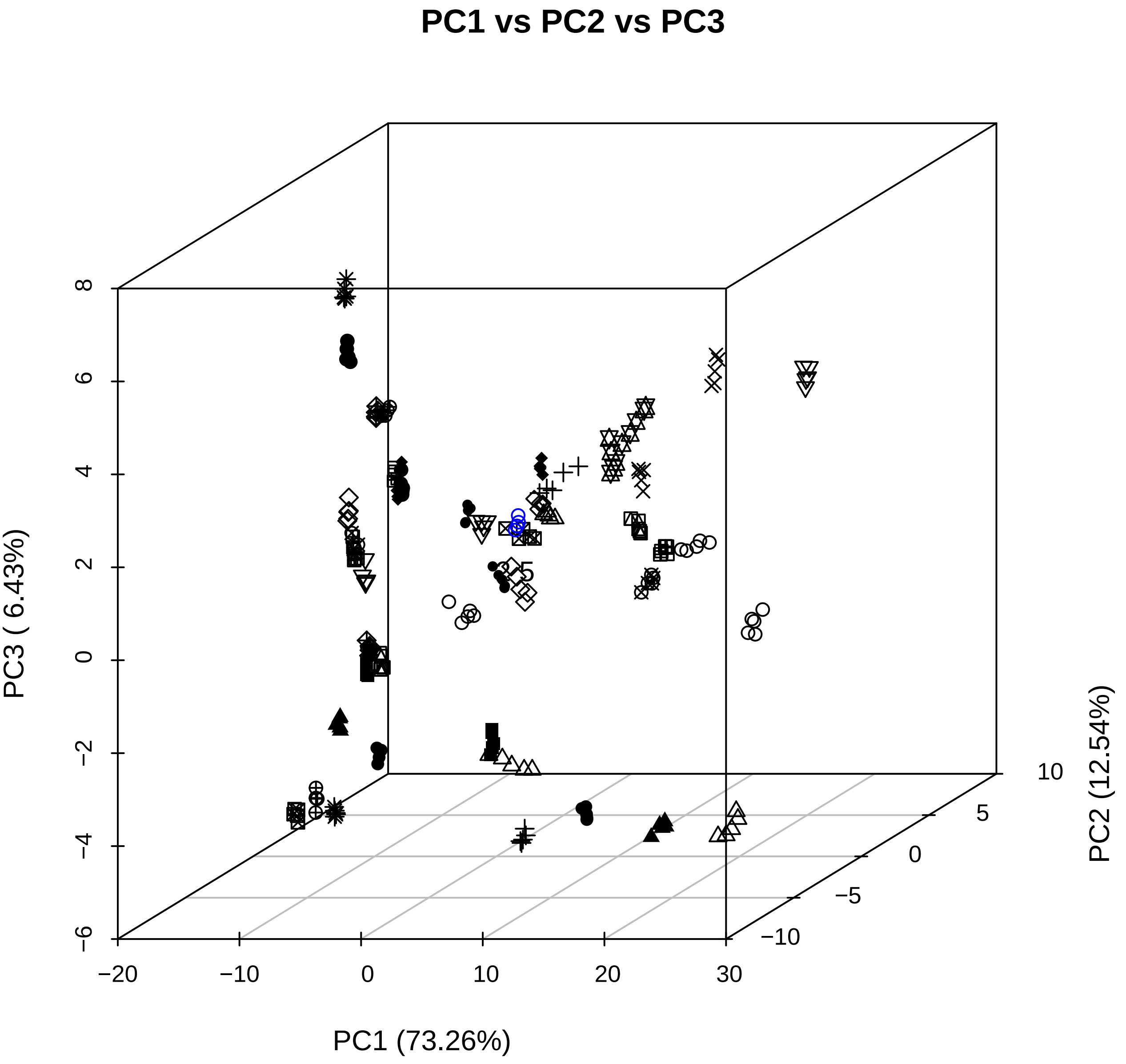
PC1 vs PC2 vs PC3
PC1 (73.26%)
PC2 (12.54%)
PC3 (6.43%)
−20
−10
0
10
20
30
−10
−5
0
5
10
−6
−4
−2
0
2
4
6
8

PC2 vs PC3 vs PC4

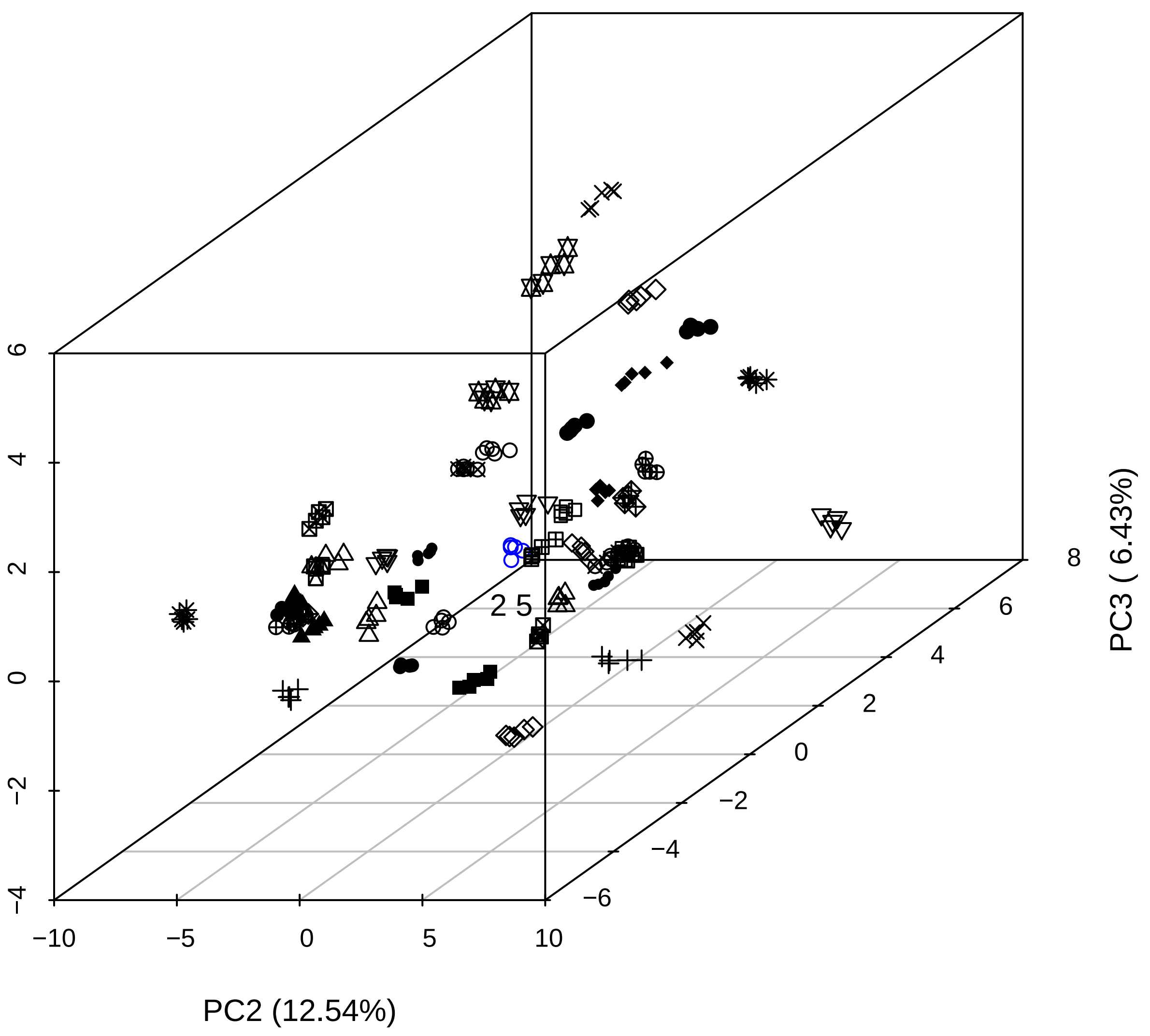

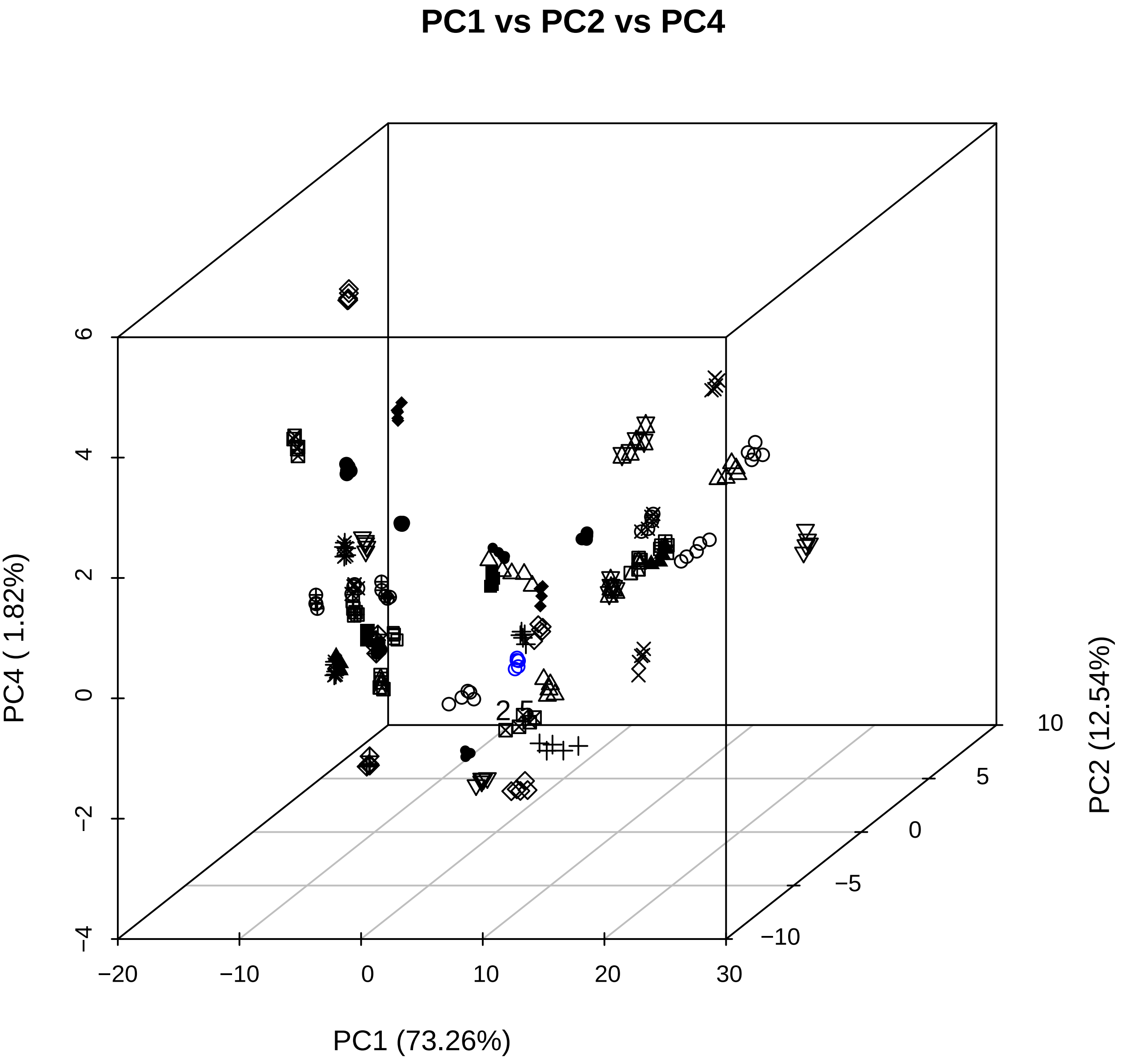
PC1 vs PC2 vs PC4
PC1 (73.26%)
PC2 (12.54%)
PC4 (1.82%)
−20
−10
0
10
20
30
−10
−5
0
5
10
−4
−2
0
2
4
6
2.5

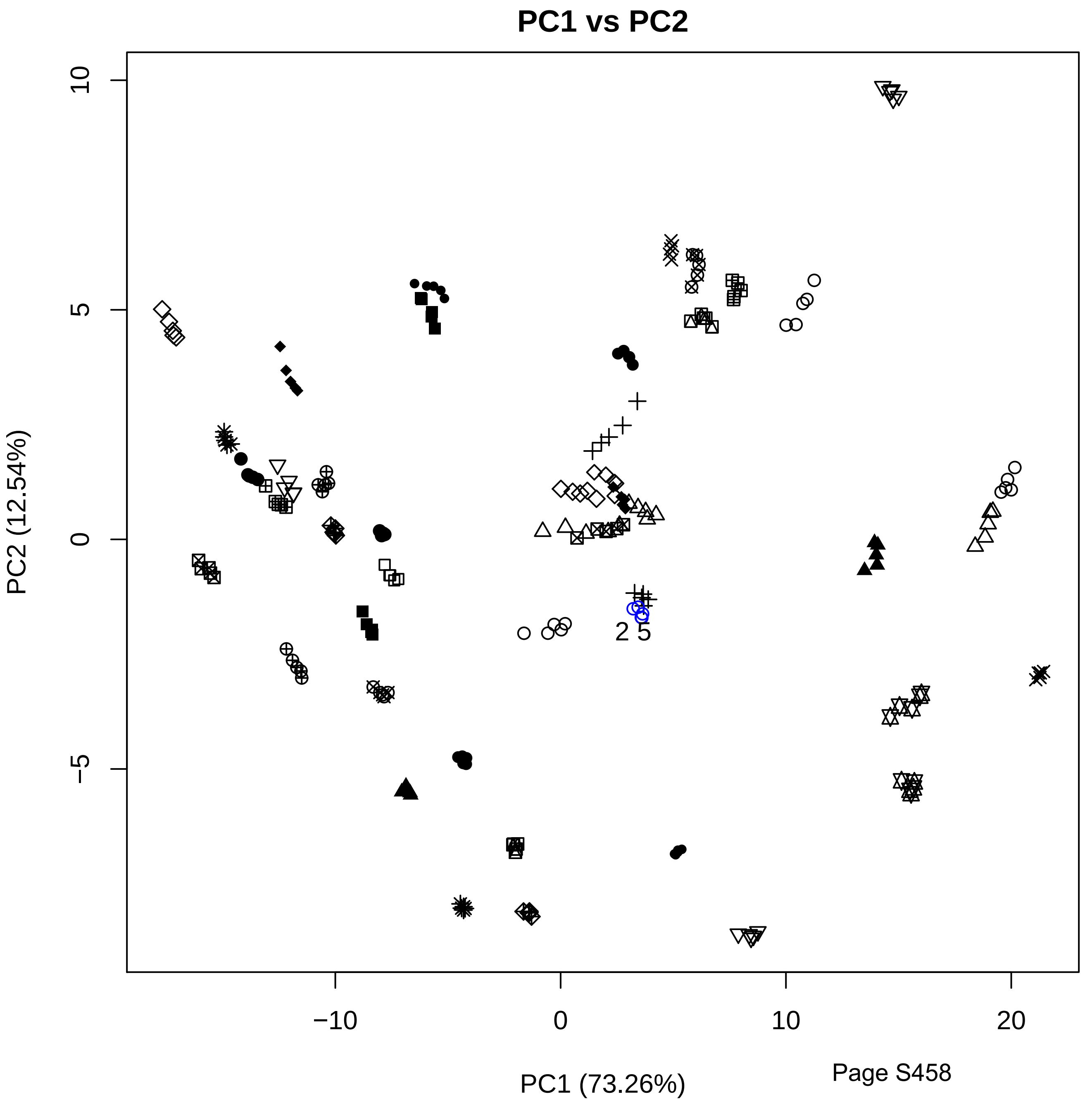

PC1 vs PC2
PC2 (12.54%)
PC1 (73.26%)
10
5
0
−5
−10
0
10
20
2 5

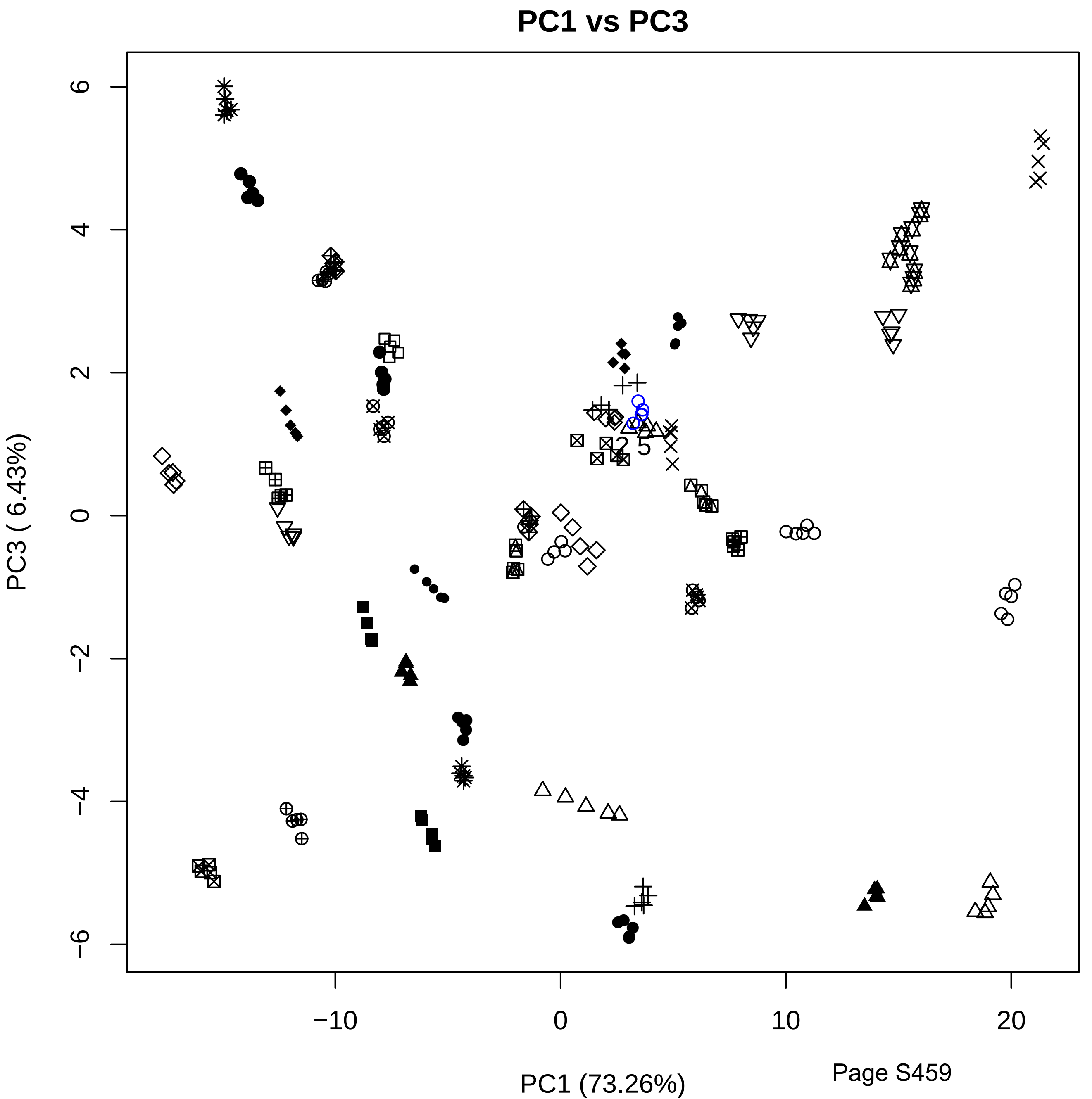
PC1 vs PC3
PC3 (6.43%)
PC1 (73.26%)
6
4
2
0
−2
−4
−6
−10
0
10
20
2
5

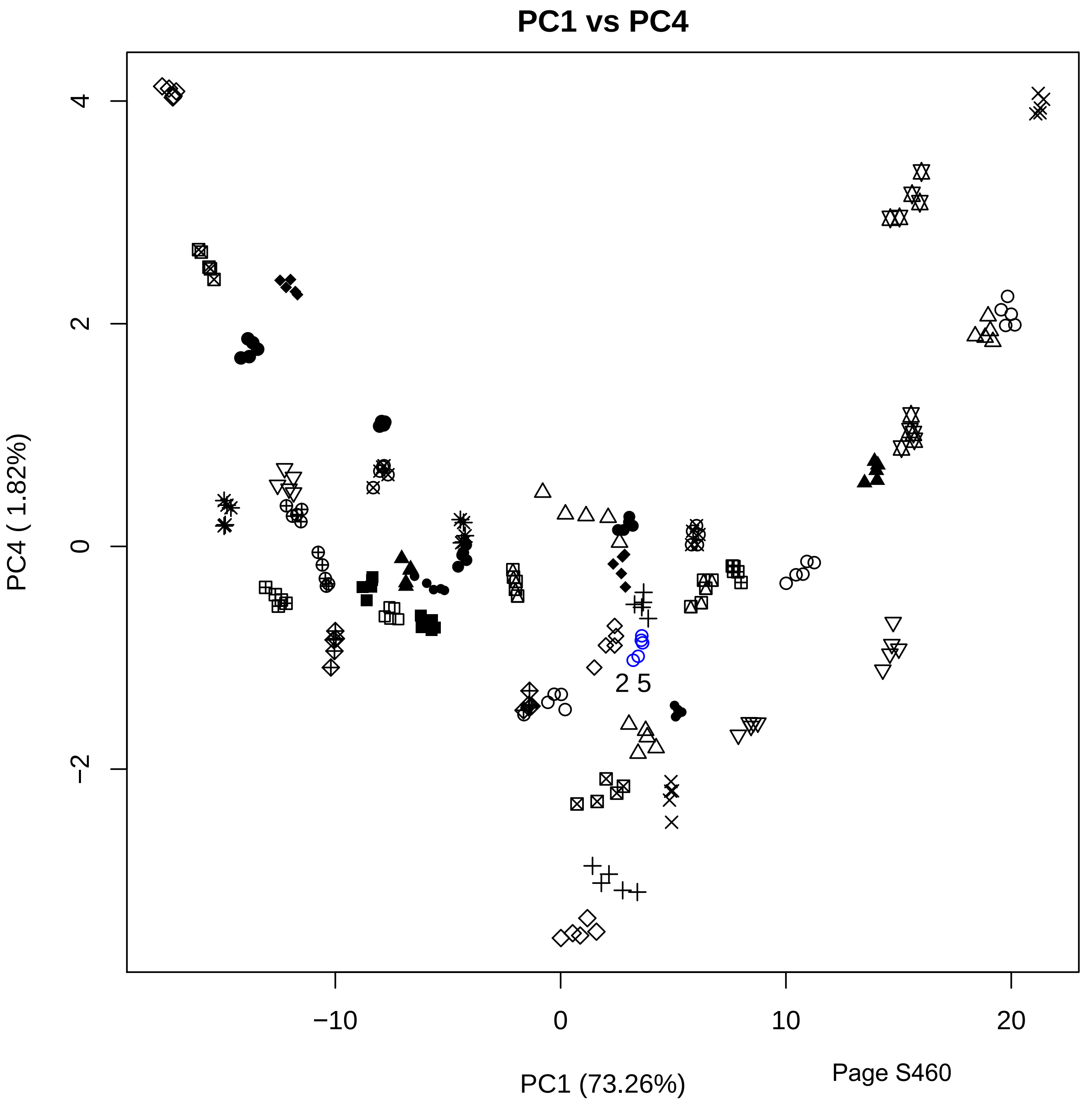
PC1 vs PC4
PC4 (1.82%)
PC1 (73.26%)
4
2
0
−2
−10
0
10
20
2 5

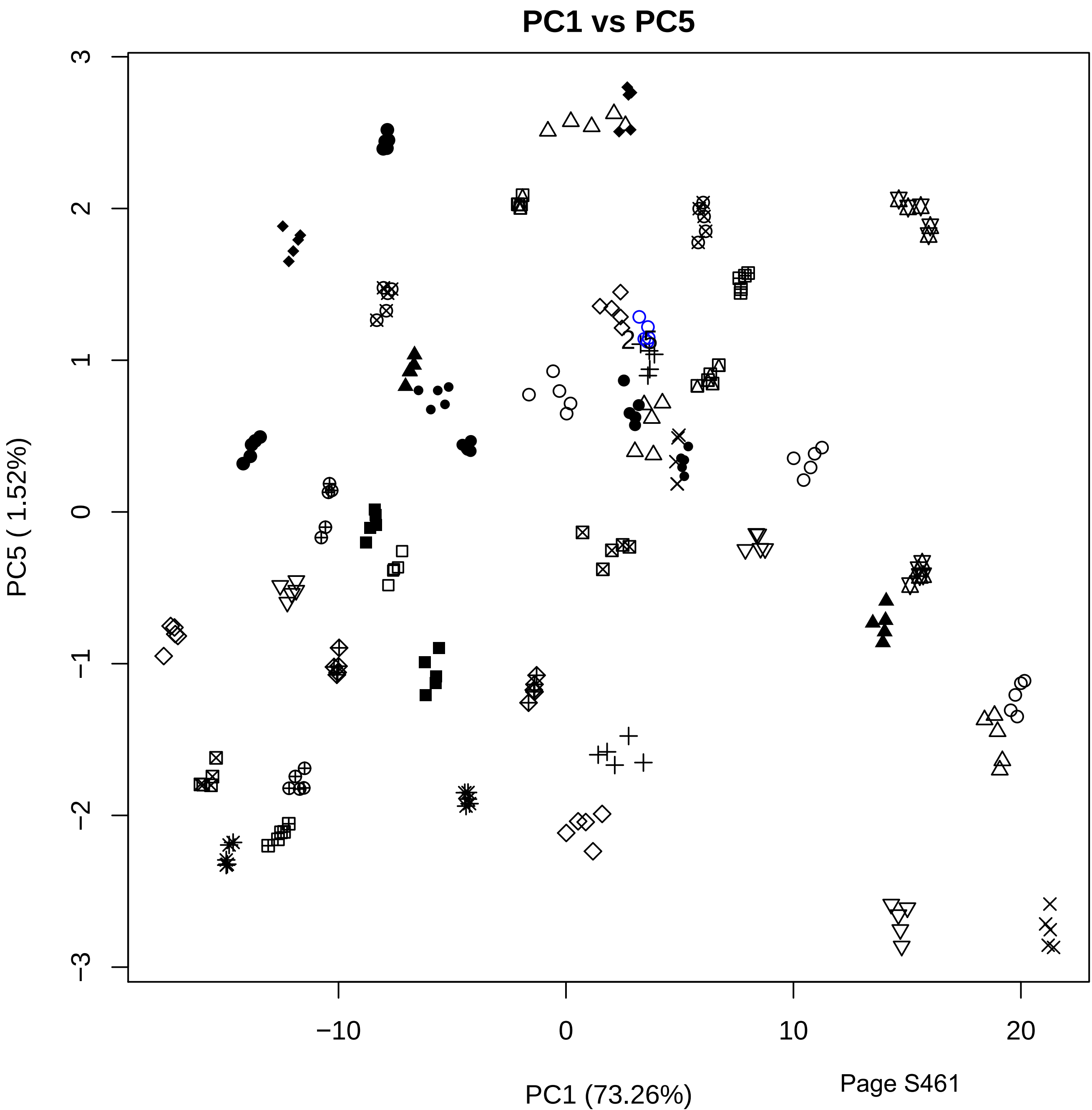

PC1 vs PC5
PC5 (1.52%)
PC1 (73.26%)
3
2
1
0
−1
−2
−3
−10
0
10
20
2

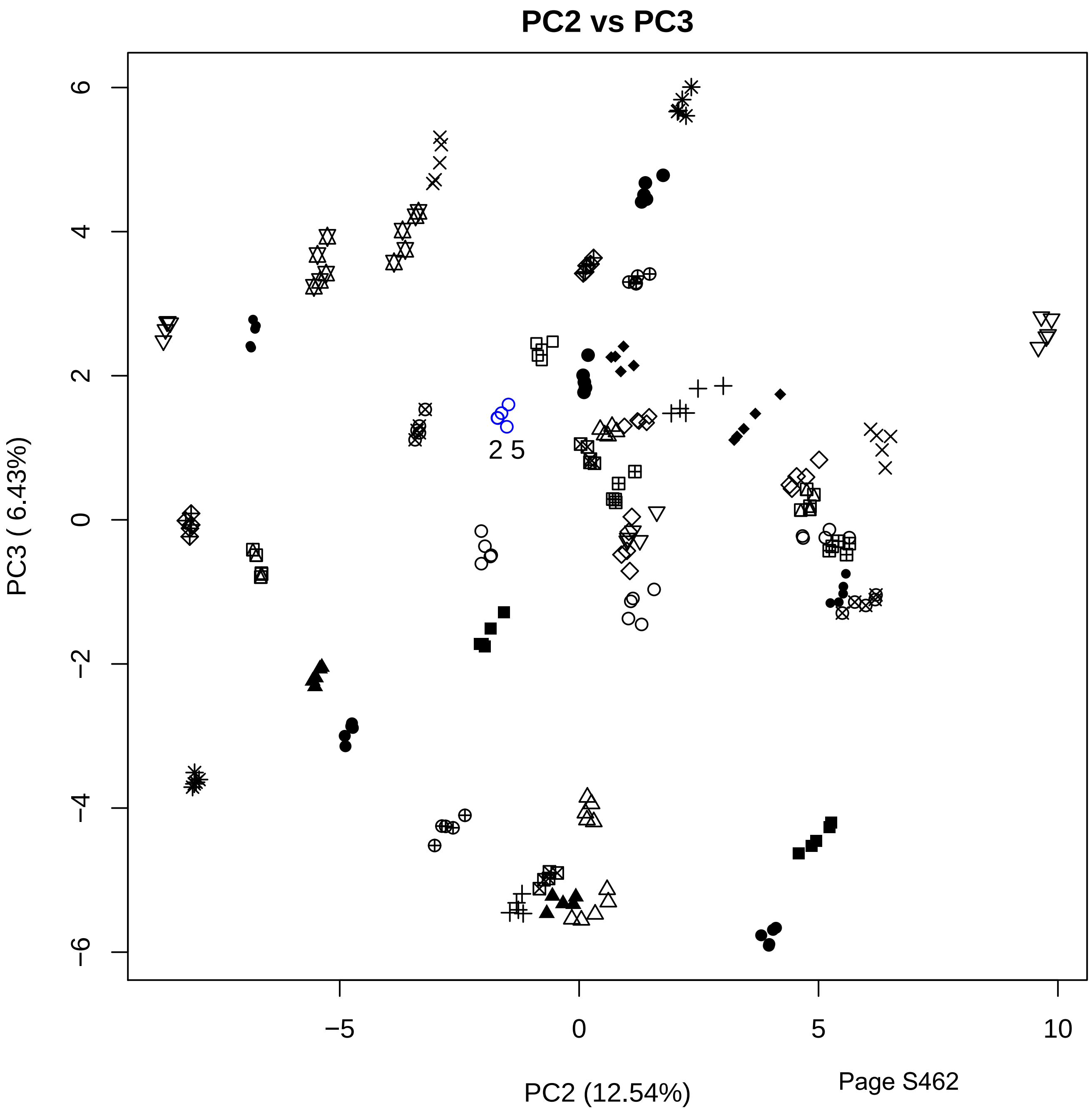
PC2 vs PC3
PC3 (6.43%)
PC2 (12.54%)
6
4
2
0
−2
−4
−6
−5
0
5
10
2 5

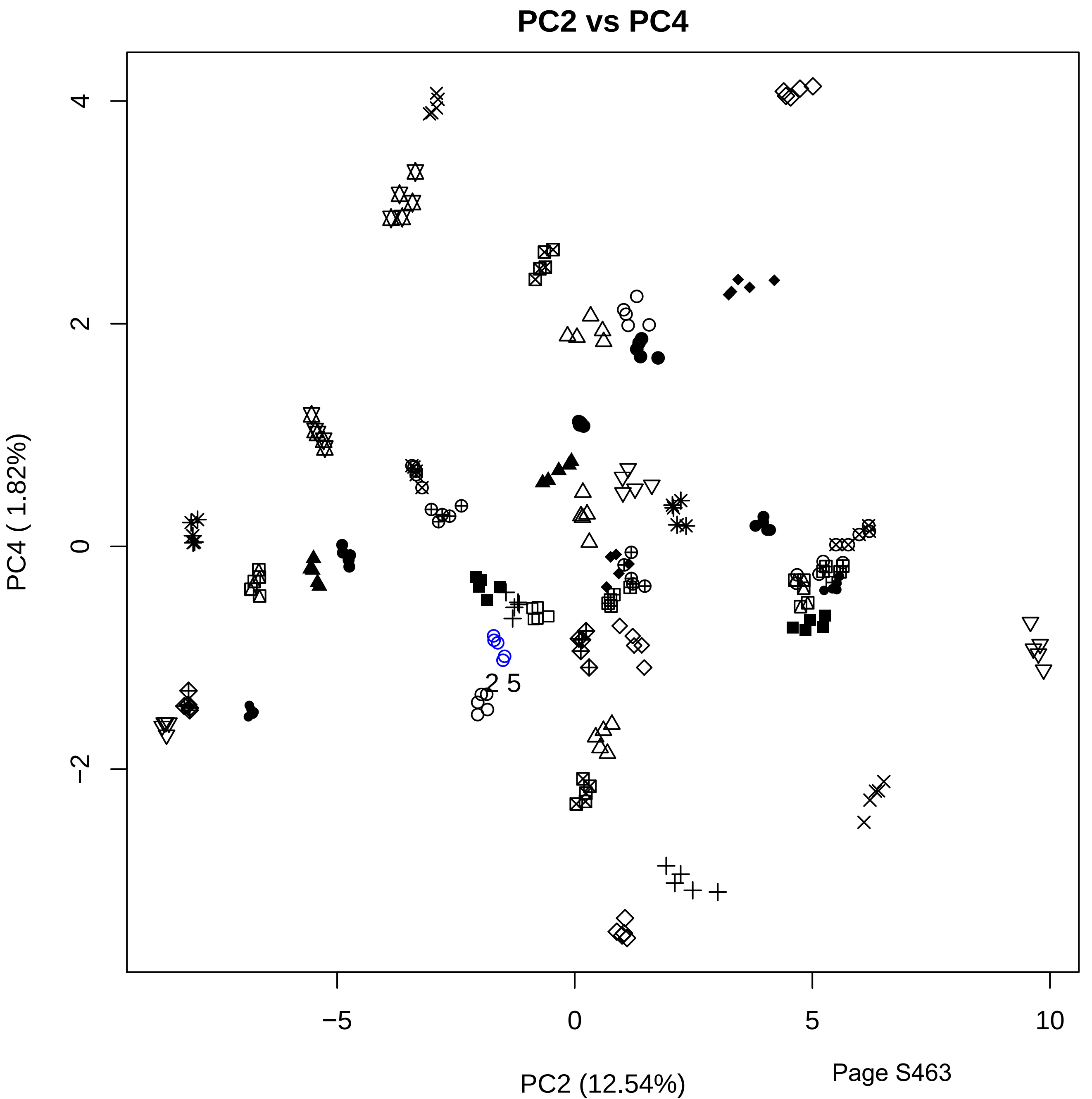
PC2 vs PC4
PC4 (1.82%)
PC2 (12.54%)
4
2
0
−2
−5
0
5
10
2 5

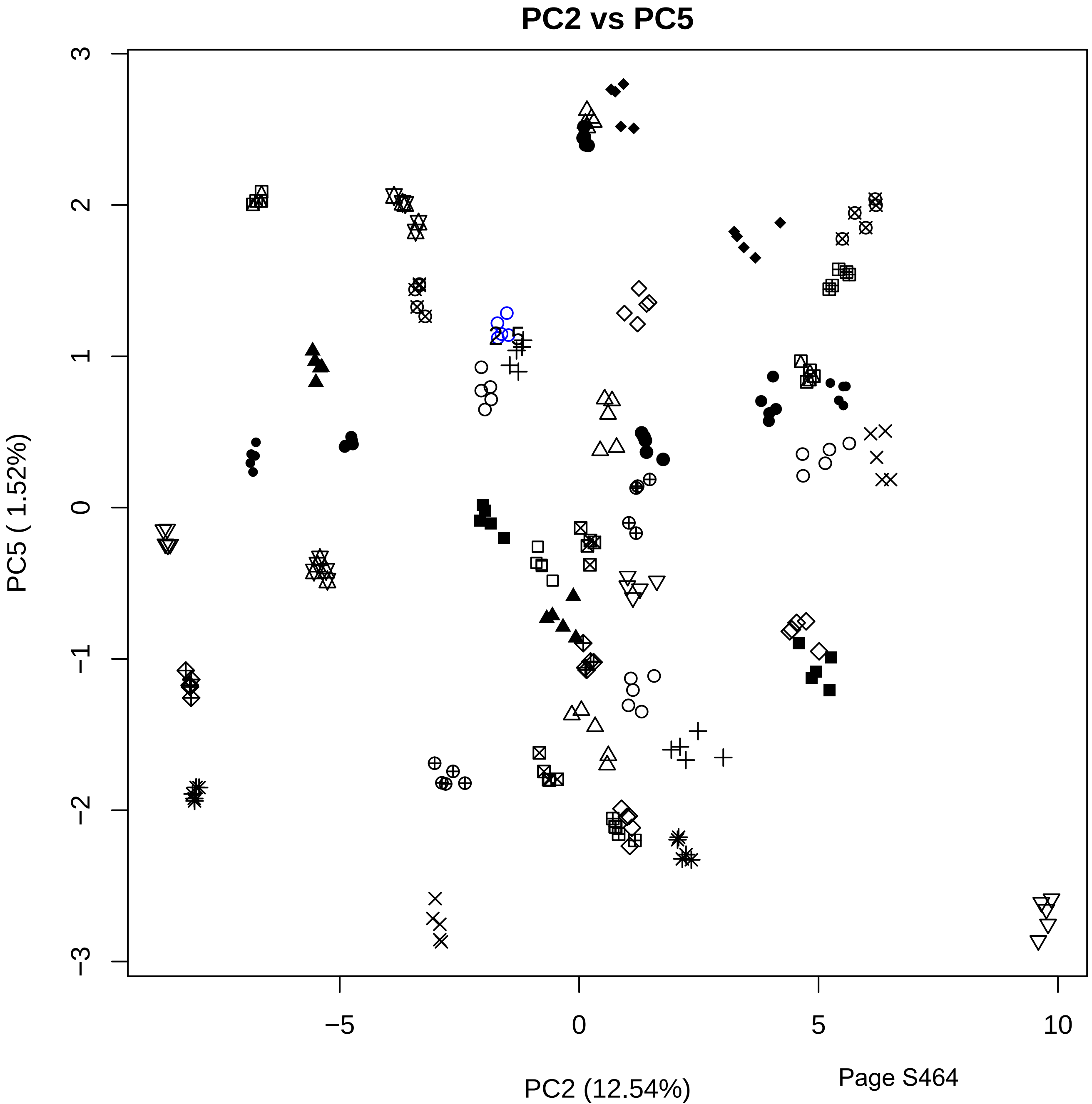
PC2 vs PC5
PC5 (1.52%)
PC2 (12.54%)
3
2
1
0
−1
−2
−3
−5
0
5
10

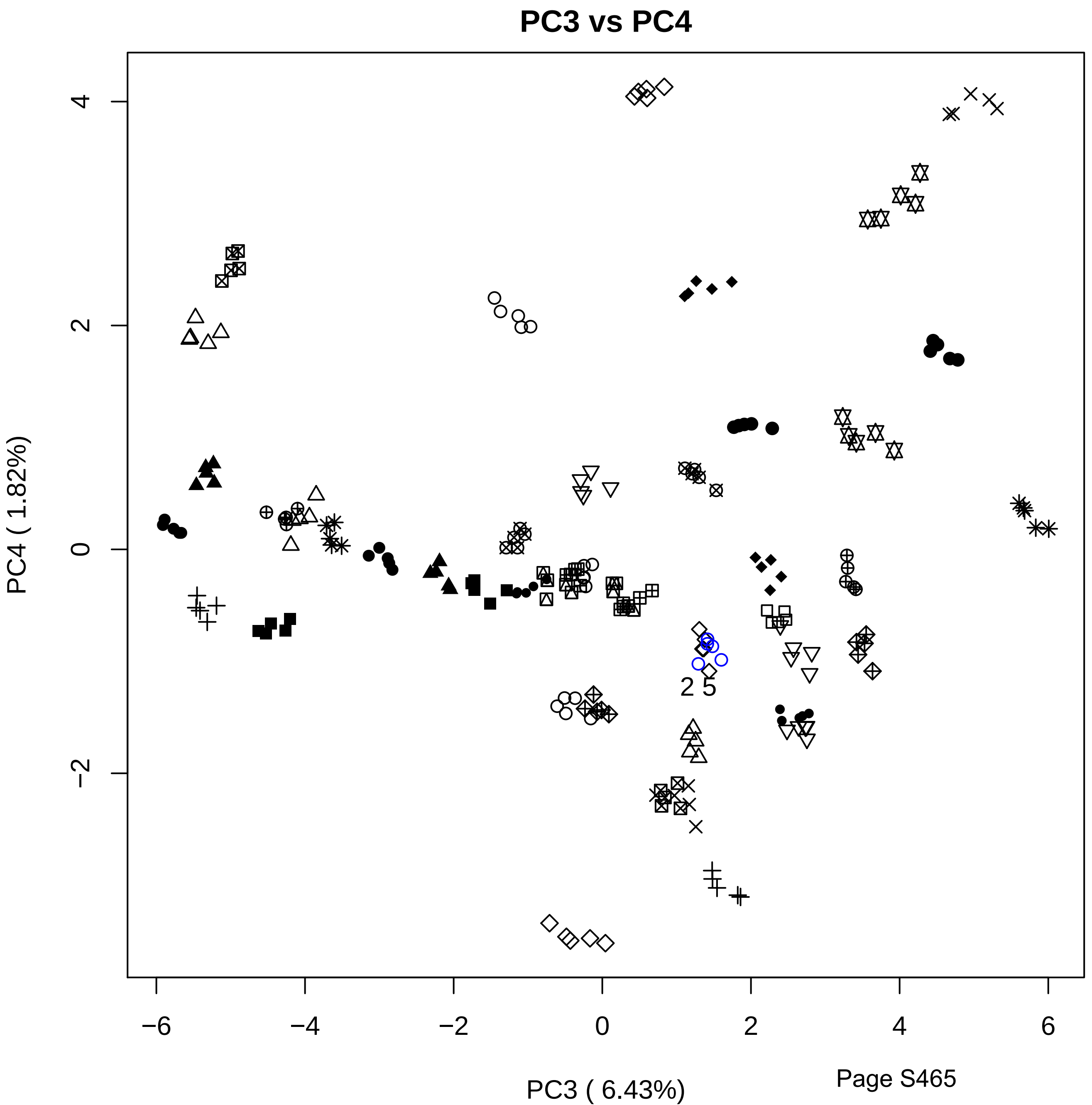
PC3 vs PC4
PC4 (1.82%)
PC3 (6.43%)
4
2
0
−2
−6
−4
−2
0
2
4
6
2 5

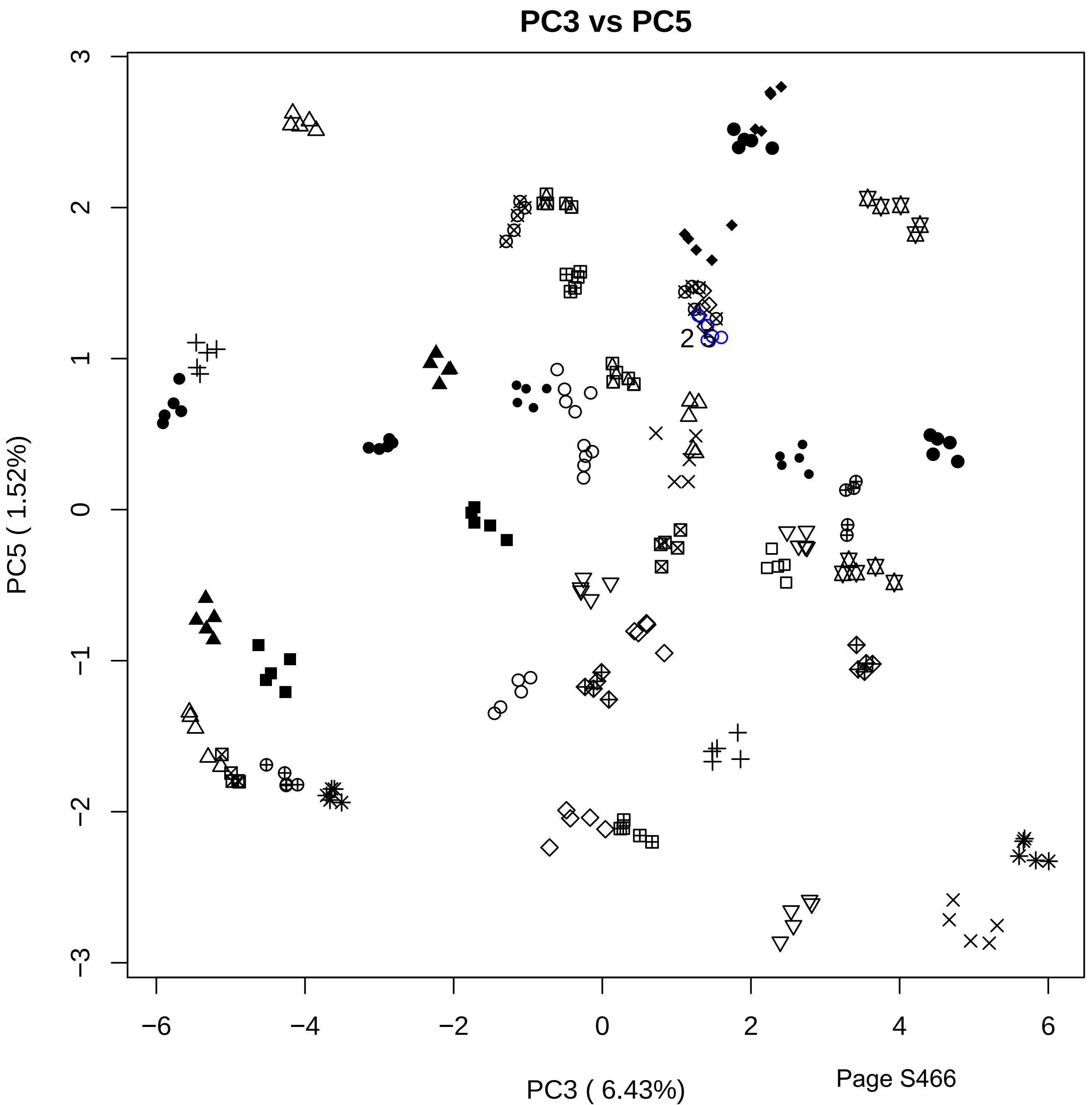
PC3 vs PC5
3
2
1
0
−1
−2
−3
PC5 (1.52%)
2 5
−6
−4
−2
0
2
4
6
PC3 (6.43%)

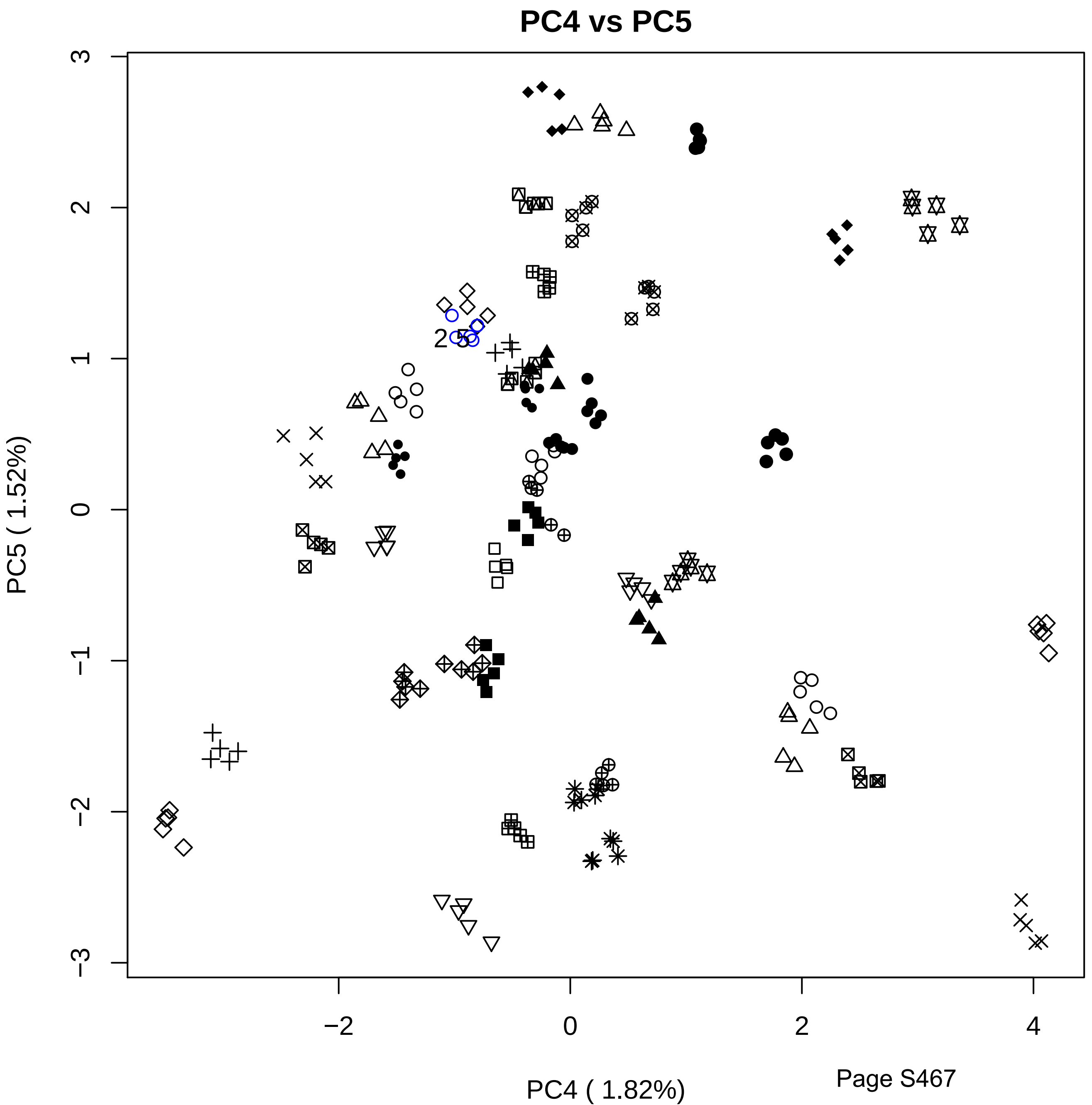
PC4 vs PC5
PC5 (1.52%)
PC4 (1.82%)
3
2
1
0
−1
−2
−3
−2
0
2
4
25

PCA of 5% OSAS vs PMH

Scaled Matrix PCAed

7th order polynomial baseline correction performed

Raw spectra smoothed to 21 Neighbours, 2nd order PN, Savtisky Golay

Normalisation between 0 and 100

D2 spectra smoothed to 41 Neighbours, 2nd order PN, Savtisky Golay

Regions Analysed: 3601−2501 and 2001−700cm−1

Samples Analysed: 1 Contam's 0 OSAS's 45 PMH's

Keele University AD Sun Dec 09 16:29:07 2018

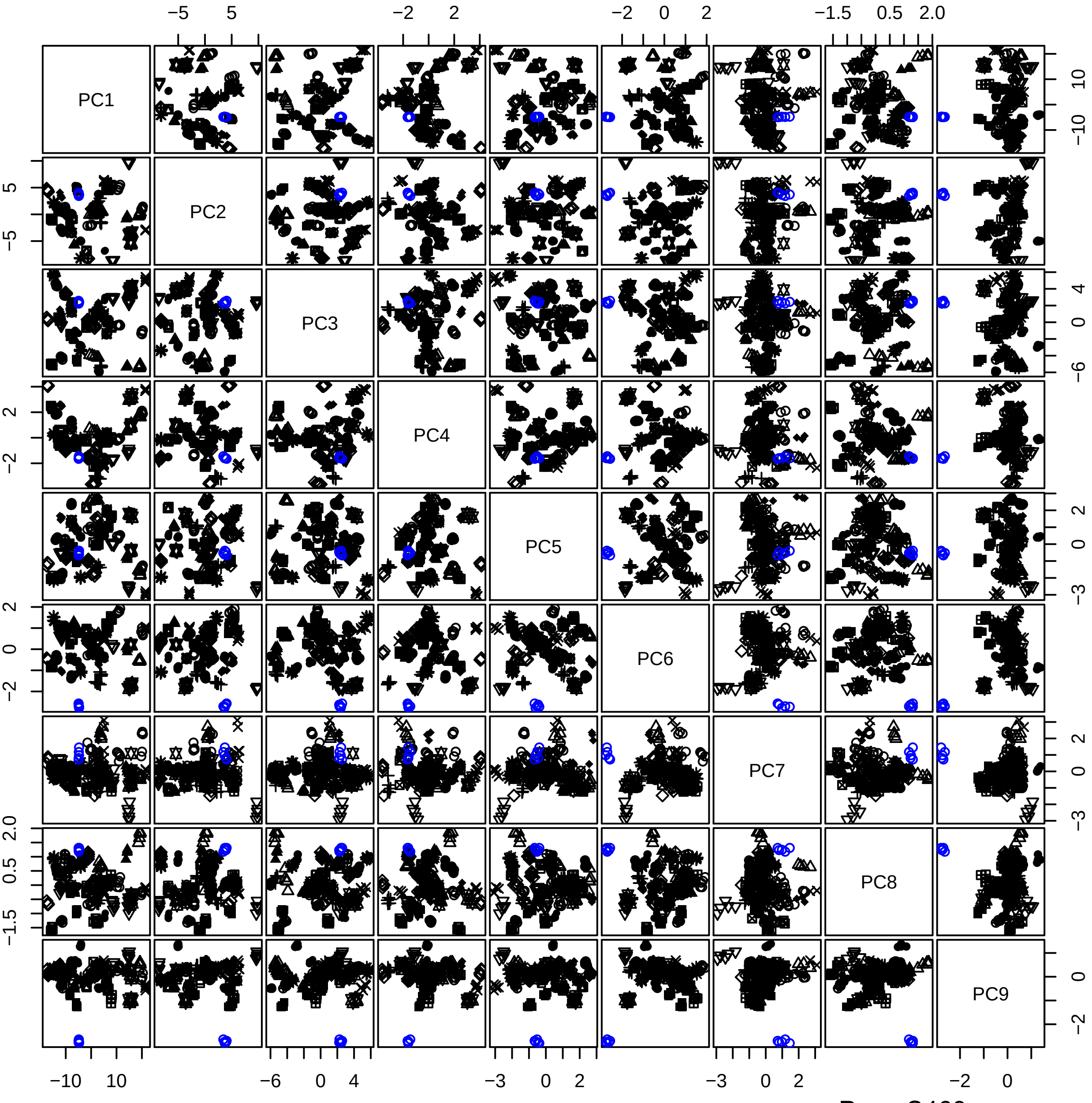
PC1
PC2
PC3
PC4
PC5
PC6
PC7
PC8
PC9
−5
5
−2
2
−2
0
2
−1.5
0.5
2.0
10
−10
5
−5
4
0
−6
2
−2
2
0
−3
2
0
−2
2
0
−3
2.0
0.5
−1.5
0
−2
−10
10
−6
0
4
−3
0
2
−3
0
2
−2
0

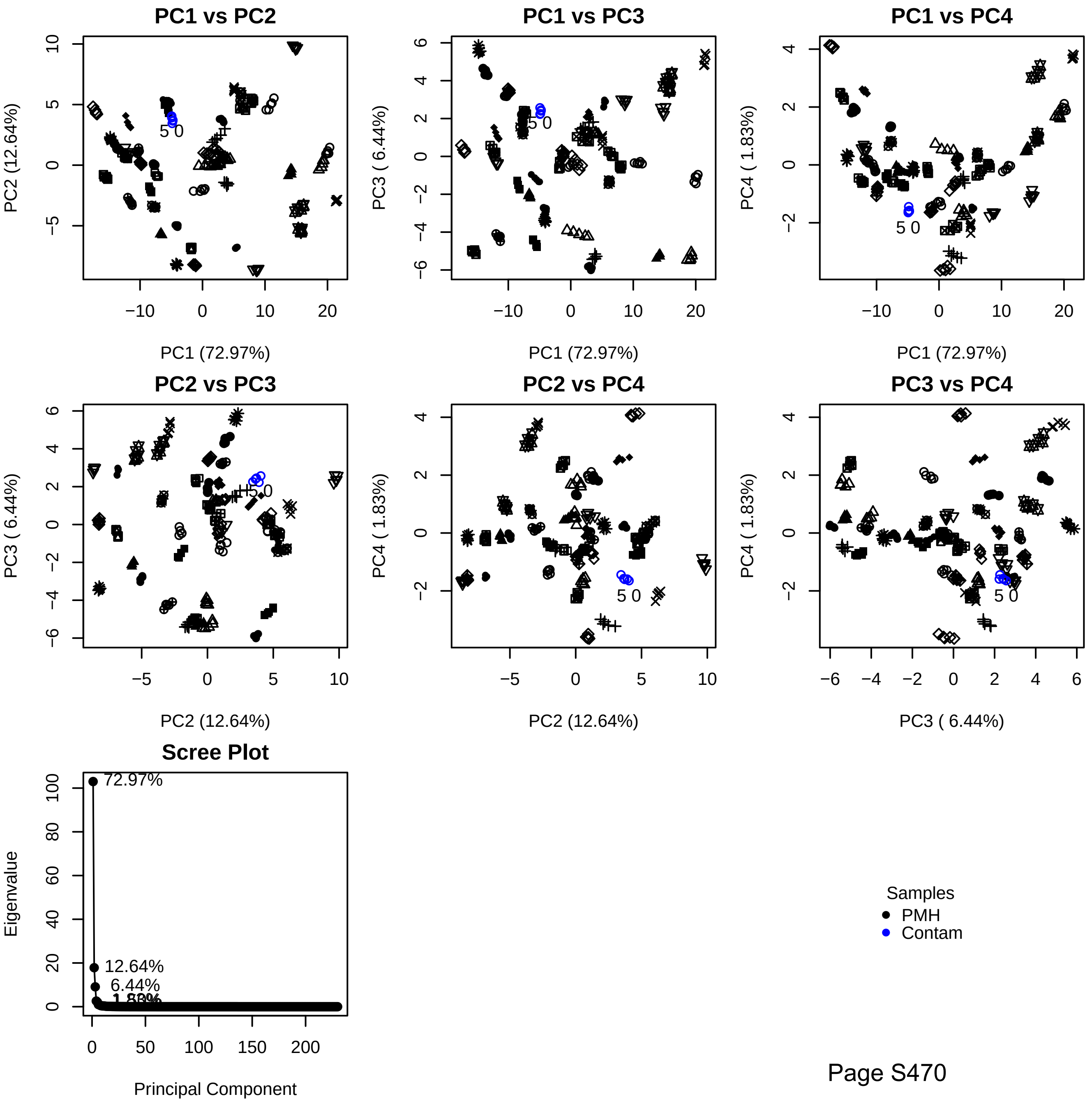
PC1 vs PC2
PC1 vs PC3
PC1 vs PC4
PC2 vs PC3
PC2 vs PC4
PC3 vs PC4
Scree Plot
PC1 (72.97%)
PC2 (12.64%)
PC3 (6.44%)
PC4 (1.83%)
5 0
Eigenvalue
Principal Component
72.97%
12.64%
6.44%
Samples
PMH
Contam

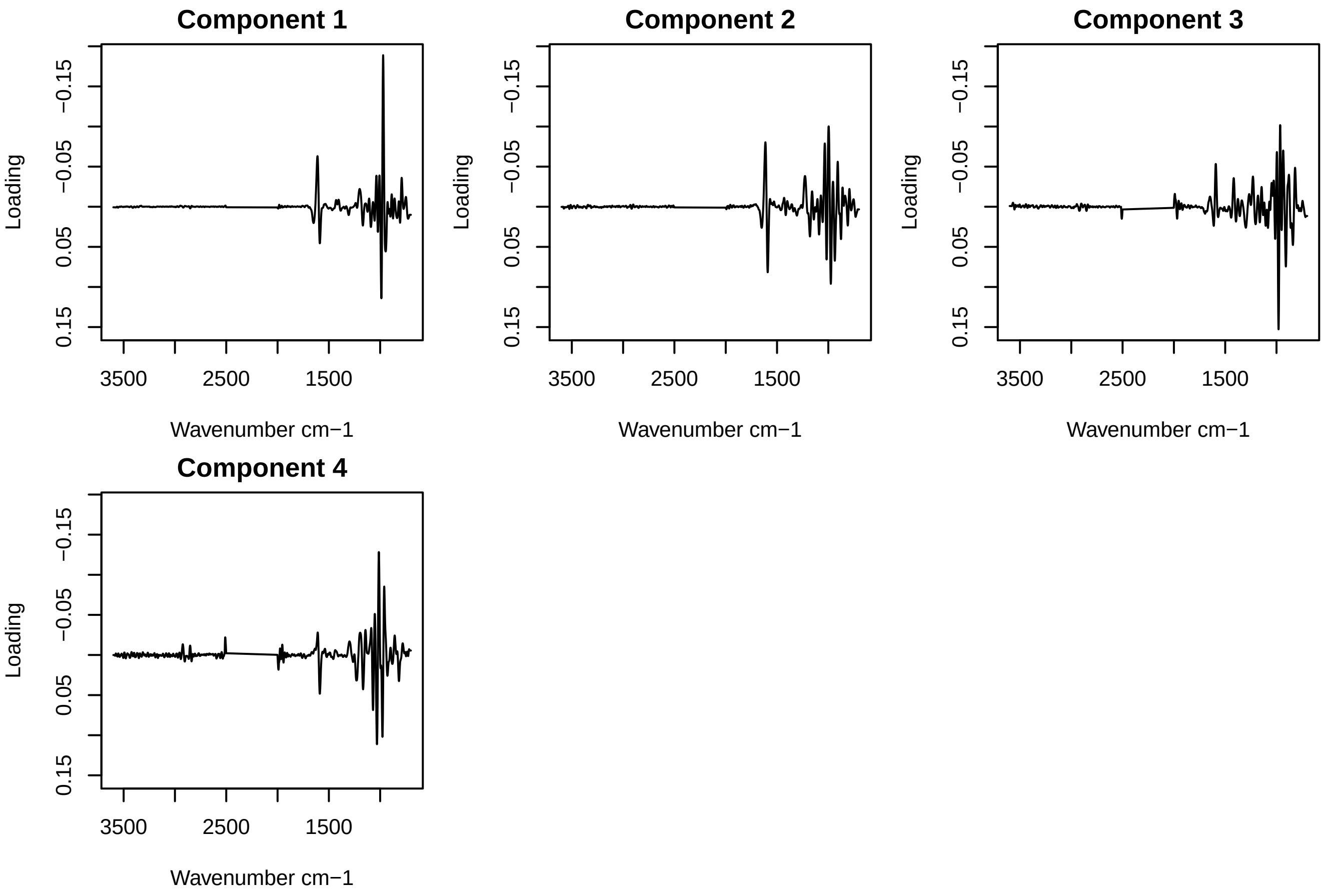
Component 1
Component 2
Component 3
Component 4
Loading
−0.15
−0.05
0.05
0.15
3500
2500
1500
Wavenumber cm−1

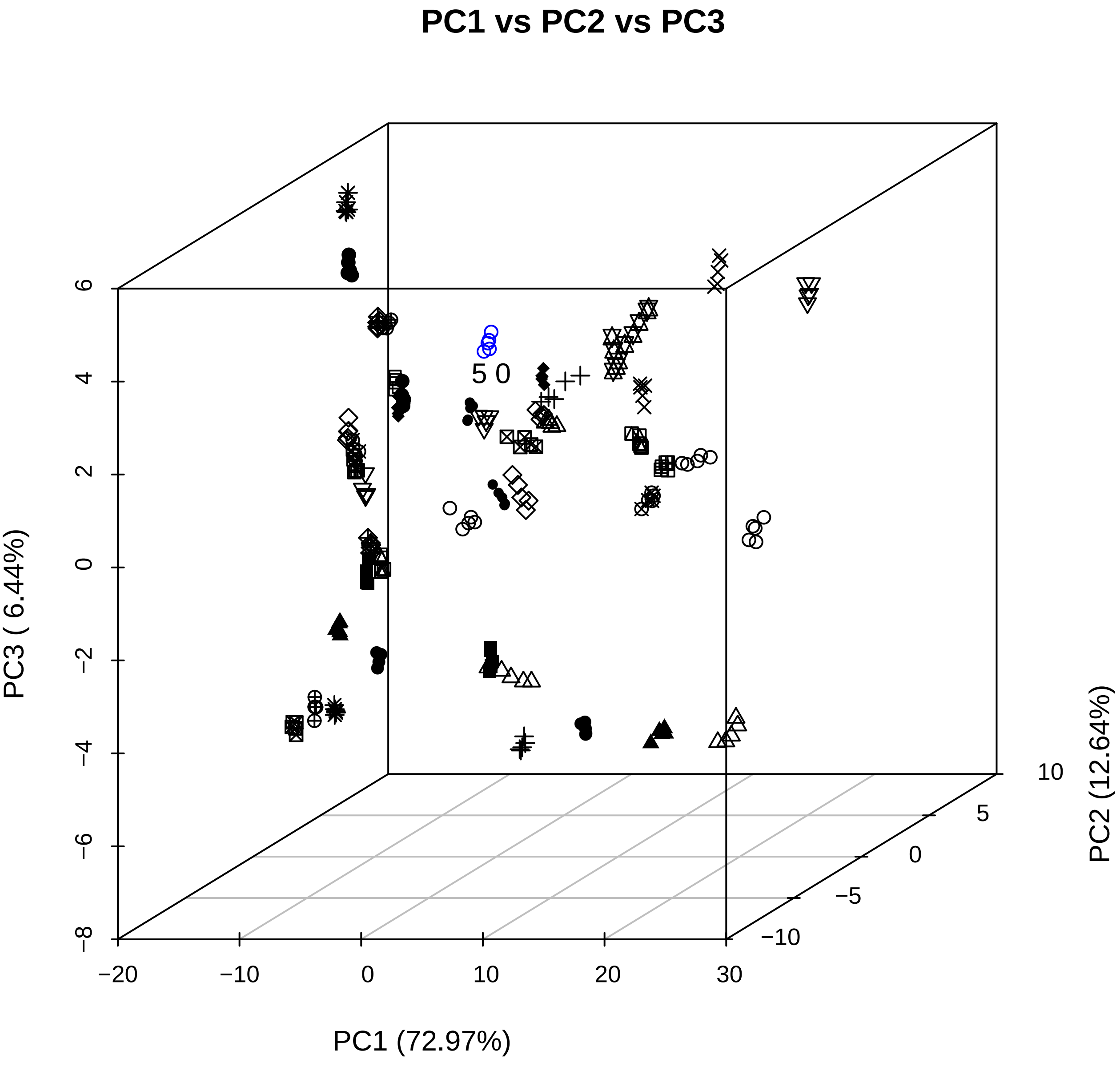

PC1 vs PC2 vs PC3
PC1 (72.97%)
PC2 (12.64%)
PC3 (6.44%)
−20
−10
0
10
20
30
−10
−5
0
5
10
−8
−6
−4
−2
0
2
4
6
5 0

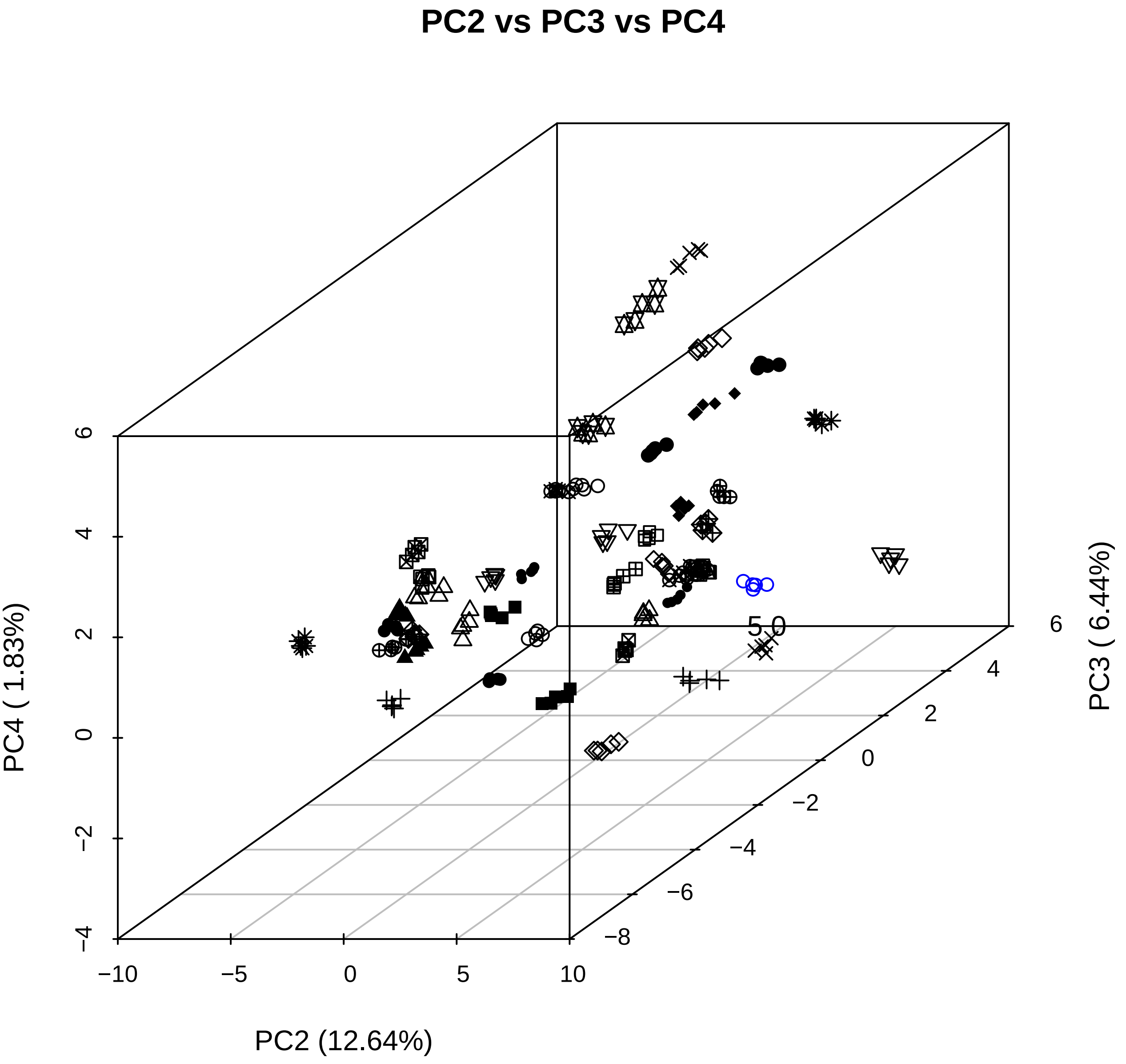
PC2 vs PC3 vs PC4
PC2 (12.64%)
PC4 (1.83%)
PC3 (6.44%)
50
−10
−5
0
5
10
−4
−2
0
2
4
6
−8
−6
−4
−2
0
2
4
6

PC1 vs PC2 vs PC4

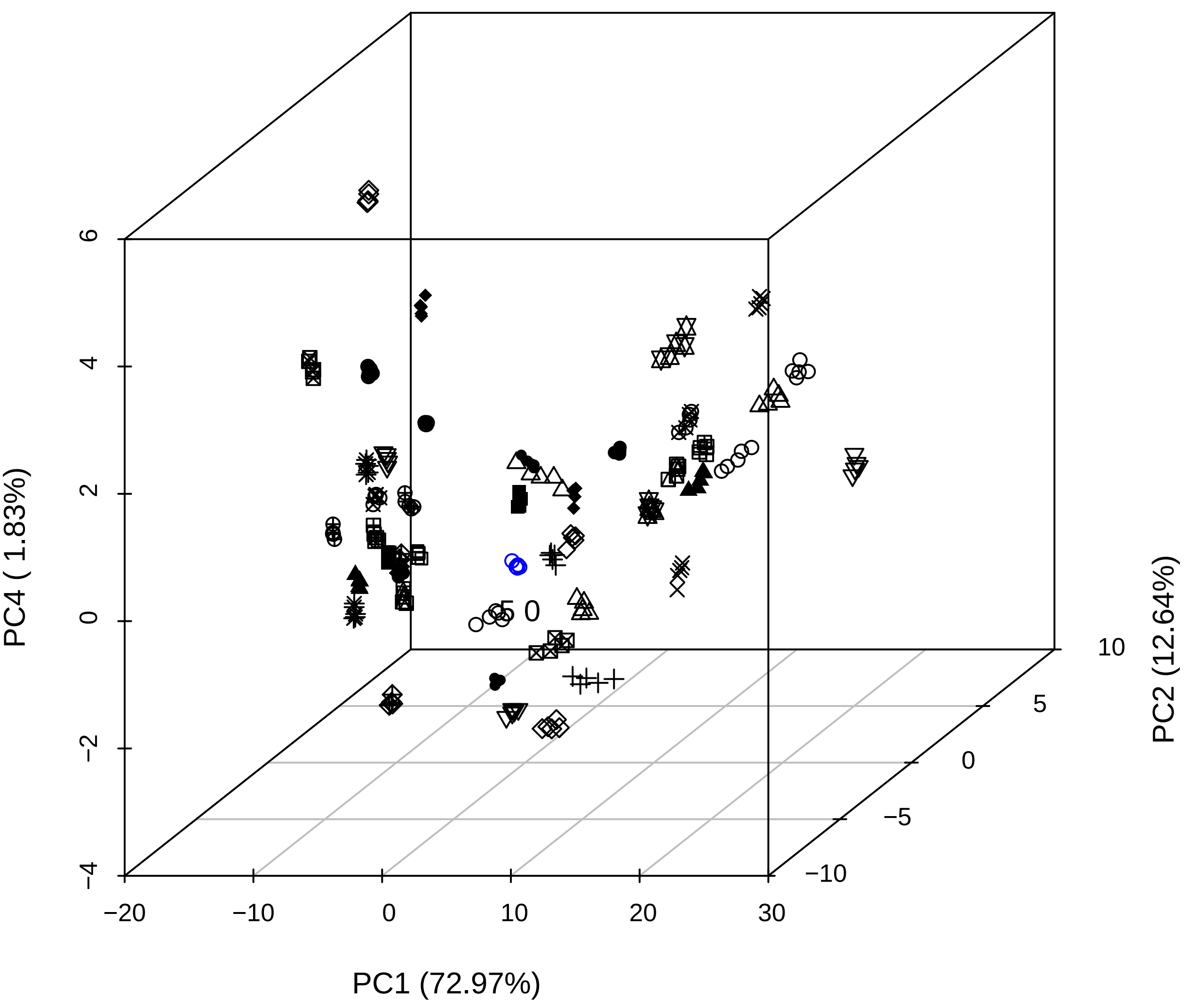

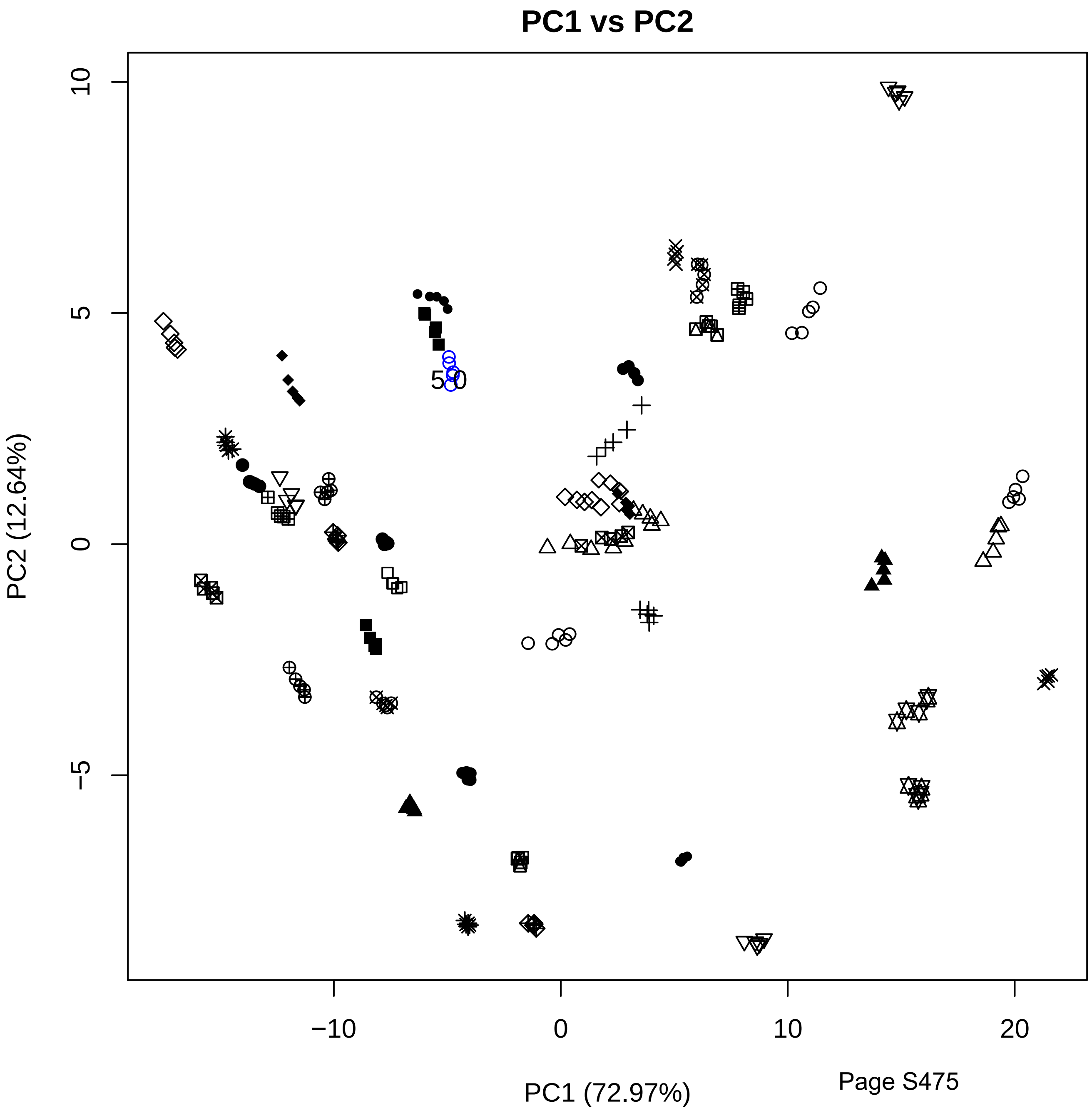
PC1 vs PC2
PC2 (12.64%)
PC1 (72.97%)
10
5
0
−5
−10
0
10
20
50

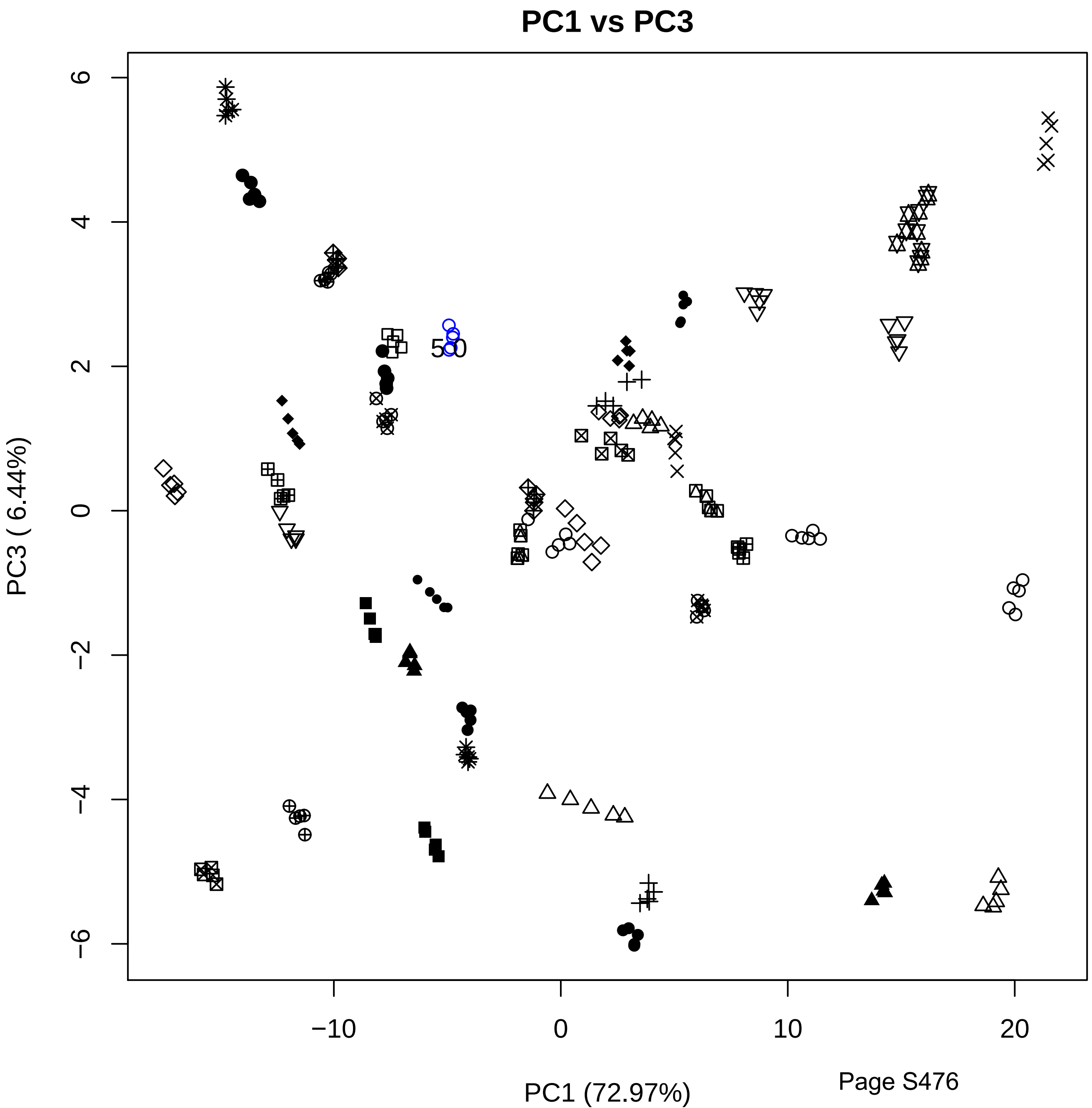

PC1 vs PC3
PC3 (6.44%)
PC1 (72.97%)
6
4
2
0
−2
−4
−6
−10
0
10
20
50

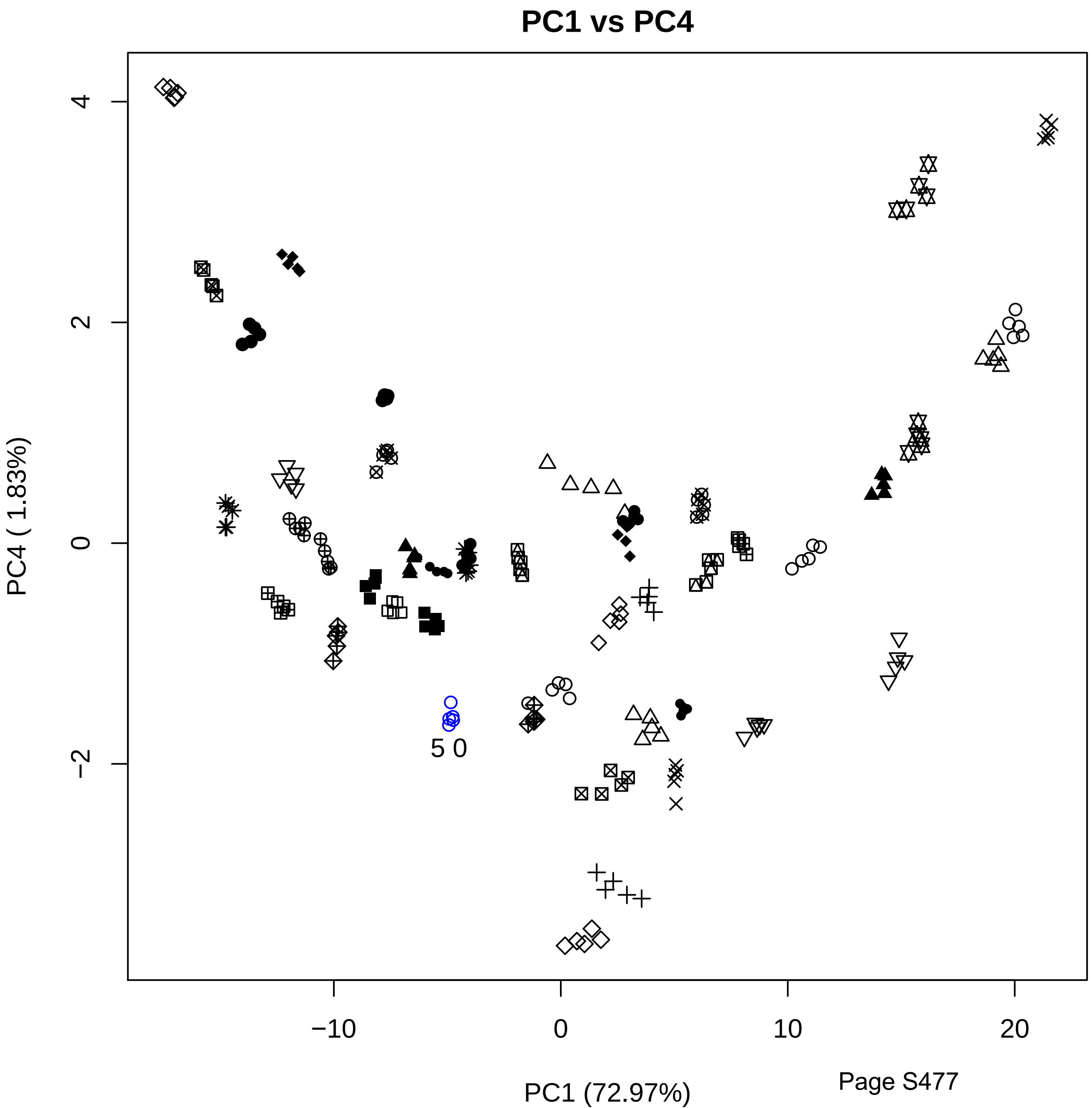
PC1 vs PC4
4
2
0
−2
PC4 (1.83%)
−10
0
10
20
PC1 (72.97%)
5 0

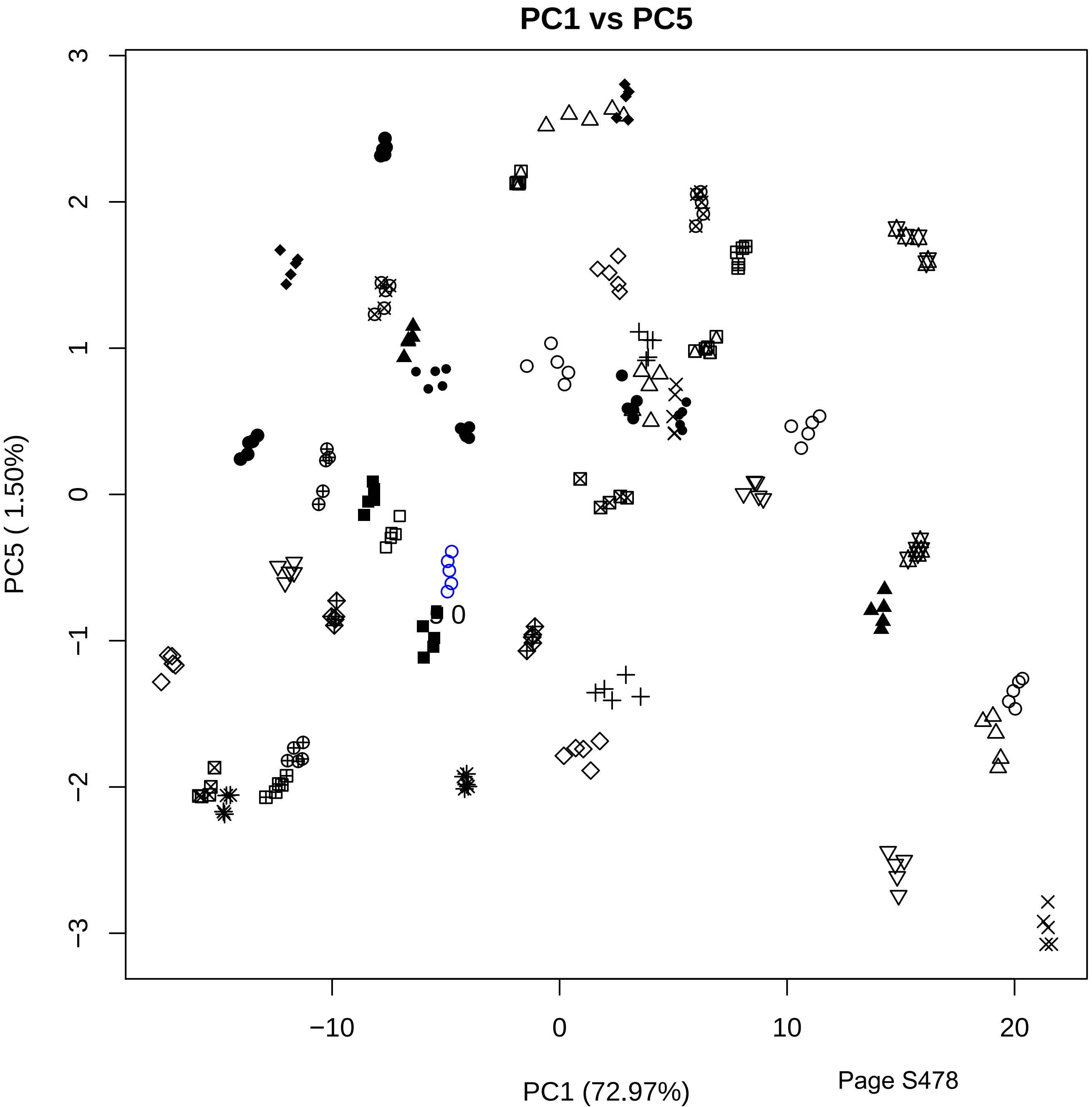
PC1 vs PC5
PC5 (1.50%)
PC1 (72.97%)
3
2
1
0
−1
−2
−3
−10
0
10
20

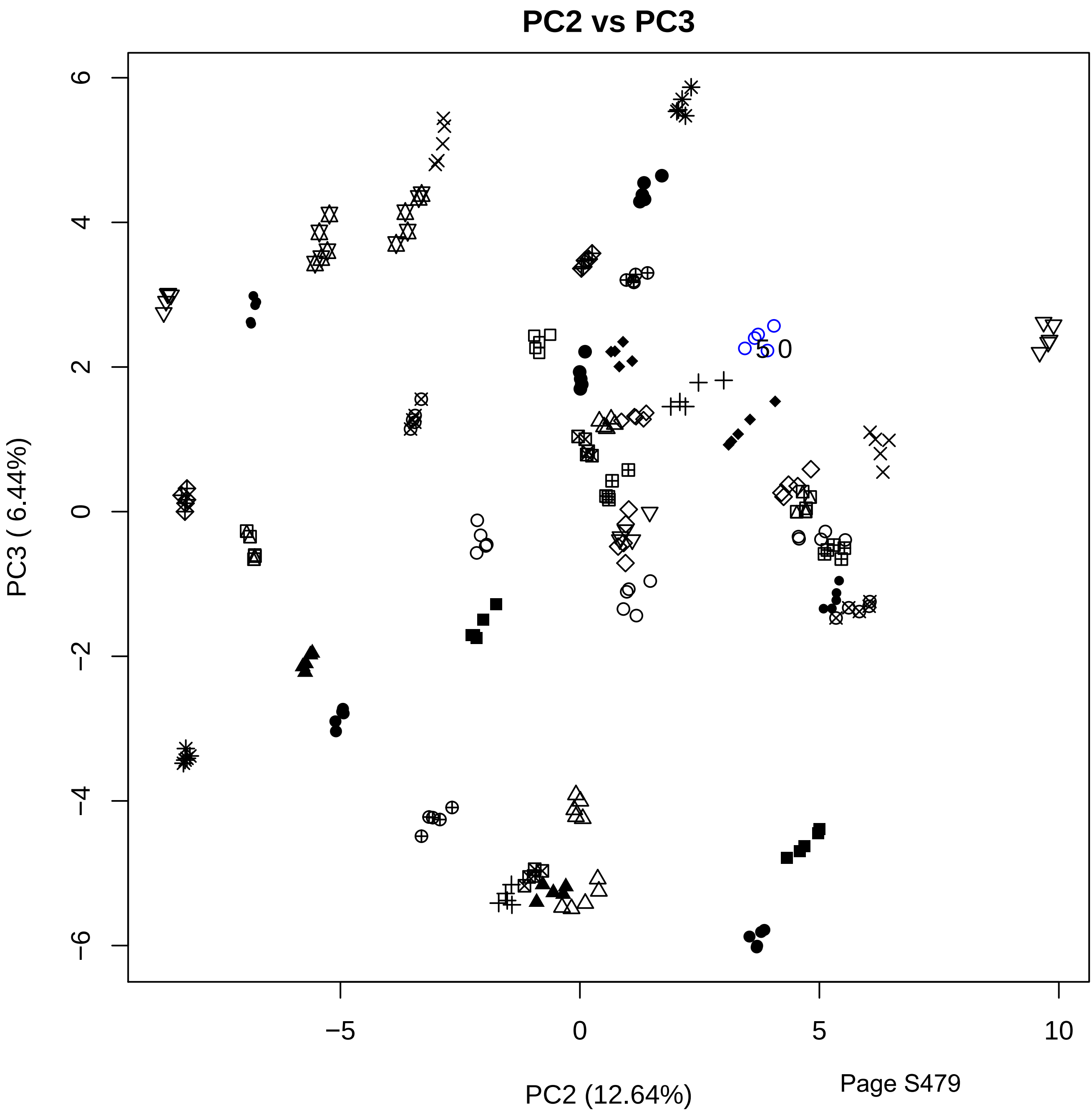
PC2 vs PC3
50
PC3 (6.44%)
PC2 (12.64%)
6
4
2
0
−2
−4
−6
−5
0
5
10

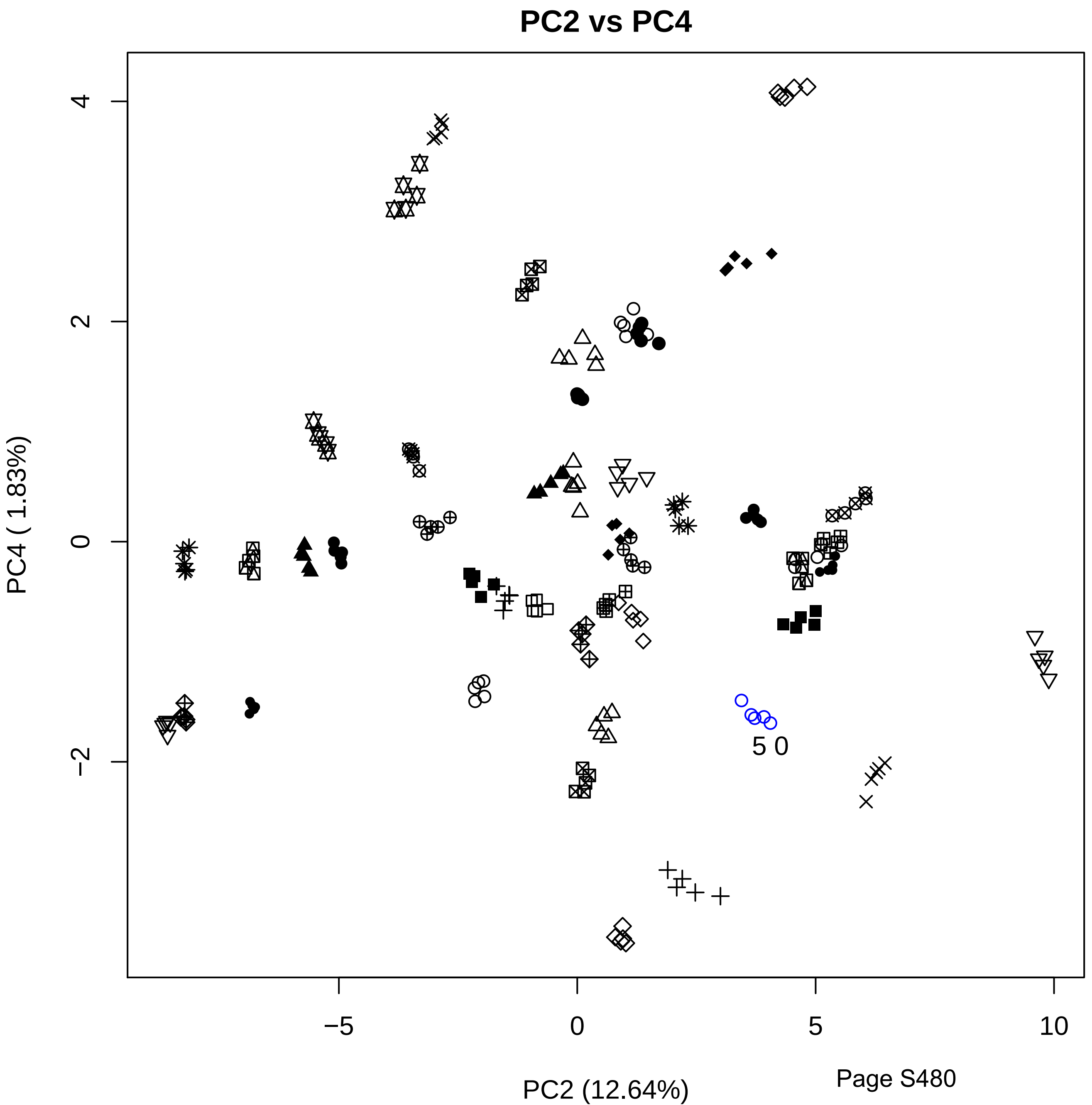
PC2 vs PC4
4
2
0
−2
PC4 (1.83%)
5 0
−5
0
5
10
PC2 (12.64%)

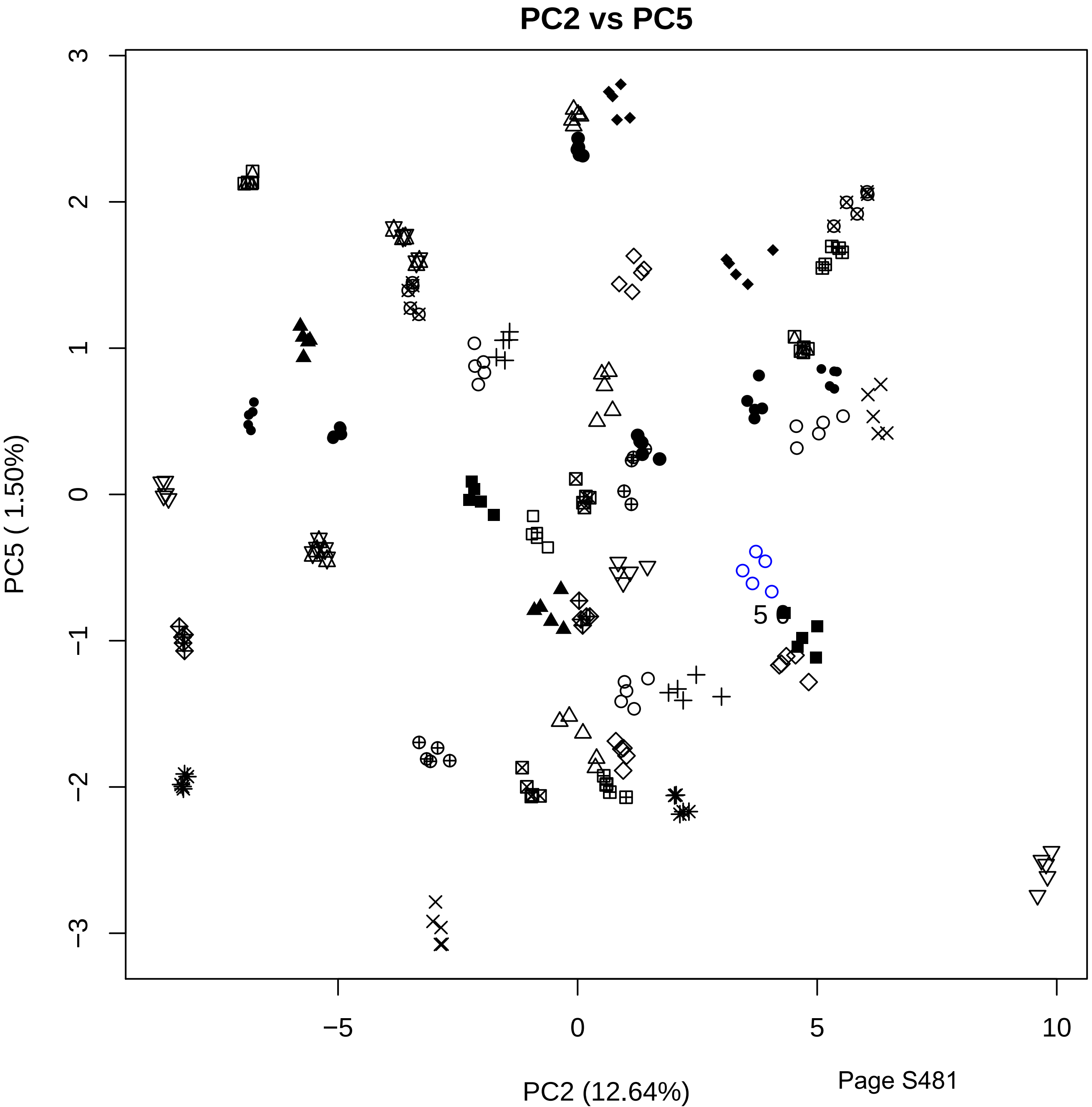
PC2 vs PC5
PC5 (1.50%)
PC2 (12.64%)
3
2
1
0
−1
−2
−3
−5
0
5
10
5

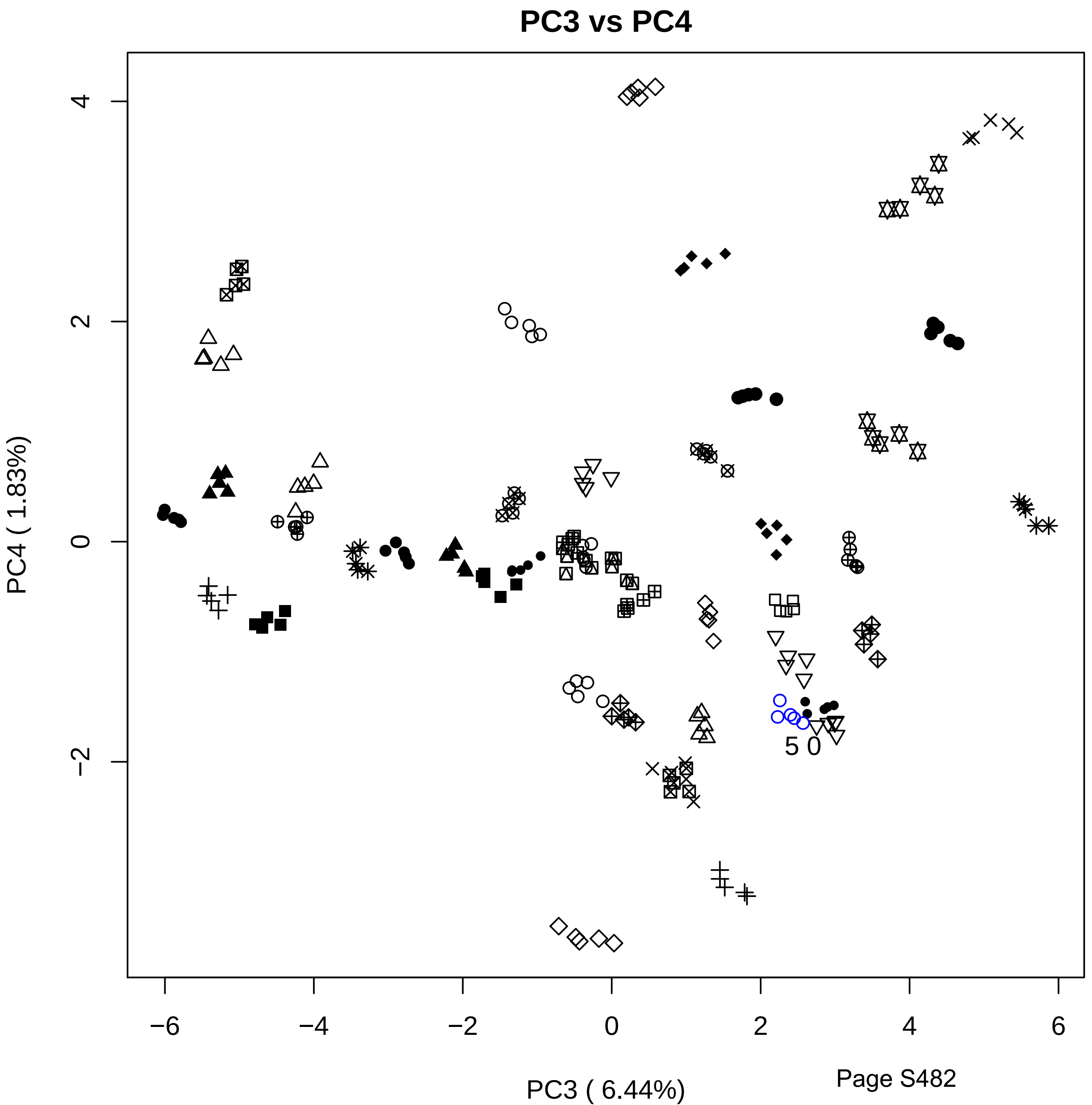
PC3 vs PC4
PC4 (1.83%)
PC3 (6.44%)
4
2
0
−2
−6
−4
−2
0
2
4
6
5 0

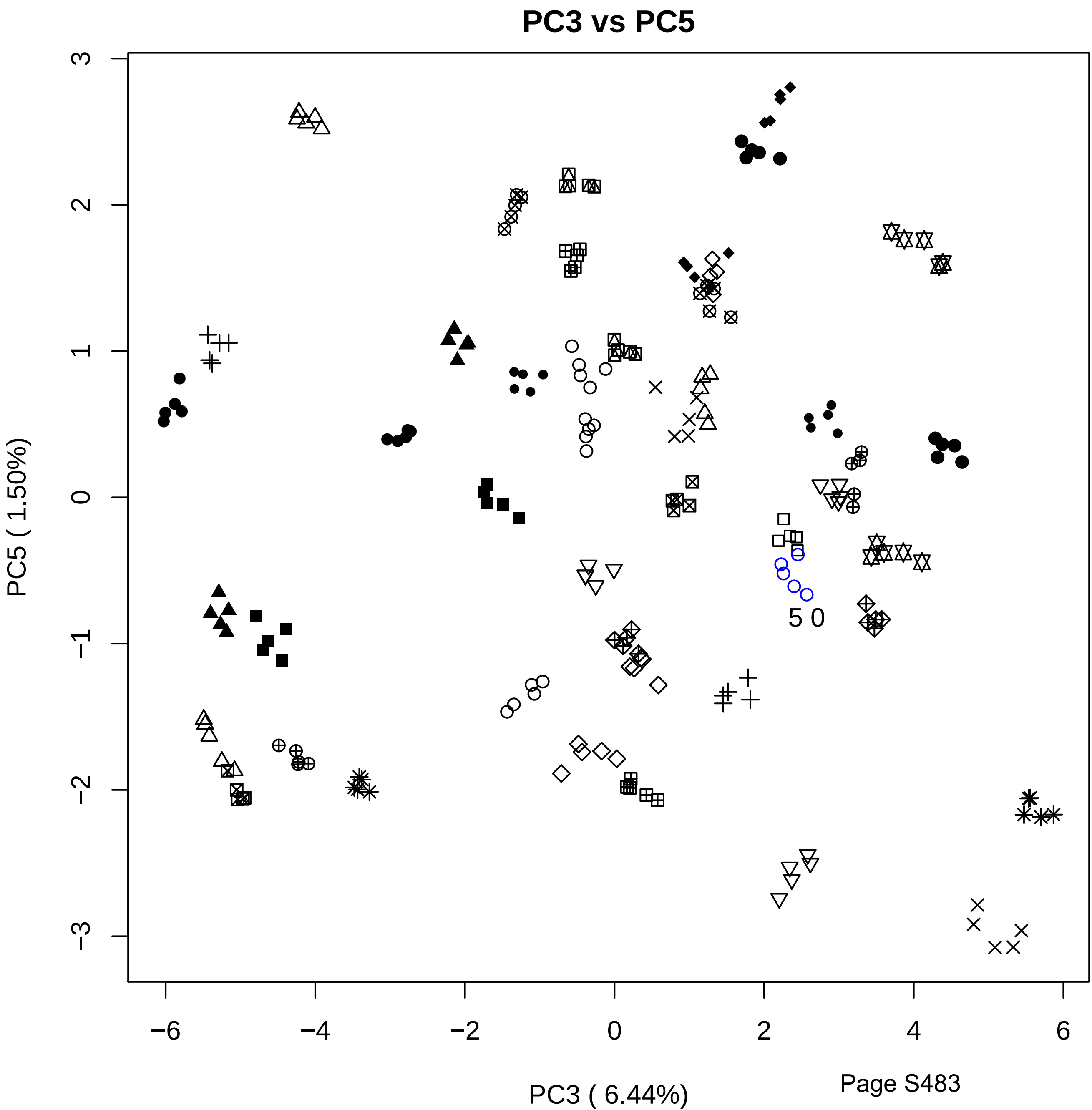

PC3 vs PC5
PC5 (1.50%)
PC3 (6.44%)
3
2
1
0
−1
−2
−3
−6
−4
−2
0
2
4
6
5 0

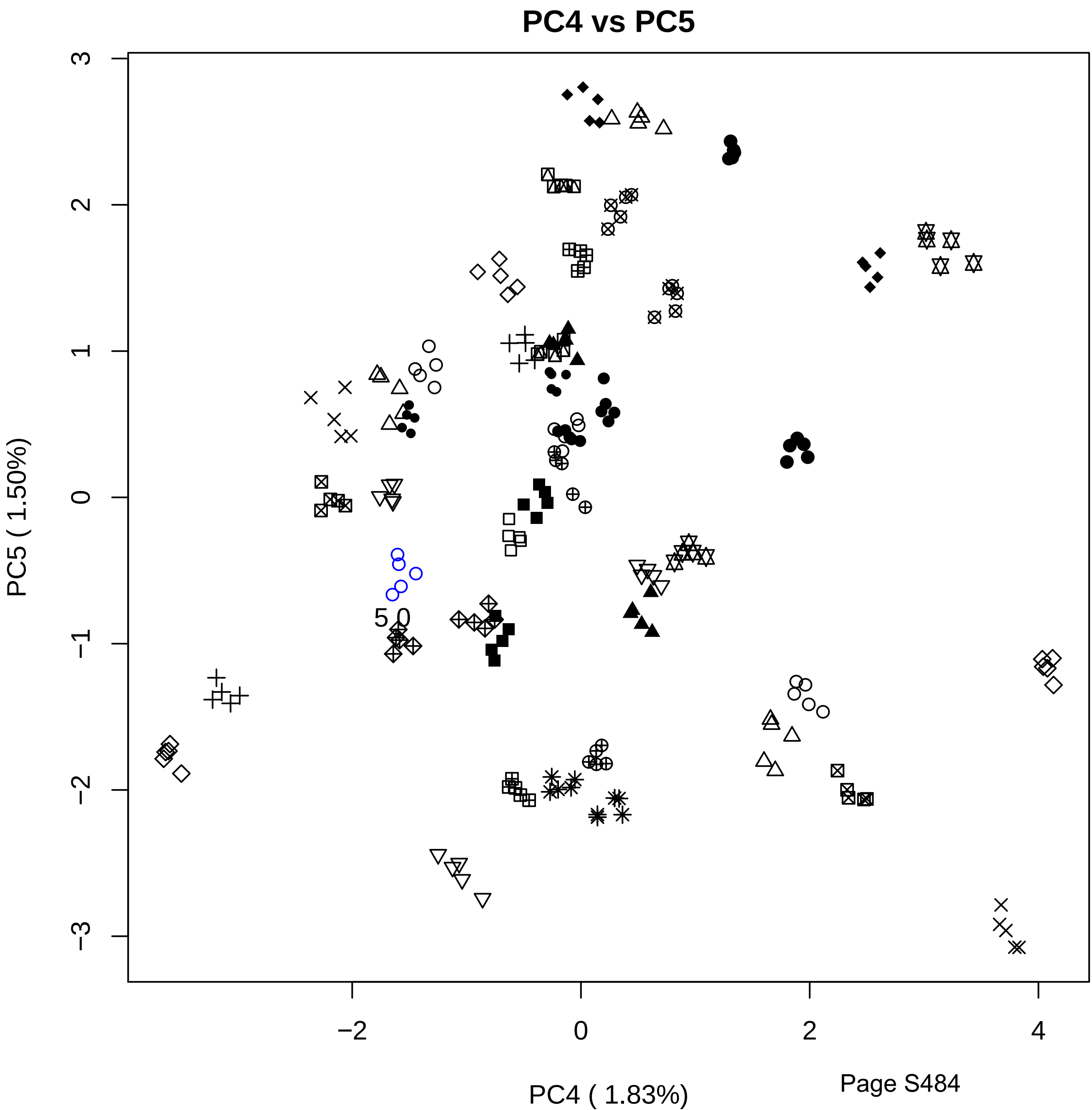
PC4 vs PC5
PC5 (1.50%)
PC4 (1.83%)
3
2
1
0
−1
−2
−3
−2
0
2
4
50

PCA of 7.5% OSAS vs PMH

Scaled Matrix PCAed

7th order polynomial baseline correction performed

Raw spectra smoothed to 21 Neighbours, 2nd order PN, Savtisky Golay

Normalisation between 0 and 100

D2 spectra smoothed to 41 Neighbours, 2nd order PN, Savtisky Golay

Regions Analysed: 3601−2501 and 2001−700cm−1

Samples Analysed: 1 Contam's 0 OSAS's 45 PMH's

Keele University AD   Sun Dec 09 16:29:13 2018

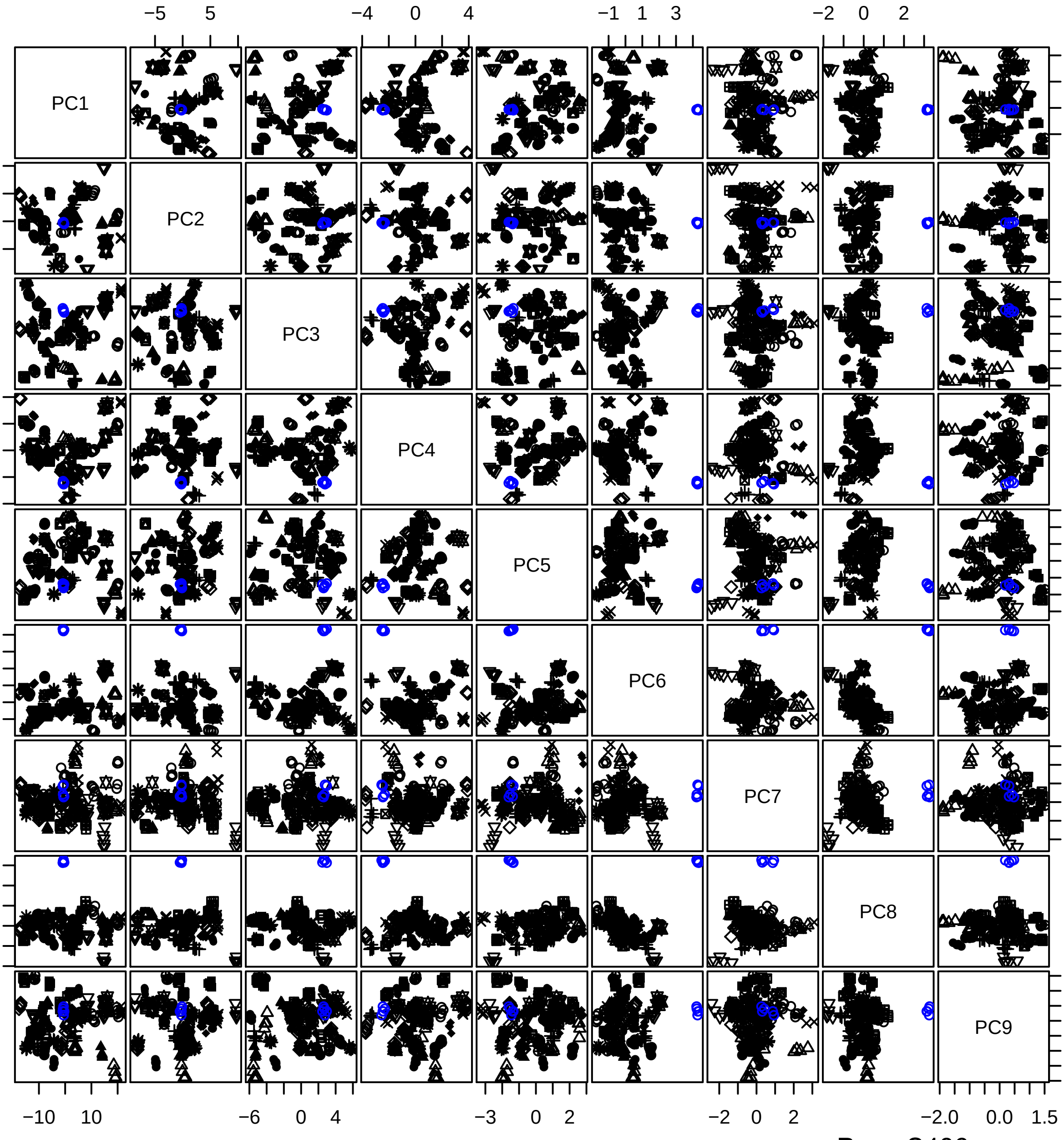
PC1
PC2
PC3
PC4
PC5
PC6
PC7
PC8
PC9

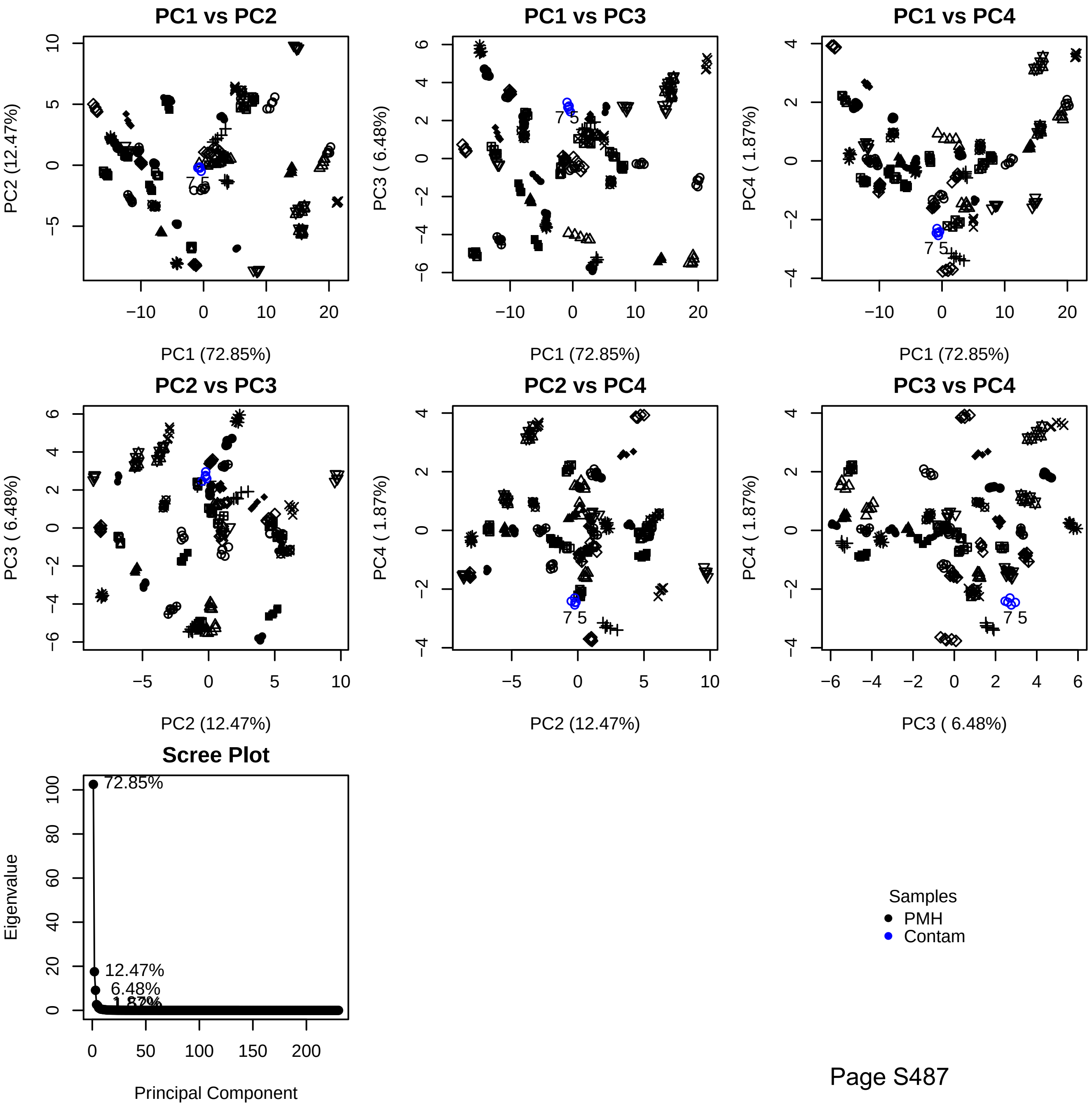
PC1 vs PC2
PC1 vs PC3
PC1 vs PC4
PC2 vs PC3
PC2 vs PC4
PC3 vs PC4
Scree Plot
PC1 (72.85%)
PC2 (12.47%)
PC3 (6.48%)
PC4 (1.87%)
Eigenvalue
Principal Component
72.85%
12.47%
6.48%
1.87%
7 5
Samples
PMH
Contam

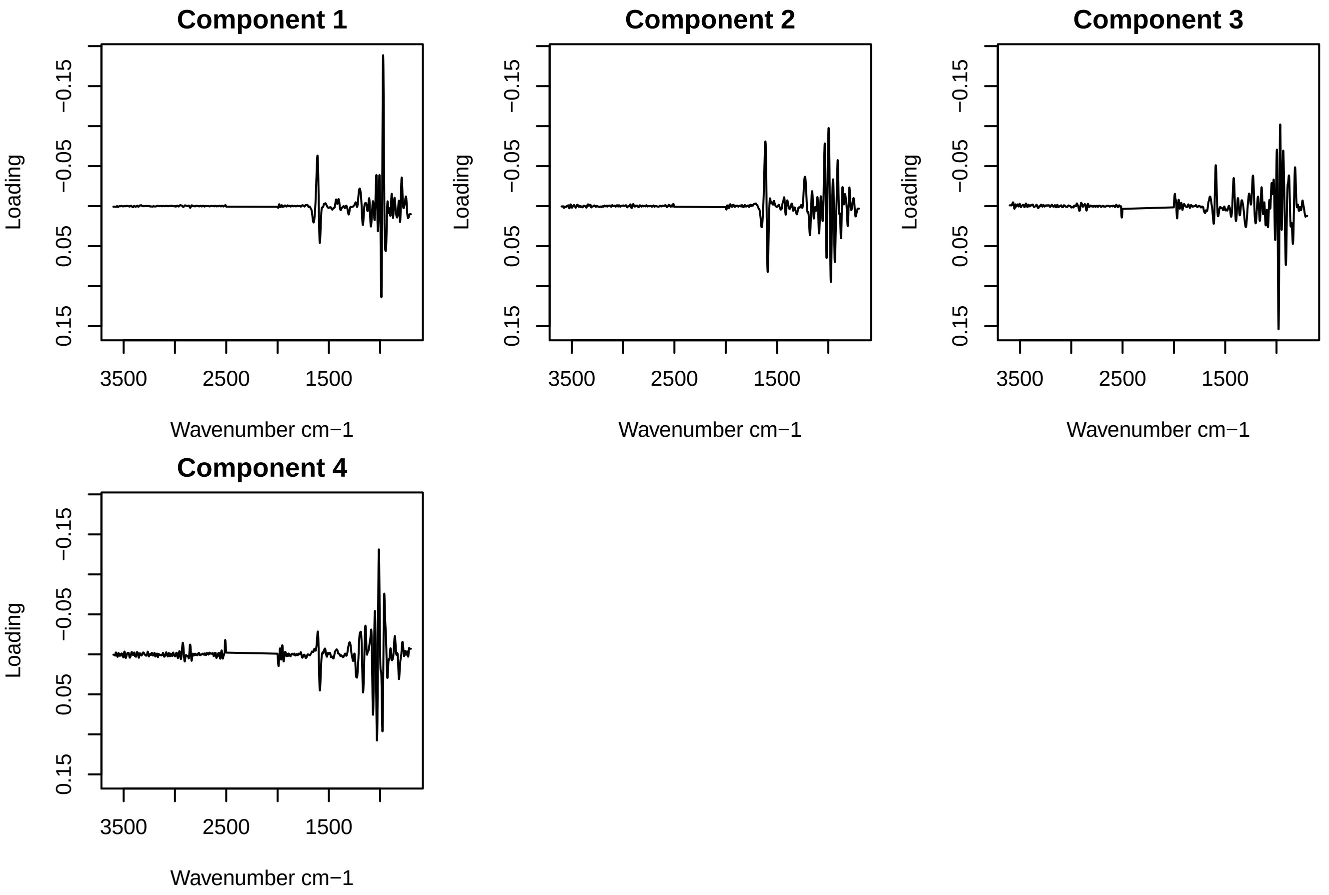

Component 1
Component 2
Component 3
Component 4
Loading
−0.15
−0.05
0.05
0.15
3500
2500
1500
Wavenumber cm−1

PC1 vs PC2 vs PC3

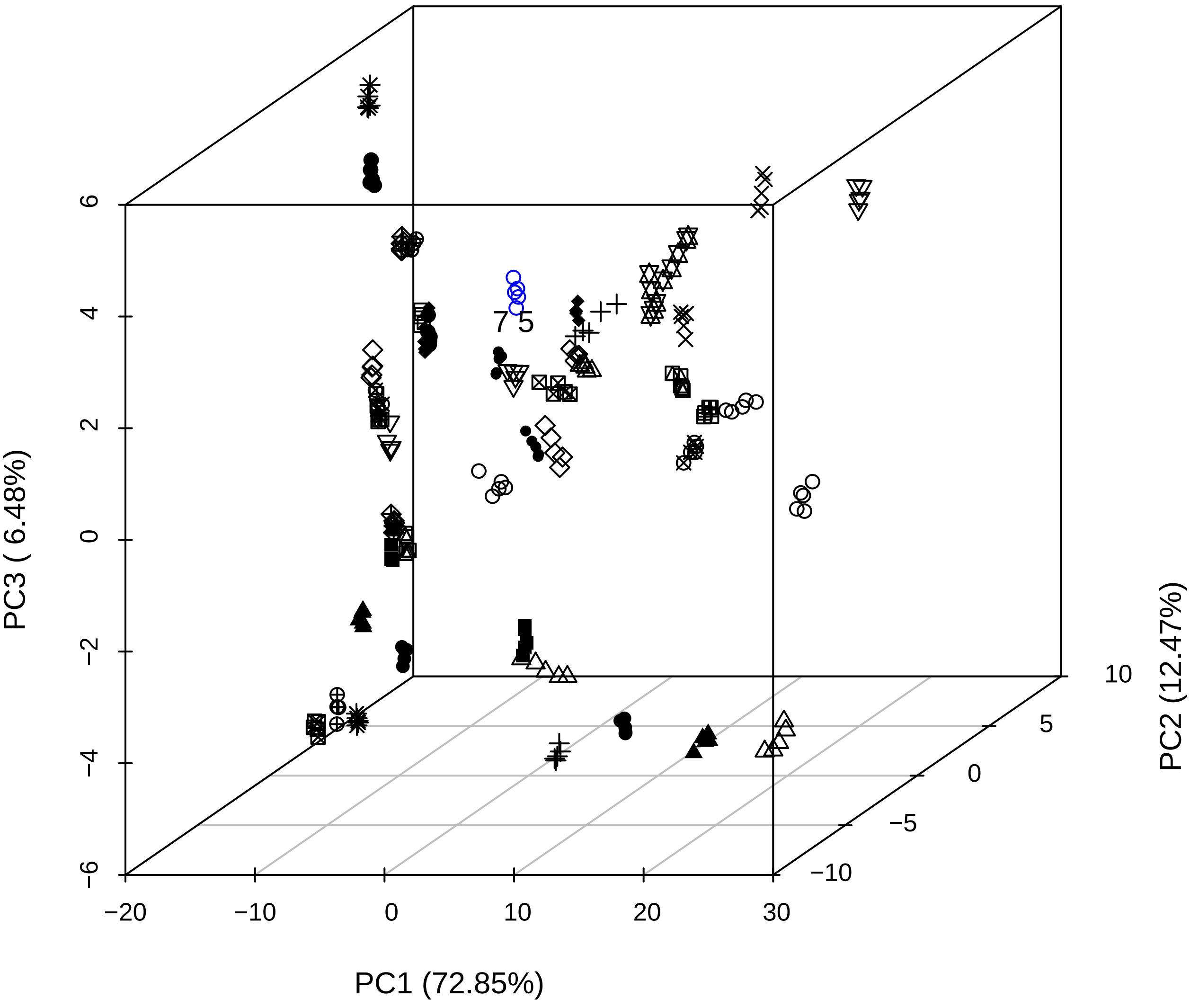

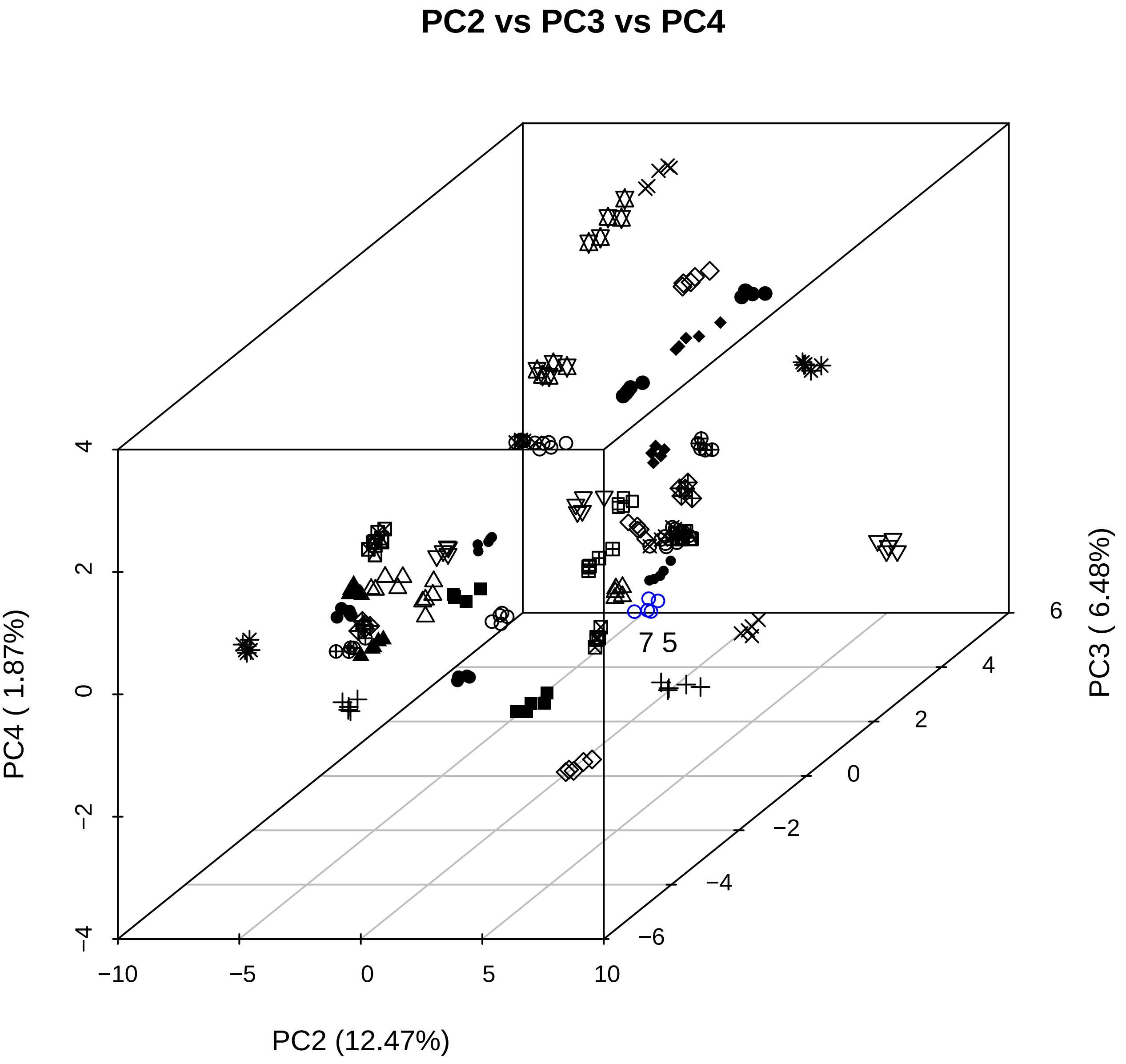

PC2 vs PC3 vs PC4
PC4 (1.87%)
PC2 (12.47%)
PC3 (6.48%)
−10
−5
0
5
10
−4
−2
0
2
4
−6
−4
−2
0
2
4
6
7 5

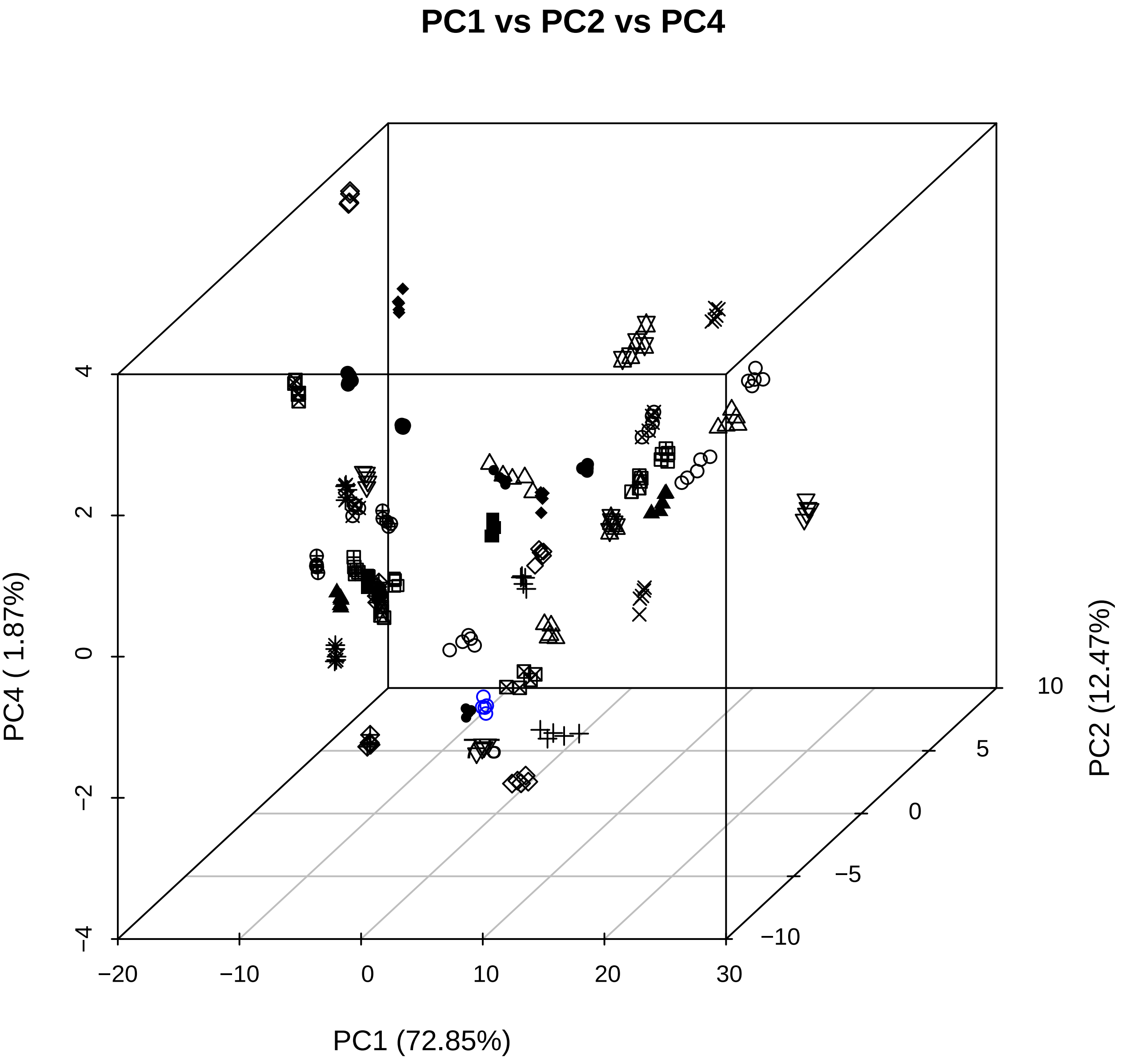

PC1 vs PC2 vs PC4
PC1 (72.85%)
PC2 (12.47%)
PC4 (1.87%)
−20
−10
0
10
20
30
−10
−5
0
5
10
−4
−2
0
2
4

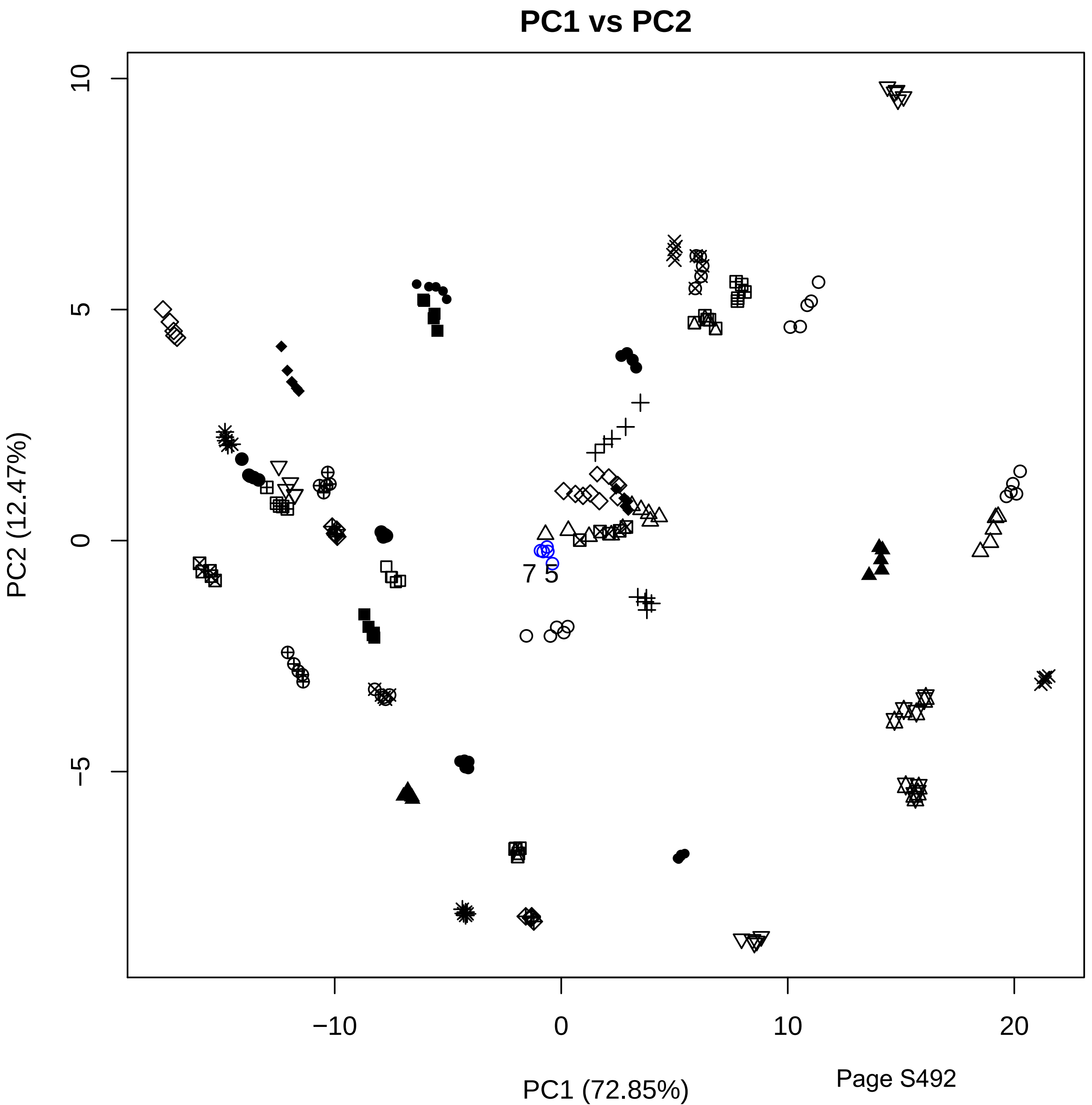
PC1 vs PC2
PC2 (12.47%)
PC1 (72.85%)
10
5
0
−5
−10
0
10
20
7
5

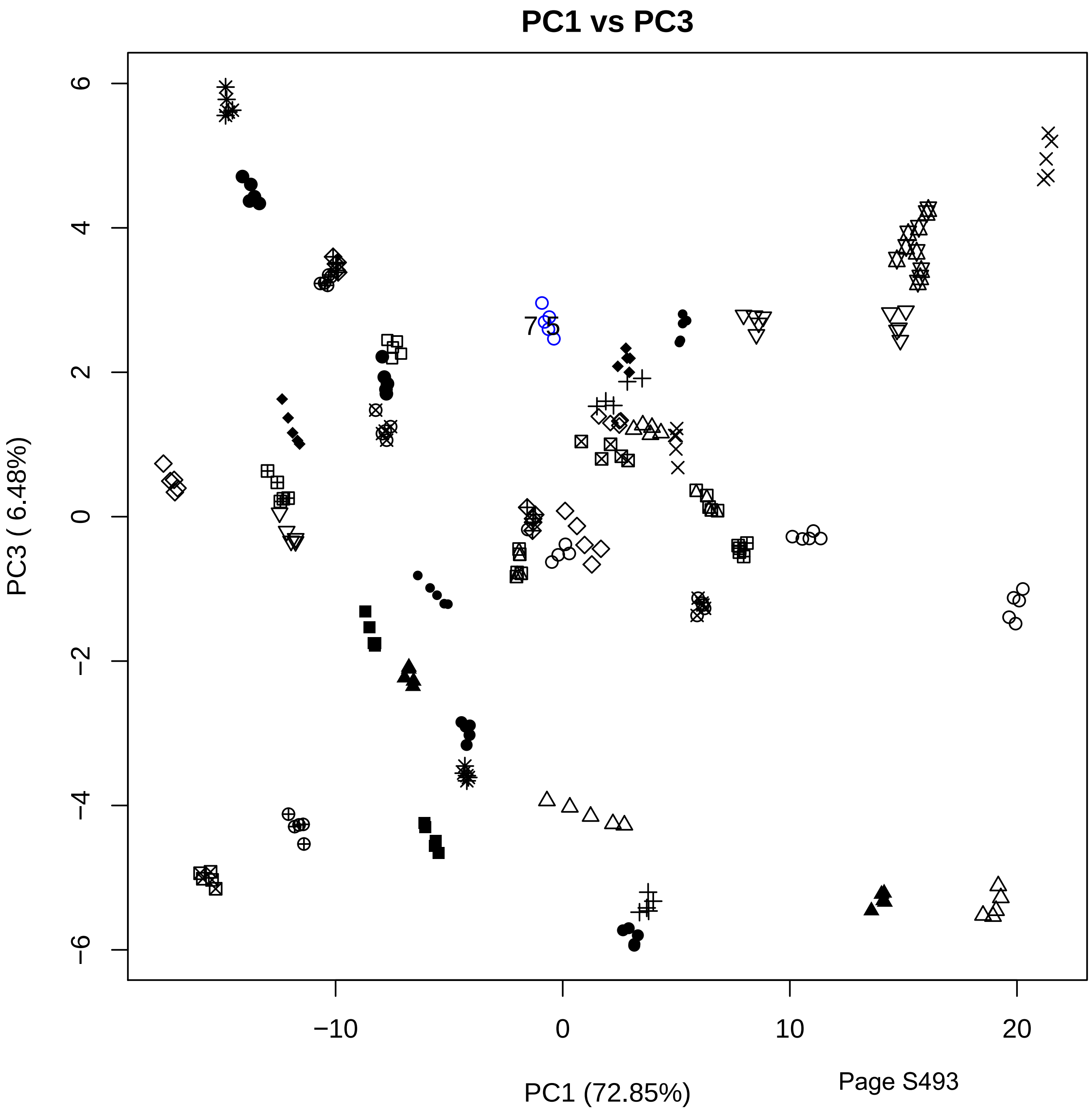

PC1 vs PC3
PC3 (6.48%)
PC1 (72.85%)
6
4
2
0
−2
−4
−6
−10
0
10
20
75

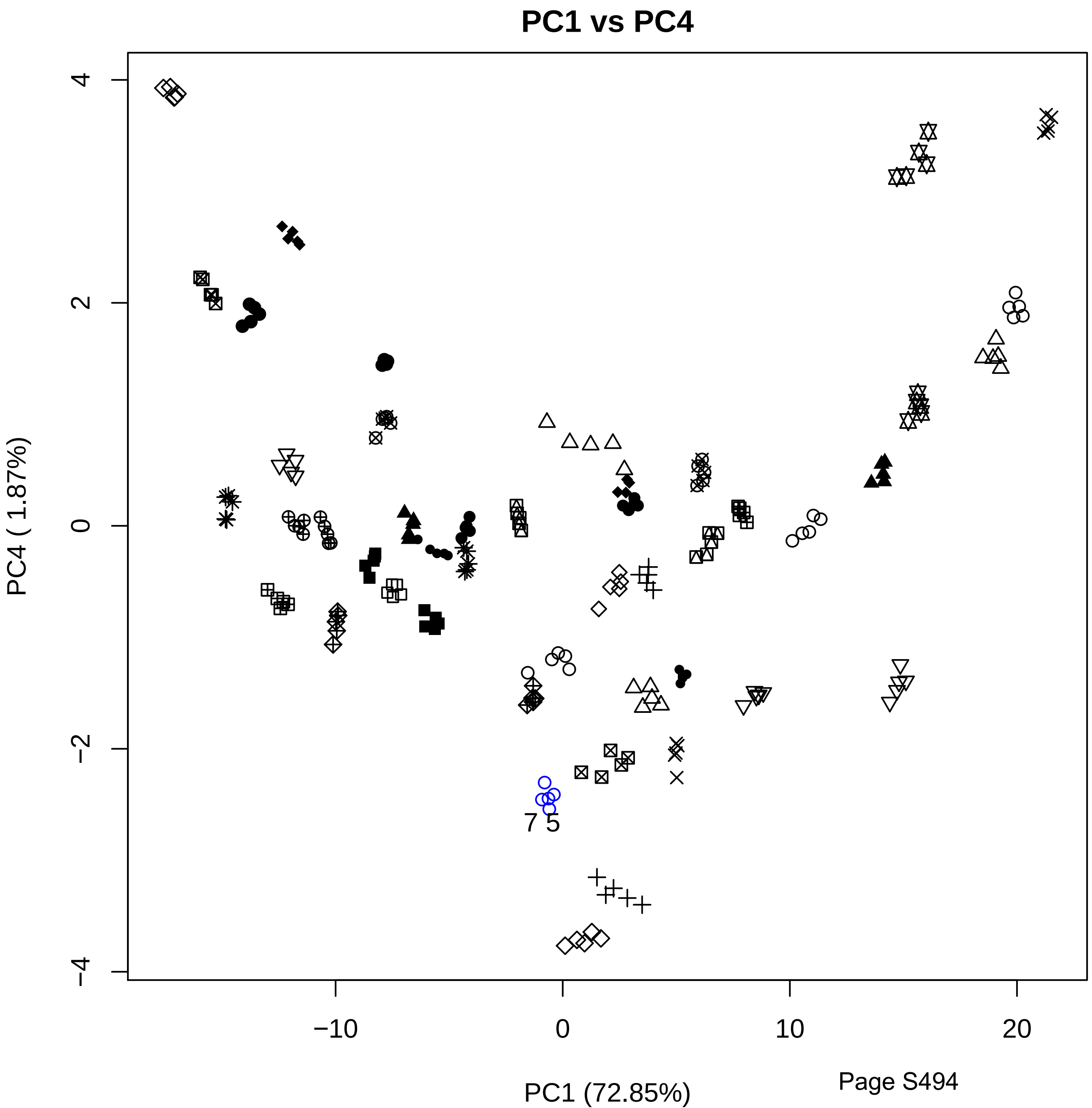
PC1 vs PC4
PC4 (1.87%)
PC1 (72.85%)
4
2
0
−2
−4
−10
0
10
20
7 5

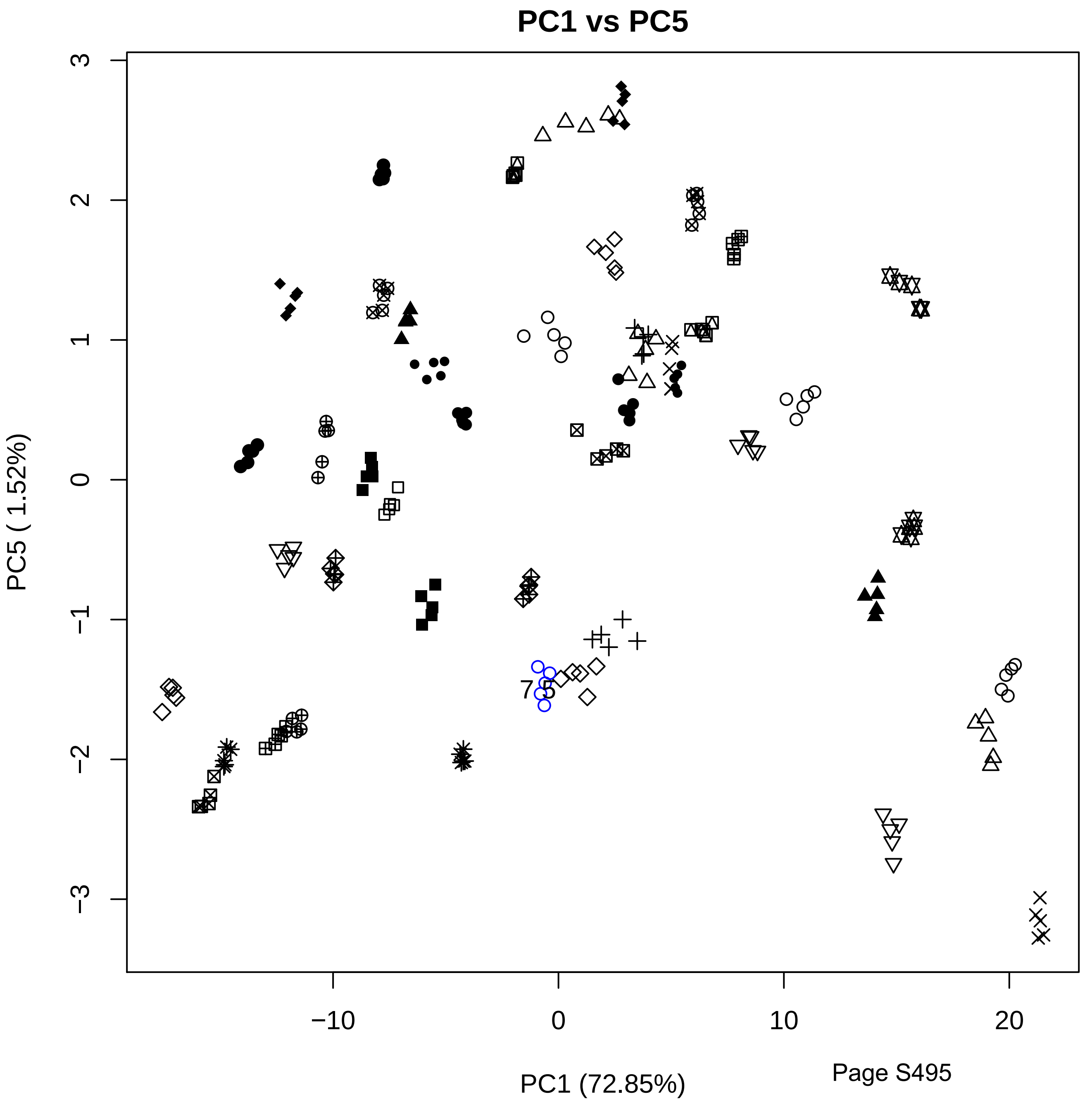
PC1 vs PC5
PC5 (1.52%)
PC1 (72.85%)
3
2
1
0
−1
−2
−3
−10
0
10
20
75

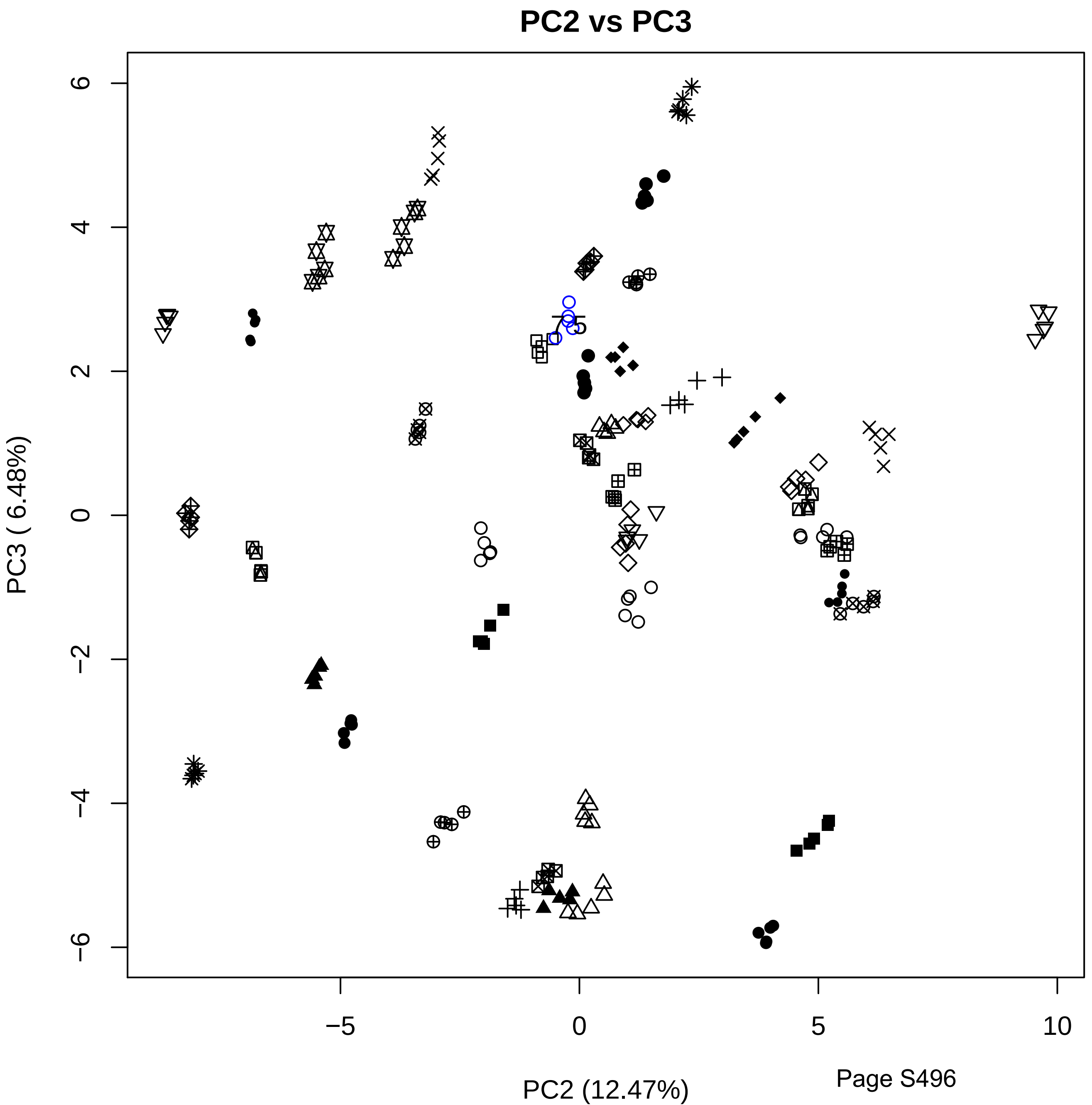
PC2 vs PC3
PC3 (6.48%)
PC2 (12.47%)
6
4
2
0
−2
−4
−6
−5
0
5
10

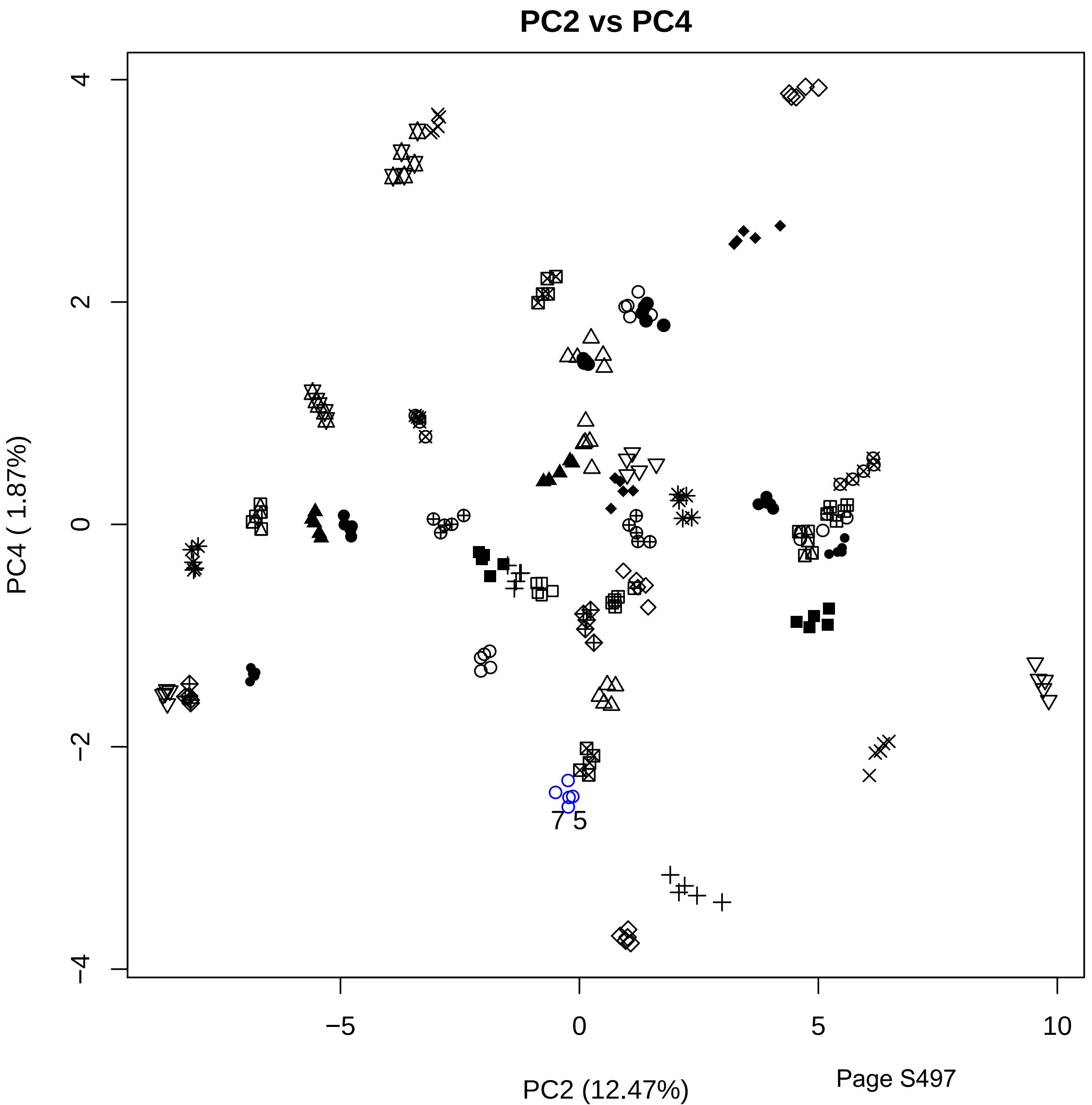

PC2 vs PC4
PC4 (1.87%)
PC2 (12.47%)
4
2
0
−2
−4
−5
0
5
10
7 5

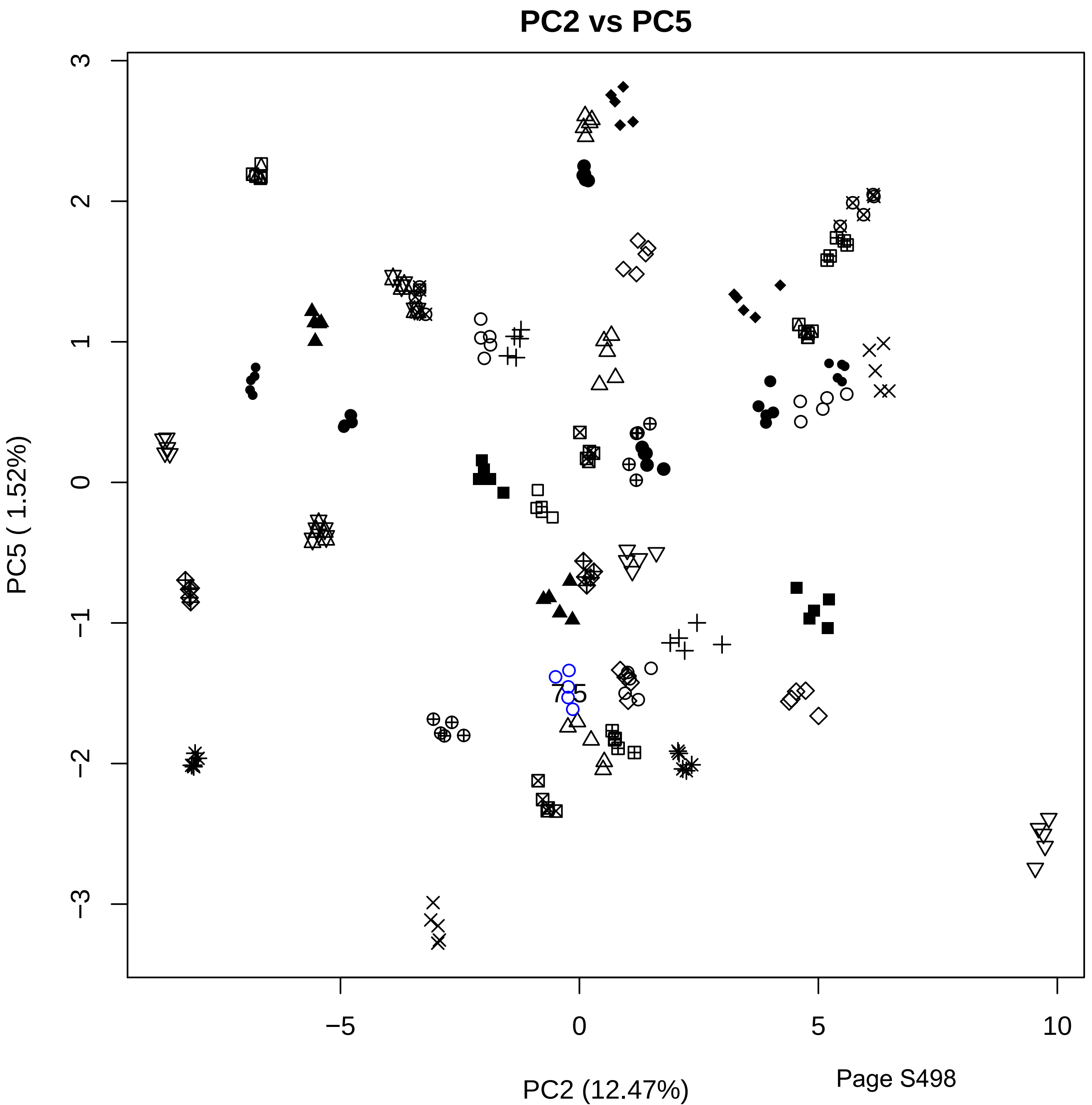
PC2 vs PC5
PC5 (1.52%)
PC2 (12.47%)
3
2
1
0
−1
−2
−3
−5
0
5
10
75

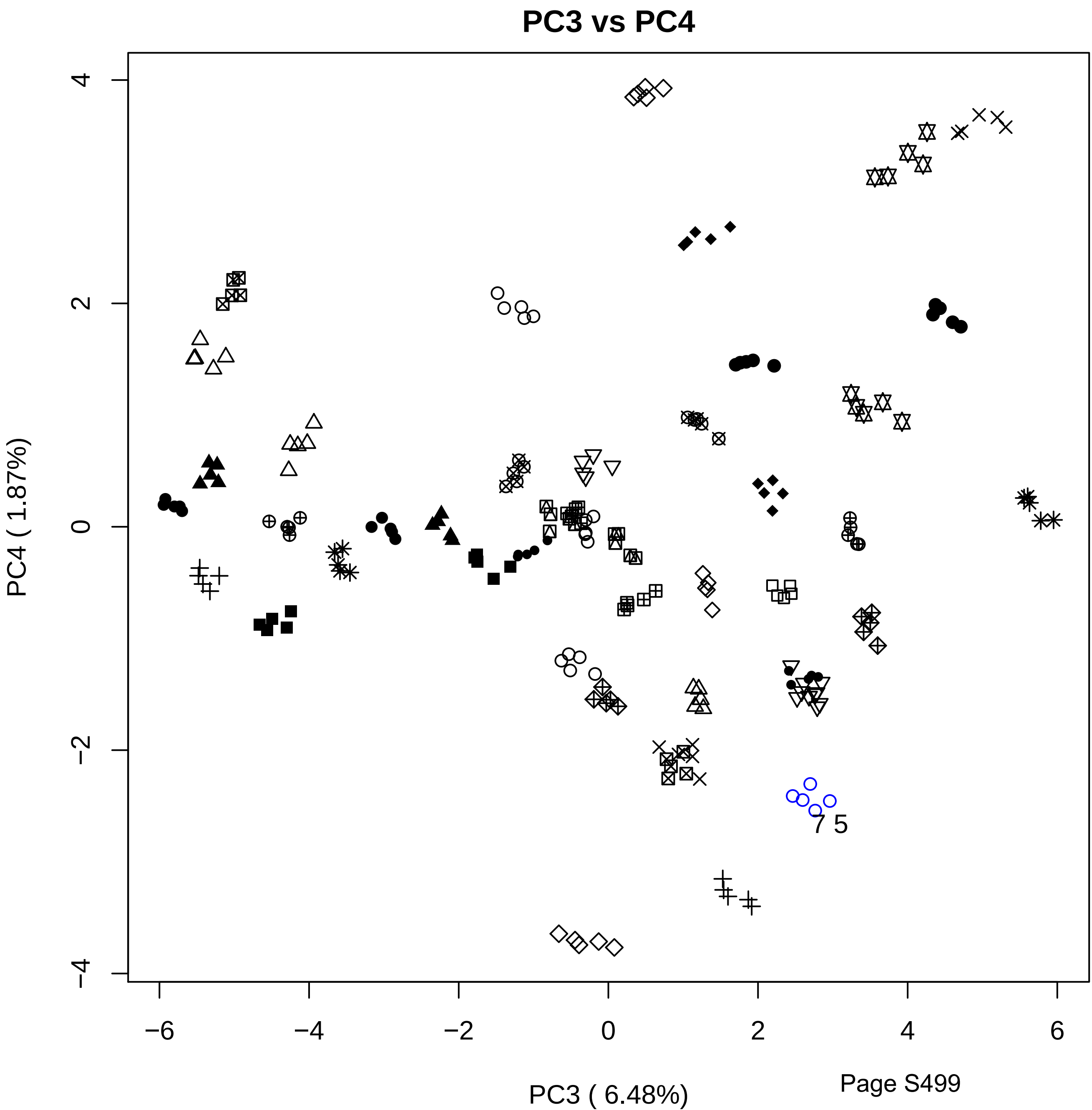

PC3 vs PC4
4
2
0
−2
−4
PC4 (1.87%)
−6
−4
−2
0
2
4
6
PC3 (6.48%)
7 5

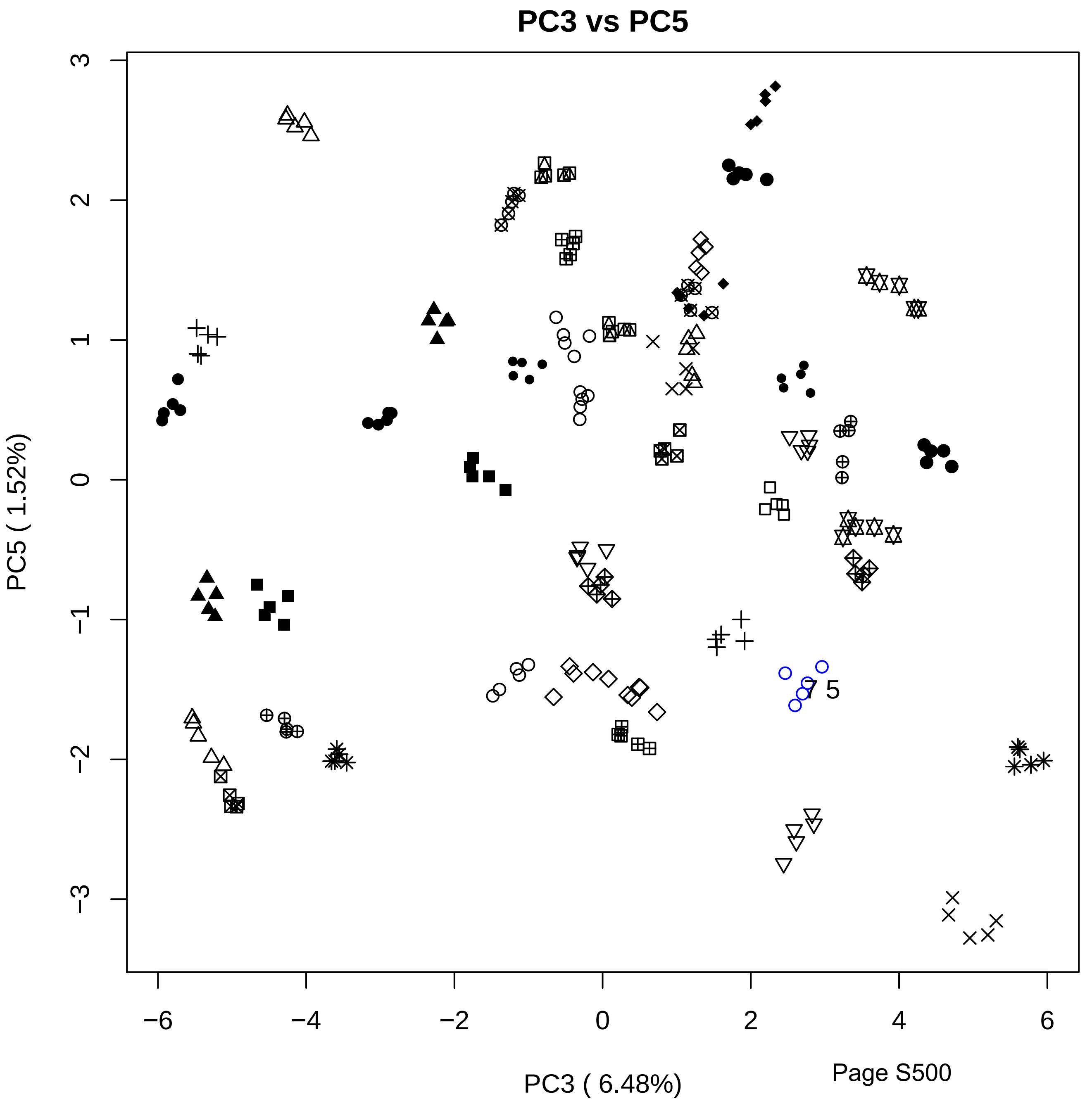

PC3 vs PC5
PC5 (1.52%)
PC3 (6.48%)
3
2
1
0
−1
−2
−3
−6
−4
−2
0
2
4
6
7 5

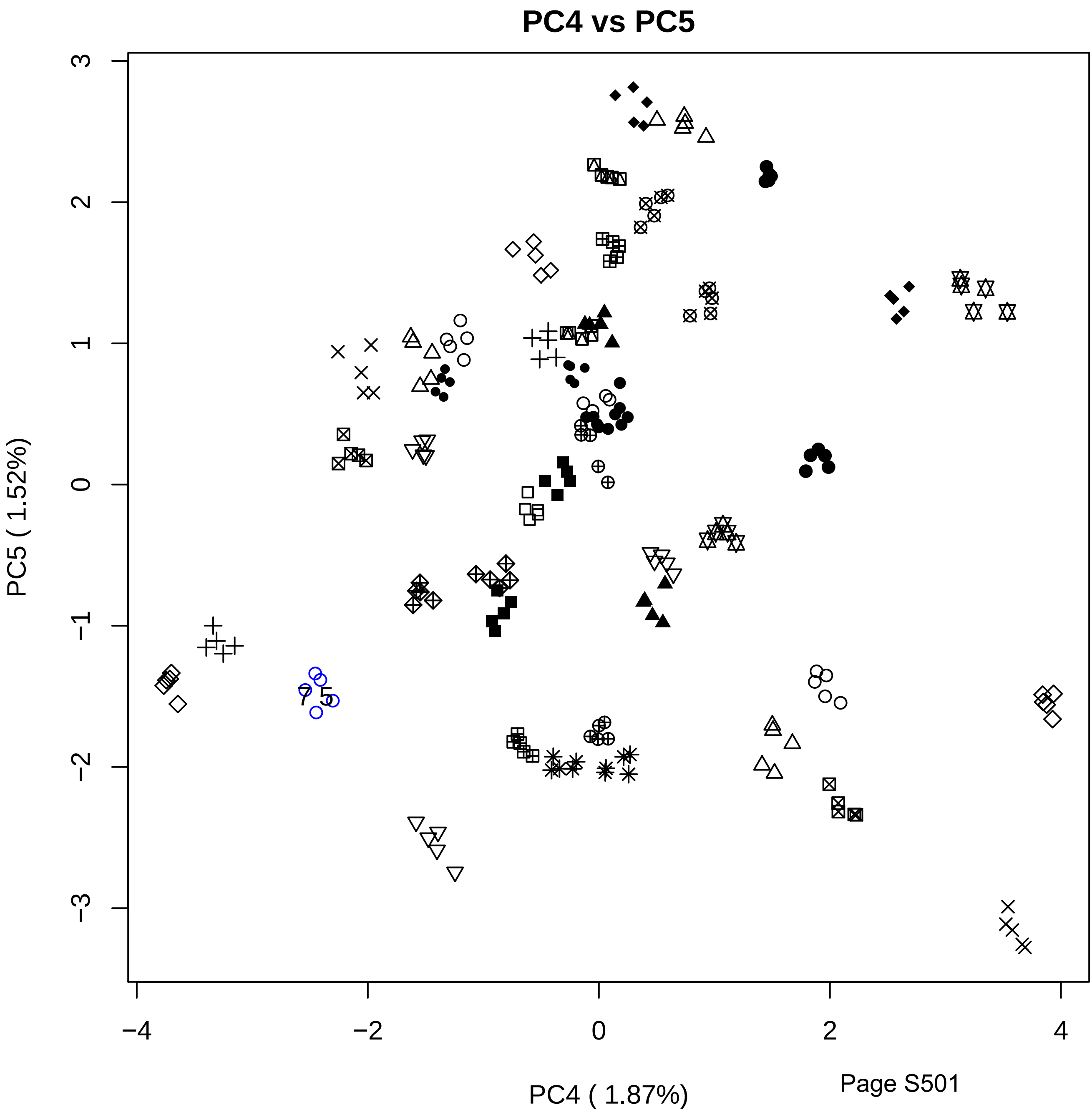

PC4 vs PC5
PC5 (1.52%)
PC4 (1.87%)
3
2
1
0
−1
−2
−3
−4
−2
0
2
4
7 5

PCA of 10% OSAS vs PMH

Scaled Matrix PCAed

7th order polynomial baseline correction performed

Raw spectra smoothed to 21 Neighbours, 2nd order PN, Savtisky Golay

Normalisation between 0 and 100

D2 spectra smoothed to 41 Neighbours, 2nd order PN, Savtisky Golay

Regions Analysed: 3601−2501 and 2001−700cm−1

Samples Analysed: 1 Contam's 0 OSAS's 45 PMH's

Keele University AD   Sun Dec 09 16:29:18 2018

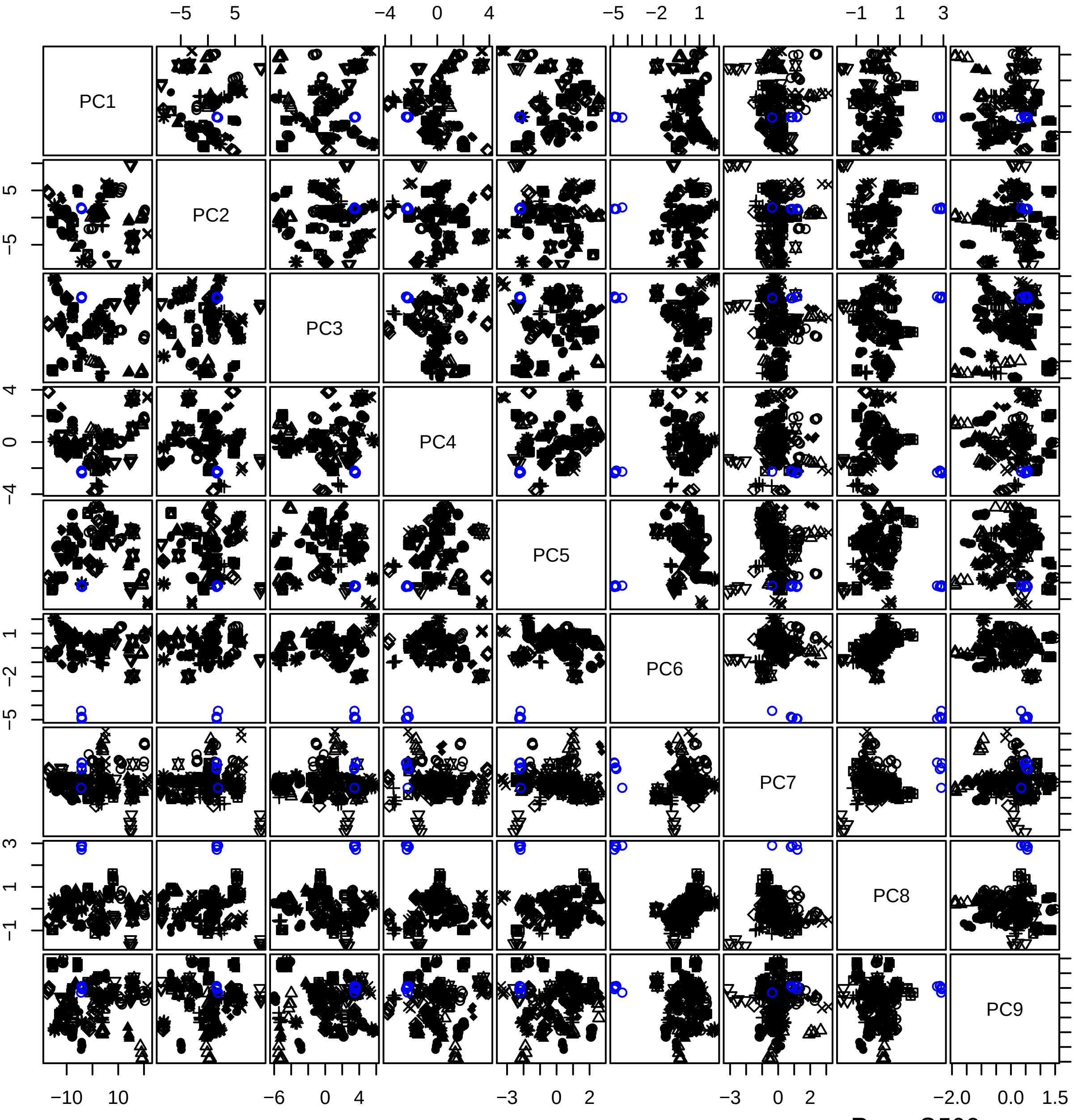
PC1
PC2
PC3
PC4
PC5
PC6
PC7
PC8
PC9

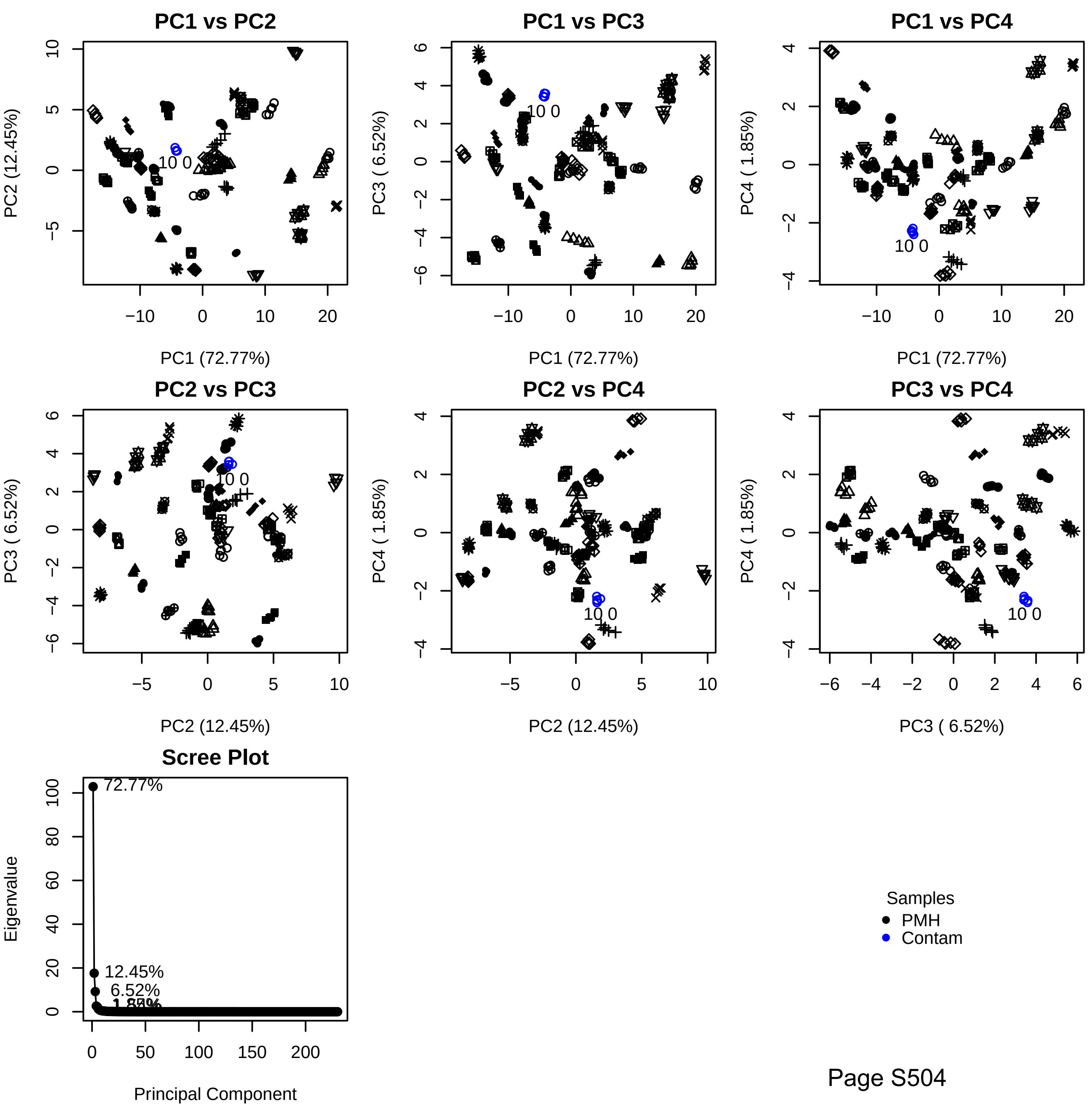
PC1 vs PC2
PC1 vs PC3
PC1 vs PC4
PC2 vs PC3
PC2 vs PC4
PC3 vs PC4
Scree Plot
PC1 (72.77%)
PC2 (12.45%)
PC3 (6.52%)
PC4 (1.85%)
10 0
Eigenvalue
Principal Component
72.77%
12.45%
6.52%
Samples
PMH
Contam

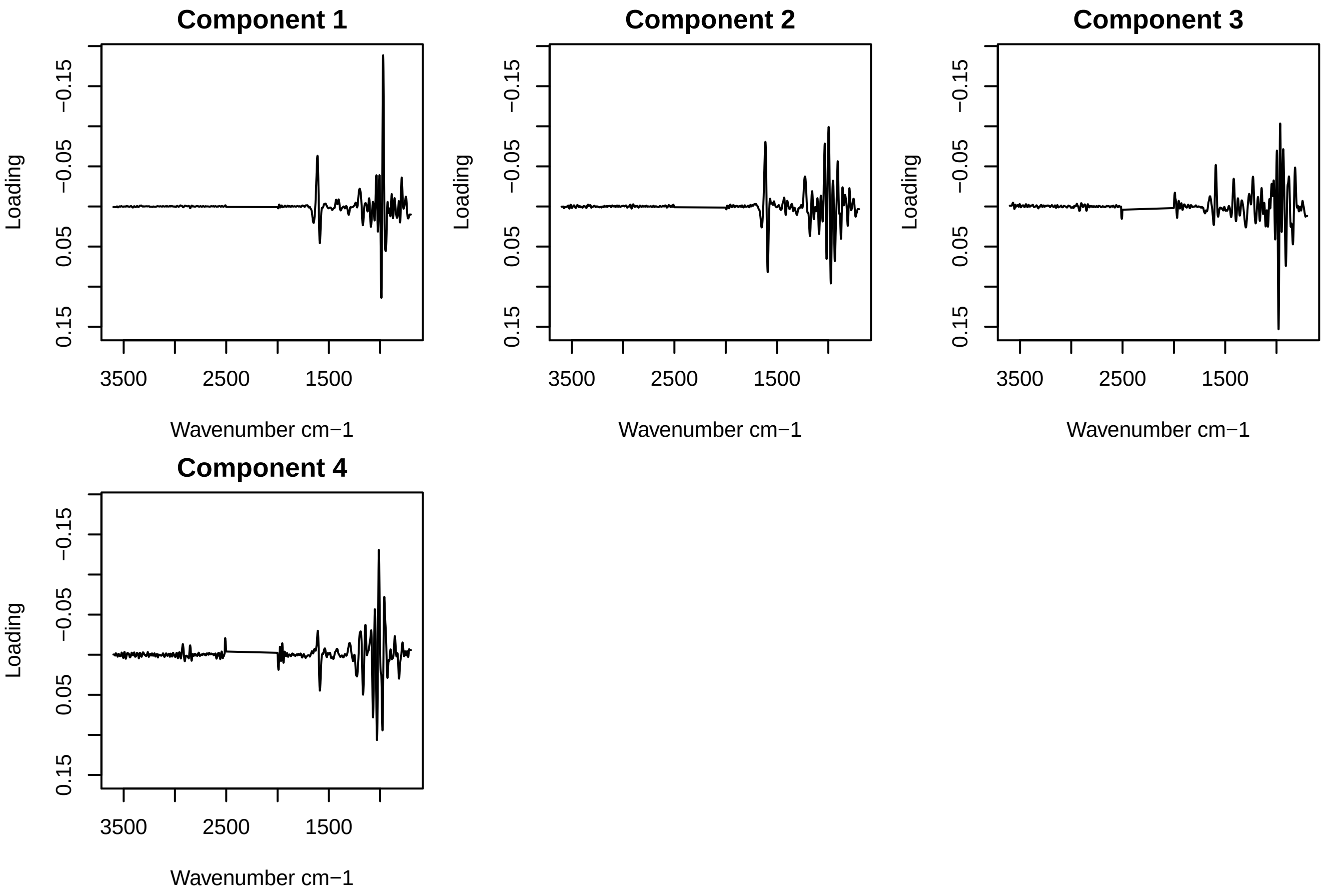
Component 1
Component 2
Component 3
Component 4
Loading
Wavenumber cm−1
3500
2500
1500
−0.15
−0.05
0.05
0.15

PC1 vs PC2 vs PC3

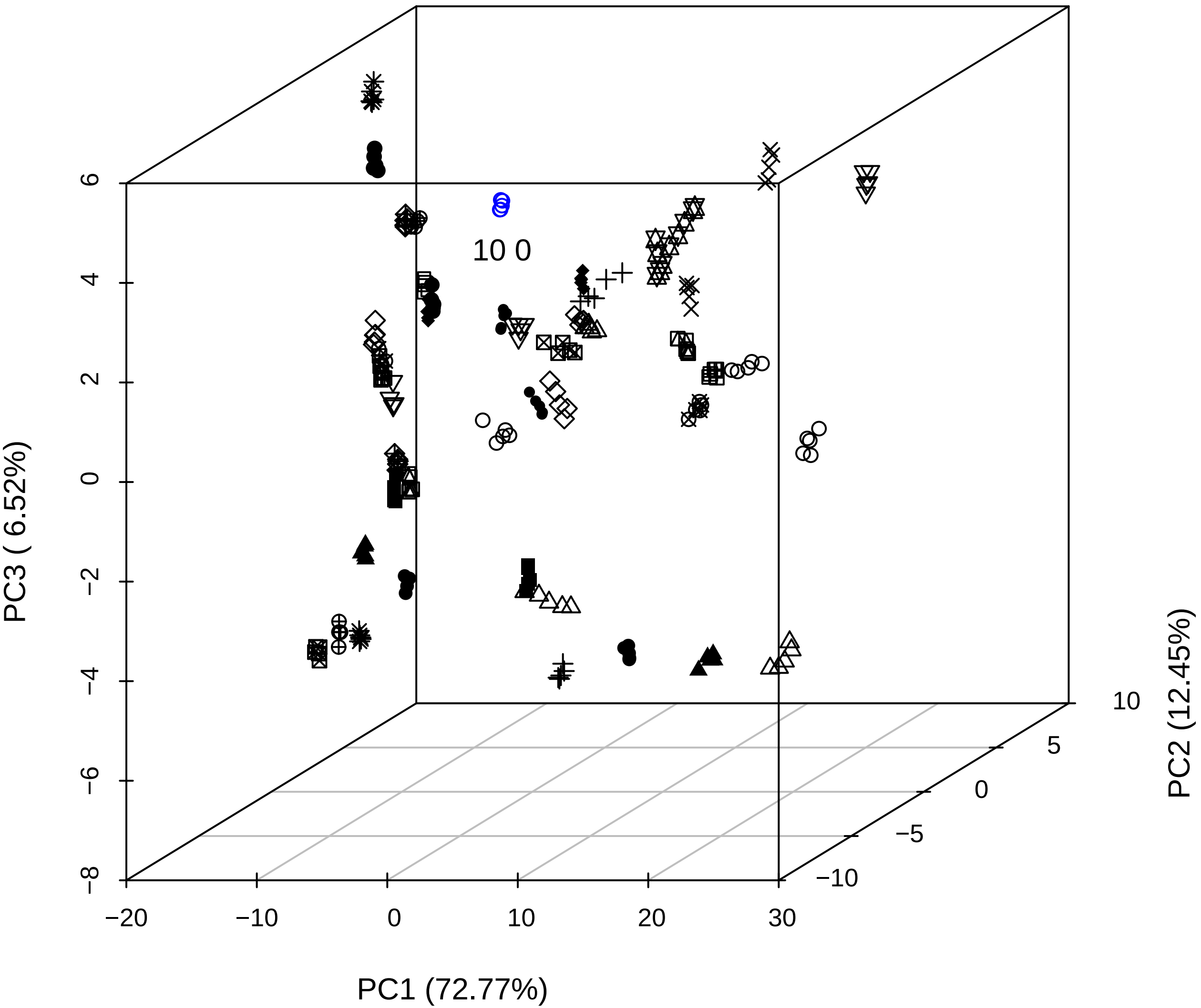

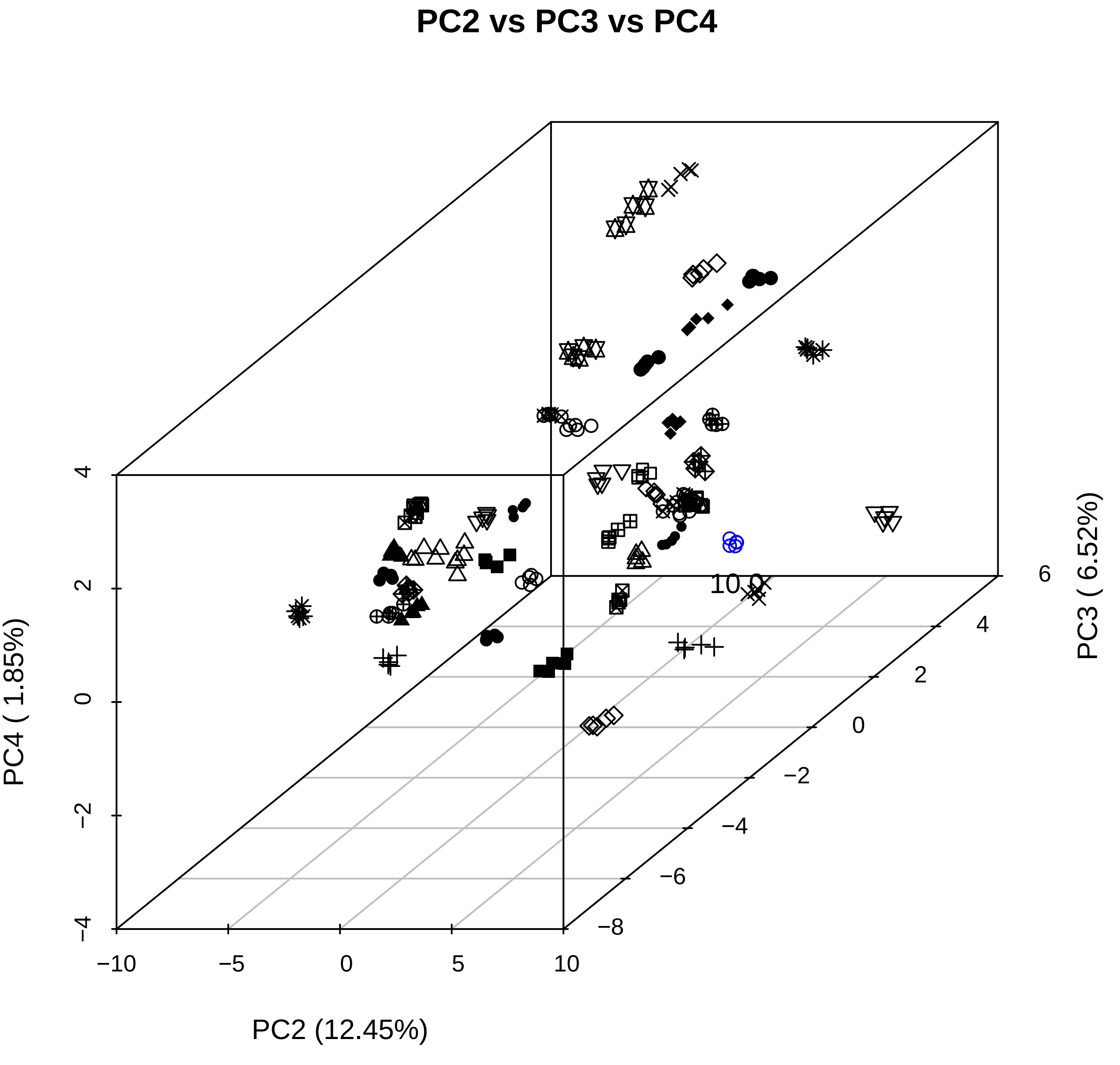
PC2 vs PC3 vs PC4
PC2 (12.45%)
PC3 (6.52%)
PC4 (1.85%)
−10
−5
0
5
10
−8
−6
−4
−2
0
2
4
6
−4
−2
0
2
4
10.0

PC1 vs PC2 vs PC4

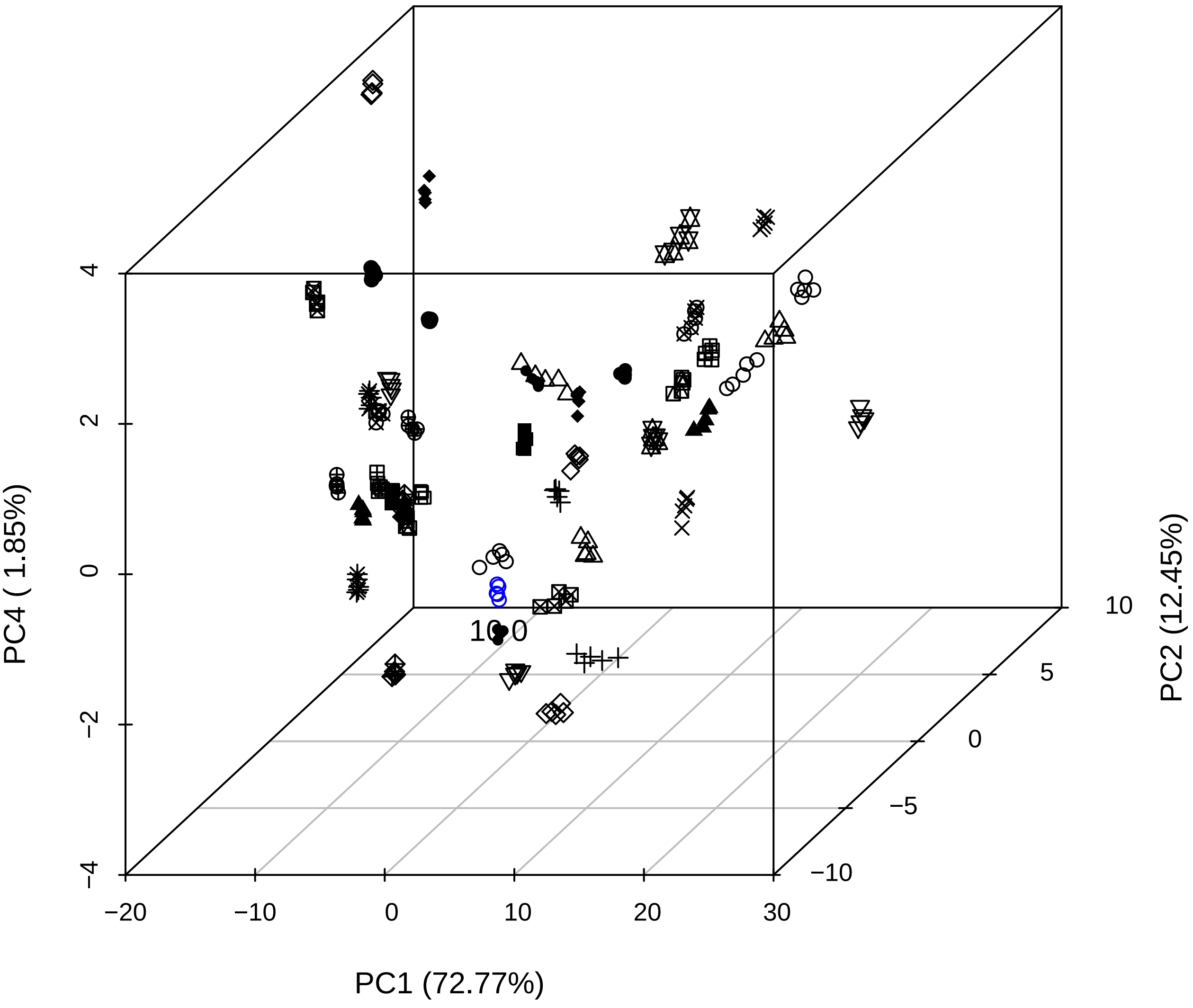

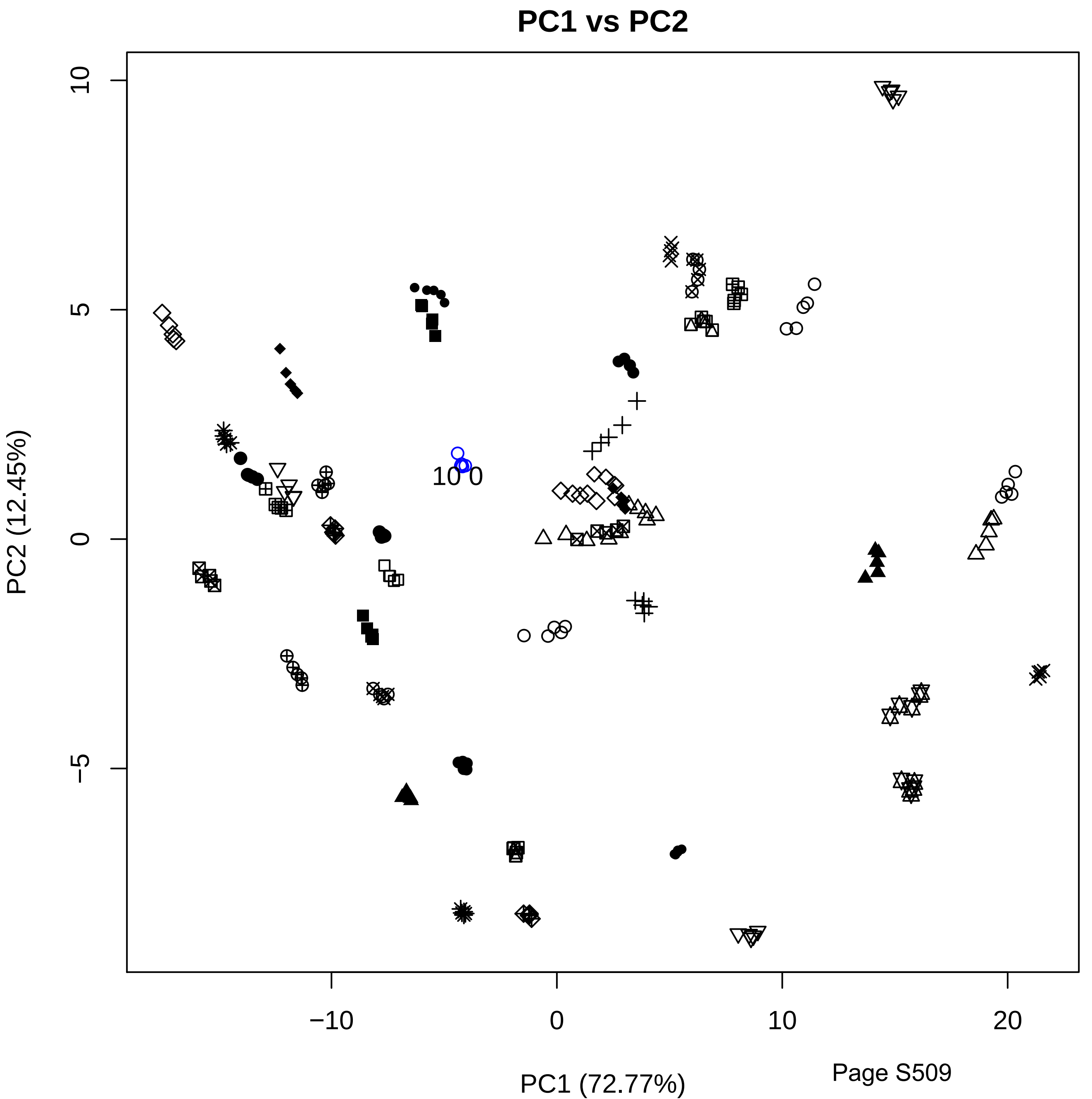
PC1 vs PC2
PC2 (12.45%)
PC1 (72.77%)
10
5
0
−5
−10
0
10
20
10 0

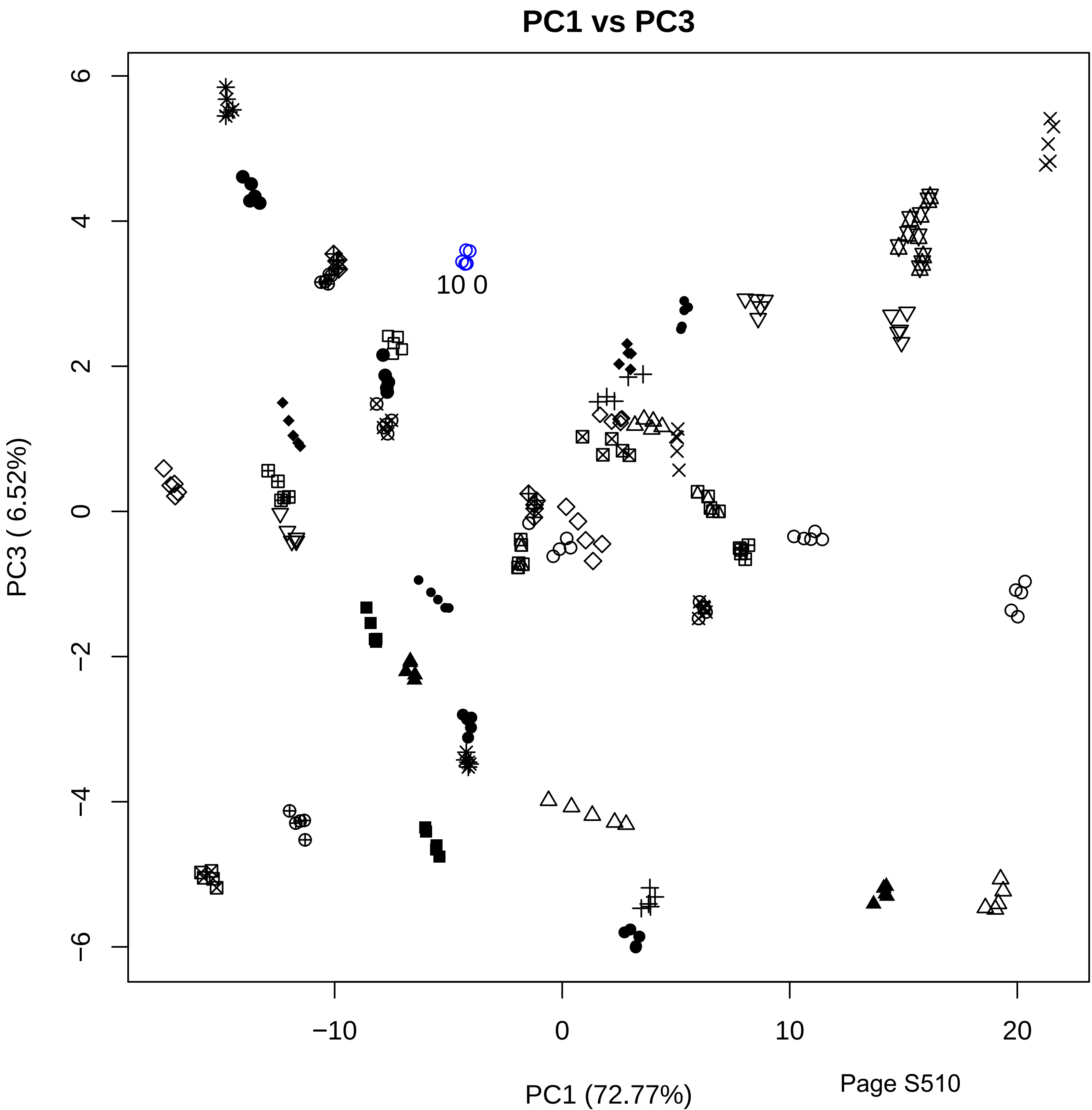
PC1 vs PC3
PC3 (6.52%)
PC1 (72.77%)
6
4
2
0
−2
−4
−6
−10
0
10
20
10 0

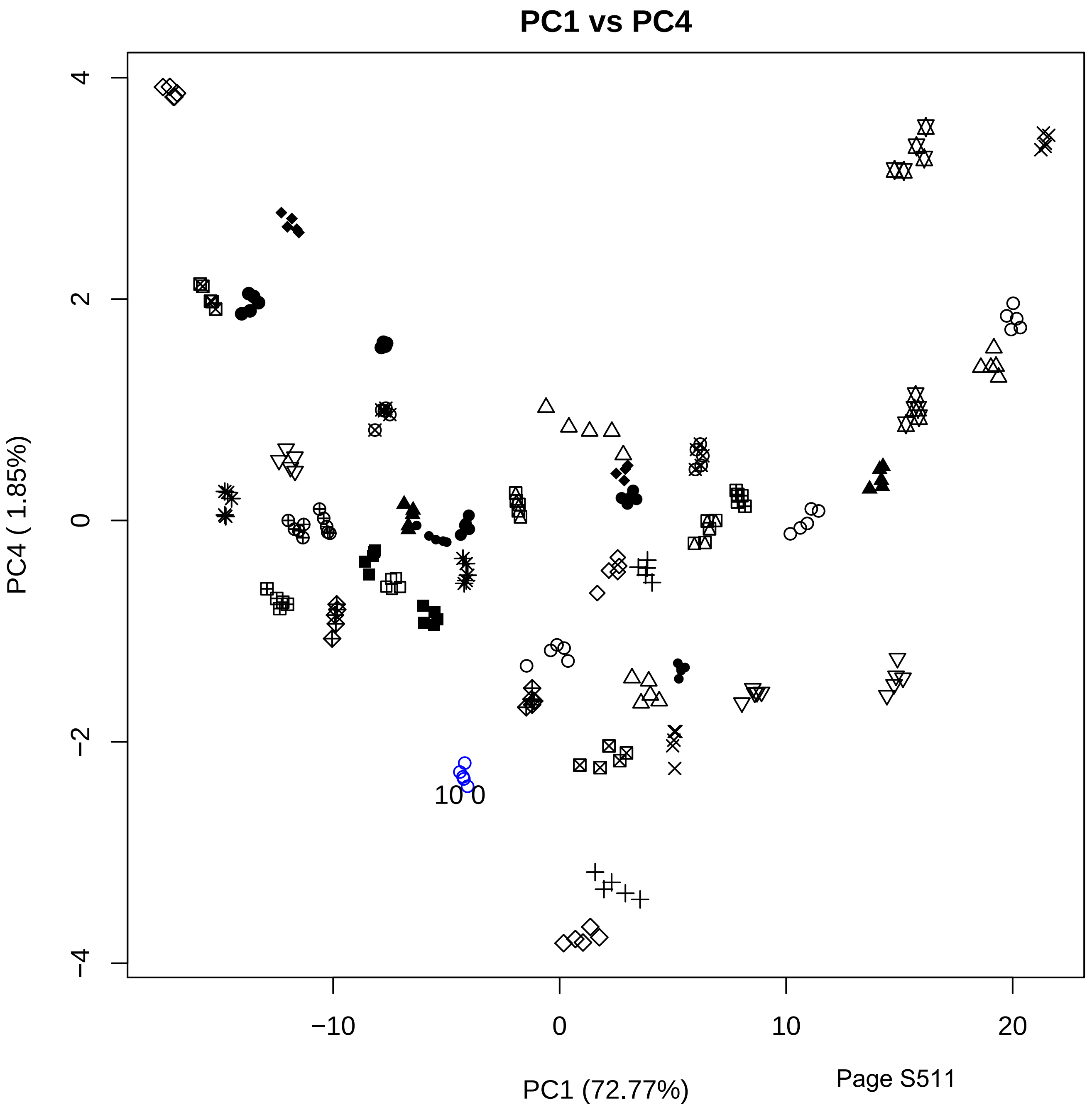
PC1 vs PC4
PC4 (1.85%)
PC1 (72.77%)
4
2
0
−2
−4
−10
0
10
20
10 0

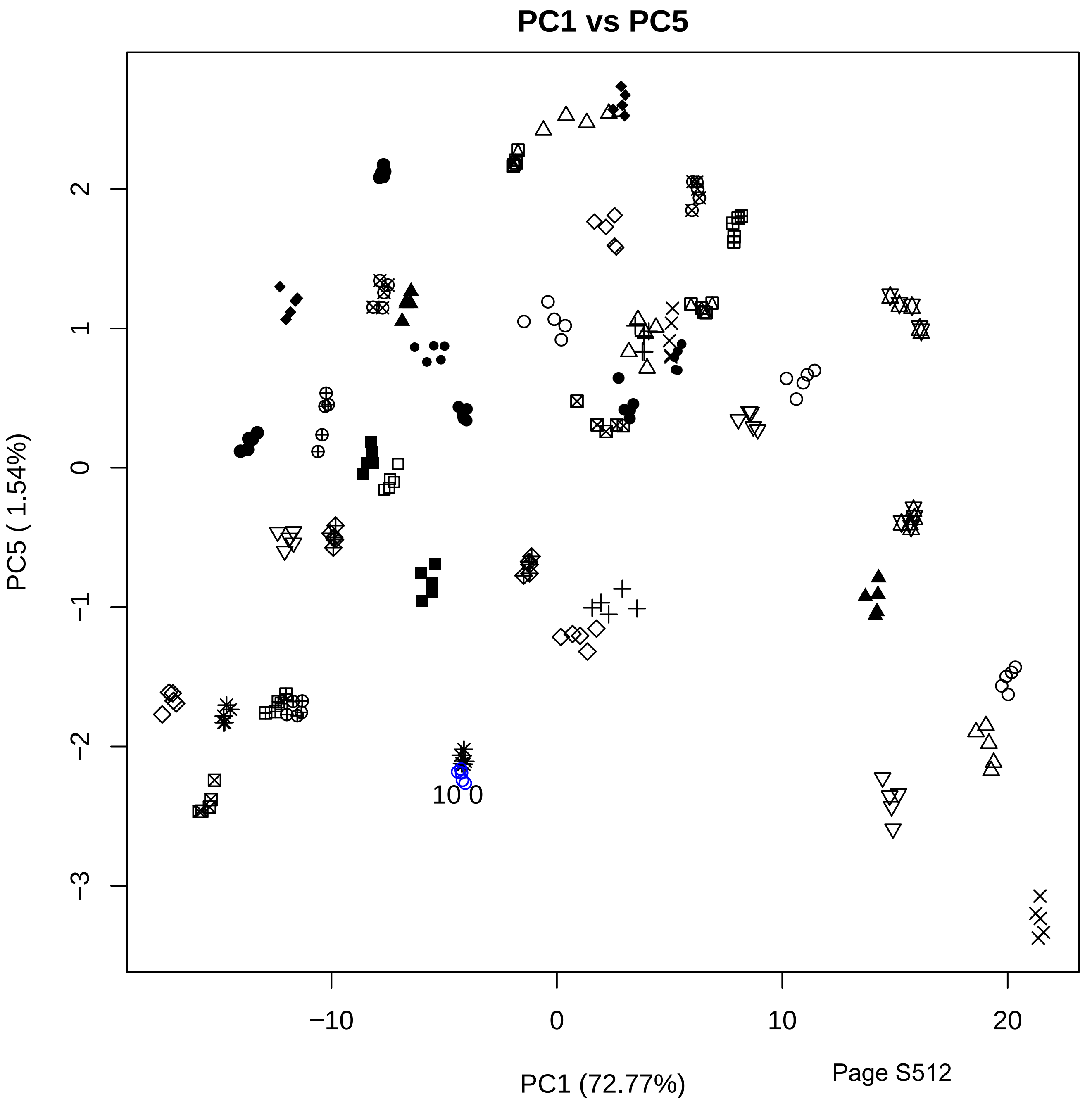

PC1 vs PC5
PC5 (1.54%)
PC1 (72.77%)
−10
0
10
20
−3
−2
−1
0
1
2
10 0

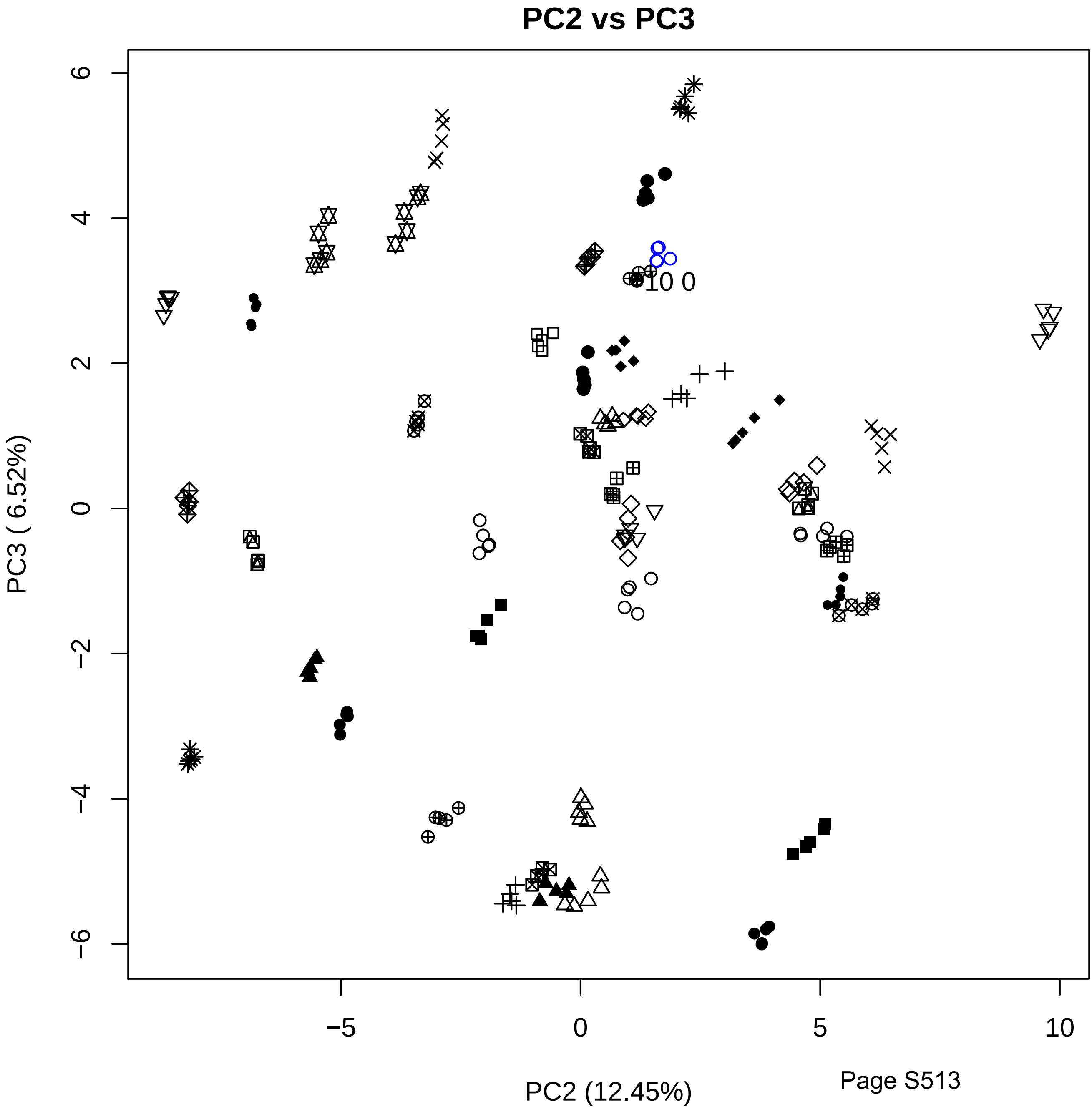
PC2 vs PC3
PC3 (6.52%)
PC2 (12.45%)
6
4
2
0
−2
−4
−6
−5
0
5
10
10 0

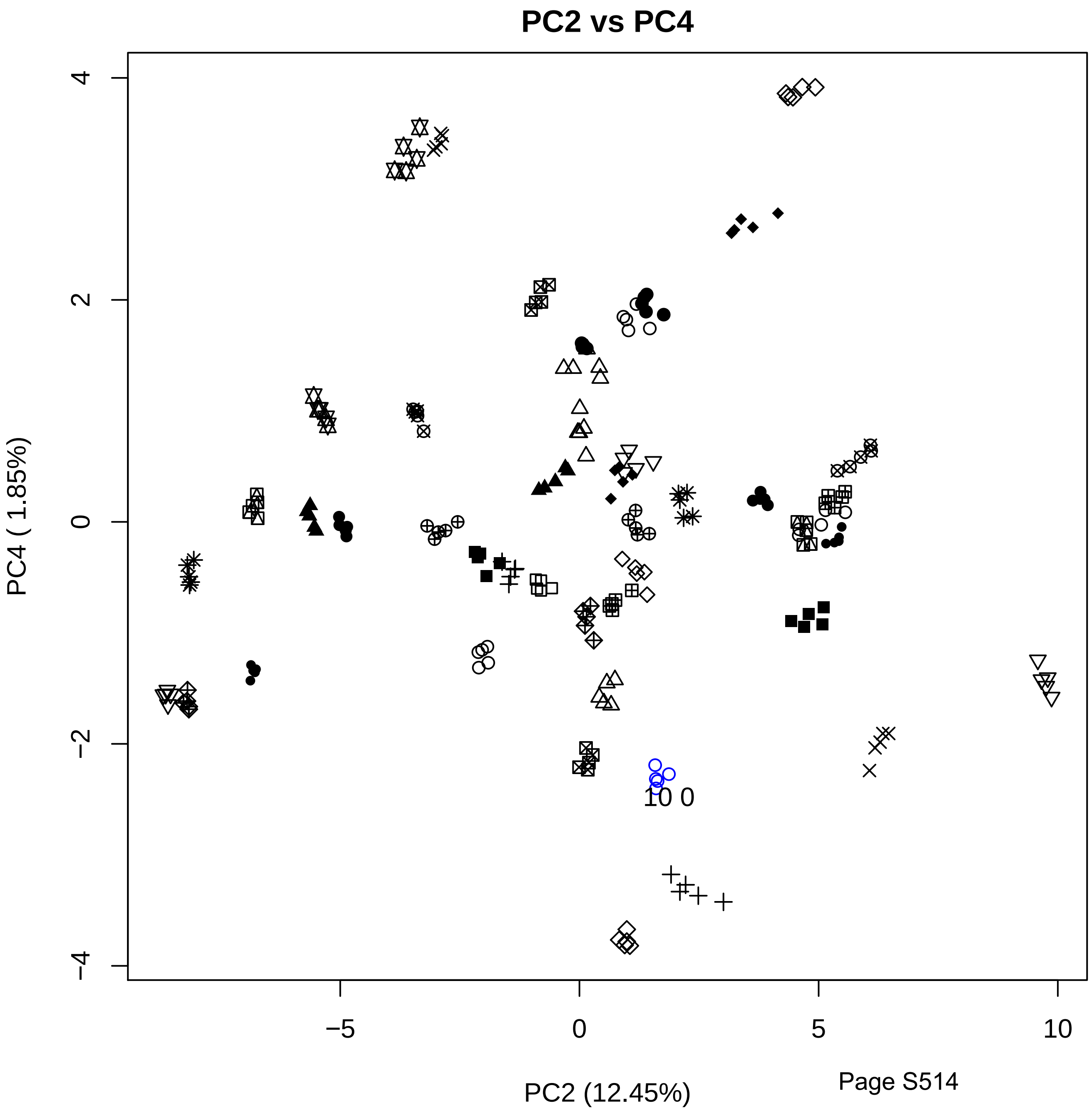
PC2 vs PC4
PC4 (1.85%)
PC2 (12.45%)
4
2
0
−2
−4
−5
0
5
10
10 0

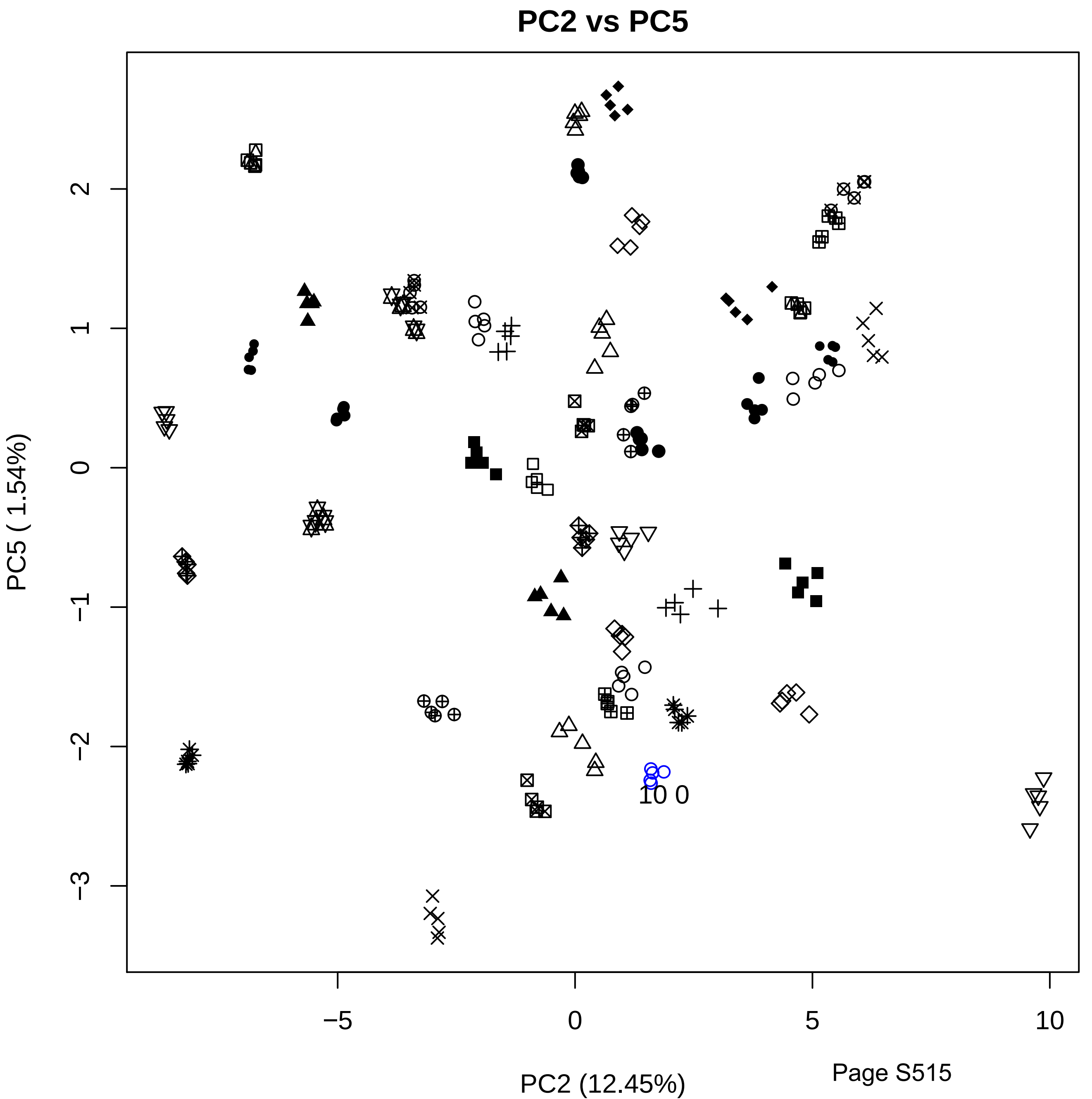
PC2 vs PC5
PC5 (1.54%)
PC2 (12.45%)
2
1
0
−1
−2
−3
−5
0
5
10
10 0

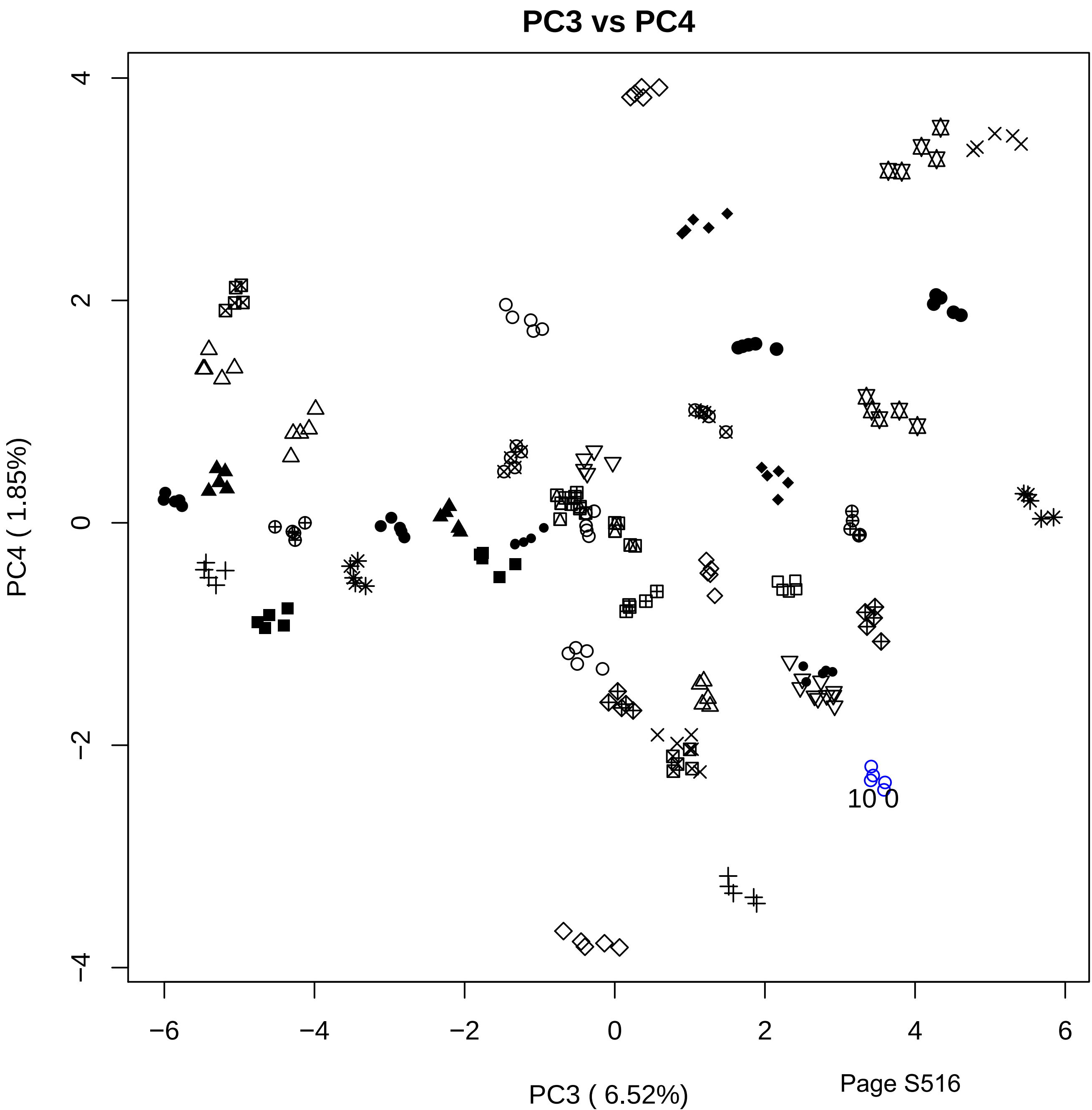
PC3 vs PC4
PC4 (1.85%)
PC3 (6.52%)
4
2
0
−2
−4
−6
−4
−2
0
2
4
6
10 0

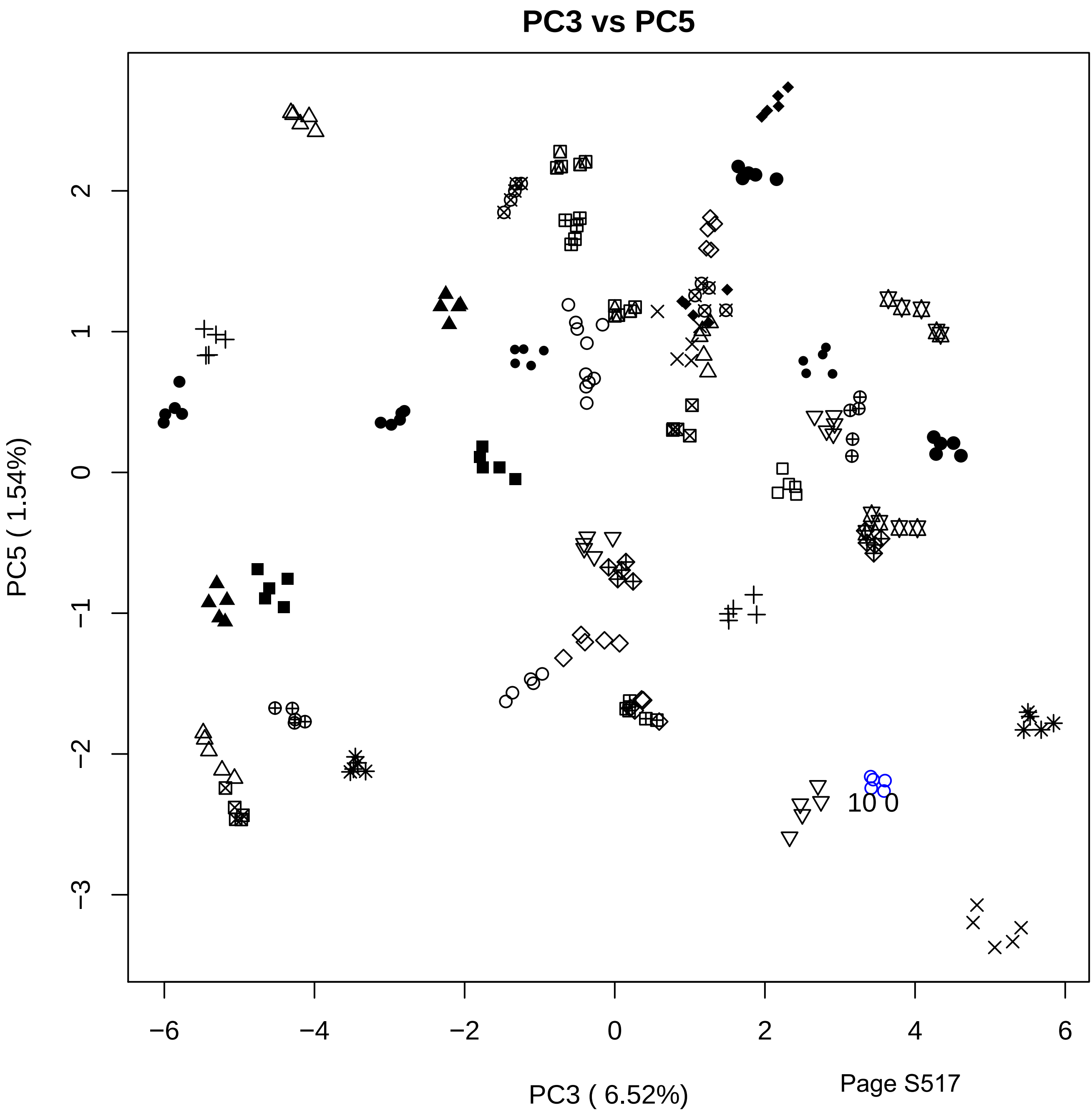

PC3 vs PC5
PC5 (1.54%)
PC3 (6.52%)
−6
−4
−2
0
2
4
6
−3
−2
−1
0
1
2
10 0

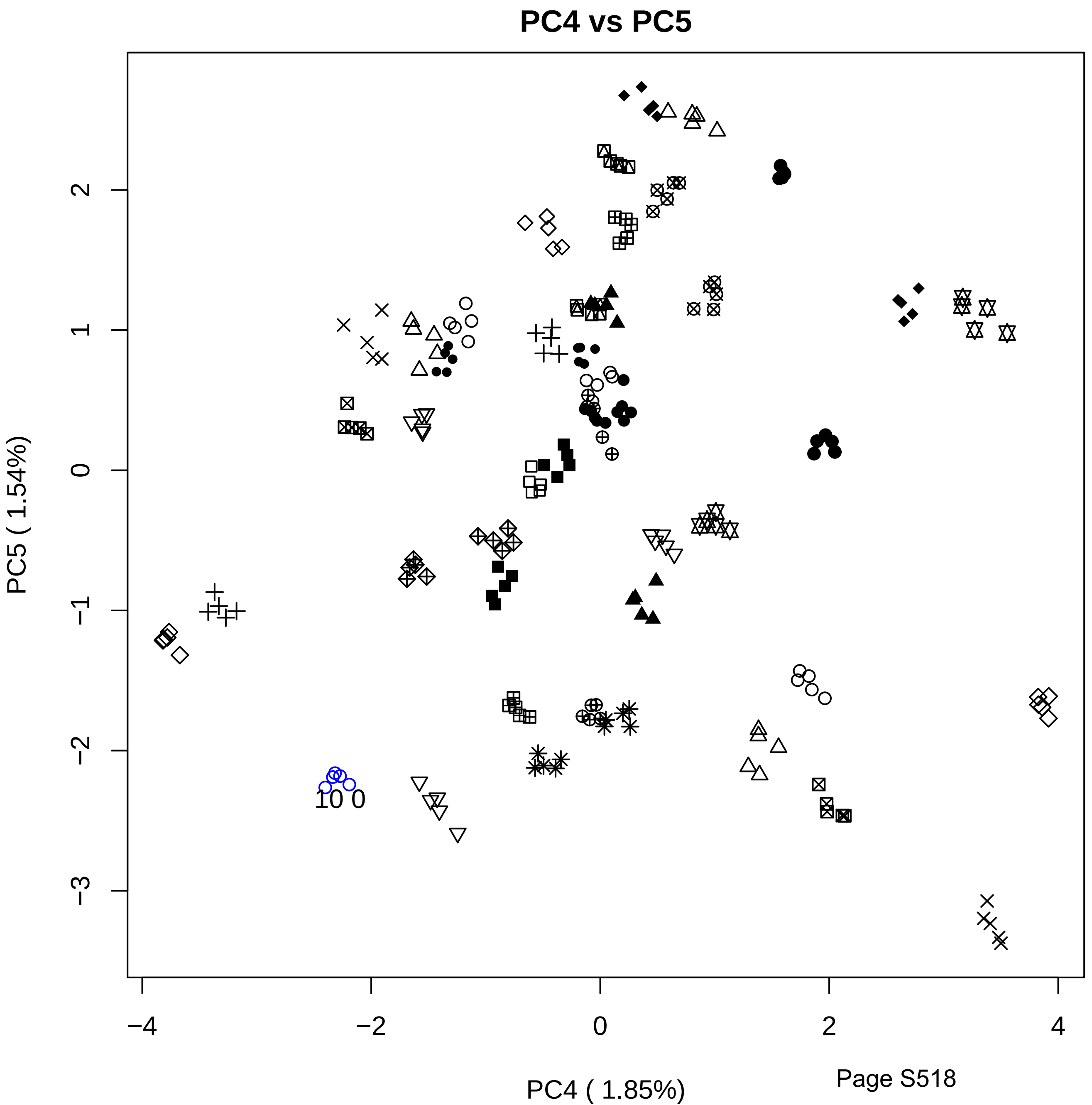
PC4 vs PC5
PC5 (1.54%)
PC4 (1.85%)
−4
−2
0
2
4
−3
−2
−1
0
1
2
10 0

PCA of 15% OSAS vs PMH

Scaled Matrix PCAed

7th order polynomial baseline correction performed

Raw spectra smoothed to 21 Neighbours, 2nd order PN, Savtisky Golay

Normalisation between 0 and 100

D2 spectra smoothed to 41 Neighbours, 2nd order PN, Savtisky Golay

Regions Analysed: 3601−2501 and 2001−700cm−1

Samples Analysed: 1 Contam's 0 OSAS's 45 PMH's

Keele University AD

Sun Dec 09 16:29:23 2018

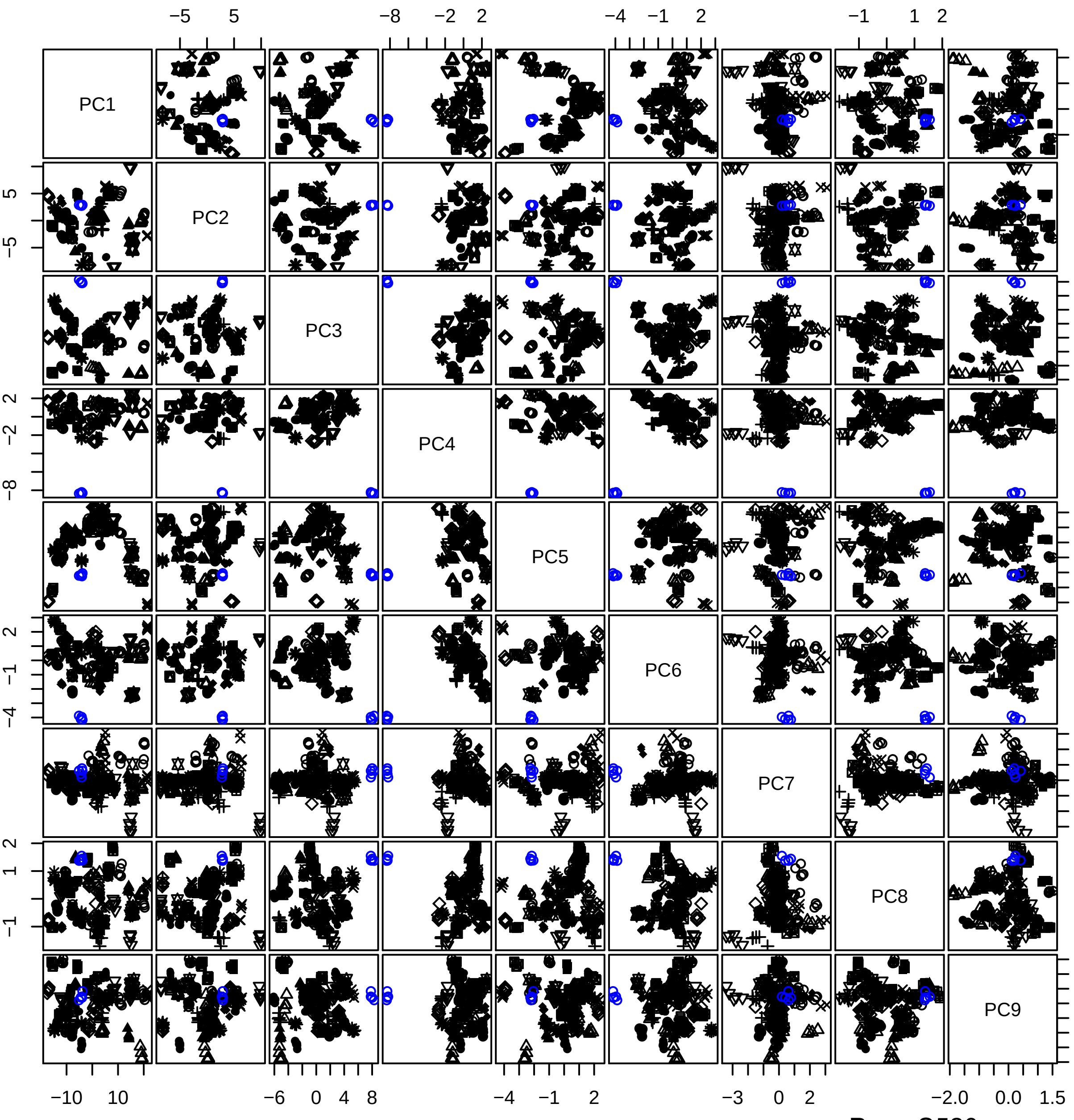

PC1
PC2
PC3
PC4
PC5
PC6
PC7
PC8
PC9

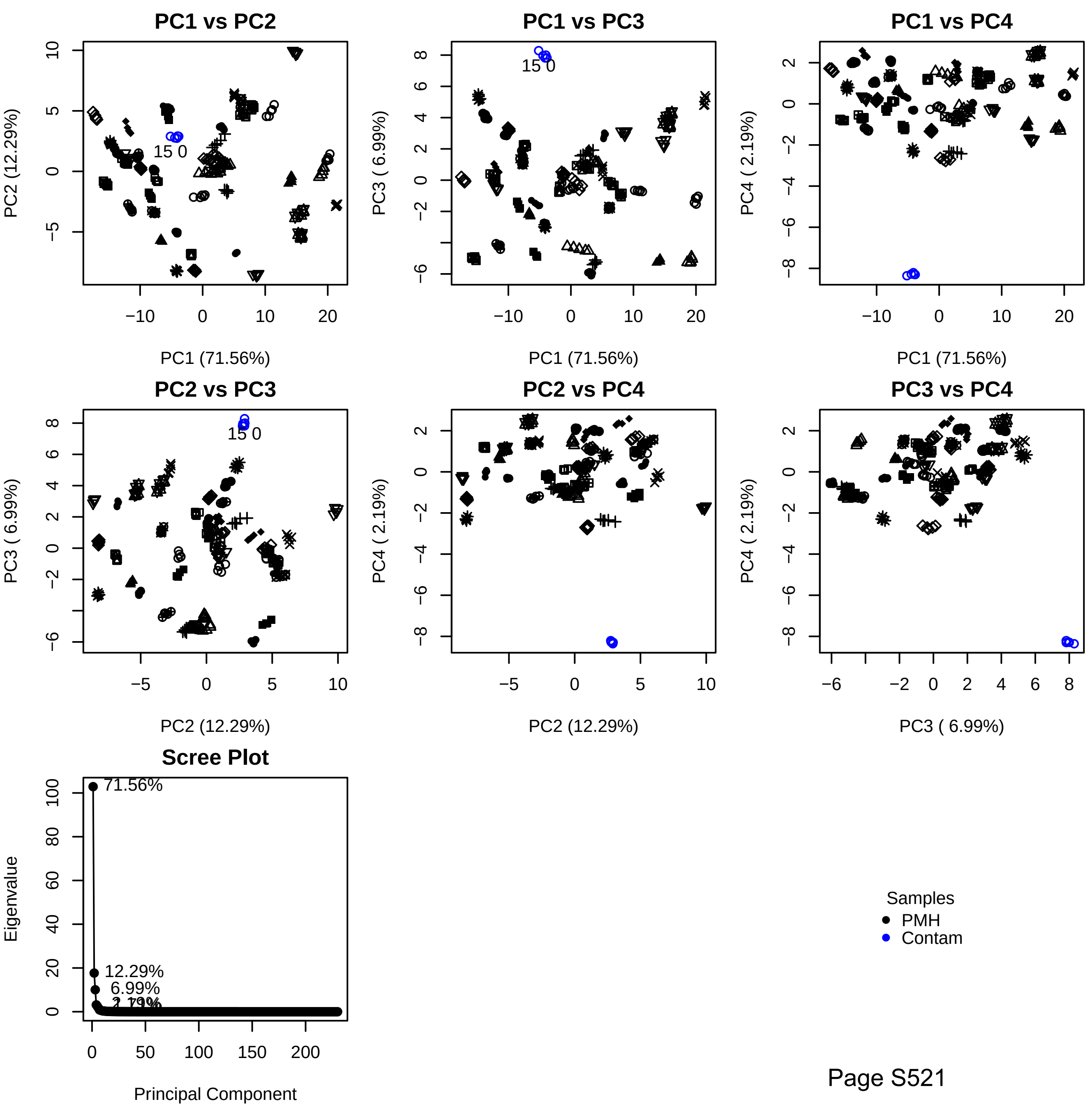
PC1 vs PC2
PC1 vs PC3
PC1 vs PC4
PC2 vs PC3
PC2 vs PC4
PC3 vs PC4
Scree Plot
PC1 (71.56%)
PC2 (12.29%)
PC3 (6.99%)
PC4 (2.19%)
15 0
Eigenvalue
Principal Component
71.56%
12.29%
6.99%
Samples
PMH
Contam

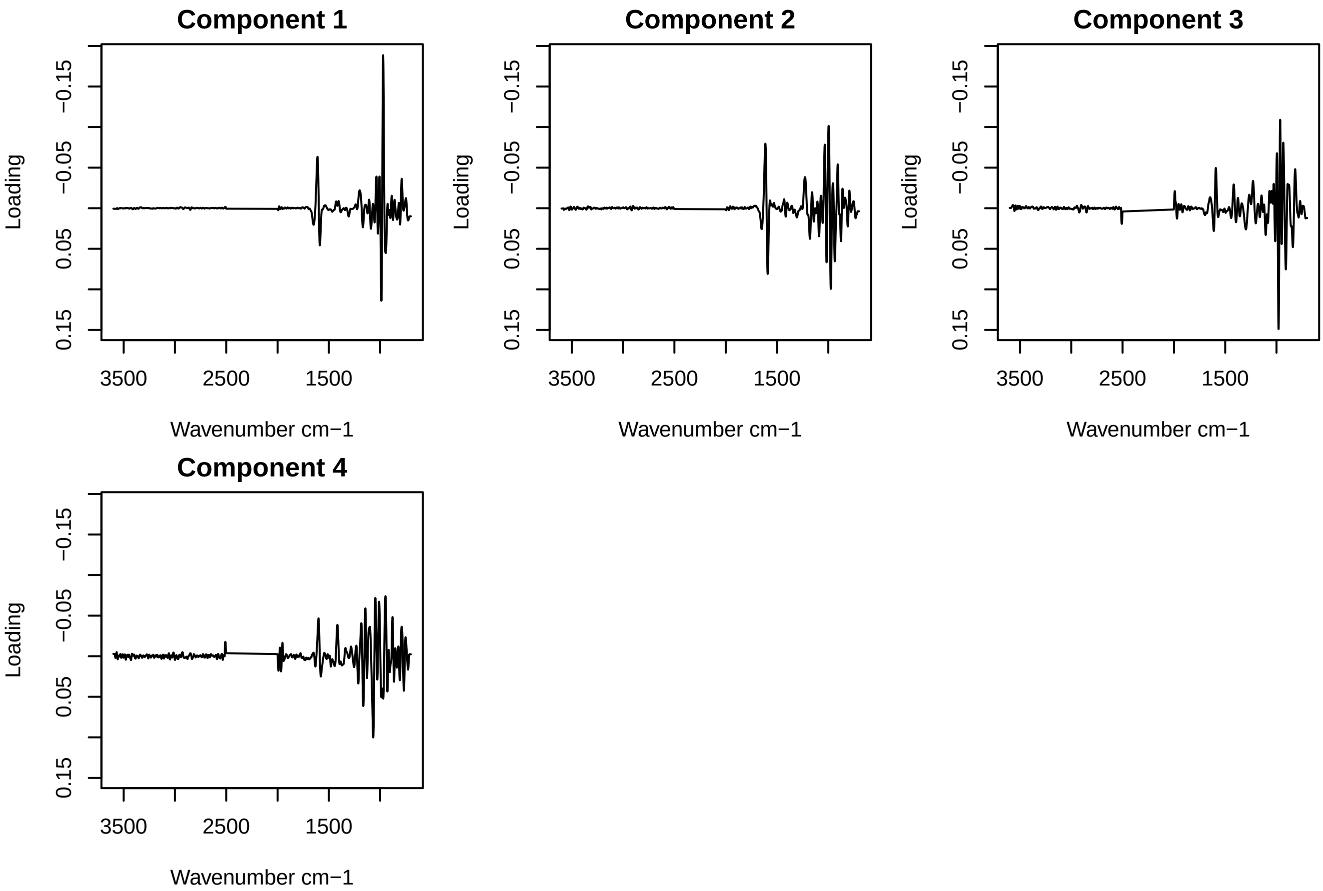
Component 1
Component 2
Component 3
Component 4
Loading
−0.15
−0.05
0.05
0.15
3500
2500
1500
Wavenumber cm−1

PC1 vs PC2 vs PC3

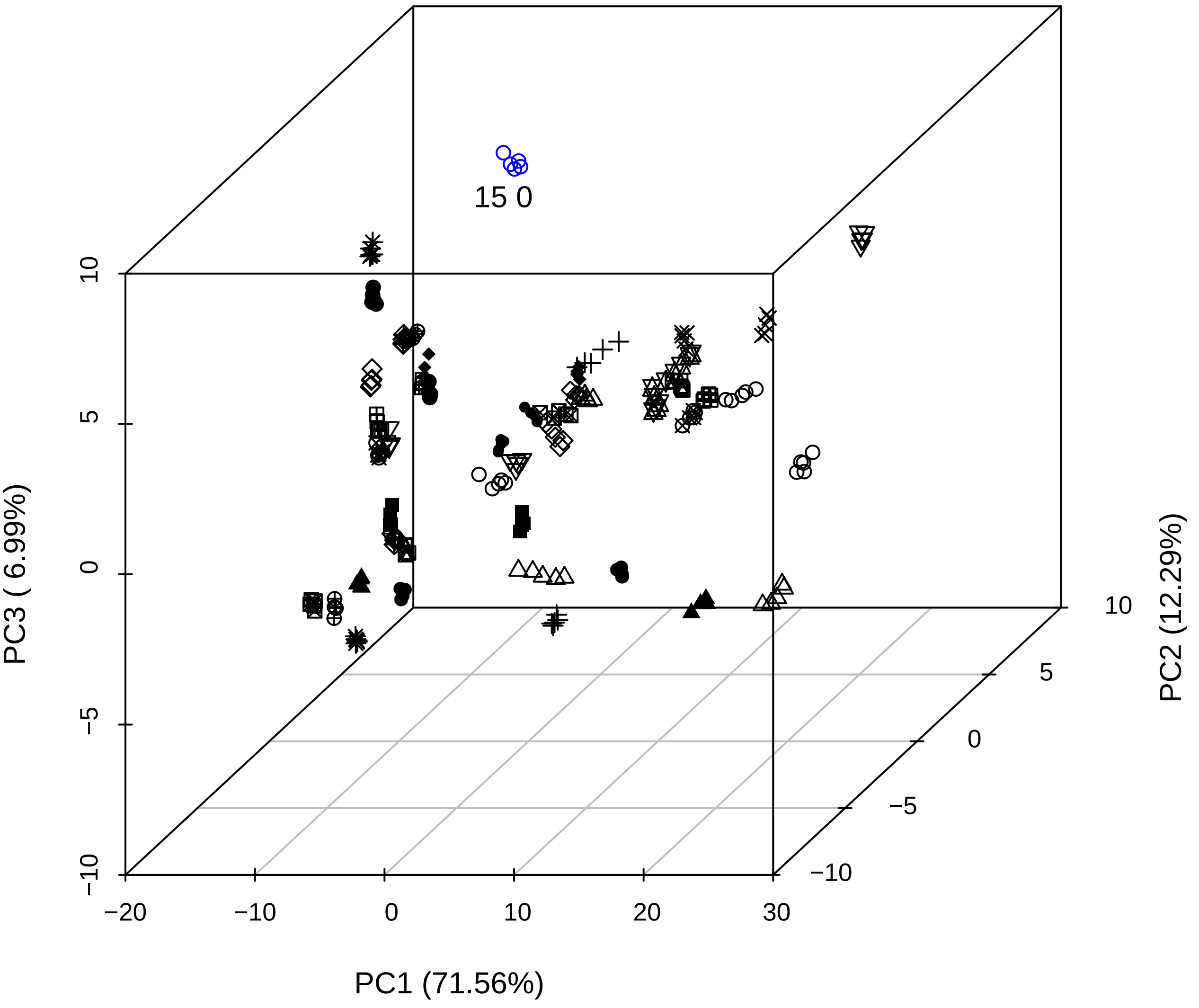

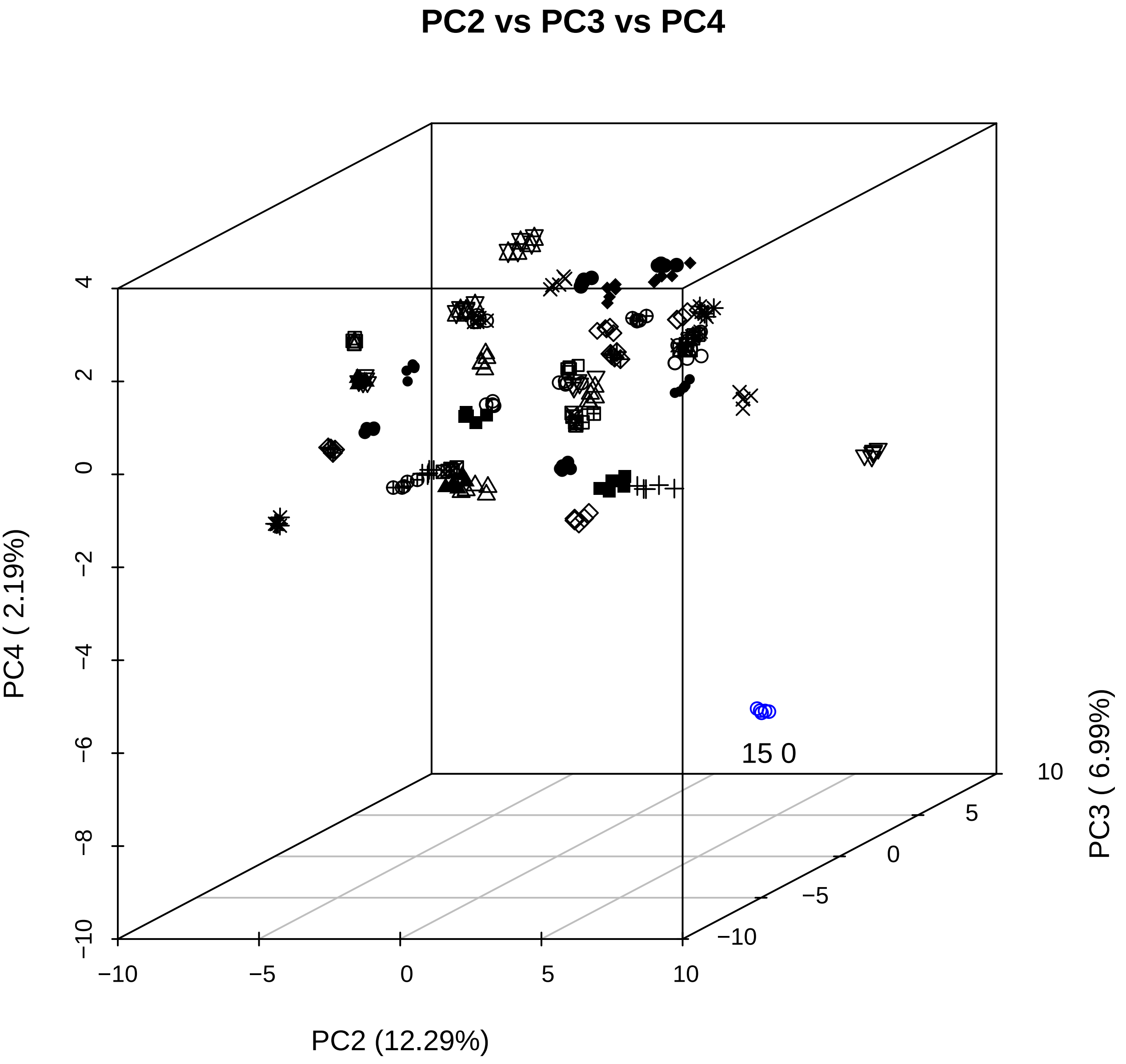
PC2 vs PC3 vs PC4
PC4 (2.19%)
4
2
0
−2
−4
−6
−8
−10
15 0
−10
−5
0
5
10
PC2 (12.29%)
10
5
0
−5
−10
PC3 (6.99%)

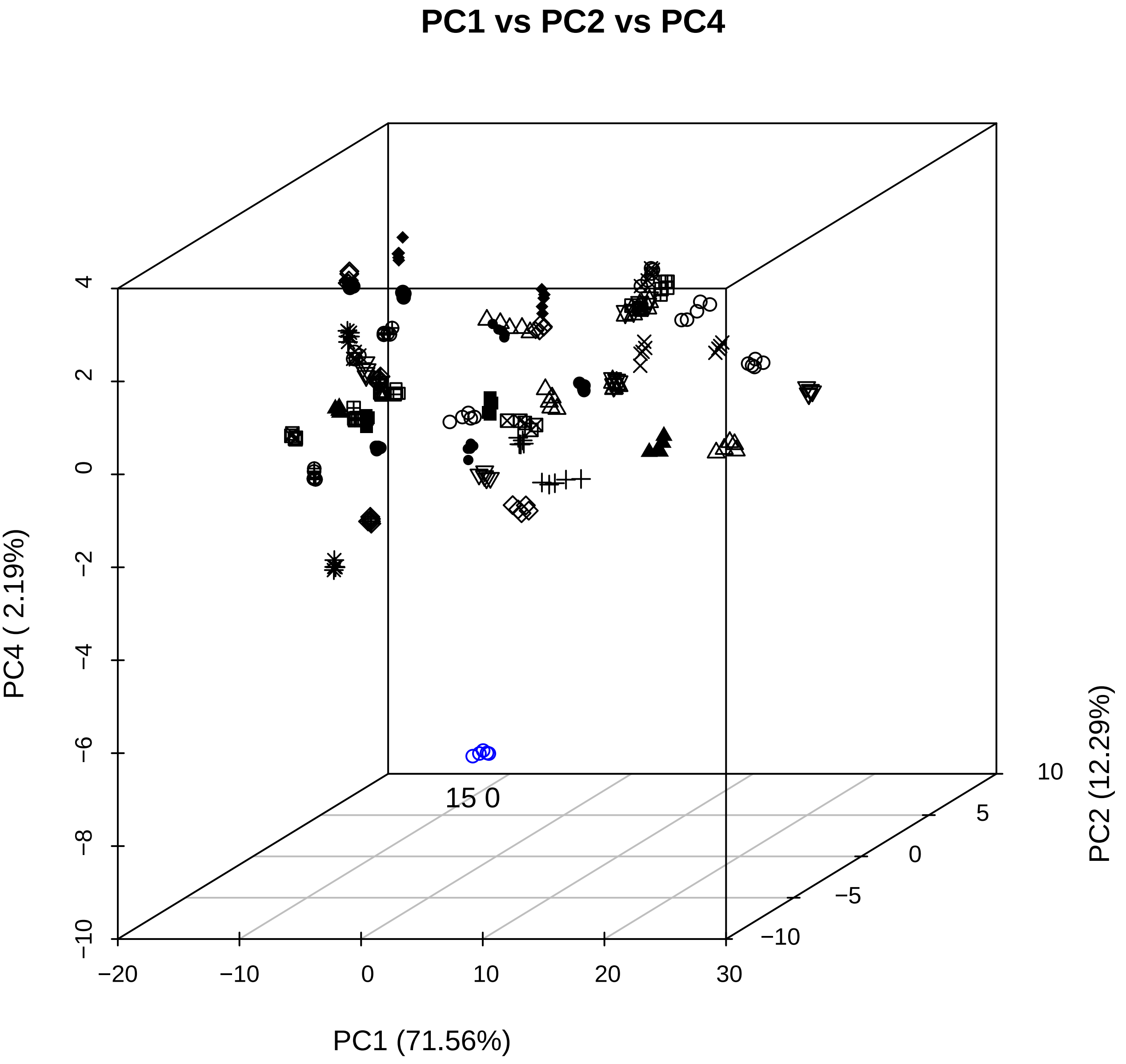
PC1 vs PC2 vs PC4
PC4 (2.19%)
4
2
0
−2
−4
−6
−8
−10
15 0
PC1 (71.56%)
−20
−10
0
10
20
30
PC2 (12.29%)
10
5
0
−5
−10

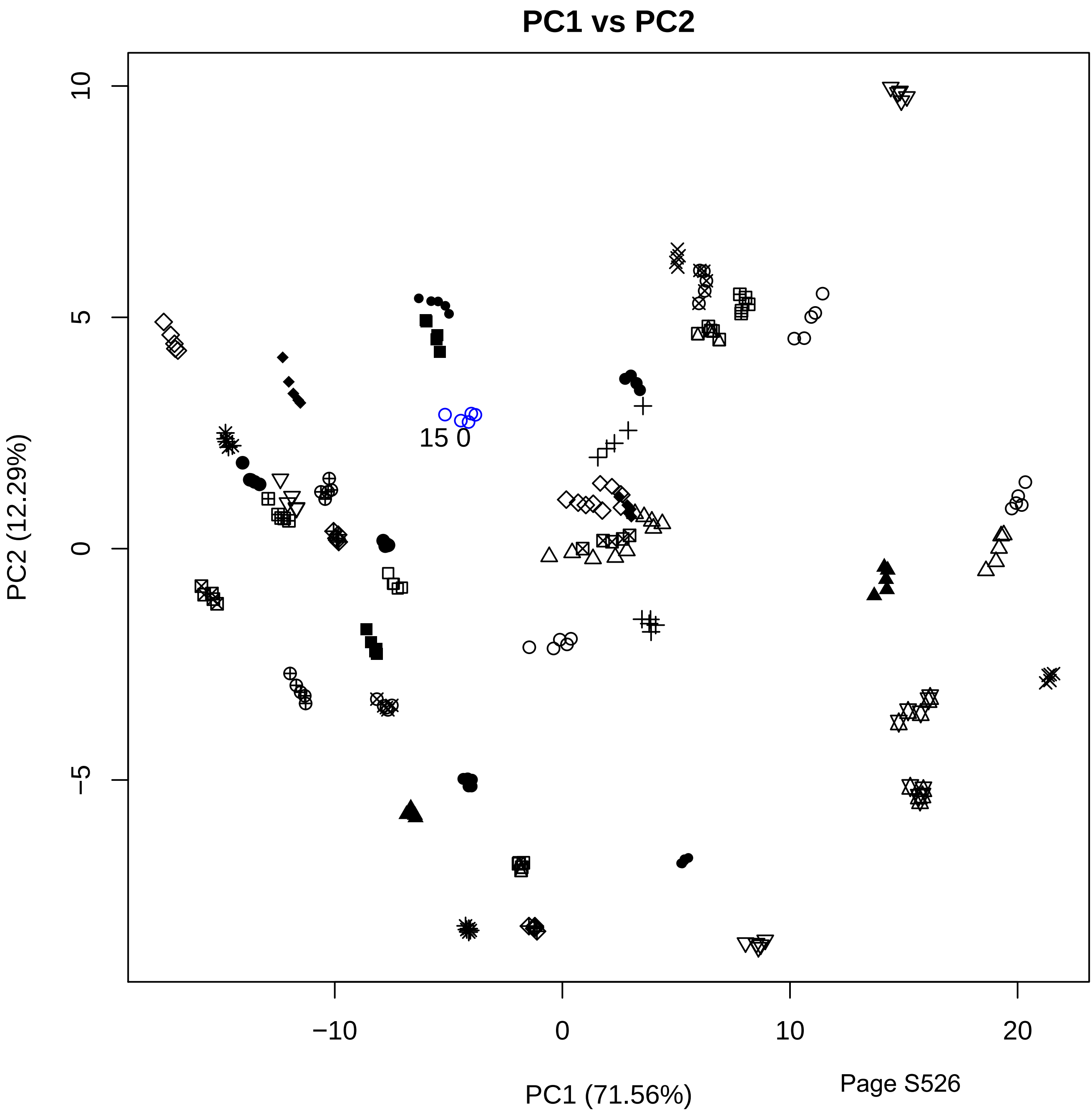

PC1 vs PC2
10
5
0
−5
PC2 (12.29%)
15 0
−10
0
10
20
PC1 (71.56%)

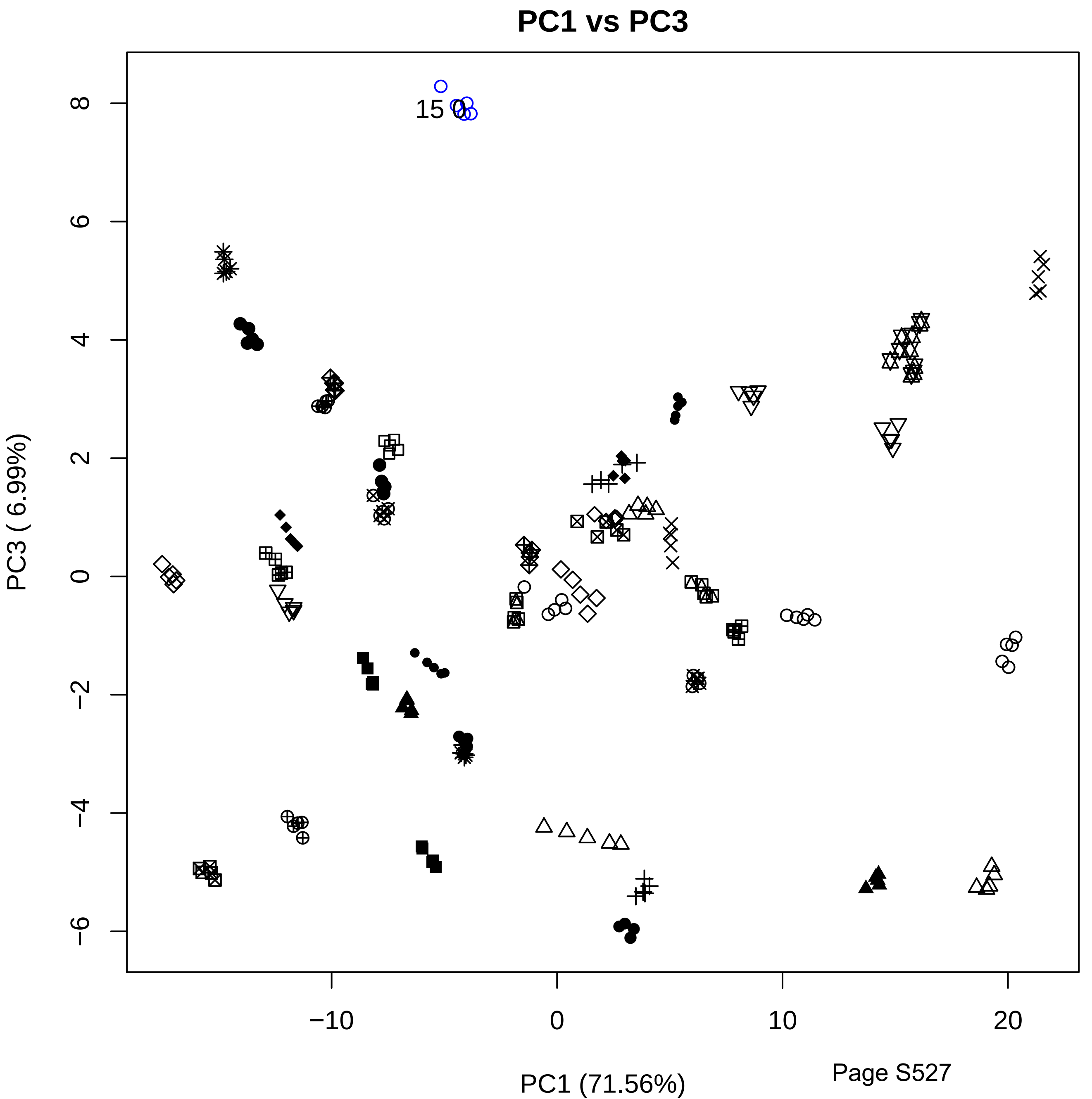

PC1 vs PC3
PC3 (6.99%)
PC1 (71.56%)
15 0
8
6
4
2
0
−2
−4
−6
−10
0
10
20

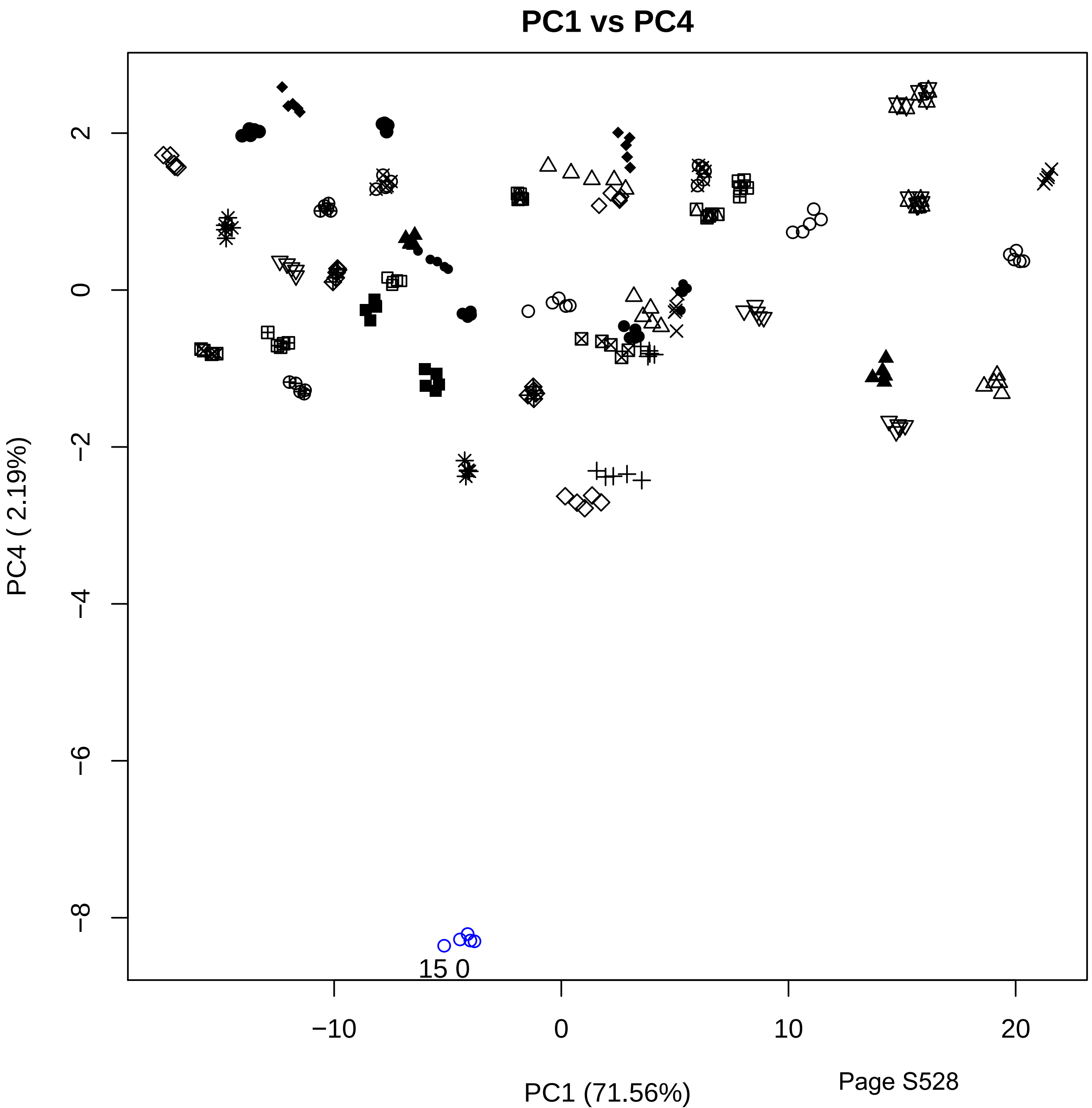

PC1 vs PC4
PC4 (2.19%)
PC1 (71.56%)
2
0
−2
−4
−6
−8
−10
0
10
20
15 0

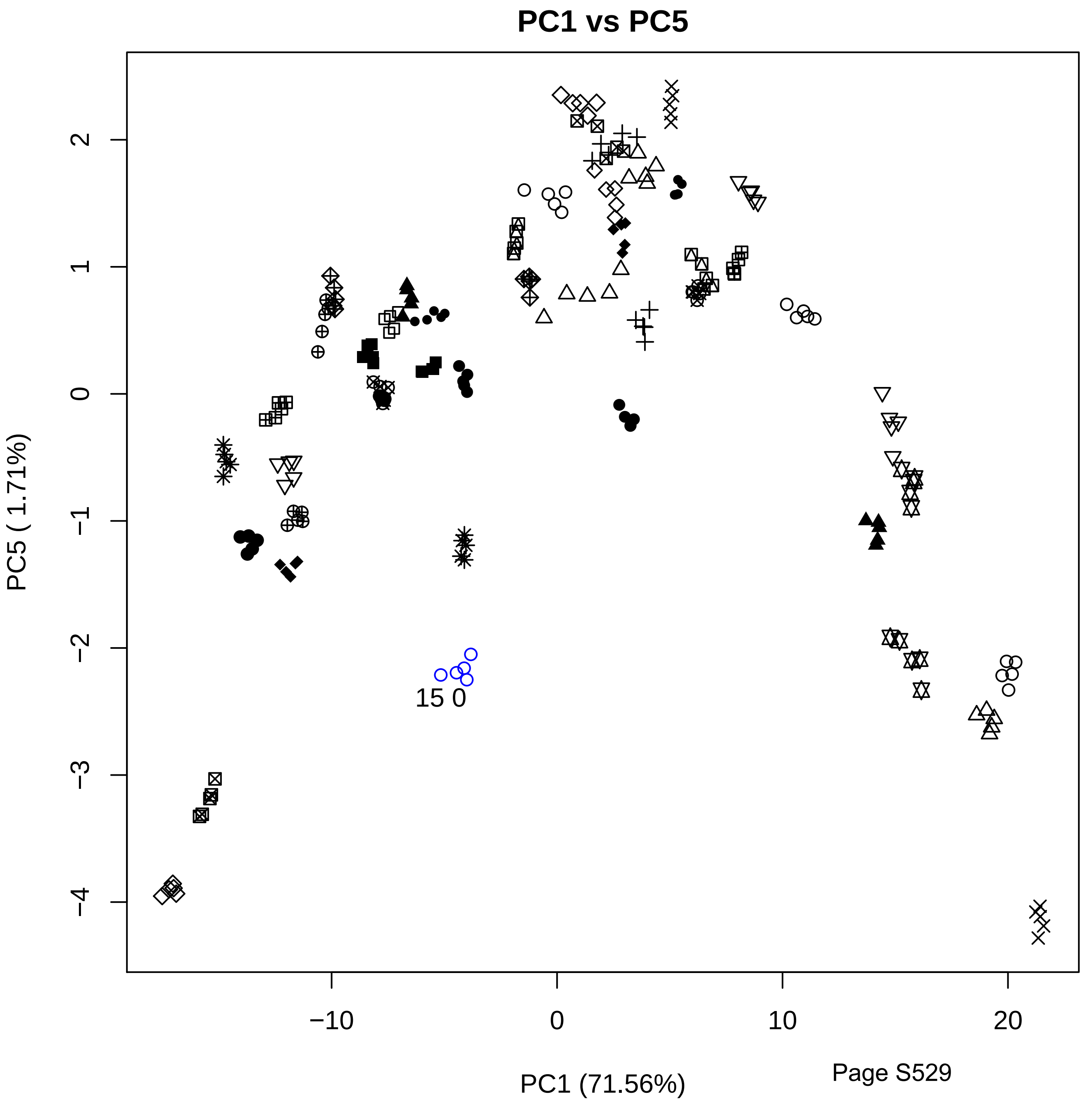
PC1 vs PC5
PC5 (1.71%)
PC1 (71.56%)
15 0
−4
−3
−2
−1
0
1
2
−10
0
10
20

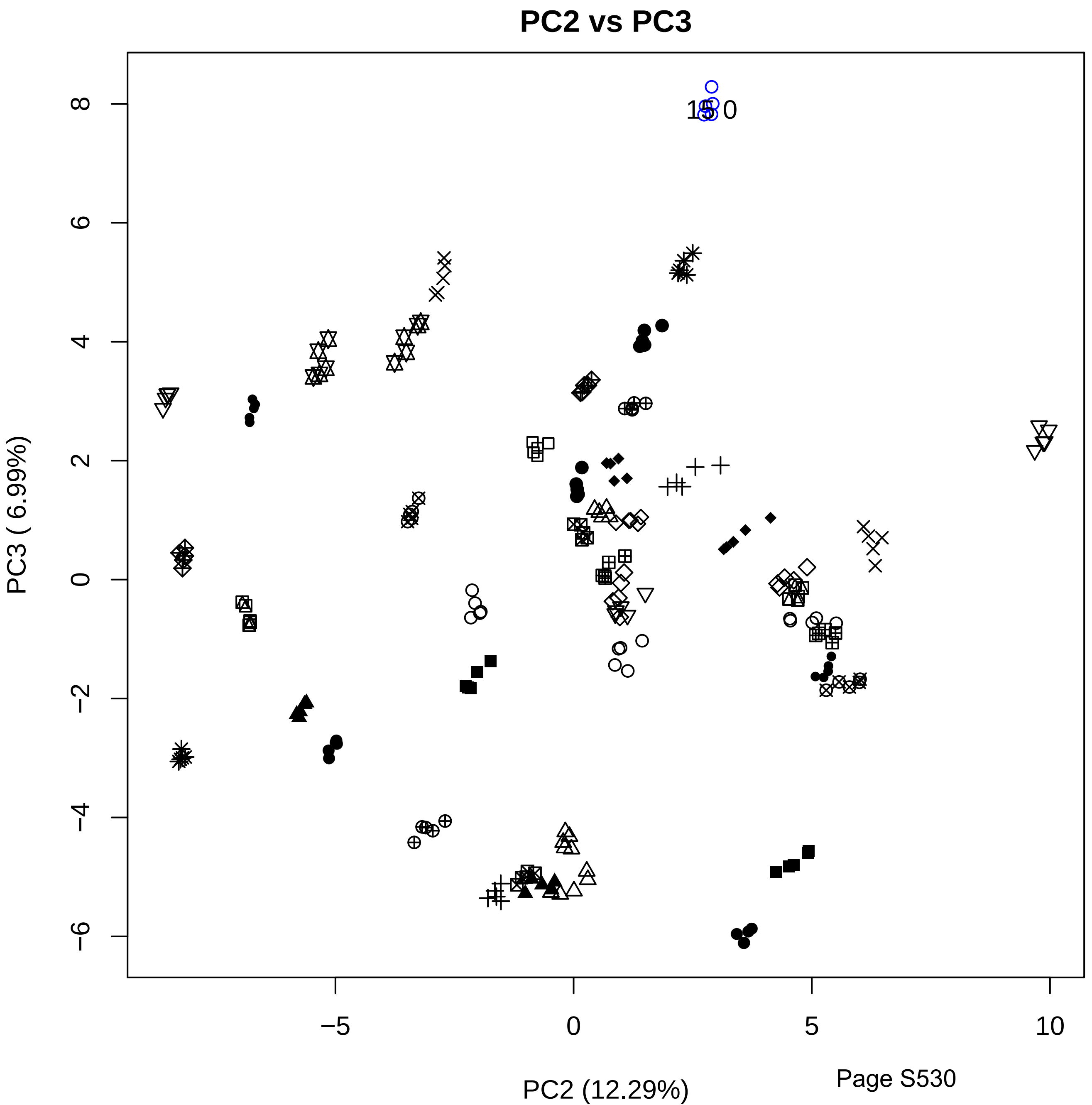

PC2 vs PC3
150
PC3 (6.99%)
PC2 (12.29%)
8
6
4
2
0
−2
−4
−6
−5
0
5
10

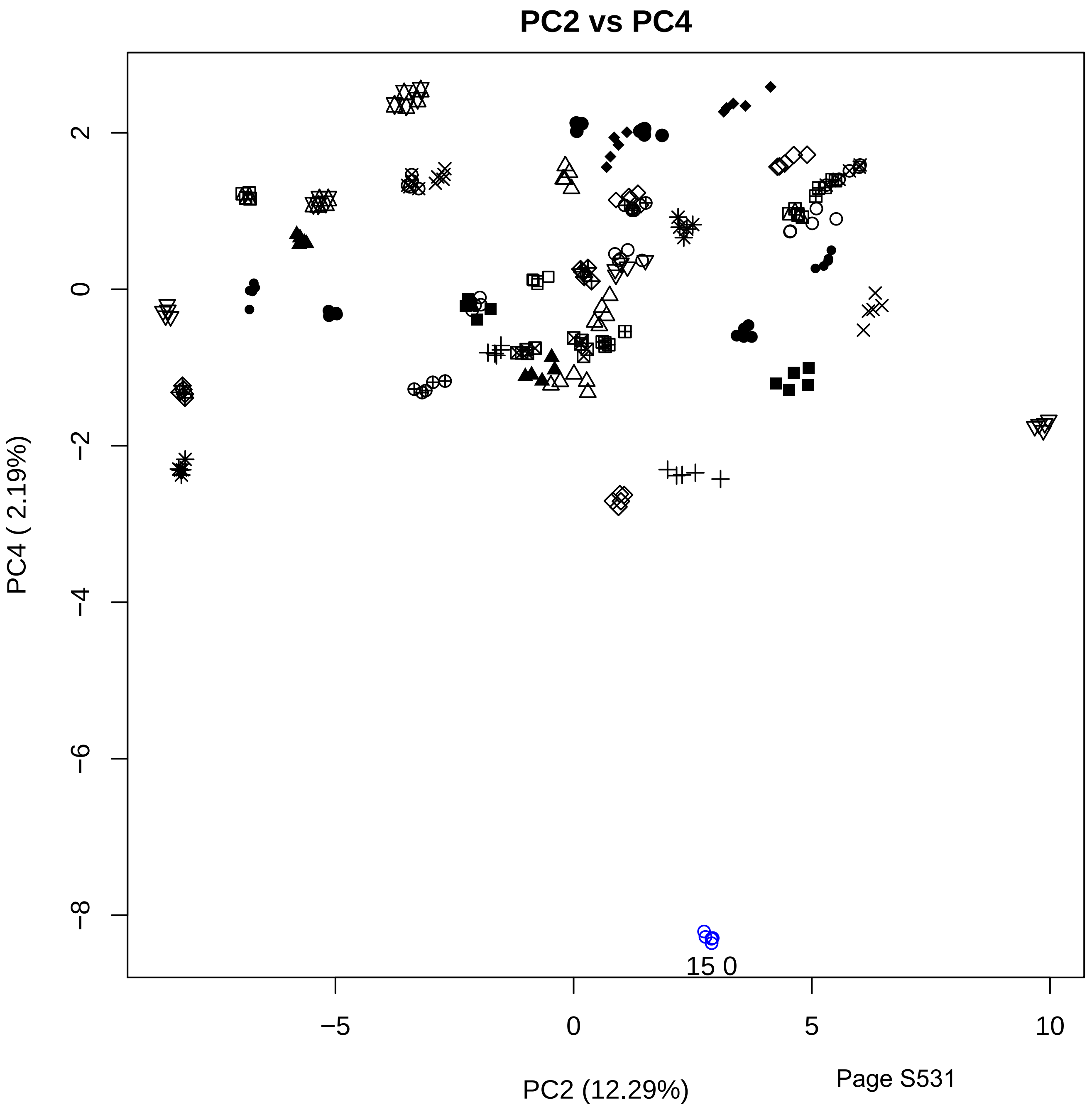
PC2 vs PC4
PC4 (2.19%)
PC2 (12.29%)
2
0
−2
−4
−6
−8
−5
0
5
10
15 0

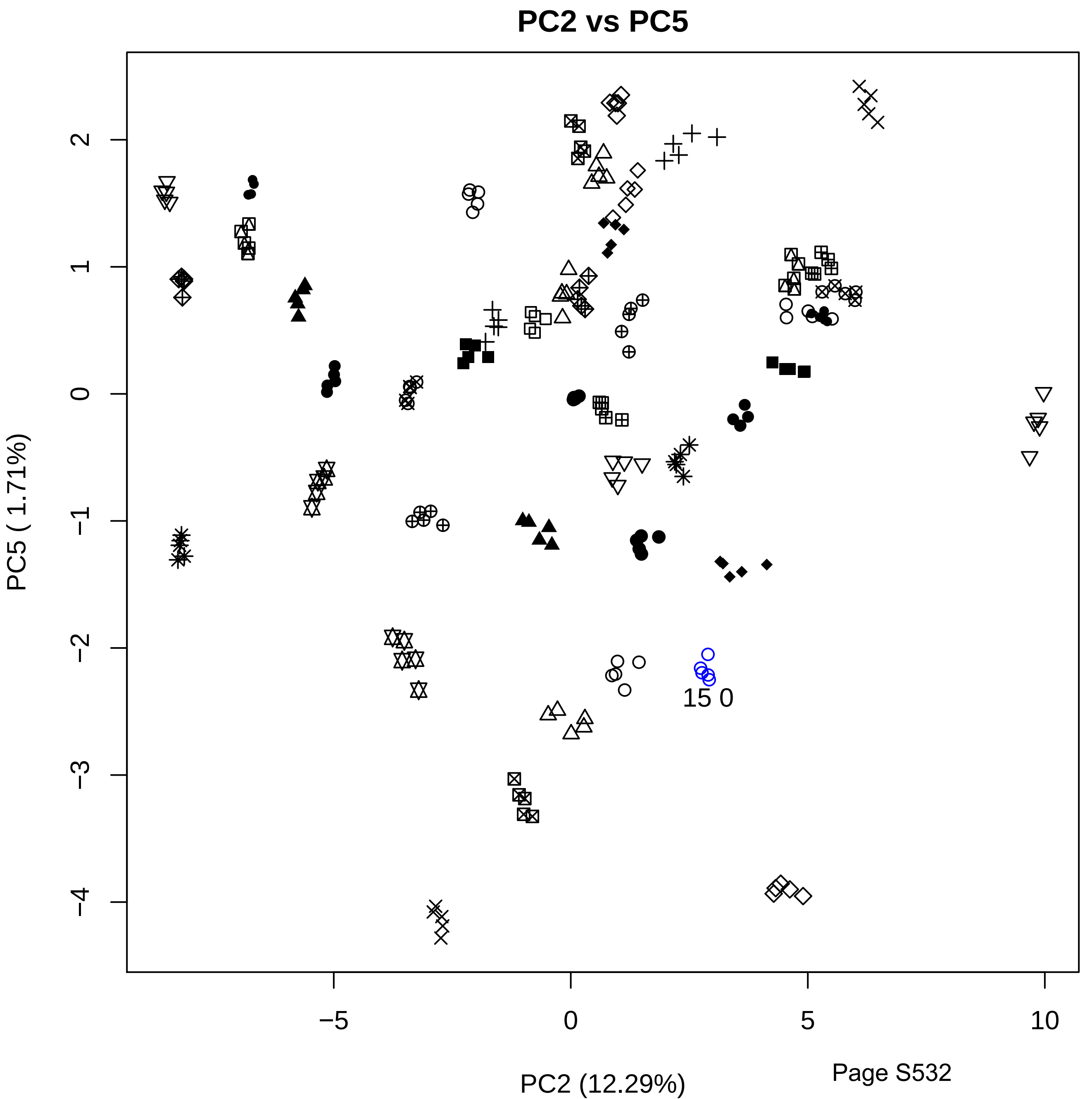
PC2 vs PC5
PC5 (1.71%)
PC2 (12.29%)
15 0
−5
0
5
10
2
1
0
−1
−2
−3
−4

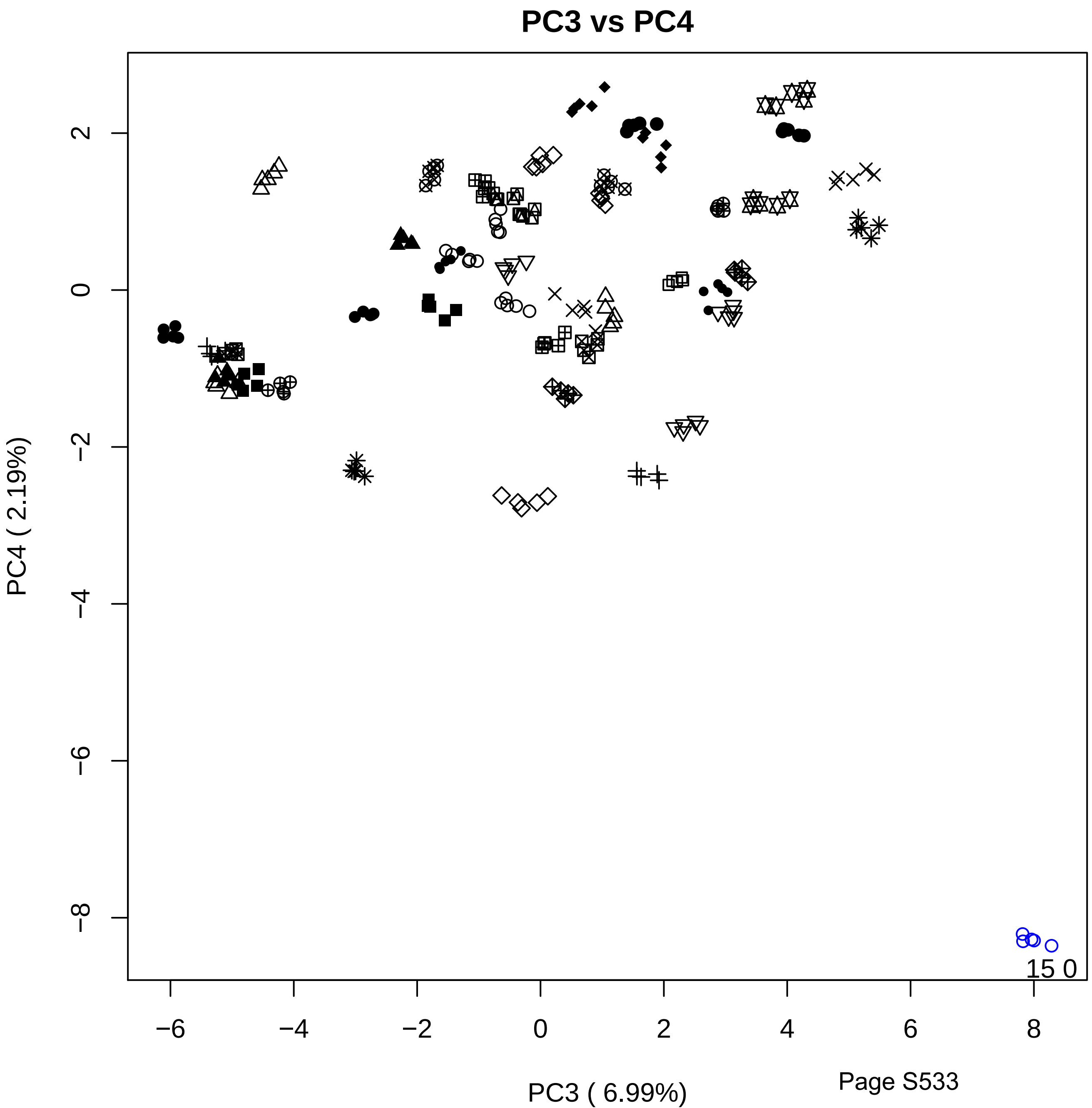
PC3 vs PC4
PC4 (2.19%)
PC3 (6.99%)
2
0
−2
−4
−6
−8
−6
−4
−2
0
2
4
6
8
15 0

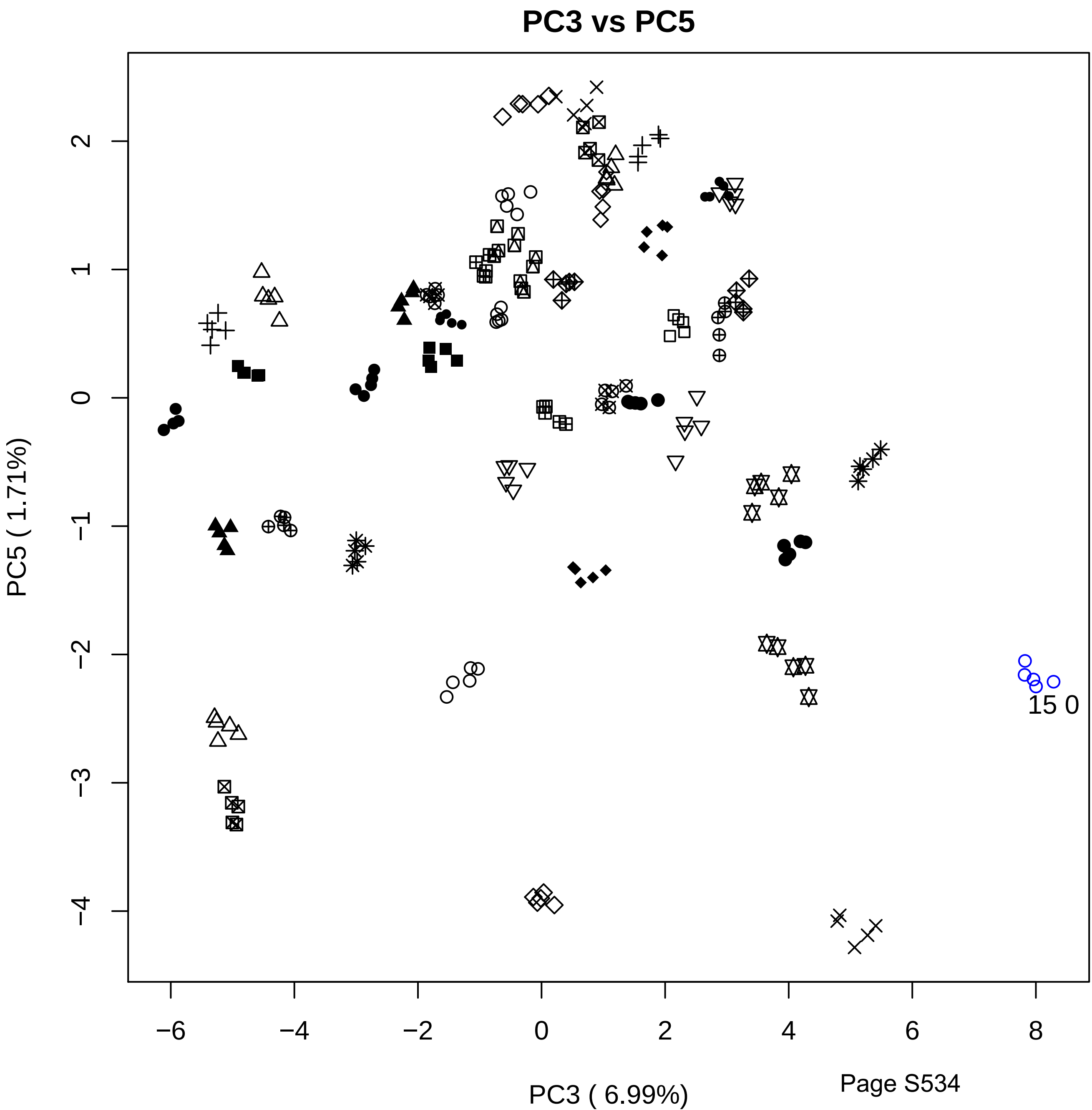
PC3 vs PC5
PC5 (1.71%)
PC3 (6.99%)
2
1
0
−1
−2
−3
−4
−6
−4
−2
0
2
4
6
8
15 0

PC4 vs PC5

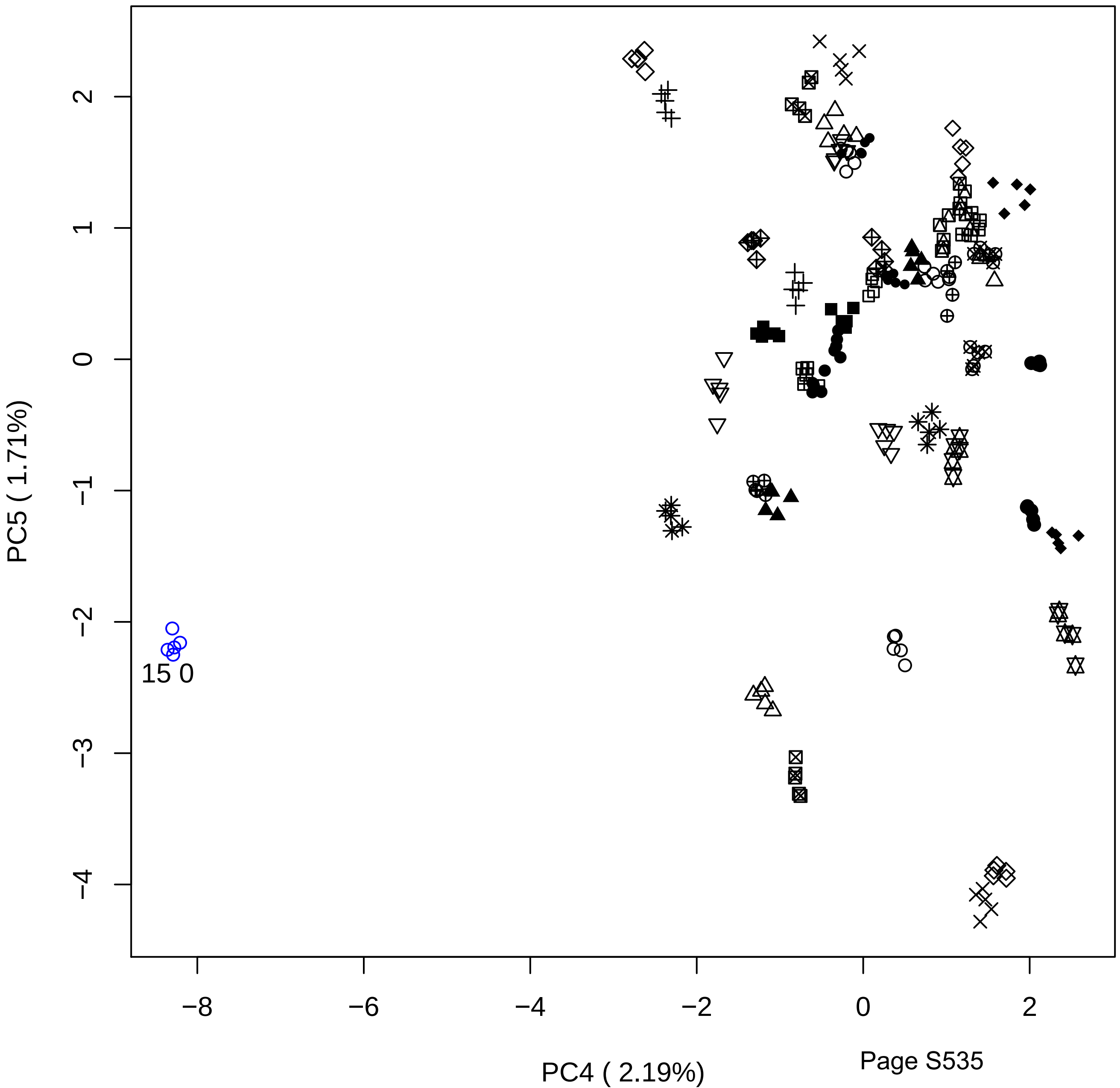

PCA of 20% OSAS vs PMH

Scaled Matrix PCAed

7th order polynomial baseline correction performed

Raw spectra smoothed to 21 Neighbours, 2nd order PN, Savtisky Golay

Normalisation between 0 and 100

D2 spectra smoothed to 41 Neighbours, 2nd order PN, Savtisky Golay

Regions Analysed: 3601−2501 and 2001−700cm−1

Samples Analysed: 1 Contam's 0 OSAS's 45 PMH's

Keele University AD　　　　Sun Dec 09 16:29:28 2018

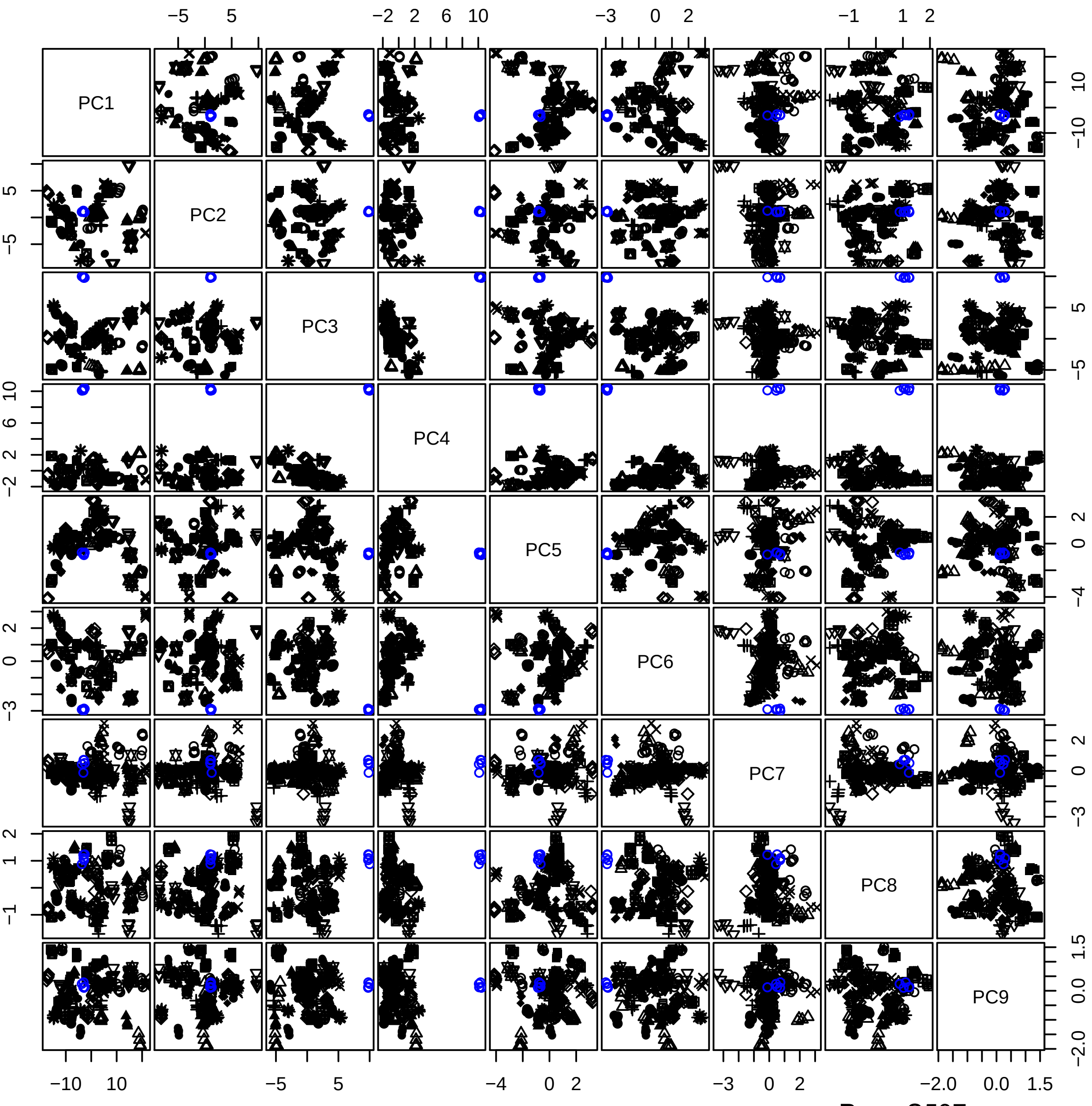
PC1
PC2
PC3
PC4
PC5
PC6
PC7
PC8
PC9

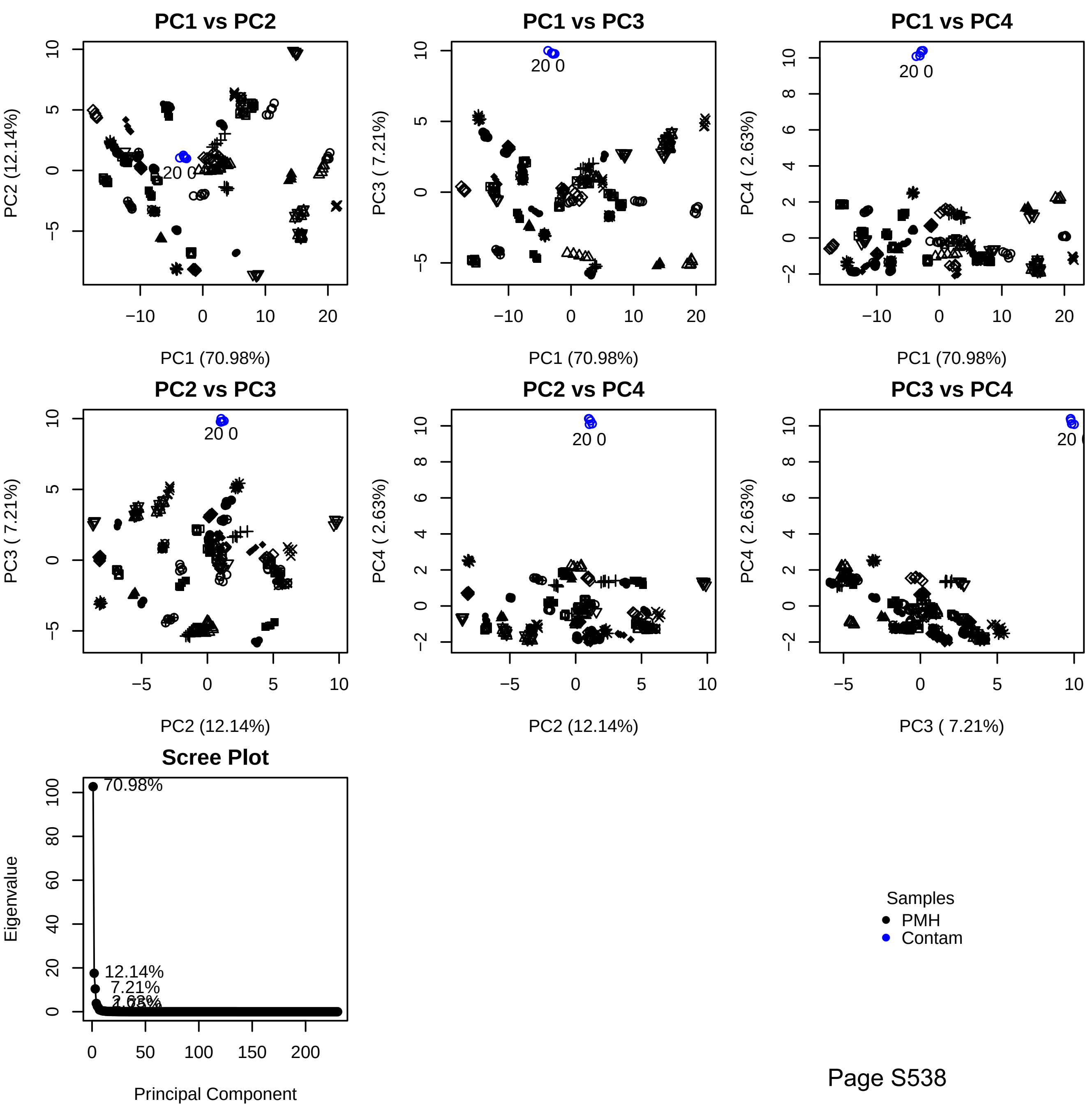

PC1 vs PC2
PC1 vs PC3
PC1 vs PC4
PC2 vs PC3
PC2 vs PC4
PC3 vs PC4
Scree Plot
PC1 (70.98%)
PC2 (12.14%)
PC3 (7.21%)
PC4 (2.63%)
Eigenvalue
Principal Component
70.98%
12.14%
7.21%
20 0
Samples
PMH
Contam

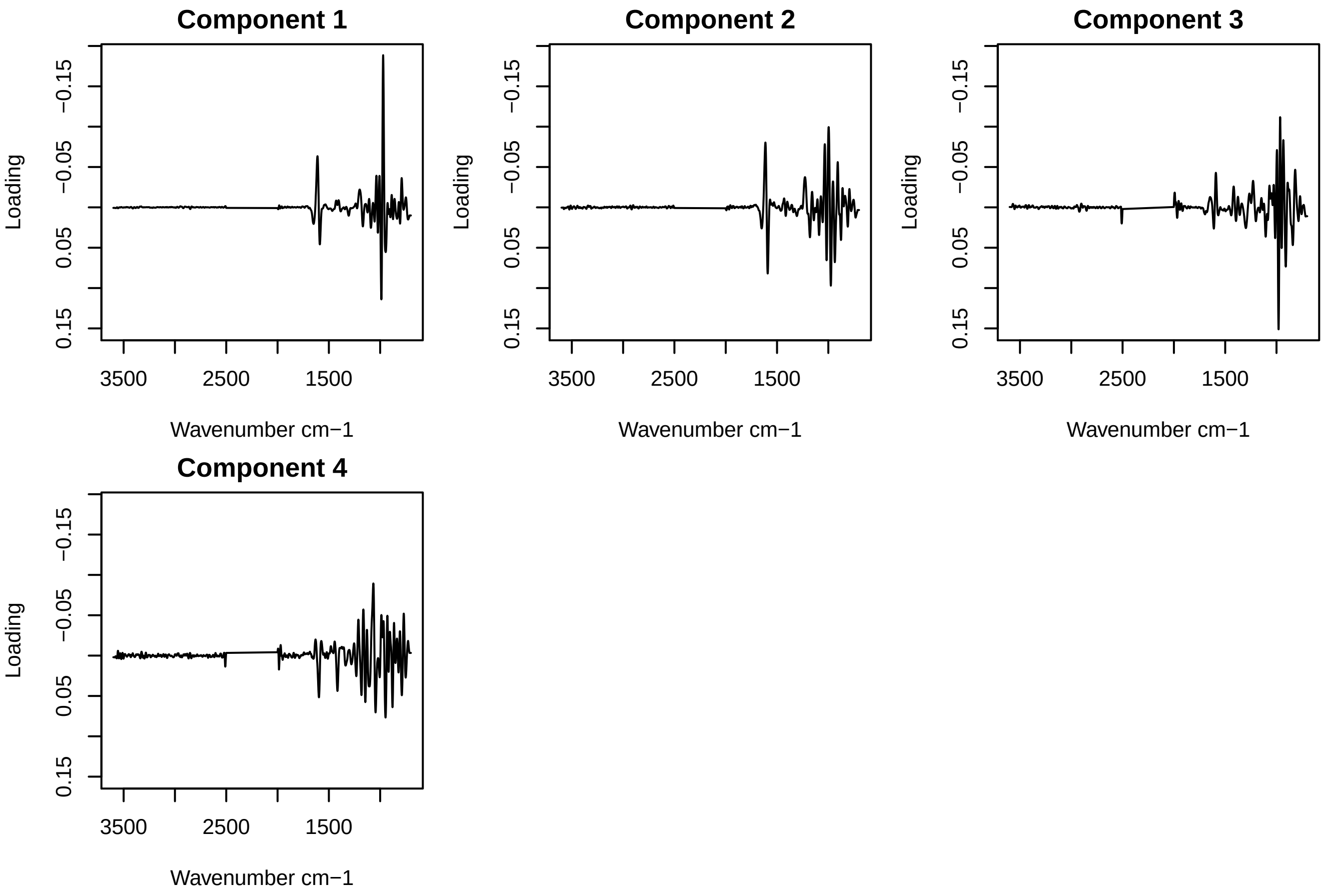
Component 1
Component 2
Component 3
Component 4
Loading
−0.15
−0.05
0.05
0.15
3500
2500
1500
Wavenumber cm−1

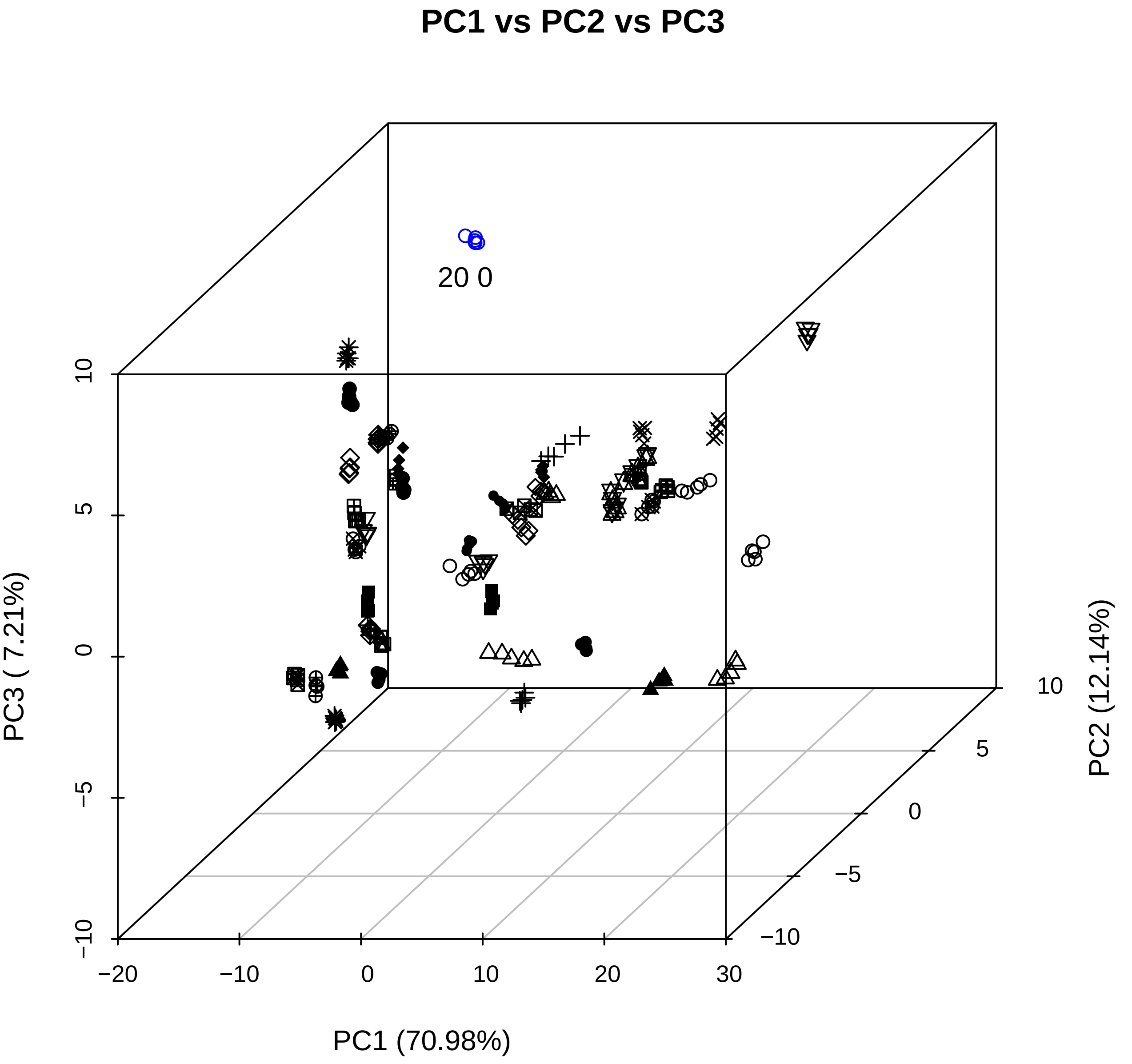
PC1 vs PC2 vs PC3
20 0
PC3 (7.21%)
PC2 (12.14%)
PC1 (70.98%)
10
5
0
−5
−10
−20
−10
0
10
20
30

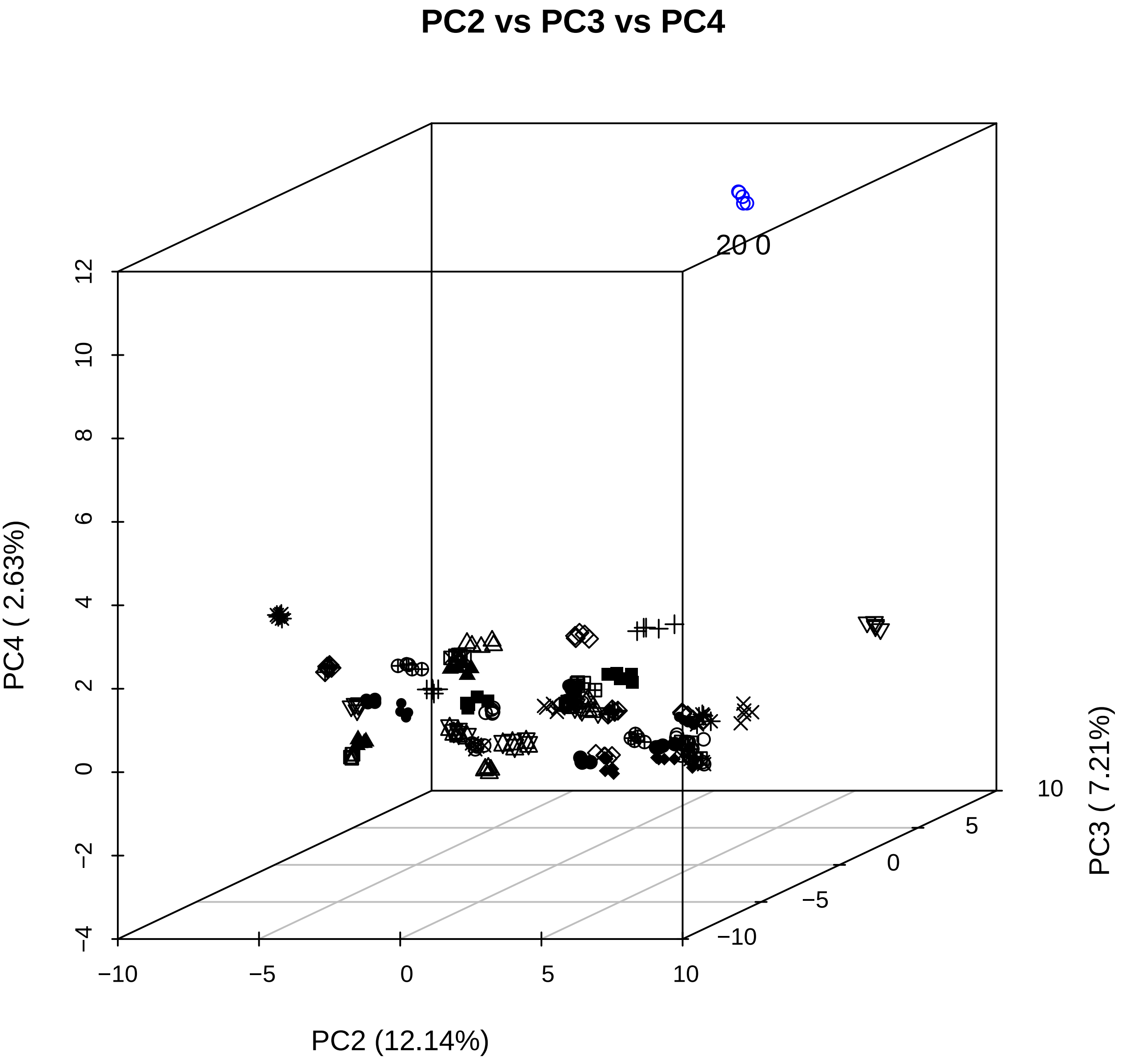
PC2 vs PC3 vs PC4
PC4 (2.63%)
PC2 (12.14%)
PC3 (7.21%)
12
10
8
6
4
2
0
−2
−4
−10
−5
0
5
10
10
5
0
−5
−10
20 0

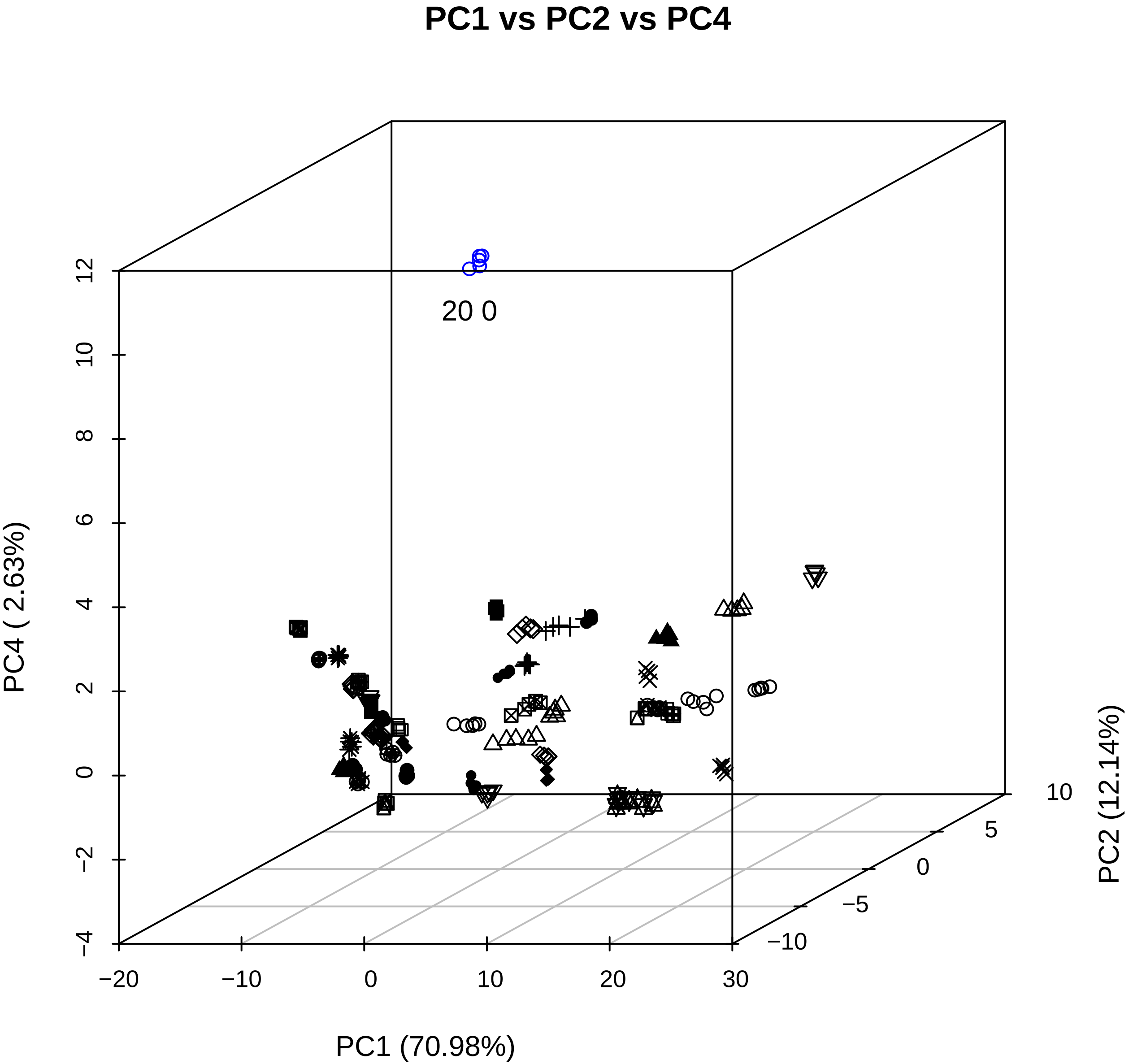
PC1 vs PC2 vs PC4
20 0
PC4 (2.63%)
PC1 (70.98%)
PC2 (12.14%)
12
10
8
6
4
2
0
−2
−4
−20
−10
0
10
20
30
10
5
0
−5
−10

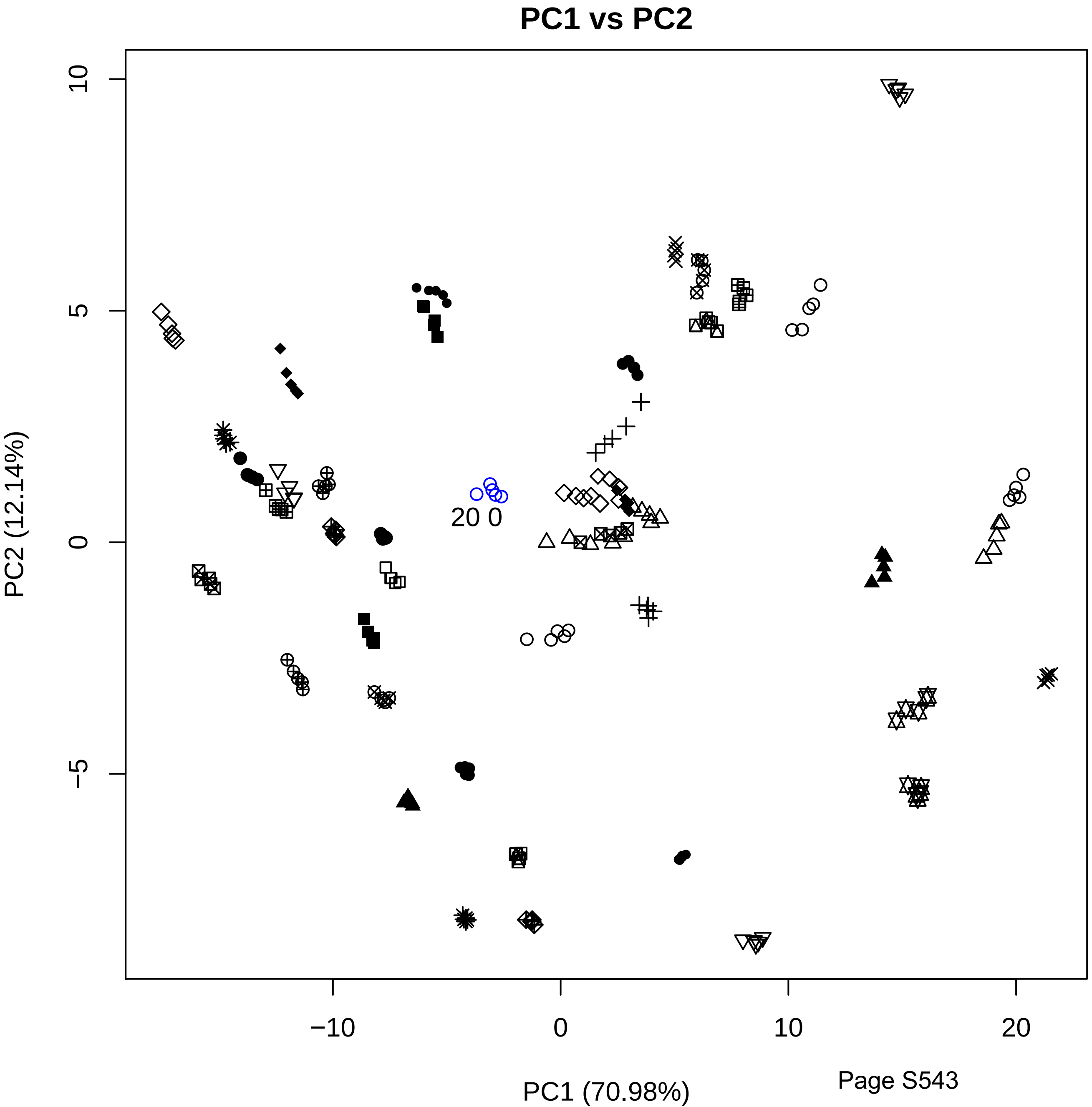
PC1 vs PC2
PC2 (12.14%)
PC1 (70.98%)
10
5
0
−5
−10
0
10
20
20 0

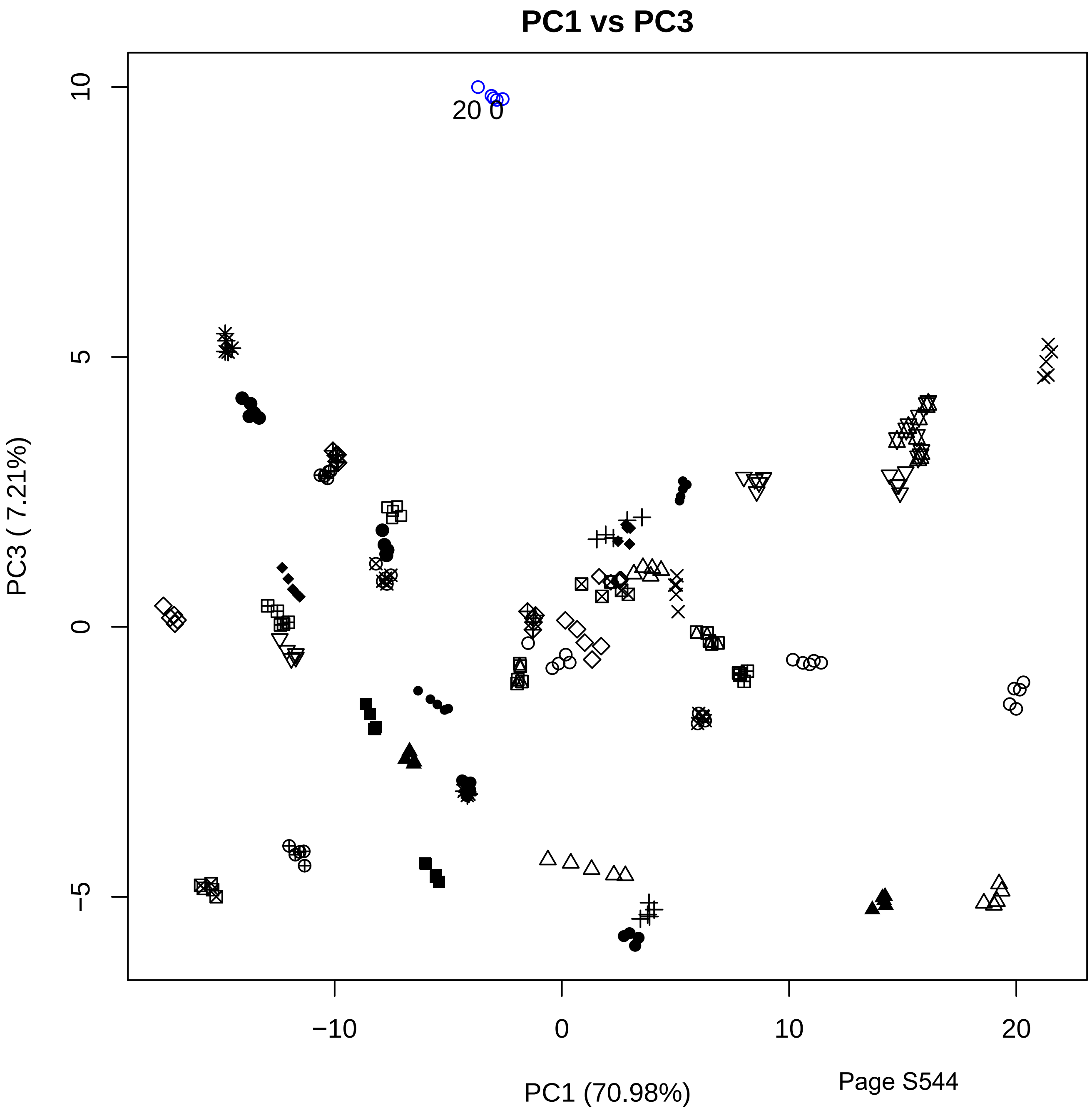
PC1 vs PC3
20 0
PC3 (7.21%)
PC1 (70.98%)
10
5
0
−5
−10
0
10
20

PC1 vs PC4

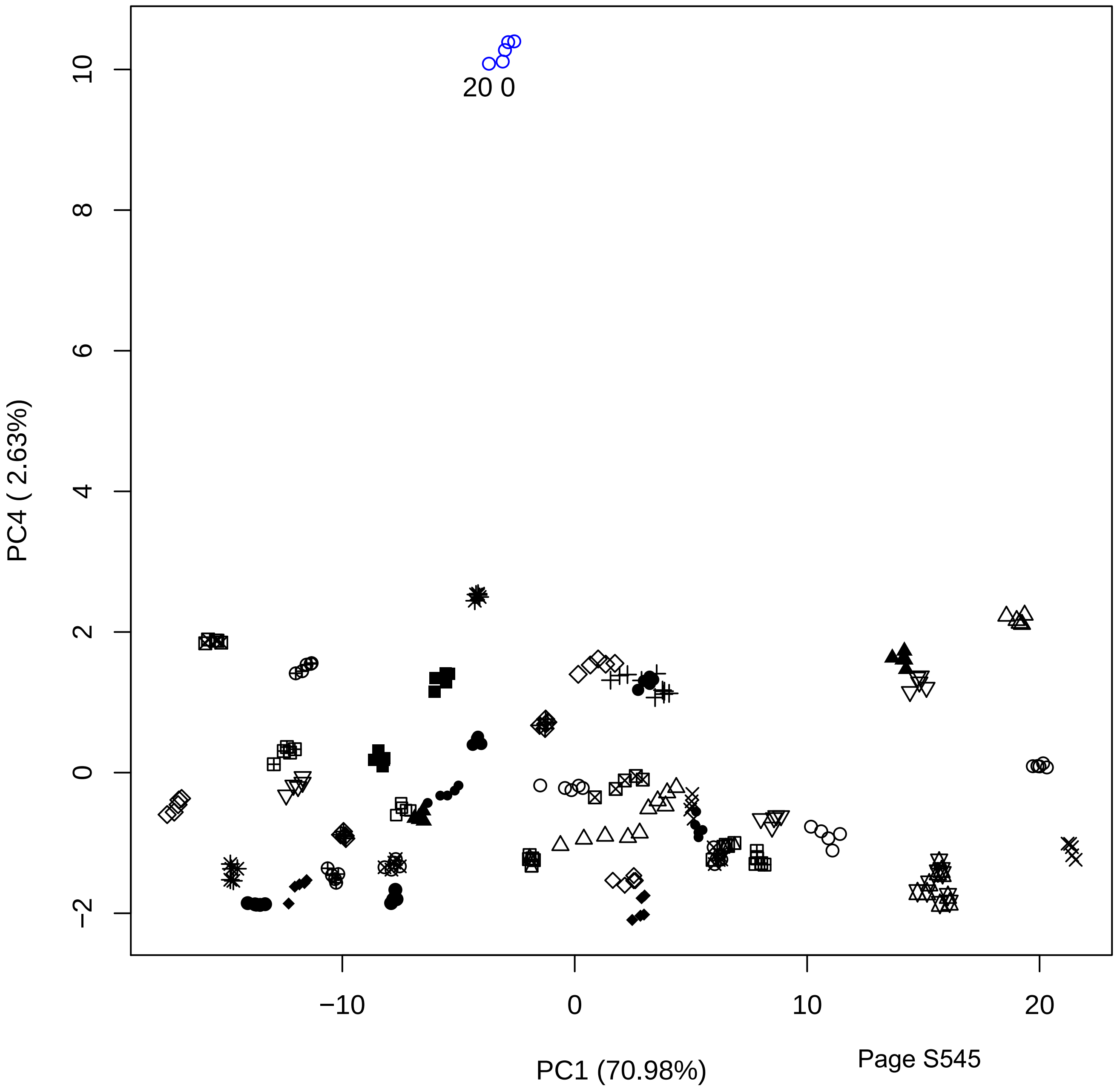

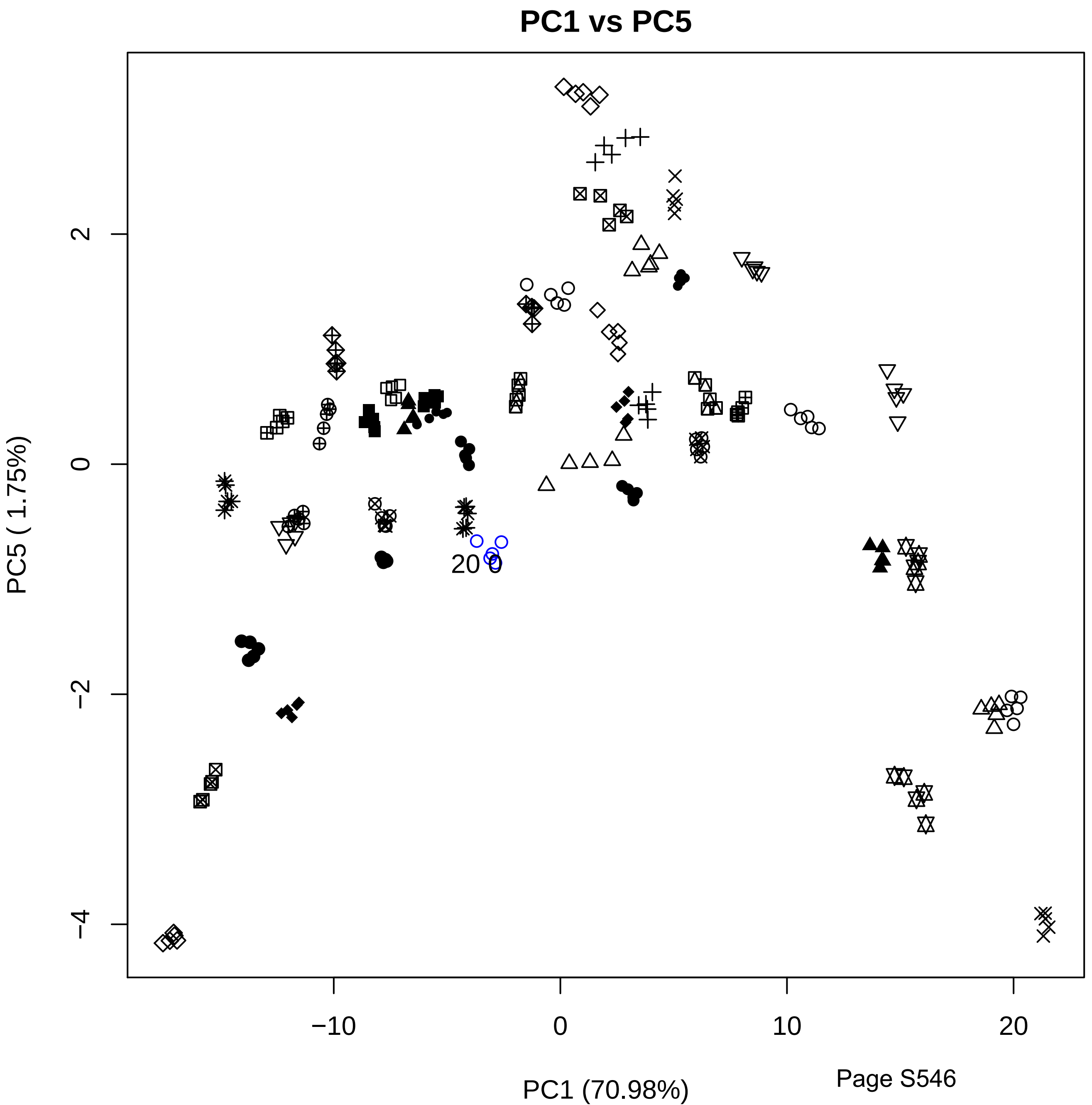

PC1 vs PC5
PC5 (1.75%)
PC1 (70.98%)
2
0
−2
−4
−10
0
10
20
20 0

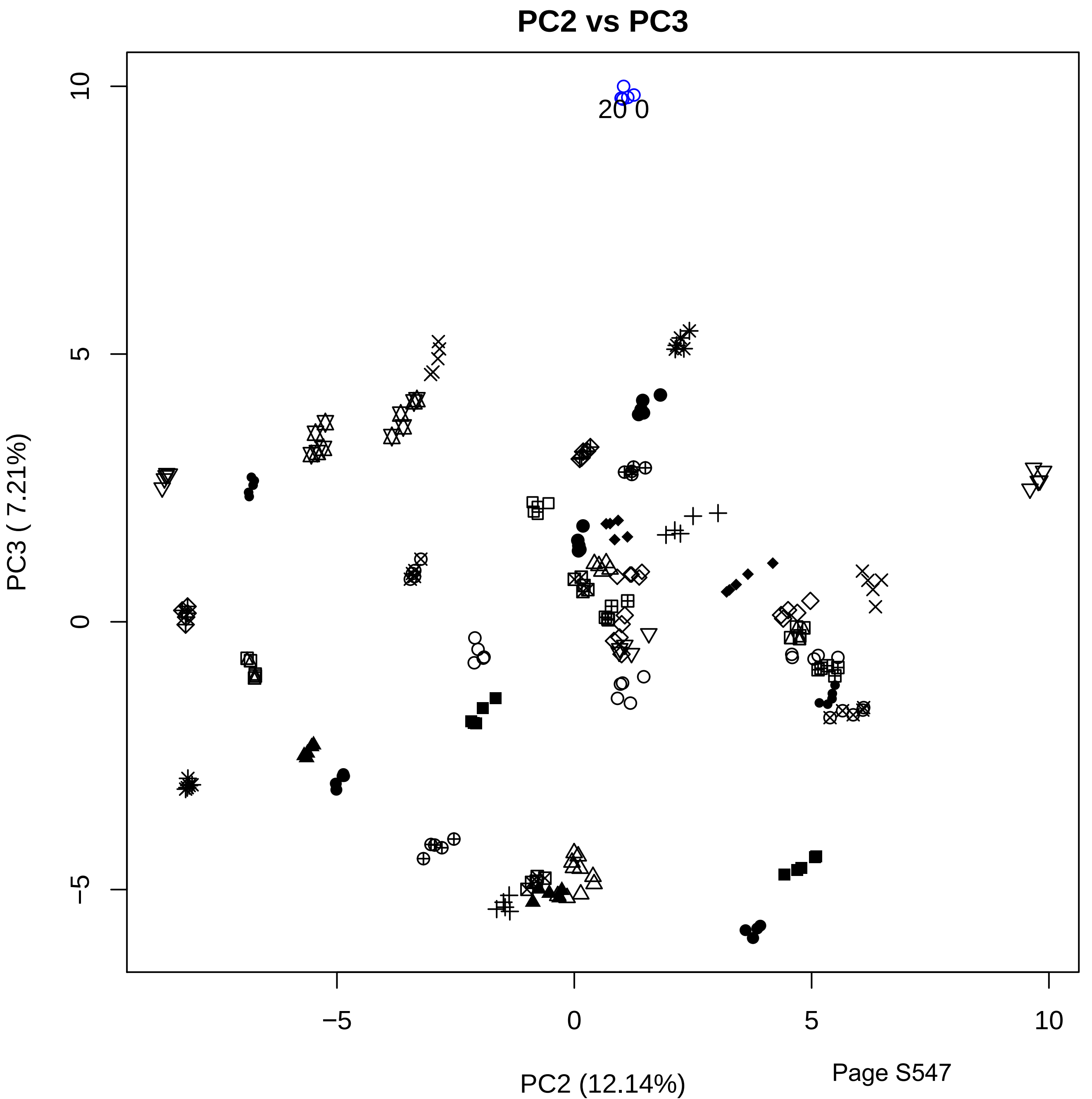
PC2 vs PC3
PC3 (7.21%)
PC2 (12.14%)
10
5
0
−5
−5
0
5
10
20 0

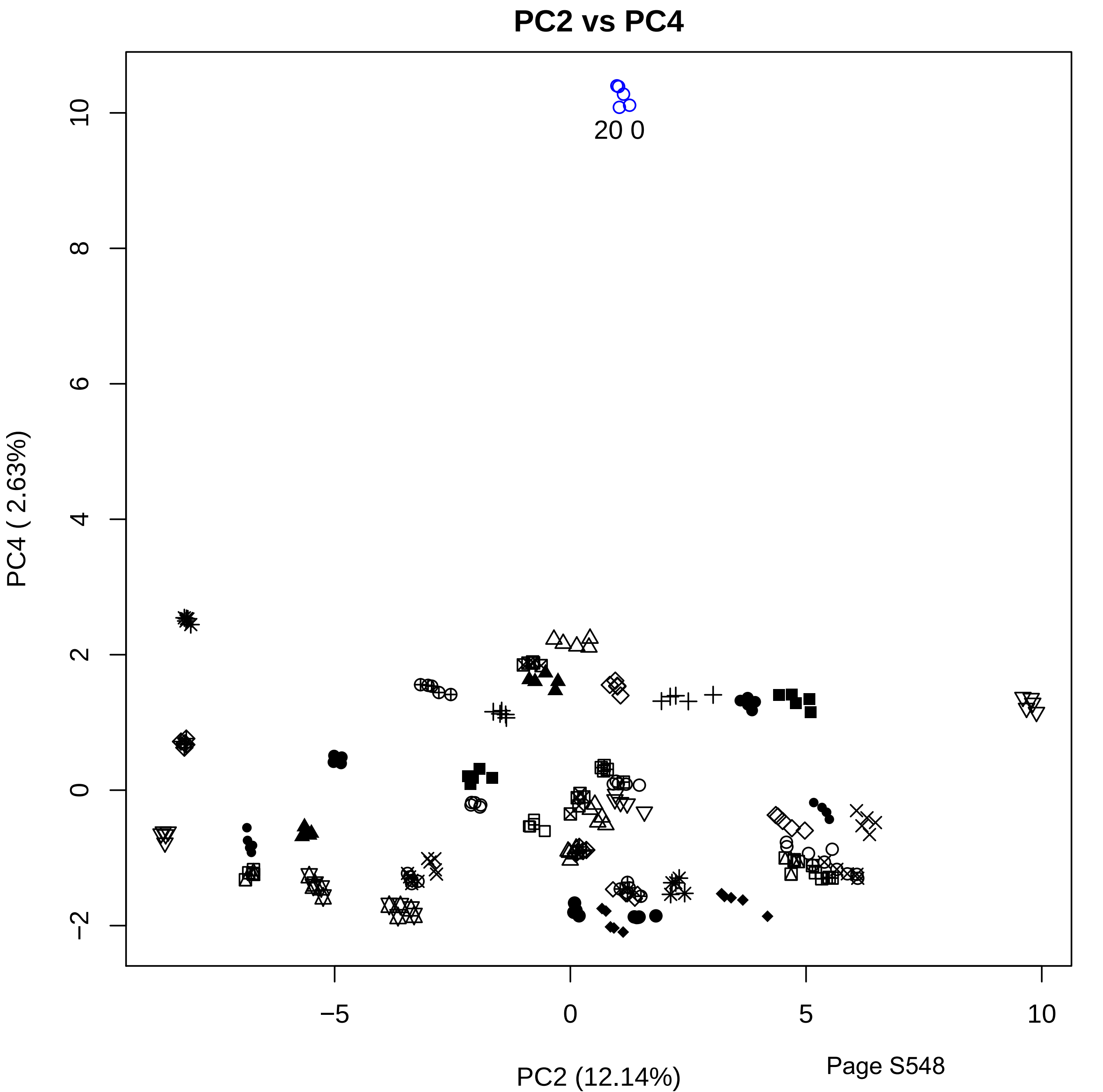
PC2 vs PC4
20 0
10
8
6
4
2
0
−2
PC4 (2.63%)
−5
0
5
10
PC2 (12.14%)

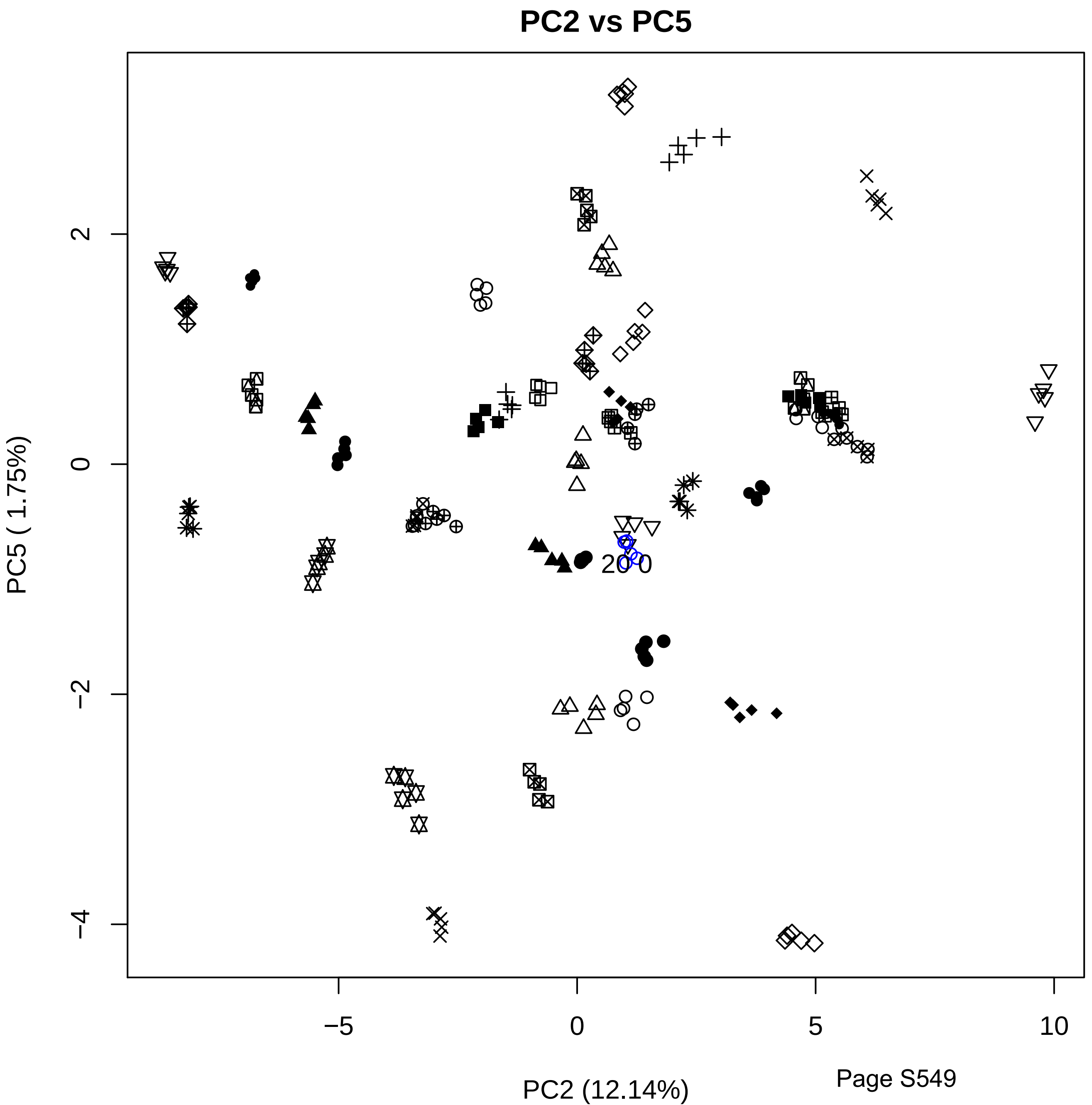

PC2 vs PC5
PC5 (1.75%)
PC2 (12.14%)
2
0
−2
−4
−5
0
5
10
20
0

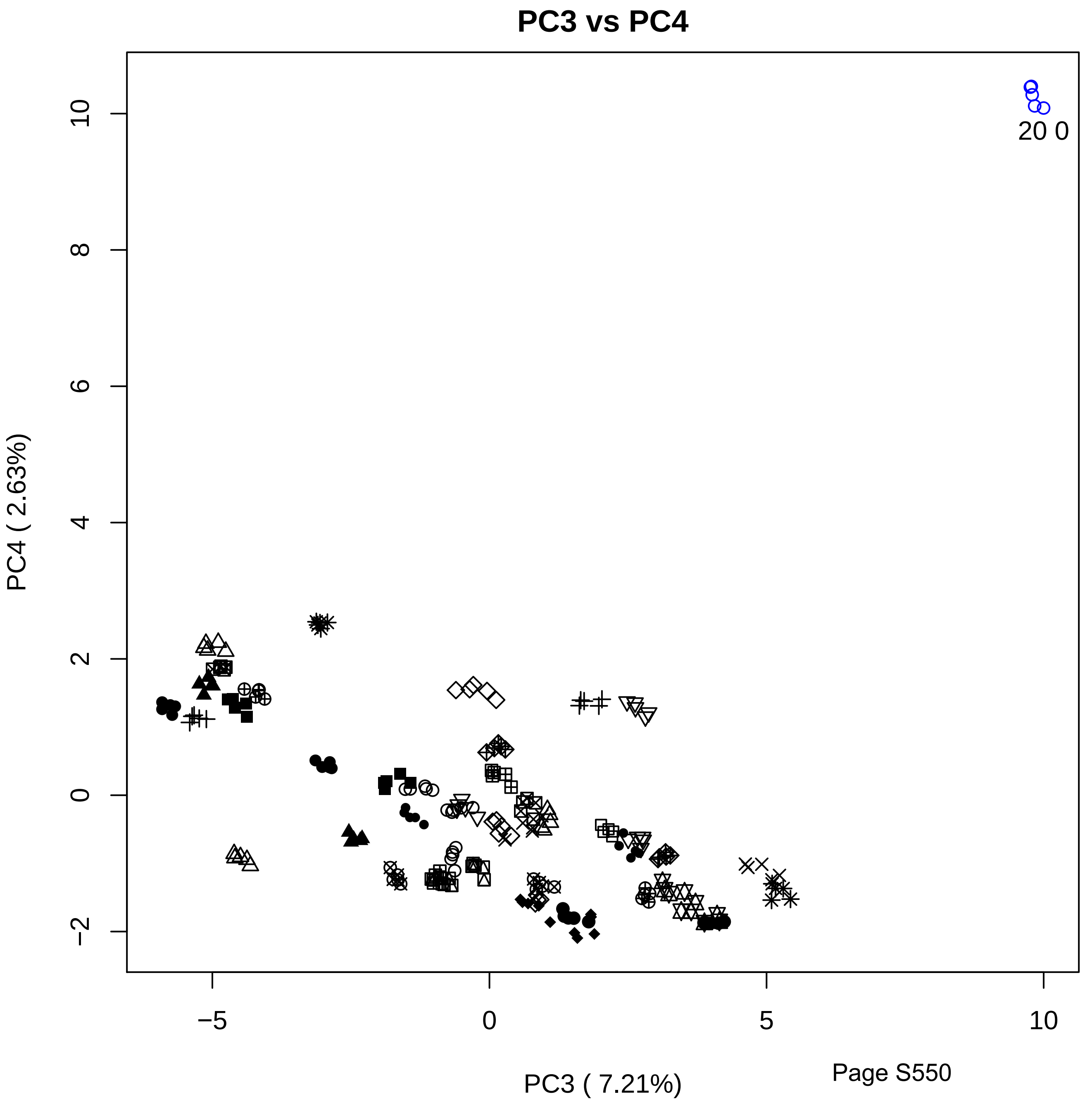

PC3 vs PC4
PC4 (2.63%)
PC3 (7.21%)
20 0
10
8
6
4
2
0
−2
−5
0
5
10

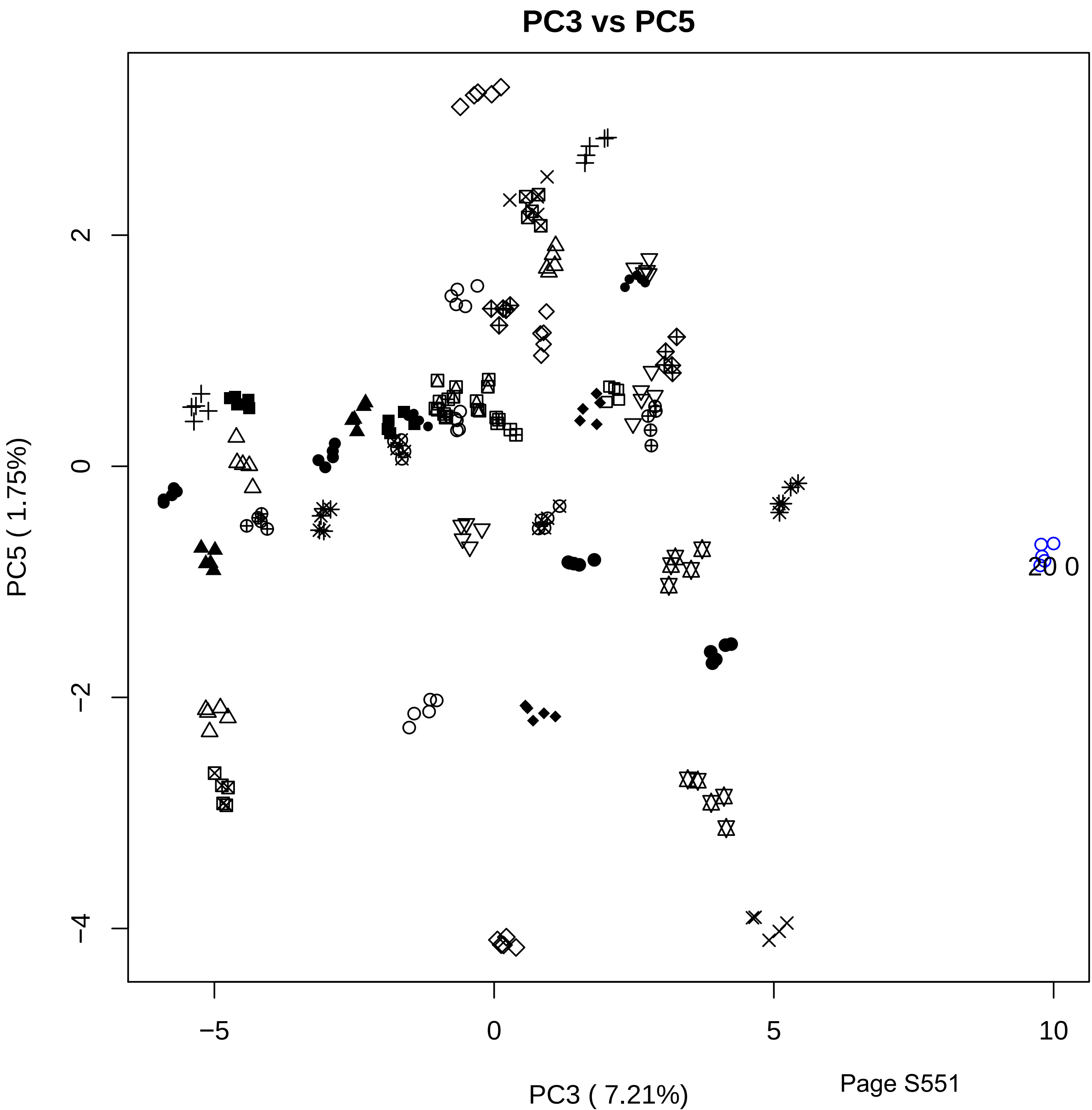
PC3 vs PC5
PC5 (1.75%)
PC3 (7.21%)
2
0
−2
−4
−5
0
5
10
20 0

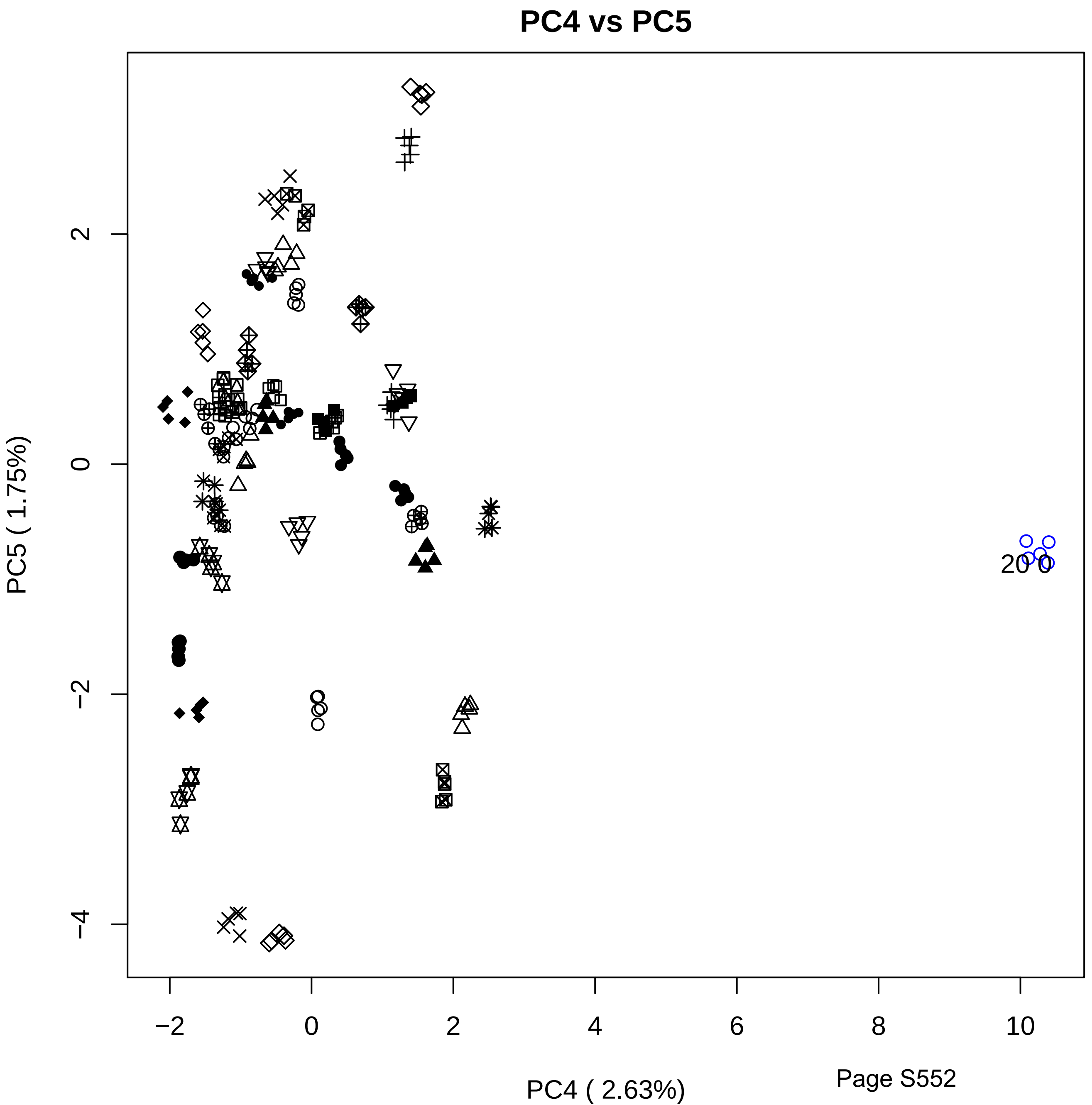
PC4 vs PC5
PC5 (1.75%)
PC4 (2.63%)
2
0
−2
−4
−2
0
2
4
6
8
10
20 0

PCA of 40% OSAS vs PMH

Scaled Matrix PCAed

7th order polynomial baseline correction performed

Raw spectra smoothed to 21 Neighbours, 2nd order PN, Savtisky Golay

Normalisation between 0 and 100

D2 spectra smoothed to 41 Neighbours, 2nd order PN, Savtisky Golay

Regions Analysed: 3601−2501 and 2001−700cm−1

Samples Analysed: 1 Contam's 0 OSAS's 45 PMH's

Keele University AD

Sun Dec 09 16:29:33 2018

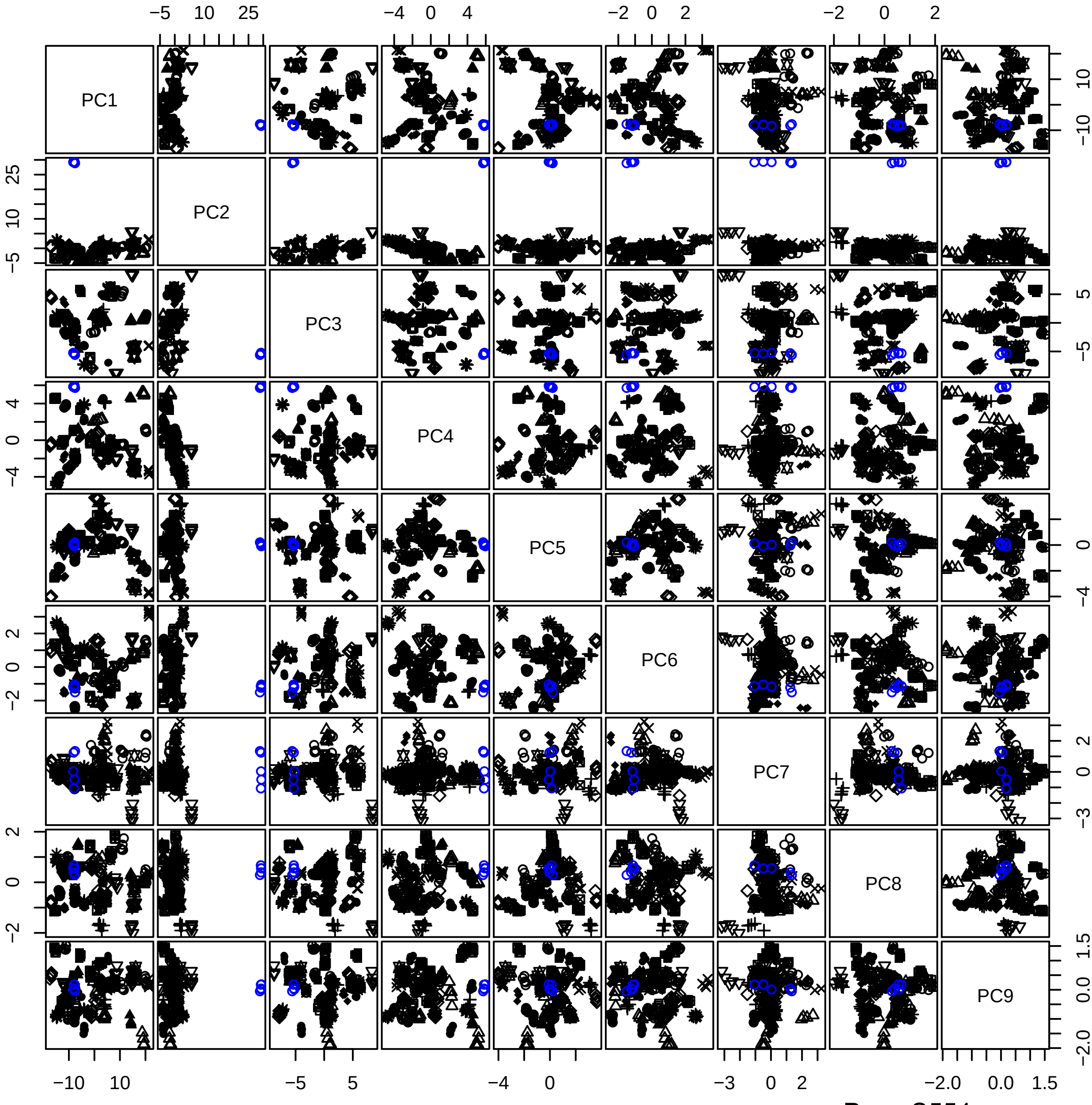
PC1
PC2
PC3
PC4
PC5
PC6
PC7
PC8
PC9
−5 10 25
−4 0 4
−2 0 2
−2 0 2
−10 10
−5 5
−4 0
−3 0 2
−2.0 0.0 1.5

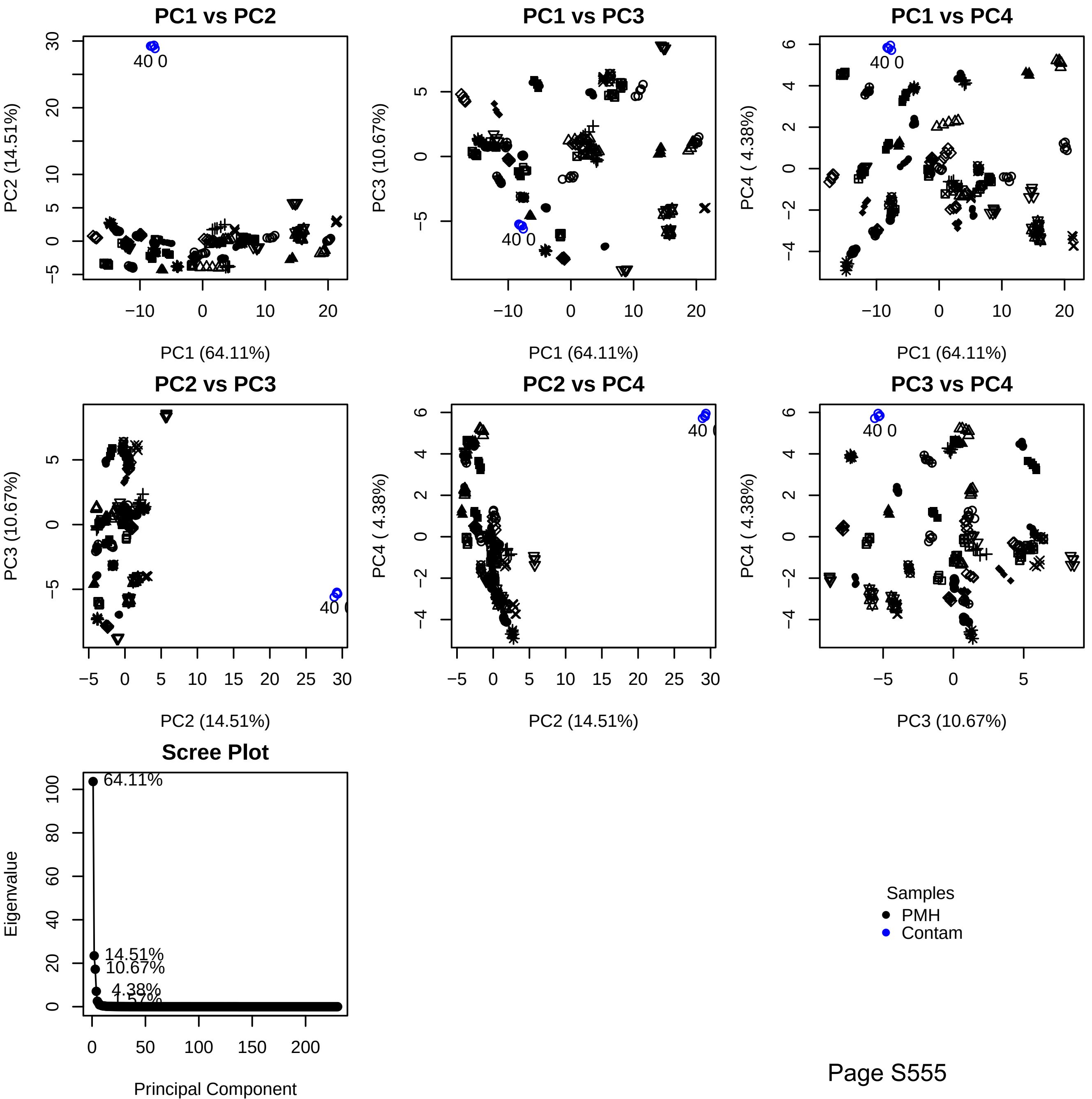

PC1 vs PC2
PC1 vs PC3
PC1 vs PC4
PC2 vs PC3
PC2 vs PC4
PC3 vs PC4
Scree Plot
PC1 (64.11%)
PC2 (14.51%)
PC3 (10.67%)
PC4 (4.38%)
40 0
Eigenvalue
Principal Component
64.11%
14.51%
10.67%
4.38%
Samples
PMH
Contam

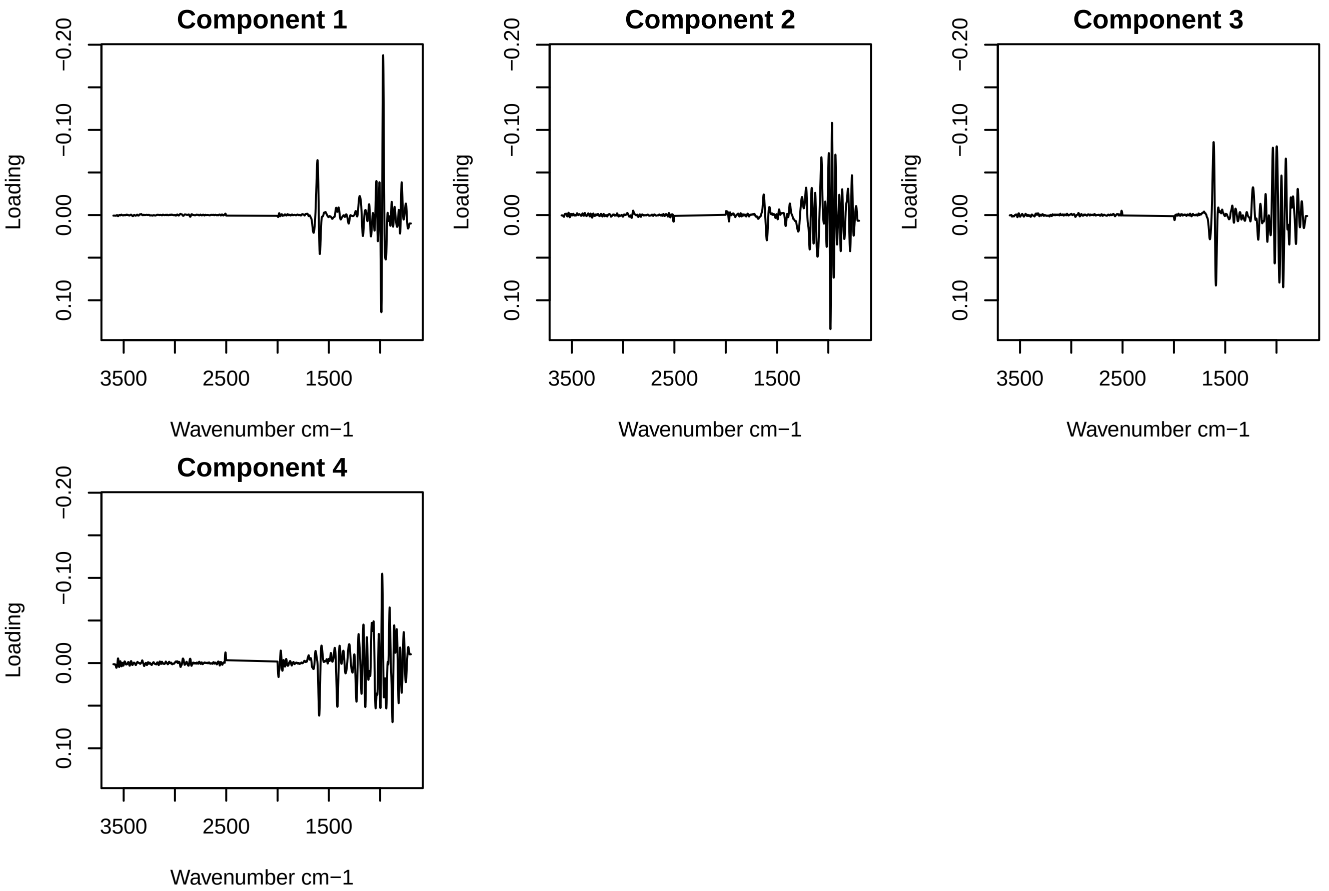
Component 1
Component 2
Component 3
Component 4
Loading
−0.20
−0.10
0.00
0.10
3500
2500
1500
Wavenumber cm−1

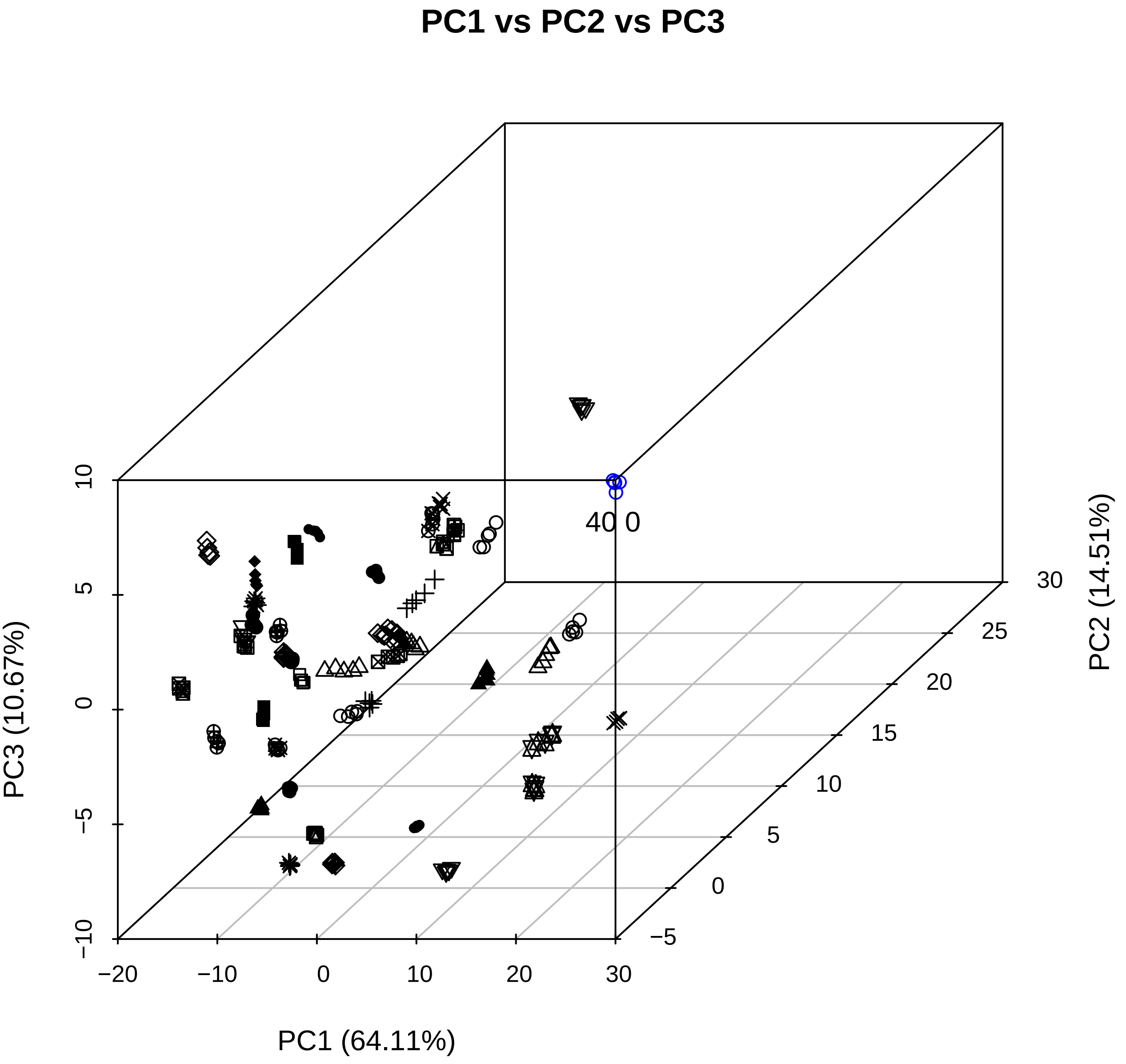

PC1 vs PC2 vs PC3
PC1 (64.11%)
PC2 (14.51%)
PC3 (10.67%)
40 0
−20
−10
0
10
20
30
−5
0
5
10
15
20
25
30
−10
−5
0
5
10

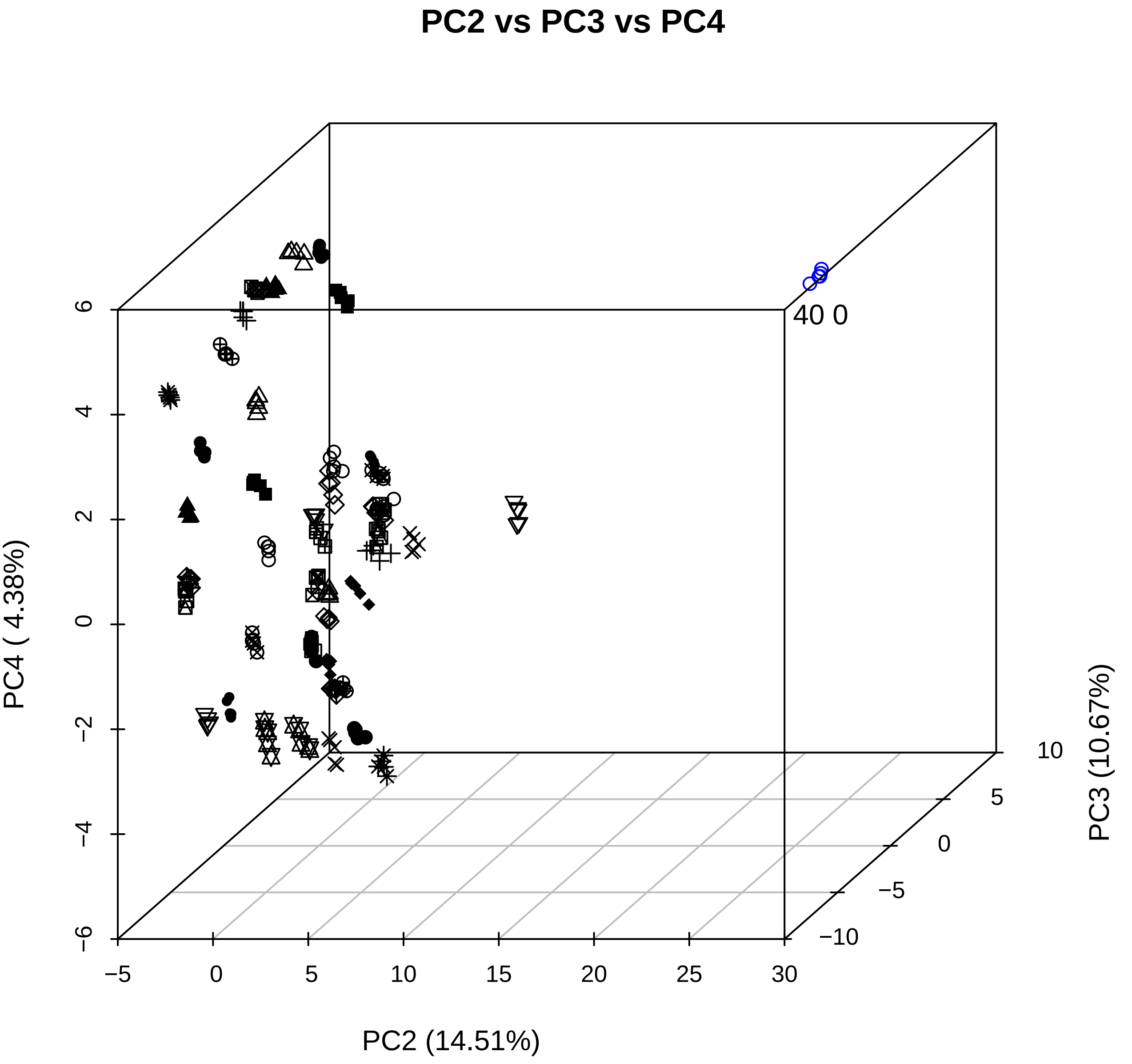
PC2 vs PC3 vs PC4
PC4 (4.38%)
PC2 (14.51%)
PC3 (10.67%)
−6
−4
−2
0
2
4
6
−5
0
5
10
15
20
25
30
−10
−5
0
5
10
40 0

PC1 vs PC2 vs PC4

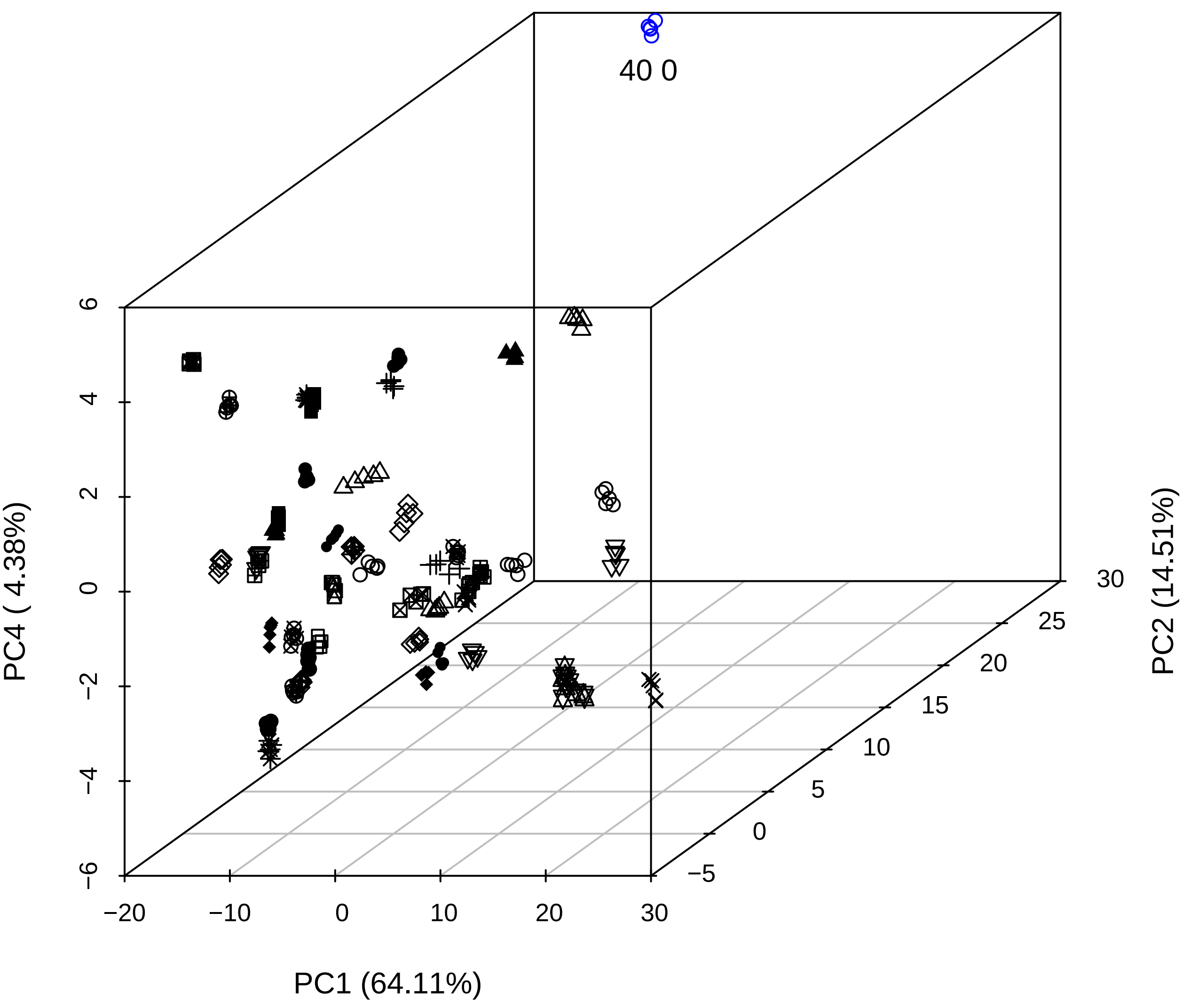

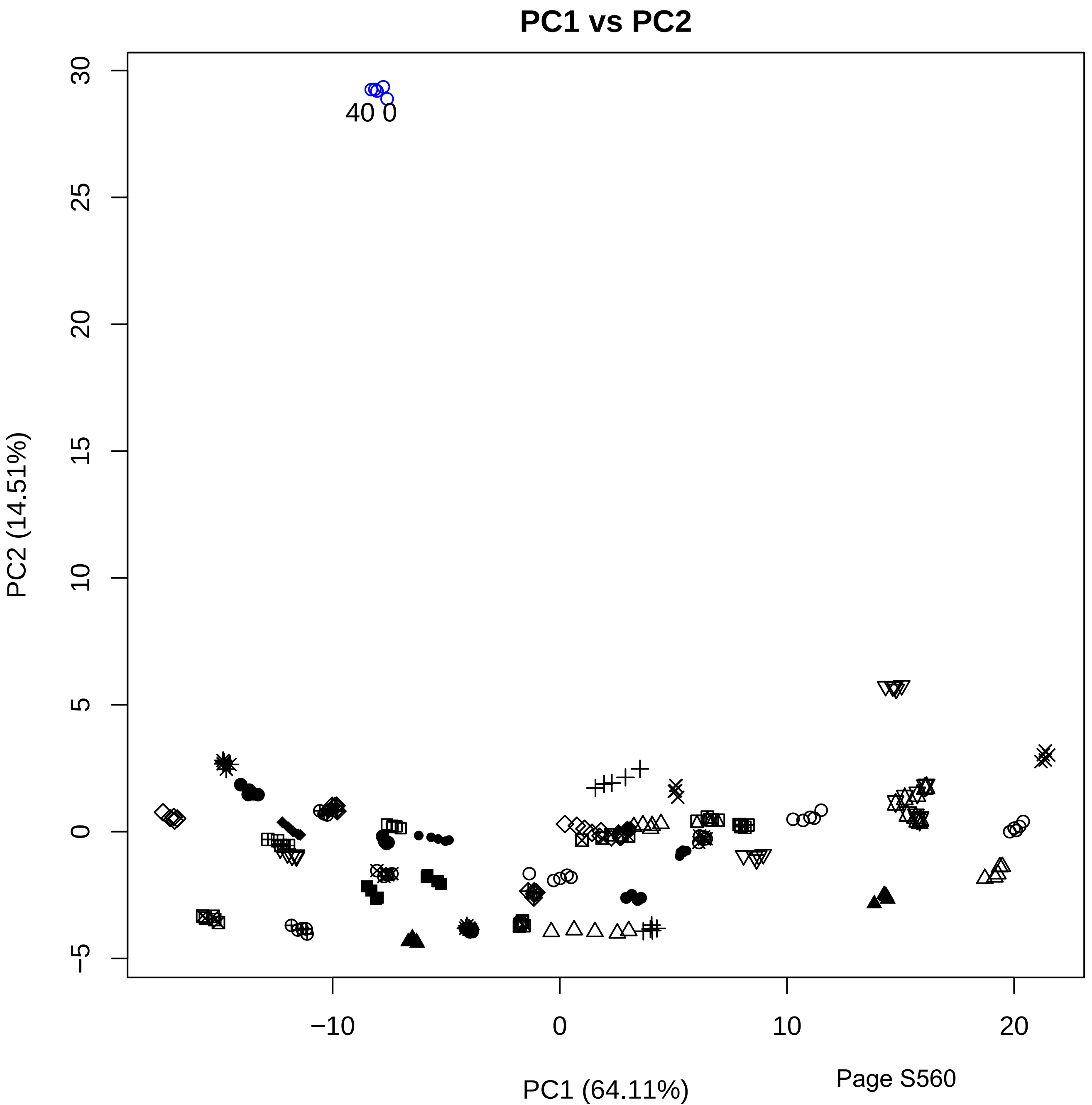
PC1 vs PC2
40 0
PC2 (14.51%)
PC1 (64.11%)
30
25
20
15
10
5
0
−5
−10
0
10
20

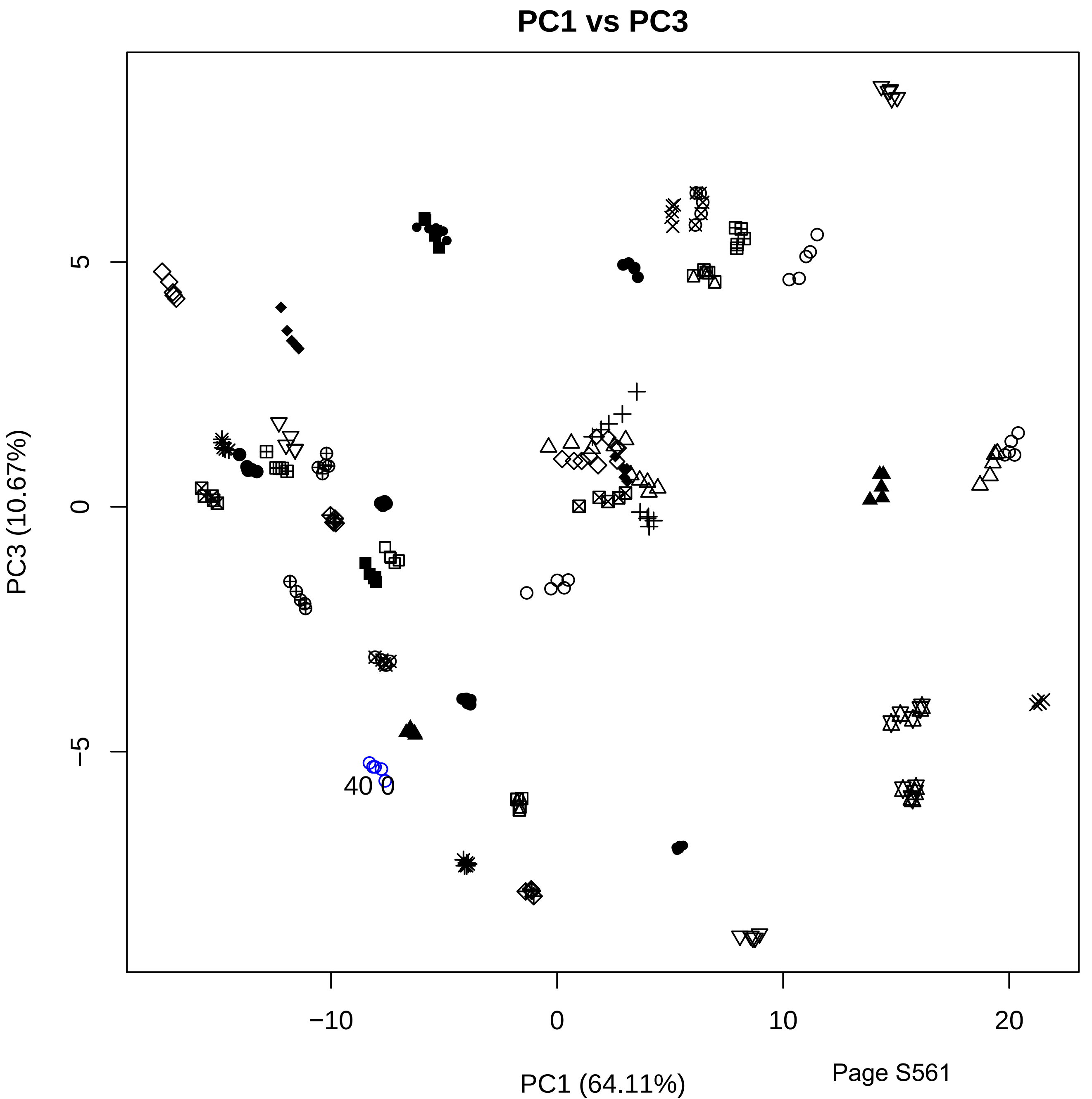

PC1 vs PC3
PC3 (10.67%)
PC1 (64.11%)
5
0
−5
−10
0
10
20
40 0

PC1 vs PC4

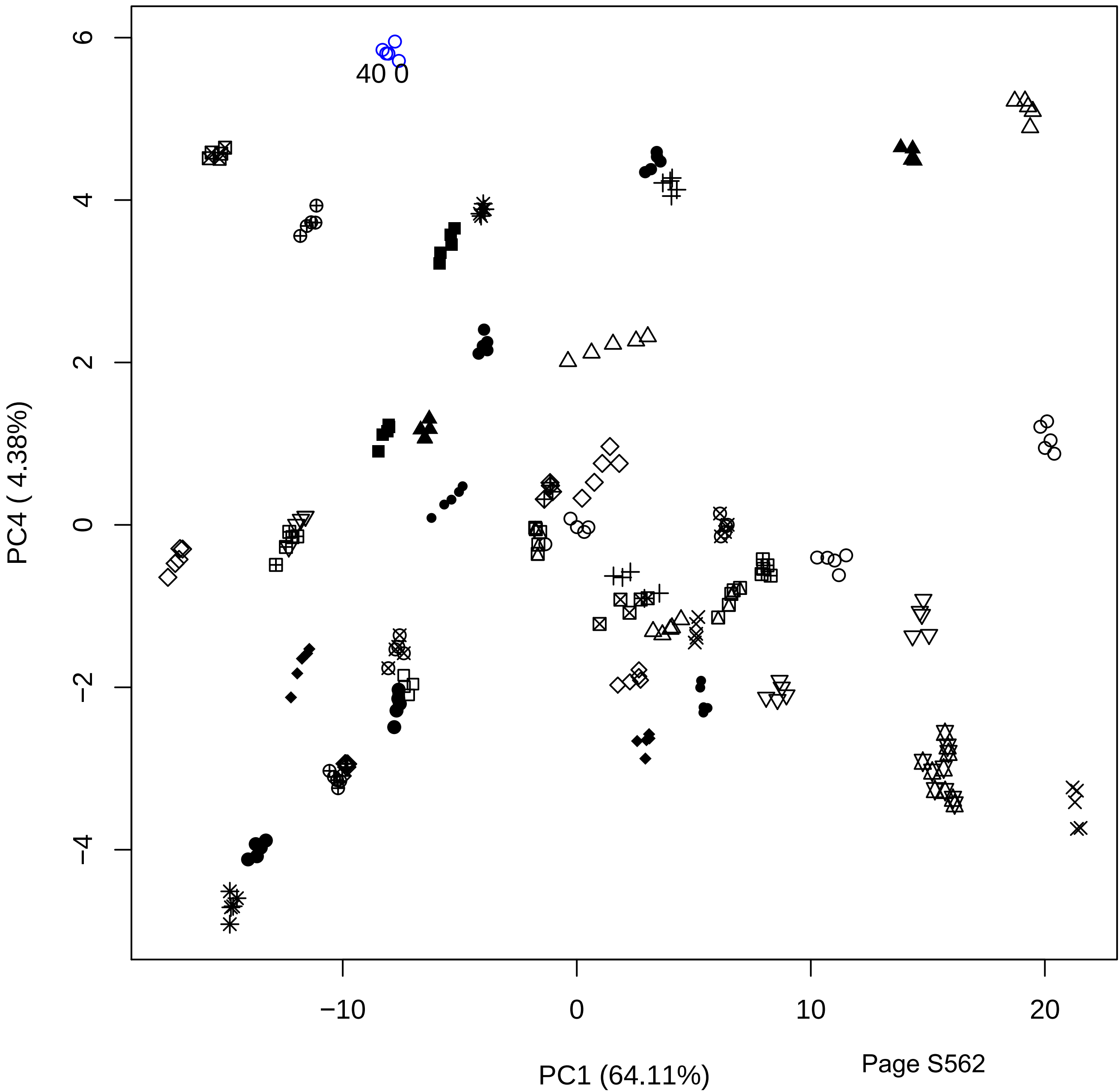

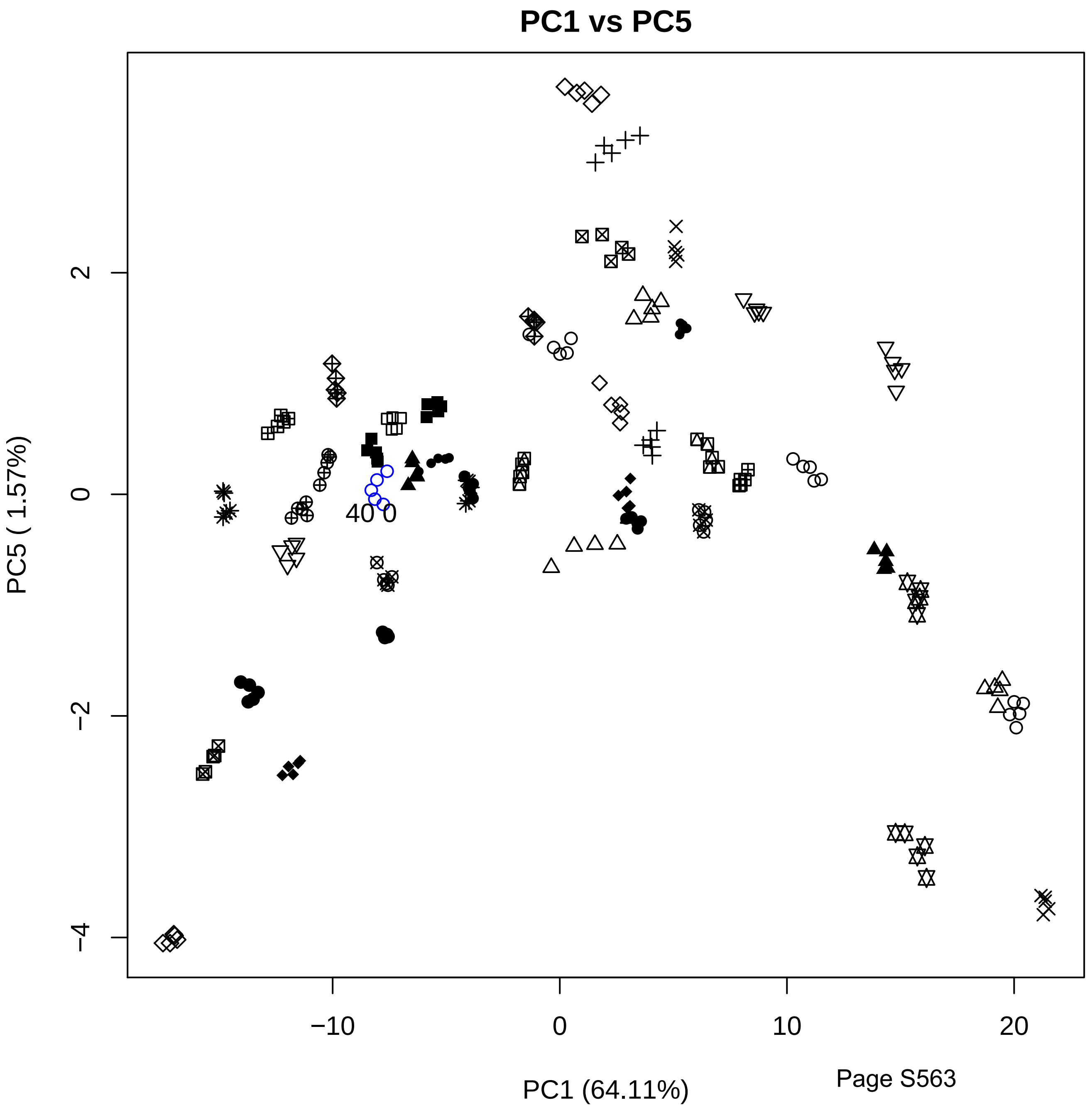

PC1 vs PC5
PC5 (1.57%)
PC1 (64.11%)
2
0
−2
−4
−10
0
10
20
40 0

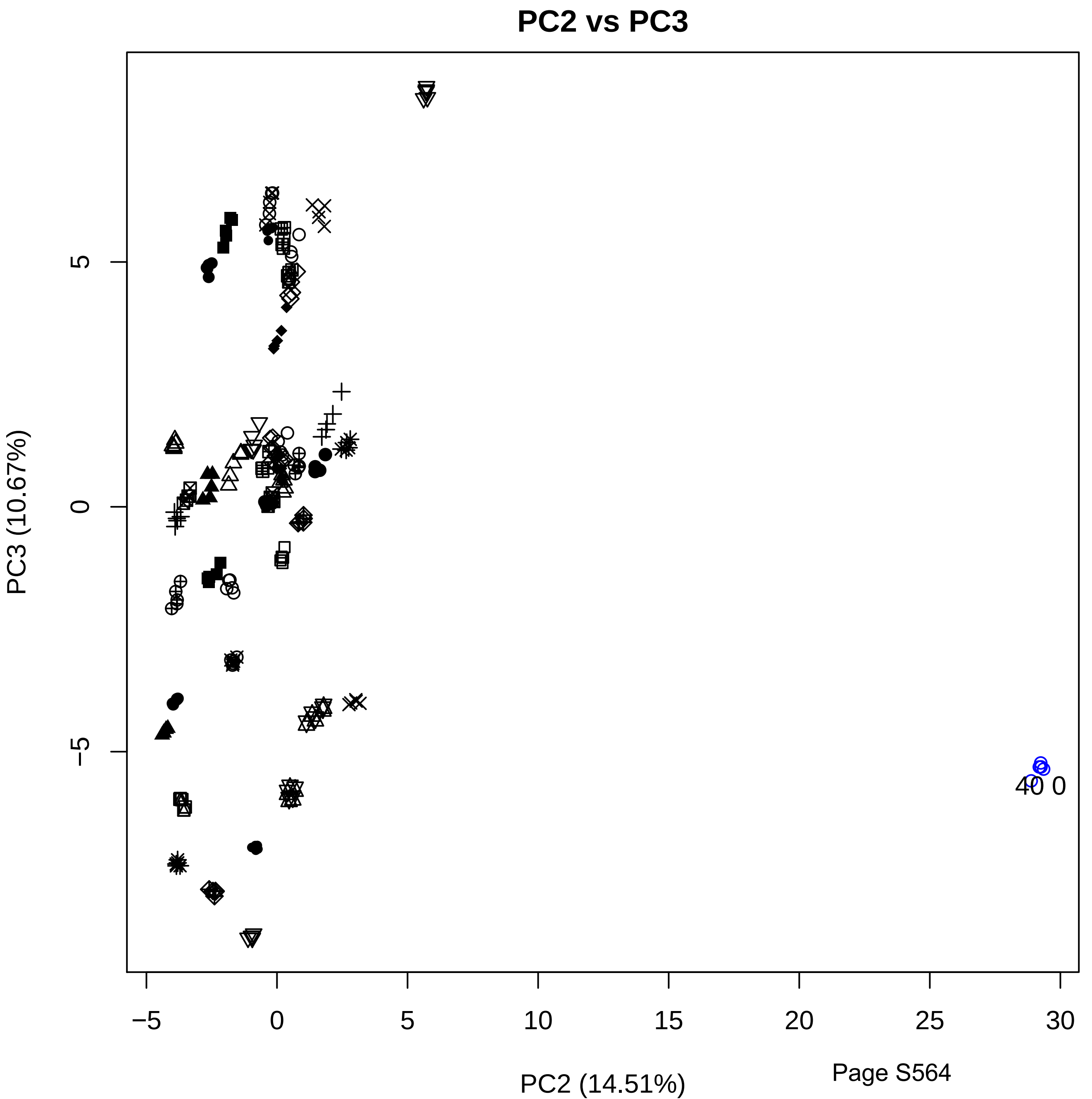

PC2 vs PC3
PC3 (10.67%)
PC2 (14.51%)
5
0
−5
−5
0
5
10
15
20
25
30
40 0

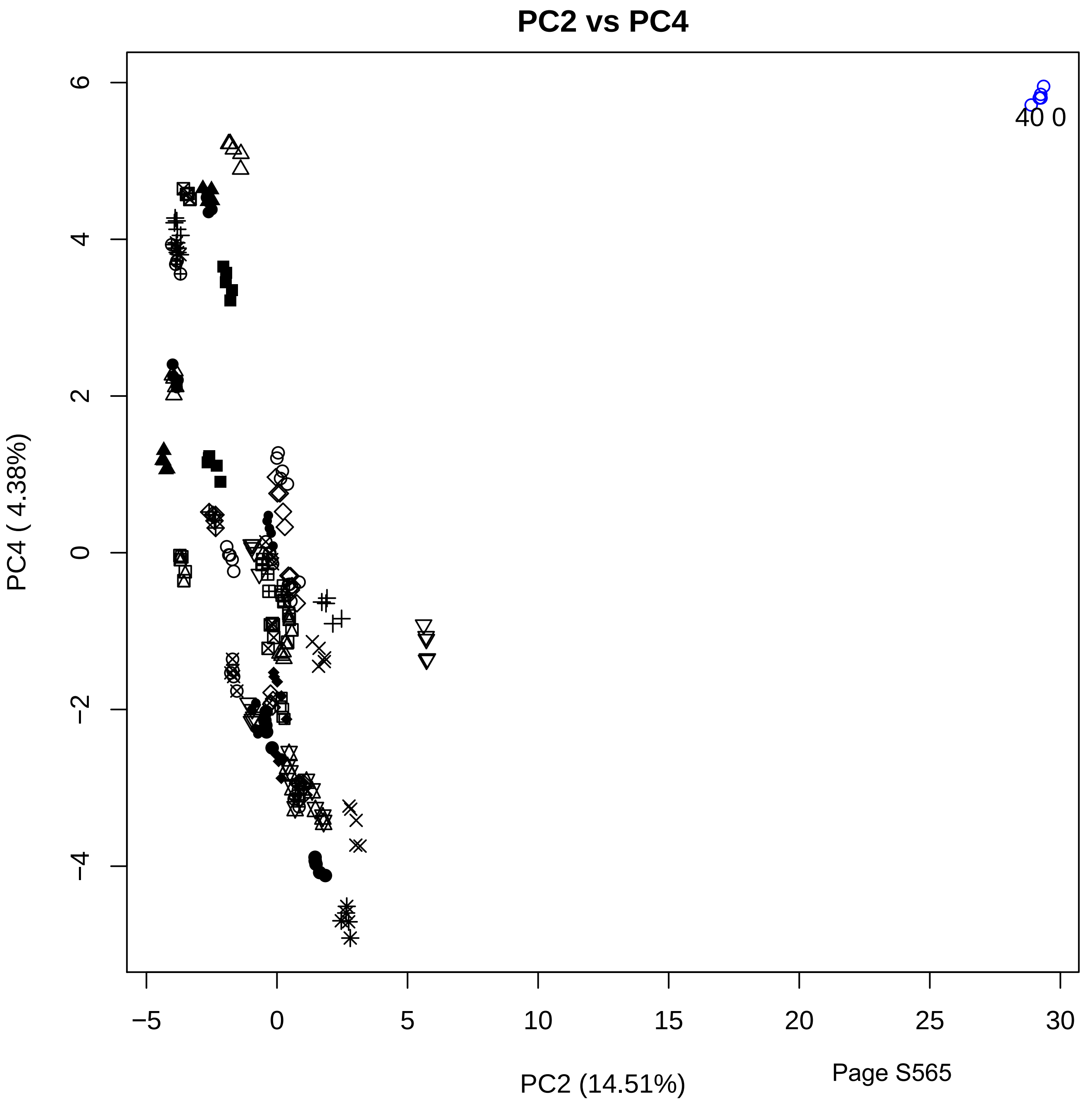

PC2 vs PC4
PC4 (4.38%)
PC2 (14.51%)
6
4
2
0
−2
−4
−5
0
5
10
15
20
25
30
40 0

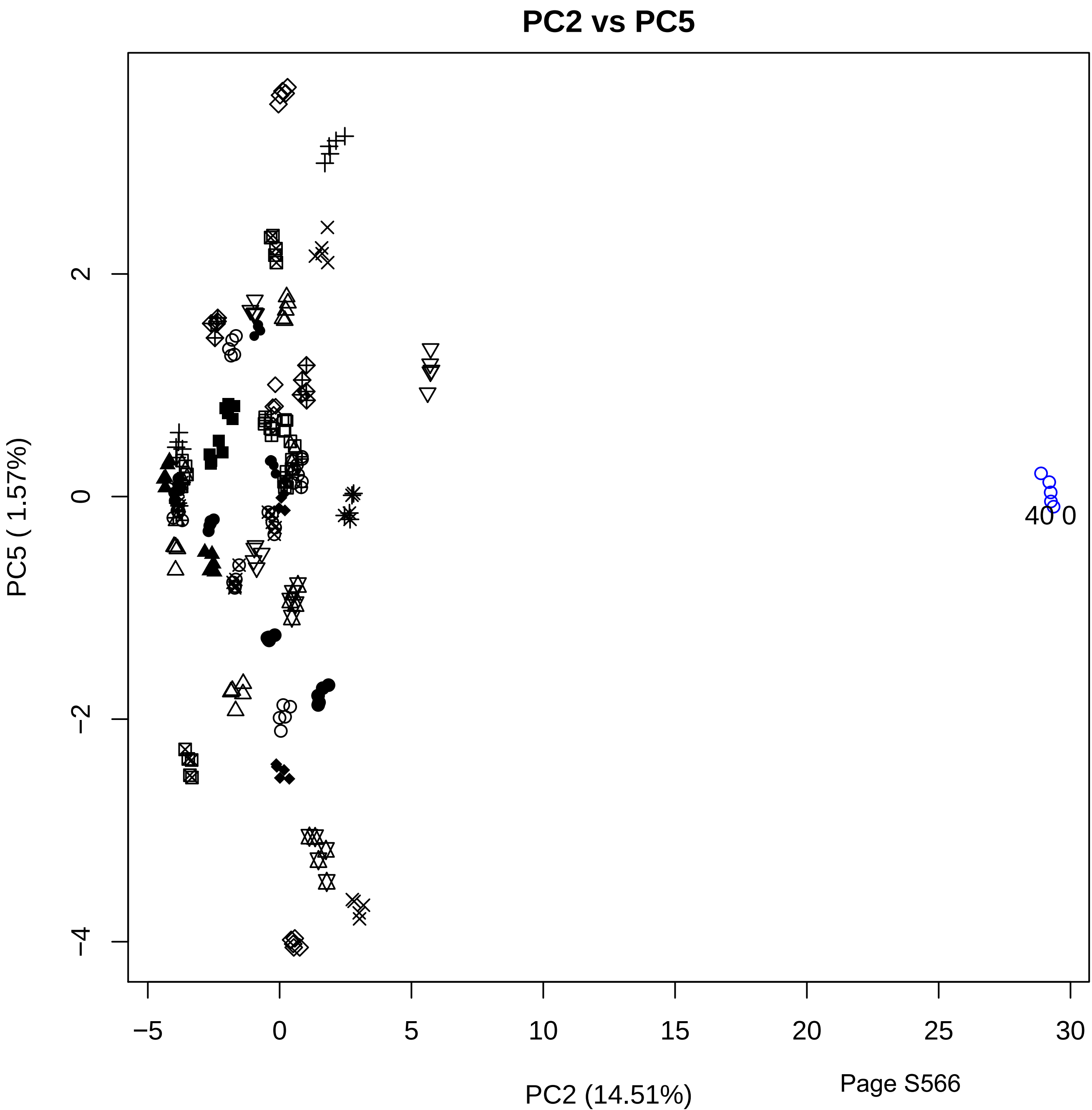
PC2 vs PC5
PC5 (1.57%)
PC2 (14.51%)
2
0
−2
−4
−5
0
5
10
15
20
25
30
40 0

PC3 vs PC4

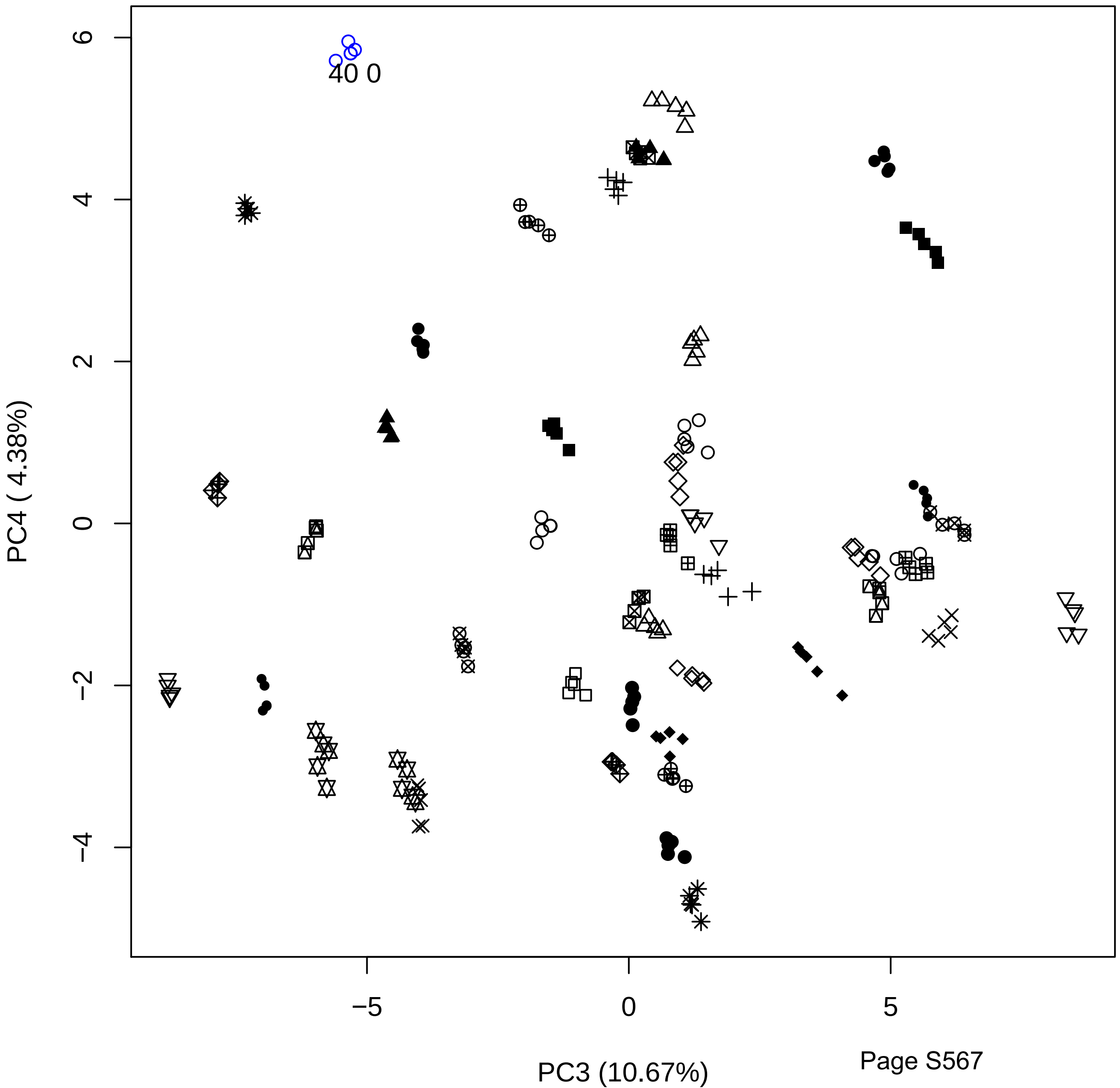

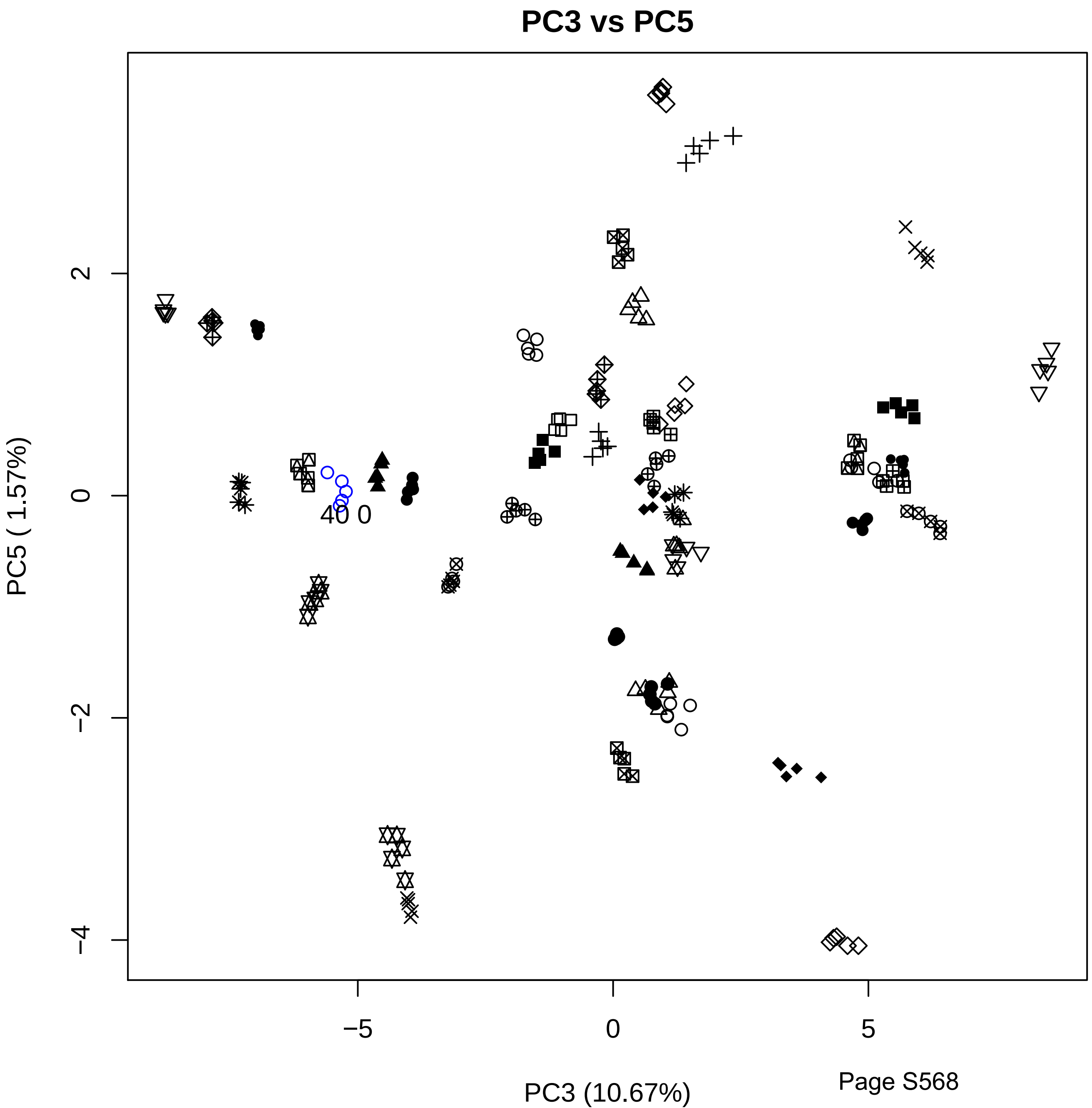

PC3 vs PC5
PC5 (1.57%)
PC3 (10.67%)
2
0
−2
−4
−5
0
5
40 0

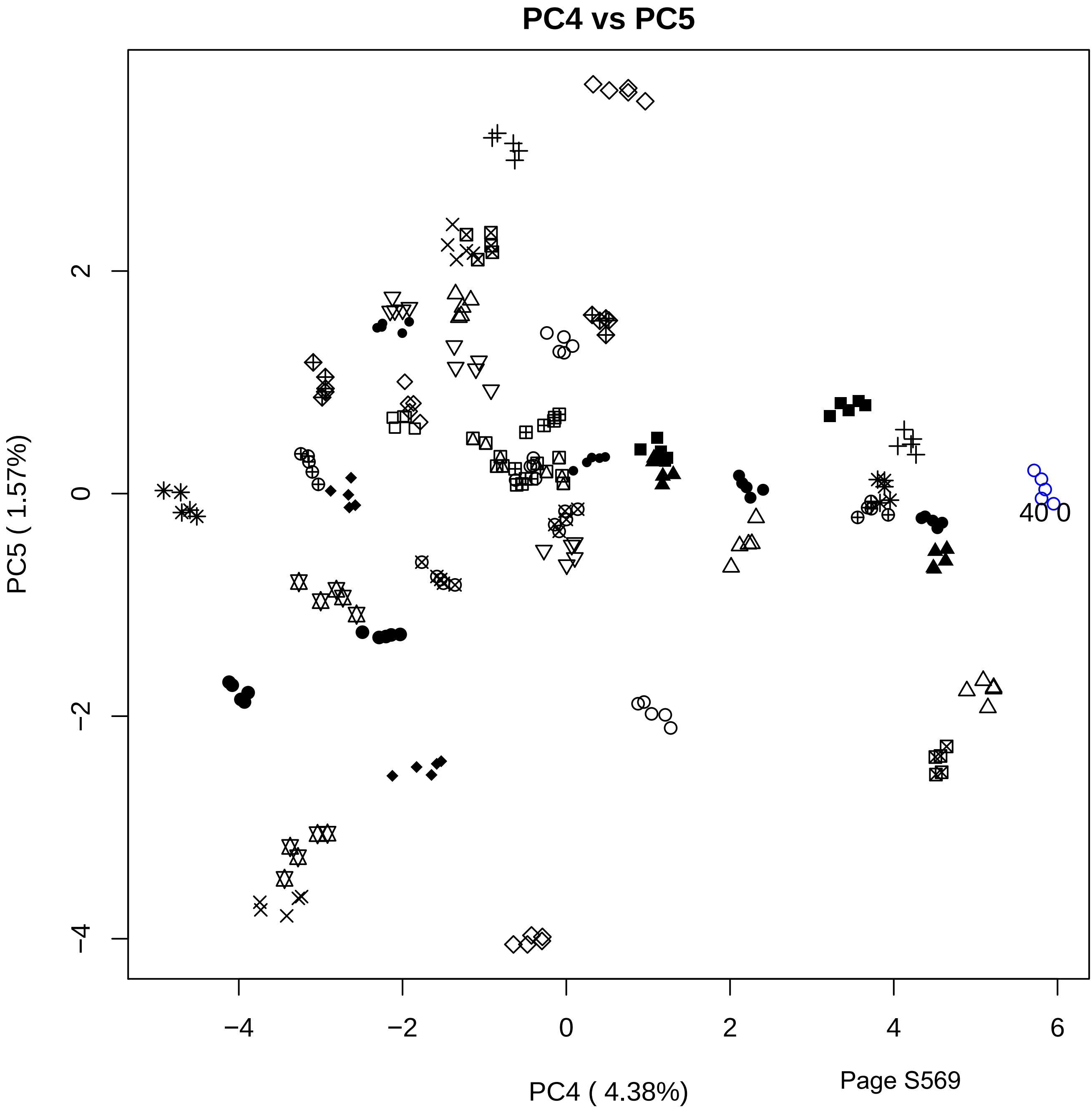
PC4 vs PC5
PC5 (1.57%)
PC4 (4.38%)
2
0
−2
−4
−4
−2
0
2
4
6
40 0

PCA of 100% OSAS vs PMH

Scaled Matrix PCAed

7th order polynomial baseline correction performed

Raw spectra smoothed to 21 Neighbours, 2nd order PN, Savtisky Golay

Normalisation between 0 and 100

D2 spectra smoothed to 41 Neighbours, 2nd order PN, Savtisky Golay

Regions Analysed: 3601−2501 and 2001−700cm−1

Samples Analysed: 0 Contam's 1 OSAS's 45 PMH's

Keele University AD

Sun Dec 09 16:29:38 2018

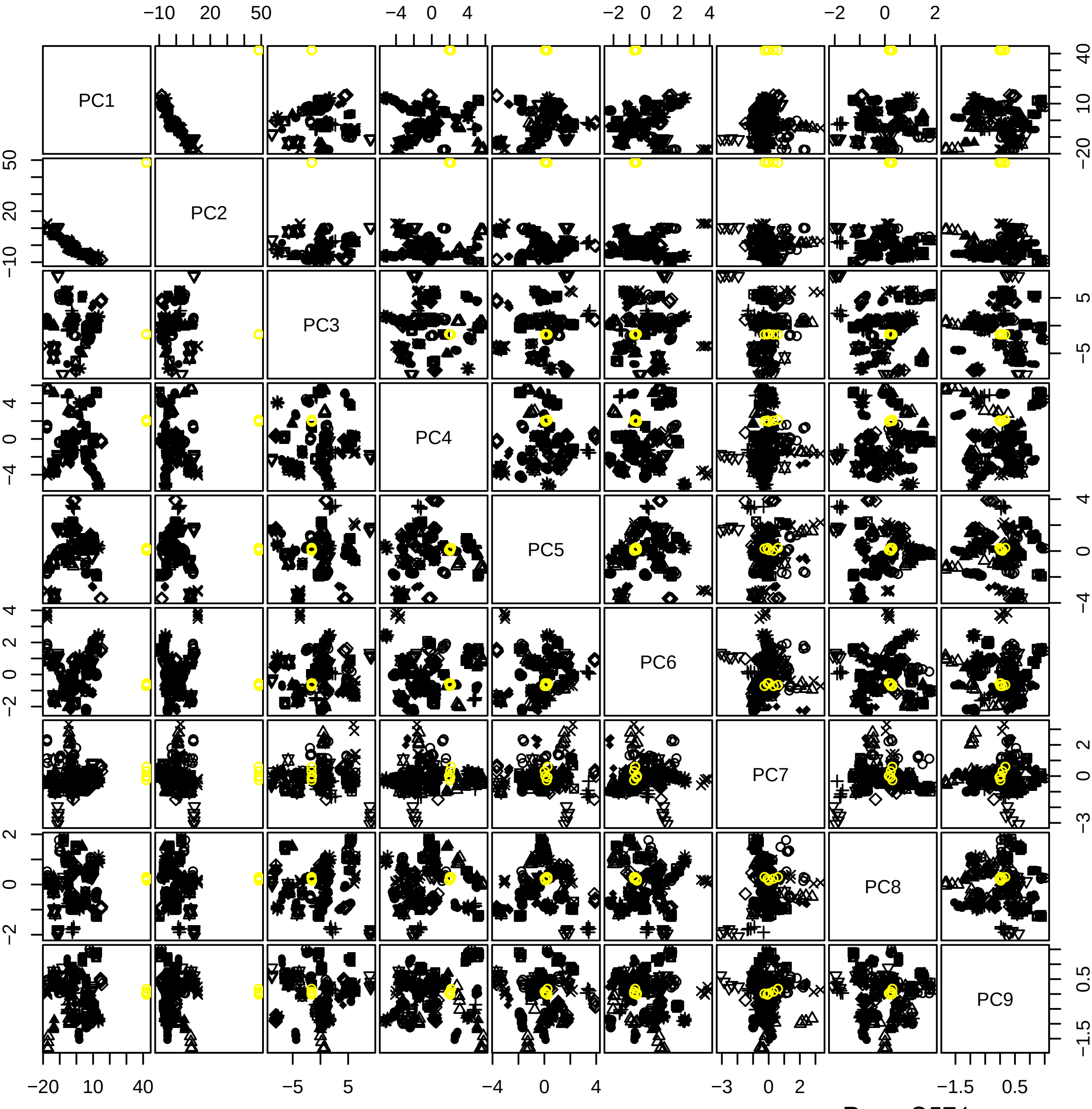
PC1
PC2
PC3
PC4
PC5
PC6
PC7
PC8
PC9

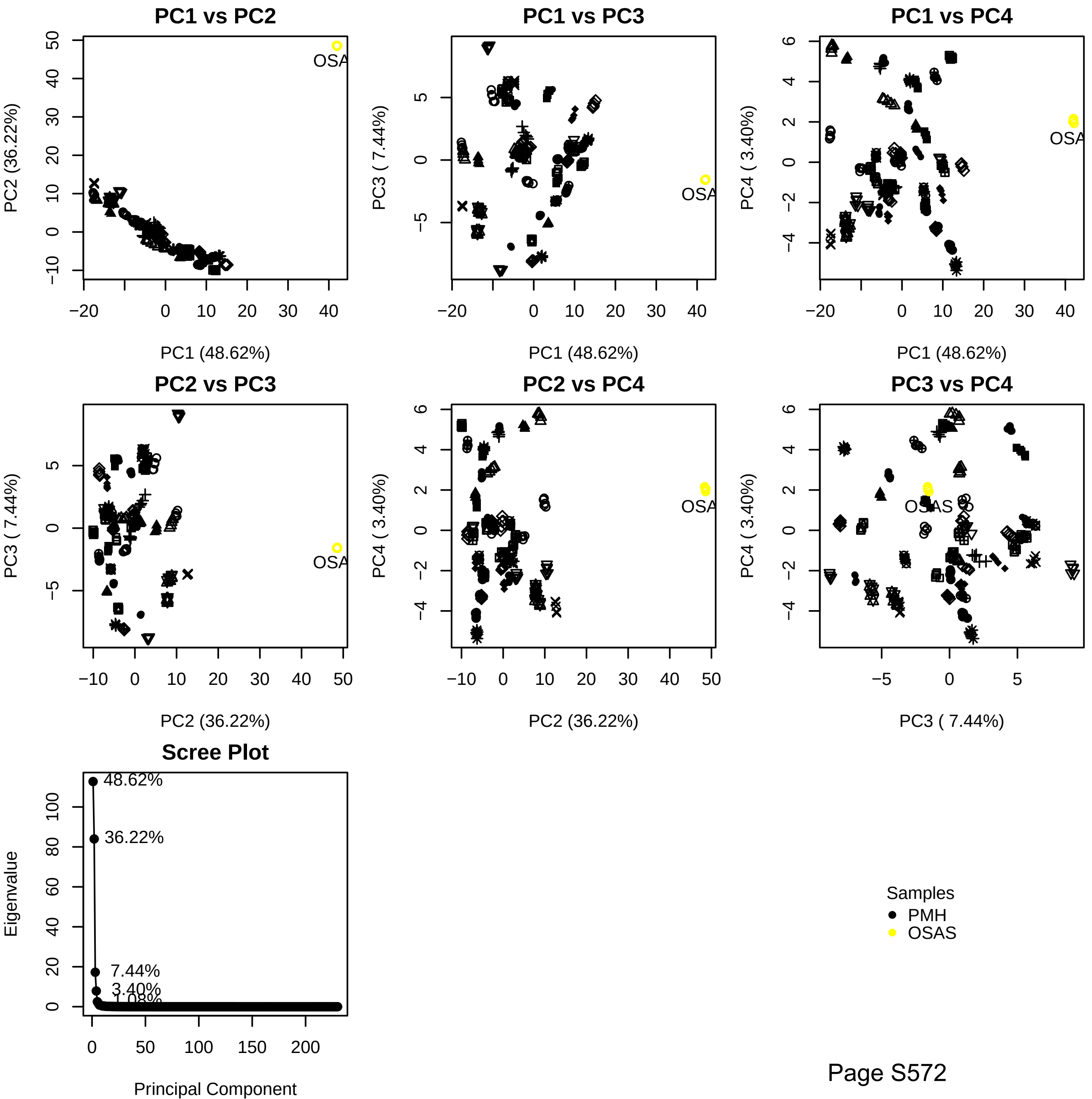
PC1 vs PC2
PC1 vs PC3
PC1 vs PC4
PC2 vs PC3
PC2 vs PC4
PC3 vs PC4
Scree Plot
PC1 (48.62%)
PC2 (36.22%)
PC3 (7.44%)
PC4 (3.40%)
OSA
OSAS
Eigenvalue
Principal Component
48.62%
36.22%
7.44%
3.40%
Samples
PMH
OSAS

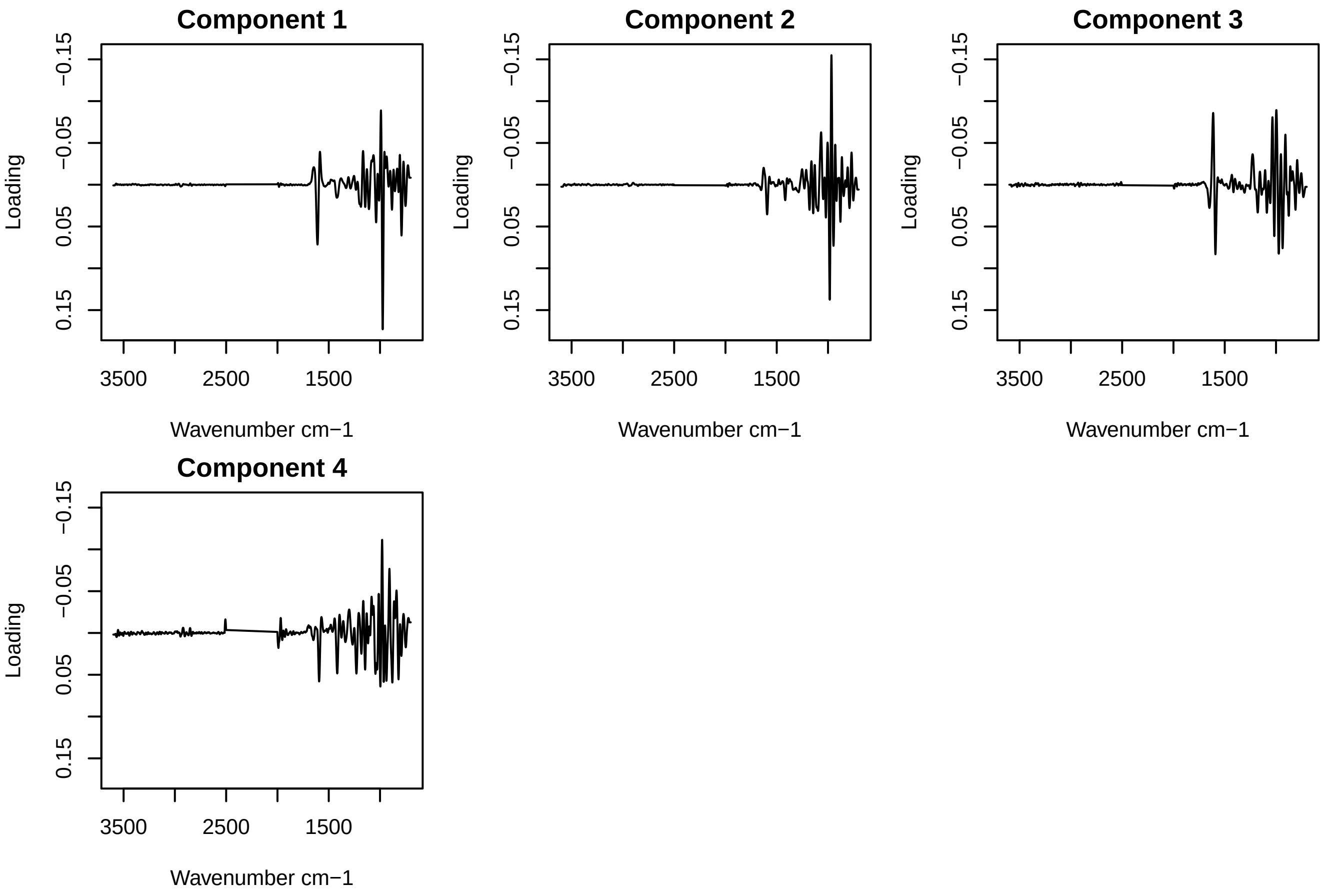
Component 1
Component 2
Component 3
Component 4
Loading
−0.15
−0.05
0.05
0.15
3500
2500
1500
Wavenumber cm−1

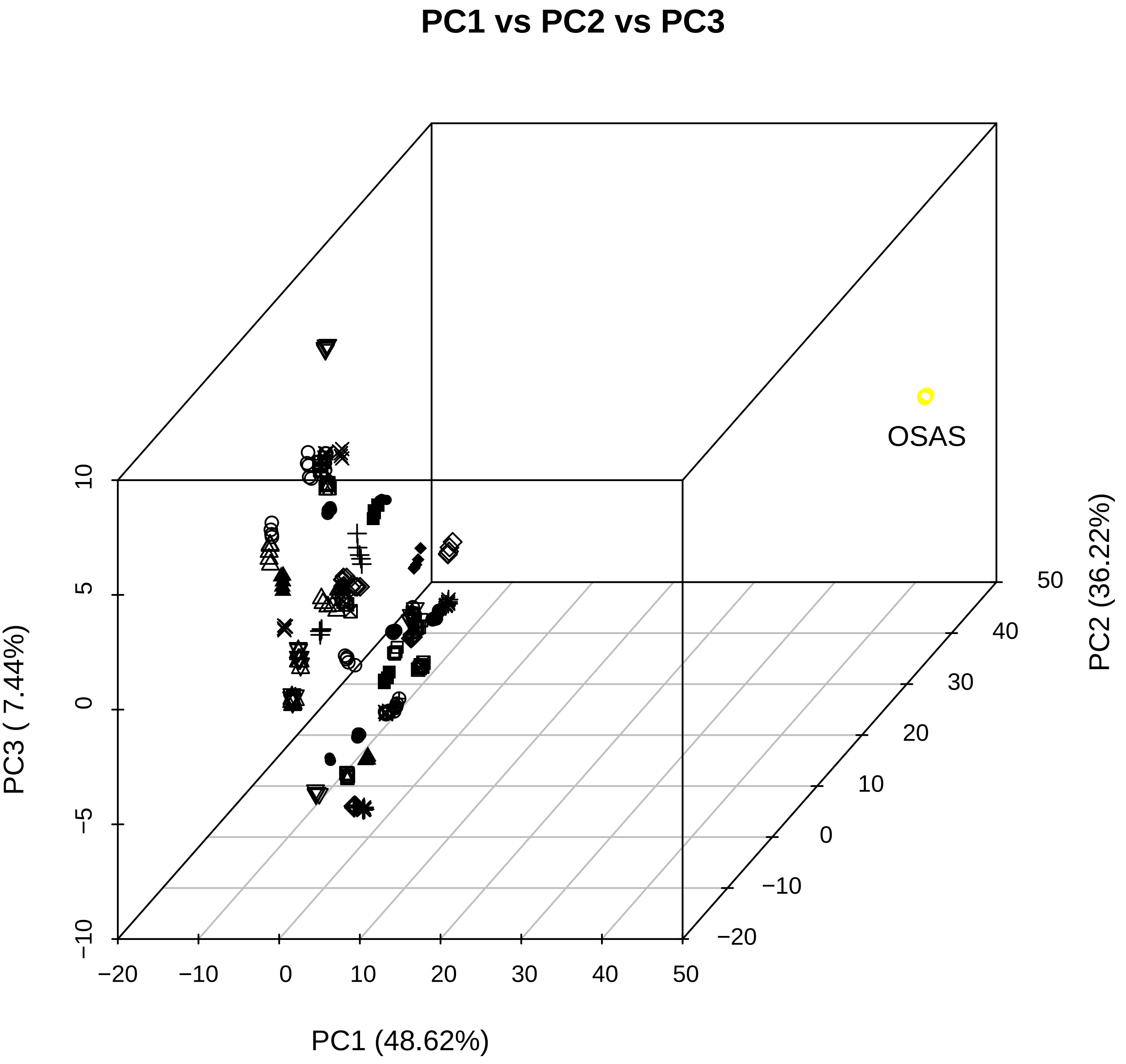
PC1 vs PC2 vs PC3
OSAS
PC1 (48.62%)
PC2 (36.22%)
PC3 (7.44%)
−20
−10
0
10
20
30
40
50
−10
−5
0
5
10

PC2 vs PC3 vs PC4

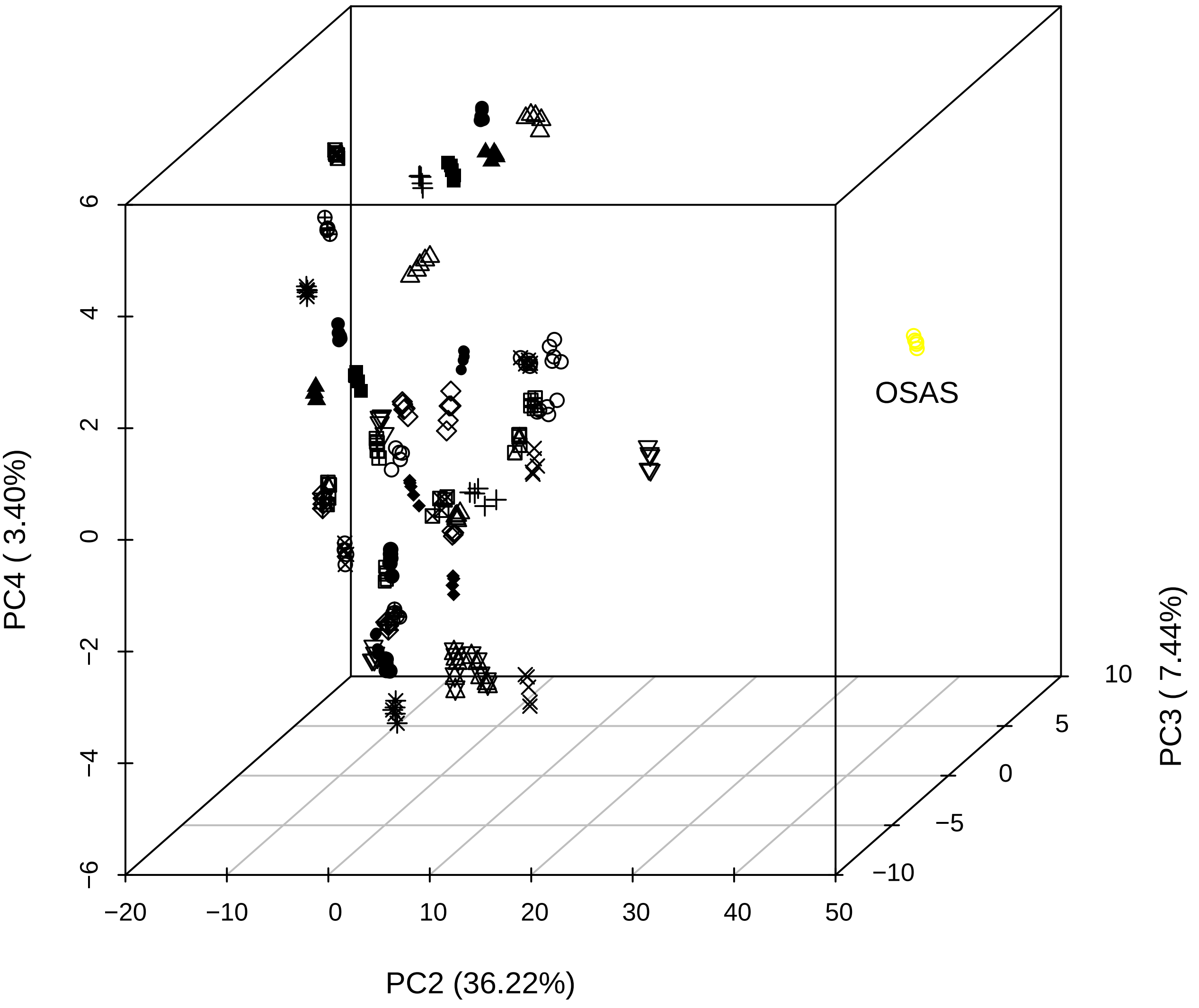

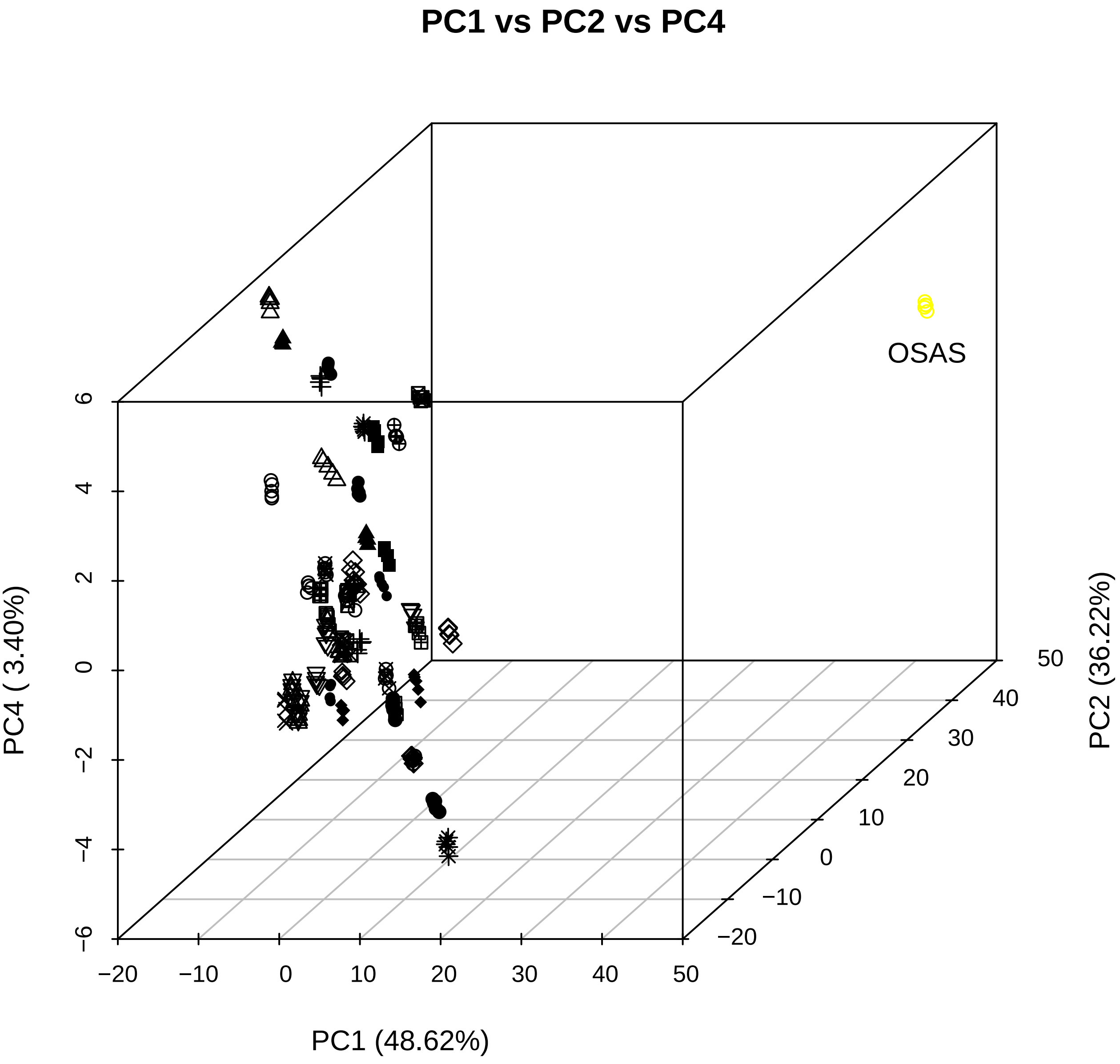
PC1 vs PC2 vs PC4
OSAS
PC1 (48.62%)
PC2 (36.22%)
PC4 (3.40%)
−20
−10
0
10
20
30
40
50
−6
−4
−2
0
2
4
6

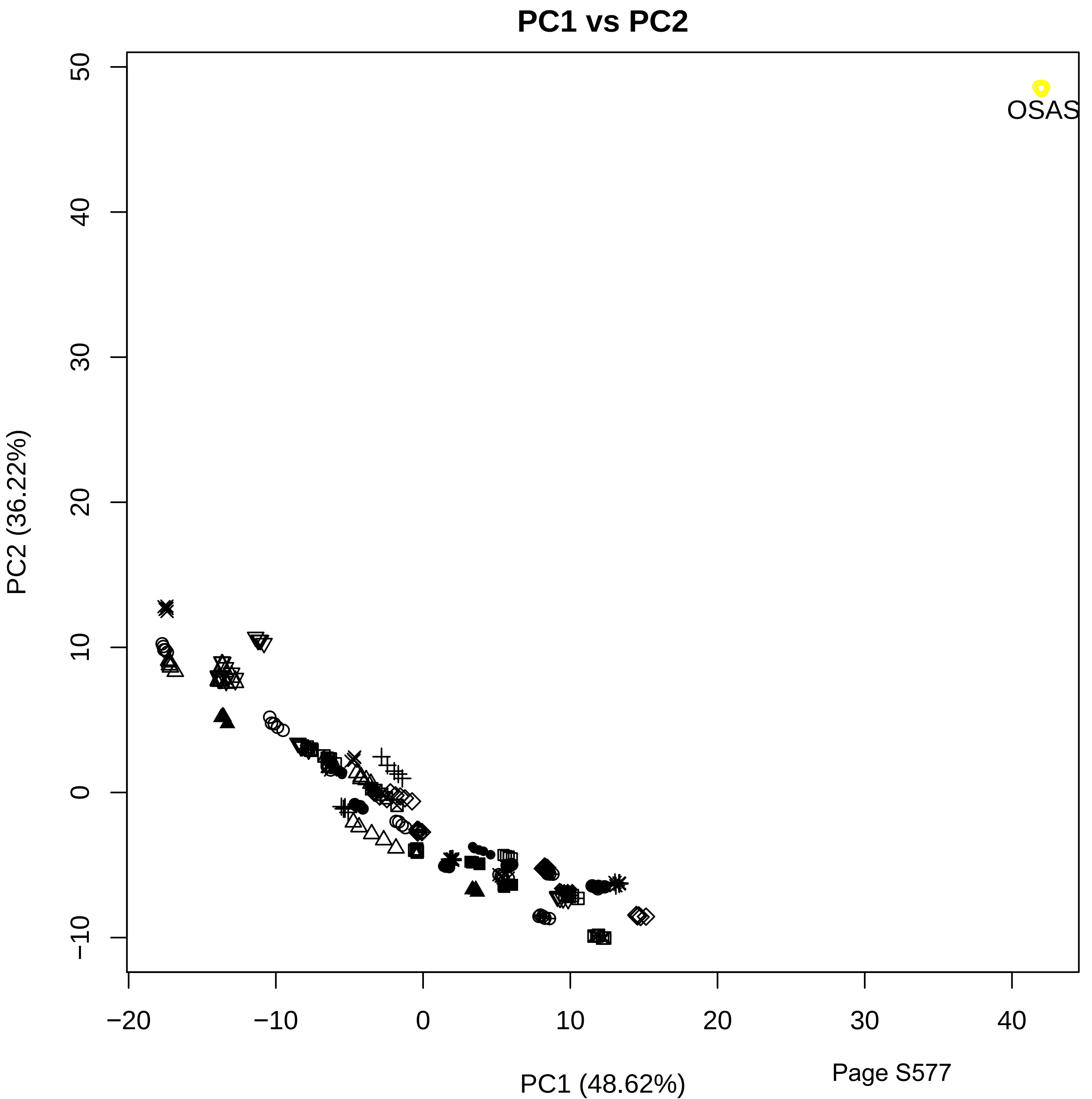
PC1 vs PC2
OSAS
PC2 (36.22%)
PC1 (48.62%)
50
40
30
20
10
0
−10
−20
−10
0
10
20
30
40

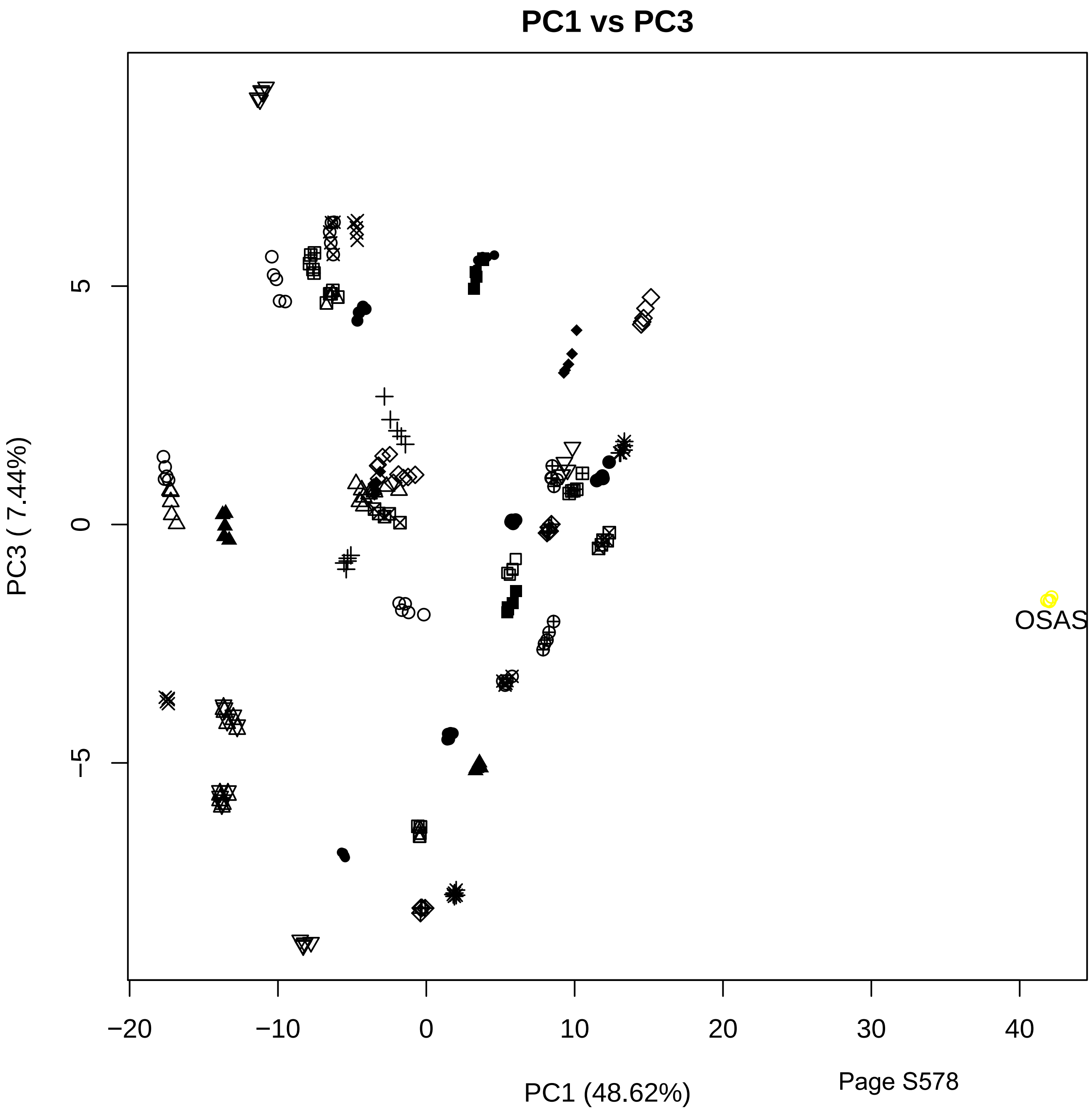
PC1 vs PC3
PC3 (7.44%)
PC1 (48.62%)
5
0
−5
−20
−10
0
10
20
30
40
OSAS

PC1 vs PC4

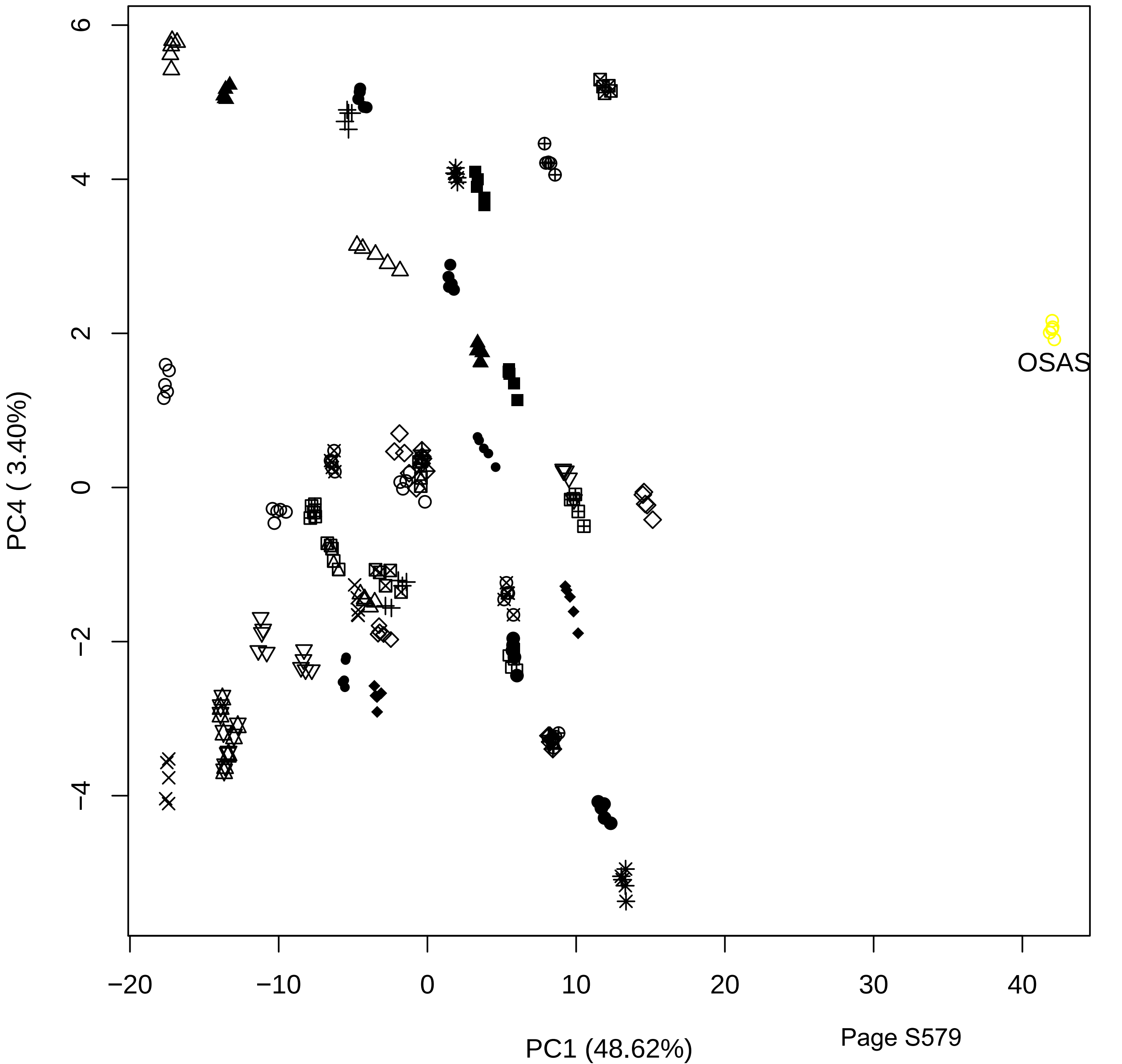

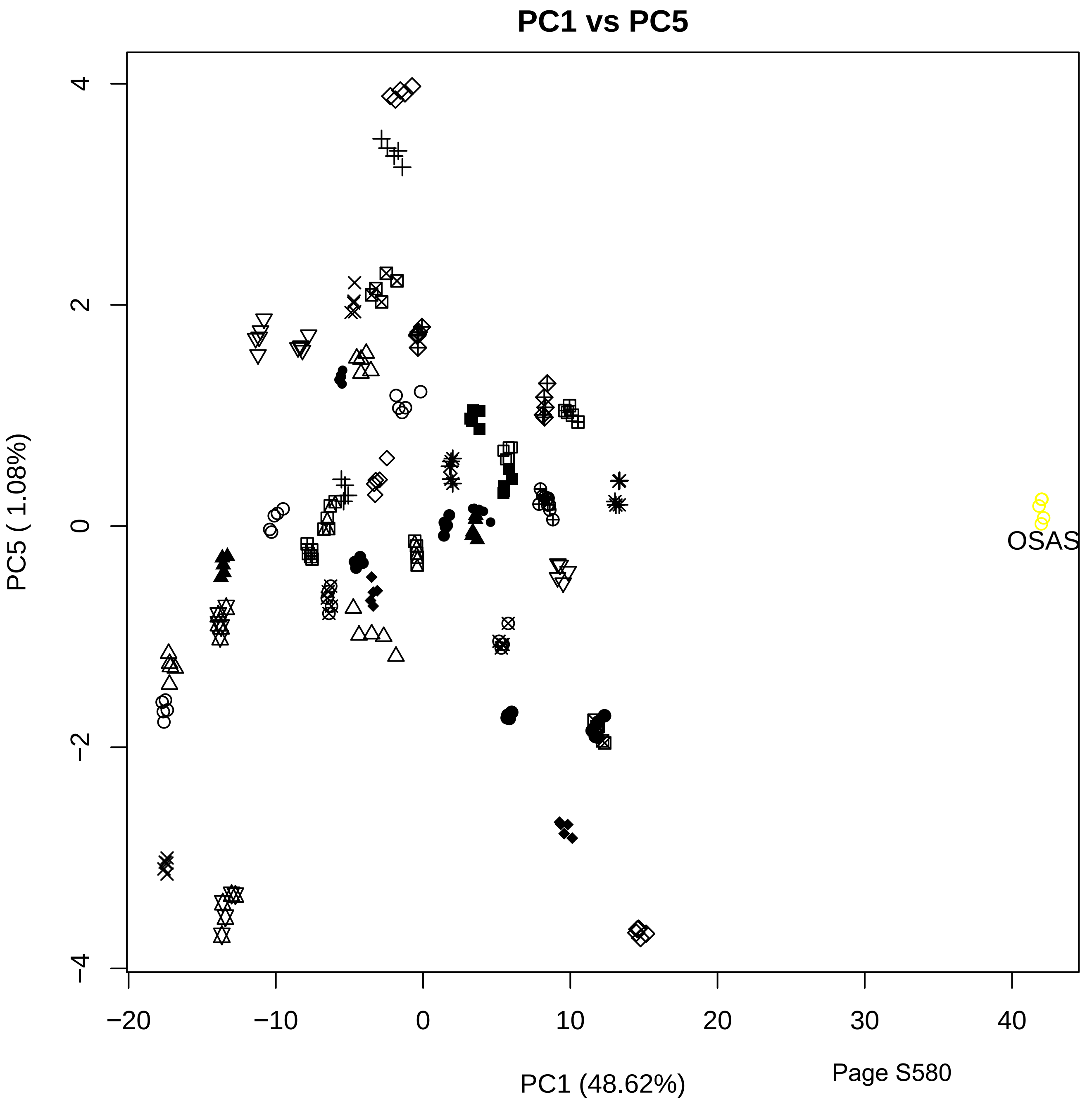
PC1 vs PC5
PC5 (1.08%)
PC1 (48.62%)
OSAS
4
2
0
−2
−4
−20
−10
0
10
20
30
40

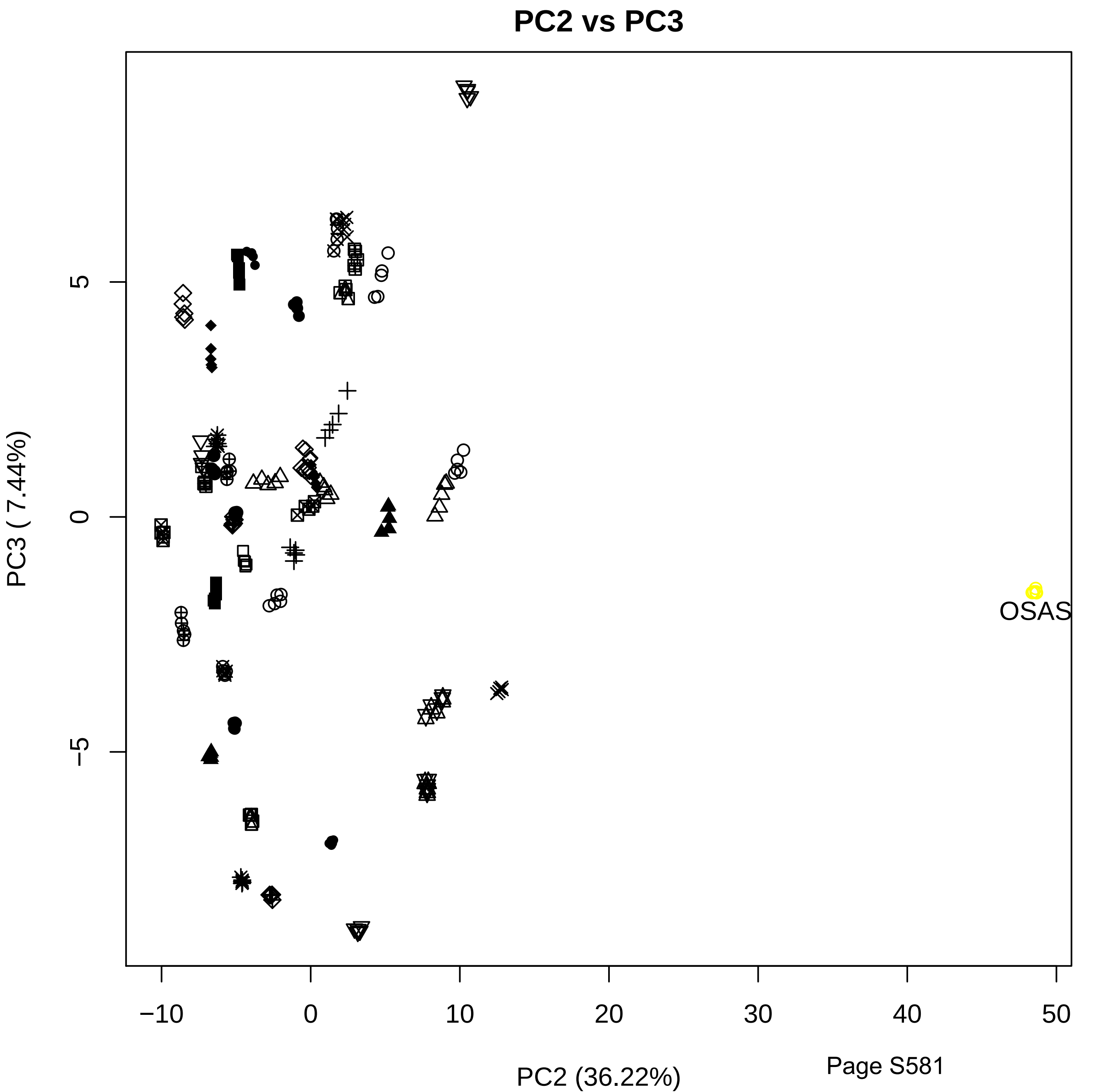
PC2 vs PC3
PC3 (7.44%)
PC2 (36.22%)
5
0
−5
−10
0
10
20
30
40
50
OSAS

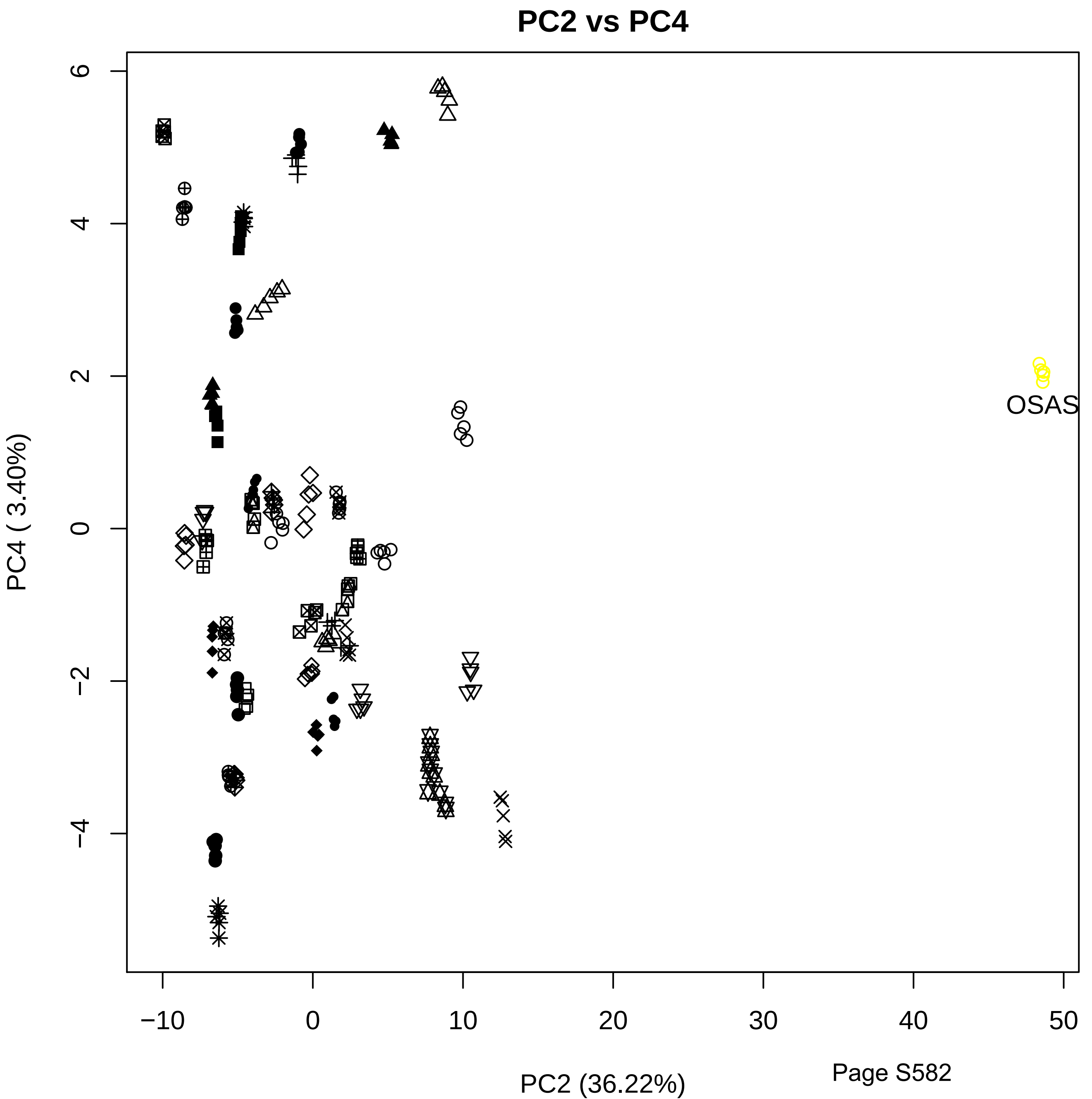
PC2 vs PC4
PC4 (3.40%)
PC2 (36.22%)
OSAS
6
4
2
0
−2
−4
−10
0
10
20
30
40
50

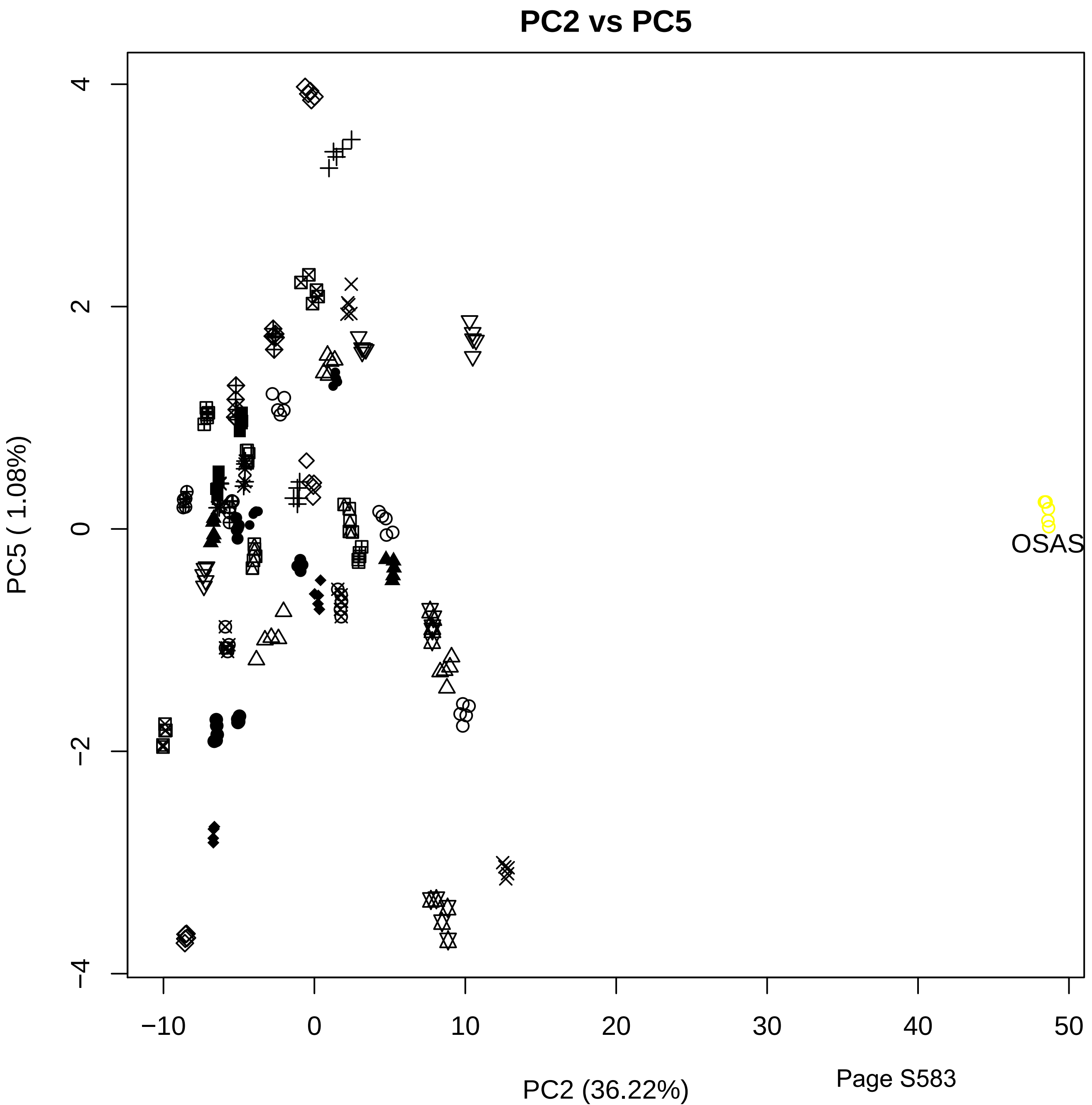
PC2 vs PC5
4
2
0
−2
−4
PC5 (1.08%)
OSAS
−10
0
10
20
30
40
50
PC2 (36.22%)

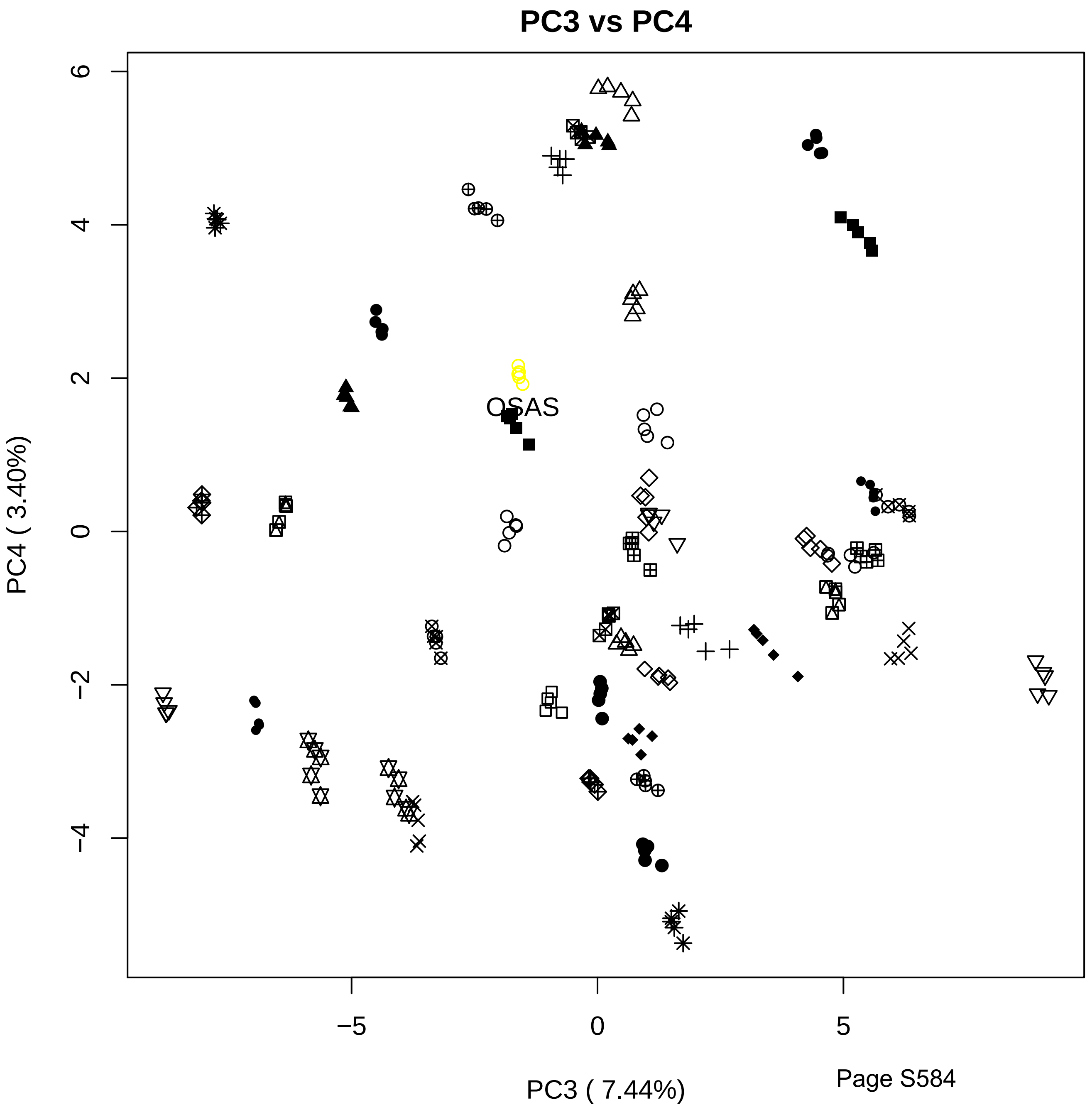

PC3 vs PC4
OSAS
PC4 (3.40%)
PC3 (7.44%)
6
4
2
0
−2
−4
−5
0
5

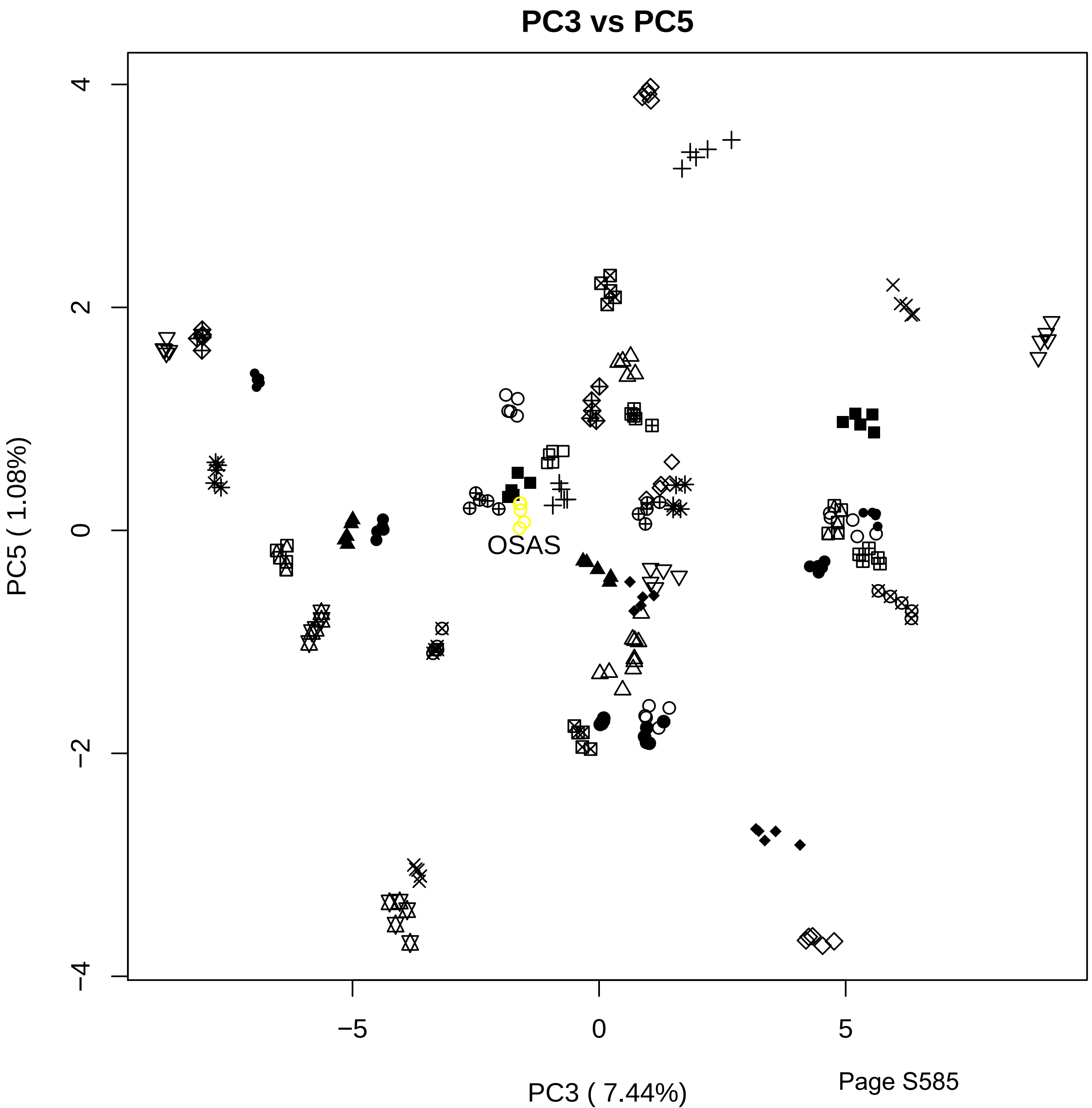
PC3 vs PC5
PC5 (1.08%)
PC3 (7.44%)
4
2
0
−2
−4
−5
0
5
OSAS

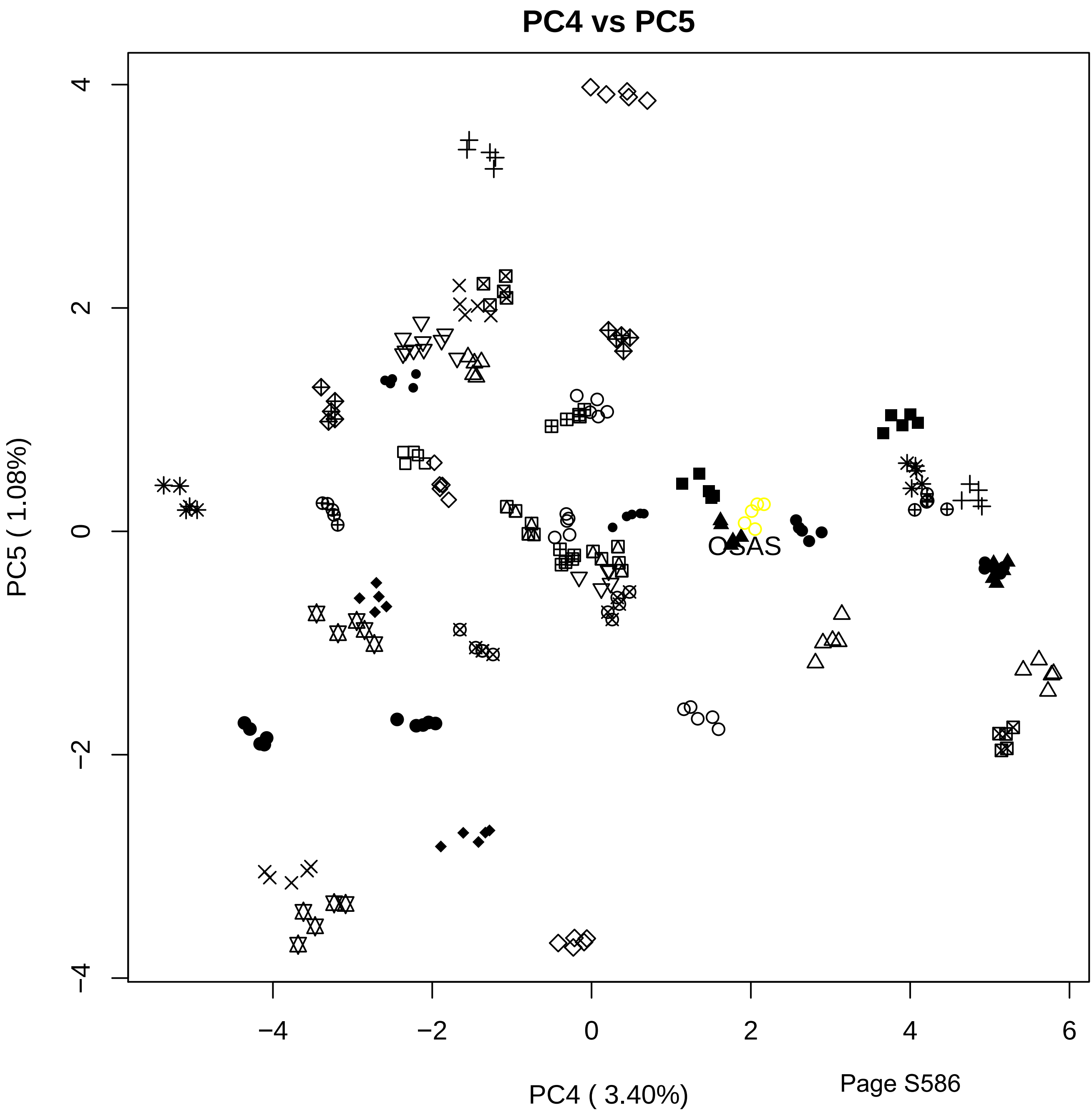
PC4 vs PC5
PC5 (1.08%)
PC4 (3.40%)
OSAS
4
2
0
−2
−4
−4
−2
0
2
4
6

DeS Contam 7th

Anthony Devlin, Keele University

PCA of All DeS vs PMH

Scaled Matrix PCAed

7th order polynomial baseline correction performed

Raw spectra smoothed to 21 Neighbours, 2nd order PN, Savtisky Golay

Normalisation between 0 and 100

D2 spectra smoothed to 41 Neighbours, 2nd order PN, Savtisky Golay

Regions Analysed: 3601−2501 and 2001−700cm−1

Samples Analysed: 12 Contam's 1 DeS's 45 PMH's

Keele University AD Sun Dec 09 17:03:33 2018

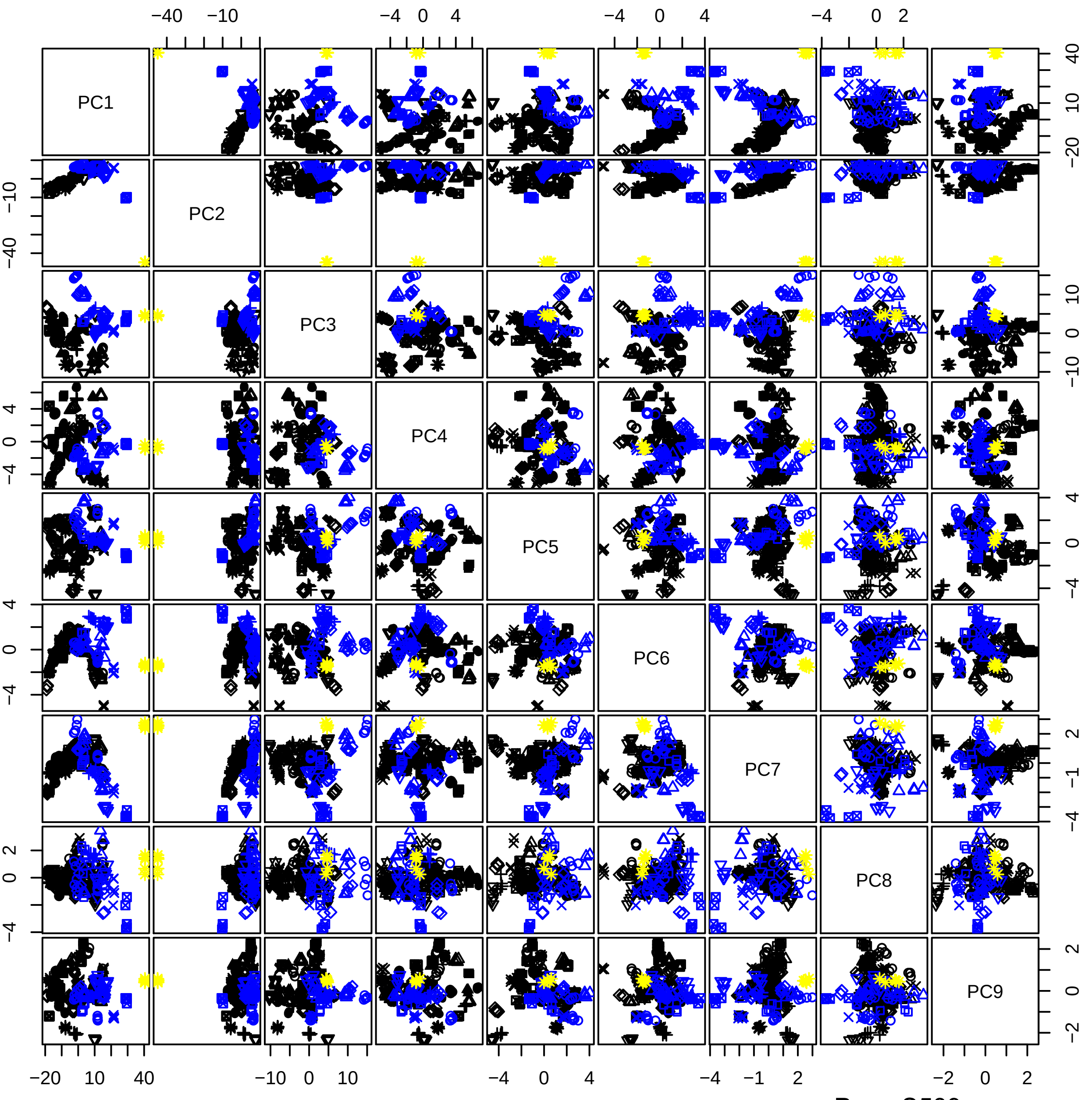

PC1
PC2
PC3
PC4
PC5
PC6
PC7
PC8
PC9

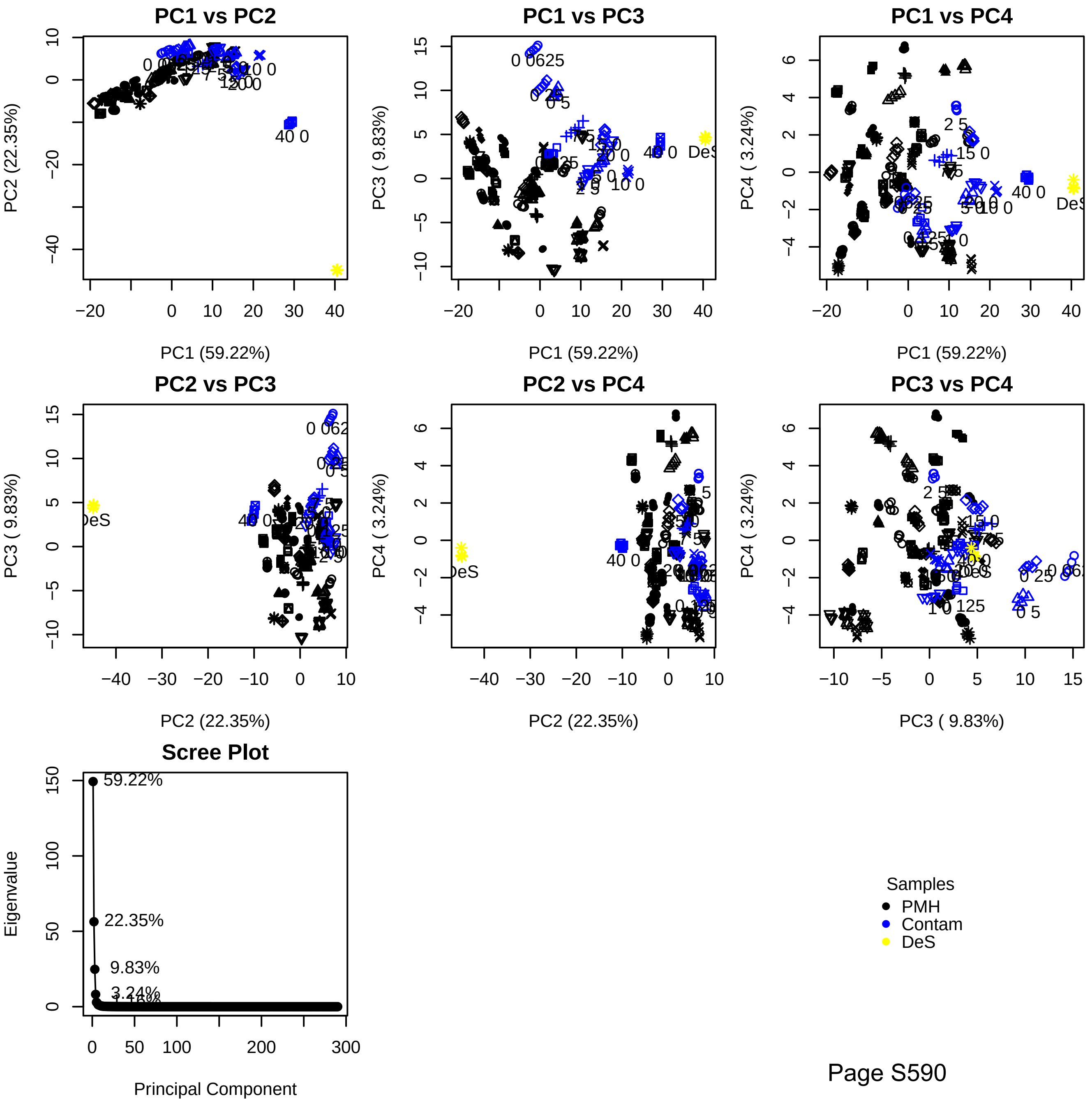
PC1 vs PC2
PC1 vs PC3
PC1 vs PC4
PC2 vs PC3
PC2 vs PC4
PC3 vs PC4
PC1 (59.22%)
PC2 (22.35%)
PC3 (9.83%)
PC4 (3.24%)
Scree Plot
Eigenvalue
Principal Component
59.22%
22.35%
9.83%
3.24%
Samples
PMH
Contam
DeS

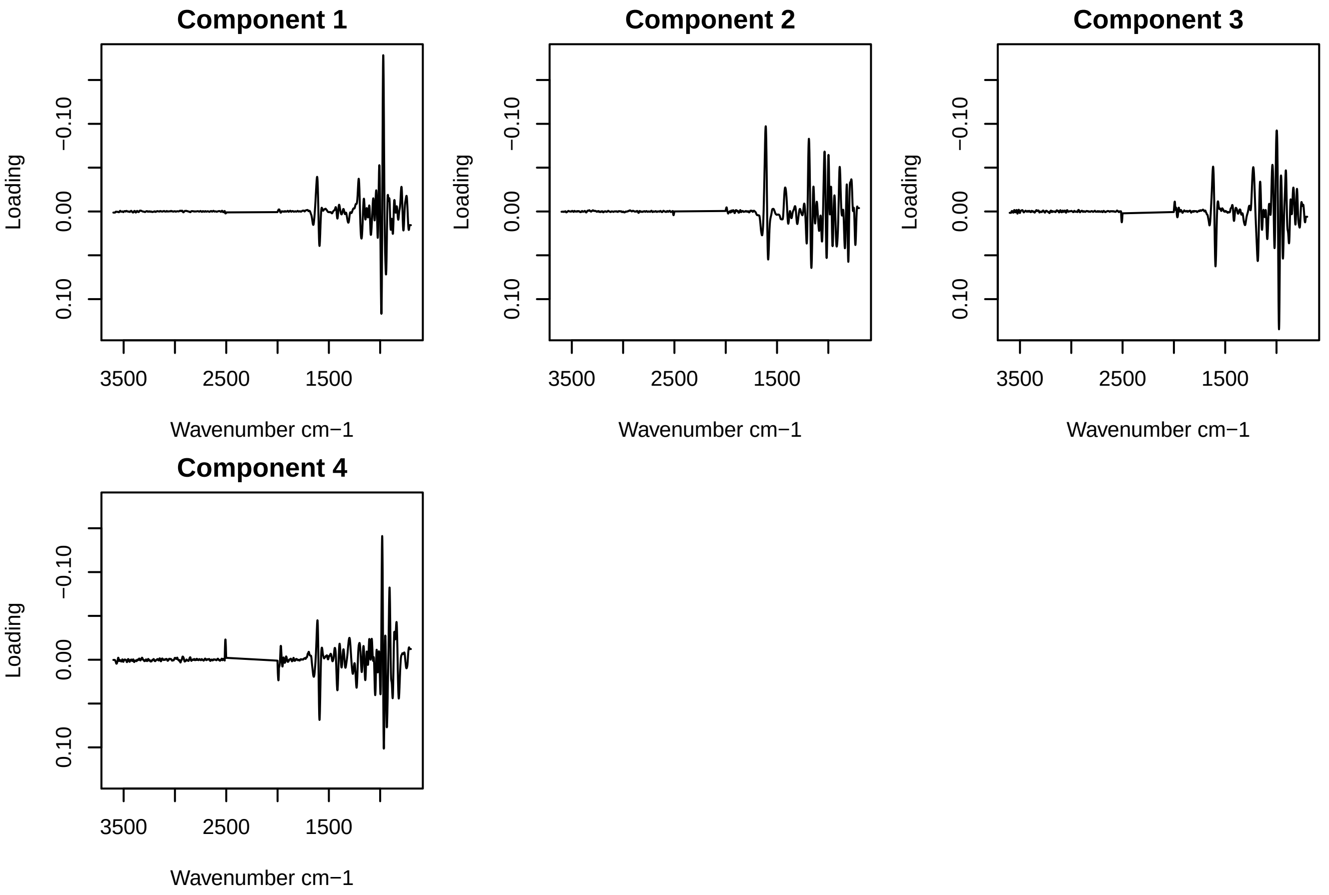
Component 1
Component 2
Component 3
Component 4
Loading
−0.10
0.00
0.10
3500
2500
1500
Wavenumber cm−1

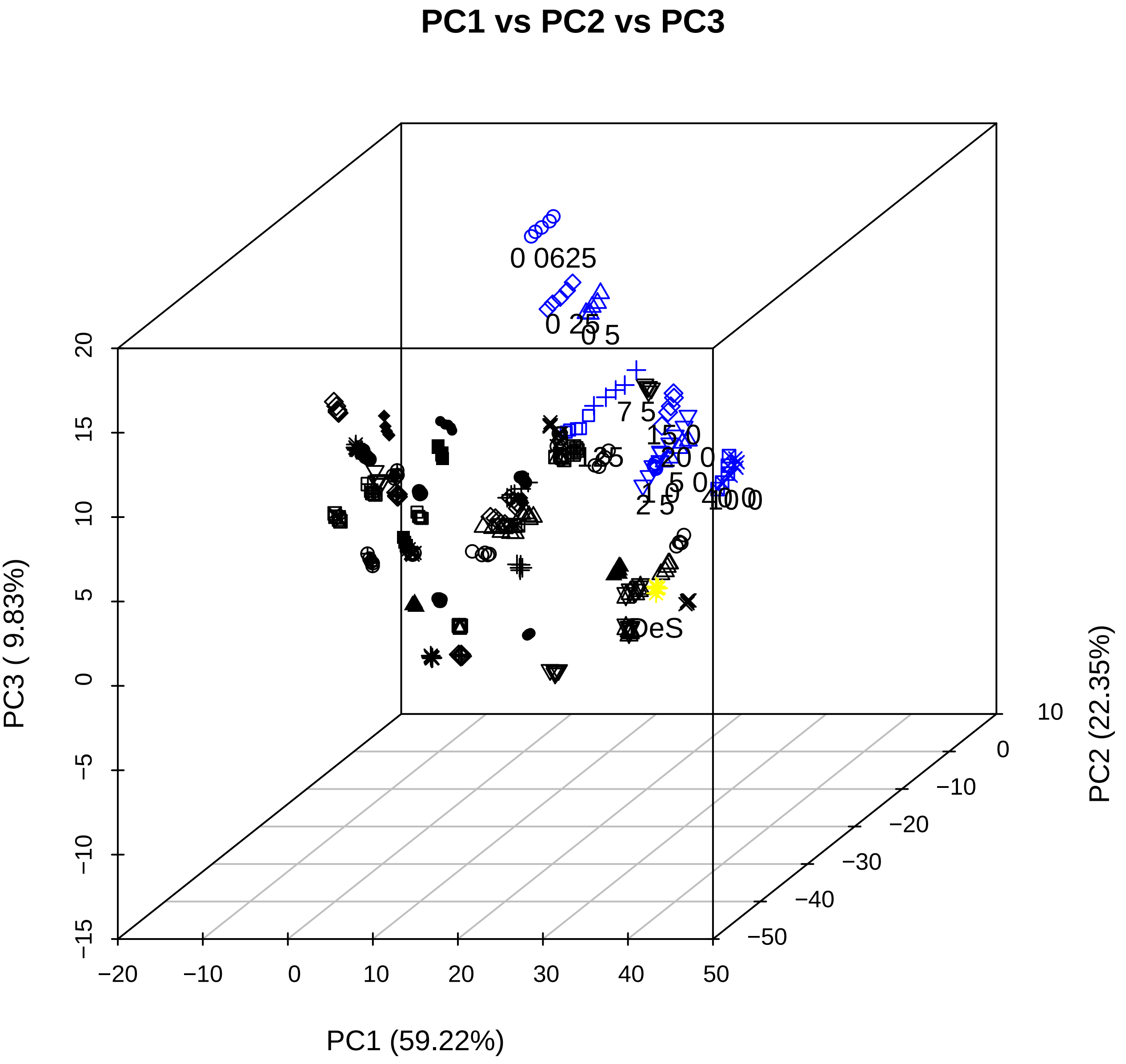
PC1 vs PC2 vs PC3
0 0625
0 25
0 5
7 5
DeS
PC1 (59.22%)
PC2 (22.35%)
PC3 (9.83%)
−20
−10
0
10
20
30
40
50
−50
−40
−30
−15
−5
5
15

PC2 vs PC3 vs PC4

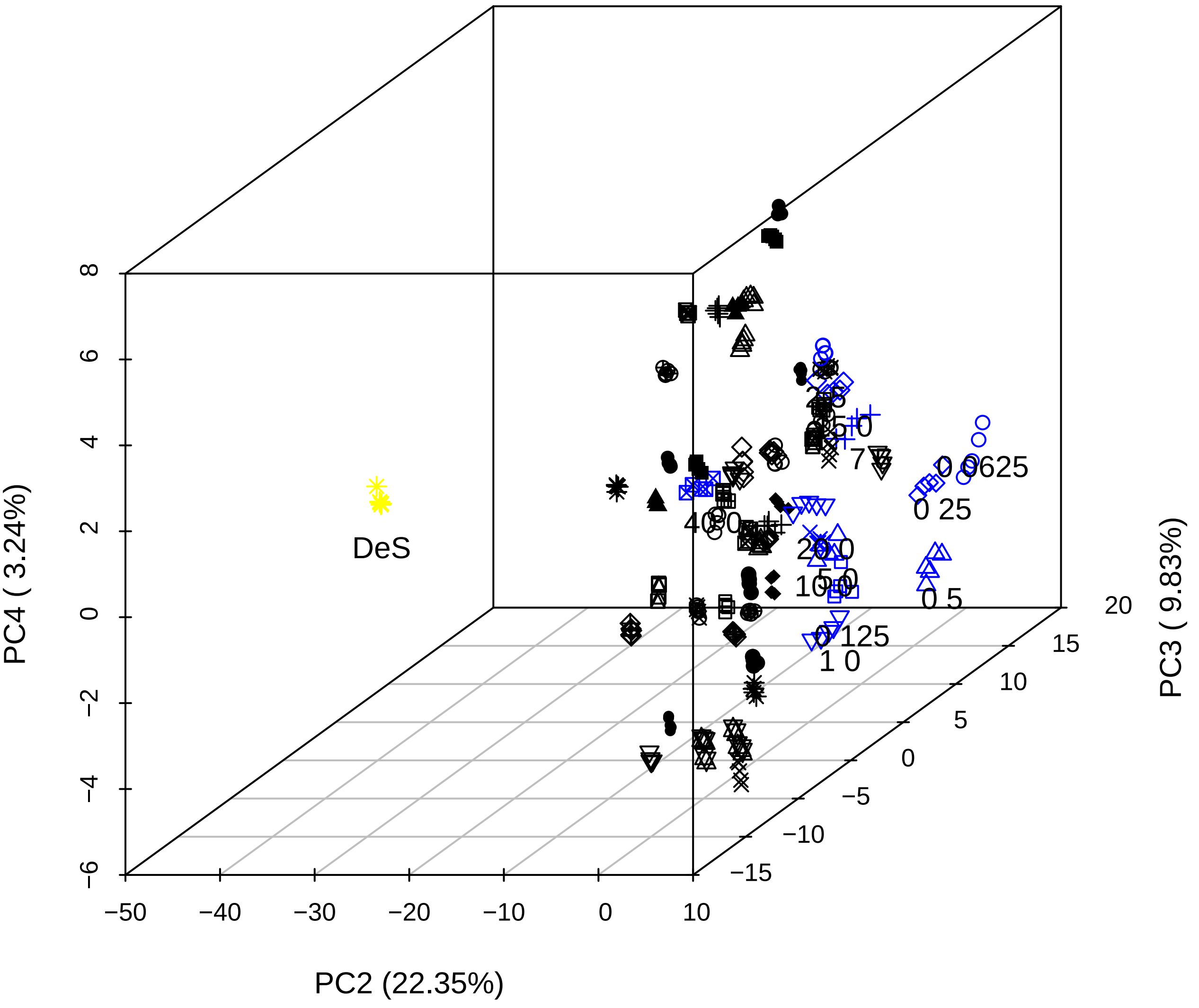

PC1 vs PC2 vs PC4

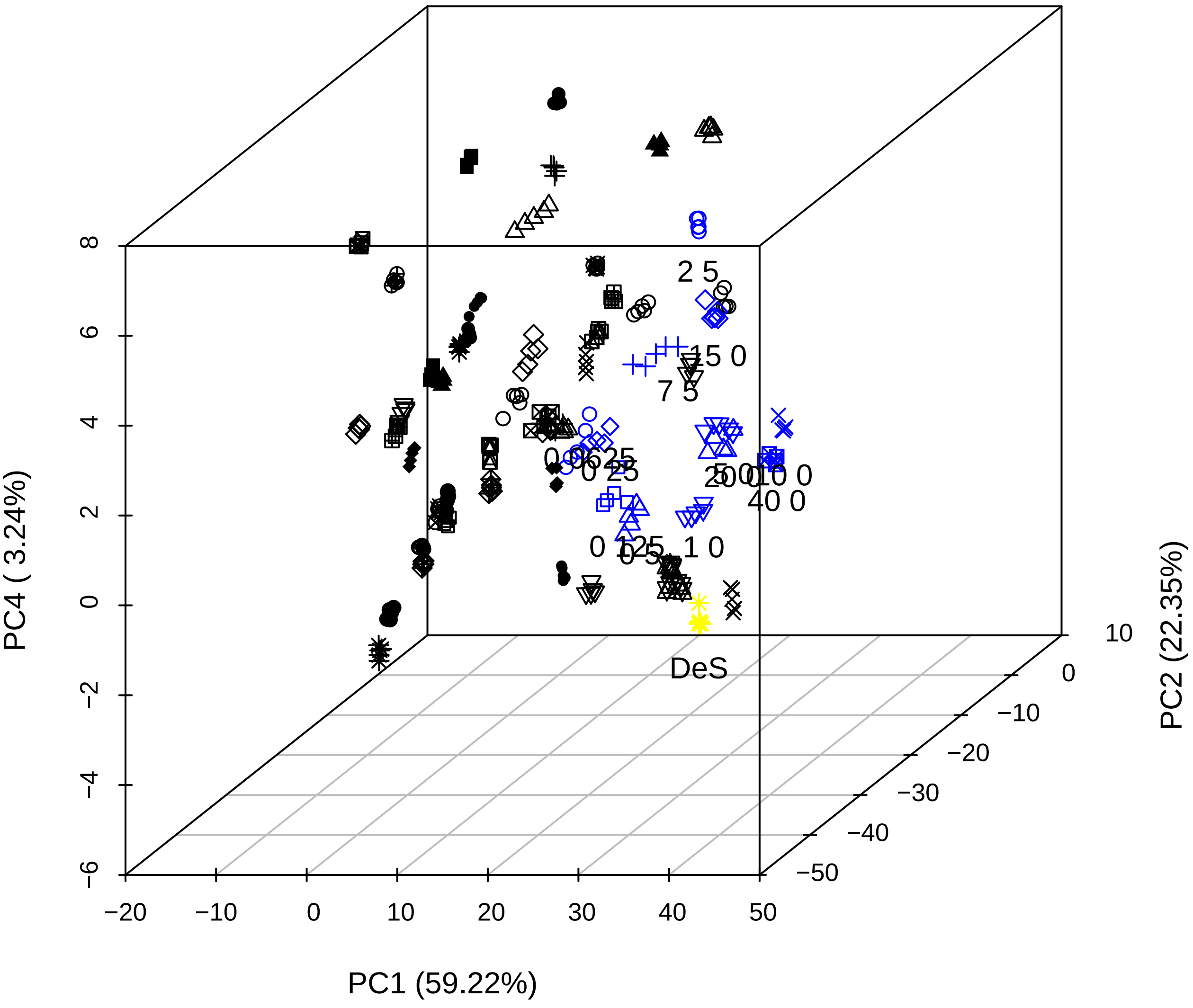

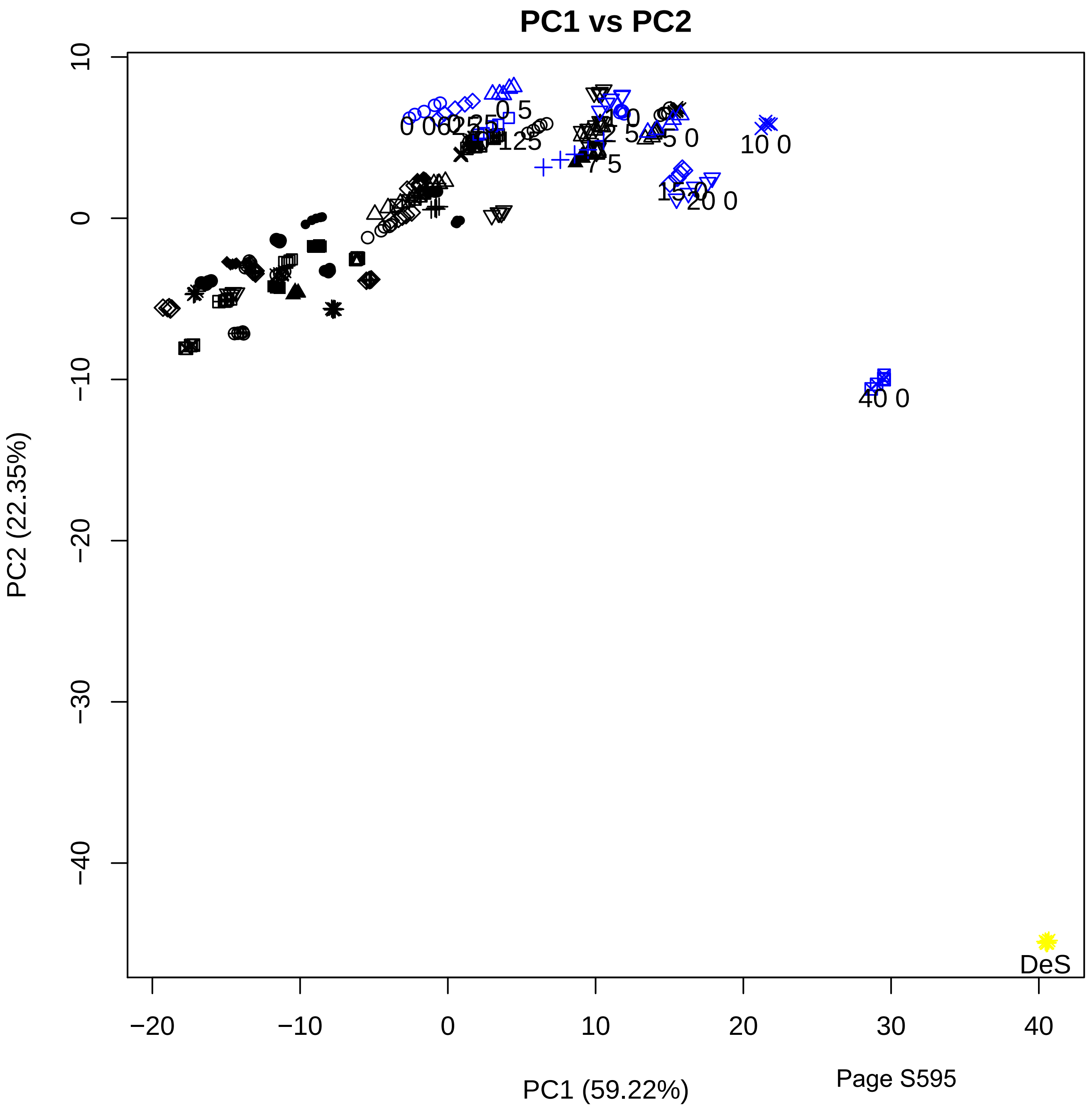
PC1 vs PC2
PC2 (22.35%)
PC1 (59.22%)
10
0
−10
−20
−30
−40
−20
−10
0
10
20
30
40
0 5
2 5
5 0
10 0
7 5
20 0
40 0
DeS

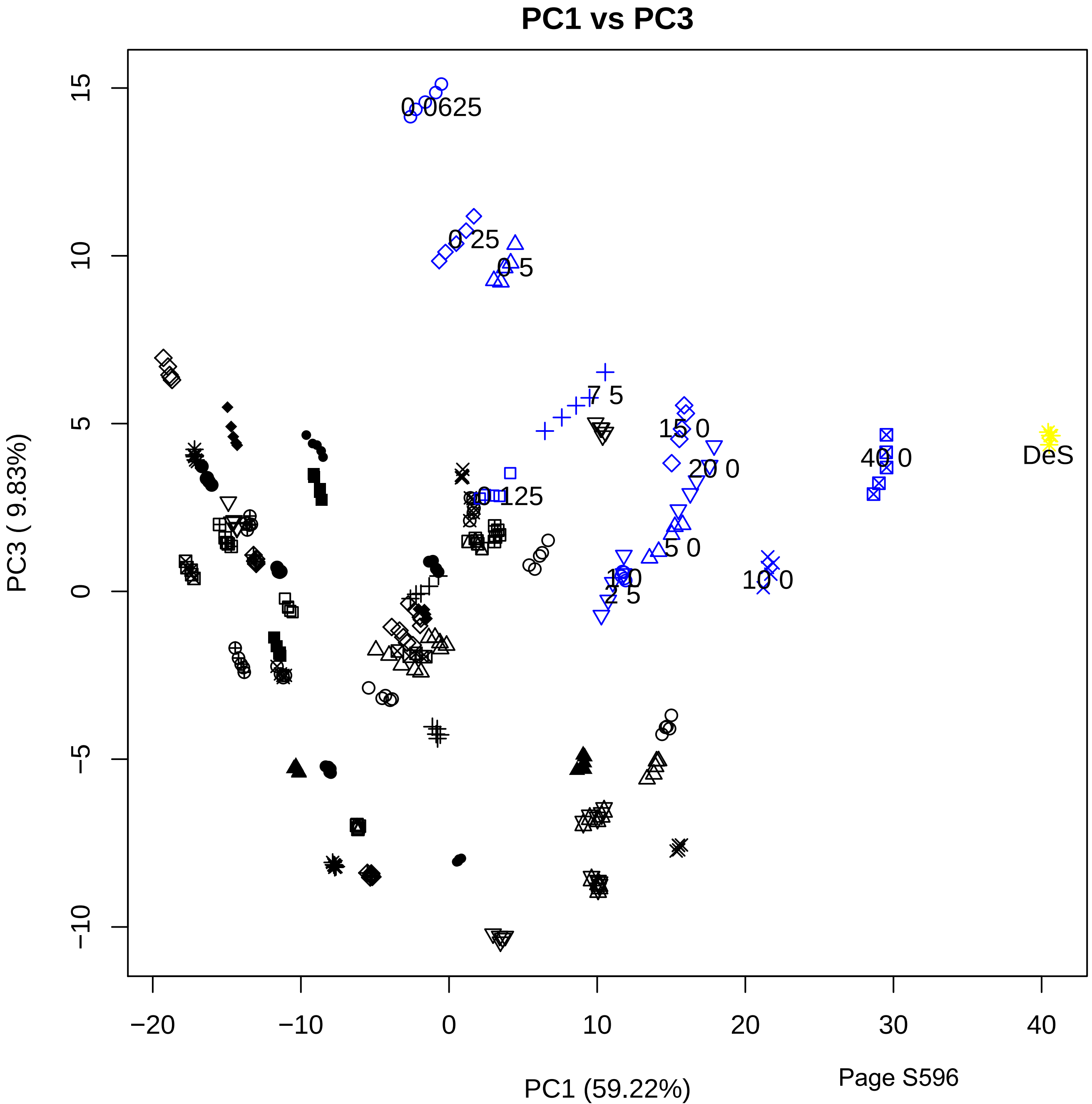

PC1 vs PC3
PC3 (9.83%)
PC1 (59.22%)
15
10
5
0
−5
−10
−20
−10
0
10
20
30
40
0 0625
0 25
0 5
7 5
15 0
20 0
40 0
DeS
0 125
5 0
1 0
2 5
10 0

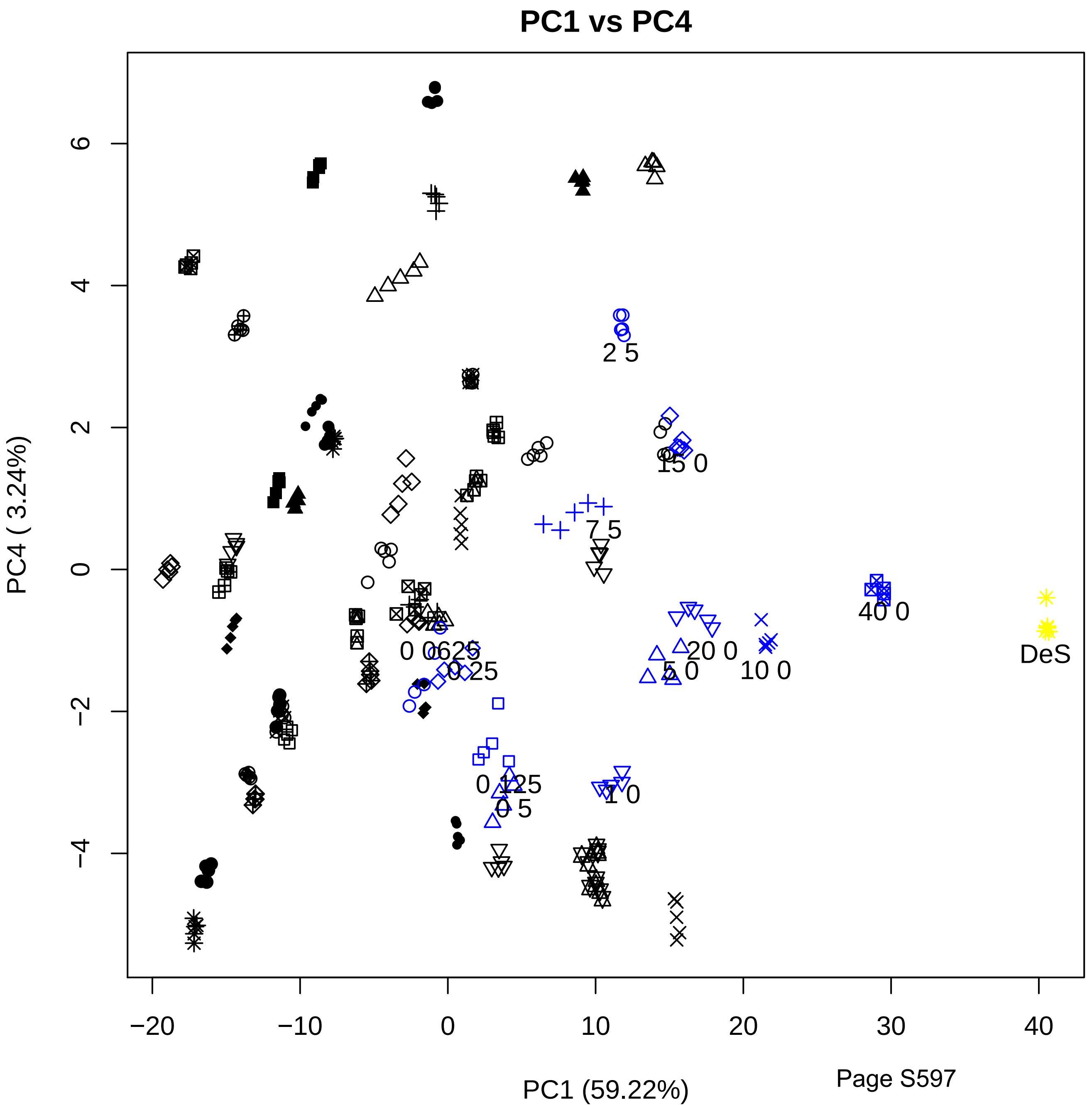

PC1 vs PC4
PC4 (3.24%)
PC1 (59.22%)
6
4
2
0
−2
−4
−20
−10
0
10
20
30
40
2 5
15 0
7 5
40 0
DeS
20 0
5 0
10 0
0 0625
0 25
0 125
0 5
1 0

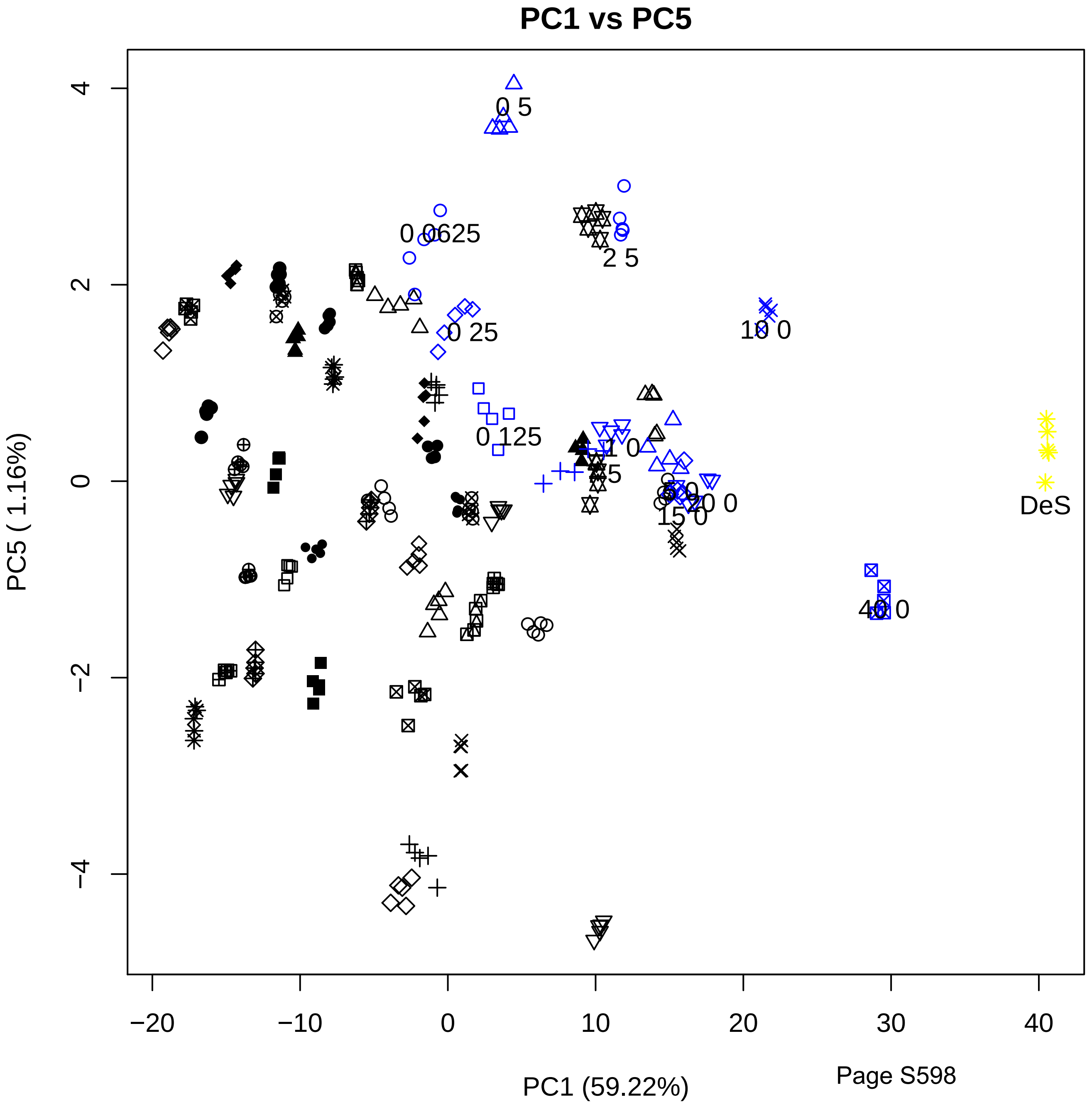
PC1 vs PC5
PC5 (1.16%)
PC1 (59.22%)
4
2
0
−2
−4
−20
−10
0
10
20
30
40
0 5
0 0625
2 5
0 25
10 0
0 125
1 0
5
5 0
20 0
15 0
40 0
DeS

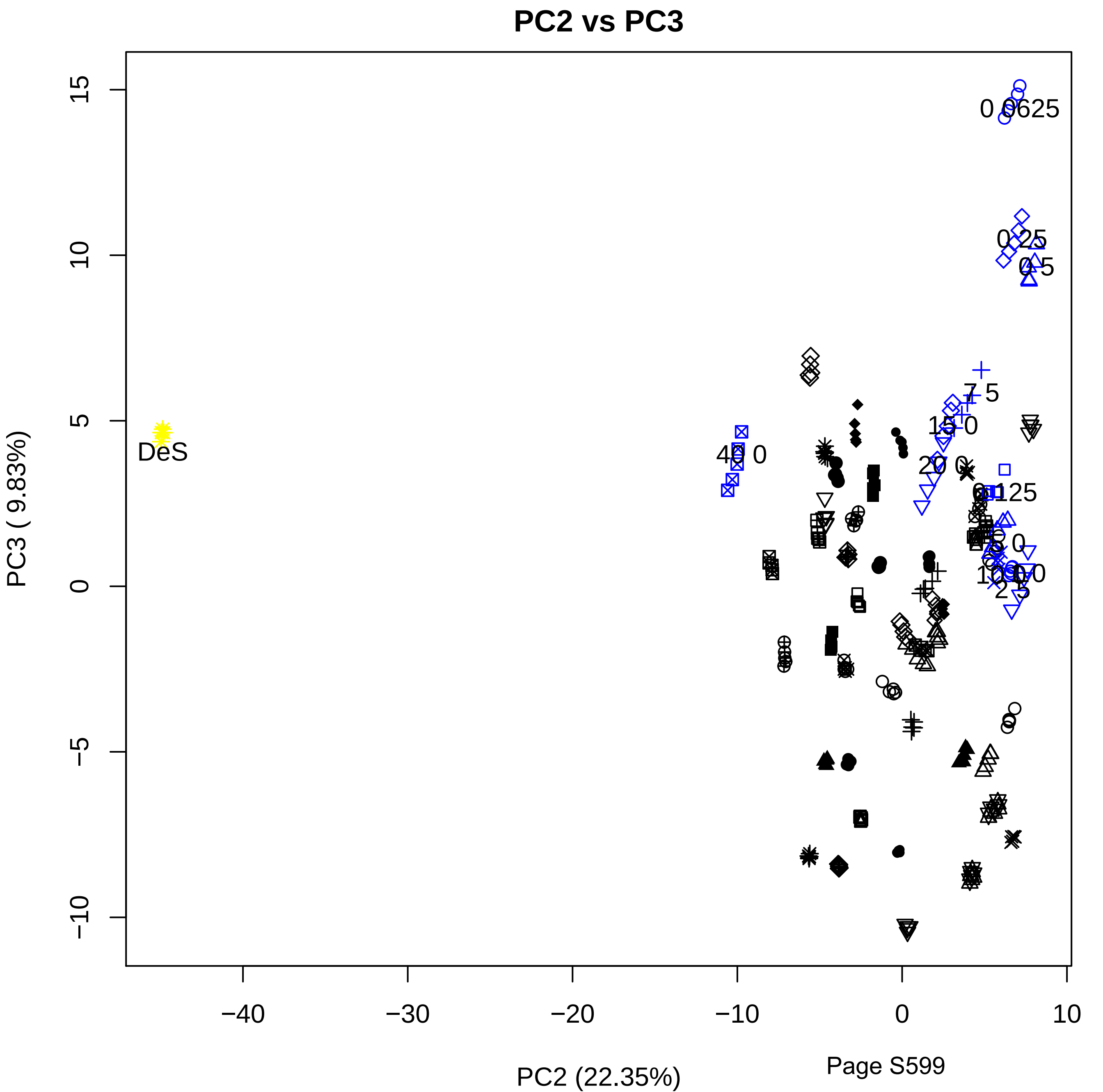
PC2 vs PC3
PC3 (9.83%)
PC2 (22.35%)
15
10
5
0
−5
−10
−40
−30
−20
−10
0
10
0.0625
0.25
0.5
7.5
15.0
40.0
20.0
0.125
DeS
5.0
10.0
2.5

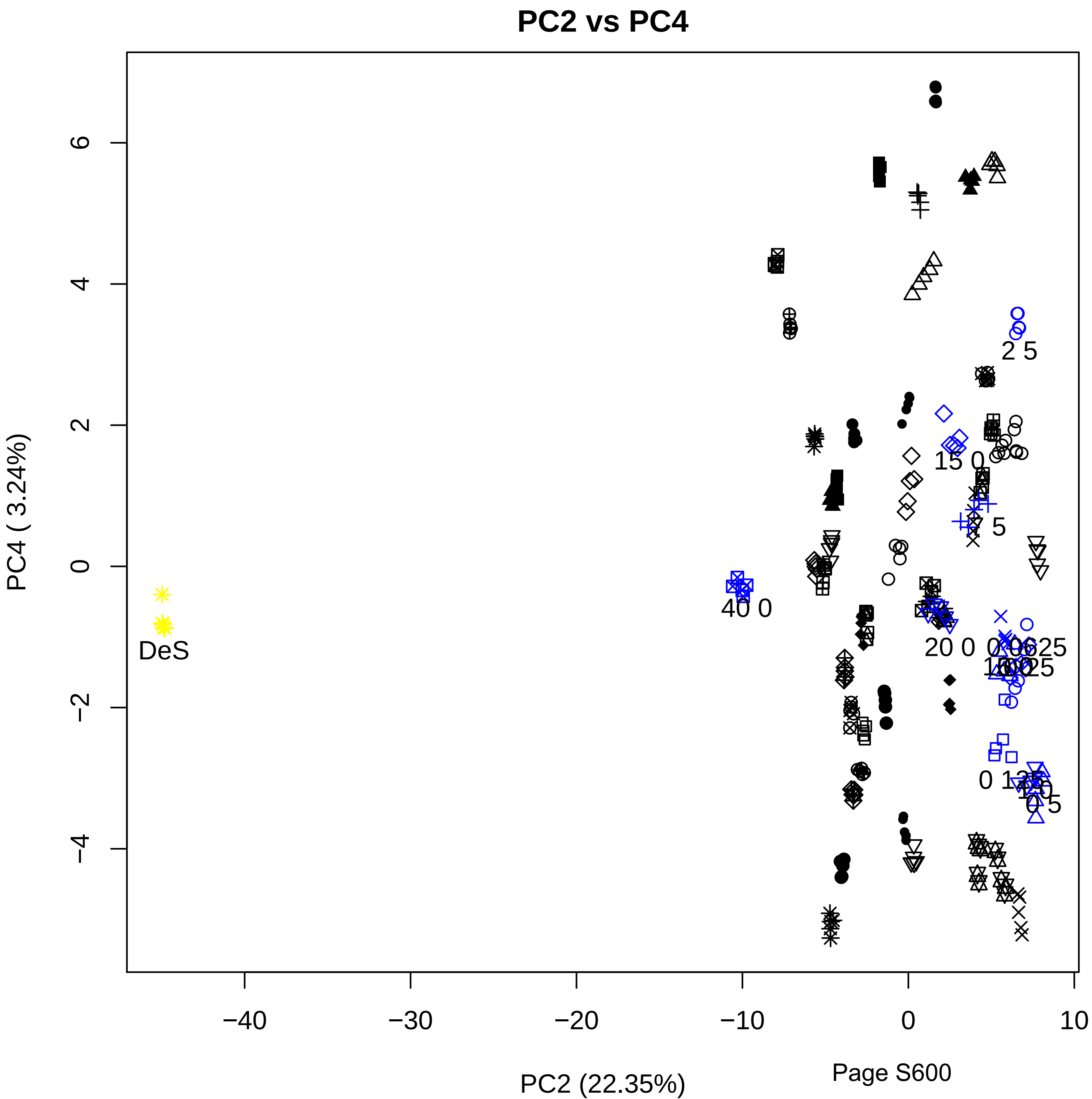
PC2 vs PC4
PC4 (3.24%)
PC2 (22.35%)
6
4
2
0
−2
−4
−40
−30
−20
−10
0
10
2 5
15 0
7 5
40 0
DeS
20 0

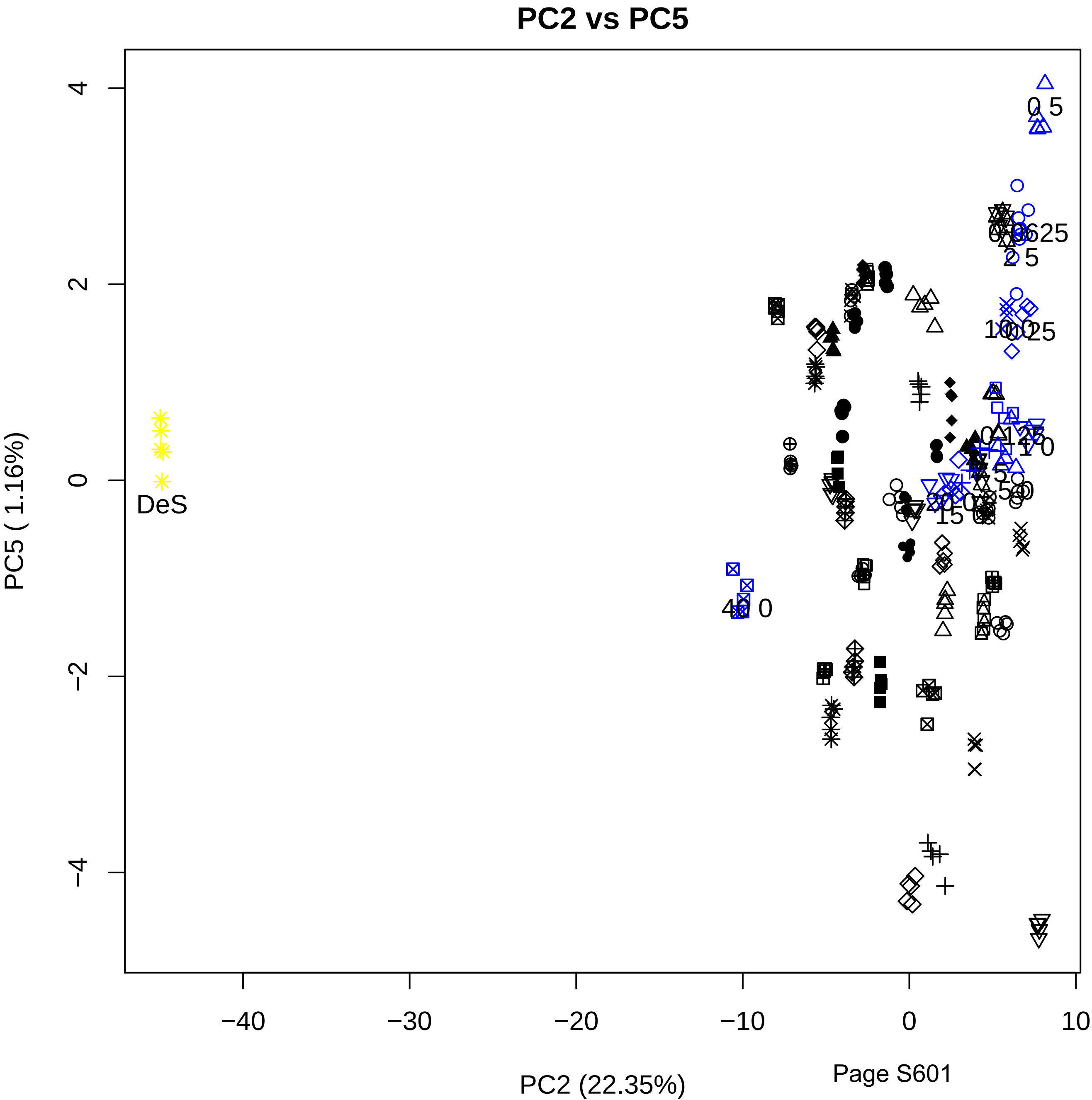
PC2 vs PC5
PC5 (1.16%)
PC2 (22.35%)
4
2
0
−2
−4
−40
−30
−20
−10
0
10
DeS

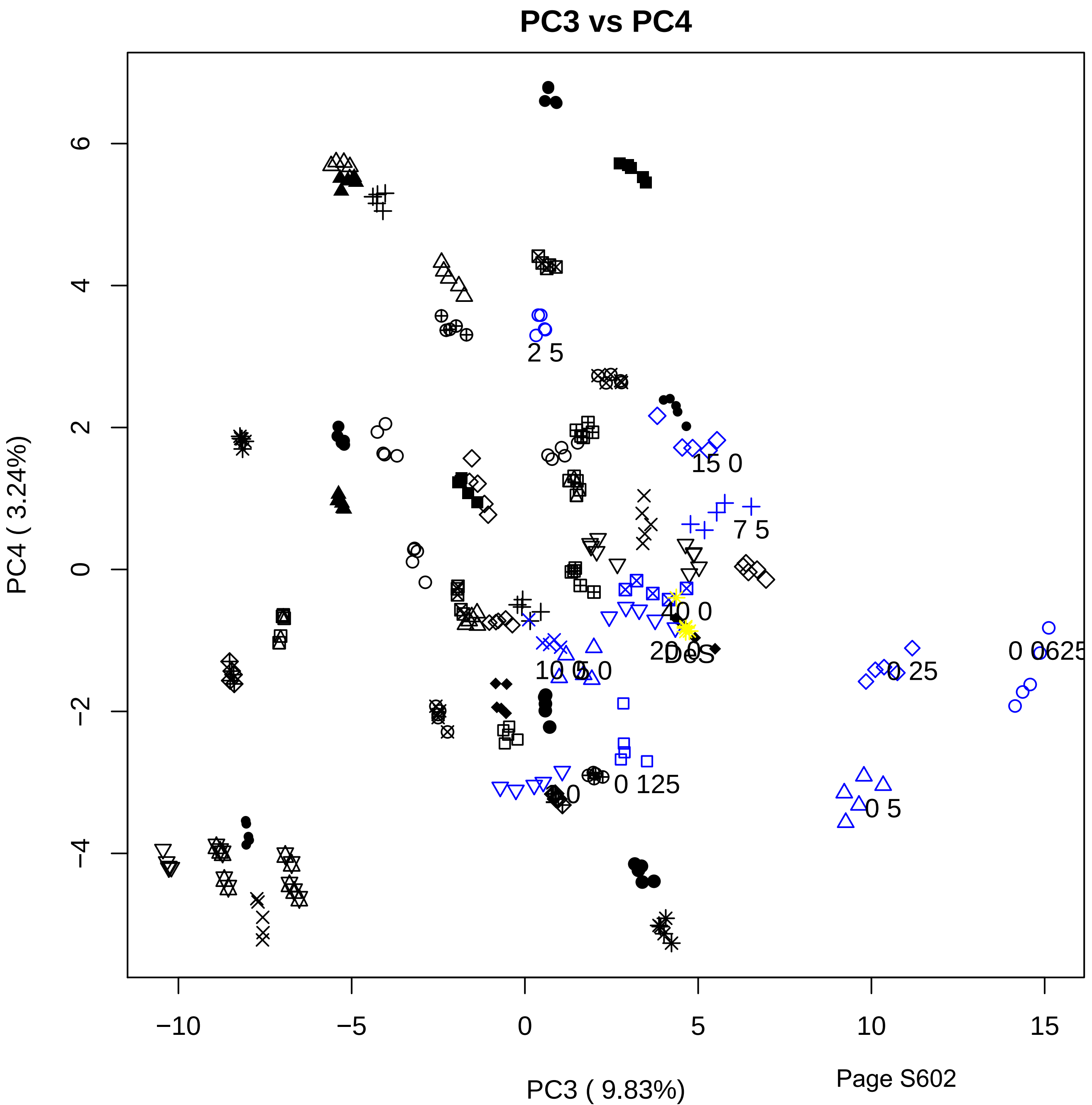
PC3 vs PC4
PC4 (3.24%)
PC3 (9.83%)
6
4
2
0
−2
−4
−10
−5
0
5
10
15
2 5
15 0
7 5
10 0
20 0
DeS
10 0
5 0
0 25
0 0625
0 125
0 5

PC3 vs PC5

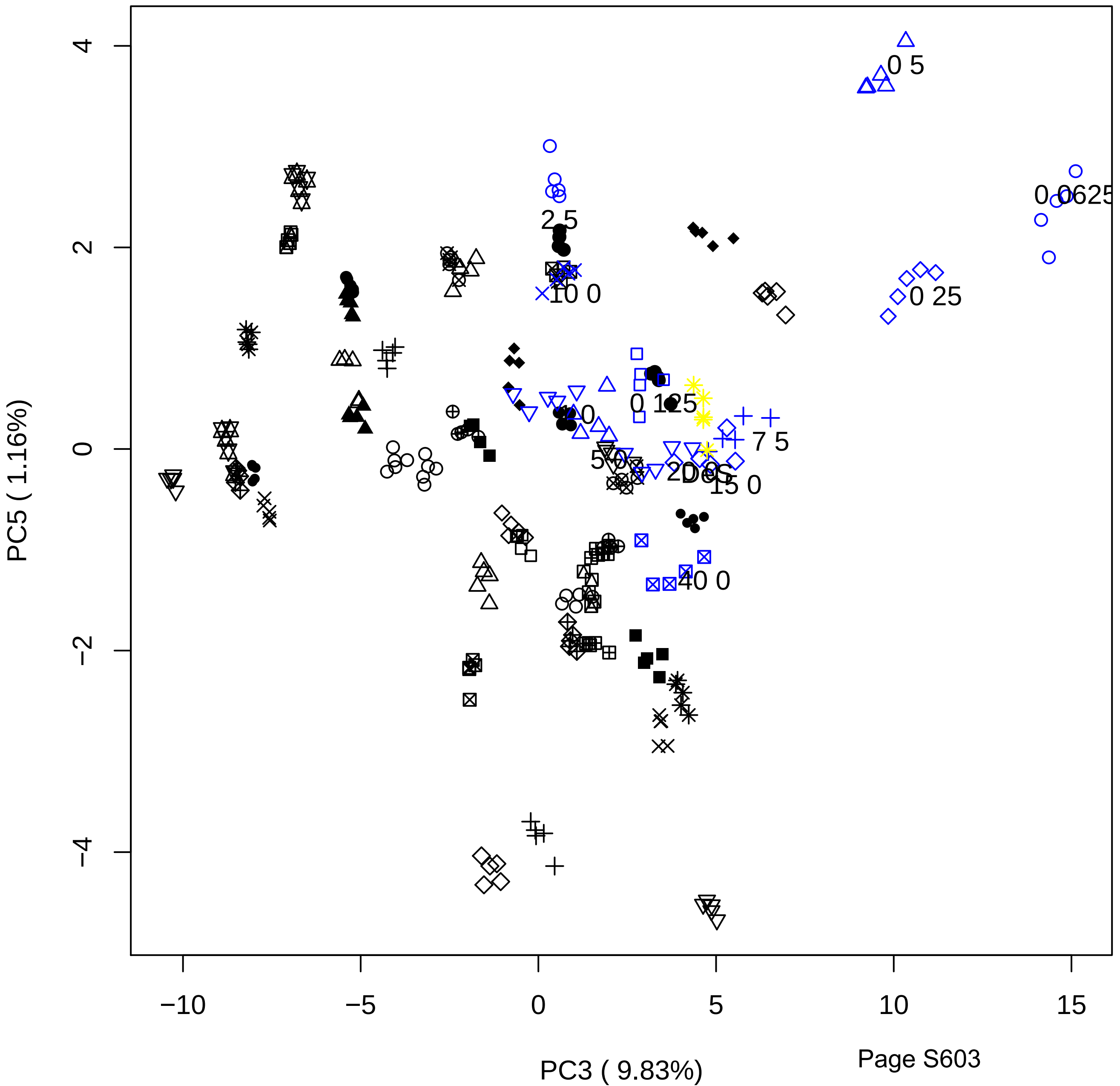
PC3 vs PC5
0 5
0 0625
2 5
10 0
0 25
0 125
7 5
5 0
2DeS
15 0
40 0
PC5 (1.16%)
PC3 (9.83%)
4
2
0
−2
−4
−10
−5
0
5
10
15

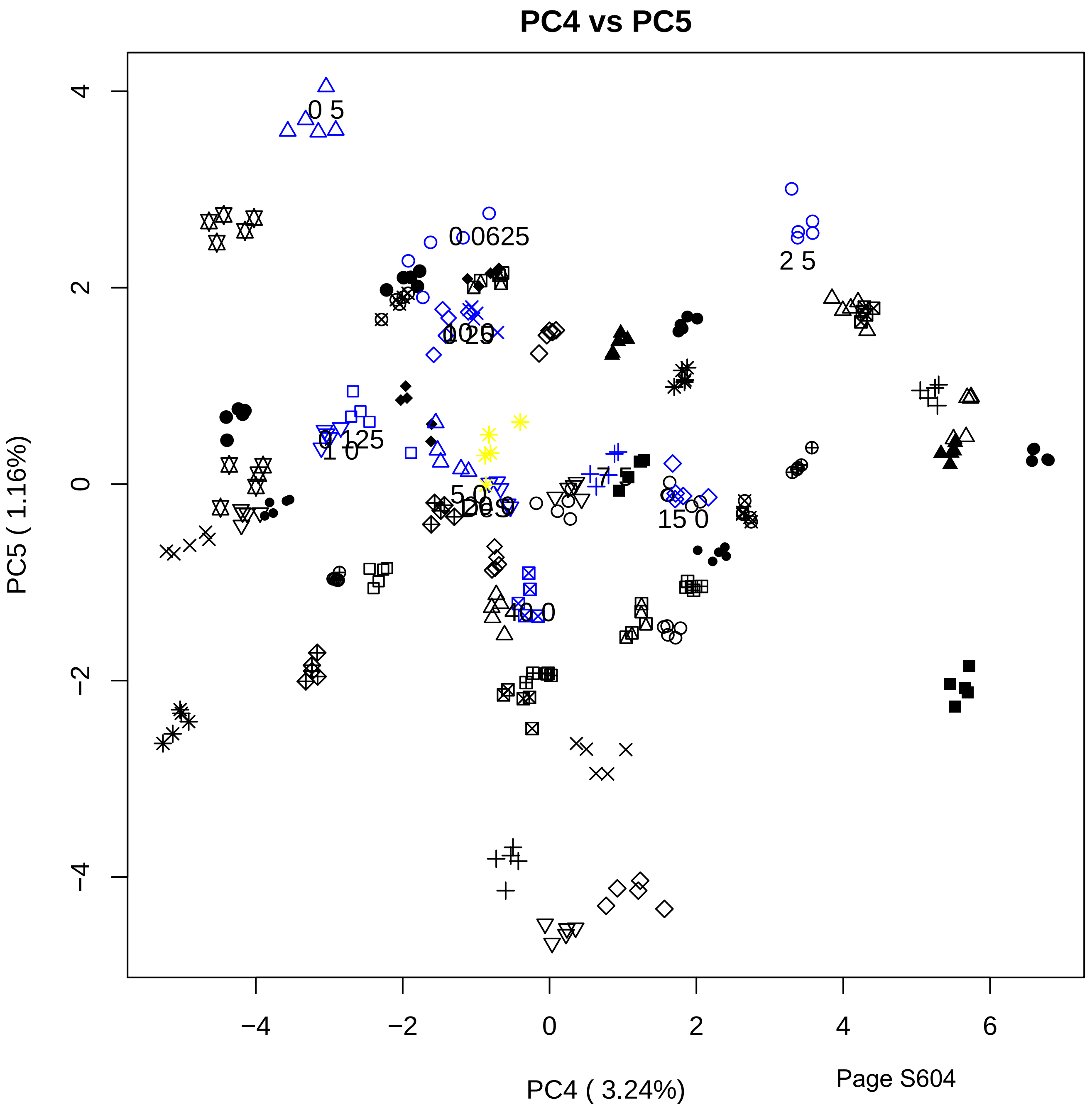
PC4 vs PC5
PC5 (1.16%)
PC4 (3.24%)
4
2
0
−2
−4
−4
−2
0
2
4
6
0 5
0 0625
2 5
0 25
0 125
1 0
5 0
DeS
7 5
15 0
40 0

PCA of 0.06% DeS vs PMH

Scaled Matrix PCAed

7th order polynomial baseline correction performed

Raw spectra smoothed to 21 Neighbours, 2nd order PN, Savtisky Golay

Normalisation between 0 and 100

D2 spectra smoothed to 41 Neighbours, 2nd order PN, Savtisky Golay

Regions Analysed: 3601−2501 and 2001−700cm−1

Samples Analysed: 1 Contam's 0 DeS's 45 PMH's

Keele University AD   Sun Dec 09 17:03:40 2018

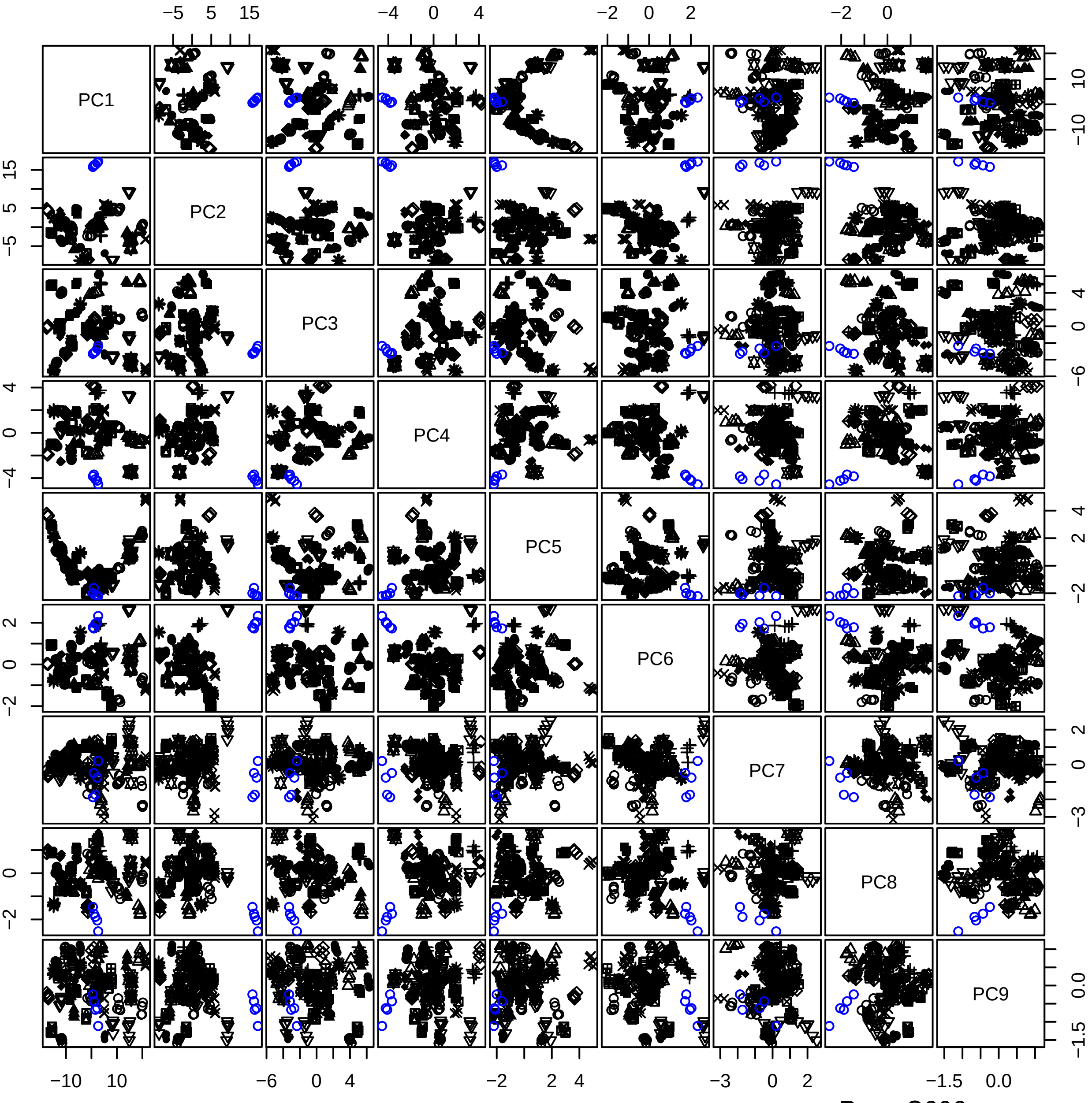
PC1
PC2
PC3
PC4
PC5
PC6
PC7
PC8
PC9

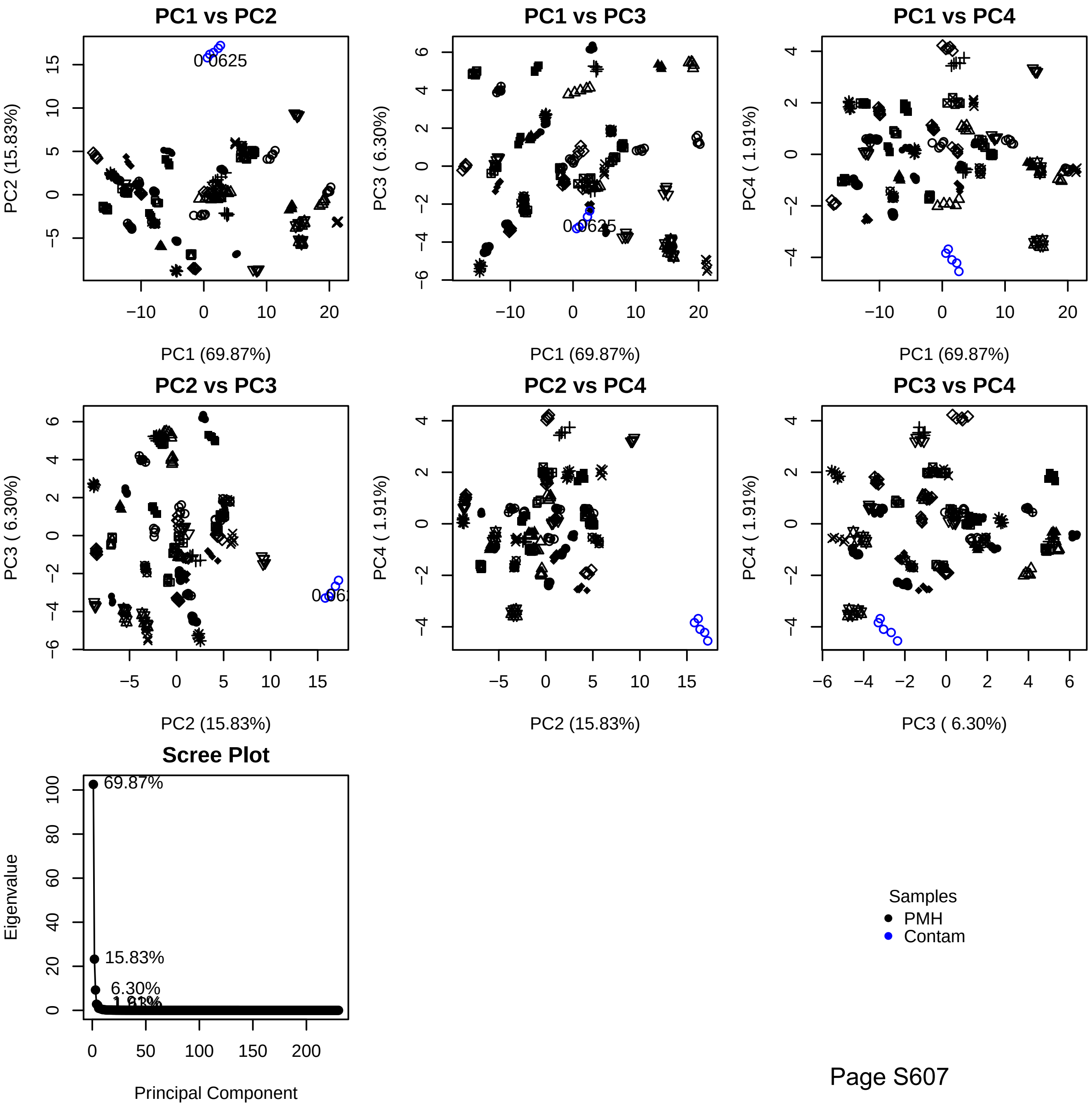
PC1 vs PC2
PC1 vs PC3
PC1 vs PC4
PC2 vs PC3
PC2 vs PC4
PC3 vs PC4
Scree Plot
PC1 (69.87%)
PC2 (15.83%)
PC3 (6.30%)
PC4 (1.91%)
0.0625
Eigenvalue
Principal Component
69.87%
15.83%
6.30%
Samples
PMH
Contam

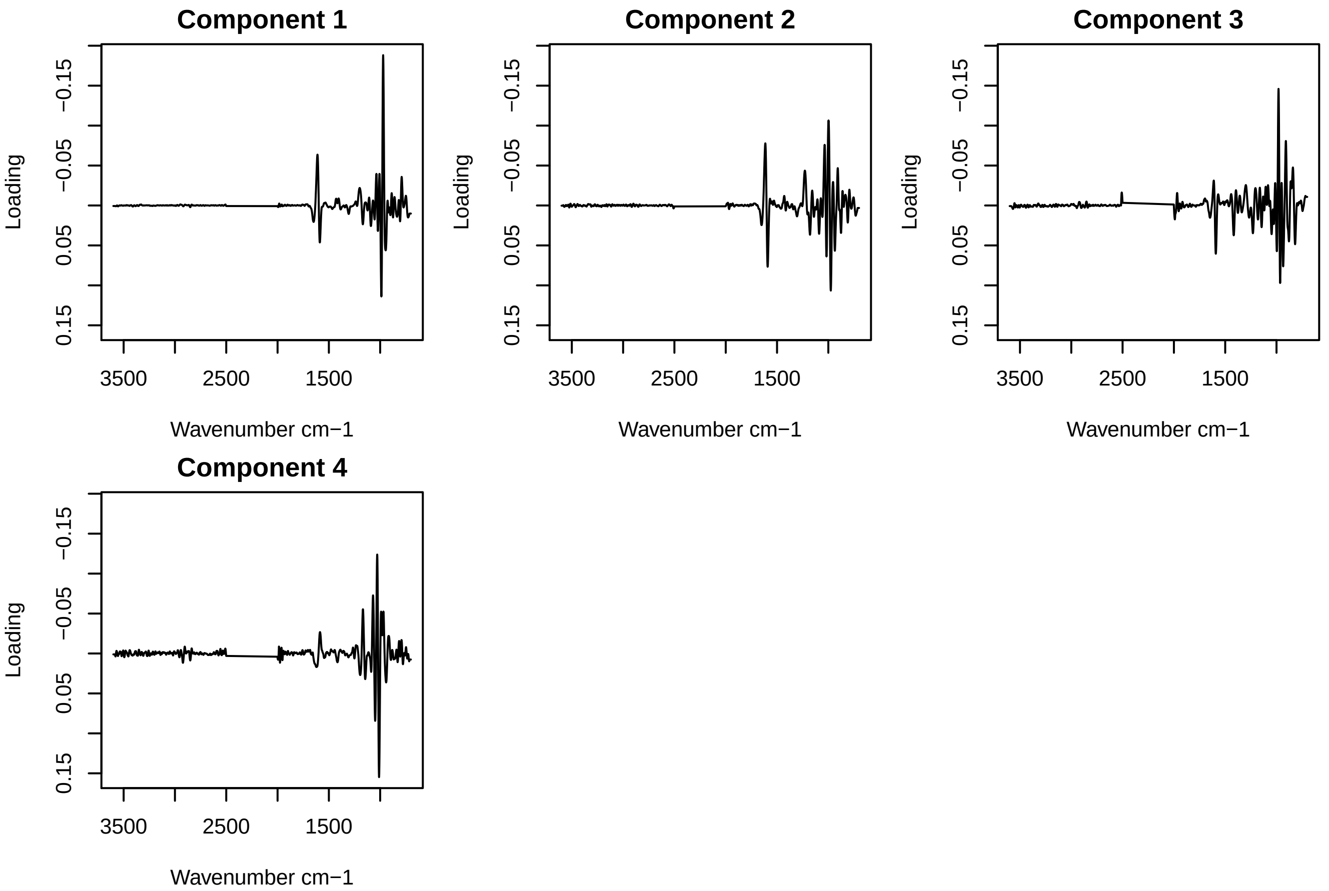

Component 1
Component 2
Component 3
Component 4
Loading
−0.15
−0.05
0.05
0.15
3500
2500
1500
Wavenumber cm−1

PC1 vs PC2 vs PC3

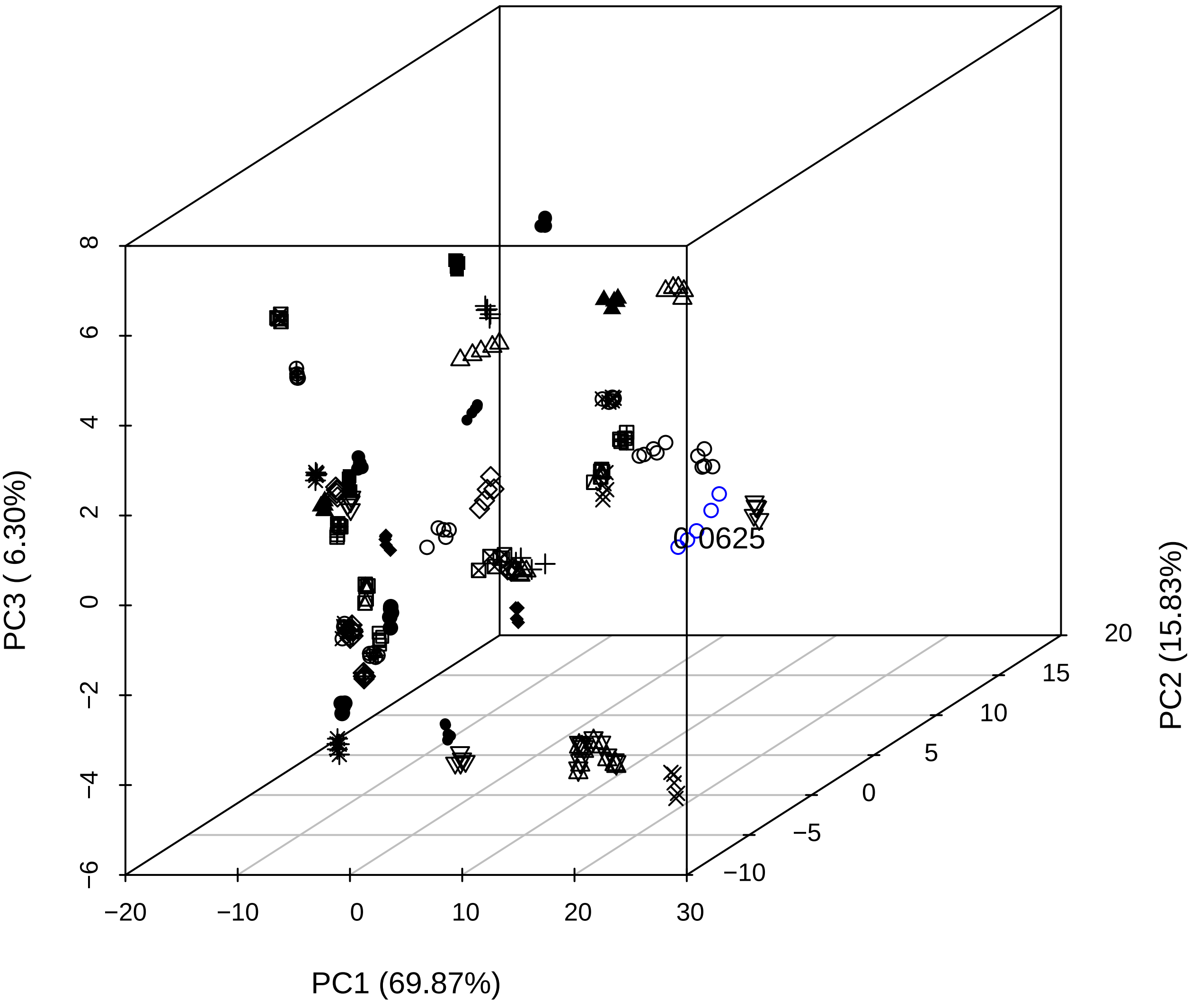

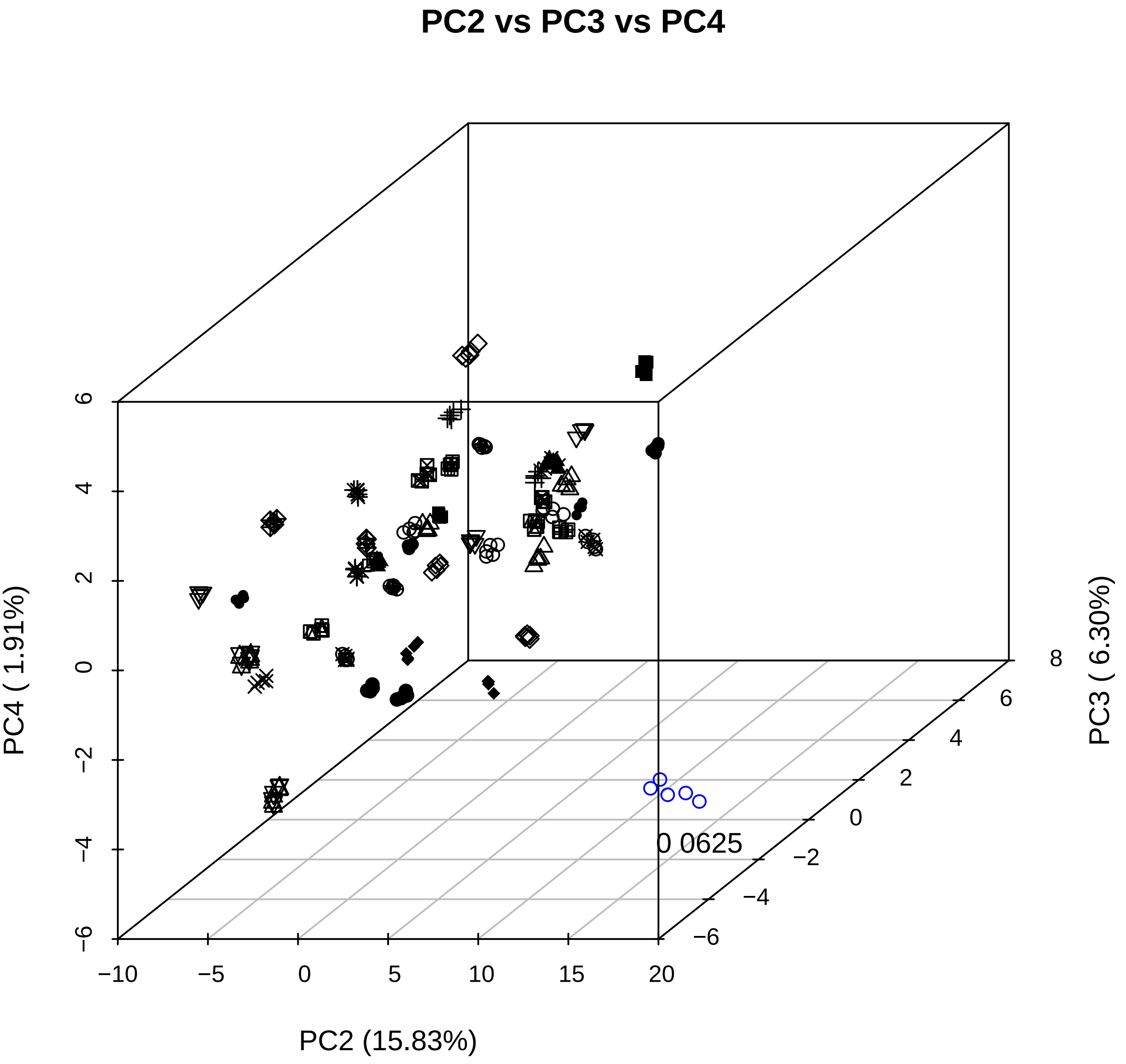
PC2 vs PC3 vs PC4
PC4 (1.91%)
PC2 (15.83%)
PC3 (6.30%)
0 0625
−10
−5
0
5
10
15
20
−6
−4
−2
0
2
4
6
8

PC1 vs PC2 vs PC4

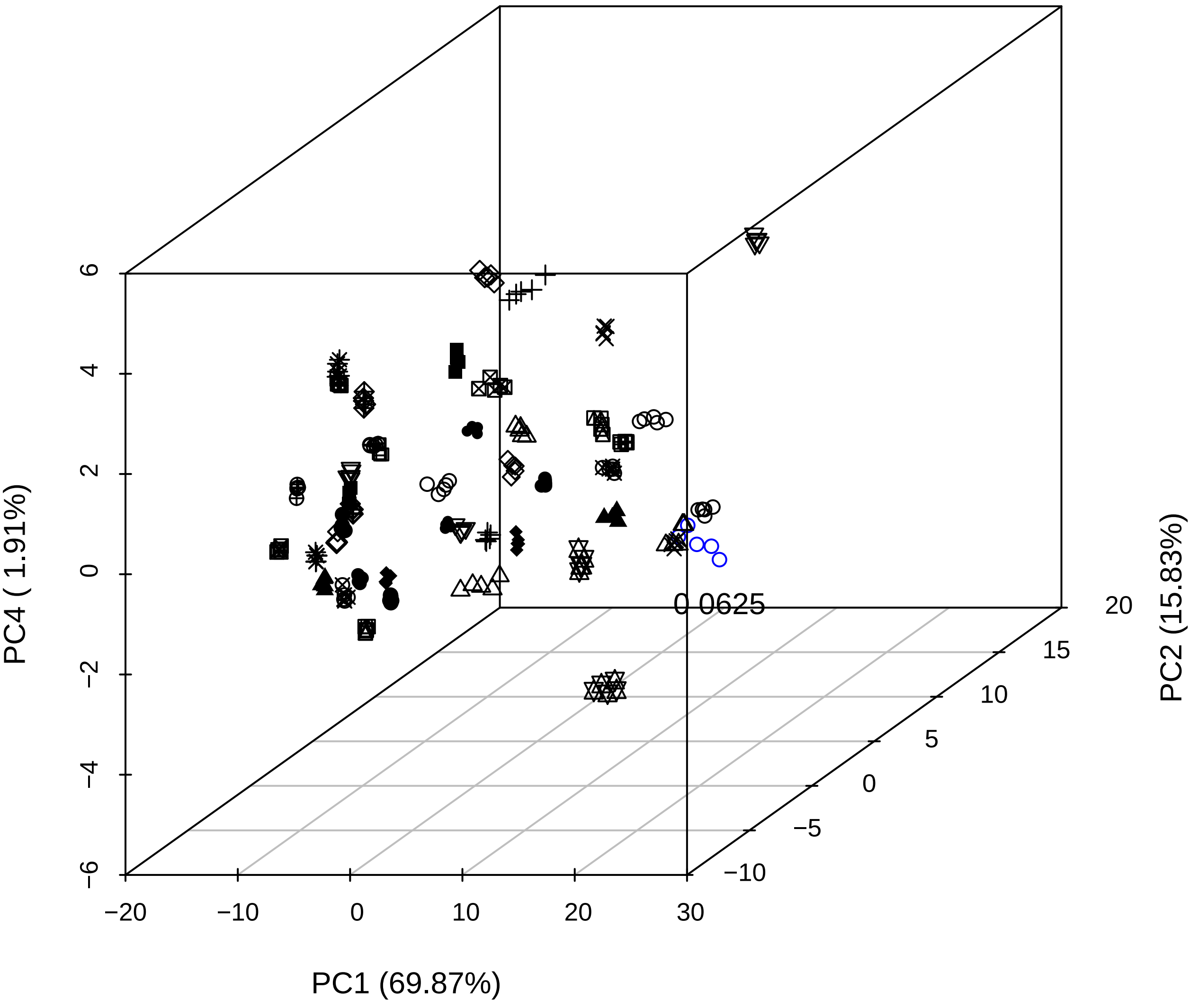

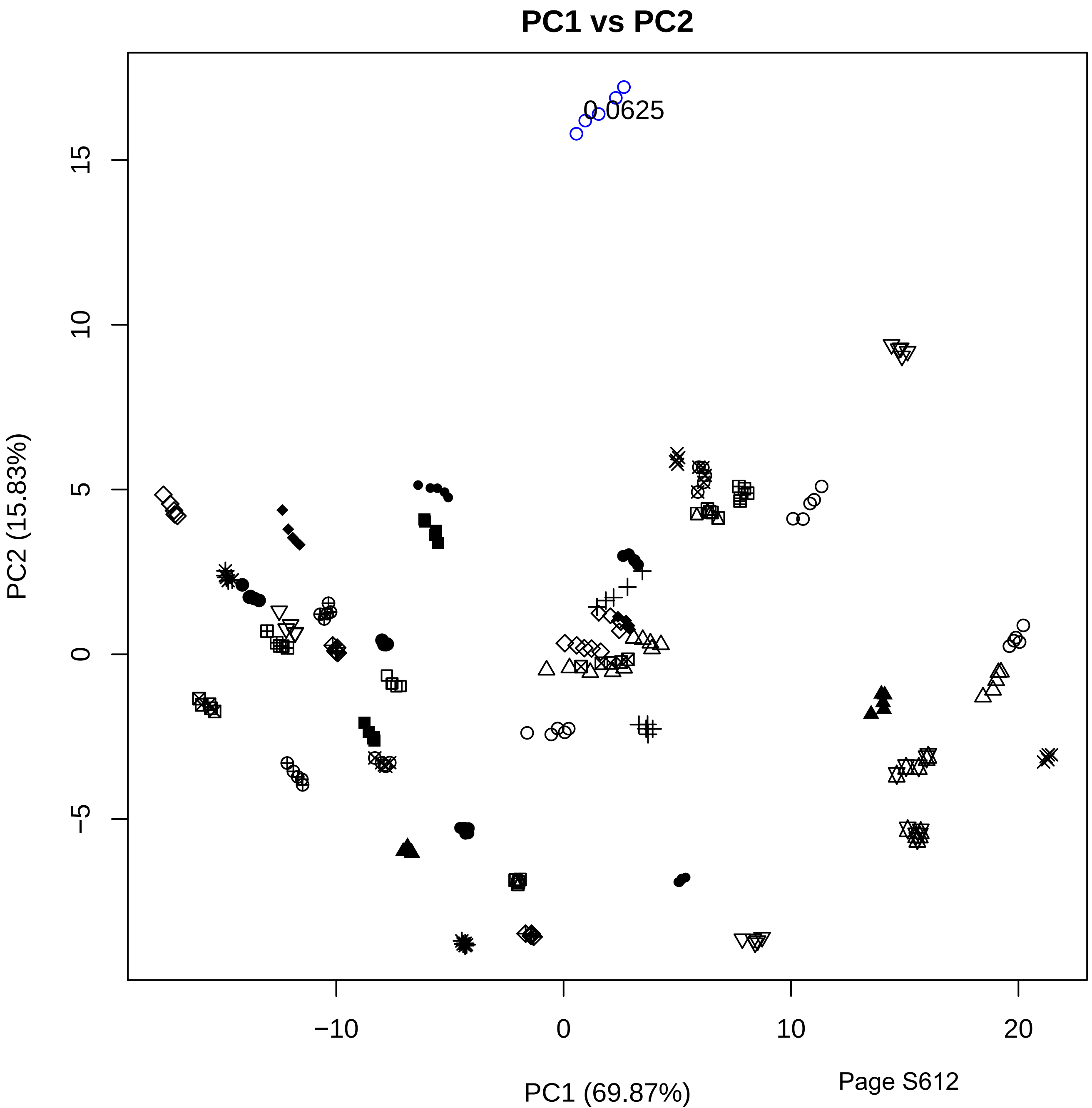
PC1 vs PC2
0.0625
PC2 (15.83%)
PC1 (69.87%)
15
10
5
0
−5
−10
0
10
20

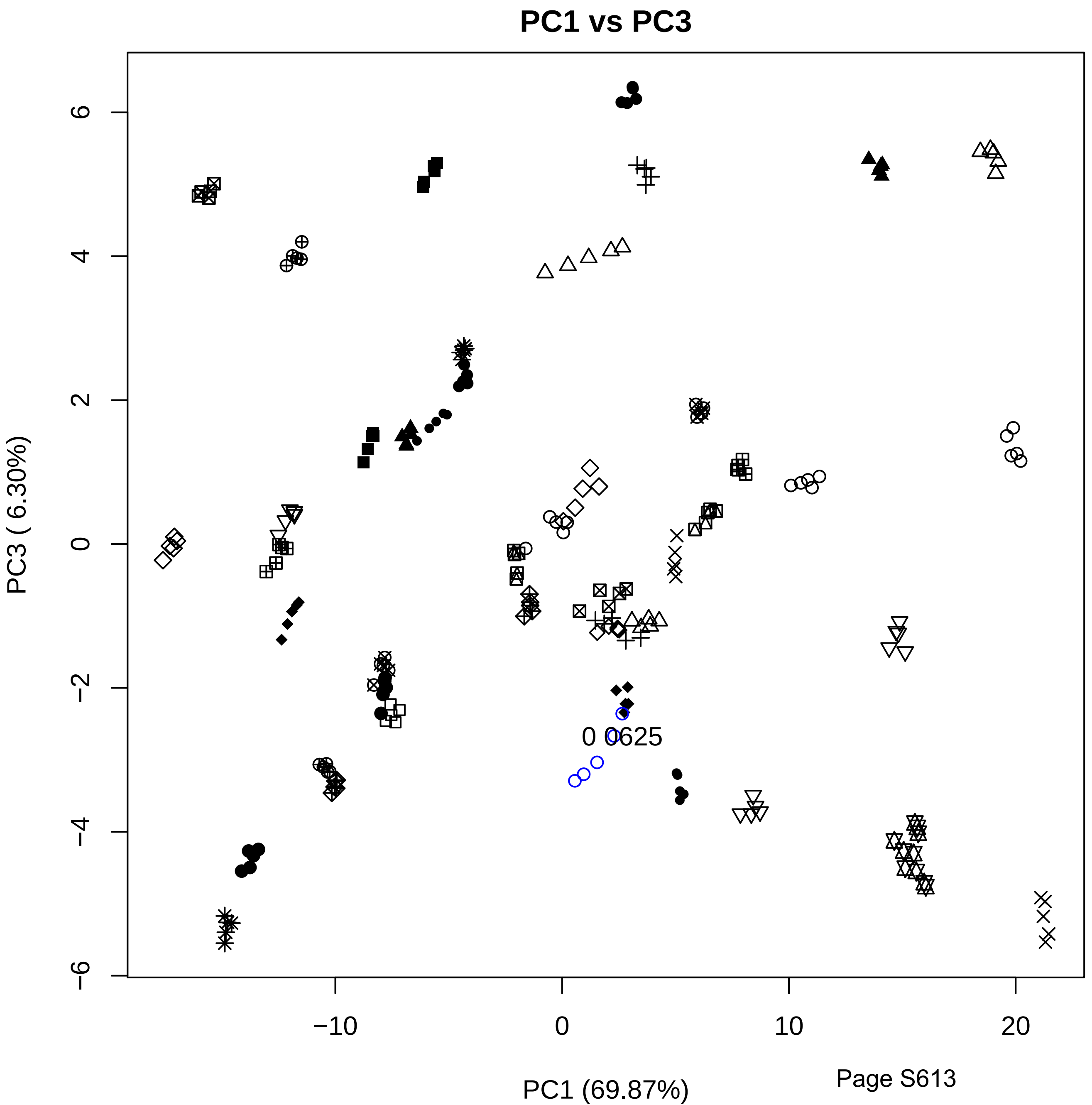

PC1 vs PC3
PC3 (6.30%)
PC1 (69.87%)
0 0625
6
4
2
0
−2
−4
−6
−10
0
10
20

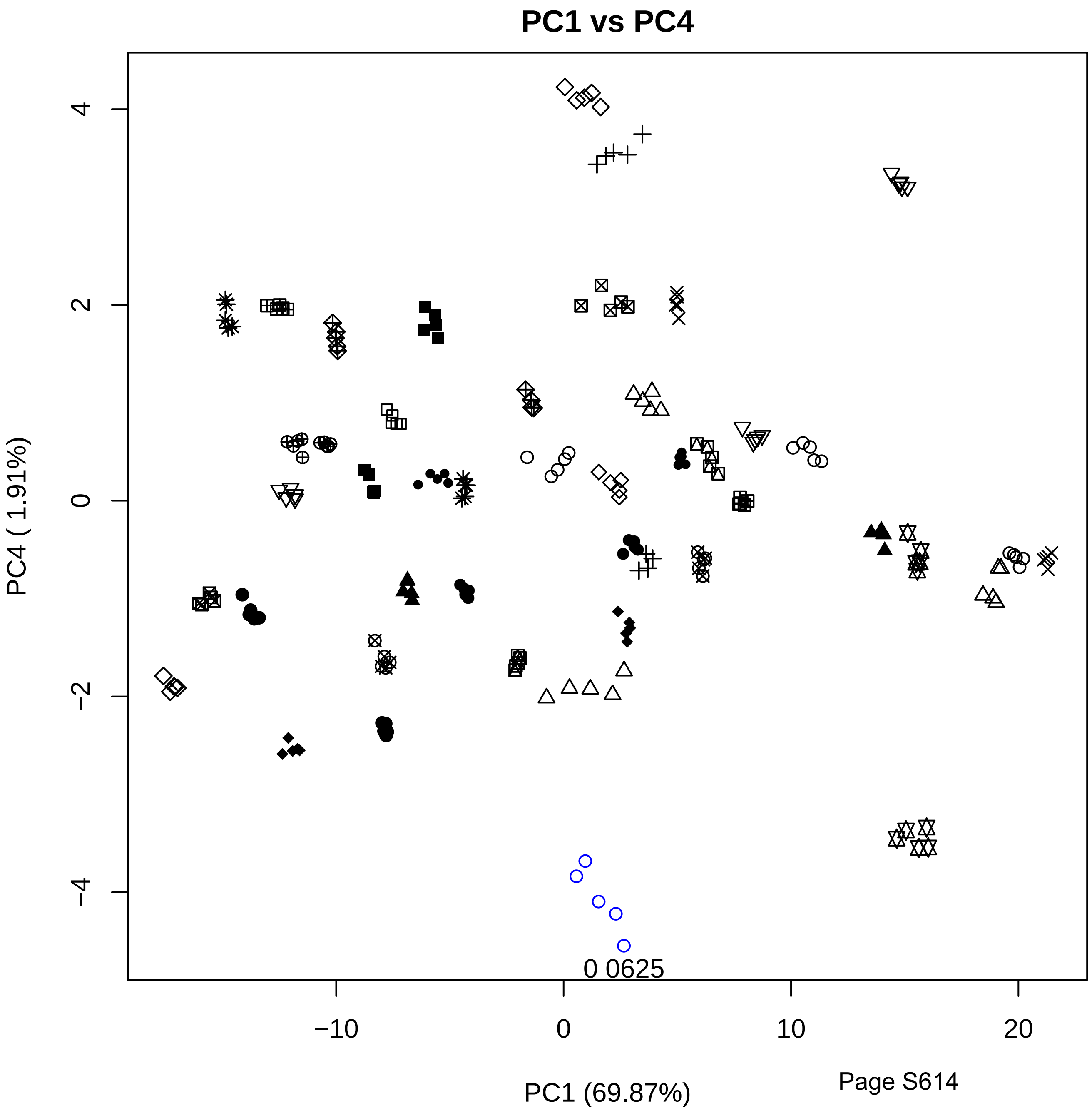

PC1 vs PC4
PC4 (1.91%)
PC1 (69.87%)
4
2
0
−2
−4
−10
0
10
20
0 0625

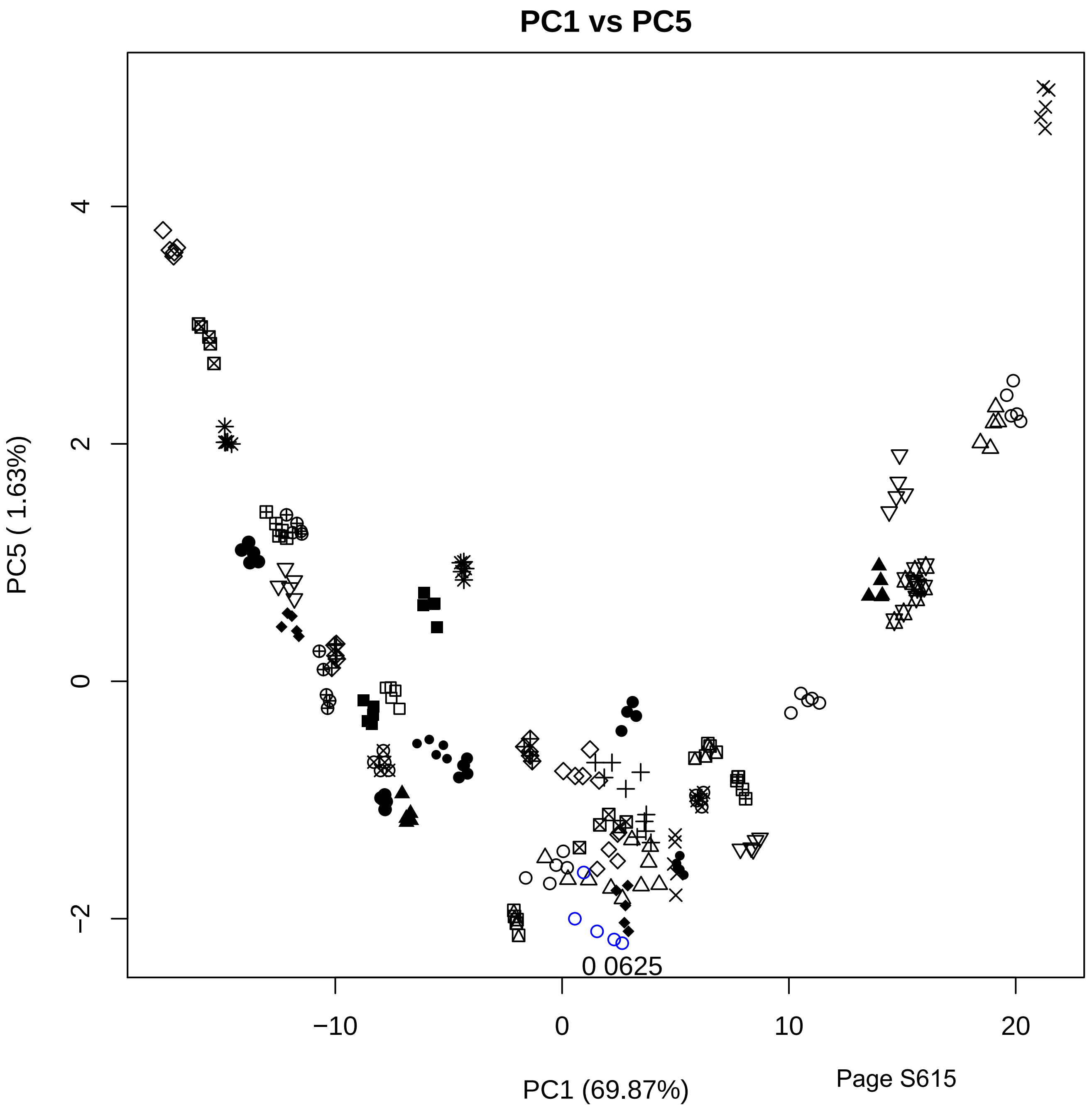

PC1 vs PC5
PC5 (1.63%)
4
2
0
−2
0 0625
−10
0
10
20
PC1 (69.87%)

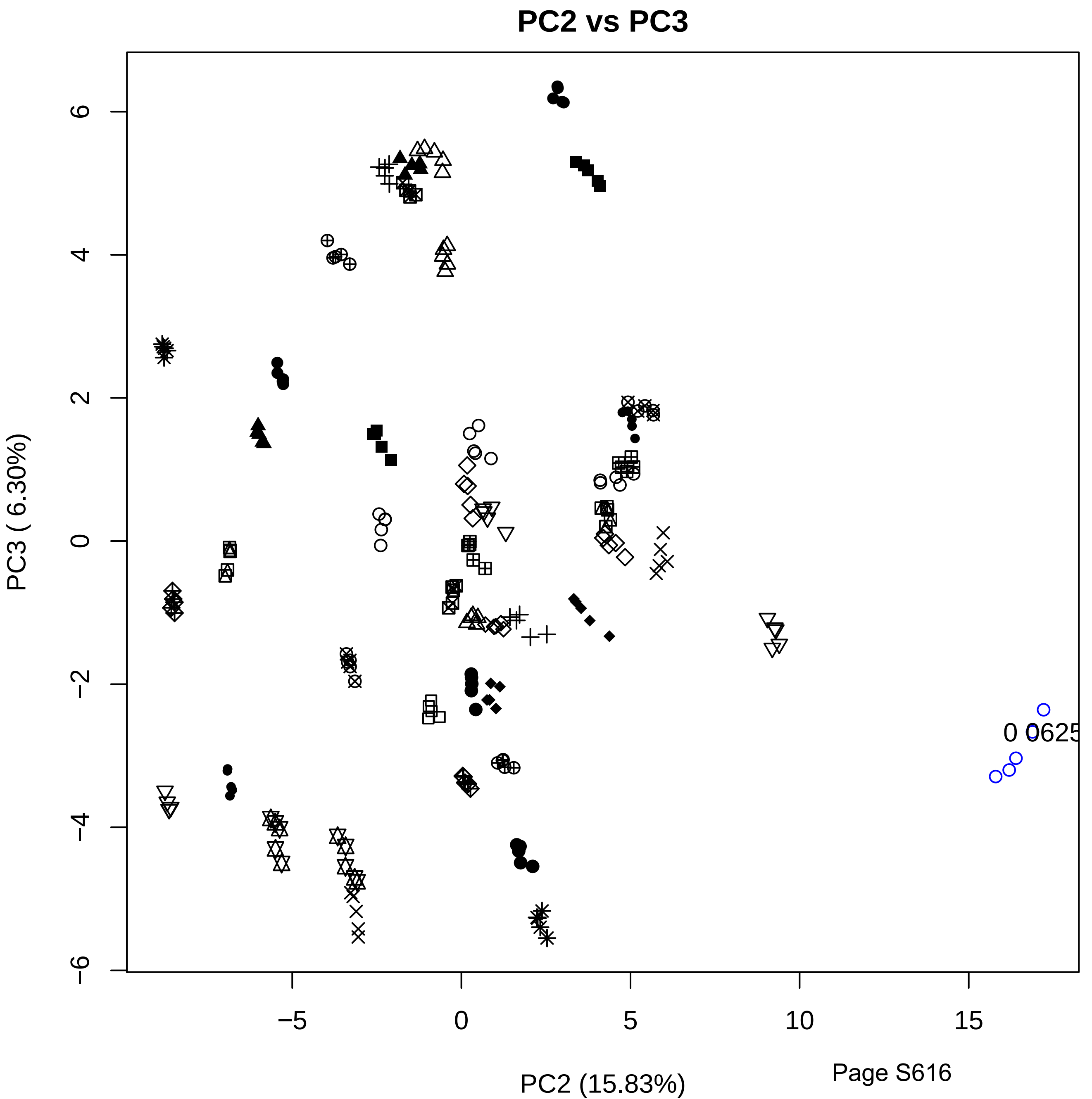
PC2 vs PC3
PC3 (6.30%)
PC2 (15.83%)
6
4
2
0
−2
−4
−6
−5
0
5
10
15
0 0625

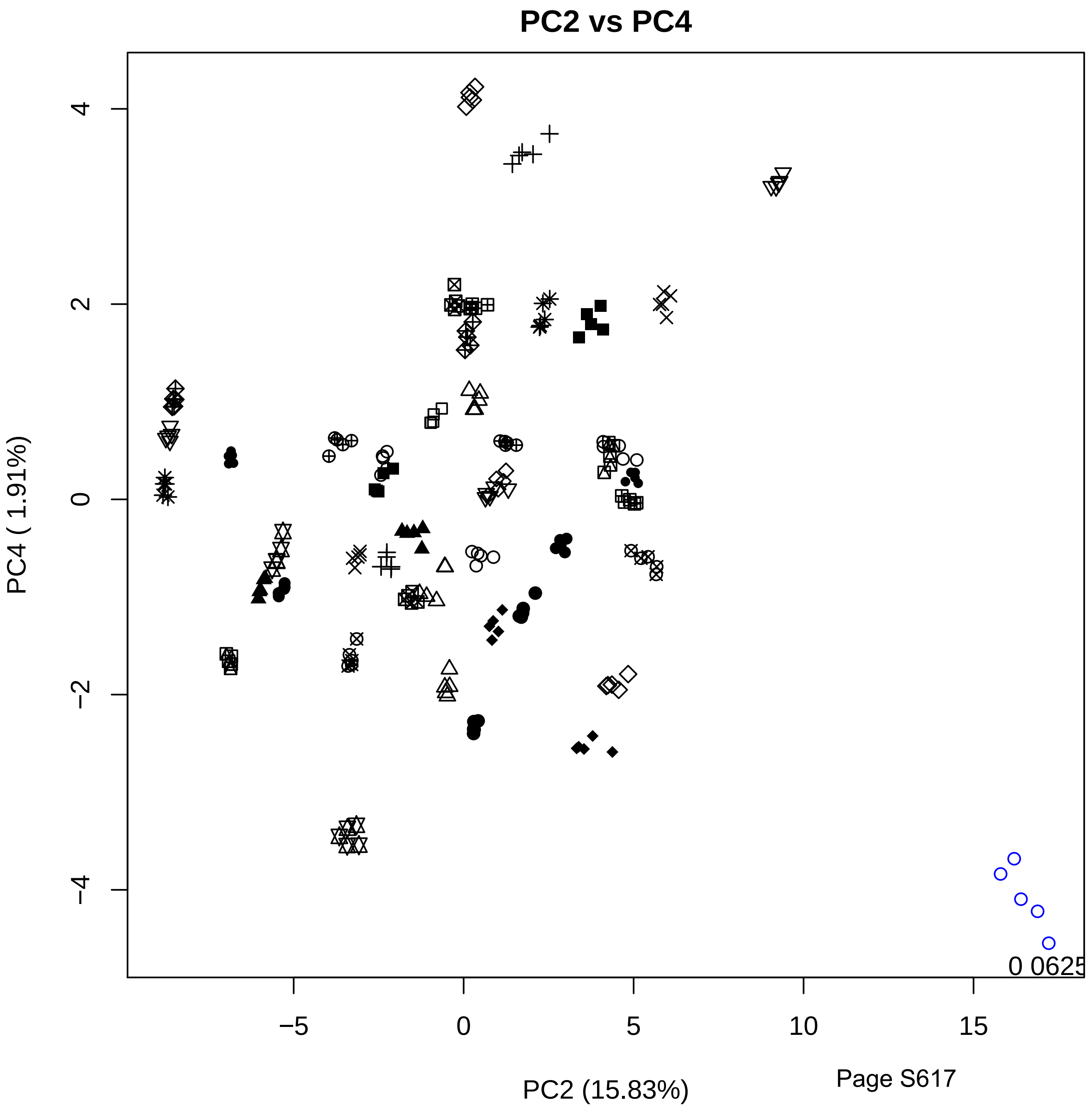
PC2 vs PC4
PC4 (1.91%)
PC2 (15.83%)
4
2
0
−2
−4
−5
0
5
10
15
0 0625

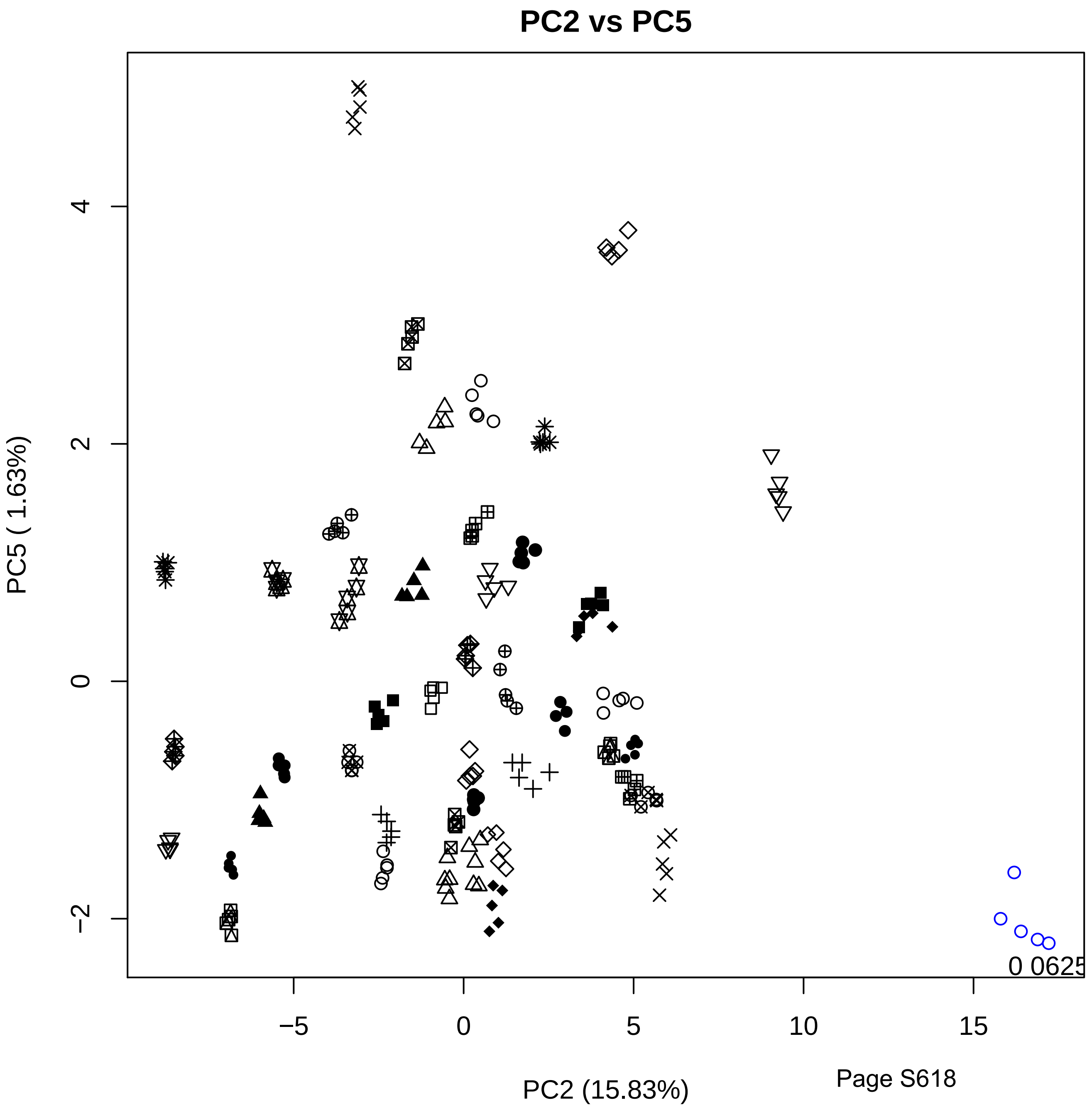

PC2 vs PC5
PC5 (1.63%)
PC2 (15.83%)
4
2
0
−2
−5
0
5
10
15
0 0625

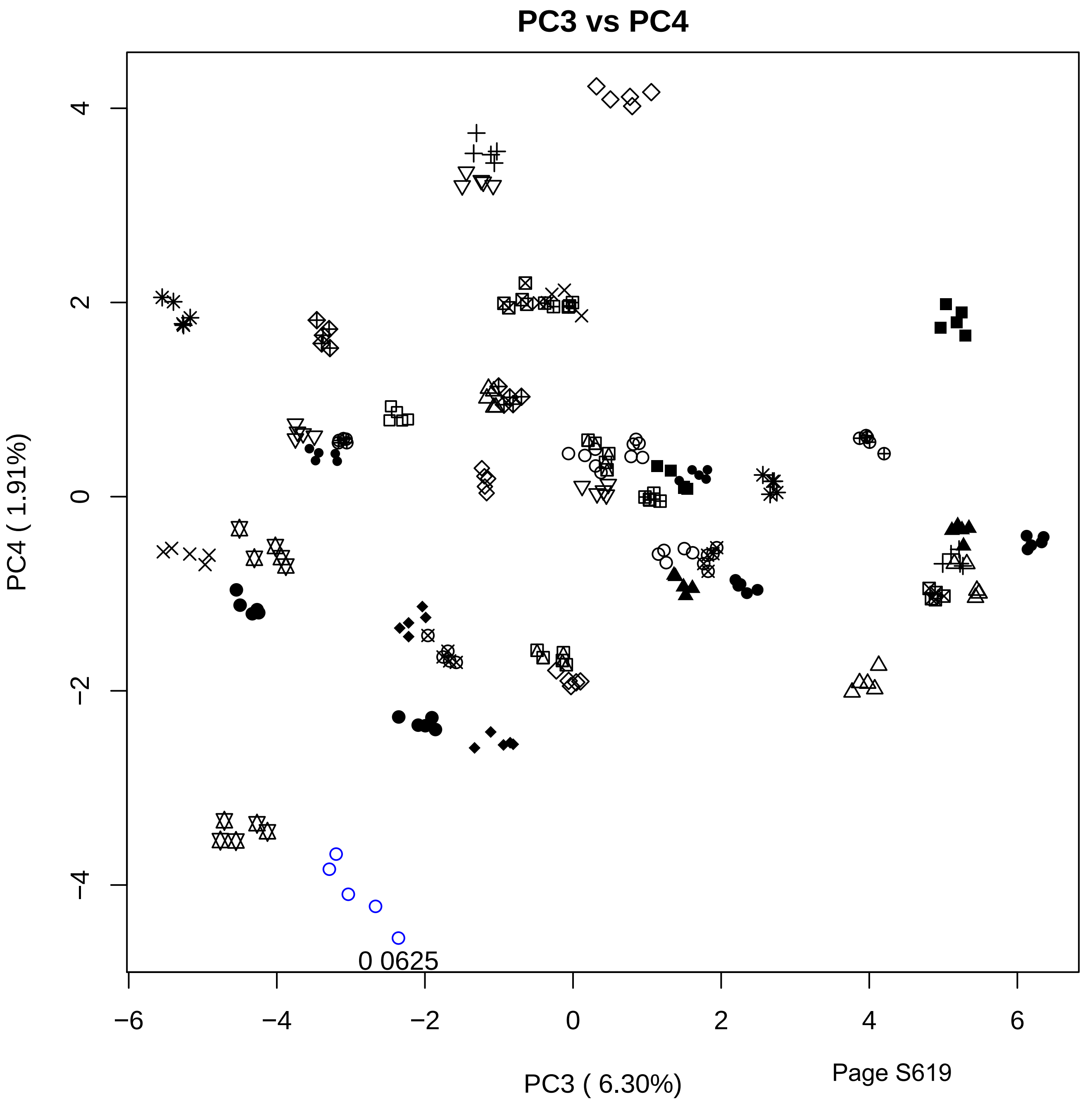
PC3 vs PC4
PC4 (1.91%)
PC3 (6.30%)
0 0625
4
2
0
−2
−4
−6
−4
−2
0
2
4
6

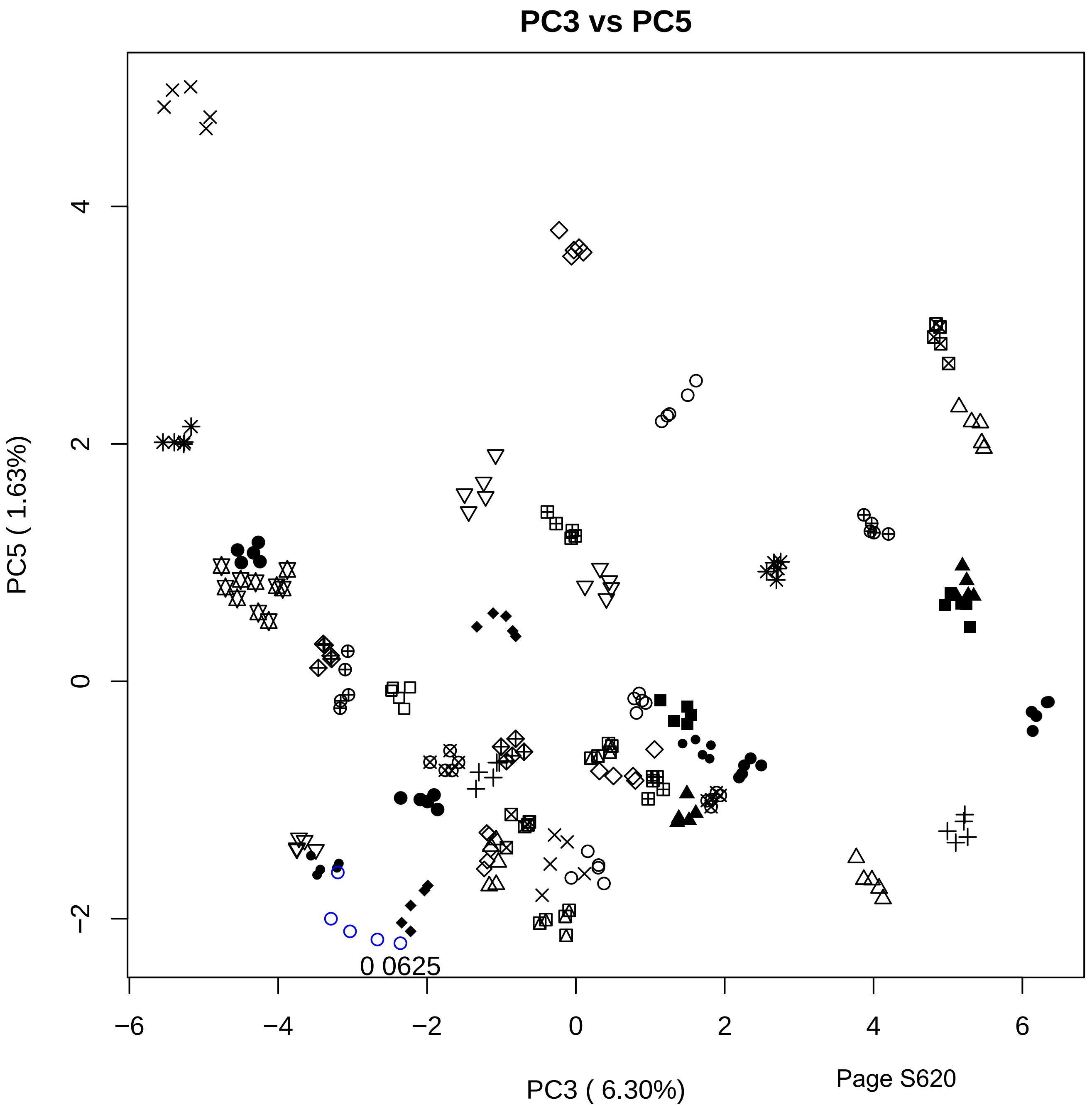

PC3 vs PC5
PC5 (1.63%)
PC3 (6.30%)
−6
−4
−2
0
2
4
6
−2
0
2
4
0 0625

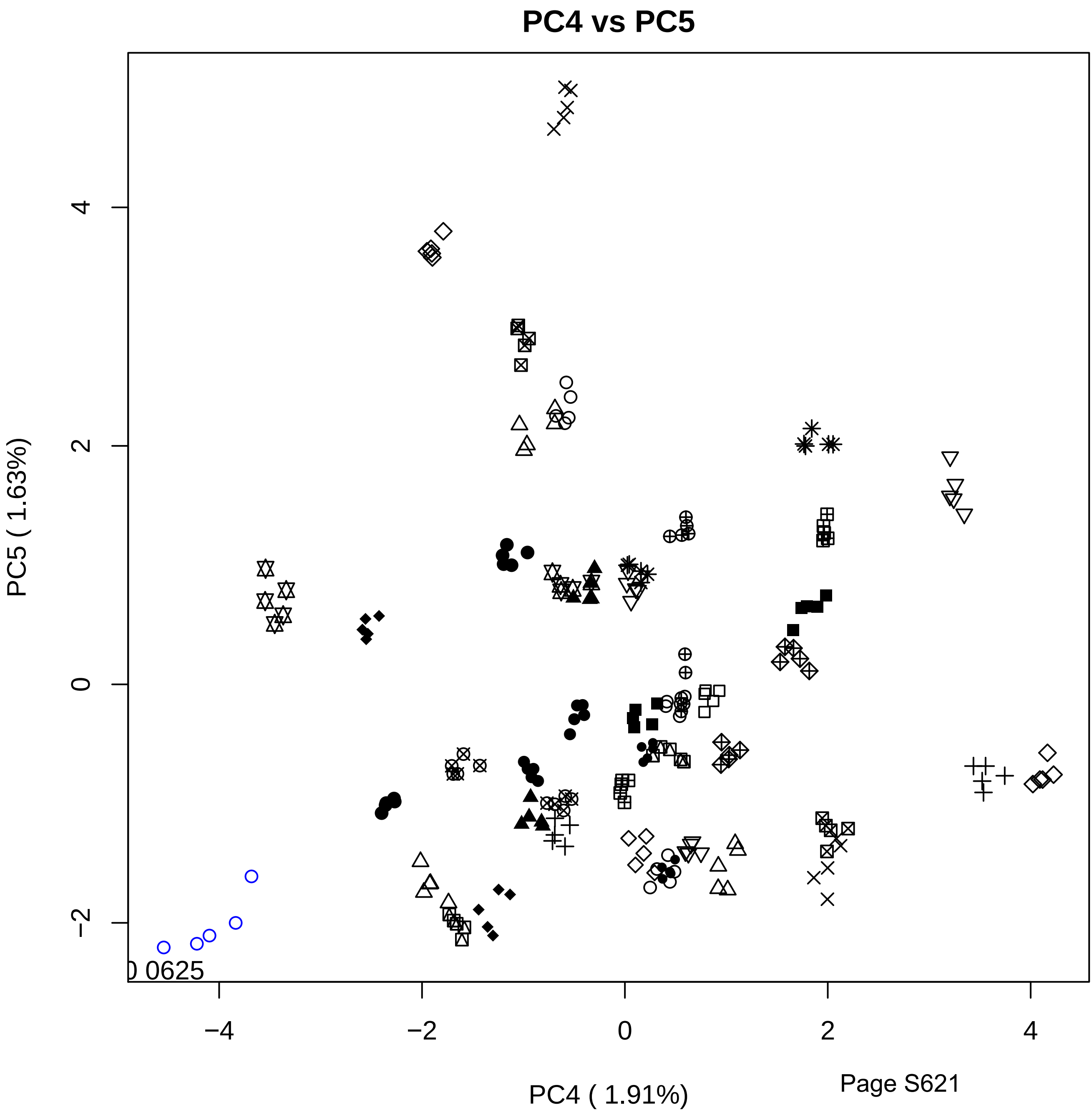

PC4 vs PC5
PC5 (1.63%)
PC4 (1.91%)
4
2
0
−2
−4
0 0625

PCA of 0.12% DeS vs PMH

Scaled Matrix PCAed

7th order polynomial baseline correction performed

Raw spectra smoothed to 21 Neighbours, 2nd order PN, Savtisky Golay

Normalisation between 0 and 100

D2 spectra smoothed to 41 Neighbours, 2nd order PN, Savtisky Golay

Regions Analysed: 3601−2501 and 2001−700cm−1

Samples Analysed: 1 Contam's 0 DeS's 45 PMH's

Keele University AD   Sun Dec 09 17:03:46 2018

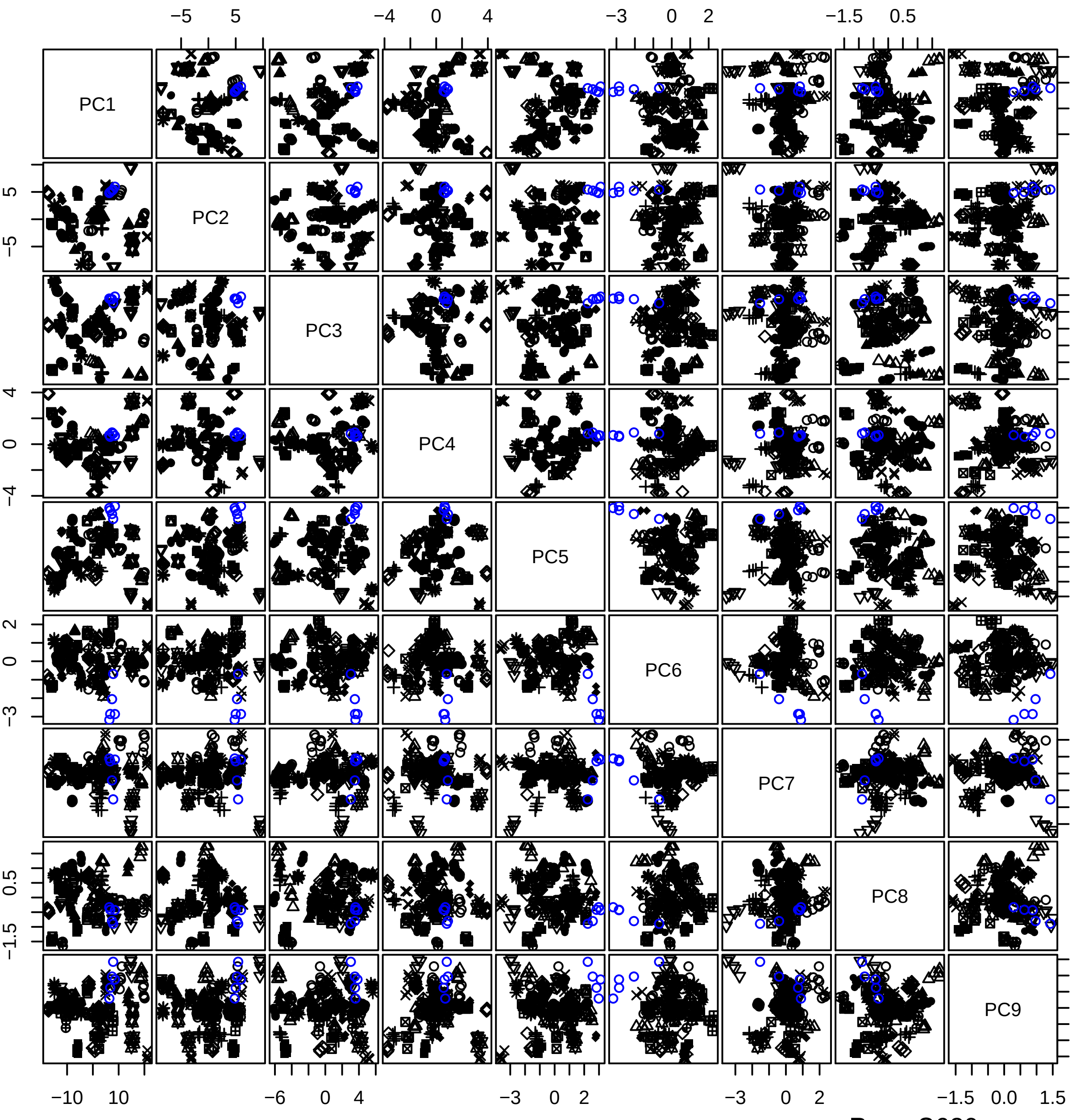

PC1
PC2
PC3
PC4
PC5
PC6
PC7
PC8
PC9

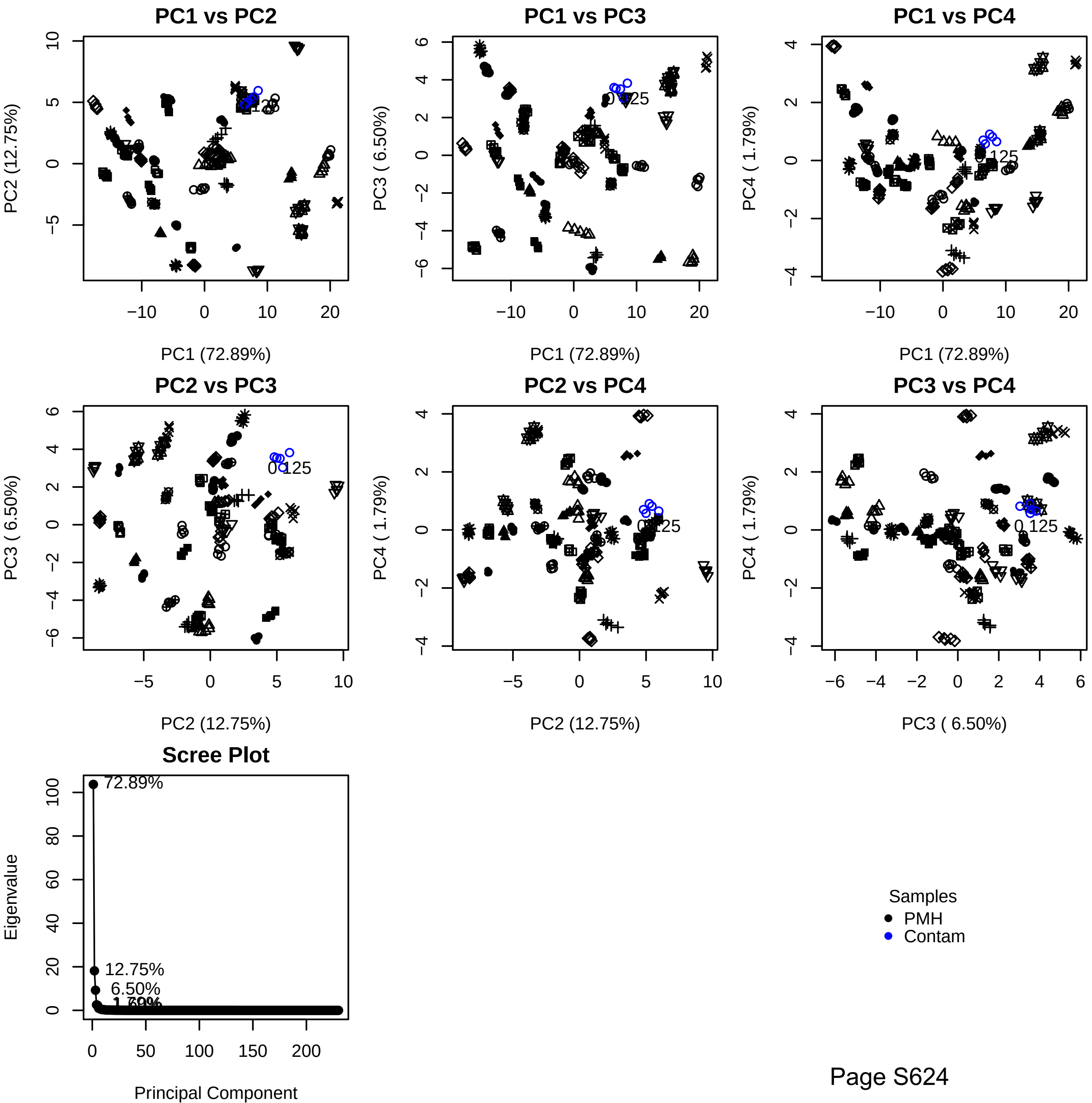
PC1 vs PC2
PC1 vs PC3
PC1 vs PC4
PC2 vs PC3
PC2 vs PC4
PC3 vs PC4
Scree Plot
PC1 (72.89%)
PC2 (12.75%)
PC3 (6.50%)
PC4 (1.79%)
0.125
72.89%
12.75%
6.50%
Eigenvalue
Principal Component
Samples
PMH
Contam

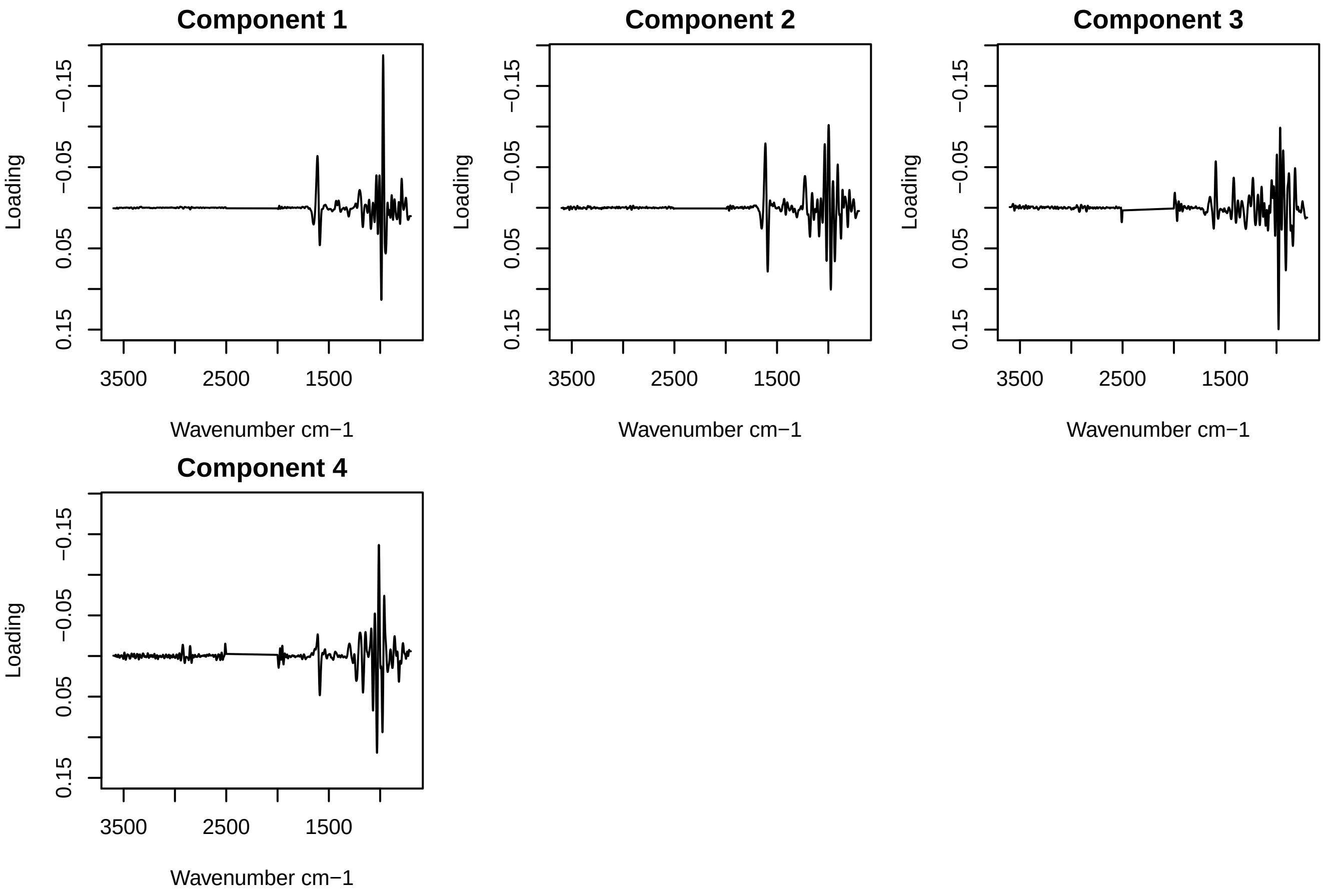
Component 1
Component 2
Component 3
Component 4
Loading
−0.15
−0.05
0.05
0.15
3500
2500
1500
Wavenumber cm−1

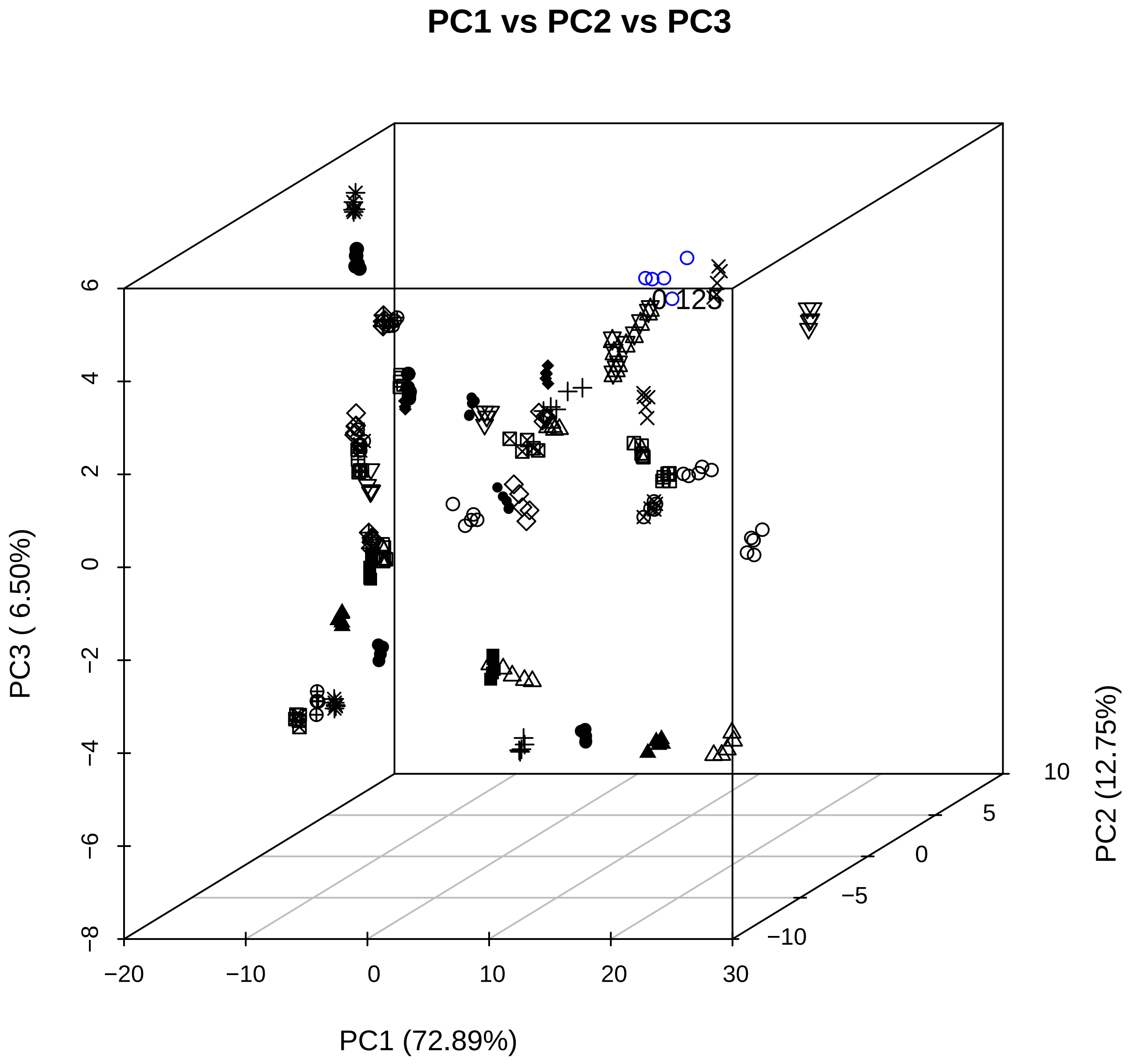
PC1 vs PC2 vs PC3
PC1 (72.89%)
PC2 (12.75%)
PC3 (6.50%)
0125

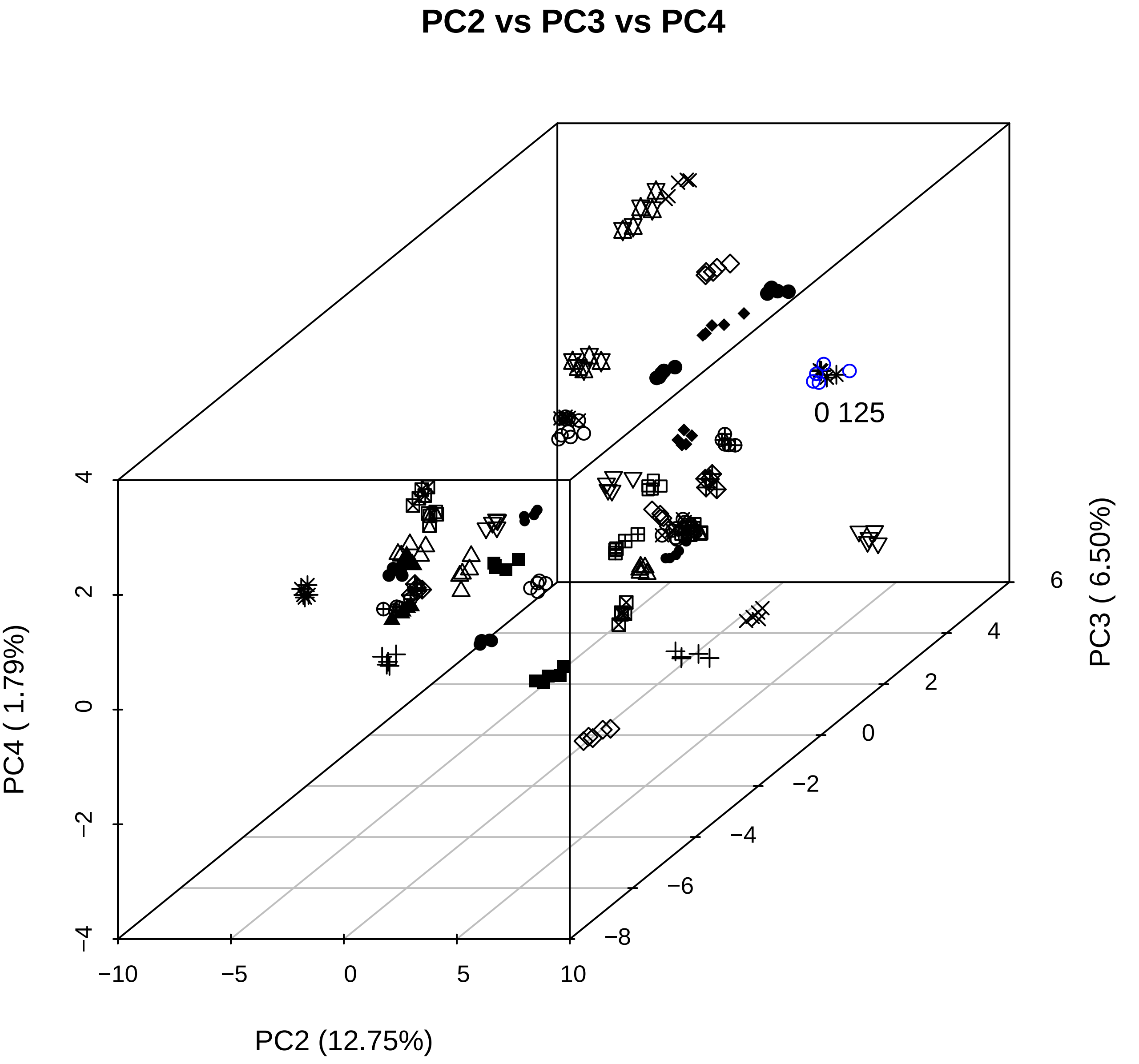
PC2 vs PC3 vs PC4
0 125
PC2 (12.75%)
PC3 (6.50%)
PC4 (1.79%)
−10
−5
0
5
10
−8
−6
−4
−2
0
2
4
6
−4
−2
0
2
4

PC1 vs PC2 vs PC4

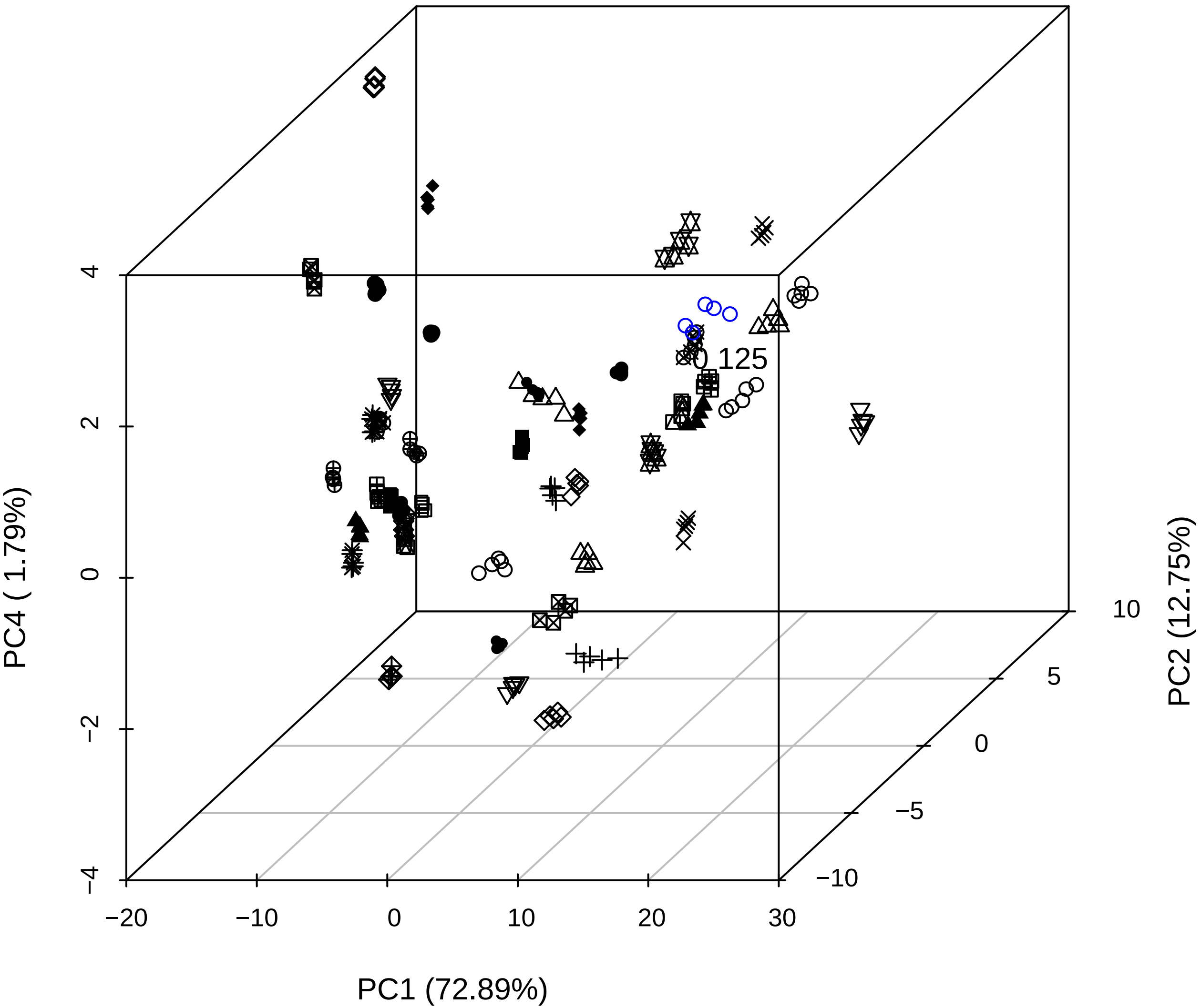

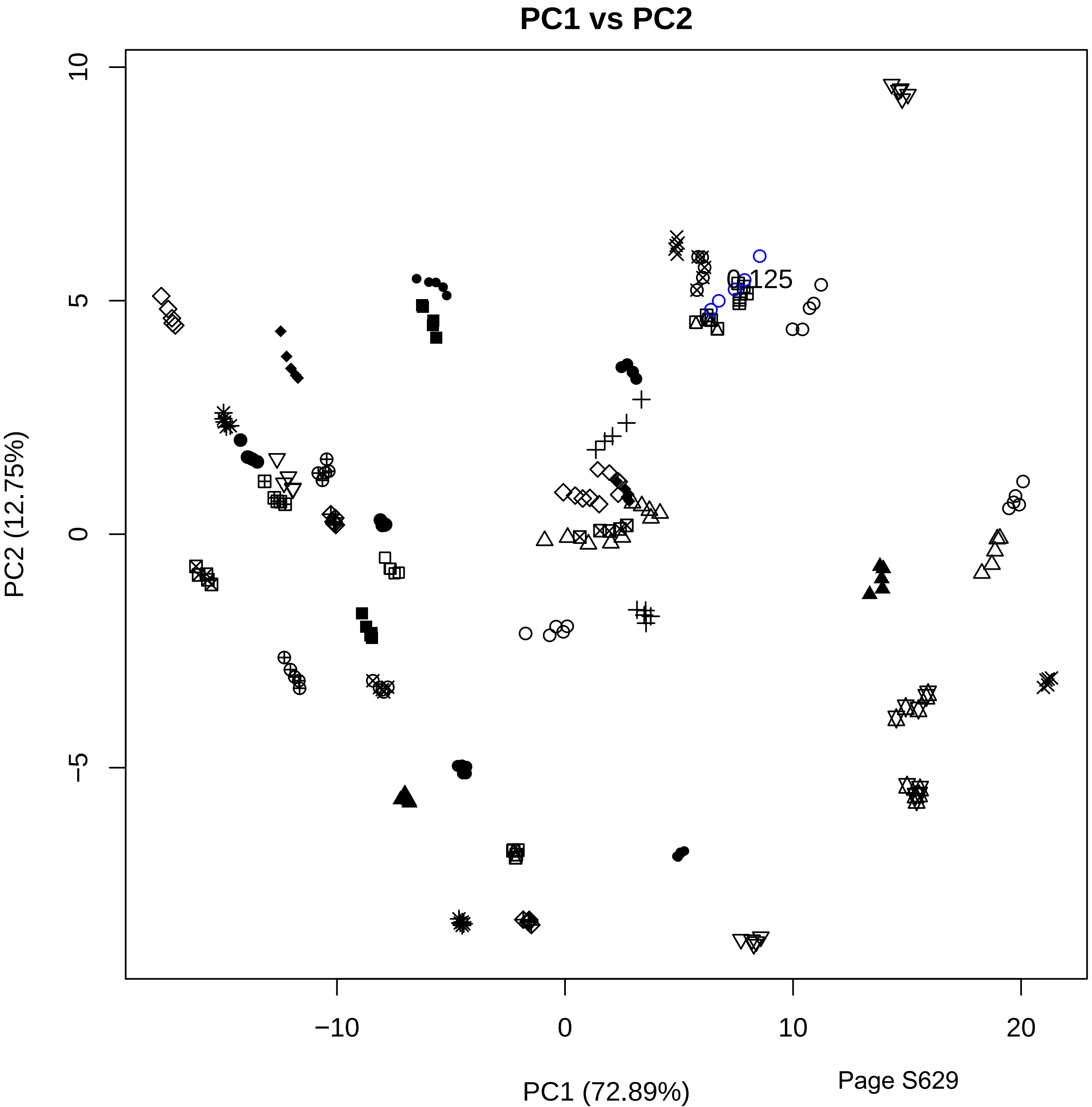
PC1 vs PC2
PC2 (12.75%)
PC1 (72.89%)
10
5
0
−5
−10
0
10
20
0.125

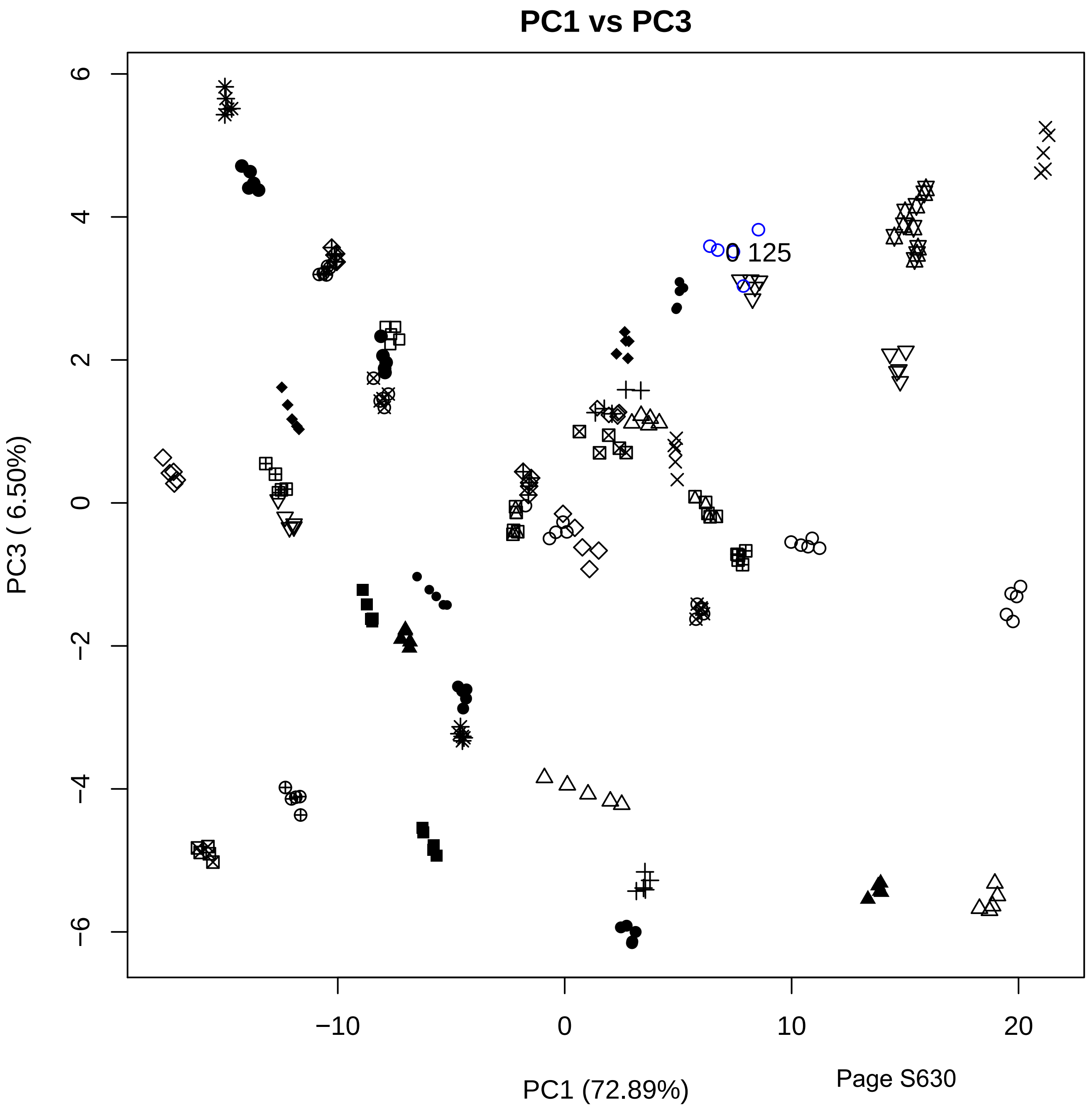

PC1 vs PC3
0 125
PC3 (6.50%)
PC1 (72.89%)
6
4
2
0
−2
−4
−6
−10
0
10
20

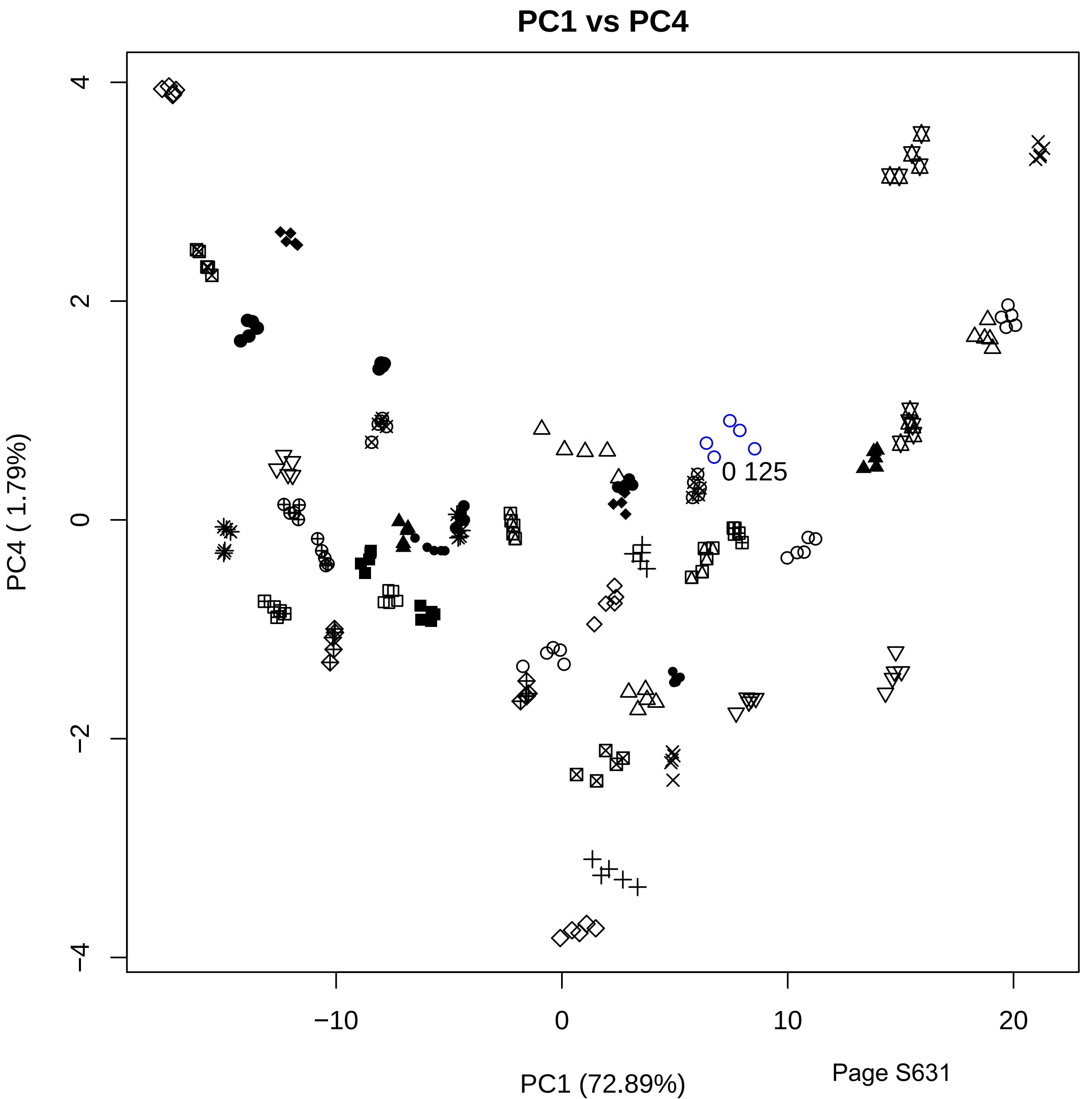
PC1 vs PC4
PC4 (1.79%)
PC1 (72.89%)
4
2
0
−2
−4
−10
0
10
20
0 125

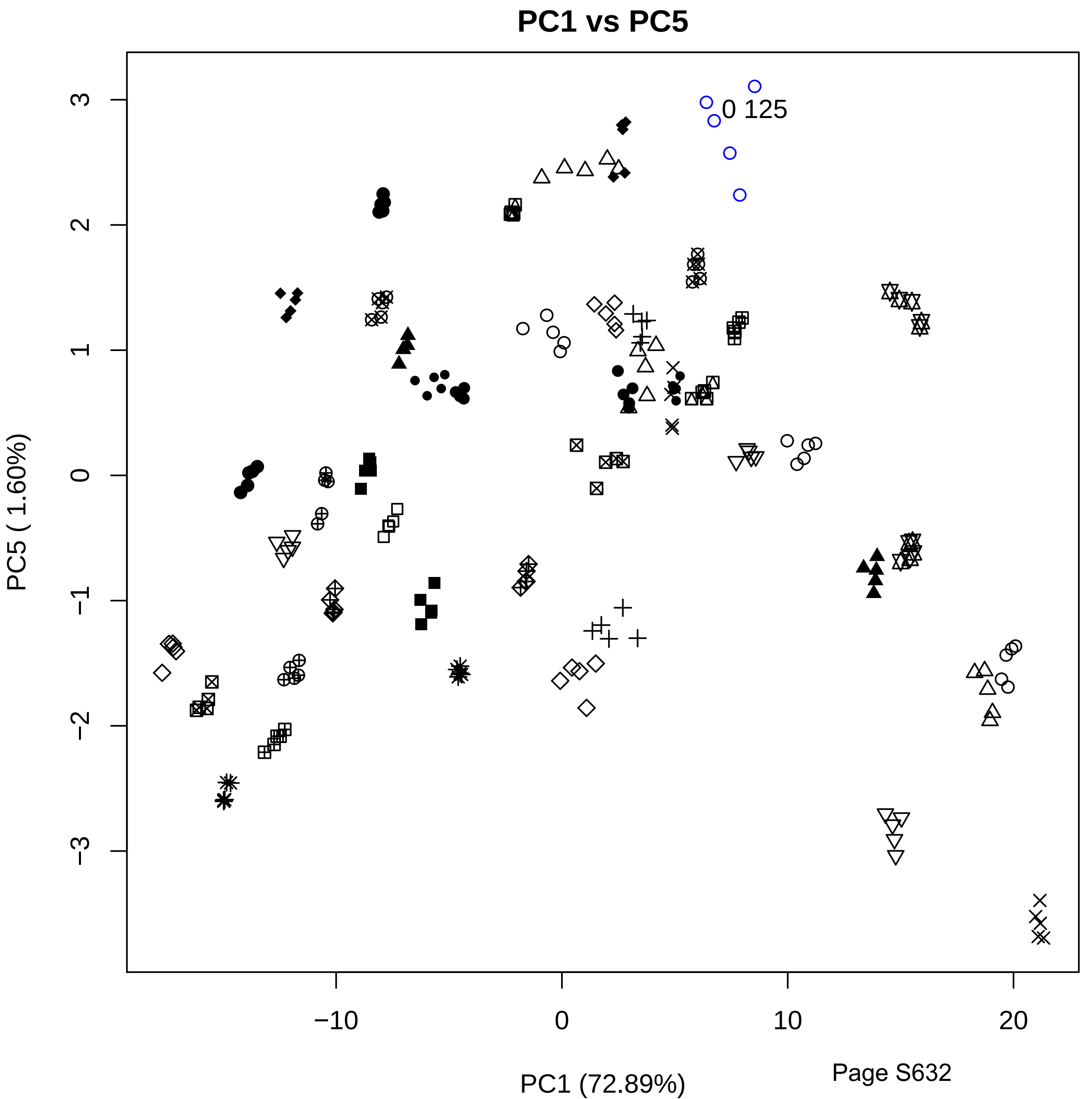

PC1 vs PC5
0 125
PC5 (1.60%)
PC1 (72.89%)
3
2
1
0
−1
−2
−3
−10
0
10
20

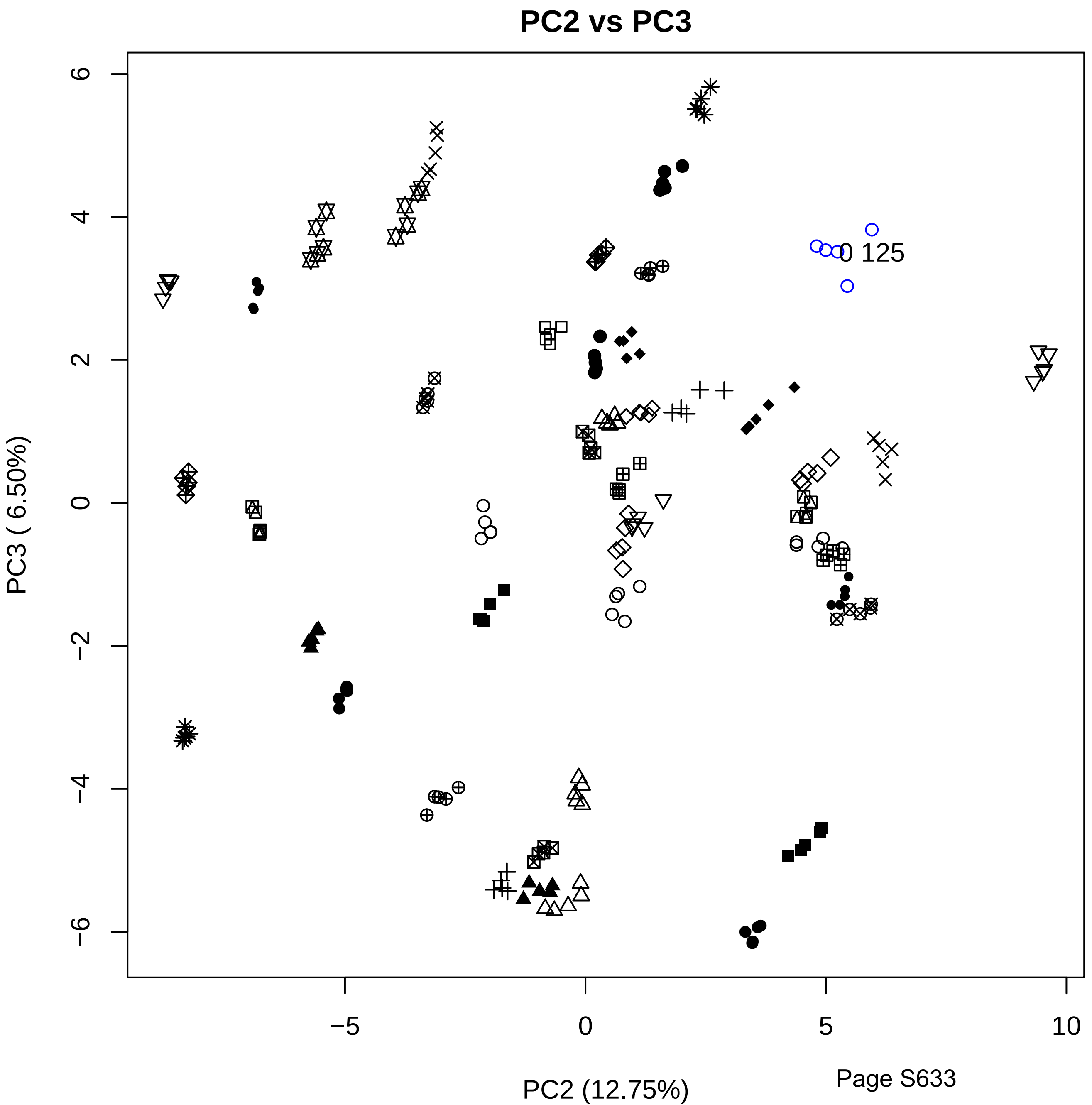
PC2 vs PC3
PC3 (6.50%)
PC2 (12.75%)
−5
0
5
10
6
4
2
0
−2
−4
−6
0 125

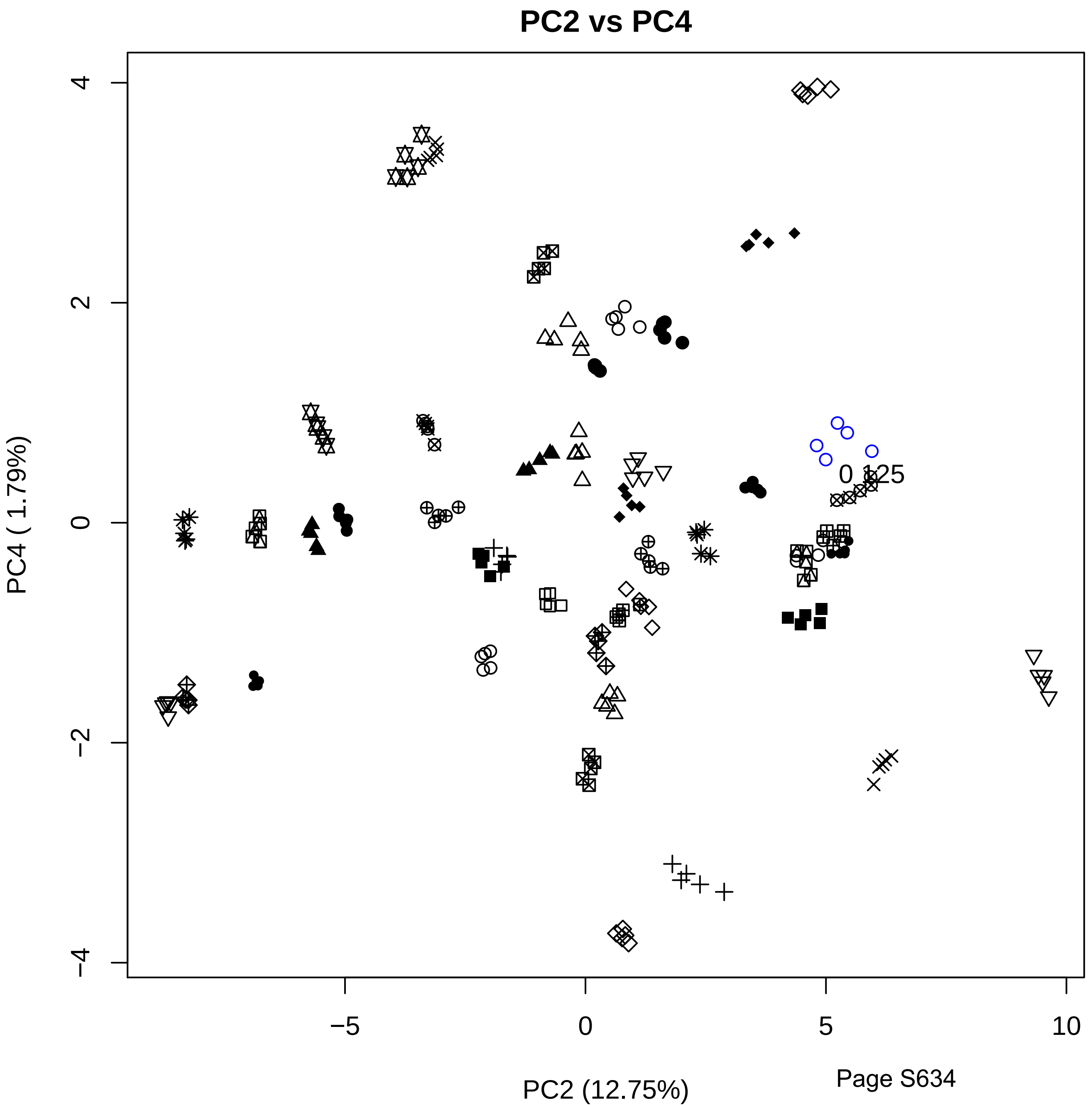
PC2 vs PC4
PC4 (1.79%)
PC2 (12.75%)
4
2
0
−2
−4
−5
0
5
10
0.125

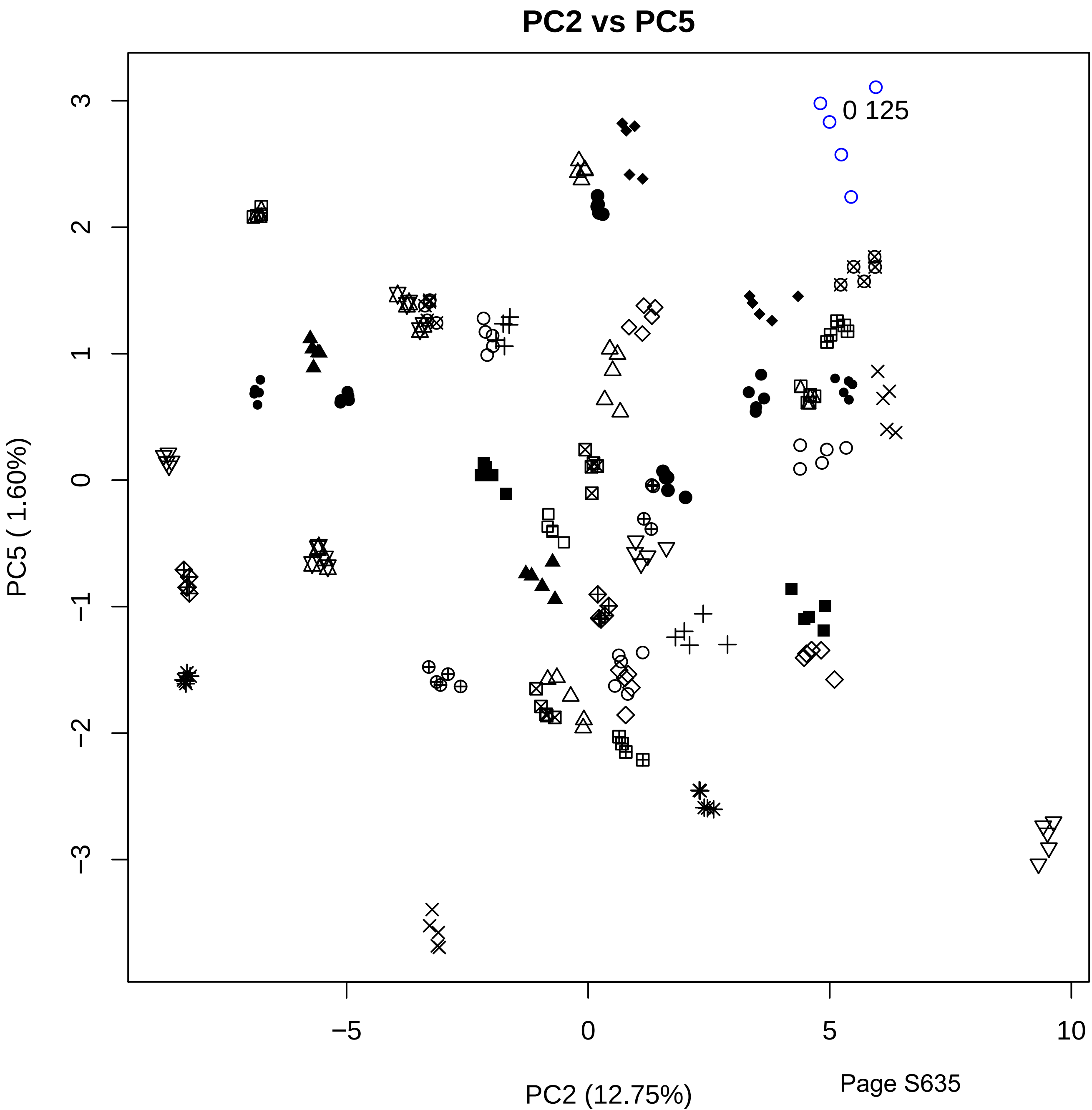
PC2 vs PC5
0 125
PC5 (1.60%)
PC2 (12.75%)
3
2
1
0
−1
−2
−3
−5
0
5
10

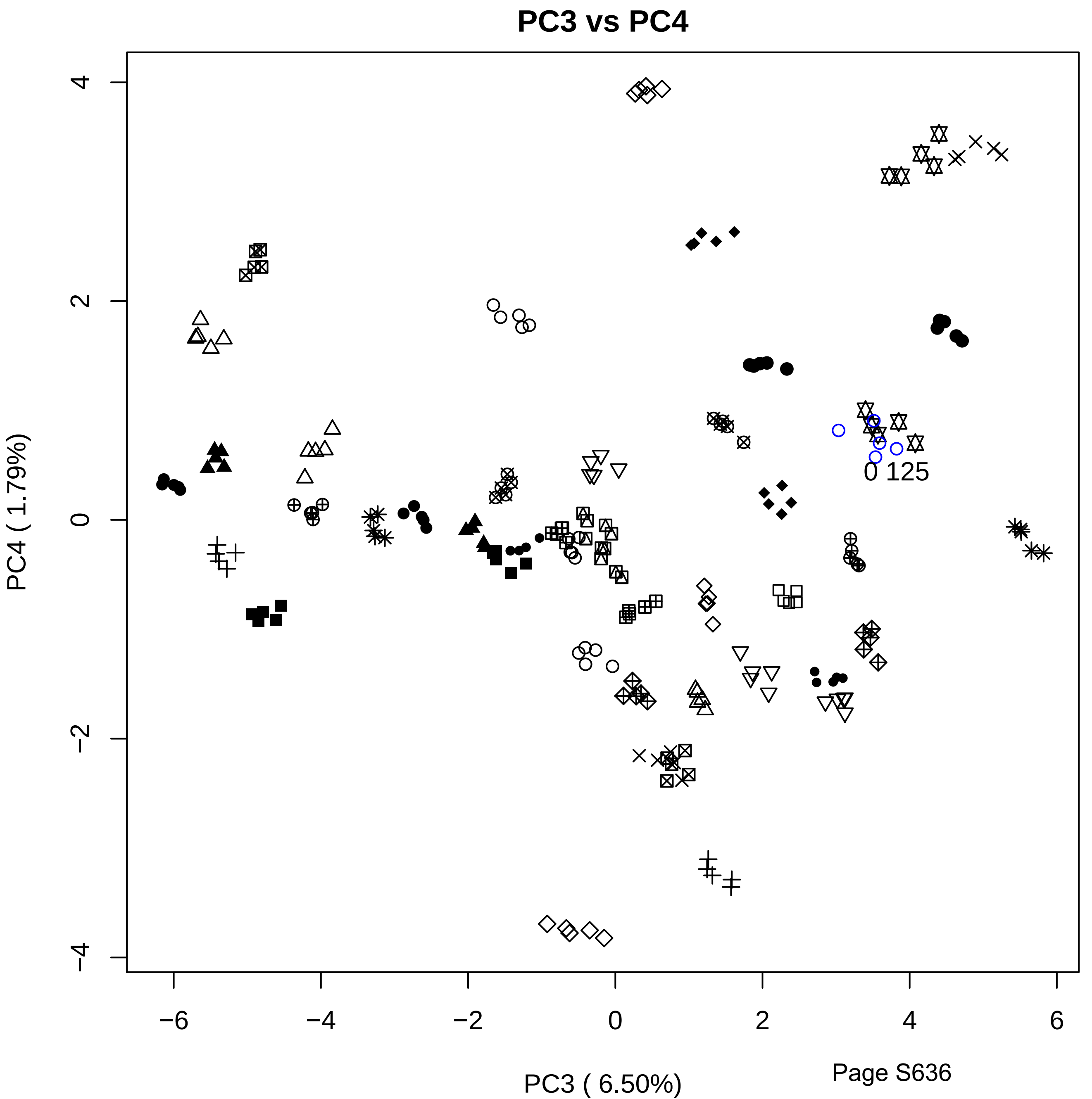
PC3 vs PC4
PC4 (1.79%)
PC3 (6.50%)
0 125
4
2
0
−2
−4
−6
−4
−2
0
2
4
6

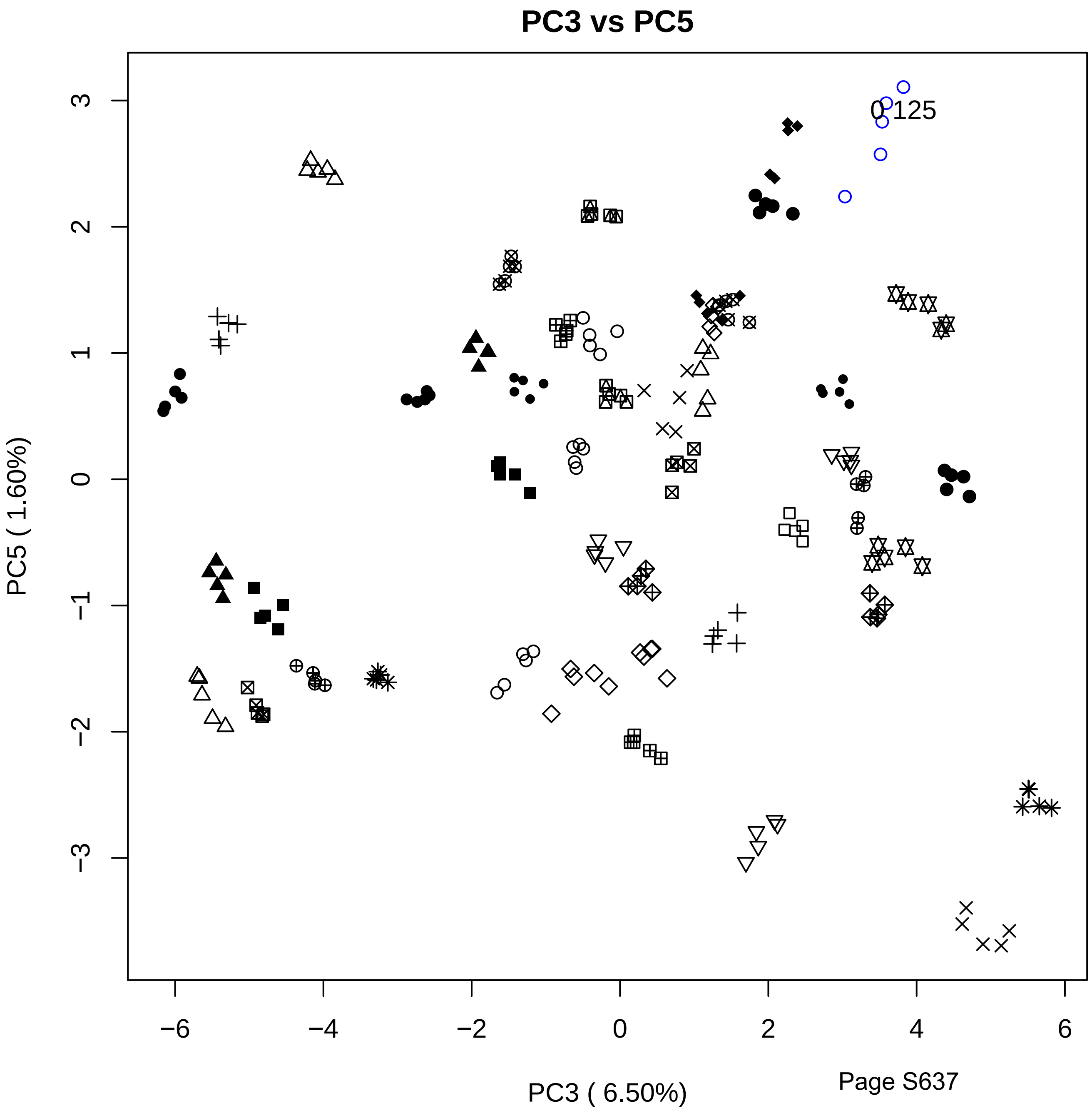

PC3 vs PC5
0.125
PC5 (1.60%)
PC3 (6.50%)
3
2
1
0
−1
−2
−3
−6
−4
−2
0
2
4
6

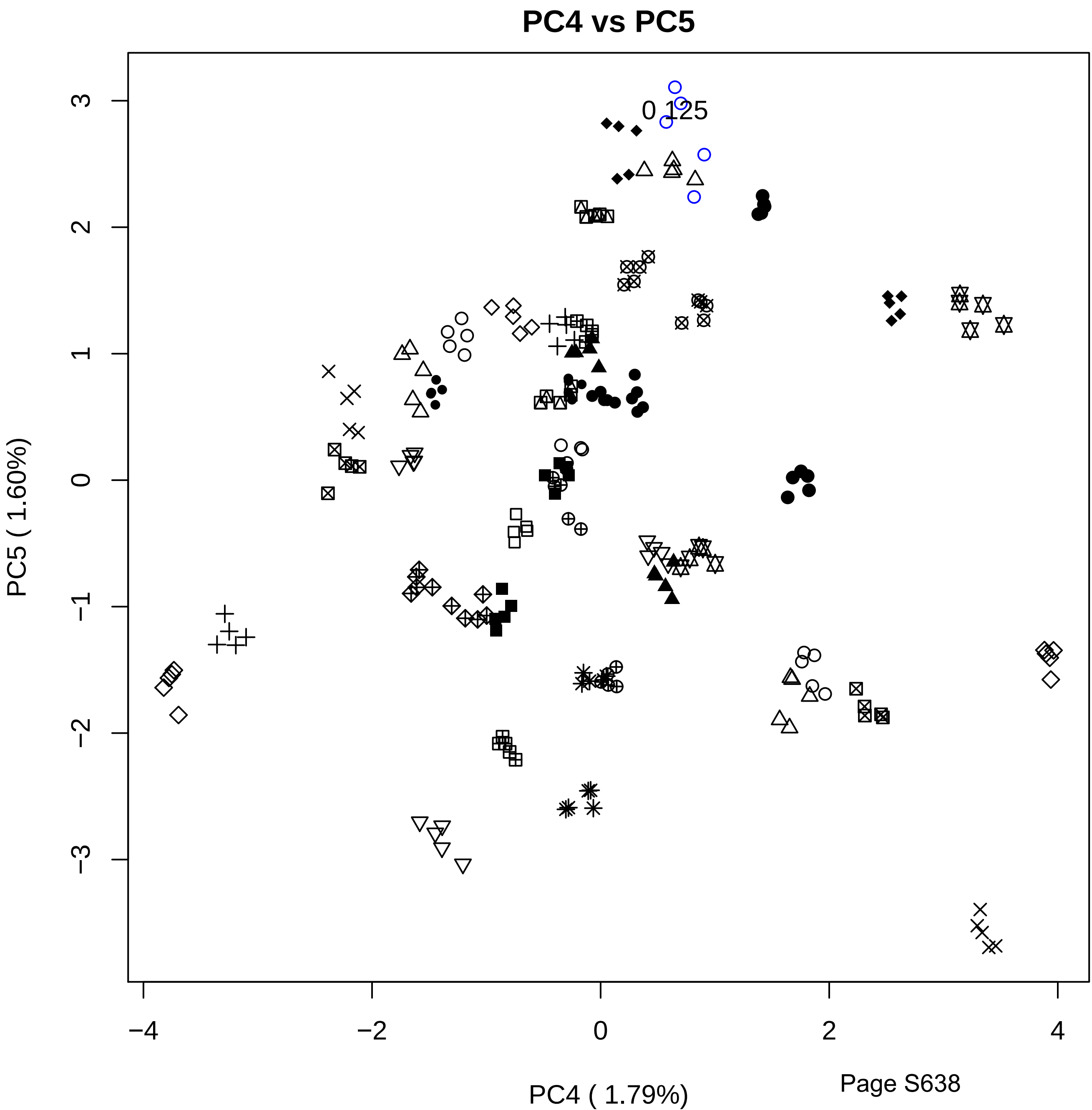

PC4 vs PC5
0.125
PC5 (1.60%)
PC4 (1.79%)
3
2
1
0
−1
−2
−3
−4
−2
0
2
4

PCA of 0.25% DeS vs PMH

Scaled Matrix PCAed

7th order polynomial baseline correction performed

Raw spectra smoothed to 21 Neighbours, 2nd order PN, Savtisky Golay

Normalisation between 0 and 100

D2 spectra smoothed to 41 Neighbours, 2nd order PN, Savtisky Golay

Regions Analysed: 3601−2501 and 2001−700cm−1

Samples Analysed: 1 Contam's 0 DeS's 45 PMH's

Keele University AD   Sun Dec 09 17:03:51 2018

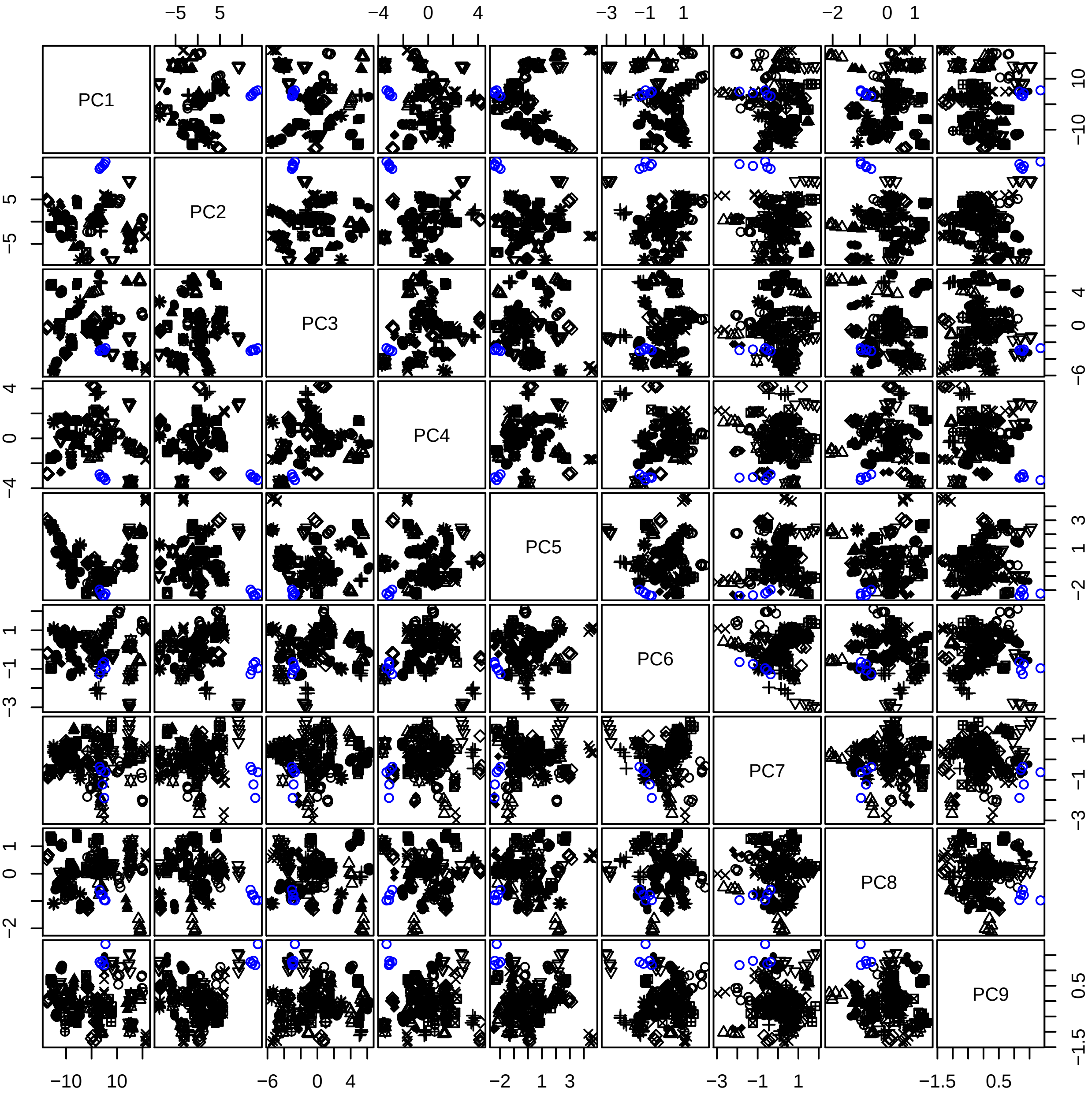
PC1
PC2
PC3
PC4
PC5
PC6
PC7
PC8
PC9

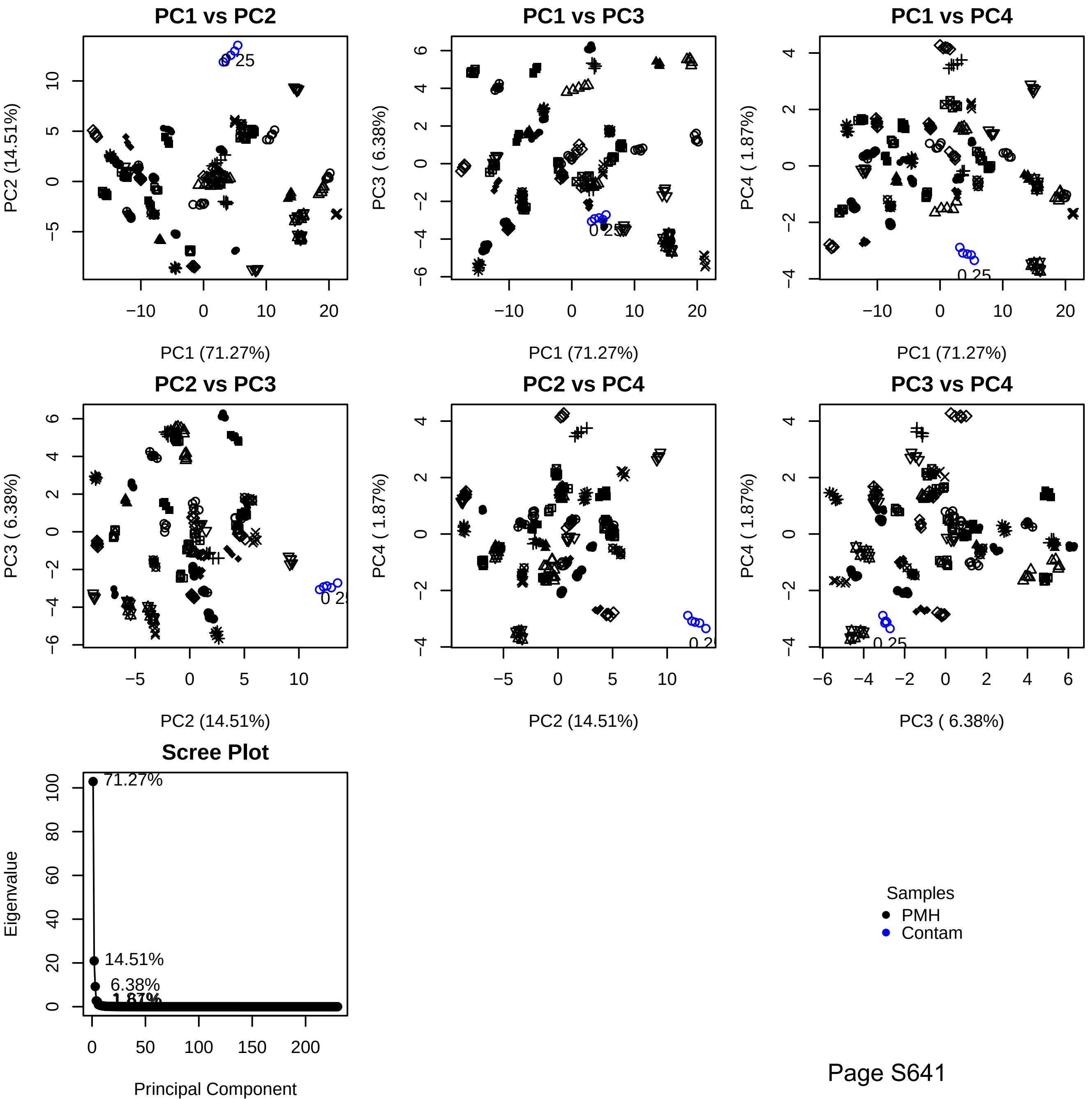
PC1 vs PC2
PC1 vs PC3
PC1 vs PC4
PC2 vs PC3
PC2 vs PC4
PC3 vs PC4
Scree Plot
PC1 (71.27%)
PC2 (14.51%)
PC3 (6.38%)
PC4 (1.87%)
0.25
Eigenvalue
Principal Component
71.27%
14.51%
6.38%
1.87%
Samples
PMH
Contam

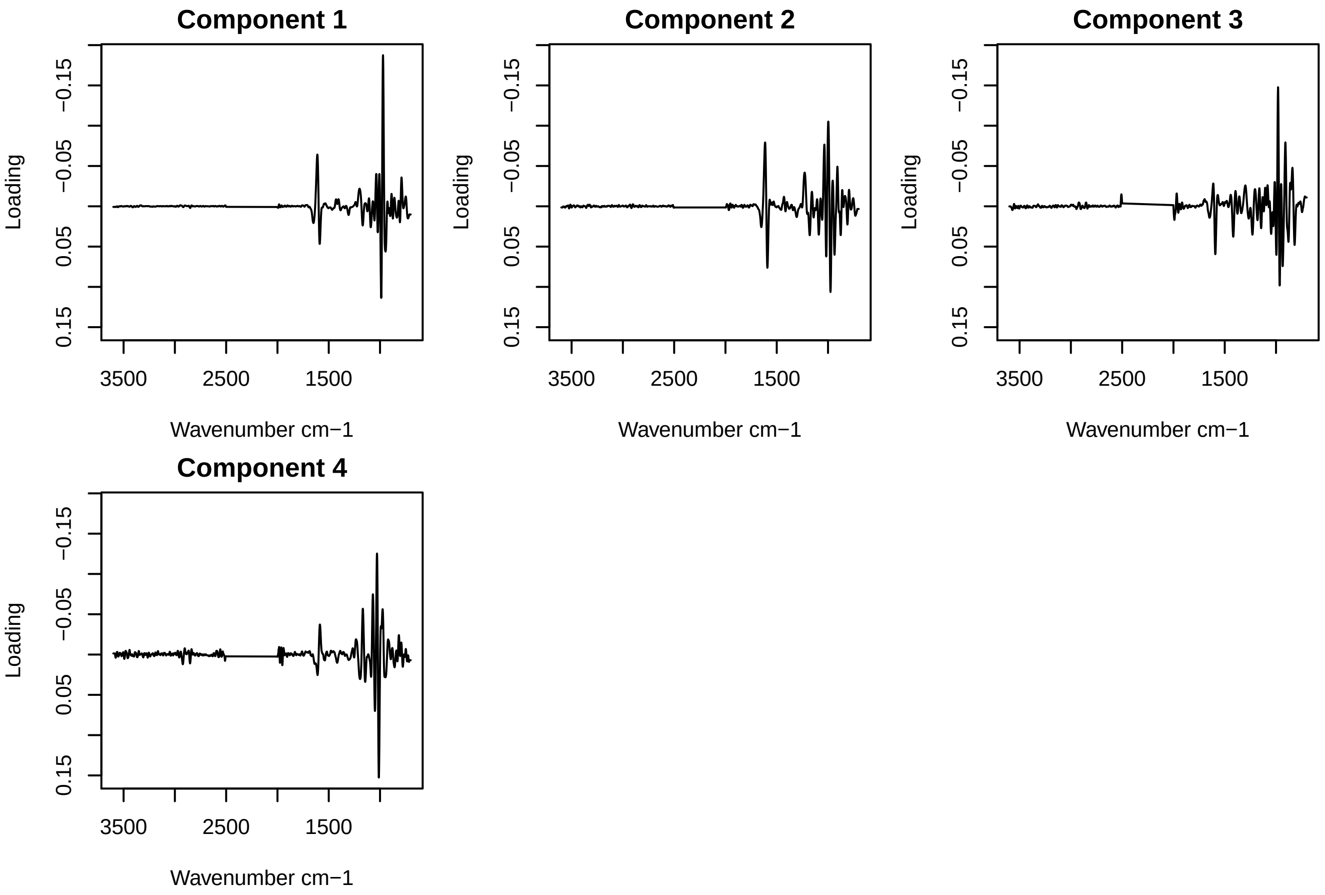

Component 1
Component 2
Component 3
Component 4
Loading
−0.15
−0.05
0.05
0.15
3500
2500
1500
Wavenumber cm−1

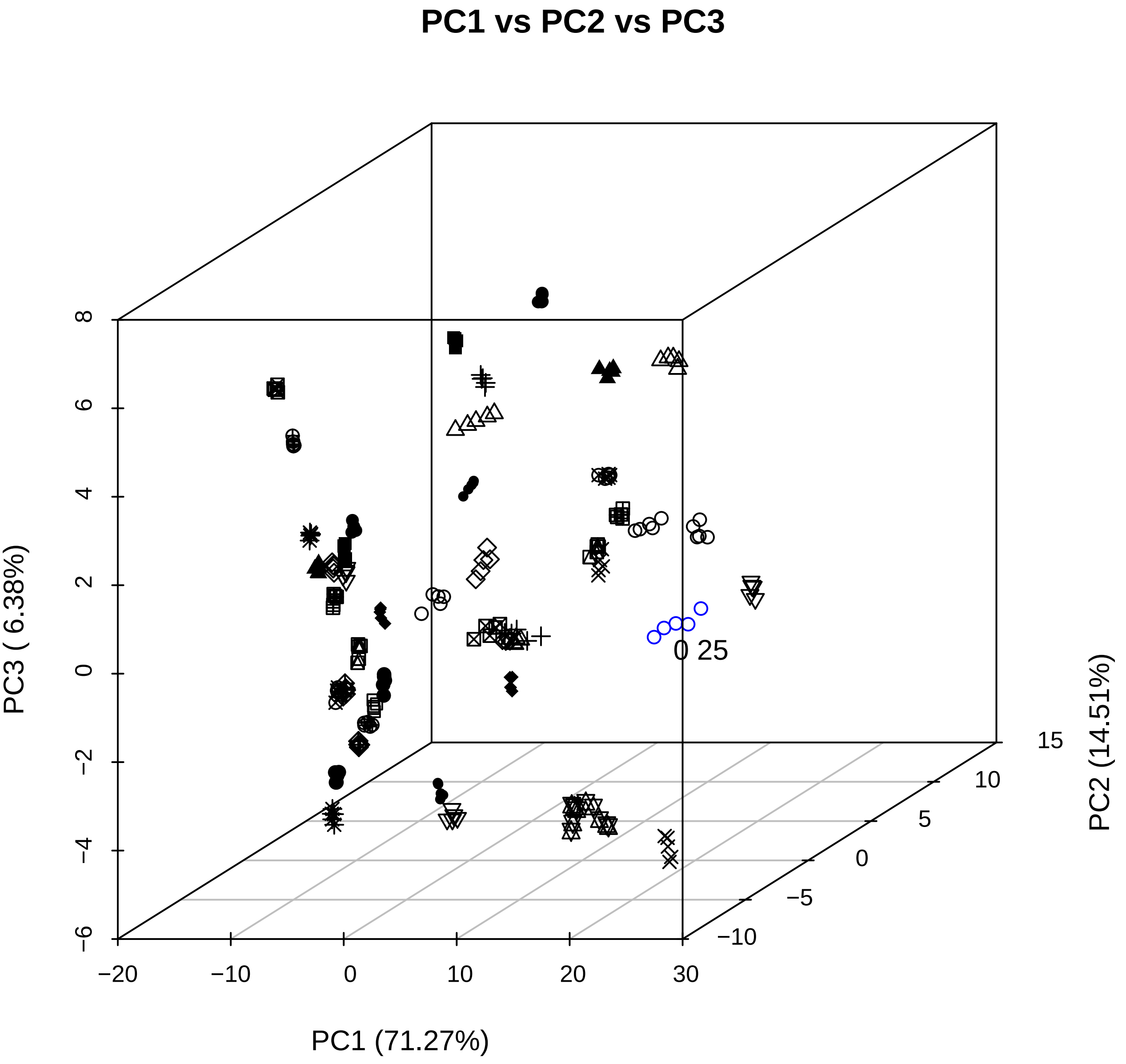

PC1 vs PC2 vs PC3
PC1 (71.27%)
PC2 (14.51%)
PC3 (6.38%)
0 25
−20
−10
0
10
20
30
−10
−5
0
5
10
15
−6
−4
−2
0
2
4
6
8

PC2 vs PC3 vs PC4

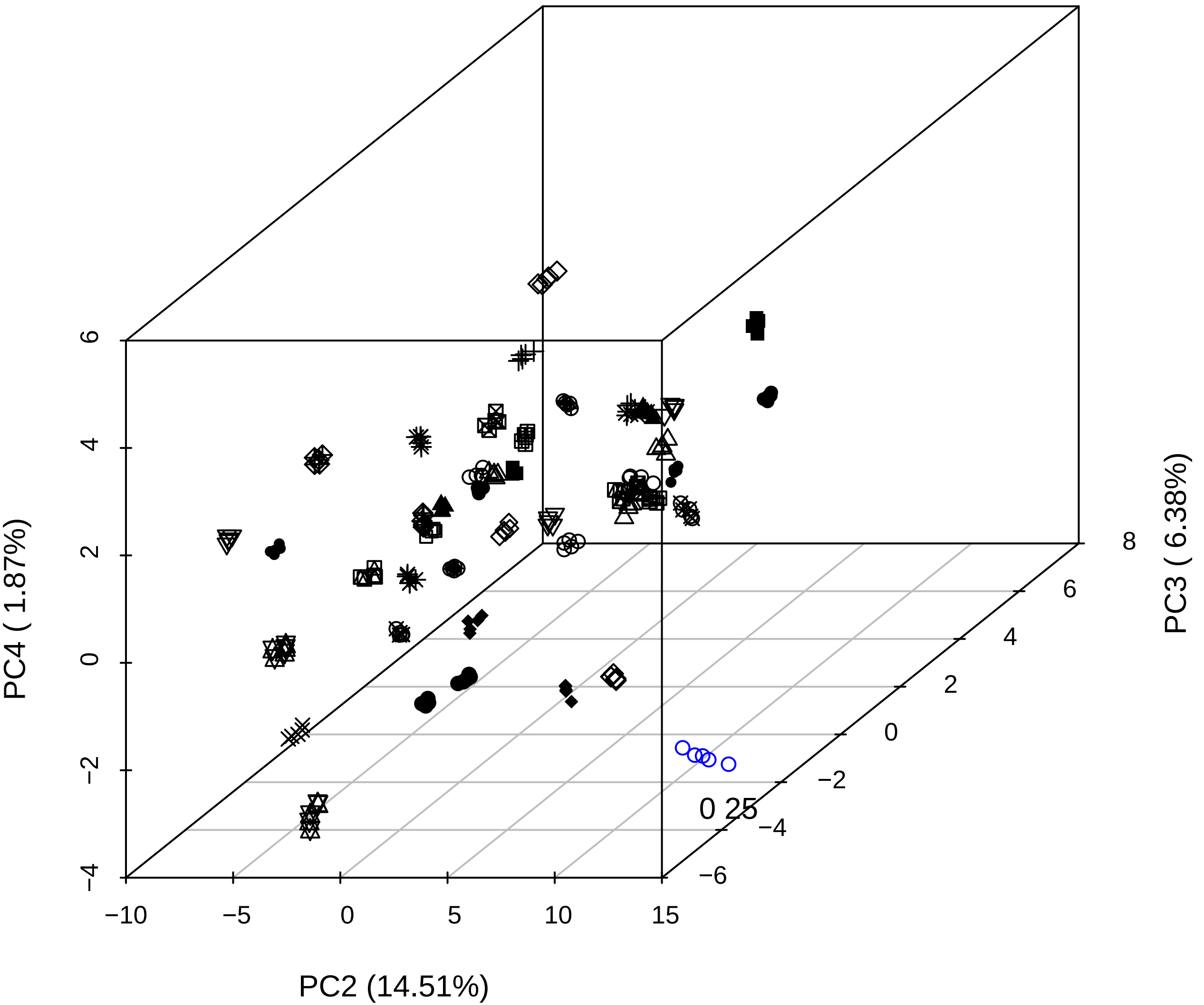

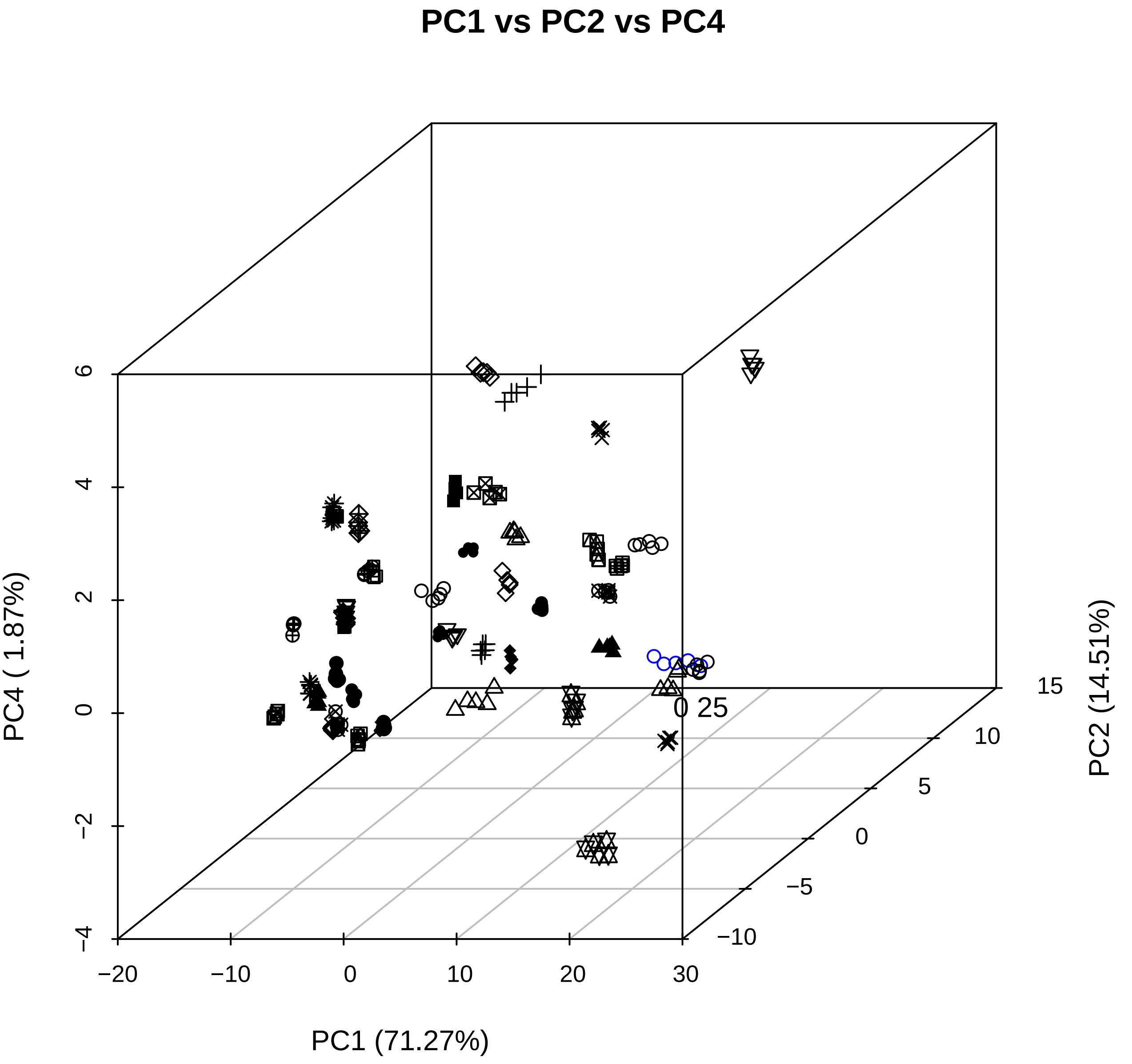
PC1 vs PC2 vs PC4
PC1 (71.27%)
PC2 (14.51%)
PC4 (1.87%)
−20
−10
0
10
20
30
−10
−5
0
5
10
15
−4
−2
0
2
4
6
0 25

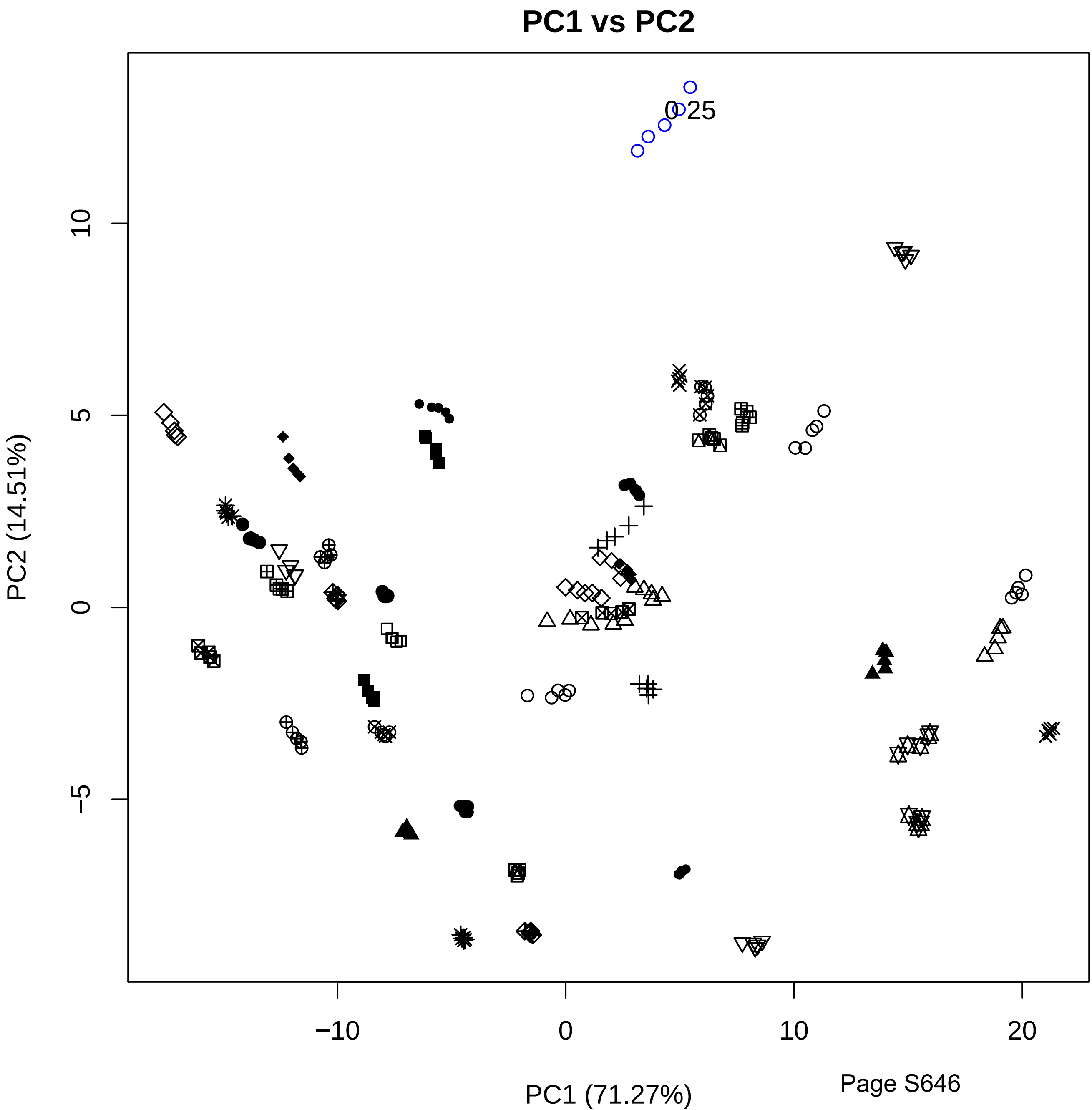

PC1 vs PC2
0.25
10
5
0
−5
PC2 (14.51%)
−10
0
10
20
PC1 (71.27%)

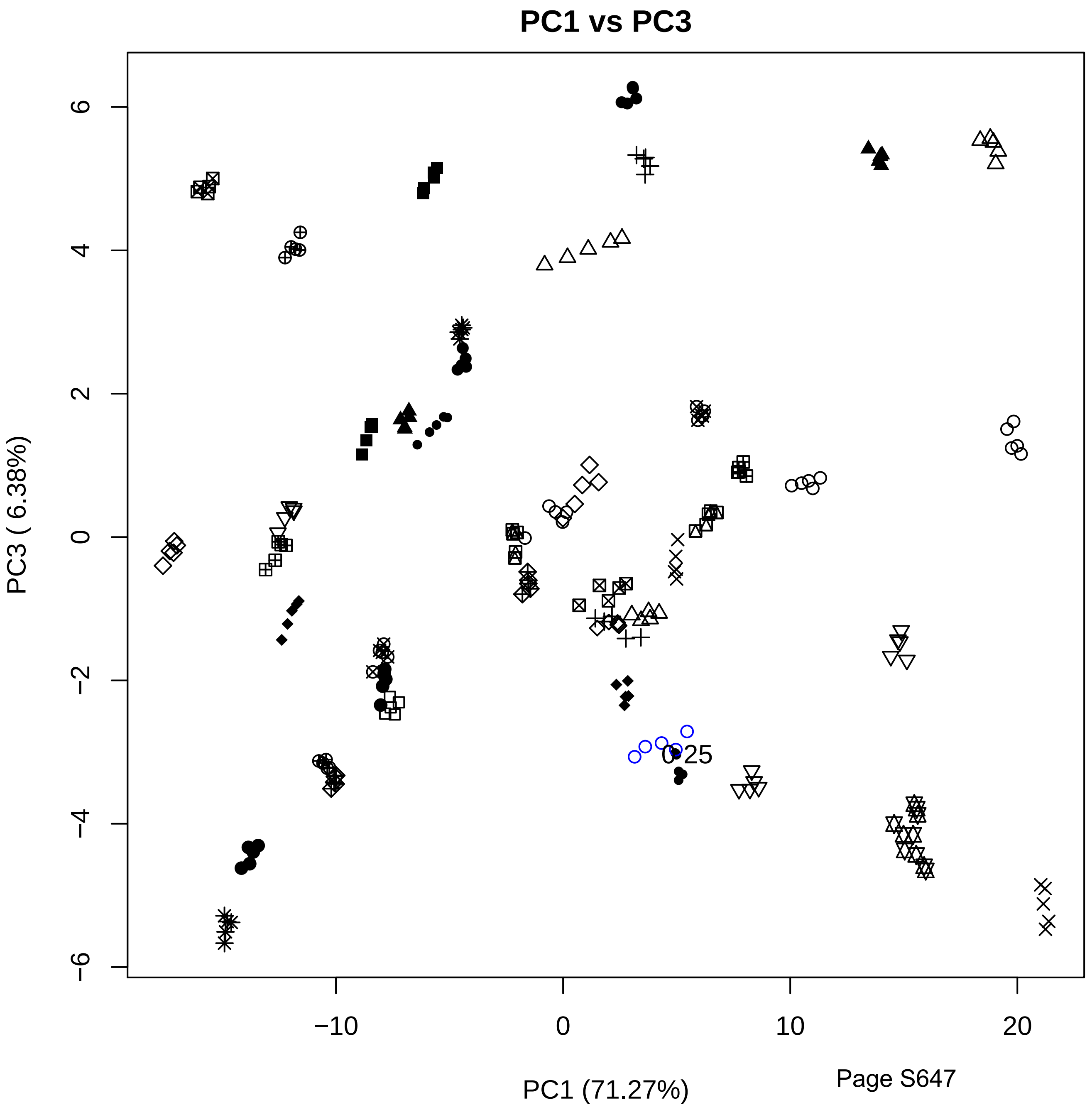
PC1 vs PC3
PC3 (6.38%)
PC1 (71.27%)
6
4
2
0
−2
−4
−6
−10
0
10
20
0.25

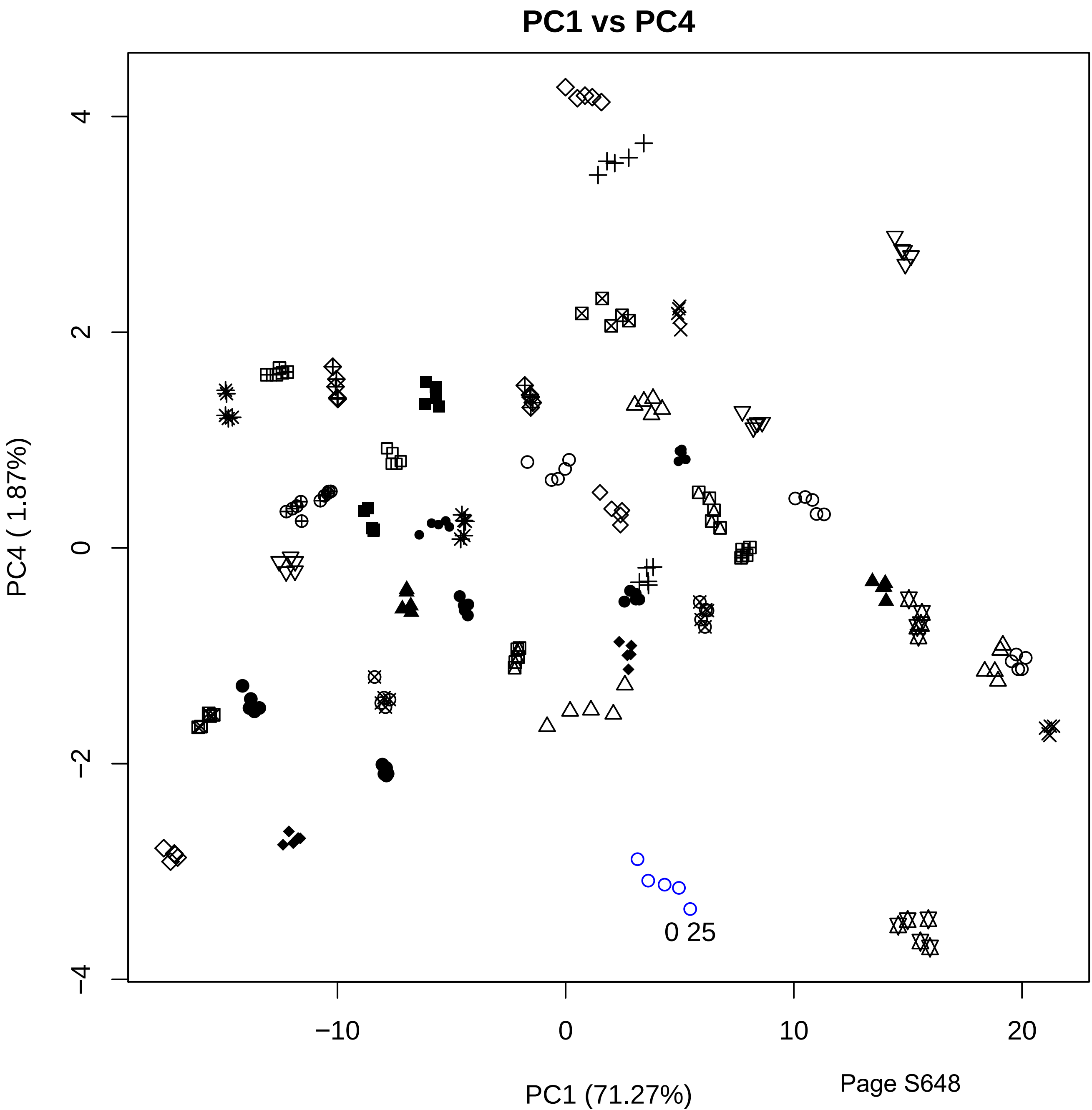
PC1 vs PC4
PC4 (1.87%)
PC1 (71.27%)
4
2
0
−2
−4
−10
0
10
20
0 25

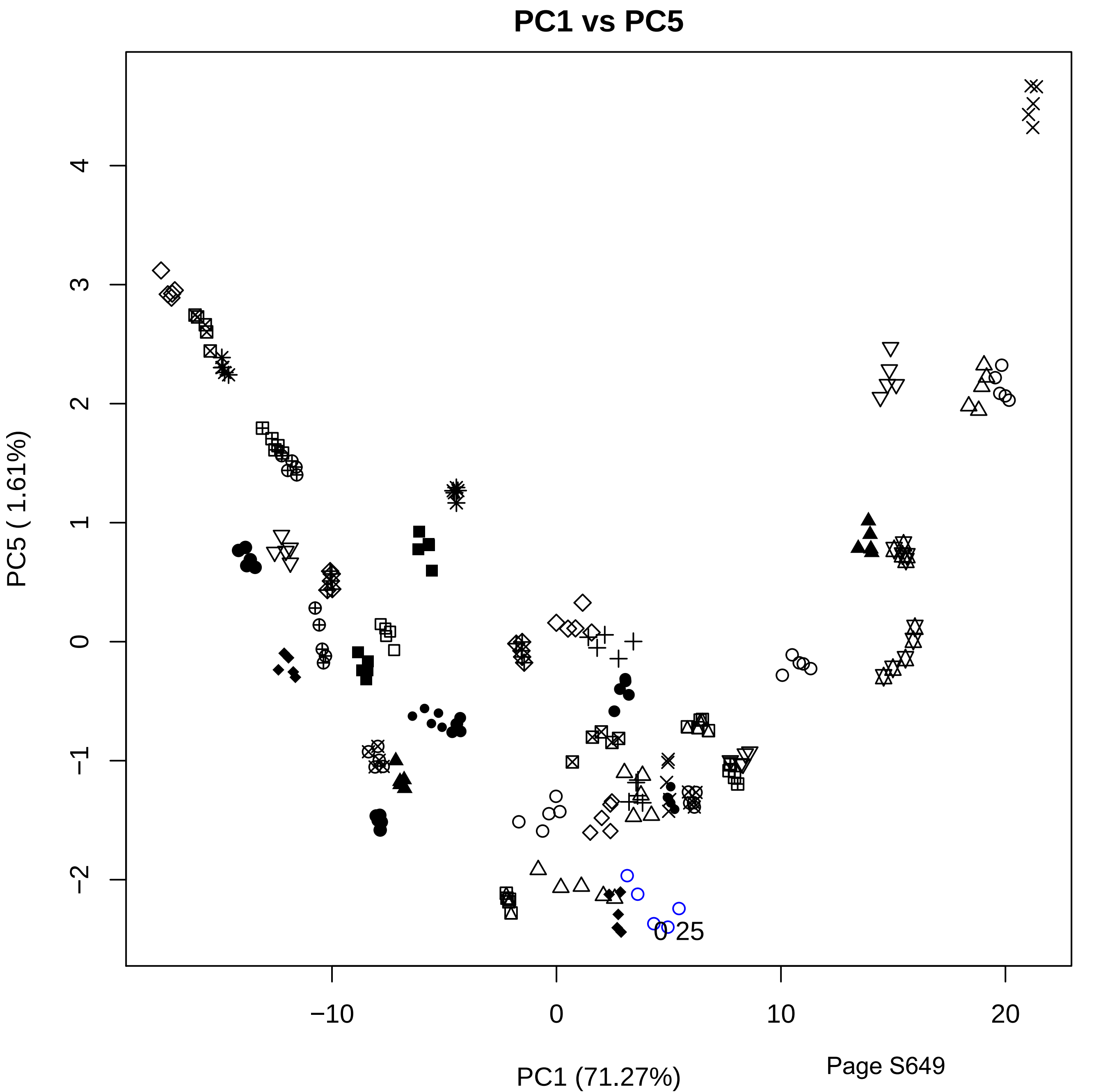
PC1 vs PC5
PC5 (1.61%)
PC1 (71.27%)
4
3
2
1
0
−1
−2
−10
0
10
20
0 25

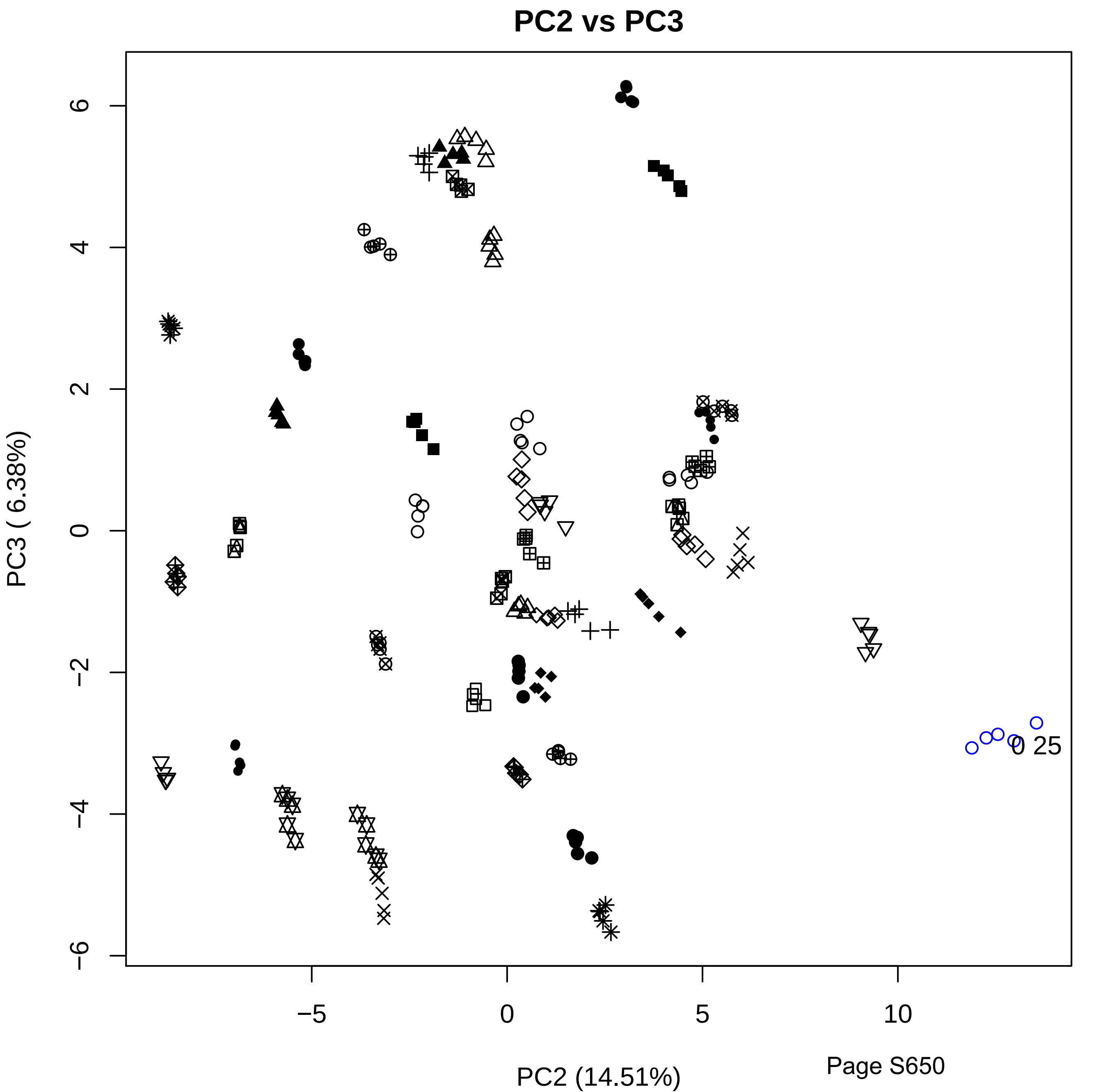

PC2 vs PC3
PC3 (6.38%)
PC2 (14.51%)
6
4
2
0
−2
−4
−6
−5
0
5
10
0 25

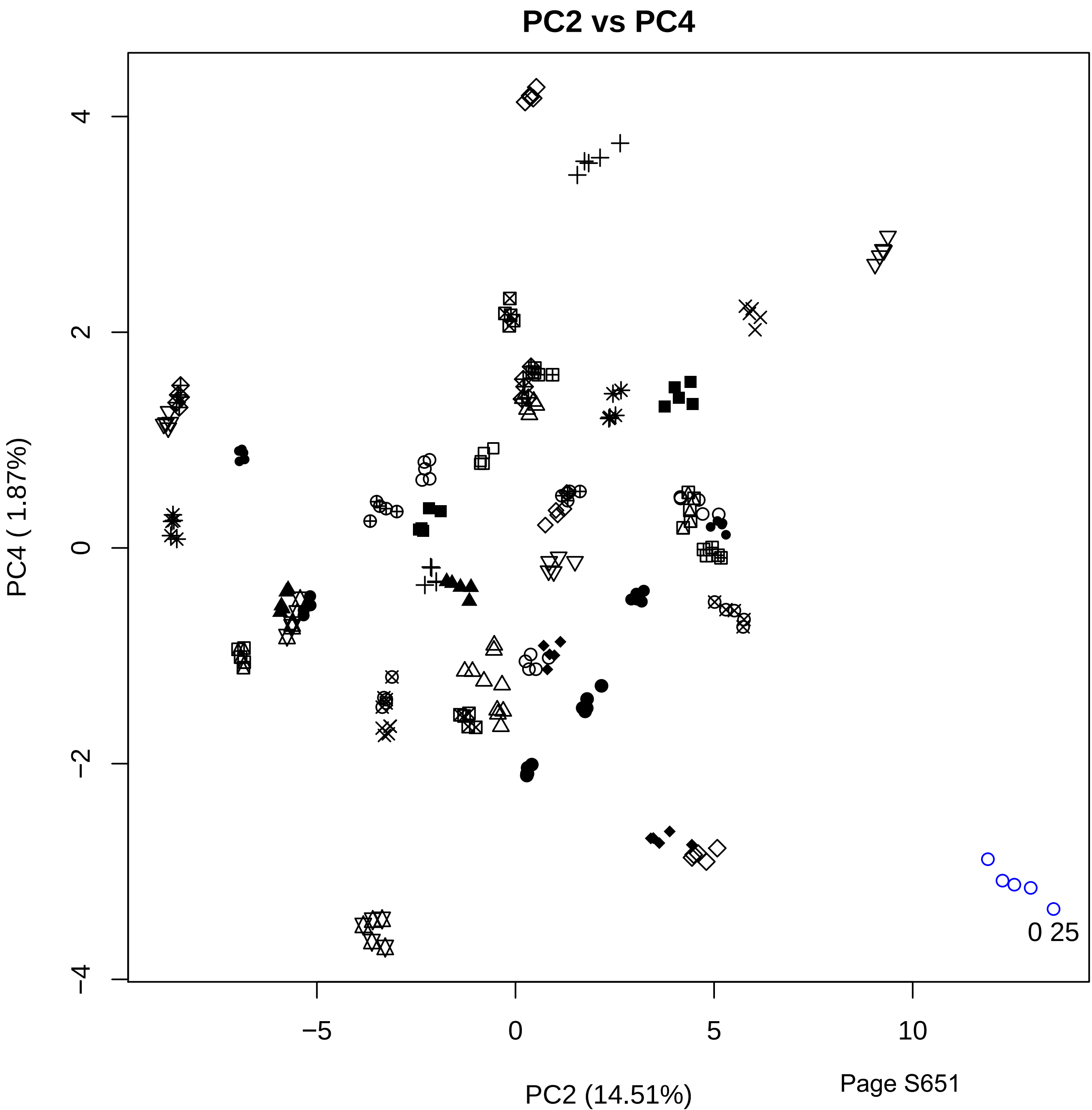
PC2 vs PC4
PC4 (1.87%)
PC2 (14.51%)
4
2
0
−2
−4
−5
0
5
10
0 25

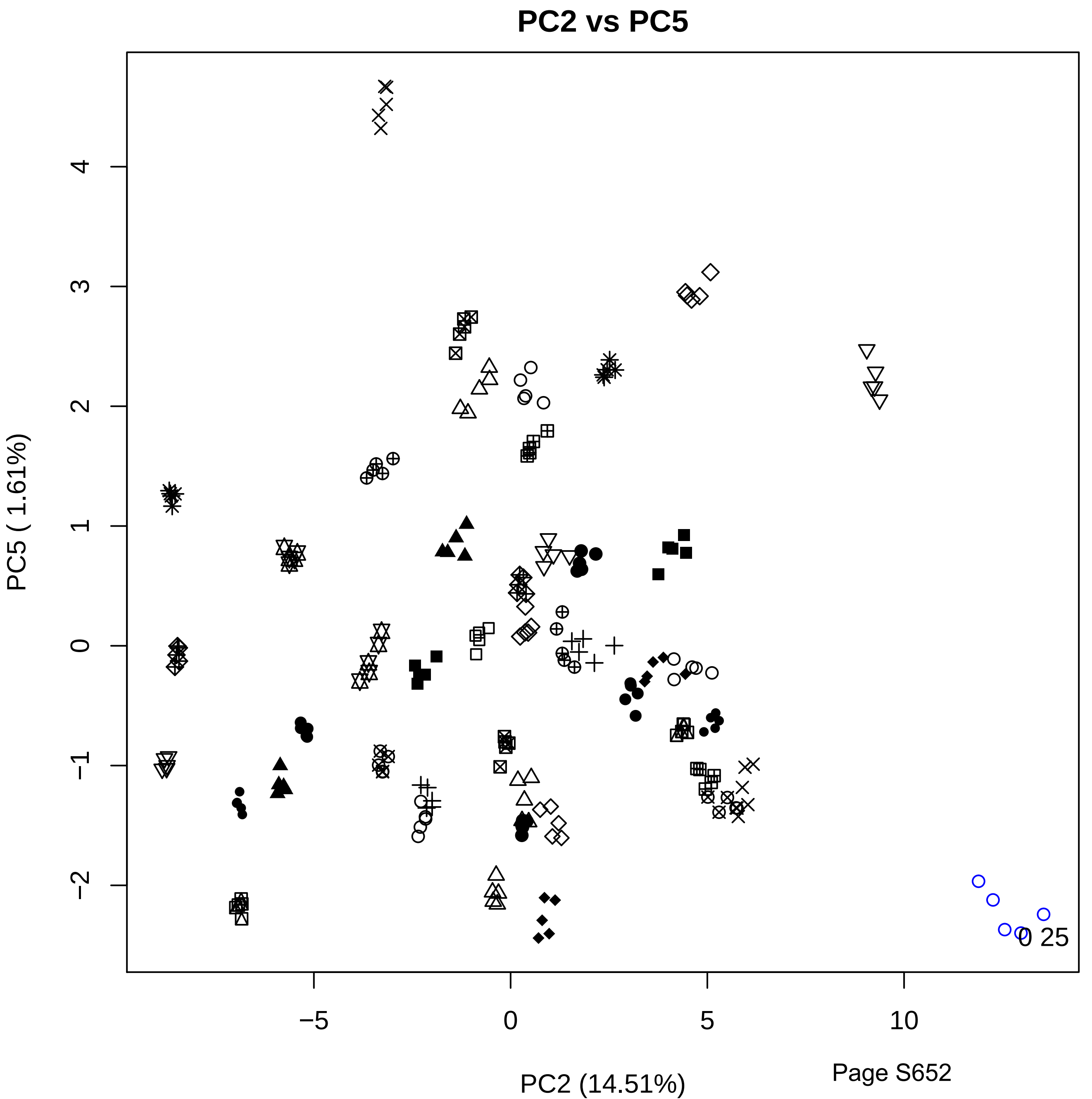
PC2 vs PC5
PC5 (1.61%)
PC2 (14.51%)
4
3
2
1
0
−1
−2
−5
0
5
10
0 25

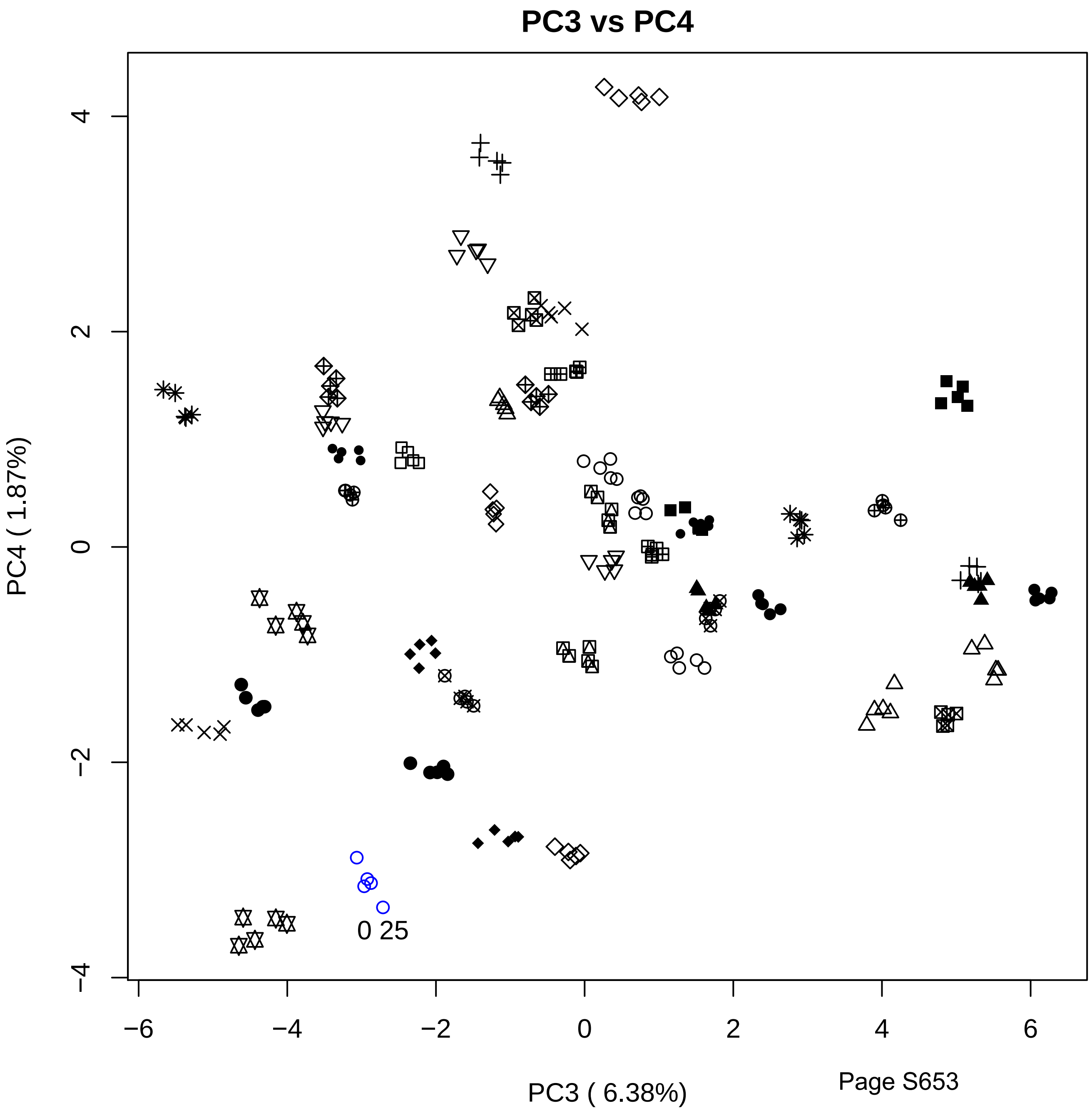
PC3 vs PC4
PC4 (1.87%)
PC3 (6.38%)
−6
−4
−2
0
2
4
6
0 25

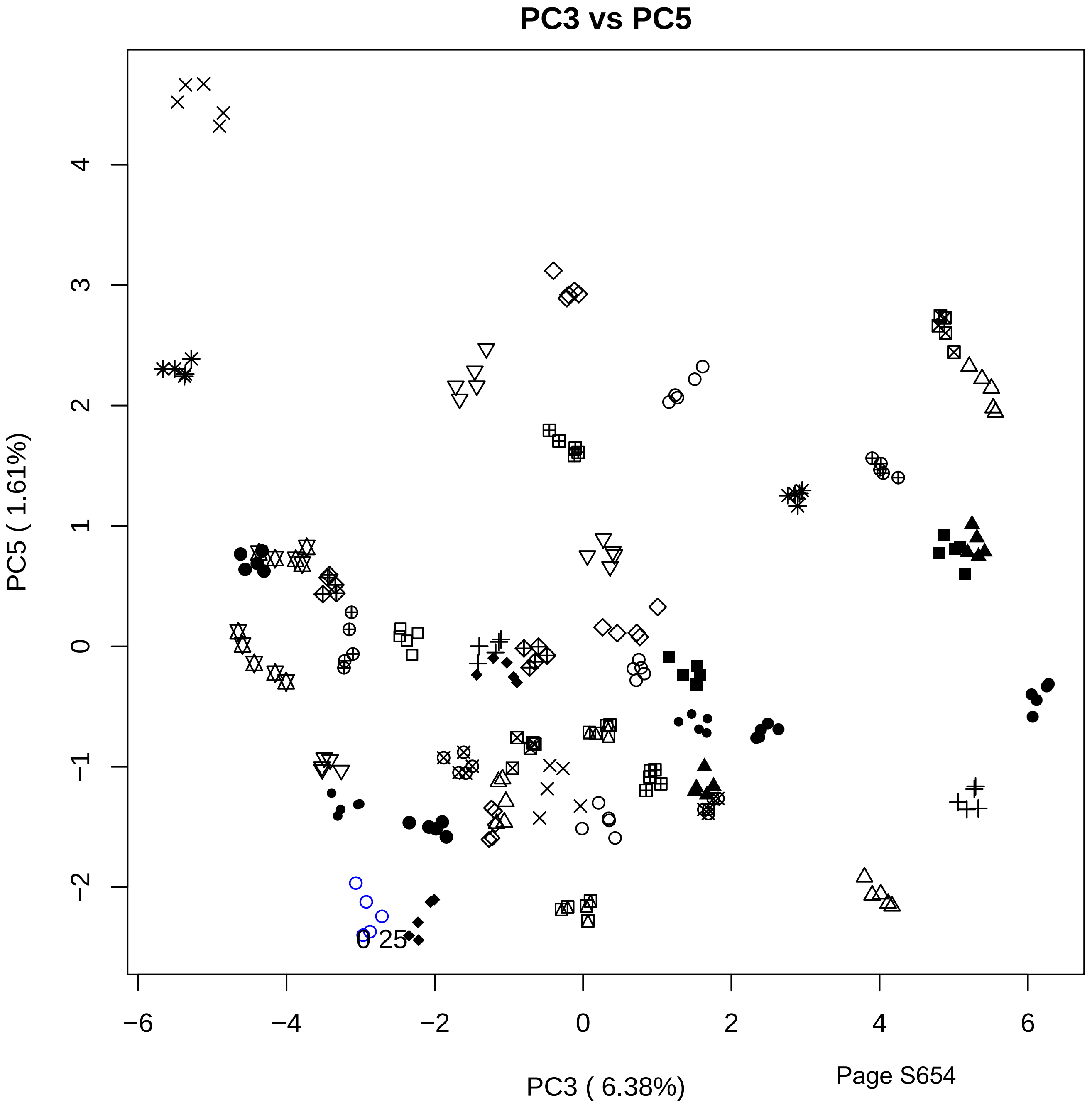
PC3 vs PC5
PC5 (1.61%)
PC3 (6.38%)
−6
−4
−2
0
2
4
6
−2
−1
0
1
2
3
4
0 25

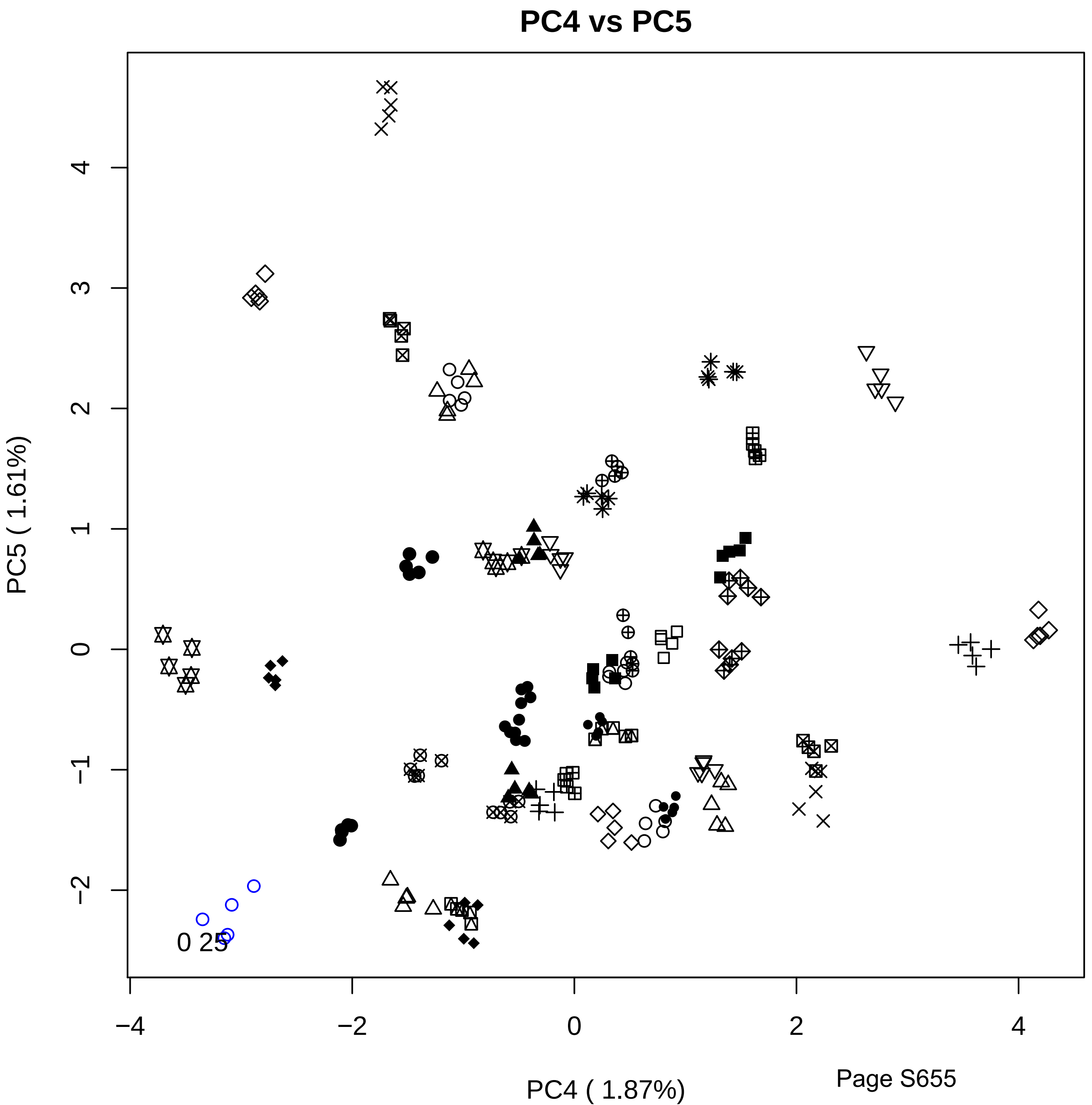
PC4 vs PC5
PC5 (1.61%)
PC4 (1.87%)
0 25

PCA of 0.5% DeS vs PMH

Scaled Matrix PCAed

7th order polynomial baseline correction performed

Raw spectra smoothed to 21 Neighbours, 2nd order PN, Savtisky Golay

Normalisation between 0 and 100

D2 spectra smoothed to 41 Neighbours, 2nd order PN, Savtisky Golay

Regions Analysed: 3601−2501 and 2001−700cm−1

Samples Analysed: 1 Contam's 0 DeS's 45 PMH's

Keele University AD   Sun Dec 09 17:03:56 2018

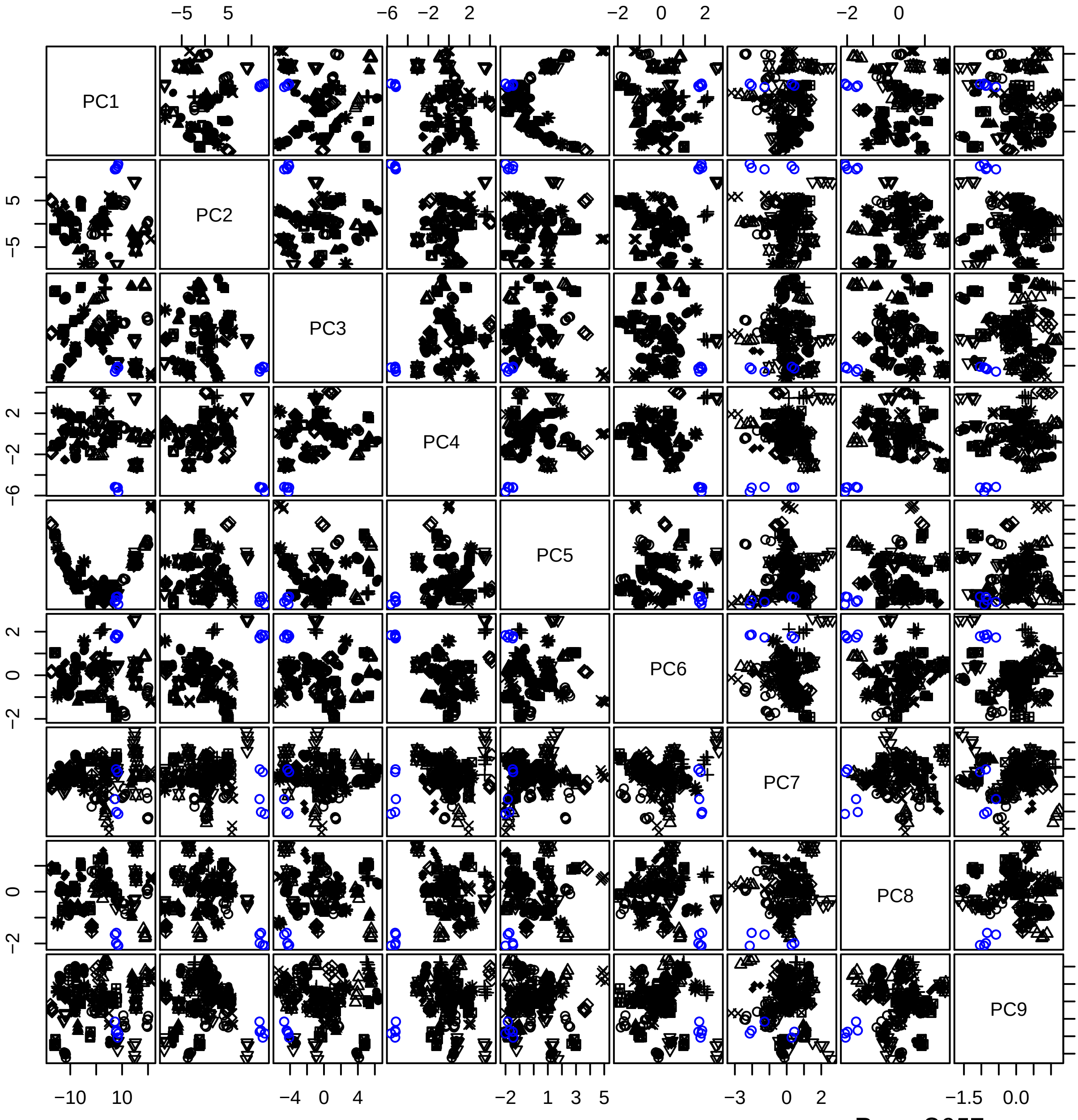
PC1
PC2
PC3
PC4
PC5
PC6
PC7
PC8
PC9

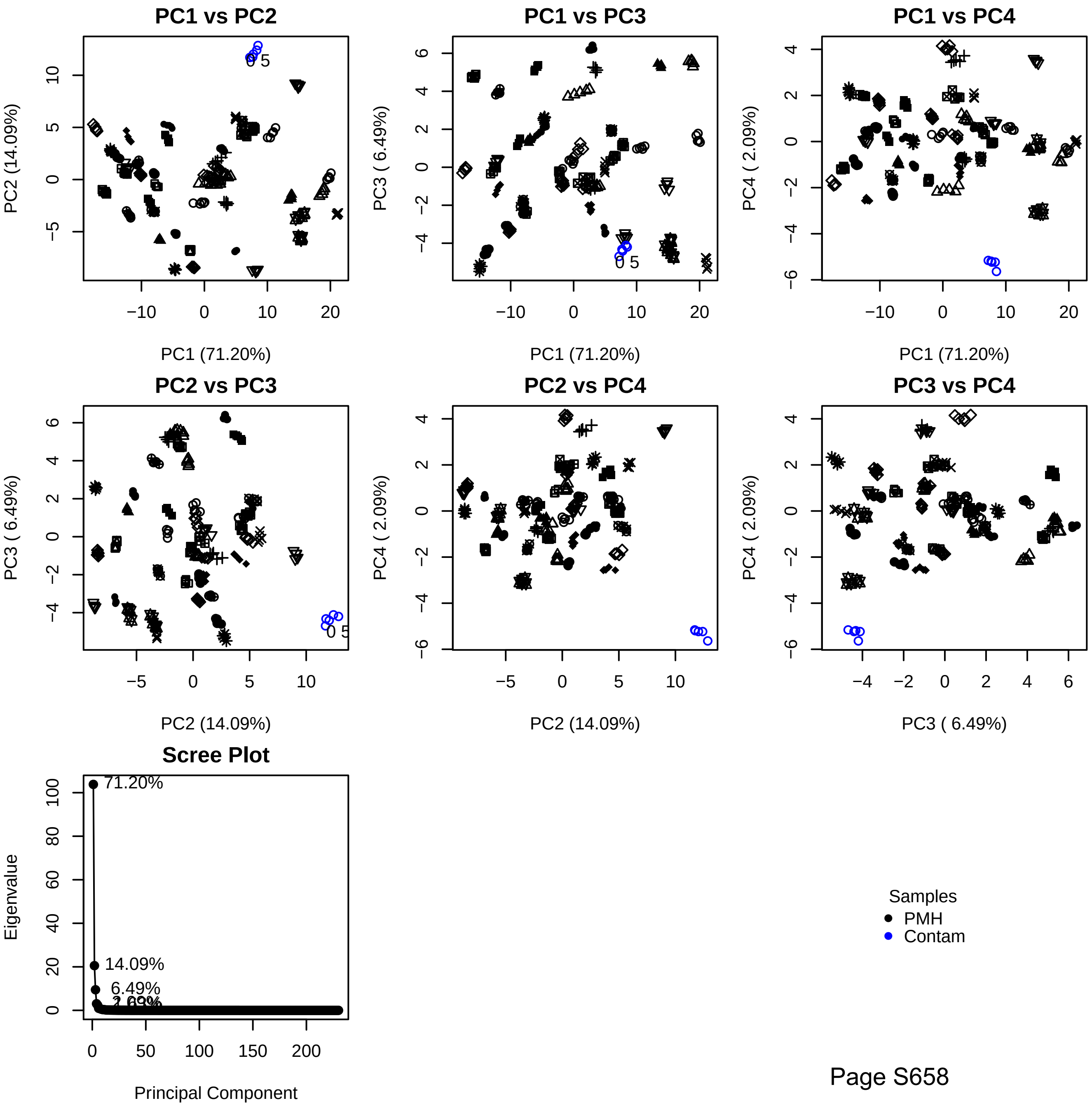
PC1 vs PC2
PC1 vs PC3
PC1 vs PC4
PC2 vs PC3
PC2 vs PC4
PC3 vs PC4
Scree Plot
PC1 (71.20%)
PC2 (14.09%)
PC3 (6.49%)
PC4 (2.09%)
0 5
Eigenvalue
Principal Component
71.20%
14.09%
6.49%
Samples
PMH
Contam

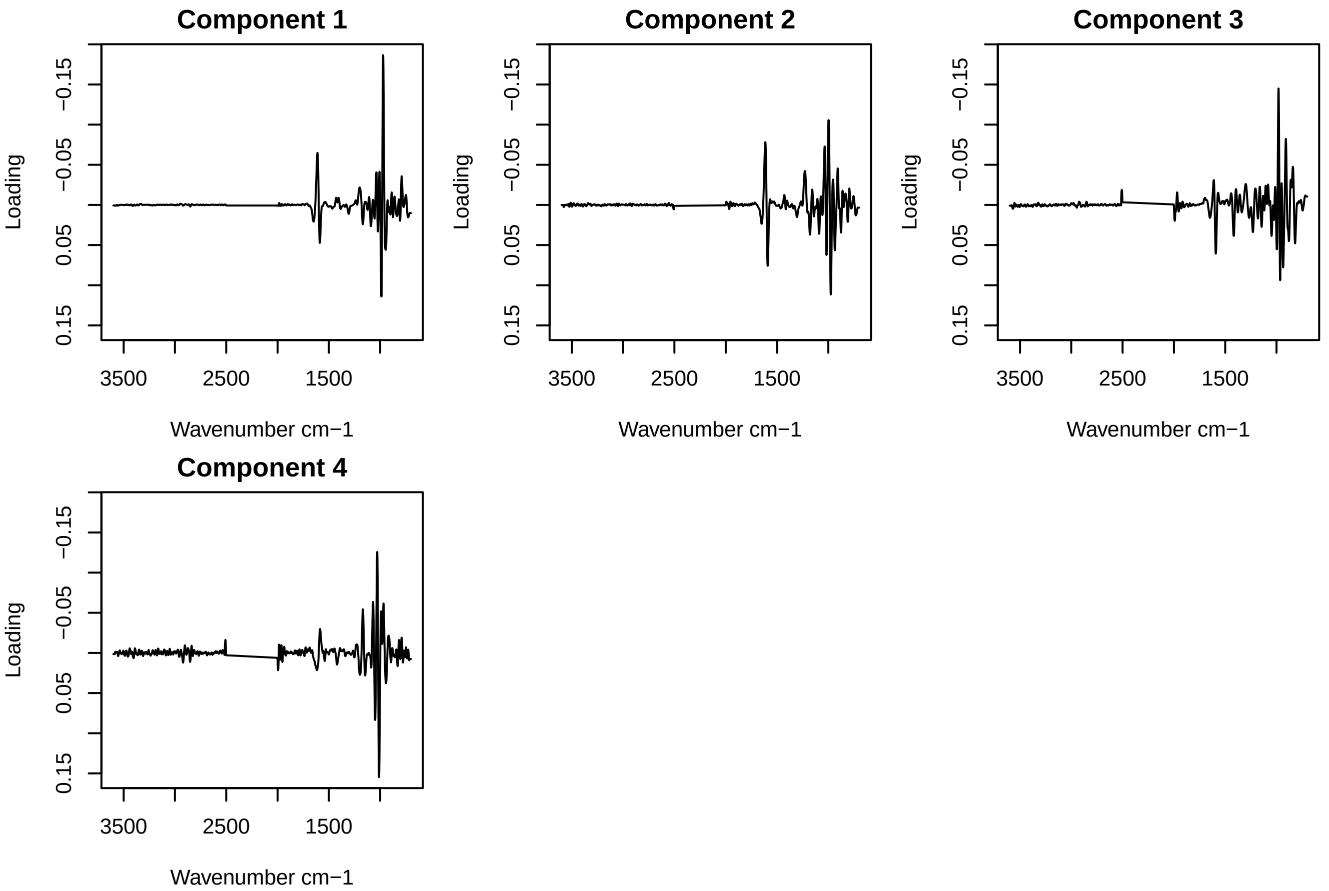
Component 1
Component 2
Component 3
Component 4
Loading
−0.15
−0.05
0.05
0.15
3500
2500
1500
Wavenumber cm−1

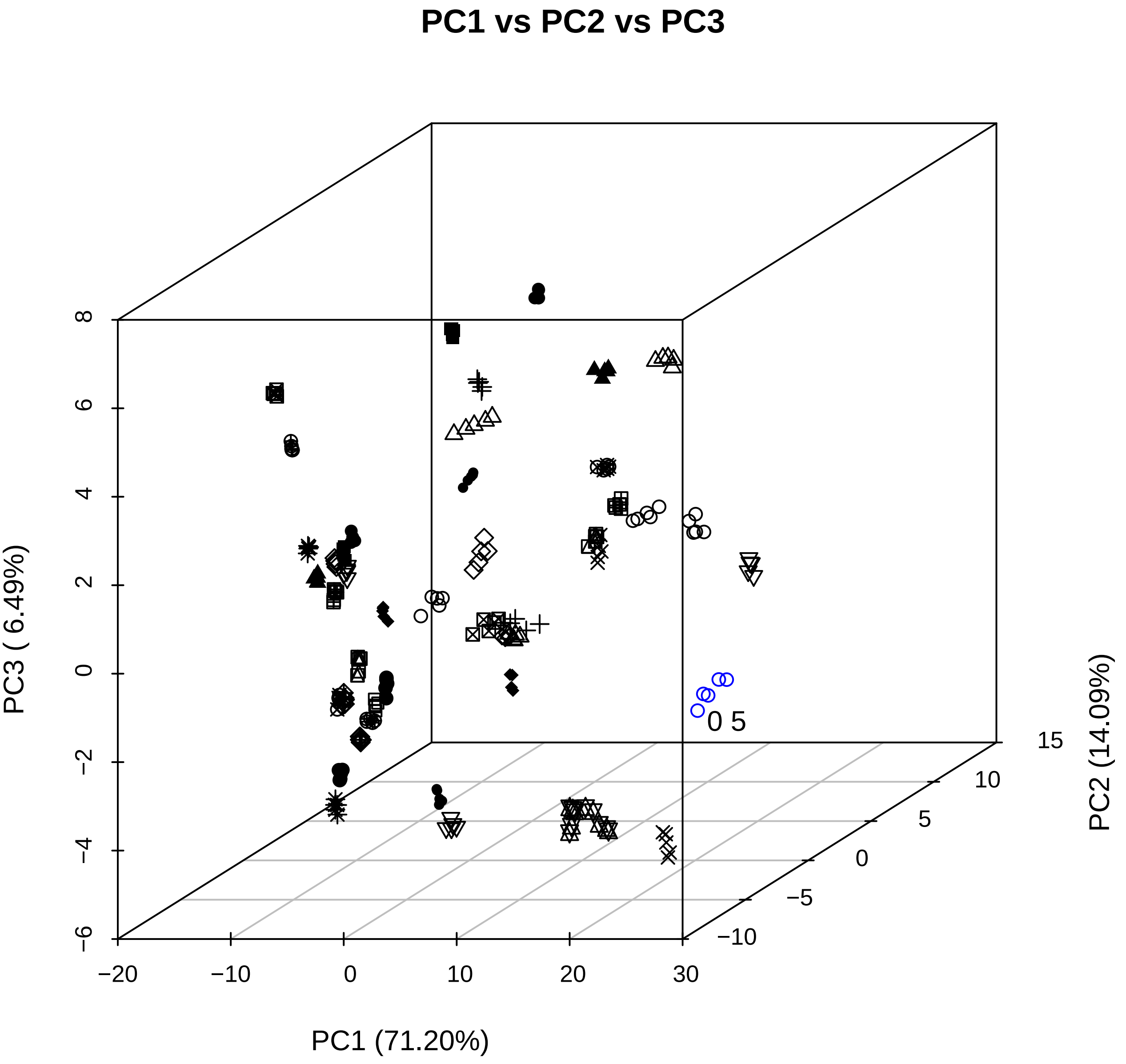

PC1 vs PC2 vs PC3
PC1 (71.20%)
PC2 (14.09%)
PC3 (6.49%)
−20
−10
0
10
20
30
−10
−5
0
5
10
15
−6
−4
−2
0
2
4
6
8
0 5

PC2 vs PC3 vs PC4

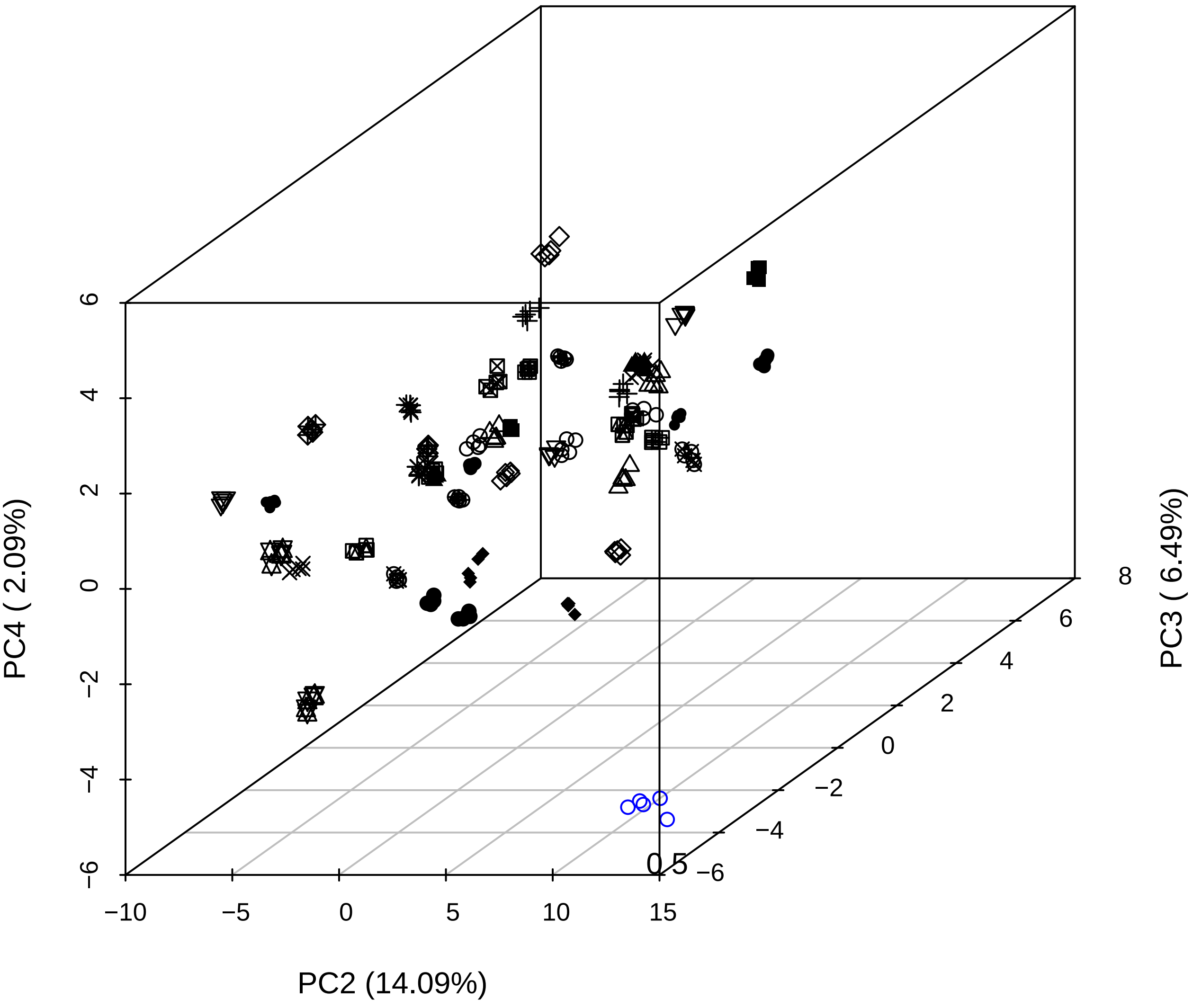

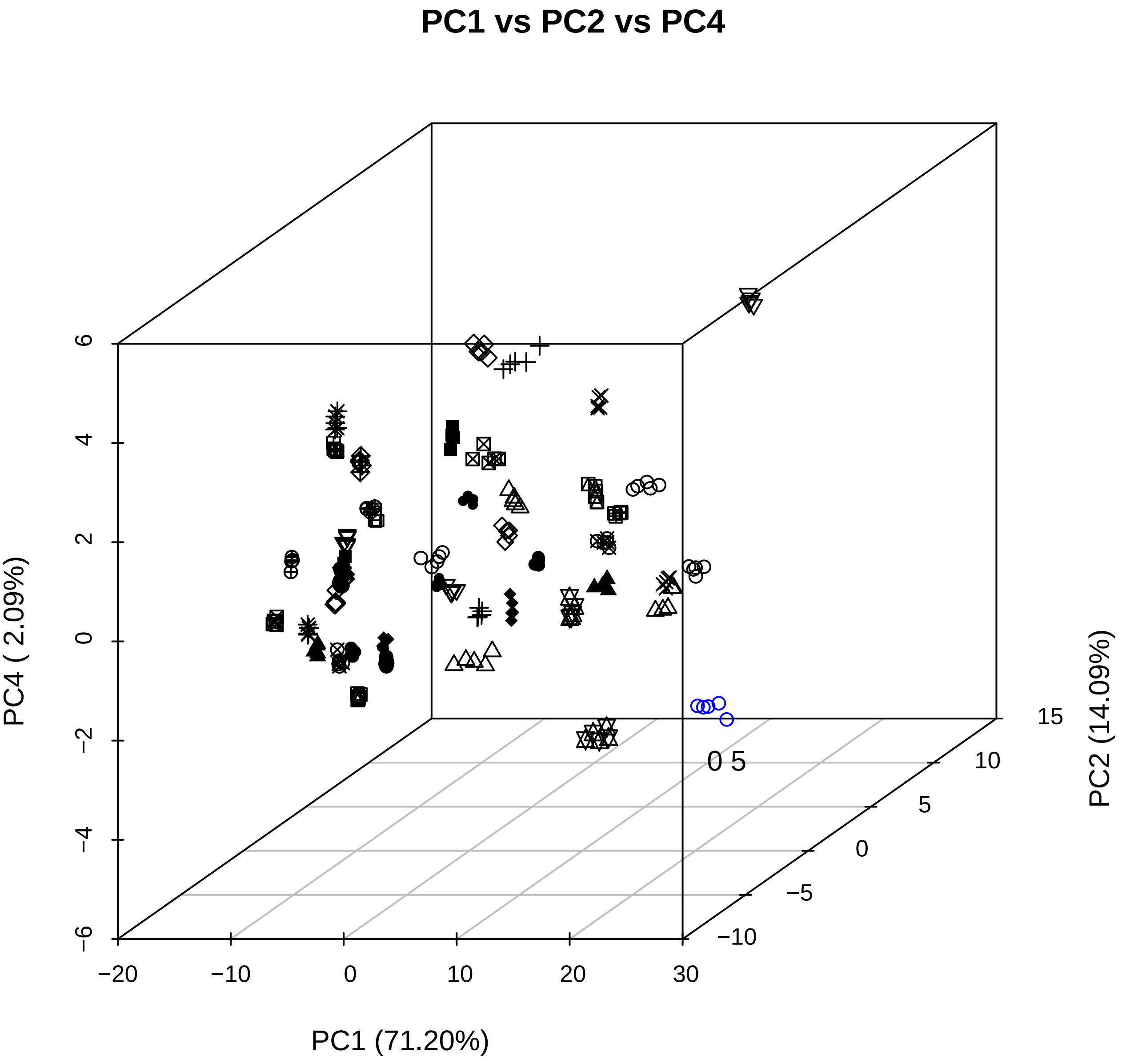

PC1 vs PC2 vs PC4
PC4 (2.09%)
6
4
2
0
−2
−4
−6
PC1 (71.20%)
−20
−10
0
10
20
30
PC2 (14.09%)
15
10
5
0
−5
−10
0 5

PC1 vs PC2

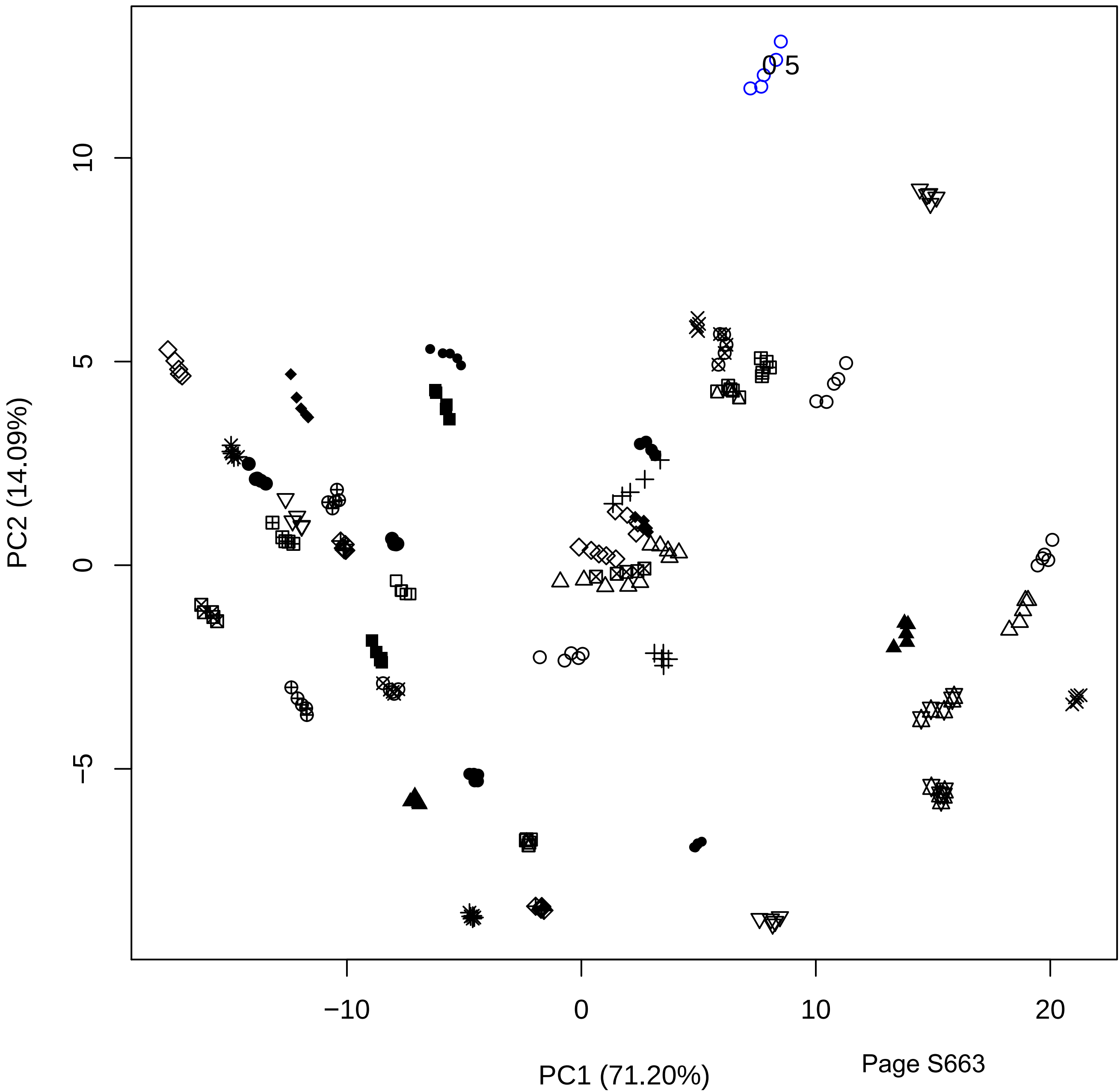

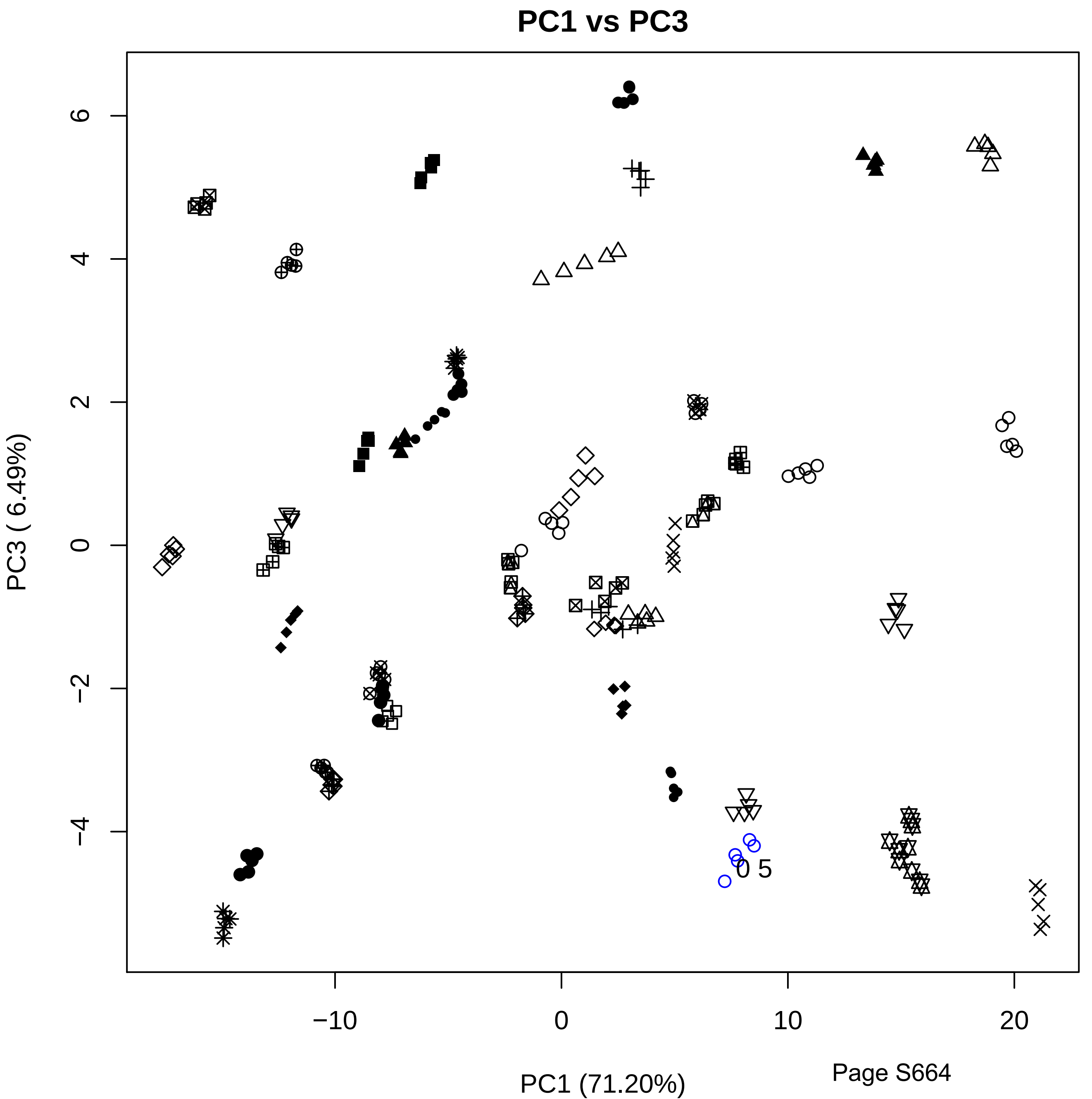
PC1 vs PC3
PC3 (6.49%)
PC1 (71.20%)
6
4
2
0
−2
−4
−10
0
10
20
0 5

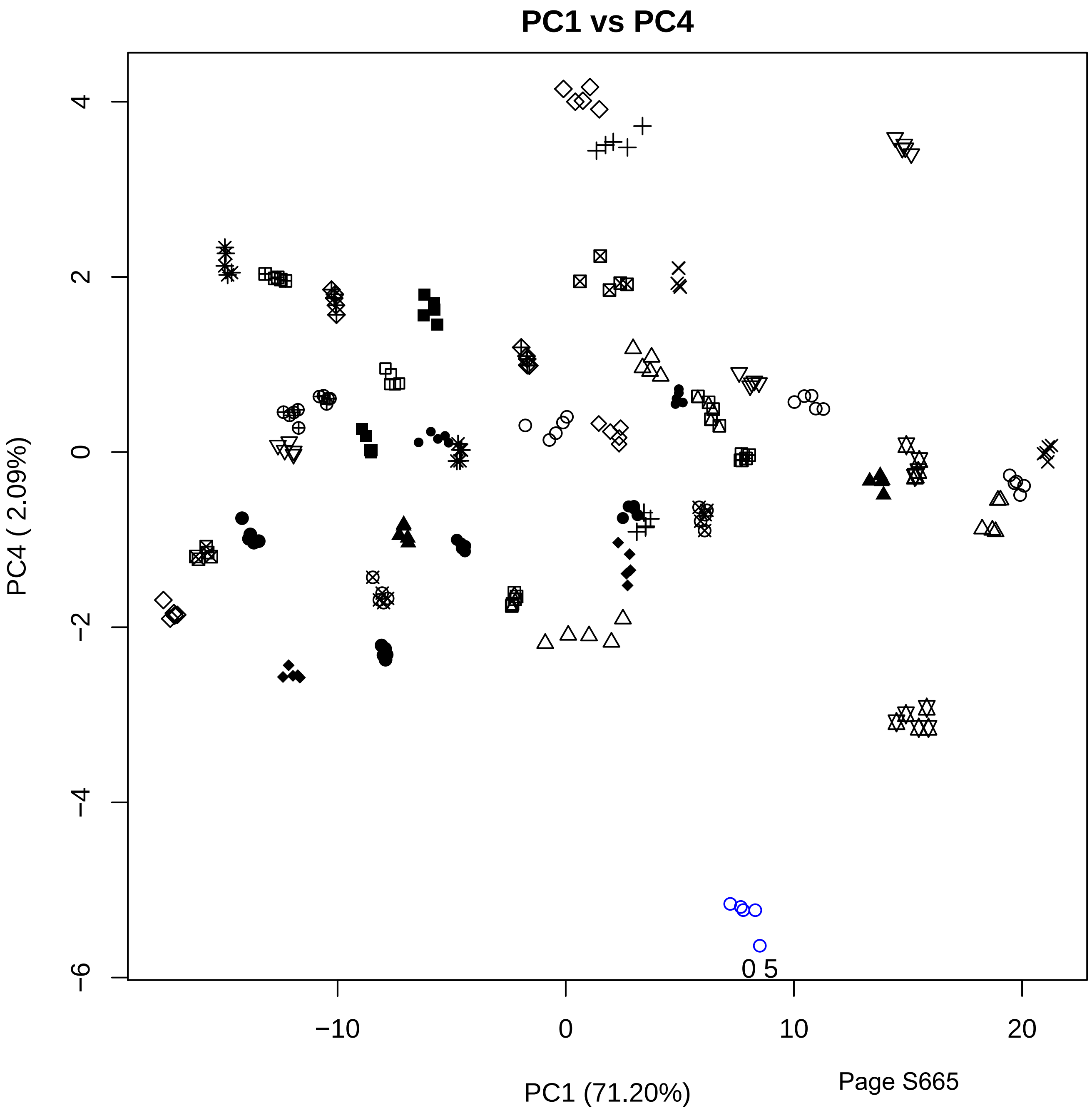
PC1 vs PC4
PC4 (2.09%)
PC1 (71.20%)
4
2
0
−2
−4
−6
−10
0
10
20
0 5

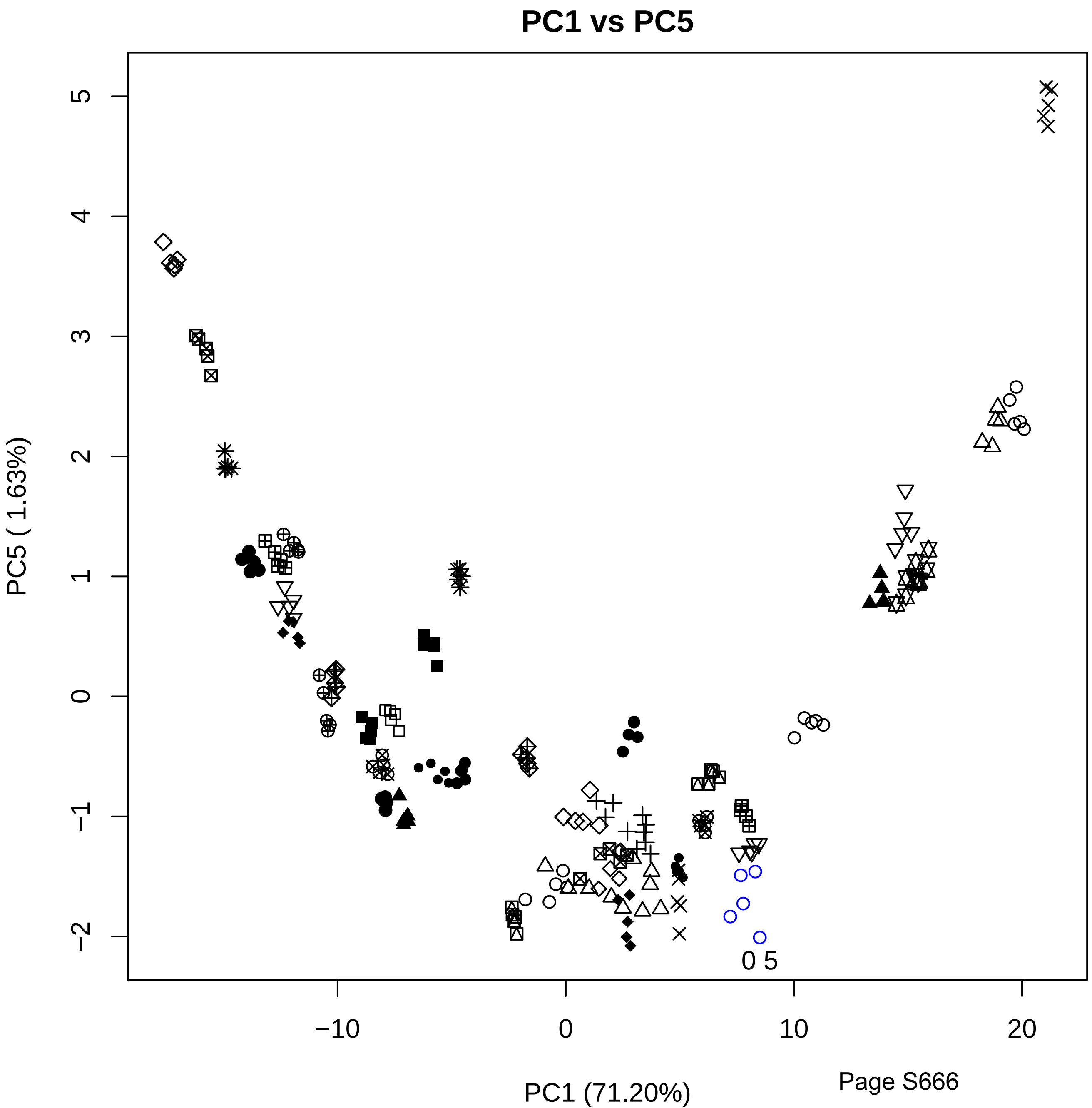
PC1 vs PC5
PC5 (1.63%)
PC1 (71.20%)
5
4
3
2
1
0
−1
−2
−10
0
10
20
0 5

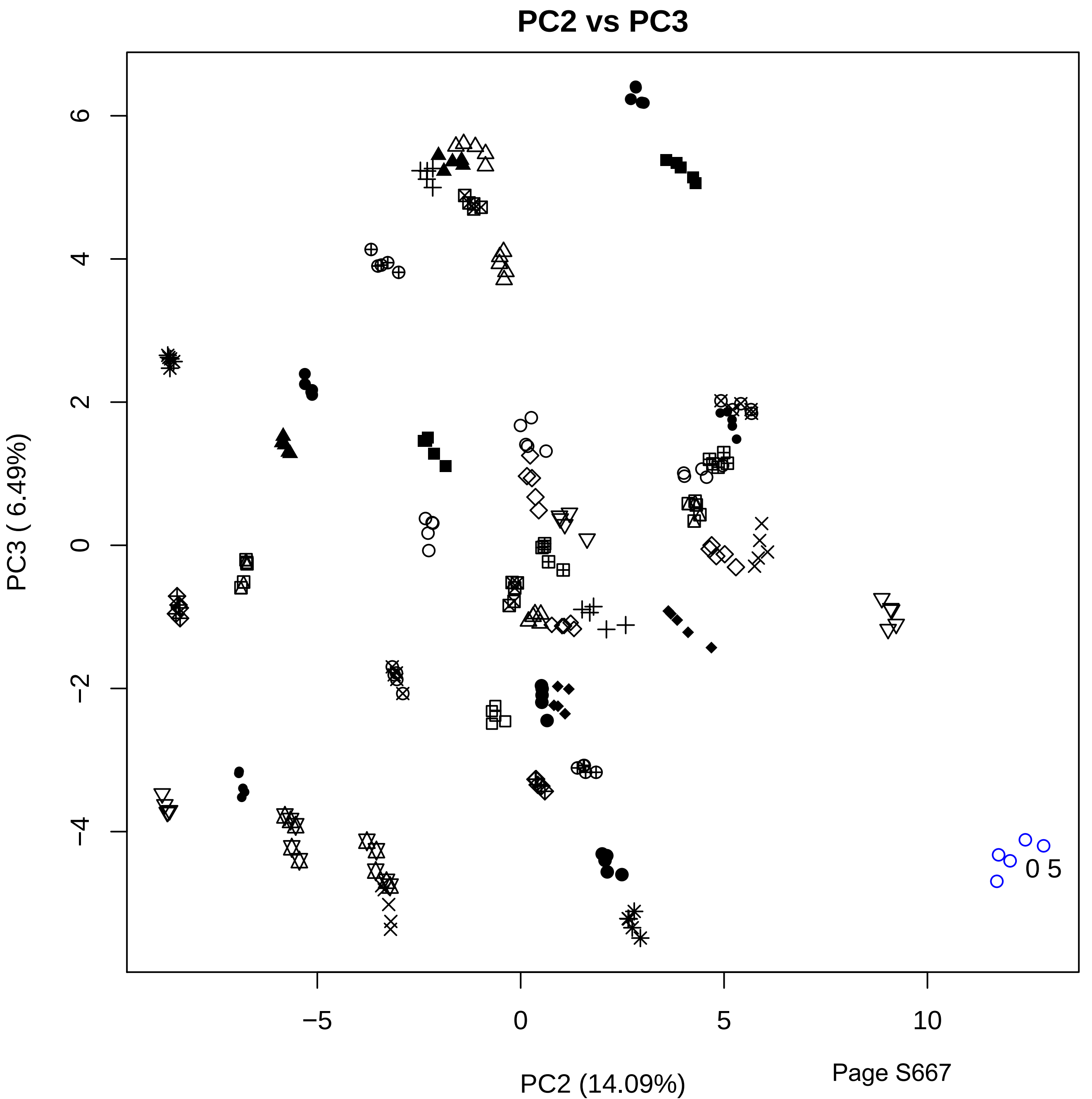
PC2 vs PC3
PC3 (6.49%)
PC2 (14.09%)
6
4
2
0
−2
−4
−5
0
5
10
0 5

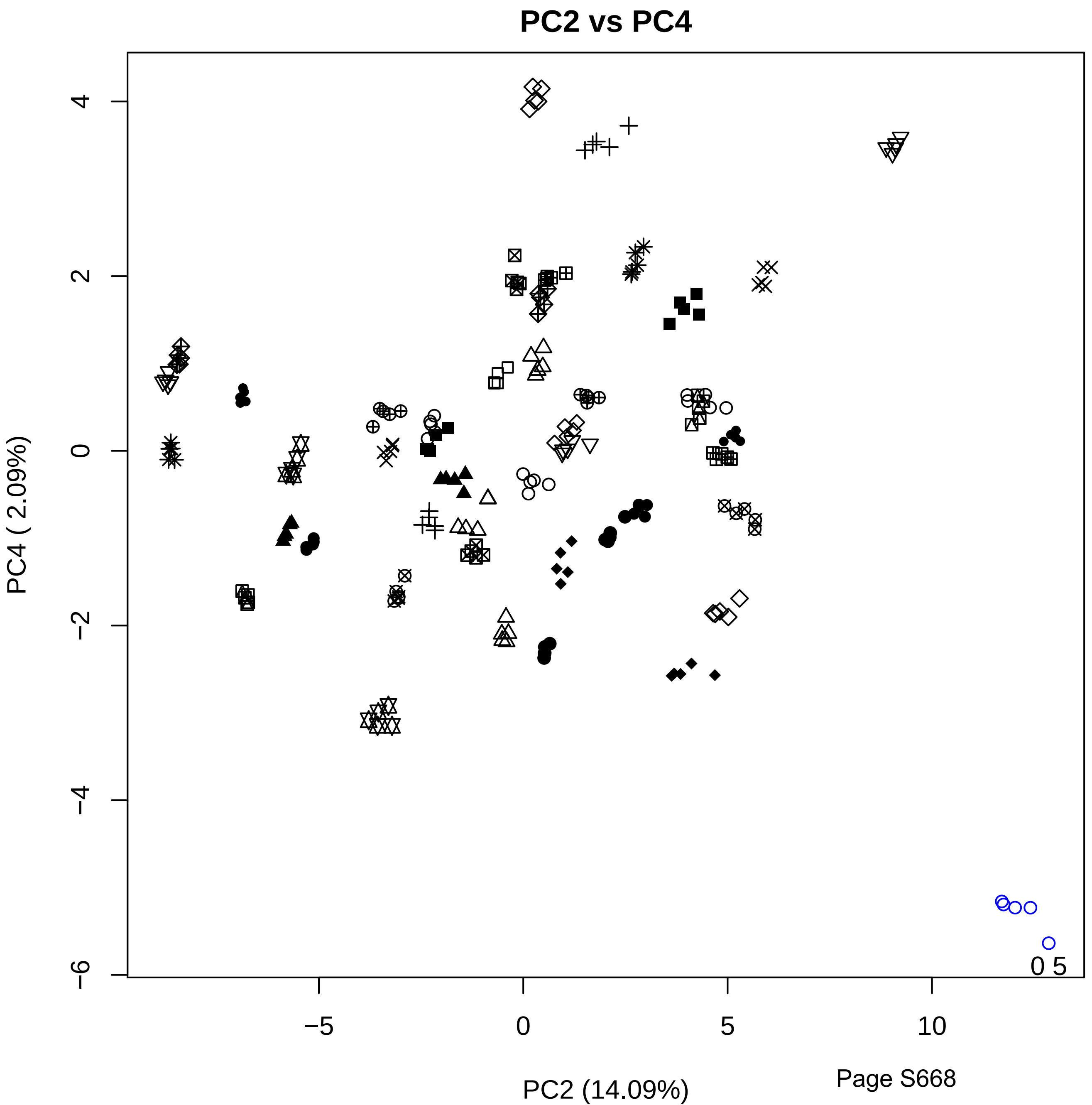
PC2 vs PC4
PC4 (2.09%)
PC2 (14.09%)
4
2
0
−2
−4
−6
−5
0
5
10
0 5

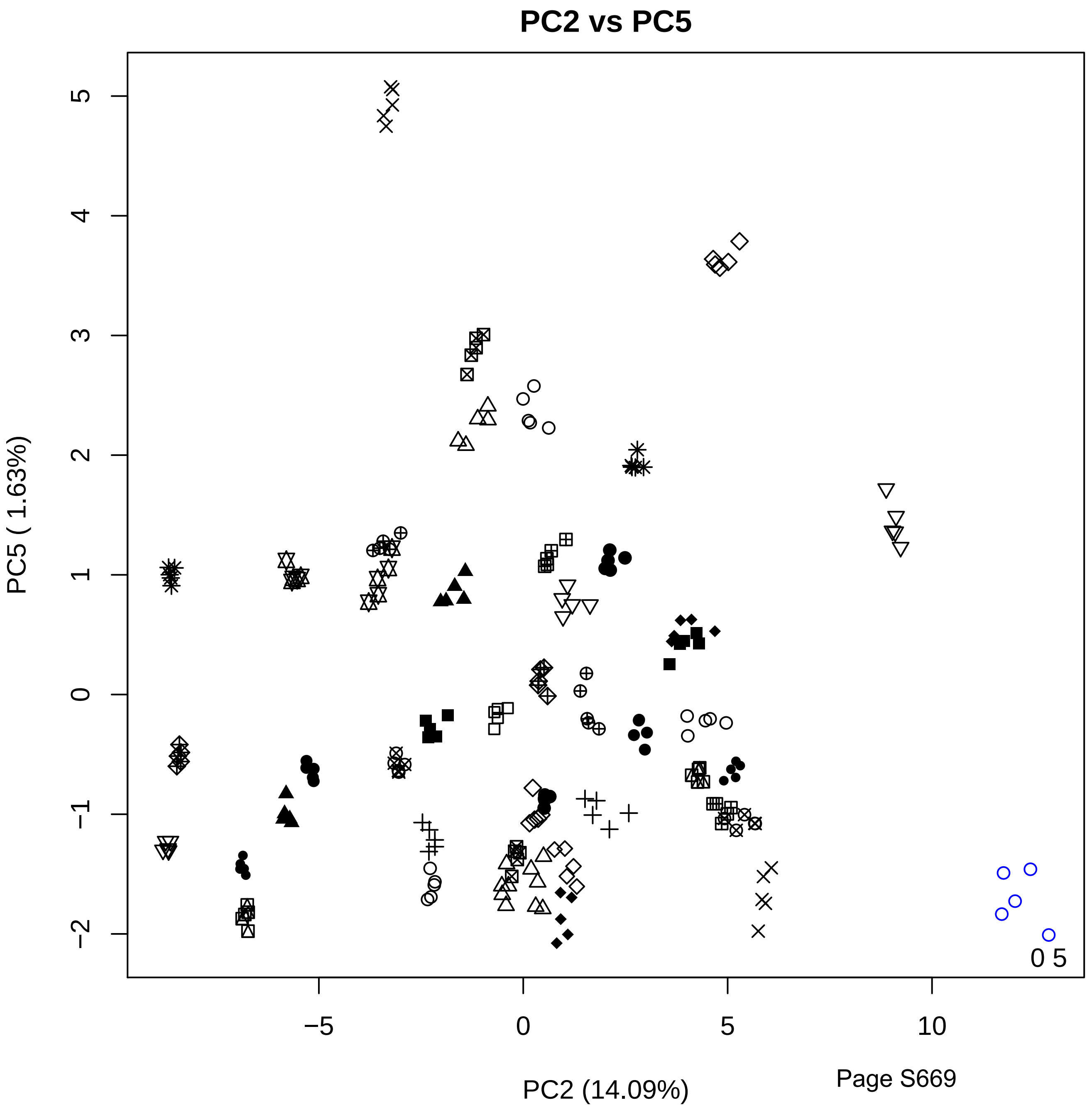

PC2 vs PC5
PC5 (1.63%)
PC2 (14.09%)
5
4
3
2
1
0
−1
−2
−5
0
5
10
0 5

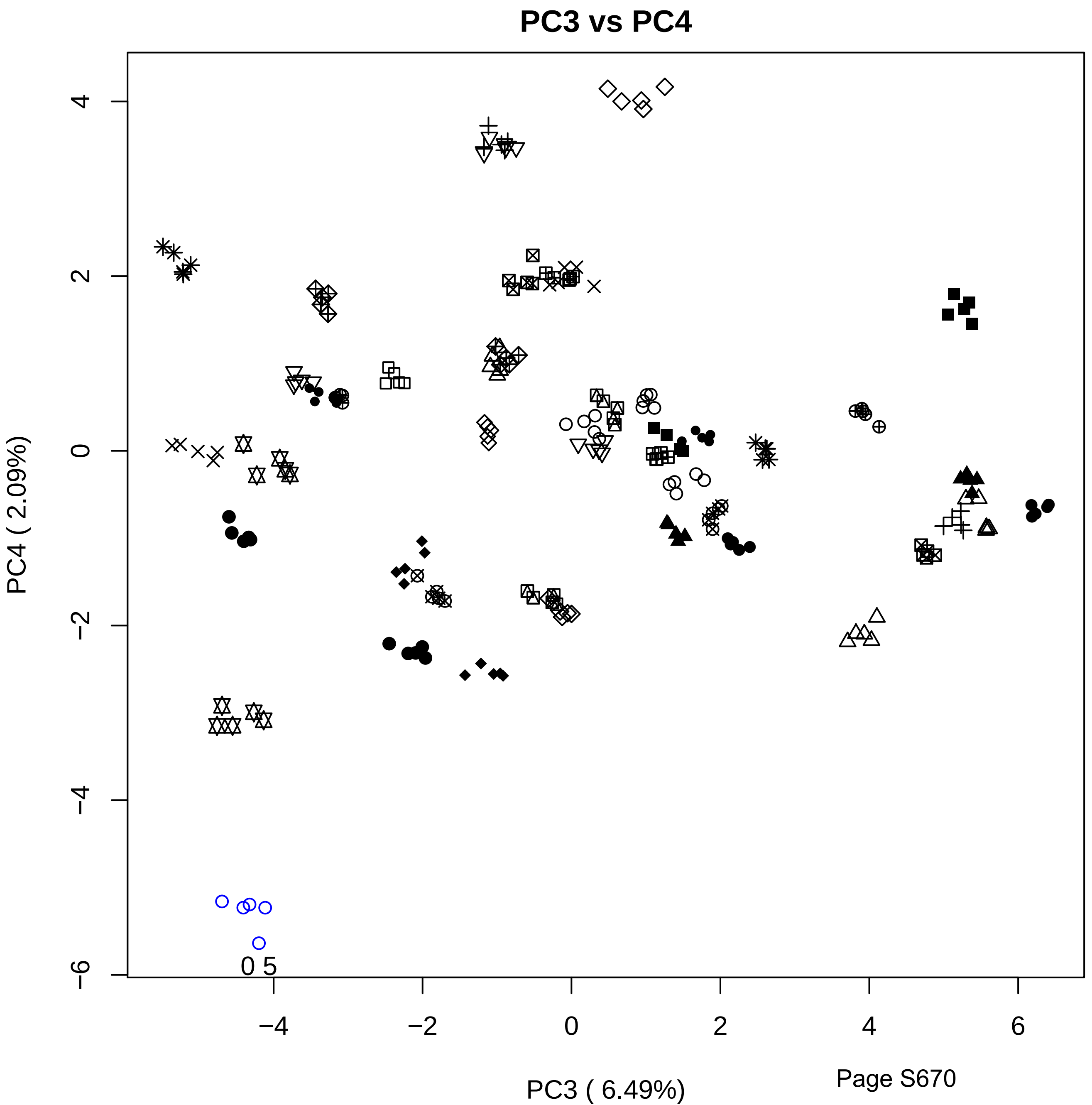

PC3 vs PC4
PC4 (2.09%)
PC3 (6.49%)
4
2
0
−2
−4
−6
−4
−2
0
2
4
6
0 5

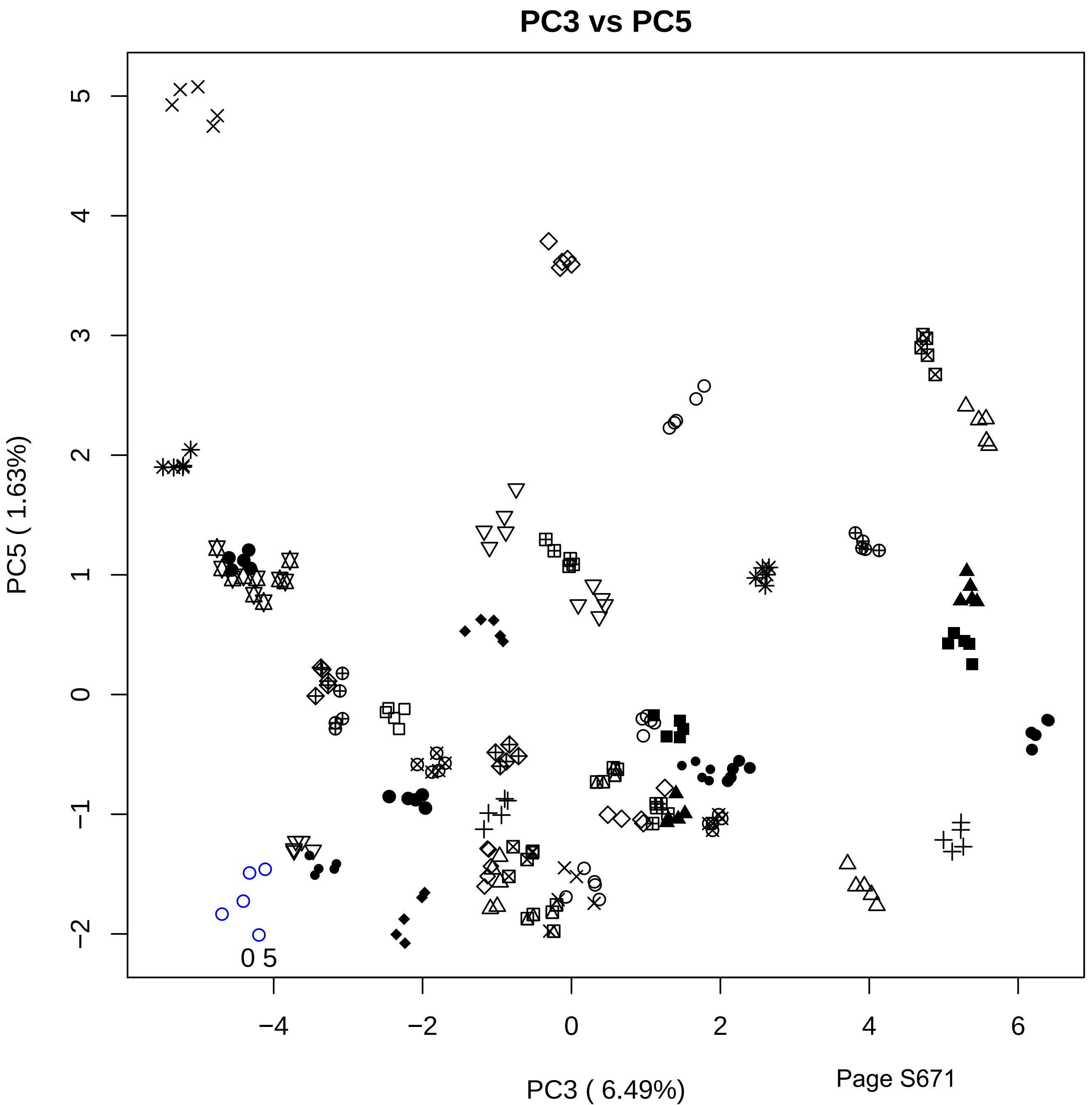
PC3 vs PC5
PC5 (1.63%)
PC3 (6.49%)
0 5
5
4
3
2
1
0
−1
−2
−4
−2
0
2
4
6

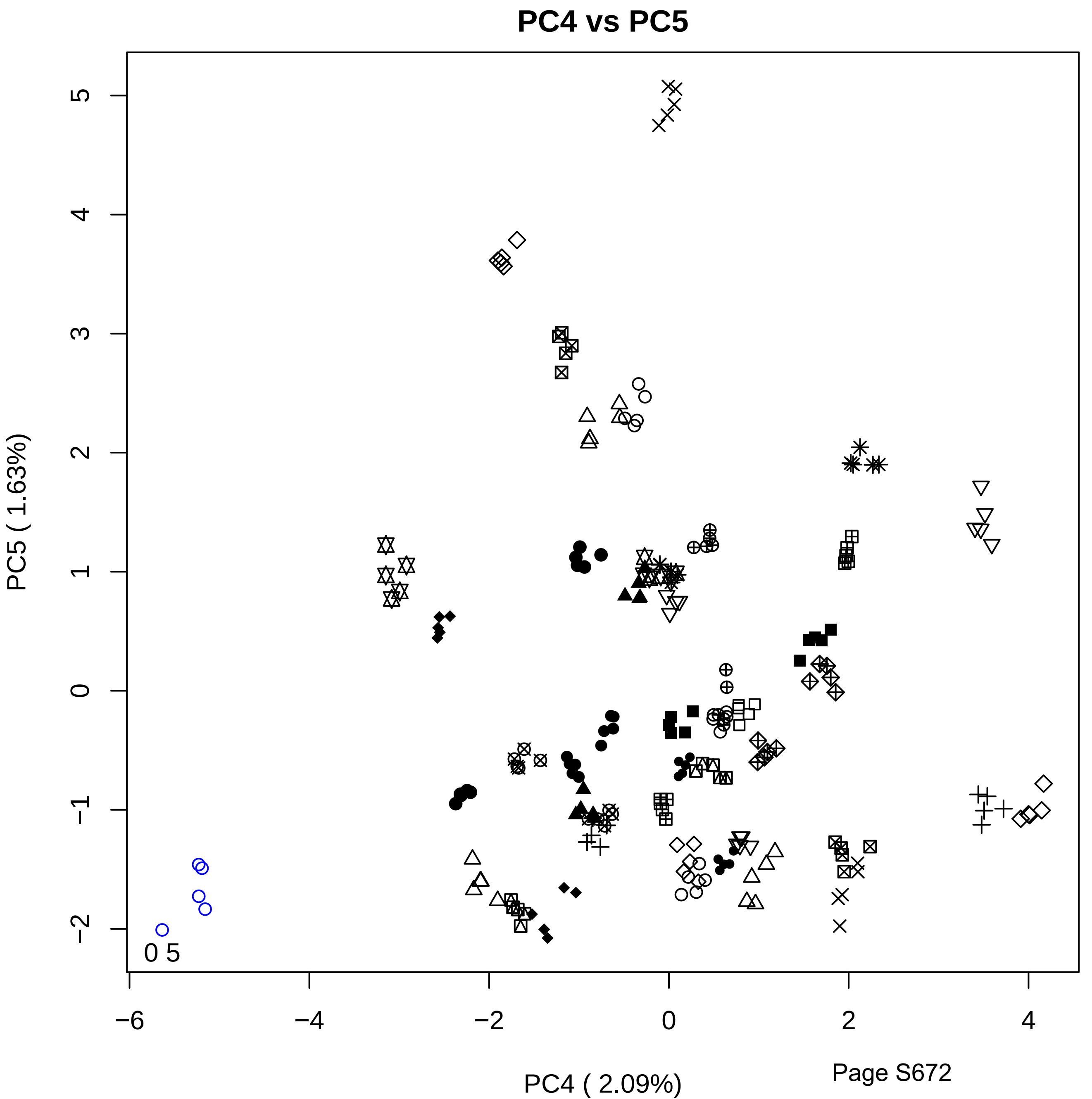
PC4 vs PC5
PC5 (1.63%)
PC4 (2.09%)
0 5
−6
−4
−2
0
2
4
−2
−1
0
1
2
3
4
5

PCA of 1% DeS vs PMH

Scaled Matrix PCAed

7th order polynomial baseline correction performed

Raw spectra smoothed to 21 Neighbours, 2nd order PN, Savtisky Golay

Normalisation between 0 and 100

D2 spectra smoothed to 41 Neighbours, 2nd order PN, Savtisky Golay

Regions Analysed: 3601−2501 and 2001−700cm−1

Samples Analysed: 1 Contam's 0 DeS's 45 PMH's

Keele University AD   Sun Dec 09 17:04:01 2018

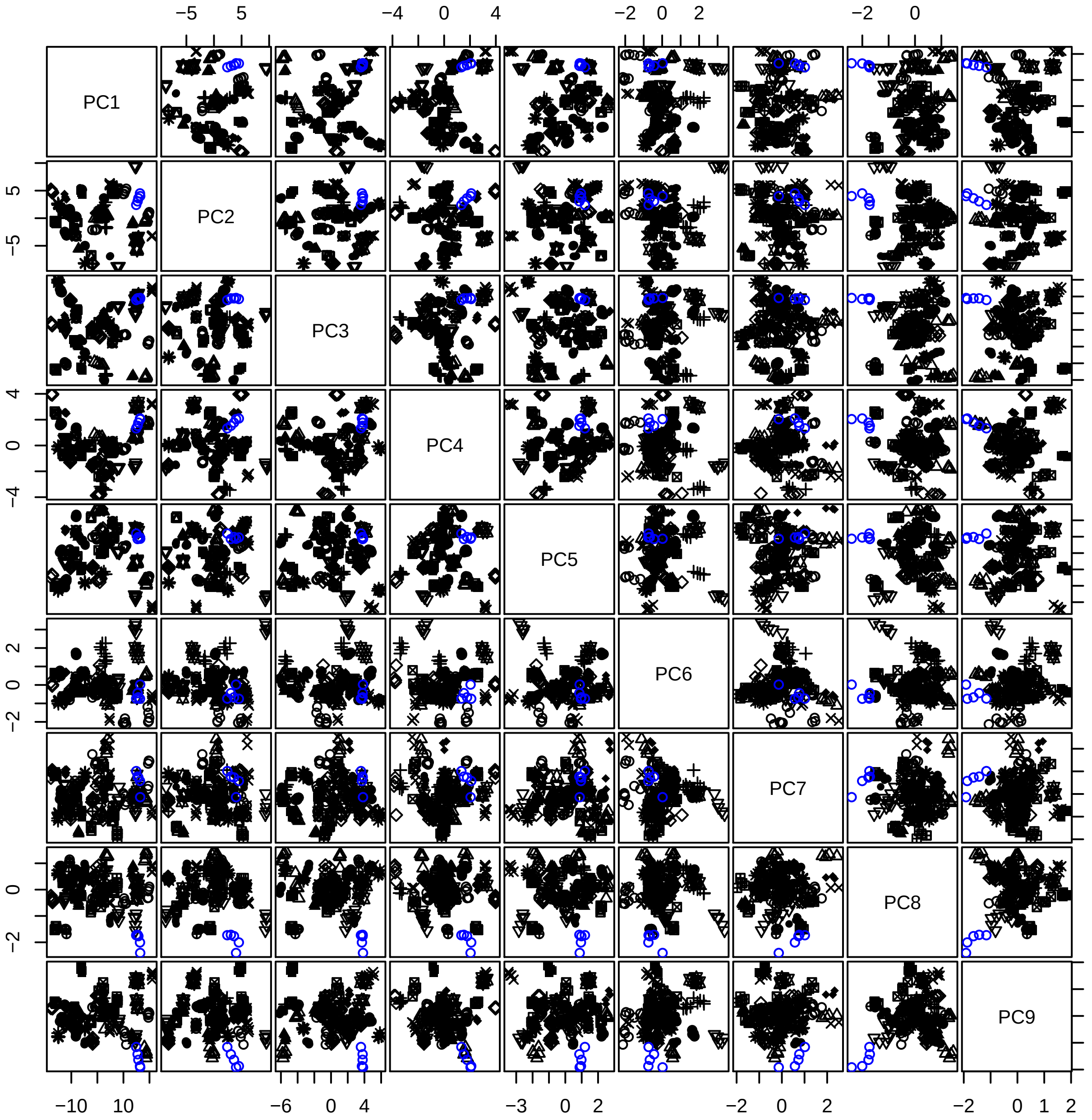

PC1
PC2
PC3
PC4
PC5
PC6
PC7
PC8
PC9

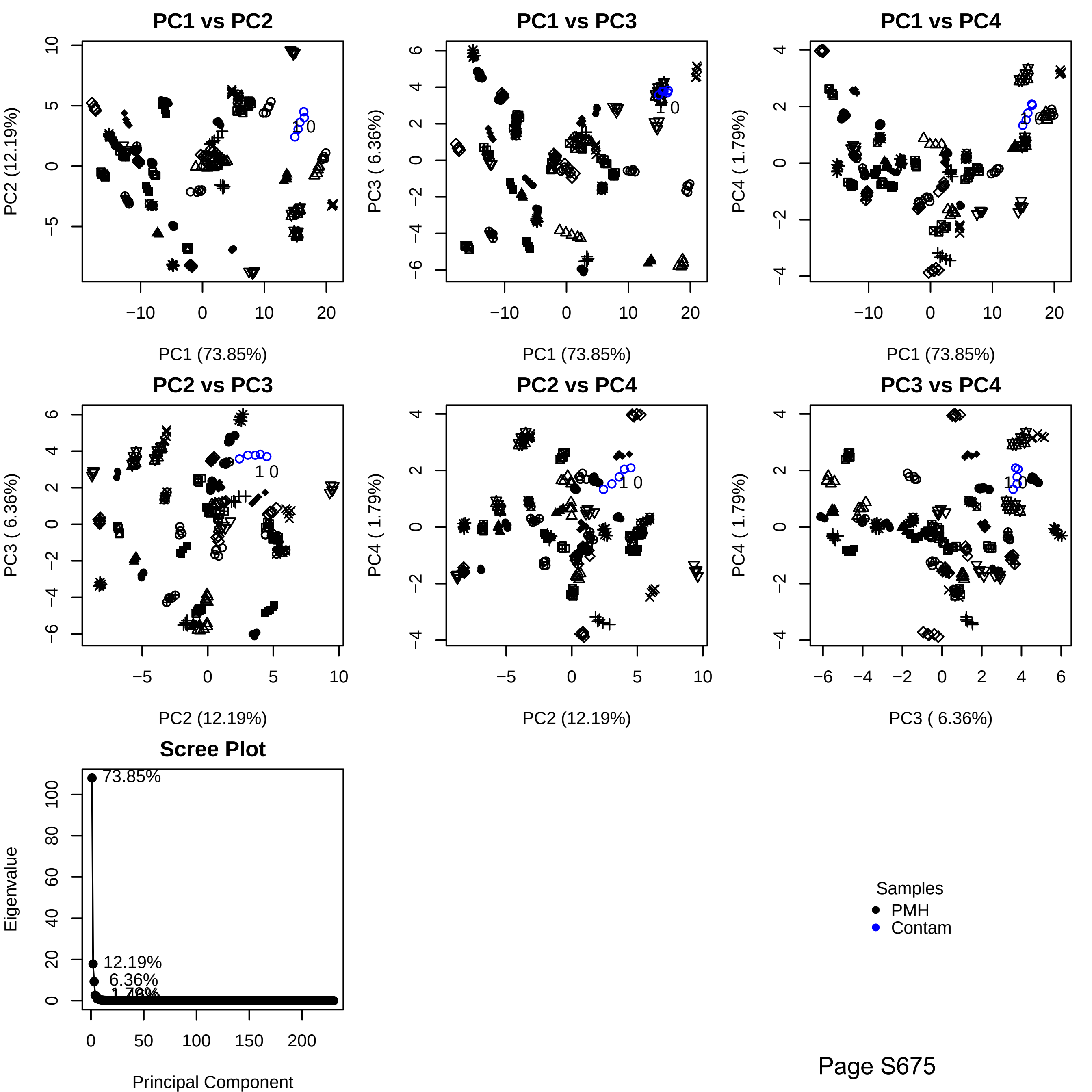
PC1 vs PC2
PC1 vs PC3
PC1 vs PC4
PC2 vs PC3
PC2 vs PC4
PC3 vs PC4
Scree Plot
PC1 (73.85%)
PC2 (12.19%)
PC3 (6.36%)
PC4 (1.79%)
Eigenvalue
Principal Component
73.85%
12.19%
6.36%
Samples
PMH
Contam

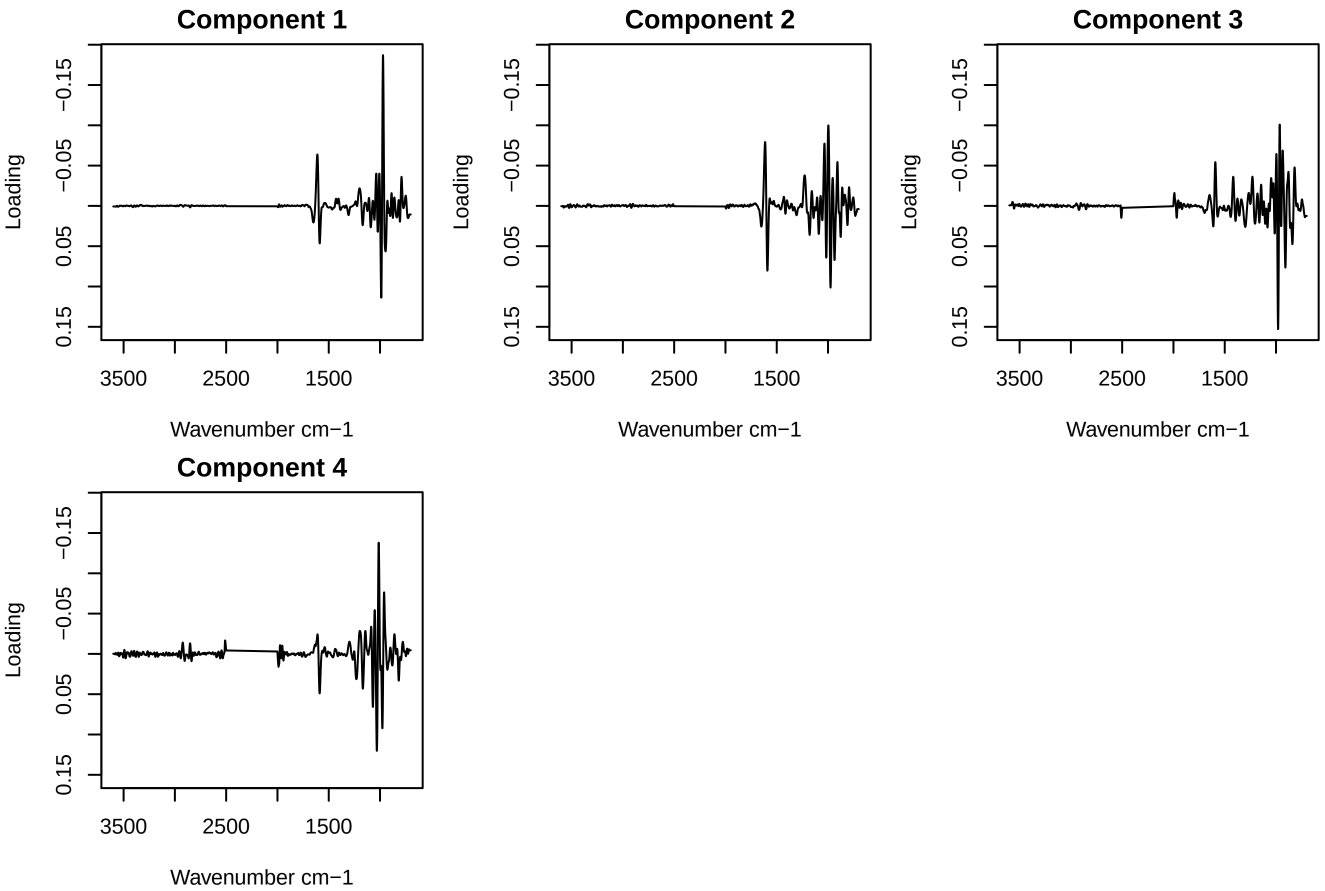
Component 1
Component 2
Component 3
Component 4
Loading
−0.15
−0.05
0.05
0.15
3500
2500
1500
Wavenumber cm−1

PC1 vs PC2 vs PC3

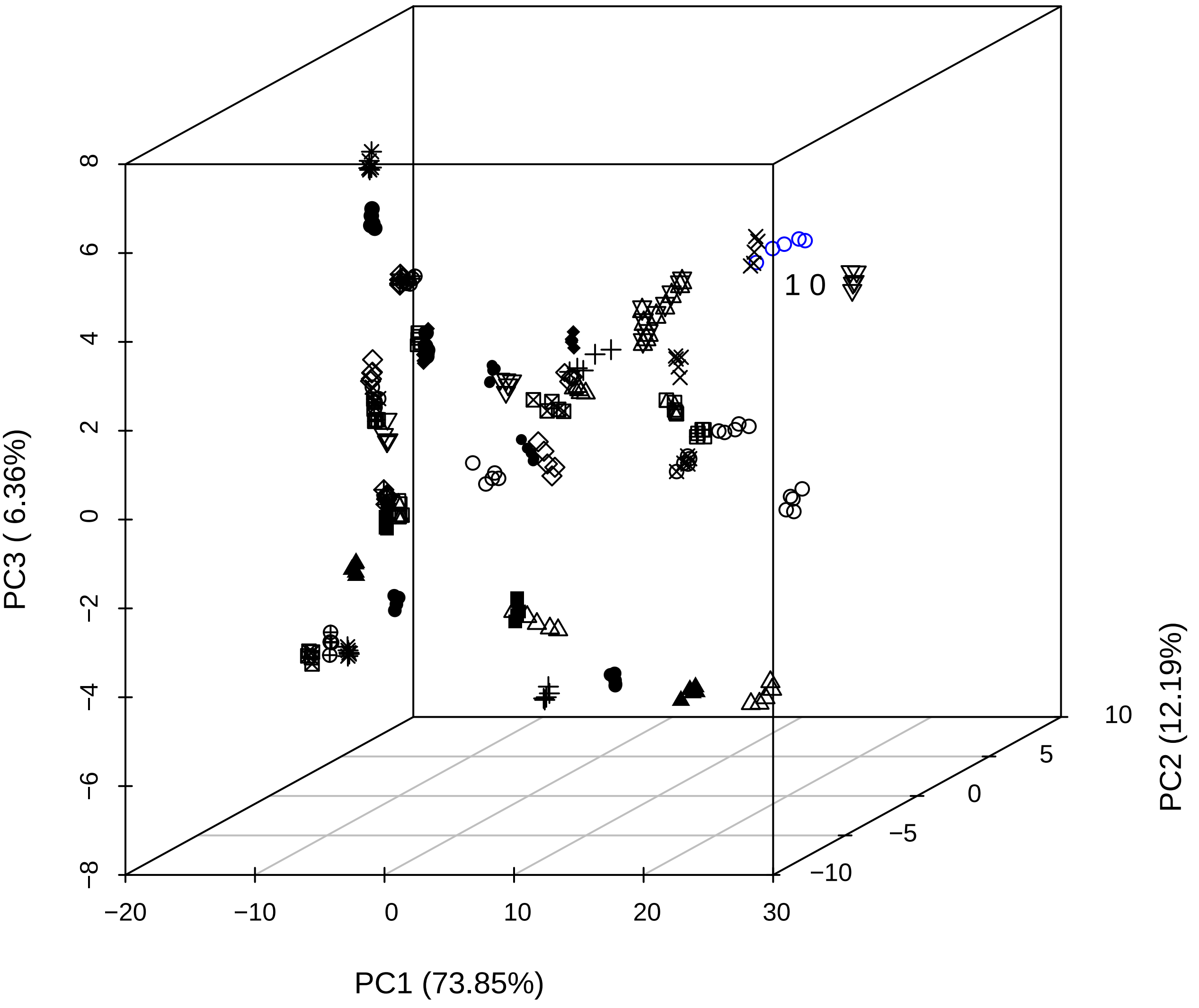

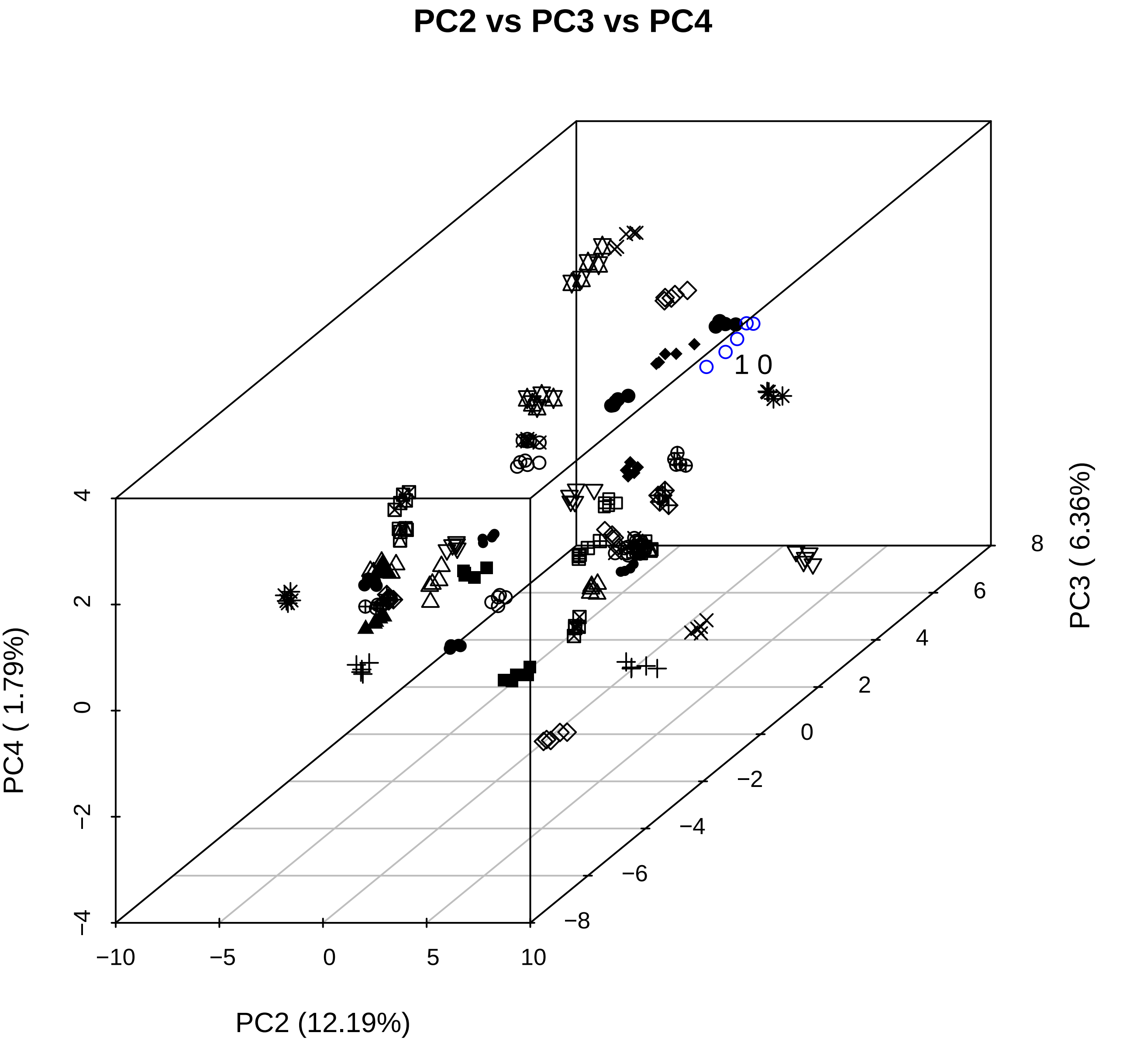
PC2 vs PC3 vs PC4
PC2 (12.19%)
PC4 (1.79%)
PC3 (6.36%)
−10
−5
0
5
10
−4
−2
0
2
4
−8
−6
−4
−2
0
2
4
6
8
1 0

PC1 vs PC2 vs PC4

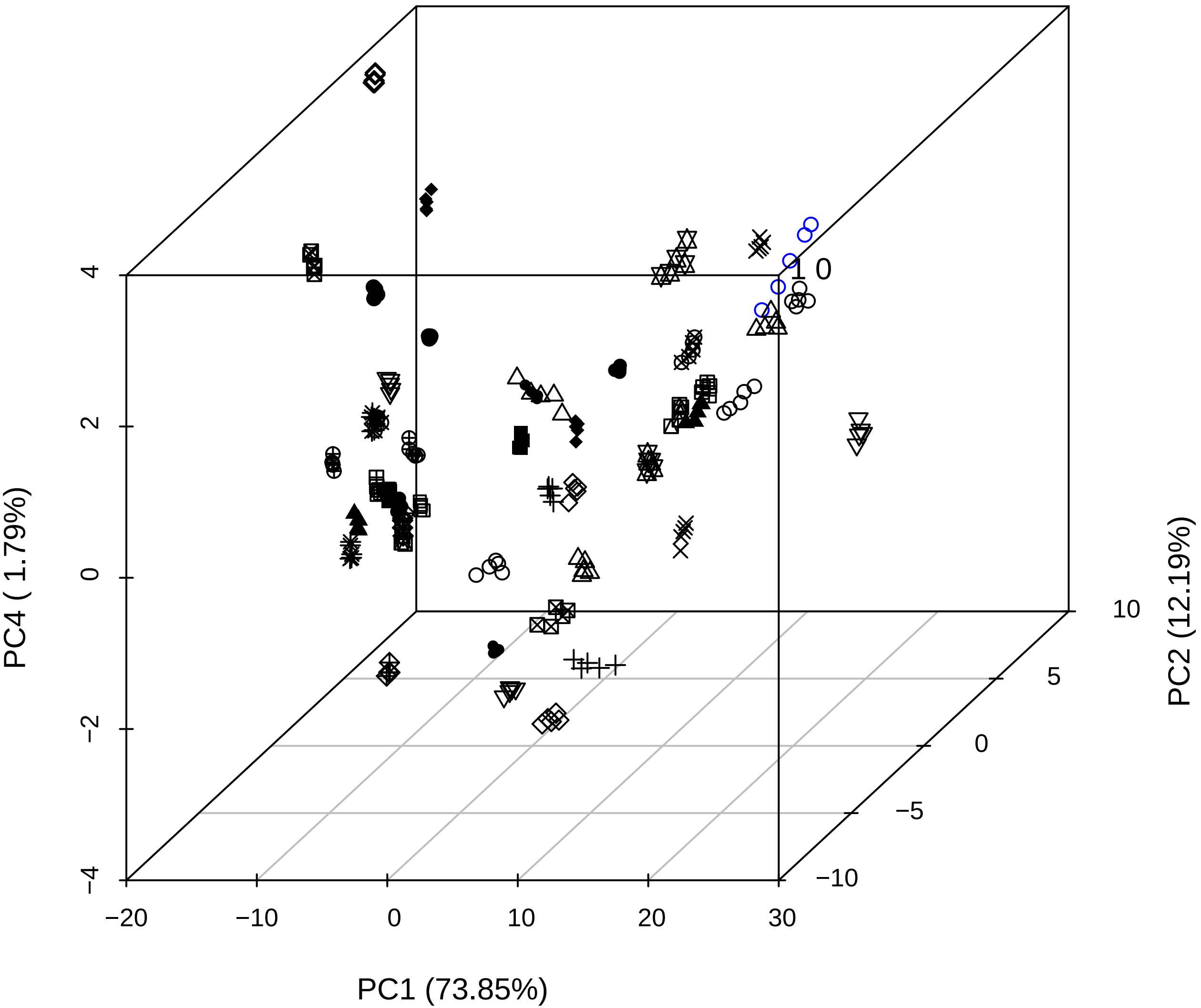

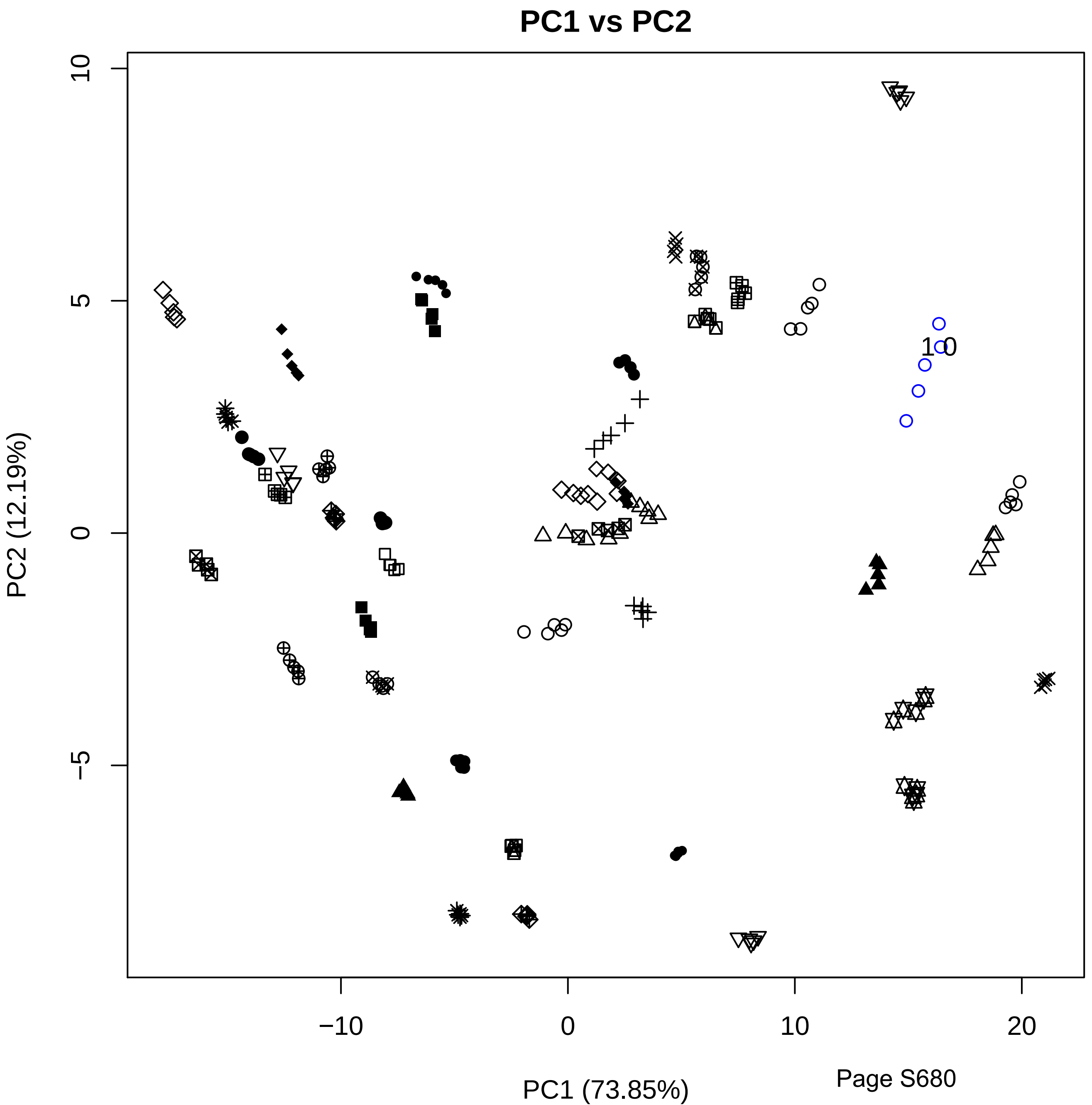

PC1 vs PC2
PC2 (12.19%)
PC1 (73.85%)
10
5
0
−5
−10
0
10
20
10

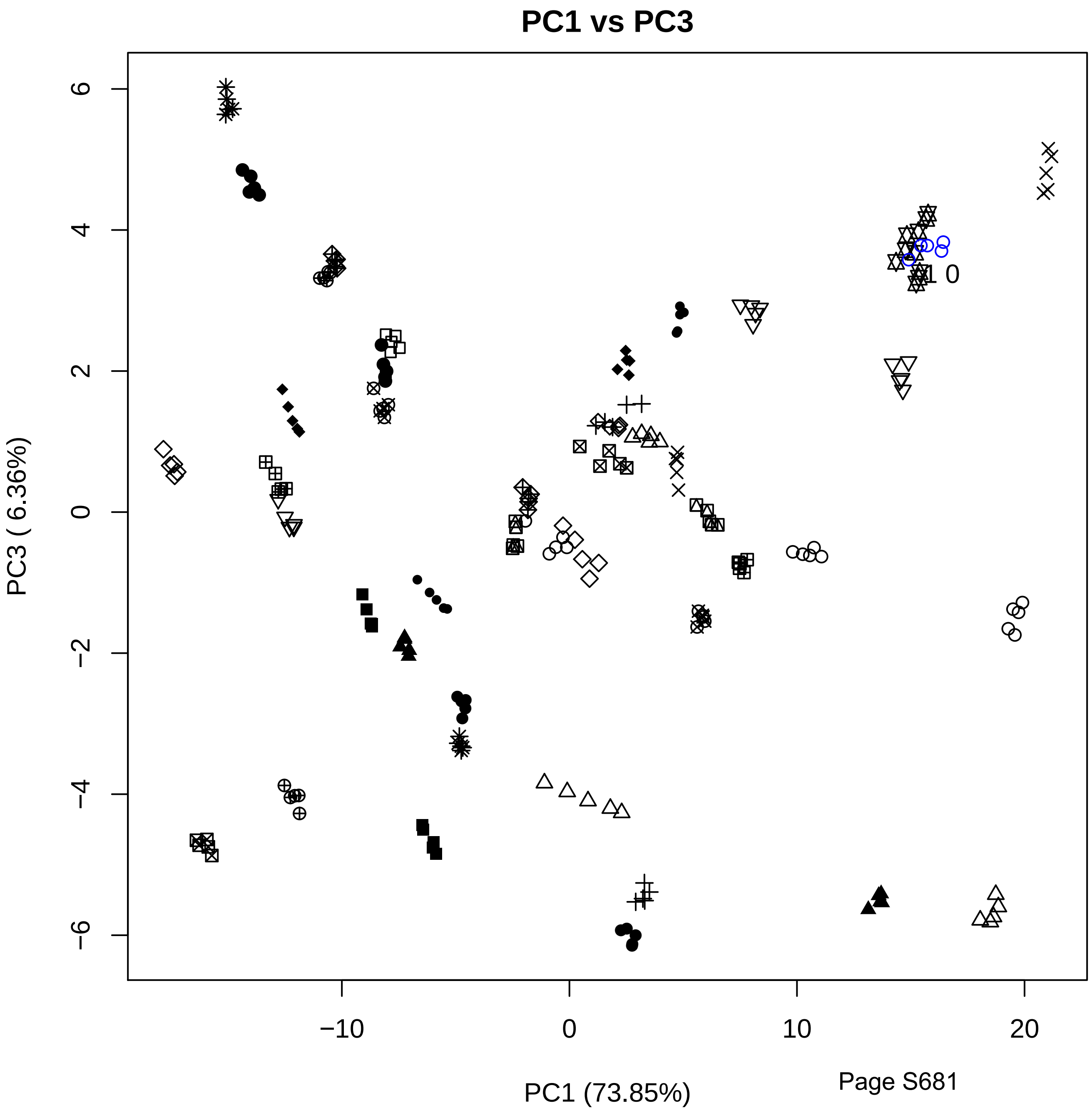
PC1 vs PC3
PC3 (6.36%)
PC1 (73.85%)
6
4
2
0
−2
−4
−6
−10
0
10
20
1 0

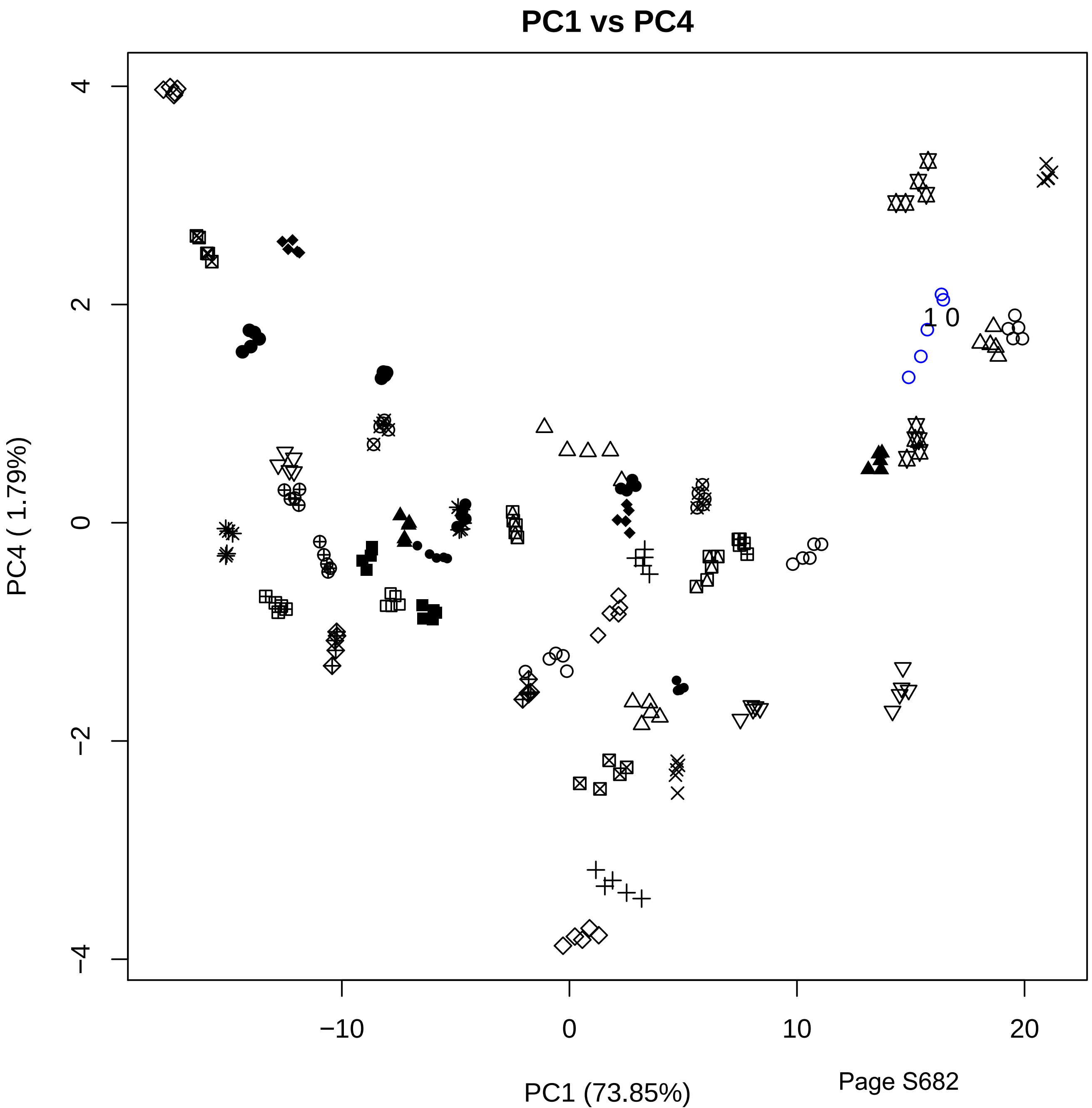
PC1 vs PC4
PC4 (1.79%)
PC1 (73.85%)
4
2
0
−2
−4
−10
0
10
20
1 0

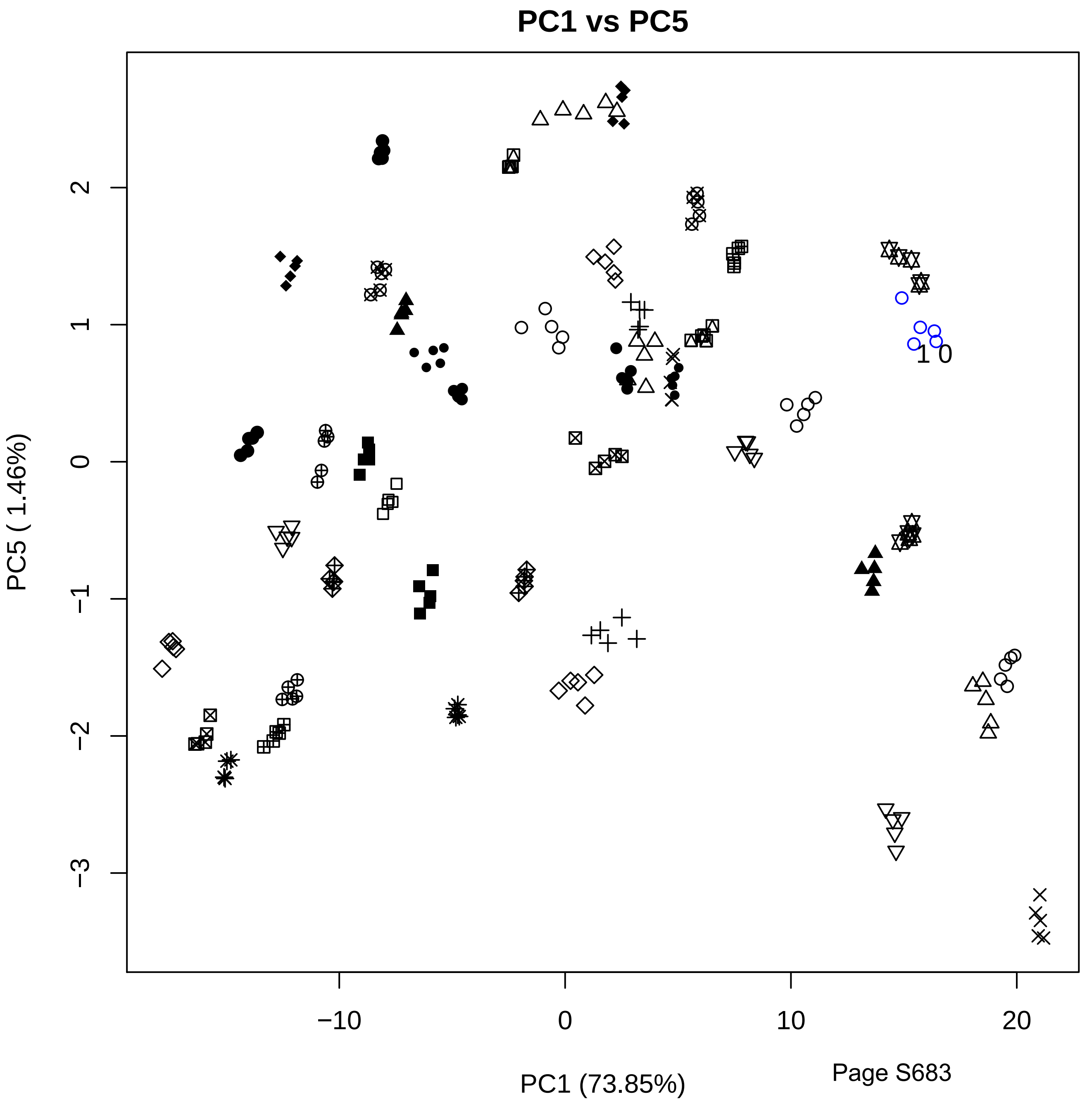
PC1 vs PC5
PC5 (1.46%)
PC1 (73.85%)
−3
−2
−1
0
1
2
−10
0
10
20
10

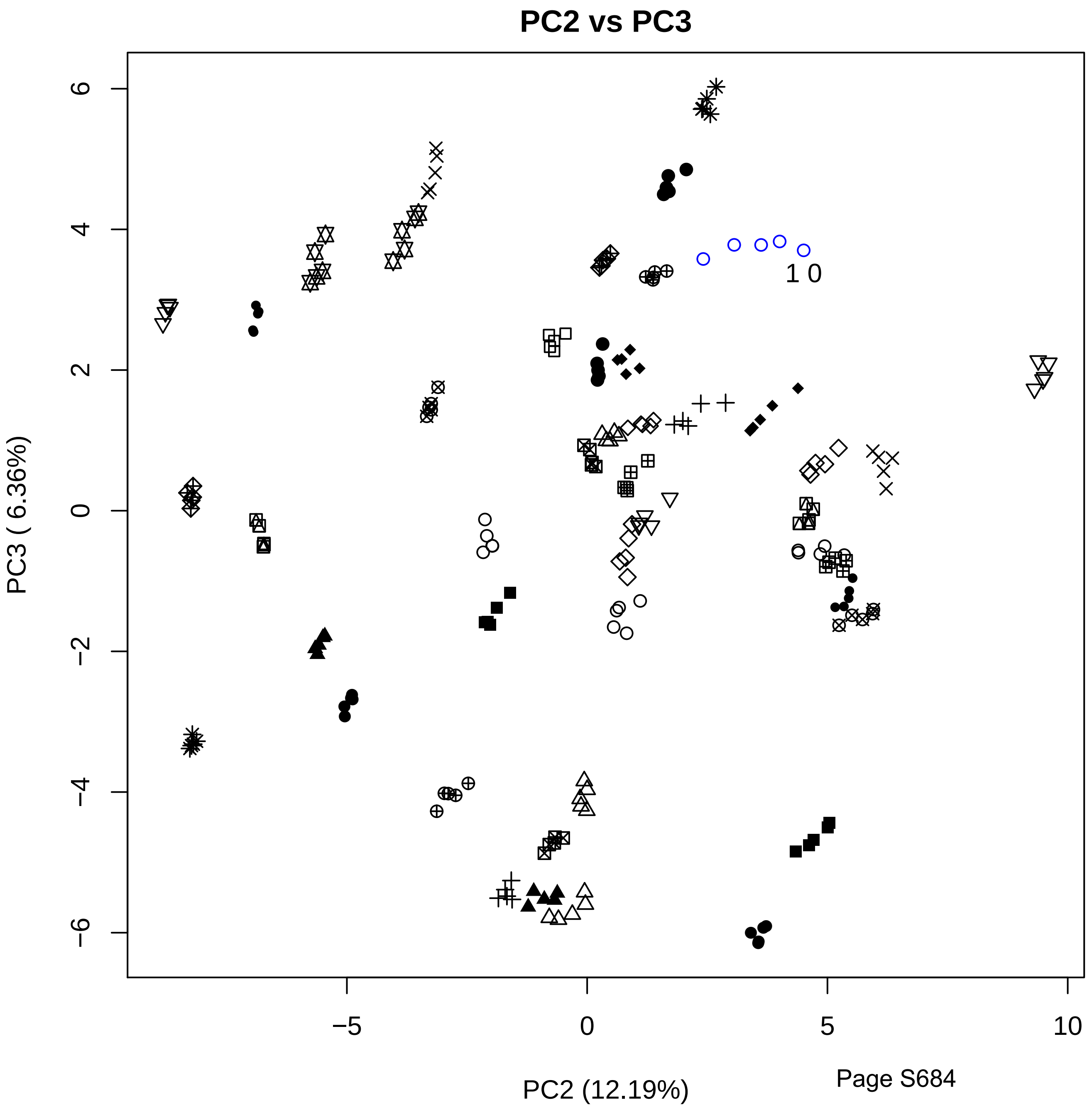
PC2 vs PC3
PC3 (6.36%)
PC2 (12.19%)
6
4
2
0
−2
−4
−6
−5
0
5
10
1 0

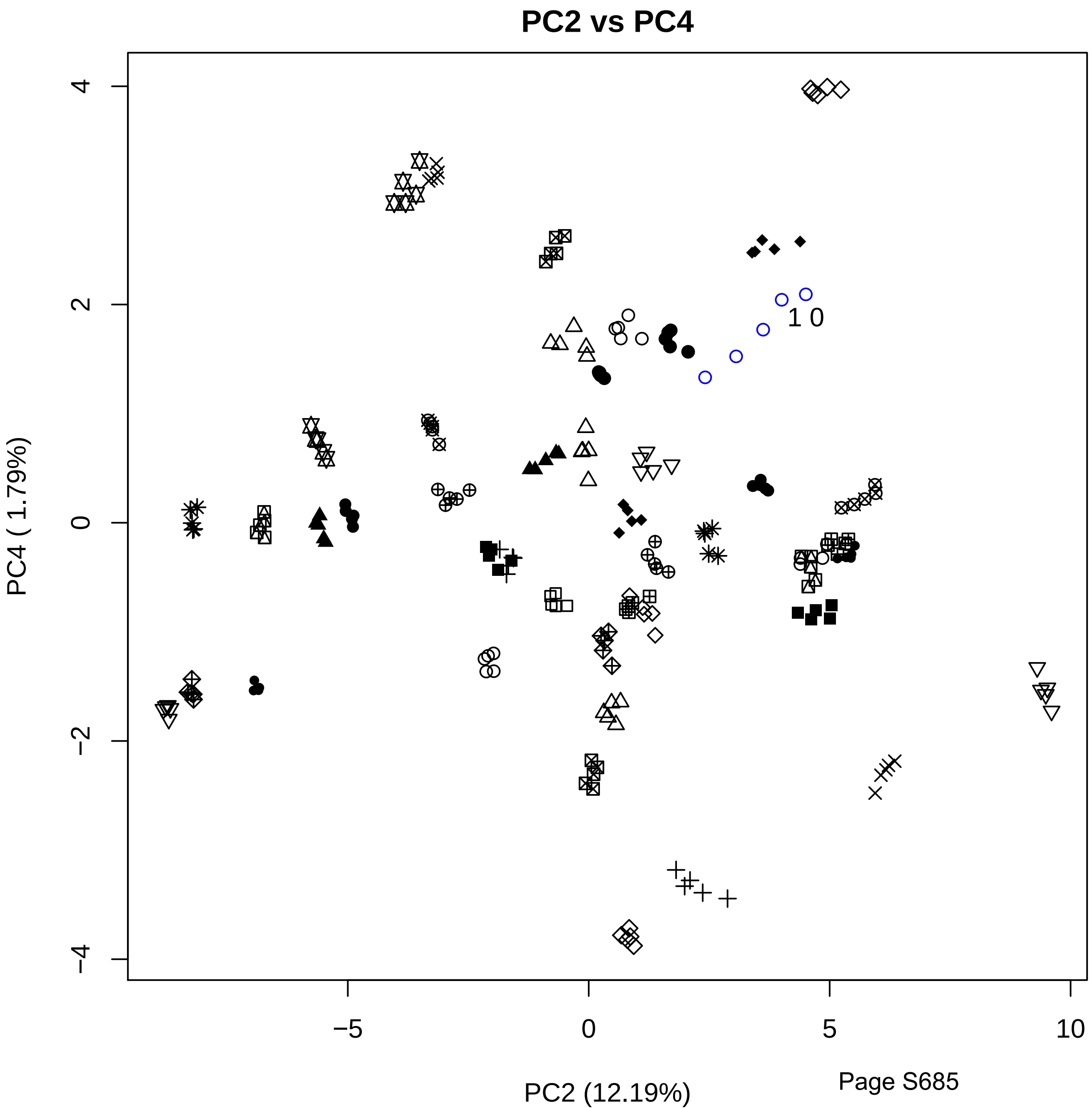

PC2 vs PC4
4
2
0
−2
−4
PC4 (1.79%)
10
−5
0
5
10
PC2 (12.19%)

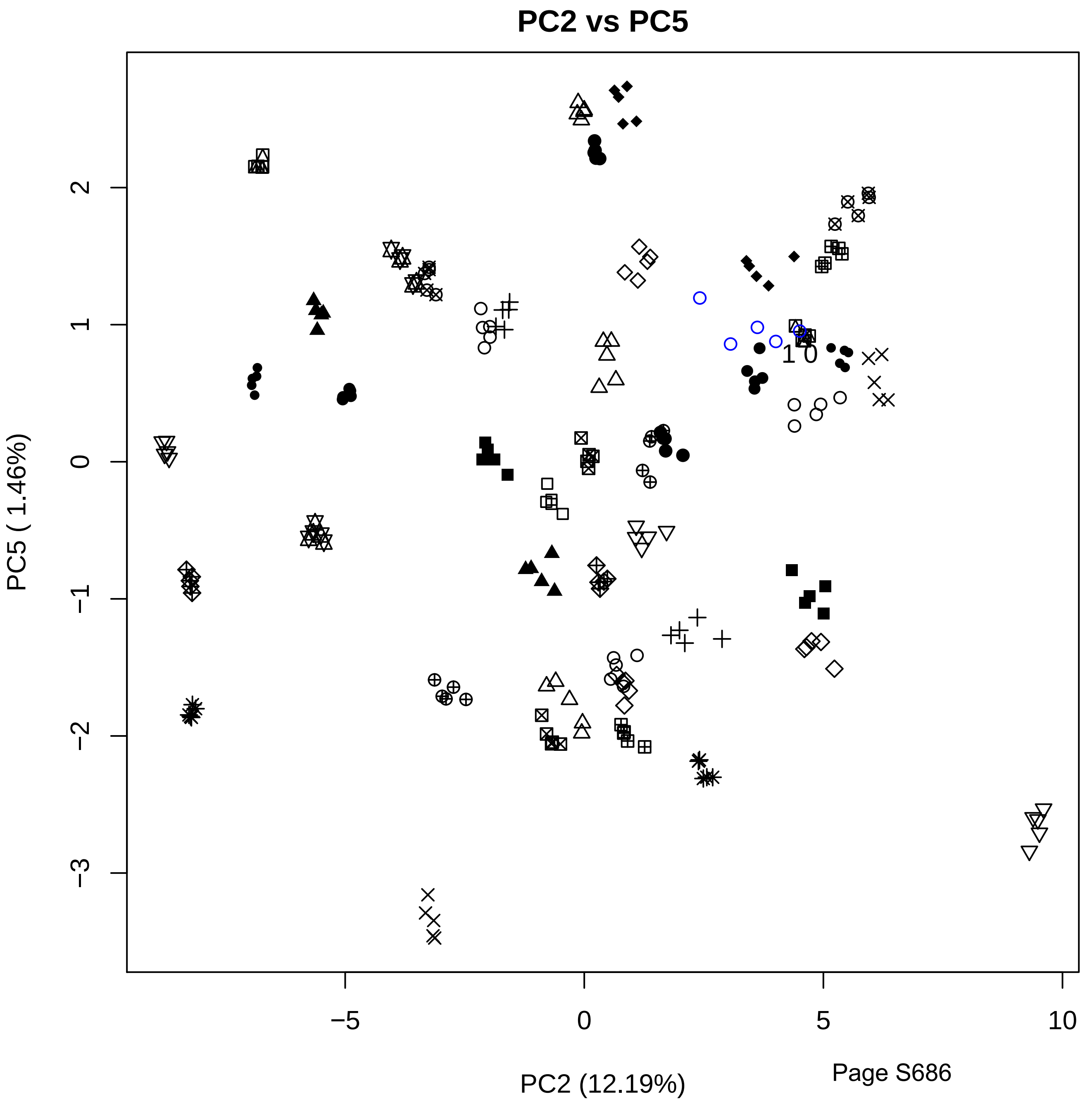

PC2 vs PC5
PC5 (1.46%)
PC2 (12.19%)
−5
0
5
10
2
1
0
−1
−2
−3
10

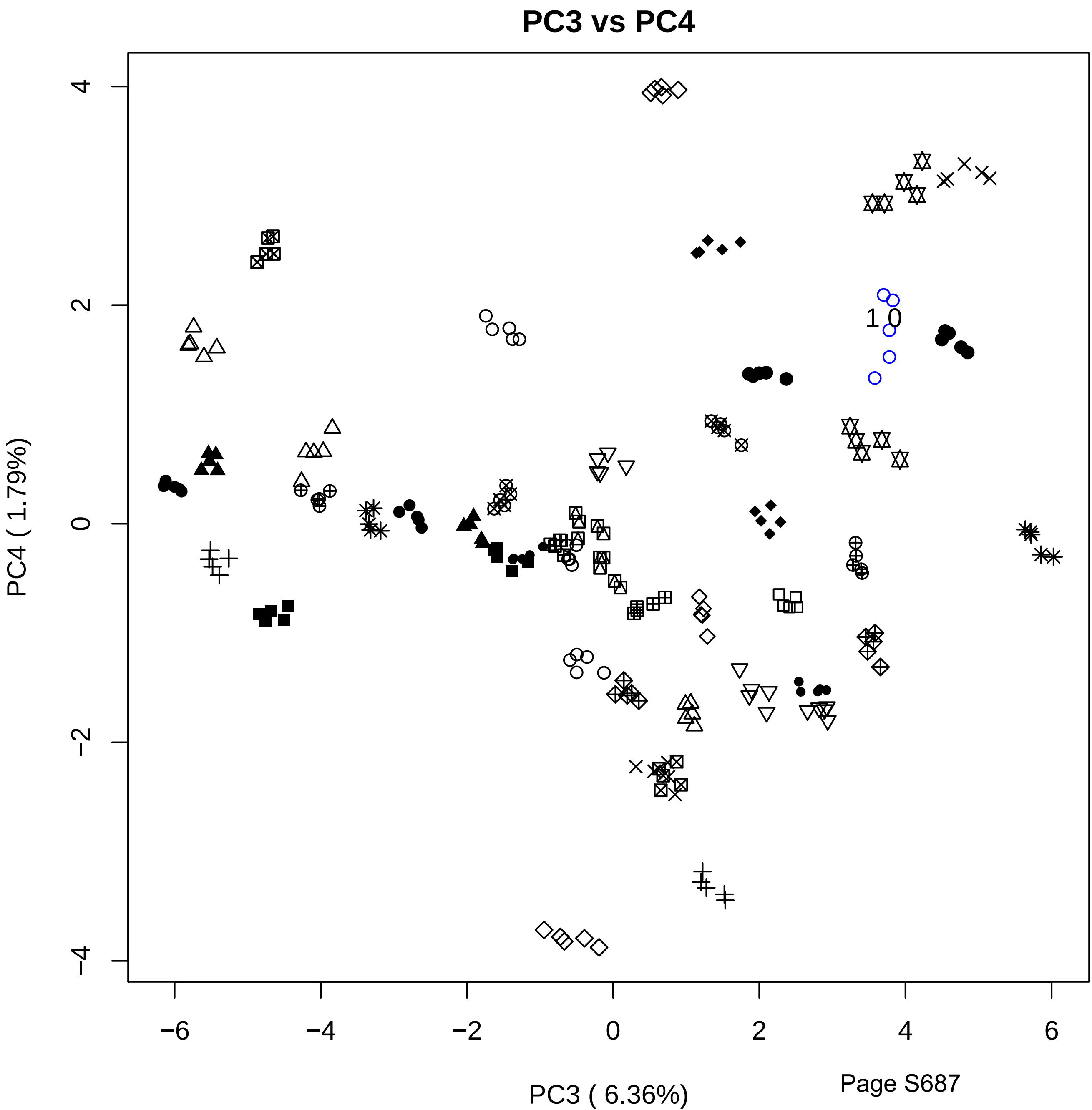
PC3 vs PC4
PC4 (1.79%)
PC3 (6.36%)
10
-6
-4
-2
0
2
4
6

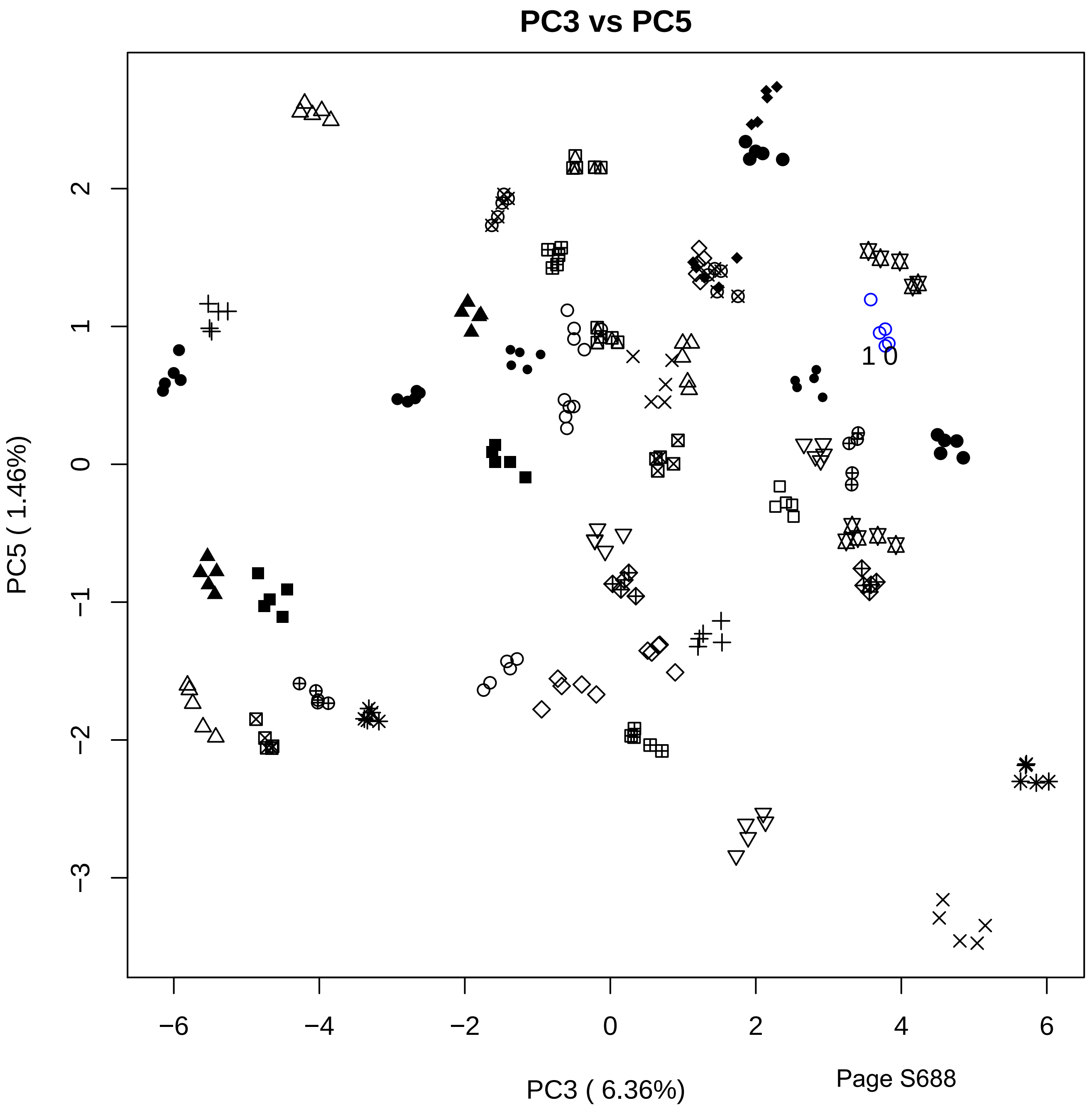
PC3 vs PC5
PC5 (1.46%)
PC3 (6.36%)
−6
−4
−2
0
2
4
6
−3
−2
−1
0
1
2
1 0

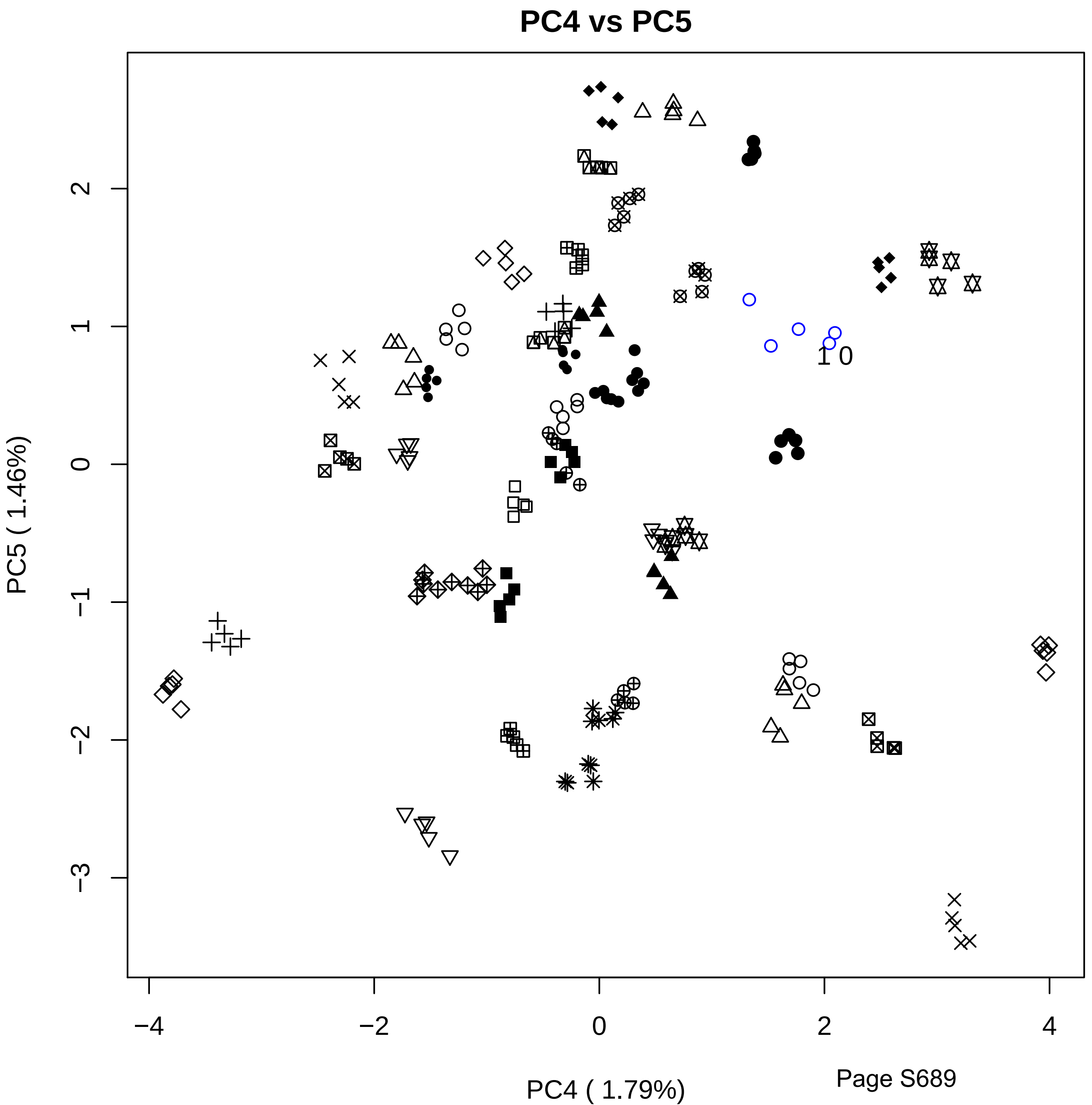

PC4 vs PC5
PC5 (1.46%)
PC4 (1.79%)
−4
−2
0
2
4
−3
−2
−1
0
1
2
10

PCA of 2.5% DeS vs PMH

Scaled Matrix PCAed

7th order polynomial baseline correction performed

Raw spectra smoothed to 21 Neighbours, 2nd order PN, Savtisky Golay

Normalisation between 0 and 100

D2 spectra smoothed to 41 Neighbours, 2nd order PN, Savtisky Golay

Regions Analysed: 3601−2501 and 2001−700cm−1

Samples Analysed: 1 Contam's 0 DeS's 45 PMH's

Keele University AD　　　　Sun Dec 09 17:04:06 2018

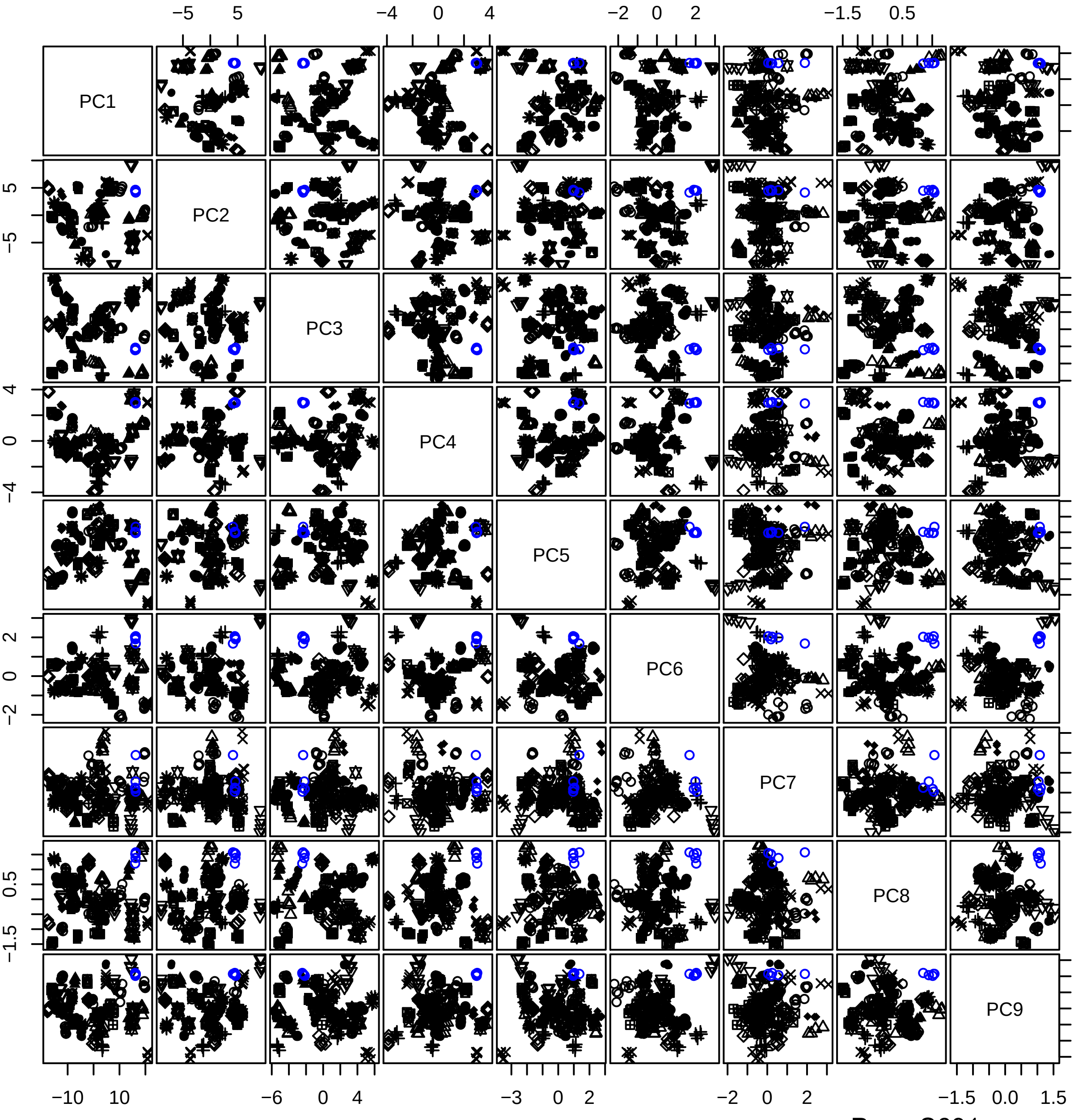
PC1
PC2
PC3
PC4
PC5
PC6
PC7
PC8
PC9

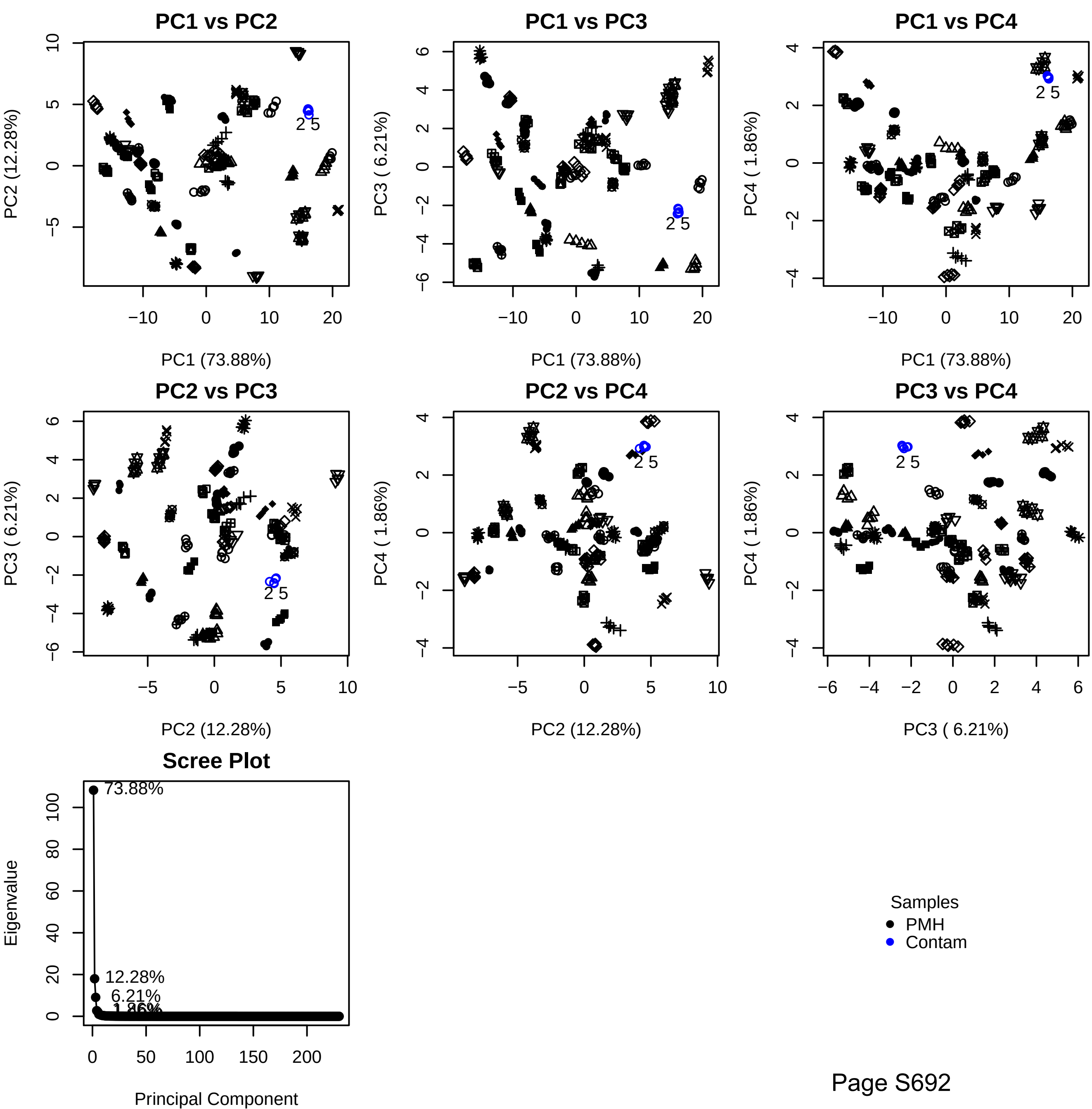
PC1 vs PC2
PC1 vs PC3
PC1 vs PC4
PC2 vs PC3
PC2 vs PC4
PC3 vs PC4
Scree Plot
PC1 (73.88%)
PC2 (12.28%)
PC3 (6.21%)
PC4 (1.86%)
2 5
Eigenvalue
Principal Component
73.88%
12.28%
6.21%
Samples
PMH
Contam

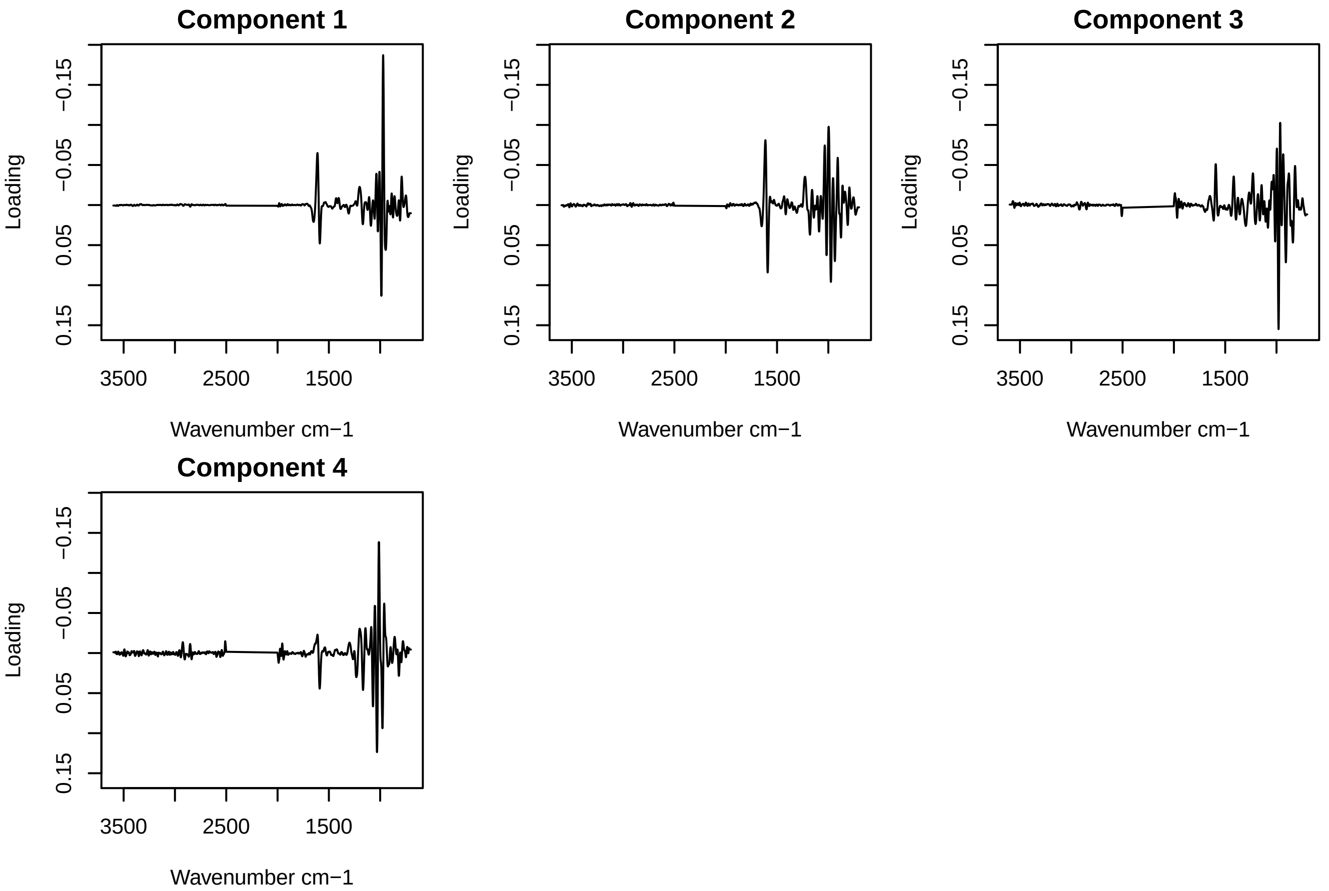

Component 1
Component 2
Component 3
Component 4
Loading
−0.15
−0.05
0.05
0.15
3500
2500
1500
Wavenumber cm−1

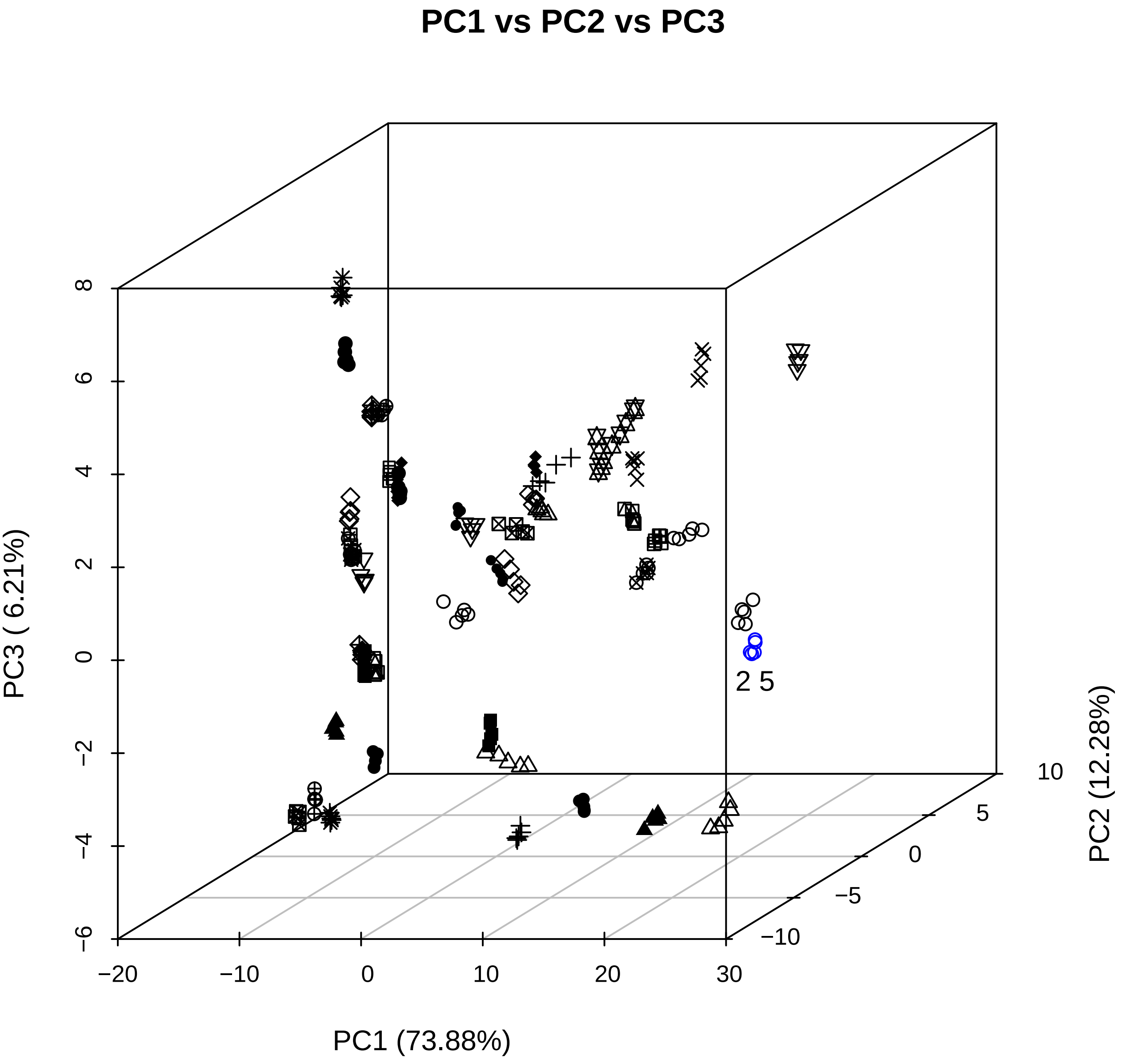
PC1 vs PC2 vs PC3
PC3 (6.21%)
8
6
4
2
0
−2
−4
−6
2 5
PC1 (73.88%)
−20
−10
0
10
20
30
PC2 (12.28%)
10
5
0
−5
−10

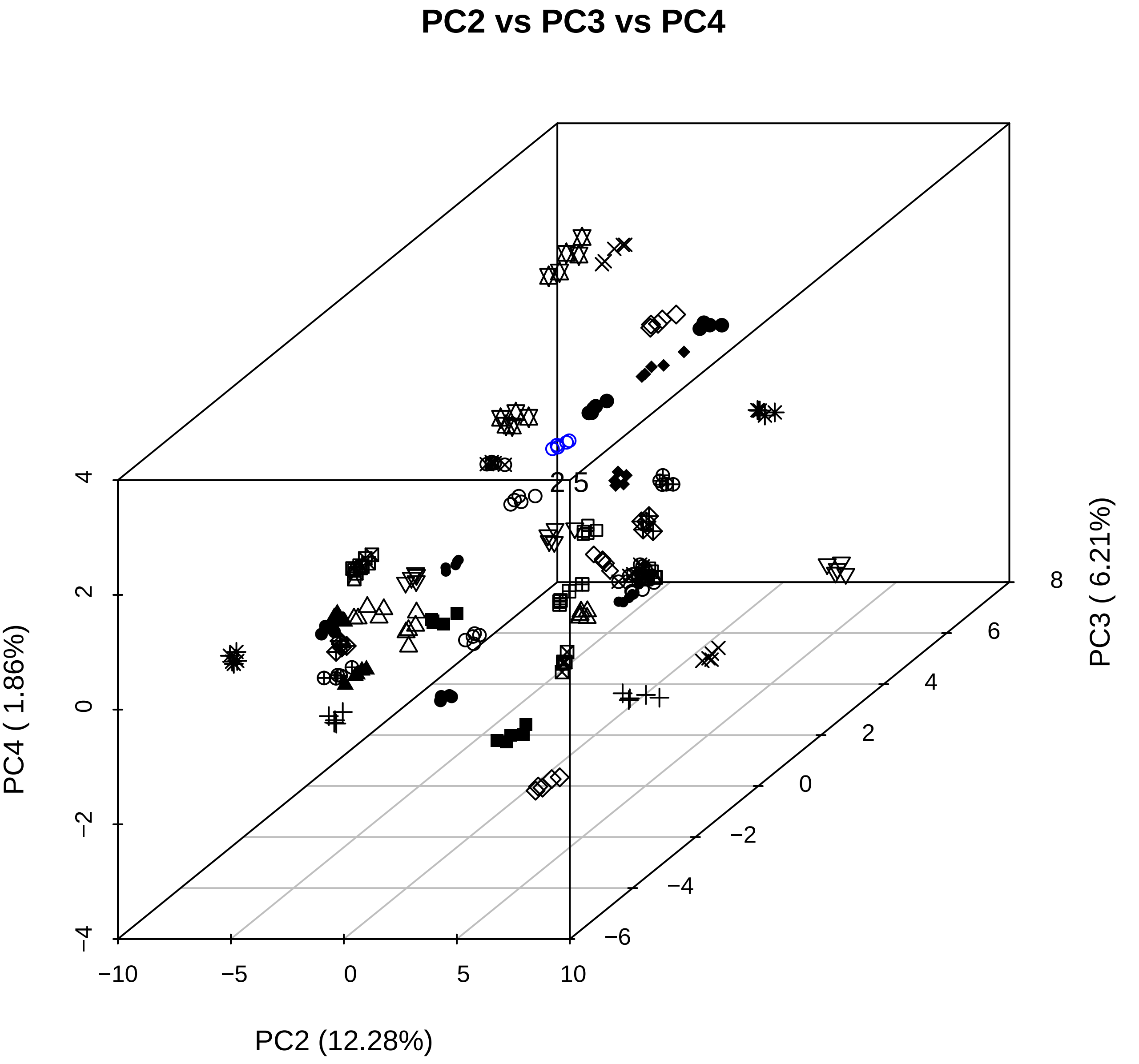
PC2 vs PC3 vs PC4
PC4 (1.86%)
PC2 (12.28%)
PC3 (6.21%)
−4
−2
0
2
4
−10
−5
0
5
10
−6
−4
−2
0
2
4
6
8
2.5

PC1 vs PC2 vs PC4

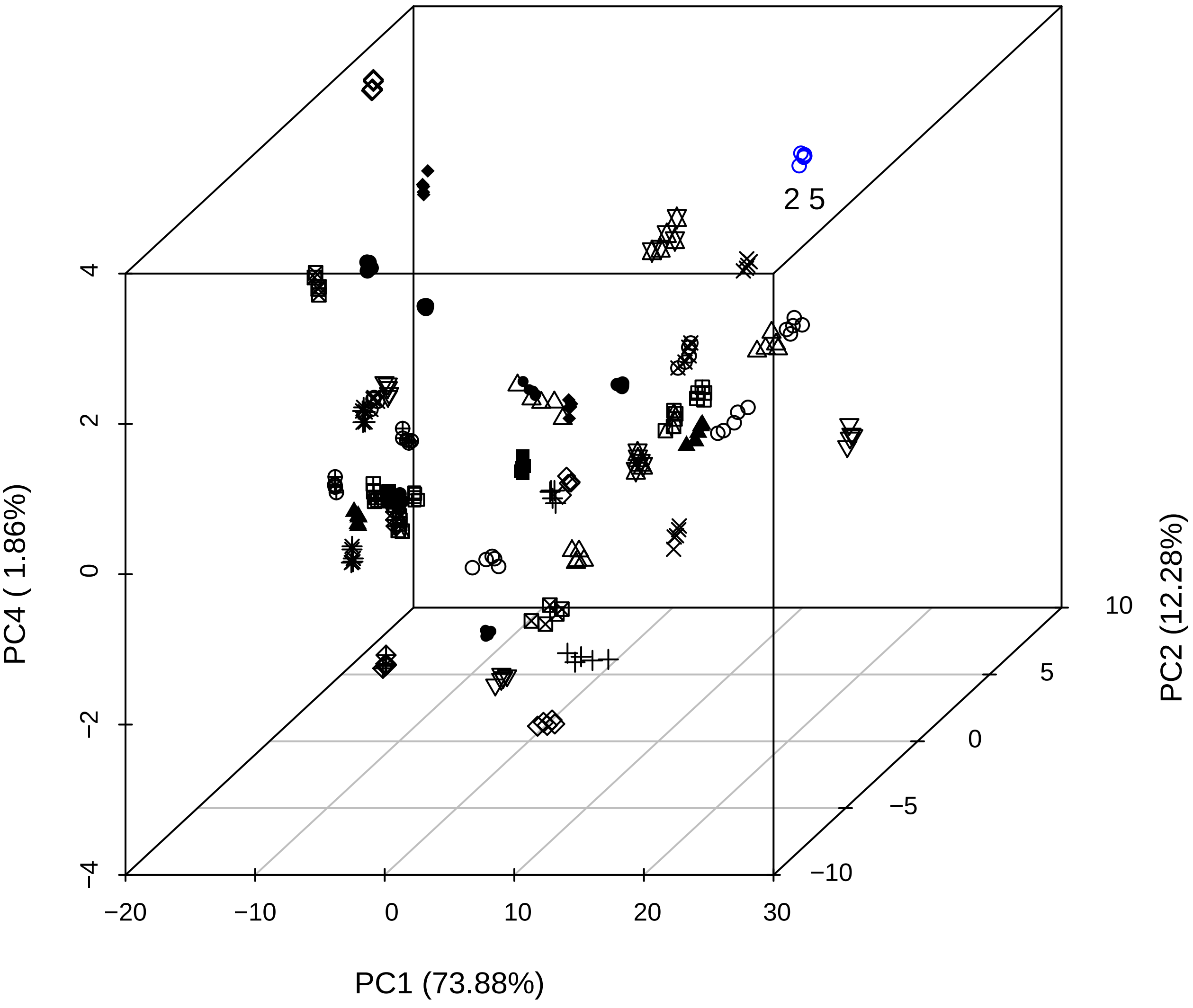

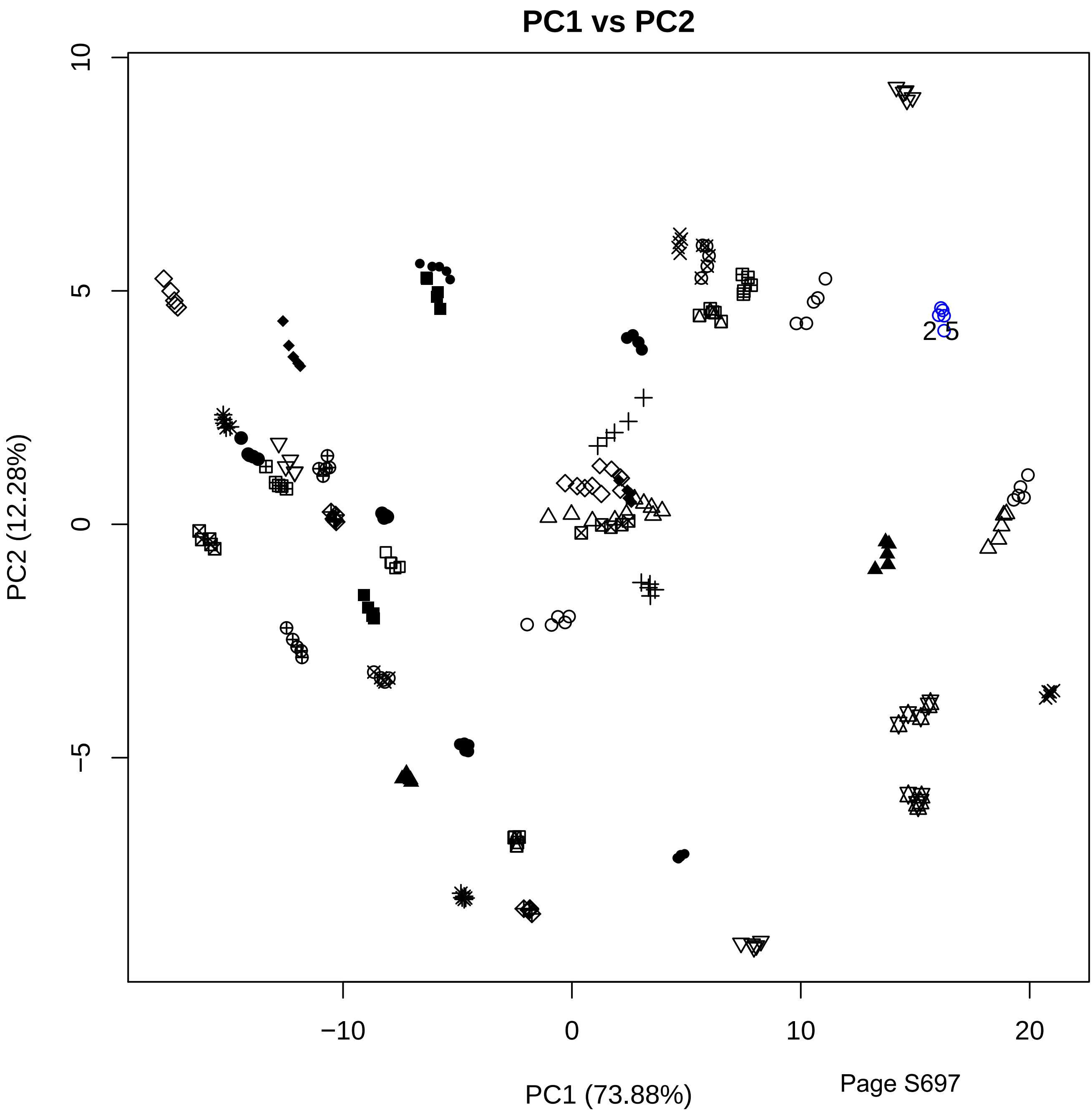
PC1 vs PC2
PC2 (12.28%)
PC1 (73.88%)
10
5
0
−5
−10
0
10
20
25

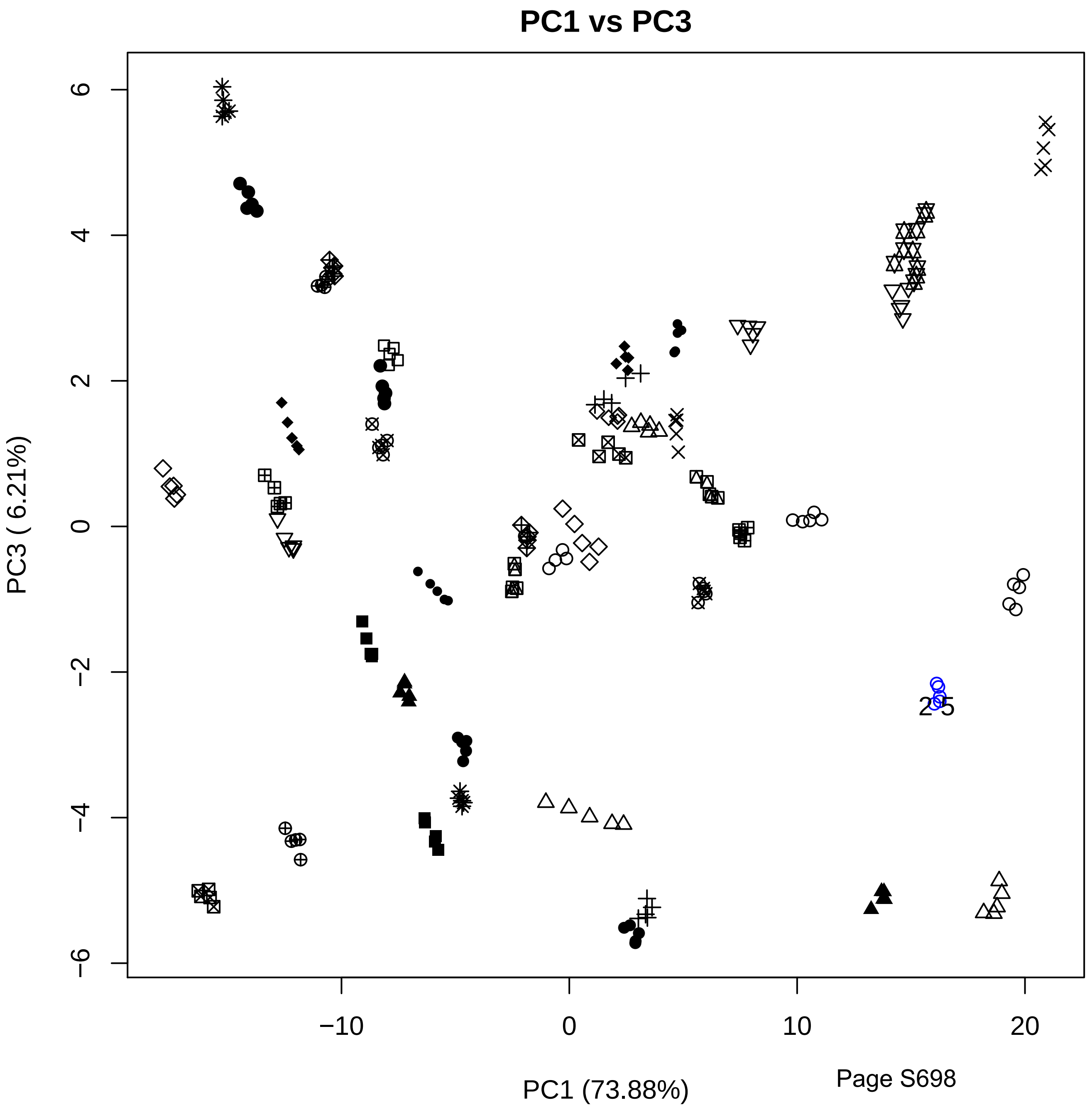
PC1 vs PC3
PC3 (6.21%)
PC1 (73.88%)
6
4
2
0
−2
−4
−6
−10
0
10
20
25

PC1 vs PC4

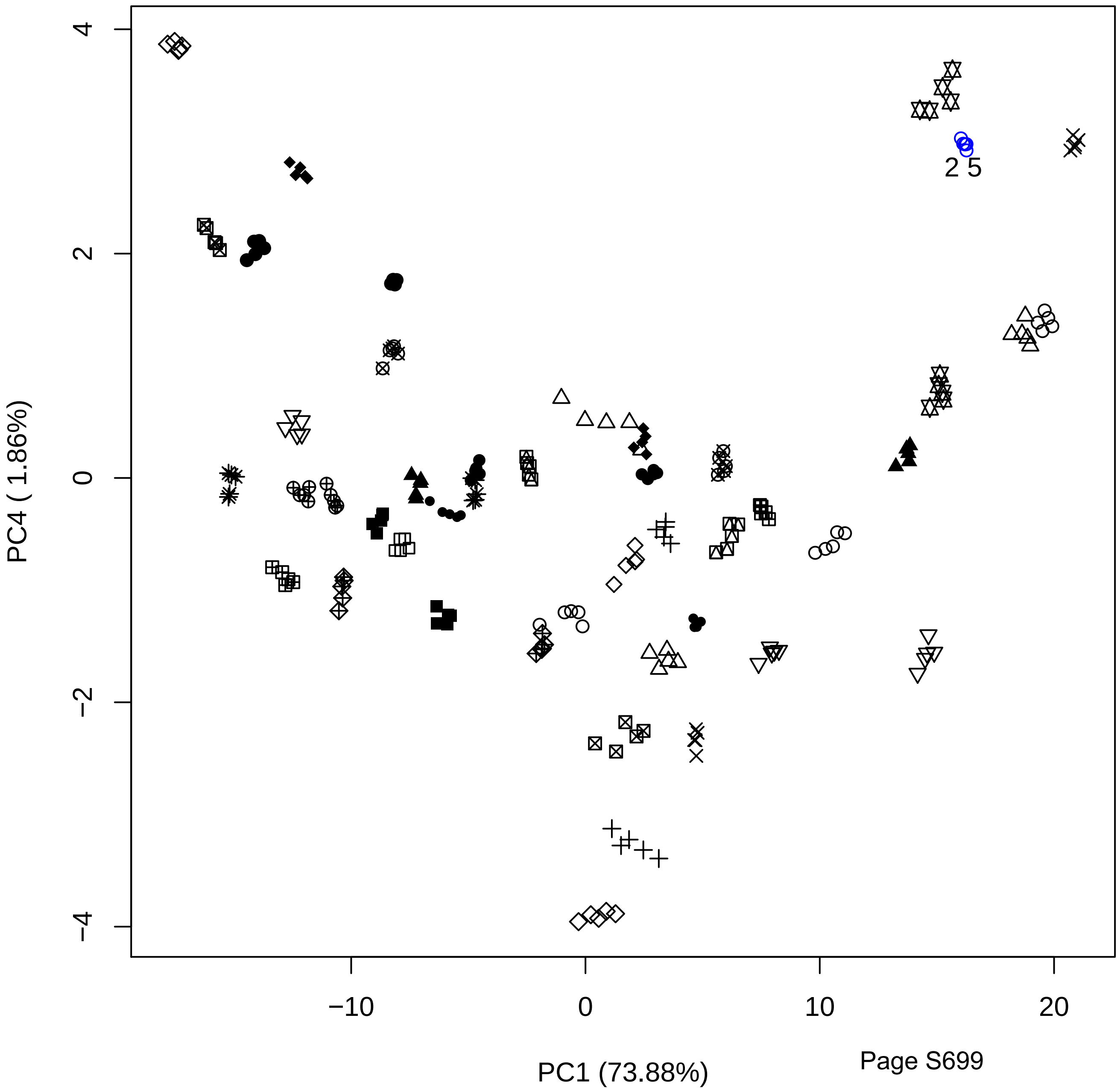

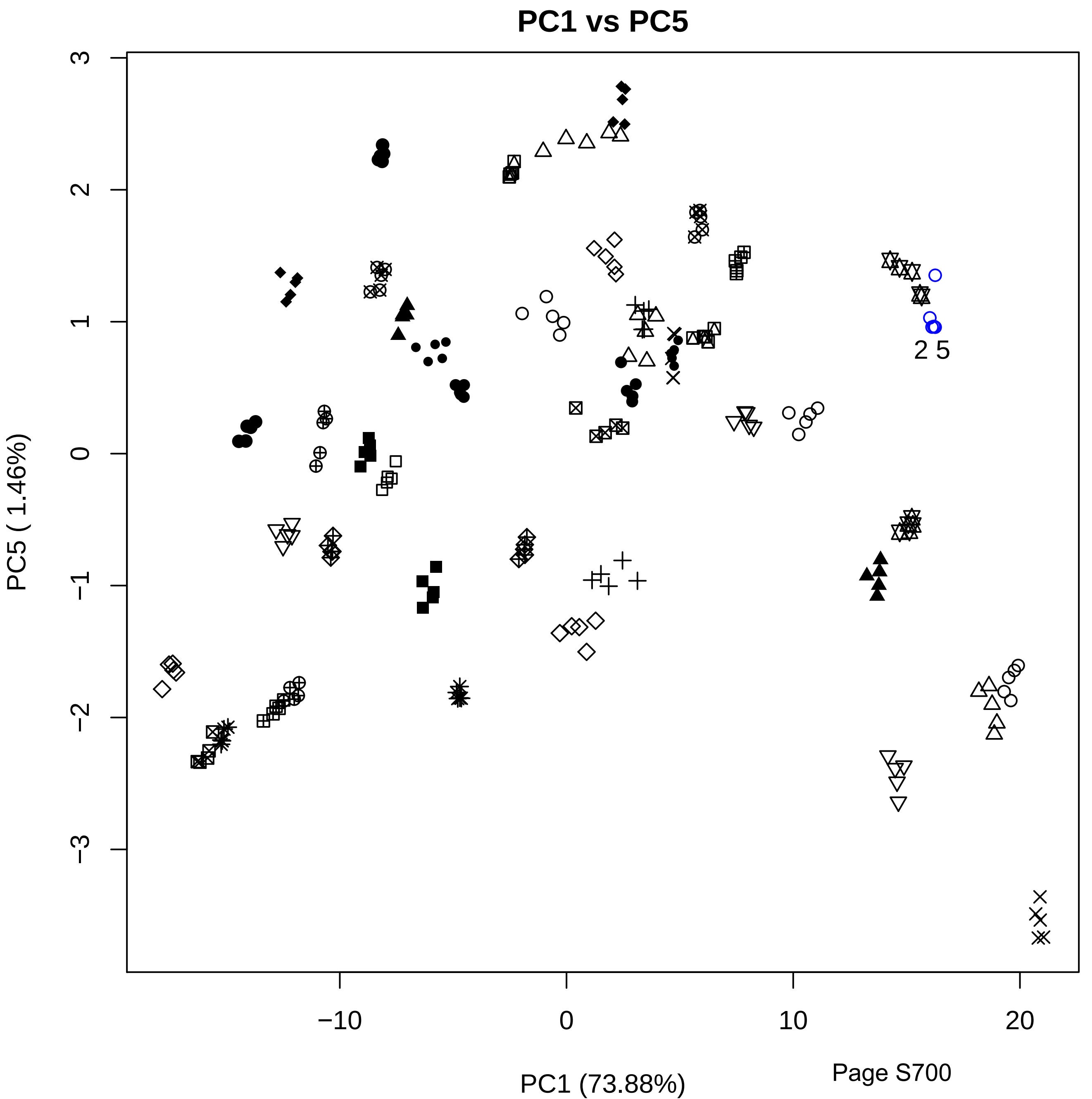
PC1 vs PC5
PC5 (1.46%)
PC1 (73.88%)
3
2
1
0
−1
−2
−3
−10
0
10
20
2 5

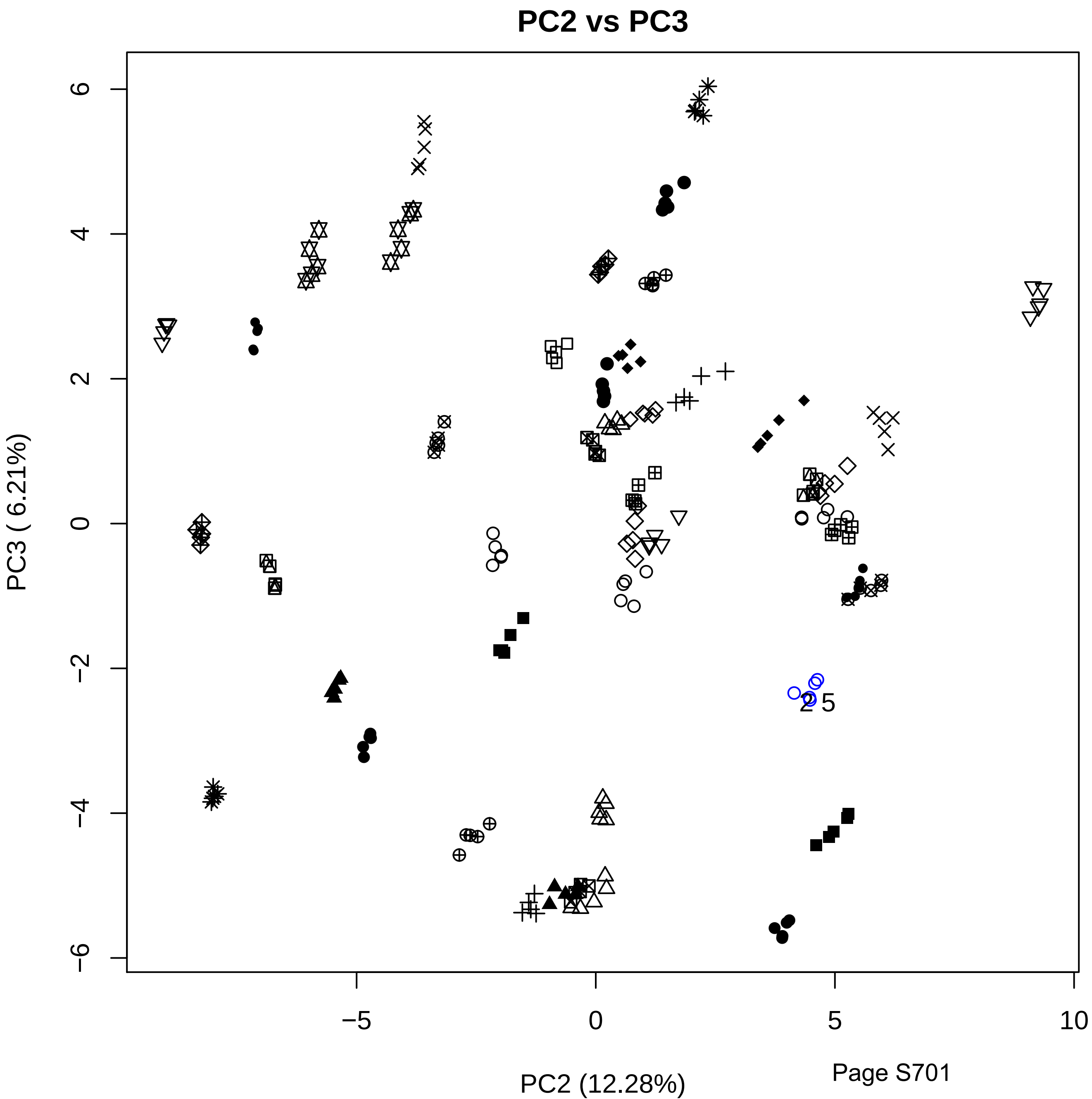
PC2 vs PC3
PC3 (6.21%)
PC2 (12.28%)
6
4
2
0
−2
−4
−6
−5
0
5
10
2 5

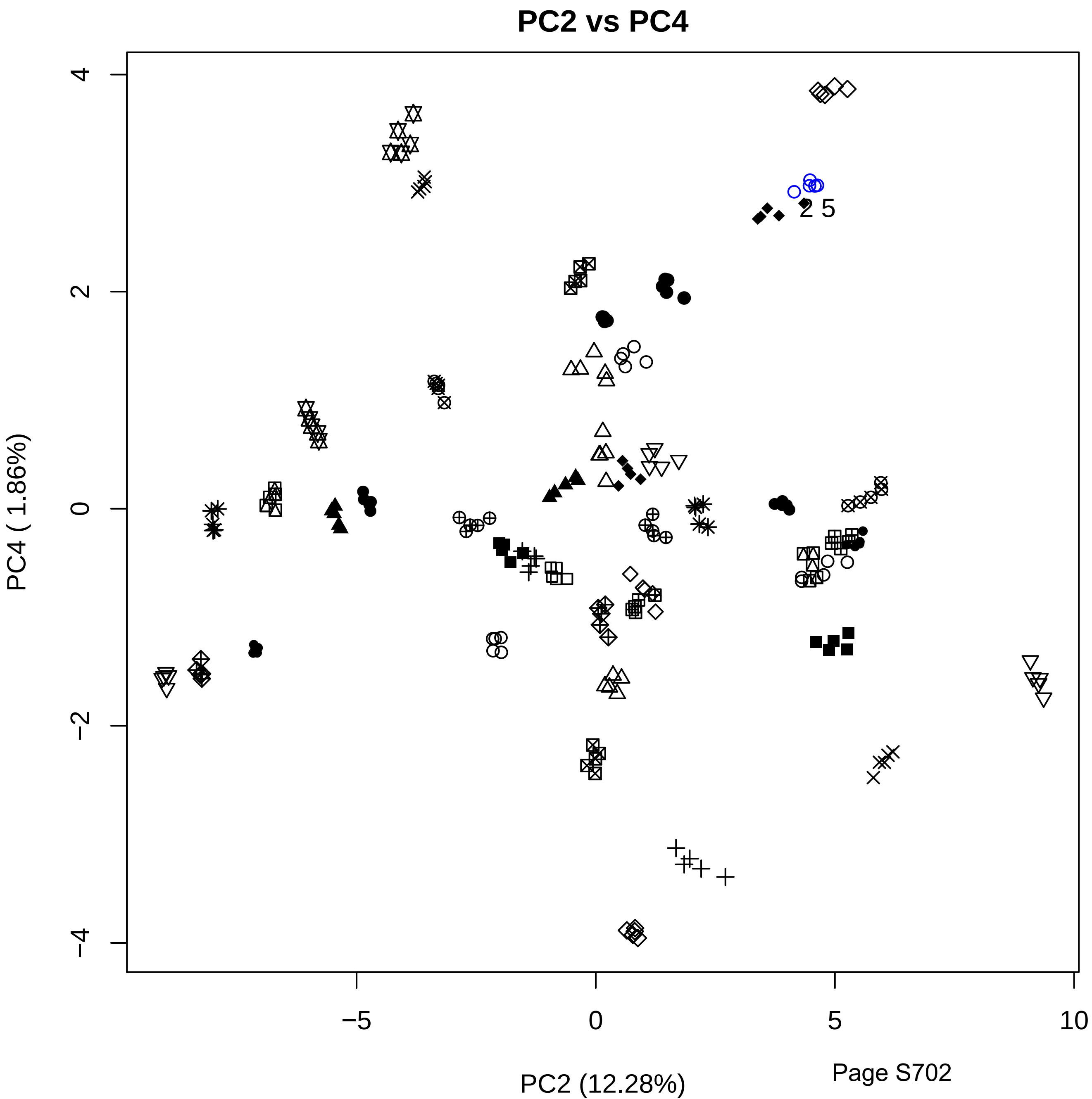

PC2 vs PC4
PC4 (1.86%)
PC2 (12.28%)
4
2
0
−2
−4
−5
0
5
10
2 5

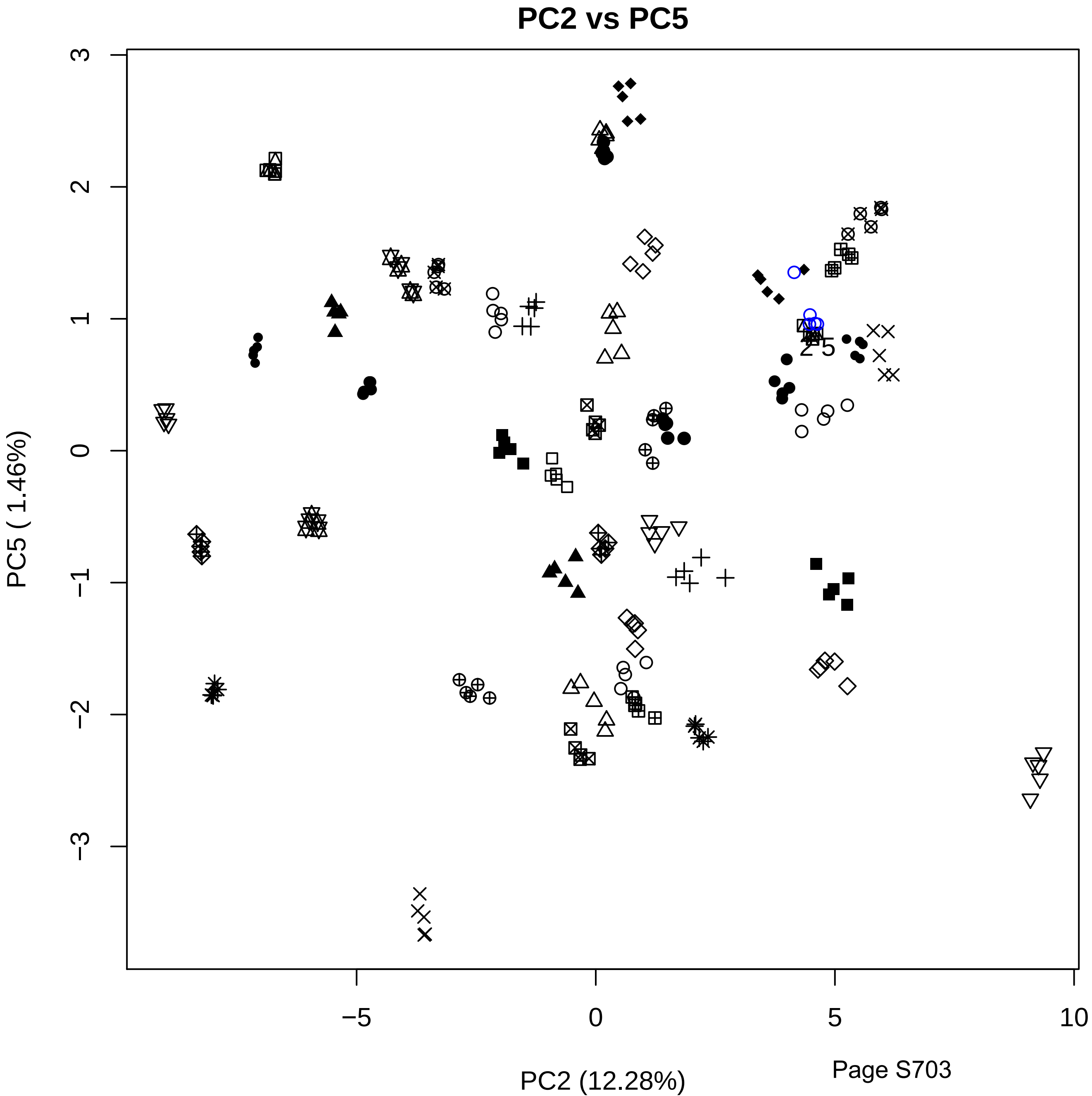
PC2 vs PC5
PC5 (1.46%)
PC2 (12.28%)
3
2
1
0
−1
−2
−3
−5
0
5
10

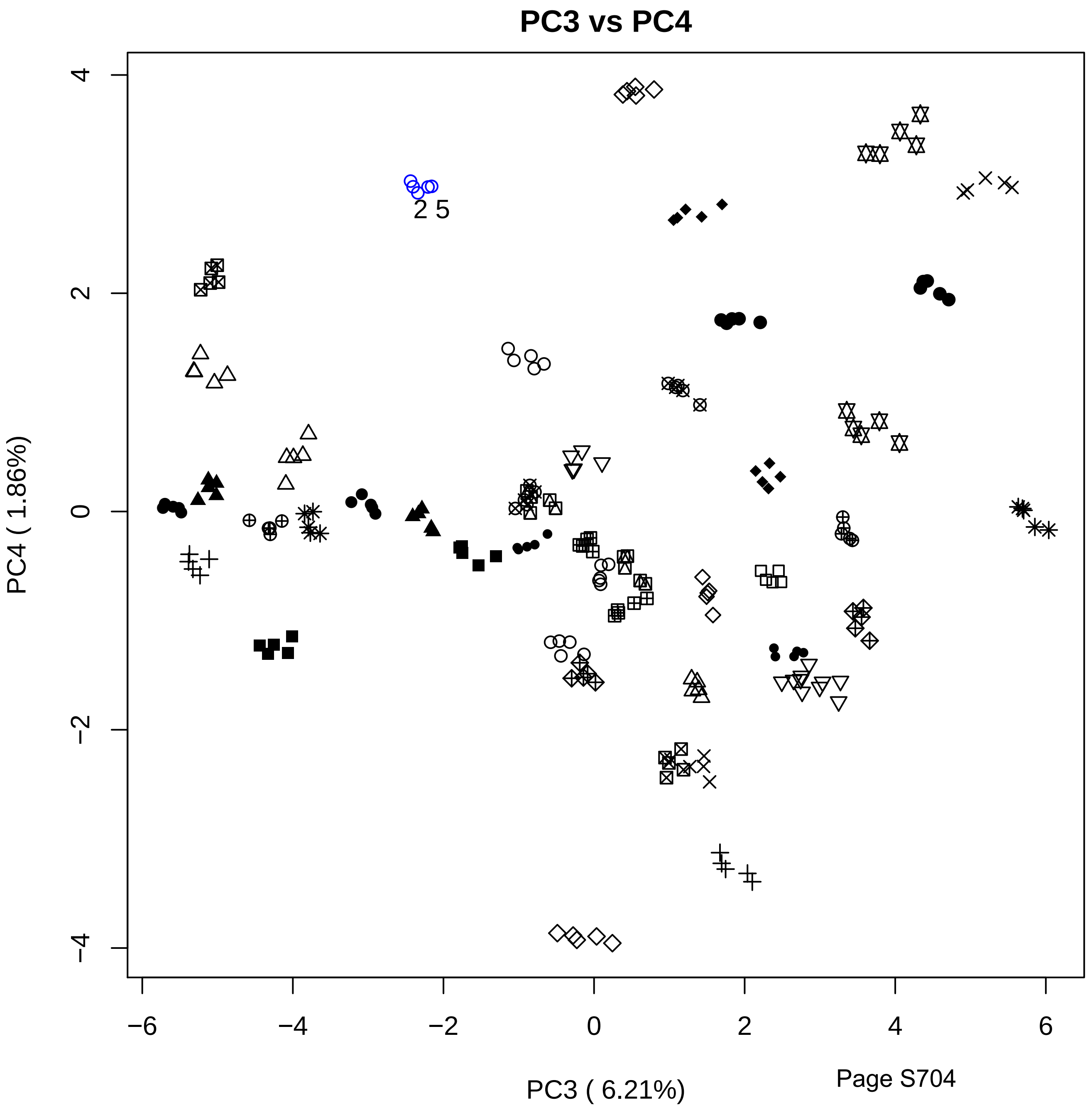
PC3 vs PC4
PC4 (1.86%)
PC3 (6.21%)
2 5
4
2
0
−2
−4
−6
−4
−2
0
2
4
6

PC3 vs PC5

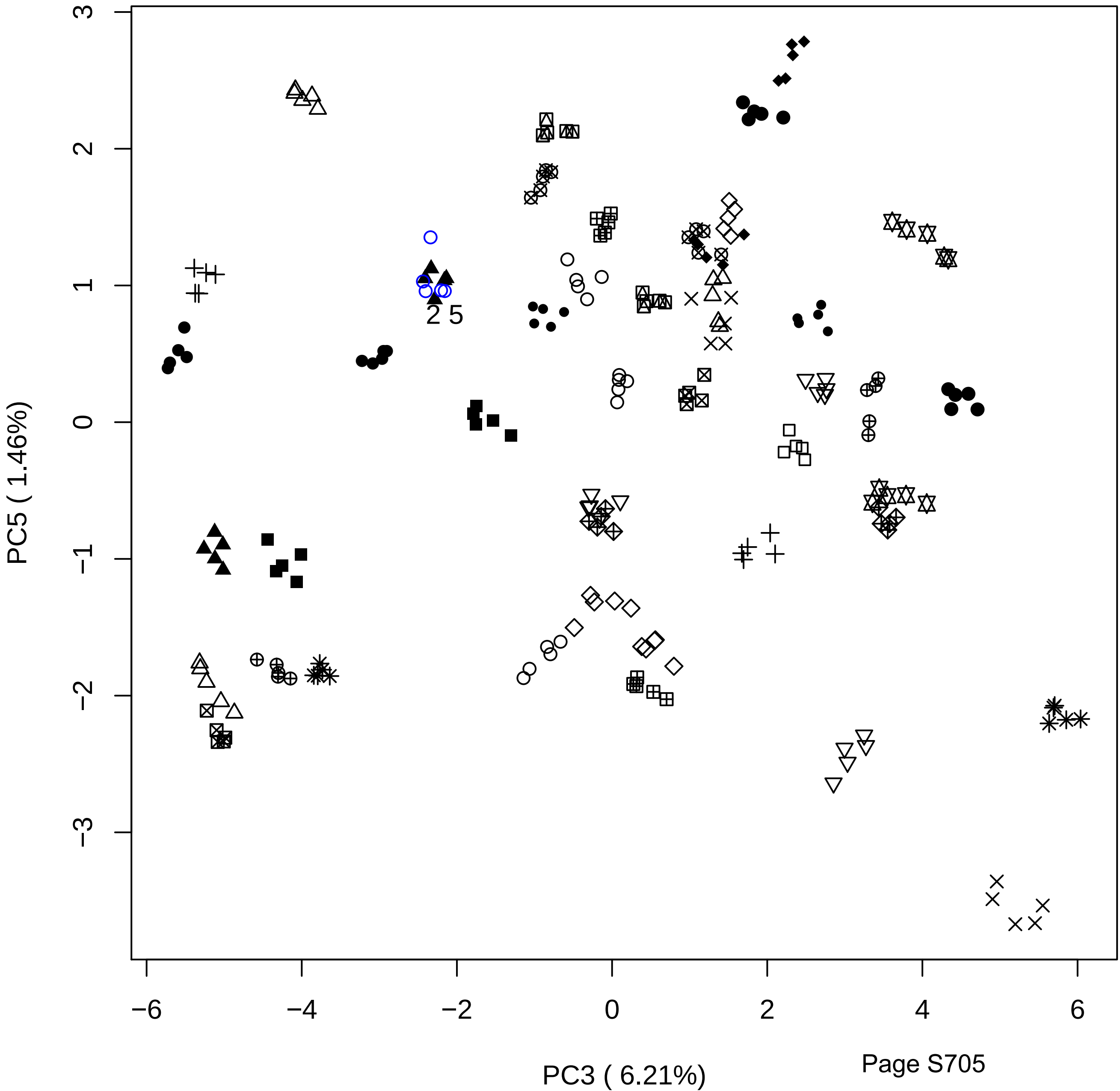

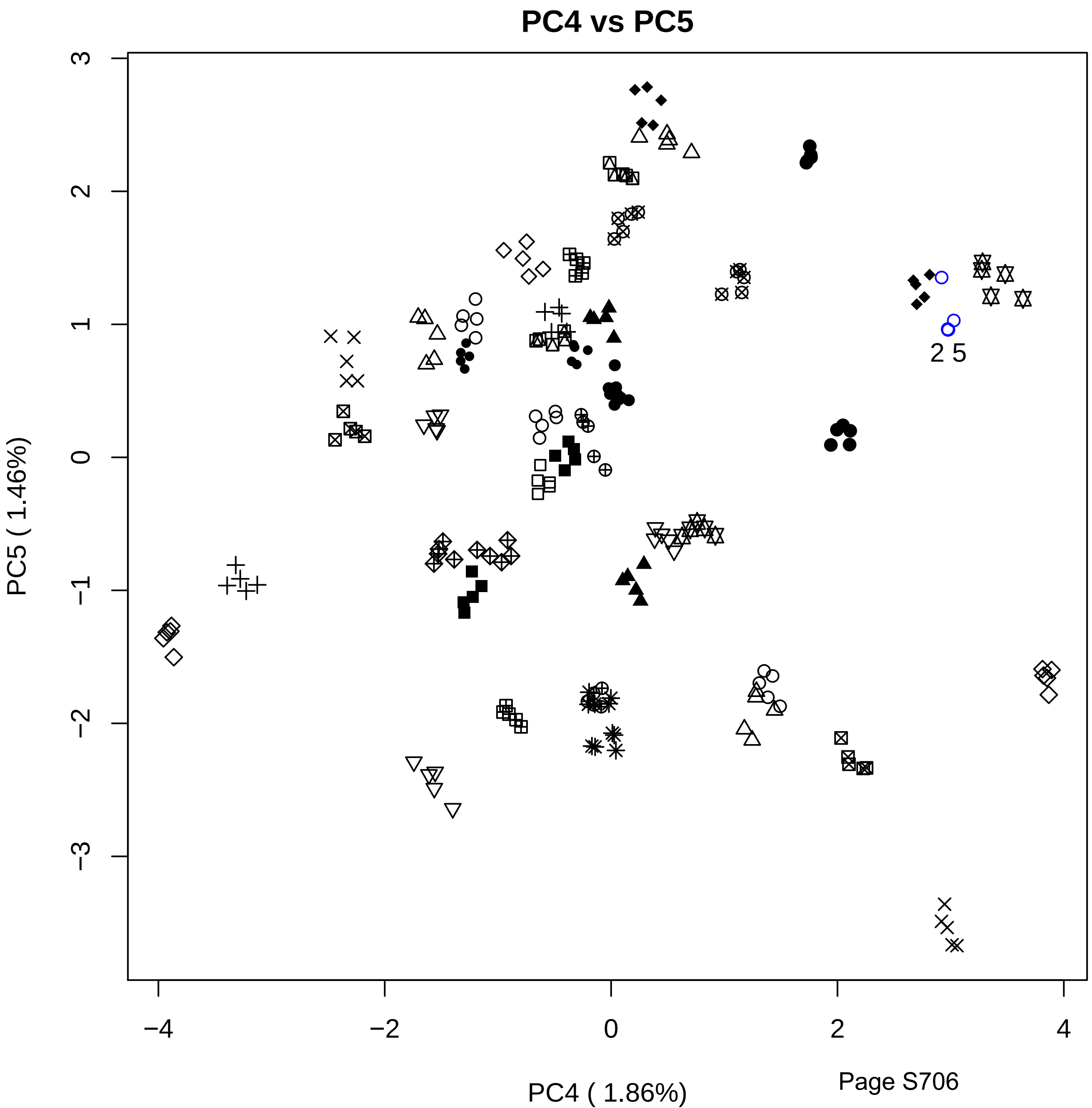

PC4 vs PC5
3
2
1
0
−1
−2
−3
PC5 (1.46%)
2 5
−4
−2
0
2
4
PC4 (1.86%)

PCA of 5% DeS vs PMH

Scaled Matrix PCAed

7th order polynomial baseline correction performed

Raw spectra smoothed to 21 Neighbours, 2nd order PN, Savtisky Golay

Normalisation between 0 and 100

D2 spectra smoothed to 41 Neighbours, 2nd order PN, Savtisky Golay

Regions Analysed: 3601−2501 and 2001−700cm−1

Samples Analysed: 1 Contam's 0 DeS's 45 PMH's

Keele University AD   Sun Dec 09 17:04:11 2018

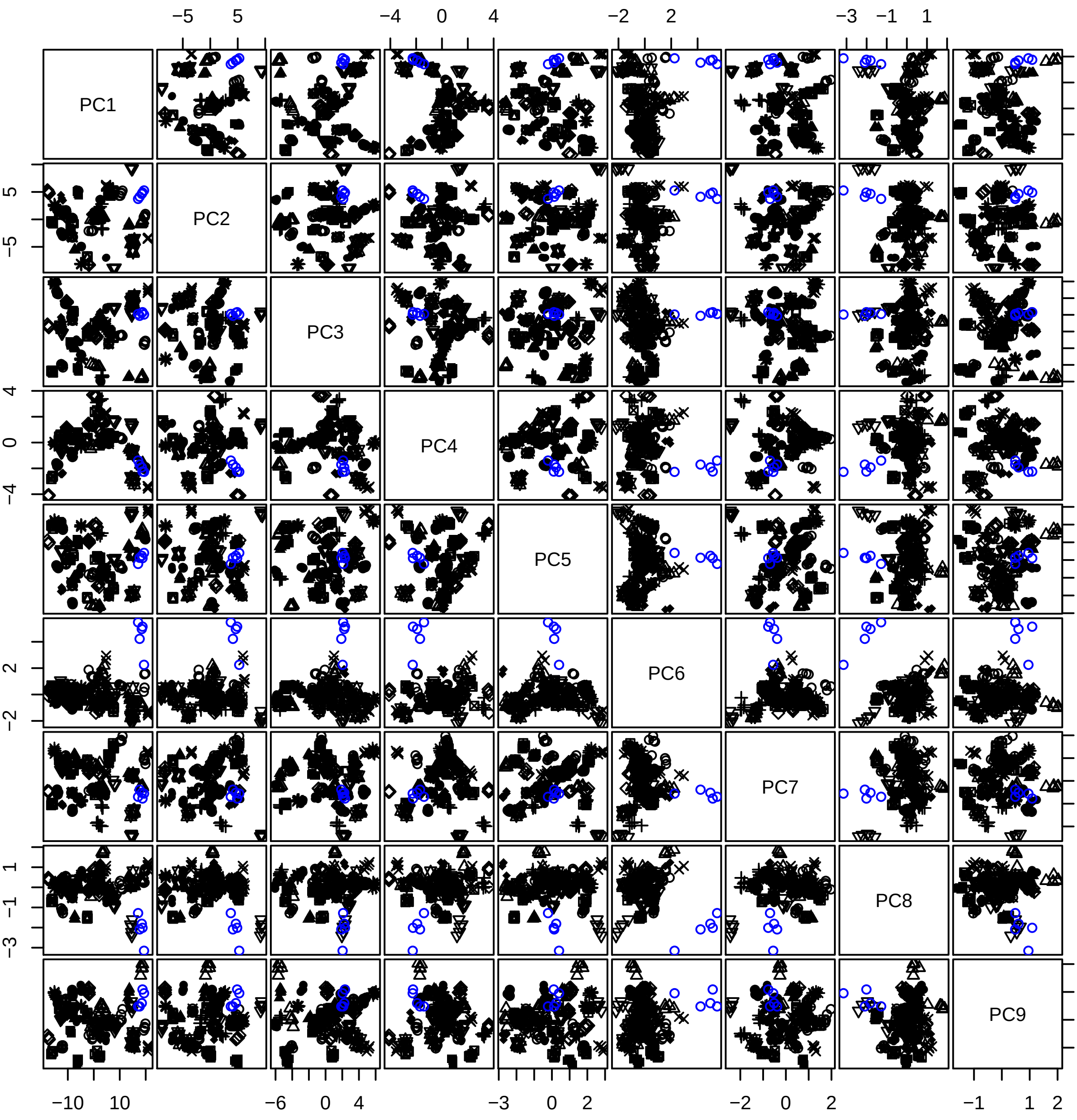
PC1
PC2
PC3
PC4
PC5
PC6
PC7
PC8
PC9

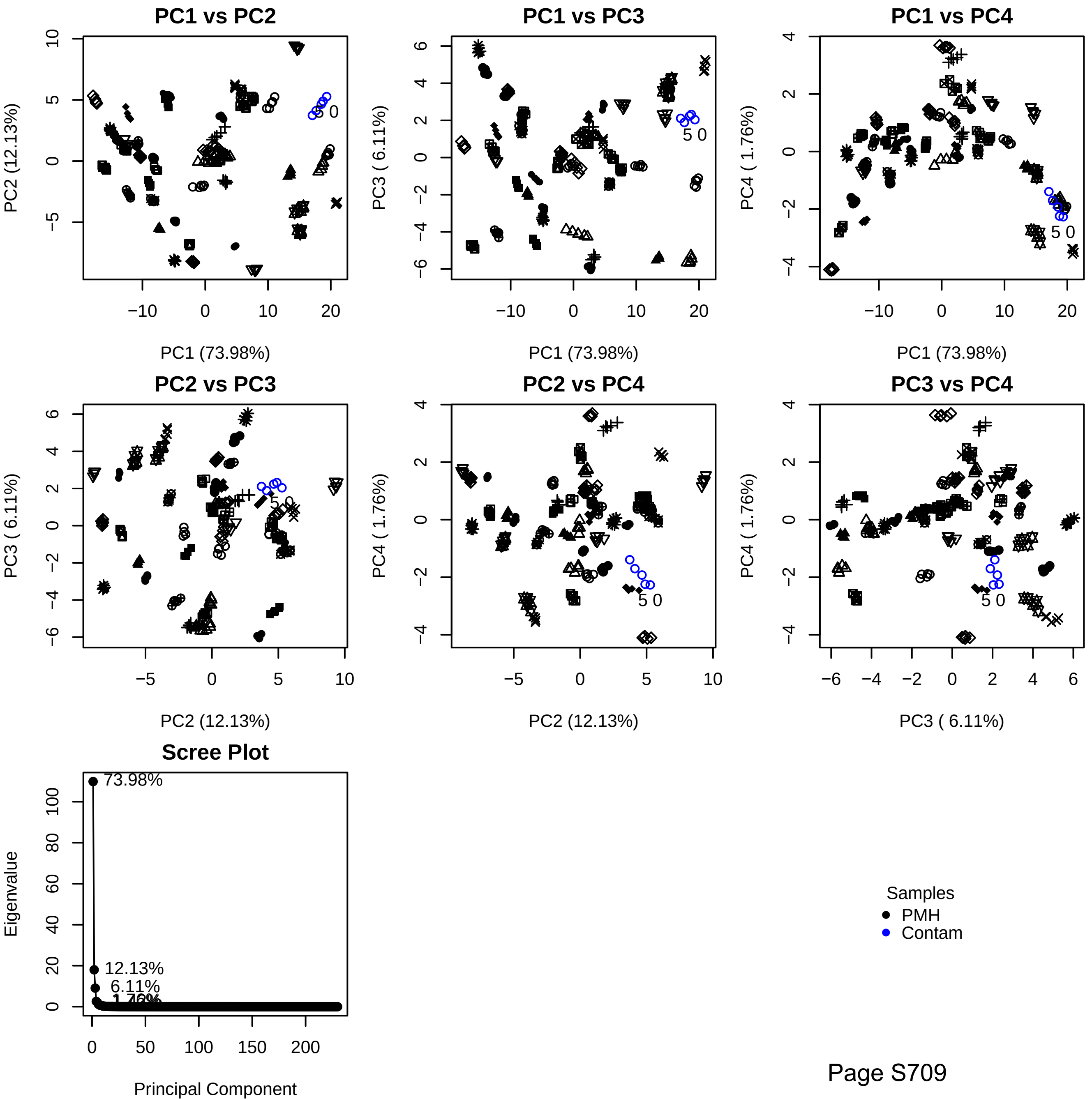

PC1 vs PC2
PC1 vs PC3
PC1 vs PC4
PC1 (73.98%)
PC2 (12.13%)
PC3 (6.11%)
PC4 (1.76%)
PC2 vs PC3
PC2 vs PC4
PC3 vs PC4
Scree Plot
Eigenvalue
Principal Component
73.98%
12.13%
6.11%
Samples
PMH
Contam

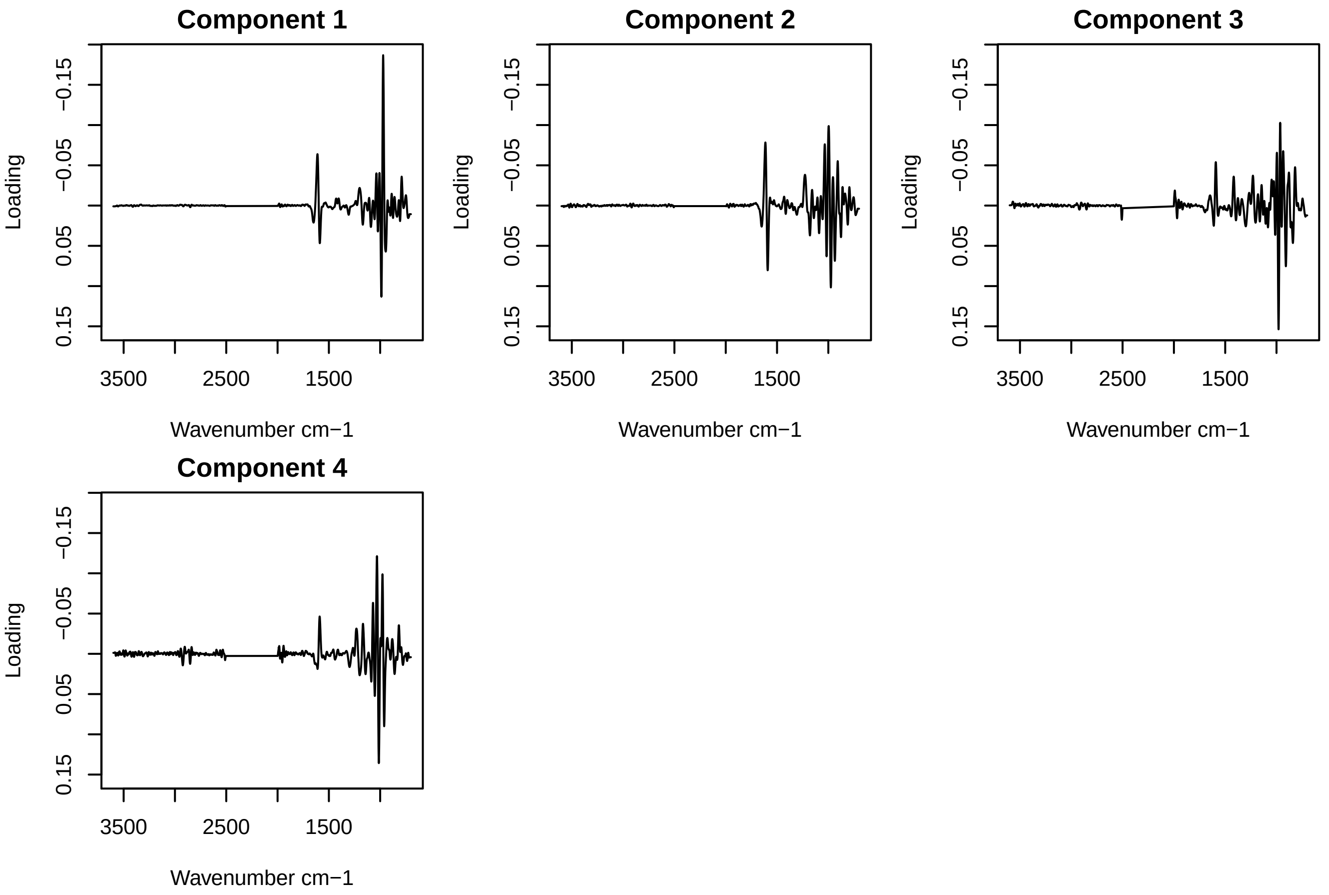
Component 1
Component 2
Component 3
Component 4
Loading
−0.15
−0.05
0.05
0.15
3500
2500
1500
Wavenumber cm−1

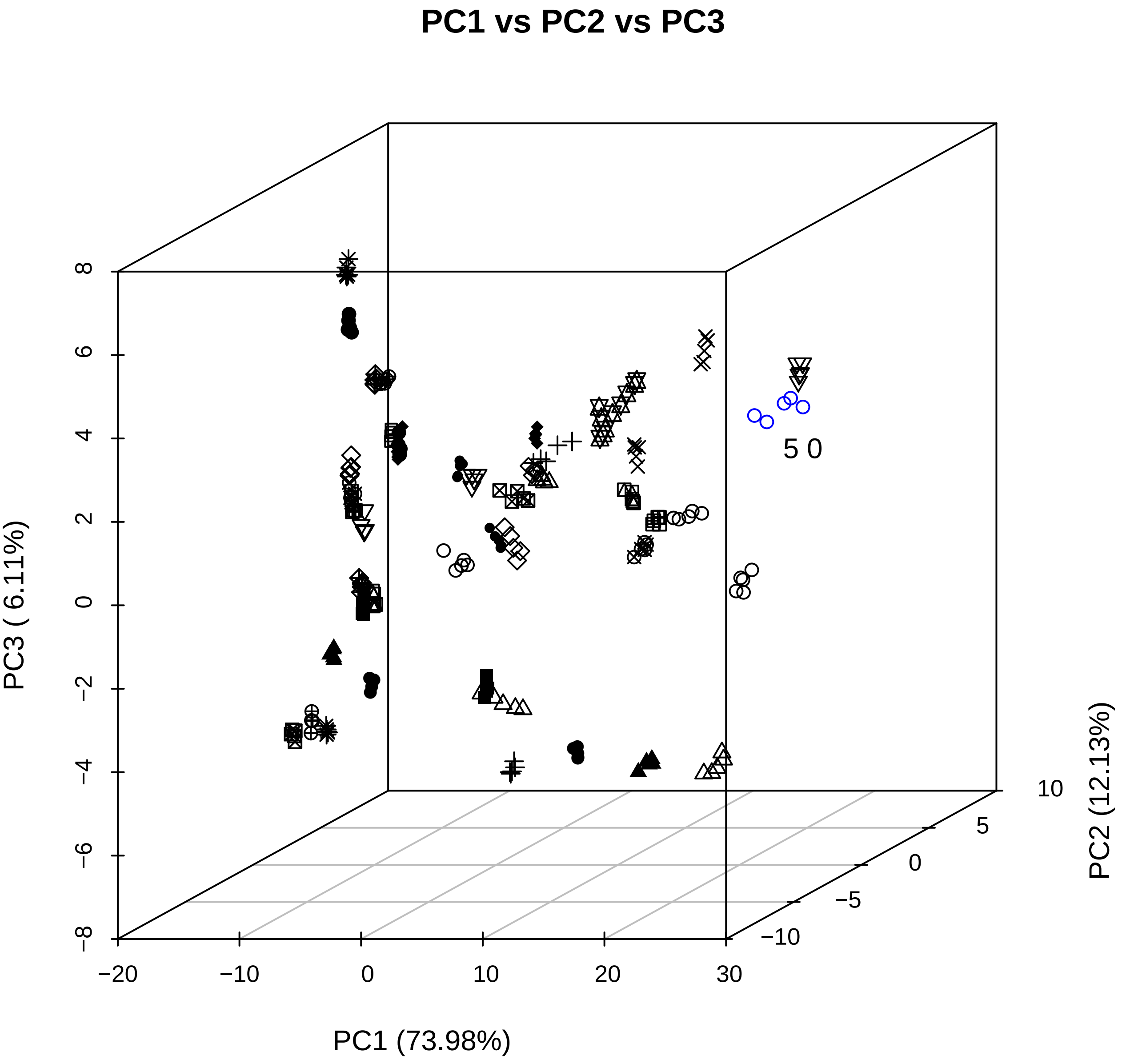
PC1 vs PC2 vs PC3
PC3 (6.11%)
8
6
4
2
0
−2
−4
−6
−8
5 0
PC1 (73.98%)
−20
−10
0
10
20
30
PC2 (12.13%)
10
5
0
−5
−10

PC2 vs PC3 vs PC4

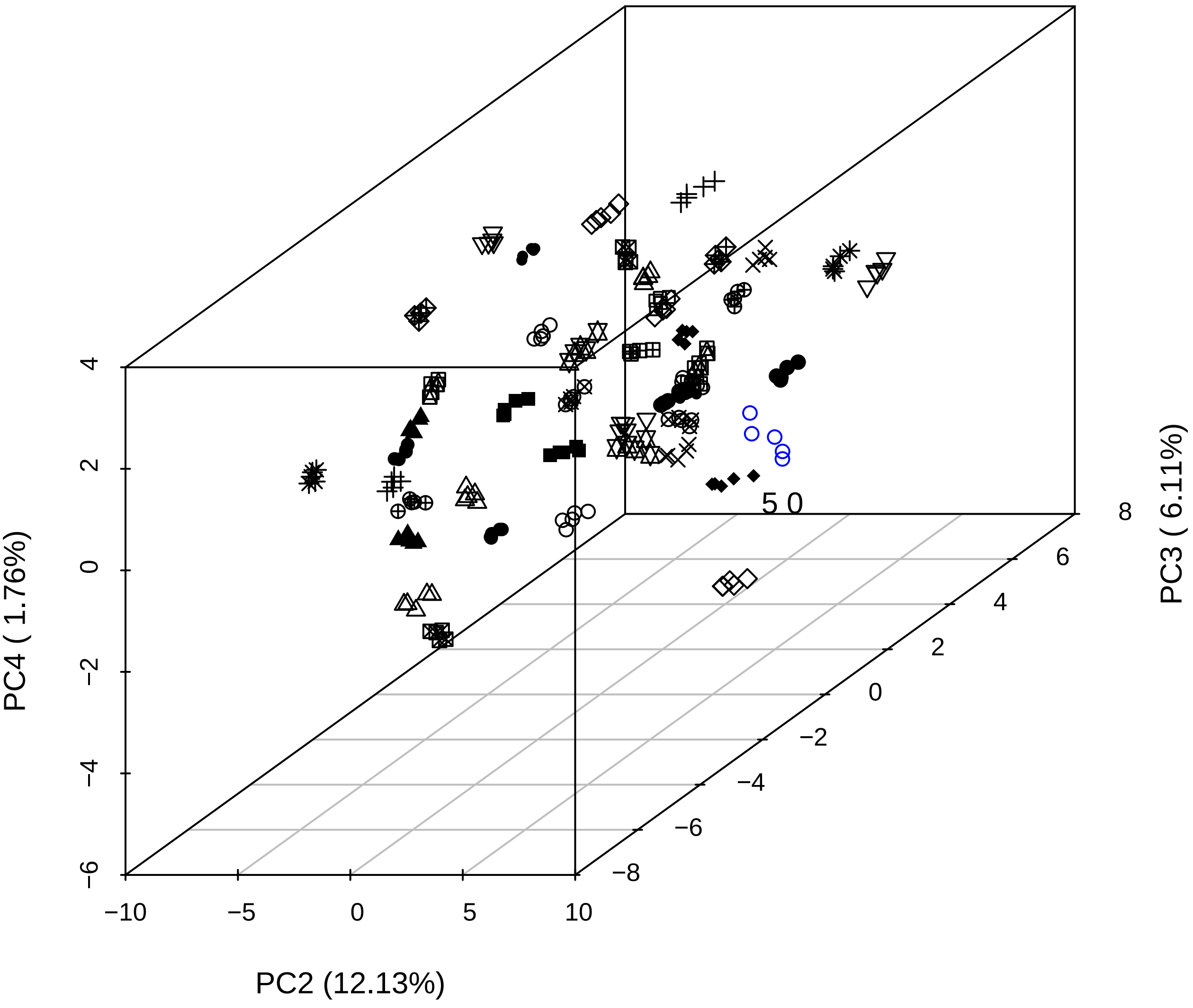

PC1 vs PC2 vs PC4

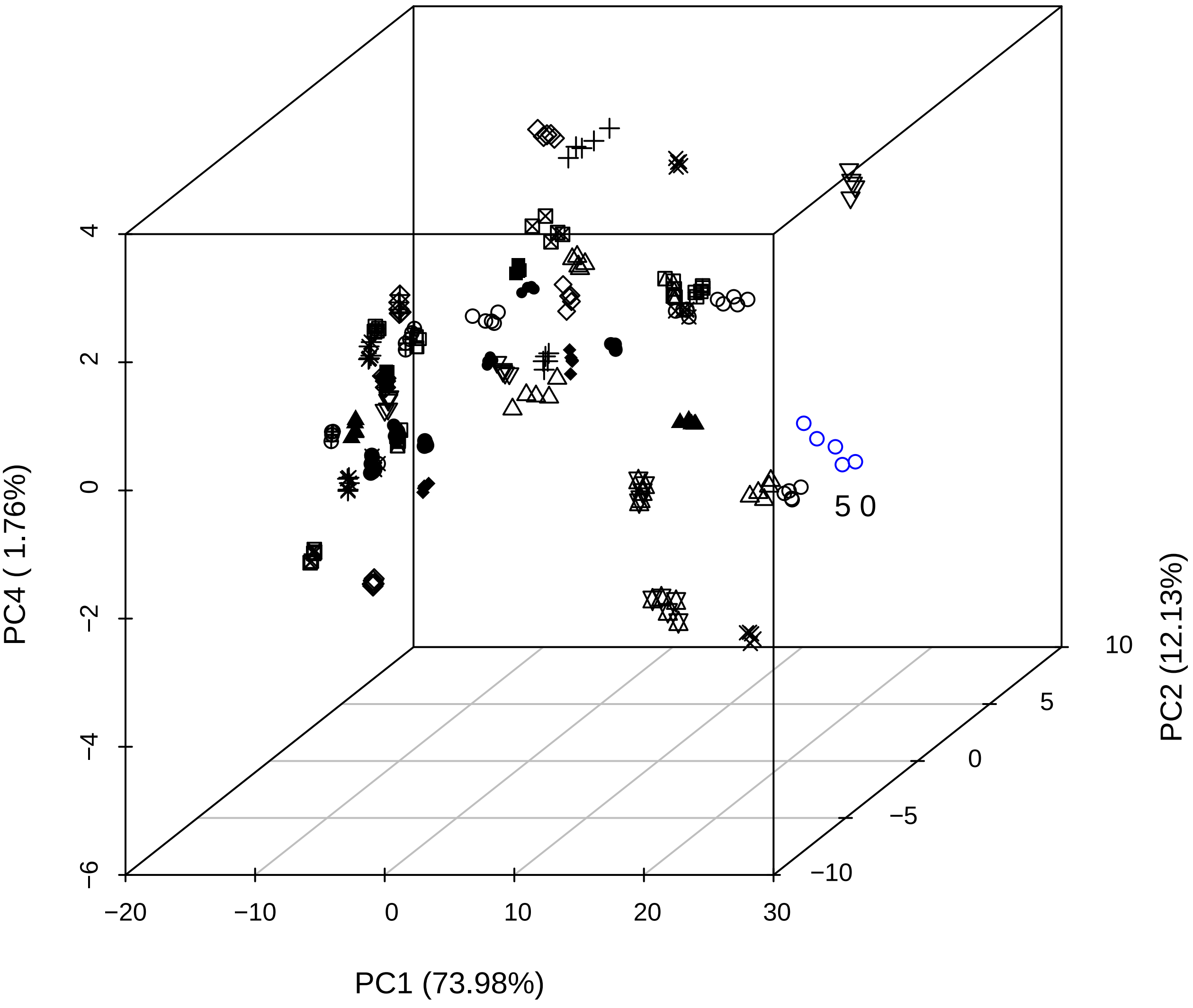

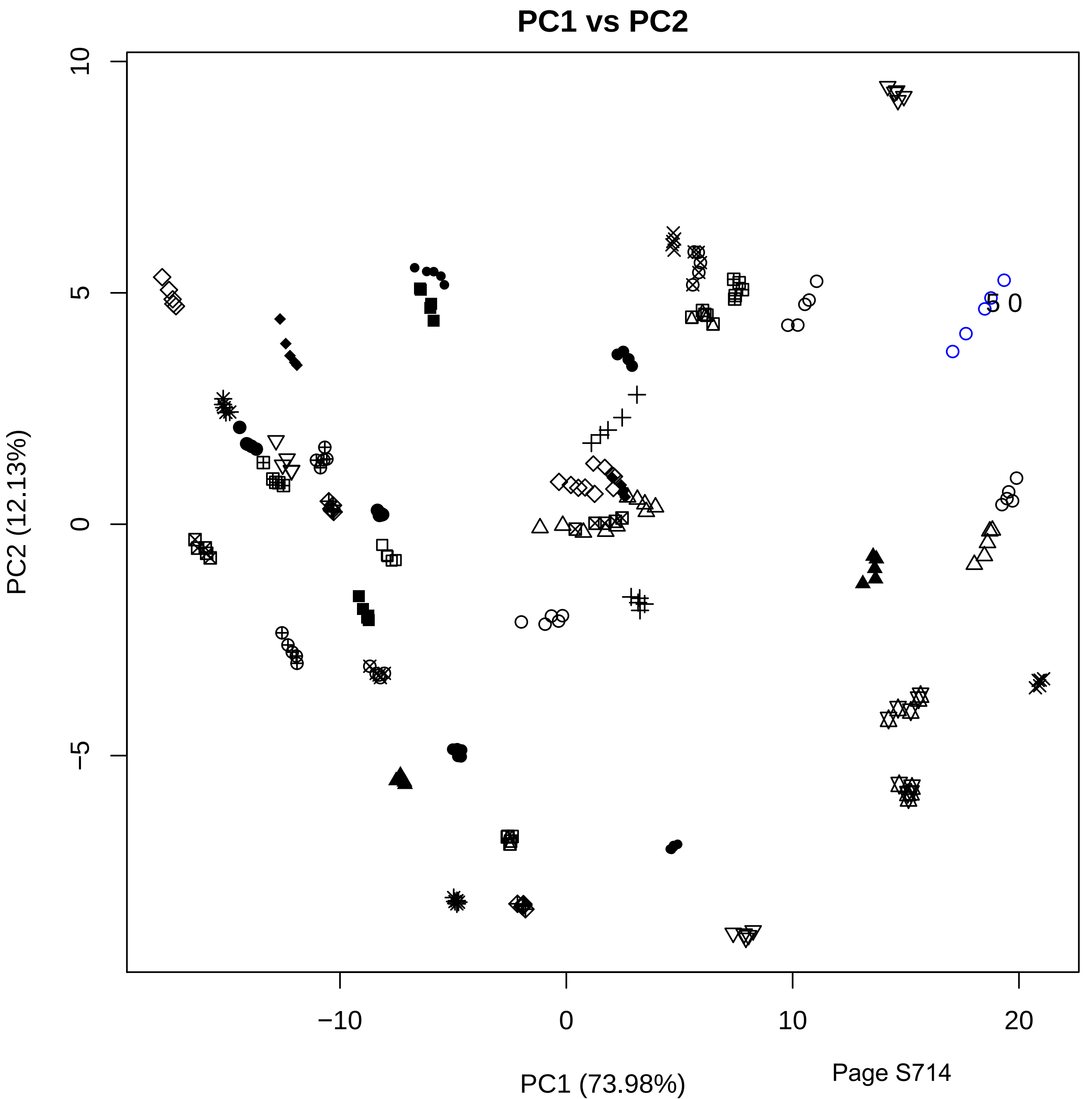

PC1 vs PC2
PC2 (12.13%)
PC1 (73.98%)
10
5
0
−5
−10
0
10
20
5 0

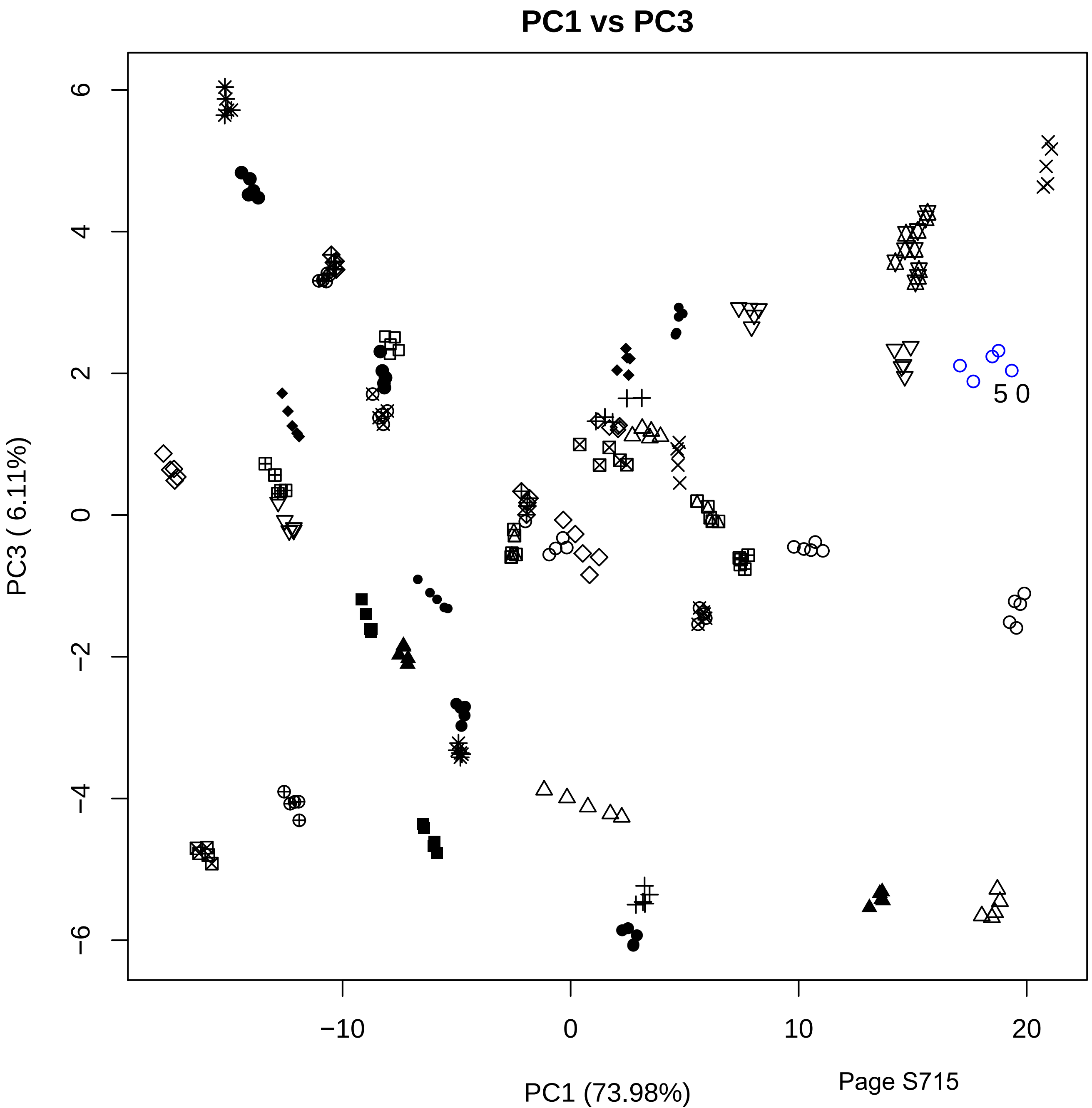

PC1 vs PC3
PC3 (6.11%)
PC1 (73.98%)
6
4
2
0
−2
−4
−6
−10
0
10
20
5 0

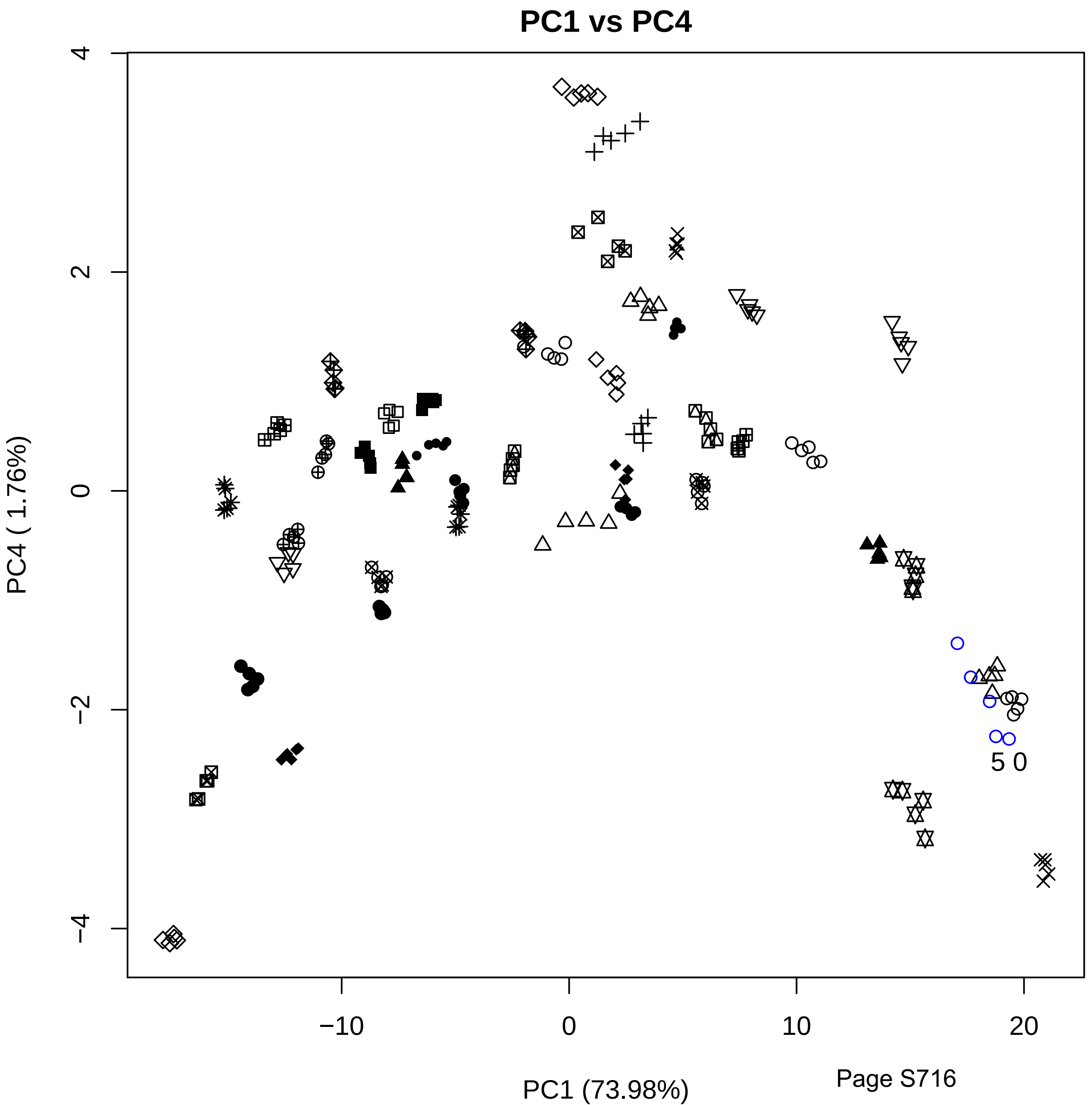

PC1 vs PC4
PC4 (1.76%)
PC1 (73.98%)
4
2
0
−2
−4
−10
0
10
20
5 0

PC1 vs PC5

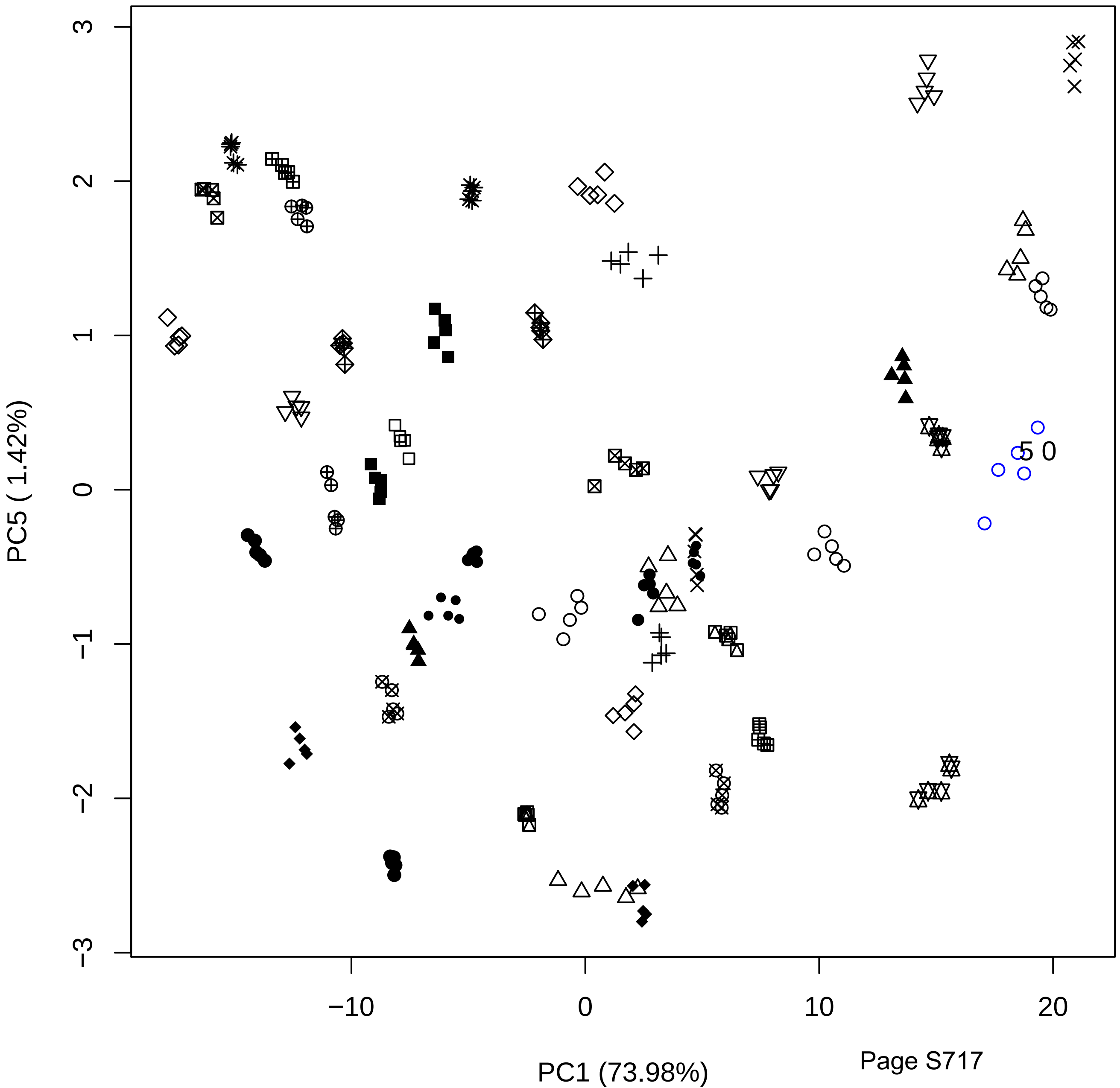

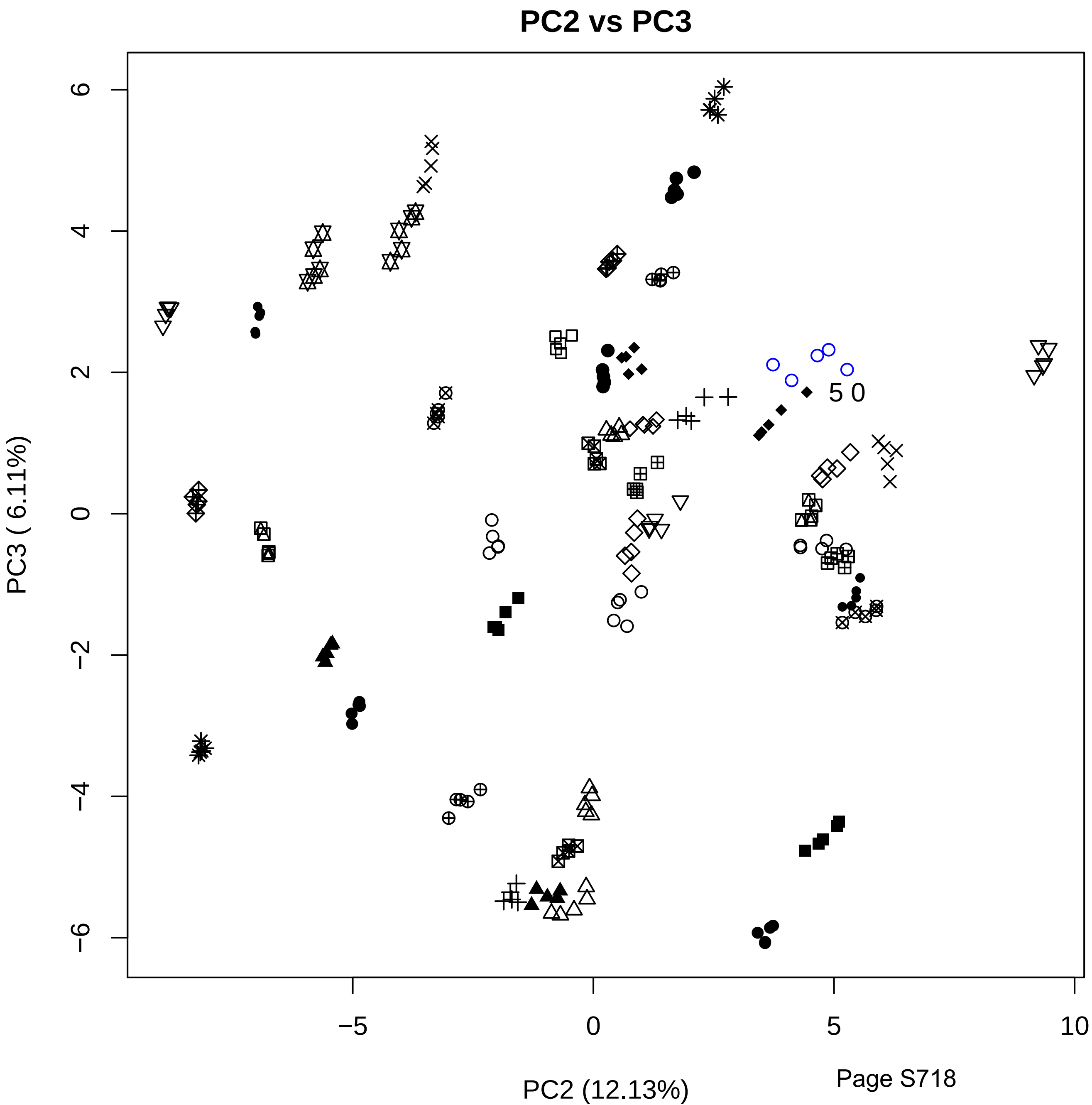

PC2 vs PC3
PC3 (6.11%)
PC2 (12.13%)
6
4
2
0
−2
−4
−6
−5
0
5
10
5 0

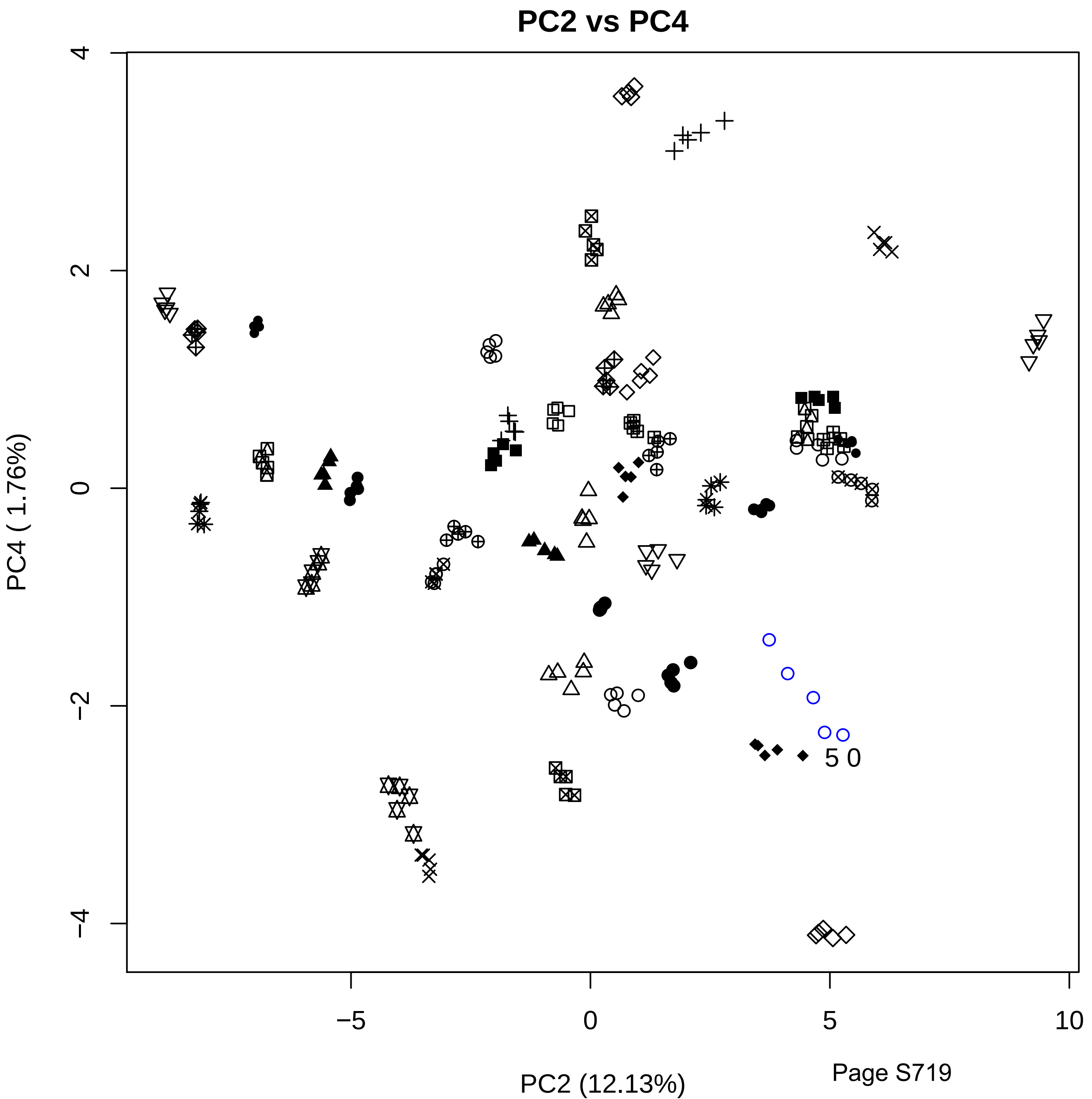
PC2 vs PC4
PC4 (1.76%)
PC2 (12.13%)
4
2
0
−2
−4
−5
0
5
10
5 0

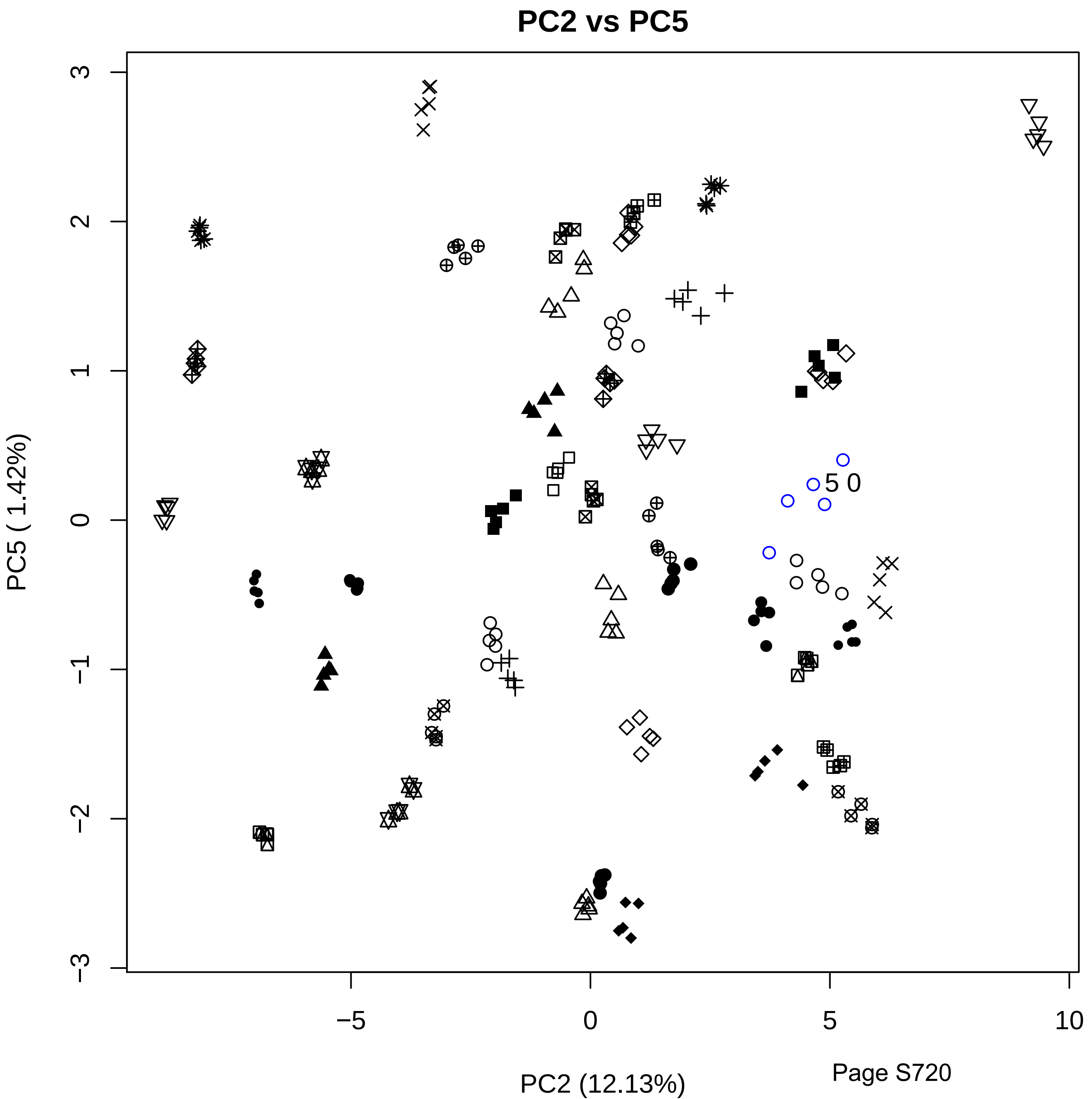
PC2 vs PC5
PC5 (1.42%)
PC2 (12.13%)
3
2
1
0
−1
−2
−3
−5
0
5
10
5 0

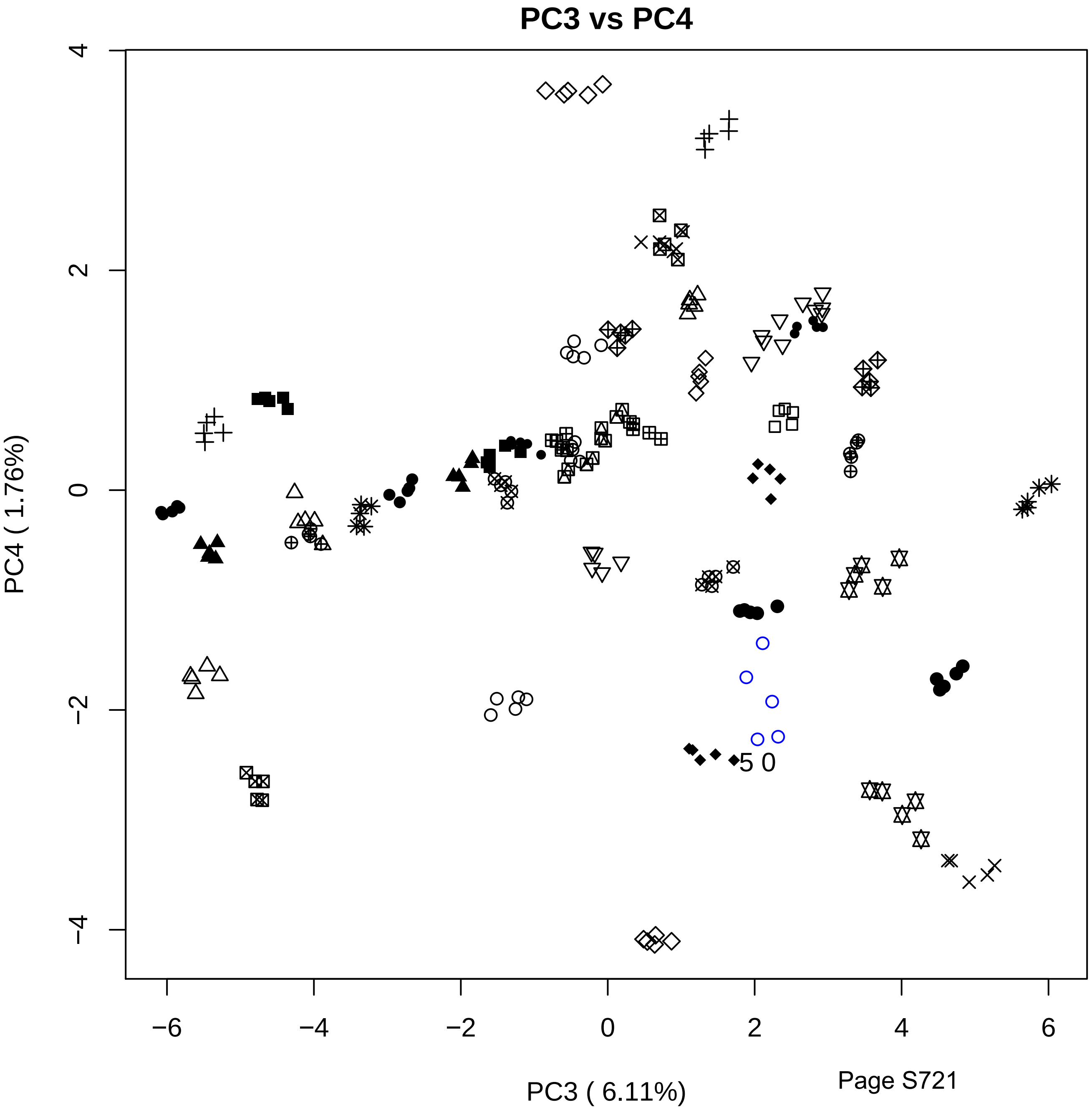
PC3 vs PC4
PC4 (1.76%)
PC3 (6.11%)
4
2
0
−2
−4
−6
6
5 0

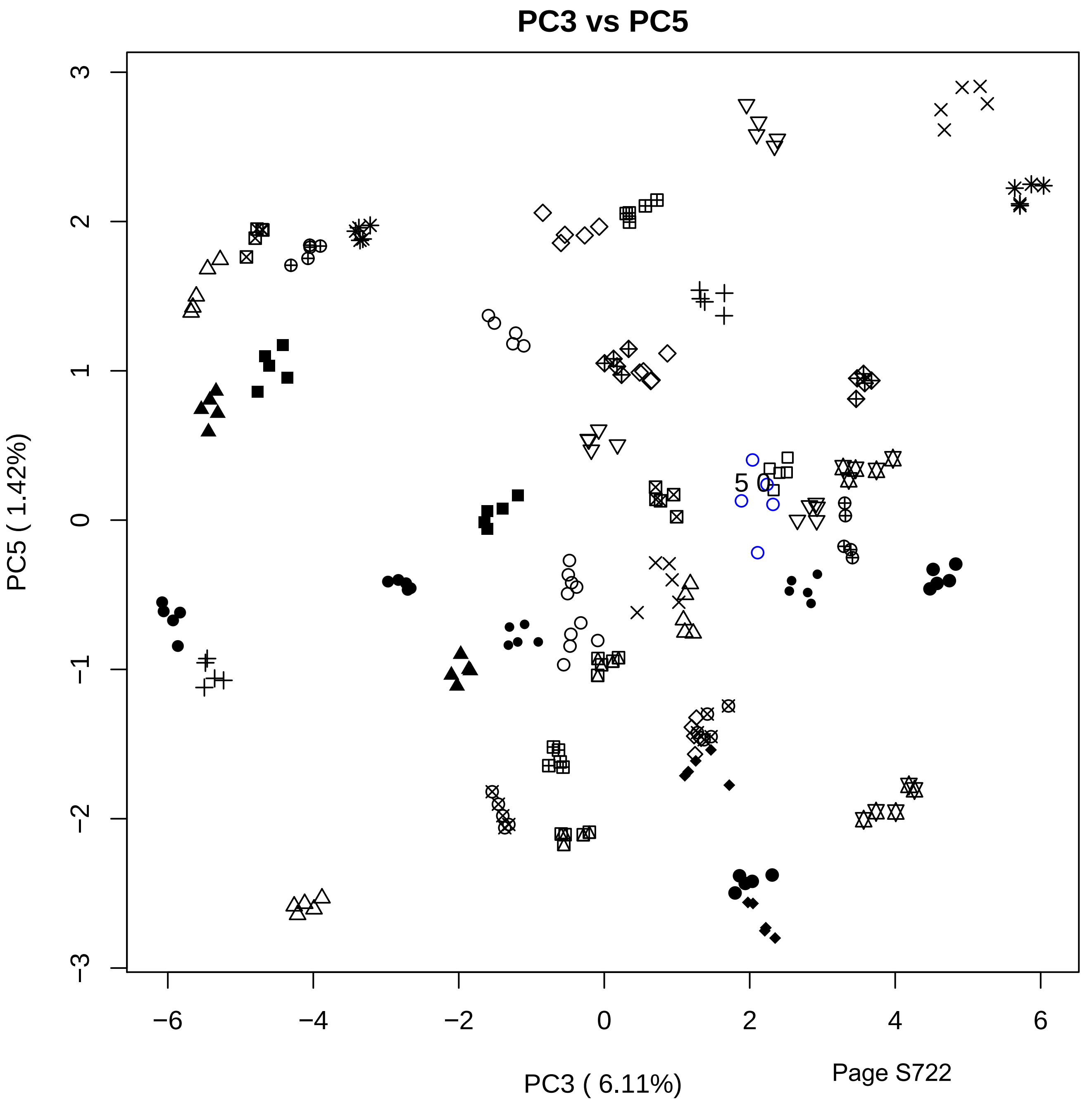
PC3 vs PC5
PC5 (1.42%)
PC3 (6.11%)
3
2
1
0
−1
−2
−3
−6
−4
−2
0
2
4
6
50

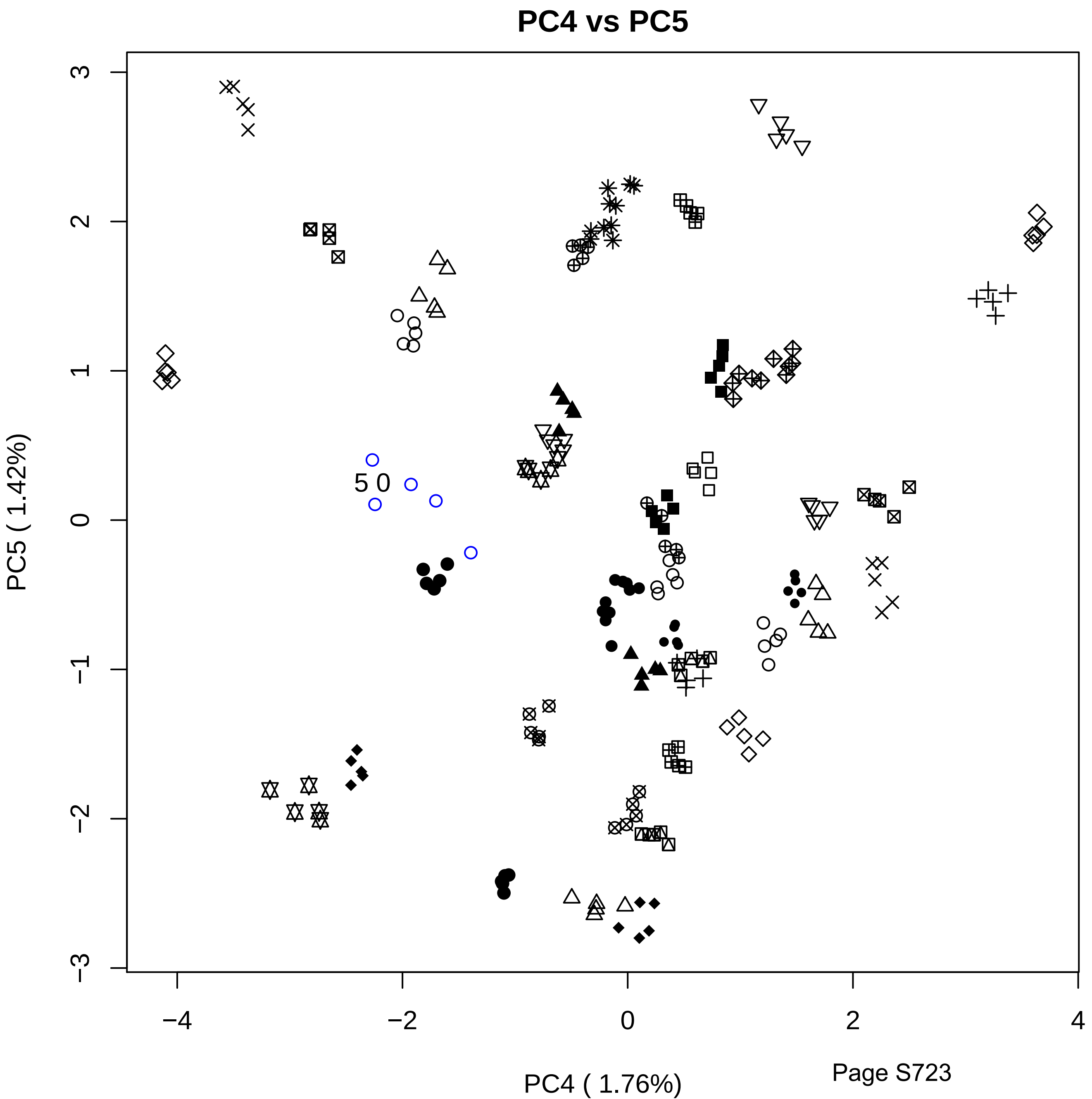
PC4 vs PC5
PC5 (1.42%)
PC4 (1.76%)
50
3
2
1
0
−1
−2
−3
−4
−2
0
2
4

PCA of 7.5% DeS vs PMH

Scaled Matrix PCAed

7th order polynomial baseline correction performed

Raw spectra smoothed to 21 Neighbours, 2nd order PN, Savtisky Golay

Normalisation between 0 and 100

D2 spectra smoothed to 41 Neighbours, 2nd order PN, Savtisky Golay

Regions Analysed: 3601−2501 and 2001−700cm−1

Samples Analysed: 1 Contam's 0 DeS's 45 PMH's

Keele University AD

Sun Dec 09 17:04:17 2018

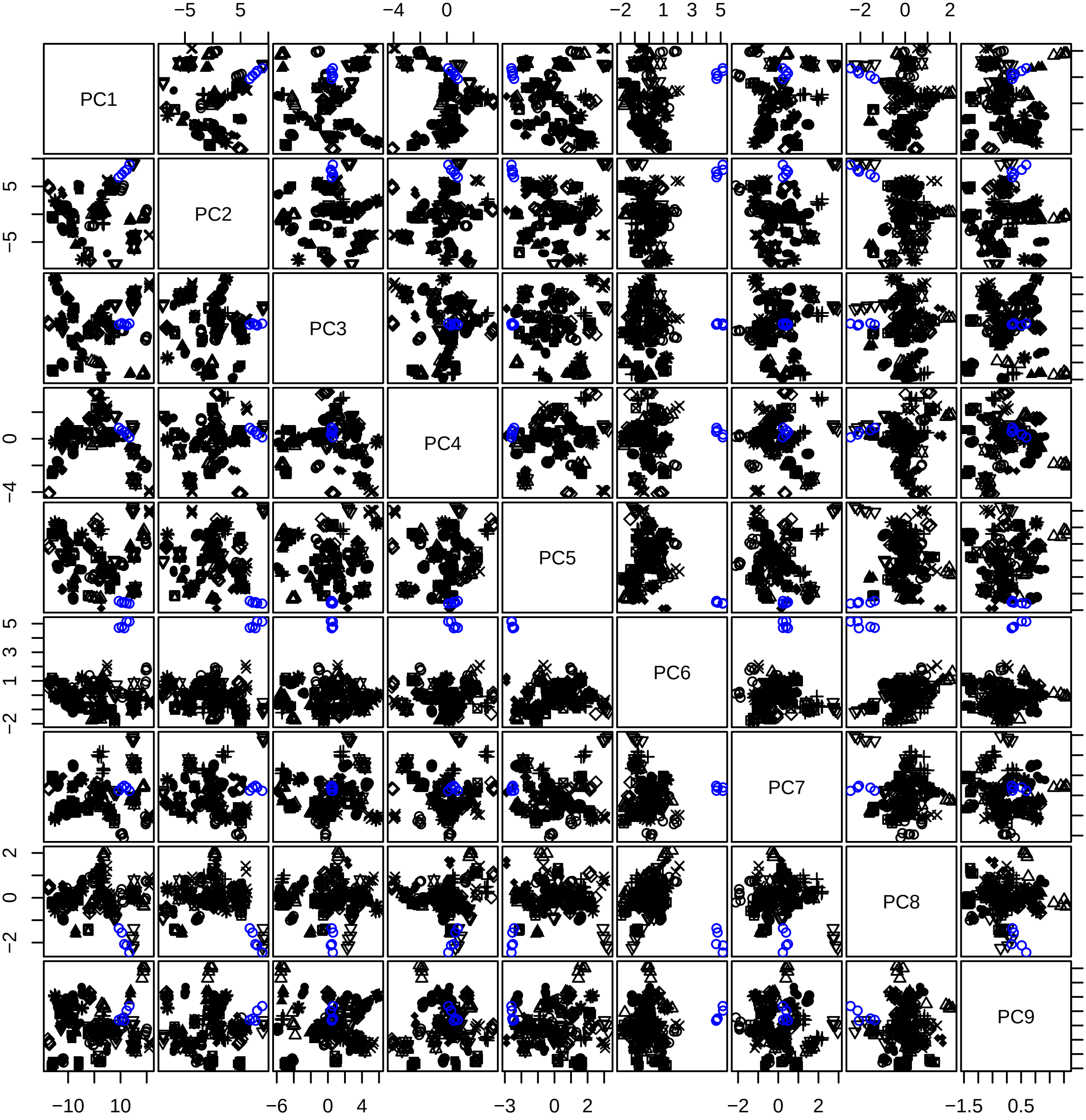
PC1
PC2
PC3
PC4
PC5
PC6
PC7
PC8
PC9
−5 5
−4 0
−2 1 3 5
−2 0 2
−10 10
−6 0 4
−3 0 2
−2 0 2
−1.5 0.5

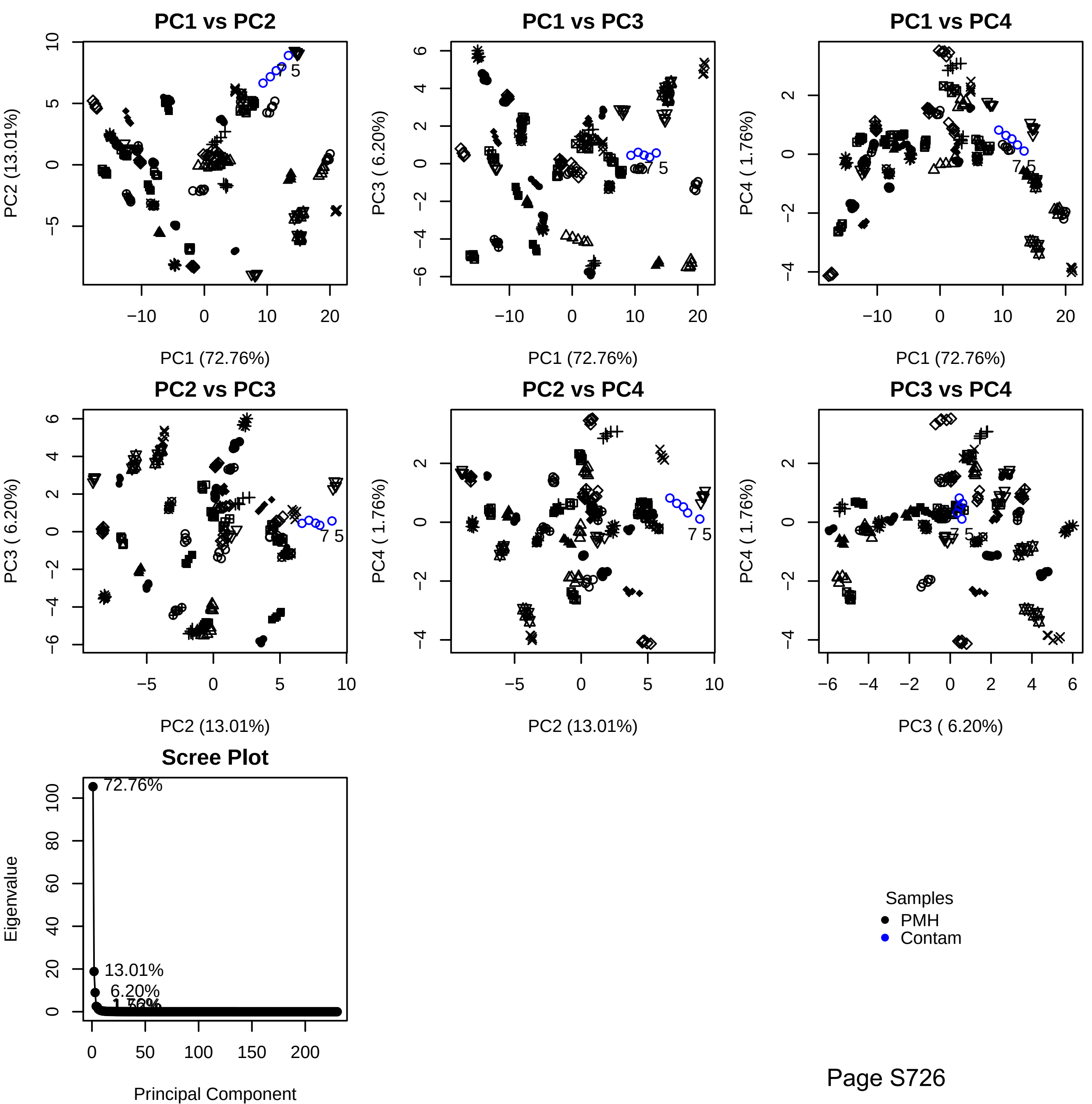
PC1 vs PC2
PC1 vs PC3
PC1 vs PC4
PC2 vs PC3
PC2 vs PC4
PC3 vs PC4
Scree Plot
PC1 (72.76%)
PC2 (13.01%)
PC3 (6.20%)
PC4 (1.76%)
Eigenvalue
Principal Component
72.76%
13.01%
6.20%
7 5
Samples
PMH
Contam

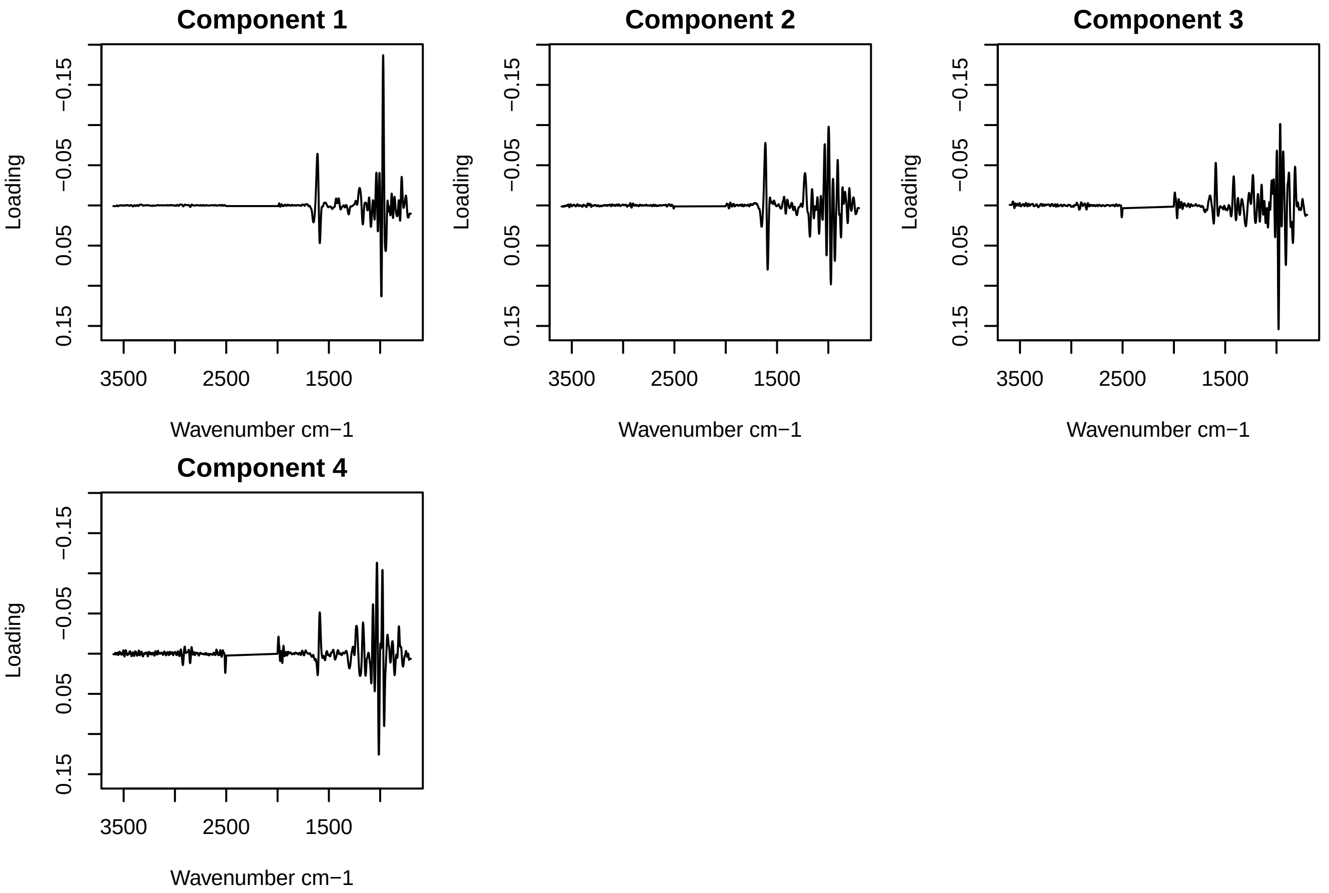
Component 1
Component 2
Component 3
Component 4
Loading
−0.15
−0.05
0.05
0.15
3500
2500
1500
Wavenumber cm−1

PC1 vs PC2 vs PC3

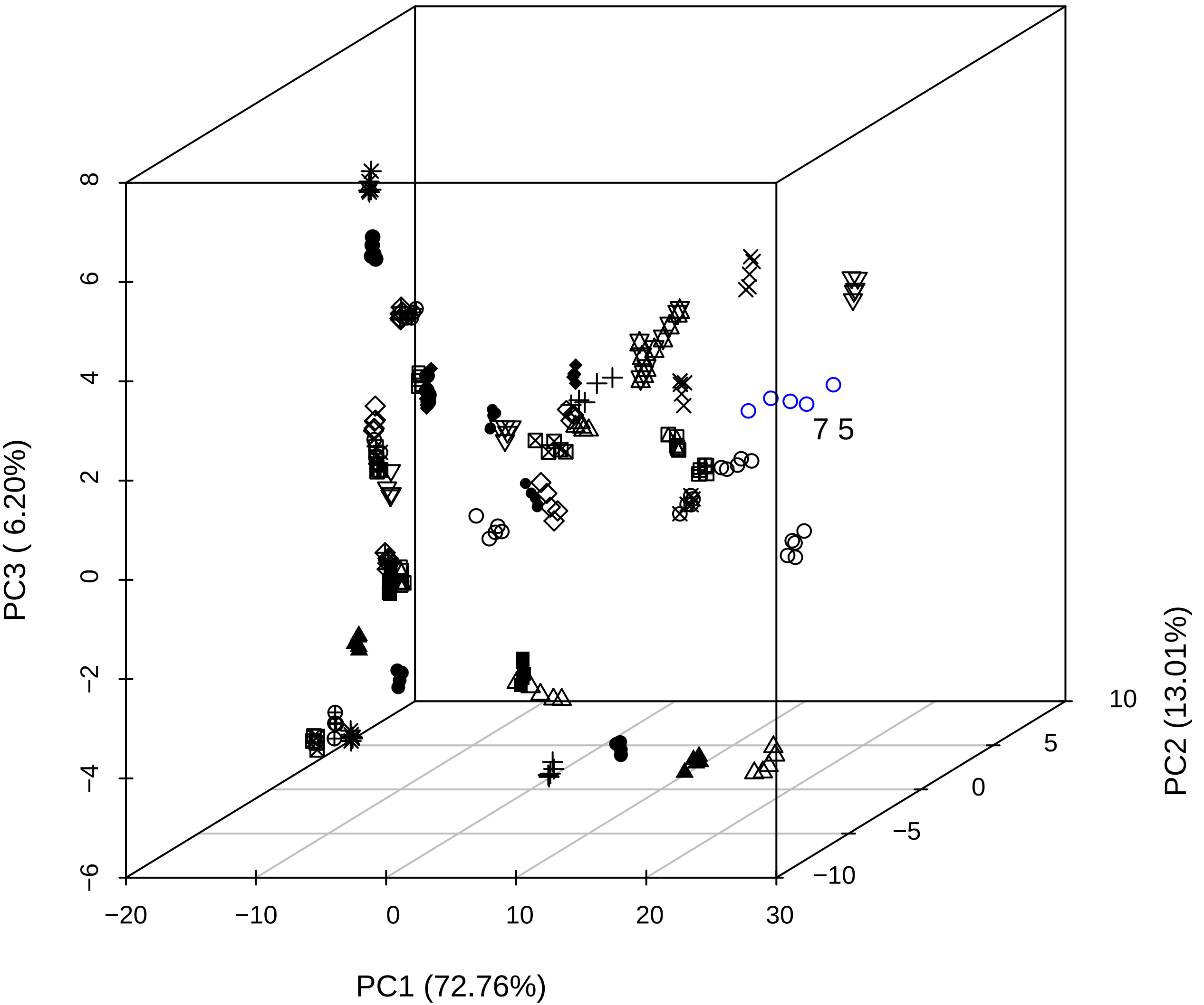

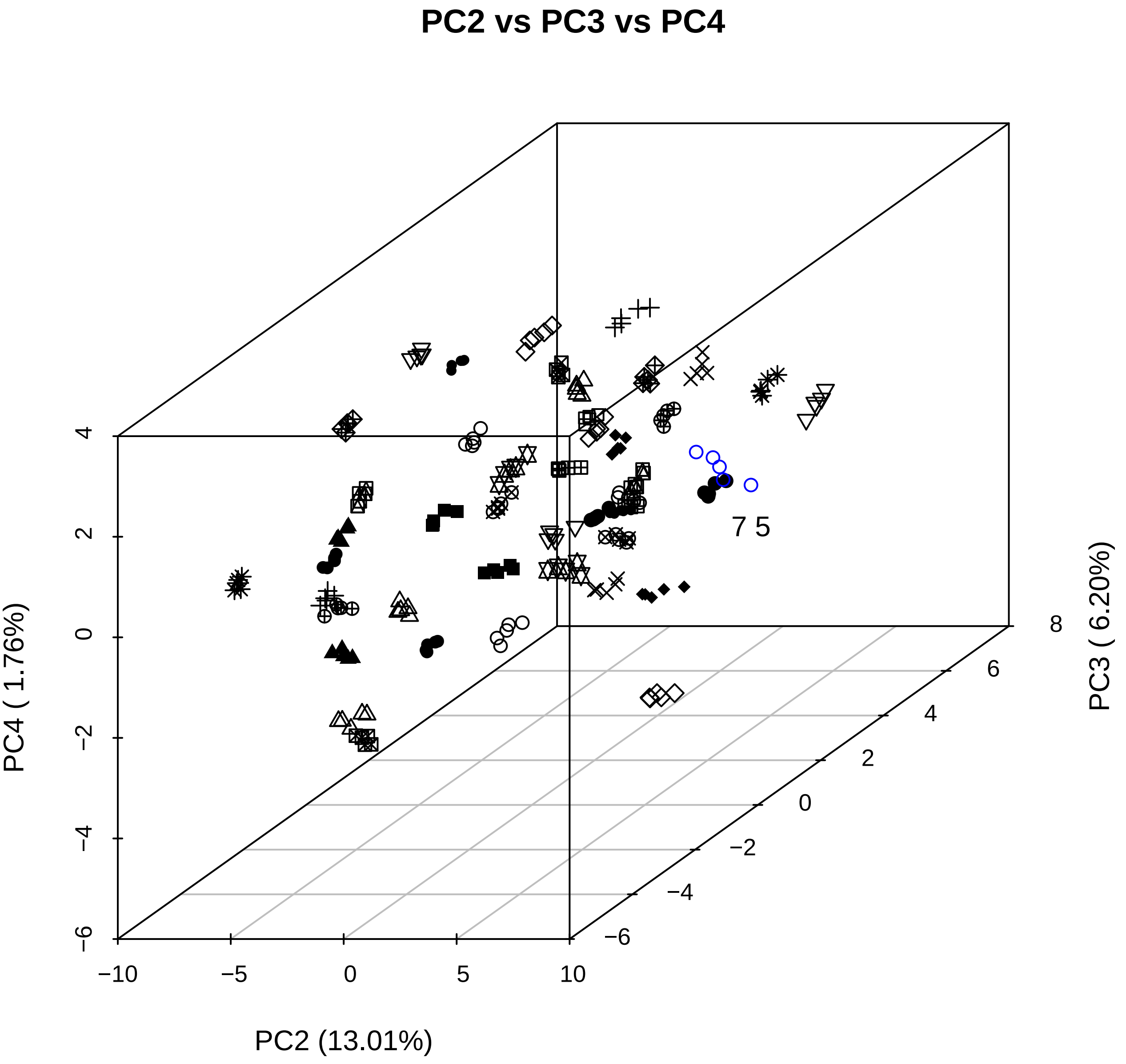

PC2 vs PC3 vs PC4
PC4 (1.76%)
PC3 (6.20%)
PC2 (13.01%)
7 5
−10
−5
0
5
10
−6
−4
−2
0
2
4
6
8

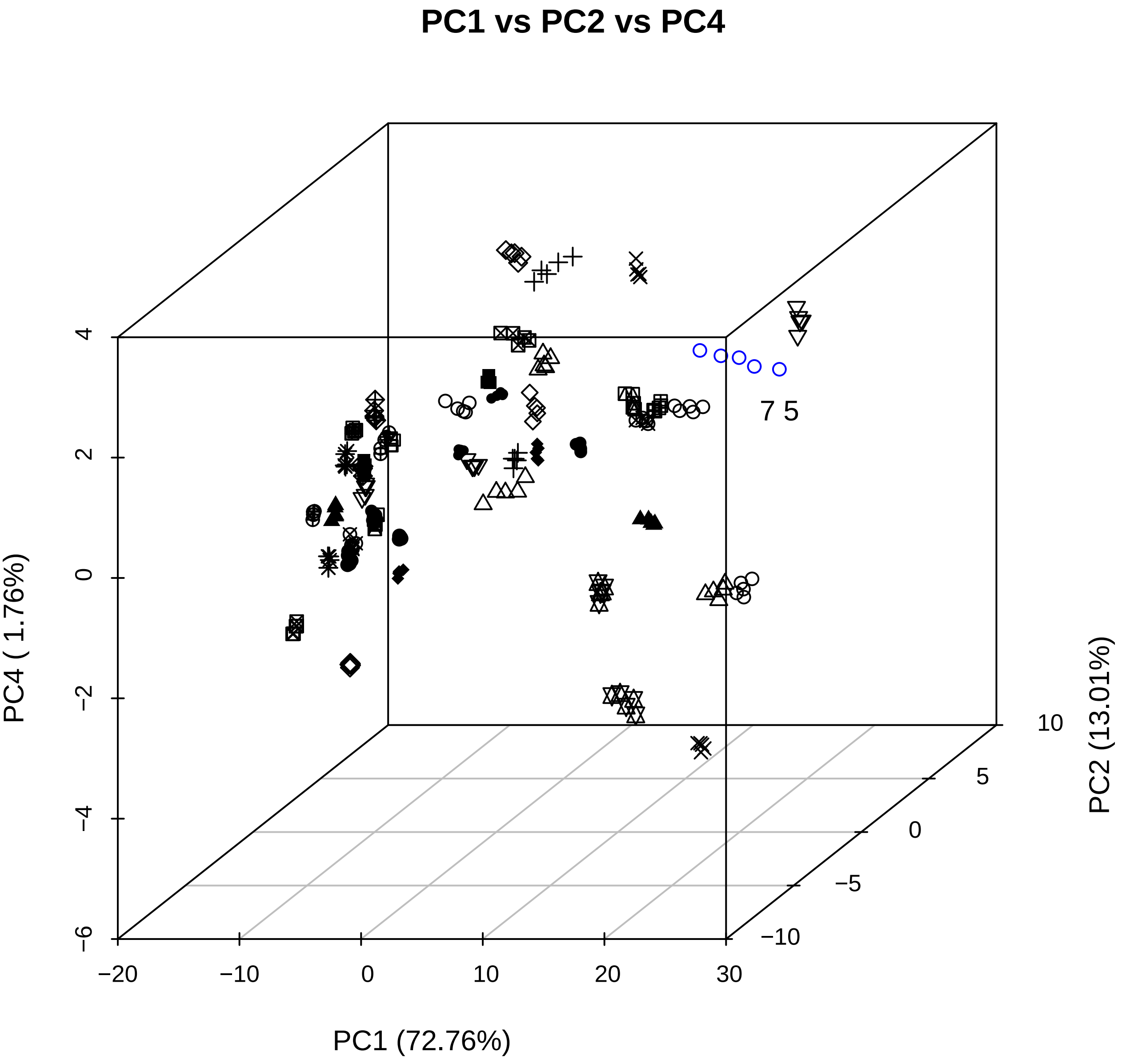

PC1 vs PC2 vs PC4
7 5
PC4 (1.76%)
4
2
0
−2
−4
−6
−20
−10
0
10
20
30
PC1 (72.76%)
10
5
0
−5
−10
PC2 (13.01%)

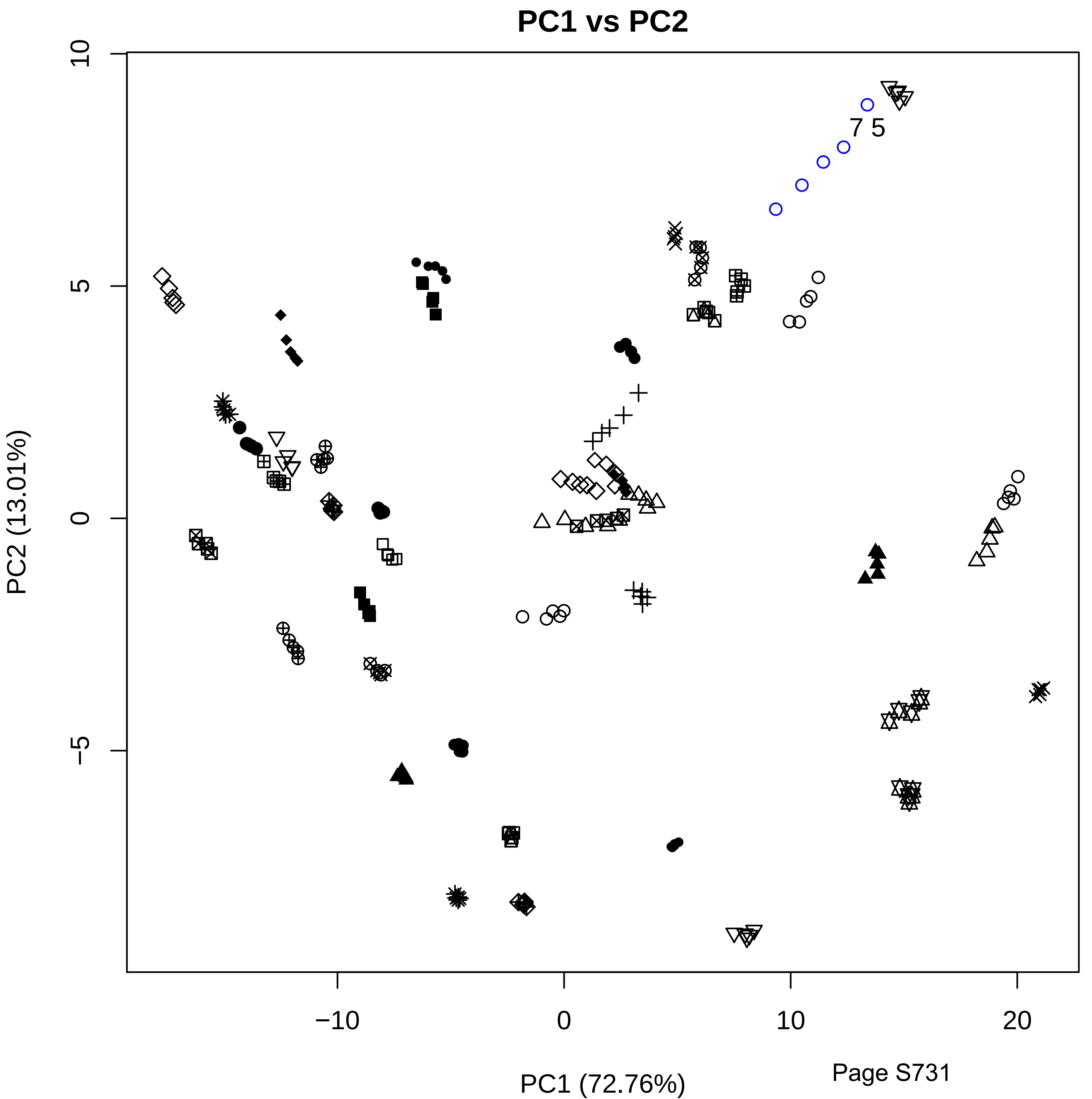
PC1 vs PC2
PC2 (13.01%)
PC1 (72.76%)
10
5
0
−5
−10
0
10
20
7 5

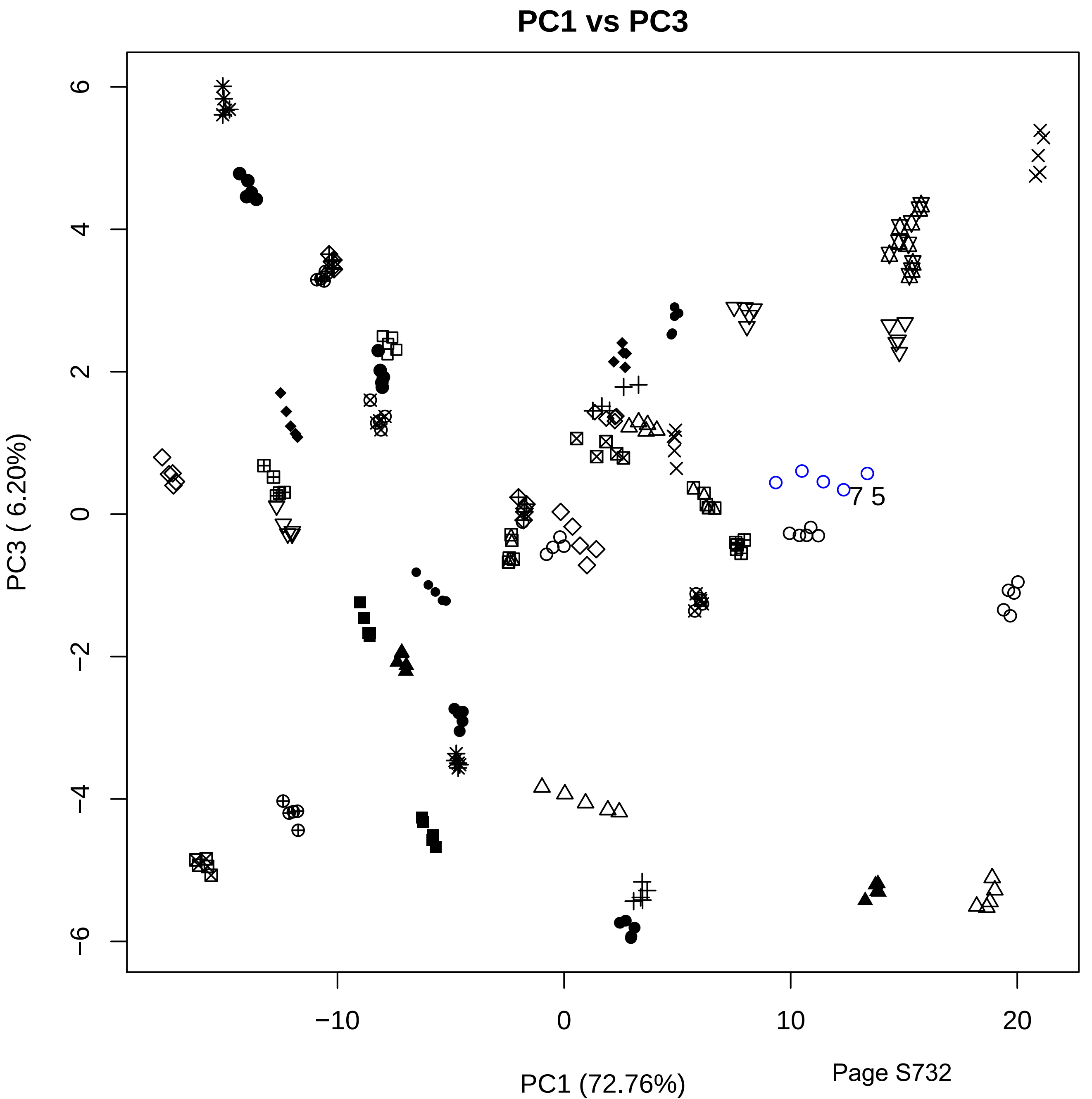
PC1 vs PC3
PC3 (6.20%)
PC1 (72.76%)
6
4
2
0
−2
−4
−6
−10
0
10
20
7 5

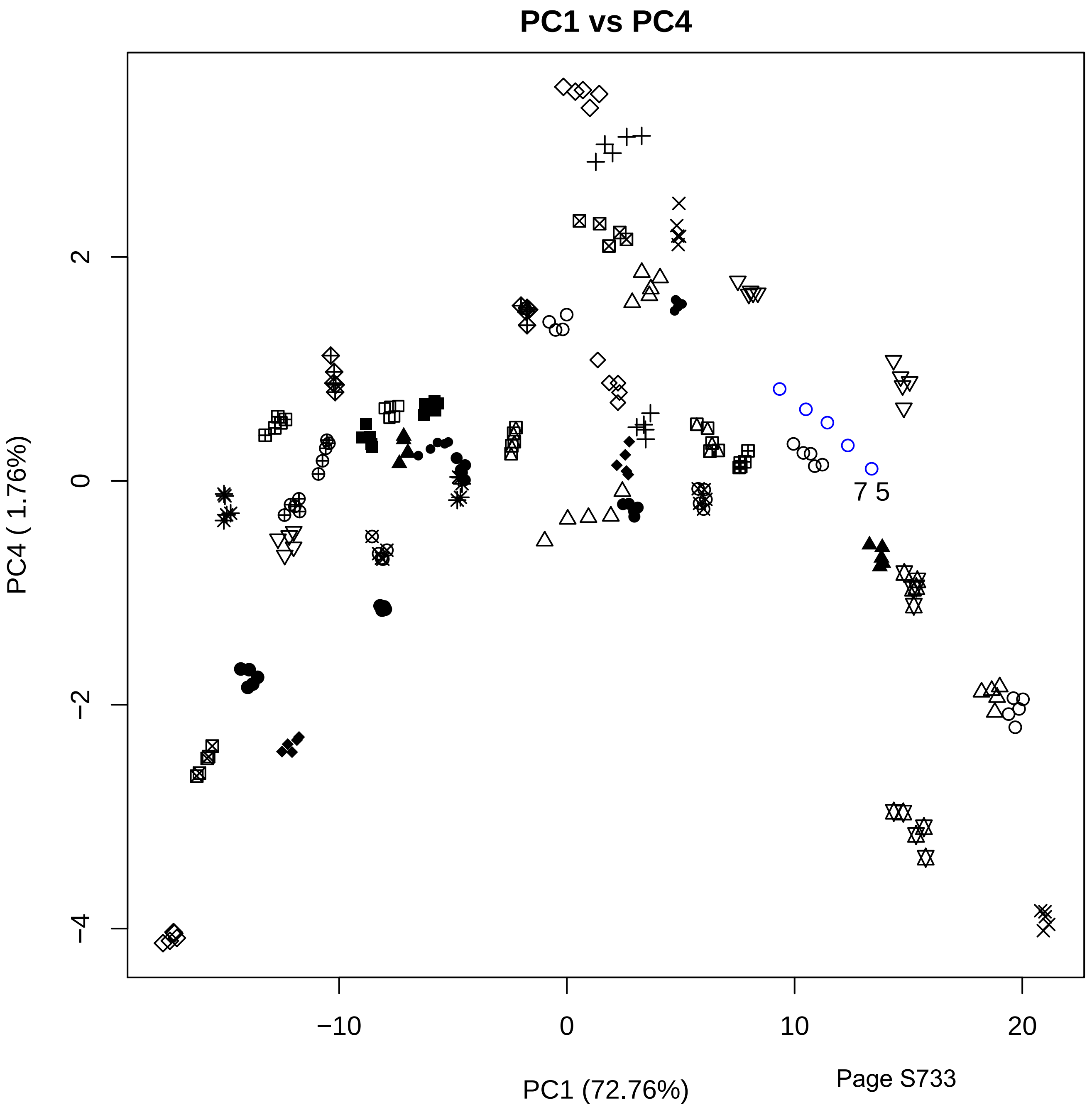
PC1 vs PC4
PC4 (1.76%)
PC1 (72.76%)
2
0
−2
−4
−10
0
10
20
7 5

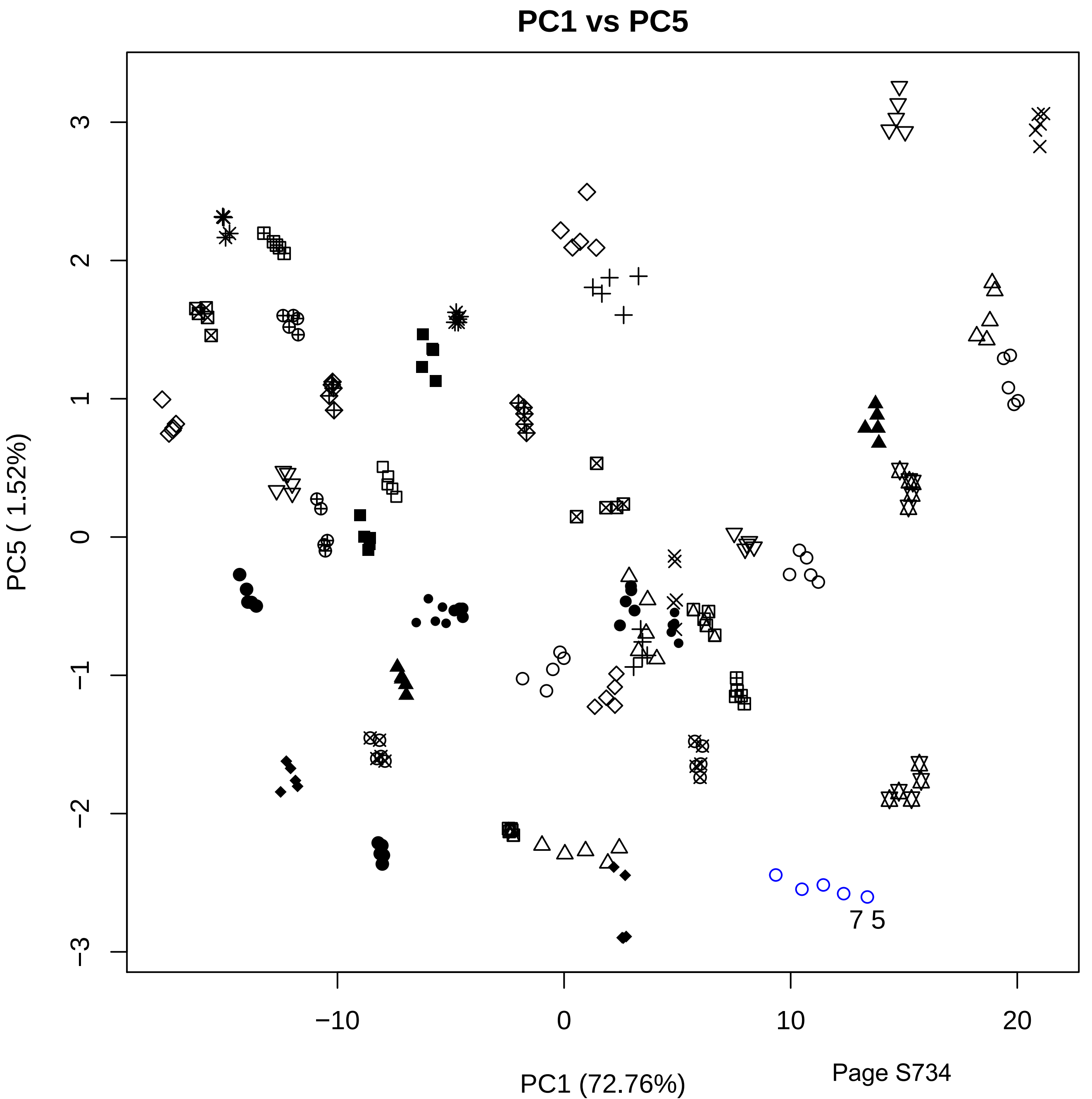

PC1 vs PC5
PC5 (1.52%)
PC1 (72.76%)
3
2
1
0
−1
−2
−3
−10
0
10
20
7 5

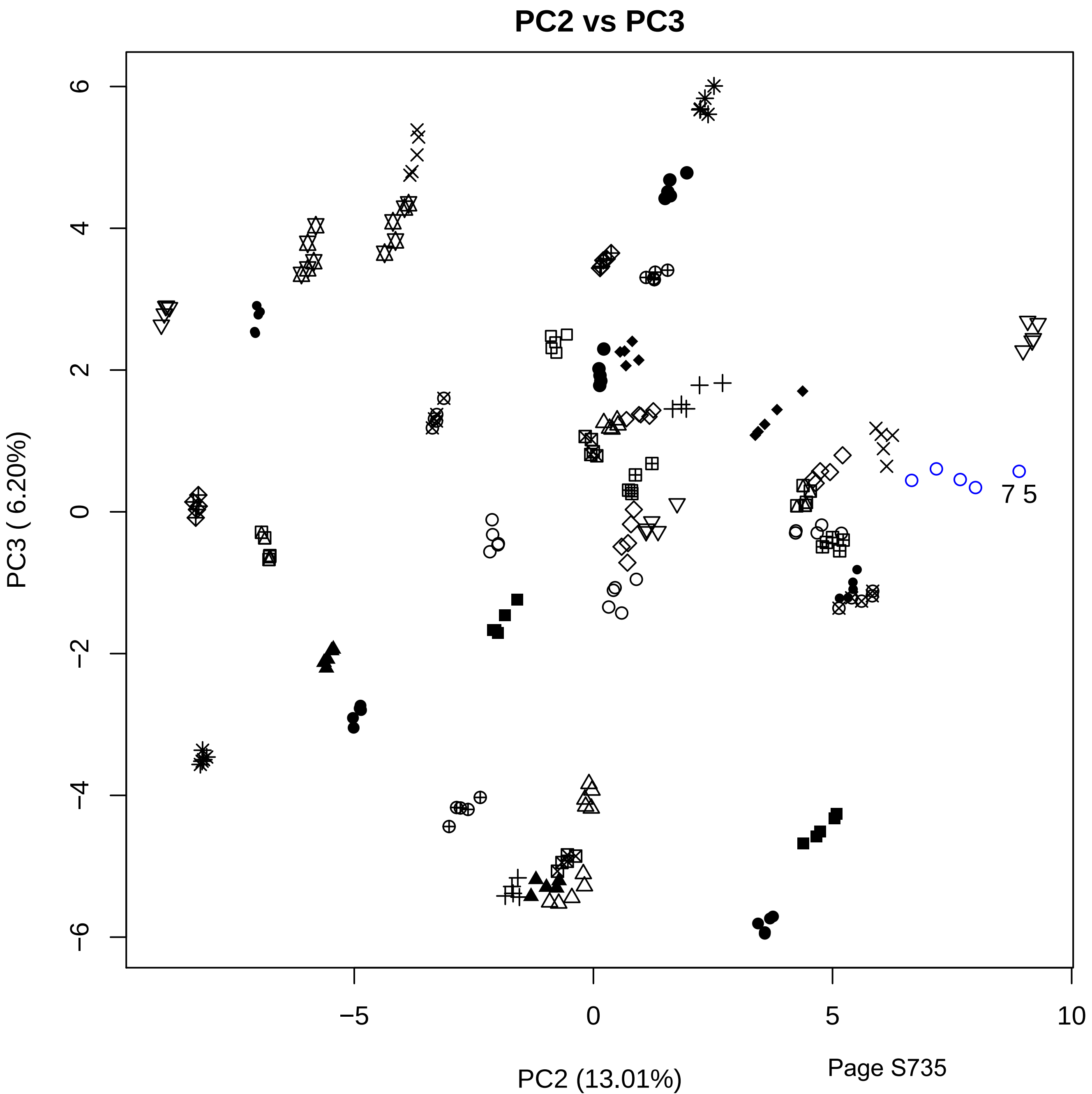

PC2 vs PC3
PC3 (6.20%)
PC2 (13.01%)
7 5
6
4
2
0
−2
−4
−6
−5
0
5
10

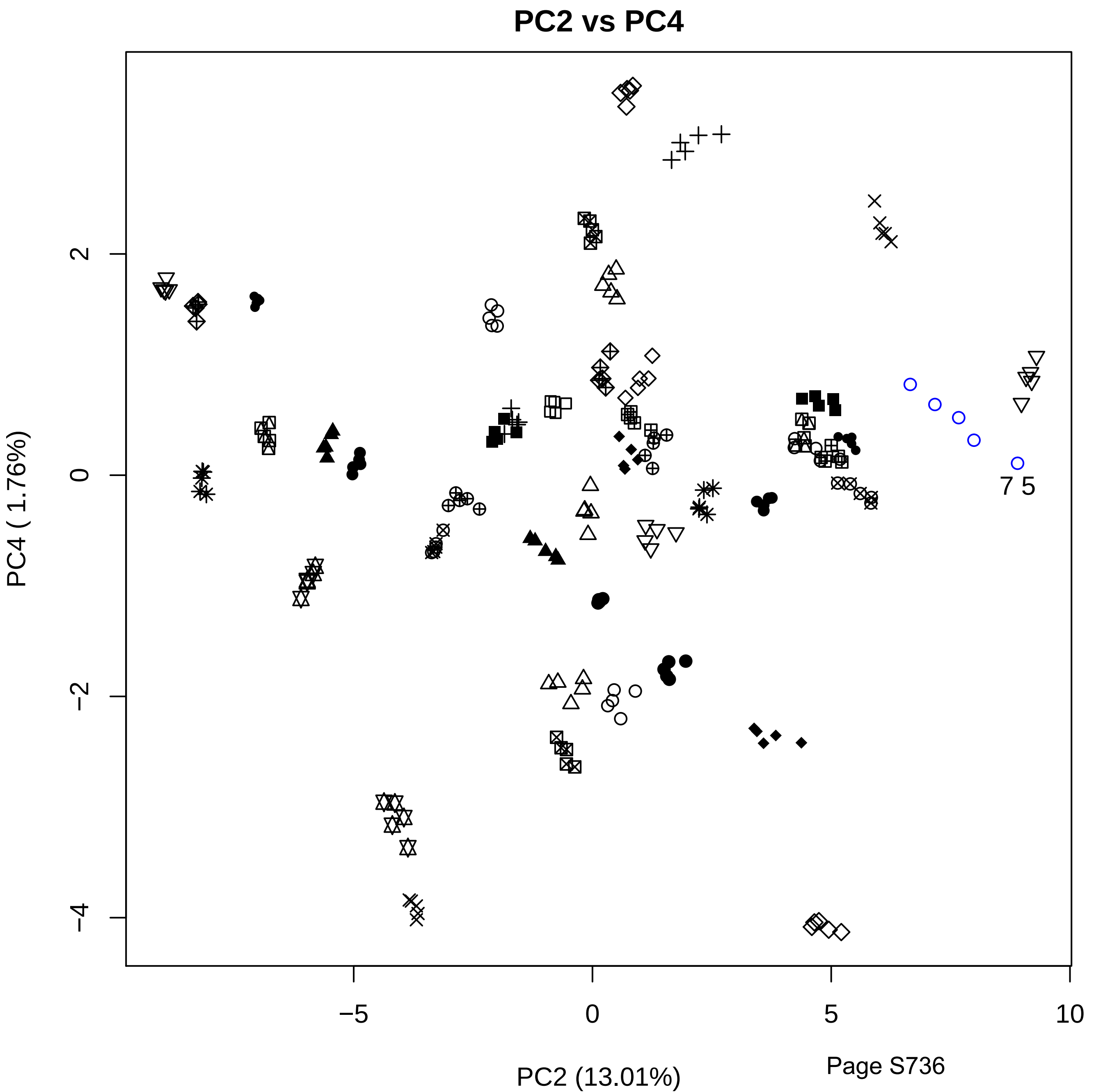
PC2 vs PC4
PC4 (1.76%)
PC2 (13.01%)
−5
0
5
10
2
0
−2
−4
7 5

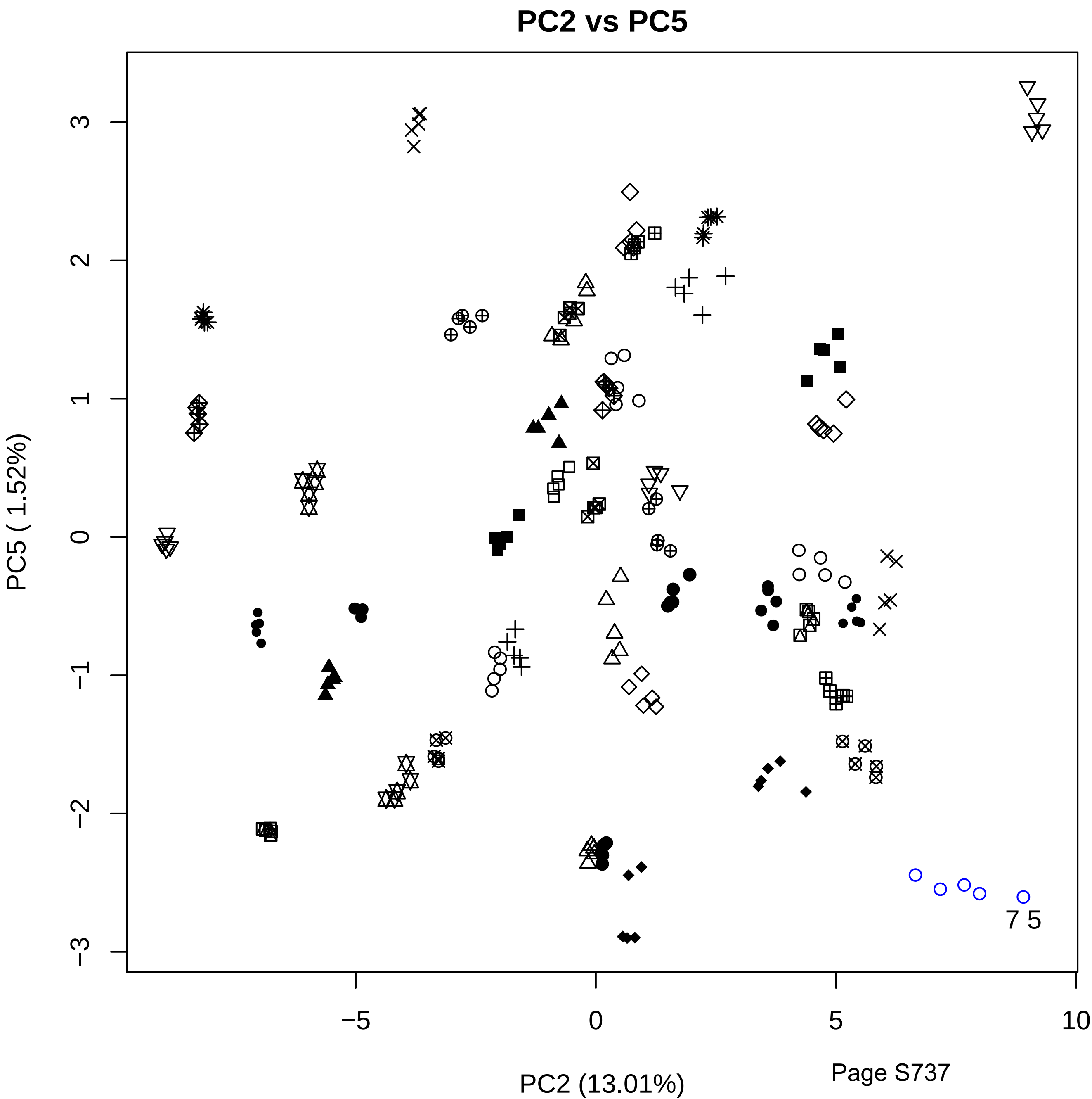
PC2 vs PC5
PC5 (1.52%)
PC2 (13.01%)
3
2
1
0
−1
−2
−3
−5
0
5
10
7 5

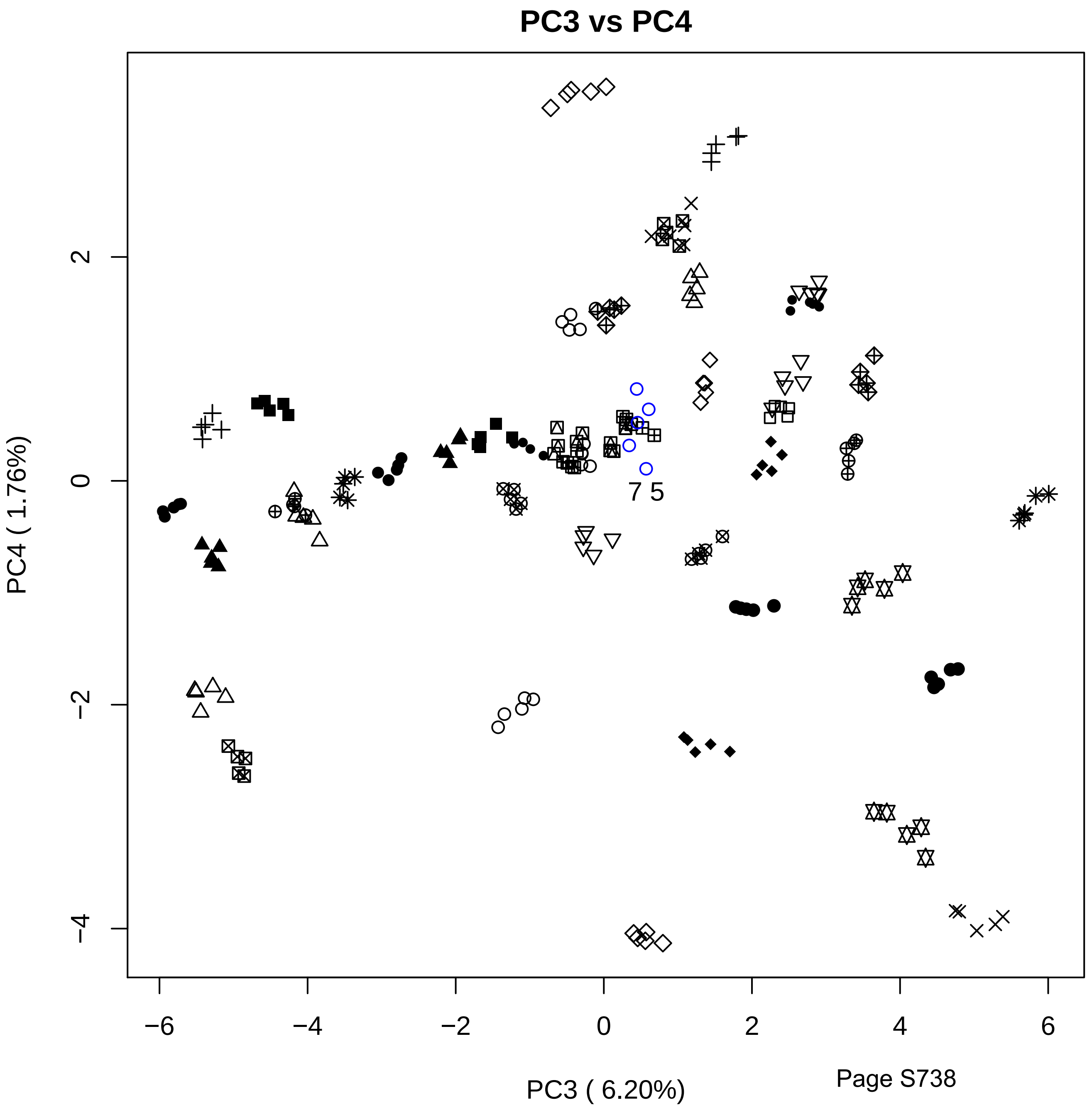
PC3 vs PC4
PC4 (1.76%)
PC3 (6.20%)
2
0
−2
−4
−6
−4
−2
0
2
4
6
7 5

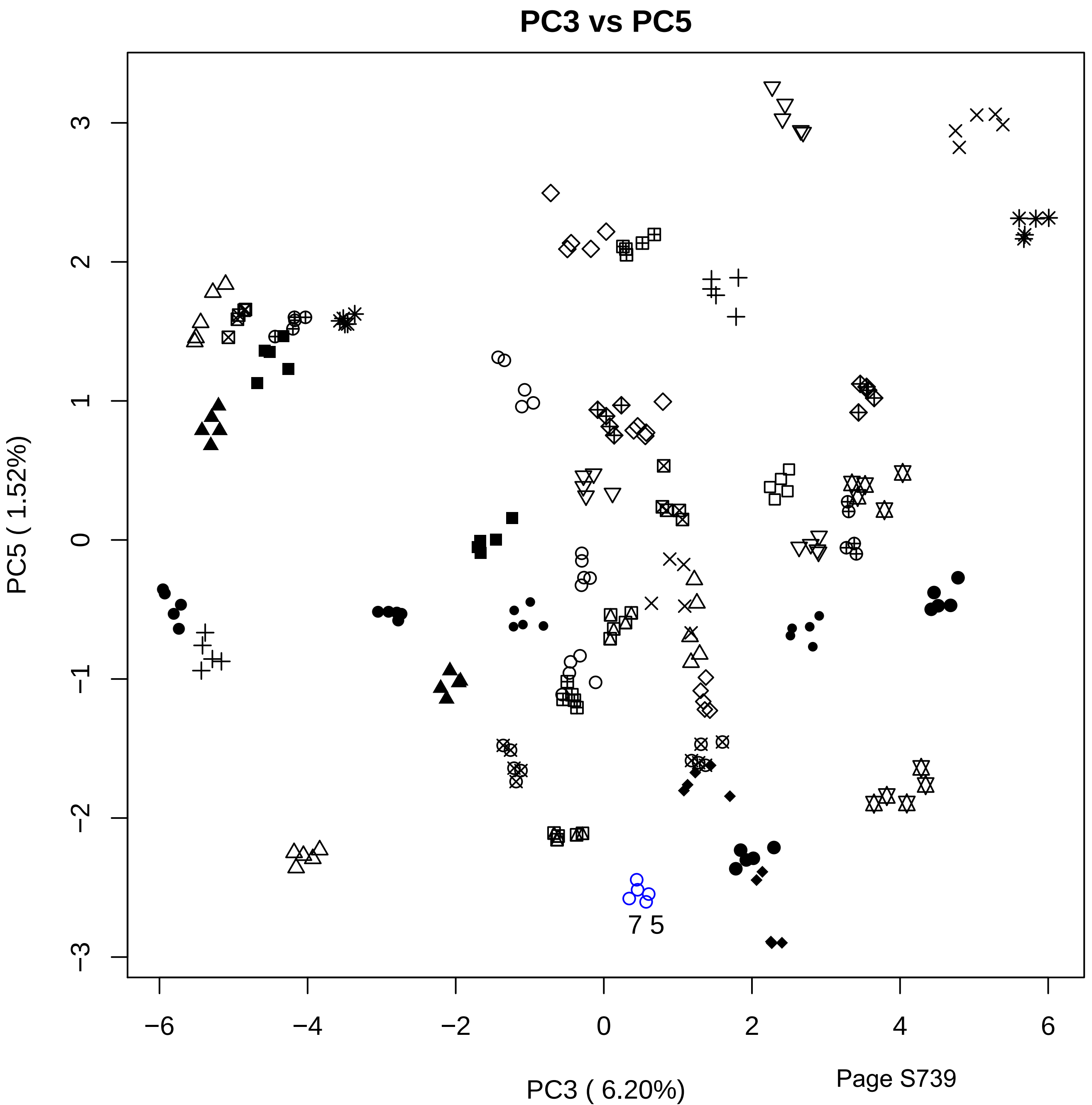
PC3 vs PC5
PC5 (1.52%)
PC3 (6.20%)
−6
−4
−2
0
2
4
6
−3
−2
−1
0
1
2
3
7 5

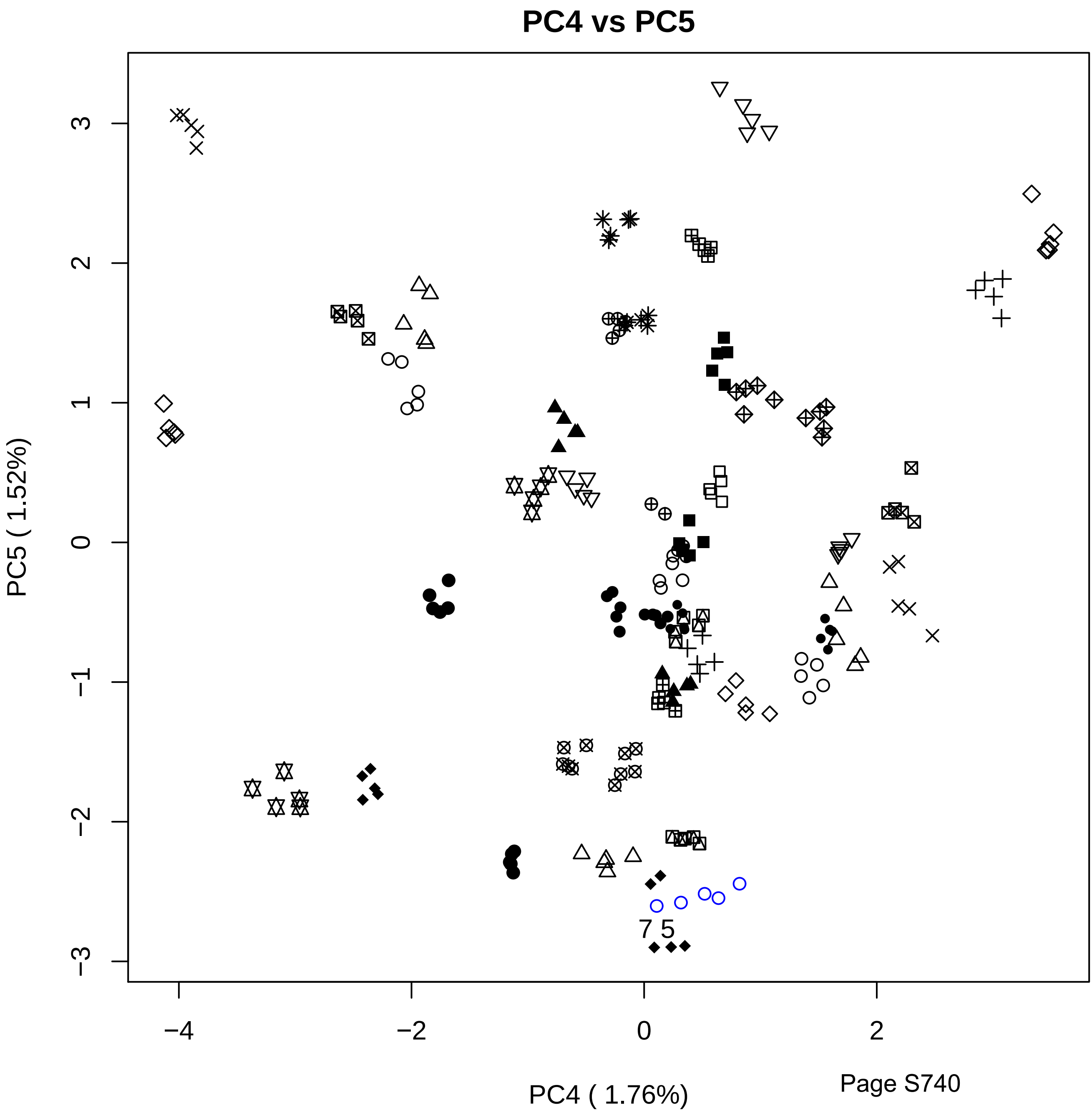
PC4 vs PC5
PC5 (1.52%)
PC4 (1.76%)
3
2
1
0
−1
−2
−3
−4
−2
0
2
7 5

PCA of 10% DeS vs PMH

Scaled Matrix PCAed

7th order polynomial baseline correction performed

Raw spectra smoothed to 21 Neighbours, 2nd order PN, Savtisky Golay

Normalisation between 0 and 100

D2 spectra smoothed to 41 Neighbours, 2nd order PN, Savtisky Golay

Regions Analysed: 3601−2501 and 2001−700cm−1

Samples Analysed: 1 Contam's 0 DeS's 45 PMH's

Keele University AD　　　Sun Dec 09 17:04:22 2018

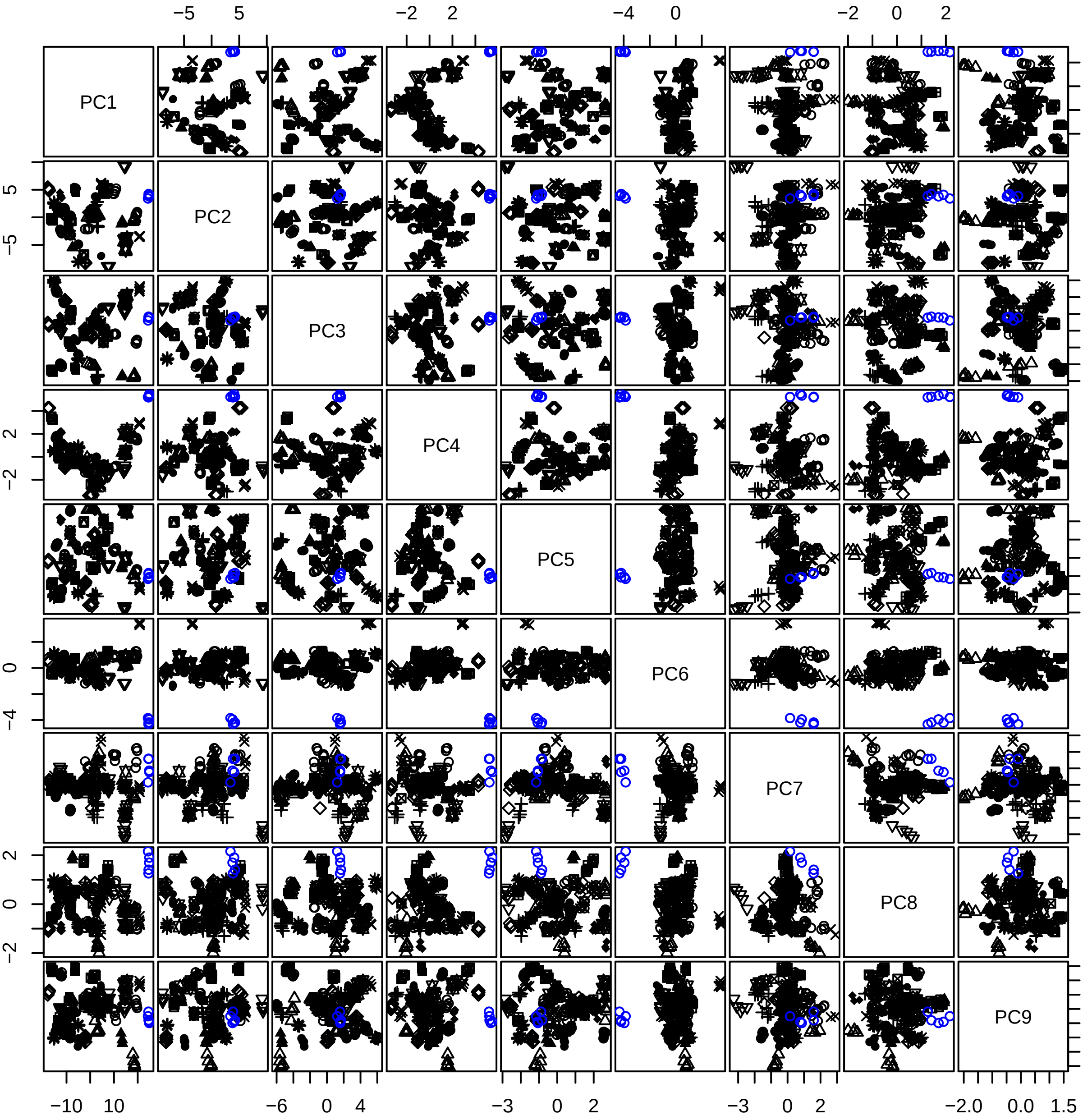
PC1
PC2
PC3
PC4
PC5
PC6
PC7
PC8
PC9
−5 5
−2 2
−4 0
−2 0 2
10
−10
5
−5
4
0
−6
2
−2
2
0
−3
0
−4
2
0
−3
2
0
−2
1.5
0.0
−2.0
−10 10
−6 0 4
−3 0 2
−3 0 2
−2.0 0.0 1.5

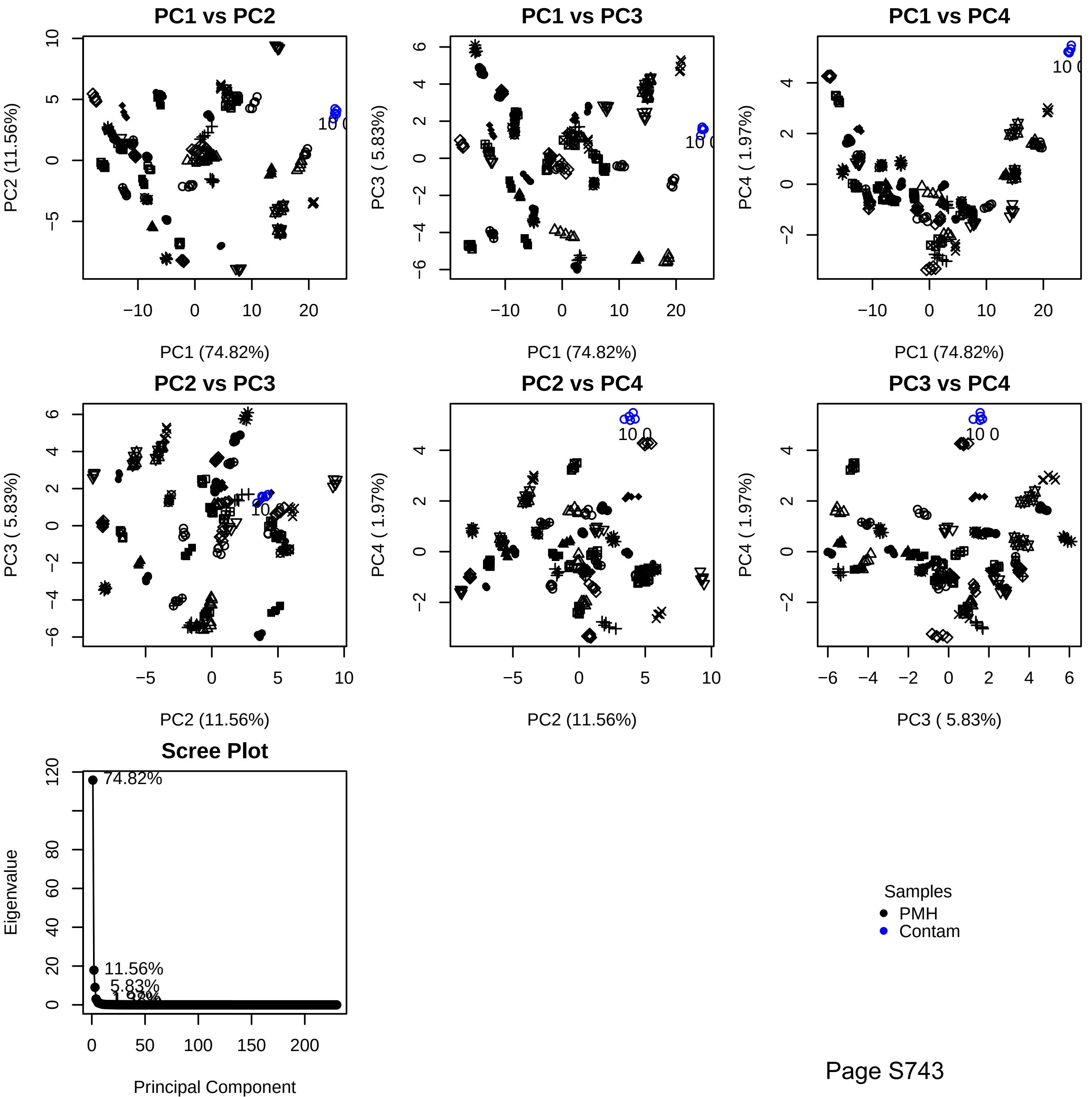
PC1 vs PC2
PC2 (11.56%)
PC1 (74.82%)
PC1 vs PC3
PC3 (5.83%)
PC1 (74.82%)
PC1 vs PC4
PC4 (1.97%)
PC1 (74.82%)
PC2 vs PC3
PC3 (5.83%)
PC2 (11.56%)
PC2 vs PC4
PC4 (1.97%)
PC2 (11.56%)
PC3 vs PC4
PC4 (1.97%)
PC3 (5.83%)
Scree Plot
Eigenvalue
Principal Component
74.82%
11.56%
5.83%
Samples
PMH
Contam

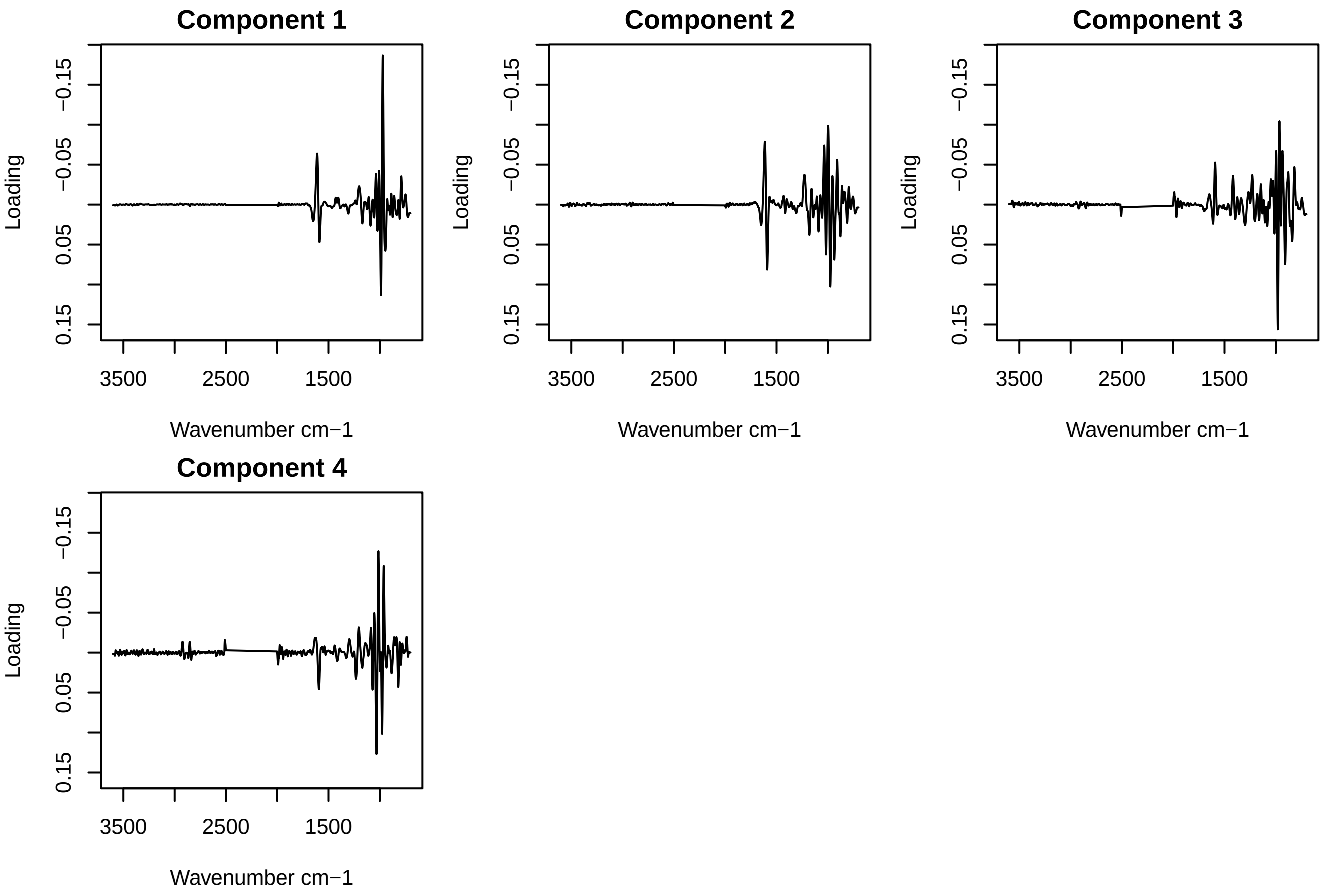
Component 1
Component 2
Component 3
Component 4
Loading
−0.15
−0.05
0.05
0.15
3500
2500
1500
Wavenumber cm−1

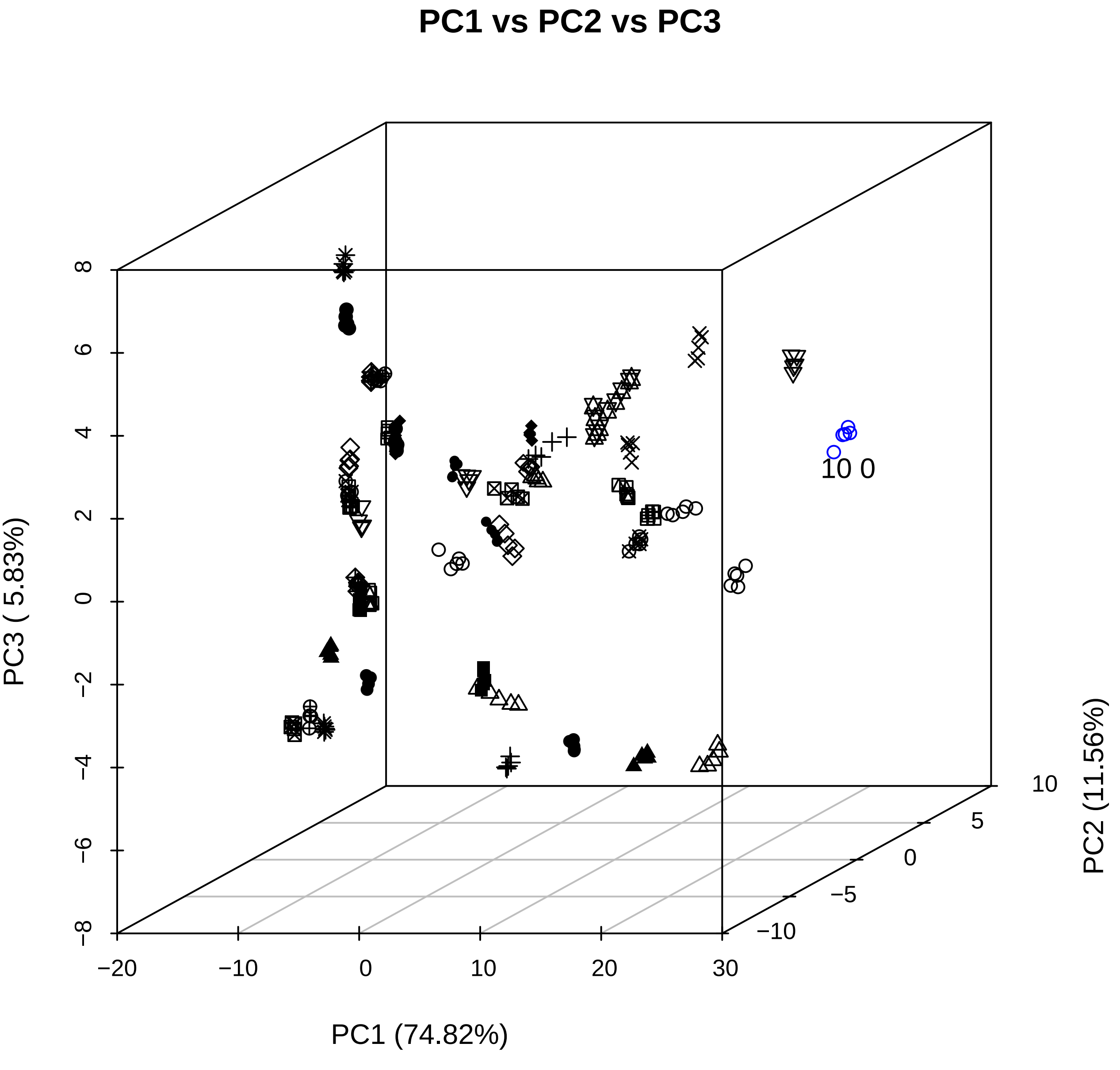

PC1 vs PC2 vs PC3
PC3 (5.83%)
PC1 (74.82%)
PC2 (11.56%)
10 0
−20
−10
0
10
20
30
−8
−6
−4
−2
0
2
4
6
8
−10
−5
0
5
10

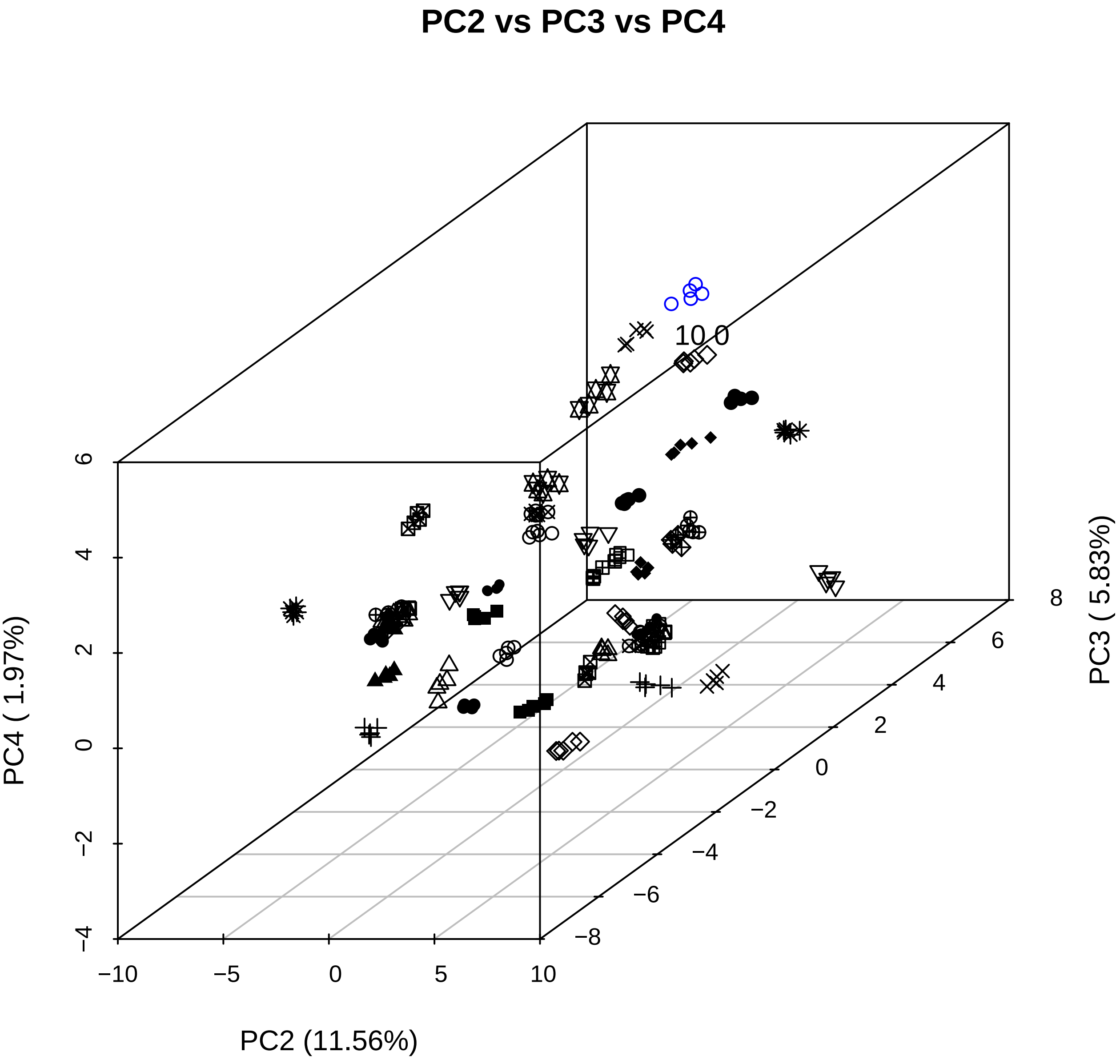
PC2 vs PC3 vs PC4
10.0
PC2 (11.56%)
PC3 (5.83%)
PC4 (1.97%)
−10
−5
0
5
10
−8
−6
−4
−2
0
2
4
6
8
−4
−2
0
2
4
6

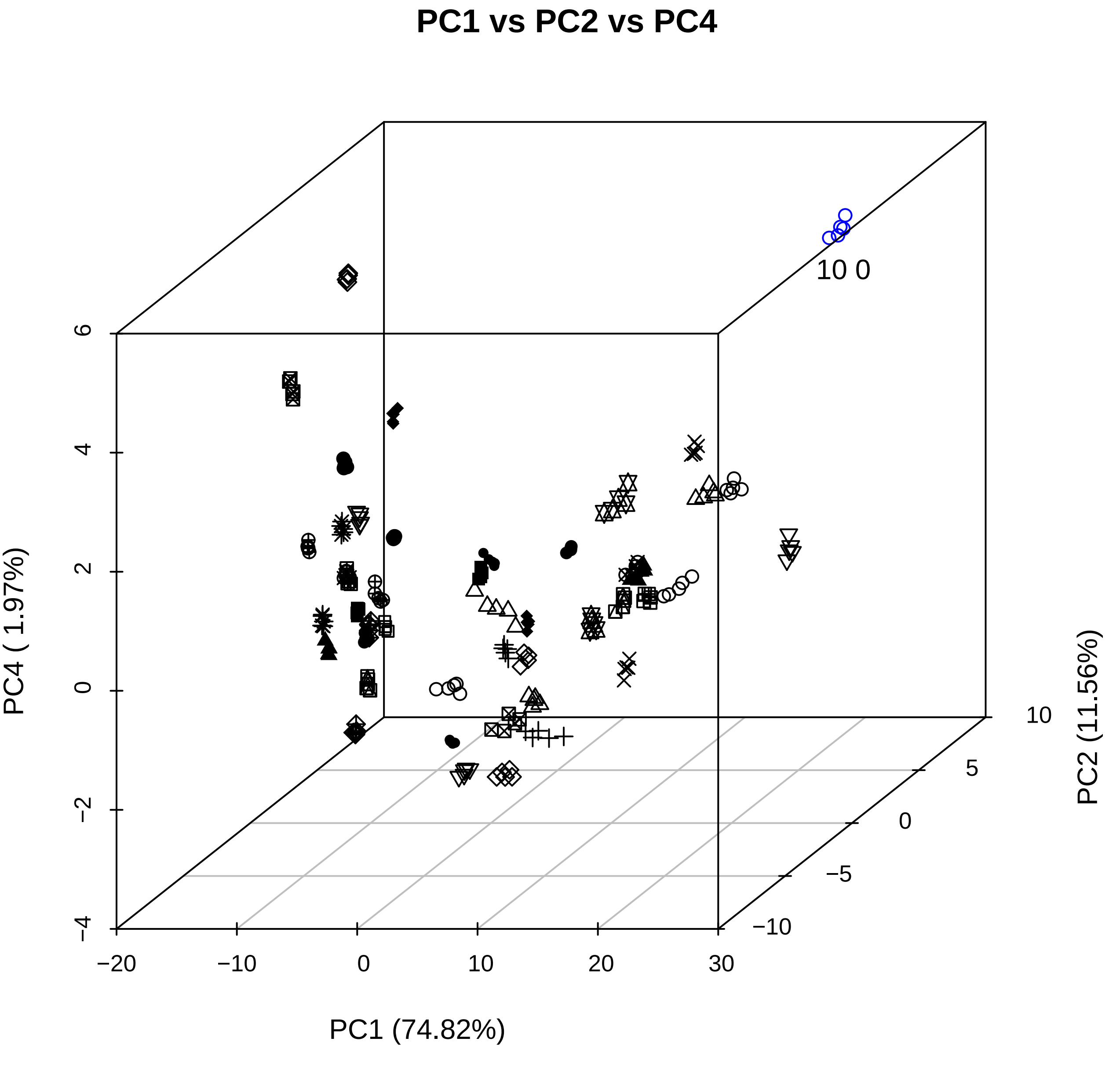

PC1 vs PC2 vs PC4
10 0
PC4 (1.97%)
−4
−2
0
2
4
6
PC1 (74.82%)
−20
−10
0
10
20
30
PC2 (11.56%)
−10
−5
0
5
10

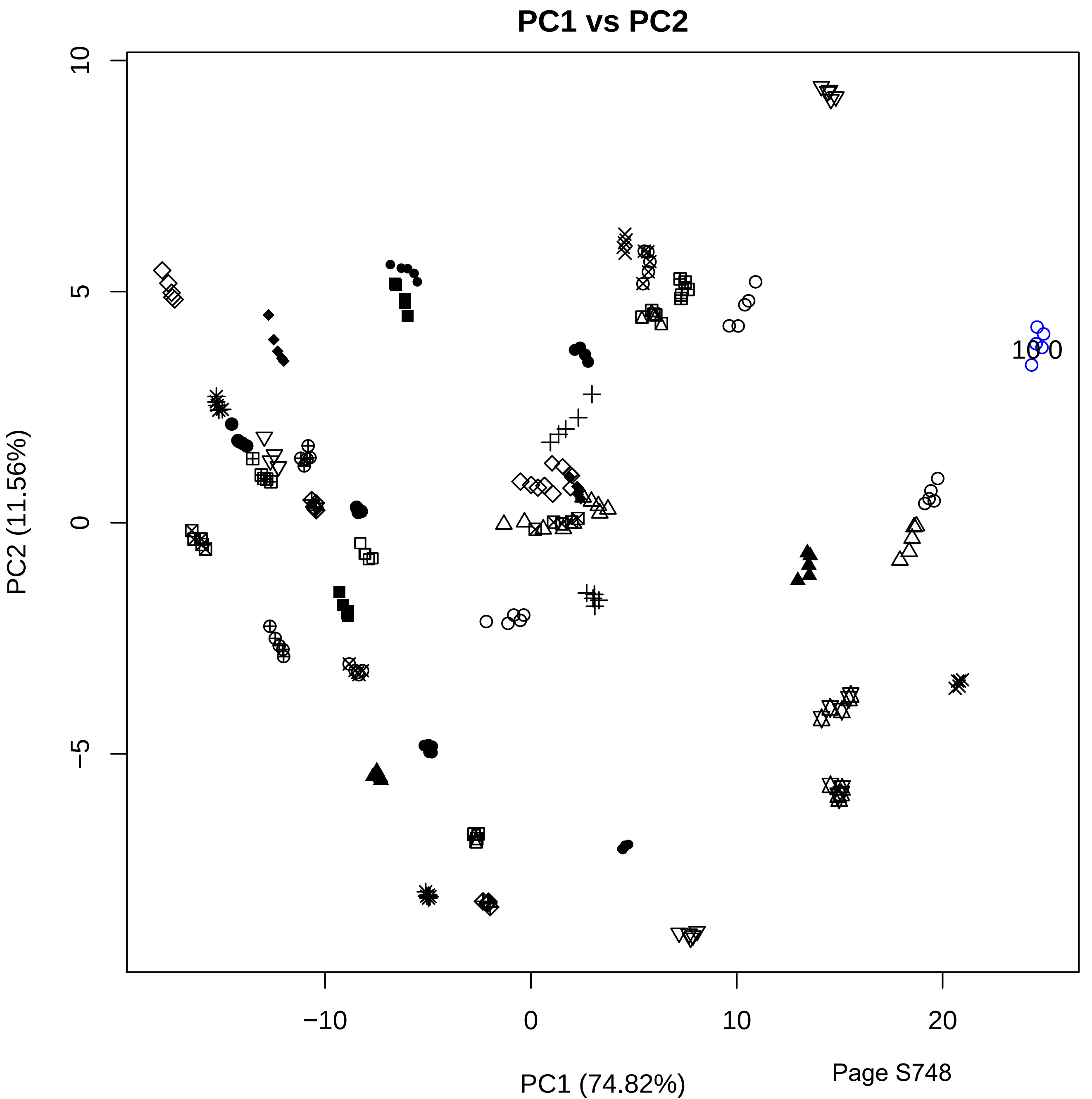
PC1 vs PC2
10
5
0
−5
PC2 (11.56%)
−10
0
10
20
PC1 (74.82%)
100

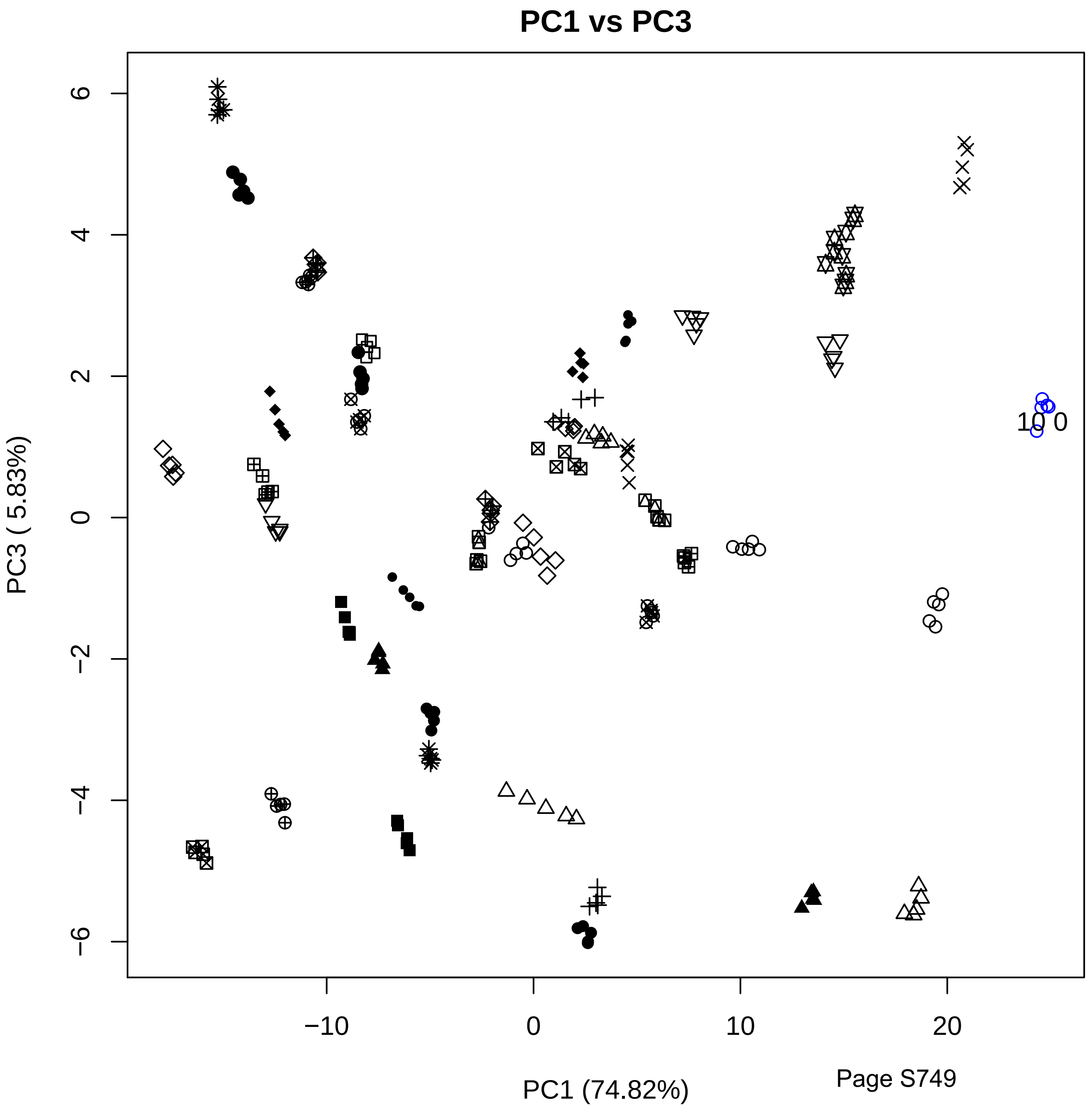
PC1 vs PC3
PC3 (5.83%)
PC1 (74.82%)
6
4
2
0
−2
−4
−6
−10
0
10
20
10 0

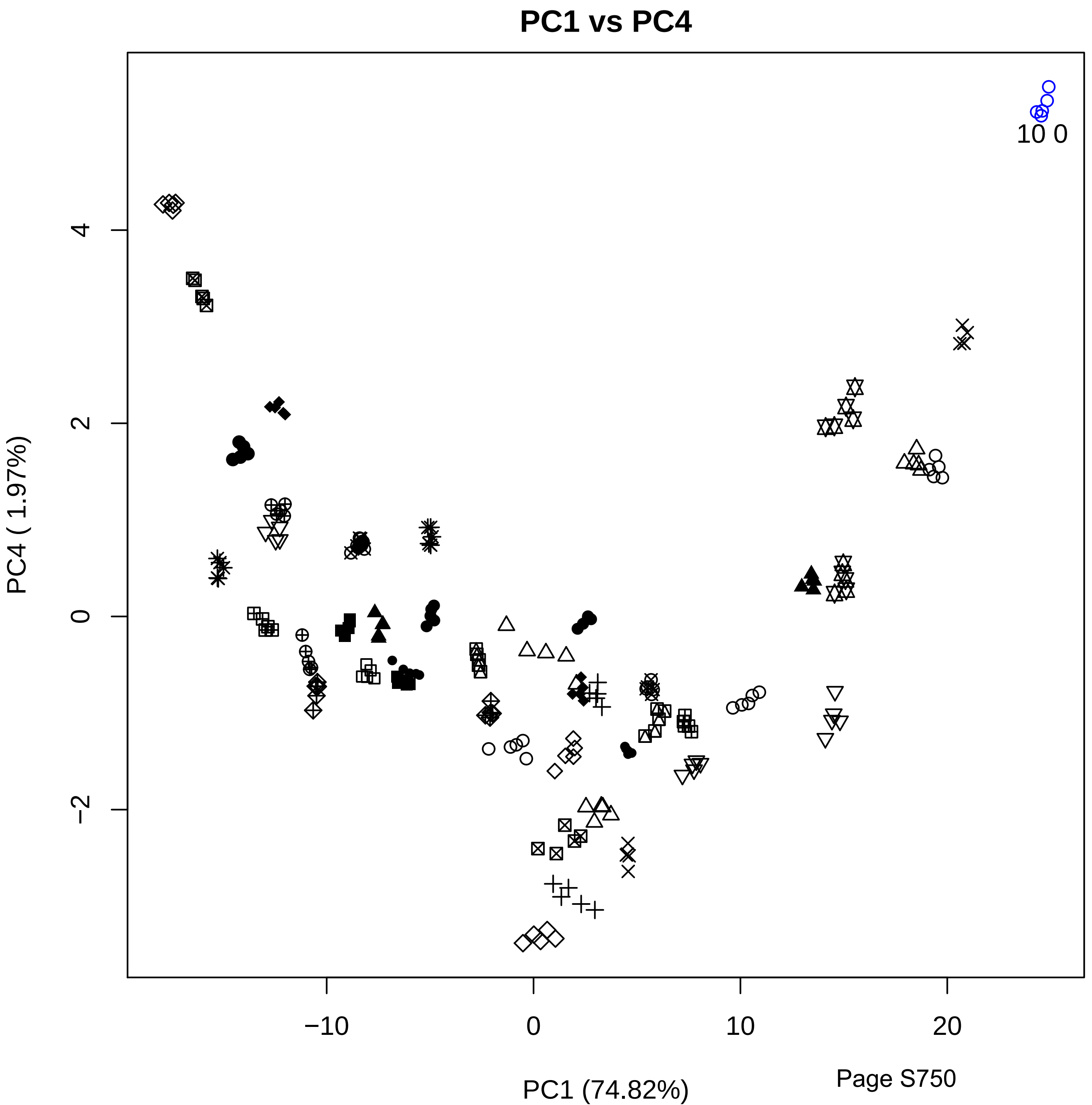
PC1 vs PC4
10 0
4
2
0
−2
PC4 (1.97%)
−10
0
10
20
PC1 (74.82%)

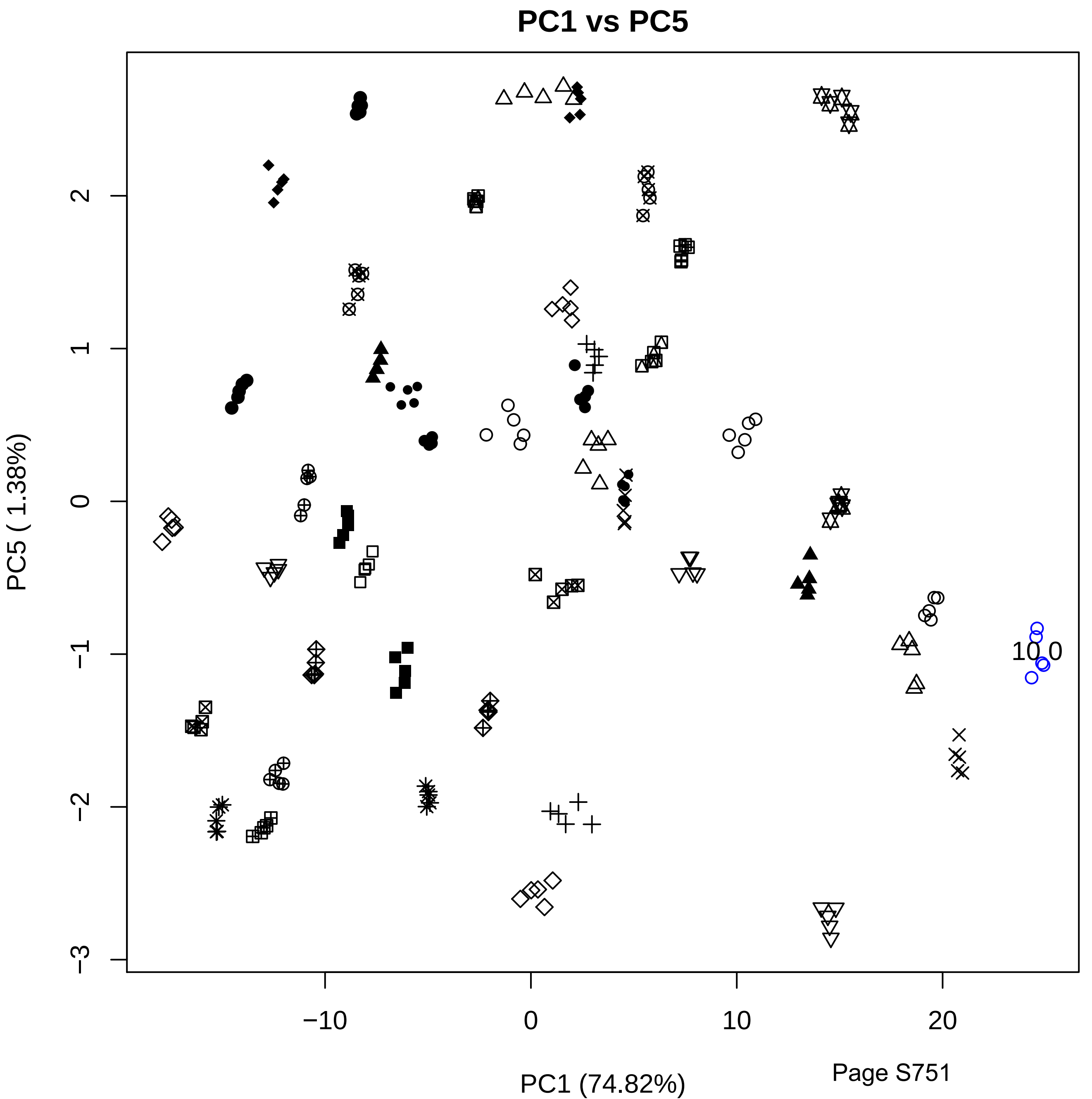
PC1 vs PC5
PC5 (1.38%)
PC1 (74.82%)
10.0
−10
0
10
20
−3
−2
−1
0
1
2

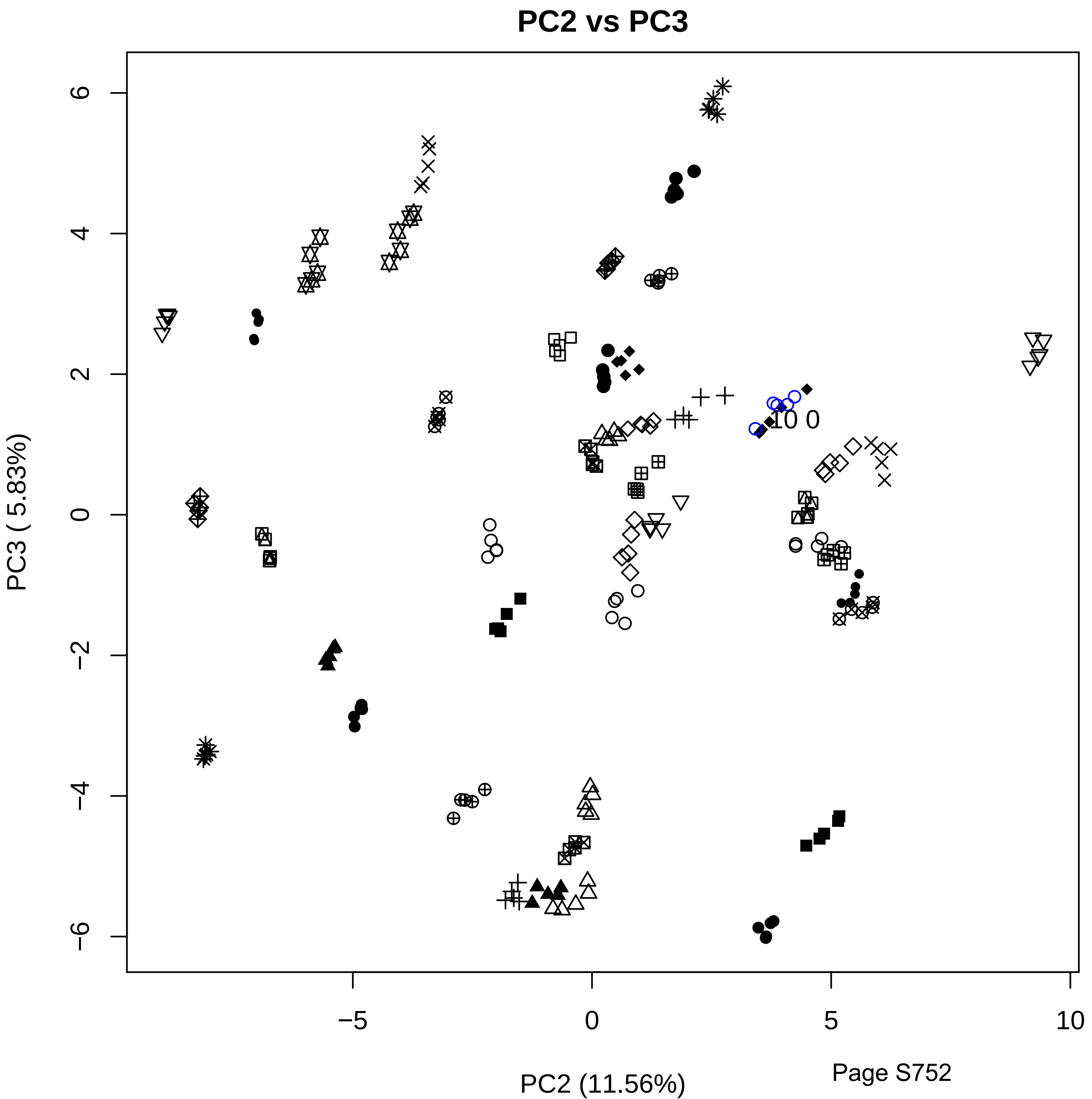
PC2 vs PC3
PC3 (5.83%)
PC2 (11.56%)
6
4
2
0
−2
−4
−6
−5
0
5
10
10 0

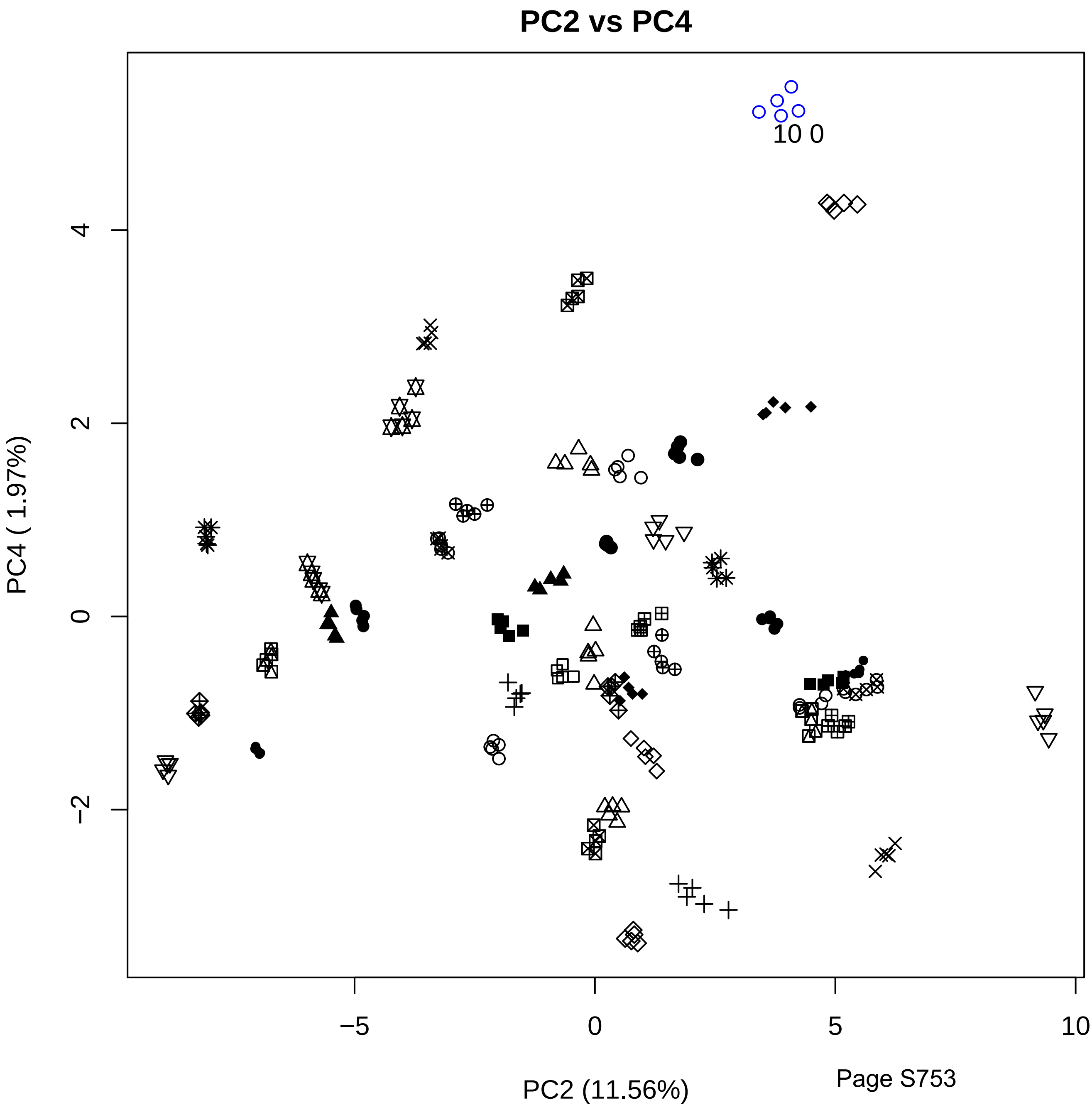

PC2 vs PC4
10 0
4
2
0
−2
PC4 (1.97%)
−5
0
5
10
PC2 (11.56%)

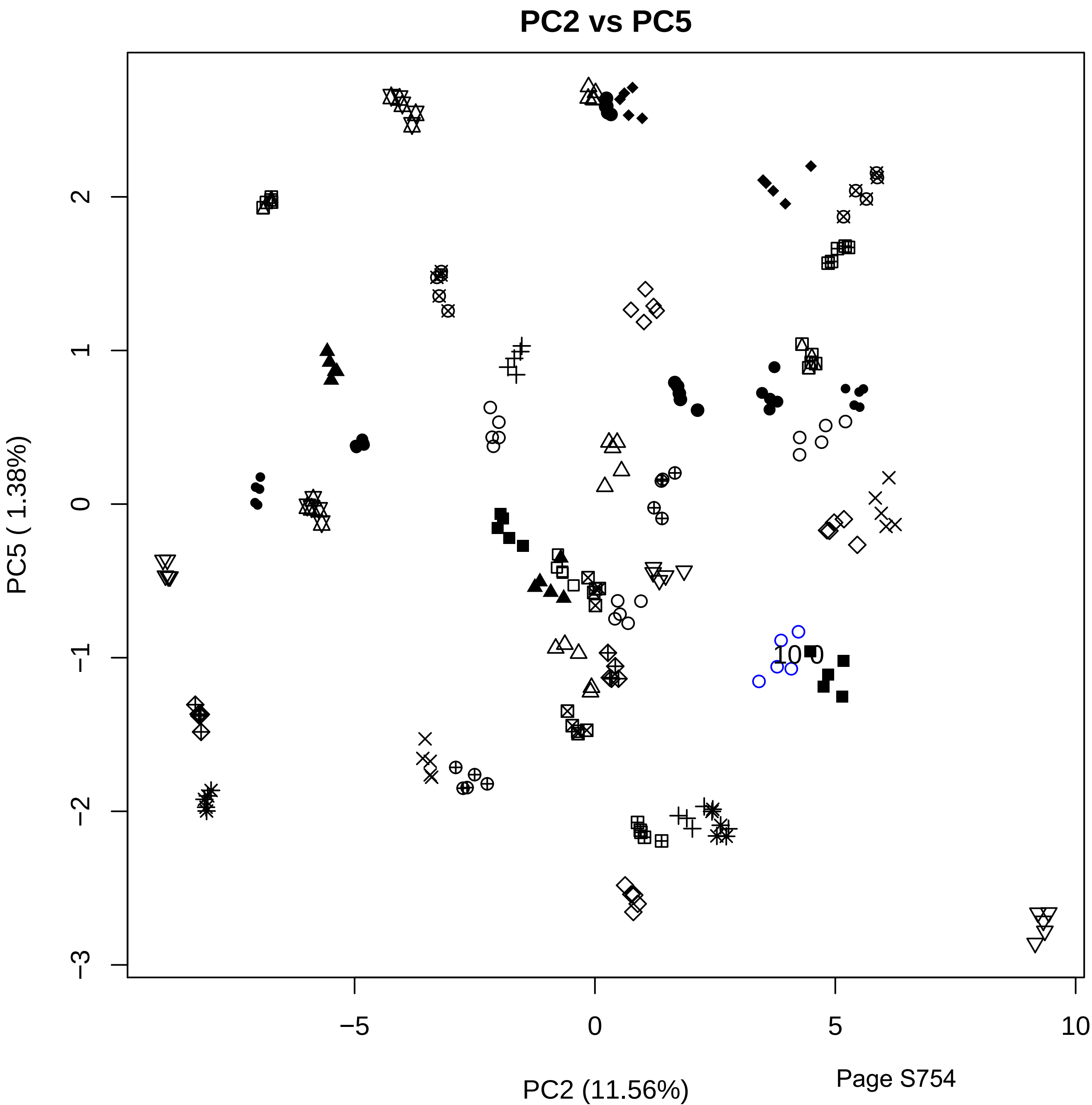
PC2 vs PC5
PC5 (1.38%)
PC2 (11.56%)
−3
−2
−1
0
1
2
−5
0
5
10
100

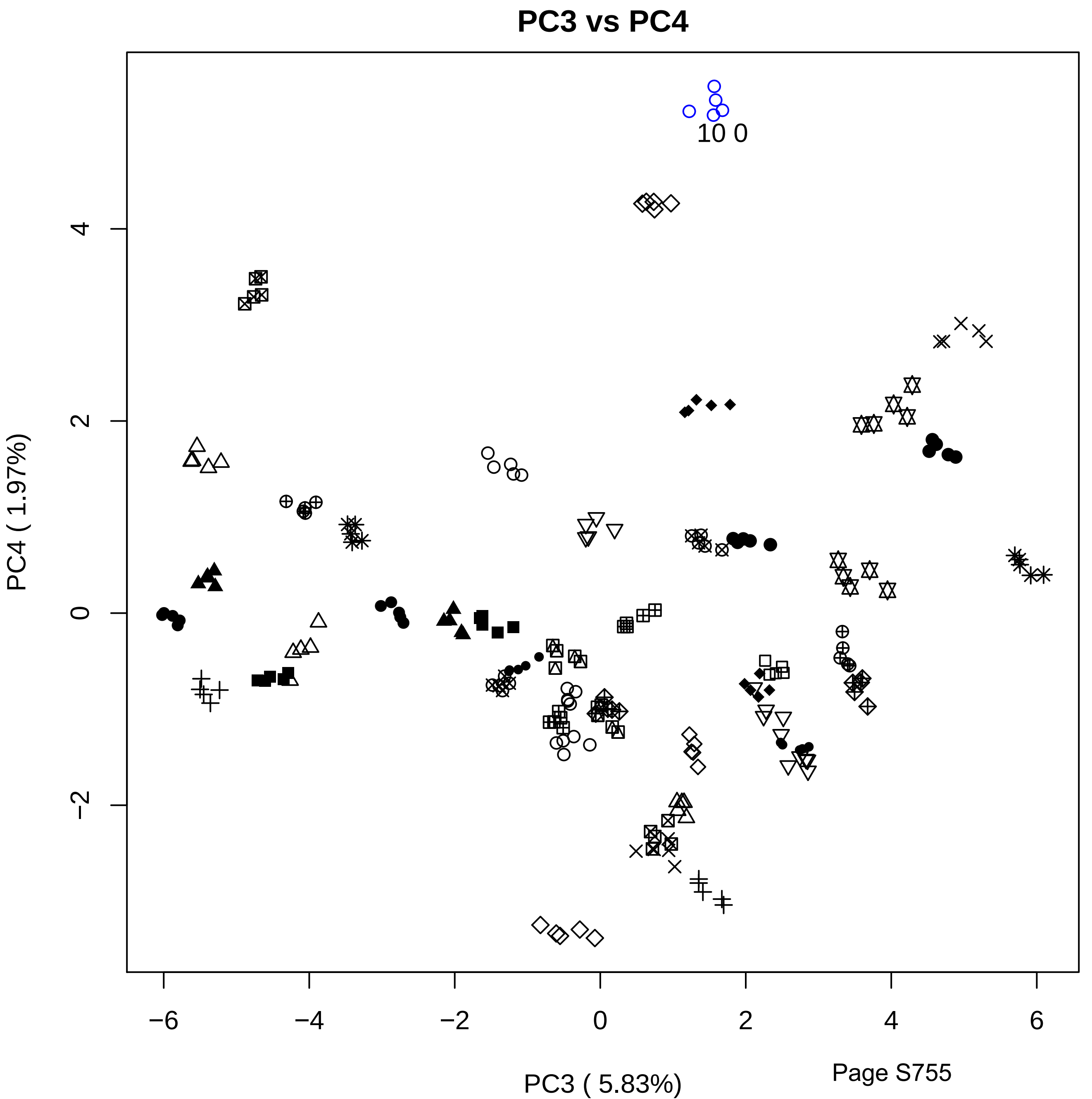
PC3 vs PC4
10 0
PC4 (1.97%)
PC3 (5.83%)
−6
−4
−2
0
2
4
6
−2
0
2
4

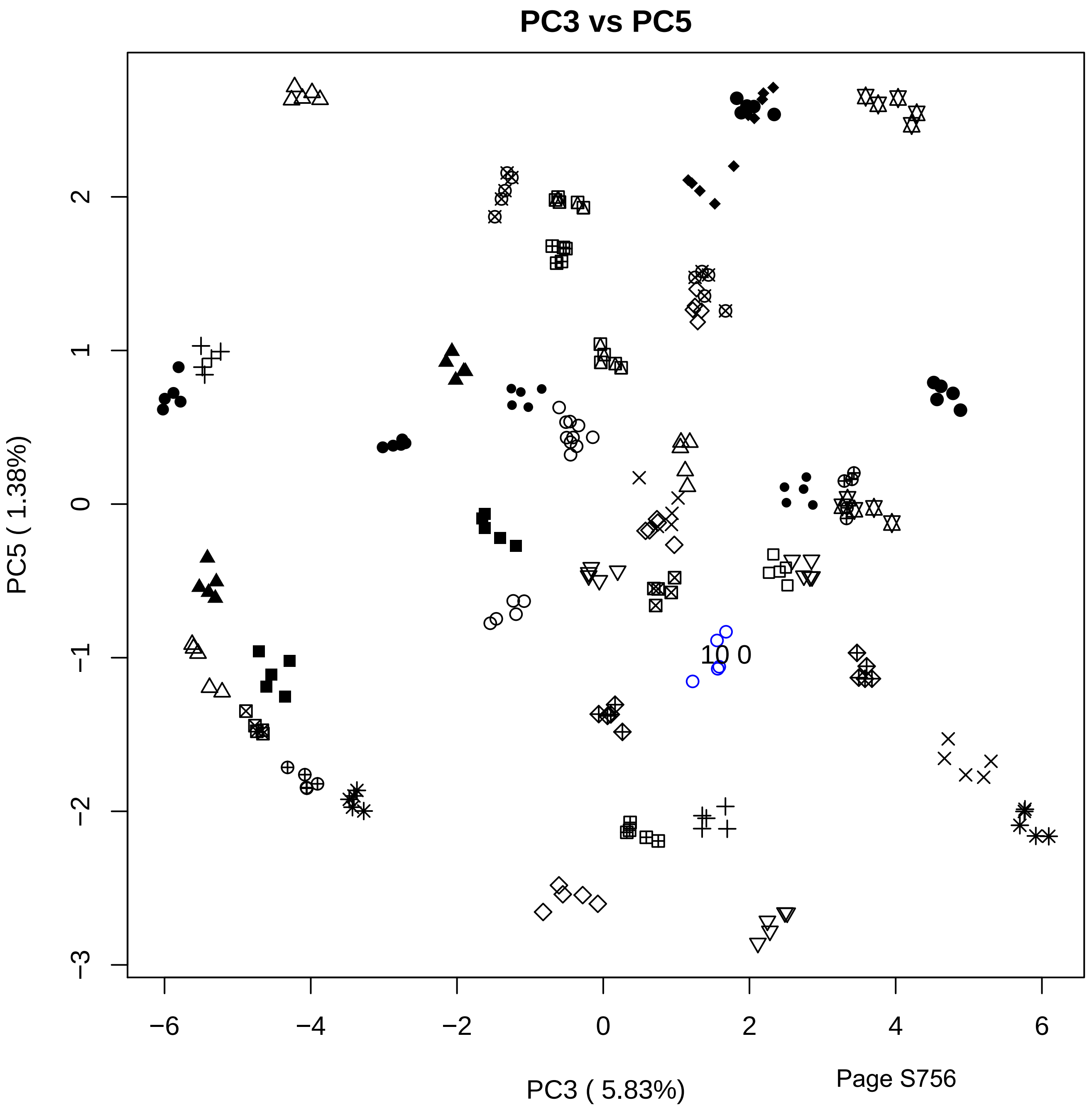

PC3 vs PC5
PC5 (1.38%)
PC3 (5.83%)
2
1
0
−1
−2
−3
−6
−4
−2
0
2
4
6
10 0

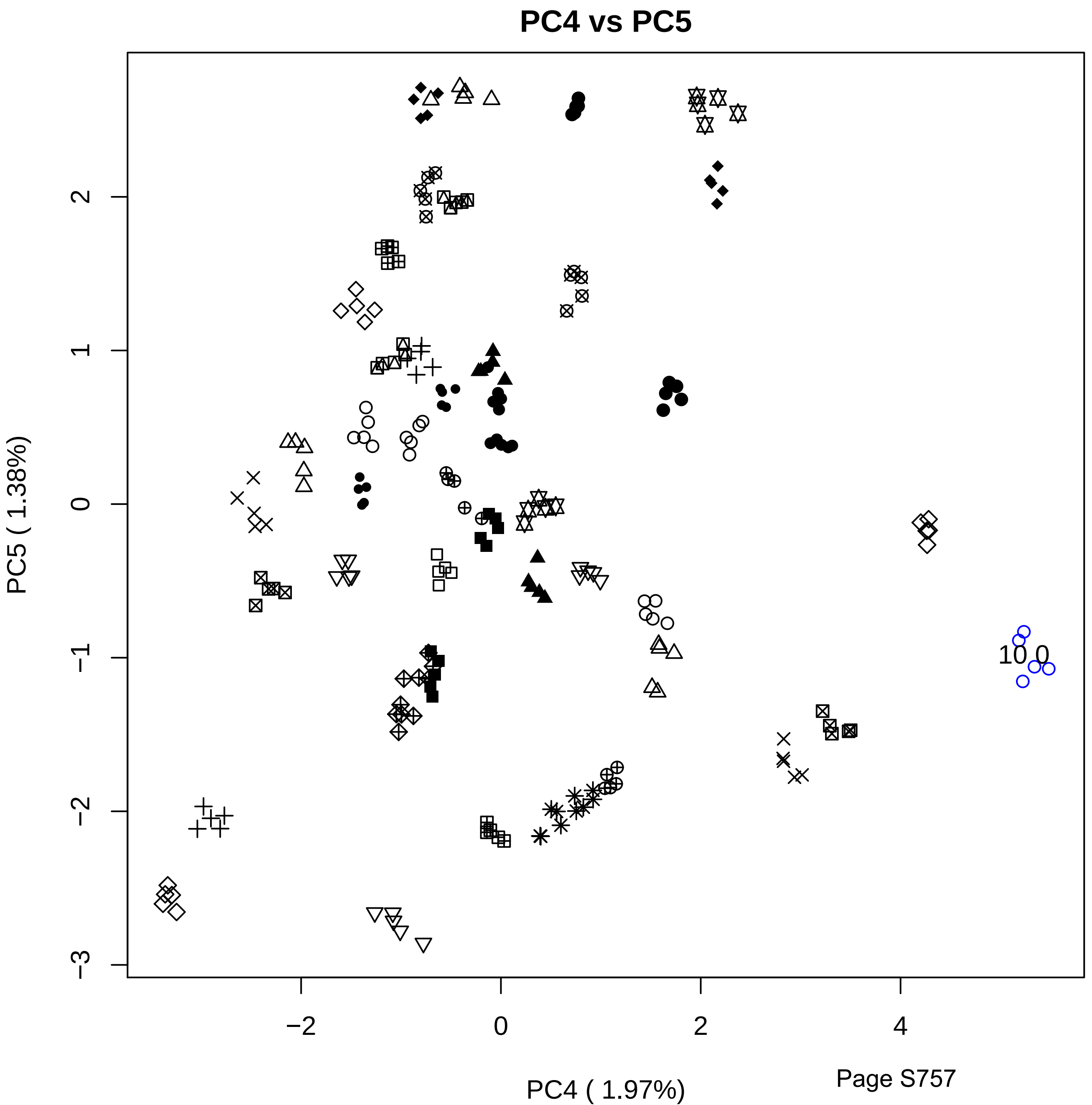
PC4 vs PC5
PC5 (1.38%)
PC4 (1.97%)
2
1
0
−1
−2
−3
−2
0
2
4
10.0

PCA of 15% DeS vs PMH

Scaled Matrix PCAed

7th order polynomial baseline correction performed

Raw spectra smoothed to 21 Neighbours, 2nd order PN, Savtisky Golay

Normalisation between 0 and 100

D2 spectra smoothed to 41 Neighbours, 2nd order PN, Savtisky Golay

Regions Analysed: 3601−2501 and 2001−700cm−1

Samples Analysed: 1 Contam's 0 DeS's 45 PMH's

Keele University AD

Sun Dec 09 17:04:27 2018

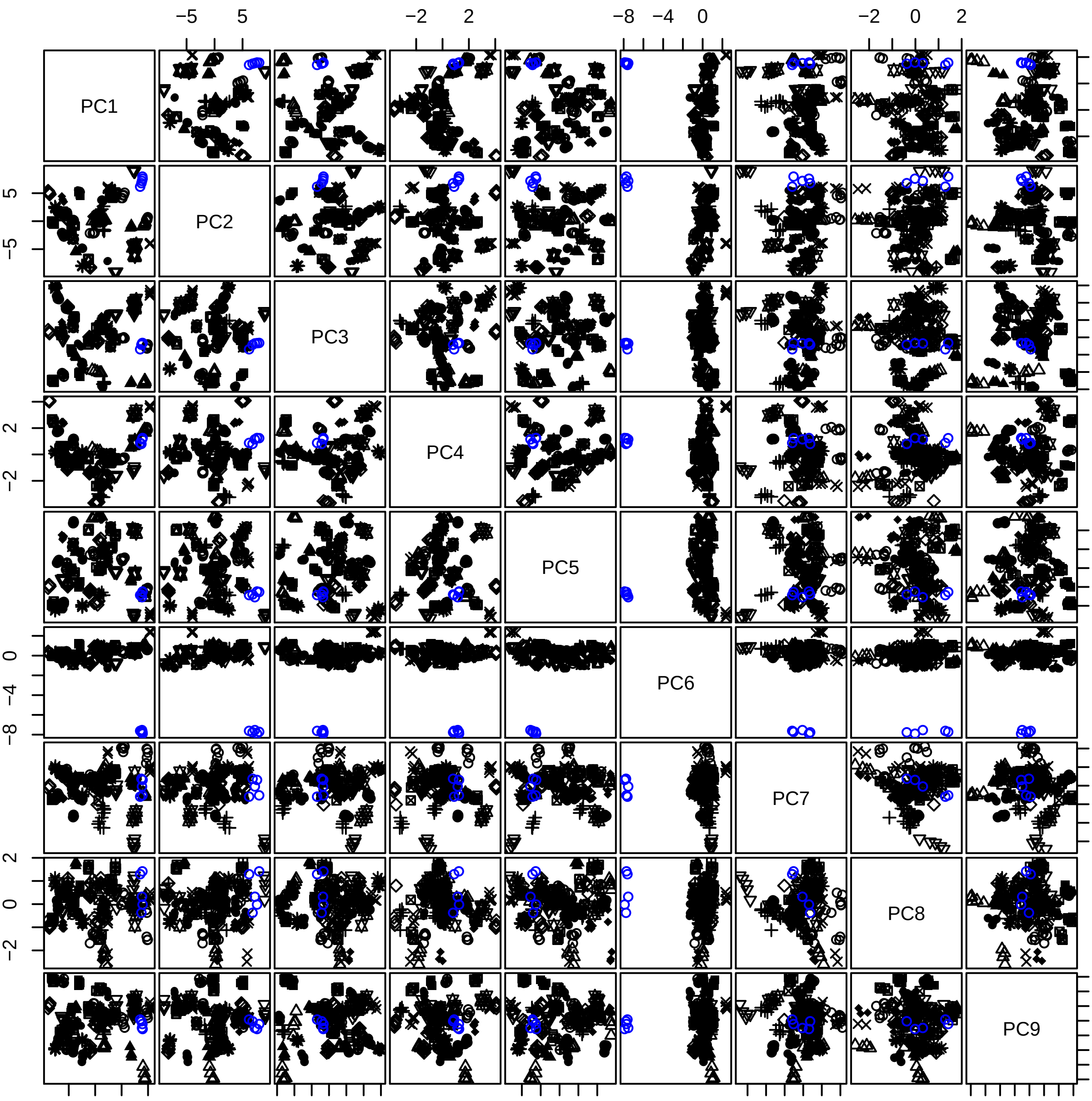
PC1
PC2
PC3
PC4
PC5
PC6
PC7
PC8
PC9
−5
5
−2
2
−8
−4
0
10
−10
−6
4
−3
−2.0
0.0
1.5

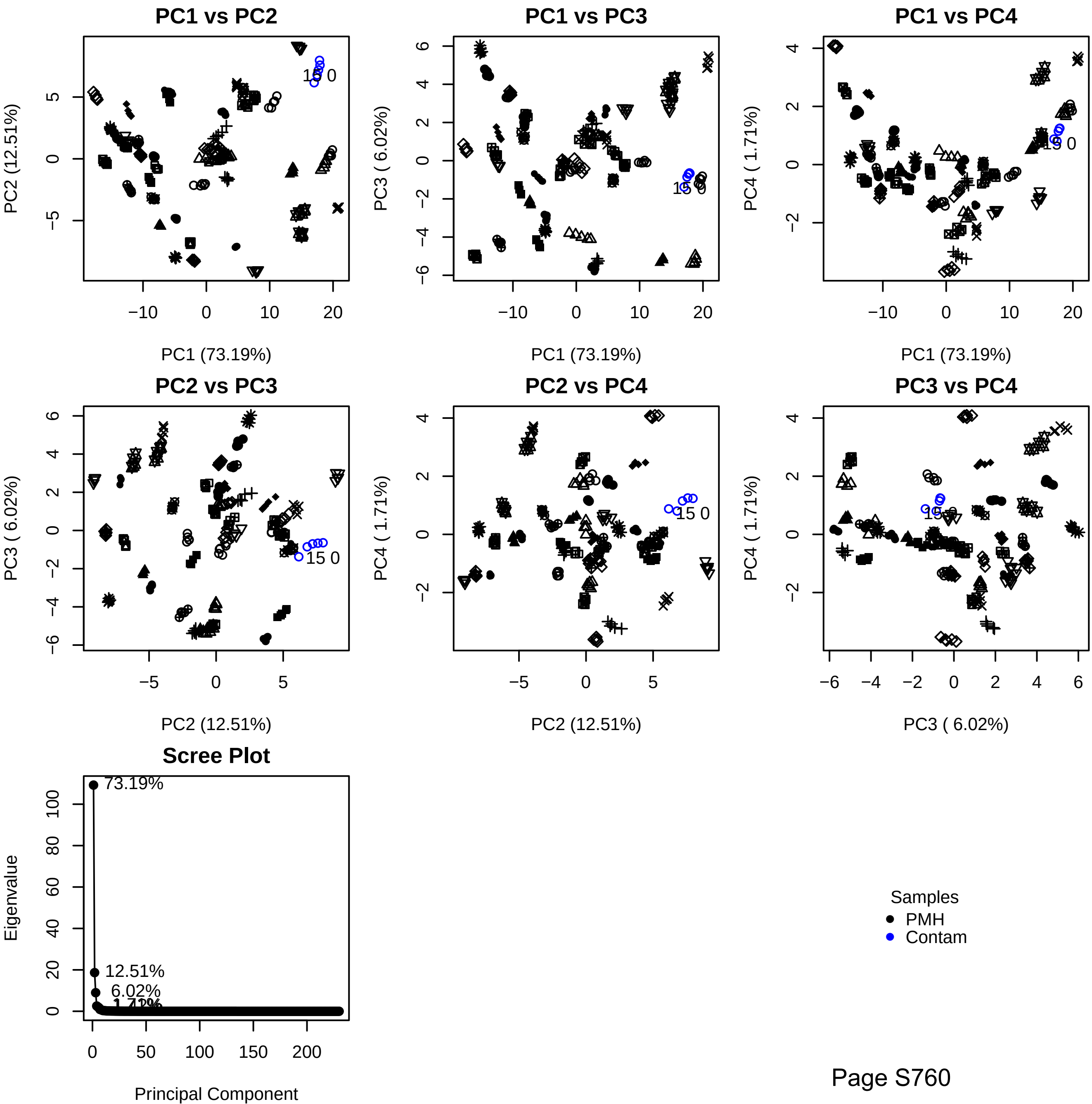
PC1 vs PC2
PC1 vs PC3
PC1 vs PC4
PC2 vs PC3
PC2 vs PC4
PC3 vs PC4
PC1 (73.19%)
PC2 (12.51%)
PC3 (6.02%)
PC4 (1.71%)
15 0
Scree Plot
73.19%
12.51%
6.02%
1.71%
Eigenvalue
Principal Component
Samples
PMH
Contam

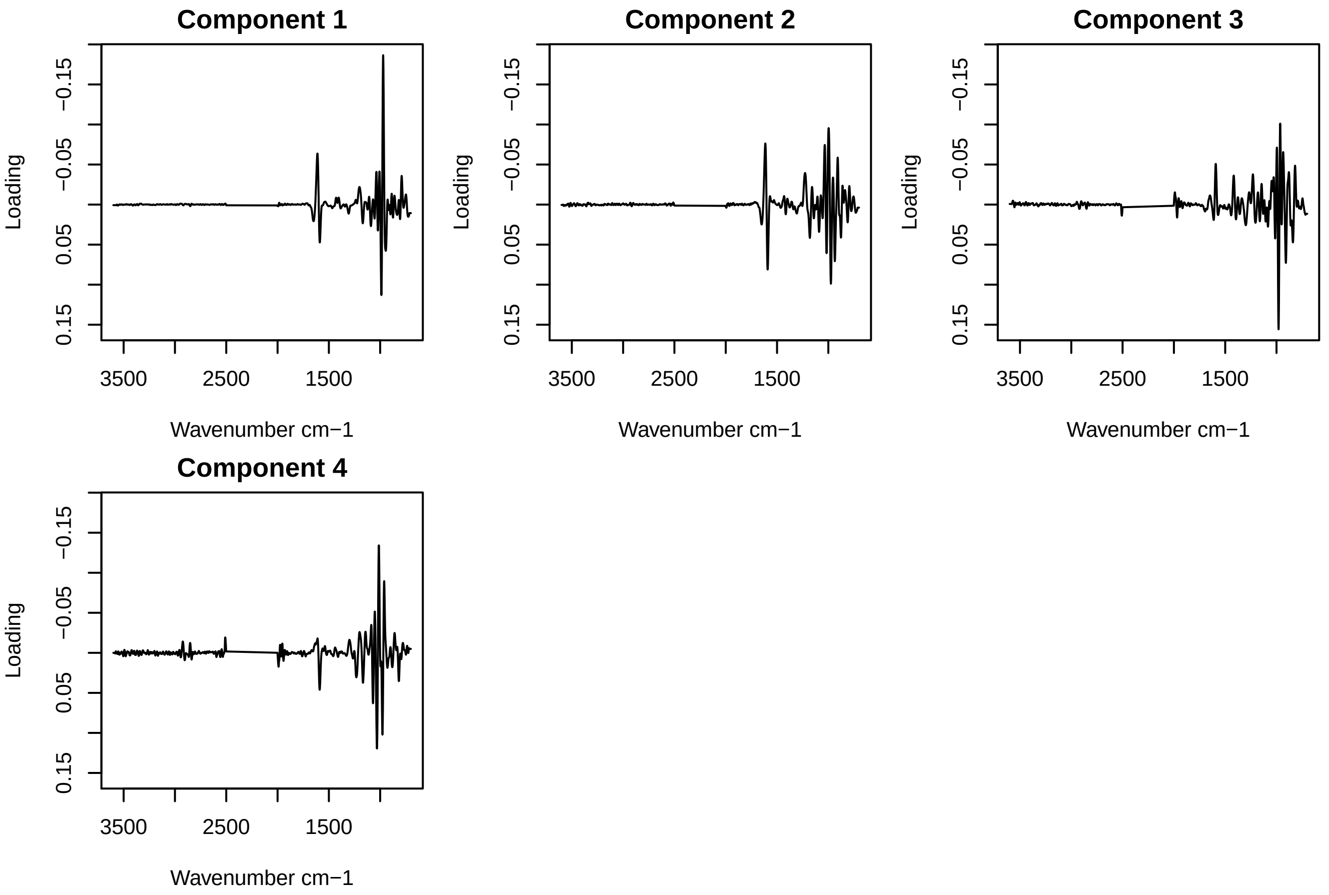
Component 1
Component 2
Component 3
Component 4
Loading
−0.15
−0.05
0.05
0.15
3500
2500
1500
Wavenumber cm−1

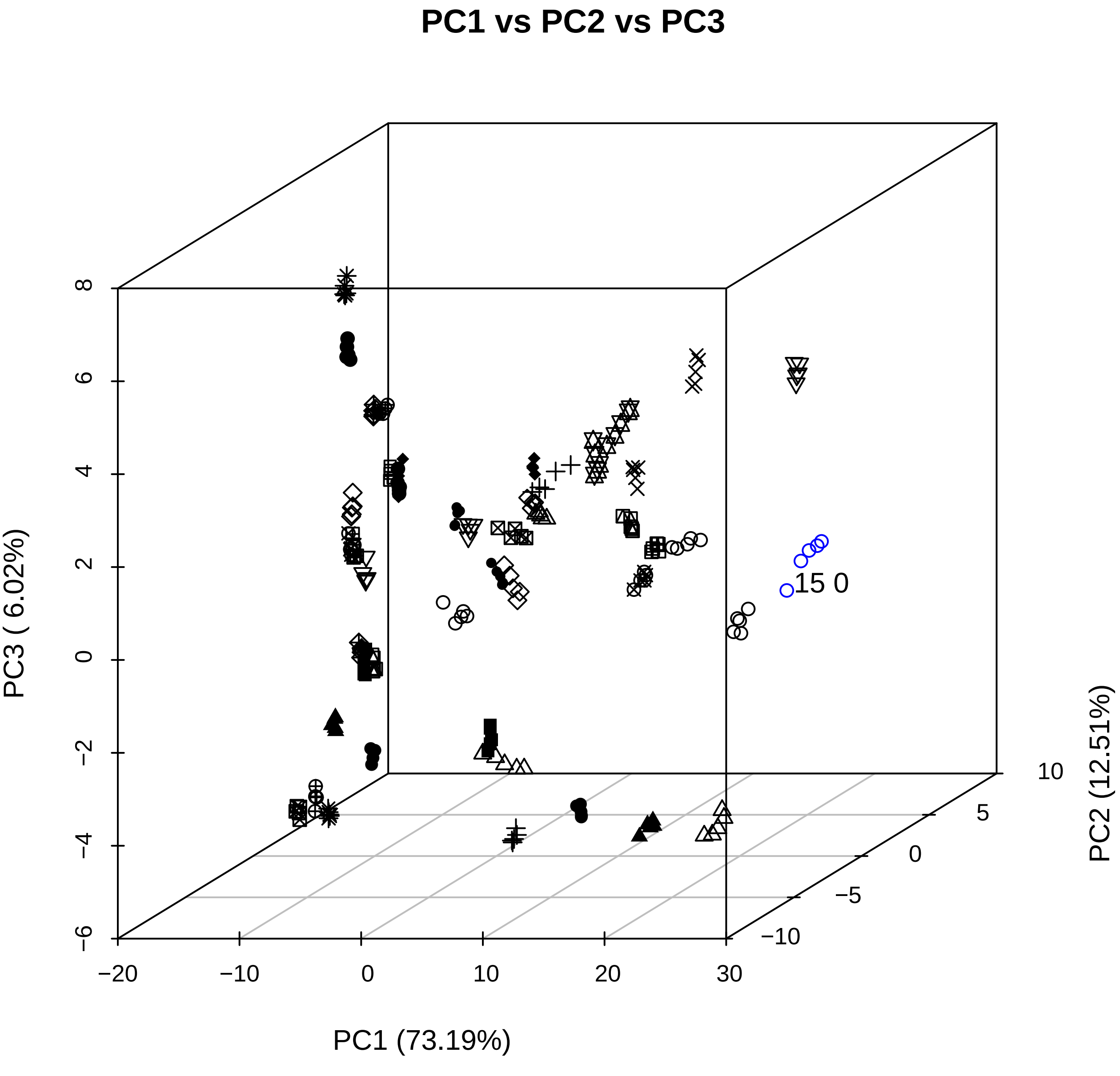

PC1 vs PC2 vs PC3
8
6
4
2
0
−2
−4
−6
PC3 (6.02%)
−20
−10
0
10
20
30
PC1 (73.19%)
10
5
0
−5
−10
PC2 (12.51%)
15 0

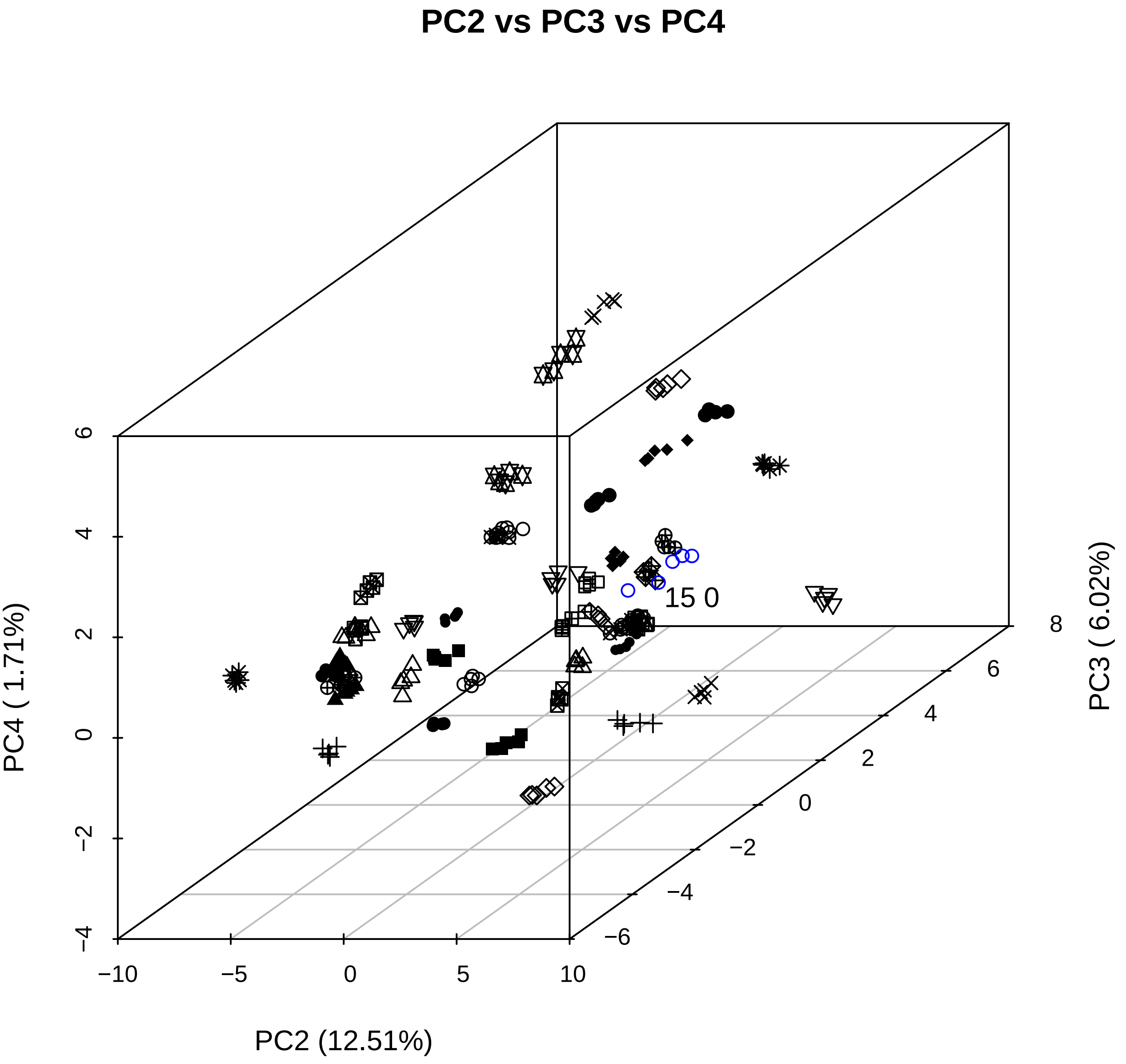
PC2 vs PC3 vs PC4
PC2 (12.51%)
PC3 (6.02%)
PC4 (1.71%)
15 0
−10
−5
0
5
10
−6
−4
−2
0
2
4
6
8
−4
−2
0
2
4
6

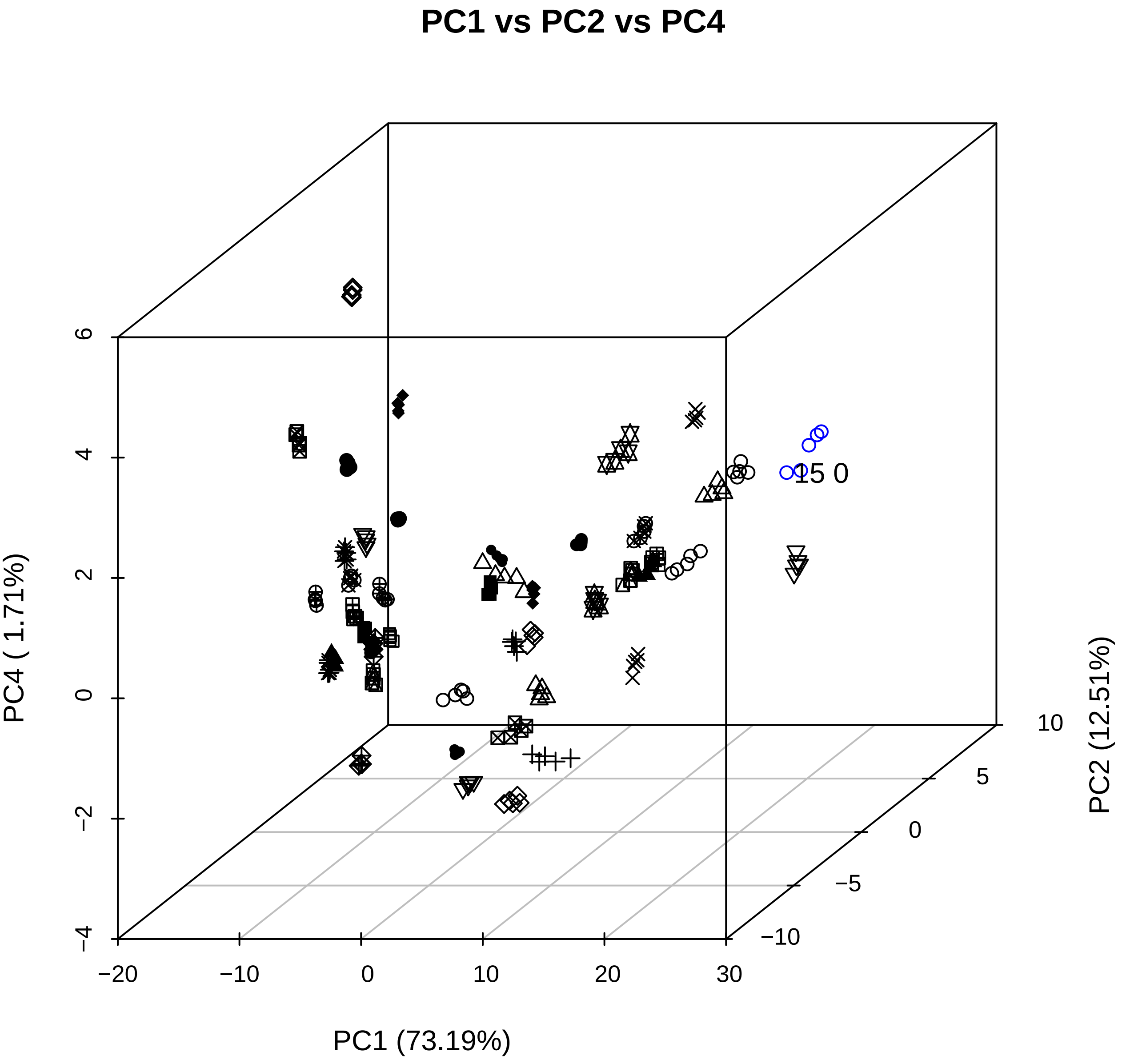

PC1 vs PC2 vs PC4
PC1 (73.19%)
PC2 (12.51%)
PC4 (1.71%)
−20
−10
0
10
20
30
−10
−5
0
5
10
−4
−2
0
2
4
6
15 0

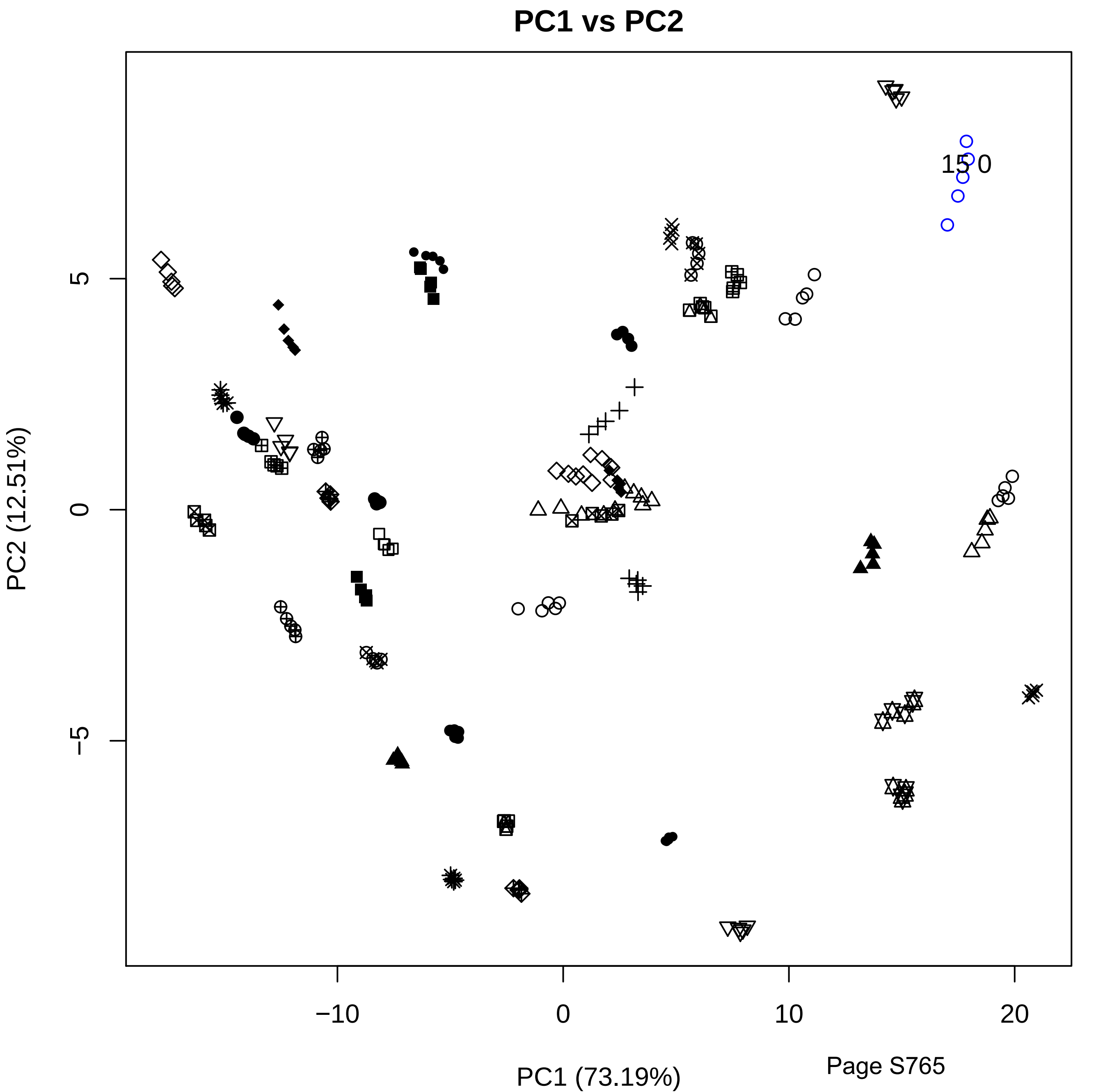
PC1 vs PC2
15 0
PC2 (12.51%)
5
0
−5
−10
0
10
20
PC1 (73.19%)

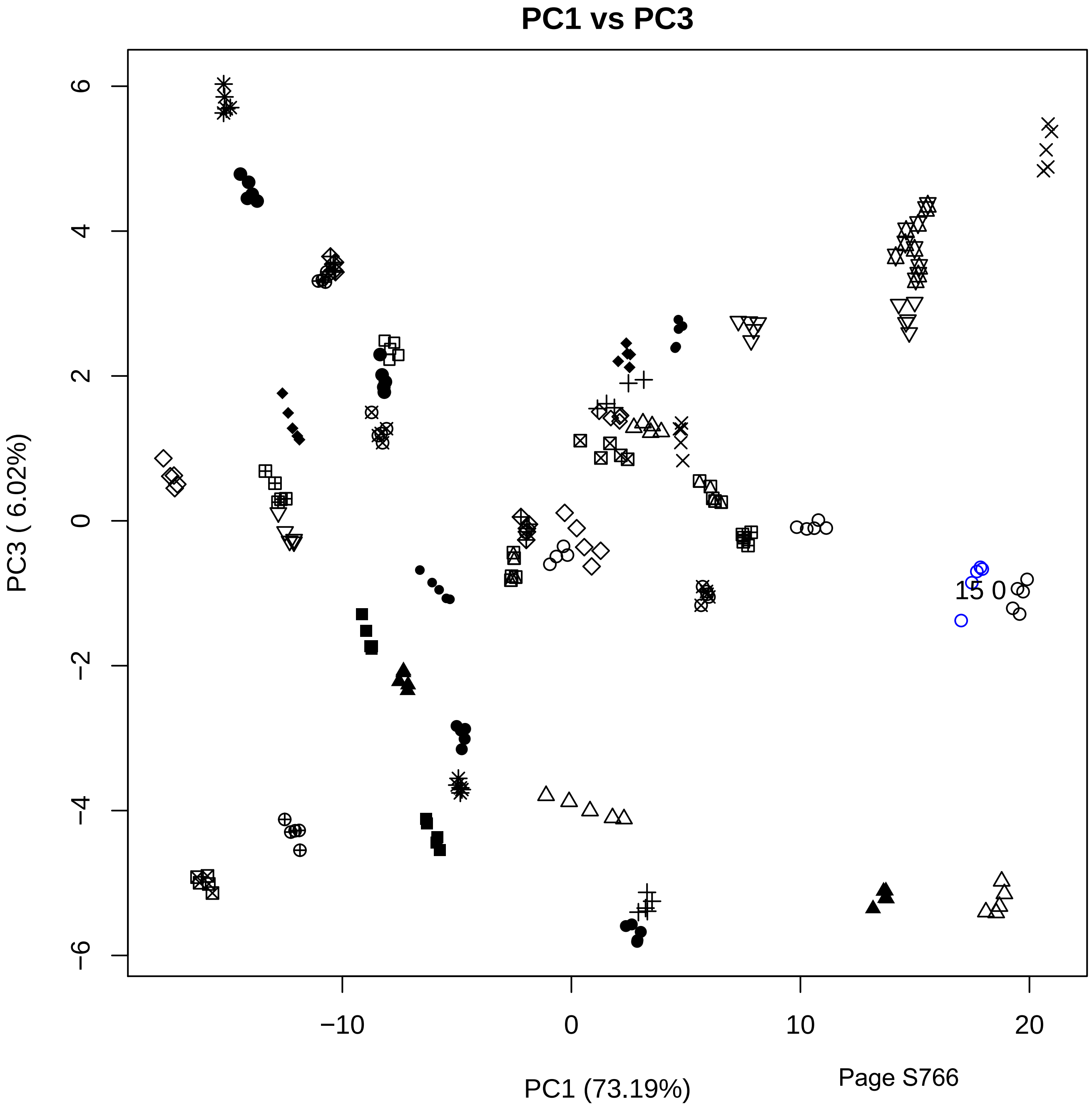
PC1 vs PC3
PC3 (6.02%)
PC1 (73.19%)
15 0

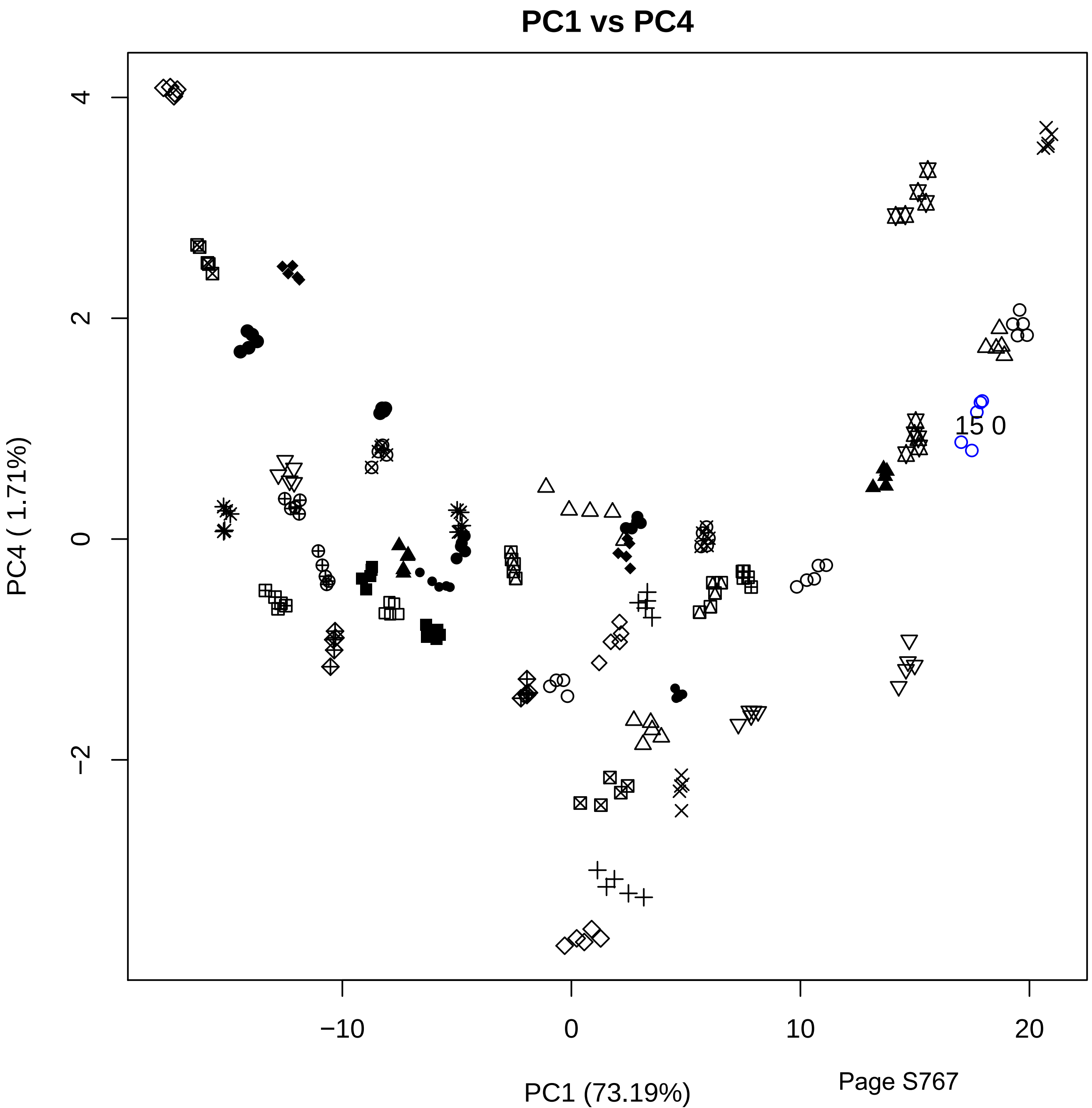

PC1 vs PC4
4
2
0
−2
PC4 (1.71%)
15 0
−10
0
10
20
PC1 (73.19%)

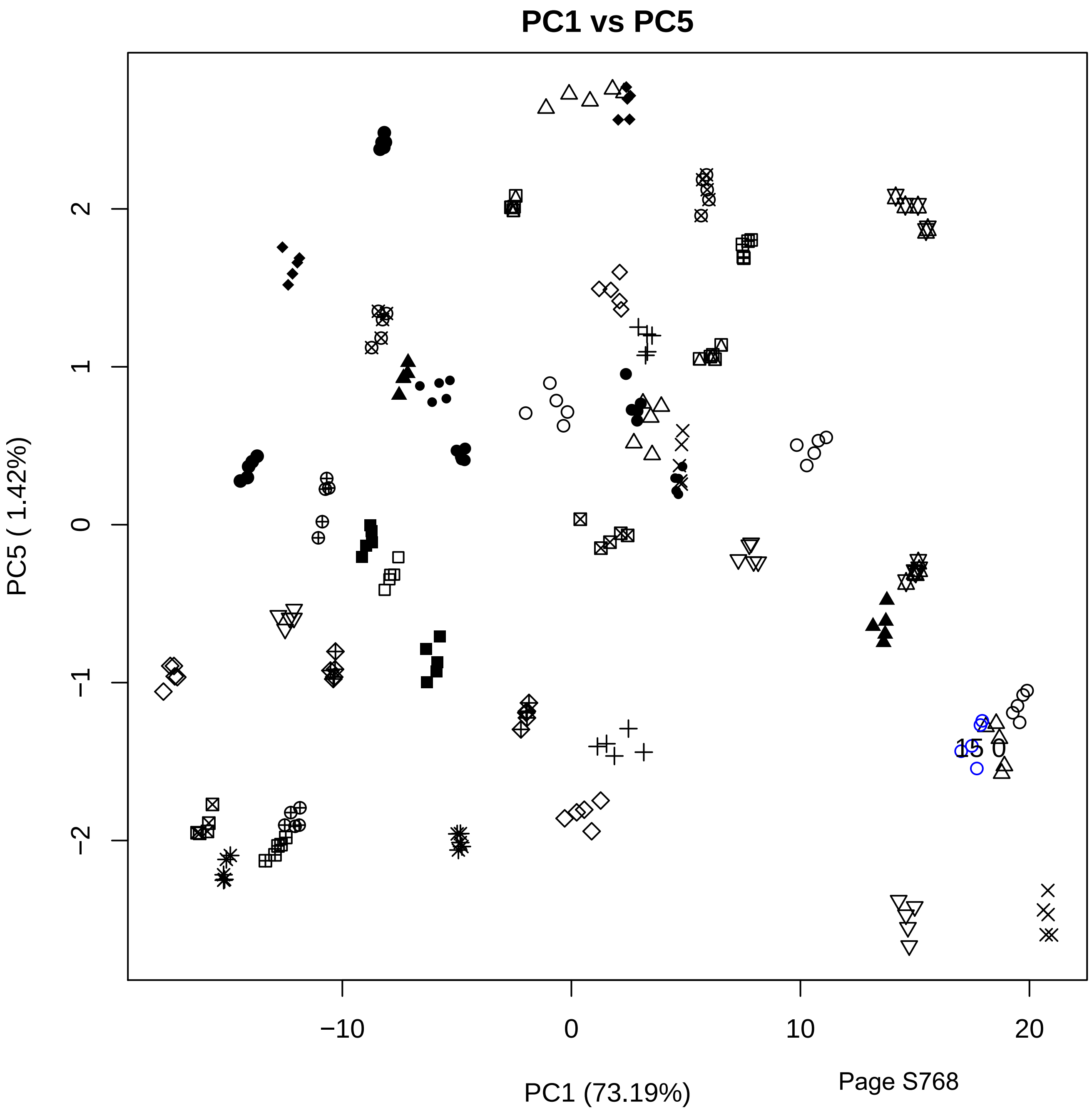
PC1 vs PC5
PC5 (1.42%)
PC1 (73.19%)
2
1
0
−1
−2
−10
0
10
20
15 0

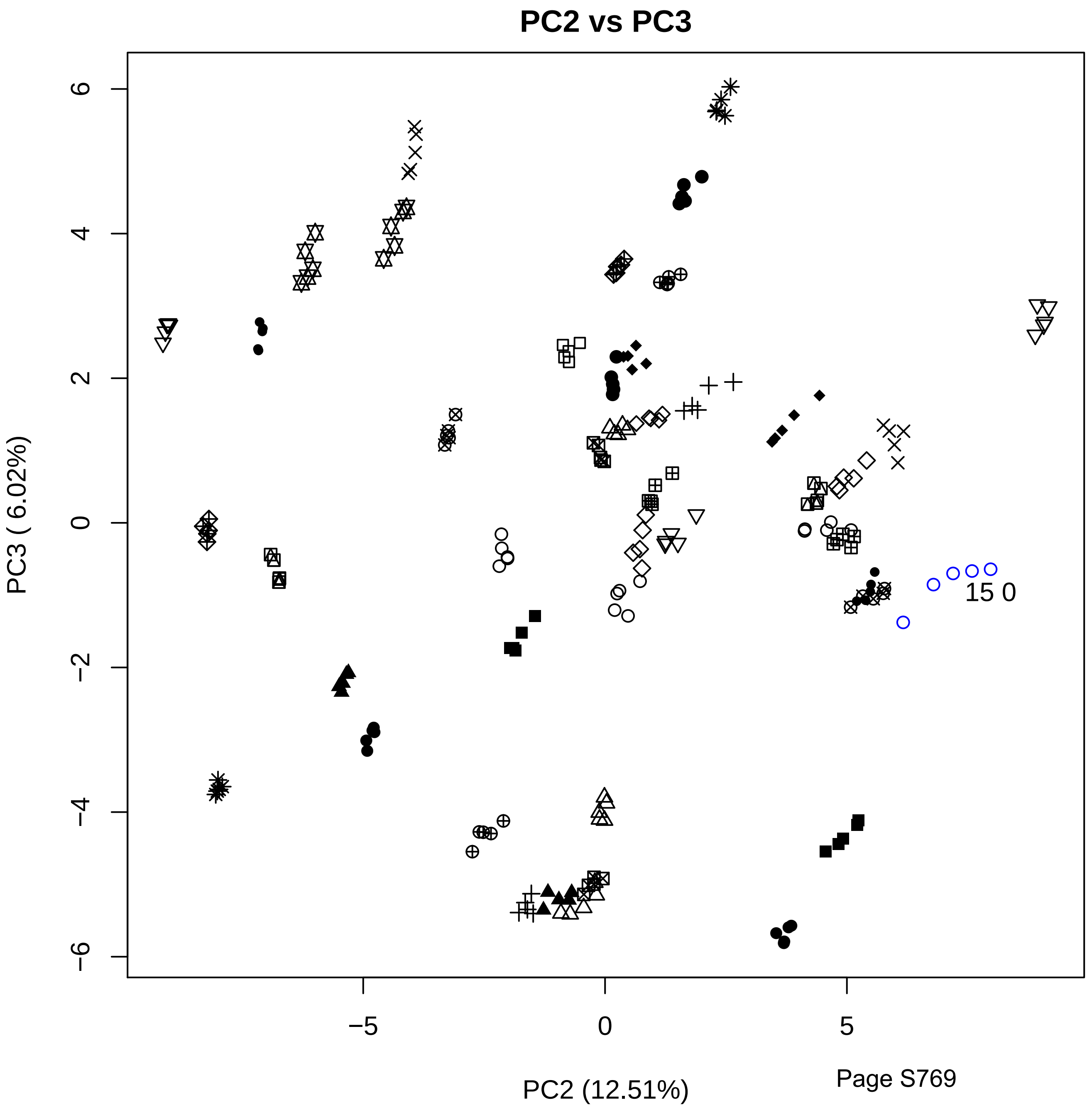
PC2 vs PC3
PC3 (6.02%)
PC2 (12.51%)
15 0

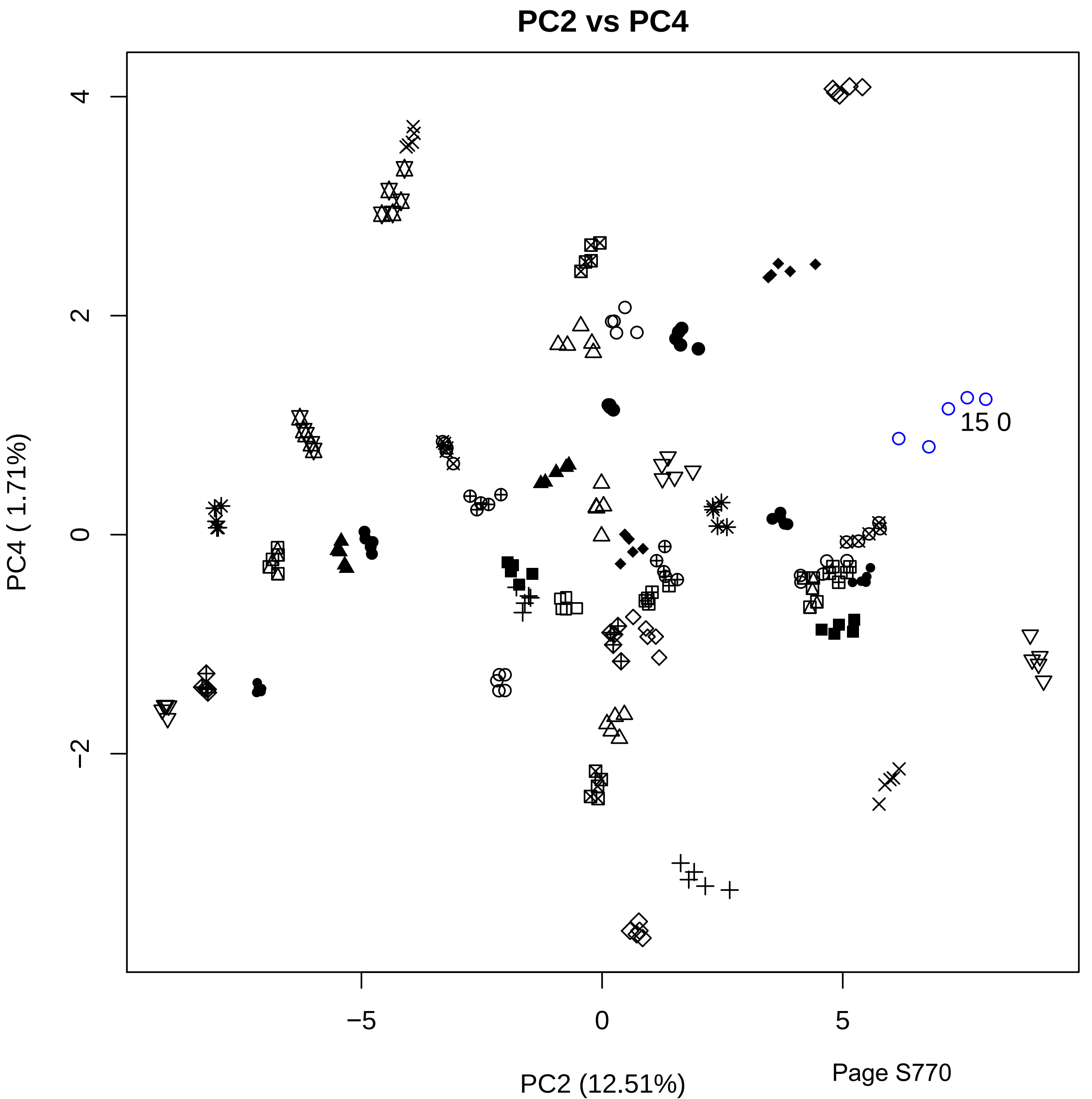
PC2 vs PC4
4
2
0
−2
PC4 (1.71%)
−5
0
5
PC2 (12.51%)
15 0

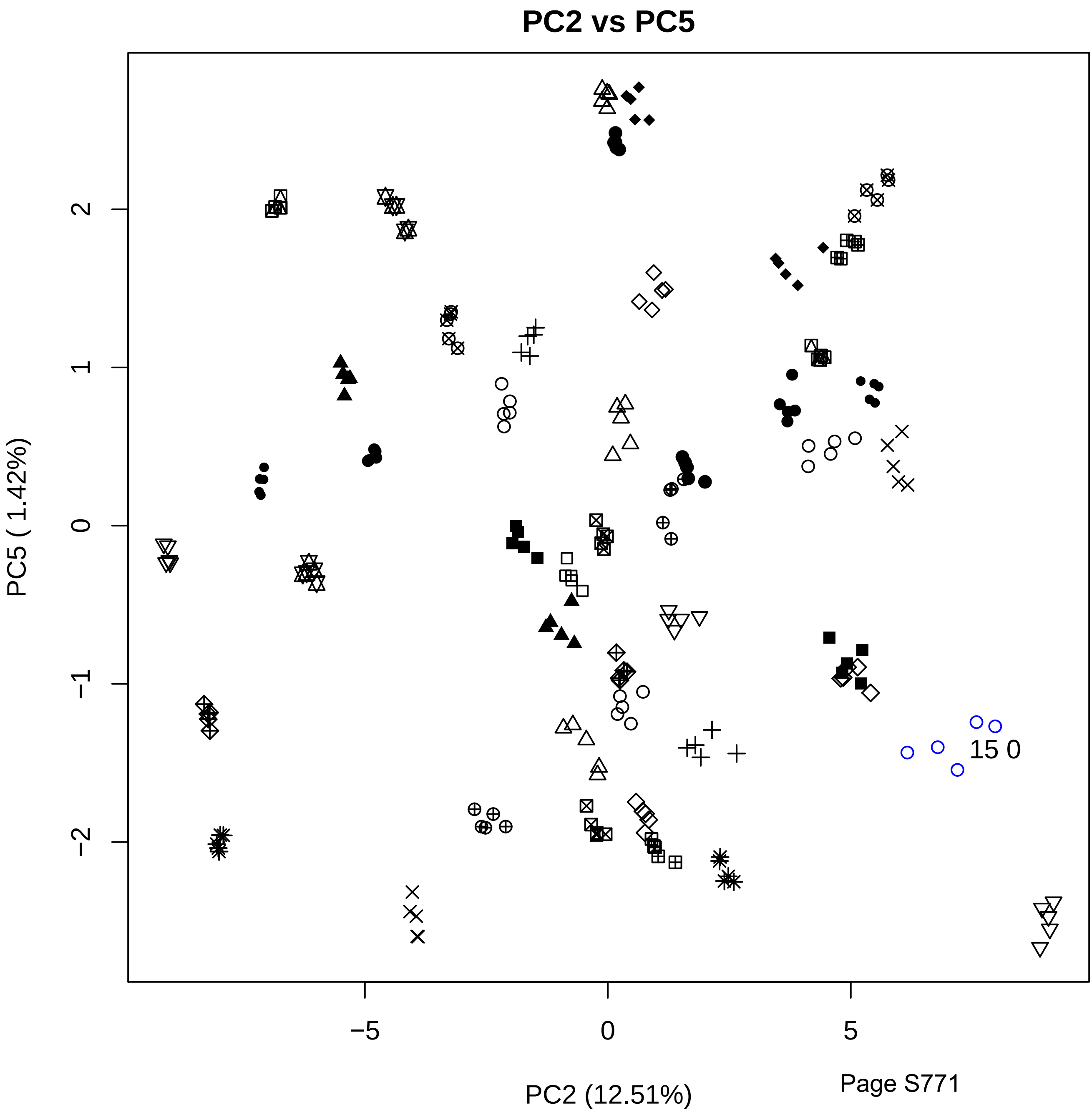
PC2 vs PC5
PC5 (1.42%)
PC2 (12.51%)
2
1
0
−1
−2
−5
0
5
15 0

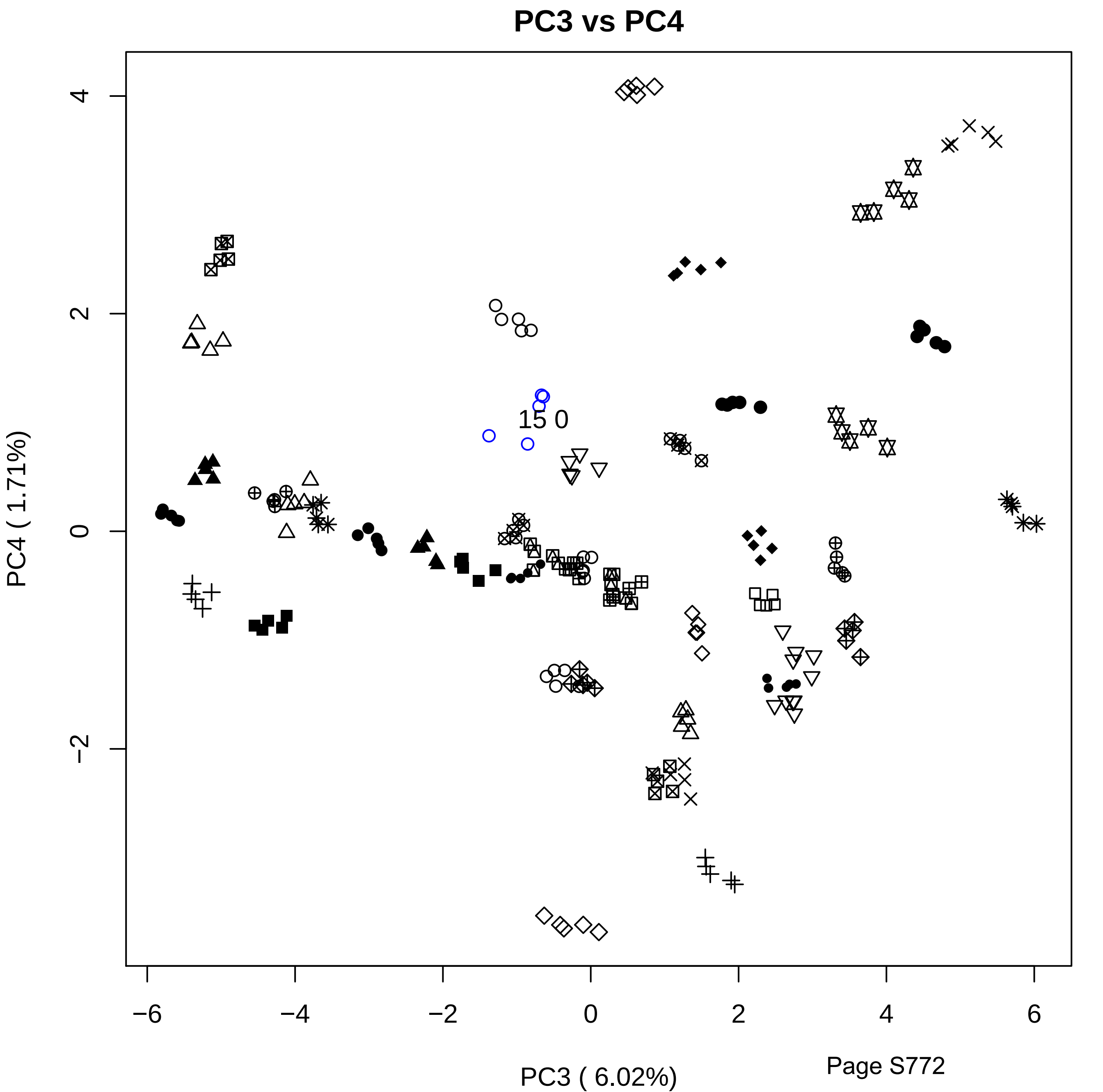
PC3 vs PC4
PC4 (1.71%)
PC3 (6.02%)
4
2
0
−2
−6
−4
−2
0
2
4
6
15 0

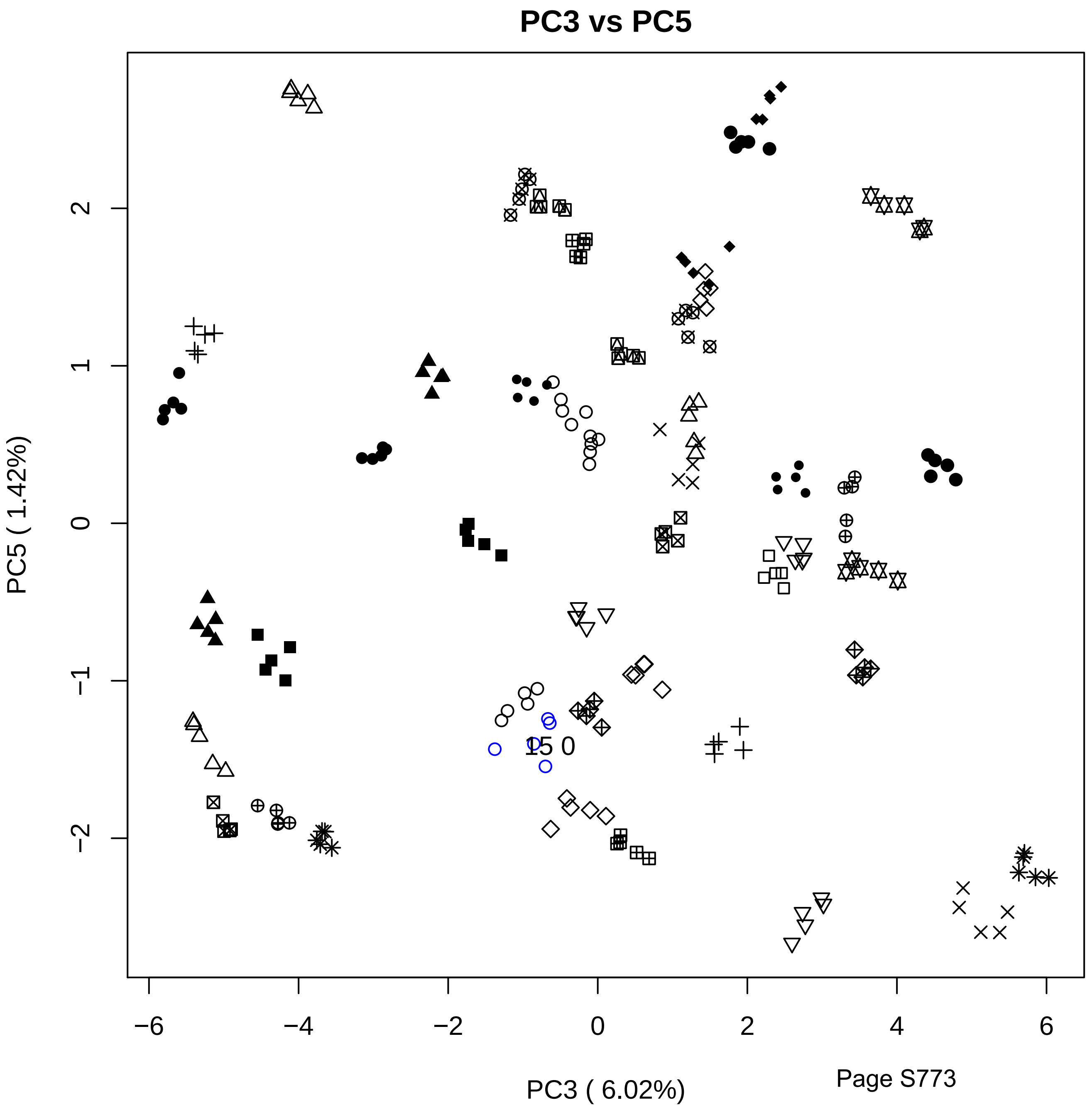
PC3 vs PC5
PC5 (1.42%)
PC3 (6.02%)
−6
−4
−2
0
2
4
6
−2
−1
0
1
2
15 0

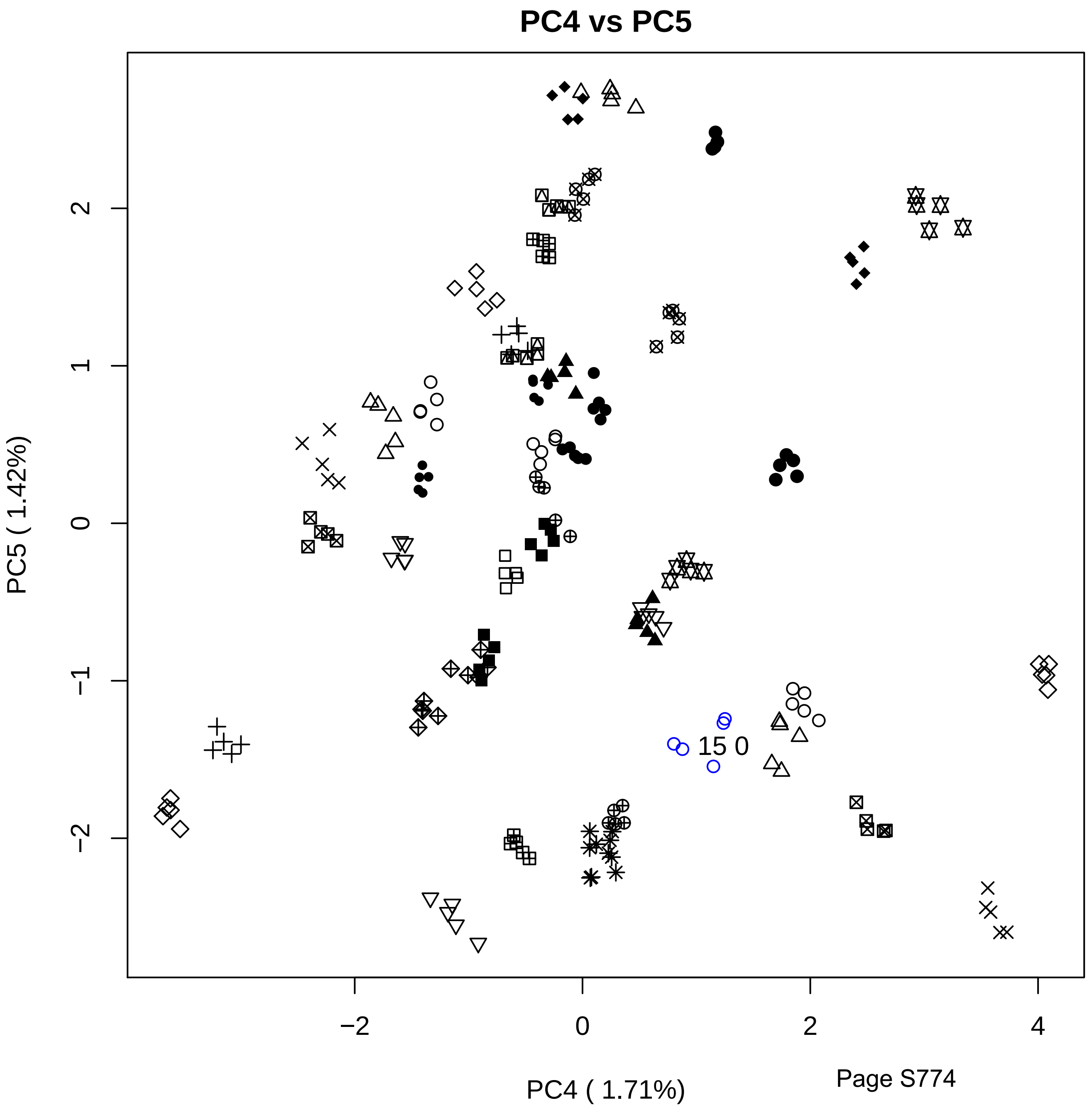
PC4 vs PC5
PC5 (1.42%)
PC4 (1.71%)
2
1
0
−1
−2
−2
0
2
4
15 0

PCA of 20% DeS vs PMH

Scaled Matrix PCAed

7th order polynomial baseline correction performed

Raw spectra smoothed to 21 Neighbours, 2nd order PN, Savtisky Golay

Normalisation between 0 and 100

D2 spectra smoothed to 41 Neighbours, 2nd order PN, Savtisky Golay

Regions Analysed: 3601−2501 and 2001−700cm−1

Samples Analysed: 1 Contam's 0 DeS's 45 PMH's

Keele University AD Sun Dec 09 17:04:32 2018

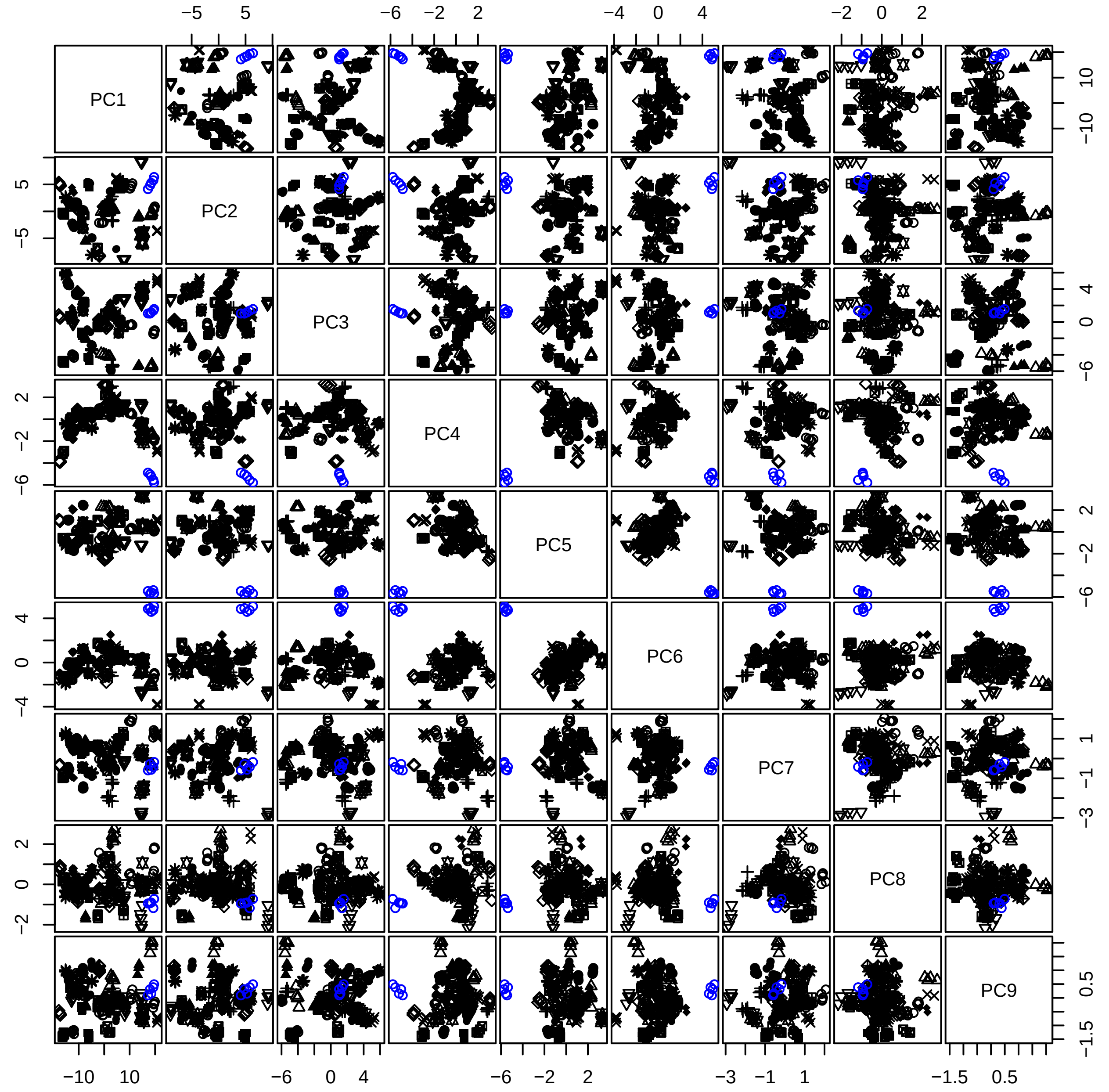
PC1
PC2
PC3
PC4
PC5
PC6
PC7
PC8
PC9

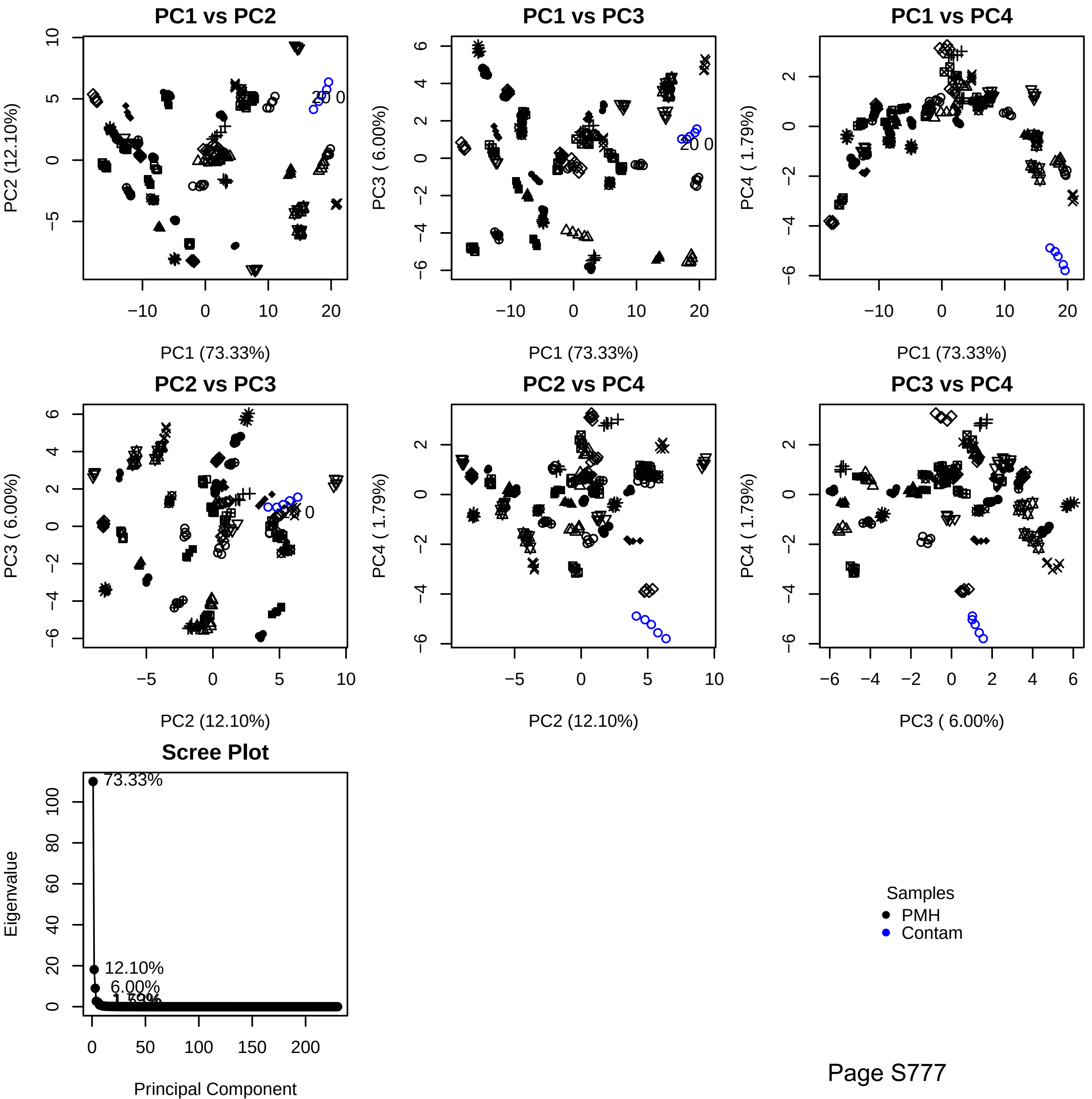

PC1 vs PC2
PC1 vs PC3
PC1 vs PC4
PC2 vs PC3
PC2 vs PC4
PC3 vs PC4
Scree Plot
PC1 (73.33%)
PC2 (12.10%)
PC3 (6.00%)
PC4 (1.79%)
Eigenvalue
Principal Component
73.33%
12.10%
6.00%
Samples
PMH
Contam

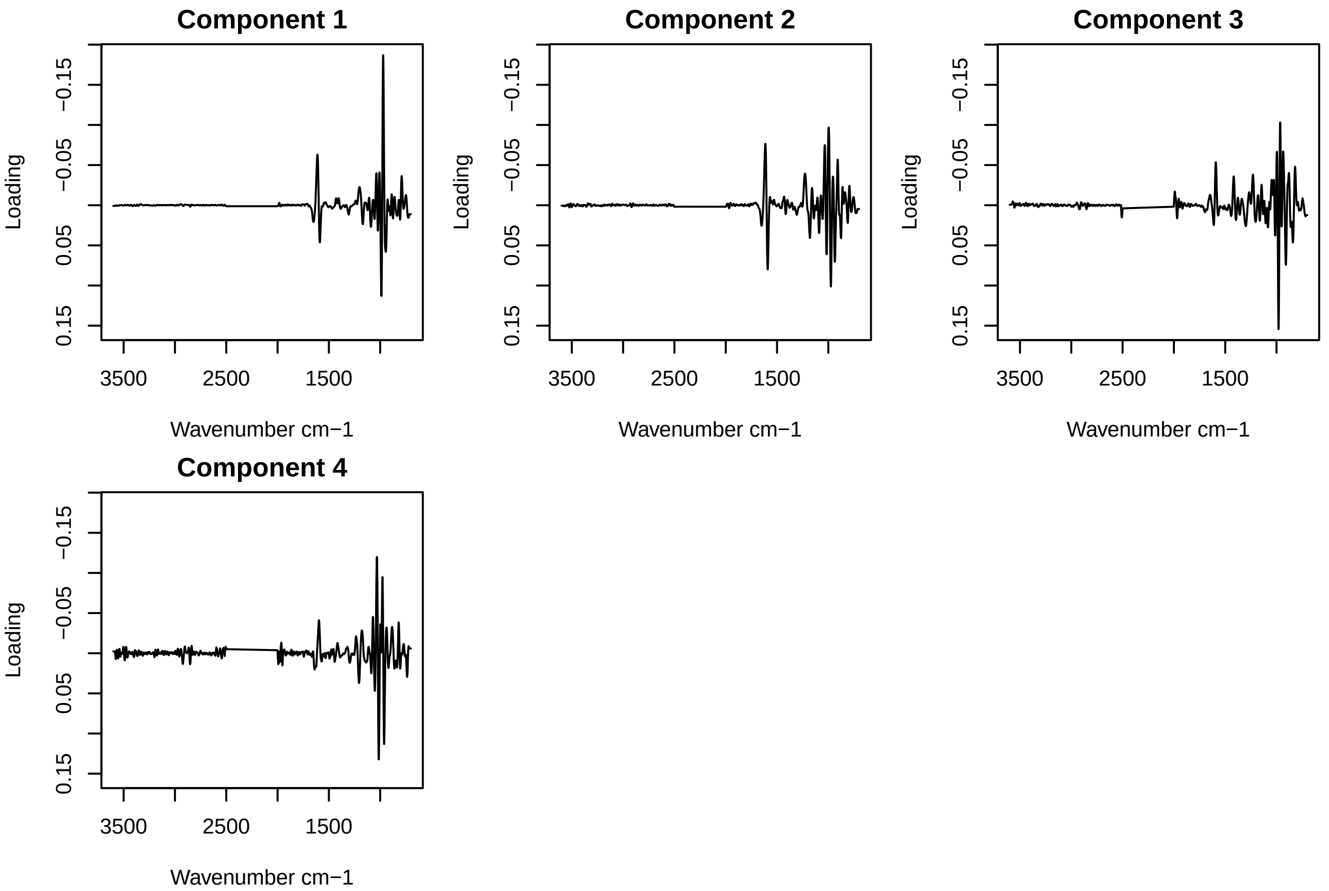
Component 1
Component 2
Component 3
Component 4
Loading
−0.15
−0.05
0.05
0.15
3500
2500
1500
Wavenumber cm−1

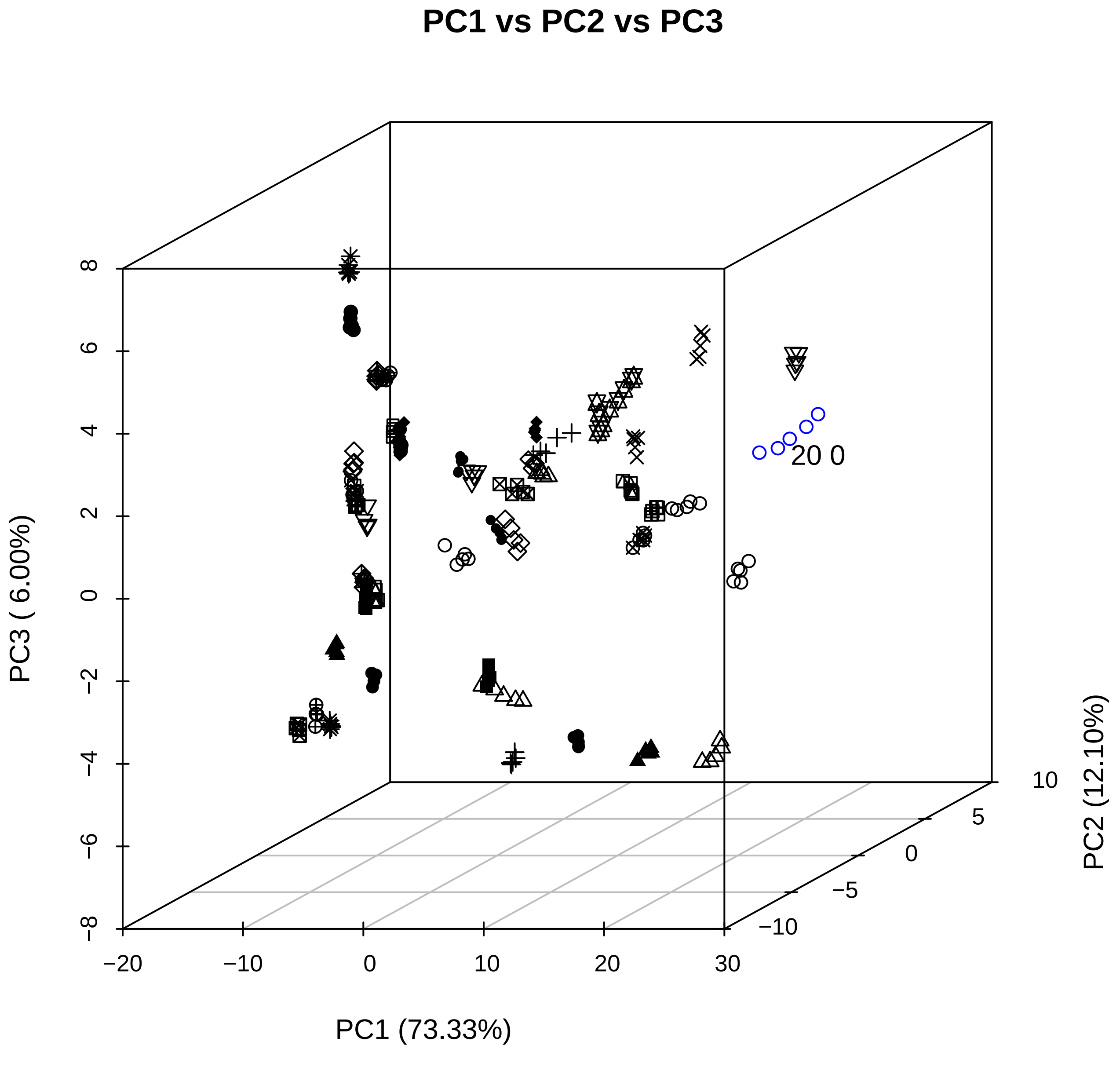

PC1 vs PC2 vs PC3
PC3 (6.00%)
PC1 (73.33%)
PC2 (12.10%)
20 0
−20
−10
0
10
20
30
−8
−6
−4
−2
0
2
4
6
8
−10
−5
0
5
10

PC2 vs PC3 vs PC4

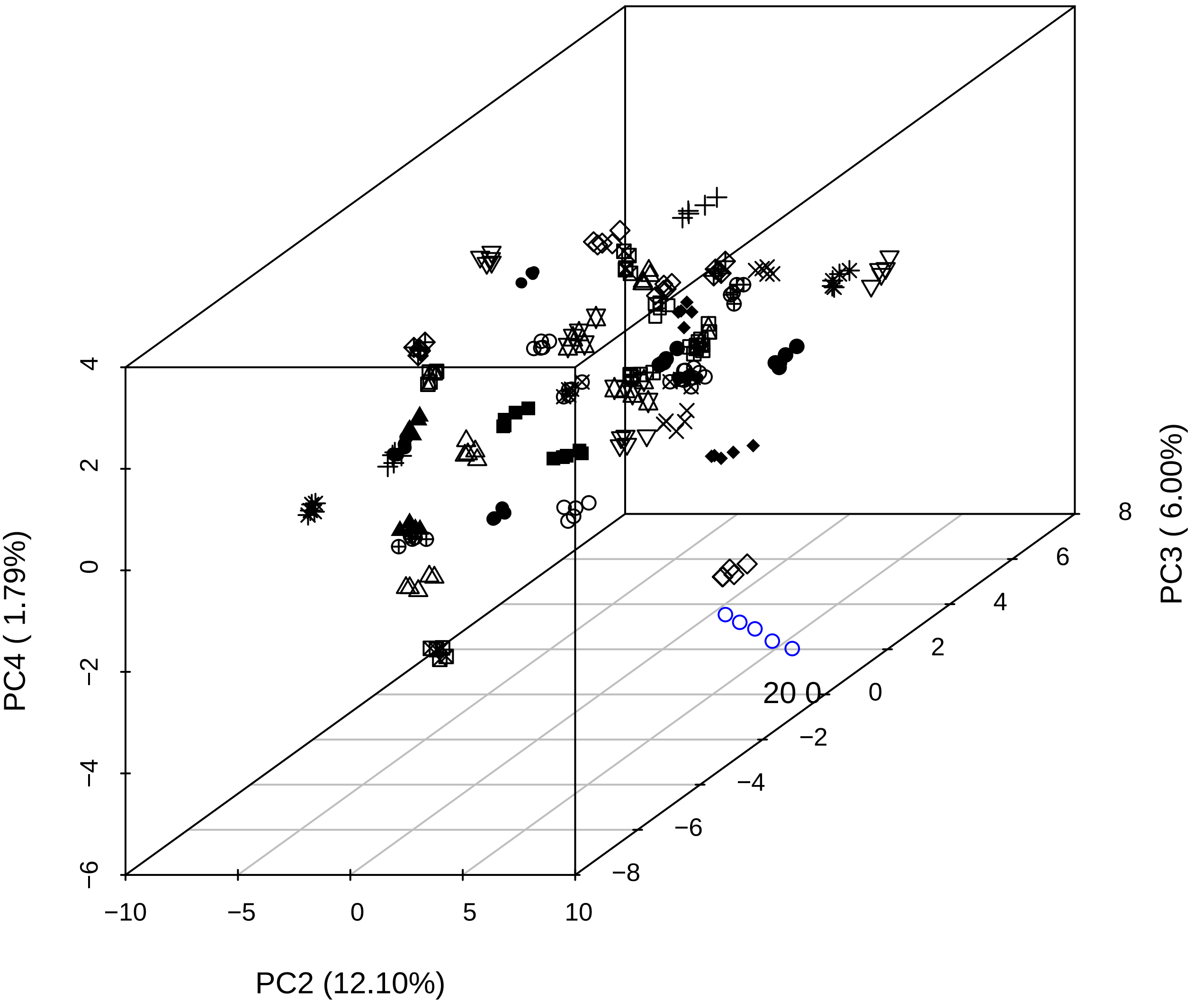

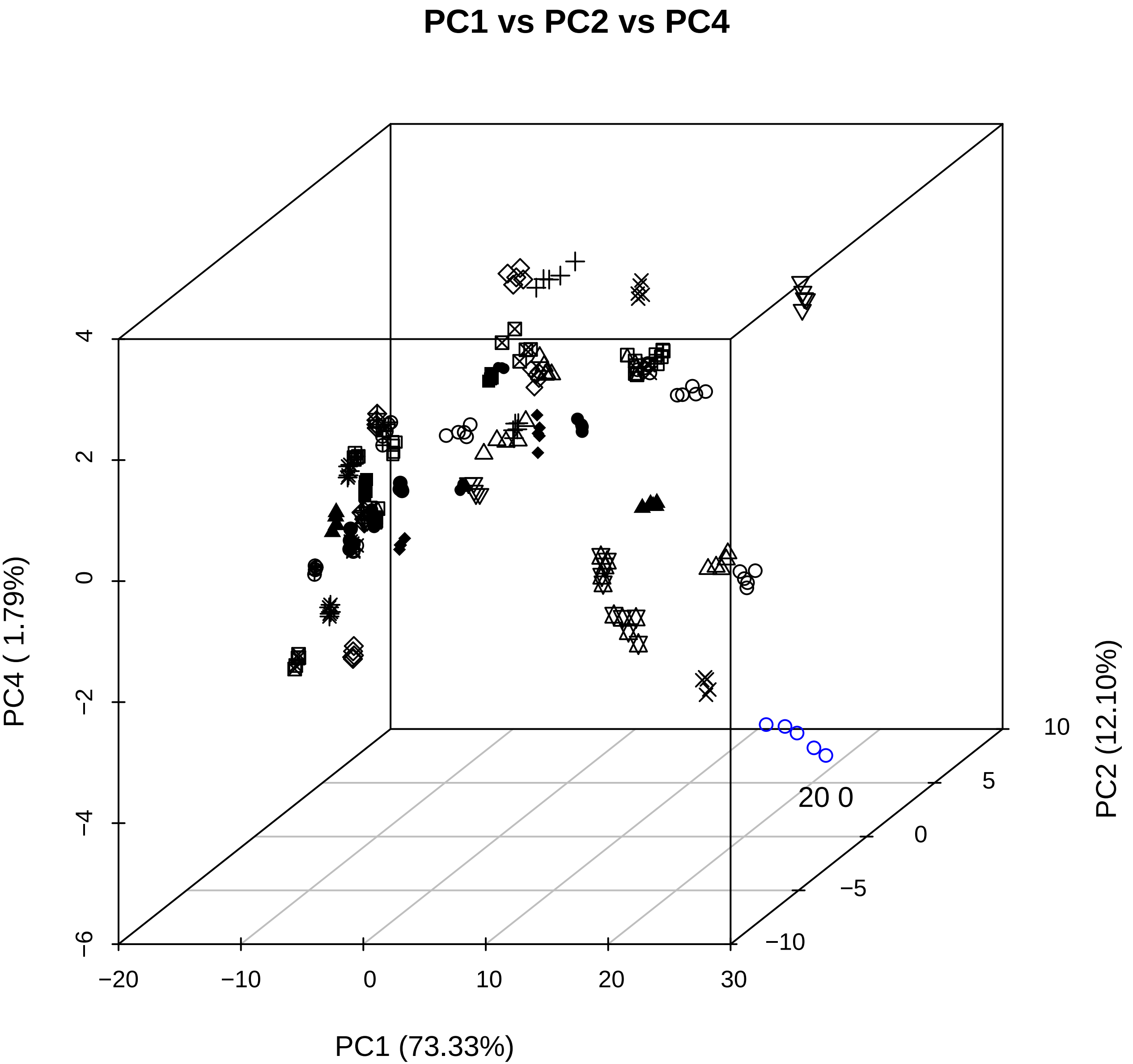

PC1 vs PC2 vs PC4
PC4 (1.79%)
PC1 (73.33%)
PC2 (12.10%)
−6
−4
−2
0
2
4
−20
−10
0
10
20
30
−10
−5
0
5
10
20 0

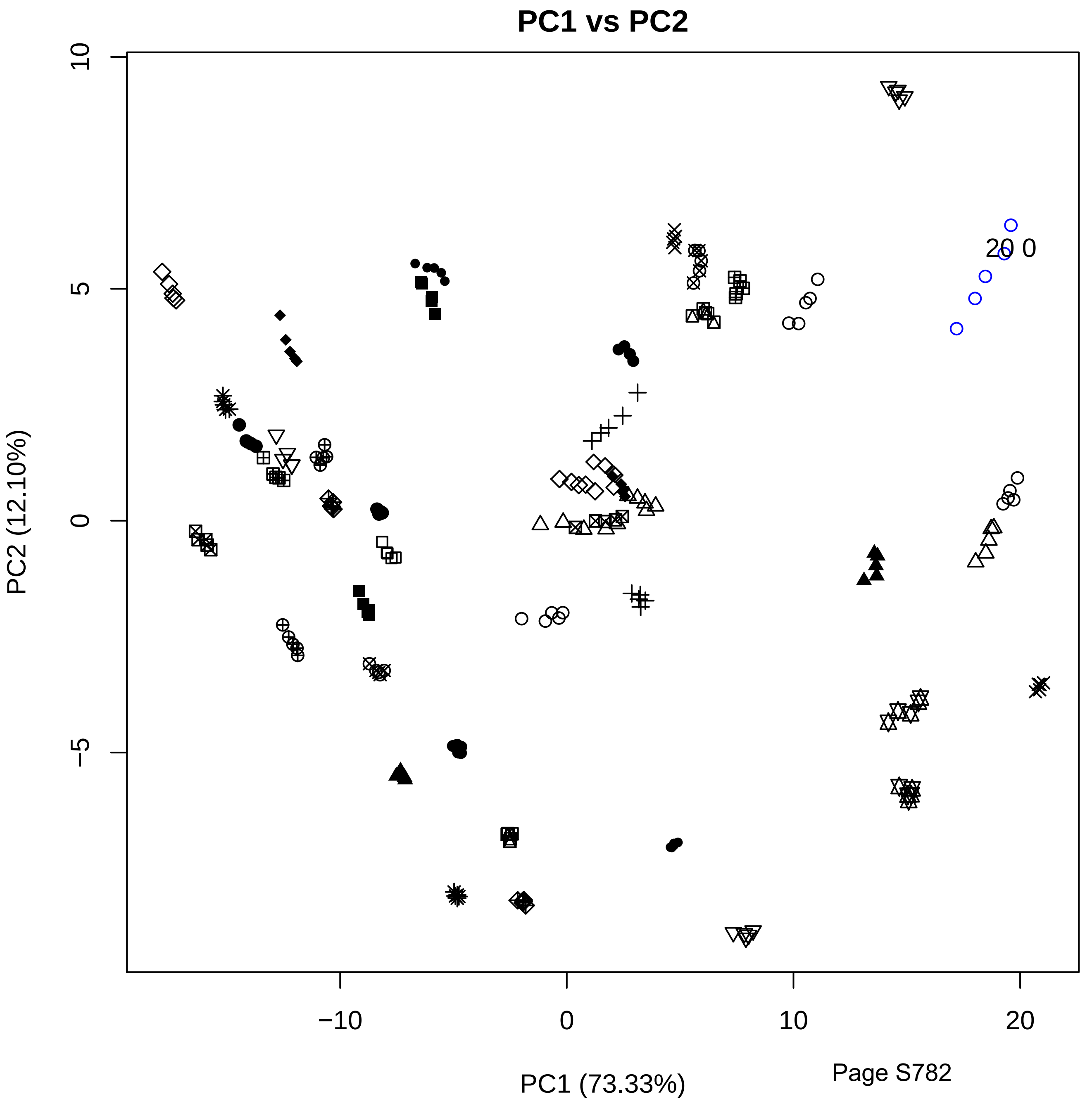
PC1 vs PC2
20 0
PC2 (12.10%)
10
5
0
−5
−10
0
10
20
PC1 (73.33%)

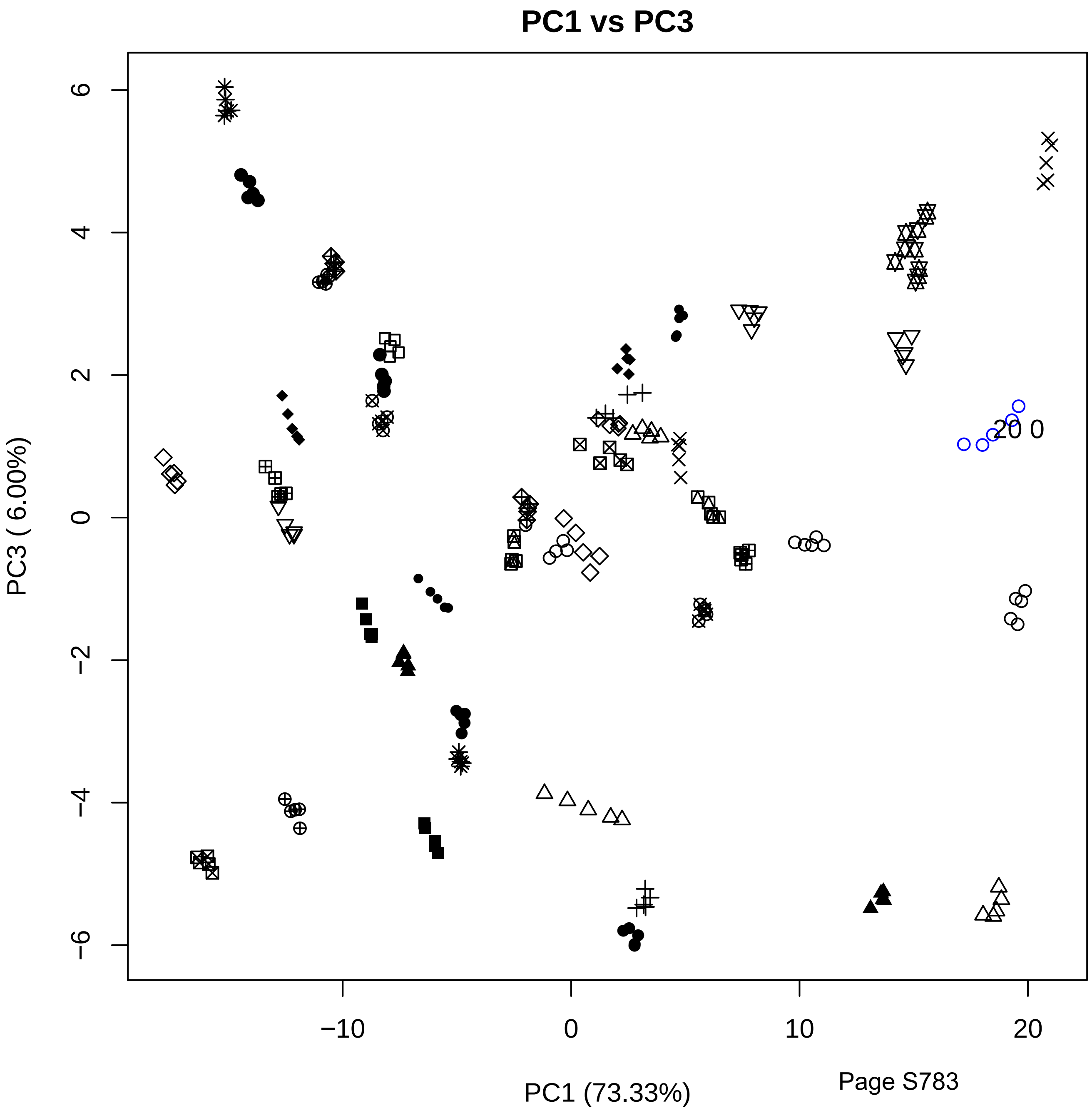
PC1 vs PC3
PC3 (6.00%)
PC1 (73.33%)
6
4
2
0
−2
−4
−6
−10
0
10
20
20 0

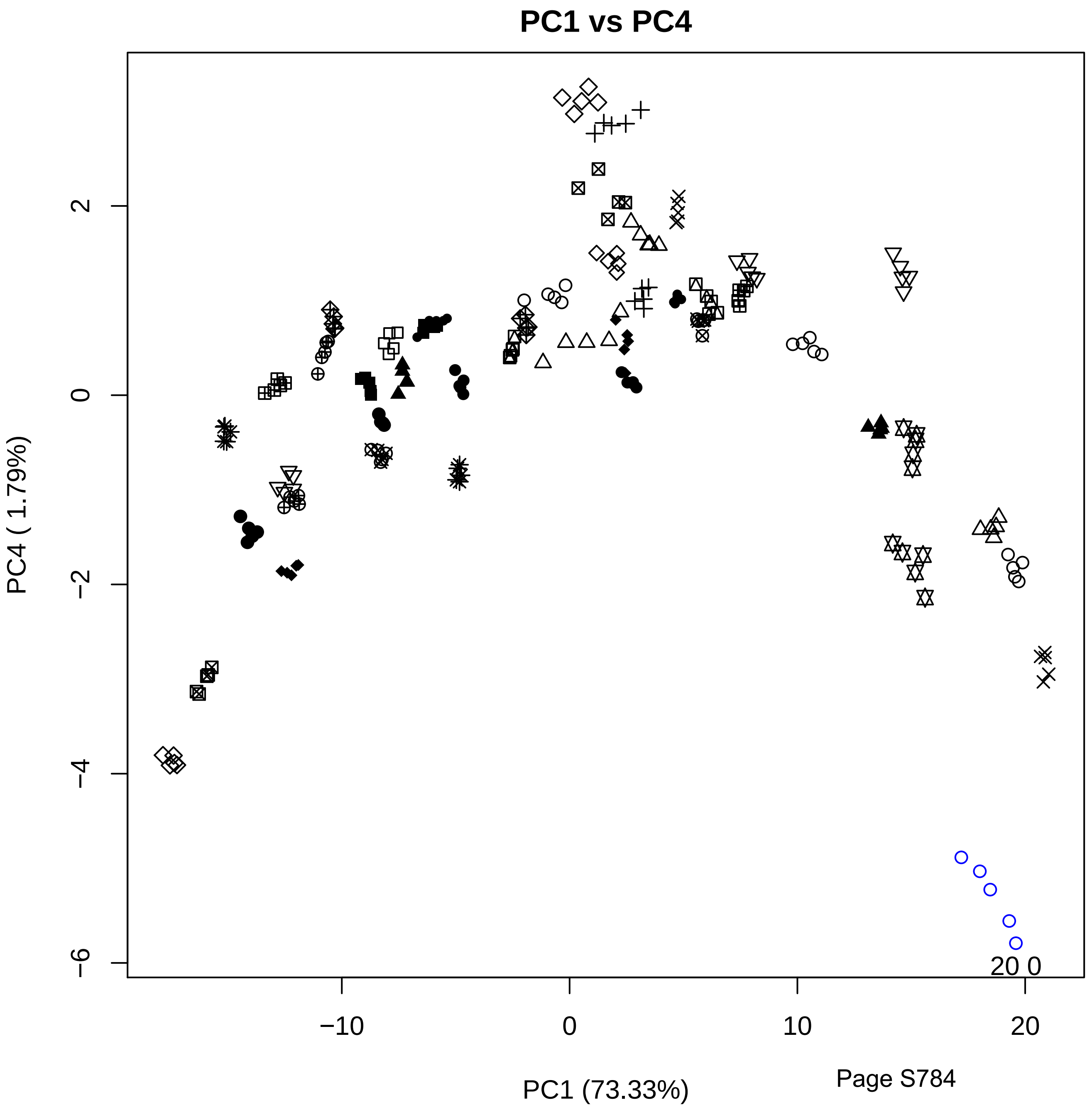
PC1 vs PC4
PC4 (1.79%)
PC1 (73.33%)
2
0
−2
−4
−6
−10
0
10
20
20 0

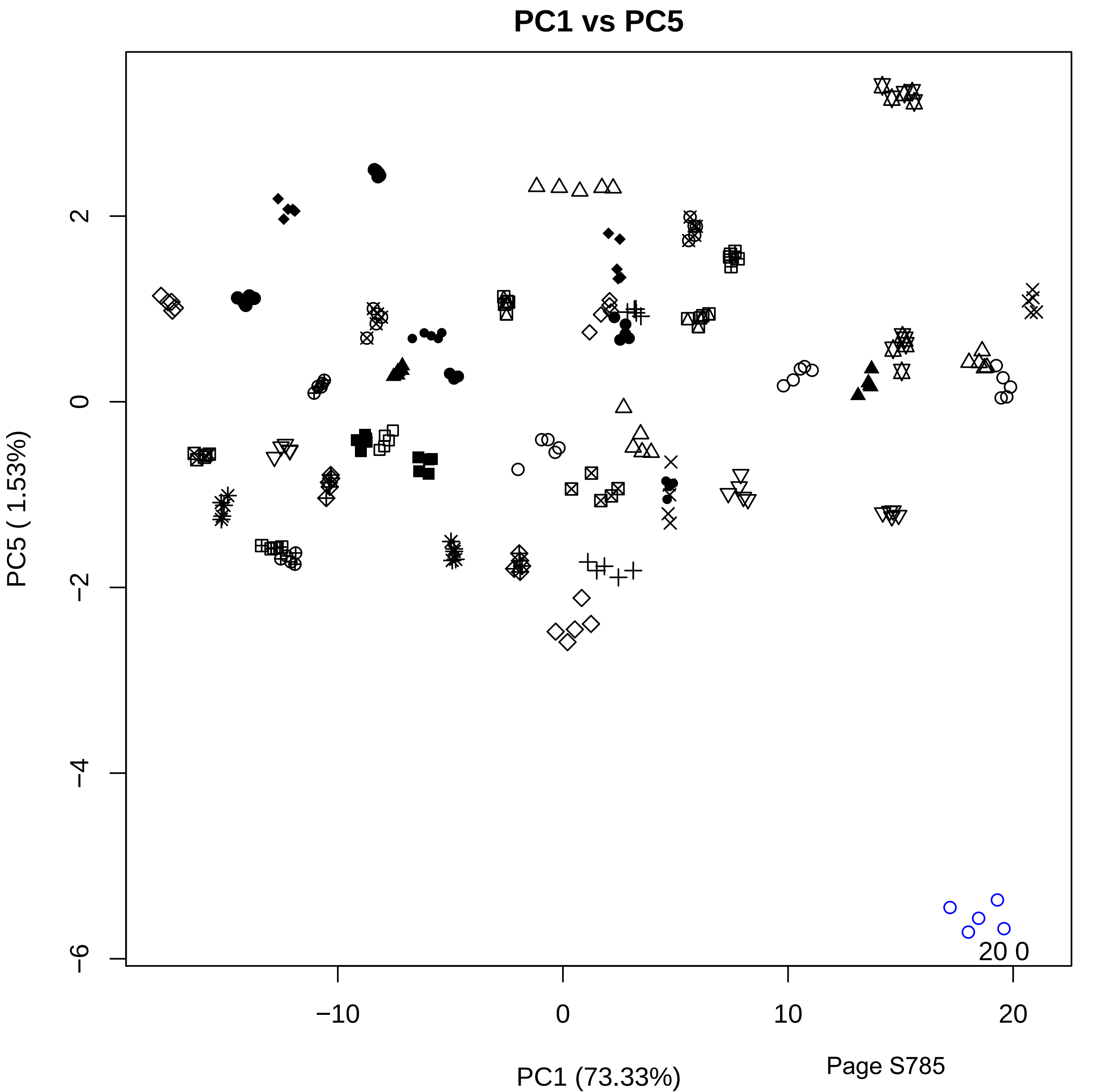
PC1 vs PC5
PC5 (1.53%)
PC1 (73.33%)
2
0
−2
−4
−6
−10
0
10
20
20 0

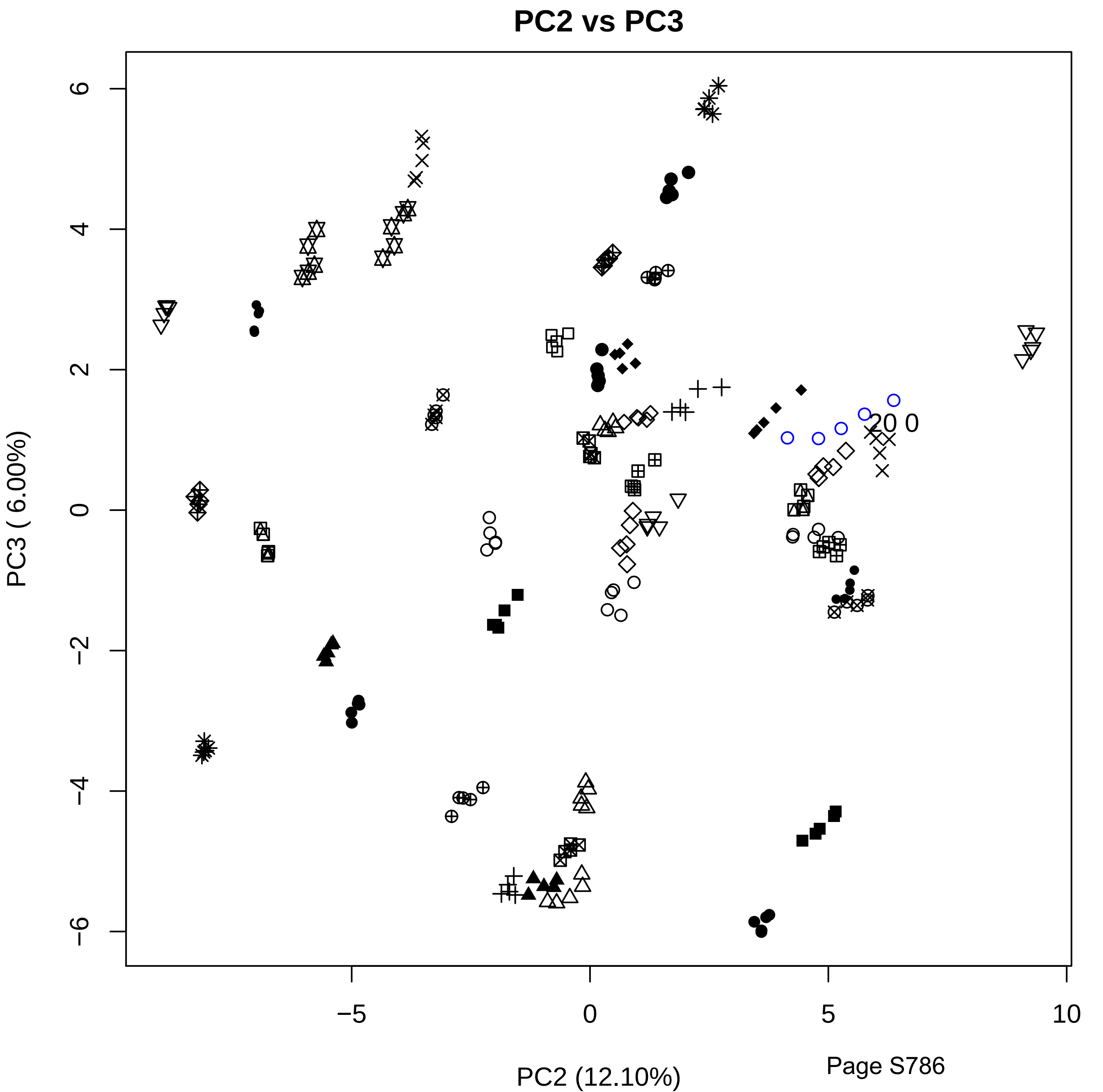
PC2 vs PC3
PC3 (6.00%)
PC2 (12.10%)
6
4
2
0
−2
−4
−6
−5
0
5
10
20 0

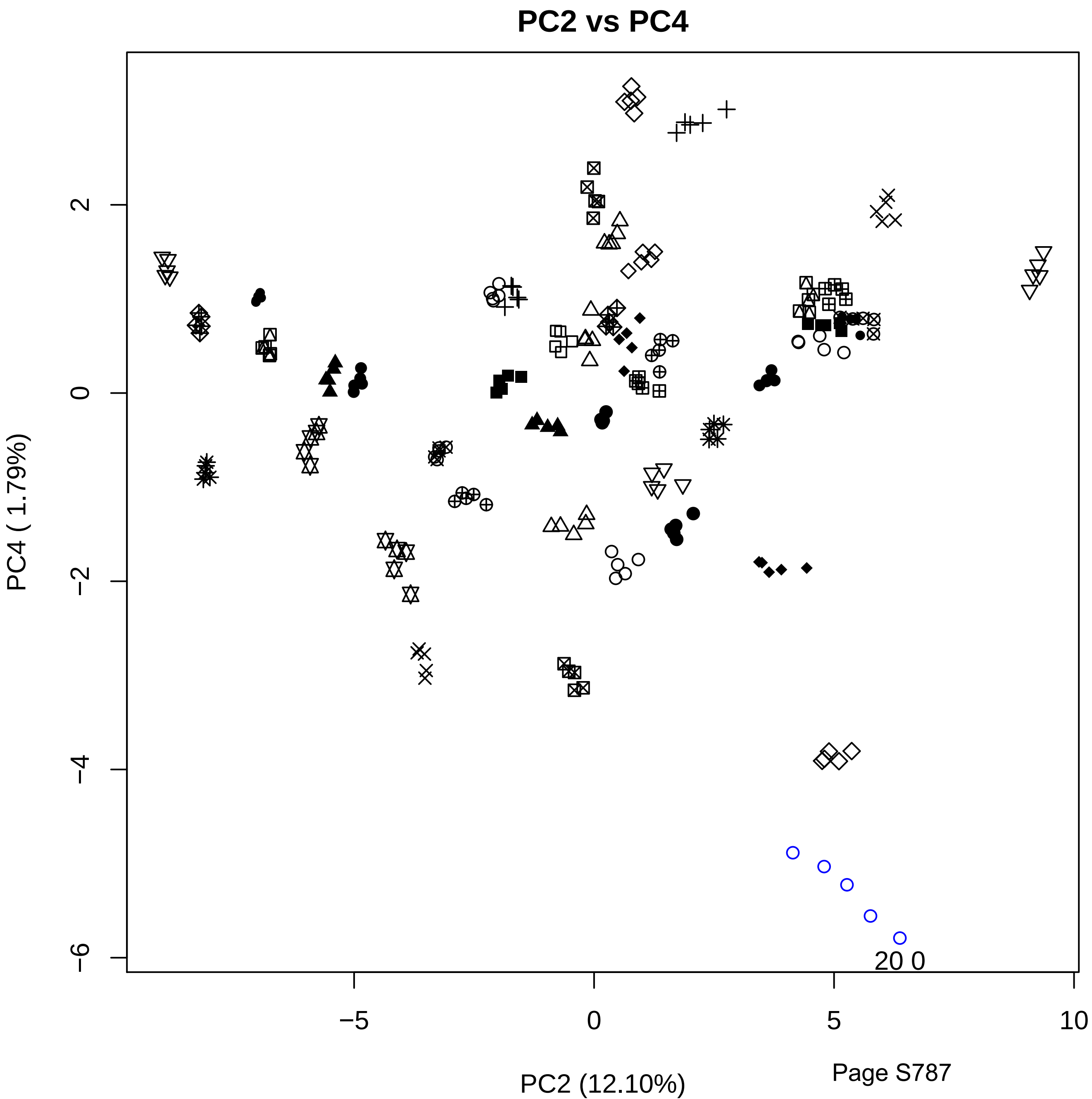
PC2 vs PC4
PC4 (1.79%)
PC2 (12.10%)
2
0
−2
−4
−6
−5
0
5
10
20 0

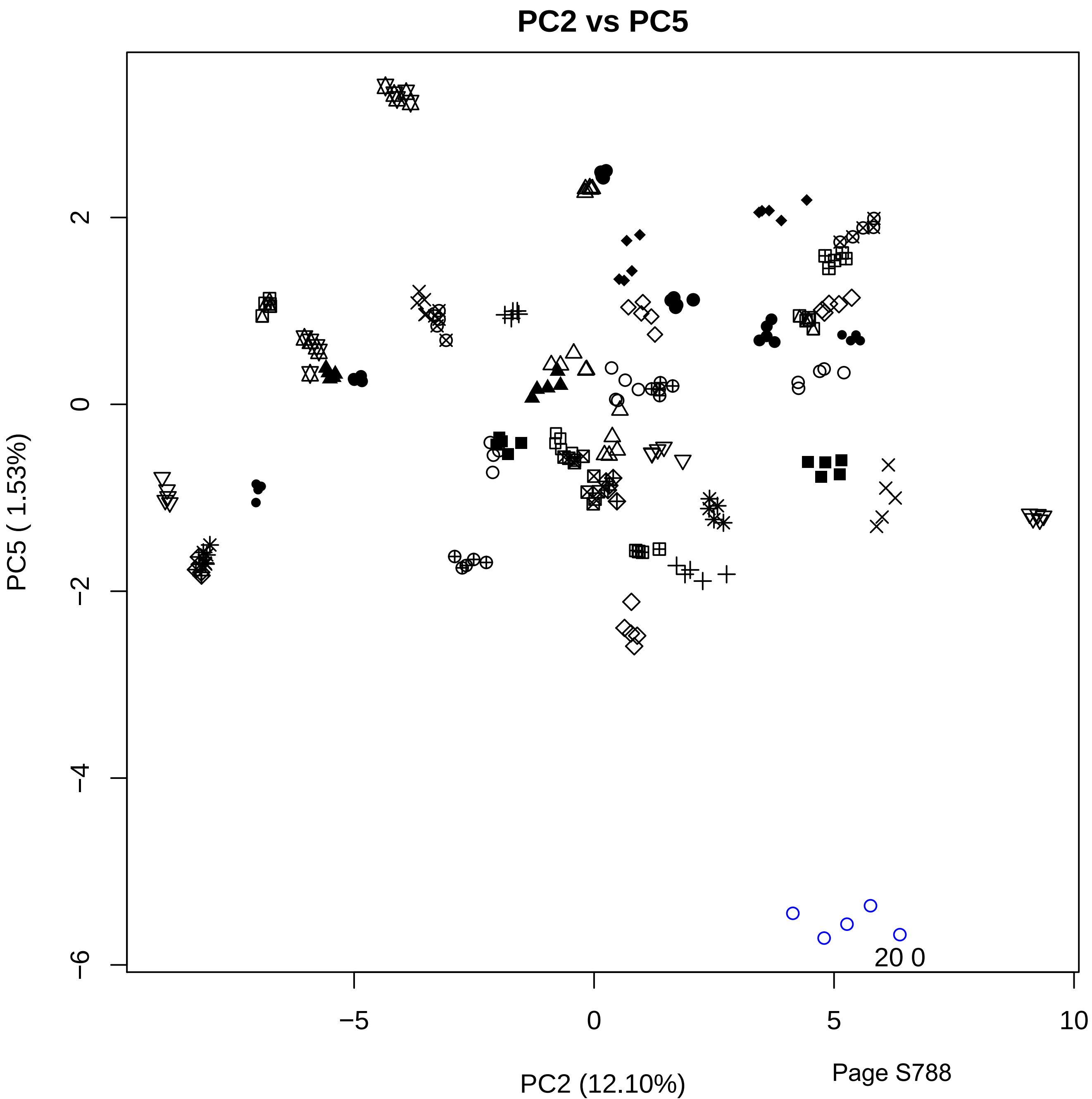
PC2 vs PC5
PC5 (1.53%)
PC2 (12.10%)
2
0
−2
−4
−6
−5
0
5
10
20 0

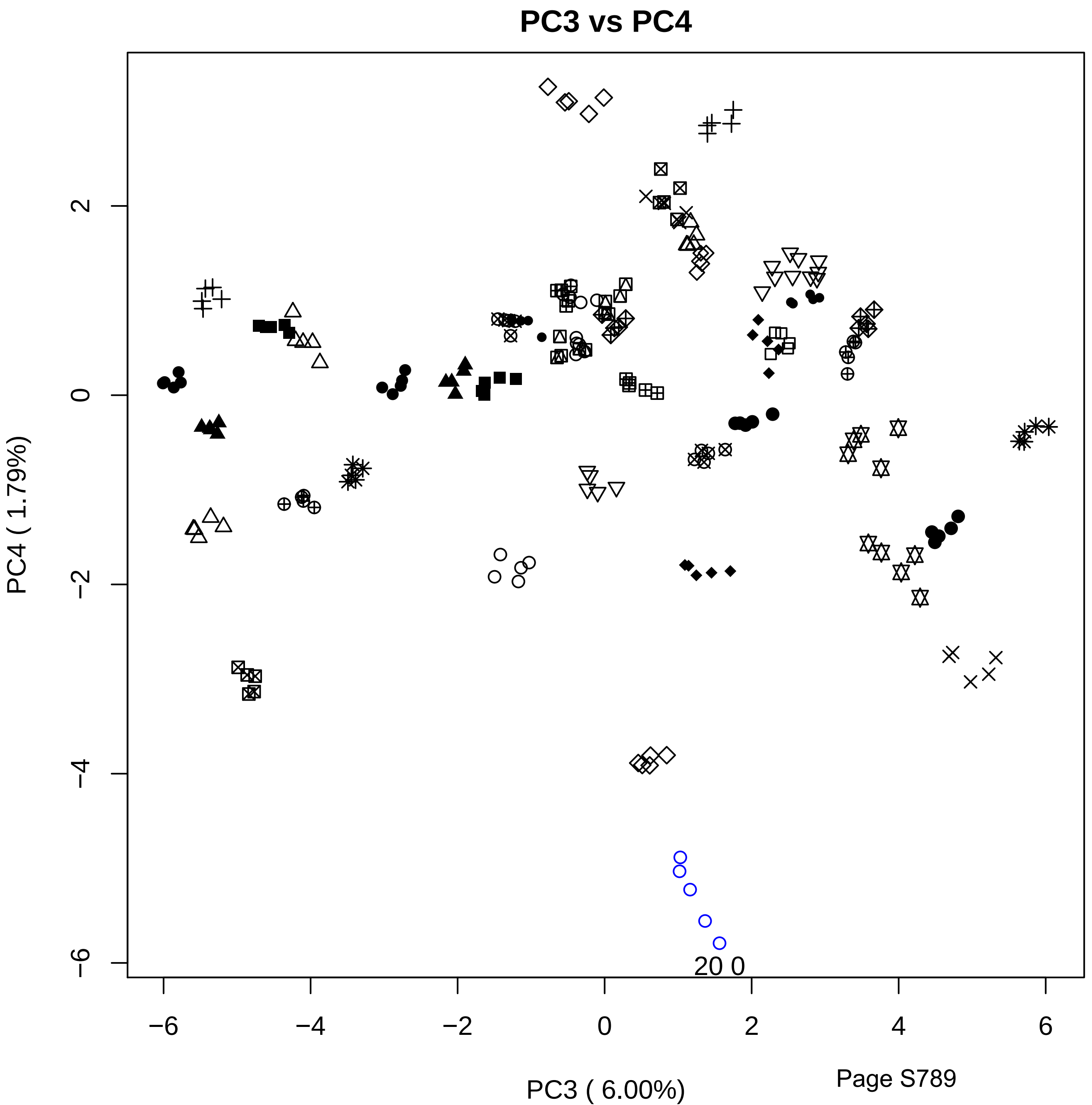
PC3 vs PC4
2
0
−2
−4
−6
PC4 (1.79%)
−6
−4
−2
0
2
4
6
PC3 (6.00%)
20 0

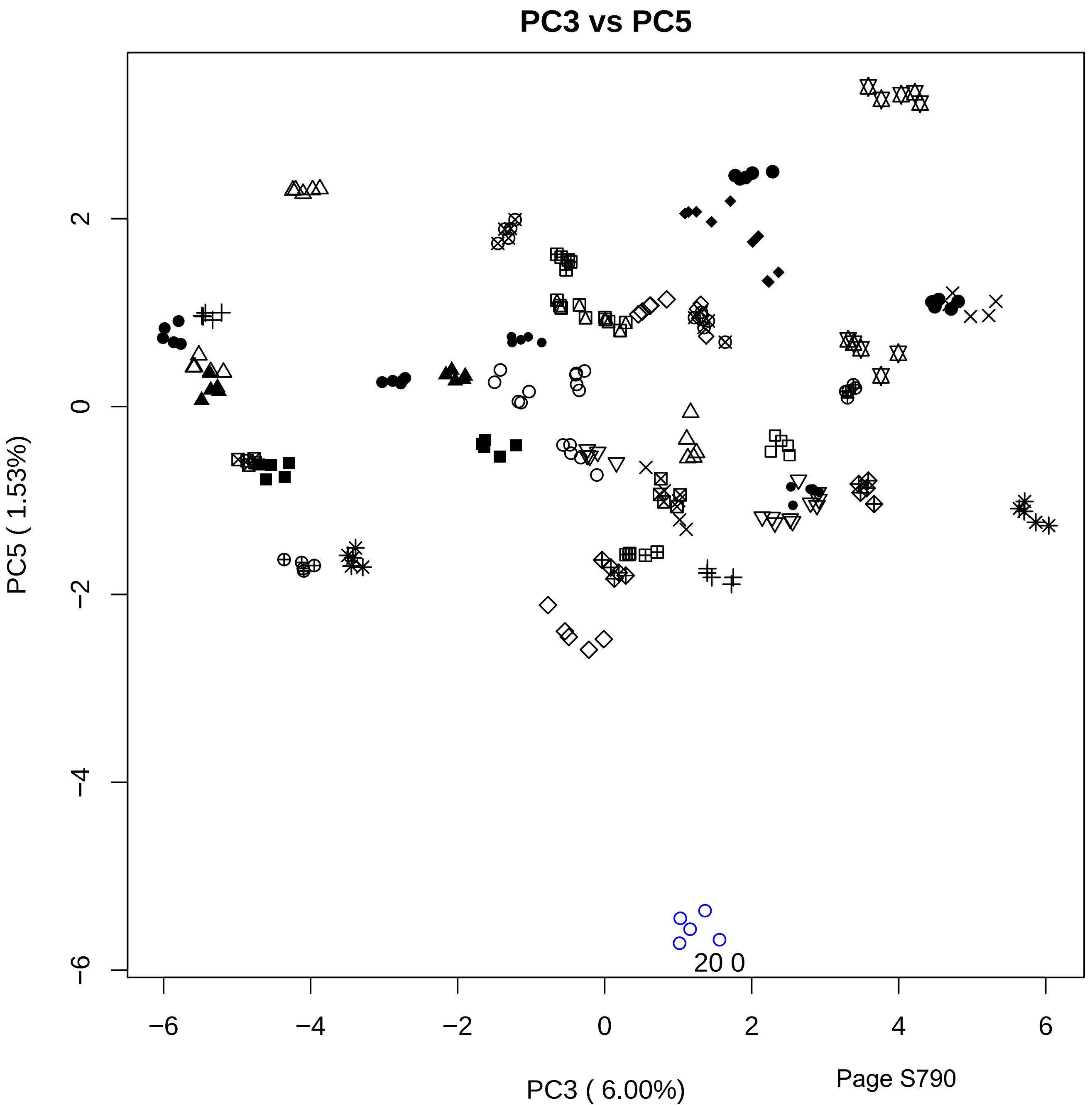

PC3 vs PC5
PC5 (1.53%)
PC3 (6.00%)
2
0
−2
−4
−6
−6
−4
−2
0
2
4
6
20 0

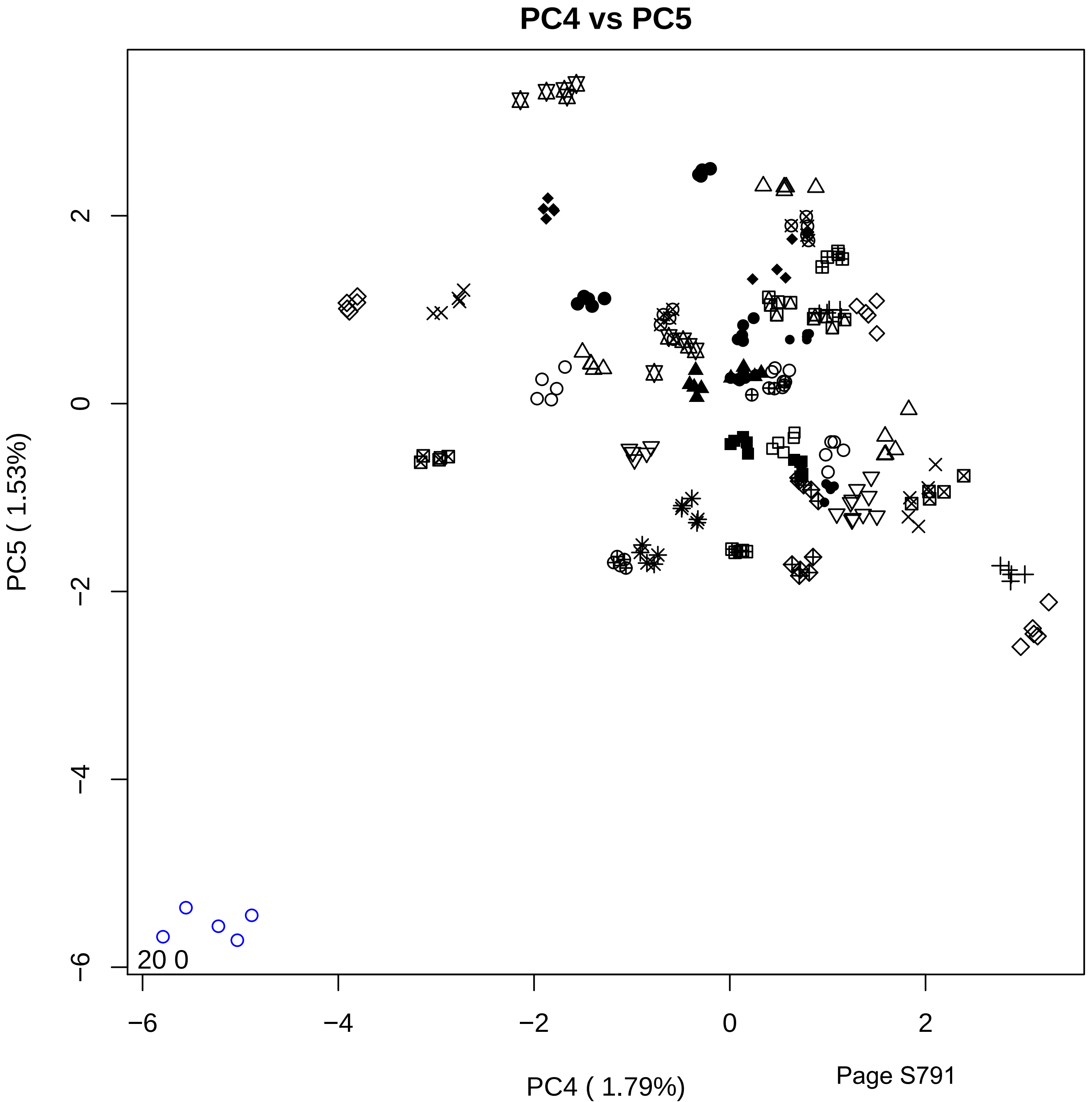
PC4 vs PC5
PC5 (1.53%)
PC4 (1.79%)
2
0
−2
−4
−6
−6
−4
−2
0
2
20 0

PCA of 40% DeS vs PMH

Scaled Matrix PCAed

7th order polynomial baseline correction performed

Raw spectra smoothed to 21 Neighbours, 2nd order PN, Savtisky Golay

Normalisation between 0 and 100

D2 spectra smoothed to 41 Neighbours, 2nd order PN, Savtisky Golay

Regions Analysed: 3601−2501 and 2001−700cm−1

Samples Analysed: 1 Contam's 0 DeS's 45 PMH's

Keele University AD

Sun Dec 09 17:04:37 2018

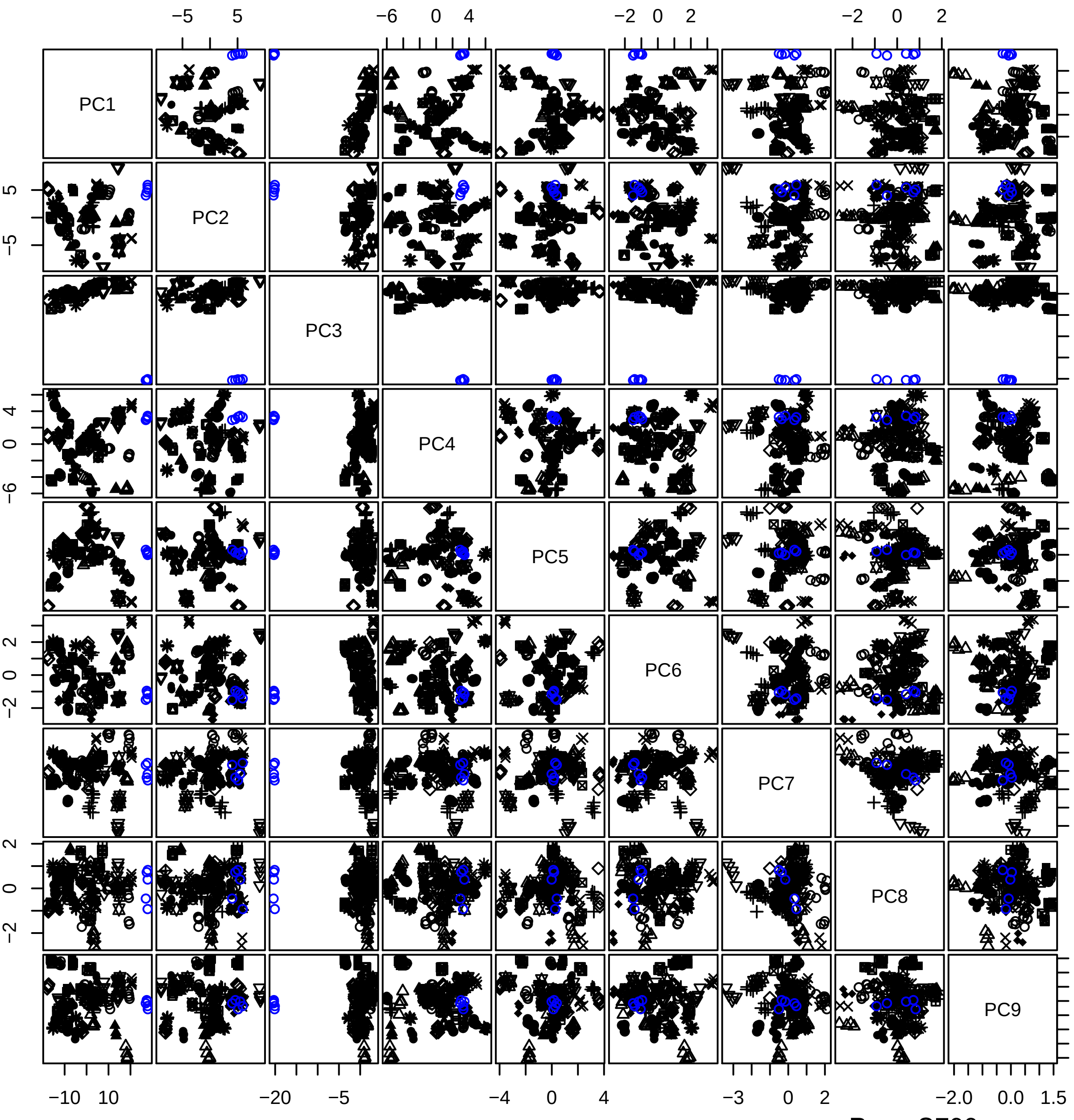

PC1
PC2
PC3
PC4
PC5
PC6
PC7
PC8
PC9

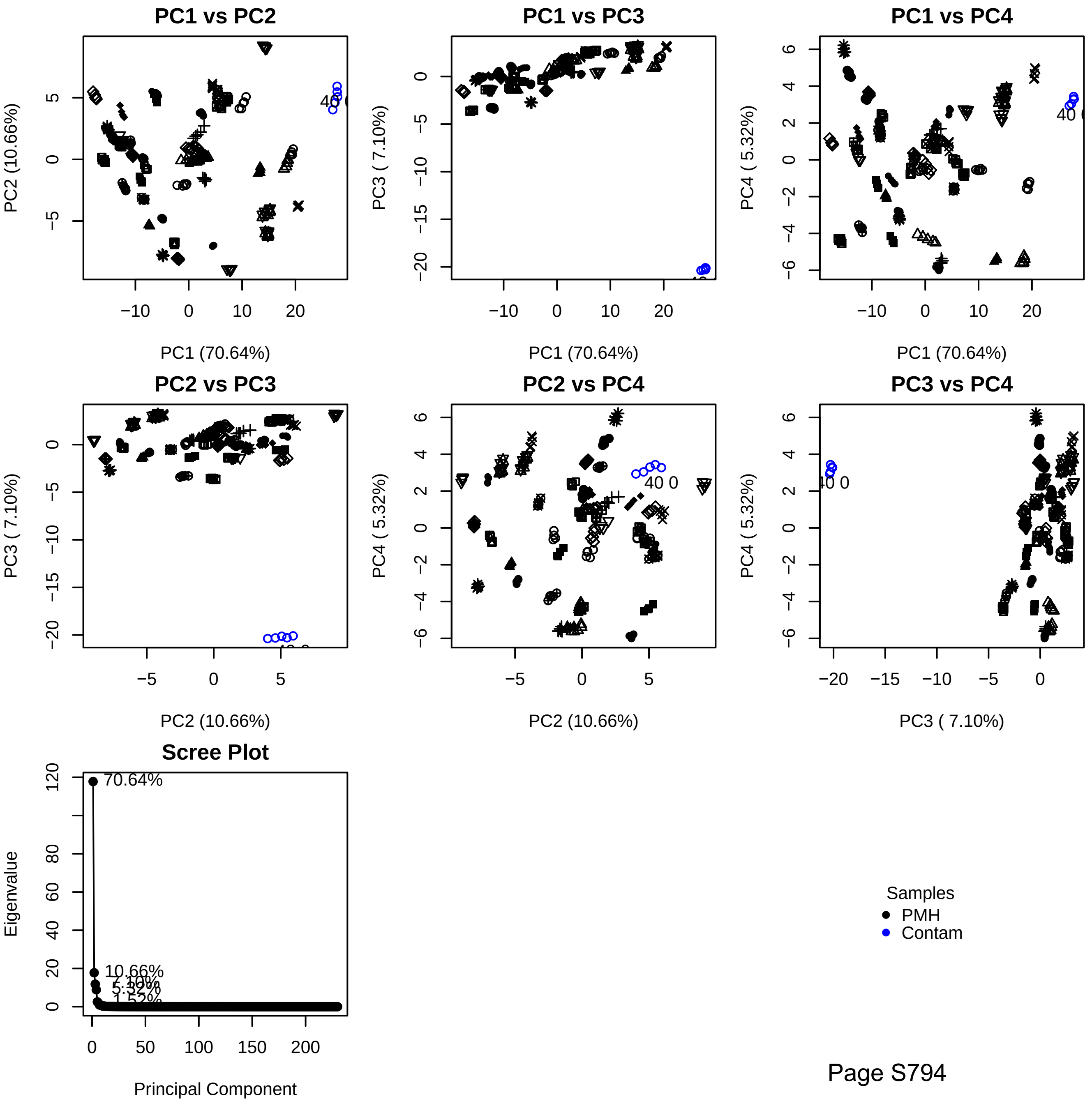
PC1 vs PC2
PC1 vs PC3
PC1 vs PC4
PC2 vs PC3
PC2 vs PC4
PC3 vs PC4
PC1 (70.64%)
PC2 (10.66%)
PC3 (7.10%)
PC4 (5.32%)
Scree Plot
70.64%
10.66%
5.32%
Eigenvalue
Principal Component
Samples
PMH
Contam

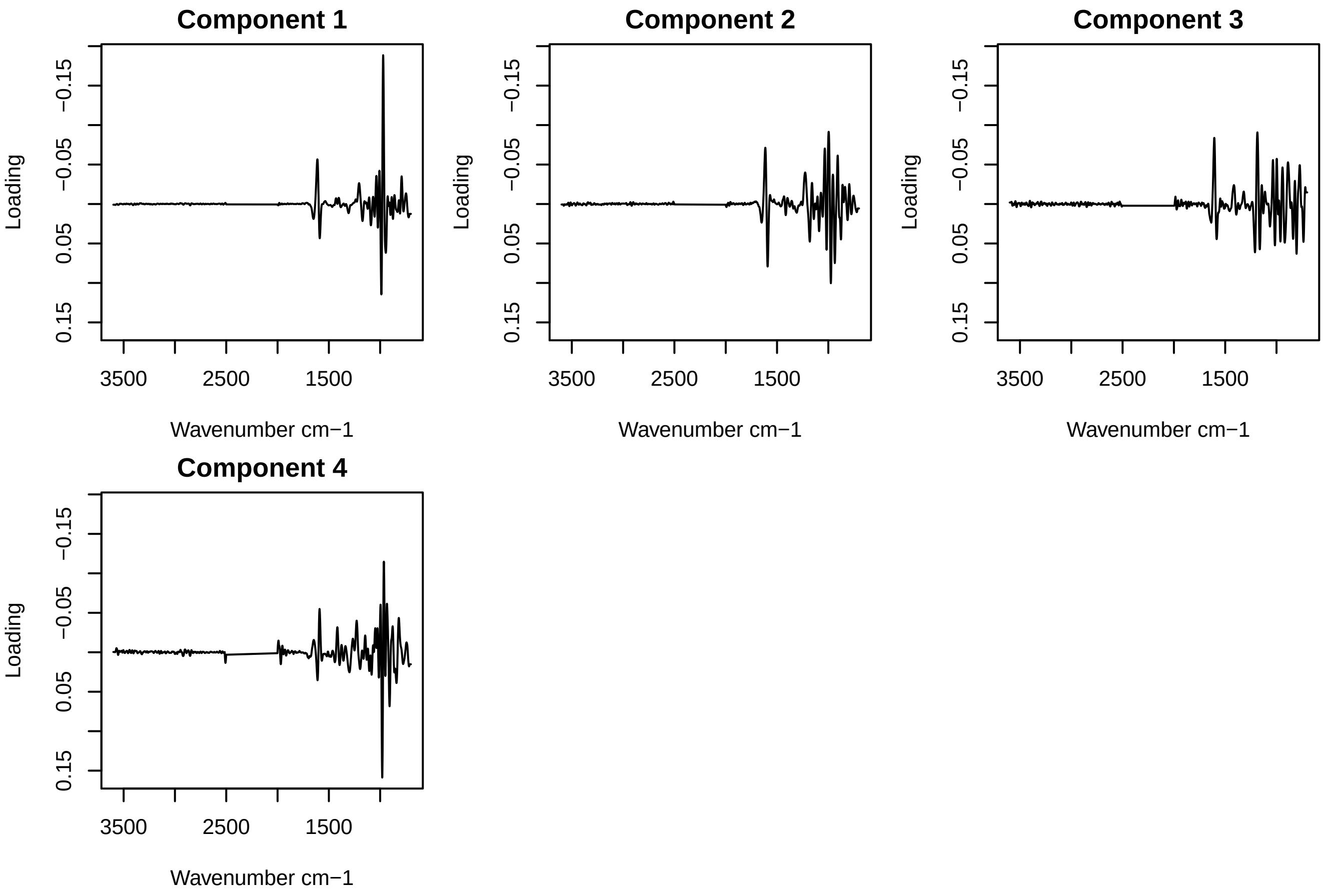
Component 1
Component 2
Component 3
Component 4
Loading
−0.15
−0.05
0.05
0.15
3500
2500
1500
Wavenumber cm−1

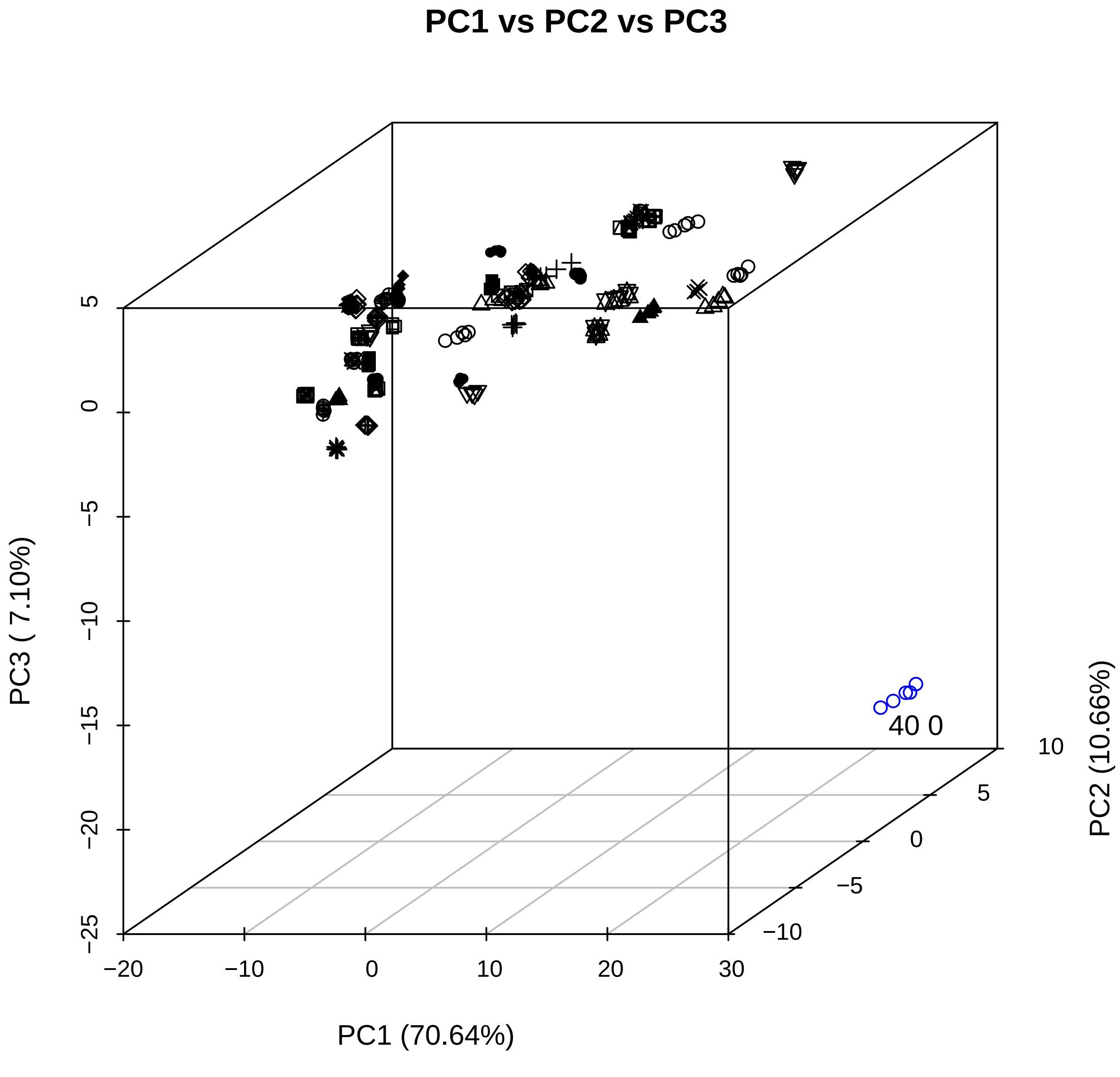
PC1 vs PC2 vs PC3
PC1 (70.64%)
PC2 (10.66%)
PC3 (7.10%)
−20
−10
0
10
20
30
−25
−20
−15
−10
−5
0
5
−10
−5
0
5
10
40 0

PC2 vs PC3 vs PC4

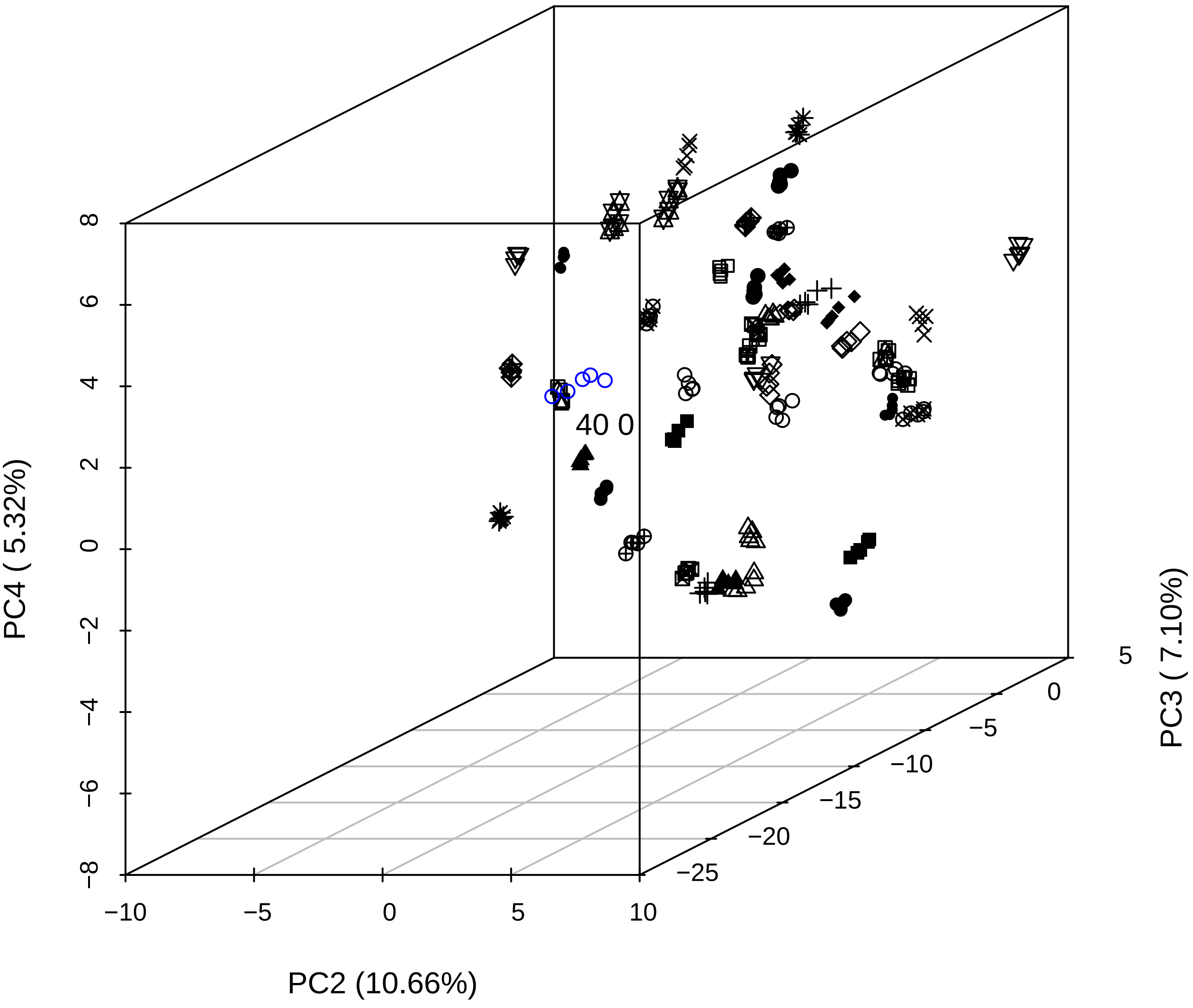

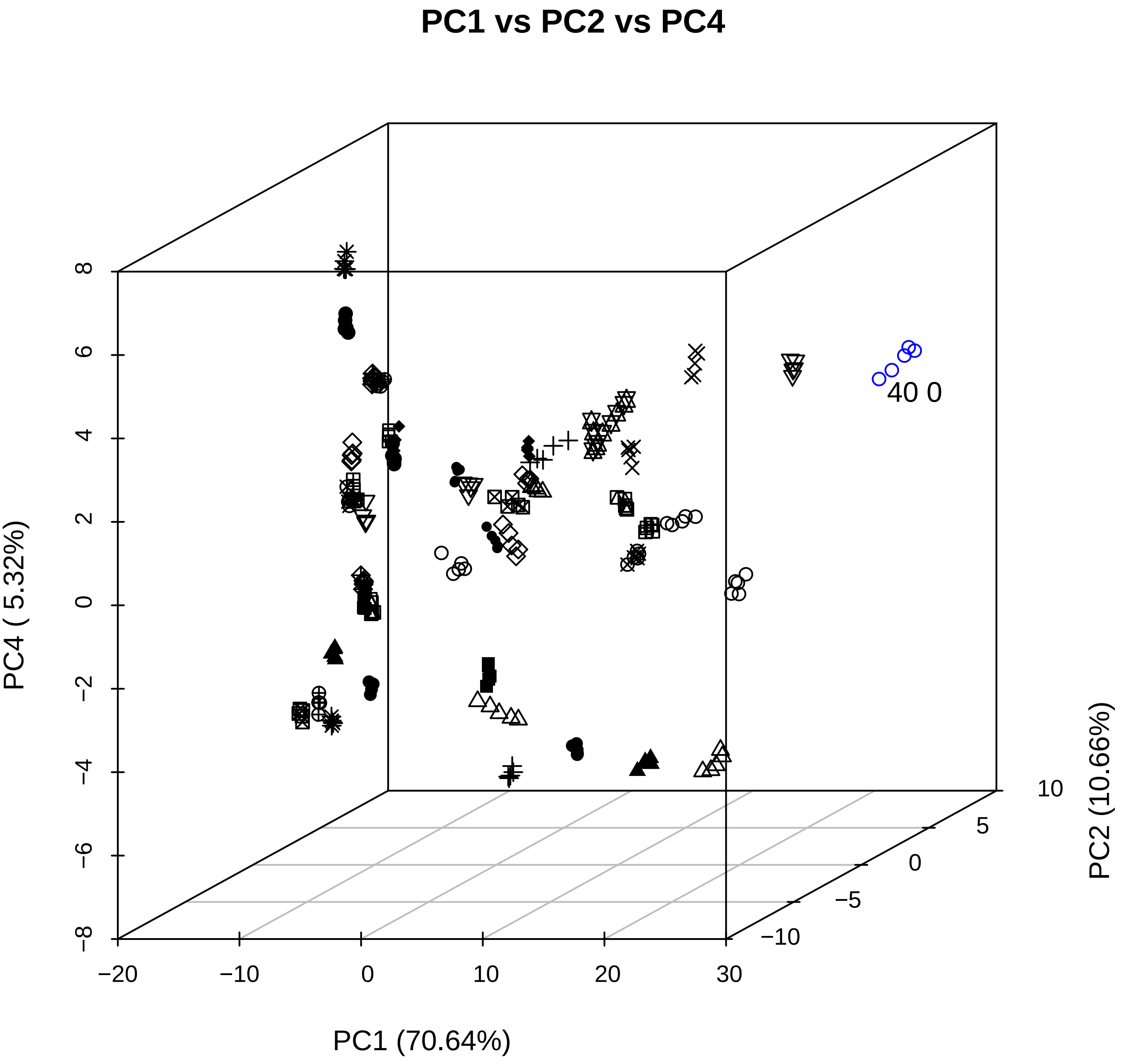
PC1 vs PC2 vs PC4
PC4 (5.32%)
PC1 (70.64%)
PC2 (10.66%)
40 0
8
6
4
2
0
−2
−4
−6
−8
−20
−10
0
10
20
30
10
5
0
−5
−10

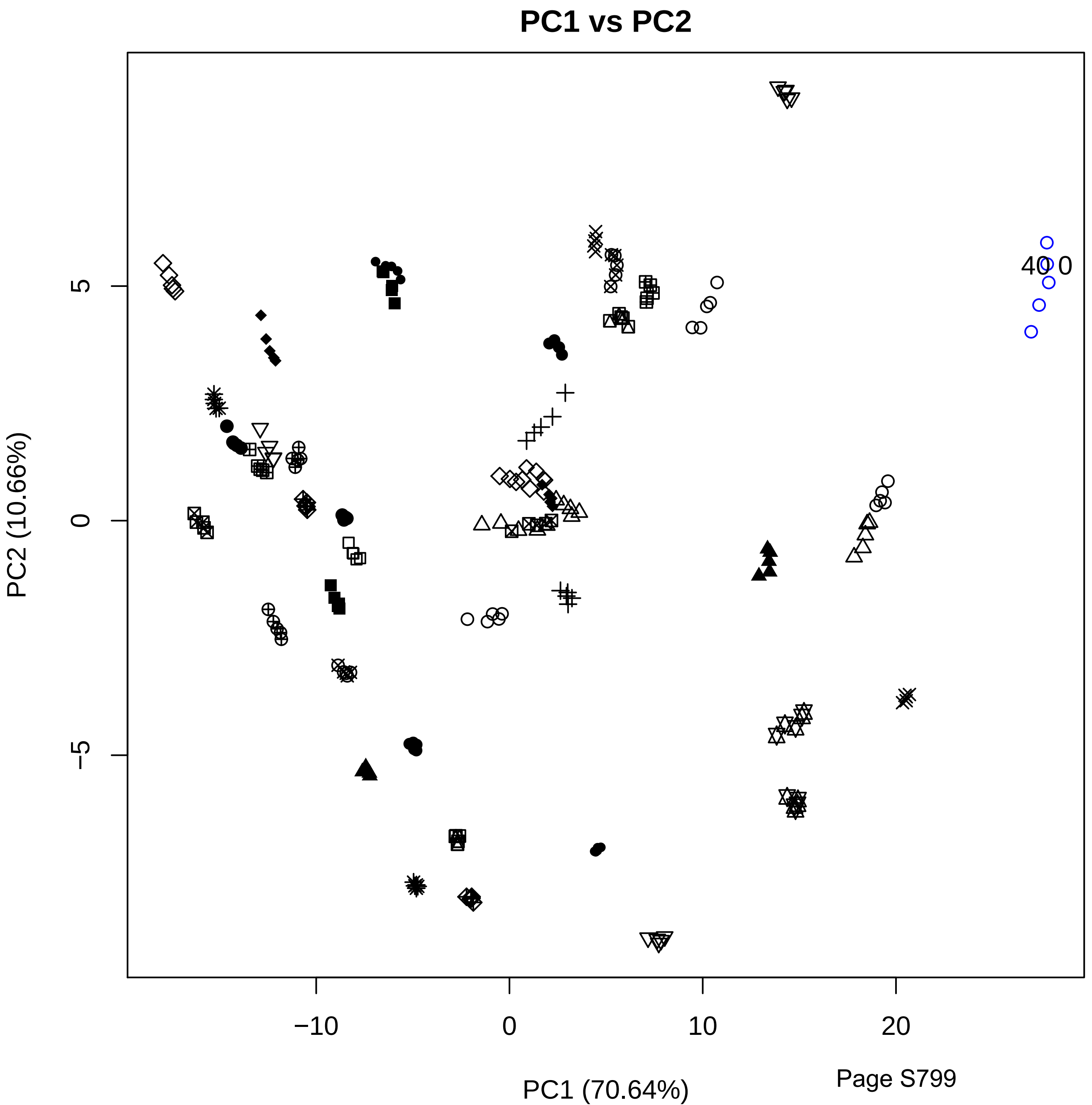
PC1 vs PC2
PC2 (10.66%)
PC1 (70.64%)
5
0
−5
−10
0
10
20
40
0

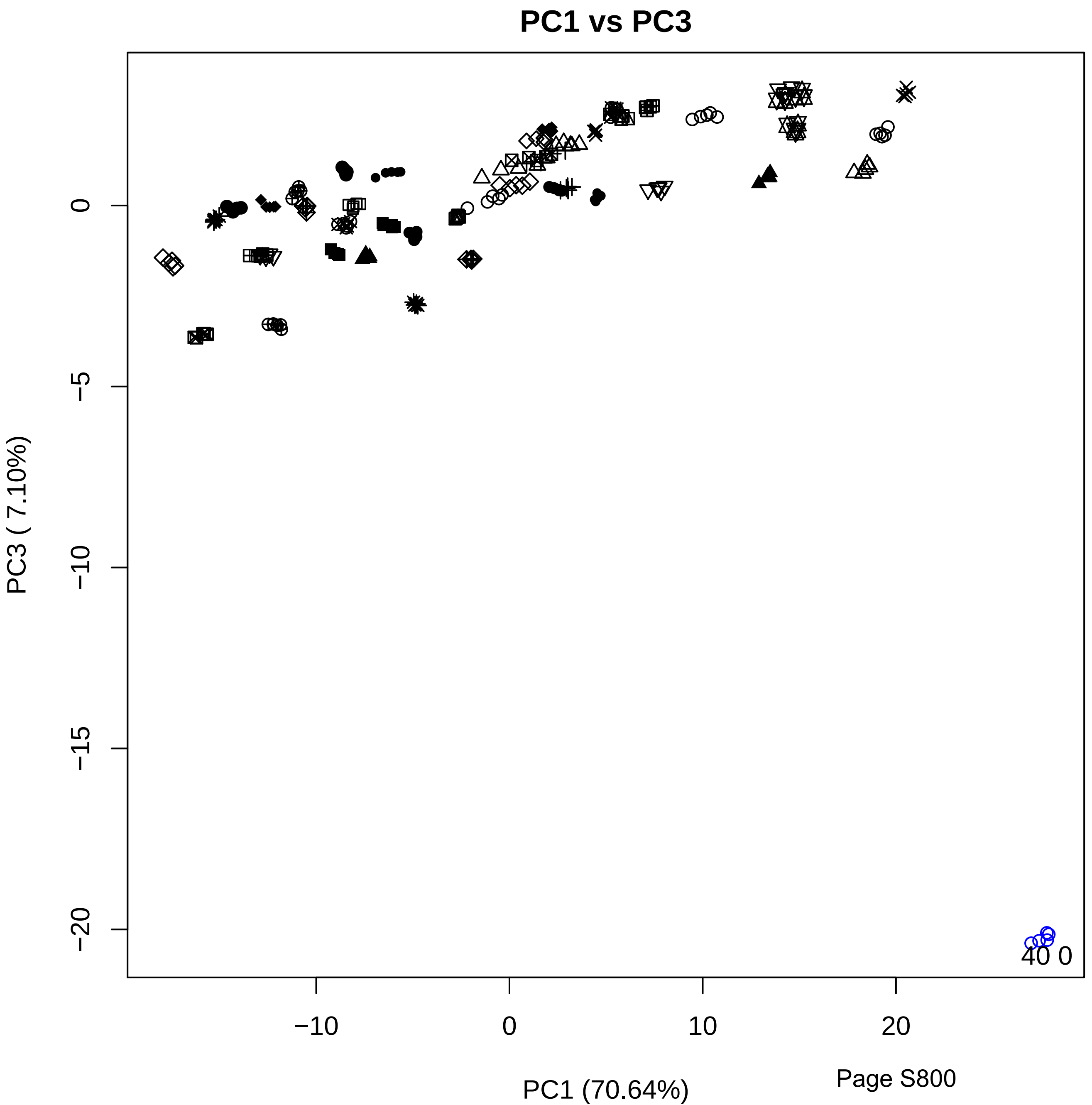
PC1 vs PC3
PC3 (7.10%)
0
−5
−10
−15
−20
−10
0
10
20
PC1 (70.64%)
40 0

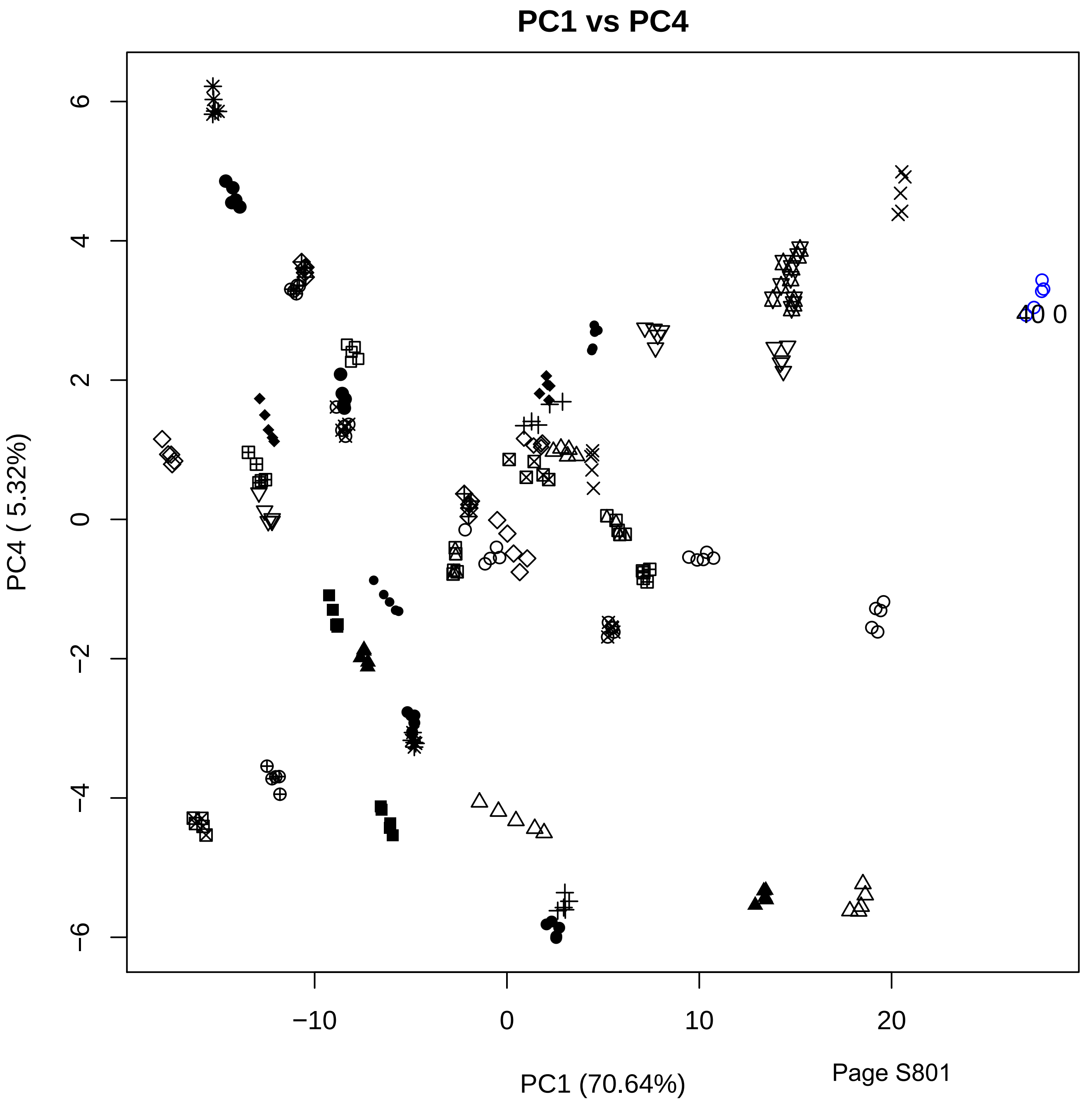
PC1 vs PC4
PC4 (5.32%)
PC1 (70.64%)
6
4
2
0
−2
−4
−6
−10
0
10
20
40 0

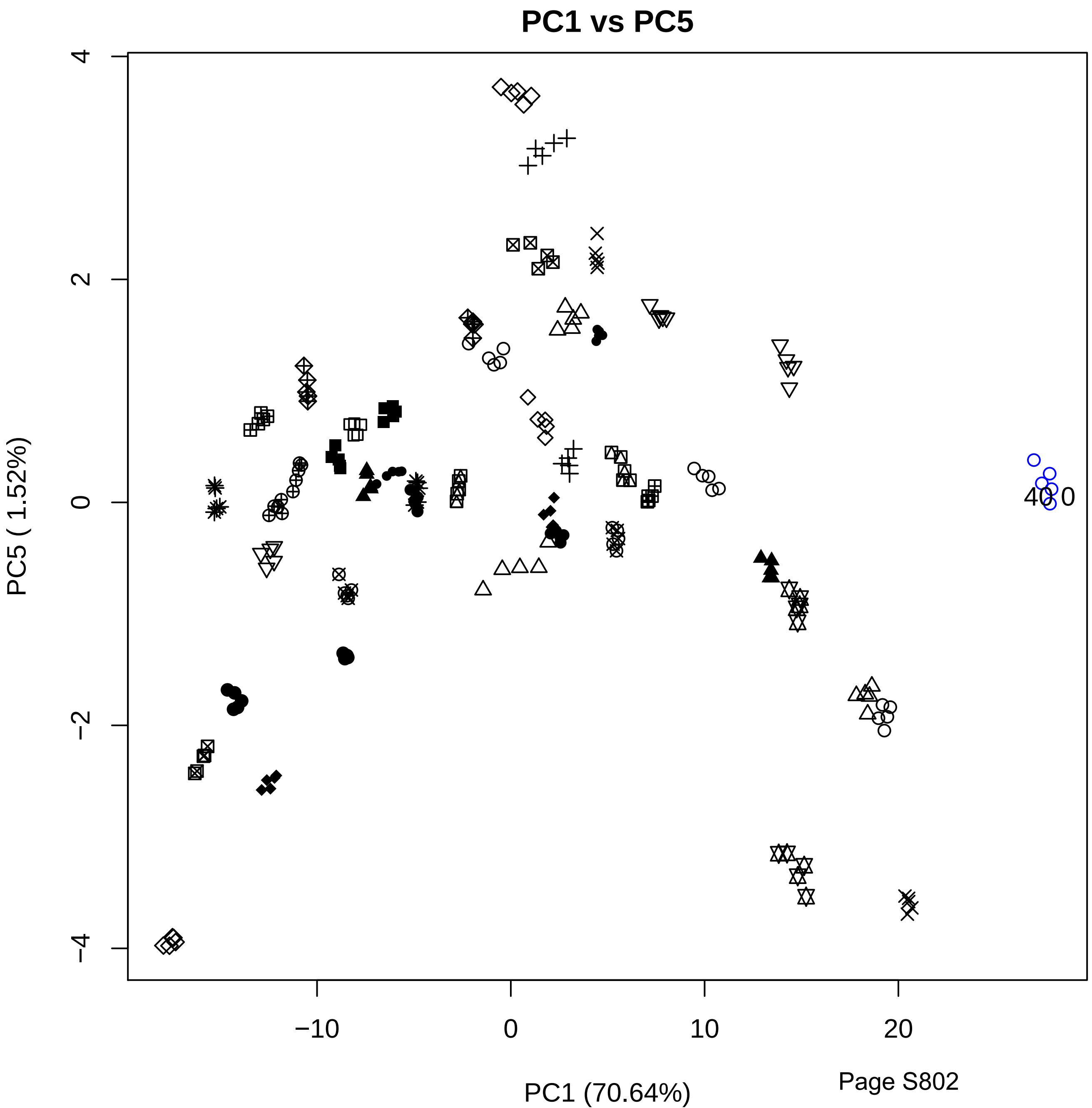
PC1 vs PC5
PC5 (1.52%)
PC1 (70.64%)
4
2
0
−2
−4
−10
0
10
20
400

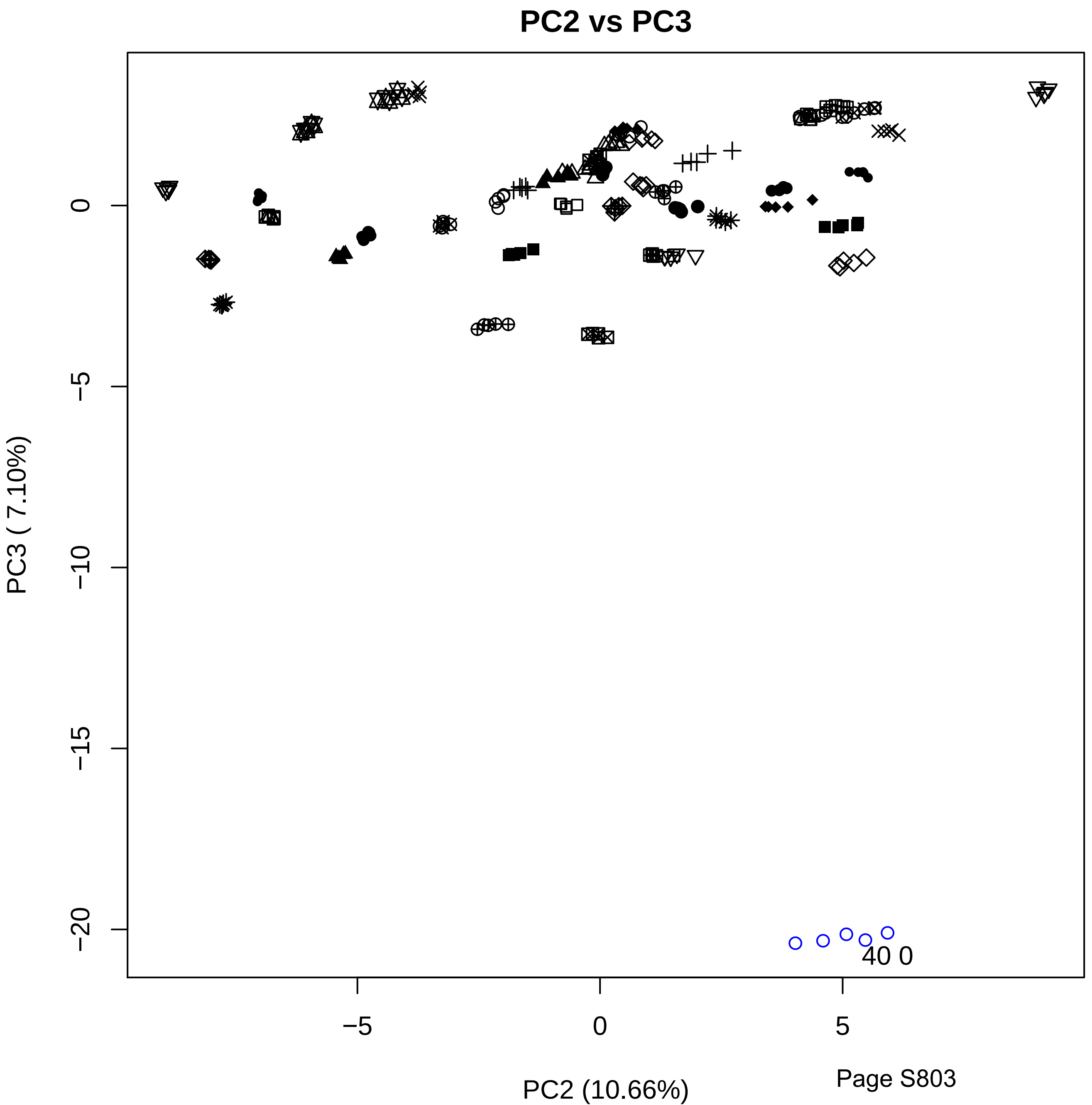
PC2 vs PC3
0
−5
−10
−15
−20
PC3 (7.10%)
40 0
−5
0
5
PC2 (10.66%)

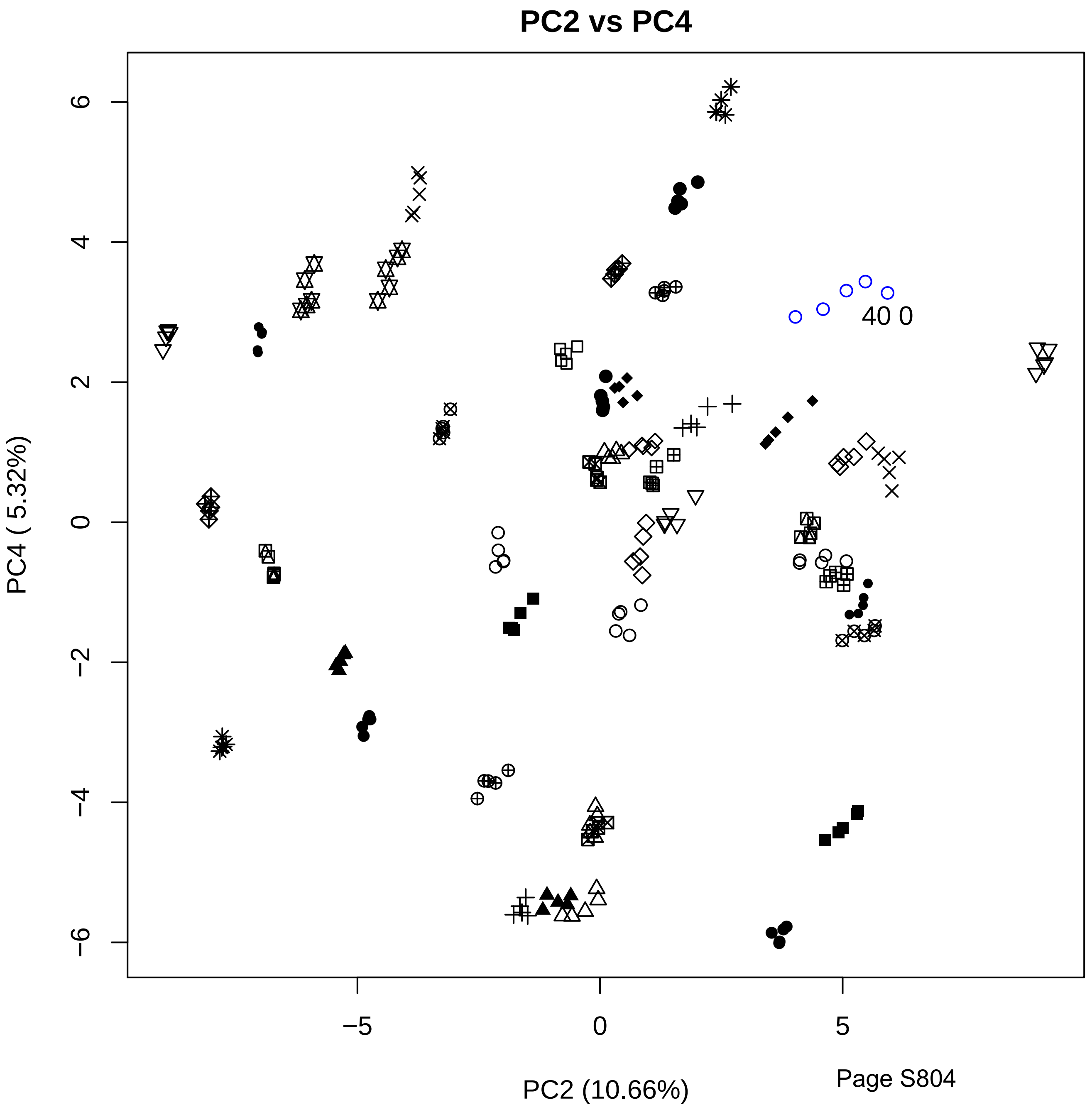
PC2 vs PC4
PC4 (5.32%)
PC2 (10.66%)
6
4
2
0
−2
−4
−6
−5
0
5
40 0

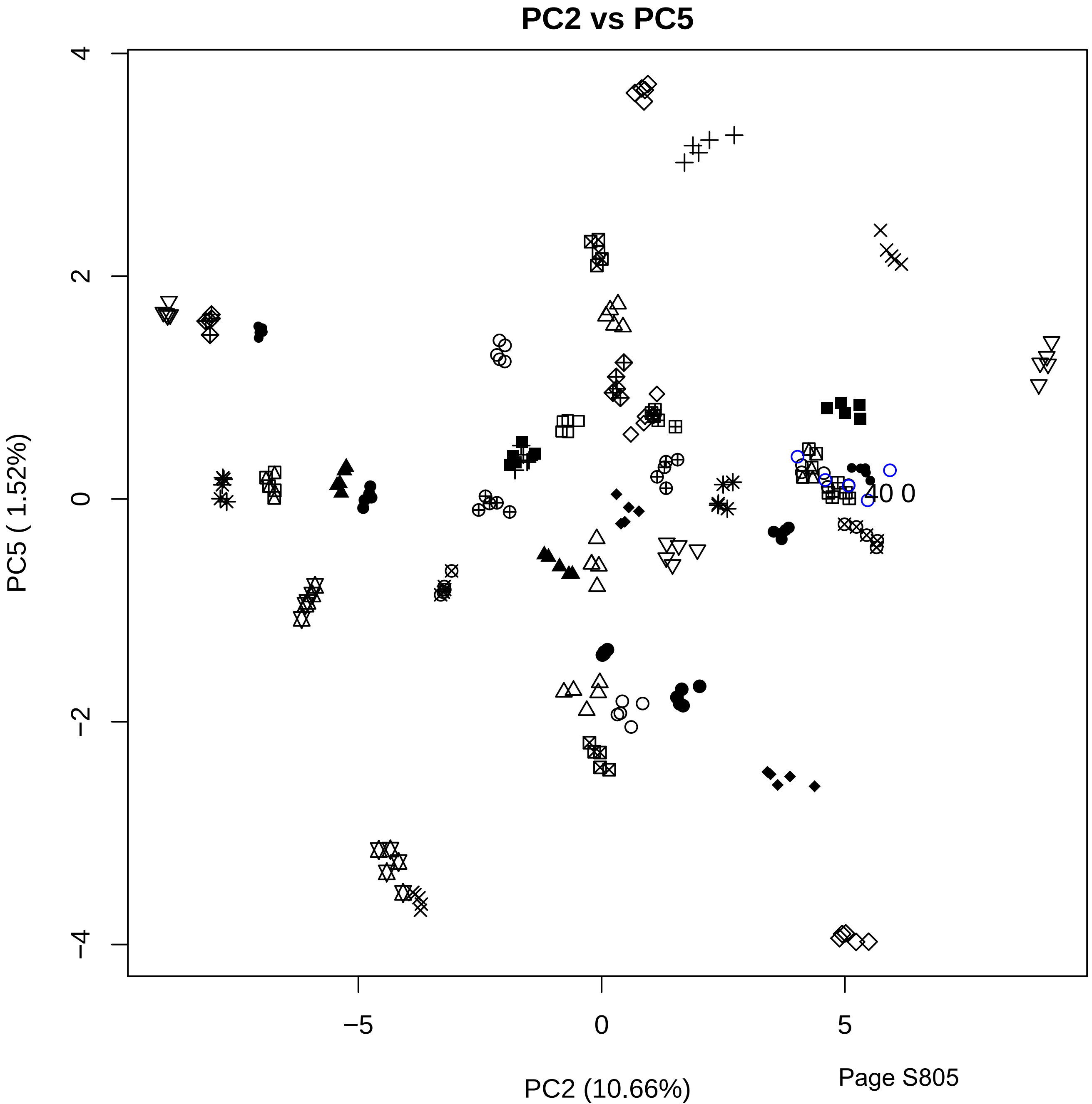
PC2 vs PC5
4
2
0
−2
−4
−5
0
5
40 0
PC5 (1.52%)
PC2 (10.66%)

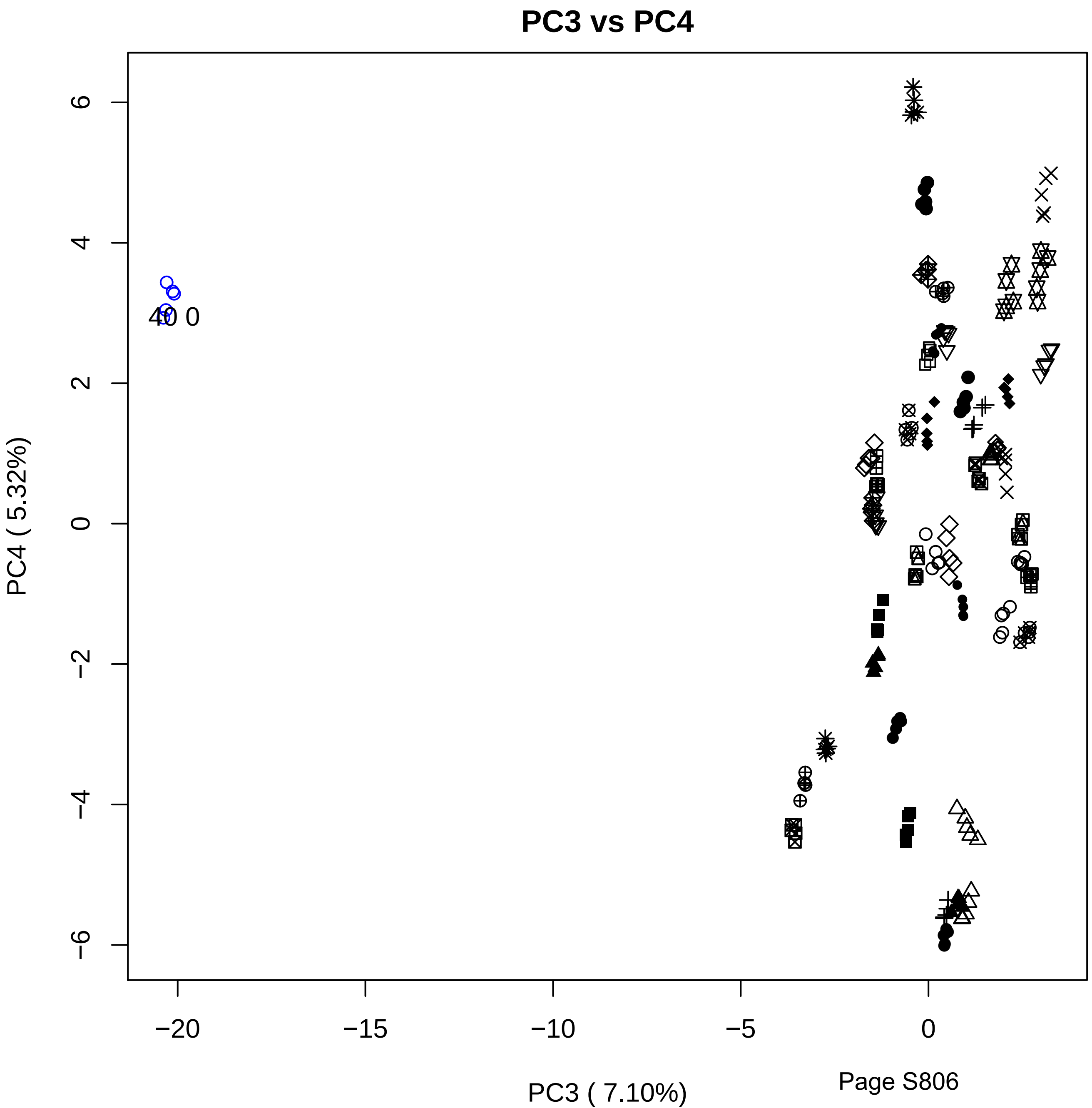
PC3 vs PC4
PC4 (5.32%)
PC3 (7.10%)
6
4
2
0
−2
−4
−6
−20
−15
−10
−5
0
40 0

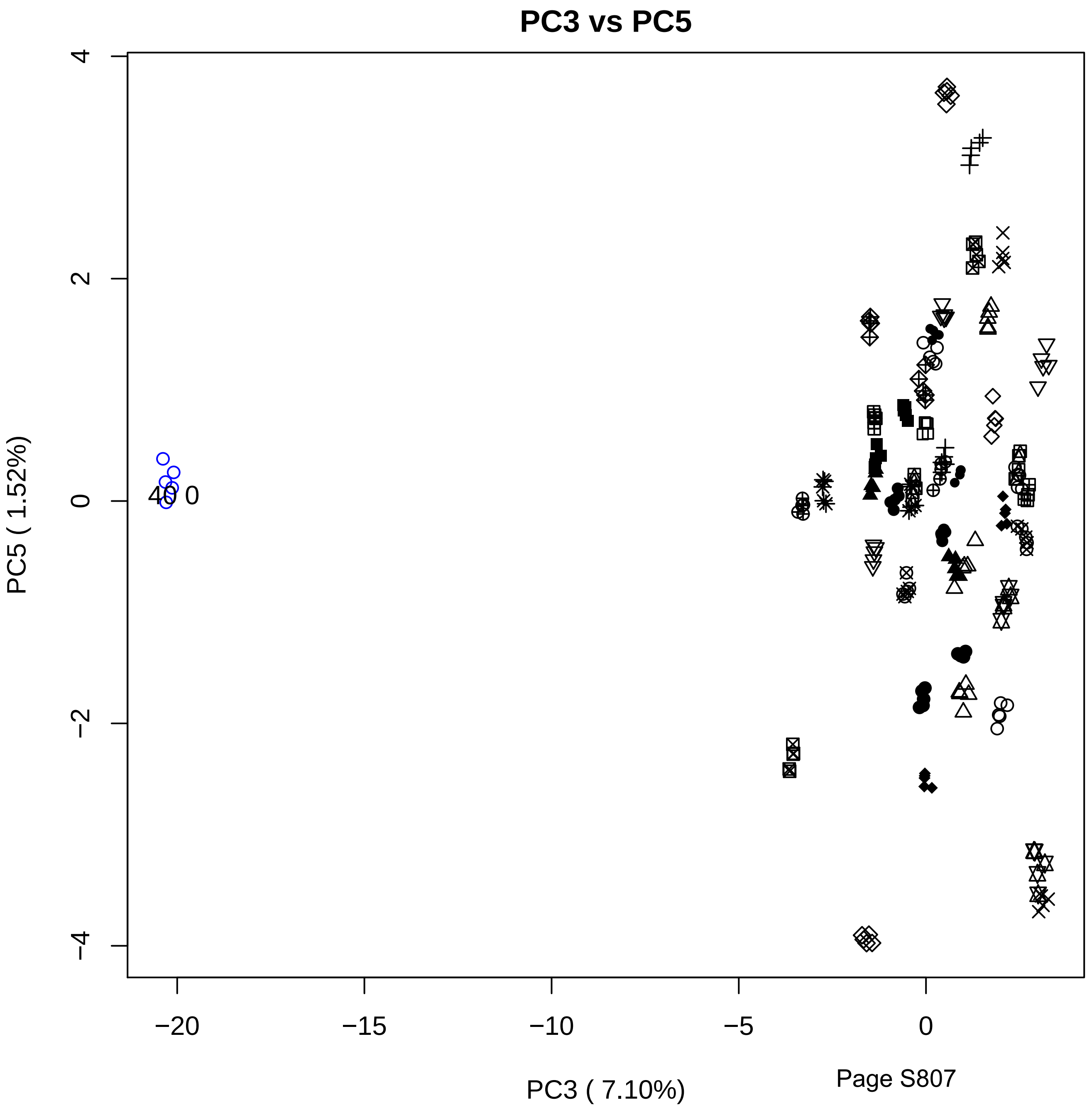
PC3 vs PC5
4
2
0
−2
−4
PC5 (1.52%)
40 0
−20
−15
−10
−5
0
PC3 (7.10%)

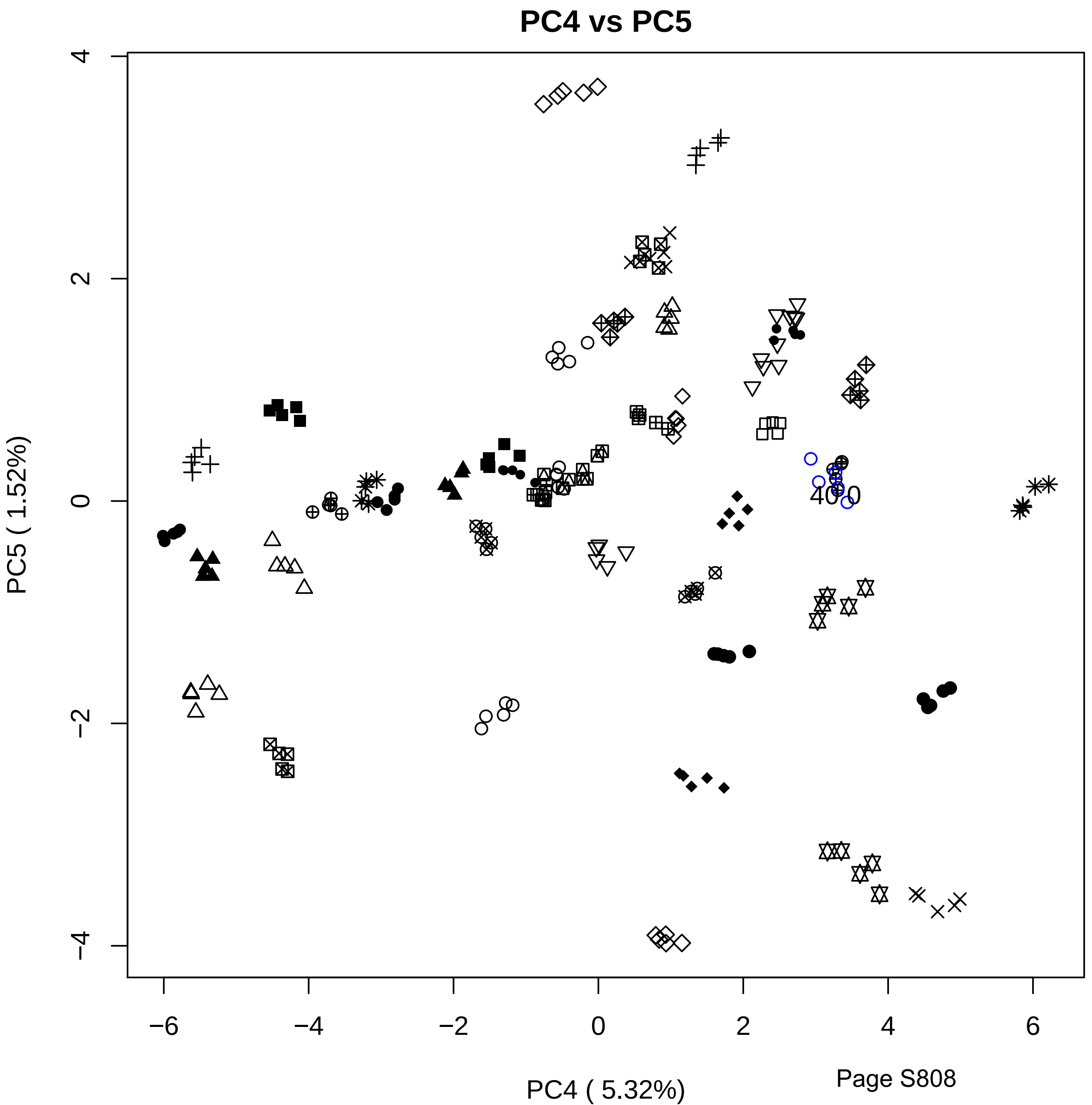
PC4 vs PC5
PC5 (1.52%)
PC4 (5.32%)
4
2
0
−2
−4
−6
6
400

PCA of 100% DeS vs PMH

Scaled Matrix PCAed

7th order polynomial baseline correction performed

Raw spectra smoothed to 21 Neighbours, 2nd order PN, Savtisky Golay

Normalisation between 0 and 100

D2 spectra smoothed to 41 Neighbours, 2nd order PN, Savtisky Golay

Regions Analysed: 3601−2501 and 2001−700cm−1

Samples Analysed: 0 Contam's 1 DeS's 45 PMH's

Keele University AD

Sun Dec 09 17:04:42 2018

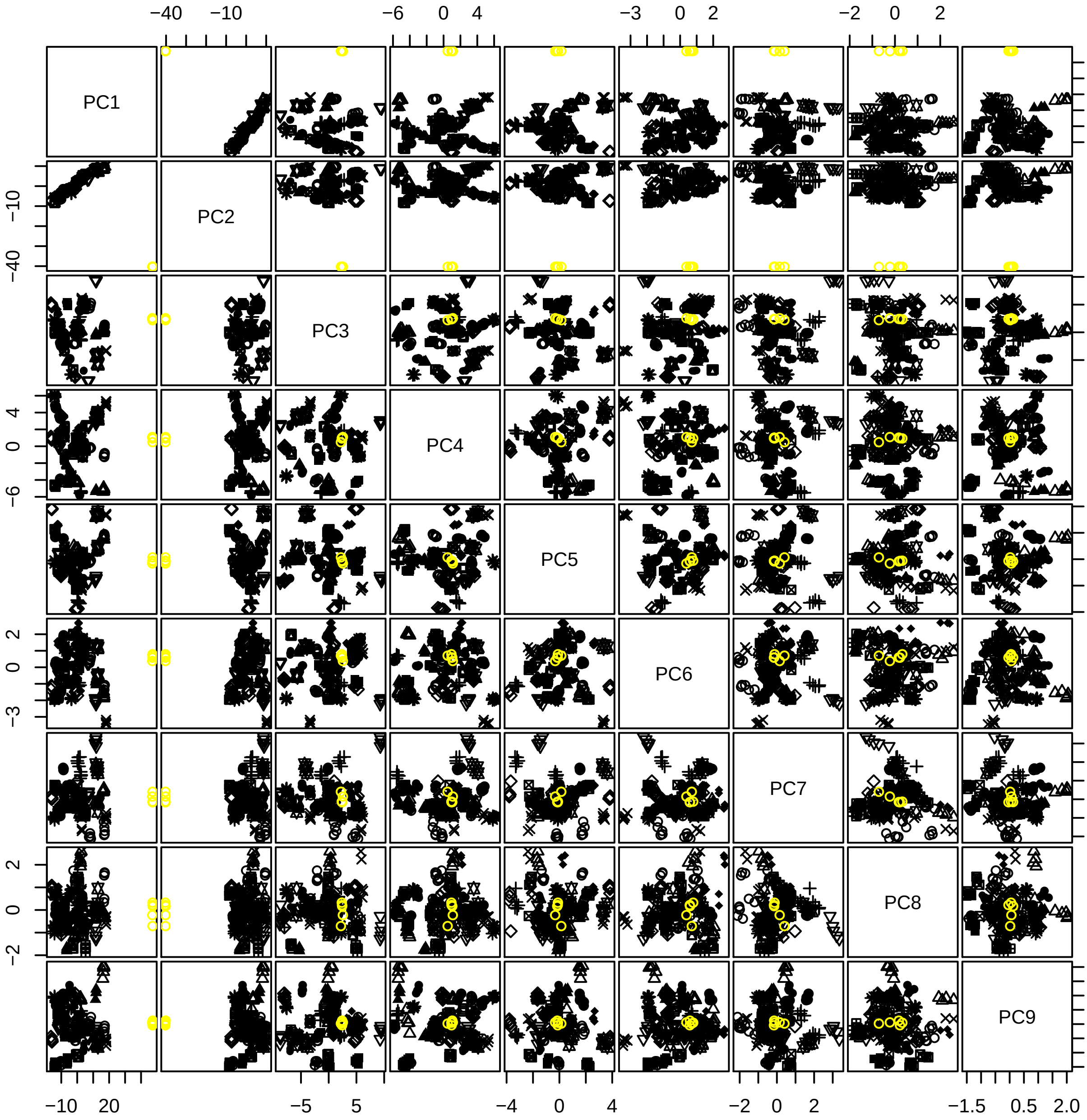

PC1
PC2
PC3
PC4
PC5
PC6
PC7
PC8
PC9

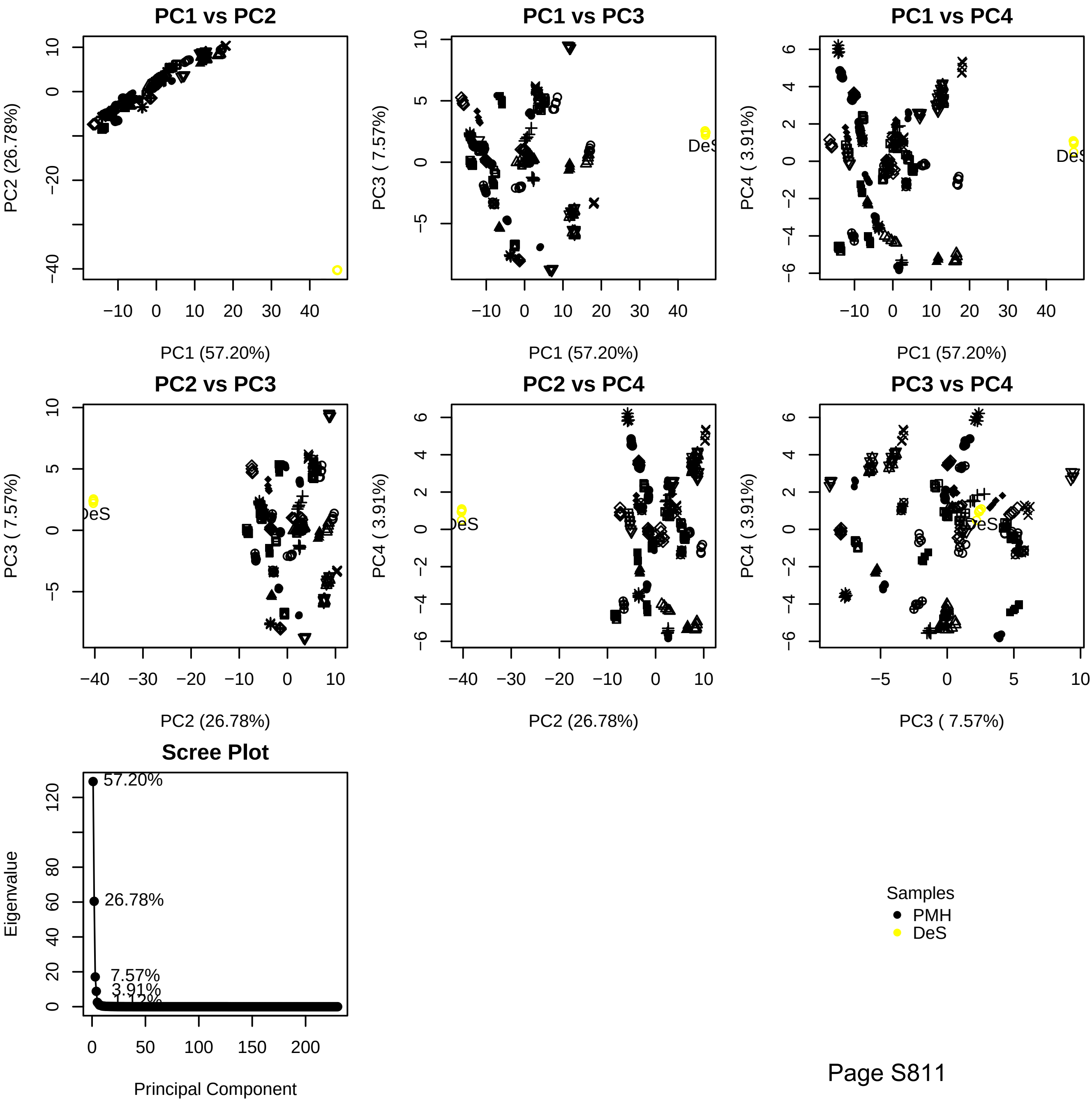
PC1 vs PC2
PC1 vs PC3
PC1 vs PC4
PC2 vs PC3
PC2 vs PC4
PC3 vs PC4
Scree Plot
PC1 (57.20%)
PC2 (26.78%)
PC3 (7.57%)
PC4 (3.91%)
DeS
Eigenvalue
Principal Component
57.20%
26.78%
7.57%
3.91%
Samples
PMH
DeS

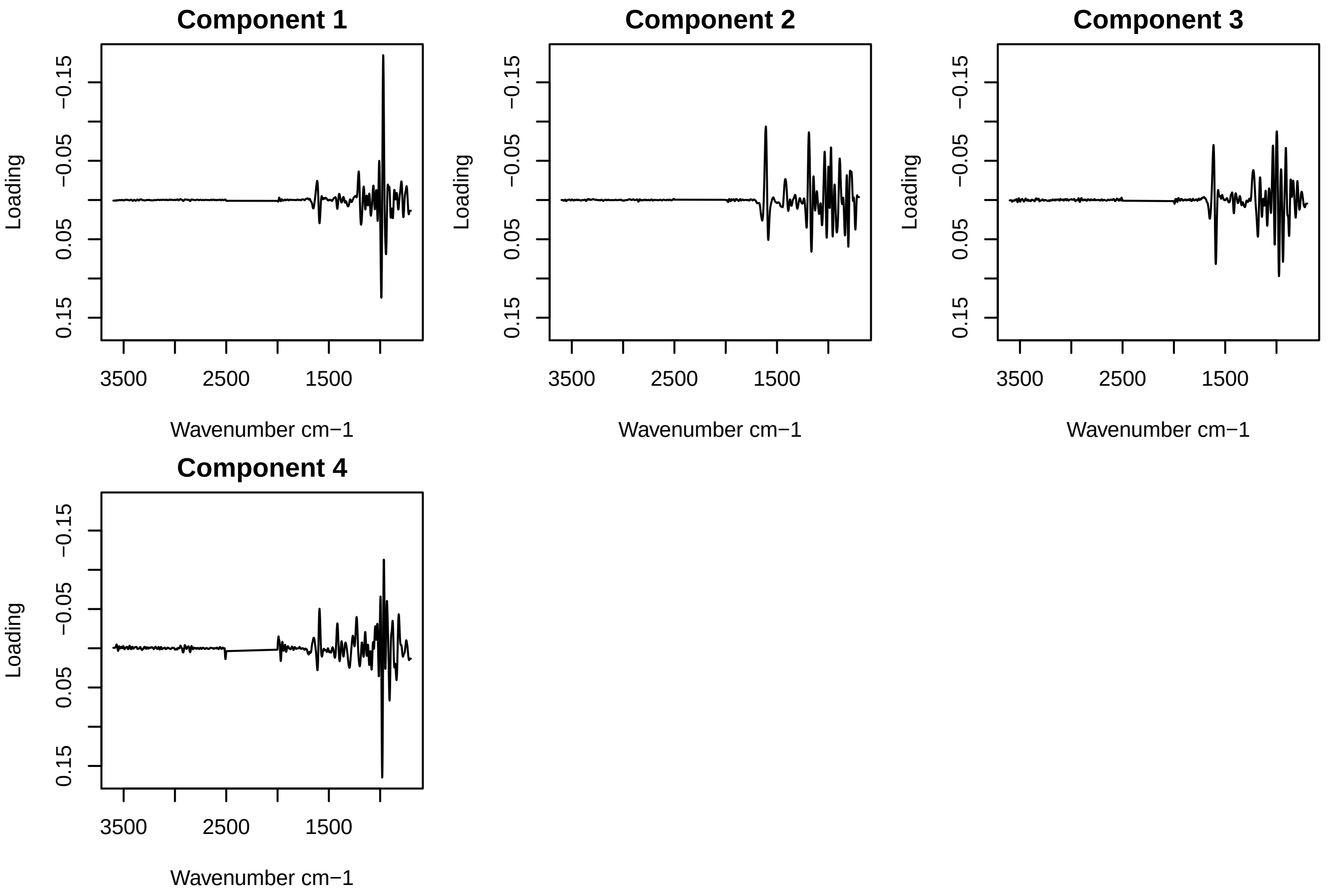
Component 1
Component 2
Component 3
Component 4
Loading
−0.15
−0.05
0.05
0.15
3500
2500
1500
Wavenumber cm−1

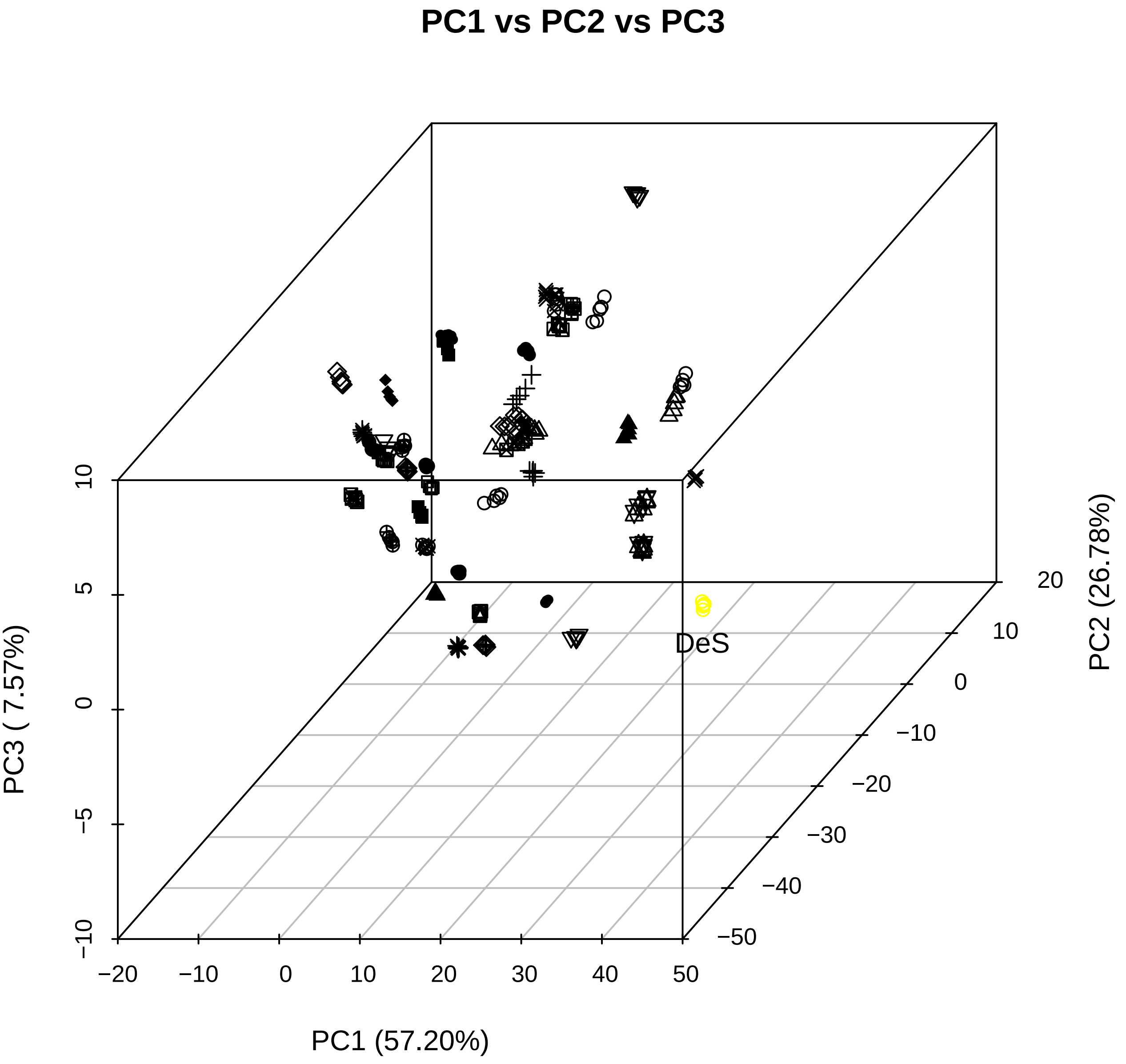
PC1 vs PC2 vs PC3
DeS
PC1 (57.20%)
PC2 (26.78%)
PC3 (7.57%)
−20
−10
0
10
20
30
40
50
20
10
0
−10
−20
−30
−40
−50
10
5
0
−5
−10

PC2 vs PC3 vs PC4

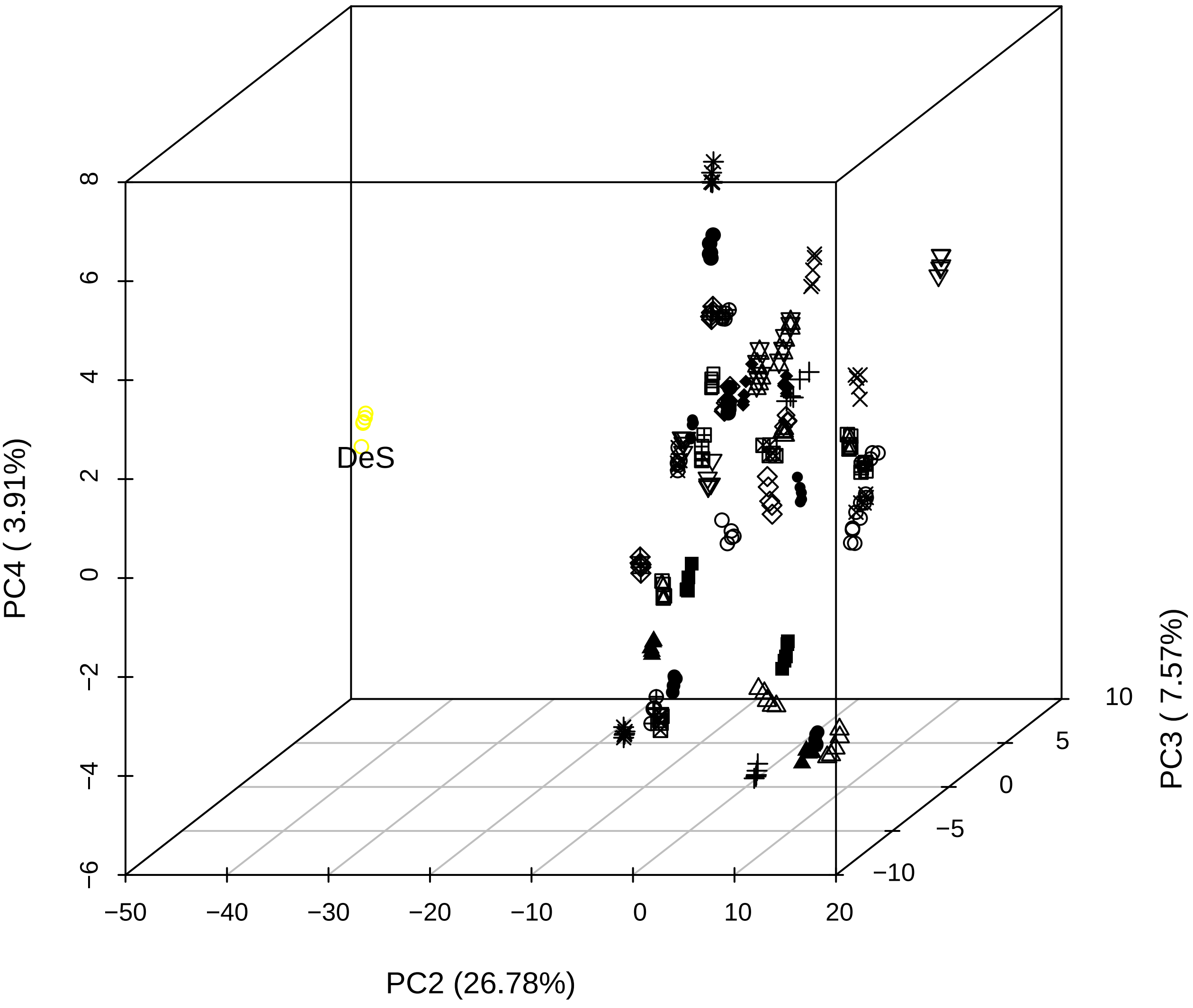

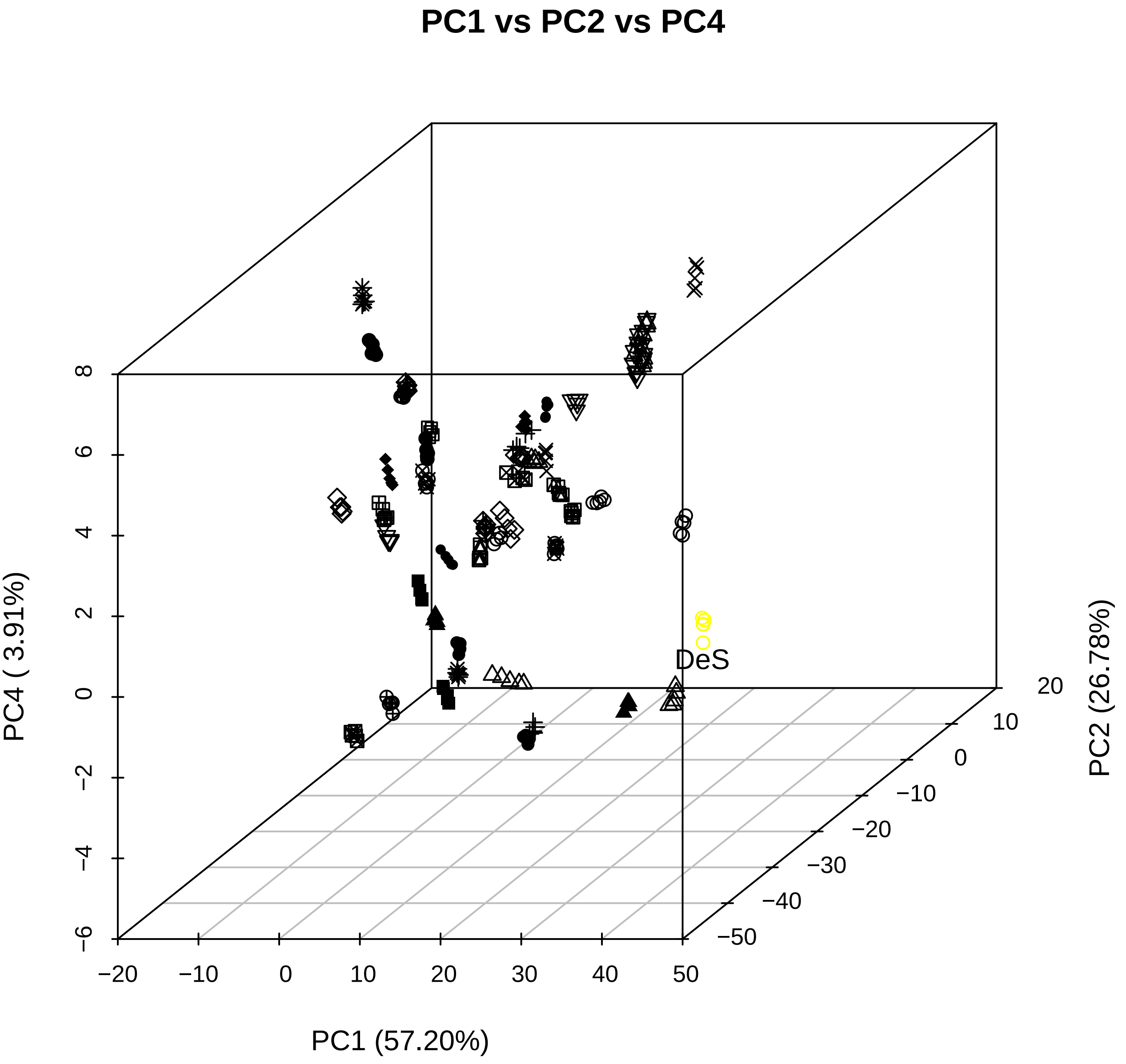
PC1 vs PC2 vs PC4
PC4 (3.91%)
PC1 (57.20%)
PC2 (26.78%)
DeS
8
6
4
2
0
−2
−4
−6
−20
−10
0
10
20
30
40
50
20
10
0
−10
−20
−30
−40
−50

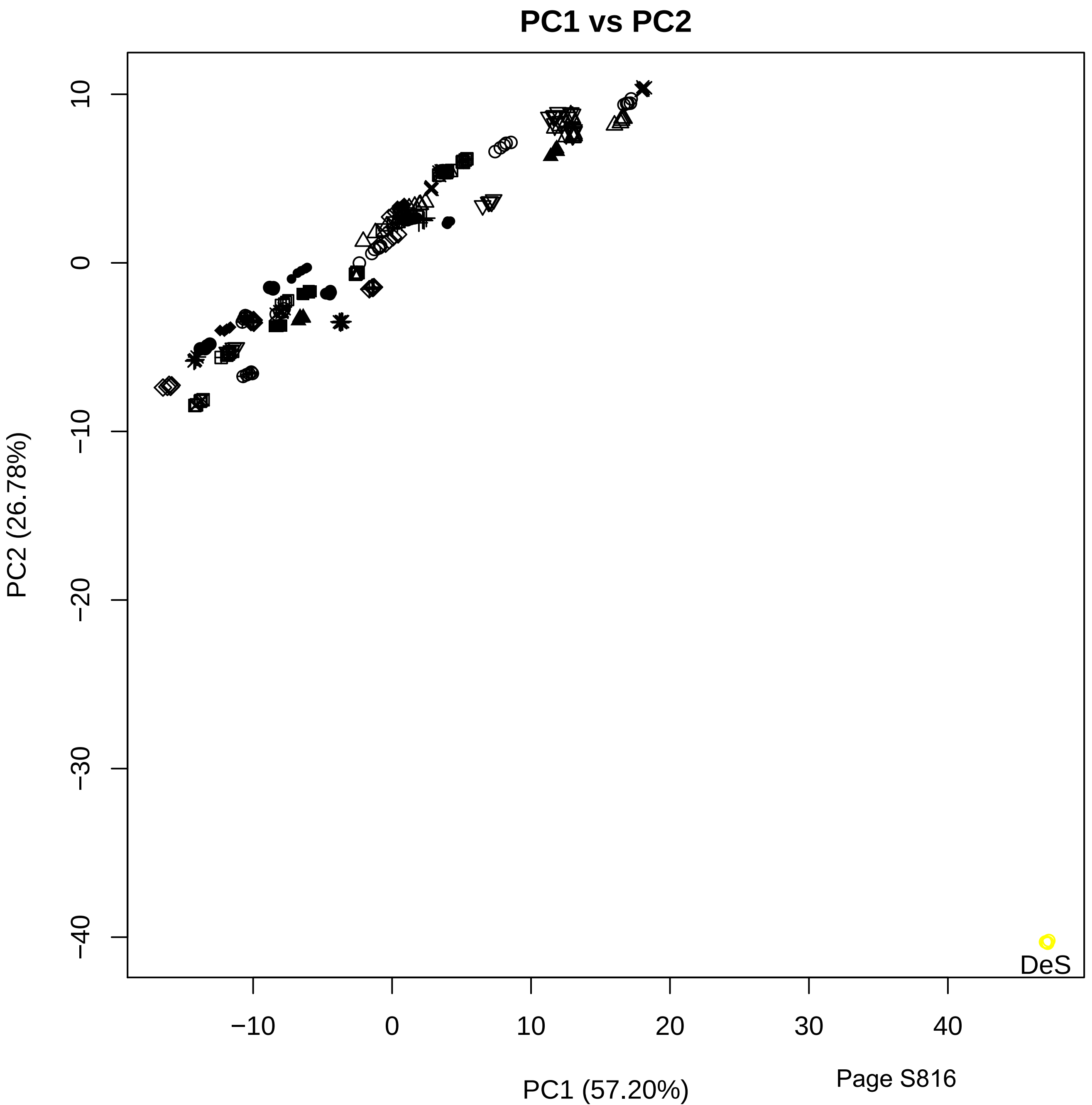
PC1 vs PC2
PC2 (26.78%)
PC1 (57.20%)
10
0
−10
−20
−30
−40
−10
0
10
20
30
40
DeS

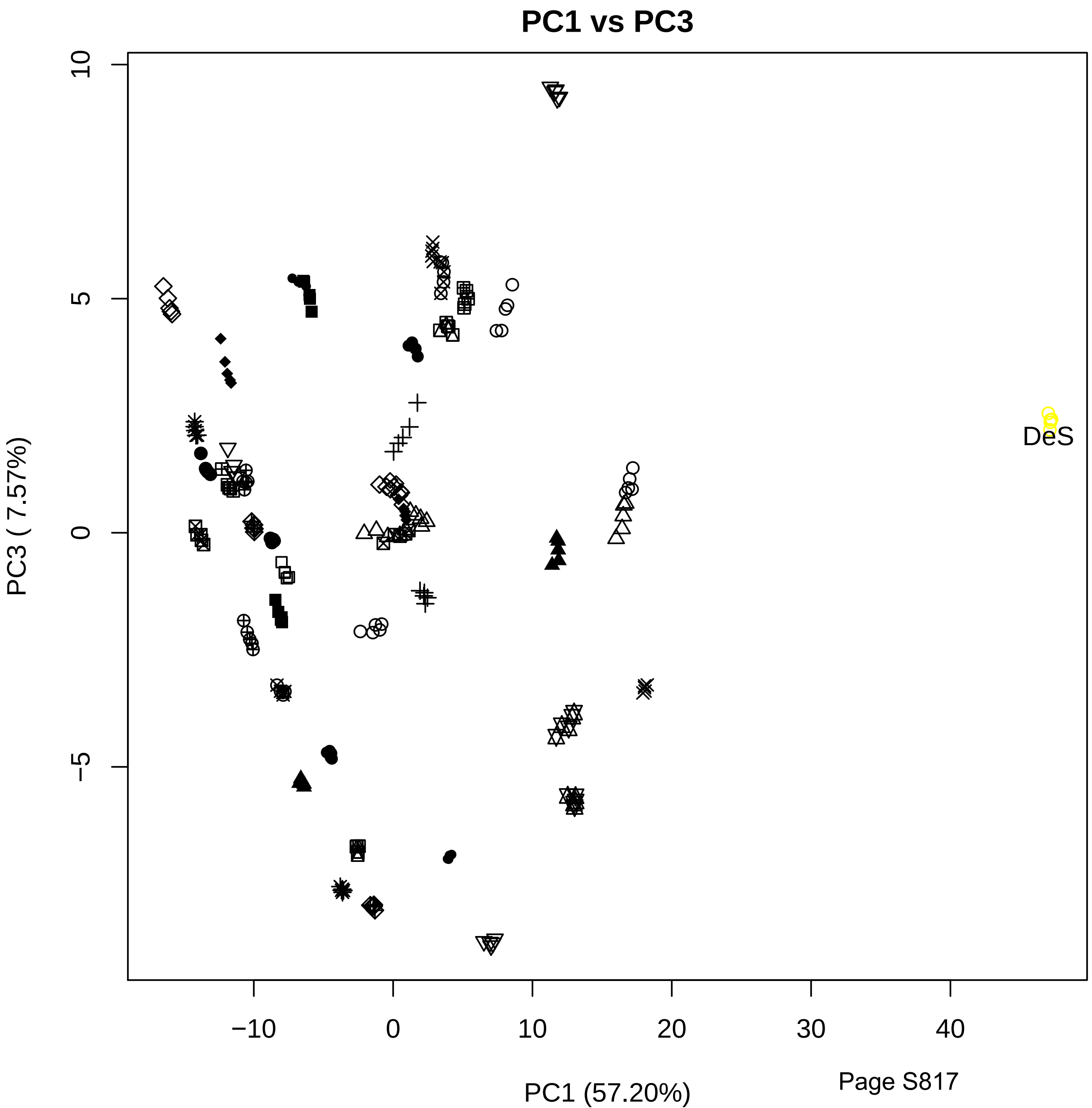

PC1 vs PC3
PC3 (7.57%)
PC1 (57.20%)
10
5
0
−5
−10
0
10
20
30
40
DeS

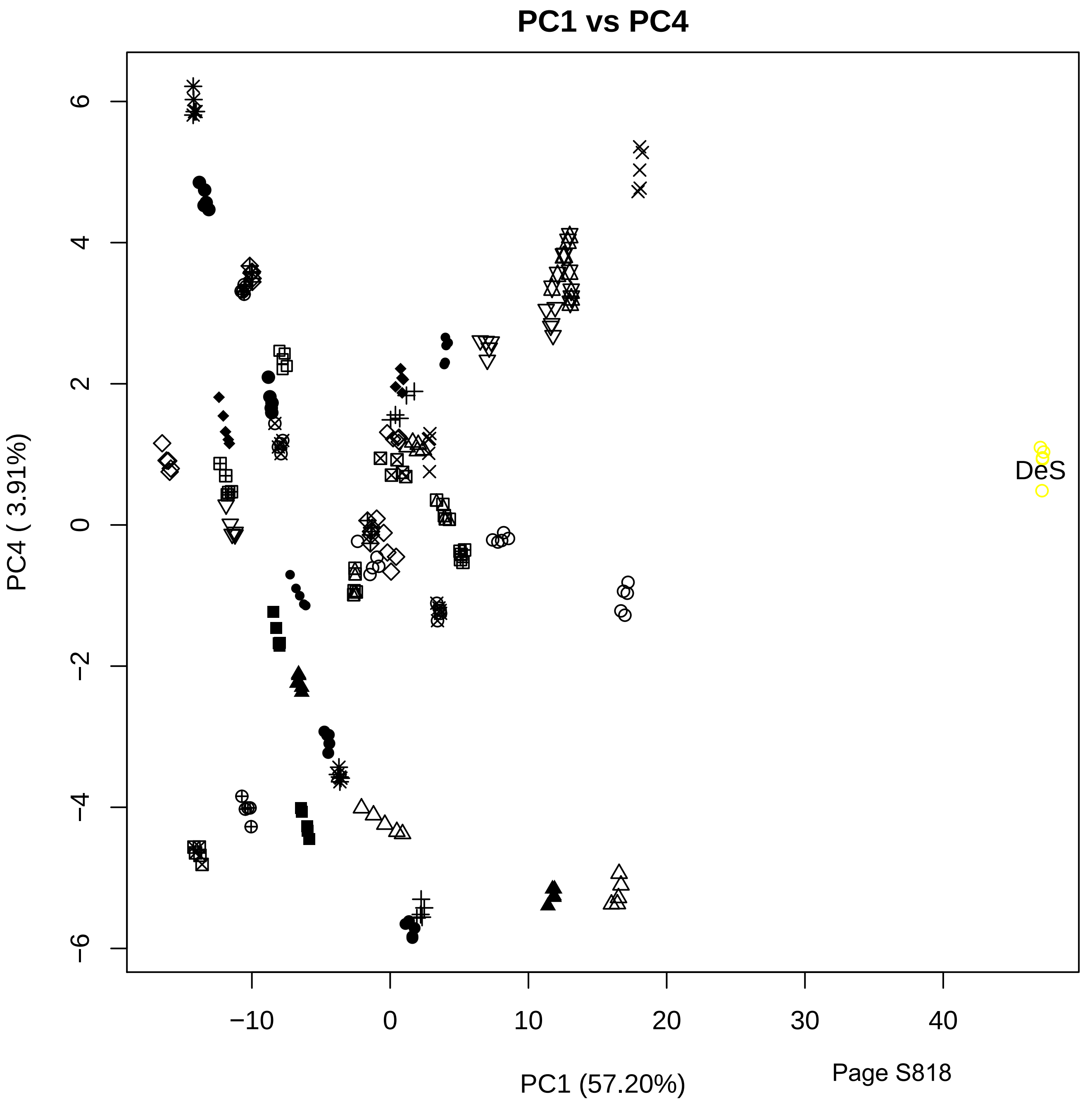
PC1 vs PC4
PC4 (3.91%)
PC1 (57.20%)
DeS
6
4
2
0
−2
−4
−6
−10
0
10
20
30
40

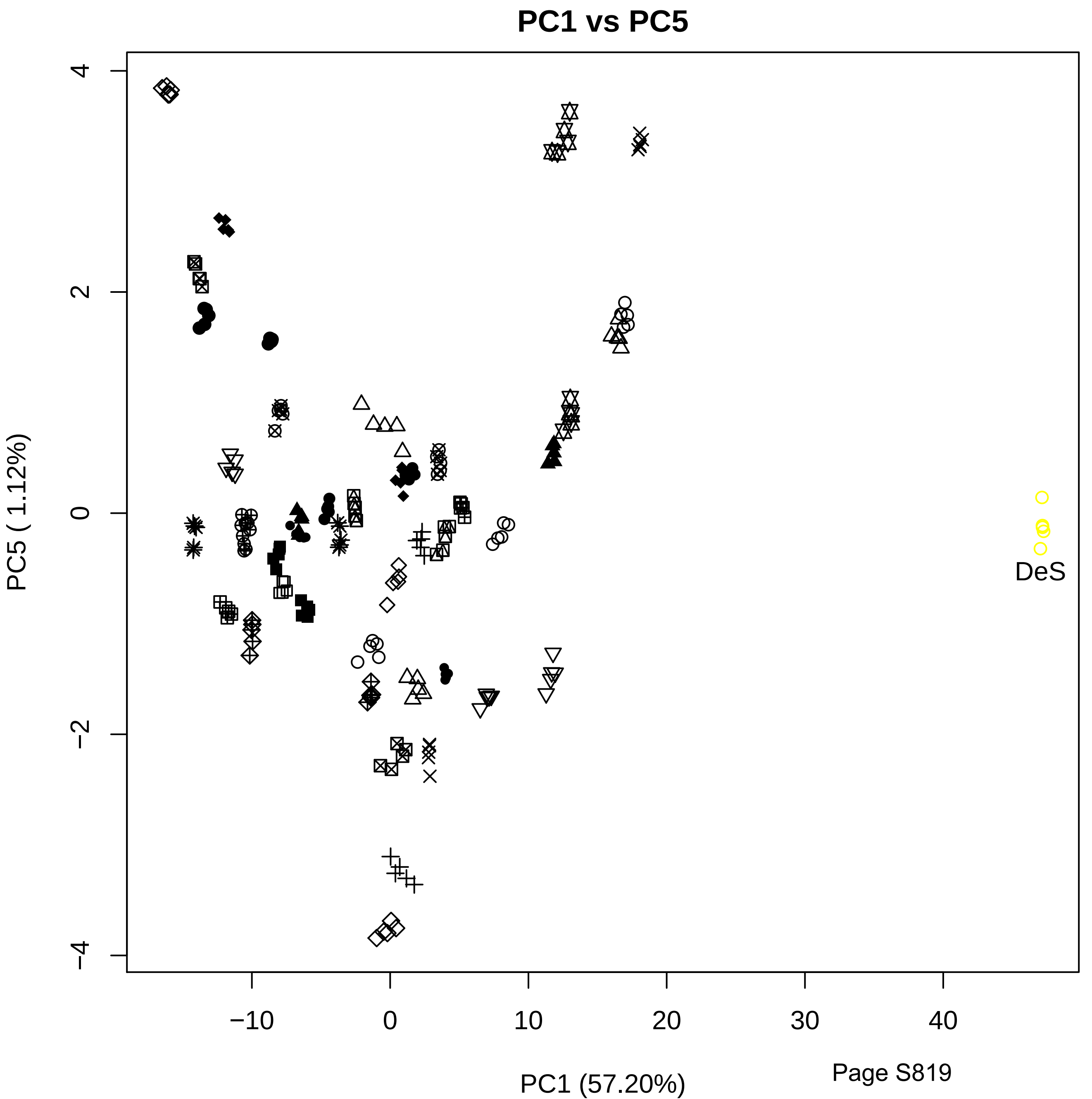
PC1 vs PC5
PC5 (1.12%)
PC1 (57.20%)
DeS
4
2
0
−2
−4
−10
0
10
20
30
40

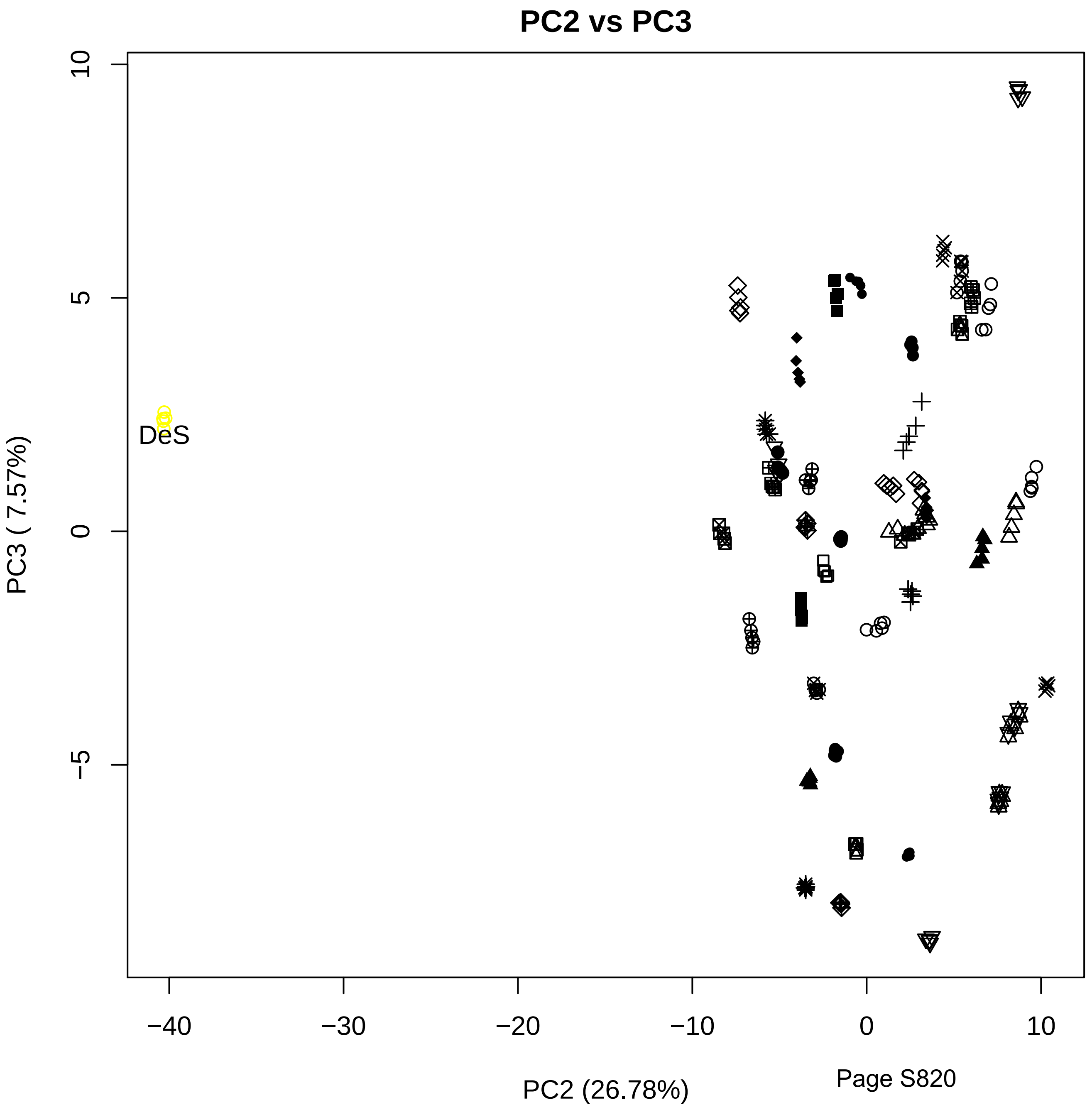
PC2 vs PC3
PC3 (7.57%)
10
5
0
−5
−40
−30
−20
−10
0
10
PC2 (26.78%)
DeS

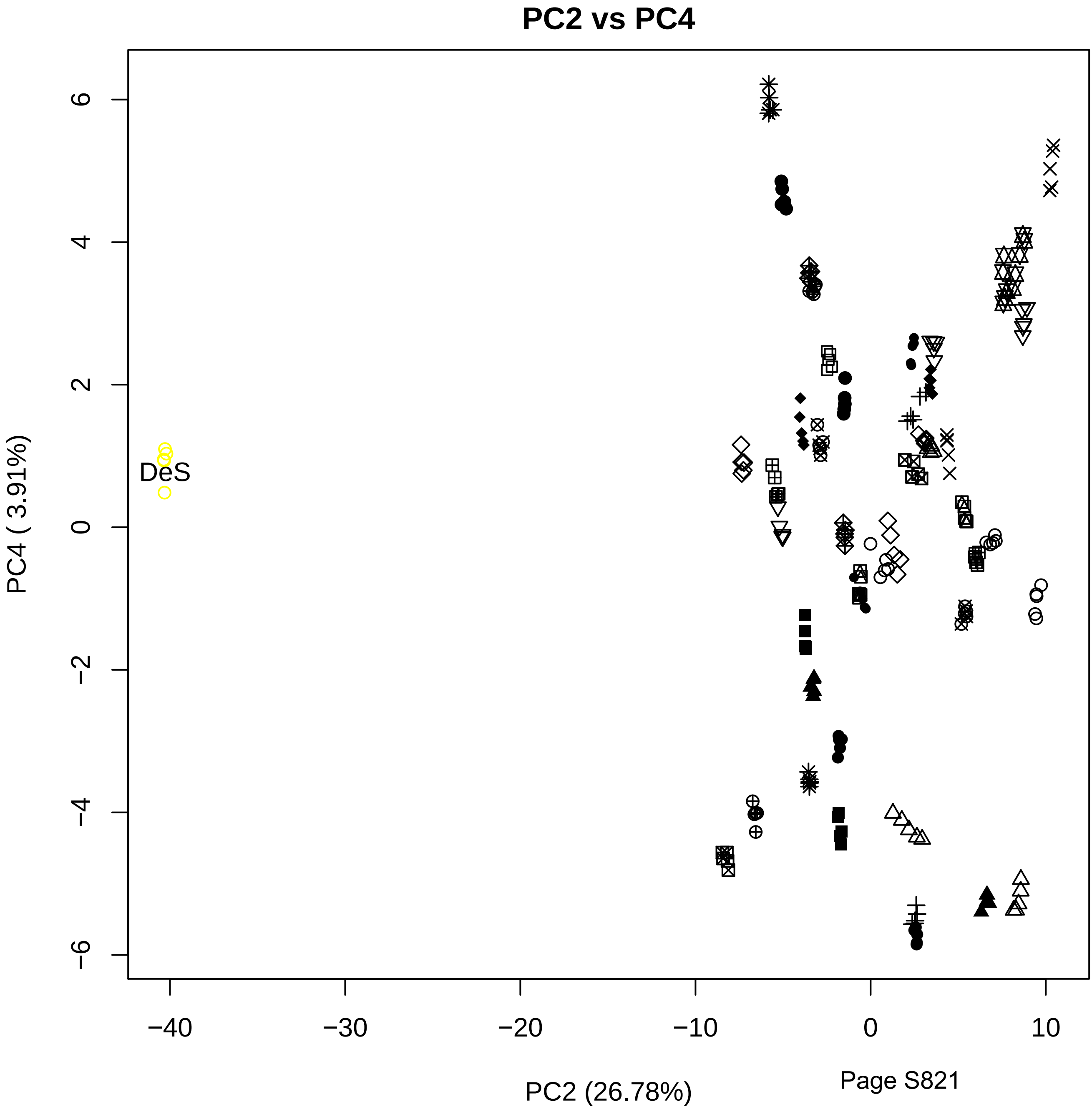

PC2 vs PC4
PC4 (3.91%)
PC2 (26.78%)
DeS
6
4
2
0
−2
−4
−6
−40
−30
−20
−10
0
10

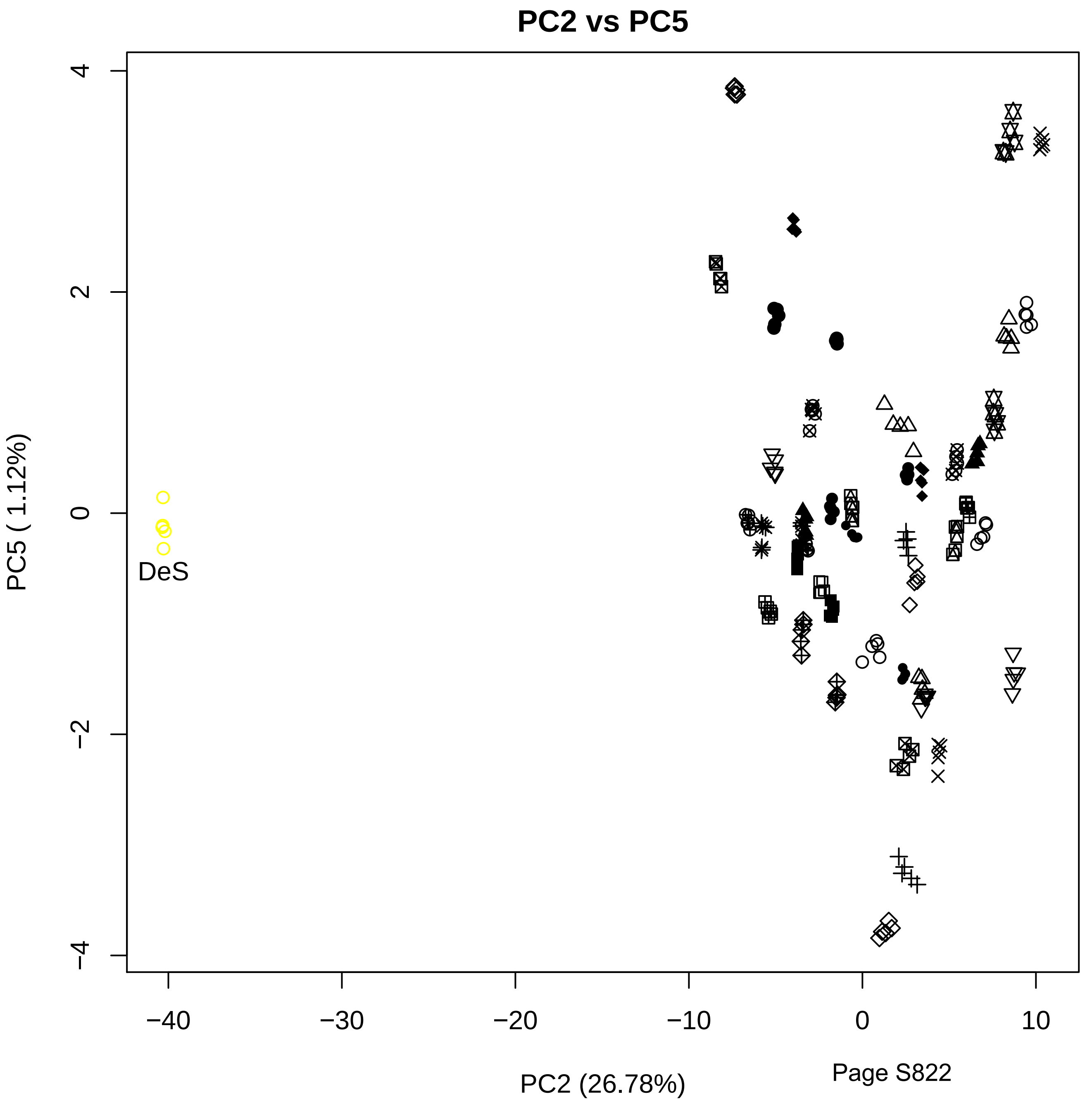
PC2 vs PC5
PC5 (1.12%)
PC2 (26.78%)
DeS
4
2
0
−2
−4
−40
−30
−20
−10
0
10

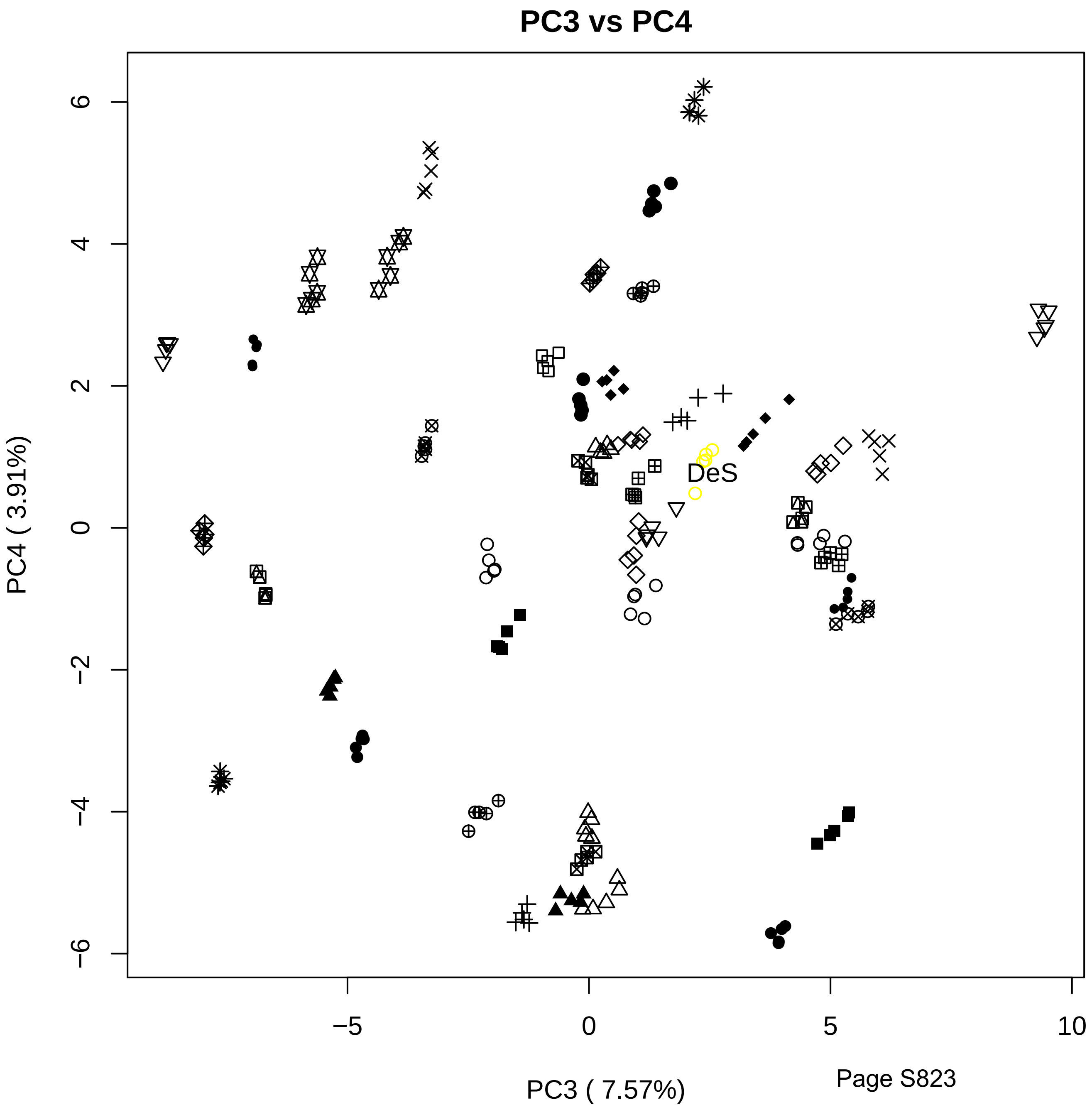
PC3 vs PC4
PC4 (3.91%)
PC3 (7.57%)
DeS
6
4
2
0
−2
−4
−6
−5
0
5
10

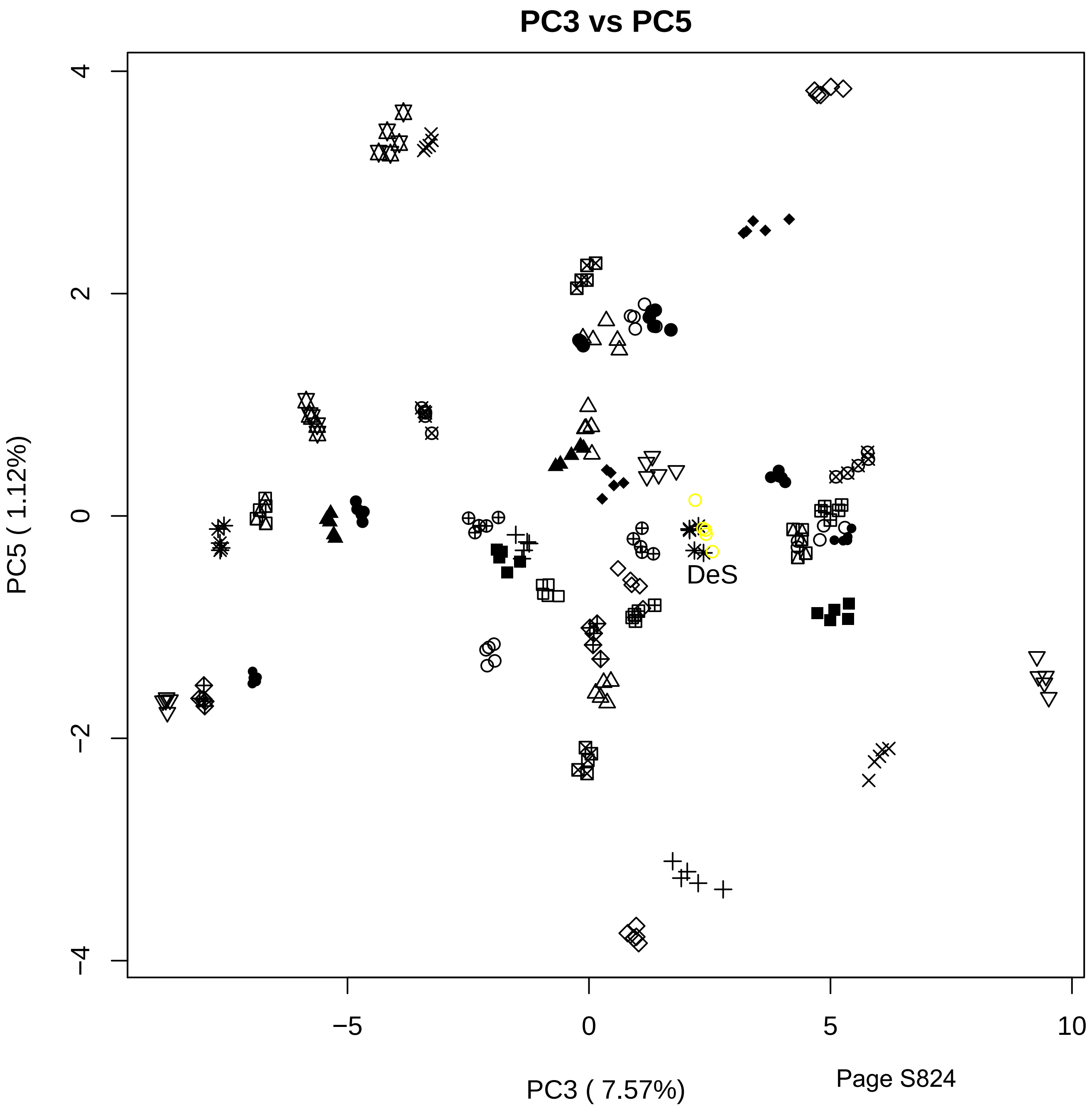
PC3 vs PC5
4
2
0
−2
−4
PC5 (1.12%)
−5
0
5
10
PC3 (7.57%)
DeS

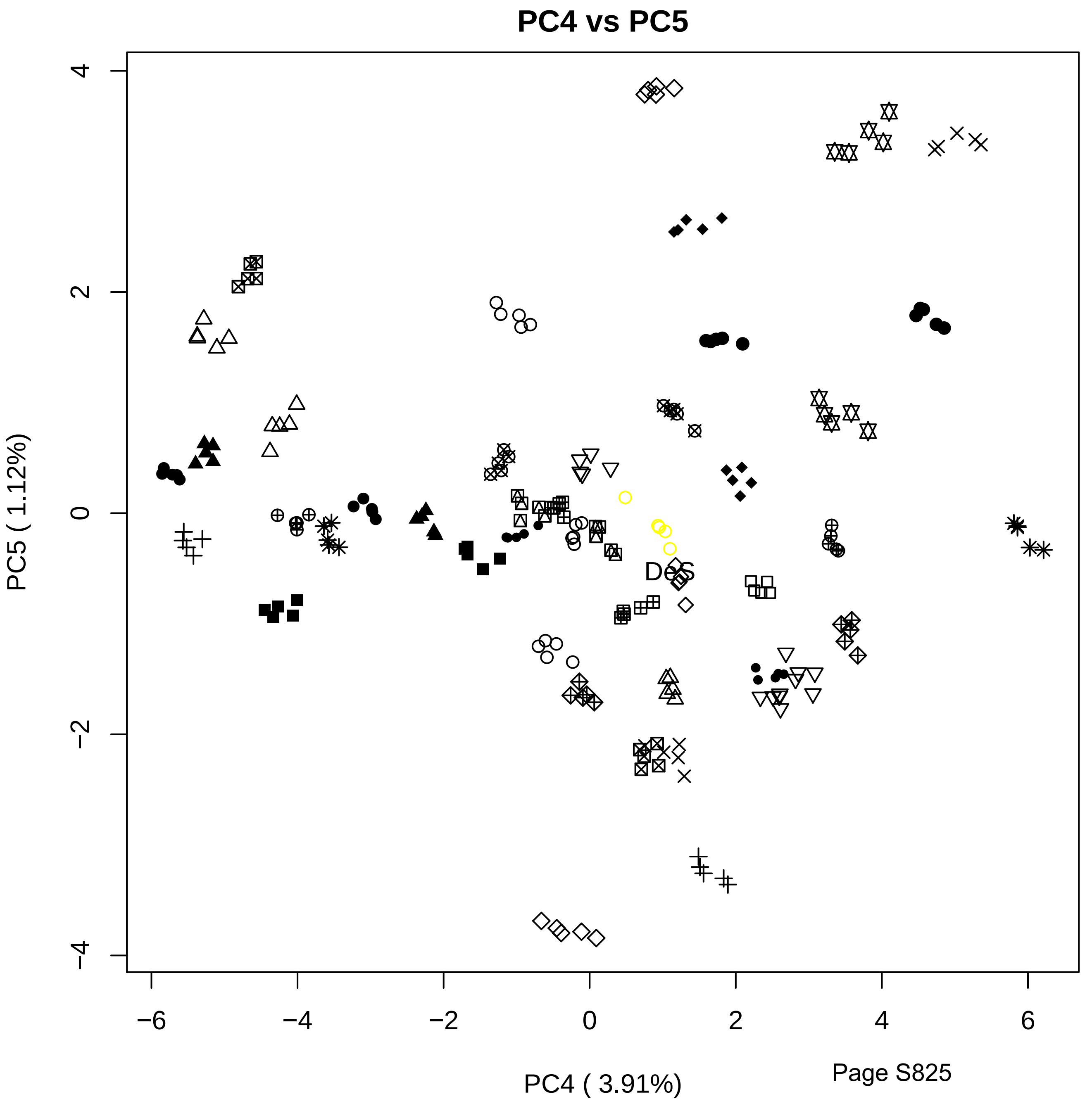

PC4 vs PC5
PC5 (1.12%)
PC4 (3.91%)
4
2
0
−2
−4
−6
−4
−2
0
2
4
6
DeS

HpSp for paper new Prep

Anthony Devlin, Keele University

PCA of All

Scaled Matrix PCAed

7th order polynomial baseline correction performed

Raw spectra smoothed to 21 Neighbours, 2nd order PN, Savtisky Golay

Normalisation between 0 and 100

D2 spectra smoothed to 41 Neighbours, 2nd order PN, Savtisky Golay

Regions Analysed: 3601−2501 and 2001−700cm−1

Samples Analysed: 0 bContam's 16 BLH's 50 BMH's 30 OMH's 0 pContam's 0 PLH's 60 PMH's

Keele University AD Fri Dec 14 13:22:01 2018

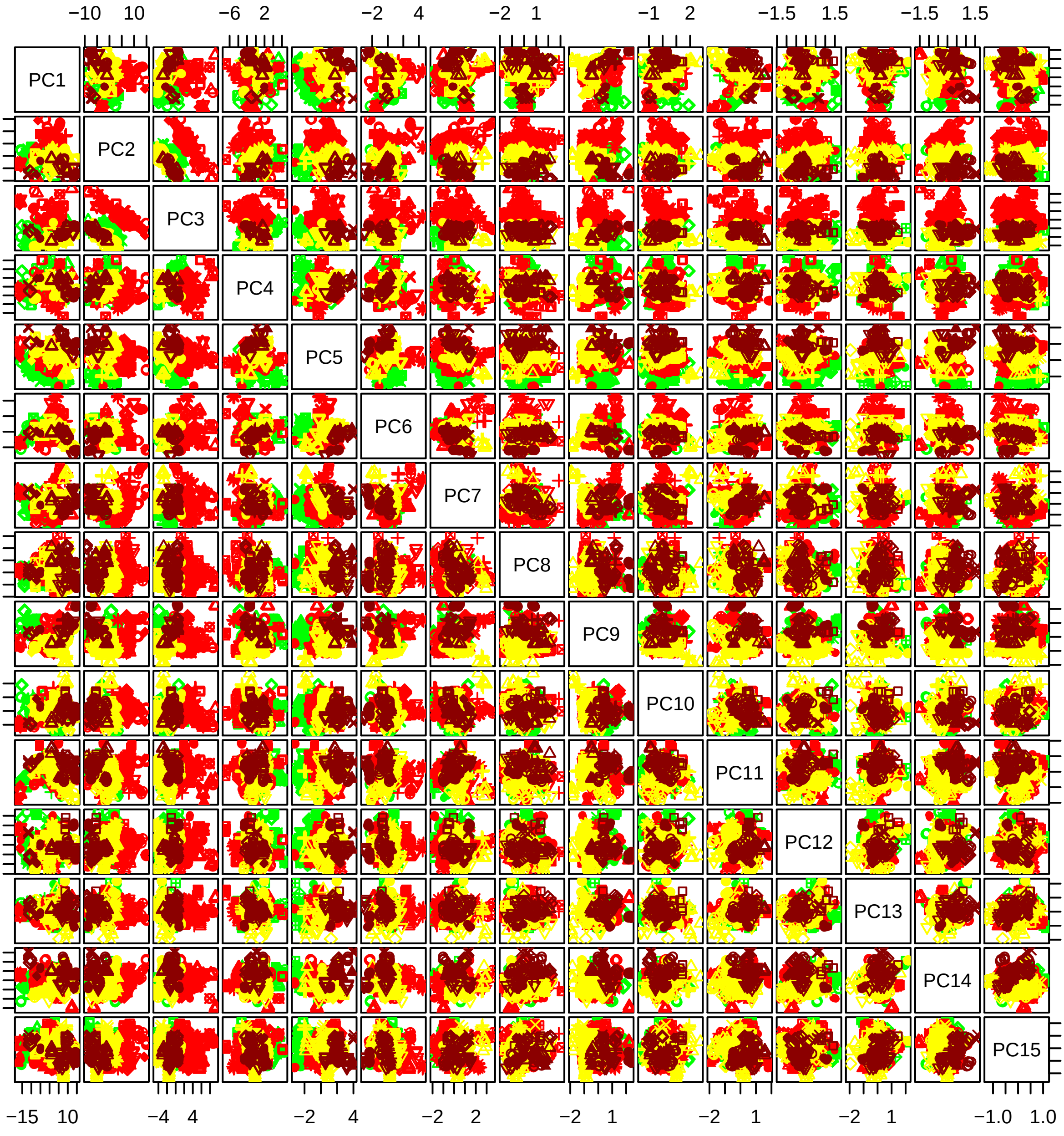

PC1
PC2
PC3
PC4
PC5
PC6
PC7
PC8
PC9
PC10
PC11
PC12
PC13
PC14
PC15
−10 10
−6 2
−2 4
−2 1
−1 2
−1.5 1.5
−1.5 1.5
10 −15
−10 10
4 −4
2 −6
4 −2
4 −2
2 −2
1 −2
1 −2
2 −1
1 −2
1.5 −1.5
1 −2
1.5 −1.5
1.0 −1.0
−15 10
−4 4
−2 4
−2 2
−2 1
−2 1
−2 1
−1.0 1.0

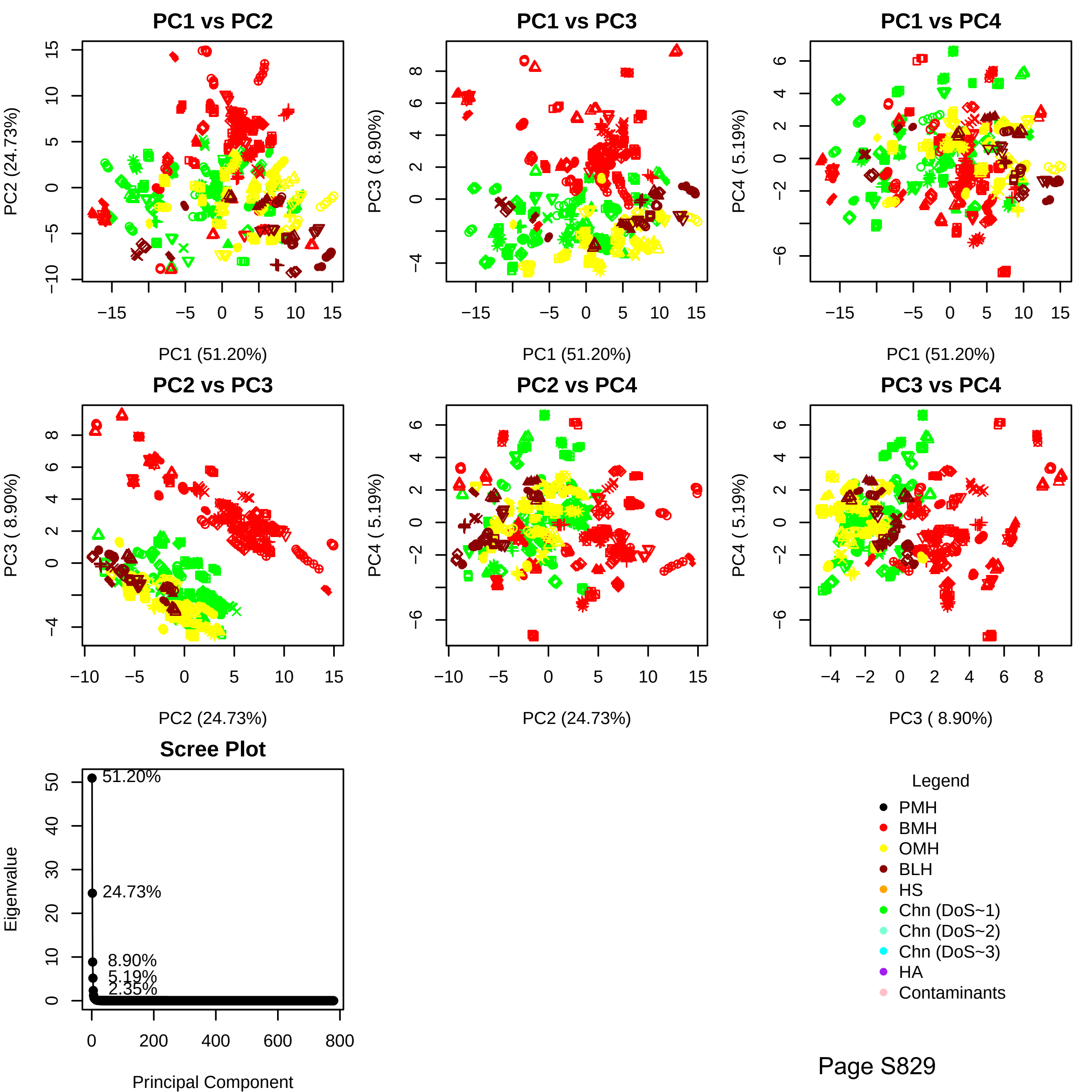
PC1 vs PC2
PC1 vs PC3
PC1 vs PC4
PC2 vs PC3
PC2 vs PC4
PC3 vs PC4
PC1 (51.20%)
PC2 (24.73%)
PC3 (8.90%)
PC4 (5.19%)
Scree Plot
Eigenvalue
Principal Component
51.20%
24.73%
8.90%
5.19%
2.35%
Legend
PMH
BMH
OMH
BLH
HS
Chn (DoS~1)
Chn (DoS~2)
Chn (DoS~3)
HA
Contaminants

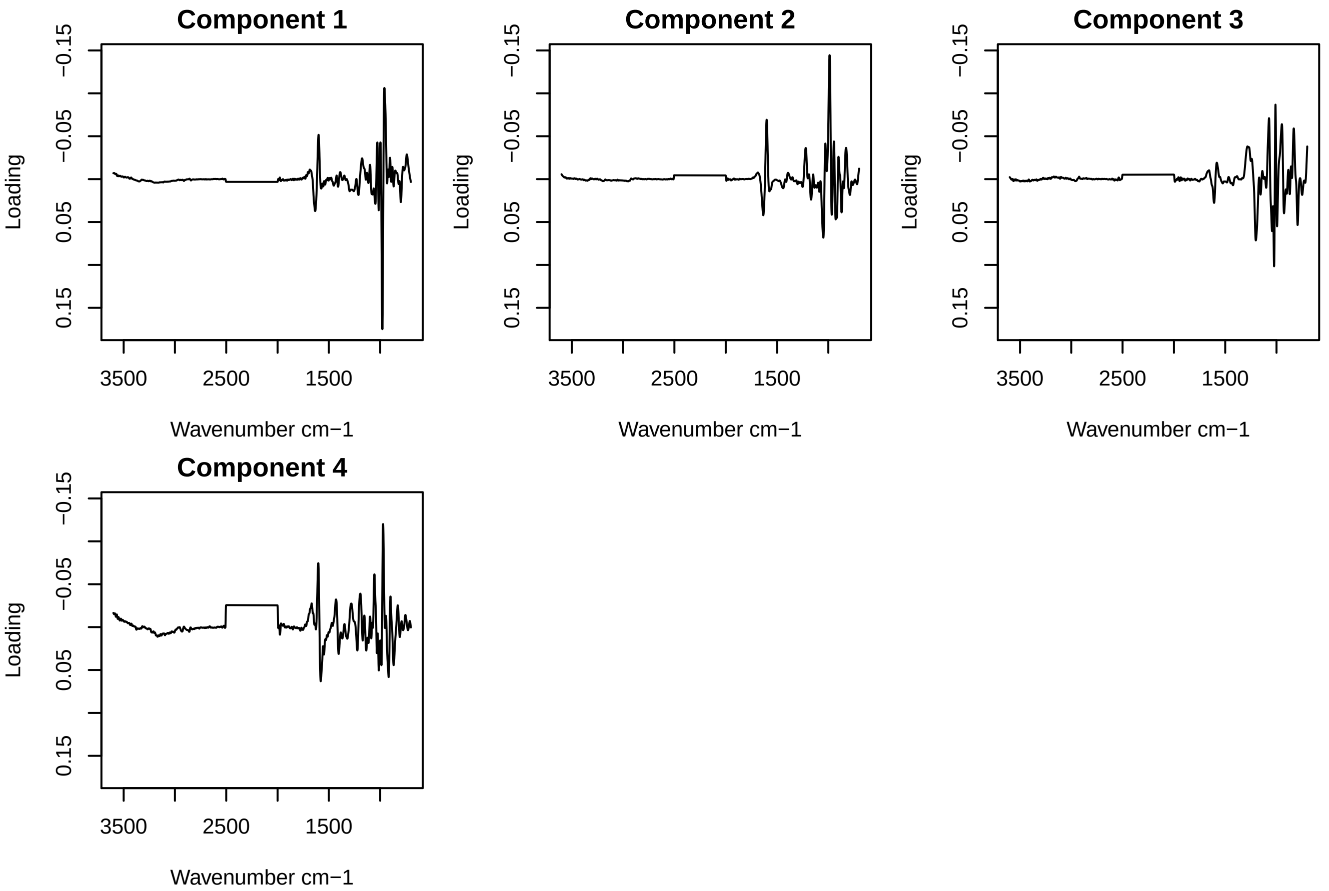
Component 1
Component 2
Component 3
Component 4
Loading
Wavenumber cm−1
−0.15
−0.05
0.05
0.15
3500
2500
1500

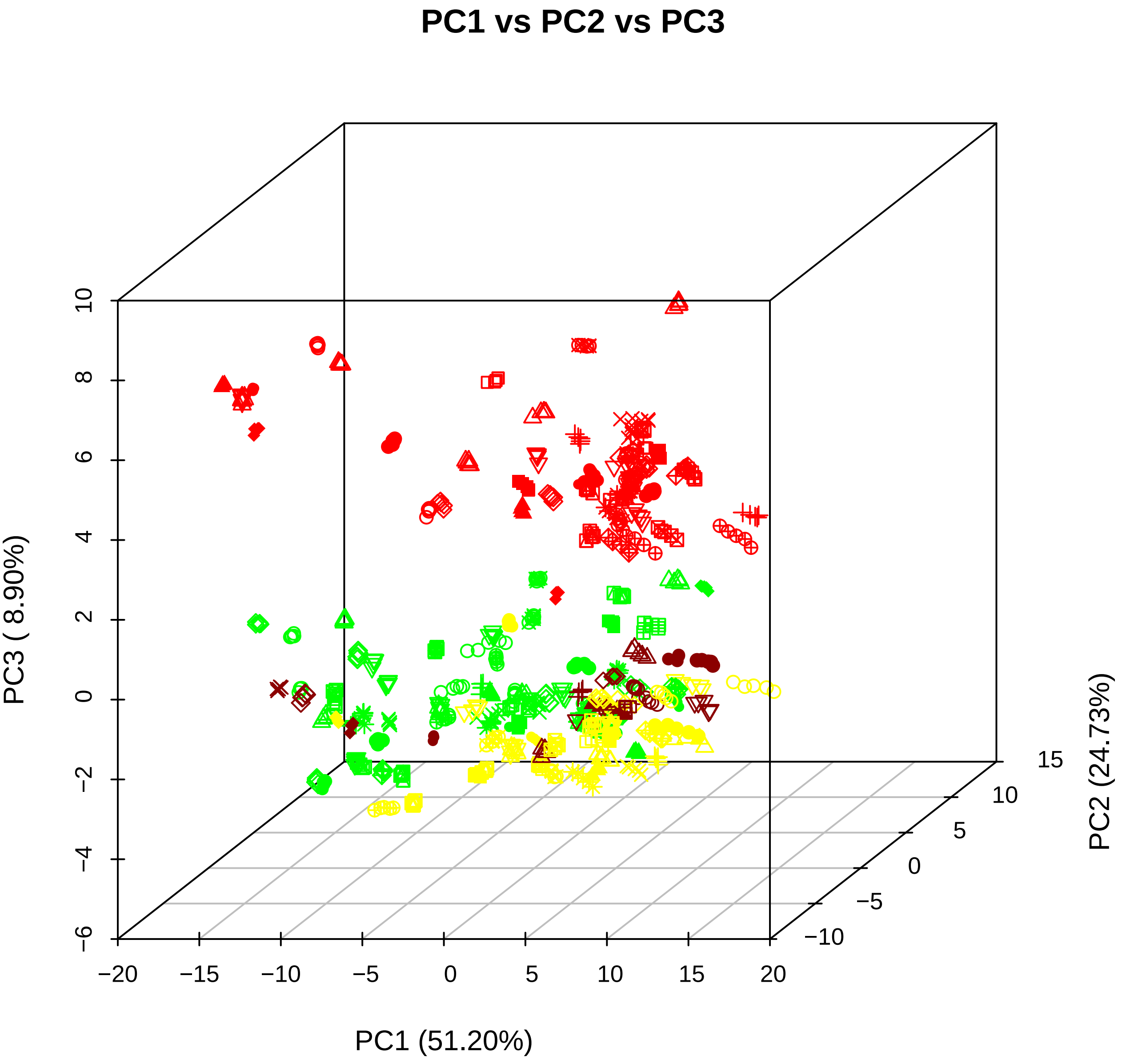

PC1 vs PC2 vs PC3
PC3 (8.90%)
PC1 (51.20%)
PC2 (24.73%)
10
8
6
4
2
0
−2
−4
−6
−20
−15
−10
−5
0
5
10
15
20
15
10
5
0
−5
−10

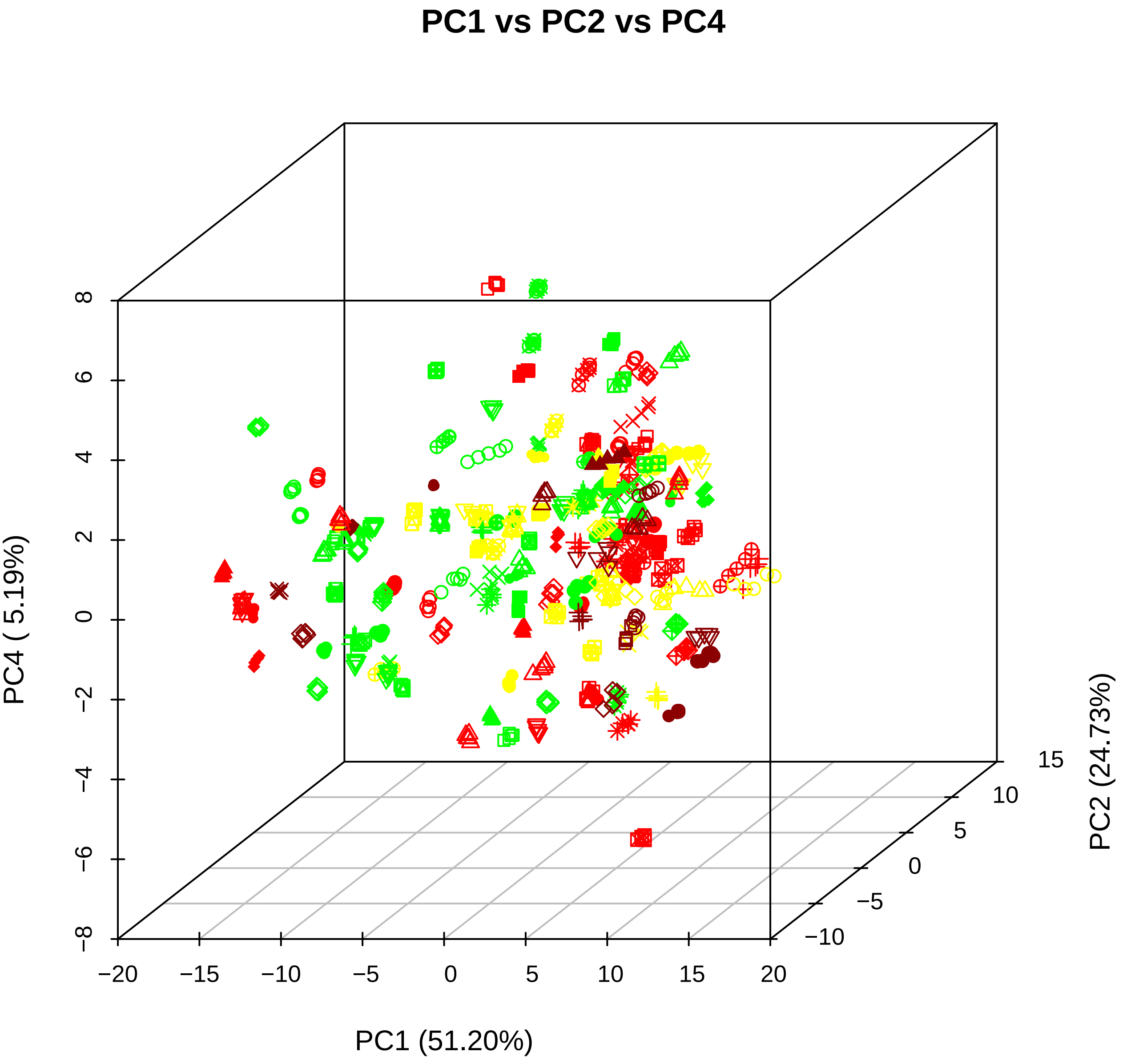

PC1 vs PC2 vs PC4
8
6
4
2
0
−2
−4
−6
−8
PC4 (5.19%)
−20
−15
−10
−5
0
5
10
15
20
PC1 (51.20%)
15
10
5
0
−5
−10
PC2 (24.73%)

PC1 vs PC3 vs PC4

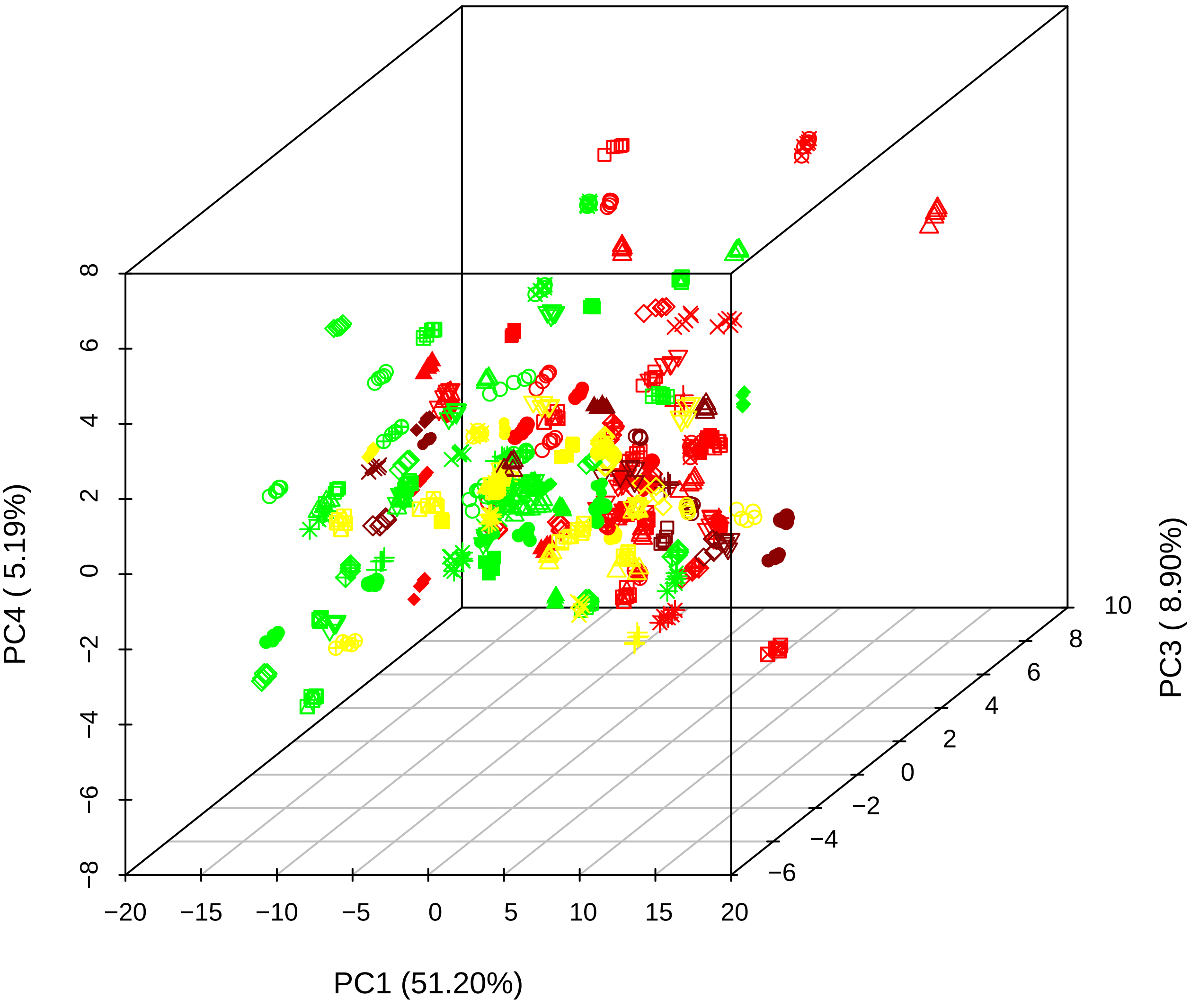

PC2 vs PC3 vs PC5

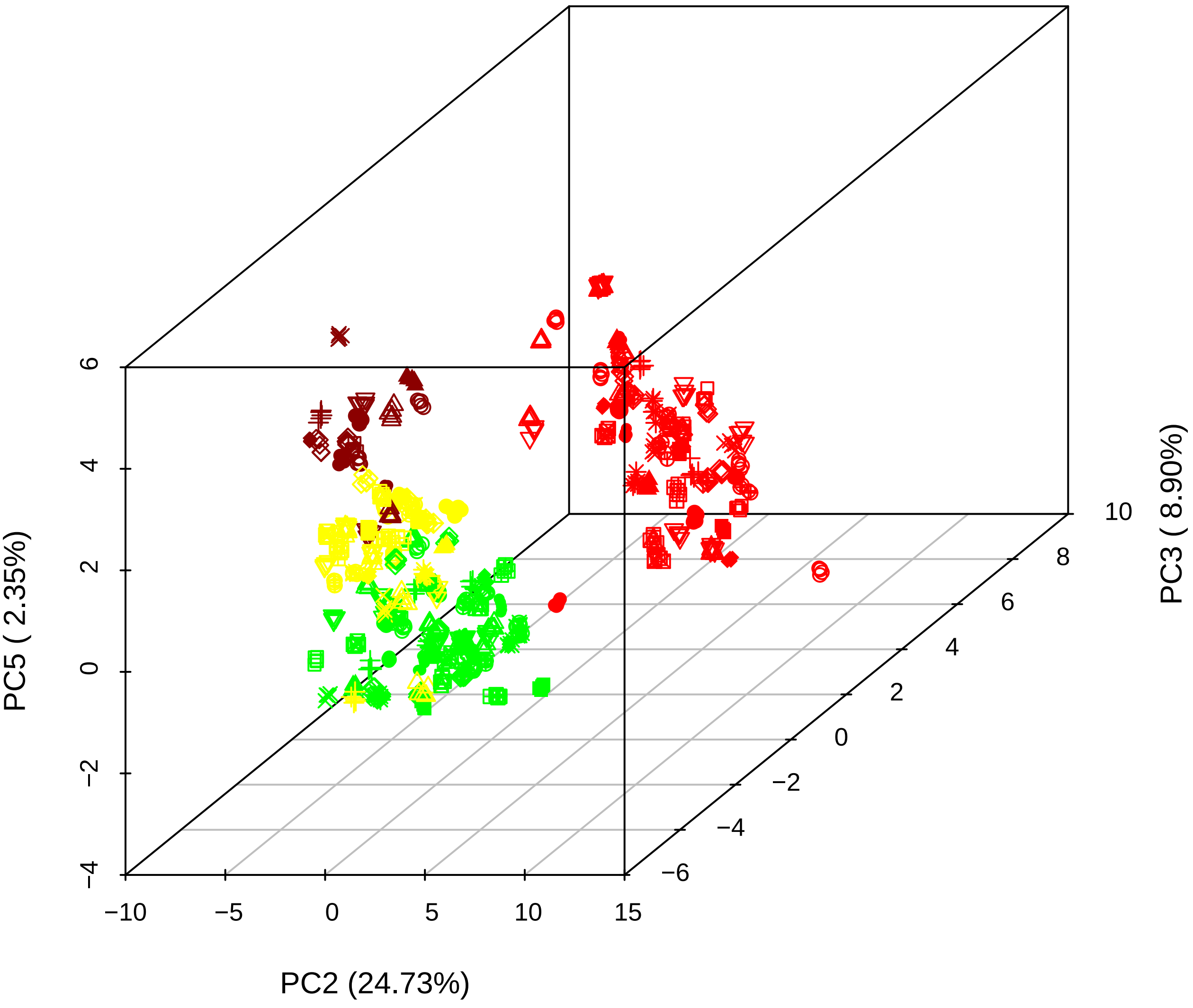

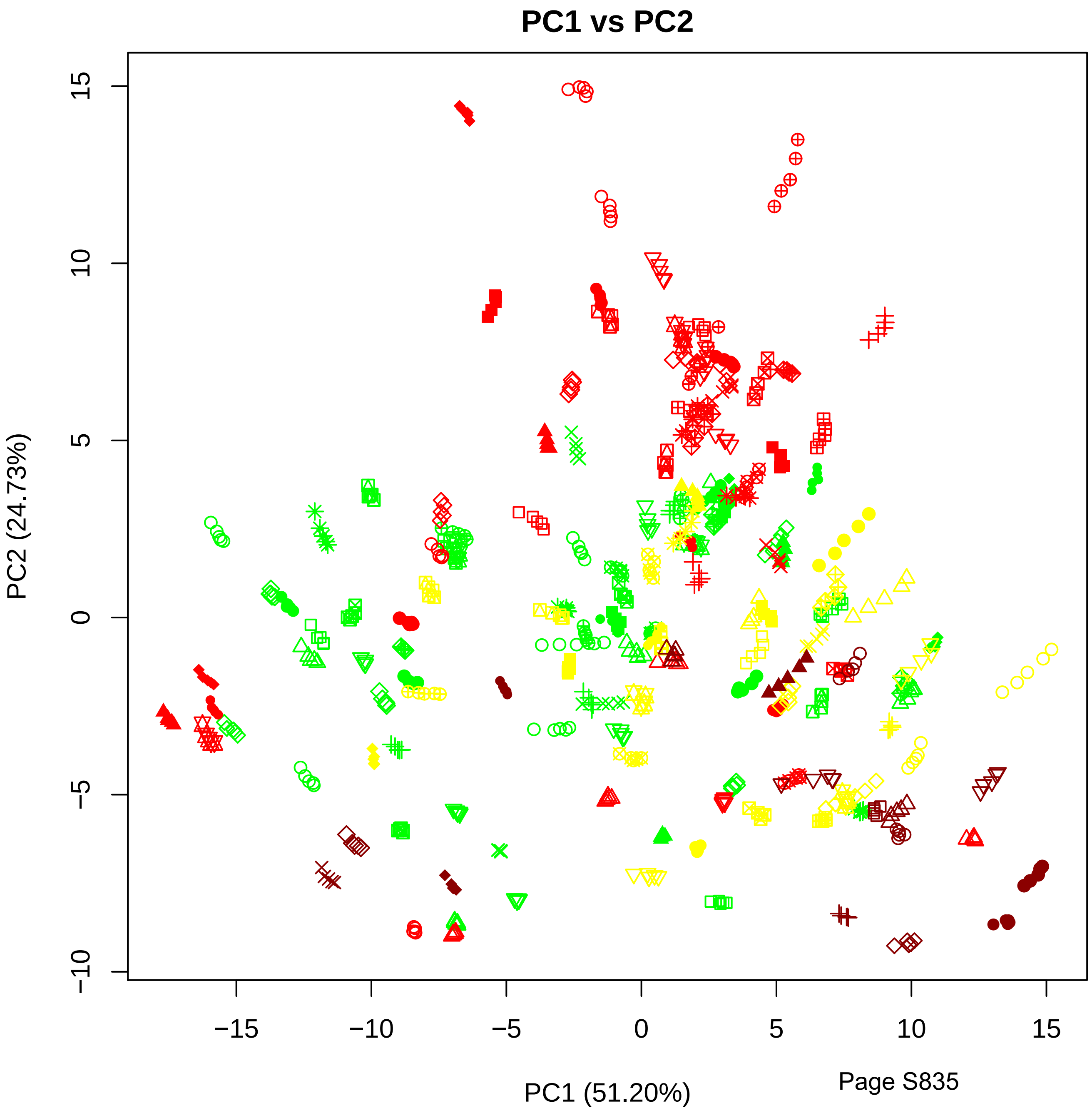
PC1 vs PC2
PC2 (24.73%)
PC1 (51.20%)
15
10
5
0
−5
−10
−15
−10
−5
0
5
10
15

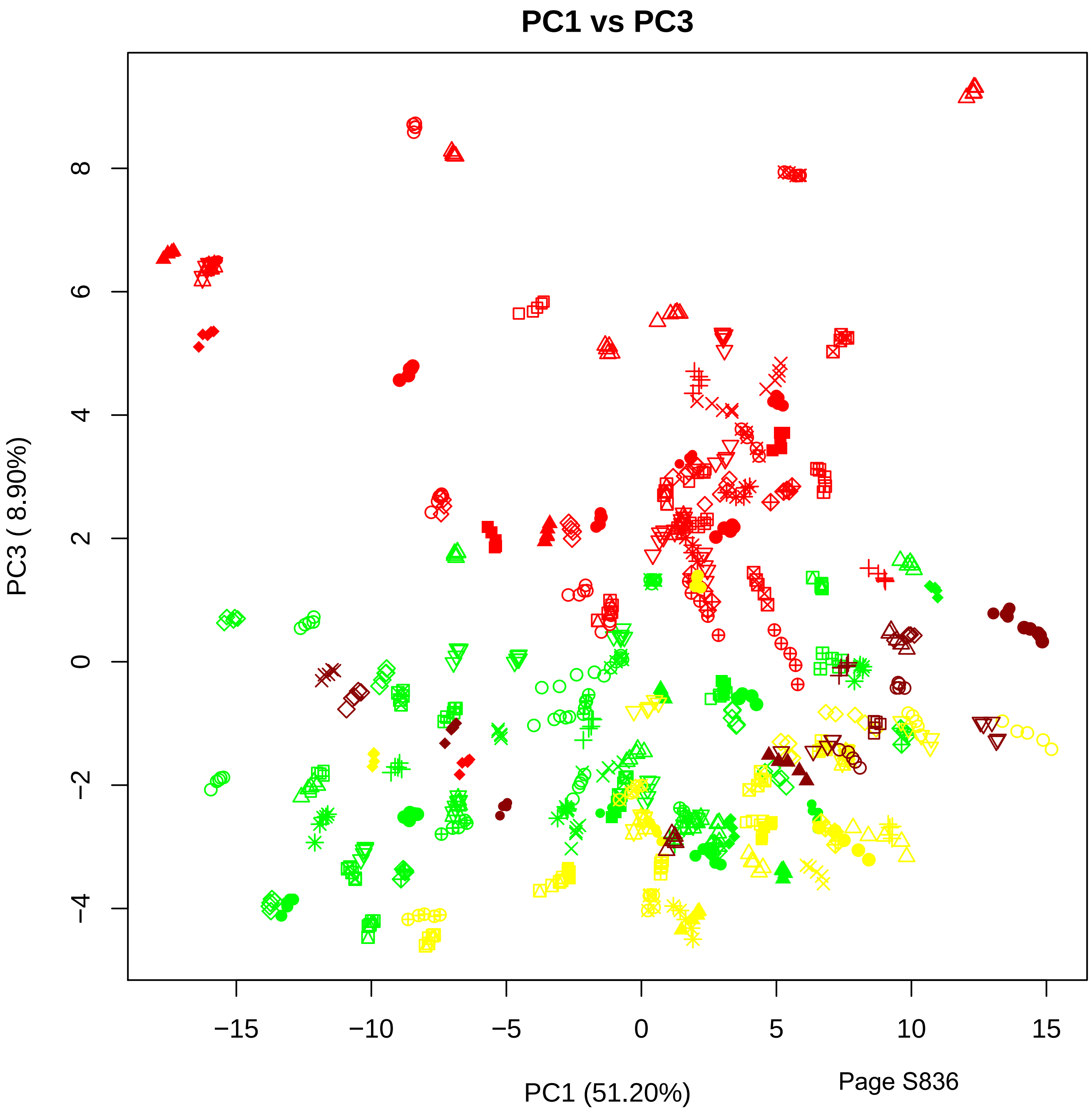
PC1 vs PC3
PC3 (8.90%)
PC1 (51.20%)
8
6
4
2
0
−2
−4
−15
−10
−5
0
5
10
15

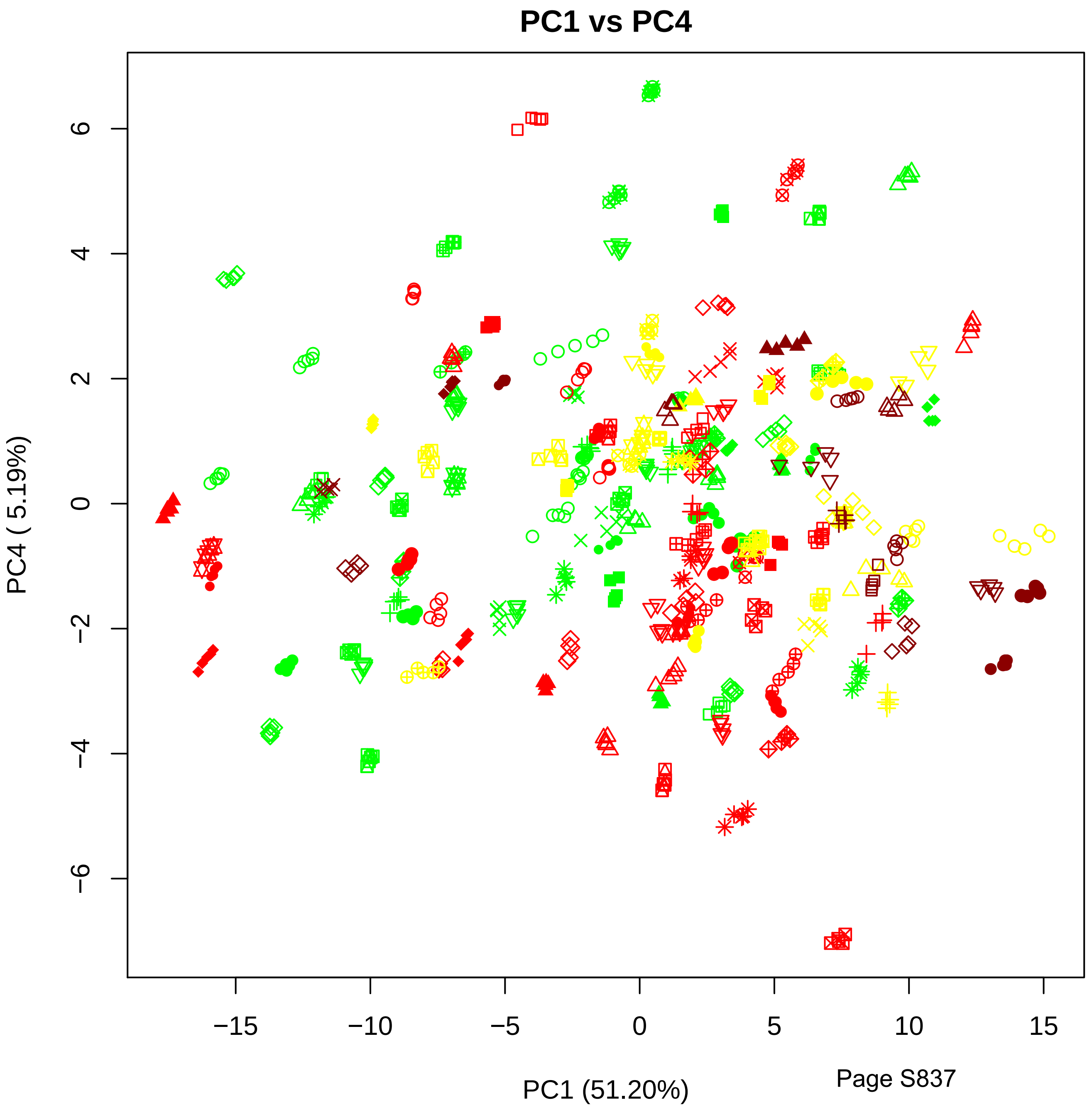
PC1 vs PC4
PC4 (5.19%)
PC1 (51.20%)
6
4
2
0
−2
−4
−6
−15
−10
−5
0
5
10
15

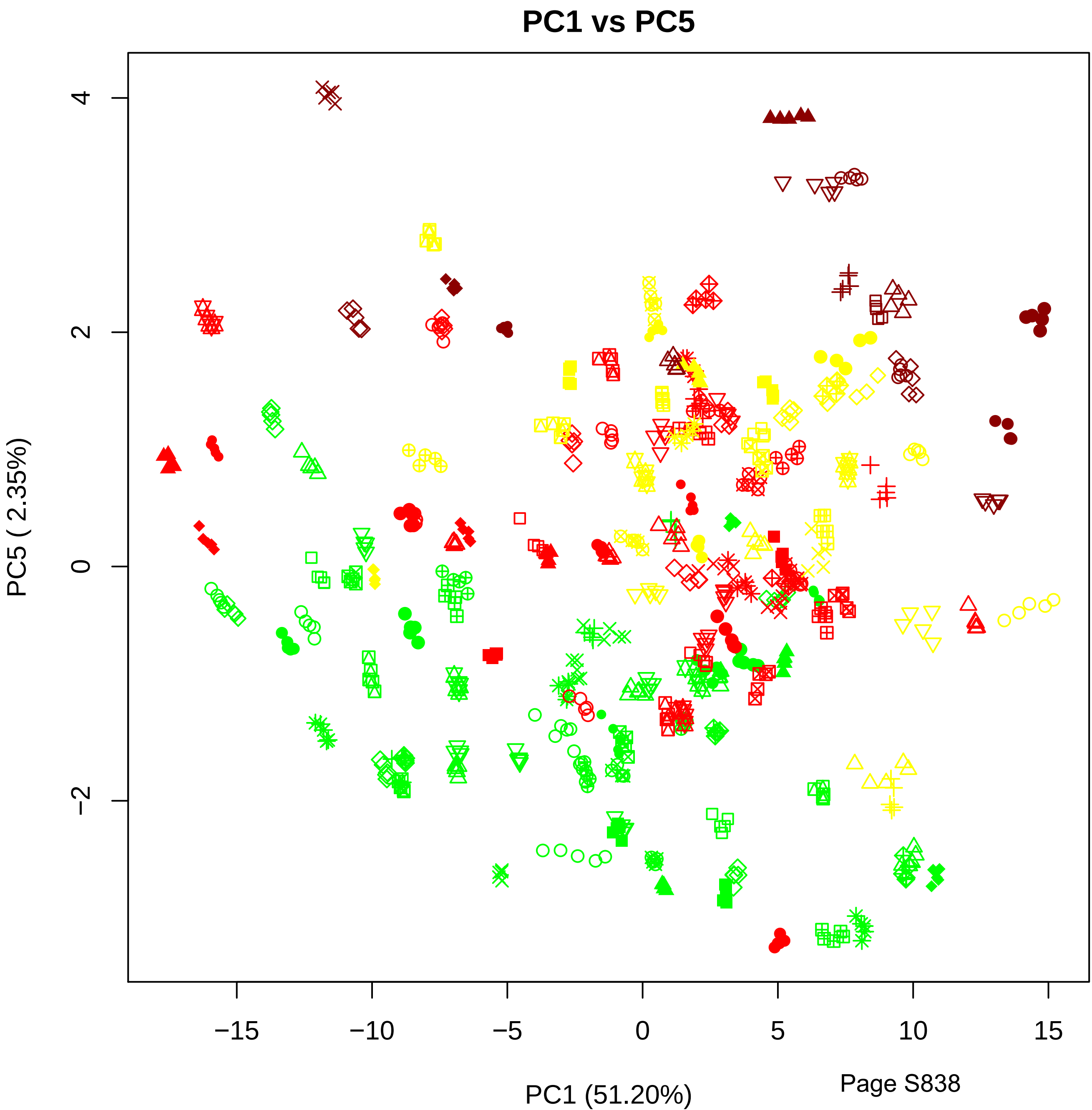

PC1 vs PC5
PC5 (2.35%)
PC1 (51.20%)
4
2
0
−2
−15
−10
−5
0
5
10
15

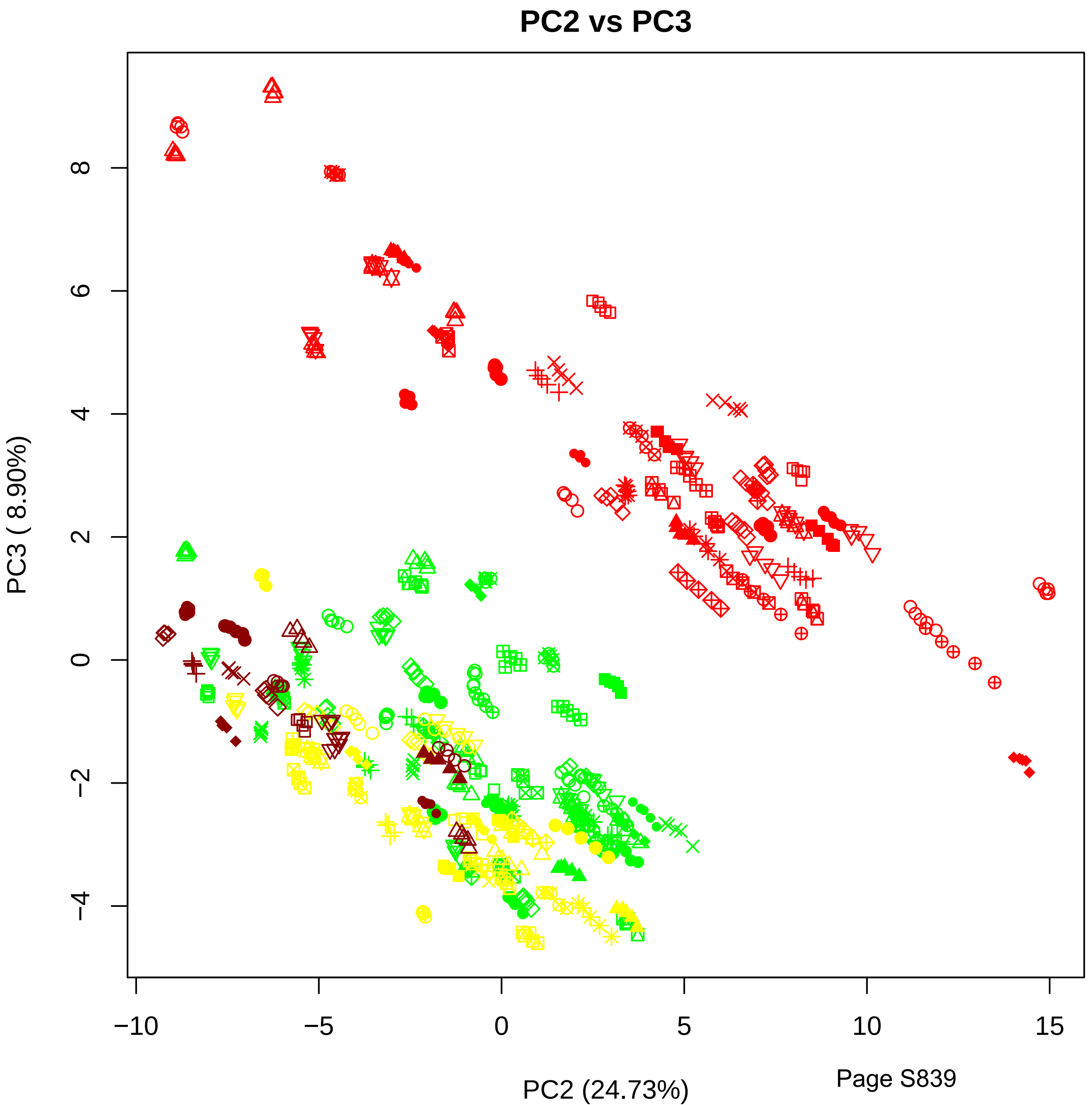
PC2 vs PC3
PC3 (8.90%)
PC2 (24.73%)
8
6
4
2
0
−2
−4
−10
−5
0
5
10
15

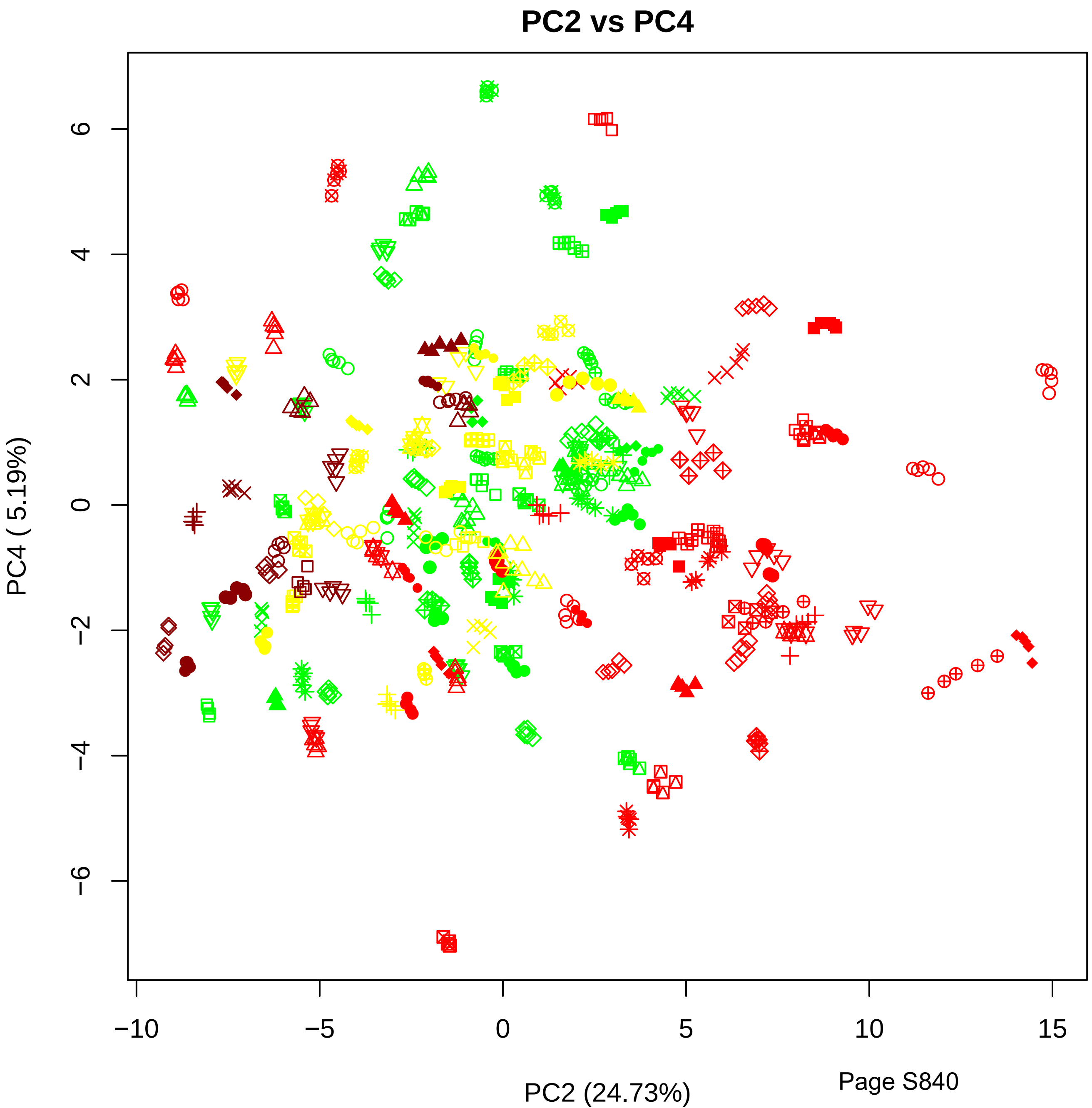
PC2 vs PC4
PC4 (5.19%)
PC2 (24.73%)
6
4
2
0
−2
−4
−6
−10
−5
0
5
10
15

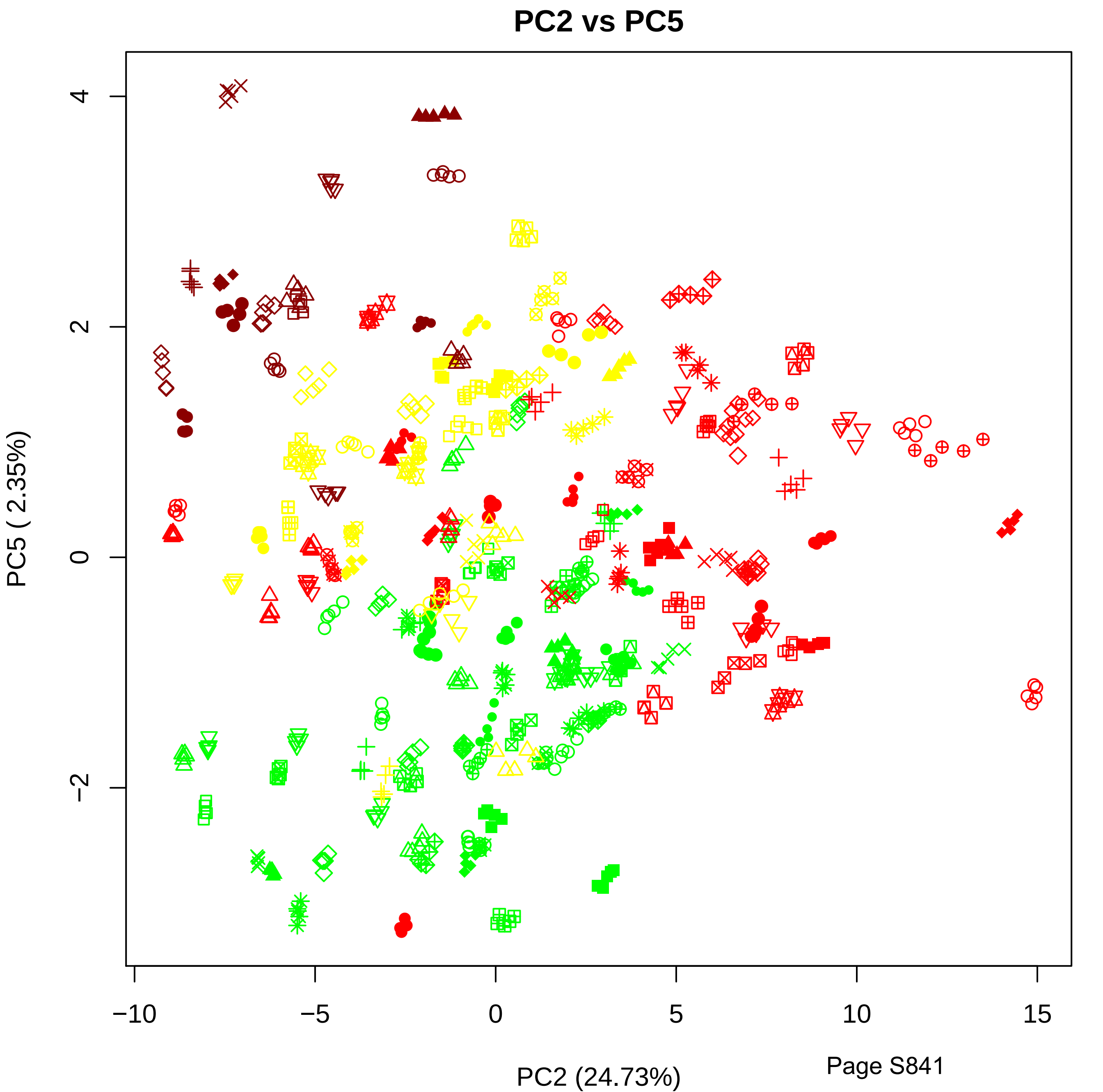
PC2 vs PC5
PC5 (2.35%)
PC2 (24.73%)
4
2
0
−2
−10
−5
0
5
10
15

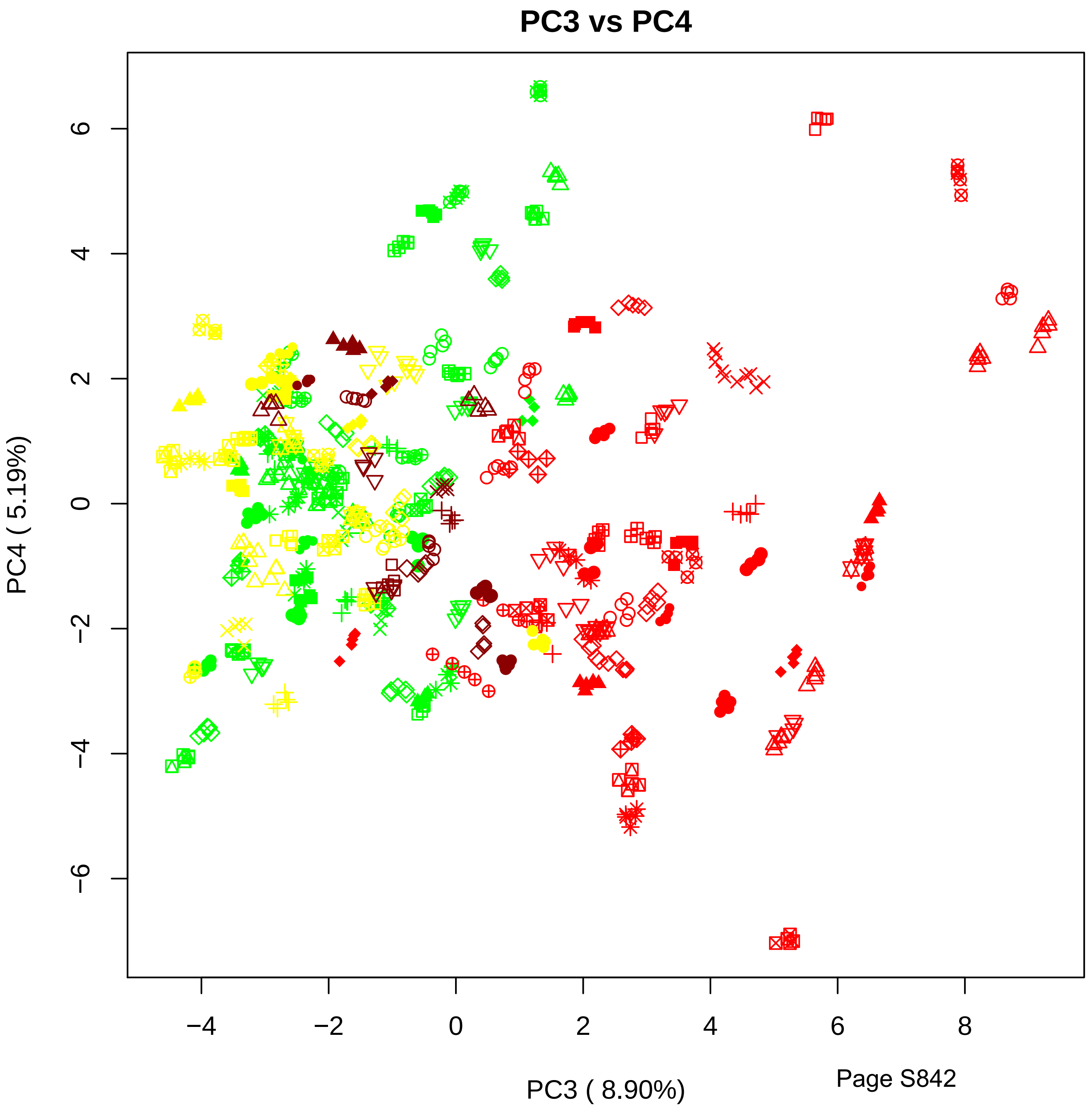
PC3 vs PC4
PC4 (5.19%)
PC3 (8.90%)
6
4
2
0
−2
−4
−6
−4
−2
0
2
4
6
8

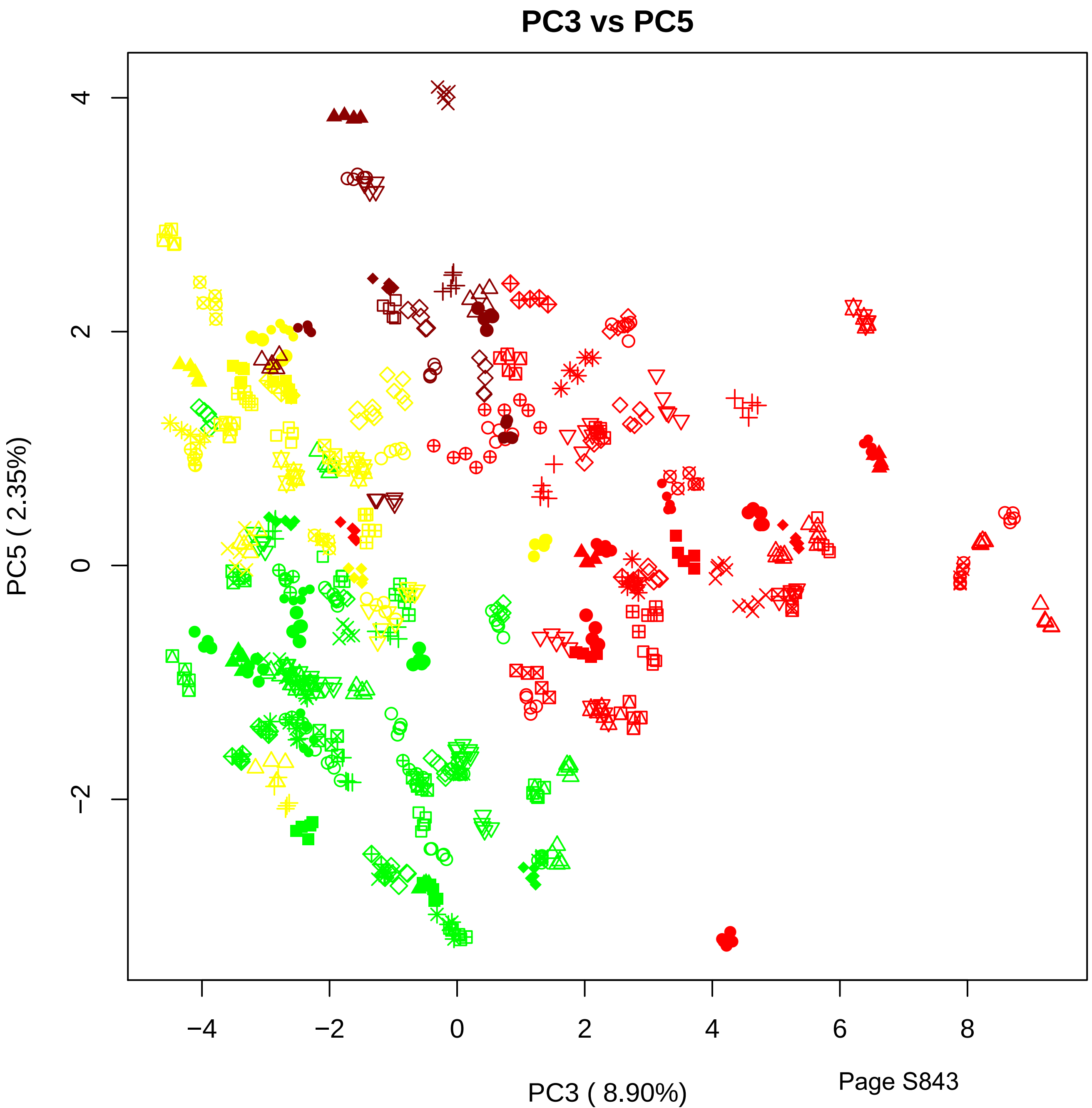
PC3 vs PC5
PC5 (2.35%)
4
2
0
−2
−4
−2
0
2
4
6
8
PC3 (8.90%)

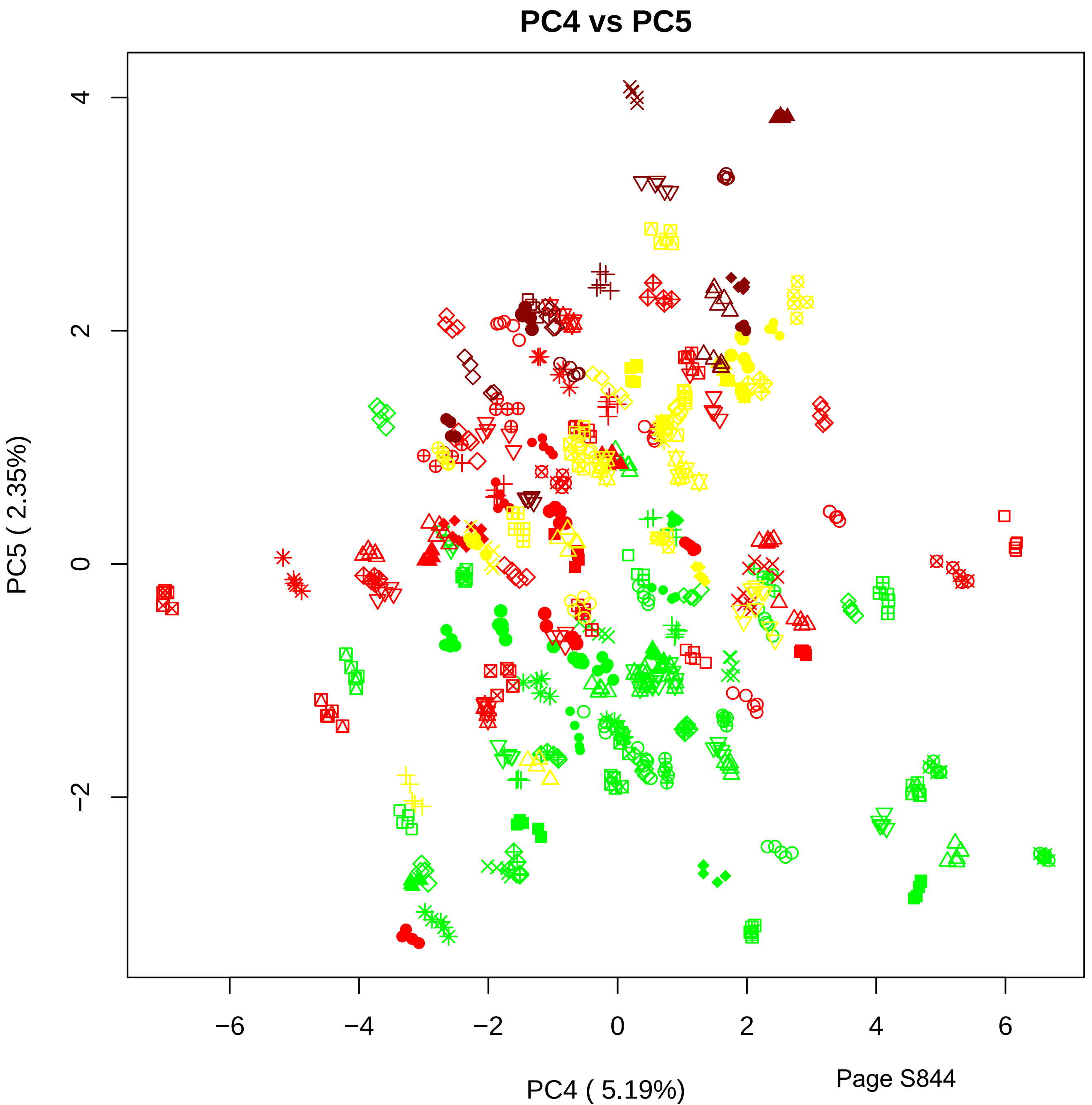
PC4 vs PC5
PC5 (2.35%)
PC4 (5.19%)
4
2
0
−2
−6
−4
−2
0
2
4
6

PCA of PMH vs OMH

Scaled Matrix PCAed

7th order polynomial baseline correction performed

Raw spectra smoothed to 21 Neighbours, 2nd order PN, Savtisky Golay

Normalisation between 0 and 100

D2 spectra smoothed to 41 Neighbours, 2nd order PN, Savtisky Golay

Regions Analysed: 3601−2501 and 2001−700cm−1

Samples Analysed: 0 bContam's 0 BLH's 0 BMH's 30 OMH's 0 pContam's 0 PLH's 60 PMH's

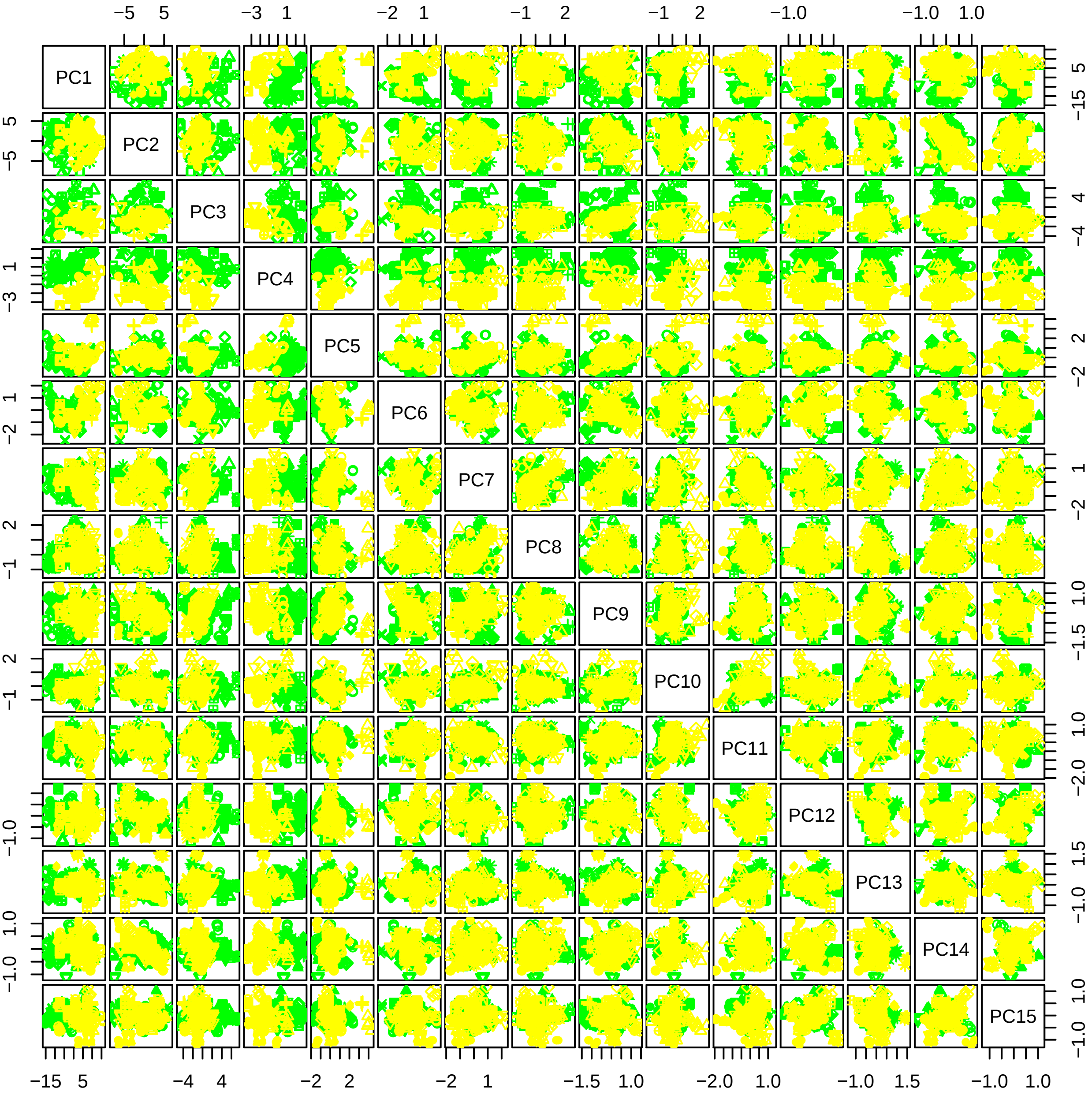

PC1
PC2
PC3
PC4
PC5
PC6
PC7
PC8
PC9
PC10
PC11
PC12
PC13
PC14
PC15

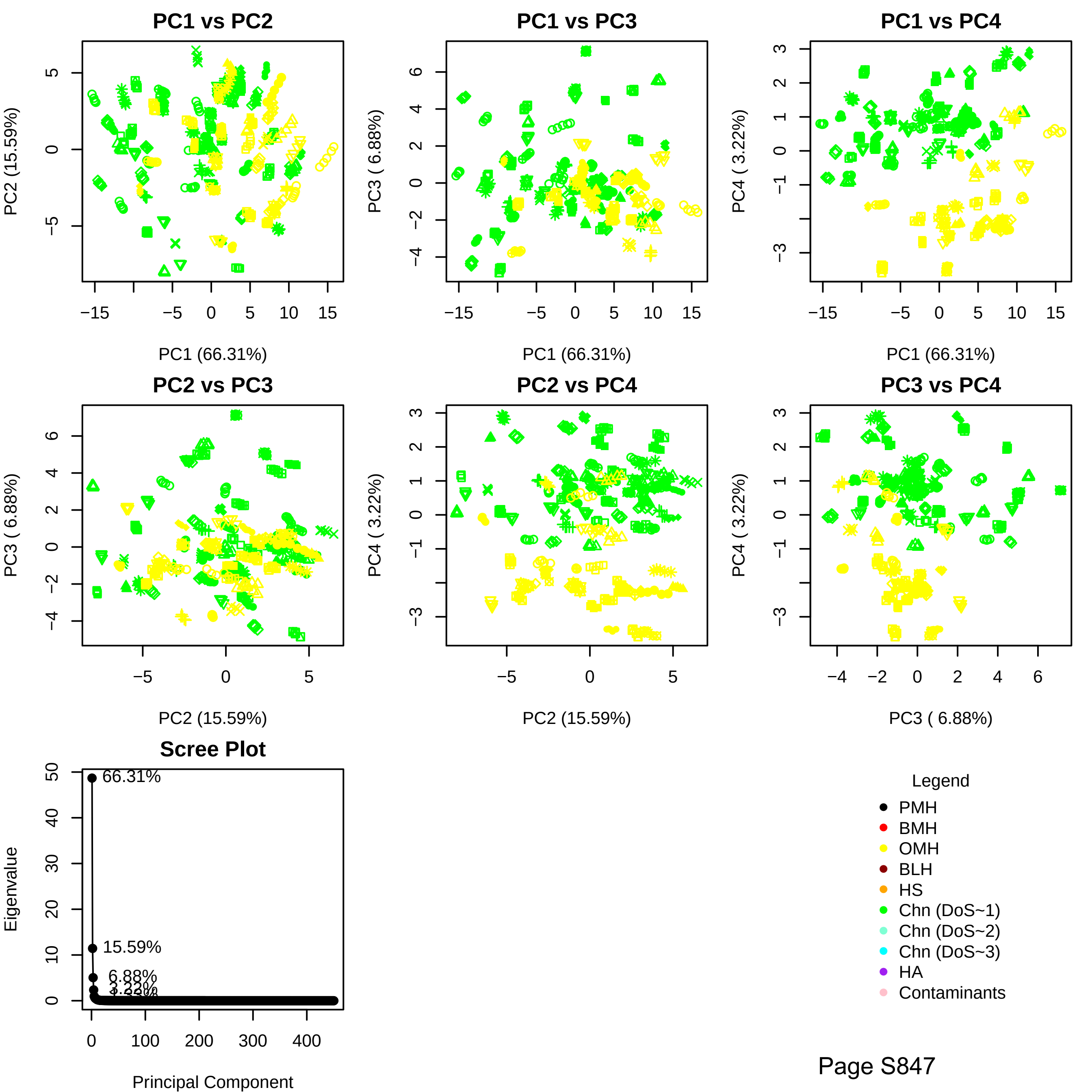

PC1 vs PC2
PC1 vs PC3
PC1 vs PC4
PC2 vs PC3
PC2 vs PC4
PC3 vs PC4
Scree Plot
PC1 (66.31%)
PC2 (15.59%)
PC3 (6.88%)
PC4 (3.22%)
Eigenvalue
Principal Component
66.31%
15.59%
6.88%
Legend
PMH
BMH
OMH
BLH
HS
Chn (DoS~1)
Chn (DoS~2)
Chn (DoS~3)
HA
Contaminants

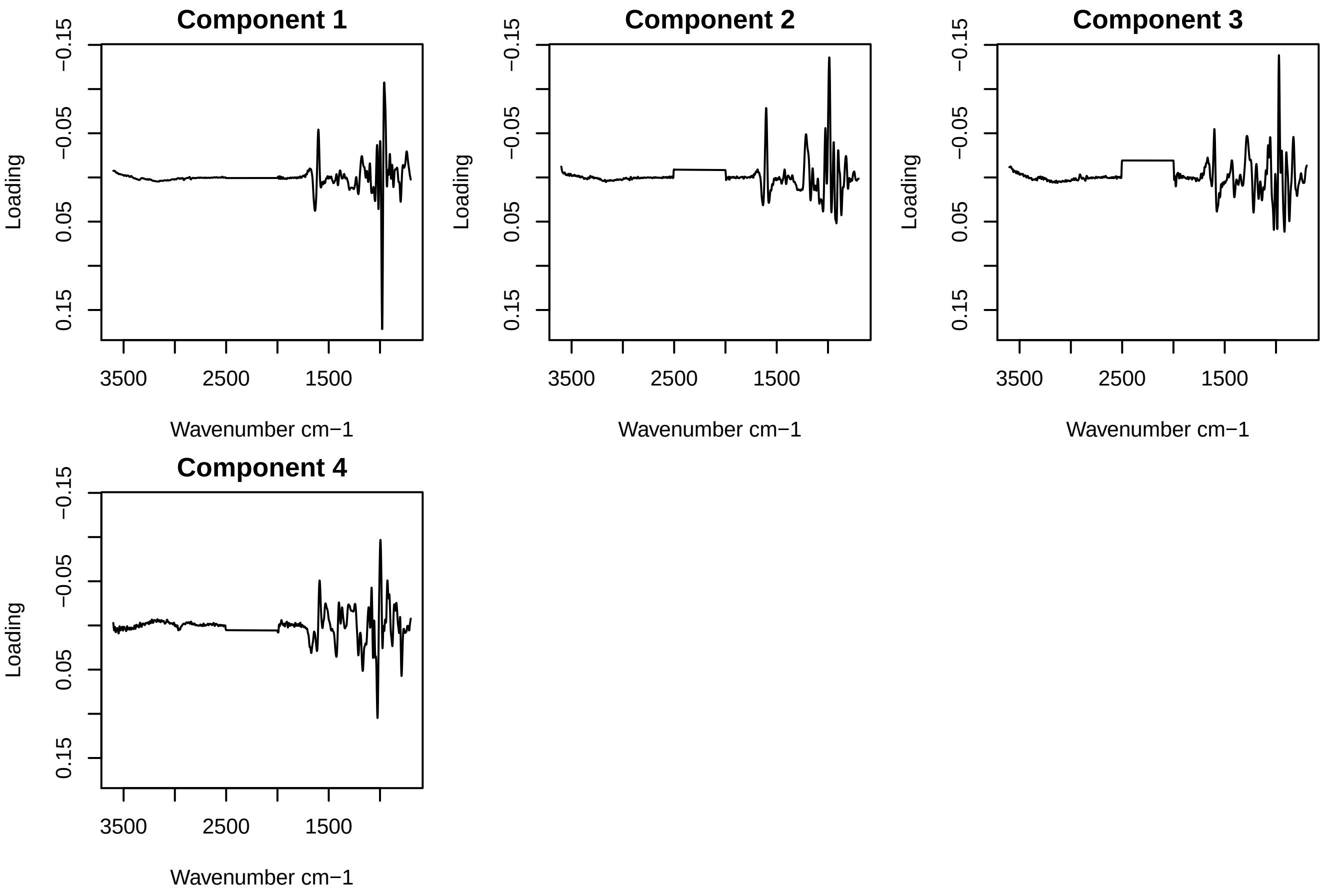
Component 1
Component 2
Component 3
Component 4
Loading
−0.15
−0.05
0.05
0.15
3500
2500
1500
Wavenumber cm−1

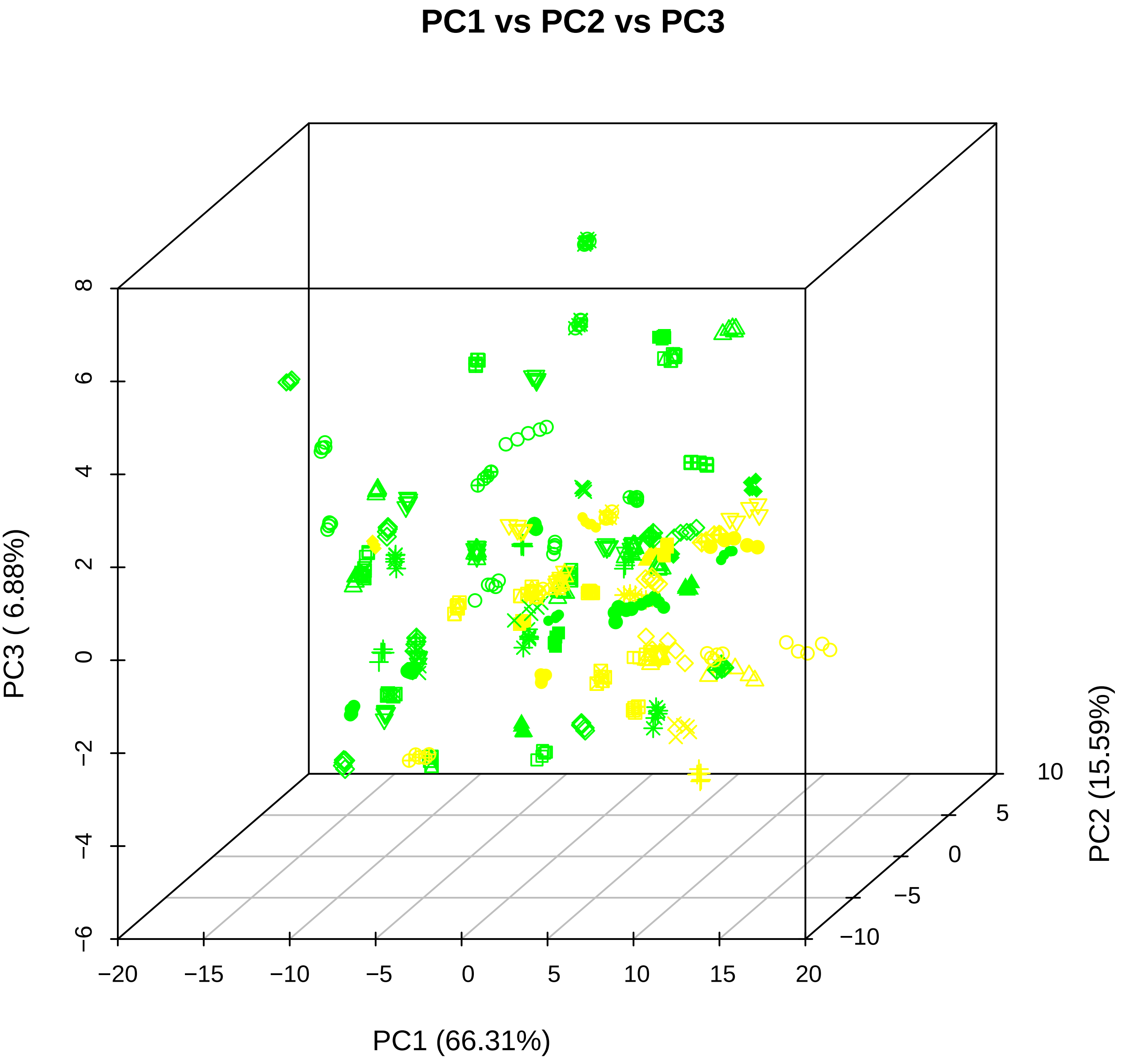

PC1 vs PC2 vs PC3
PC3 (6.88%)
PC1 (66.31%)
PC2 (15.59%)
8
6
4
2
0
−2
−4
−6
−20
−15
−10
−5
0
5
10
15
20
10
5
0
−5
−10

PC1 vs PC2 vs PC4

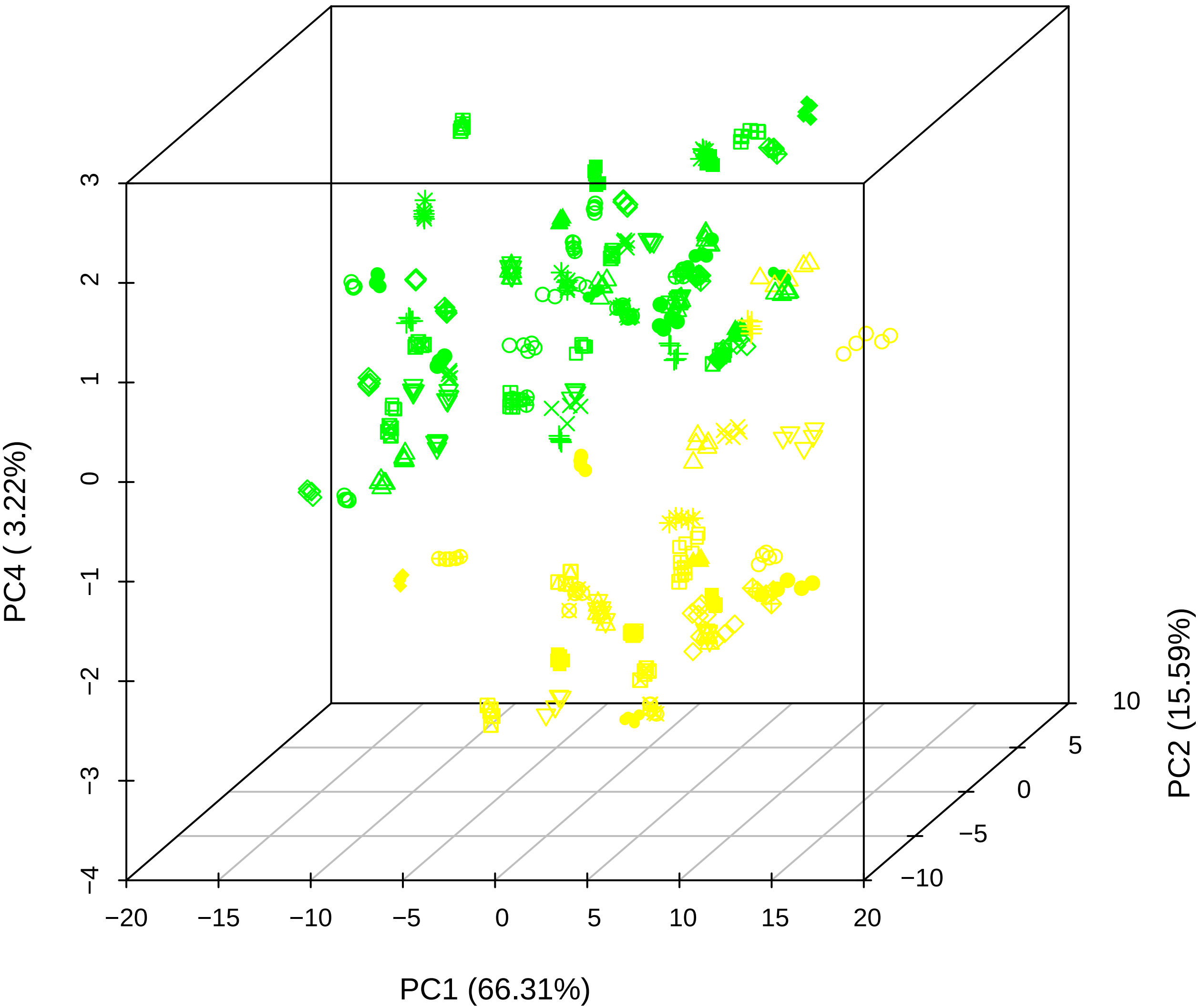

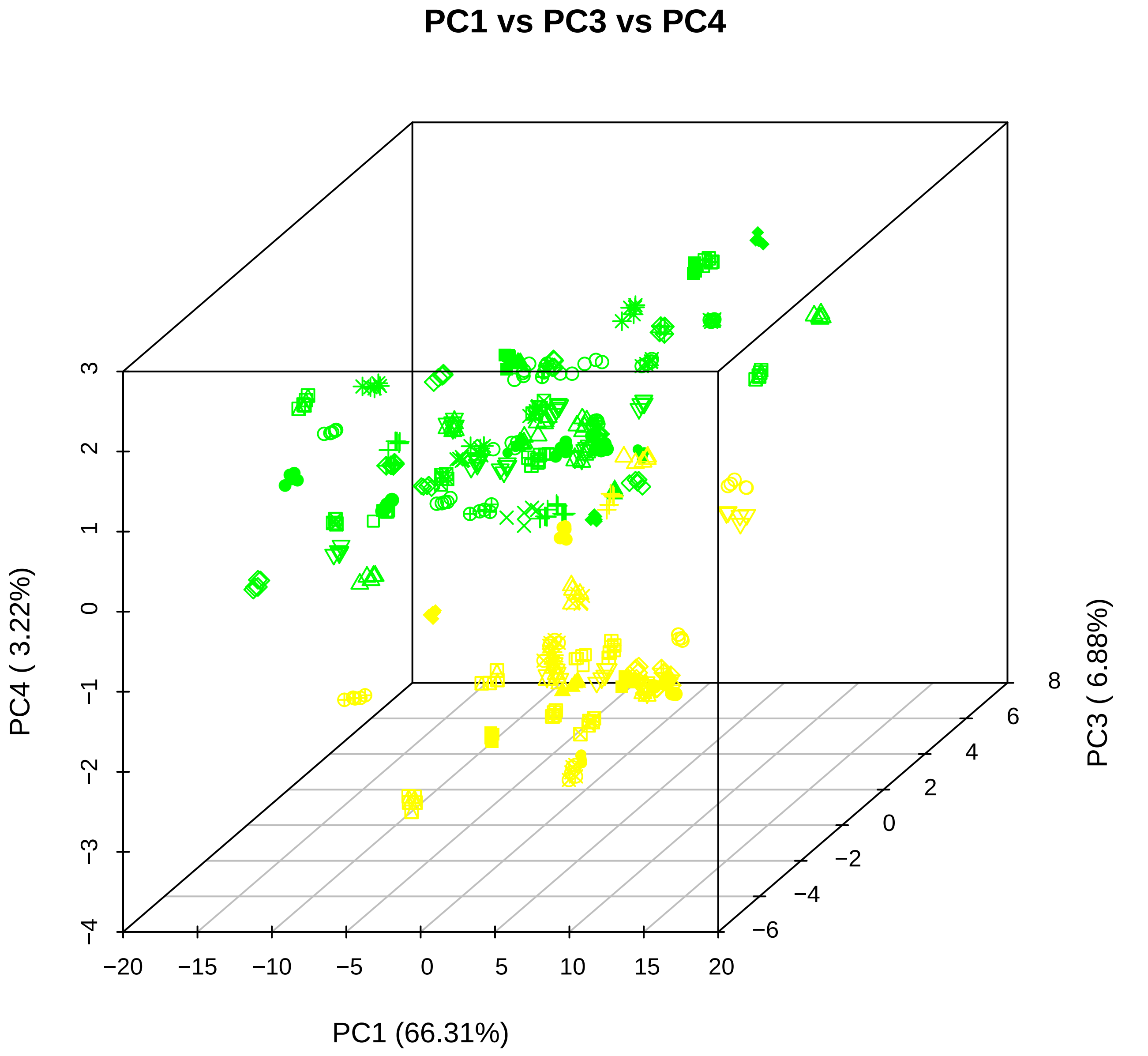

PC1 vs PC3 vs PC4
PC1 (66.31%)
PC3 (6.88%)
PC4 (3.22%)
−20 −15 −10 −5 0 5 10 15 20
−6 −4 −2 0 2 4 6 8
−4 −3 −2 −1 0 1 2 3

PC2 vs PC3 vs PC5

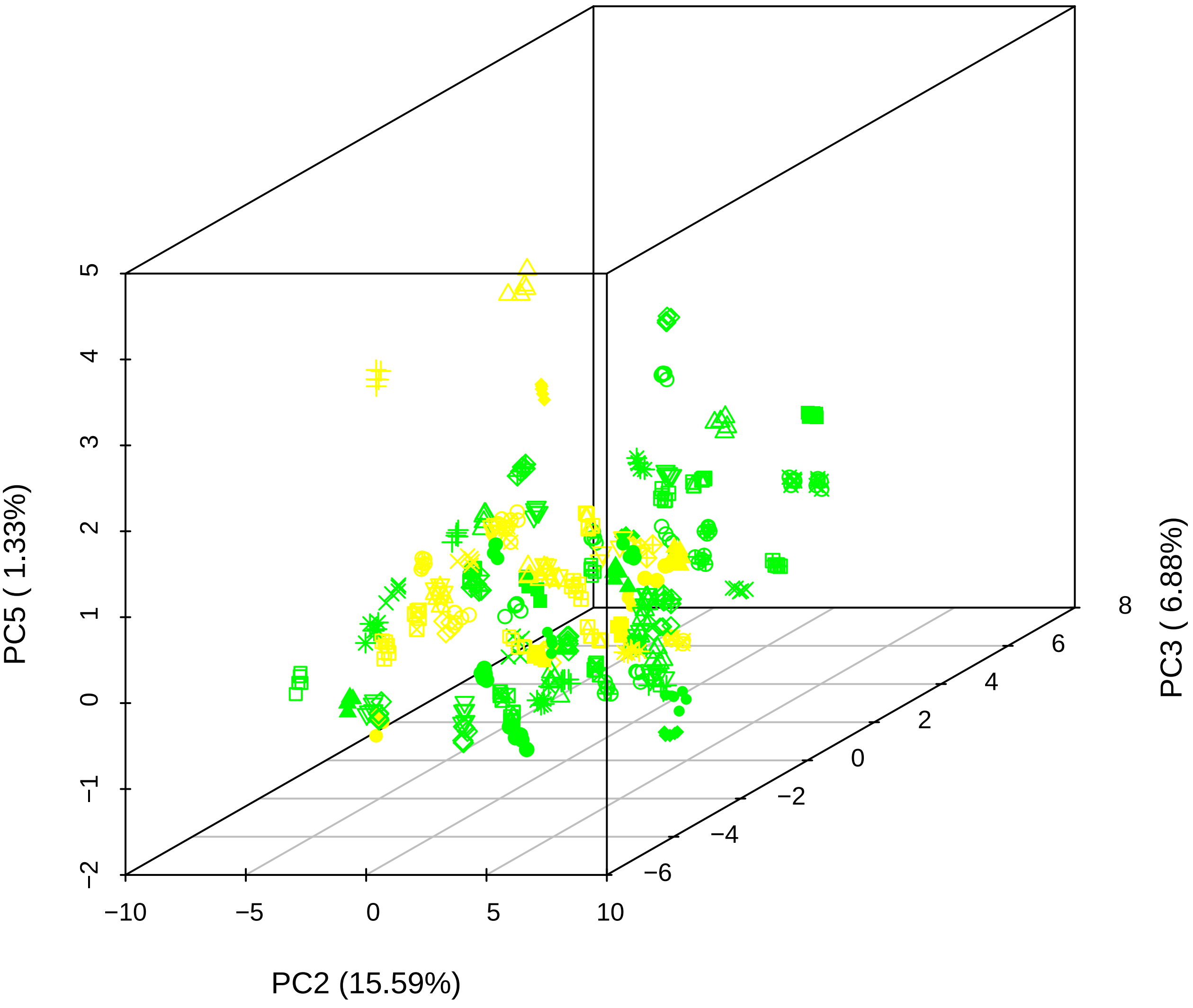

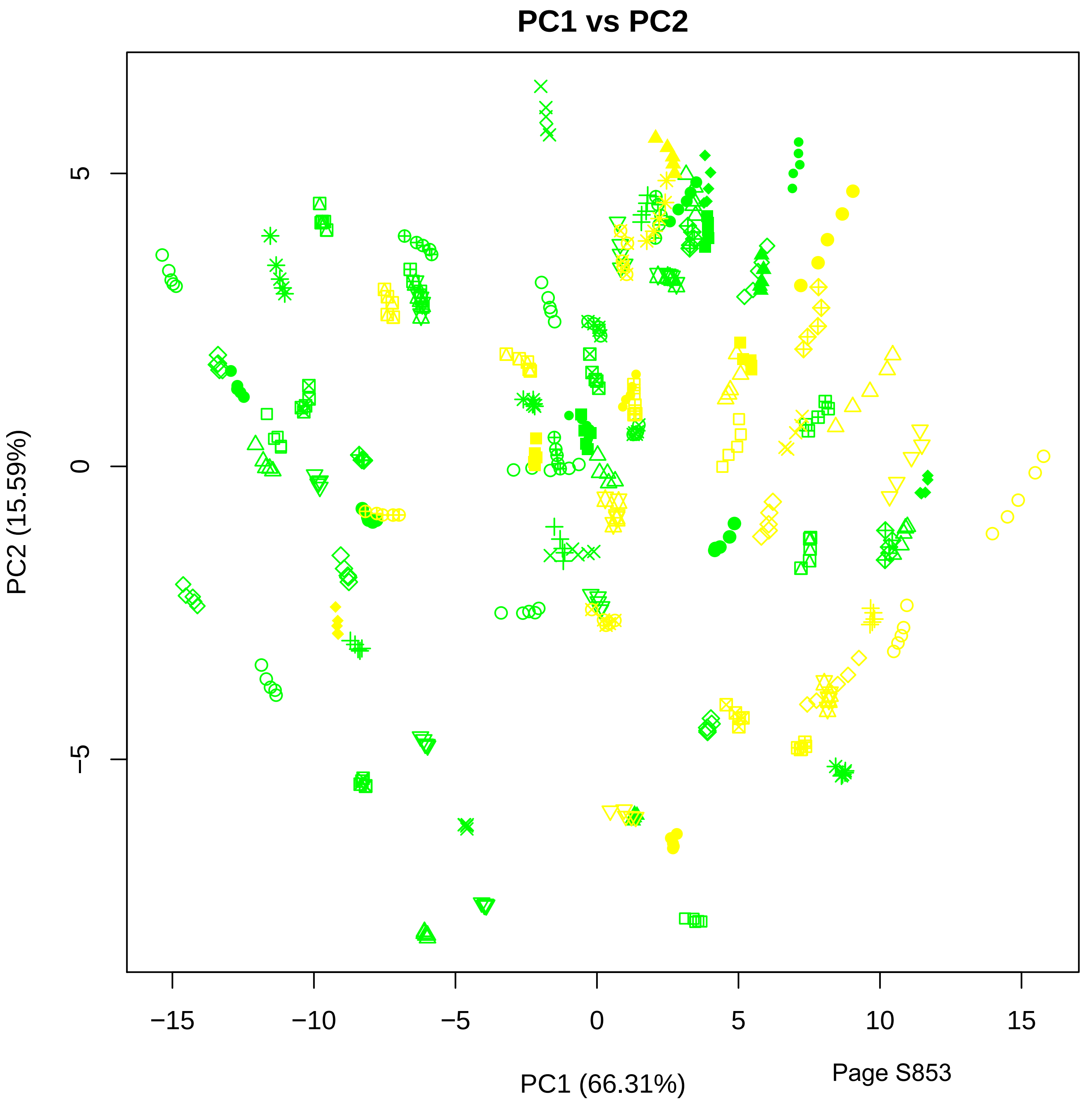
PC1 vs PC2
PC2 (15.59%)
5
0
−5
−15
−10
−5
0
5
10
15
PC1 (66.31%)

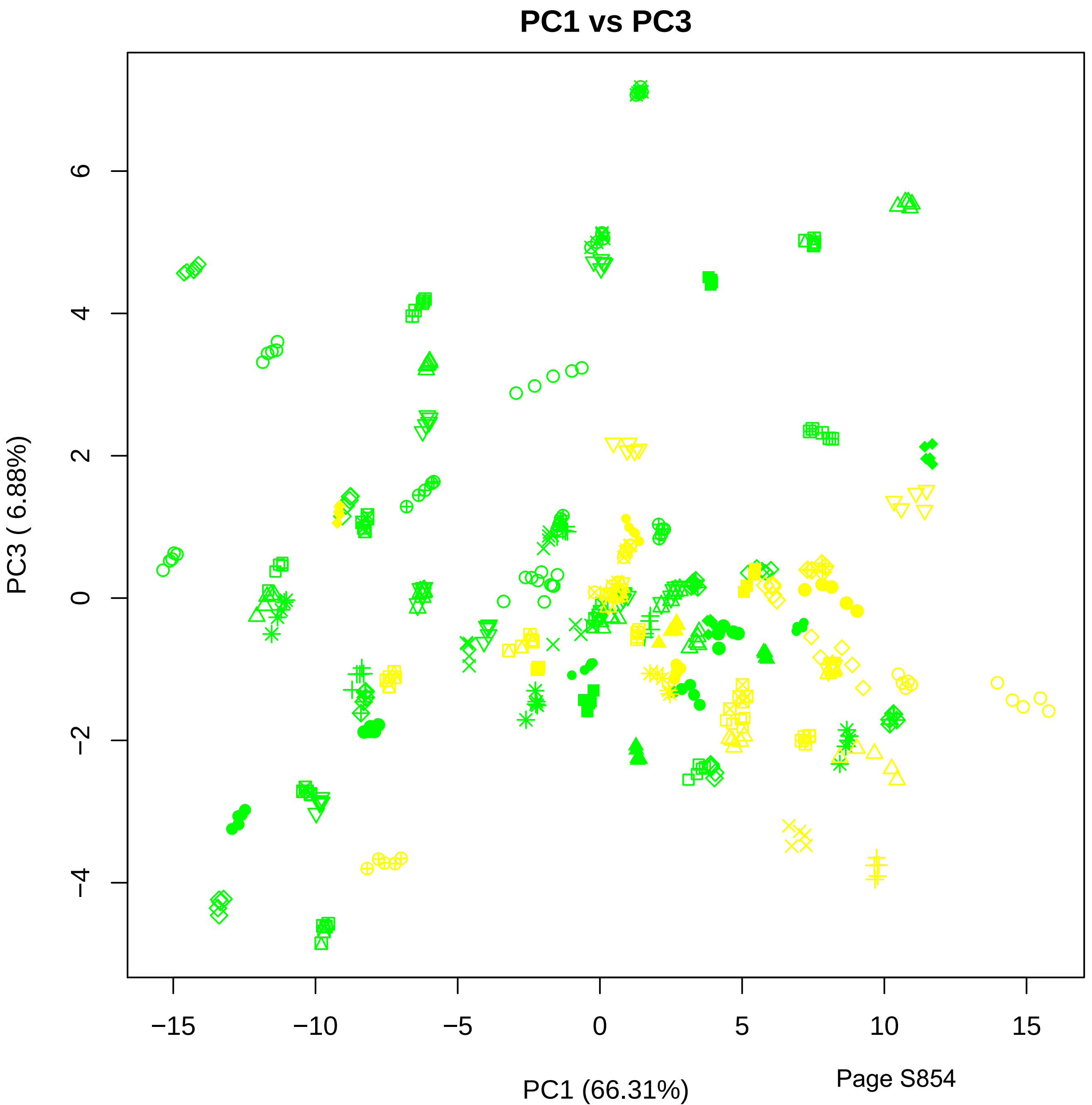
PC1 vs PC3
PC3 (6.88%)
PC1 (66.31%)
6
4
2
0
−2
−4
−15
−10
−5
0
5
10
15

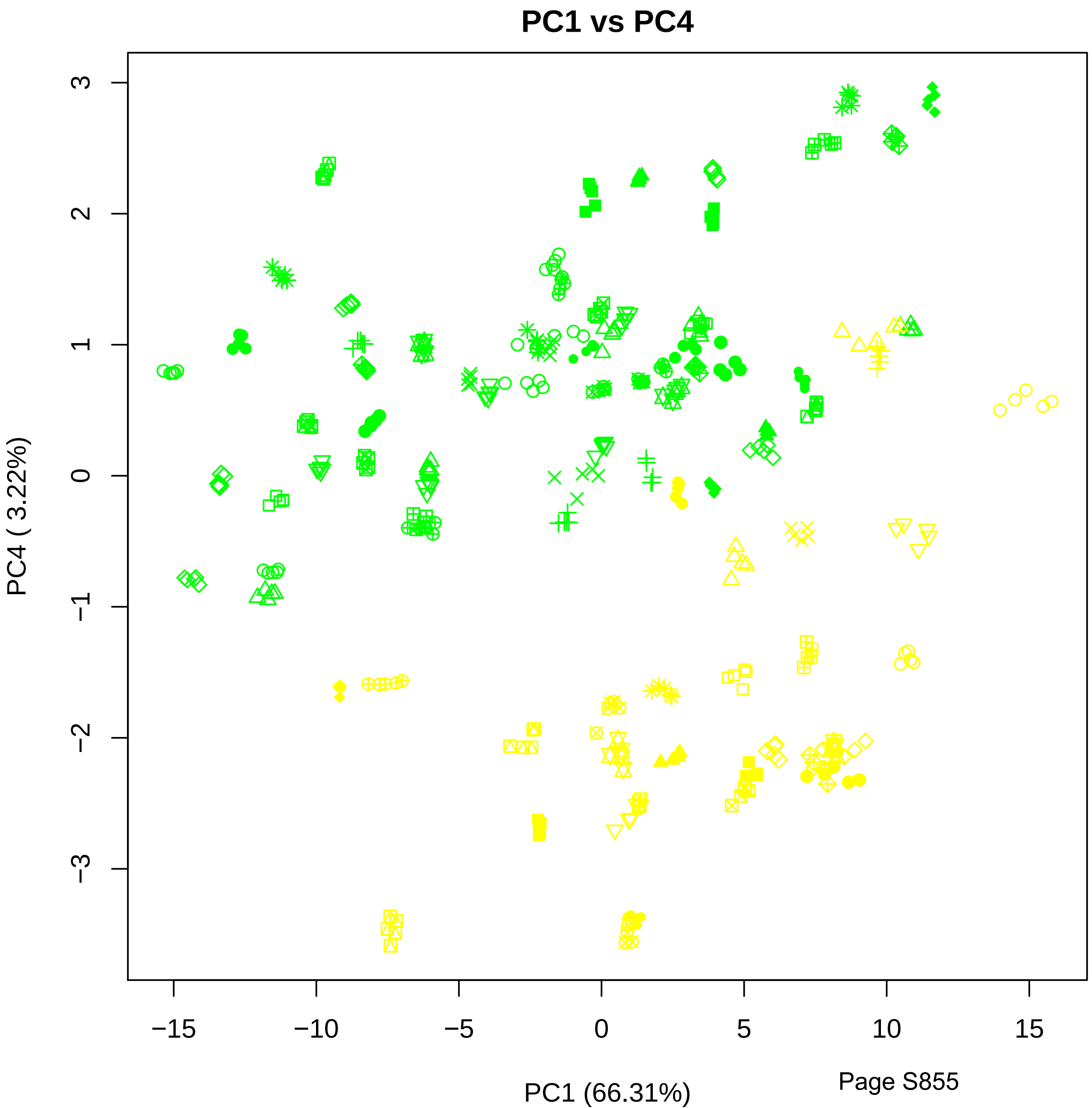
PC1 vs PC4
PC4 (3.22%)
PC1 (66.31%)
3
2
1
0
−1
−2
−3
−15
−10
−5
0
5
10
15

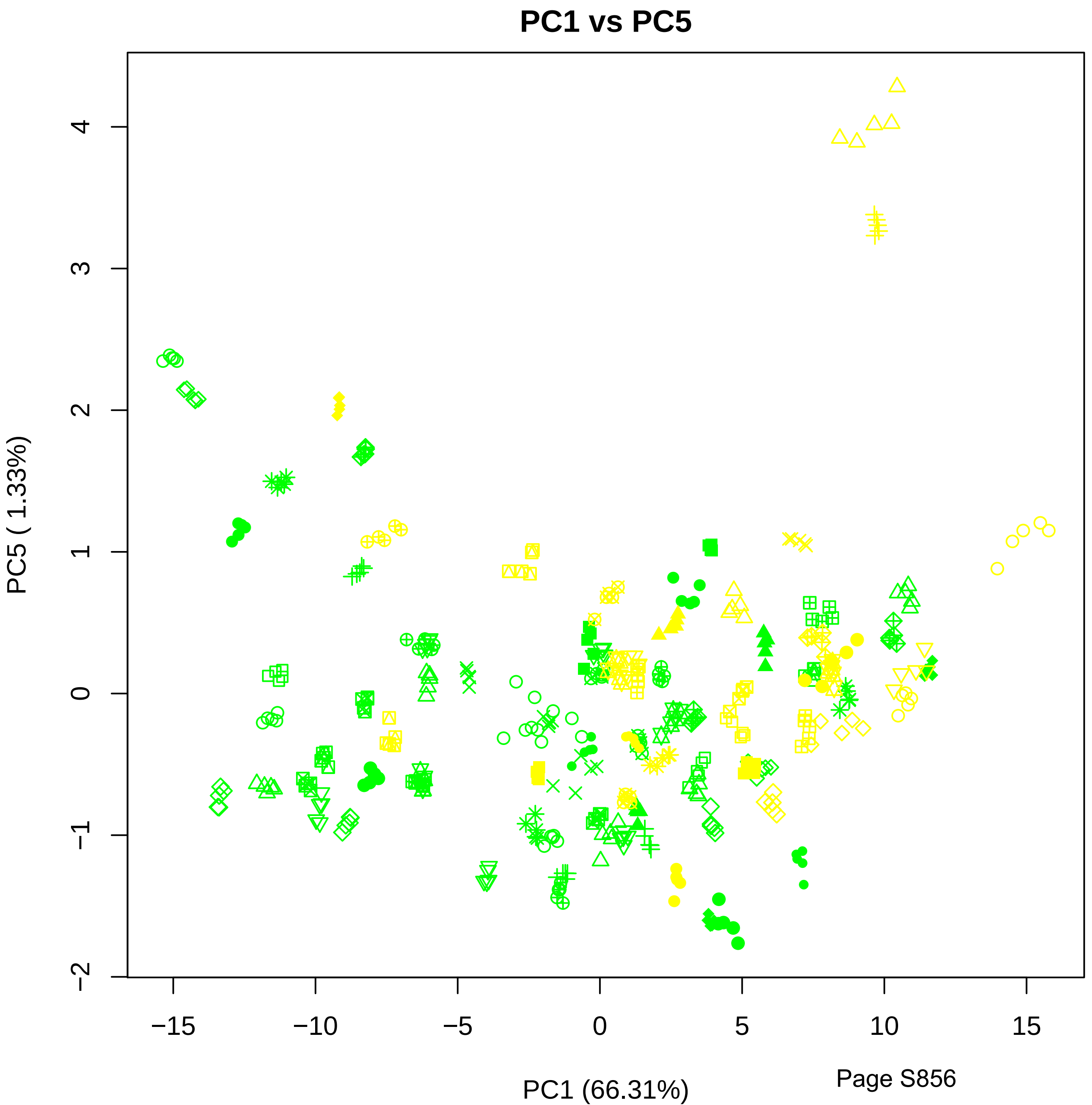

PC1 vs PC5
PC5 (1.33%)
PC1 (66.31%)
−2
−1
0
1
2
3
4
−15
−10
−5
0
5
10
15

PC2 vs PC3

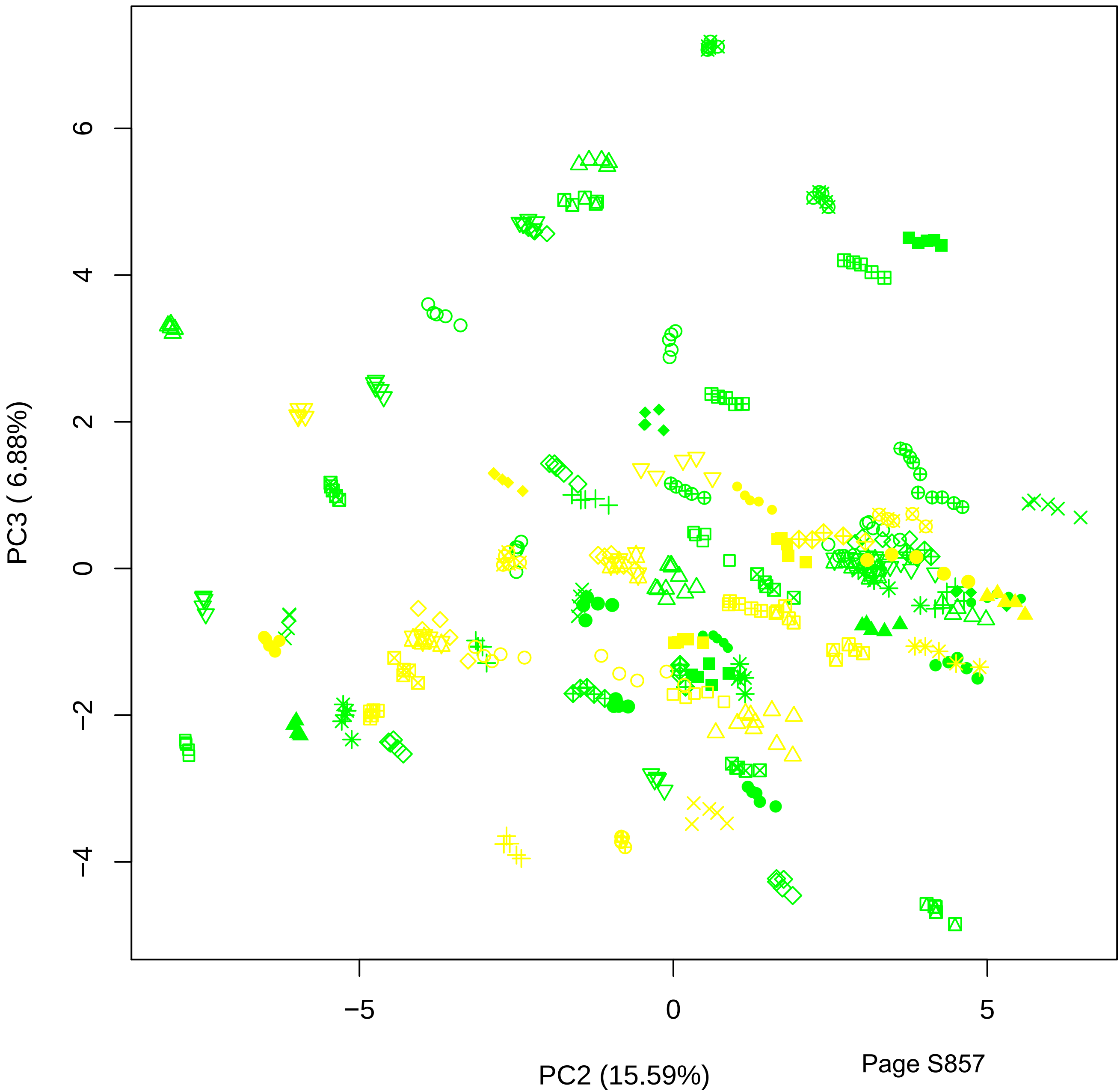

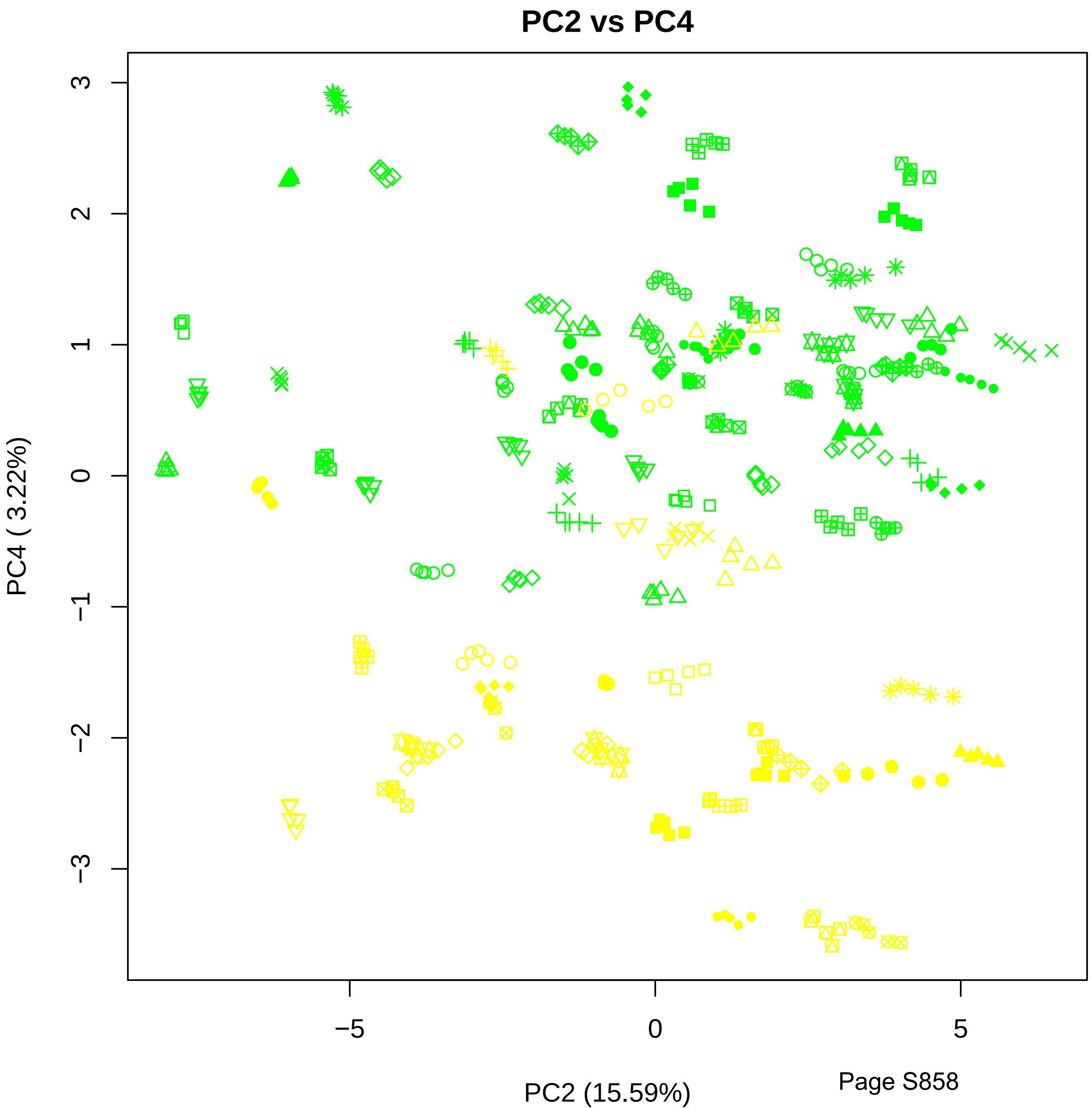
PC2 vs PC4
PC4 (3.22%)
PC2 (15.59%)
3
2
1
0
−1
−2
−3
−5
0
5

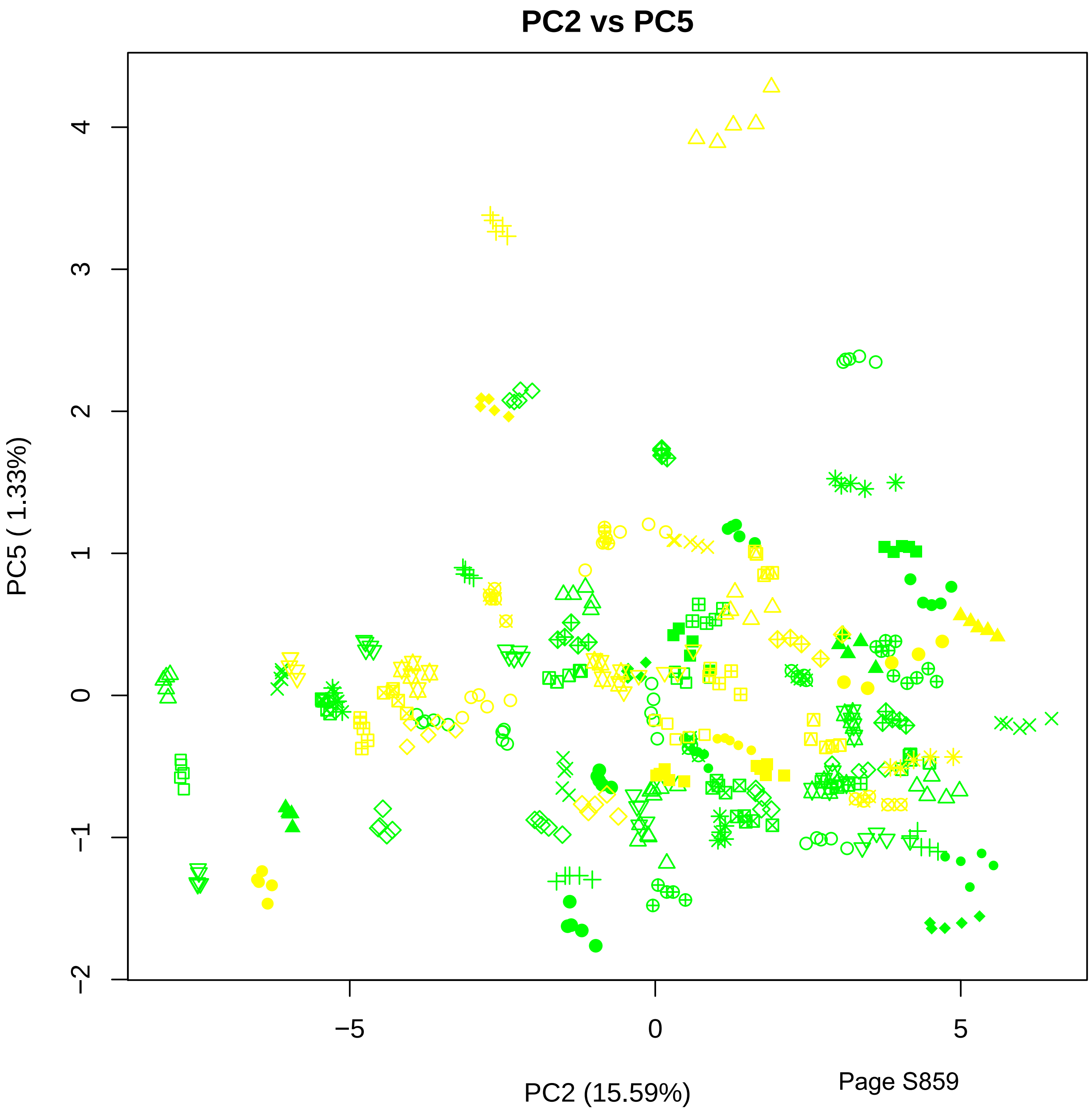
PC2 vs PC5
PC5 (1.33%)
PC2 (15.59%)
4
3
2
1
0
−1
−2
−5
0
5

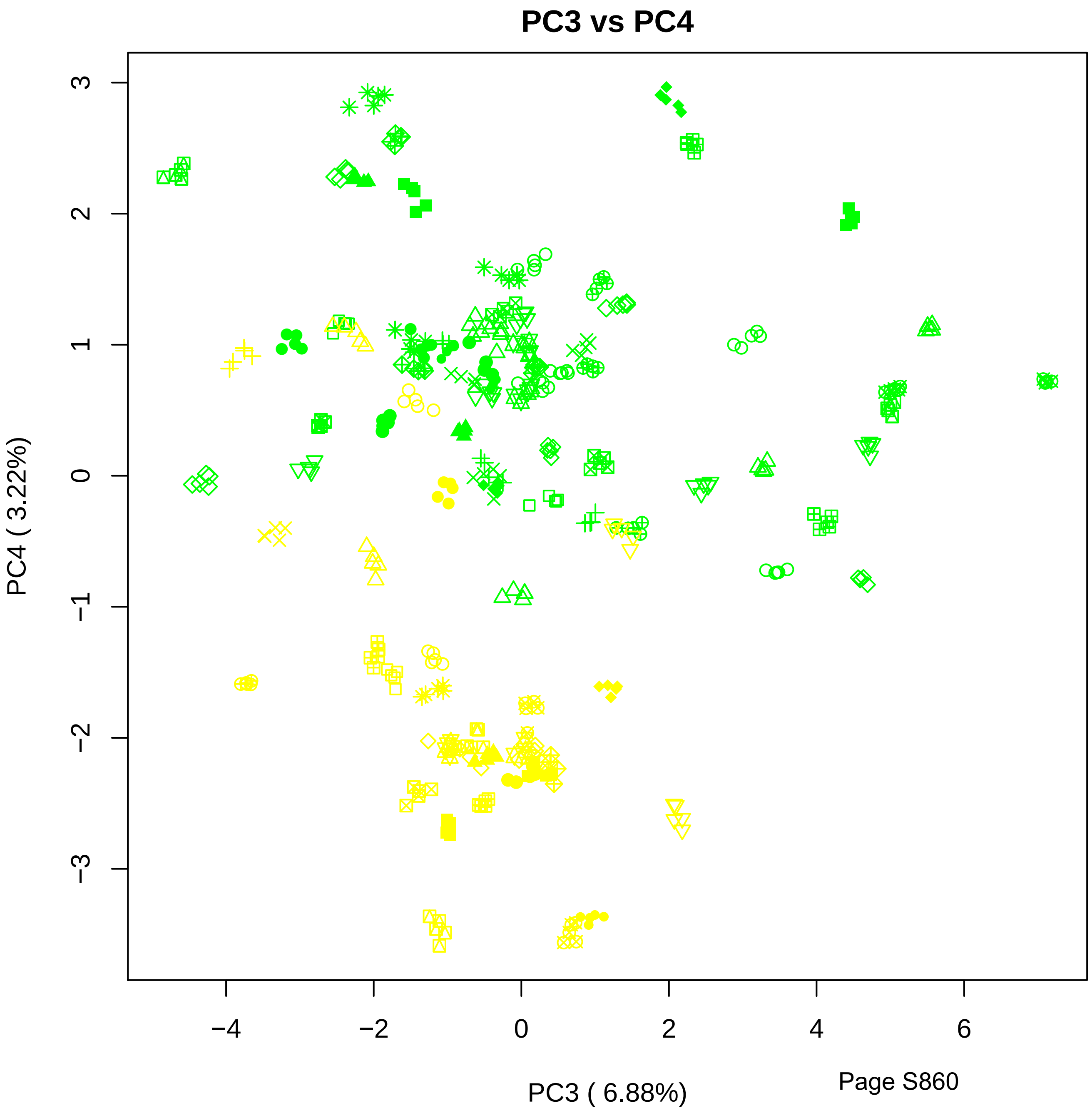
PC3 vs PC4
PC4 (3.22%)
PC3 (6.88%)
3
2
1
0
−1
−2
−3
−4
−2
0
2
4
6

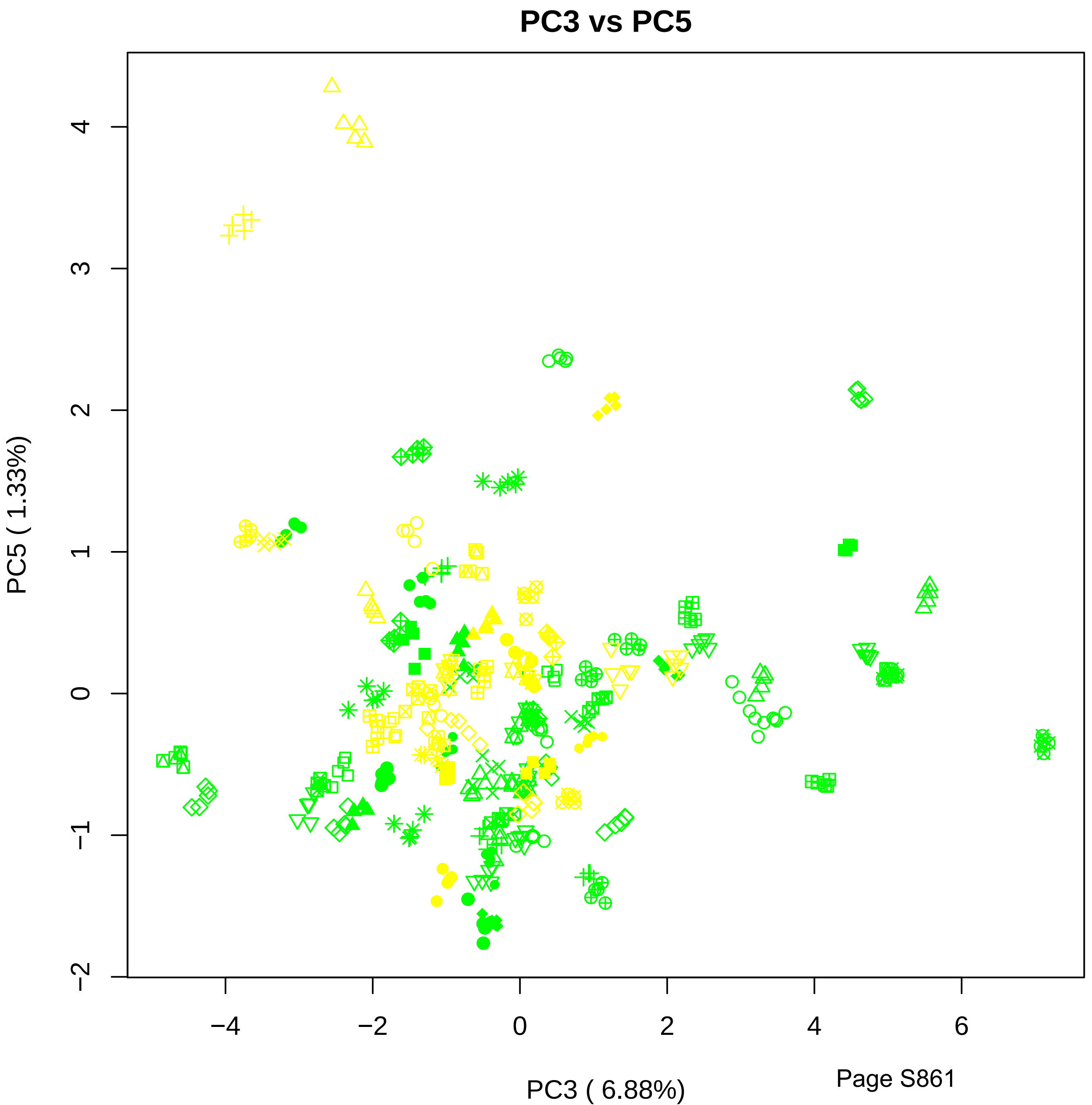
PC3 vs PC5
PC5 (1.33%)
PC3 (6.88%)
−2
−1
0
1
2
3
4
−4
−2
0
2
4
6

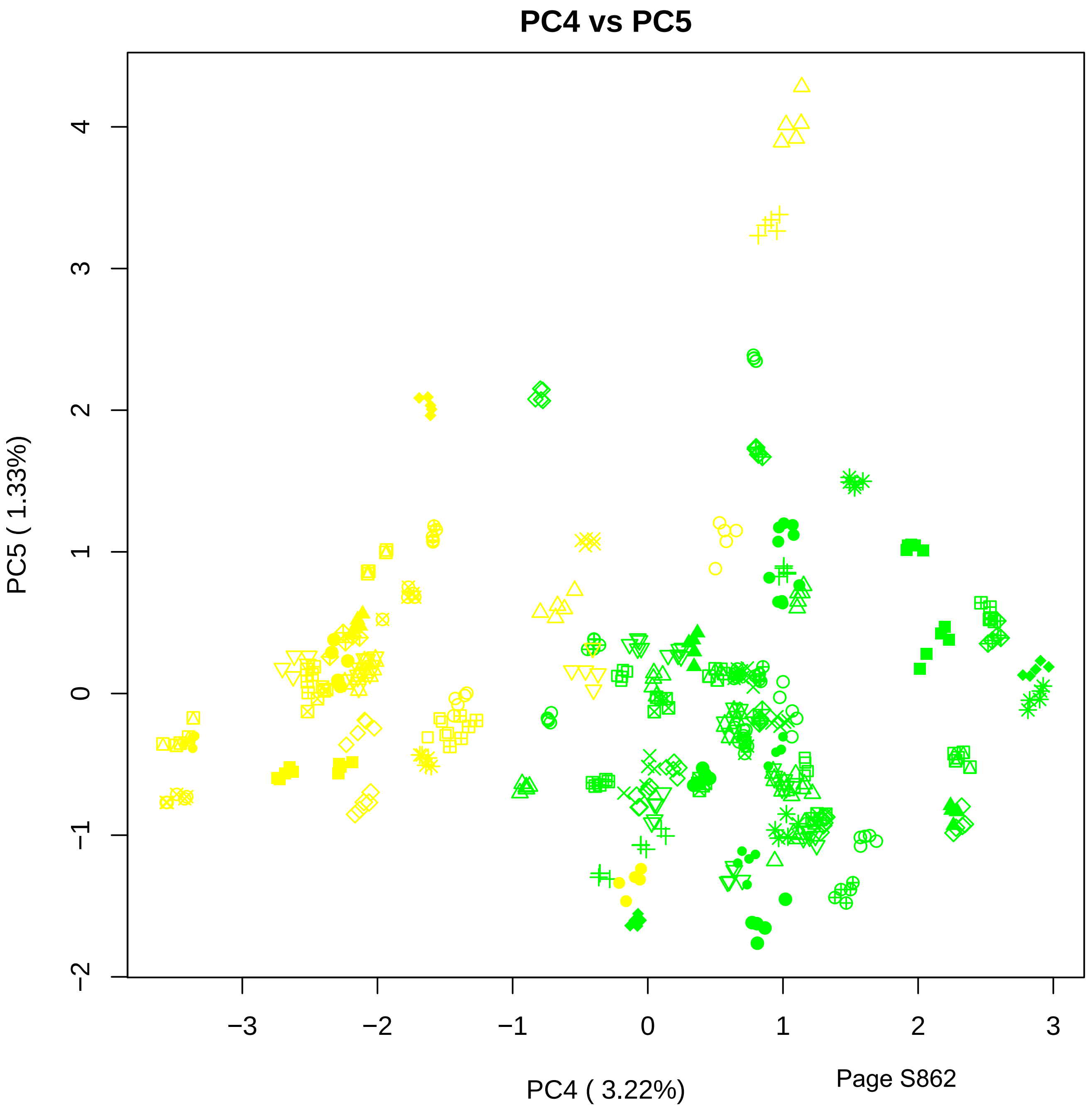
PC4 vs PC5
PC5 (1.33%)
PC4 (3.22%)
−2
−1
0
1
2
3
4
−3
−2
−1
0
1
2
3

PCA of PMH vs BLH

Scaled Matrix PCAed

7th order polynomial baseline correction performed

Raw spectra smoothed to 21 Neighbours, 2nd order PN, Savtisky Golay

Normalisation between 0 and 100

D2 spectra smoothed to 41 Neighbours, 2nd order PN, Savtisky Golay

Regions Analysed: 3601−2501 and 2001−700cm−1

Samples Analysed: 0 bContam's 16 BLH's 0 BMH's 0 OMH's 0 pContam's 0 PLH's 60 PMH's

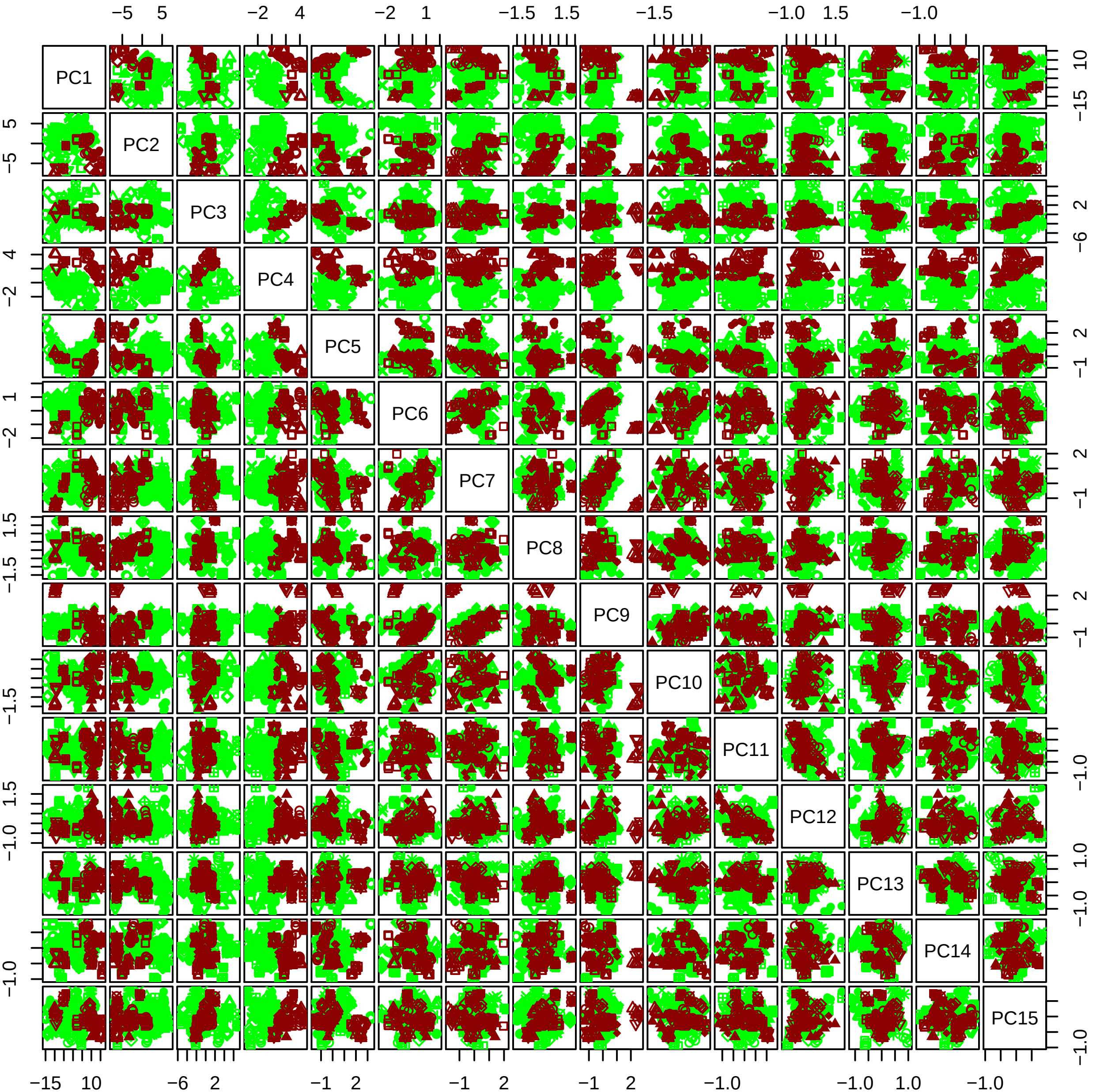
PC1
PC2
PC3
PC4
PC5
PC6
PC7
PC8
PC9
PC10
PC11
PC12
PC13
PC14
PC15

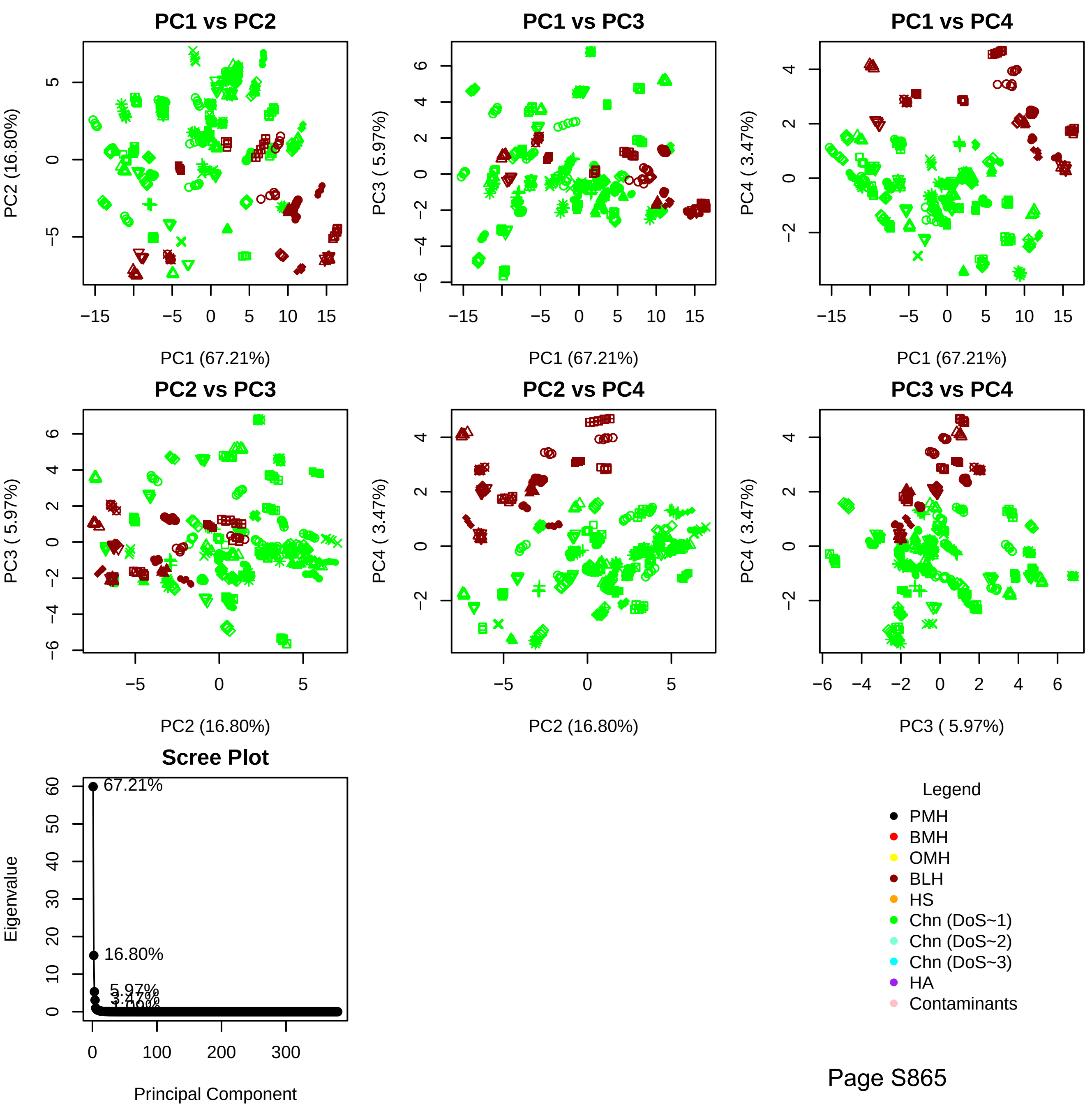
PC1 vs PC2
PC1 vs PC3
PC1 vs PC4
PC2 vs PC3
PC2 vs PC4
PC3 vs PC4
PC1 (67.21%)
PC2 (16.80%)
PC3 (5.97%)
PC4 (3.47%)
Scree Plot
67.21%
16.80%
5.97%
Eigenvalue
Principal Component
Legend
PMH
BMH
OMH
BLH
HS
Chn (DoS~1)
Chn (DoS~2)
Chn (DoS~3)
HA
Contaminants

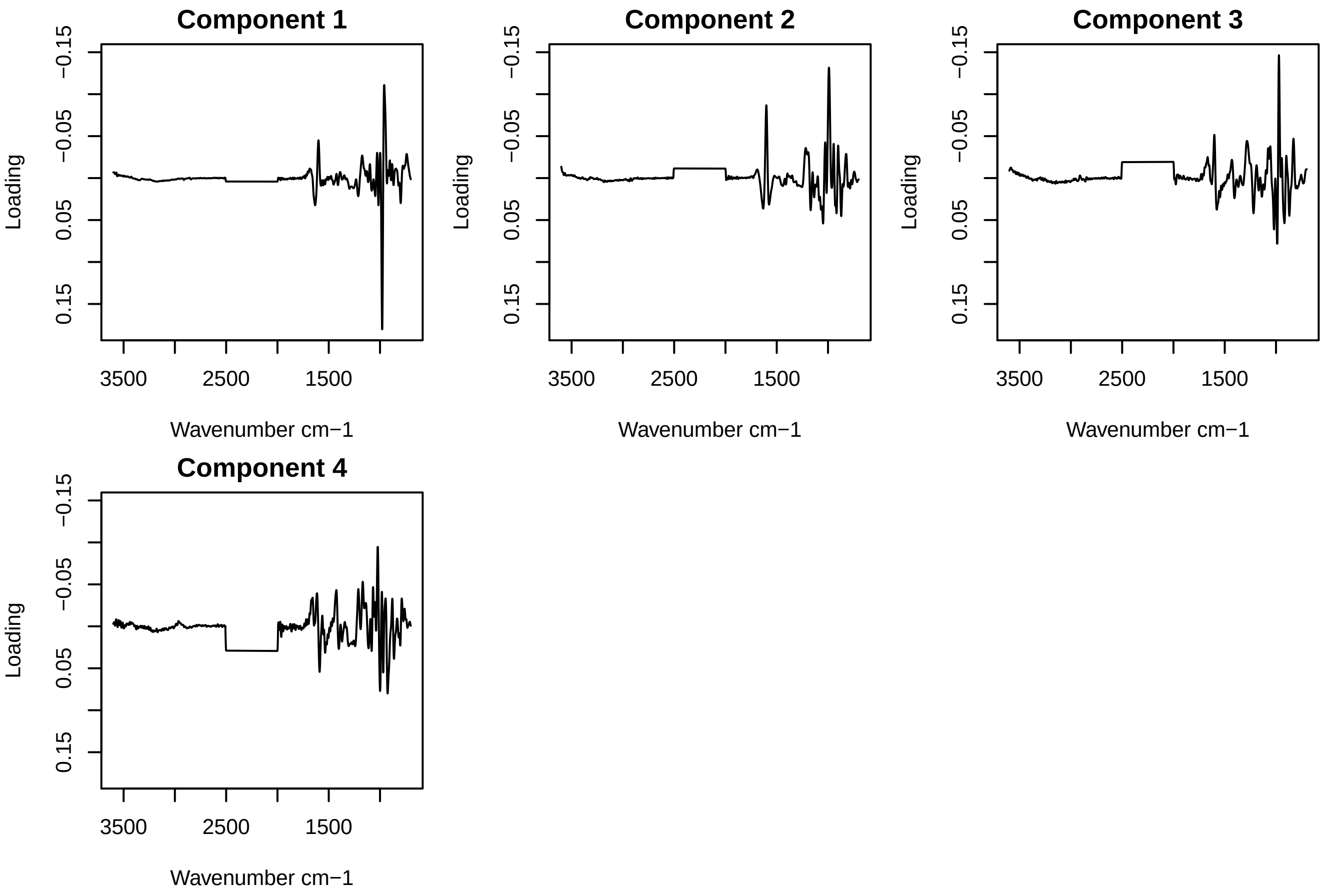

Component 1
Component 2
Component 3
Component 4
Loading
−0.15
−0.05
0.05
0.15
3500
2500
1500
Wavenumber cm−1

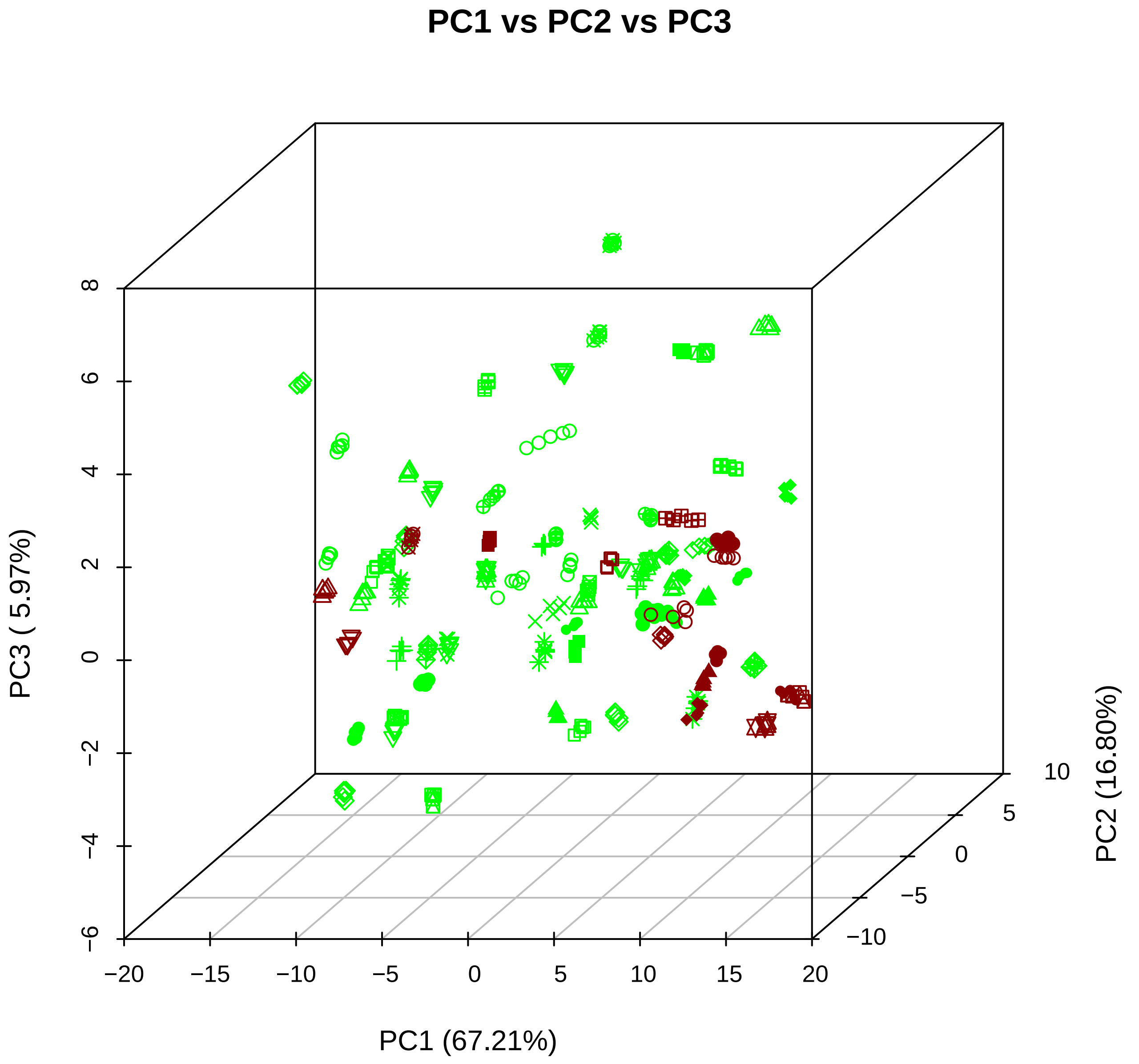
PC1 vs PC2 vs PC3
PC1 (67.21%)
PC2 (16.80%)
PC3 (5.97%)
−20
−15
−10
−5
0
5
10
15
20
−6
−4
−2
0
2
4
6
8
−10
−5
0
5
10

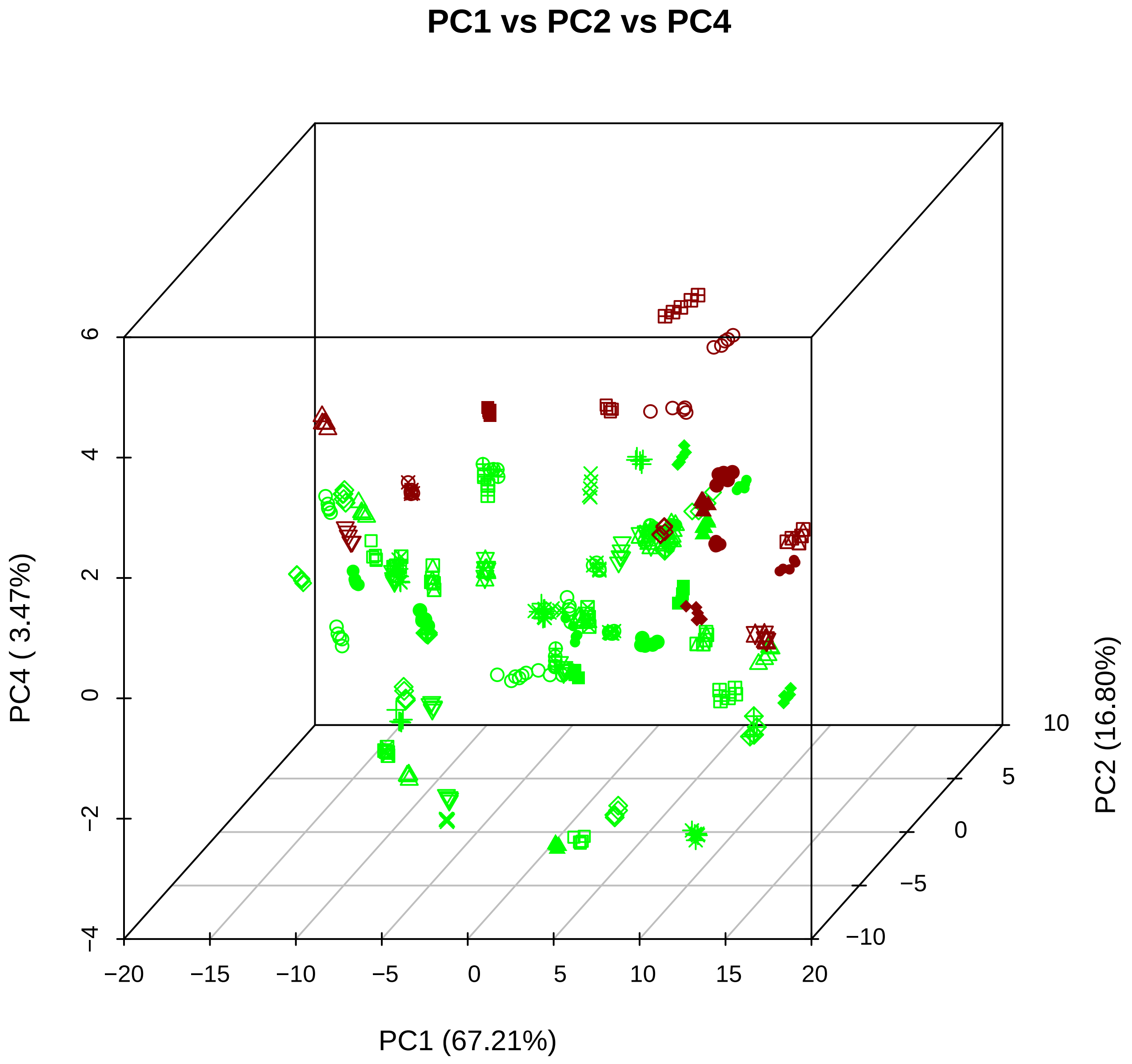
PC1 vs PC2 vs PC4
PC4 (3.47%)
6
4
2
0
−2
−4
−20
−15
−10
−5
0
5
10
15
20
PC1 (67.21%)
PC2 (16.80%)
10
5
0
−5
−10

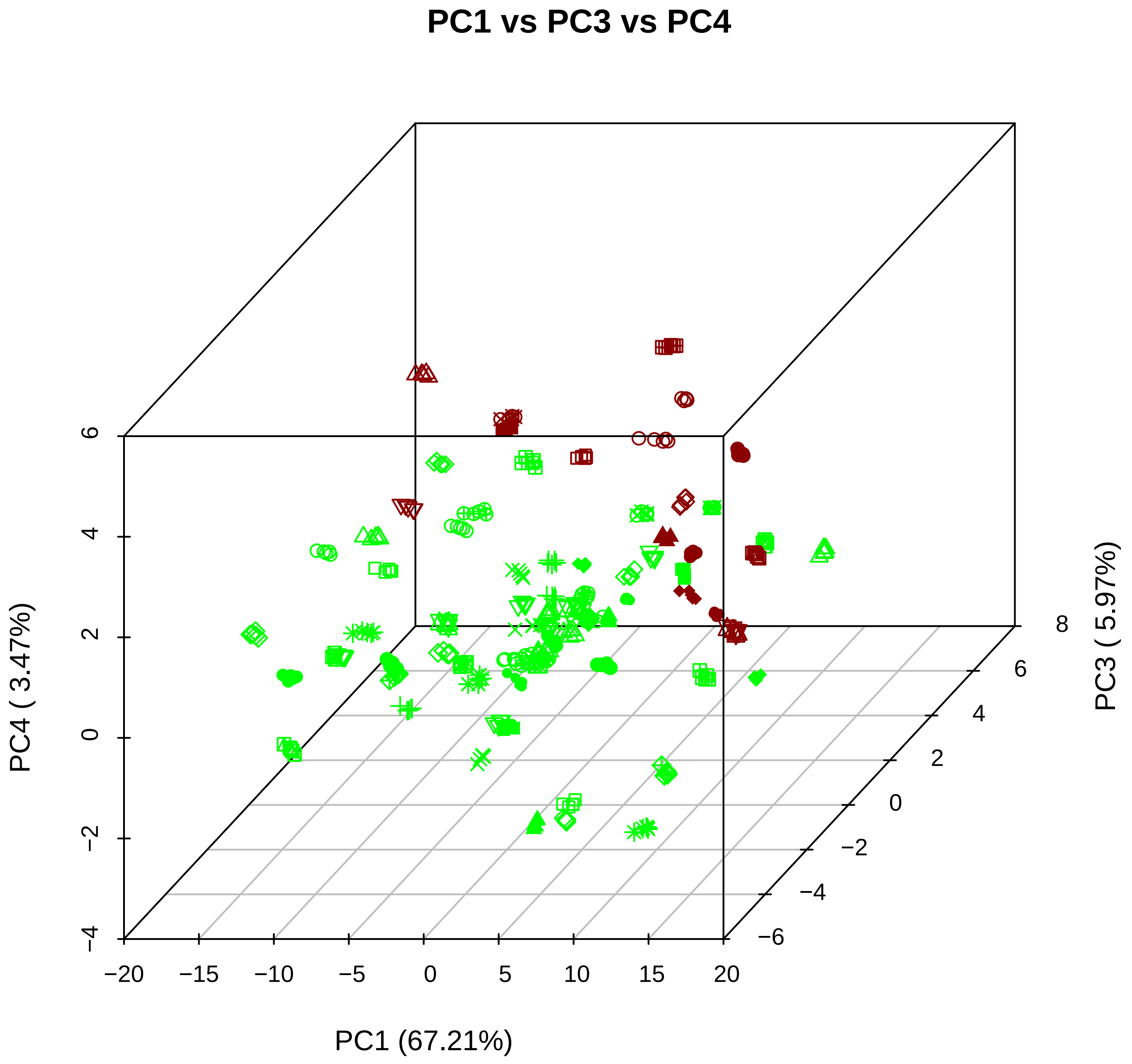

PC1 vs PC3 vs PC4
PC4 (3.47%)
PC1 (67.21%)
PC3 (5.97%)
−4
−2
0
2
4
6
−20
−15
−10
−5
0
5
10
15
20
−6
−4
−2
0
2
4
6
8

PC2 vs PC3 vs PC5

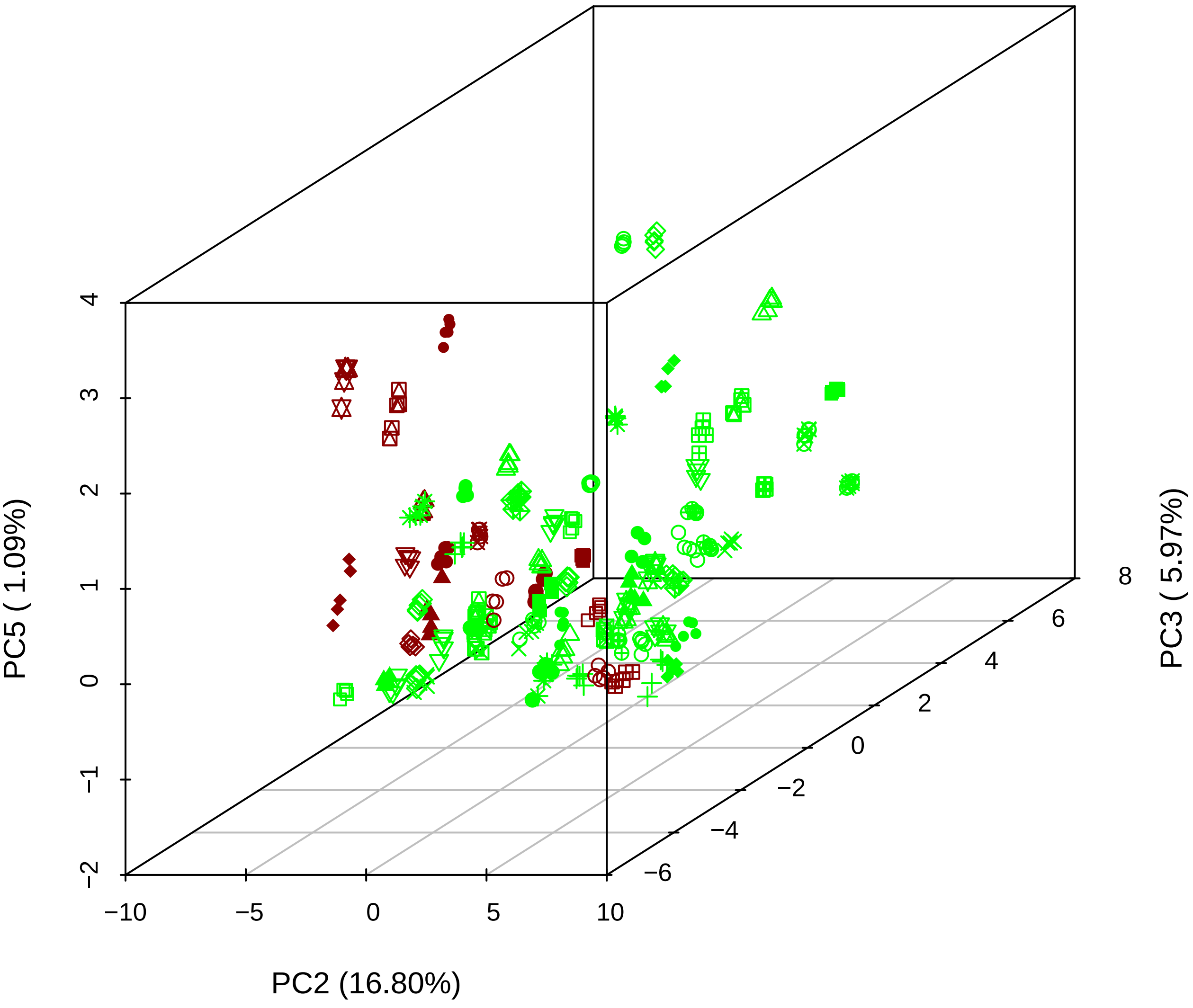

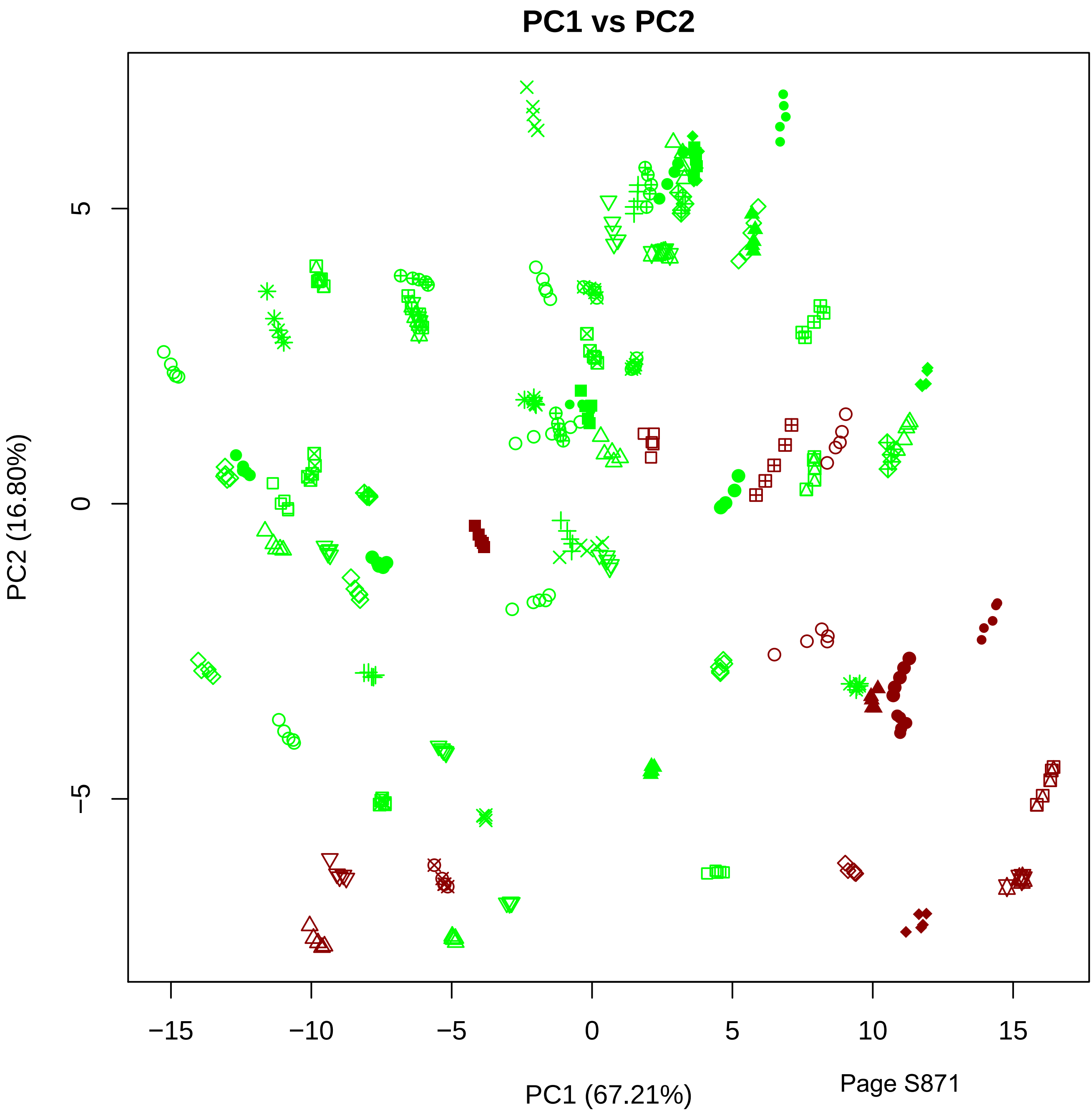

PC1 vs PC2
PC2 (16.80%)
PC1 (67.21%)
5
0
−5
−15
−10
−5
0
5
10
15

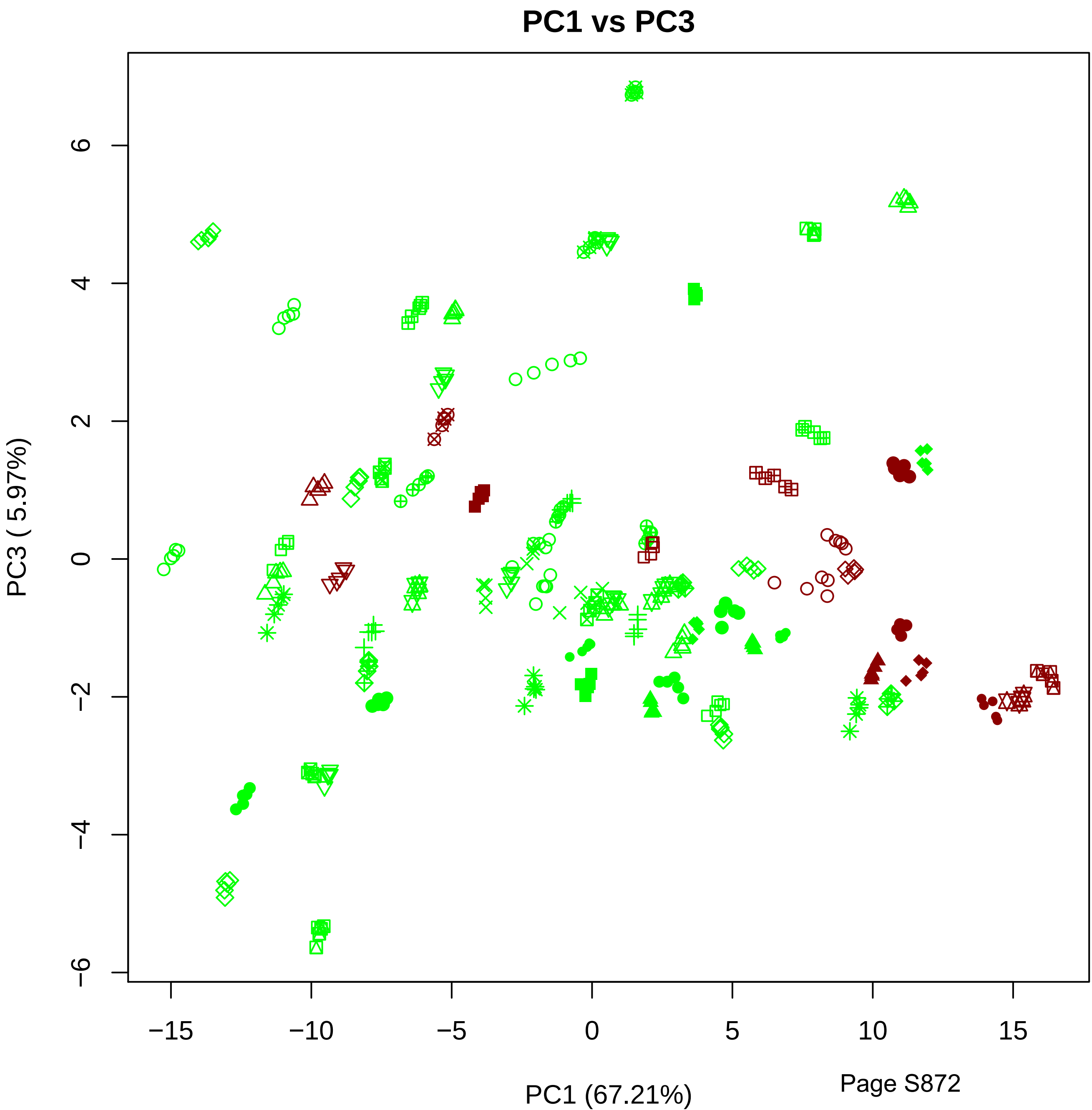

PC1 vs PC3
PC3 (5.97%)
PC1 (67.21%)
6
4
2
0
−2
−4
−6
−15
−10
−5
0
5
10
15

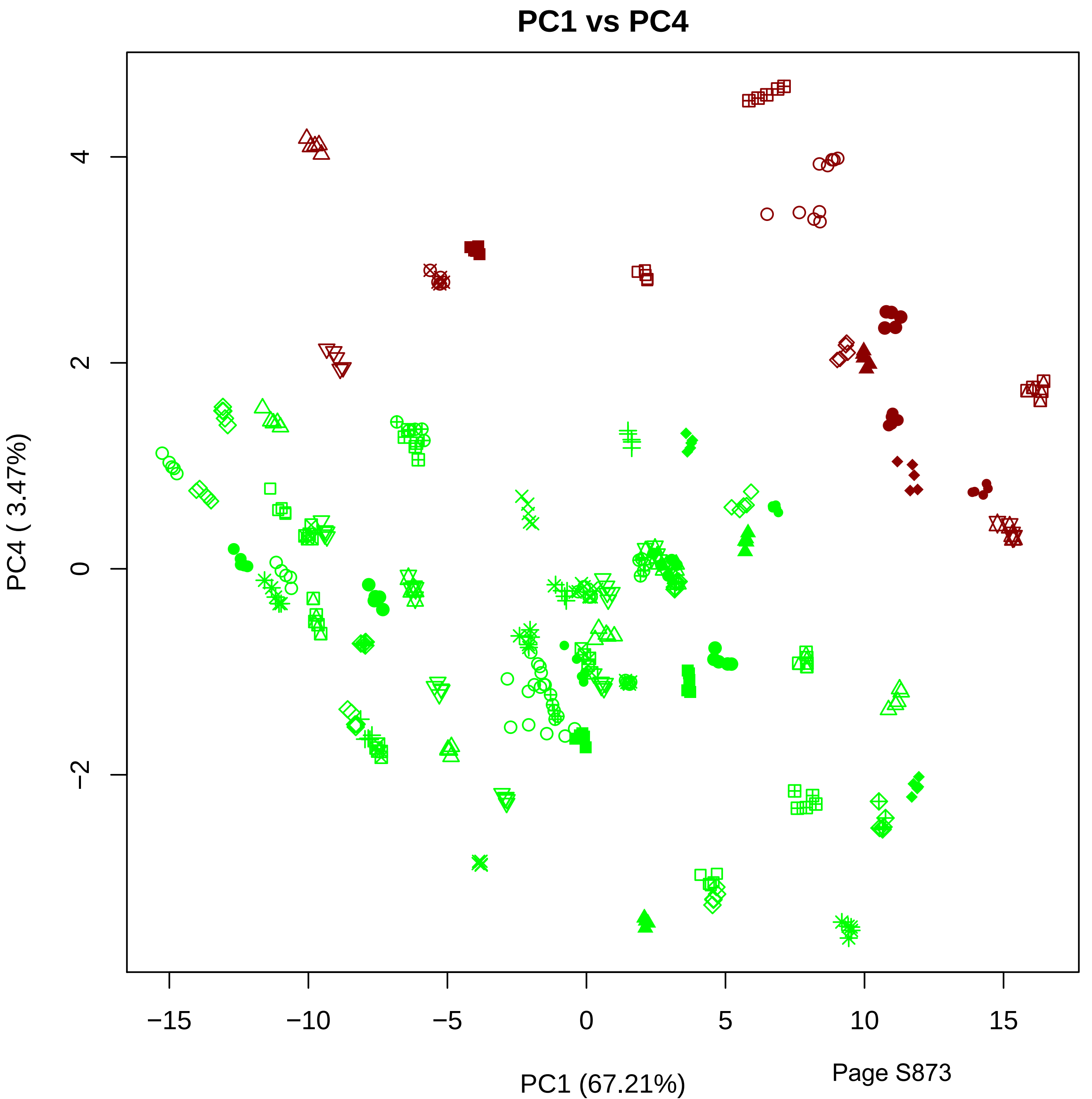
PC1 vs PC4
PC4 (3.47%)
4
2
0
−2
−15
−10
−5
0
5
10
15
PC1 (67.21%)

PC1 vs PC5

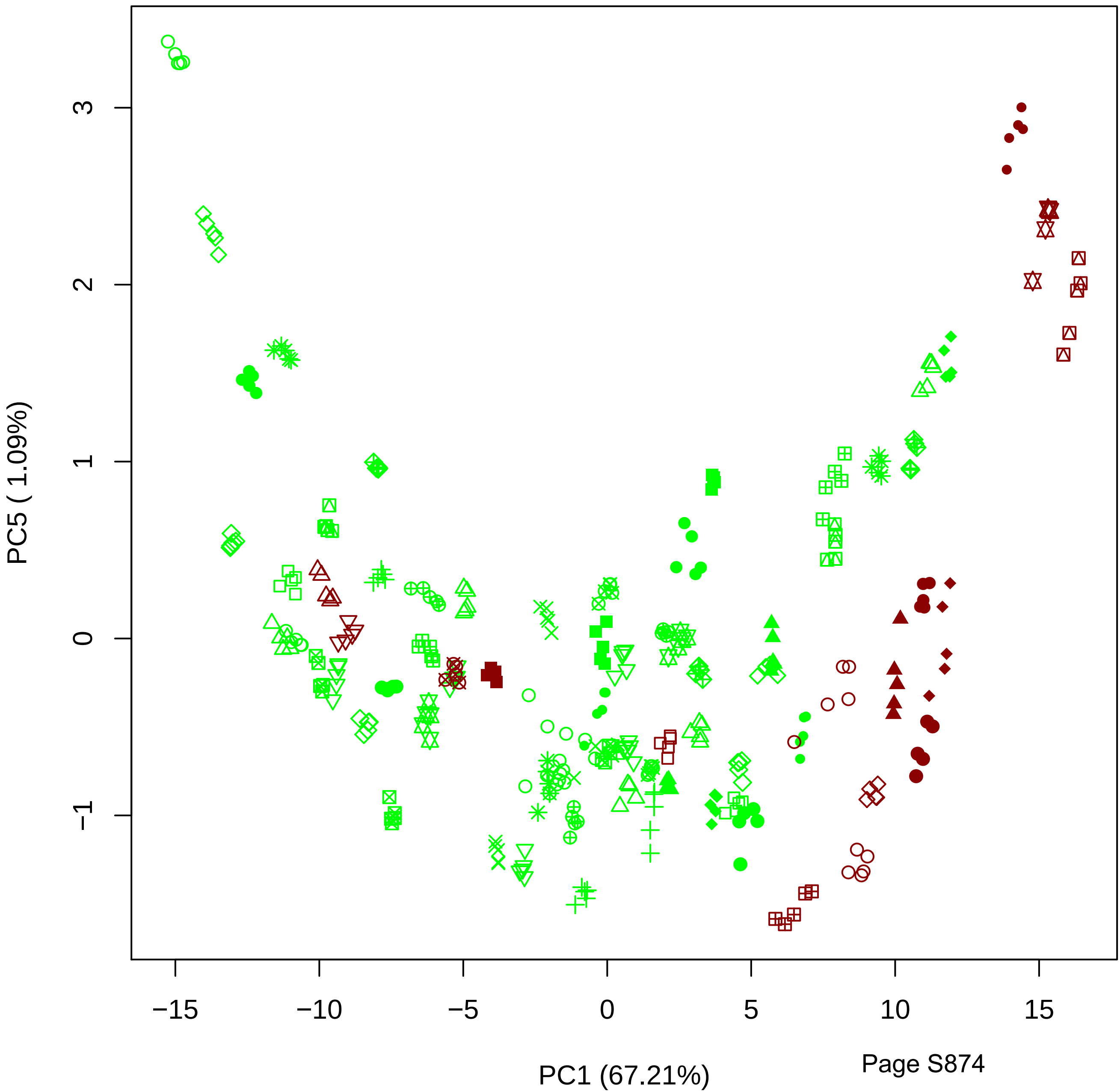

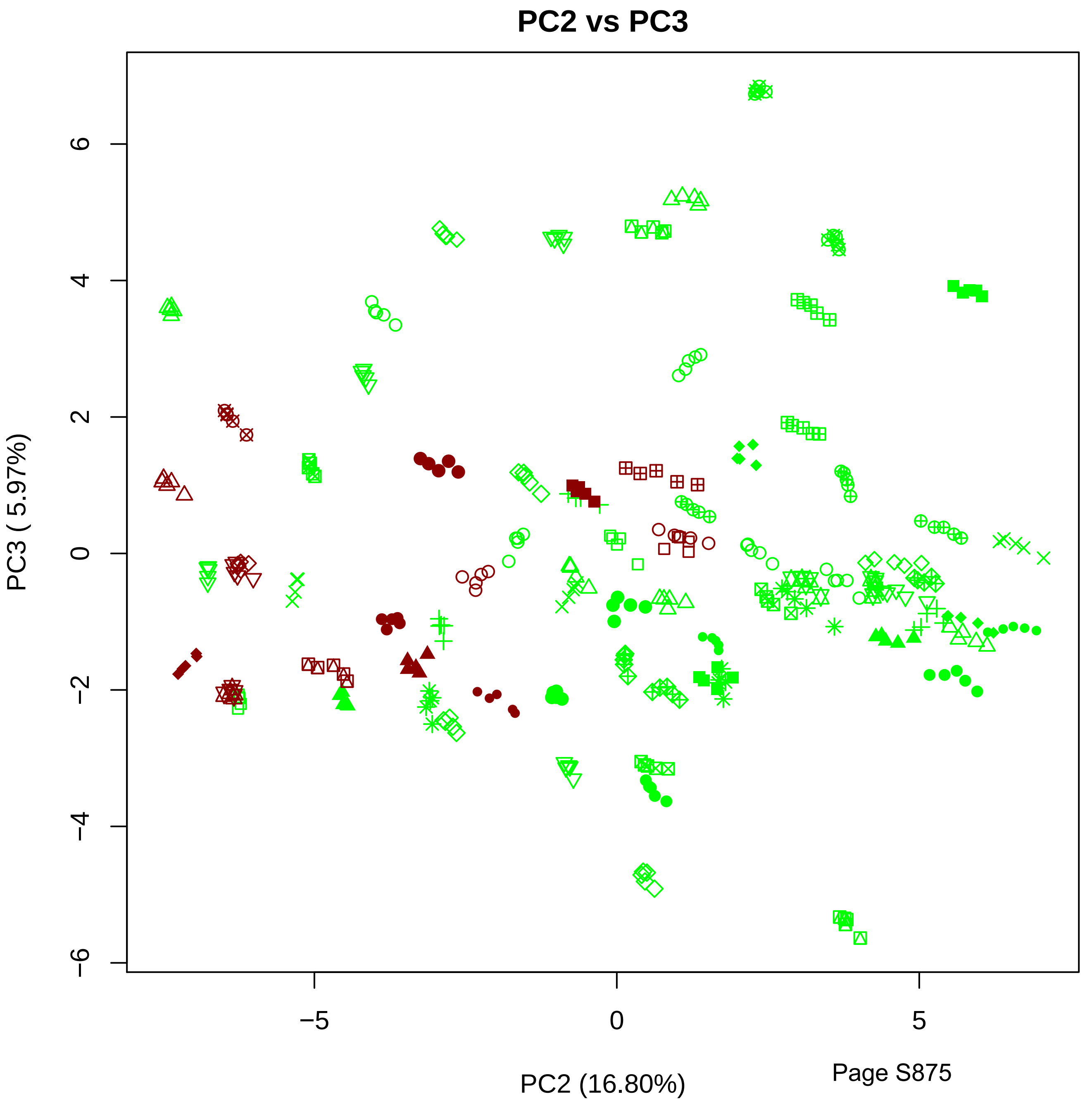

PC2 vs PC3
PC3 (5.97%)
PC2 (16.80%)
6
4
2
0
−2
−4
−6
−5
0
5

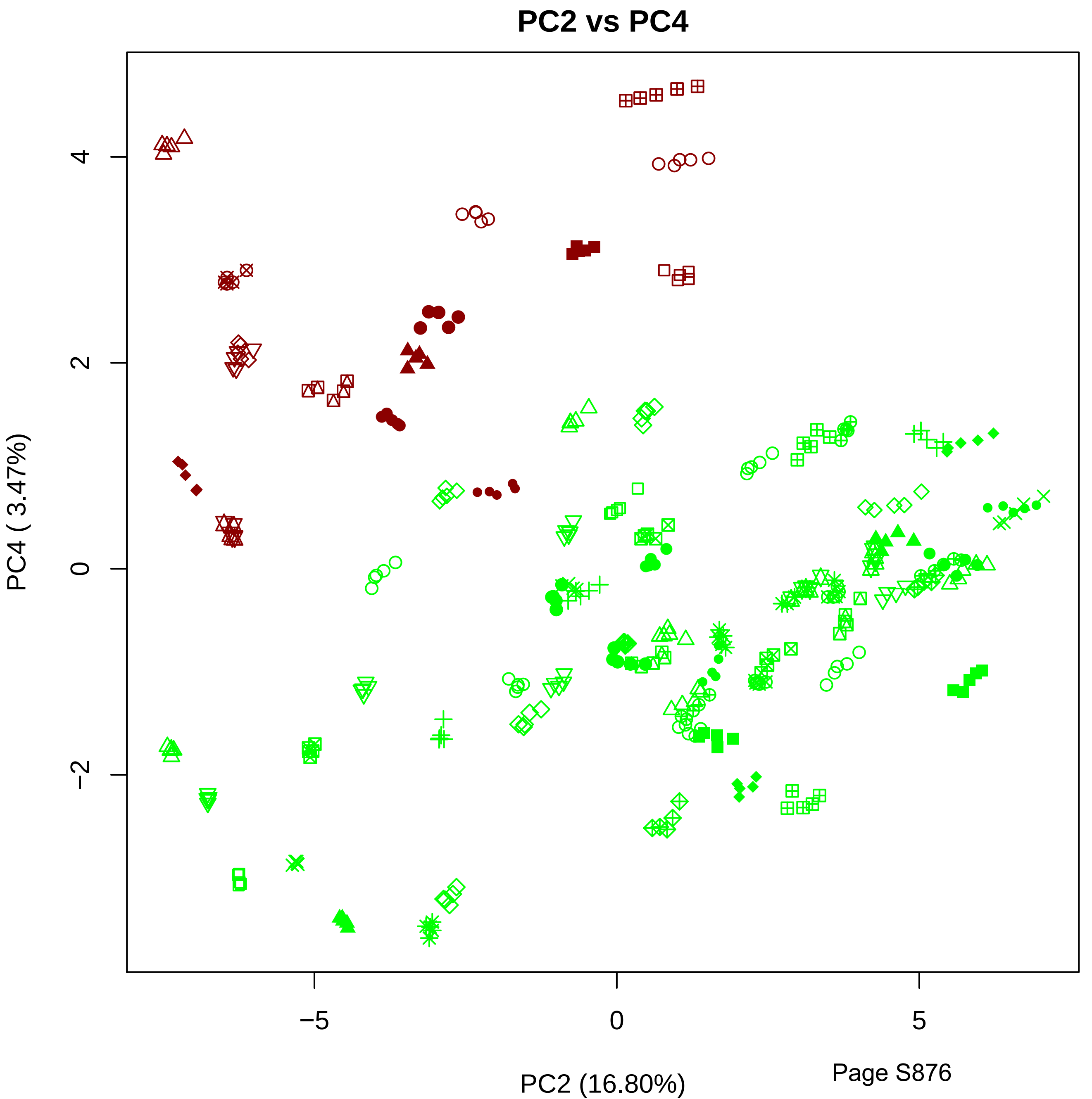
PC2 vs PC4
4
2
0
−2
PC4 (3.47%)
−5
0
5
PC2 (16.80%)

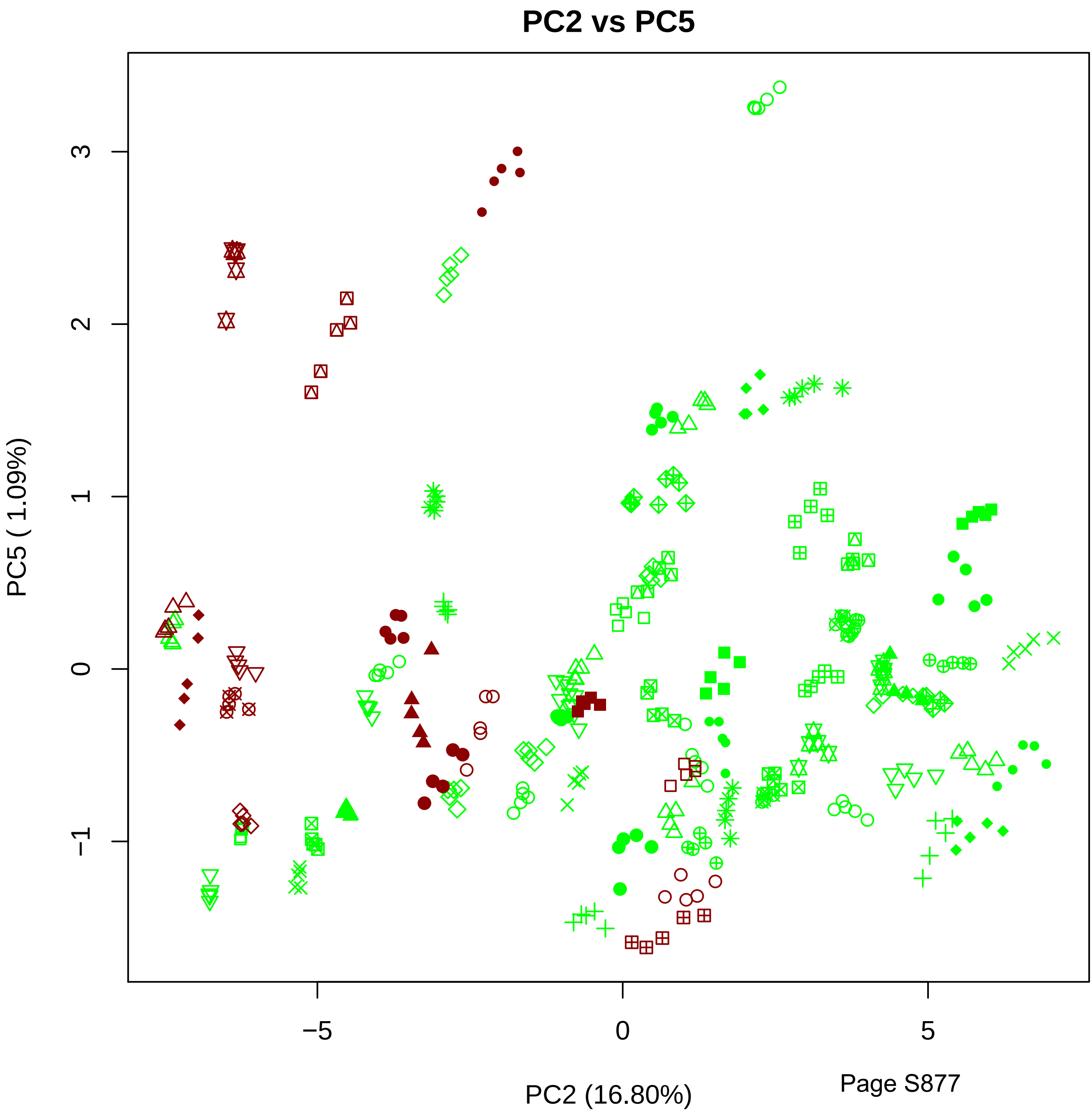
PC2 vs PC5
PC5 (1.09%)
PC2 (16.80%)
3
2
1
0
−1
−5
0
5

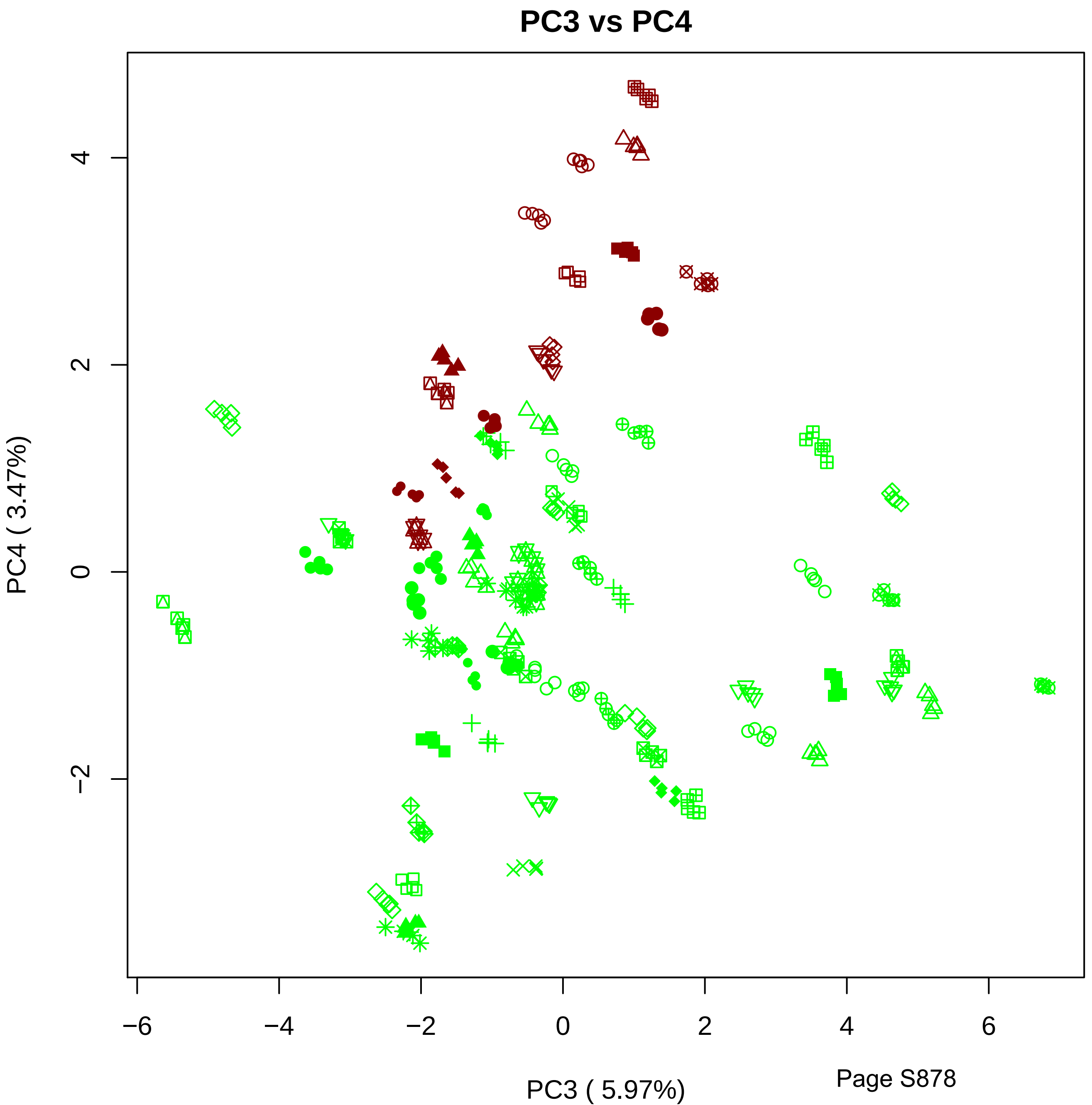
PC3 vs PC4
PC4 (3.47%)
PC3 (5.97%)
4
2
0
−2
−6
−4
−2
0
2
4
6

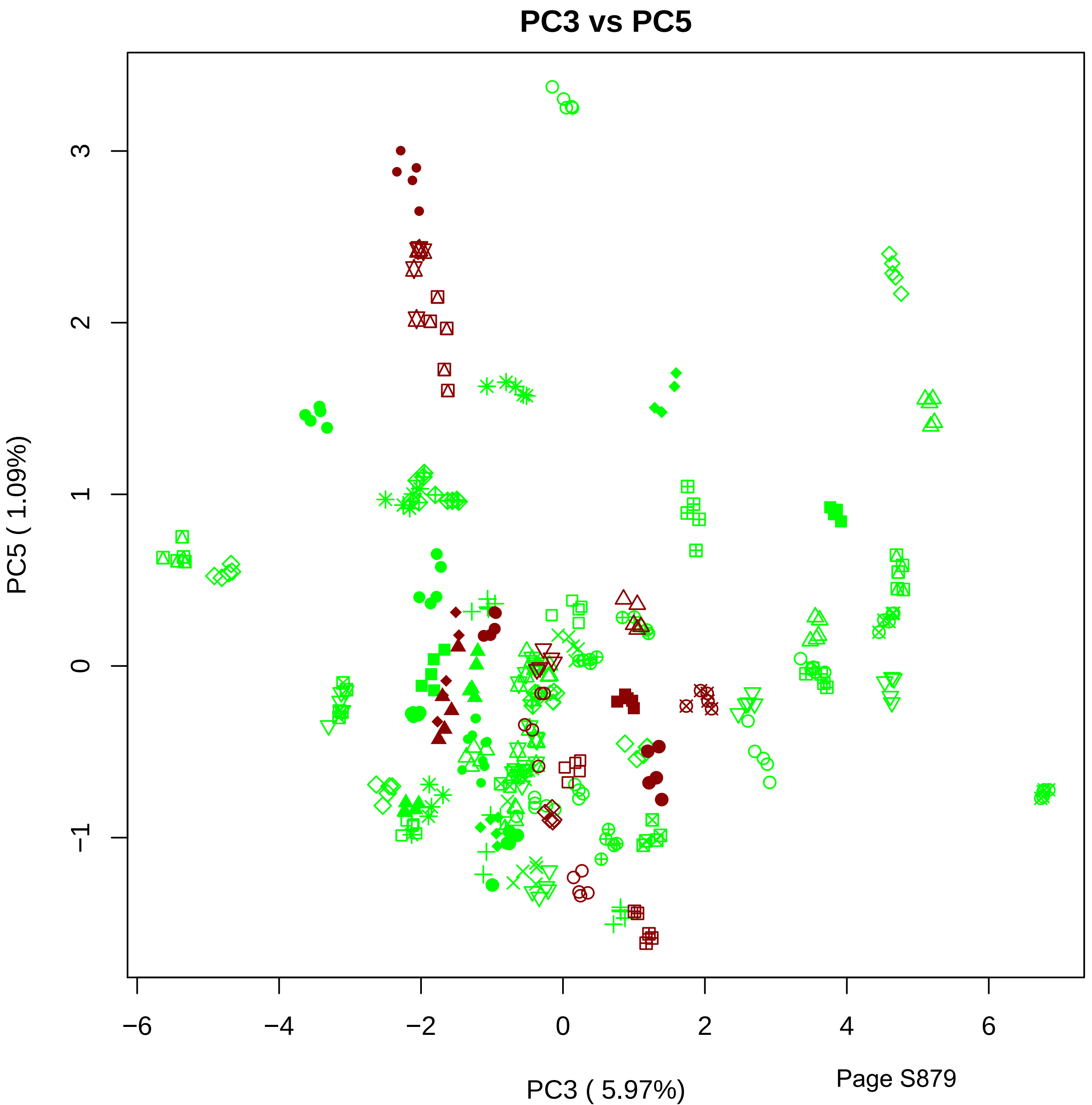
PC3 vs PC5
PC5 (1.09%)
PC3 (5.97%)
−6
−4
−2
0
2
4
6
−1
0
1
2
3

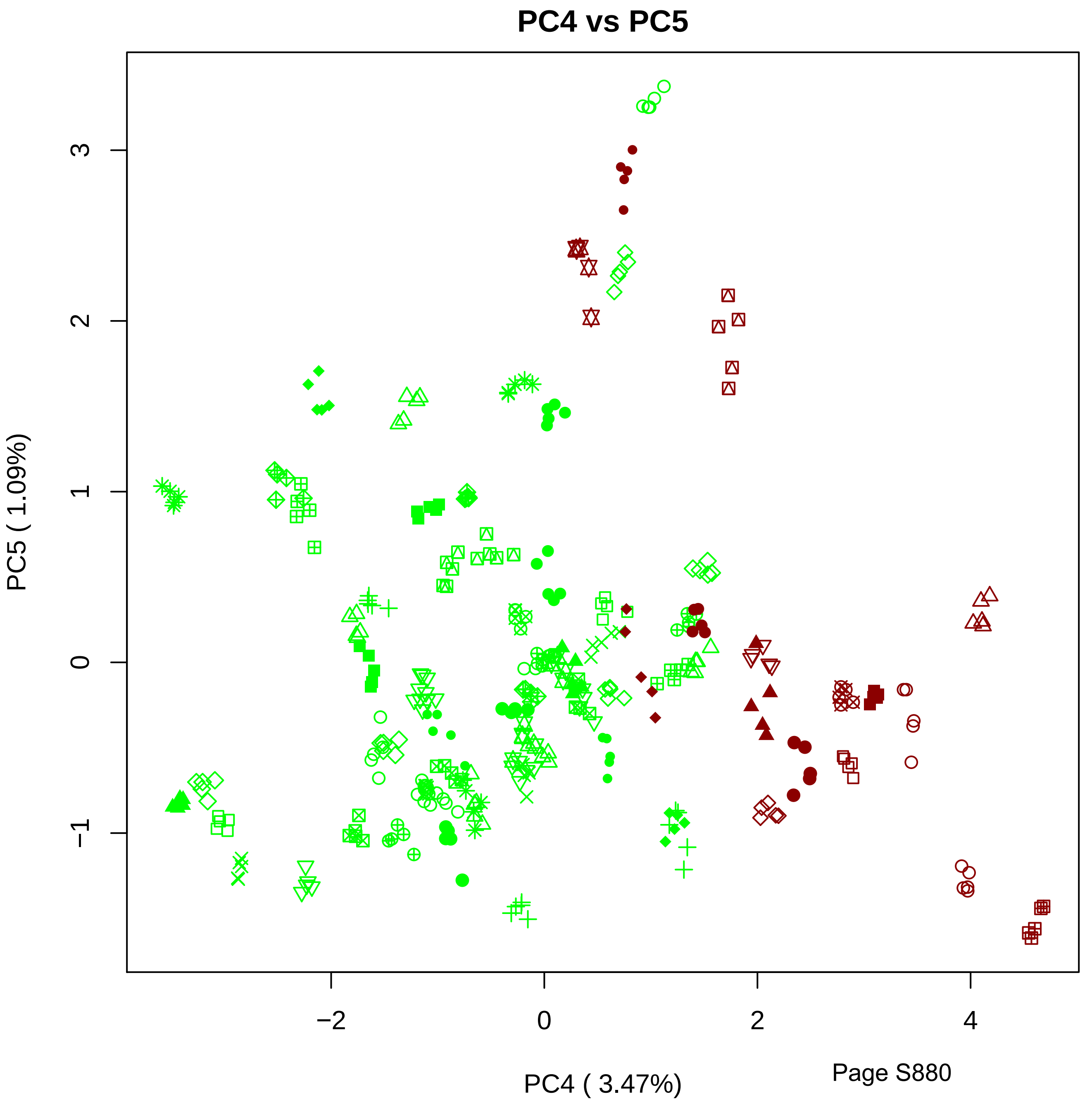
PC4 vs PC5
PC5 (1.09%)
PC4 (3.47%)
3
2
1
0
−1
−2
0
2
4

PCA of All BMH(a) vs PMH(a)

Scaled Matrix PCAed

7th order polynomial baseline correction performed

Raw spectra smoothed to 21 Neighbours, 2nd order PN, Savtisky Golay

Normalisation between 0 and 100

D2 spectra smoothed to 41 Neighbours, 2nd order PN, Savtisky Golay

Regions Analysed: 3601−2501 and 2001−700cm−1

Samples Analysed: 8 bContam's 0 BLH's 0 BMH's 0 OMH's 0 pContam's 0 PLH's 60 PMH's

Keele University AD

Fri Dec 14 14:19:45 2018

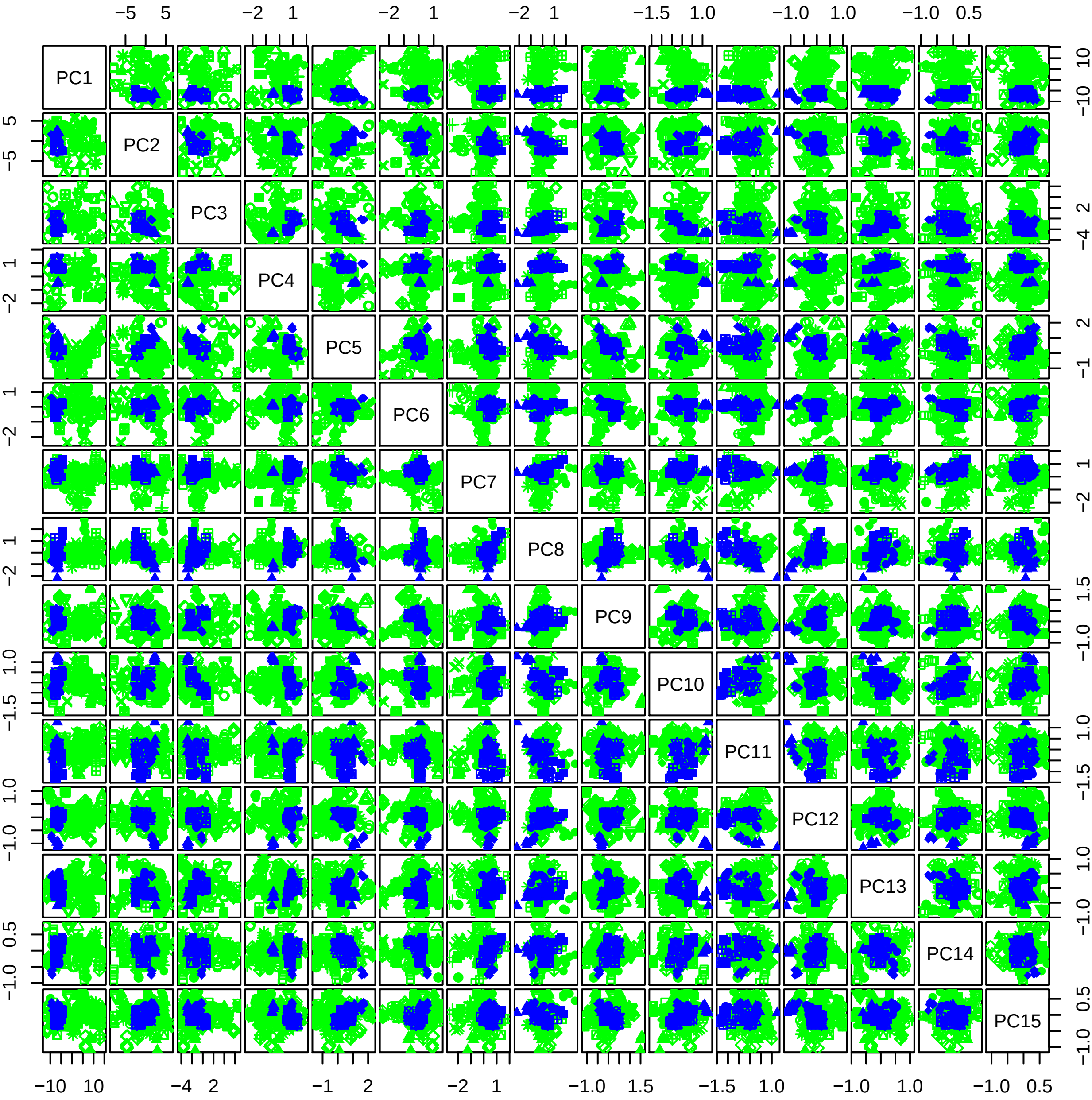
PC1
PC2
PC3
PC4
PC5
PC6
PC7
PC8
PC9
PC10
PC11
PC12
PC13
PC14
PC15

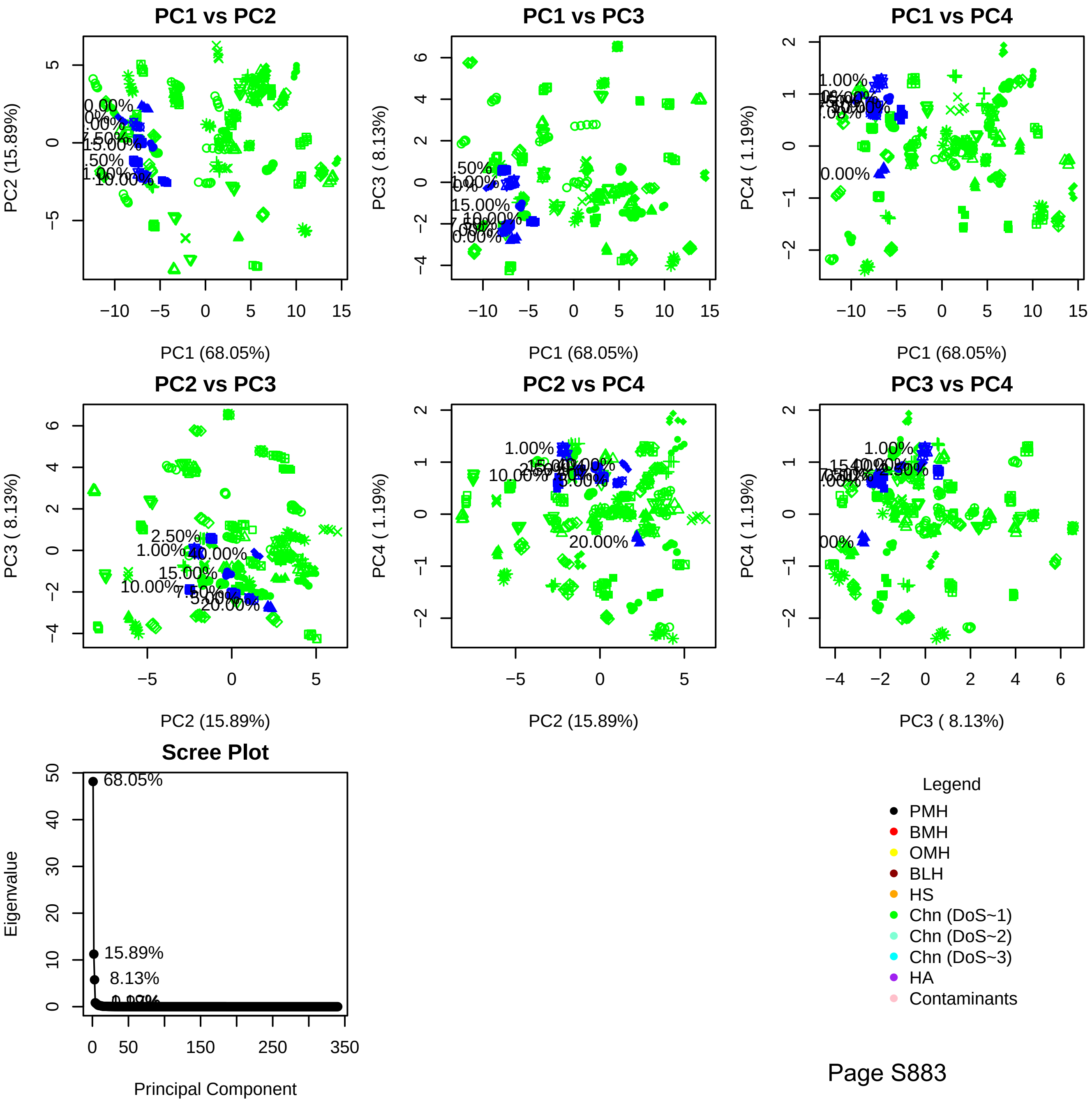
PC1 vs PC2
PC1 vs PC3
PC1 vs PC4
PC2 vs PC3
PC2 vs PC4
PC3 vs PC4
Scree Plot
PC1 (68.05%)
PC2 (15.89%)
PC3 (8.13%)
PC4 (1.19%)
Eigenvalue
Principal Component
68.05%
15.89%
8.13%
Legend
PMH
BMH
OMH
BLH
HS
Chn (DoS~1)
Chn (DoS~2)
Chn (DoS~3)
HA
Contaminants

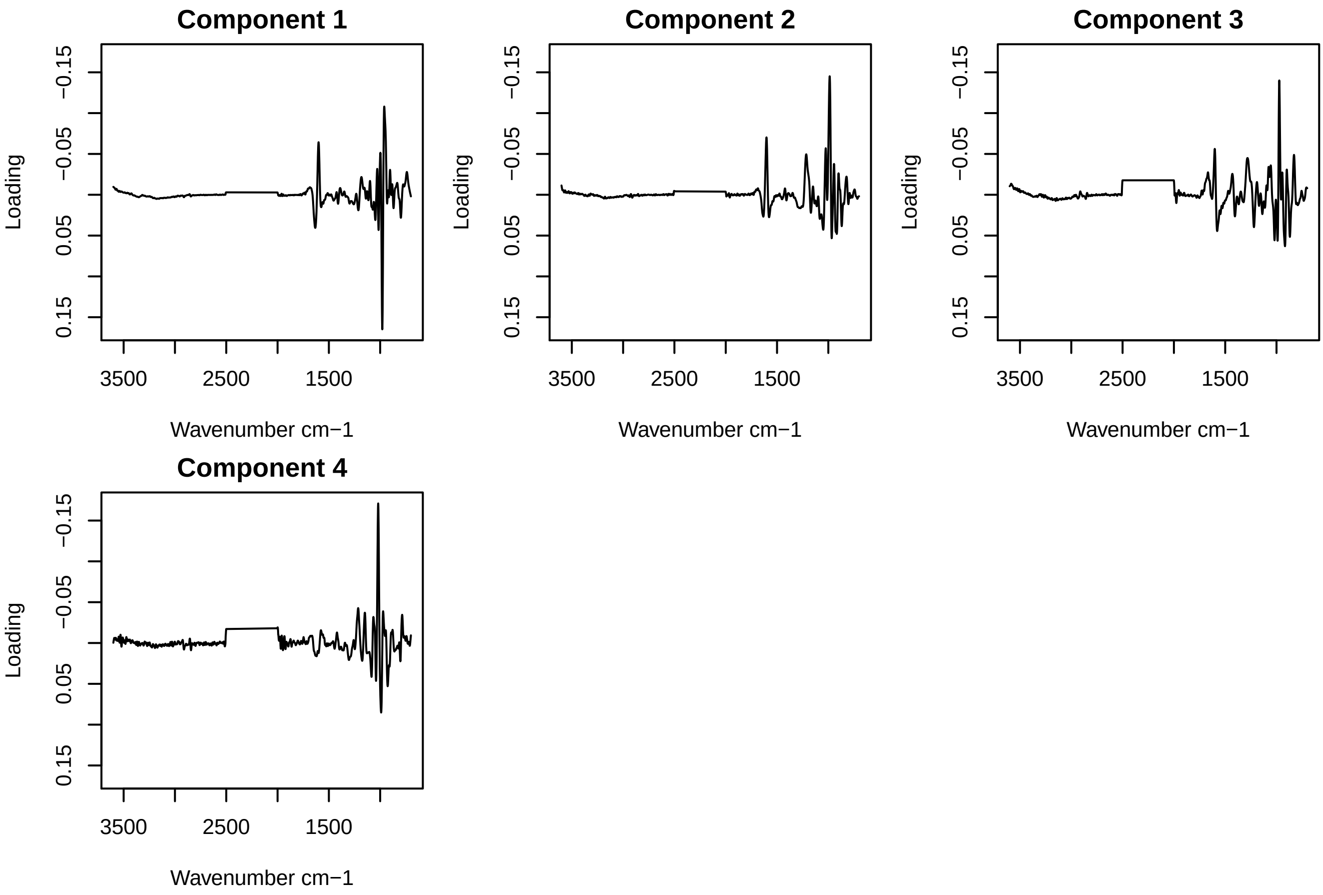
Component 1
Component 2
Component 3
Component 4
Loading
−0.15
−0.05
0.05
0.15
3500
2500
1500
Wavenumber cm−1

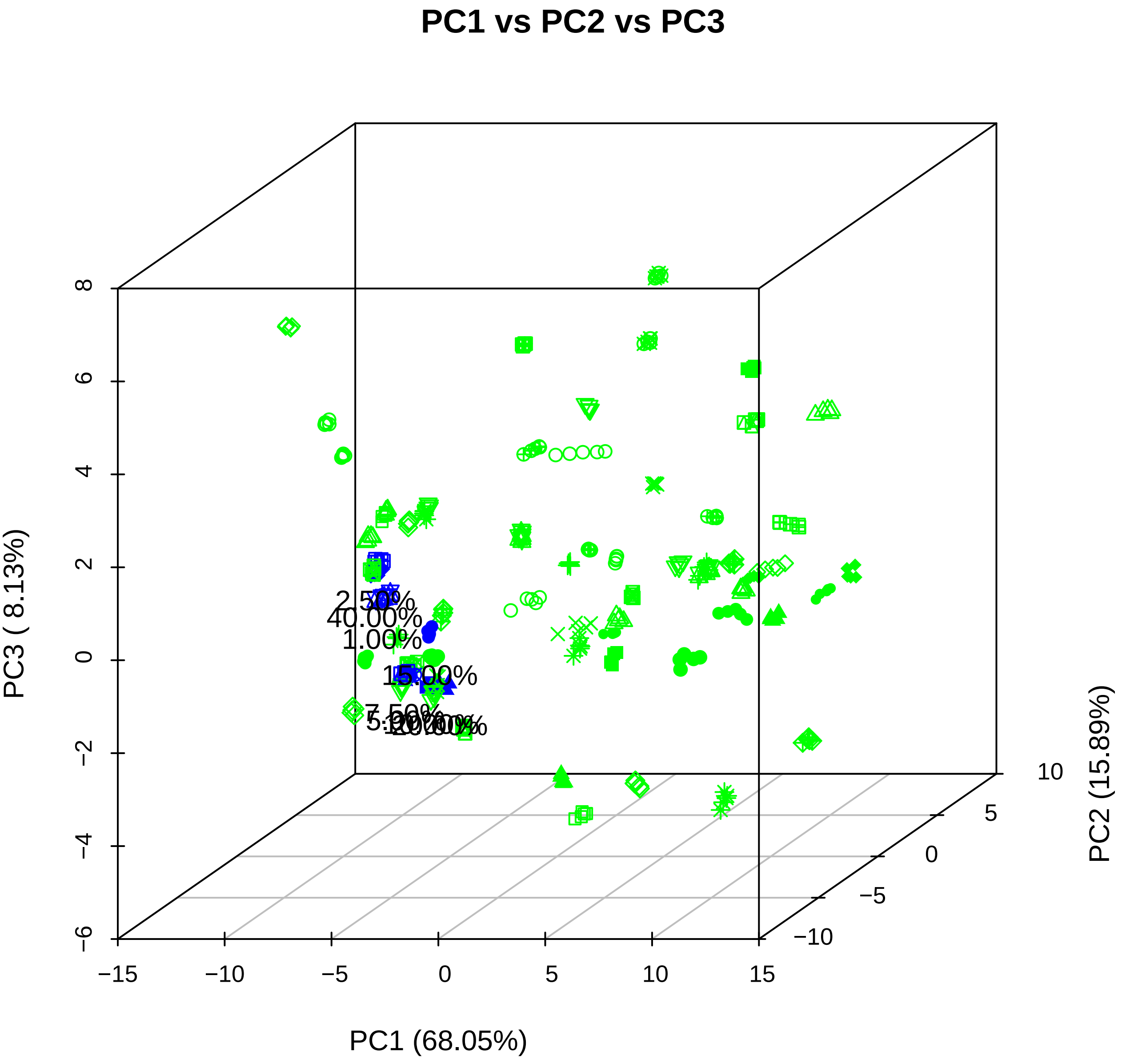
PC1 vs PC2 vs PC3
PC1 (68.05%)
PC2 (15.89%)
PC3 (8.13%)
−15
−10
−5
0
5
10
15
−10
−5
0
5
10
−6
−4
−2
0
2
4
6
8
2.50%
40.00%
1.00%
15.00%
7.50%

PC1 vs PC2 vs PC4

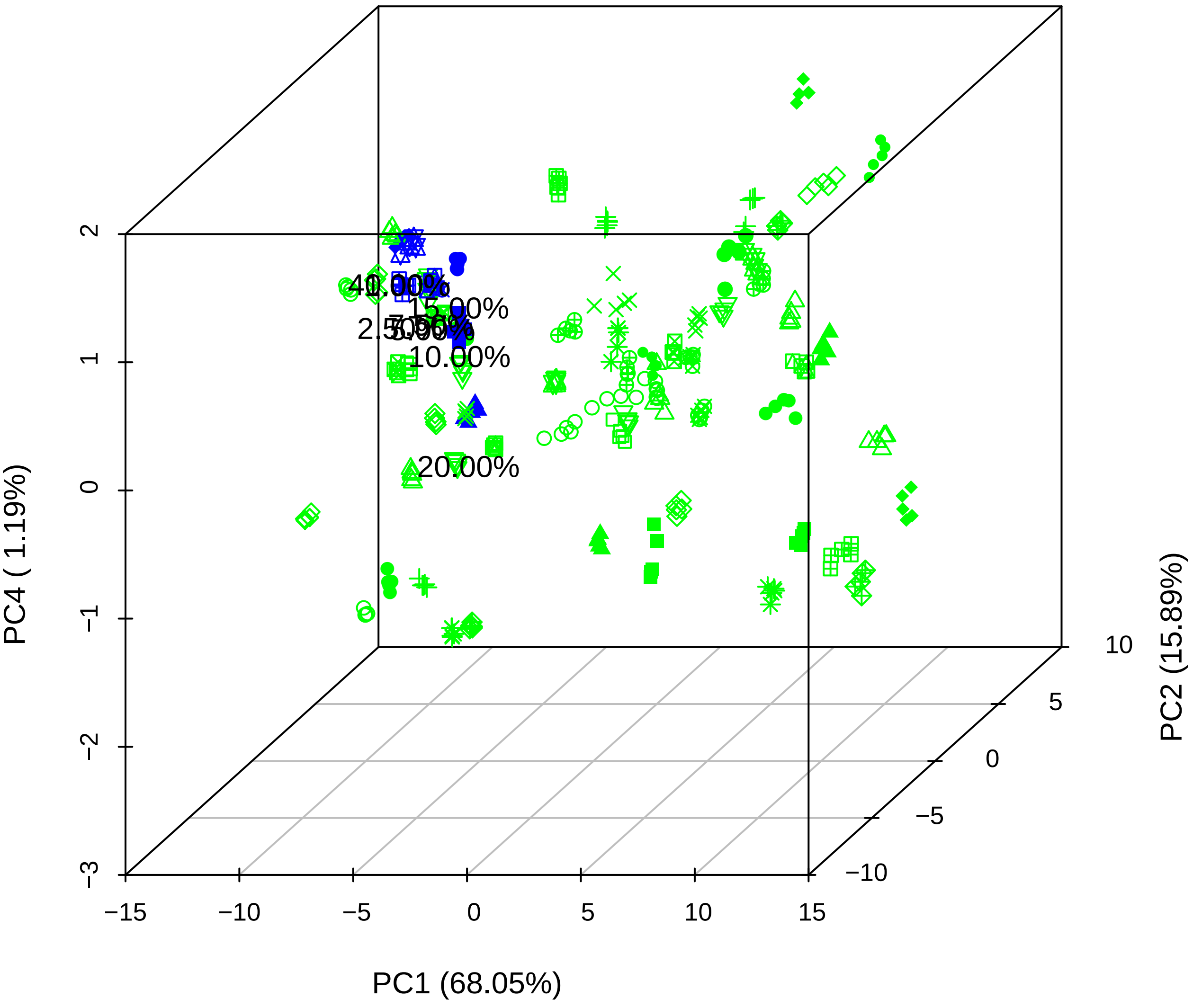

PC1 vs PC3 vs PC4

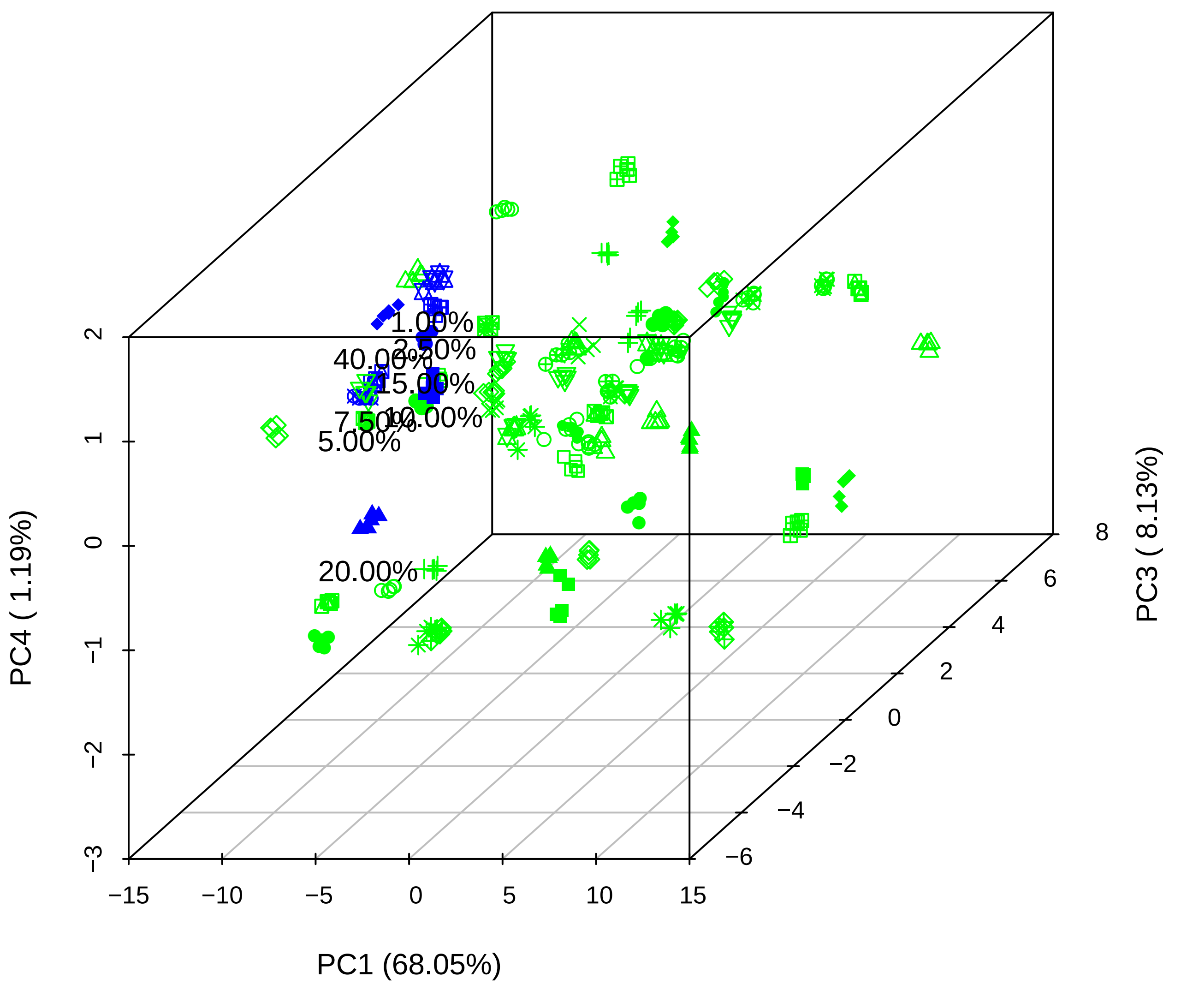

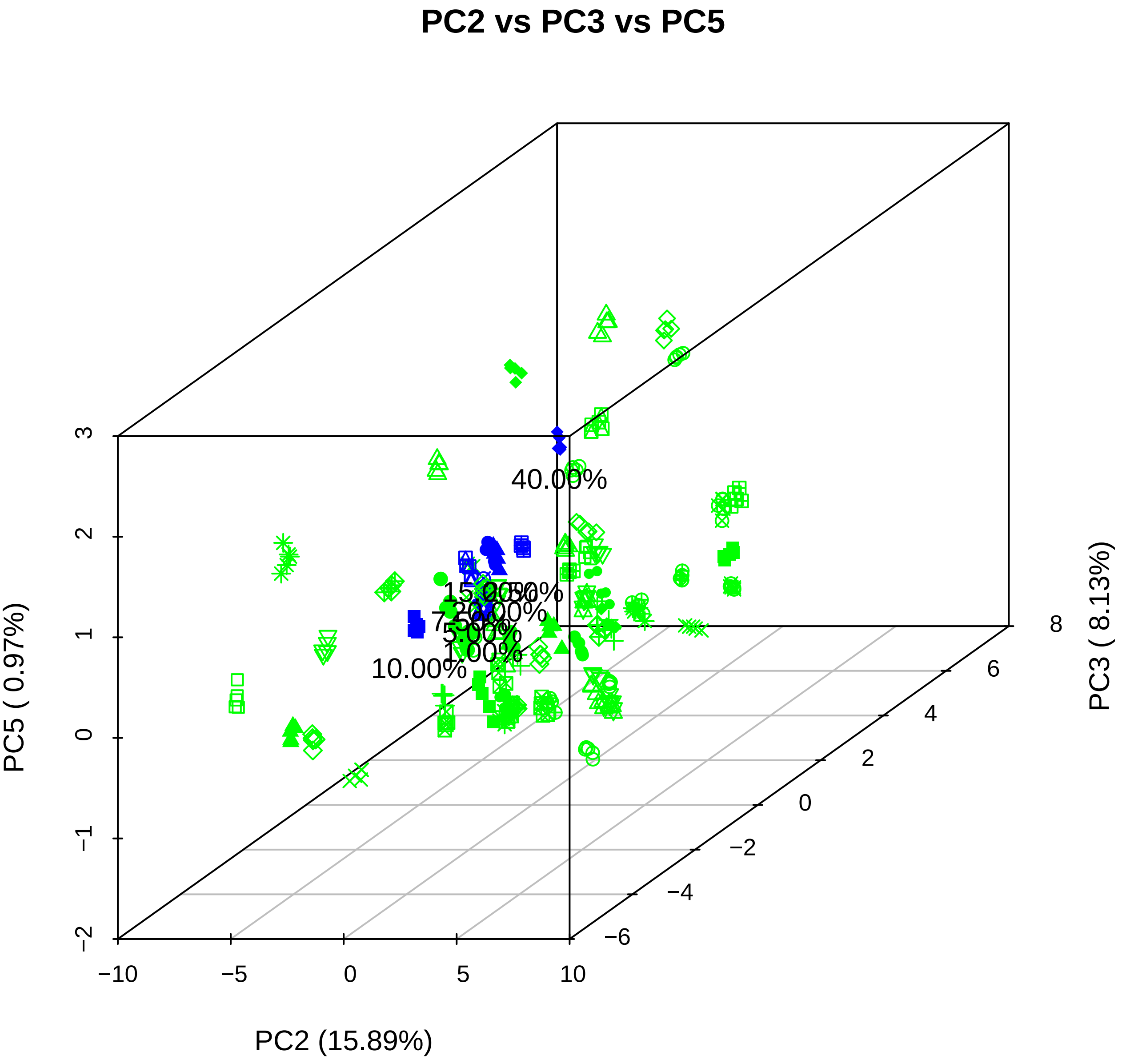

PC2 vs PC3 vs PC5
PC2 (15.89%)
PC3 (8.13%)
PC5 (0.97%)
−10
−5
0
5
10
−6
−4
−2
0
2
4
6
8
−2
−1
0
1
2
3
40.00%
10.00%

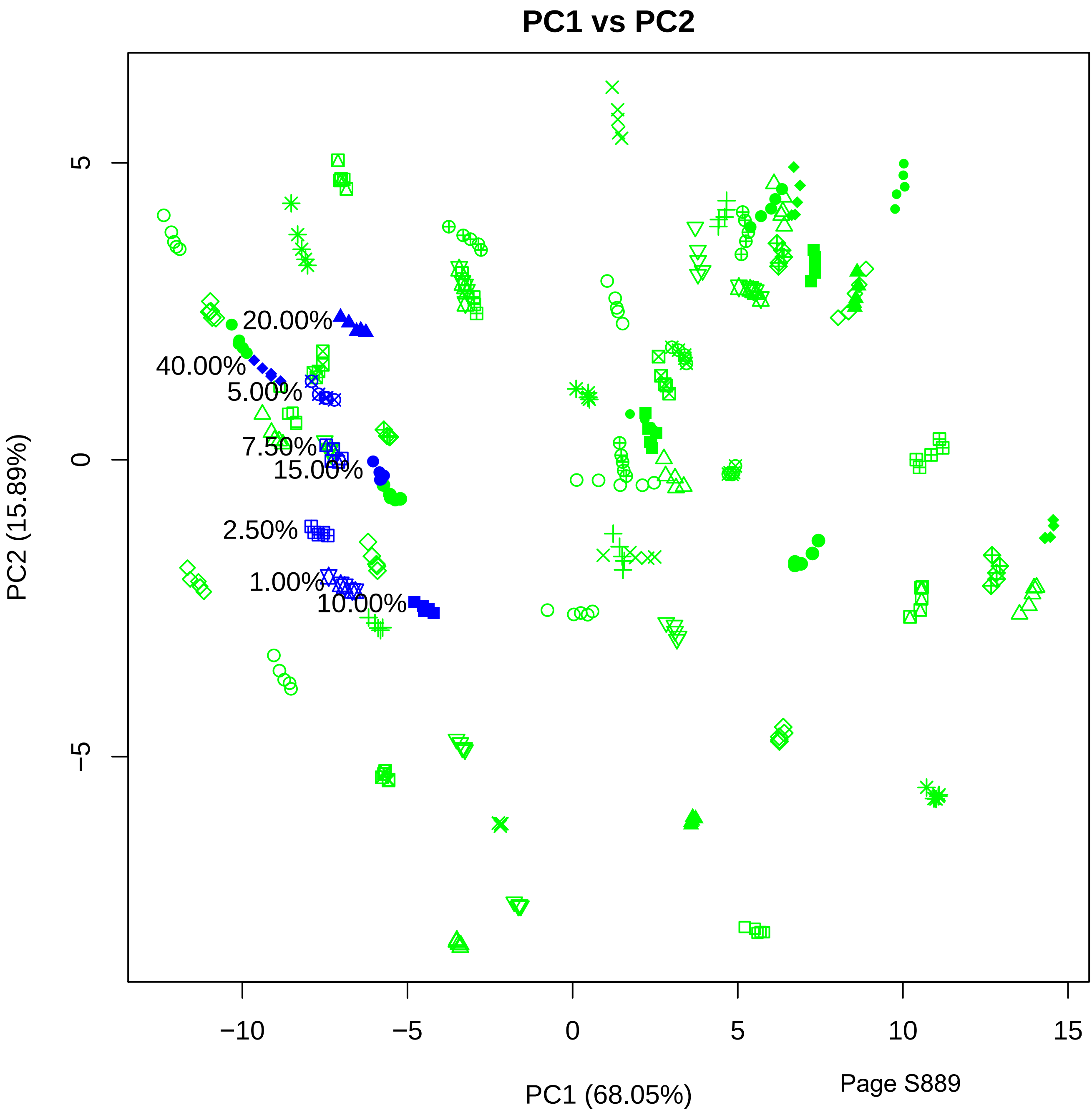

PC1 vs PC2
20.00%
40.00%
5.00%
7.50%
15.00%
2.50%
1.00%
10.00%
PC2 (15.89%)
PC1 (68.05%)
5
0
−5
−10
10
15

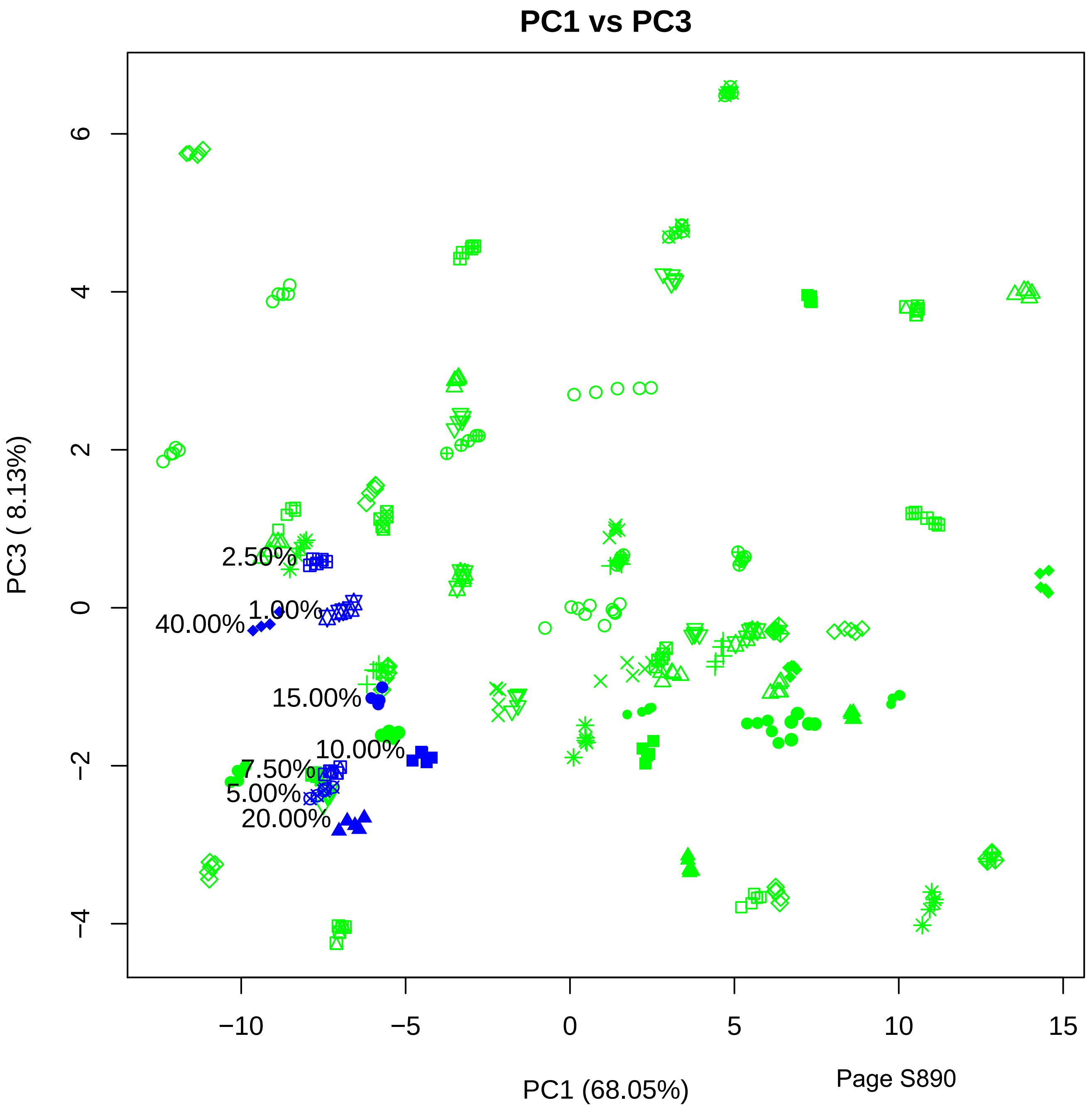
PC1 vs PC3
PC3 (8.13%)
PC1 (68.05%)
6
4
2
0
−2
−4
−10
−5
0
5
10
15
2.50%
1.00%
40.00%
15.00%
10.00%
7.50%
5.00%
20.00%

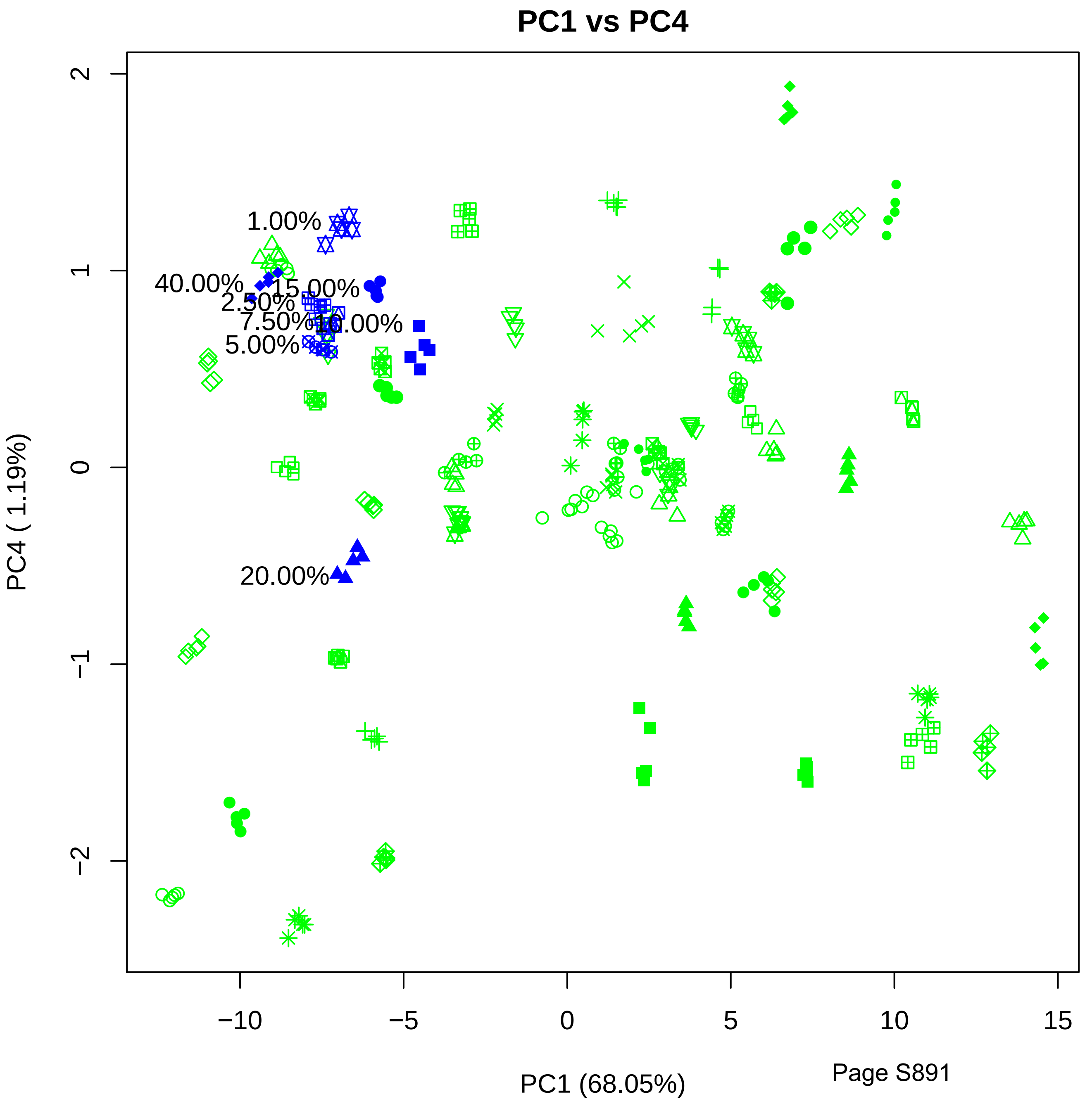

PC1 vs PC4
1.00%
40.00%
15.00%
2.50%
7.50%
10.00%
5.00%
20.00%
PC4 (1.19%)
PC1 (68.05%)
2
1
0
−1
−2
−10
−5
0
5
10
15

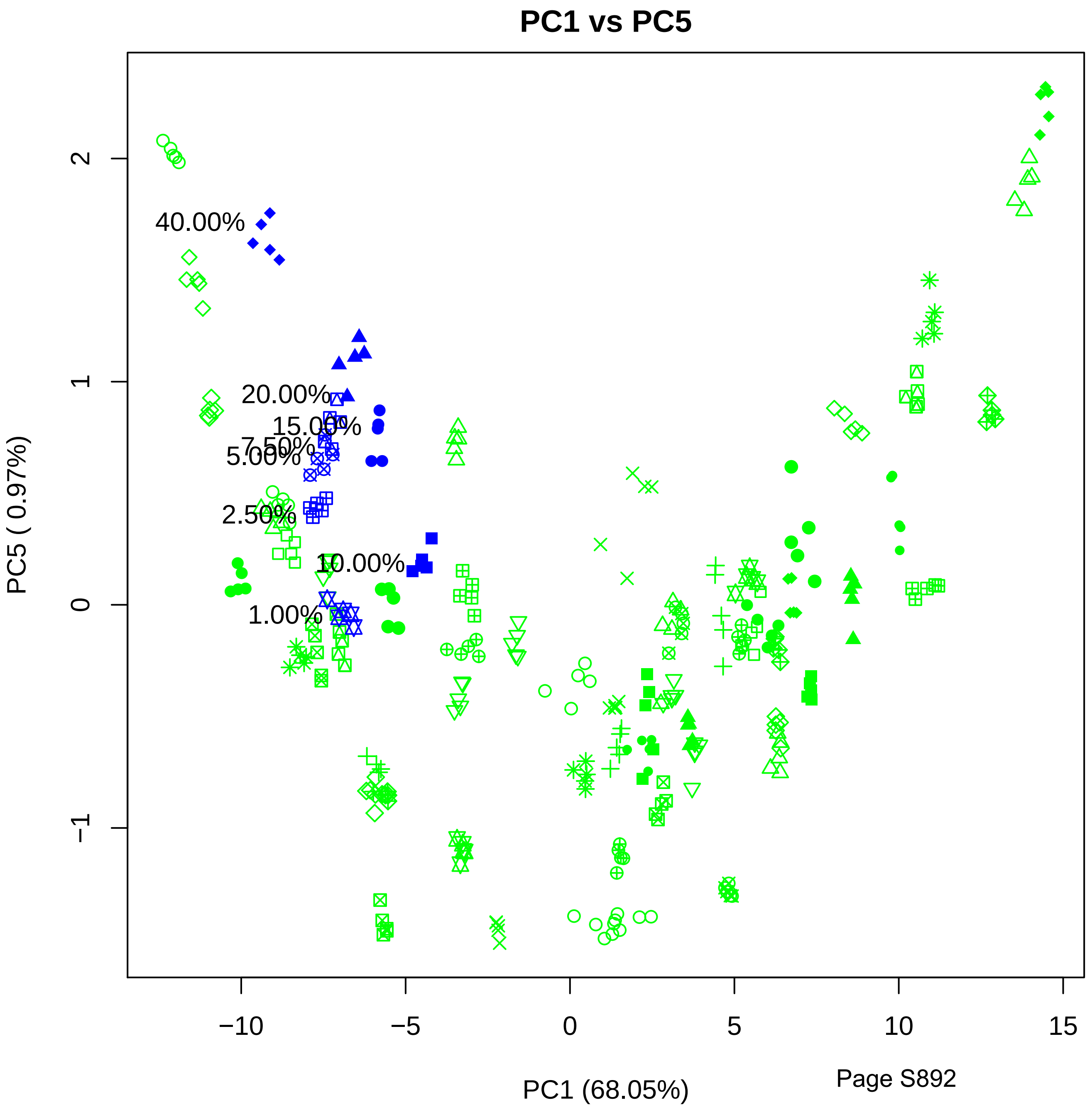
PC1 vs PC5
40.00%
20.00%
15.00%
7.50%
5.00%
2.50%
10.00%
1.00%
PC5 (0.97%)
PC1 (68.05%)
−10
−5
0
5
10
15
−1
0
1
2

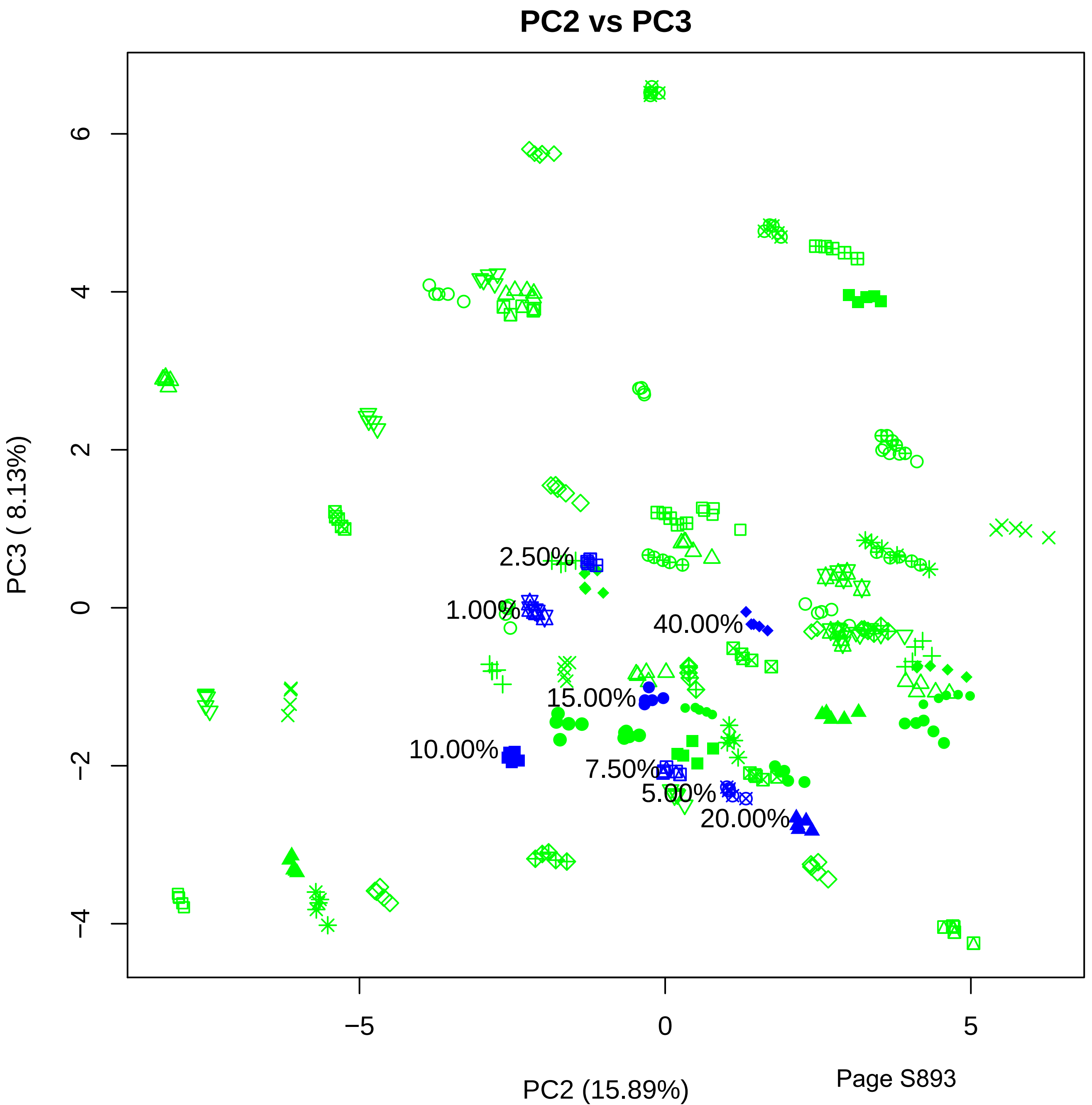
PC2 vs PC3
PC3 (8.13%)
PC2 (15.89%)
6
4
2
0
−2
−4
−5
0
5
2.50%
1.00%
40.00%
15.00%
10.00%
7.50%
5.00%
20.00%

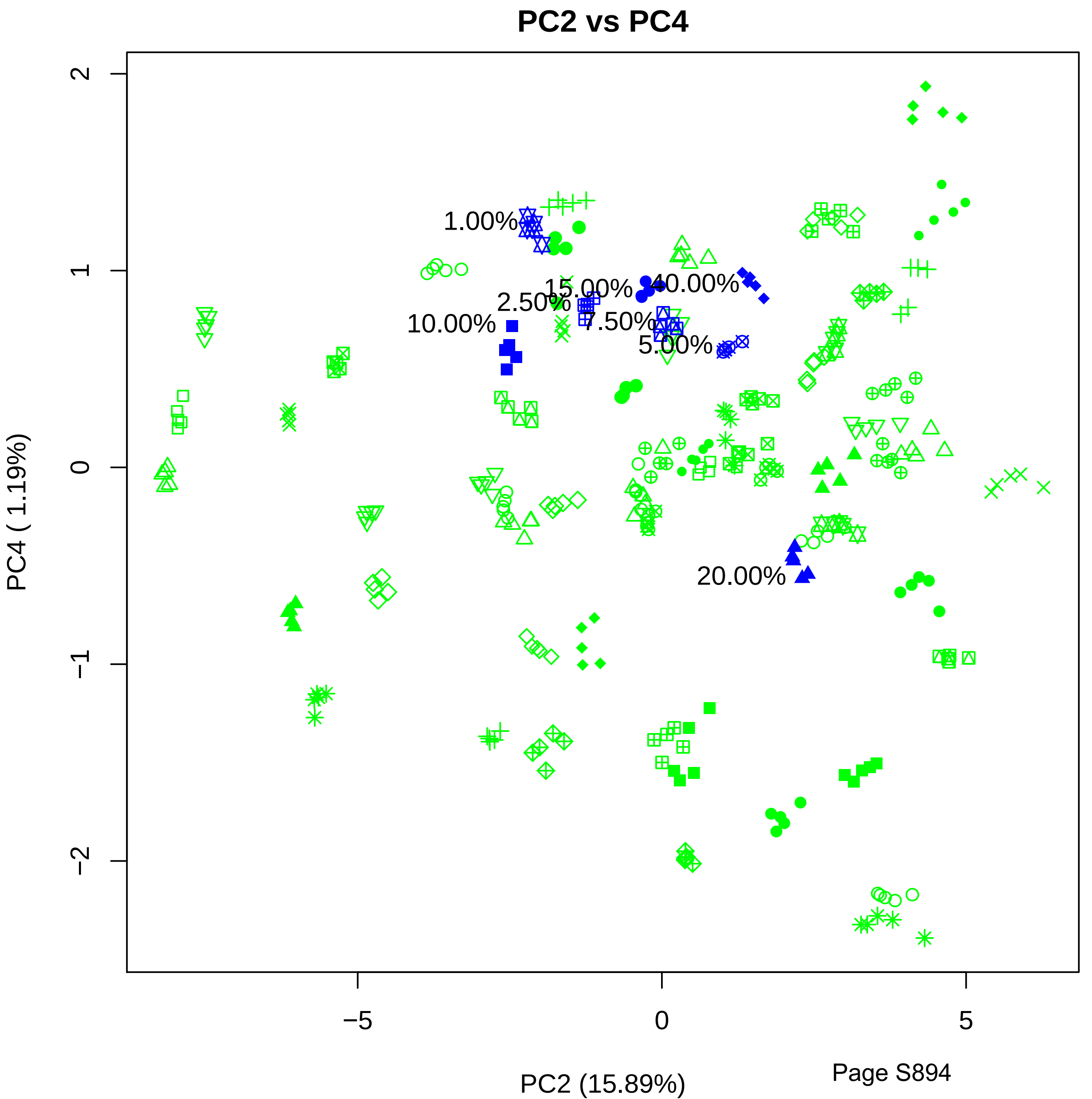
PC2 vs PC4
1.00%
15.00%
40.00%
2.50%
10.00%
7.50%
5.00%
20.00%
2
1
0
−1
−2
−5
0
5
PC4 (1.19%)
PC2 (15.89%)

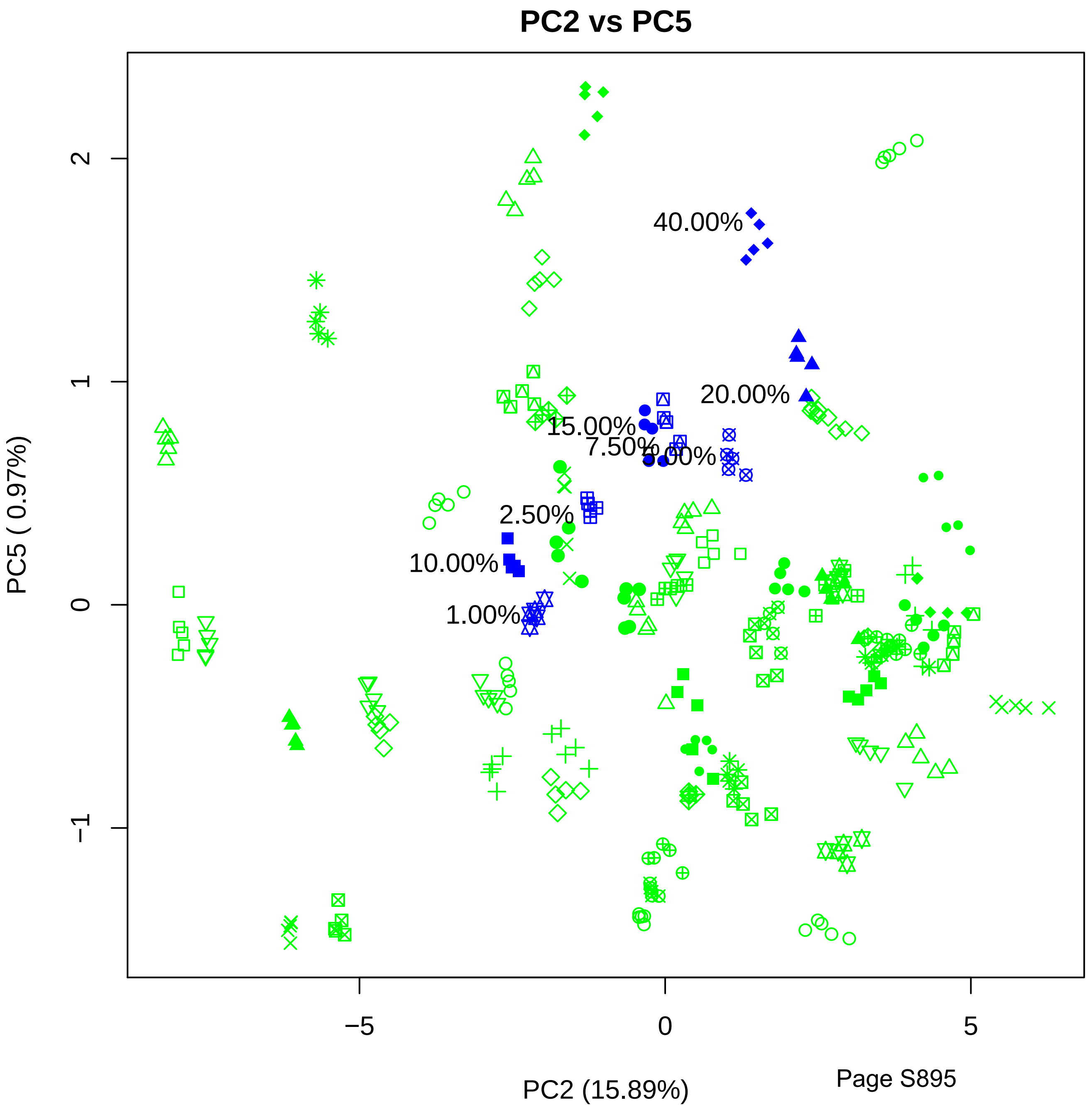
PC2 vs PC5
40.00%
20.00%
15.00%
7.50%
5.00%
2.50%
10.00%
1.00%
2
1
0
−1
−5
0
5
PC5 (0.97%)
PC2 (15.89%)

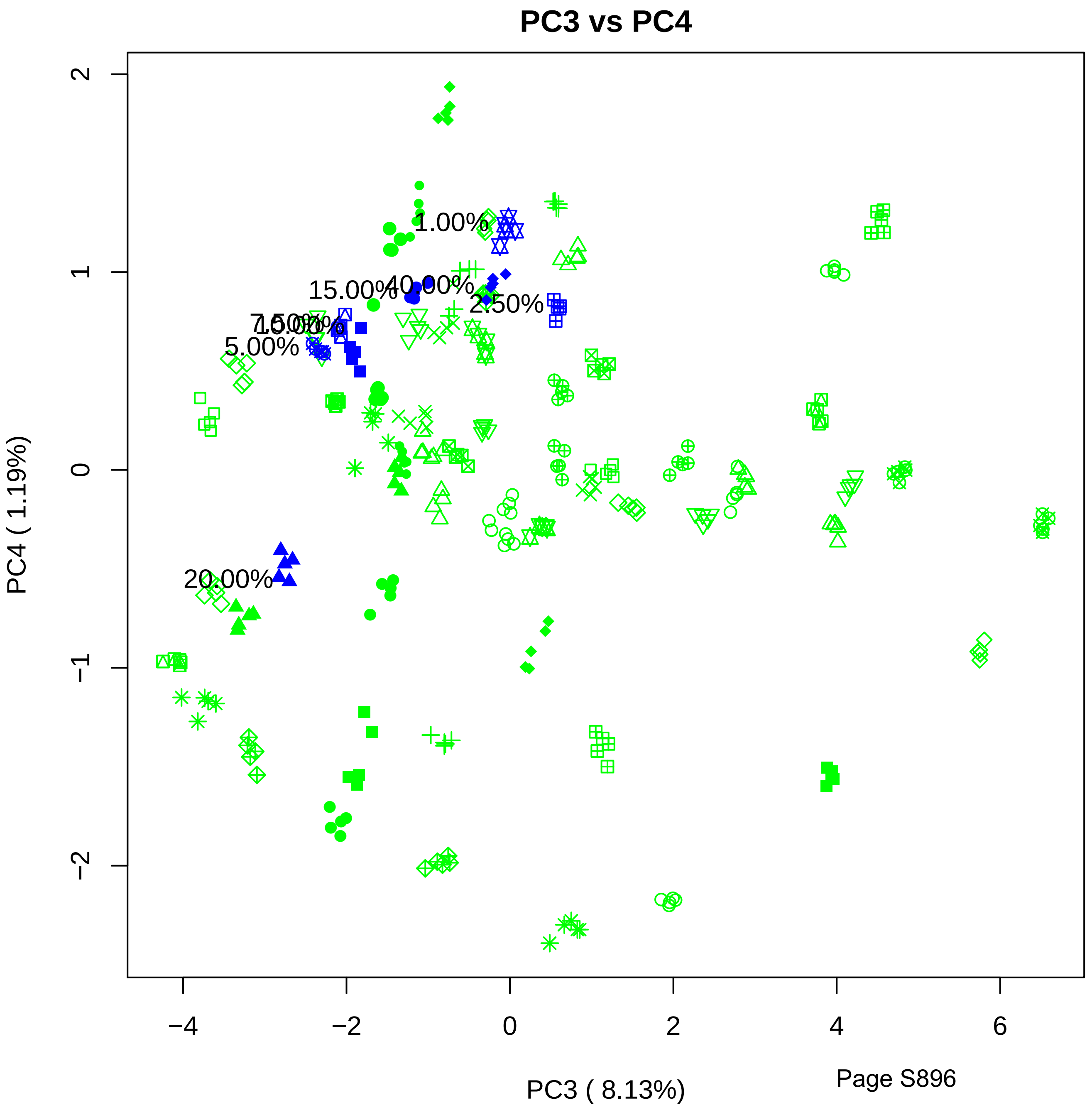
PC3 vs PC4
PC4 (1.19%)
PC3 (8.13%)
1.00%
15.00%
40.00%
2.50%
7.50%
10.00%
5.00%
20.00%
2
1
0
−1
−2
−4
−2
0
2
4
6

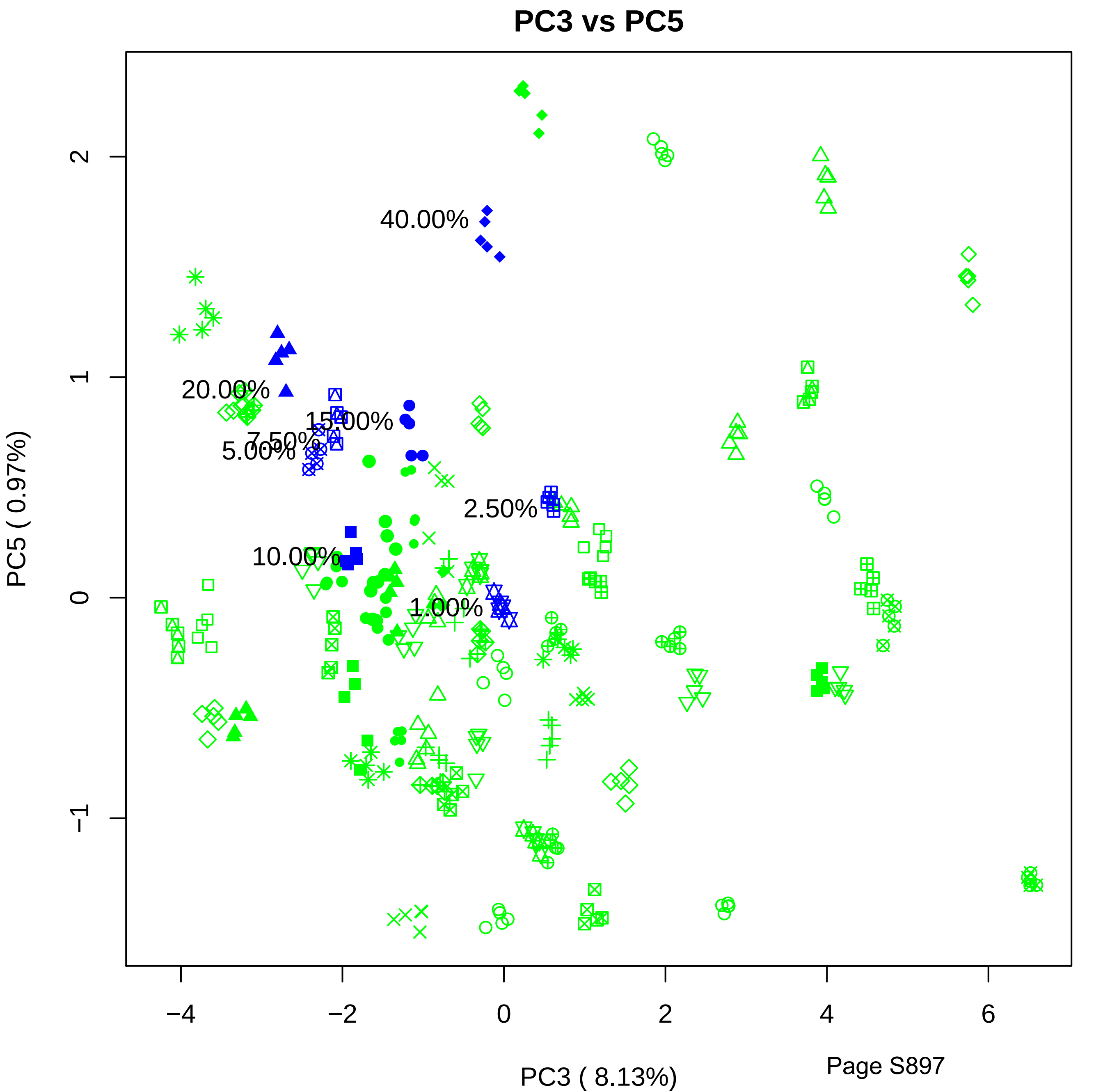
PC3 vs PC5
PC5 (0.97%)
PC3 (8.13%)
40.00%
20.00%
15.00%
7.50%
5.00%
2.50%
10.00%
1.00%
2
1
0
−1
−4
−2
0
2
4
6

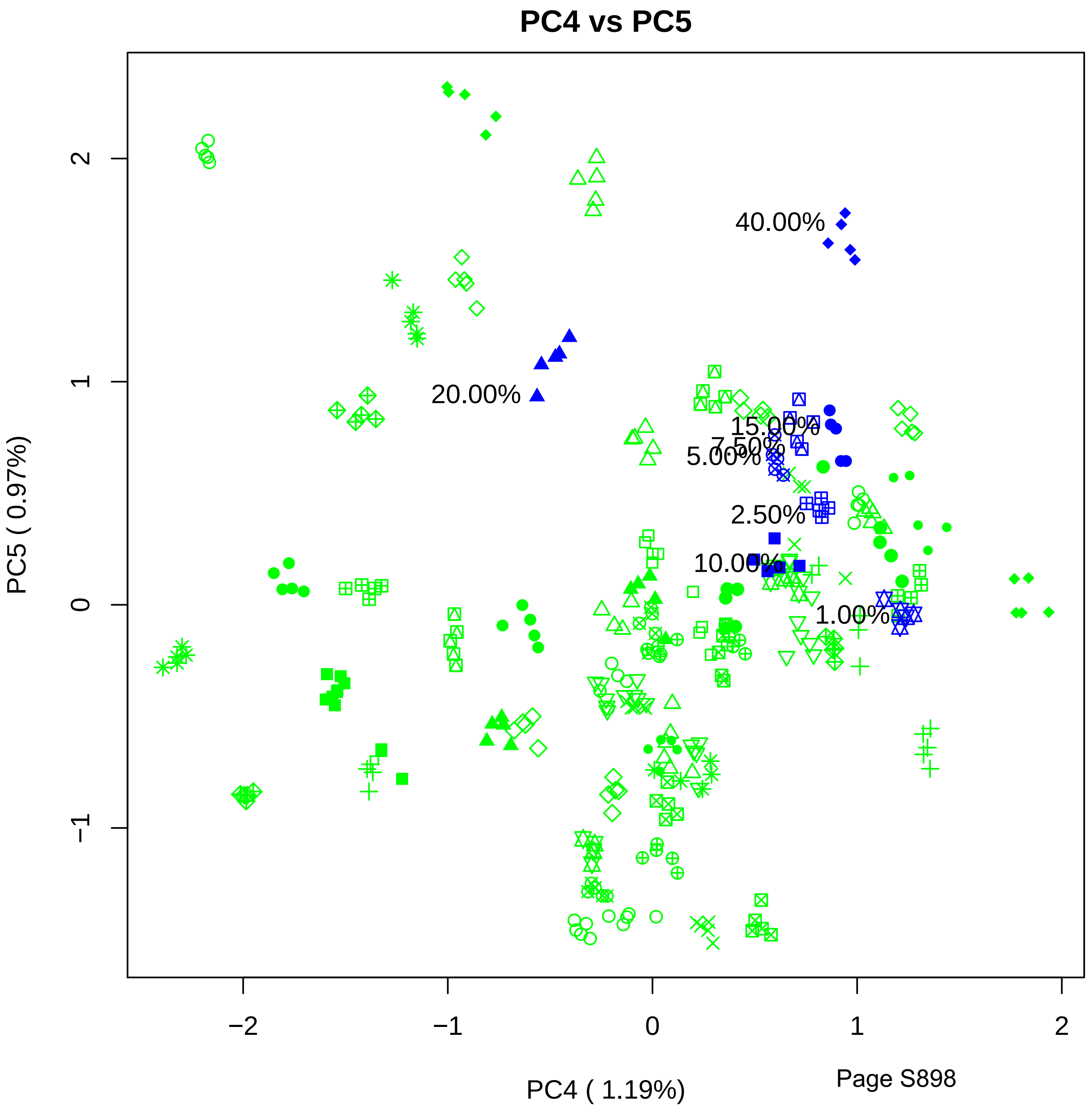
PC4 vs PC5
40.00%
20.00%
15.00%
7.50%
5.00%
2.50%
10.00%
1.00%
PC5 (0.97%)
PC4 (1.19%)
−2
−1
0
1
2

PCA of 1% BMH(a) vs PMH(a)

Scaled Matrix PCAed

7th order polynomial baseline correction performed

Raw spectra smoothed to 21 Neighbours, 2nd order PN, Savtisky Golay

Normalisation between 0 and 100

D2 spectra smoothed to 41 Neighbours, 2nd order PN, Savtisky Golay

Regions Analysed: 3601−2501 and 2001−700cm−1

Samples Analysed: 1 bContam's 0 BLH's 0 BMH's 0 OMH's 0 pContam's 0 PLH's 60 PMH's

Keele University AD Fri Dec 14 14:19:54 2018

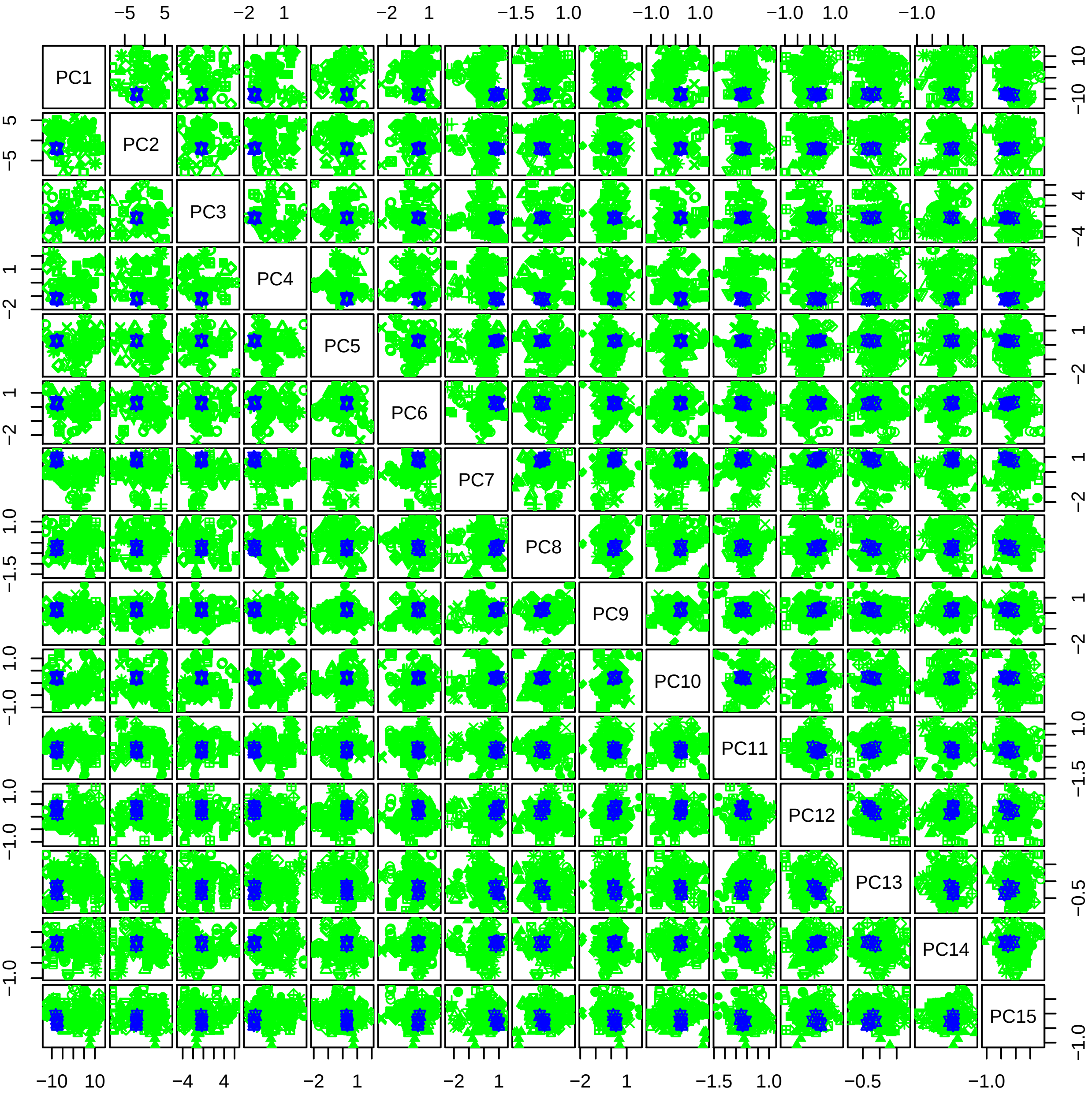
PC1
PC2
PC3
PC4
PC5
PC6
PC7
PC8
PC9
PC10
PC11
PC12
PC13
PC14
PC15

Page S900

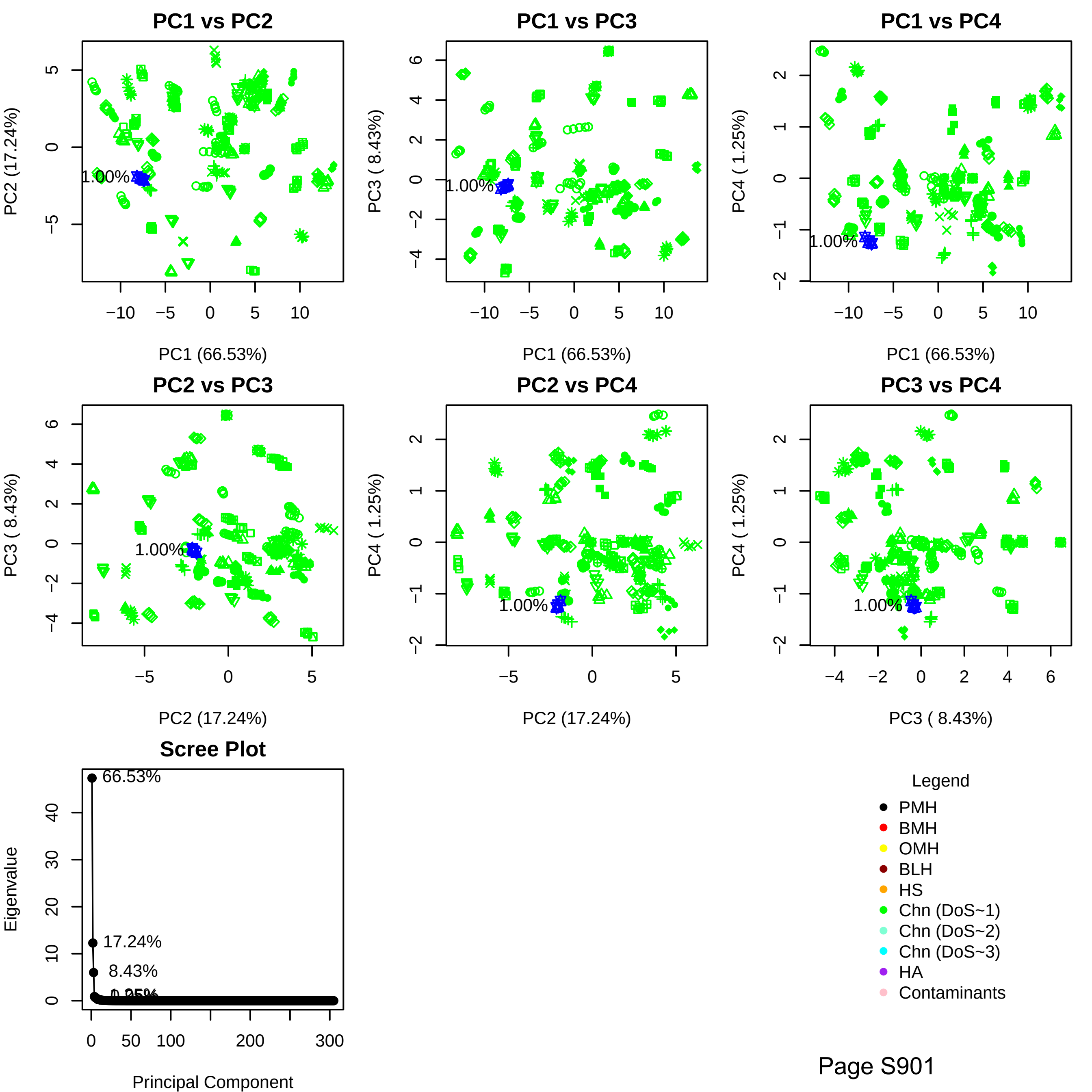

PC1 vs PC2
PC1 vs PC3
PC1 vs PC4
PC2 vs PC3
PC2 vs PC4
PC3 vs PC4
Scree Plot
PC1 (66.53%)
PC2 (17.24%)
PC3 (8.43%)
PC4 (1.25%)
1.00%
66.53%
17.24%
8.43%
Eigenvalue
Principal Component
Legend
PMH
BMH
OMH
BLH
HS
Chn (DoS~1)
Chn (DoS~2)
Chn (DoS~3)
HA
Contaminants

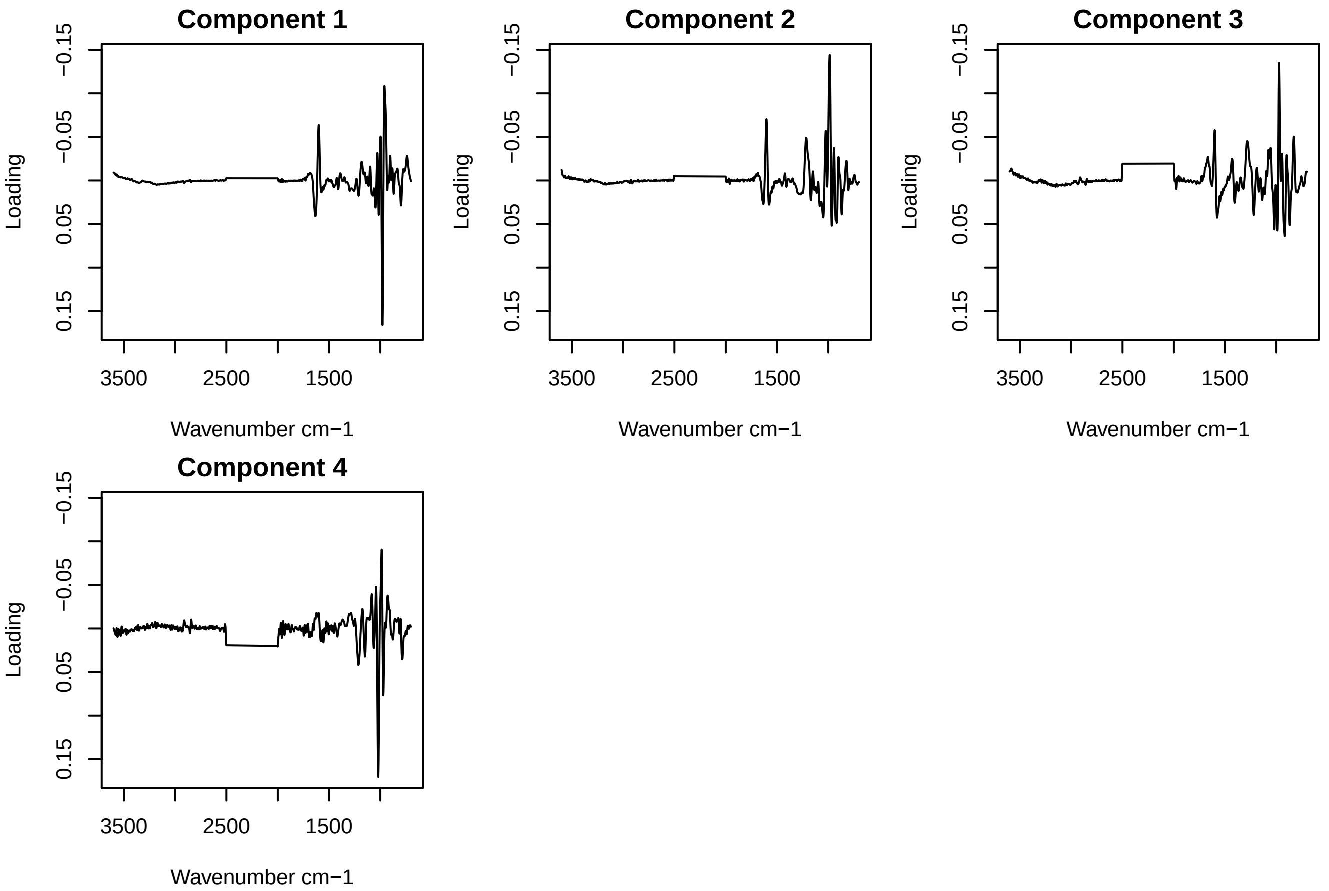

Component 1
Component 2
Component 3
Component 4
Loading
−0.15
−0.05
0.05
0.15
3500
2500
1500
Wavenumber cm−1

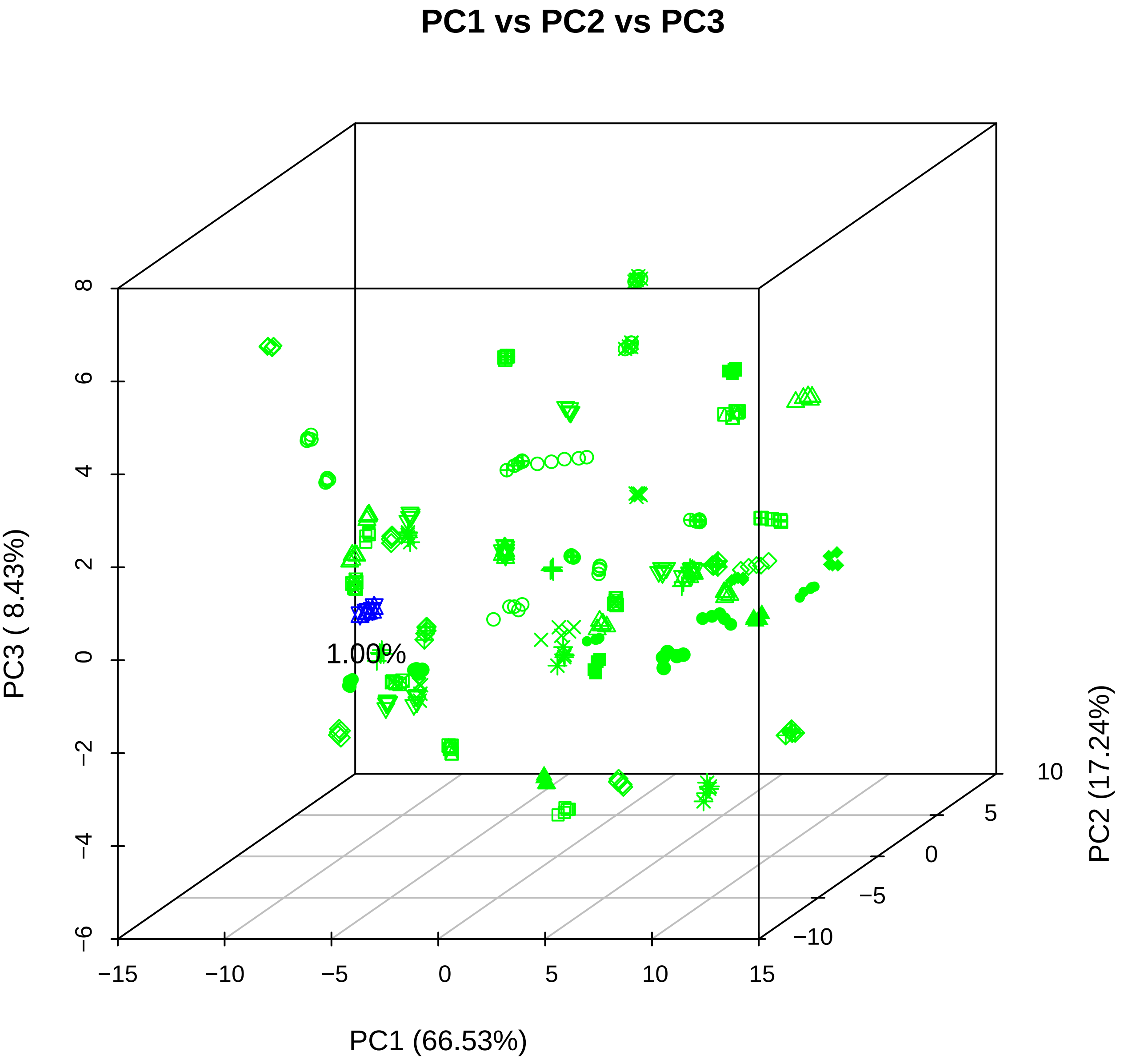
PC1 vs PC2 vs PC3
PC3 (8.43%)
PC1 (66.53%)
PC2 (17.24%)
1.00%
8
6
4
2
0
−2
−4
−6
−15
−10
−5
0
5
10
15
10
5
0
−5
−10

PC1 vs PC2 vs PC4

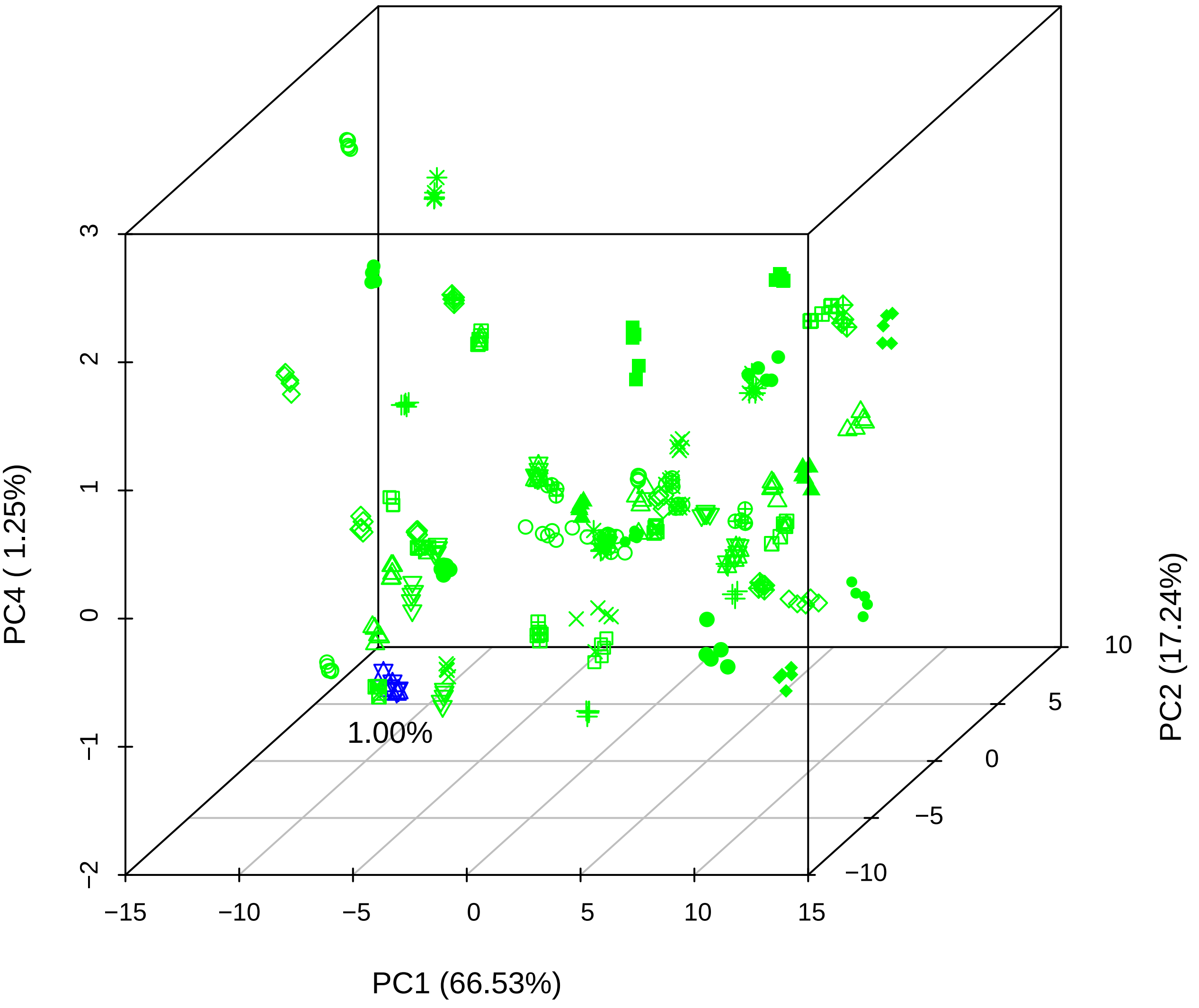

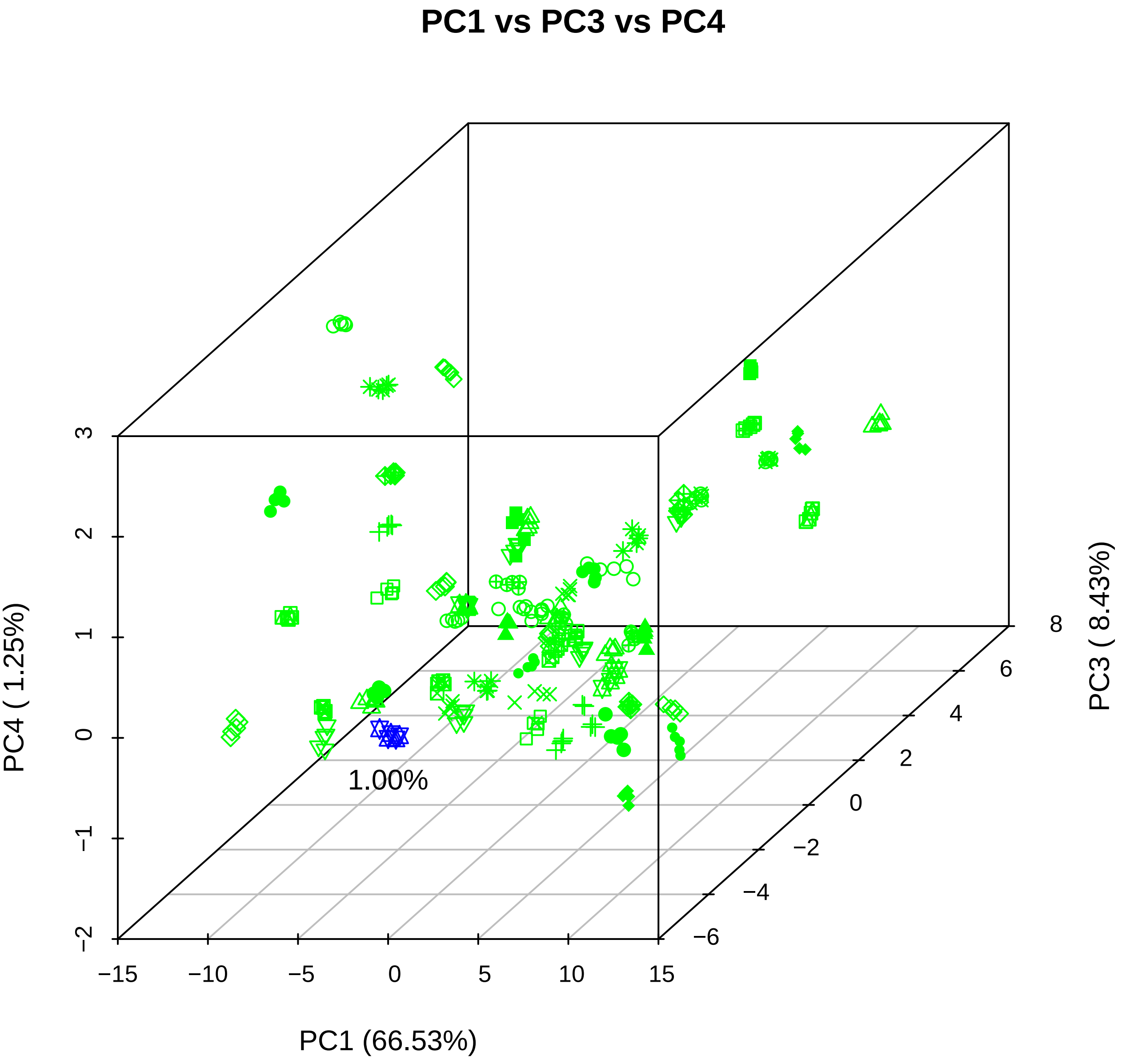
PC1 vs PC3 vs PC4
PC4 (1.25%)
PC3 (8.43%)
PC1 (66.53%)
1.00%
−2
−1
0
1
2
3
−15
−10
−5
0
5
10
15
−6
−4
−2
0
2
4
6
8

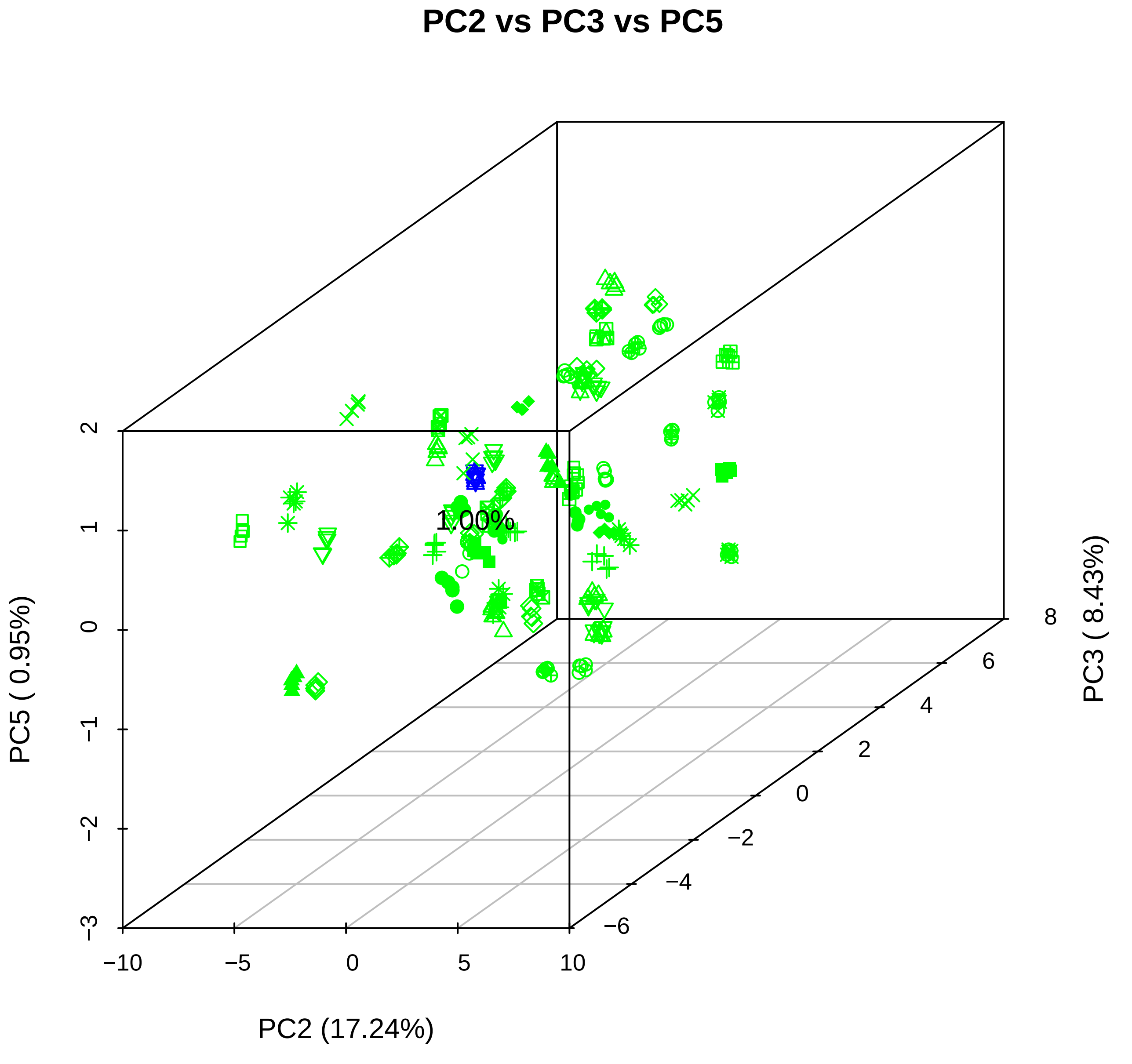

PC2 vs PC3 vs PC5
1.00%
PC2 (17.24%)
PC5 (0.95%)
PC3 (8.43%)
−10
−5
0
5
10
−3
−2
−1
0
1
2
−6
−4
−2
0
2
4
6
8

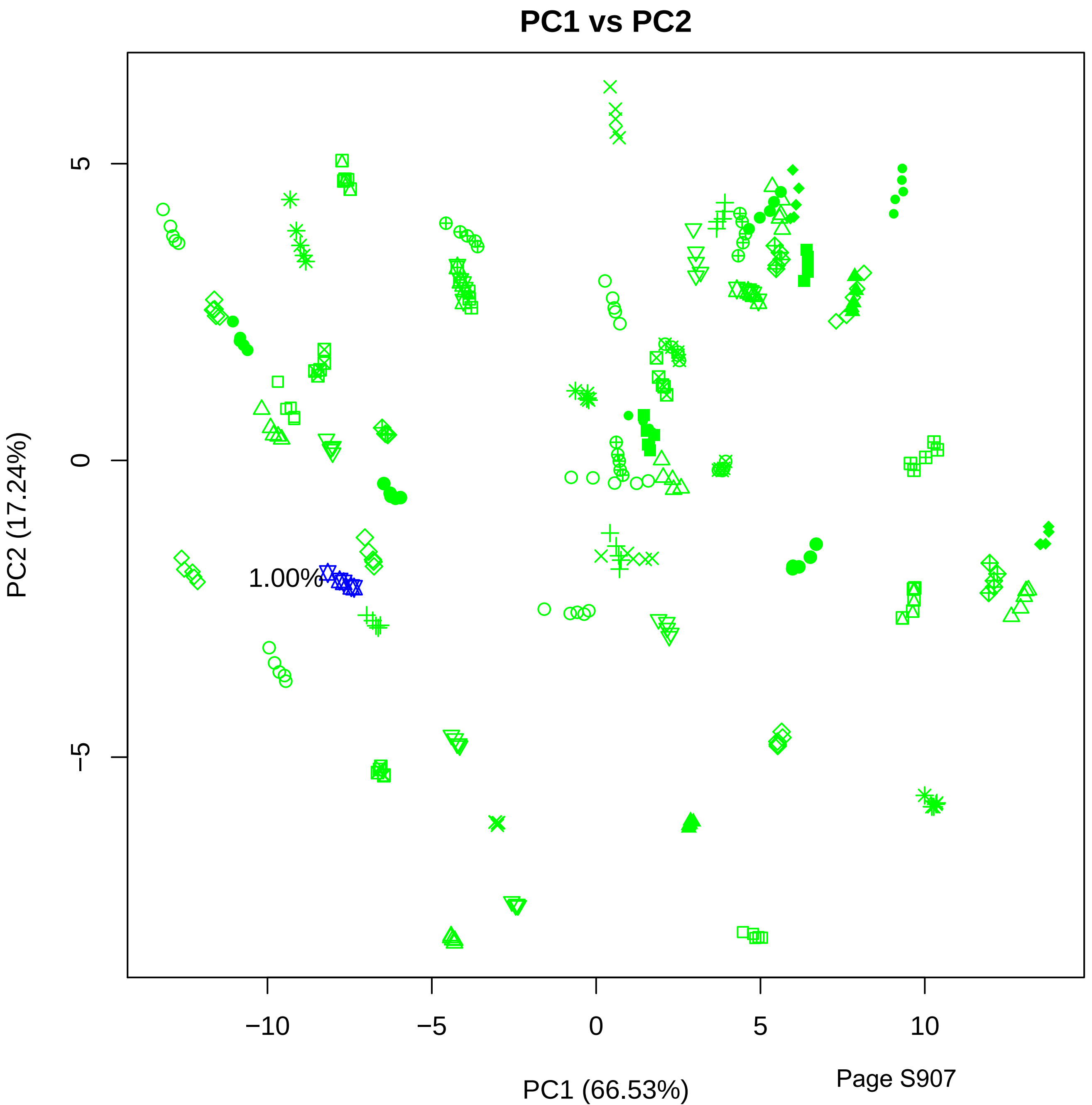

PC1 vs PC2
PC2 (17.24%)
PC1 (66.53%)
1.00%
5
0
−5
−10
−5
0
5
10

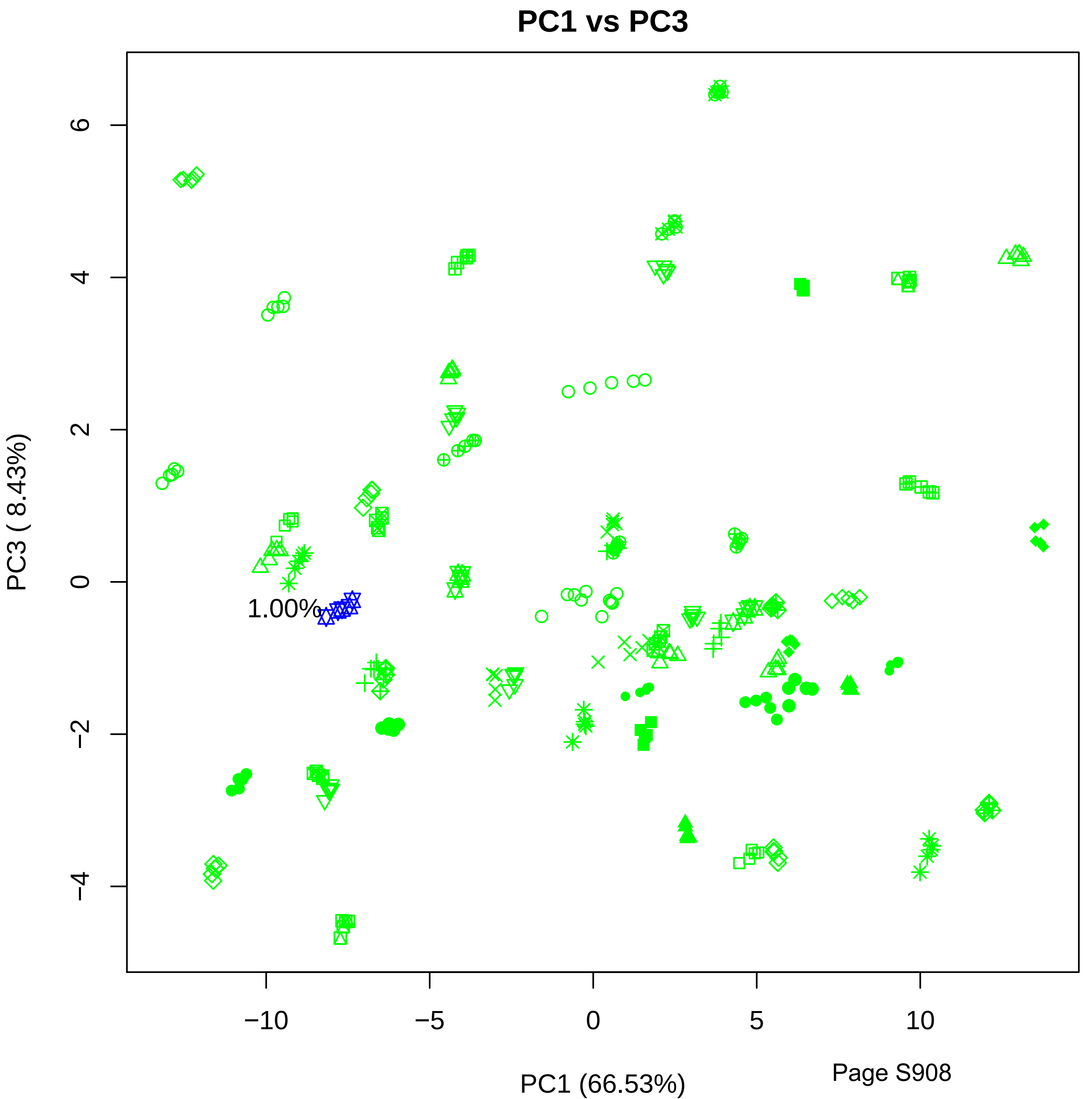
PC1 vs PC3
PC3 (8.43%)
PC1 (66.53%)
1.00%
6
4
2
0
−2
−4
−10
−5
0
5
10

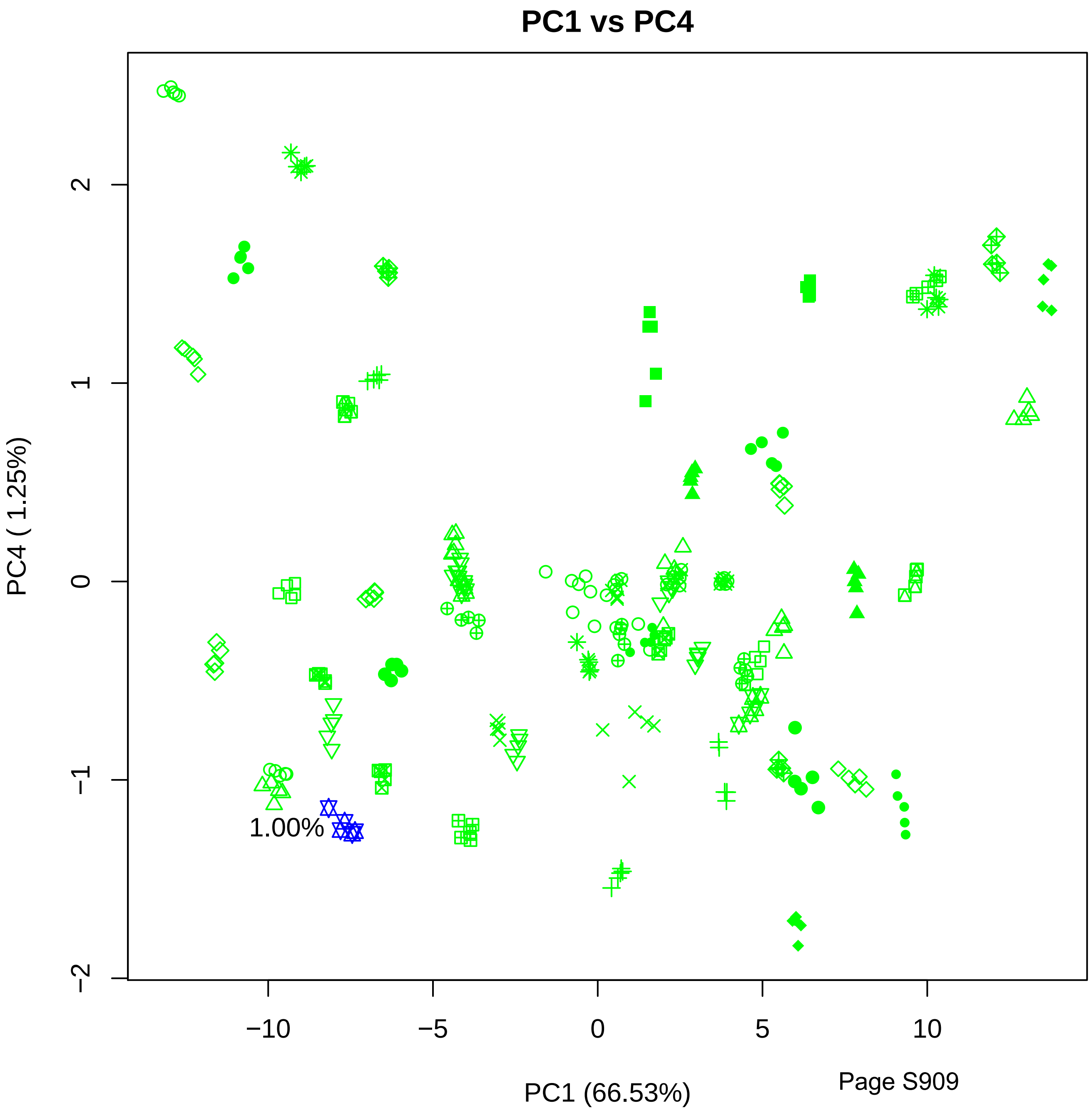
PC1 vs PC4
PC4 (1.25%)
PC1 (66.53%)
1.00%
−10
−5
0
5
10
−2
−1
0
1
2

PC1 vs PC5

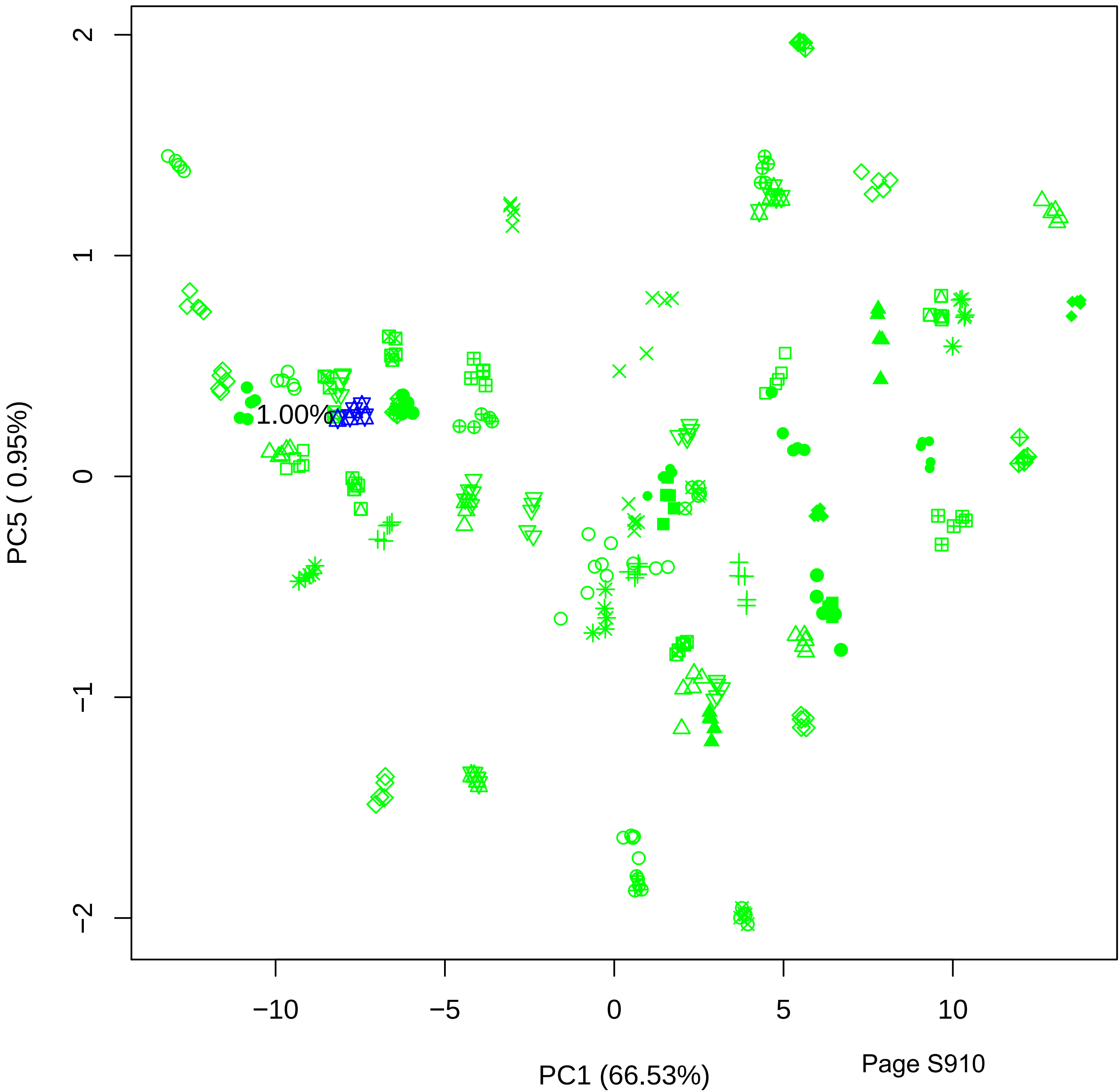

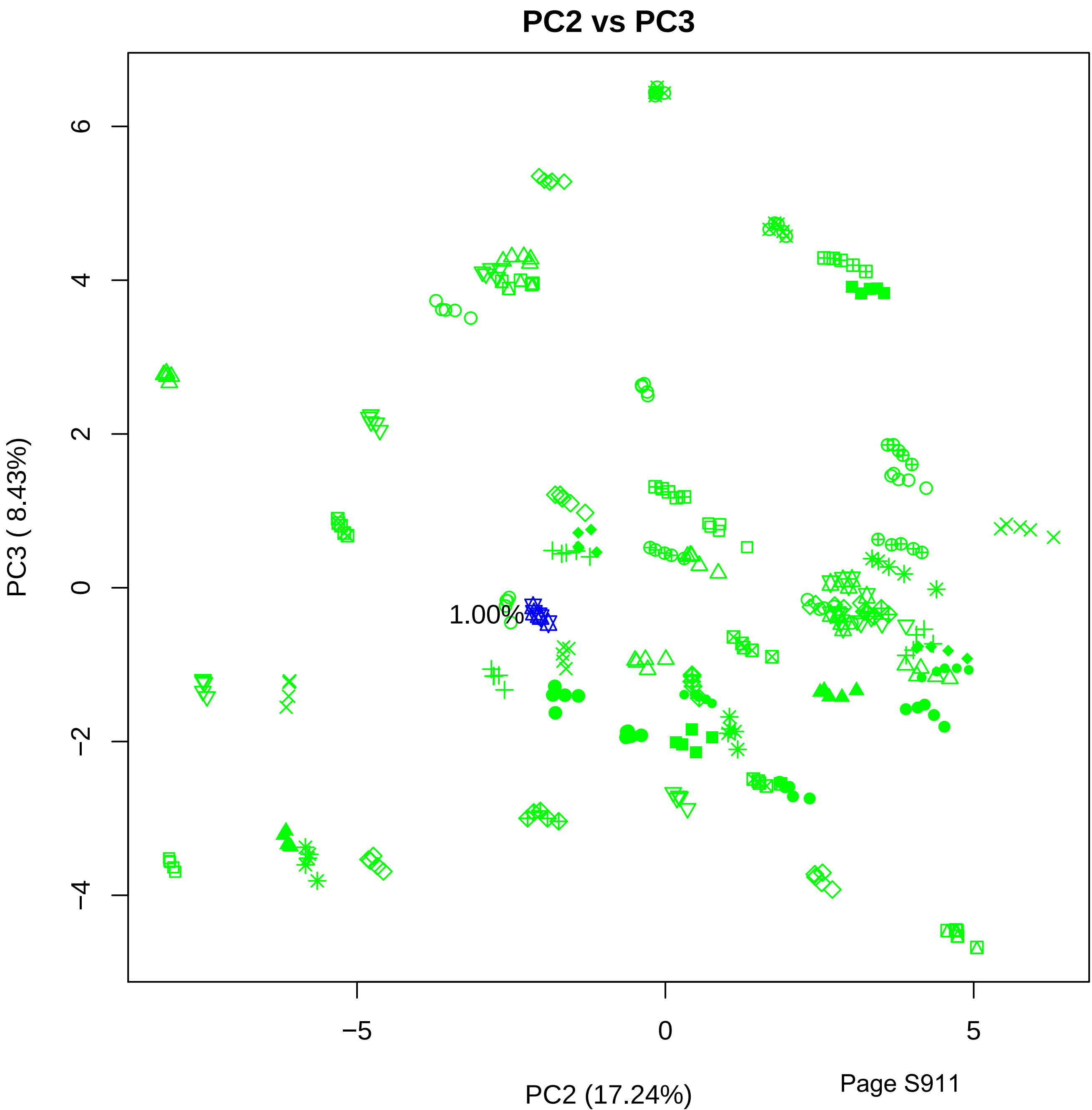

PC2 vs PC3
PC3 (8.43%)
PC2 (17.24%)
1.00%
6
4
2
0
−2
−4
−5
0
5

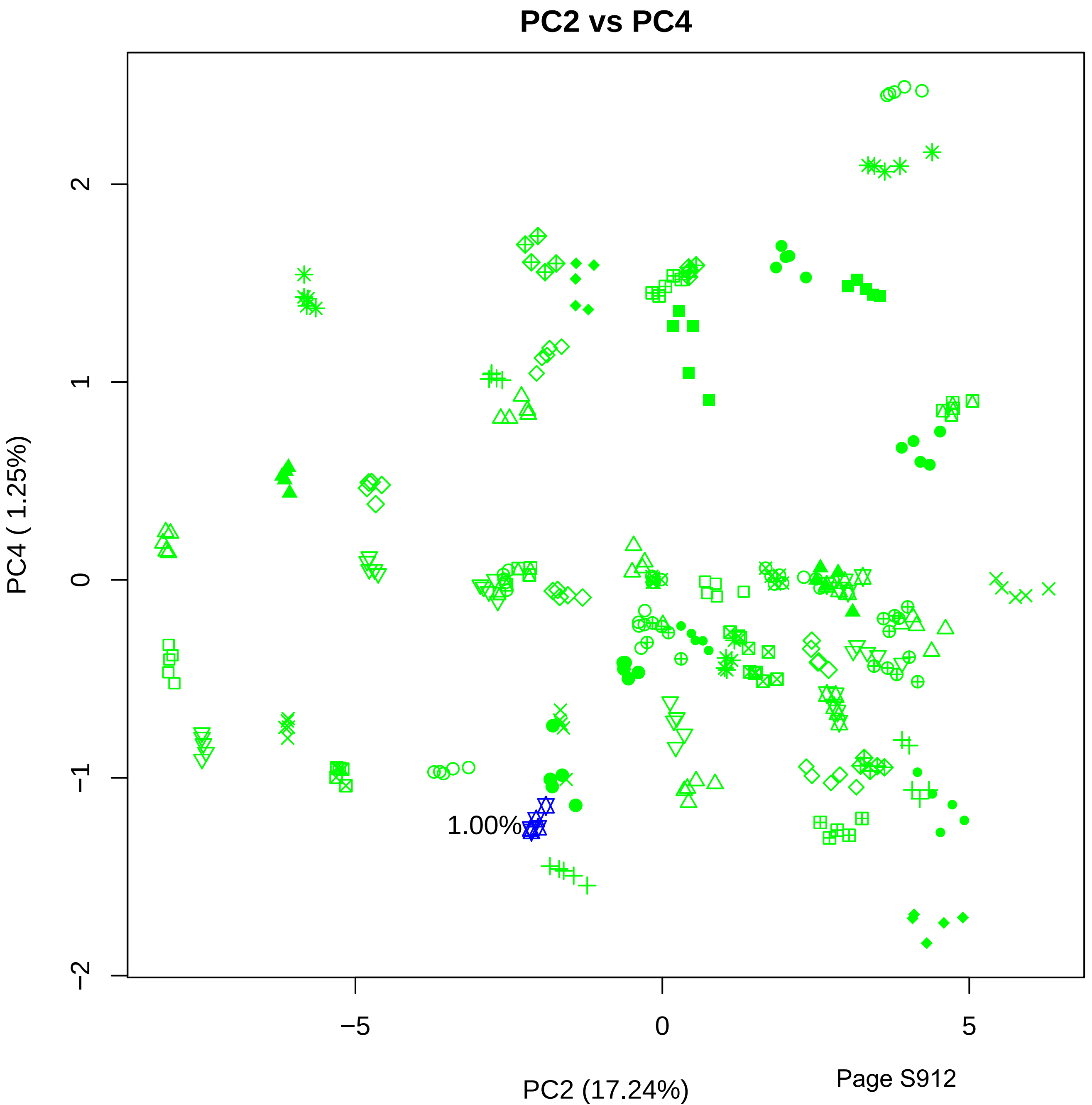
PC2 vs PC4
PC4 (1.25%)
PC2 (17.24%)
1.00%
2
1
0
−1
−2
−5
0
5

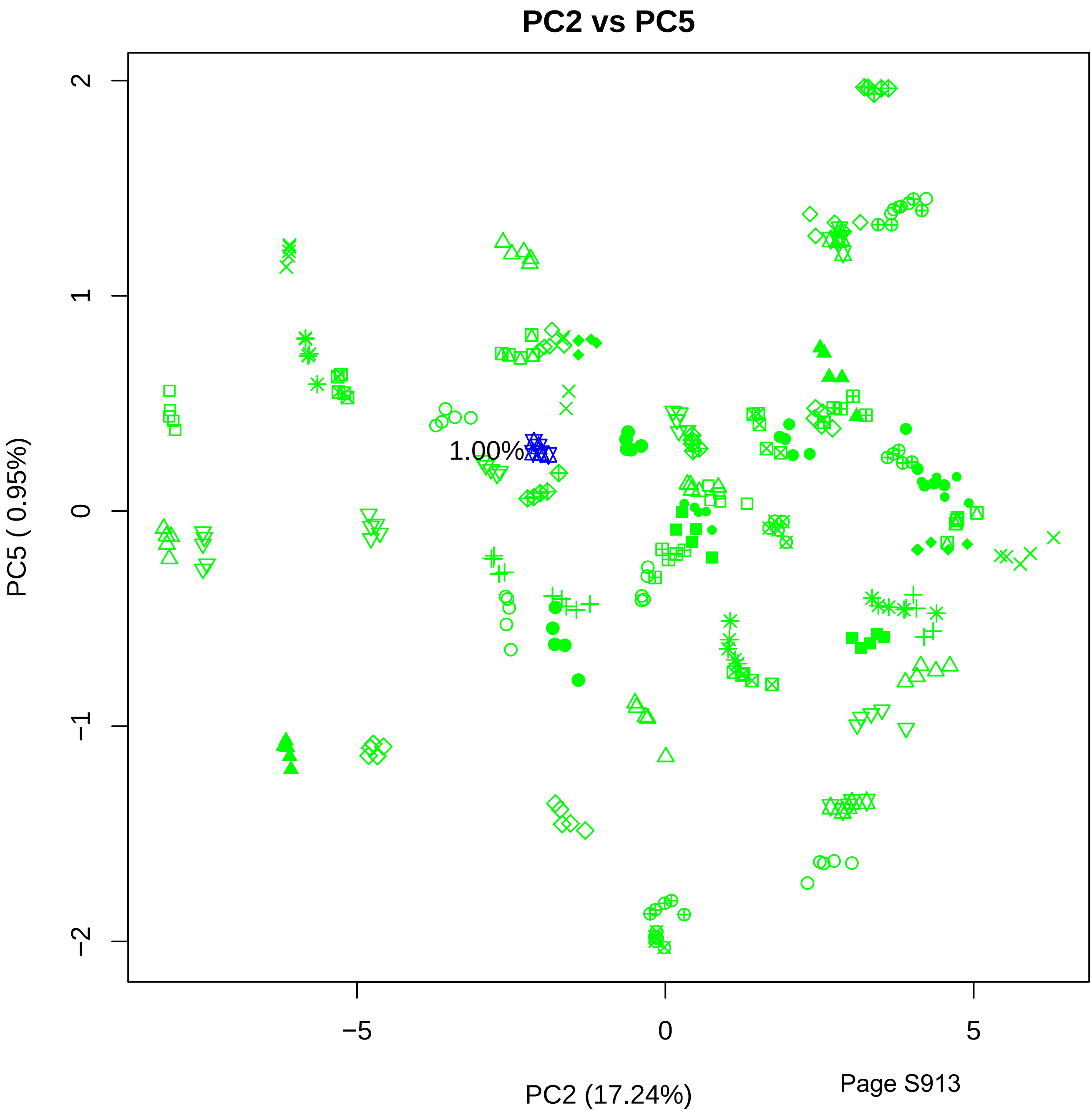

PC2 vs PC5
1.00%
PC5 (0.95%)
PC2 (17.24%)
2
1
0
−1
−2
−5
0
5

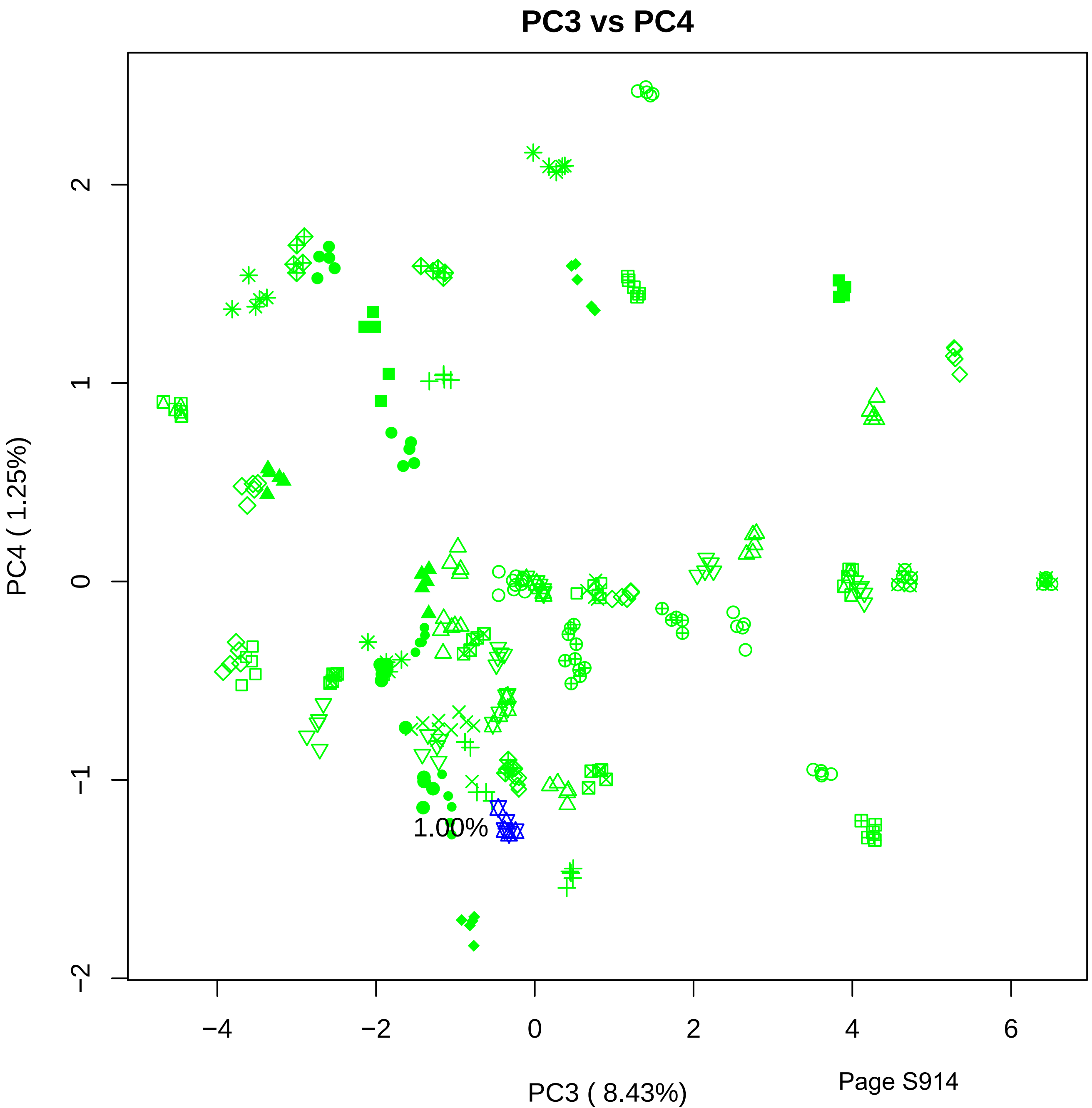

PC3 vs PC4
PC4 (1.25%)
PC3 (8.43%)
2
1
0
−1
−2
−4
−2
0
2
4
6
1.00%

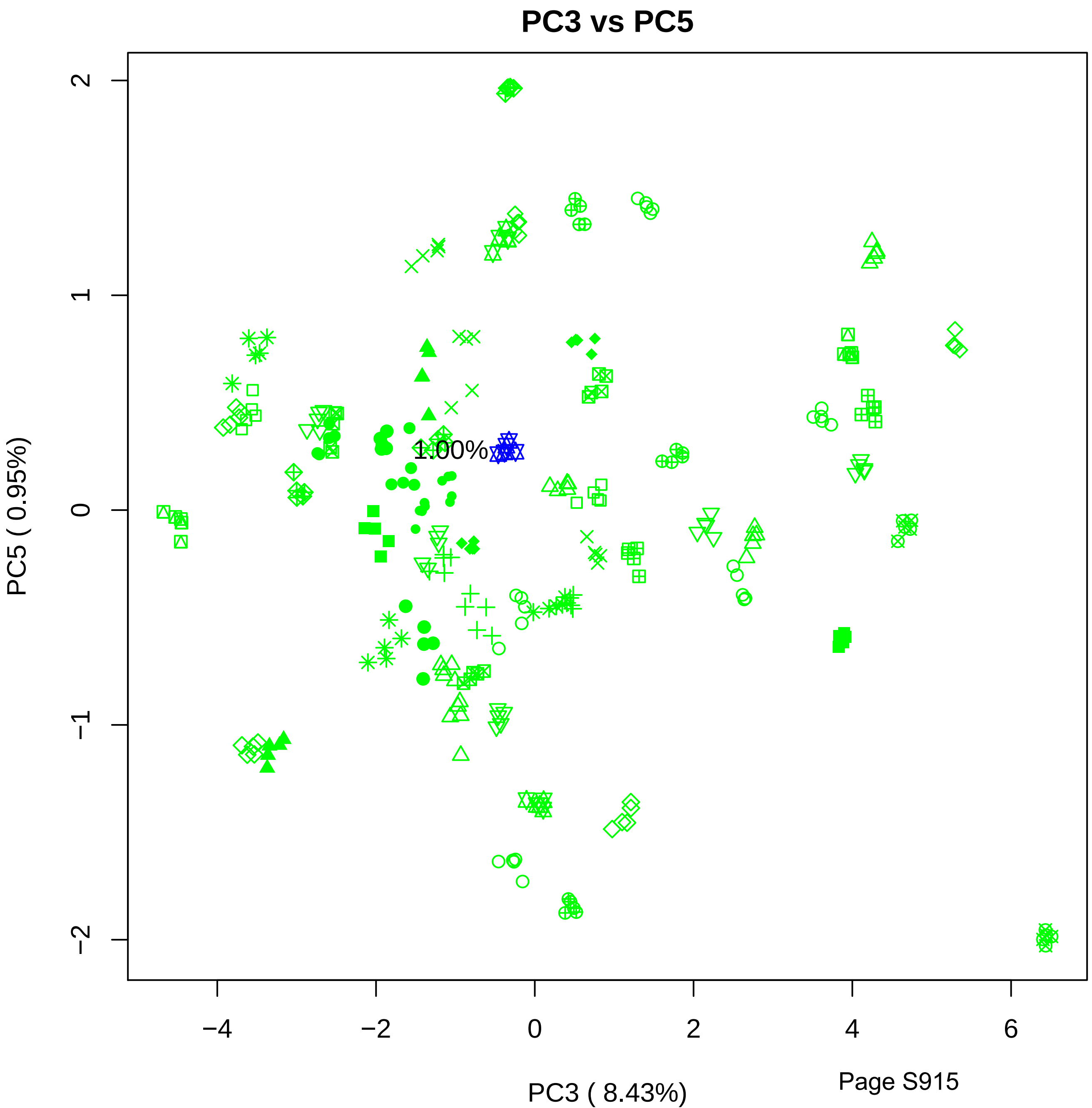
PC3 vs PC5
PC5 (0.95%)
PC3 (8.43%)
1.00%
2
1
0
−1
−2
−4
−2
0
2
4
6

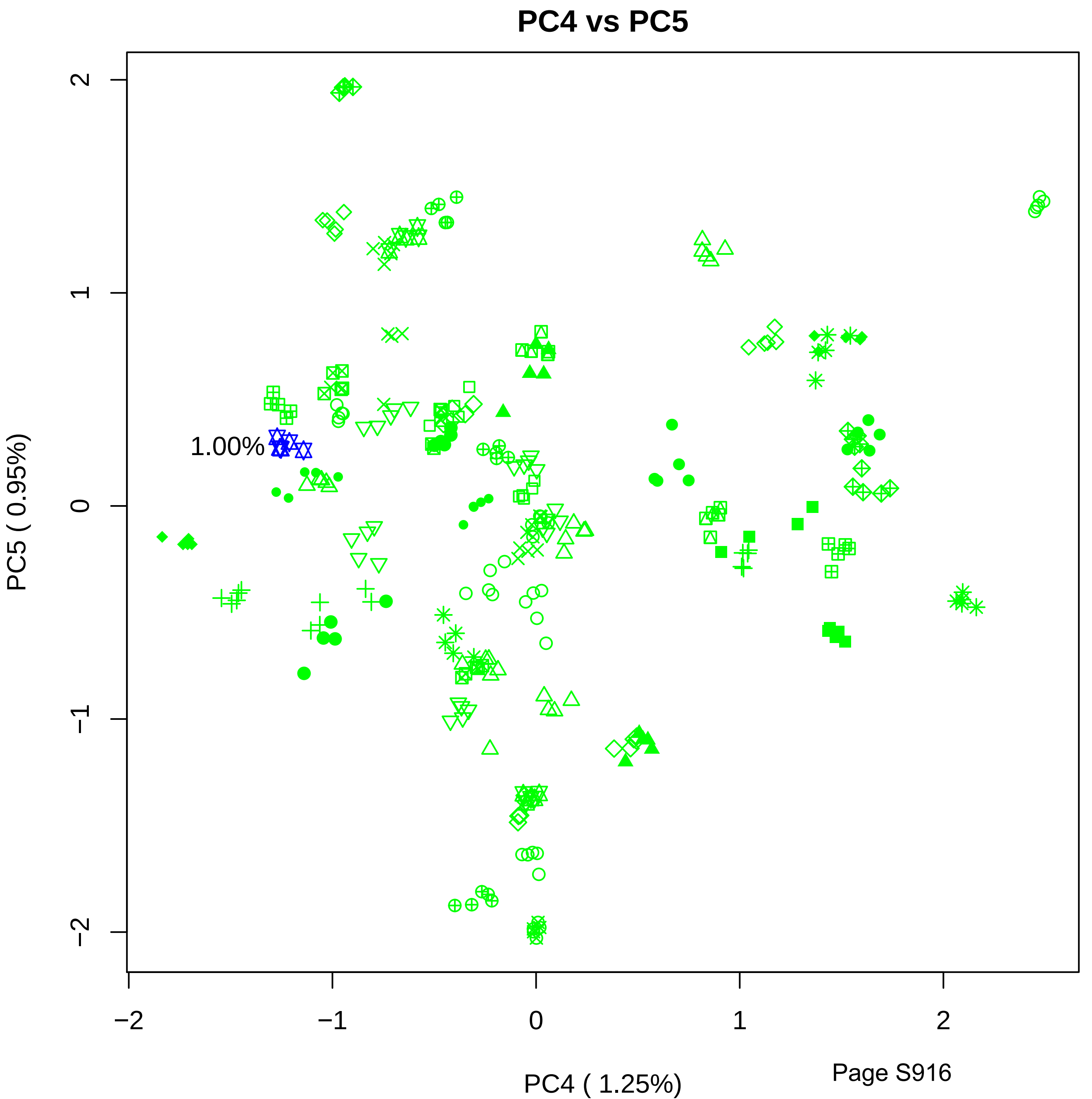
PC4 vs PC5
1.00%
PC5 (0.95%)
PC4 (1.25%)
2
1
0
−1
−2
−2
−1
0
1
2

PCA of 2.5% BMH(a) vs PMH (a)

Scaled Matrix PCAed

7th order polynomial baseline correction performed

Raw spectra smoothed to 21 Neighbours, 2nd order PN, Savtisky Golay

Normalisation between 0 and 100

D2 spectra smoothed to 41 Neighbours, 2nd order PN, Savtisky Golay

Regions Analysed: 3601−2501 and 2001−700cm−1

Samples Analysed: 1 bContam's 0 BLH's 0 BMH's 0 OMH's 0 pContam's 0 PLH's 60 PMH's

Keele University AD

Fri Dec 14 14:20:03 2018

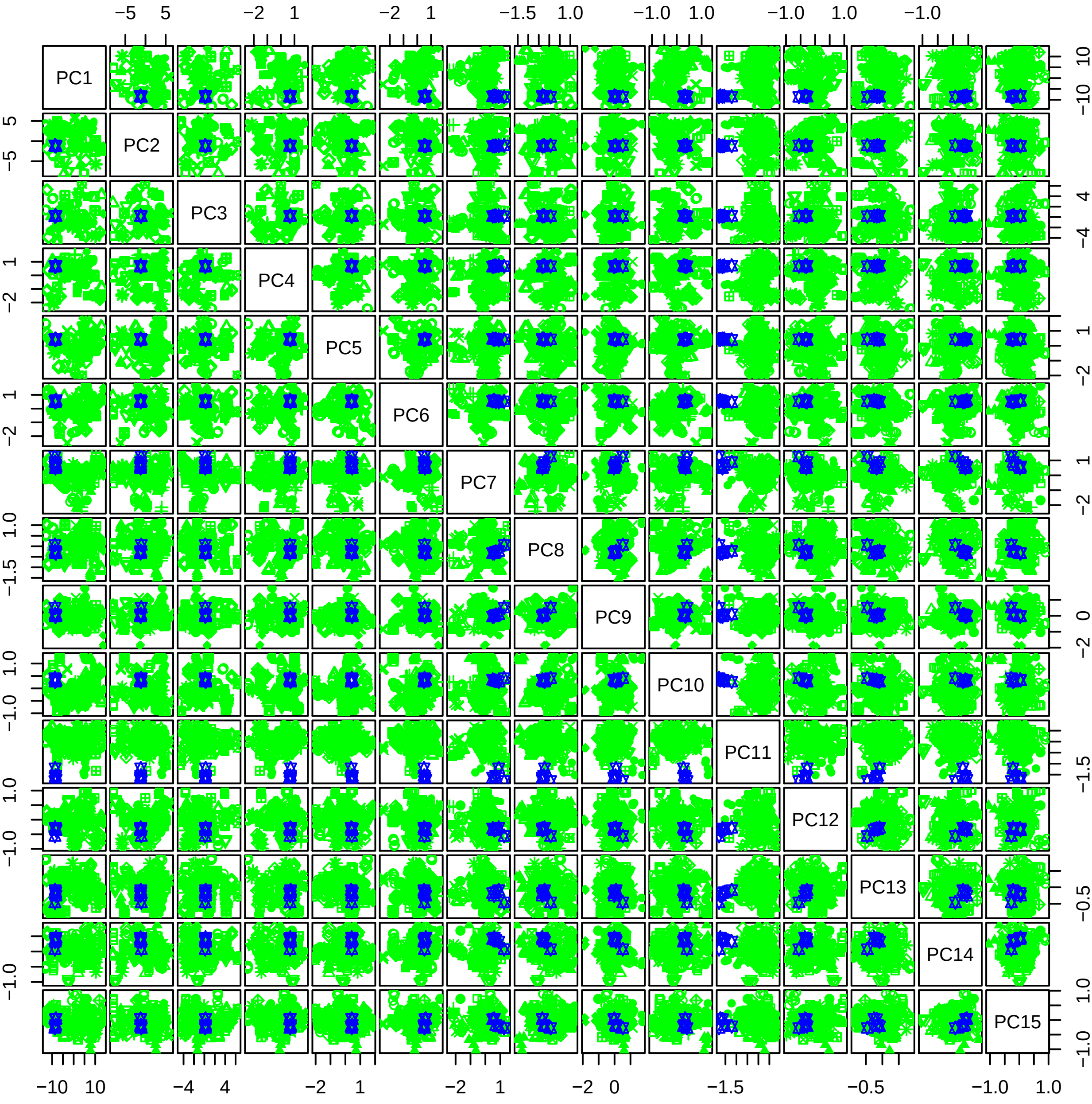
PC1
PC2
PC3
PC4
PC5
PC6
PC7
PC8
PC9
PC10
PC11
PC12
PC13
PC14
PC15

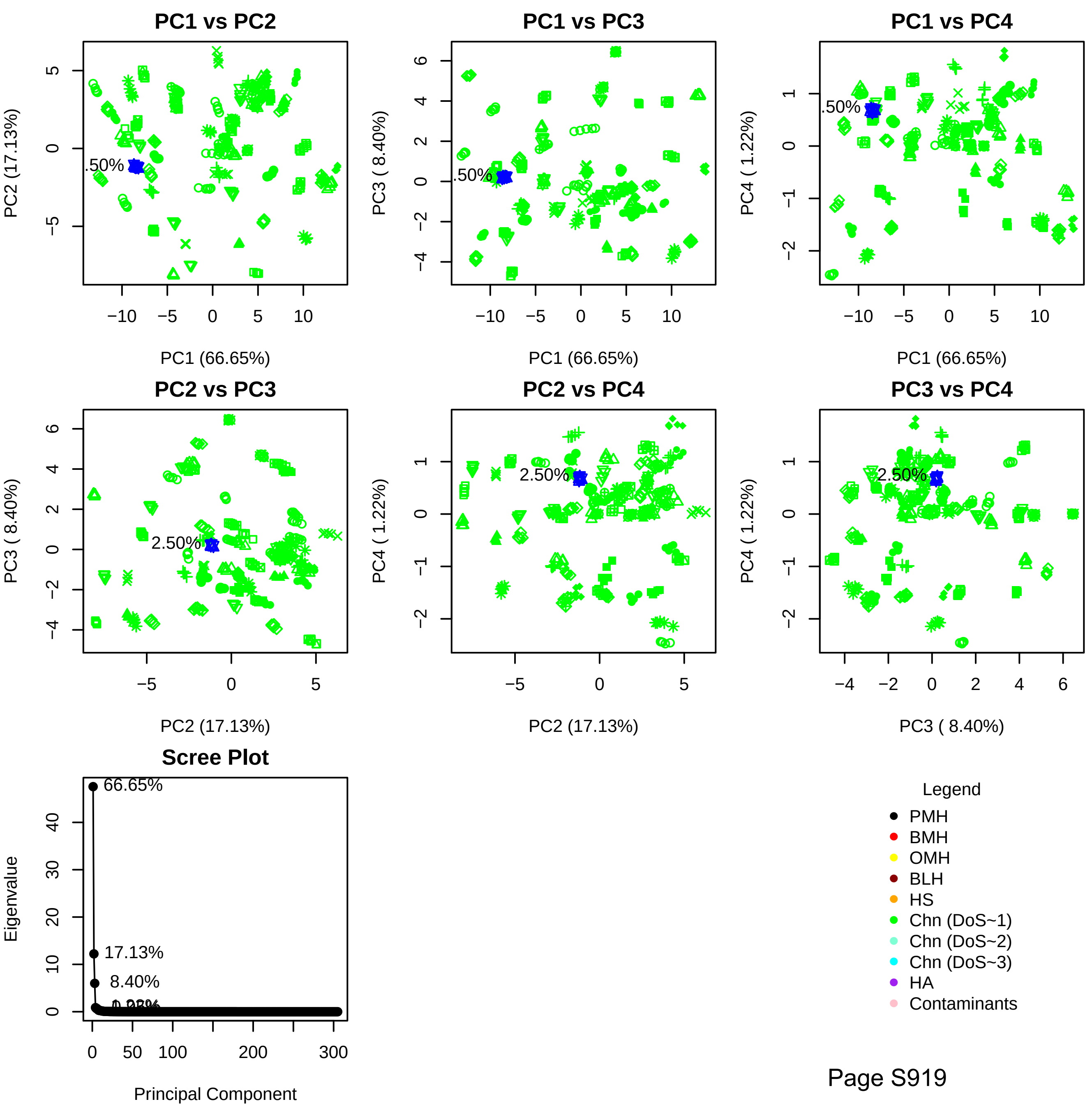
PC1 vs PC2
PC1 vs PC3
PC1 vs PC4
PC2 vs PC3
PC2 vs PC4
PC3 vs PC4
Scree Plot
PC1 (66.65%)
PC2 (17.13%)
PC3 (8.40%)
PC4 (1.22%)
.50%
2.50%
Eigenvalue
Principal Component
66.65%
17.13%
8.40%
Legend
PMH
BMH
OMH
BLH
HS
Chn (DoS~1)
Chn (DoS~2)
Chn (DoS~3)
HA
Contaminants

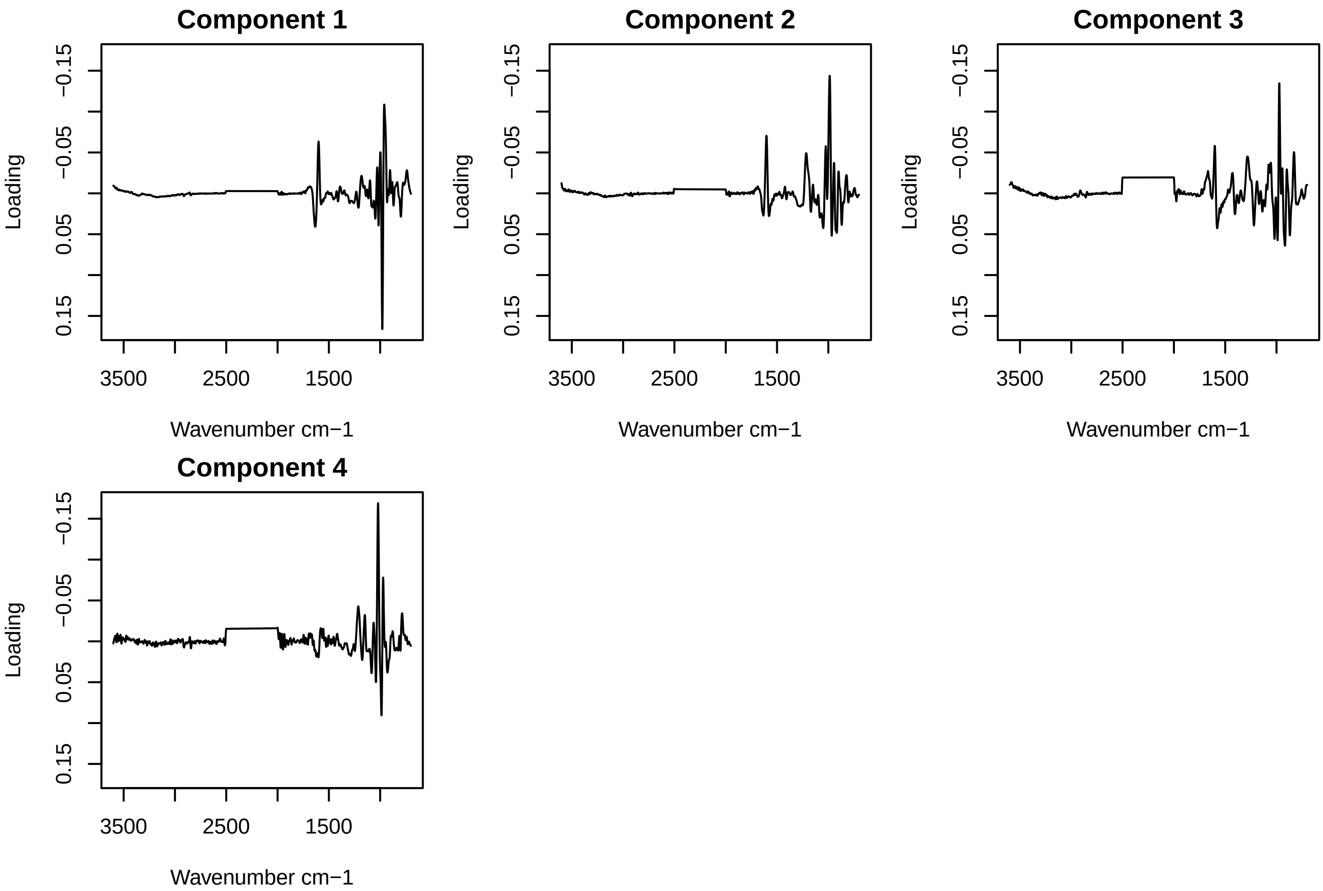
Component 1
Component 2
Component 3
Component 4
Loading
−0.15
−0.05
0.05
0.15
3500
2500
1500
Wavenumber cm−1

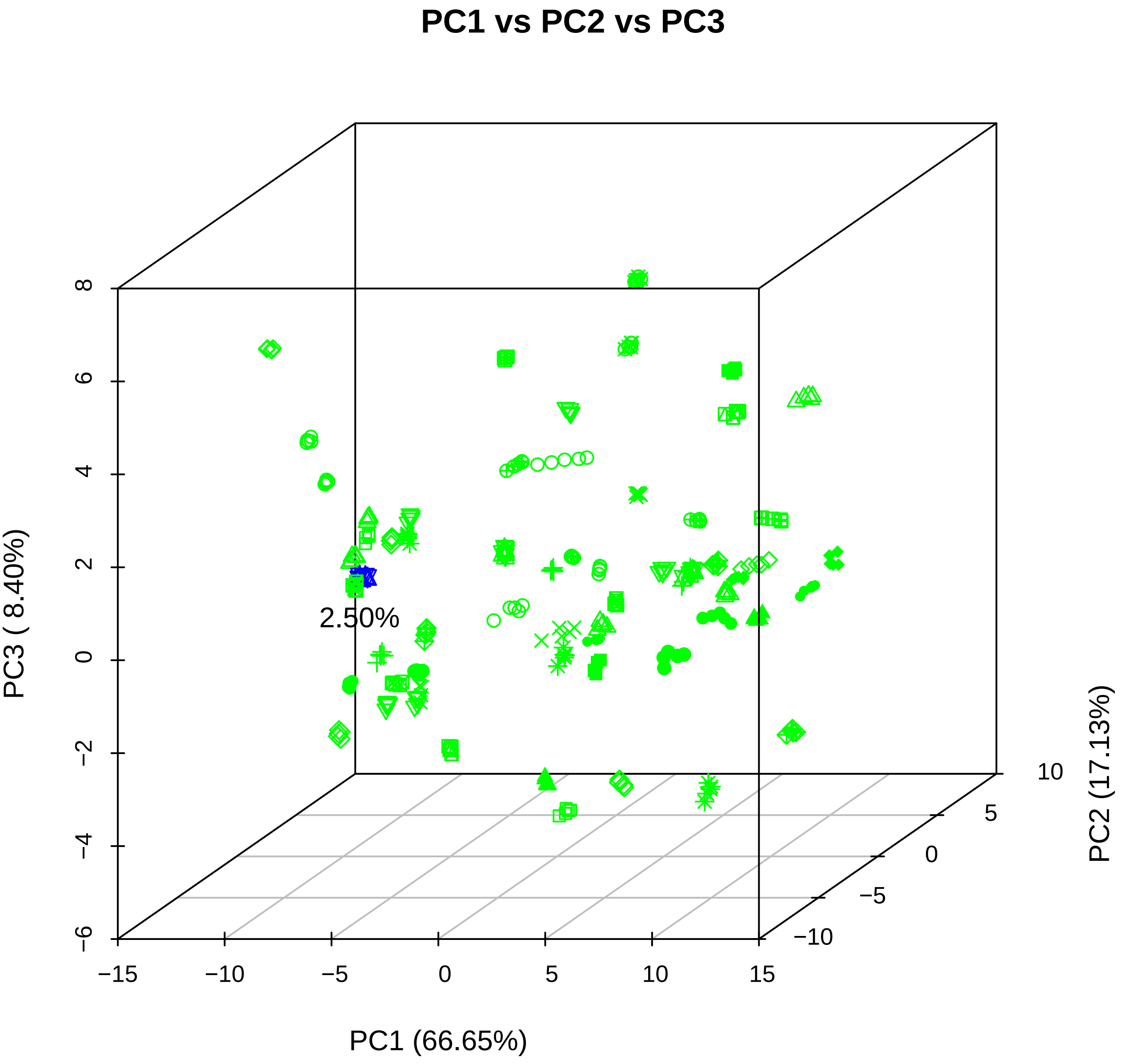
PC1 vs PC2 vs PC3
PC3 (8.40%)
PC1 (66.65%)
PC2 (17.13%)
2.50%
8
6
4
2
0
−2
−4
−6
−15
−10
−5
0
5
10
15
10
5
0
−5
−10

PC1 vs PC2 vs PC4

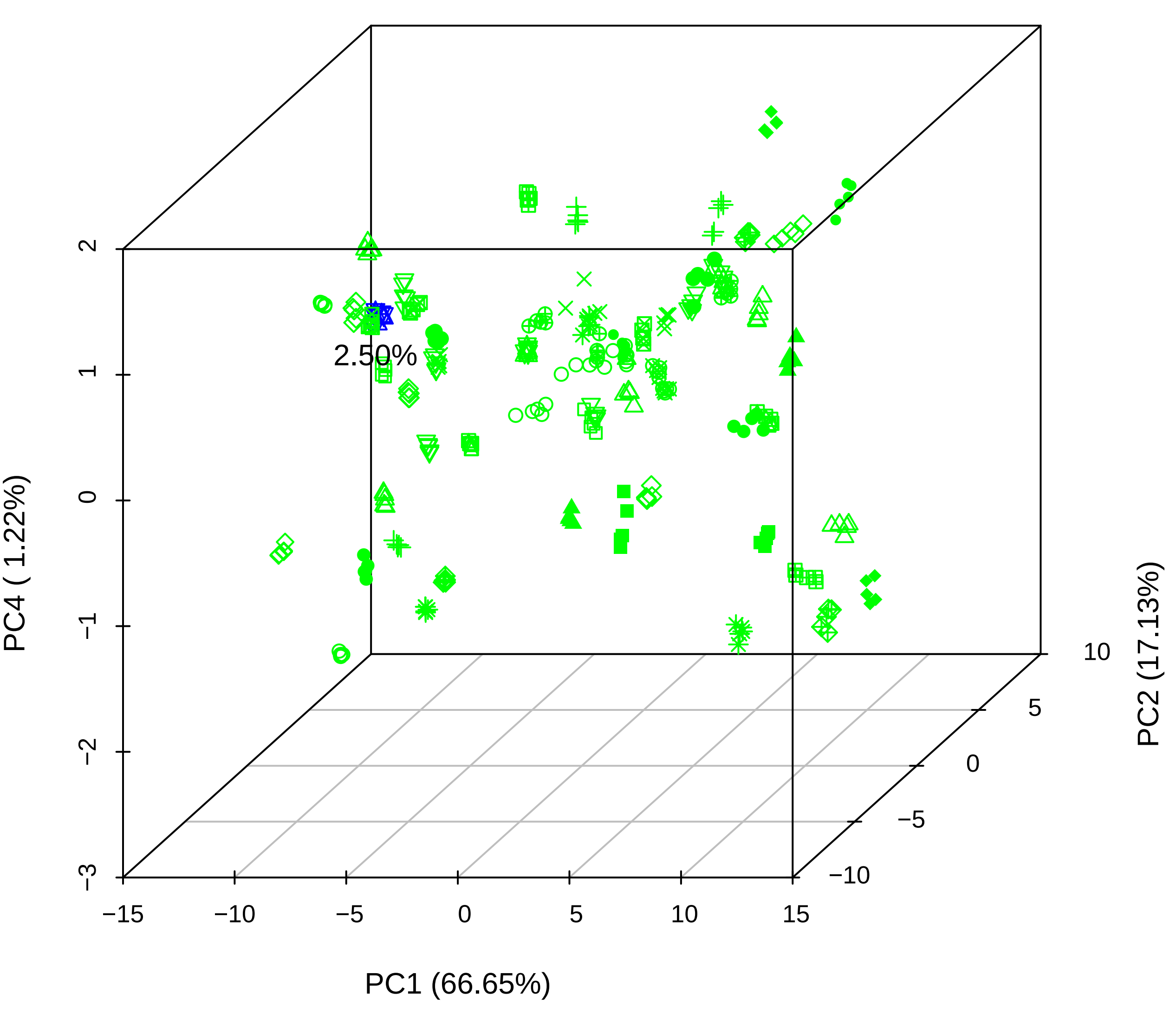

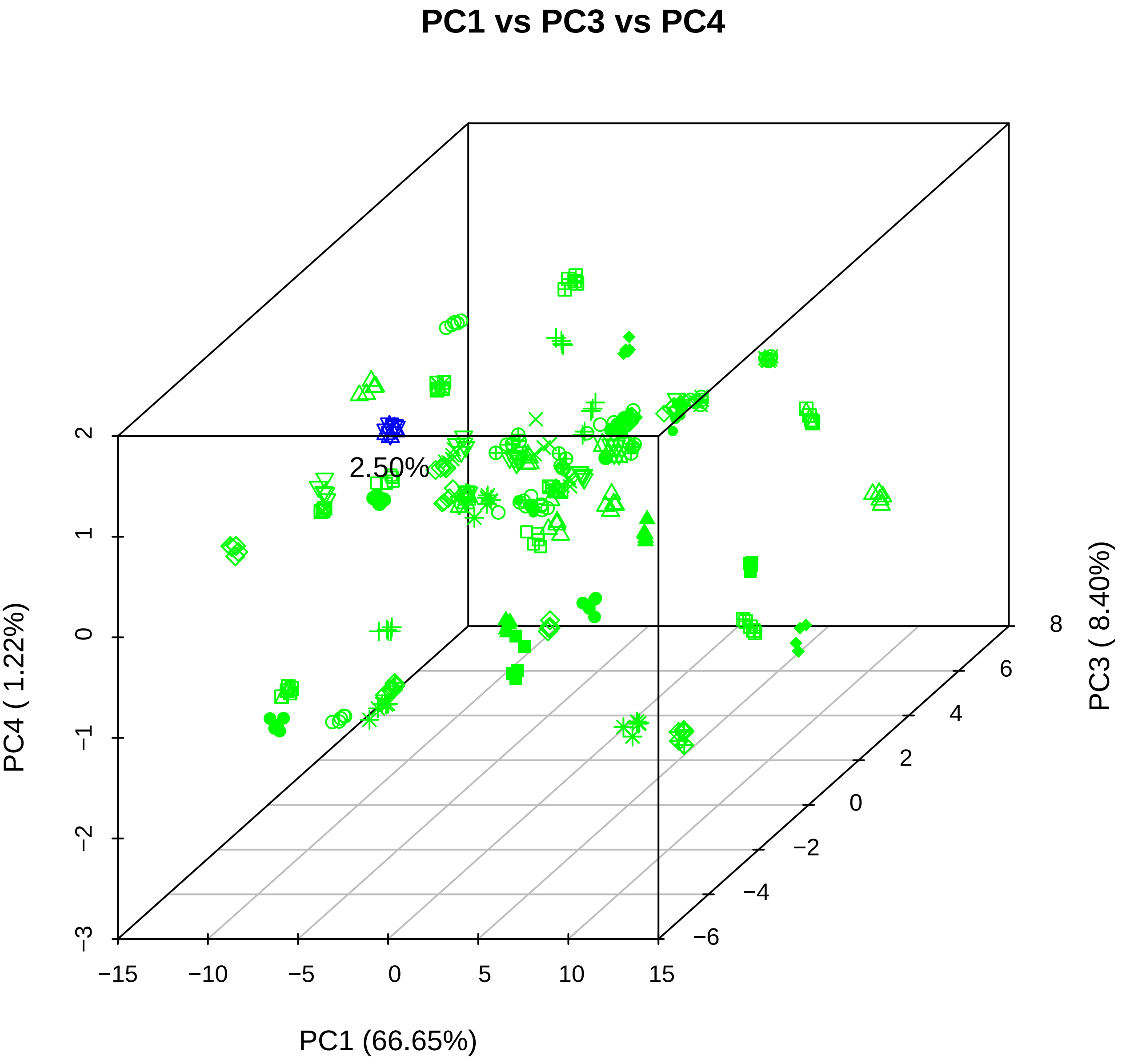
PC1 vs PC3 vs PC4
2.50%
PC1 (66.65%)
PC4 (1.22%)
PC3 (8.40%)
−15
−10
−5
0
5
10
15
−3
−2
−1
0
1
2
−6
−4
−2
0
2
4
6
8

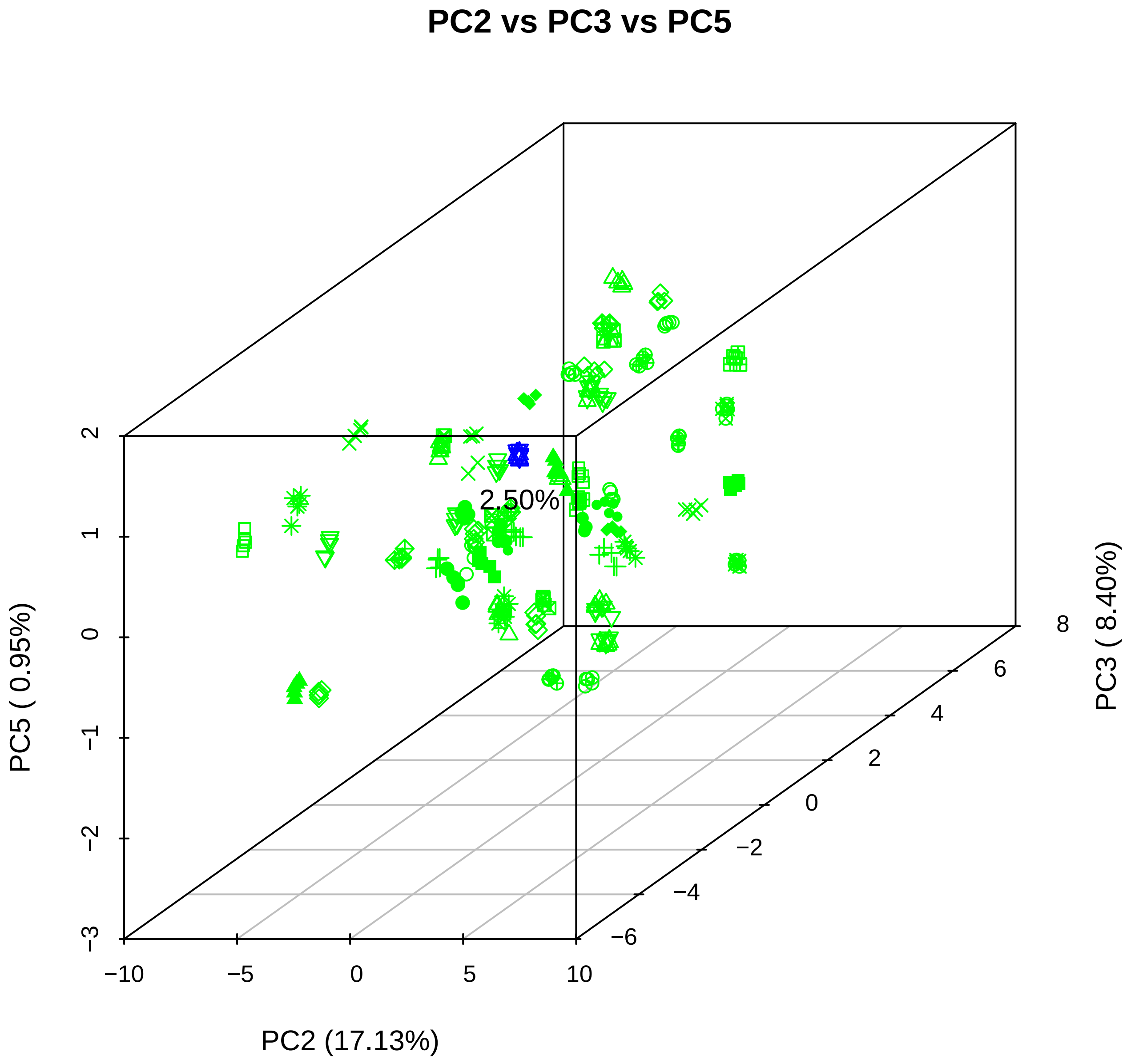
PC2 vs PC3 vs PC5
2.50%
PC2 (17.13%)
PC5 (0.95%)
PC3 (8.40%)
−10
−5
0
5
10
−3
−2
−1
0
1
2
−6
−4
−2
0
2
4
6
8

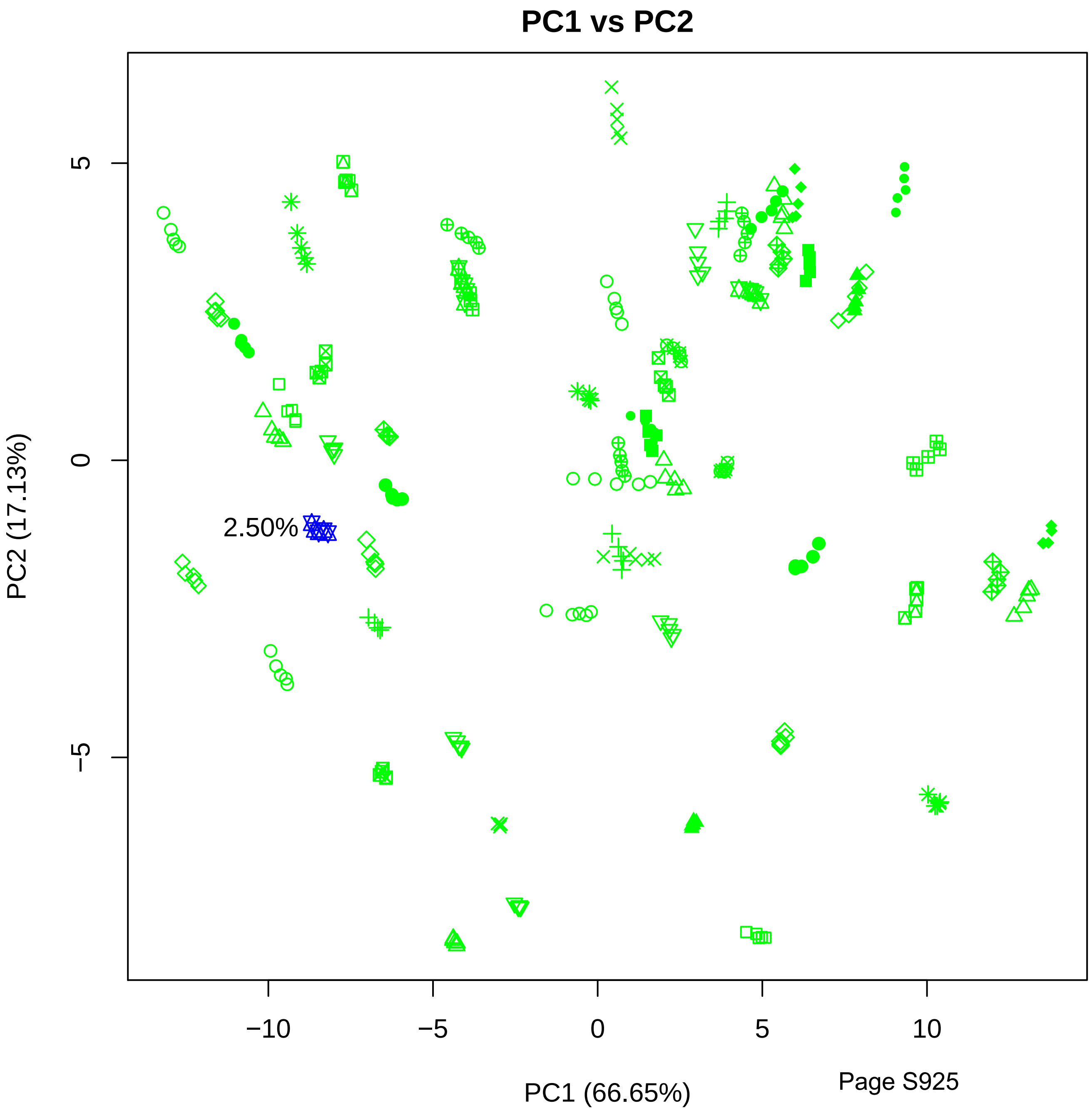
PC1 vs PC2
5
0
−5
−10
−5
0
5
10
2.50%
PC2 (17.13%)
PC1 (66.65%)

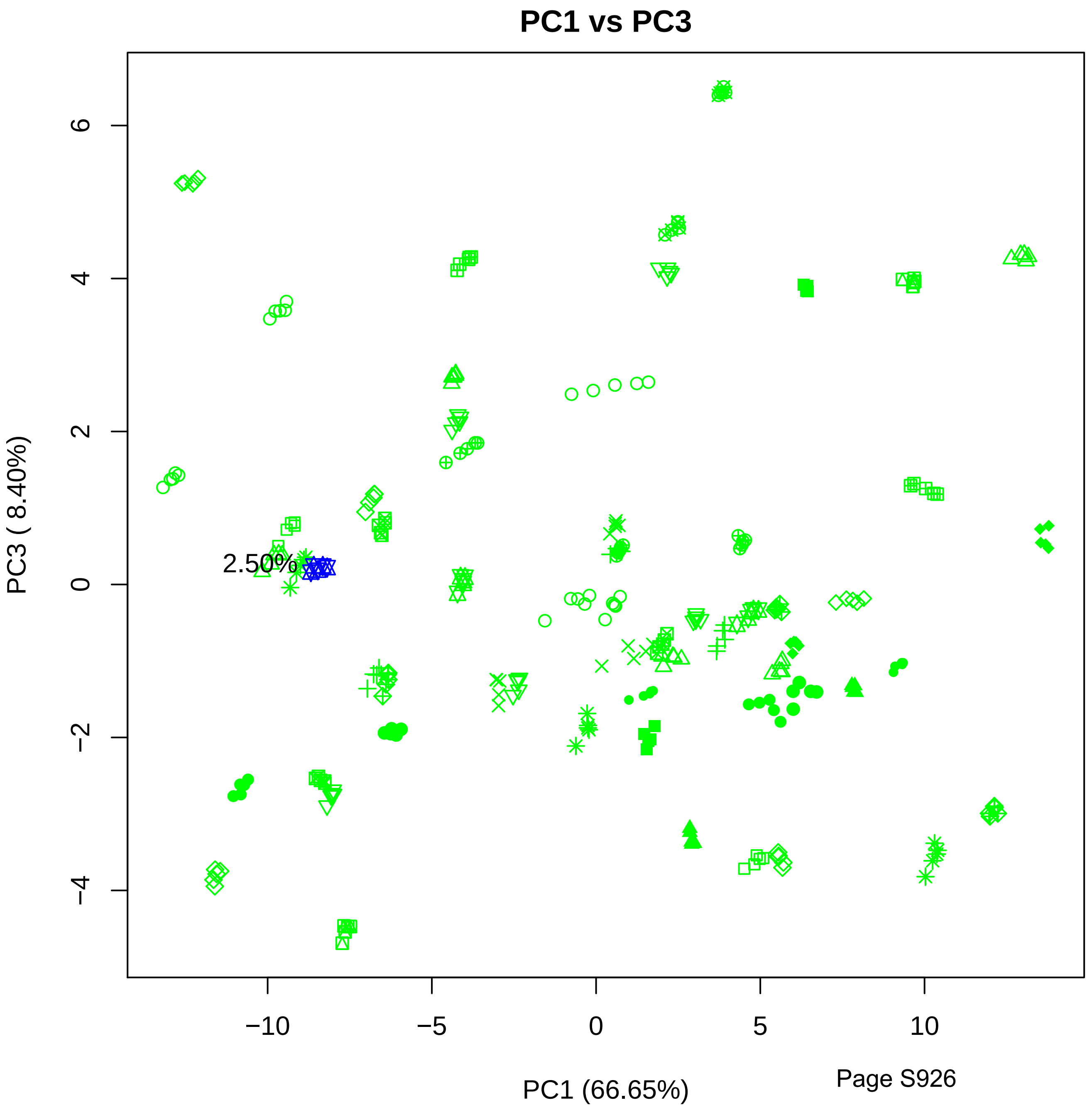

PC1 vs PC3
PC3 (8.40%)
PC1 (66.65%)
2.50%
6
4
2
0
−2
−4
−10
−5
0
5
10

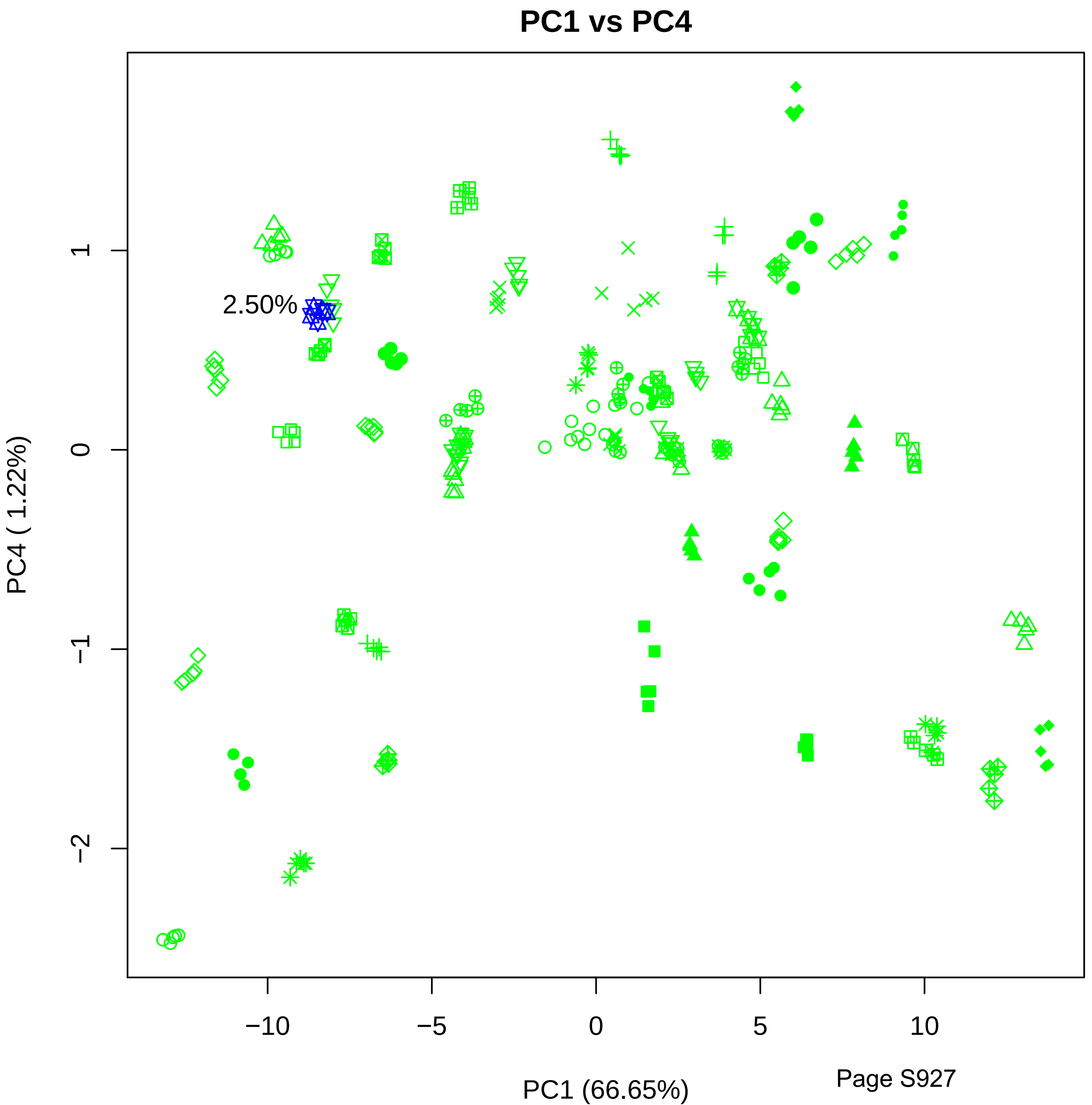

PC1 vs PC4
2.50%
PC4 (1.22%)
PC1 (66.65%)
1
0
−1
−2
−10
−5
0
5
10

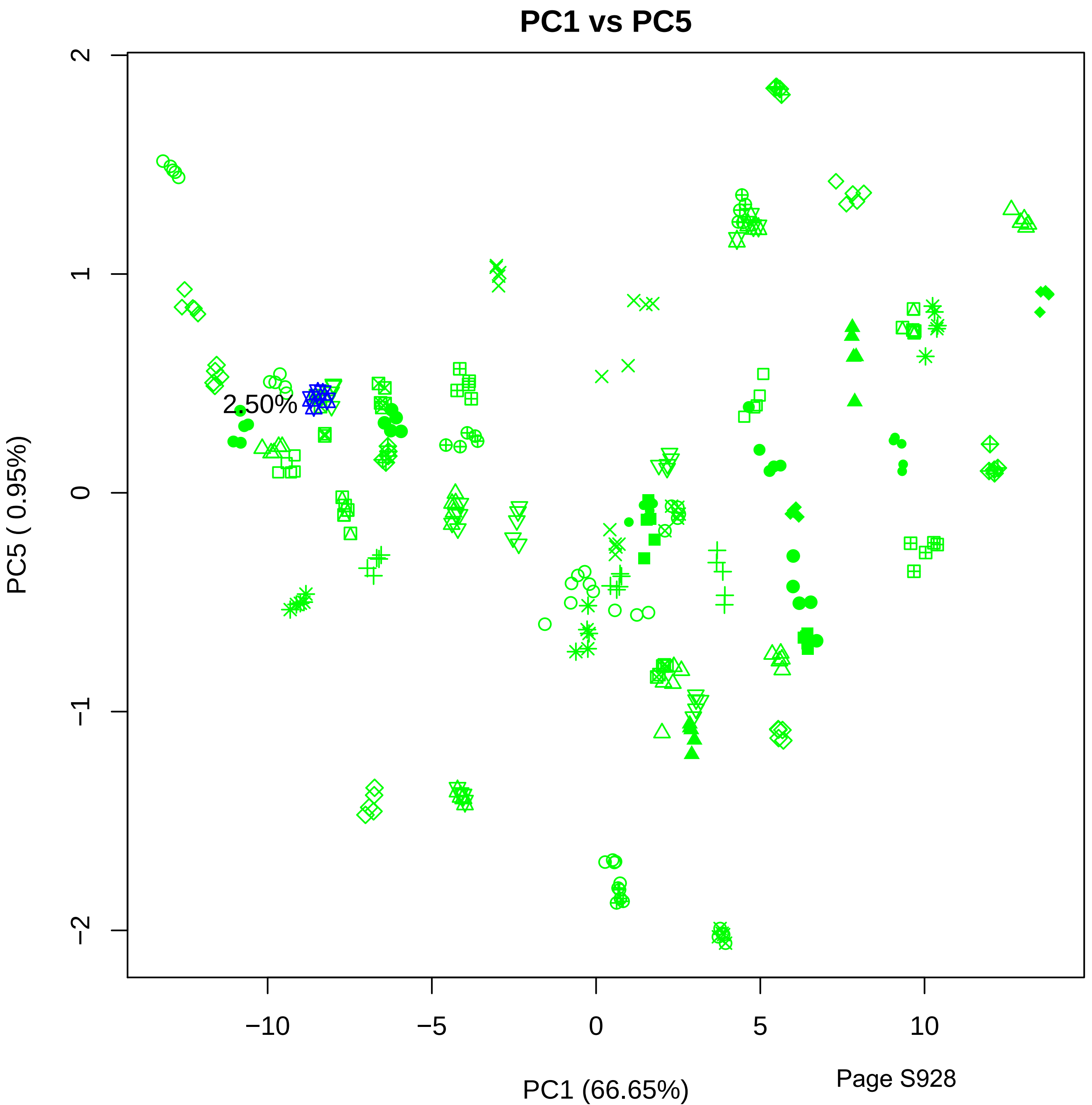
PC1 vs PC5
2
1
0
−1
−2
PC5 (0.95%)
−10
−5
0
5
10
PC1 (66.65%)
2.50%

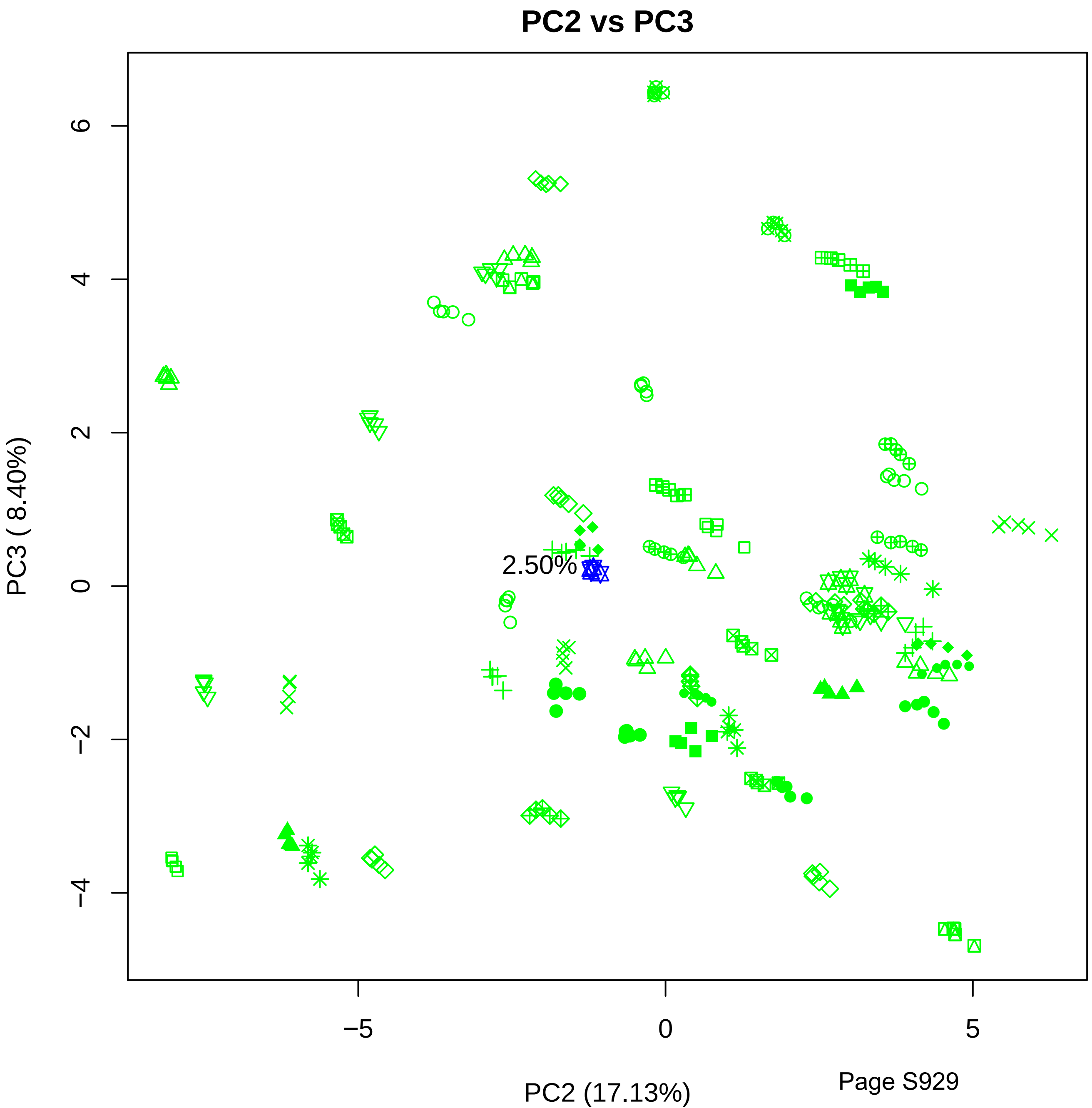
PC2 vs PC3
PC3 (8.40%)
PC2 (17.13%)
2.50%
6
4
2
0
−2
−4
−5
0
5

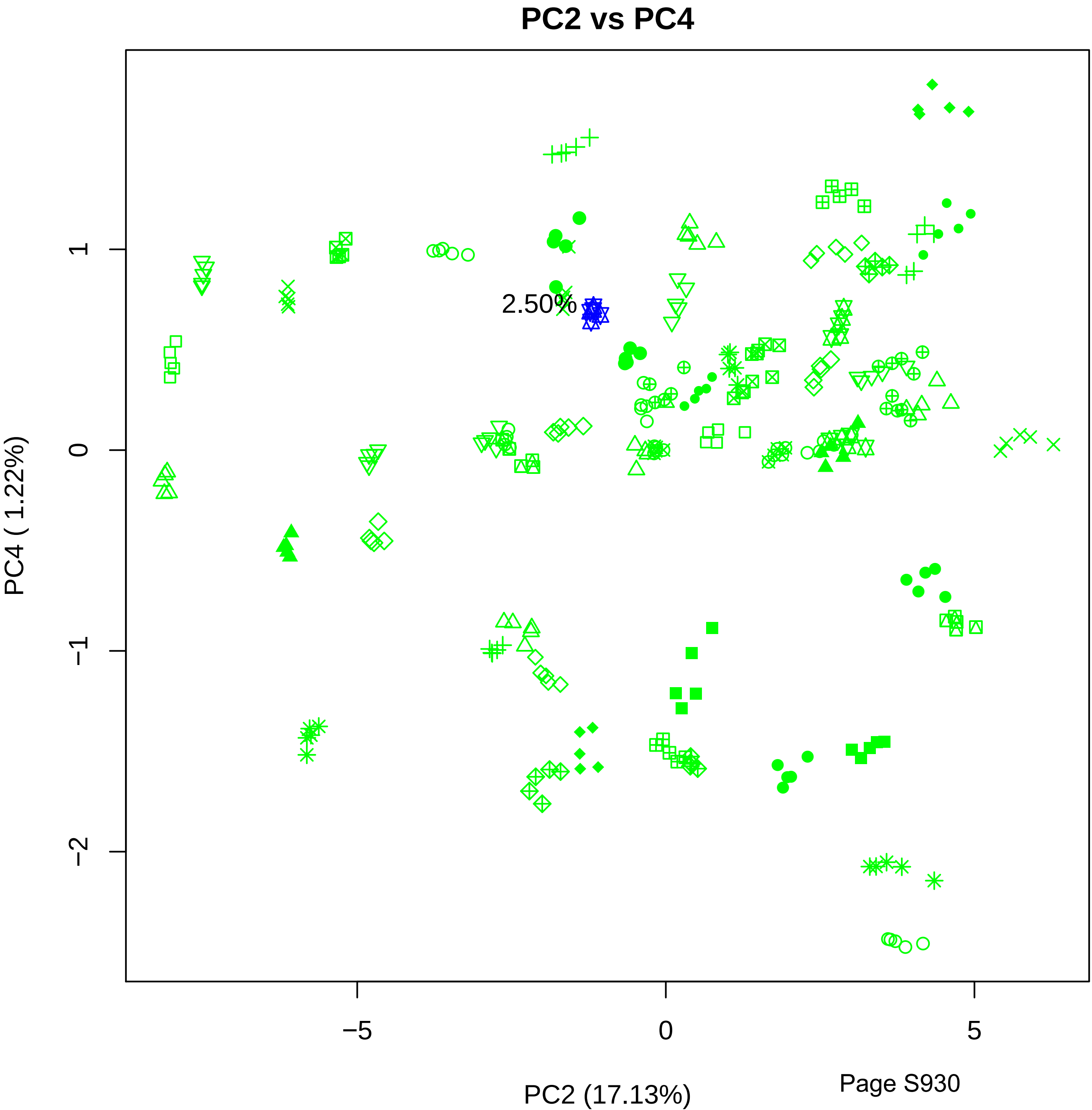
PC2 vs PC4
2.50%
PC4 (1.22%)
PC2 (17.13%)
1
0
−1
−2
−5
0
5

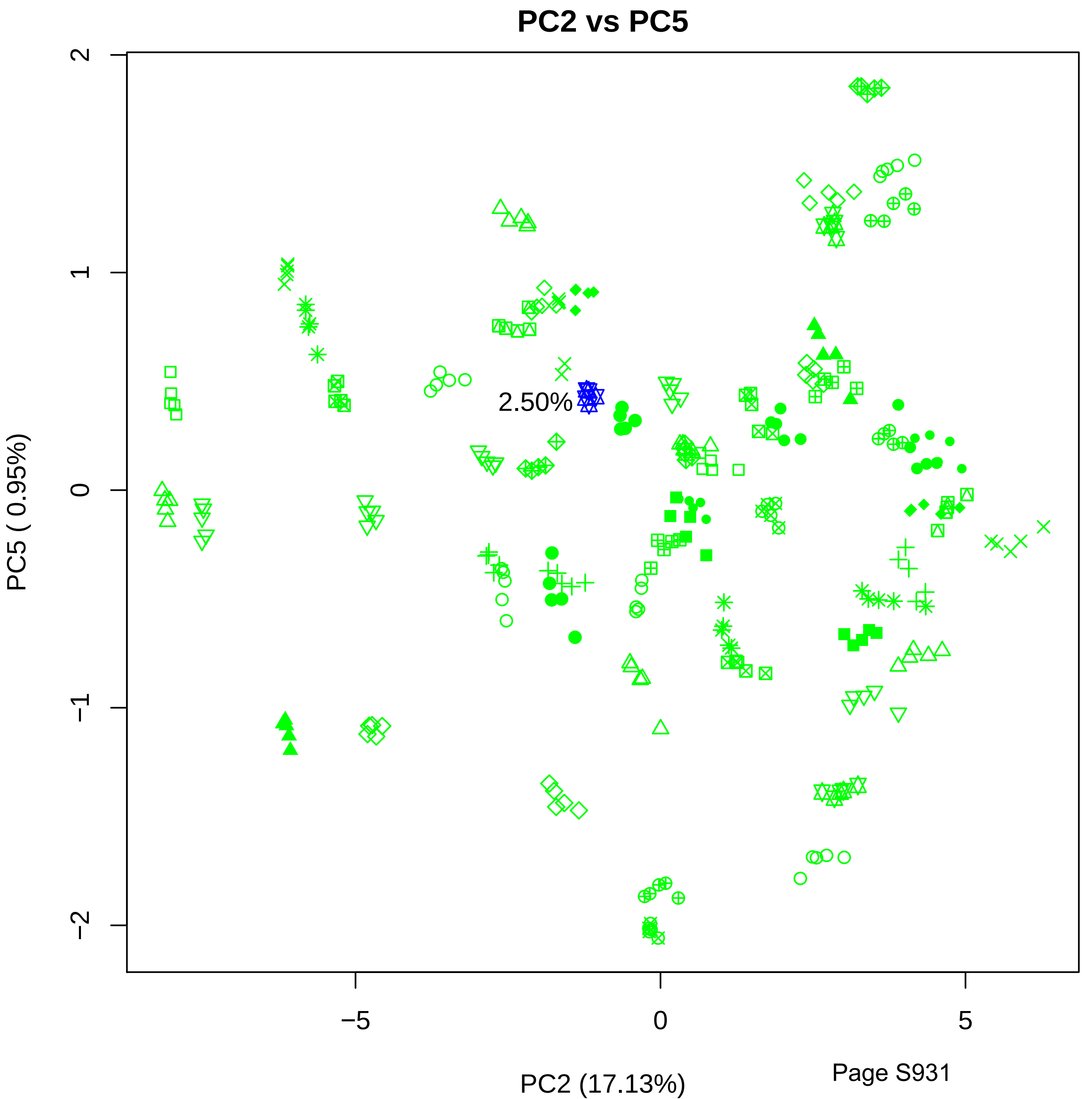
PC2 vs PC5
2
1
0
−1
−2
PC5 (0.95%)
2.50%
−5
0
5
PC2 (17.13%)

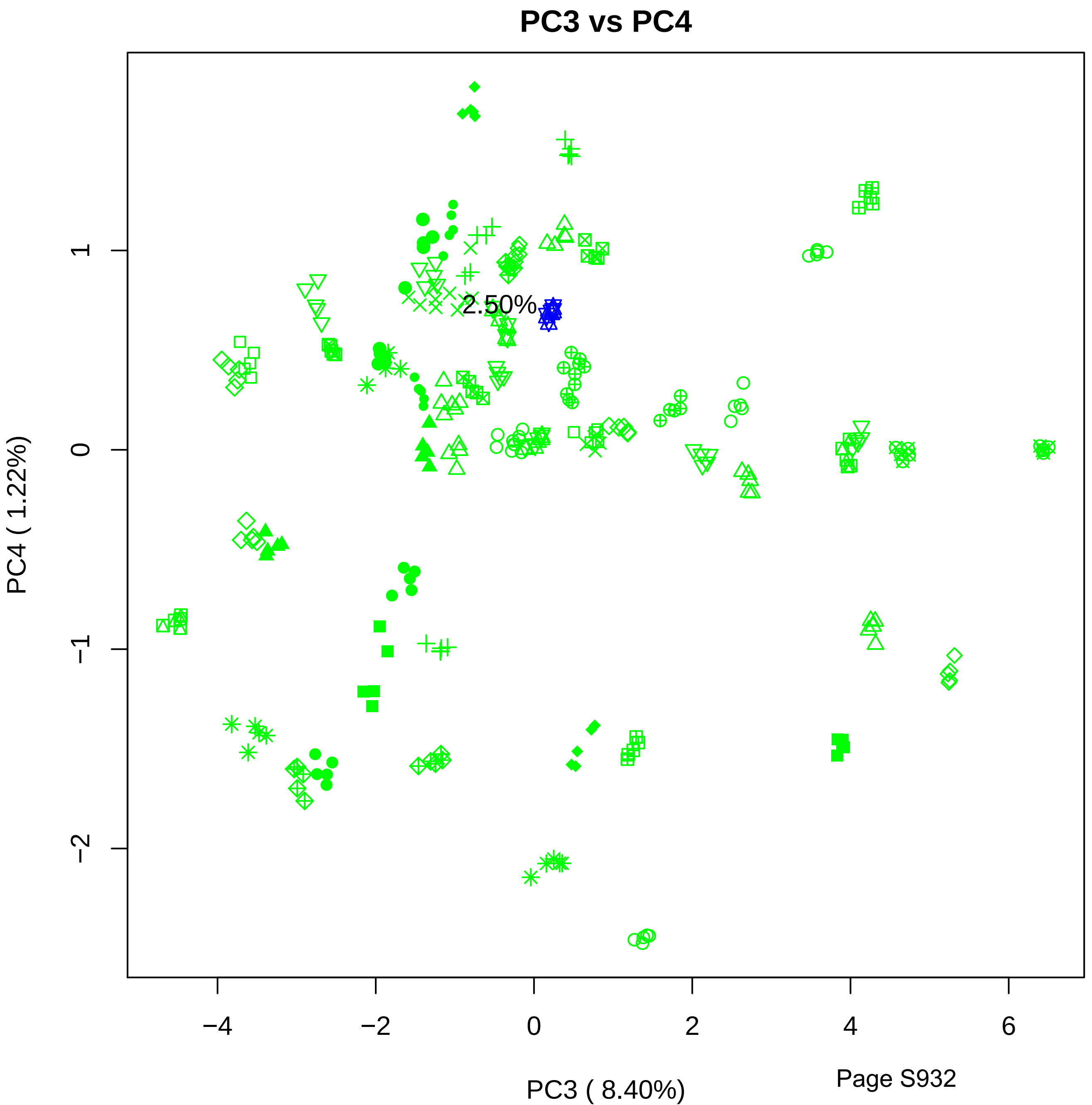
PC3 vs PC4
2.50%
PC4 (1.22%)
PC3 (8.40%)
−4
−2
0
2
4
6
−2
−1
0
1

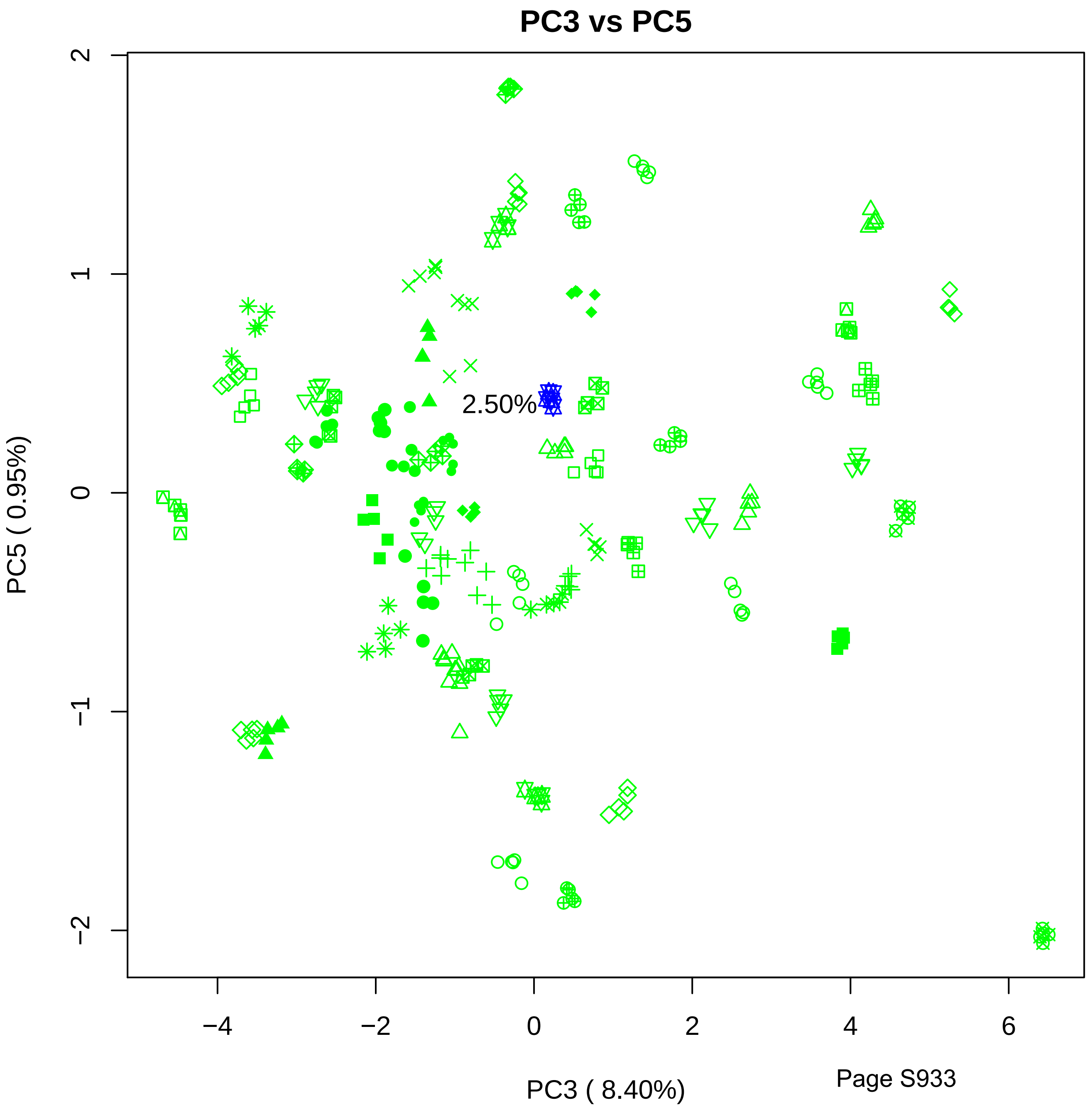

PC3 vs PC5
2.50%
PC5 (0.95%)
PC3 (8.40%)
2
1
0
−1
−2
−4
−2
0
2
4
6

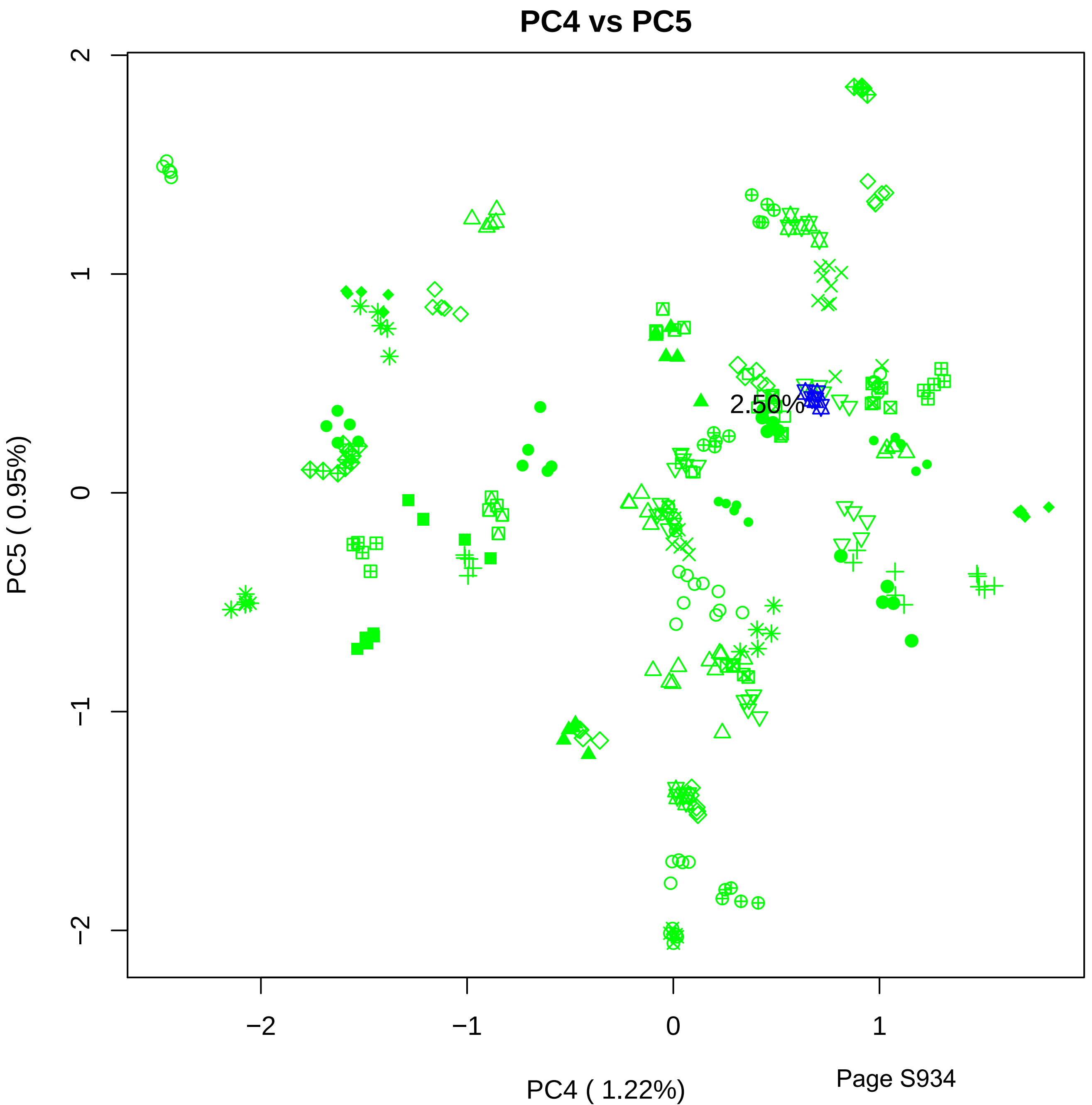
PC4 vs PC5
PC5 (0.95%)
PC4 (1.22%)
2.50%
2
1
0
−1
−2
−2
−1
0
1

PCA of 5% BMH (a) vs PMH (a)

Scaled Matrix PCAed

7th order polynomial baseline correction performed

Raw spectra smoothed to 21 Neighbours, 2nd order PN, Savtisky Golay

Normalisation between 0 and 100

D2 spectra smoothed to 41 Neighbours, 2nd order PN, Savtisky Golay

Regions Analysed: 3601−2501 and 2001−700cm−1

Samples Analysed: 1 bContam's 0 BLH's 0 BMH's 0 OMH's 0 pContam's 0 PLH's 60 PMH's

Keele University AD

Fri Dec 14 14:20:11 2018

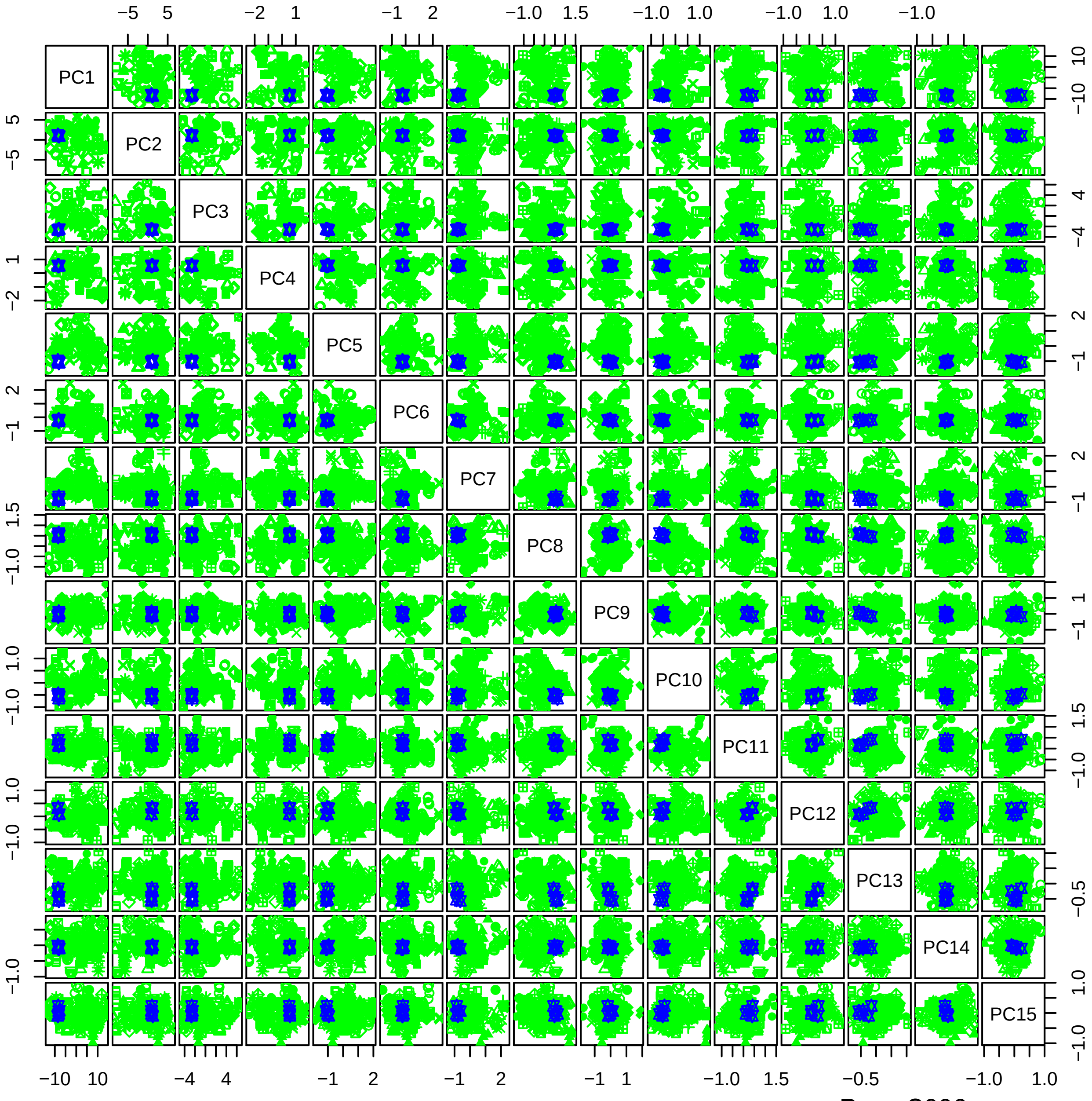

PC1
PC2
PC3
PC4
PC5
PC6
PC7
PC8
PC9
PC10
PC11
PC12
PC13
PC14
PC15

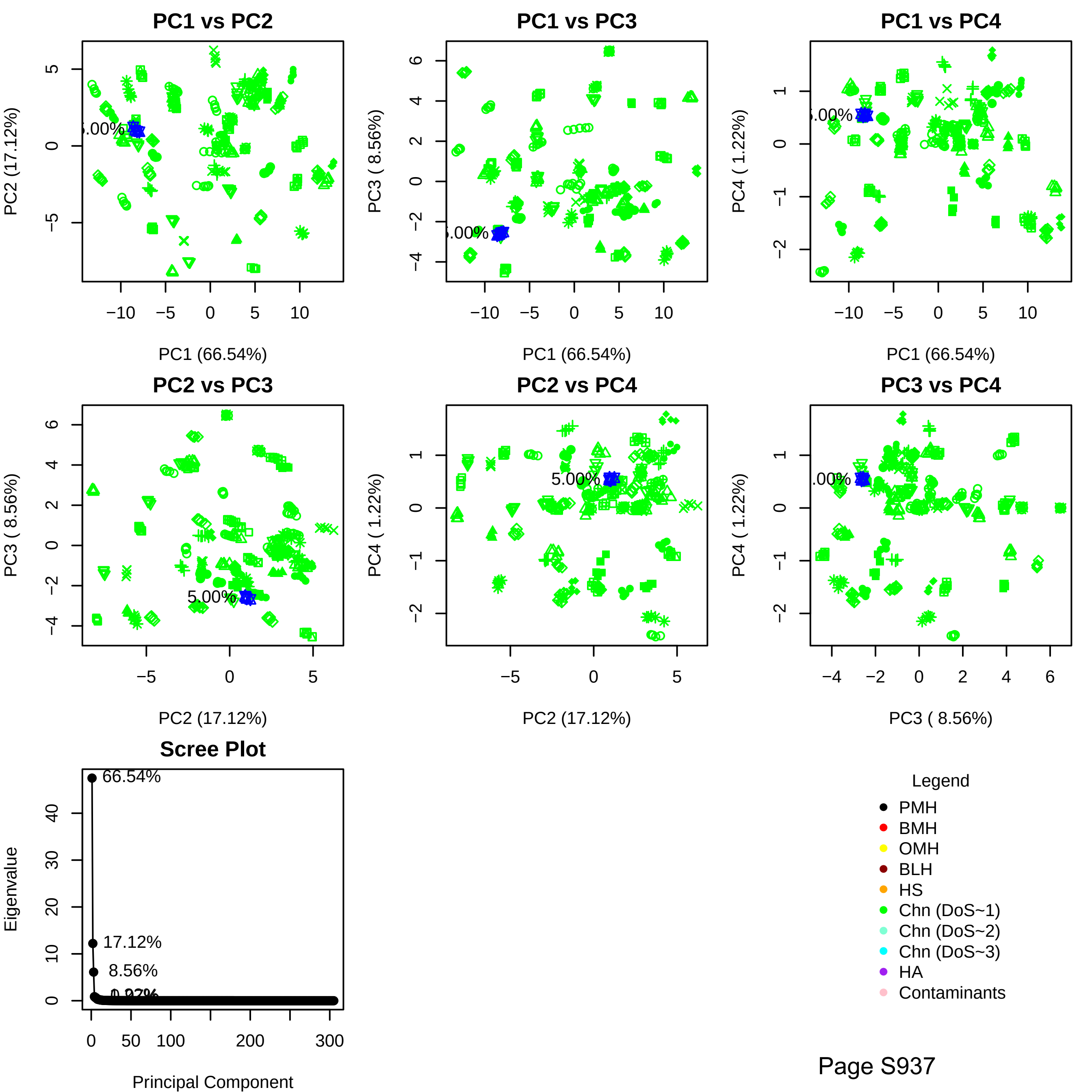
PC1 vs PC2
PC1 vs PC3
PC1 vs PC4
PC2 vs PC3
PC2 vs PC4
PC3 vs PC4
Scree Plot
PC1 (66.54%)
PC2 (17.12%)
PC3 (8.56%)
PC4 (1.22%)
5.00%
Eigenvalue
Principal Component
66.54%
17.12%
8.56%
Legend
PMH
BMH
OMH
BLH
HS
Chn (DoS~1)
Chn (DoS~2)
Chn (DoS~3)
HA
Contaminants

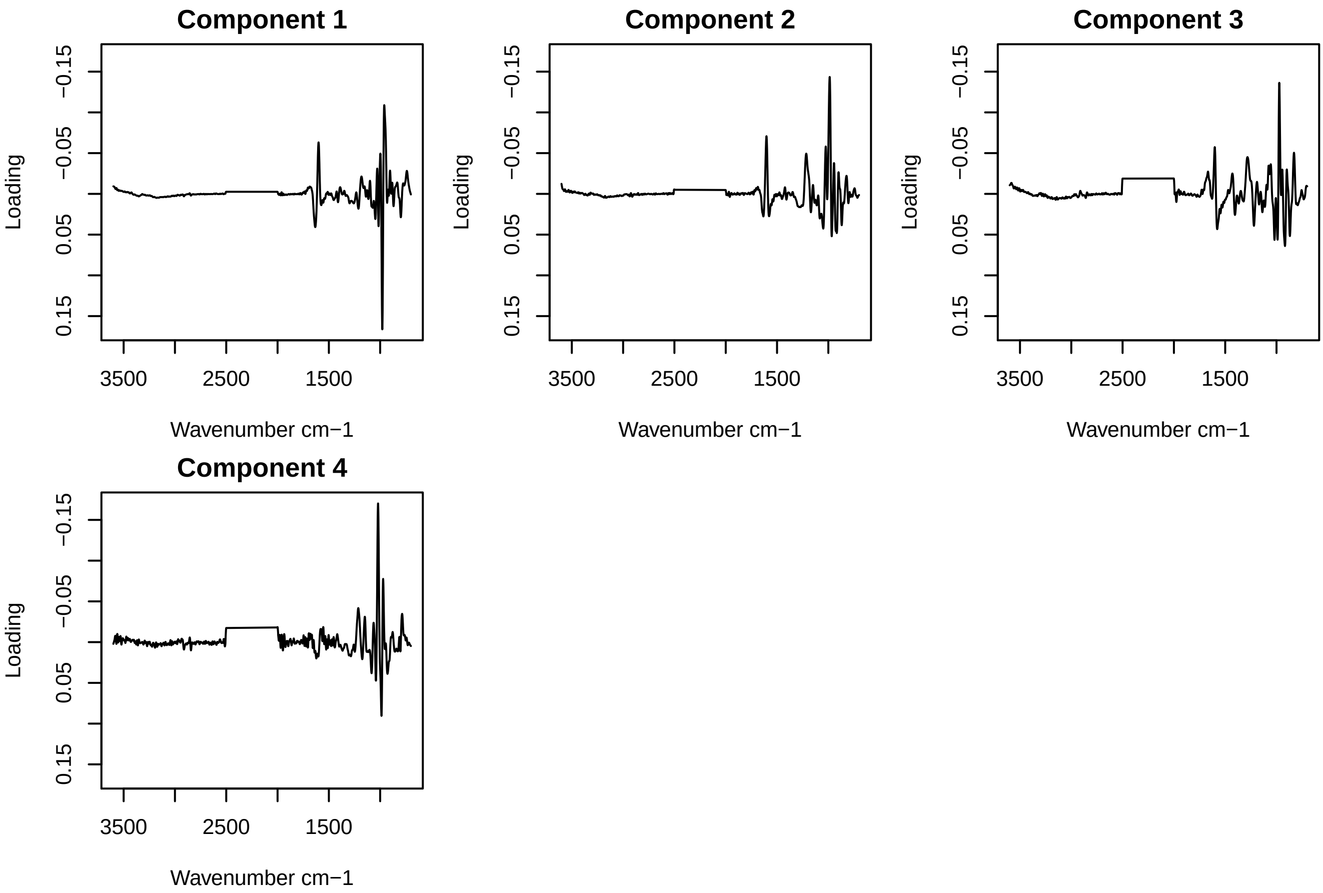
Component 1
Component 2
Component 3
Component 4
Loading
−0.15
−0.05
0.05
0.15
3500
2500
1500
Wavenumber cm−1

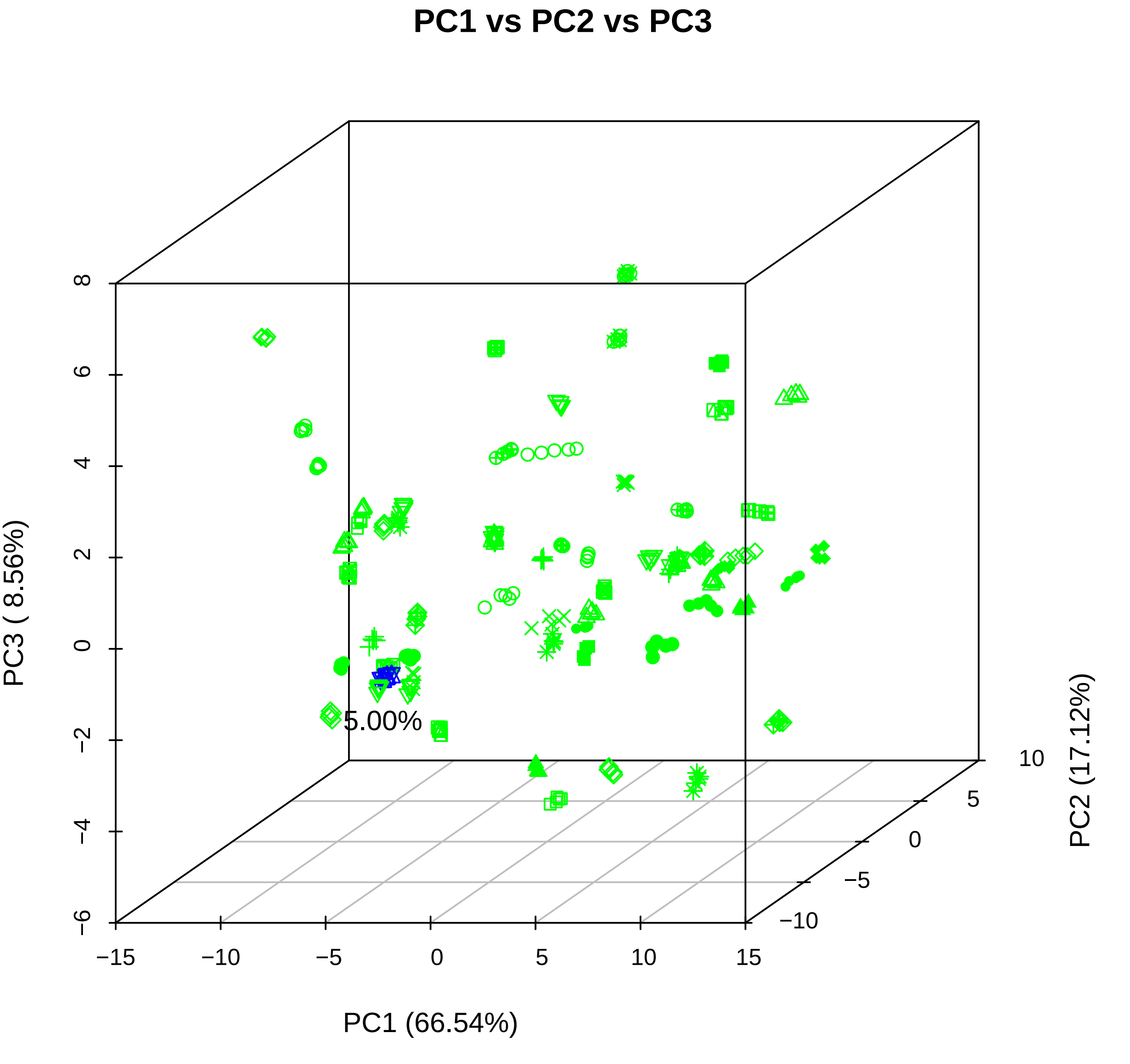
PC1 vs PC2 vs PC3
PC1 (66.54%)
PC2 (17.12%)
PC3 (8.56%)
5.00%

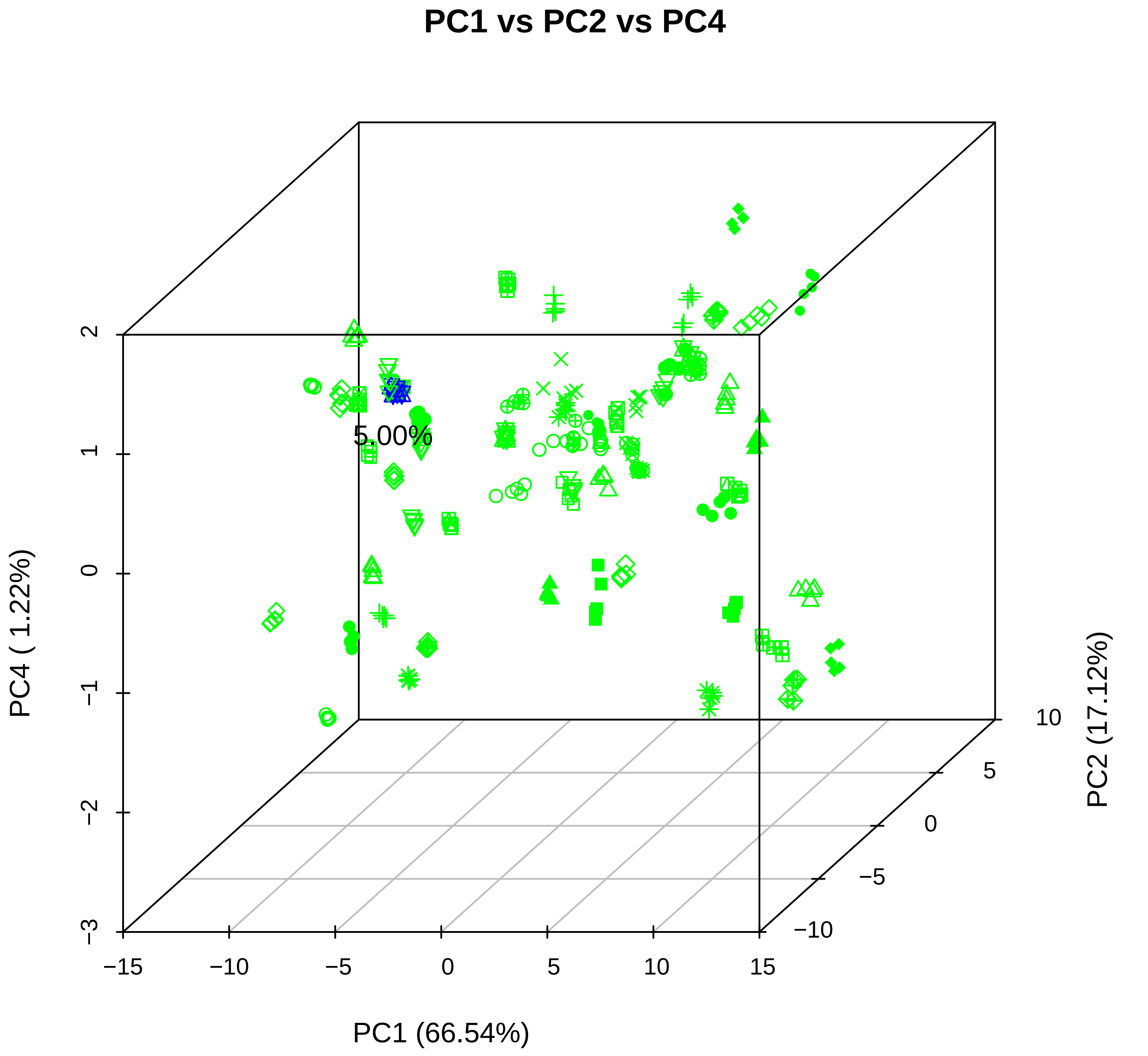
PC1 vs PC2 vs PC4
5.00%
PC4 (1.22%)
PC2 (17.12%)
PC1 (66.54%)
−15
−10
−5
0
5
10
15
−3
−2
−1
0
1
2
−10
−5
0
5
10

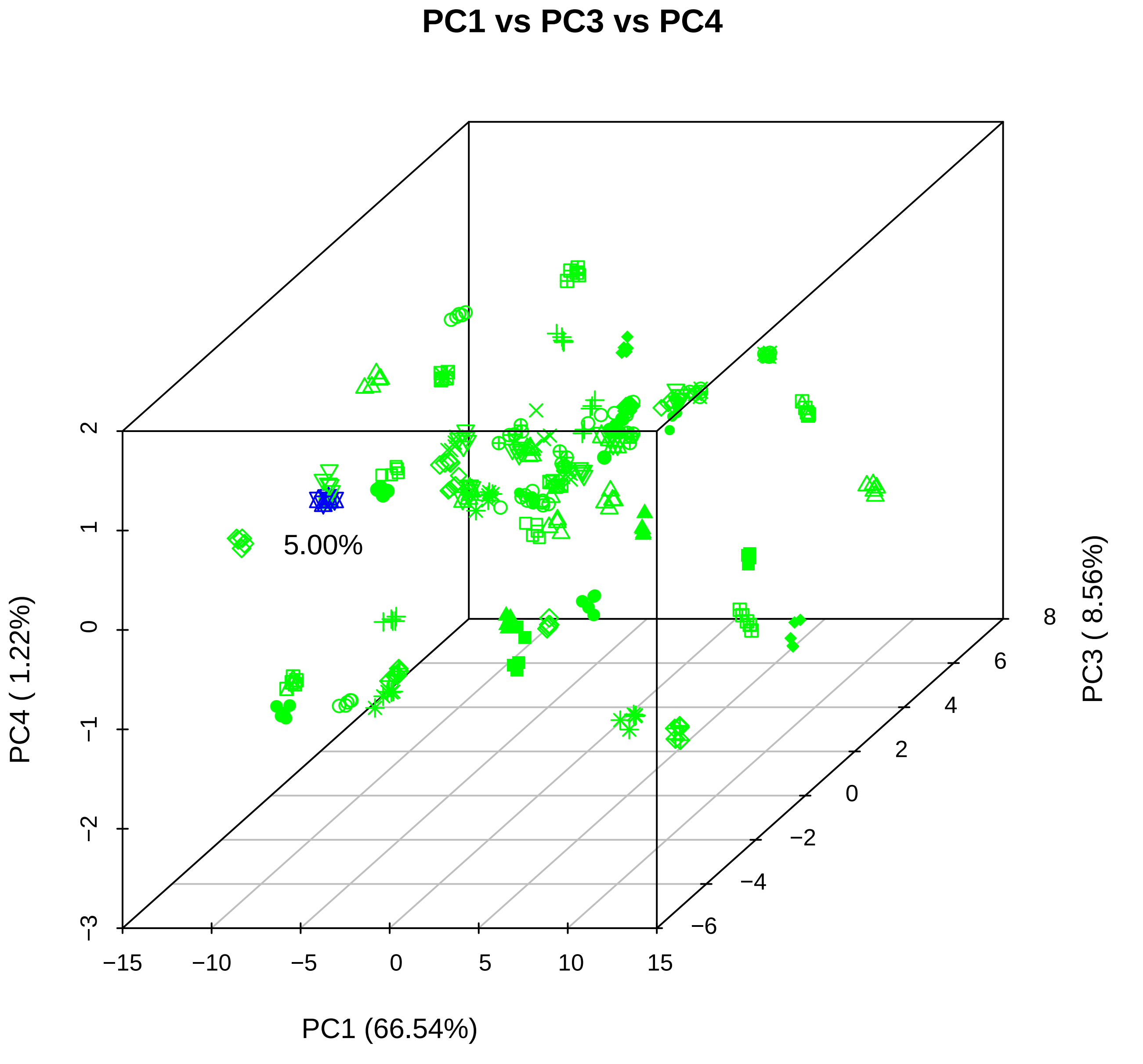
PC1 vs PC3 vs PC4
5.00%
PC1 (66.54%)
PC4 (1.22%)
PC3 (8.56%)
−15
−10
−5
0
5
10
15
−3
−2
−1
0
1
2
−6
−4
−2
0
2
4
6
8

PC2 vs PC3 vs PC5

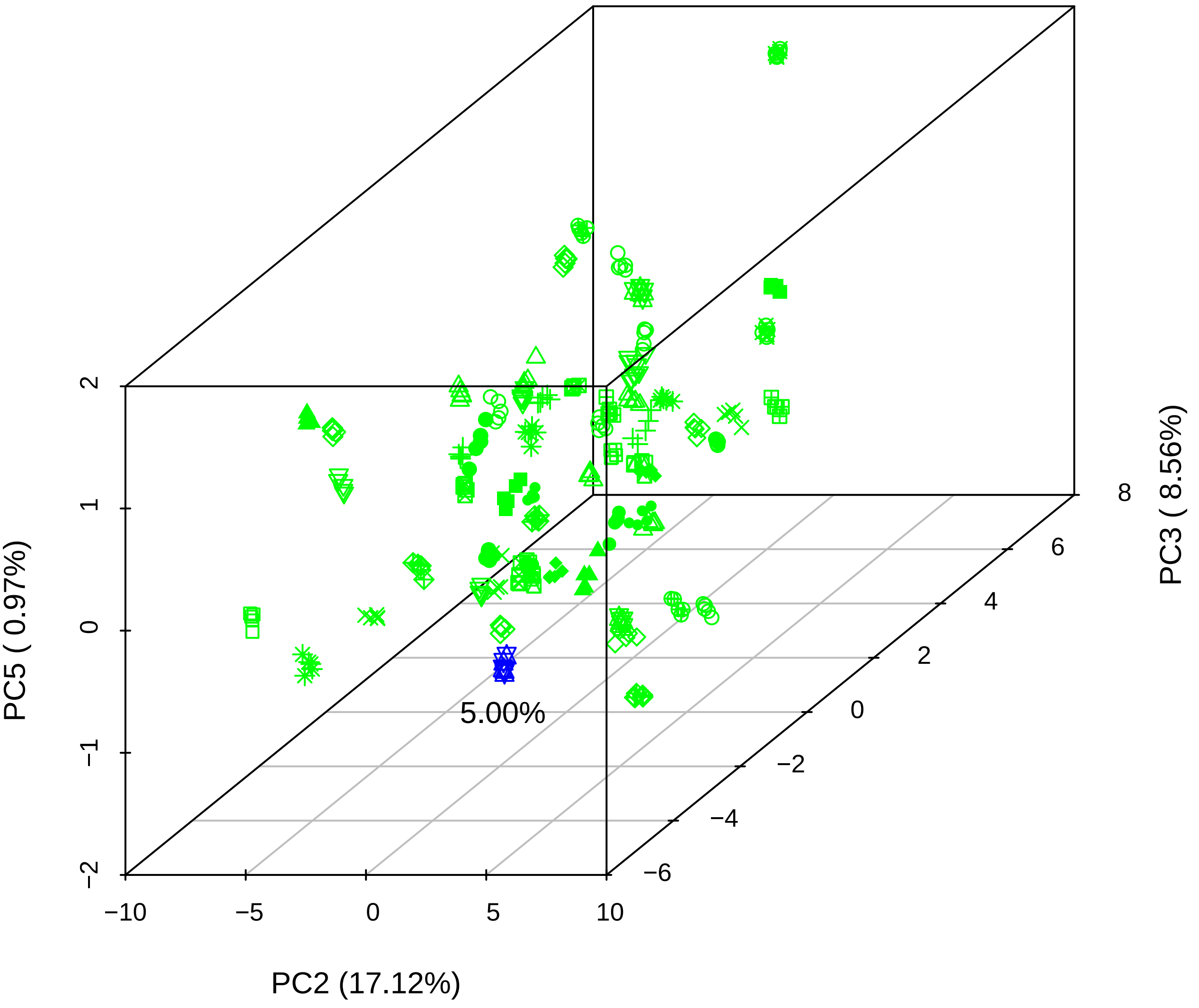

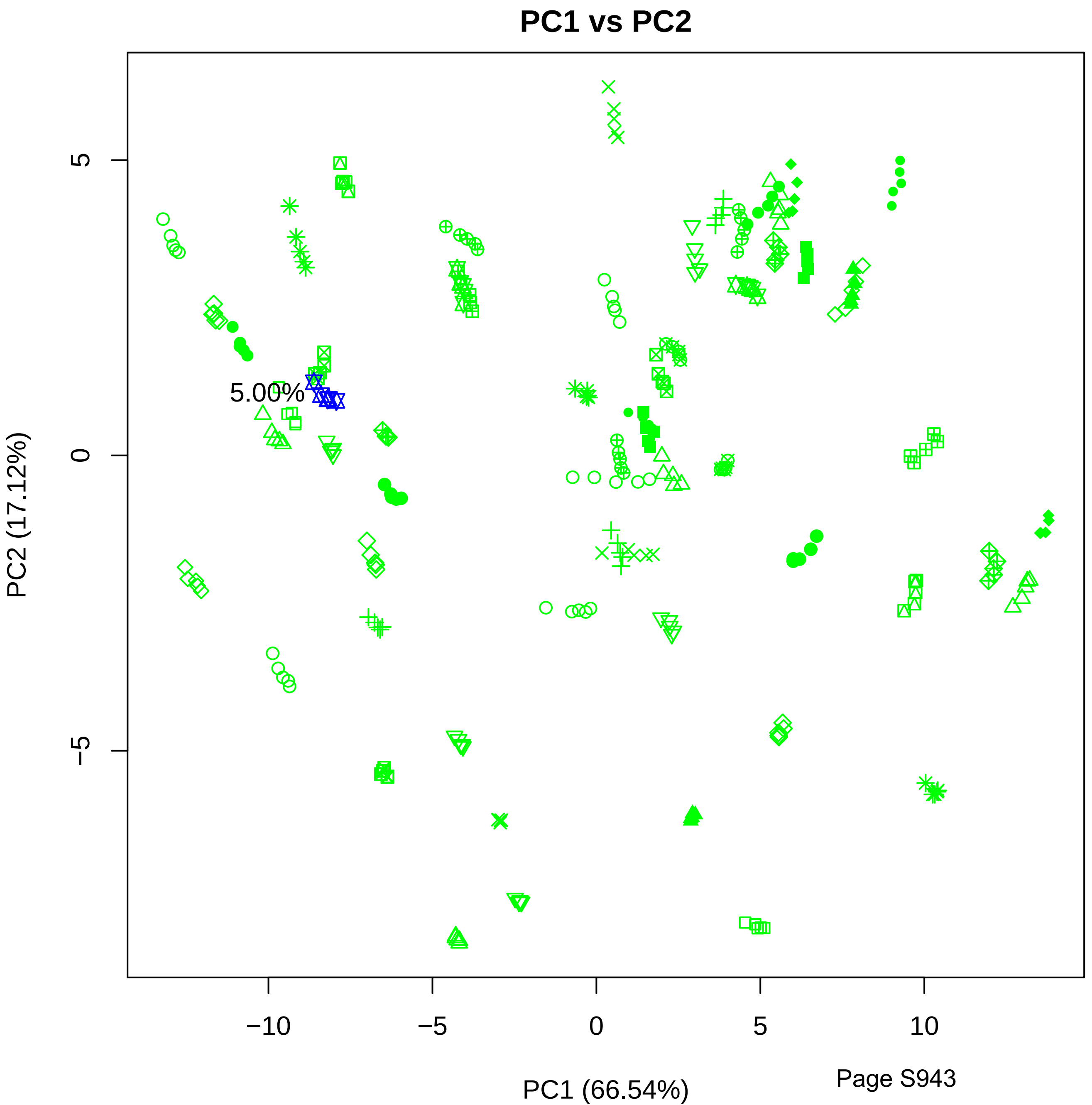
PC1 vs PC2
5.00%
5
0
−5
−10
−5
0
5
10
PC1 (66.54%)
PC2 (17.12%)

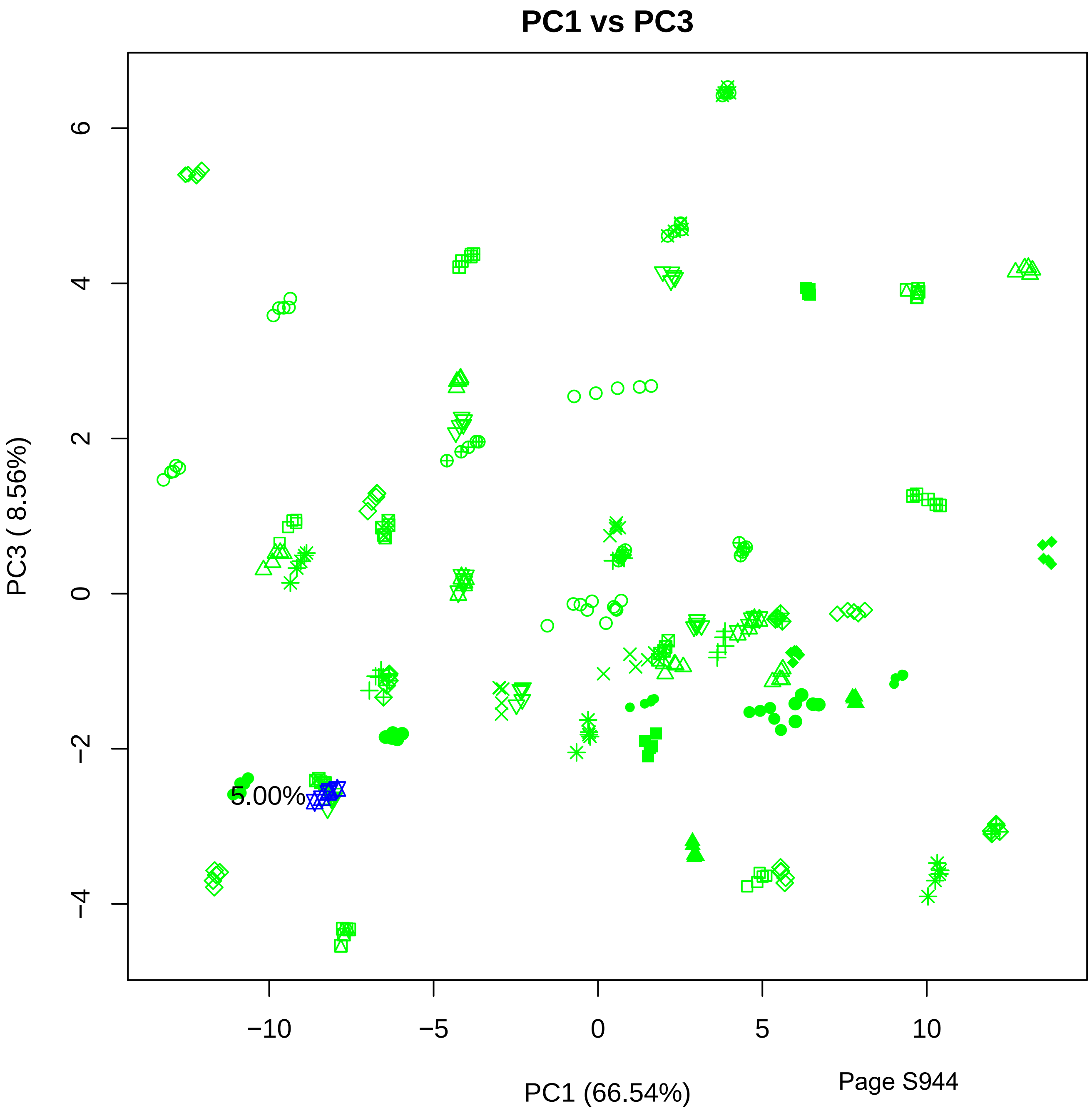
PC1 vs PC3
PC3 (8.56%)
6
4
2
0
−2
−4
5.00%
−10
−5
0
5
10
PC1 (66.54%)

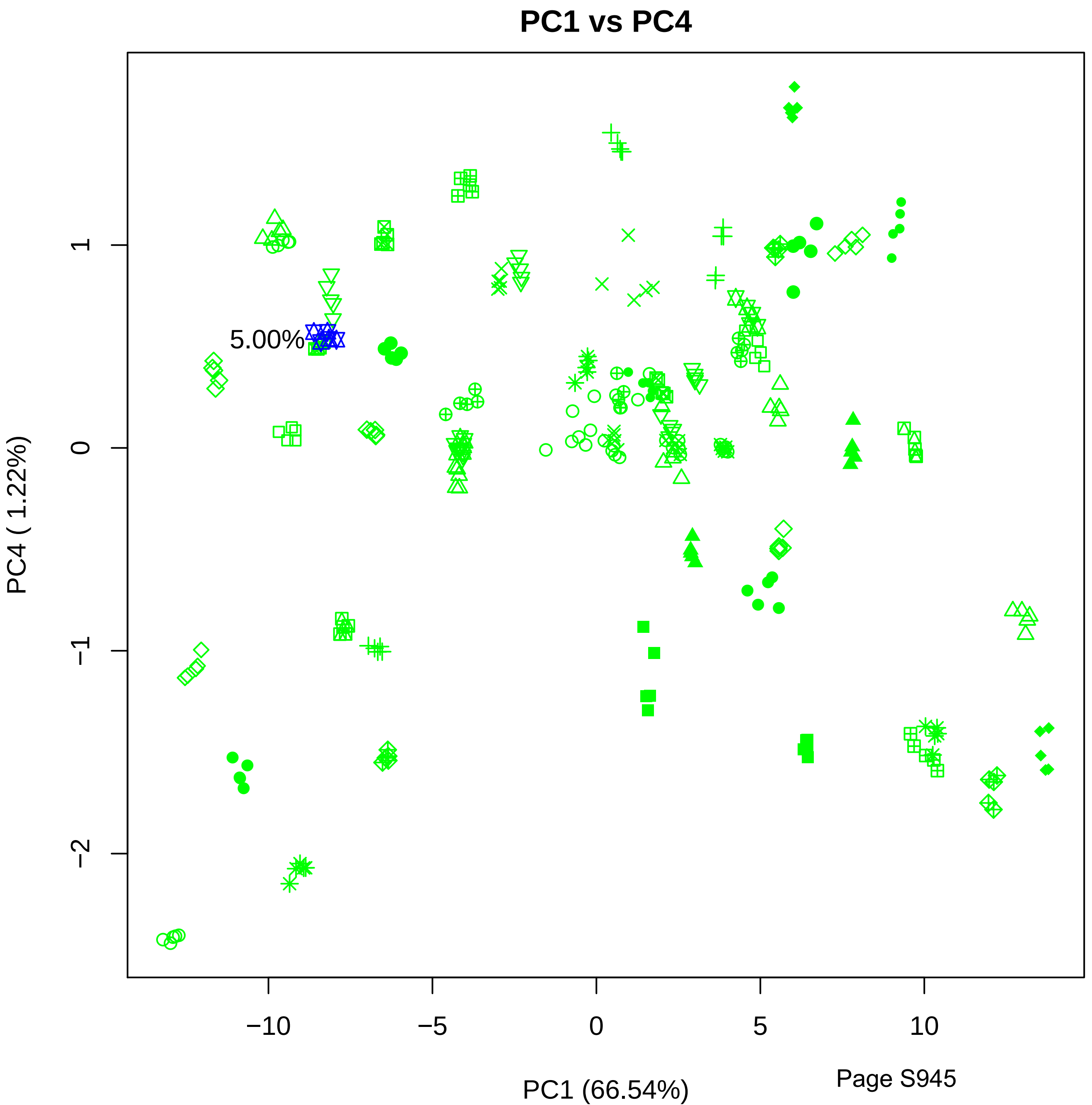

PC1 vs PC4
5.00%
PC4 (1.22%)
PC1 (66.54%)
1
0
−1
−2
−10
−5
0
5
10

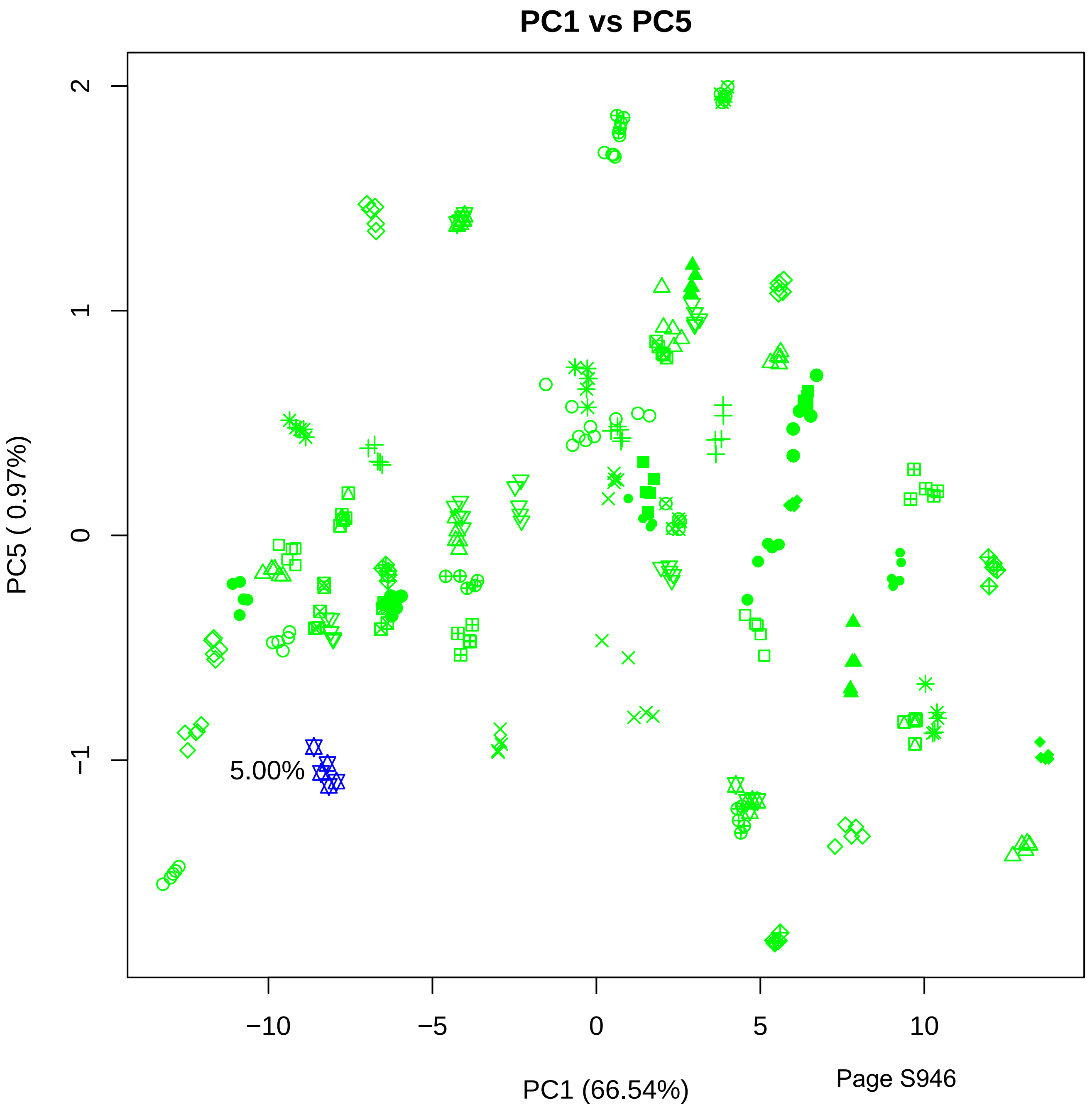

PC1 vs PC5
PC5 (0.97%)
PC1 (66.54%)
2
1
0
−1
−10
−5
0
5
10
5.00%

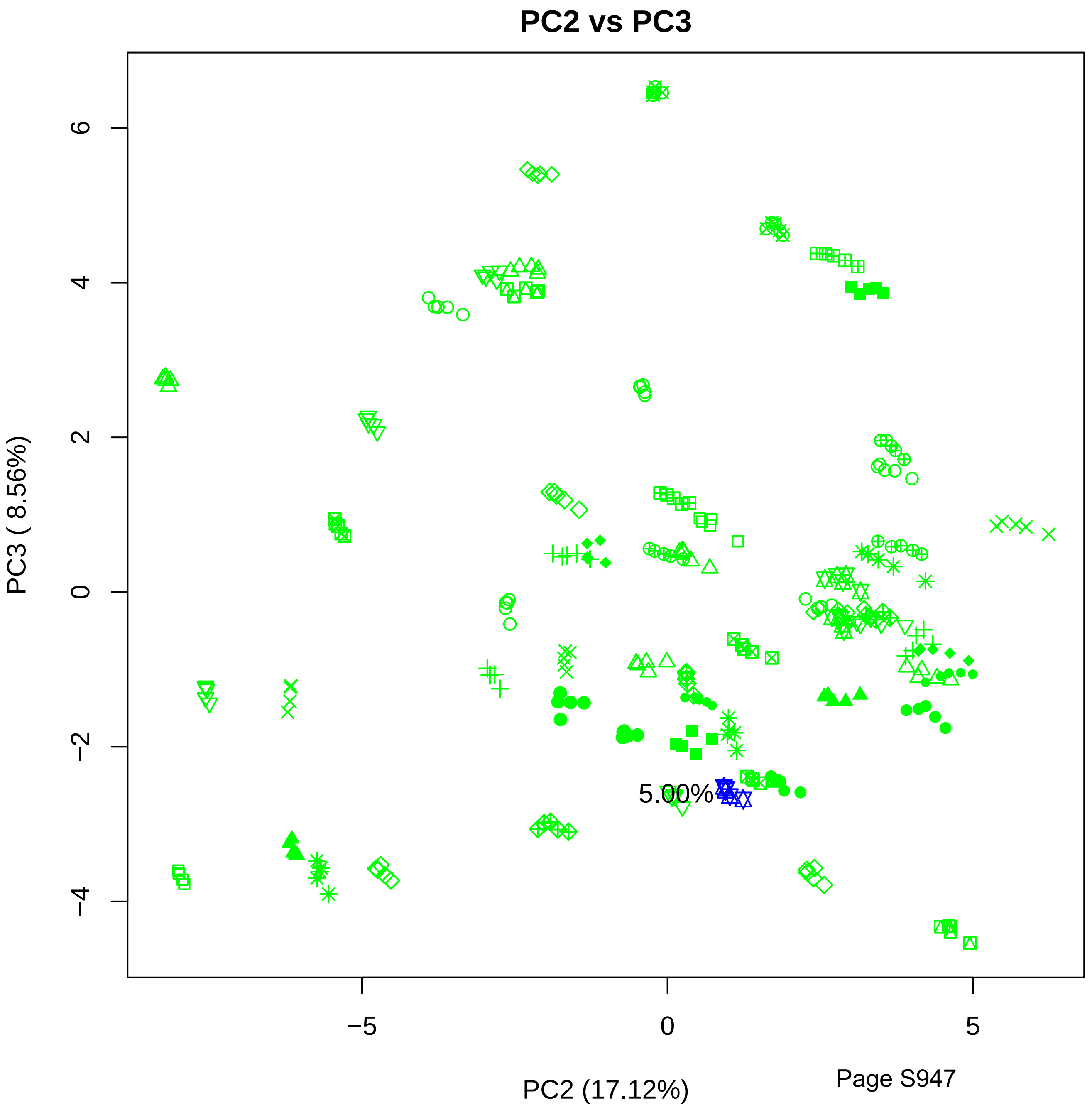
PC2 vs PC3
PC3 (8.56%)
PC2 (17.12%)
−5
0
5
−4
−2
0
2
4
6
5.00%

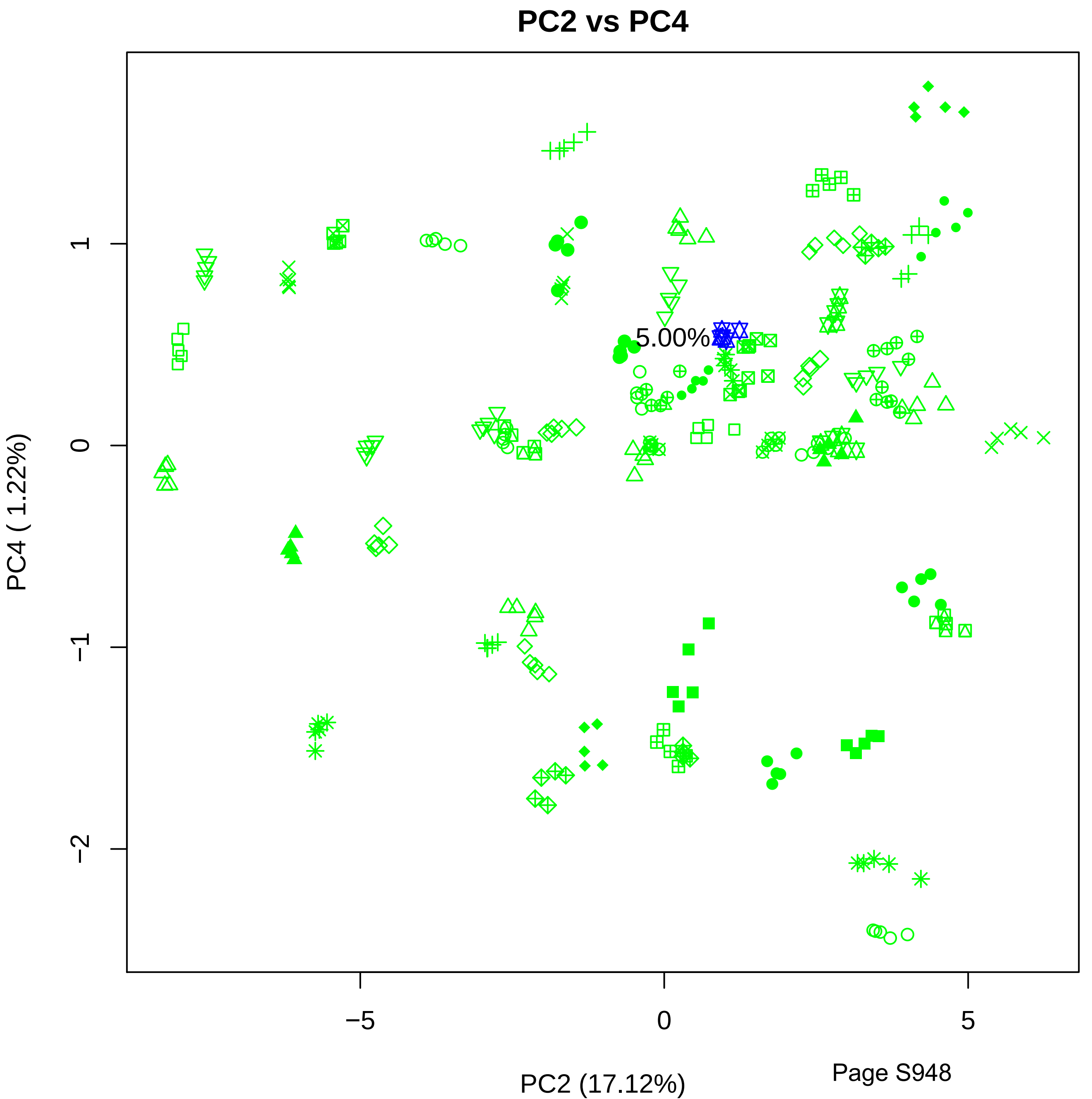

PC2 vs PC4
5.00%
1
0
−1
−2
PC4 (1.22%)
−5
0
5
PC2 (17.12%)

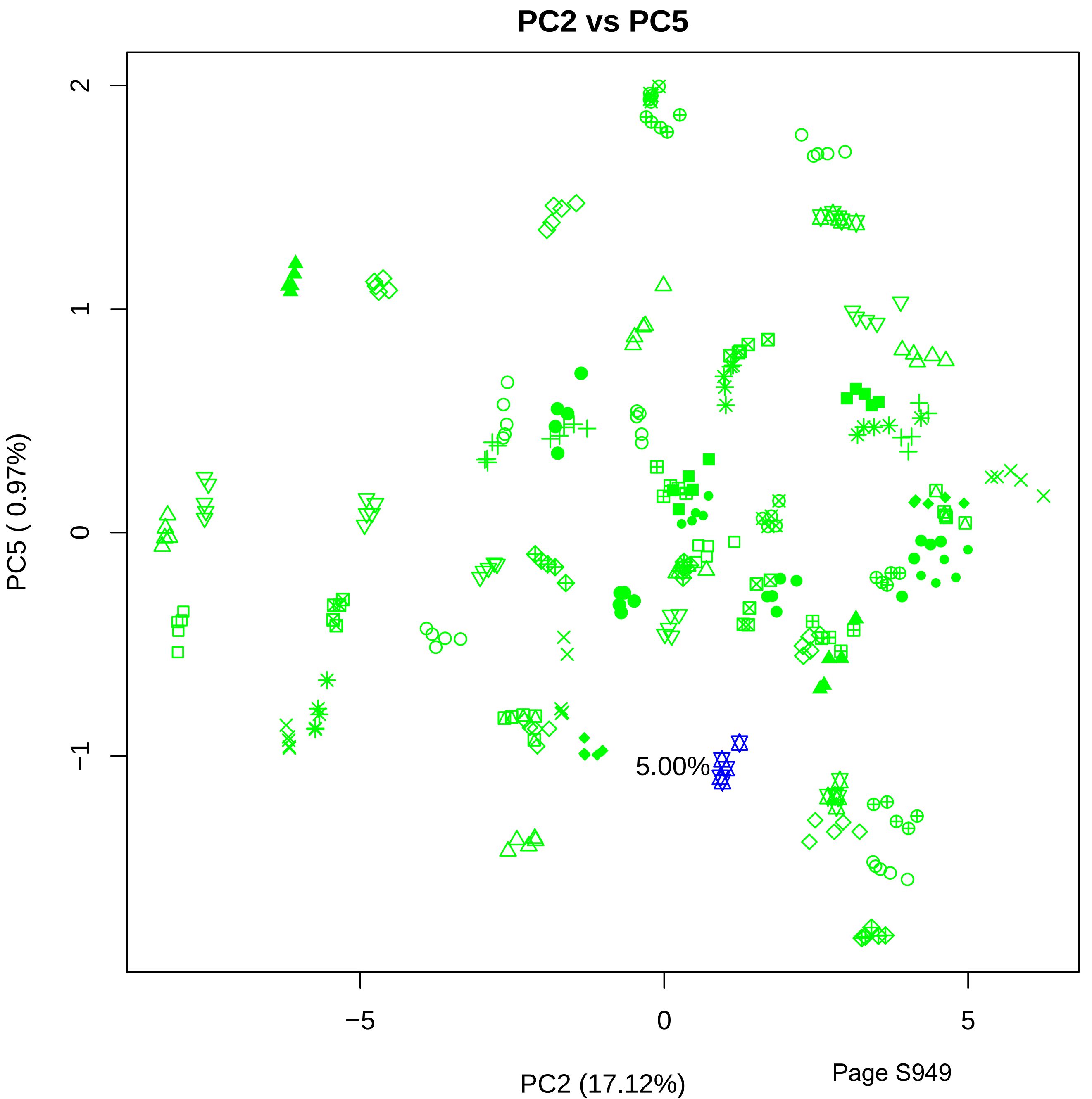
PC2 vs PC5
2
1
0
−1
PC5 (0.97%)
−5
0
5
PC2 (17.12%)
5.00%

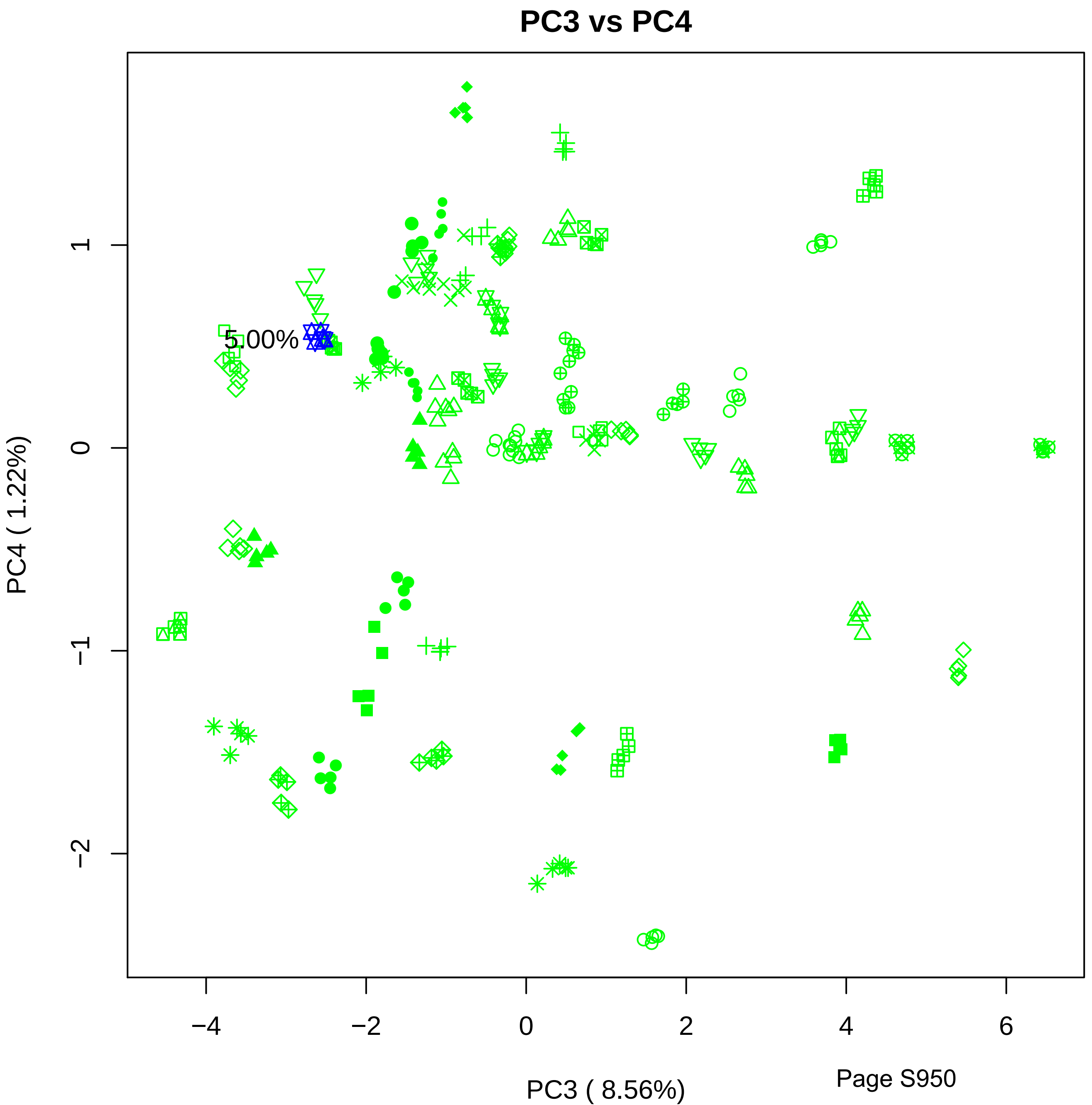

PC3 vs PC4
5.00%
PC4 (1.22%)
PC3 (8.56%)
−2
−1
0
1
−4
−2
0
2
4
6

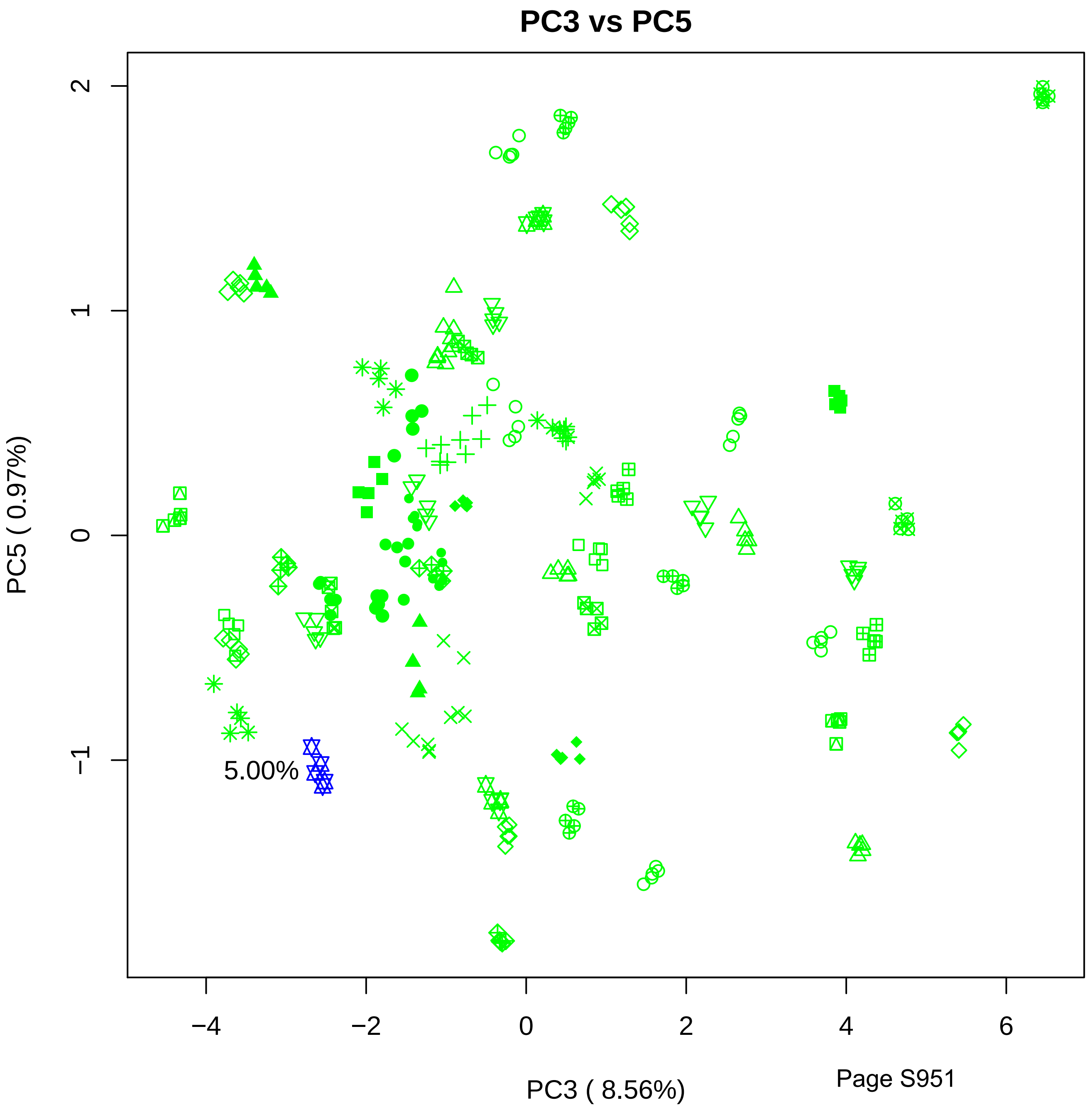

PC3 vs PC5
2
1
0
−1
PC5 (0.97%)
5.00%
−4
−2
0
2
4
6
PC3 (8.56%)

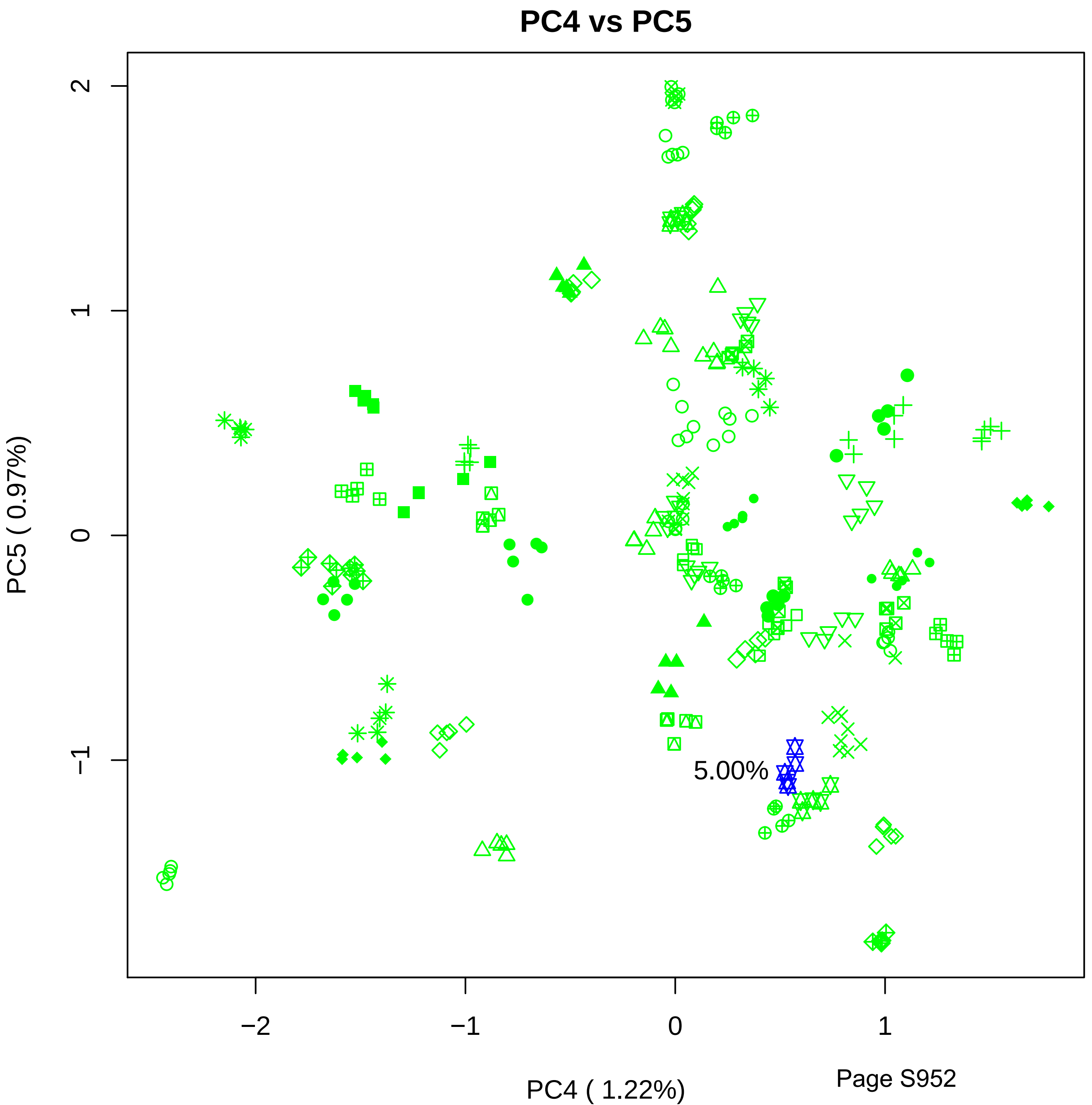
PC4 vs PC5
2
1
0
−1
PC5 (0.97%)
5.00%
−2
−1
0
1
PC4 (1.22%)

PCA of 7.5% BMH(a) vs PMH(a)

Scaled Matrix PCAed

7th order polynomial baseline correction performed

Raw spectra smoothed to 21 Neighbours, 2nd order PN, Savtisky Golay

Normalisation between 0 and 100

D2 spectra smoothed to 41 Neighbours, 2nd order PN, Savtisky Golay

Regions Analysed: 3601−2501 and 2001−700cm−1

Samples Analysed: 1 bContam's 0 BLH's 0 BMH's 0 OMH's 0 pContam's 0 PLH's 60 PMH's

Keele University AD

Fri Dec 14 14:20:19 2018

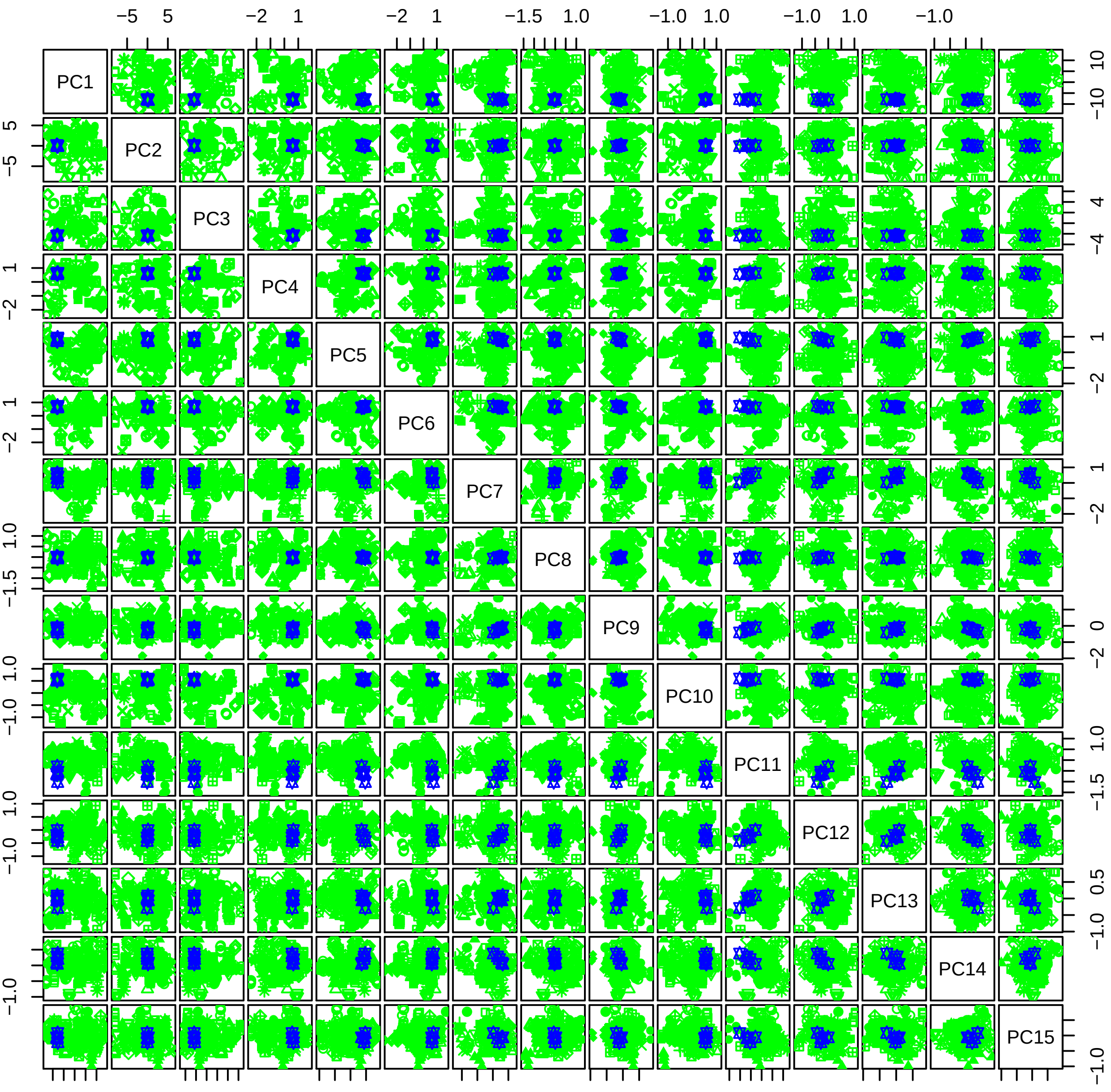
PC1
PC2
PC3
PC4
PC5
PC6
PC7
PC8
PC9
PC10
PC11
PC12
PC13
PC14
PC15
−5 5
−2 1
−2 1
−1.5 1.0
−1.0 1.0
−1.0 1.0
−1.0
−10 10
−4 4
−2 1
−2 1
−2 0
−1.5 1.0
−1.0 0.5
−1.0
10 −10
5 −5
4 −4
1 −2
1 −2
1 −2
1 −2
1.0 −1.5
0 −2
1.0 −1.0
1.0 −1.5
1.0 −1.0
0.5 −1.0
−1.0
−1.0

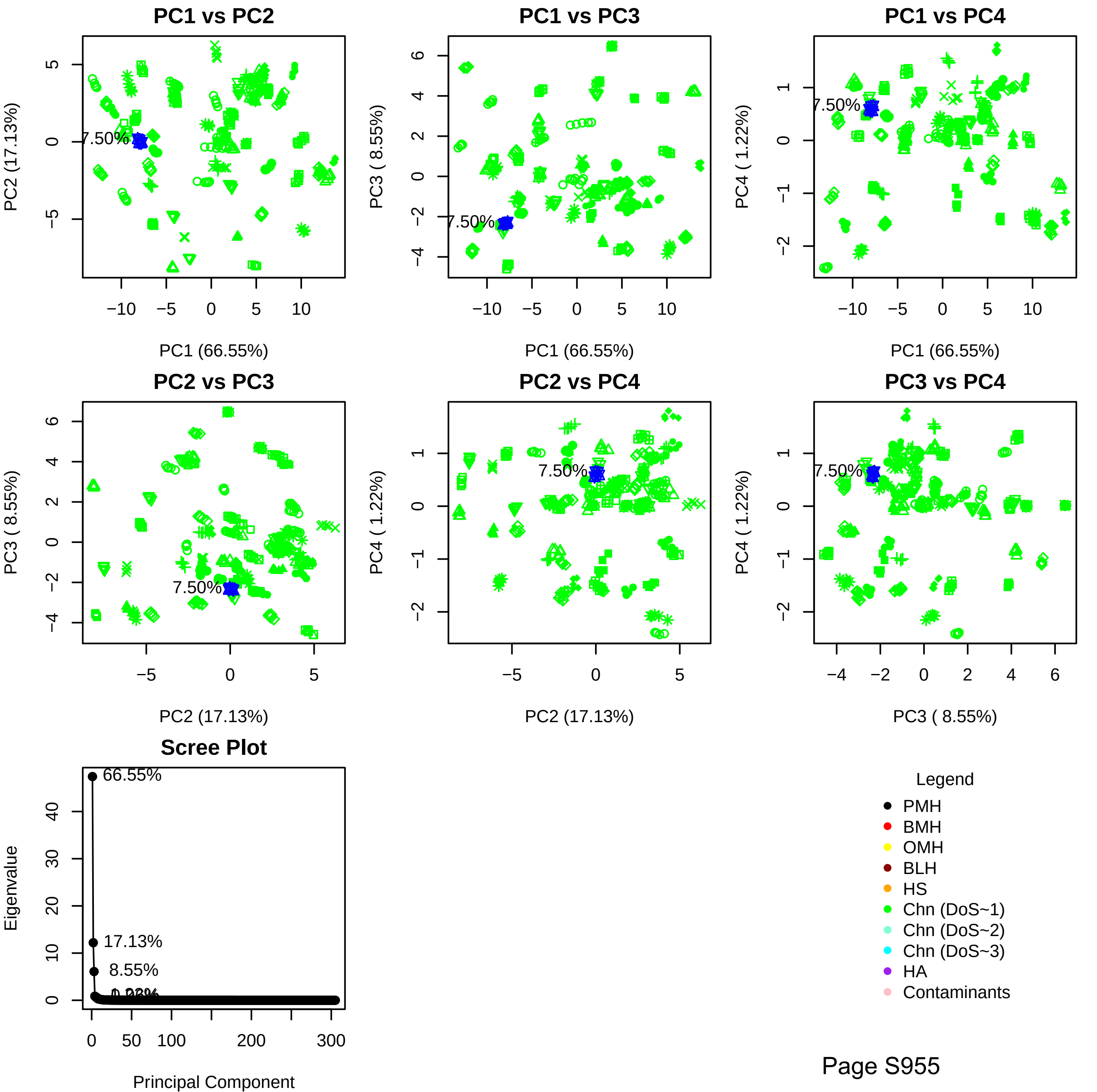
PC1 vs PC2
PC1 vs PC3
PC1 vs PC4
PC2 vs PC3
PC2 vs PC4
PC3 vs PC4
Scree Plot
PC1 (66.55%)
PC2 (17.13%)
PC3 (8.55%)
PC4 (1.22%)
7.50%
Eigenvalue
Principal Component
66.55%
17.13%
8.55%
Legend
PMH
BMH
OMH
BLH
HS
Chn (DoS~1)
Chn (DoS~2)
Chn (DoS~3)
HA
Contaminants

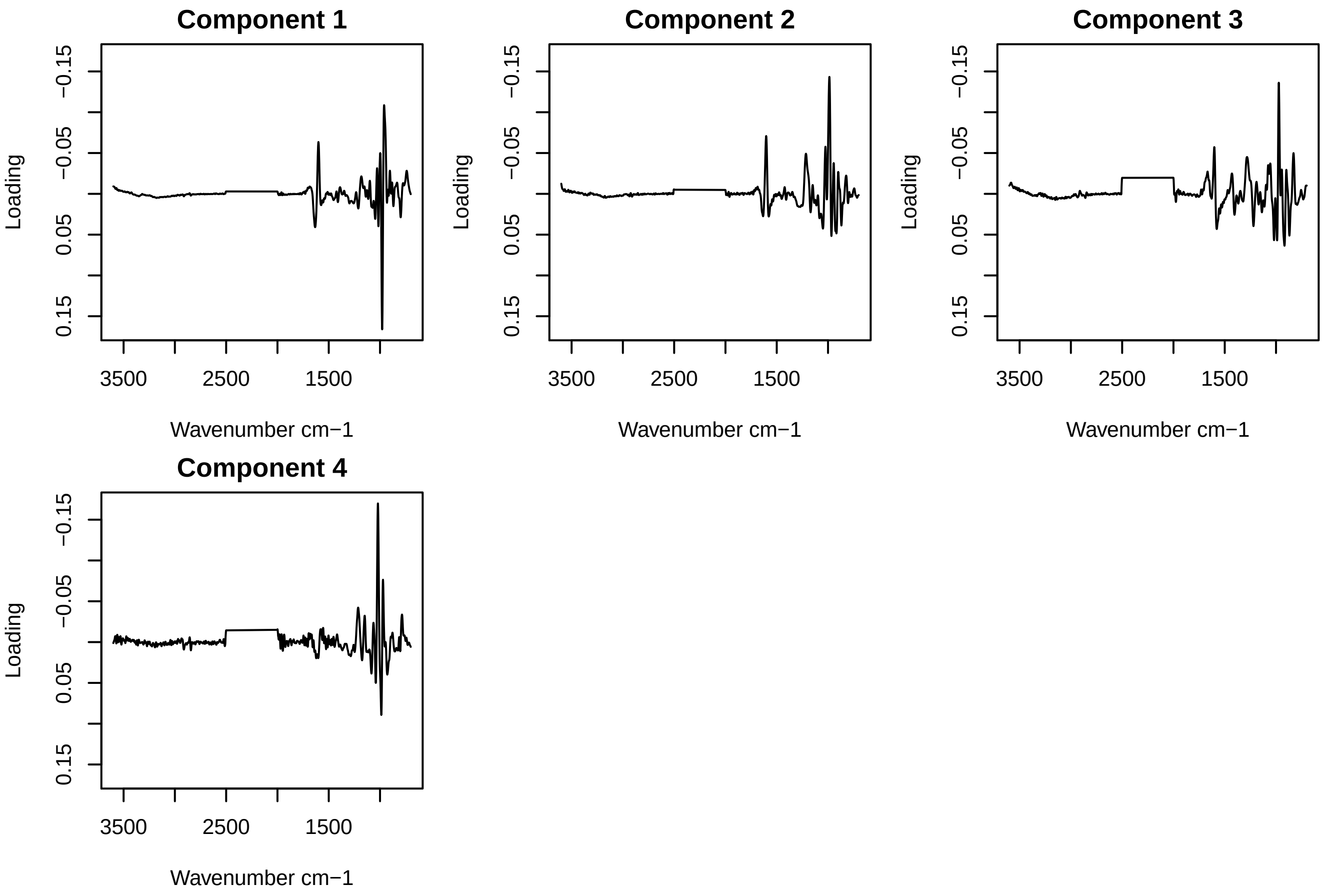
Component 1
Component 2
Component 3
Component 4
Loading
−0.15
−0.05
0.05
0.15
3500
2500
1500
Wavenumber cm−1

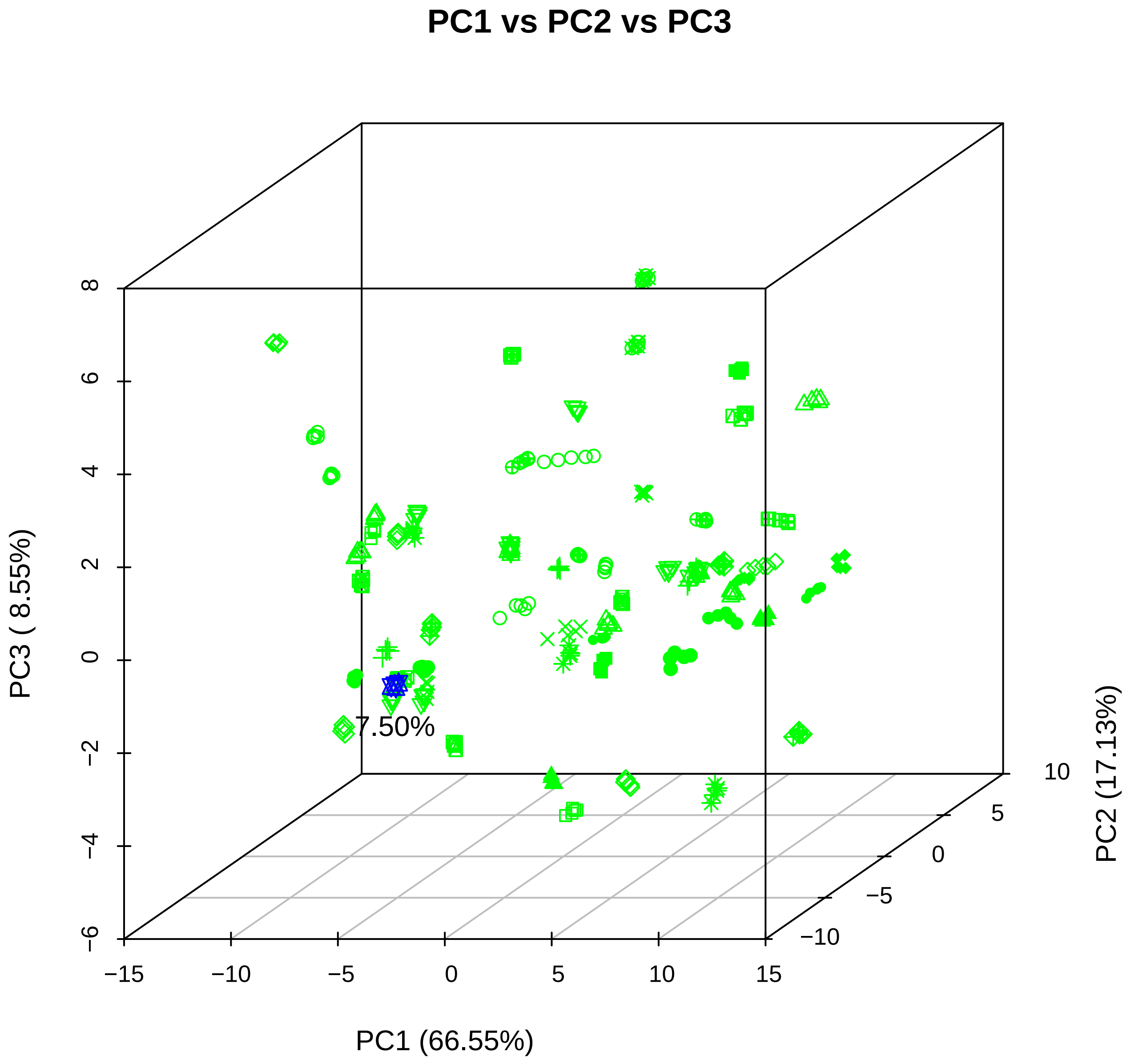

PC1 vs PC2 vs PC3
8
6
4
2
0
−2
−4
−6
PC3 (8.55%)
7.50%
−15
−10
−5
0
5
10
15
PC1 (66.55%)
10
5
0
−5
−10
PC2 (17.13%)

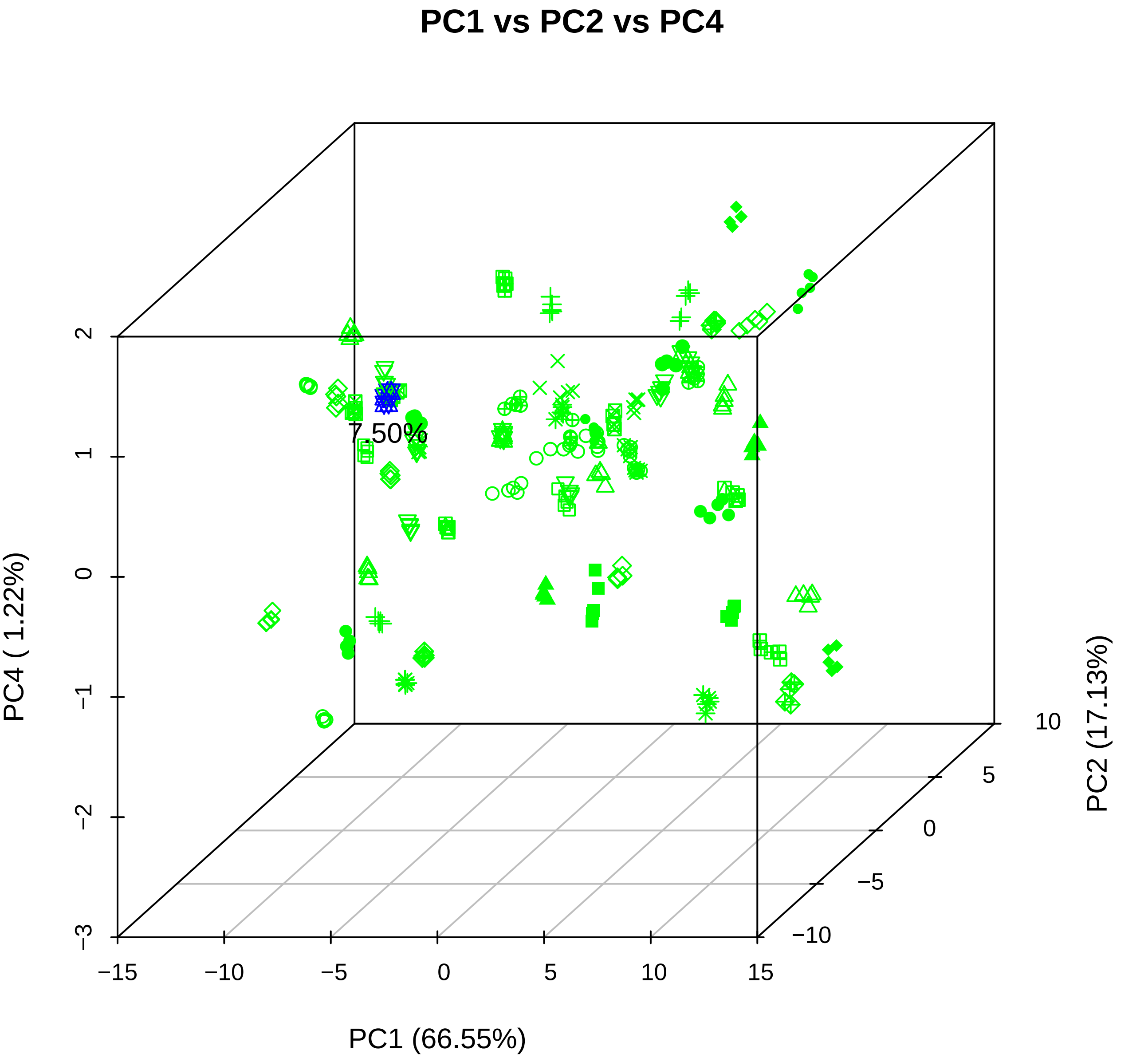
PC1 vs PC2 vs PC4
7.50%
PC4 (1.22%)
2
1
0
−1
−2
−3
−15
−10
−5
0
5
10
15
PC1 (66.55%)
10
5
0
−5
−10
PC2 (17.13%)

PC1 vs PC3 vs PC4

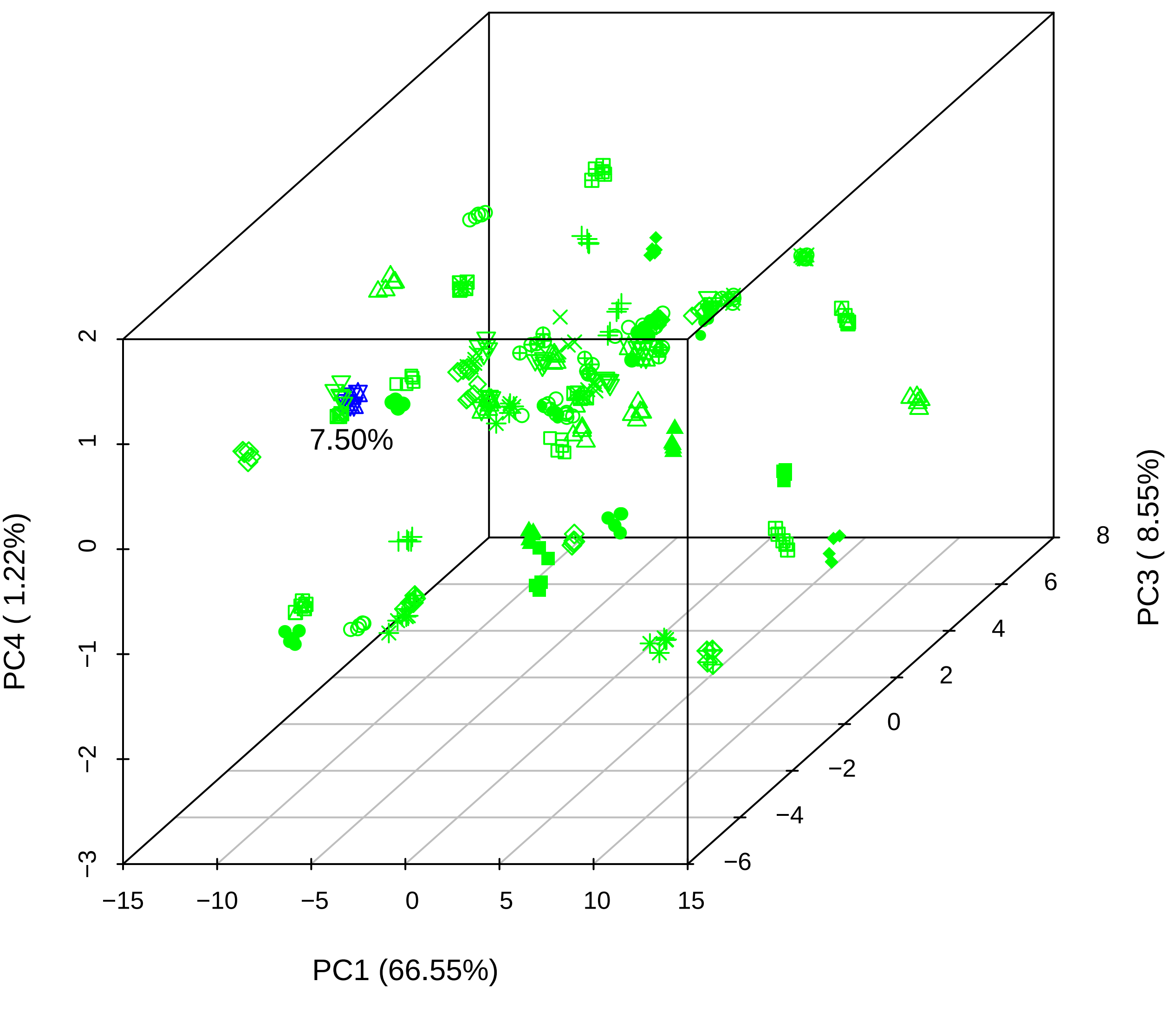

PC2 vs PC3 vs PC5

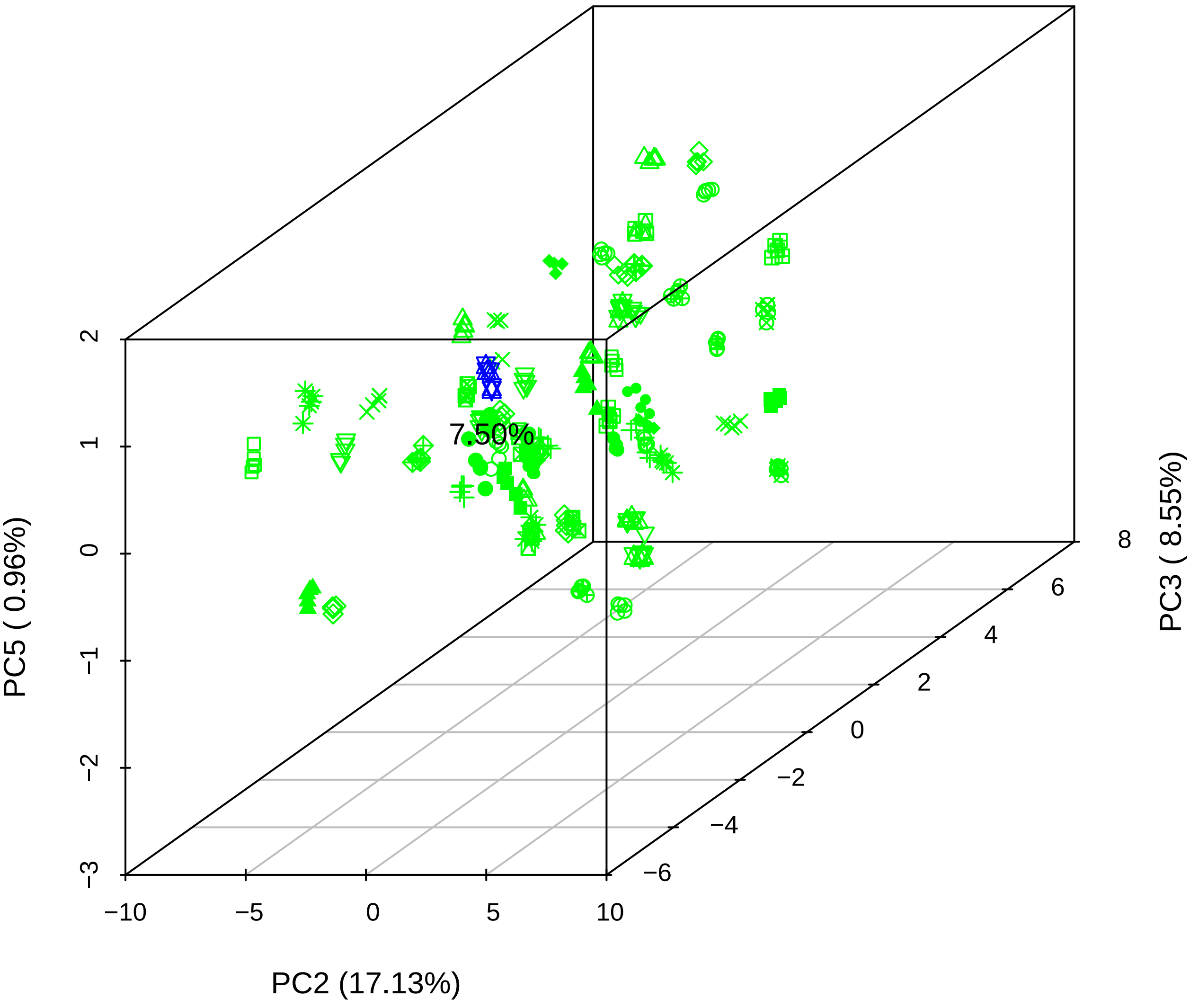

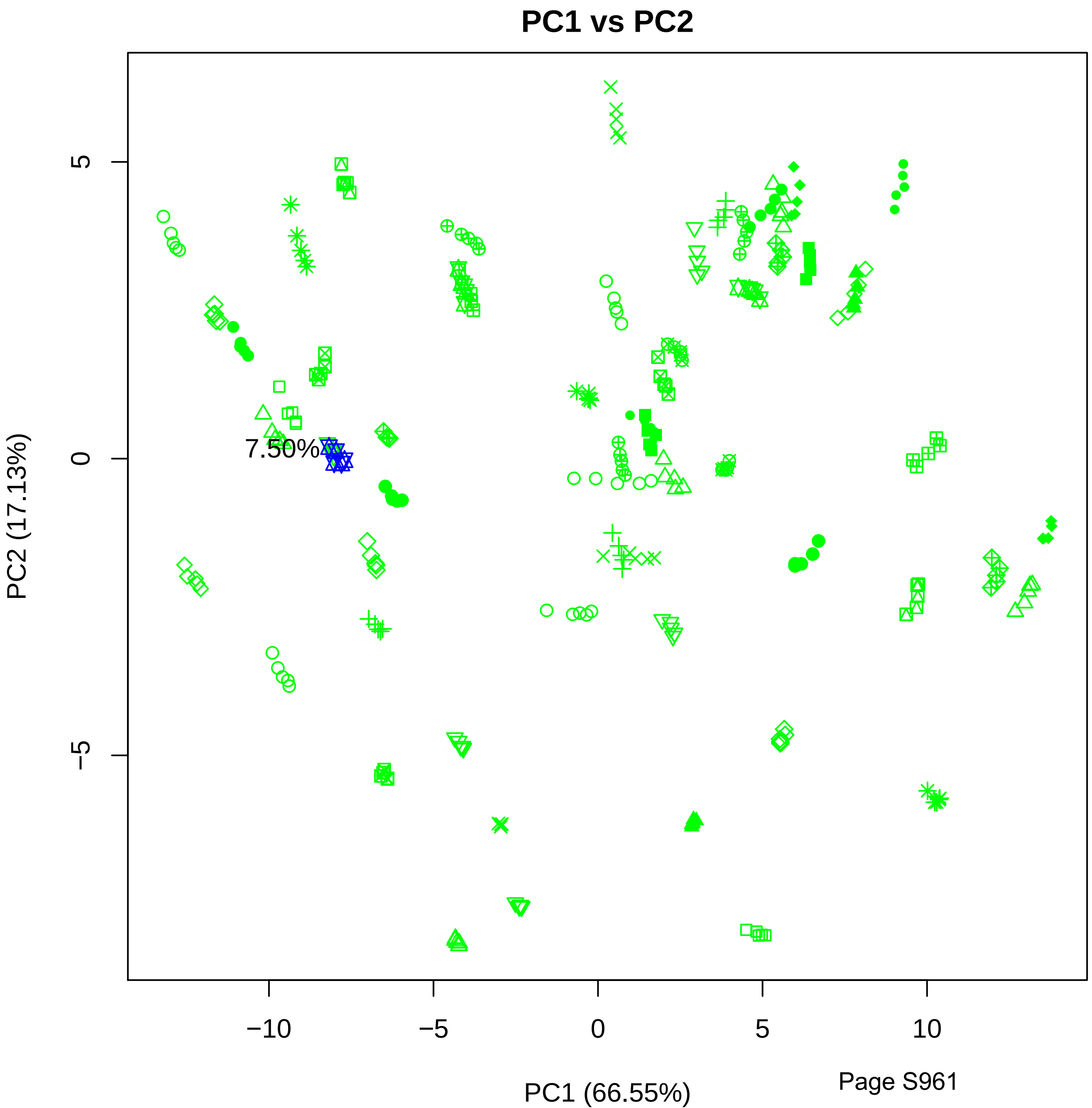
PC1 vs PC2
PC2 (17.13%)
PC1 (66.55%)
7.50%
5
0
−5
−10
−5
0
5
10

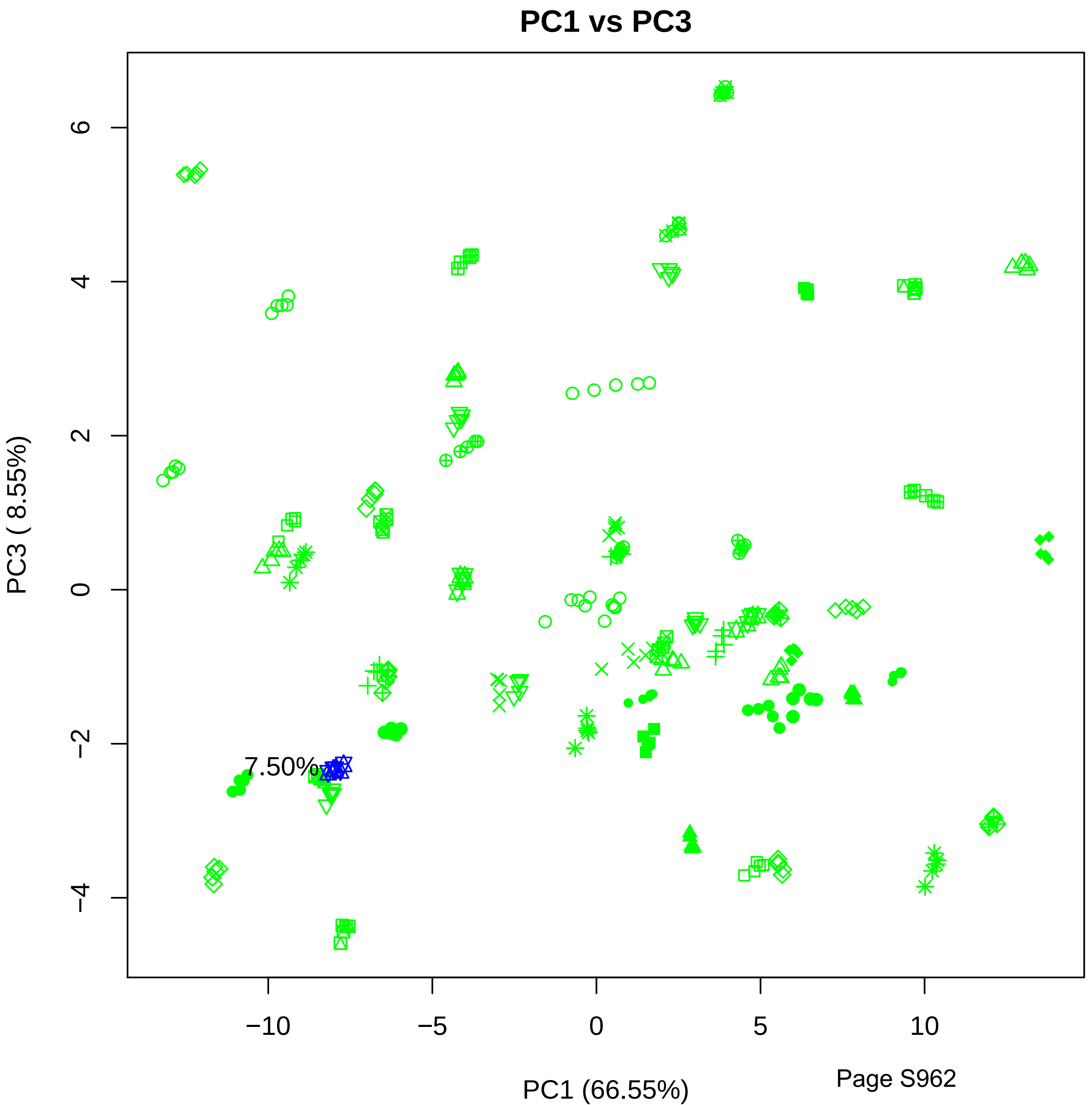
PC1 vs PC3
PC3 (8.55%)
PC1 (66.55%)
7.50%
6
4
2
0
−2
−4
−10
−5
0
5
10

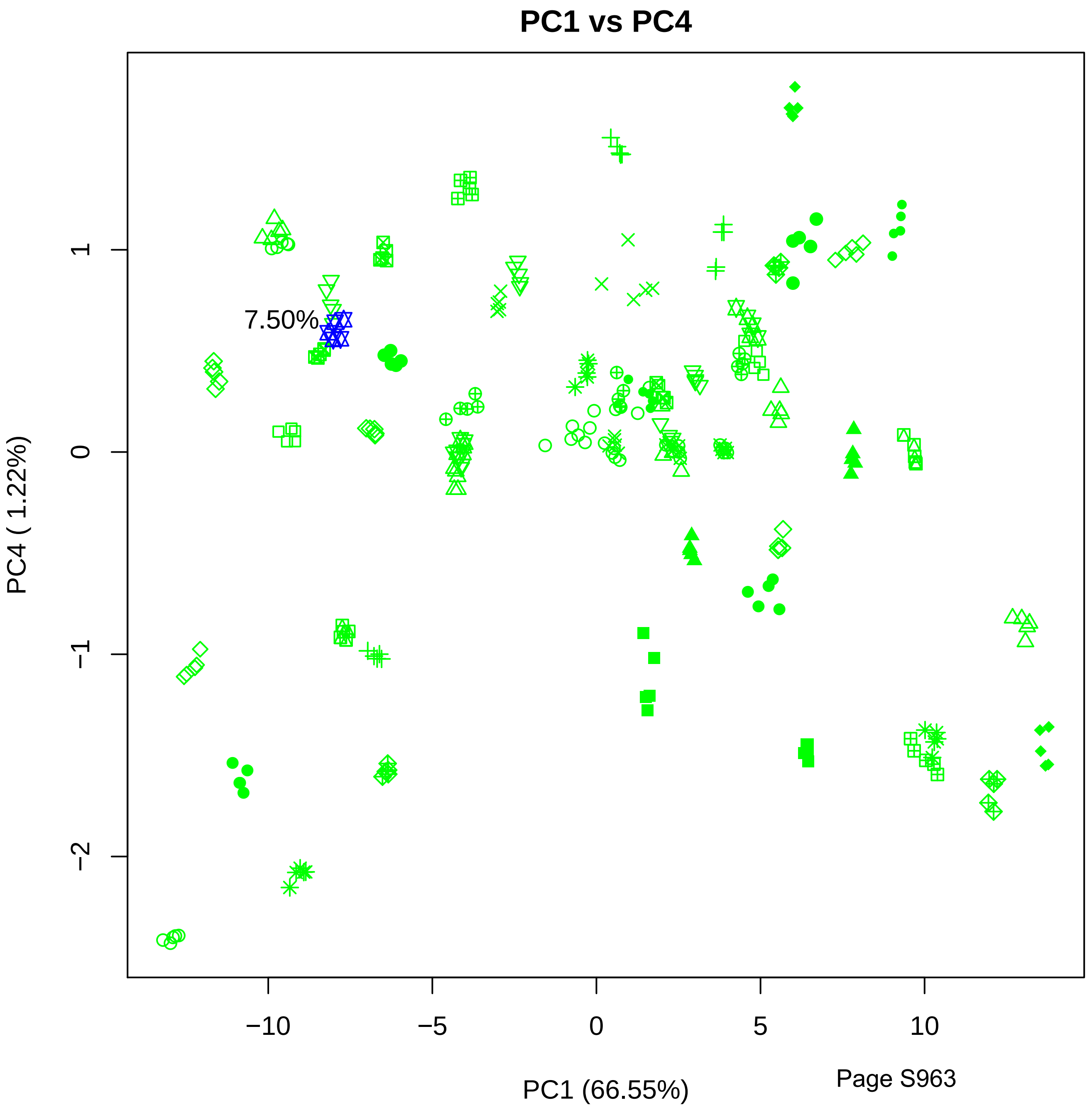
PC1 vs PC4
7.50%
PC4 (1.22%)
PC1 (66.55%)
1
0
−1
−2
−10
−5
0
5
10

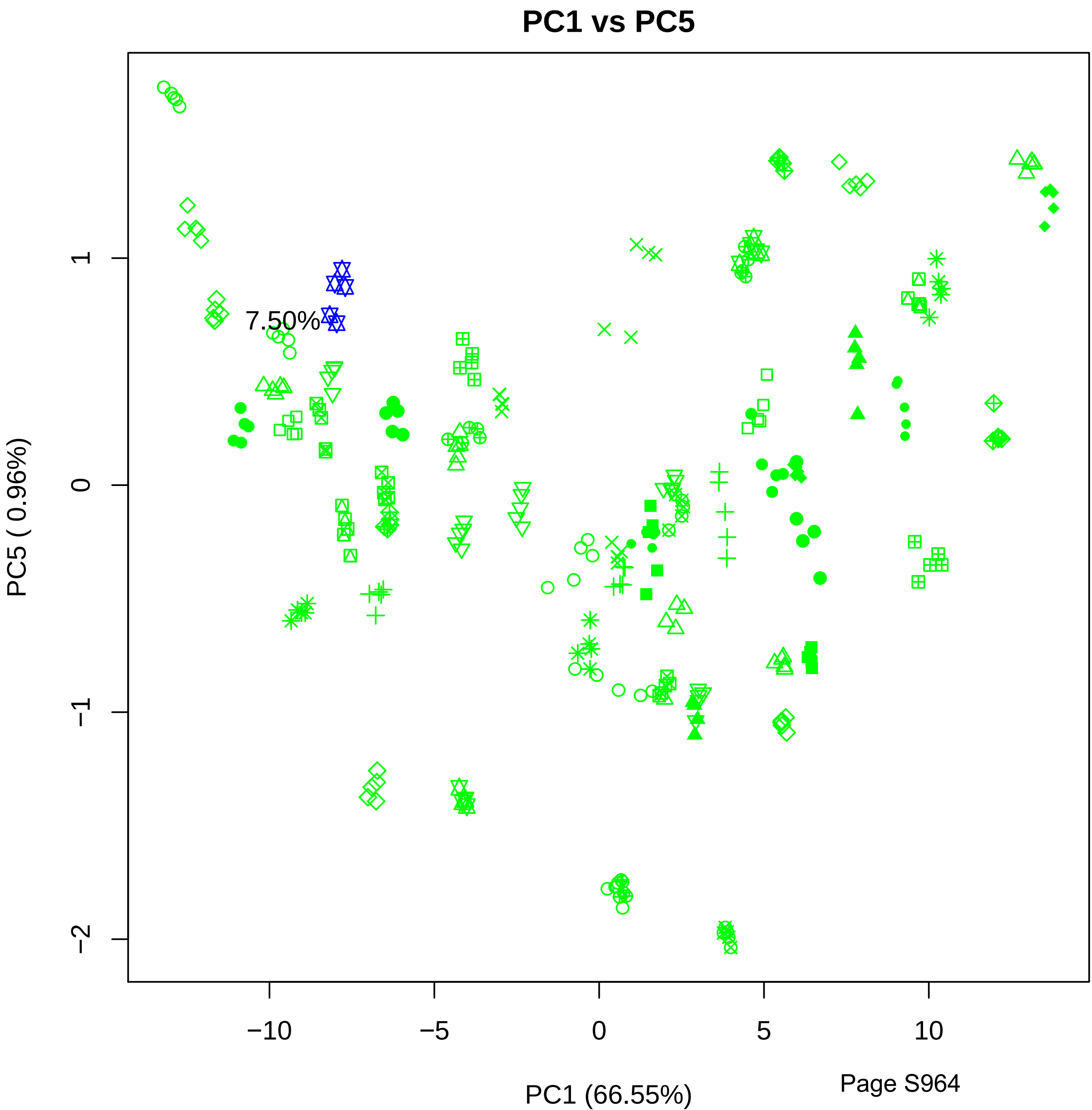

PC1 vs PC5
7.50%
1
0
−1
−2
−10
−5
0
5
10
PC5 (0.96%)
PC1 (66.55%)

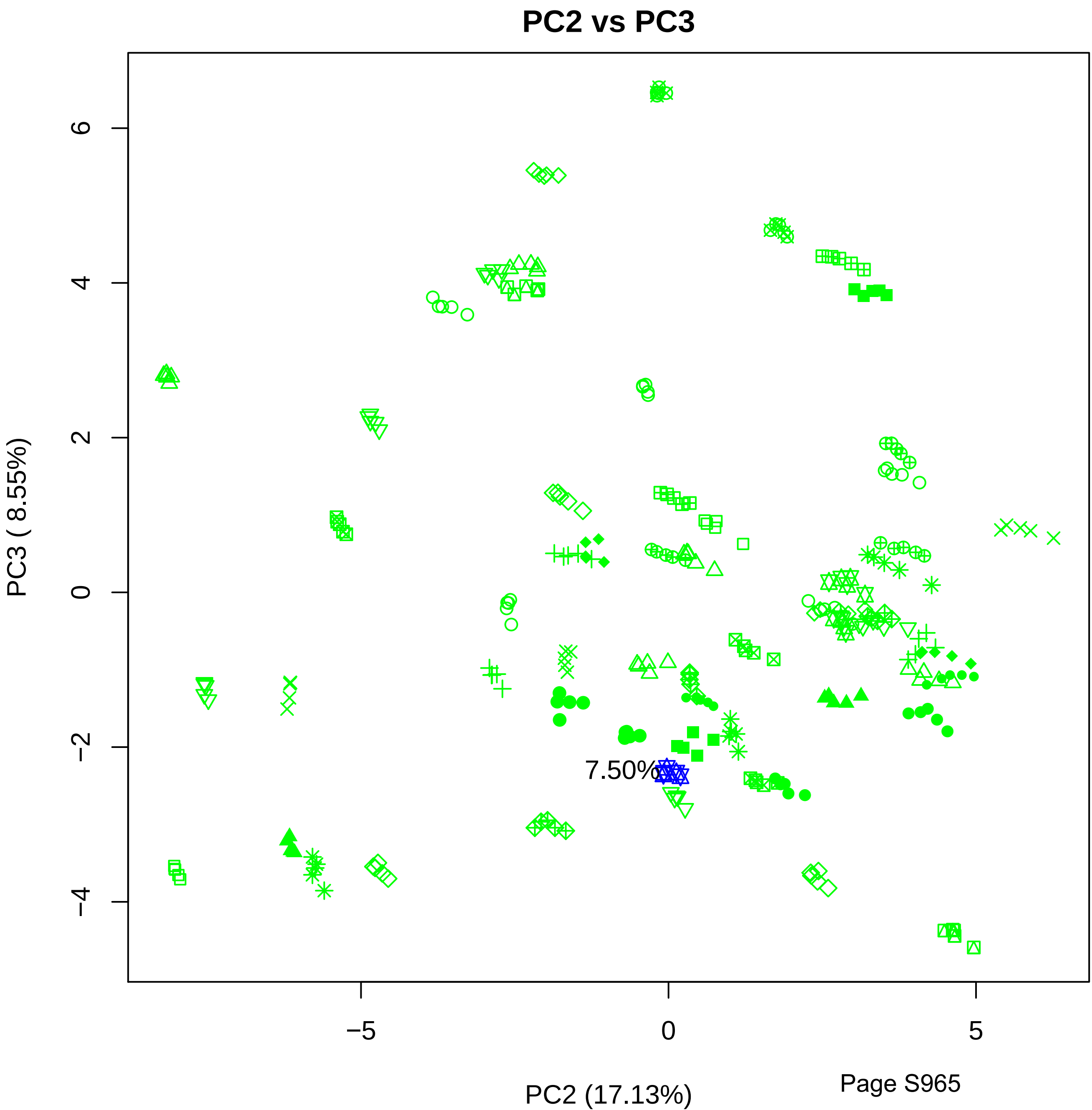
PC2 vs PC3
PC3 (8.55%)
PC2 (17.13%)
7.50%
−5
0
5
6
4
2
0
−2
−4

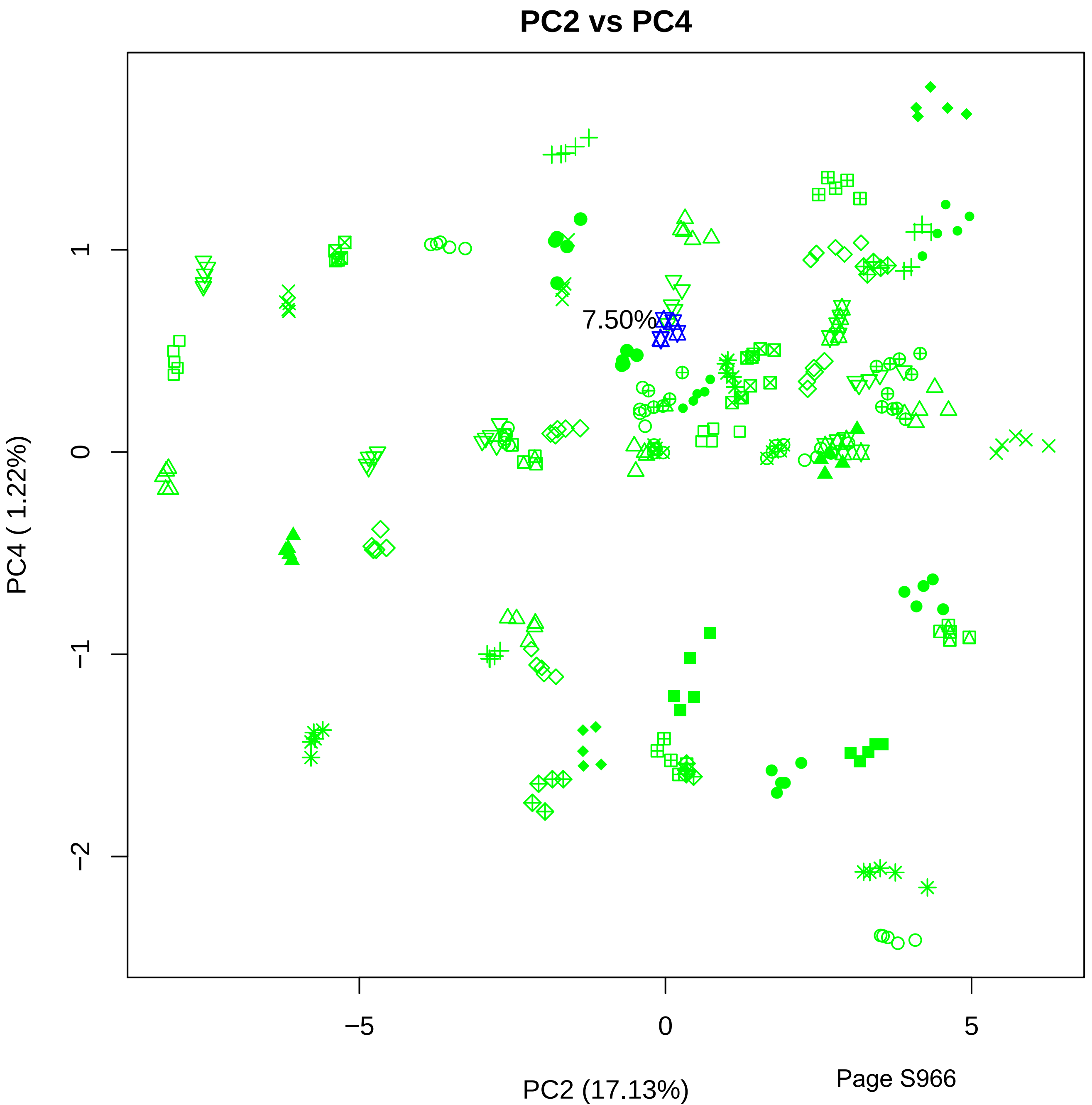

PC2 vs PC4
PC4 (1.22%)
PC2 (17.13%)
7.50%
1
0
−1
−2
−5
0
5

PC2 vs PC5

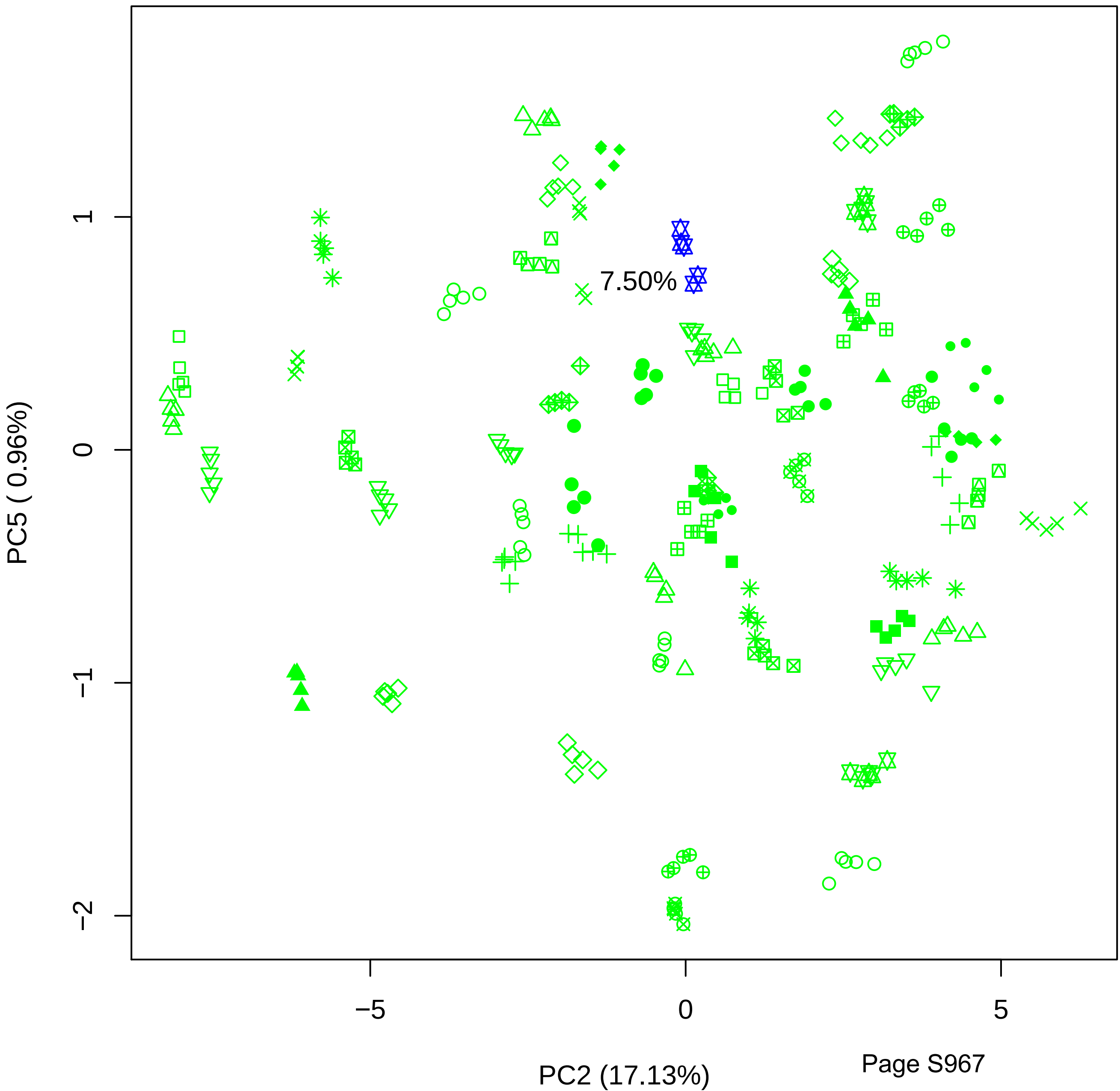

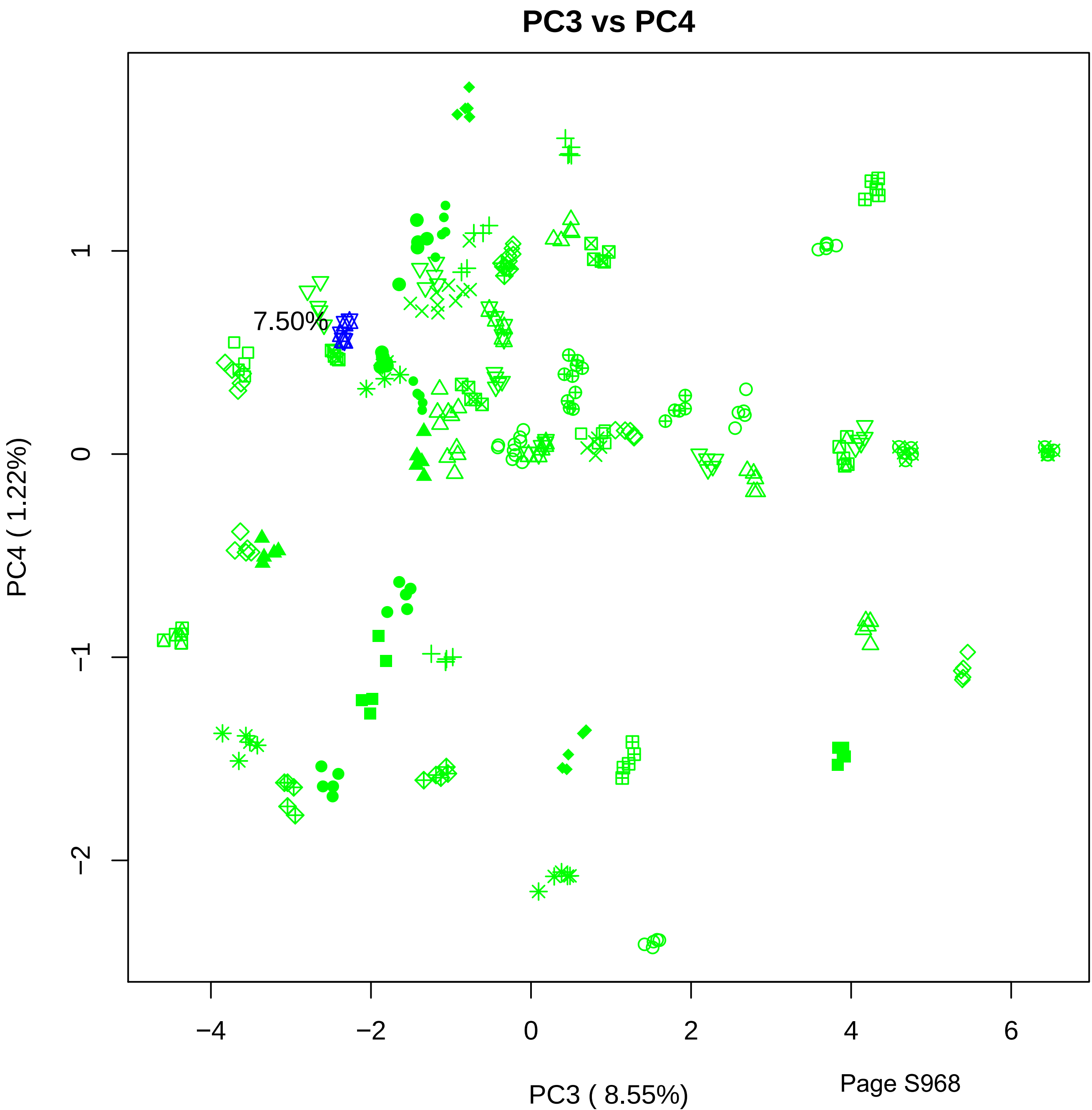

PC3 vs PC4
7.50%
PC4 (1.22%)
1
0
−1
−2
−4
−2
0
2
4
6
PC3 (8.55%)

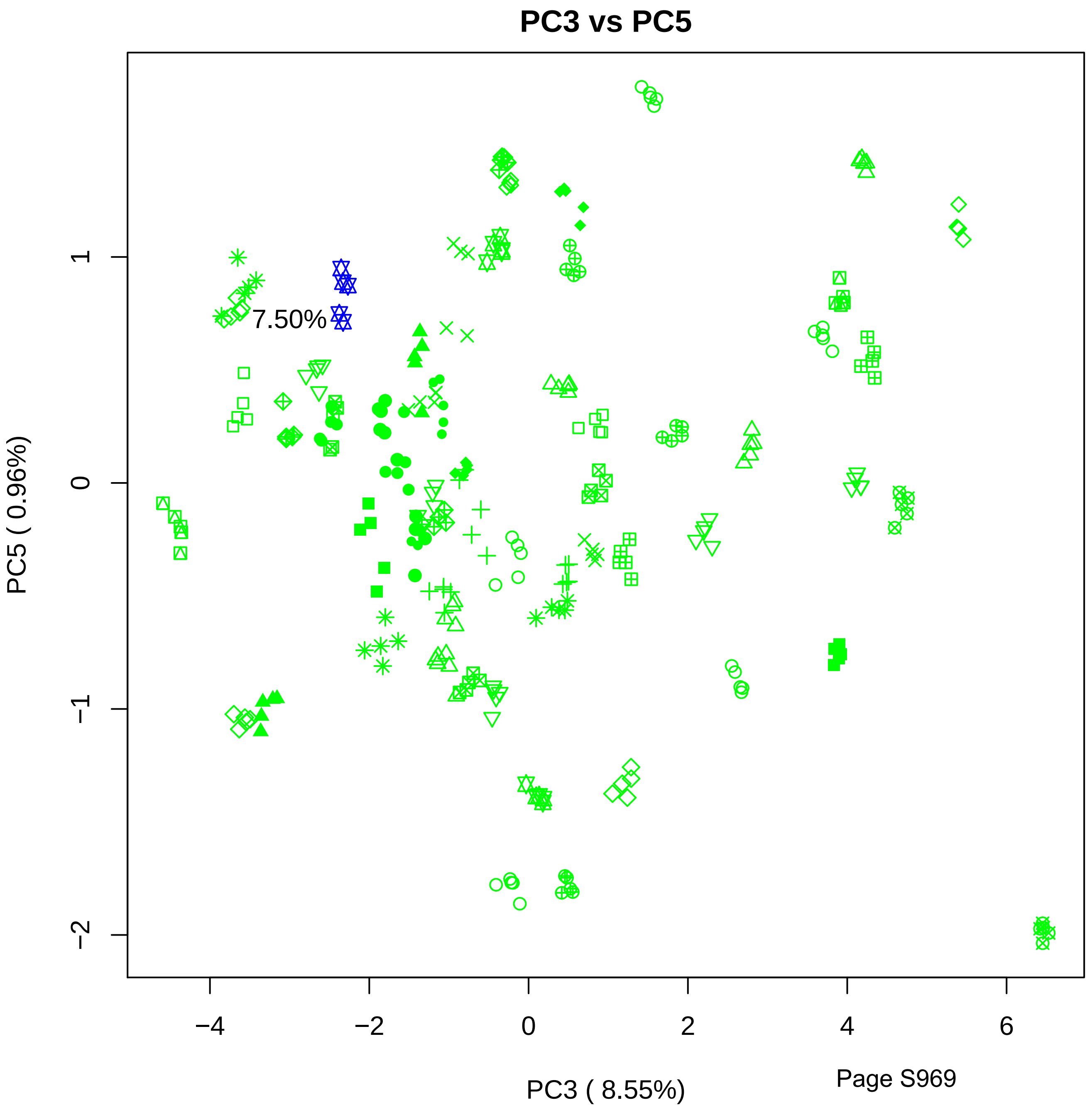
PC3 vs PC5
7.50%
PC5 (0.96%)
PC3 (8.55%)
1
0
−1
−2
−4
−2
0
2
4
6

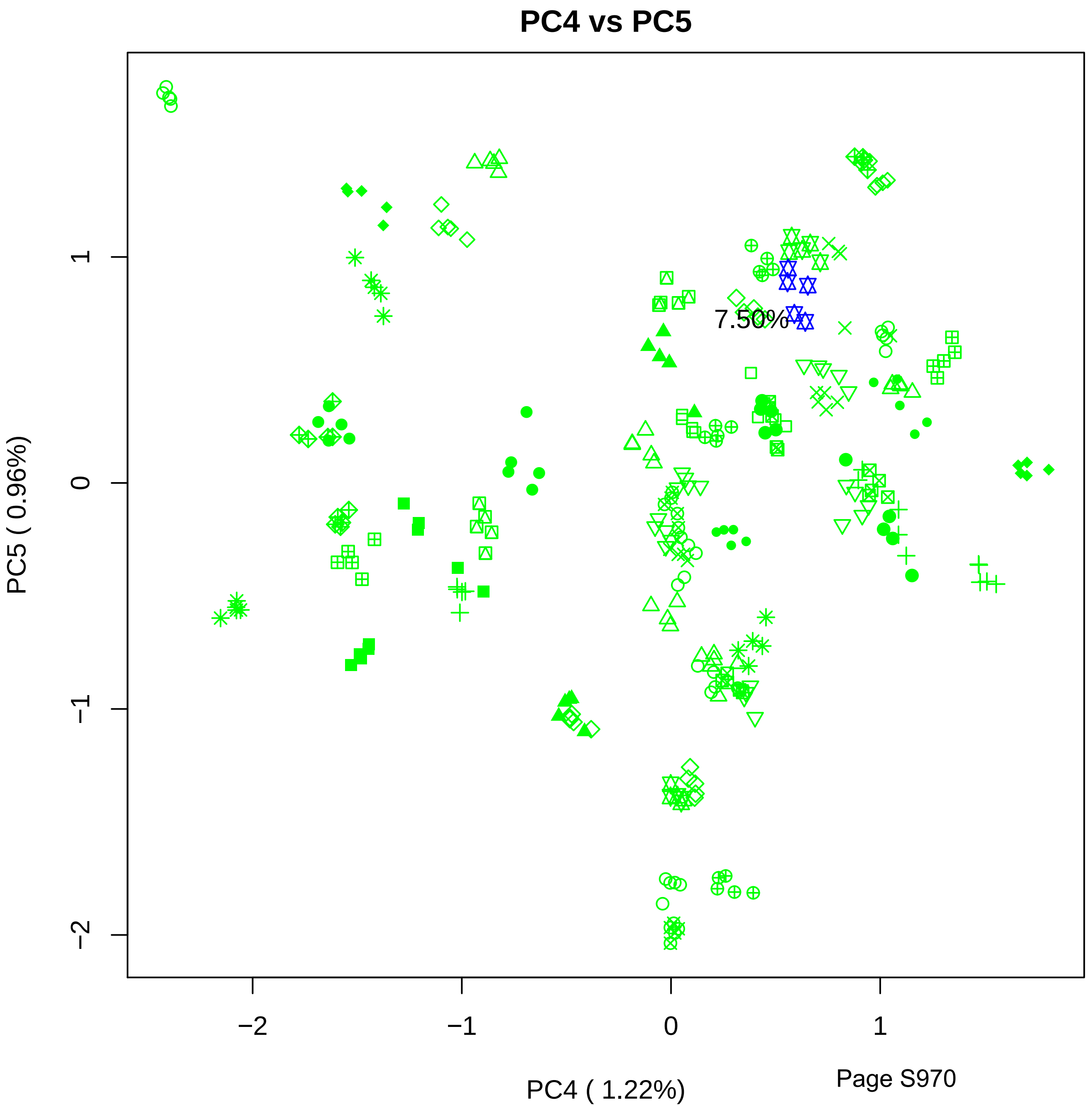

PC4 vs PC5
PC5 (0.96%)
PC4 (1.22%)
7.50%
1
0
−1
−2
−2
−1
0
1

PCA of 10% BMH(a) vs PMH (a)

Scaled Matrix PCAed

7th order polynomial baseline correction performed

Raw spectra smoothed to 21 Neighbours, 2nd order PN, Savtisky Golay

Normalisation between 0 and 100

D2 spectra smoothed to 41 Neighbours, 2nd order PN, Savtisky Golay

Regions Analysed: 3601−2501 and 2001−700cm−1

Samples Analysed: 1 bContam's 0 BLH's 0 BMH's 0 OMH's 0 pContam's 0 PLH's 60 PMH's

Keele University AD

Fri Dec 14 14:20:28 2018

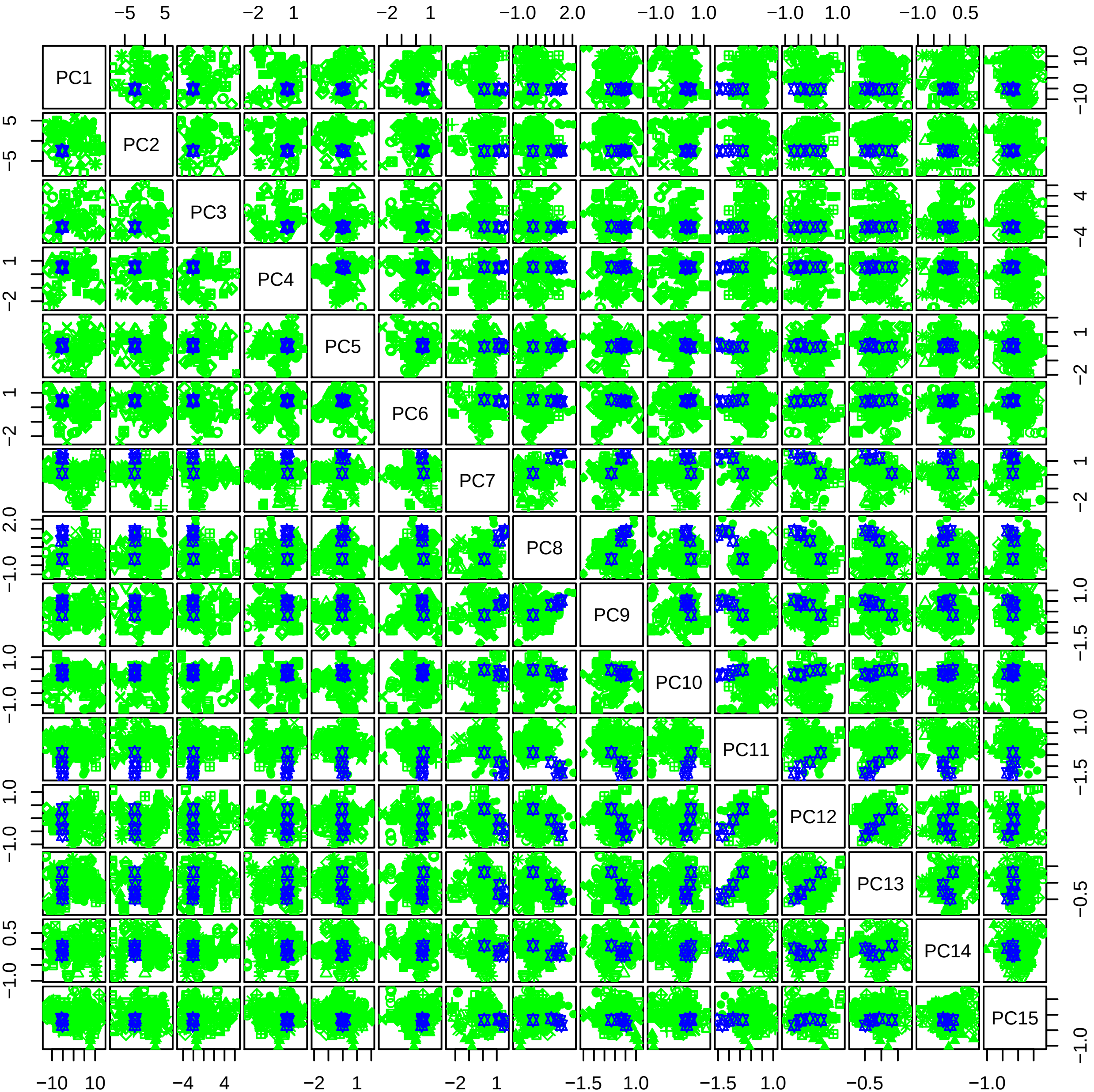

PC1
PC2
PC3
PC4
PC5
PC6
PC7
PC8
PC9
PC10
PC11
PC12
PC13
PC14
PC15

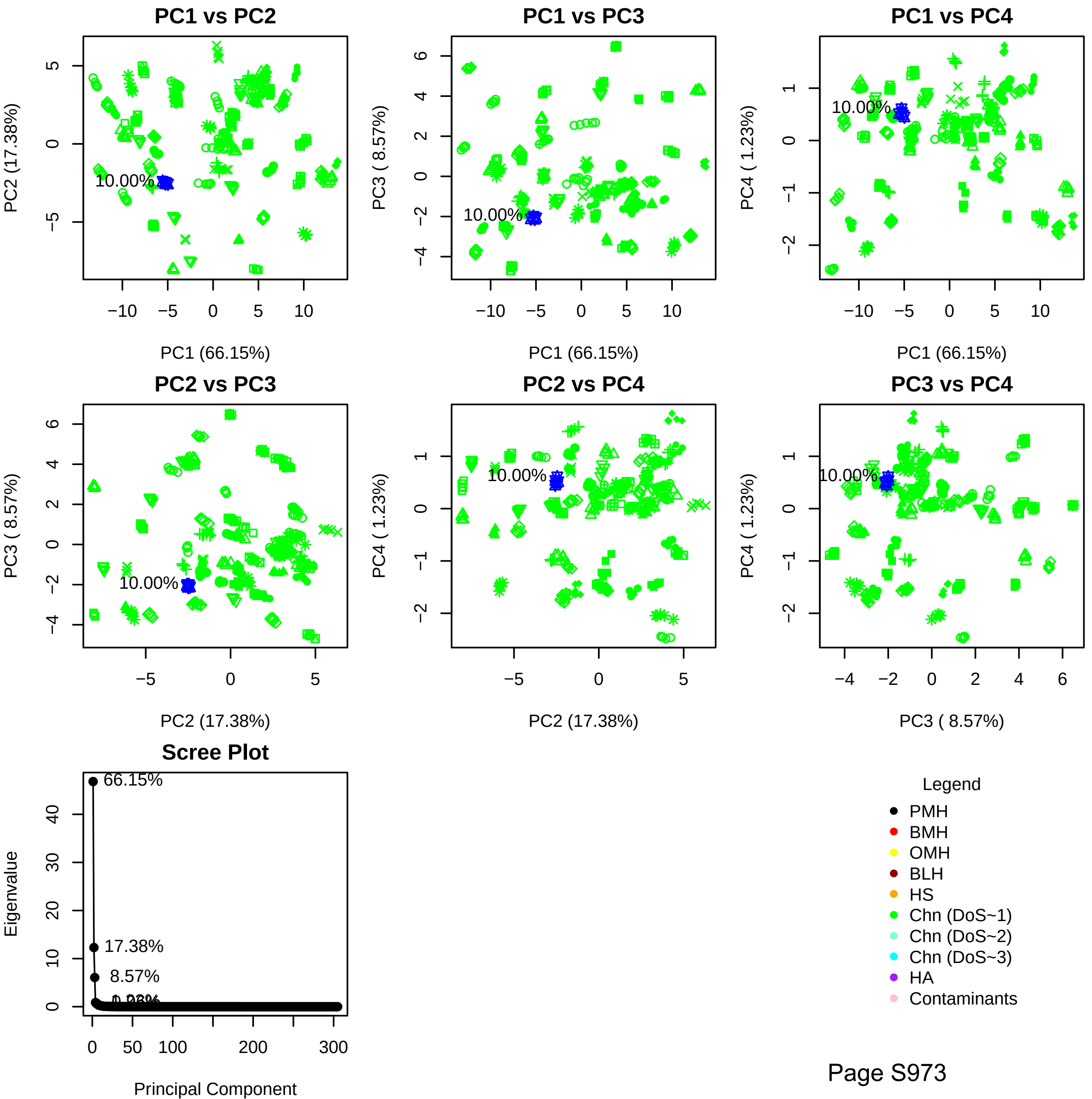
PC1 vs PC2
PC2 (17.38%)
PC1 (66.15%)
10.00%
PC1 vs PC3
PC3 (8.57%)
PC1 (66.15%)
10.00%
PC1 vs PC4
PC4 (1.23%)
PC1 (66.15%)
10.00%
PC2 vs PC3
PC3 (8.57%)
PC2 (17.38%)
10.00%
PC2 vs PC4
PC4 (1.23%)
PC2 (17.38%)
10.00%
PC3 vs PC4
PC4 (1.23%)
PC3 (8.57%)
10.00%
Scree Plot
Eigenvalue
Principal Component
66.15%
17.38%
8.57%
Legend
PMH
BMH
OMH
BLH
HS
Chn (DoS~1)
Chn (DoS~2)
Chn (DoS~3)
HA
Contaminants

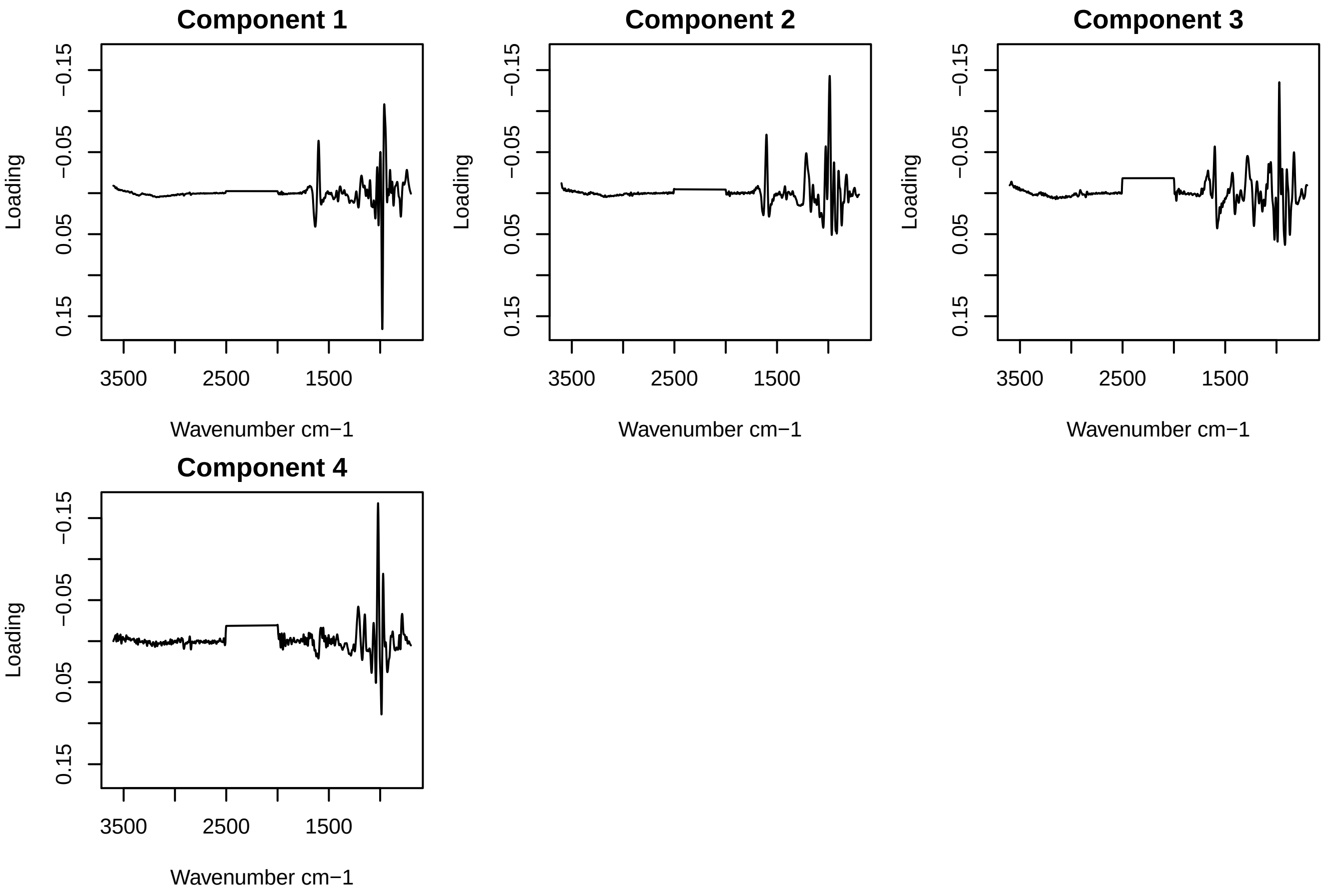
Component 1
Component 2
Component 3
Component 4
Loading
−0.15
−0.05
0.05
0.15
3500
2500
1500
Wavenumber cm−1

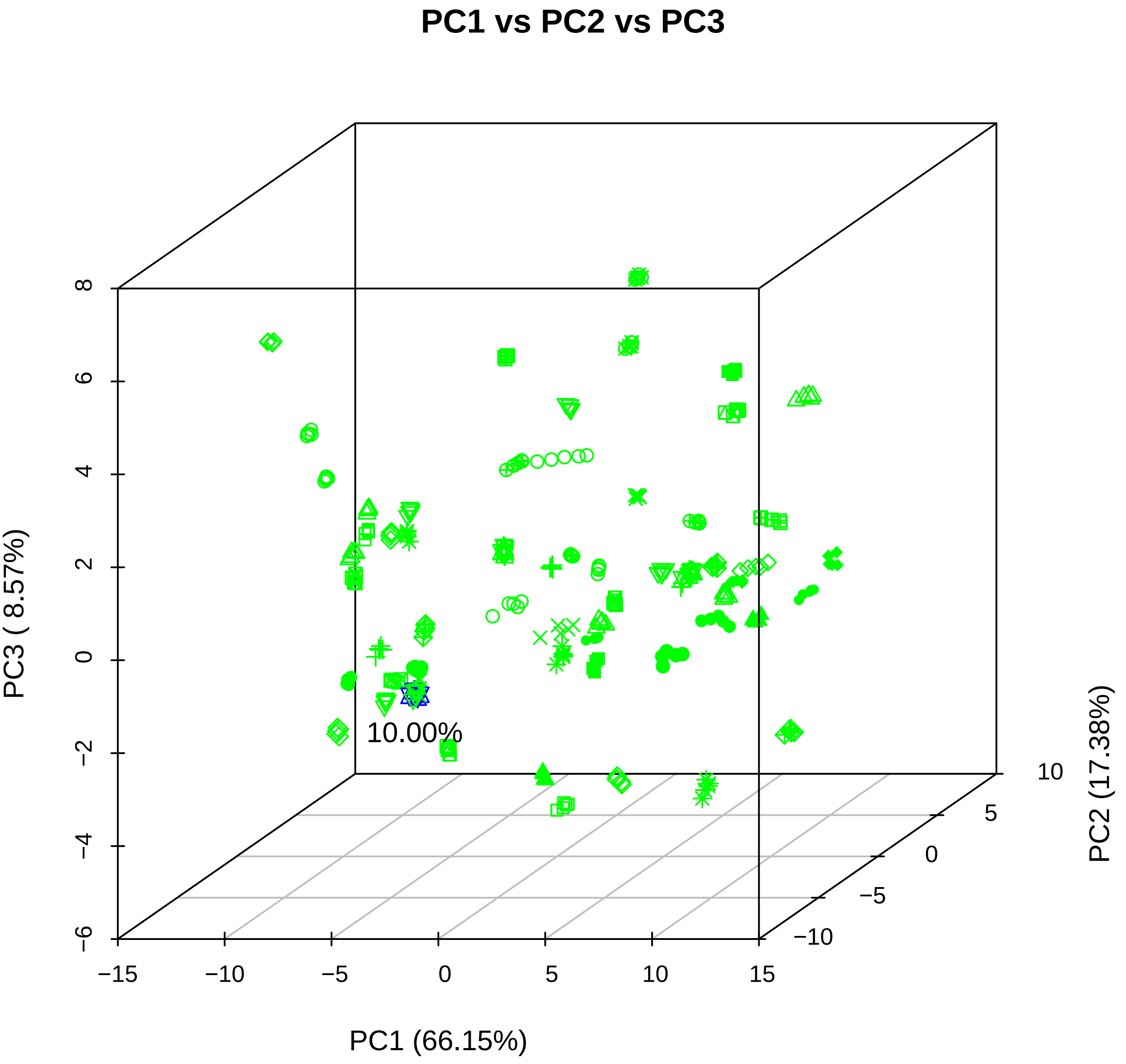
PC1 vs PC2 vs PC3
PC3 (8.57%)
8
6
4
2
0
−2
−4
−6
10.00%
PC1 (66.15%)
−15
−10
−5
0
5
10
15
PC2 (17.38%)
10
5
0
−5
−10

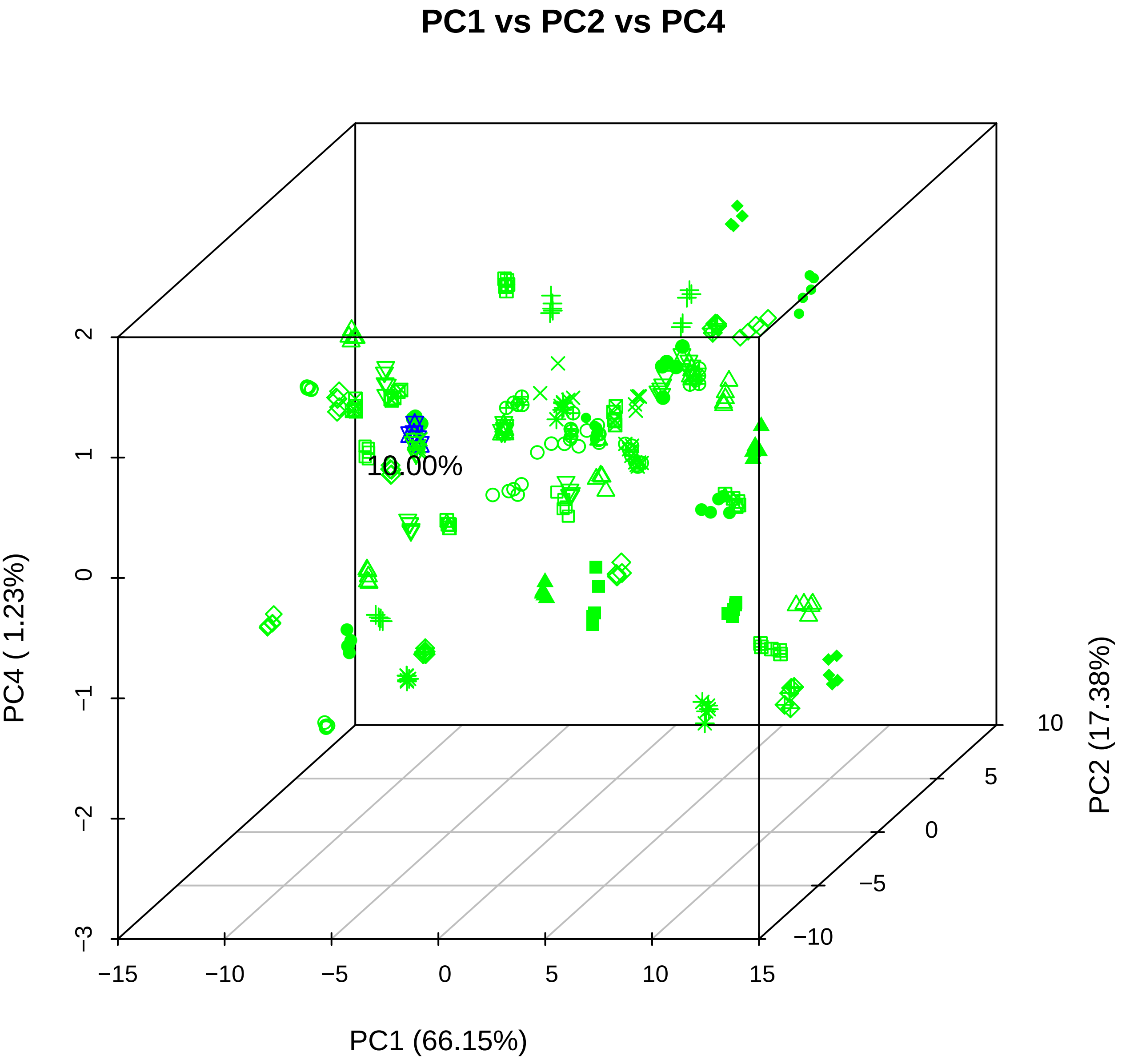
PC1 vs PC2 vs PC4
10.00%
PC4 (1.23%)
PC1 (66.15%)
PC2 (17.38%)
−3
−2
−1
0
1
2
−15
−10
−5
0
5
10
15
−10
−5
0
5
10

PC1 vs PC3 vs PC4

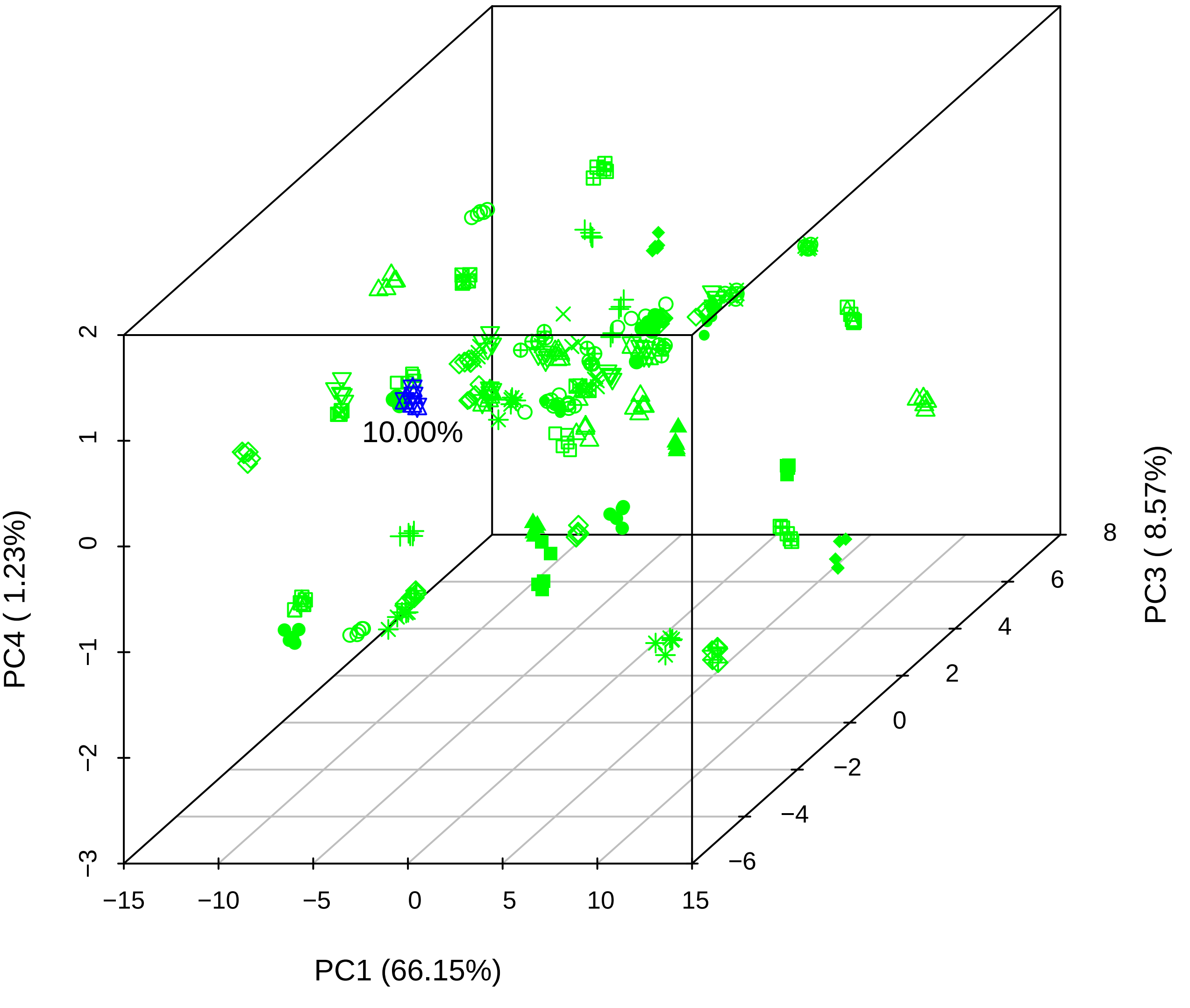

PC2 vs PC3 vs PC5

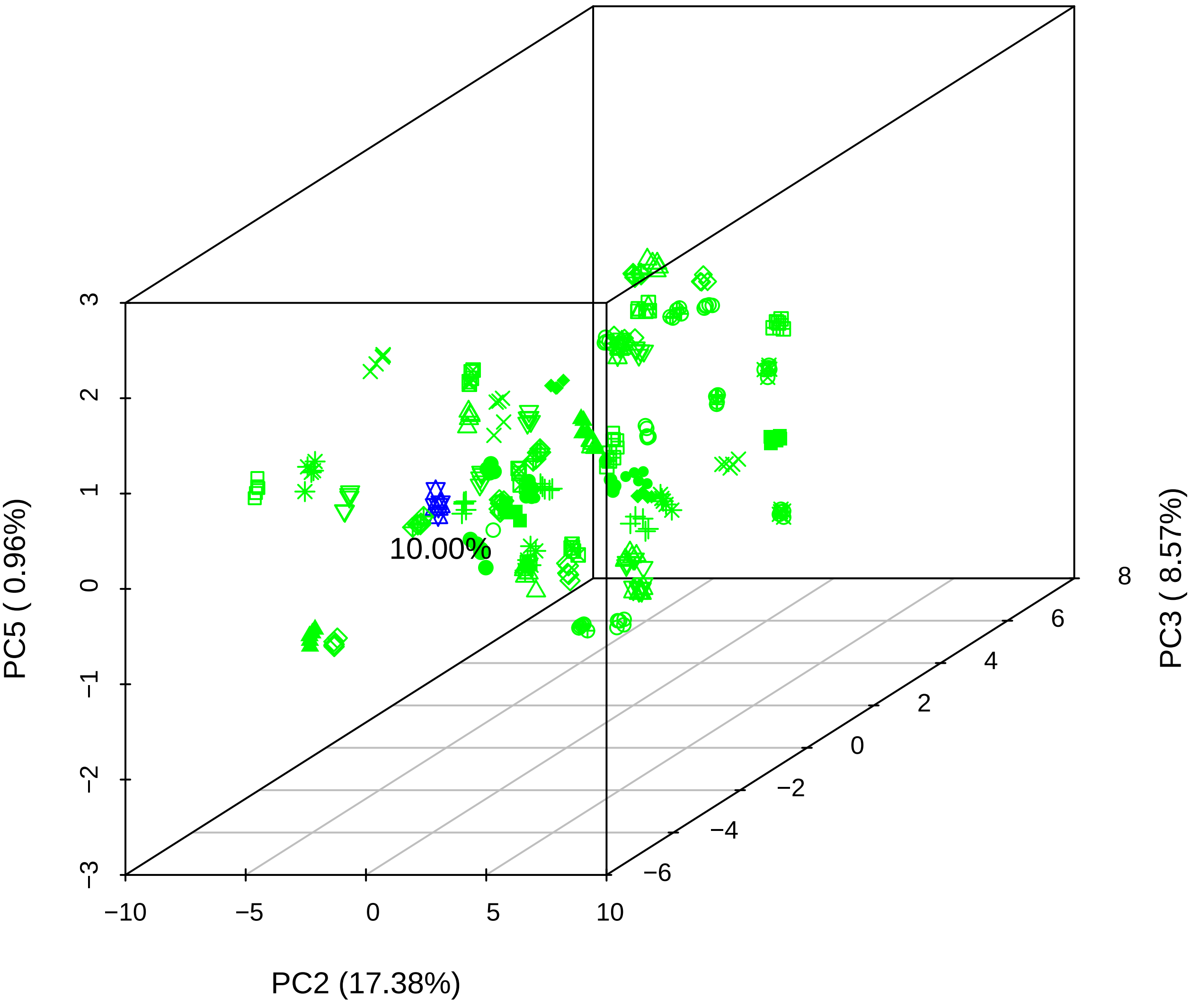

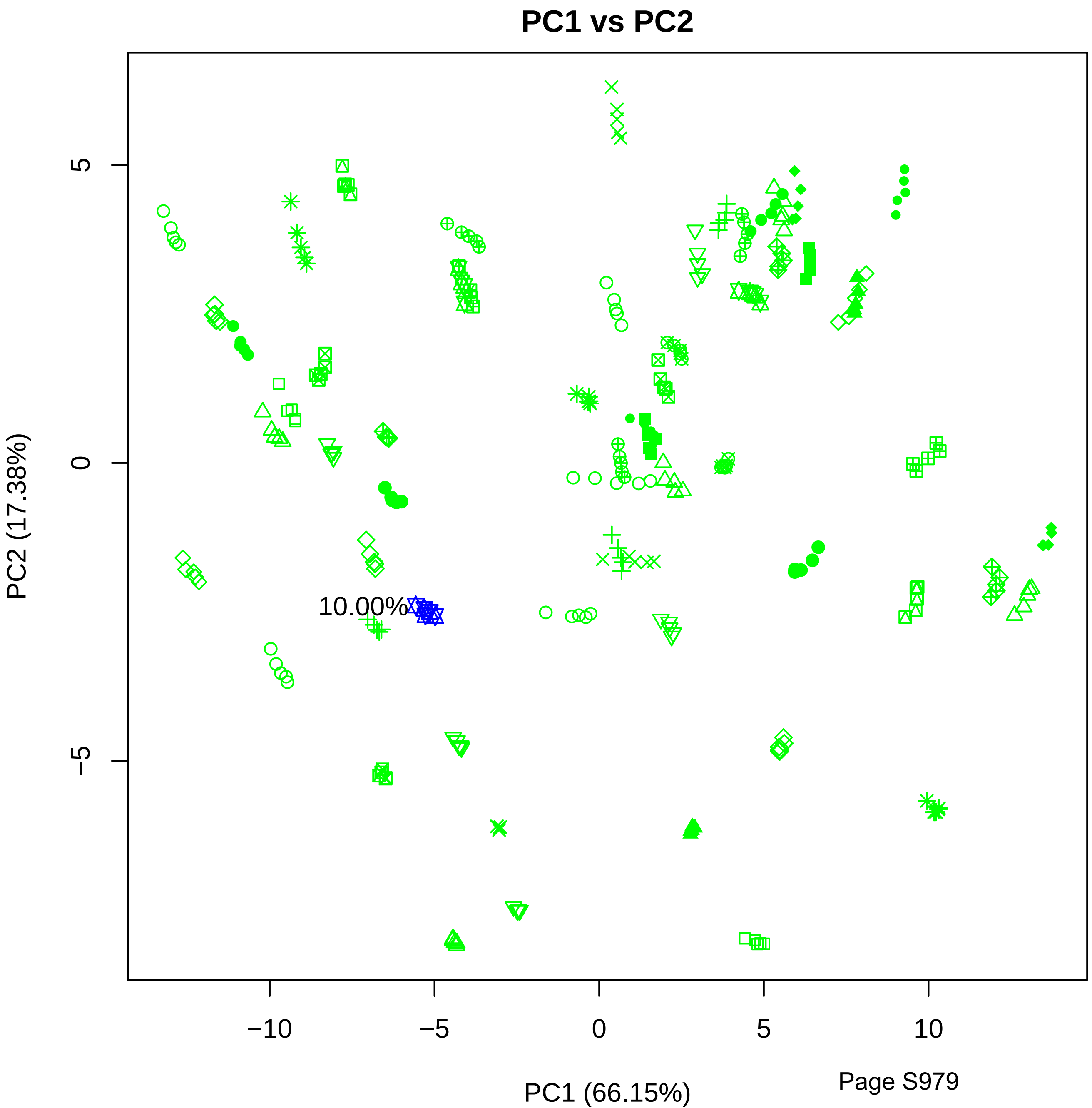

PC1 vs PC2
PC2 (17.38%)
PC1 (66.15%)
10.00%
5
0
−5
−10
−5
0
5
10

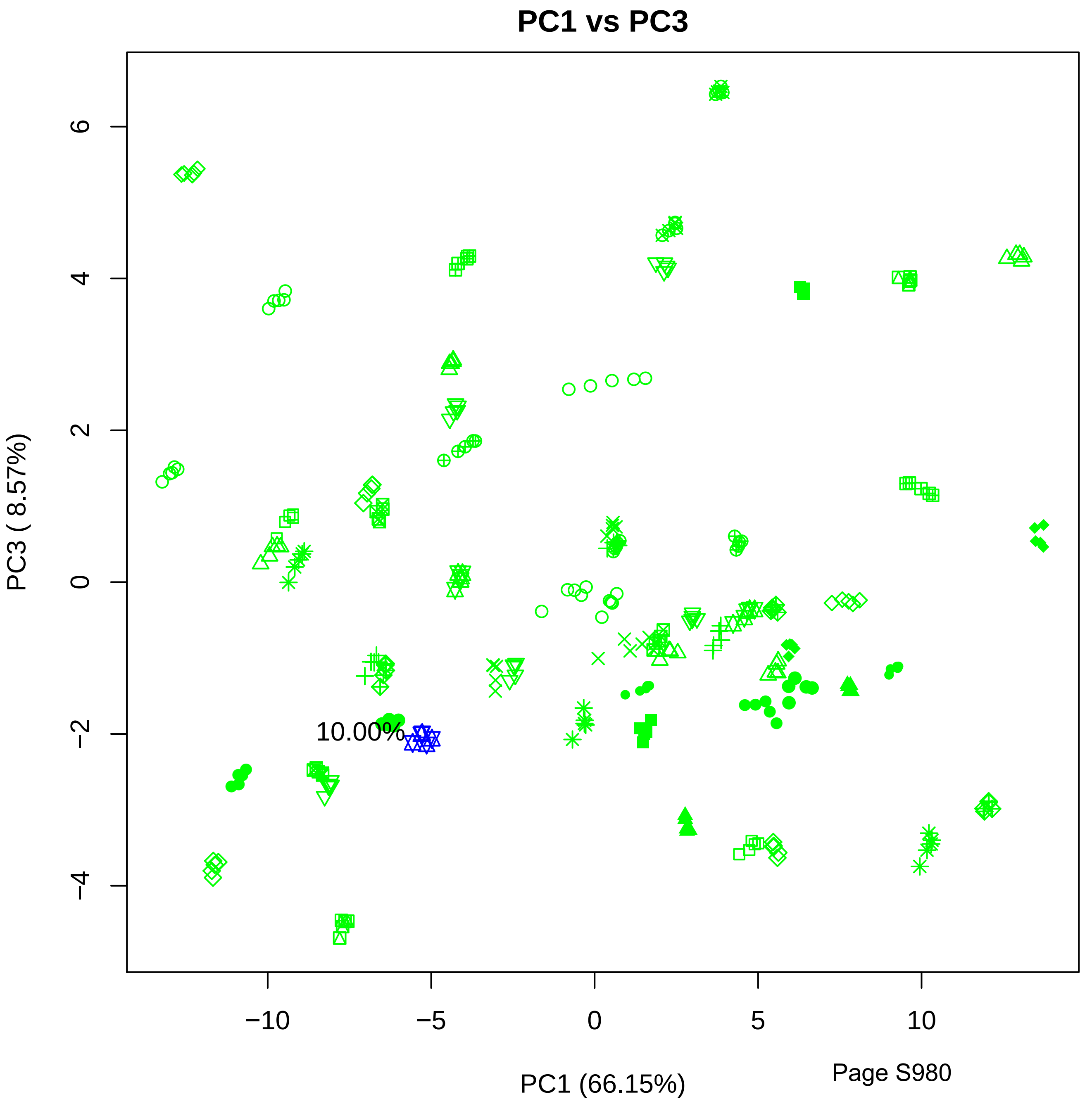
PC1 vs PC3
PC3 (8.57%)
PC1 (66.15%)
10.00%
6
4
2
0
−2
−4
−10
−5
0
5
10

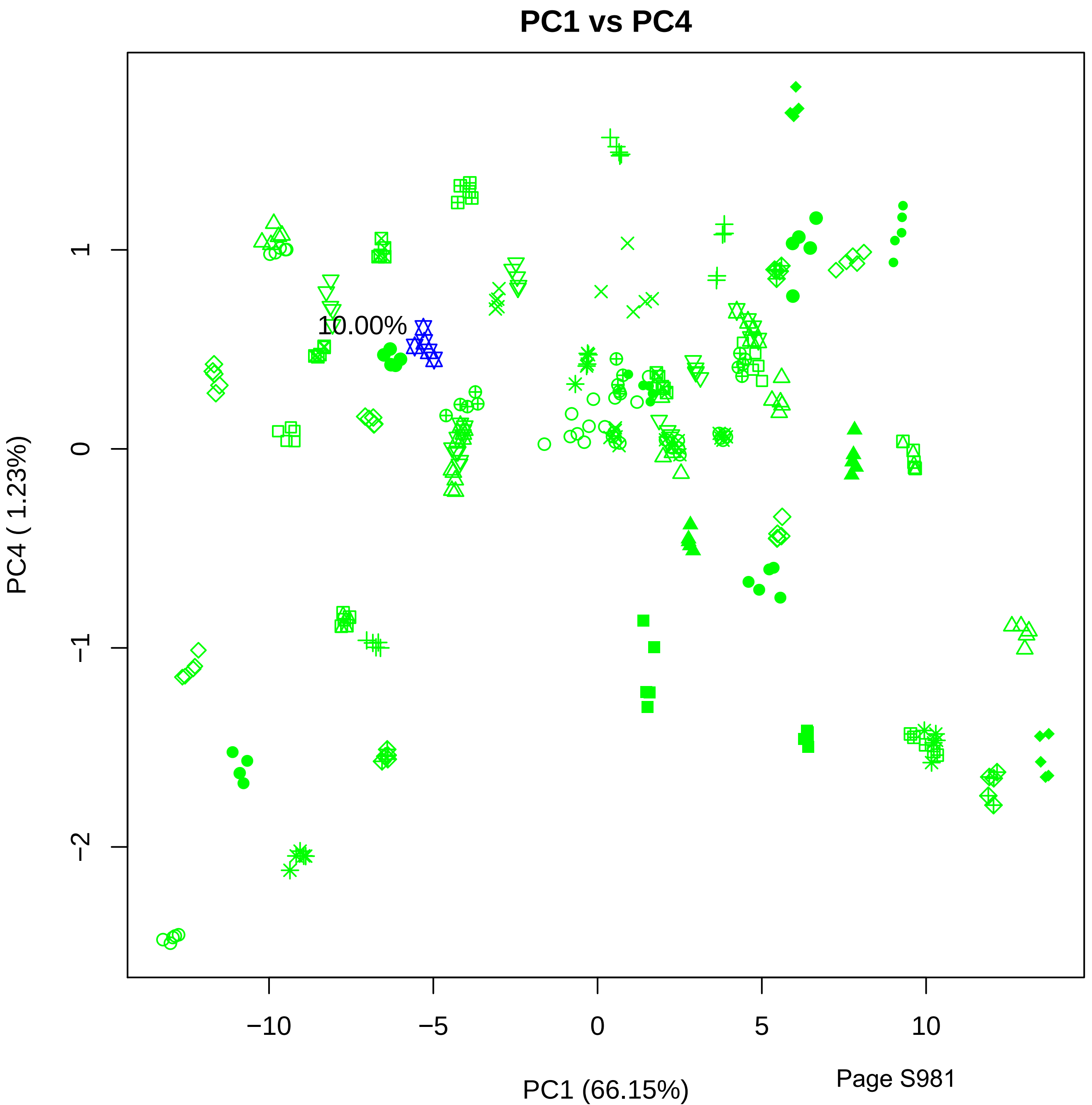
PC1 vs PC4
10.00%
1
0
−1
−2
PC4 (1.23%)
−10
−5
0
5
10
PC1 (66.15%)

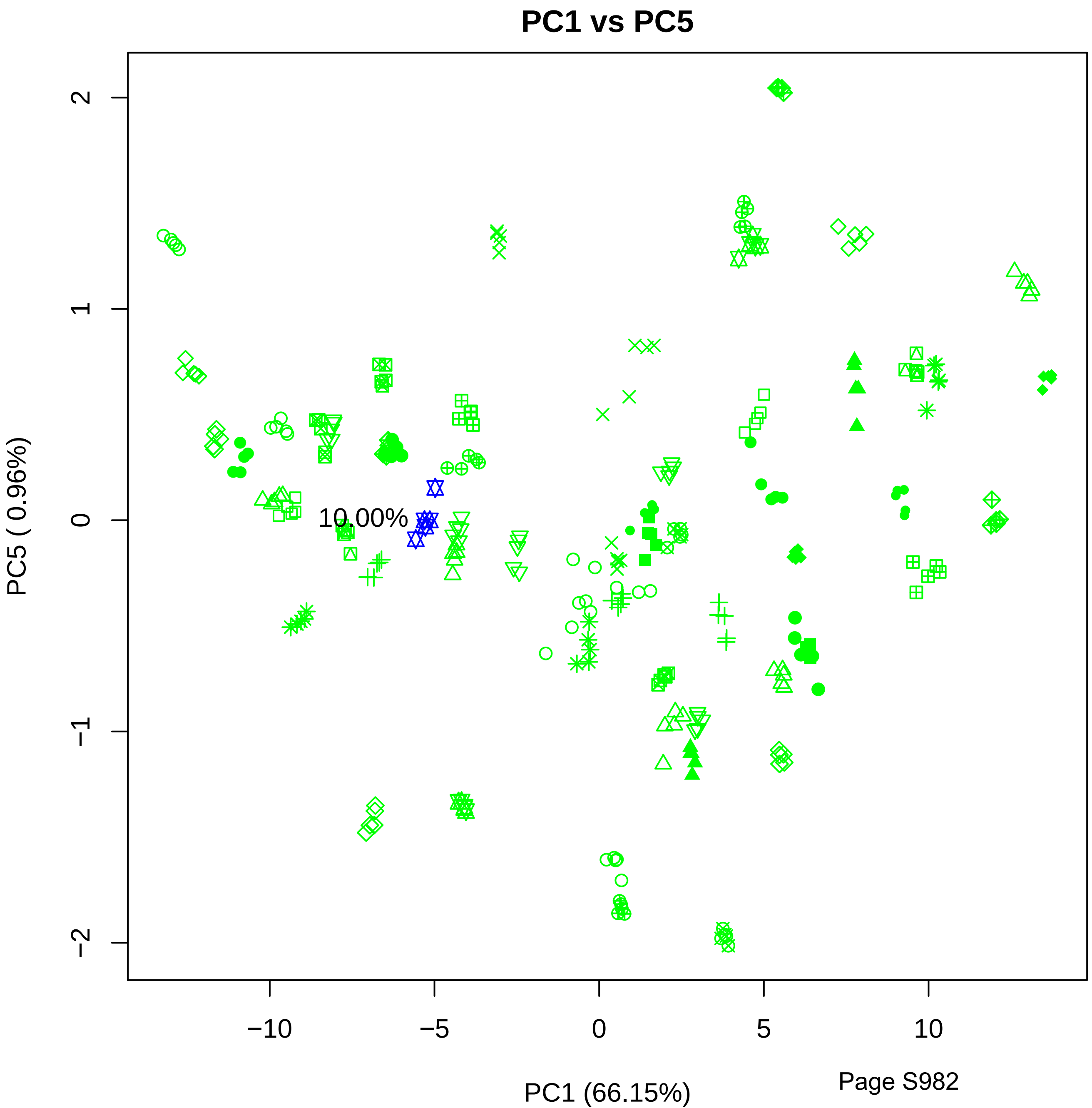
PC1 vs PC5
10.00%
2
1
0
−1
−2
PC5 (0.96%)
−10
−5
0
5
10
PC1 (66.15%)

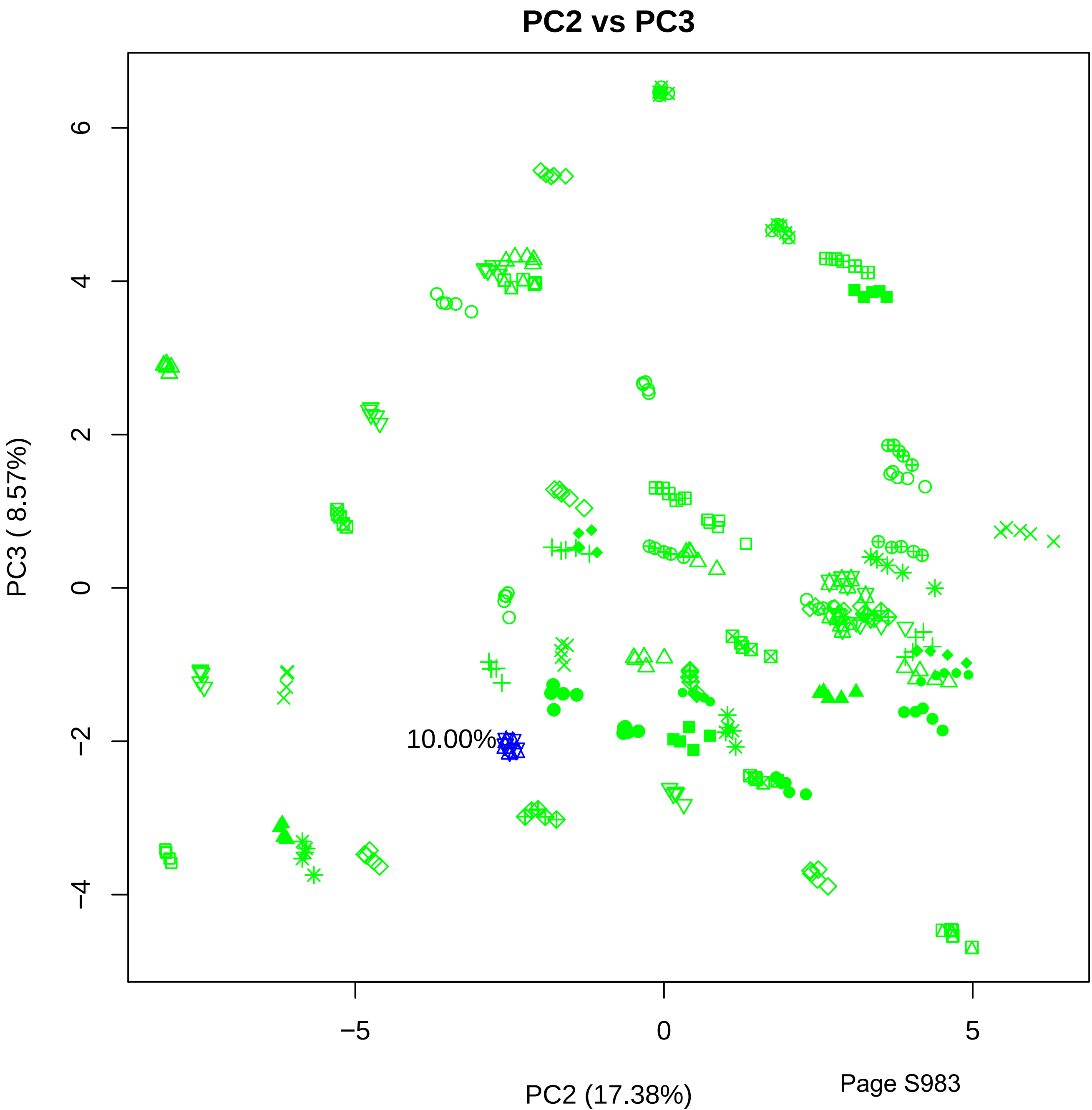
PC2 vs PC3
6
4
2
0
−2
−4
PC3 (8.57%)
10.00%
−5
0
5
PC2 (17.38%)

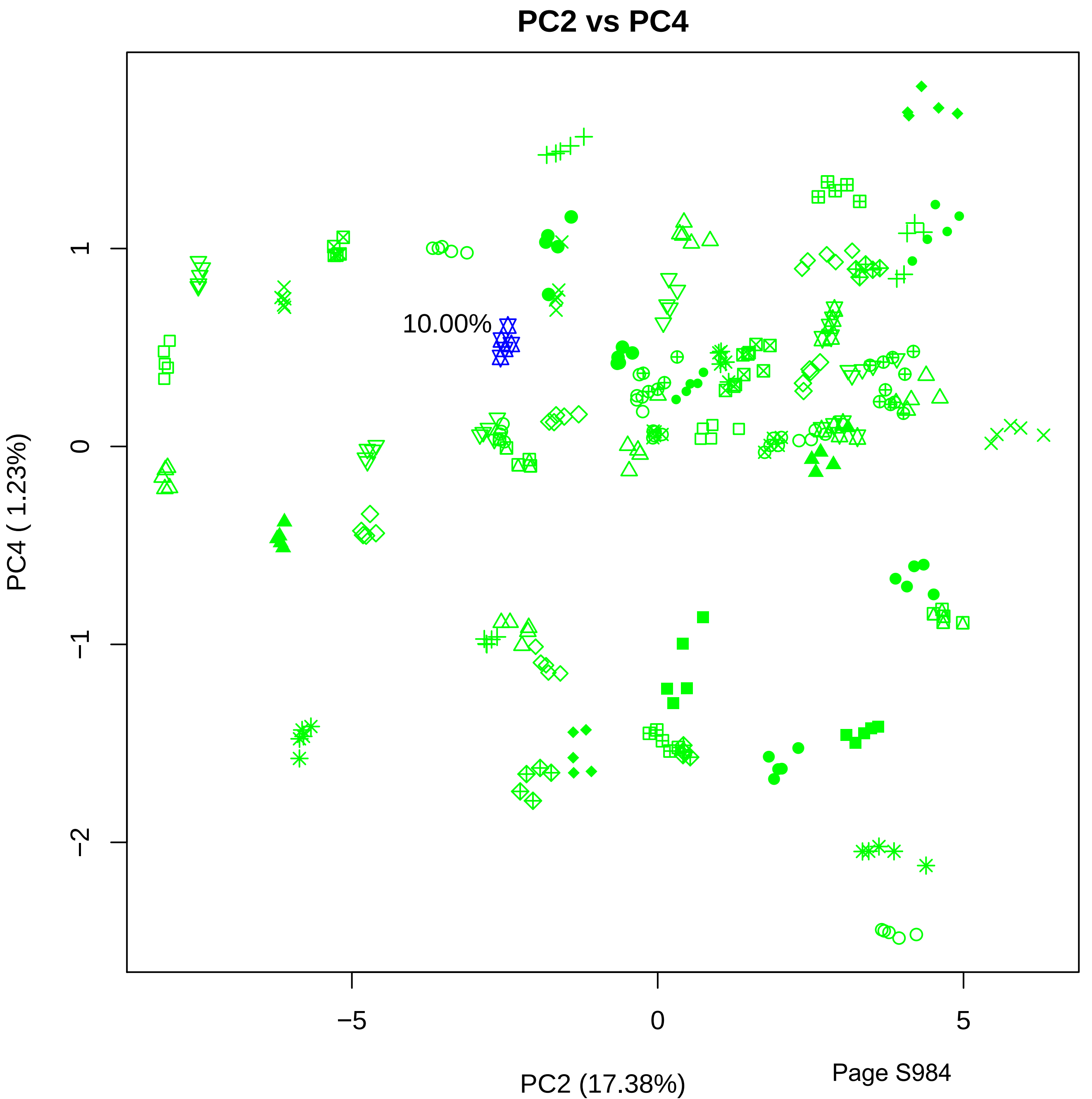
PC2 vs PC4
10.00%
PC4 (1.23%)
PC2 (17.38%)
−5
0
5
−2
−1
0
1

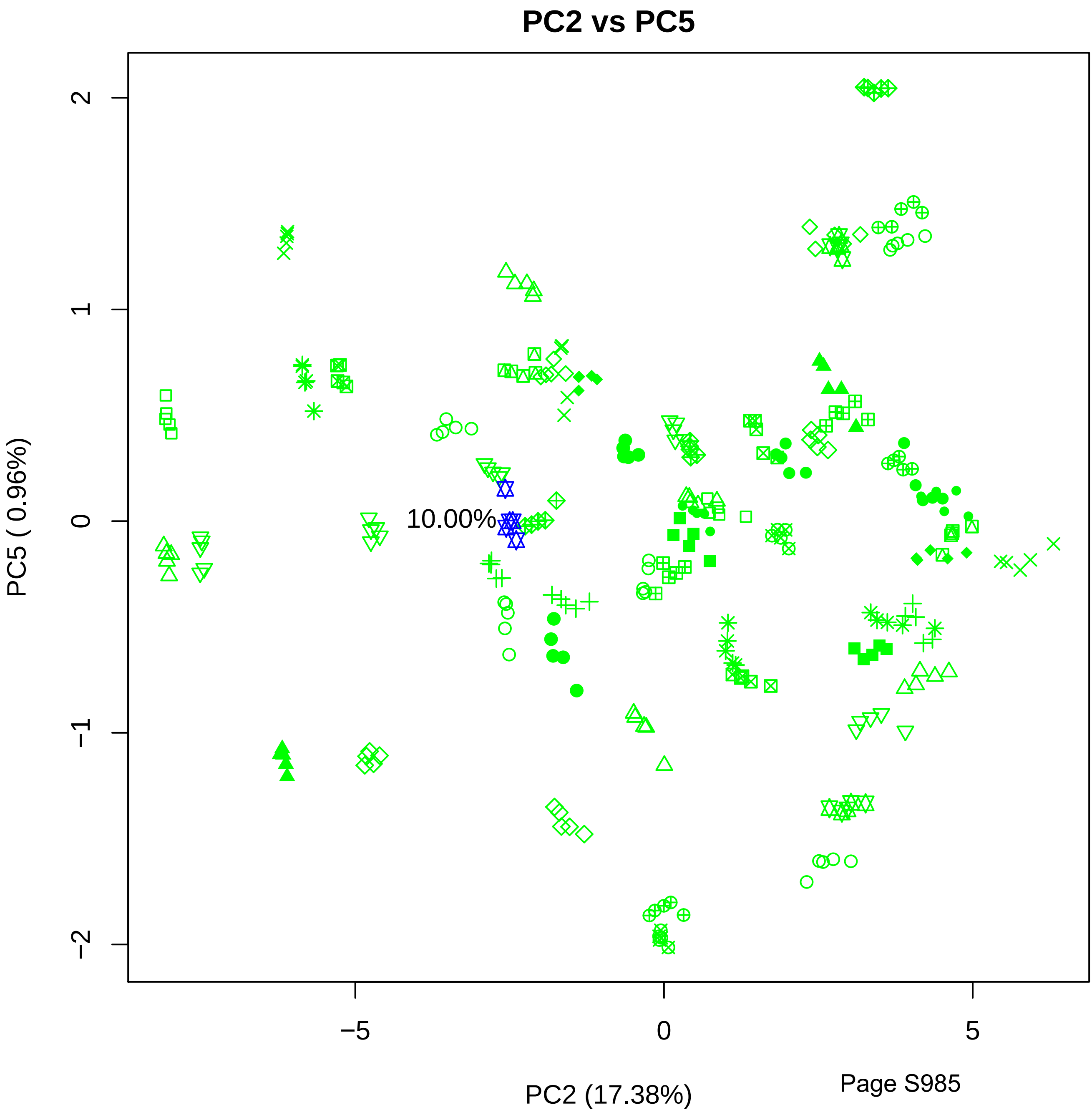

PC2 vs PC5
10.00%
PC5 (0.96%)
PC2 (17.38%)
2
1
0
−1
−2
−5
0
5

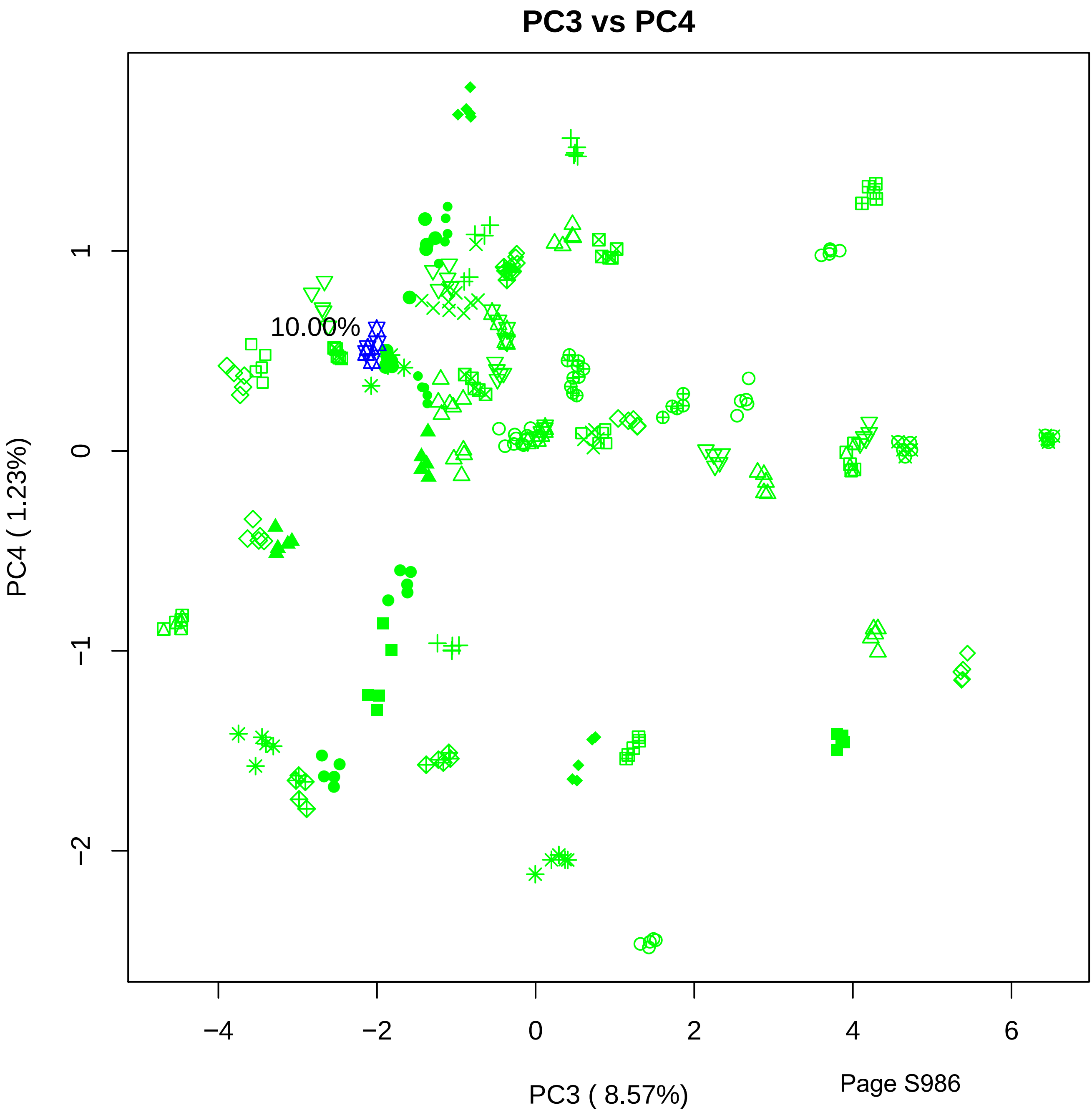
PC3 vs PC4
10.00%
1
0
−1
−2
−4
−2
0
2
4
6
PC4 (1.23%)
PC3 (8.57%)

PC3 vs PC5

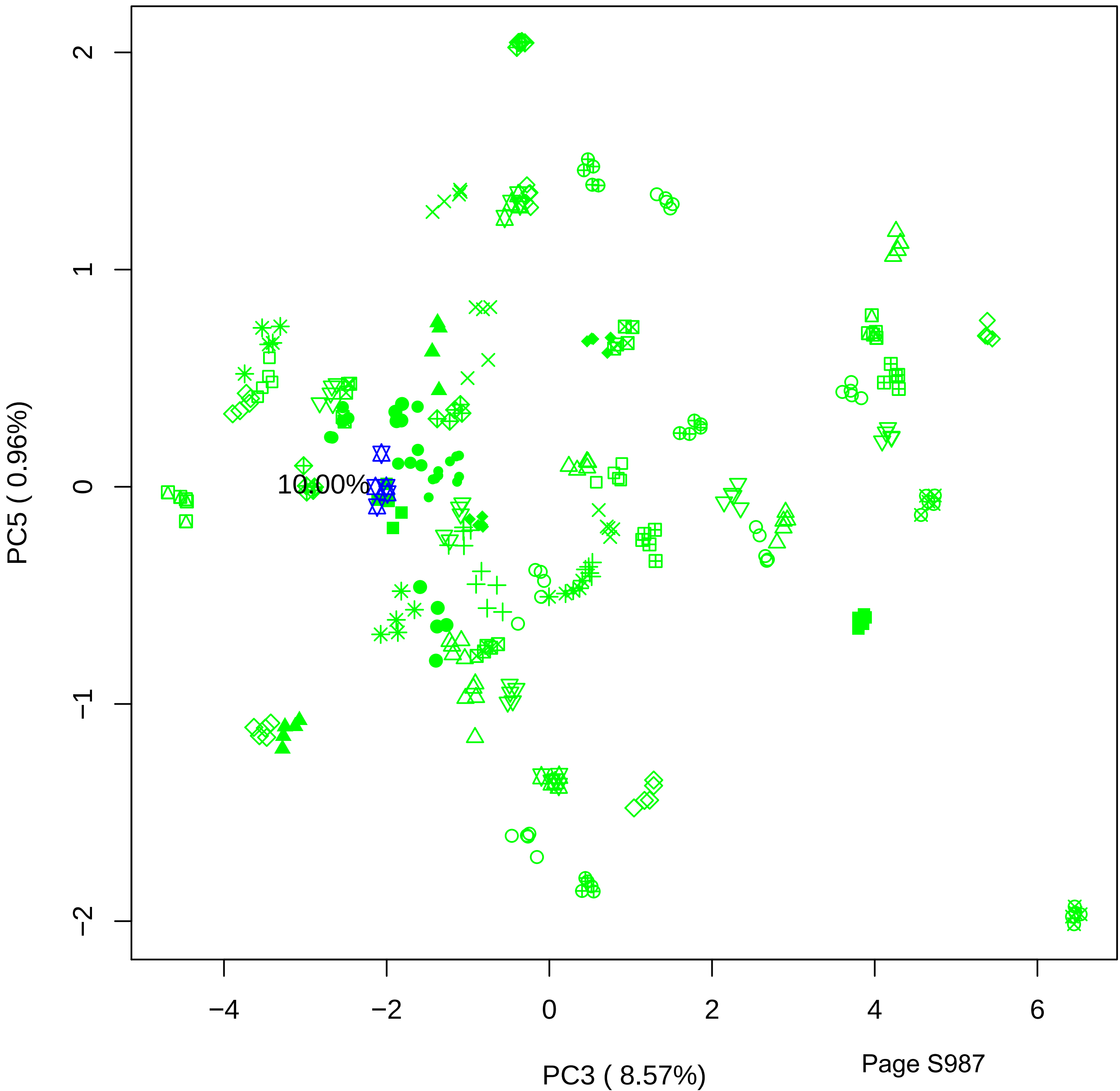

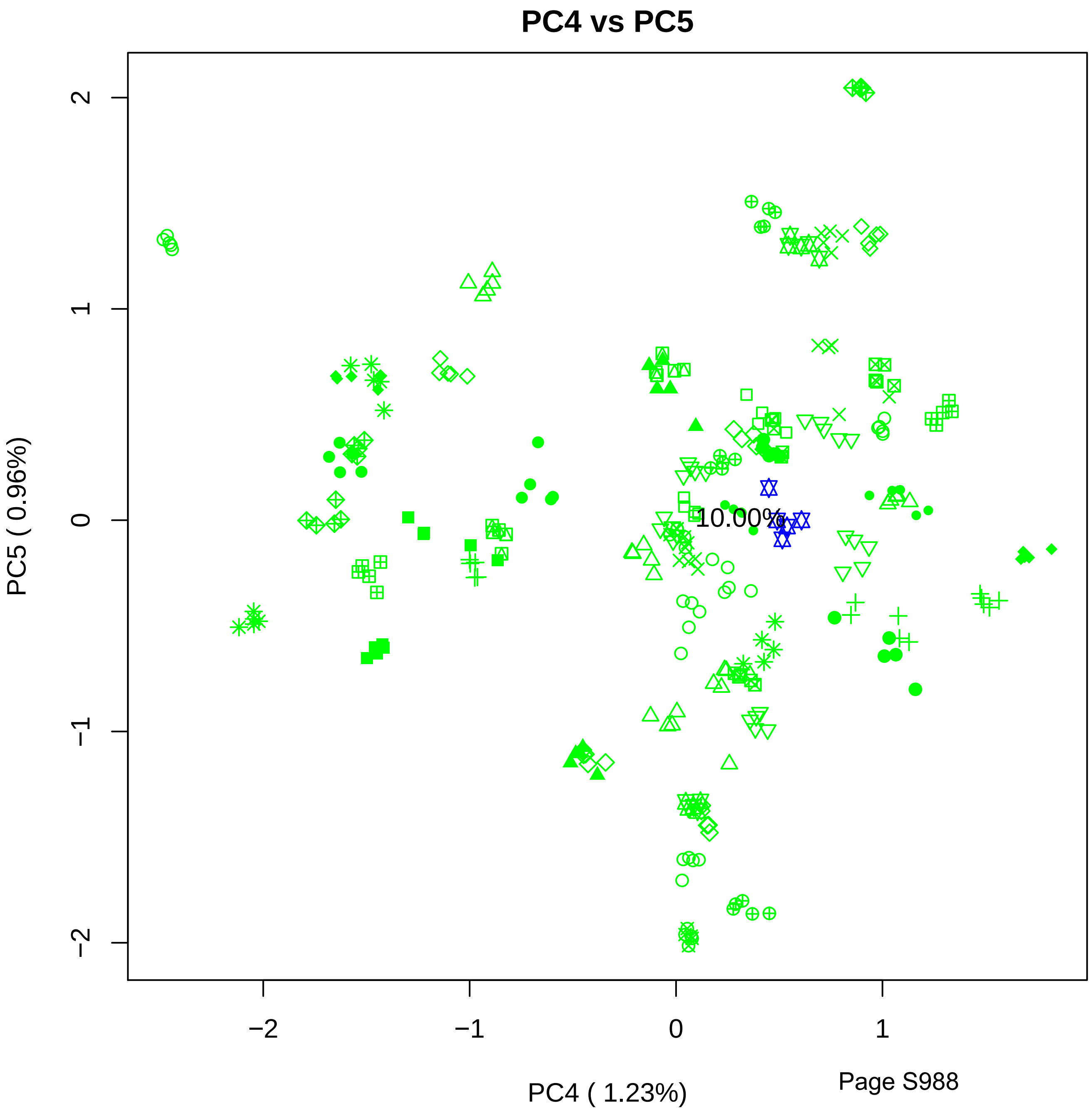
PC4 vs PC5
PC5 (0.96%)
PC4 (1.23%)
10.00%
2
1
0
−1
−2

PCA of 15% BMH(a) vs PMH(a)

Scaled Matrix PCAed

7th order polynomial baseline correction performed

Raw spectra smoothed to 21 Neighbours, 2nd order PN, Savtisky Golay

Normalisation between 0 and 100

D2 spectra smoothed to 41 Neighbours, 2nd order PN, Savtisky Golay

Regions Analysed: 3601−2501 and 2001−700cm−1

Samples Analysed: 1 bContam's 0 BLH's 0 BMH's 0 OMH's 0 pContam's 0 PLH's 60 PMH's

Keele University AD

Fri Dec 14 14:20:36 2018

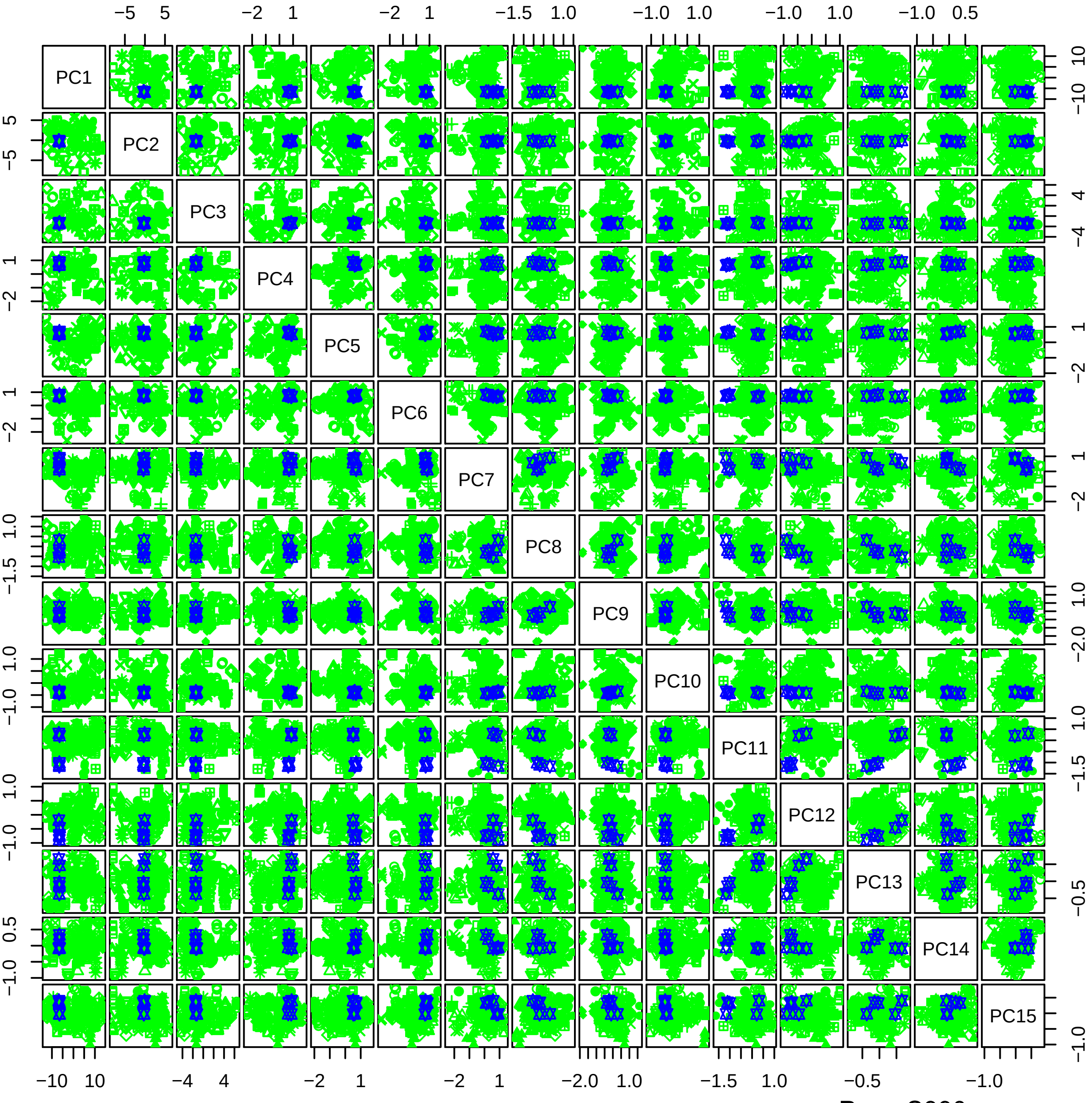

PC1
PC2
PC3
PC4
PC5
PC6
PC7
PC8
PC9
PC10
PC11
PC12
PC13
PC14
PC15

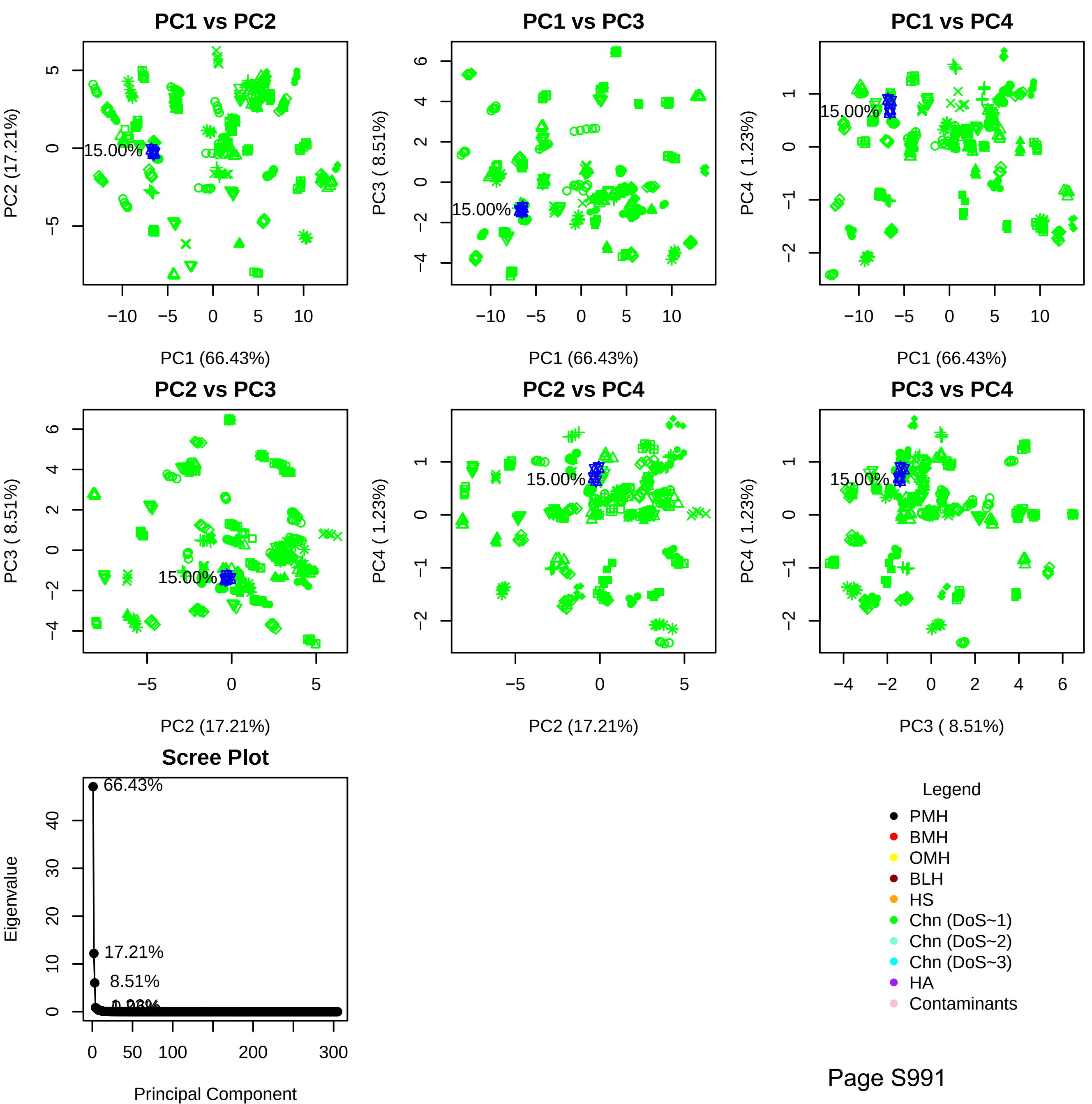
PC1 vs PC2
PC1 vs PC3
PC1 vs PC4
PC2 vs PC3
PC2 vs PC4
PC3 vs PC4
Scree Plot
PC1 (66.43%)
PC2 (17.21%)
PC3 (8.51%)
PC4 (1.23%)
15.00%
Eigenvalue
Principal Component
66.43%
17.21%
8.51%
Legend
PMH
BMH
OMH
BLH
HS
Chn (DoS~1)
Chn (DoS~2)
Chn (DoS~3)
HA
Contaminants

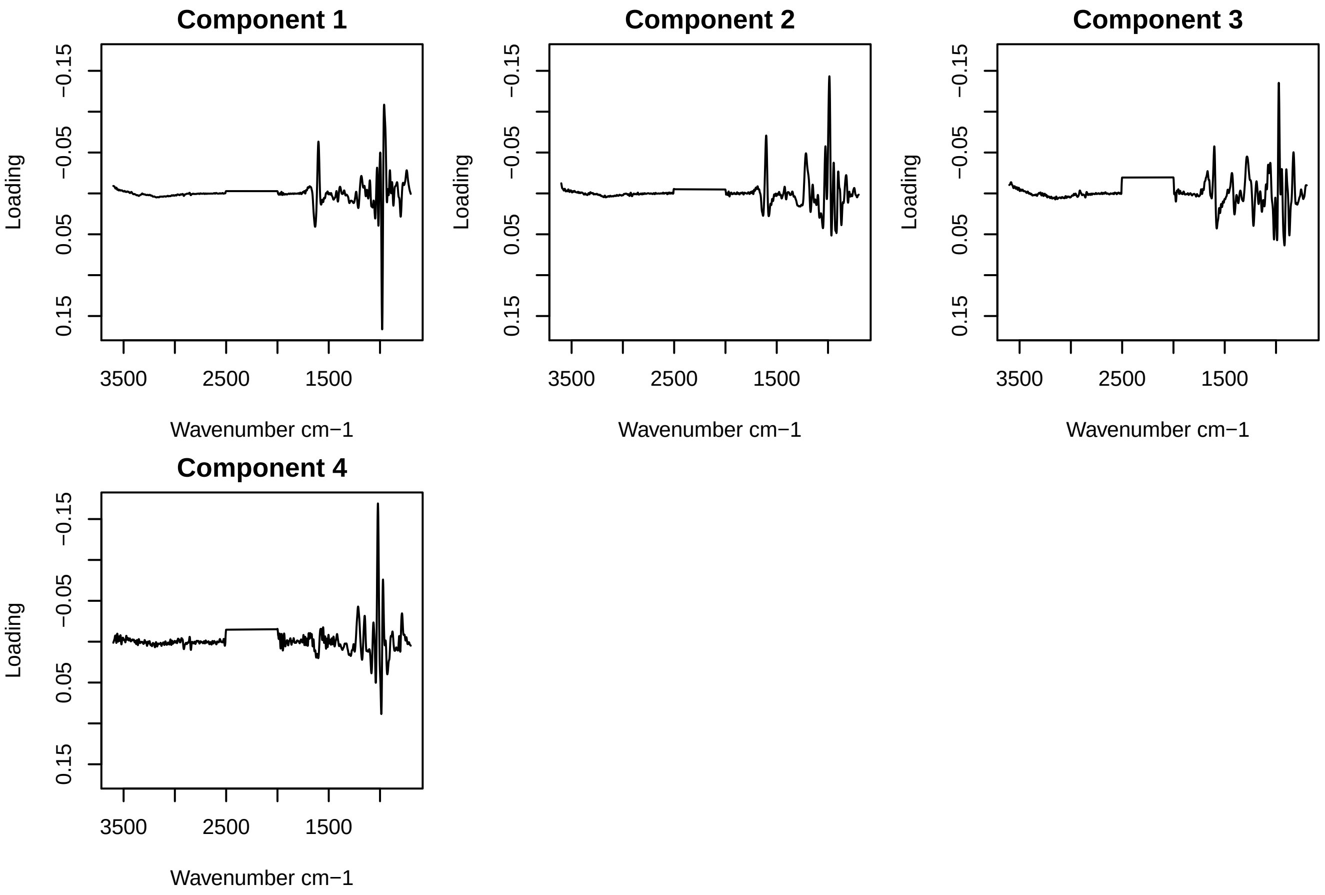
Component 1
Component 2
Component 3
Component 4
Loading
−0.15
−0.05
0.05
0.15
3500
2500
1500
Wavenumber cm−1

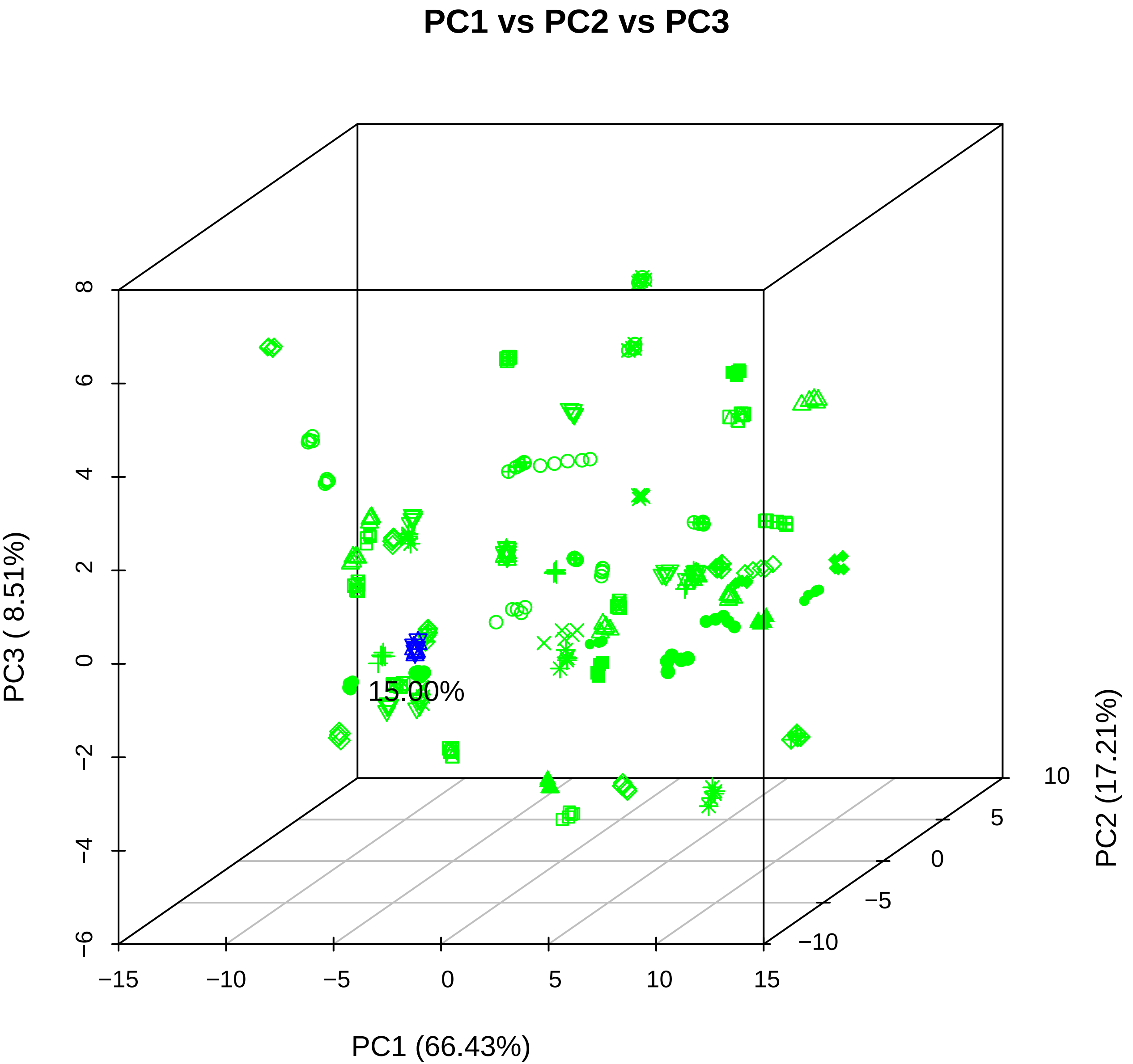

PC1 vs PC2 vs PC3
PC3 (8.51%)
PC1 (66.43%)
PC2 (17.21%)
15.00%
8
6
4
2
0
−2
−4
−6
−15
−10
−5
0
5
10
15
10
5
0
−5
−10

PC1 vs PC2 vs PC4

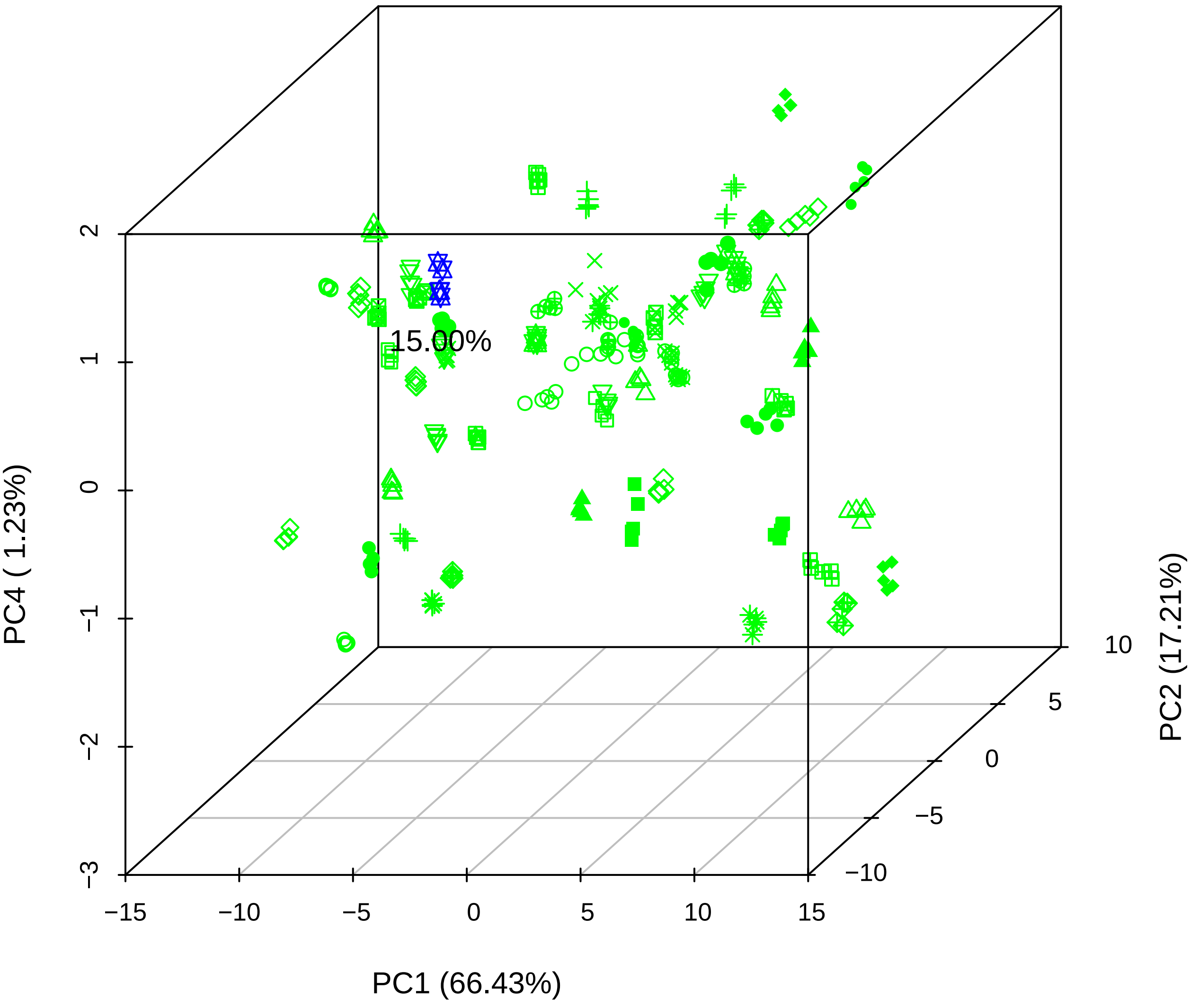

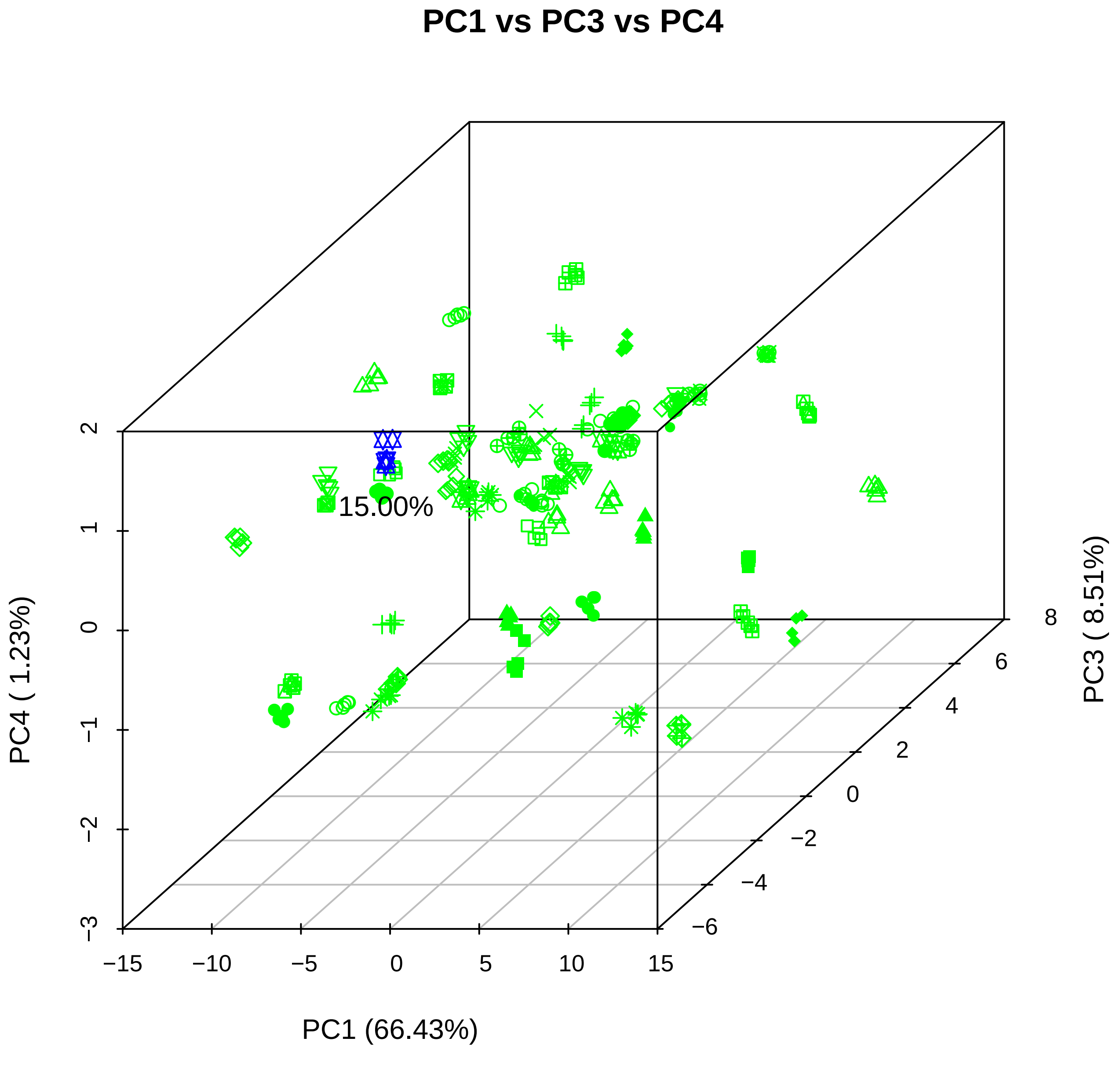

PC1 vs PC3 vs PC4
15.00%
PC4 (1.23%)
2
1
0
−1
−2
−3
−15
−10
−5
0
5
10
15
PC1 (66.43%)
PC3 (8.51%)
8
6
4
2
0
−2
−4
−6

PC2 vs PC3 vs PC5

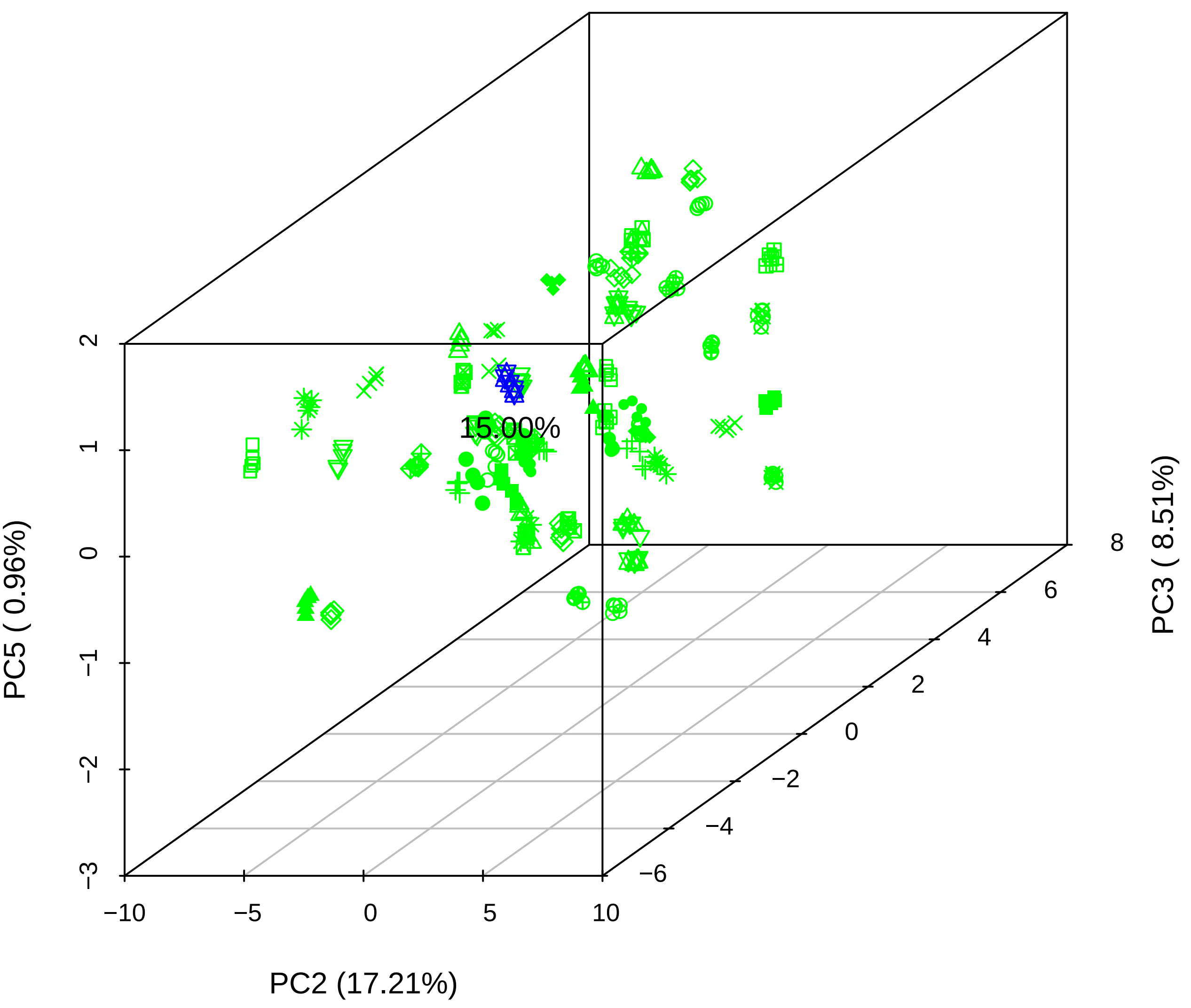

PC1 vs PC2

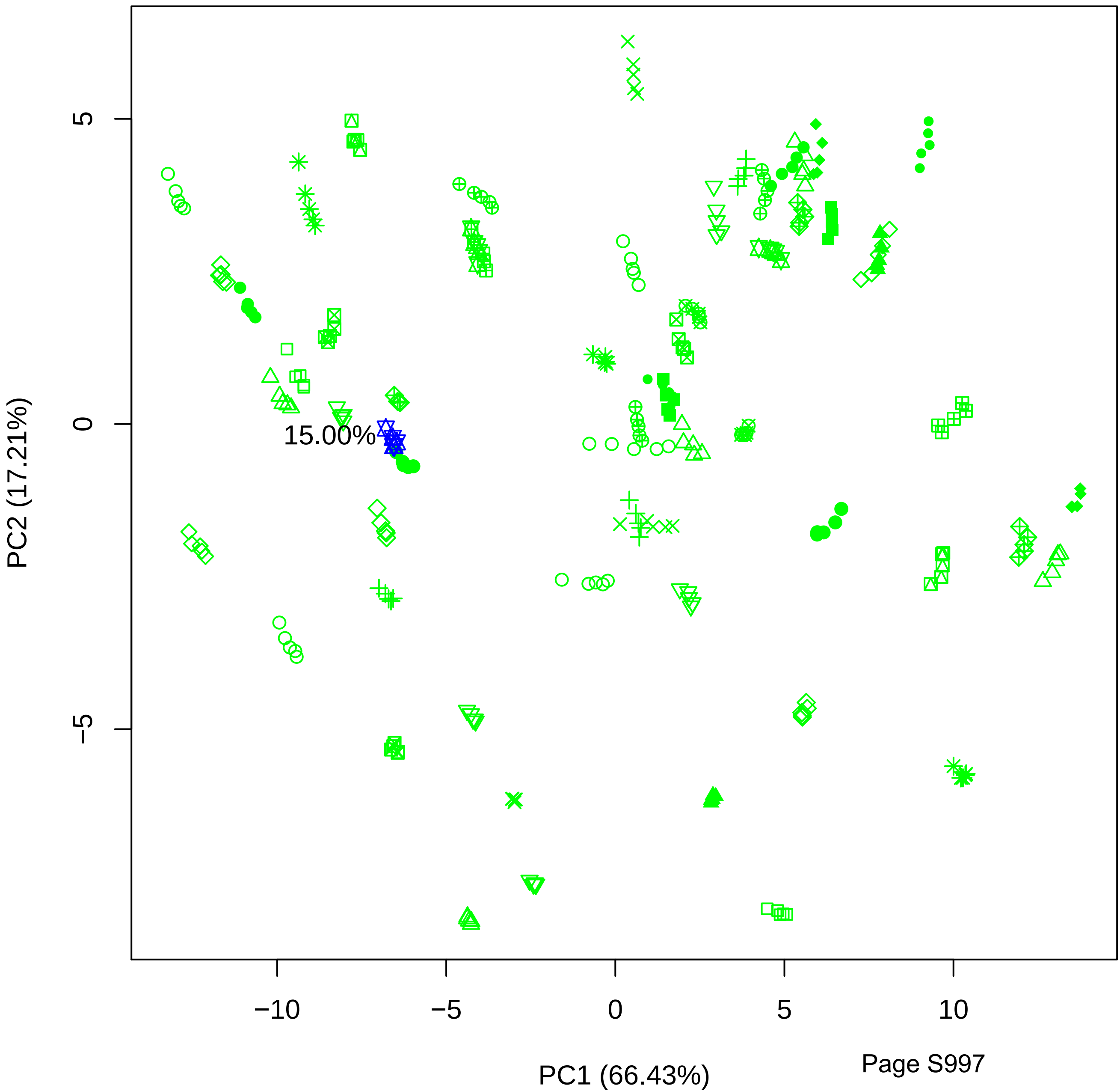

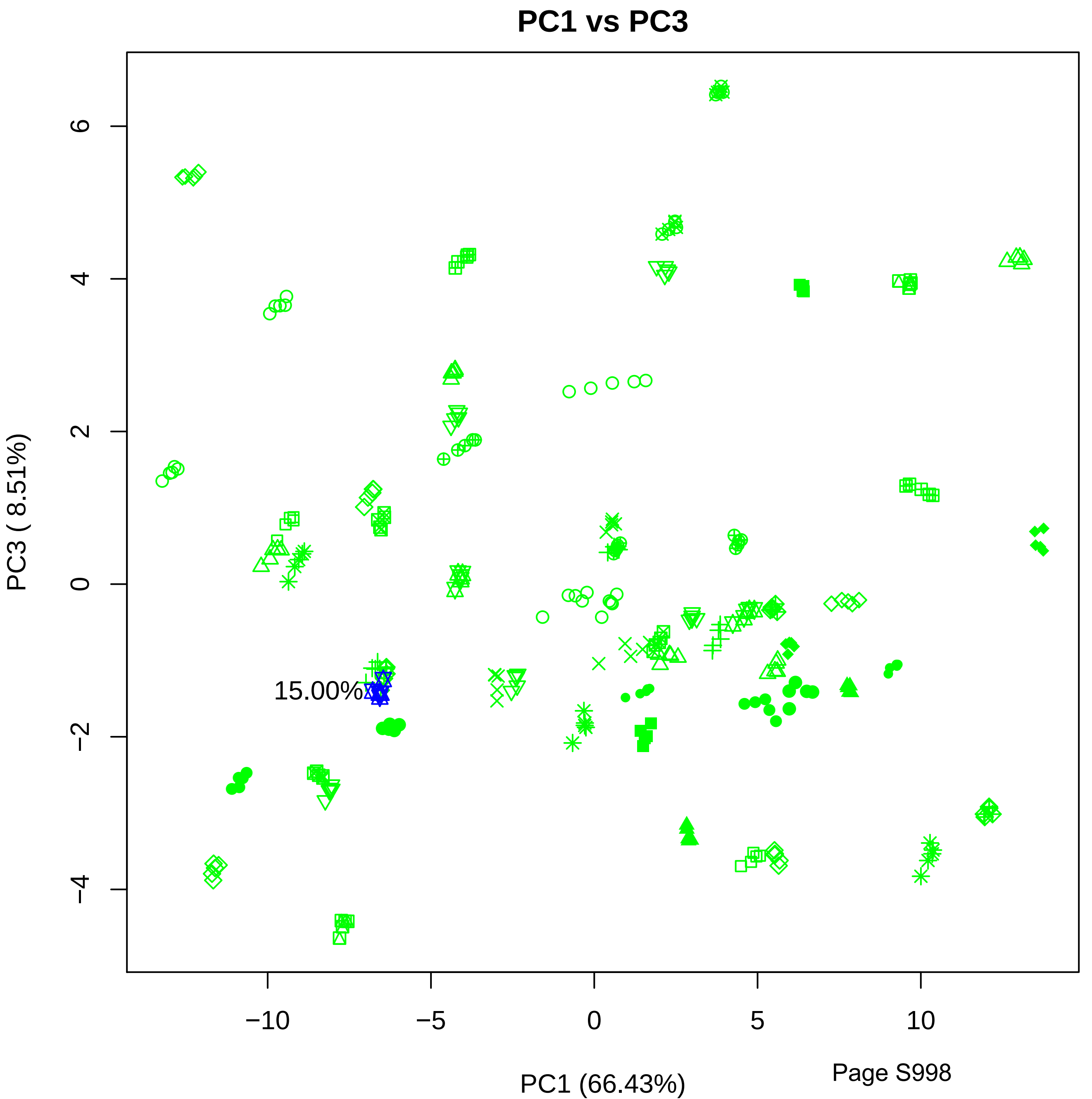
PC1 vs PC3
PC3 (8.51%)
PC1 (66.43%)
15.00%
6
4
2
0
−2
−4
−10
−5
0
5
10

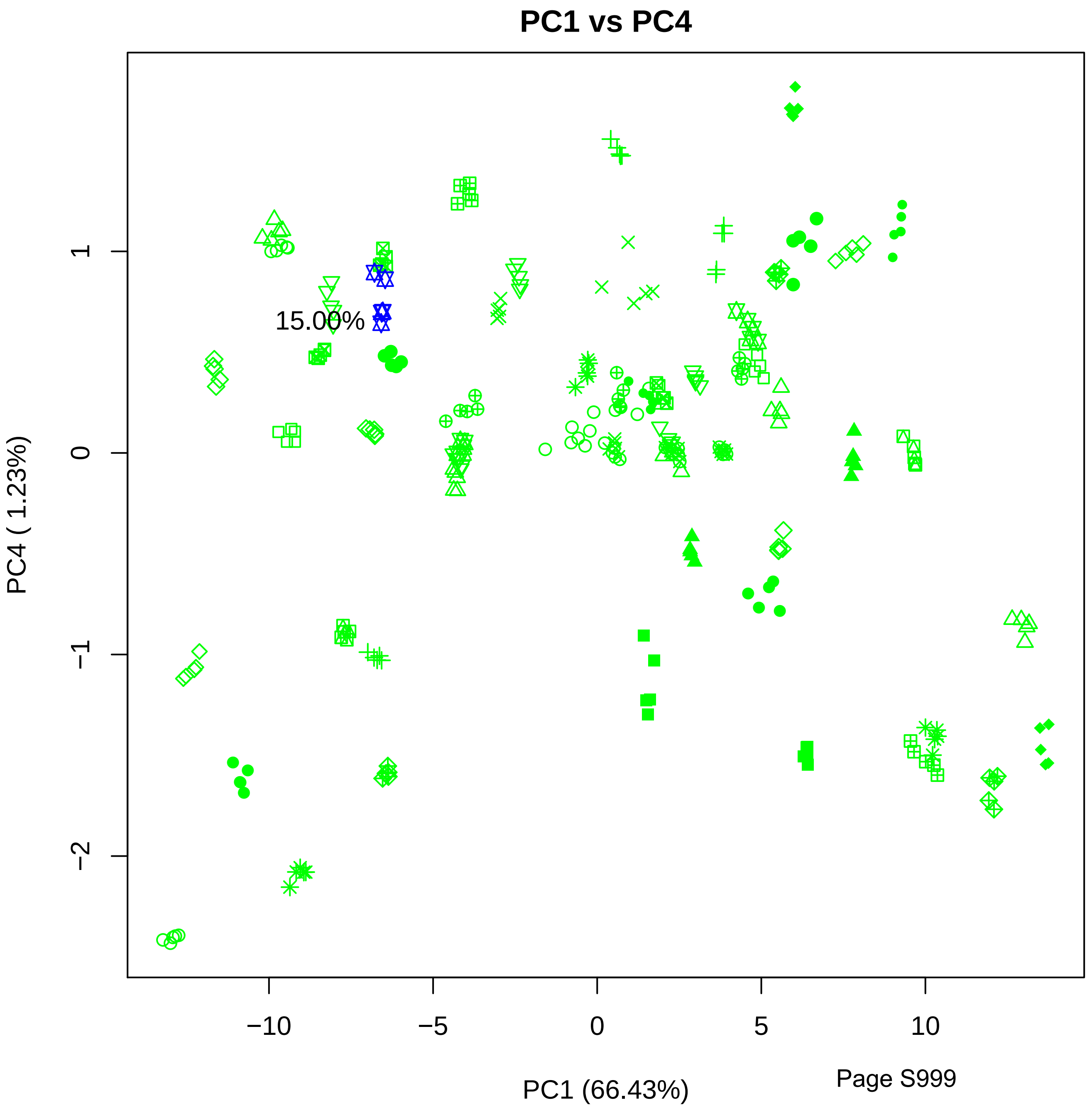
PC1 vs PC4
15.00%
PC4 (1.23%)
PC1 (66.43%)
−10
−5
0
5
10
1
0
−1
−2

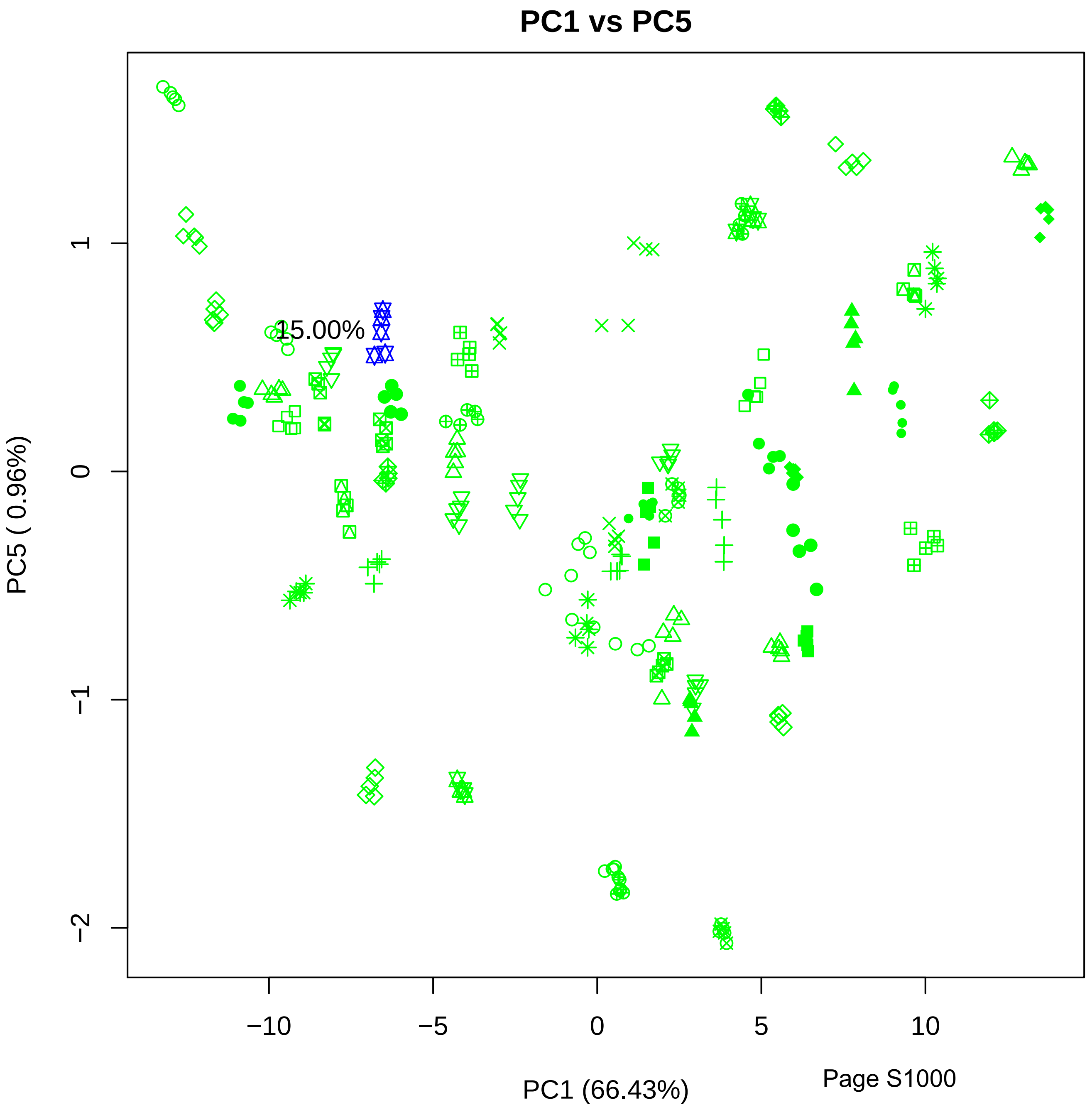
PC1 vs PC5
15.00%
PC5 (0.96%)
1
0
−1
−2
−10
−5
0
5
10
PC1 (66.43%)

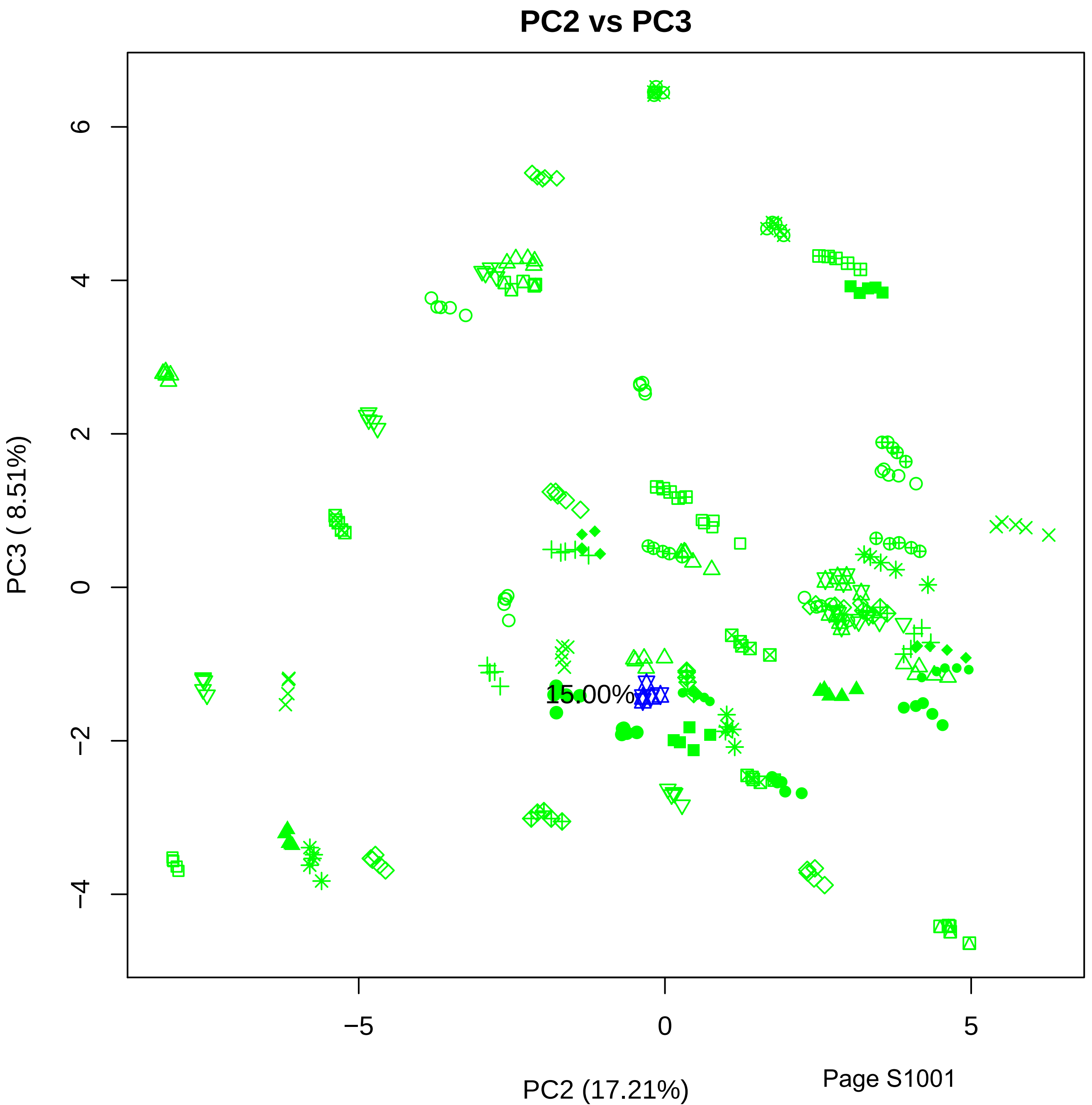
PC2 vs PC3
PC3 (8.51%)
PC2 (17.21%)
15.00%
6
4
2
0
−2
−4
−5
0
5

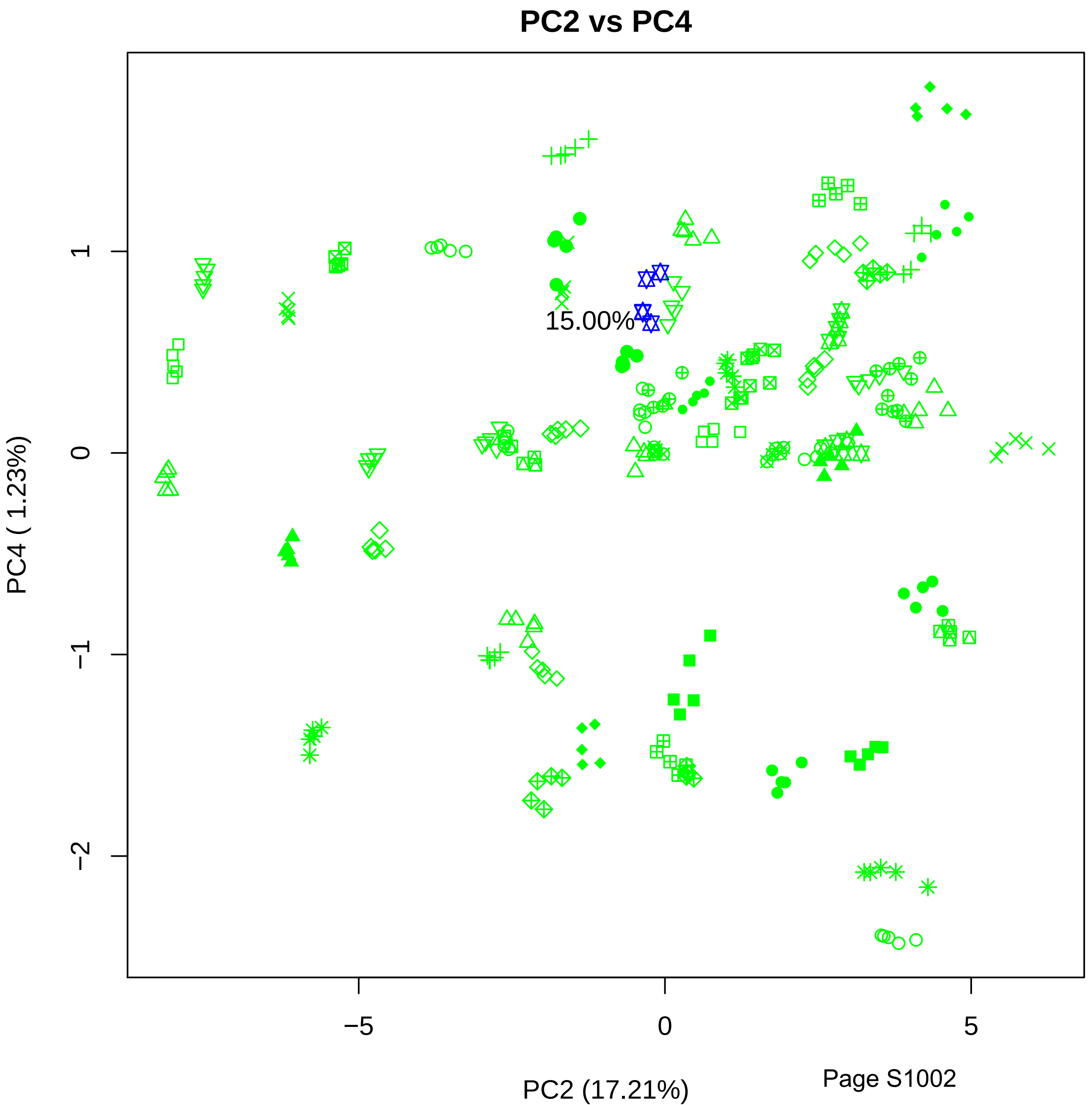
PC2 vs PC4
15.00%
1
0
−1
−2
−5
0
5
PC4 (1.23%)
PC2 (17.21%)

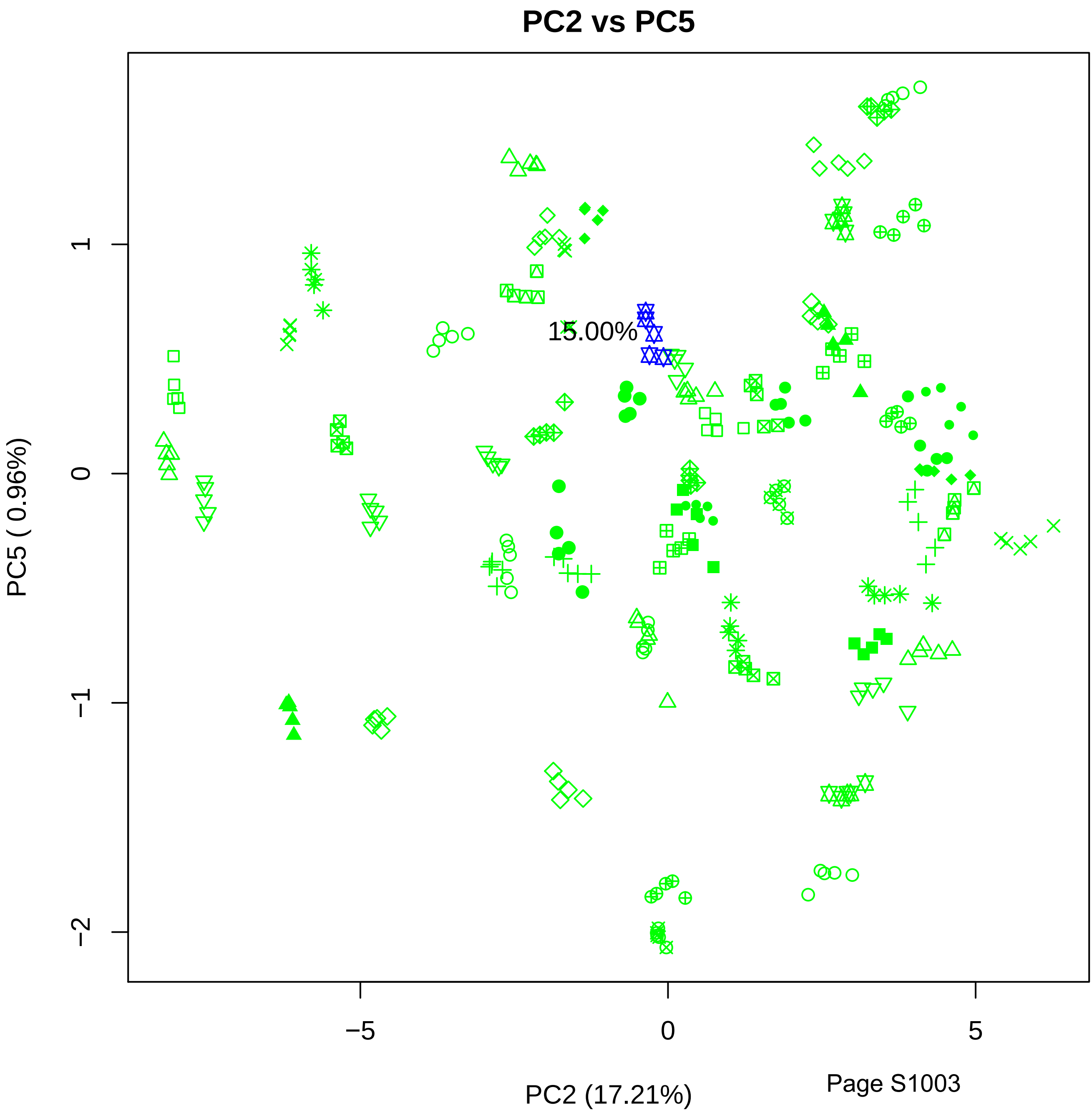
PC2 vs PC5
15.00%
1
0
−1
−2
−5
0
5
PC5 (0.96%)
PC2 (17.21%)

PC3 vs PC4

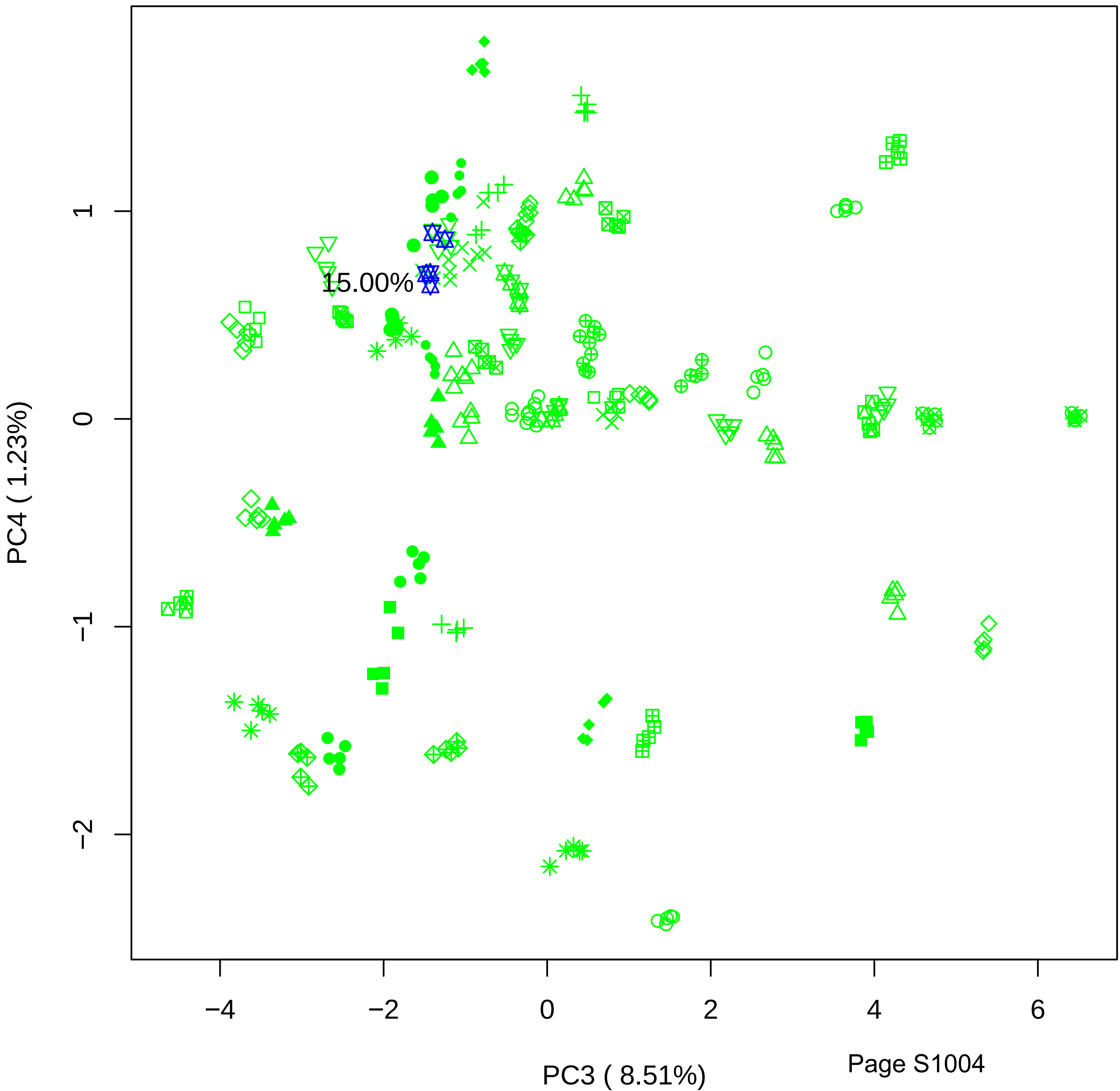

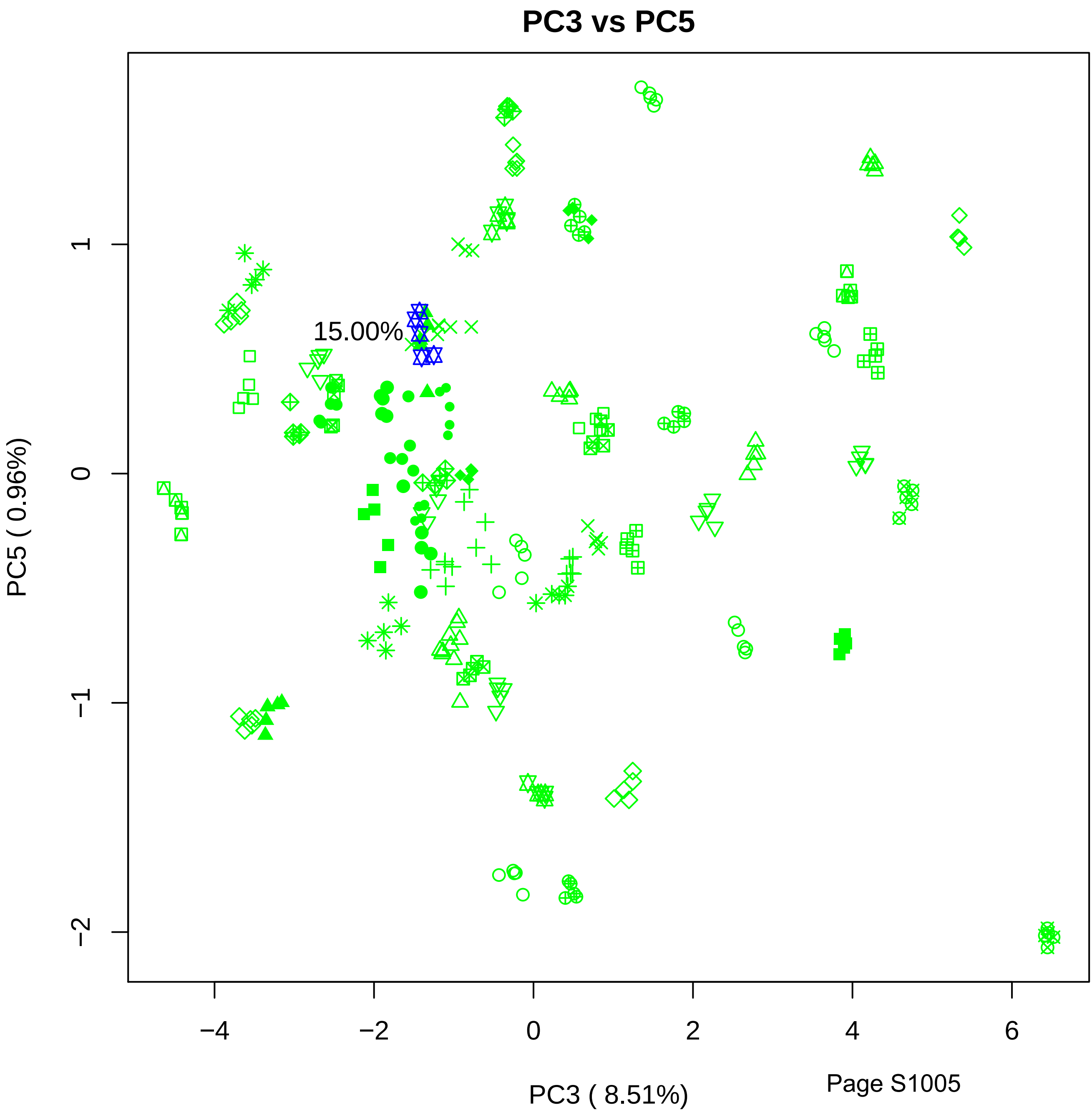
PC3 vs PC5
15.00%
PC5 (0.96%)
PC3 (8.51%)
1
0
−1
−2
−4
−2
0
2
4
6

PC4 vs PC5

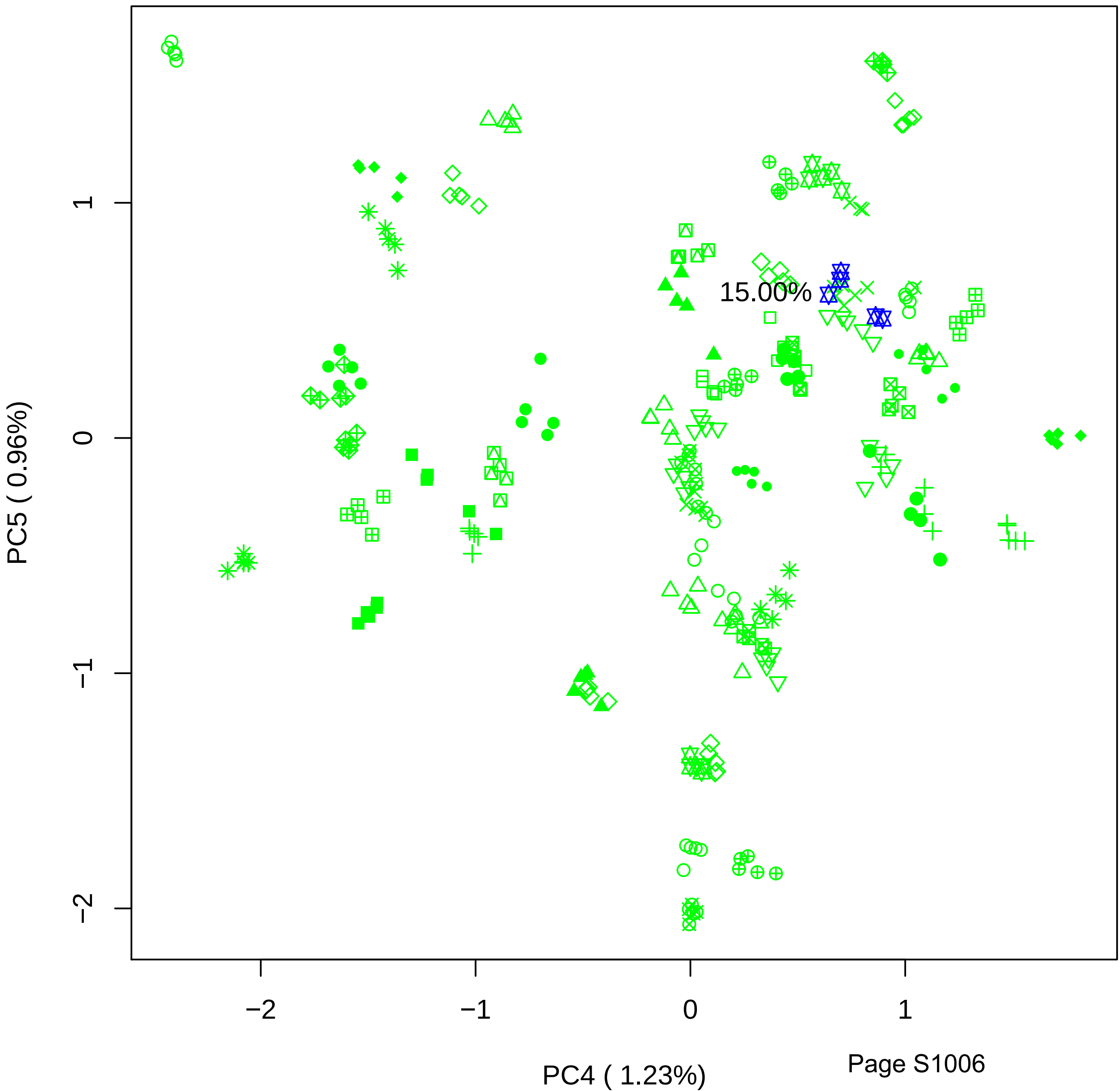

PCA of 20% BMH(a) vs PMH(a)

Scaled Matrix PCAed

7th order polynomial baseline correction performed

Raw spectra smoothed to 21 Neighbours, 2nd order PN, Savtisky Golay

Normalisation between 0 and 100

D2 spectra smoothed to 41 Neighbours, 2nd order PN, Savtisky Golay

Regions Analysed: 3601−2501 and 2001−700cm−1

Samples Analysed: 1 bContam's 0 BLH's 0 BMH's 0 OMH's 0 pContam's 0 PLH's 60 PMH's

Keele University AD

Fri Dec 14 14:20:44 2018

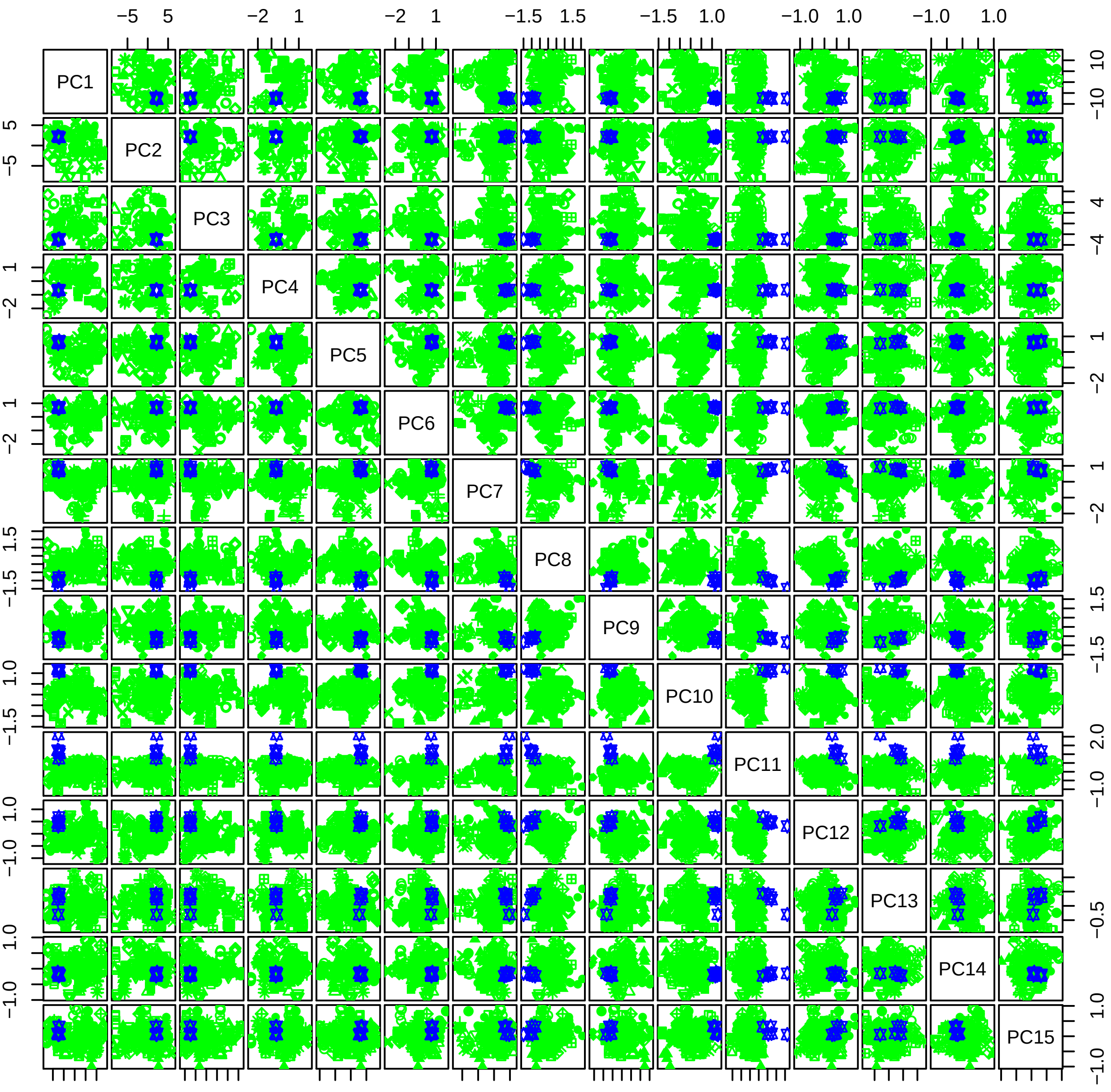

PC1
PC2
PC3
PC4
PC5
PC6
PC7
PC8
PC9
PC10
PC11
PC12
PC13
PC14
PC15

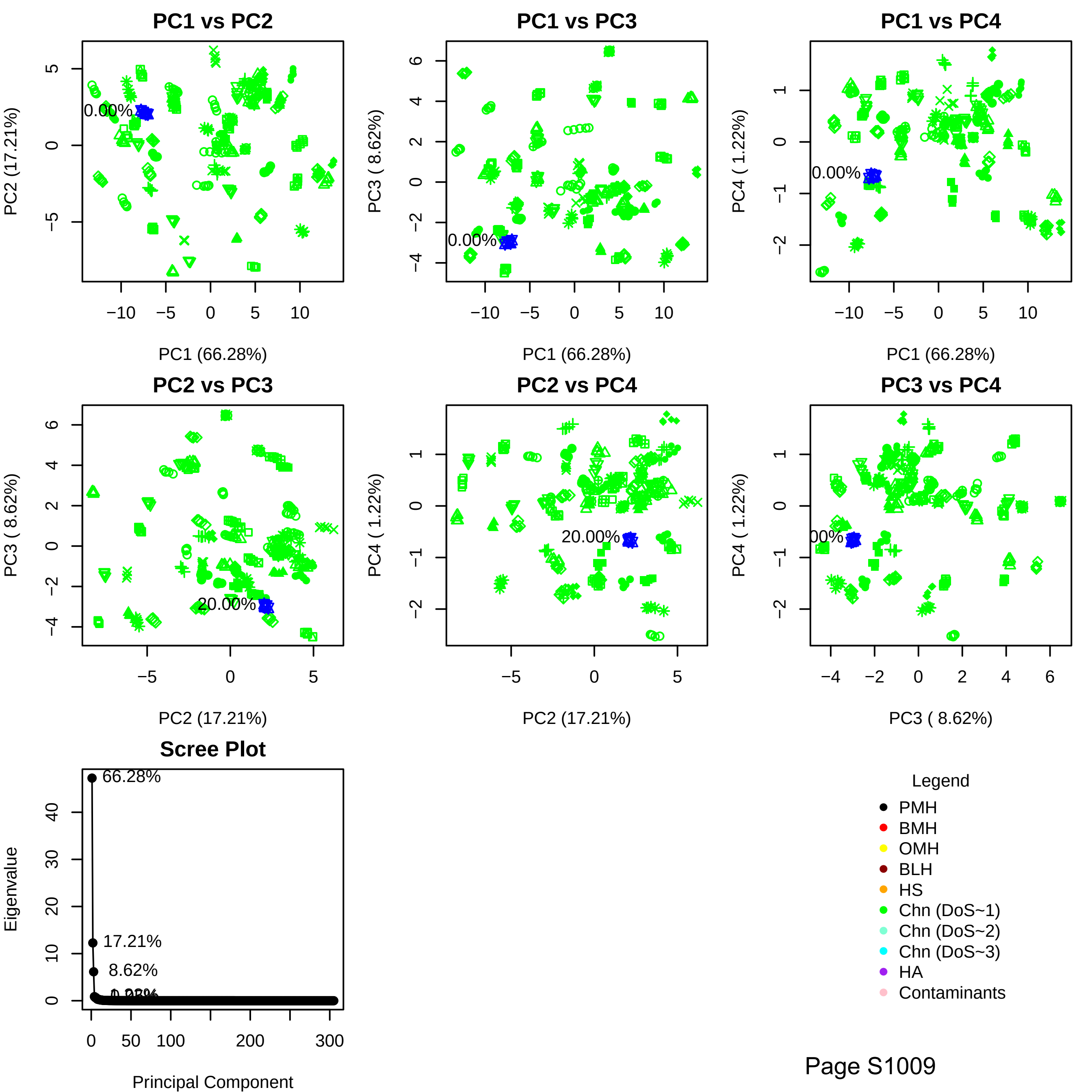
PC1 vs PC2
PC1 vs PC3
PC1 vs PC4
PC2 vs PC3
PC2 vs PC4
PC3 vs PC4
Scree Plot
PC1 (66.28%)
PC2 (17.21%)
PC3 (8.62%)
PC4 (1.22%)
0.00%
20.00%
Eigenvalue
Principal Component
66.28%
17.21%
8.62%
Legend
PMH
BMH
OMH
BLH
HS
Chn (DoS~1)
Chn (DoS~2)
Chn (DoS~3)
HA
Contaminants

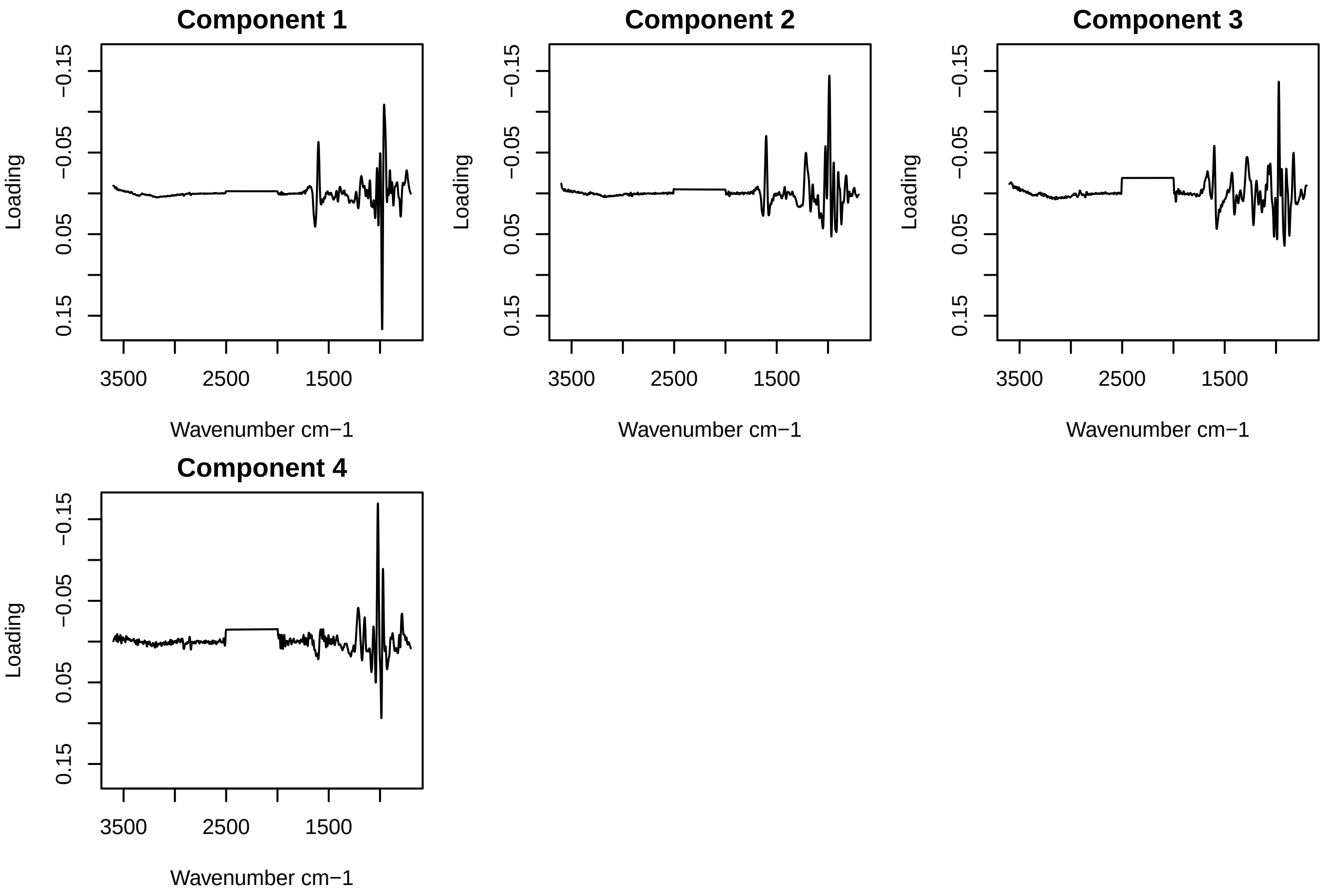
Component 1
Component 2
Component 3
Component 4
Loading
−0.15
−0.05
0.05
0.15
3500
2500
1500
Wavenumber cm−1

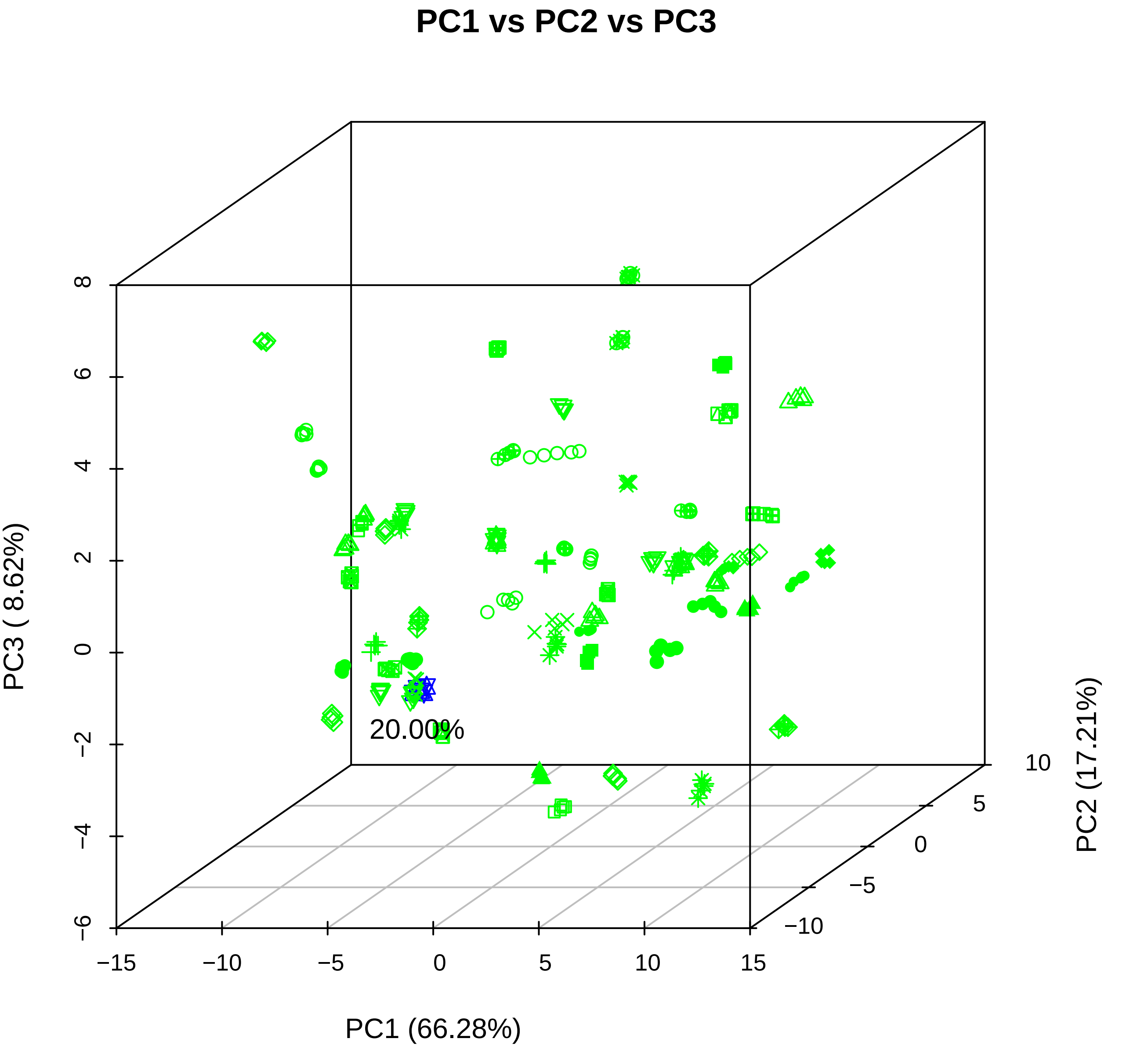

PC1 vs PC2 vs PC3
PC3 (8.62%)
PC1 (66.28%)
PC2 (17.21%)
20.00%
−6
−4
−2
0
2
4
6
8
−15
−10
−5
0
5
10
15
−10
−5
0
5
10

PC1 vs PC2 vs PC4

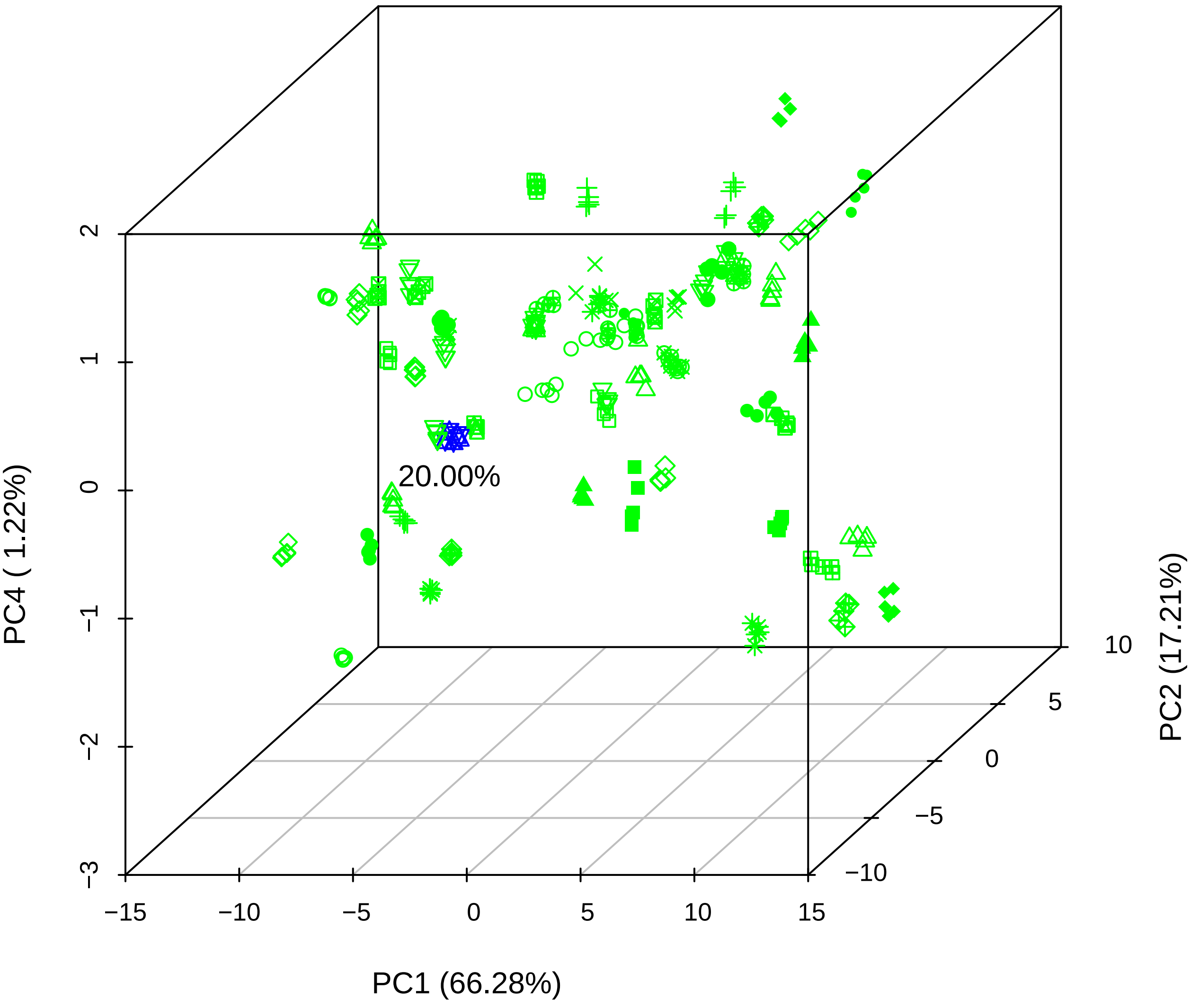

PC1 vs PC3 vs PC4

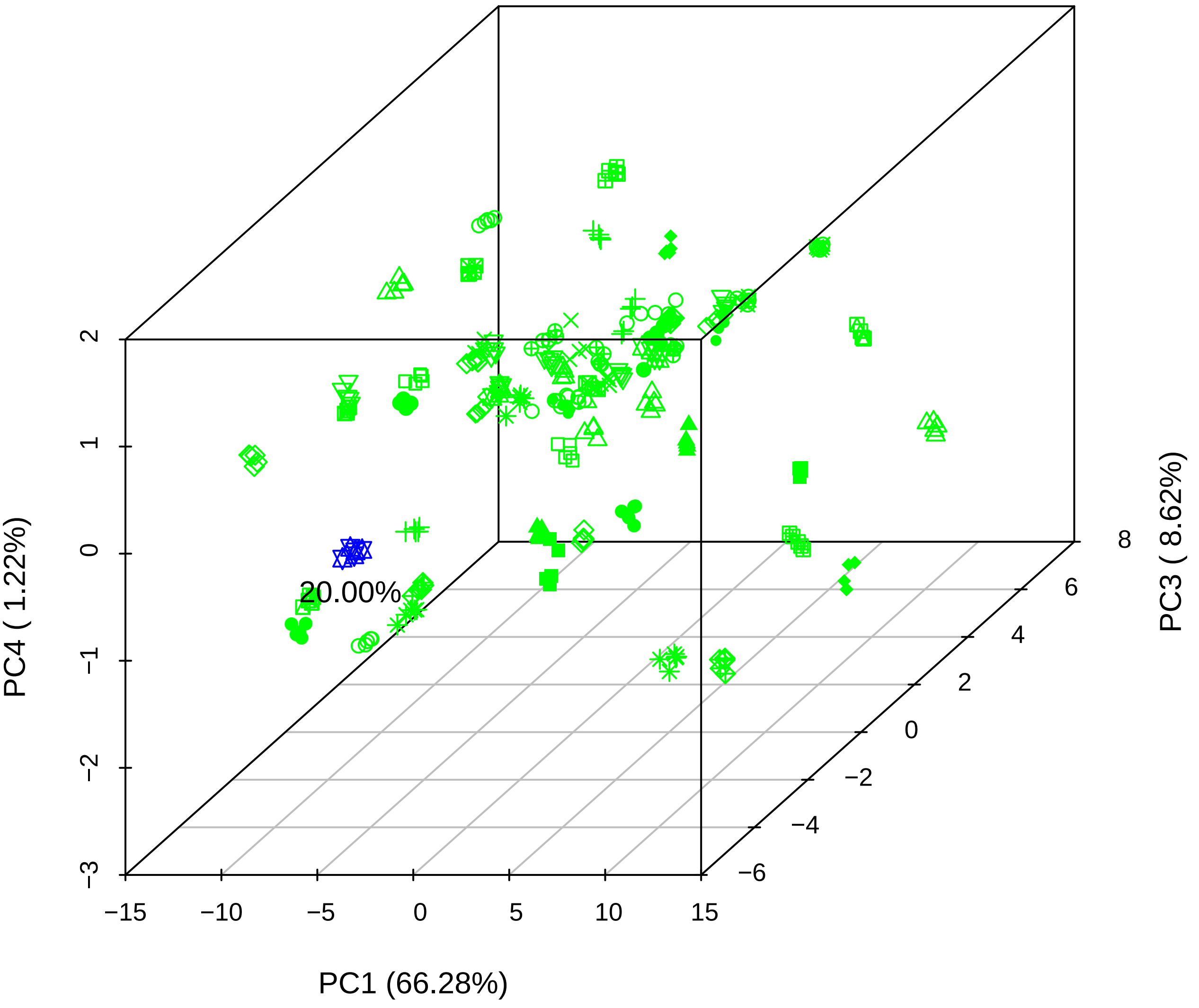

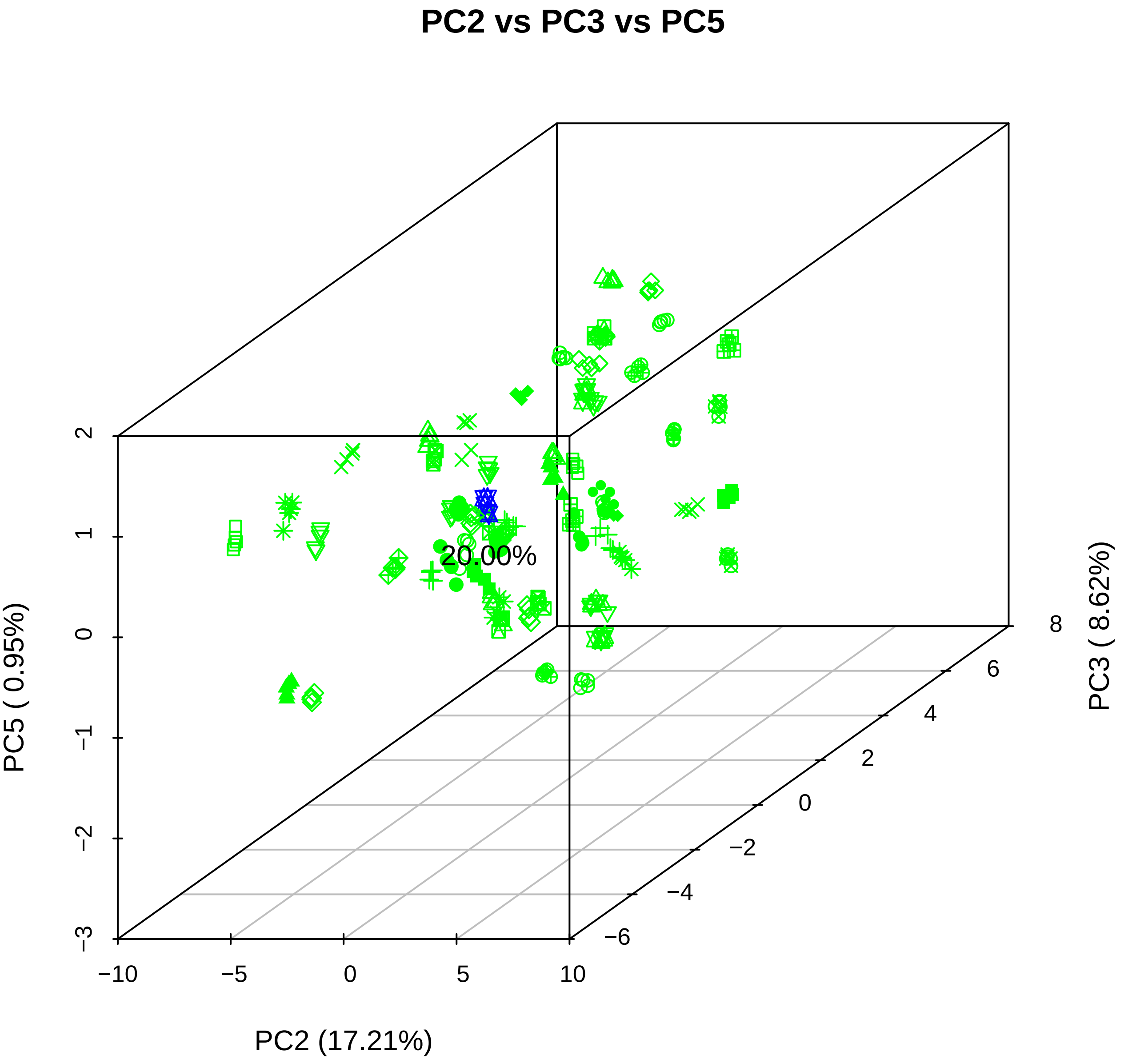

PC2 vs PC3 vs PC5
20.00%
PC5 (0.95%)
PC2 (17.21%)
PC3 (8.62%)
−10
−5
0
5
10
−3
−2
−1
0
1
2
−6
−4
−2
0
2
4
6
8

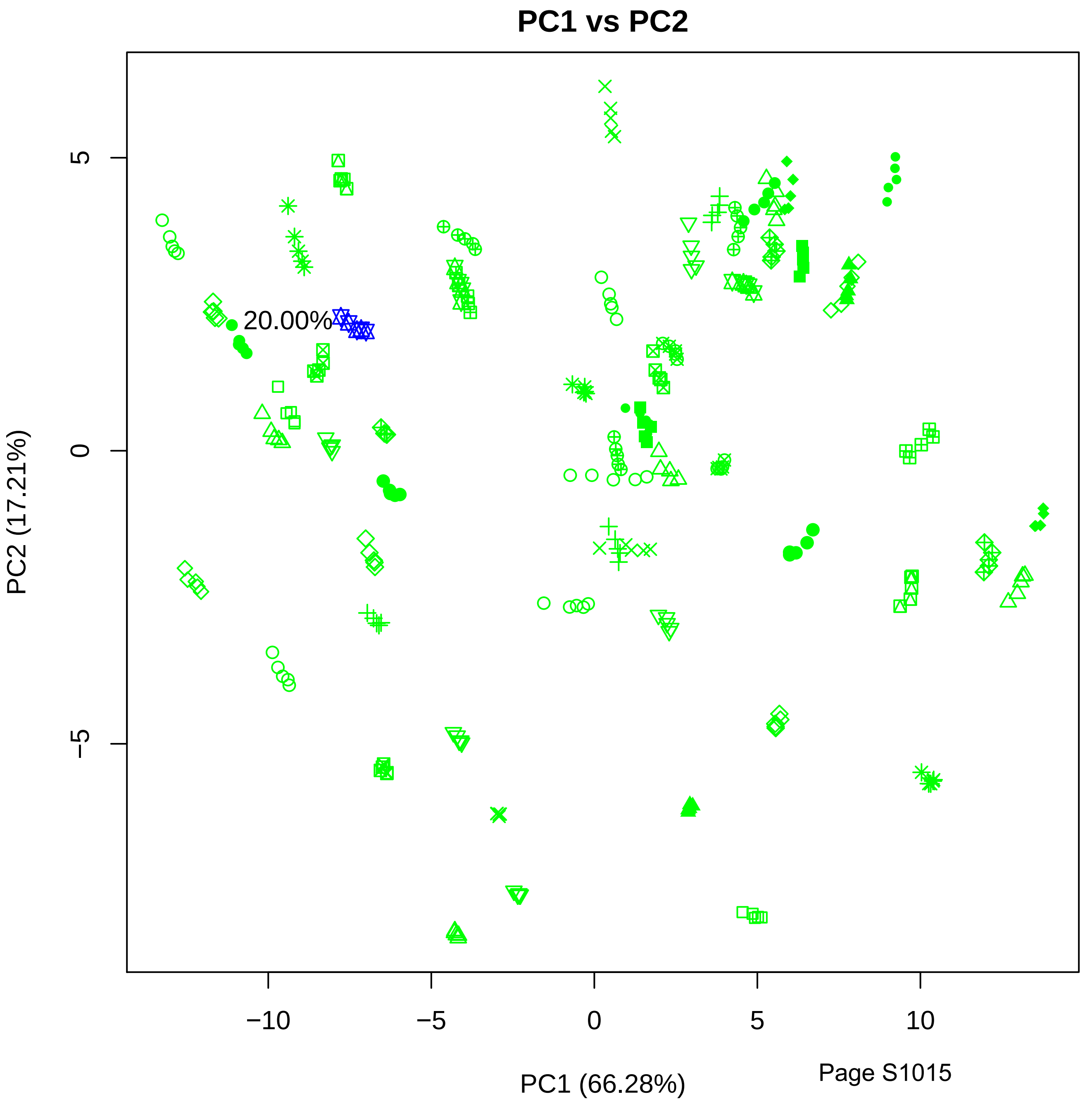
PC1 vs PC2
20.00%
5
0
−5
−10
−5
0
5
10
PC2 (17.21%)
PC1 (66.28%)

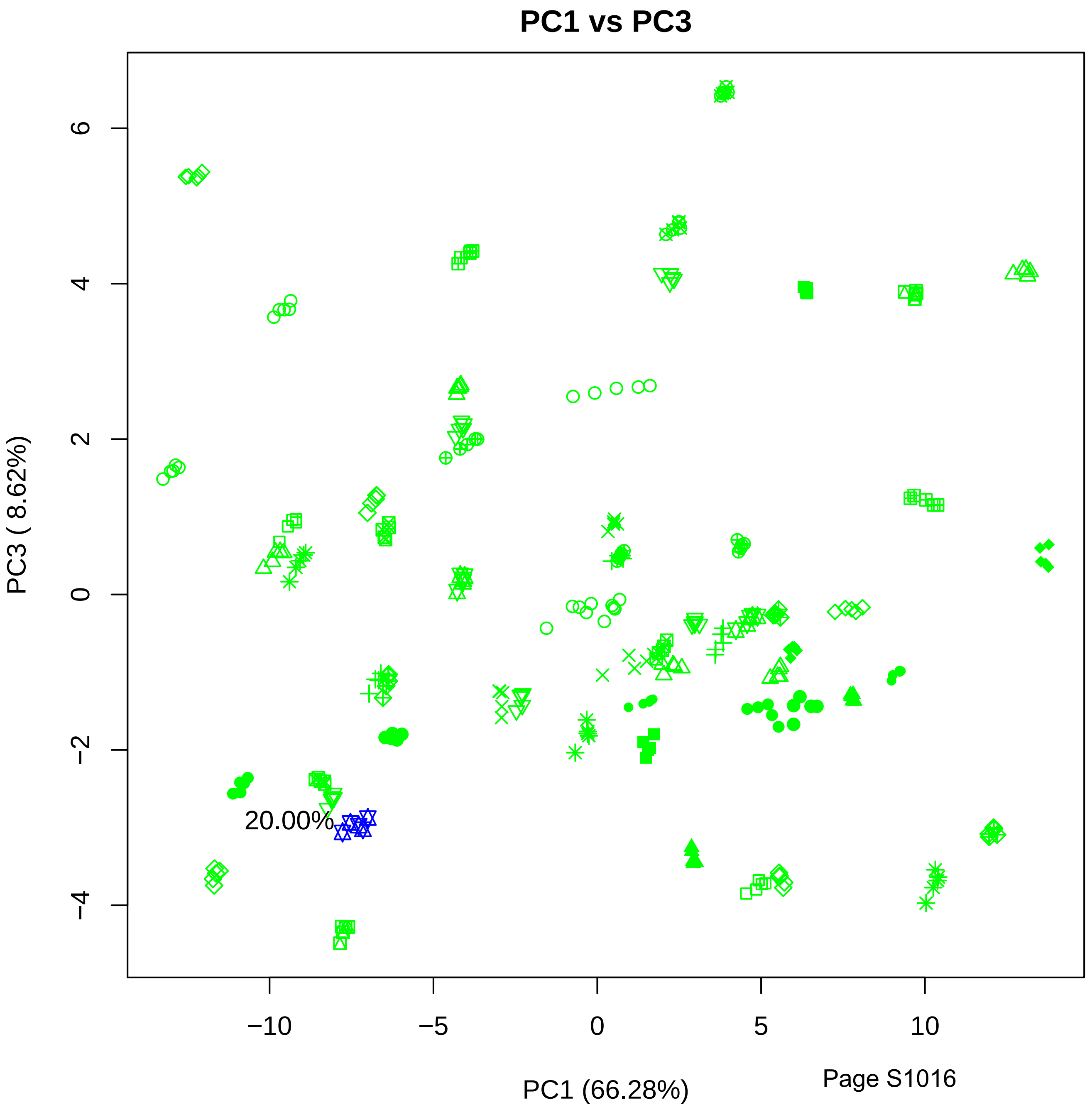
PC1 vs PC3
PC3 (8.62%)
PC1 (66.28%)
20.00%
6
4
2
0
−2
−4
−10
−5
0
5
10

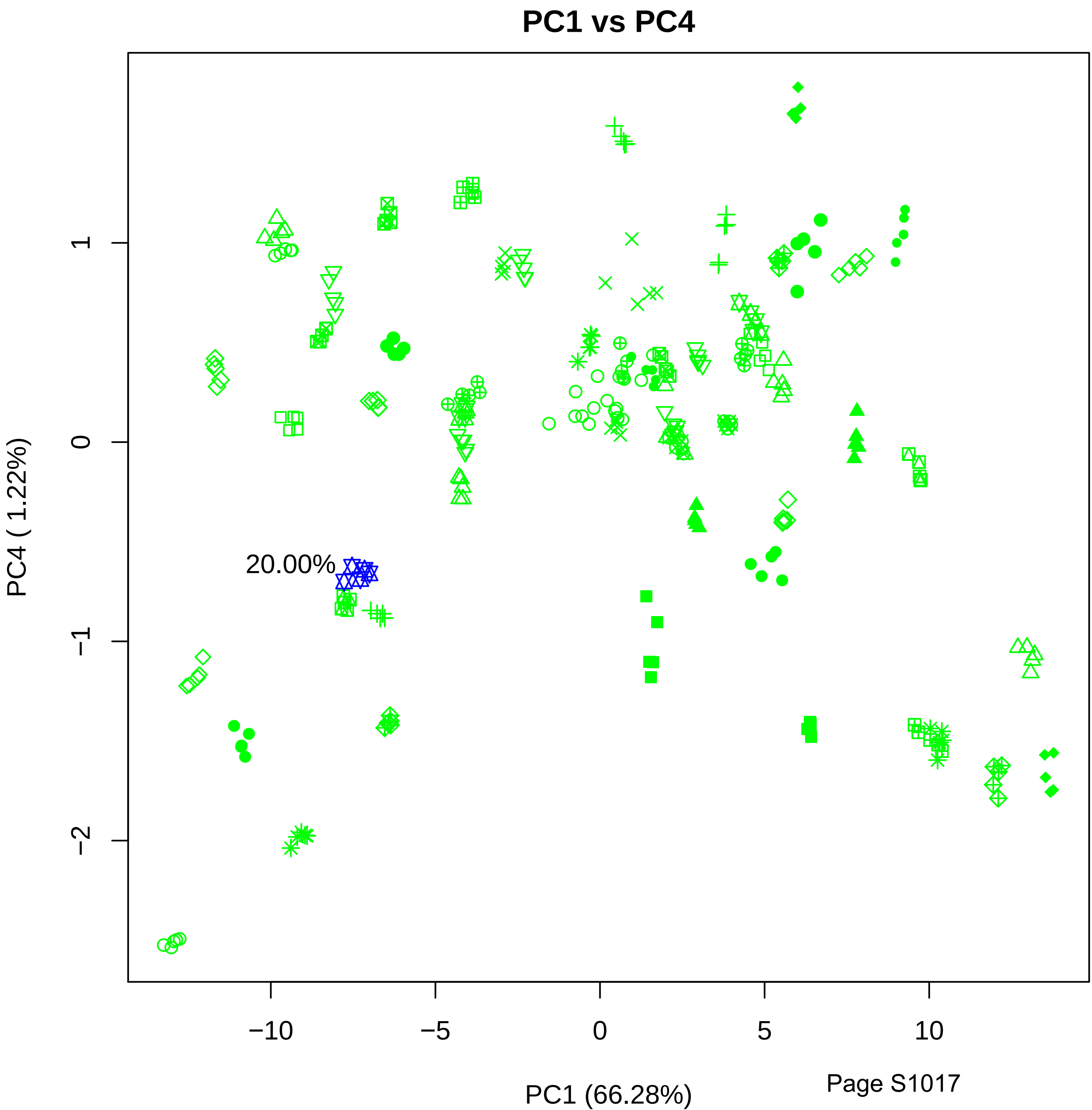

PC1 vs PC4
20.00%
1
0
−1
−2
−10
−5
0
5
10
PC4 (1.22%)
PC1 (66.28%)

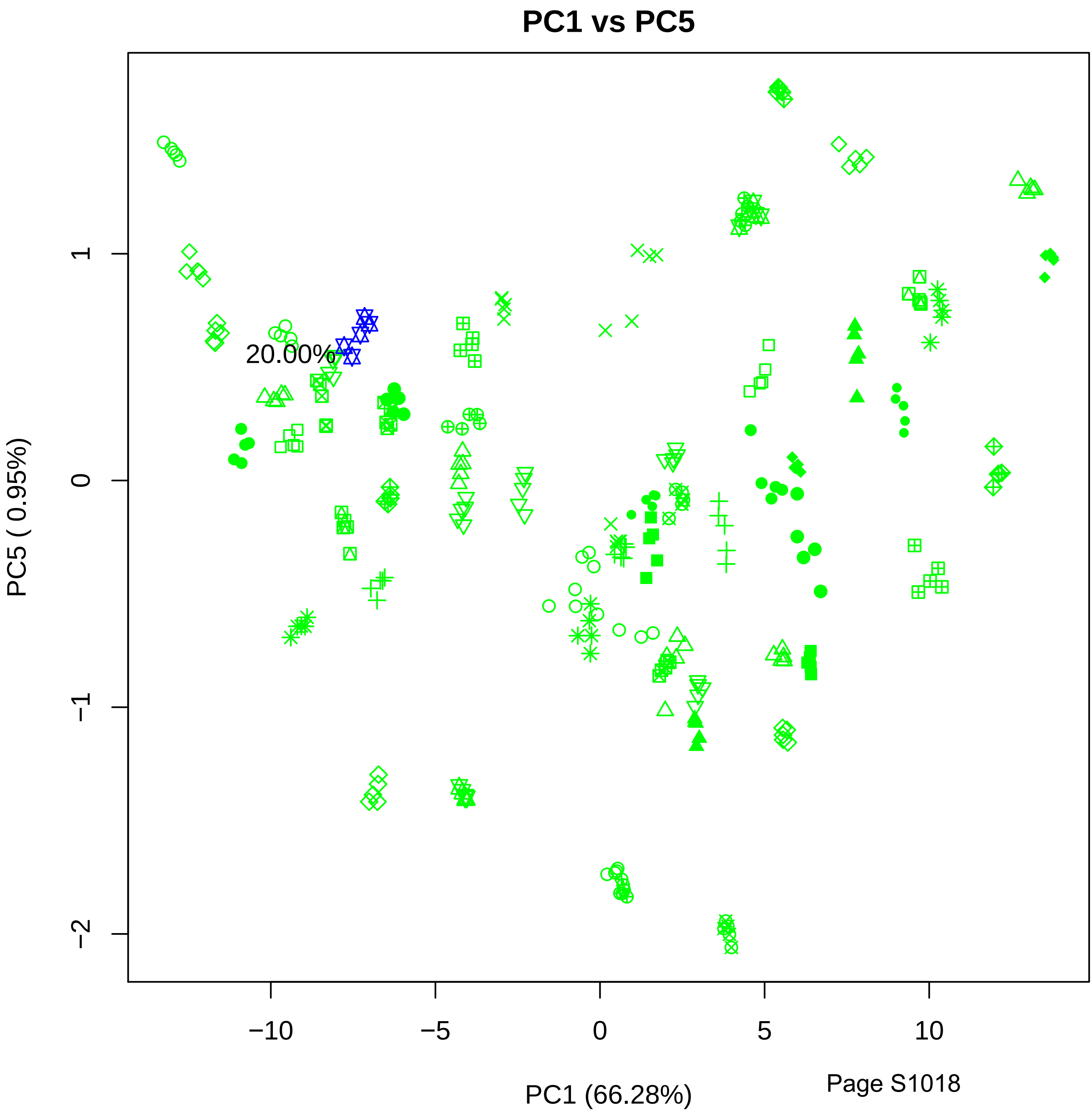
PC1 vs PC5
20.00%
PC5 (0.95%)
1
0
−1
−2
−10
−5
0
5
10
PC1 (66.28%)

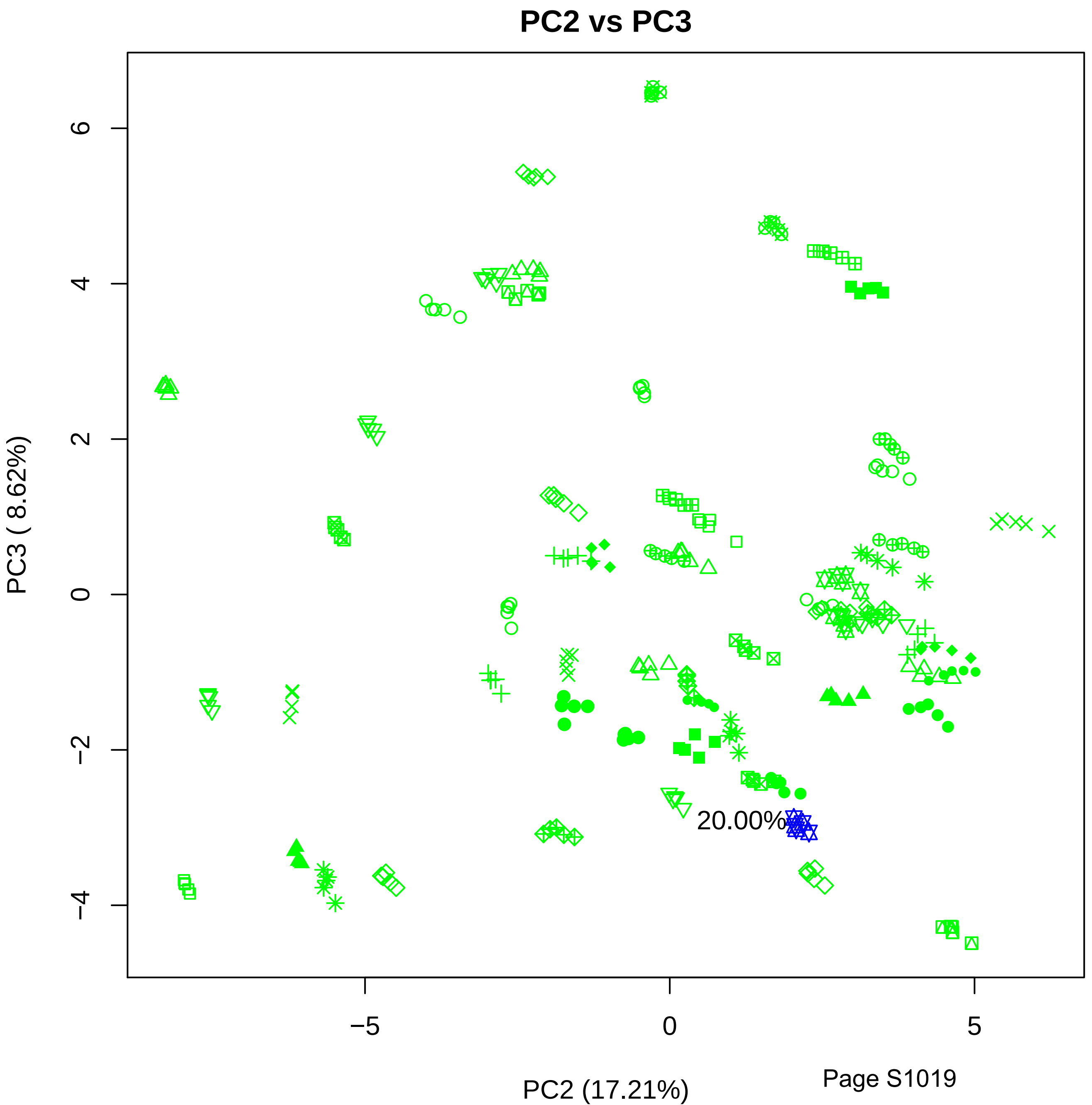

PC2 vs PC3
PC3 (8.62%)
PC2 (17.21%)
6
4
2
0
−2
−4
−5
0
5
20.00%

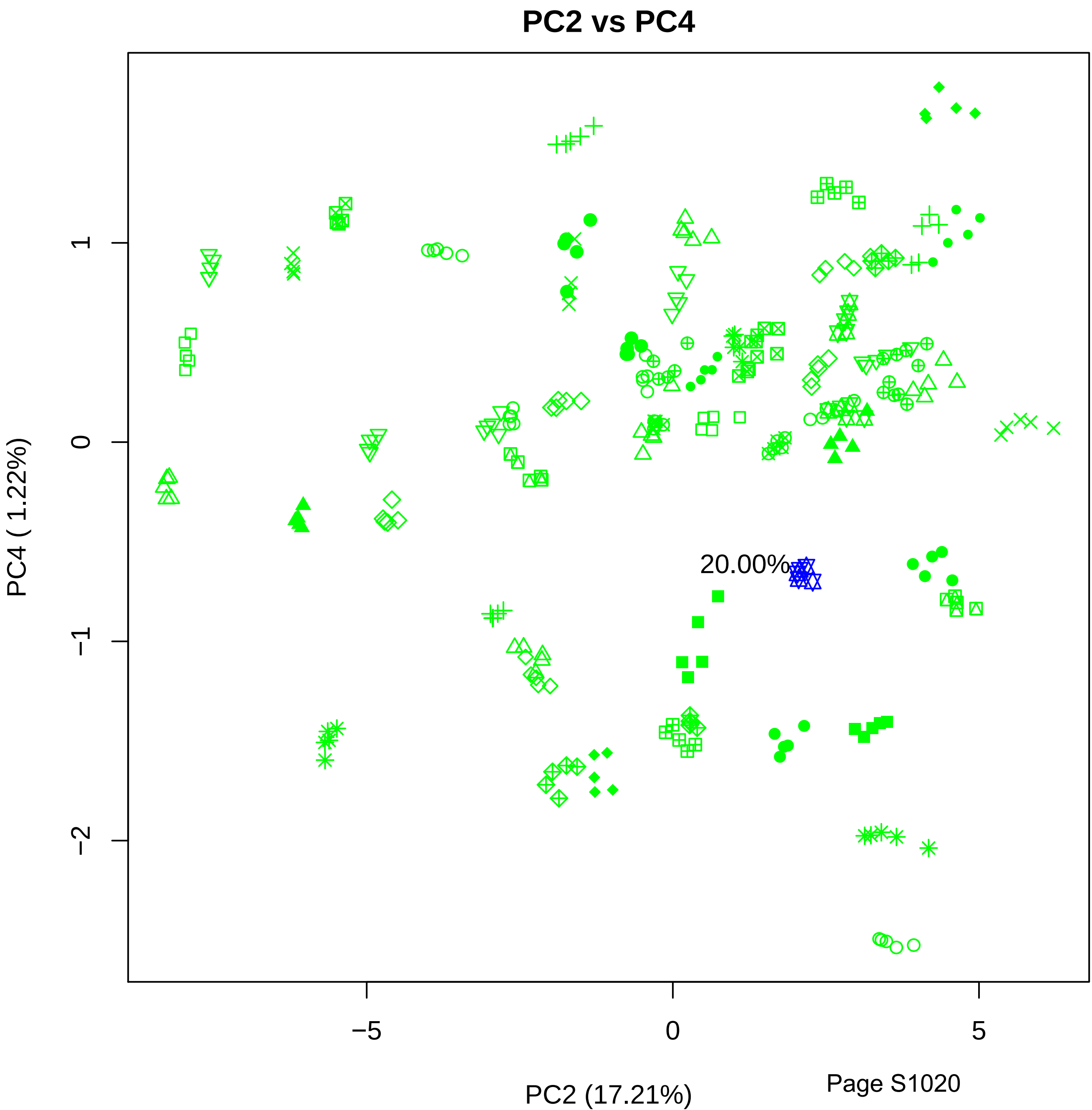
PC2 vs PC4
PC4 (1.22%)
PC2 (17.21%)
1
0
−1
−2
−5
0
5
20.00%

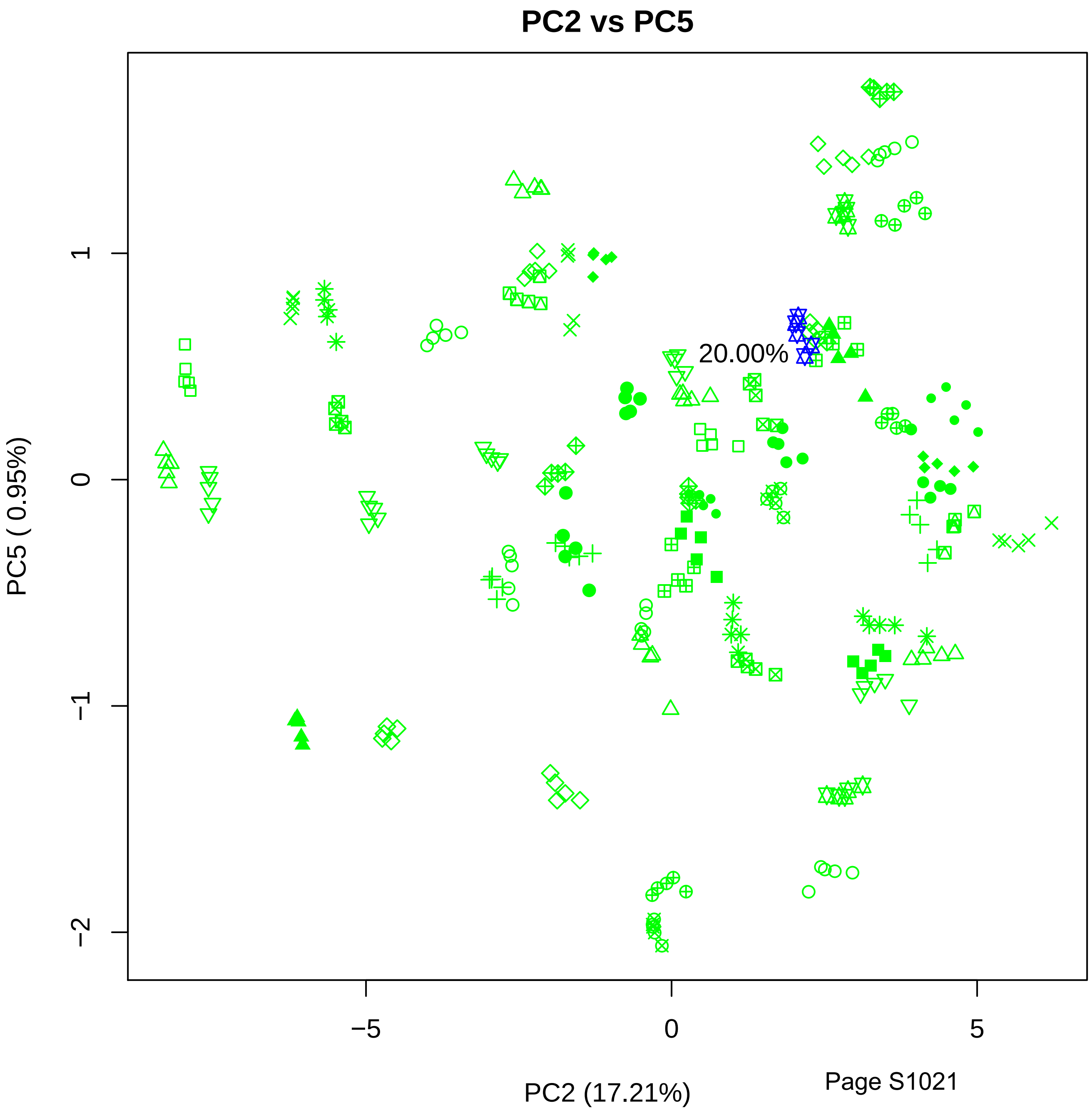

PC2 vs PC5
20.00%
PC5 (0.95%)
PC2 (17.21%)
1
0
−1
−2
−5
0
5

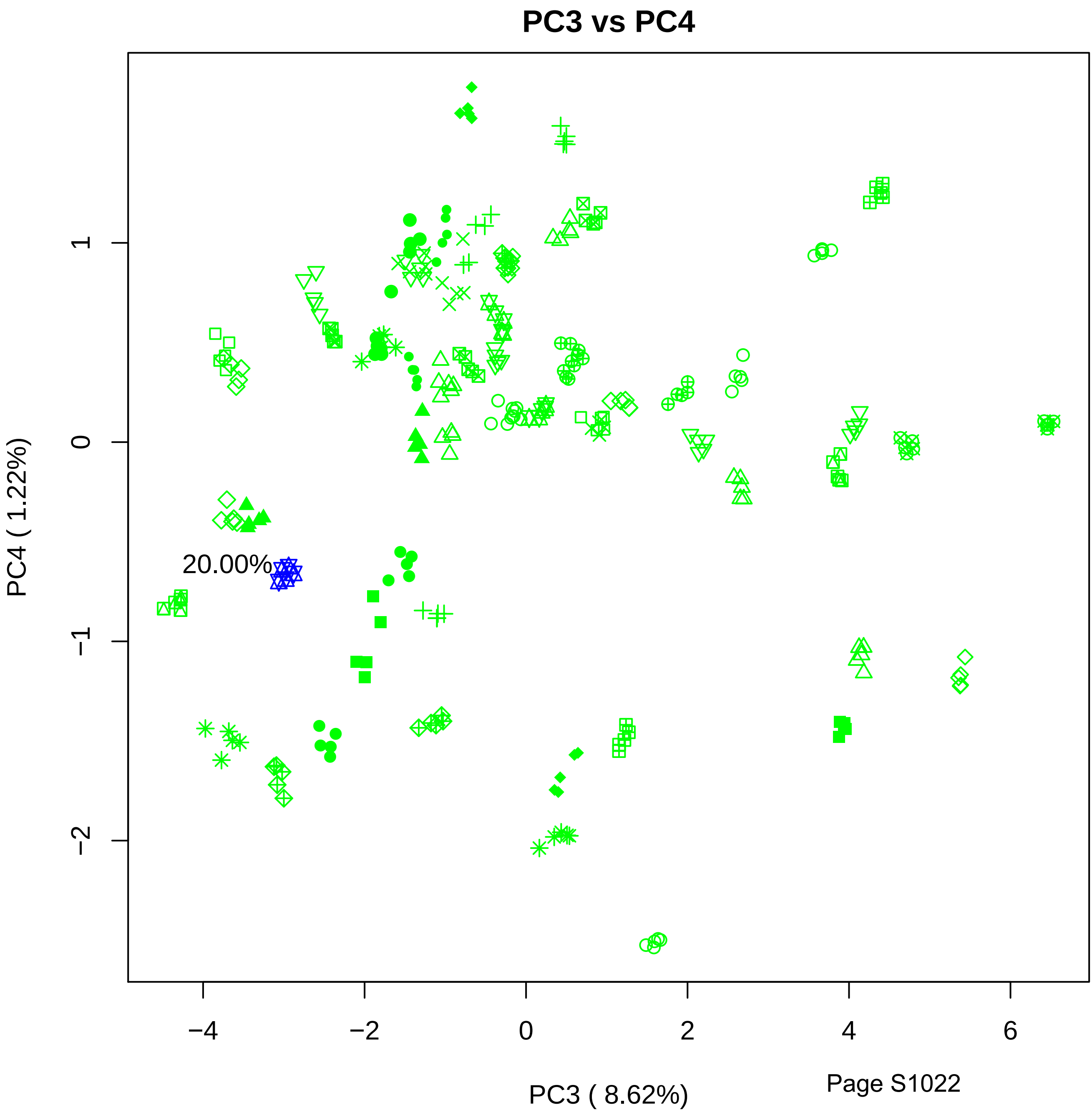

PC3 vs PC4
20.00%
PC4 (1.22%)
PC3 (8.62%)
−4
−2
0
2
4
6
−2
−1
0
1

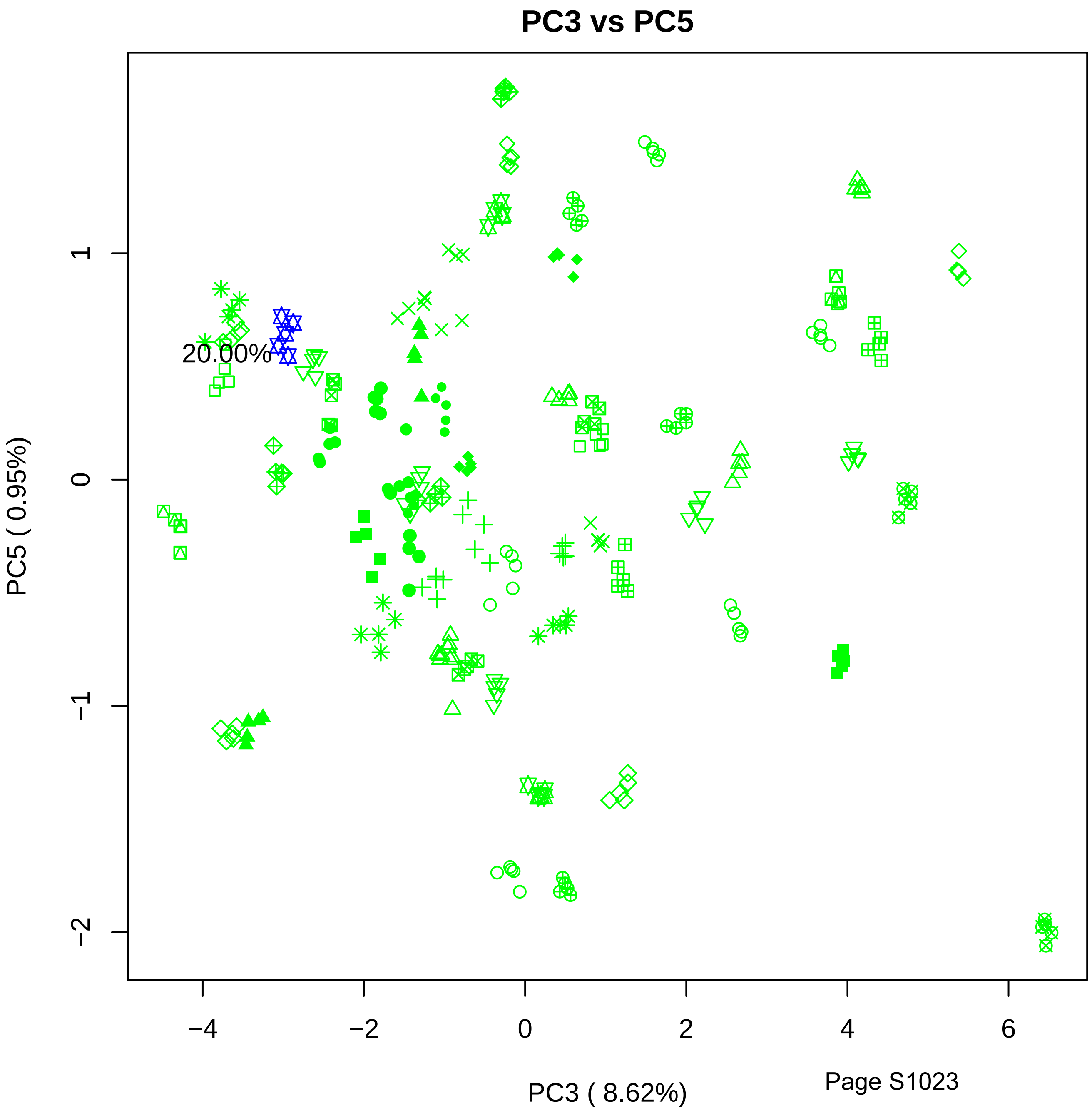

PC3 vs PC5
20.00%
PC5 (0.95%)
1
0
−1
−2
−4
−2
0
2
4
6
PC3 (8.62%)

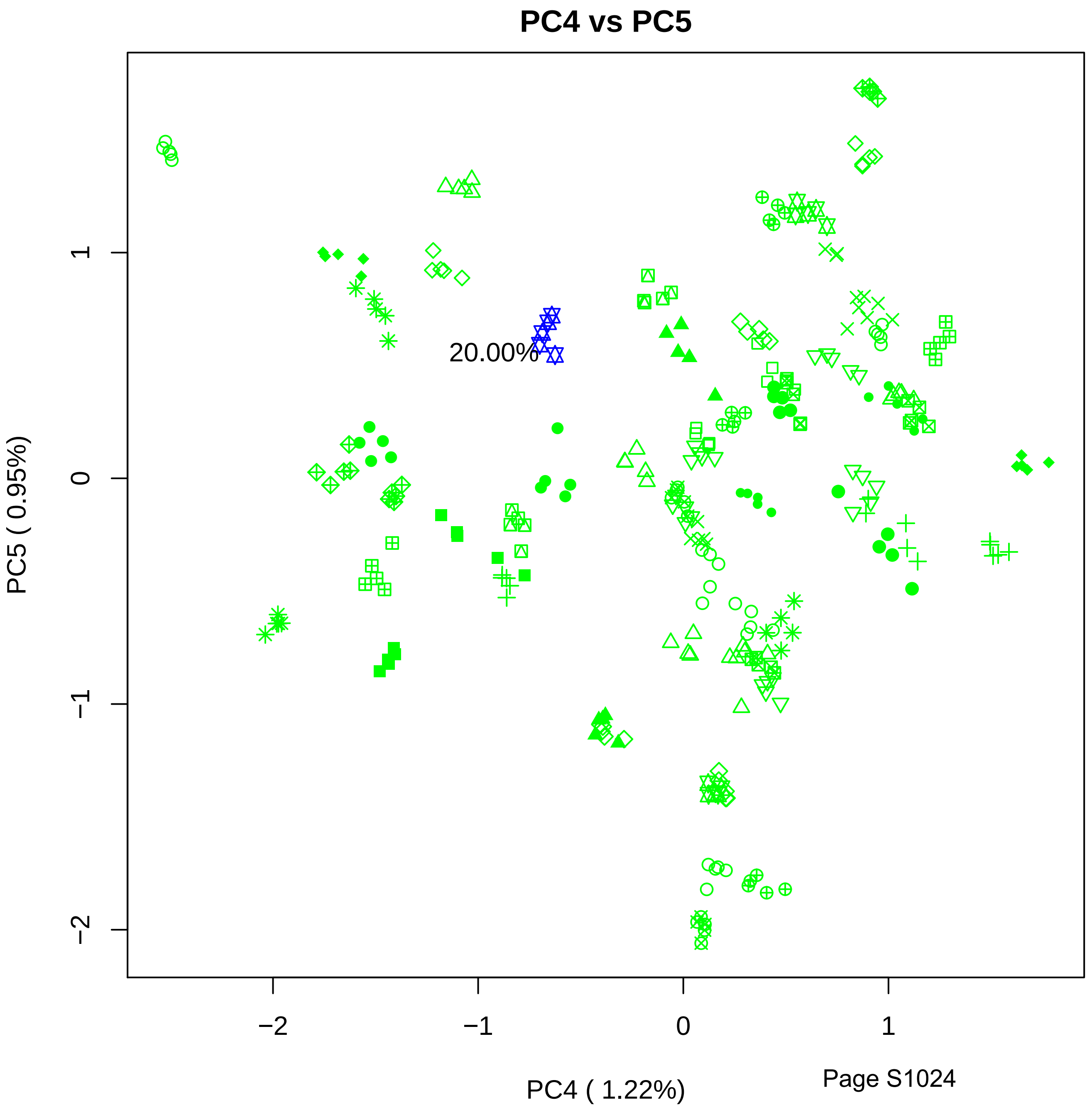
PC4 vs PC5
20.00%
PC5 (0.95%)
PC4 (1.22%)
1
0
−1
−2
−2
−1
0
1

PCA of 40% BMH(a) vs PMH(a)

Scaled Matrix PCAed

7th order polynomial baseline correction performed

Raw spectra smoothed to 21 Neighbours, 2nd order PN, Savtisky Golay

Normalisation between 0 and 100

D2 spectra smoothed to 41 Neighbours, 2nd order PN, Savtisky Golay

Regions Analysed: 3601−2501 and 2001−700cm−1

Samples Analysed: 1 bContam's 0 BLH's 0 BMH's 0 OMH's 0 pContam's 0 PLH's 60 PMH's

Keele University AD Fri Dec 14 14:20:52 2018

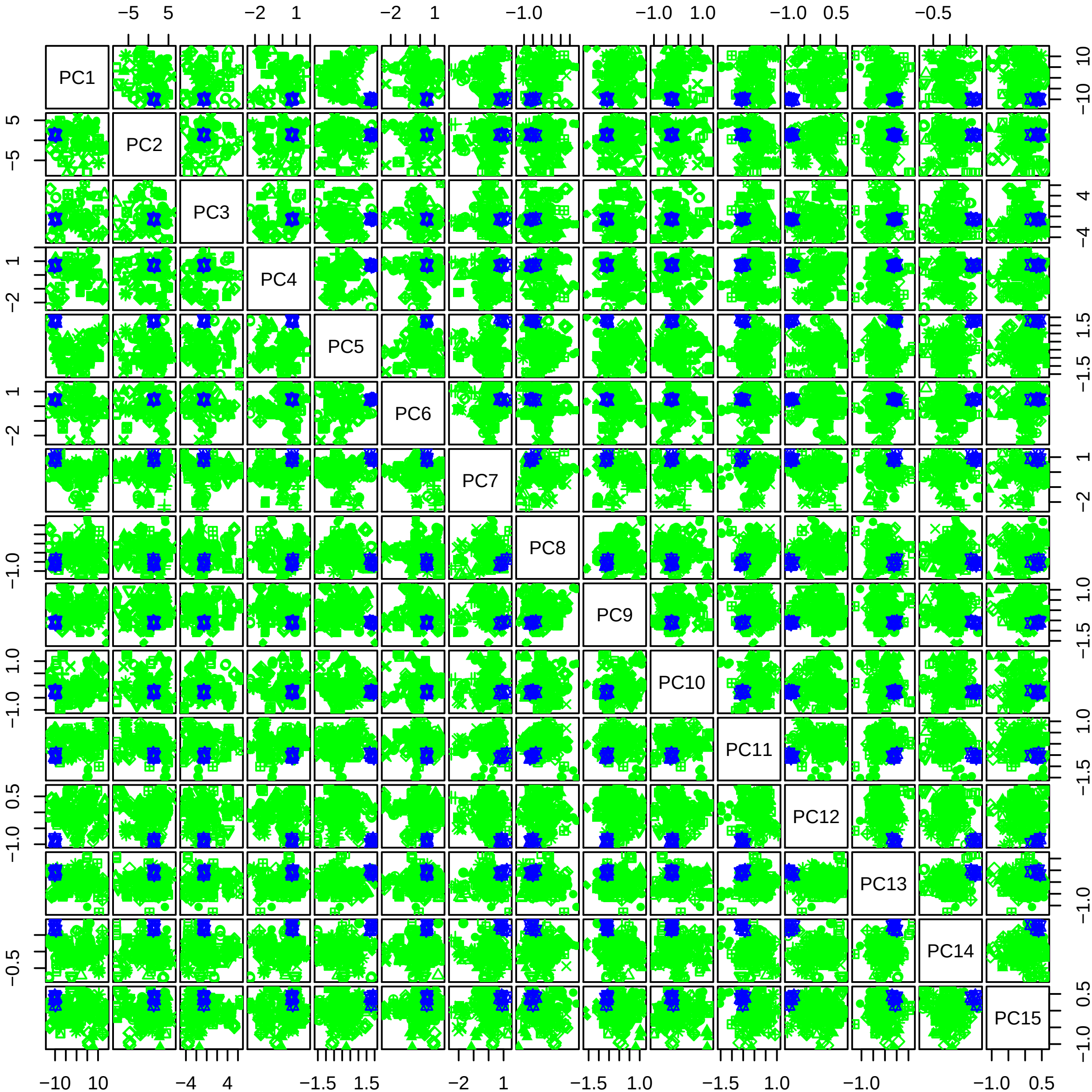
PC1
PC2
PC3
PC4
PC5
PC6
PC7
PC8
PC9
PC10
PC11
PC12
PC13
PC14
PC15

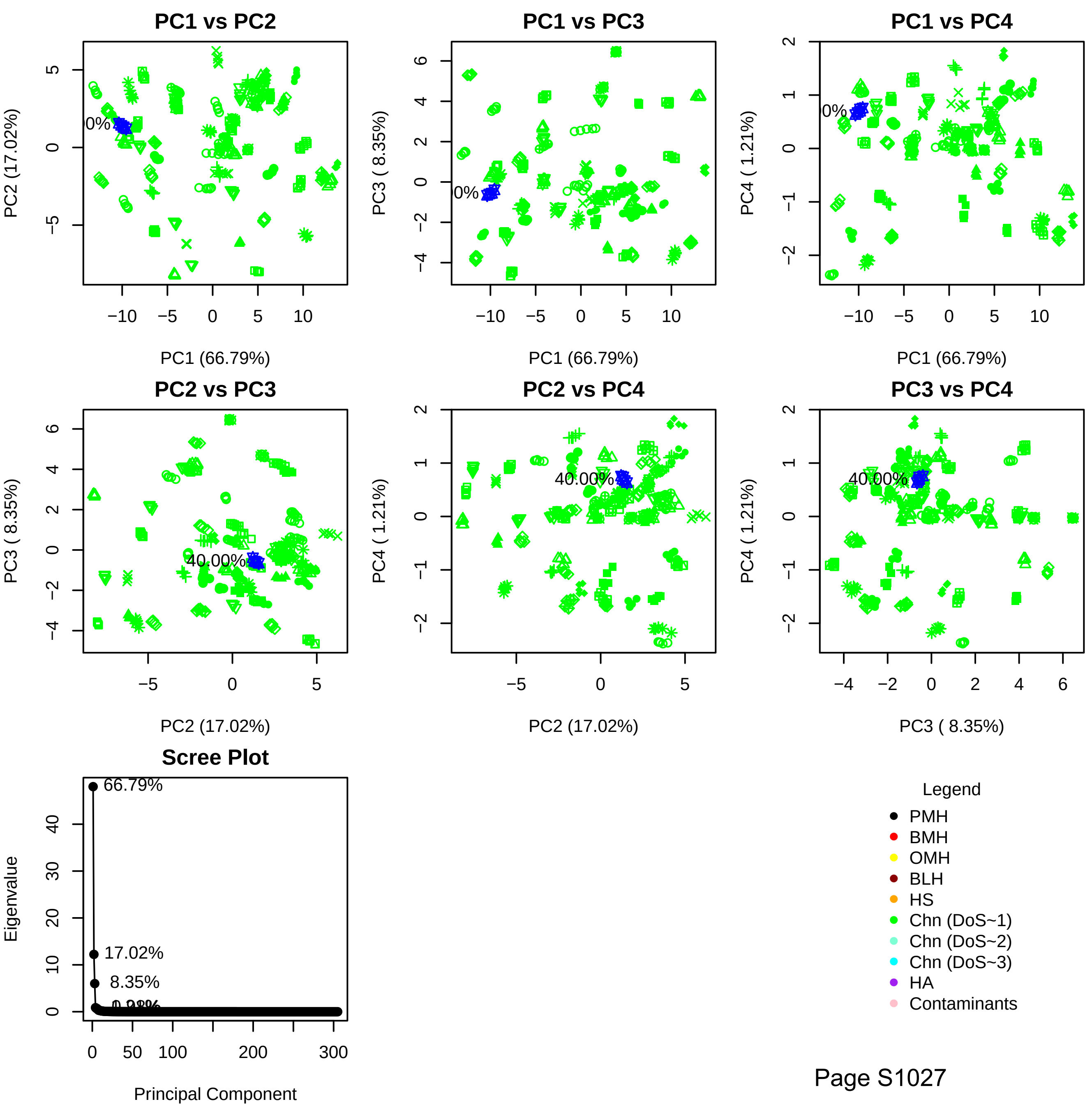
PC1 vs PC2
PC1 vs PC3
PC1 vs PC4
PC2 vs PC3
PC2 vs PC4
PC3 vs PC4
Scree Plot
PC1 (66.79%)
PC2 (17.02%)
PC3 (8.35%)
PC4 (1.21%)
0%
40.00%
Eigenvalue
Principal Component
66.79%
17.02%
8.35%
Legend
PMH
BMH
OMH
BLH
HS
Chn (DoS~1)
Chn (DoS~2)
Chn (DoS~3)
HA
Contaminants

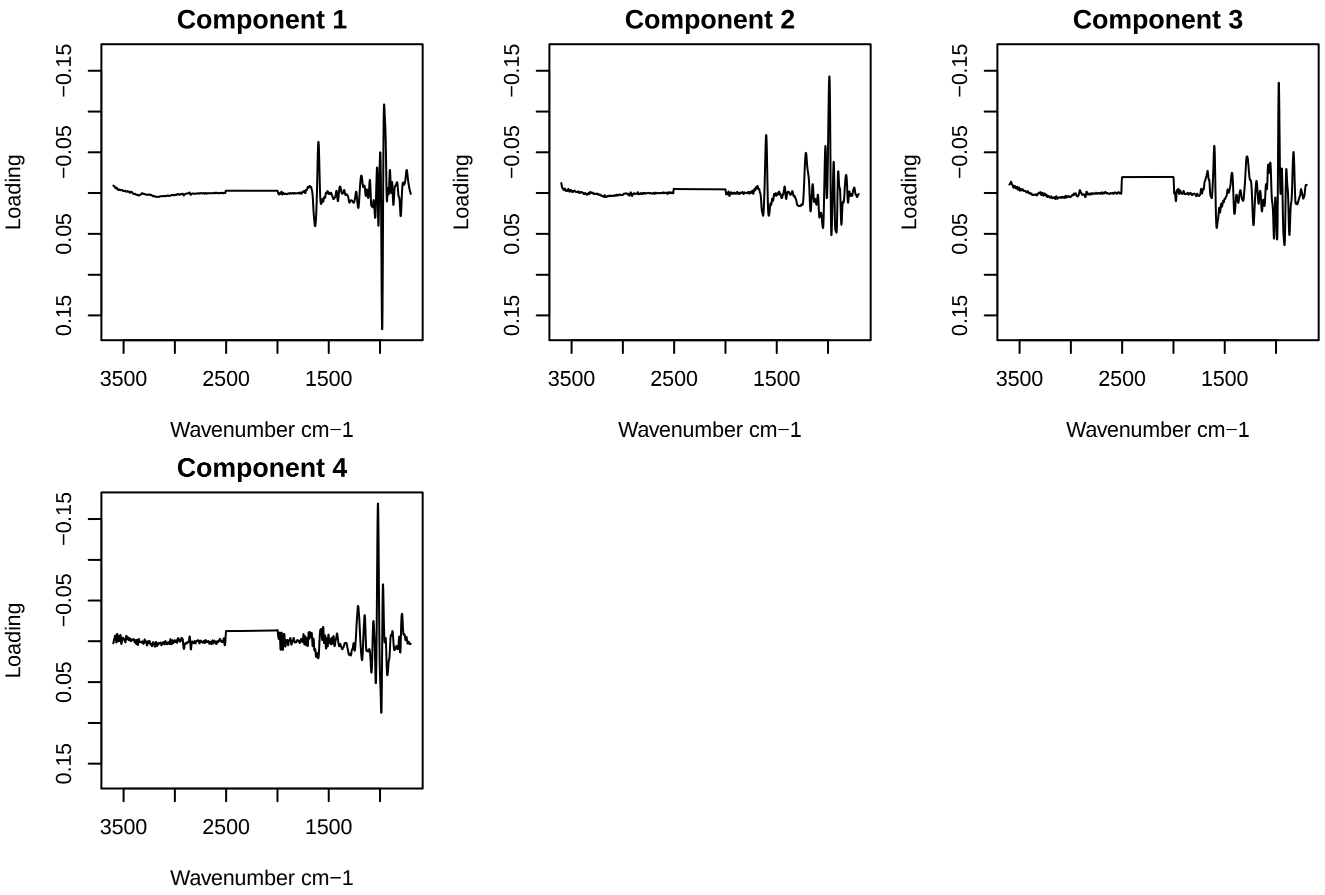
Component 1
Component 2
Component 3
Component 4
Loading
−0.15
−0.05
0.05
0.15
3500
2500
1500
Wavenumber cm−1

PC1 vs PC2 vs PC3

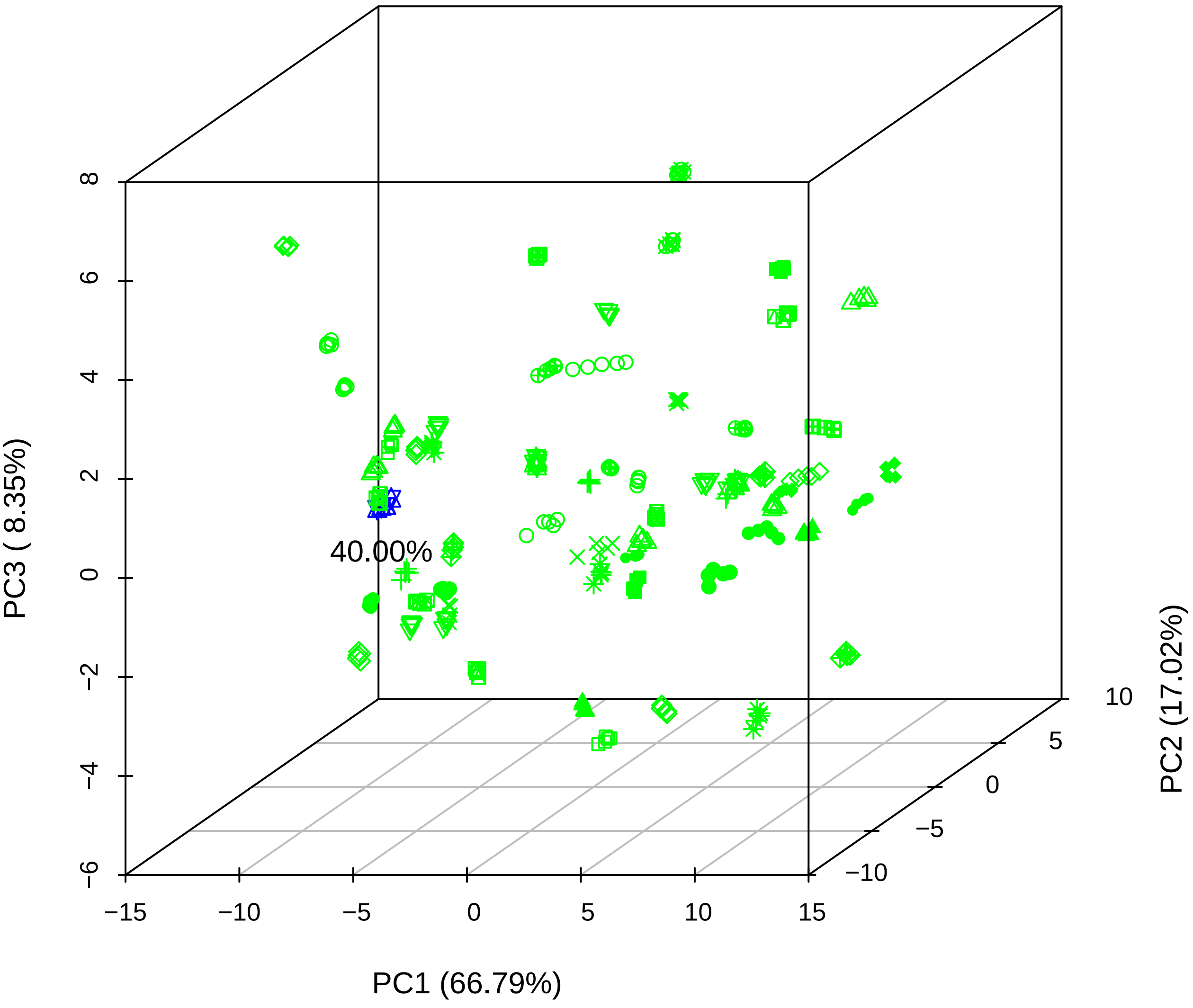

PC1 vs PC2 vs PC4

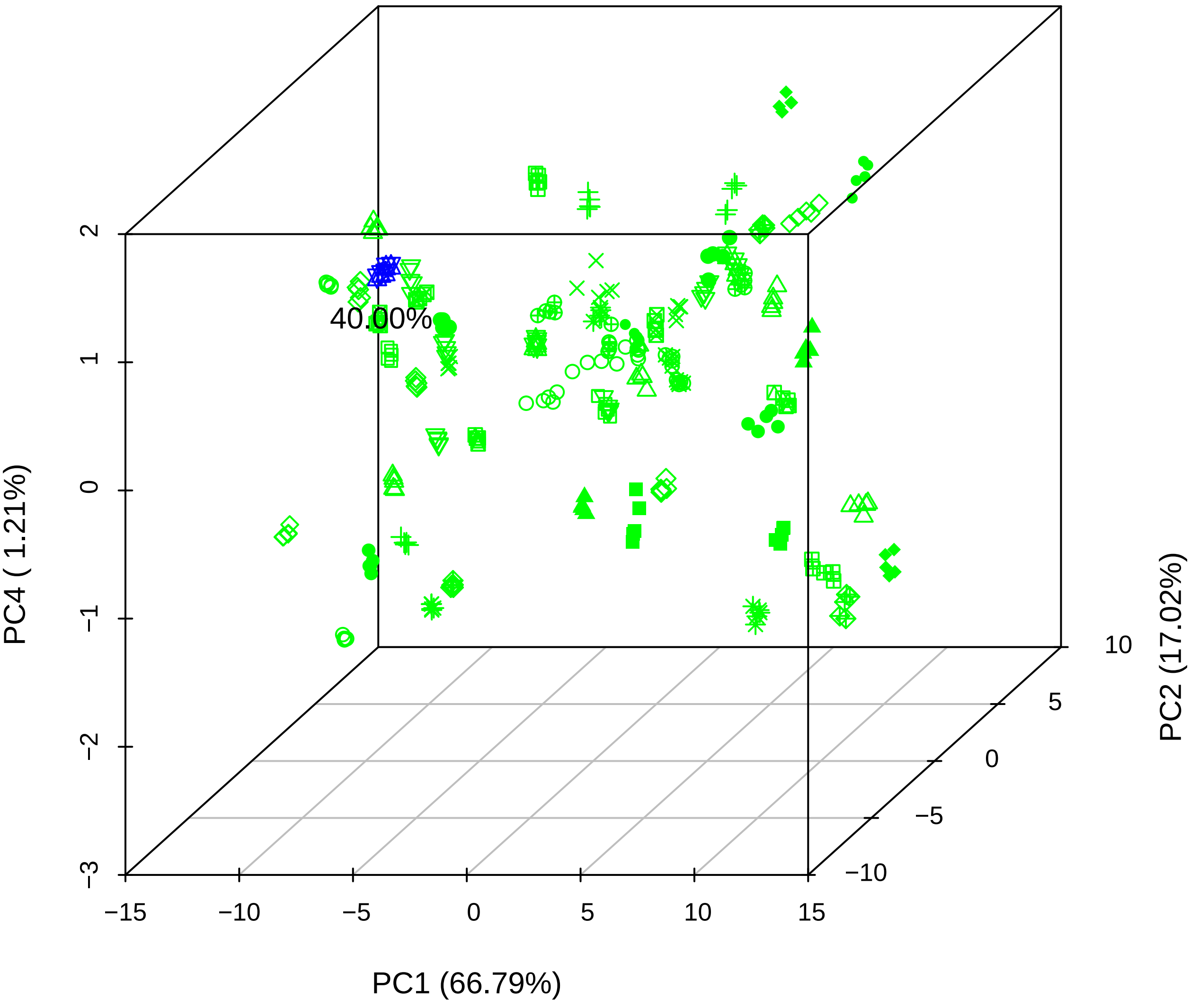

PC1 vs PC3 vs PC4

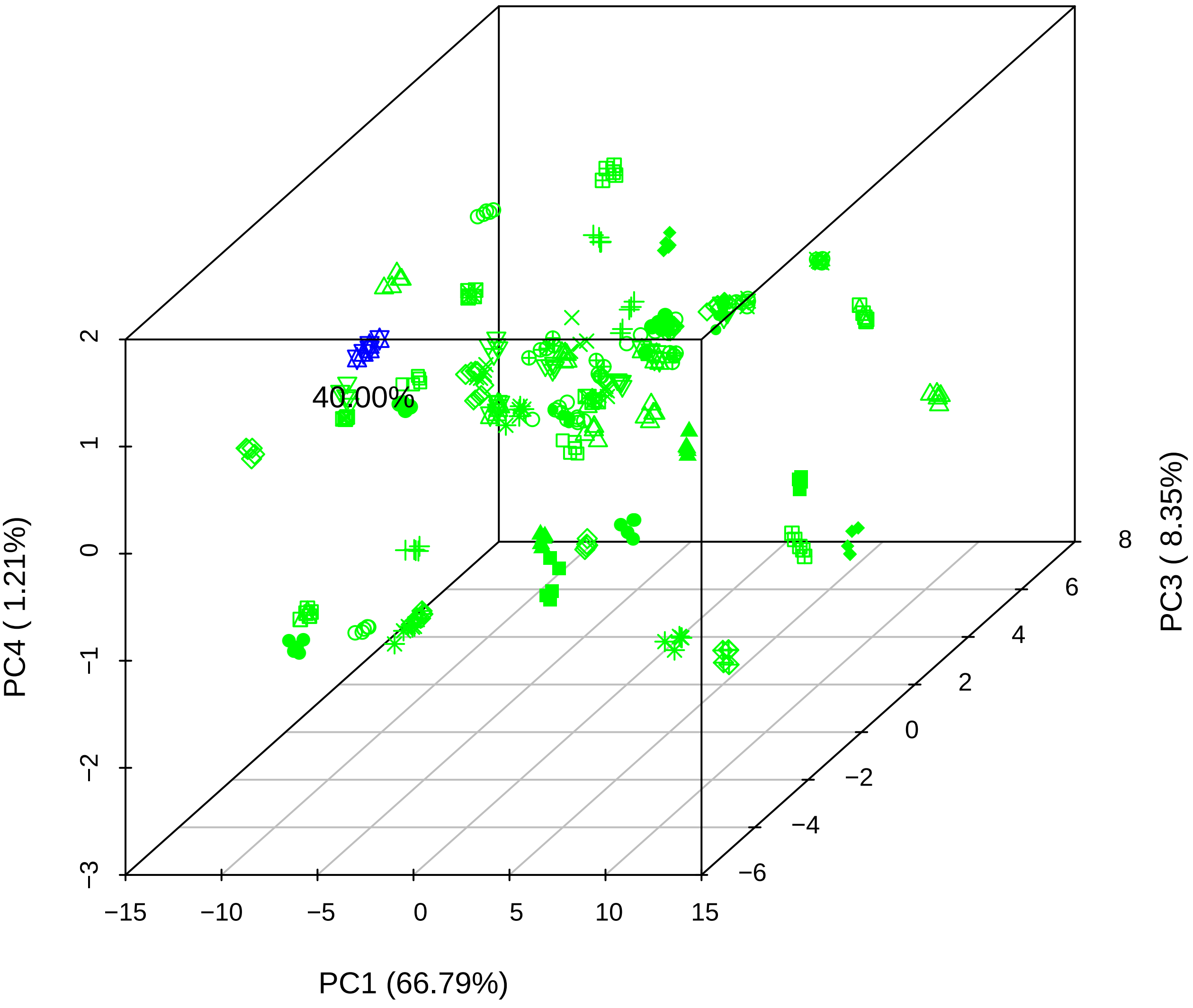

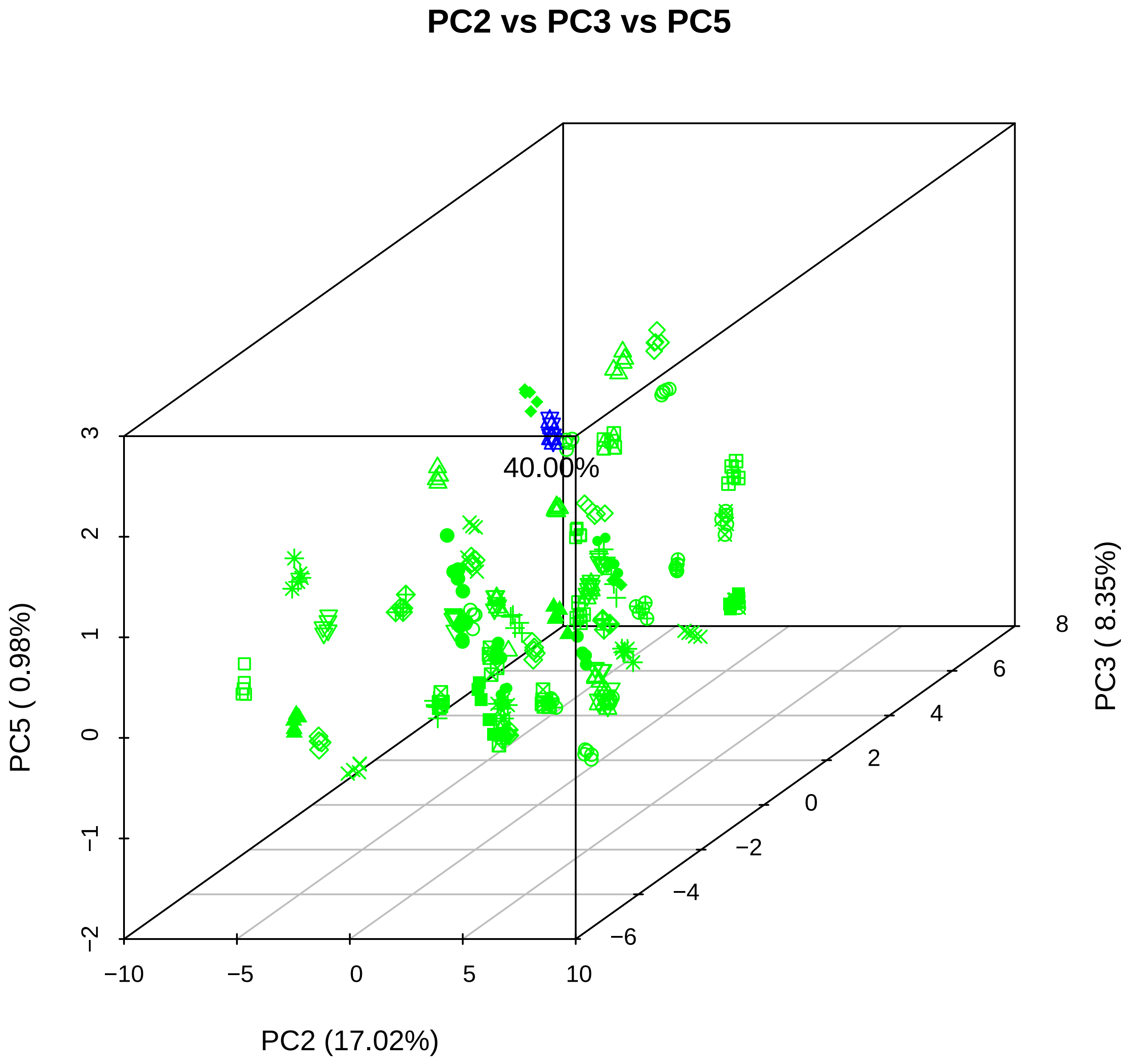

PC2 vs PC3 vs PC5
40.00%
PC5 (0.98%)
PC3 (8.35%)
PC2 (17.02%)
−2
−1
0
1
2
3
−10
−5
0
5
10
−6
−4
−2
0
2
4
6
8

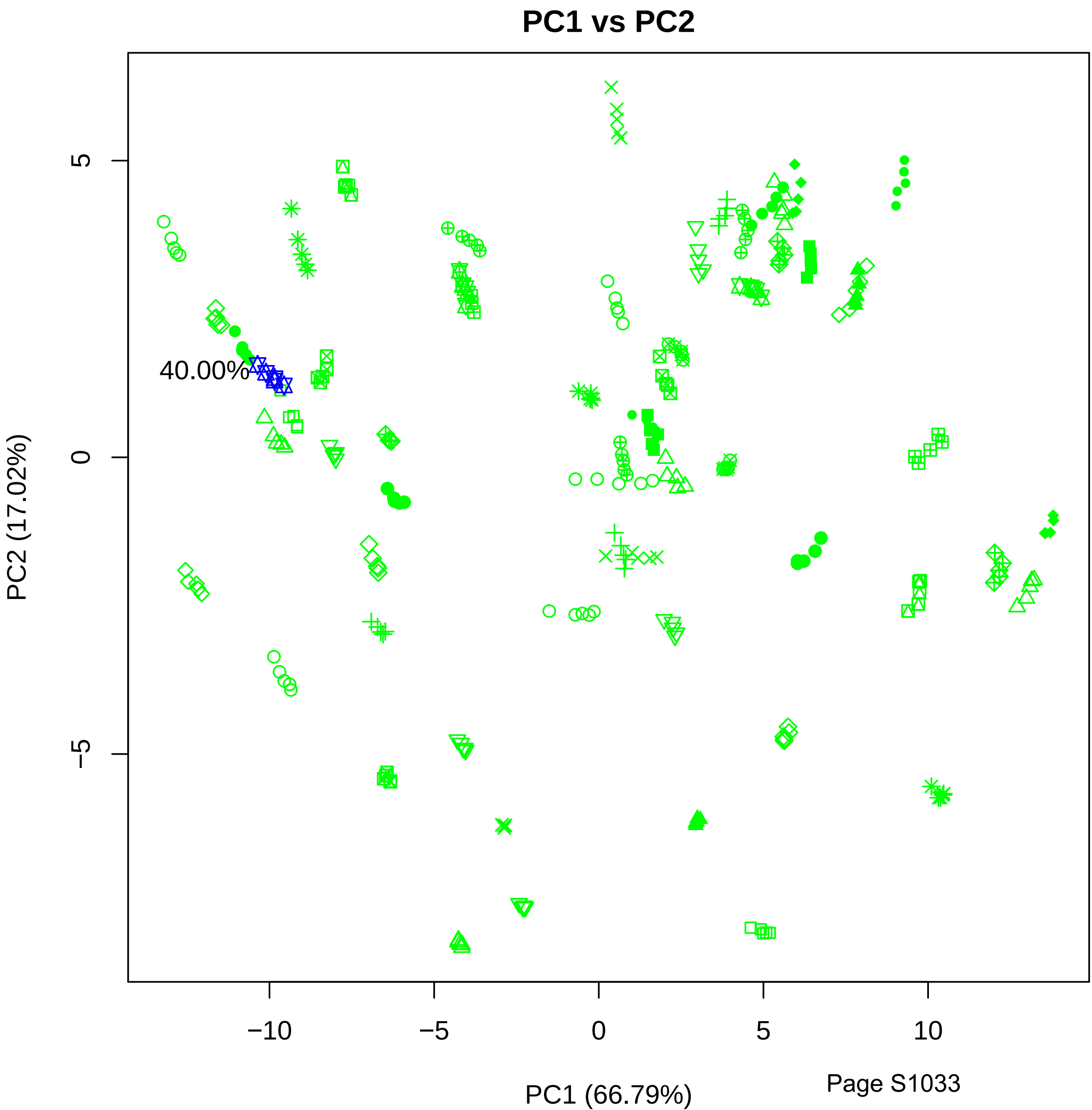

PC1 vs PC2
40.00%
5
0
−5
−10
−5
0
5
10
PC2 (17.02%)
PC1 (66.79%)

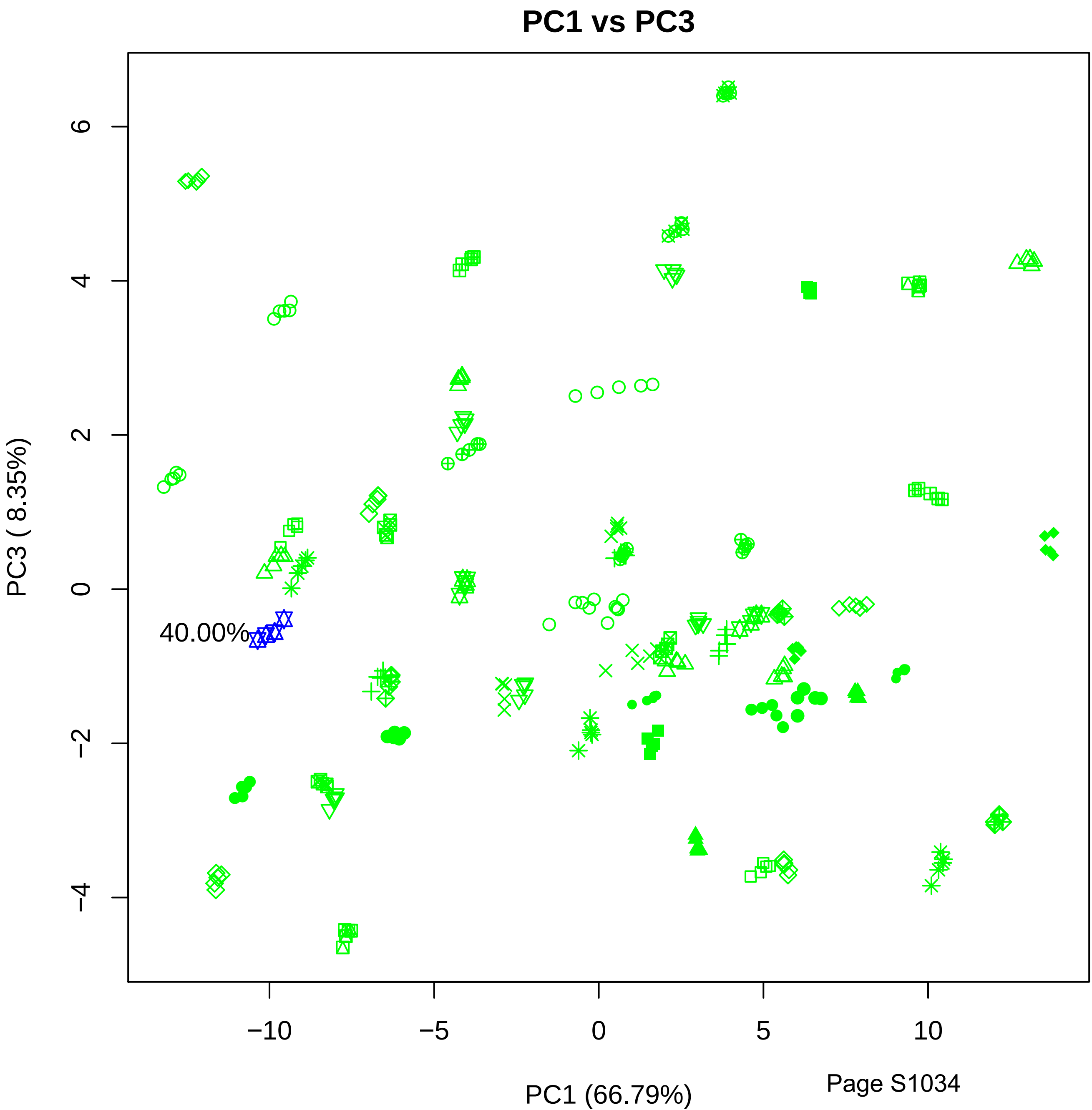

PC1 vs PC3
PC3 (8.35%)
PC1 (66.79%)
40.00%
6
4
2
0
−2
−4
−10
−5
0
5
10

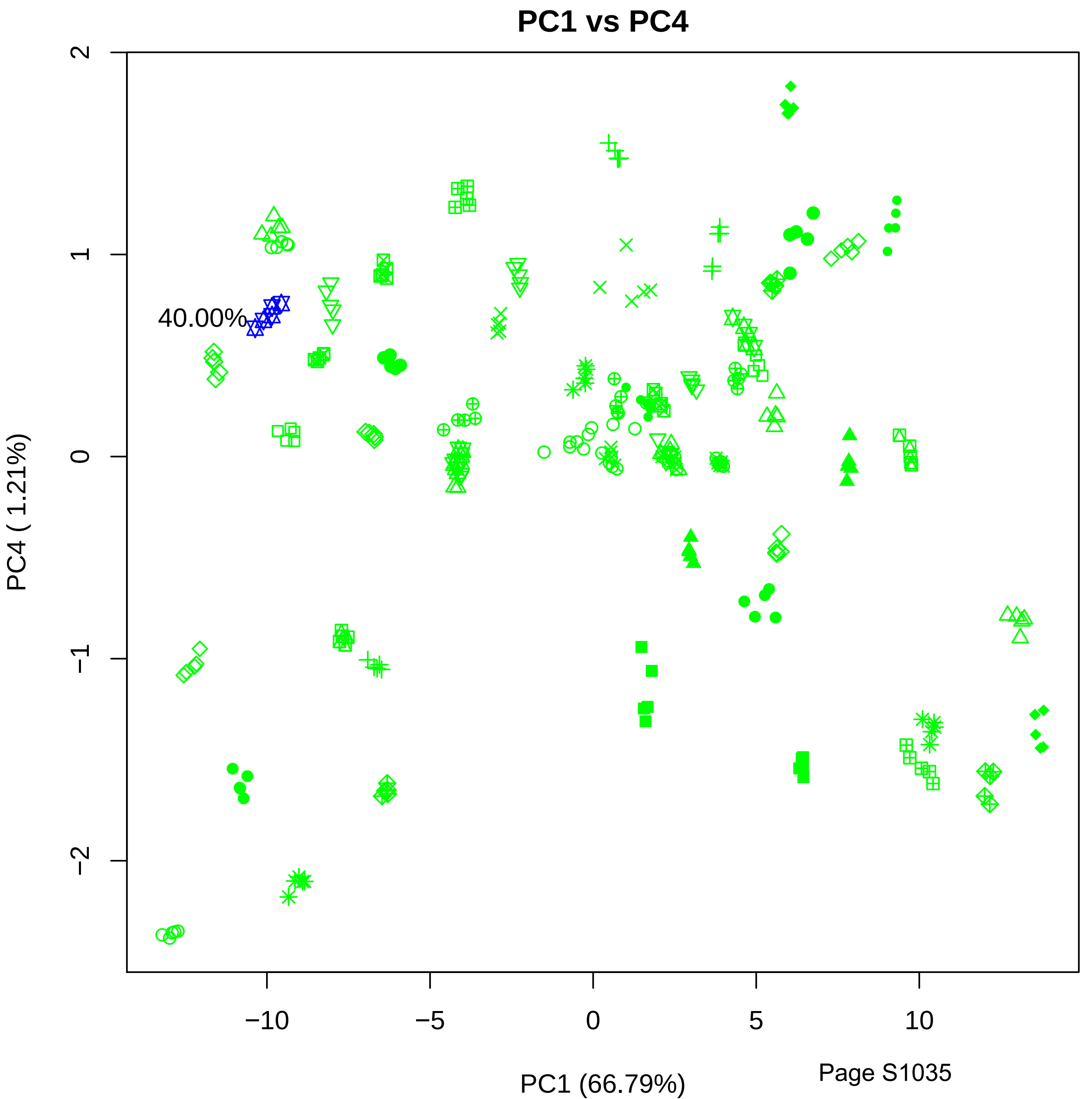
PC1 vs PC4
40.00%
PC4 (1.21%)
PC1 (66.79%)
−10
−5
0
5
10
2
1
0
−1
−2

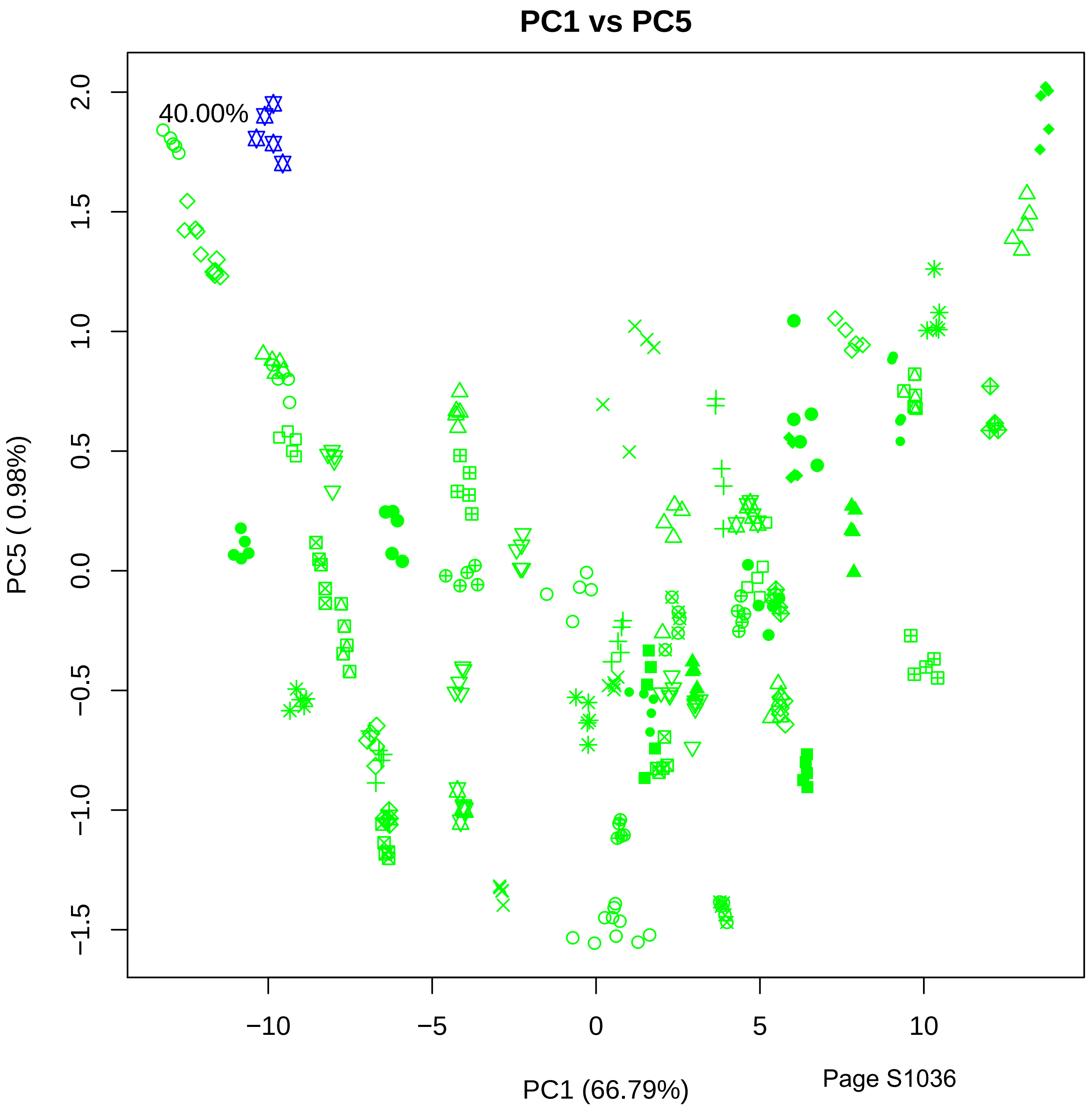

PC1 vs PC5
40.00%
PC5 (0.98%)
2.0
1.5
1.0
0.5
0.0
−0.5
−1.0
−1.5
−10
−5
0
5
10
PC1 (66.79%)

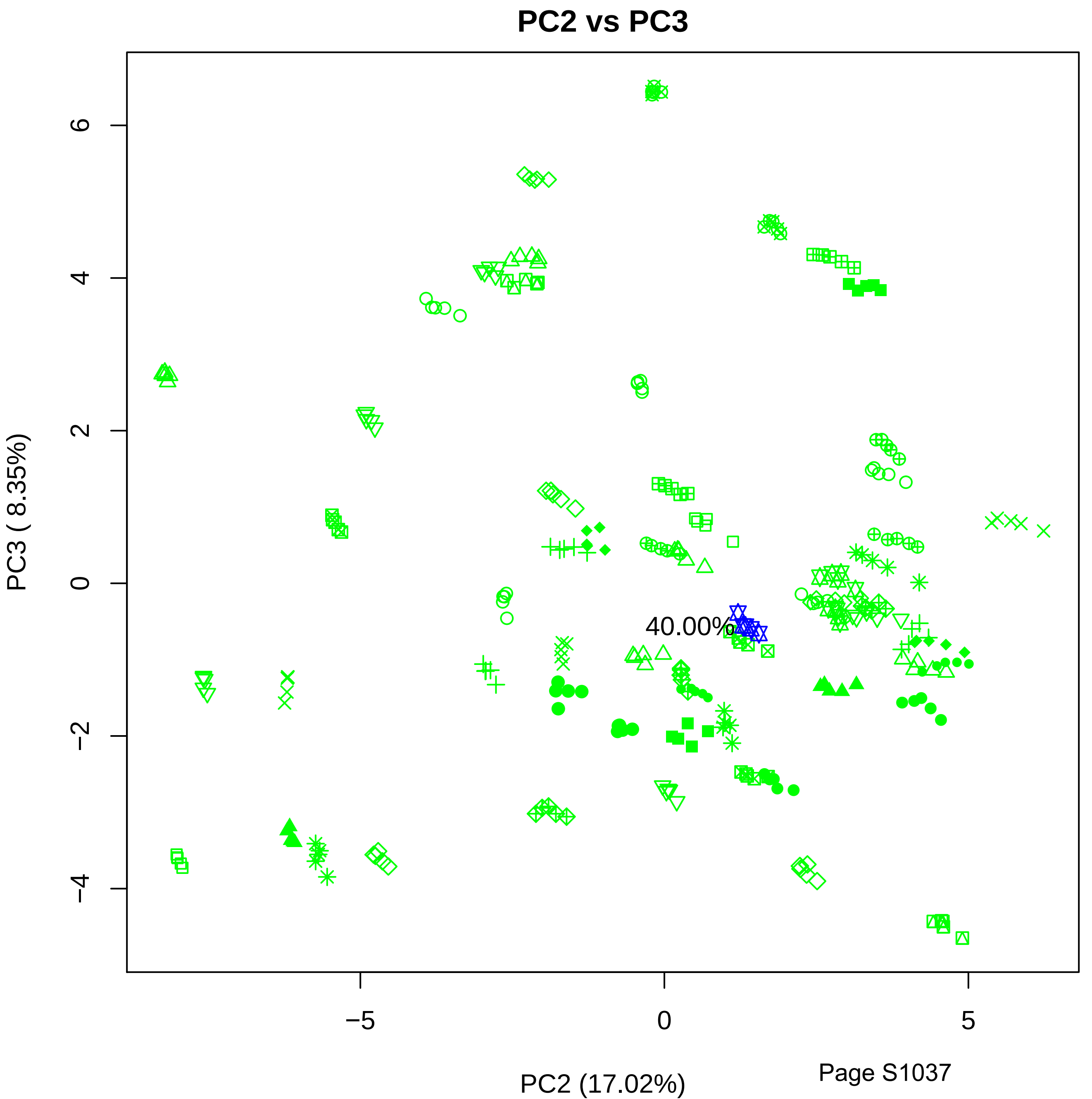

PC2 vs PC3
PC3 (8.35%)
PC2 (17.02%)
40.00%
6
4
2
0
−2
−4
−5
0
5

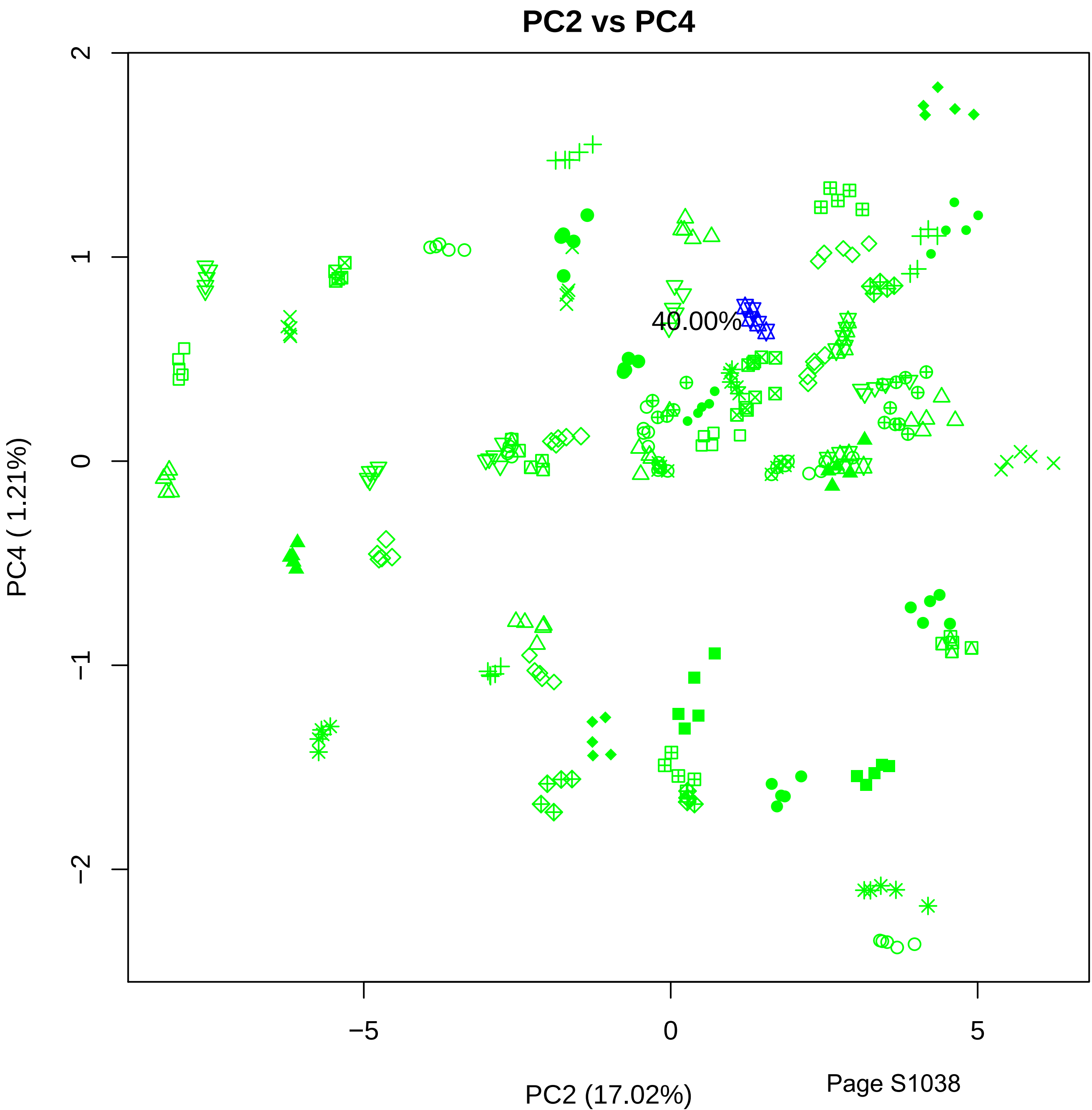
PC2 vs PC4
2
1
0
−1
−2
PC4 (1.21%)
40.00%
−5
0
5
PC2 (17.02%)

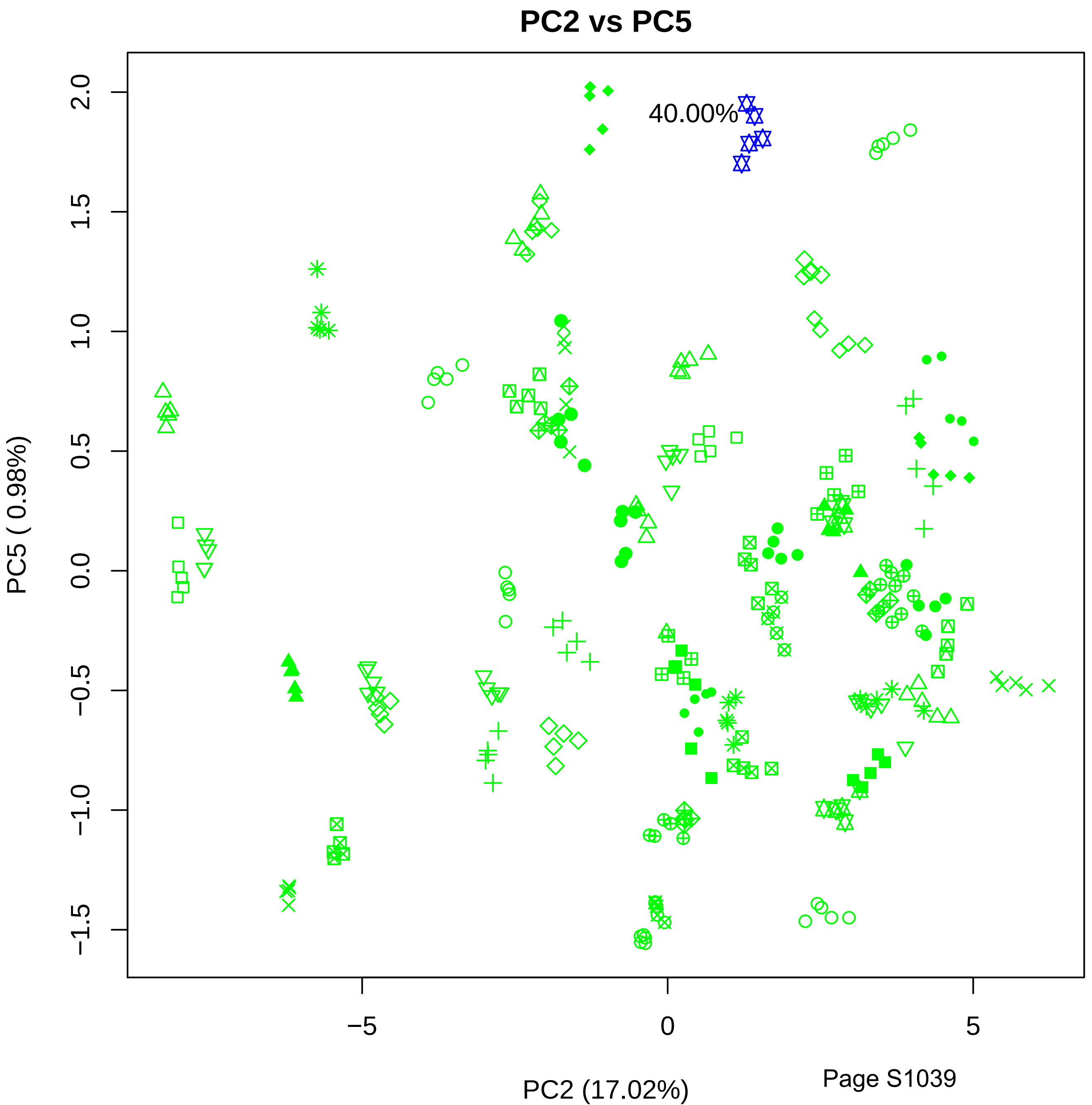
PC2 vs PC5
40.00%
PC5 (0.98%)
2.0
1.5
1.0
0.5
0.0
−0.5
−1.0
−1.5
−5
0
5
PC2 (17.02%)

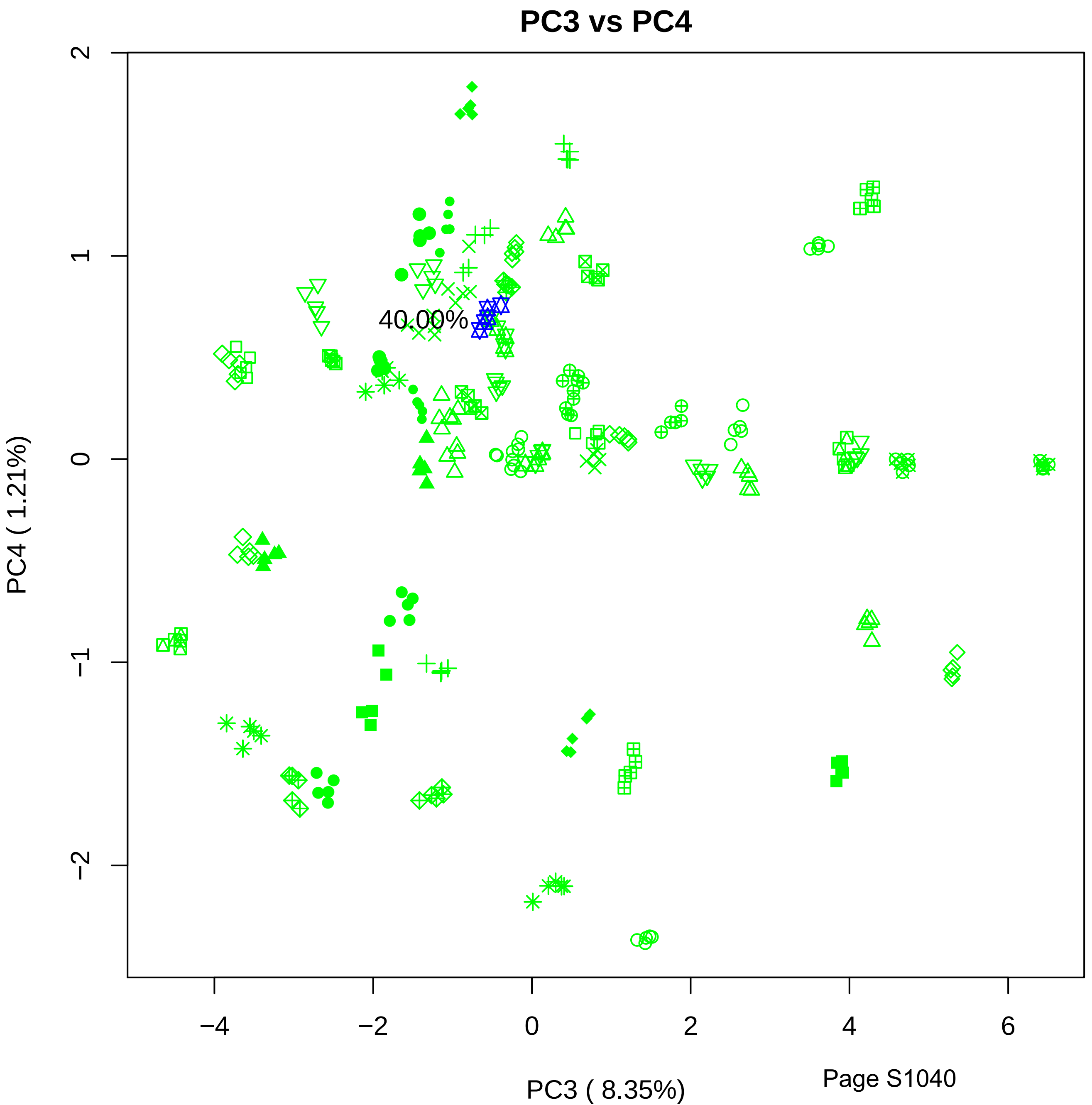

PC3 vs PC4
40.00%
PC4 (1.21%)
PC3 (8.35%)
2
1
0
−1
−2
−4
−2
0
2
4
6

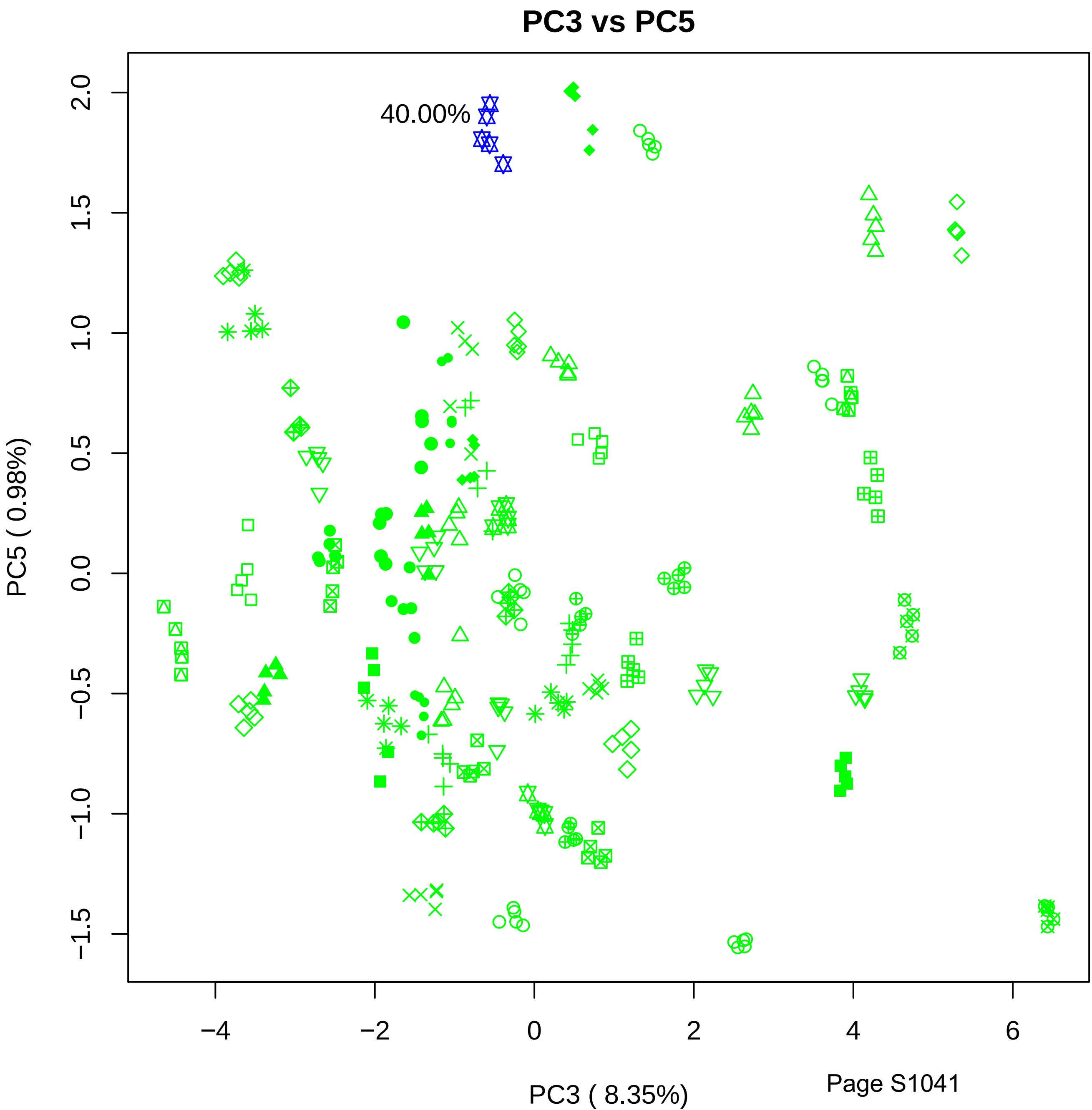
PC3 vs PC5
40.00%
2.0
1.5
1.0
0.5
0.0
−0.5
−1.0
−1.5
PC5 (0.98%)
−4
−2
0
2
4
6
PC3 (8.35%)

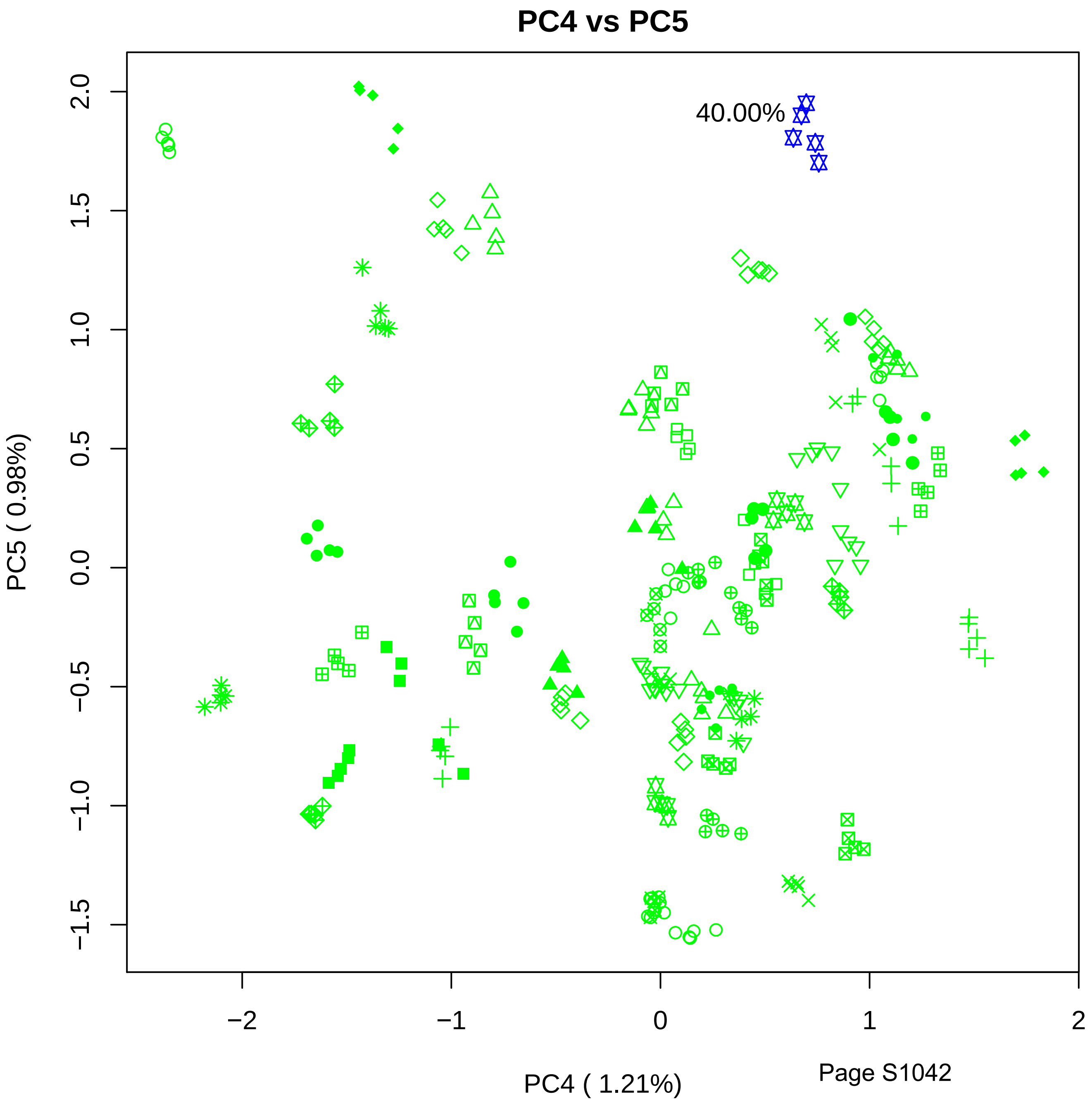
PC4 vs PC5
40.00%
2.0
1.5
1.0
0.5
0.0
−0.5
−1.0
−1.5
PC5 (0.98%)
−2
−1
0
1
2
PC4 (1.21%)

PCA of All BMH(b) vs PMH(b)

Scaled Matrix PCAed

7th order polynomial baseline correction performed

Raw spectra smoothed to 21 Neighbours, 2nd order PN, Savtisky Golay

Normalisation between 0 and 100

D2 spectra smoothed to 41 Neighbours, 2nd order PN, Savtisky Golay

Regions Analysed: 3601−2501 and 2001−700cm−1

Samples Analysed: 8 bContam's 0 BLH's 0 BMH's 0 OMH's 0 pContam's 0 PLH's 60 PMH's

Keele University AD

Fri Dec 14 14:21:00 2018

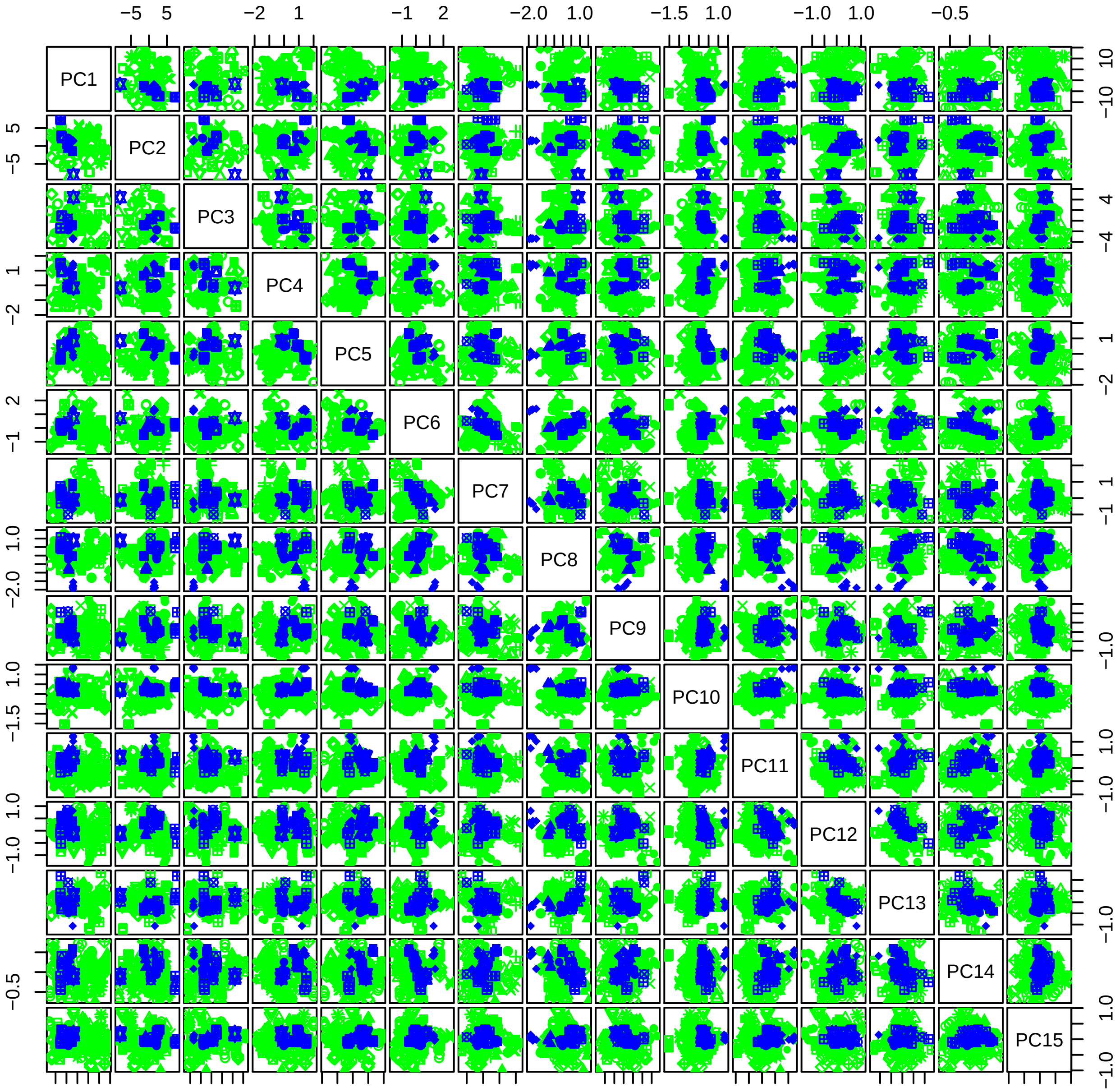
PC1
PC2
PC3
PC4
PC5
PC6
PC7
PC8
PC9
PC10
PC11
PC12
PC13
PC14
PC15

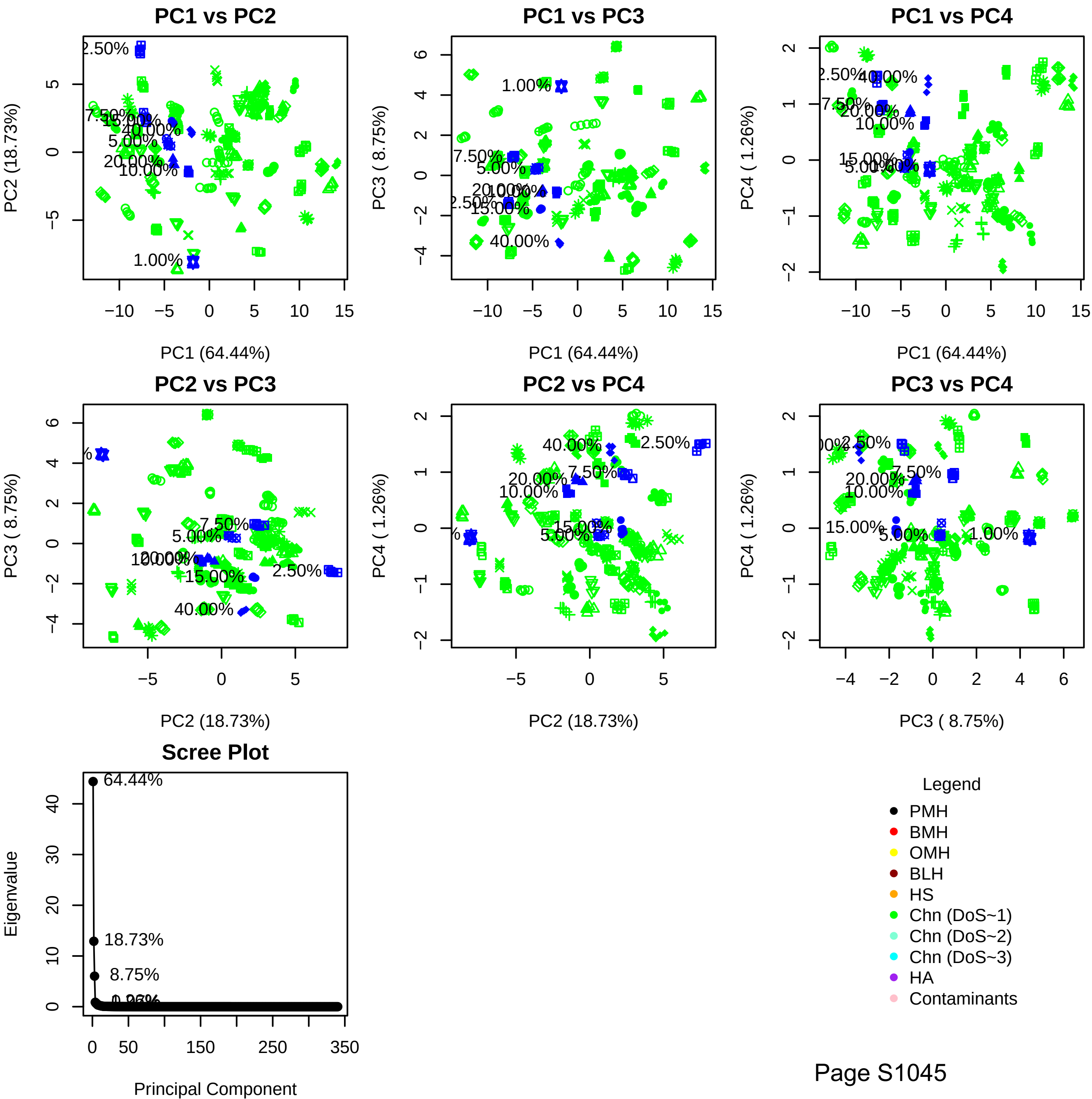
PC1 vs PC2
PC1 vs PC3
PC1 vs PC4
PC2 vs PC3
PC2 vs PC4
PC3 vs PC4
Scree Plot
PC1 (64.44%)
PC2 (18.73%)
PC3 (8.75%)
PC4 (1.26%)
Eigenvalue
Principal Component
64.44%
18.73%
8.75%
Legend
PMH
BMH
OMH
BLH
HS
Chn (DoS~1)
Chn (DoS~2)
Chn (DoS~3)
HA
Contaminants

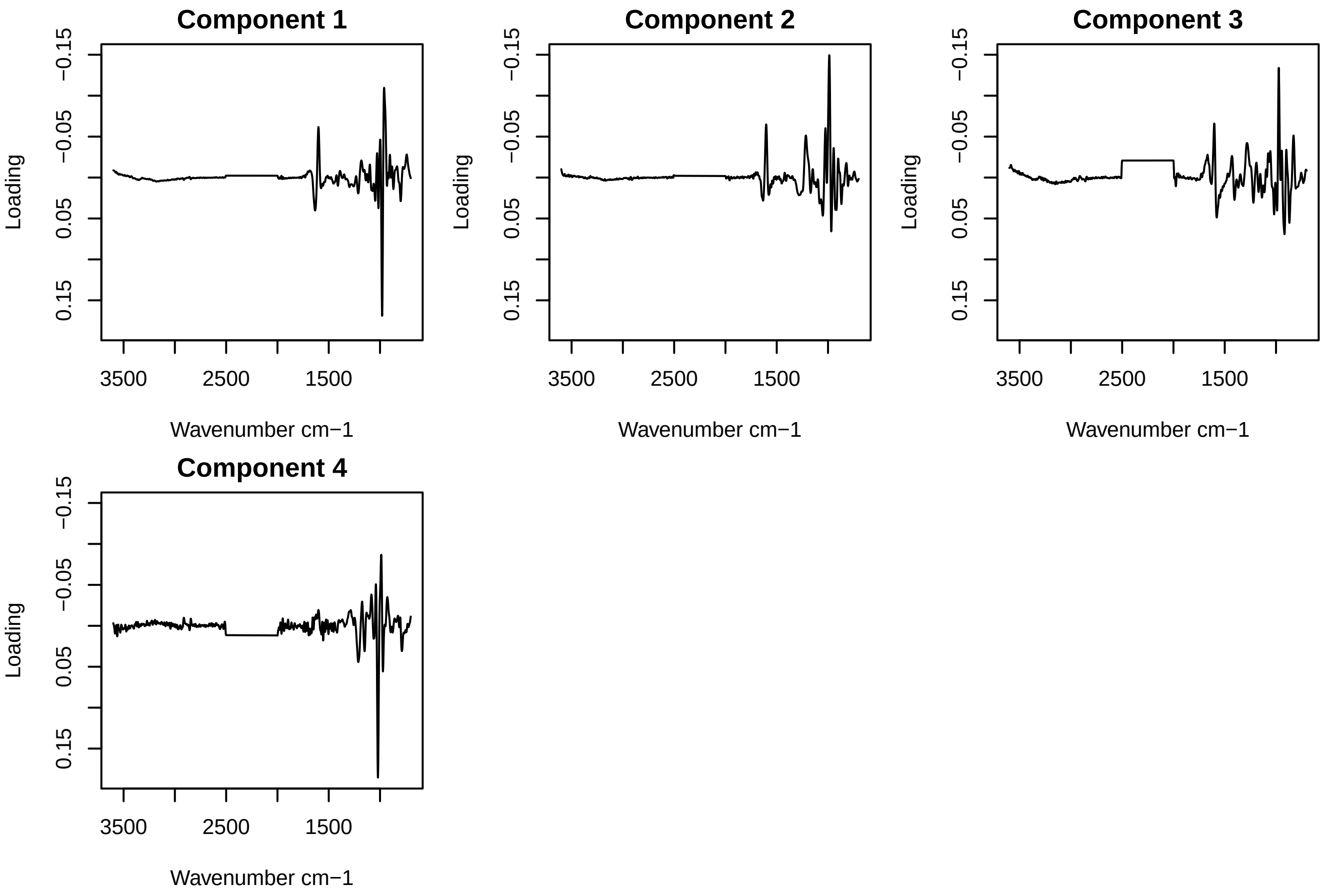
Component 1
Component 2
Component 3
Component 4
Loading
−0.15
−0.05
0.05
0.15
3500
2500
1500
Wavenumber cm−1

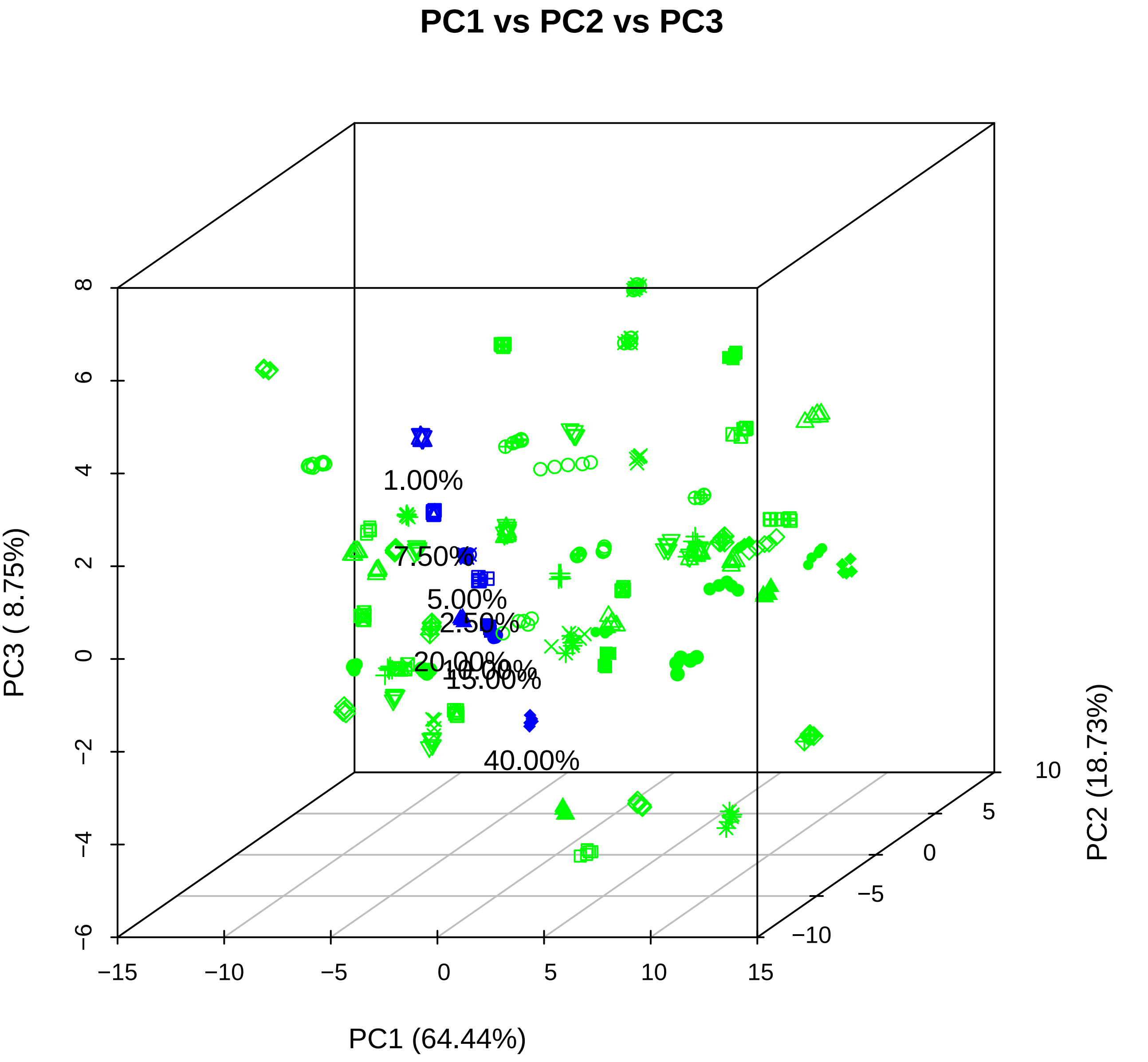

PC1 vs PC2 vs PC3
PC1 (64.44%)
PC2 (18.73%)
PC3 (8.75%)
−15
−10
−5
0
5
10
15
−10
−5
0
5
10
−6
−4
−2
0
2
4
6
8
1.00%
7.50%
5.00%
2.50%
20.00%
10.00%
15.00%
40.00%

PC1 vs PC2 vs PC4

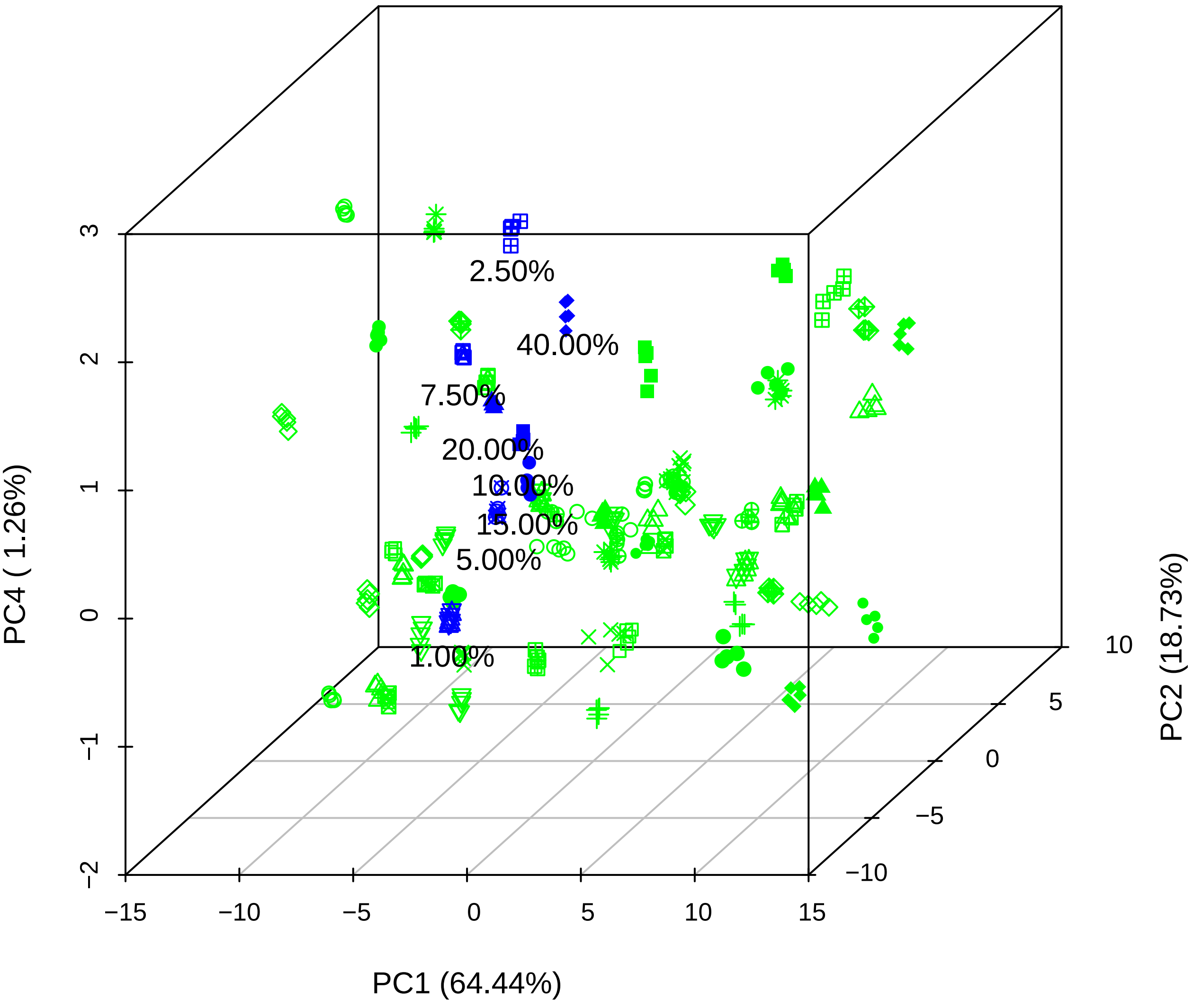

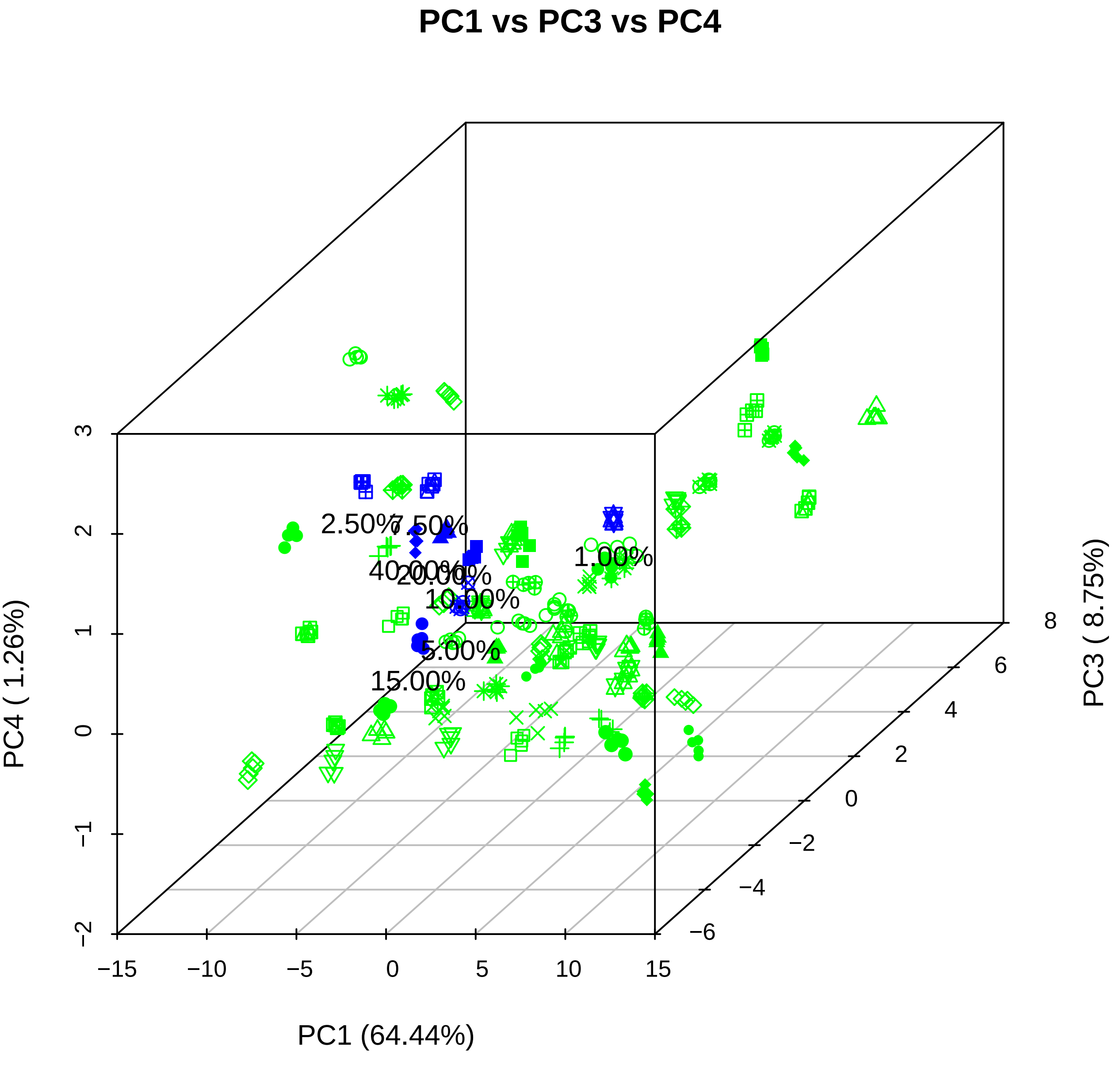

PC1 vs PC3 vs PC4
PC1 (64.44%)
PC4 (1.26%)
PC3 (8.75%)
−15
−10
−5
0
5
10
15
−2
−1
0
1
2
3
−6
−4
−2
0
2
4
6
8
2.50%
7.50%
40.00%
20.00%
10.00%
1.00%
5.00%
15.00%

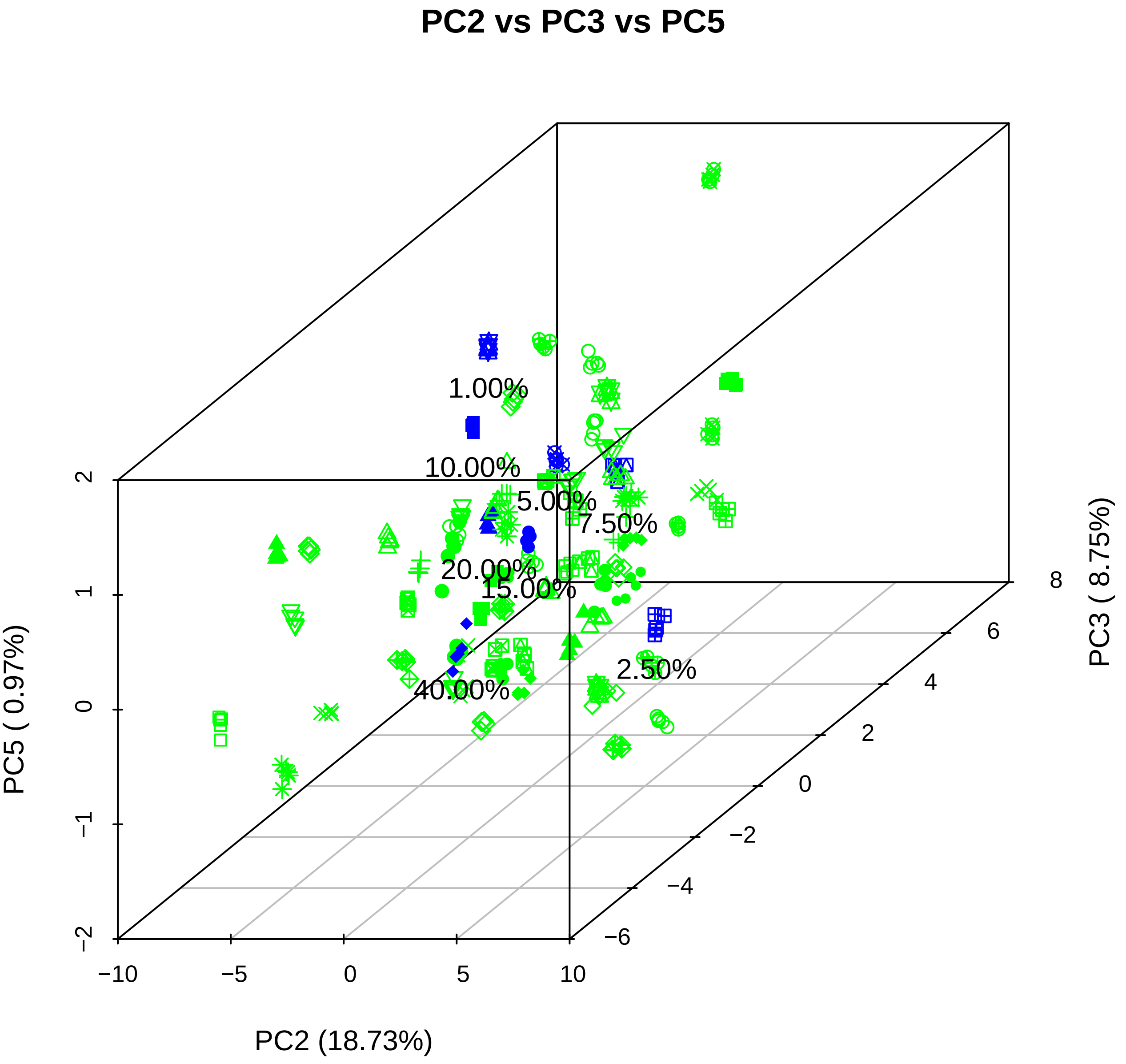
PC2 vs PC3 vs PC5
1.00%
10.00%
5.00%
7.50%
20.00%
15.00%
2.50%
40.00%
PC5 (0.97%)
−2
−1
0
1
2
−10
−5
0
5
10
PC2 (18.73%)
PC3 (8.75%)
−6
−4
−2
0
2
4
6
8

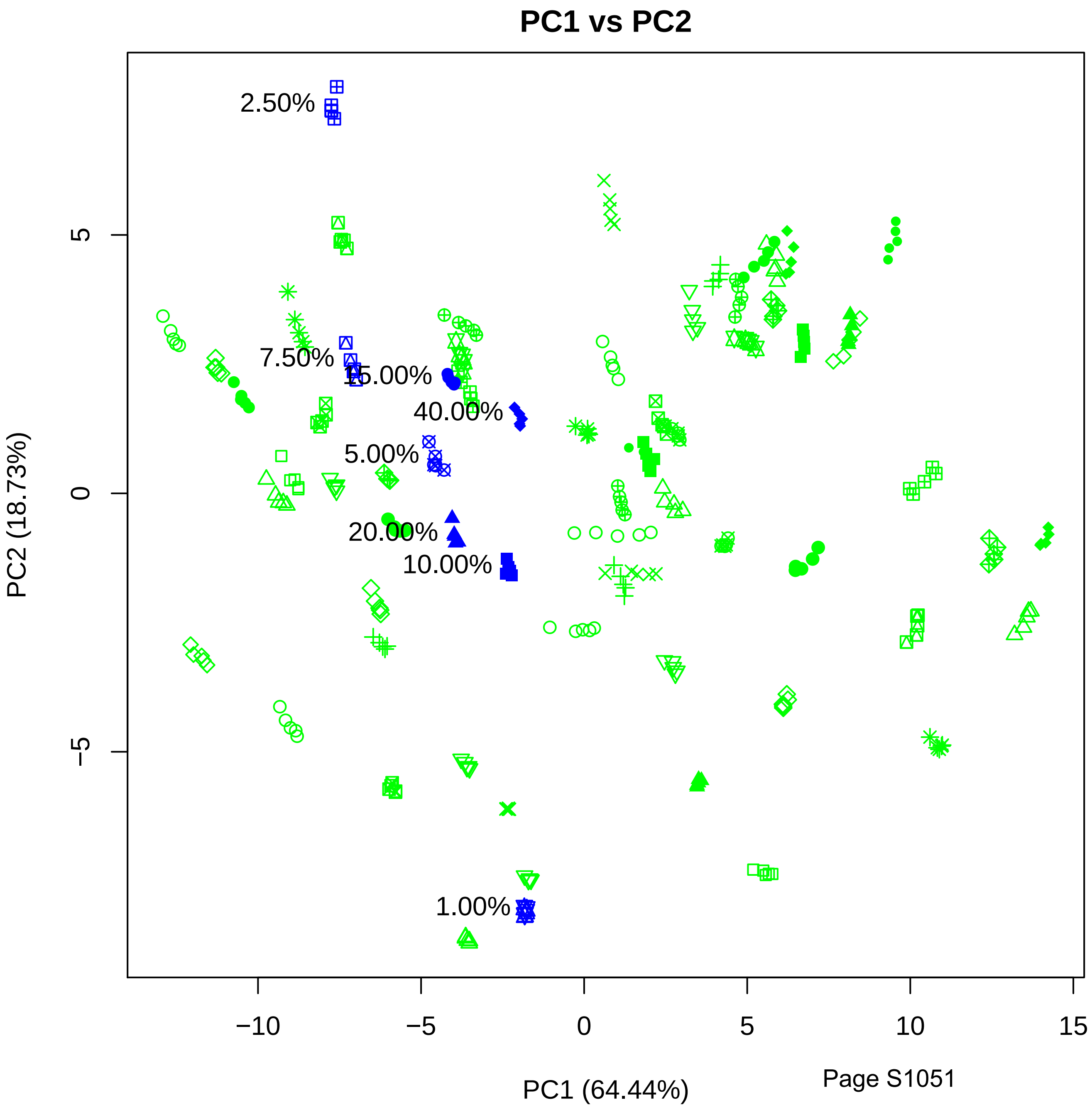
PC1 vs PC2
2.50%
7.50%
15.00%
40.00%
5.00%
20.00%
10.00%
1.00%
5
0
−5
−10
−5
0
5
10
15
PC2 (18.73%)
PC1 (64.44%)

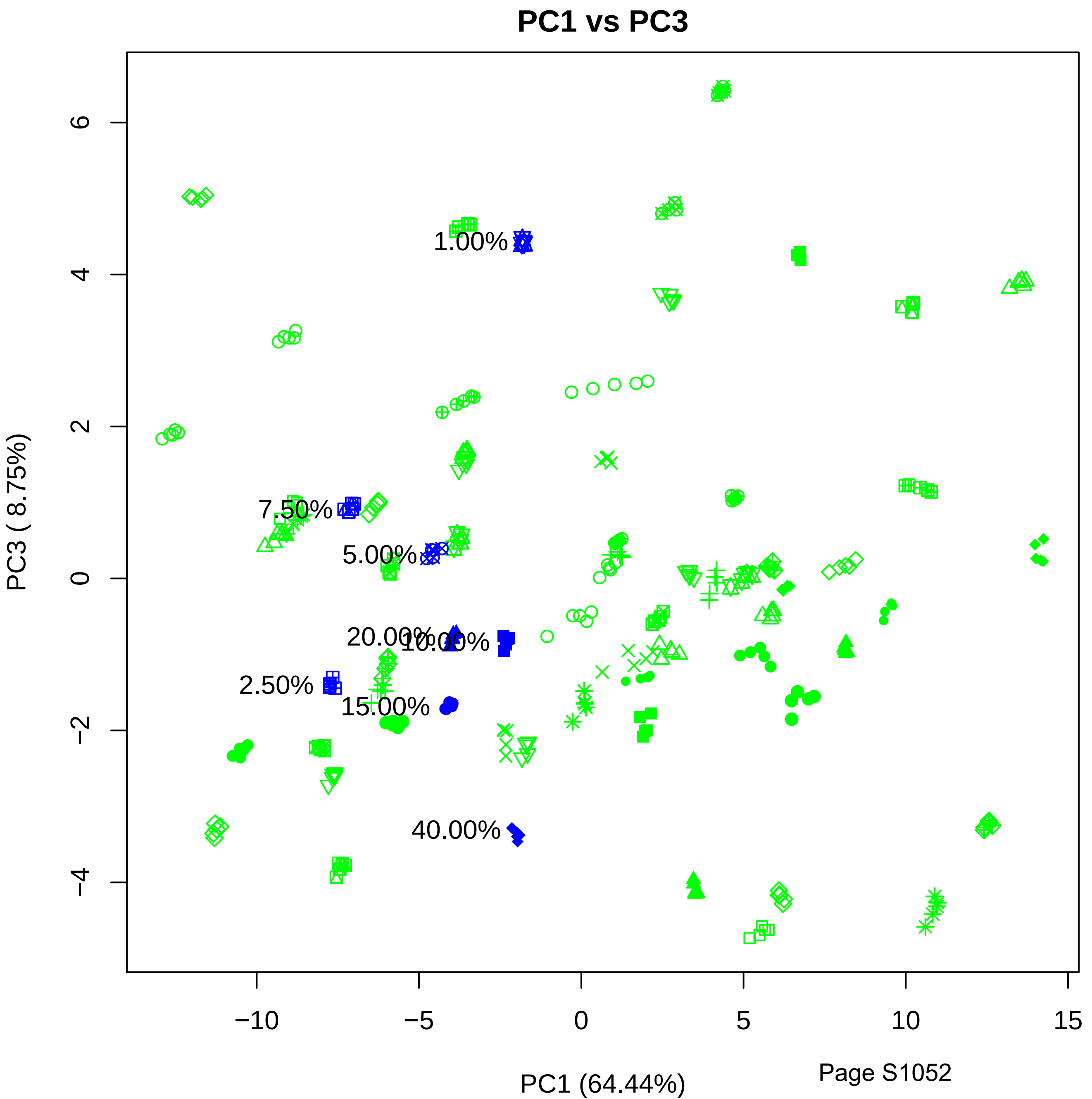

PC1 vs PC3
PC3 (8.75%)
PC1 (64.44%)
6
4
2
0
−2
−4
−10
−5
0
5
10
15
1.00%
7.50%
5.00%
20.00%
10.00%
2.50%
15.00%
40.00%

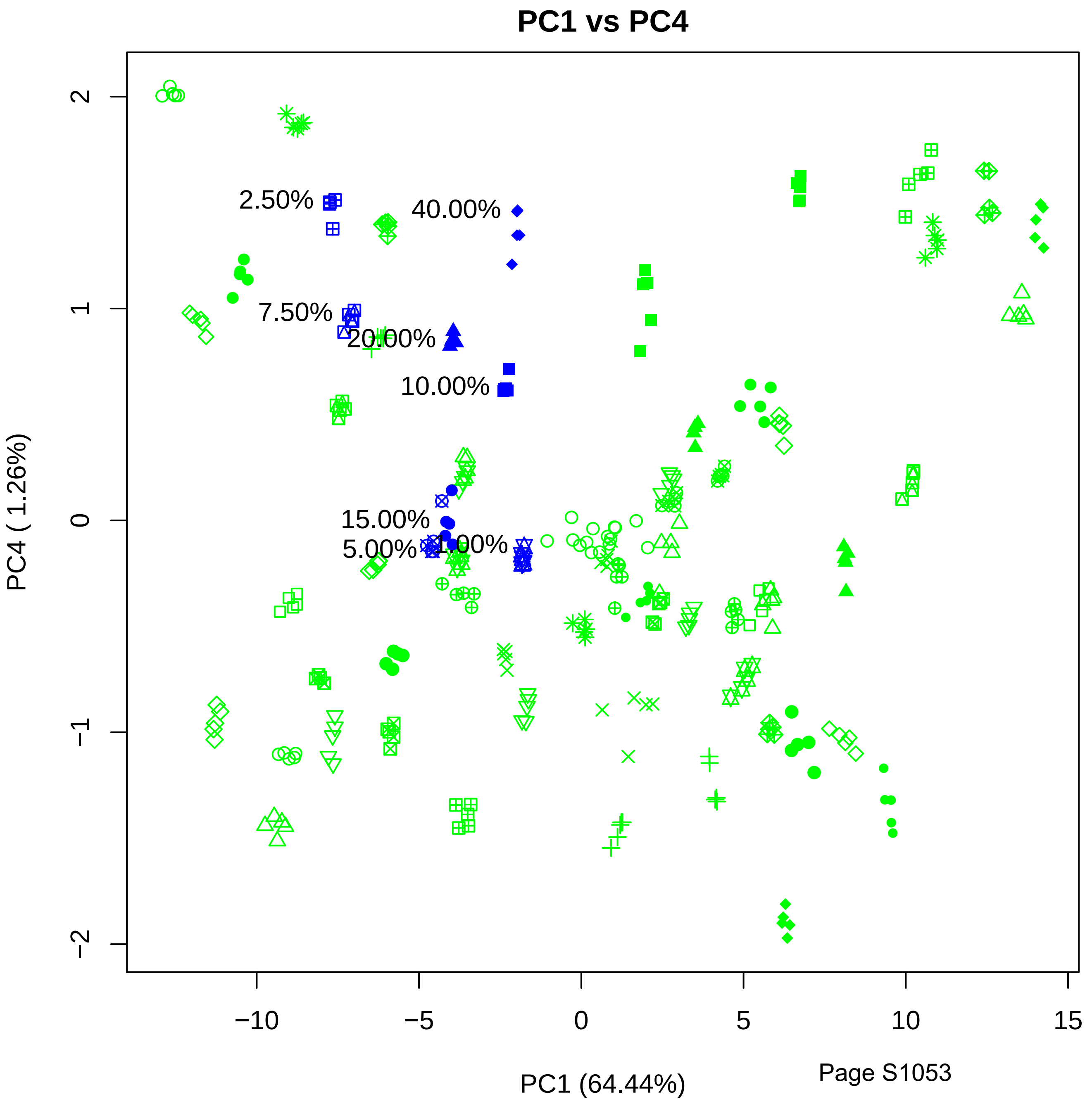

PC1 vs PC4
2.50%
40.00%
7.50%
20.00%
10.00%
15.00%
5.00%
1.00%
PC4 (1.26%)
PC1 (64.44%)
2
1
0
−1
−2
−10
−5
0
5
10
15

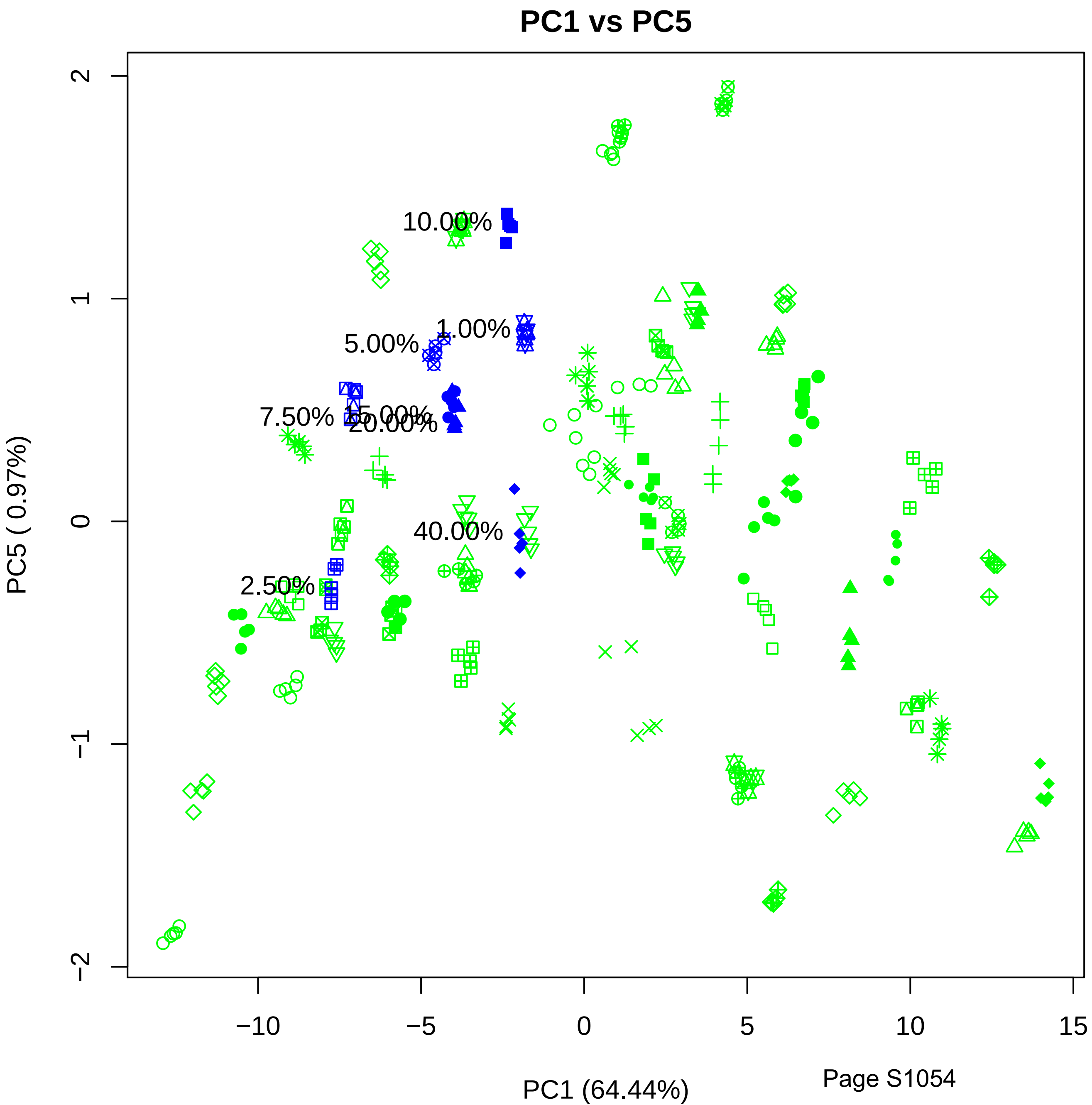
PC1 vs PC5
10.00%
1.00%
5.00%
7.50%
15.00%
20.00%
40.00%
2.50%
PC5 (0.97%)
PC1 (64.44%)
2
1
0
−1
−2
−10
−5
0
5
10
15

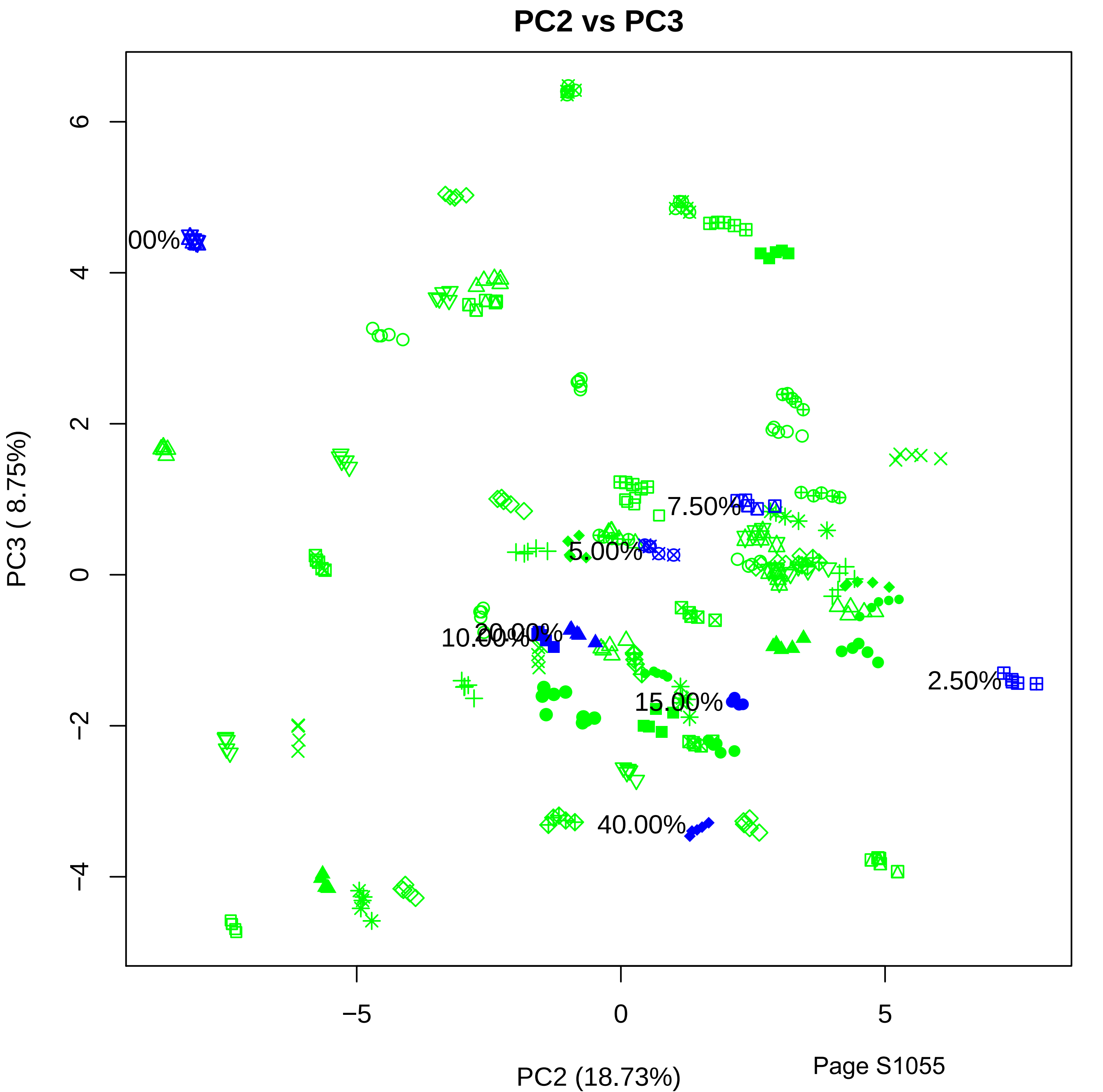
PC2 vs PC3
PC3 (8.75%)
PC2 (18.73%)
6
4
2
0
−2
−4
−5
0
5
00%
7.50%
5.00%
10.00%
20.00%
15.00%
2.50%
40.00%

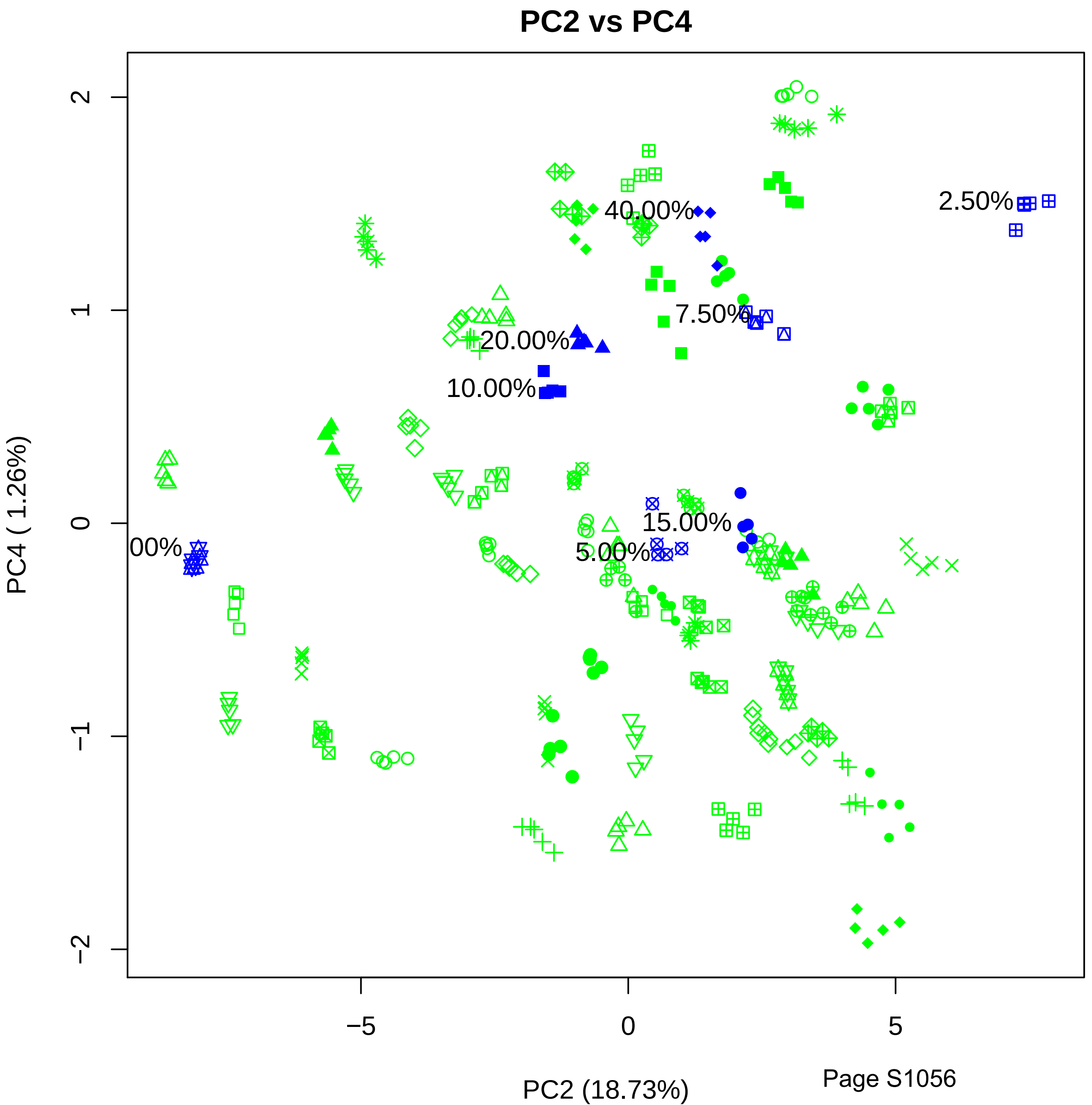
PC2 vs PC4
PC4 (1.26%)
PC2 (18.73%)
2
1
0
−1
−2
−5
0
5
2.50%
40.00%
7.50%
20.00%
10.00%
15.00%
5.00%
00%

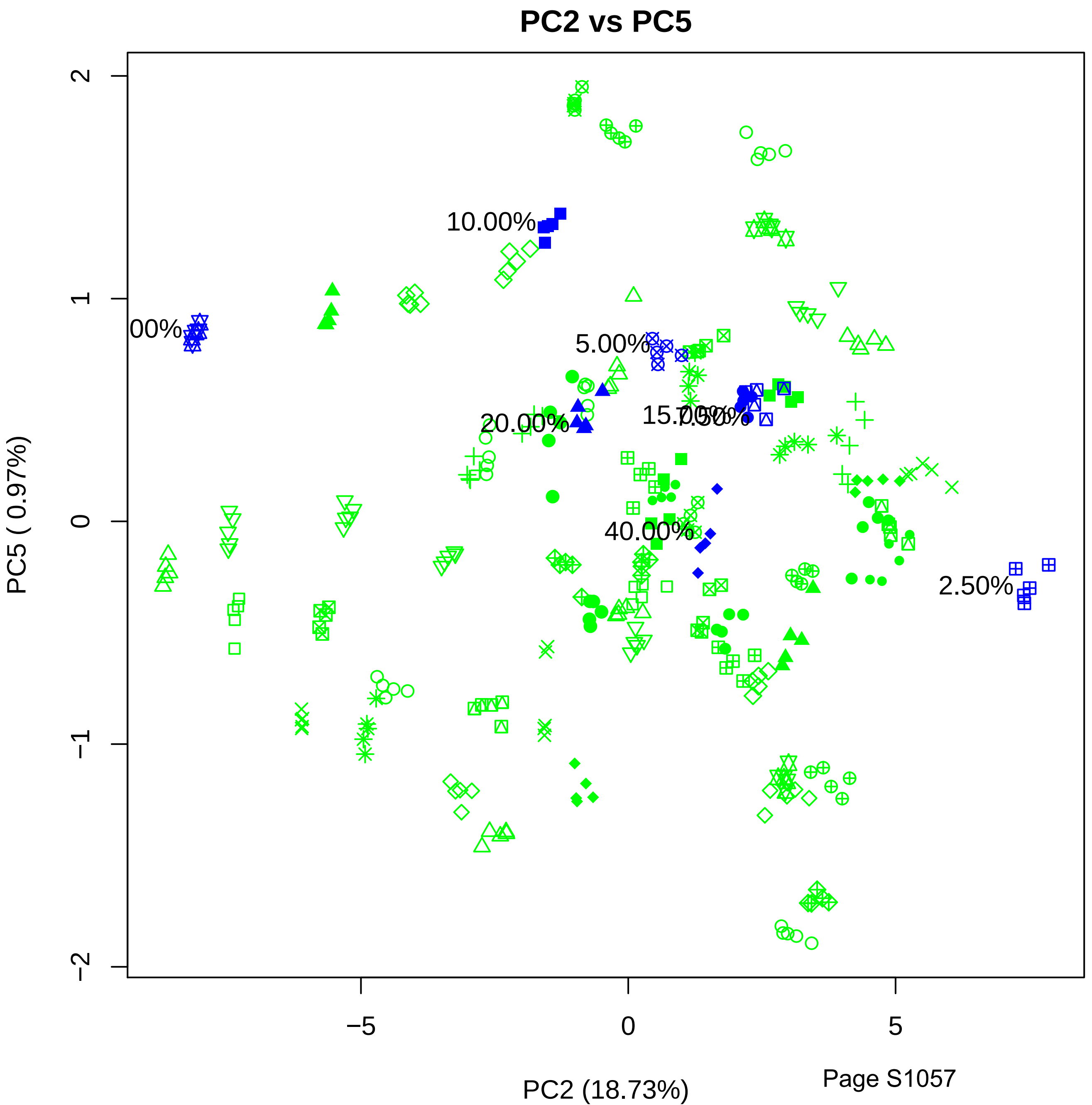
PC2 vs PC5
PC5 (0.97%)
PC2 (18.73%)
2
1
0
−1
−2
−5
0
5
10.00%
00%
5.00%
20.00%
15.00%
7.50%
40.00%
2.50%

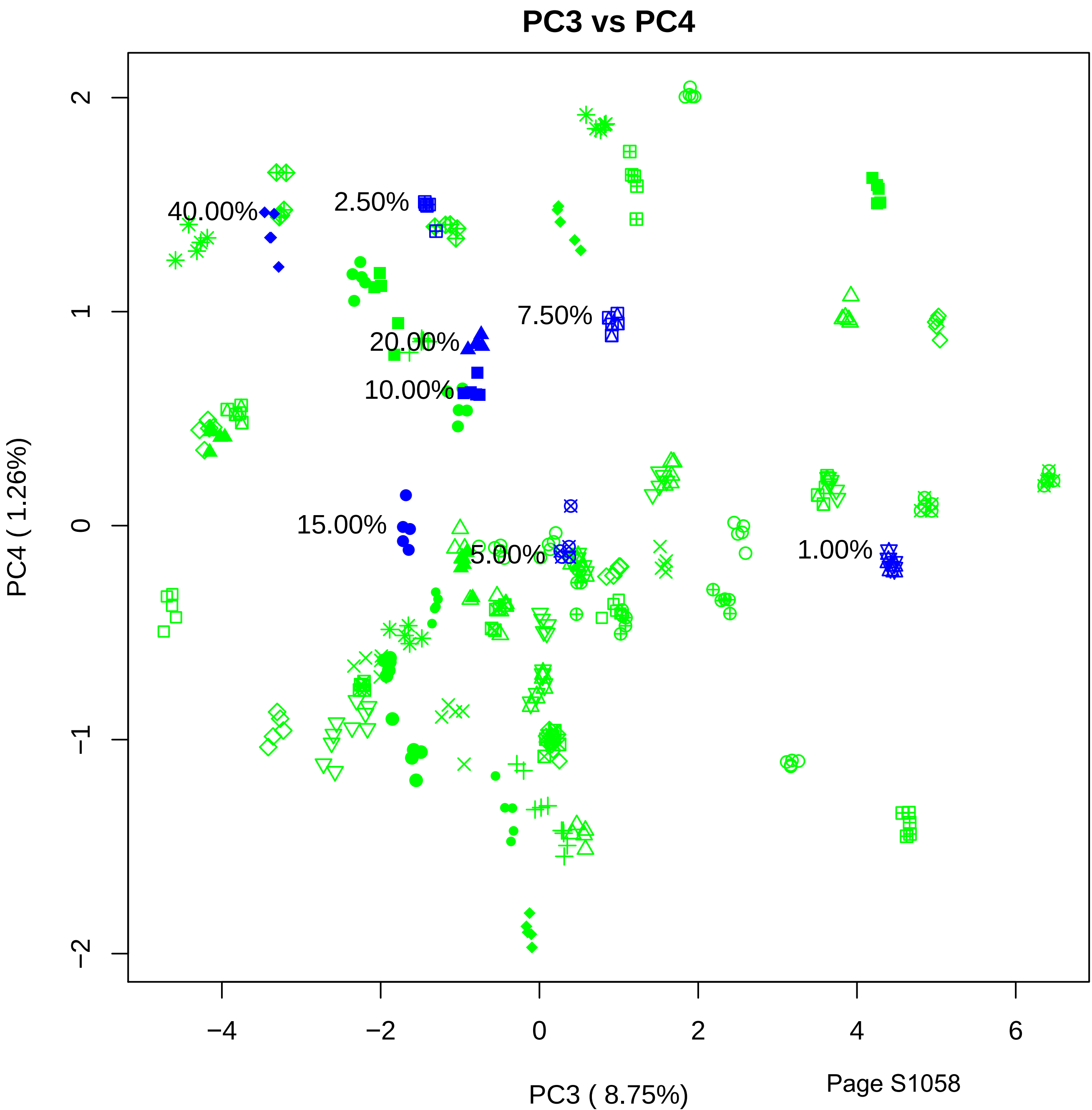
PC3 vs PC4
PC4 (1.26%)
PC3 (8.75%)
2
1
0
−1
−2
−4
−2
0
2
4
6
40.00%
2.50%
7.50%
20.00%
10.00%
15.00%
5.00%
1.00%

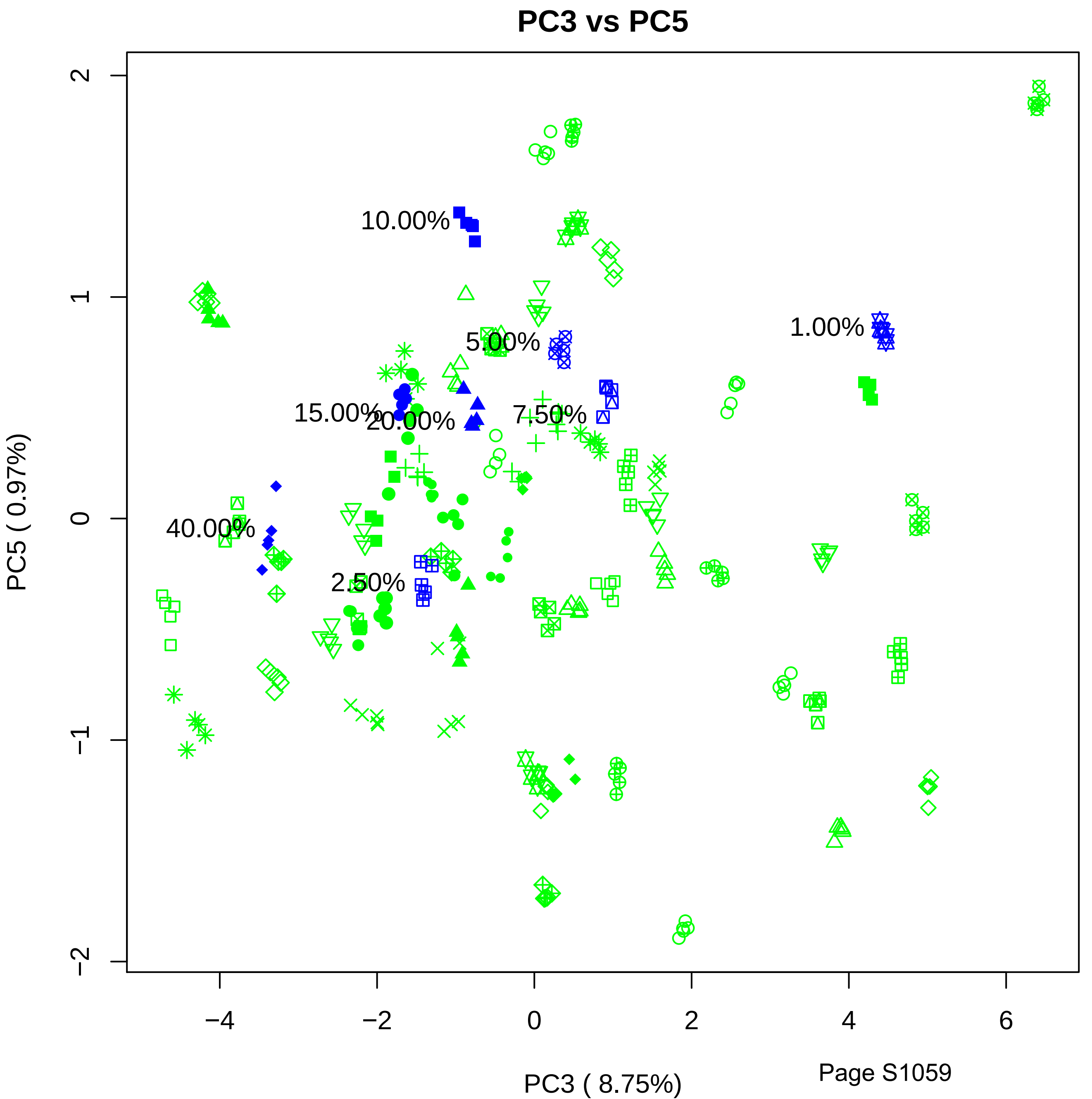

PC3 vs PC5
PC5 (0.97%)
PC3 (8.75%)
−2
−1
0
1
2
−4
−2
0
2
4
6
10.00%
1.00%
5.00%
15.00%
20.00%
7.50%
40.00%
2.50%

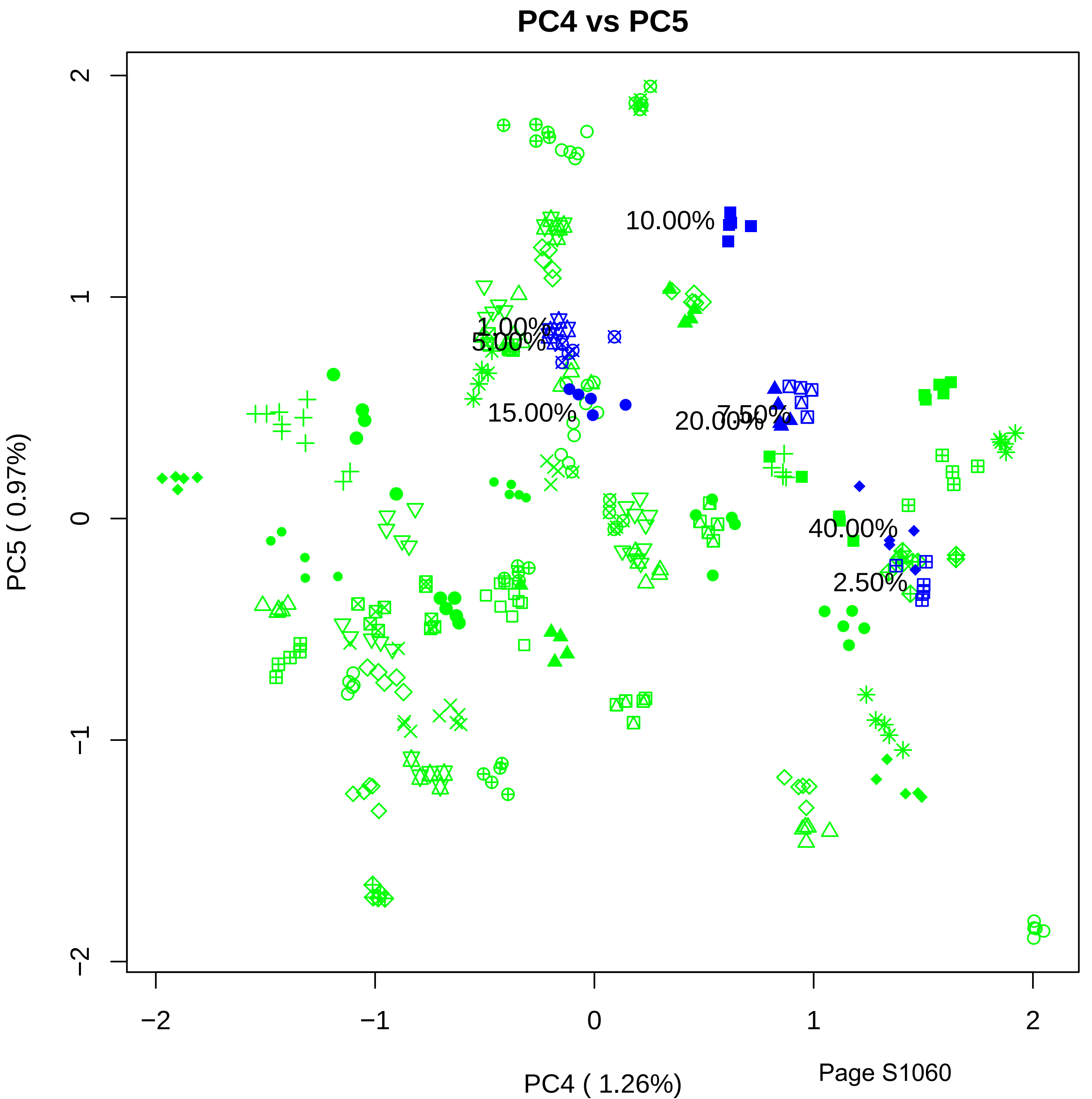
PC4 vs PC5
PC5 (0.97%)
PC4 (1.26%)
2
1
0
−1
−2
−2
−1
0
1
2
10.00%
1.00%
5.00%
15.00%
20.00%
7.50%
40.00%
2.50%

PCA of 1% BMH(b) vs PMH(b)

Scaled Matrix PCAed

7th order polynomial baseline correction performed

Raw spectra smoothed to 21 Neighbours, 2nd order PN, Savtisky Golay

Normalisation between 0 and 100

D2 spectra smoothed to 41 Neighbours, 2nd order PN, Savtisky Golay

Regions Analysed: 3601−2501 and 2001−700cm−1

Samples Analysed: 1 bContam's 0 BLH's 0 BMH's 0 OMH's 0 pContam's 0 PLH's 60 PMH's

Keele University AD

Fri Dec 14 14:21:09 2018

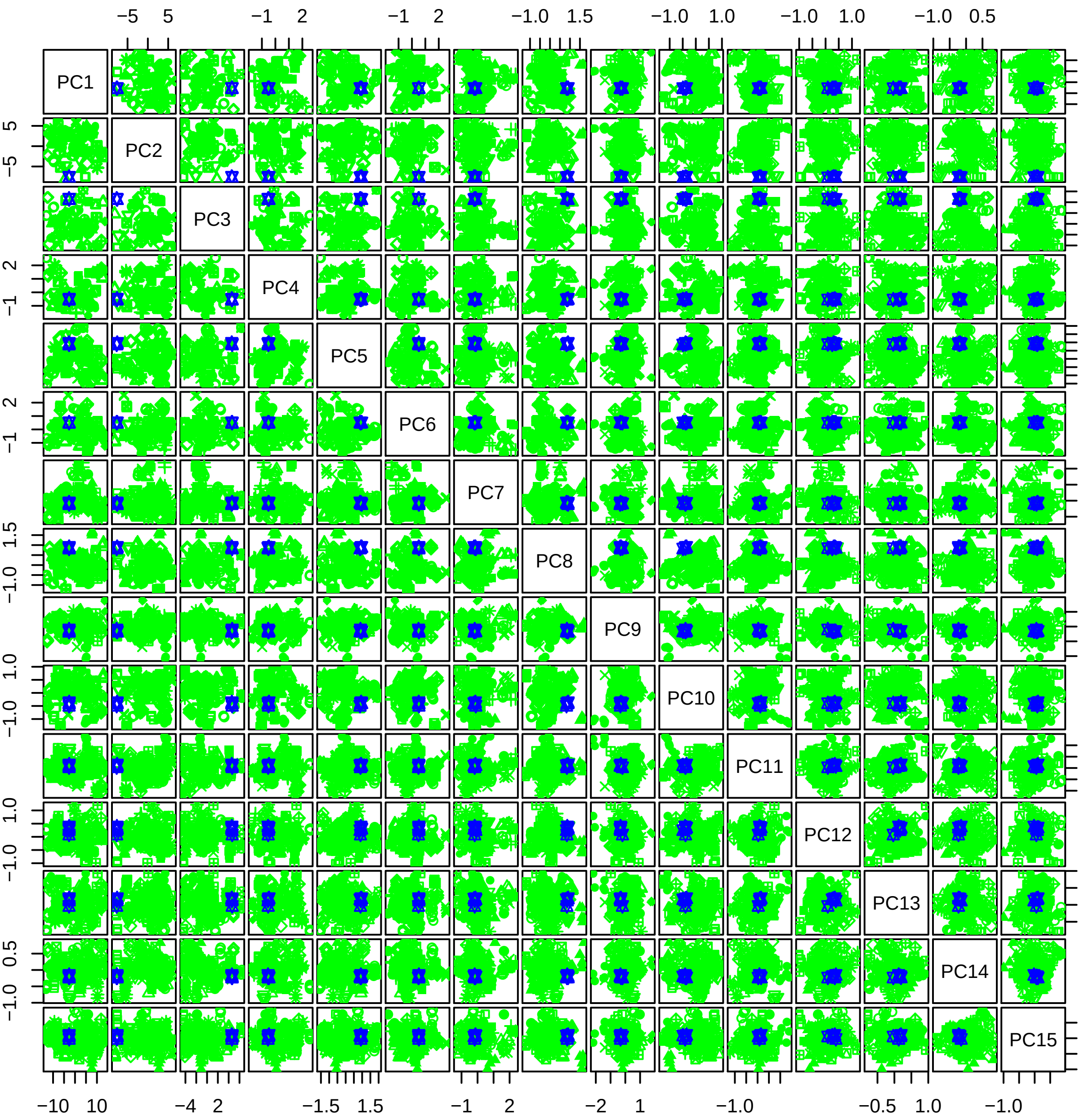

PC1
PC2
PC3
PC4
PC5
PC6
PC7
PC8
PC9
PC10
PC11
PC12
PC13
PC14
PC15

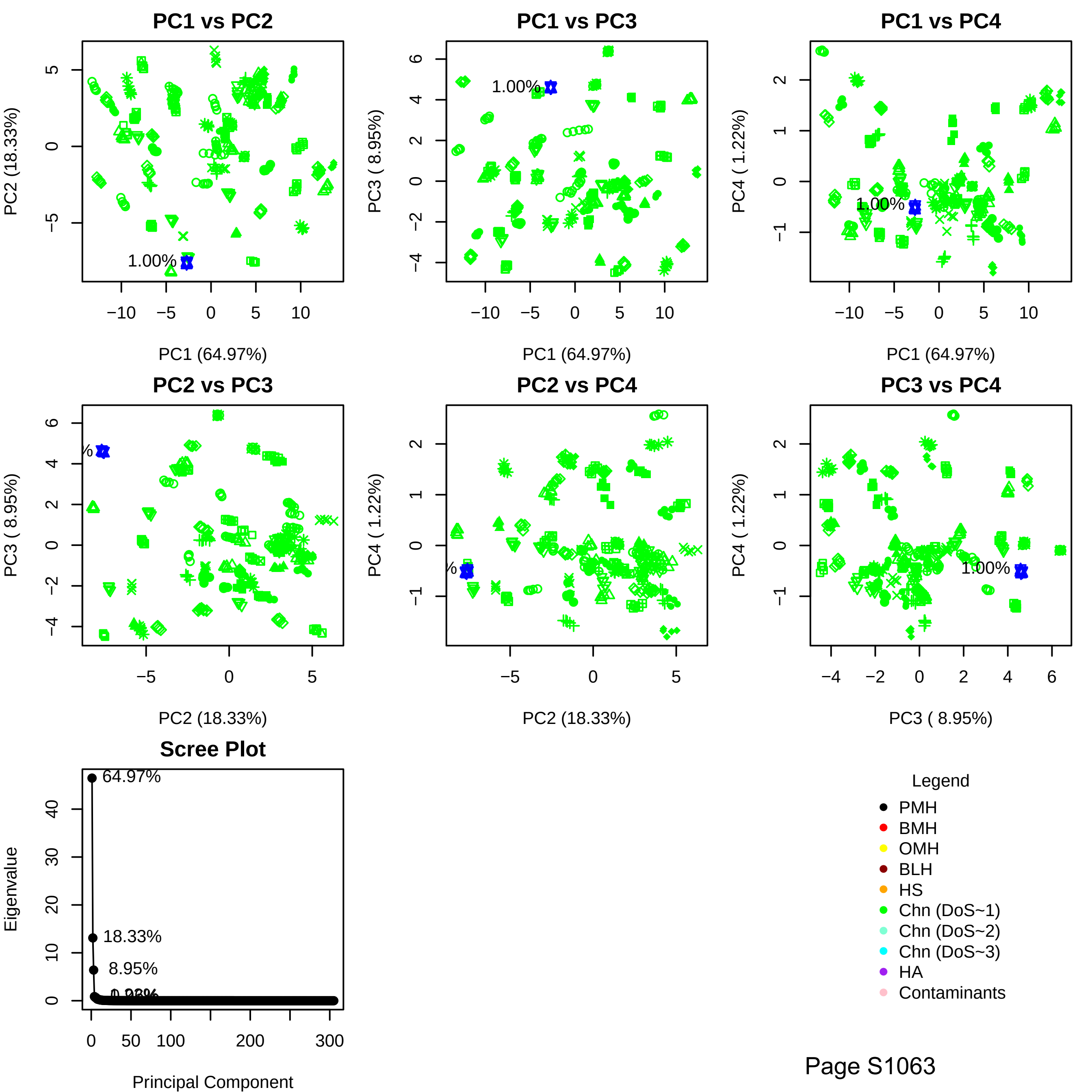
PC1 vs PC2
PC1 vs PC3
PC1 vs PC4
PC2 vs PC3
PC2 vs PC4
PC3 vs PC4
Scree Plot
PC1 (64.97%)
PC2 (18.33%)
PC3 (8.95%)
PC4 (1.22%)
1.00%
Eigenvalue
Principal Component
64.97%
18.33%
8.95%
Legend
PMH
BMH
OMH
BLH
HS
Chn (DoS~1)
Chn (DoS~2)
Chn (DoS~3)
HA
Contaminants

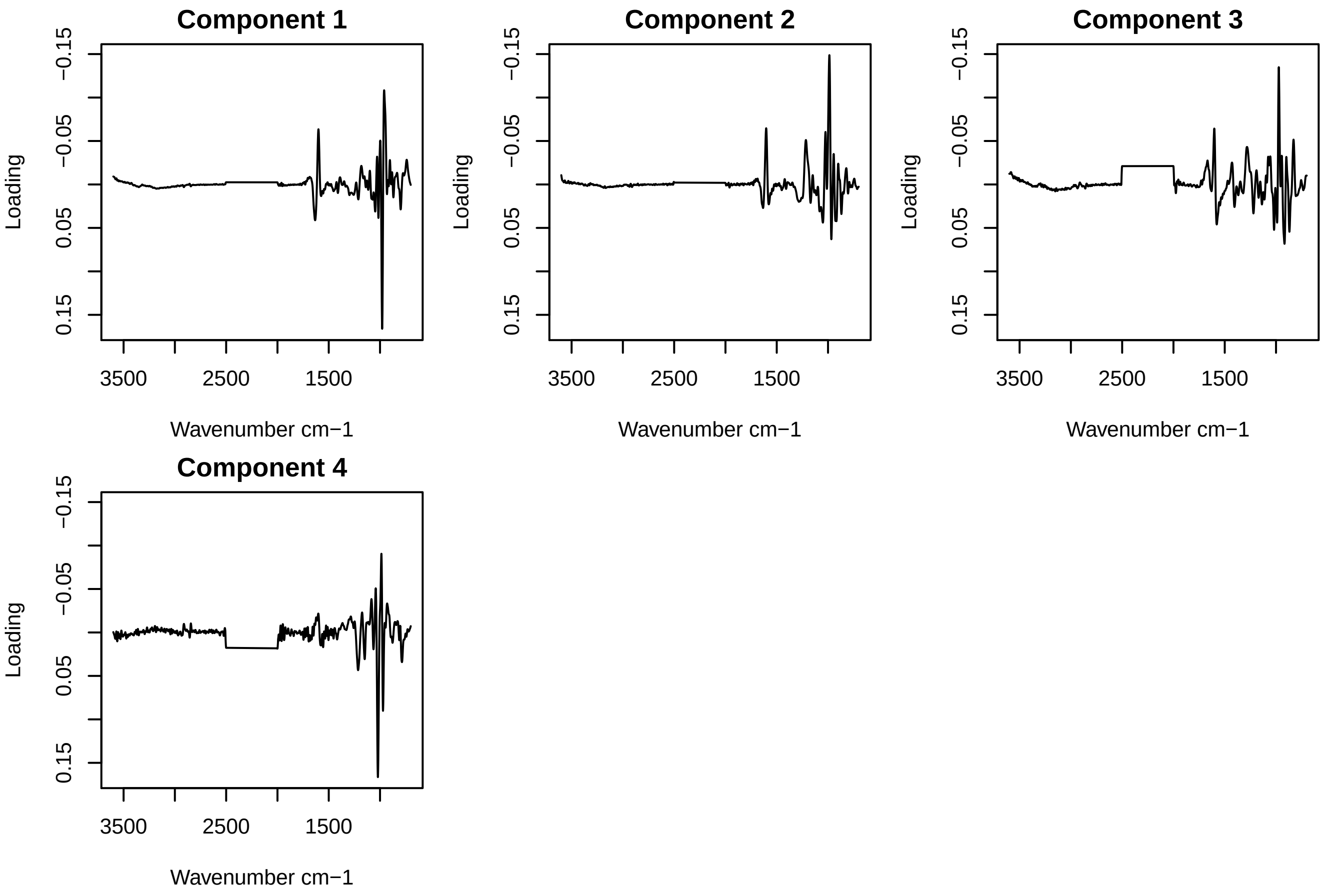
Component 1
Component 2
Component 3
Component 4
Loading
−0.15
−0.05
0.05
0.15
3500
2500
1500
Wavenumber cm−1

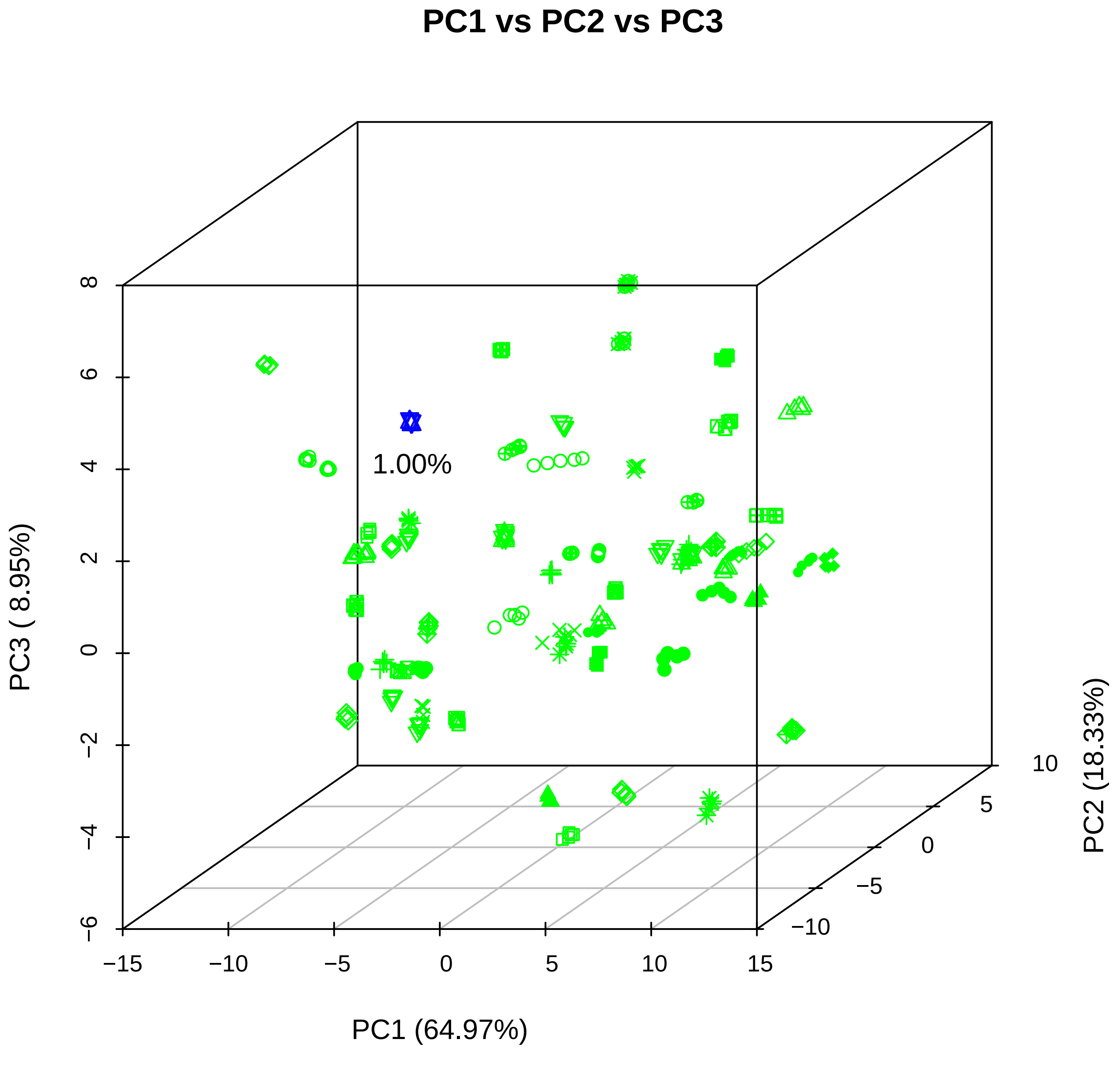

PC1 vs PC2 vs PC3
1.00%
PC3 (8.95%)
PC1 (64.97%)
PC2 (18.33%)
−15
−10
−5
0
5
10
15
−6
−4
−2
0
2
4
6
8
−10
−5
0
5
10

PC1 vs PC2 vs PC4

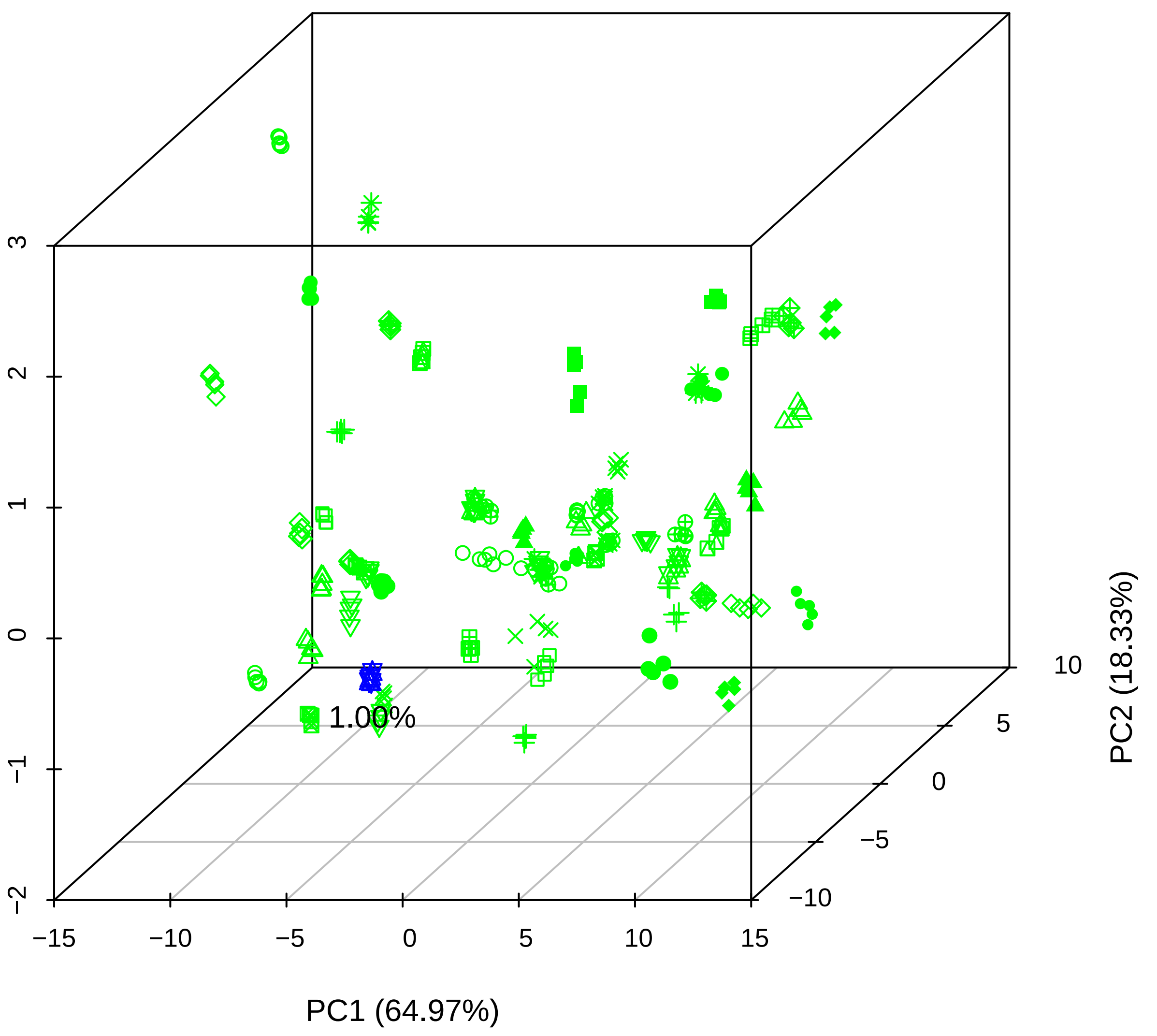

PC1 vs PC3 vs PC4

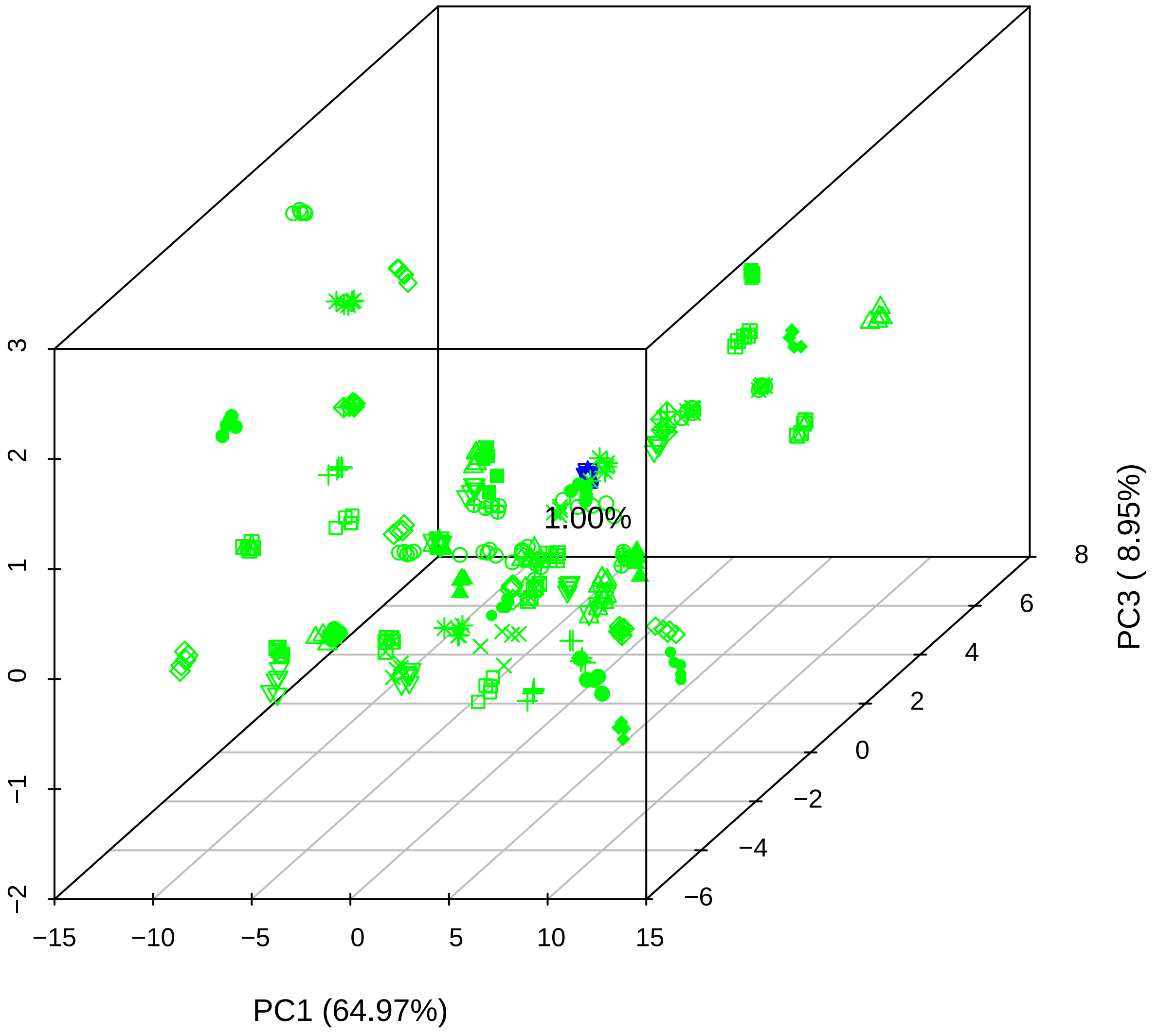

PC2 vs PC3 vs PC5

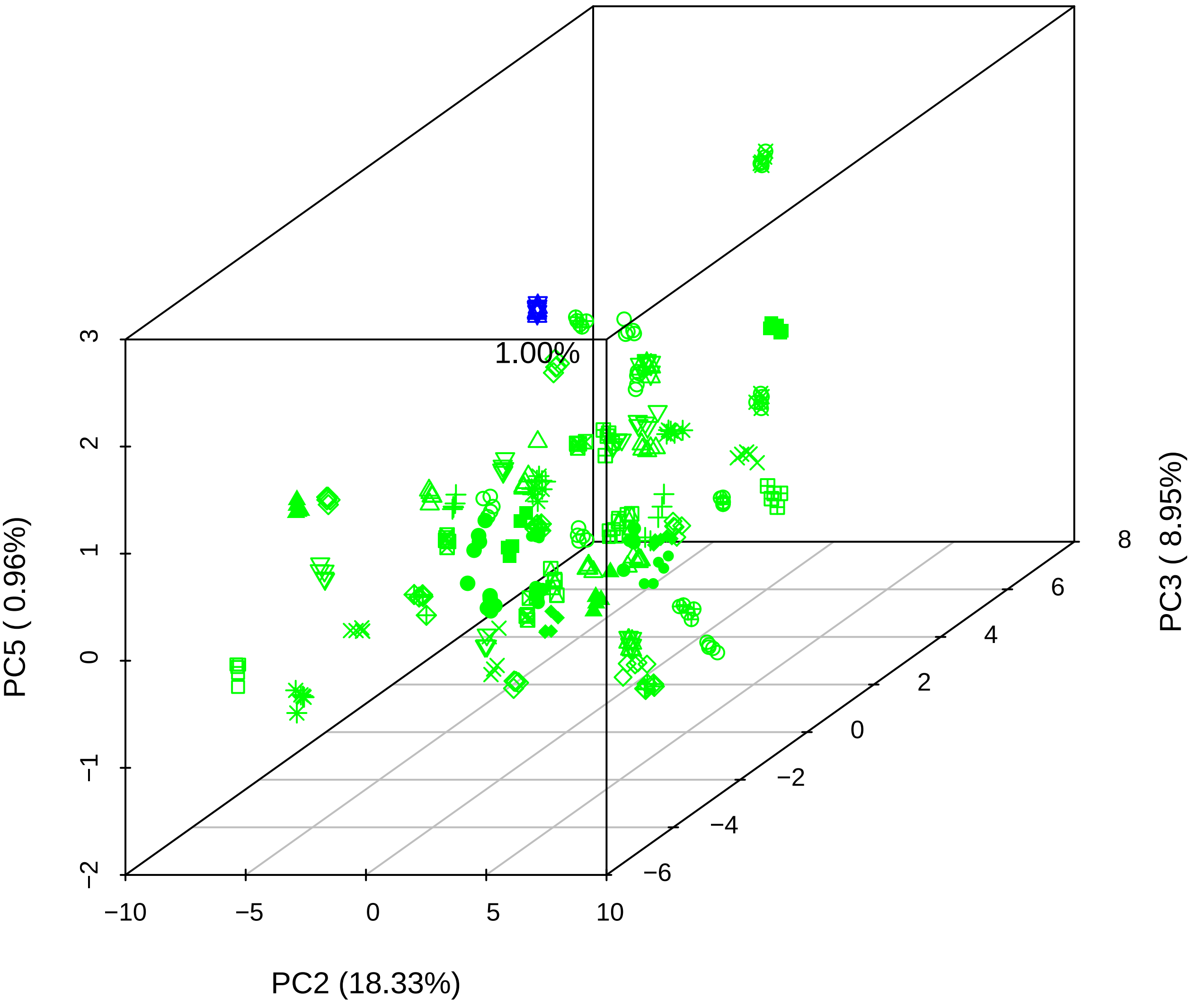

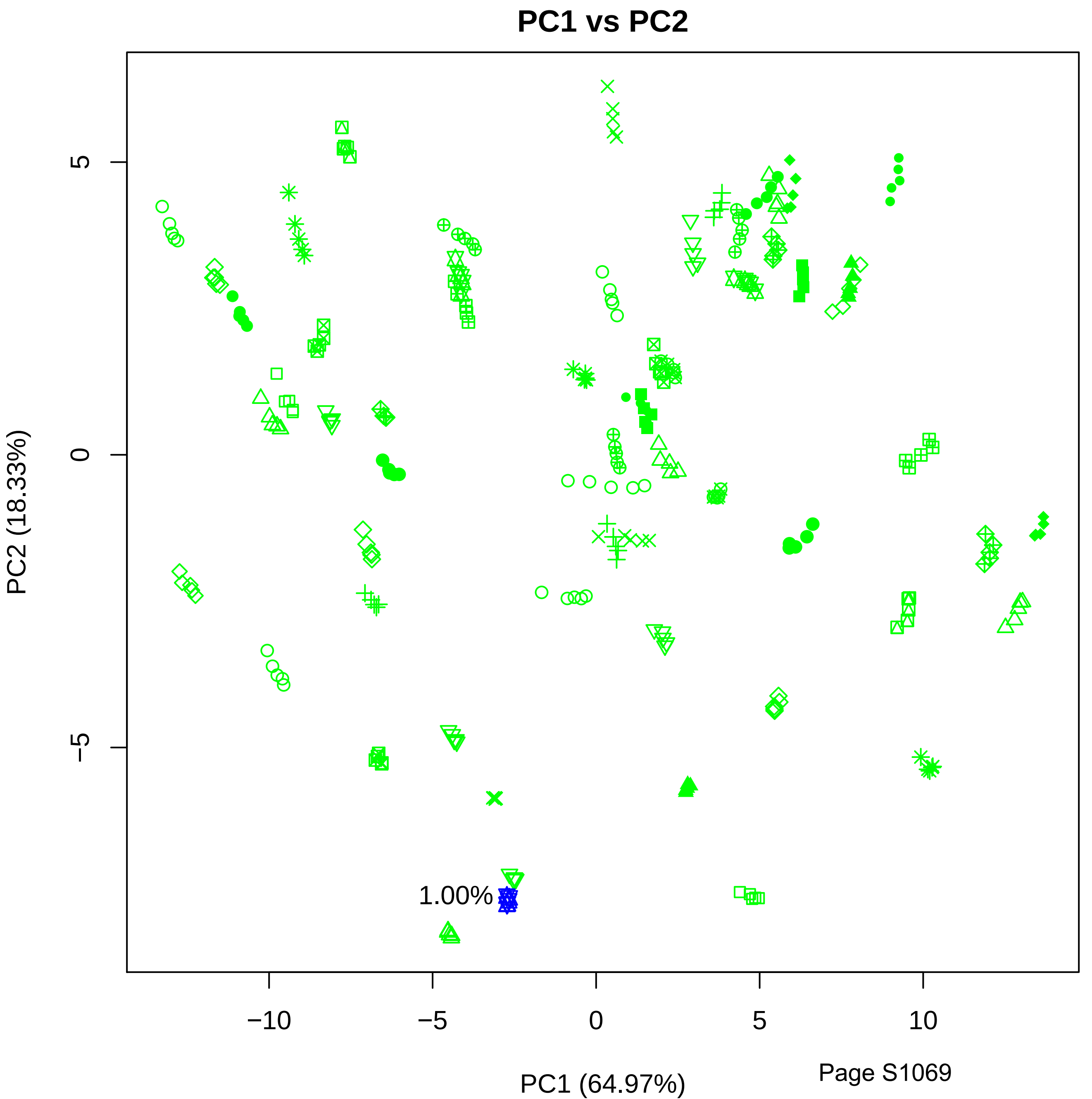
PC1 vs PC2
PC2 (18.33%)
PC1 (64.97%)
5
0
−5
−10
−5
0
5
10
1.00%

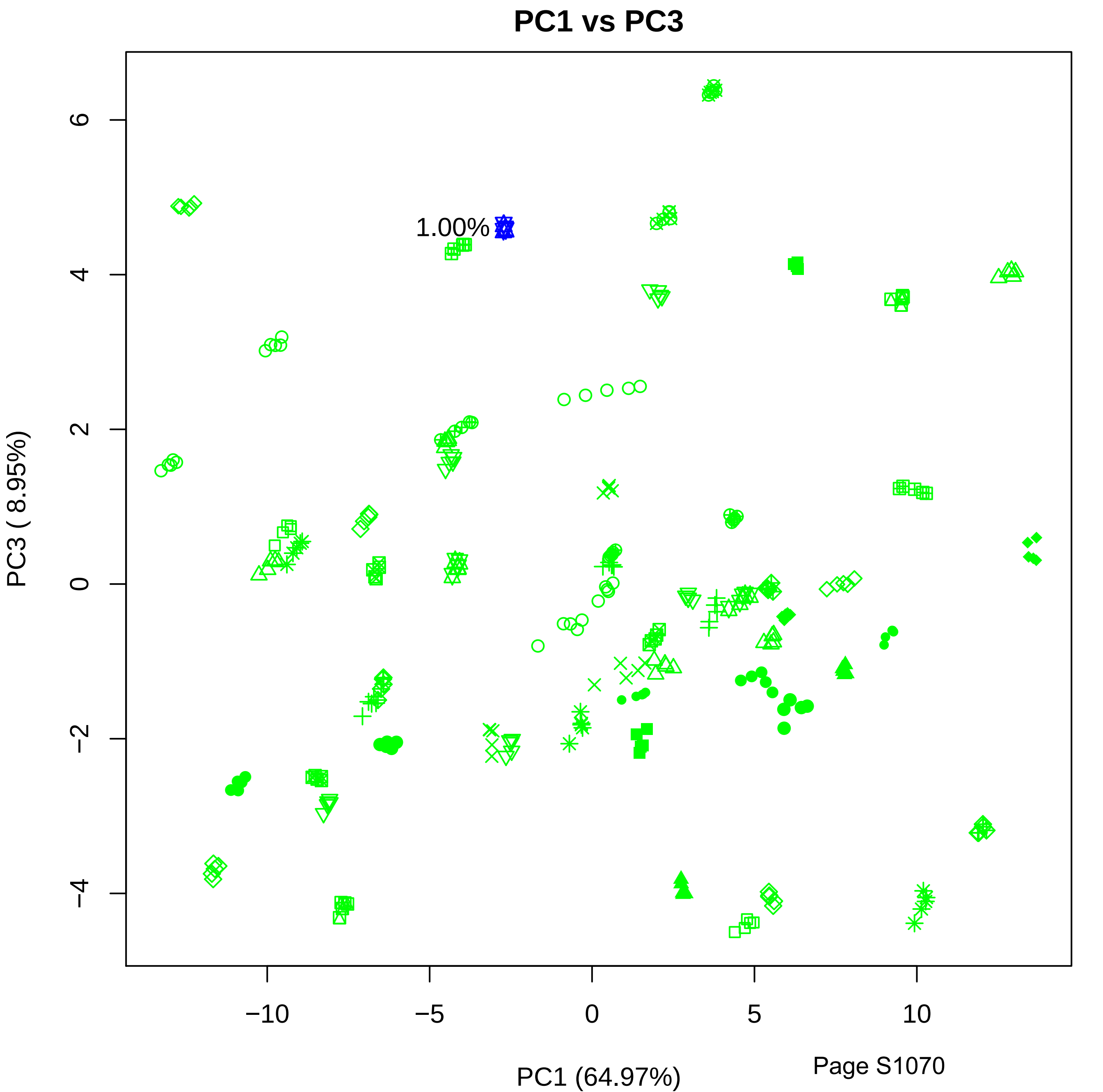

PC1 vs PC3
1.00%
PC3 (8.95%)
PC1 (64.97%)
6
4
2
0
−2
−4
−10
−5
0
5
10

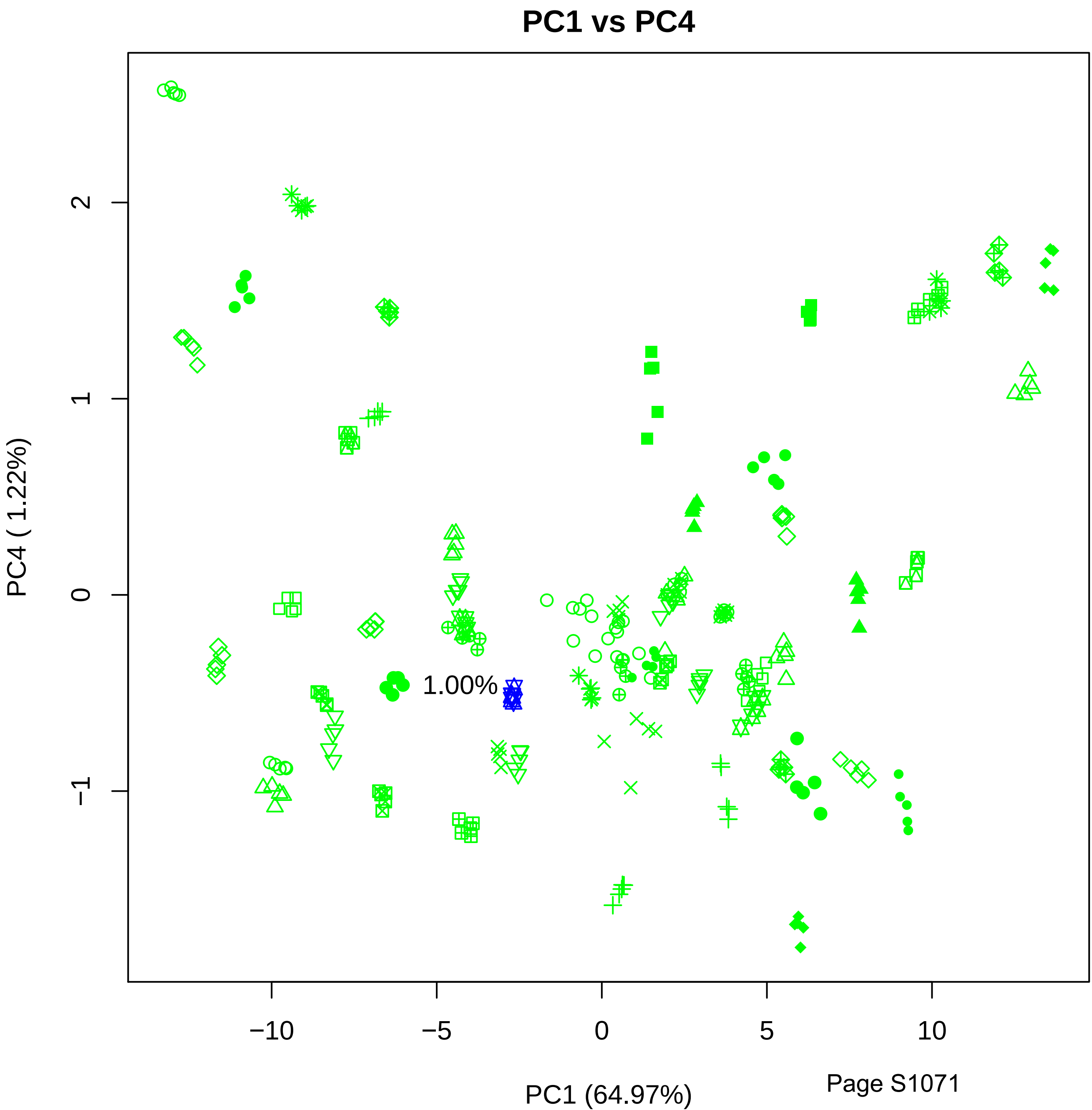

PC1 vs PC4
PC4 (1.22%)
PC1 (64.97%)
1.00%
2
1
0
−1
−10
−5
0
5
10

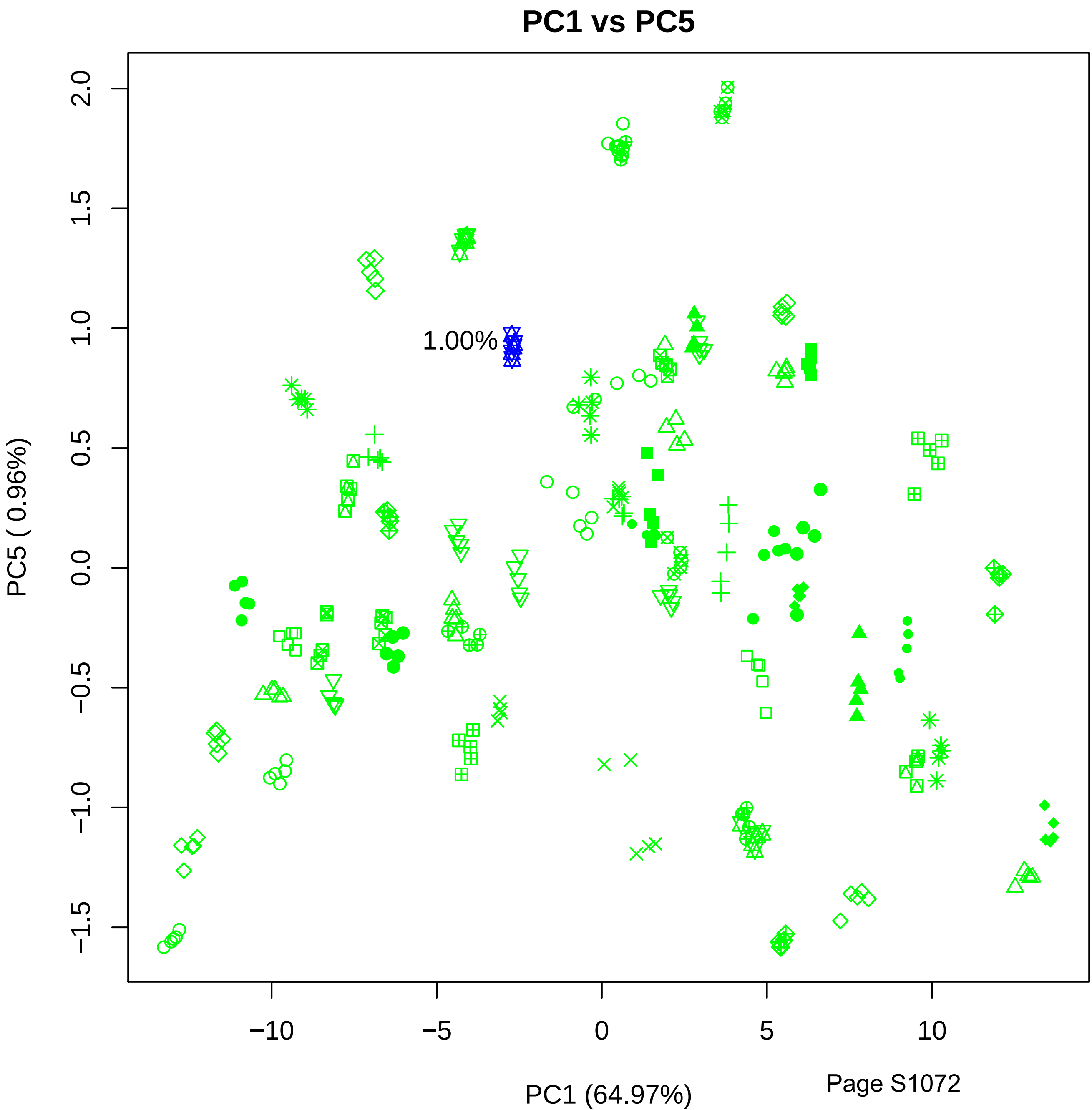

PC1 vs PC5
1.00%
PC5 (0.96%)
PC1 (64.97%)
2.0
1.5
1.0
0.5
0.0
−0.5
−1.0
−1.5
−10
−5
0
5
10

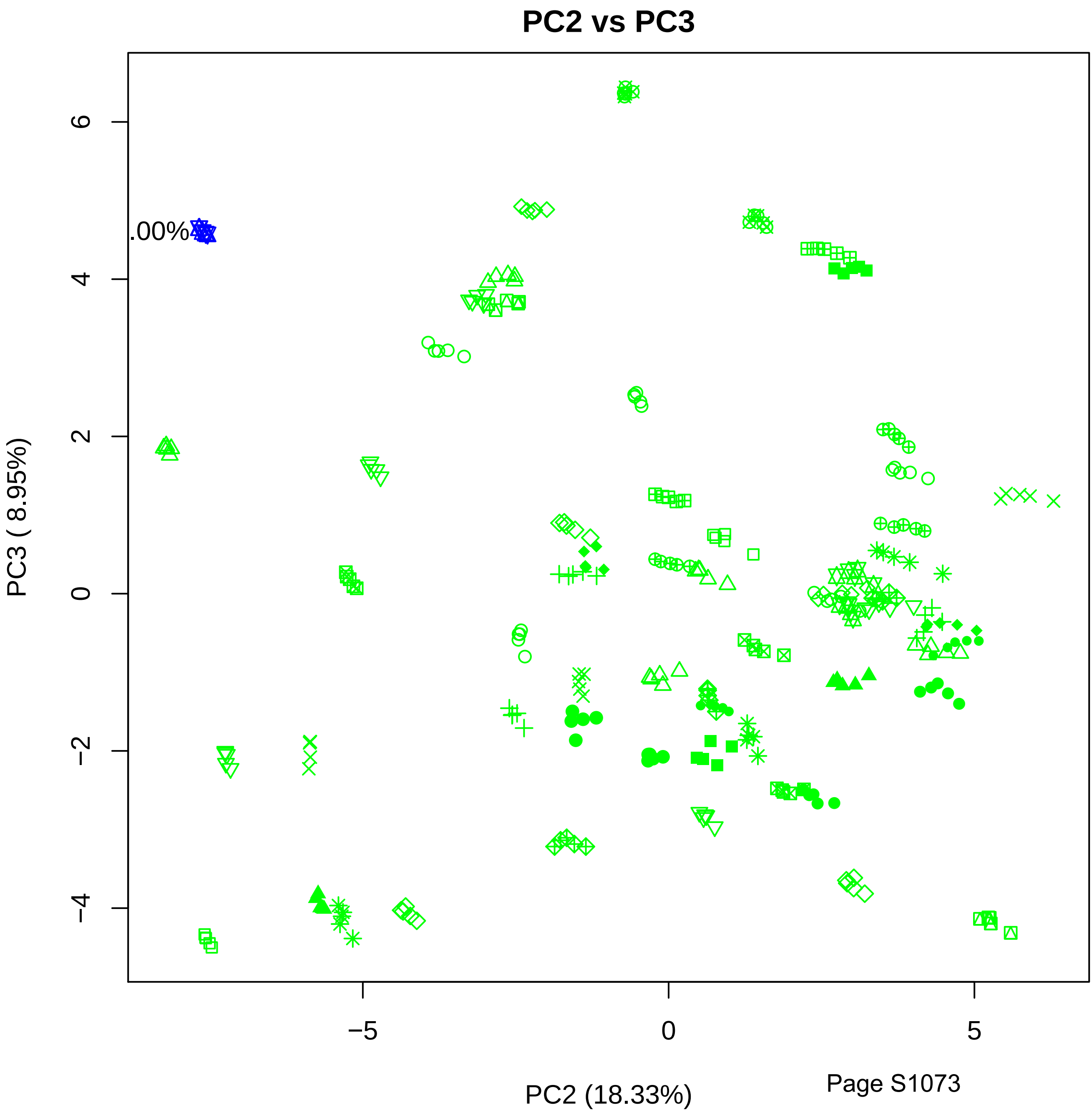

PC2 vs PC3
.00%
PC3 (8.95%)
PC2 (18.33%)
6
4
2
0
−2
−4
−5
0
5

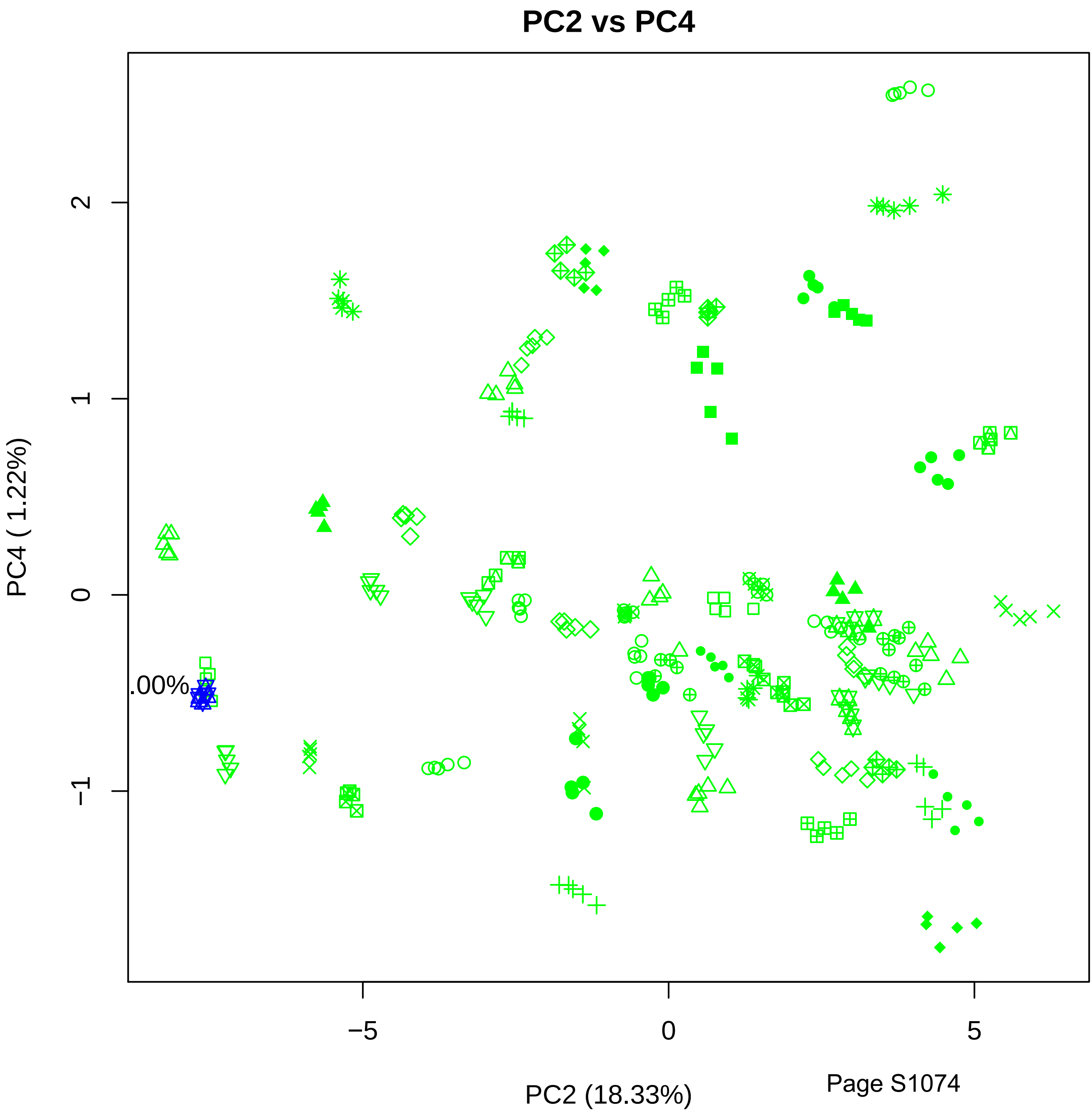

PC2 vs PC4
PC4 (1.22%)
PC2 (18.33%)
.00%
2
1
0
−1
−5
0
5

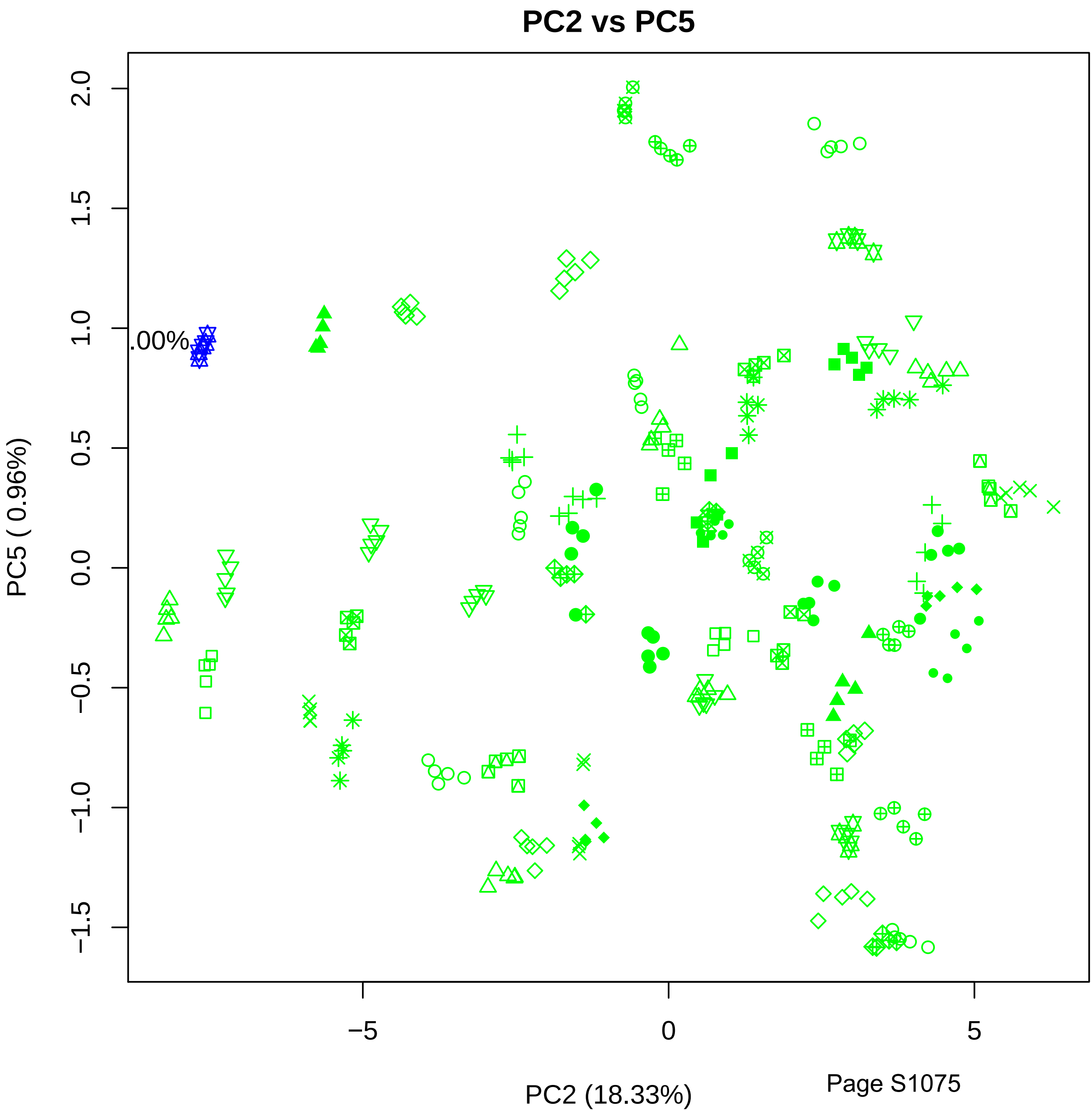
PC2 vs PC5
2.0
1.5
1.0
0.5
0.0
−0.5
−1.0
−1.5
.00%
PC5 (0.96%)
−5
0
5
PC2 (18.33%)

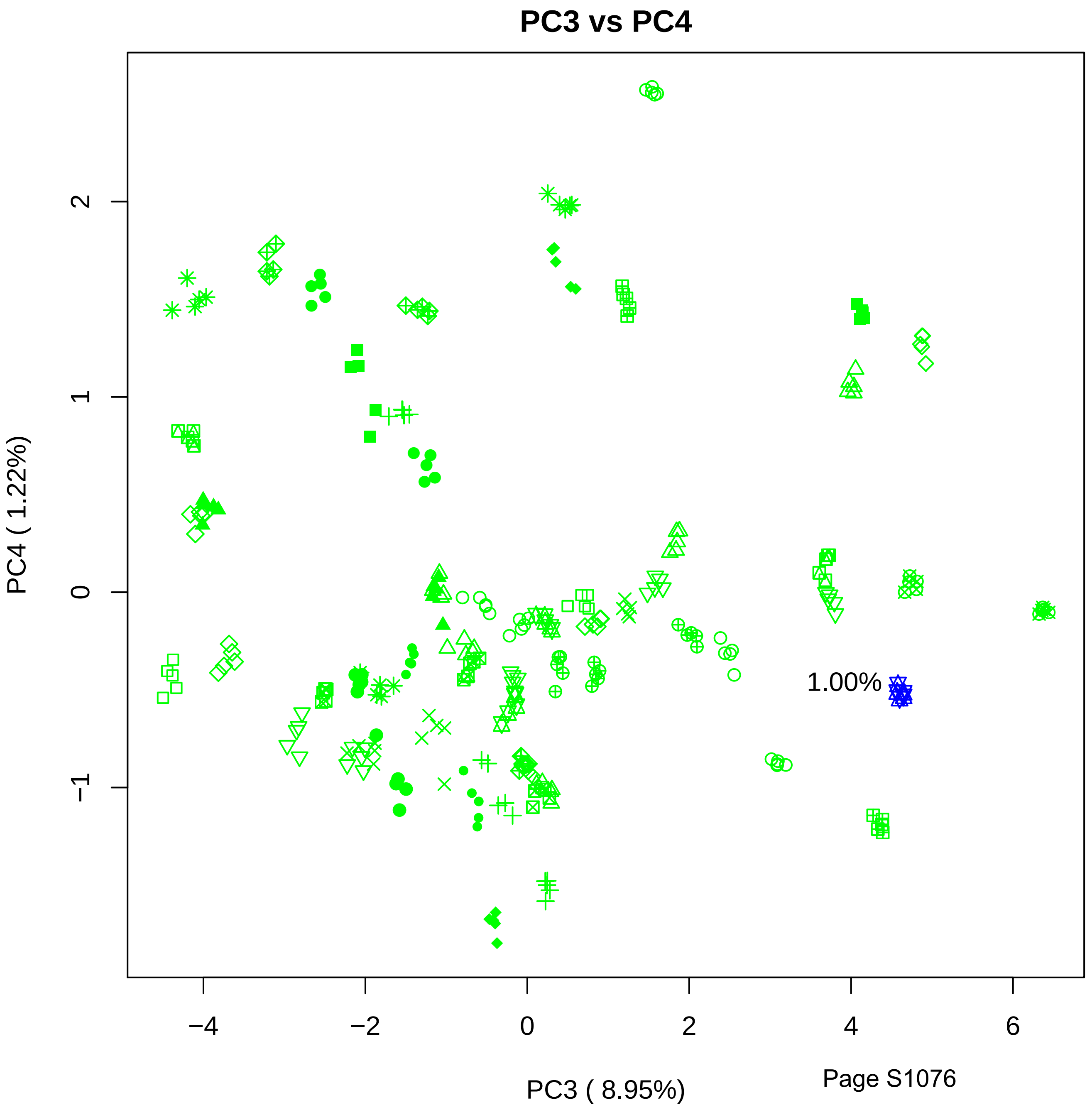
PC3 vs PC4
PC4 (1.22%)
PC3 (8.95%)
1.00%
2
1
0
−1
−4
−2
0
2
4
6

PC3 vs PC5

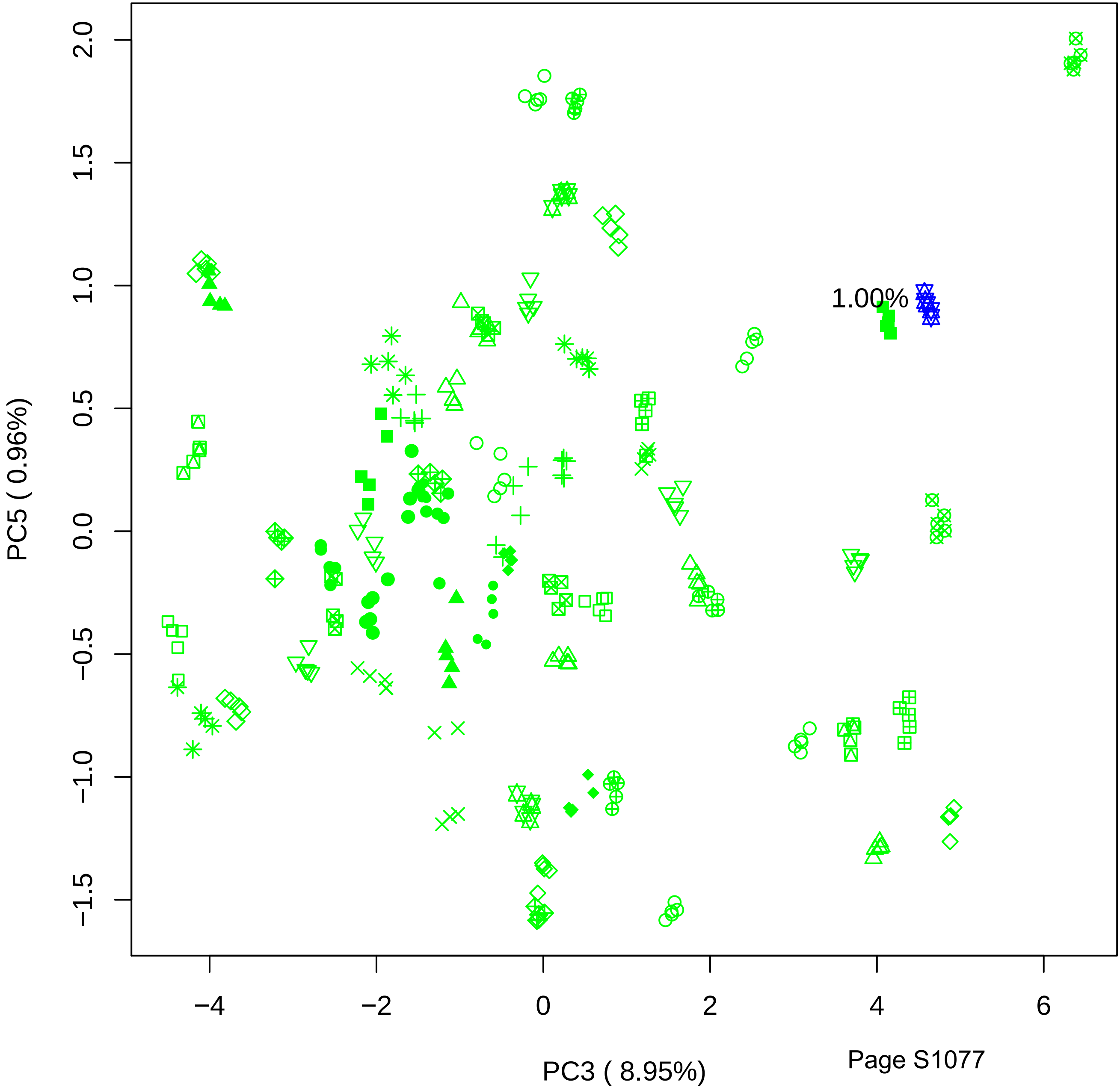

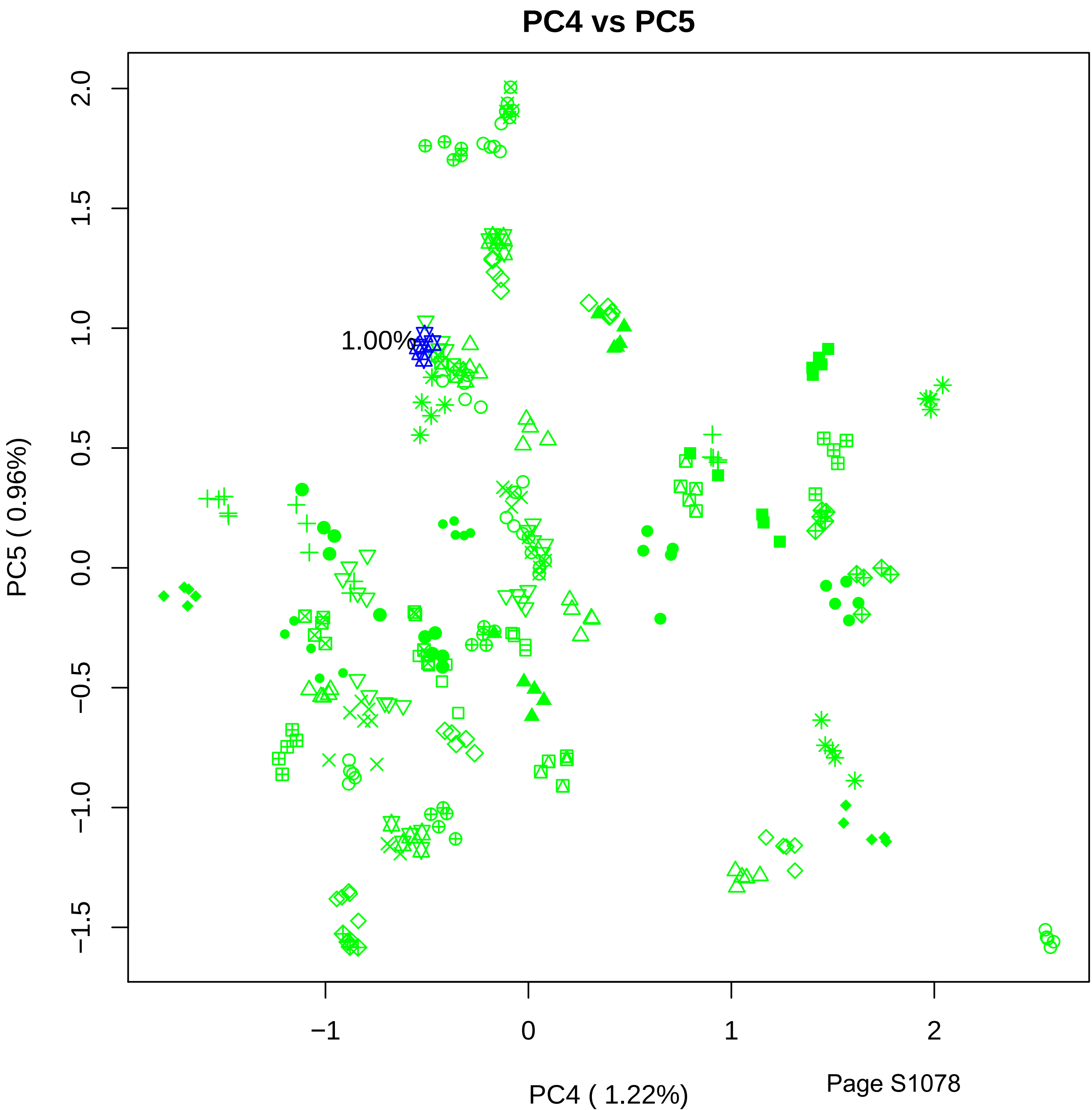
PC4 vs PC5
PC5 (0.96%)
PC4 (1.22%)
1.00%
2.0
1.5
1.0
0.5
0.0
−0.5
−1.0
−1.5
−1
0
1
2

PCA of 2.5% BMH (b) vs PMH (b)

Scaled Matrix PCAed

7th order polynomial baseline correction performed

Raw spectra smoothed to 21 Neighbours, 2nd order PN, Savtisky Golay

Normalisation between 0 and 100

D2 spectra smoothed to 41 Neighbours, 2nd order PN, Savtisky Golay

Regions Analysed: 3601−2501 and 2001−700cm−1

Samples Analysed: 1 bContam's 0 BLH's 0 BMH's 0 OMH's 0 pContam's 0 PLH's 60 PMH's

Keele University AD

Fri Dec 14 14:21:17 2018

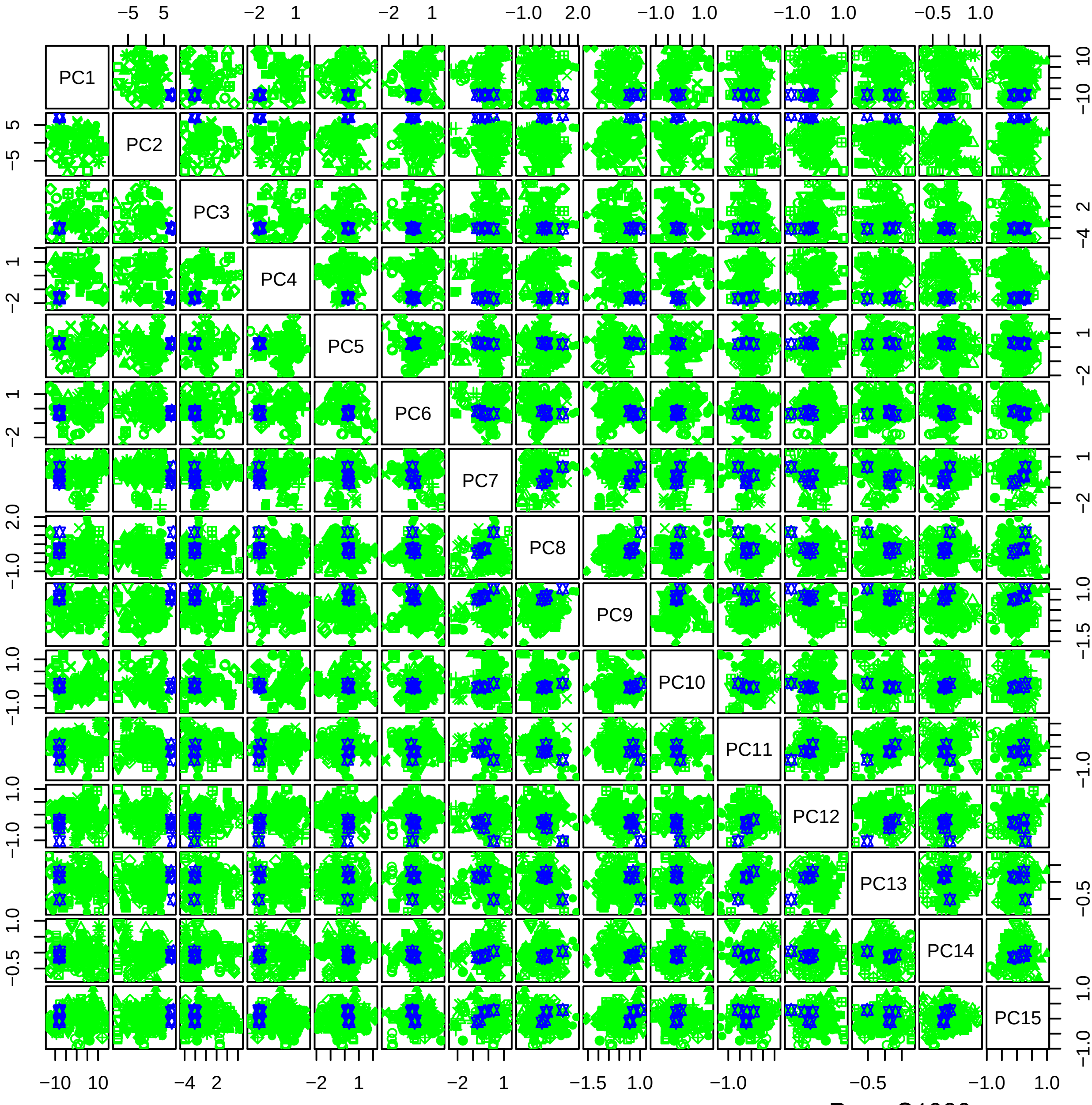
PC1
PC2
PC3
PC4
PC5
PC6
PC7
PC8
PC9
PC10
PC11
PC12
PC13
PC14
PC15

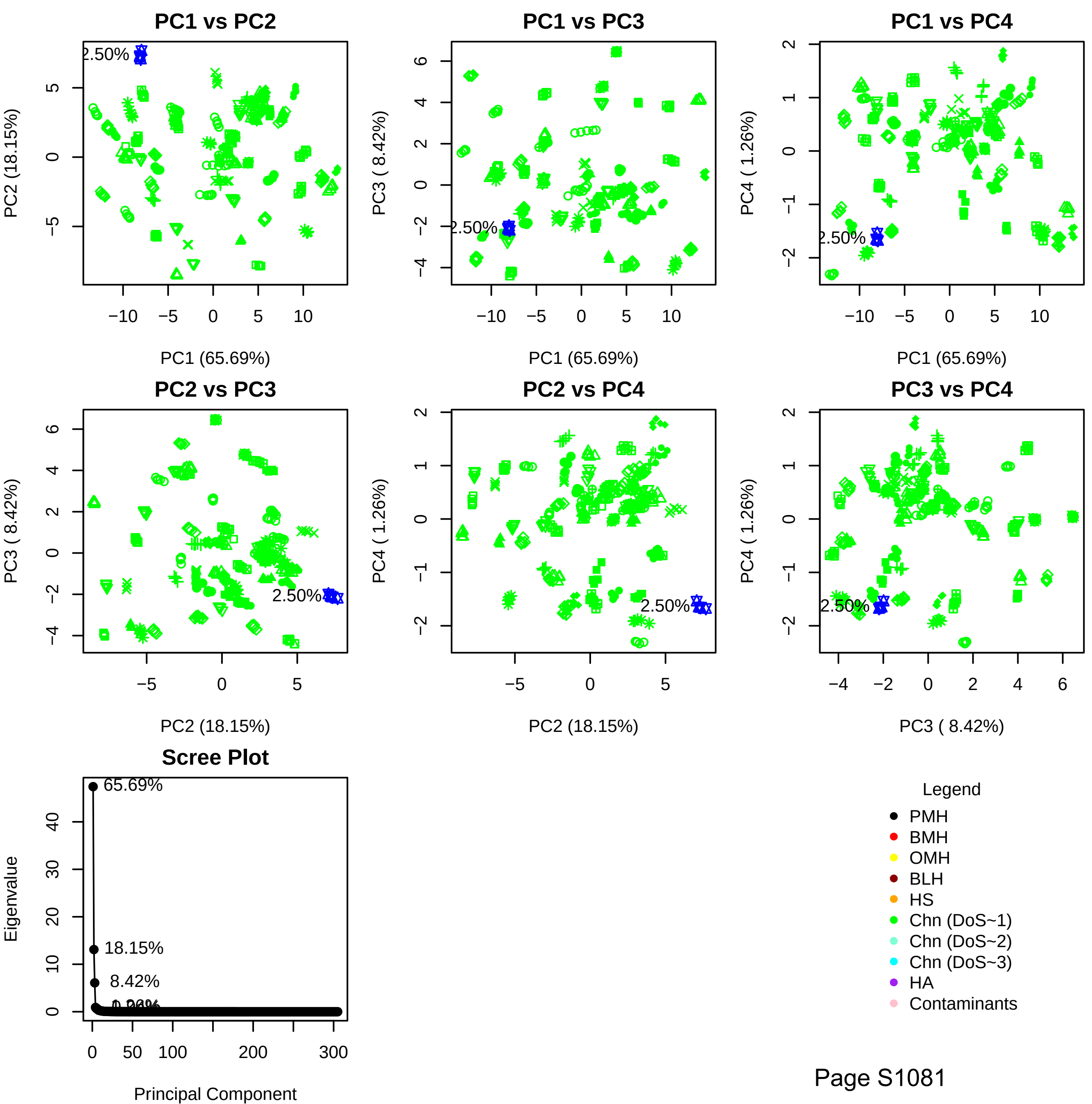
PC1 vs PC2
PC1 vs PC3
PC1 vs PC4
PC2 vs PC3
PC2 vs PC4
PC3 vs PC4
Scree Plot
PC1 (65.69%)
PC2 (18.15%)
PC3 (8.42%)
PC4 (1.26%)
2.50%
65.69%
18.15%
8.42%
Eigenvalue
Principal Component
Legend
PMH
BMH
OMH
BLH
HS
Chn (DoS~1)
Chn (DoS~2)
Chn (DoS~3)
HA
Contaminants

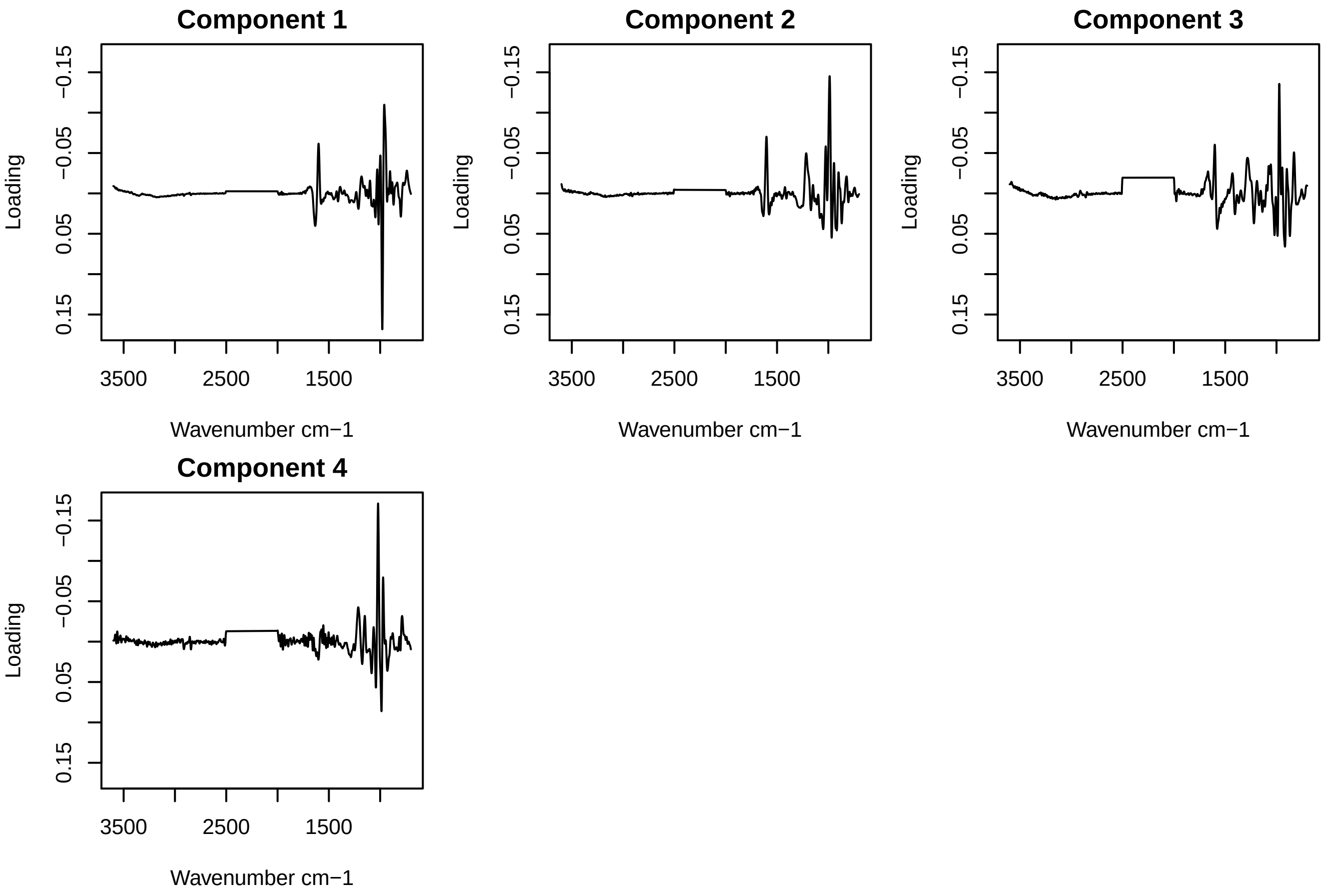
Component 1
Component 2
Component 3
Component 4
Loading
−0.15
−0.05
0.05
0.15
3500
2500
1500
Wavenumber cm−1

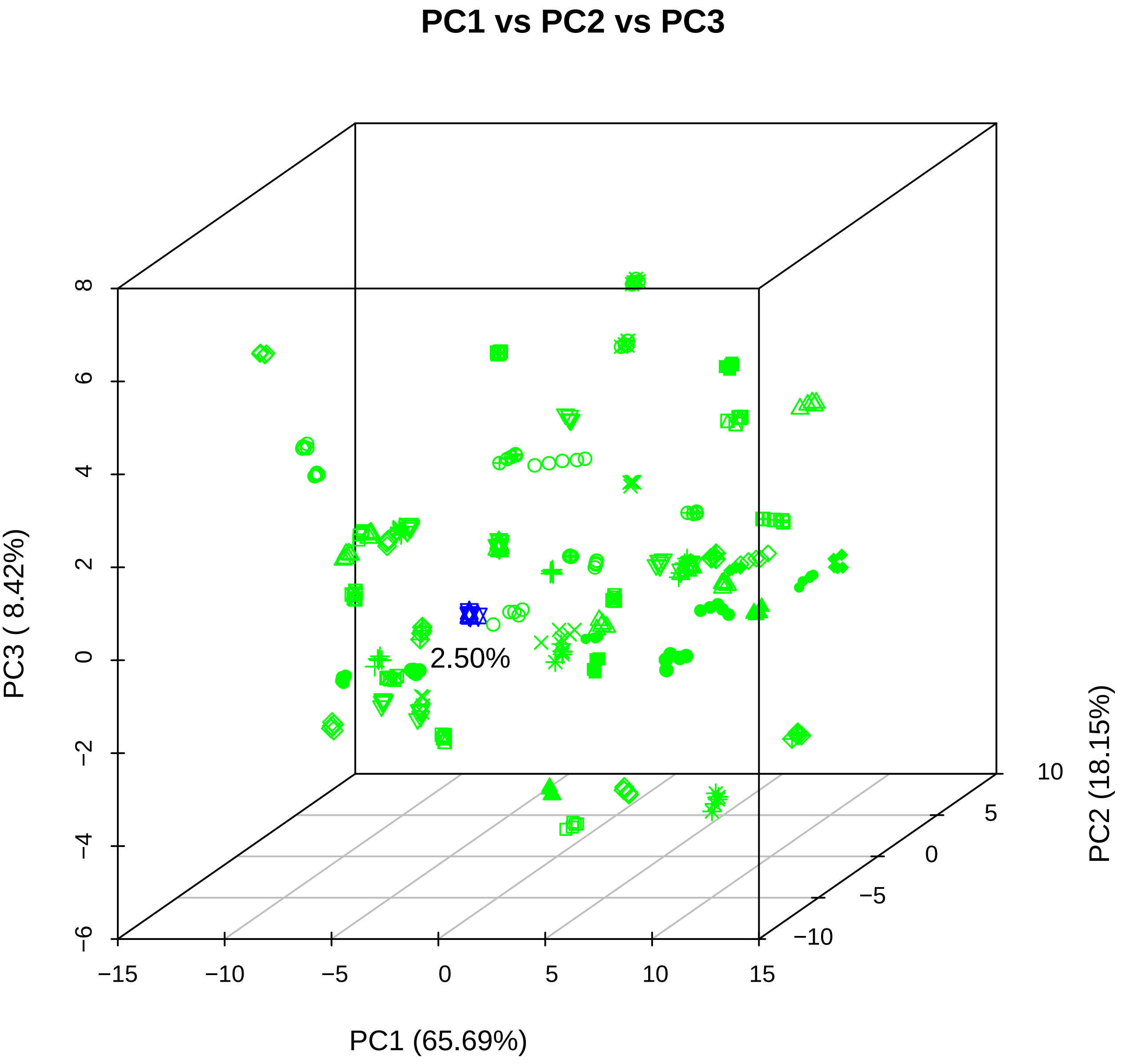
PC1 vs PC2 vs PC3
PC1 (65.69%)
PC2 (18.15%)
PC3 (8.42%)
2.50%
−15
−10
−5
0
5
10
15
−6
−4
−2
0
2
4
6
8
−10
−5
0
5
10

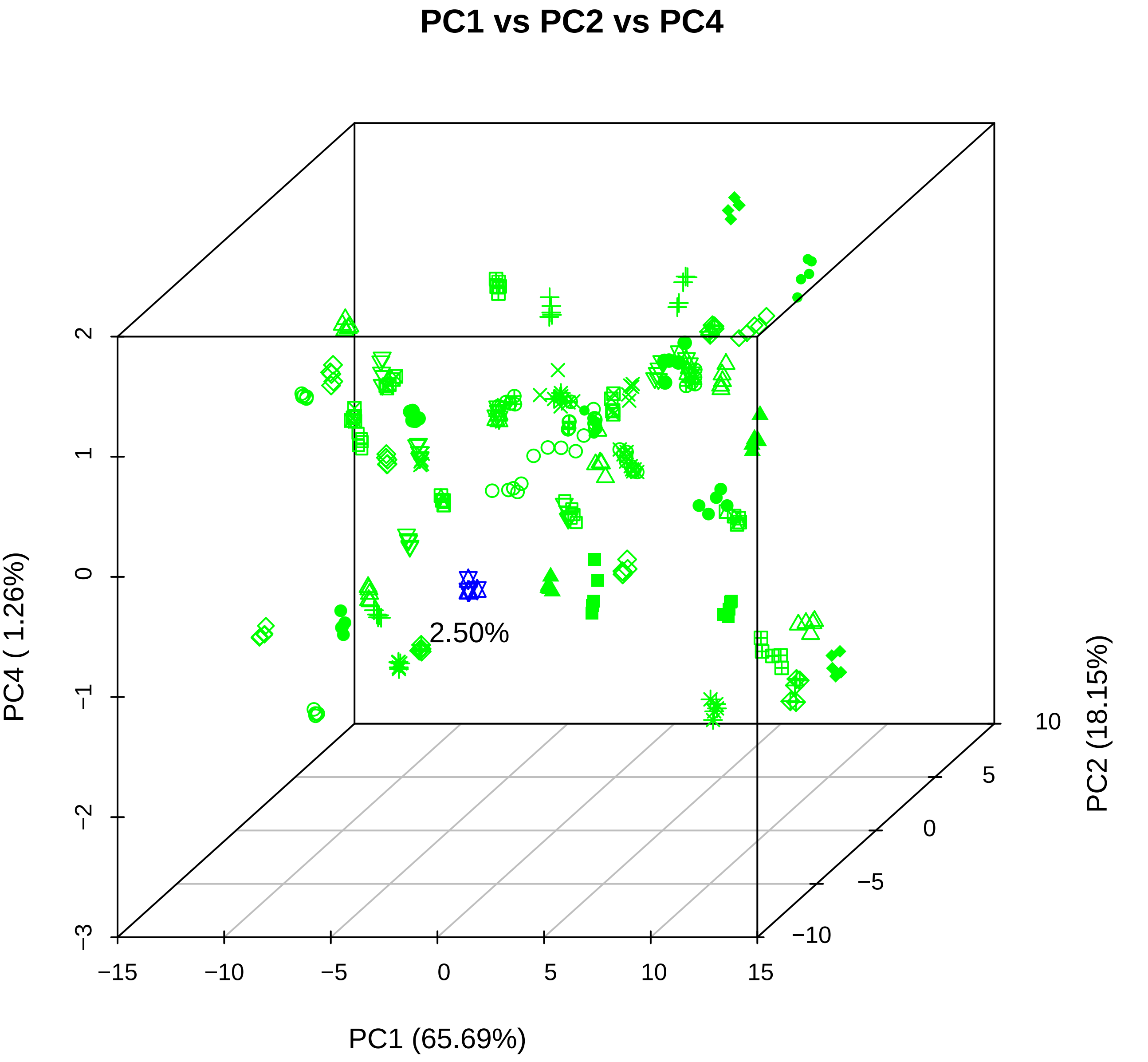
PC1 vs PC2 vs PC4
PC4 (1.26%)
2
1
0
−1
−2
−3
2.50%
−15
−10
−5
0
5
10
15
PC1 (65.69%)
10
5
0
−5
−10
PC2 (18.15%)

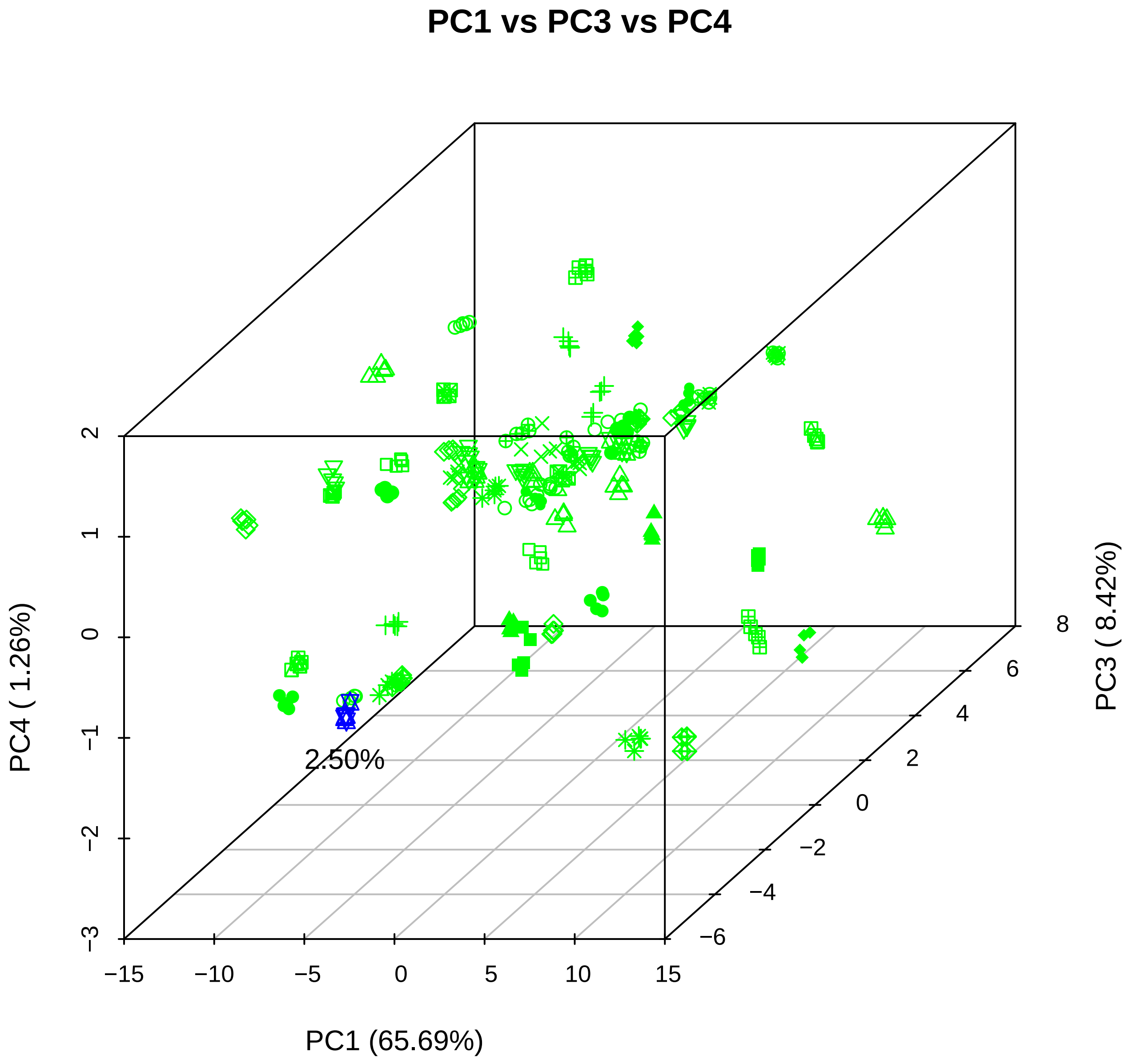

PC1 vs PC3 vs PC4
PC4 (1.26%)
2
1
0
−1
−2
−3
−15
−10
−5
0
5
10
15
PC1 (65.69%)
PC3 (8.42%)
8
6
4
2
0
−2
−4
−6
2.50%

PC2 vs PC3 vs PC5

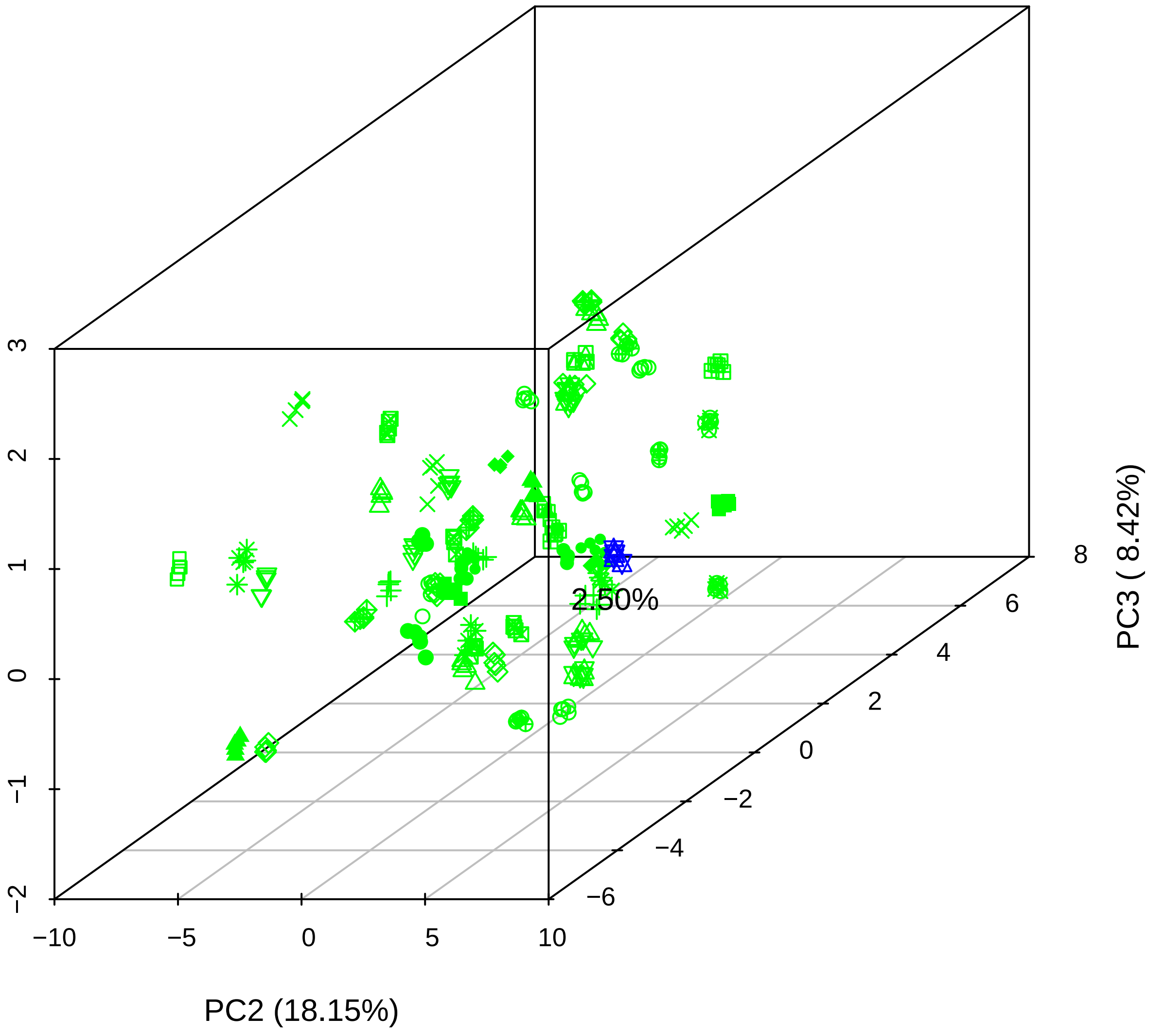

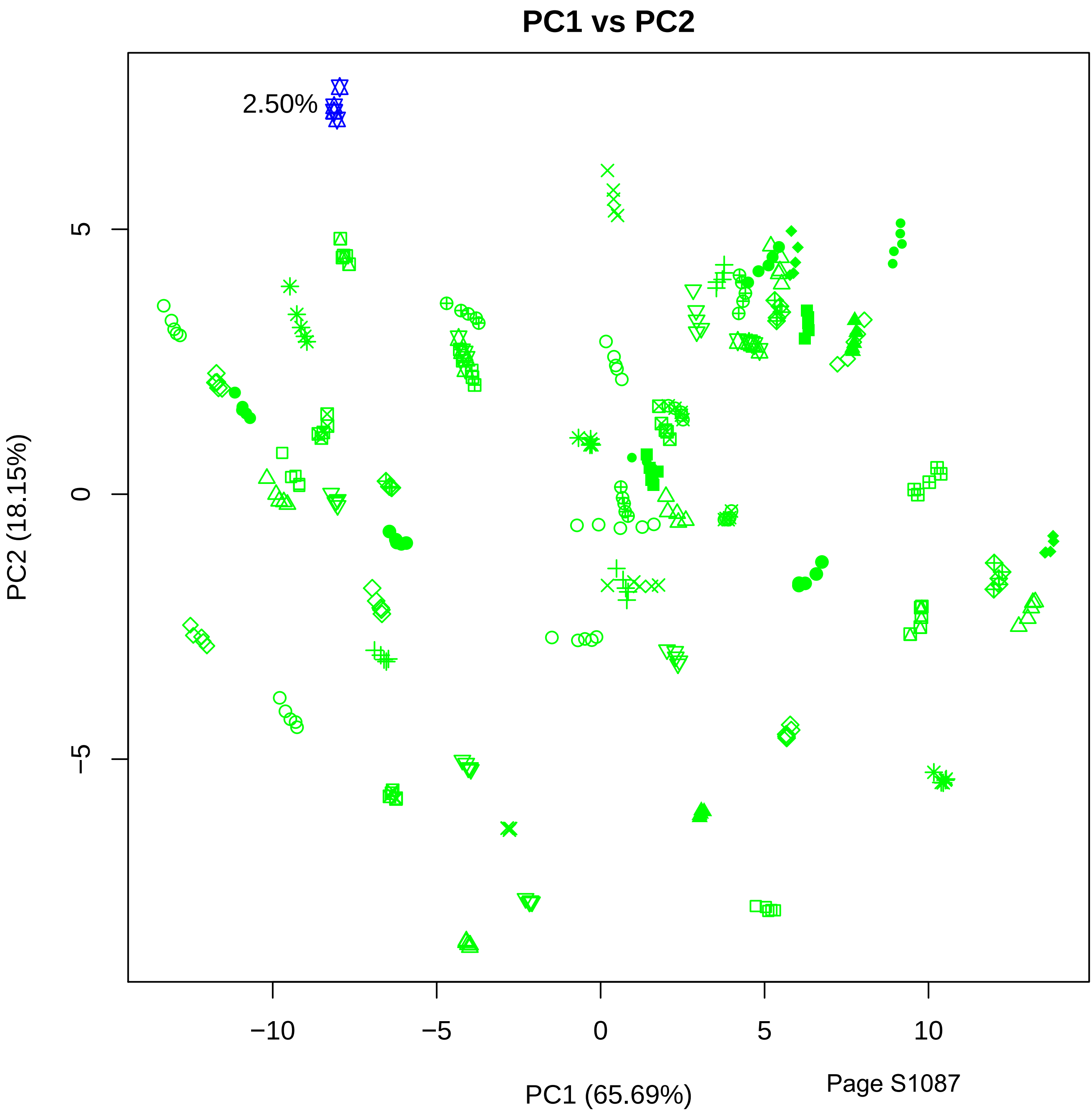

PC1 vs PC2
2.50%
5
0
−5
PC2 (18.15%)
−10
−5
0
5
10
PC1 (65.69%)

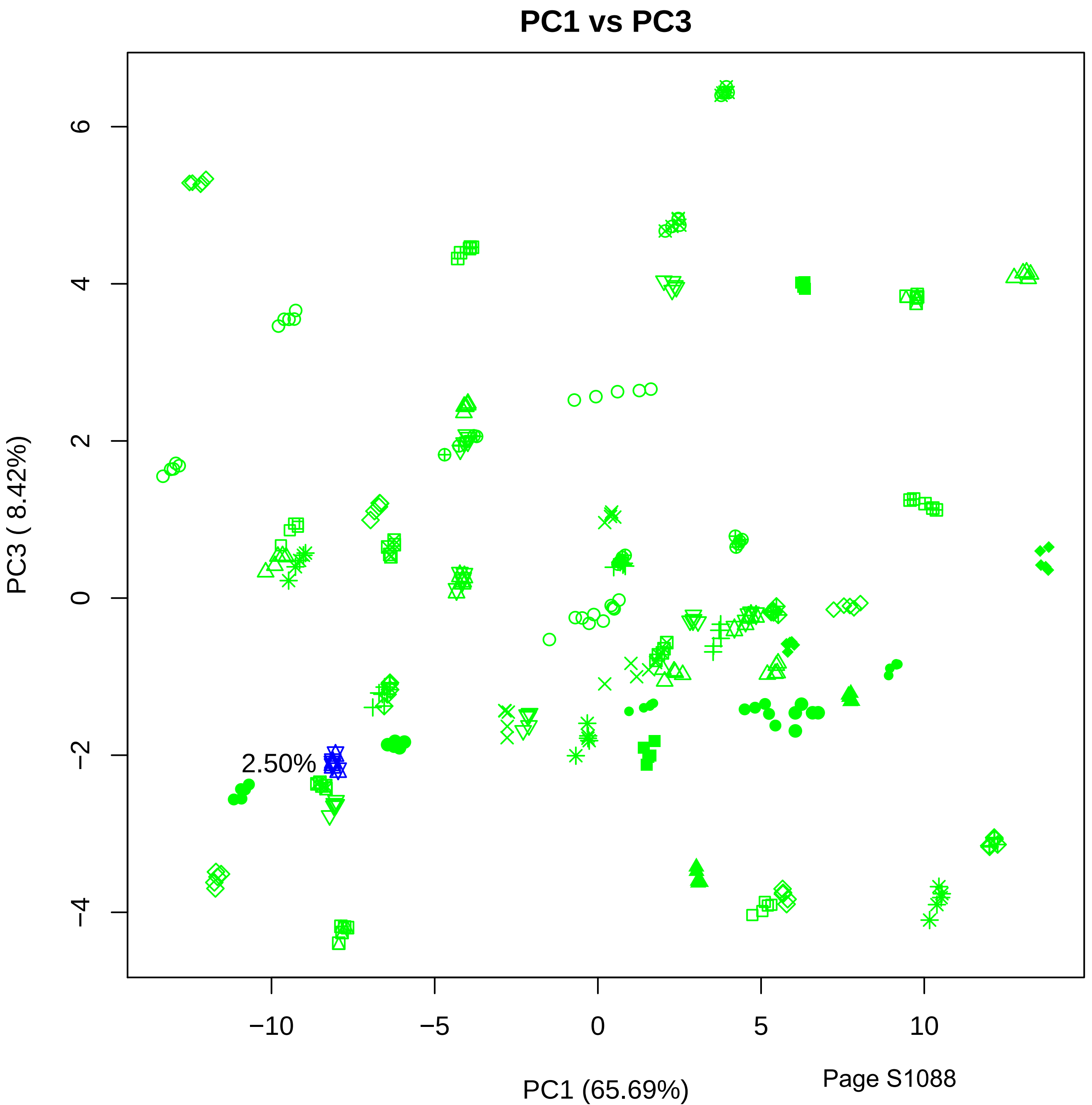

PC1 vs PC3
PC3 (8.42%)
PC1 (65.69%)
2.50%
6
4
2
0
−2
−4
−10
−5
0
5
10

PC1 vs PC4

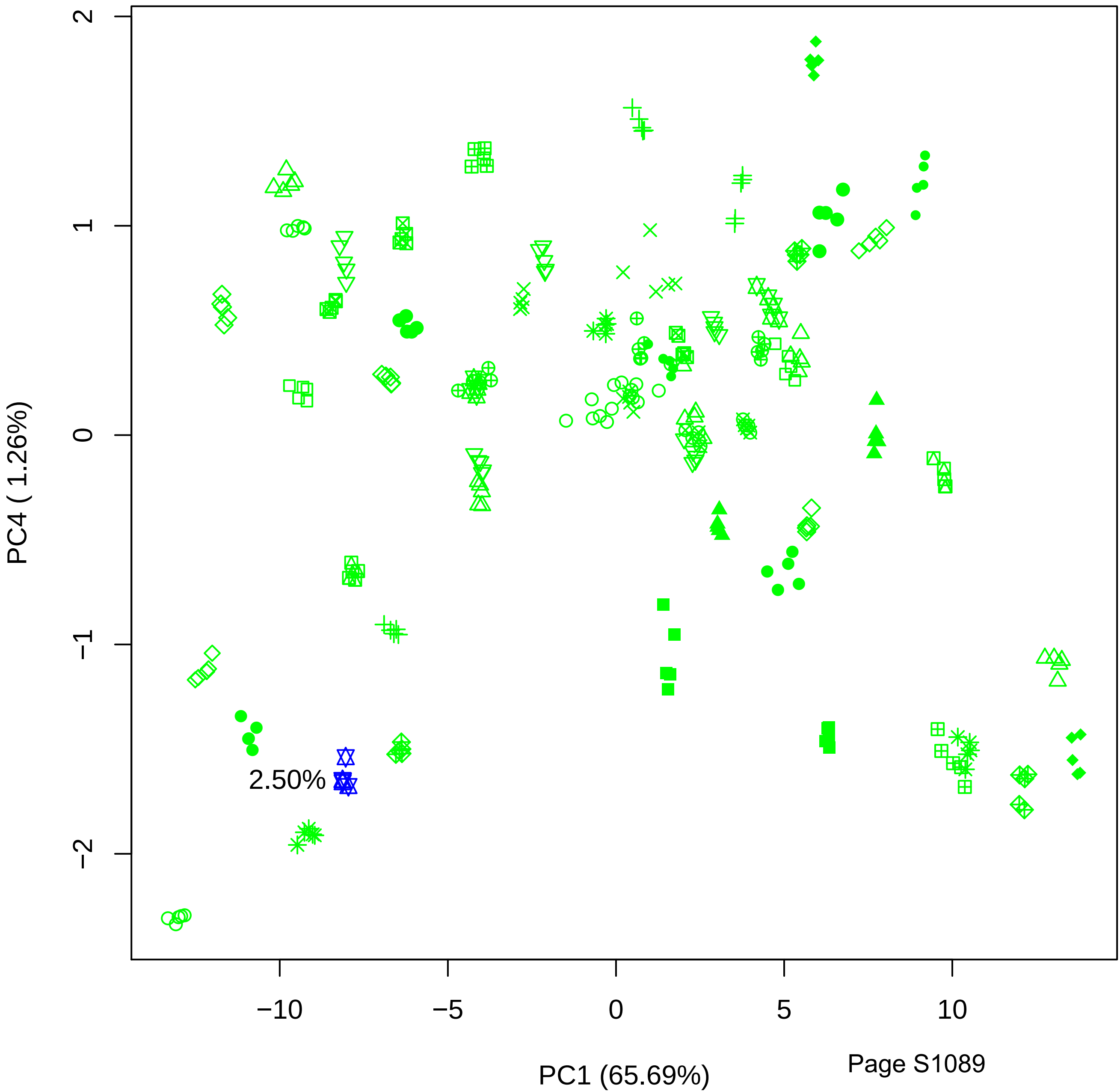

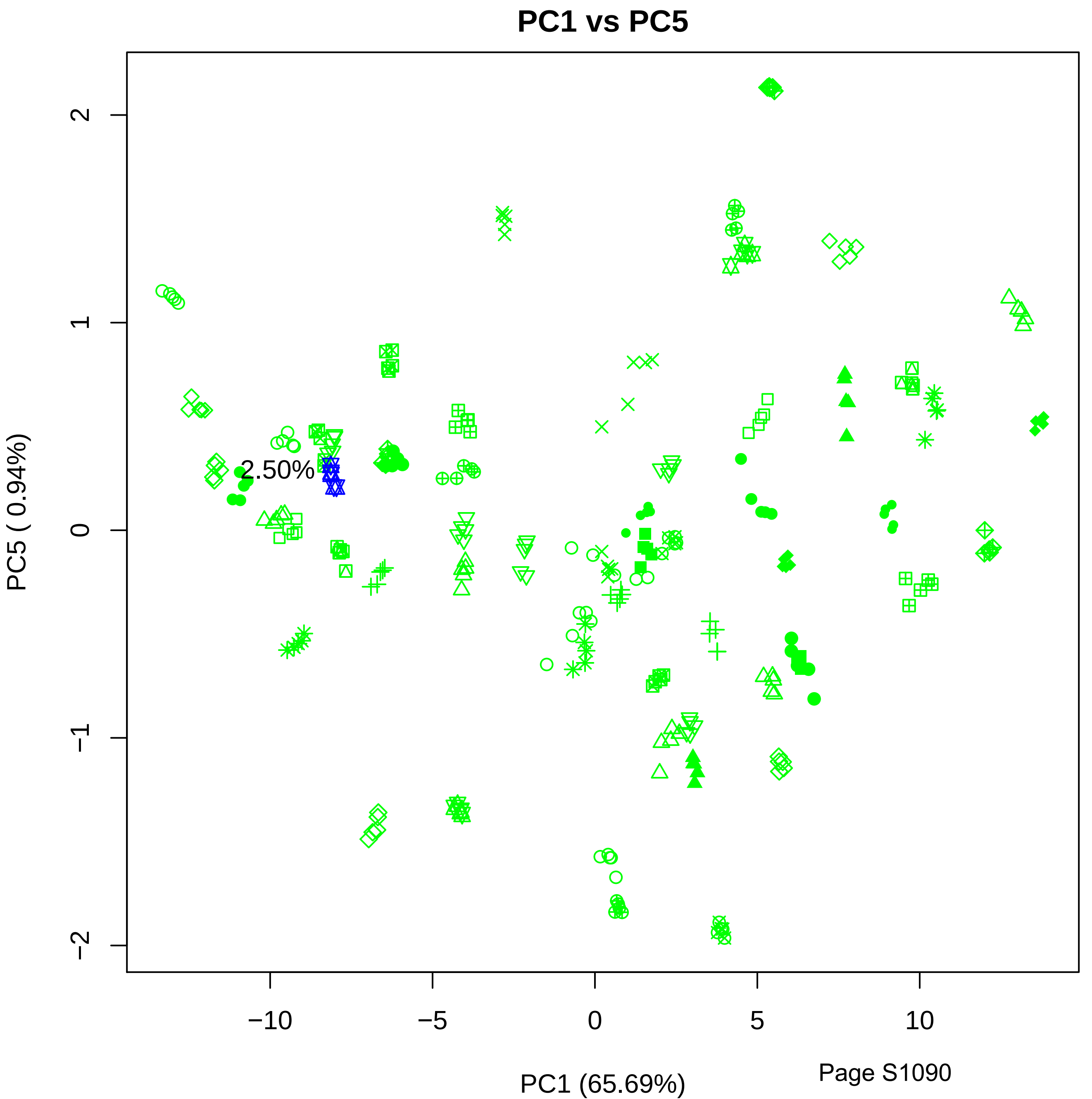
PC1 vs PC5
PC5 (0.94%)
PC1 (65.69%)
2.50%
2
1
0
−1
−2
−10
−5
0
5
10

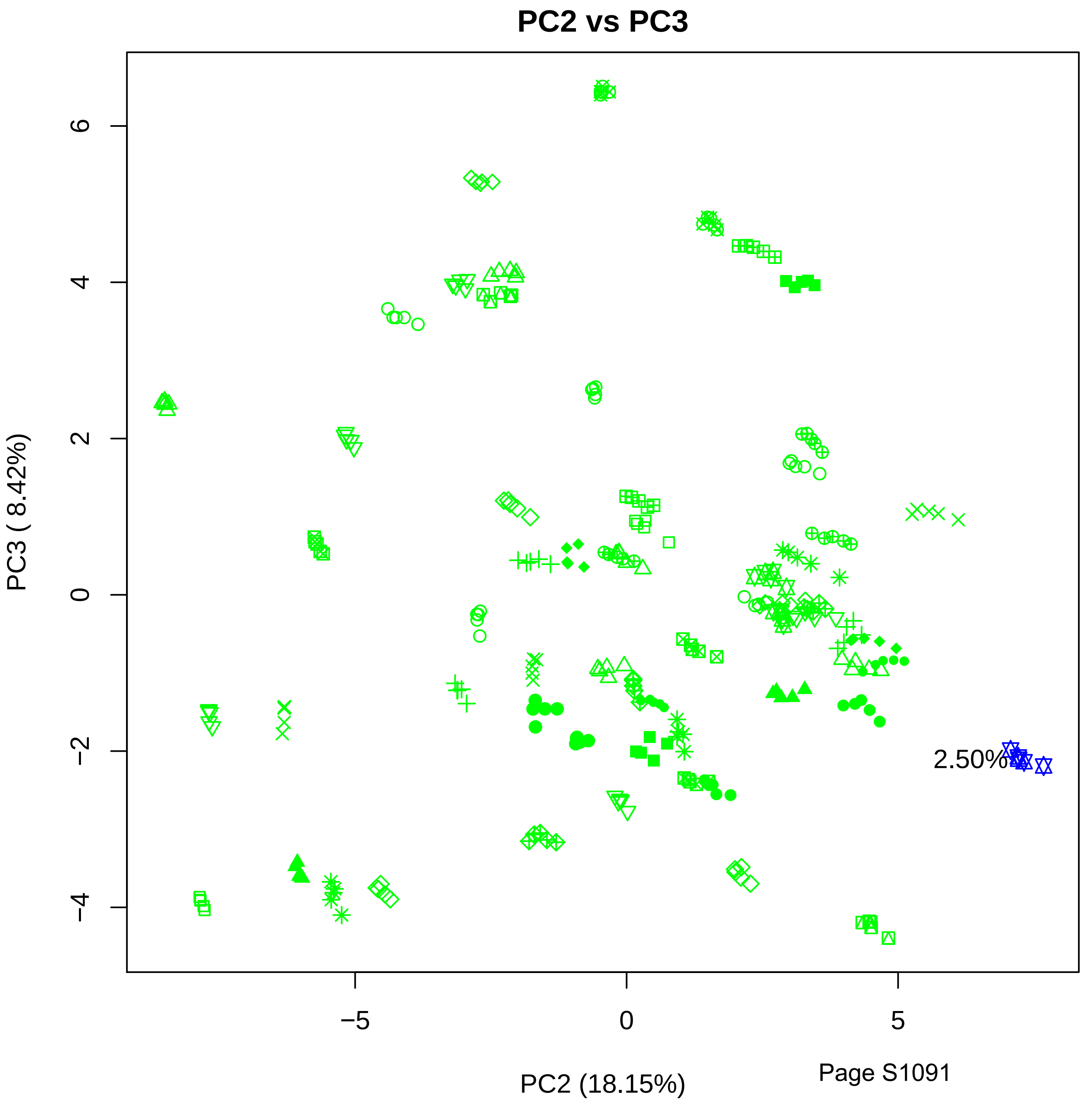
PC2 vs PC3
PC3 (8.42%)
PC2 (18.15%)
6
4
2
0
−2
−4
−5
0
5
2.50%

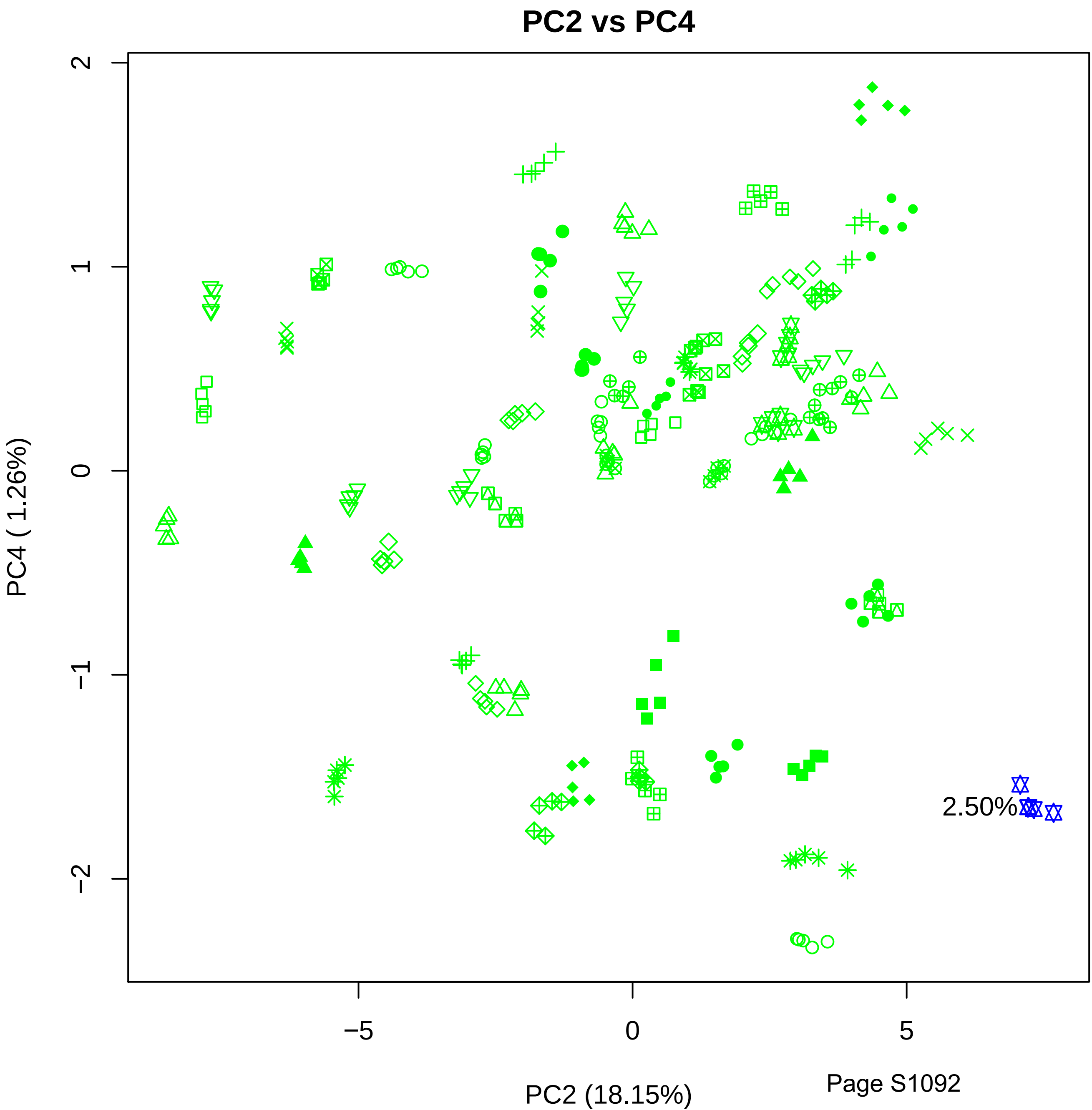
PC2 vs PC4
PC4 (1.26%)
PC2 (18.15%)
2.50%
2
1
0
−1
−2
−5
0
5

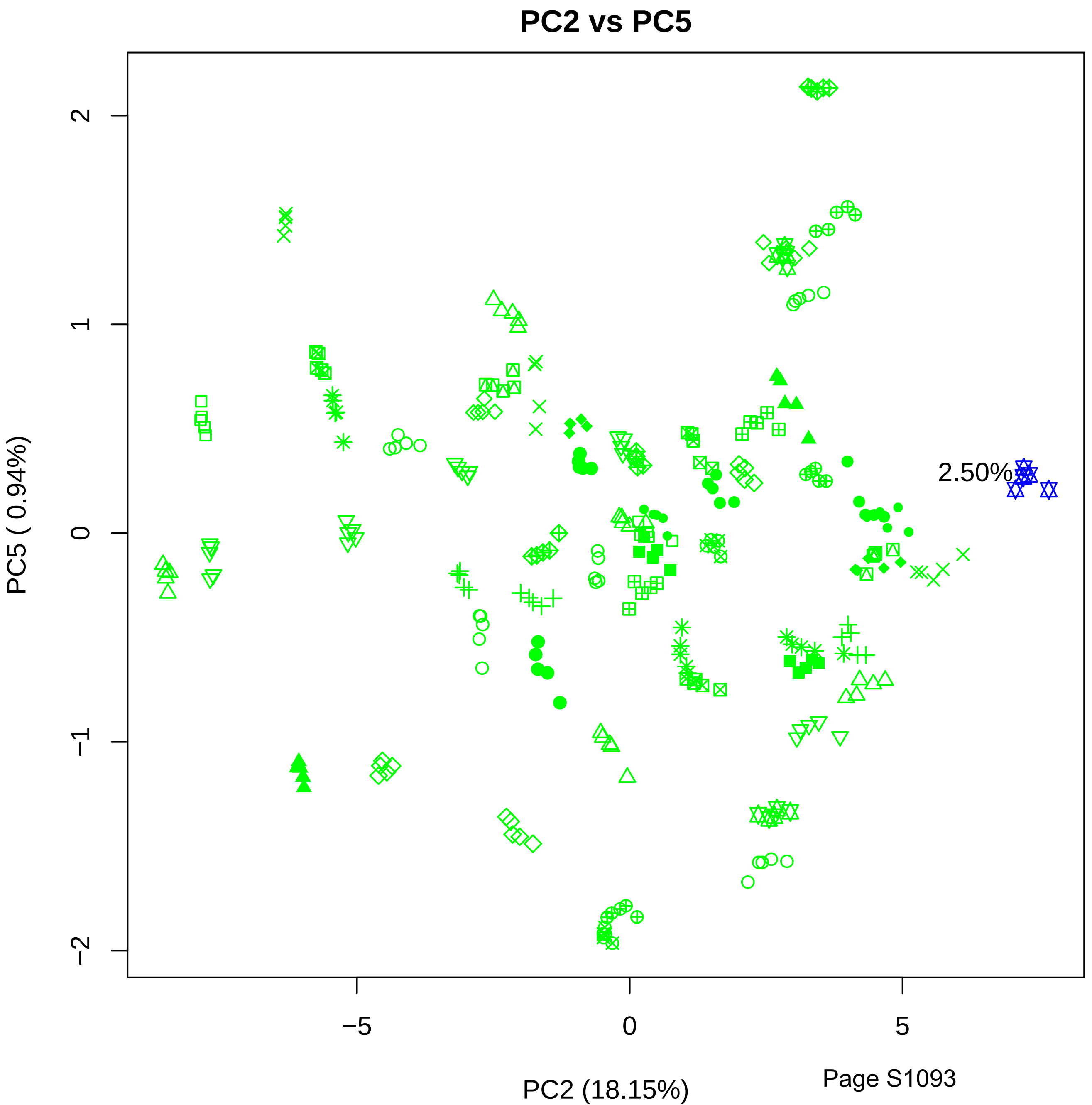

PC2 vs PC5
2.50%
PC5 (0.94%)
PC2 (18.15%)
2
1
0
−1
−2
−5
0
5

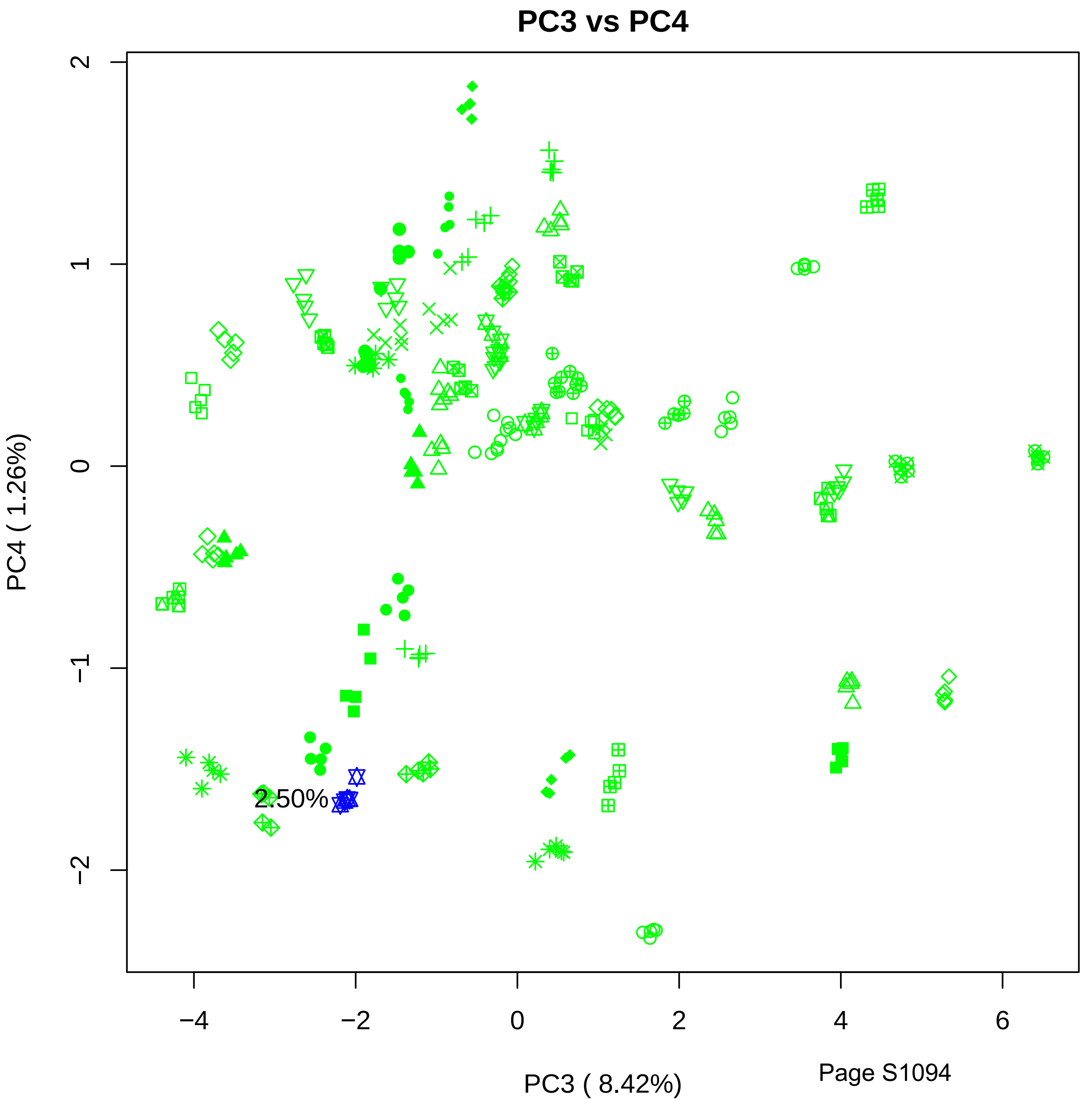
PC3 vs PC4
PC4 (1.26%)
PC3 (8.42%)
2.50%
2
1
0
−1
−2
−4
−2
0
2
4
6

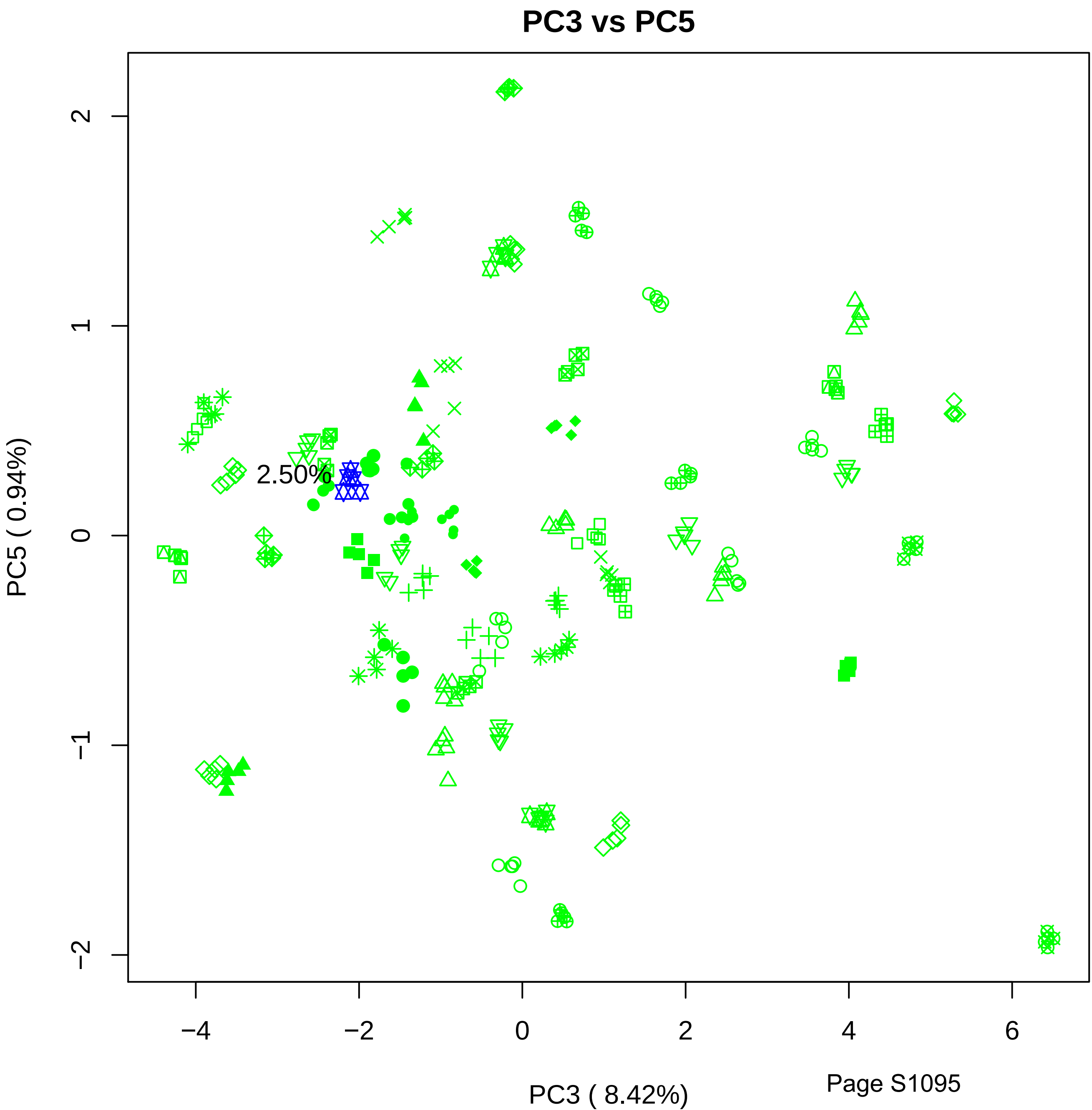
PC3 vs PC5
2.50%
PC5 (0.94%)
PC3 (8.42%)
2
1
0
−1
−2
−4
−2
0
2
4
6

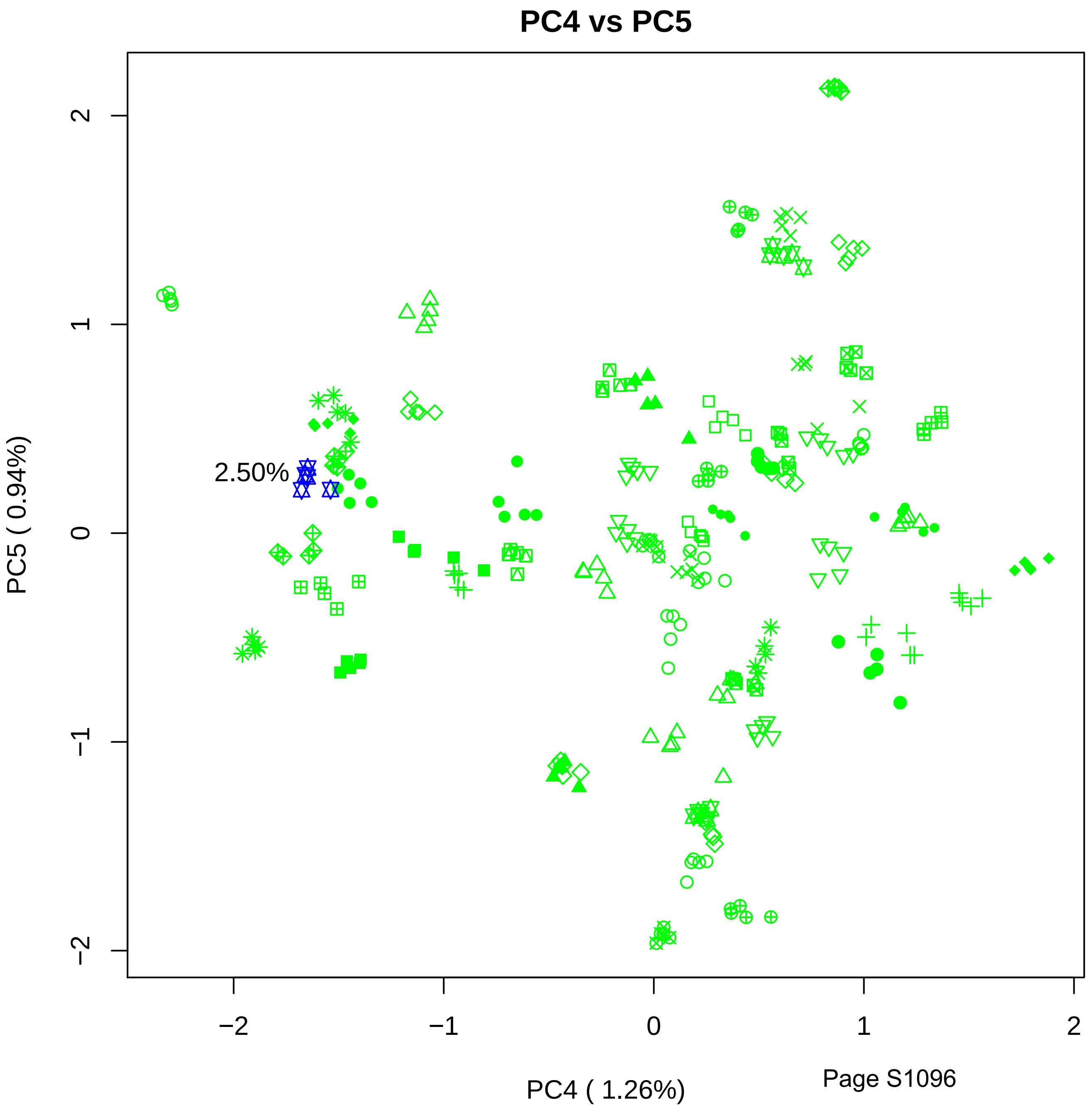

PC4 vs PC5
2.50%
PC5 (0.94%)
PC4 (1.26%)
2
1
0
−1
−2
−2
−1
0
1
2

PCA of 5% BMH(b) vs PMH(b)

Scaled Matrix PCAed

7th order polynomial baseline correction performed

Raw spectra smoothed to 21 Neighbours, 2nd order PN, Savtisky Golay

Normalisation between 0 and 100

D2 spectra smoothed to 41 Neighbours, 2nd order PN, Savtisky Golay

Regions Analysed: 3601−2501 and 2001−700cm−1

Samples Analysed: 1 bContam's 0 BLH's 0 BMH's 0 OMH's 0 pContam's 0 PLH's 60 PMH's

Keele University AD   Fri Dec 14 14:21:25 2018

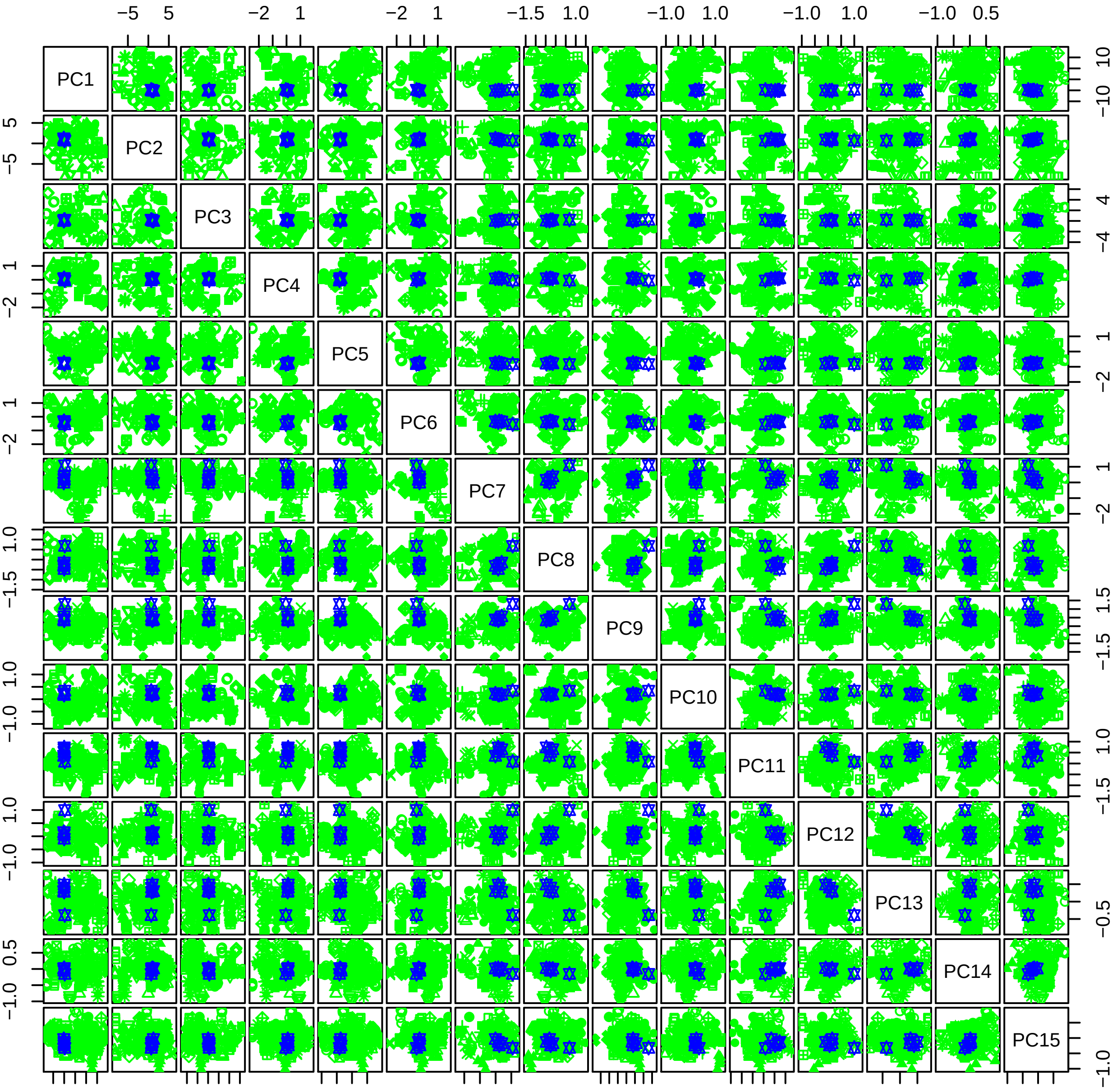
PC1
PC2
PC3
PC4
PC5
PC6
PC7
PC8
PC9
PC10
PC11
PC12
PC13
PC14
PC15
−5 5
−2 1
−2 1
−1.5 1.0
−1.0 1.0
−1.0 1.0
−1.0 0.5
−10 10
−4 4
−2 1
−2 1
−1.5 1.5
−1.5 1.0
−0.5
−1.0

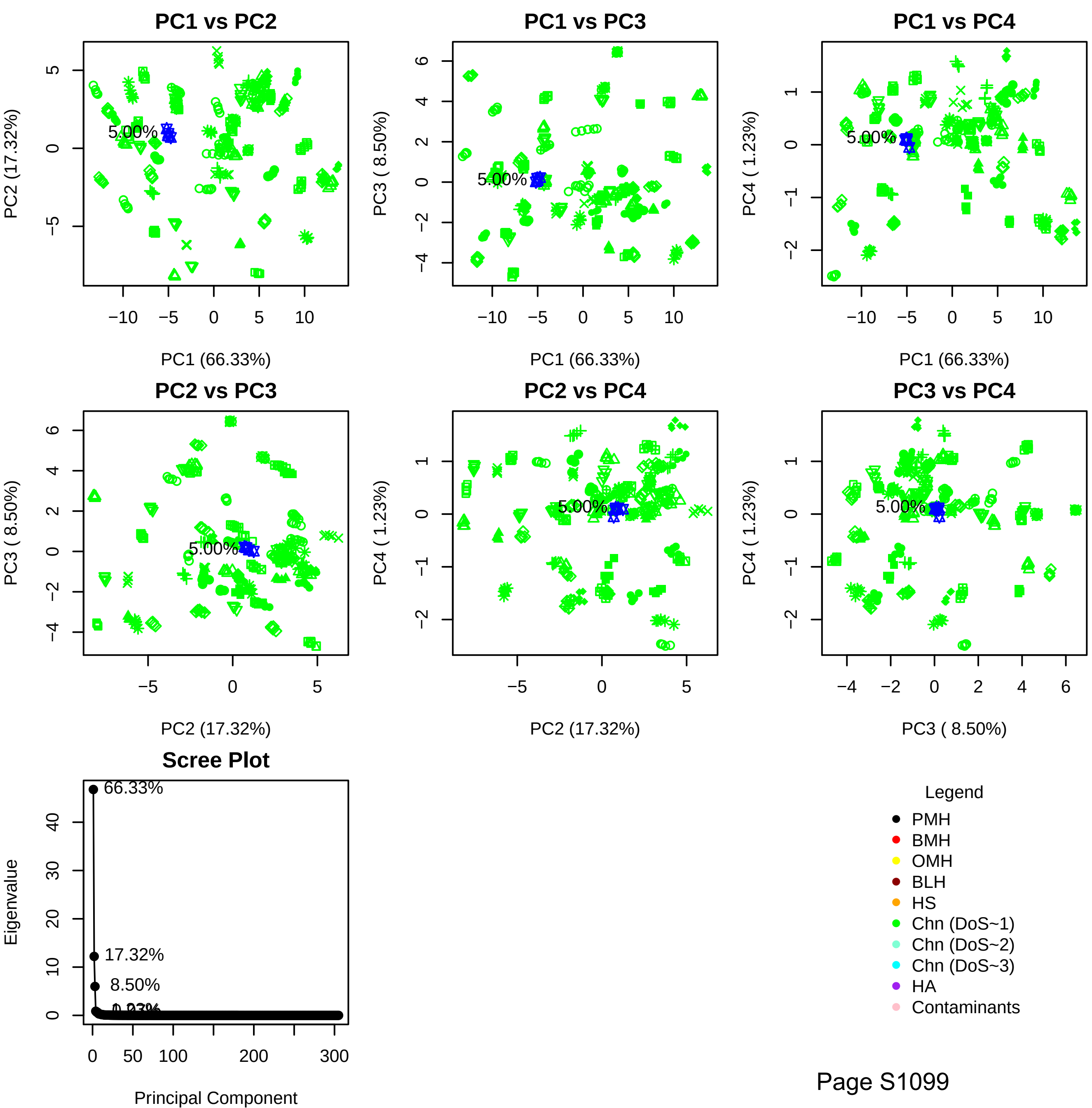
PC1 vs PC2
PC1 vs PC3
PC1 vs PC4
PC2 vs PC3
PC2 vs PC4
PC3 vs PC4
Scree Plot
PC1 (66.33%)
PC2 (17.32%)
PC3 (8.50%)
PC4 (1.23%)
5.00%
Eigenvalue
Principal Component
66.33%
17.32%
8.50%
Legend
PMH
BMH
OMH
BLH
HS
Chn (DoS~1)
Chn (DoS~2)
Chn (DoS~3)
HA
Contaminants

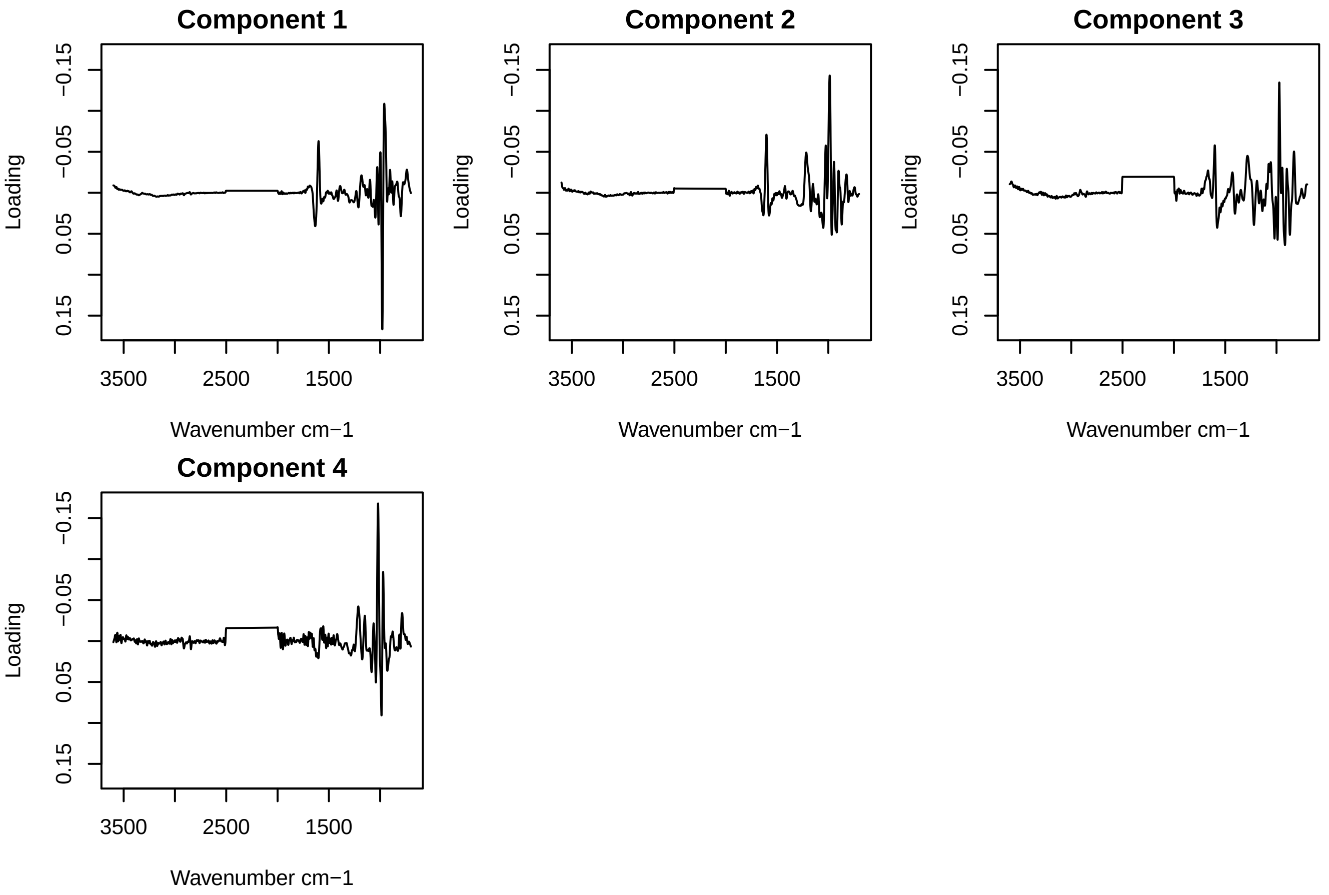
Component 1
Component 2
Component 3
Component 4
Loading
−0.15
−0.05
0.05
0.15
3500
2500
1500
Wavenumber cm−1

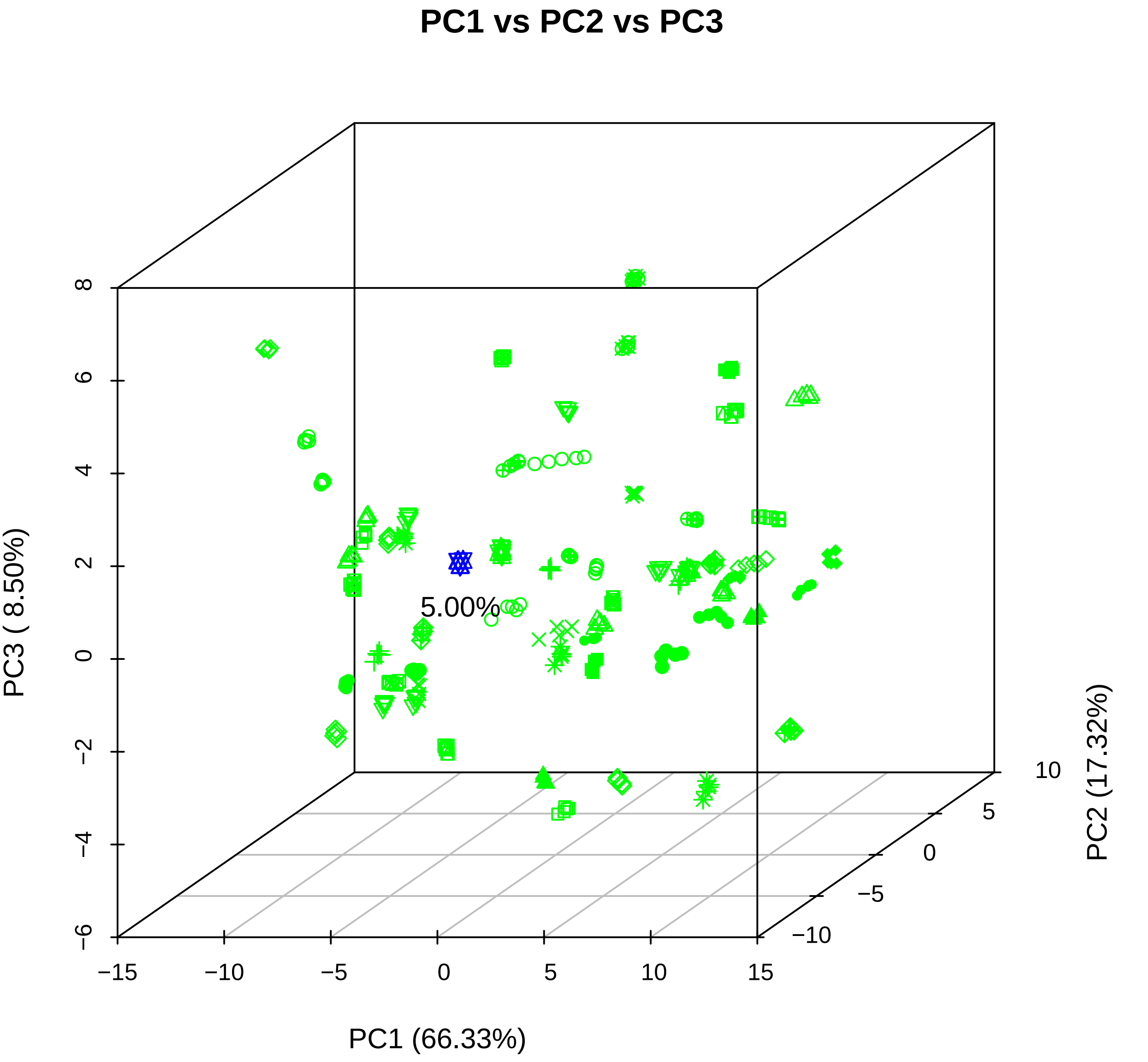
PC1 vs PC2 vs PC3
5.00%
PC3 (8.50%)
8
6
4
2
0
−2
−4
−6
−15
−10
−5
0
5
10
15
PC1 (66.33%)
PC2 (17.32%)
10
5
0
−5
−10

PC1 vs PC2 vs PC4

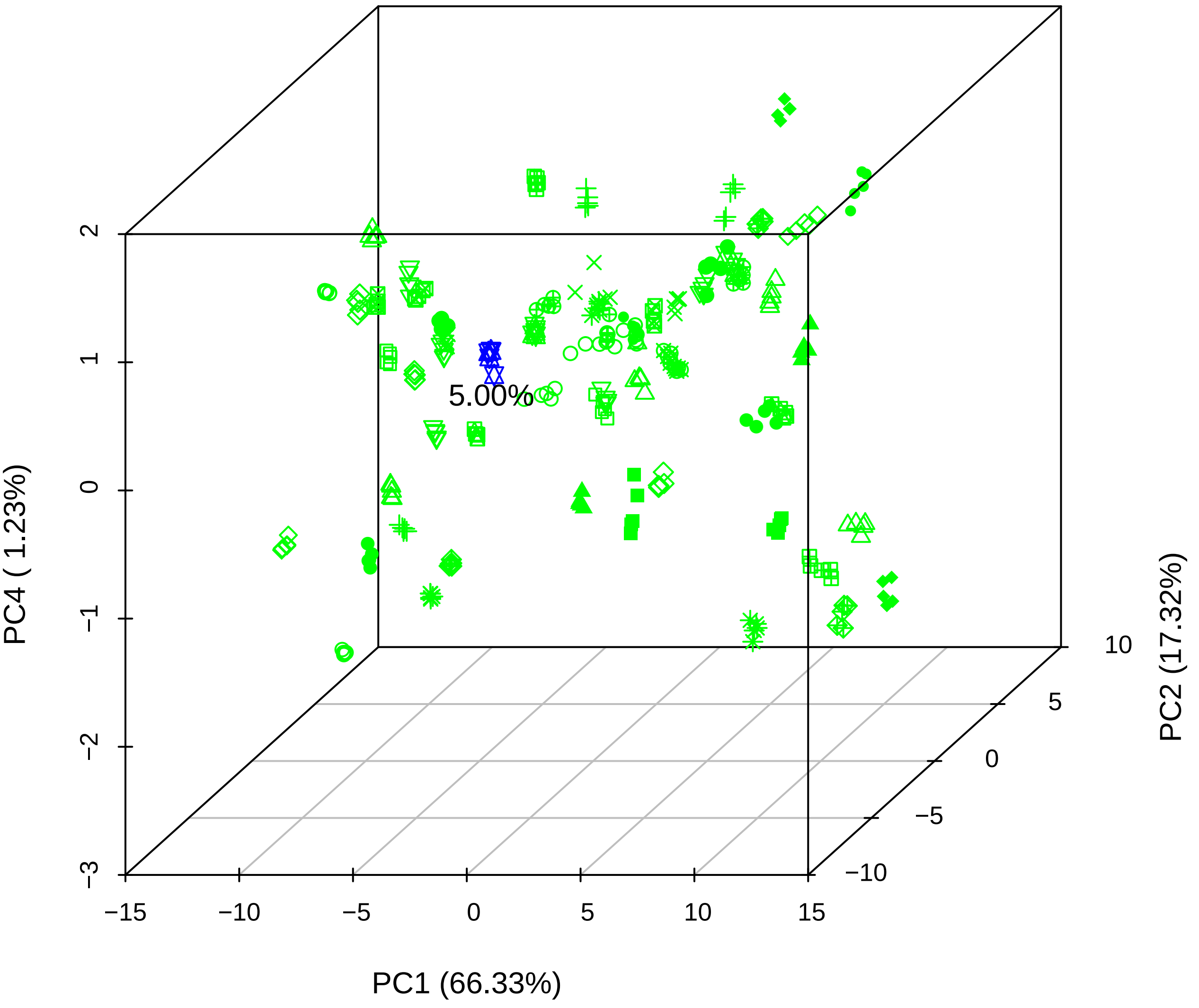

PC1 vs PC3 vs PC4

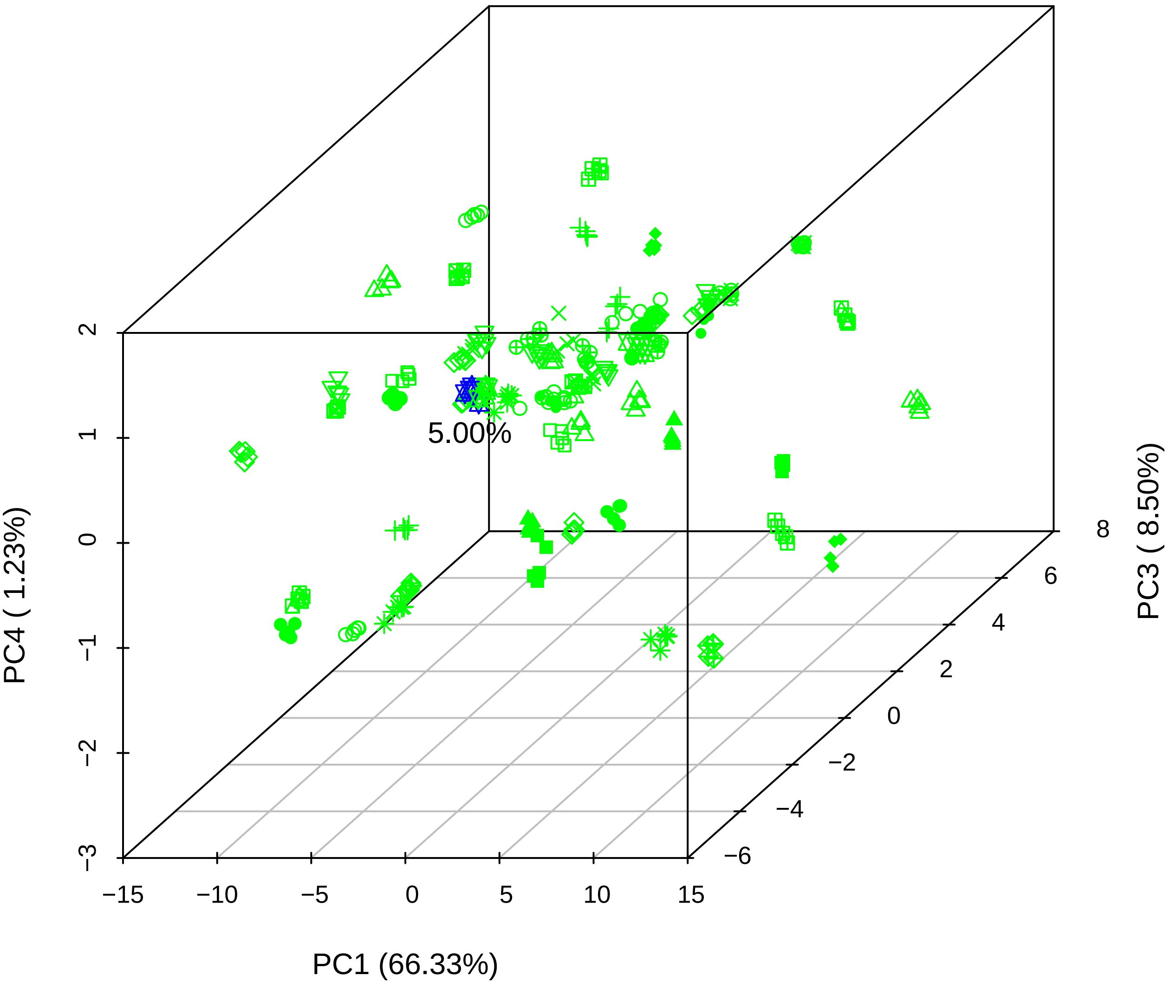

PC2 vs PC3 vs PC5

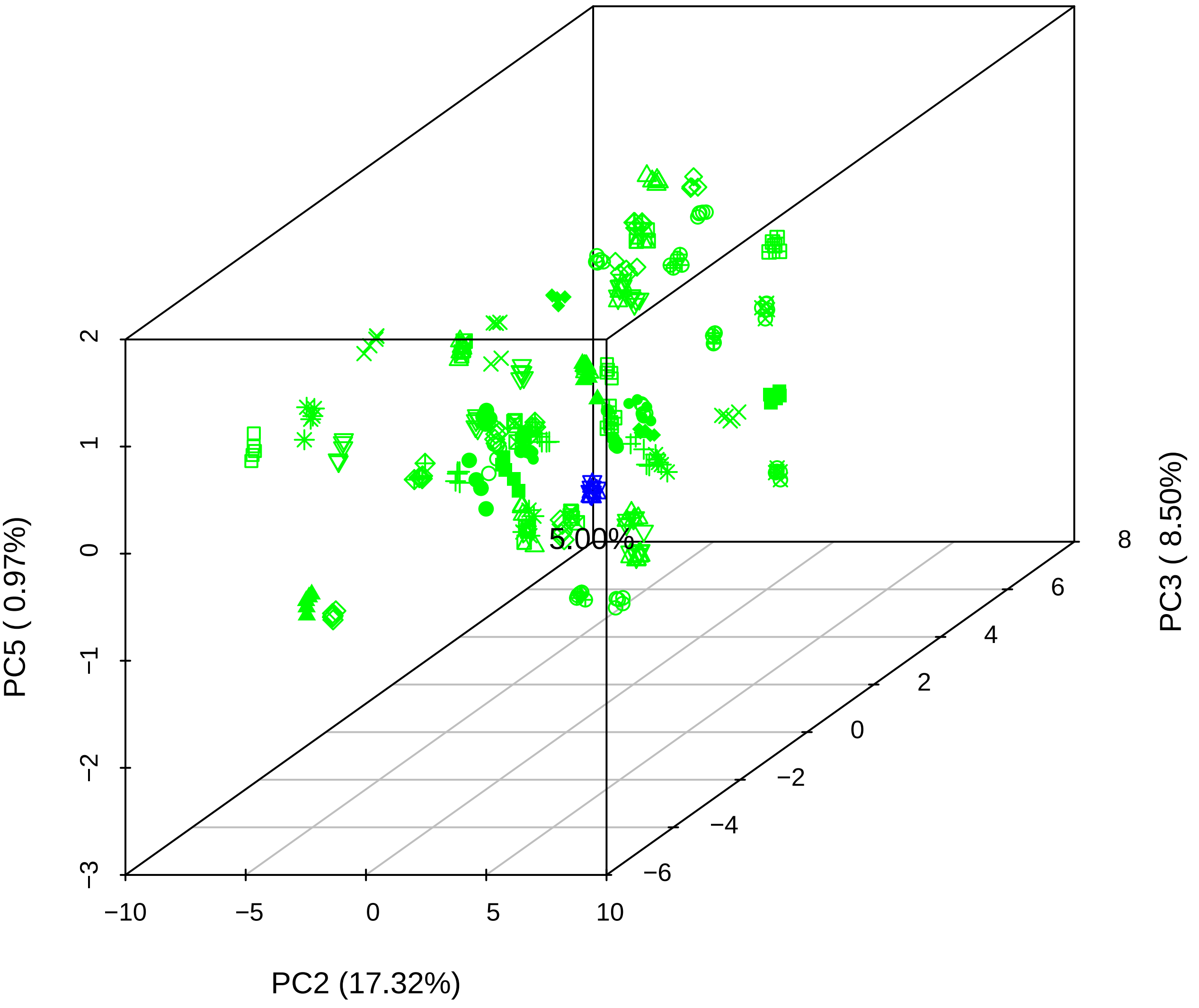

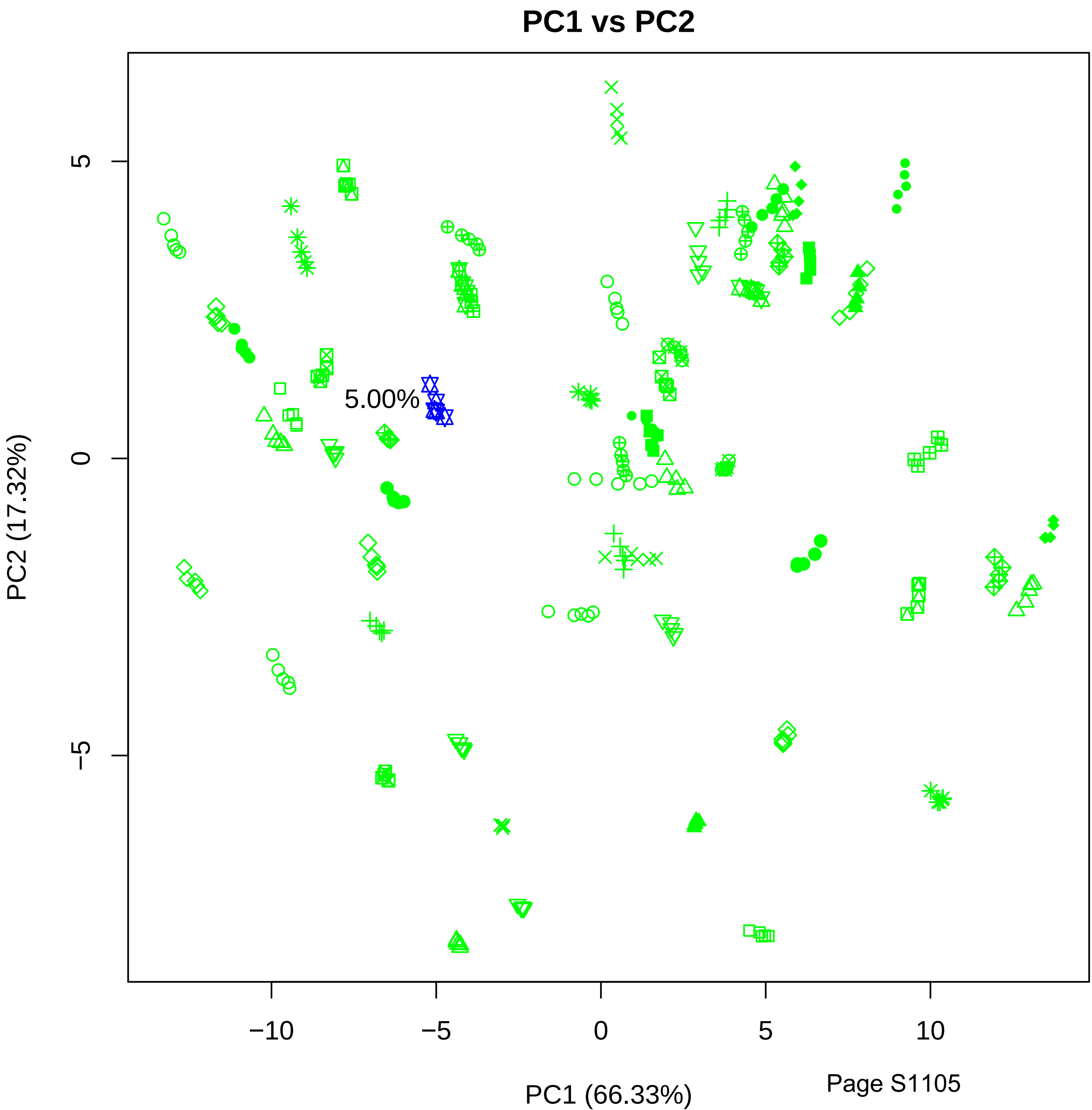
PC1 vs PC2
5.00%
PC1 (66.33%)
PC2 (17.32%)
−10
−5
0
5
10
−5
0
5

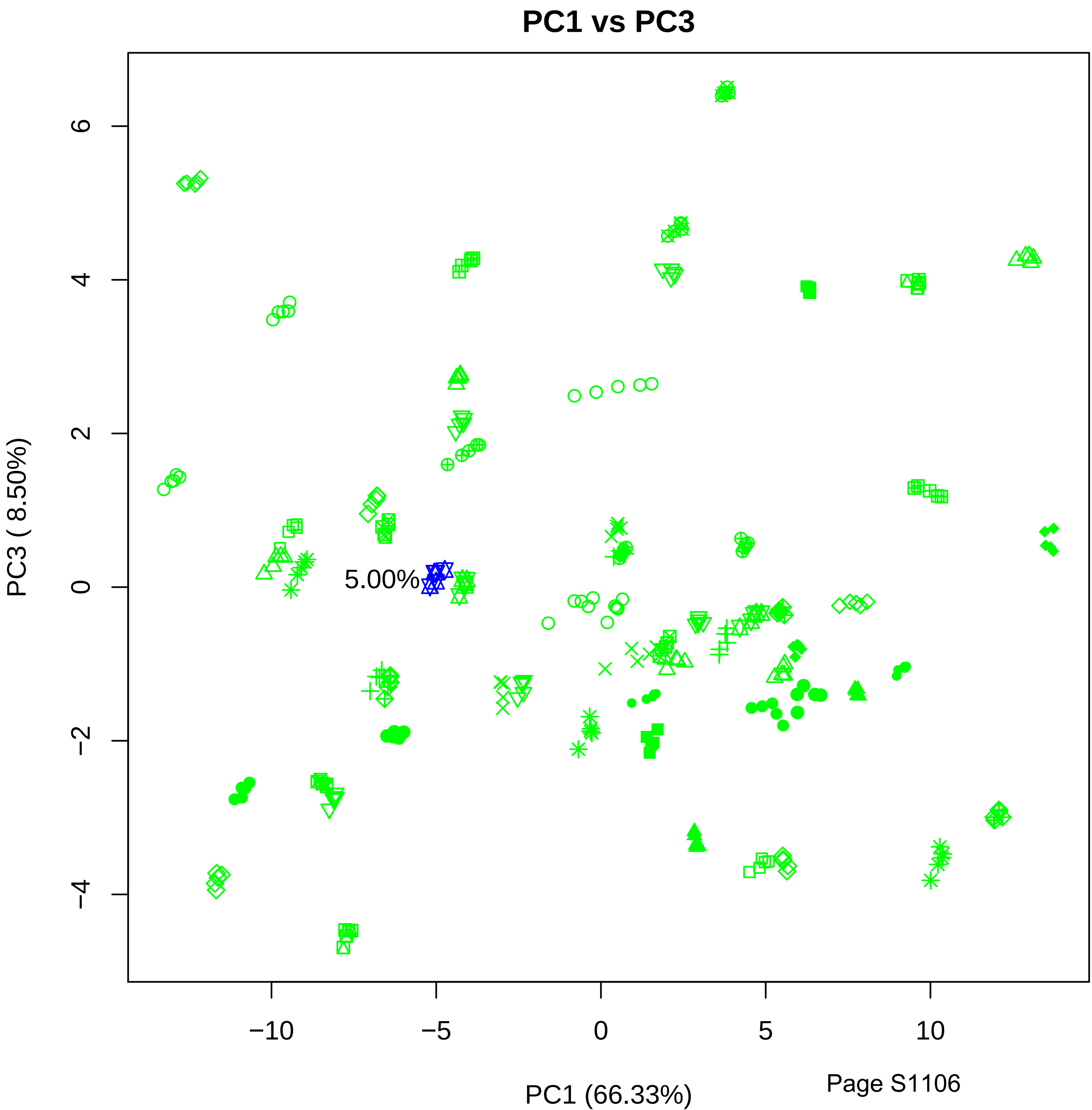

PC1 vs PC3
PC3 (8.50%)
PC1 (66.33%)
5.00%
−10
−5
0
5
10
−4
−2
0
2
4
6

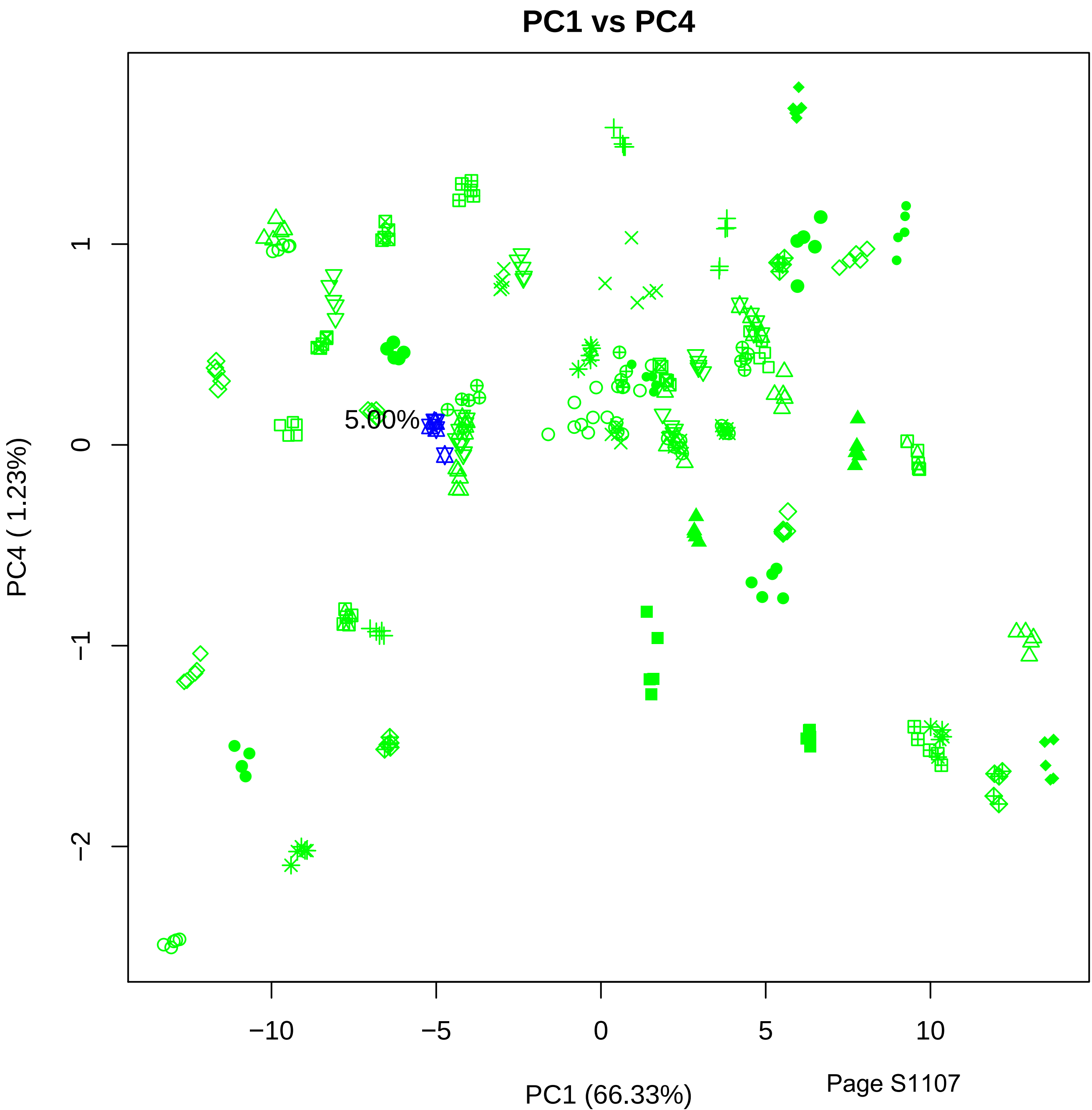

PC1 vs PC4
5.00%
1
0
−1
−2
PC4 (1.23%)
−10
−5
0
5
10
PC1 (66.33%)

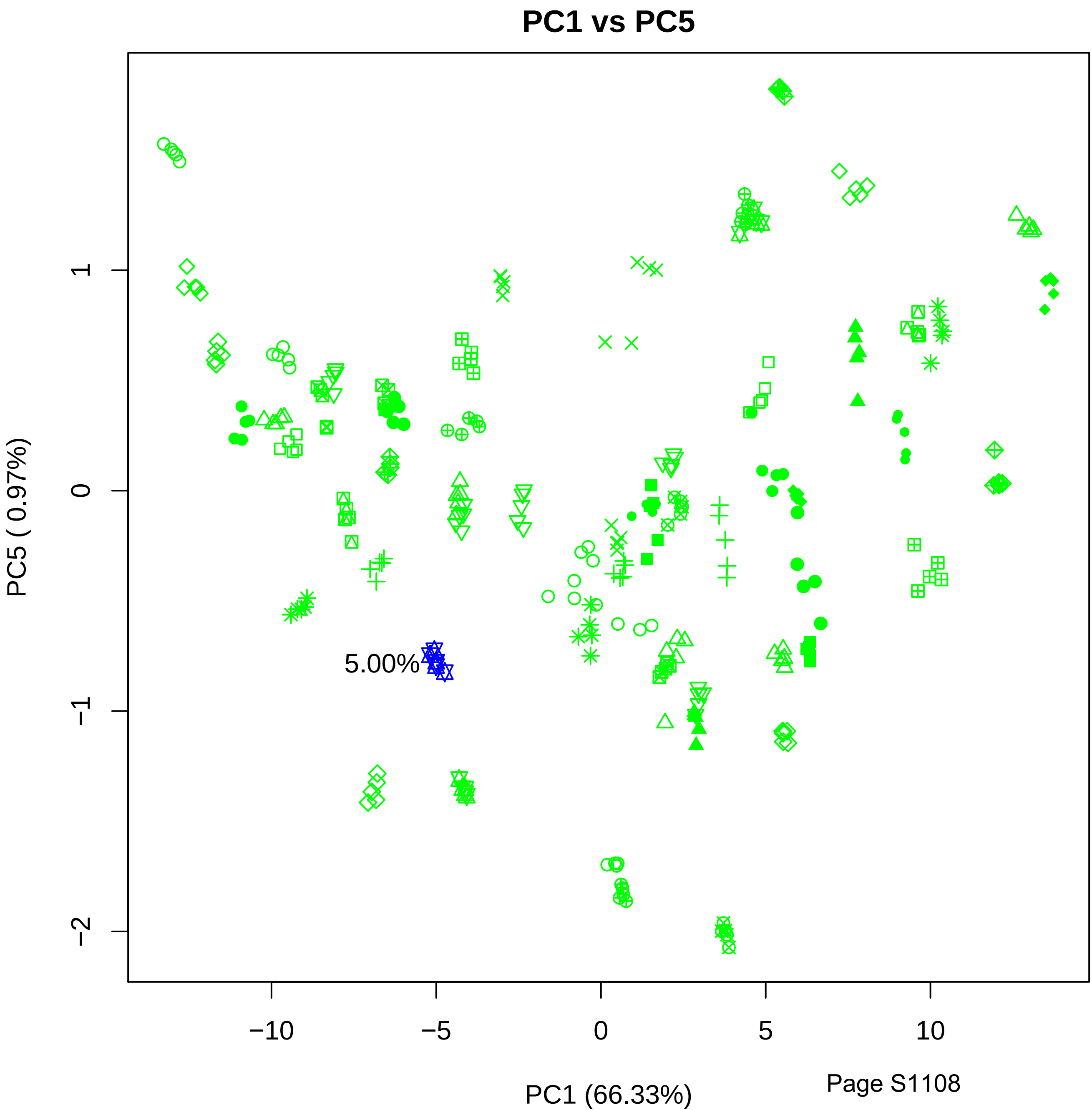
PC1 vs PC5
PC5 (0.97%)
PC1 (66.33%)
5.00%
1
0
−1
−2
−10
−5
0
5
10

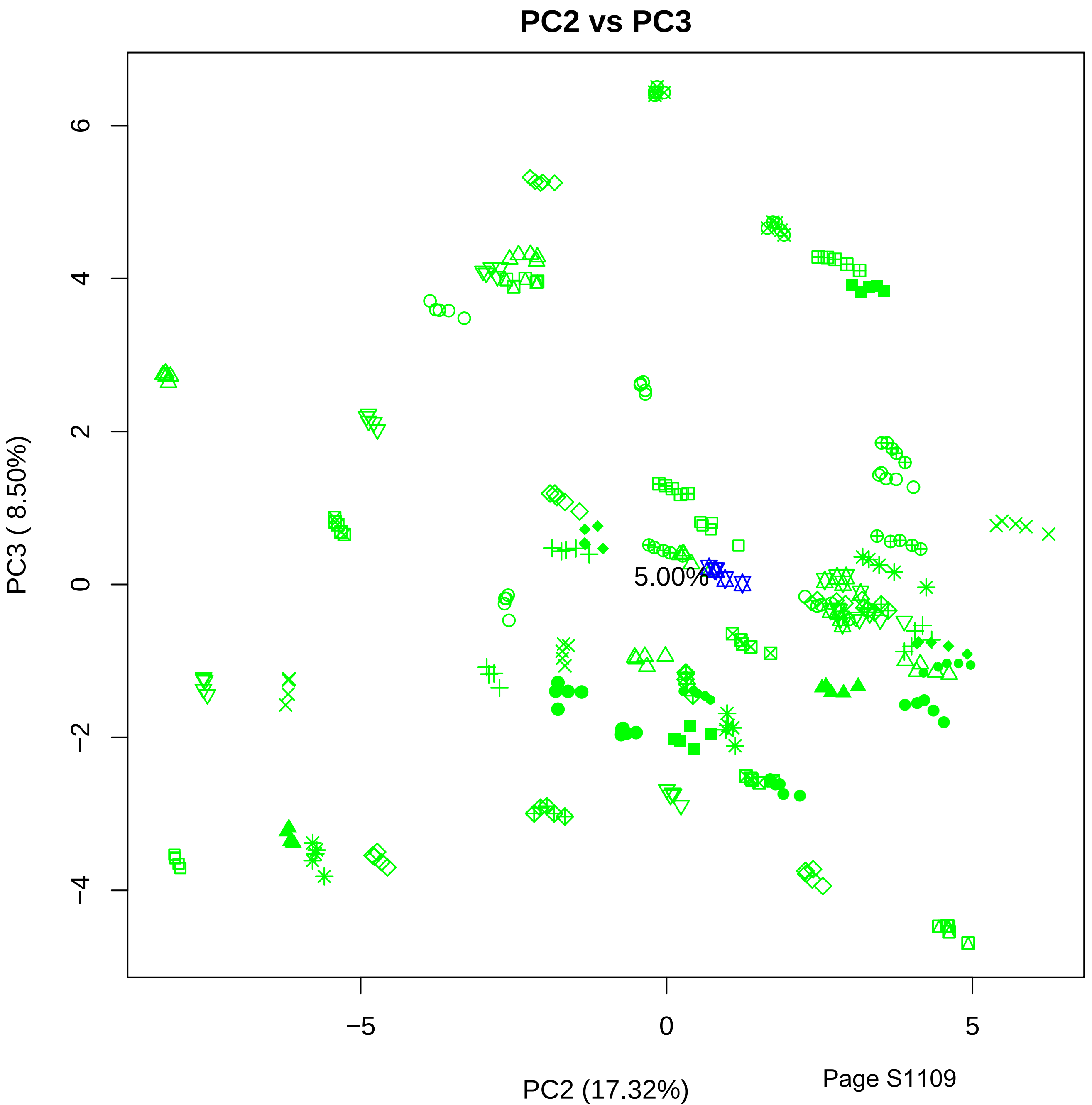

PC2 vs PC3
PC3 (8.50%)
PC2 (17.32%)
6
4
2
0
−2
−4
−5
0
5
5.00%

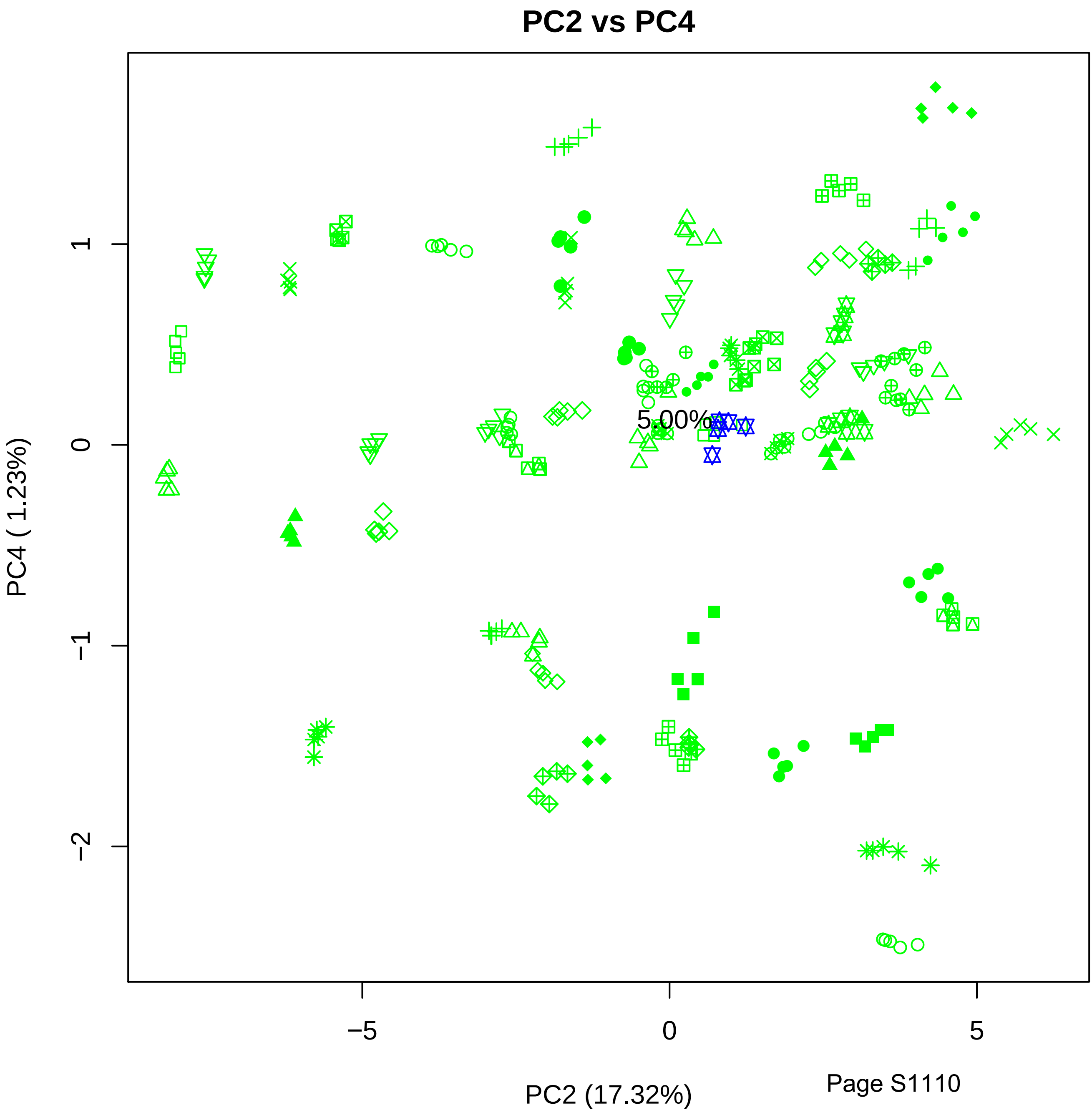
PC2 vs PC4
PC4 (1.23%)
PC2 (17.32%)
1
0
−1
−2
−5
0
5
5.00%

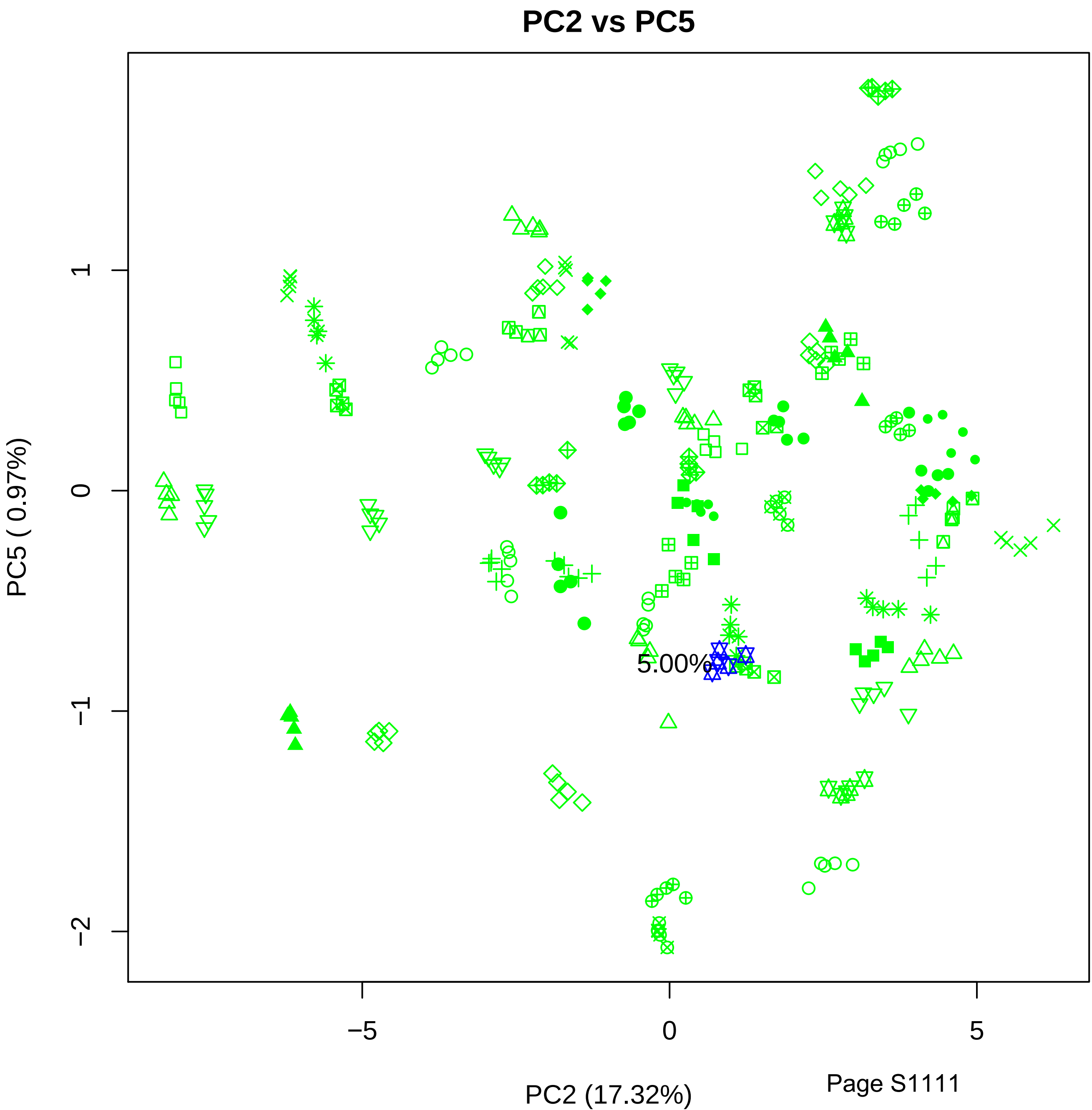
PC2 vs PC5
PC5 (0.97%)
1
0
−1
−2
−5
0
5
5.00%
PC2 (17.32%)

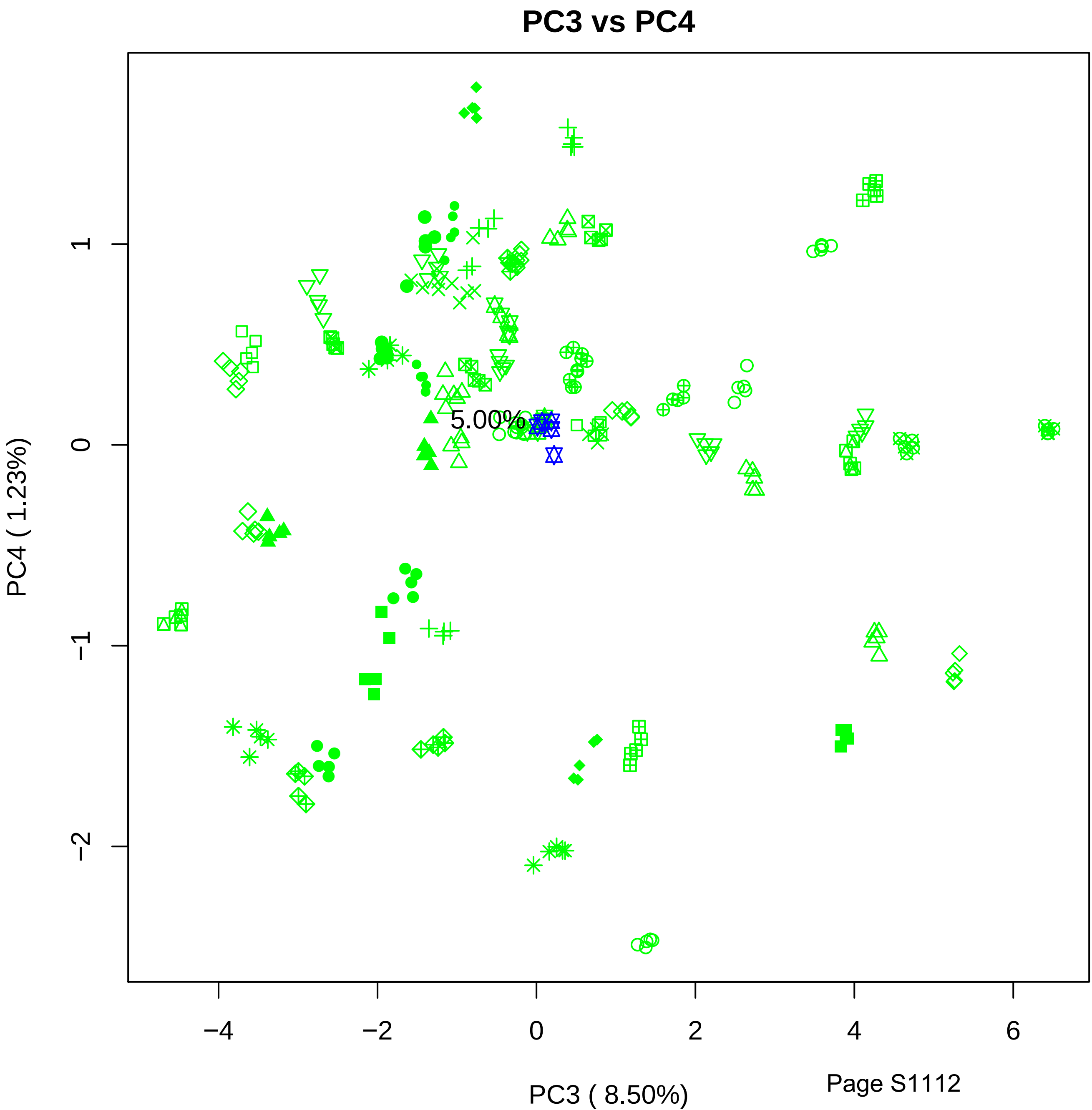
PC3 vs PC4
5.00%
PC4 (1.23%)
PC3 (8.50%)
1
0
−1
−2
−4
−2
0
2
4
6

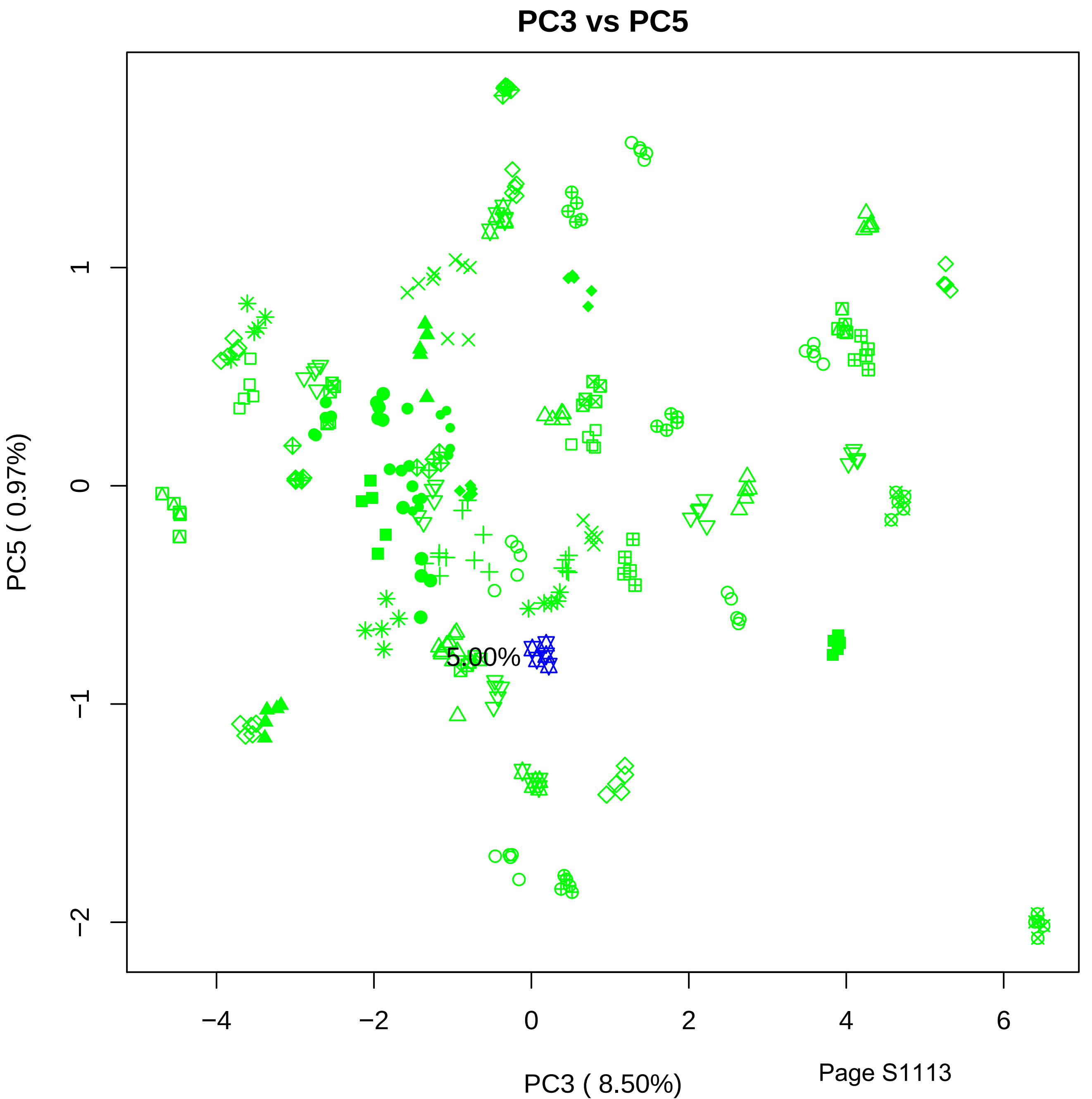

PC3 vs PC5
PC5 (0.97%)
PC3 (8.50%)
5.00%
−4
−2
0
2
4
6
1
0
−1
−2

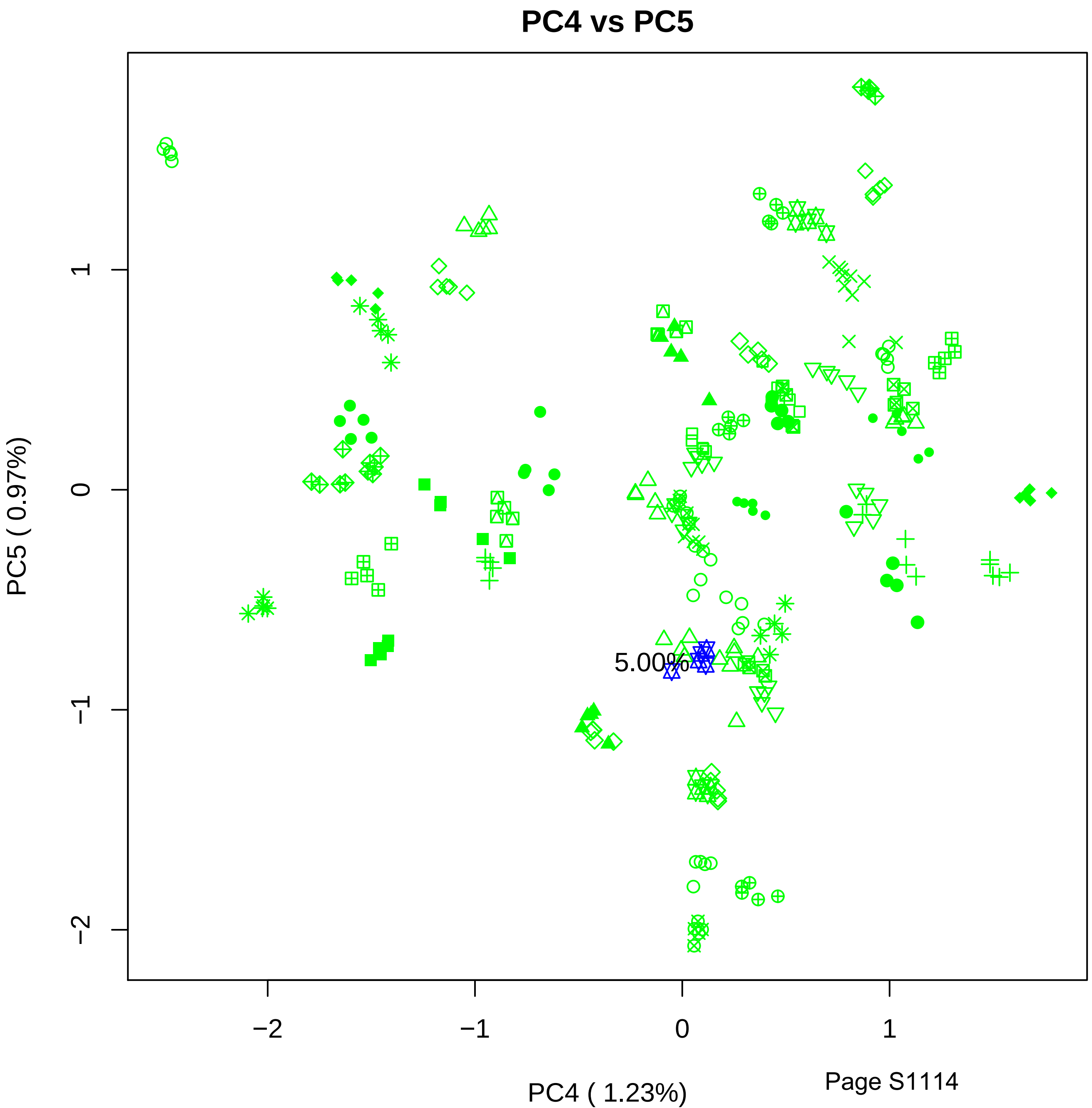

PC4 vs PC5
PC5 (0.97%)
PC4 (1.23%)
5.00%
1
0
−1
−2
−2
−1
0
1

PCA of 7.5% BMH(b) vs PMH(b)

Scaled Matrix PCAed

7th order polynomial baseline correction performed

Raw spectra smoothed to 21 Neighbours, 2nd order PN, Savtisky Golay

Normalisation between 0 and 100

D2 spectra smoothed to 41 Neighbours, 2nd order PN, Savtisky Golay

Regions Analysed: 3601−2501 and 2001−700cm−1

Samples Analysed: 1 bContam's 0 BLH's 0 BMH's 0 OMH's 0 pContam's 0 PLH's 60 PMH's

Keele University AD

Fri Dec 14 14:21:34 2018

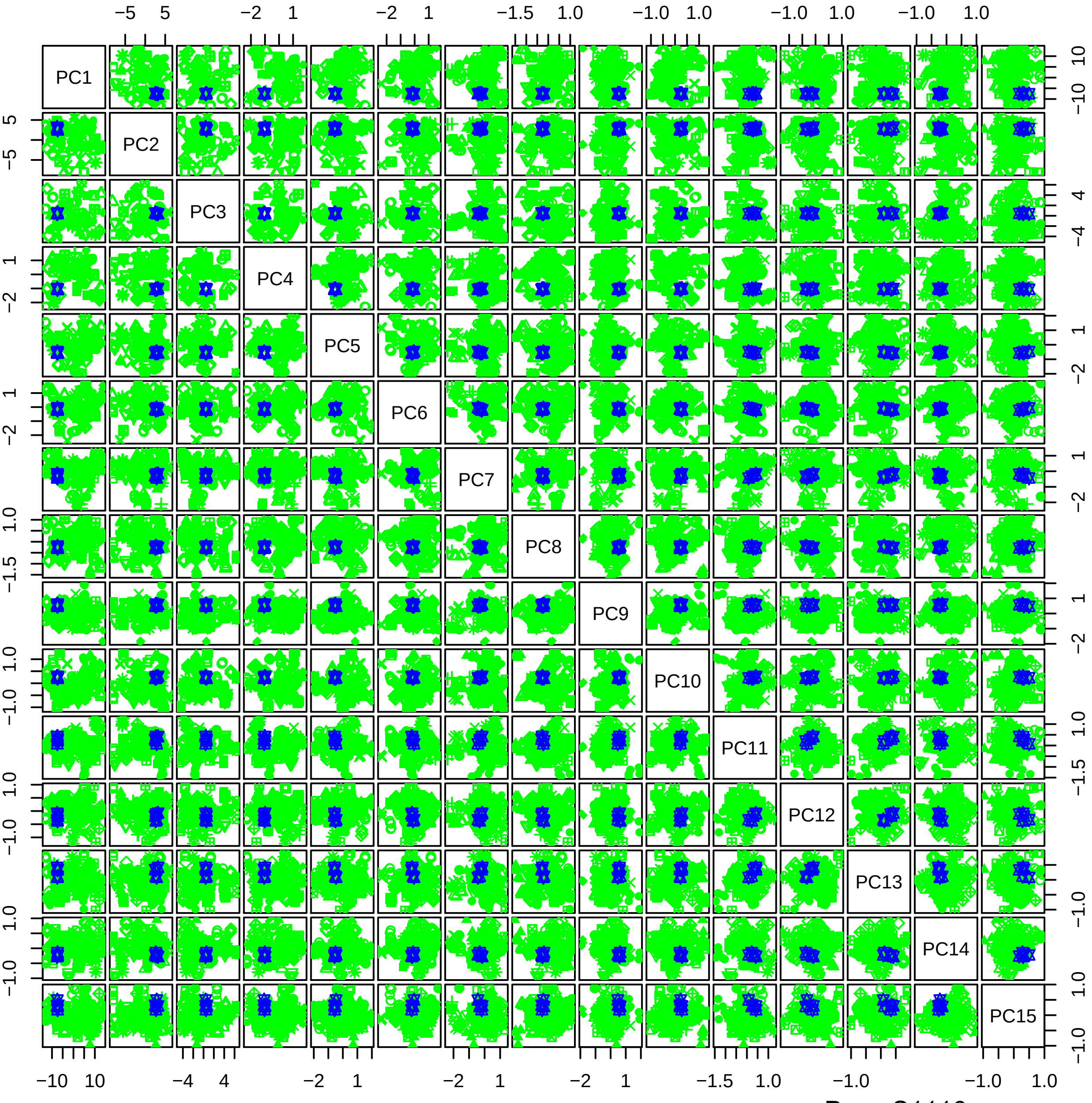

PC1
PC2
PC3
PC4
PC5
PC6
PC7
PC8
PC9
PC10
PC11
PC12
PC13
PC14
PC15

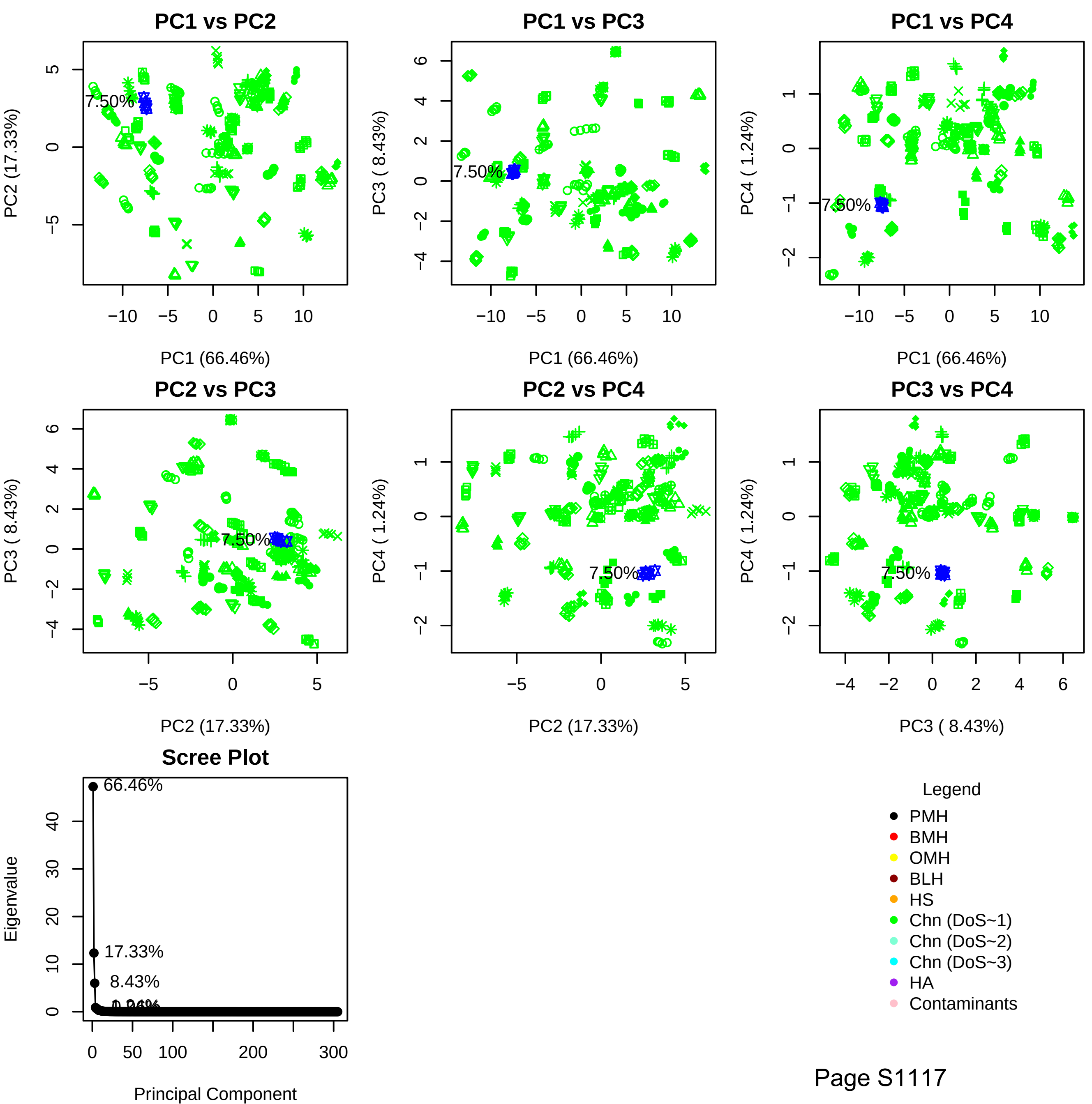
PC1 vs PC2
PC1 vs PC3
PC1 vs PC4
PC2 vs PC3
PC2 vs PC4
PC3 vs PC4
Scree Plot
PC1 (66.46%)
PC2 (17.33%)
PC3 (8.43%)
PC4 (1.24%)
7.50%
Eigenvalue
Principal Component
66.46%
17.33%
8.43%
Legend
PMH
BMH
OMH
BLH
HS
Chn (DoS~1)
Chn (DoS~2)
Chn (DoS~3)
HA
Contaminants

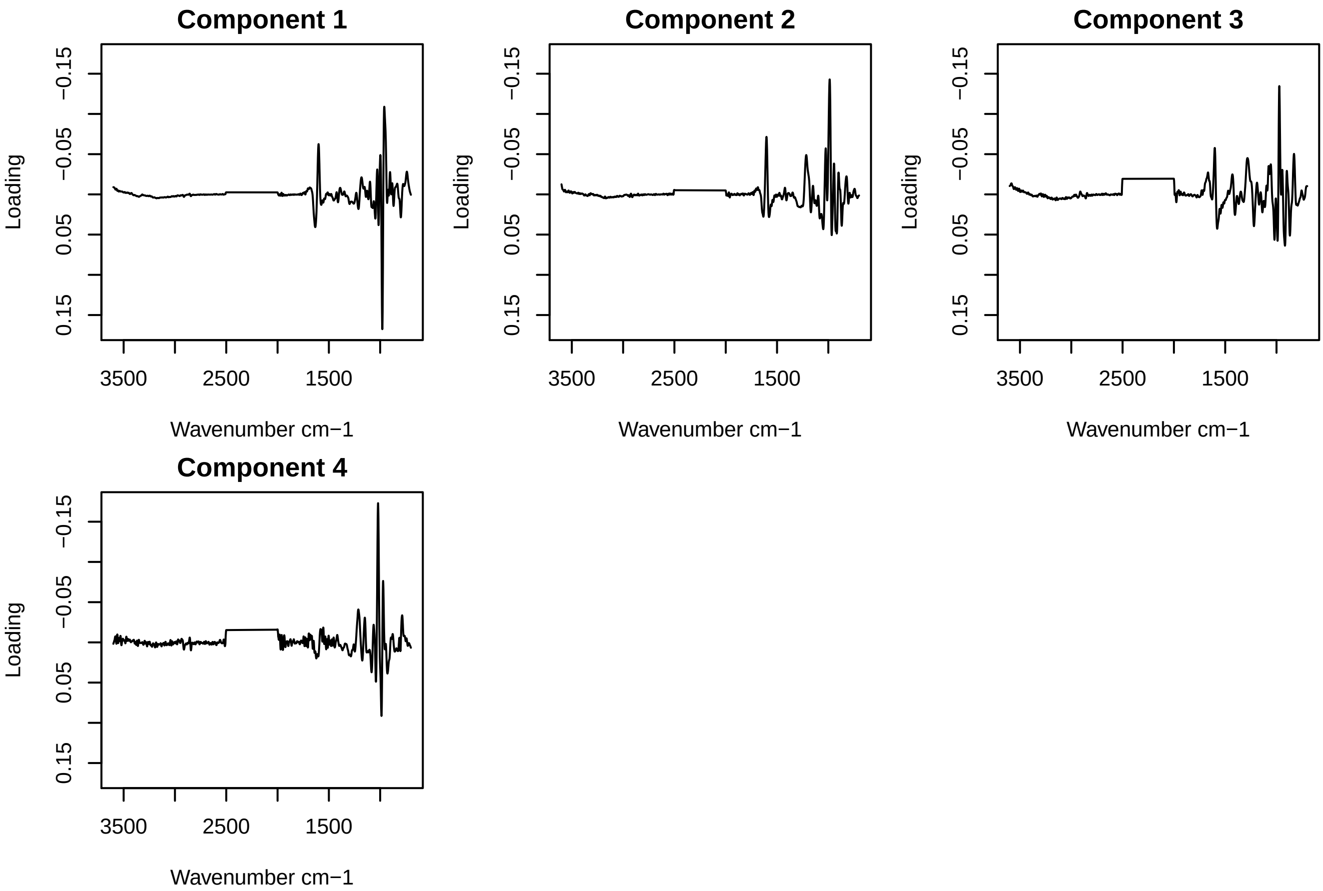

Component 1
Component 2
Component 3
Component 4
Loading
−0.15
−0.05
0.05
0.15
3500
2500
1500
Wavenumber cm−1

PC1 vs PC2 vs PC3

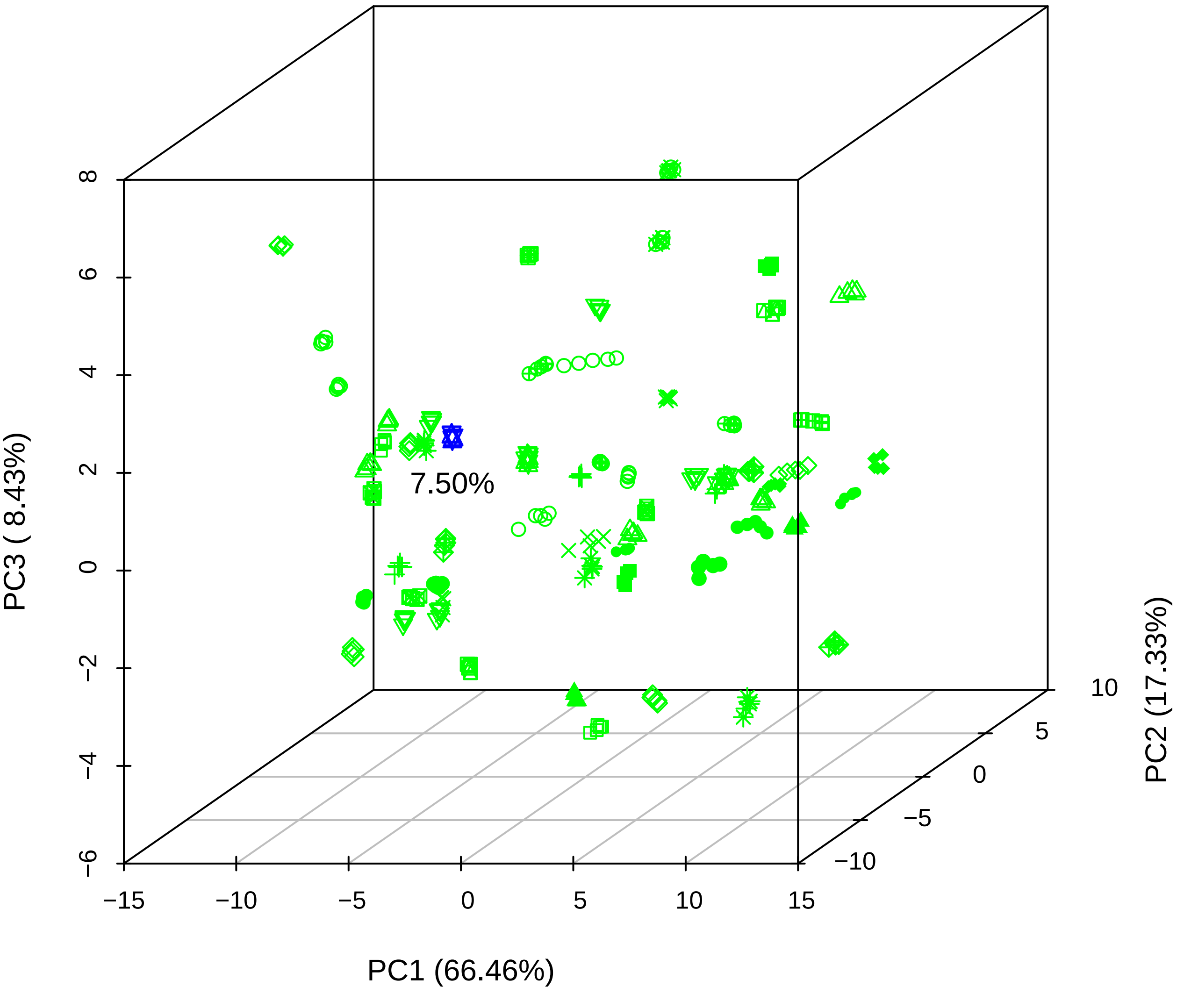

PC1 vs PC2 vs PC4

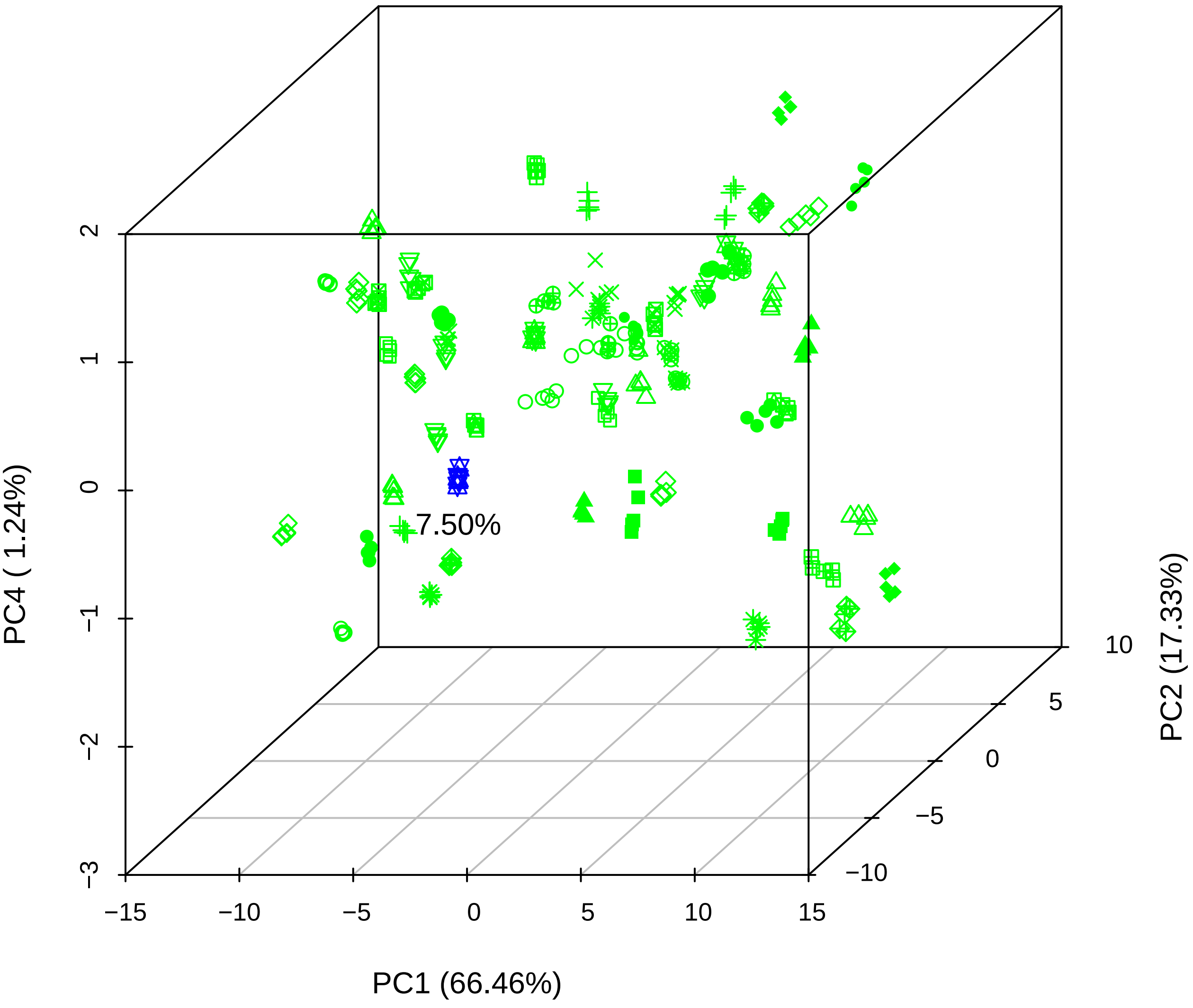

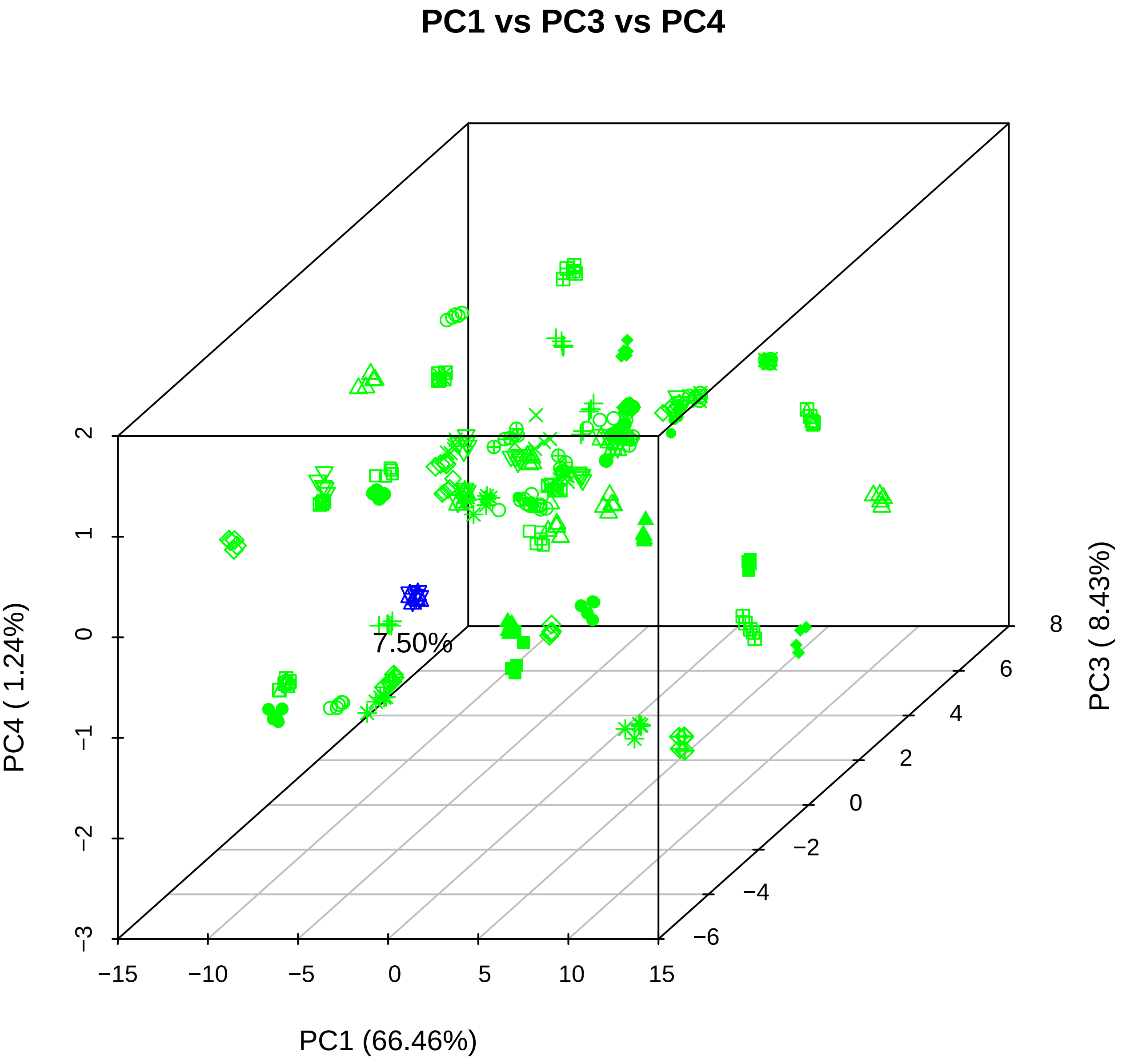
PC1 vs PC3 vs PC4
PC4 (1.24%)
PC1 (66.46%)
PC3 (8.43%)
7.50%
−15
−10
−5
0
5
10
15
−3
−2
−1
0
1
2
−6
−4
−2
0
2
4
6
8

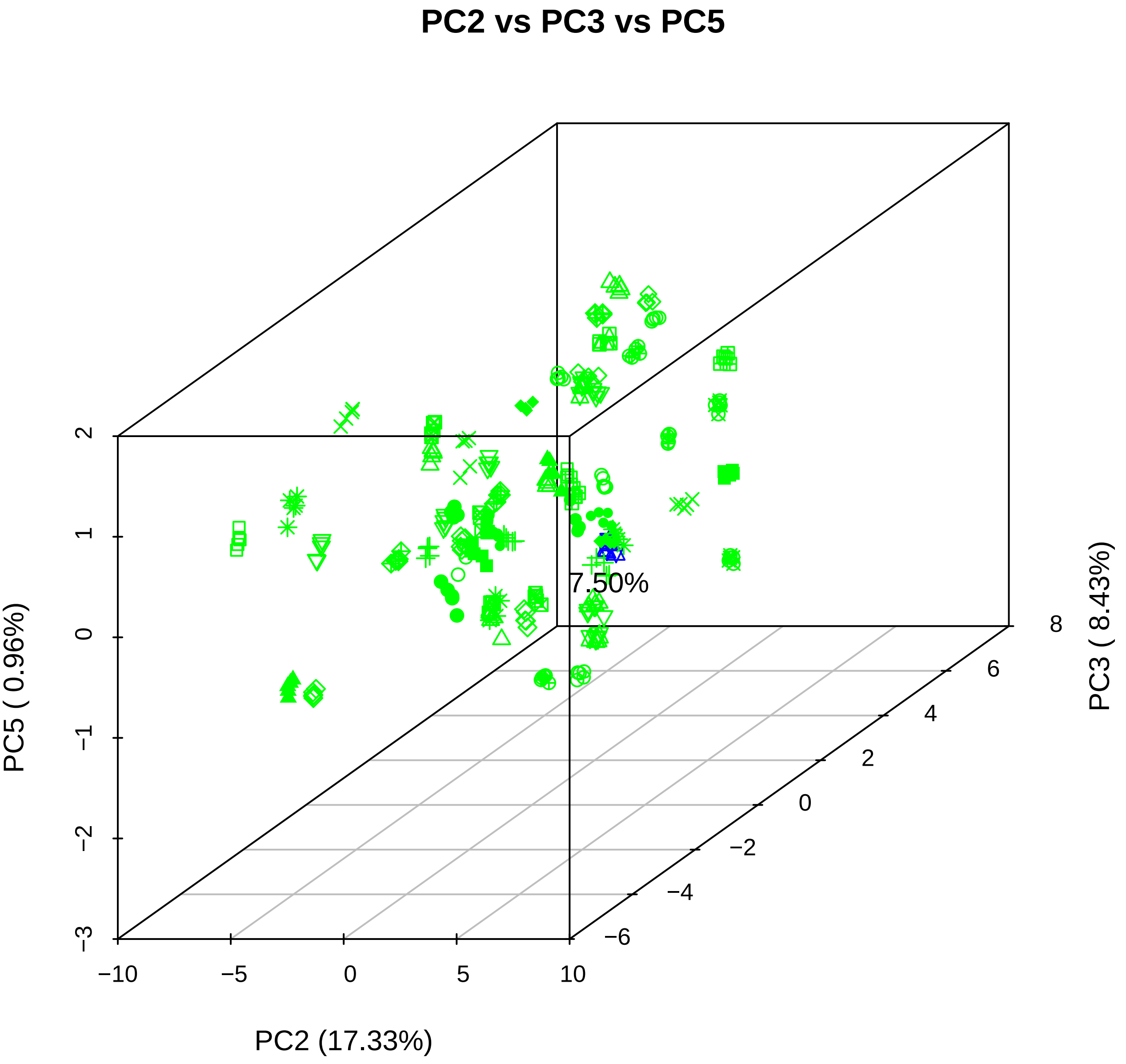
PC2 vs PC3 vs PC5
PC2 (17.33%)
PC5 (0.96%)
PC3 (8.43%)
7.50%
−10
−5
0
5
10
−3
−2
−1
0
1
2
−6
−4
−2
0
2
4
6
8

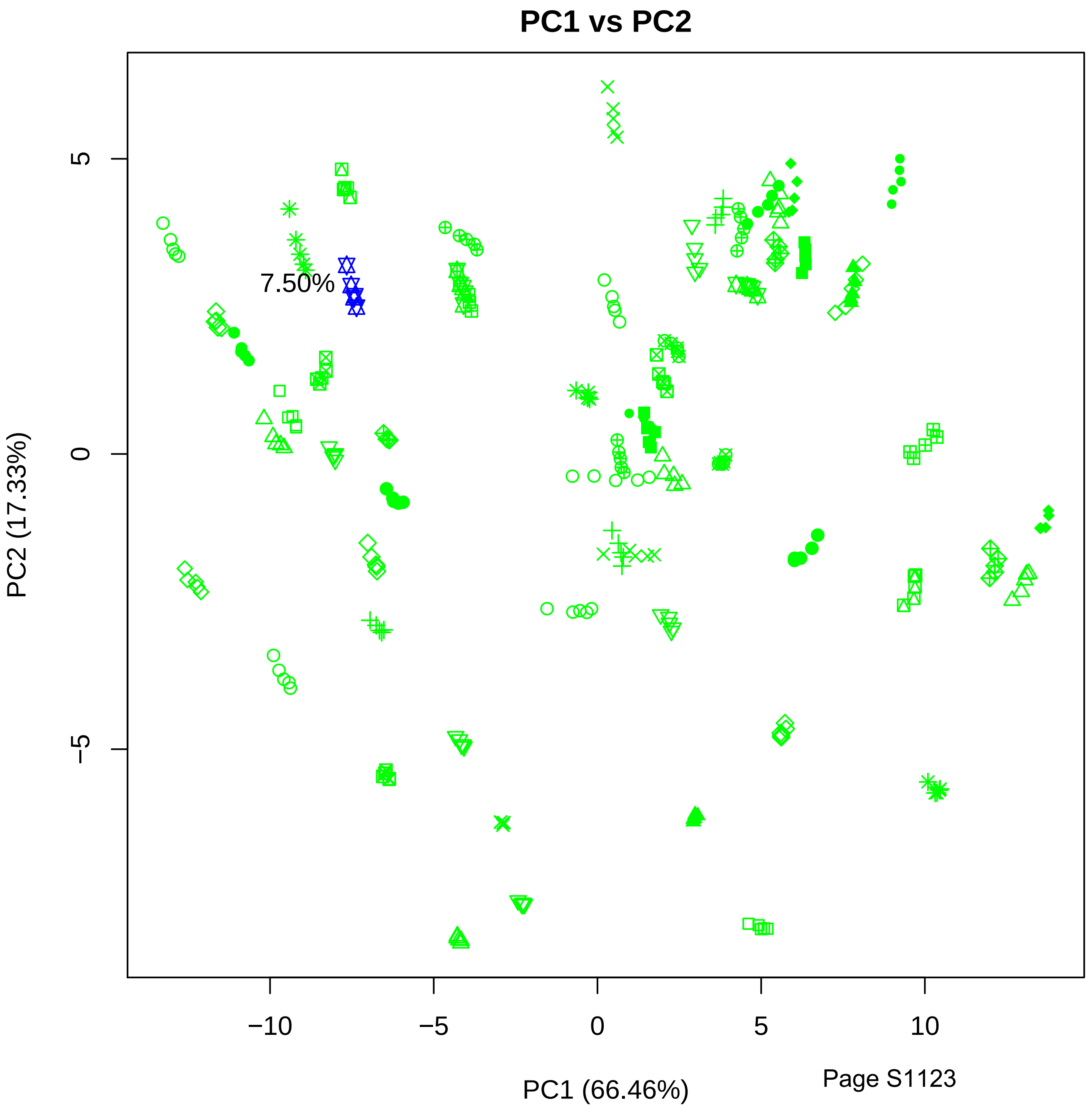
PC1 vs PC2
7.50%
PC2 (17.33%)
PC1 (66.46%)
5
0
−5
−10
−5
0
5
10

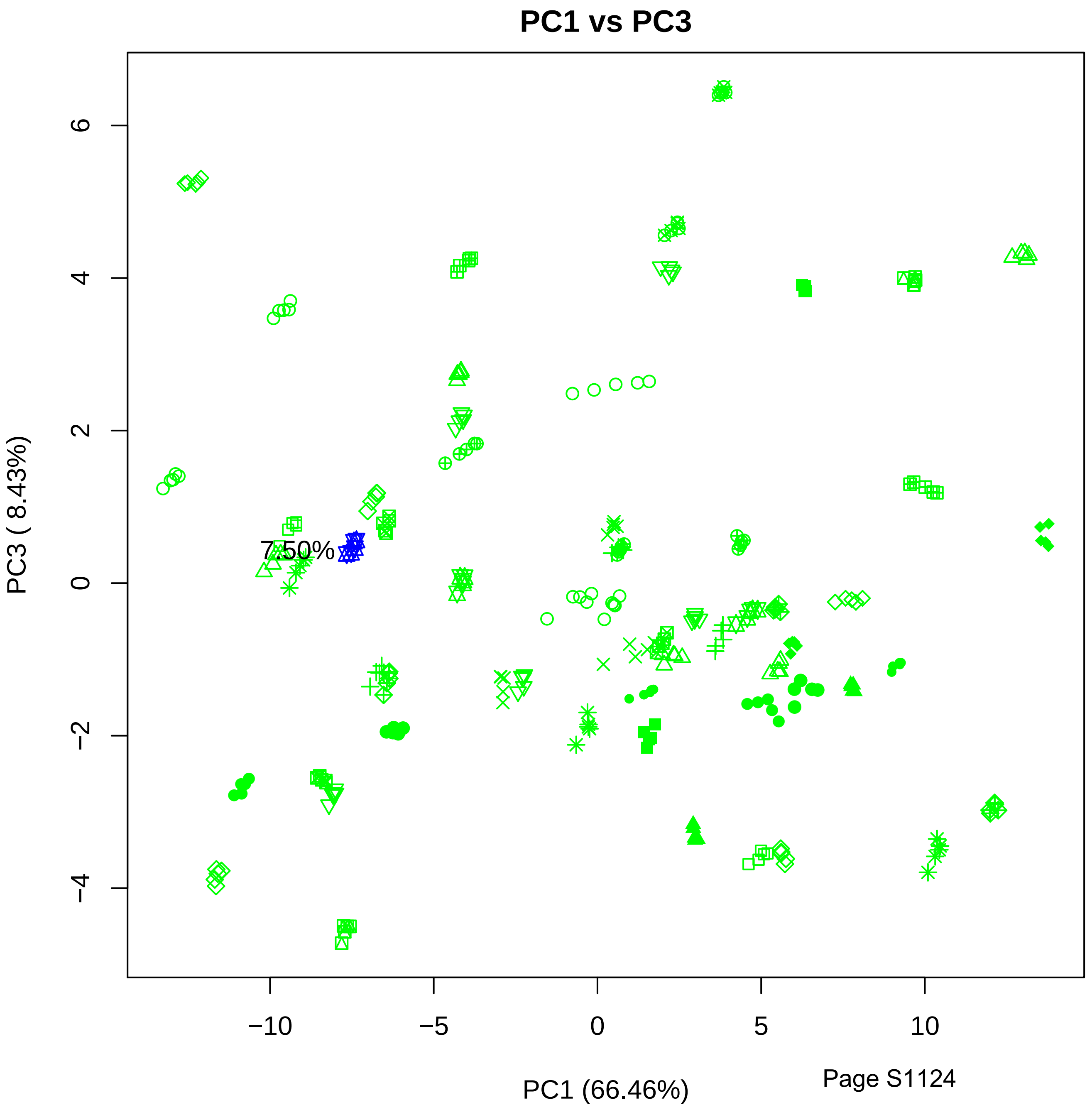
PC1 vs PC3
PC3 (8.43%)
PC1 (66.46%)
7.50%
6
4
2
0
−2
−4
−10
−5
0
5
10

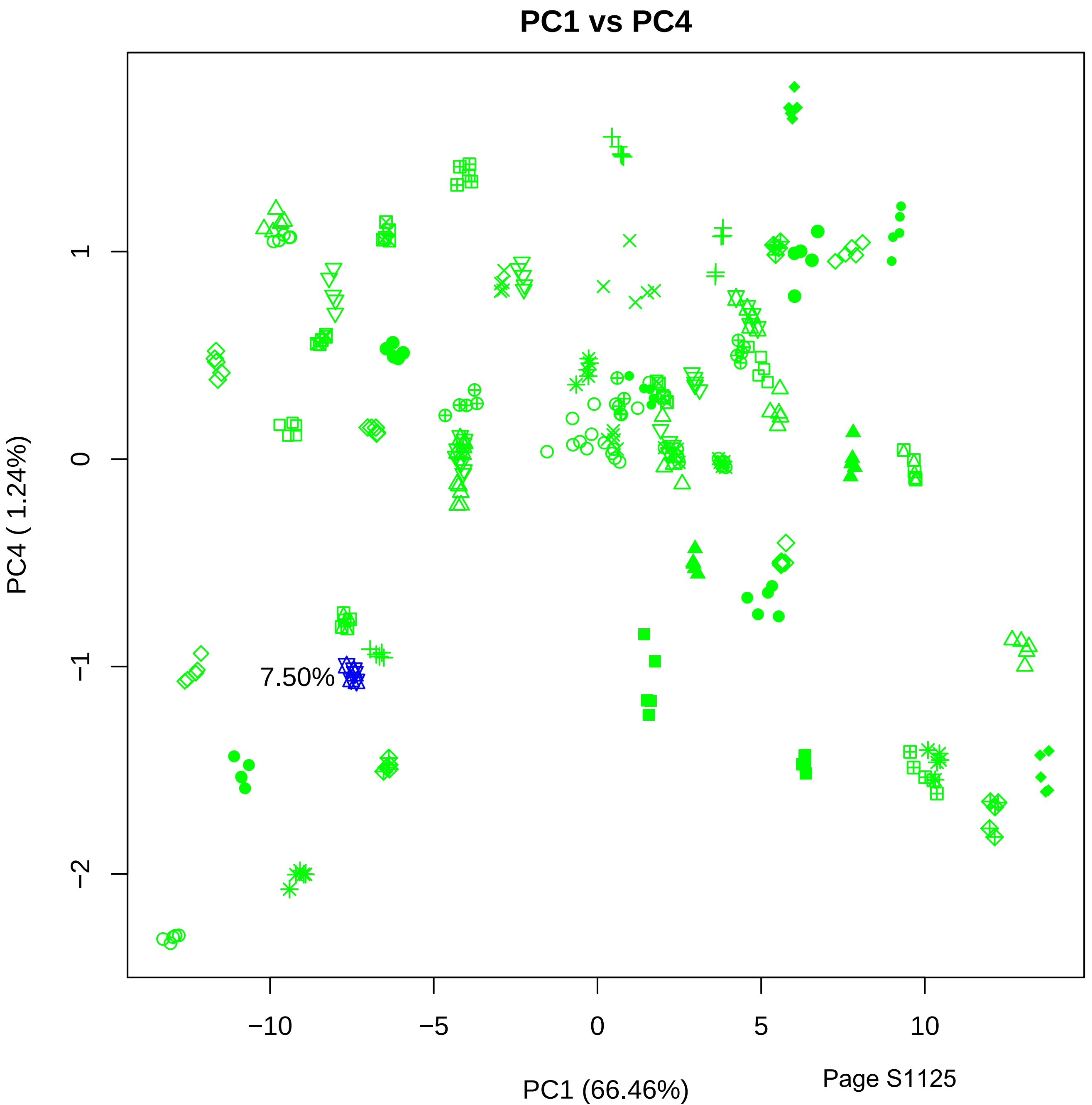
PC1 vs PC4
PC4 (1.24%)
PC1 (66.46%)
7.50%
1
0
−1
−2
−10
−5
0
5
10

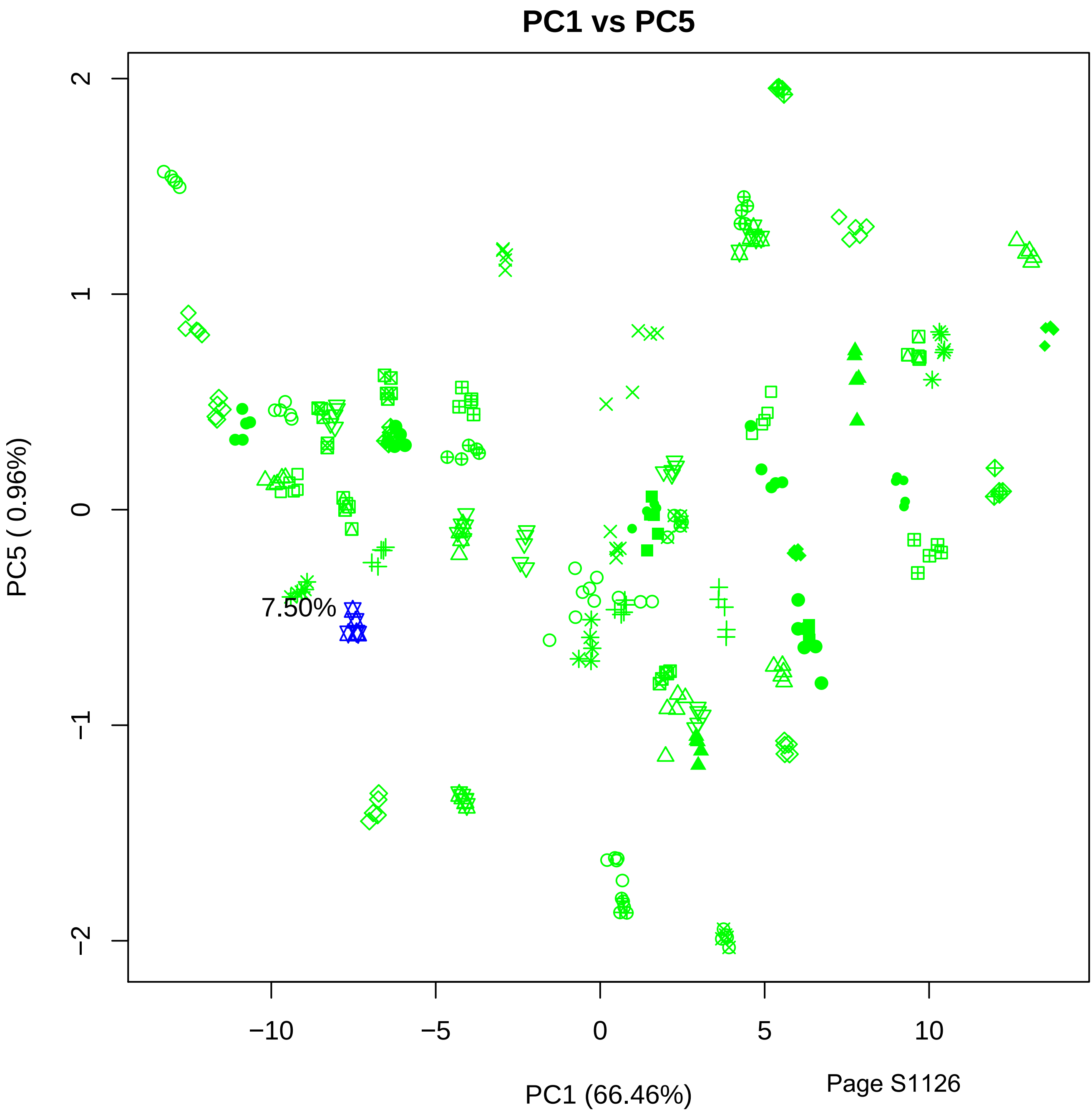

PC1 vs PC5
PC5 (0.96%)
PC1 (66.46%)
7.50%
2
1
0
−1
−2
−10
−5
0
5
10

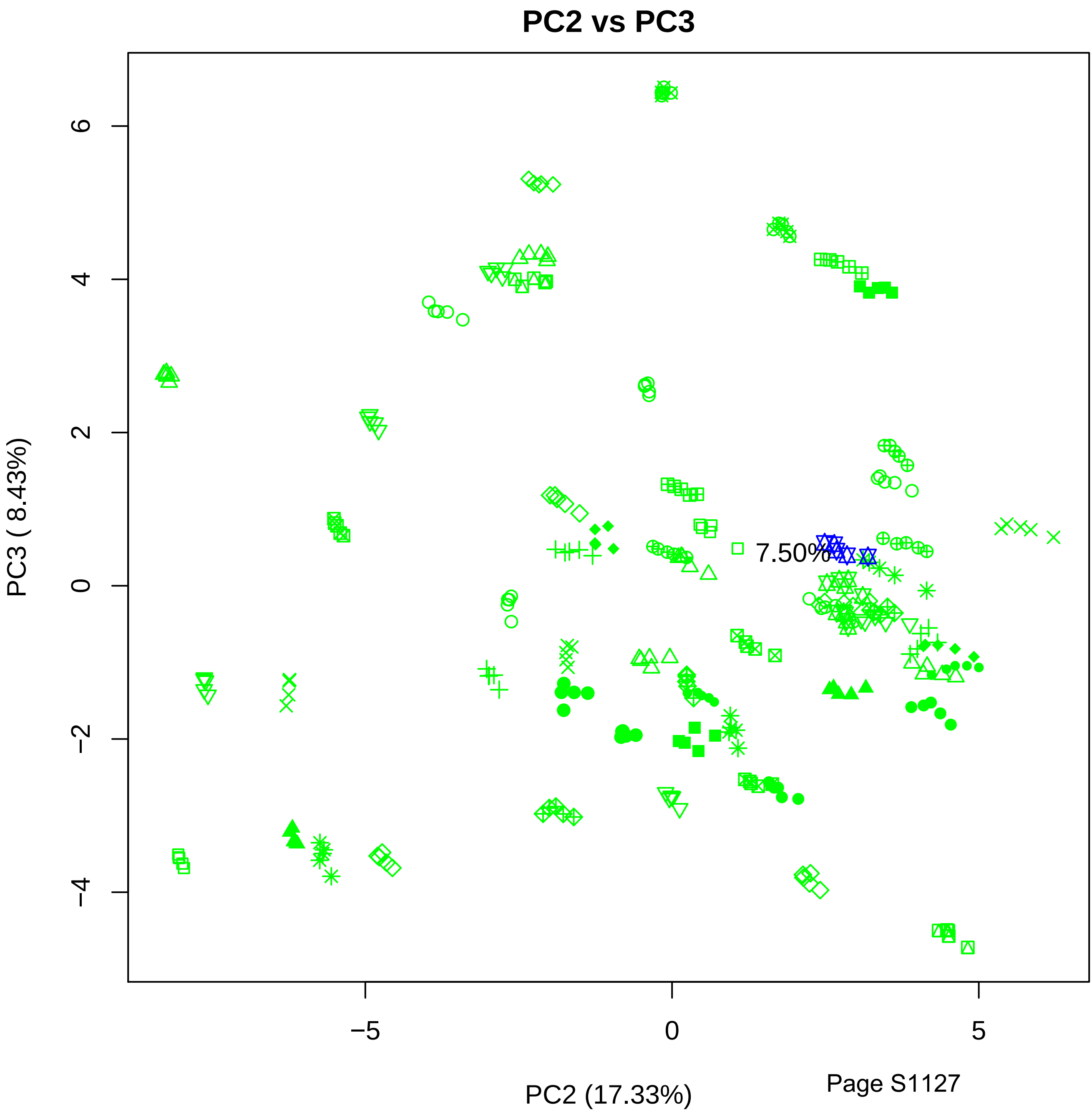

PC2 vs PC3
6
4
2
0
−2
−4
PC3 (8.43%)
7.50%
−5
0
5
PC2 (17.33%)

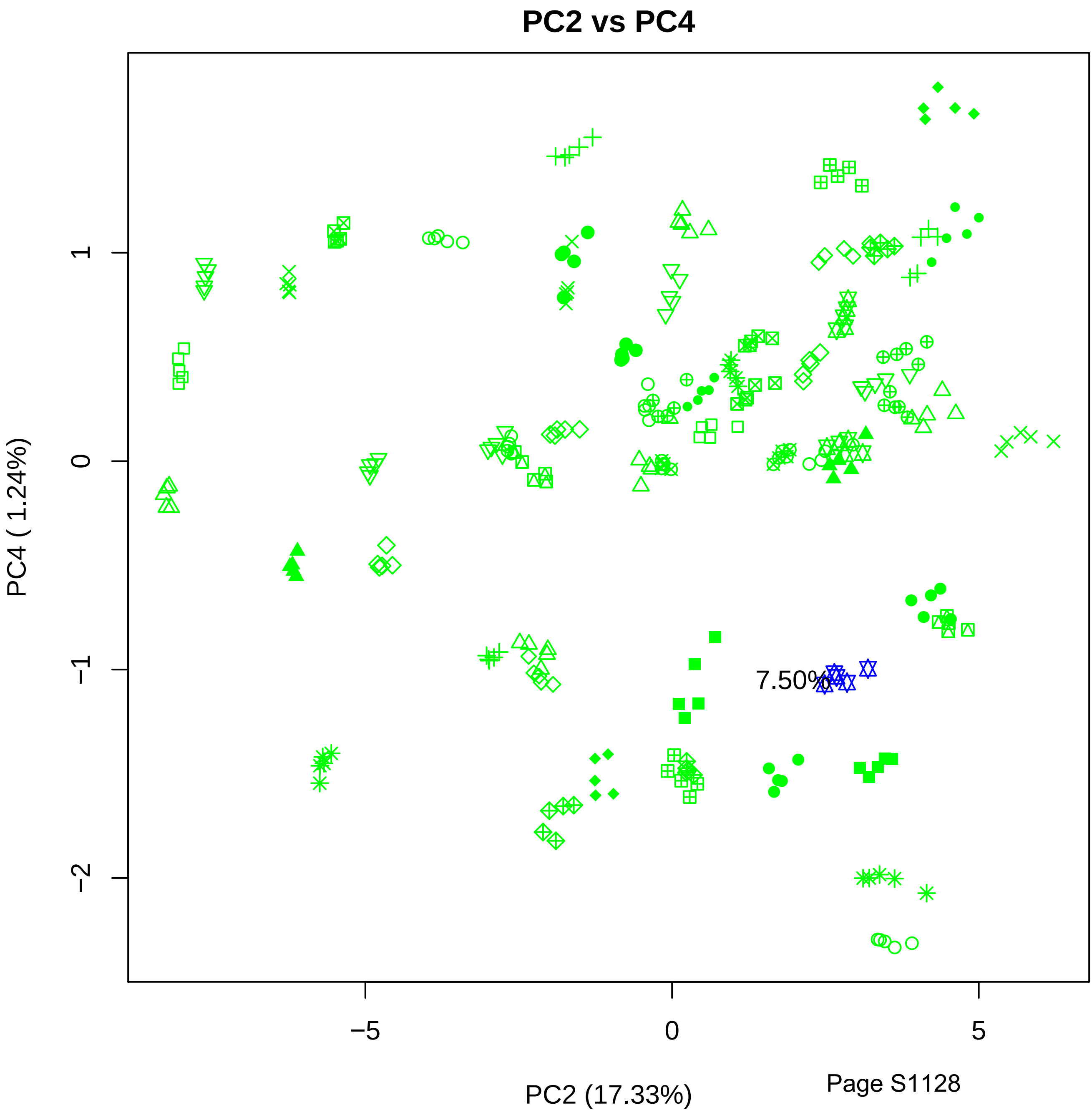
PC2 vs PC4
PC4 (1.24%)
PC2 (17.33%)
1
0
−1
−2
−5
0
5
7.50%

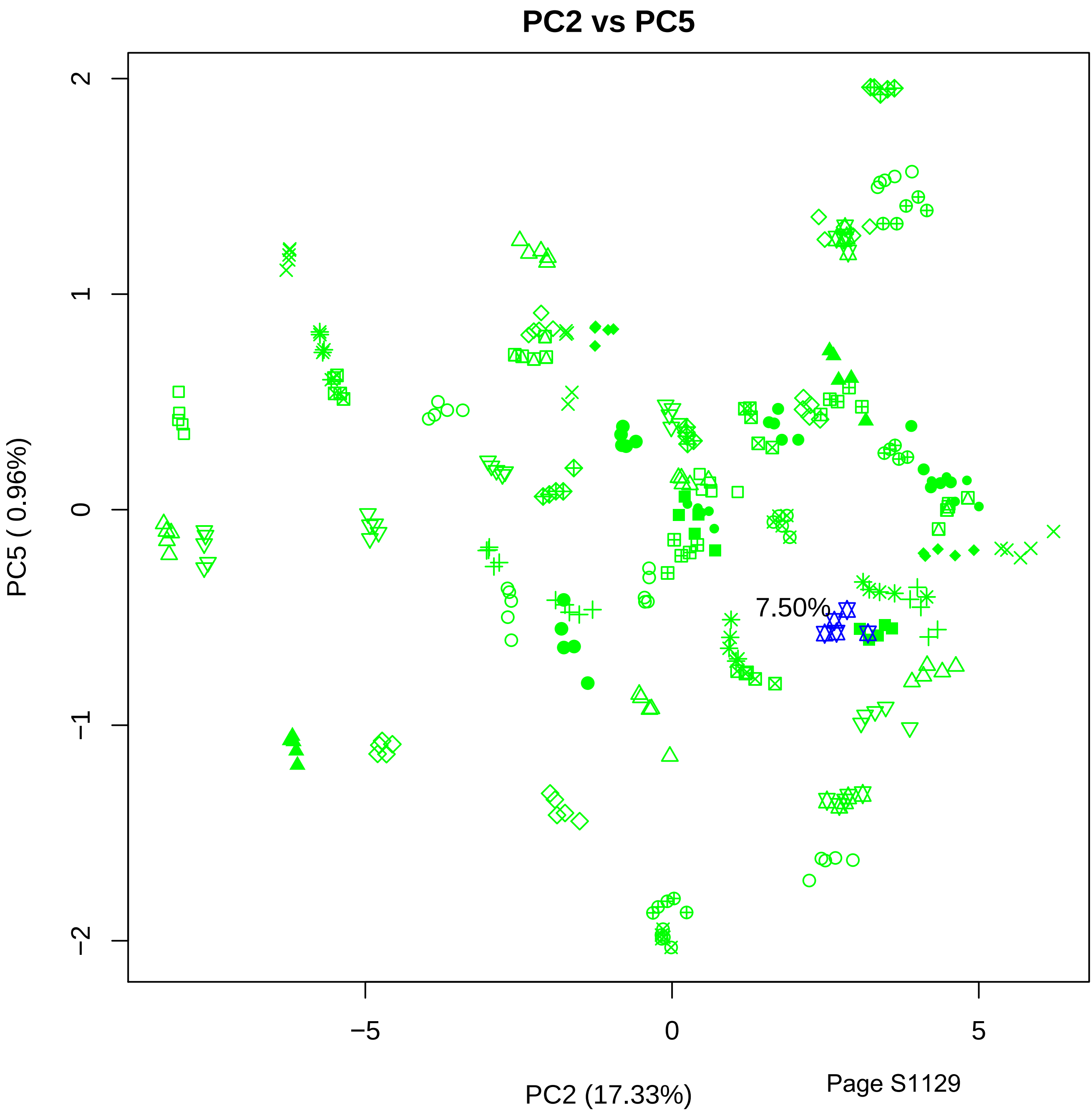
PC2 vs PC5
PC5 (0.96%)
PC2 (17.33%)
2
1
0
−1
−2
−5
0
5
7.50%

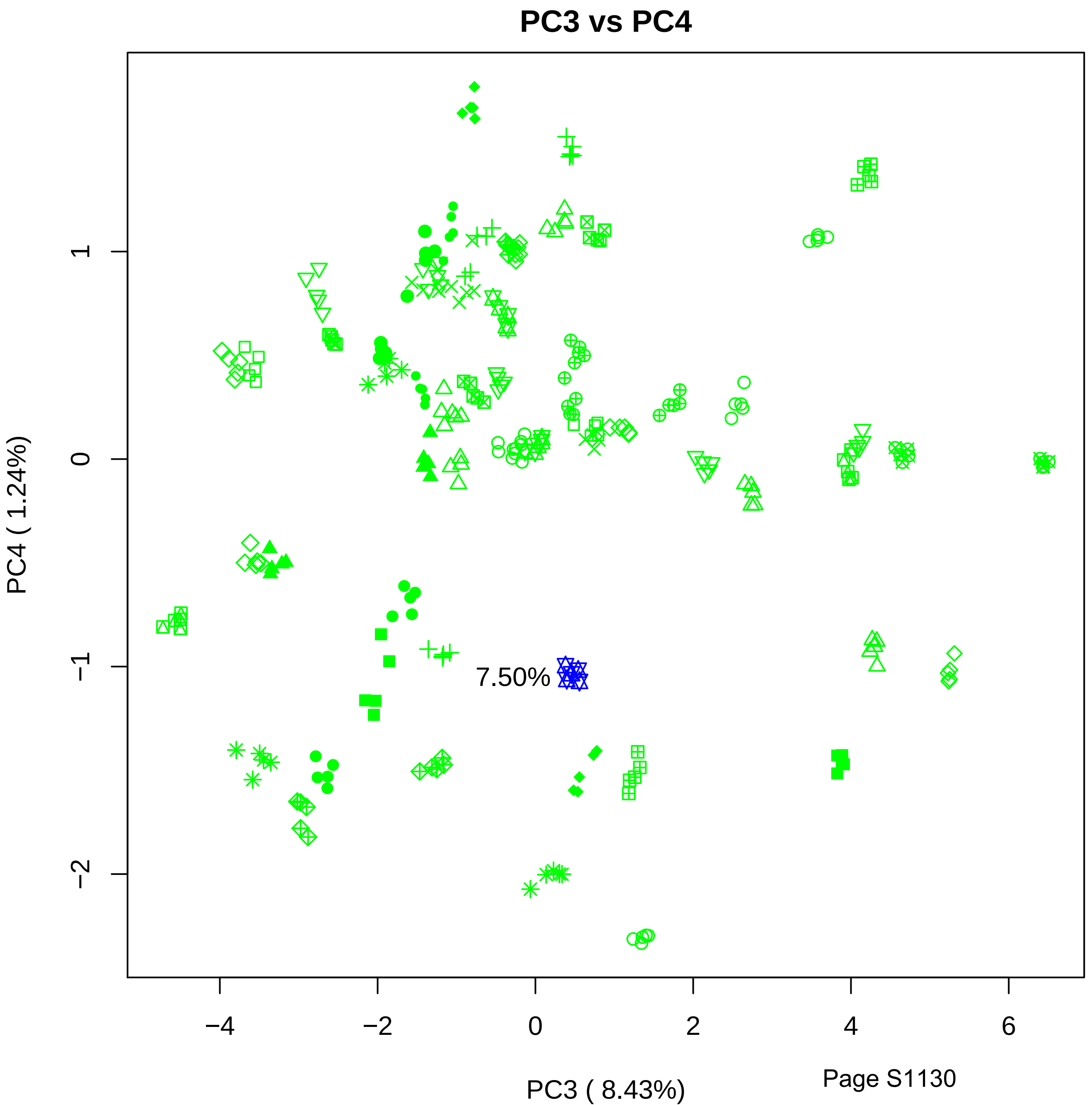
PC3 vs PC4
PC4 (1.24%)
PC3 (8.43%)
7.50%
−4
−2
0
2
4
6
1
0
−1
−2

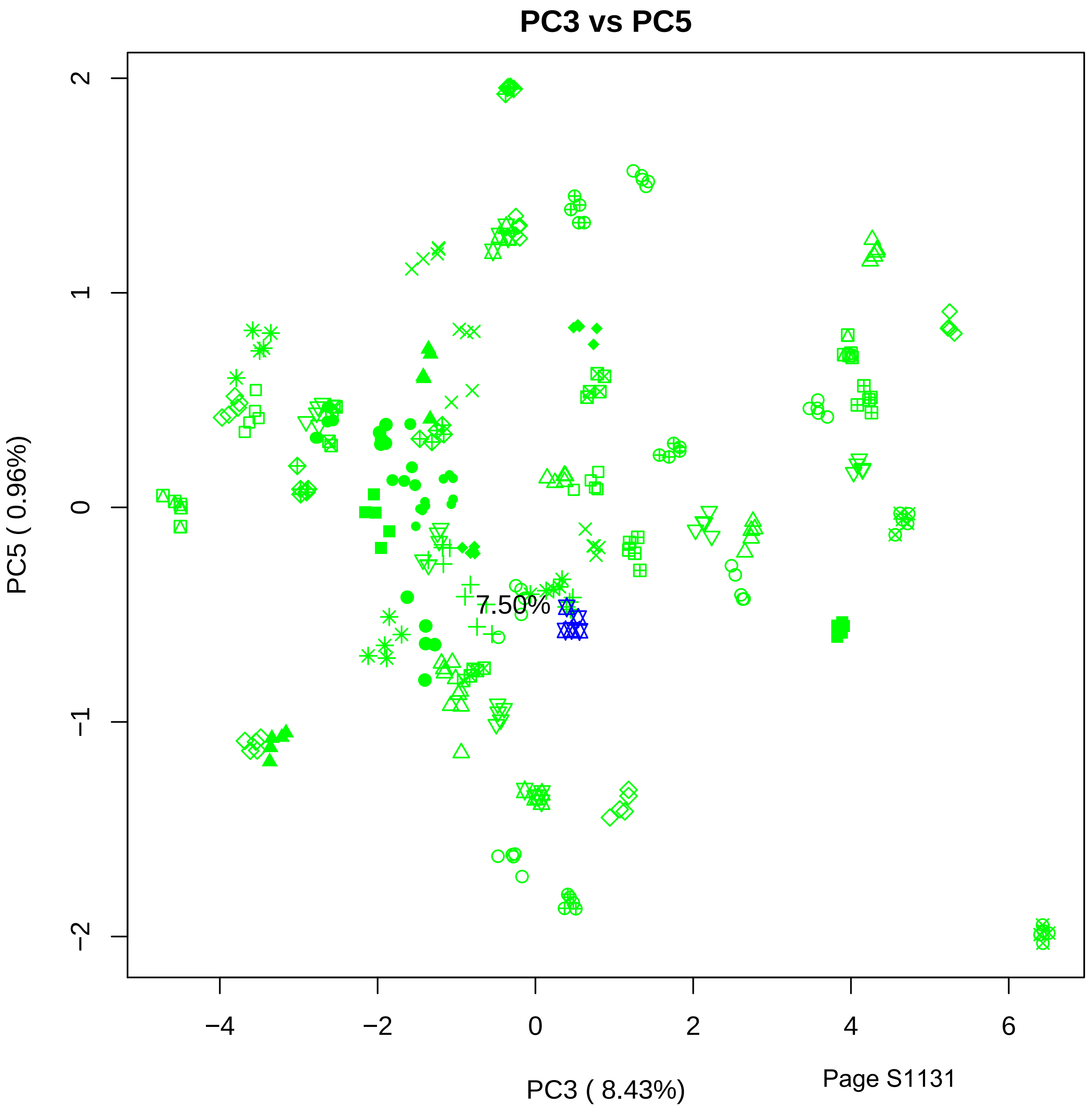
PC3 vs PC5
2
1
0
−1
−2
PC5 (0.96%)
−4
−2
0
2
4
6
PC3 (8.43%)
7.50%

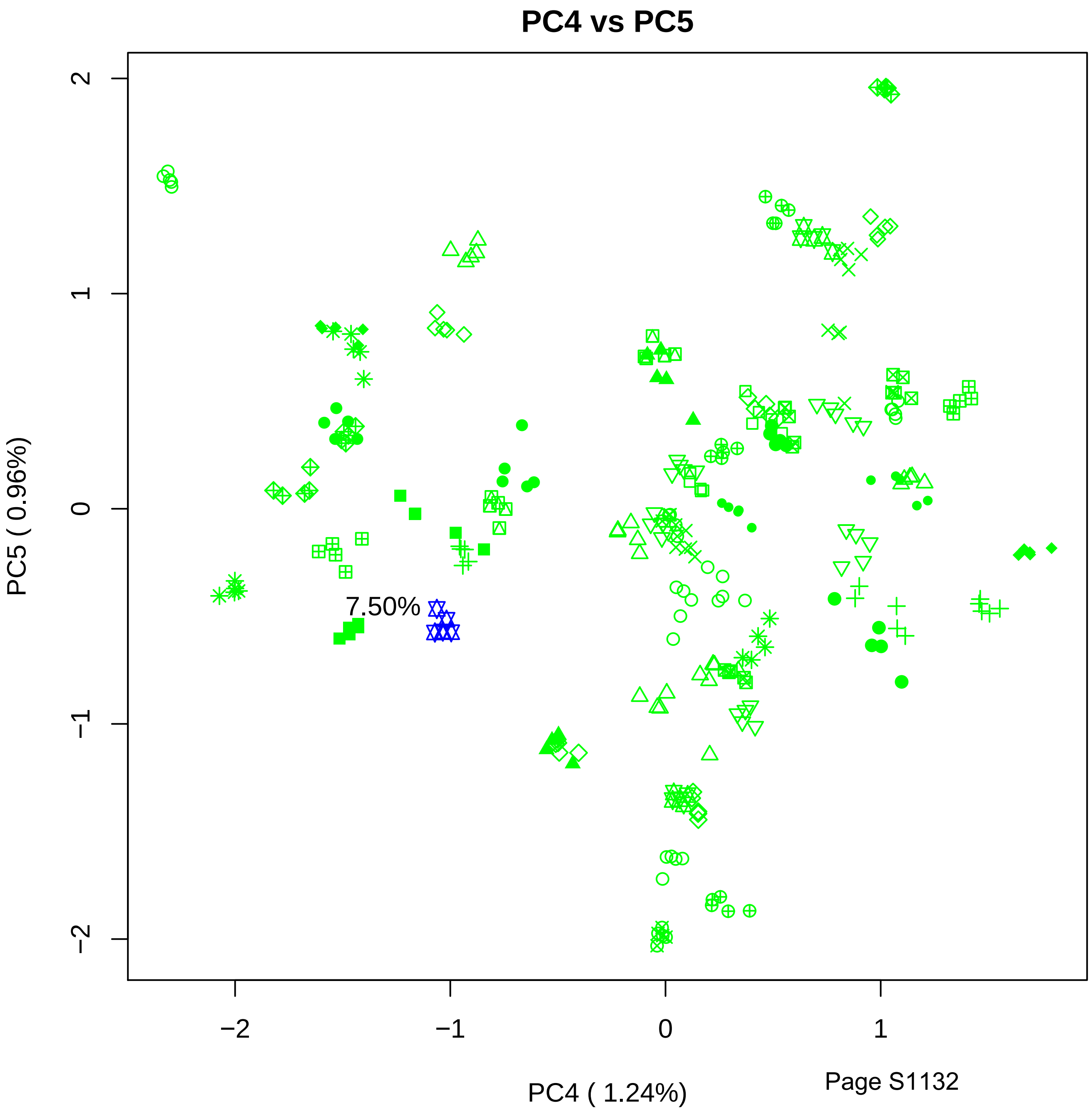

PC4 vs PC5
PC5 (0.96%)
PC4 (1.24%)
7.50%
2
1
0
−1
−2
−2
−1
0
1

PCA of 10% BMH(b) vs PMH(b)

Scaled Matrix PCAed

7th order polynomial baseline correction performed

Raw spectra smoothed to 21 Neighbours, 2nd order PN, Savtisky Golay

Normalisation between 0 and 100

D2 spectra smoothed to 41 Neighbours, 2nd order PN, Savtisky Golay

Regions Analysed: 3601−2501 and 2001−700cm−1

Samples Analysed: 1 bContam's 0 BLH's 0 BMH's 0 OMH's 0 pContam's 0 PLH's 60 PMH's

Keele University AD

Fri Dec 14 14:21:41 2018

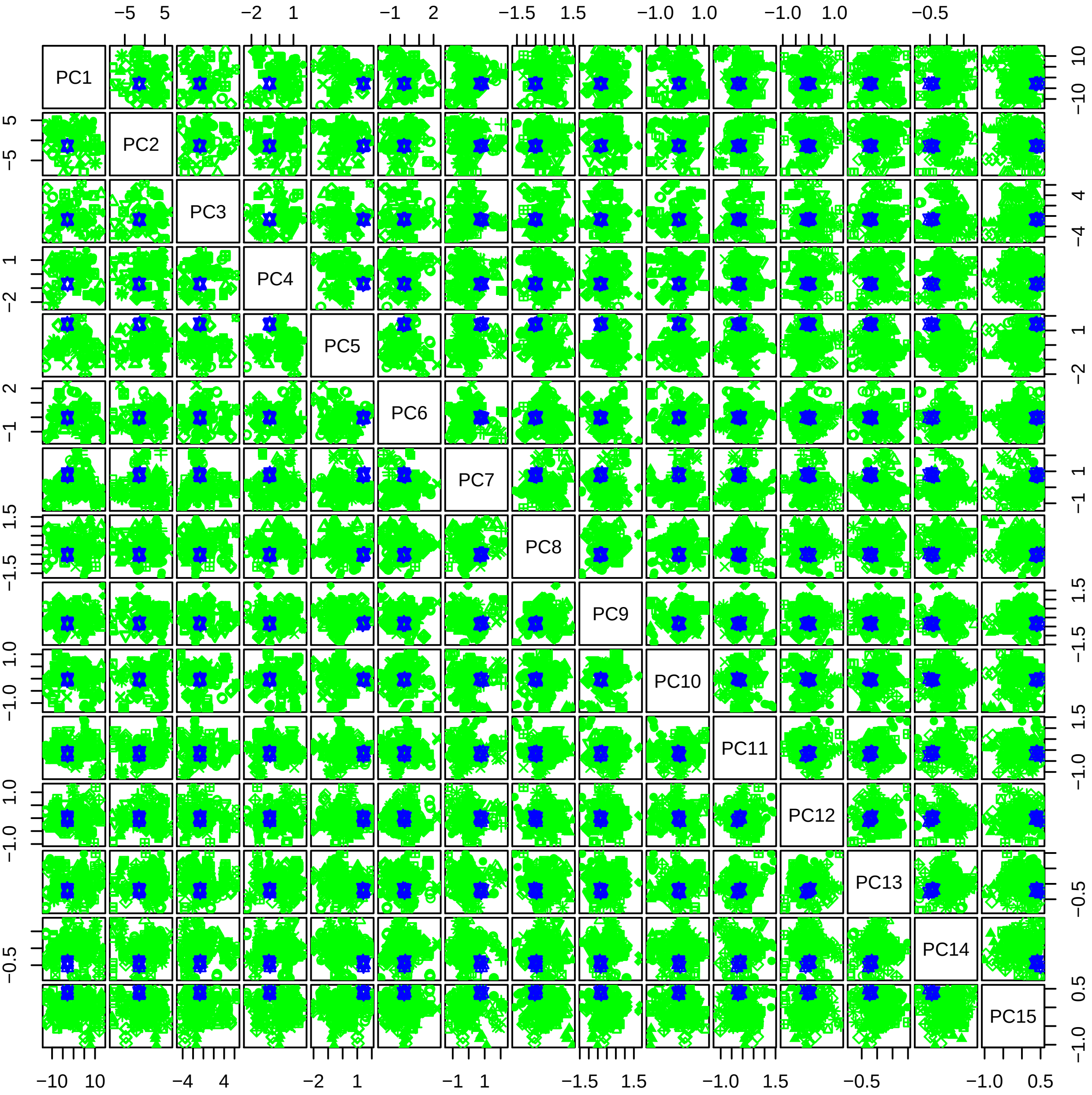
PC1
PC2
PC3
PC4
PC5
PC6
PC7
PC8
PC9
PC10
PC11
PC12
PC13
PC14
PC15

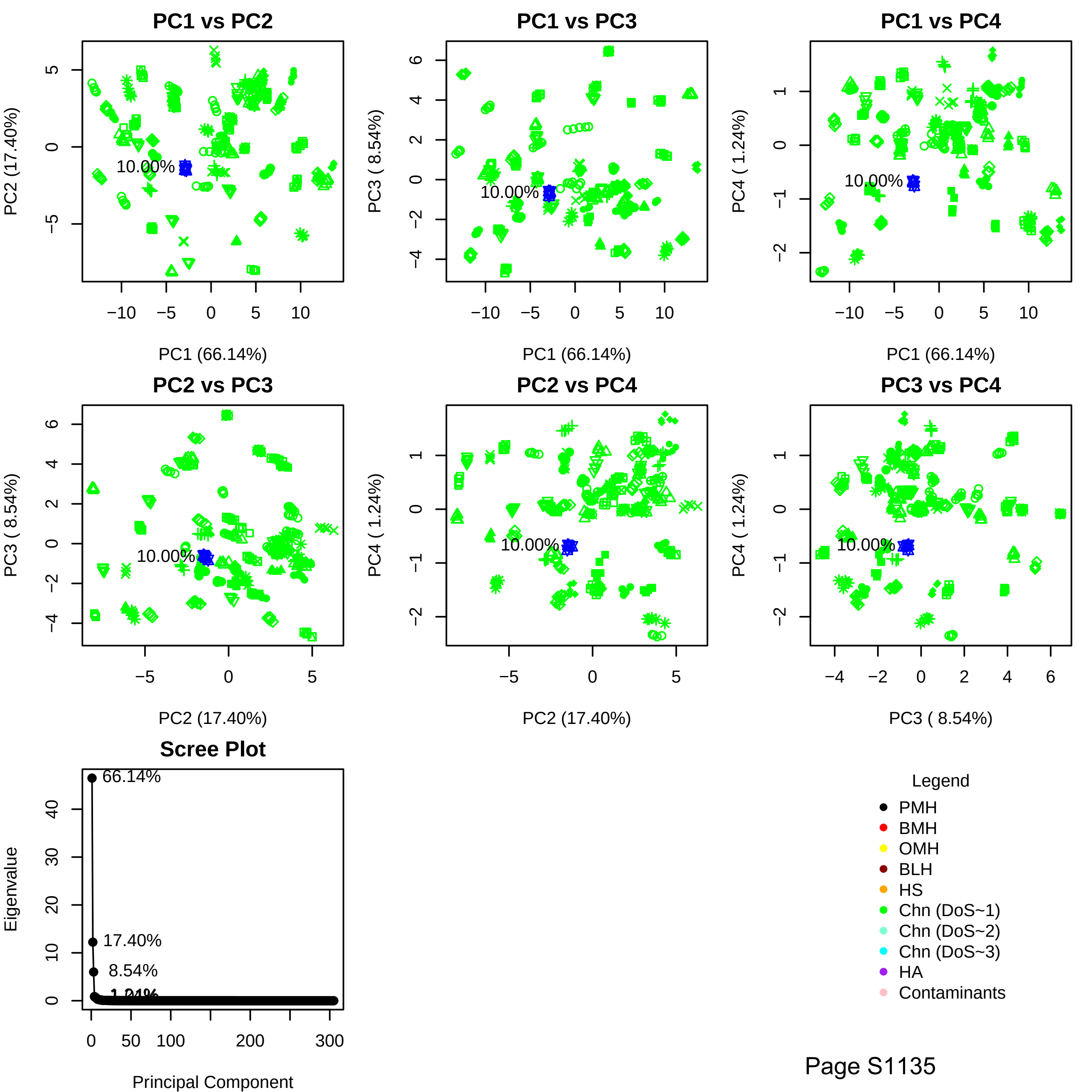
PC1 vs PC2
PC1 vs PC3
PC1 vs PC4
PC2 vs PC3
PC2 vs PC4
PC3 vs PC4
PC1 (66.14%)
PC2 (17.40%)
PC3 (8.54%)
PC4 (1.24%)
10.00%
Scree Plot
Eigenvalue
Principal Component
66.14%
17.40%
8.54%
Legend
PMH
BMH
OMH
BLH
HS
Chn (DoS~1)
Chn (DoS~2)
Chn (DoS~3)
HA
Contaminants

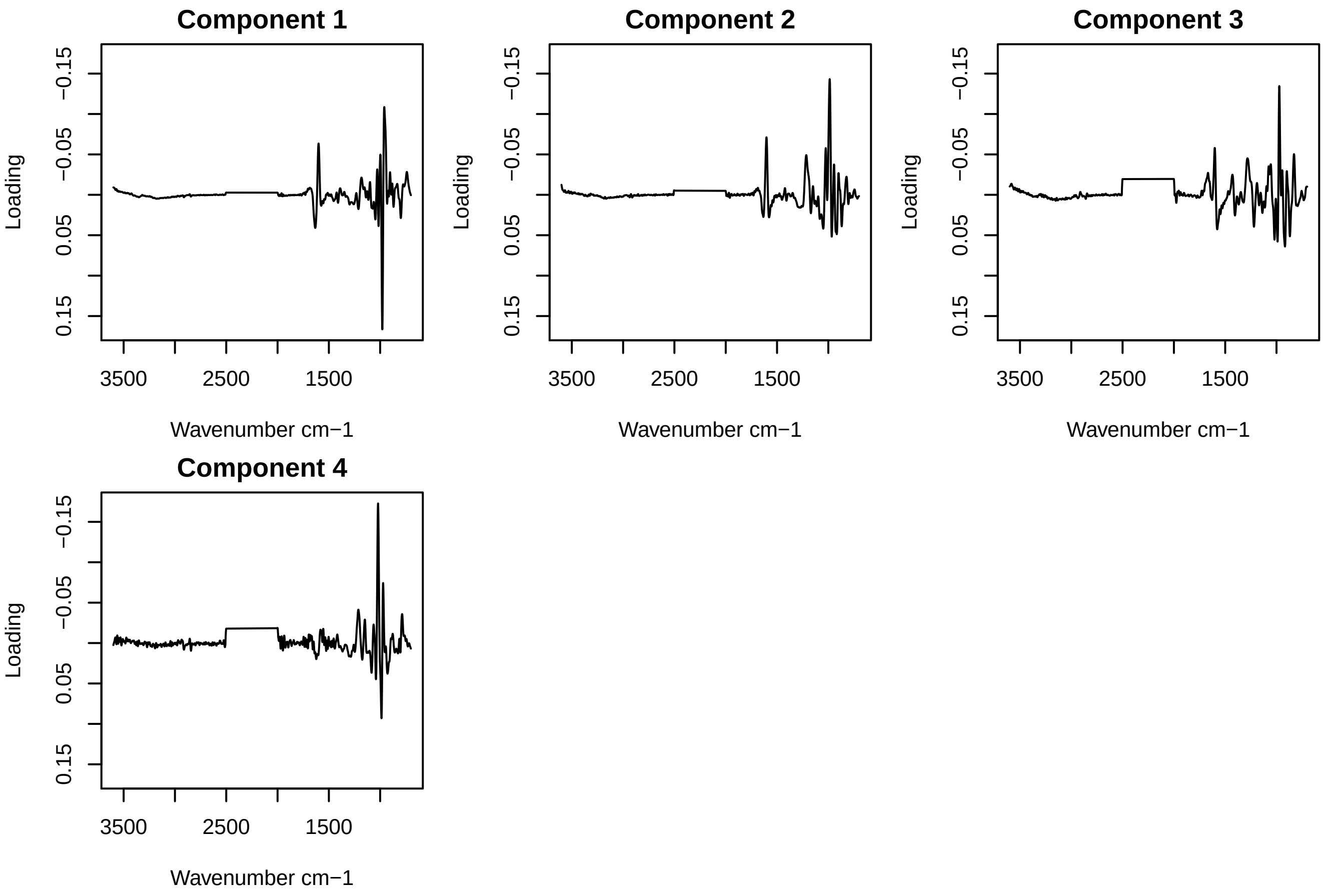

Component 1
Component 2
Component 3
Component 4
Loading
−0.15
−0.05
0.05
0.15
3500
2500
1500
Wavenumber cm−1

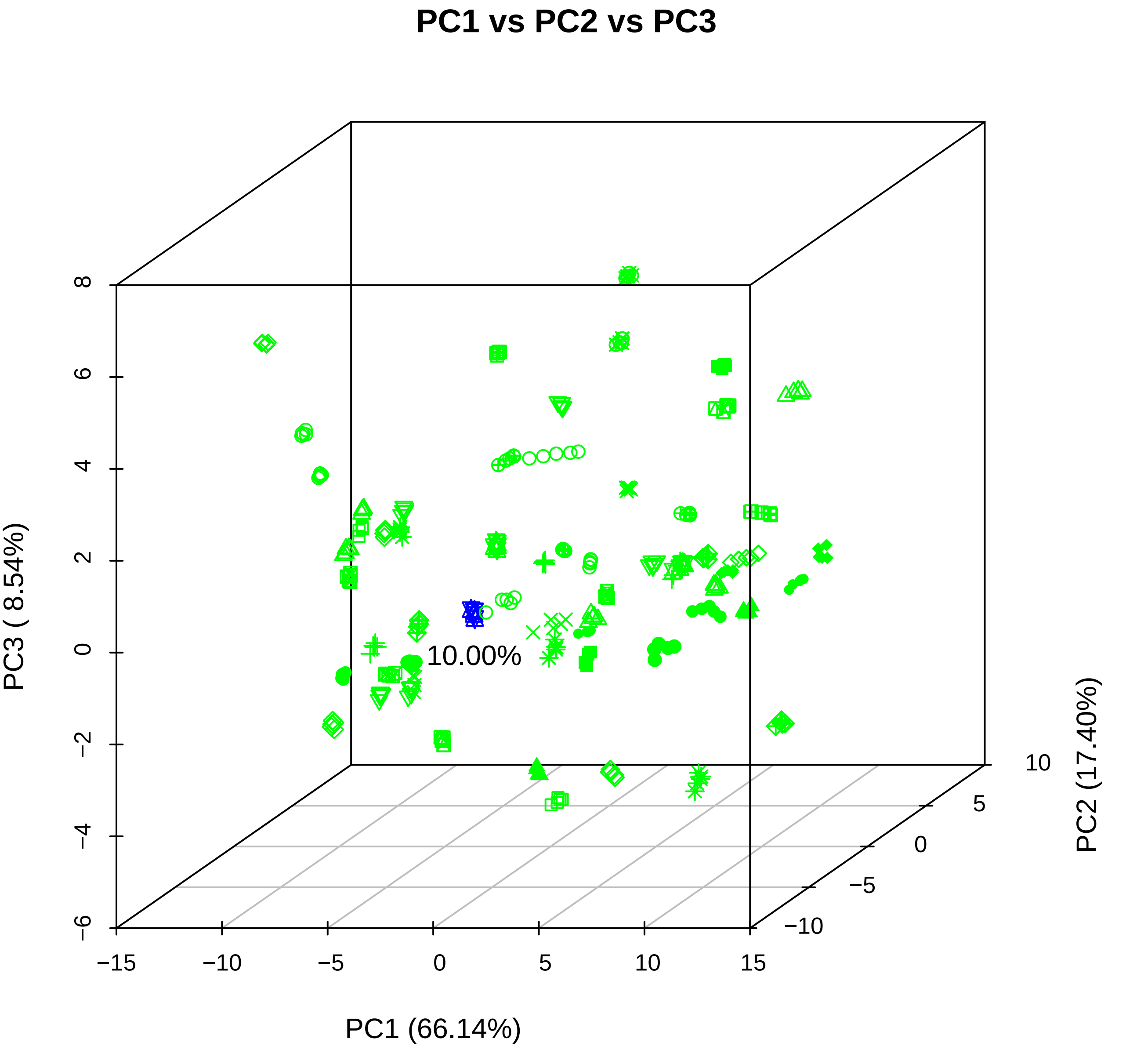
PC1 vs PC2 vs PC3
PC3 (8.54%)
8
6
4
2
0
−2
−4
−6
10.00%
−15
−10
−5
0
5
10
15
PC1 (66.14%)
PC2 (17.40%)
10
5
0
−5
−10

PC1 vs PC2 vs PC4

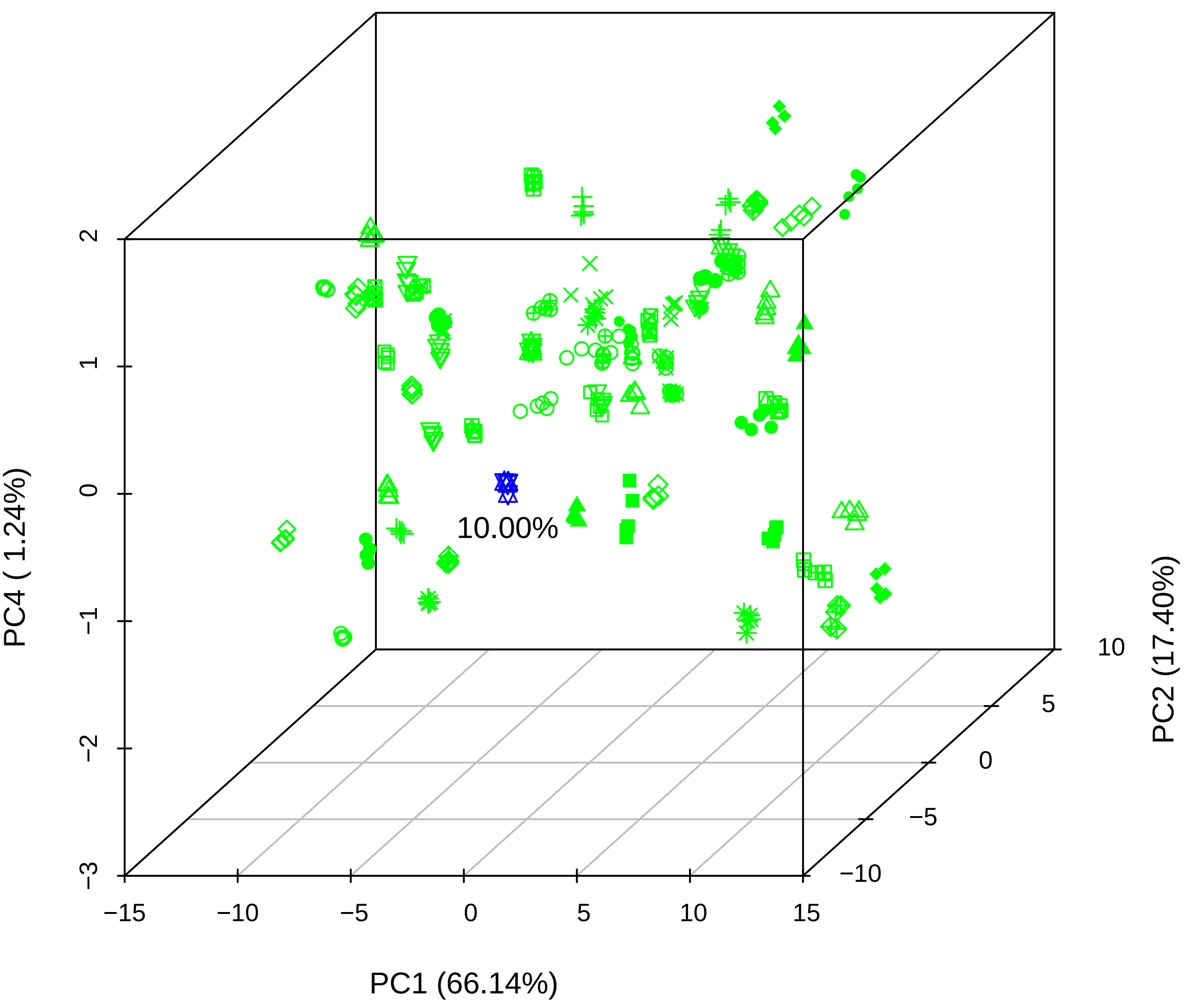

PC1 vs PC3 vs PC4

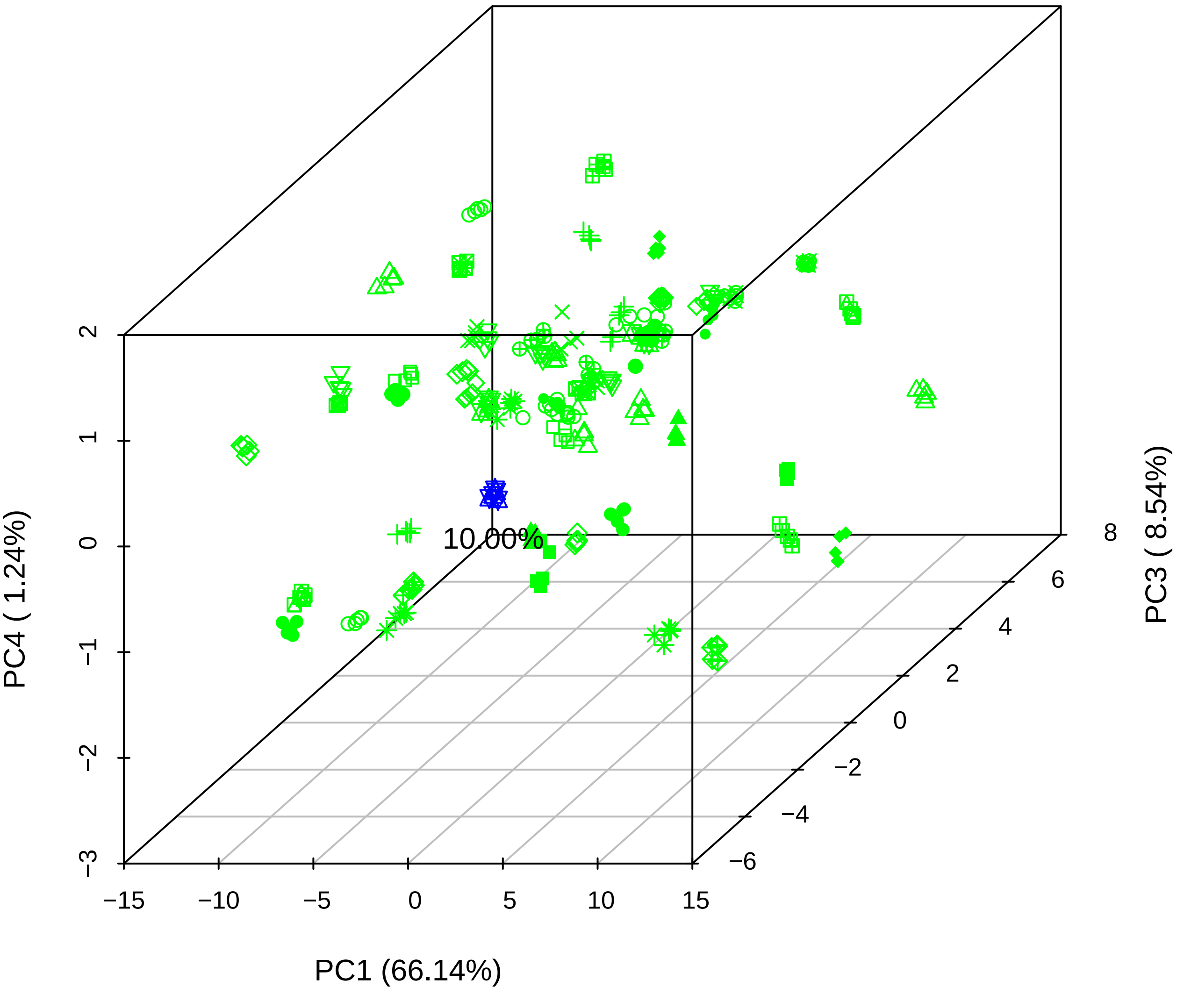

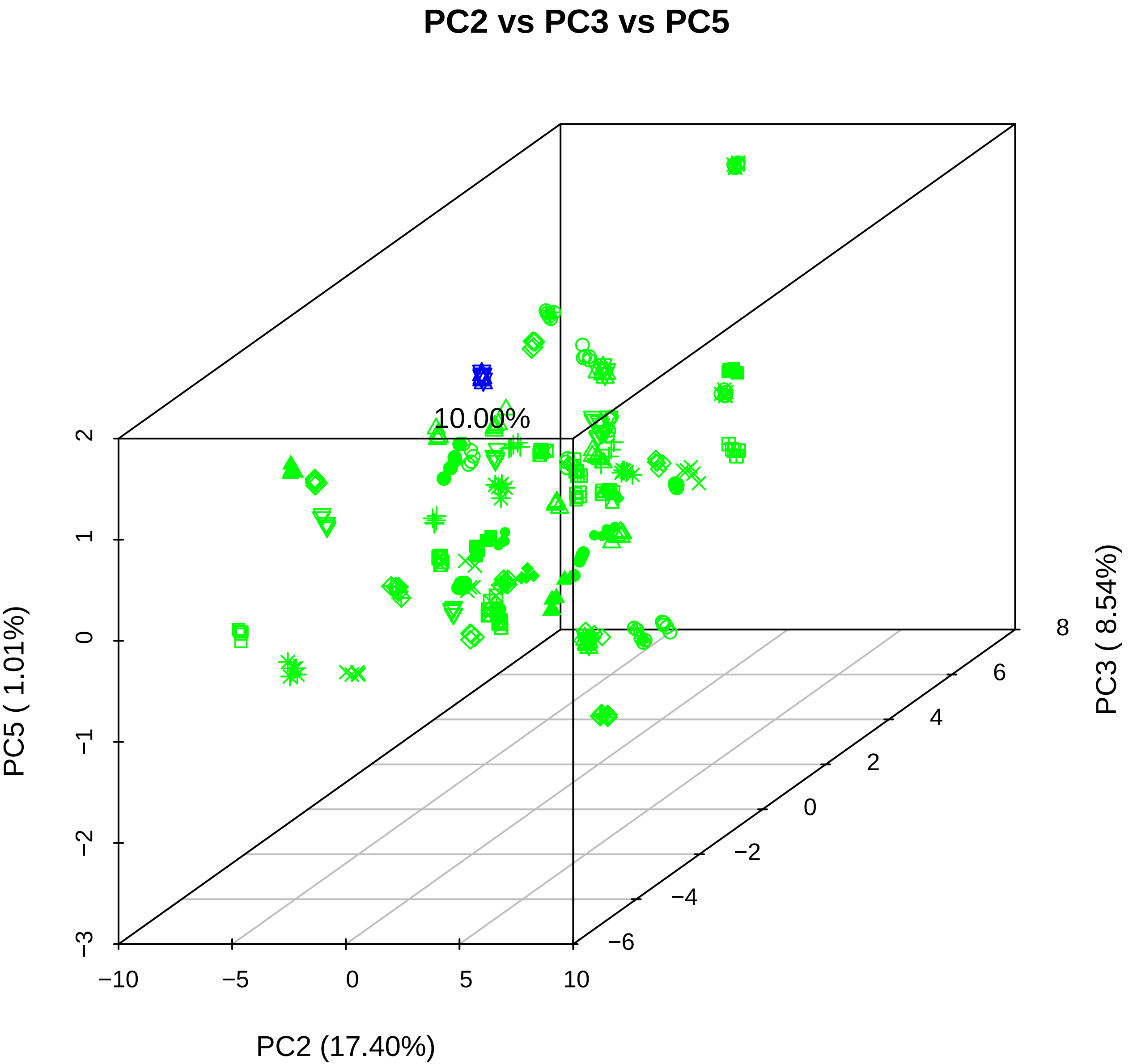

PC2 vs PC3 vs PC5
10.00%
PC2 (17.40%)
PC5 (1.01%)
PC3 (8.54%)
−10
−5
0
5
10
2
1
0
−1
−2
−3
8
6
4
2
0
−2
−4
−6

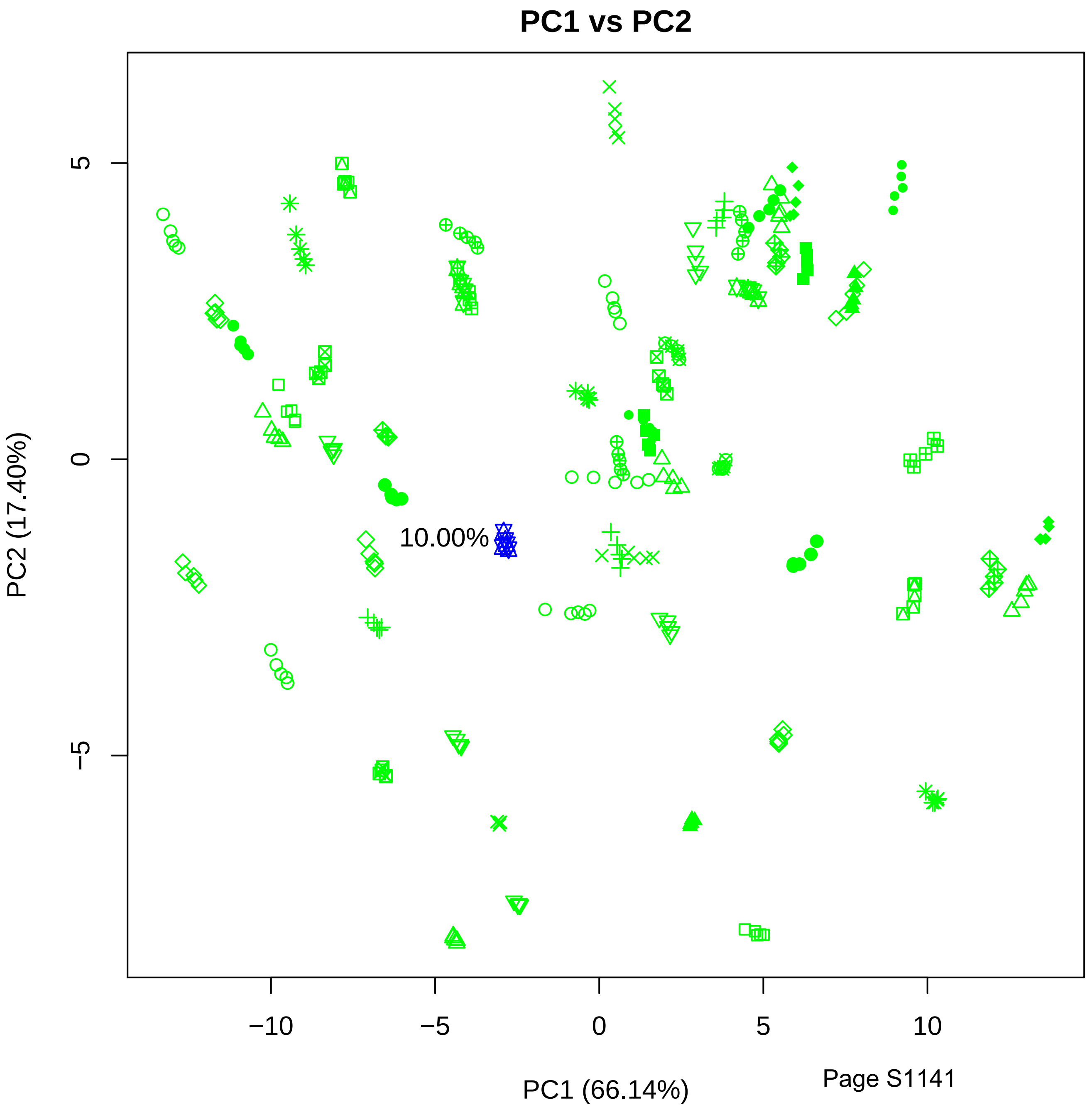
PC1 vs PC2
10.00%
PC2 (17.40%)
PC1 (66.14%)
5
0
−5
−10
−5
0
5
10

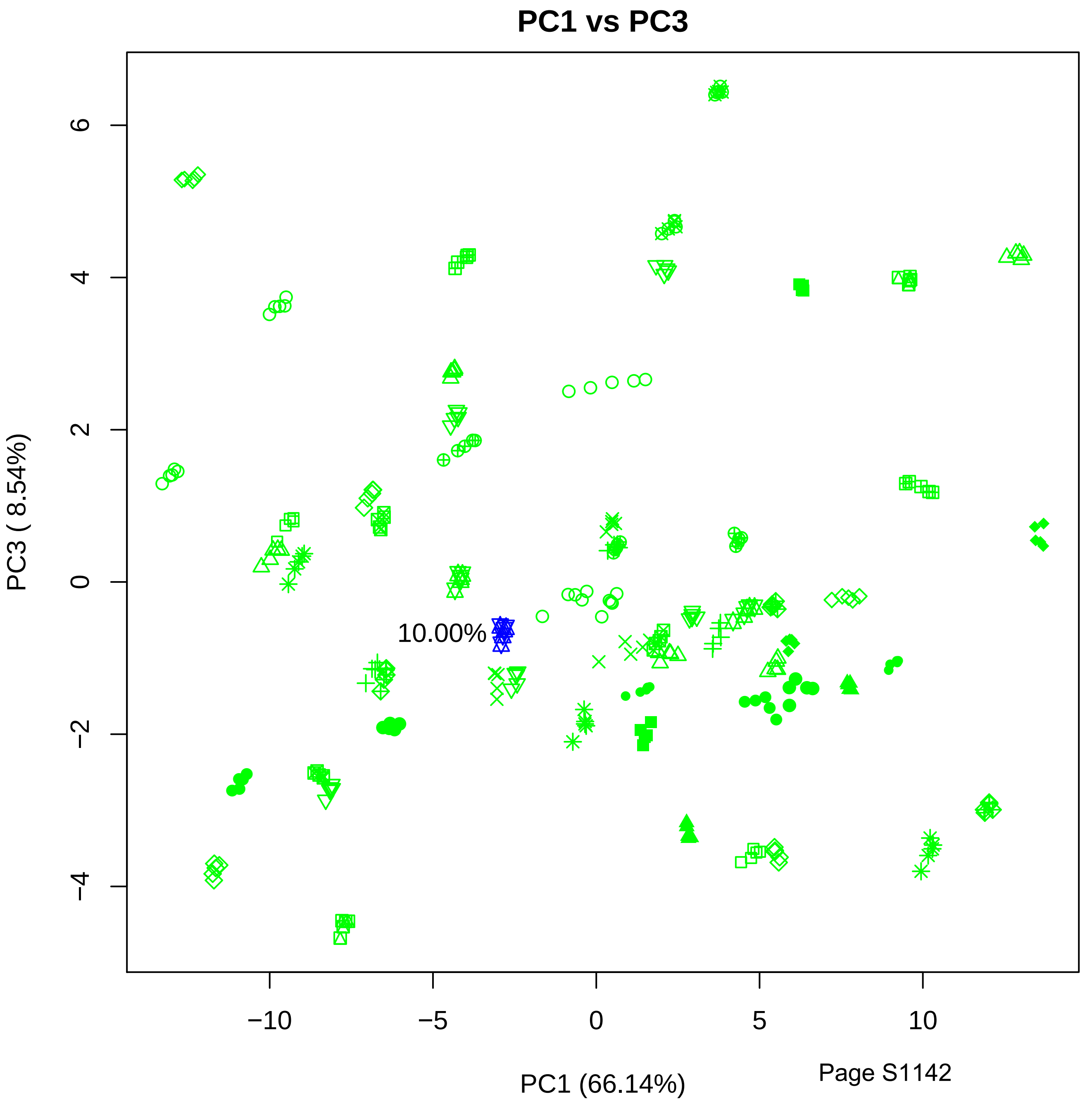

PC1 vs PC3
PC3 (8.54%)
PC1 (66.14%)
10.00%
6
4
2
0
−2
−4
−10
−5
0
5
10

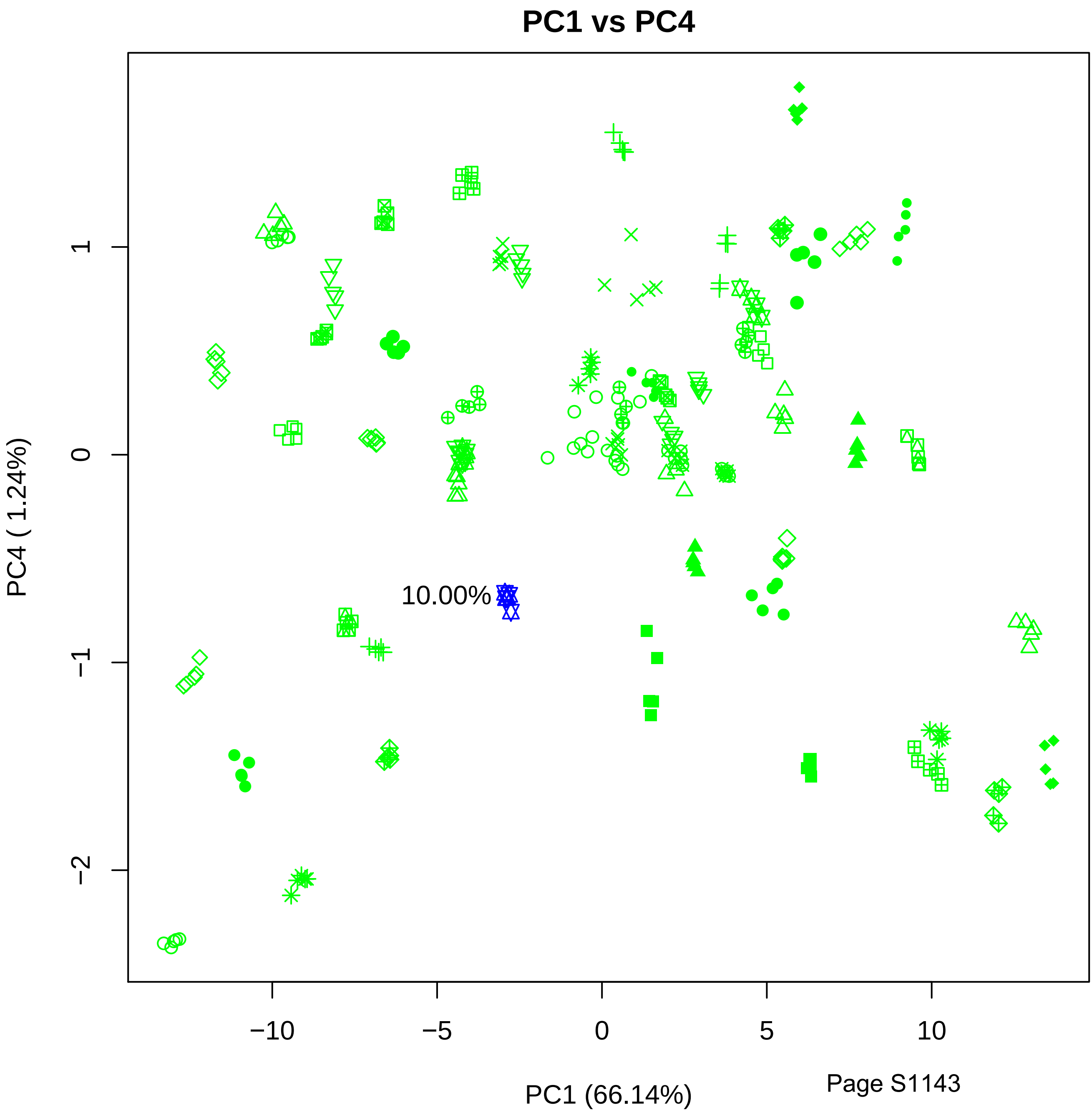

PC1 vs PC4
PC4 (1.24%)
PC1 (66.14%)
10.00%
−10
−5
0
5
10
−2
−1
0
1

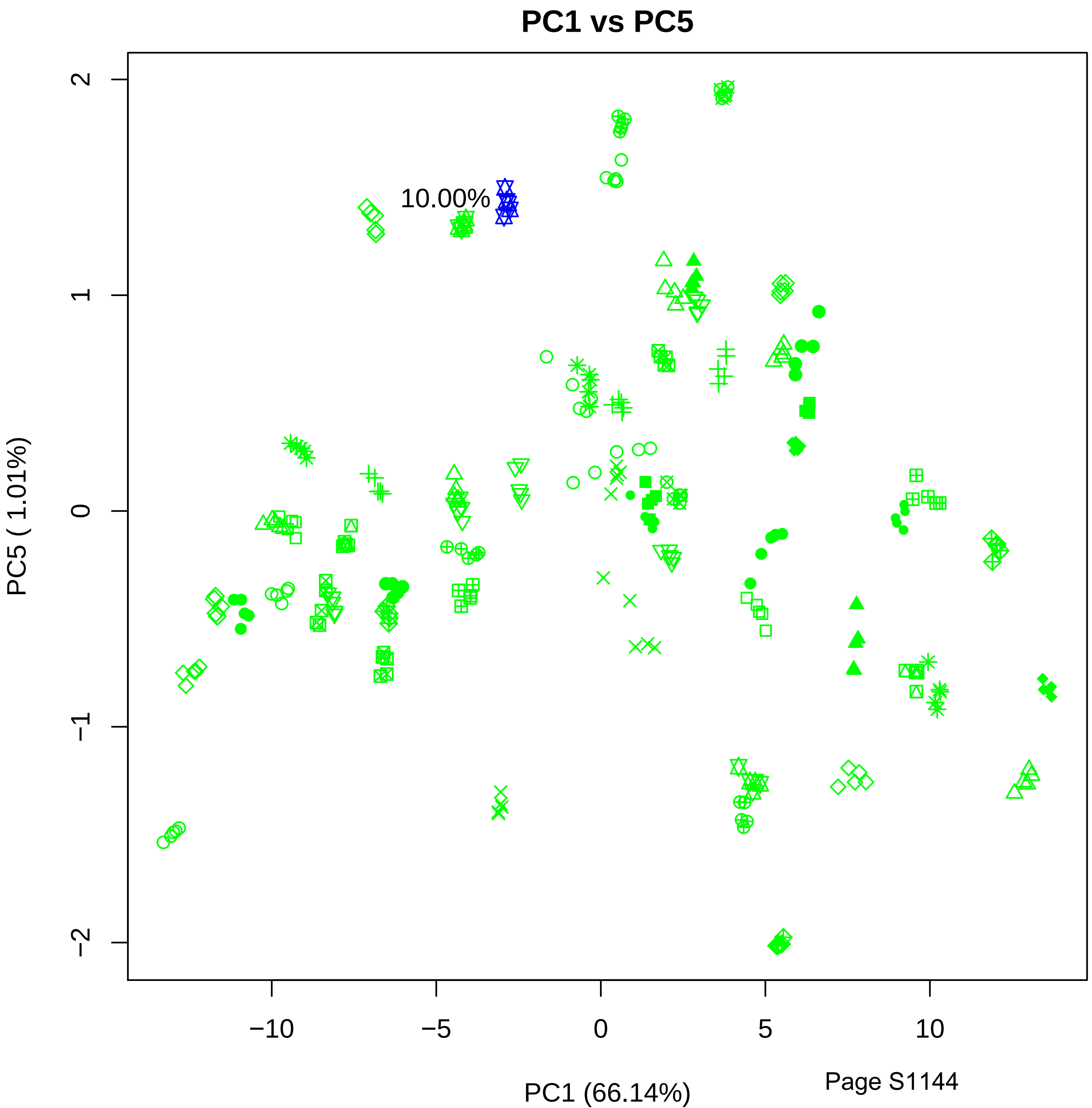
PC1 vs PC5
10.00%
PC5 (1.01%)
PC1 (66.14%)
2
1
0
−1
−2
−10
−5
0
5
10

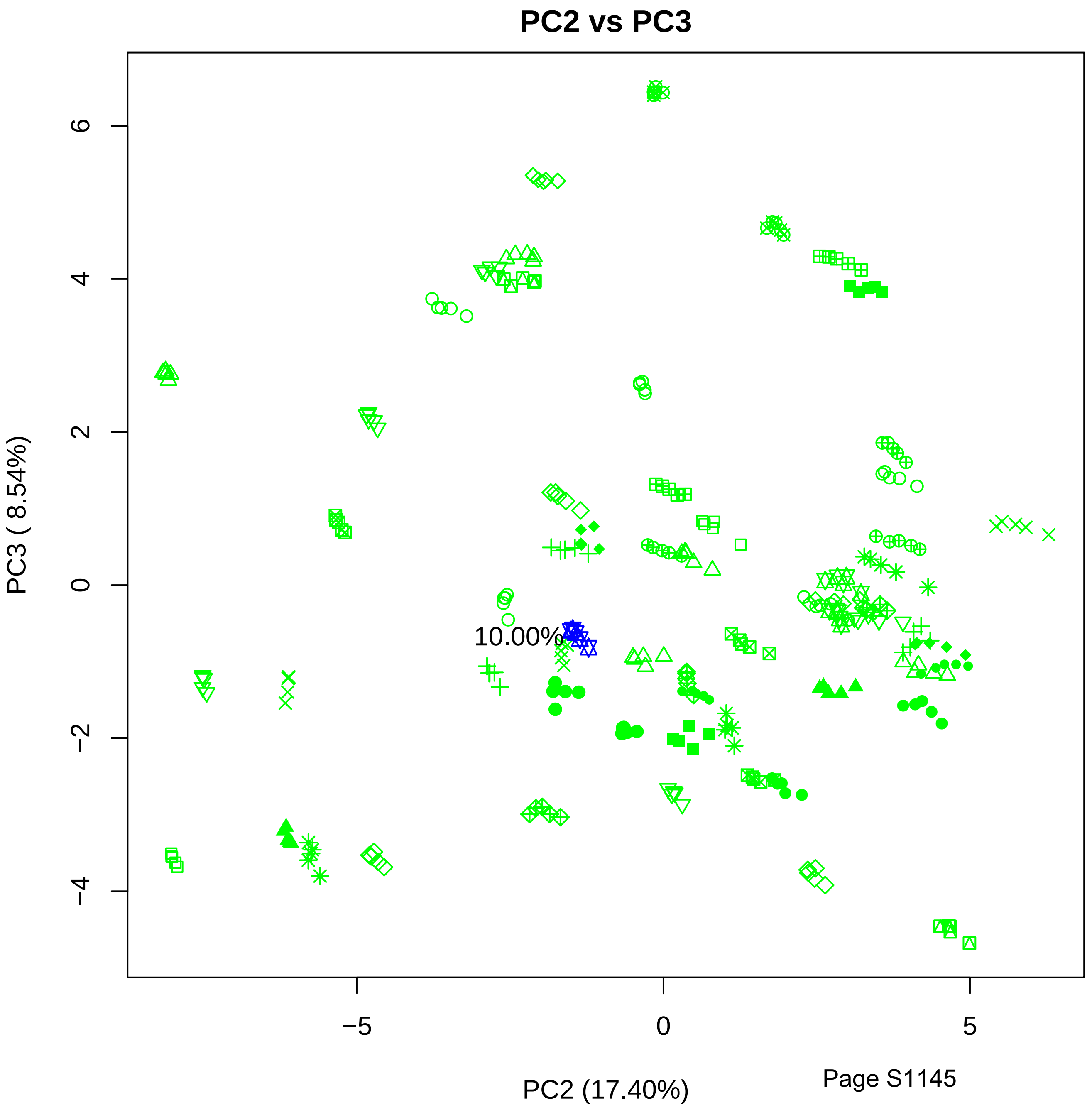

PC2 vs PC3
PC3 (8.54%)
PC2 (17.40%)
10.00%
6
4
2
0
−2
−4
−5
0
5

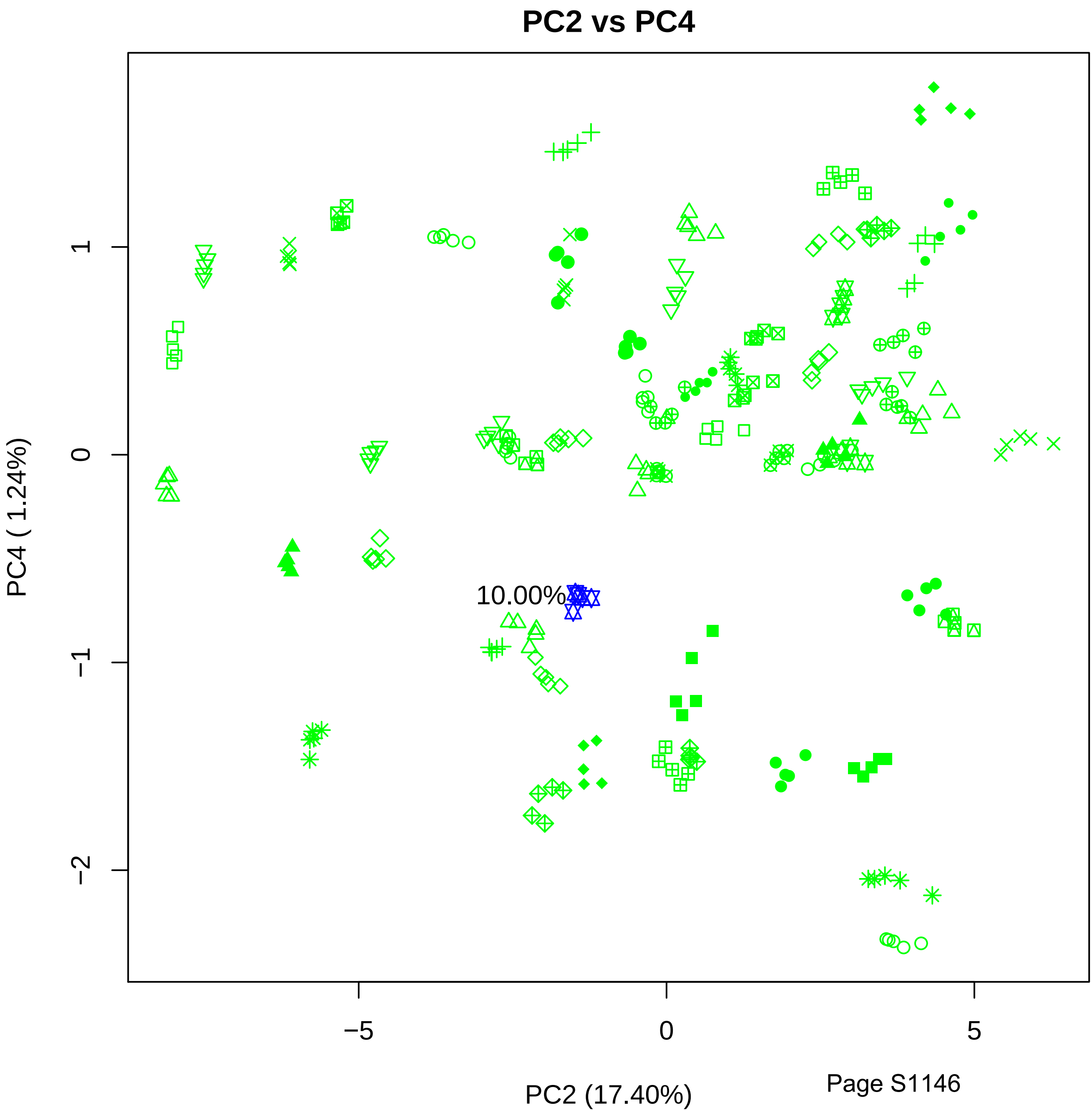

PC2 vs PC4
PC4 (1.24%)
PC2 (17.40%)
10.00%
1
0
−1
−2
−5
0
5

PC2 vs PC5

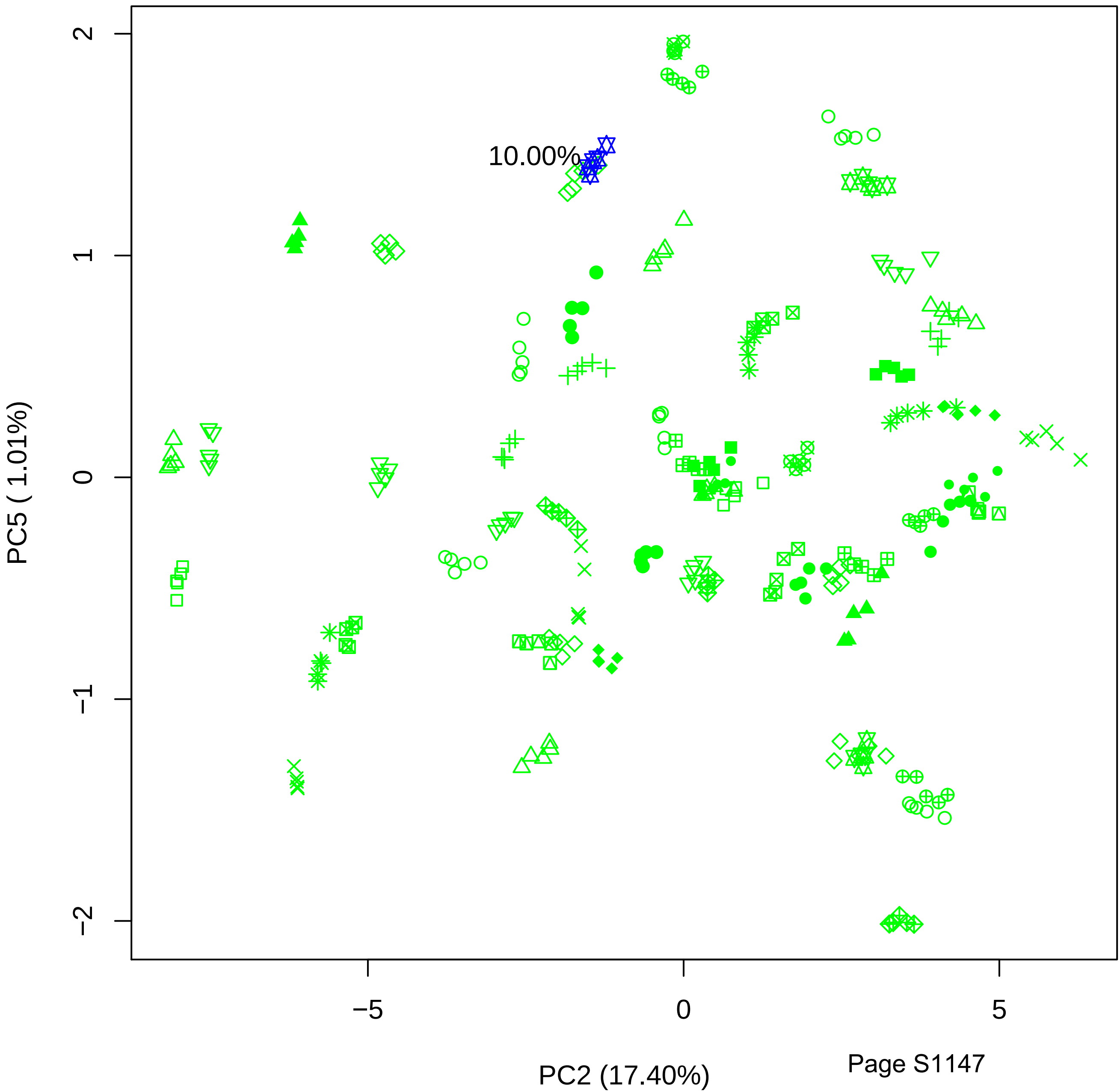

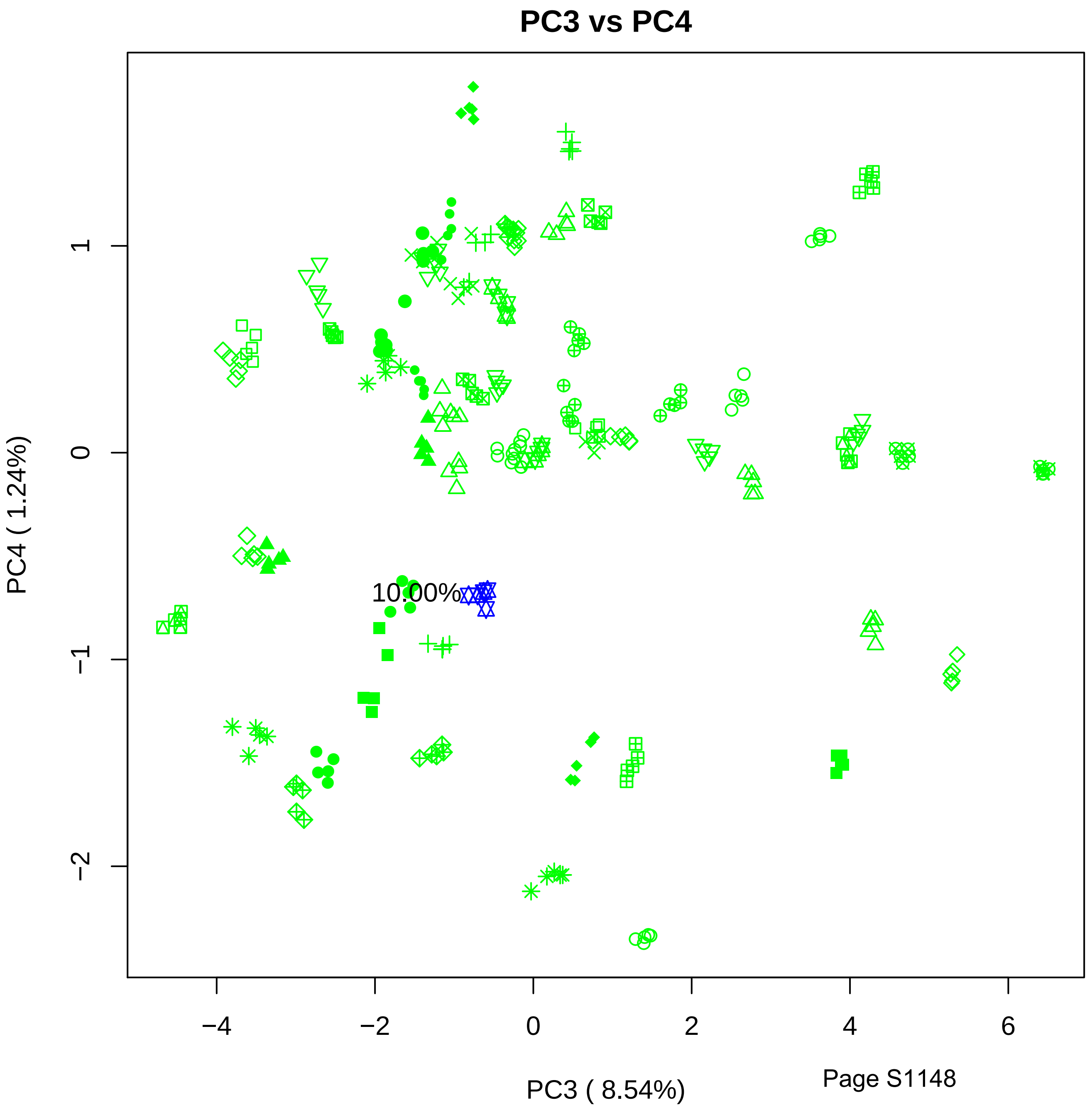

PC3 vs PC4
PC4 (1.24%)
PC3 (8.54%)
10.00%
1
0
−1
−2
−4
−2
0
2
4
6

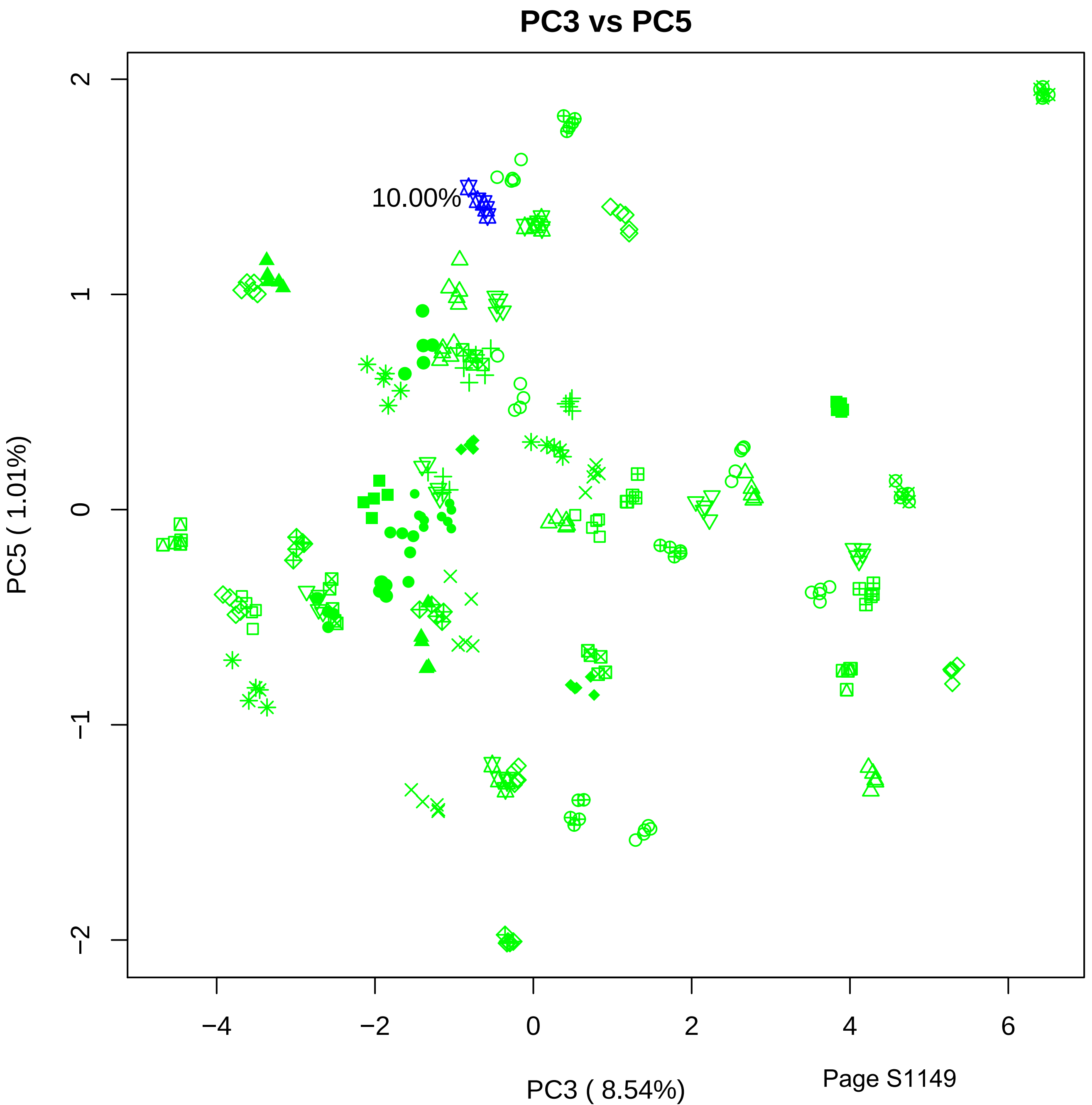
PC3 vs PC5
10.00%
PC5 (1.01%)
PC3 (8.54%)
2
1
0
−1
−2
−4
−2
0
2
4
6

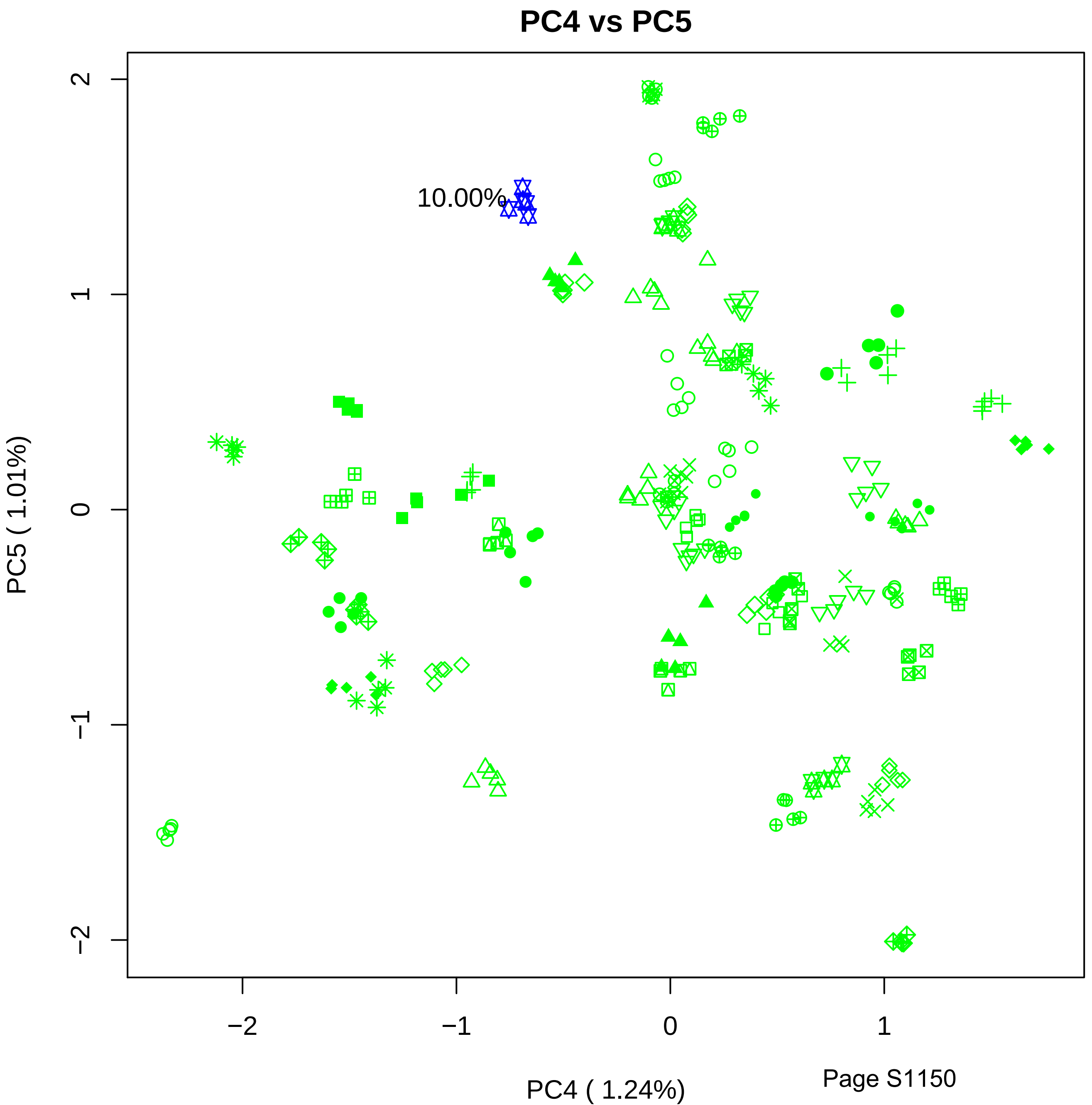

PC4 vs PC5
10.00%
PC5 (1.01%)
PC4 (1.24%)
2
1
0
−1
−2
−2
−1
0
1

PCA of 15% BMH(b) vs PMH(b)

Scaled Matrix PCAed

7th order polynomial baseline correction performed

Raw spectra smoothed to 21 Neighbours, 2nd order PN, Savtisky Golay

Normalisation between 0 and 100

D2 spectra smoothed to 41 Neighbours, 2nd order PN, Savtisky Golay

Regions Analysed: 3601−2501 and 2001−700cm−1

Samples Analysed: 1 bContam's 0 BLH's 0 BMH's 0 OMH's 0 pContam's 0 PLH's 60 PMH's

Keele University AD Fri Dec 14 14:21:49 2018

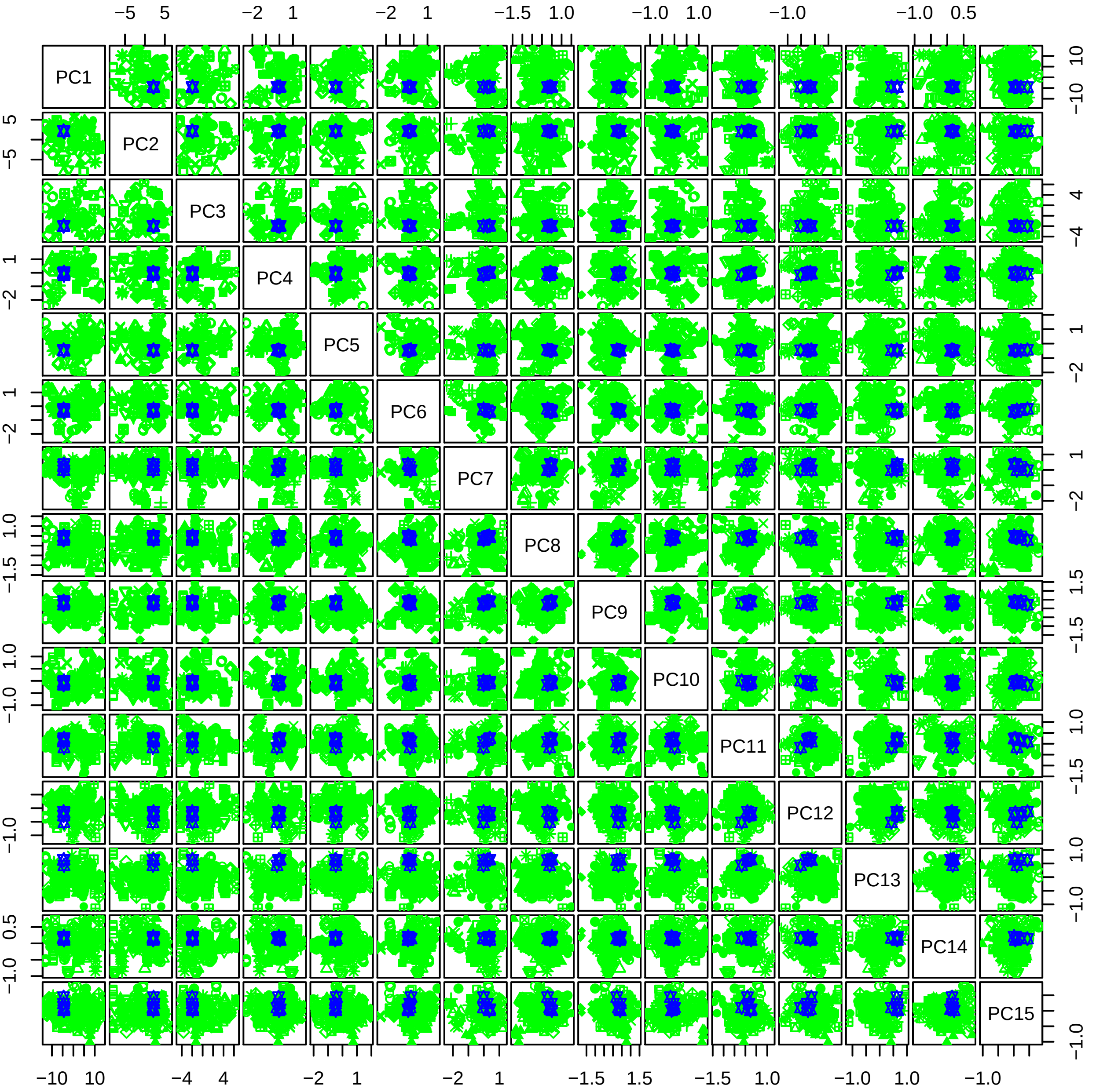
PC1
PC2
PC3
PC4
PC5
PC6
PC7
PC8
PC9
PC10
PC11
PC12
PC13
PC14
PC15

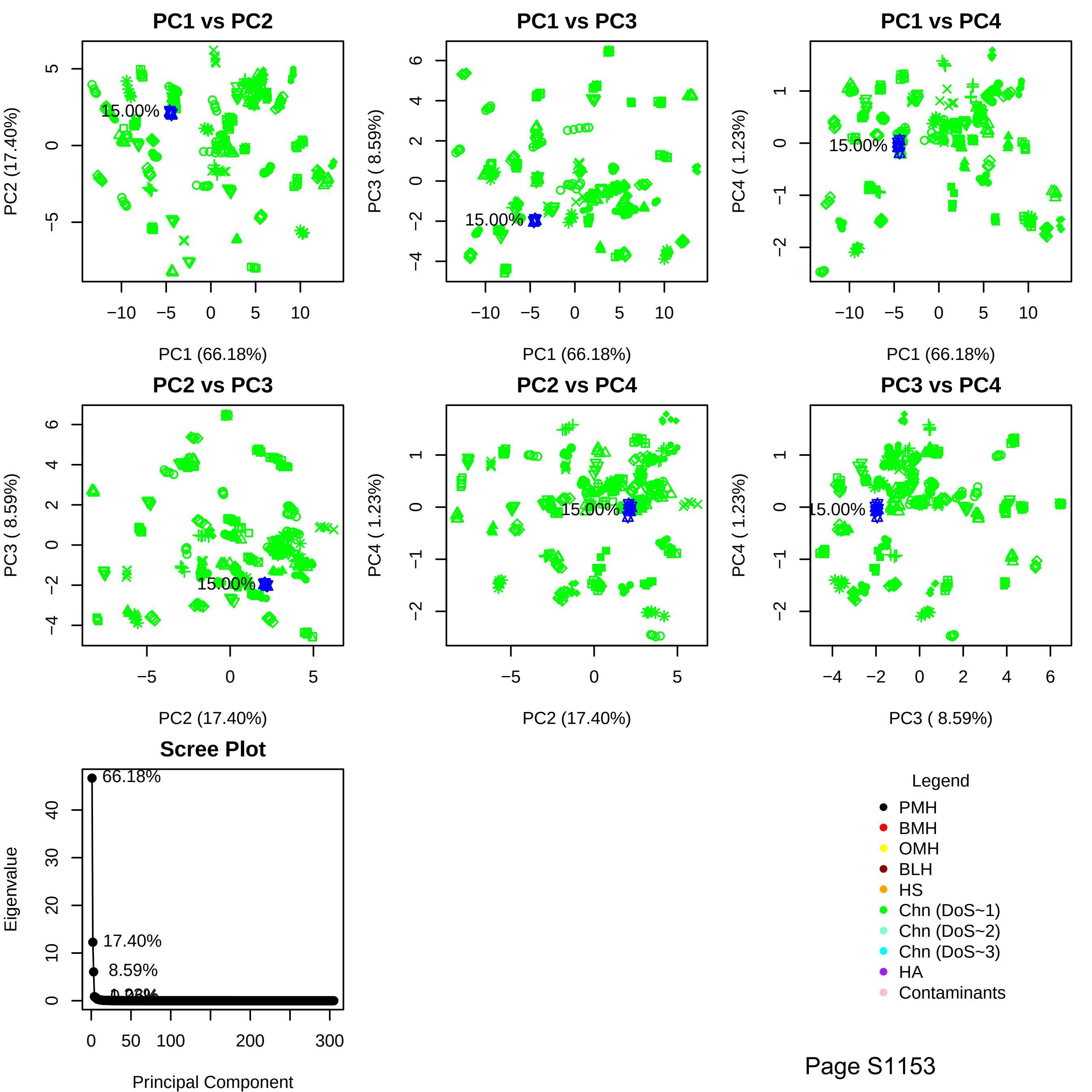
PC1 vs PC2
PC1 vs PC3
PC1 vs PC4
PC2 vs PC3
PC2 vs PC4
PC3 vs PC4
15.00%
PC1 (66.18%)
PC2 (17.40%)
PC3 (8.59%)
PC4 (1.23%)
Scree Plot
66.18%
17.40%
8.59%
Eigenvalue
Principal Component
Legend
PMH
BMH
OMH
BLH
HS
Chn (DoS~1)
Chn (DoS~2)
Chn (DoS~3)
HA
Contaminants

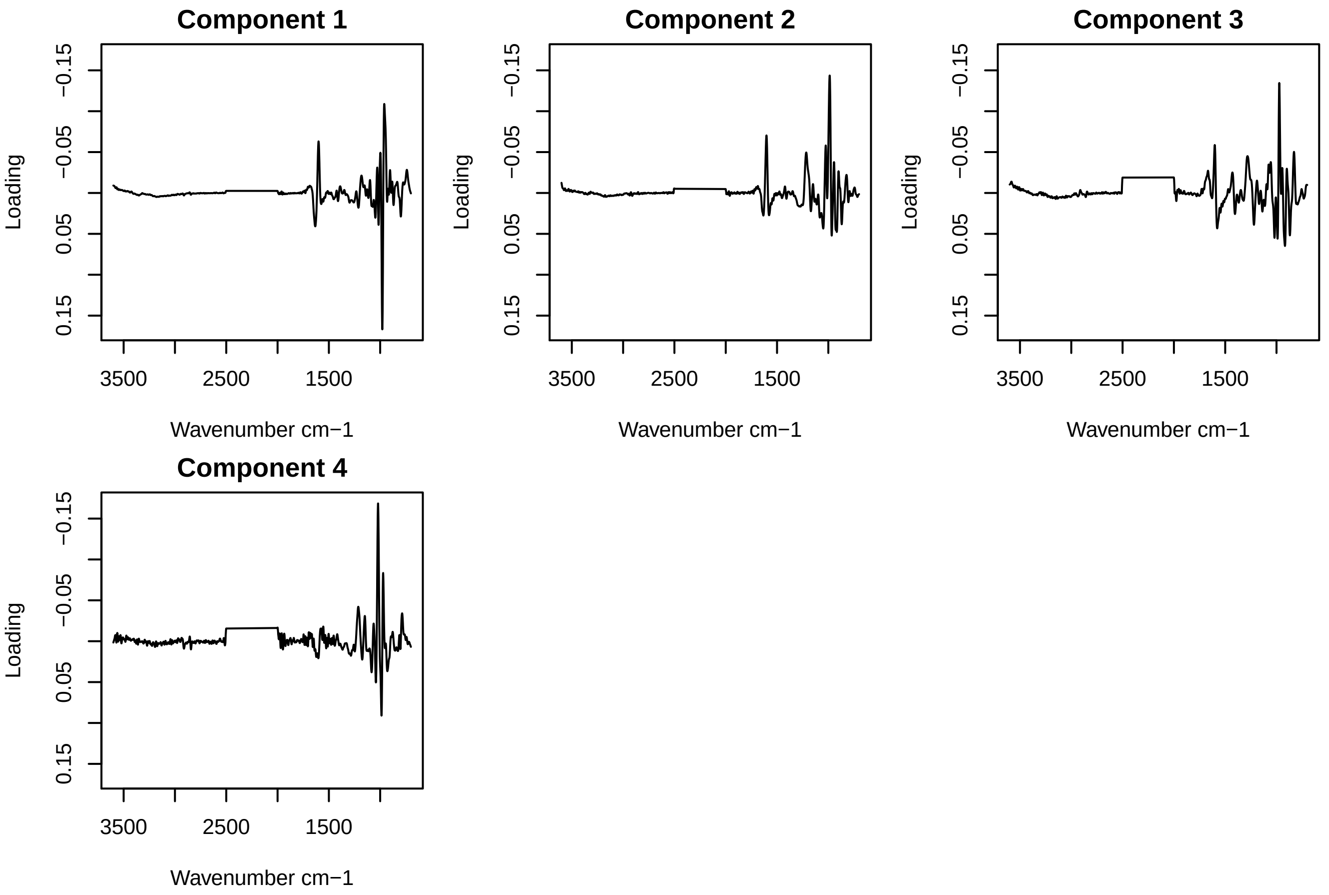
Component 1
Component 2
Component 3
Component 4
Loading
−0.15
−0.05
0.05
0.15
3500
2500
1500
Wavenumber cm−1

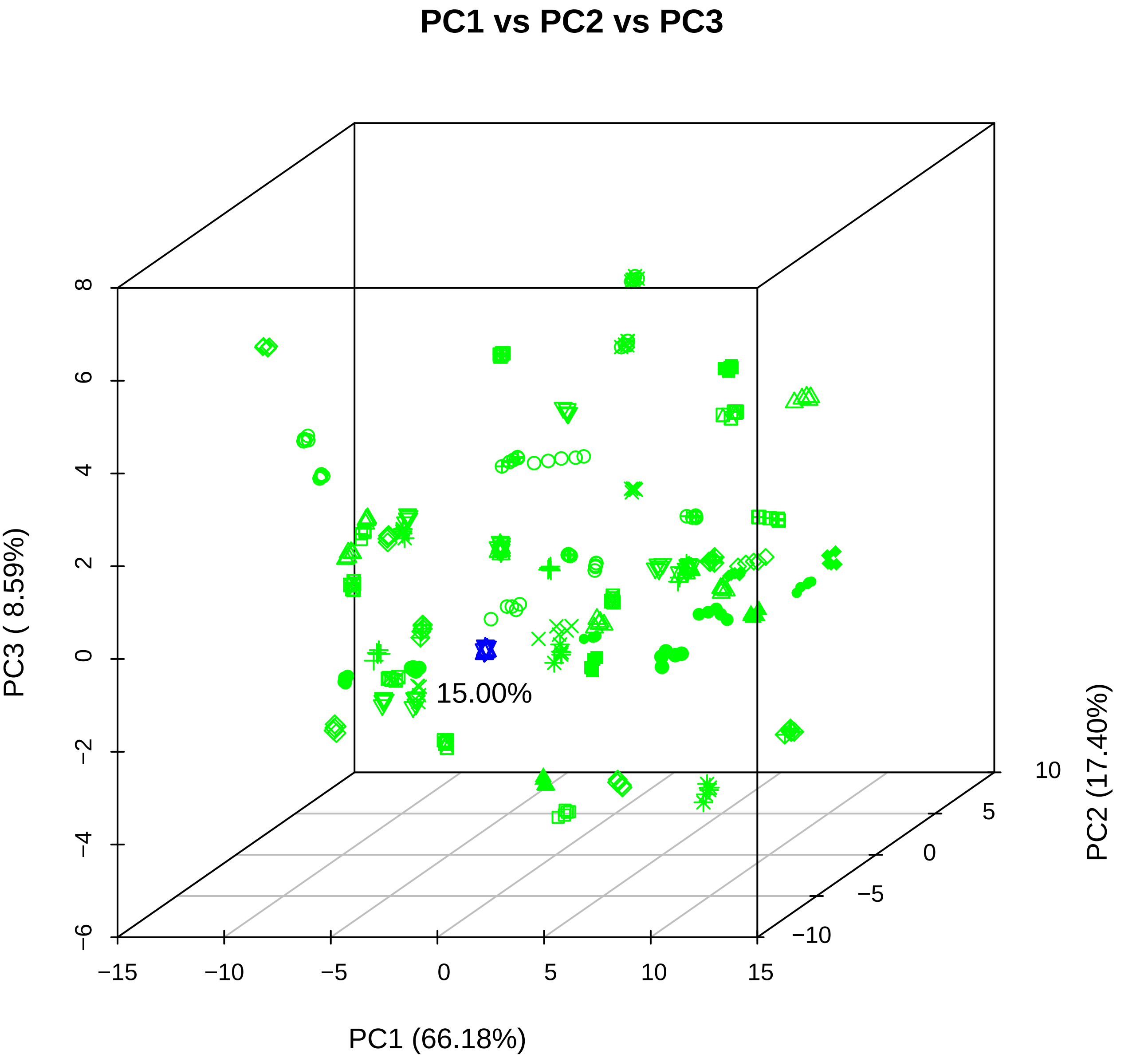

PC1 vs PC2 vs PC3
15.00%
PC1 (66.18%)
PC2 (17.40%)
PC3 (8.59%)
−15
−10
−5
0
5
10
15
−6
−4
−2
0
2
4
6
8
−10
−5
0
5
10

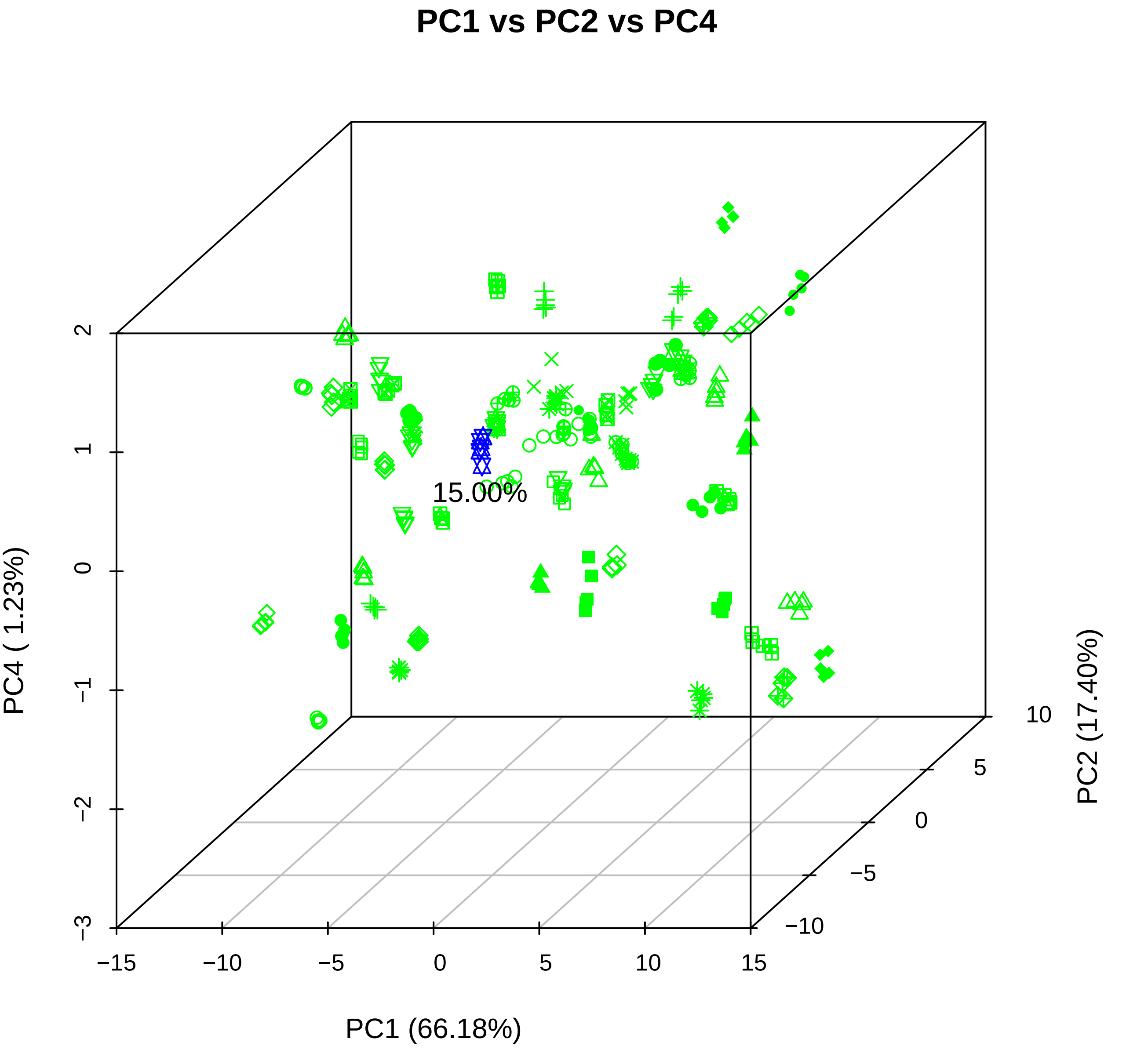

PC1 vs PC2 vs PC4
15.00%
PC1 (66.18%)
PC2 (17.40%)
PC4 (1.23%)
−15
−10
−5
0
5
10
15
−3
−2
−1
0
1
2

PC1 vs PC3 vs PC4

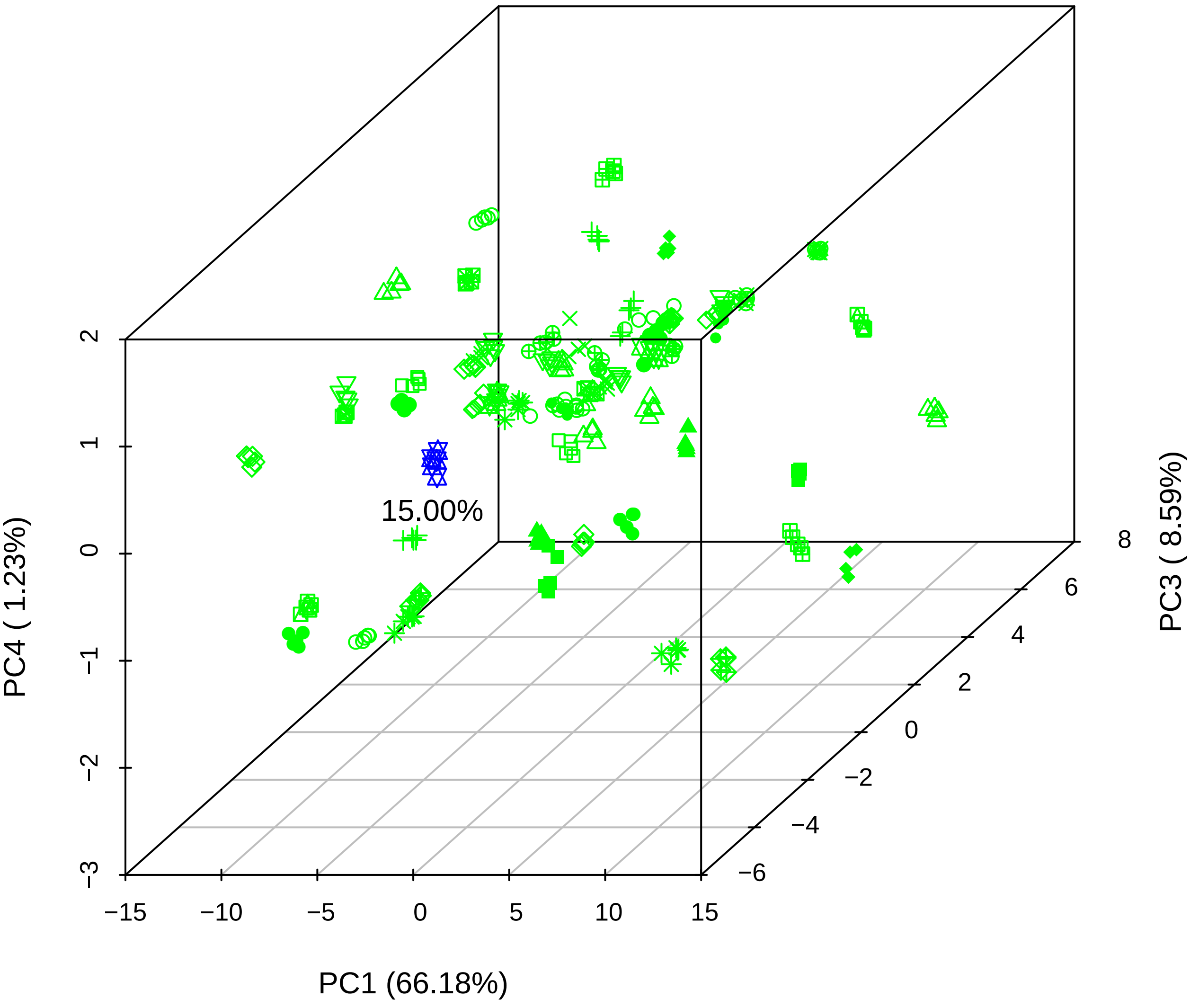

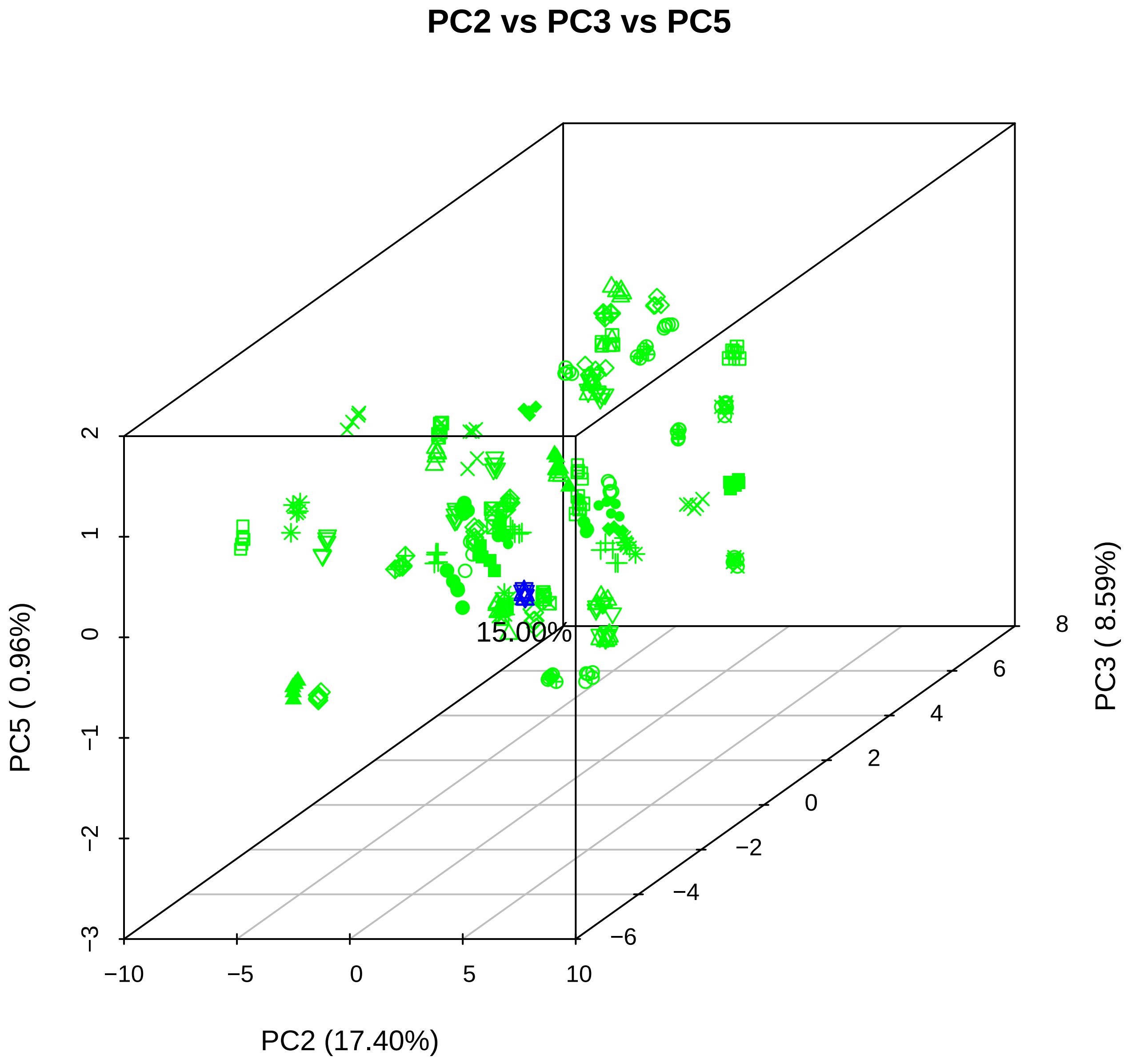

PC2 vs PC3 vs PC5
15.00%
PC5 (0.96%)
PC2 (17.40%)
PC3 (8.59%)
−10
−5
0
5
10
−3
−2
−1
0
1
2
−6
−4
−2
0
2
4
6
8

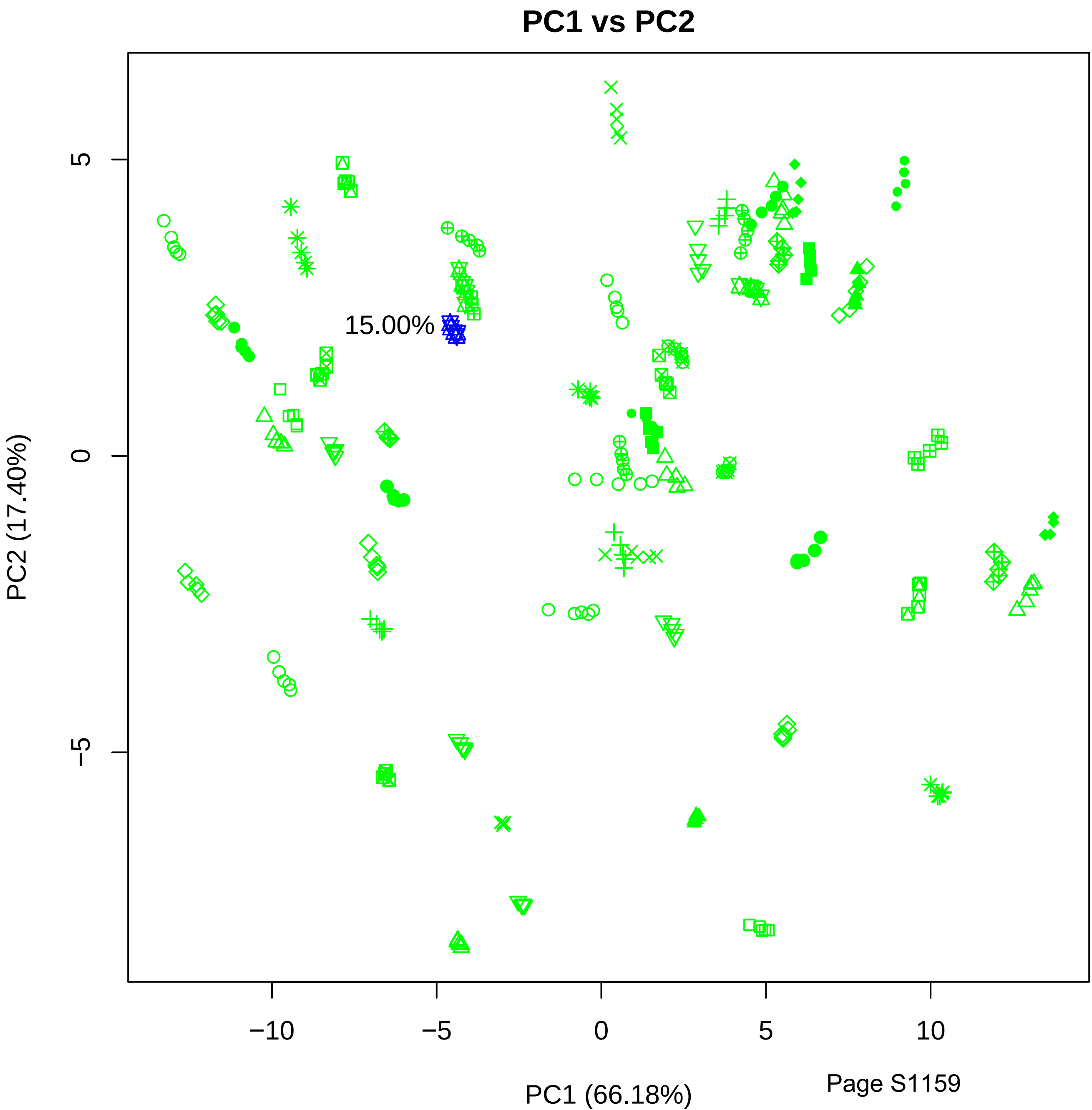

PC1 vs PC2
15.00%
5
0
−5
−10
−5
0
5
10
PC2 (17.40%)
PC1 (66.18%)

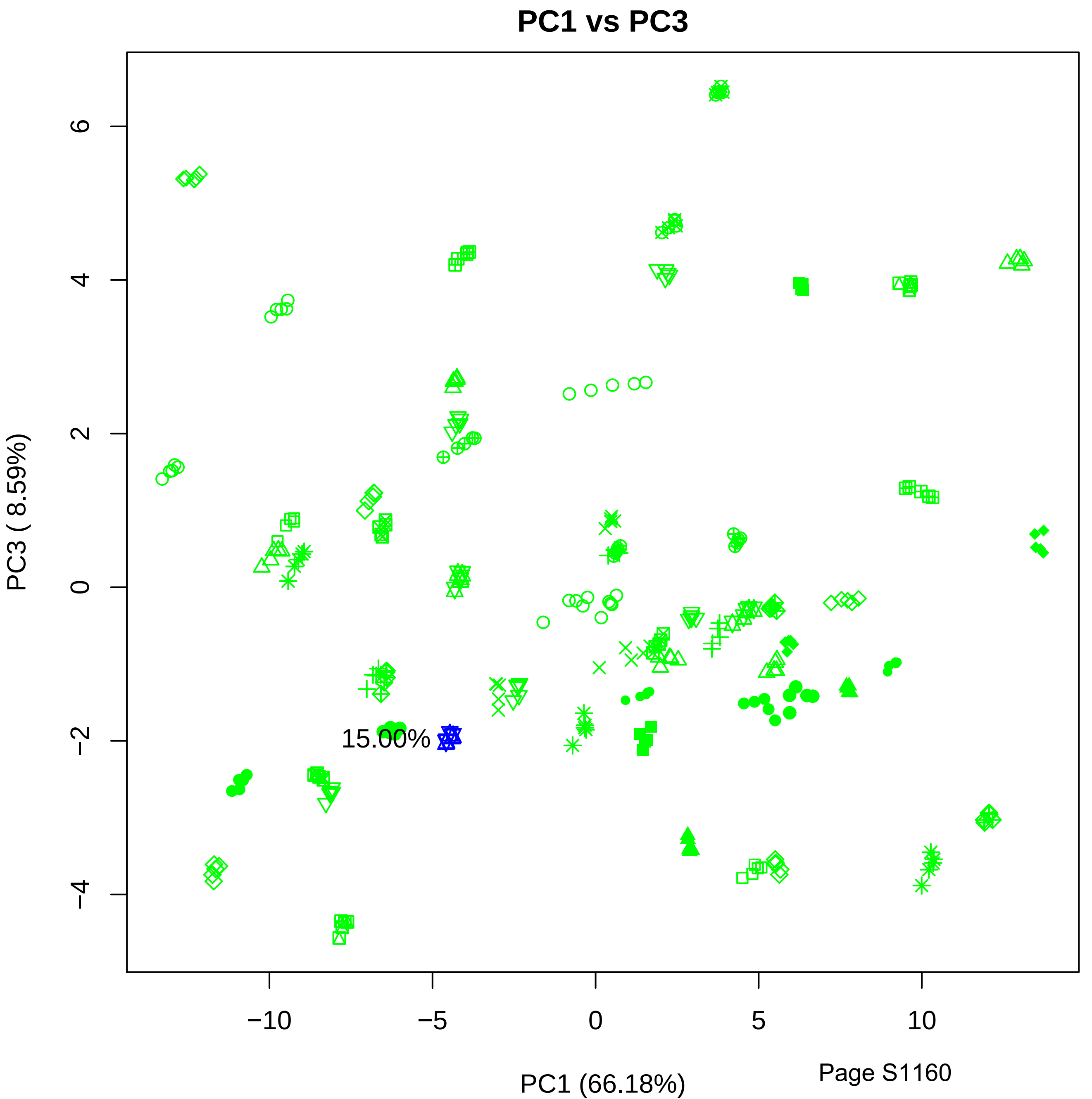

PC1 vs PC3
PC3 (8.59%)
PC1 (66.18%)
15.00%
6
4
2
0
−2
−4
−10
−5
0
5
10

PC1 vs PC4

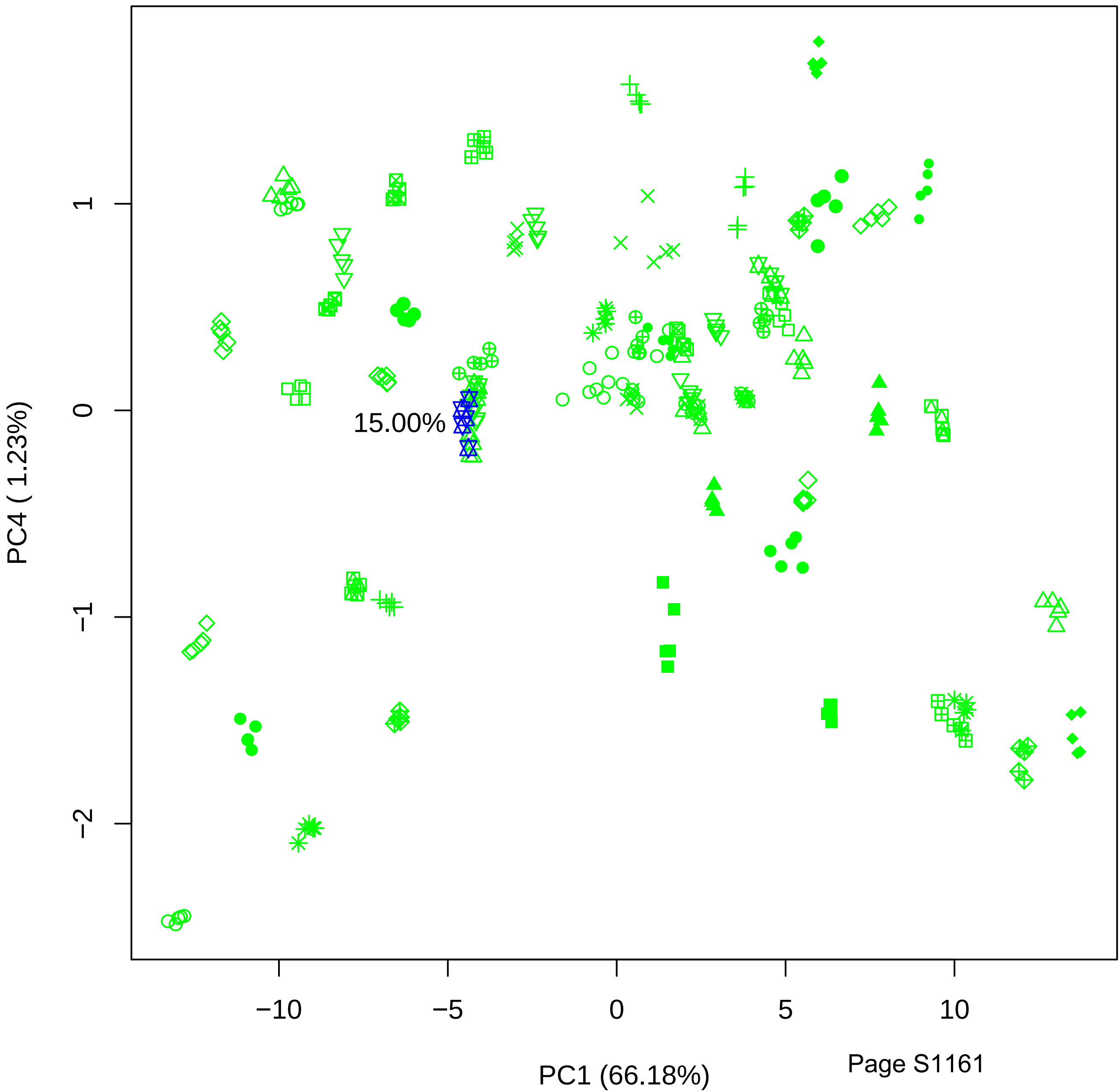

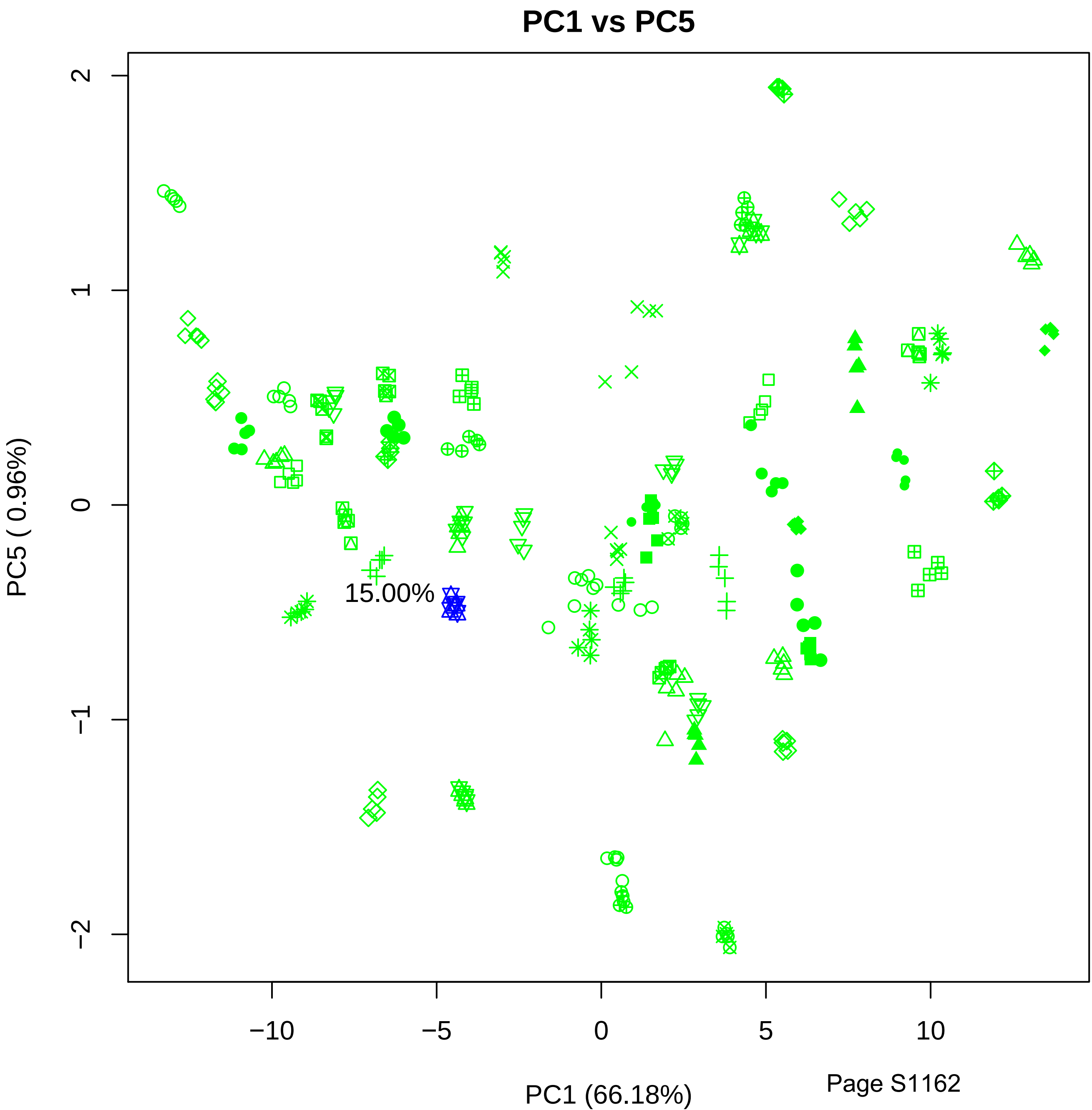
PC1 vs PC5
PC5 (0.96%)
PC1 (66.18%)
15.00%
2
1
0
−1
−2
−10
−5
0
5
10

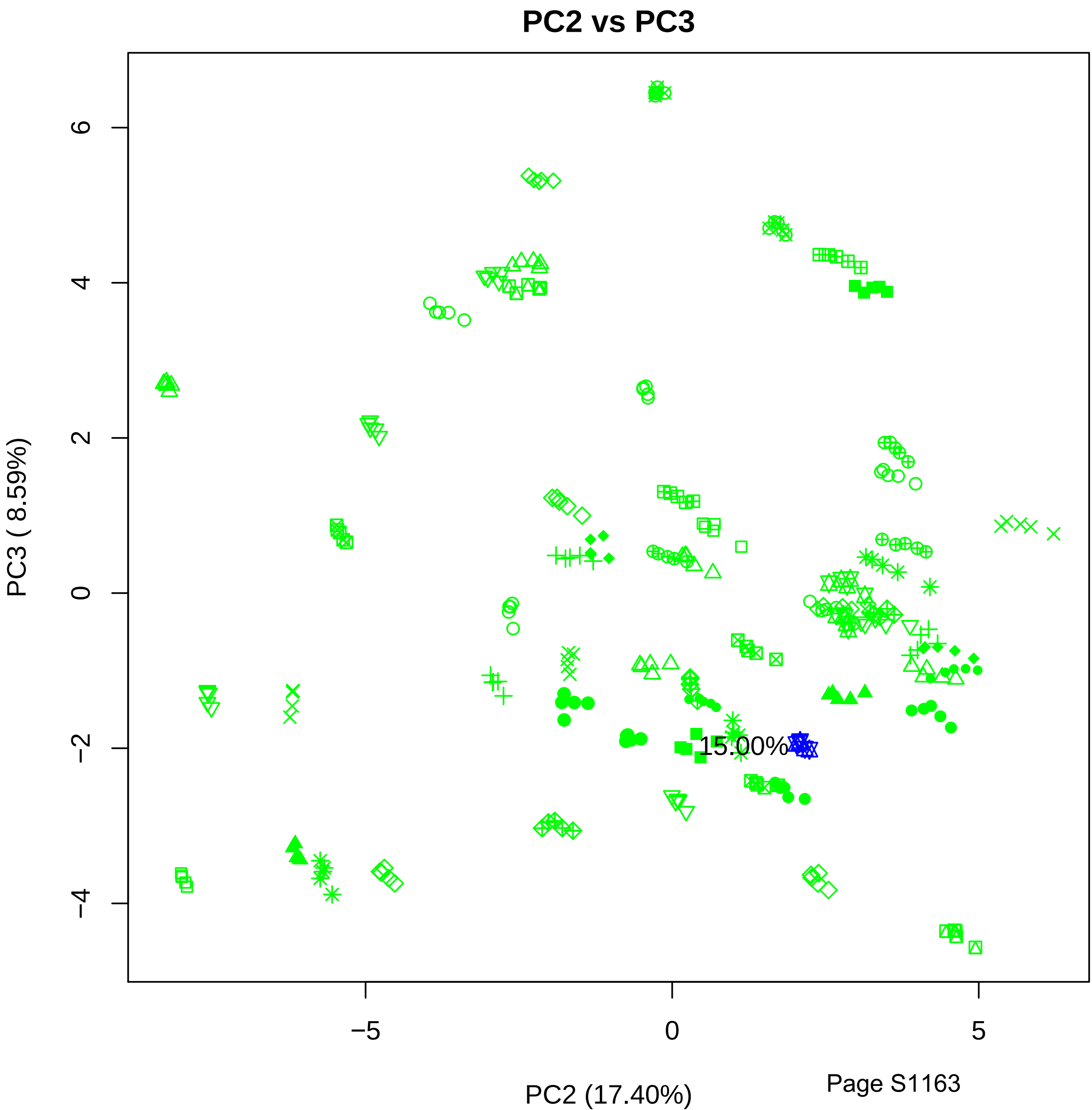

PC2 vs PC3
6
4
2
0
−2
−4
PC3 (8.59%)
15.00%
−5
0
5
PC2 (17.40%)

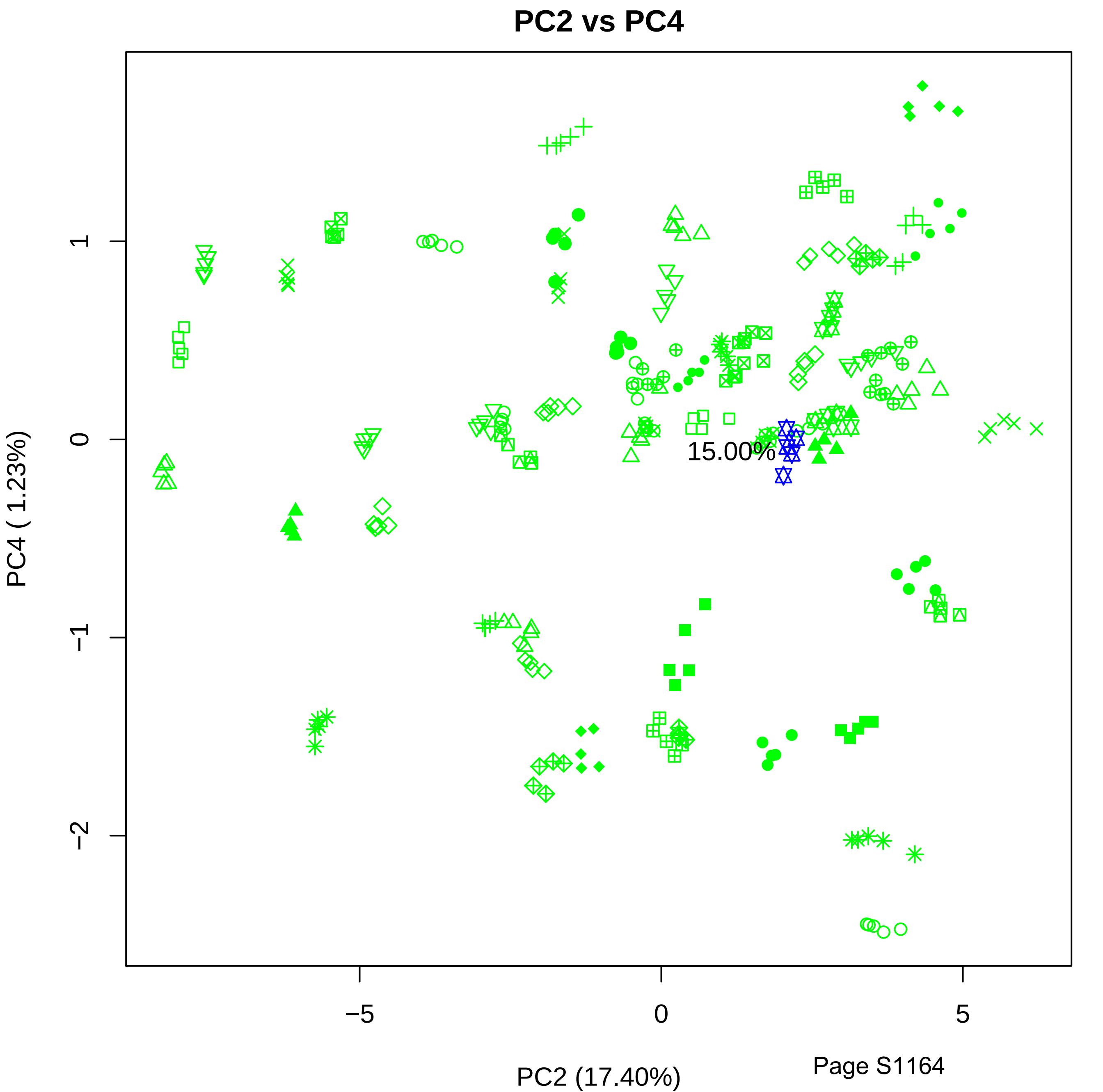
PC2 vs PC4
PC4 (1.23%)
PC2 (17.40%)
1
0
−1
−2
−5
0
5
15.00%

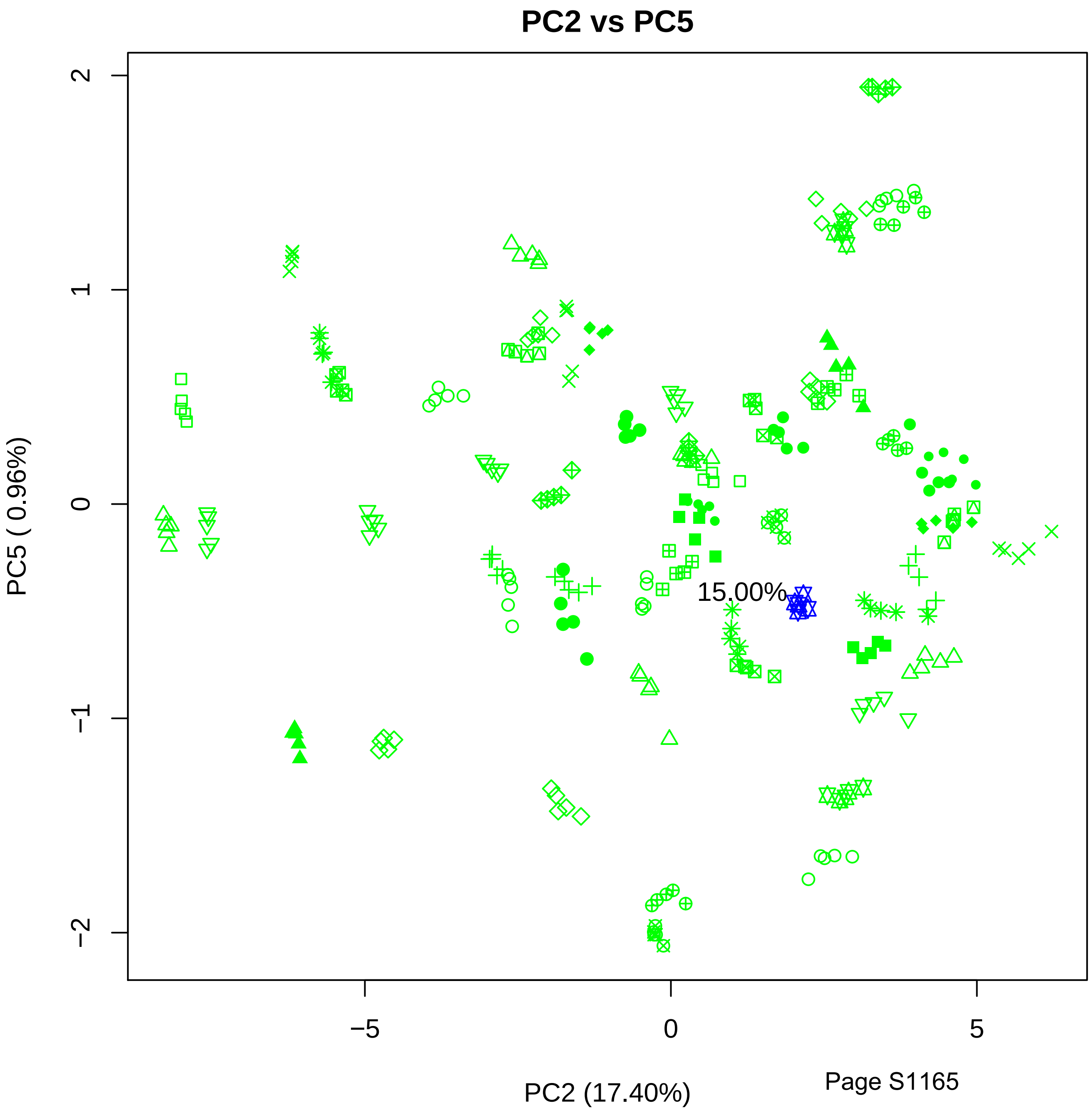
PC2 vs PC5
PC5 (0.96%)
PC2 (17.40%)
2
1
0
−1
−2
−5
0
5
15.00%

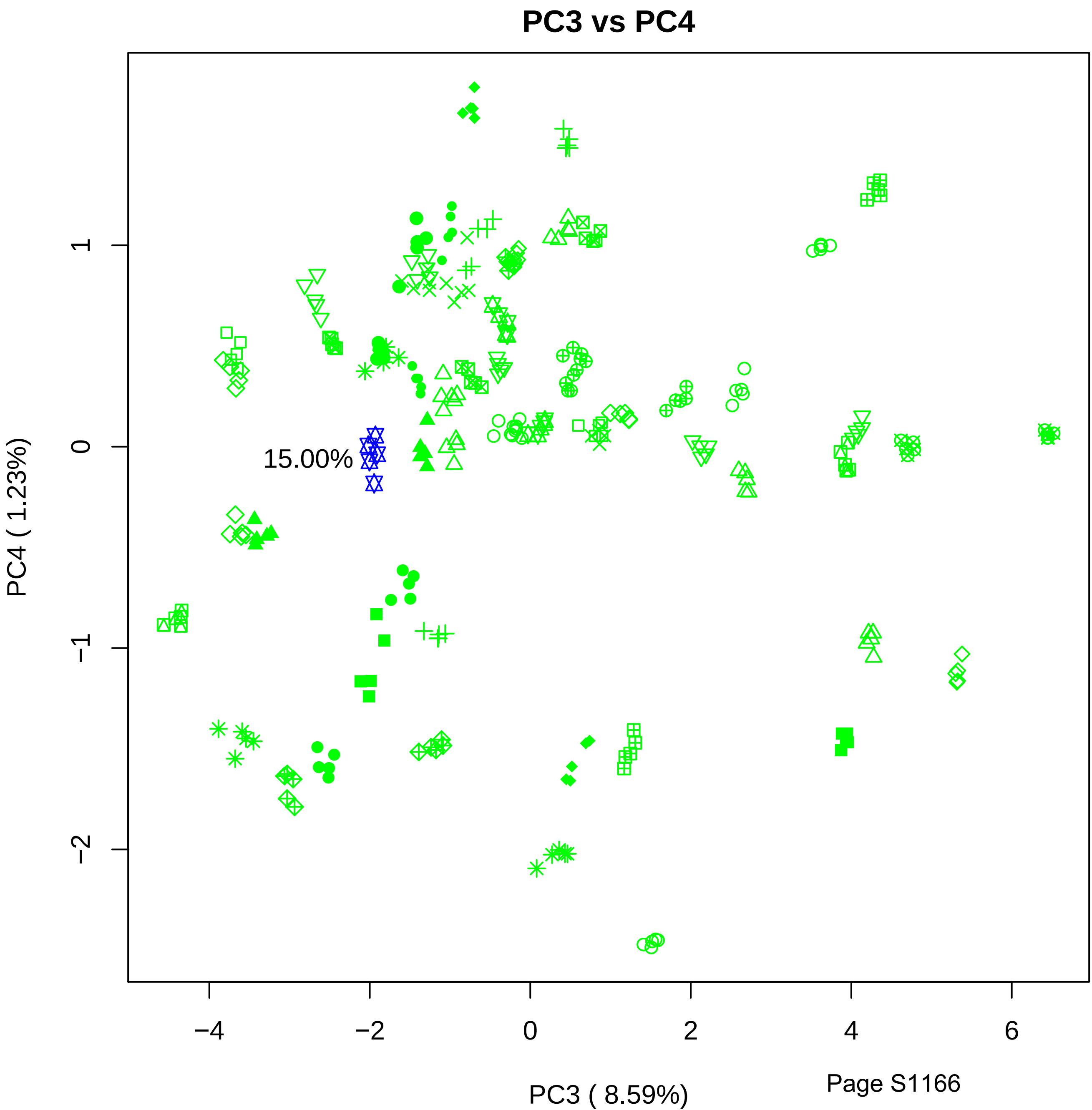

PC3 vs PC4
15.00%
PC4 (1.23%)
PC3 (8.59%)
−4
−2
0
2
4
6
1
0
−1
−2

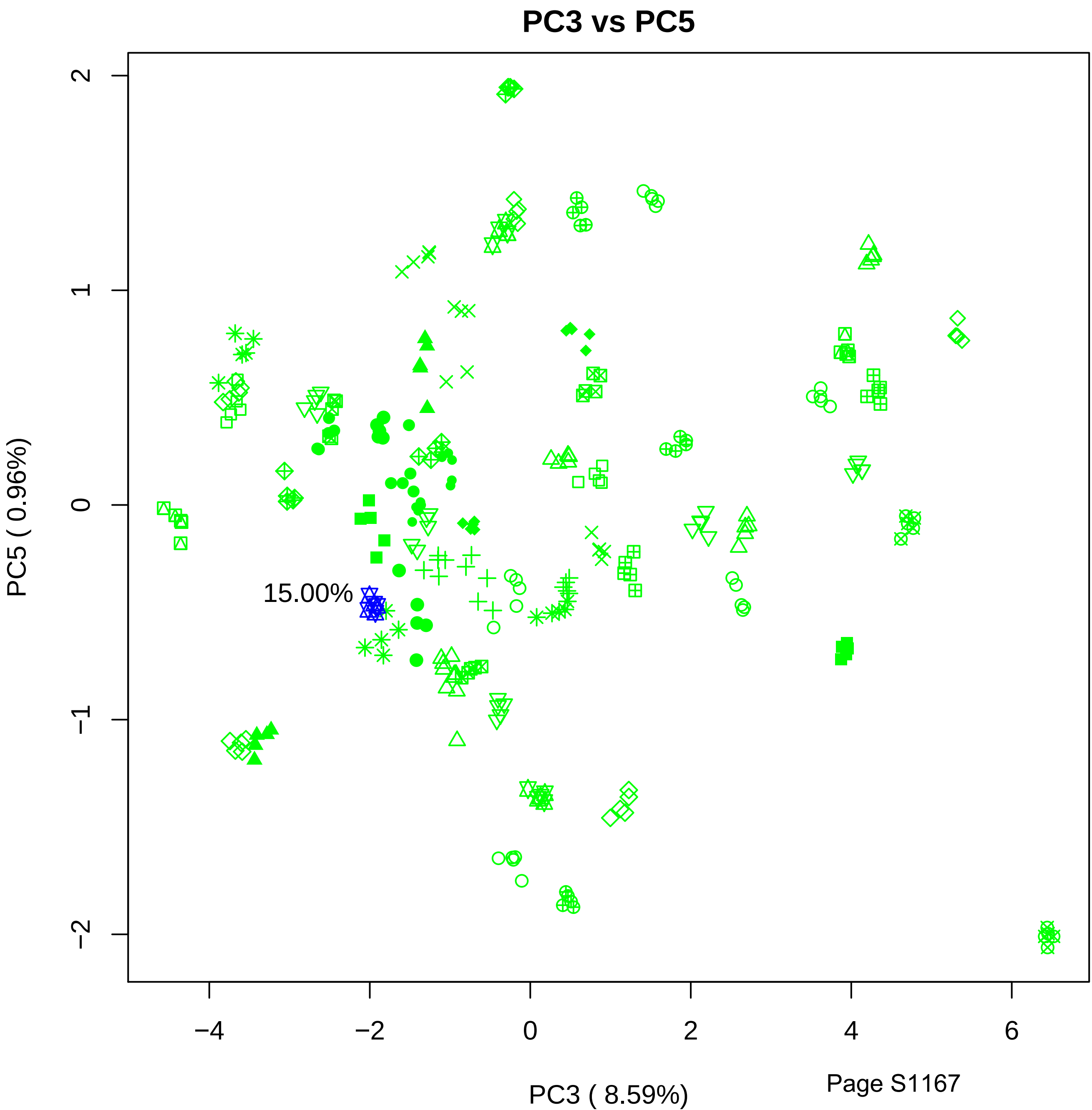
PC3 vs PC5
PC5 (0.96%)
PC3 (8.59%)
15.00%
2
1
0
−1
−2
−4
−2
0
2
4
6

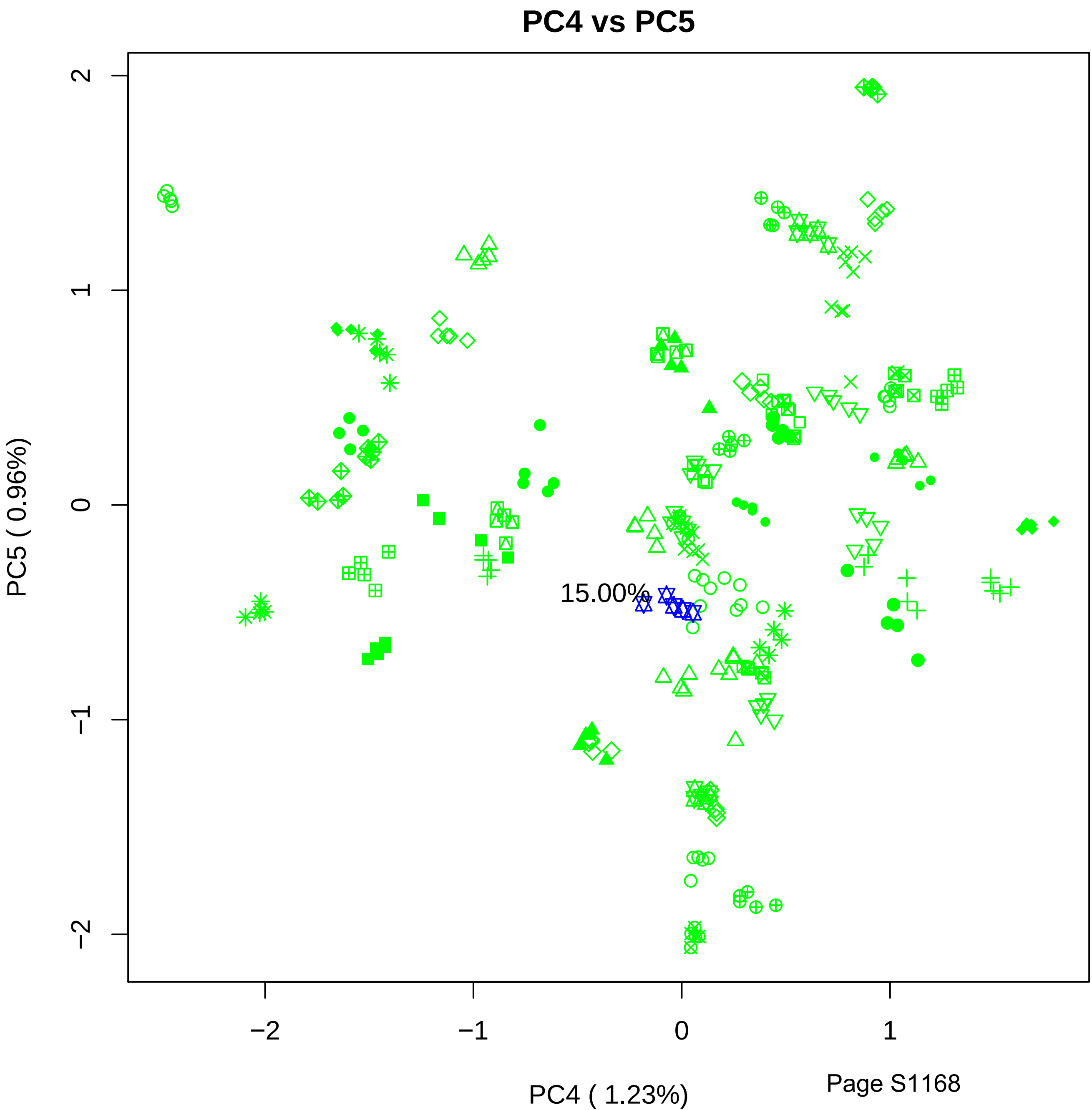

PC4 vs PC5
PC5 (0.96%)
PC4 (1.23%)
15.00%
2
1
0
−1
−2
−2
−1
0
1

PCA of 20% BMH(b) vs PMH(b)

Scaled Matrix PCAed

7th order polynomial baseline correction performed

Raw spectra smoothed to 21 Neighbours, 2nd order PN, Savtisky Golay

Normalisation between 0 and 100

D2 spectra smoothed to 41 Neighbours, 2nd order PN, Savtisky Golay

Regions Analysed: 3601−2501 and 2001−700cm−1

Samples Analysed: 1 bContam's 0 BLH's 0 BMH's 0 OMH's 0 pContam's 0 PLH's 60 PMH's

Keele University AD

Fri Dec 14 14:21:58 2018

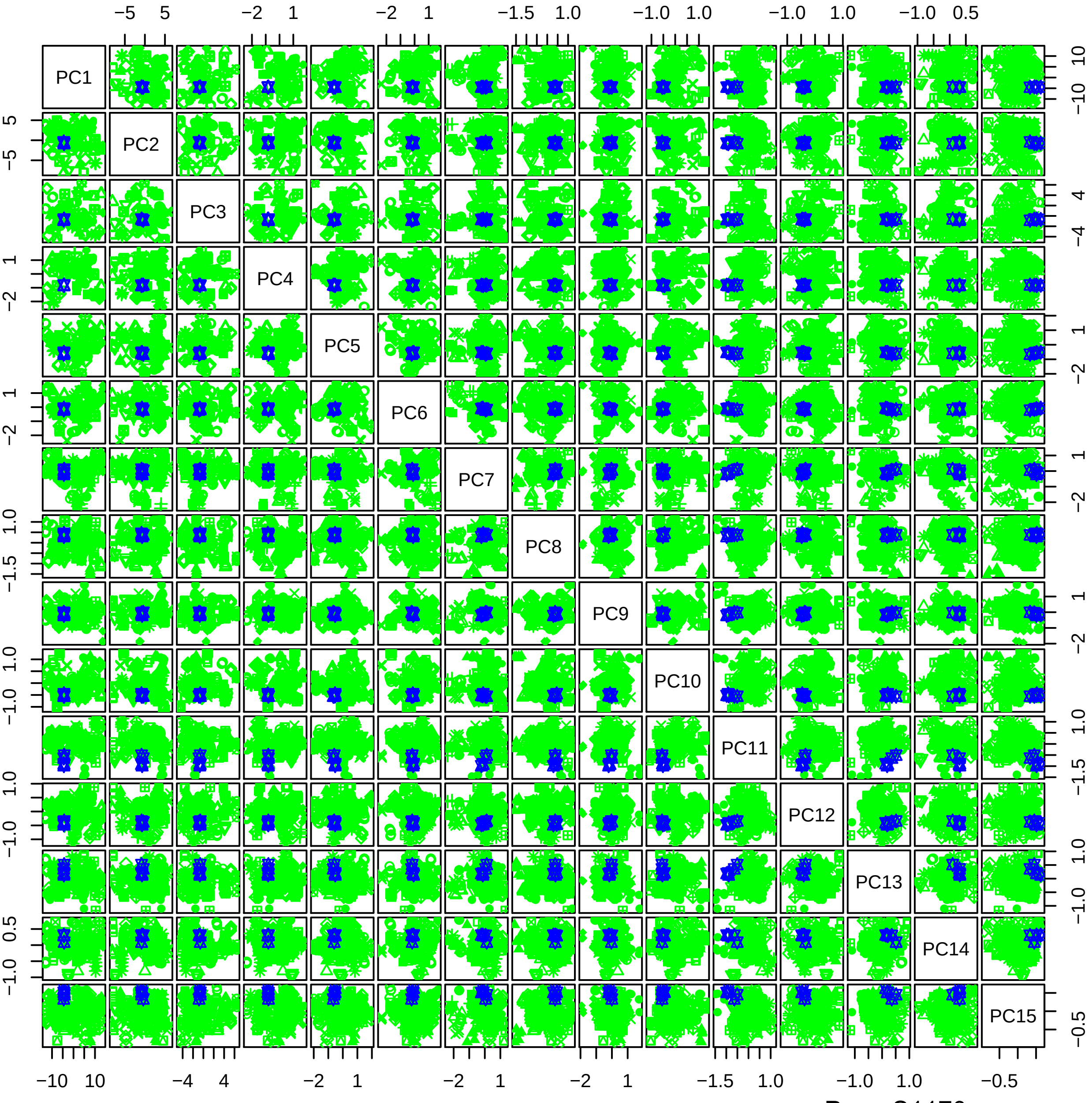
PC1
PC2
PC3
PC4
PC5
PC6
PC7
PC8
PC9
PC10
PC11
PC12
PC13
PC14
PC15

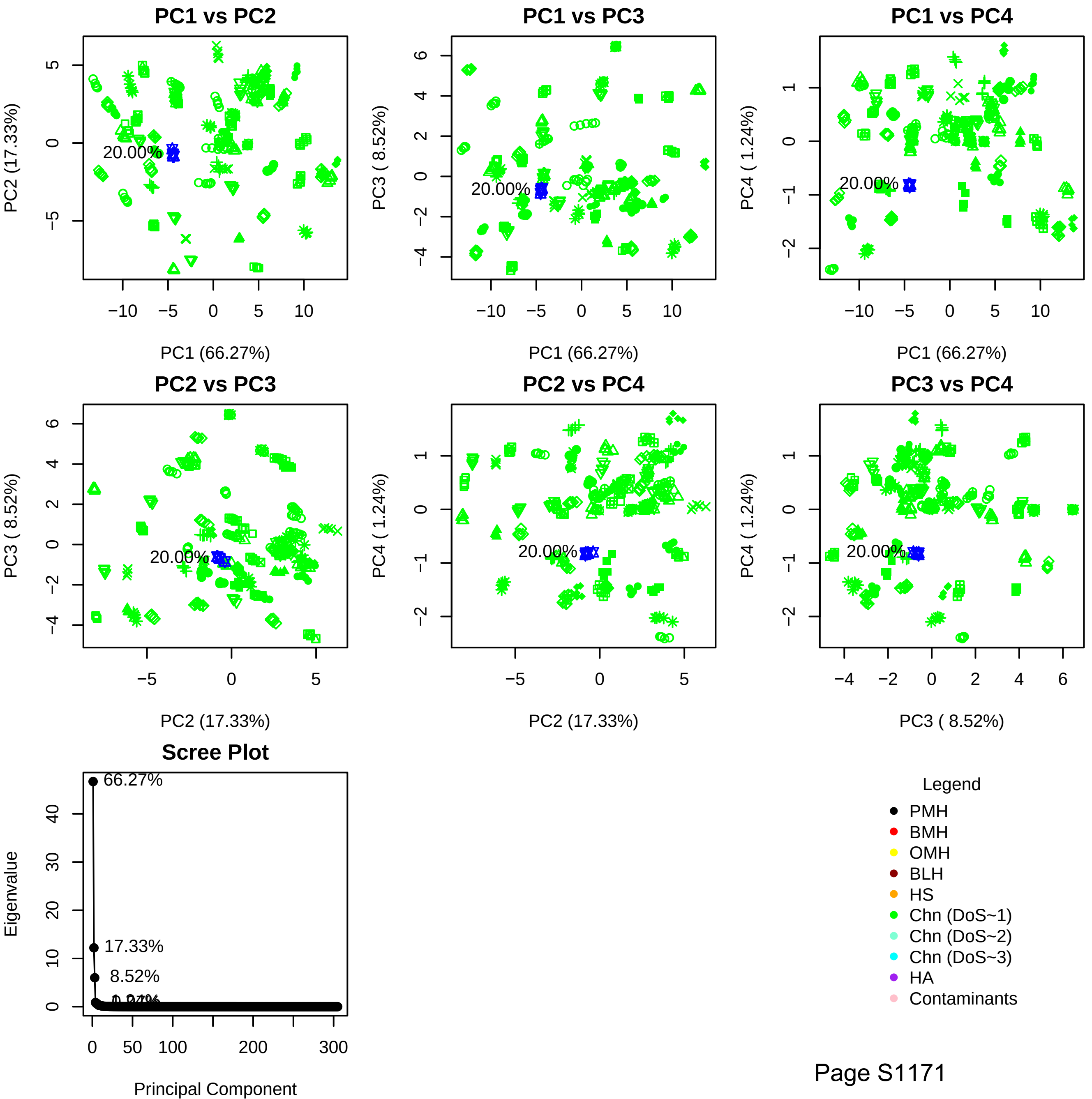
PC1 vs PC2
PC1 vs PC3
PC1 vs PC4
PC2 vs PC3
PC2 vs PC4
PC3 vs PC4
Scree Plot
PC1 (66.27%)
PC2 (17.33%)
PC3 (8.52%)
PC4 (1.24%)
20.00%
66.27%
17.33%
8.52%
Eigenvalue
Principal Component
Legend
PMH
BMH
OMH
BLH
HS
Chn (DoS~1)
Chn (DoS~2)
Chn (DoS~3)
HA
Contaminants

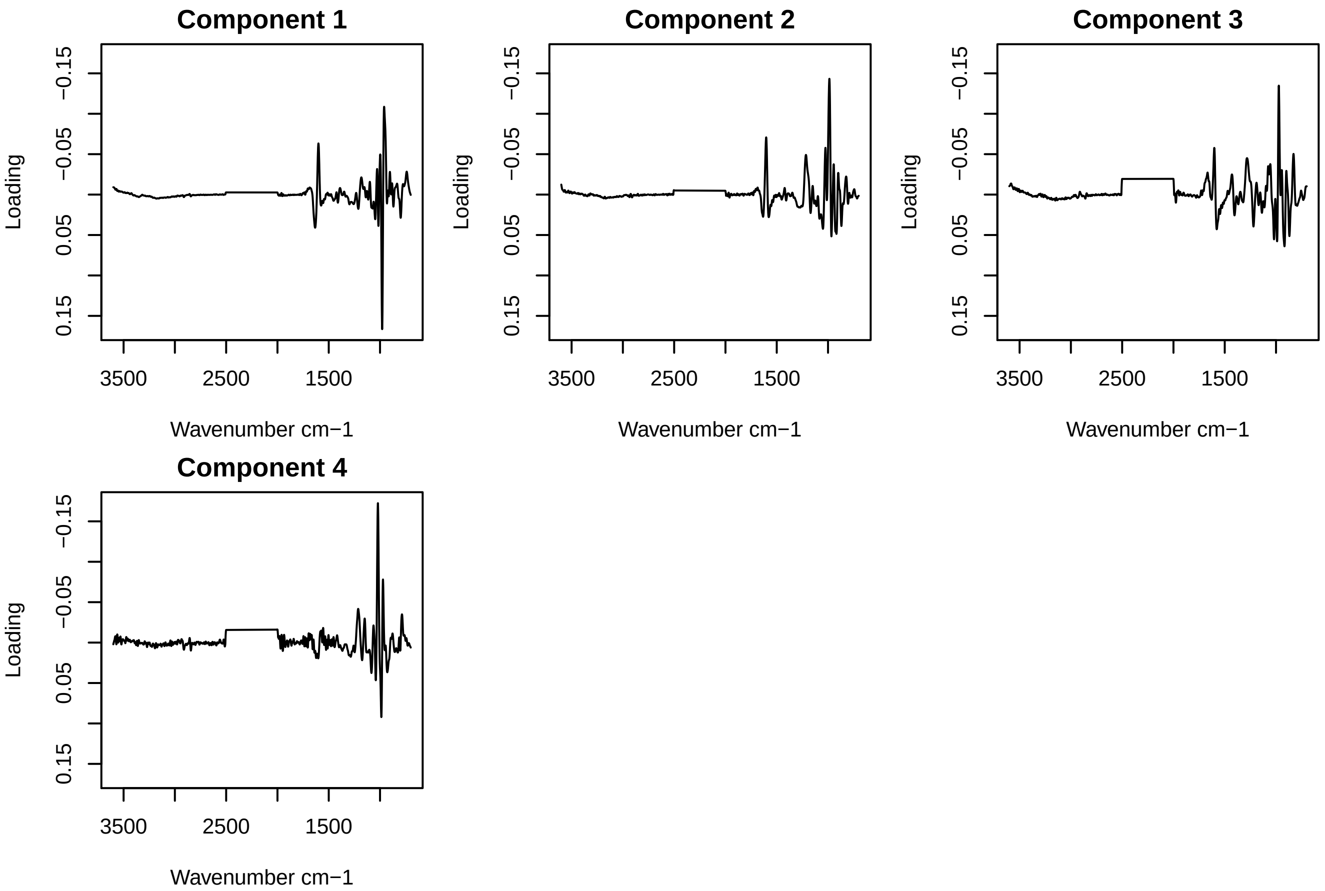
Component 1
Component 2
Component 3
Component 4
Loading
−0.15
−0.05
0.05
0.15
3500
2500
1500
Wavenumber cm−1

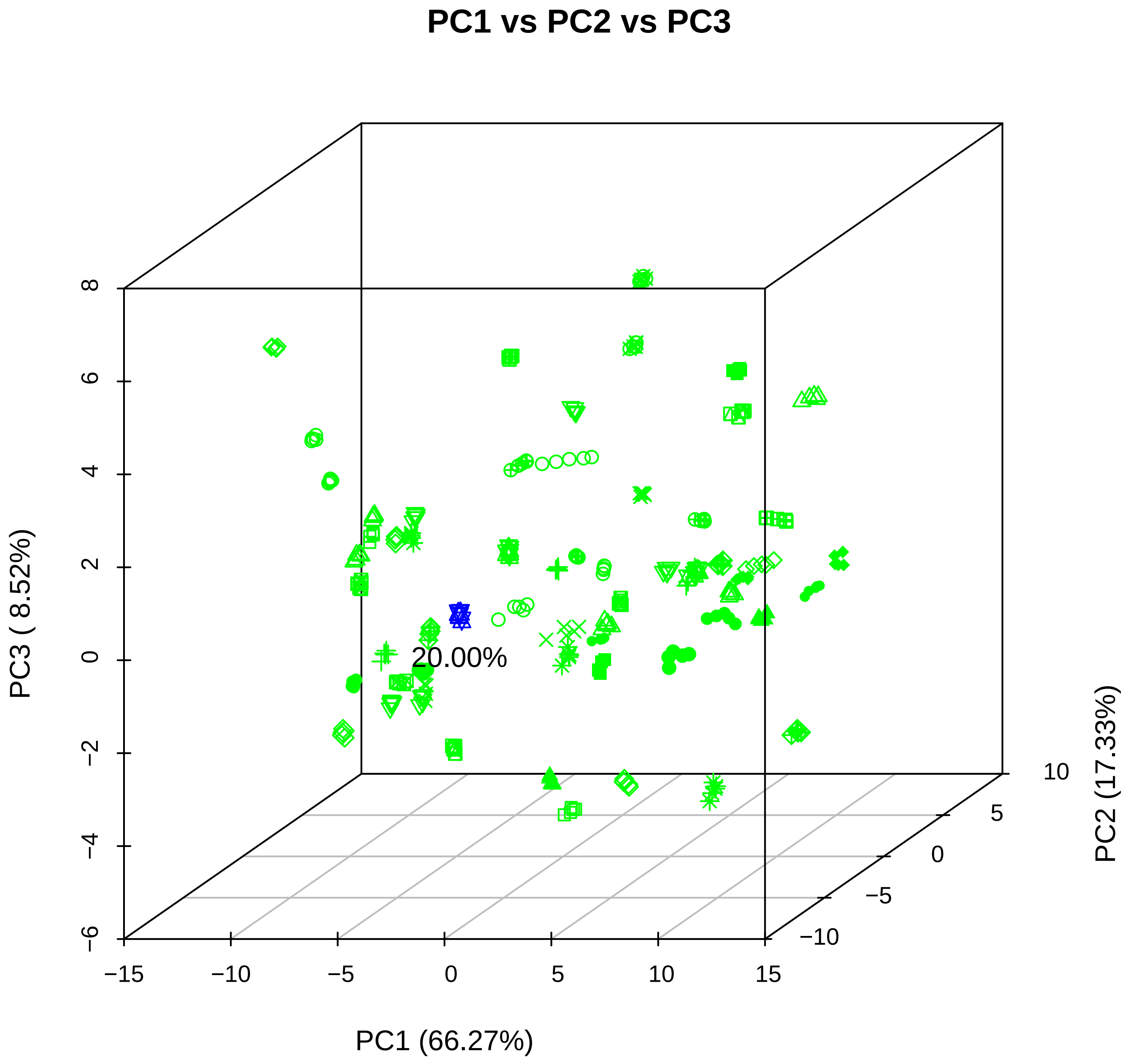
PC1 vs PC2 vs PC3
PC1 (66.27%)
PC2 (17.33%)
PC3 (8.52%)
20.00%
−15
−10
−5
0
5
10
15
−6
−4
−2
0
2
4
6
8
−10
−5
0
5
10

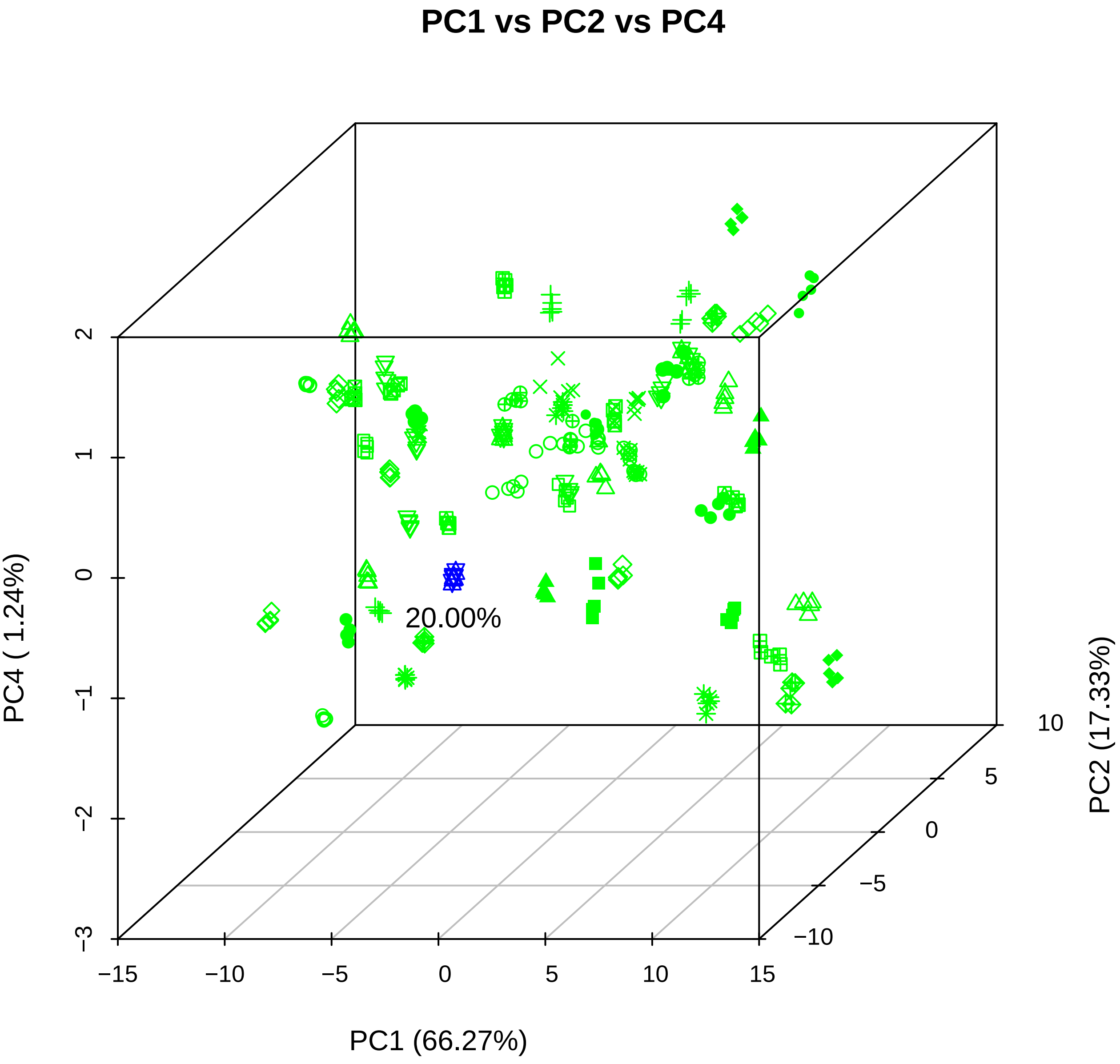

PC1 vs PC2 vs PC4
20.00%
PC1 (66.27%)
PC2 (17.33%)
PC4 (1.24%)
−15
−10
−5
0
5
10
15
−3
−2
−1
0
1
2
−10
−5
0
5
10

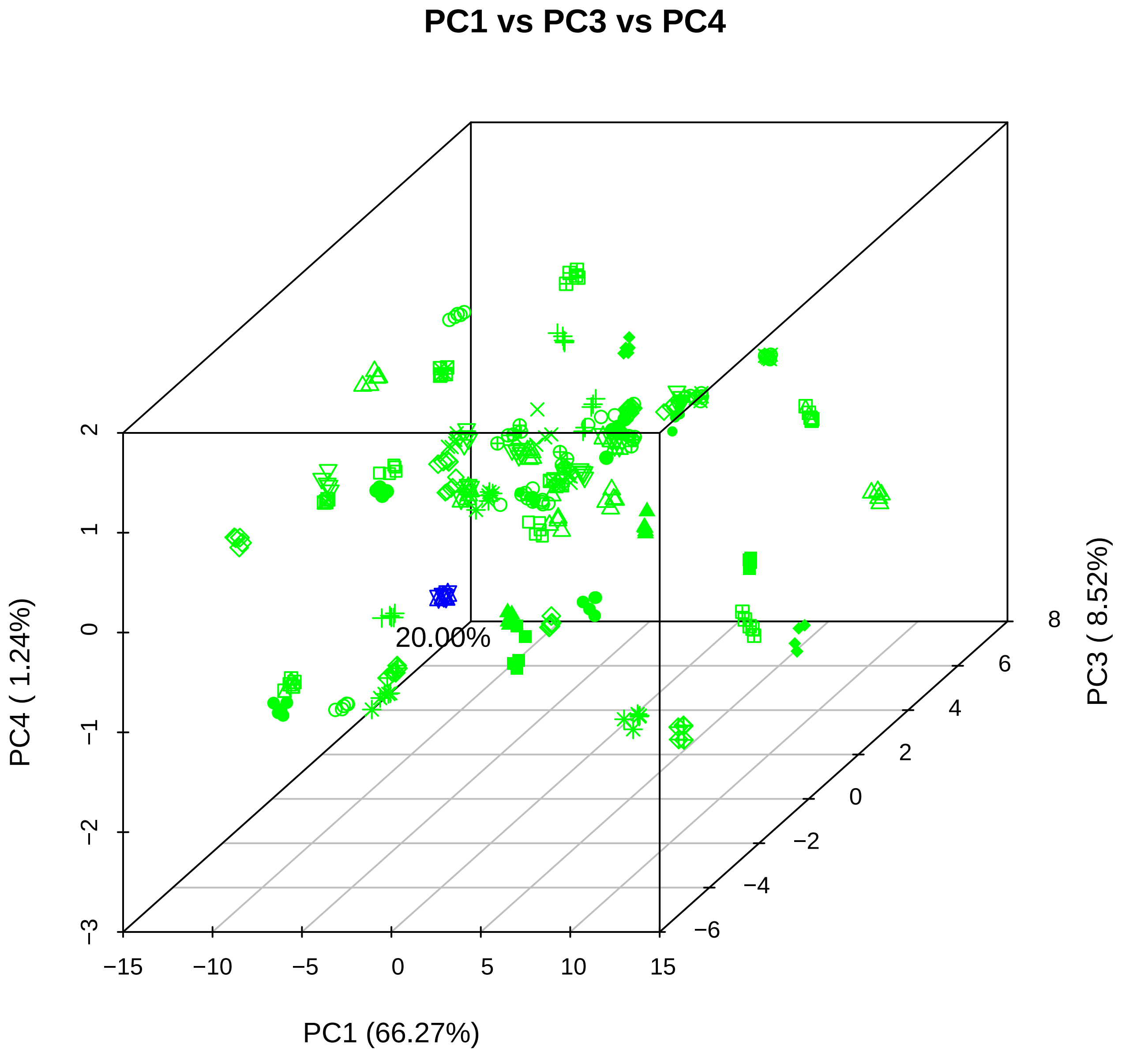

PC1 vs PC3 vs PC4
20.00%
PC1 (66.27%)
PC4 (1.24%)
PC3 (8.52%)
−15
−10
−5
0
5
10
15
−3
−2
−1
0
1
2
−6
−4
−2
0
2
4
6
8

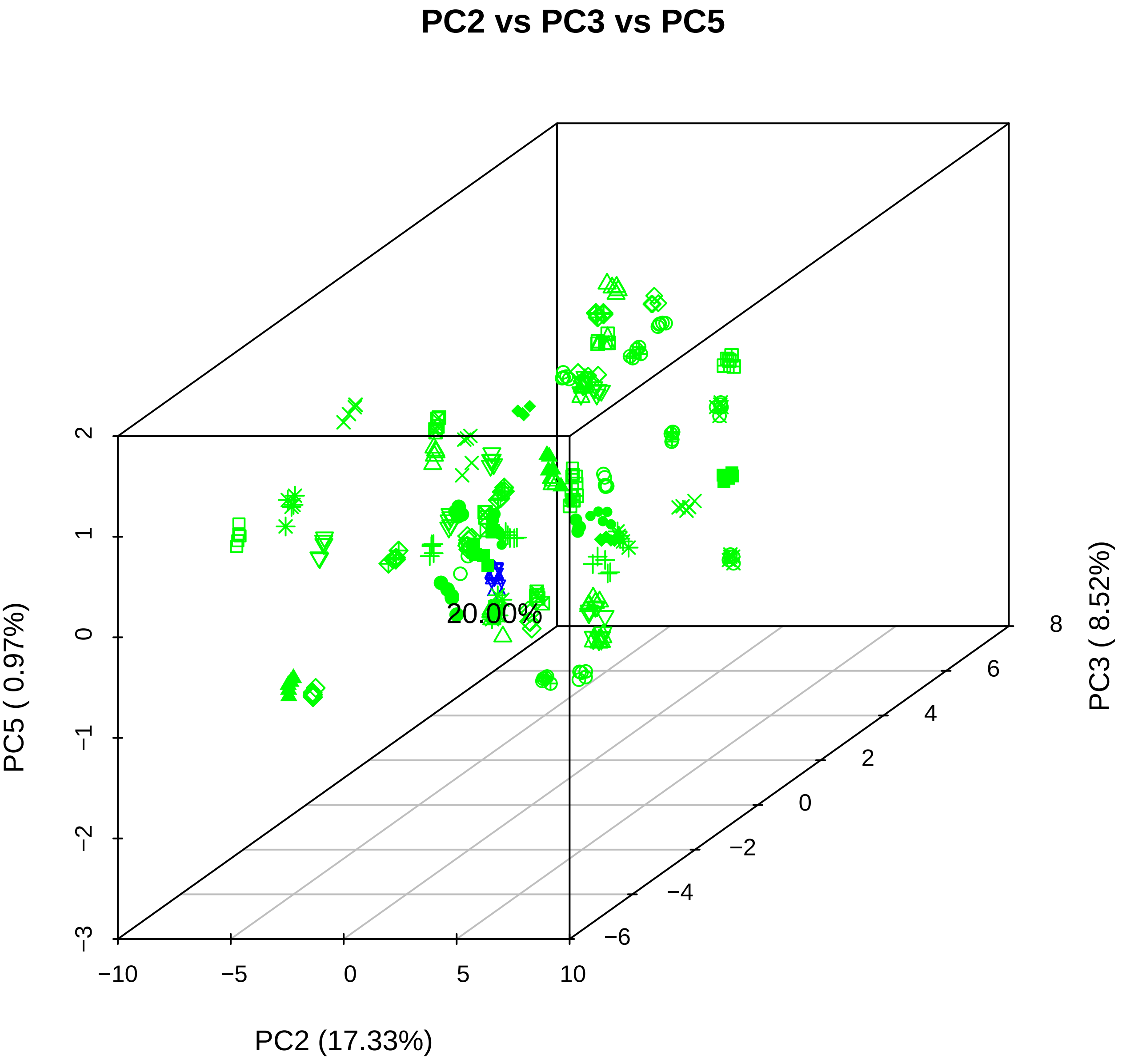

PC2 vs PC3 vs PC5
20.00%
PC2 (17.33%)
PC3 (8.52%)
PC5 (0.97%)
−10
−5
0
5
10
−6
−4
−2
0
2
4
6
8
−3
−2
−1
0
1
2

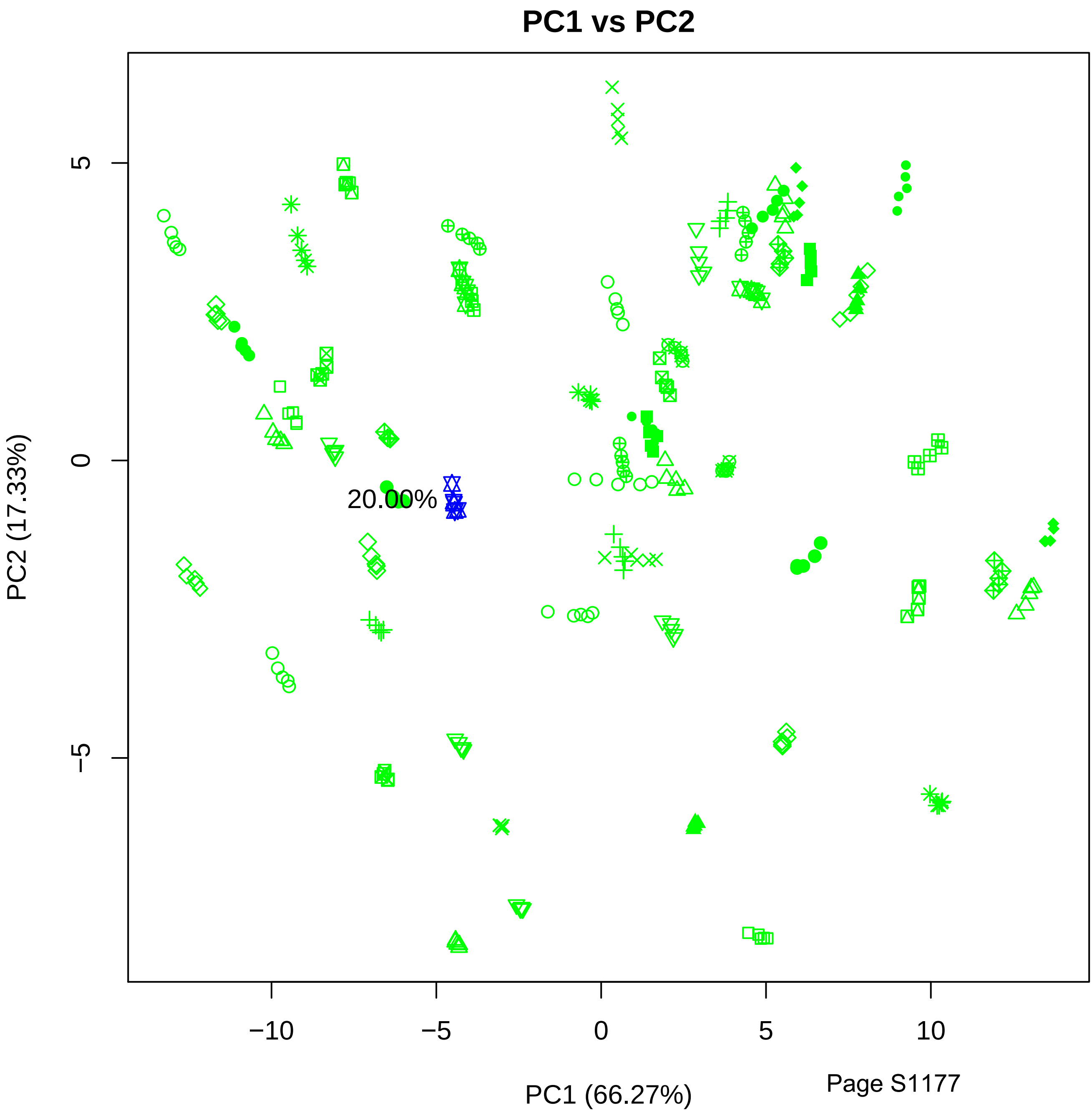
PC1 vs PC2
PC2 (17.33%)
PC1 (66.27%)
20.00%
5
0
−5
−10
−5
0
5
10

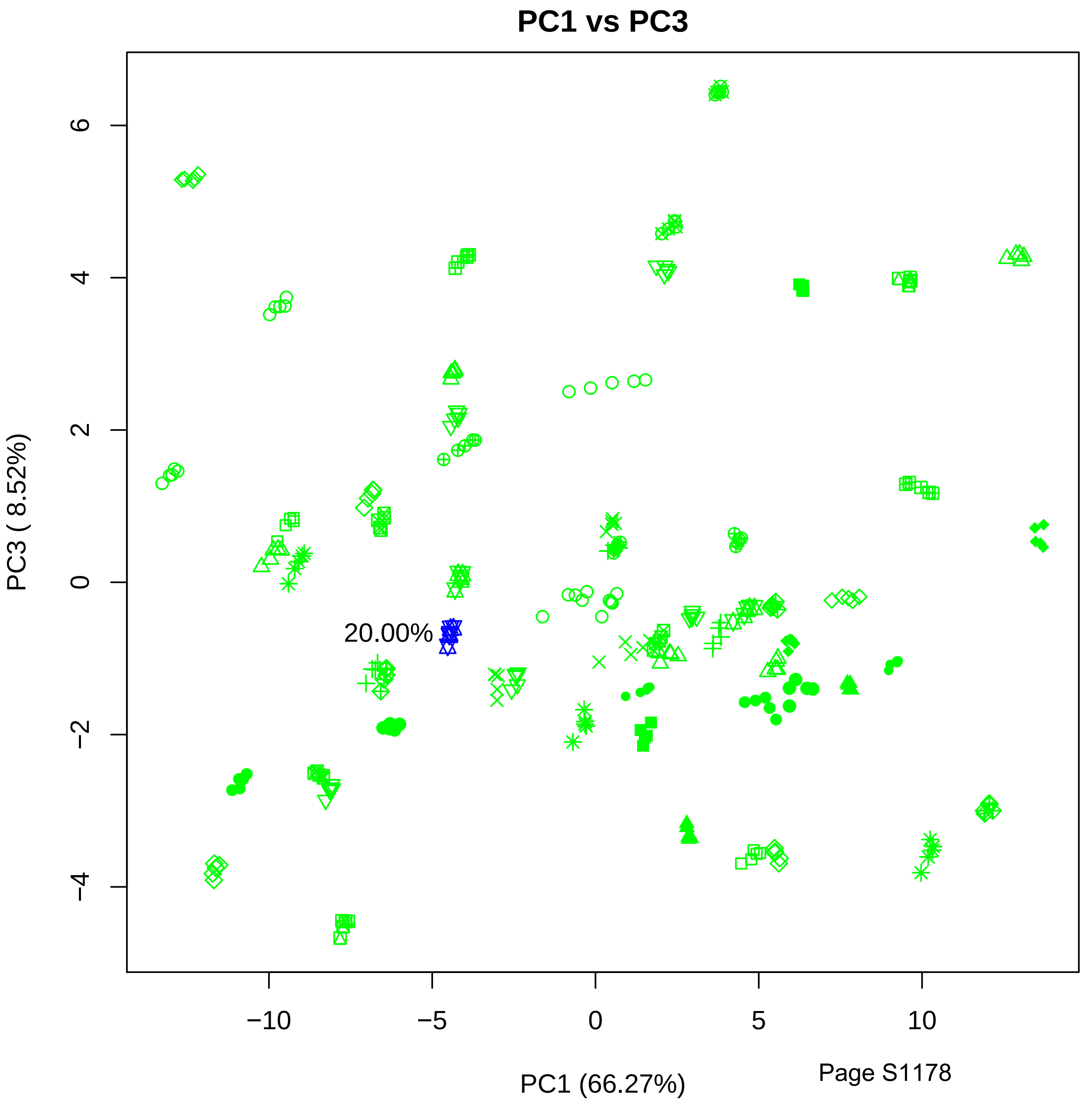
PC1 vs PC3
PC3 (8.52%)
PC1 (66.27%)
20.00%
−10
−5
0
5
10
−4
−2
0
2
4
6

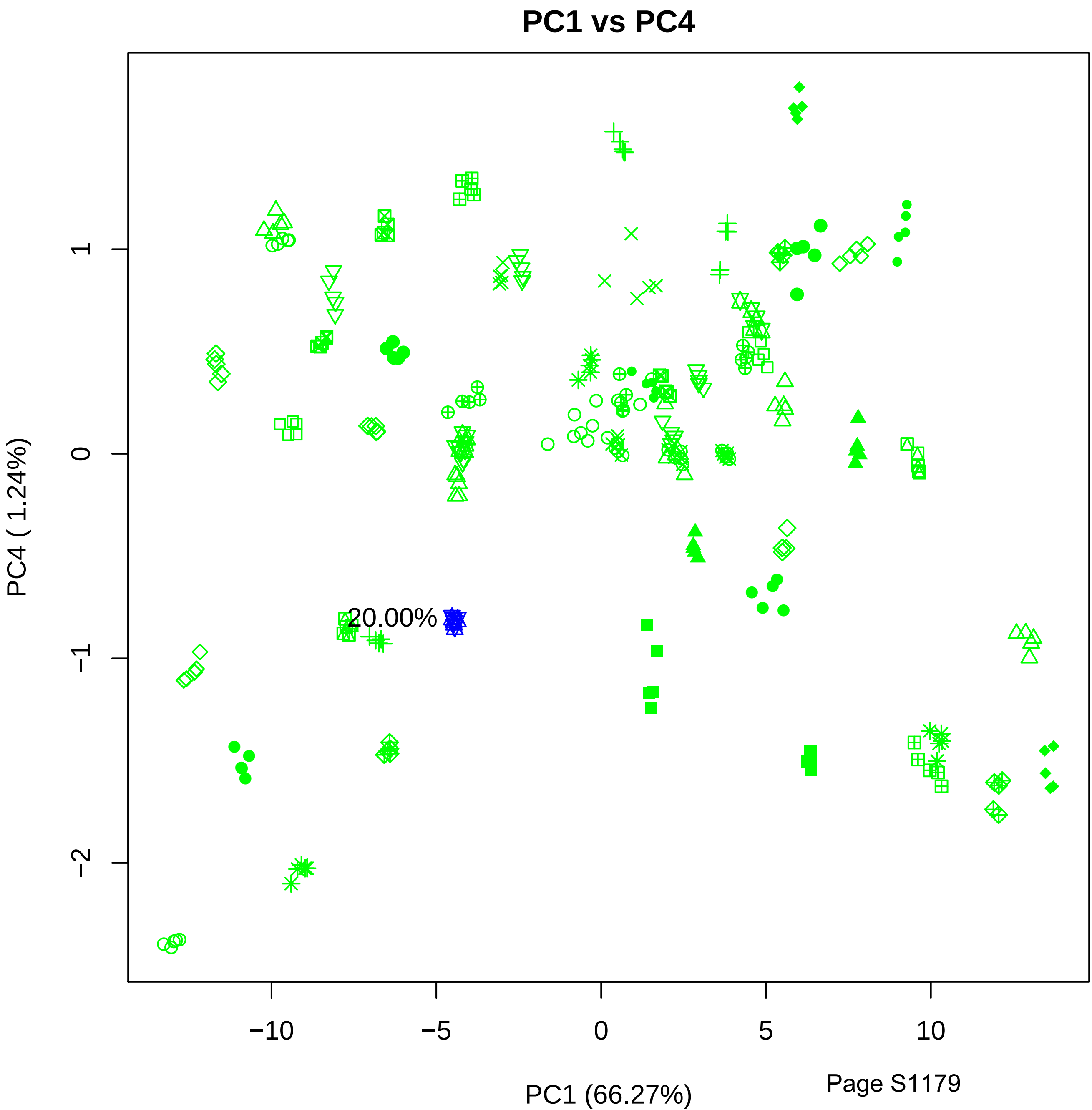

PC1 vs PC4
20.00%
PC4 (1.24%)
PC1 (66.27%)
−10
−5
0
5
10
−2
−1
0
1

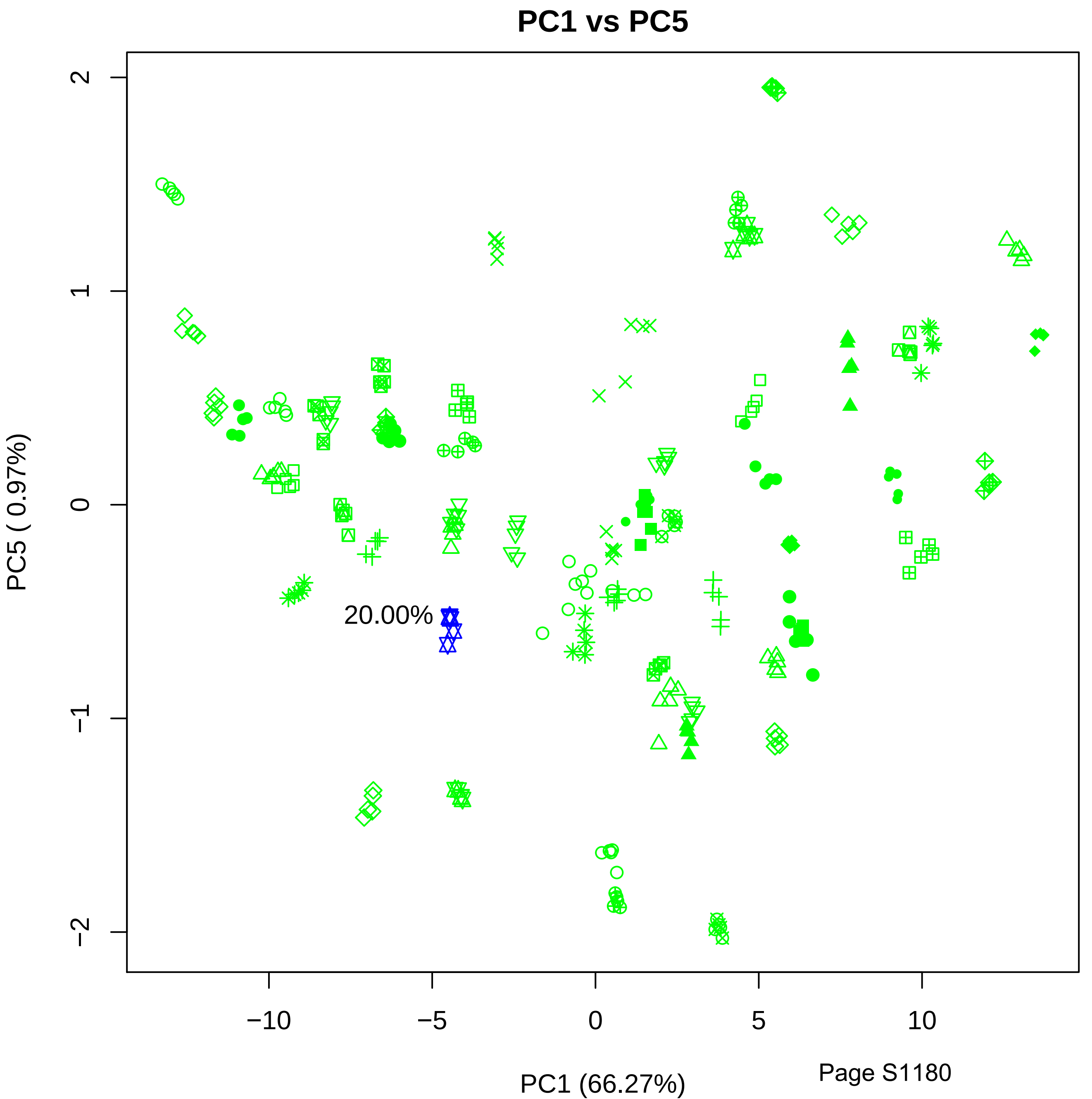
PC1 vs PC5
PC5 (0.97%)
PC1 (66.27%)
20.00%
−10
−5
0
5
10
2
1
0
−1
−2

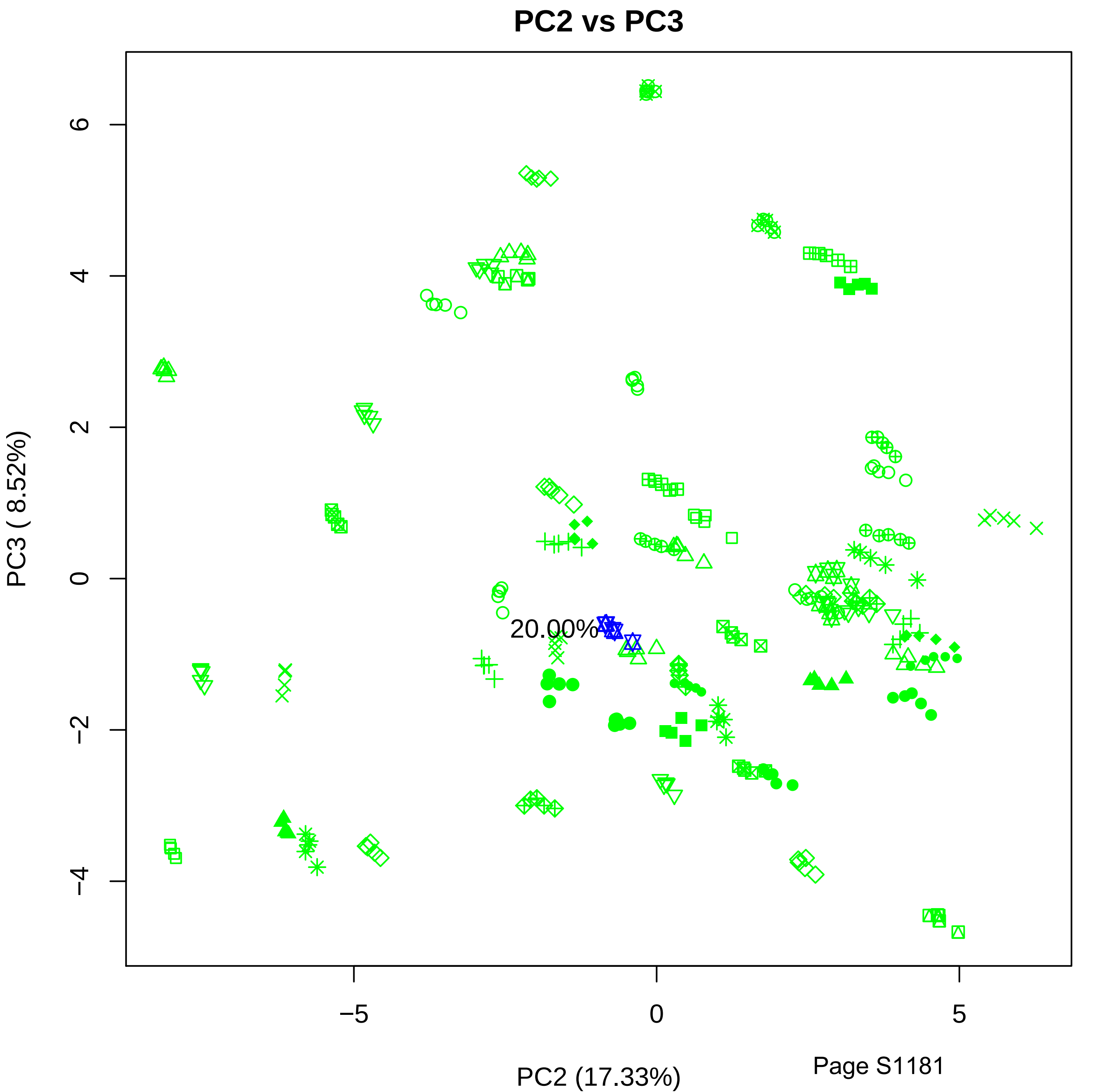

PC2 vs PC3
PC3 (8.52%)
PC2 (17.33%)
20.00%
6
4
2
0
−2
−4
−5
0
5

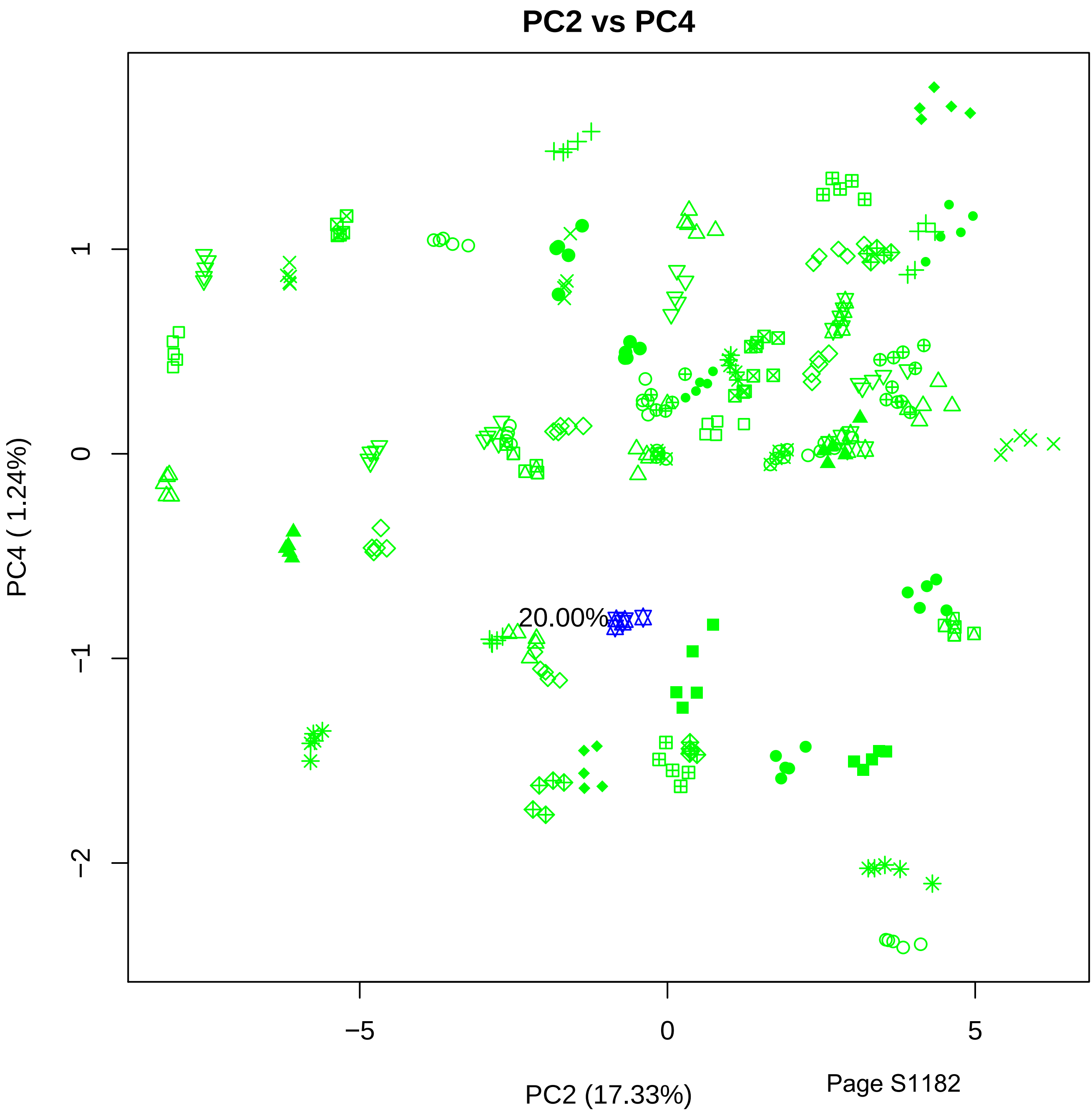

PC2 vs PC4
PC4 (1.24%)
1
0
−1
−2
−5
0
5
20.00%
PC2 (17.33%)

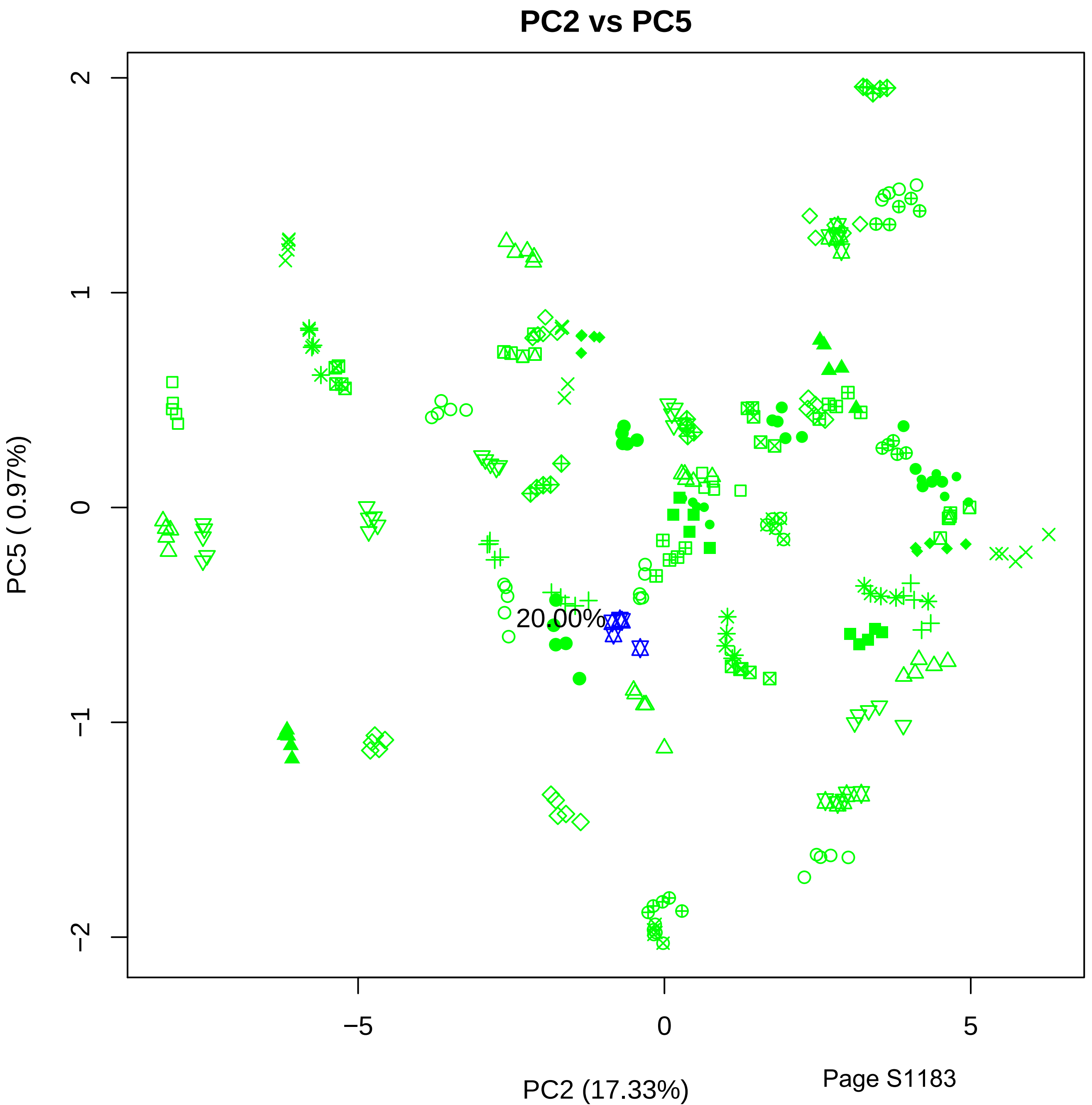

PC2 vs PC5
PC5 (0.97%)
PC2 (17.33%)
20.00%
2
1
0
−1
−2
−5
0
5

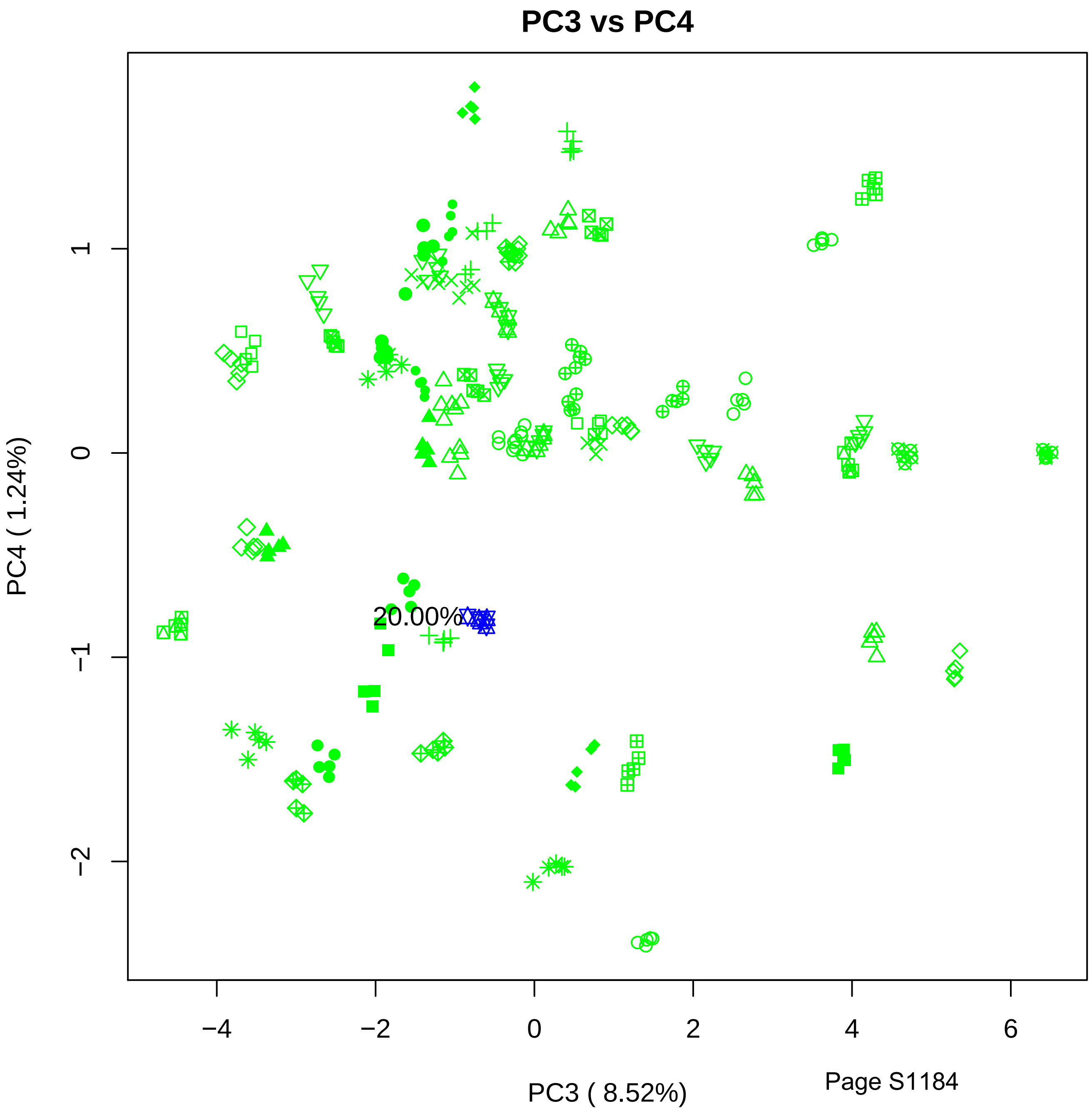

PC3 vs PC4
20.00%
PC4 (1.24%)
PC3 (8.52%)
1
0
−1
−2
−4
−2
0
2
4
6

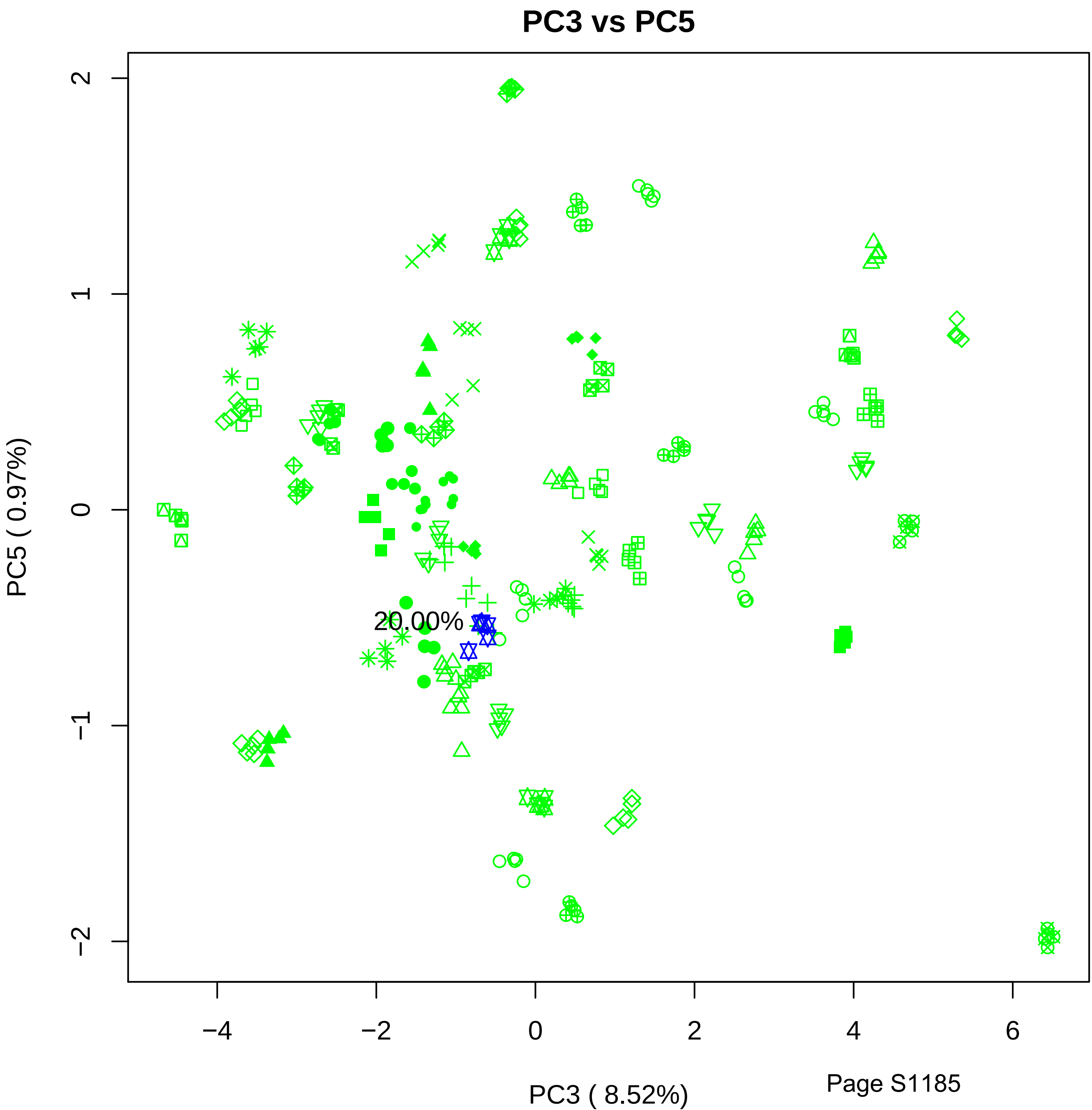
PC3 vs PC5
PC5 (0.97%)
PC3 (8.52%)
20.00%
2
1
0
−1
−2
−4
−2
0
2
4
6

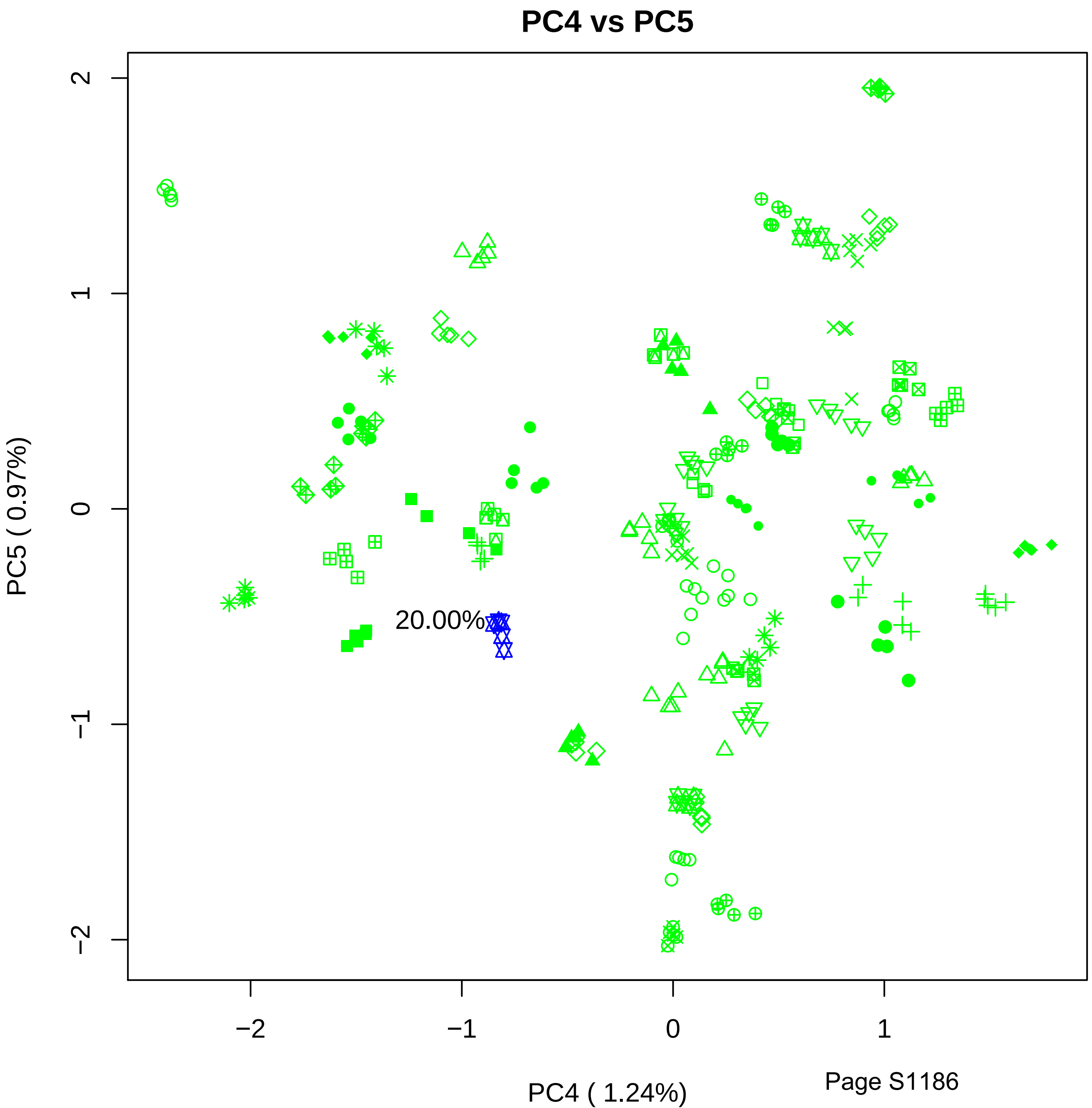

PC4 vs PC5
PC5 (0.97%)
PC4 (1.24%)
20.00%
2
1
0
−1
−2
−2
−1
0
1

PCA of 40% BMH(b) vs PMH(b)

Scaled Matrix PCAed

7th order polynomial baseline correction performed

Raw spectra smoothed to 21 Neighbours, 2nd order PN, Savtisky Golay

Normalisation between 0 and 100

D2 spectra smoothed to 41 Neighbours, 2nd order PN, Savtisky Golay

Regions Analysed: 3601−2501 and 2001−700cm−1

Samples Analysed: 1 bContam's 0 BLH's 0 BMH's 0 OMH's 0 pContam's 0 PLH's 60 PMH's

Keele University AD

Fri Dec 14 14:22:07 2018

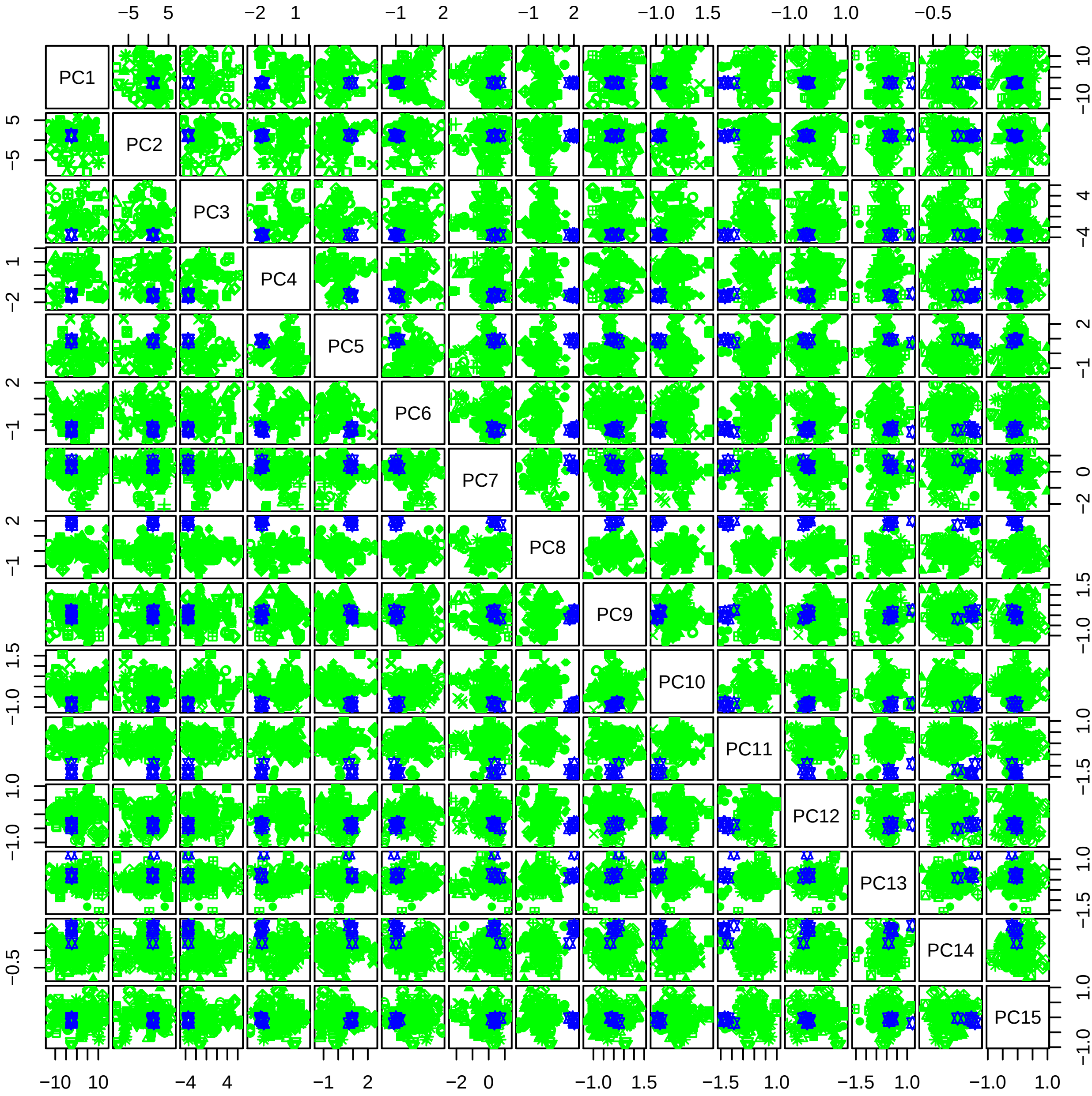
PC1
PC2
PC3
PC4
PC5
PC6
PC7
PC8
PC9
PC10
PC11
PC12
PC13
PC14
PC15

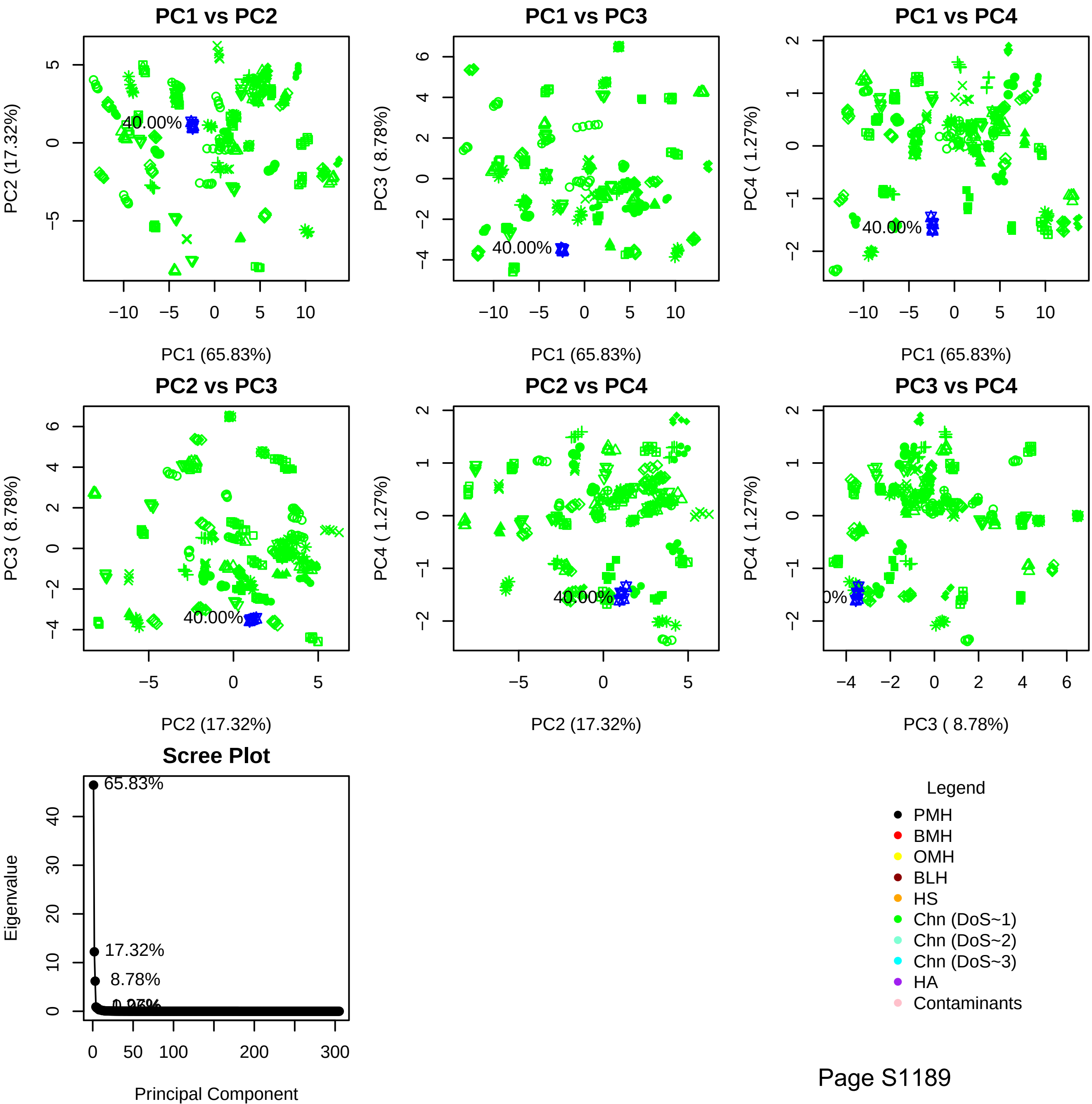
PC1 vs PC2
PC1 vs PC3
PC1 vs PC4
PC2 vs PC3
PC2 vs PC4
PC3 vs PC4
Scree Plot
PC1 (65.83%)
PC2 (17.32%)
PC3 (8.78%)
PC4 (1.27%)
40.00%
65.83%
17.32%
8.78%
Eigenvalue
Principal Component
Legend
PMH
BMH
OMH
BLH
HS
Chn (DoS~1)
Chn (DoS~2)
Chn (DoS~3)
HA
Contaminants

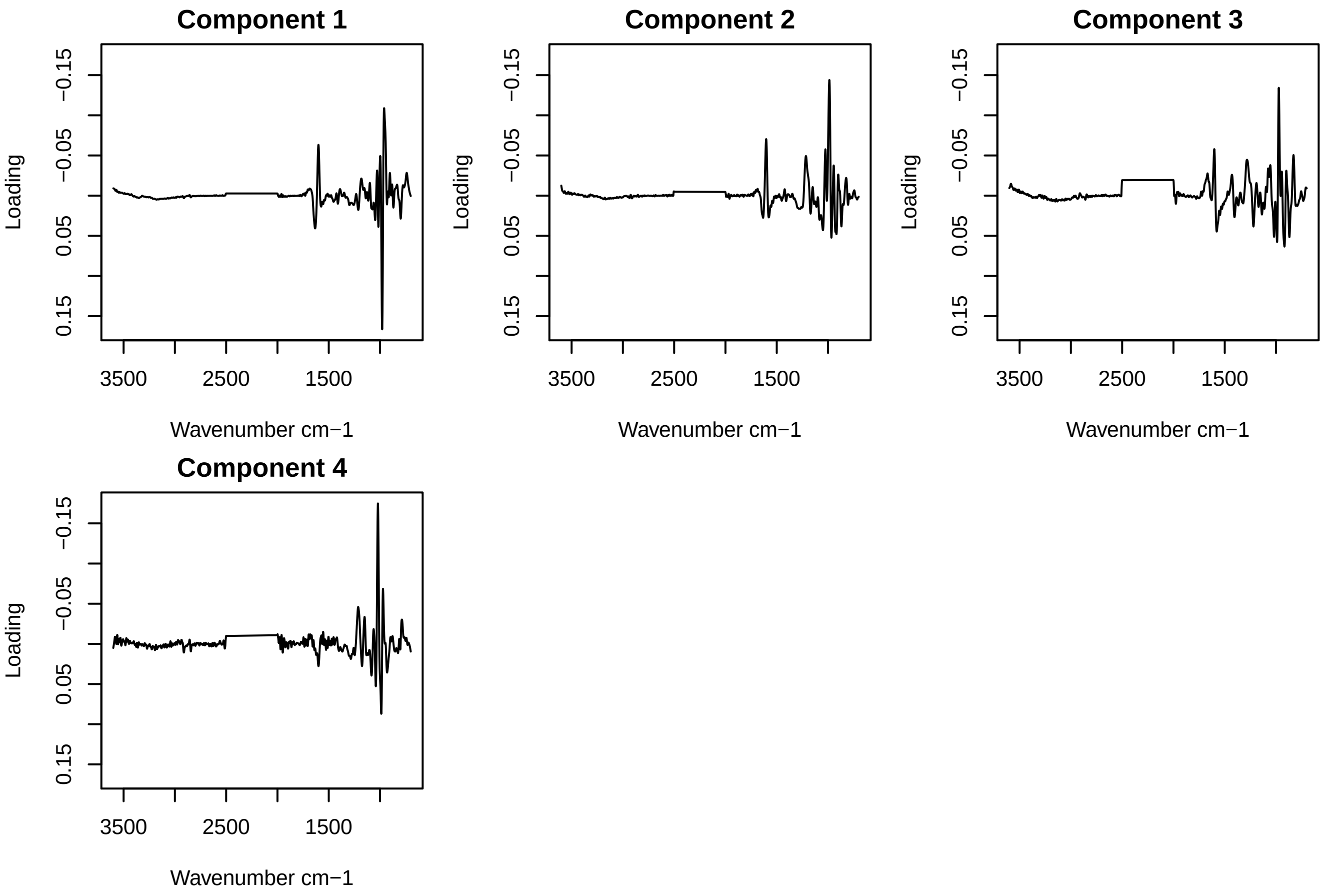

Component 1
Component 2
Component 3
Component 4
Loading
−0.15
−0.05
0.05
0.15
3500
2500
1500
Wavenumber cm−1

PC1 vs PC2 vs PC3

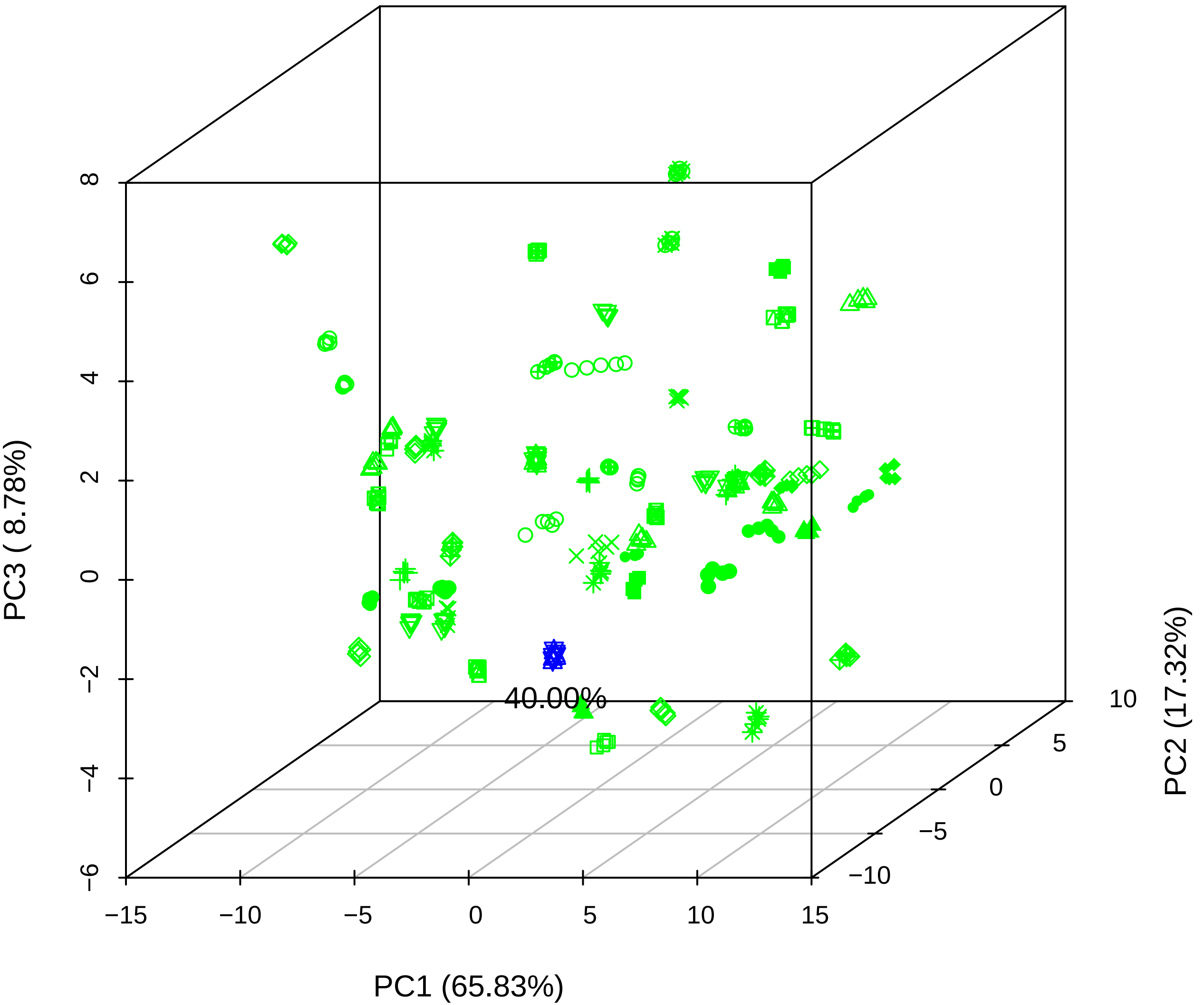

PC1 vs PC2 vs PC4

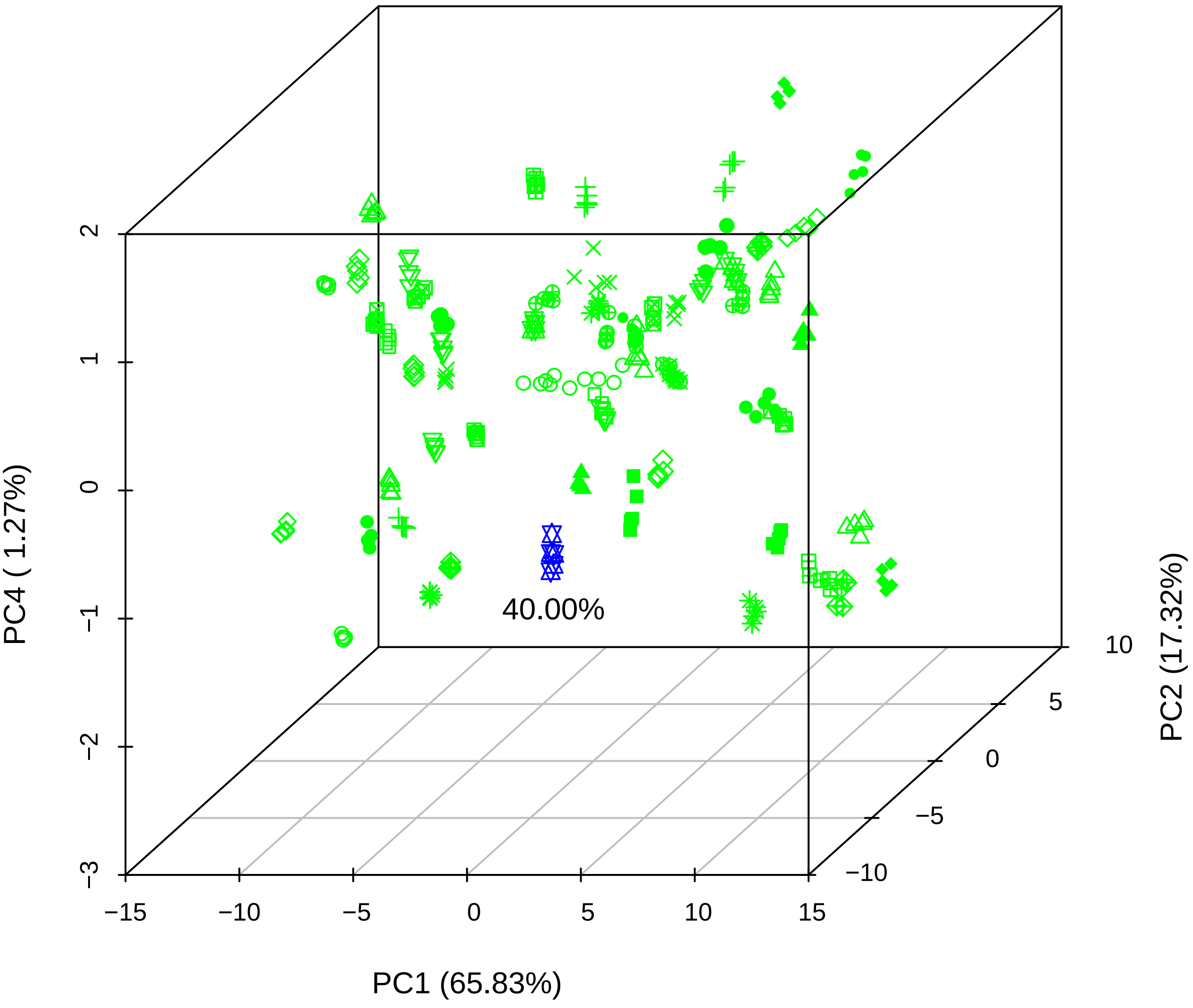

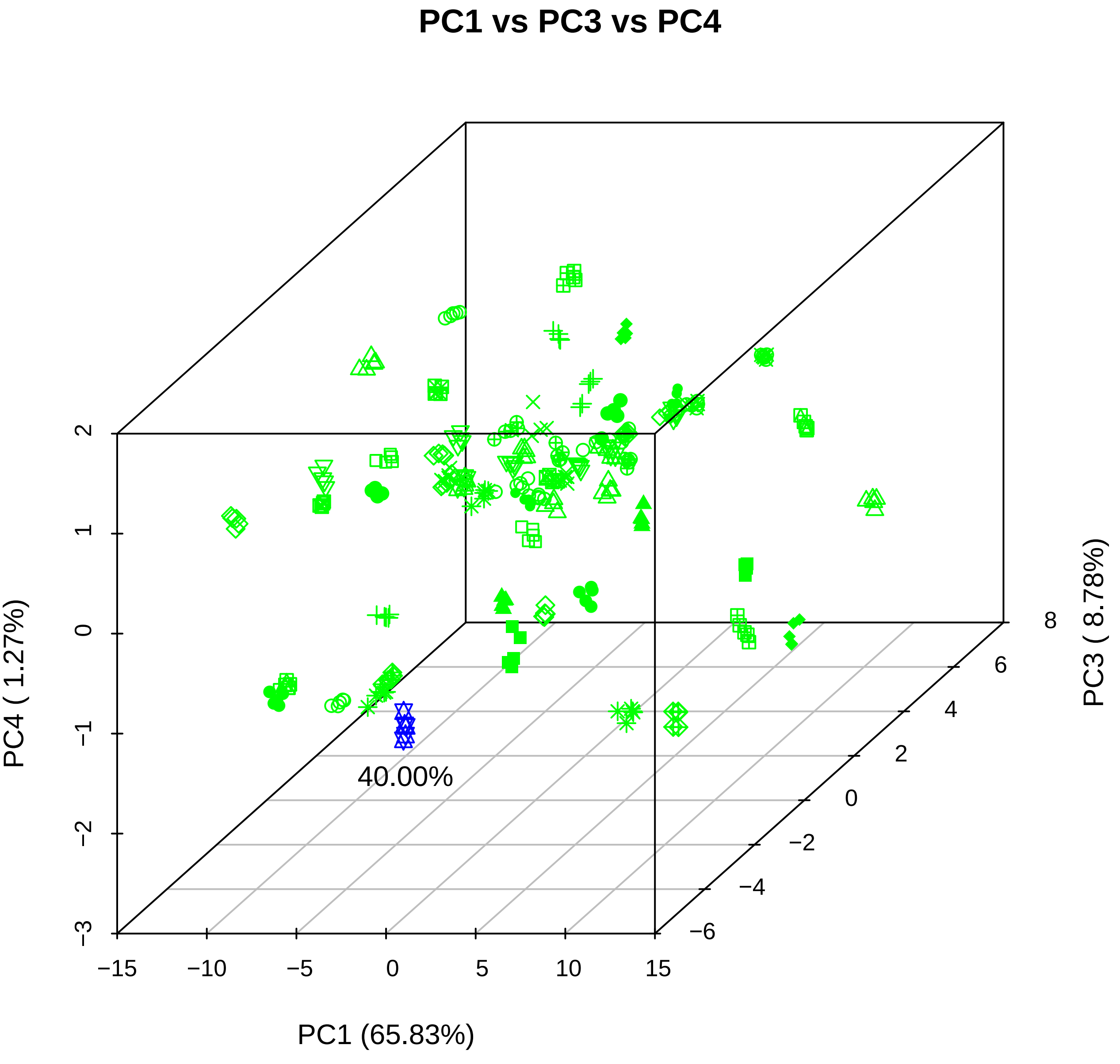

PC1 vs PC3 vs PC4
PC4 (1.27%)
PC3 (8.78%)
PC1 (65.83%)
40.00%
−15
−10
−5
0
5
10
15
−3
−2
−1
0
1
2
−6
−4
−2
0
2
4
6
8

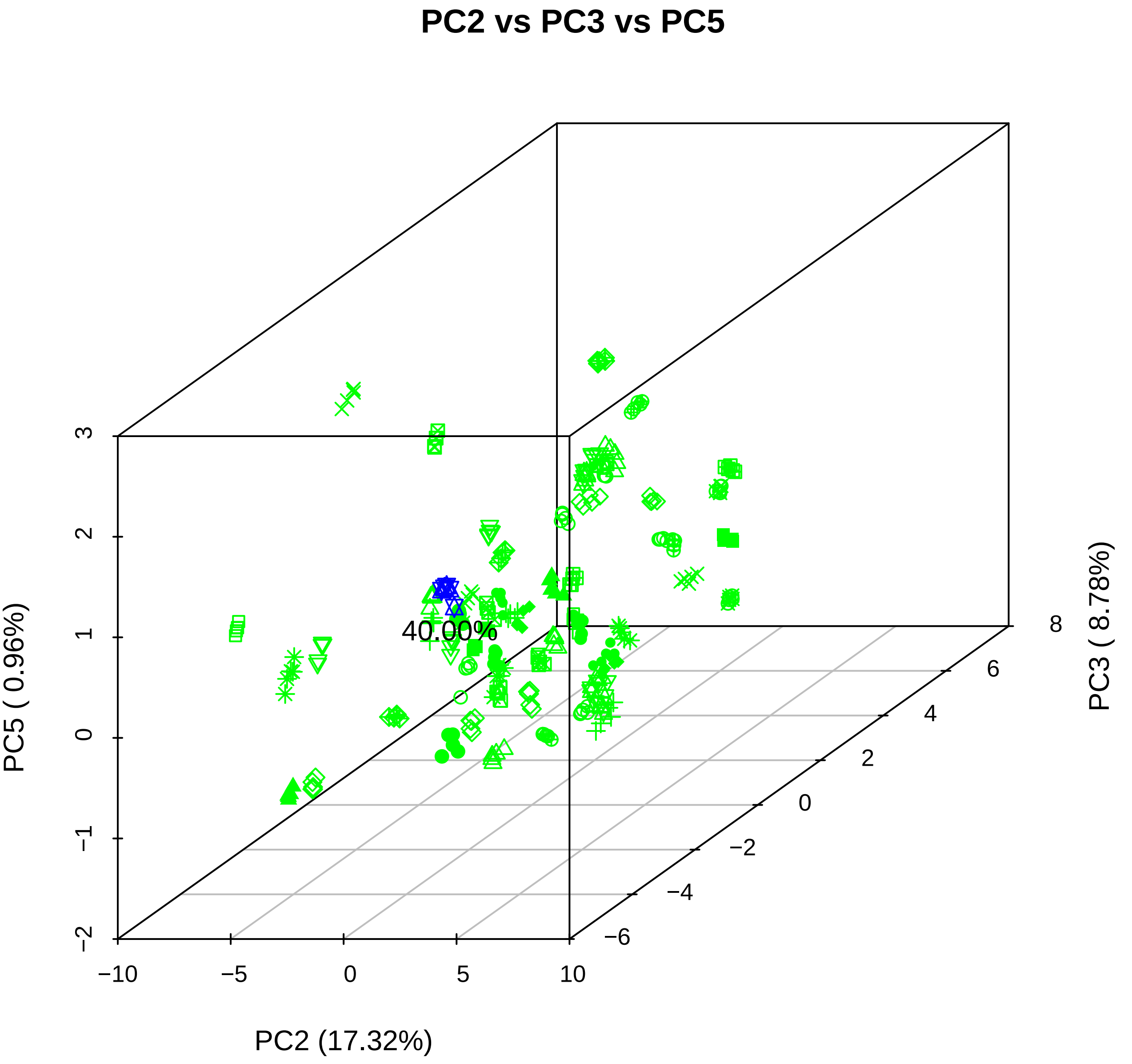
PC2 vs PC3 vs PC5
40.00%
PC2 (17.32%)
PC5 (0.96%)
PC3 (8.78%)
−10
−5
0
5
10
−2
−1
0
1
2
3
−6
−4
−2
0
2
4
6
8

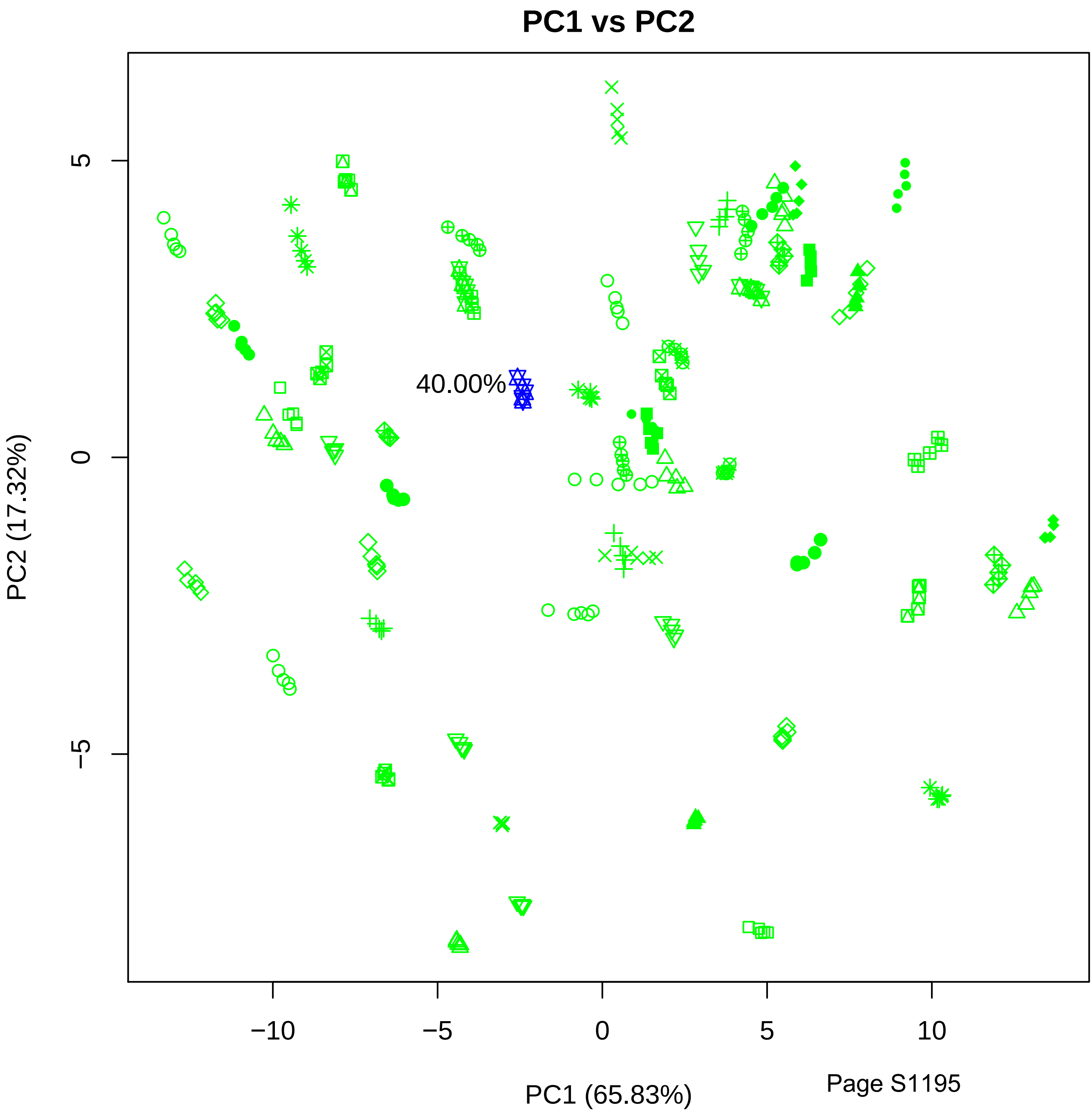

PC1 vs PC2
40.00%
5
0
−5
−10
−5
0
5
10
PC2 (17.32%)
PC1 (65.83%)

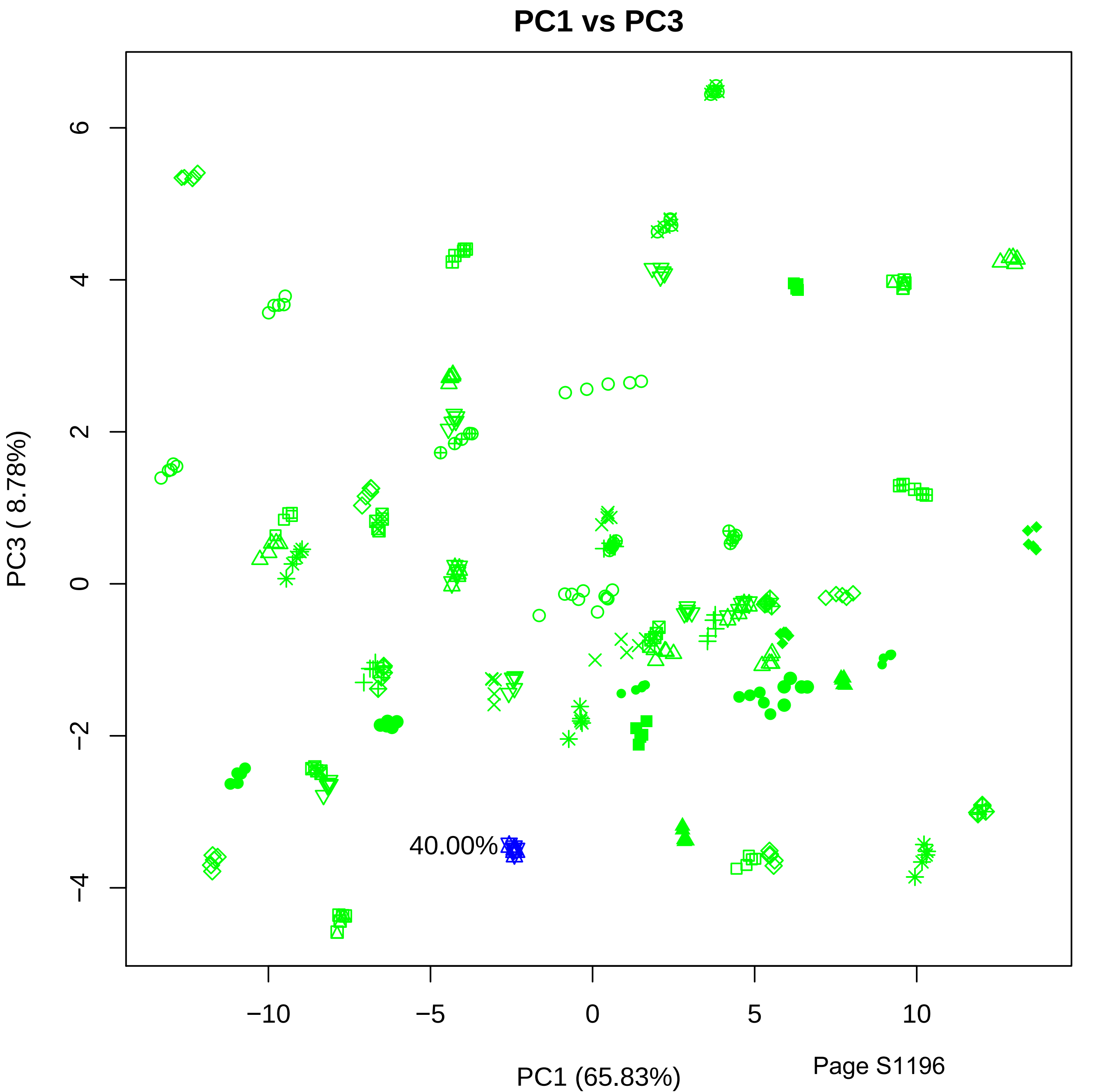
PC1 vs PC3
PC3 (8.78%)
PC1 (65.83%)
40.00%
6
4
2
0
−2
−4
−10
−5
0
5
10

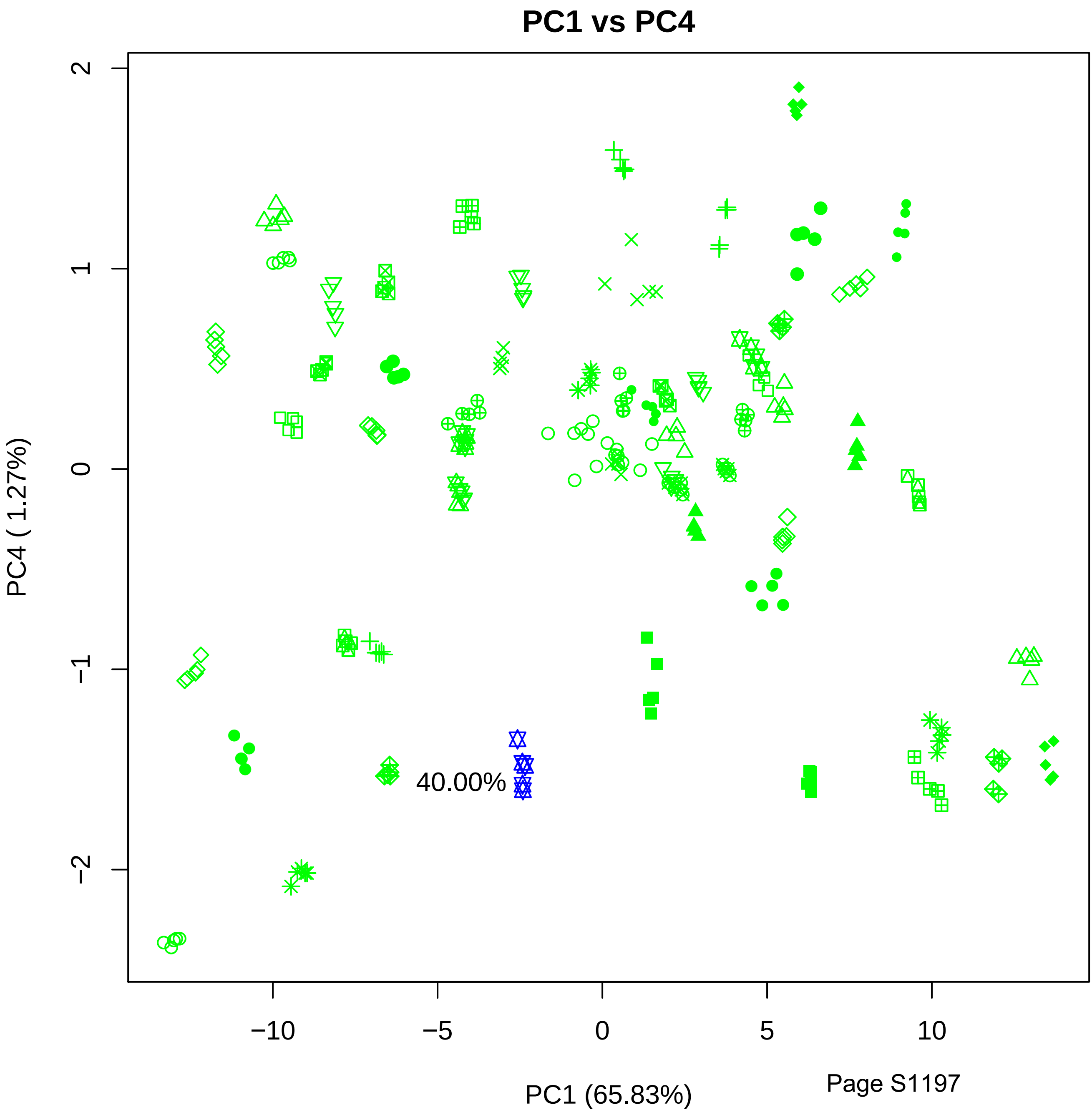
PC1 vs PC4
PC4 (1.27%)
PC1 (65.83%)
40.00%
−10
−5
0
5
10
2
1
0
−1
−2

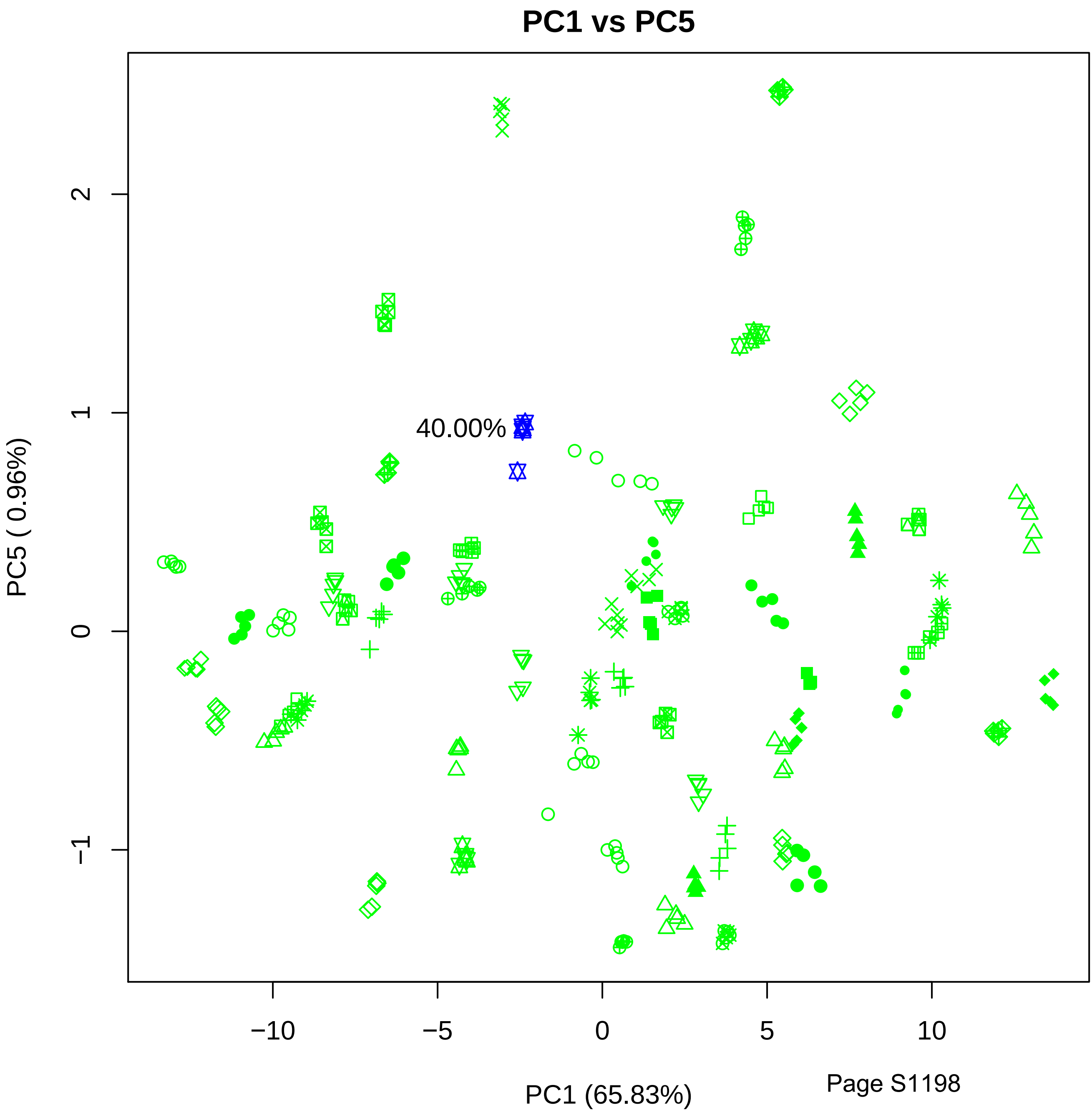

PC1 vs PC5
2
1
0
−1
PC5 (0.96%)
40.00%
−10
−5
0
5
10
PC1 (65.83%)

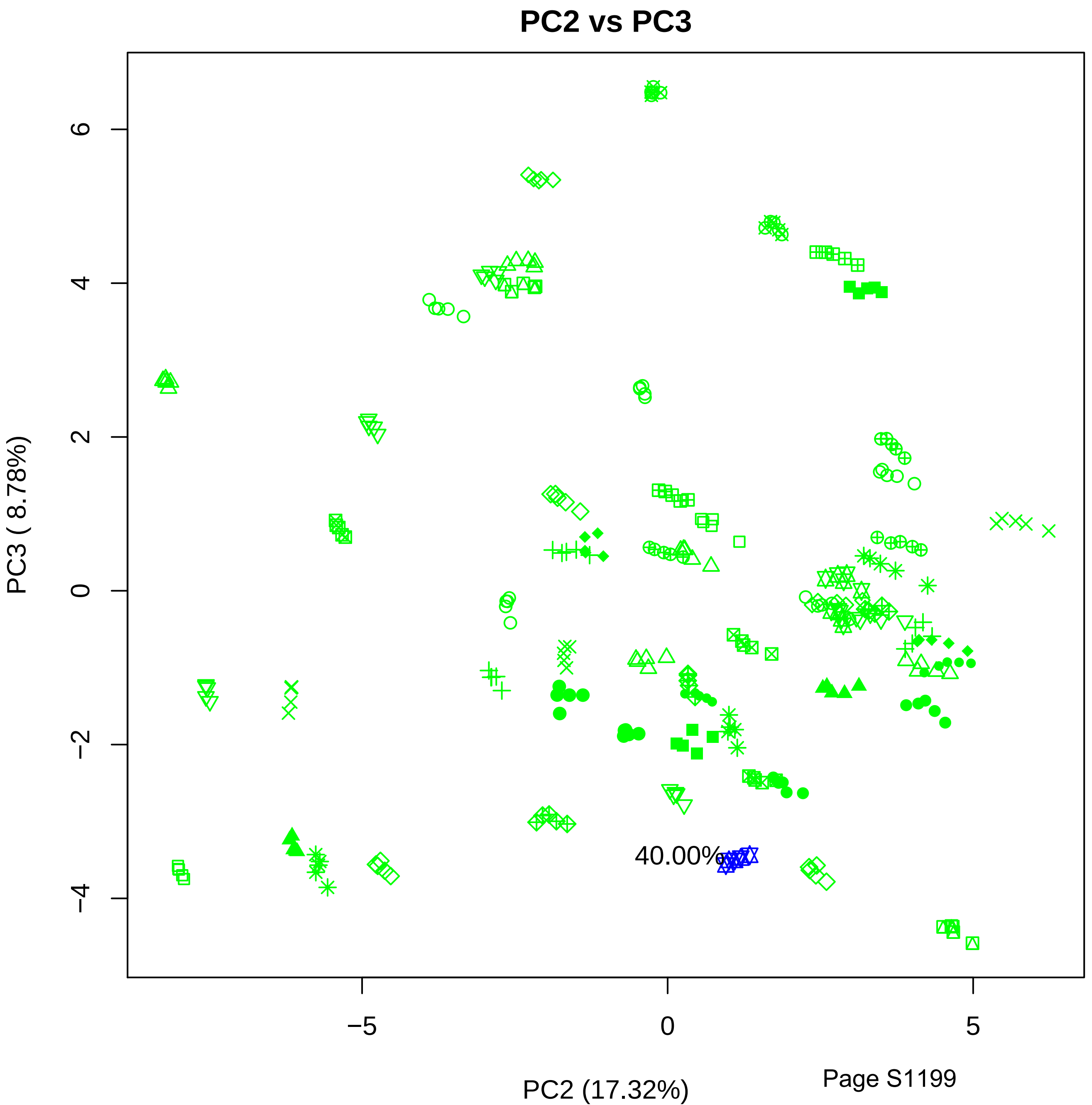
PC2 vs PC3
PC3 (8.78%)
PC2 (17.32%)
6
4
2
0
−2
−4
−5
0
5
40.00%

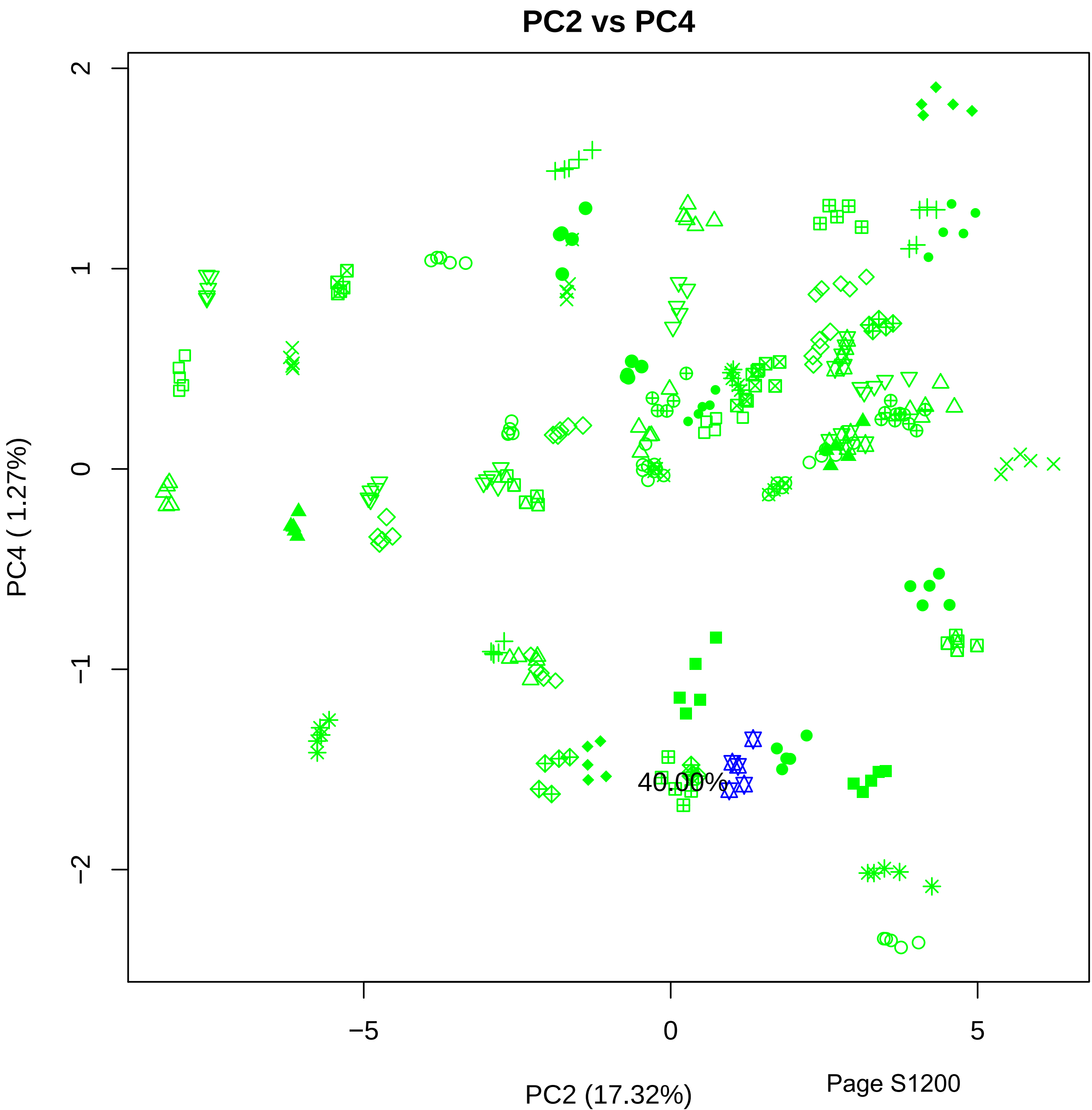

PC2 vs PC4
PC4 (1.27%)
PC2 (17.32%)
40.00%
2
1
0
−1
−2
−5
0
5

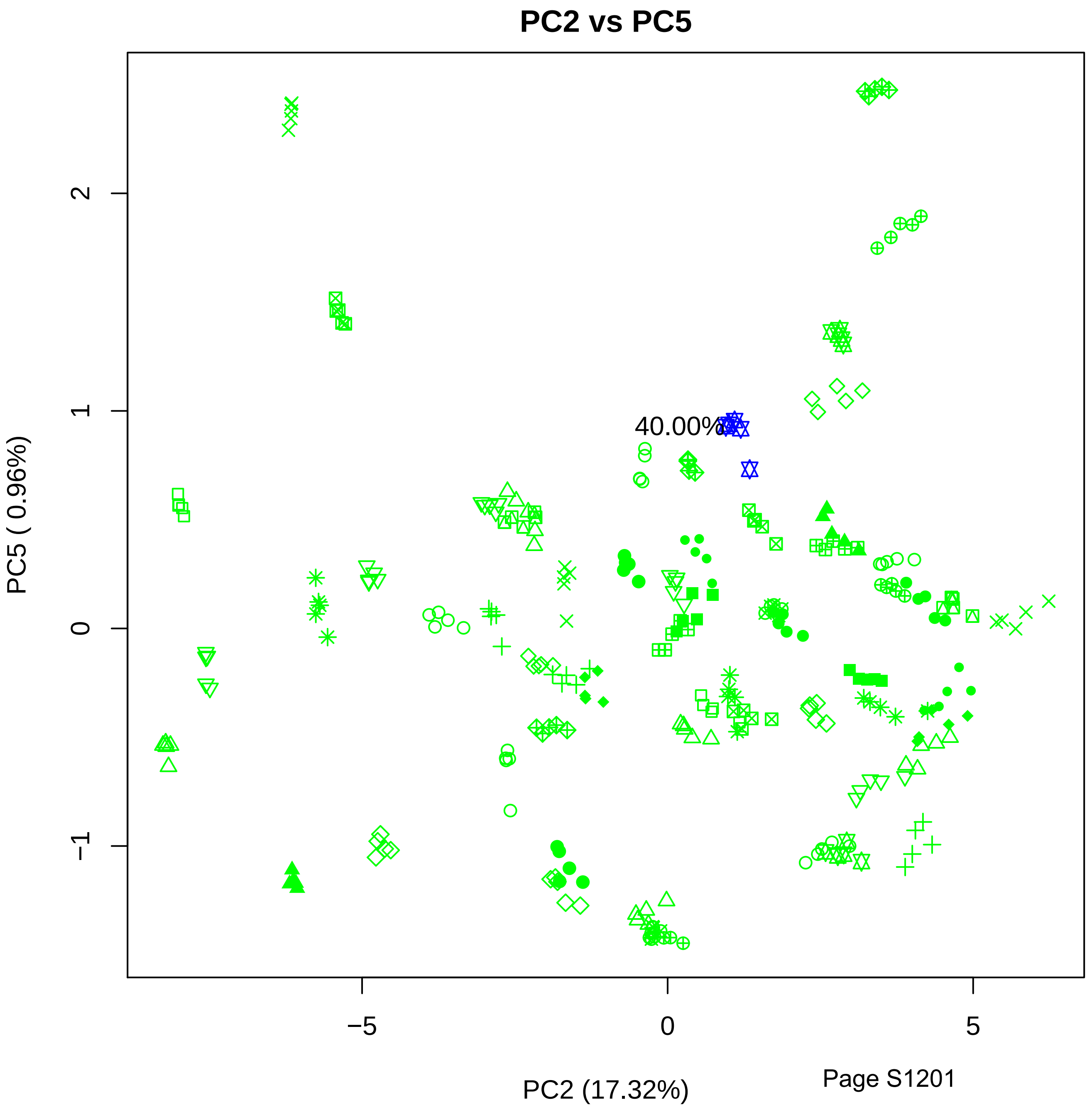
PC2 vs PC5
40.00%
PC5 (0.96%)
PC2 (17.32%)
−1
0
1
2
−5
0
5

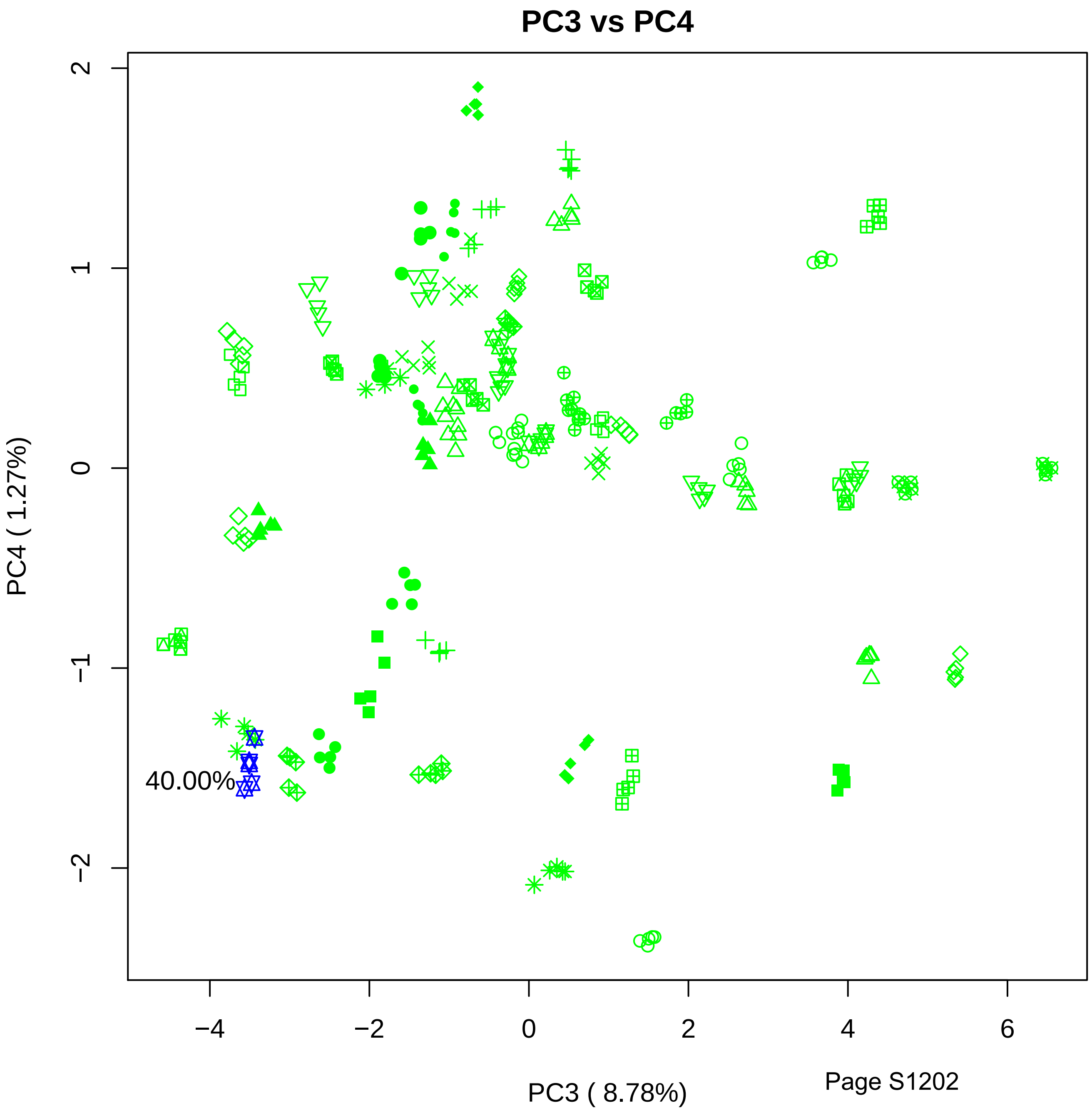
PC3 vs PC4
PC4 (1.27%)
PC3 (8.78%)
40.00%
2
1
0
−1
−2
−4
−2
0
2
4
6

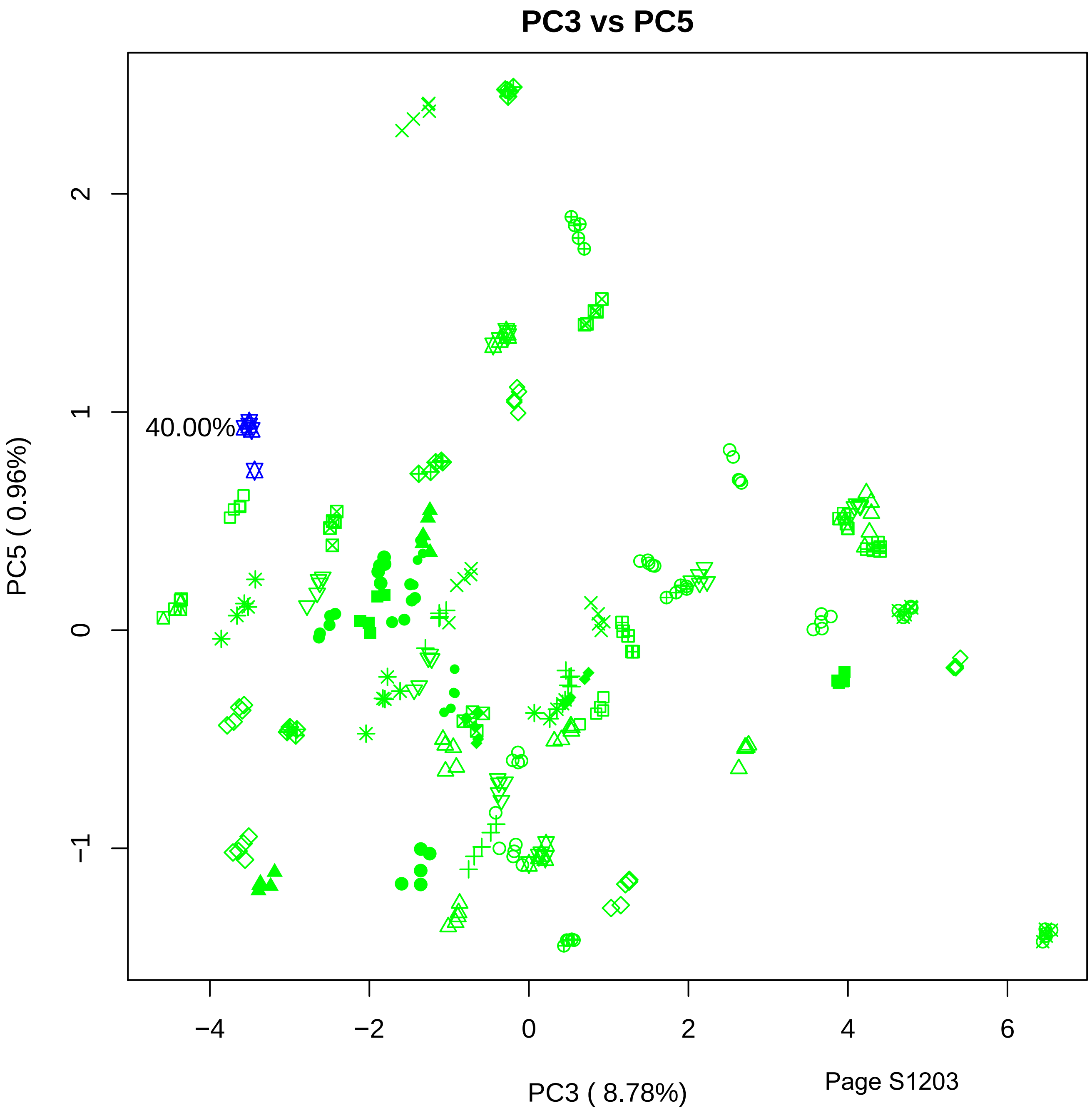
PC3 vs PC5
40.00%
PC5 (0.96%)
PC3 (8.78%)
2
1
0
−1
−4
−2
0
2
4
6

PC4 vs PC5

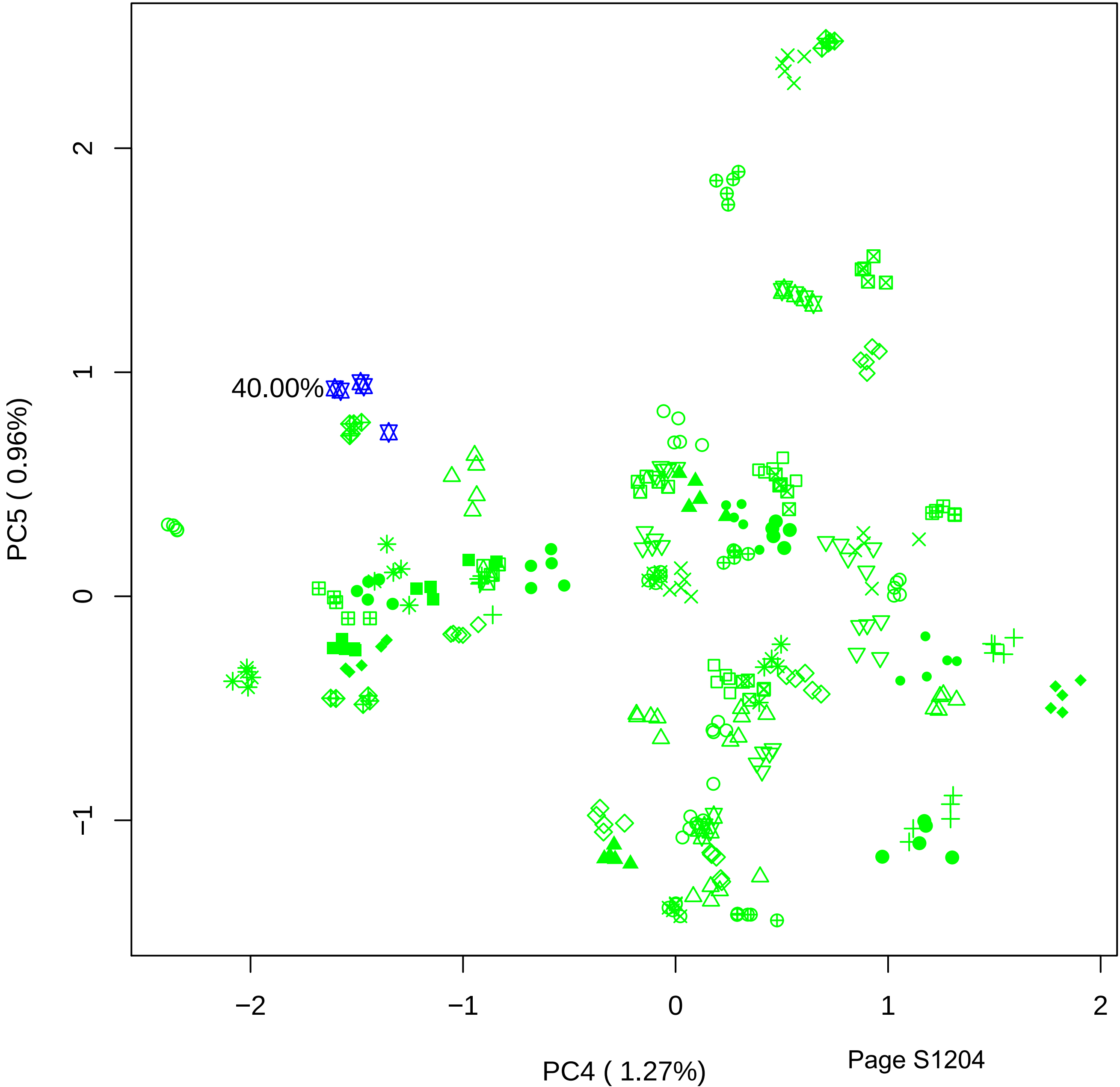

PCA of PMH vs PMH(c) contaminated with BMH(c)

Scaled Matrix PCAed

7th order polynomial baseline correction performed

Raw spectra smoothed to 21 Neighbours, 2nd order PN, Savtisky Golay

Normalisation between 0 and 100

D2 spectra smoothed to 41 Neighbours, 2nd order PN, Savtisky Golay

Regions Analysed: 3601−2501 and 2001−700cm−1

Samples Analysed: 8 bContam's 0 BLH's 0 BMH's 0 OMH's 0 pContam's 0 PLH's 60 PMH's

Keele University AD

Fri Dec 14 14:18:29 2018

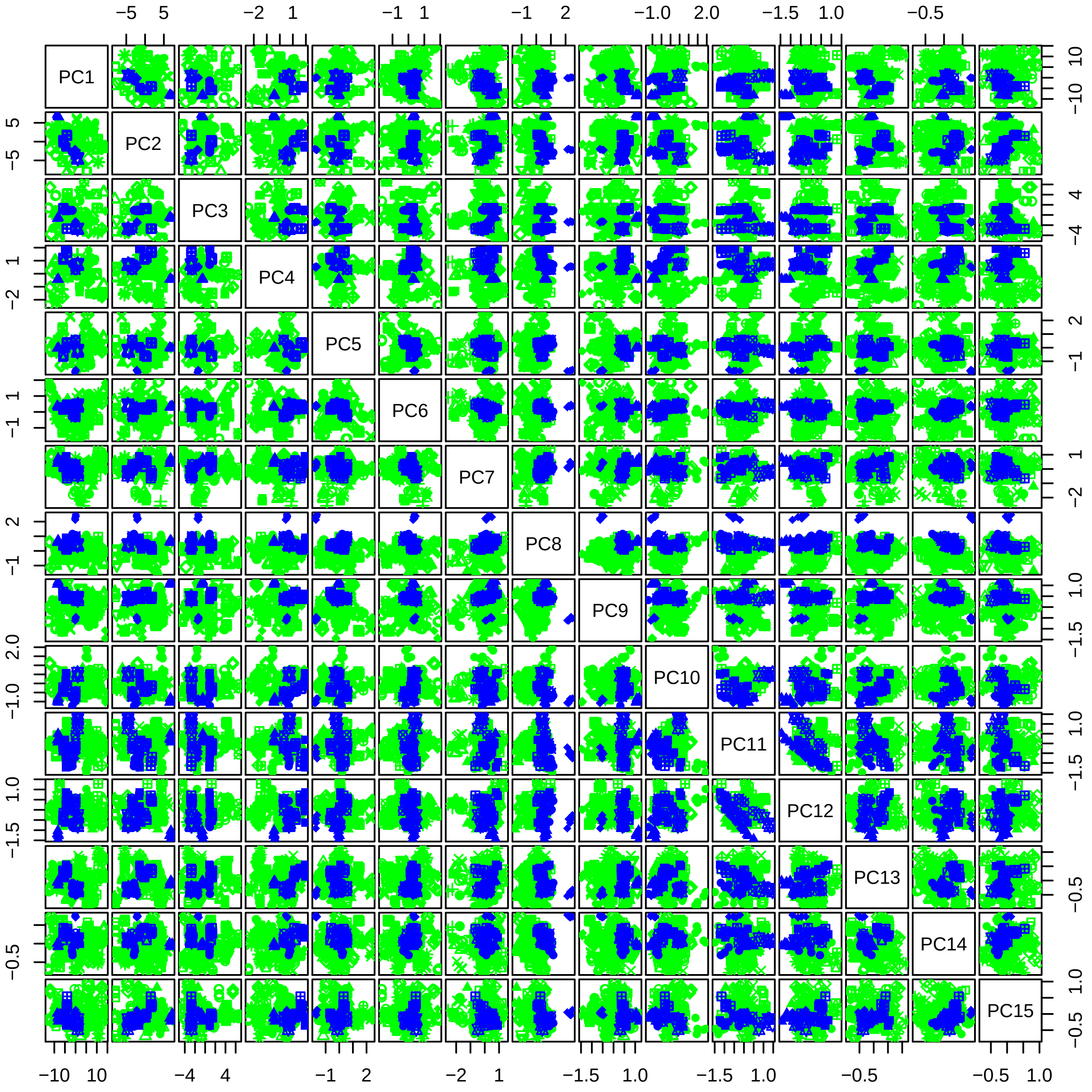
PC1
PC2
PC3
PC4
PC5
PC6
PC7
PC8
PC9
PC10
PC11
PC12
PC13
PC14
PC15

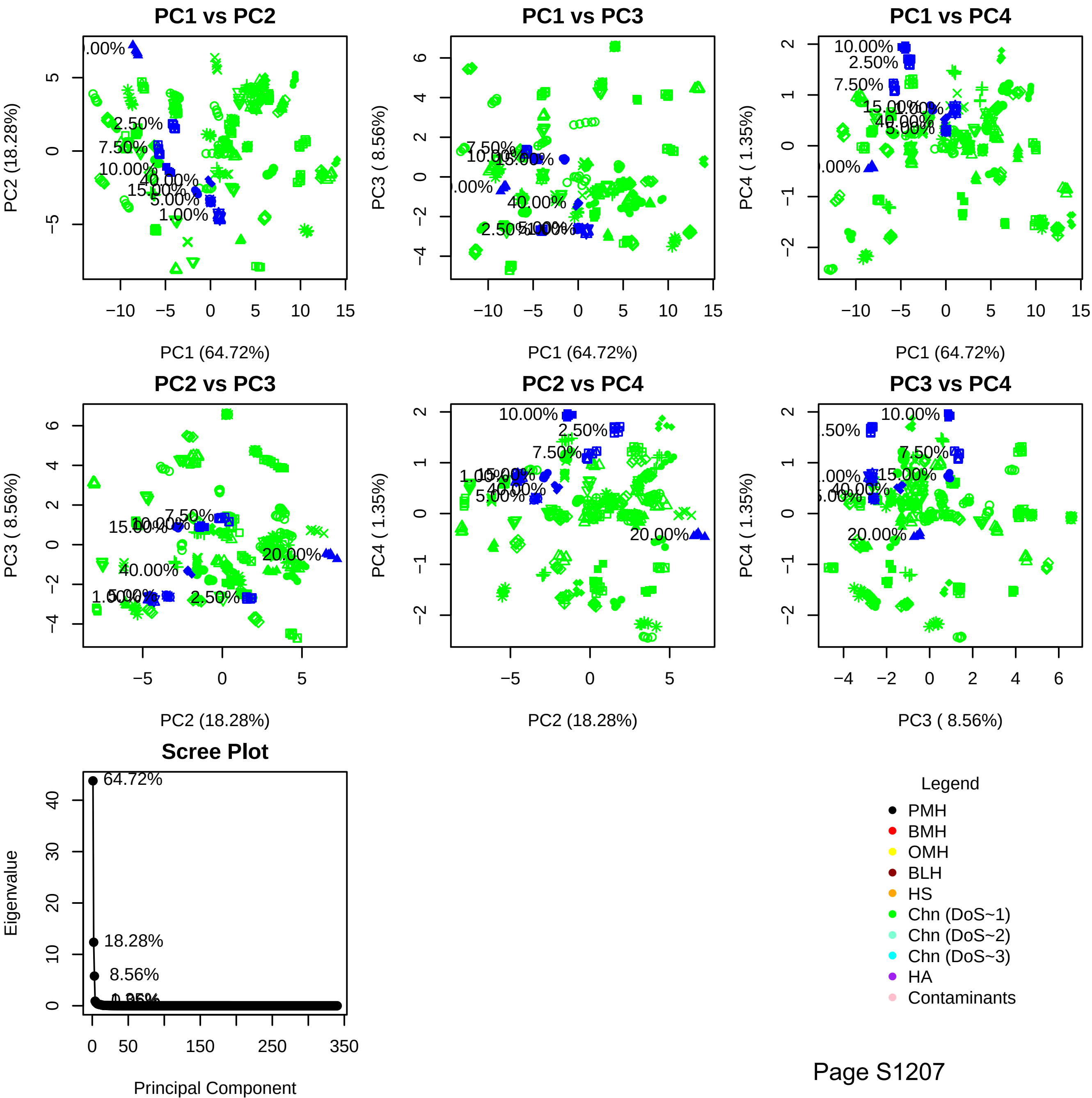
PC1 vs PC2
PC1 vs PC3
PC1 vs PC4
PC2 vs PC3
PC2 vs PC4
PC3 vs PC4
PC1 (64.72%)
PC2 (18.28%)
PC3 (8.56%)
PC4 (1.35%)
Scree Plot
Eigenvalue
Principal Component
64.72%
18.28%
8.56%
Legend
PMH
BMH
OMH
BLH
HS
Chn (DoS~1)
Chn (DoS~2)
Chn (DoS~3)
HA
Contaminants

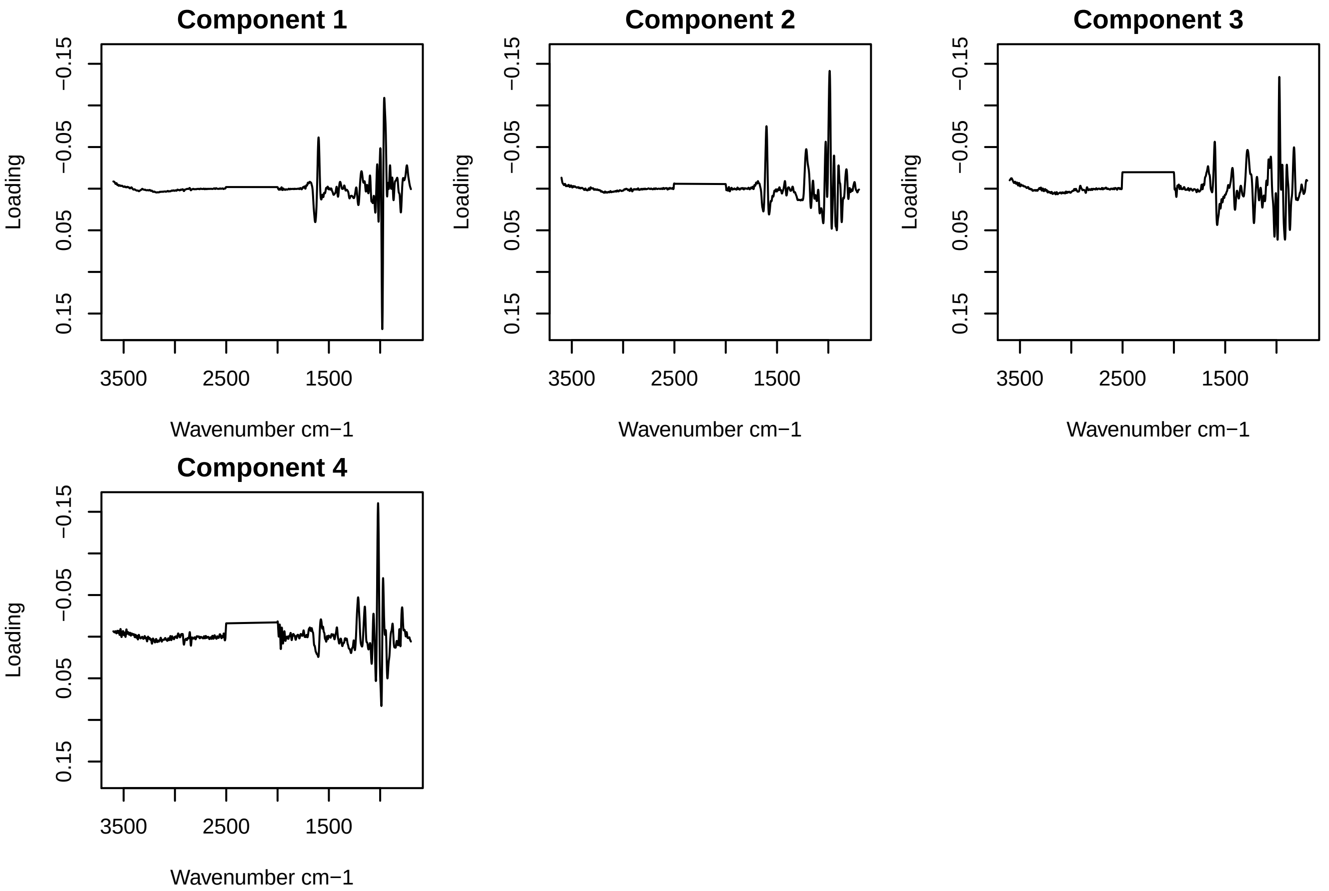
Component 1
Component 2
Component 3
Component 4
Loading
−0.15
−0.05
0.05
0.15
3500
2500
1500
Wavenumber cm−1

PC1 vs PC2 vs PC3

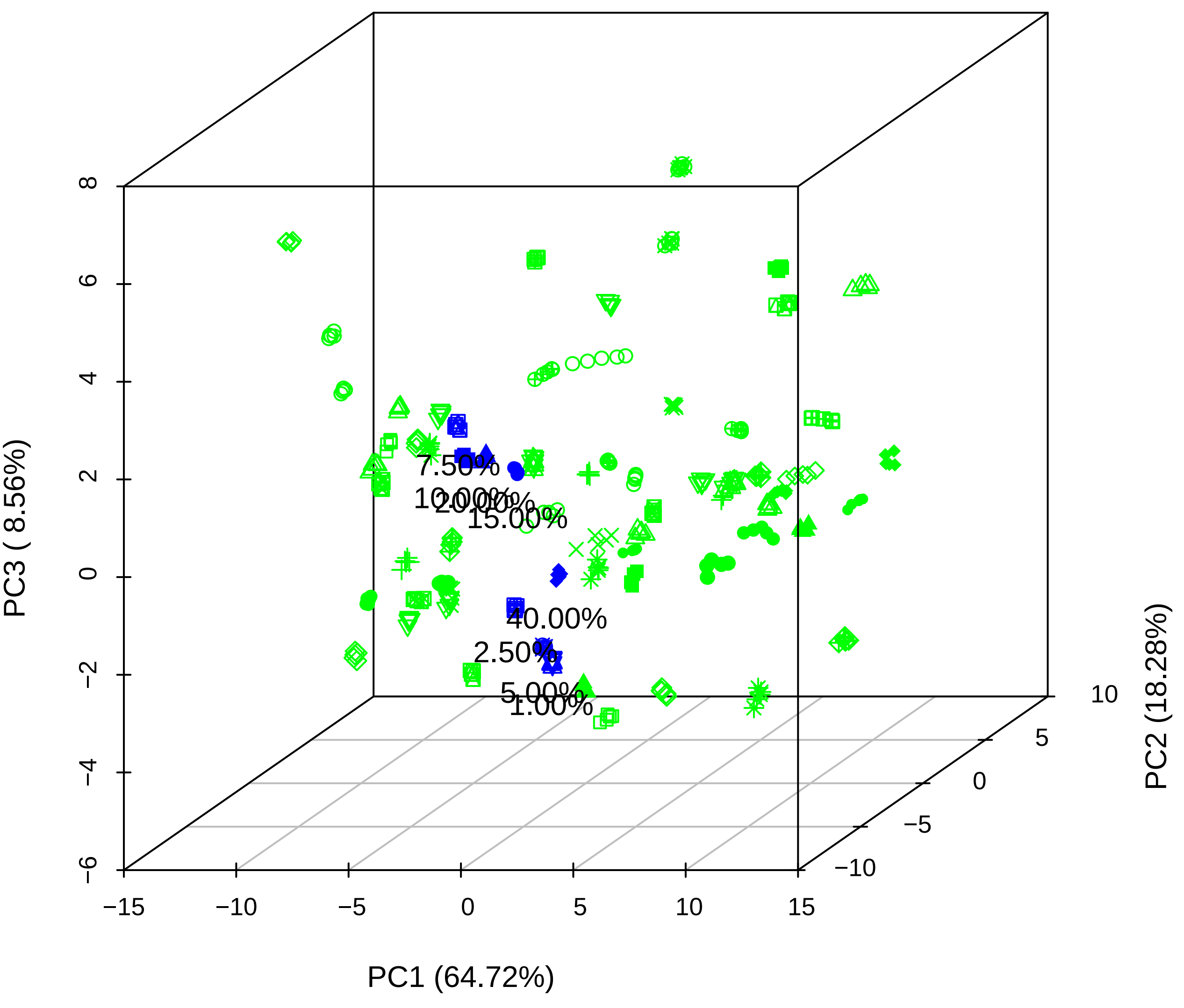

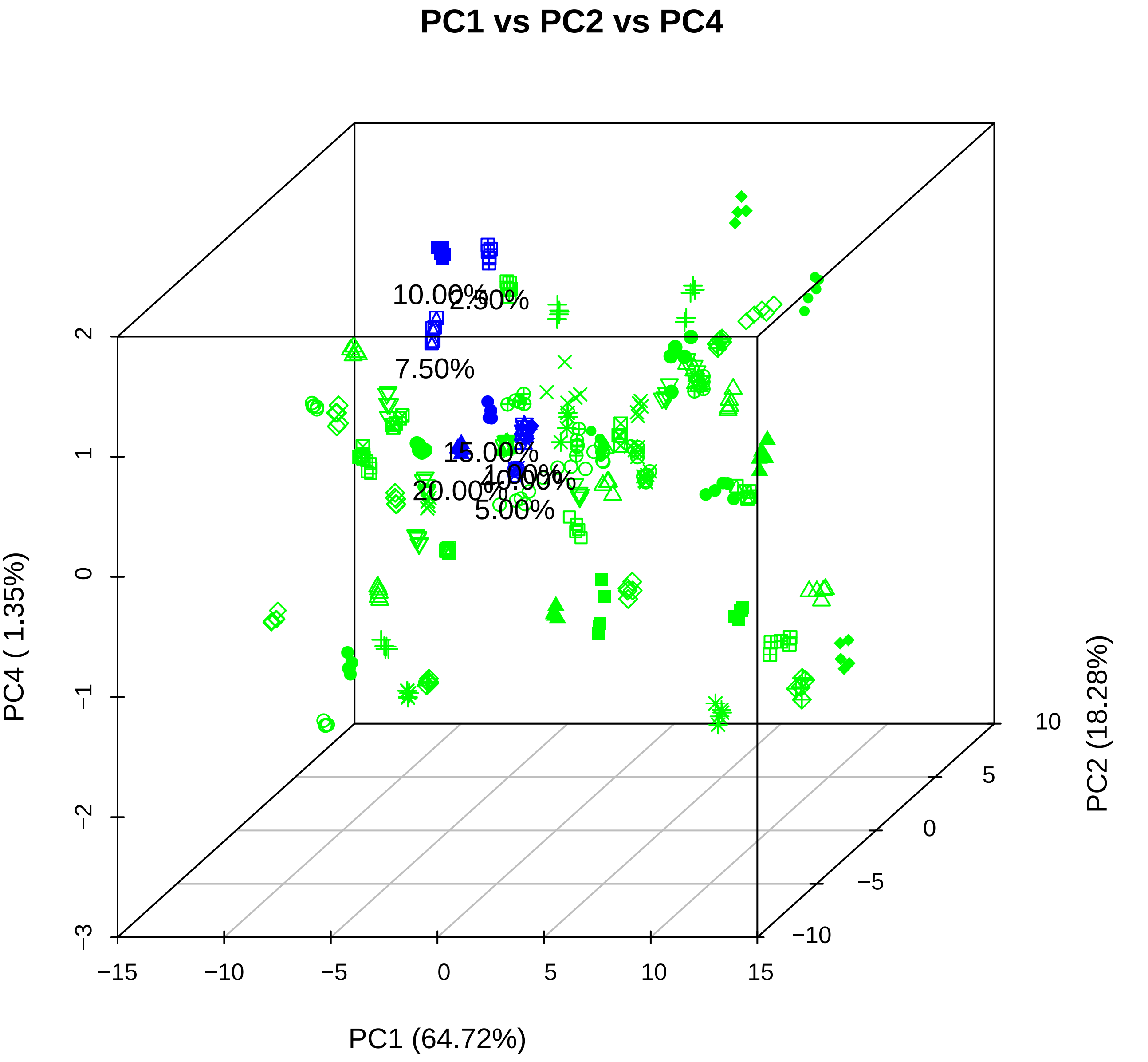
PC1 vs PC2 vs PC4
PC4 (1.35%)
PC1 (64.72%)
PC2 (18.28%)
10.00%
2.50%
7.50%
15.00%
20.00%
5.00%
−15
−10
−5
0
5
10
15
−3
−2
−1
0
1
2
−10
−5
0
5
10

PC1 vs PC3 vs PC4

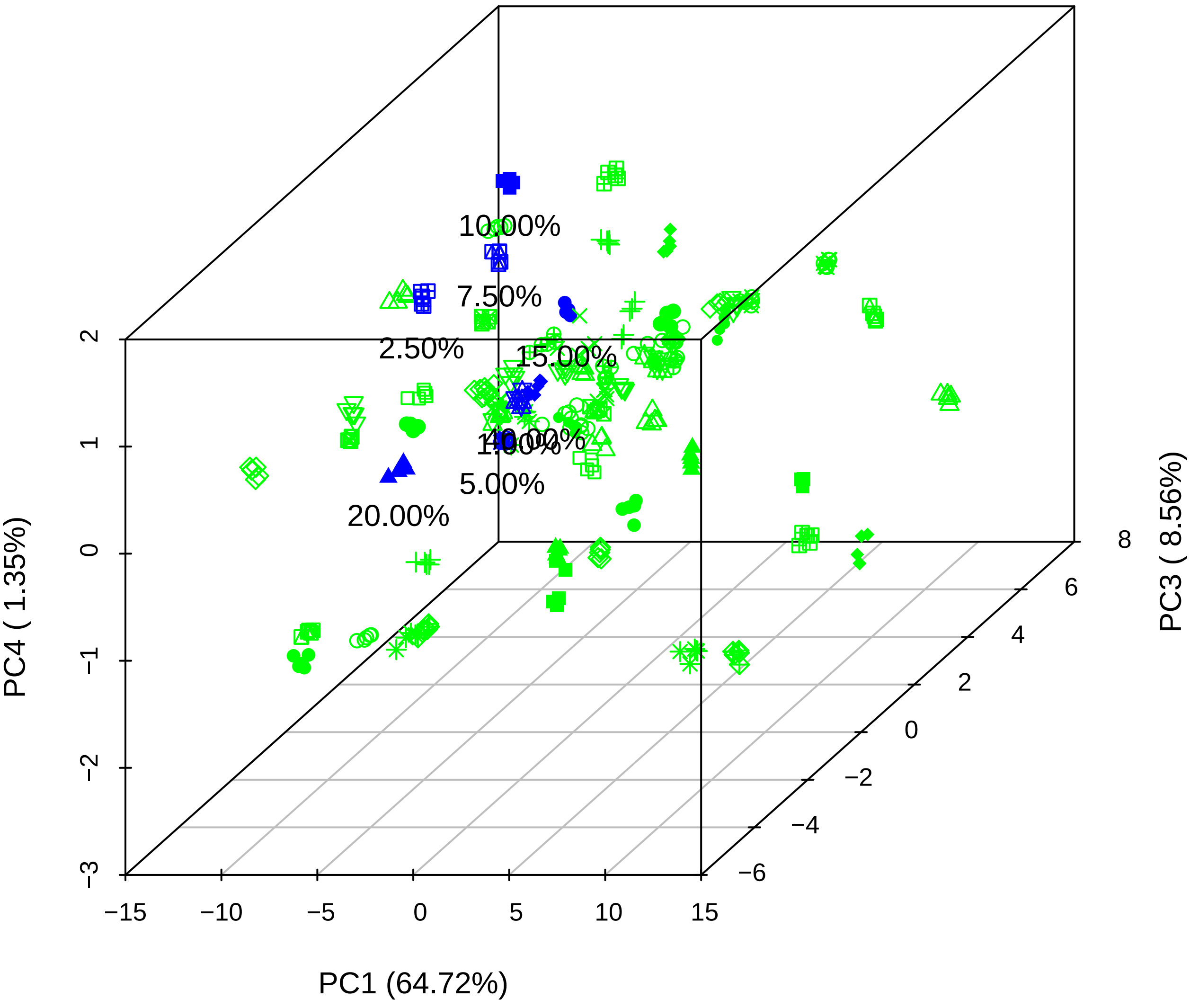

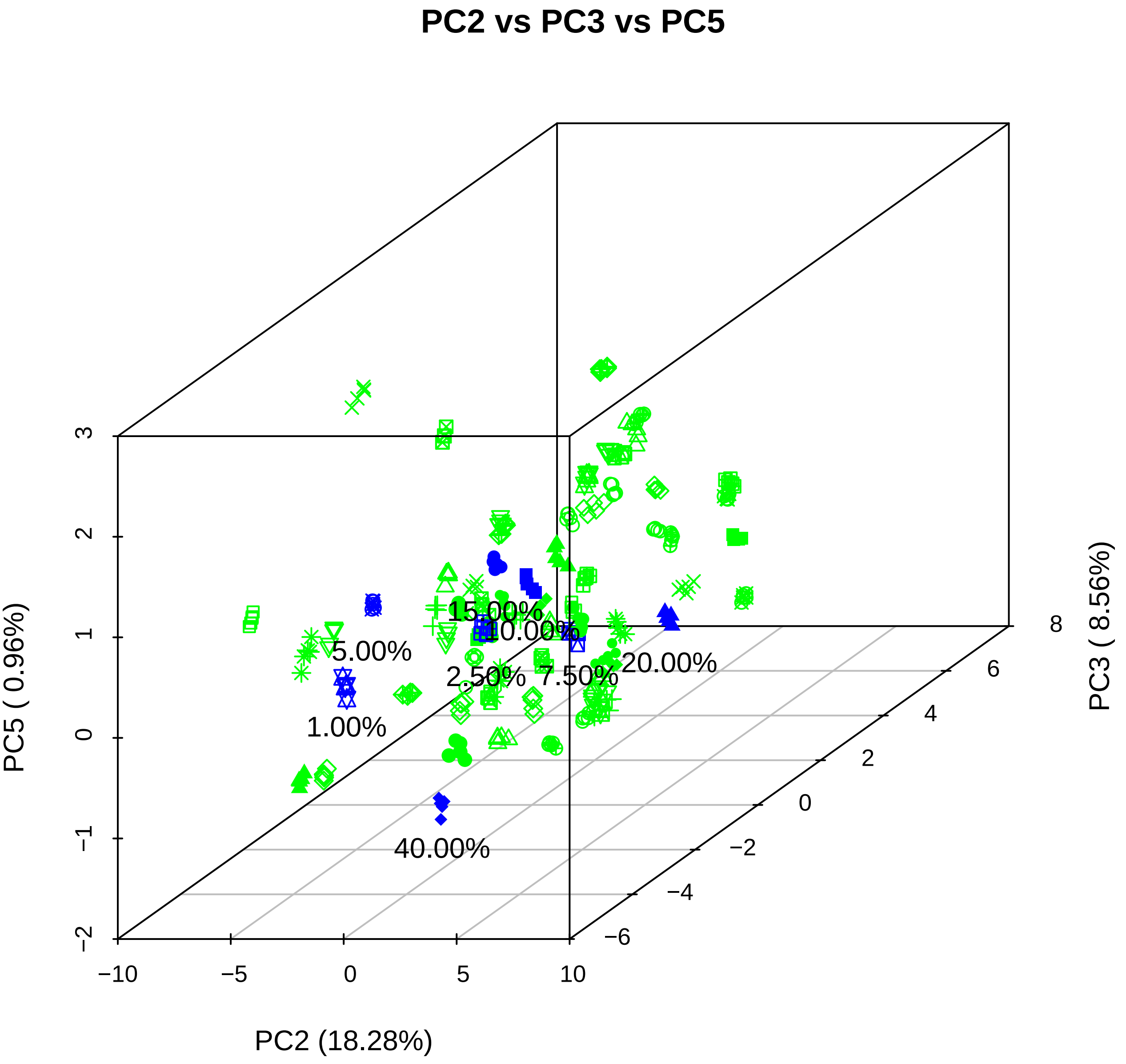

PC2 vs PC3 vs PC5
PC5 (0.96%)
PC2 (18.28%)
PC3 (8.56%)
−2
−1
0
1
2
3
−10
−5
0
5
10
−6
−4
−2
0
2
4
6
8
1.00%
2.50%
5.00%
7.50%
10.00%
15.00%
20.00%
40.00%

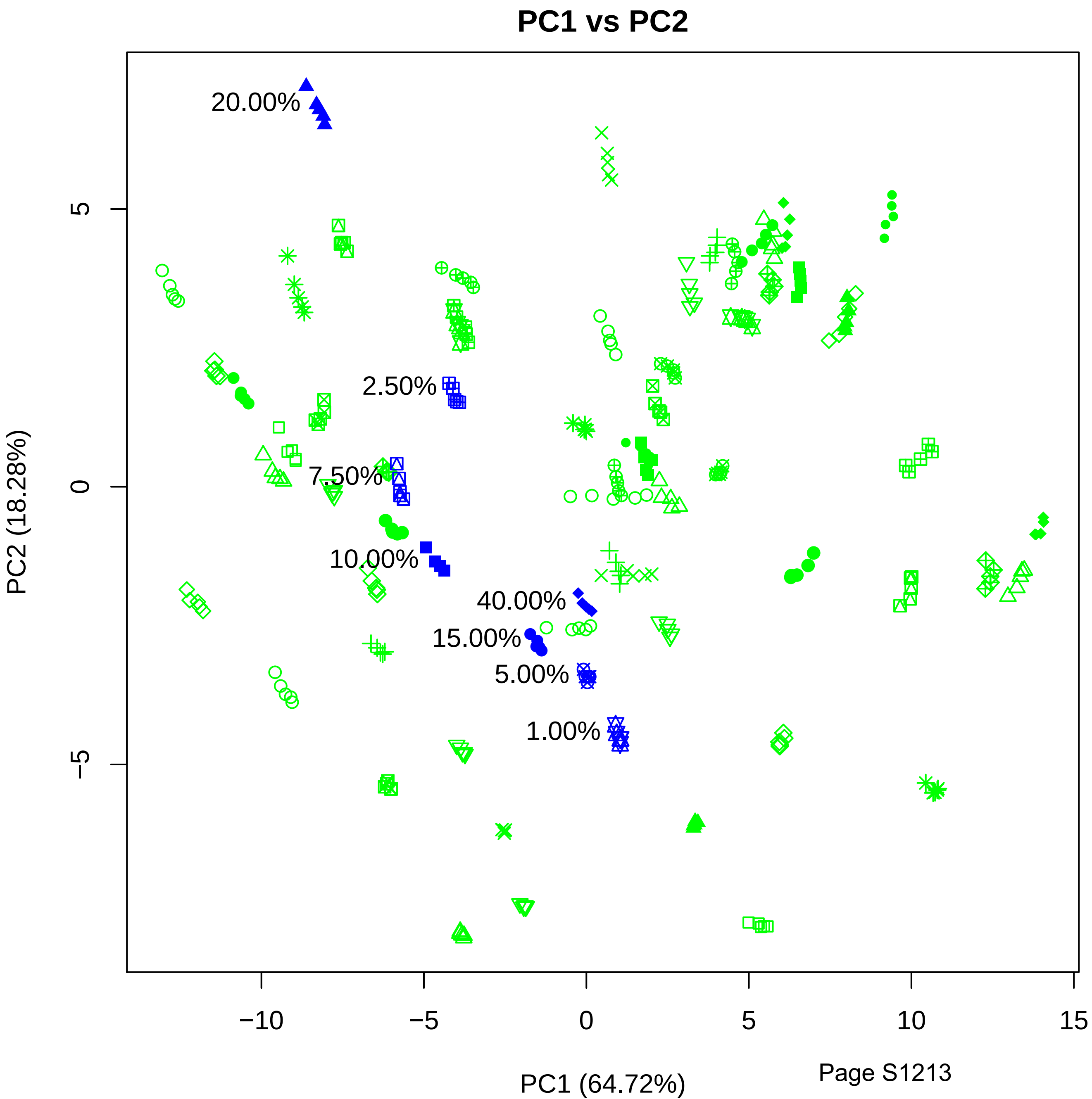
PC1 vs PC2
20.00%
2.50%
7.50%
10.00%
40.00%
15.00%
5.00%
1.00%
5
0
−5
−10
−5
0
5
10
15
PC2 (18.28%)
PC1 (64.72%)

PC1 vs PC3

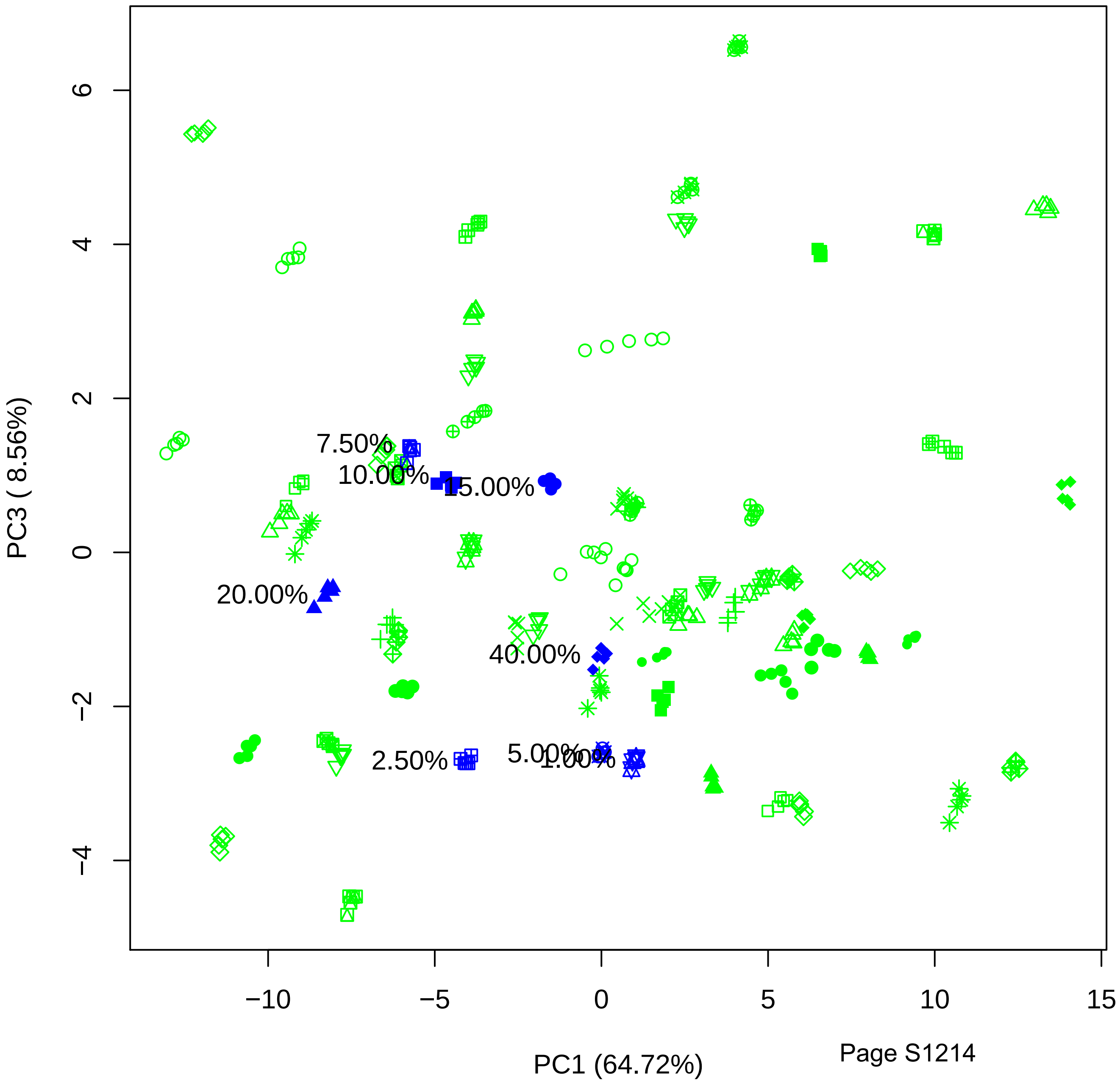

PC3 (8.56%)
PC1 (64.72%)
6
4
2
0
−2
−4
−10
−5
0
5
10
15
7.50%
10.00%
15.00%
20.00%
40.00%
2.50%
5.00%
1.00%

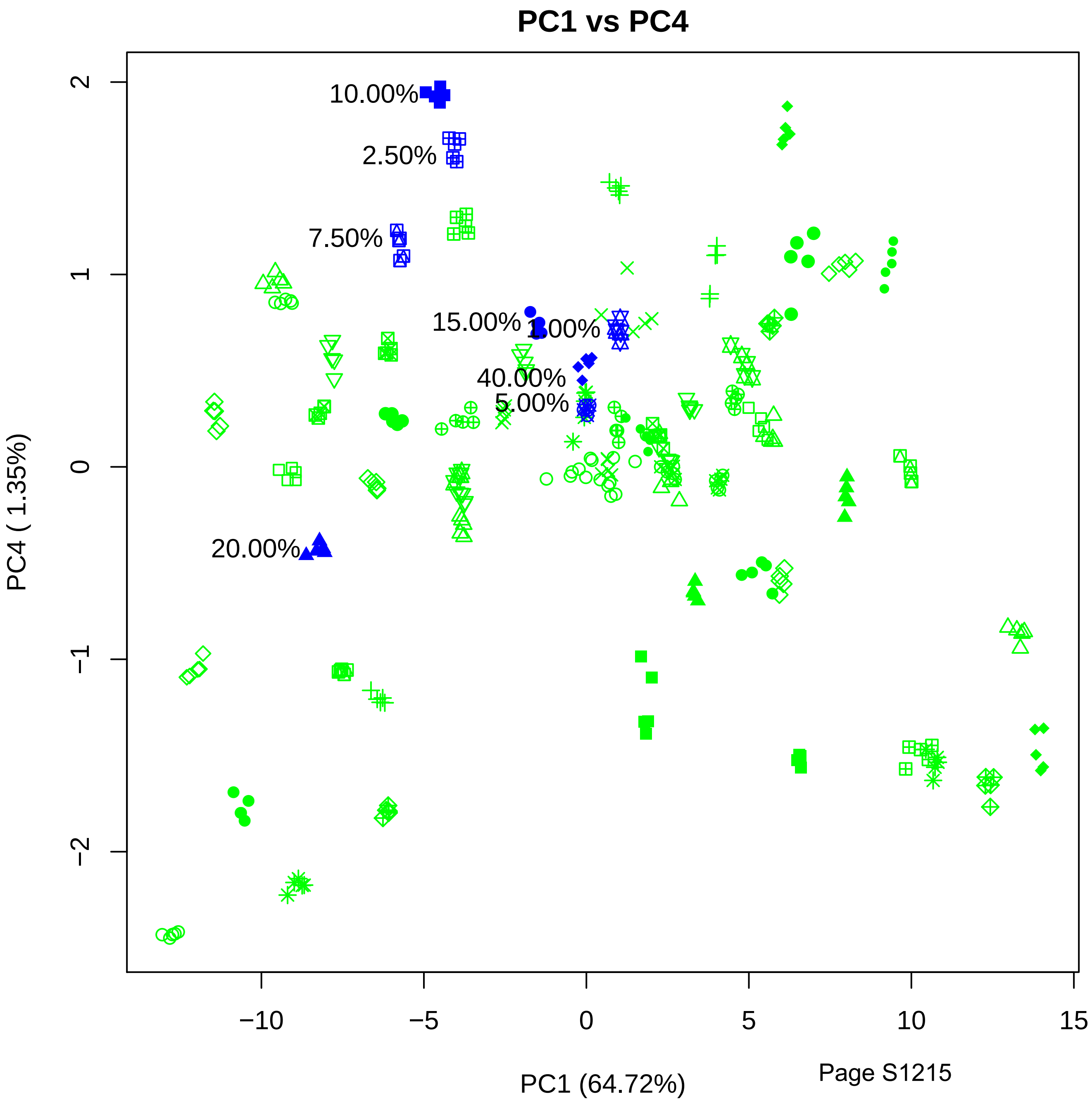
PC1 vs PC4
10.00%
2.50%
7.50%
15.00%
1.00%
40.00%
5.00%
20.00%
PC4 (1.35%)
PC1 (64.72%)
2
1
0
−1
−2
−10
−5
0
5
10
15

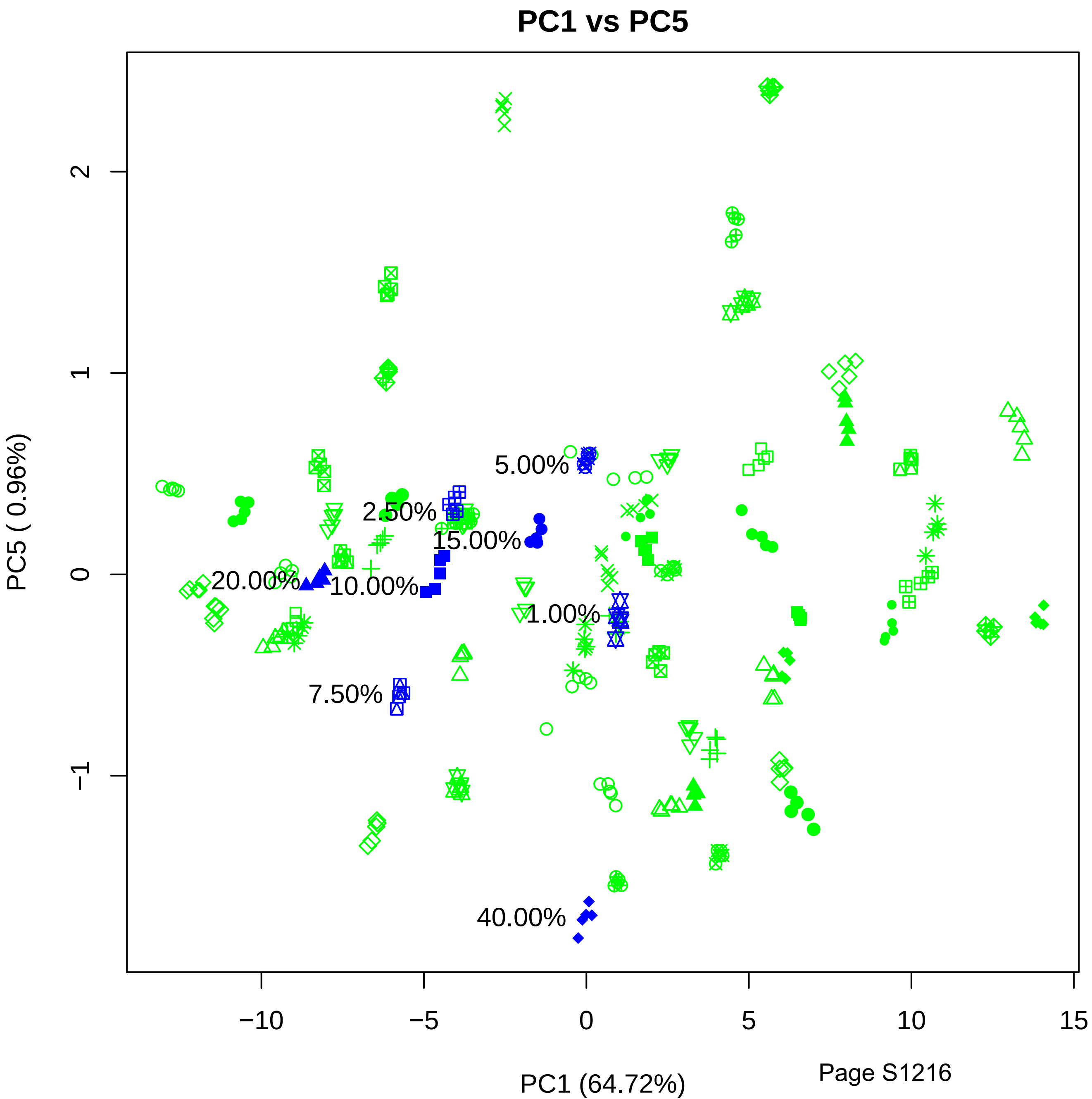

PC1 vs PC5
PC5 (0.96%)
PC1 (64.72%)
2
1
0
−1
−10
−5
0
5
10
15
5.00%
2.50%
15.00%
20.00%
10.00%
1.00%
7.50%
40.00%

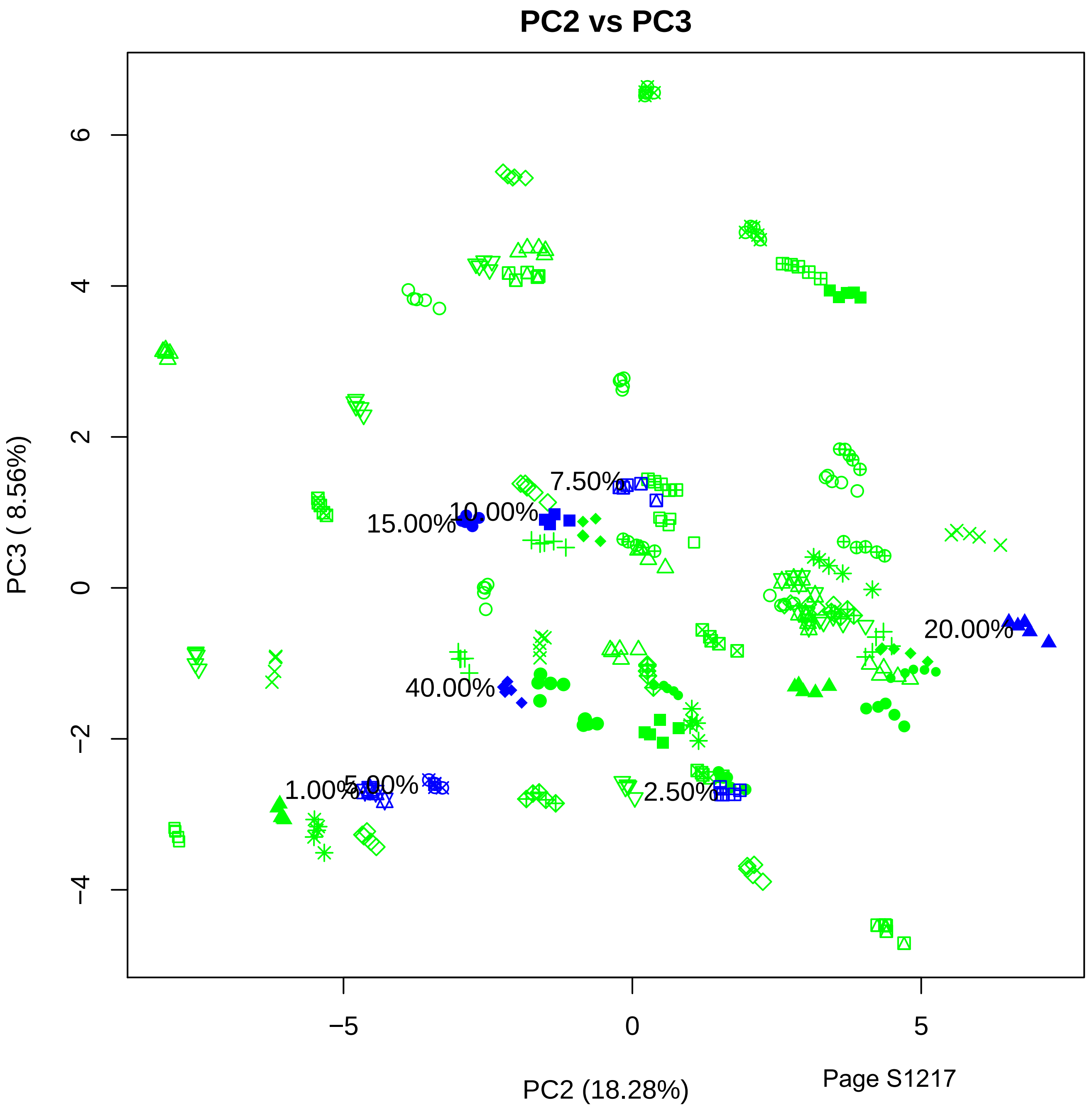
PC2 vs PC3
PC3 (8.56%)
PC2 (18.28%)
6
4
2
0
−2
−4
−5
0
5
7.50%
10.00%
15.00%
20.00%
40.00%
1.00%
5.00%
2.50%

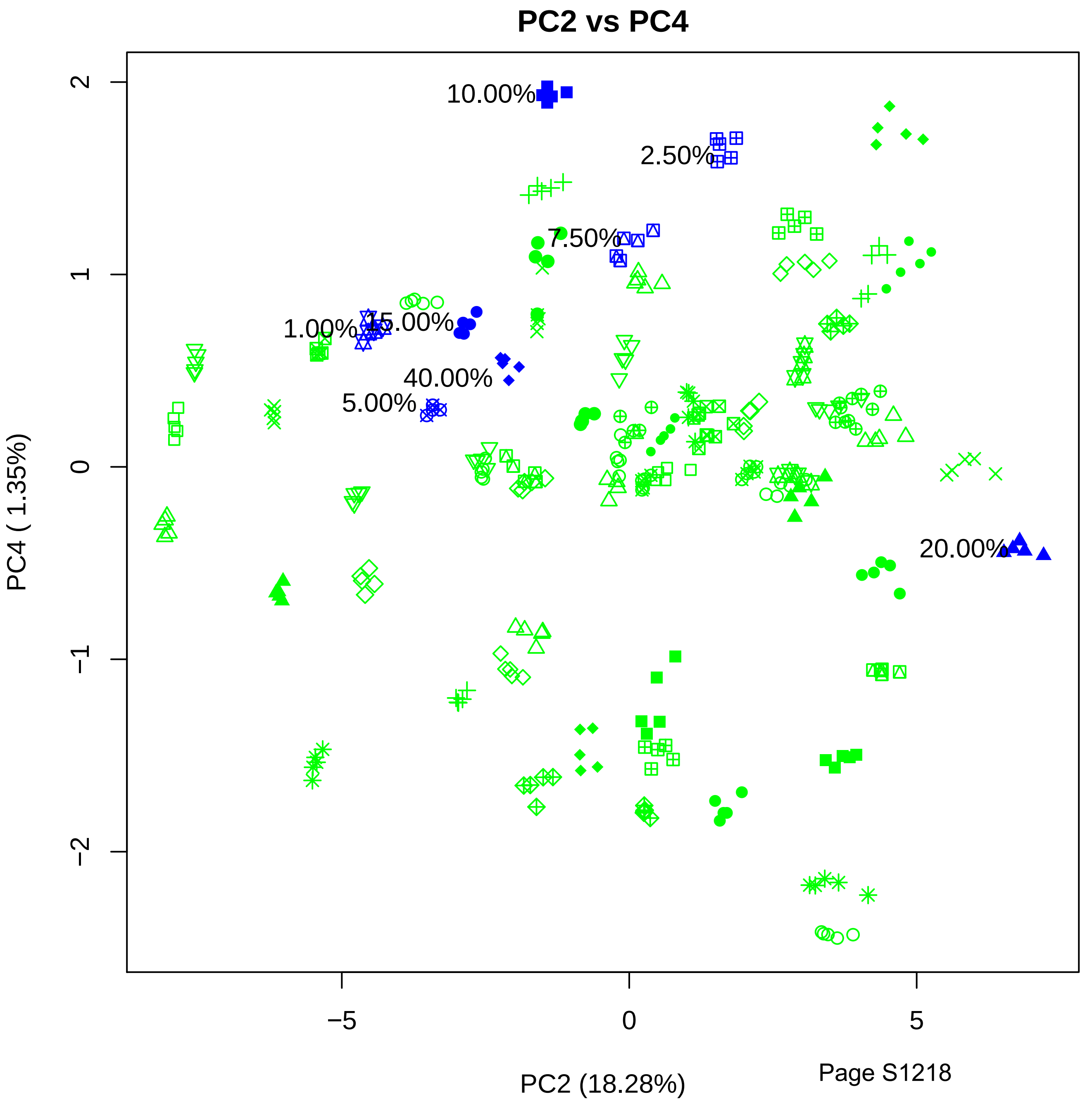
PC2 vs PC4
10.00%
2.50%
7.50%
1.00%
15.00%
40.00%
5.00%
20.00%
2
1
0
−1
−2
−5
0
5
PC4 (1.35%)
PC2 (18.28%)

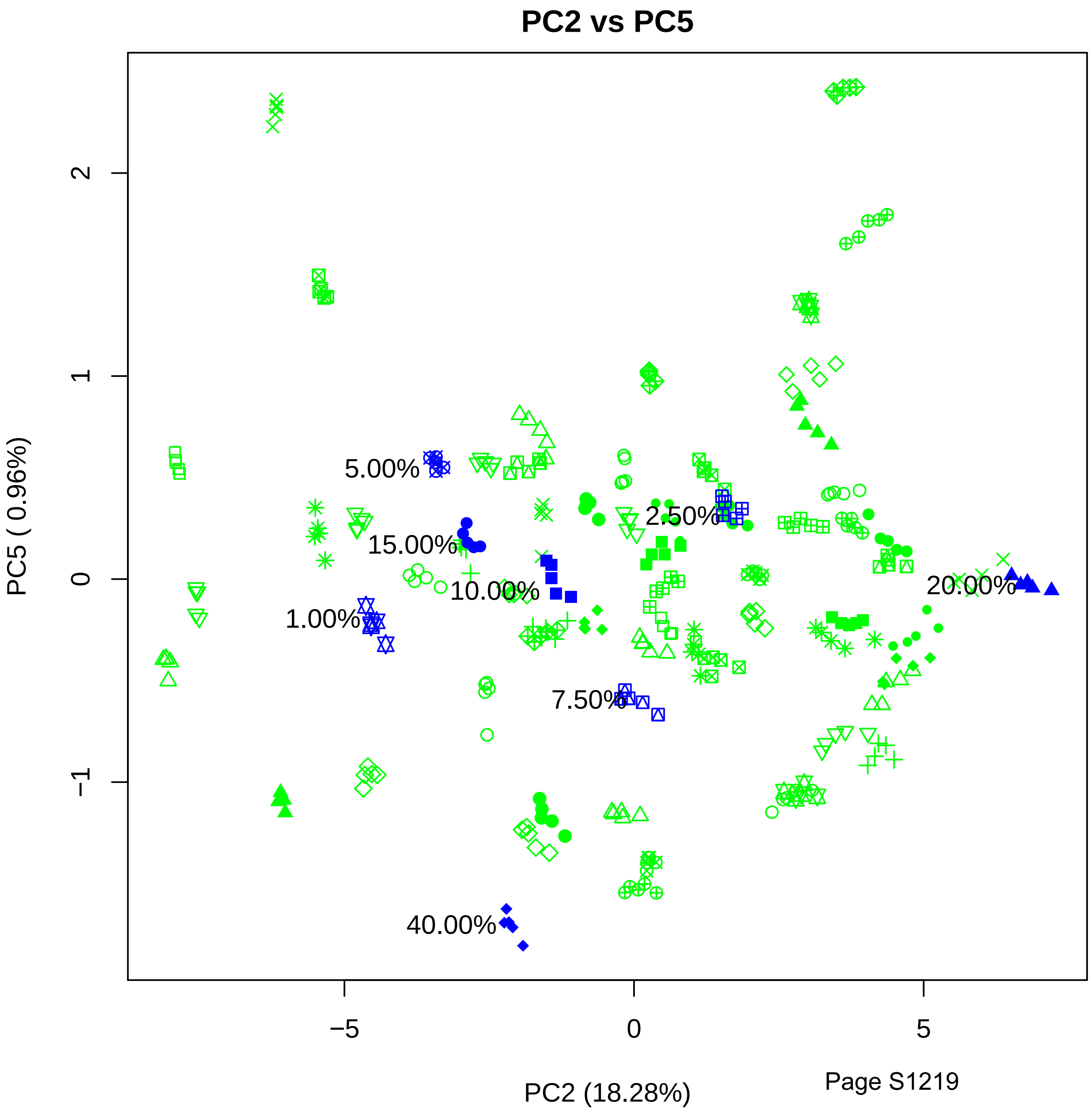
PC2 vs PC5
PC5 (0.96%)
PC2 (18.28%)
2
1
0
−1
−5
0
5
5.00%
15.00%
10.00%
1.00%
2.50%
20.00%
7.50%
40.00%

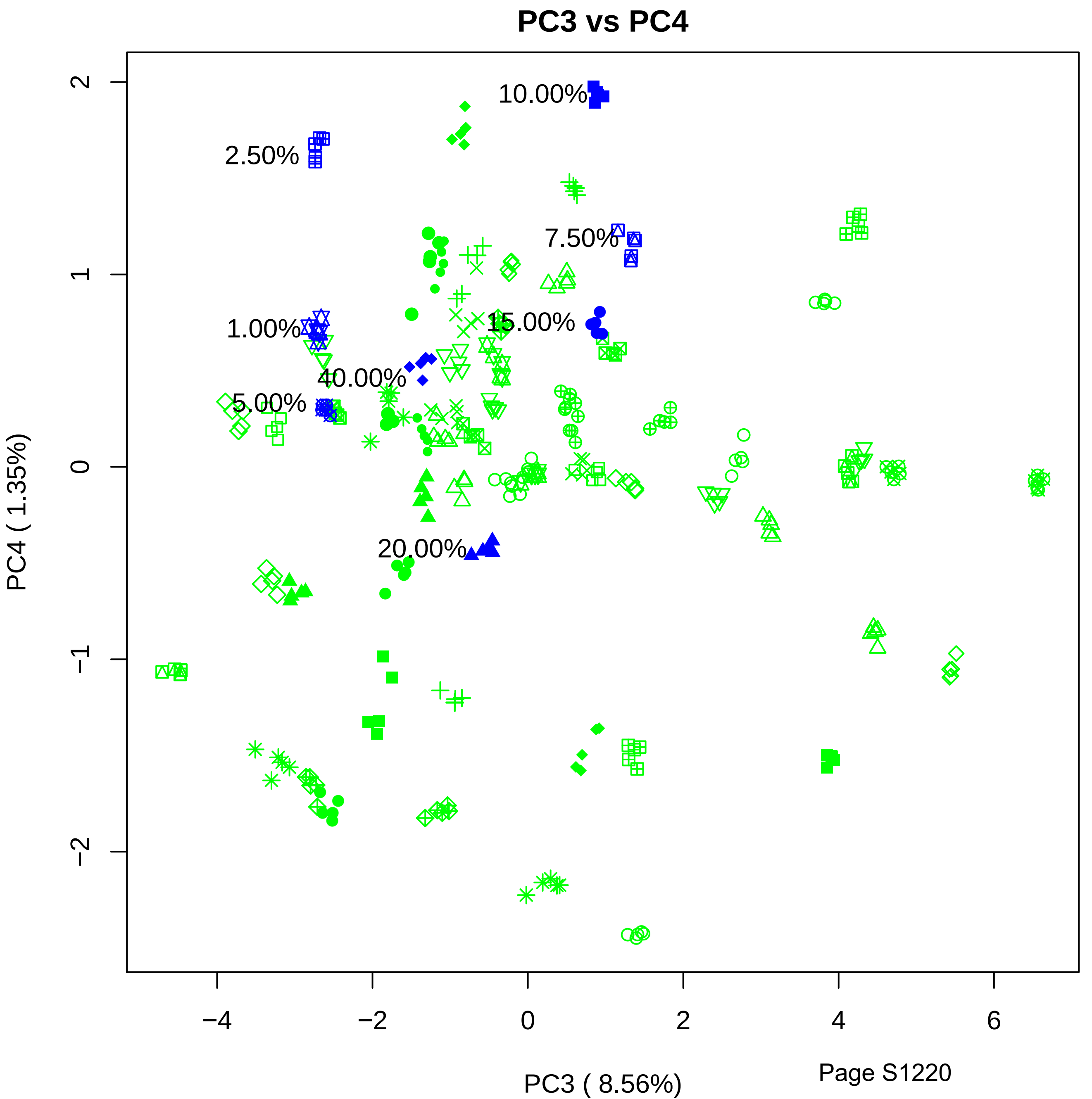
PC3 vs PC4
PC4 (1.35%)
PC3 (8.56%)
10.00%
2.50%
7.50%
1.00%
15.00%
40.00%
5.00%
20.00%
2
1
0
−1
−2
−4
−2
0
2
4
6

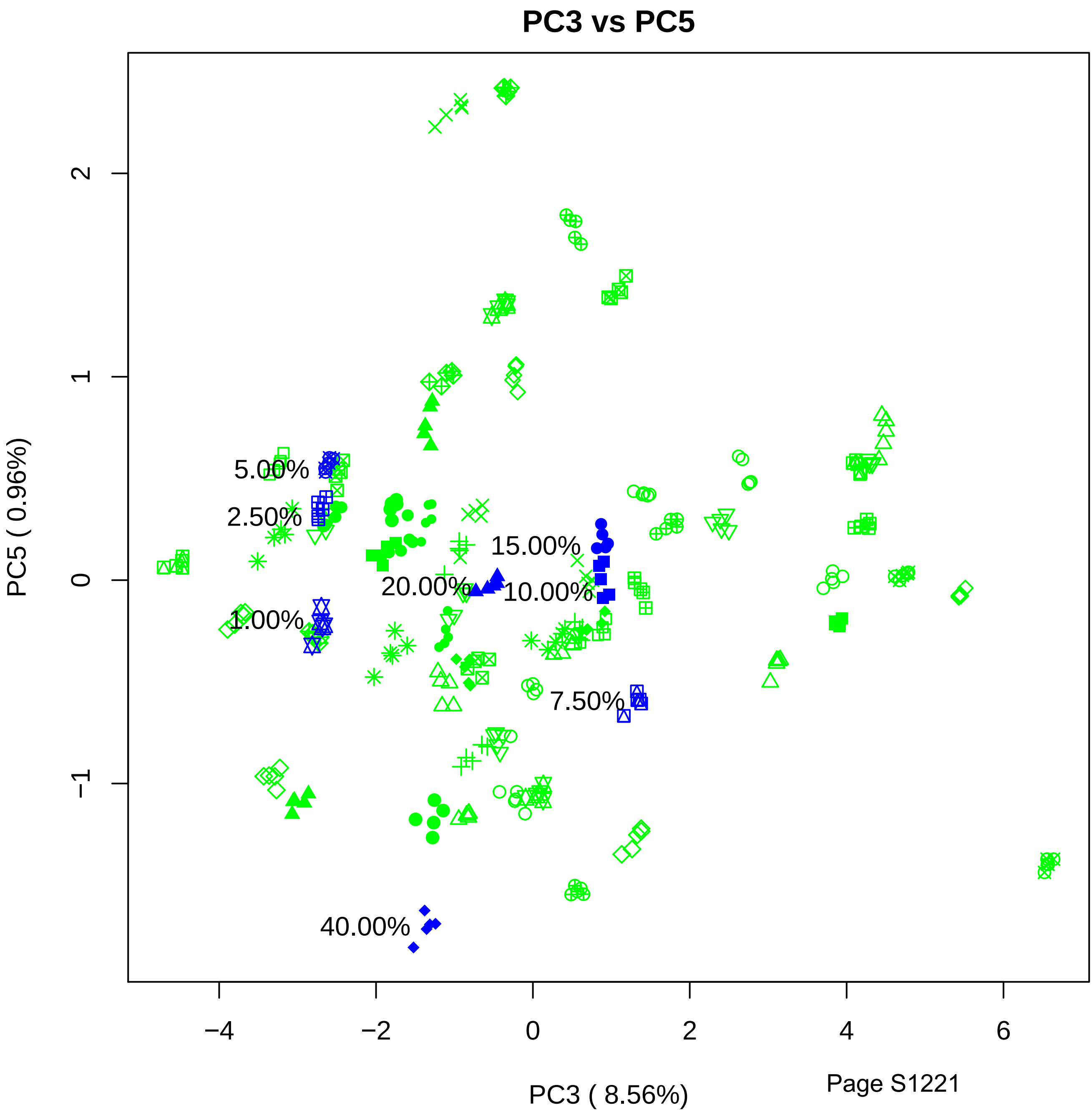

PC3 vs PC5
PC5 (0.96%)
PC3 (8.56%)
2
1
0
−1
−4
−2
0
2
4
6
5.00%
2.50%
15.00%
20.00%
10.00%
1.00%
7.50%
40.00%

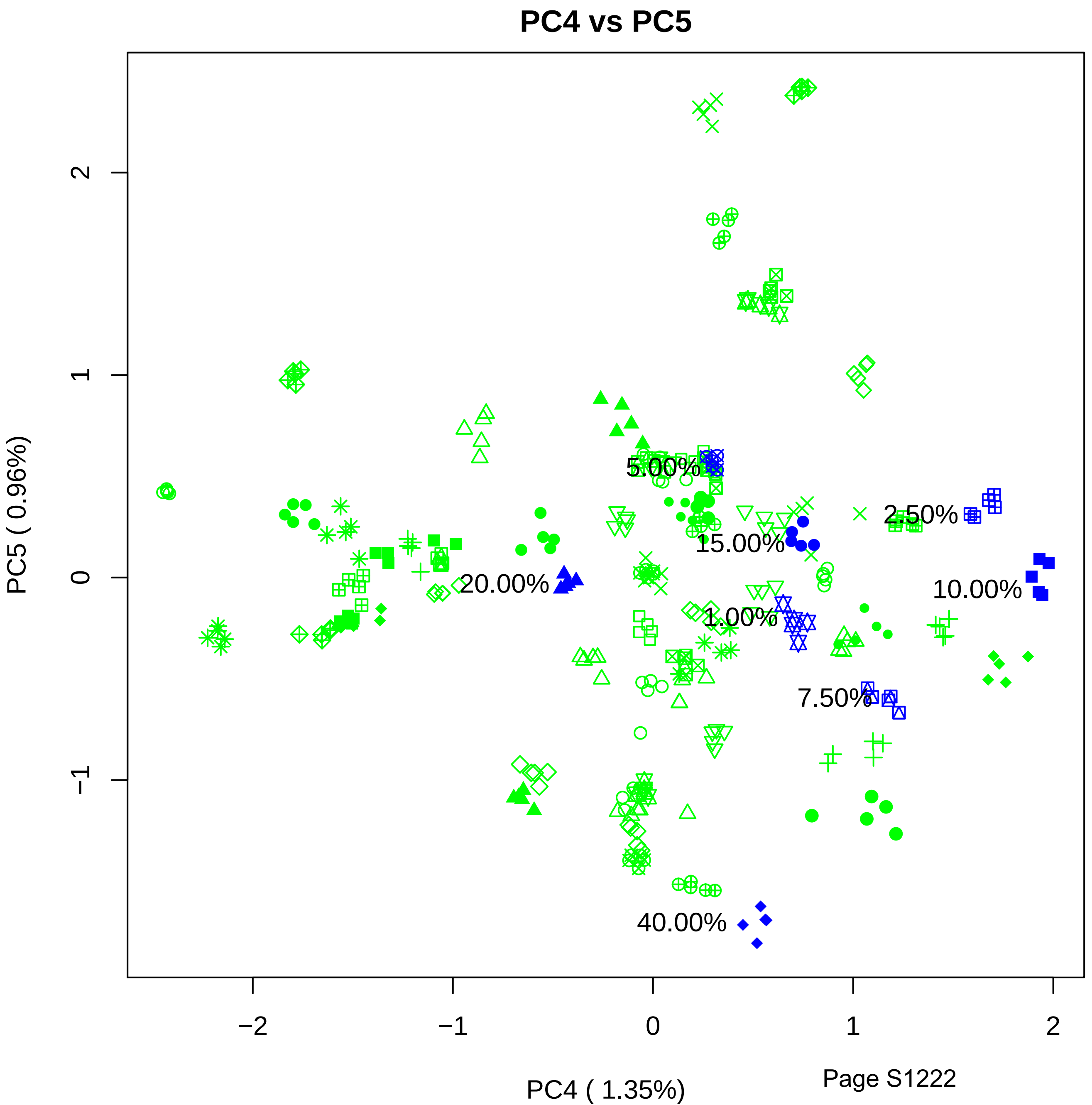
PC4 vs PC5
PC5 (0.96%)
PC4 (1.35%)
−2
−1
0
1
2
5.00%
2.50%
15.00%
20.00%
10.00%
1.00%
7.50%
40.00%

PCA of 1% BMH (c) vs PMH (c)

Scaled Matrix PCAed

7th order polynomial baseline correction performed

Raw spectra smoothed to 21 Neighbours, 2nd order PN, Savtisky Golay

Normalisation between 0 and 100

D2 spectra smoothed to 41 Neighbours, 2nd order PN, Savtisky Golay

Regions Analysed: 3601−2501 and 2001−700cm−1

Samples Analysed: 1 bContam's 0 BLH's 0 BMH's 0 OMH's 0 pContam's 0 PLH's 60 PMH's

Keele University AD

Fri Dec 14 14:18:39 2018

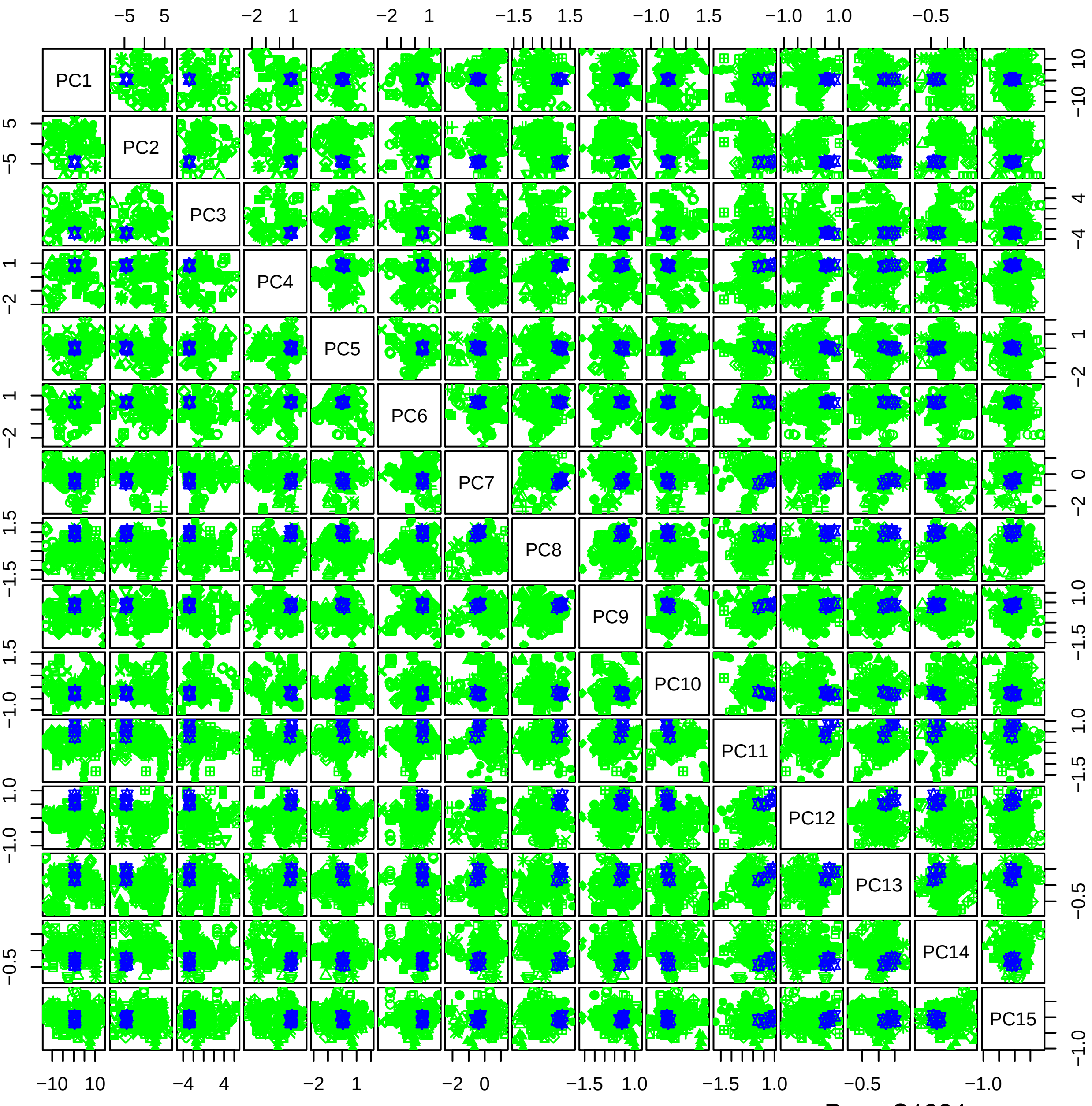
PC1
PC2
PC3
PC4
PC5
PC6
PC7
PC8
PC9
PC10
PC11
PC12
PC13
PC14
PC15

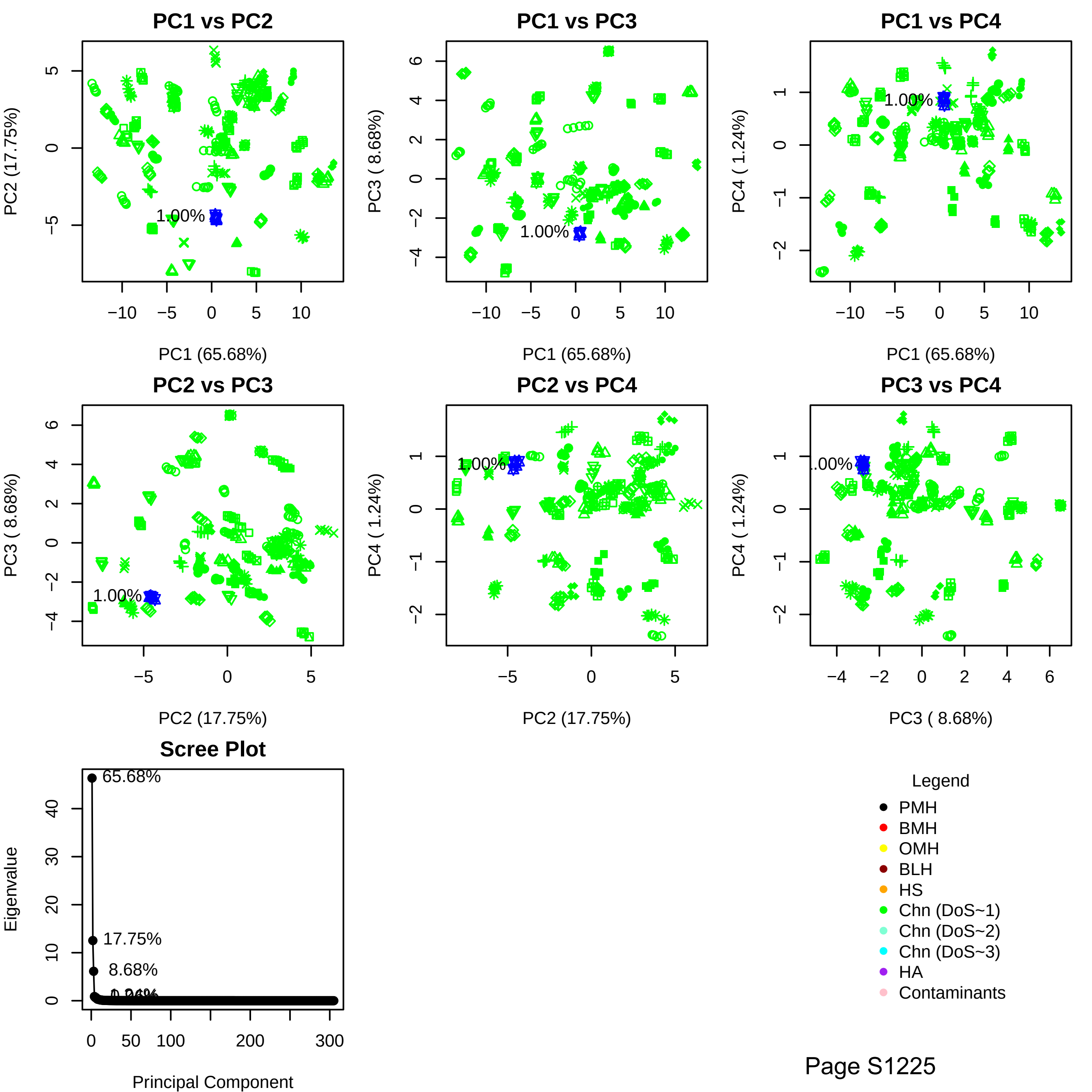

PC1 vs PC2
PC1 vs PC3
PC1 vs PC4
PC2 vs PC3
PC2 vs PC4
PC3 vs PC4
Scree Plot
PC1 (65.68%)
PC2 (17.75%)
PC3 (8.68%)
PC4 (1.24%)
1.00%
Eigenvalue
Principal Component
65.68%
17.75%
8.68%
Legend
PMH
BMH
OMH
BLH
HS
Chn (DoS~1)
Chn (DoS~2)
Chn (DoS~3)
HA
Contaminants

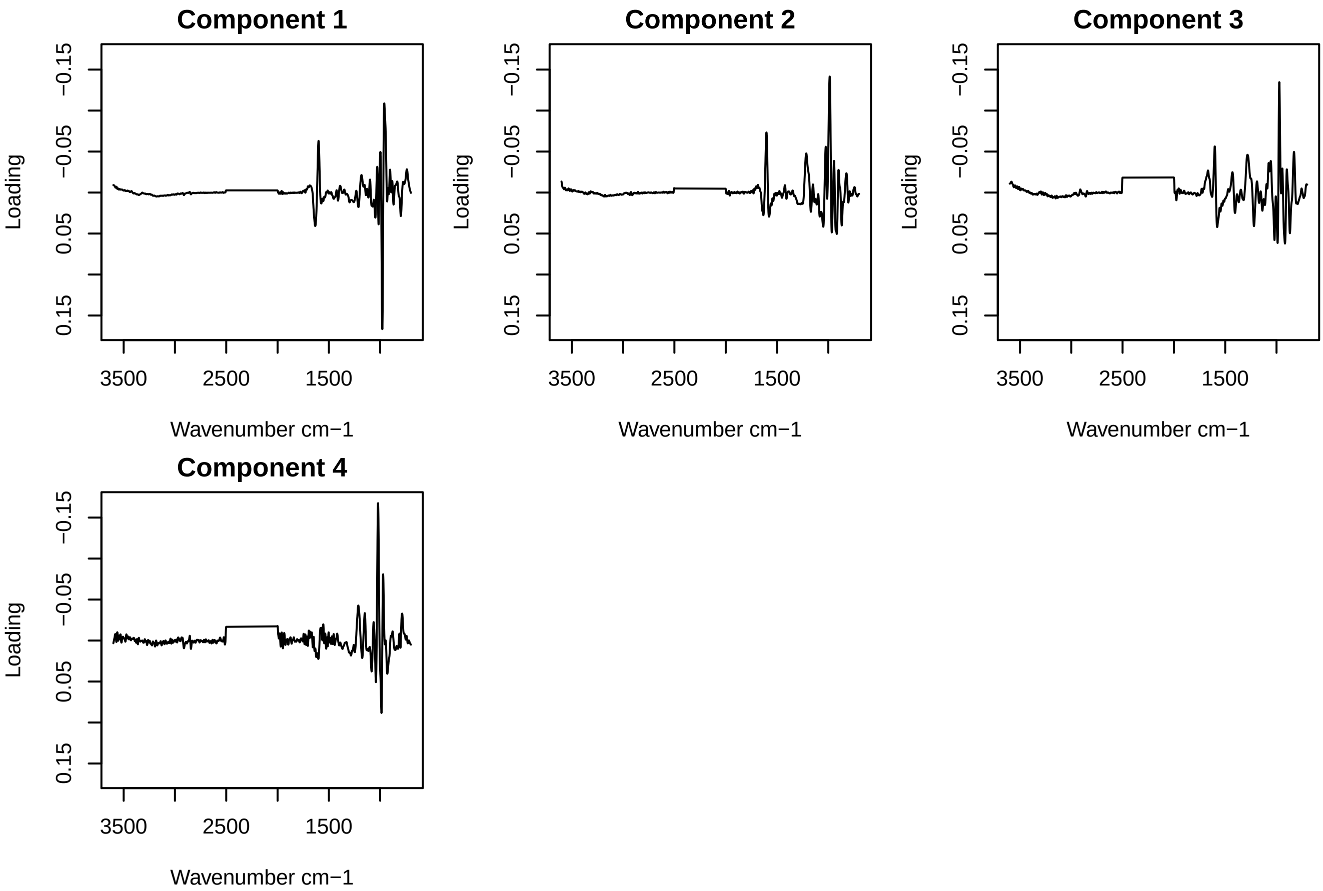
Component 1
Loading
−0.15
−0.05
0.05
0.15
3500
2500
1500
Wavenumber cm−1
Component 2
Loading
−0.15
−0.05
0.05
0.15
3500
2500
1500
Wavenumber cm−1
Component 3
Loading
−0.15
−0.05
0.05
0.15
3500
2500
1500
Wavenumber cm−1
Component 4
Loading
−0.15
−0.05
0.05
0.15
3500
2500
1500
Wavenumber cm−1

PC1 vs PC2 vs PC3

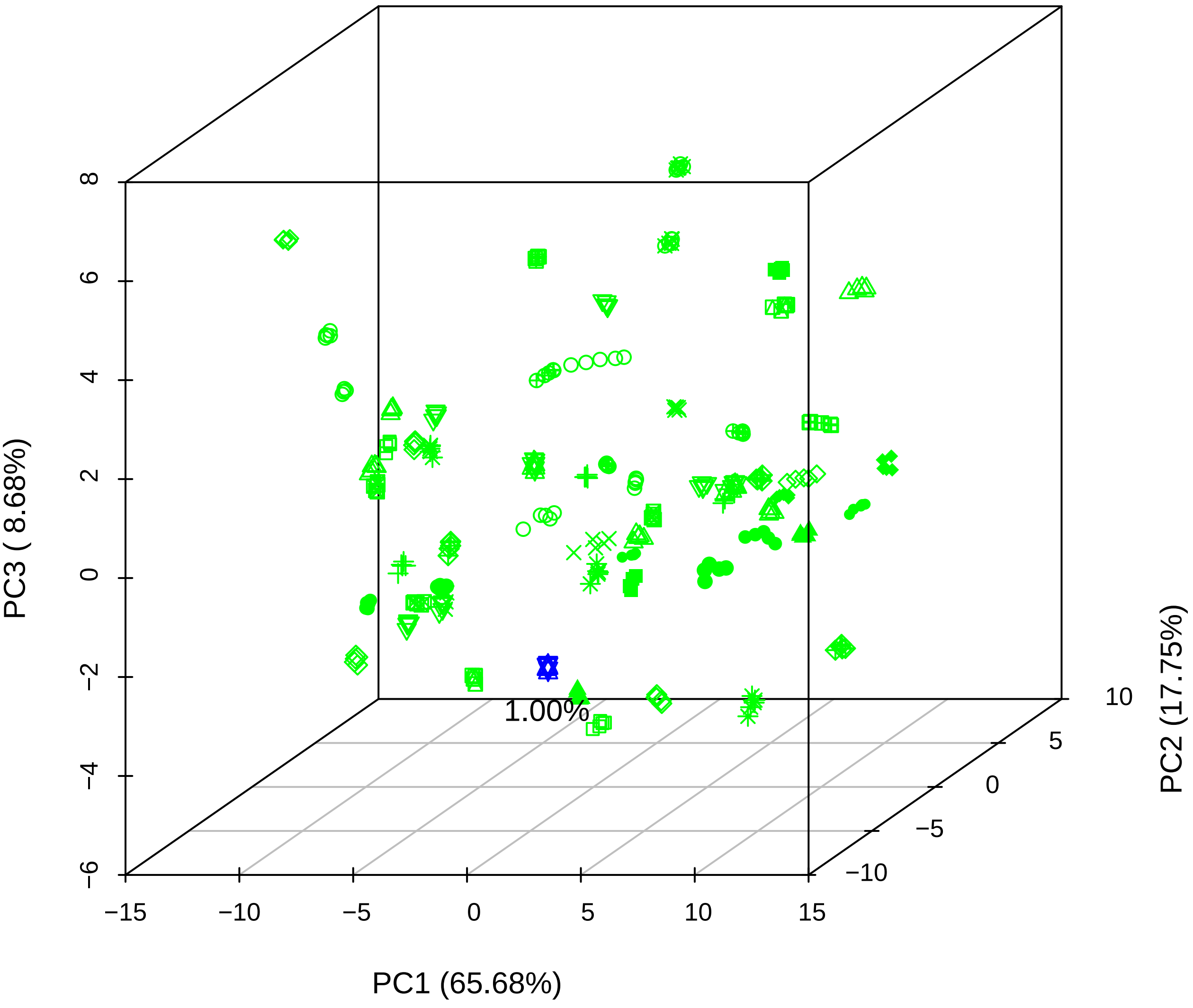

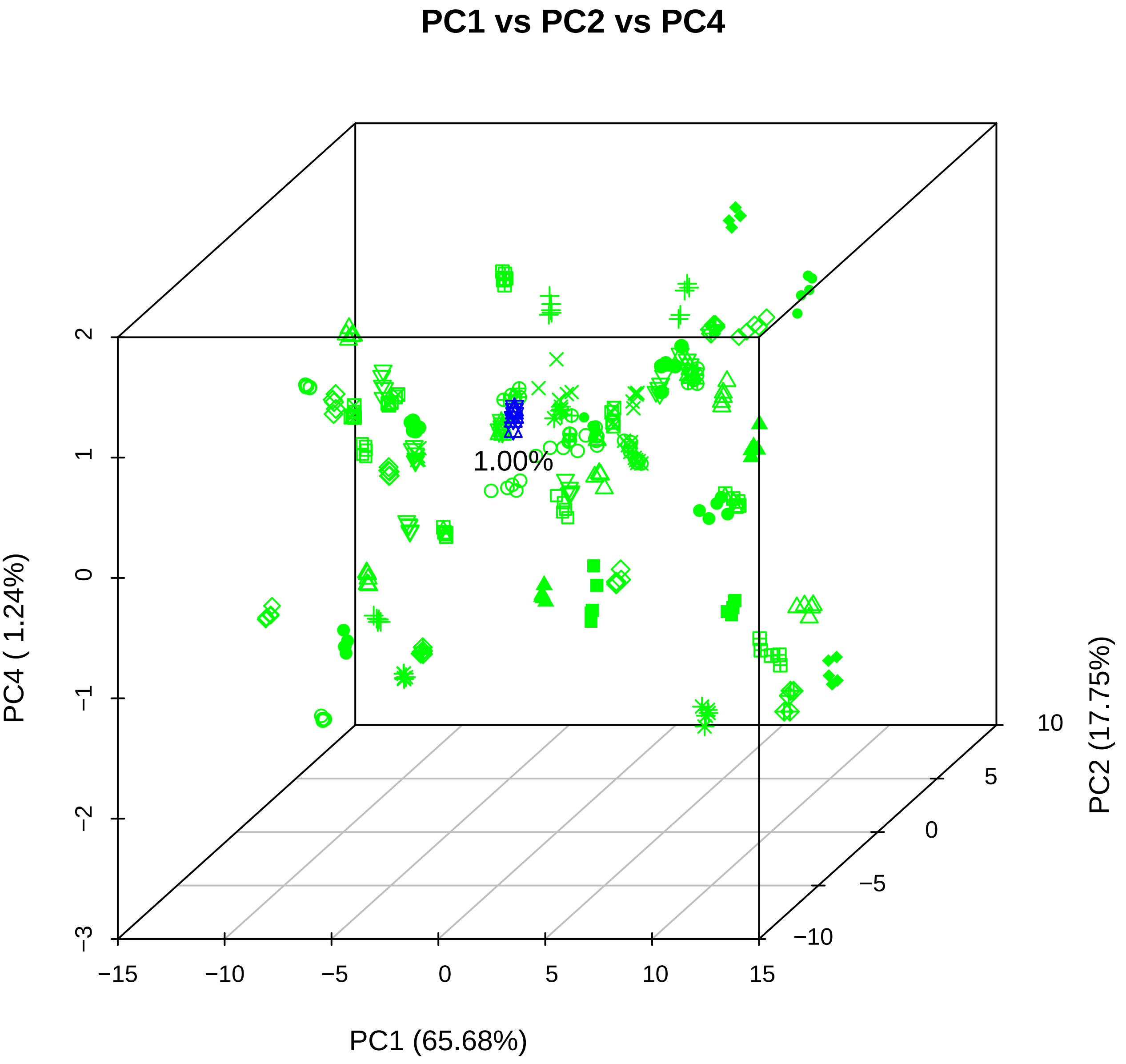

PC1 vs PC2 vs PC4
1.00%
PC1 (65.68%)
PC2 (17.75%)
PC4 (1.24%)
−15
−10
−5
0
5
10
15
−10
−5
0
5
10
−3
−2
−1
0
1
2

PC1 vs PC3 vs PC4

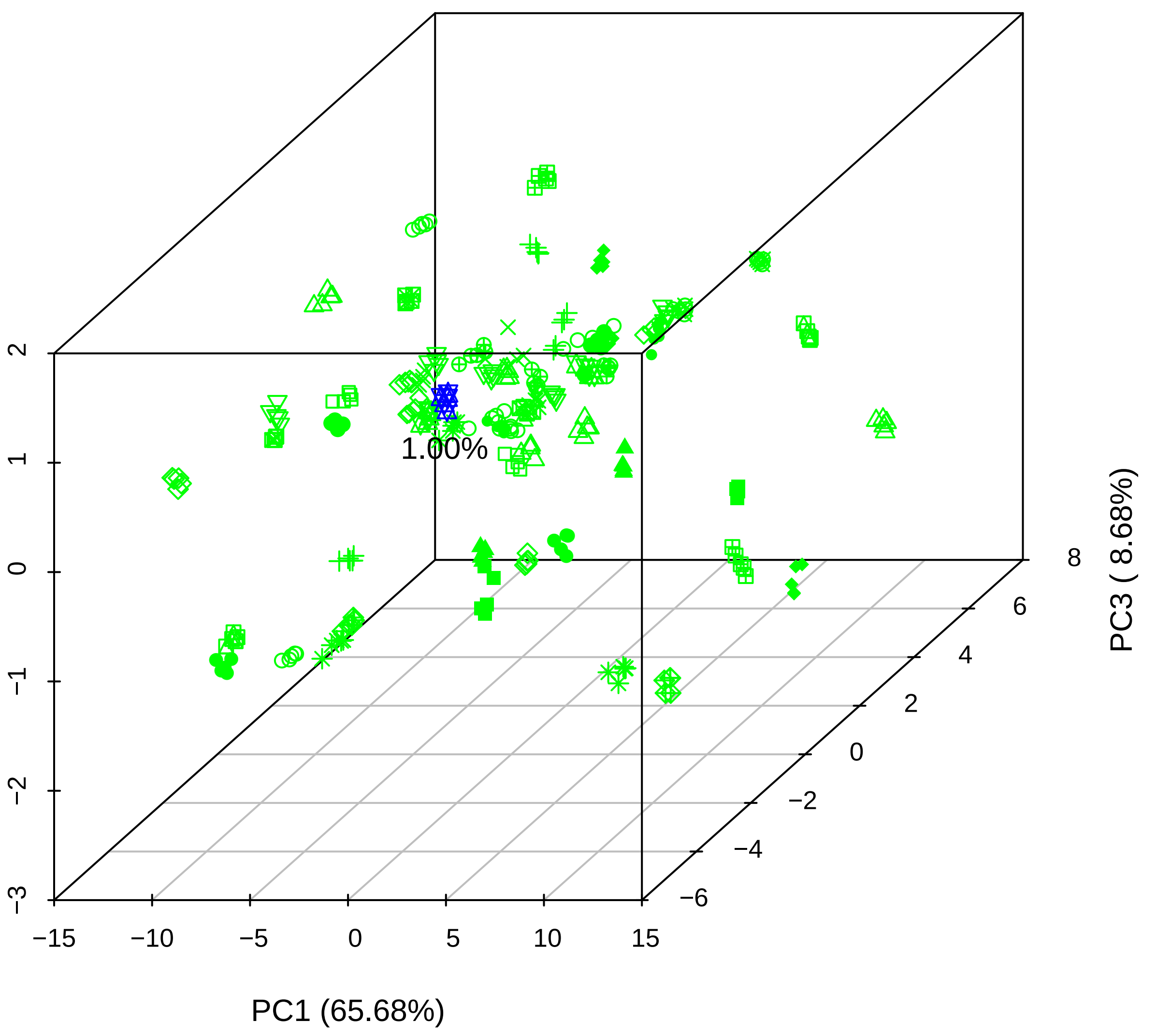

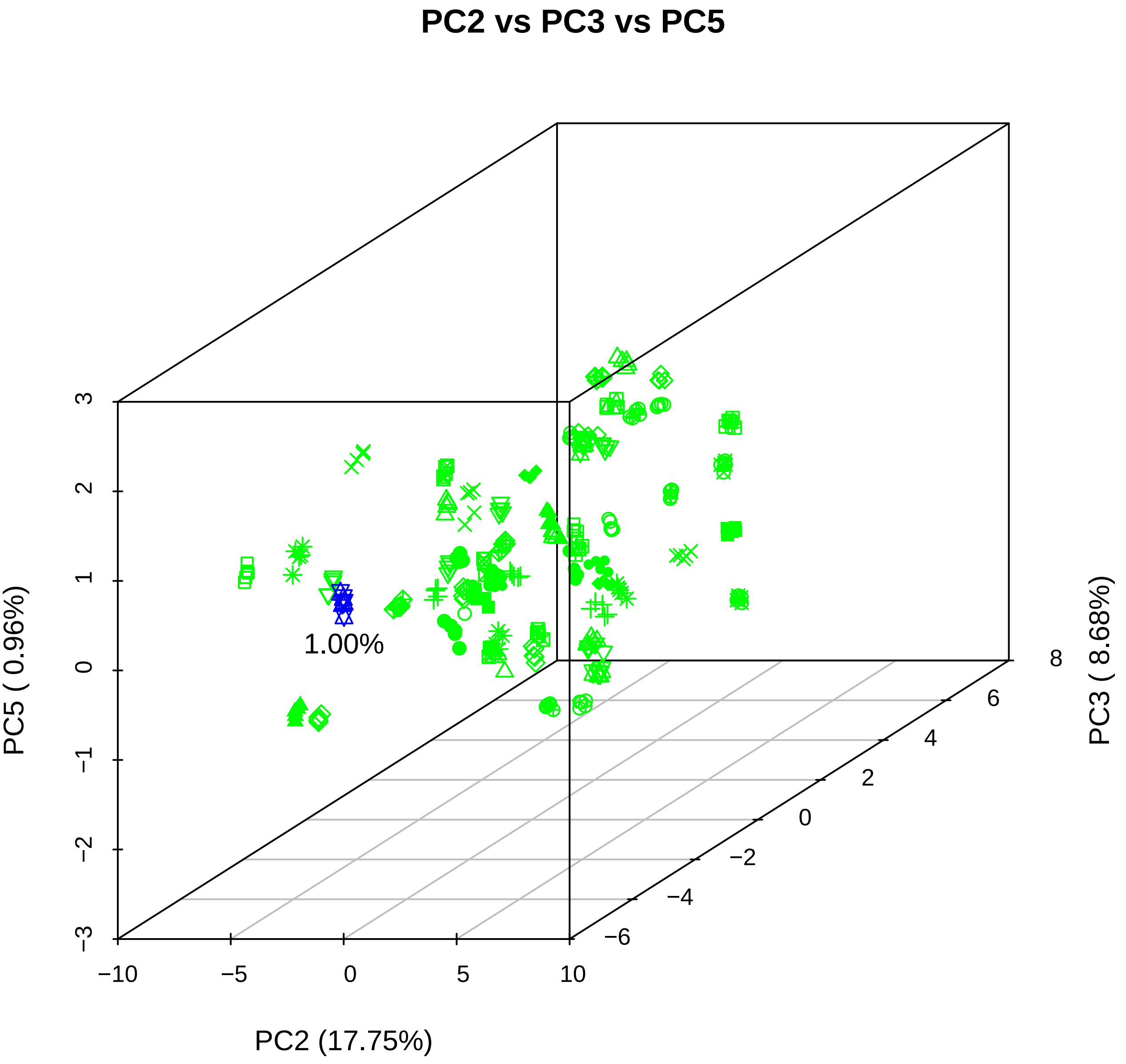

PC2 vs PC3 vs PC5
PC5 (0.96%)
PC2 (17.75%)
PC3 (8.68%)
1.00%
3
2
1
0
−1
−2
−3
−10
−5
0
5
10
8
6
4
2
0
−2
−4
−6

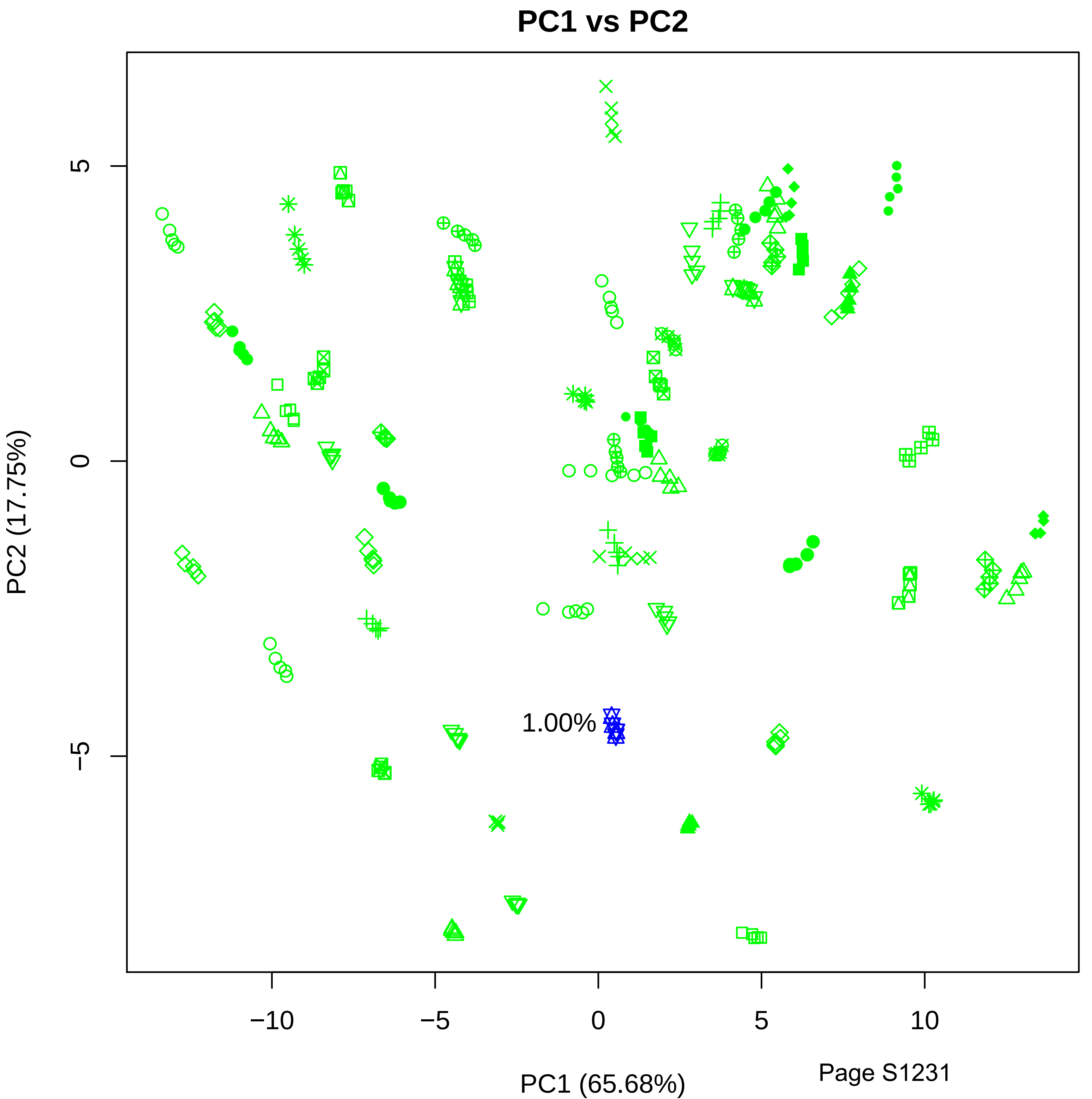

PC1 vs PC2
5
0
−5
PC2 (17.75%)
−10
−5
0
5
10
PC1 (65.68%)
1.00%

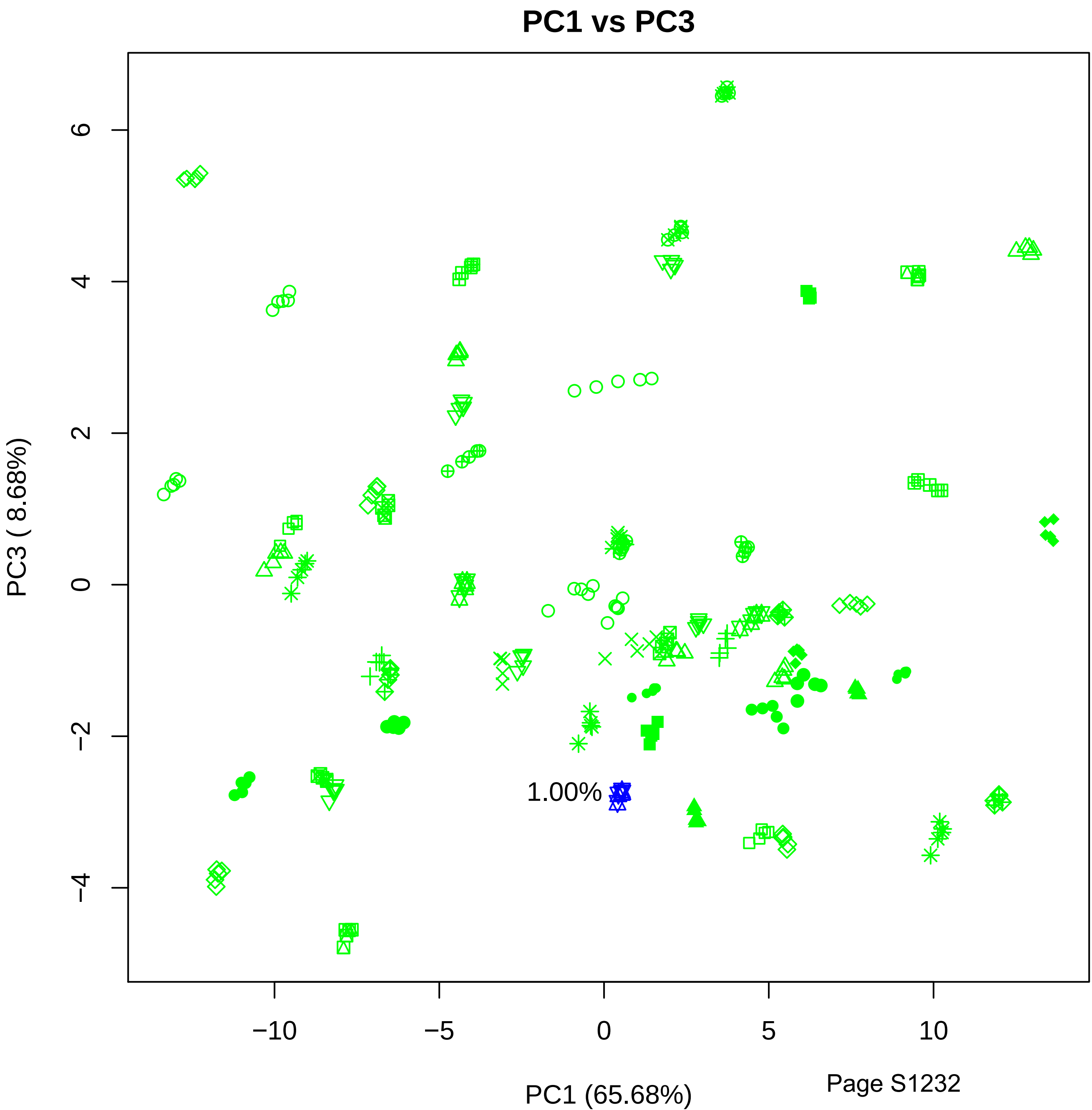

PC1 vs PC3
PC3 (8.68%)
PC1 (65.68%)
1.00%
6
4
2
0
−2
−4
−10
−5
0
5
10

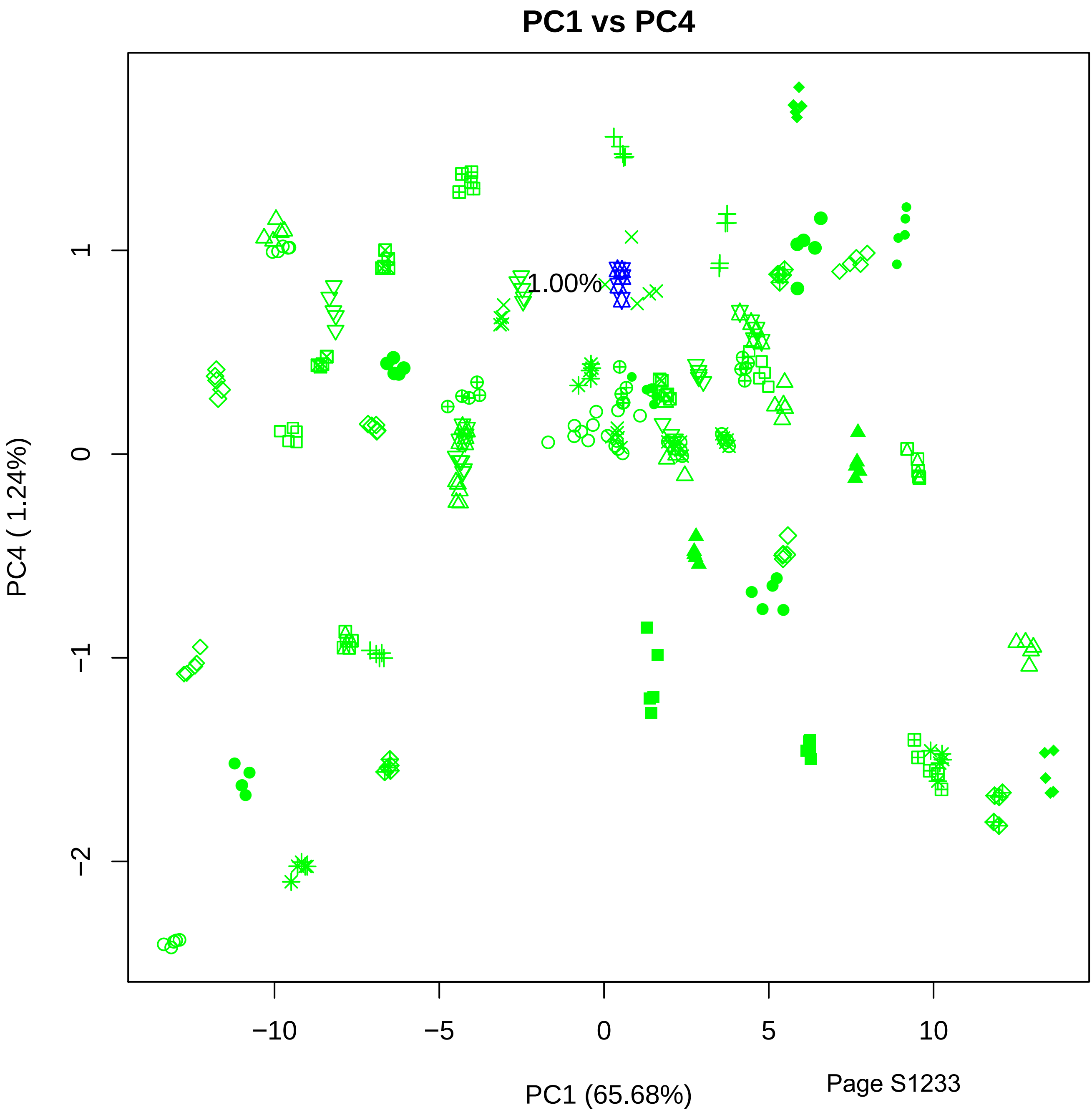

PC1 vs PC4
1.00%
1
0
−1
−2
PC4 (1.24%)
−10
−5
0
5
10
PC1 (65.68%)

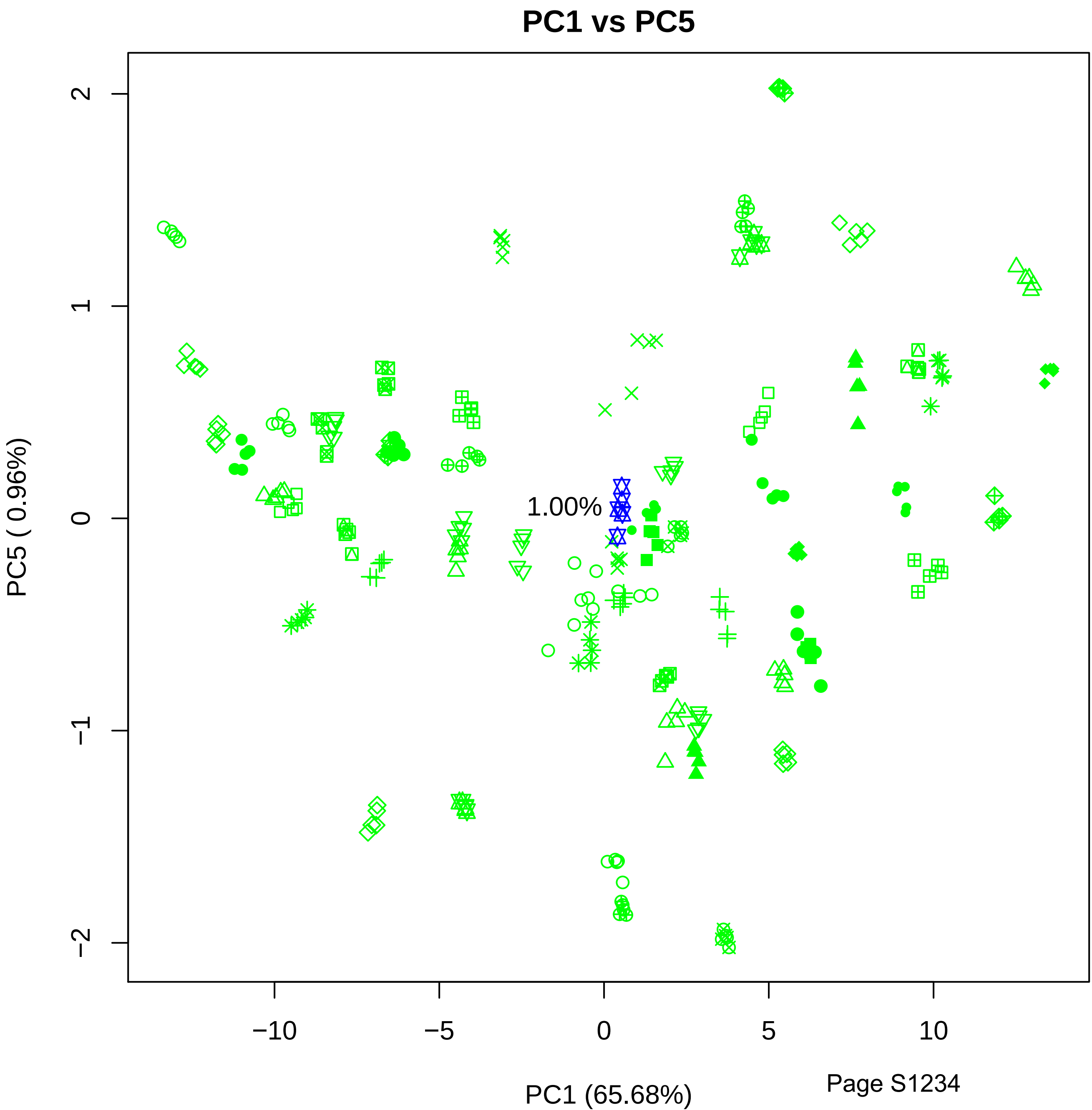

PC1 vs PC5
2
1
0
−1
−2
PC5 (0.96%)
1.00%
−10
−5
0
5
10
PC1 (65.68%)

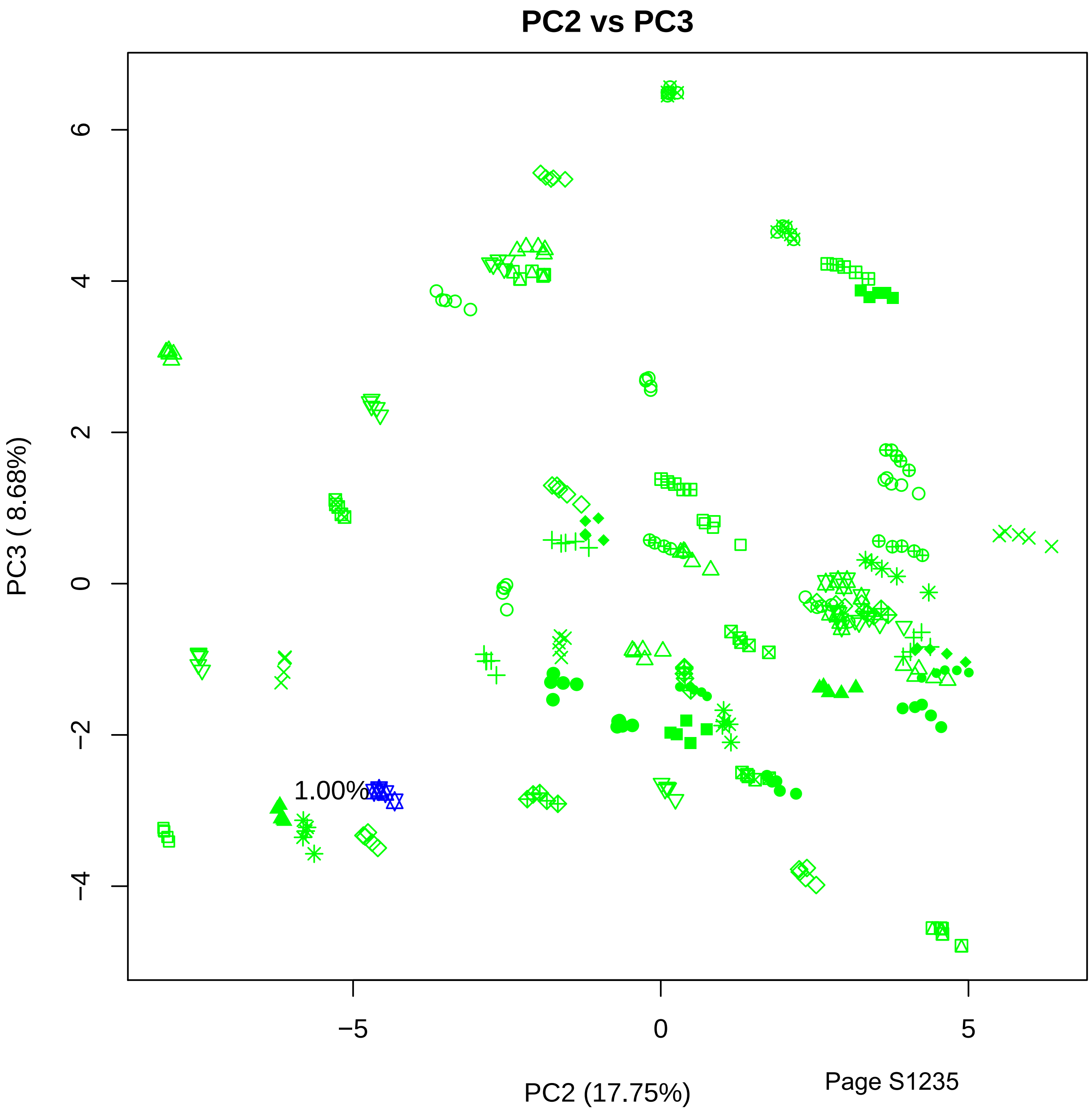
PC2 vs PC3
PC3 (8.68%)
PC2 (17.75%)
6
4
2
0
−2
−4
−5
0
5
1.00%

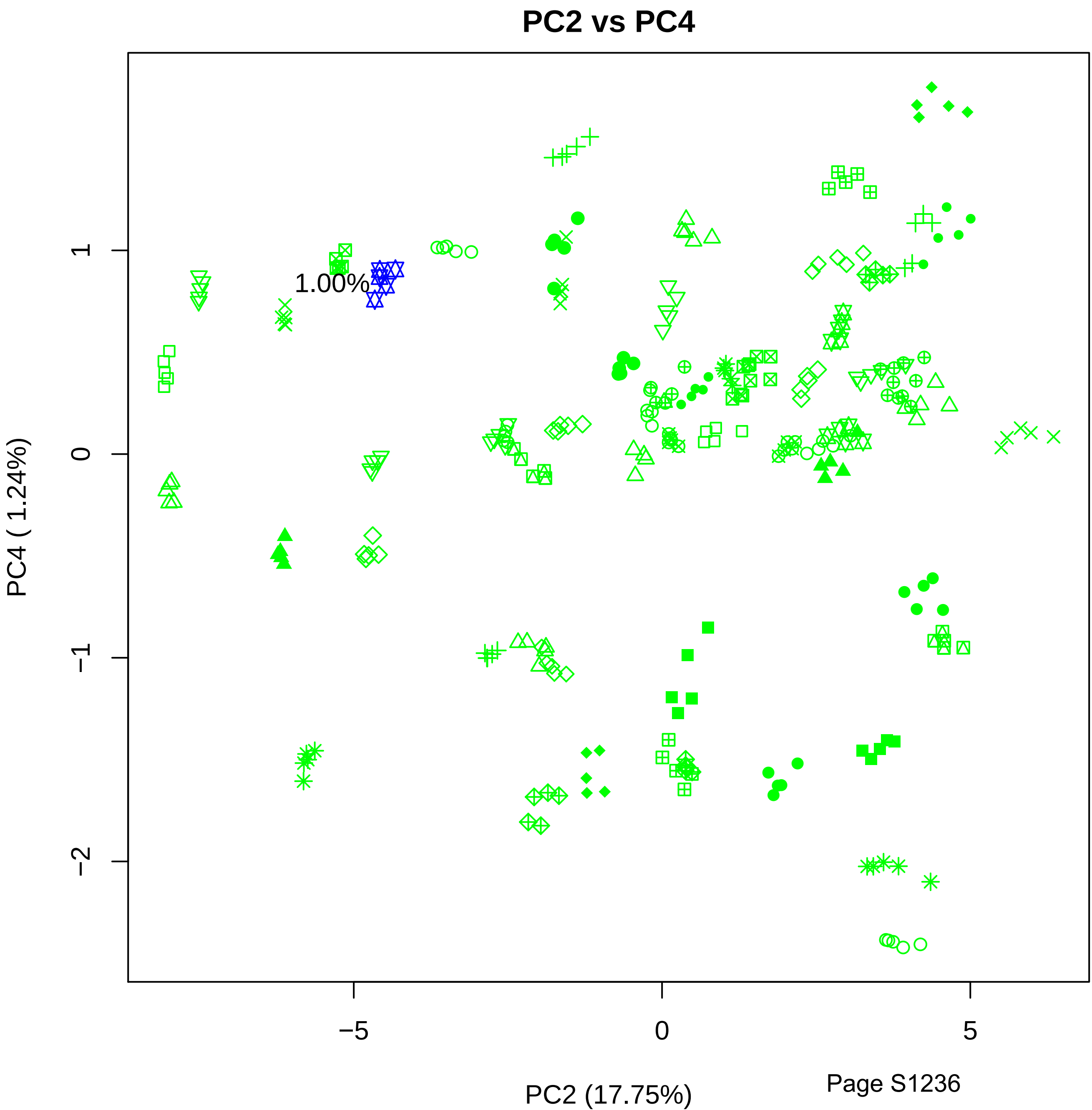
PC2 vs PC4
1.00%
1
0
−1
−2
PC4 (1.24%)
−5
0
5
PC2 (17.75%)

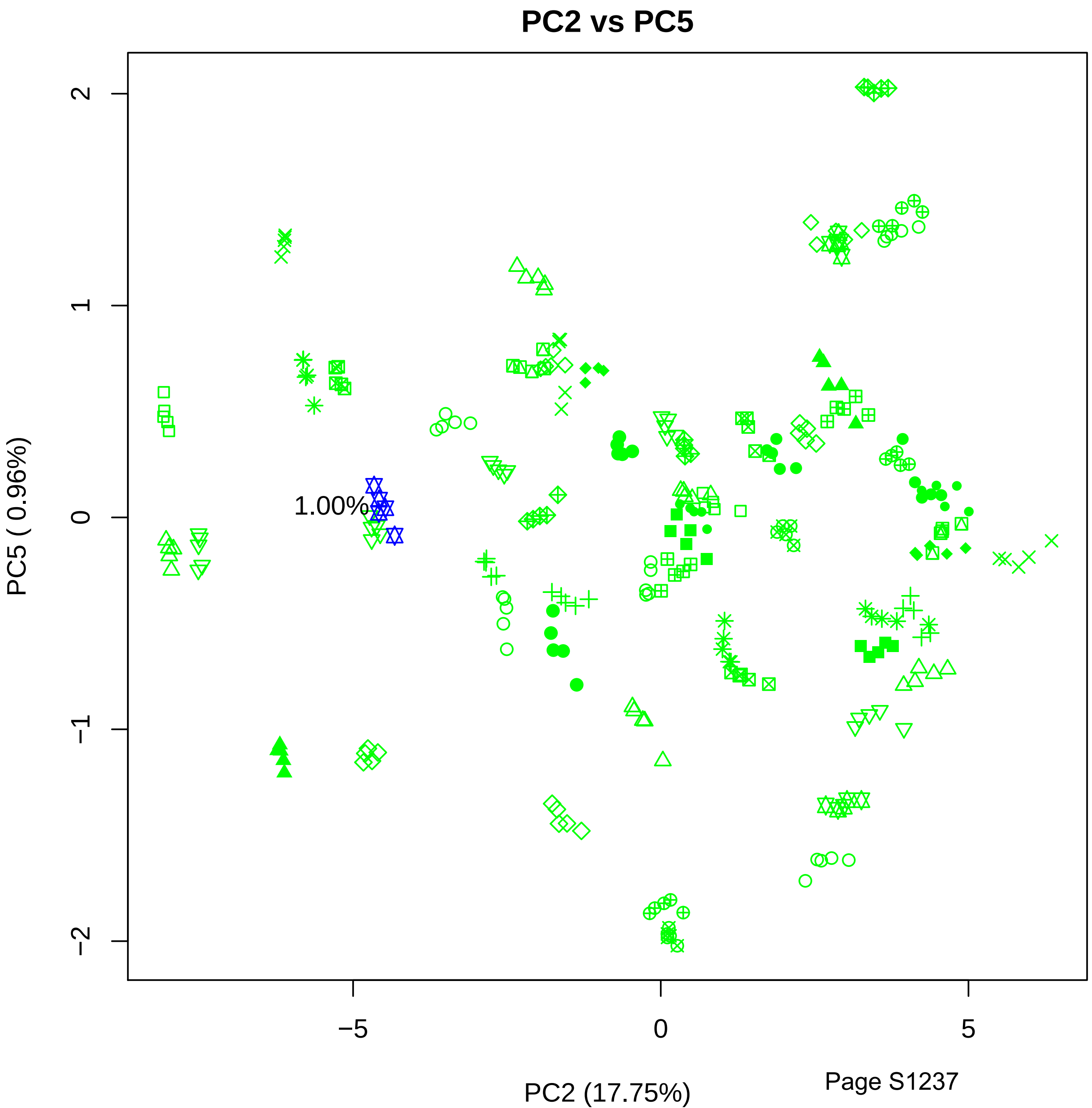

PC2 vs PC5
2
1
0
−1
−2
PC5 (0.96%)
1.00%
−5
0
5
PC2 (17.75%)

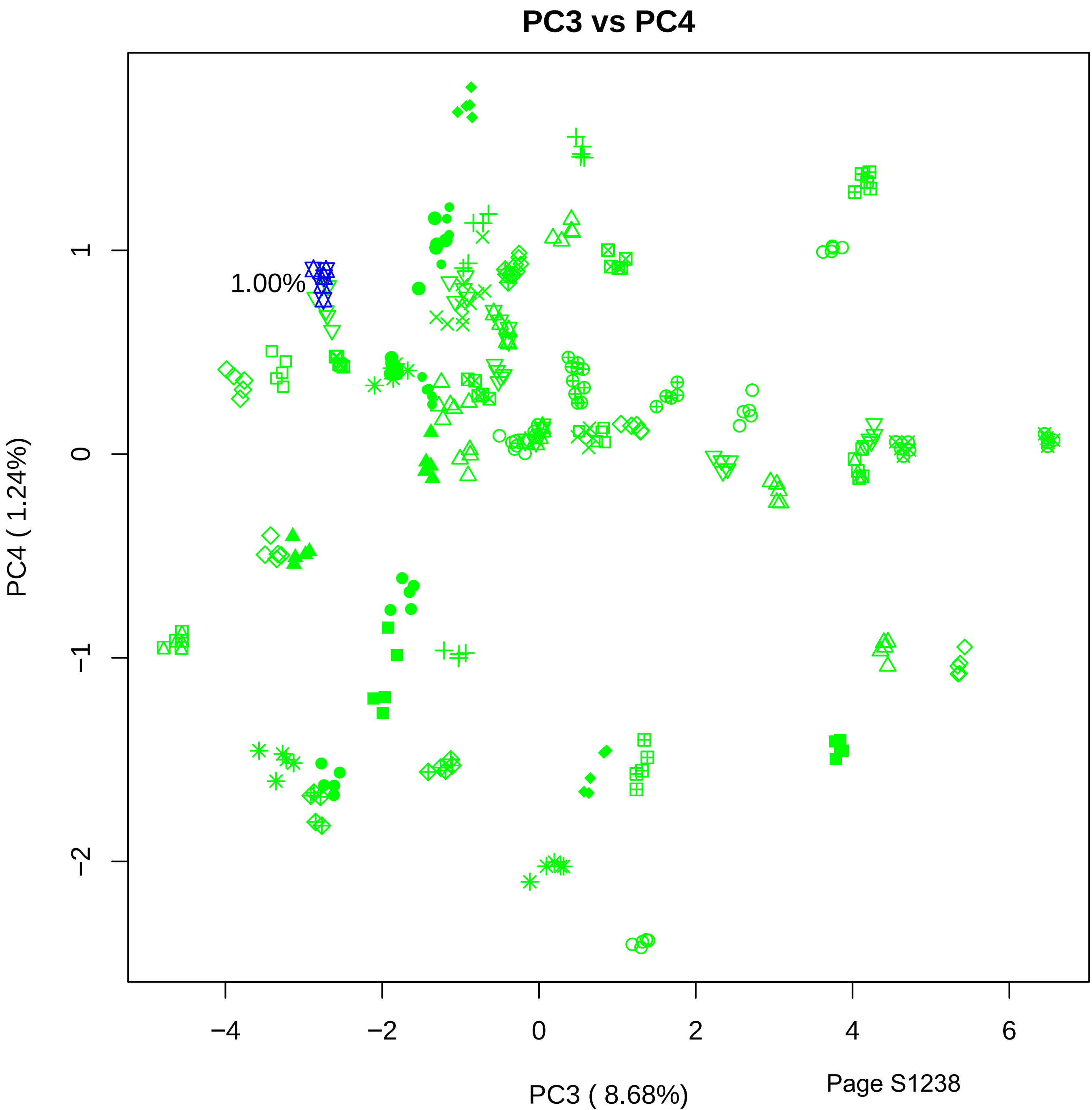

PC3 vs PC4
1.00%
PC4 (1.24%)
PC3 (8.68%)
1
0
−1
−2
−4
−2
0
2
4
6

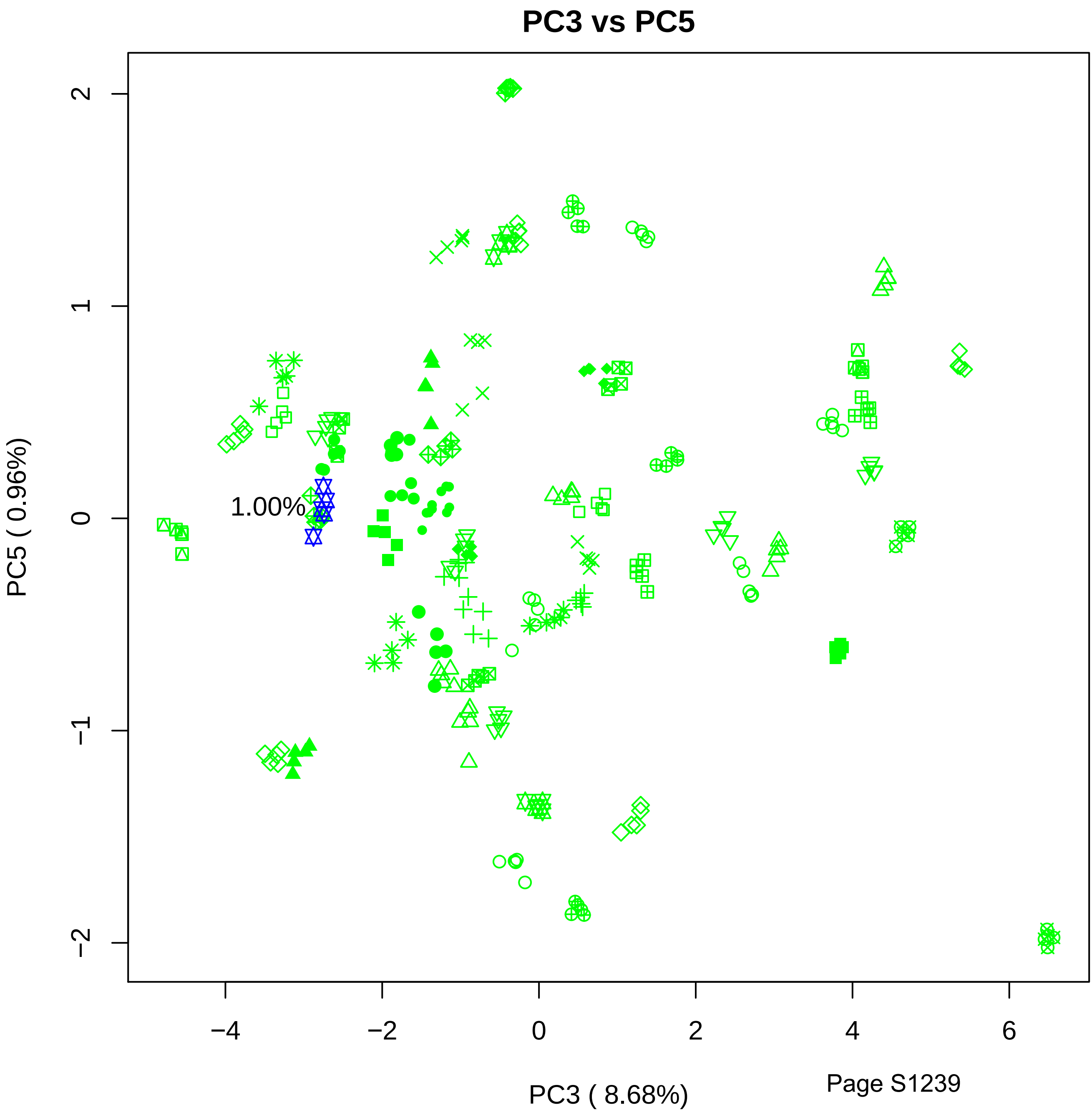

PC3 vs PC5
2
1
0
−1
−2
PC5 (0.96%)
1.00%
−4
−2
0
2
4
6
PC3 (8.68%)

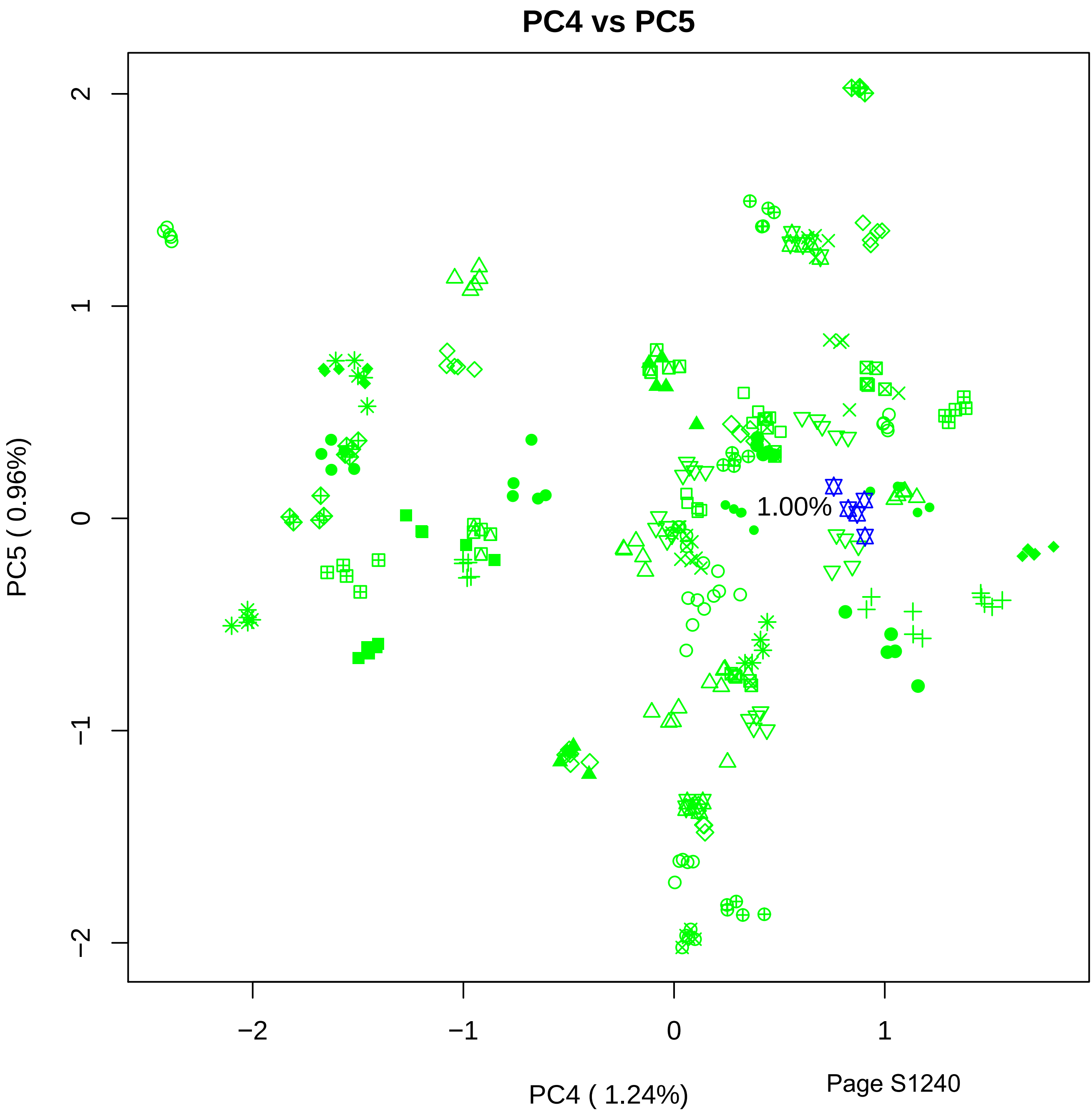

PC4 vs PC5
PC5 (0.96%)
PC4 (1.24%)
2
1
0
−1
−2
−2
−1
0
1
1.00%

PCA of 2.5% BMH (c) vs PMH (c)

Scaled Matrix PCAed

7th order polynomial baseline correction performed

Raw spectra smoothed to 21 Neighbours, 2nd order PN, Savtisky Golay

Normalisation between 0 and 100

D2 spectra smoothed to 41 Neighbours, 2nd order PN, Savtisky Golay

Regions Analysed: 3601−2501 and 2001−700cm−1

Samples Analysed: 1 bContam's 0 BLH's 0 BMH's 0 OMH's 0 pContam's 0 PLH's 60 PMH's

Keele University AD

Fri Dec 14 14:18:47 2018

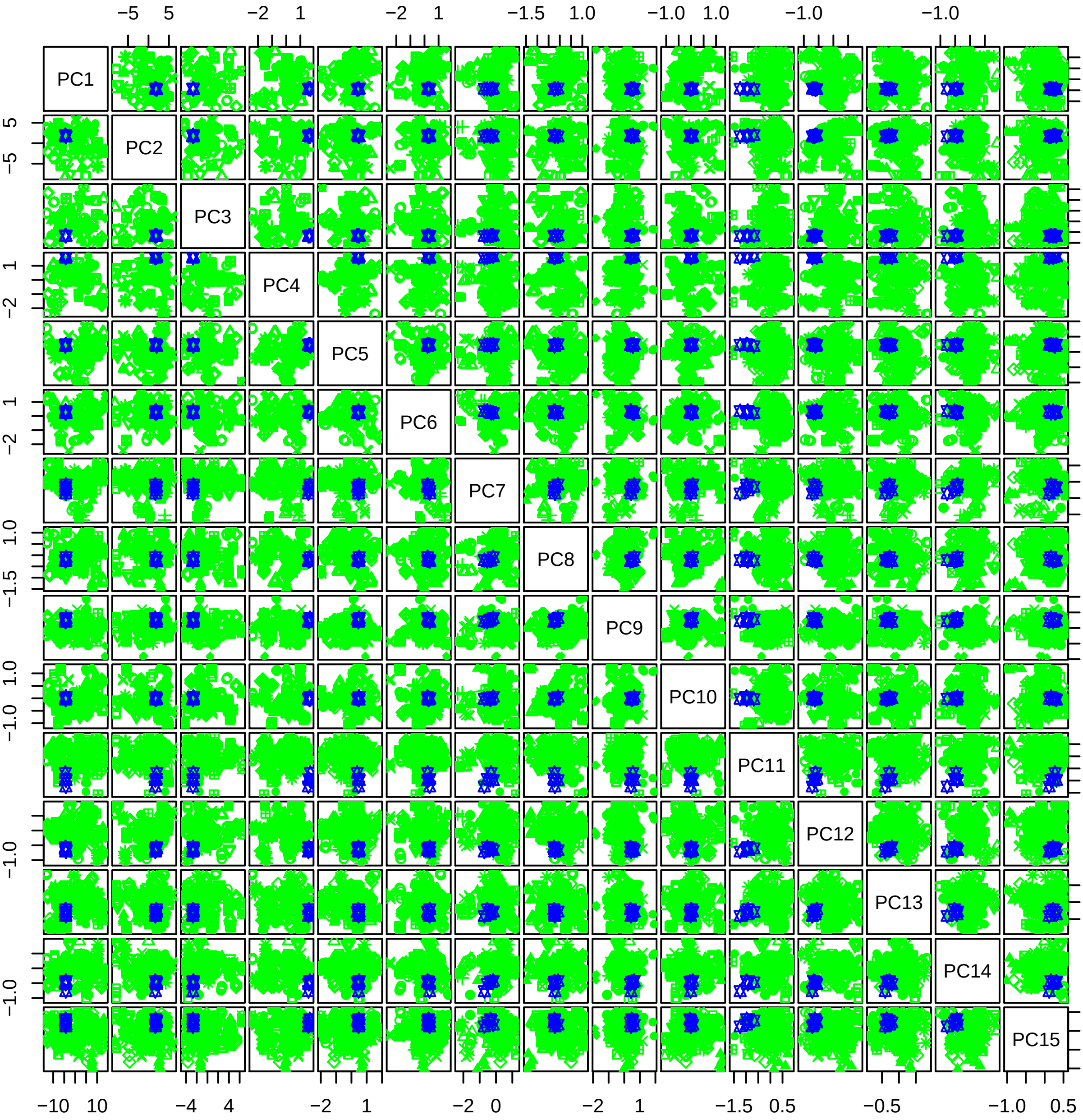
PC1
PC2
PC3
PC4
PC5
PC6
PC7
PC8
PC9
PC10
PC11
PC12
PC13
PC14
PC15

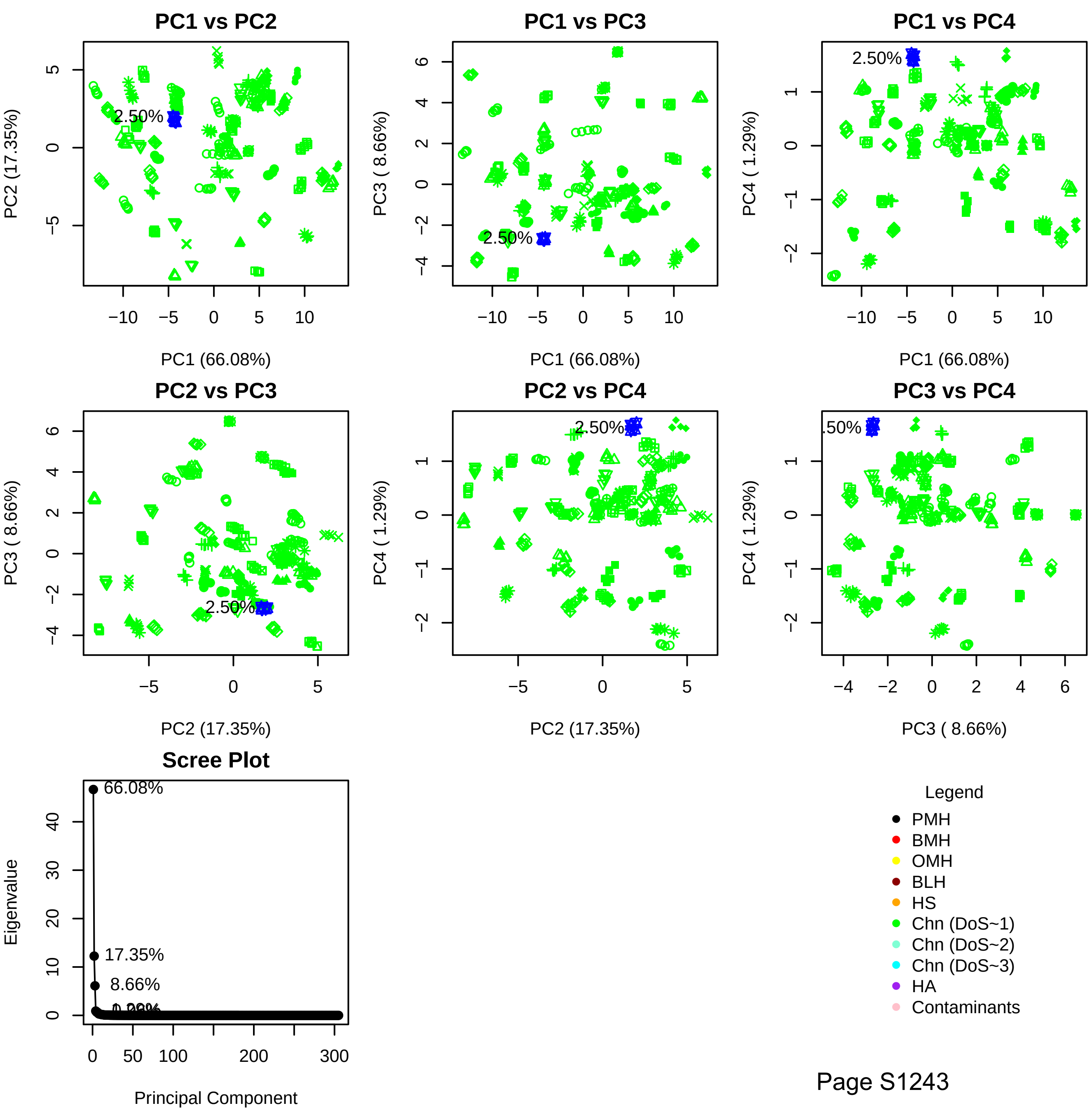
PC1 vs PC2
PC1 vs PC3
PC1 vs PC4
PC2 vs PC3
PC2 vs PC4
PC3 vs PC4
Scree Plot
PC1 (66.08%)
PC2 (17.35%)
PC3 (8.66%)
PC4 (1.29%)
2.50%
Eigenvalue
Principal Component
66.08%
17.35%
8.66%
Legend
PMH
BMH
OMH
BLH
HS
Chn (DoS~1)
Chn (DoS~2)
Chn (DoS~3)
HA
Contaminants

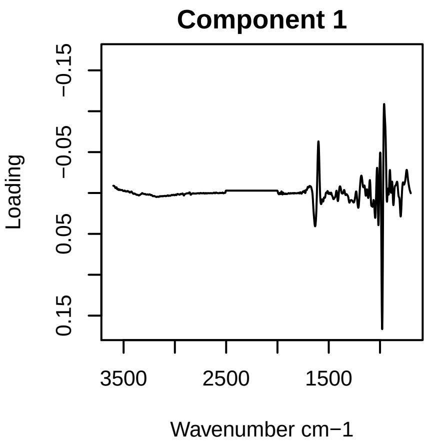
Component 1
Loading
−0.15
−0.05
0.05
0.15
3500
2500
1500
Wavenumber cm−1

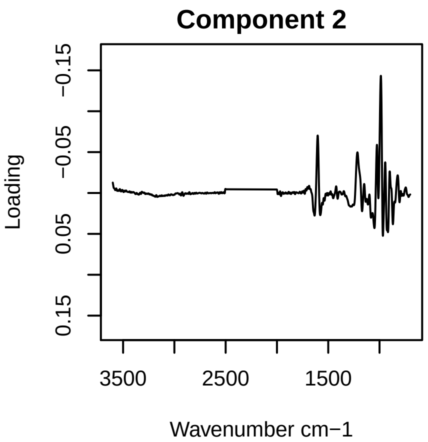
Component 2
Loading
−0.15
−0.05
0.05
0.15
3500
2500
1500
Wavenumber cm−1

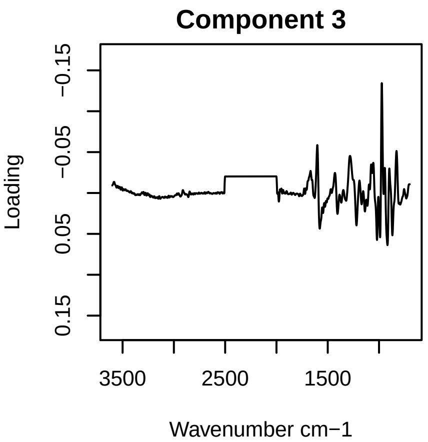
Component 3
Loading
−0.15
−0.05
0.05
0.15
3500
2500
1500
Wavenumber cm−1

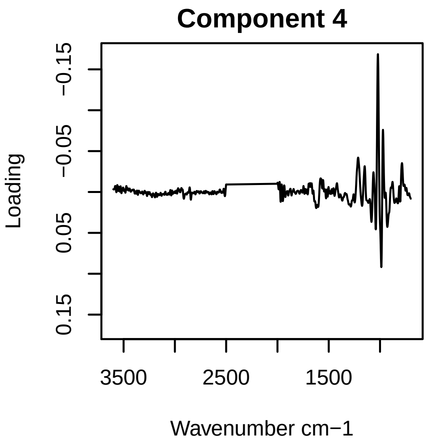
Component 4
Loading
−0.15
−0.05
0.05
0.15
3500
2500
1500
Wavenumber cm−1

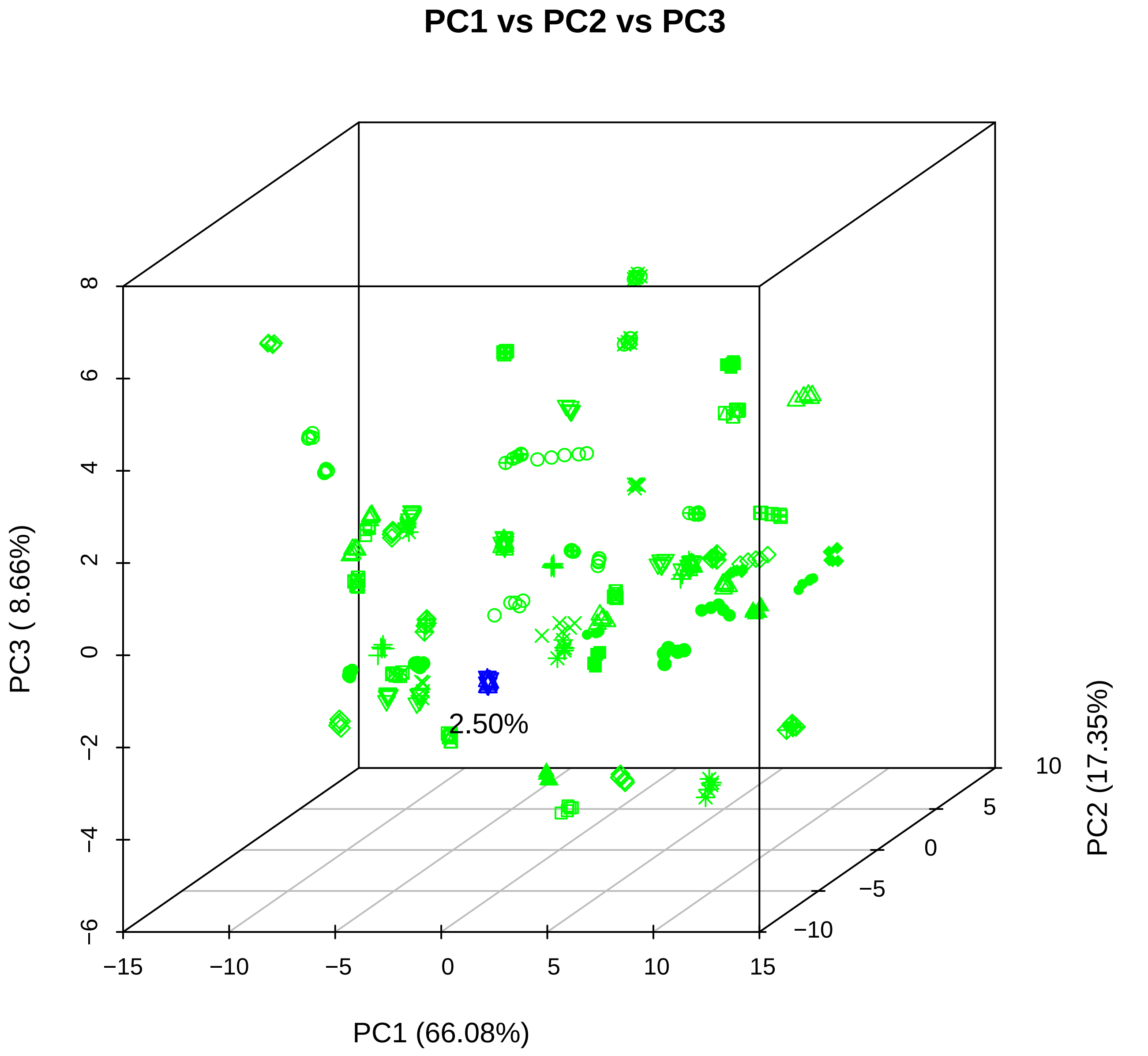
PC1 vs PC2 vs PC3
8
6
4
2
0
−2
−4
−6
PC3 (8.66%)
2.50%
−15
−10
−5
0
5
10
15
PC1 (66.08%)
10
5
0
−5
−10
PC2 (17.35%)

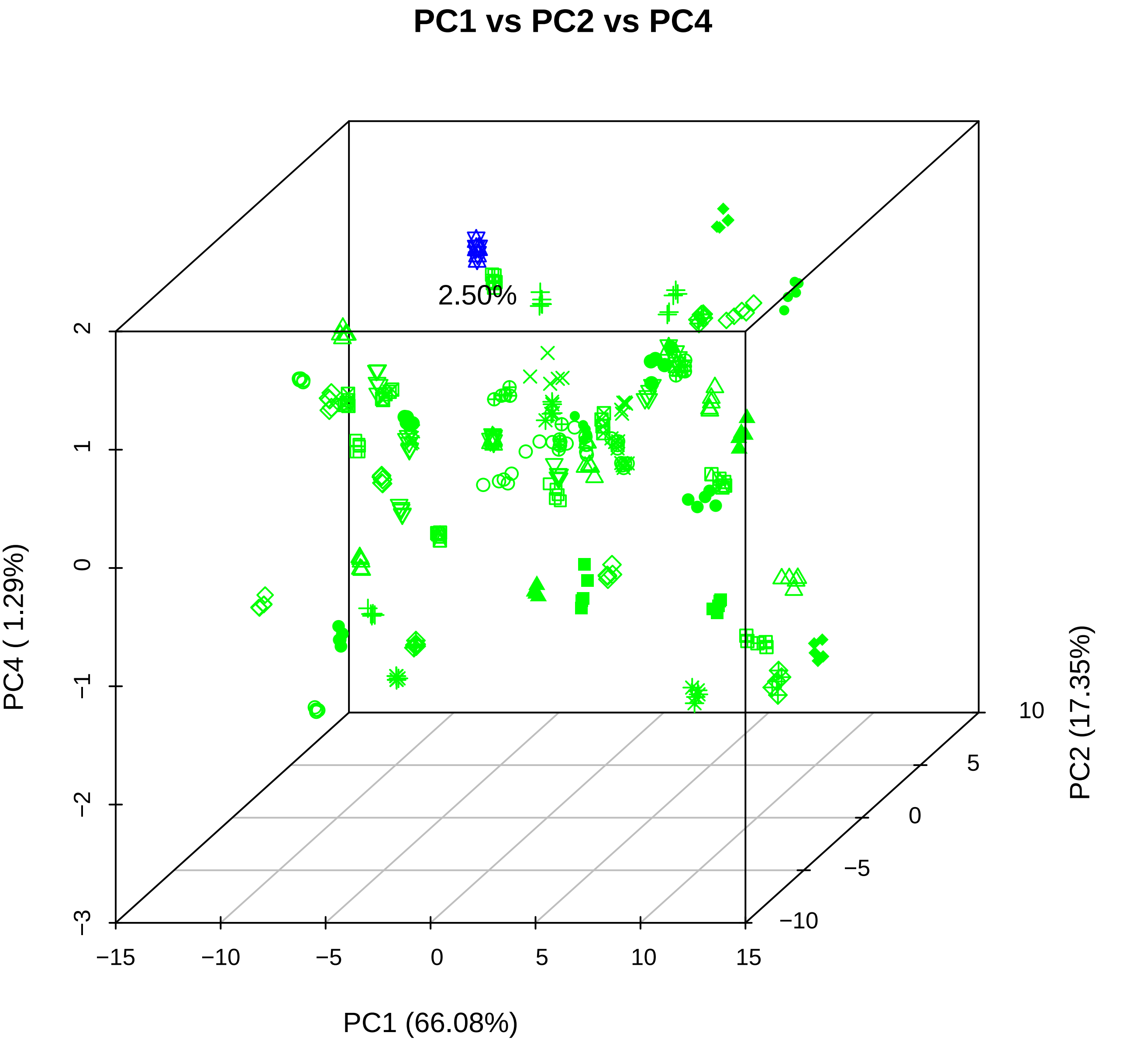

PC1 vs PC2 vs PC4
2.50%
PC4 (1.29%)
PC1 (66.08%)
PC2 (17.35%)
−15
−10
−5
0
5
10
15
−3
−2
−1
0
1
2
−10
−5
0
5
10

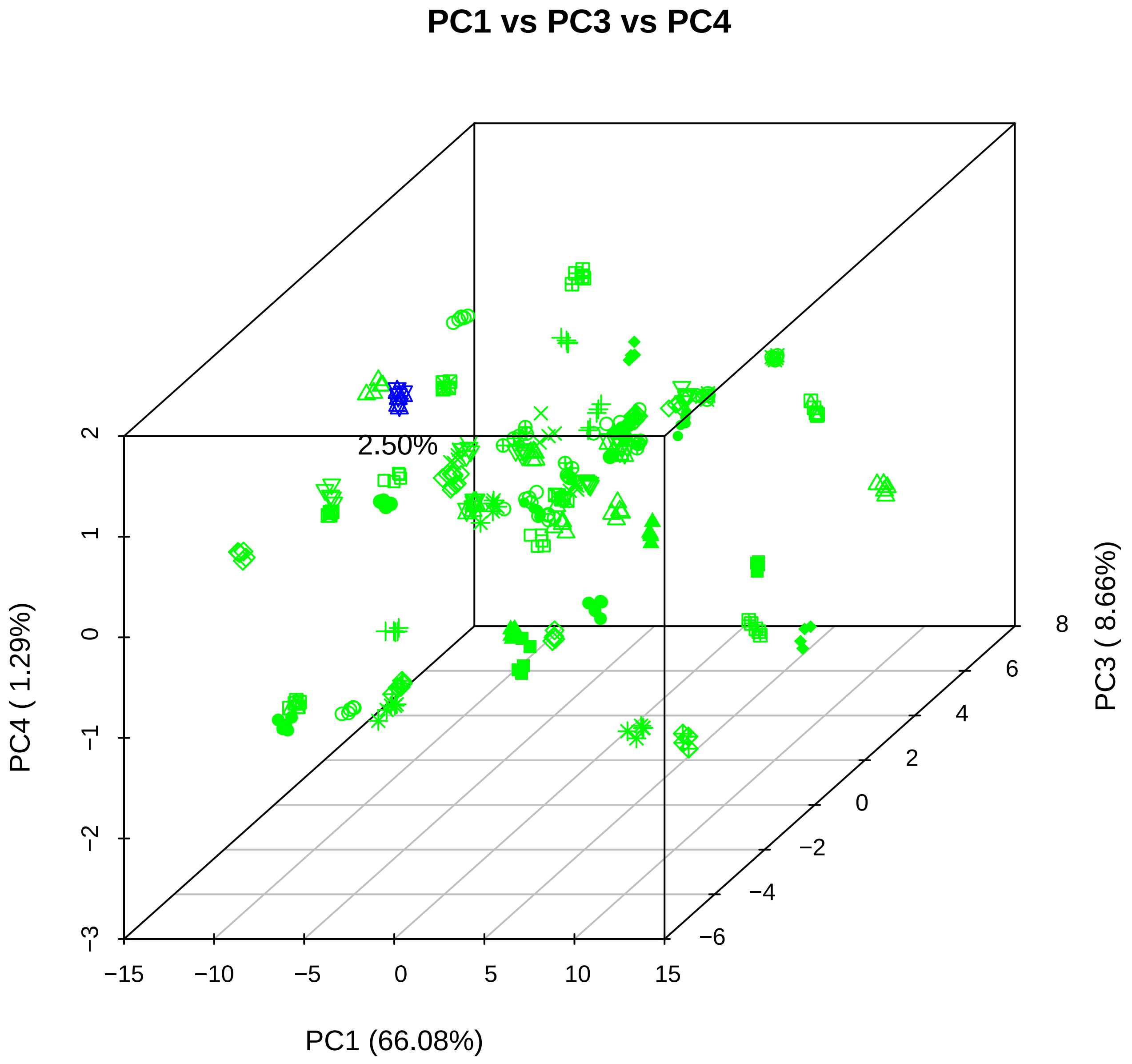

PC1 vs PC3 vs PC4
2.50%
PC4 (1.29%)
2
1
0
−1
−2
−3
−15
−10
−5
0
5
10
15
PC1 (66.08%)
PC3 (8.66%)
8
6
4
2
0
−2
−4
−6

PC2 vs PC3 vs PC5

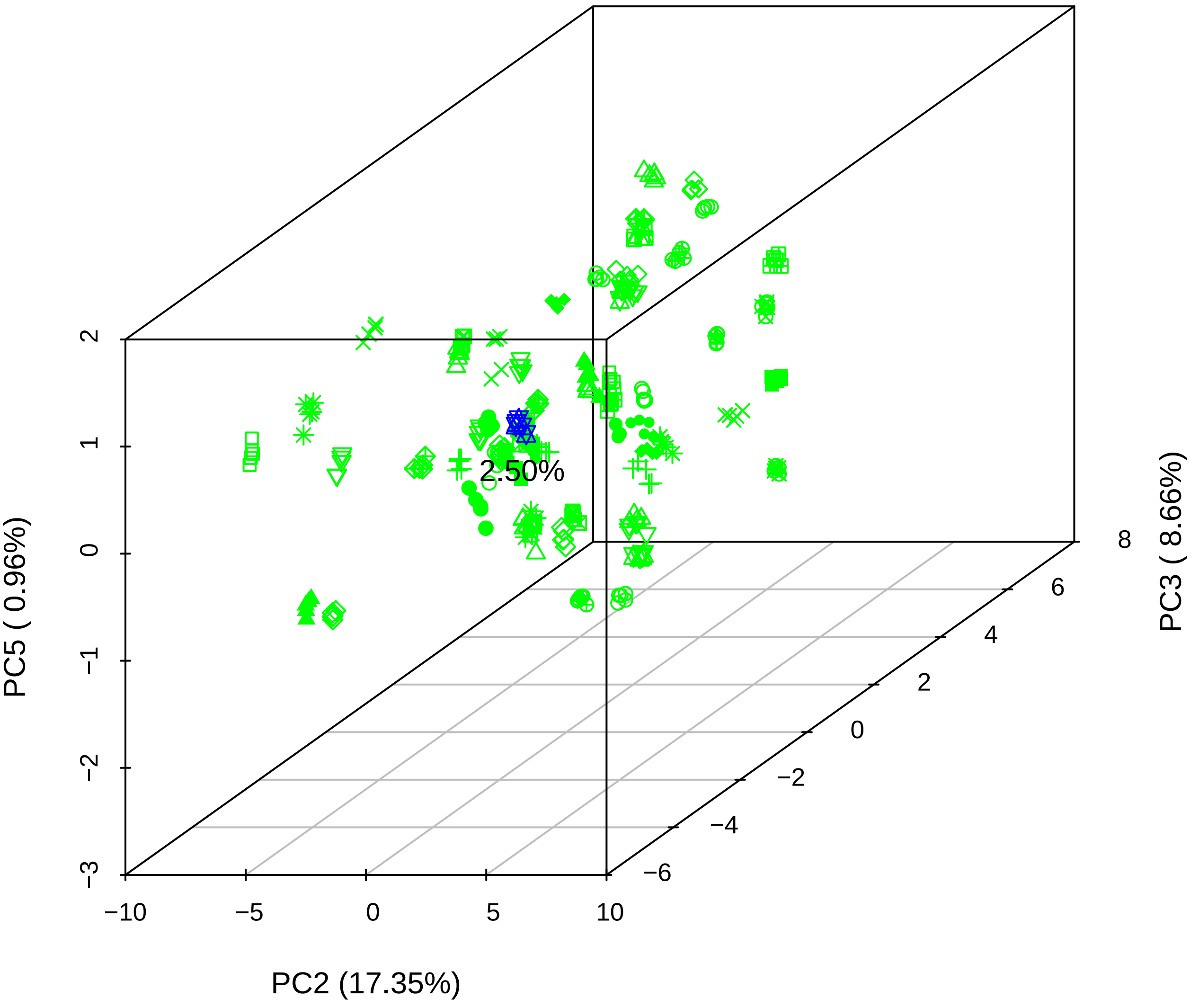

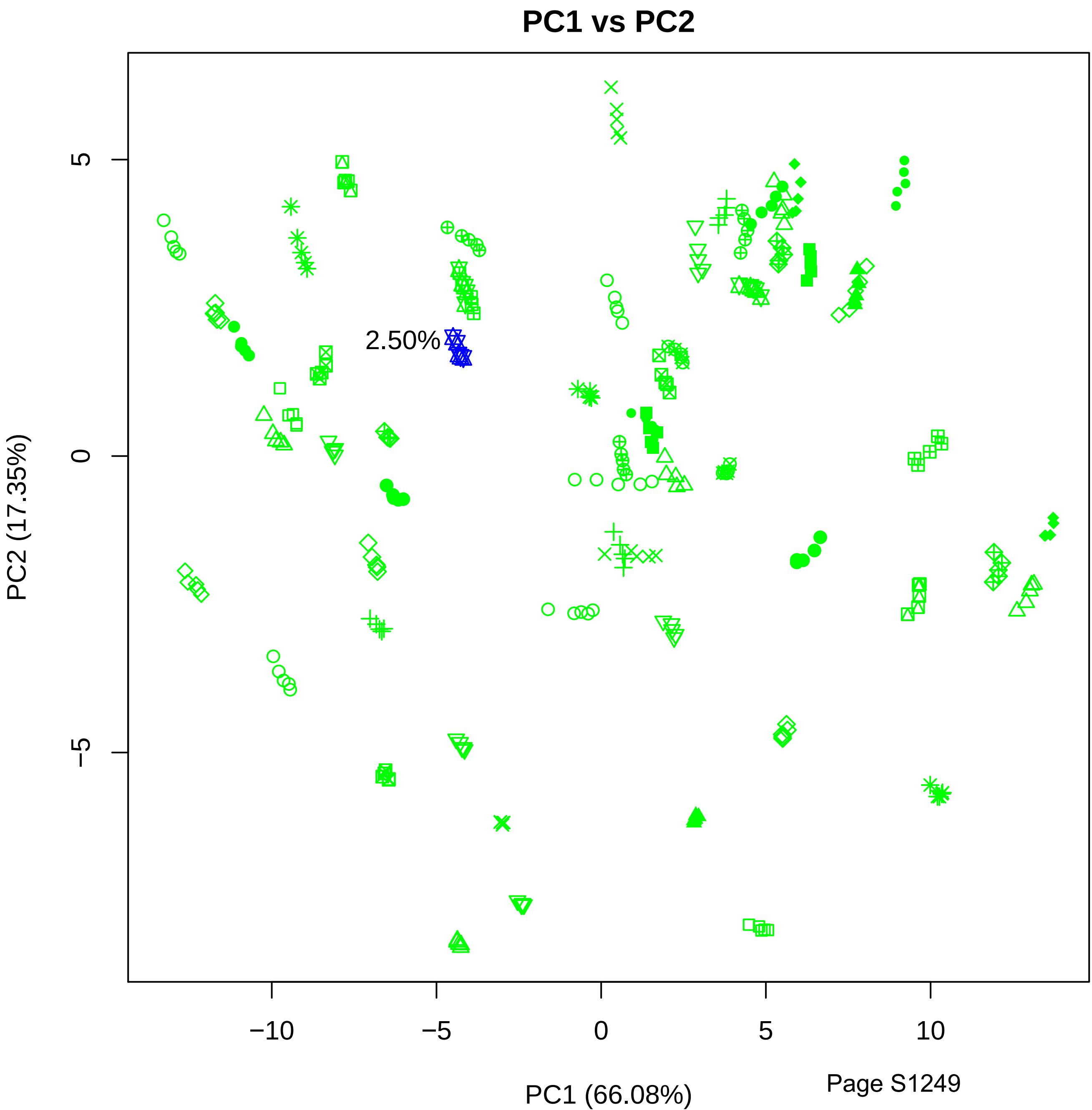
PC1 vs PC2
2.50%
PC1 (66.08%)
PC2 (17.35%)
−10
−5
0
5
10

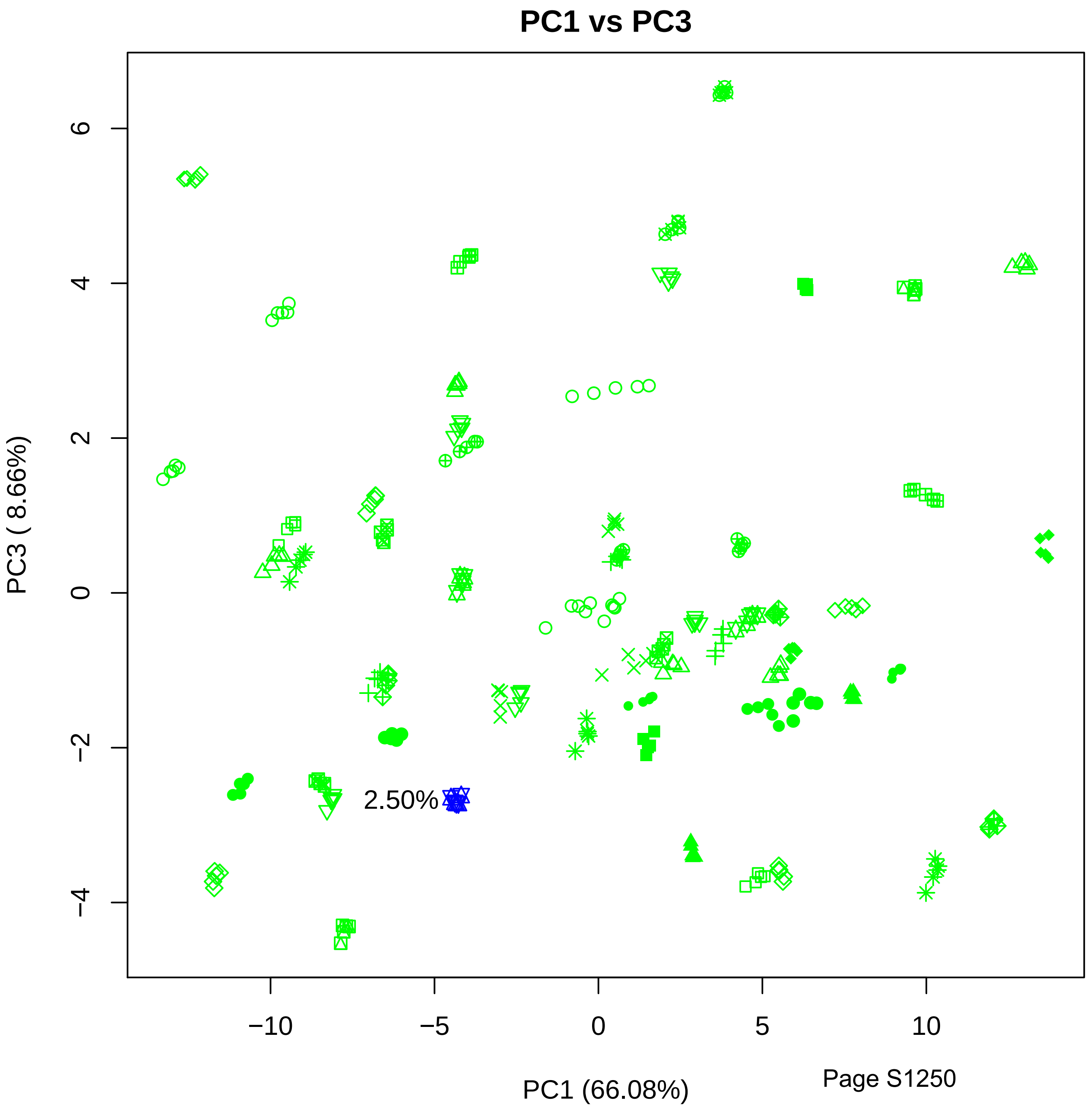

PC1 vs PC3
6
4
2
0
−2
−4
PC3 (8.66%)
−10
−5
0
5
10
PC1 (66.08%)
2.50%

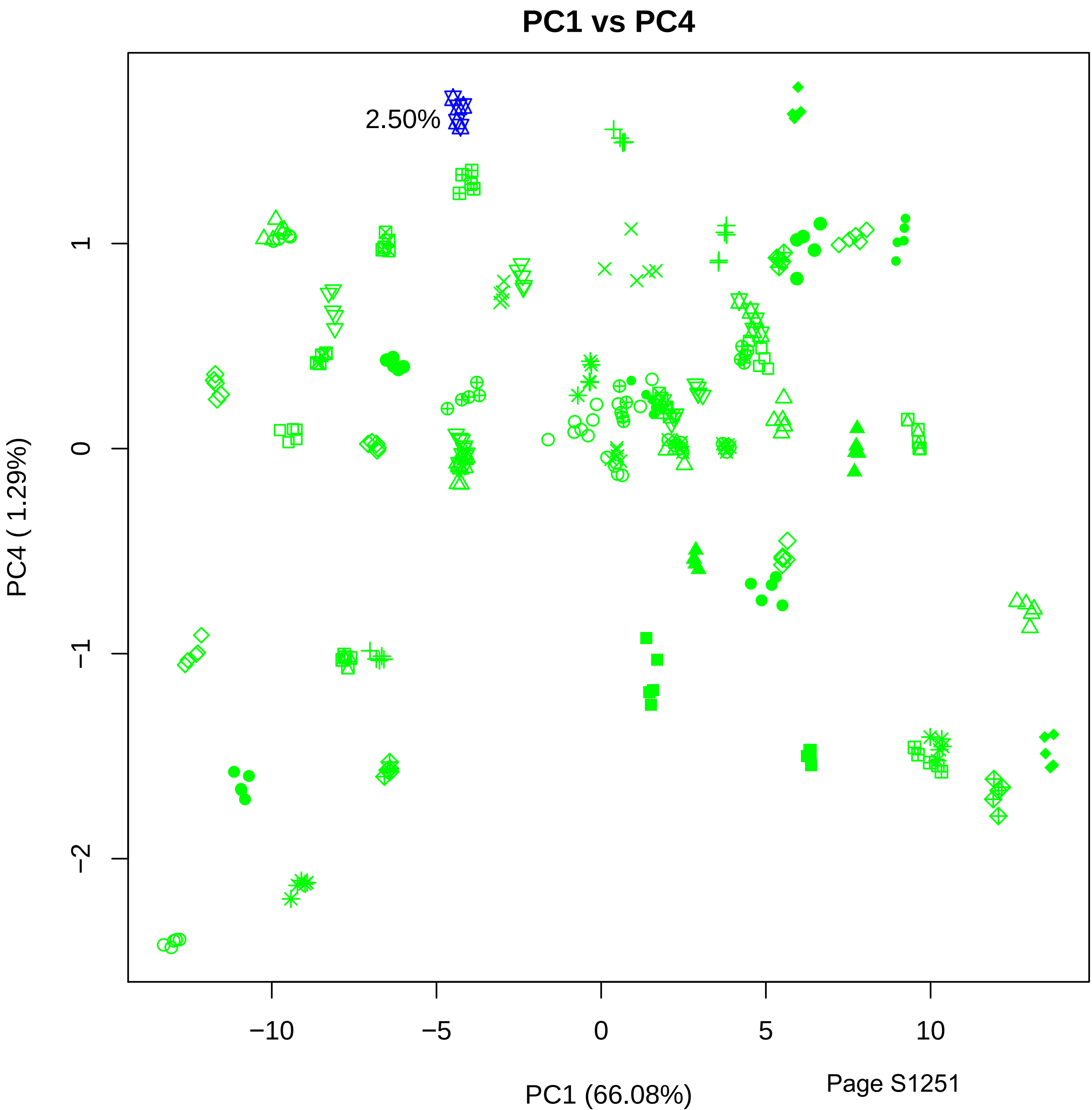

PC1 vs PC4
2.50%
PC4 (1.29%)
PC1 (66.08%)
1
0
−1
−2
−10
−5
0
5
10

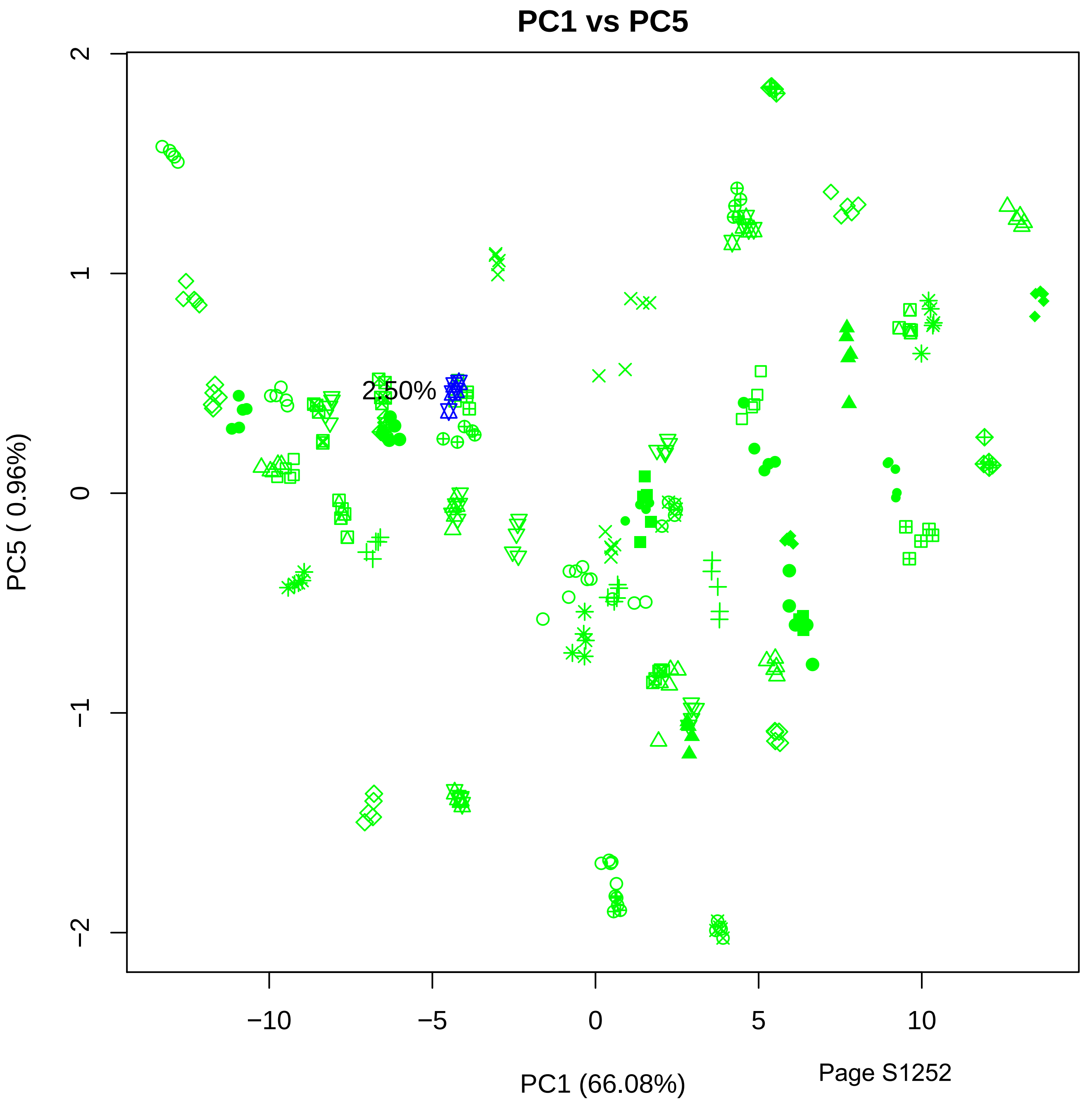

PC1 vs PC5
PC5 (0.96%)
PC1 (66.08%)
2.50%
2
1
0
−1
−2
−10
−5
0
5
10

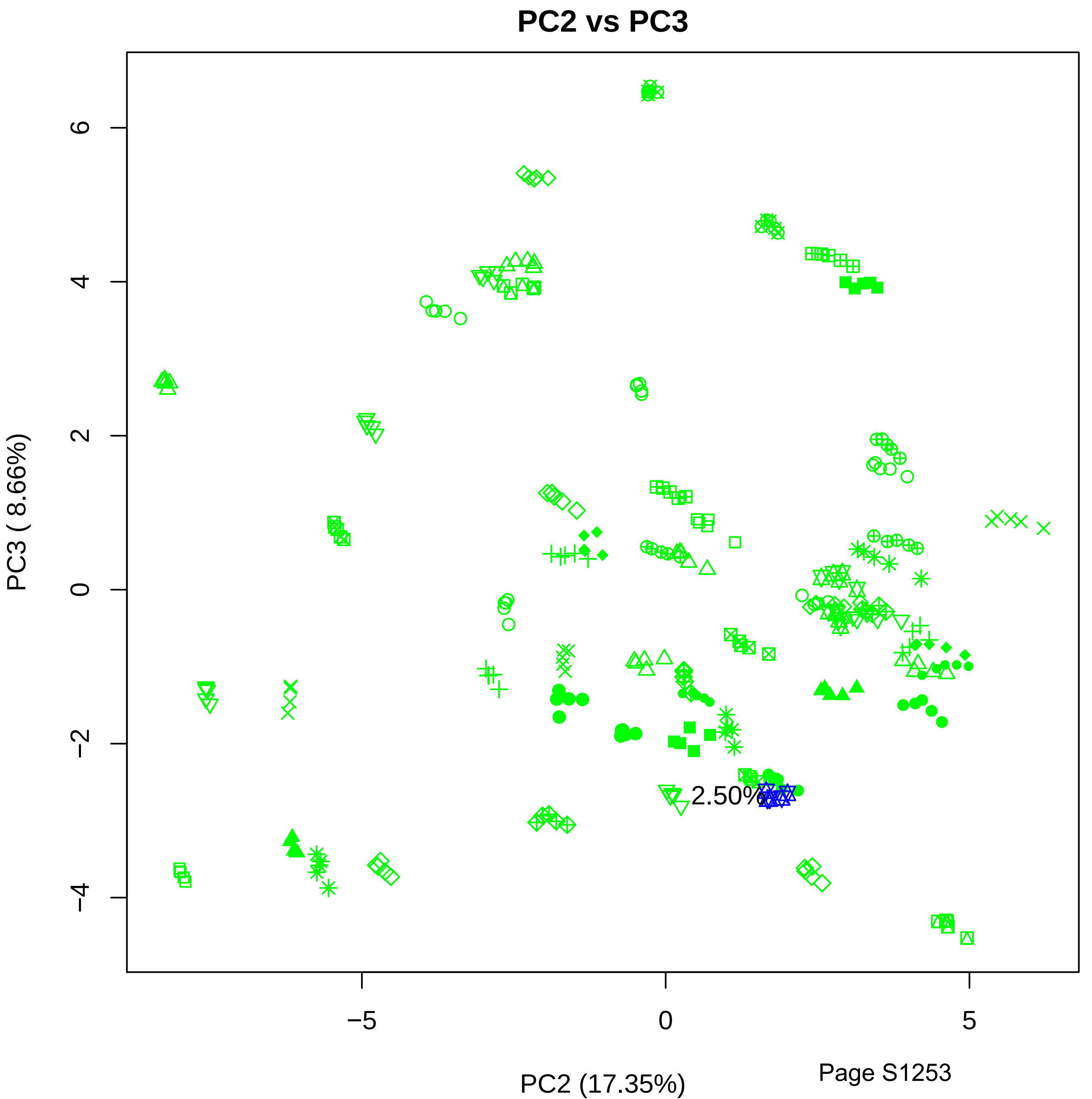
PC2 vs PC3
PC3 (8.66%)
PC2 (17.35%)
2.50%
6
4
2
0
−2
−4
−5
0
5

PC2 vs PC4

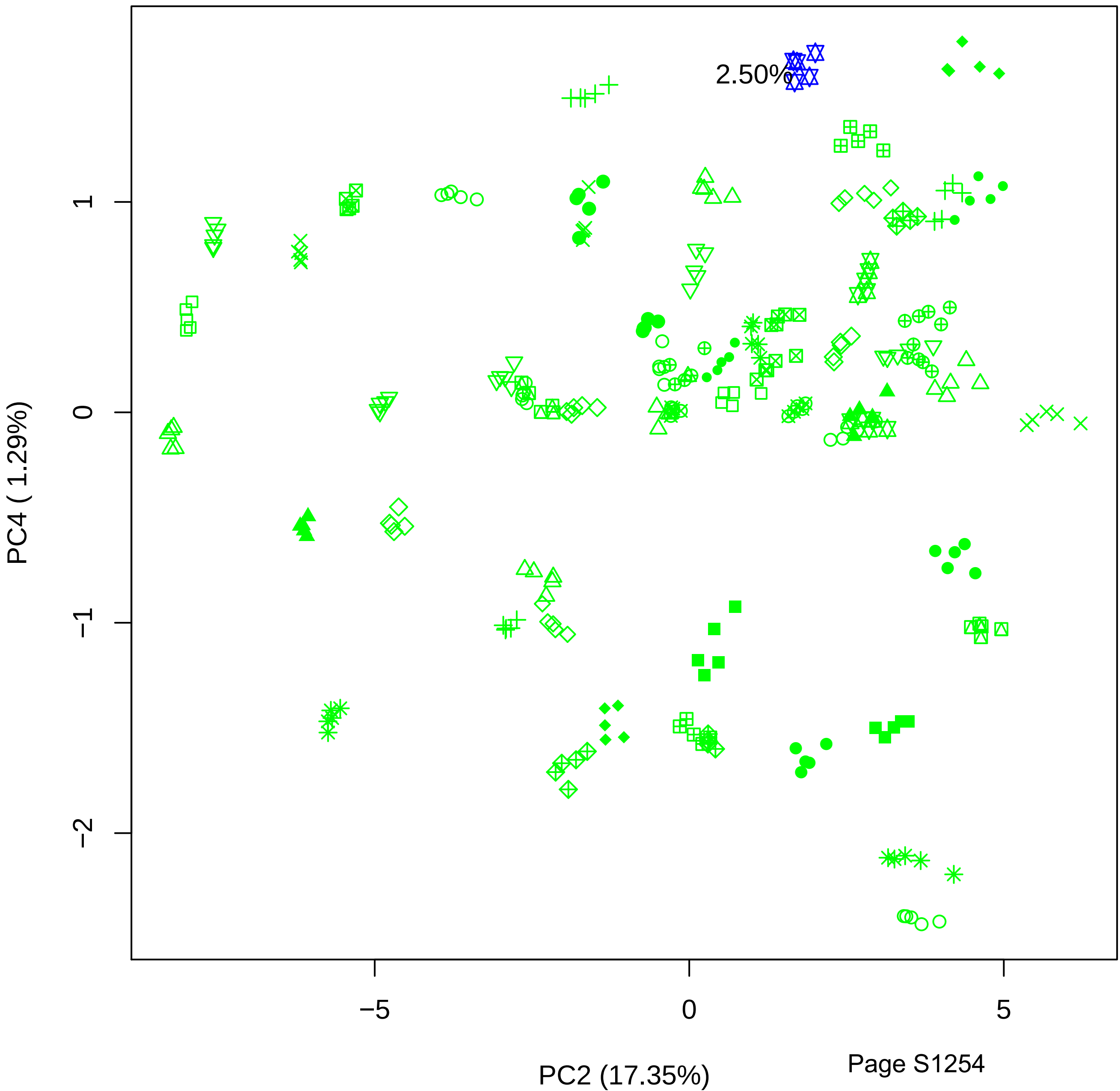

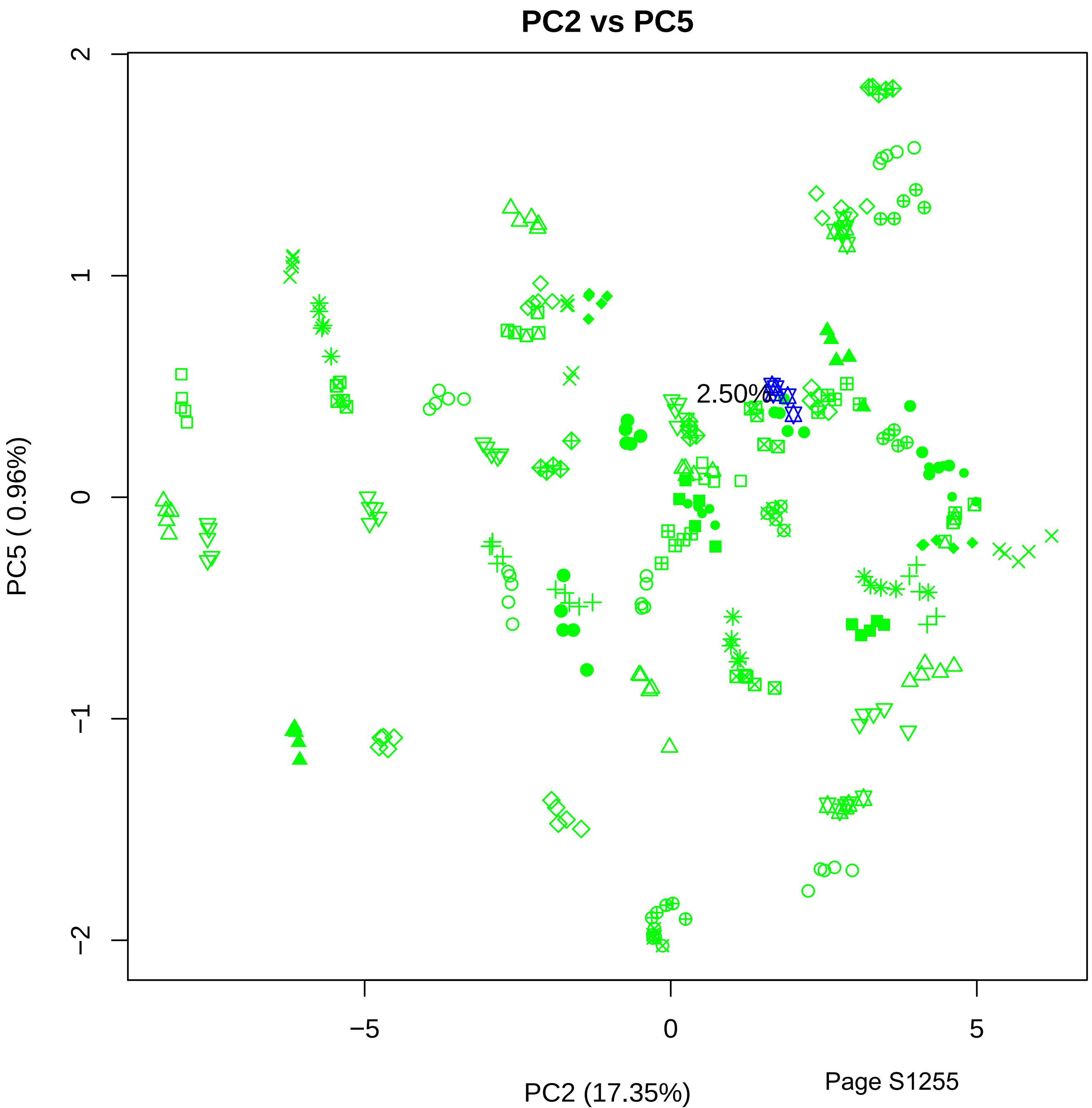

PC2 vs PC5
2
1
0
−1
−2
PC5 (0.96%)
2.50%
−5
0
5
PC2 (17.35%)

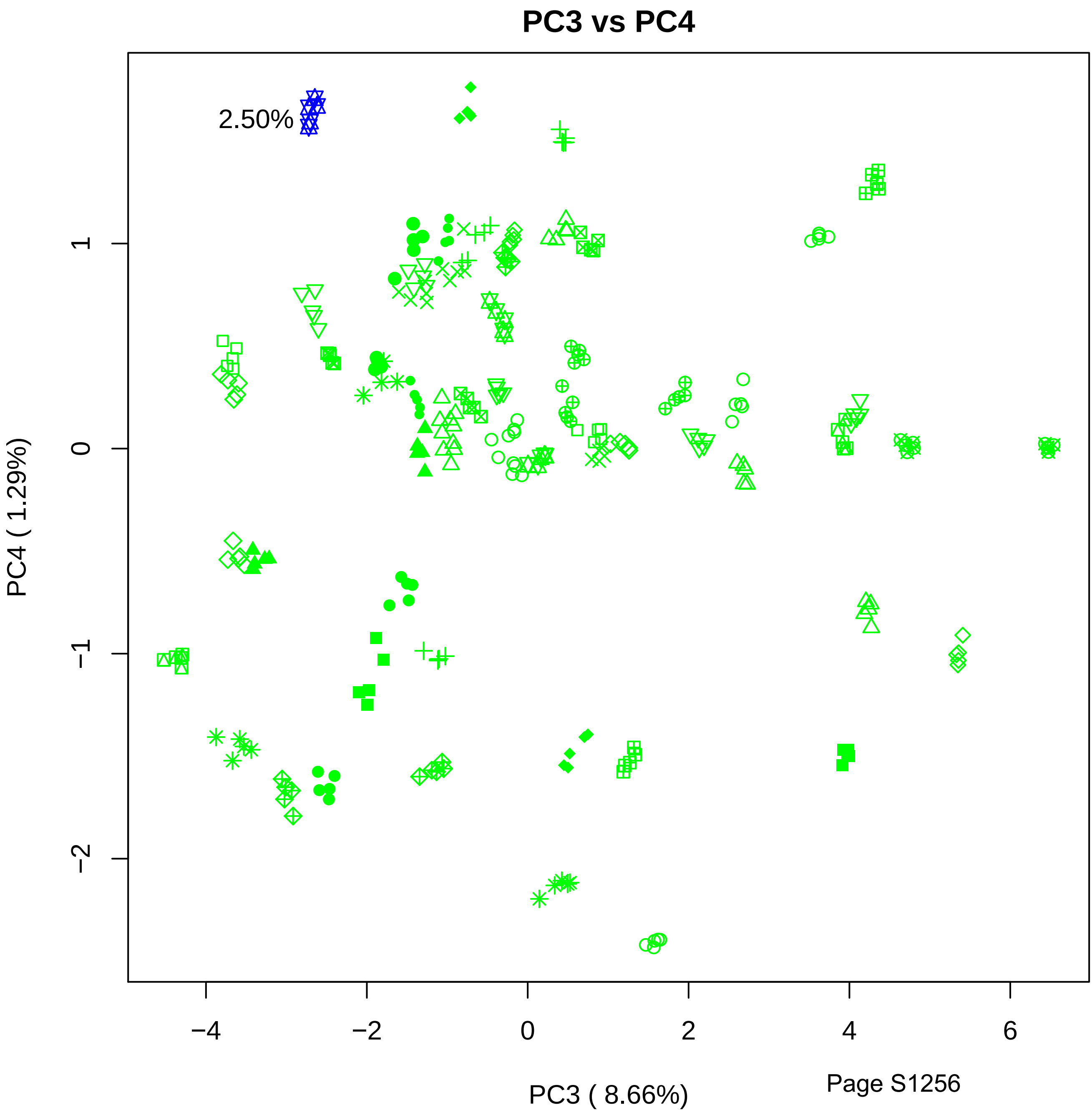
PC3 vs PC4
2.50%
PC4 (1.29%)
PC3 (8.66%)
1
0
−1
−2
−4
−2
0
2
4
6

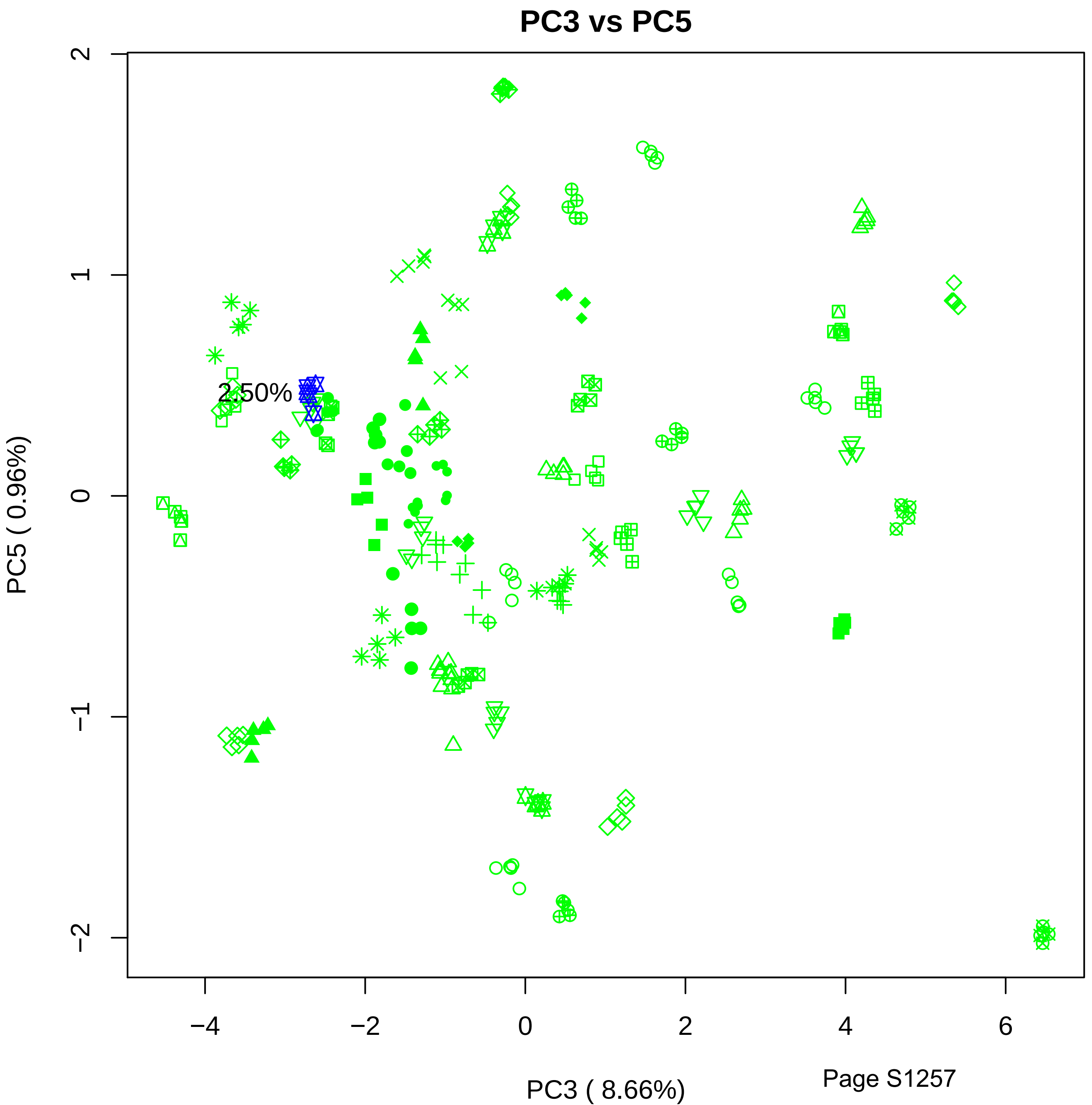

PC3 vs PC5
PC5 (0.96%)
PC3 (8.66%)
2.50%
2
1
0
−1
−2
−4
−2
0
2
4
6

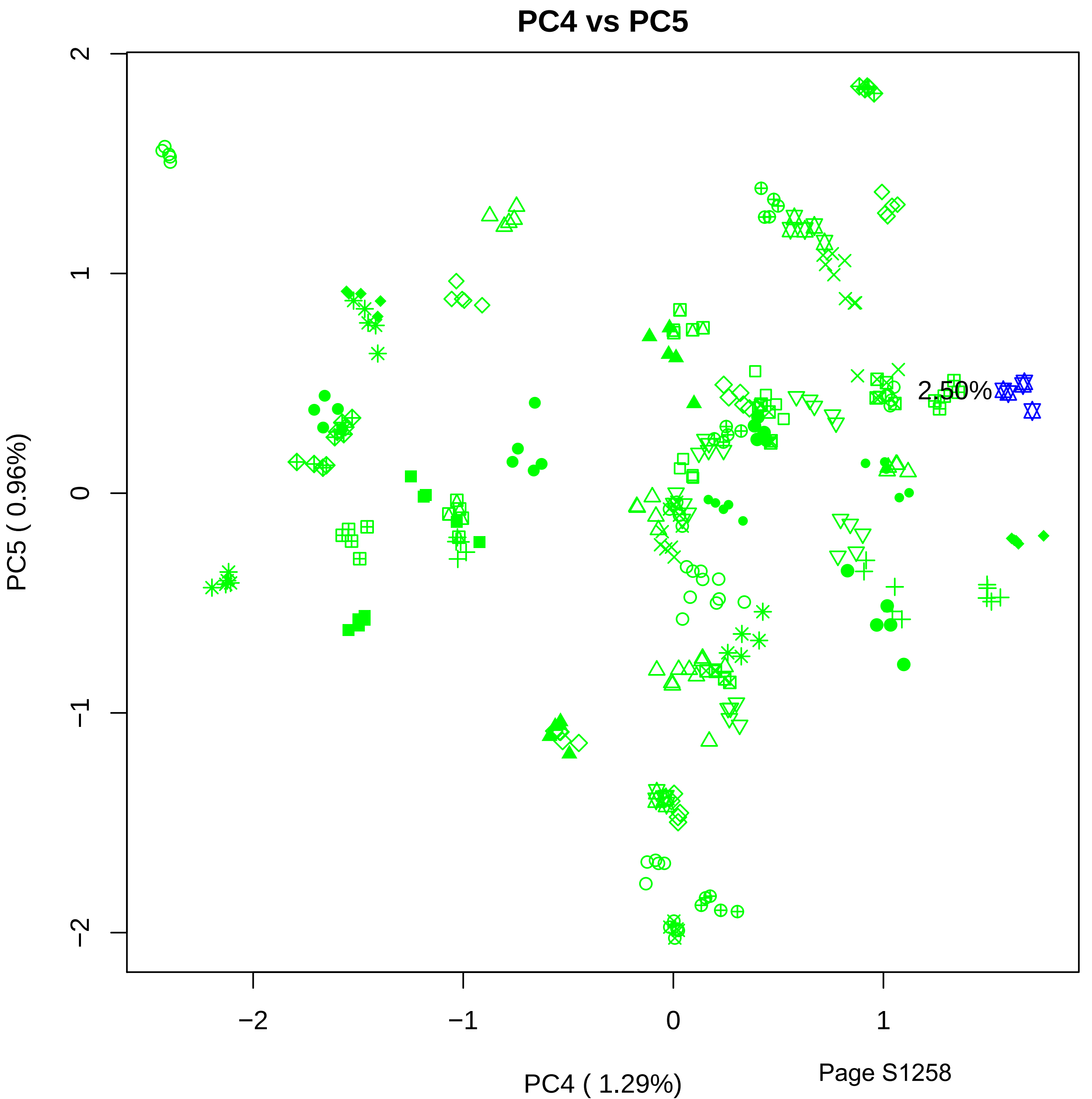
PC4 vs PC5
PC5 (0.96%)
PC4 (1.29%)
2.50%
2
1
0
−1
−2
−2
−1
0
1

PCA of 5% BMH(c) vs PMH(c)

Scaled Matrix PCAed

7th order polynomial baseline correction performed

Raw spectra smoothed to 21 Neighbours, 2nd order PN, Savtisky Golay

Normalisation between 0 and 100

D2 spectra smoothed to 41 Neighbours, 2nd order PN, Savtisky Golay

Regions Analysed: 3601−2501 and 2001−700cm−1

Samples Analysed: 1 bContam's 0 BLH's 0 BMH's 0 OMH's 0 pContam's 0 PLH's 60 PMH's

Keele University AD

Fri Dec 14 14:18:56 2018

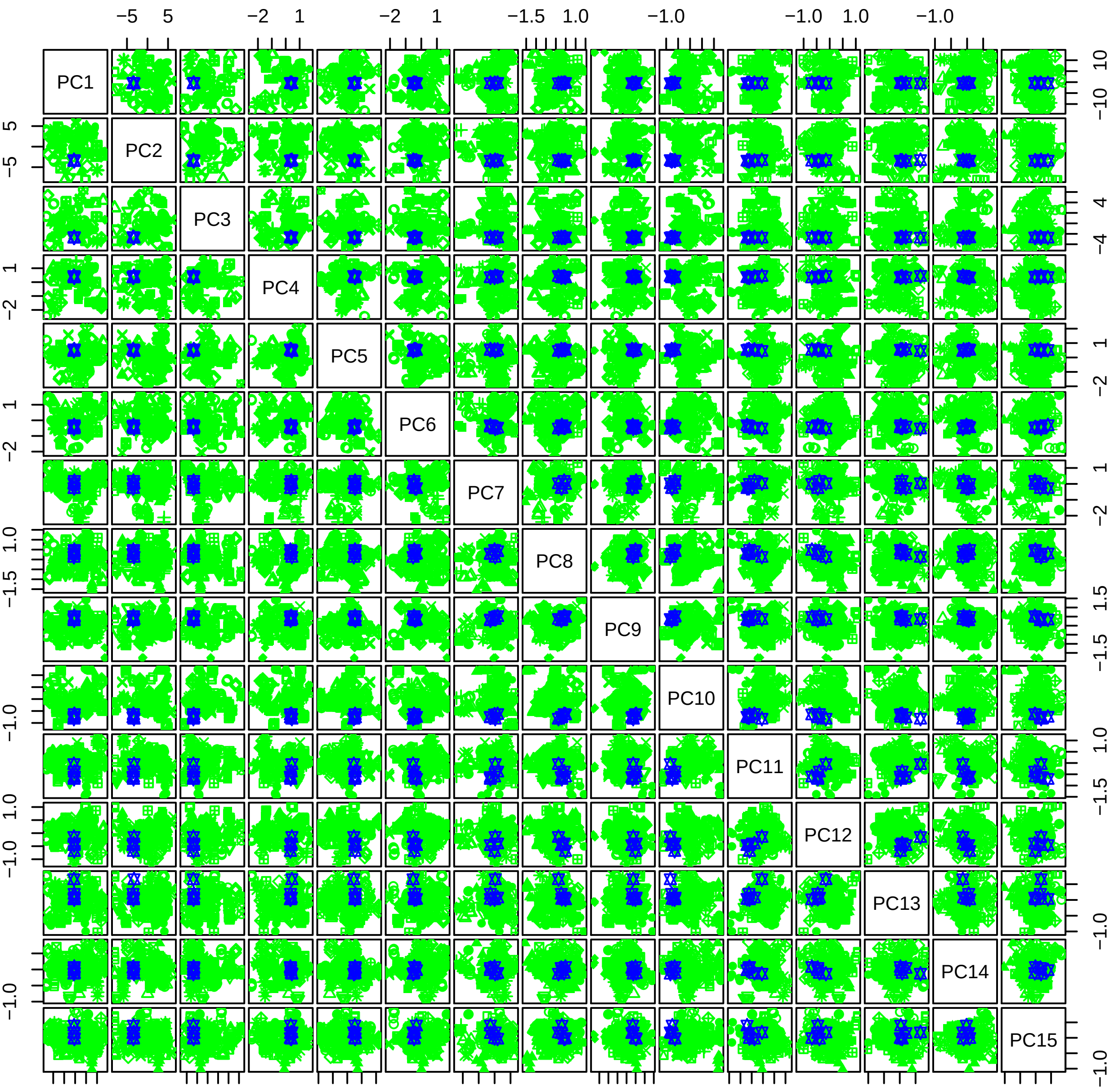

PC1
PC2
PC3
PC4
PC5
PC6
PC7
PC8
PC9
PC10
PC11
PC12
PC13
PC14
PC15

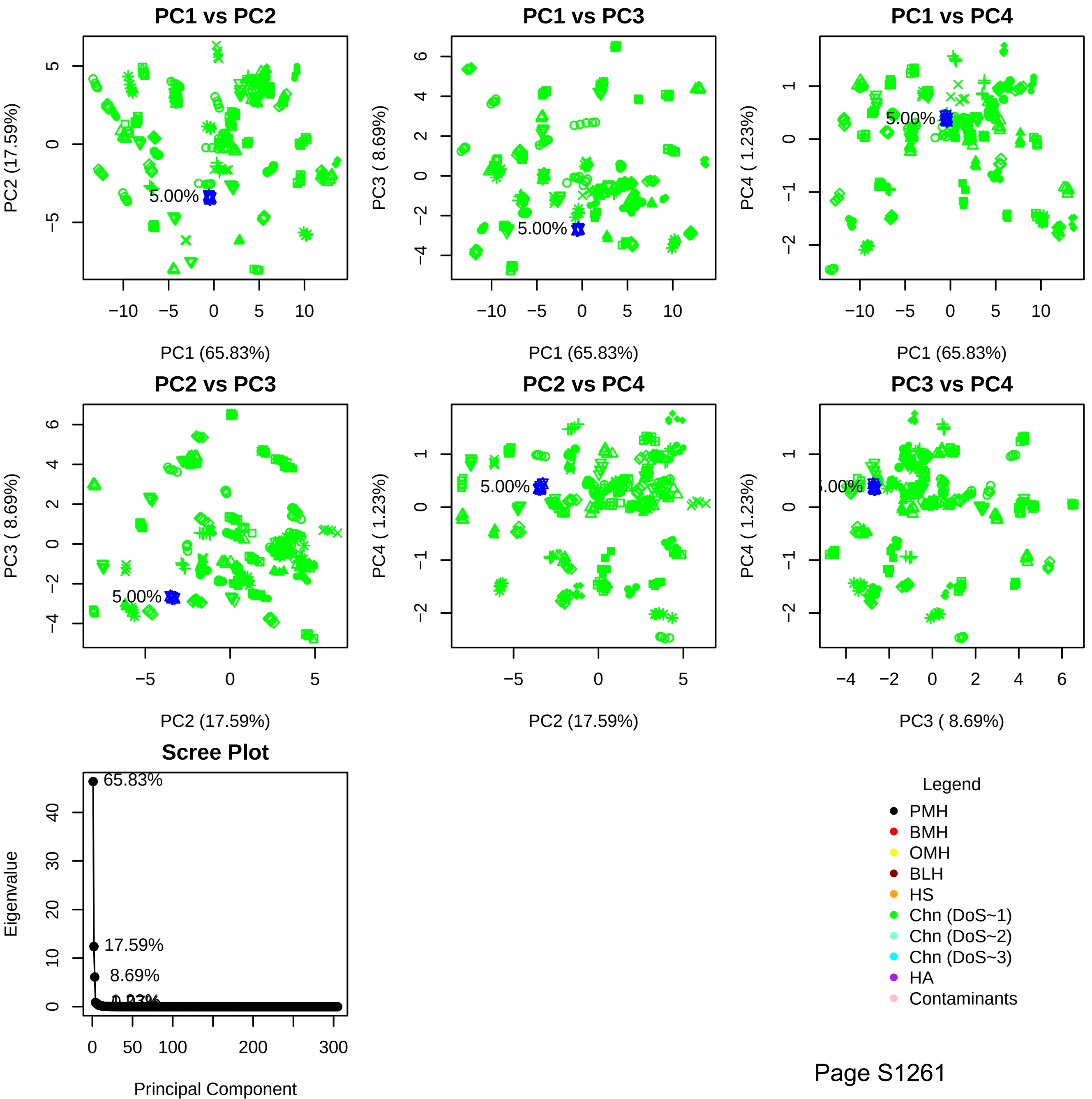
PC1 vs PC2
PC1 vs PC3
PC1 vs PC4
PC2 vs PC3
PC2 vs PC4
PC3 vs PC4
Scree Plot
PC1 (65.83%)
PC2 (17.59%)
PC3 (8.69%)
PC4 (1.23%)
5.00%
Eigenvalue
Principal Component
65.83%
17.59%
8.69%
Legend
PMH
BMH
OMH
BLH
HS
Chn (DoS~1)
Chn (DoS~2)
Chn (DoS~3)
HA
Contaminants

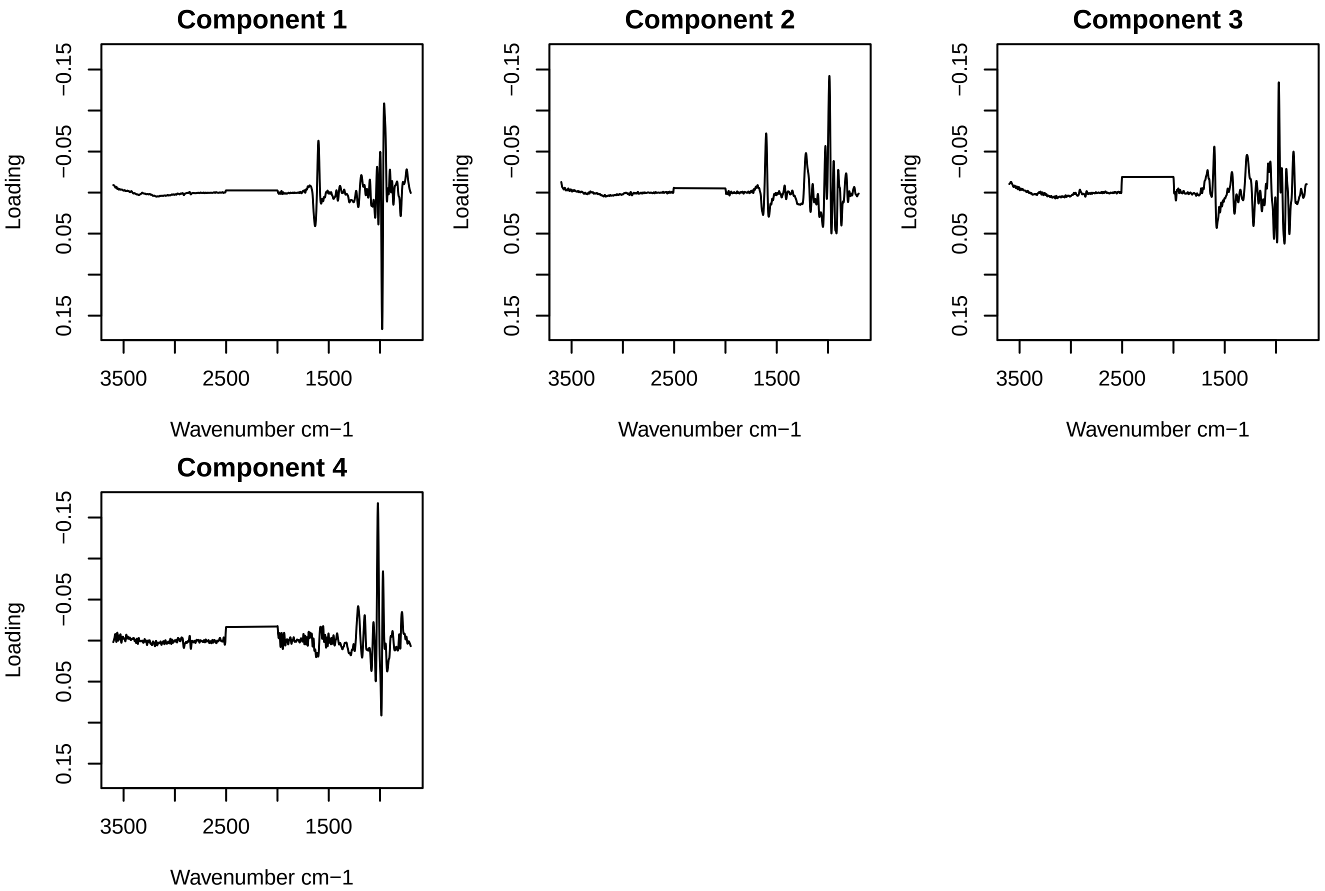
Component 1
Component 2
Component 3
Component 4
Loading
−0.15
−0.05
0.05
0.15
3500
2500
1500
Wavenumber cm−1

PC1 vs PC2 vs PC3

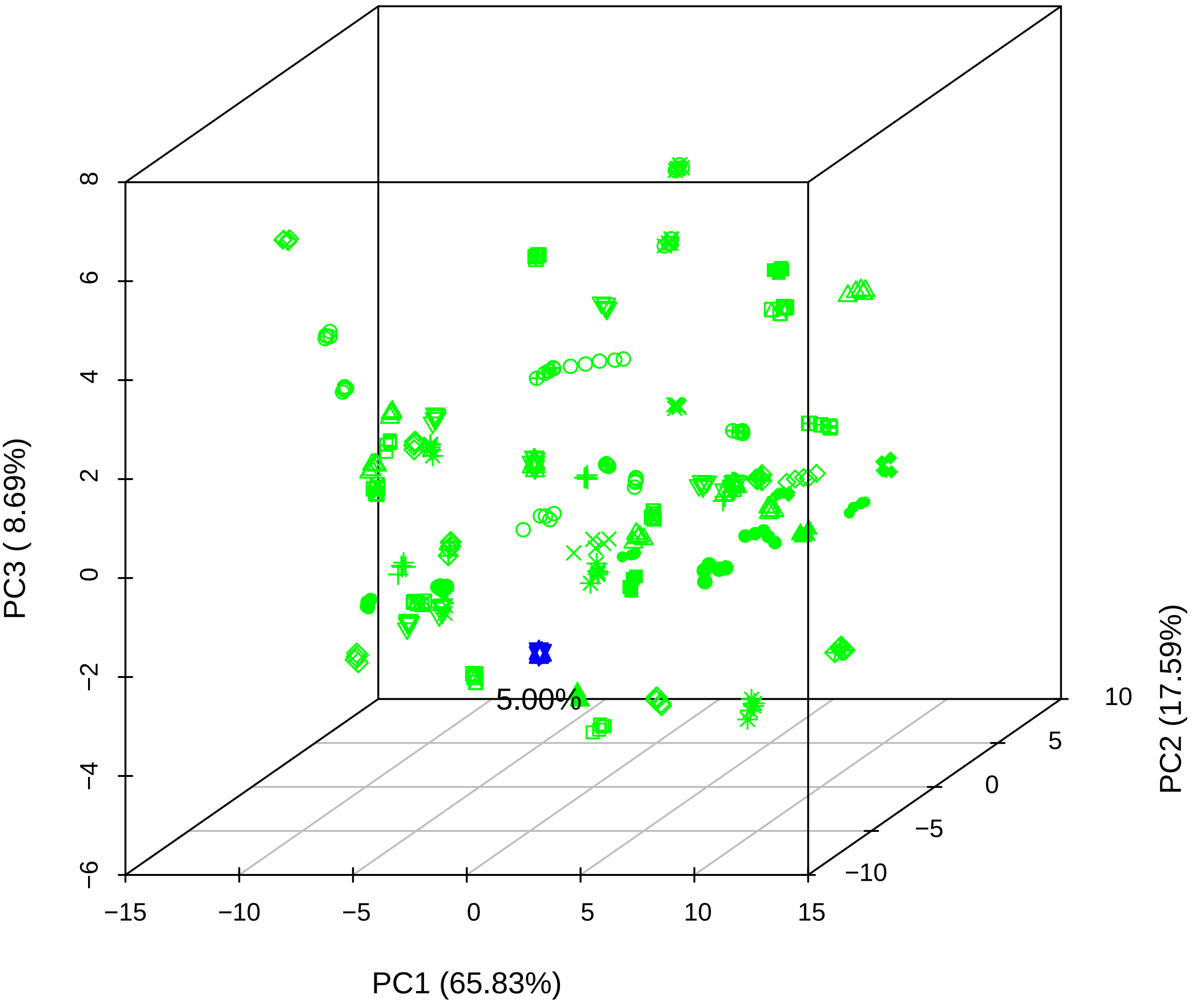

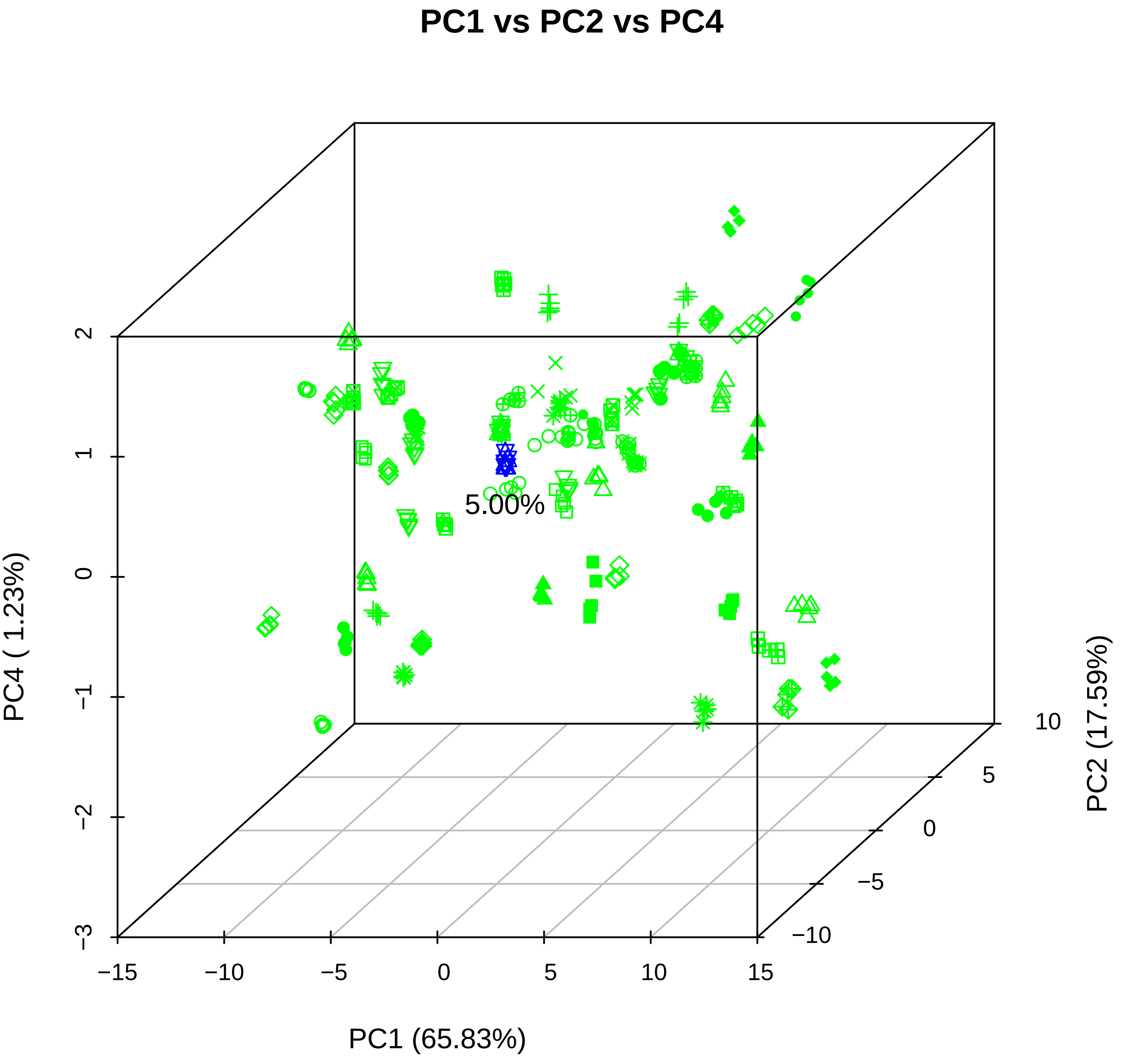

PC1 vs PC2 vs PC4
5.00%
PC4 (1.23%)
−3
−2
−1
0
1
2
−15
−10
−5
0
5
10
15
PC1 (65.83%)
PC2 (17.59%)
−10
−5
0
5
10

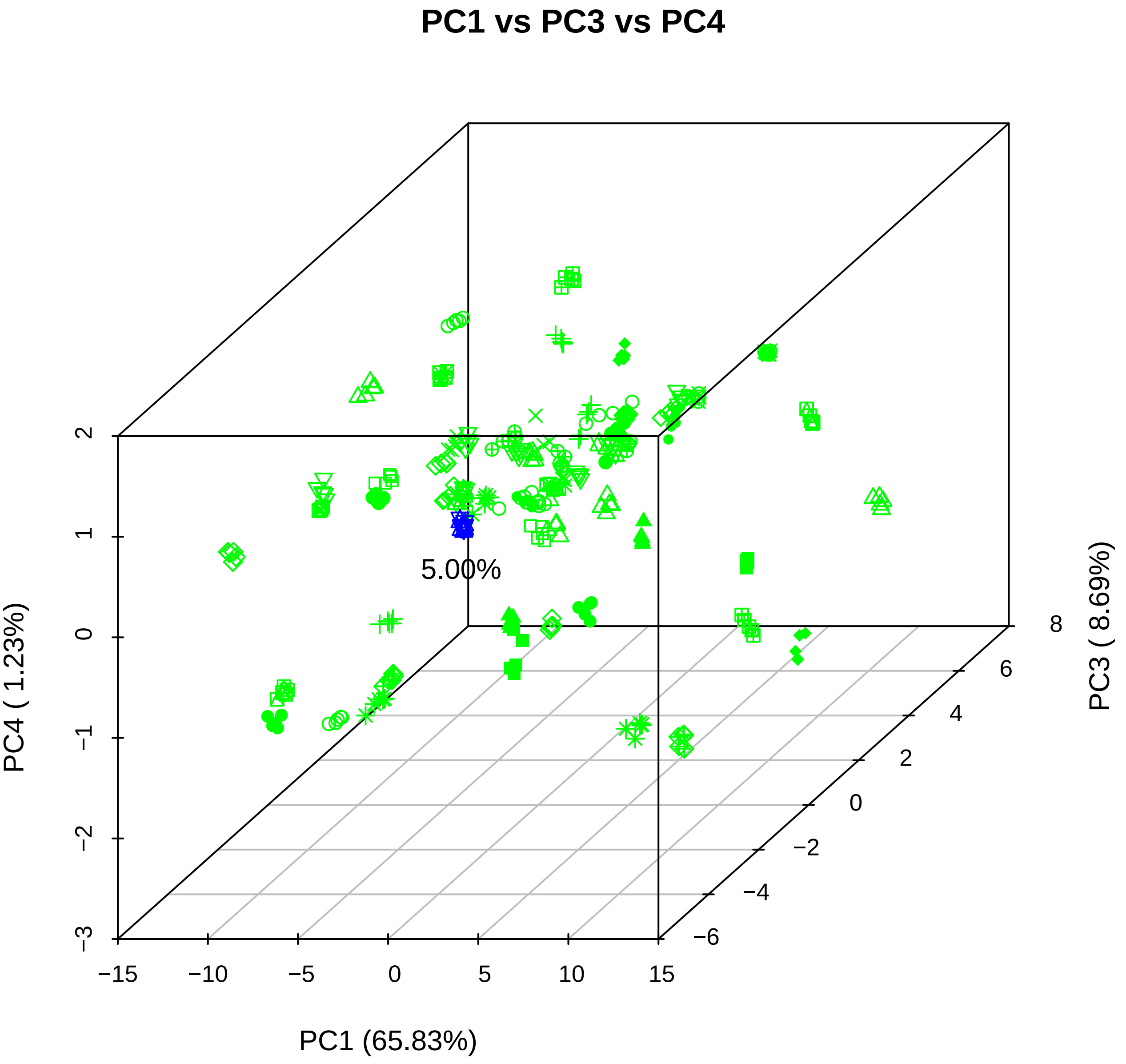

PC1 vs PC3 vs PC4
5.00%
PC1 (65.83%)
PC4 (1.23%)
PC3 (8.69%)
−15
−10
−5
0
5
10
15
−3
−2
−1
0
1
2
−6
−4
−2
0
2
4
6
8

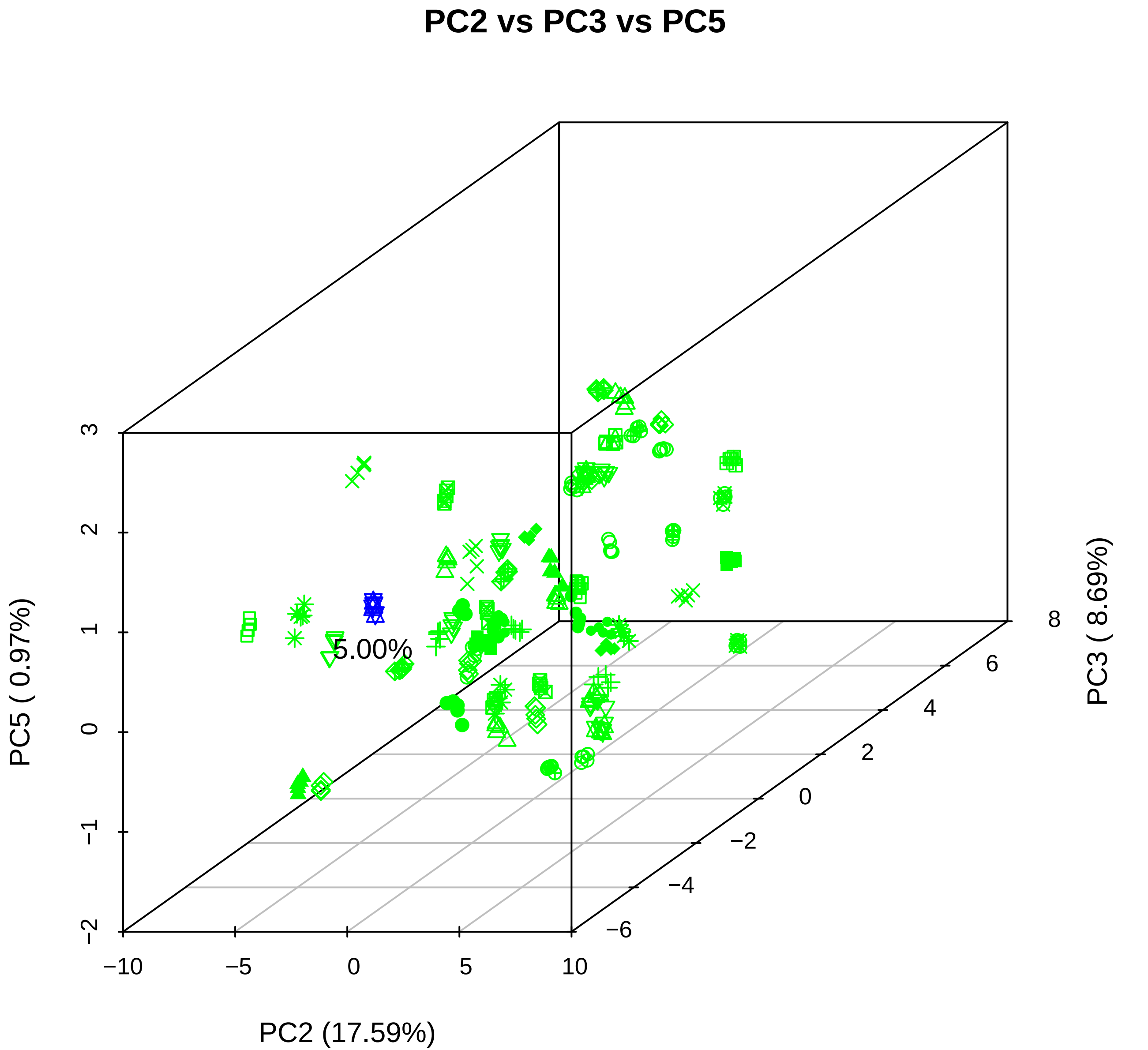
PC2 vs PC3 vs PC5
5.00%
PC2 (17.59%)
PC5 (0.97%)
PC3 (8.69%)
−10
−5
0
5
10
−2
−1
0
1
2
3
−6
−4
−2
0
2
4
6
8

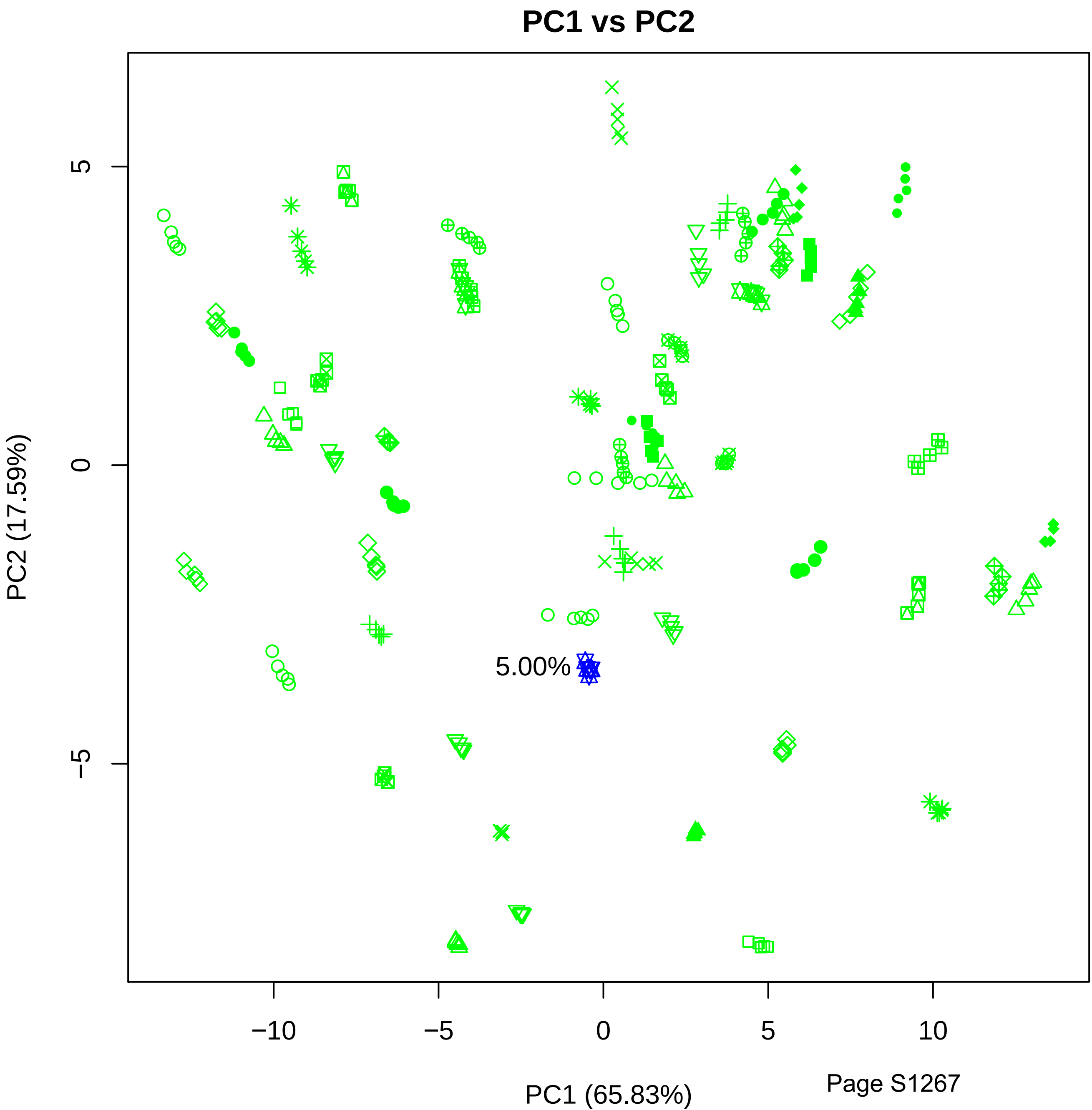
PC1 vs PC2
5
0
−5
PC2 (17.59%)
−10
−5
0
5
10
PC1 (65.83%)
5.00%

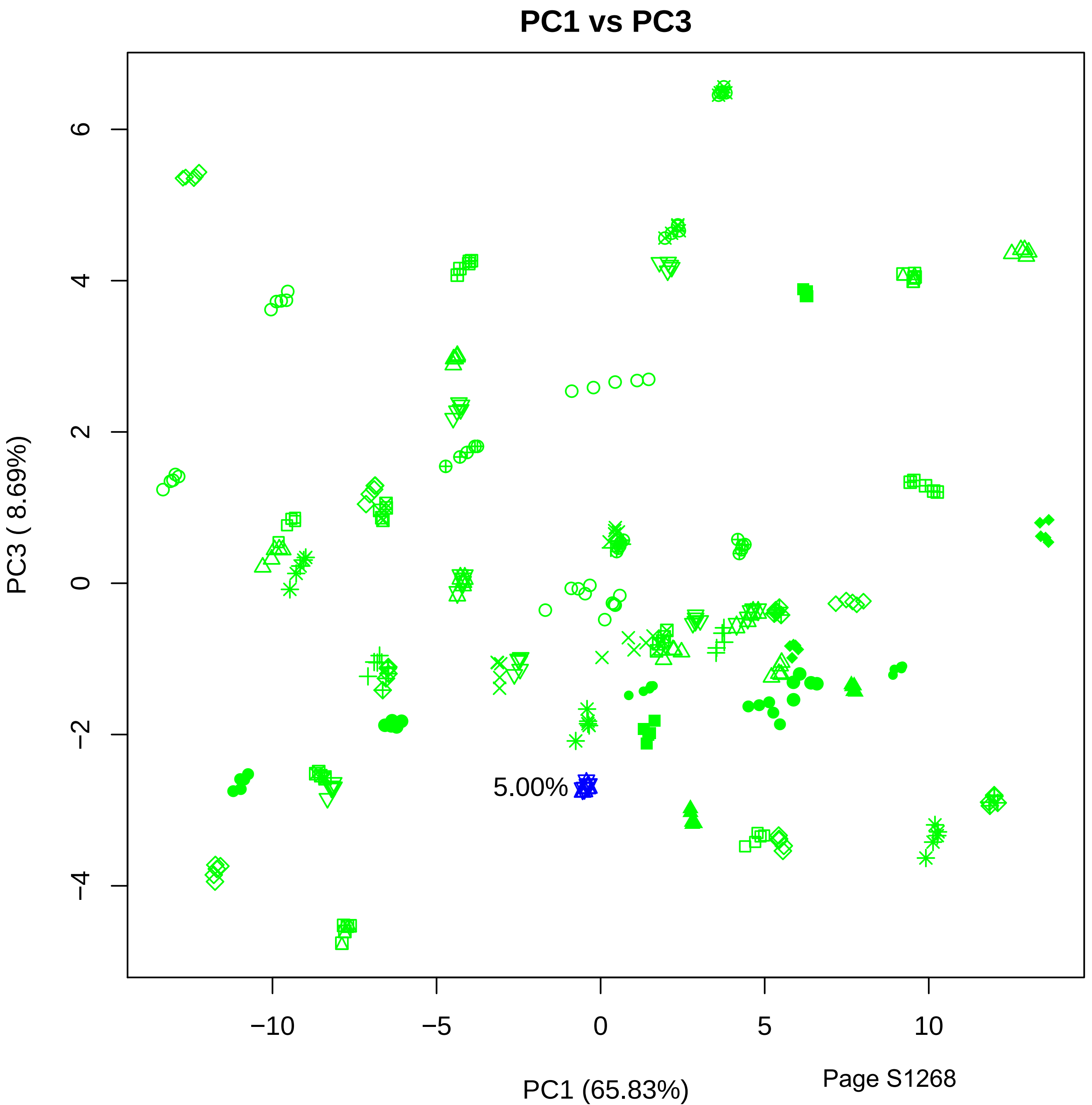
PC1 vs PC3
PC3 (8.69%)
PC1 (65.83%)
6
4
2
0
−2
−4
−10
−5
0
5
10
5.00%

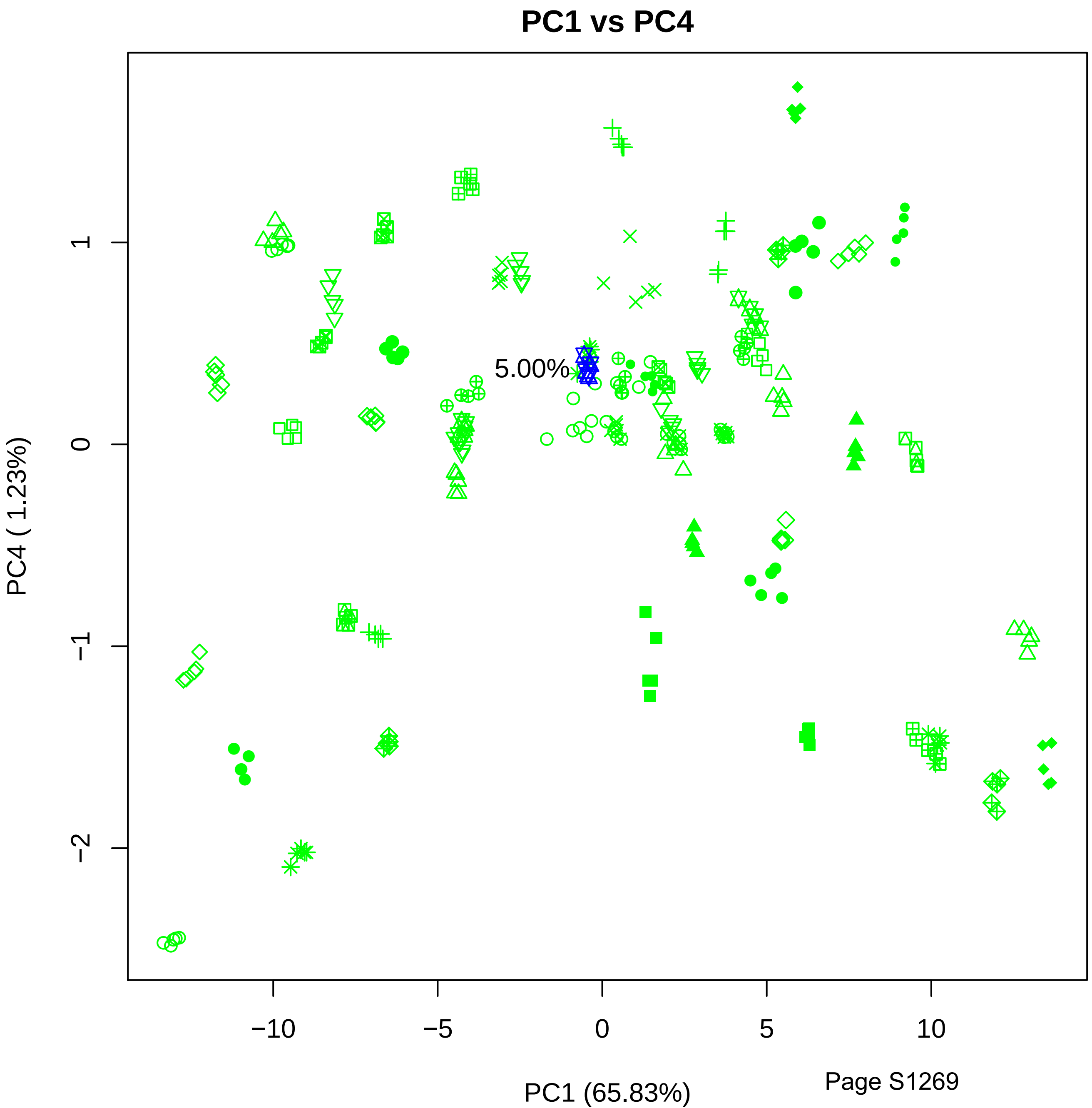

PC1 vs PC4
5.00%
1
0
−1
−2
−10
−5
0
5
10
PC4 (1.23%)
PC1 (65.83%)

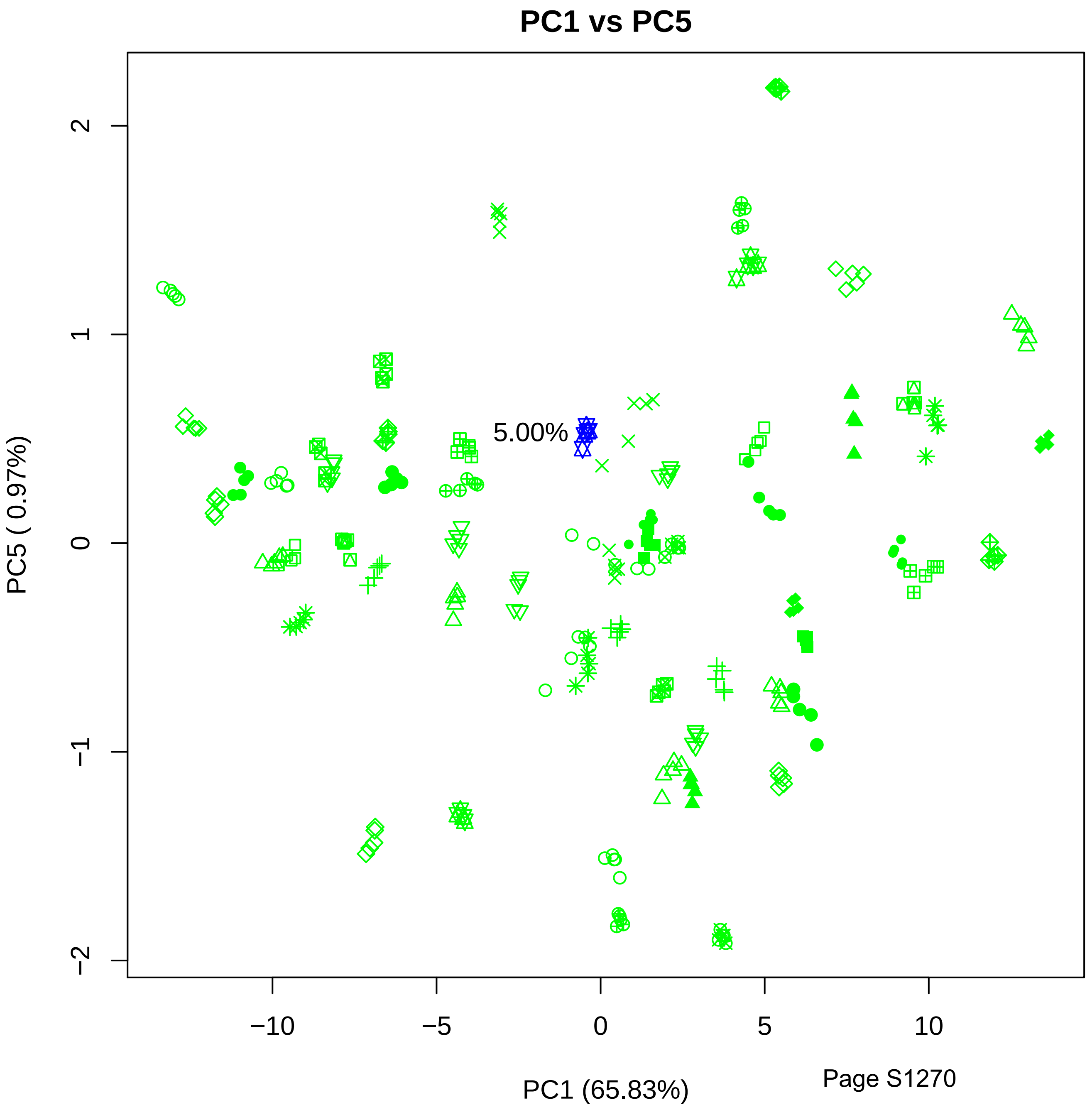
PC1 vs PC5
5.00%
PC5 (0.97%)
PC1 (65.83%)
2
1
0
−1
−2
−10
−5
0
5
10

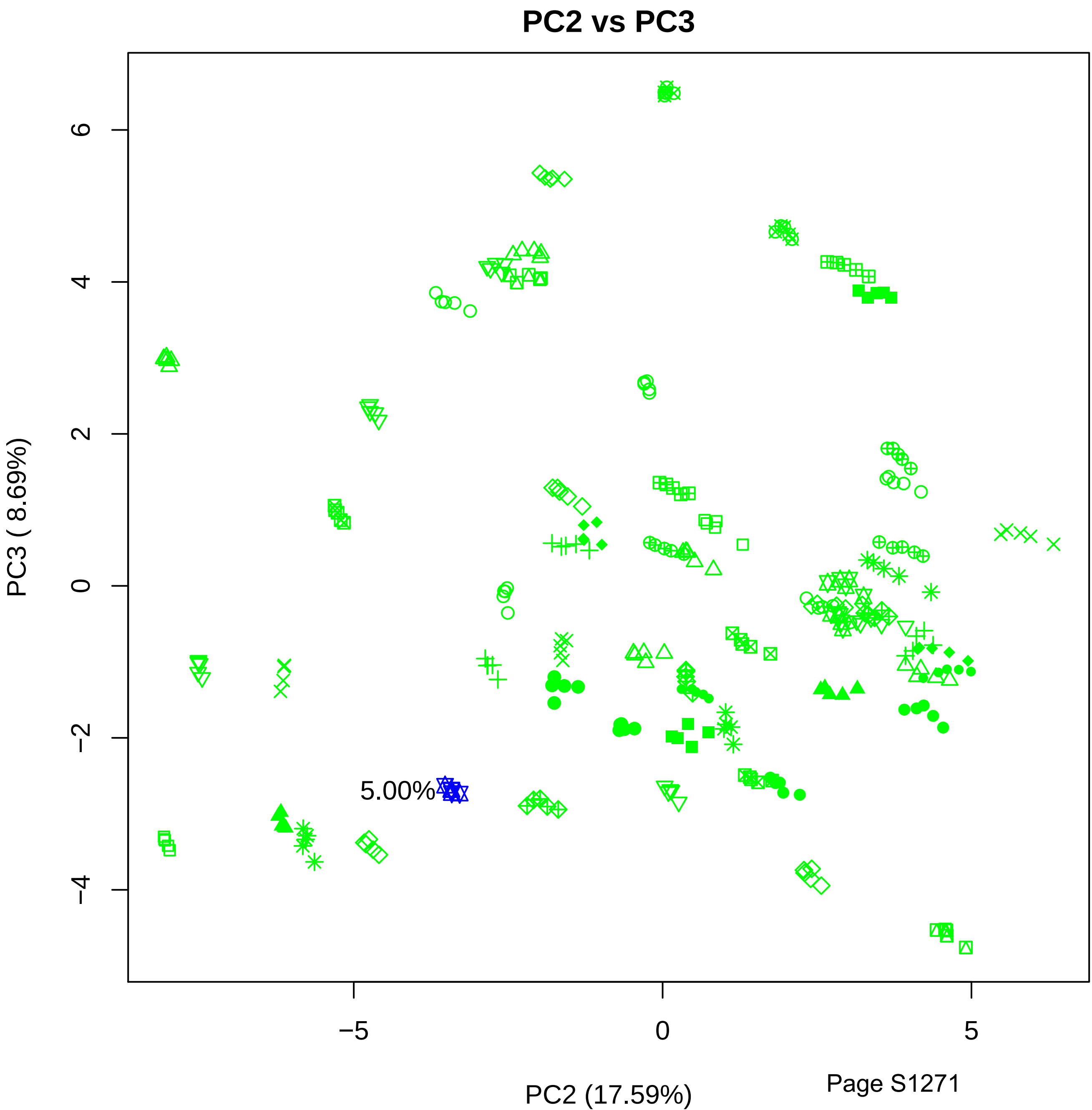
PC2 vs PC3
PC3 (8.69%)
PC2 (17.59%)
5.00%
6
4
2
0
−2
−4
−5
0
5

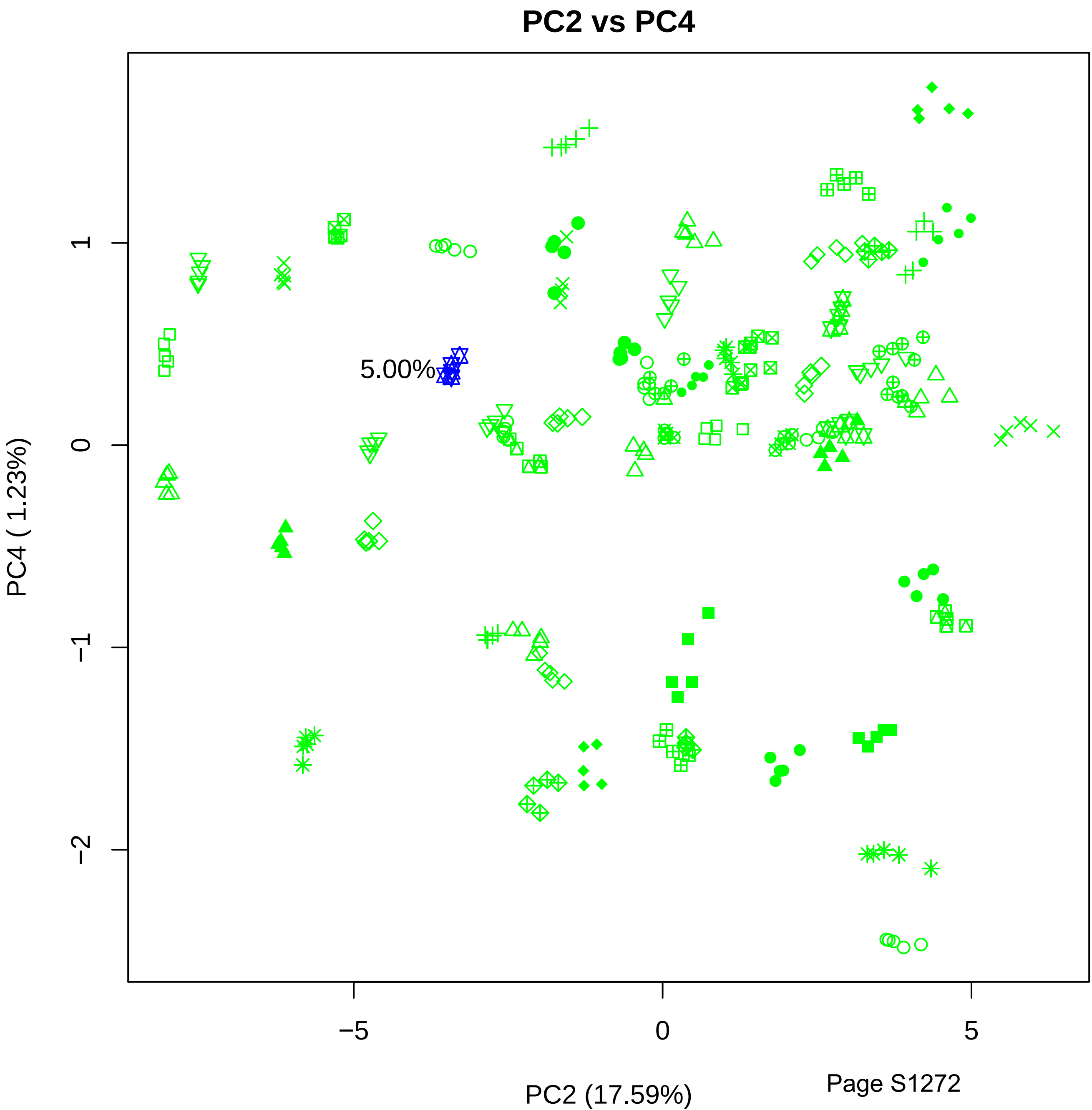
PC2 vs PC4
5.00%
PC4 (1.23%)
PC2 (17.59%)
1
0
−1
−2
−5
0
5

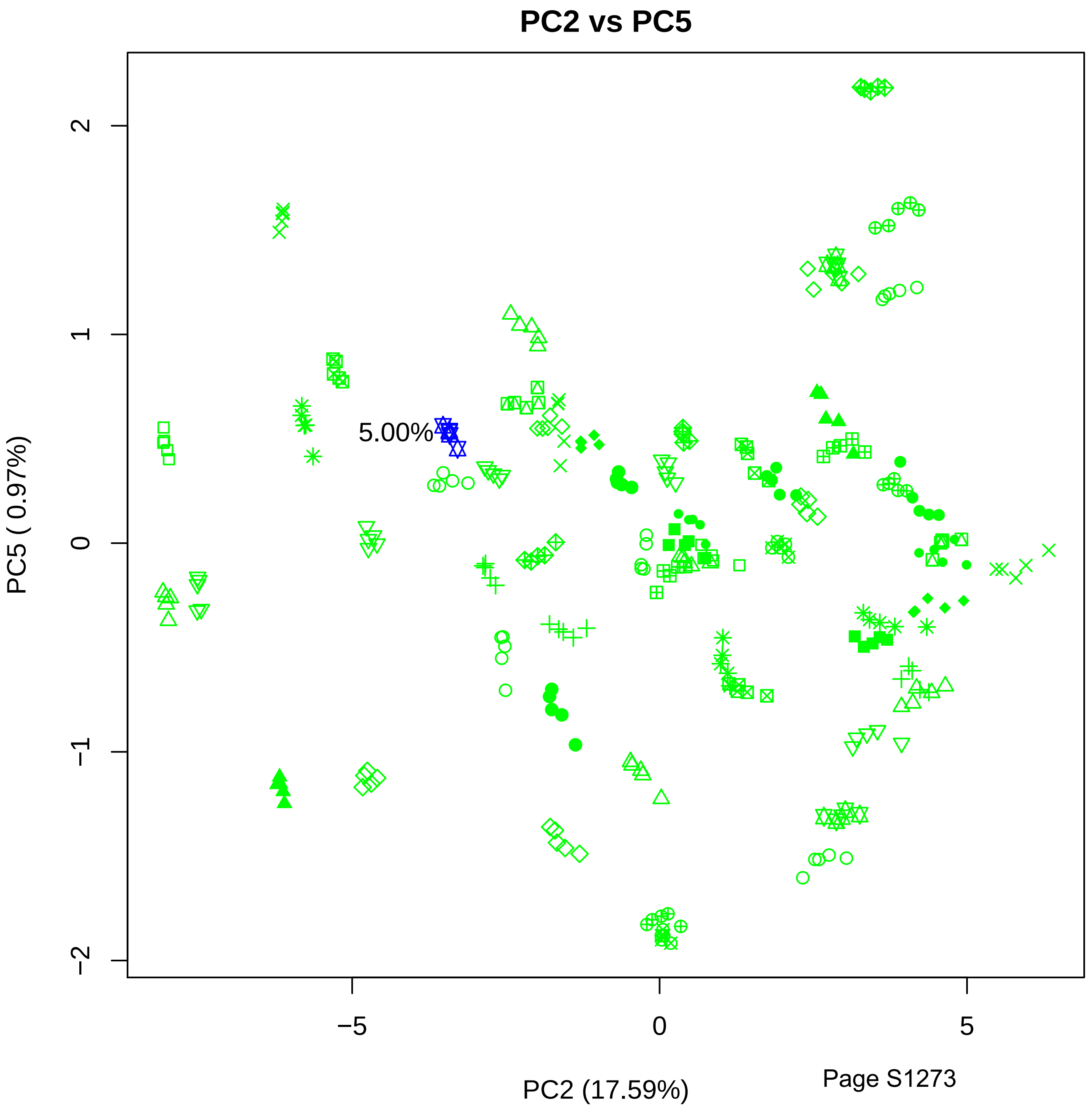
PC2 vs PC5
5.00%
PC5 (0.97%)
PC2 (17.59%)
2
1
0
−1
−2
−5
0
5

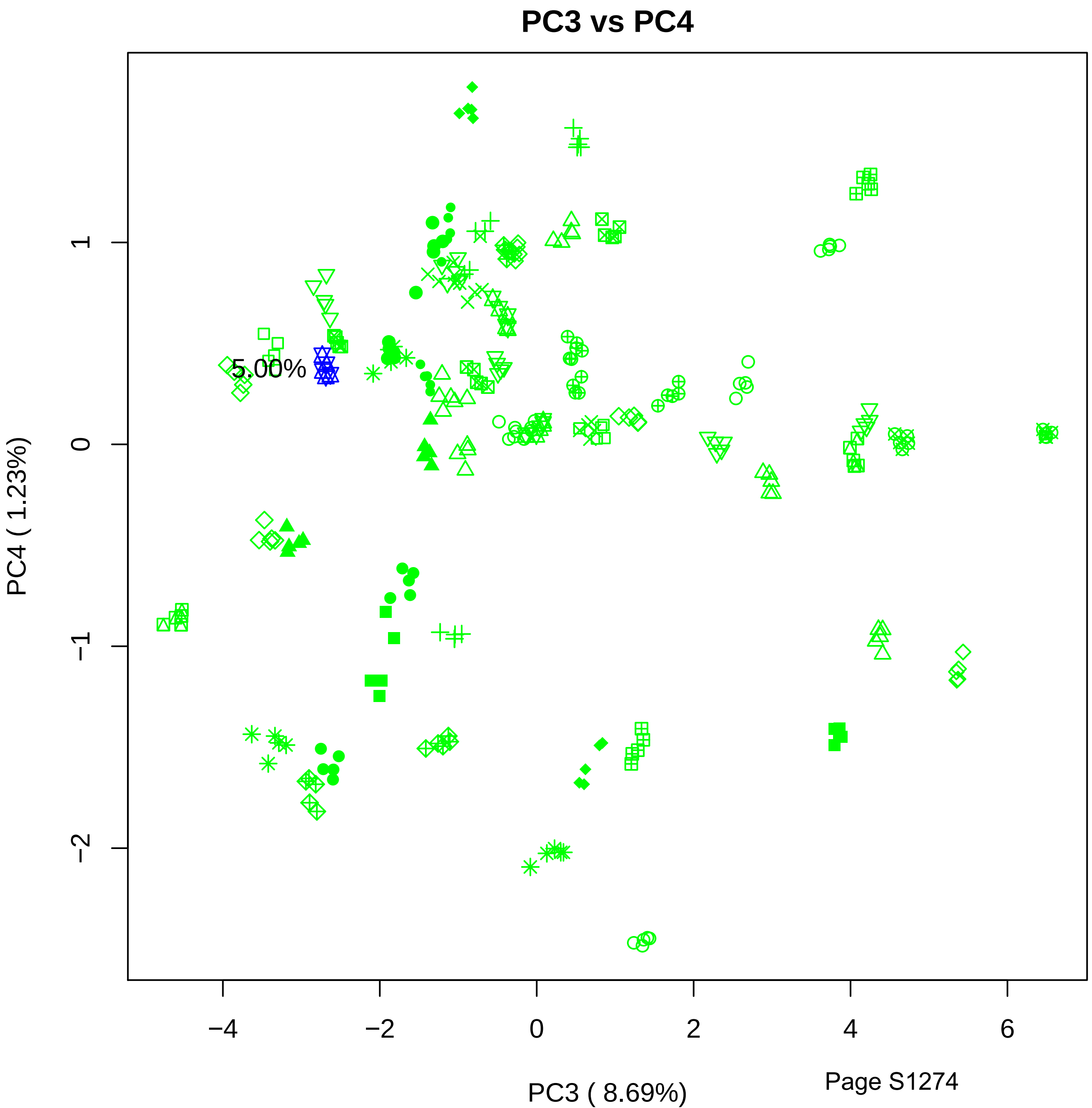

PC3 vs PC4
5.00%
PC4 (1.23%)
1
0
−1
−2
−4
−2
0
2
4
6
PC3 (8.69%)

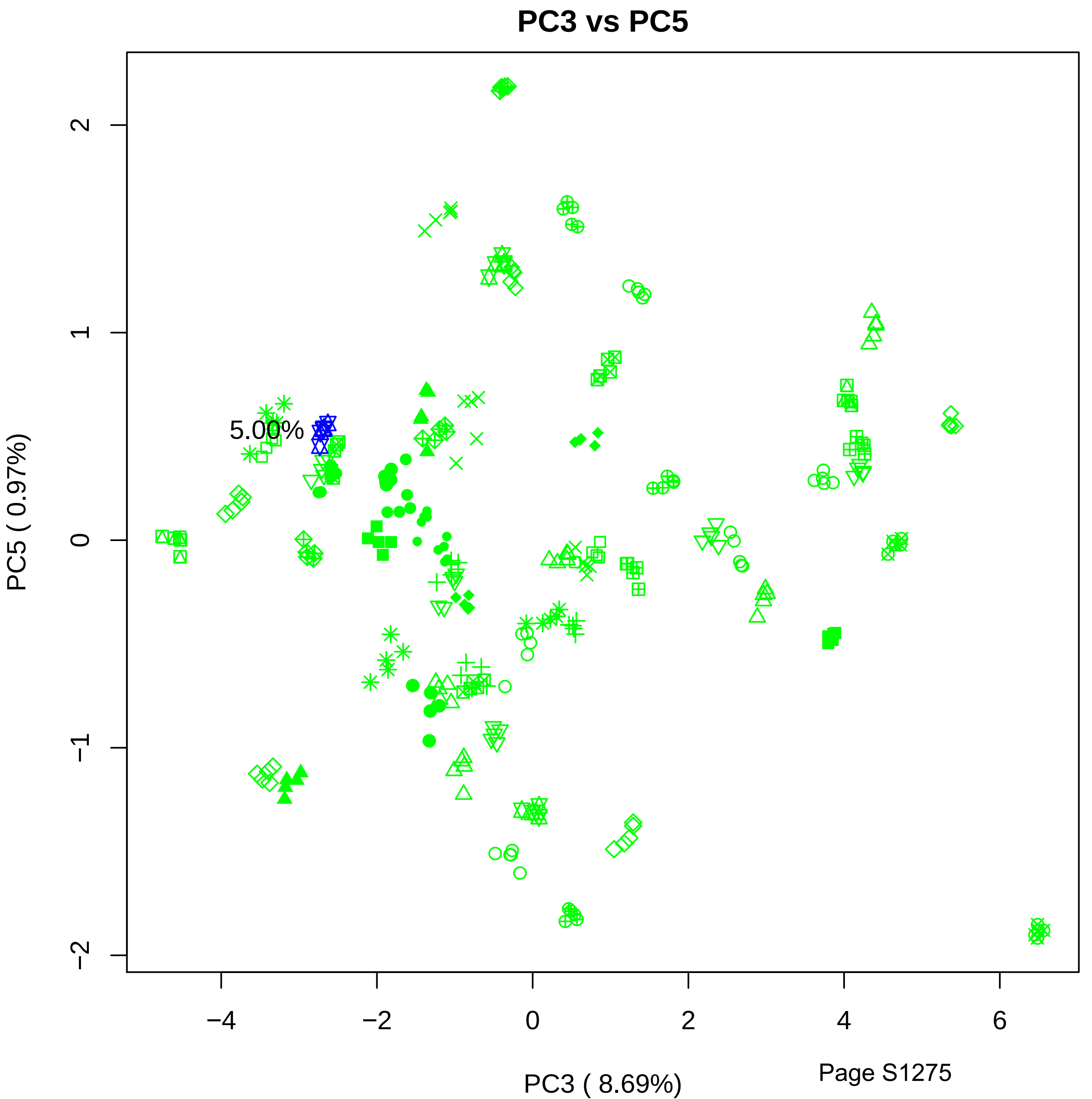

PC3 vs PC5
PC5 (0.97%)
PC3 (8.69%)
5.00%
2
1
0
−1
−2
−4
−2
0
2
4
6

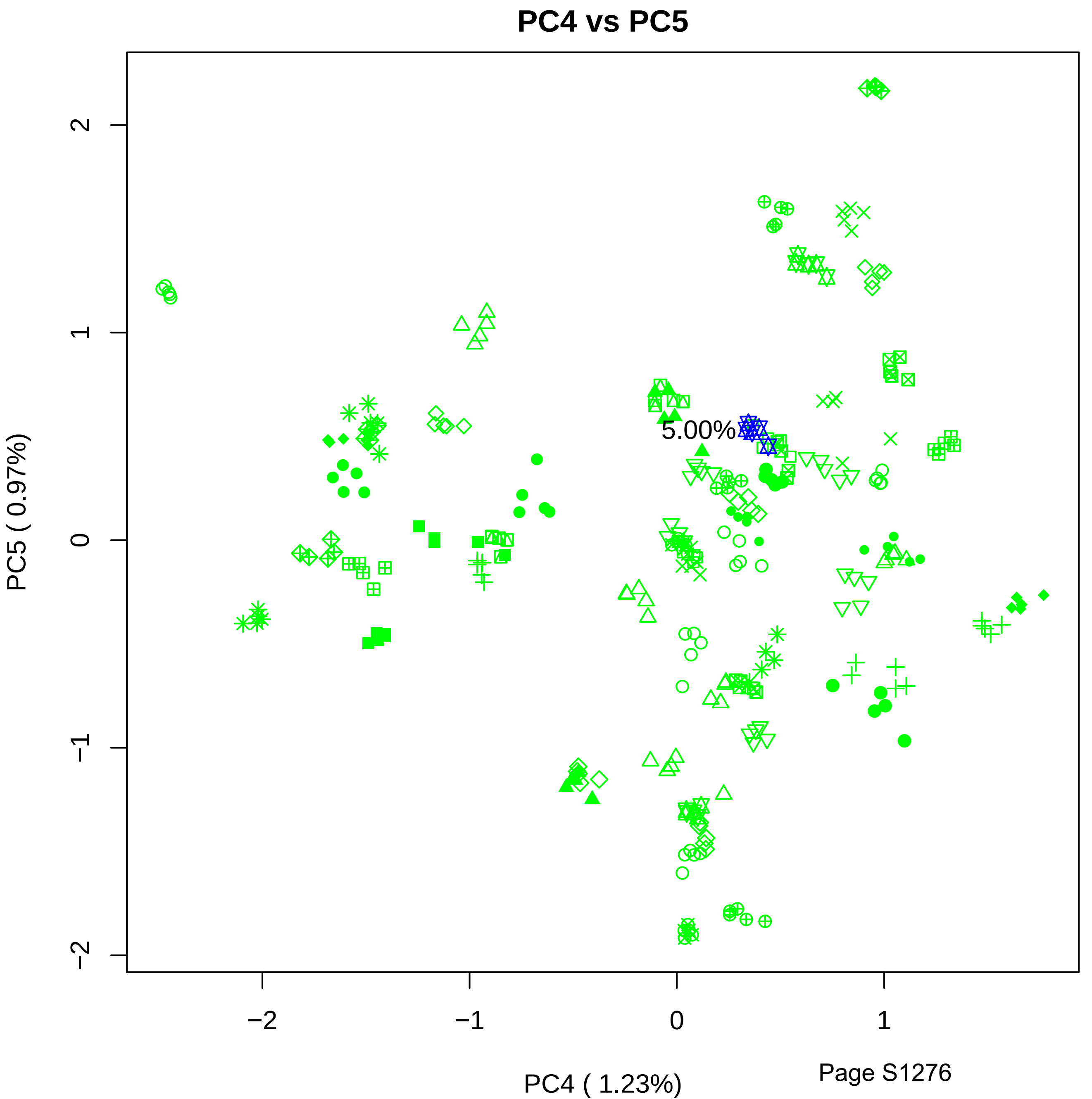

PC4 vs PC5
PC5 (0.97%)
PC4 (1.23%)
5.00%
2
1
0
−1
−2
−2
−1
0
1

PCA of 7.5% BMH(a) vs PMH(a)

Scaled Matrix PCAed

7th order polynomial baseline correction performed

Raw spectra smoothed to 21 Neighbours, 2nd order PN, Savtisky Golay

Normalisation between 0 and 100

D2 spectra smoothed to 41 Neighbours, 2nd order PN, Savtisky Golay

Regions Analysed: 3601−2501 and 2001−700cm−1

Samples Analysed: 1 bContam's 0 BLH's 0 BMH's 0 OMH's 0 pContam's 0 PLH's 60 PMH's

Keele University AD

Fri Dec 14 14:19:05 2018

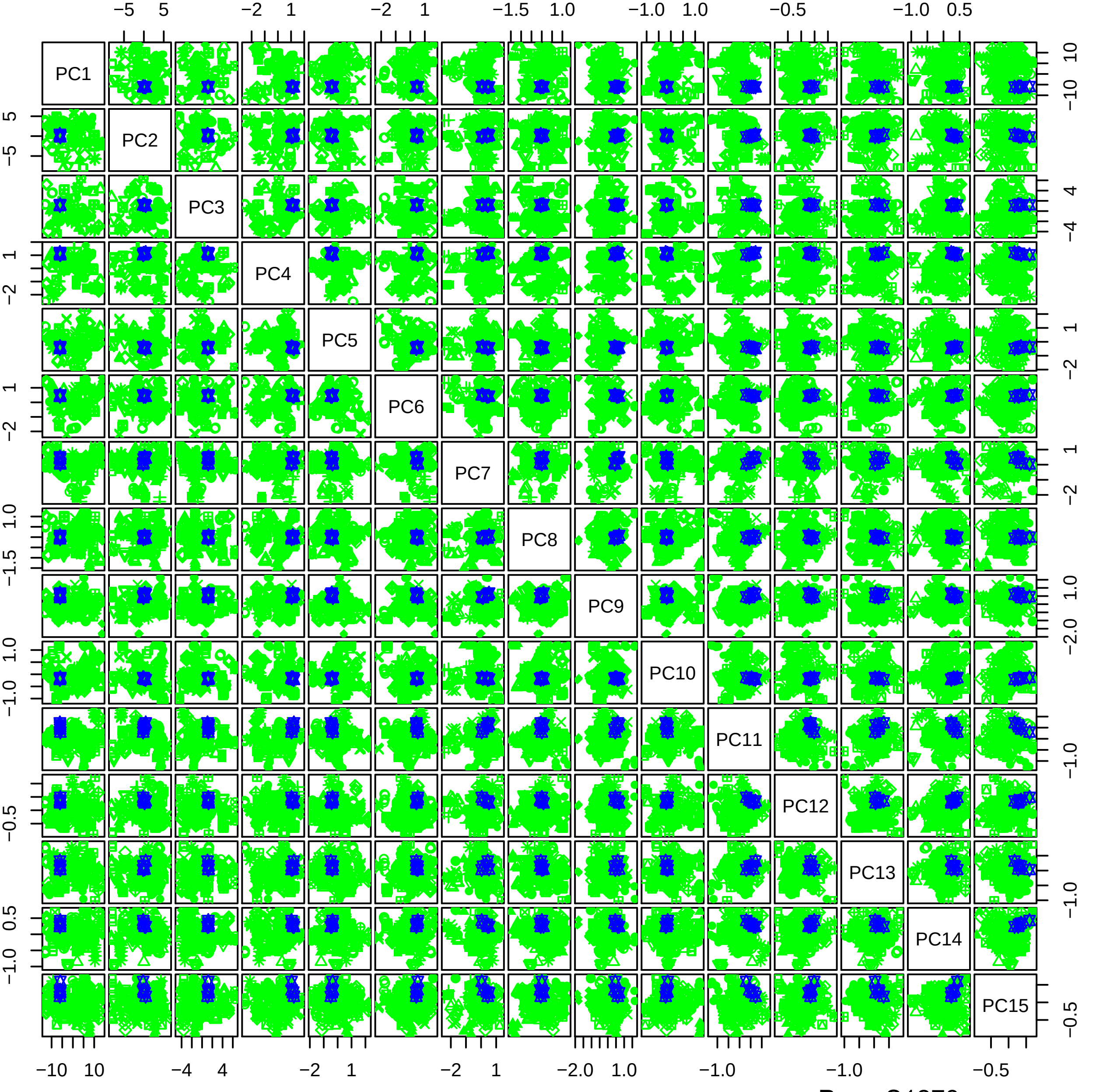
PC1
PC2
PC3
PC4
PC5
PC6
PC7
PC8
PC9
PC10
PC11
PC12
PC13
PC14
PC15

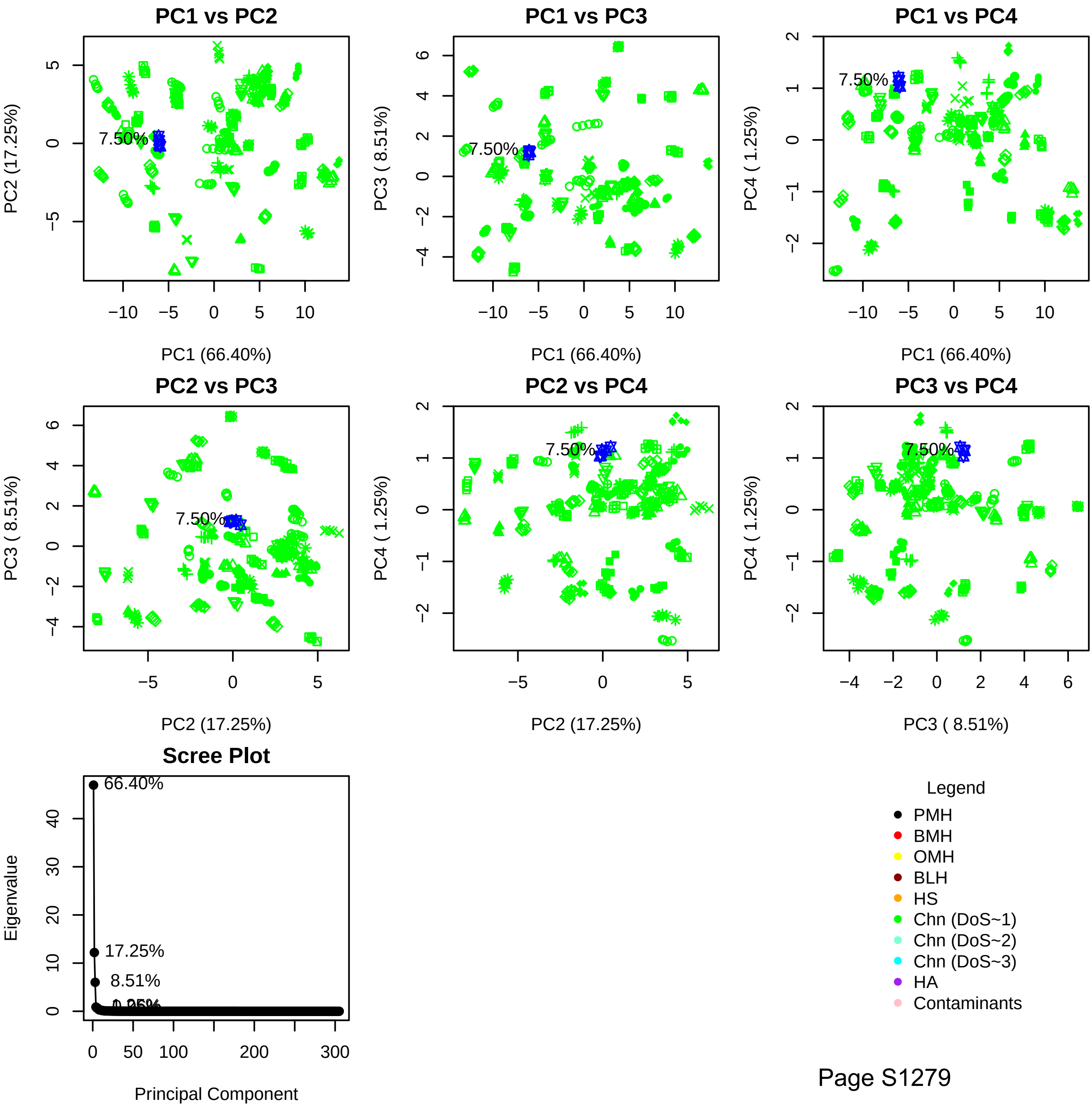
PC1 vs PC2
PC1 vs PC3
PC1 vs PC4
PC2 vs PC3
PC2 vs PC4
PC3 vs PC4
Scree Plot
PC1 (66.40%)
PC2 (17.25%)
PC3 (8.51%)
PC4 (1.25%)
7.50%
Eigenvalue
Principal Component
66.40%
17.25%
8.51%
Legend
PMH
BMH
OMH
BLH
HS
Chn (DoS~1)
Chn (DoS~2)
Chn (DoS~3)
HA
Contaminants

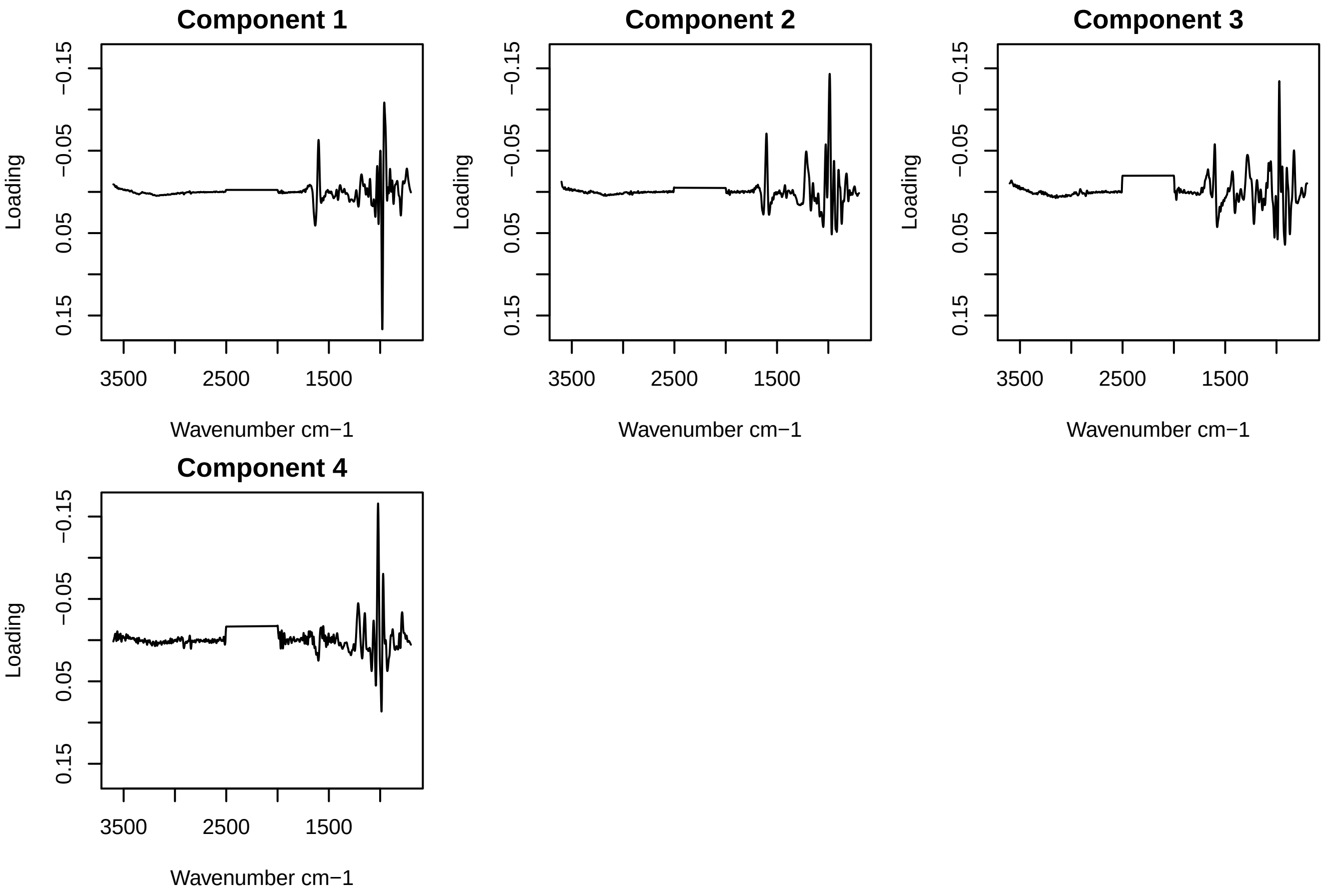

Component 1
Component 2
Component 3
Component 4
Loading
−0.15
−0.05
0.05
0.15
3500
2500
1500
Wavenumber cm−1

PC1 vs PC2 vs PC3

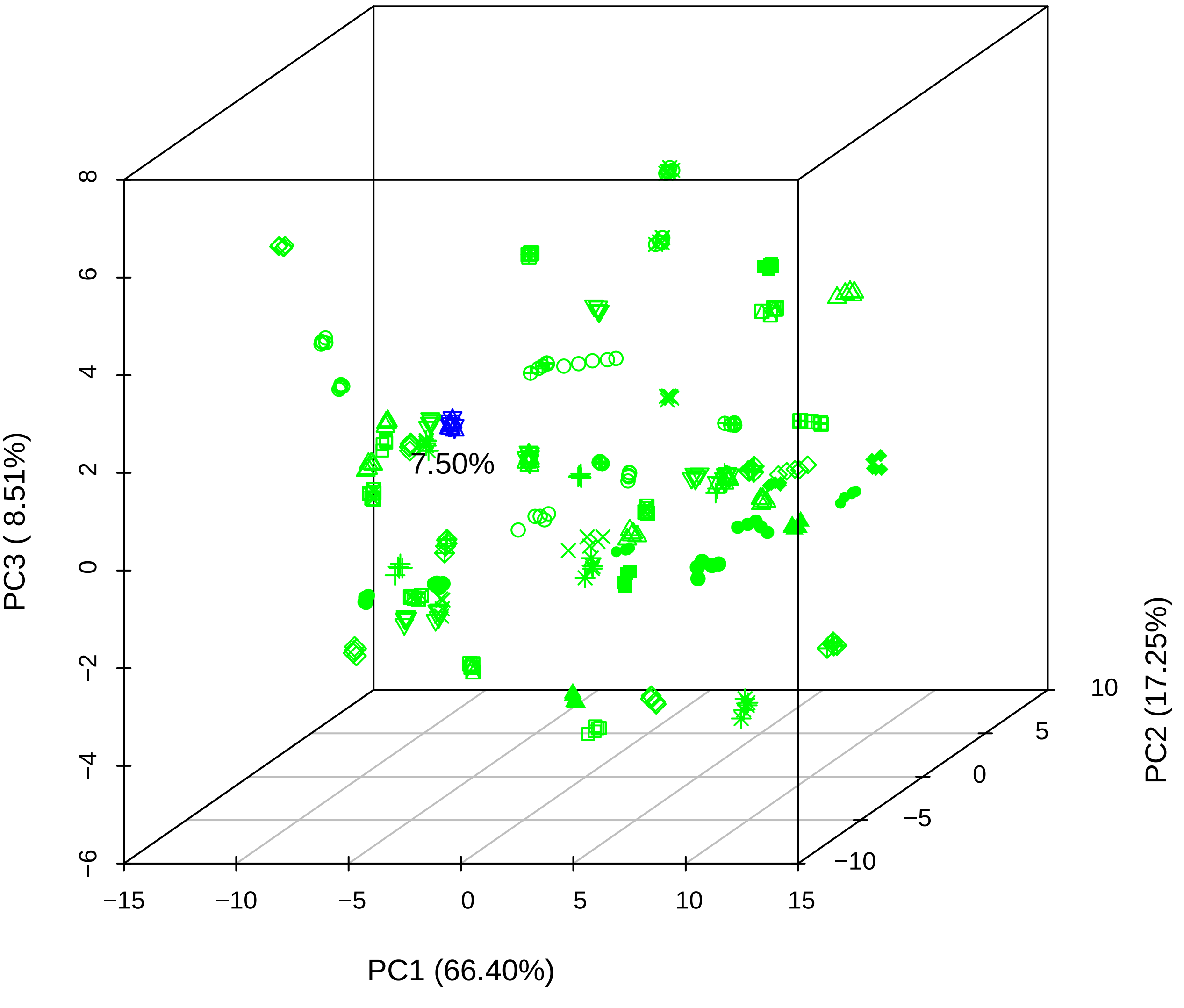

PC1 vs PC2 vs PC4

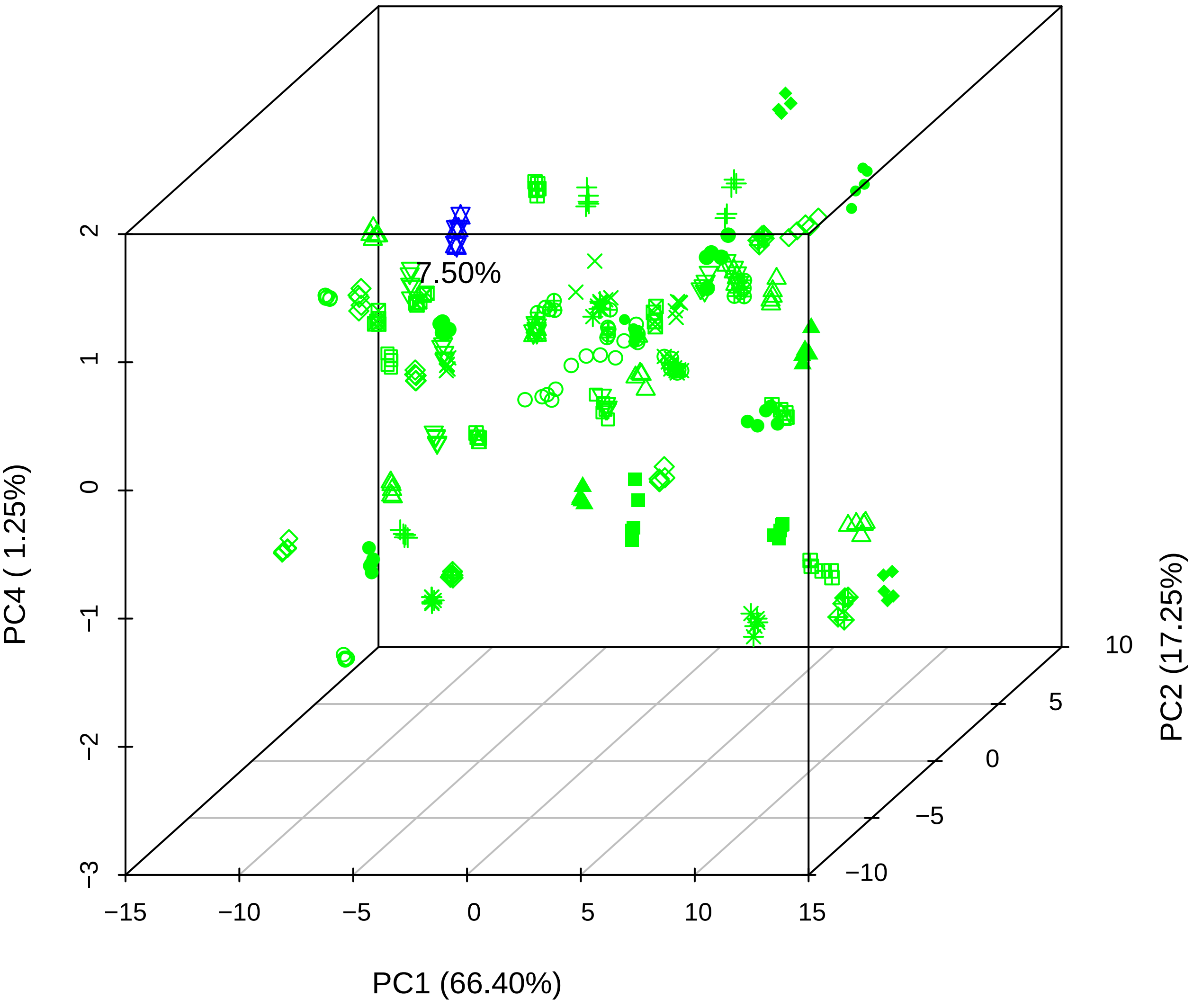

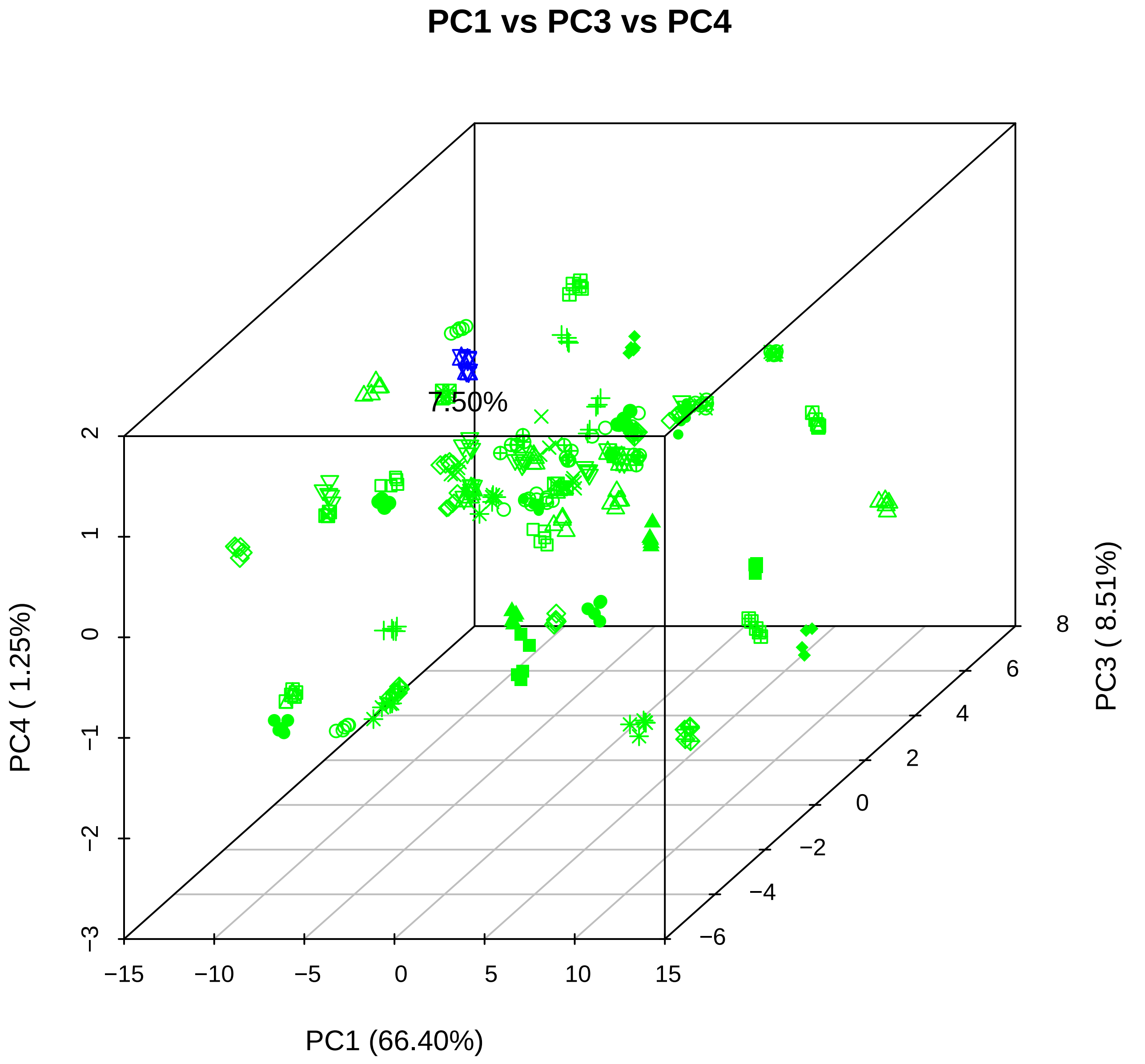
PC1 vs PC3 vs PC4
7.50%
PC1 (66.40%)
PC4 (1.25%)
PC3 (8.51%)
−15
−10
−5
0
5
10
15
−3
−2
−1
0
1
2
−6
−4
−2
0
2
4
6
8

PC2 vs PC3 vs PC5

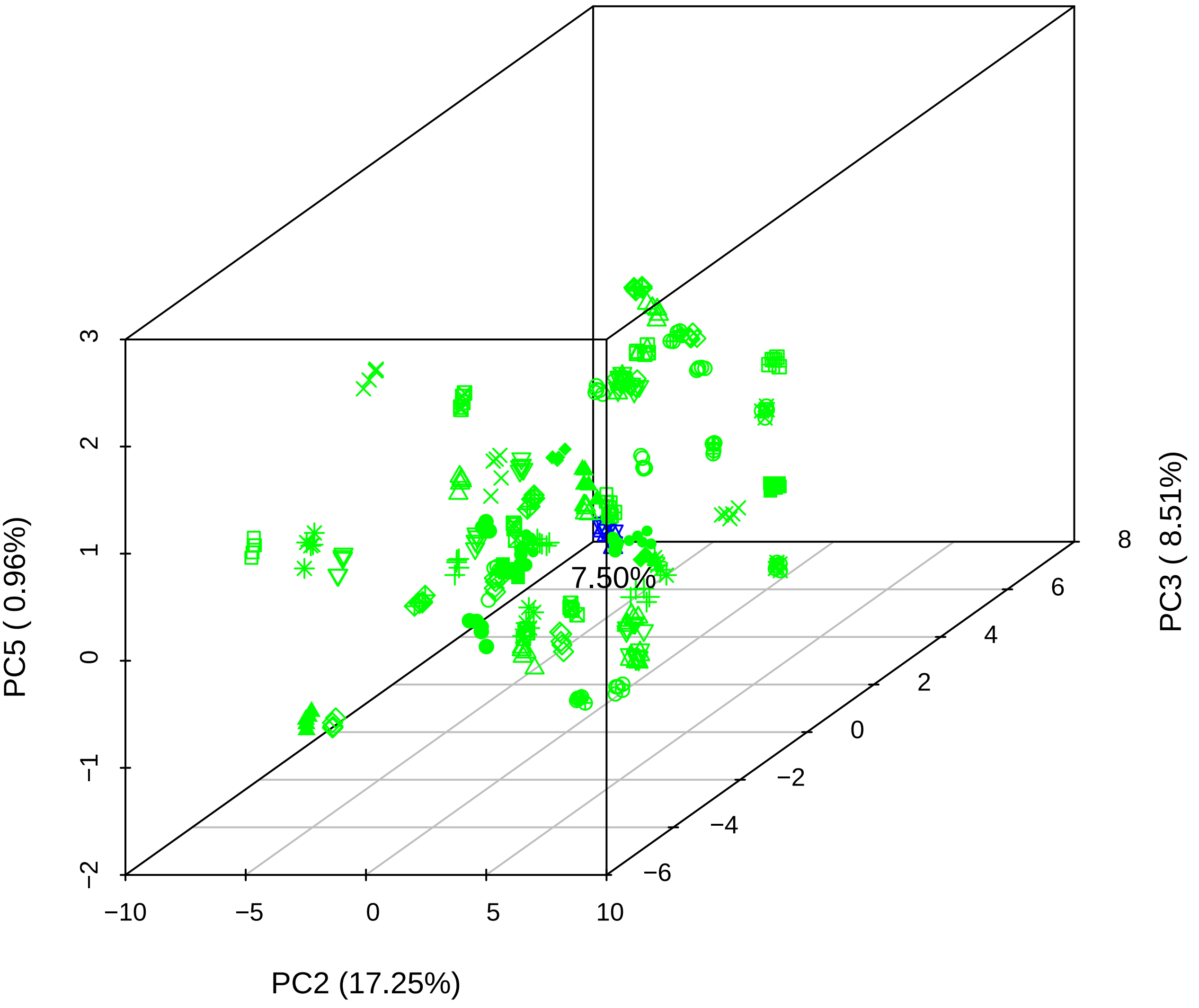

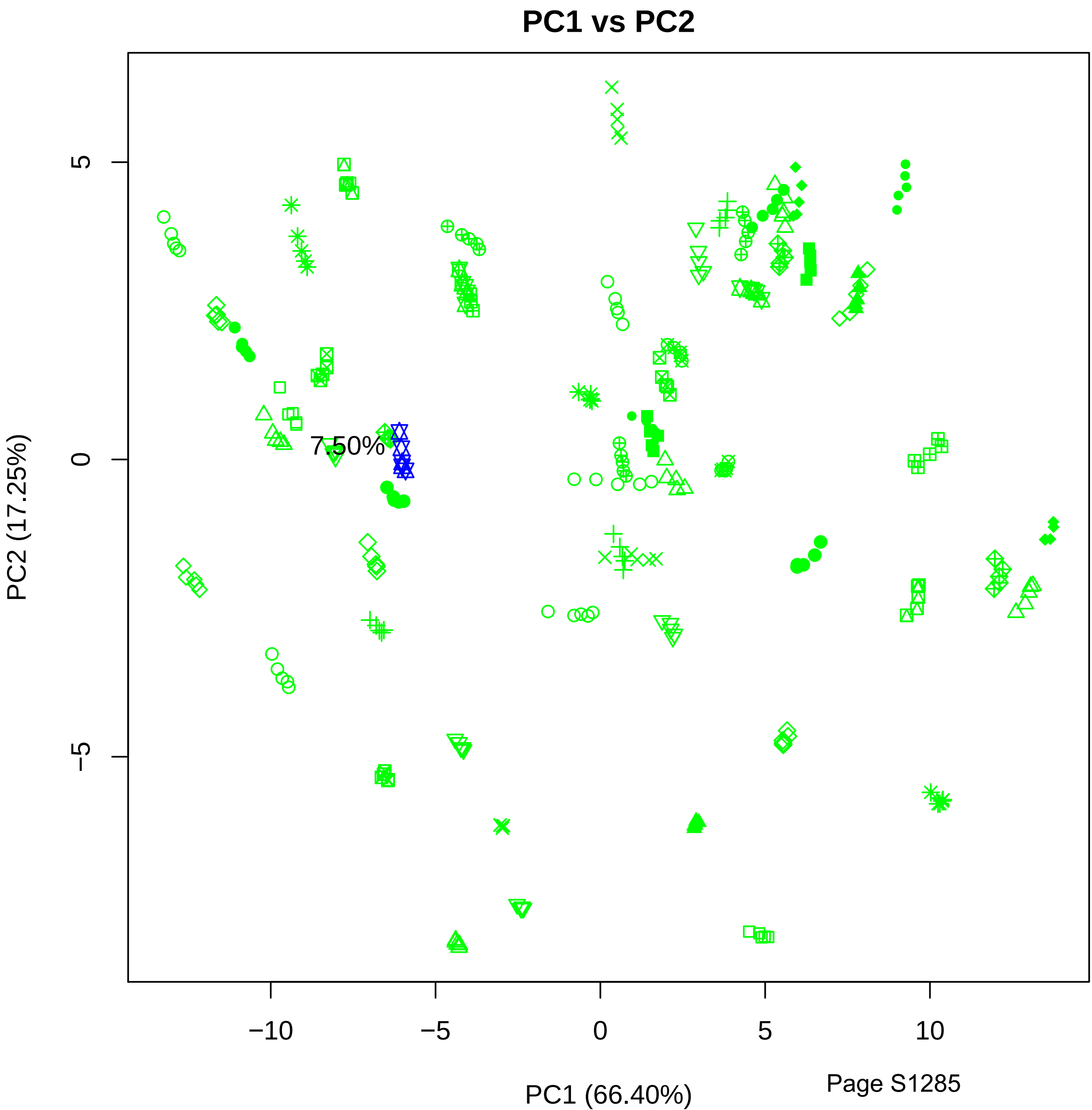

PC1 vs PC2
PC2 (17.25%)
PC1 (66.40%)
5
0
−5
−10
−5
0
5
10
7.50%

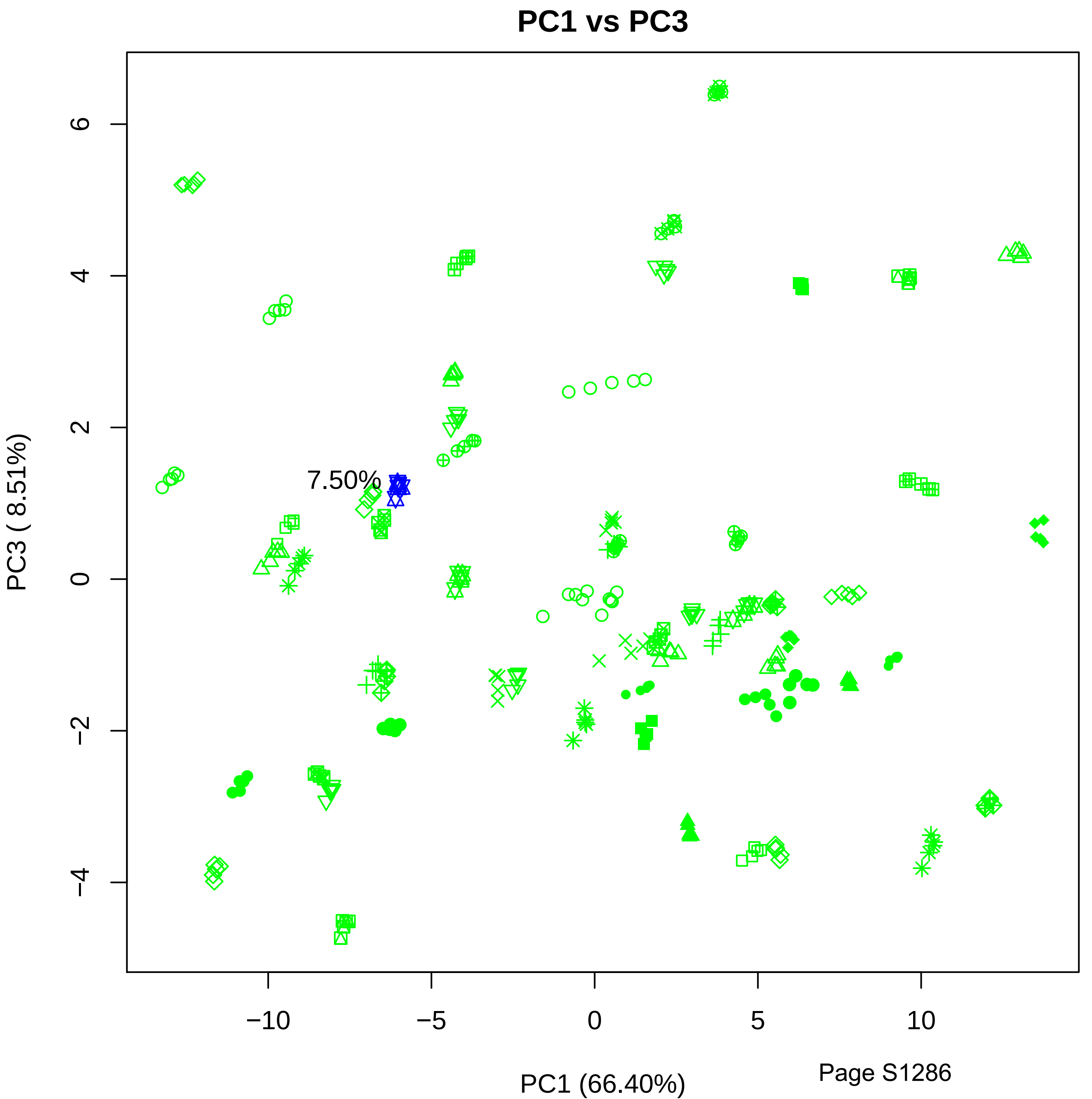
PC1 vs PC3
7.50%
PC3 (8.51%)
PC1 (66.40%)
6
4
2
0
−2
−4
−10
−5
0
5
10

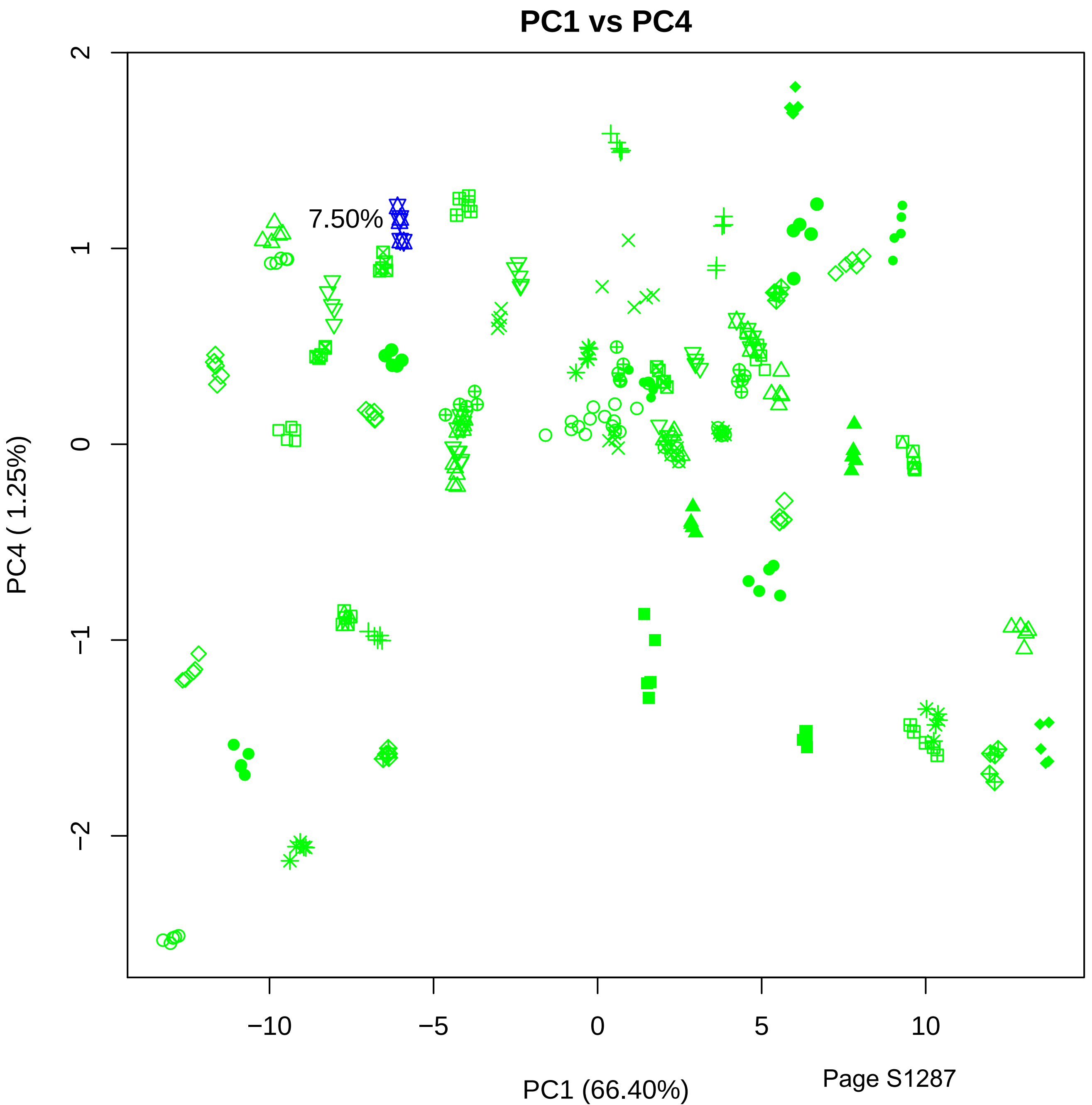

PC1 vs PC4
7.50%
2
1
0
−1
−2
PC4 (1.25%)
−10
−5
0
5
10
PC1 (66.40%)

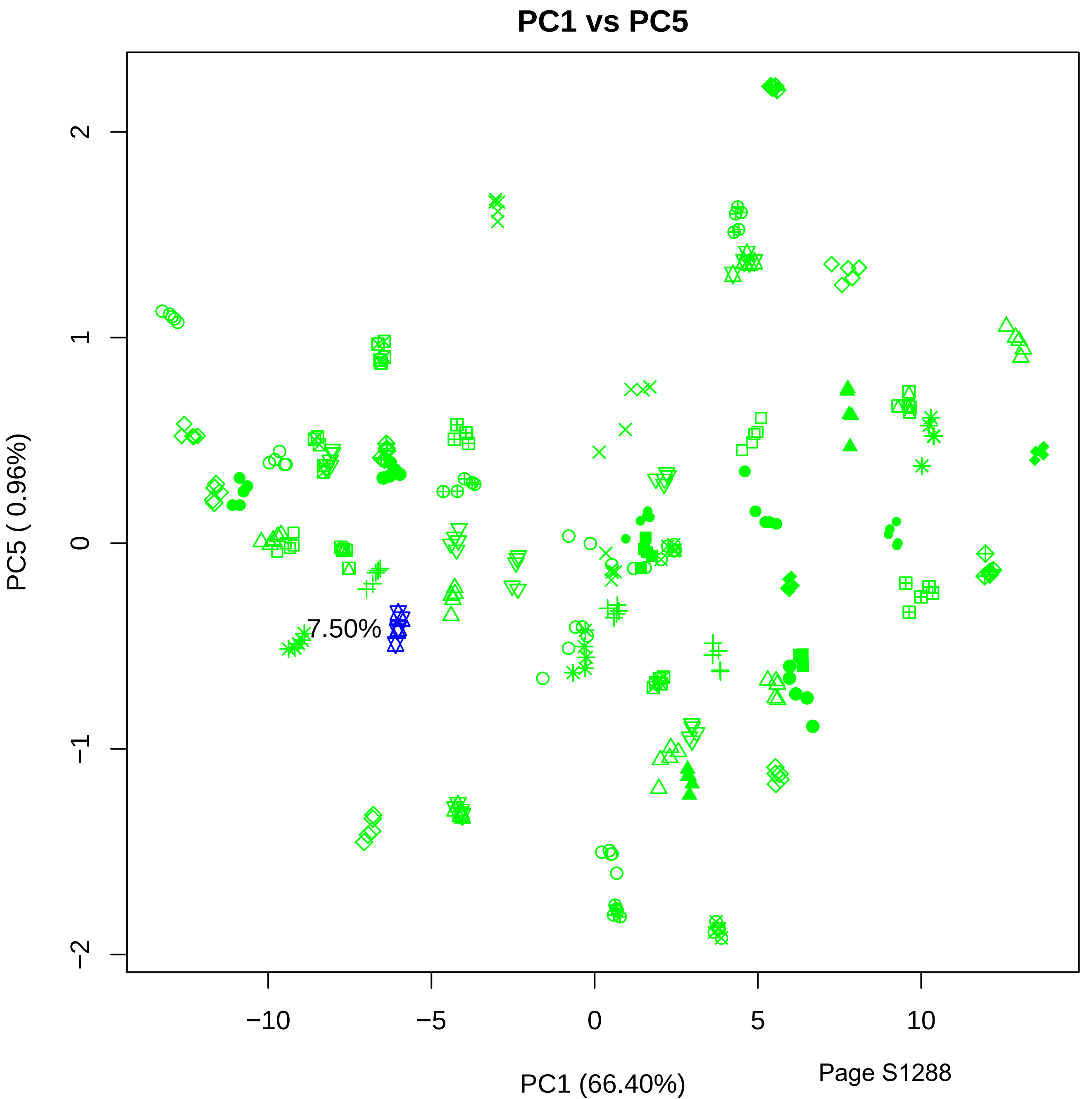
PC1 vs PC5
2
1
0
−1
−2
PC5 (0.96%)
7.50%
−10
−5
0
5
10
PC1 (66.40%)

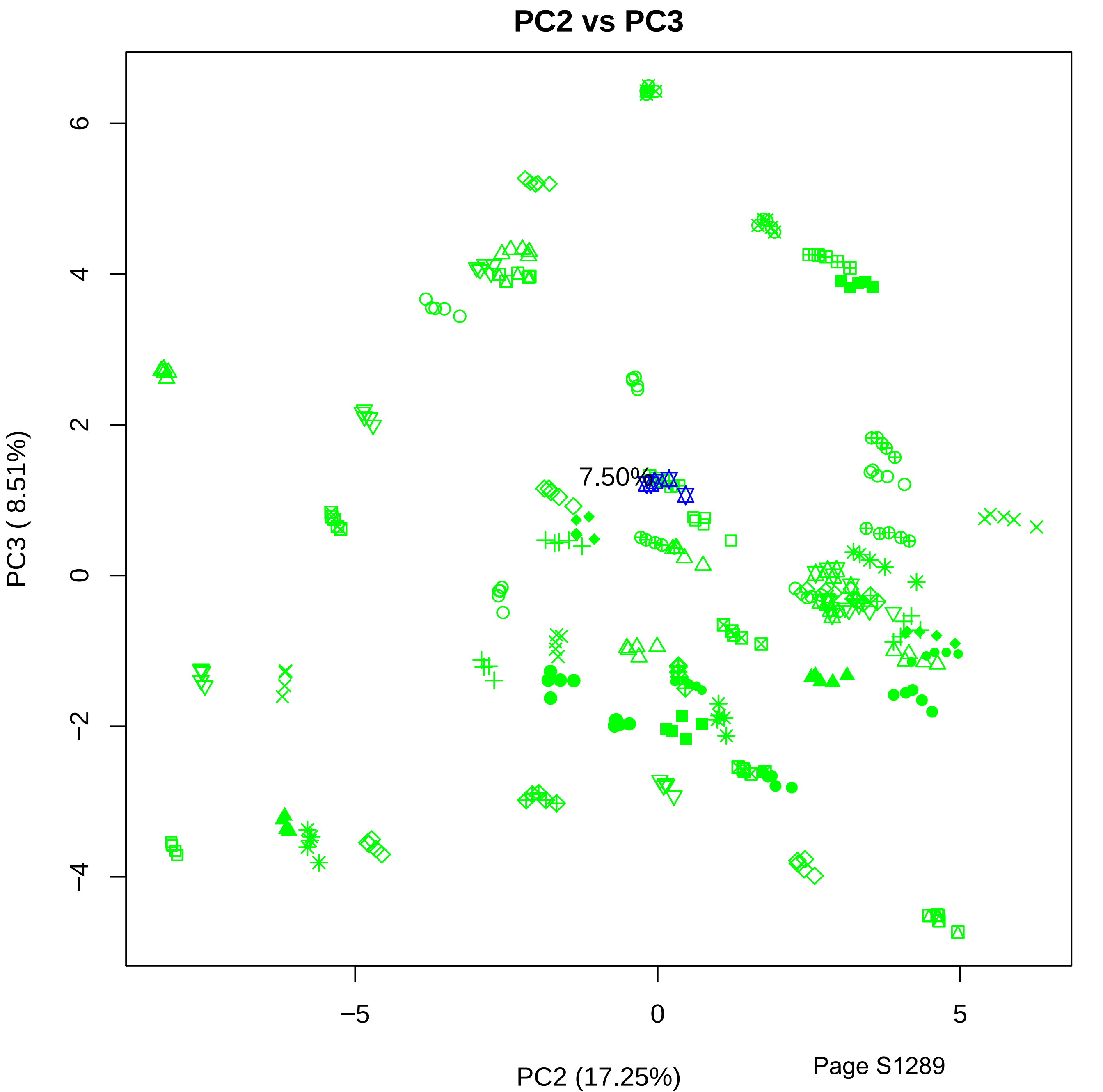
PC2 vs PC3
PC3 (8.51%)
6
4
2
0
−2
−4
7.50%
−5
0
5
PC2 (17.25%)

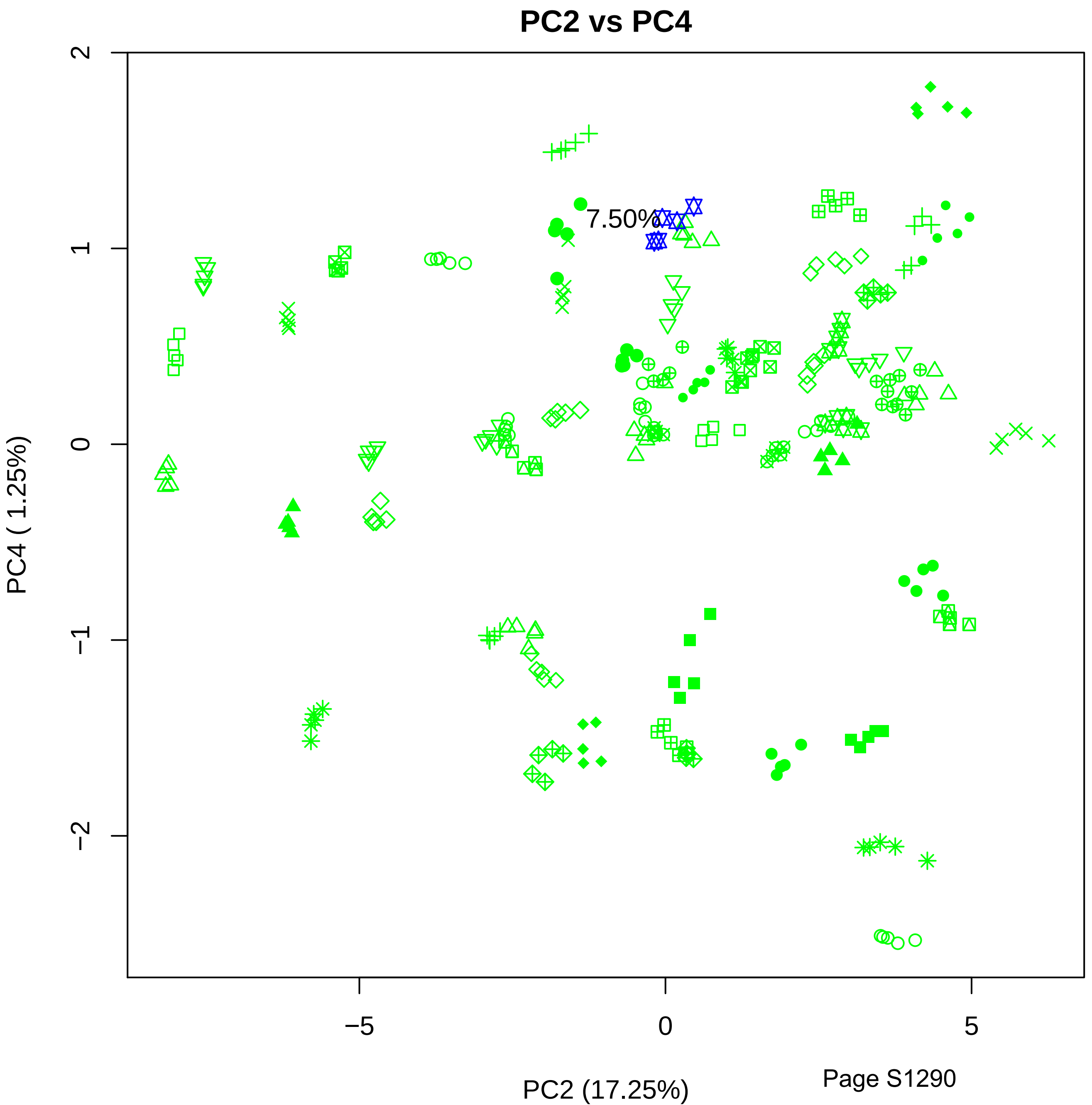
PC2 vs PC4
7.50%
PC4 (1.25%)
PC2 (17.25%)
2
1
0
−1
−2
−5
0
5

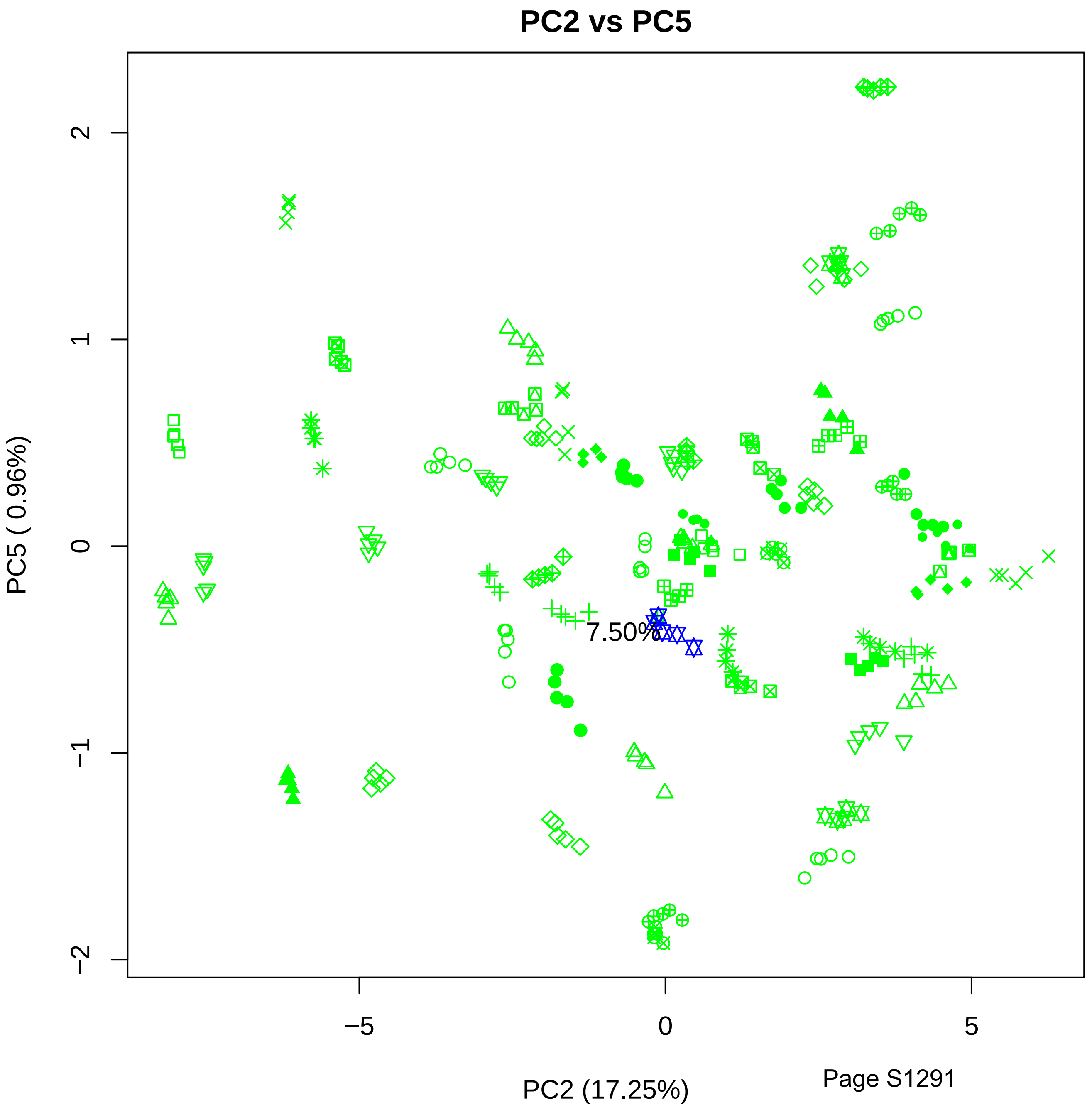

PC2 vs PC5
PC5 (0.96%)
PC2 (17.25%)
2
1
0
−1
−2
−5
0
5
7.50%

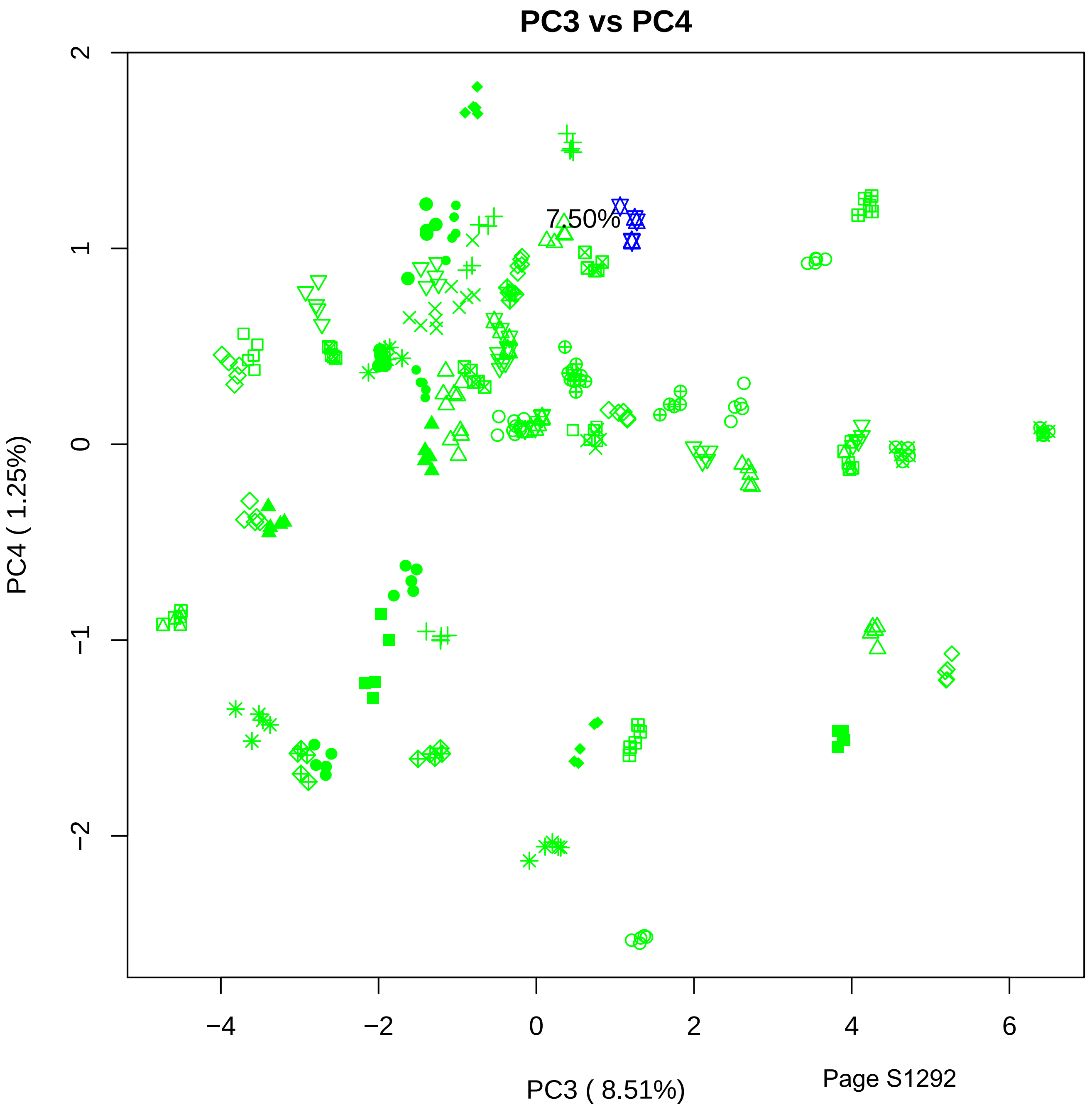

PC3 vs PC4
7.50%
PC4 (1.25%)
PC3 (8.51%)
−4
−2
0
2
4
6
−2
−1
0
1
2

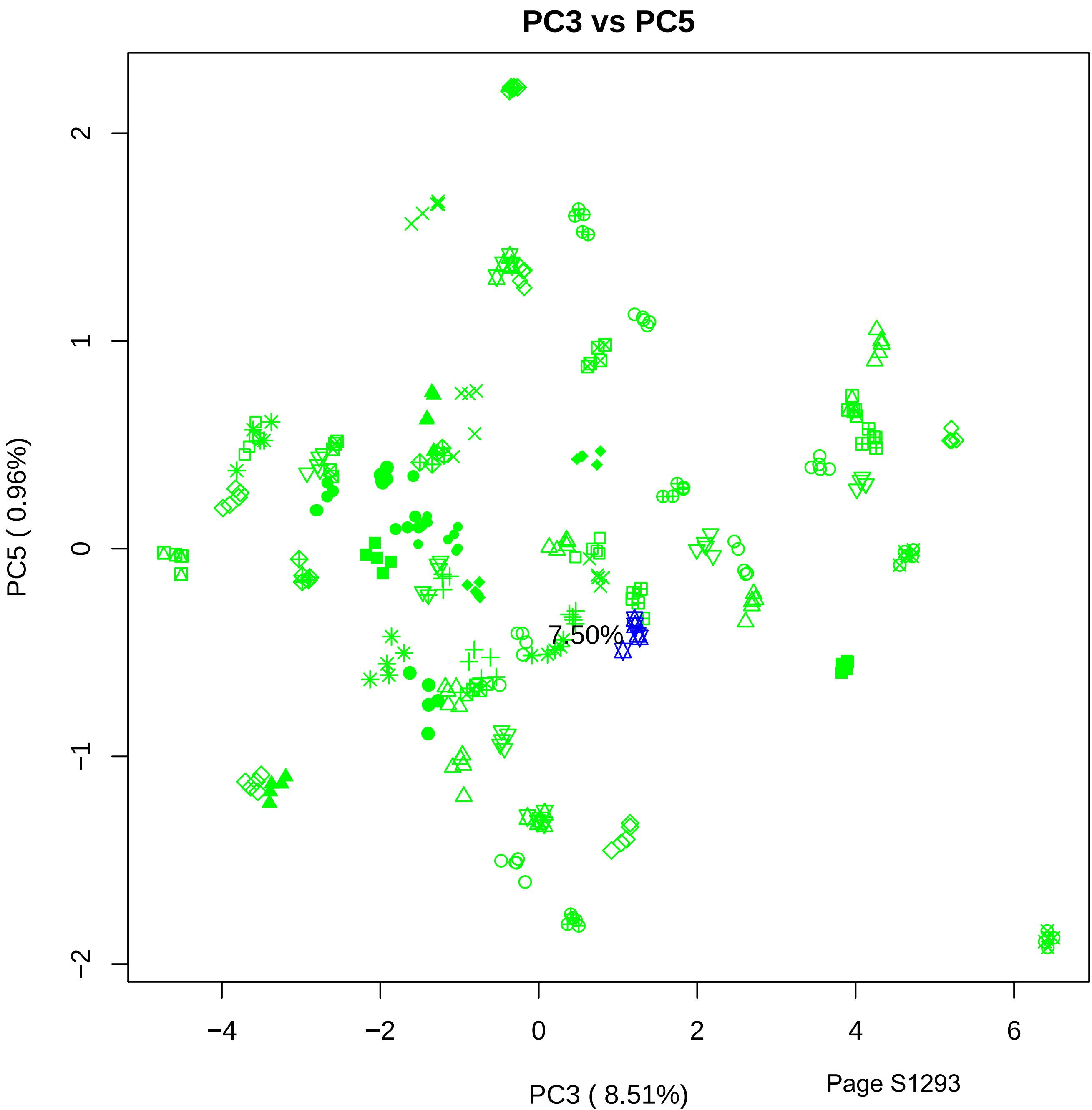
PC3 vs PC5
2
1
0
−1
−2
PC5 (0.96%)
7.50%
−4
−2
0
2
4
6
PC3 (8.51%)

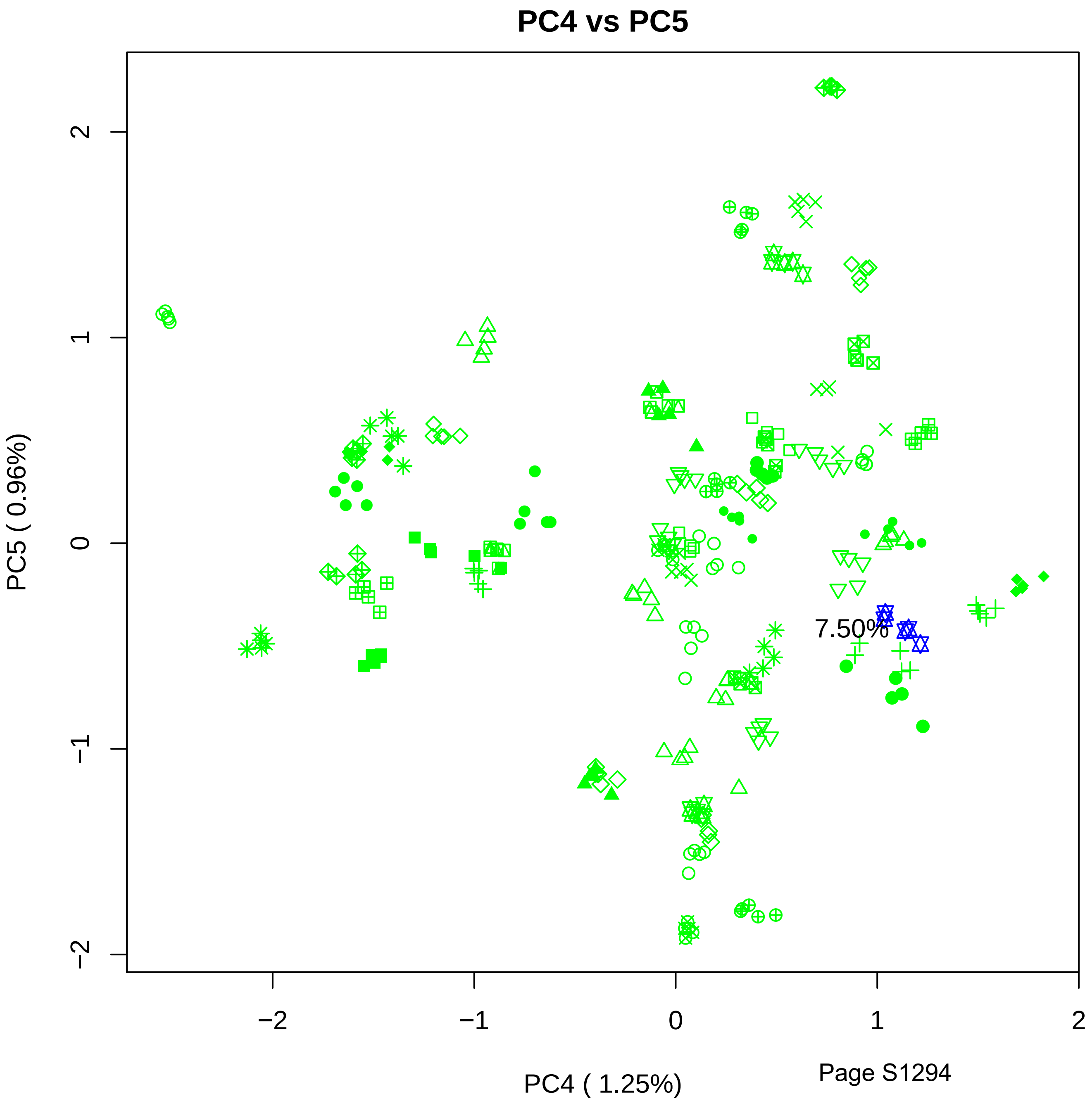

PC4 vs PC5
PC5 (0.96%)
PC4 (1.25%)
2
1
0
−1
−2
−2
−1
0
1
2
7.50%

PCA of 10% BMH(c) vs PMH(c)

Scaled Matrix PCAed

7th order polynomial baseline correction performed

Raw spectra smoothed to 21 Neighbours, 2nd order PN, Savtisky Golay

Normalisation between 0 and 100

D2 spectra smoothed to 41 Neighbours, 2nd order PN, Savtisky Golay

Regions Analysed: 3601−2501 and 2001−700cm−1

Samples Analysed: 1 bContam's 0 BLH's 0 BMH's 0 OMH's 0 pContam's 0 PLH's 60 PMH's

Keele University AD Fri Dec 14 14:19:13 2018

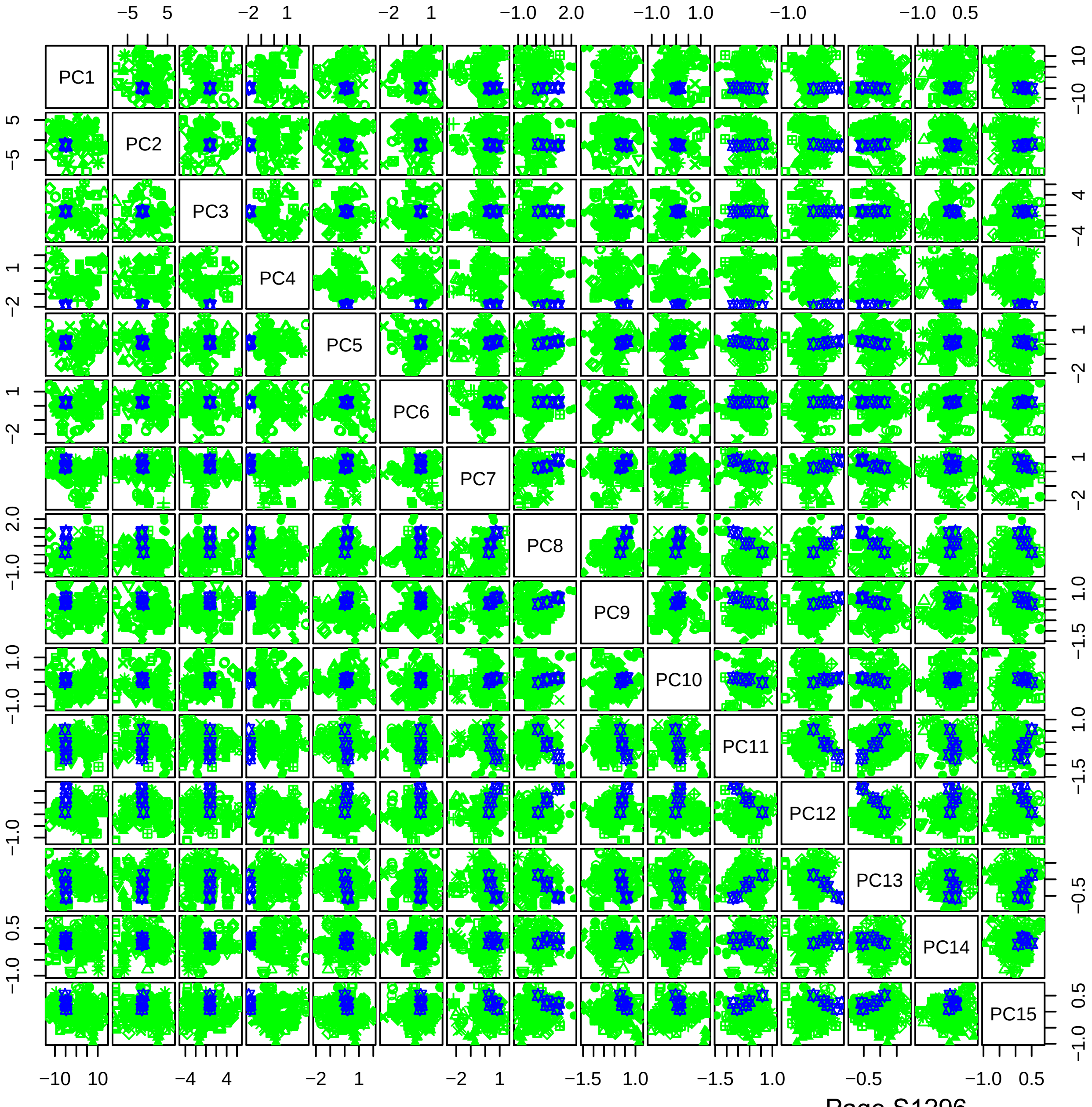
PC1
PC2
PC3
PC4
PC5
PC6
PC7
PC8
PC9
PC10
PC11
PC12
PC13
PC14
PC15

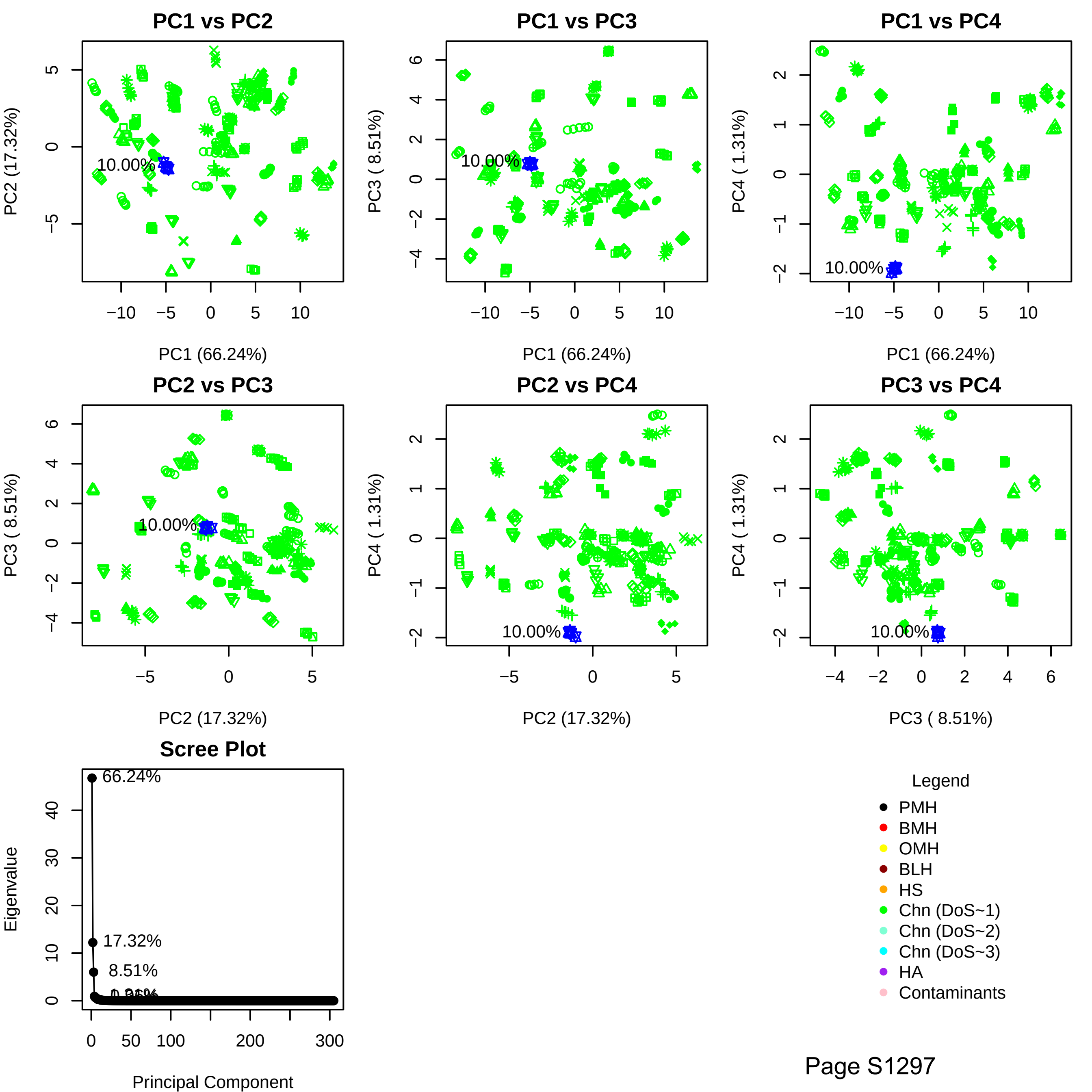

PC1 vs PC2
PC1 vs PC3
PC1 vs PC4
PC2 vs PC3
PC2 vs PC4
PC3 vs PC4
Scree Plot
PC1 (66.24%)
PC2 (17.32%)
PC3 (8.51%)
PC4 (1.31%)
10.00%
Eigenvalue
Principal Component
66.24%
17.32%
8.51%
Legend
PMH
BMH
OMH
BLH
HS
Chn (DoS~1)
Chn (DoS~2)
Chn (DoS~3)
HA
Contaminants

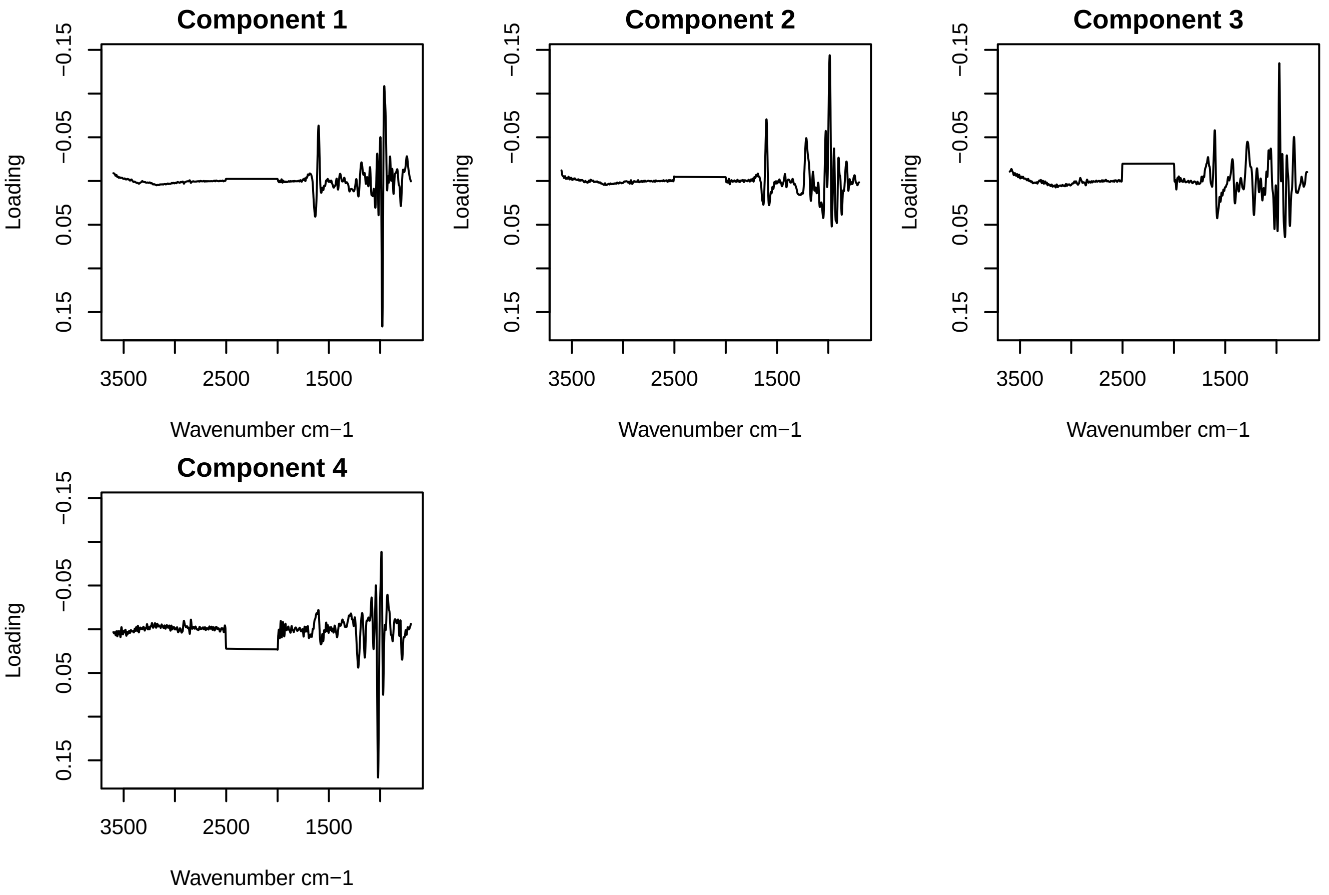
Component 1
Component 2
Component 3
Component 4
Loading
−0.15
−0.05
0.05
0.15
3500
2500
1500
Wavenumber cm−1

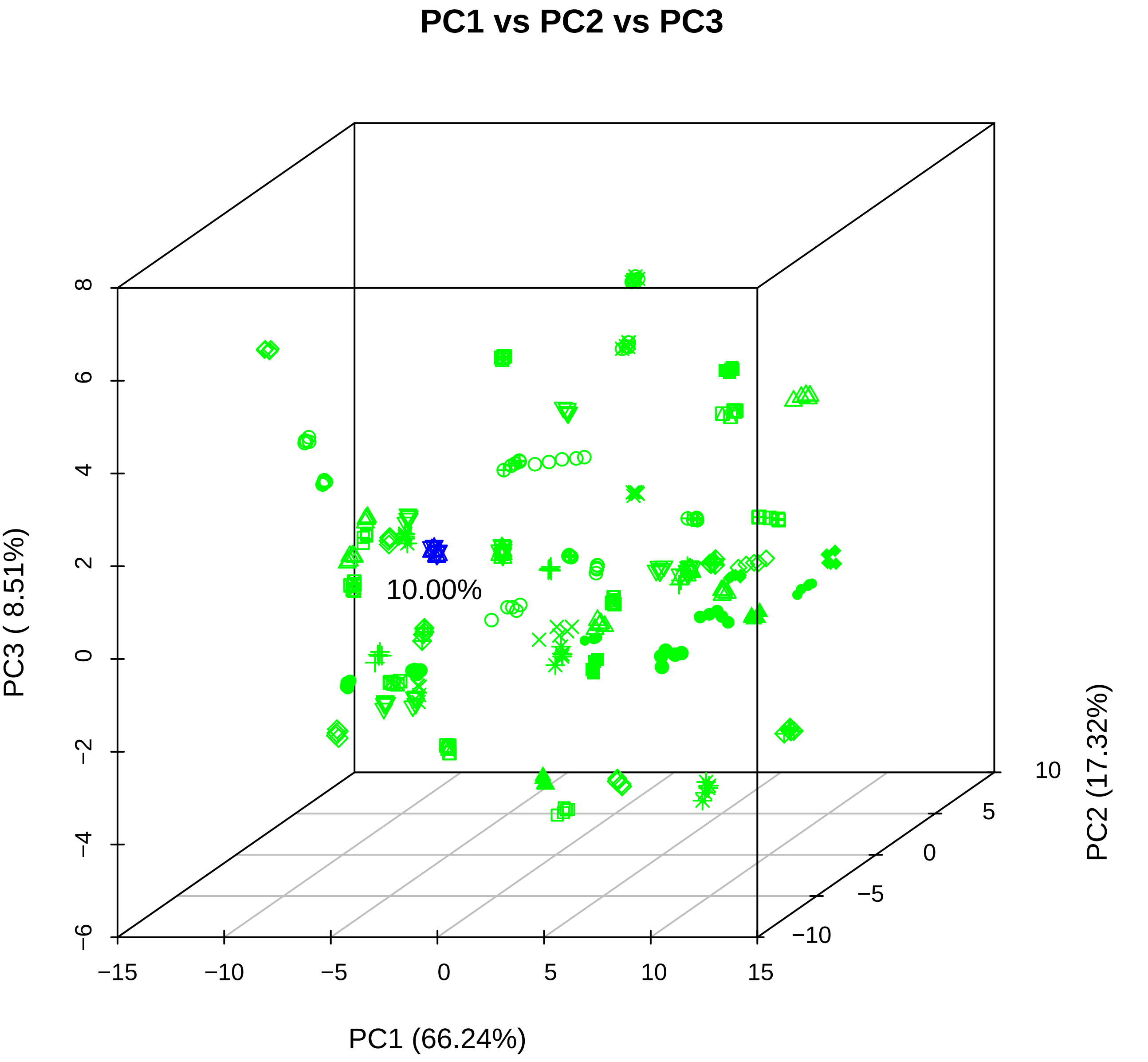
PC1 vs PC2 vs PC3
10.00%
PC1 (66.24%)
PC2 (17.32%)
PC3 (8.51%)
−15
−10
−5
0
5
10
15
−10
−5
0
5
10
−6
−4
−2
0
2
4
6
8

PC1 vs PC2 vs PC4

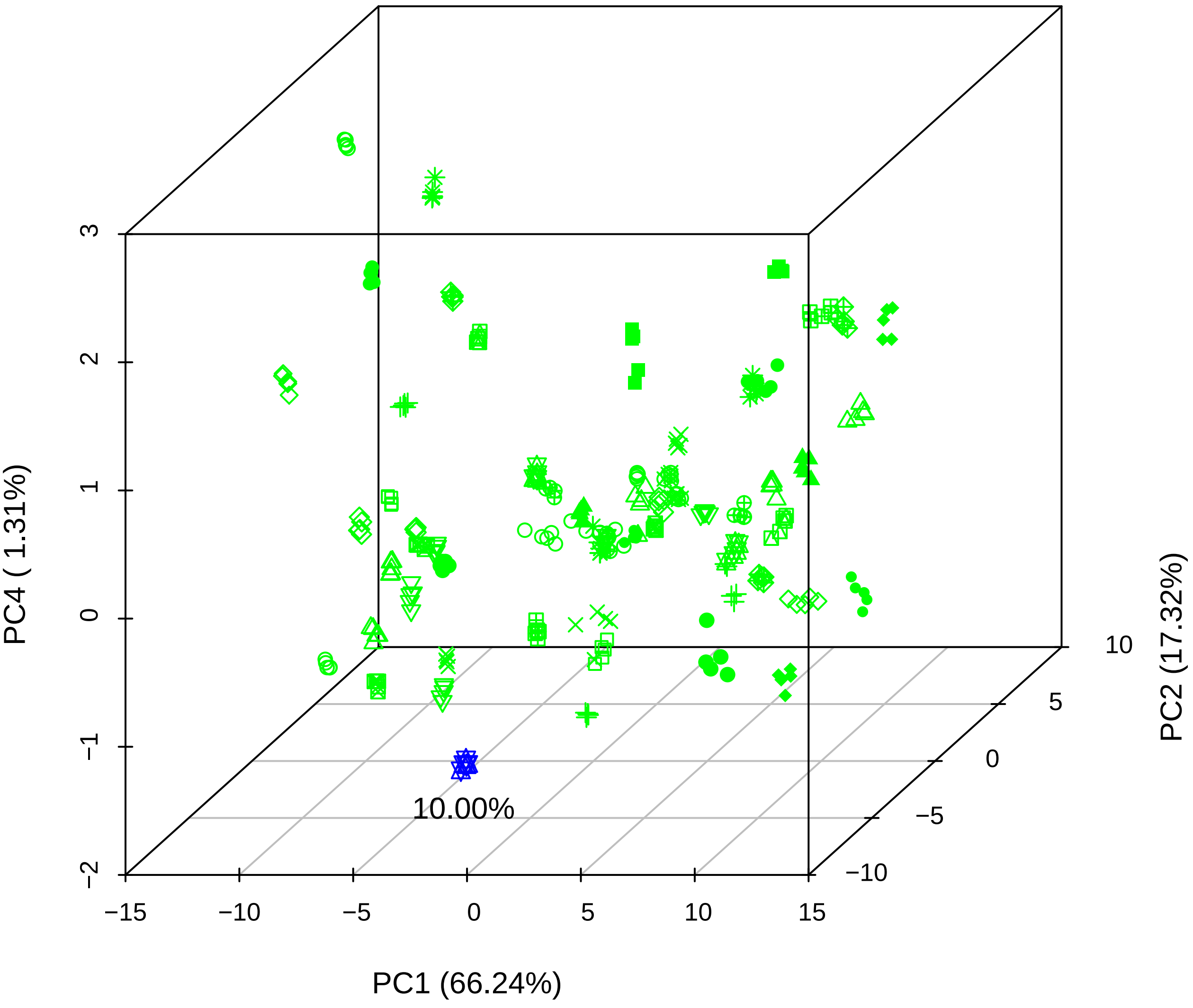

PC1 vs PC3 vs PC4

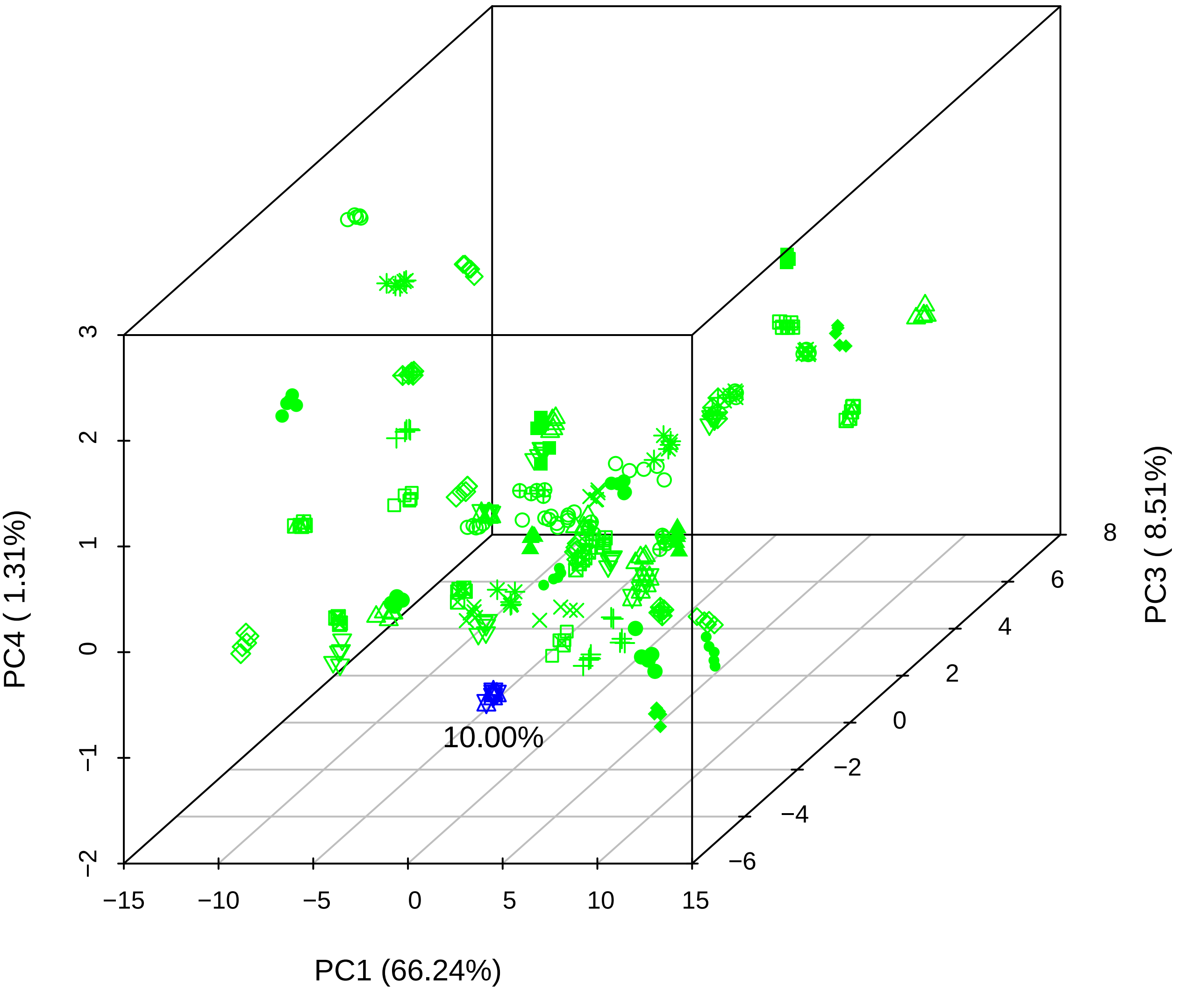

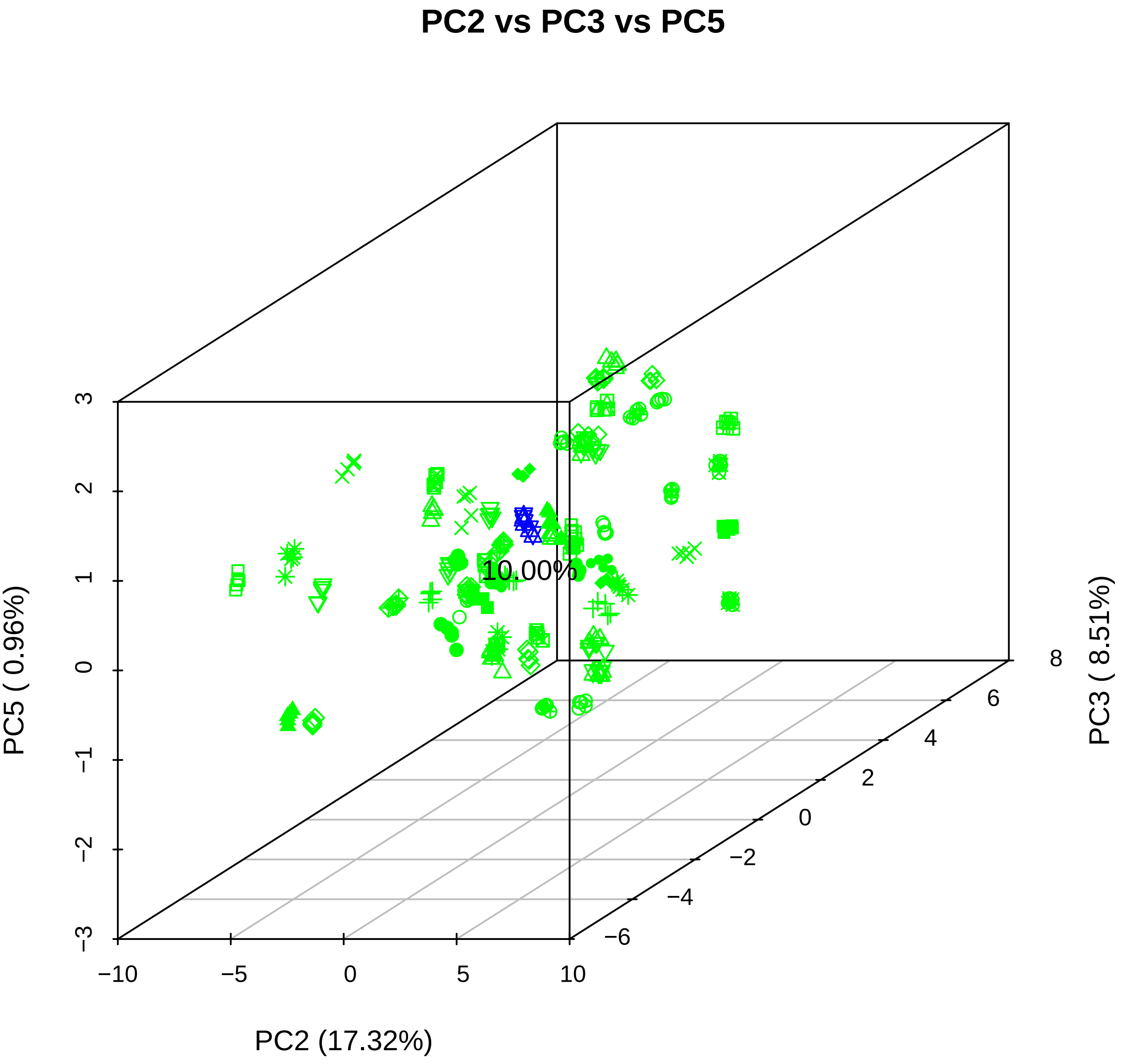

PC2 vs PC3 vs PC5
PC5 (0.96%)
3
2
1
0
−1
−2
−3
10.00%
−10
−5
0
5
10
PC2 (17.32%)
PC3 (8.51%)
8
6
4
2
0
−2
−4
−6

PC1 vs PC2

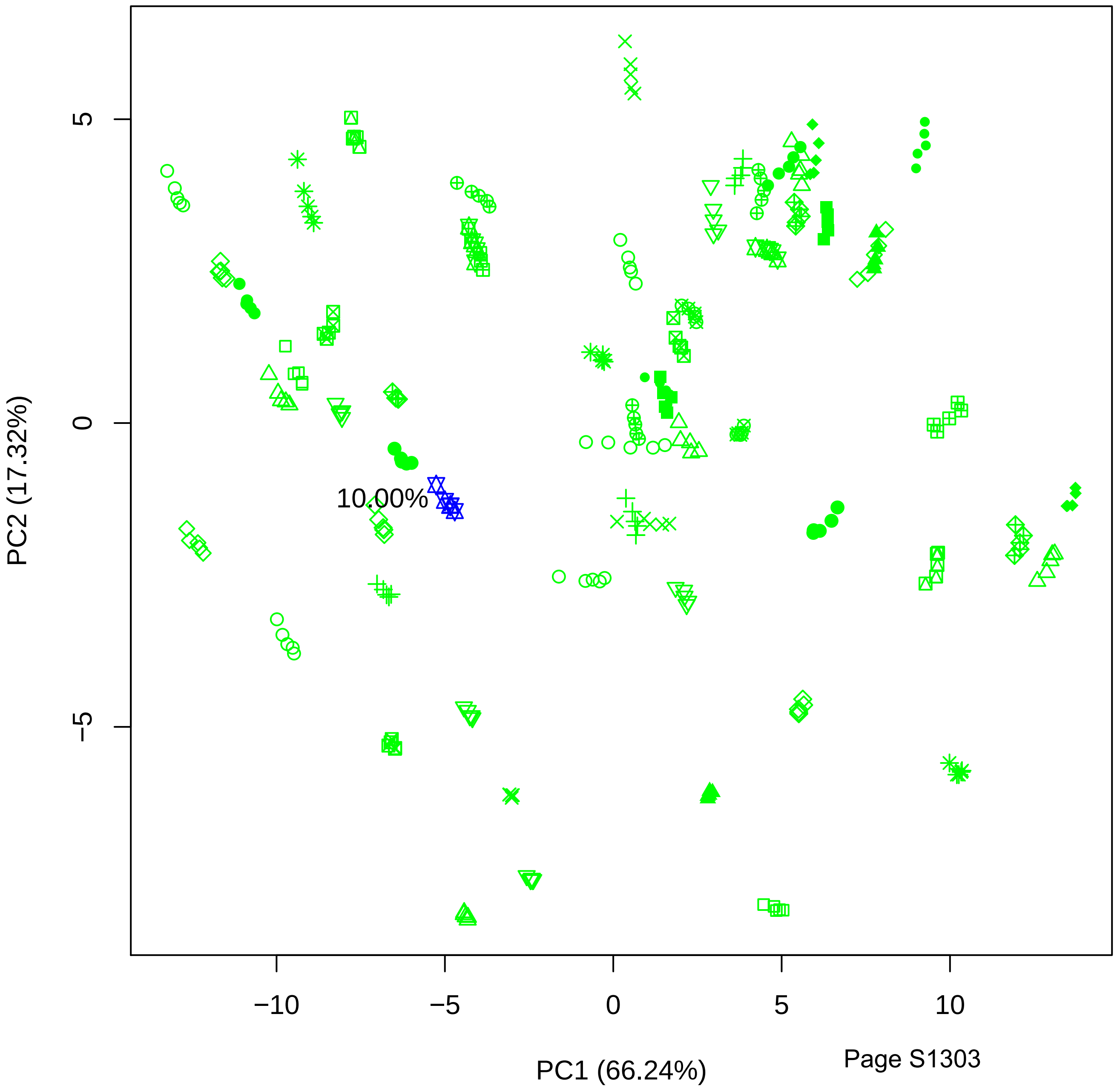

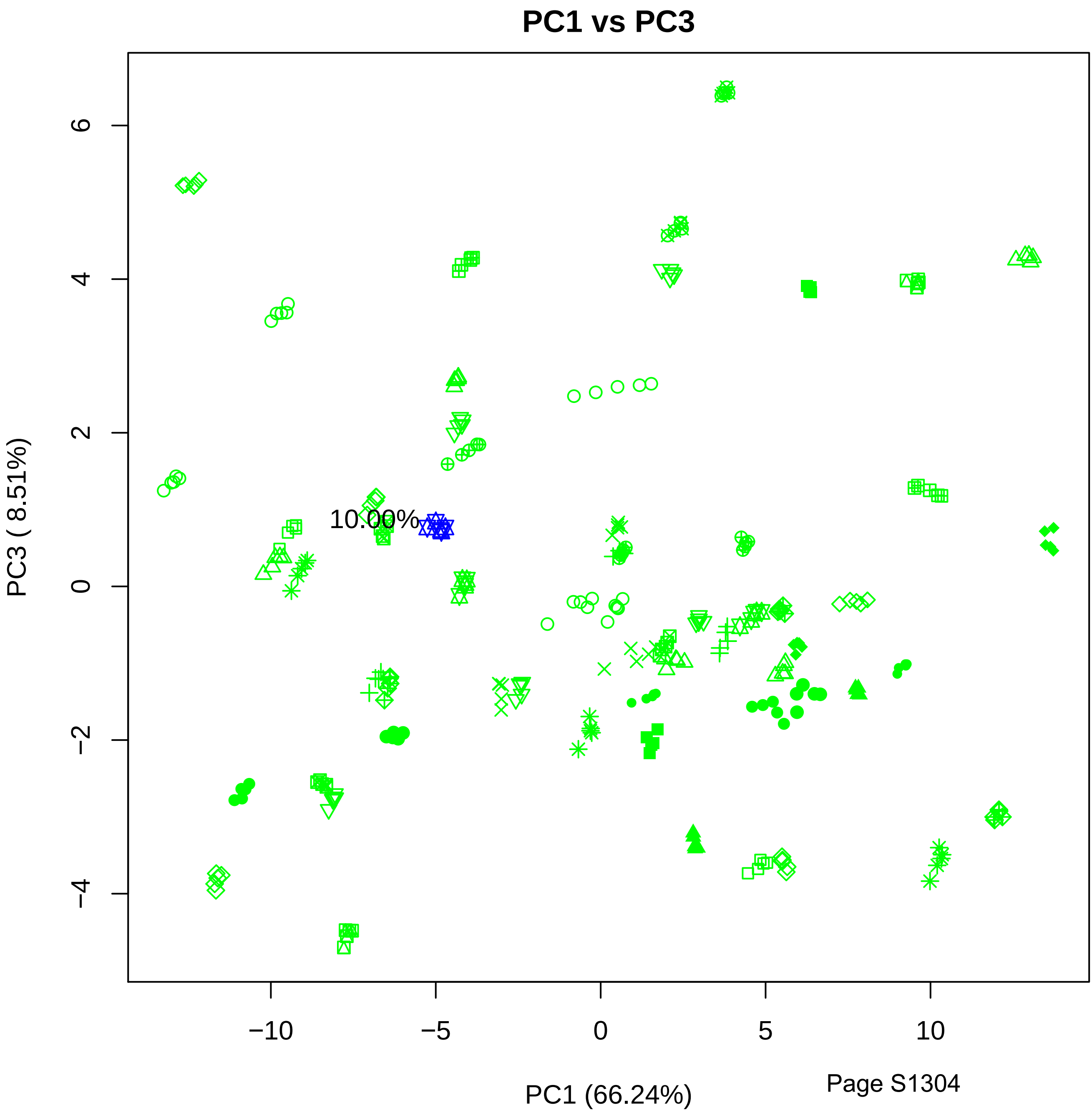

PC1 vs PC3
PC3 (8.51%)
PC1 (66.24%)
10.00%
6
4
2
0
−2
−4
−10
−5
0
5
10

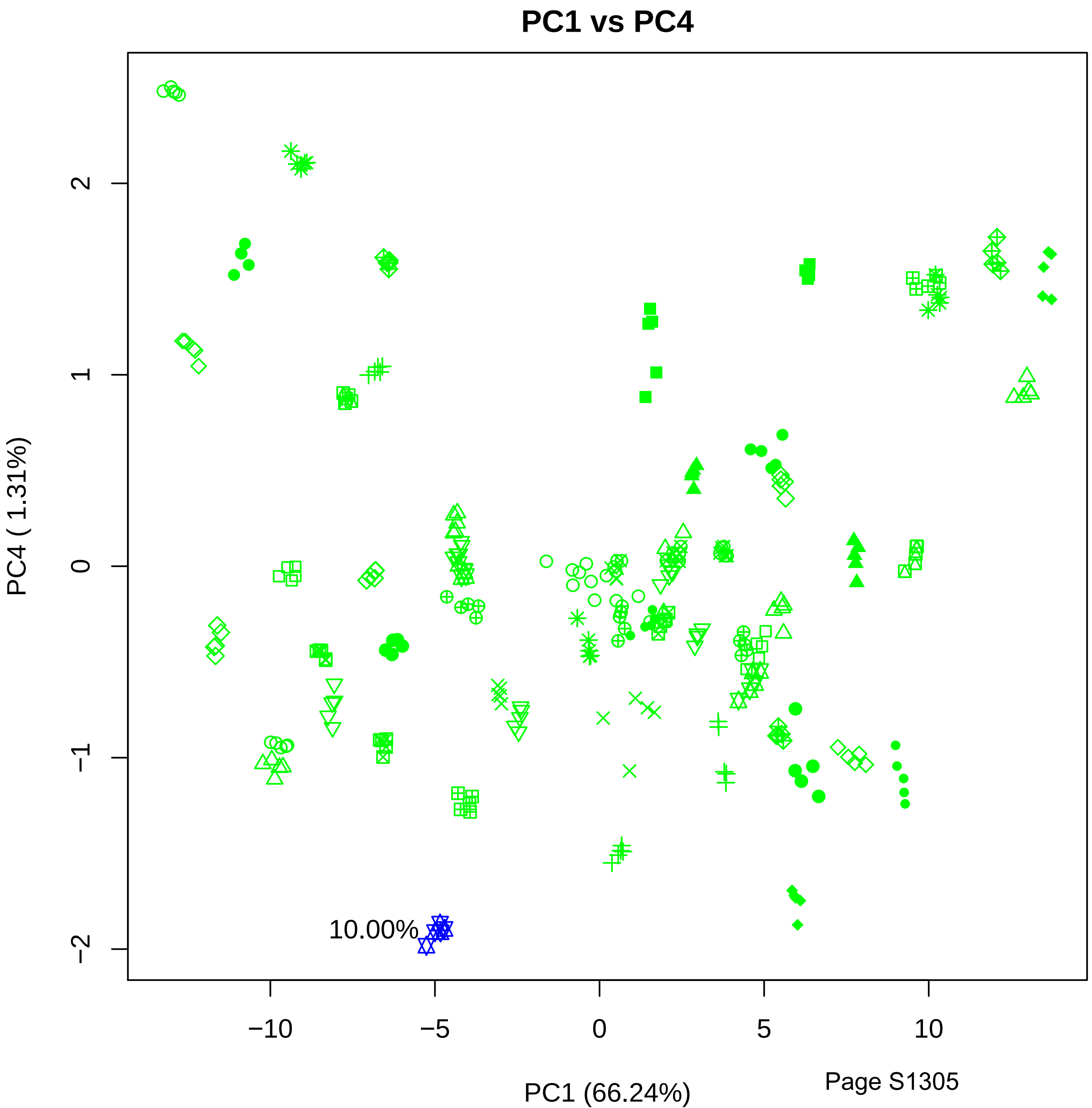
PC1 vs PC4
PC4 (1.31%)
PC1 (66.24%)
2
1
0
−1
−2
−10
−5
0
5
10
10.00%

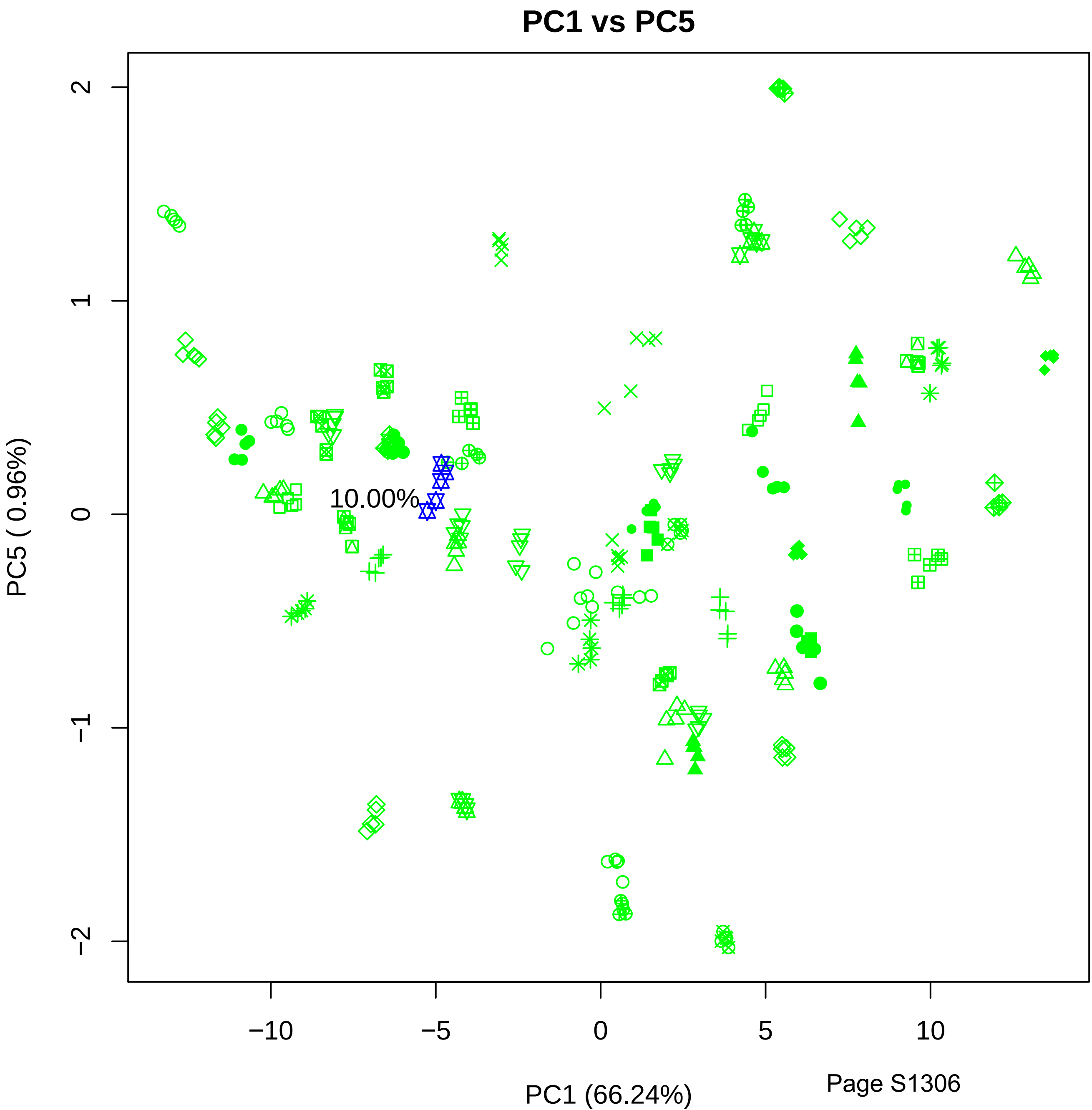
PC1 vs PC5
10.00%
PC5 (0.96%)
PC1 (66.24%)
2
1
0
−1
−2
−10
−5
0
5
10

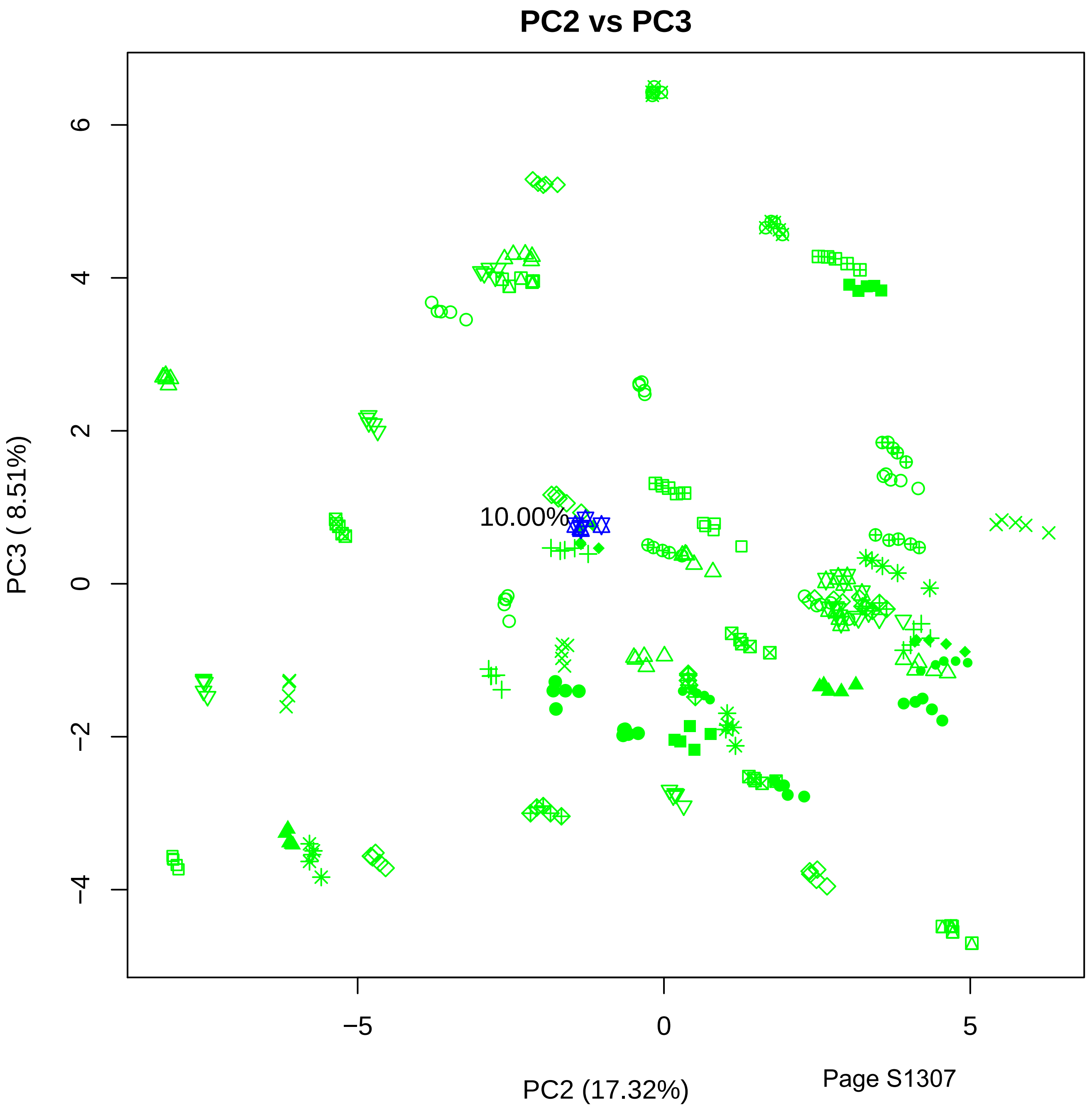

PC2 vs PC3
PC3 (8.51%)
PC2 (17.32%)
10.00%
6
4
2
0
−2
−4
−5
0
5

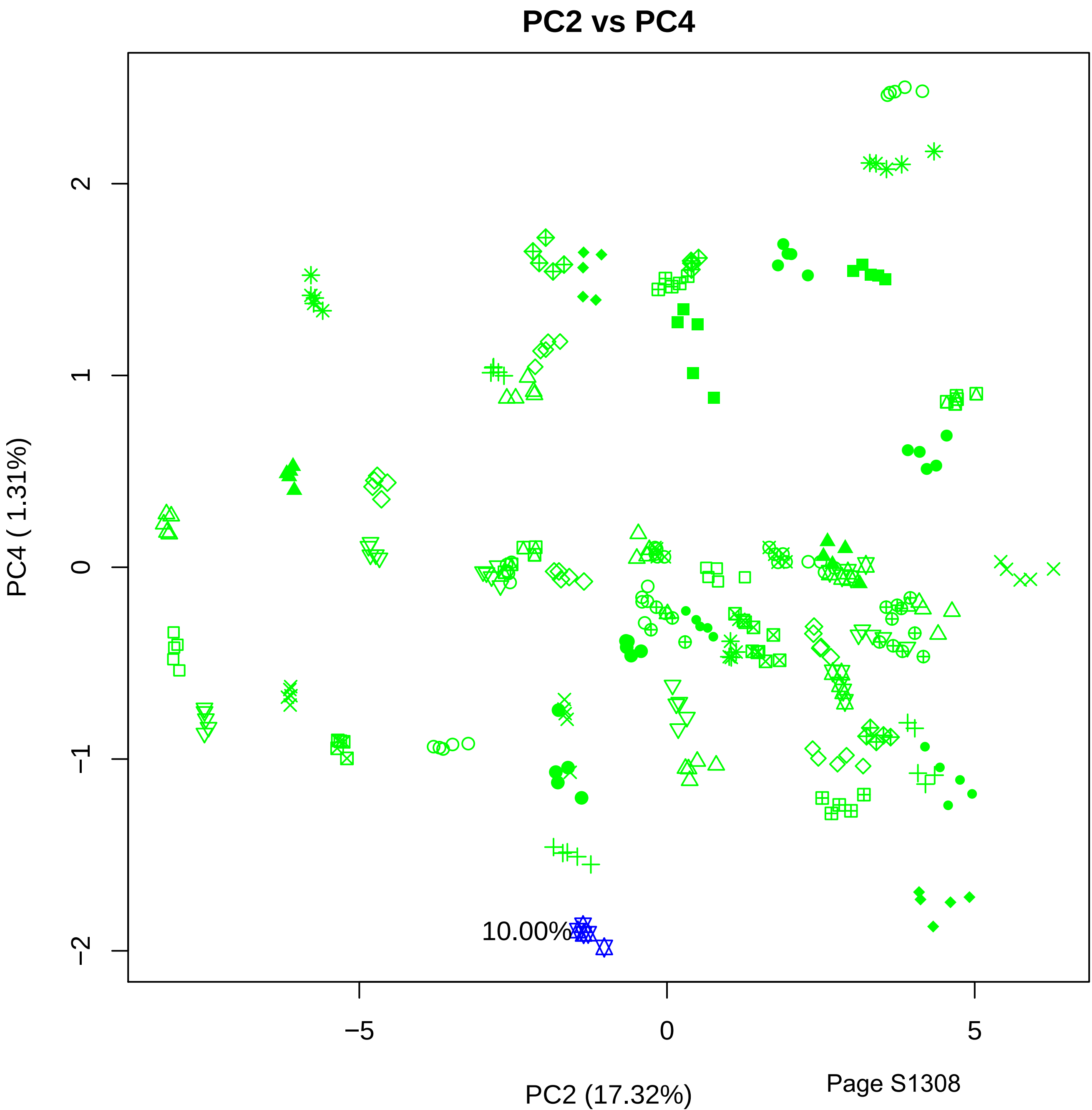

PC2 vs PC4
PC4 (1.31%)
PC2 (17.32%)
2
1
0
−1
−2
−5
0
5
10.00%

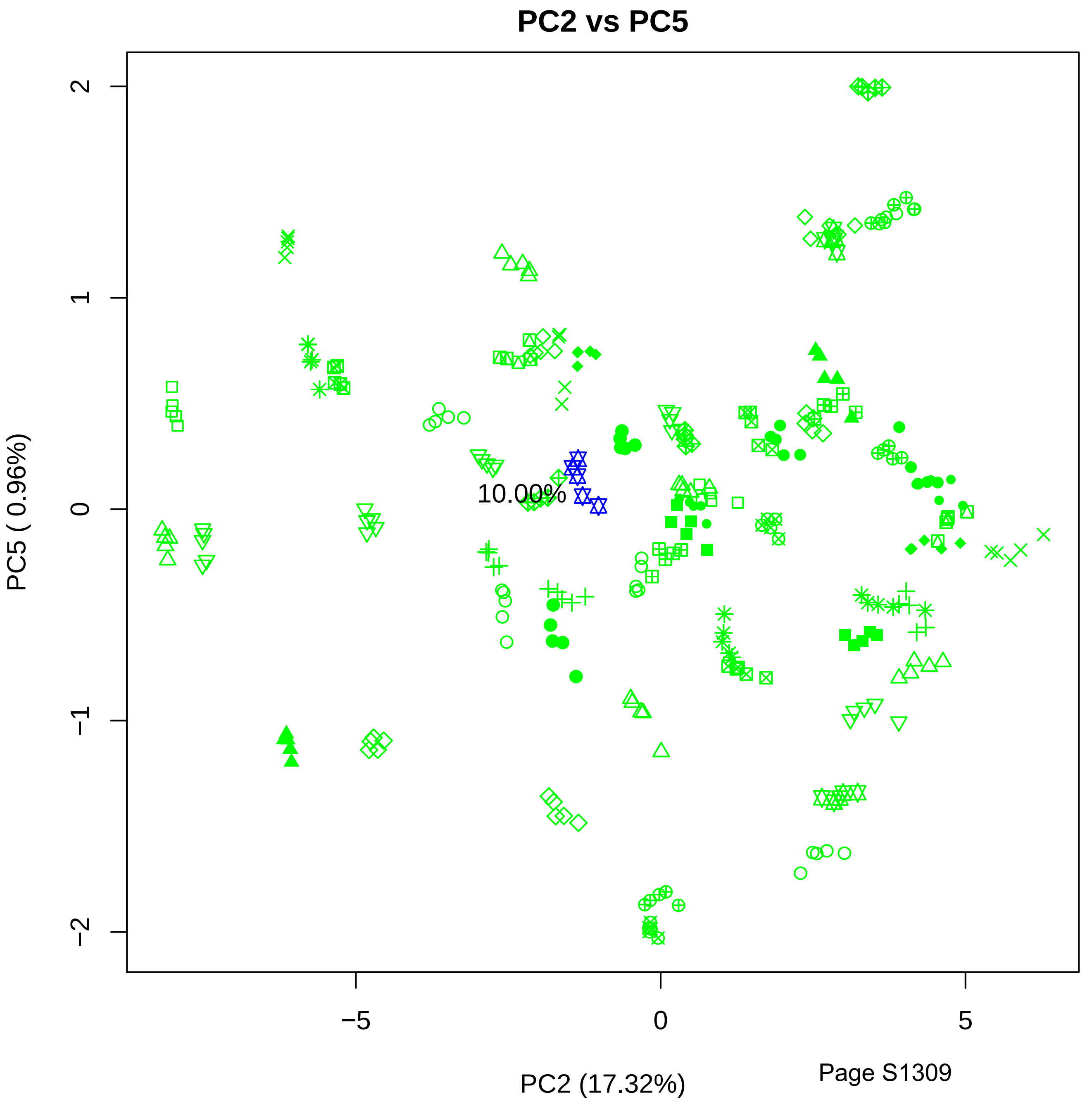

PC2 vs PC5
10.00%
PC5 (0.96%)
PC2 (17.32%)
2
1
0
−1
−2
−5
0
5

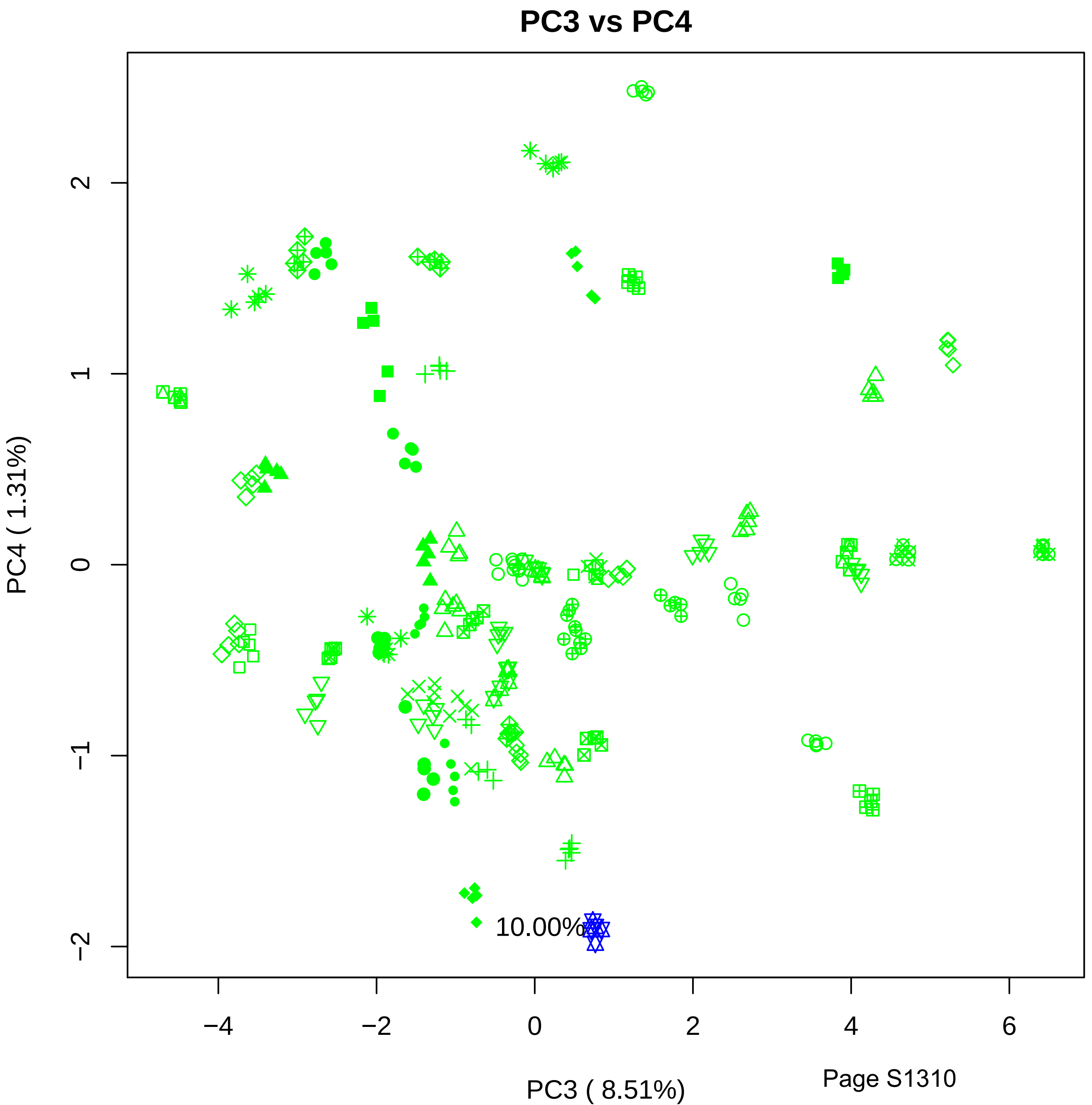

PC3 vs PC4
PC4 (1.31%)
PC3 (8.51%)
10.00%
−4
−2
0
2
4
6
−2
−1
0
1
2

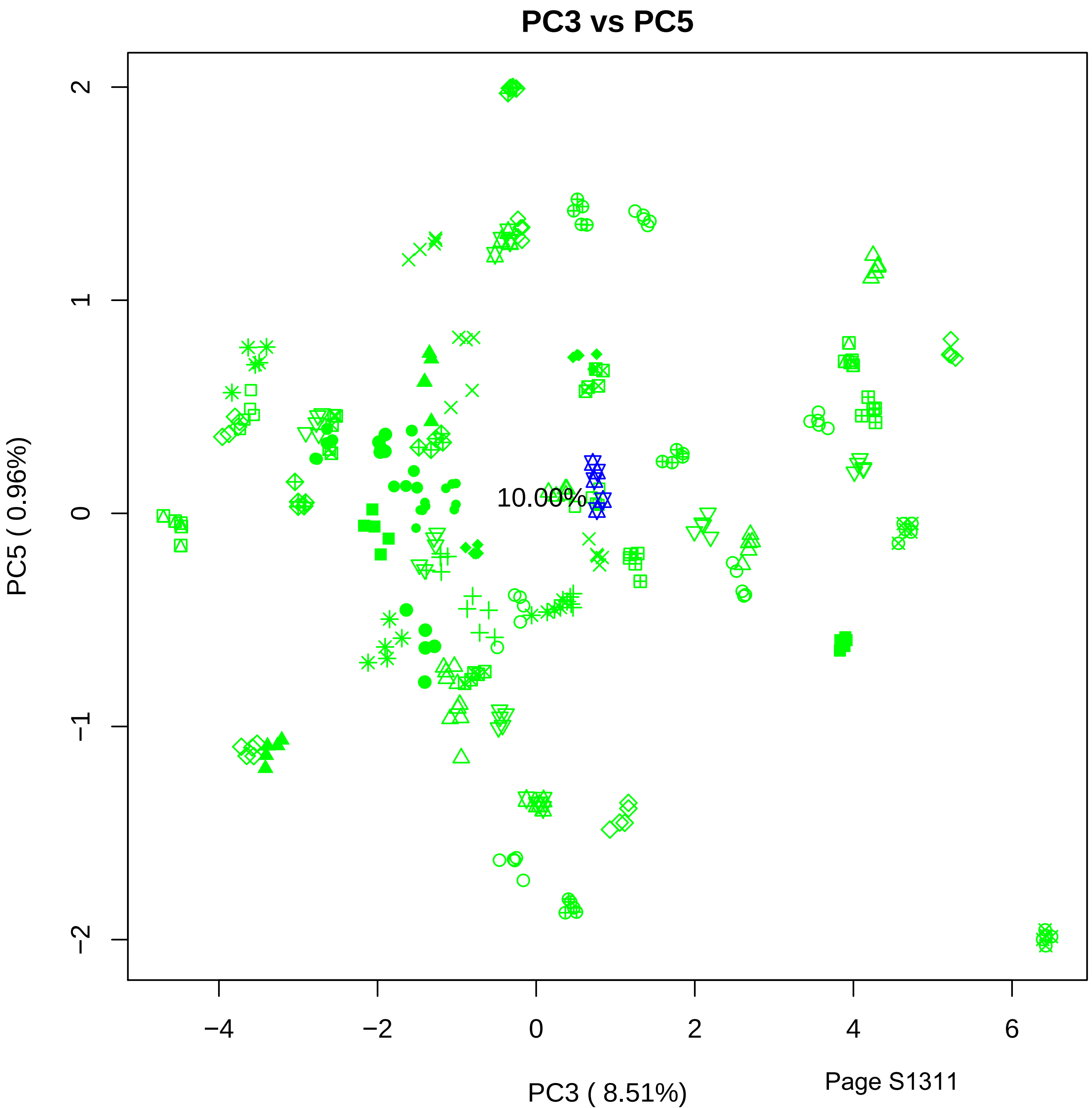
PC3 vs PC5
PC5 (0.96%)
PC3 (8.51%)
10.00%
2
1
0
−1
−2
−4
−2
0
2
4
6

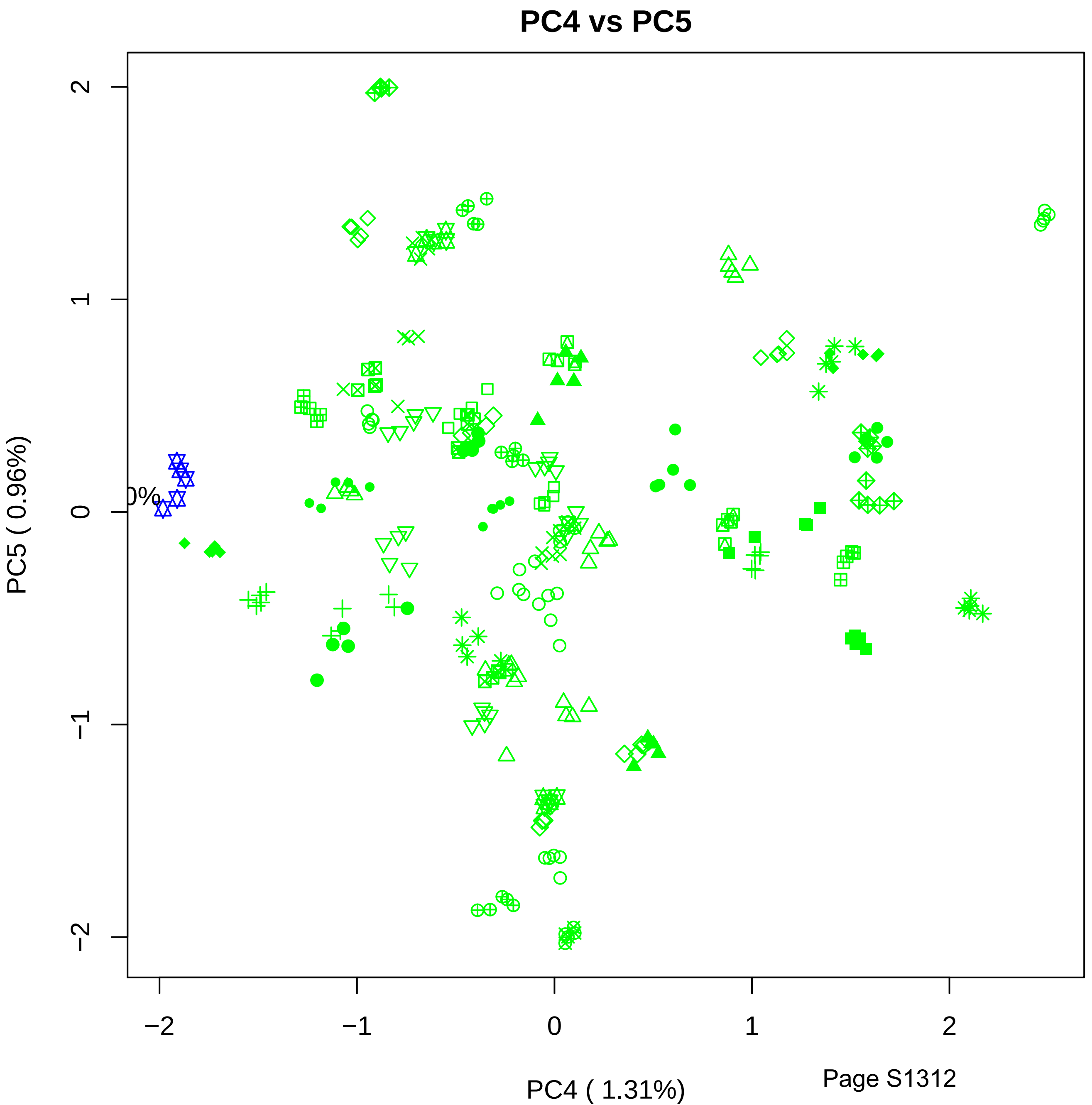
PC4 vs PC5
PC5 (0.96%)
PC4 (1.31%)
2
1
0
−1
−2
−2
−1
0
1
2

PCA of 15% BMH(c) vs PMH(c)

Scaled Matrix PCAed

7th order polynomial baseline correction performed

Raw spectra smoothed to 21 Neighbours, 2nd order PN, Savtisky Golay

Normalisation between 0 and 100

D2 spectra smoothed to 41 Neighbours, 2nd order PN, Savtisky Golay

Regions Analysed: 3601−2501 and 2001−700cm−1

Samples Analysed: 1 bContam's 0 BLH's 0 BMH's 0 OMH's 0 pContam's 0 PLH's 60 PMH's

Keele University AD   Fri Dec 14 14:19:21 2018

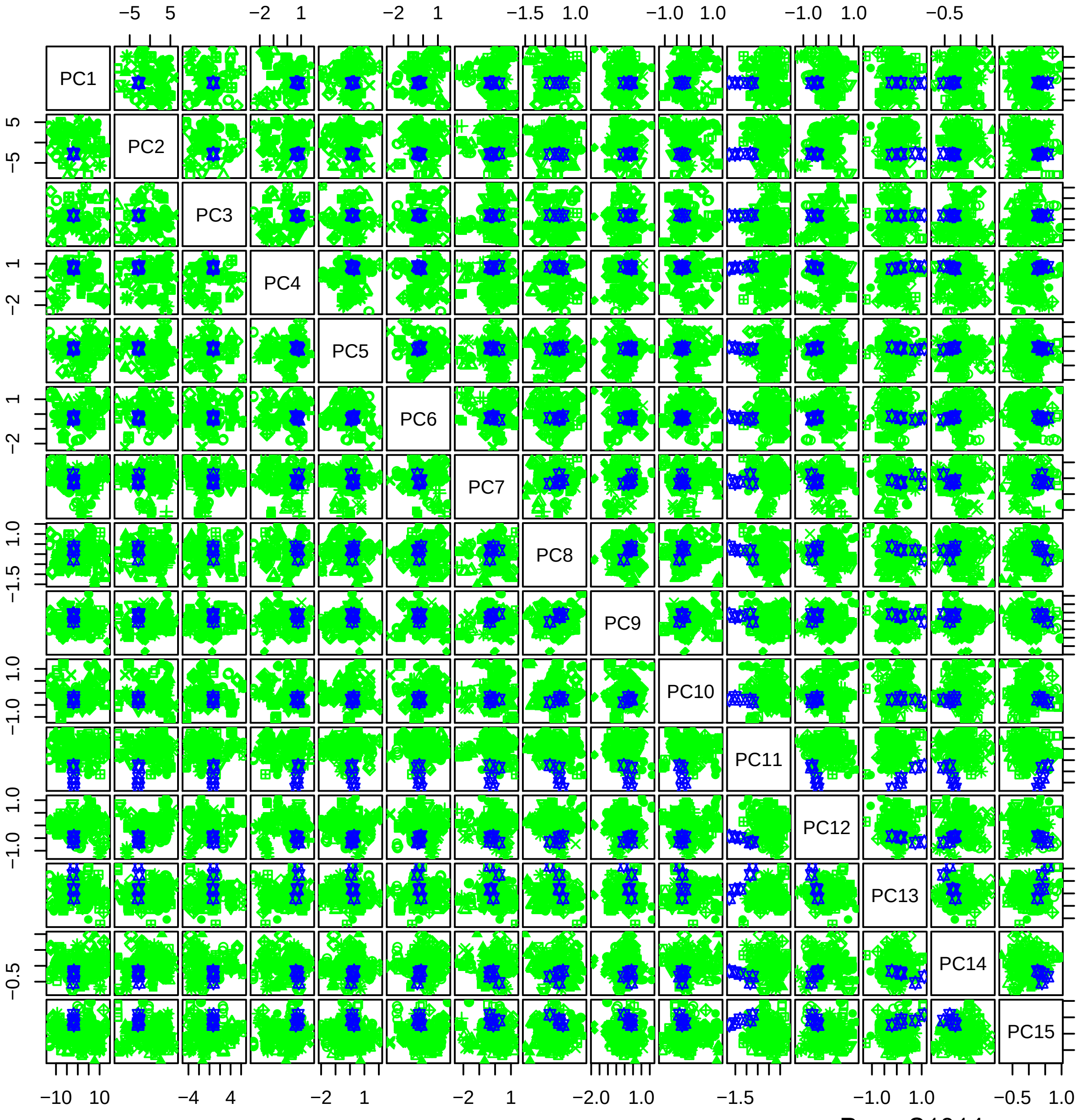
PC1
PC2
PC3
PC4
PC5
PC6
PC7
PC8
PC9
PC10
PC11
PC12
PC13
PC14
PC15

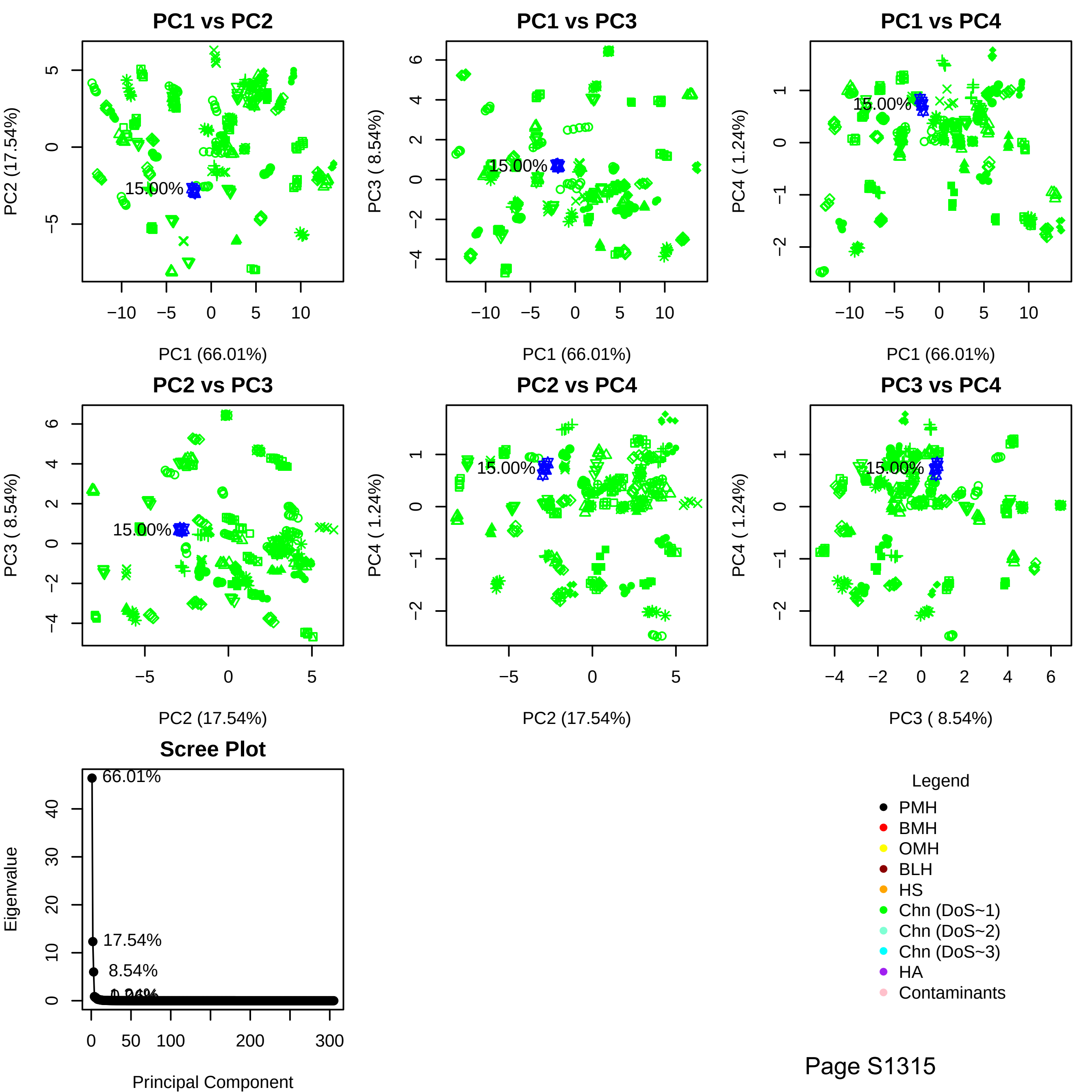
PC1 vs PC2
PC1 vs PC3
PC1 vs PC4
PC2 vs PC3
PC2 vs PC4
PC3 vs PC4
Scree Plot
PC1 (66.01%)
PC2 (17.54%)
PC3 (8.54%)
PC4 (1.24%)
15.00%
Eigenvalue
Principal Component
66.01%
17.54%
8.54%
Legend
PMH
BMH
OMH
BLH
HS
Chn (DoS~1)
Chn (DoS~2)
Chn (DoS~3)
HA
Contaminants

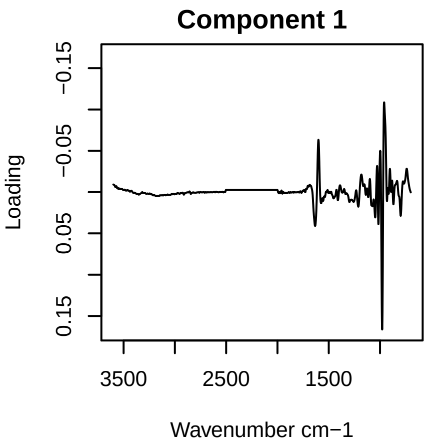
Component 1
Loading
−0.15
−0.05
0.05
0.15
3500
2500
1500
Wavenumber cm−1

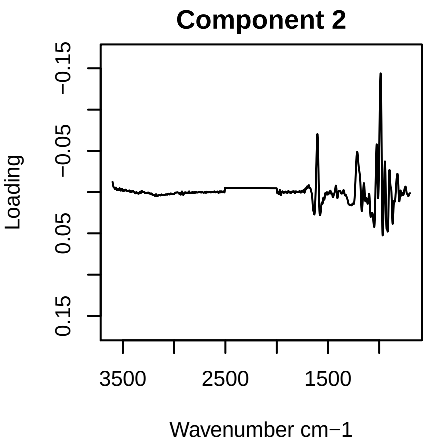
Component 2
Loading
−0.15
−0.05
0.05
0.15
3500
2500
1500
Wavenumber cm−1

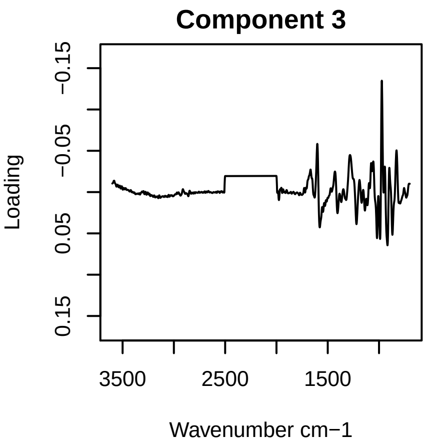
Component 3
Loading
−0.15
−0.05
0.05
0.15
3500
2500
1500
Wavenumber cm−1

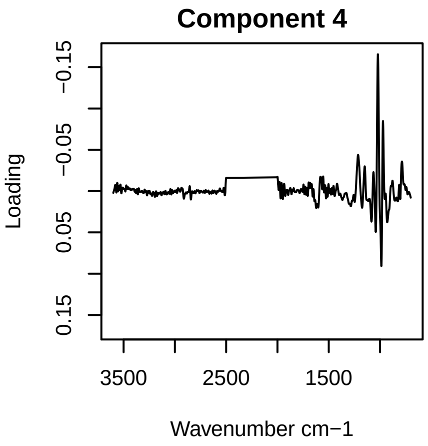
Component 4
Loading
−0.15
−0.05
0.05
0.15
3500
2500
1500
Wavenumber cm−1

PC1 vs PC2 vs PC3

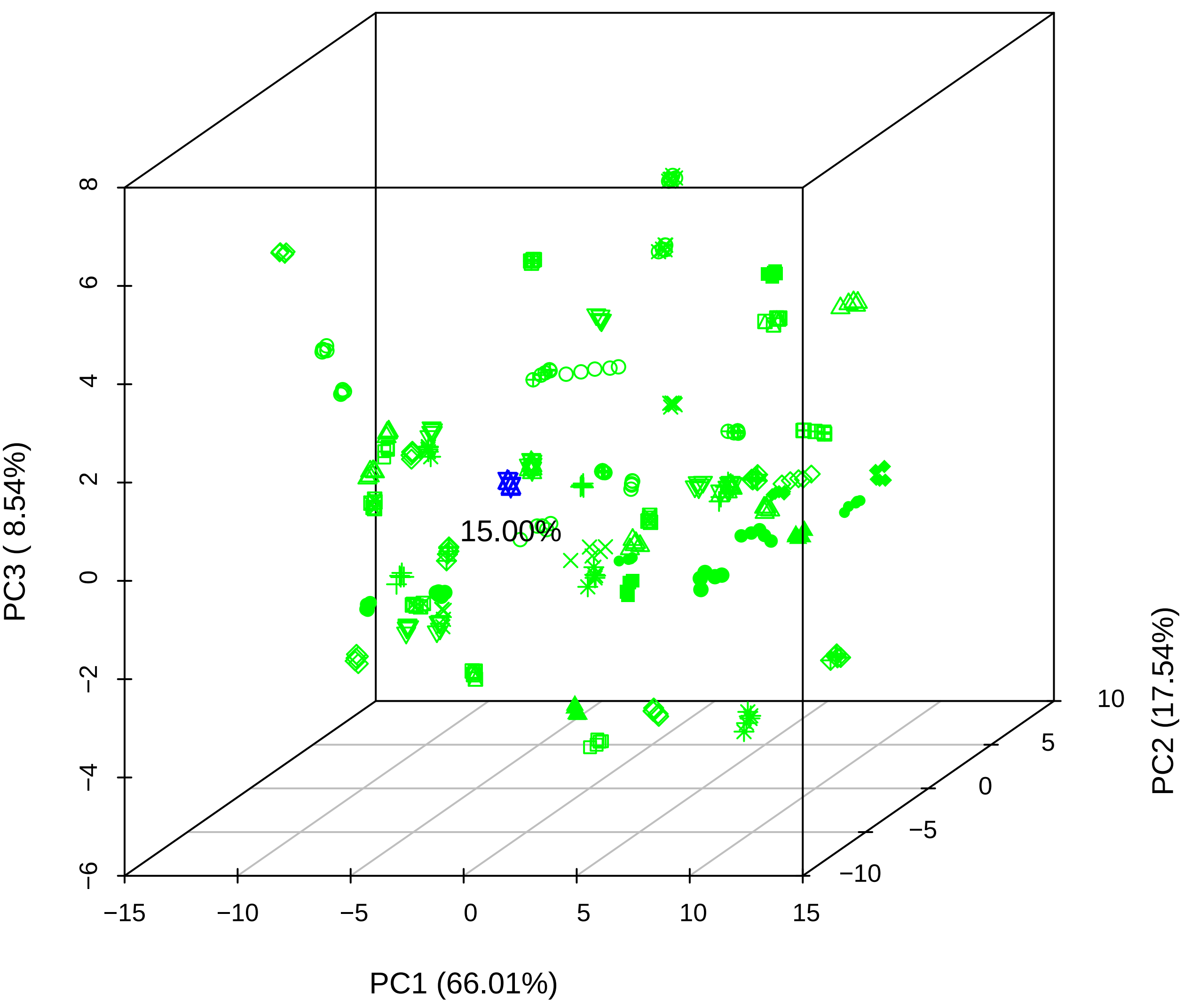

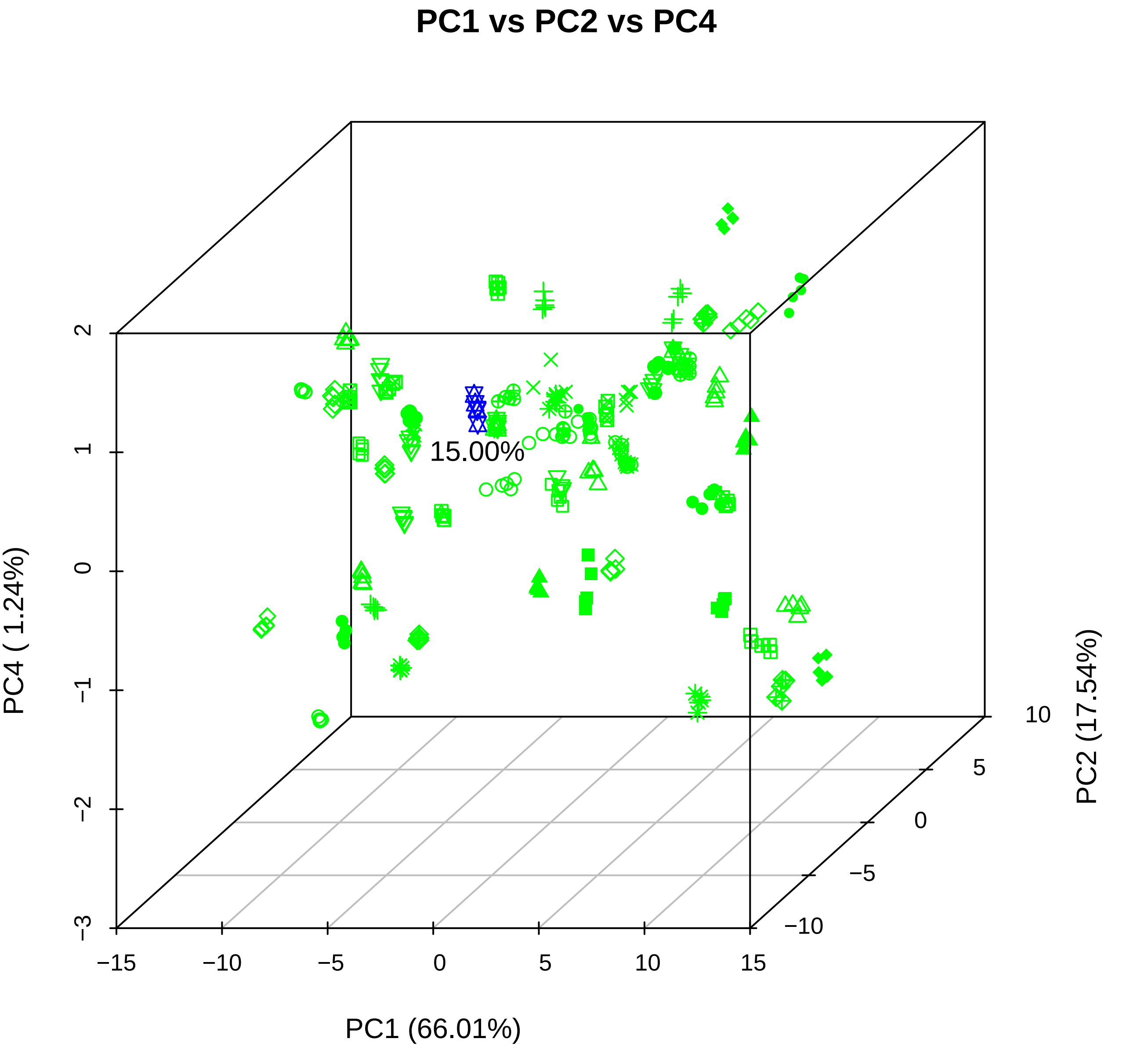

PC1 vs PC2 vs PC4
15.00%
PC1 (66.01%)
PC2 (17.54%)
PC4 (1.24%)
−15
−10
−5
0
5
10
15
−10
−5
0
5
10
−3
−2
−1
0
1
2

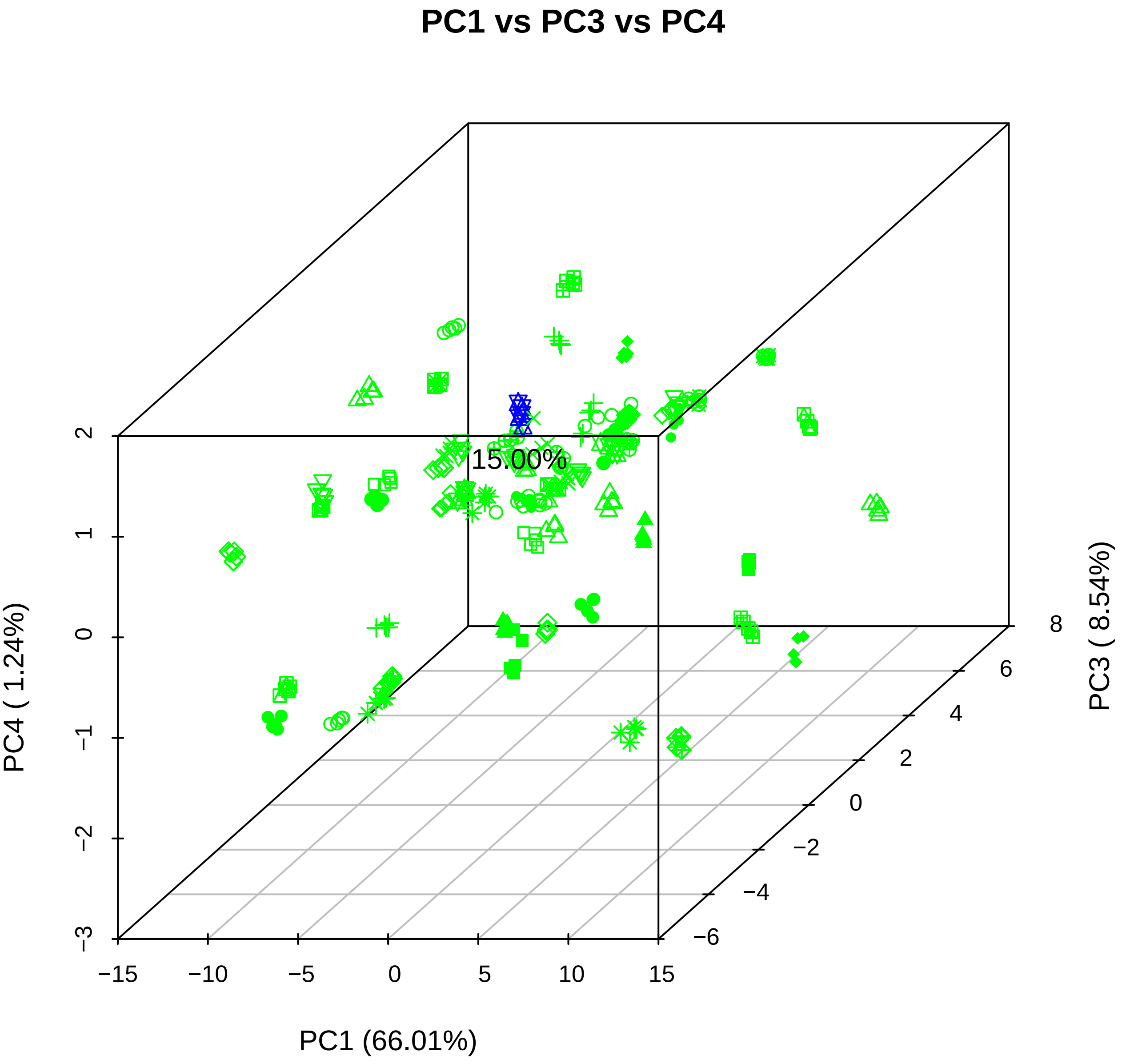

PC1 vs PC3 vs PC4
15.00%
2
1
0
−1
−2
−3
PC4 (1.24%)
−15
−10
−5
0
5
10
15
PC1 (66.01%)
8
6
4
2
0
−2
−4
−6
PC3 (8.54%)

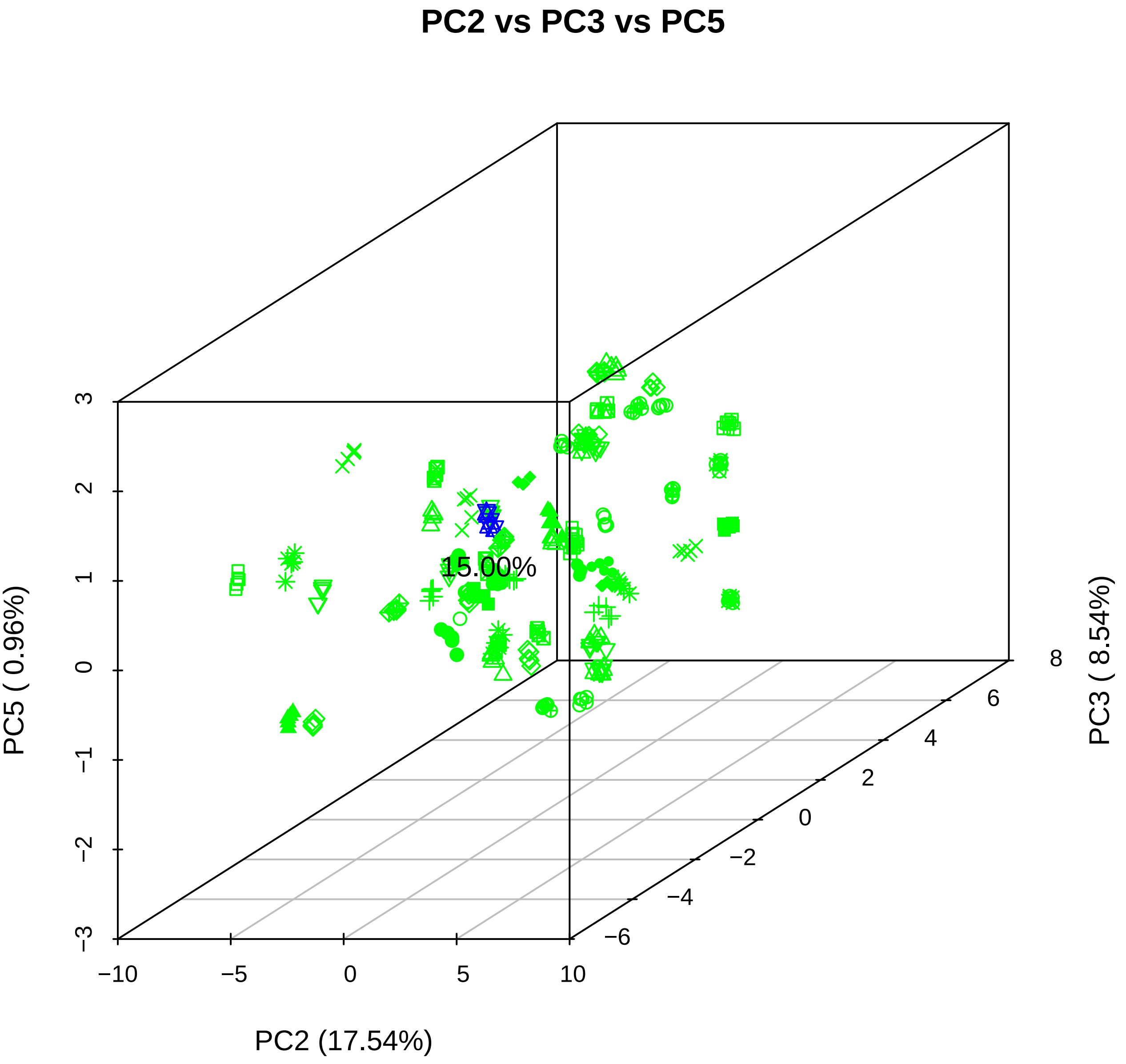

PC2 vs PC3 vs PC5
15.00%
PC5 (0.96%)
PC2 (17.54%)
PC3 (8.54%)
3
2
1
0
−1
−2
−3
−10
−5
0
5
10
8
6
4
2
0
−2
−4
−6

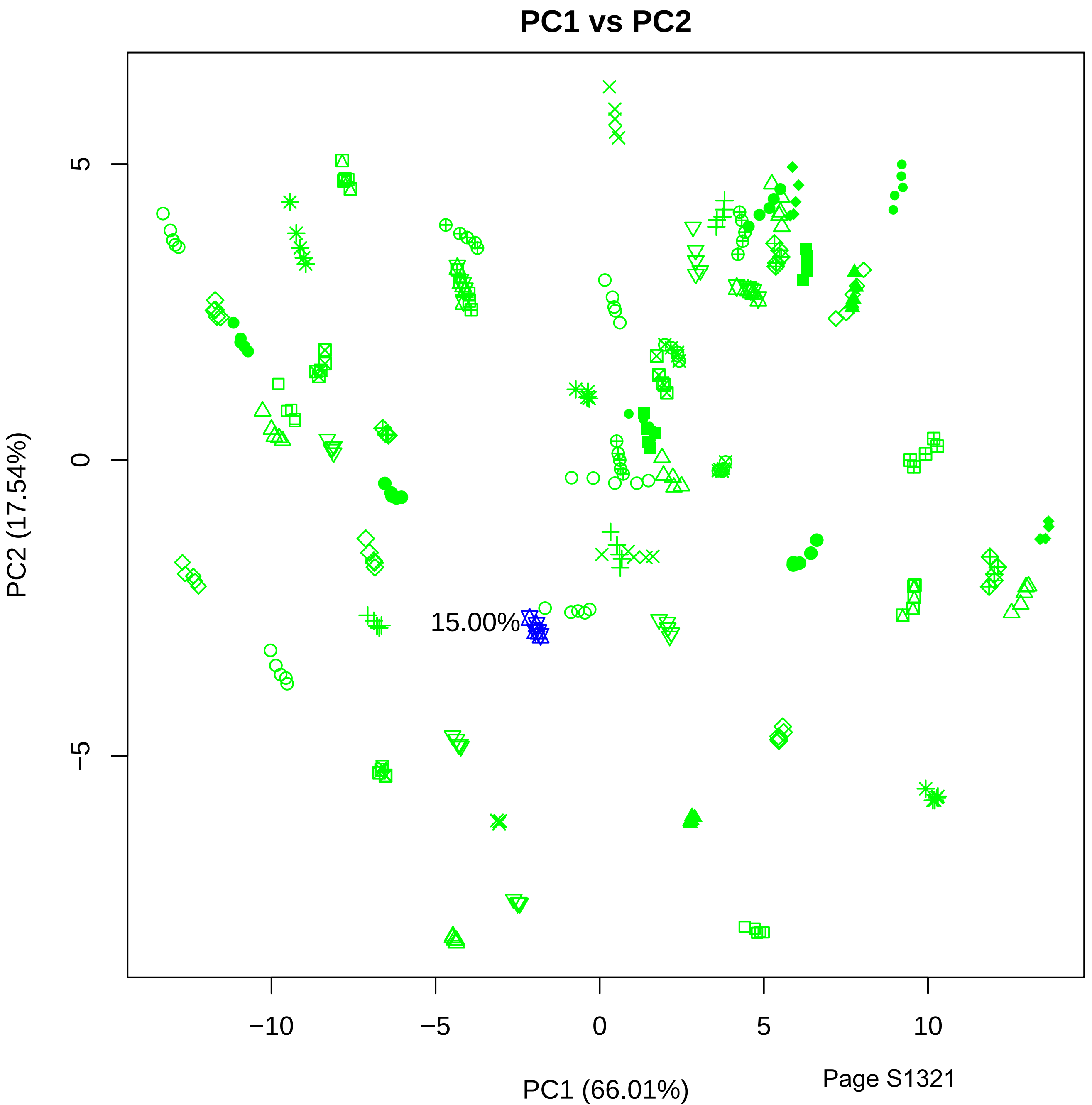
PC1 vs PC2
5
0
−5
PC2 (17.54%)
−10
−5
0
5
10
PC1 (66.01%)
15.00%

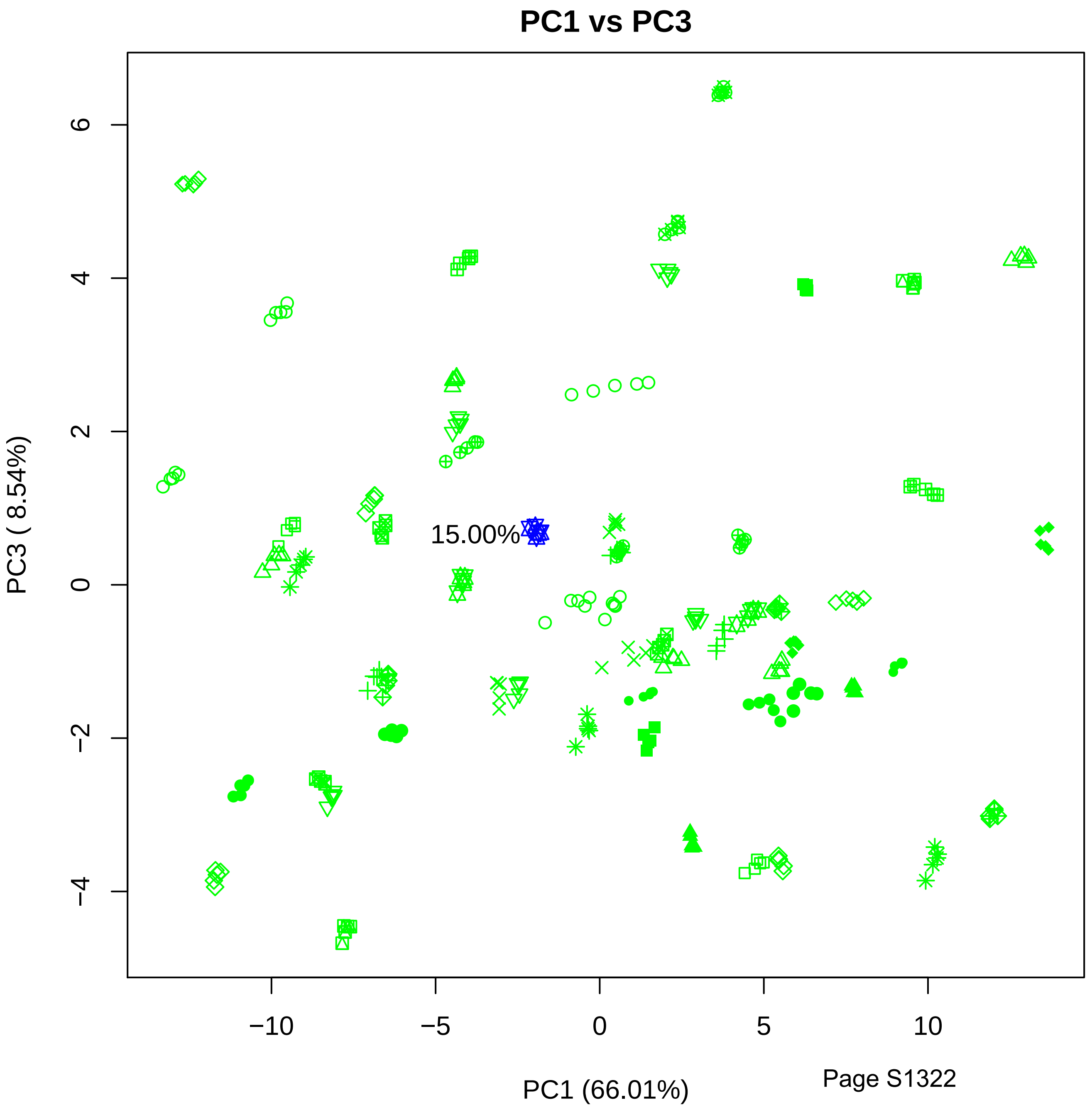

PC1 vs PC3
6
4
2
0
−2
−4
PC3 (8.54%)
15.00%
−10
−5
0
5
10
PC1 (66.01%)

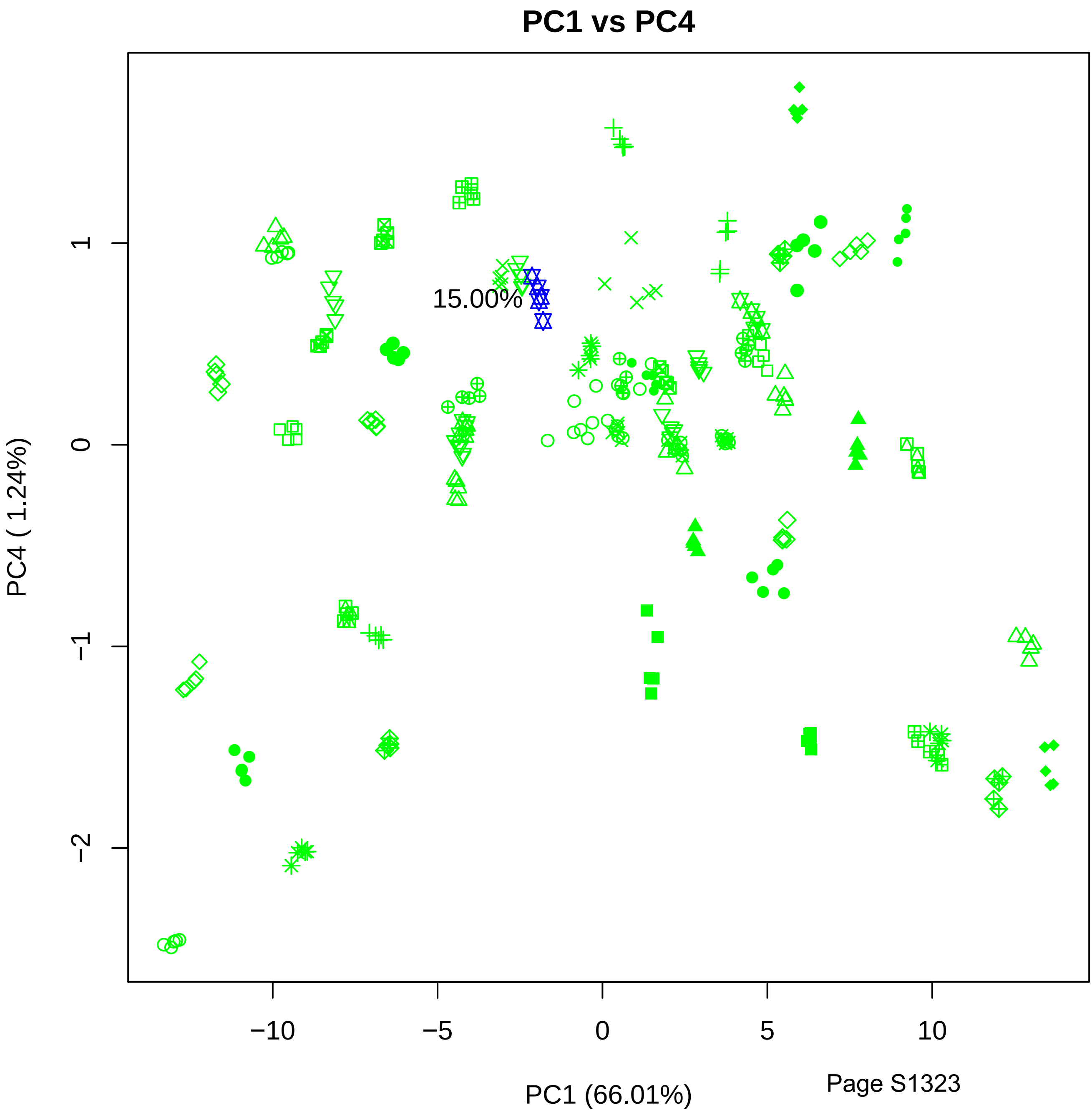

PC1 vs PC4
15.00%
1
0
−1
−2
−10
−5
0
5
10
PC4 (1.24%)
PC1 (66.01%)

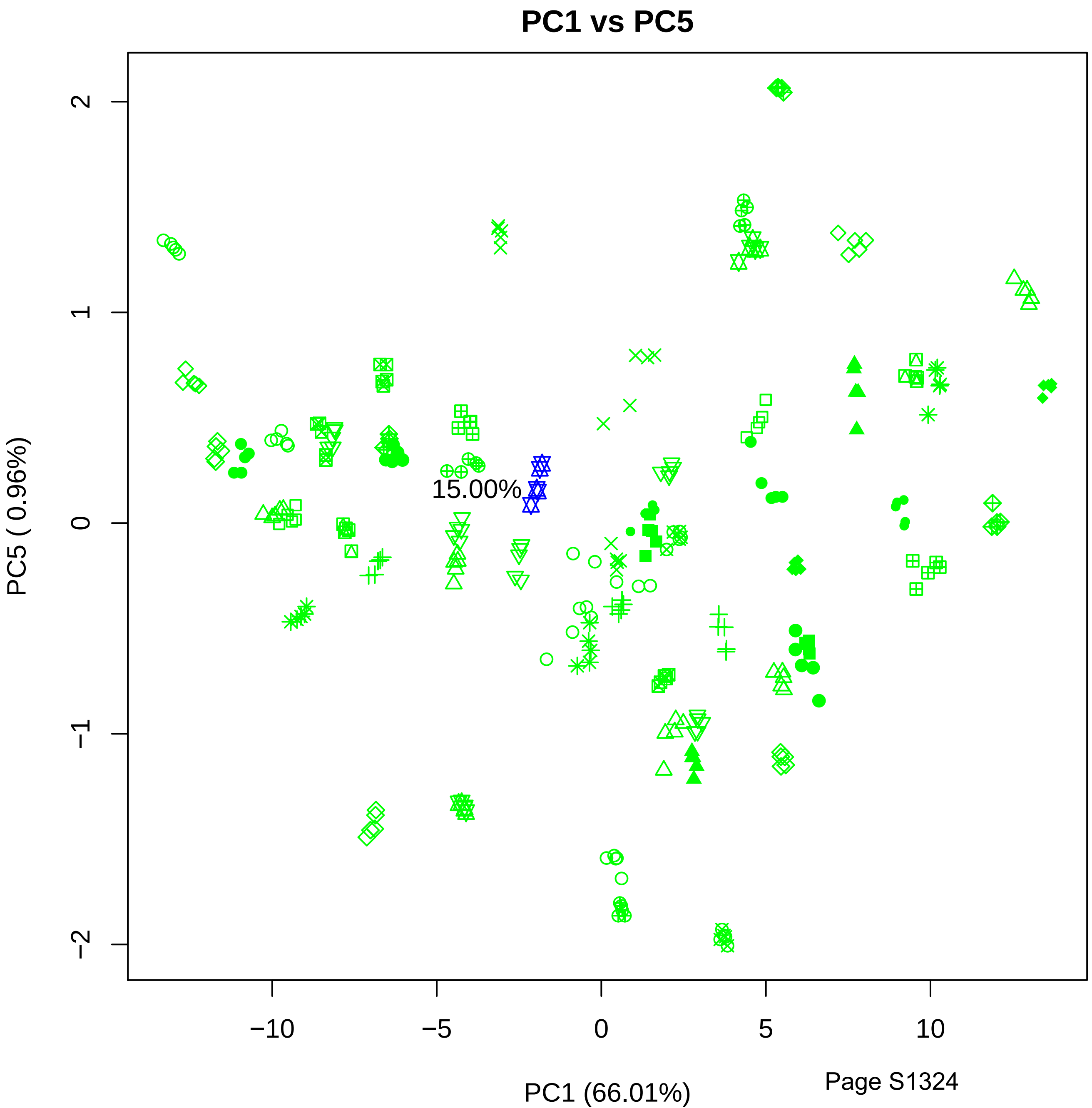

PC1 vs PC5
15.00%
PC5 (0.96%)
PC1 (66.01%)
2
1
0
−1
−2
−10
−5
0
5
10

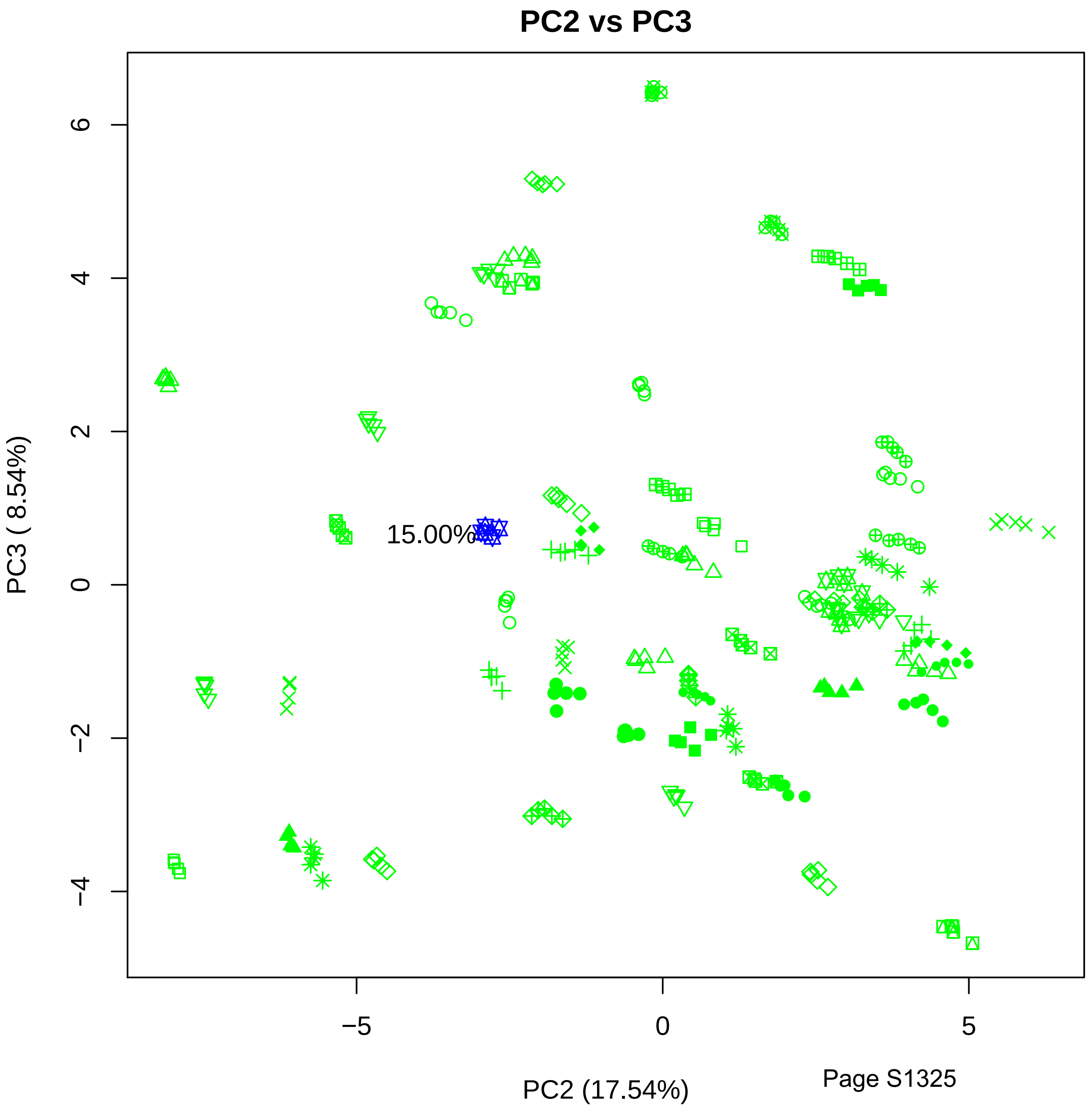
PC2 vs PC3
PC3 (8.54%)
PC2 (17.54%)
15.00%
6
4
2
0
−2
−4
−5
0
5

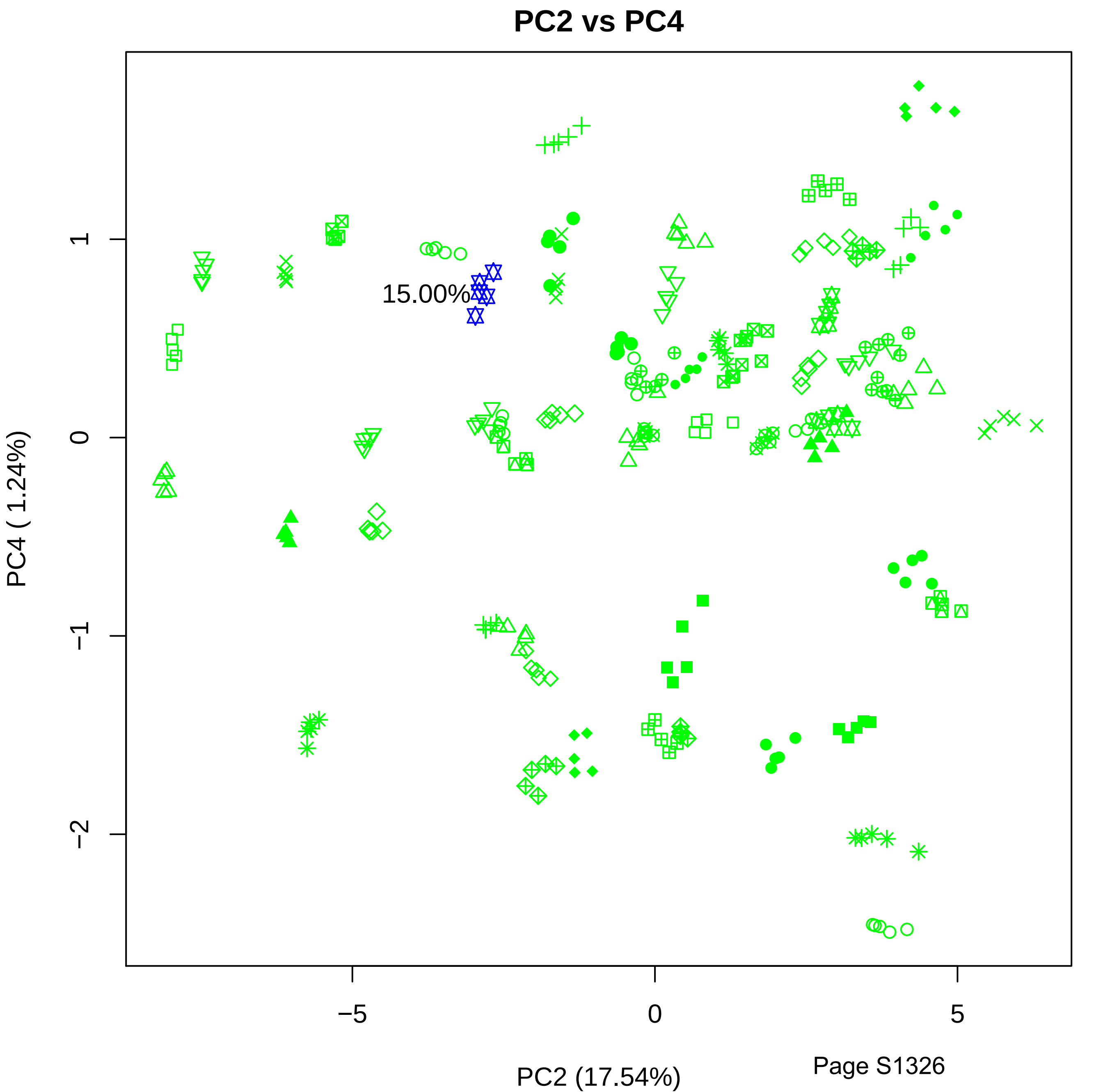

PC2 vs PC4
15.00%
PC4 (1.24%)
1
0
−1
−2
−5
0
5
PC2 (17.54%)

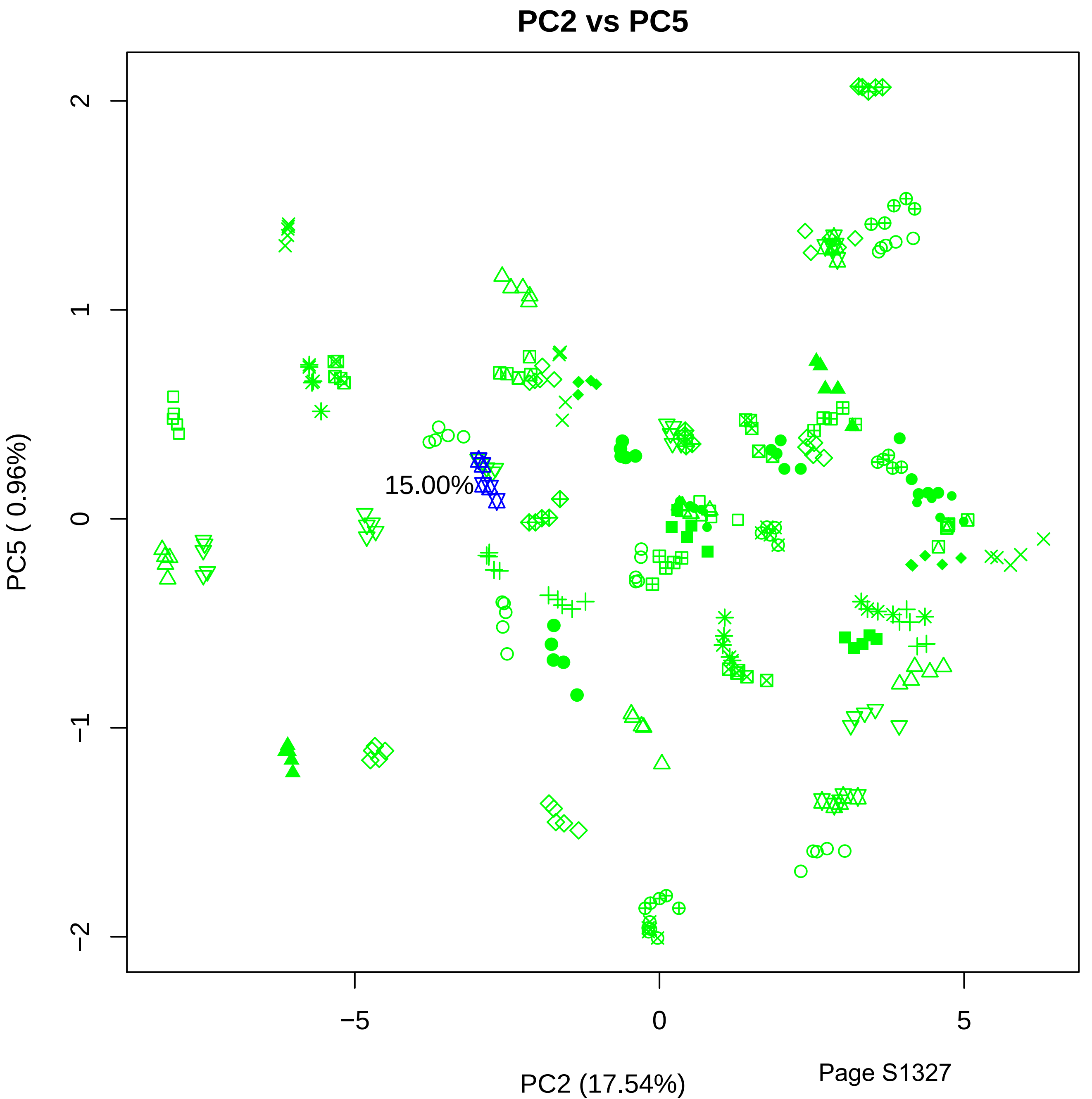
PC2 vs PC5
PC5 (0.96%)
PC2 (17.54%)
15.00%
2
1
0
−1
−2
−5
0
5

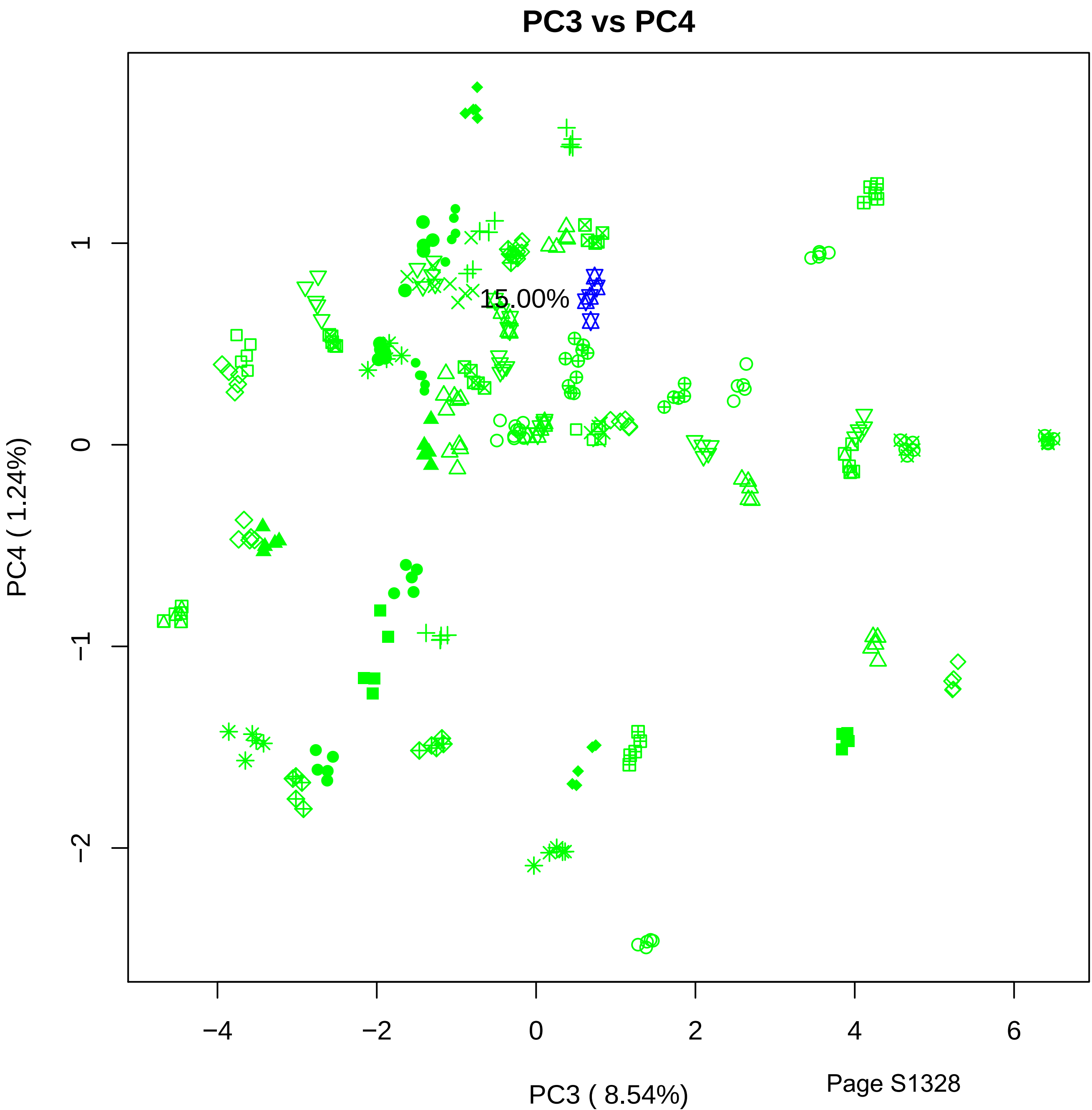
PC3 vs PC4
15.00%
1
0
−1
−2
PC4 (1.24%)
−4
−2
0
2
4
6
PC3 (8.54%)

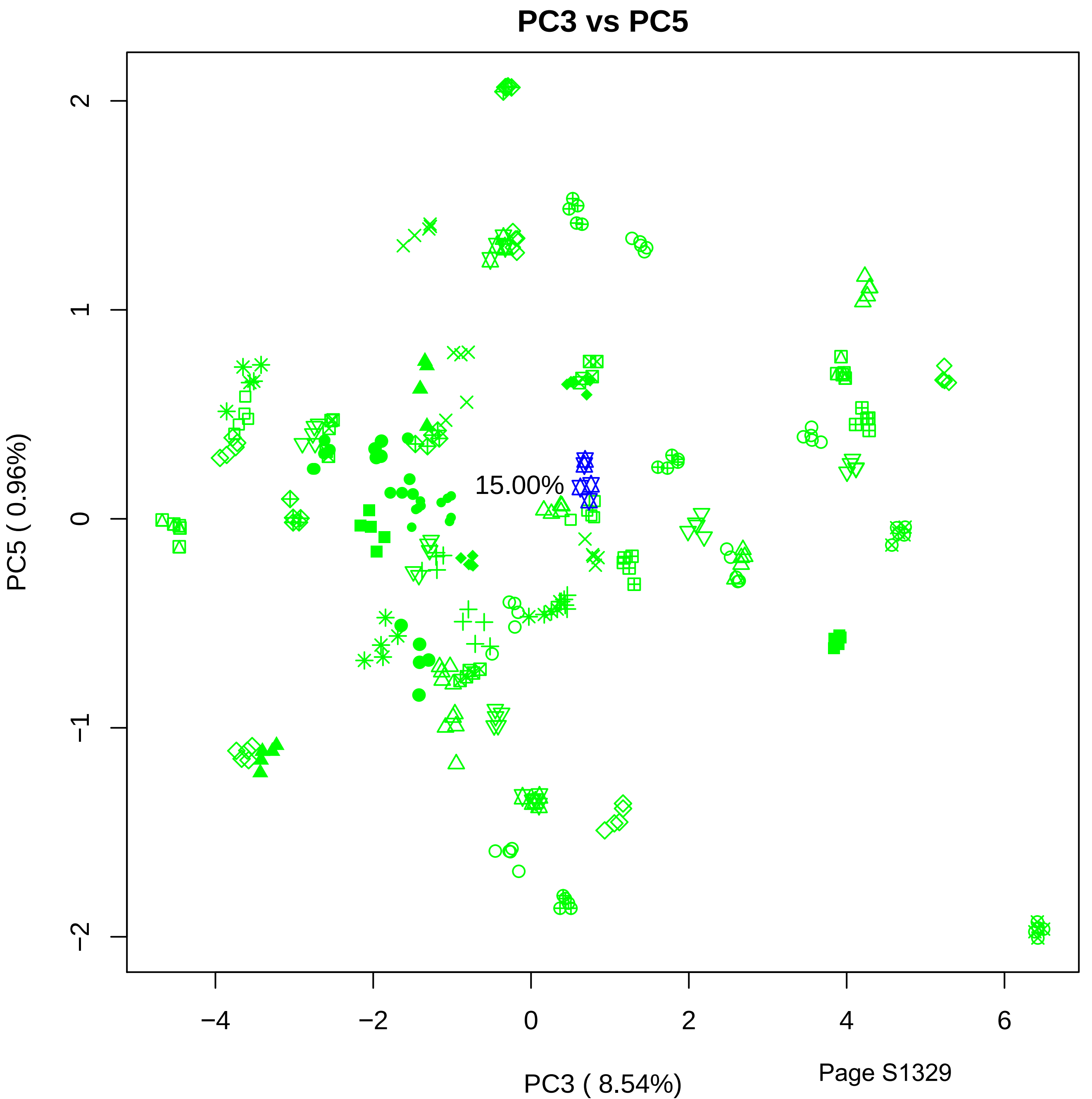

PC3 vs PC5
PC5 (0.96%)
PC3 (8.54%)
15.00%
2
1
0
−1
−2
−4
−2
0
2
4
6

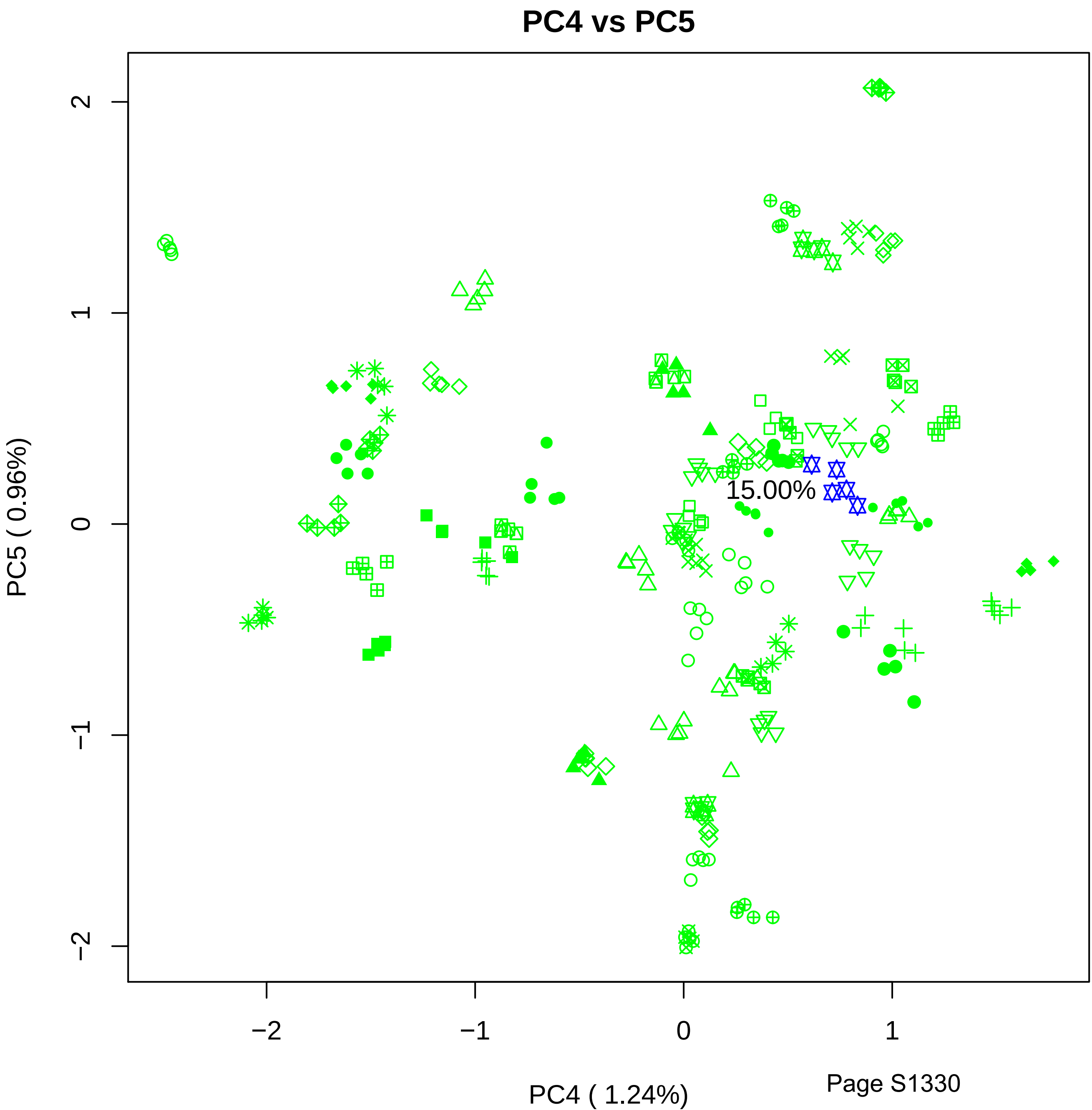

PC4 vs PC5
15.00%
PC5 (0.96%)
PC4 (1.24%)
2
1
0
−1
−2
−2
−1
0
1

PCA of 20% BMH(c) vs PMH(c)

Scaled Matrix PCAed

7th order polynomial baseline correction performed

Raw spectra smoothed to 21 Neighbours, 2nd order PN, Savtisky Golay

Normalisation between 0 and 100

D2 spectra smoothed to 41 Neighbours, 2nd order PN, Savtisky Golay

Regions Analysed: 3601−2501 and 2001−700cm−1

Samples Analysed: 1 bContam's 0 BLH's 0 BMH's 0 OMH's 0 pContam's 0 PLH's 60 PMH's

Keele University AD

Fri Dec 14 14:19:29 2018

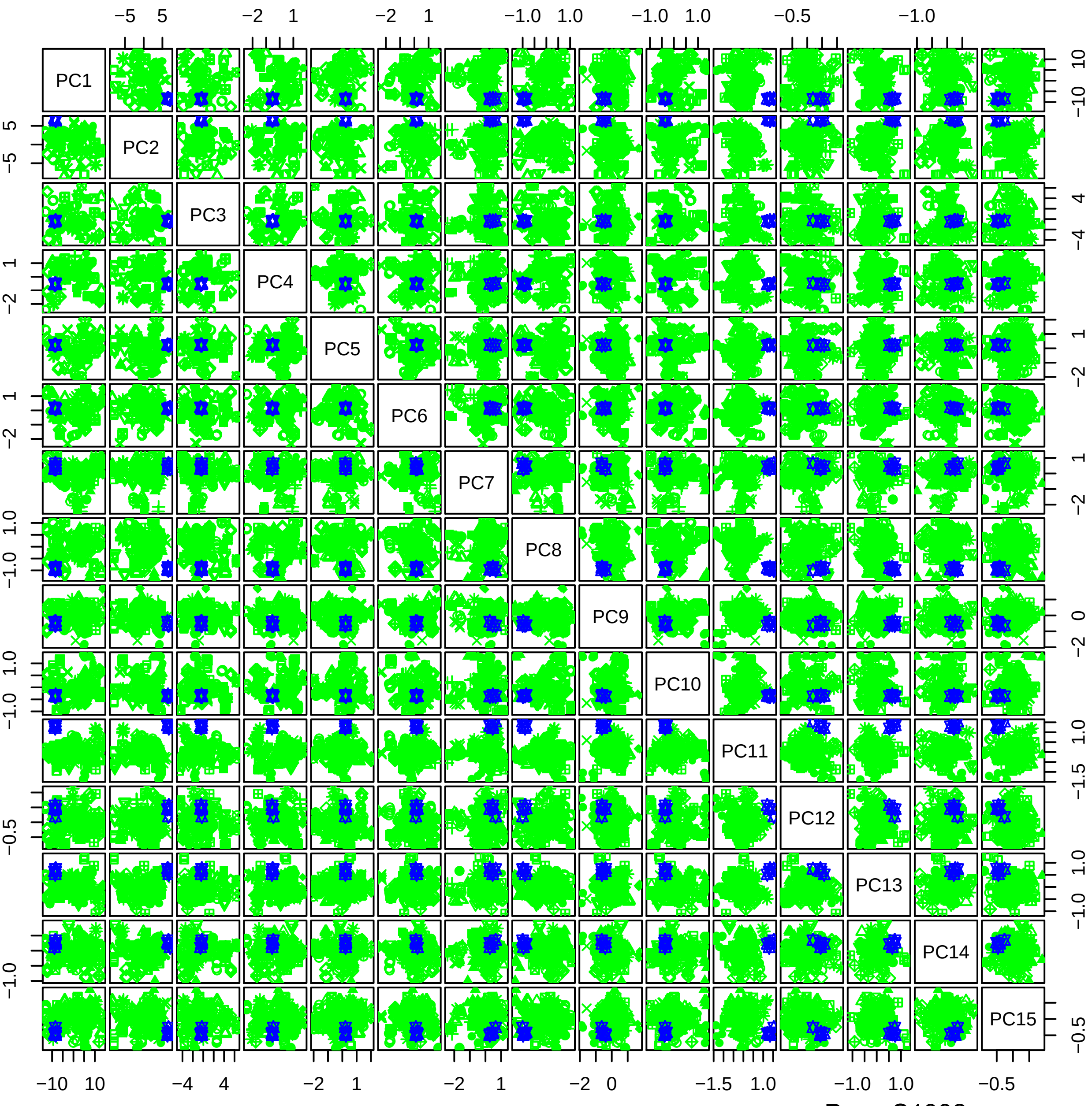

PC1
PC2
PC3
PC4
PC5
PC6
PC7
PC8
PC9
PC10
PC11
PC12
PC13
PC14
PC15
−5 5
−2 1
−2 1
−1.0 1.0
−1.0 1.0
−0.5
−1.0
−10 10
−4 4
−2 1
−2 1
−2 0
−1.5 1.0
−1.0 1.0
−0.5

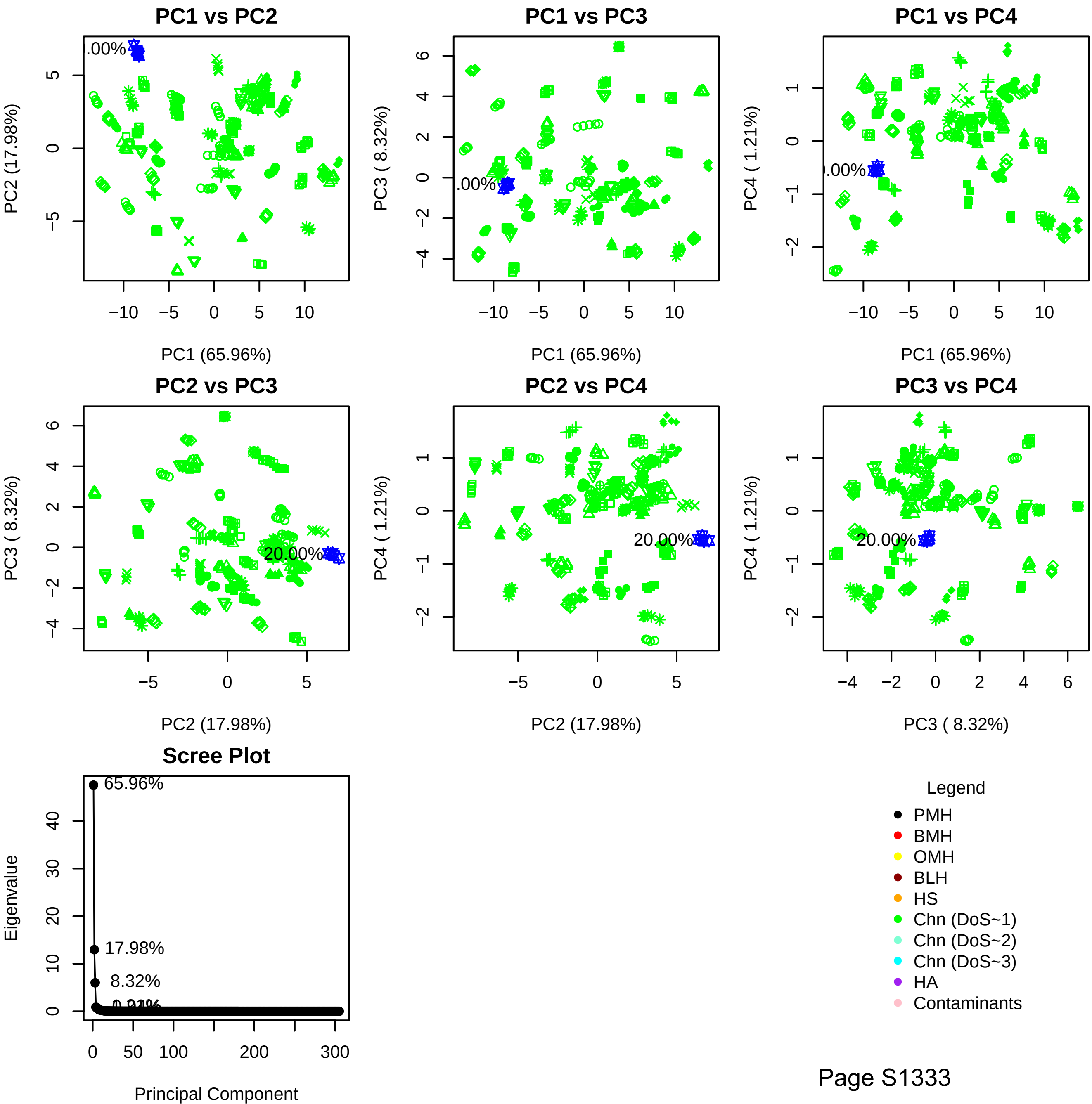
PC1 vs PC2
PC2 (17.98%)
PC1 (65.96%)
PC1 vs PC3
PC3 (8.32%)
PC1 (65.96%)
PC1 vs PC4
PC4 (1.21%)
PC1 (65.96%)
PC2 vs PC3
PC3 (8.32%)
PC2 (17.98%)
20.00%
PC2 vs PC4
PC4 (1.21%)
PC2 (17.98%)
20.00%
PC3 vs PC4
PC4 (1.21%)
PC3 (8.32%)
20.00%
Scree Plot
Eigenvalue
Principal Component
65.96%
17.98%
8.32%
Legend
PMH
BMH
OMH
BLH
HS
Chn (DoS~1)
Chn (DoS~2)
Chn (DoS~3)
HA
Contaminants

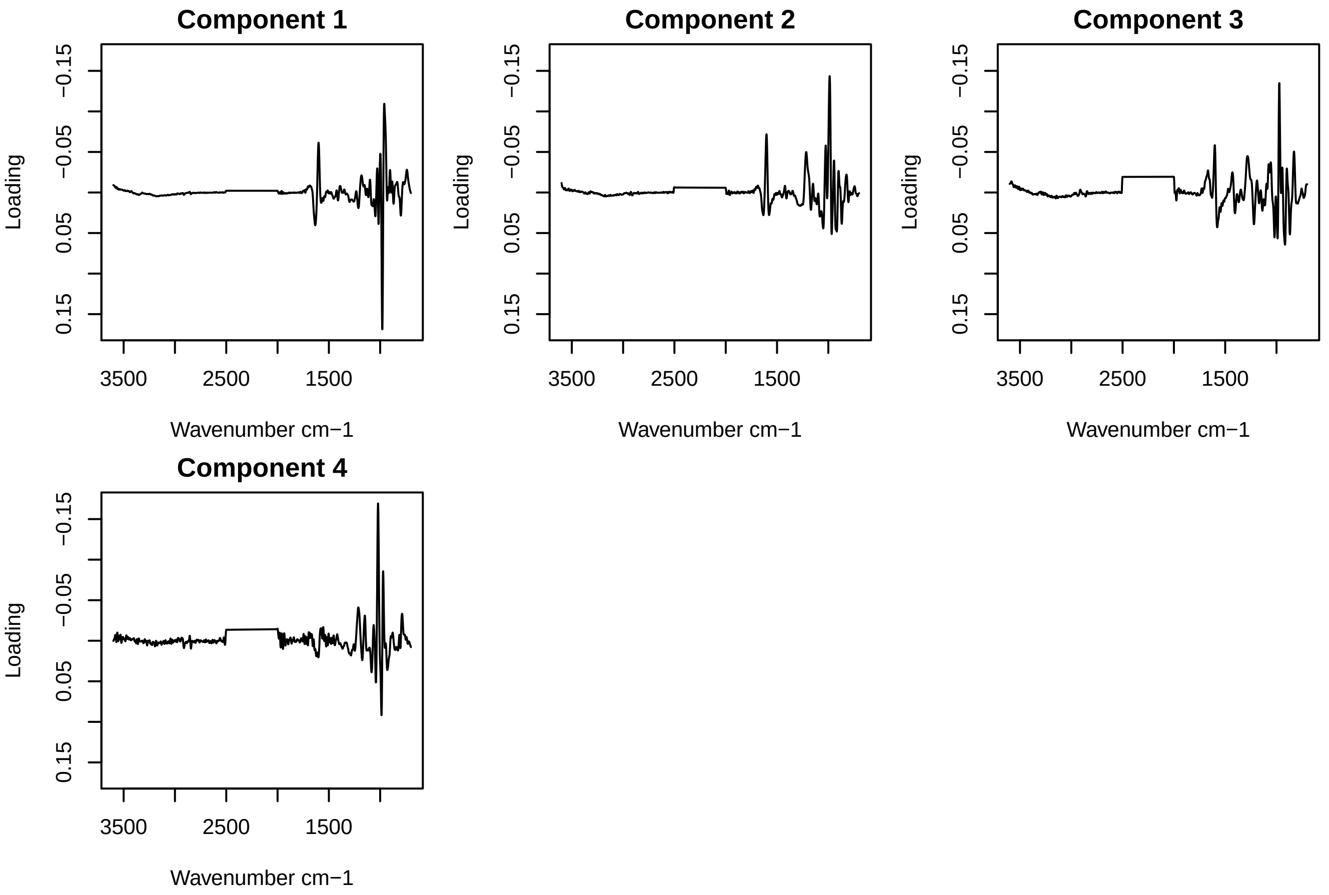
Component 1
Loading
−0.15
−0.05
0.05
0.15
3500
2500
1500
Wavenumber cm−1
Component 2
Loading
−0.15
−0.05
0.05
0.15
3500
2500
1500
Wavenumber cm−1
Component 3
Loading
−0.15
−0.05
0.05
0.15
3500
2500
1500
Wavenumber cm−1
Component 4
Loading
−0.15
−0.05
0.05
0.15
3500
2500
1500
Wavenumber cm−1

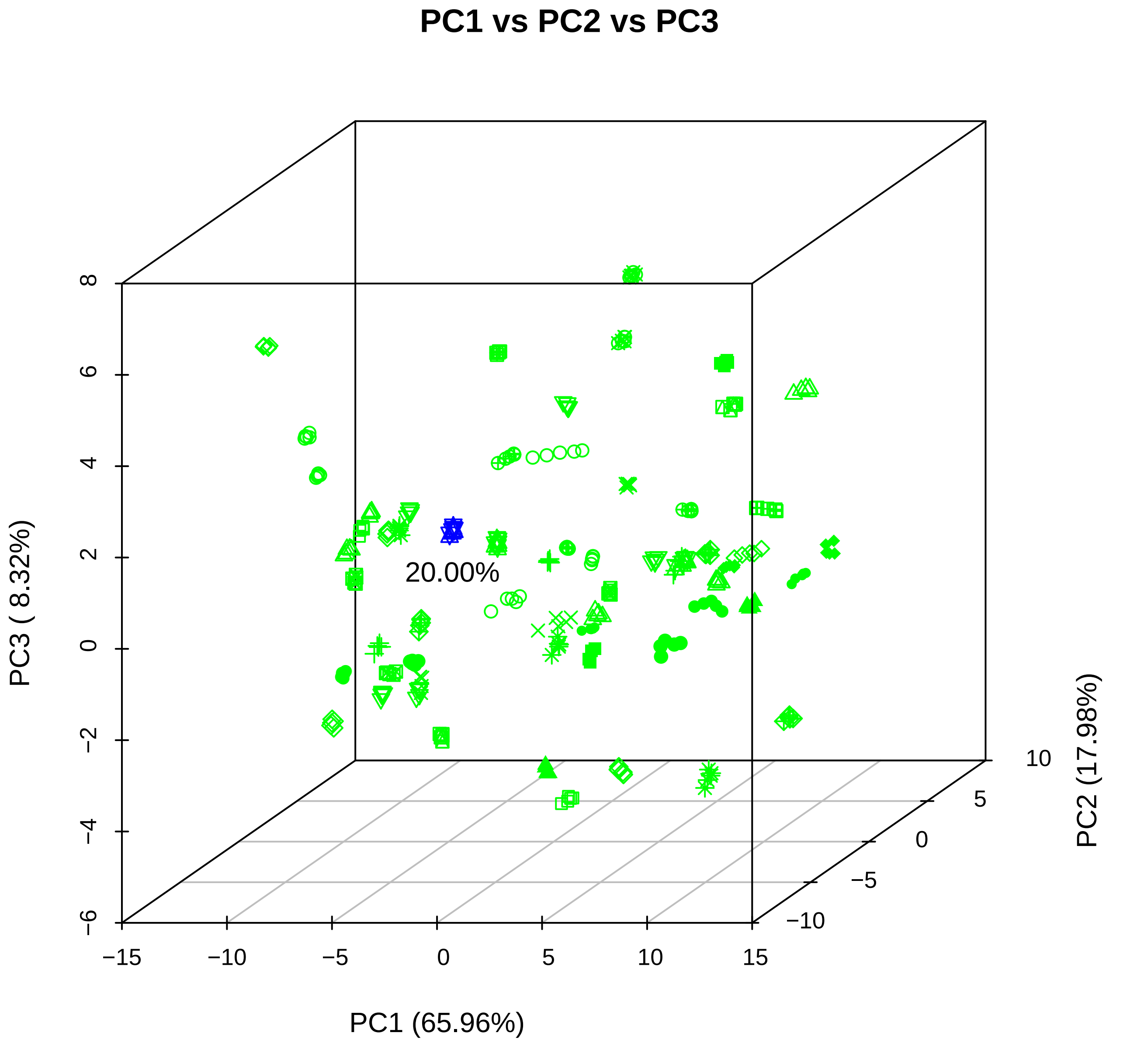

PC1 vs PC2 vs PC3
20.00%
PC3 (8.32%)
8
6
4
2
0
−2
−4
−6
−15
−10
−5
0
5
10
15
PC1 (65.96%)
PC2 (17.98%)
10
5
0
−5
−10

PC1 vs PC2 vs PC4

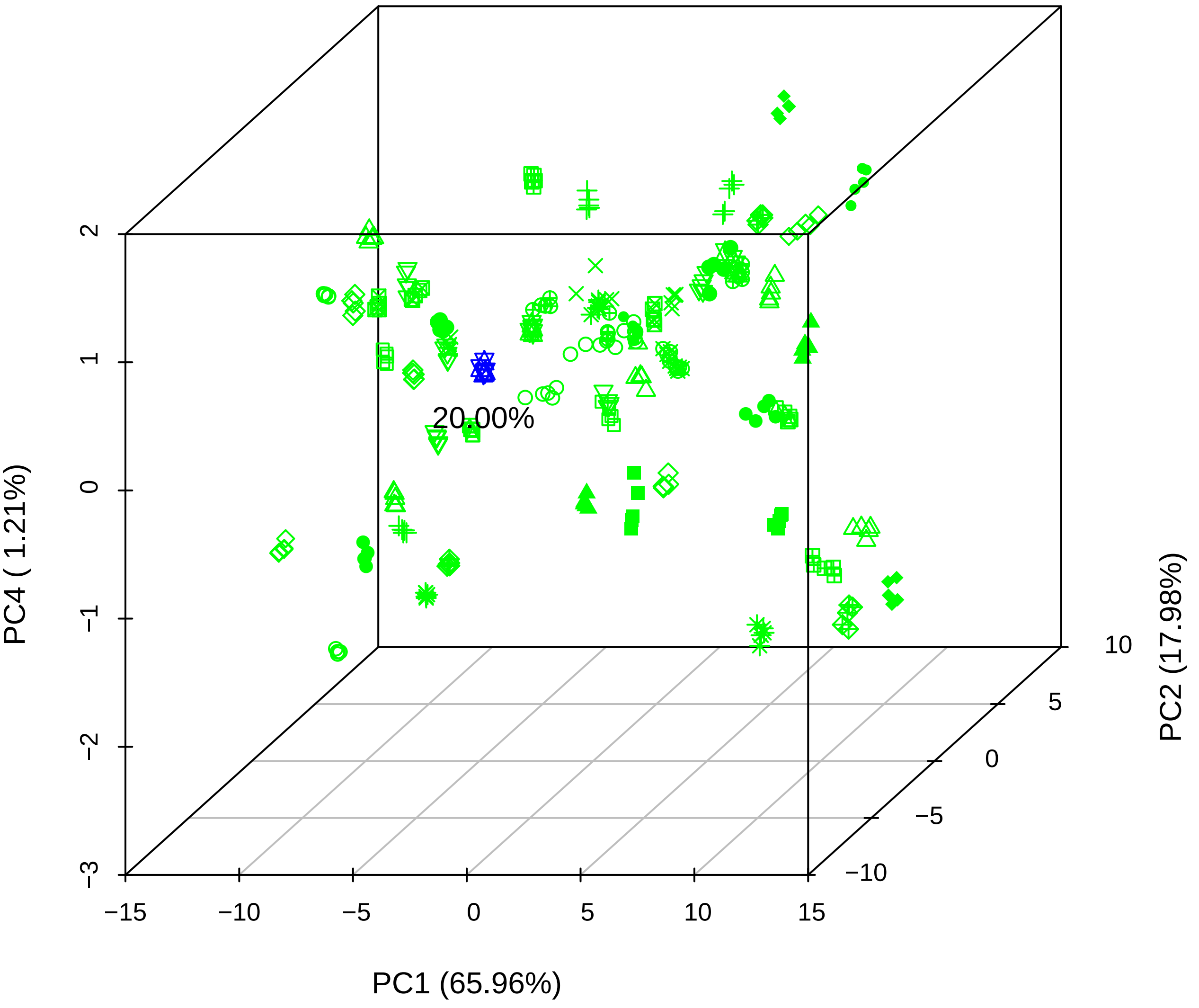

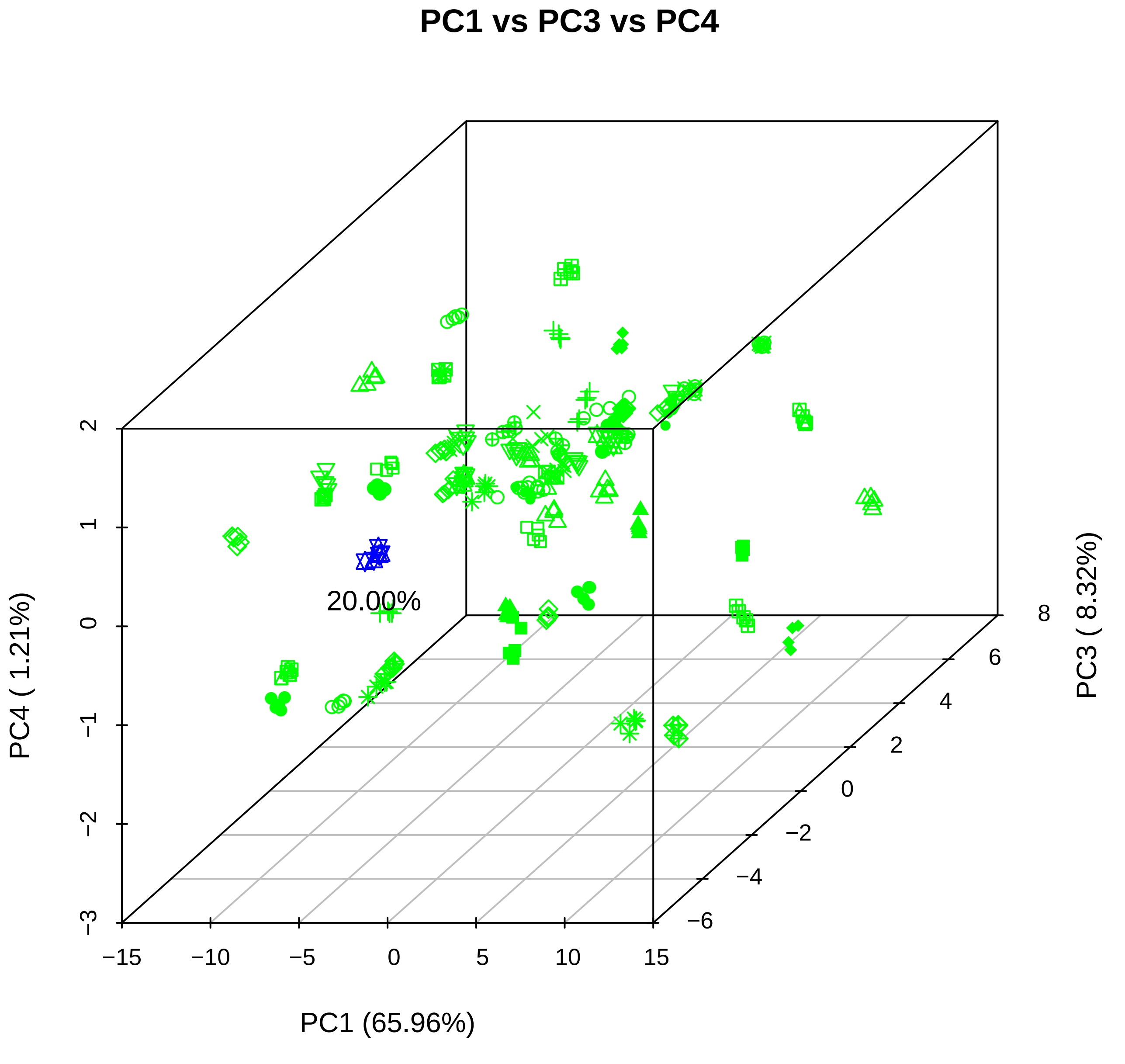
PC1 vs PC3 vs PC4
PC1 (65.96%)
PC4 (1.21%)
PC3 (8.32%)
20.00%
−15
−10
−5
0
5
10
15
−3
−2
−1
0
1
2
−6
−4
−2
0
2
4
6
8

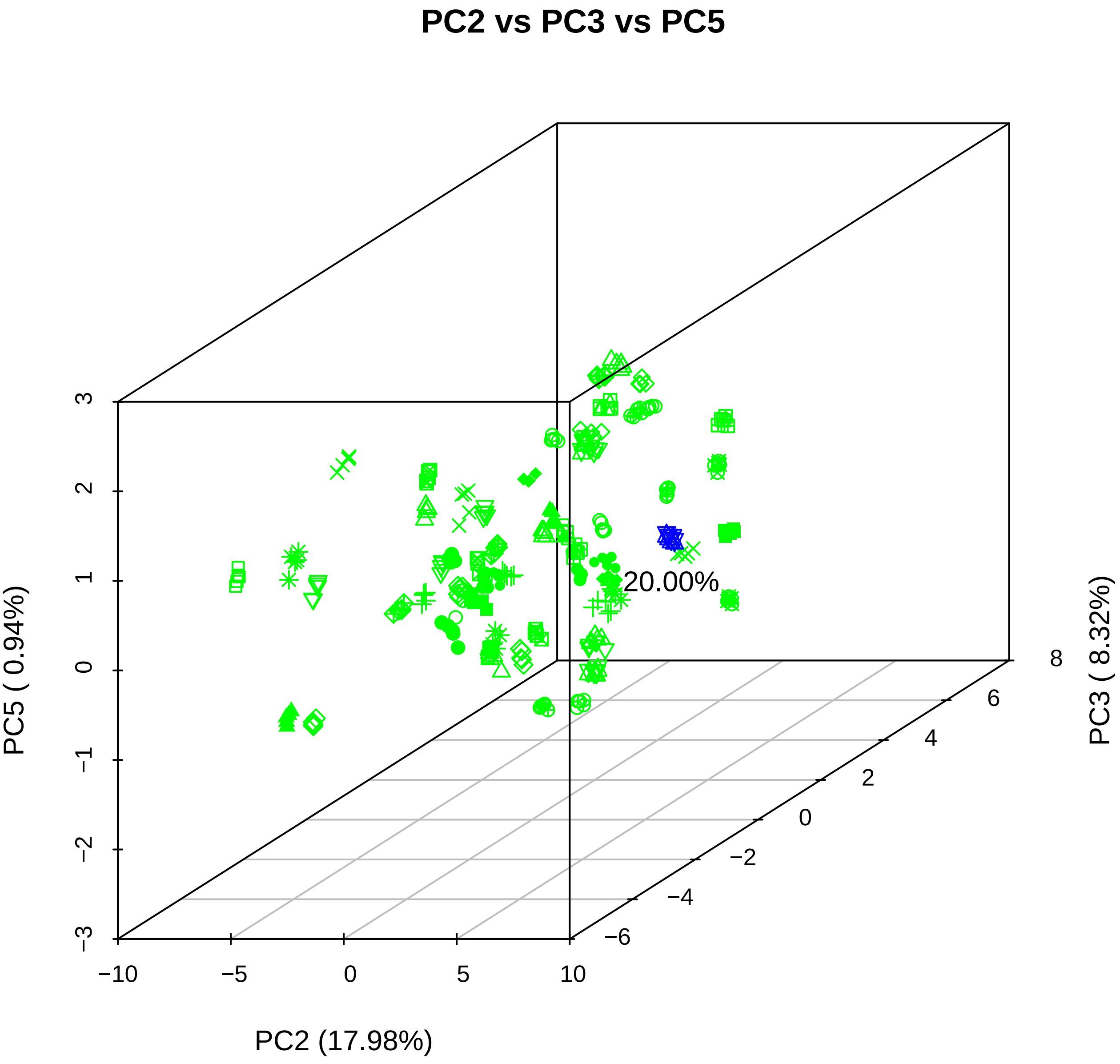
PC2 vs PC3 vs PC5
PC5 (0.94%)
PC2 (17.98%)
PC3 (8.32%)
20.00%
−10
−5
0
5
10
−3
−2
−1
0
1
2
3
−6
−4
−2
0
2
4
6
8

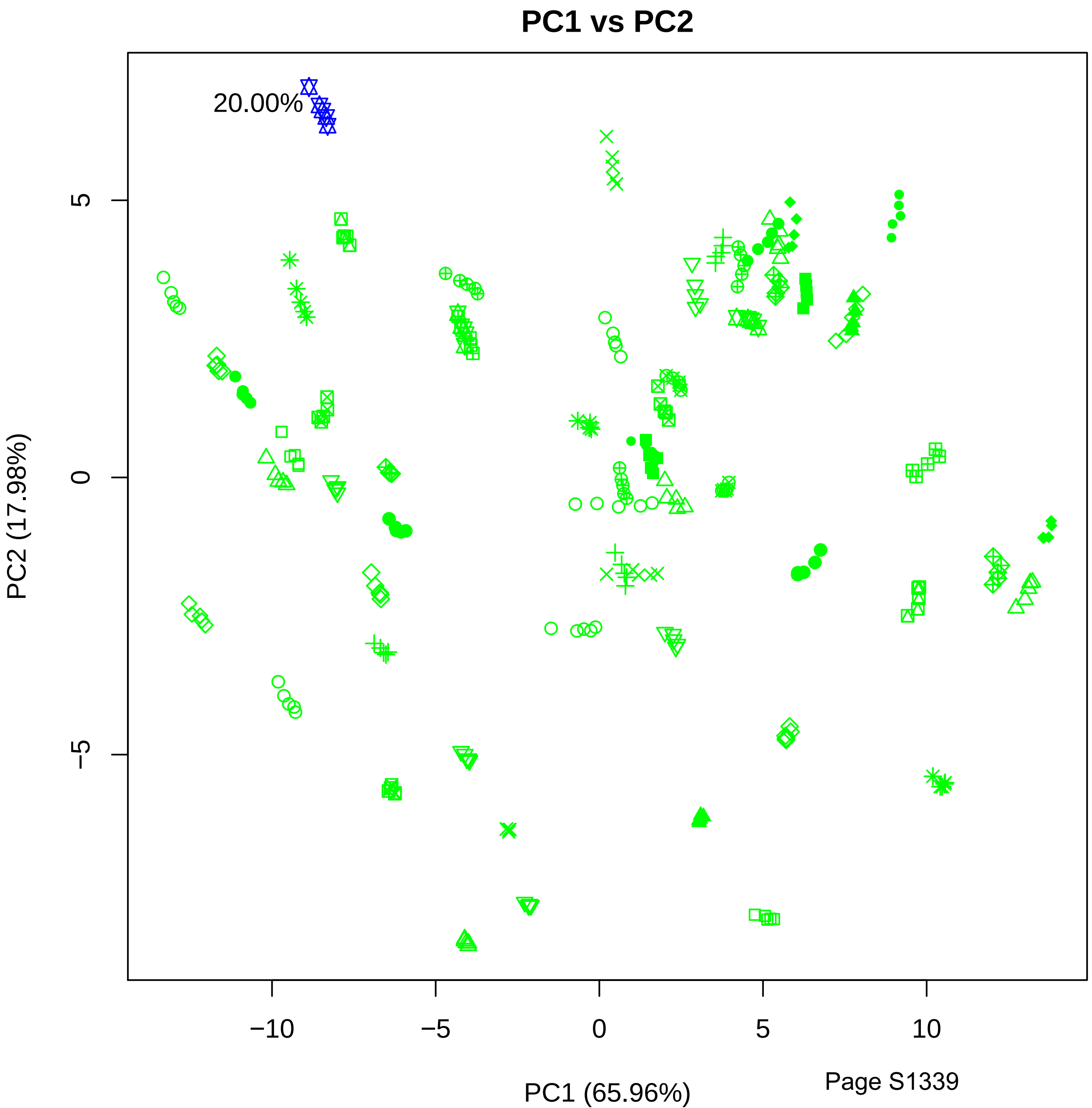

PC1 vs PC2
20.00%
PC2 (17.98%)
PC1 (65.96%)
−10
−5
0
5
10

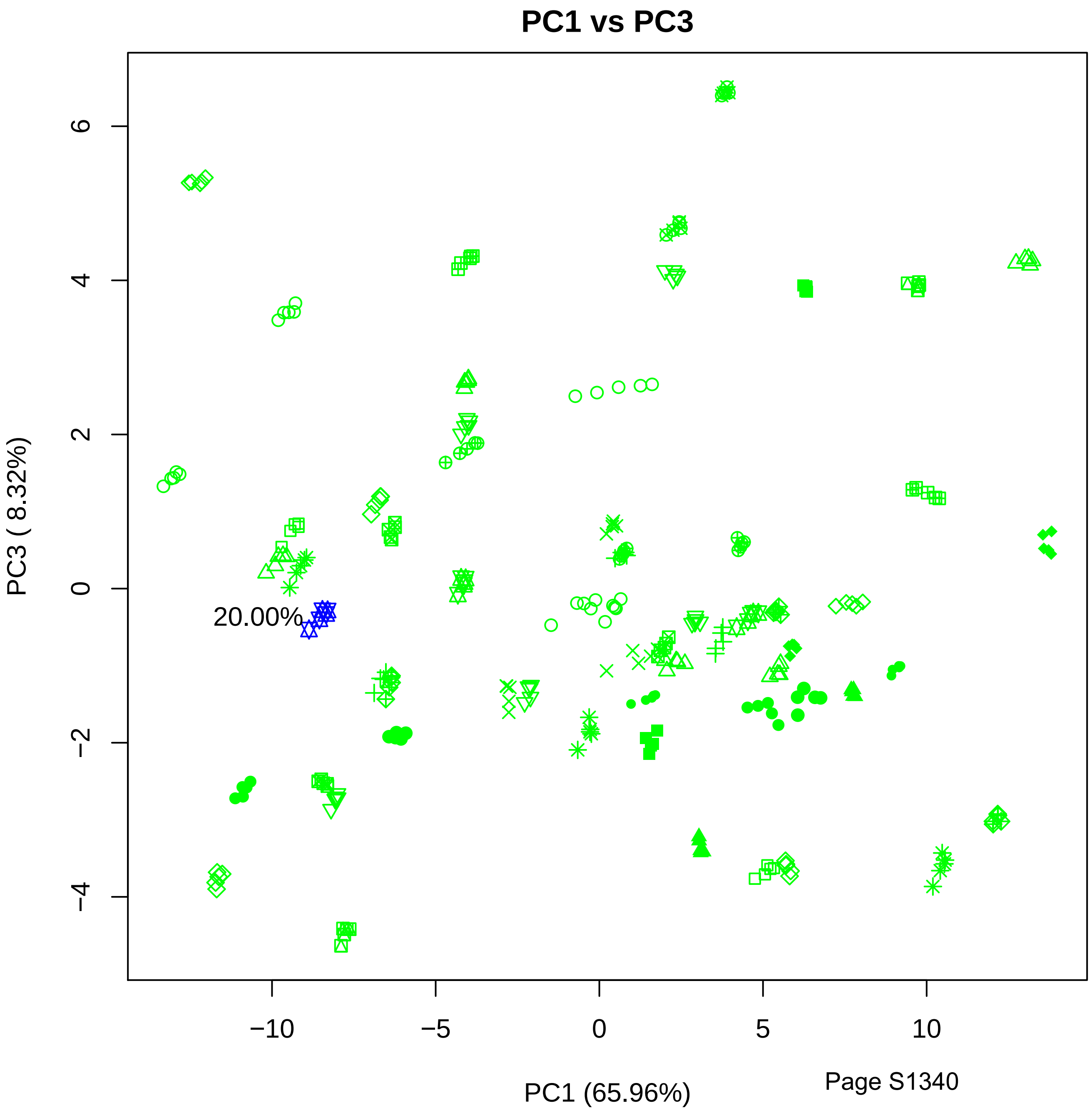

PC1 vs PC3
PC3 (8.32%)
PC1 (65.96%)
20.00%
−10
−5
0
5
10
−4
−2
0
2
4
6

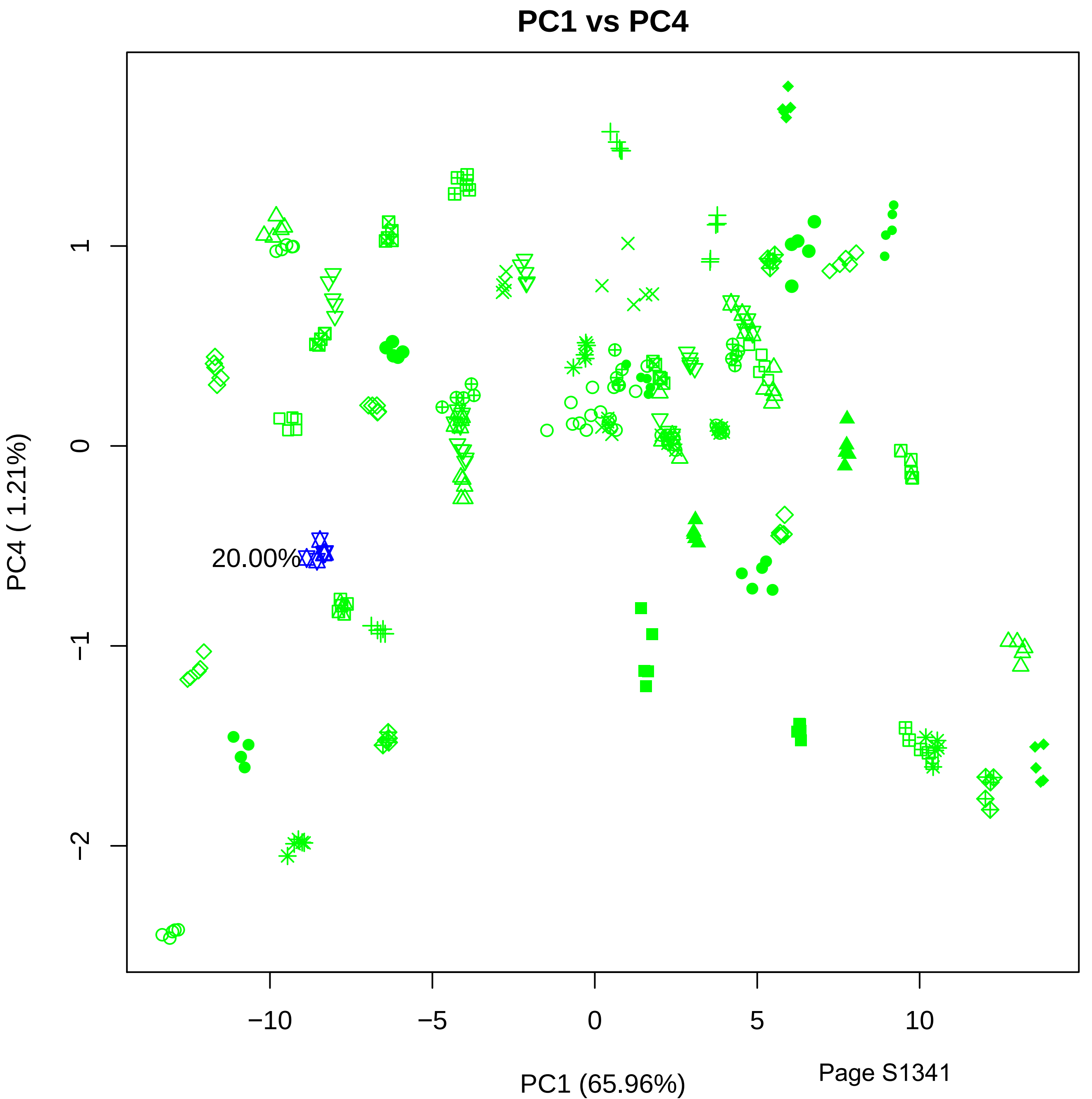

PC1 vs PC4
20.00%
PC4 (1.21%)
PC1 (65.96%)
−10
−5
0
5
10
−2
−1
0
1

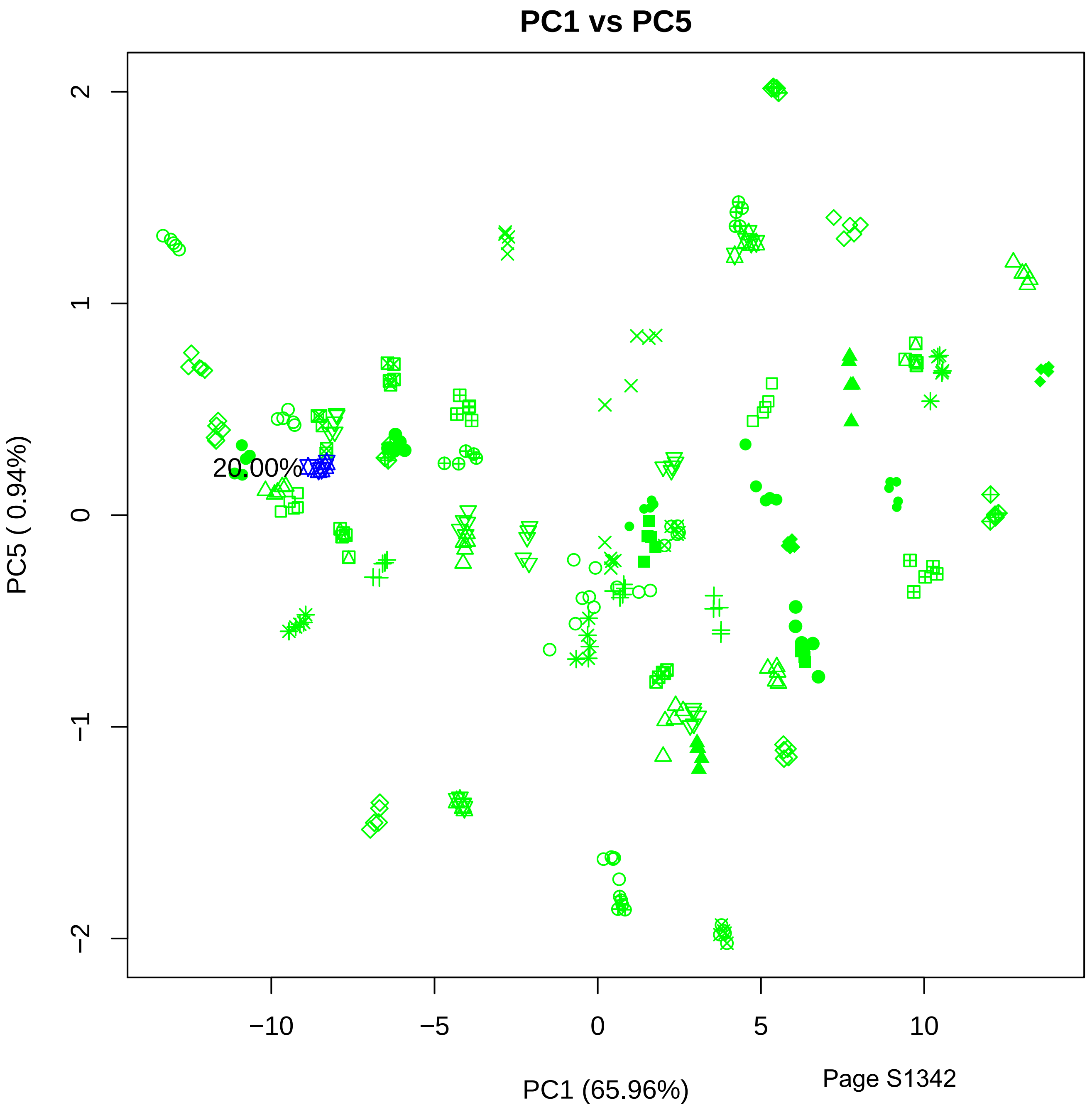

PC1 vs PC5
PC5 (0.94%)
PC1 (65.96%)
20.00%
2
1
0
−1
−2
−10
−5
0
5
10

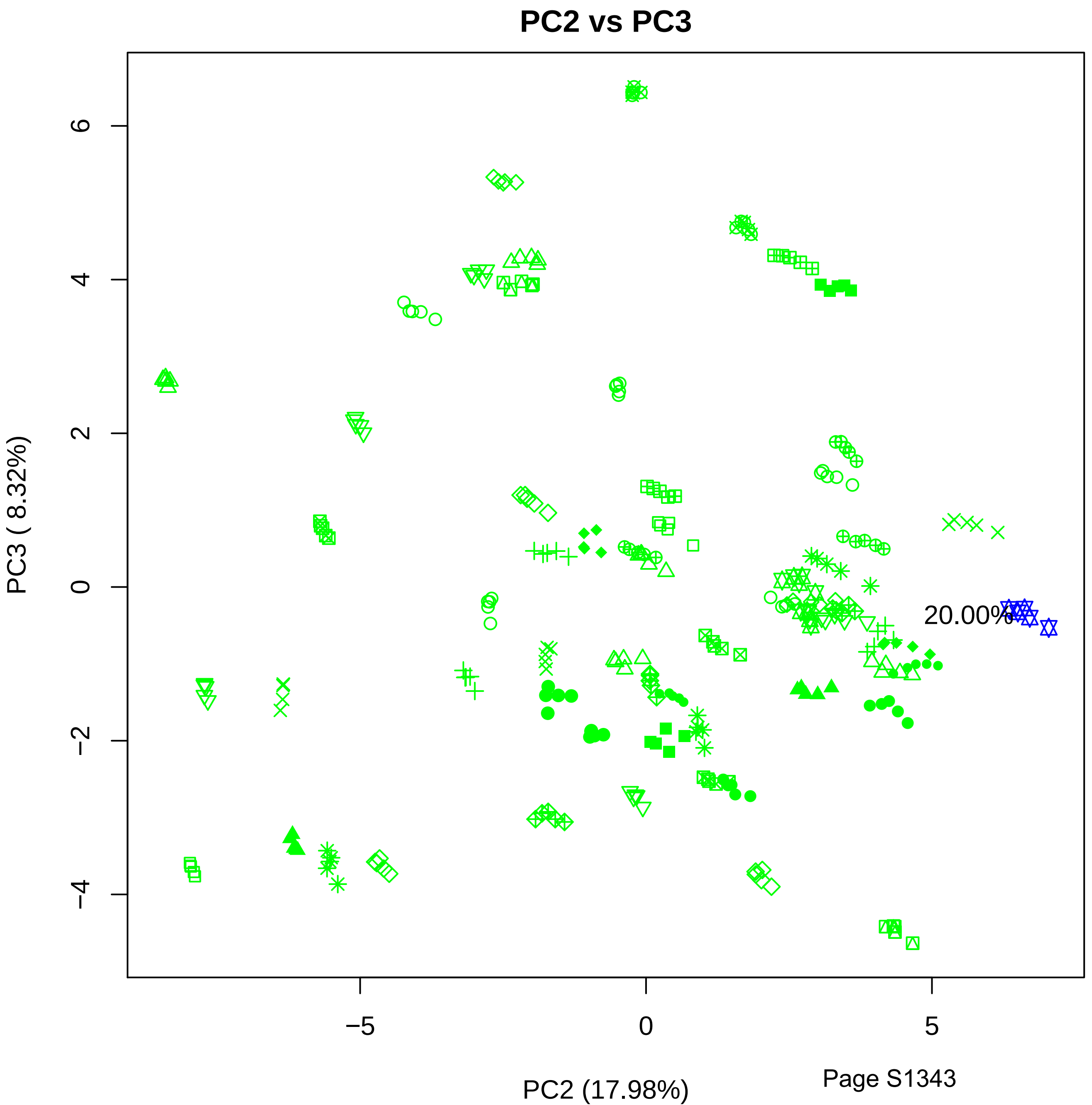
PC2 vs PC3
PC3 (8.32%)
PC2 (17.98%)
6
4
2
0
−2
−4
−5
0
5
20.00%

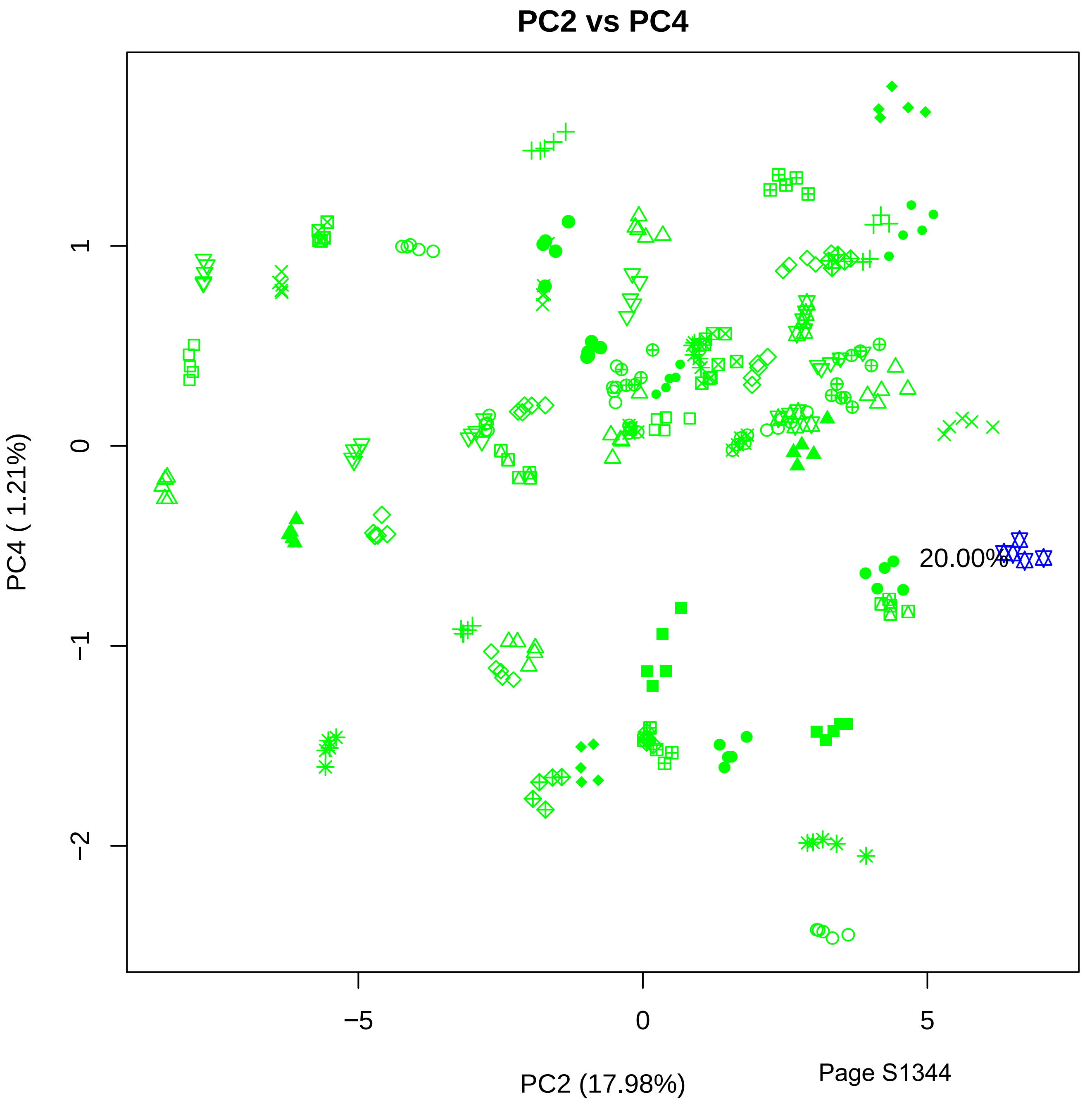
PC2 vs PC4
PC4 (1.21%)
PC2 (17.98%)
20.00%
1
0
−1
−2
−5
0
5

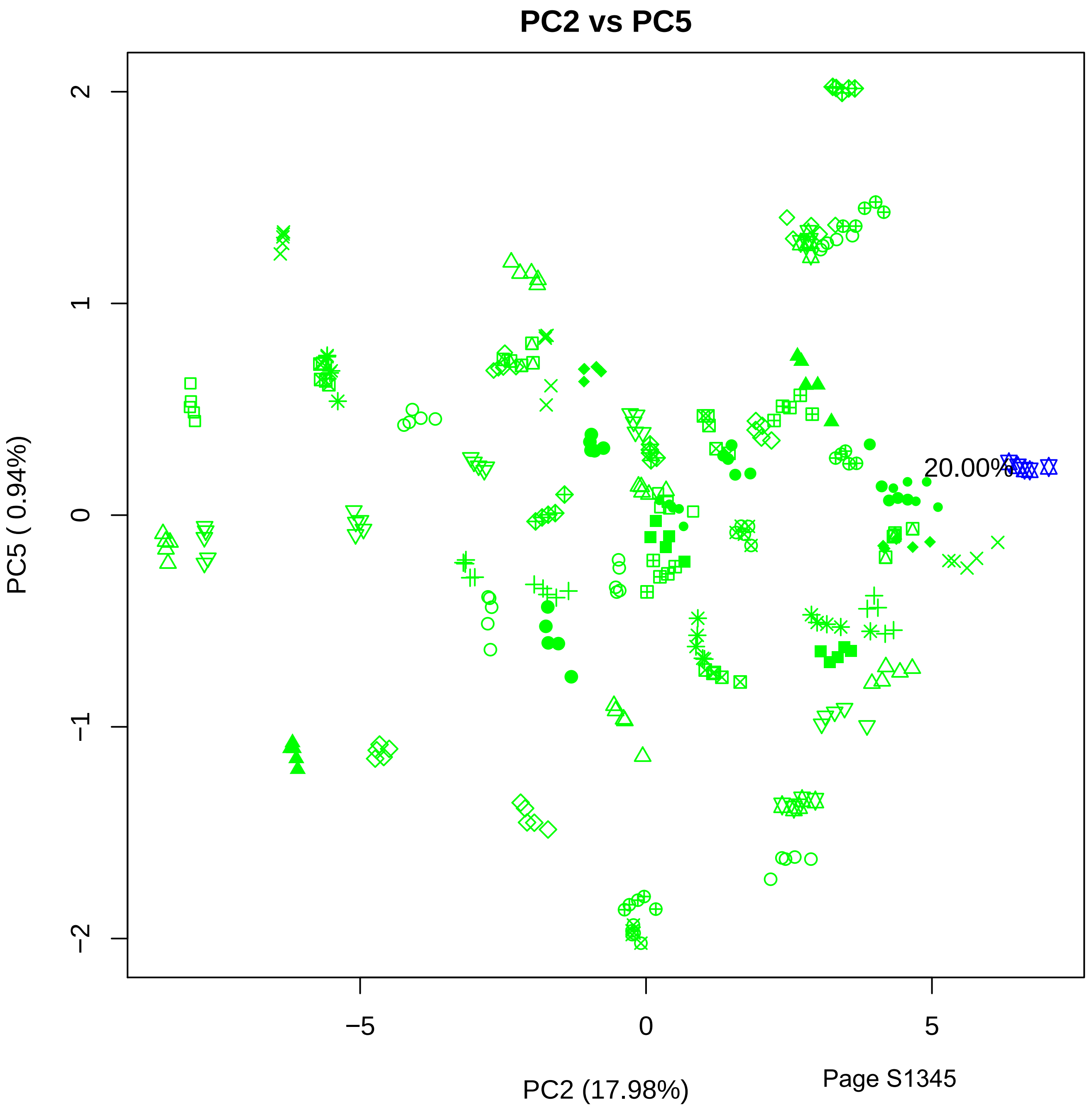

PC2 vs PC5
PC5 (0.94%)
PC2 (17.98%)
20.00%
2
1
0
−1
−2
−5
0
5

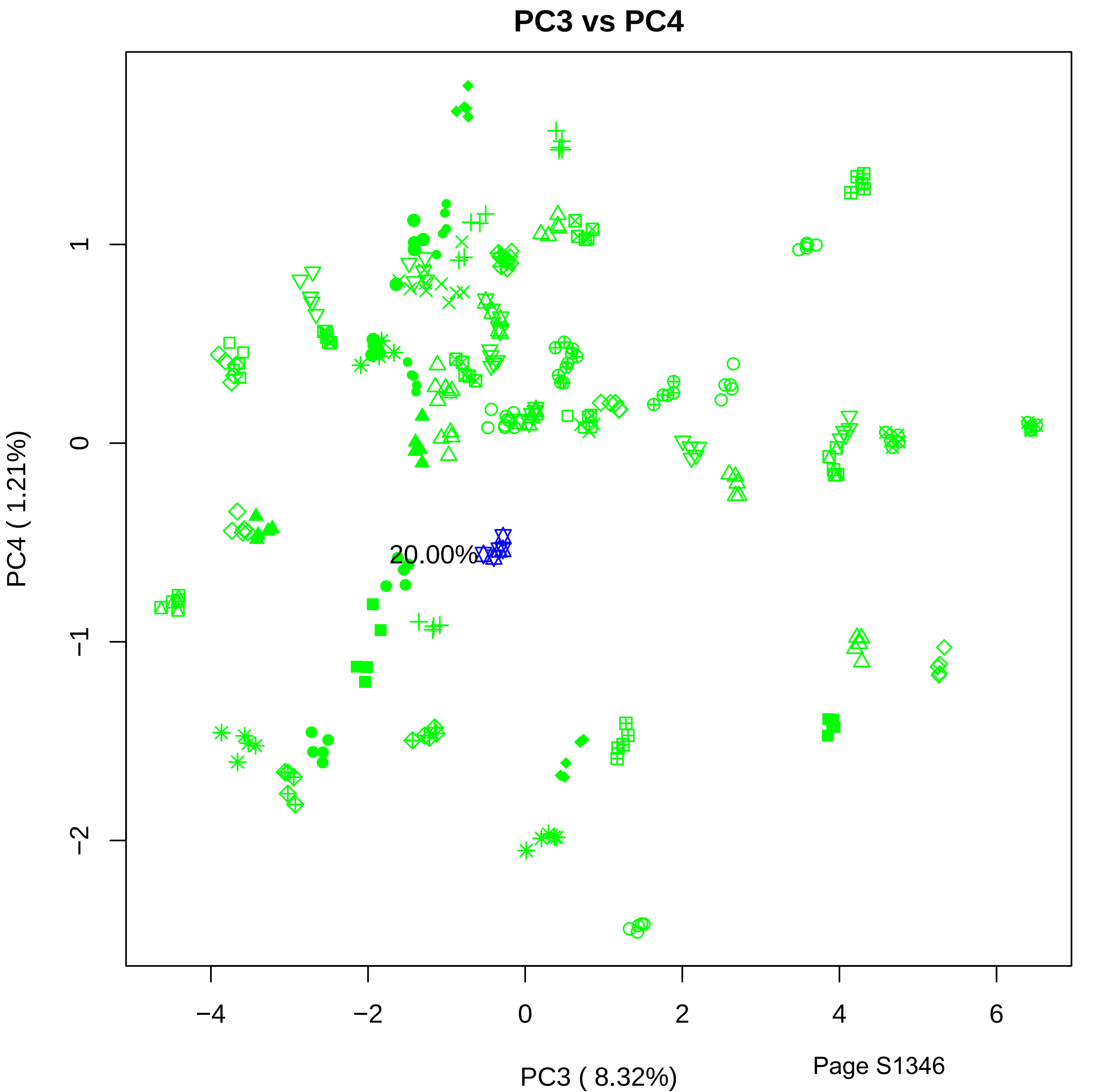

PC3 vs PC4
PC4 (1.21%)
PC3 (8.32%)
20.00%
1
0
−1
−2
−4
−2
0
2
4
6

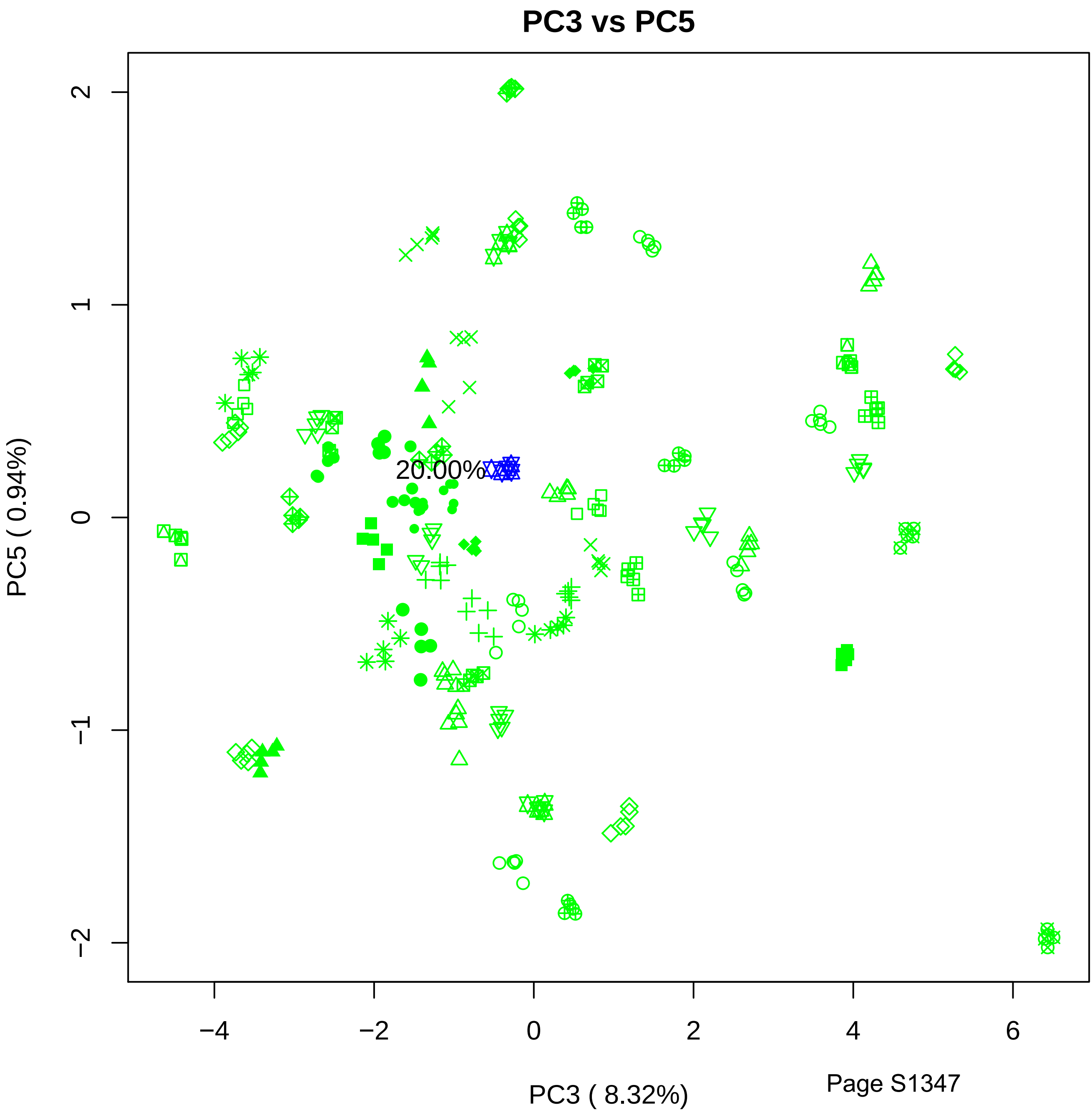

PC3 vs PC5
20.00%
PC5 (0.94%)
PC3 (8.32%)
2
1
0
−1
−2
−4
−2
0
2
4
6

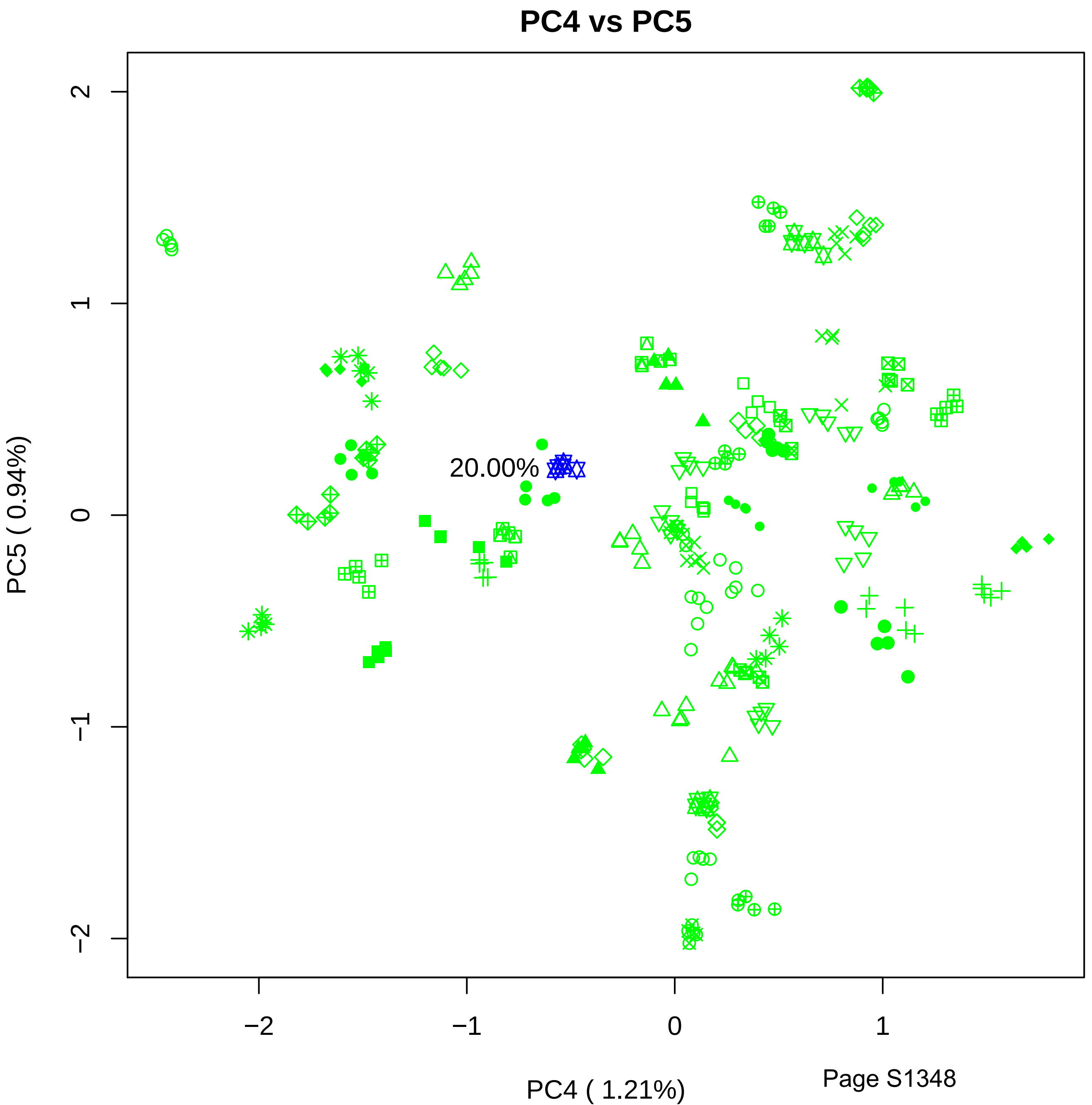

PC4 vs PC5
20.00%
PC5 (0.94%)
PC4 (1.21%)
2
1
0
−1
−2
−2
−1
0
1

PCA of 40% BMH(c) vs PMH(c)

Scaled Matrix PCAed

7th order polynomial baseline correction performed

Raw spectra smoothed to 21 Neighbours, 2nd order PN, Savtisky Golay

Normalisation between 0 and 100

D2 spectra smoothed to 41 Neighbours, 2nd order PN, Savtisky Golay

Regions Analysed: 3601−2501 and 2001−700cm−1

Samples Analysed: 1 bContam's 0 BLH's 0 BMH's 0 OMH's 0 pContam's 0 PLH's 60 PMH's

Keele University AD

Fri Dec 14 14:19:36 2018

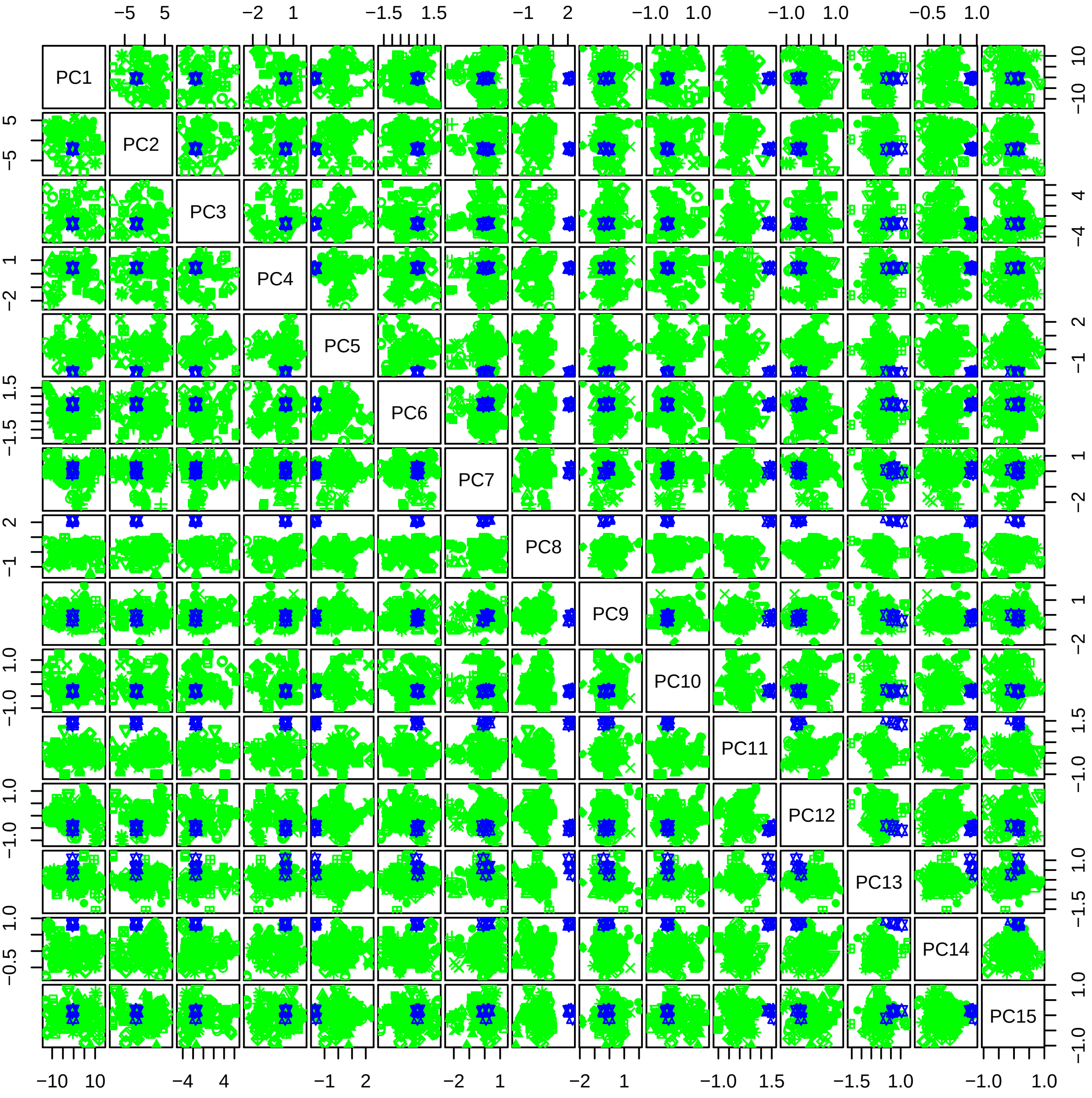
PC1
PC2
PC3
PC4
PC5
PC6
PC7
PC8
PC9
PC10
PC11
PC12
PC13
PC14
PC15

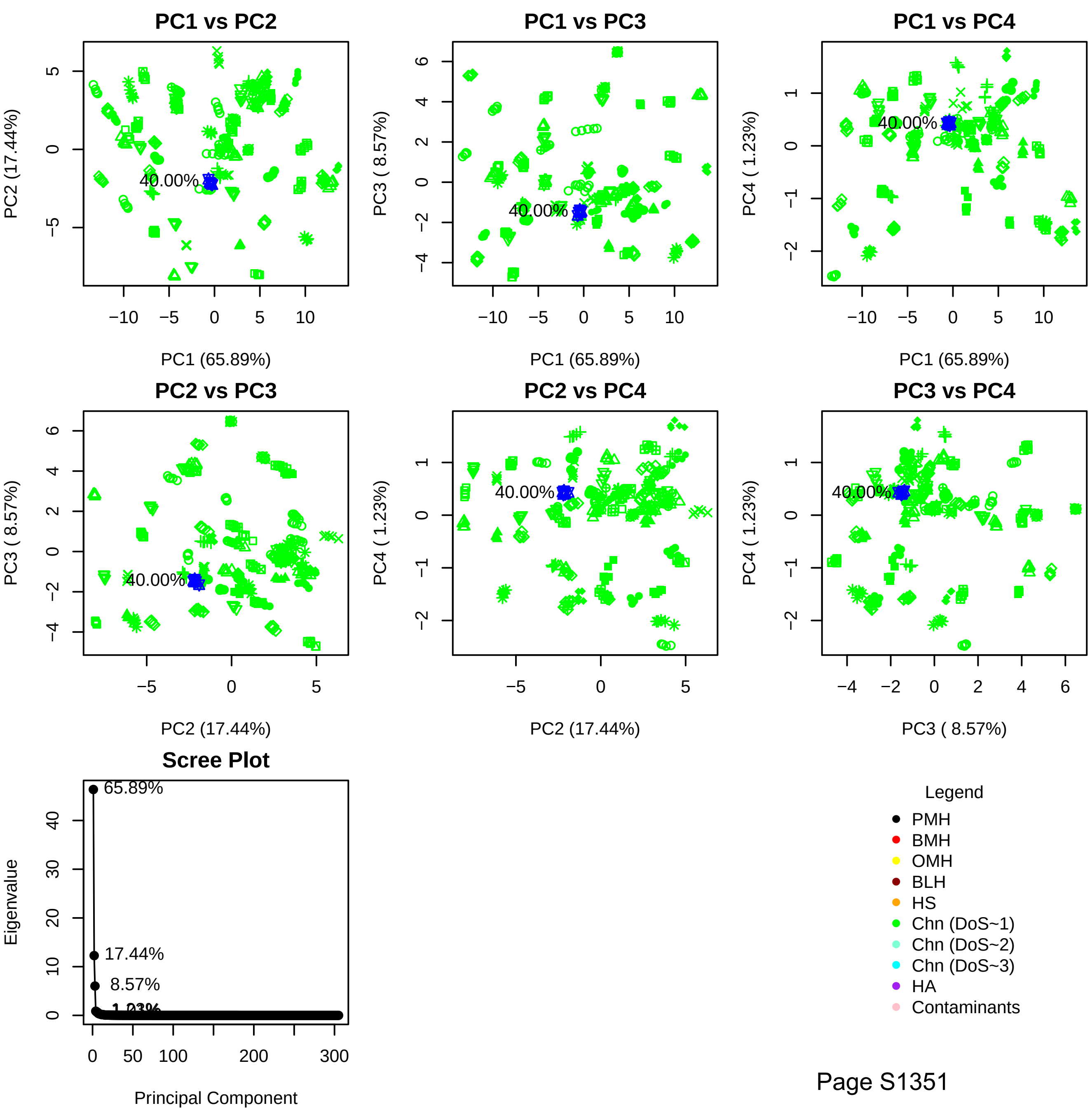

PC1 vs PC2
PC1 vs PC3
PC1 vs PC4
PC2 vs PC3
PC2 vs PC4
PC3 vs PC4
Scree Plot
PC1 (65.89%)
PC2 (17.44%)
PC3 (8.57%)
PC4 (1.23%)
40.00%
Eigenvalue
Principal Component
65.89%
17.44%
8.57%
Legend
PMH
BMH
OMH
BLH
HS
Chn (DoS~1)
Chn (DoS~2)
Chn (DoS~3)
HA
Contaminants

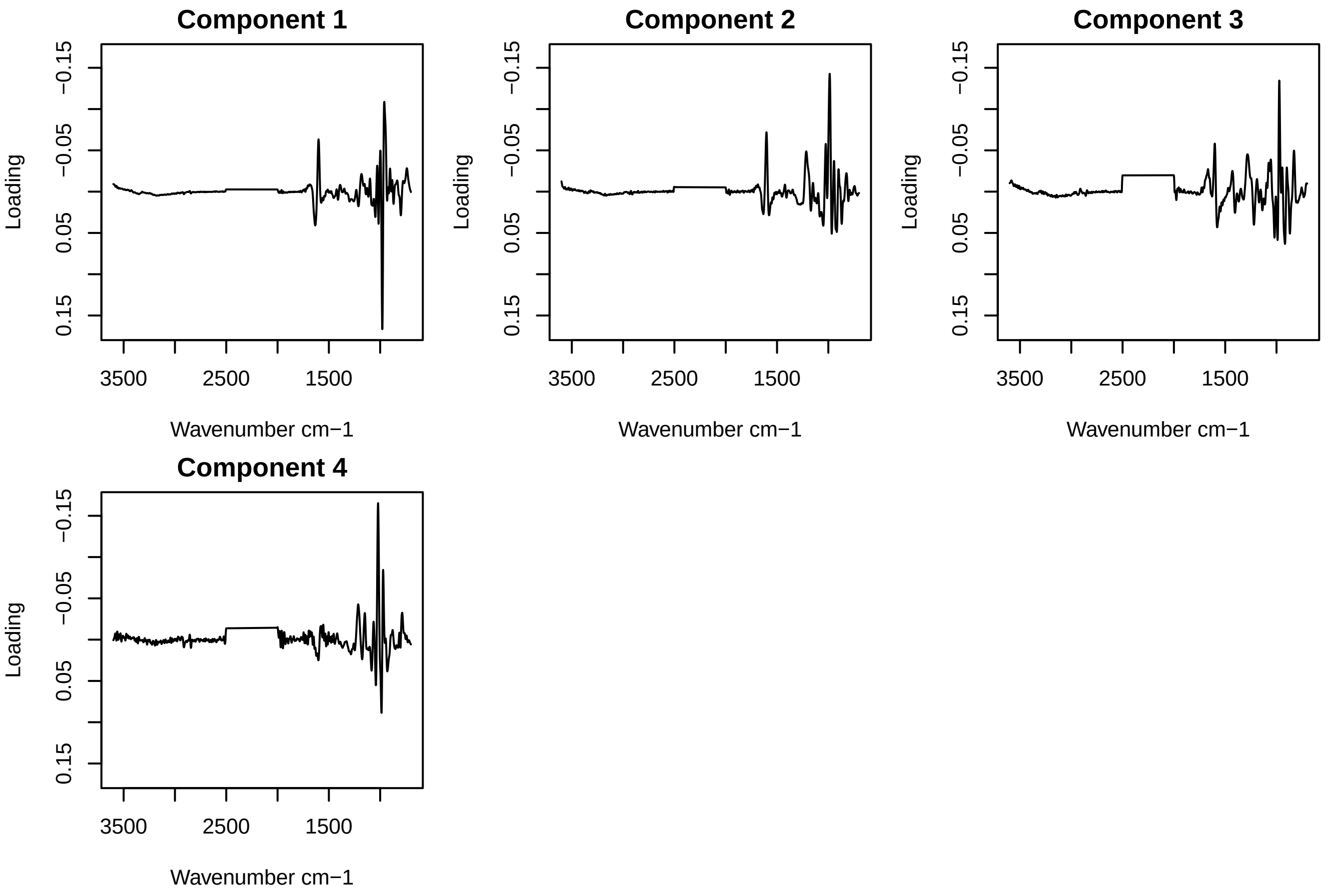
Component 1
Component 2
Component 3
Component 4
Loading
−0.15
−0.05
0.05
0.15
3500
2500
1500
Wavenumber cm−1

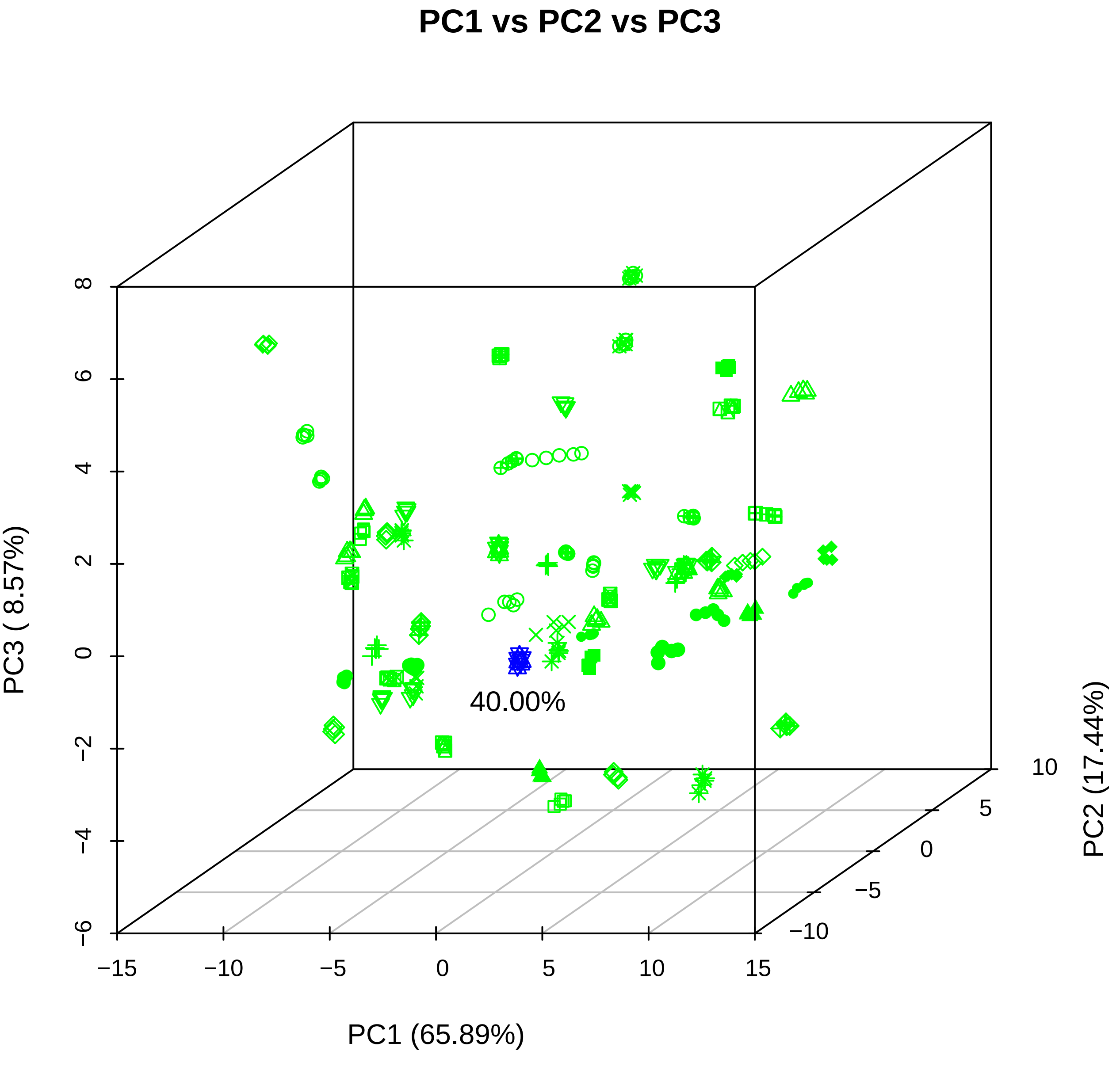
PC1 vs PC2 vs PC3
PC1 (65.89%)
PC2 (17.44%)
PC3 (8.57%)
40.00%
−15
−10
−5
0
5
10
15
−6
−4
−2
0
2
4
6
8
−10
−5
0
5
10

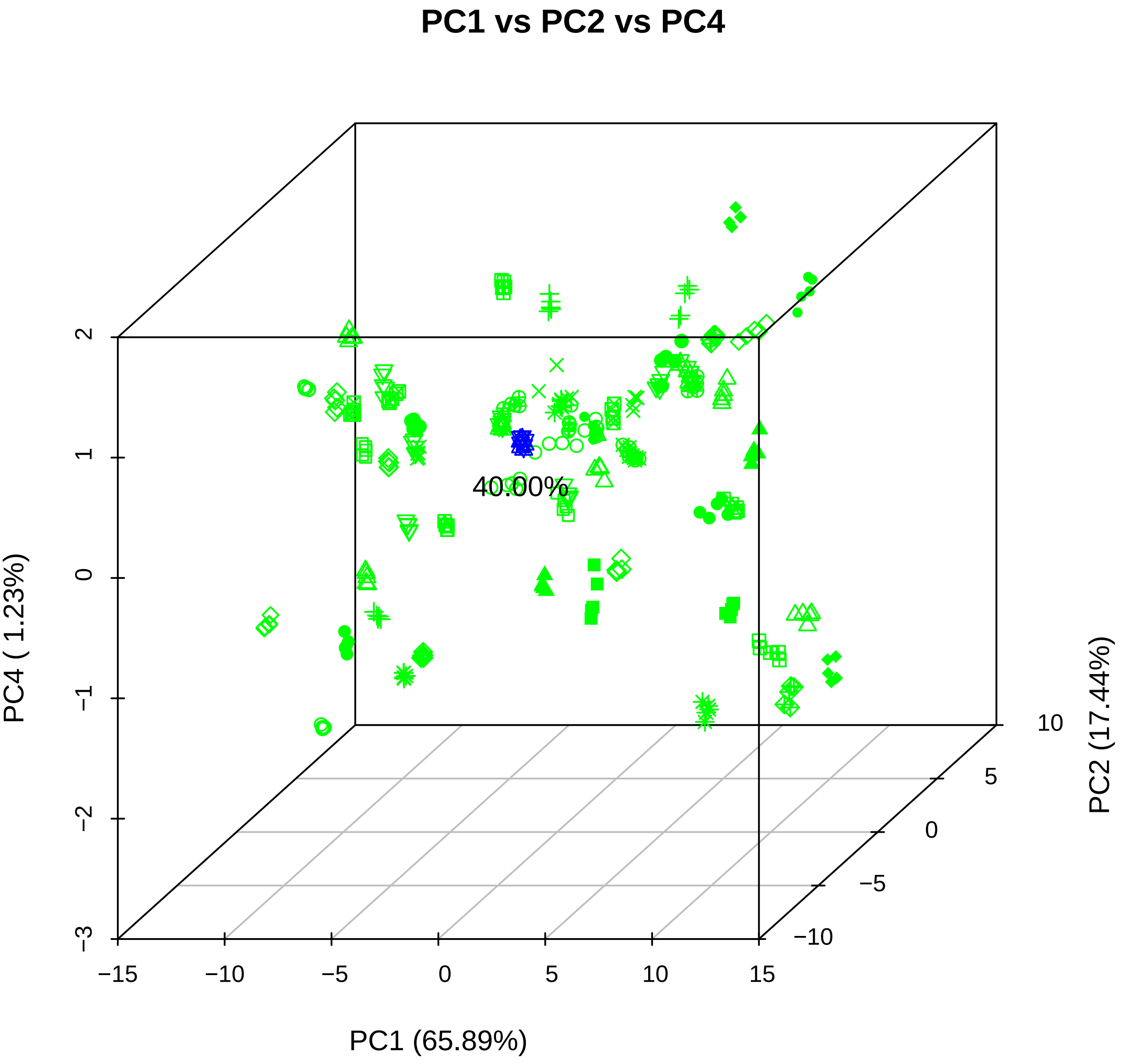

PC1 vs PC2 vs PC4
40.00%
PC1 (65.89%)
PC2 (17.44%)
PC4 (1.23%)
−15
−10
−5
0
5
10
15
−10
−5
0
5
10
−3
−2
−1
0
1
2

PC1 vs PC3 vs PC4

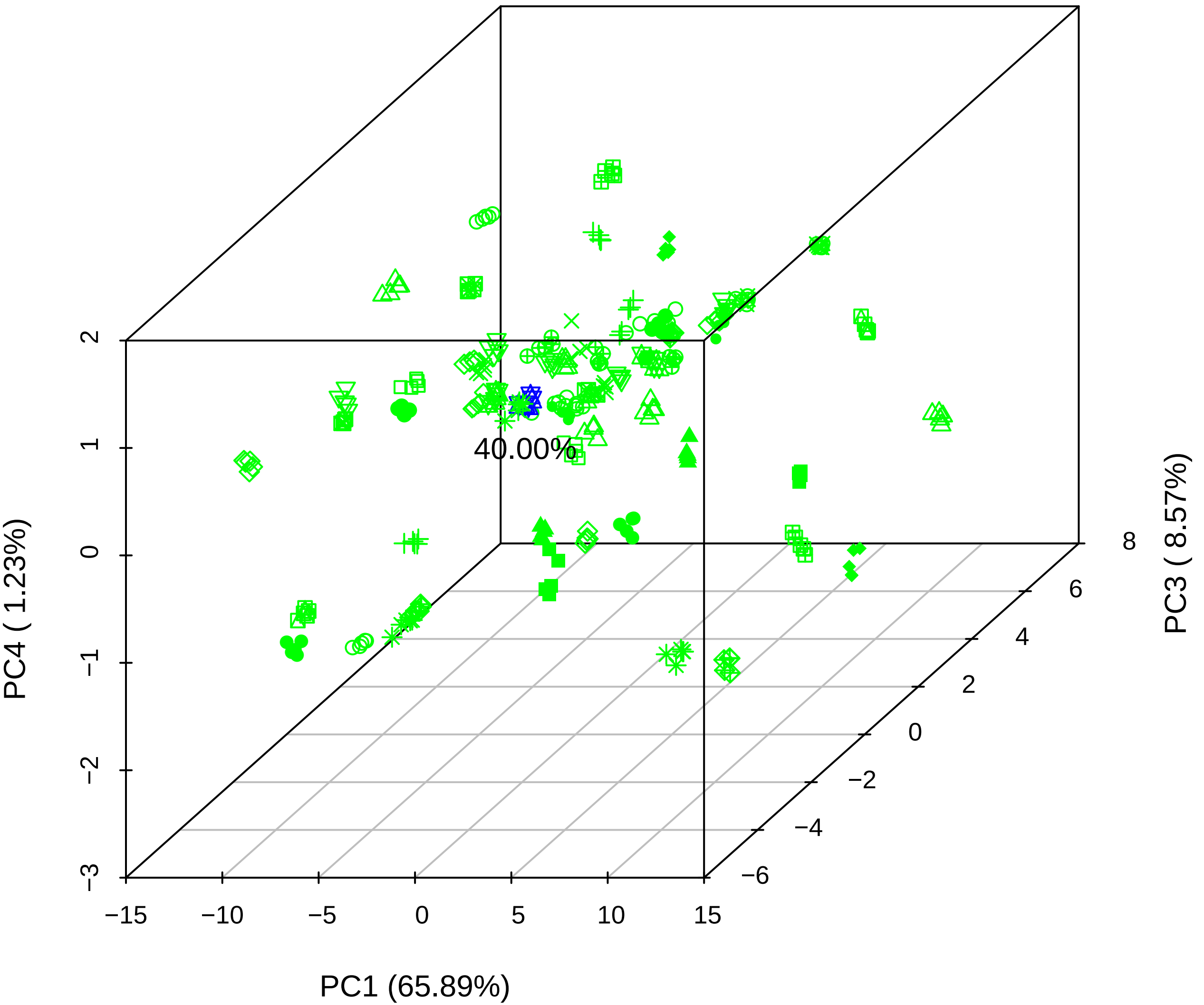

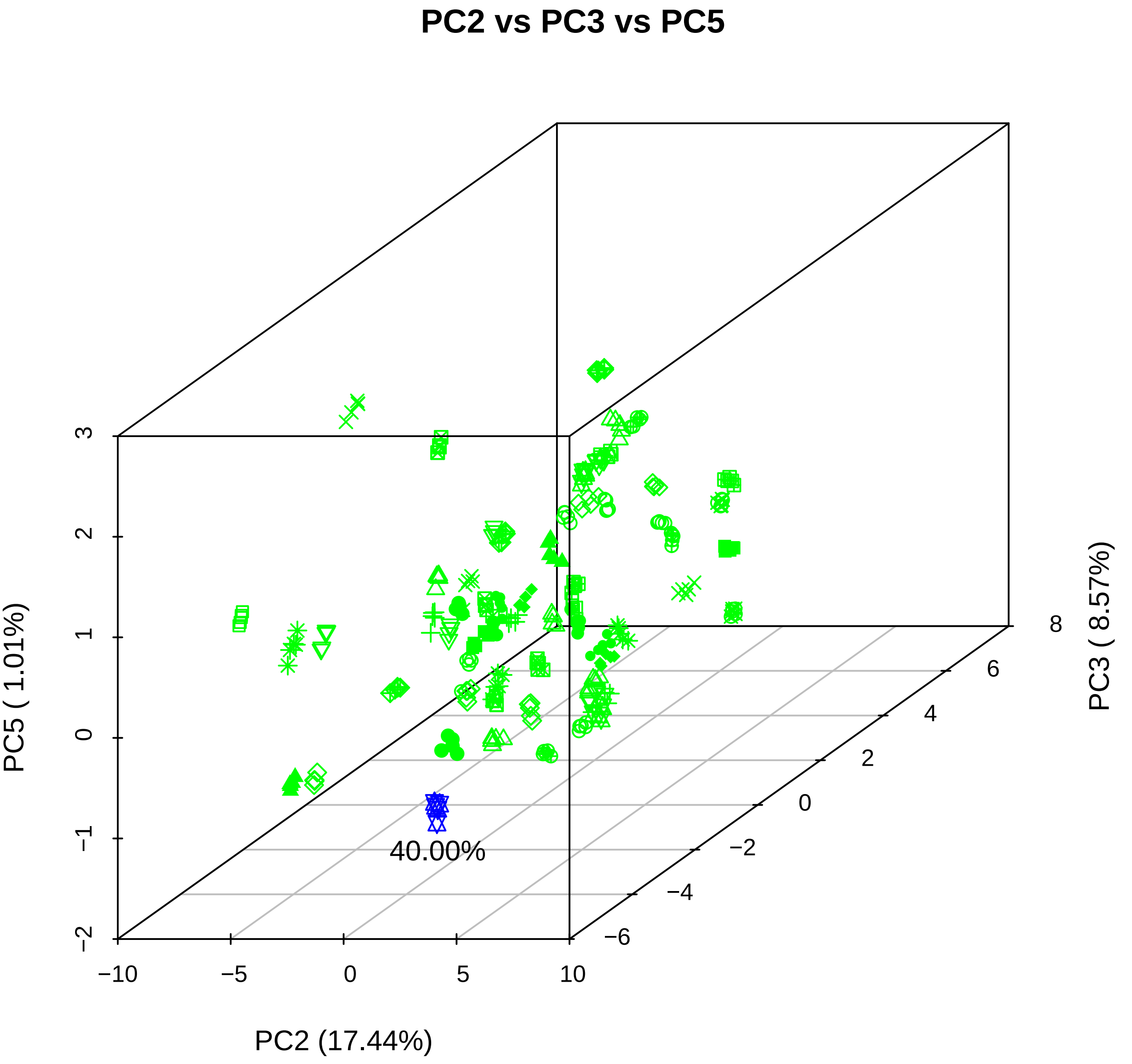

PC2 vs PC3 vs PC5
PC5 (1.01%)
PC2 (17.44%)
PC3 (8.57%)
40.00%
−10
−5
0
5
10
−2
−1
0
1
2
3
−6
−4
−2
0
2
4
6
8

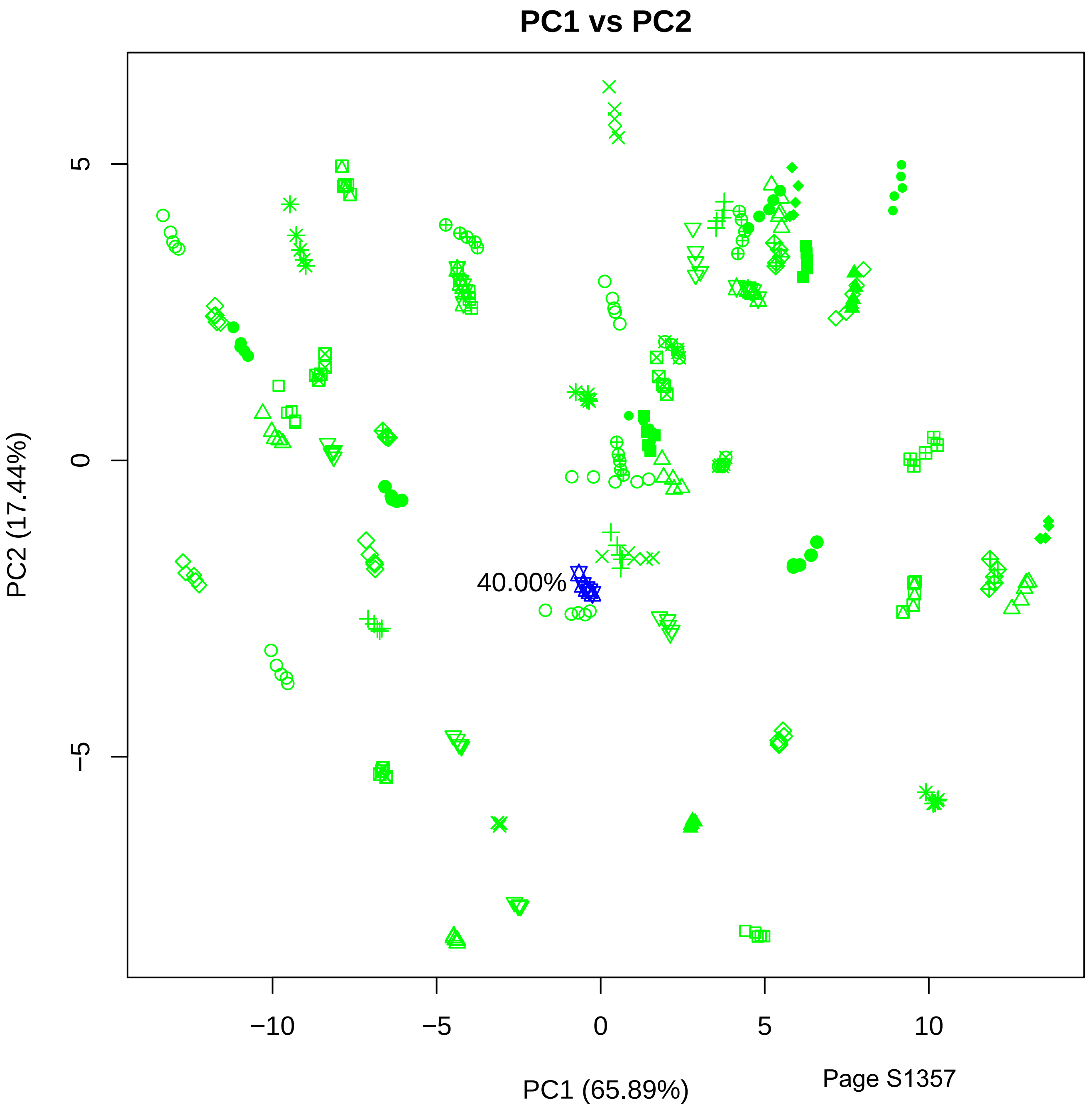
PC1 vs PC2
PC2 (17.44%)
PC1 (65.89%)
40.00%
5
0
−5
−10
−5
0
5
10

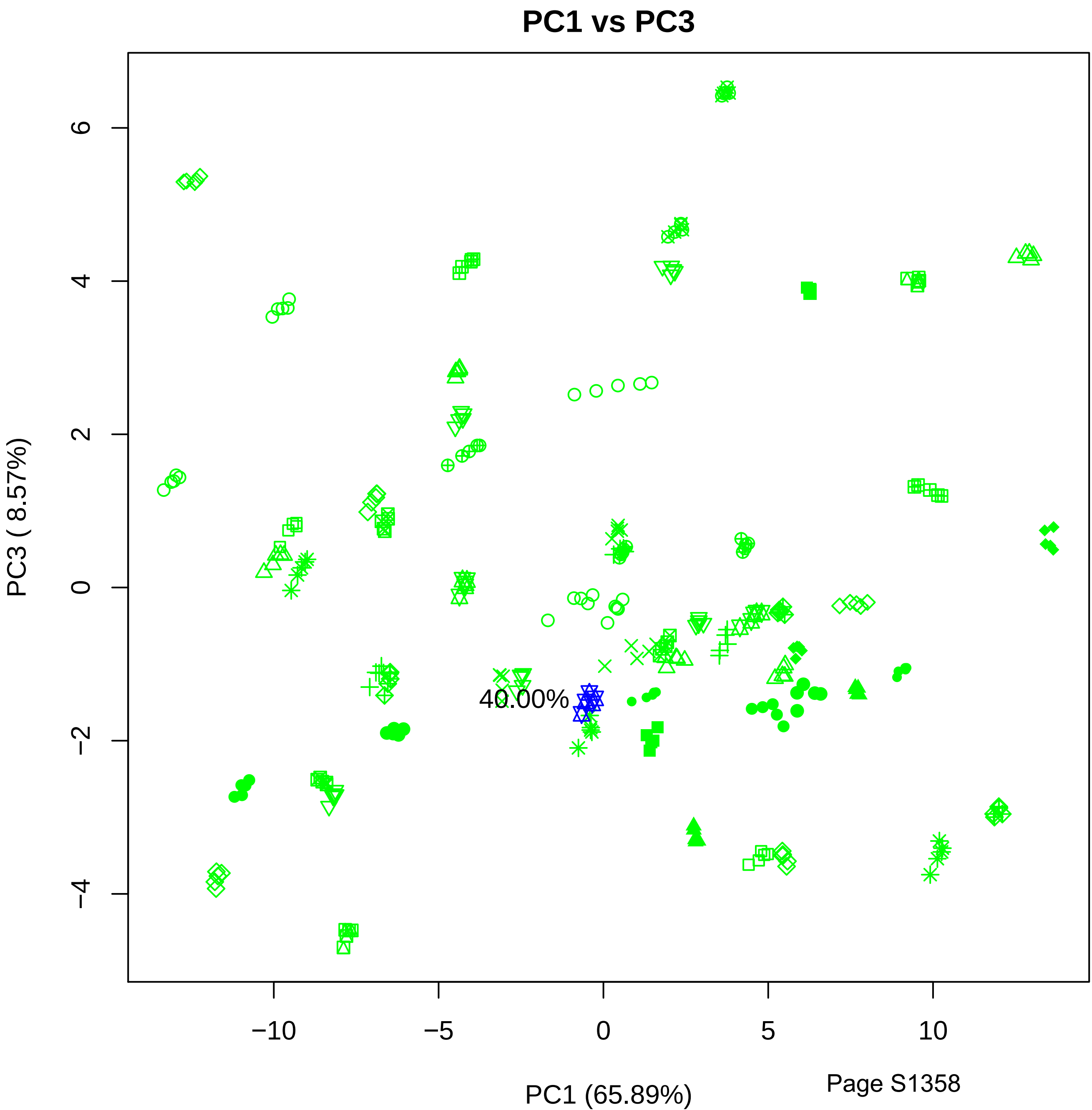

PC1 vs PC3
PC3 (8.57%)
6
4
2
0
−2
−4
40.00%
−10
−5
0
5
10
PC1 (65.89%)

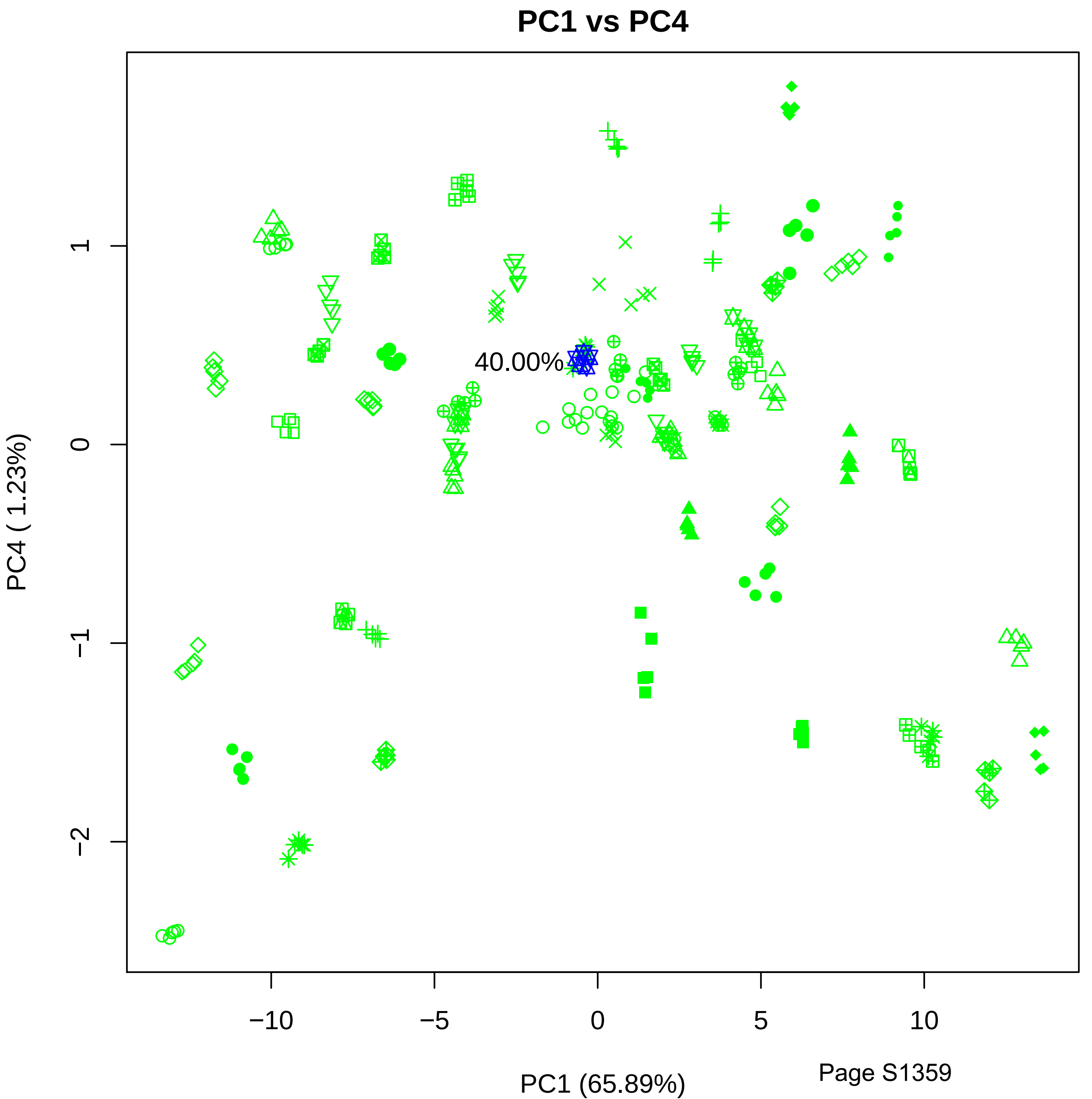
PC1 vs PC4
PC4 (1.23%)
PC1 (65.89%)
40.00%
1
0
−1
−2
−10
−5
0
5
10

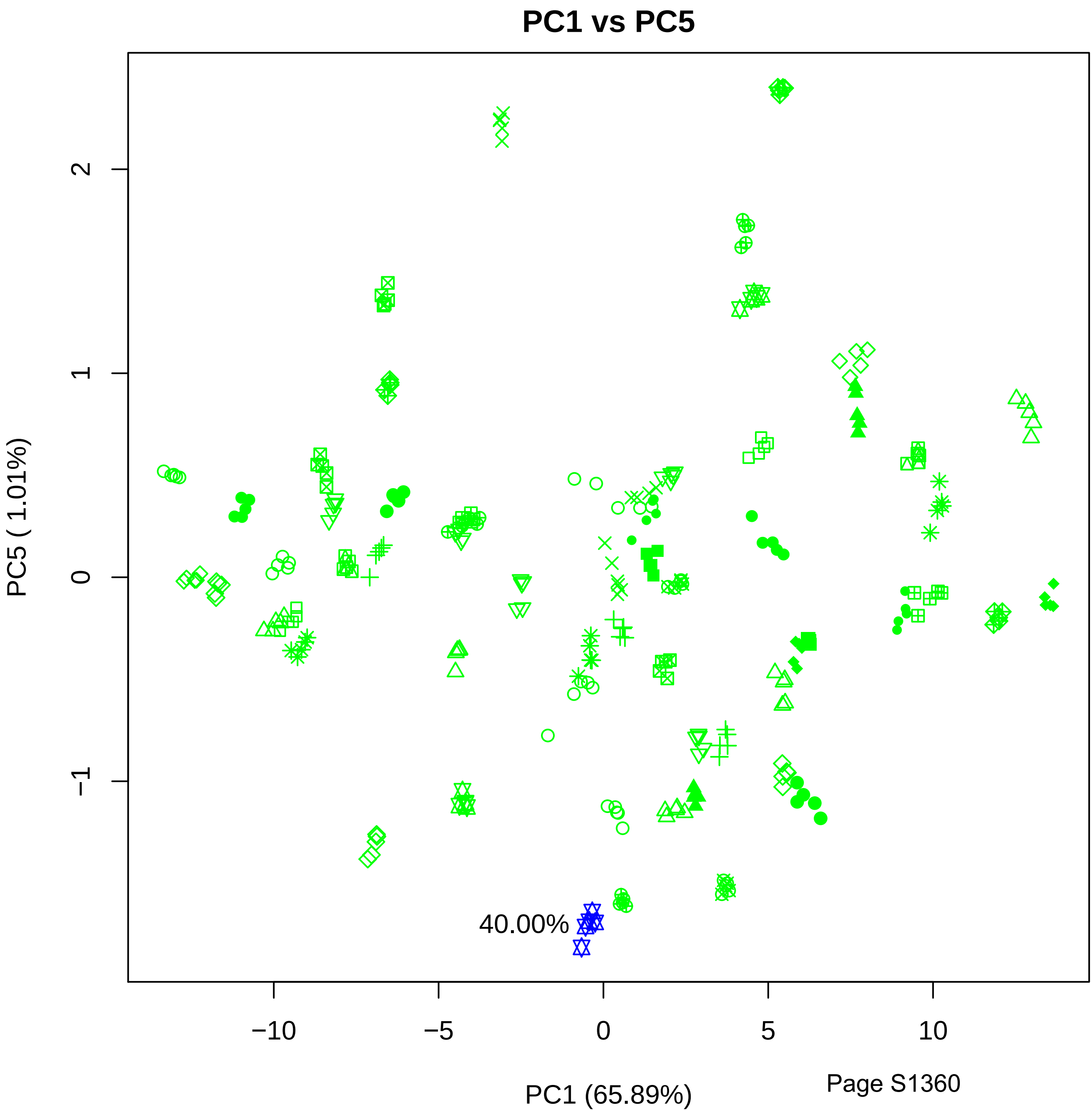

PC1 vs PC5
PC5 (1.01%)
2
1
0
−1
40.00%
−10
−5
0
5
10
PC1 (65.89%)

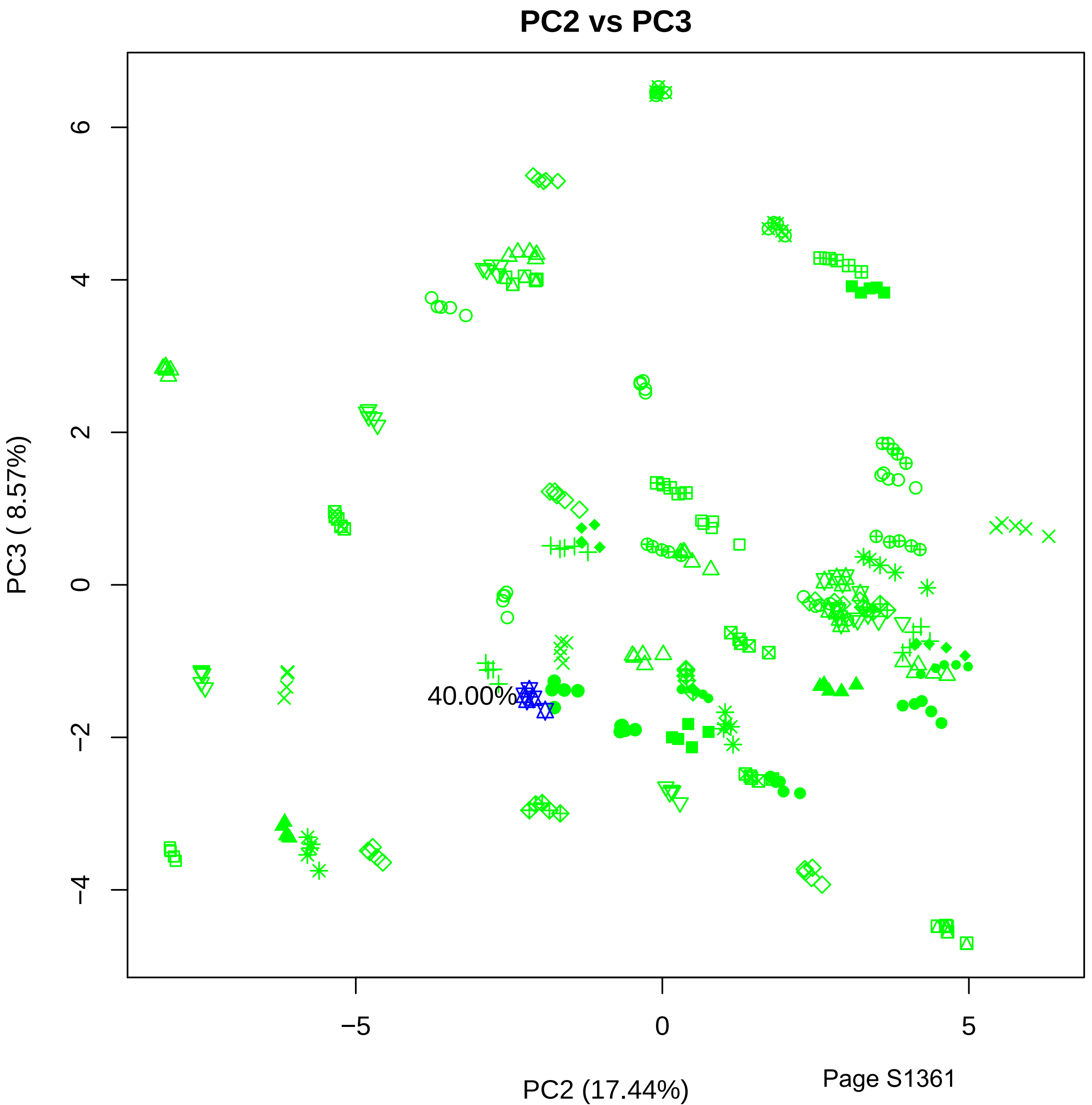
PC2 vs PC3
PC3 (8.57%)
PC2 (17.44%)
6
4
2
0
−2
−4
−5
0
5
40.00%

PC2 vs PC4

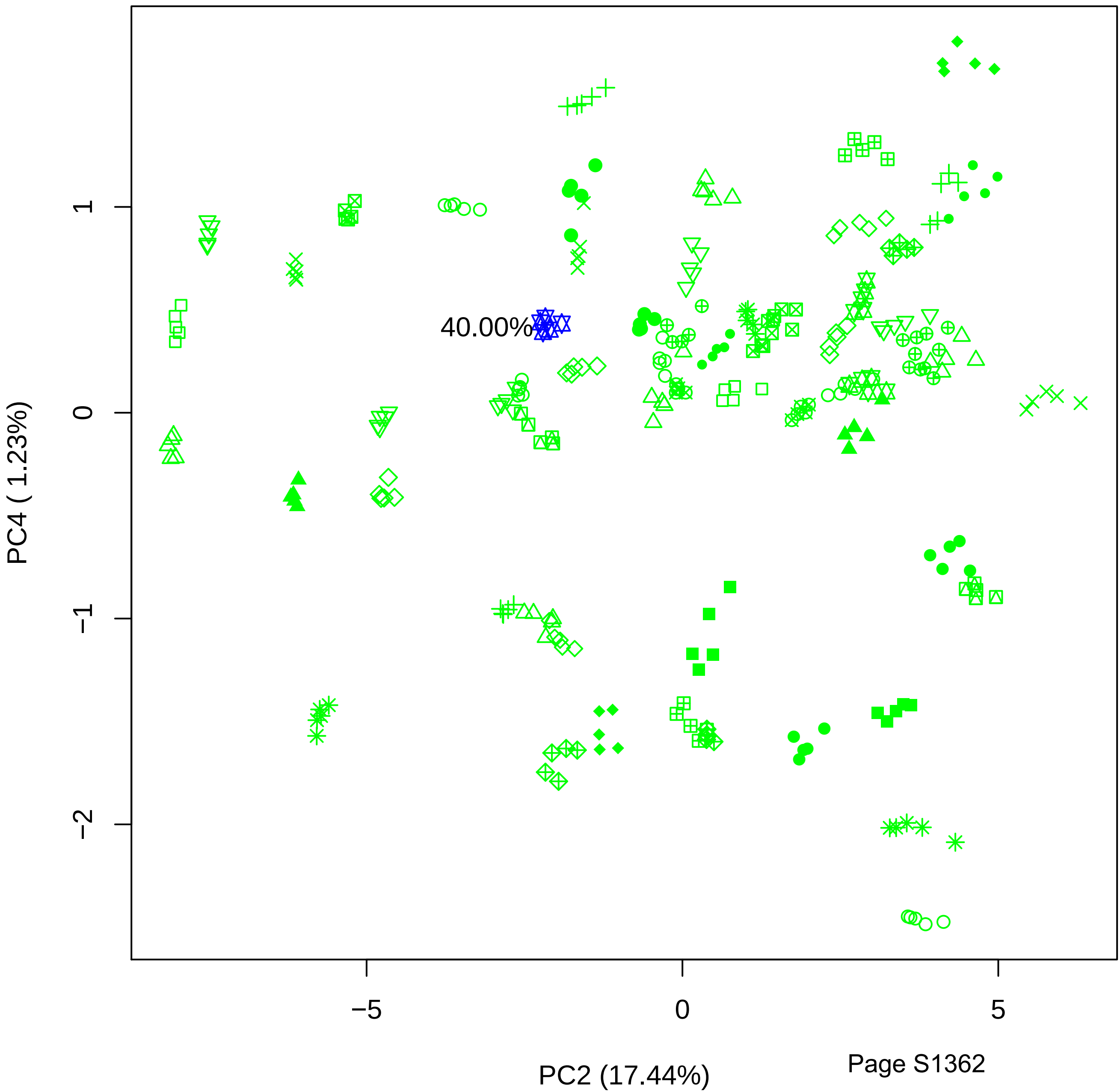

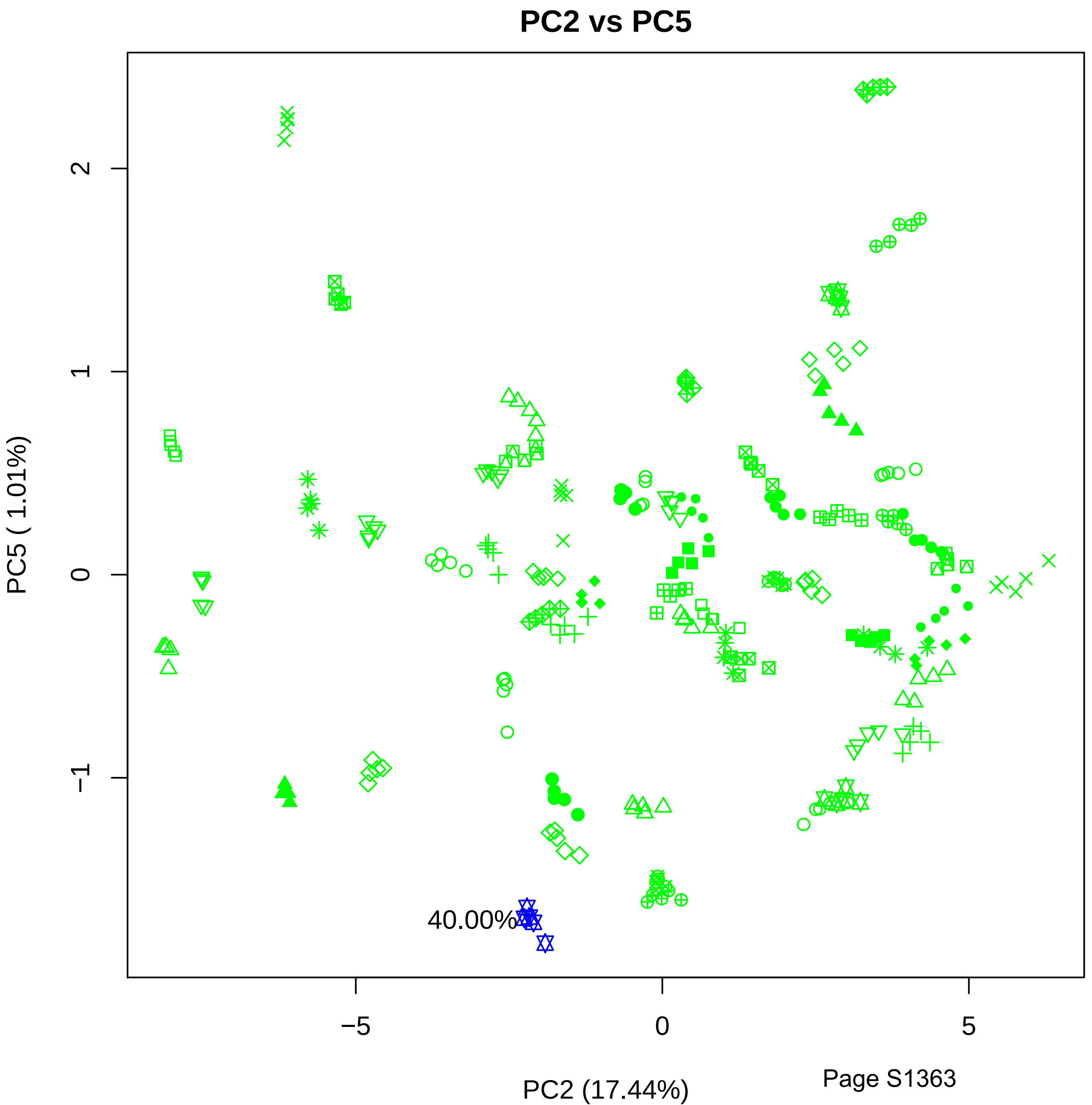
PC2 vs PC5
PC5 (1.01%)
PC2 (17.44%)
40.00%
−5
0
5
−1
0
1
2

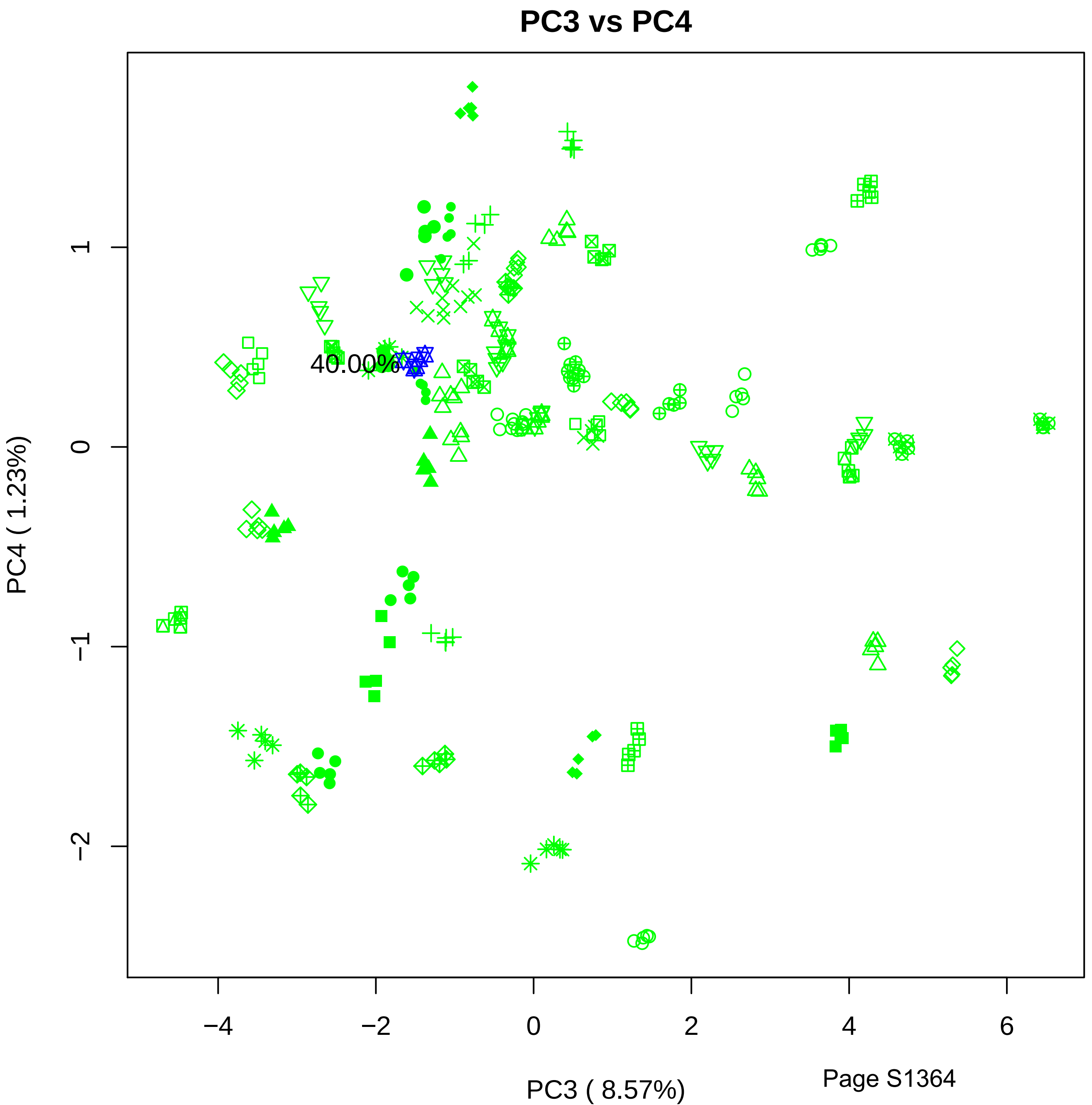

PC3 vs PC4
40.00%
PC4 (1.23%)
PC3 (8.57%)
−4
−2
0
2
4
6
1
0
−1
−2

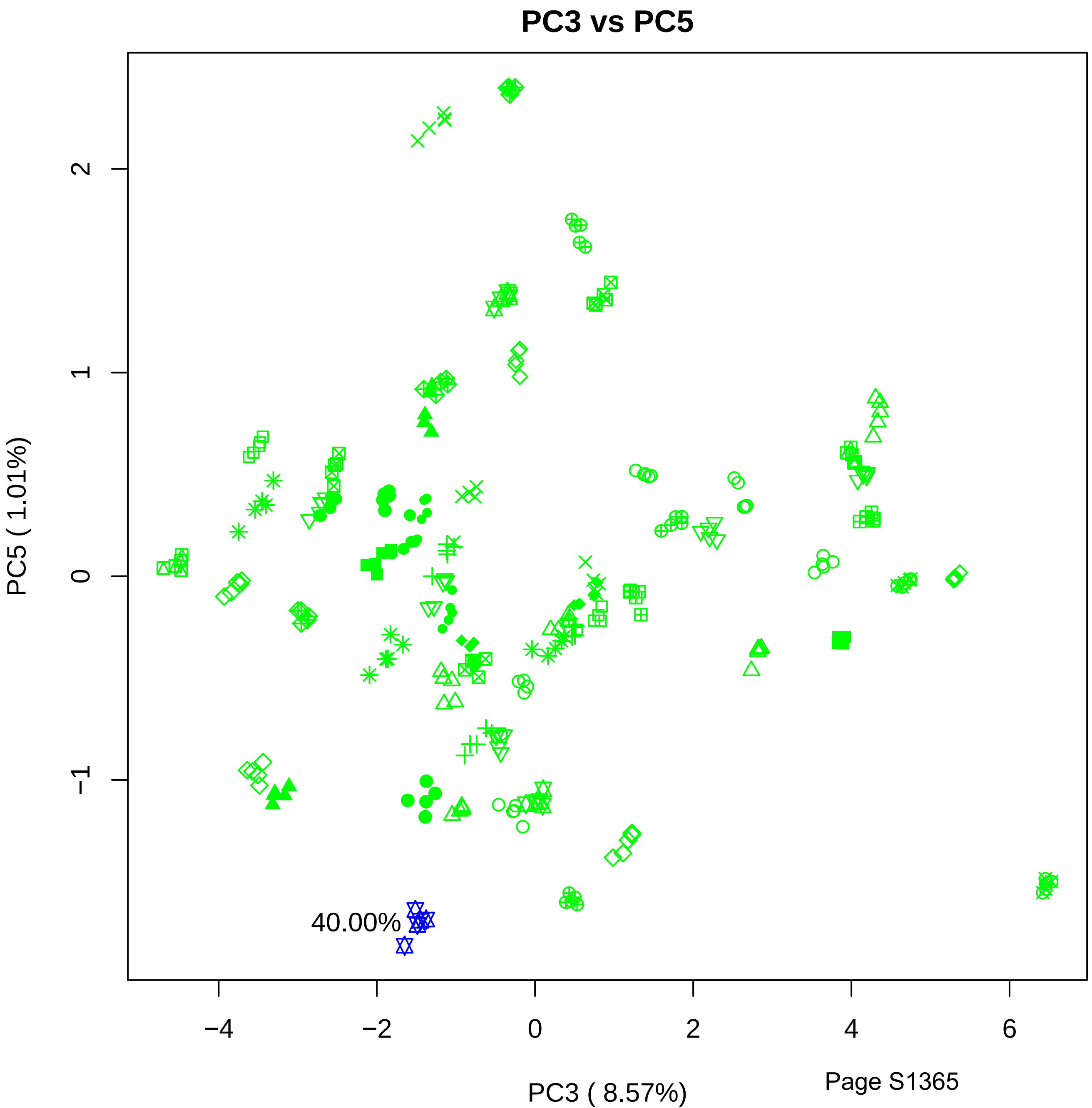
PC3 vs PC5
PC5 (1.01%)
PC3 (8.57%)
40.00%
2
1
0
−1
−4
−2
0
2
4
6

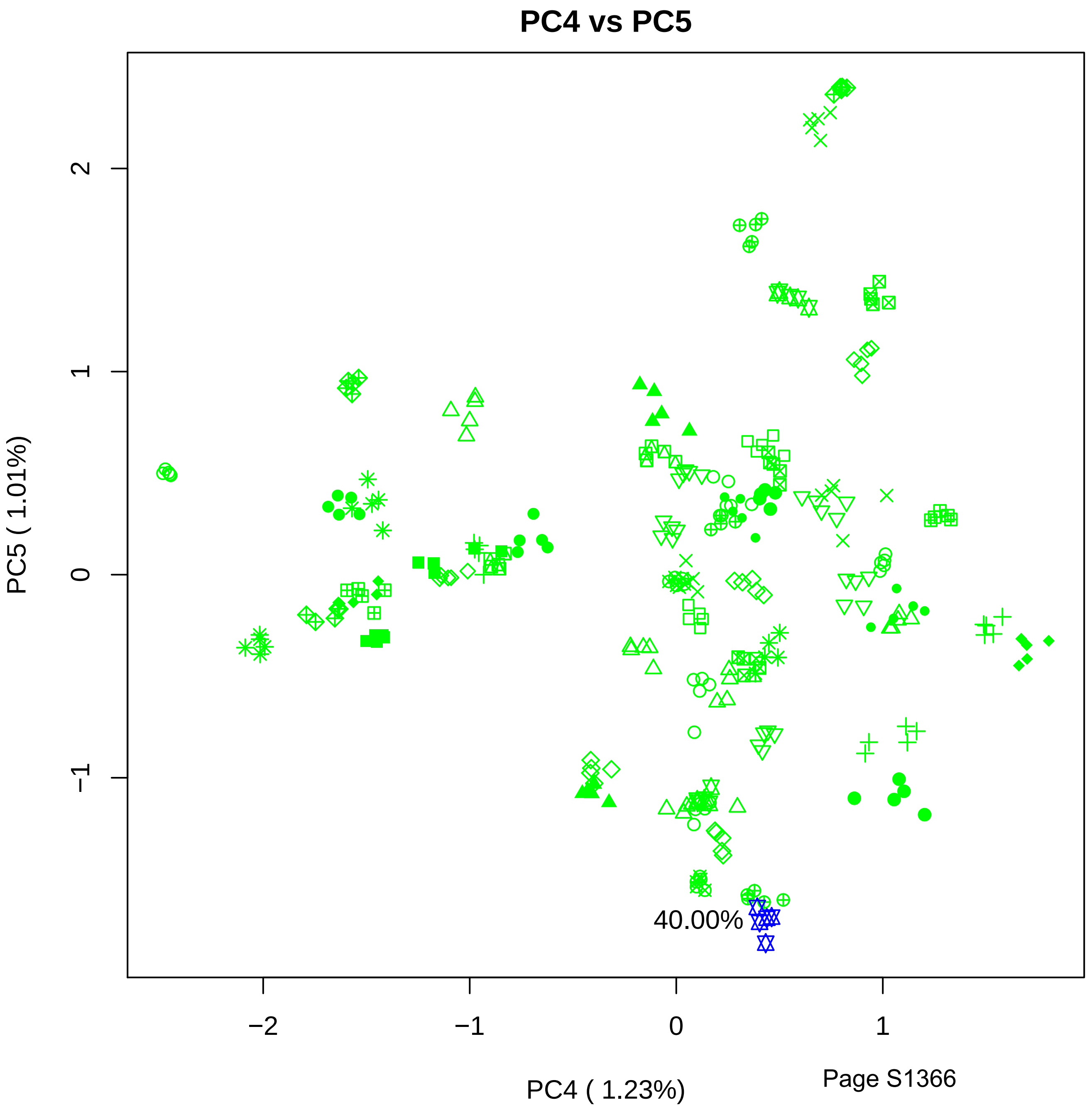
PC4 vs PC5
PC5 (1.01%)
PC4 (1.23%)
40.00%
2
1
0
−1
−2
−1
0
1

PCA of All BMH(d) vs PMH(d)

Scaled Matrix PCAed

7th order polynomial baseline correction performed

Raw spectra smoothed to 21 Neighbours, 2nd order PN, Savtisky Golay

Normalisation between 0 and 100

D2 spectra smoothed to 41 Neighbours, 2nd order PN, Savtisky Golay

Regions Analysed: 3601−2501 and 2001−700cm−1

Samples Analysed: 8 bContam's 0 BLH's 0 BMH's 0 OMH's 0 pContam's 0 PLH's 60 PMH's

Keele University AD

Fri Dec 14 14:22:15 2018

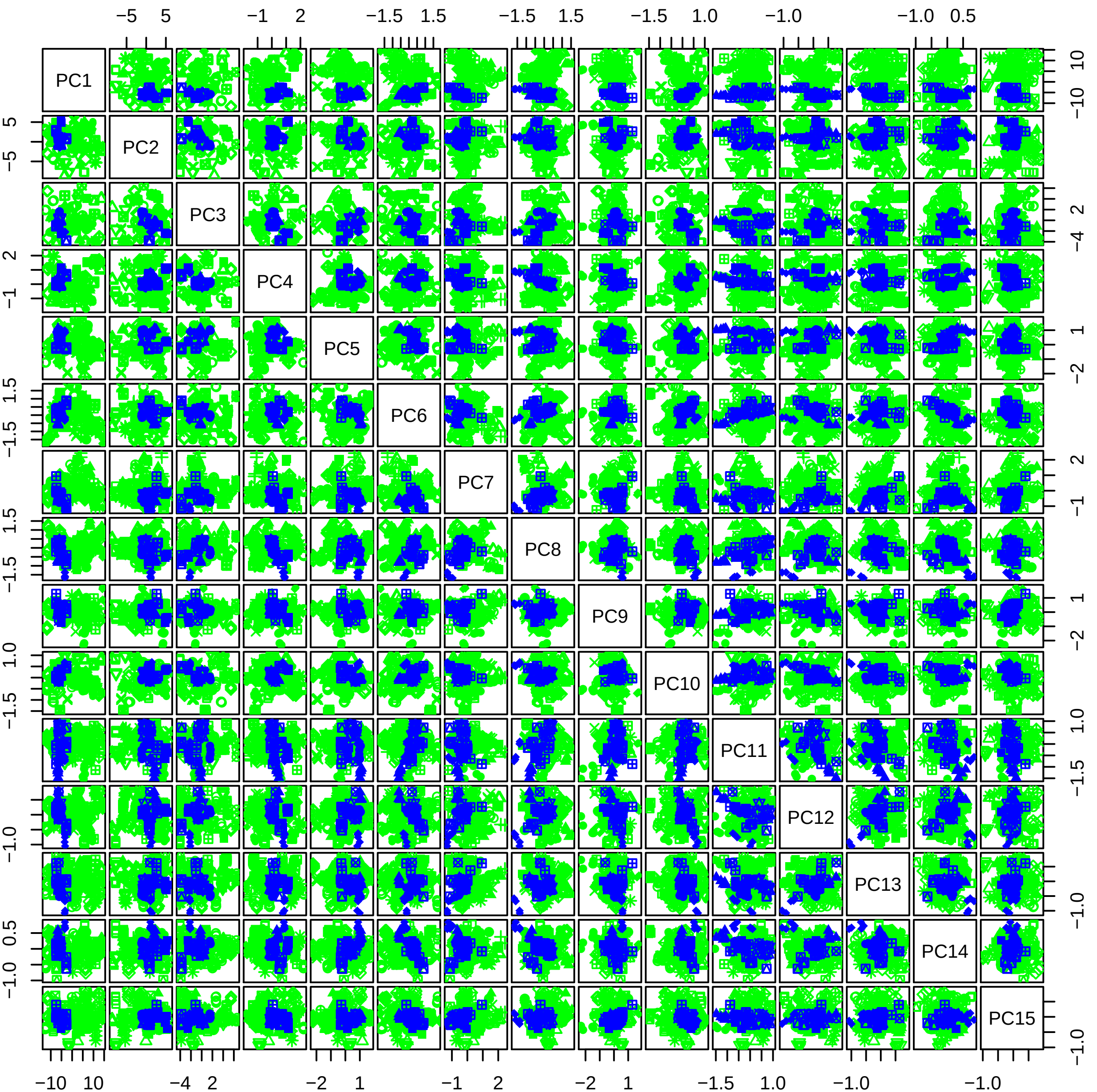
PC1
PC2
PC3
PC4
PC5
PC6
PC7
PC8
PC9
PC10
PC11
PC12
PC13
PC14
PC15
−5
5
−1
2
−1.5
1.5
−1.5
1.5
−1.5
1.0
−1.0
−1.0
0.5
10
−10
5
−5
2
−4
2
−1
1
−2
1.5
−1.5
2
−1
1.5
−1.5
1
−2
1.0
−1.5
1.0
−1.5
−1.0
−1.0
0.5
−1.0
−1.0
−10
10
−4
2
−2
1
−1
2
−2
1
−1.5
1.0
−1.0
−1.0

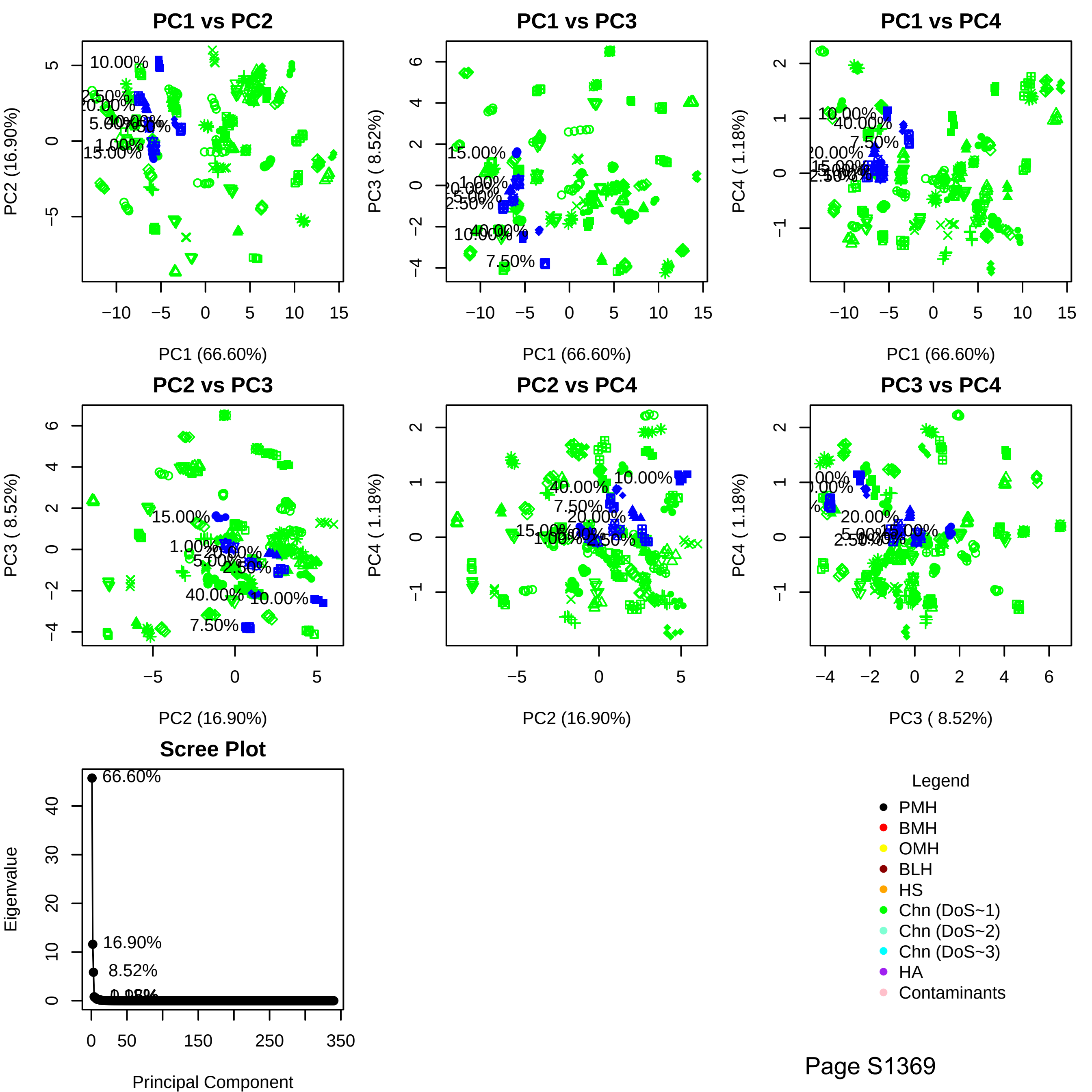

PC1 vs PC2
PC1 vs PC3
PC1 vs PC4
PC2 vs PC3
PC2 vs PC4
PC3 vs PC4
PC1 (66.60%)
PC2 (16.90%)
PC3 (8.52%)
PC4 (1.18%)
Scree Plot
66.60%
16.90%
8.52%
Eigenvalue
Principal Component
Legend
PMH
BMH
OMH
BLH
HS
Chn (DoS~1)
Chn (DoS~2)
Chn (DoS~3)
HA
Contaminants

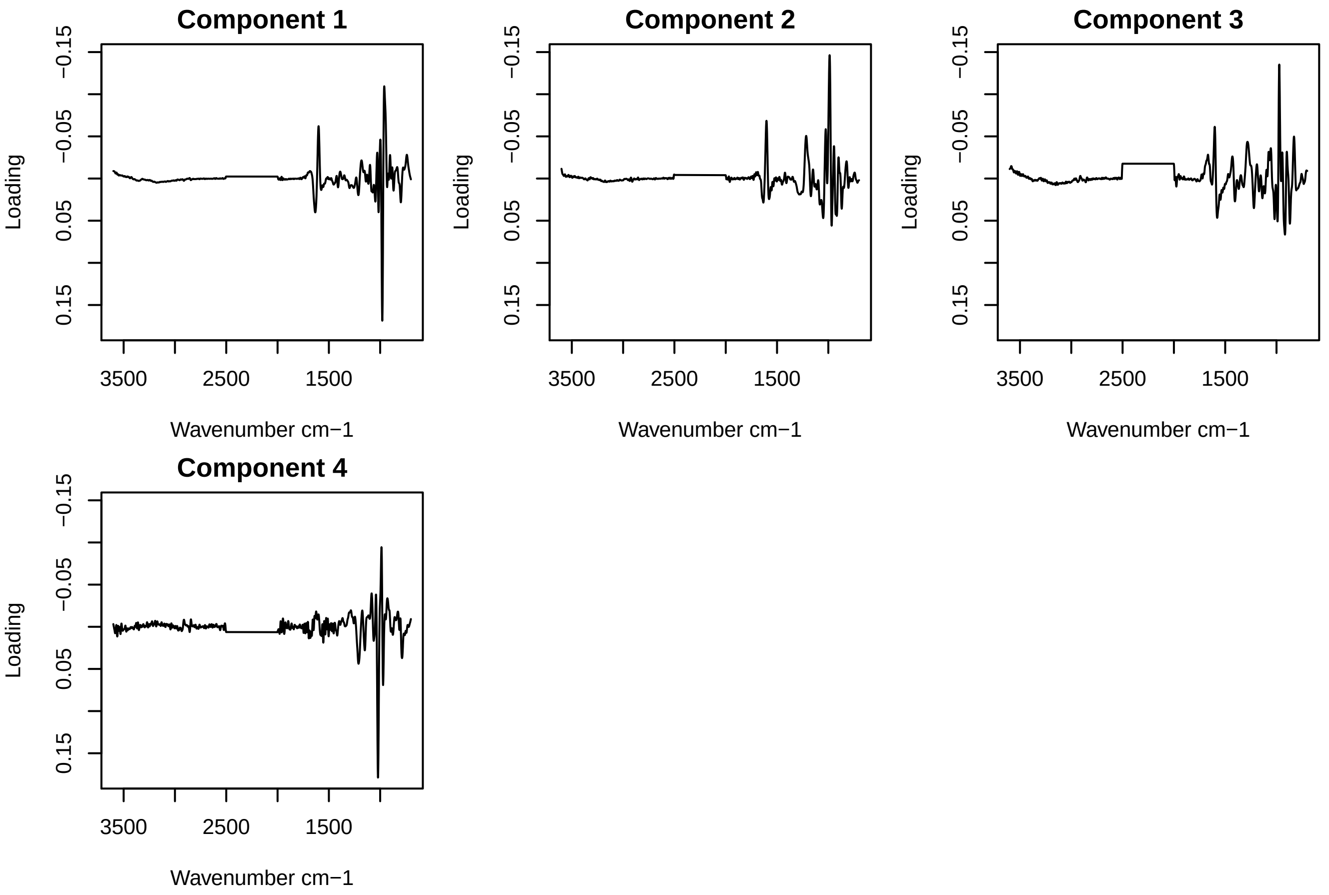
Component 1
Component 2
Component 3
Component 4
Loading
−0.15
−0.05
0.05
0.15
3500
2500
1500
Wavenumber cm−1

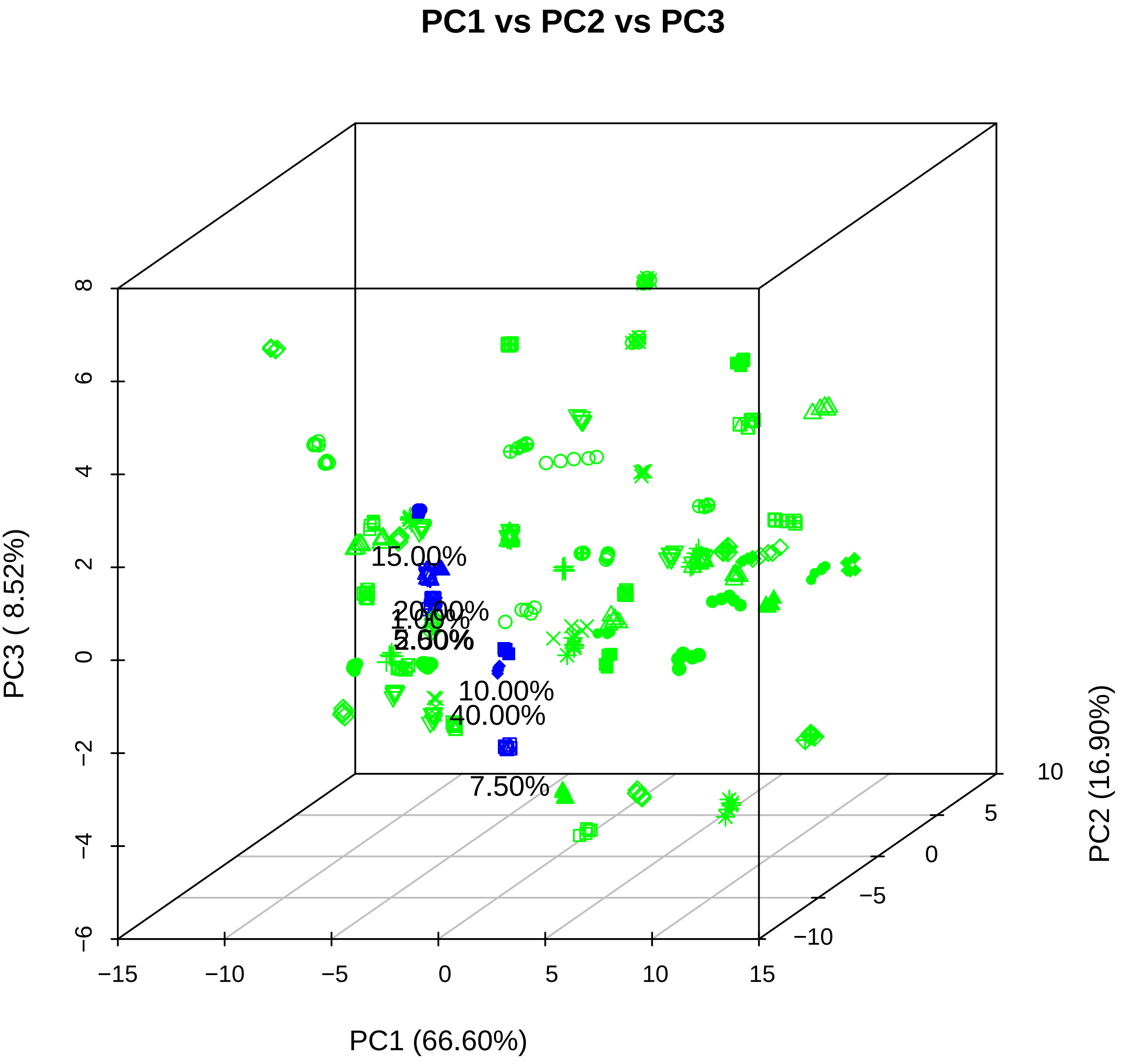
PC1 vs PC2 vs PC3
PC1 (66.60%)
PC2 (16.90%)
PC3 (8.52%)
15.00%
20.00%
1.00%
10.00%
40.00%
7.50%
−15
−10
−5
0
5
10
15
−6
−4
−2
0
2
4
6
8
−10
−5
0
5
10

PC1 vs PC2 vs PC4

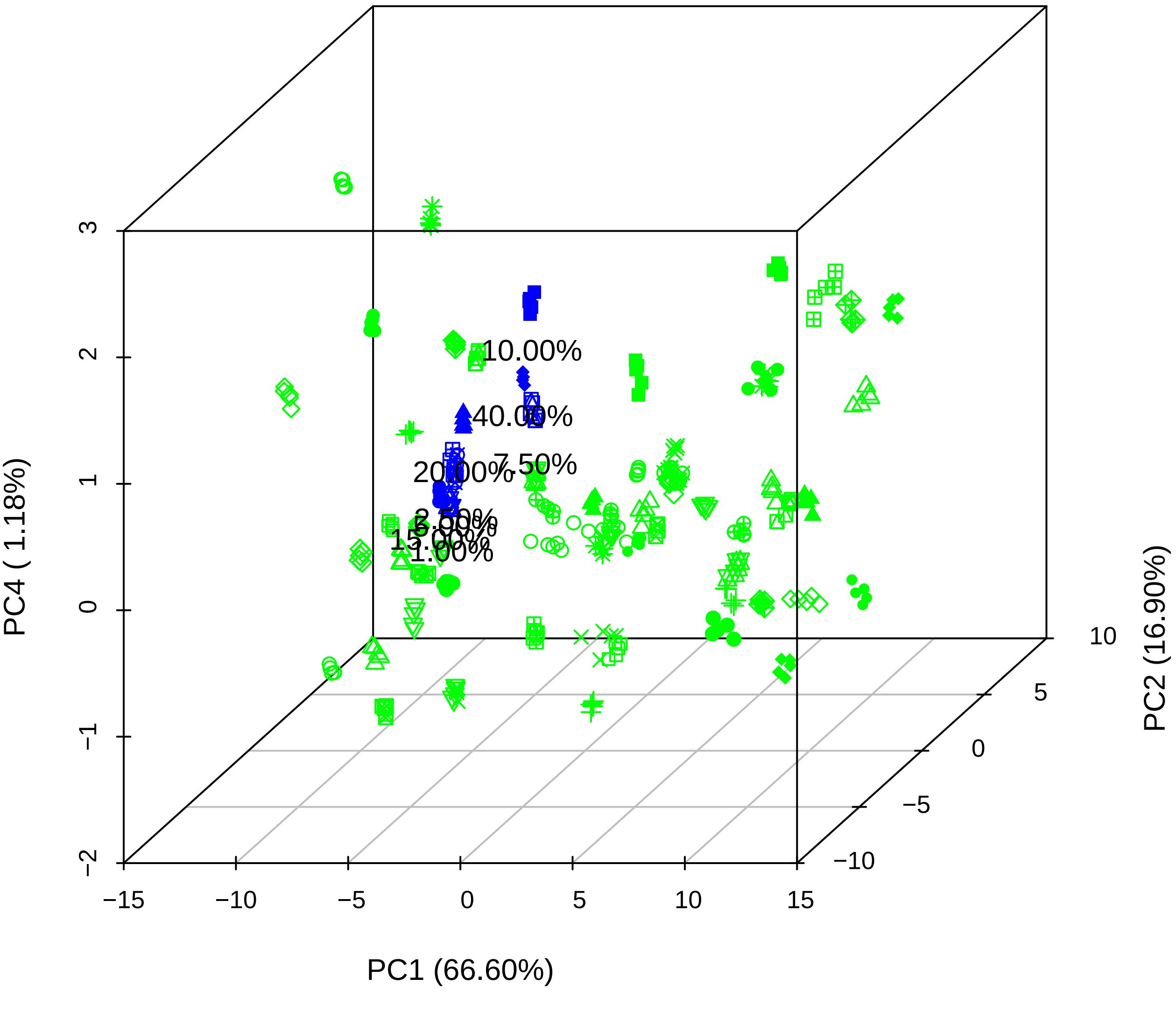

PC1 vs PC3 vs PC4

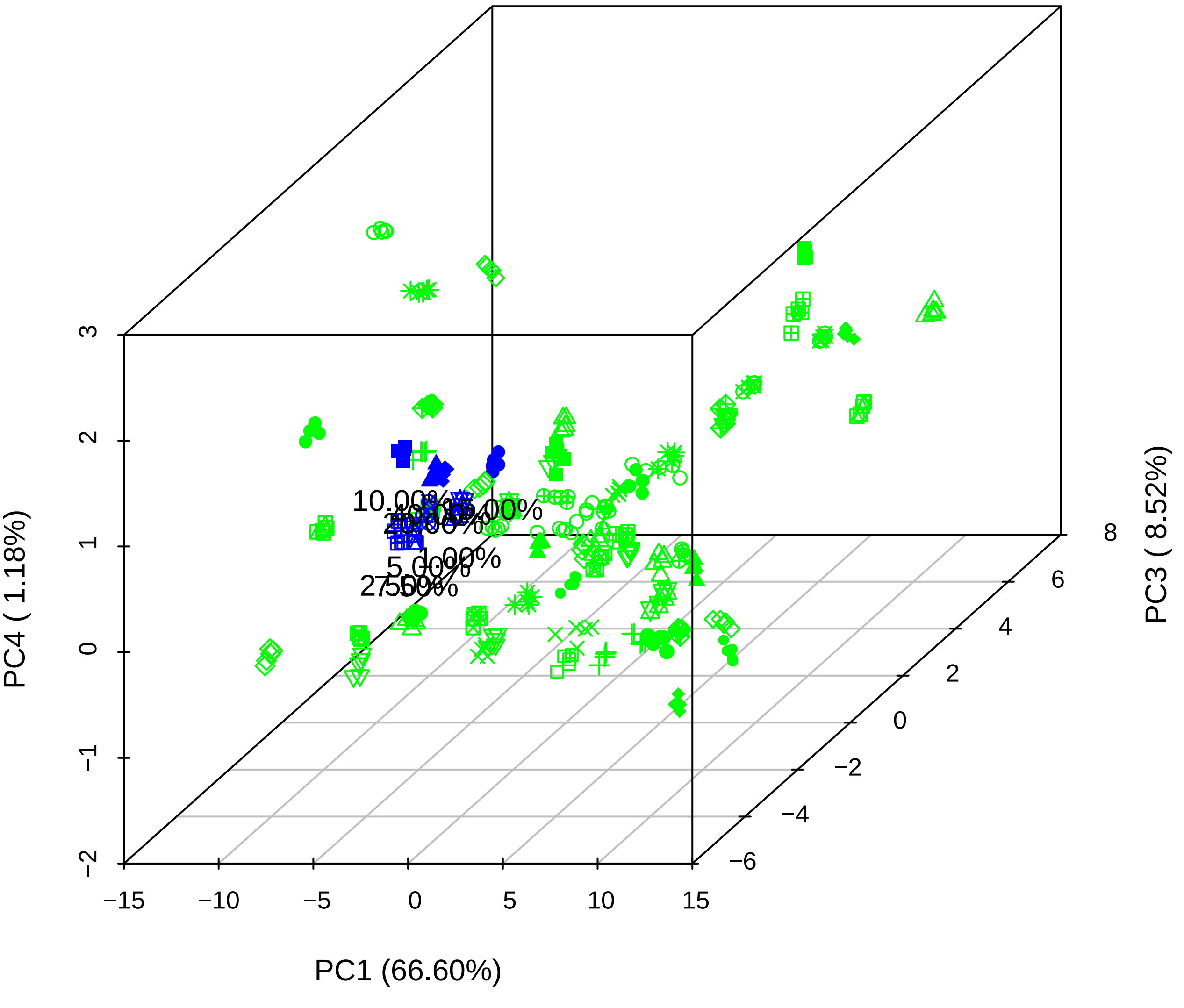

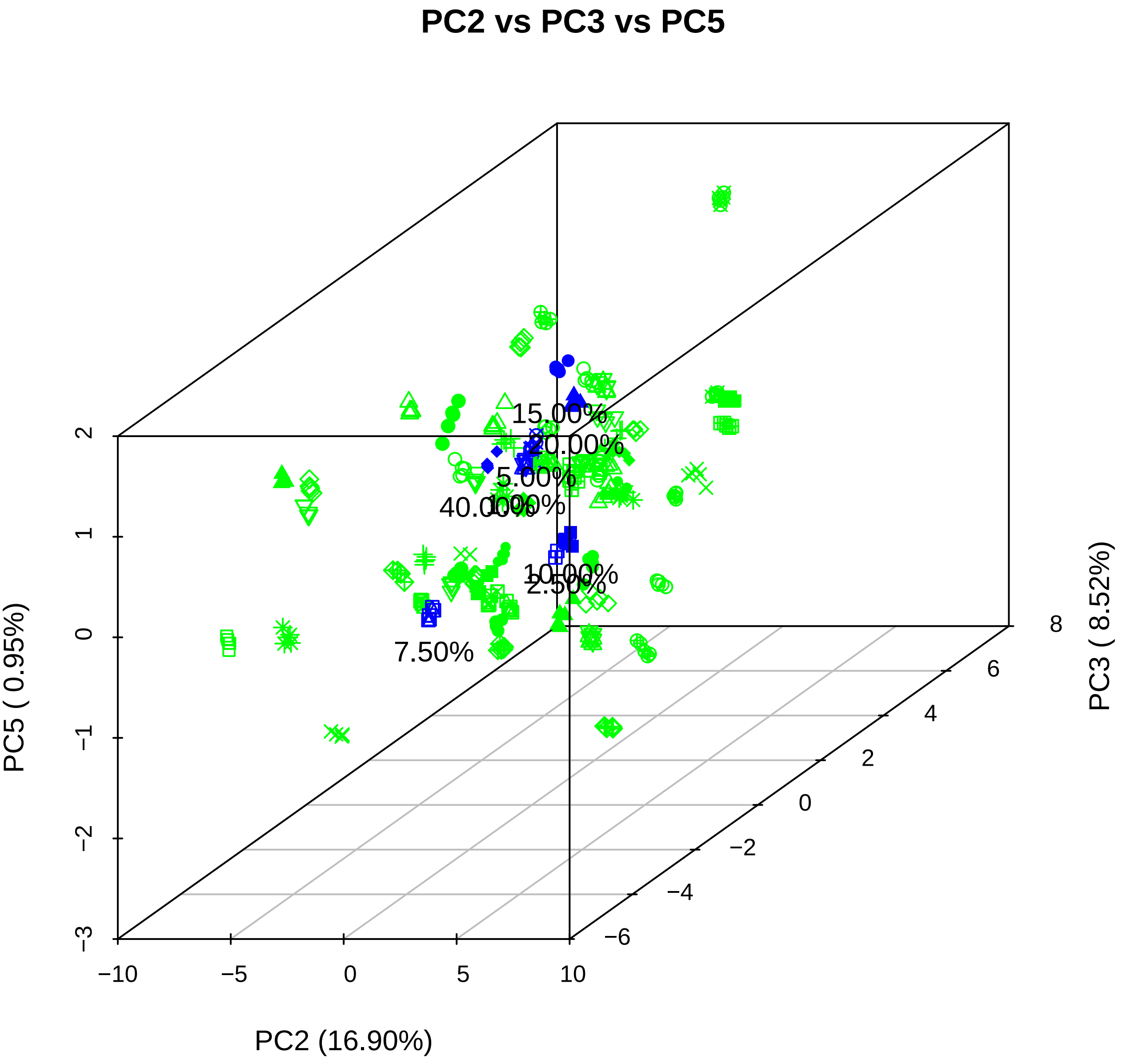

PC2 vs PC3 vs PC5
15.00%
20.00%
5.00%
40.00%
1.00%
10.00%
2.50%
7.50%
2
1
0
−1
−2
−3
PC5 (0.95%)
−10
−5
0
5
10
PC2 (16.90%)
8
6
4
2
0
−2
−4
−6
PC3 (8.52%)

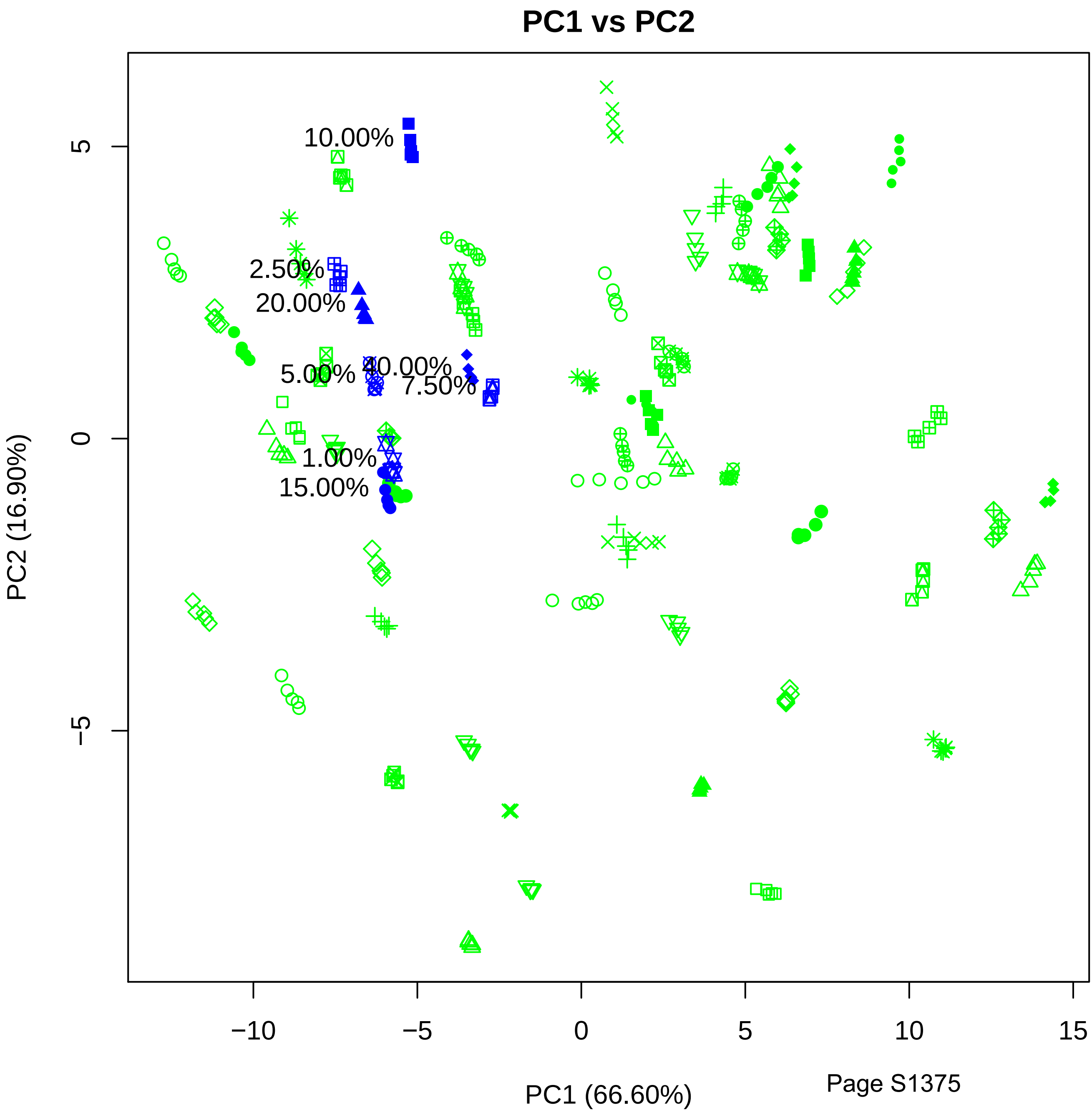

PC1 vs PC2
10.00%
2.50%
20.00%
5.00%
40.00%
7.50%
1.00%
15.00%
5
0
−5
−10
−5
0
5
10
15
PC2 (16.90%)
PC1 (66.60%)

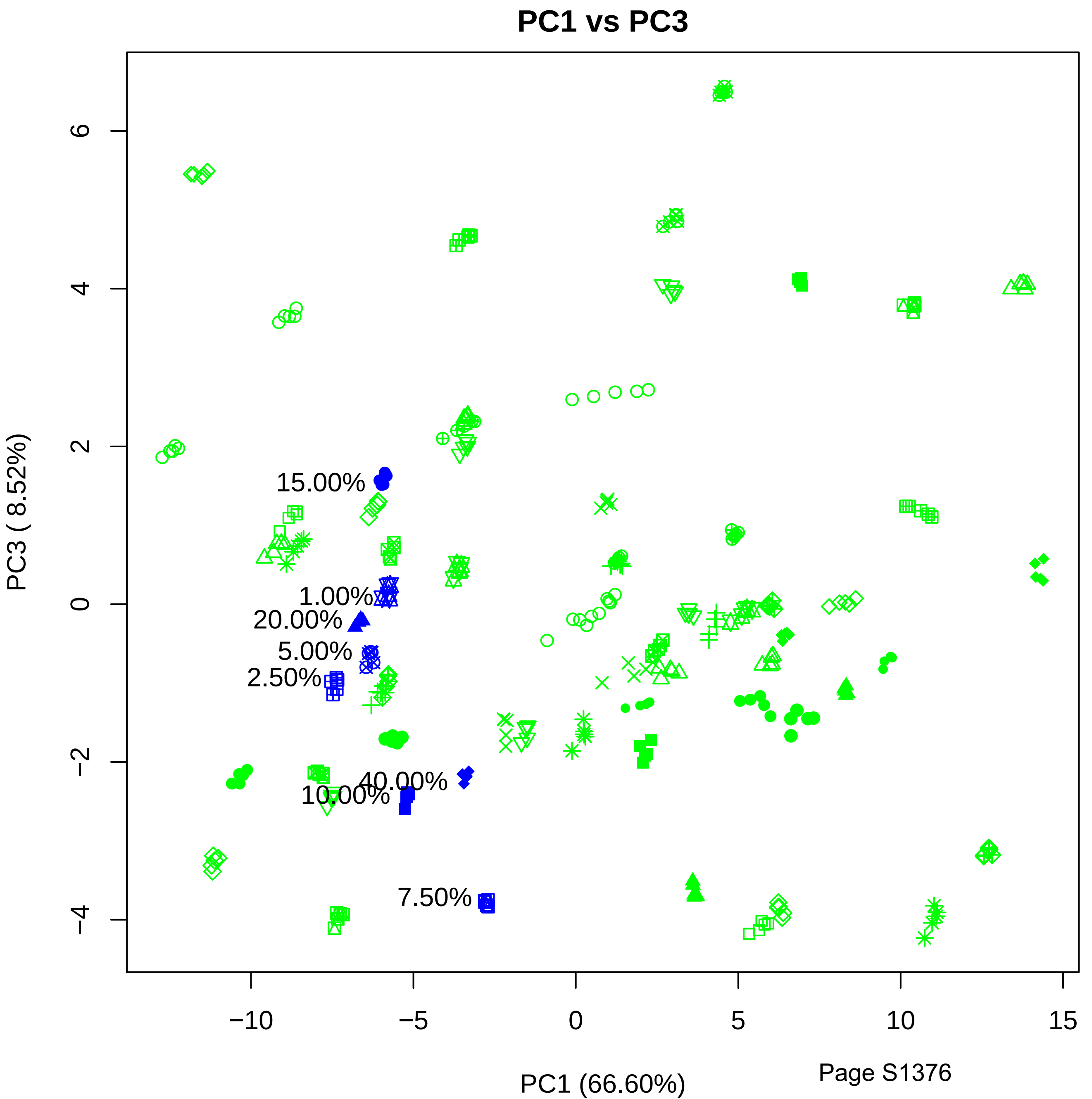

PC1 vs PC3
15.00%
1.00%
20.00%
5.00%
2.50%
40.00%
10.00%
7.50%
PC3 (8.52%)
PC1 (66.60%)
6
4
2
0
−2
−4
−10
−5
0
5
10
15

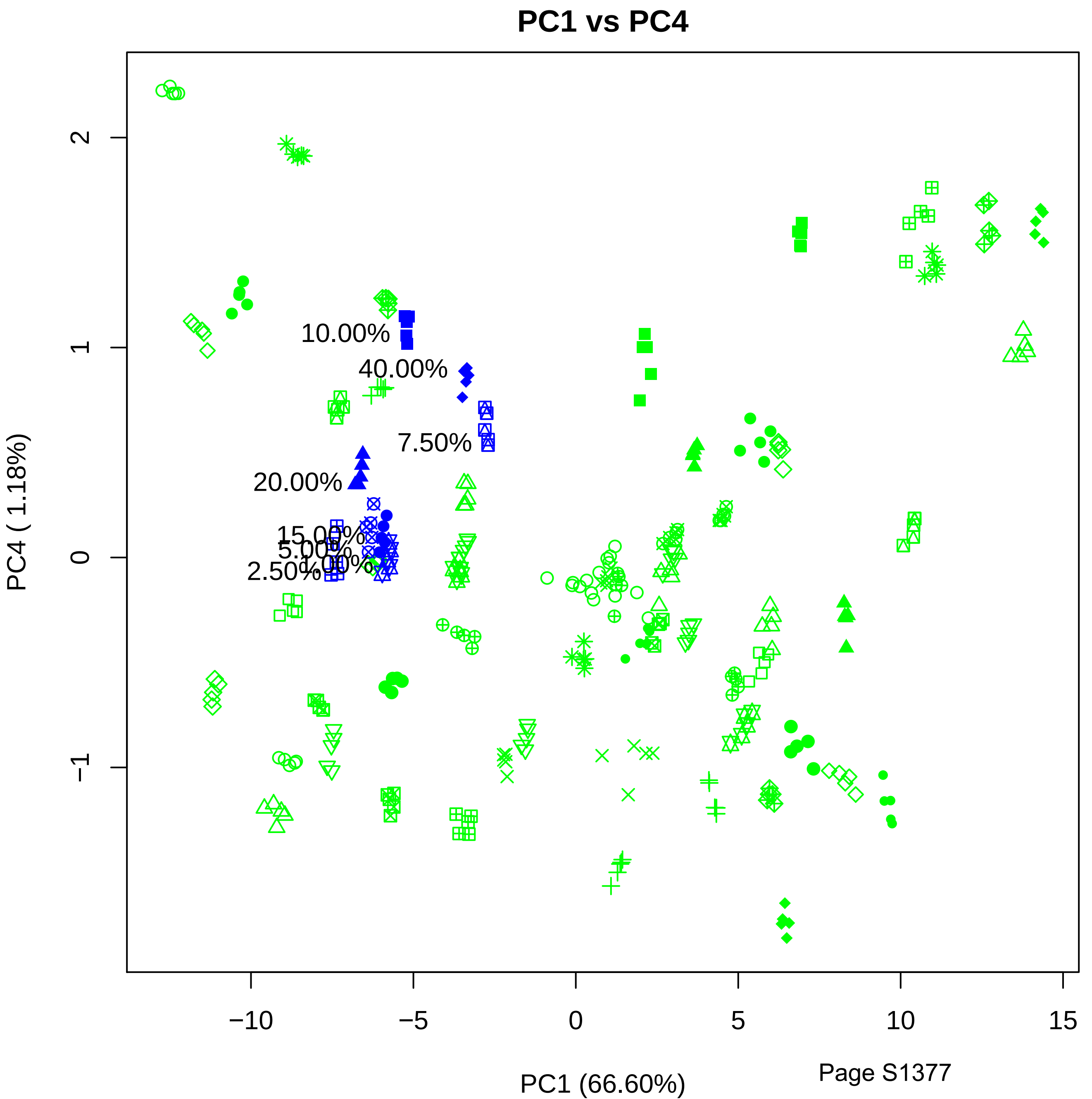

PC1 vs PC4
10.00%
40.00%
7.50%
20.00%
15.00%
5.00%
2.50%
1.00%
PC4 (1.18%)
PC1 (66.60%)
−10
−5
0
5
10
15
−1
0
1
2

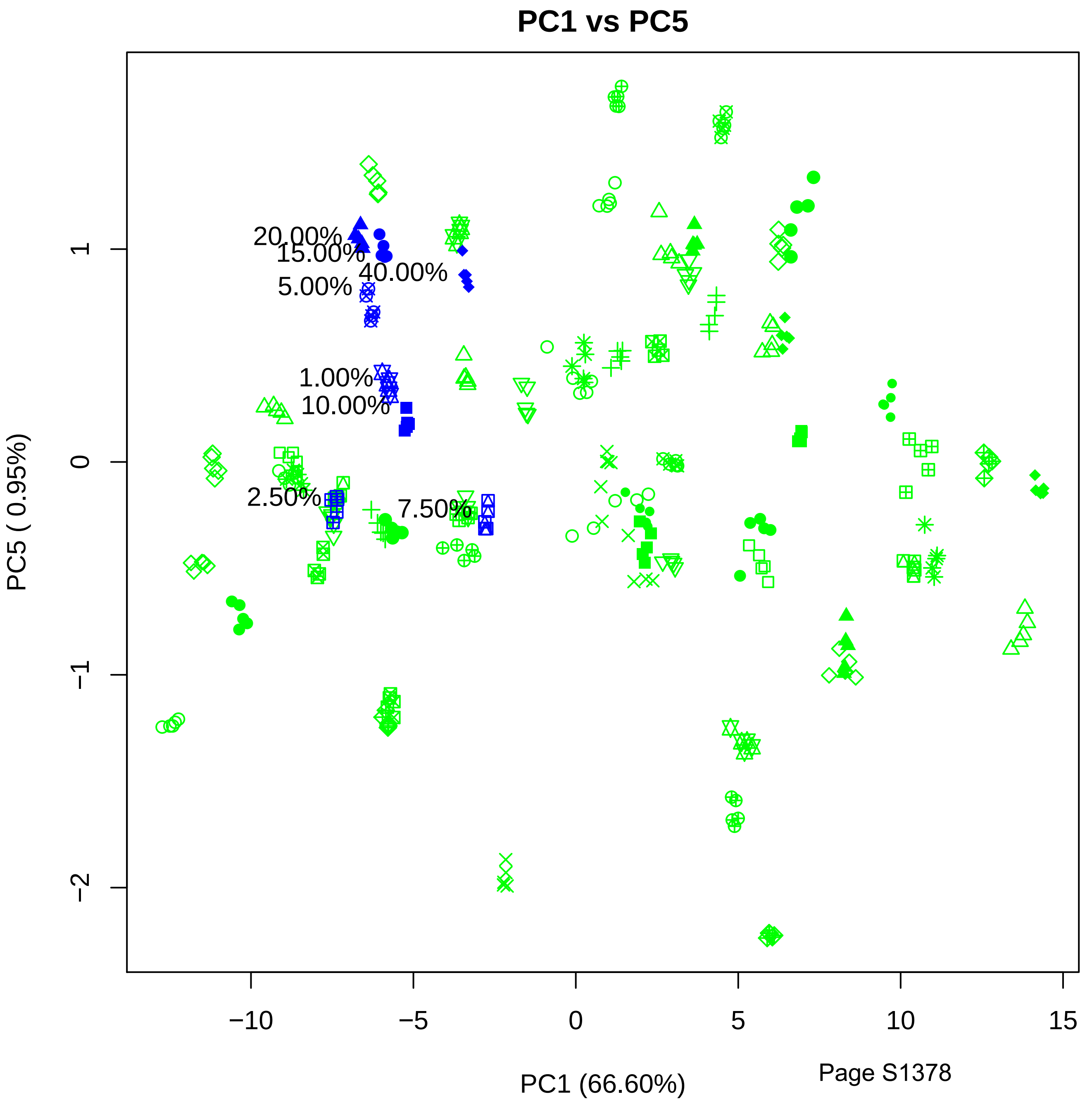
PC1 vs PC5
20.00%
15.00%
40.00%
5.00%
1.00%
10.00%
2.50%
7.50%
1
0
−1
−2
−10
−5
0
5
10
15
PC5 (0.95%)
PC1 (66.60%)

PC2 vs PC3

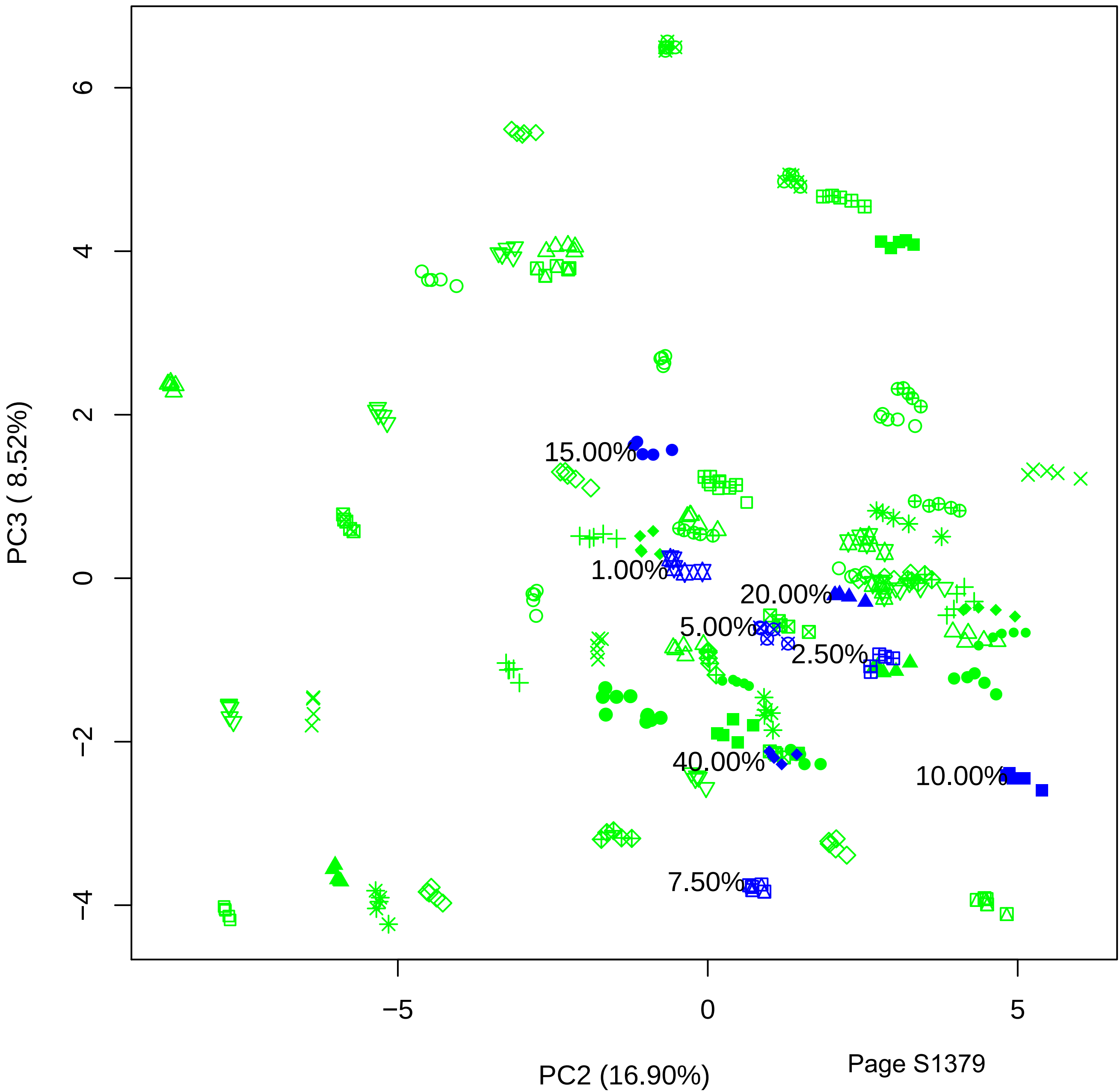

PC3 (8.52%)
PC2 (16.90%)
6
4
2
0
−2
−4
−5
0
5
15.00%
1.00%
20.00%
5.00%
2.50%
40.00%
10.00%
7.50%

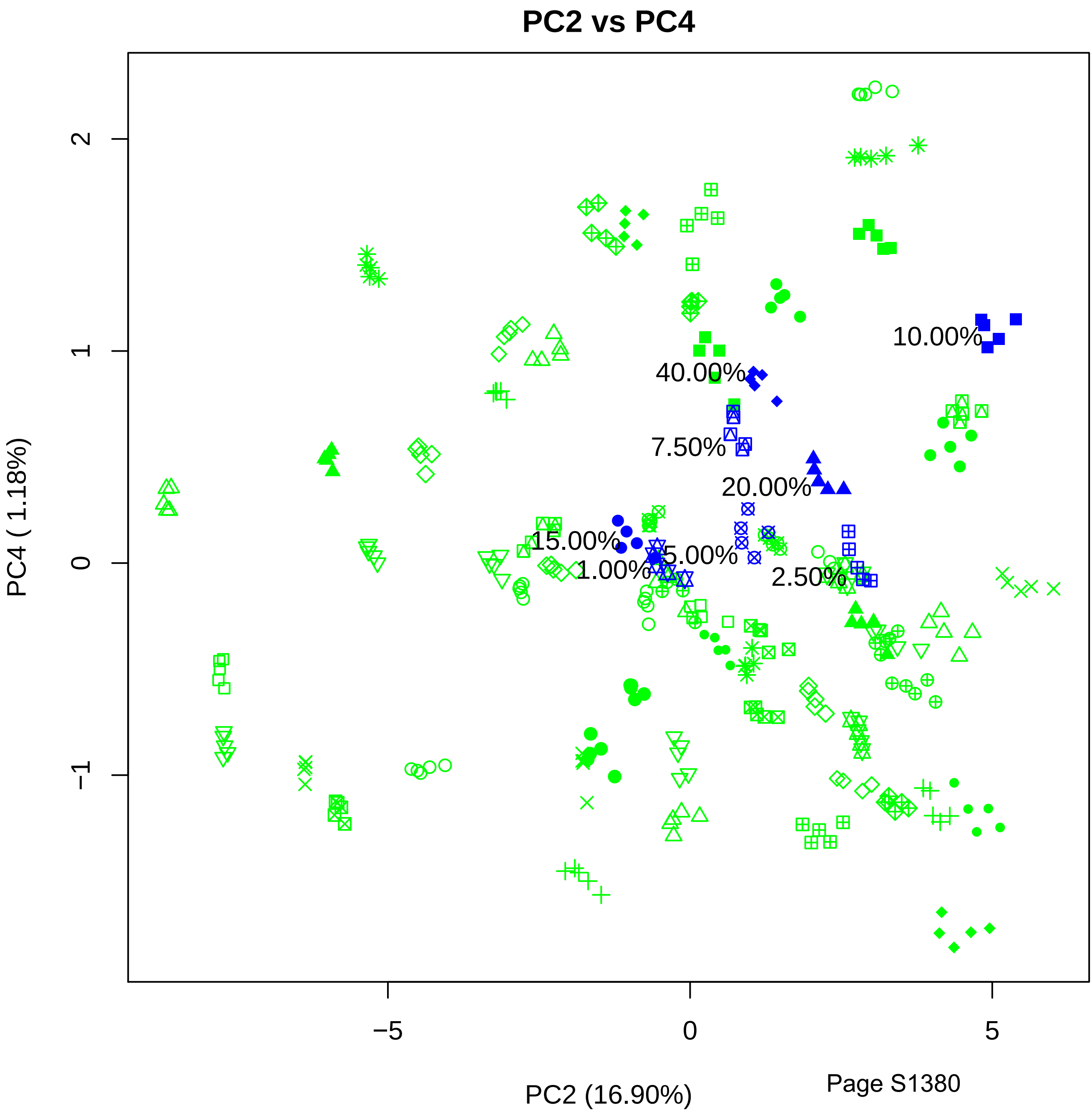
PC2 vs PC4
PC4 (1.18%)
PC2 (16.90%)
2
1
0
−1
−5
0
5
10.00%
40.00%
7.50%
20.00%
15.00%
5.00%
1.00%
2.50%

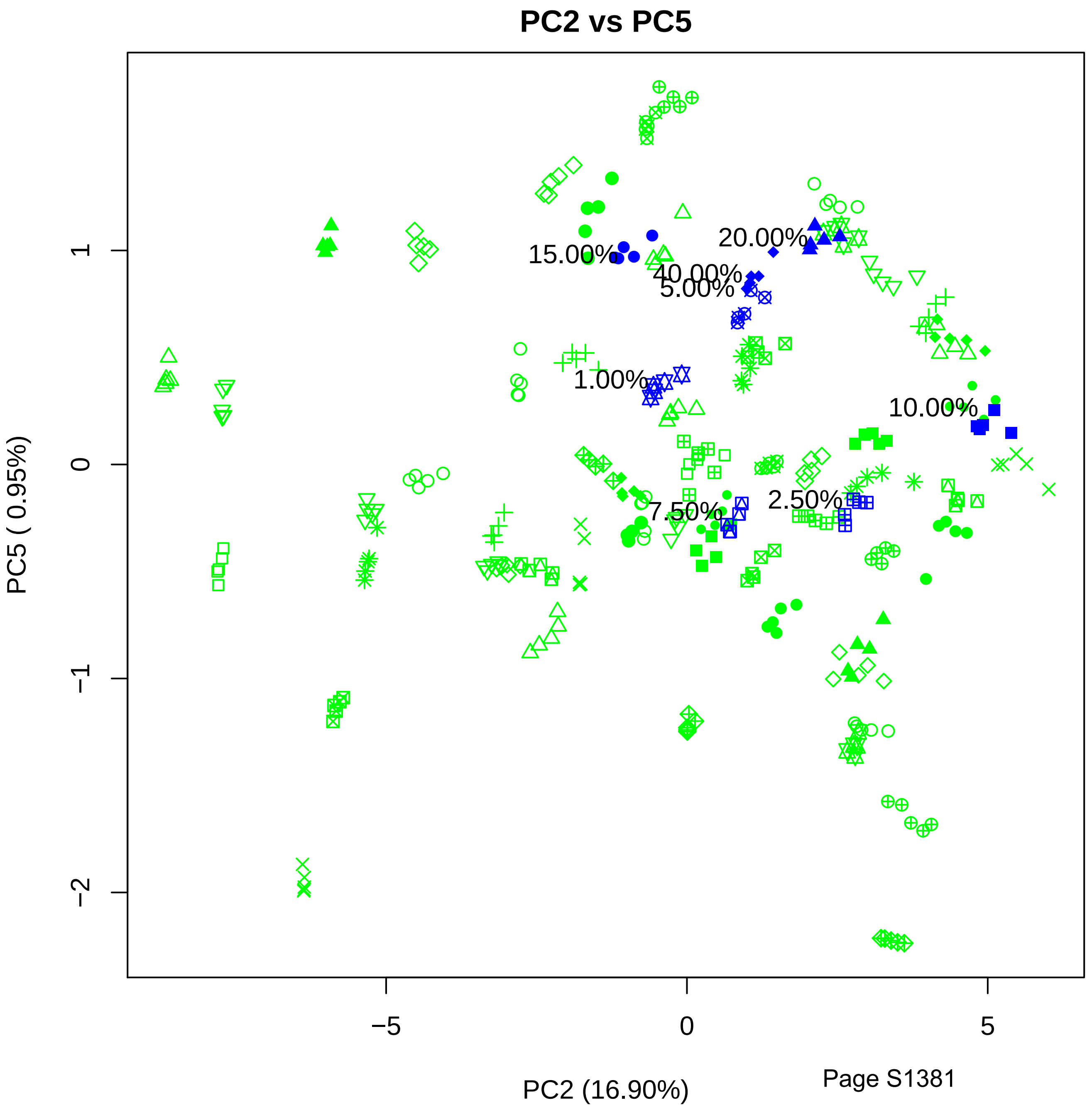
PC2 vs PC5
PC5 (0.95%)
PC2 (16.90%)
1
0
−1
−2
−5
0
5
15.00%
20.00%
40.00%
5.00%
1.00%
10.00%
7.50%
2.50%

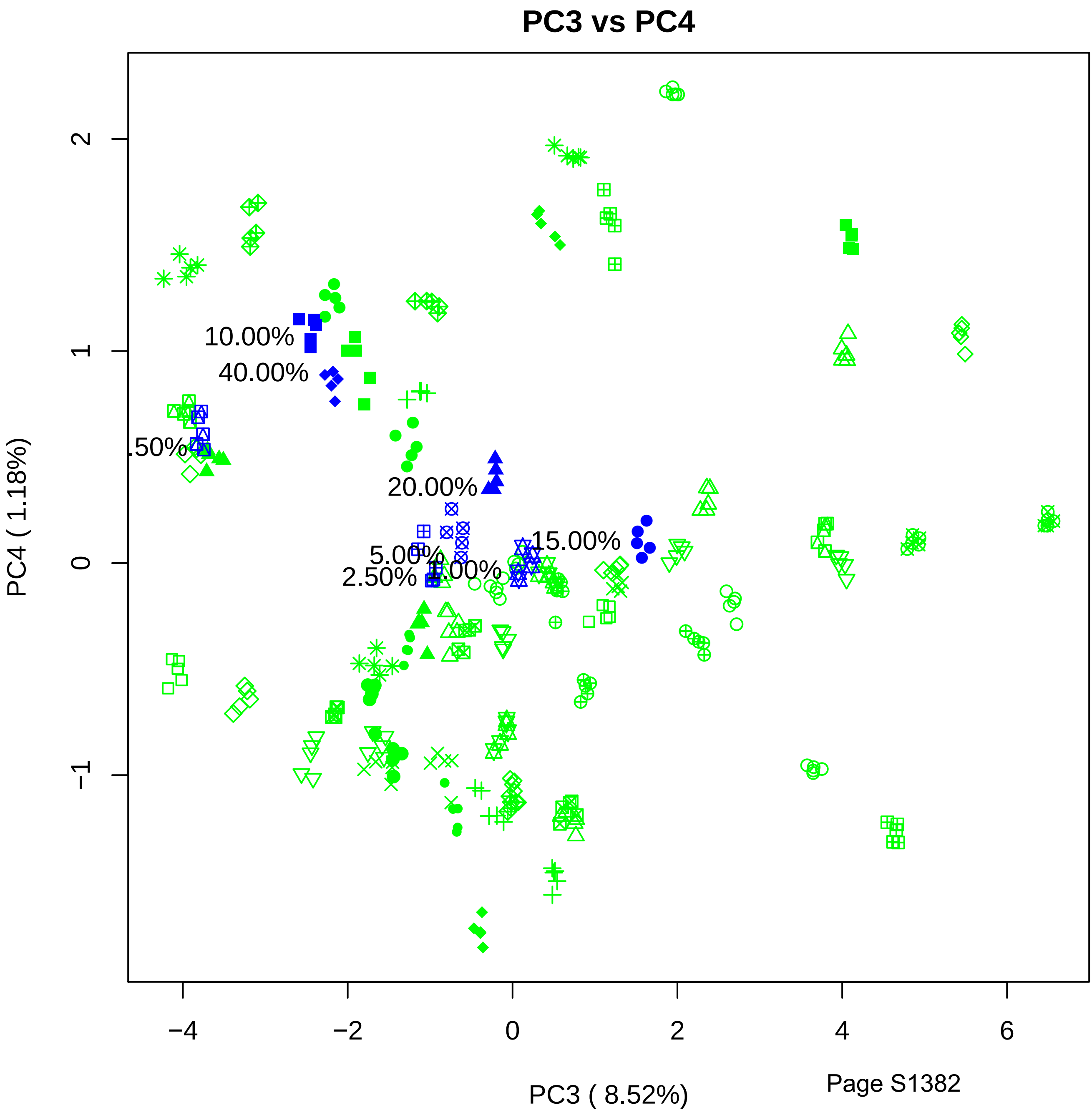

PC3 vs PC4
PC4 (1.18%)
PC3 (8.52%)
2
1
0
−1
−4
−2
0
2
4
6
10.00%
40.00%
.50%
20.00%
15.00%
5.00%
2.50%
1.00%

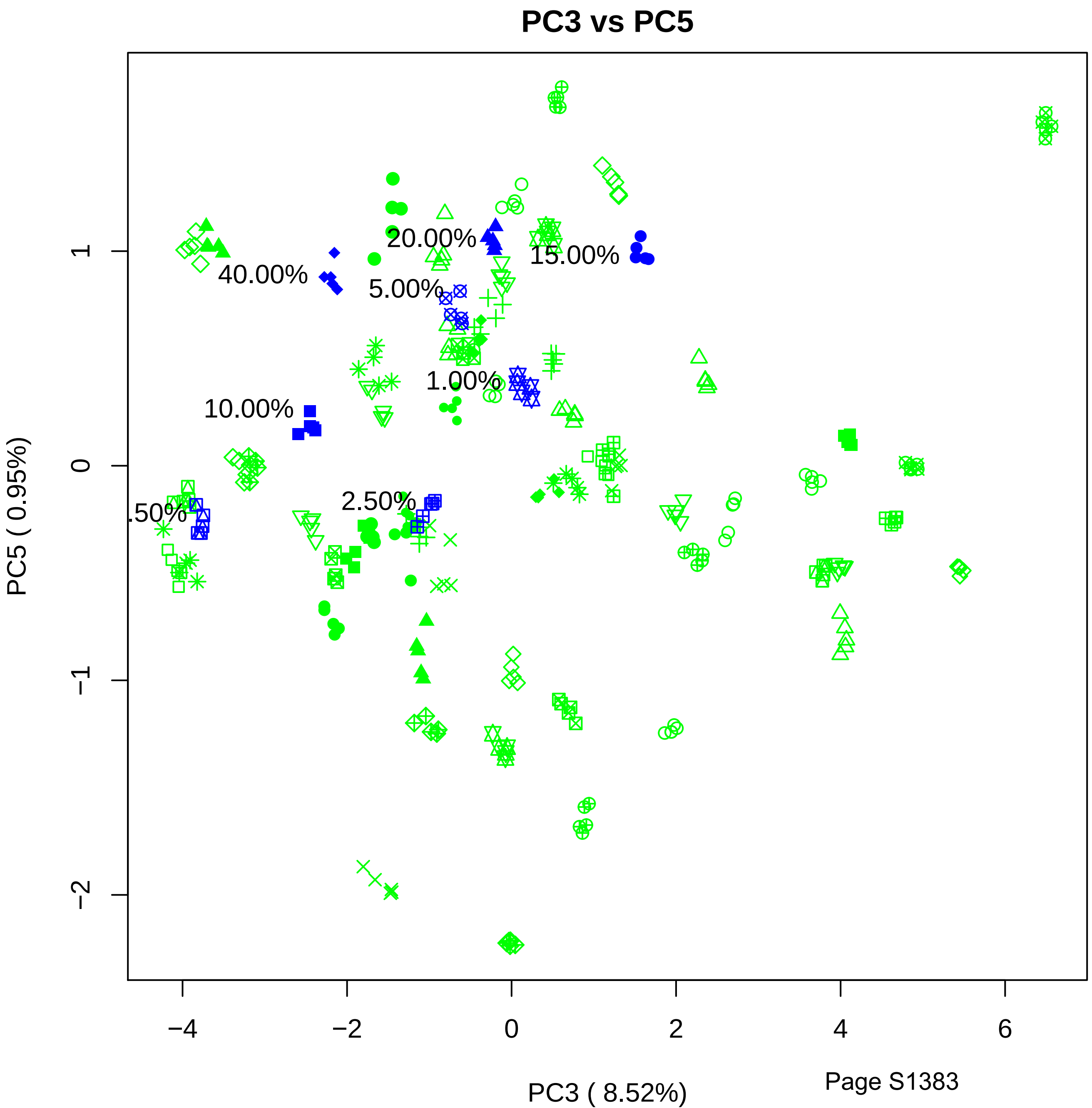

PC3 vs PC5
PC5 (0.95%)
PC3 (8.52%)
1
0
−1
−2
−4
−2
0
2
4
6
40.00%
20.00%
15.00%
5.00%
1.00%
10.00%
2.50%
.50%

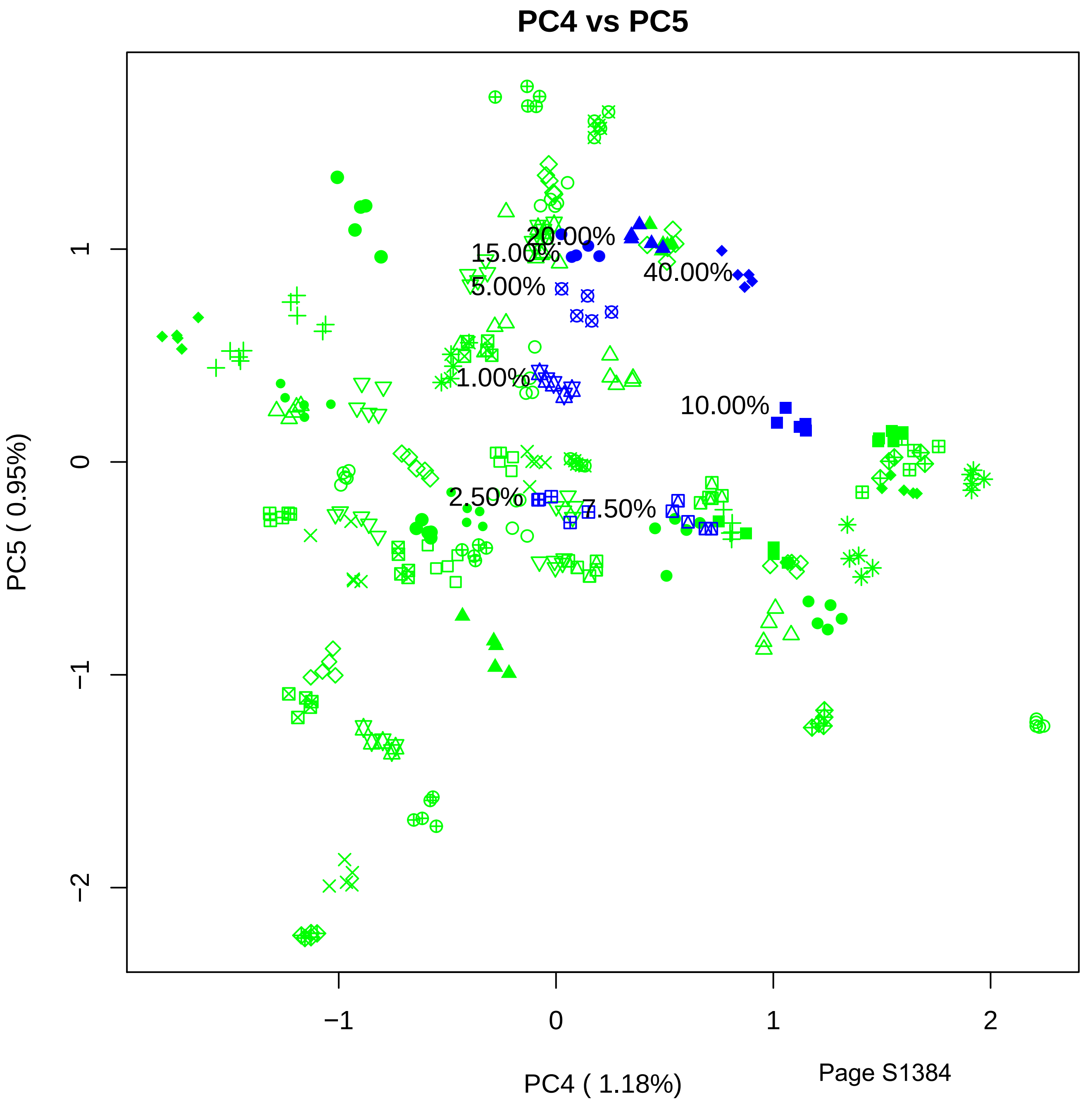

PC4 vs PC5
20.00%
15.00%
40.00%
5.00%
1.00%
10.00%
2.50%
7.50%
1
0
−1
−2
2
PC5 (0.95%)
PC4 (1.18%)

PCA of 1% BMH(d) vs PMH(d)

Scaled Matrix PCAed

7th order polynomial baseline correction performed

Raw spectra smoothed to 21 Neighbours, 2nd order PN, Savtisky Golay

Normalisation between 0 and 100

D2 spectra smoothed to 41 Neighbours, 2nd order PN, Savtisky Golay

Regions Analysed: 3601−2501 and 2001−700cm−1

Samples Analysed: 1 bContam's 0 BLH's 0 BMH's 0 OMH's 0 pContam's 0 PLH's 60 PMH's

Keele University AD

Fri Dec 14 14:22:24 2018

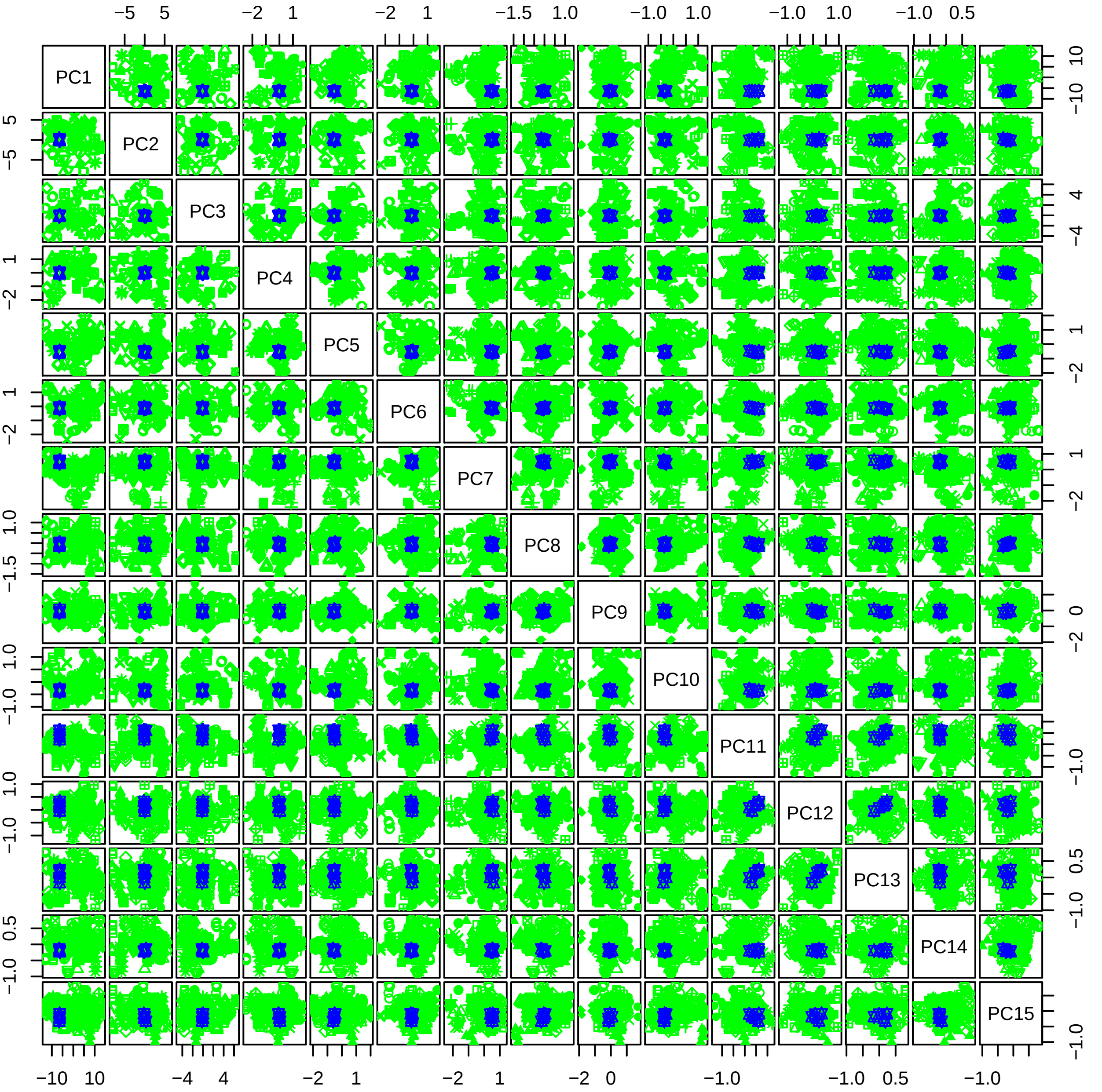
PC1
PC2
PC3
PC4
PC5
PC6
PC7
PC8
PC9
PC10
PC11
PC12
PC13
PC14
PC15

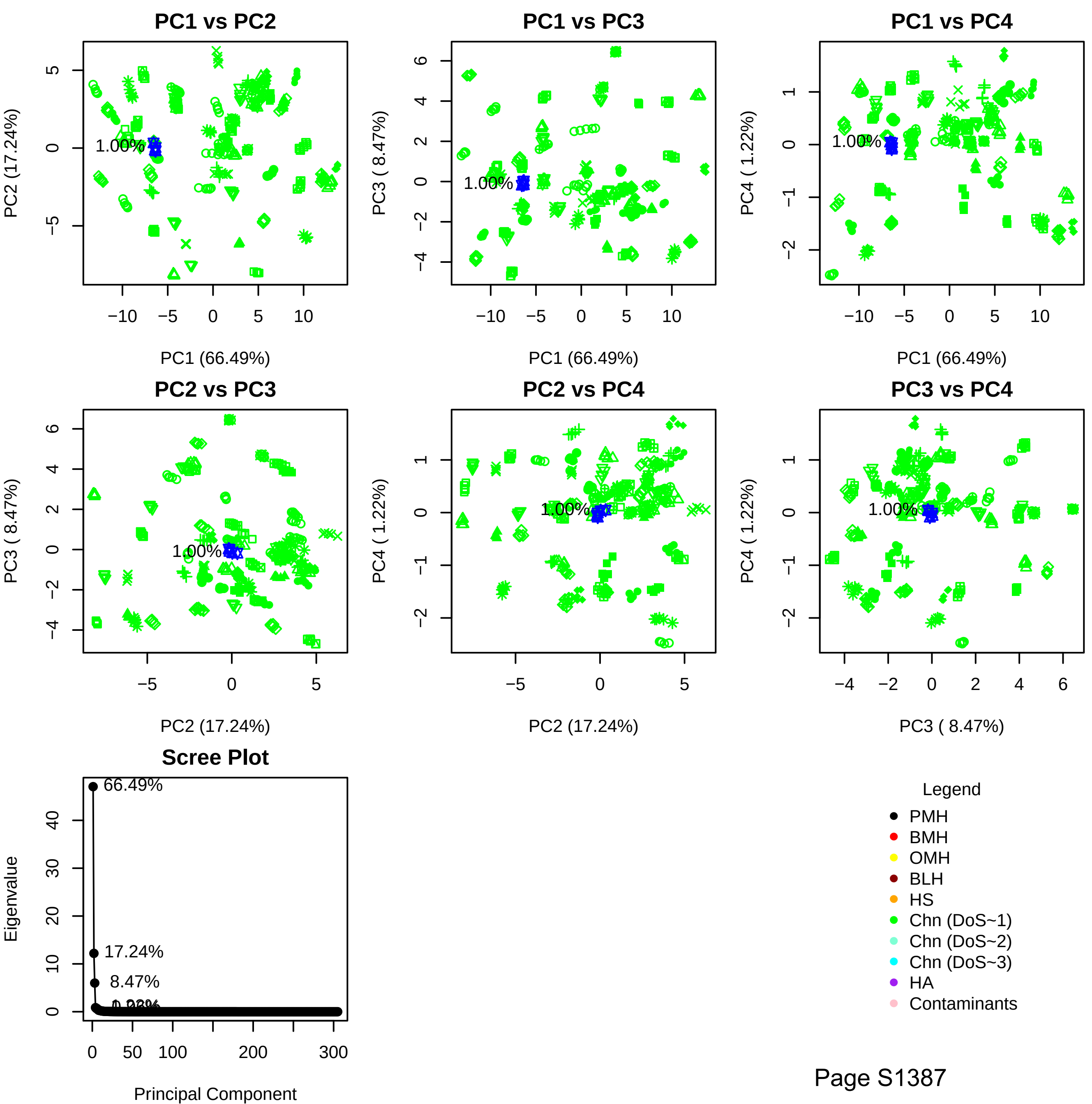
PC1 vs PC2
PC1 vs PC3
PC1 vs PC4
PC2 vs PC3
PC2 vs PC4
PC3 vs PC4
Scree Plot
PC1 (66.49%)
PC2 (17.24%)
PC3 (8.47%)
PC4 (1.22%)
1.00%
Eigenvalue
Principal Component
66.49%
17.24%
8.47%
Legend
PMH
BMH
OMH
BLH
HS
Chn (DoS~1)
Chn (DoS~2)
Chn (DoS~3)
HA
Contaminants

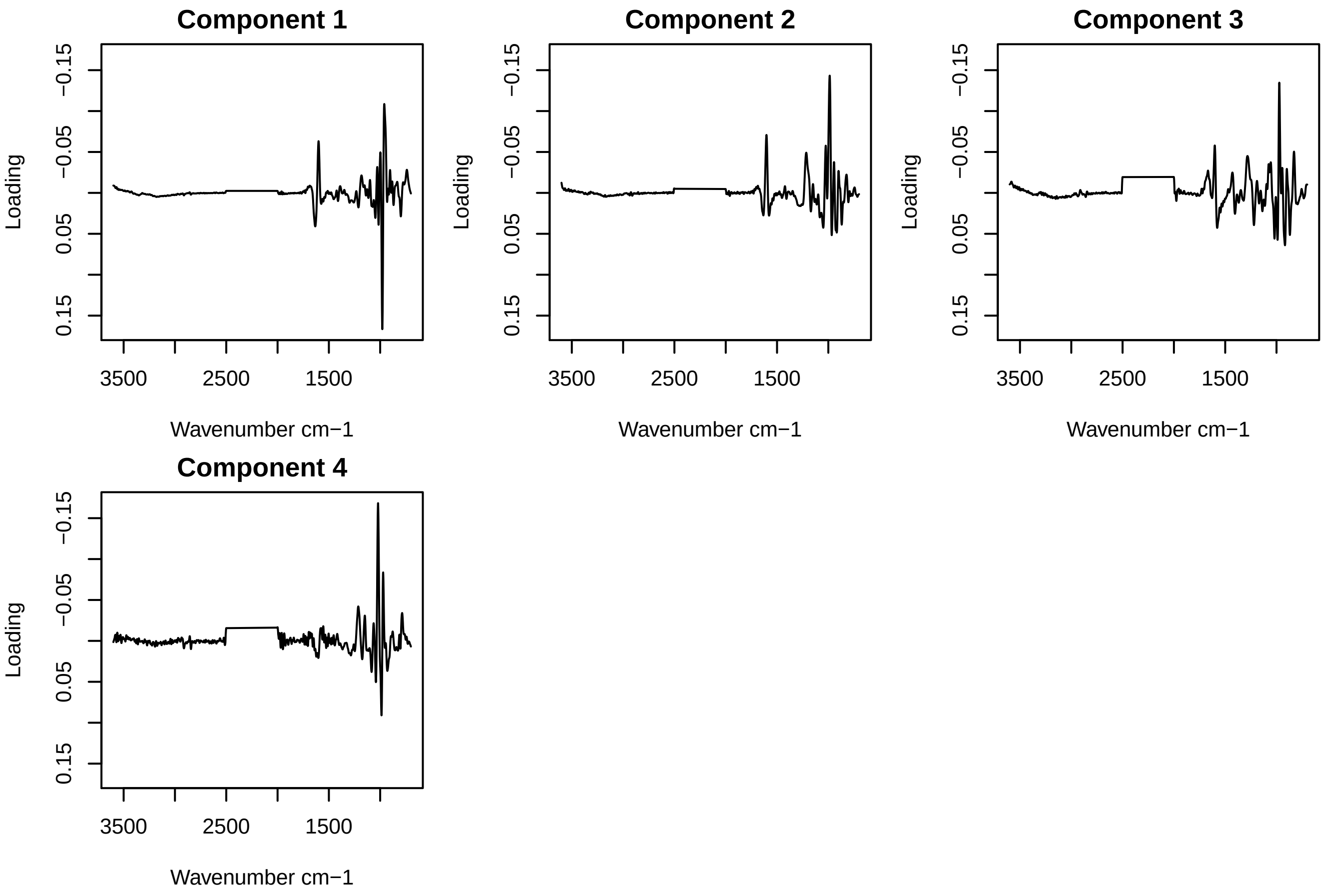

Component 1
Component 2
Component 3
Component 4
Loading
−0.15
−0.05
0.05
0.15
3500
2500
1500
Wavenumber cm−1

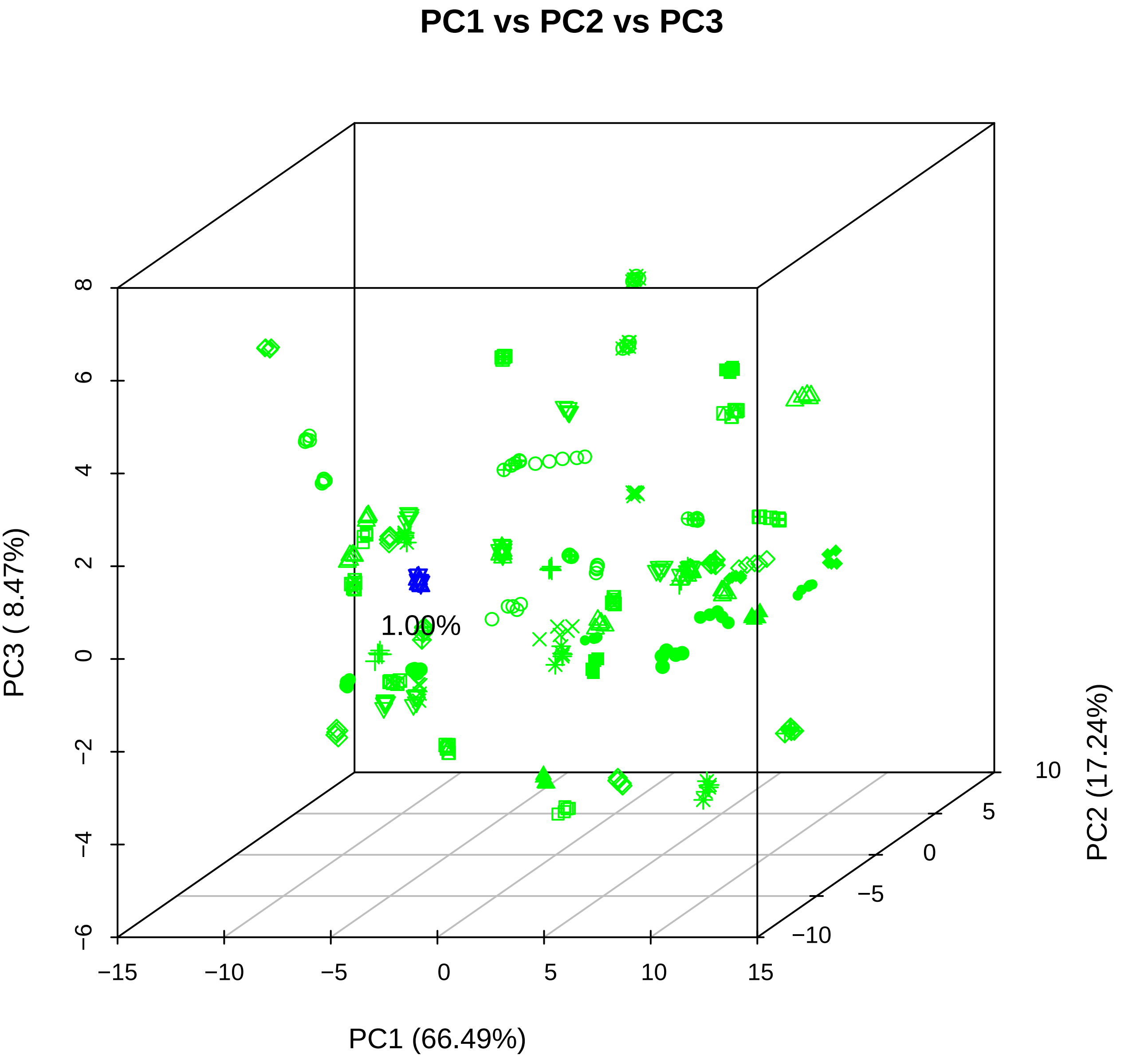

PC1 vs PC2 vs PC3
1.00%
PC1 (66.49%)
PC2 (17.24%)
PC3 (8.47%)
−15
−10
−5
0
5
10
15
−6
−4
−2
0
2
4
6
8
−10
−5
0
5
10

PC1 vs PC2 vs PC4

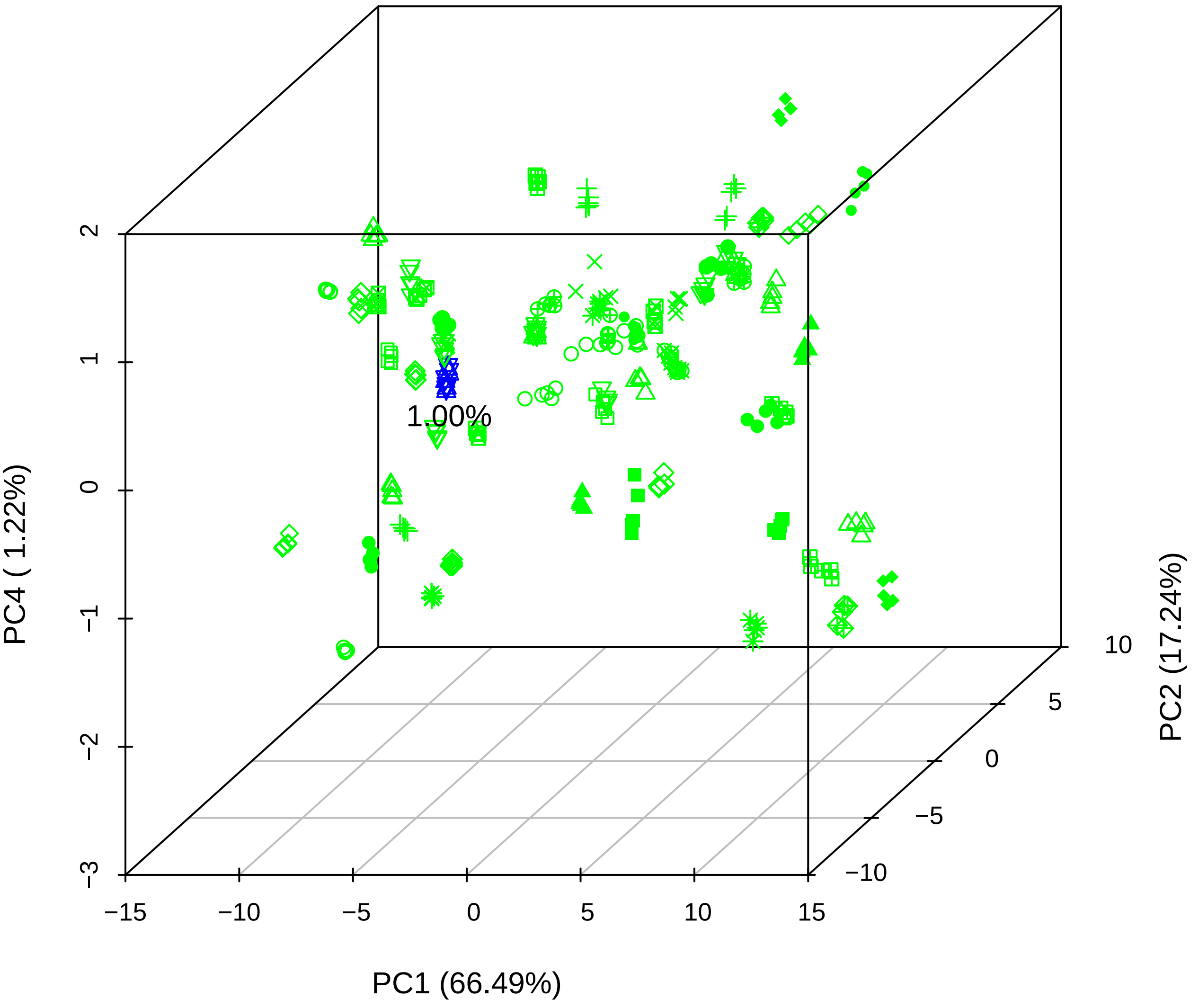

PC1 vs PC3 vs PC4

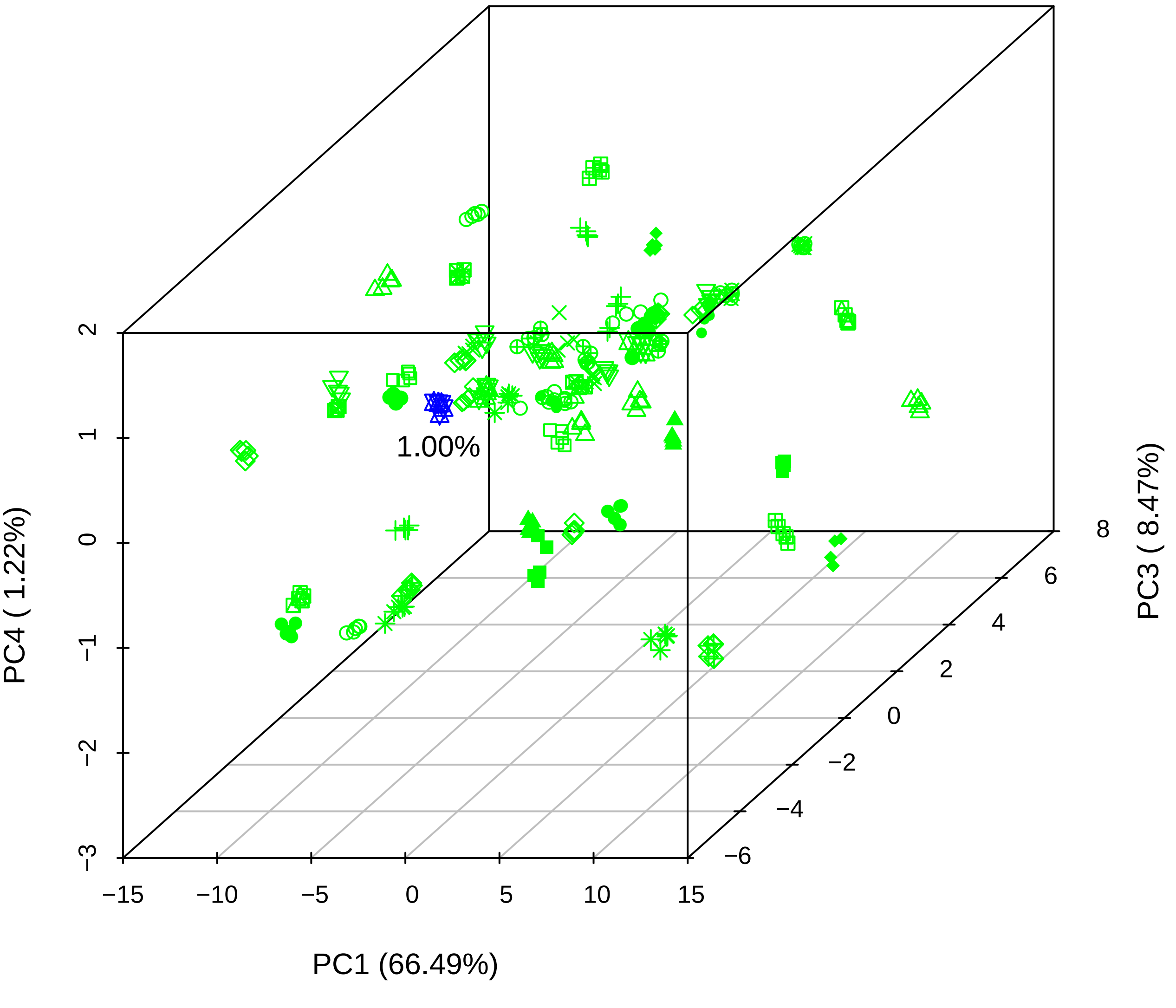

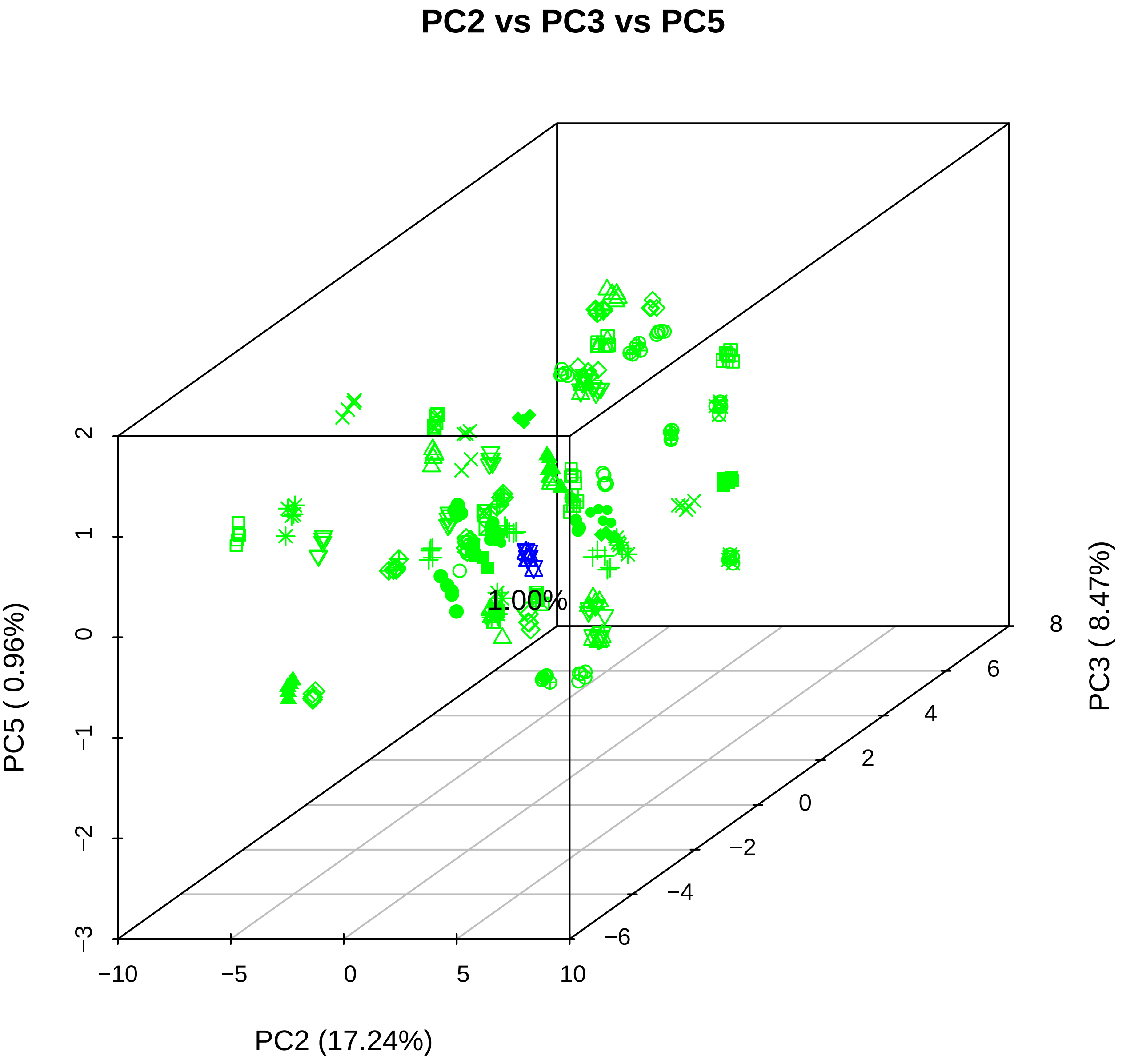
PC2 vs PC3 vs PC5
PC2 (17.24%)
PC5 (0.96%)
PC3 (8.47%)
1.00%
−10
−5
0
5
10
−3
−2
−1
0
1
2
−6
−4
−2
0
2
4
6
8

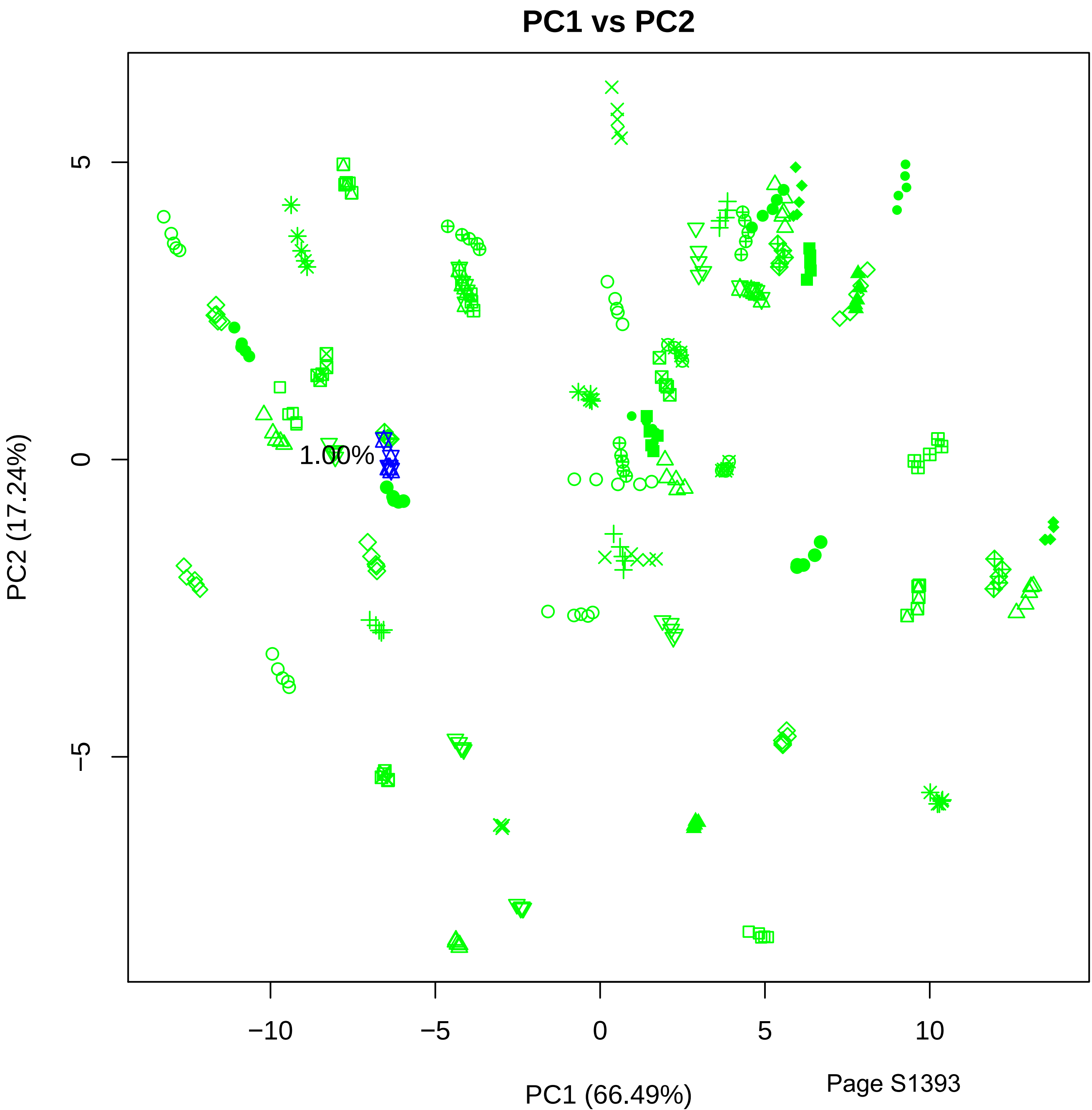

PC1 vs PC2
1.00%
5
0
−5
−10
−5
0
5
10
PC2 (17.24%)
PC1 (66.49%)

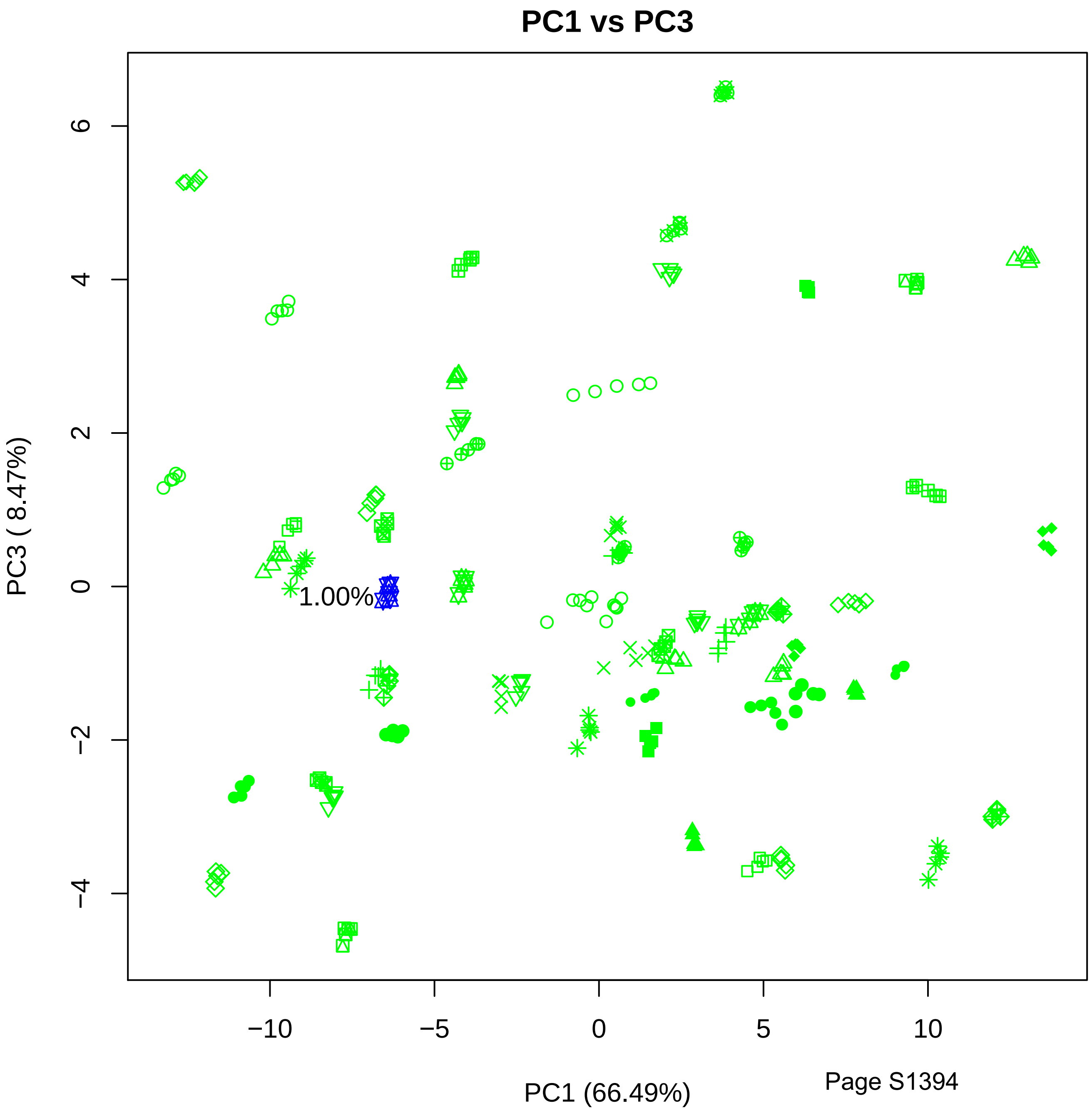
PC1 vs PC3
PC3 (8.47%)
PC1 (66.49%)
1.00%
6
4
2
0
−2
−4
−10
−5
0
5
10

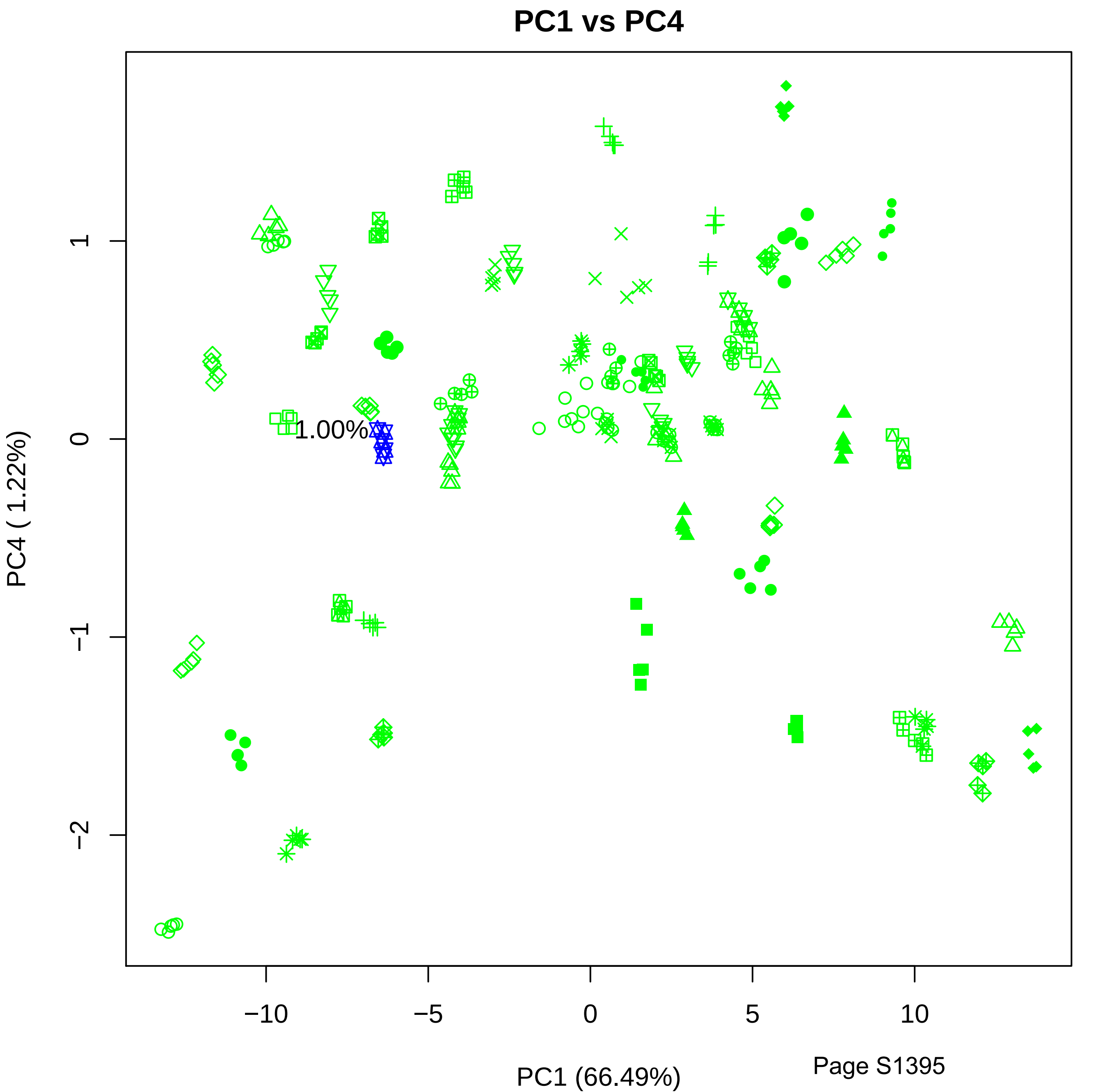
PC1 vs PC4
1.00%
PC4 (1.22%)
PC1 (66.49%)
1
0
−1
−2
−10
−5
0
5
10

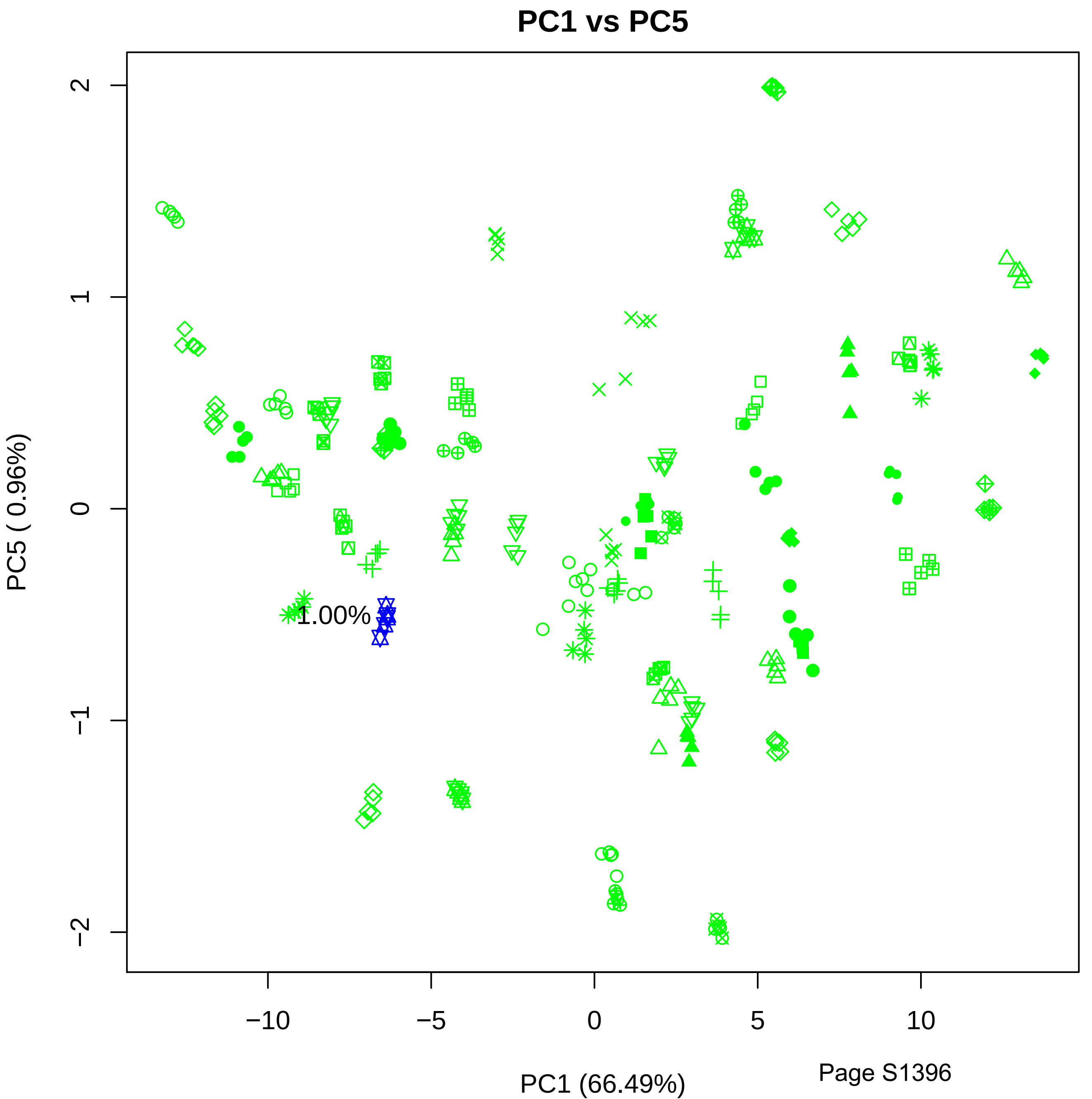
PC1 vs PC5
PC5 (0.96%)
PC1 (66.49%)
1.00%
2
1
0
−1
−2
−10
−5
0
5
10

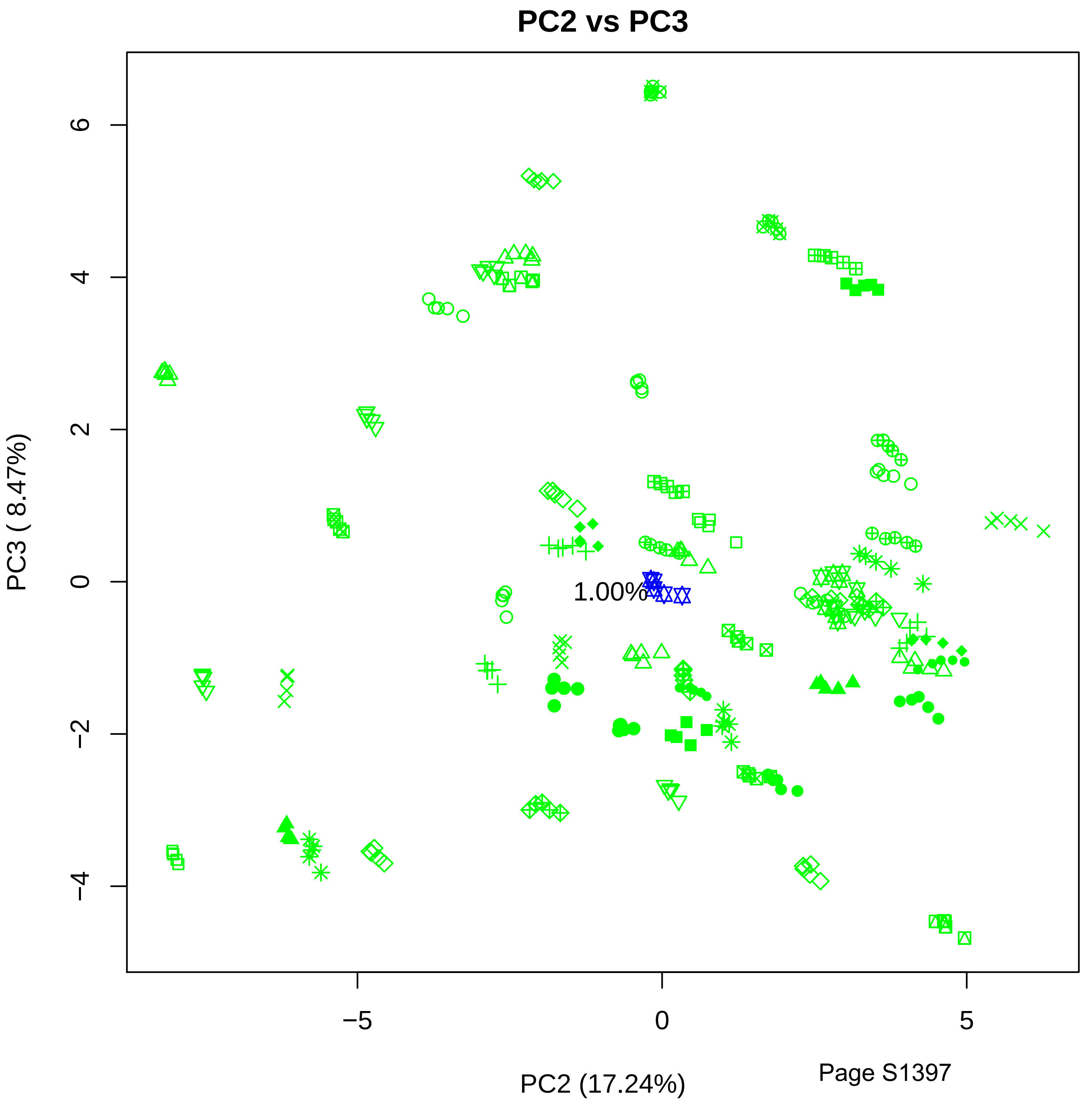
PC2 vs PC3
PC3 (8.47%)
PC2 (17.24%)
1.00%
6
4
2
0
−2
−4
−5
0
5

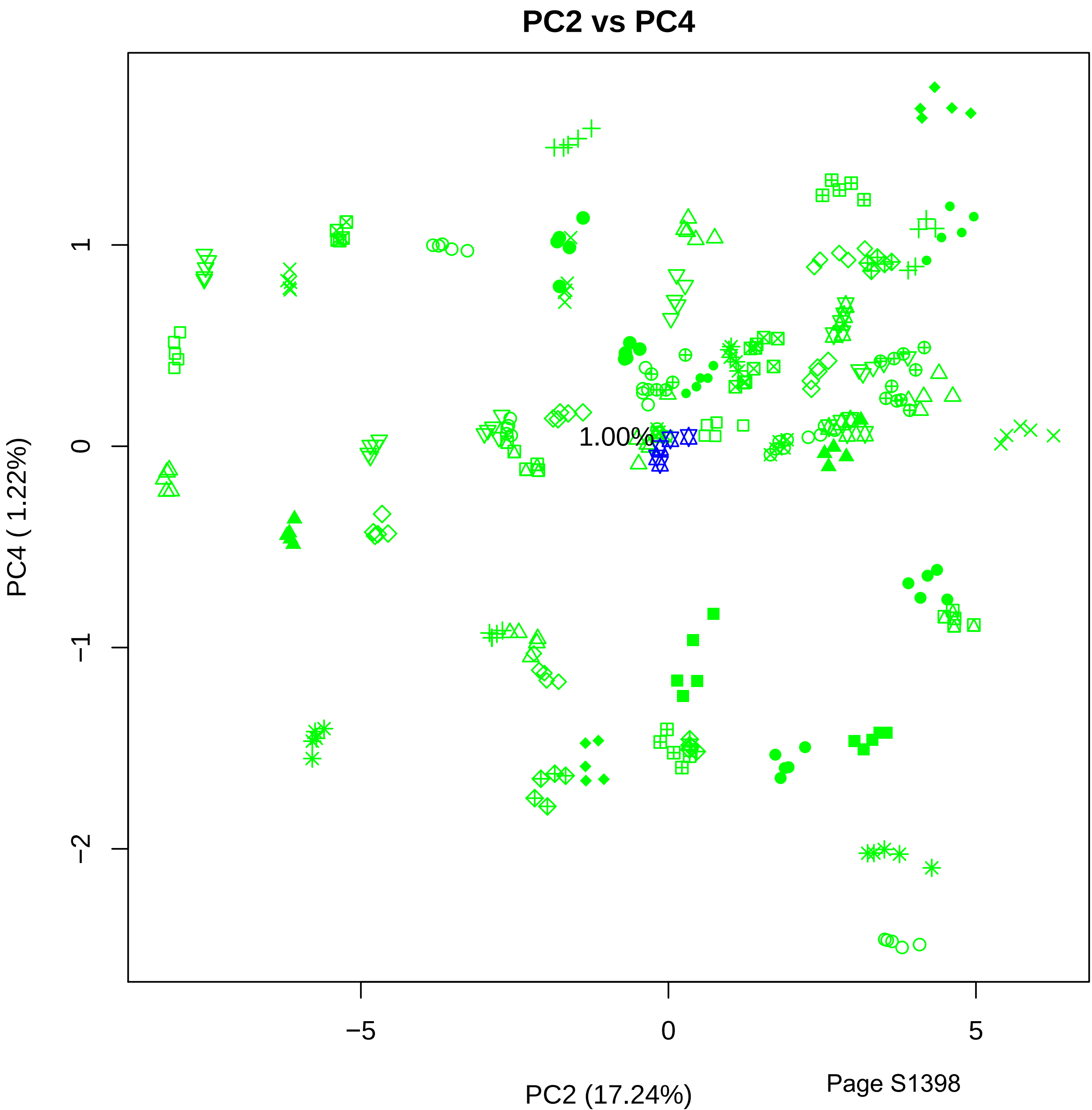

PC2 vs PC4
1.00%
PC4 (1.22%)
PC2 (17.24%)
−5
0
5
−2
−1
0
1

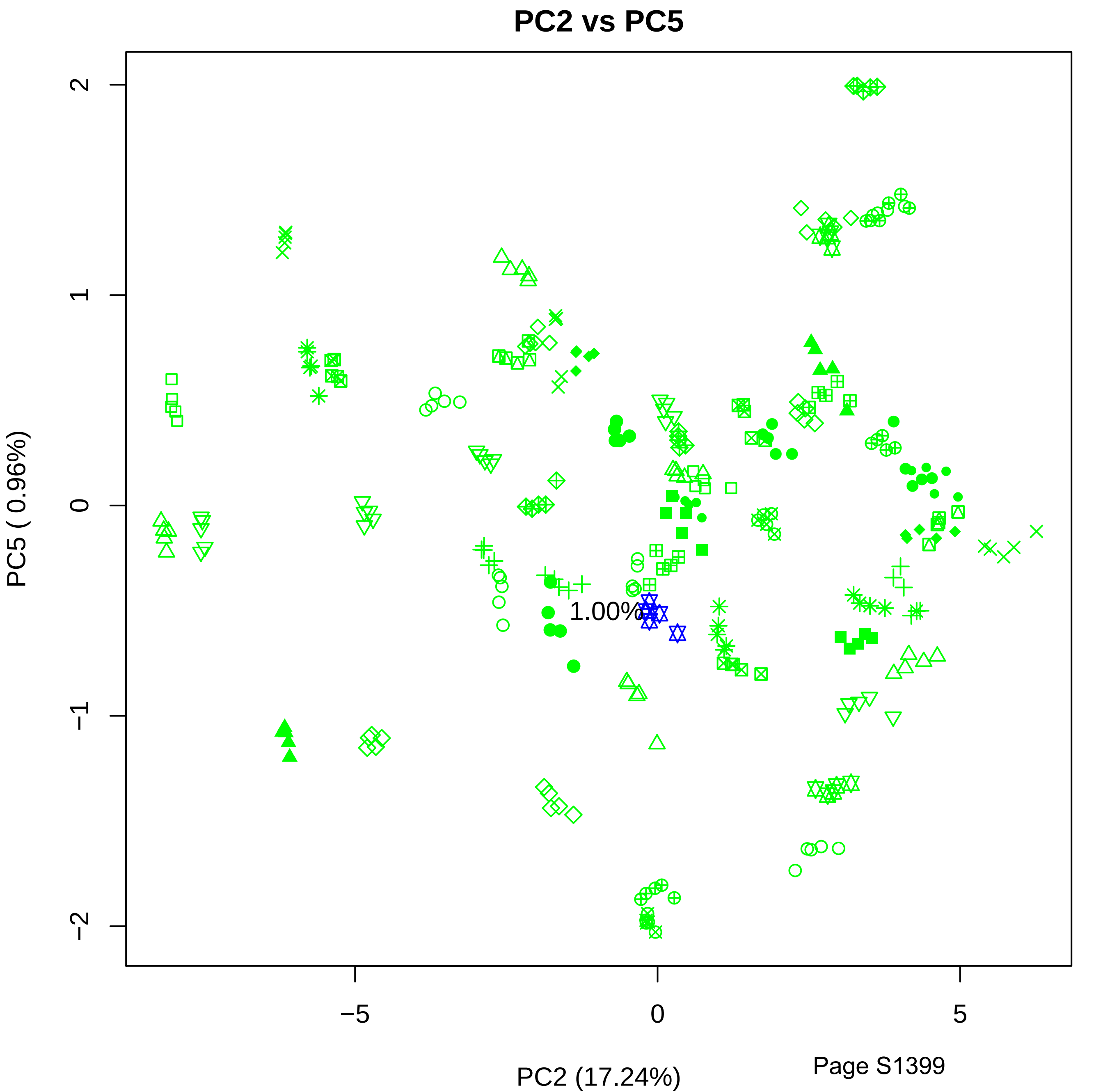

PC2 vs PC5
2
1
0
−1
−2
PC5 (0.96%)
1.00%
−5
0
5
PC2 (17.24%)

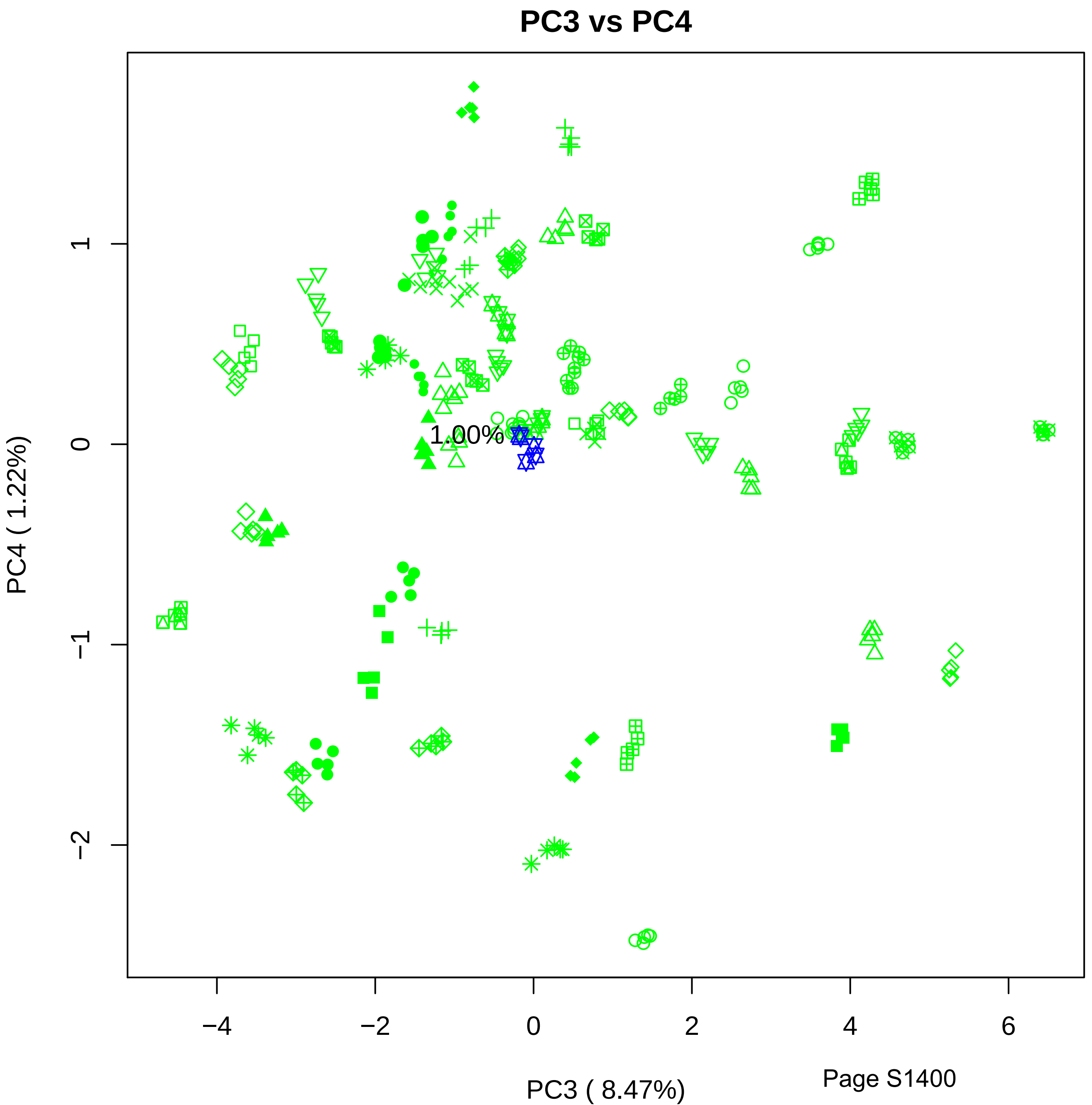

PC3 vs PC4
1.00%
PC4 (1.22%)
PC3 (8.47%)
−4
−2
0
2
4
6
−2
−1
0
1

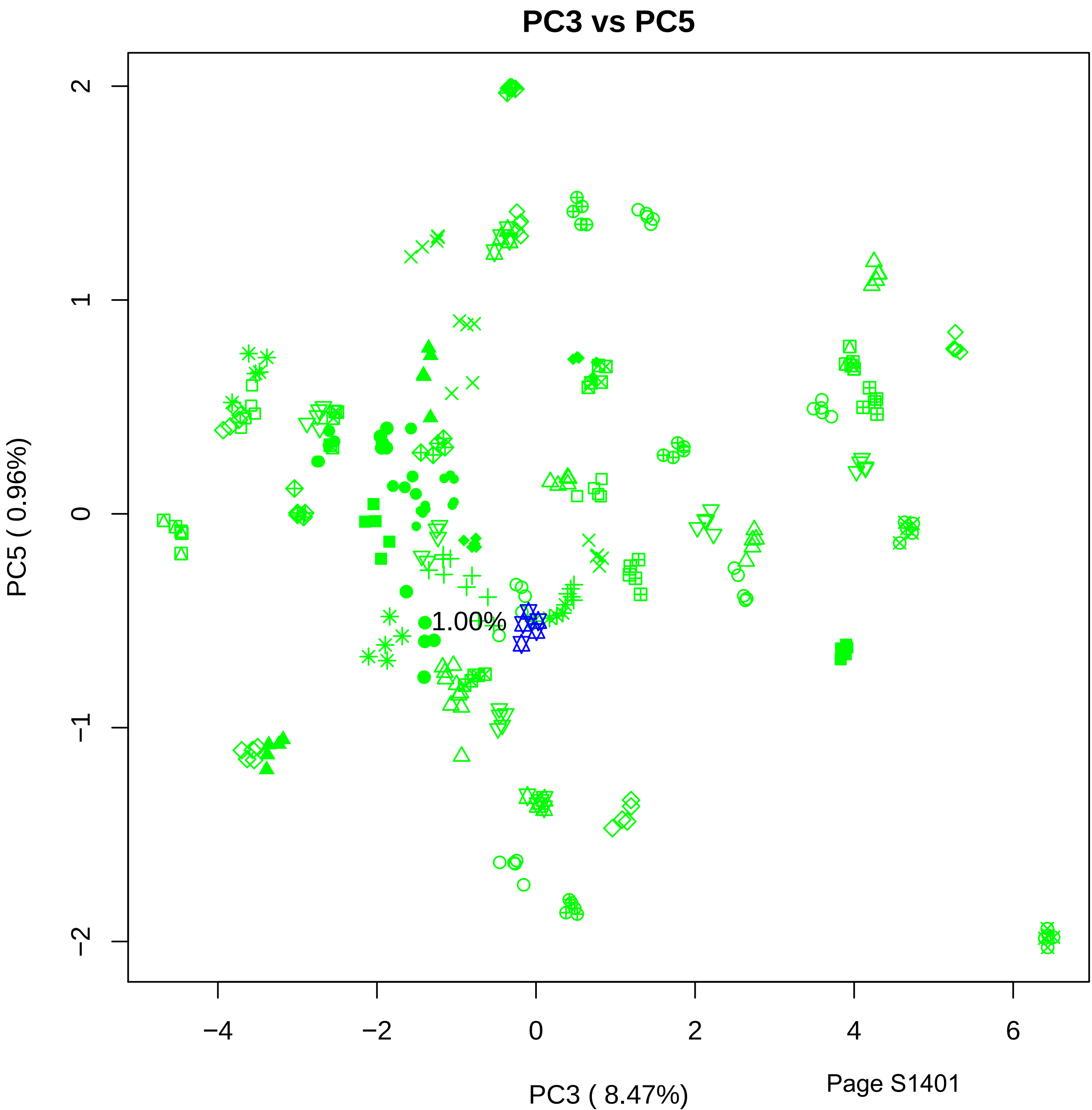
PC3 vs PC5
PC5 (0.96%)
PC3 (8.47%)
1.00%
2
1
0
−1
−2
−4
−2
0
2
4
6

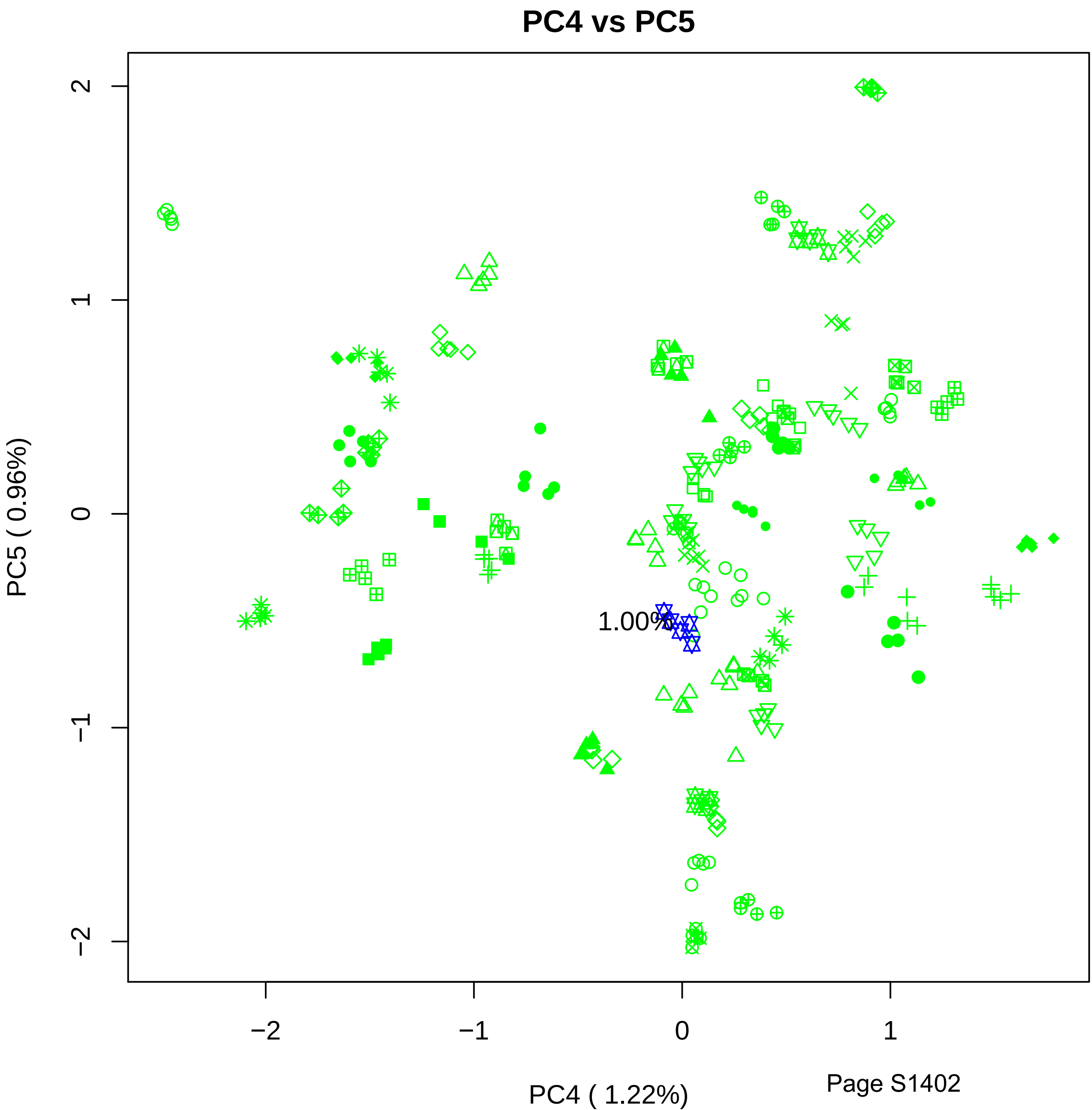

PC4 vs PC5
2
1
0
−1
−2
PC5 (0.96%)
1.00%
−2
−1
0
1
PC4 (1.22%)

PCA of 2.5% BMH(d) vs PMH(d)

Scaled Matrix PCAed

7th order polynomial baseline correction performed

Raw spectra smoothed to 21 Neighbours, 2nd order PN, Savtisky Golay

Normalisation between 0 and 100

D2 spectra smoothed to 41 Neighbours, 2nd order PN, Savtisky Golay

Regions Analysed: 3601−2501 and 2001−700cm−1

Samples Analysed: 1 bContam's 0 BLH's 0 BMH's 0 OMH's 0 pContam's 0 PLH's 60 PMH's

Keele University AD

Fri Dec 14 14:22:32 2018

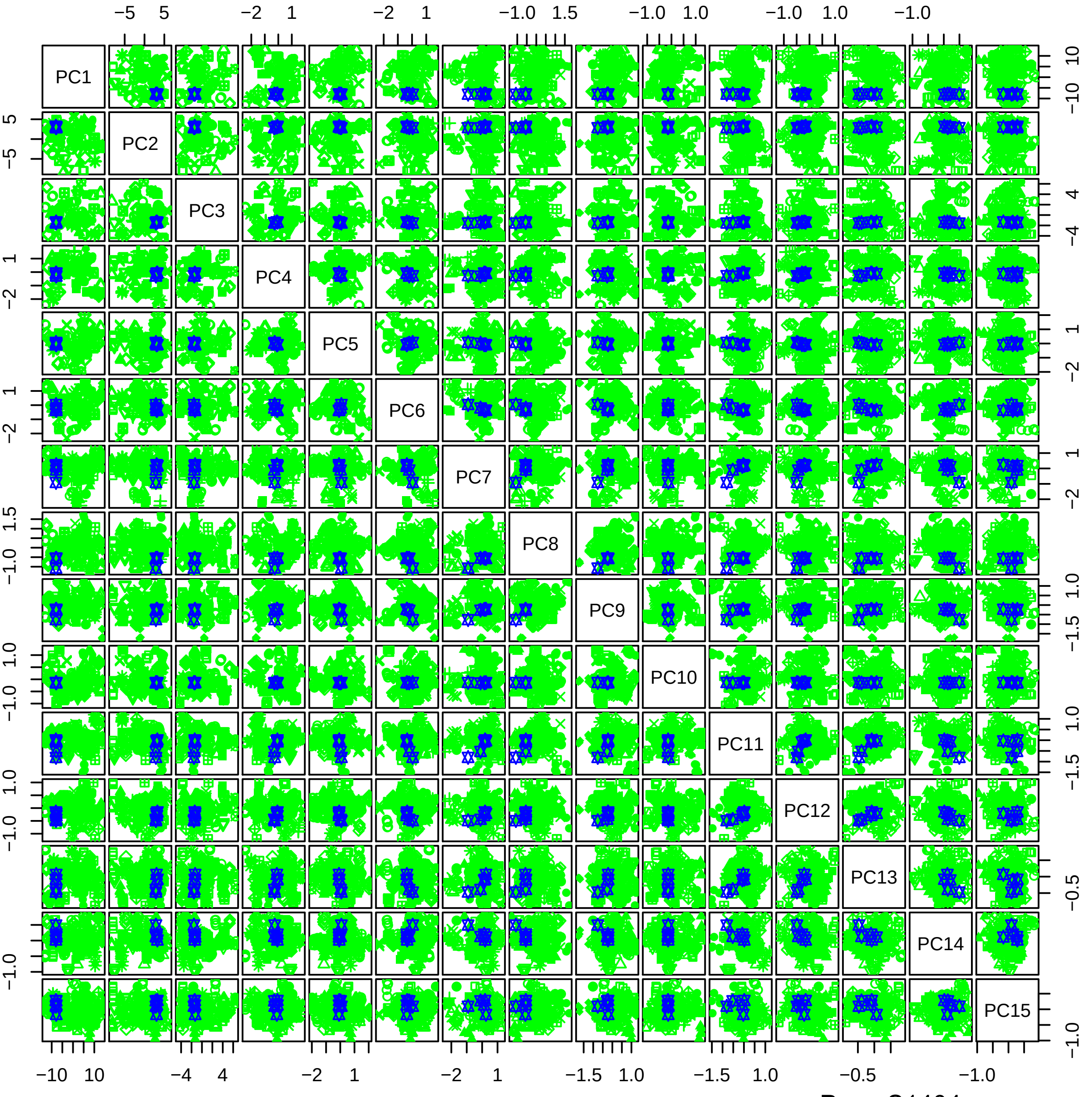
PC1
PC2
PC3
PC4
PC5
PC6
PC7
PC8
PC9
PC10
PC11
PC12
PC13
PC14
PC15

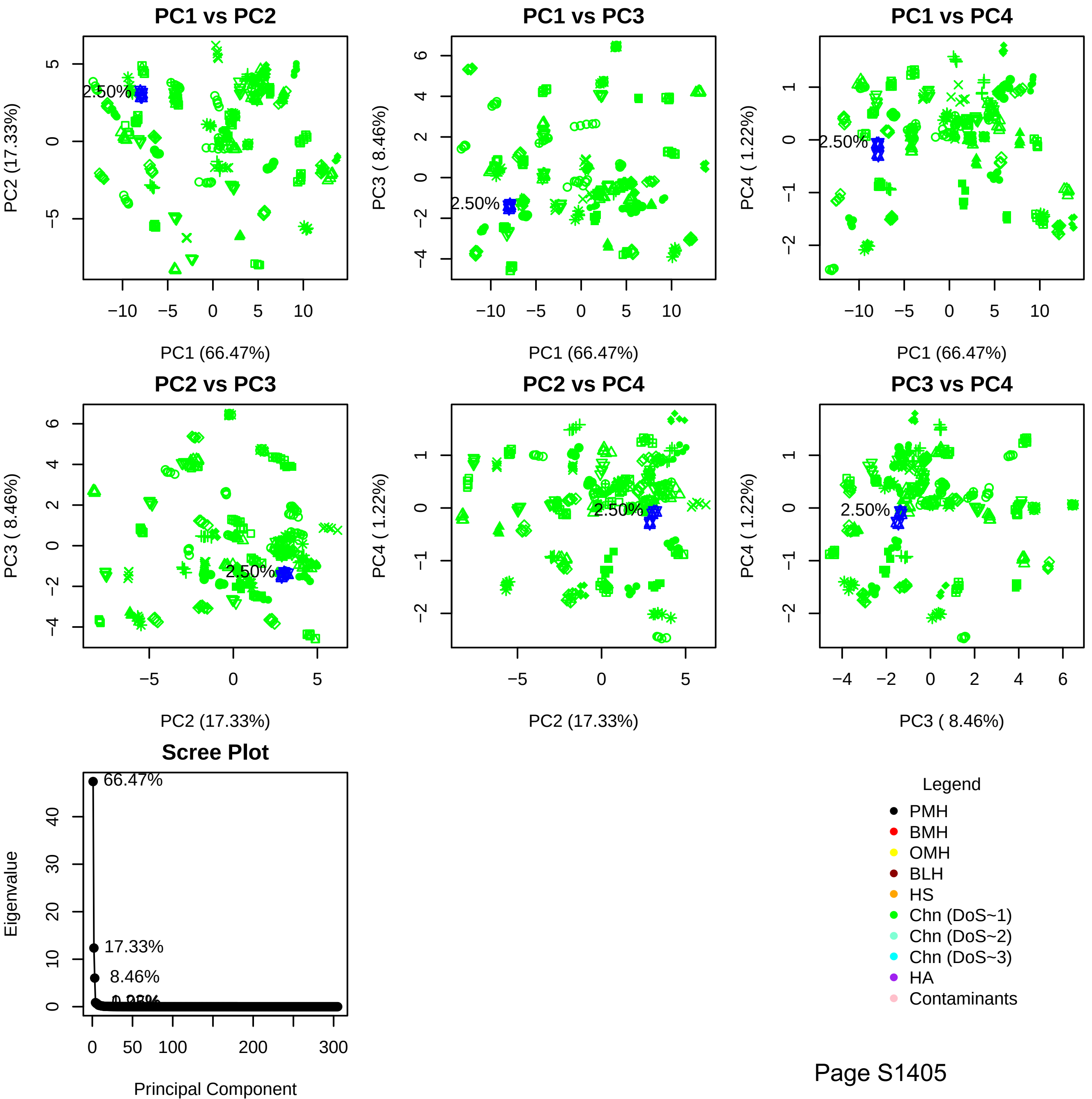
PC1 vs PC2
PC1 vs PC3
PC1 vs PC4
PC2 vs PC3
PC2 vs PC4
PC3 vs PC4
Scree Plot
PC1 (66.47%)
PC2 (17.33%)
PC3 (8.46%)
PC4 (1.22%)
2.50%
Eigenvalue
Principal Component
66.47%
17.33%
8.46%
Legend
PMH
BMH
OMH
BLH
HS
Chn (DoS~1)
Chn (DoS~2)
Chn (DoS~3)
HA
Contaminants

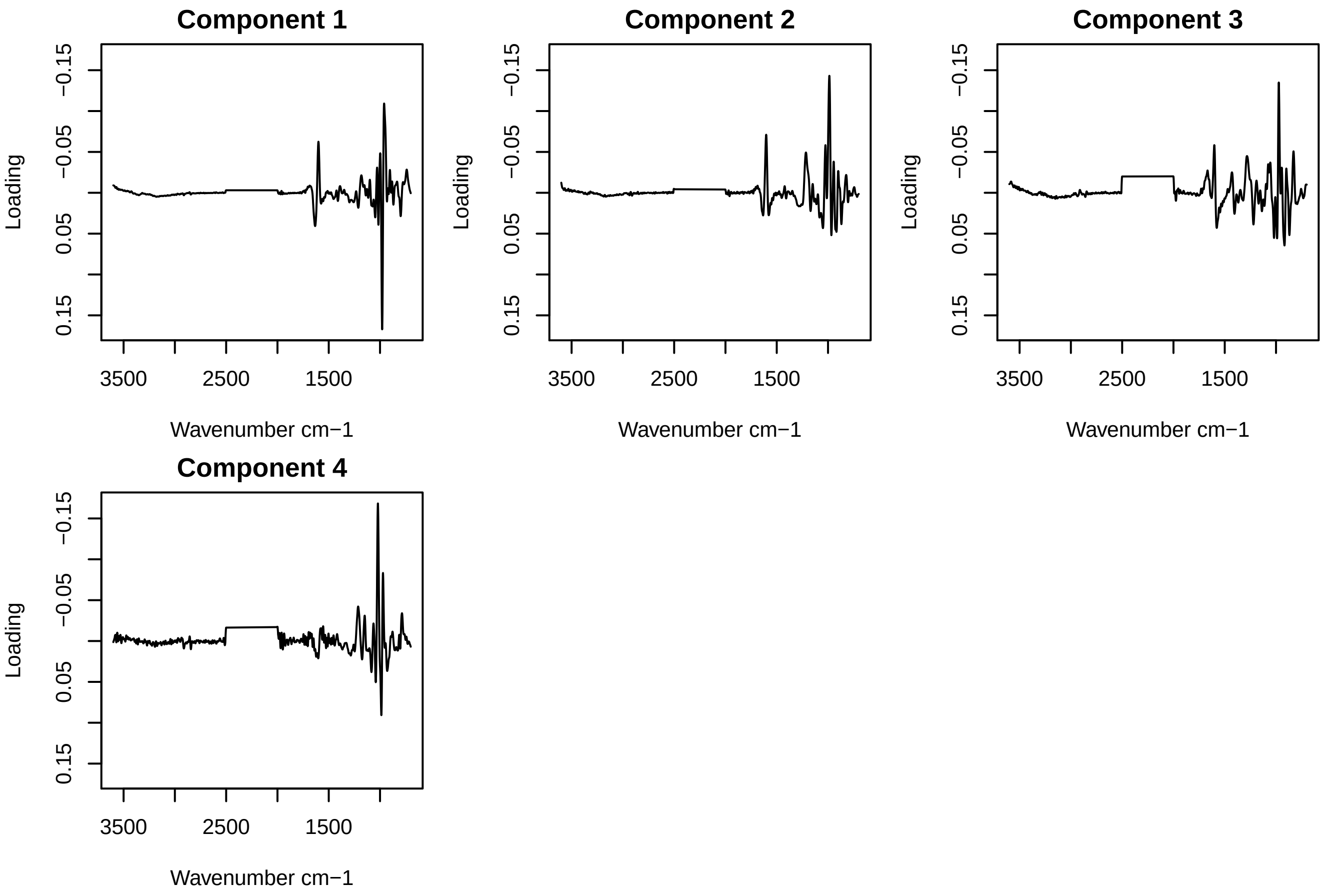
Component 1
Component 2
Component 3
Component 4
Loading
−0.15
−0.05
0.05
0.15
3500
2500
1500
Wavenumber cm−1

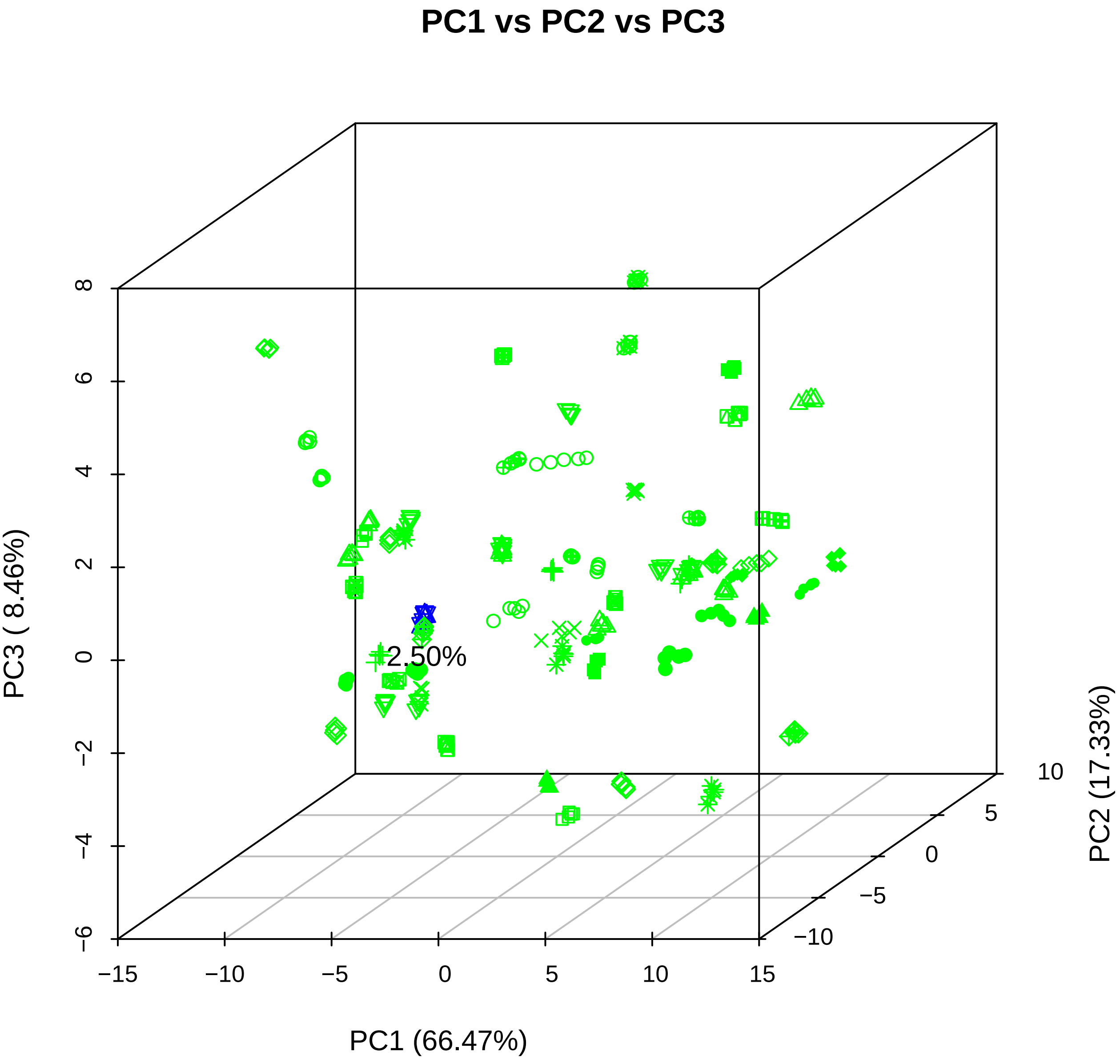

PC1 vs PC2 vs PC3
PC3 (8.46%)
8
6
4
2
0
−2
−4
−6
−15
−10
−5
0
5
10
15
PC1 (66.47%)
PC2 (17.33%)
10
5
0
−5
−10
2.50%

PC1 vs PC2 vs PC4

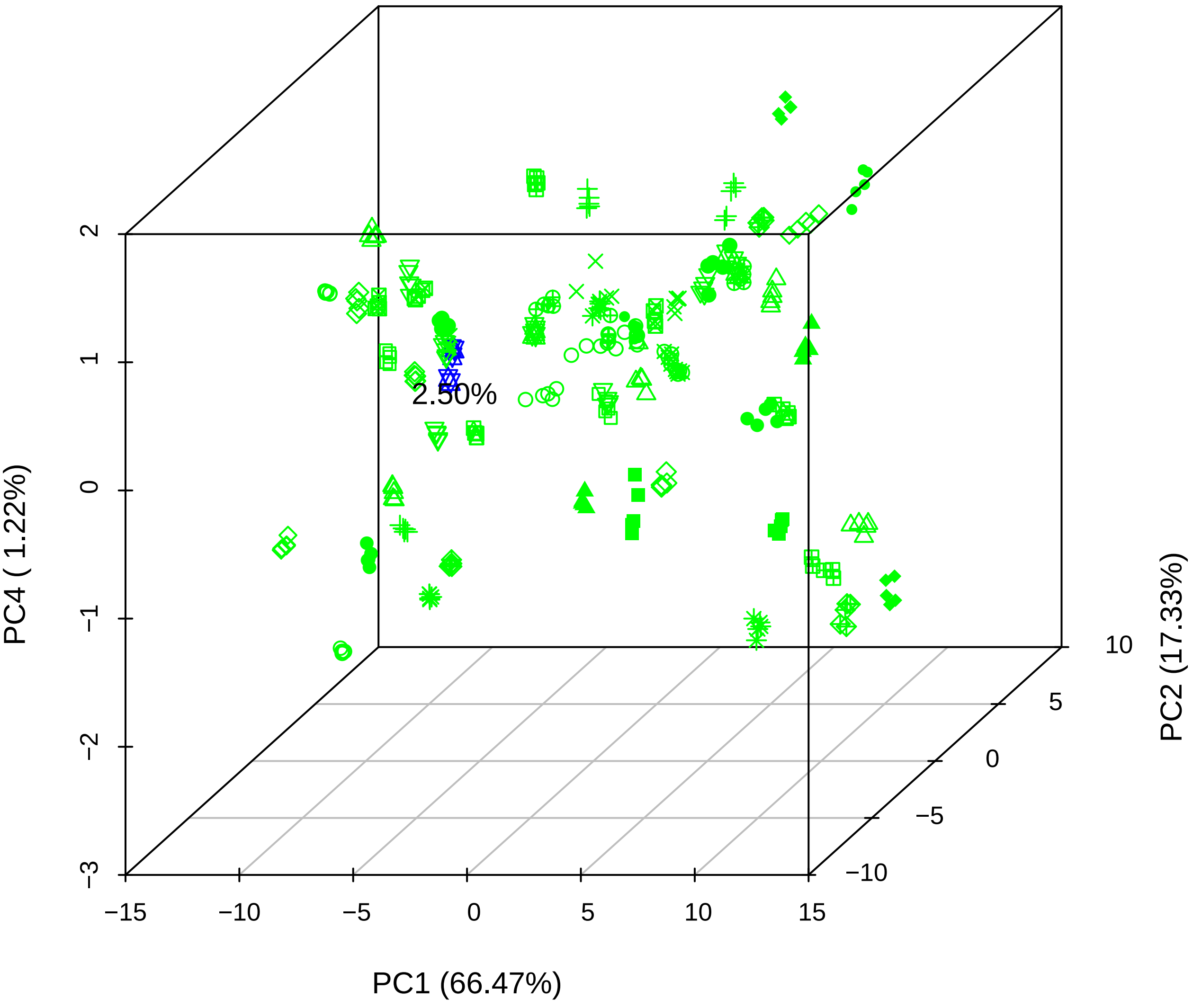

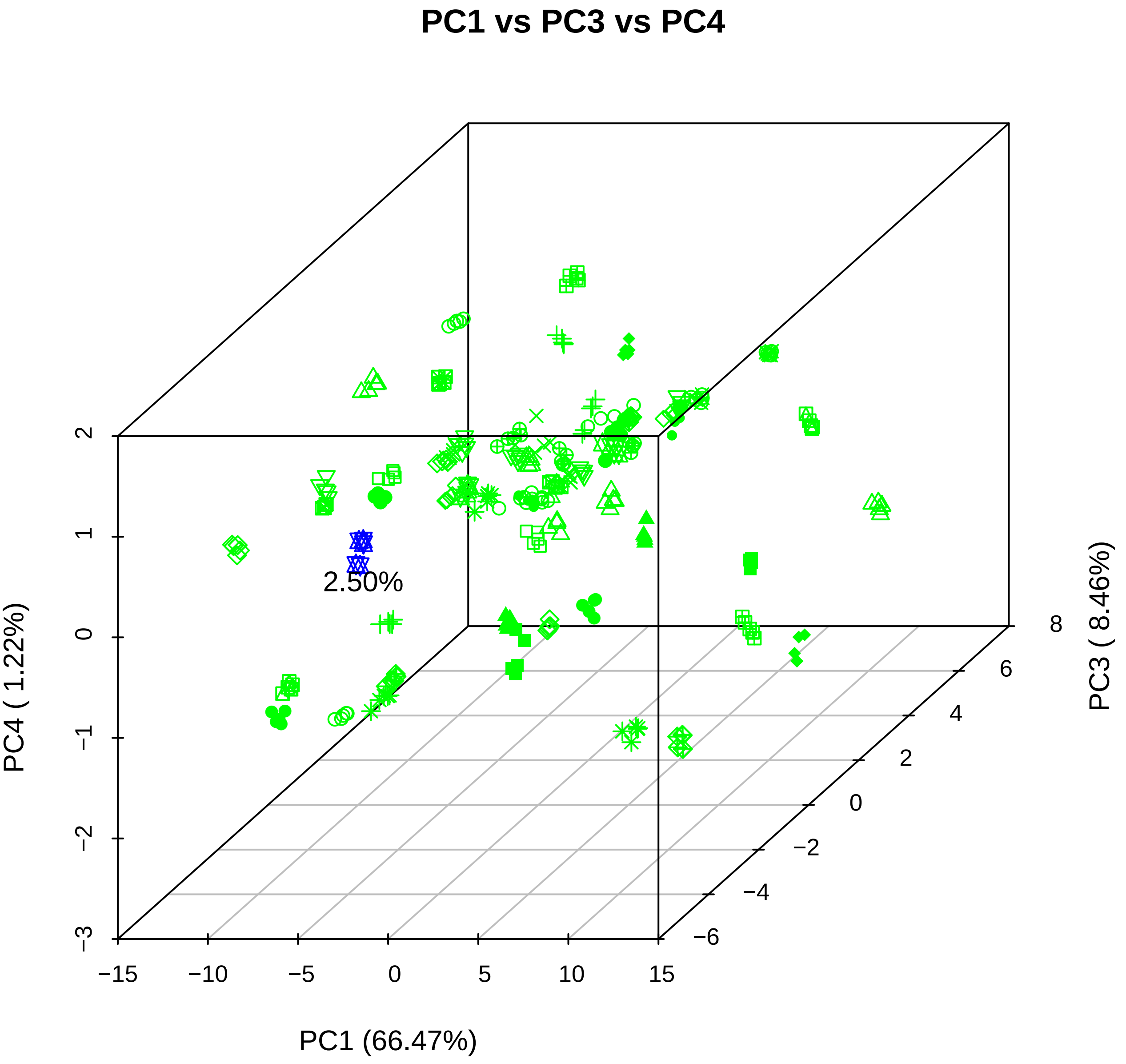

PC1 vs PC3 vs PC4
2.50%
PC1 (66.47%)
PC4 (1.22%)
PC3 (8.46%)
−15
−10
−5
0
5
10
15
−3
−2
−1
0
1
2
−6
−4
−2
0
2
4
6
8

PC2 vs PC3 vs PC5

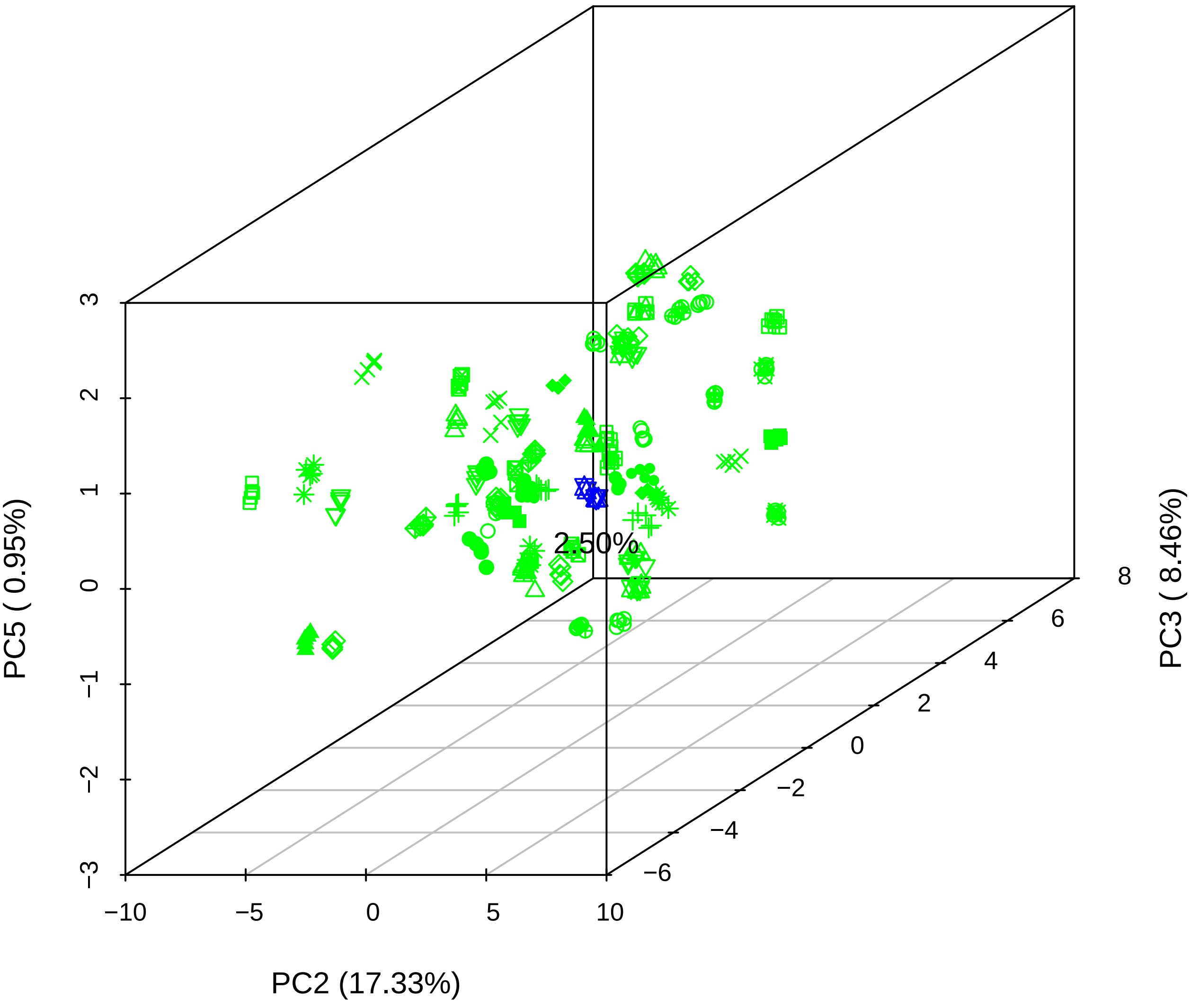

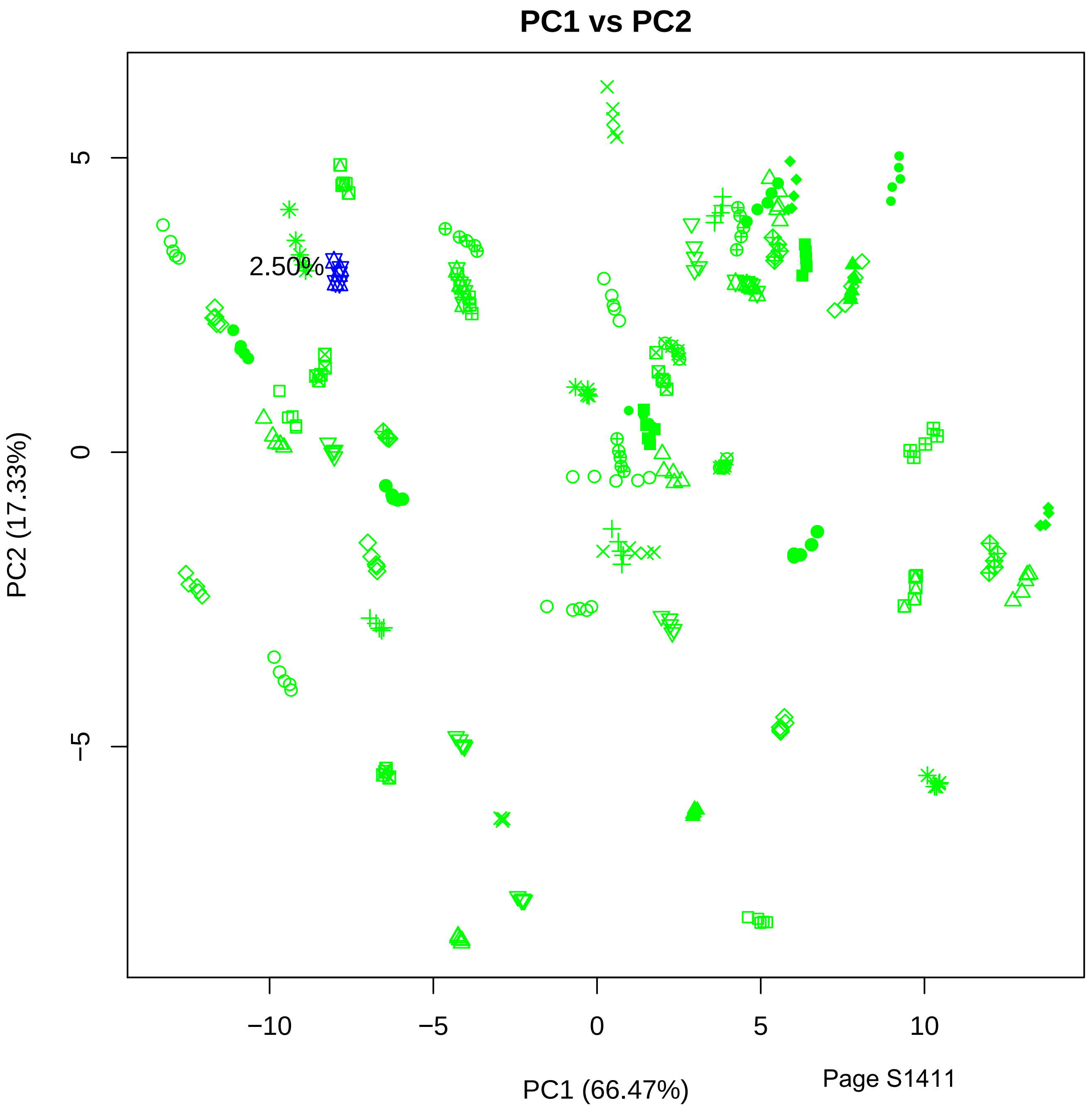

PC1 vs PC2
2.50%
5
0
−5
−10
−5
0
5
10
PC2 (17.33%)
PC1 (66.47%)

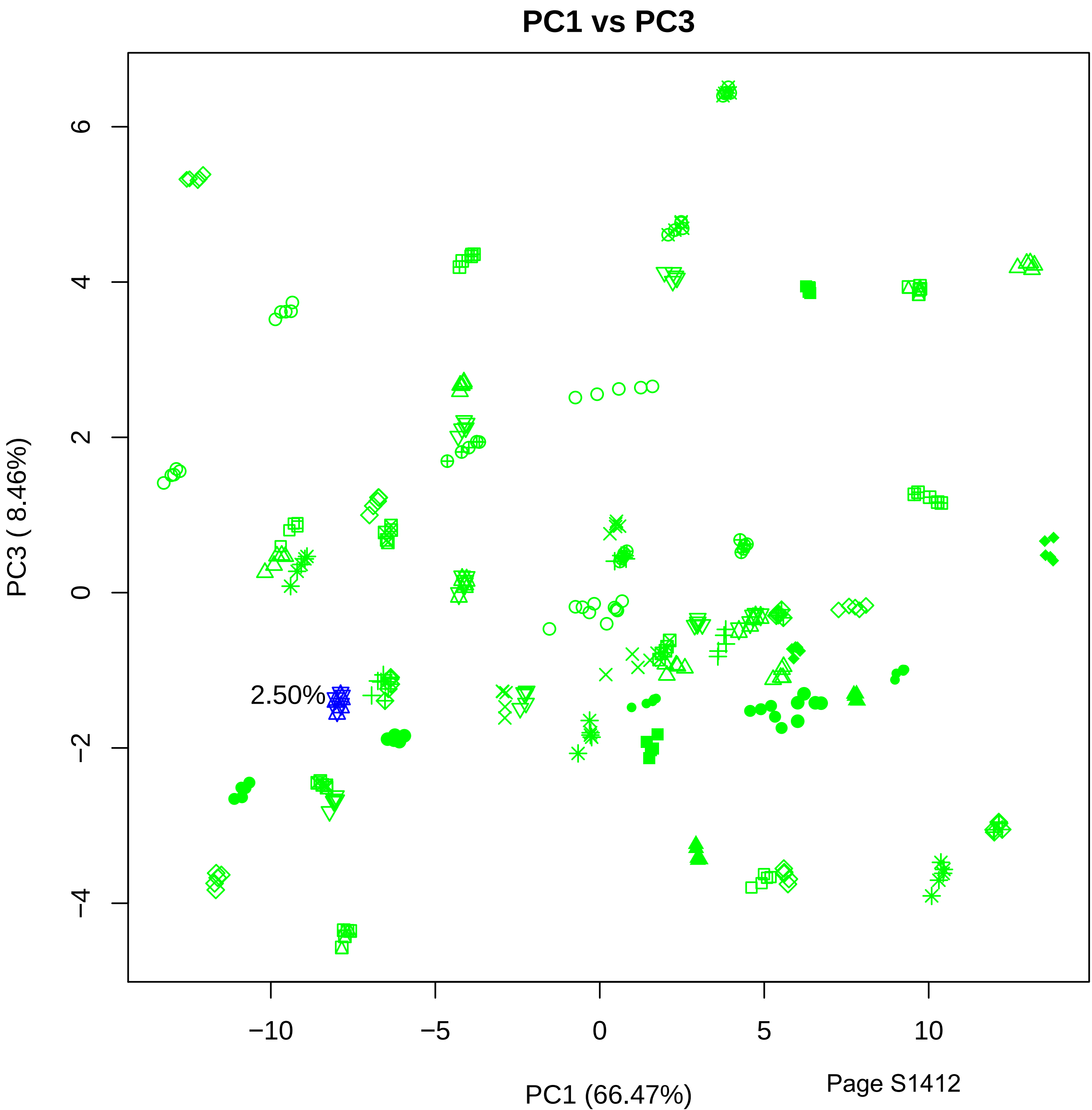
PC1 vs PC3
PC3 (8.46%)
PC1 (66.47%)
2.50%
6
4
2
0
−2
−4
−10
−5
0
5
10

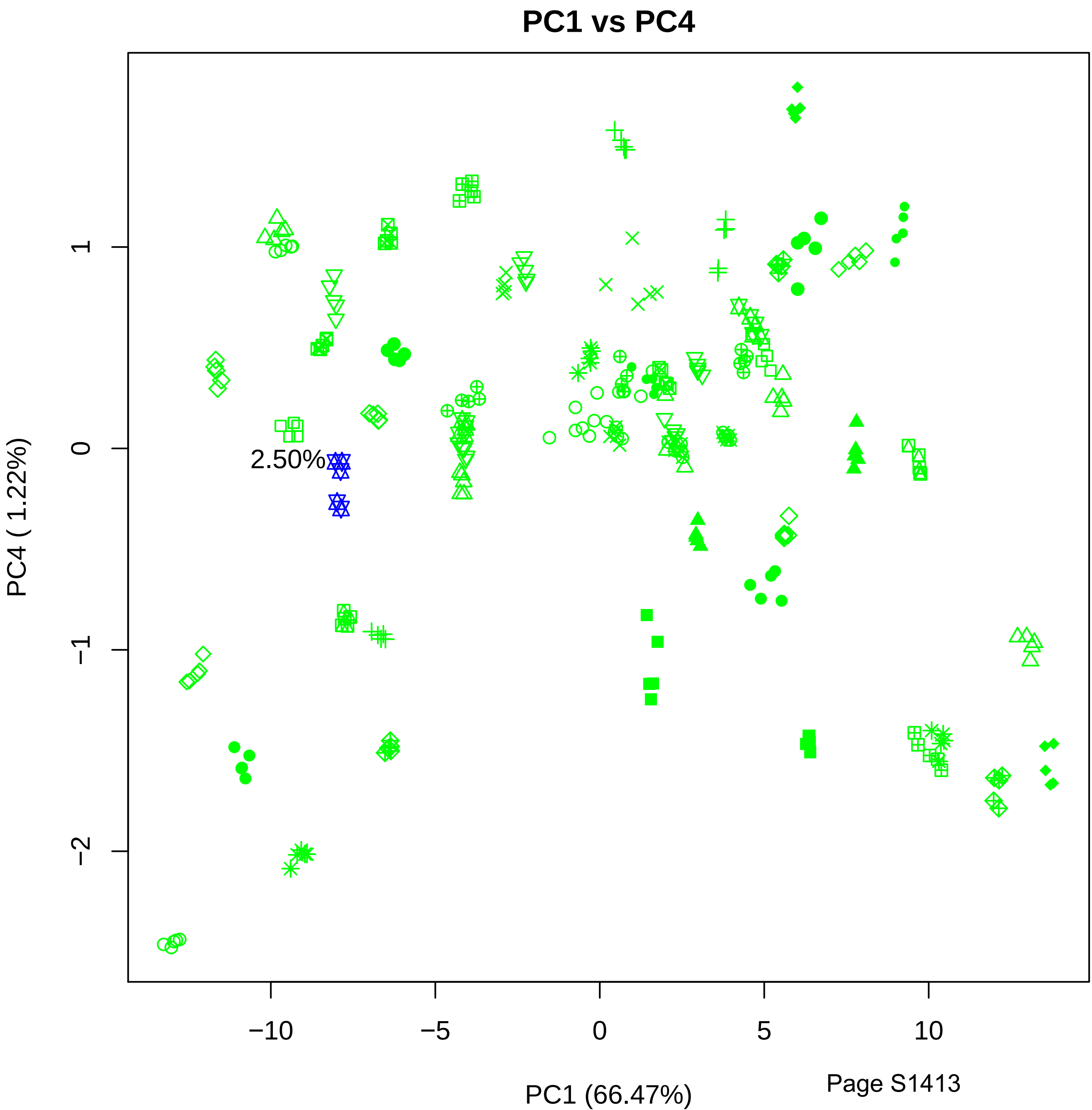

PC1 vs PC4
2.50%
PC4 (1.22%)
1
0
−1
−2
−10
−5
0
5
10
PC1 (66.47%)

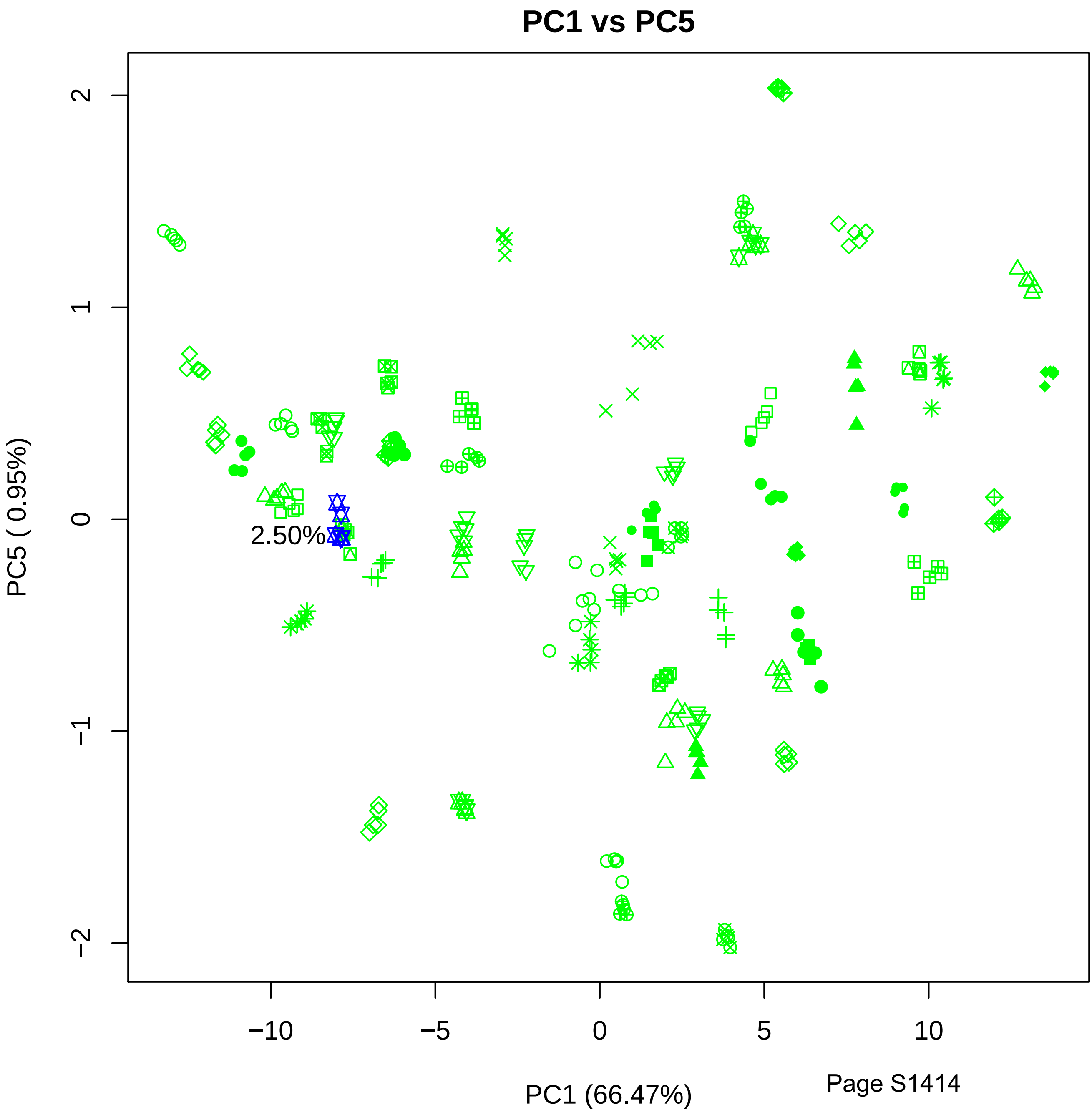
PC1 vs PC5
PC5 (0.95%)
PC1 (66.47%)
2.50%
2
1
0
−1
−2
−10
−5
0
5
10

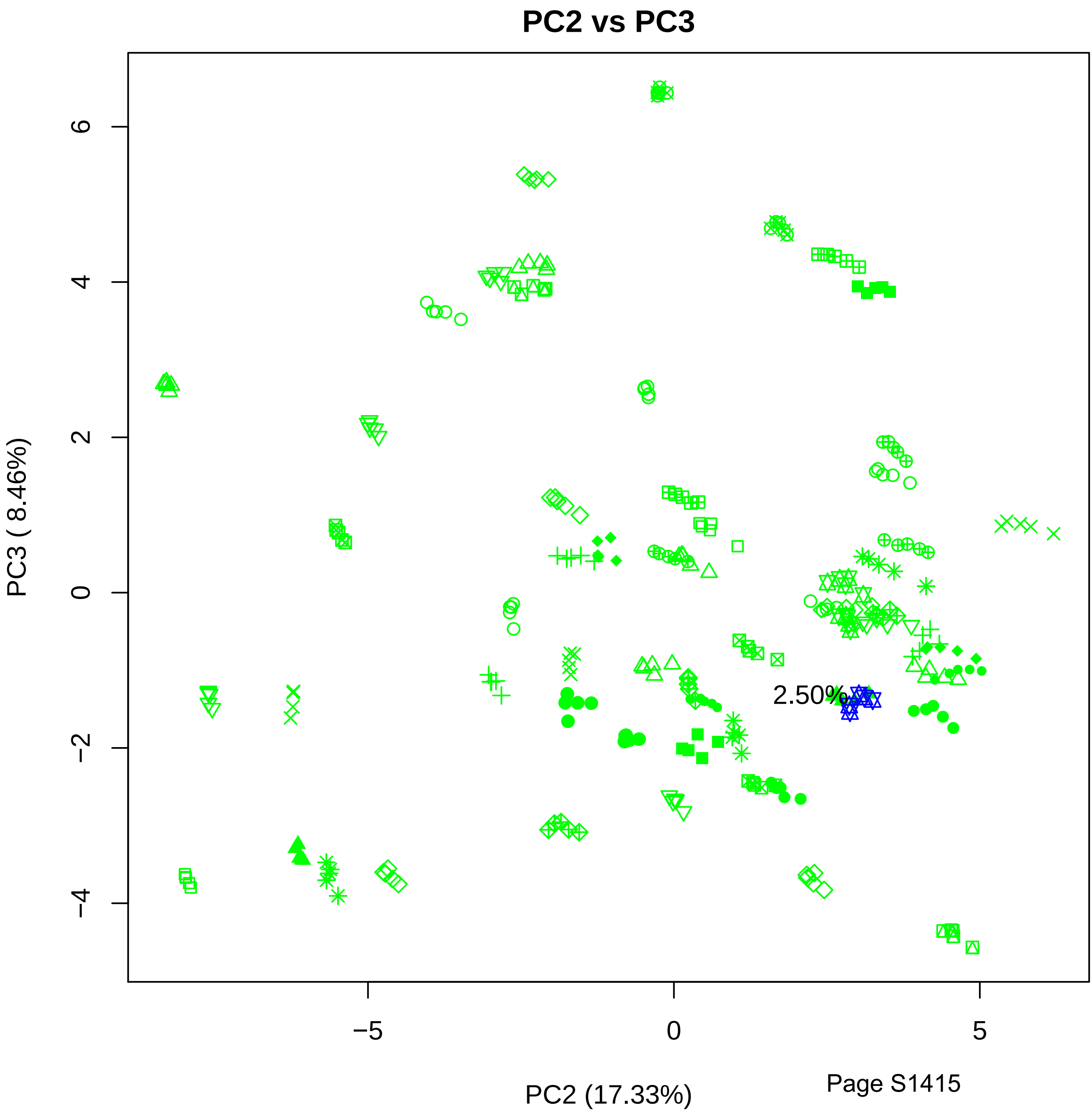
PC2 vs PC3
PC3 (8.46%)
PC2 (17.33%)
6
4
2
0
−2
−4
−5
0
5
2.50%

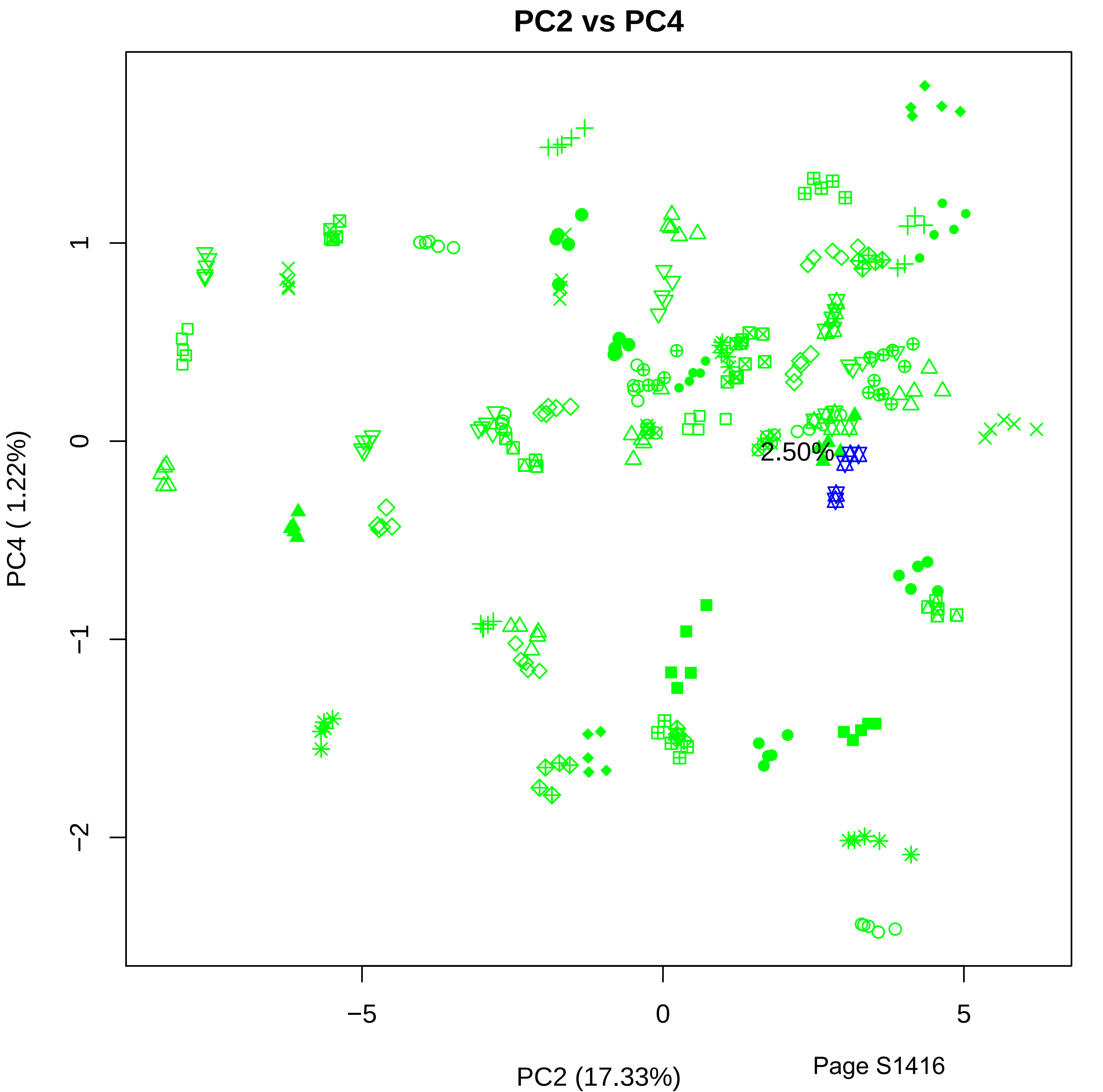

PC2 vs PC4
PC4 (1.22%)
PC2 (17.33%)
1
0
−1
−2
−5
0
5
2.50%

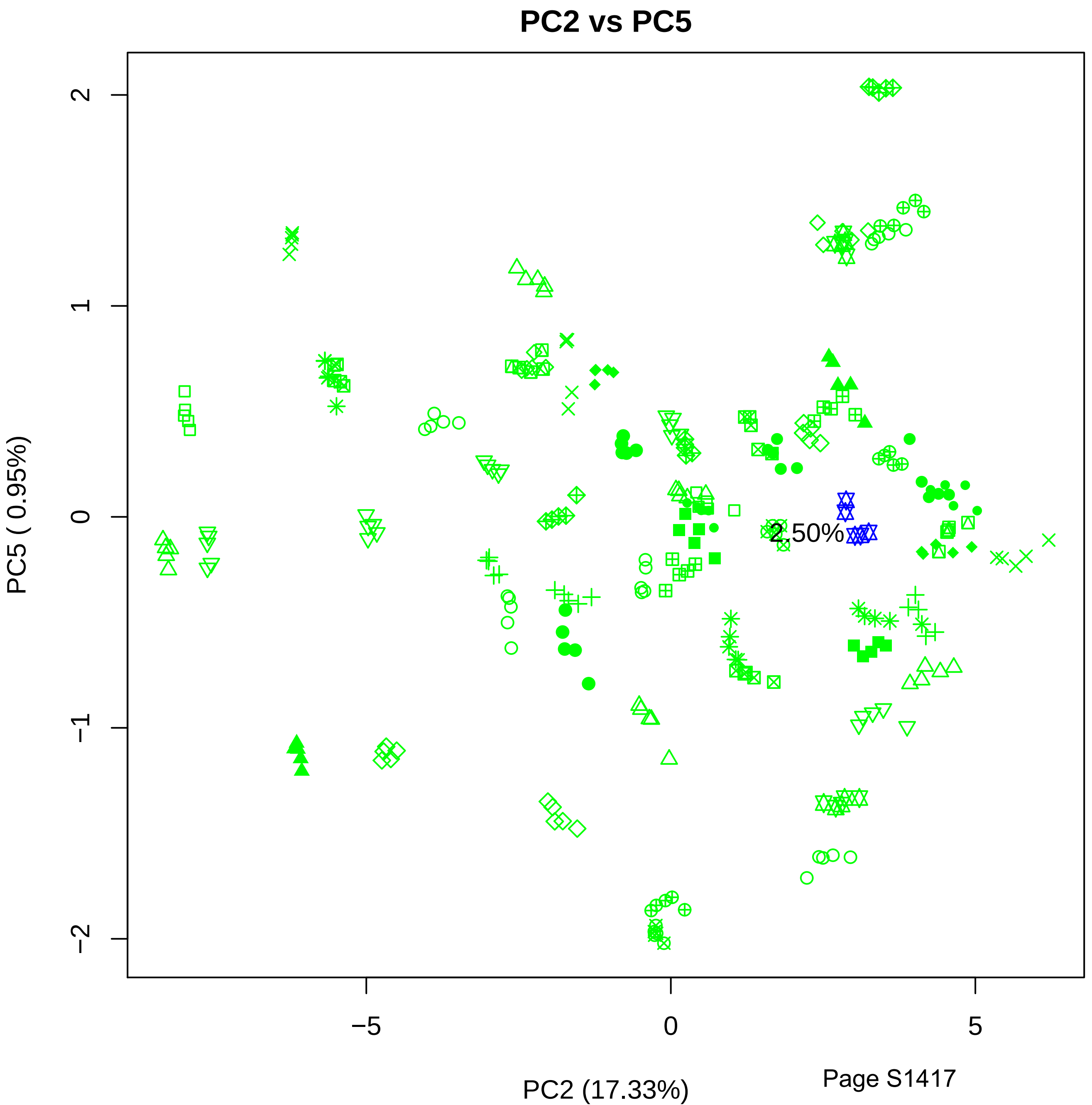
PC2 vs PC5
PC5 (0.95%)
PC2 (17.33%)
2
1
0
−1
−2
−5
0
5
2.50%

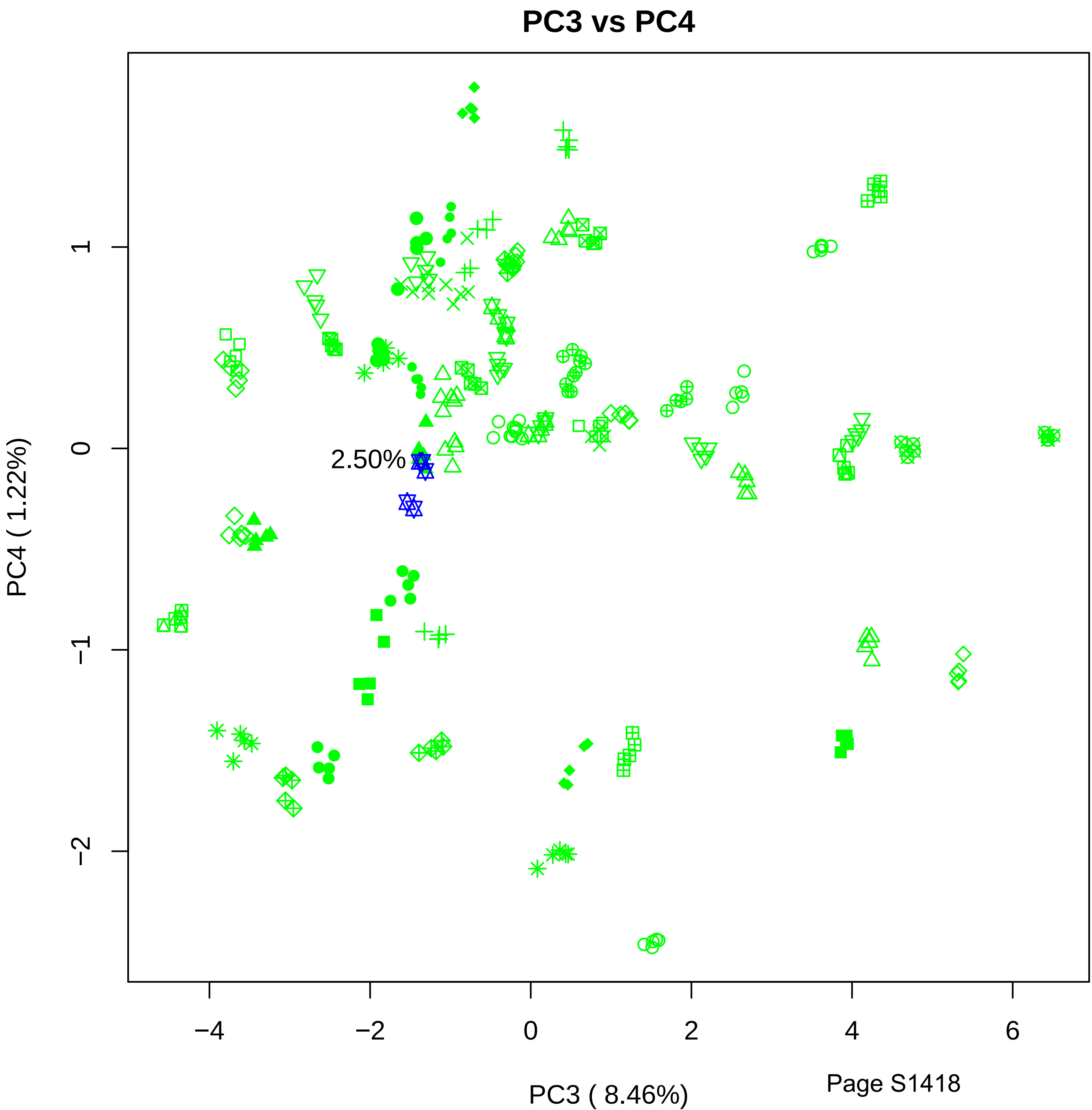

PC3 vs PC4
2.50%
PC4 (1.22%)
PC3 (8.46%)
1
0
−1
−2
−4
−2
0
2
4
6

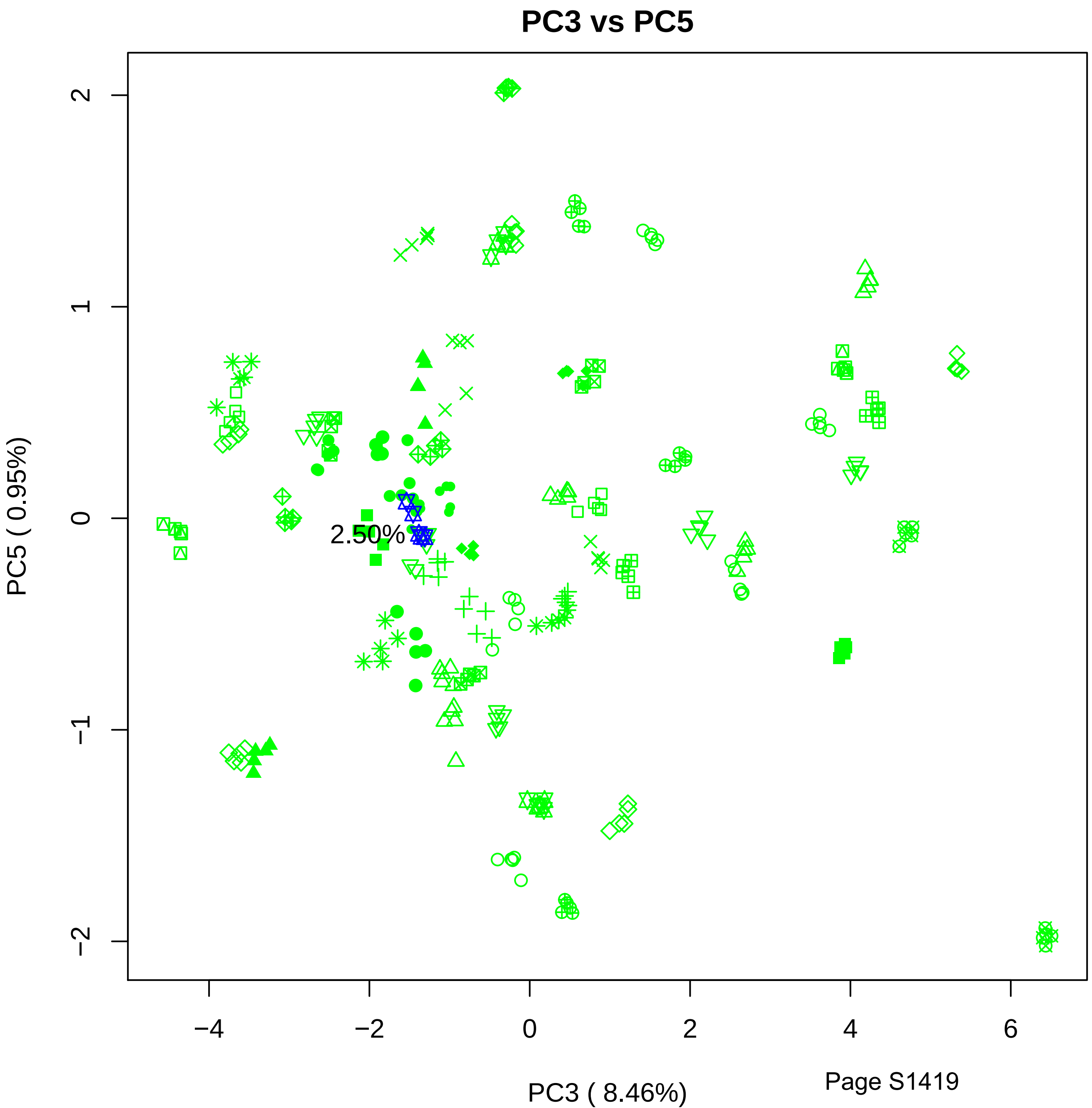

PC3 vs PC5
2
1
0
−1
−2
PC5 (0.95%)
2.50%
−4
−2
0
2
4
6
PC3 (8.46%)

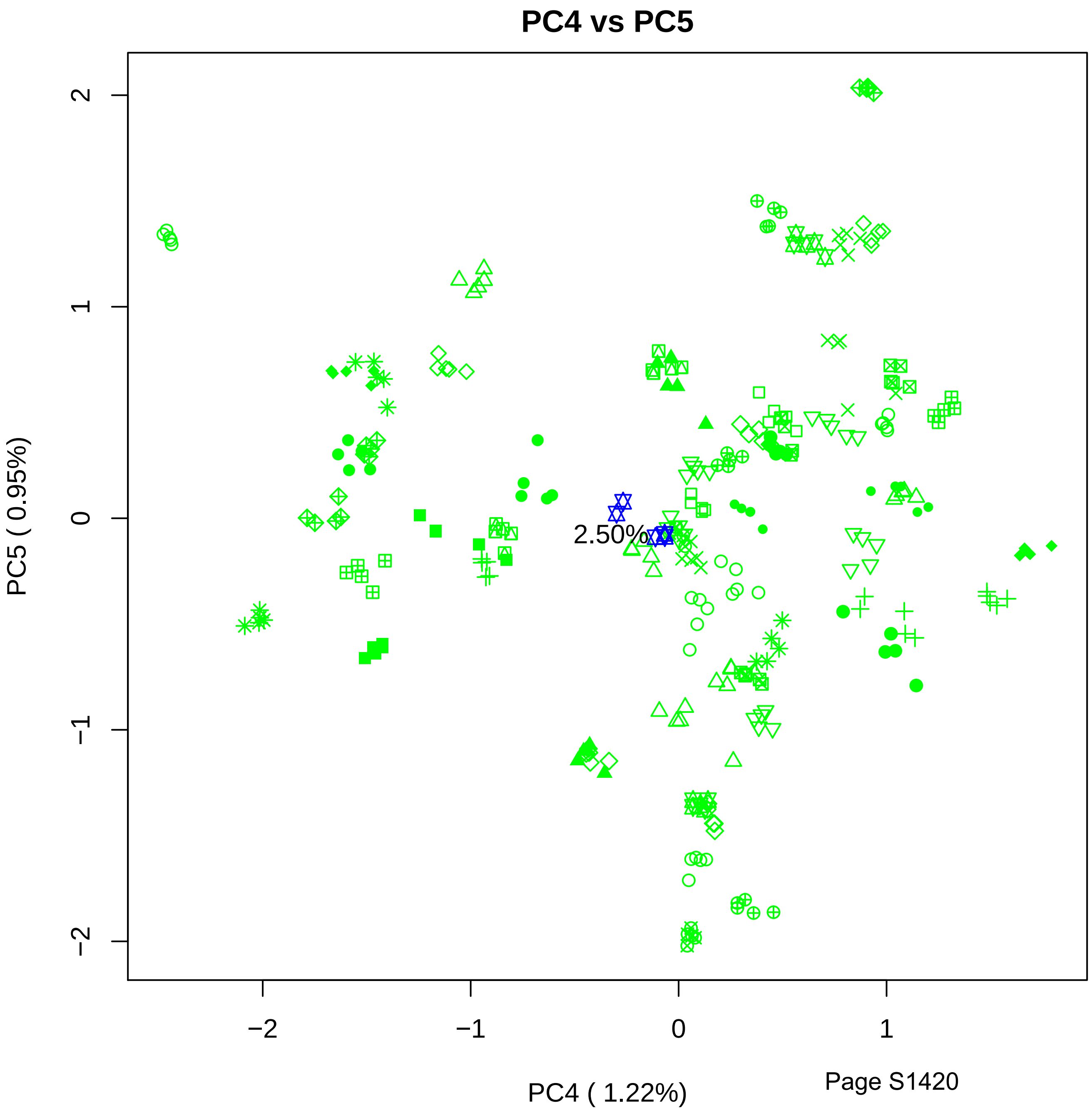

PC4 vs PC5
PC5 (0.95%)
PC4 (1.22%)
2.50%
2
1
0
−1
−2
−2
−1
0
1

PCA of 5% BMH(d) vs PMH(d)

Scaled Matrix PCAed

7th order polynomial baseline correction performed

Raw spectra smoothed to 21 Neighbours, 2nd order PN, Savtisky Golay

Normalisation between 0 and 100

D2 spectra smoothed to 41 Neighbours, 2nd order PN, Savtisky Golay

Regions Analysed: 3601−2501 and 2001−700cm−1

Samples Analysed: 1 bContam's 0 BLH's 0 BMH's 0 OMH's 0 pContam's 0 PLH's 60 PMH's

Keele University AD

Fri Dec 14 14:22:40 2018

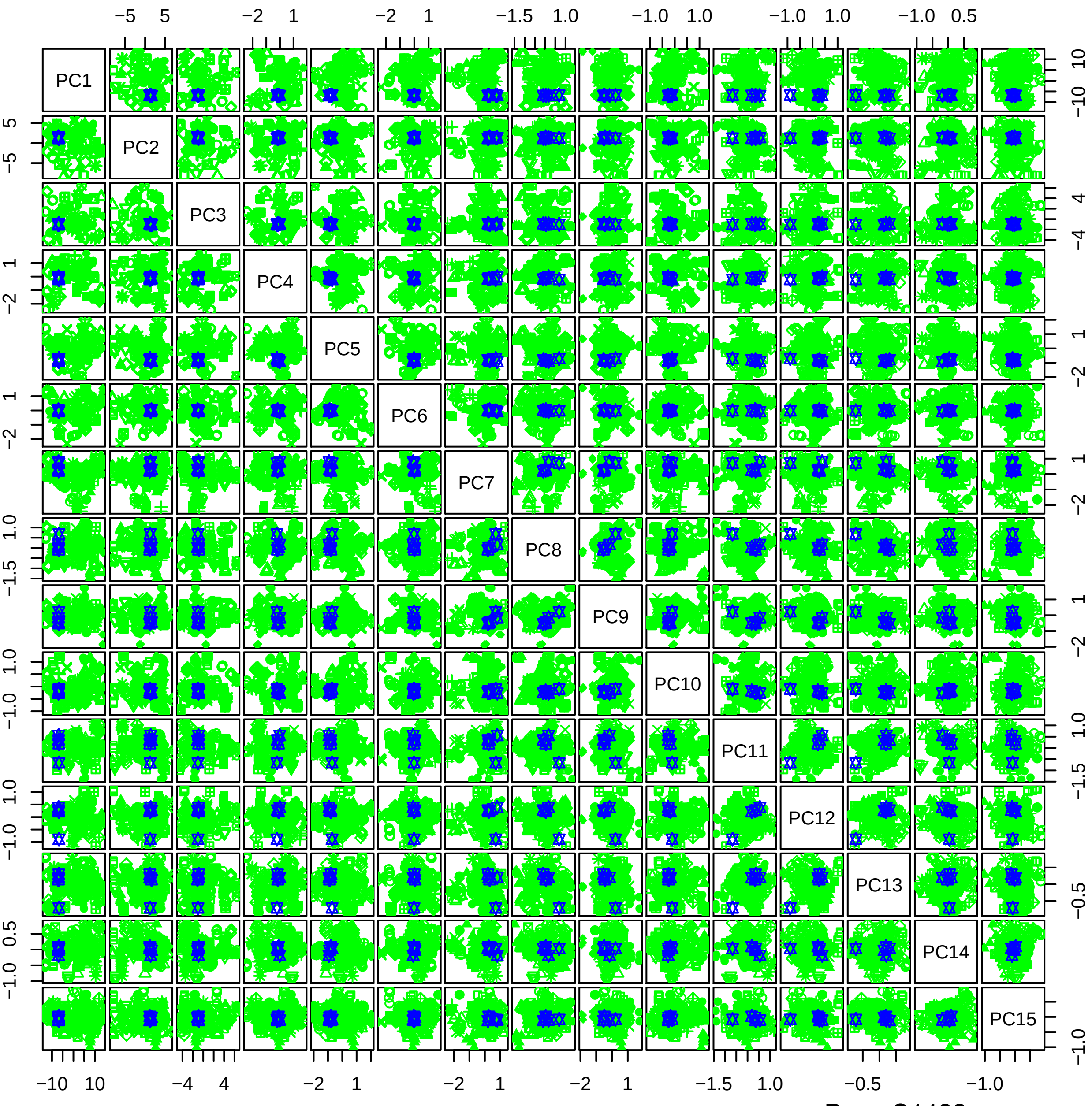
PC1
PC2
PC3
PC4
PC5
PC6
PC7
PC8
PC9
PC10
PC11
PC12
PC13
PC14
PC15

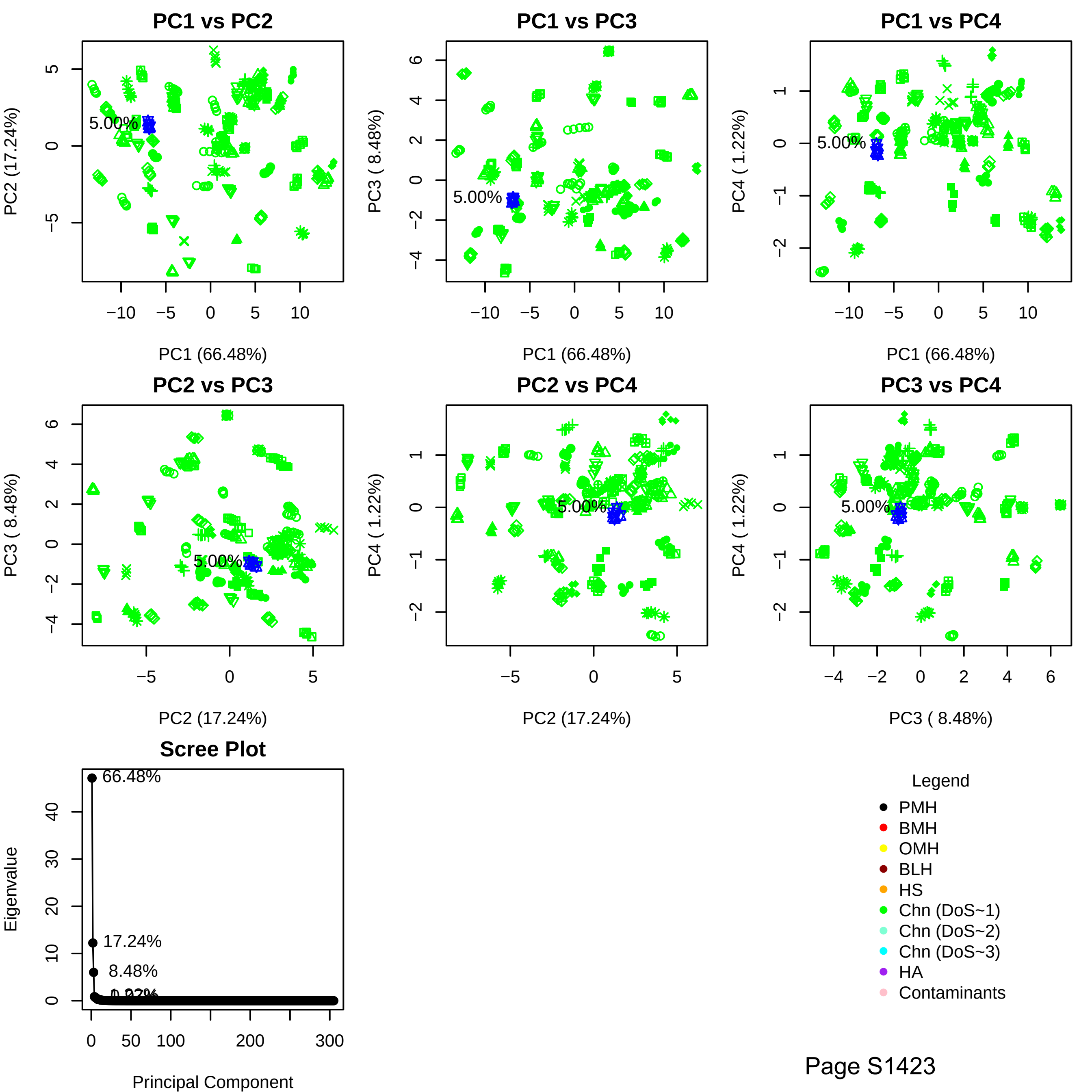
PC1 vs PC2
PC1 vs PC3
PC1 vs PC4
PC2 vs PC3
PC2 vs PC4
PC3 vs PC4
Scree Plot
PC1 (66.48%)
PC2 (17.24%)
PC3 (8.48%)
PC4 (1.22%)
5.00%
Eigenvalue
Principal Component
66.48%
17.24%
8.48%
Legend
PMH
BMH
OMH
BLH
HS
Chn (DoS~1)
Chn (DoS~2)
Chn (DoS~3)
HA
Contaminants

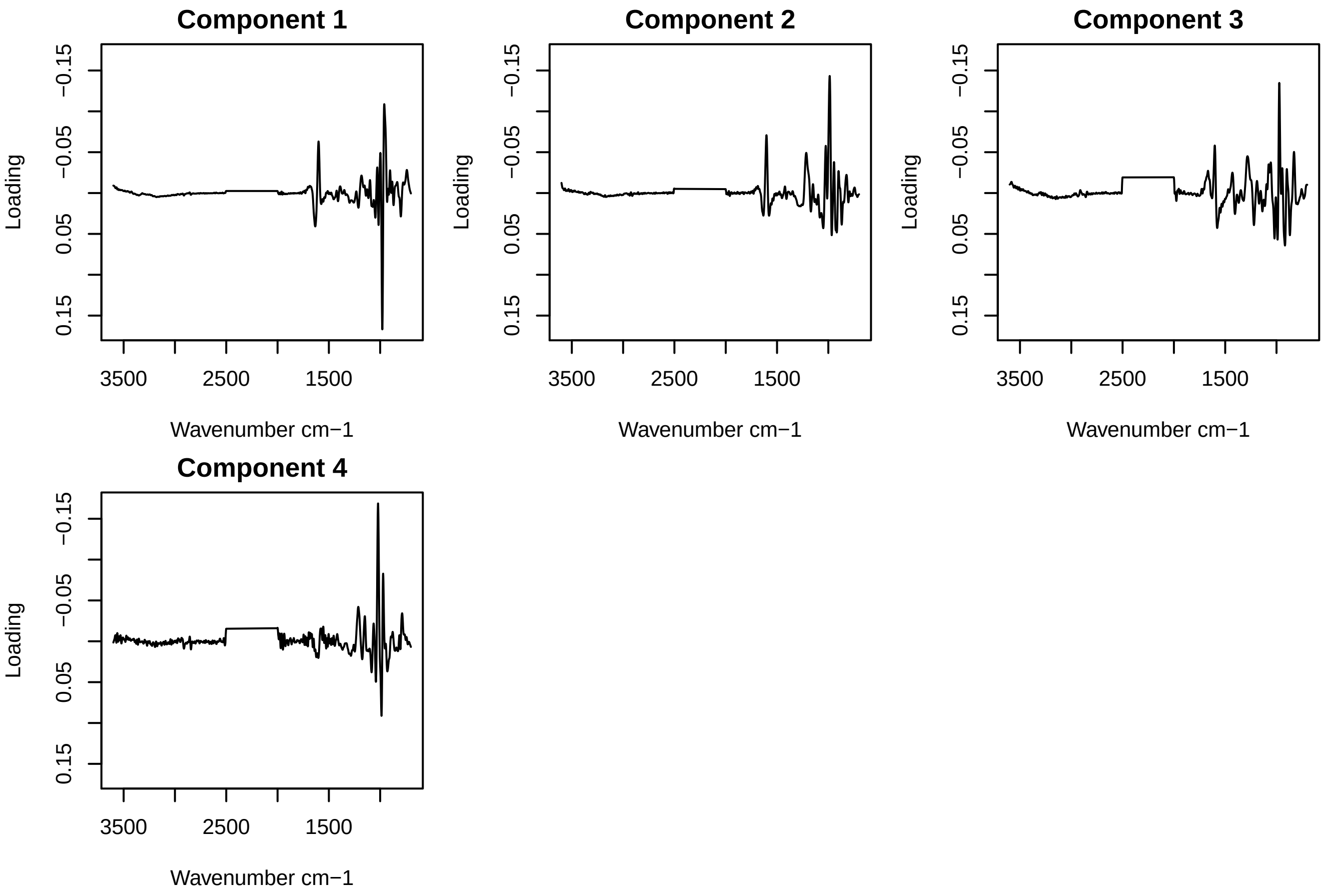

Component 1
Component 2
Component 3
Component 4
Loading
−0.15
−0.05
0.05
0.15
3500
2500
1500
Wavenumber cm−1

PC1 vs PC2 vs PC3

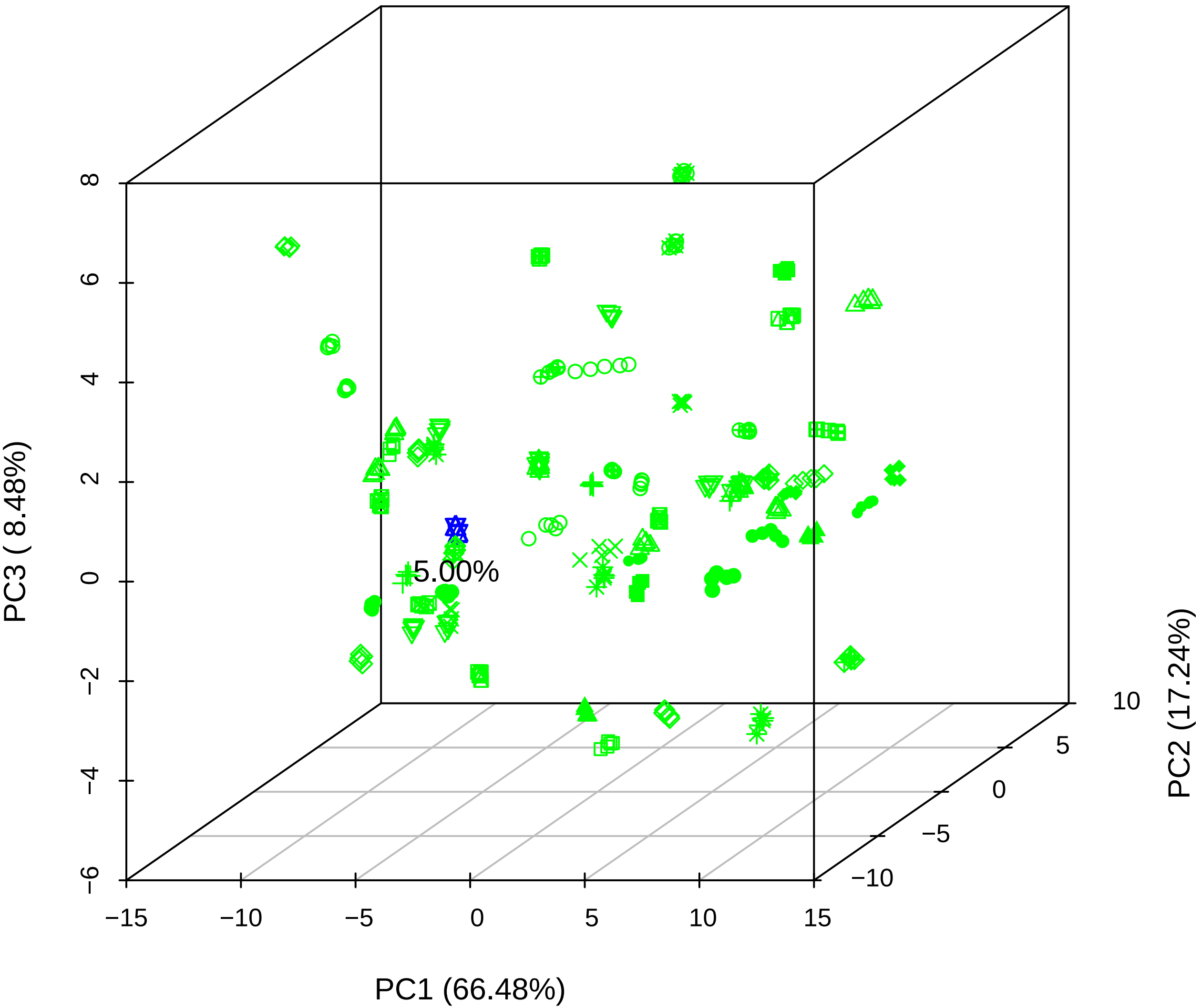

PC1 vs PC2 vs PC4

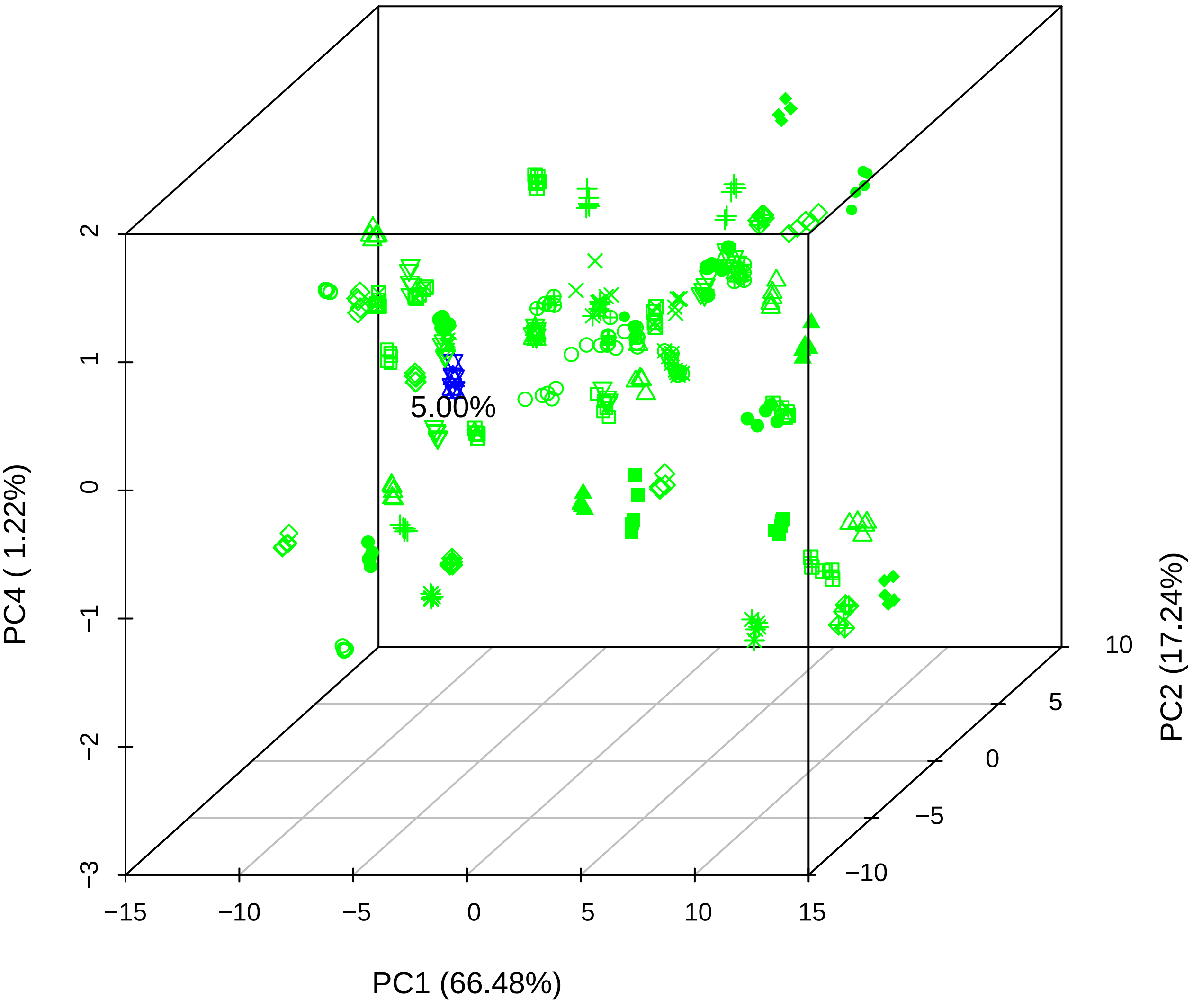

PC1 vs PC3 vs PC4

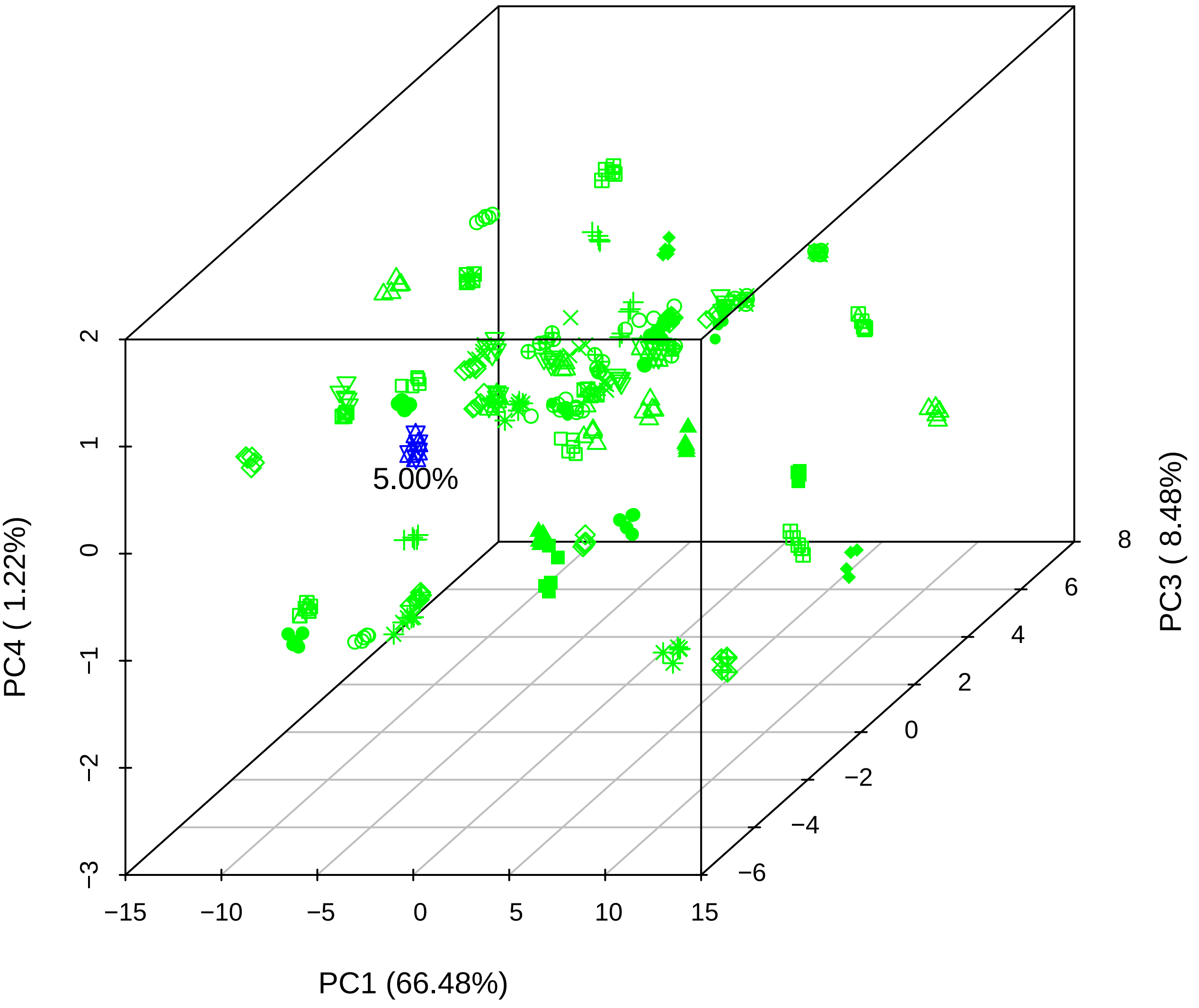

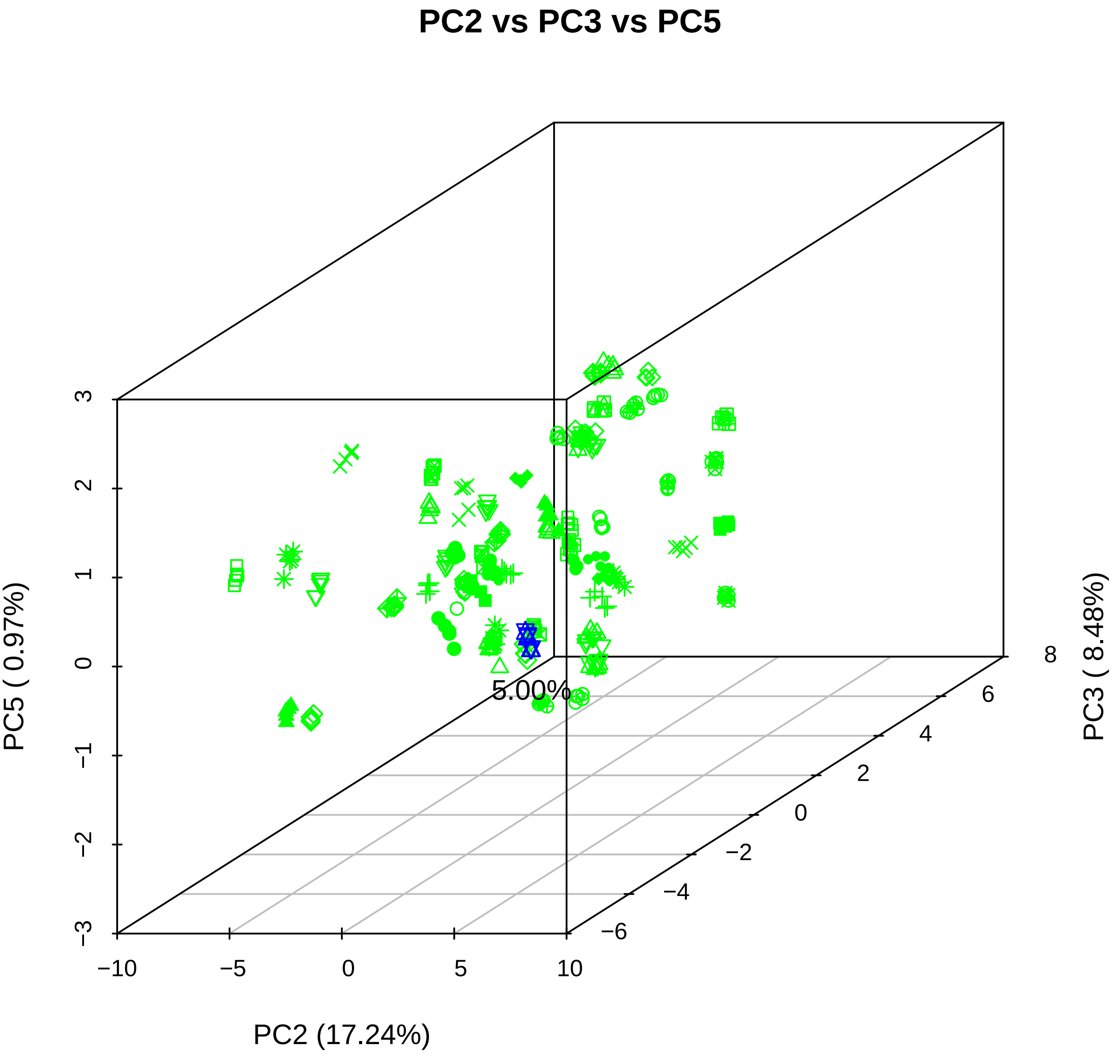
PC2 vs PC3 vs PC5
PC5 (0.97%)
PC2 (17.24%)
PC3 (8.48%)
5.00%
3
2
1
0
−1
−2
−3
−10
−5
0
5
10
8
6
4
2
0
−2
−4
−6

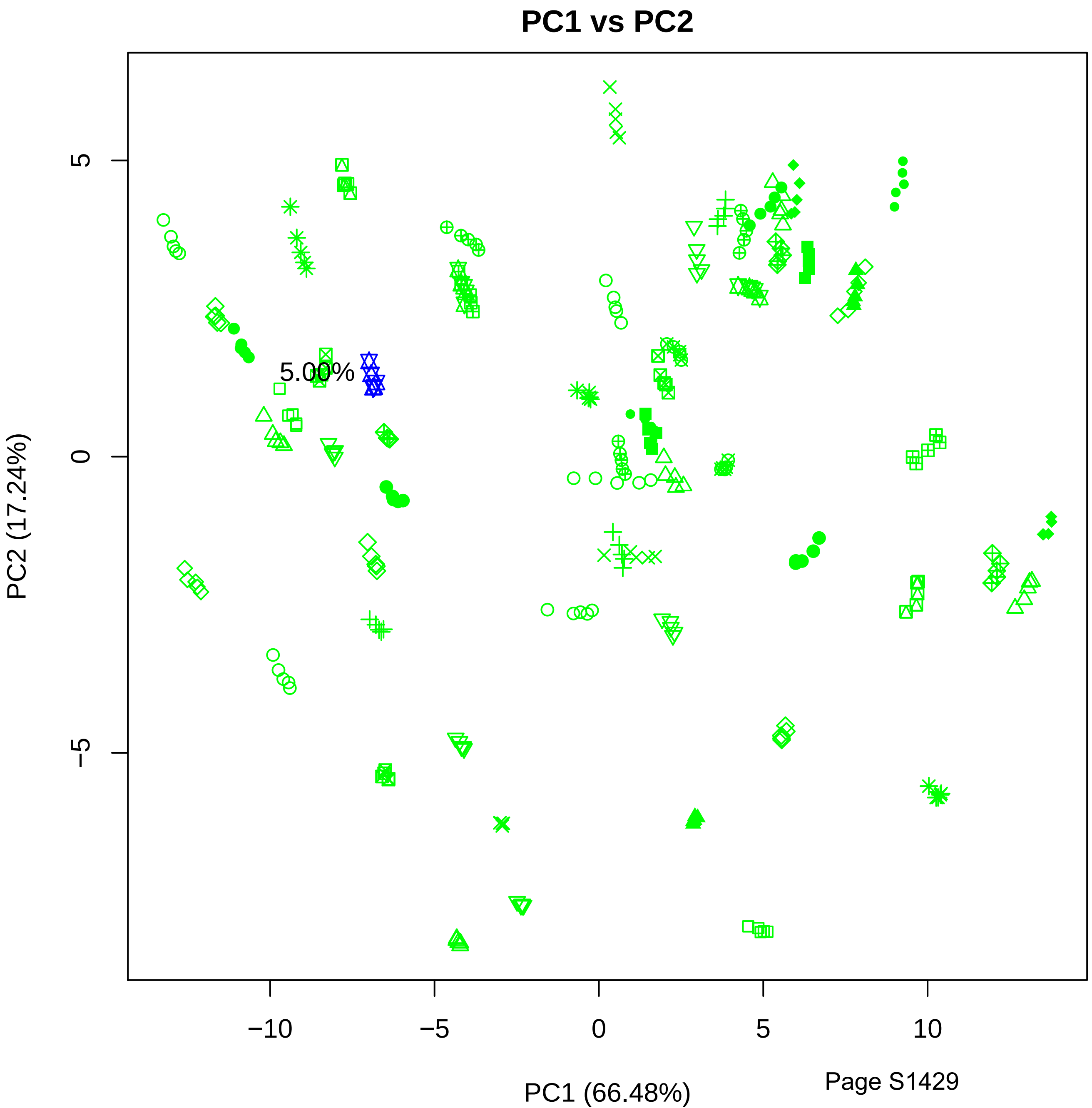

PC1 vs PC2
5.00%
5
0
−5
−10
−5
0
5
10
PC2 (17.24%)
PC1 (66.48%)

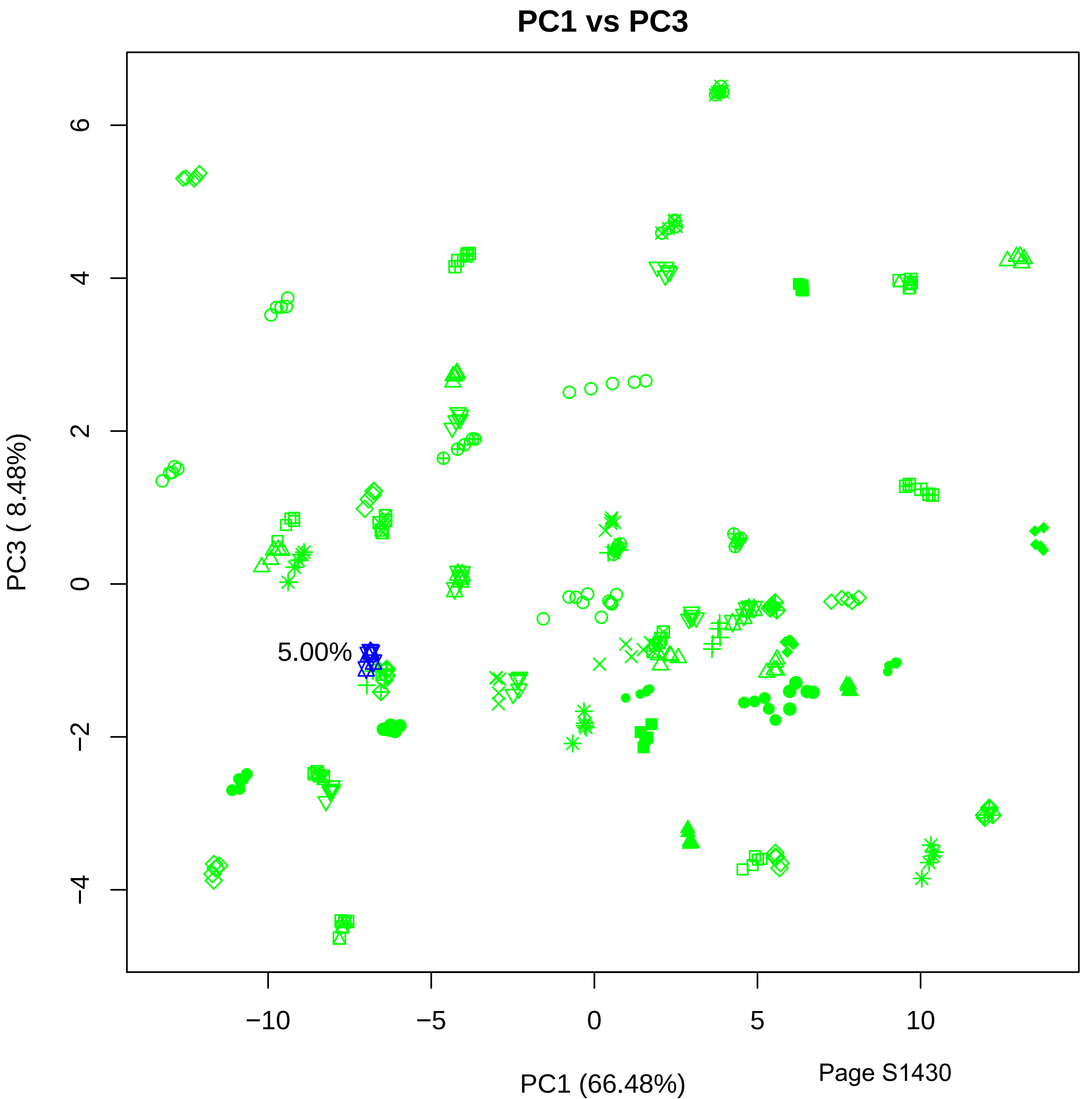

PC1 vs PC3
PC3 (8.48%)
PC1 (66.48%)
5.00%
6
4
2
0
−2
−4
−10
−5
0
5
10

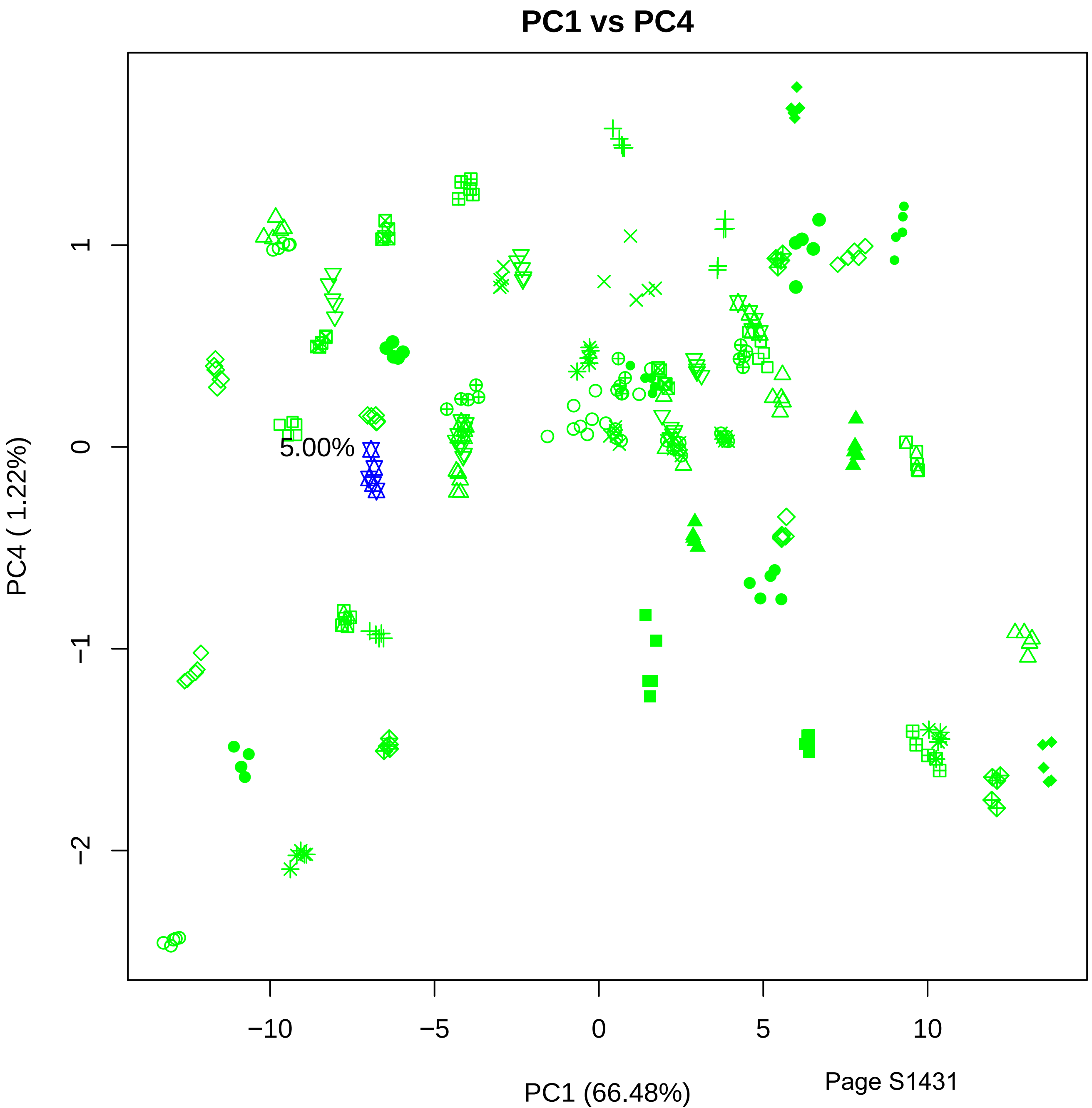

PC1 vs PC4
1
0
−1
−2
PC4 (1.22%)
5.00%
−10
−5
0
5
10
PC1 (66.48%)

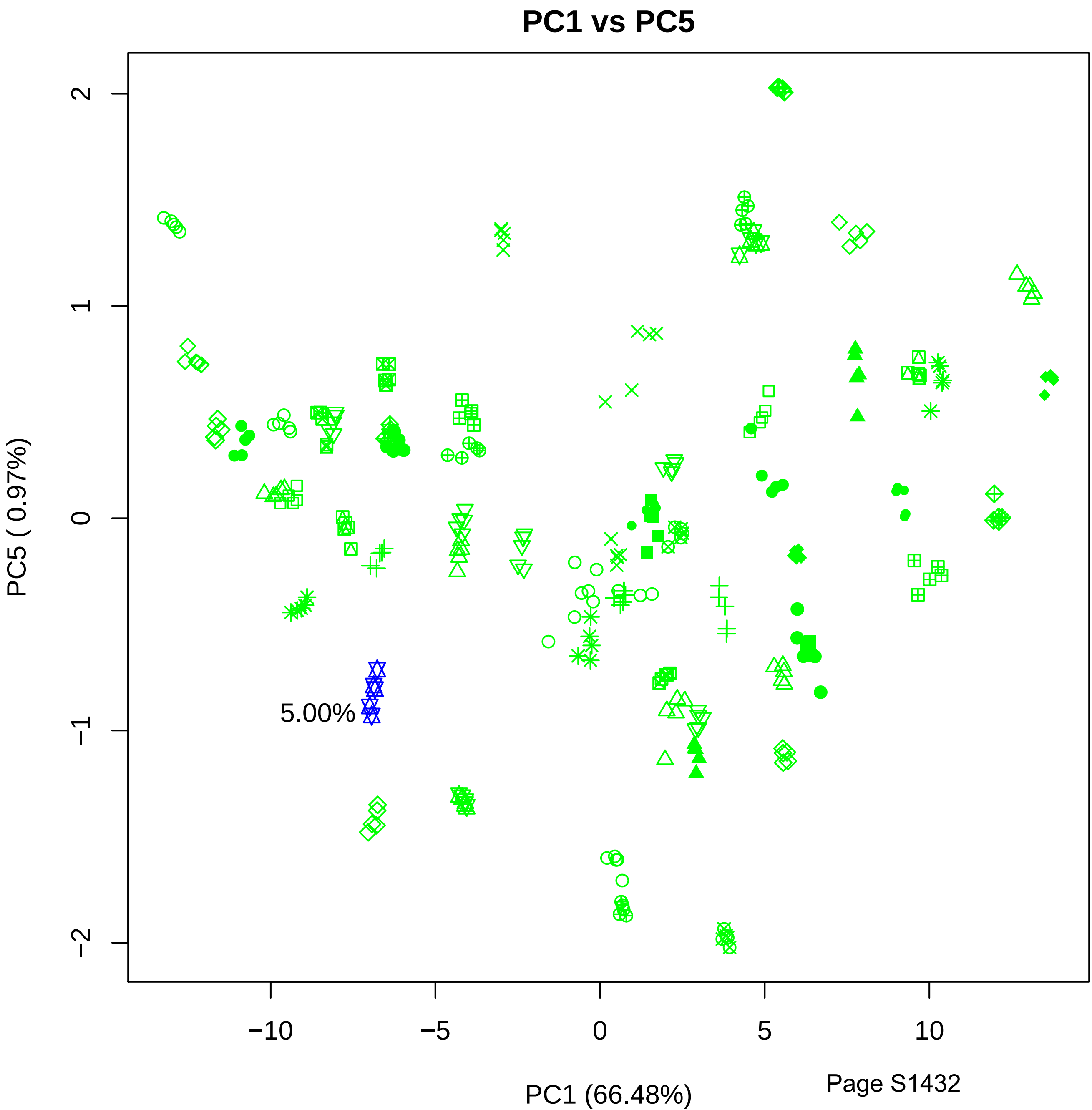
PC1 vs PC5
PC5 (0.97%)
PC1 (66.48%)
5.00%
2
1
0
−1
−2
−10
−5
0
5
10

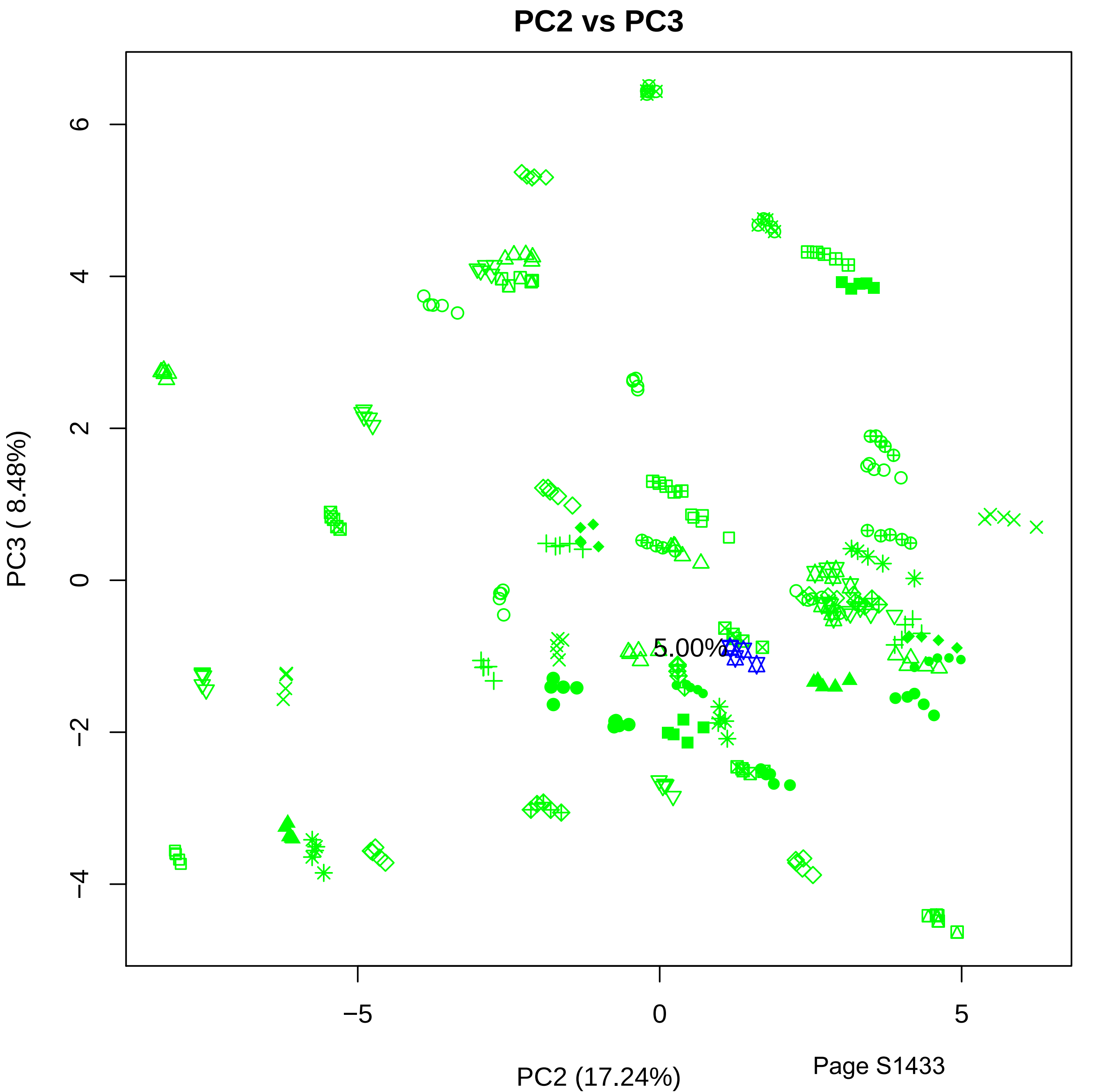
PC2 vs PC3
PC3 (8.48%)
6
4
2
0
−2
−4
5.00%
−5
0
5
PC2 (17.24%)

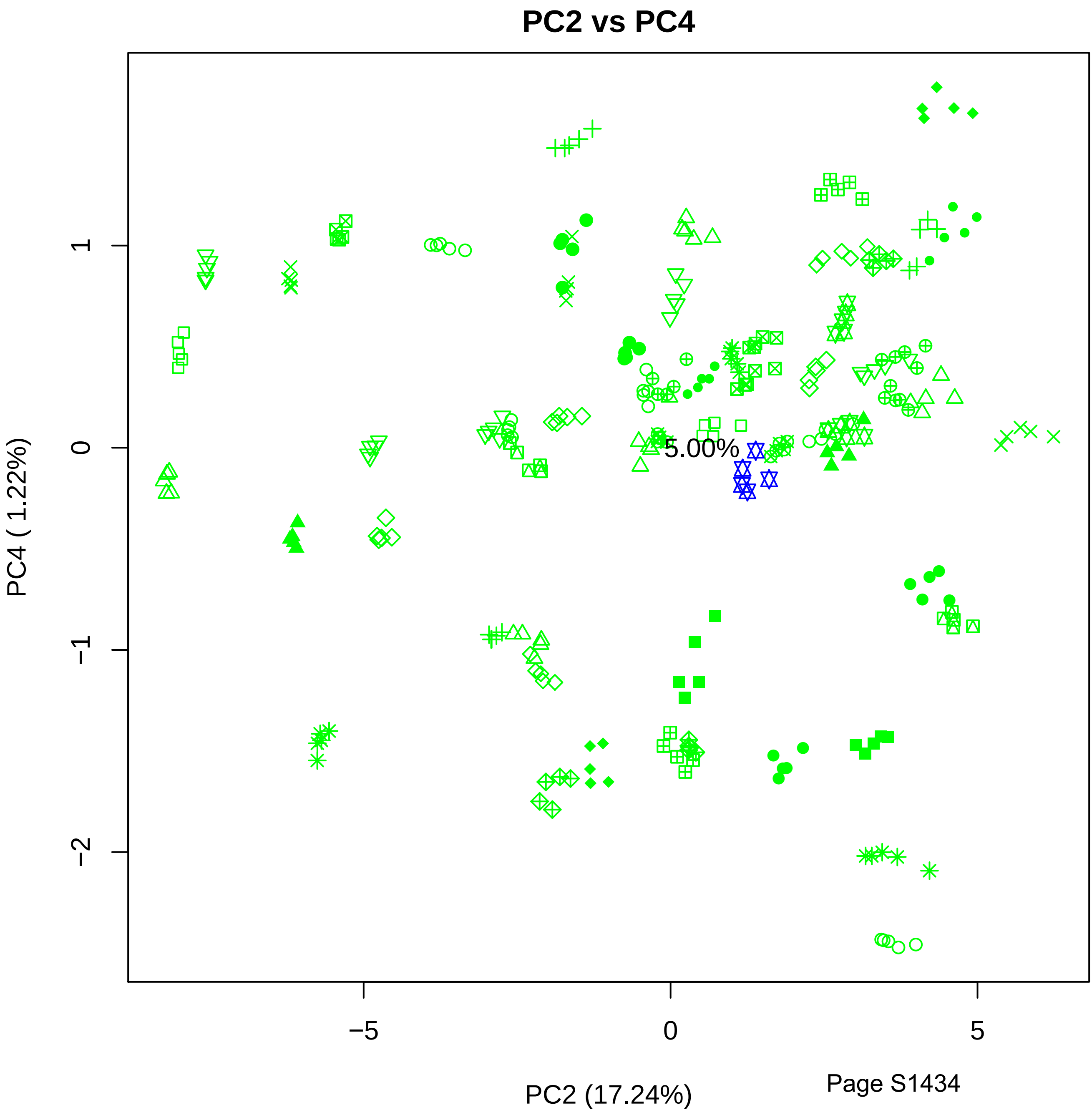

PC2 vs PC4
PC4 (1.22%)
PC2 (17.24%)
1
0
−1
−2
−5
0
5
5.00%

PC2 vs PC5

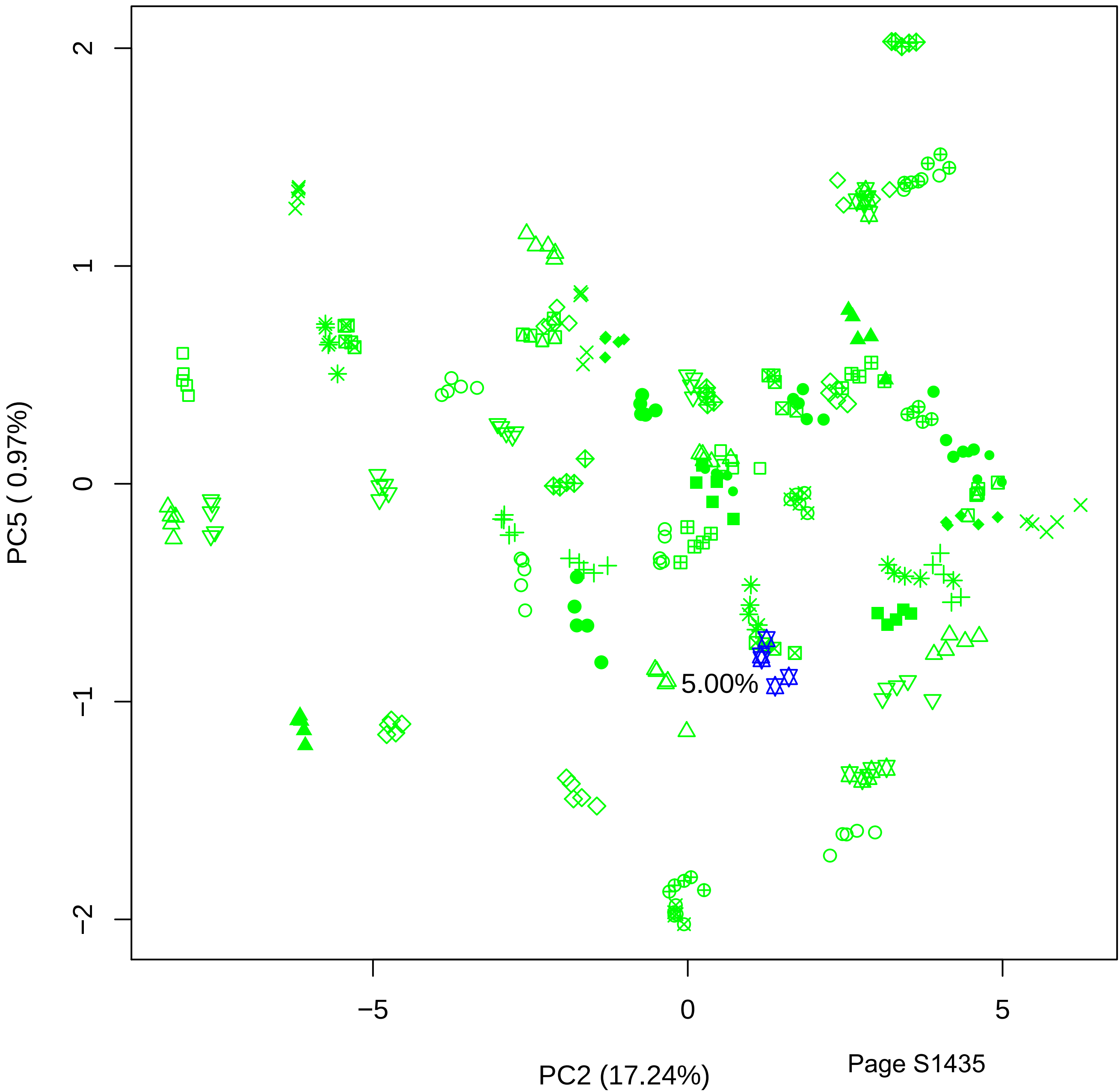

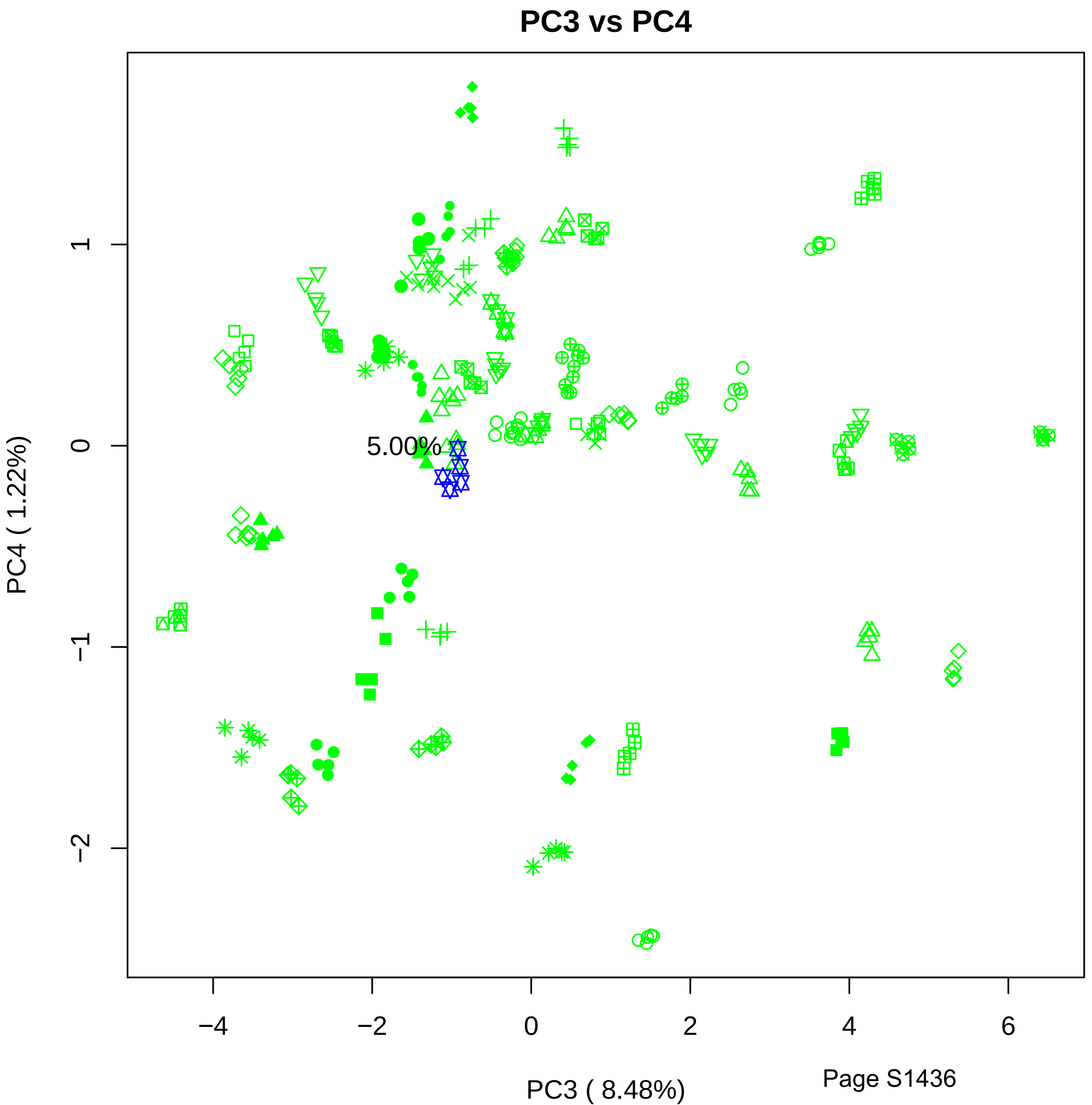

PC3 vs PC4
5.00%
PC4 (1.22%)
PC3 (8.48%)
−4
−2
0
2
4
6
−2
−1
0
1

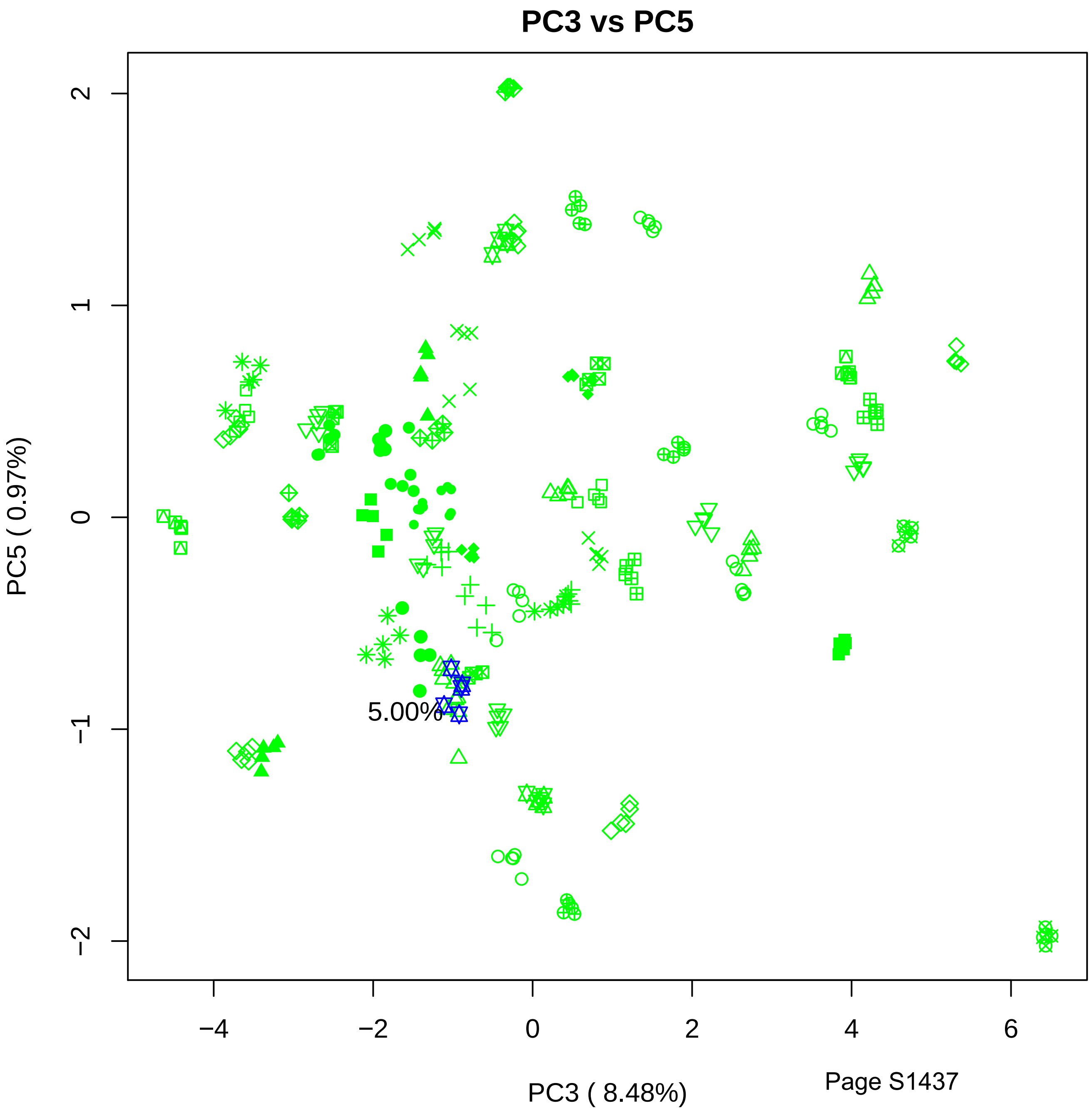
PC3 vs PC5
PC5 (0.97%)
PC3 (8.48%)
5.00%
2
1
0
−1
−2
−4
−2
0
2
4
6

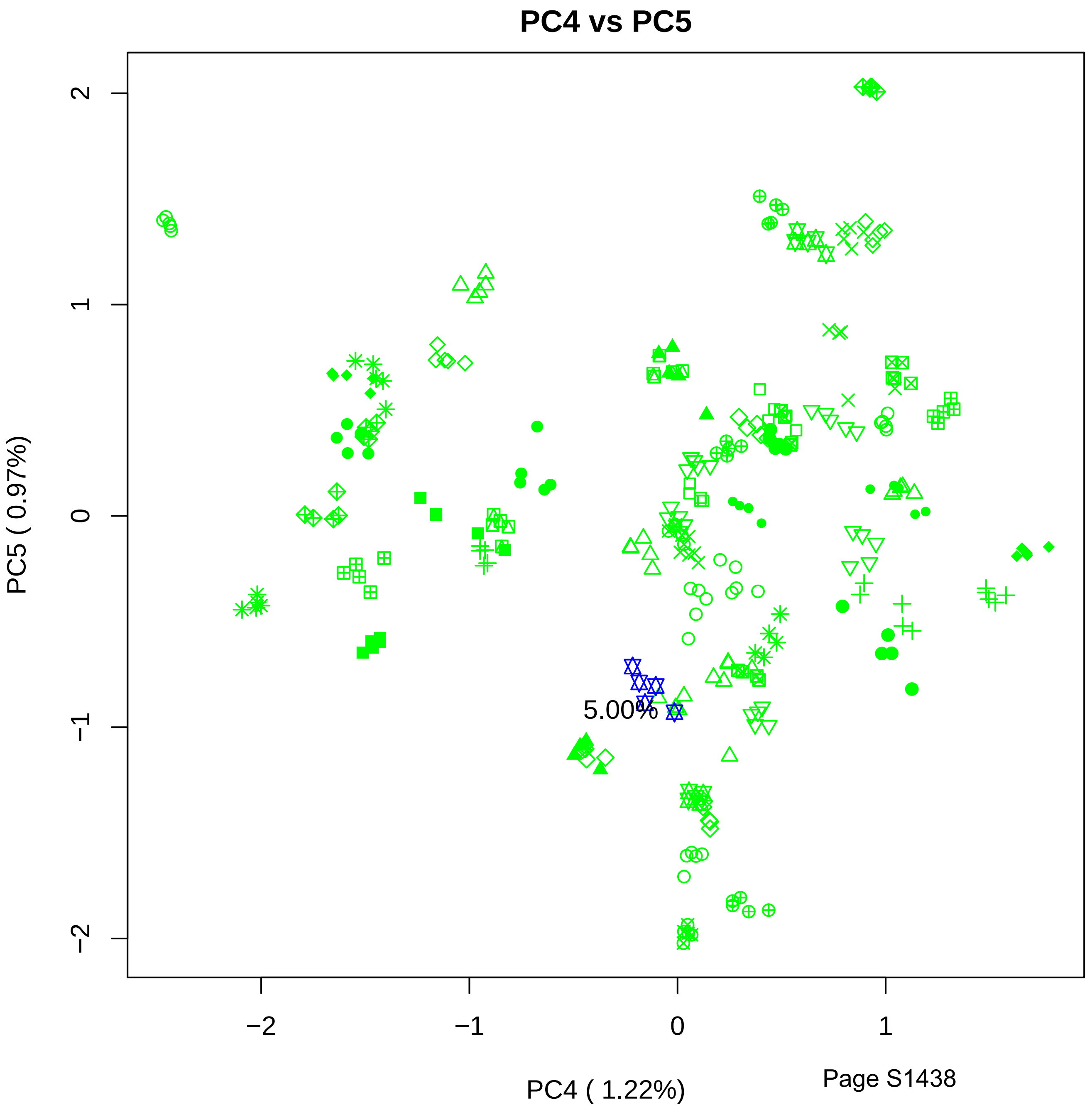

PC4 vs PC5
PC5 (0.97%)
PC4 (1.22%)
5.00%
2
1
0
−1
−2
−2
−1
0
1

PCA of 7.5% BMH(d) vs PMH(d)

Scaled Matrix PCAed

7th order polynomial baseline correction performed

Raw spectra smoothed to 21 Neighbours, 2nd order PN, Savtisky Golay

Normalisation between 0 and 100

D2 spectra smoothed to 41 Neighbours, 2nd order PN, Savtisky Golay

Regions Analysed: 3601−2501 and 2001−700cm−1

Samples Analysed: 1 bContam's 0 BLH's 0 BMH's 0 OMH's 0 pContam's 0 PLH's 60 PMH's

Keele University AD

Fri Dec 14 14:22:49 2018

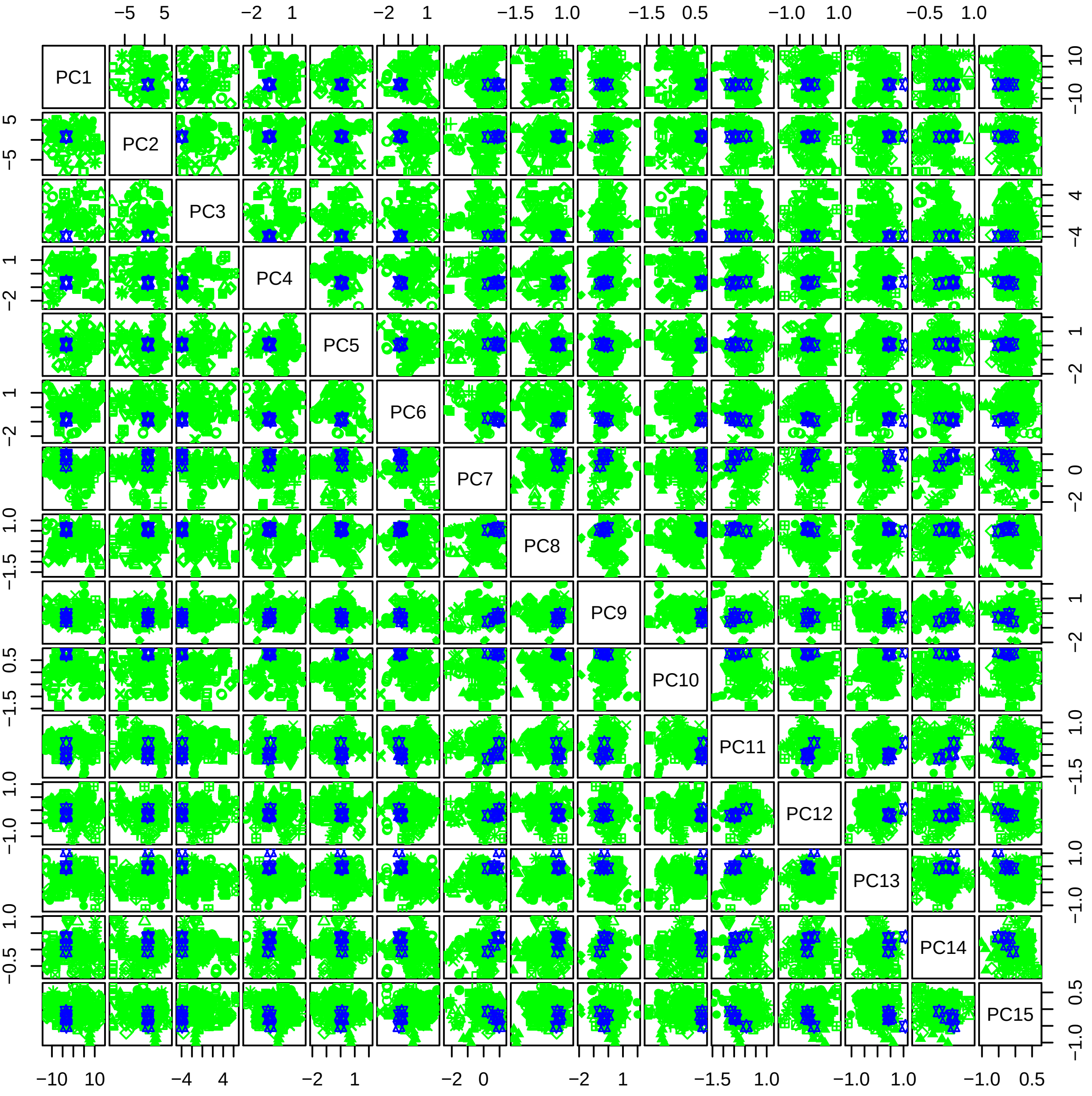
PC1
PC2
PC3
PC4
PC5
PC6
PC7
PC8
PC9
PC10
PC11
PC12
PC13
PC14
PC15

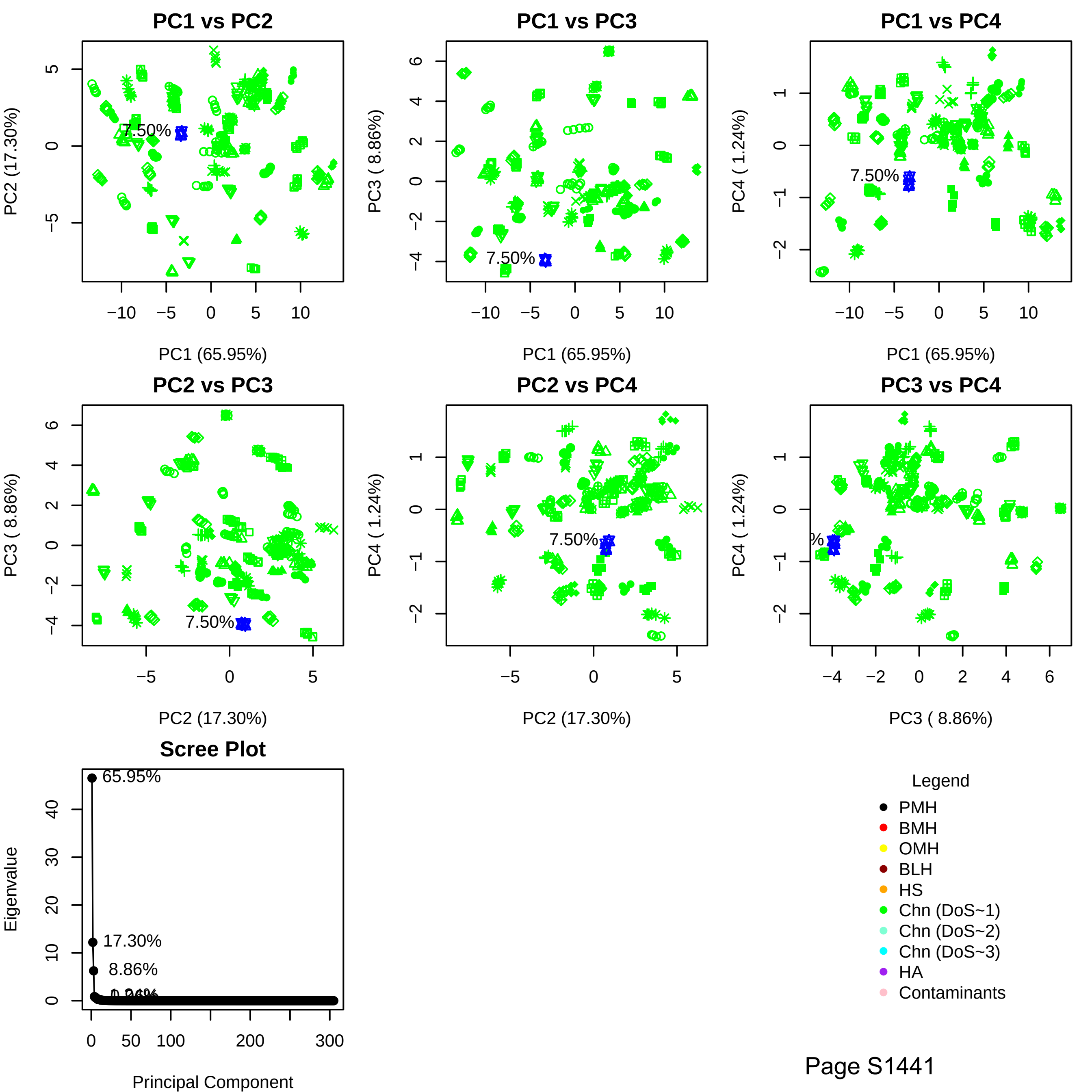

PC1 vs PC2
PC1 vs PC3
PC1 vs PC4
PC2 vs PC3
PC2 vs PC4
PC3 vs PC4
Scree Plot
PC1 (65.95%)
PC2 (17.30%)
PC3 (8.86%)
PC4 (1.24%)
7.50%
Eigenvalue
Principal Component
65.95%
17.30%
8.86%
Legend
PMH
BMH
OMH
BLH
HS
Chn (DoS~1)
Chn (DoS~2)
Chn (DoS~3)
HA
Contaminants

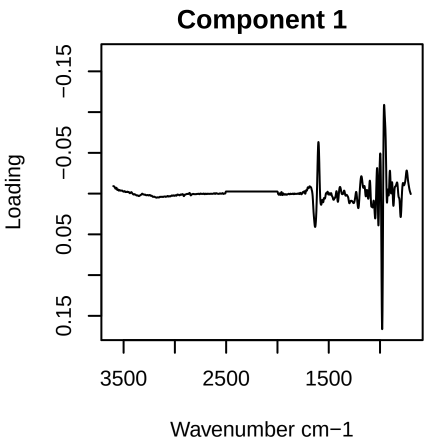

Component 1
Loading
-0.15
-0.05
0.05
0.15
3500
2500
1500
Wavenumber cm−1

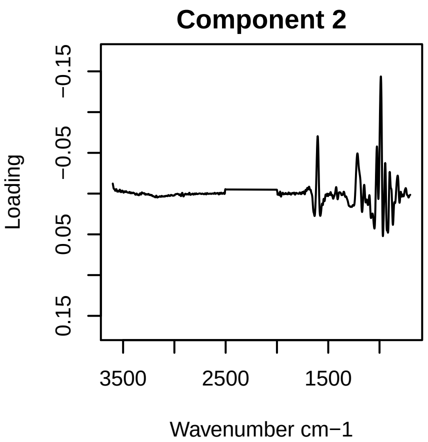

Component 2
Loading
-0.15
-0.05
0.05
0.15
3500
2500
1500
Wavenumber cm−1

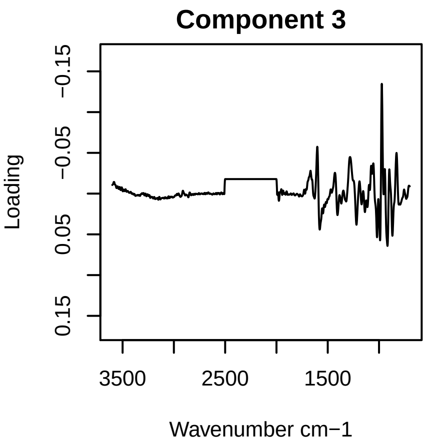

Component 3
Loading
-0.15
-0.05
0.05
0.15
3500
2500
1500
Wavenumber cm−1

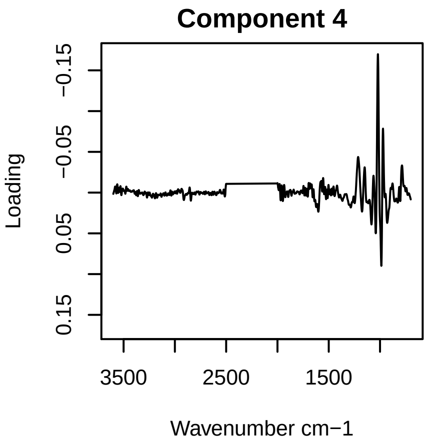

Component 4
Loading
-0.15
-0.05
0.05
0.15
3500
2500
1500
Wavenumber cm−1

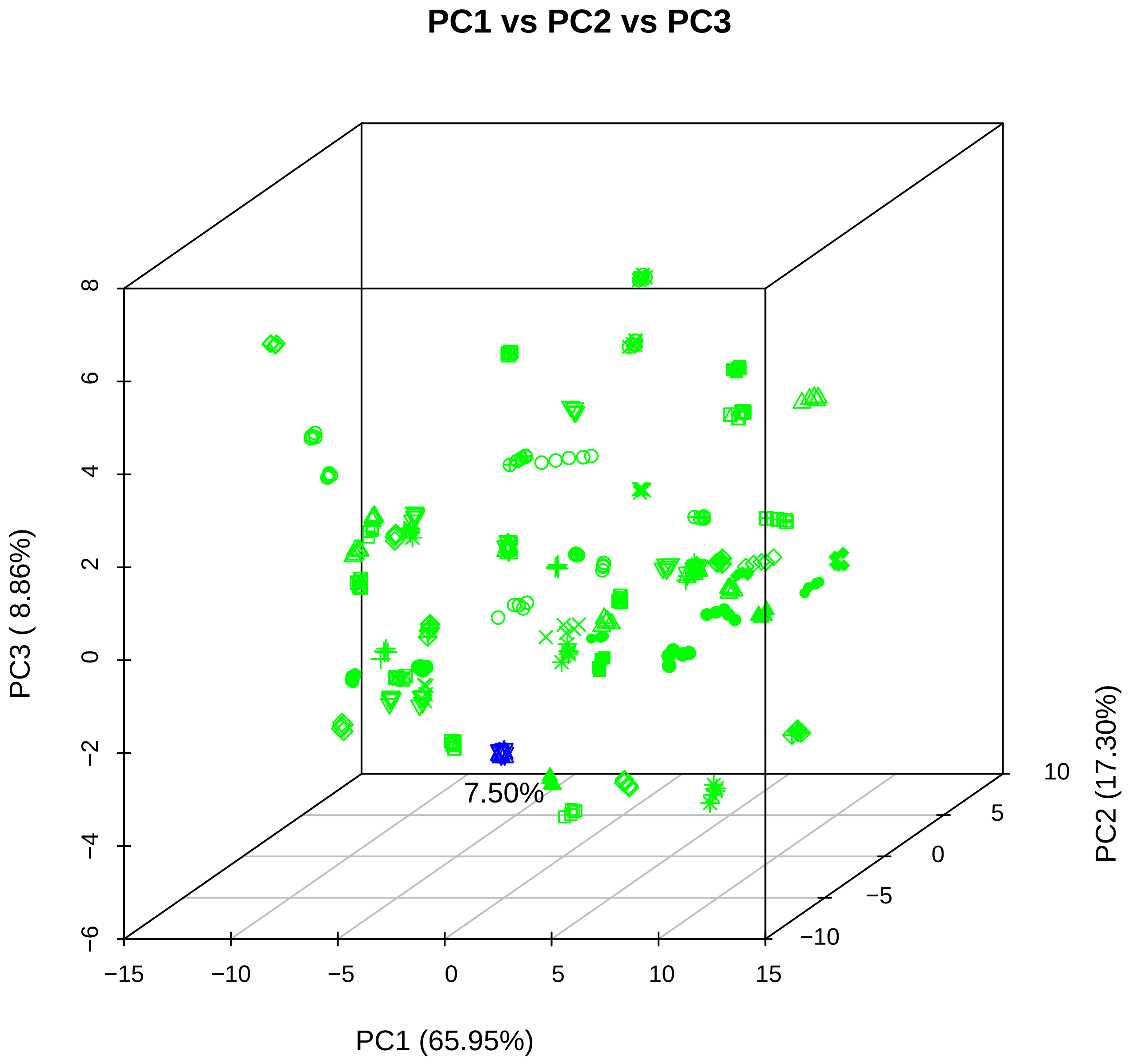
PC1 vs PC2 vs PC3
PC3 (8.86%)
PC1 (65.95%)
PC2 (17.30%)
7.50%
−15
−10
−5
0
5
10
15
−6
−4
−2
0
2
4
6
8
−10
−5
0
5
10

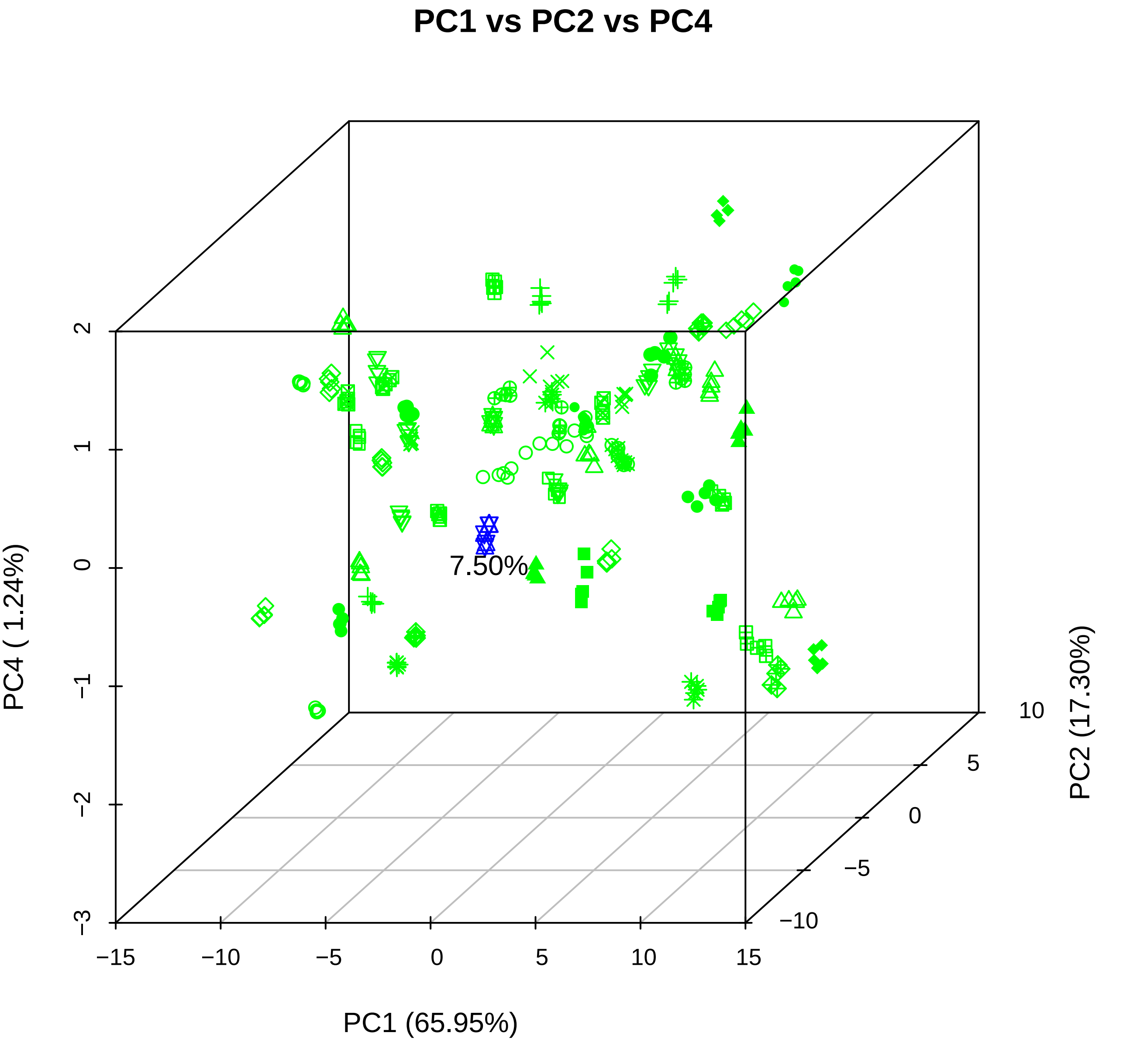

PC1 vs PC2 vs PC4
PC4 (1.24%)
PC1 (65.95%)
PC2 (17.30%)
7.50%
−15
−10
−5
0
5
10
15
−3
−2
−1
0
1
2
−10
−5
0
5
10

PC1 vs PC3 vs PC4

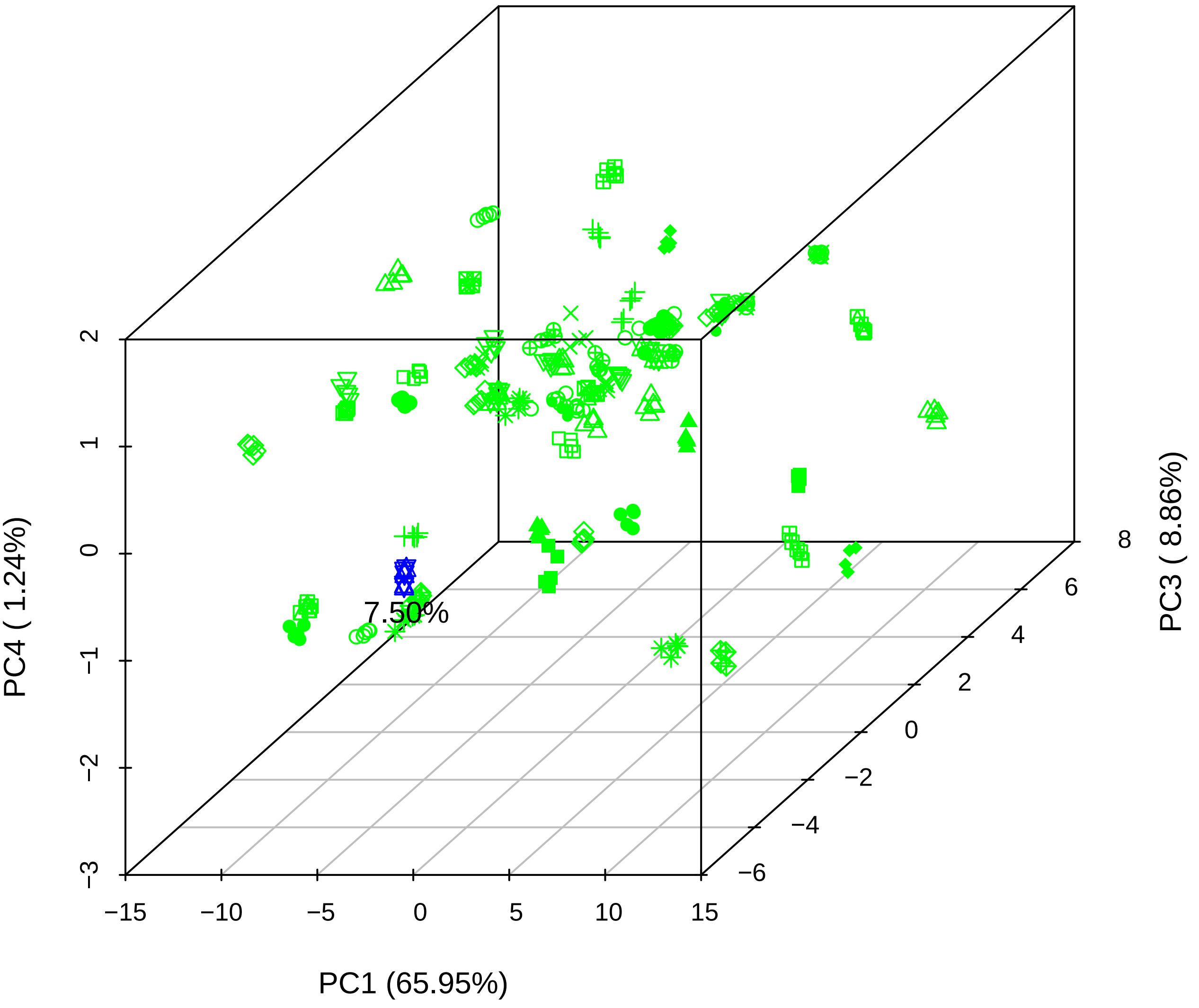

PC2 vs PC3 vs PC5

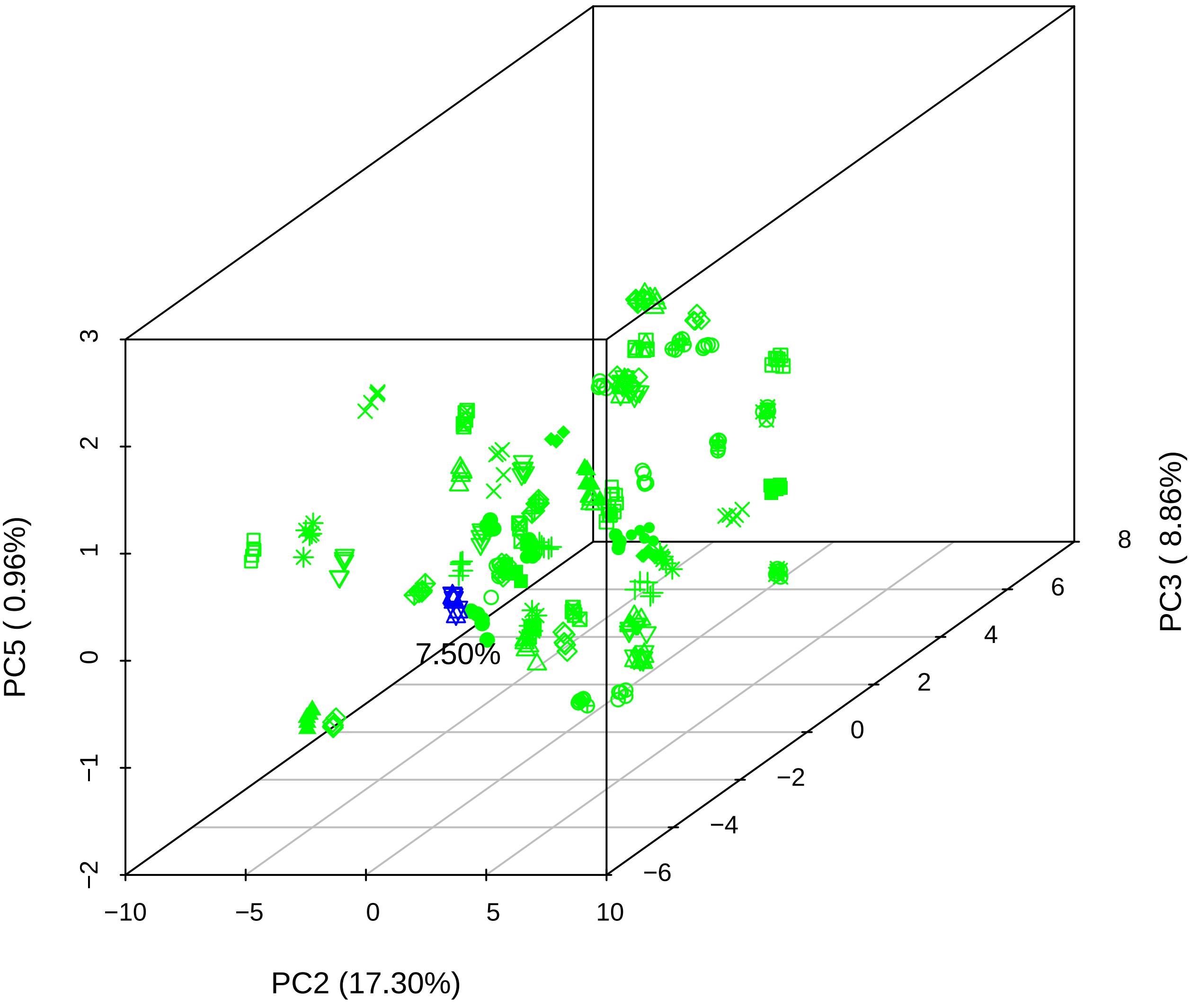

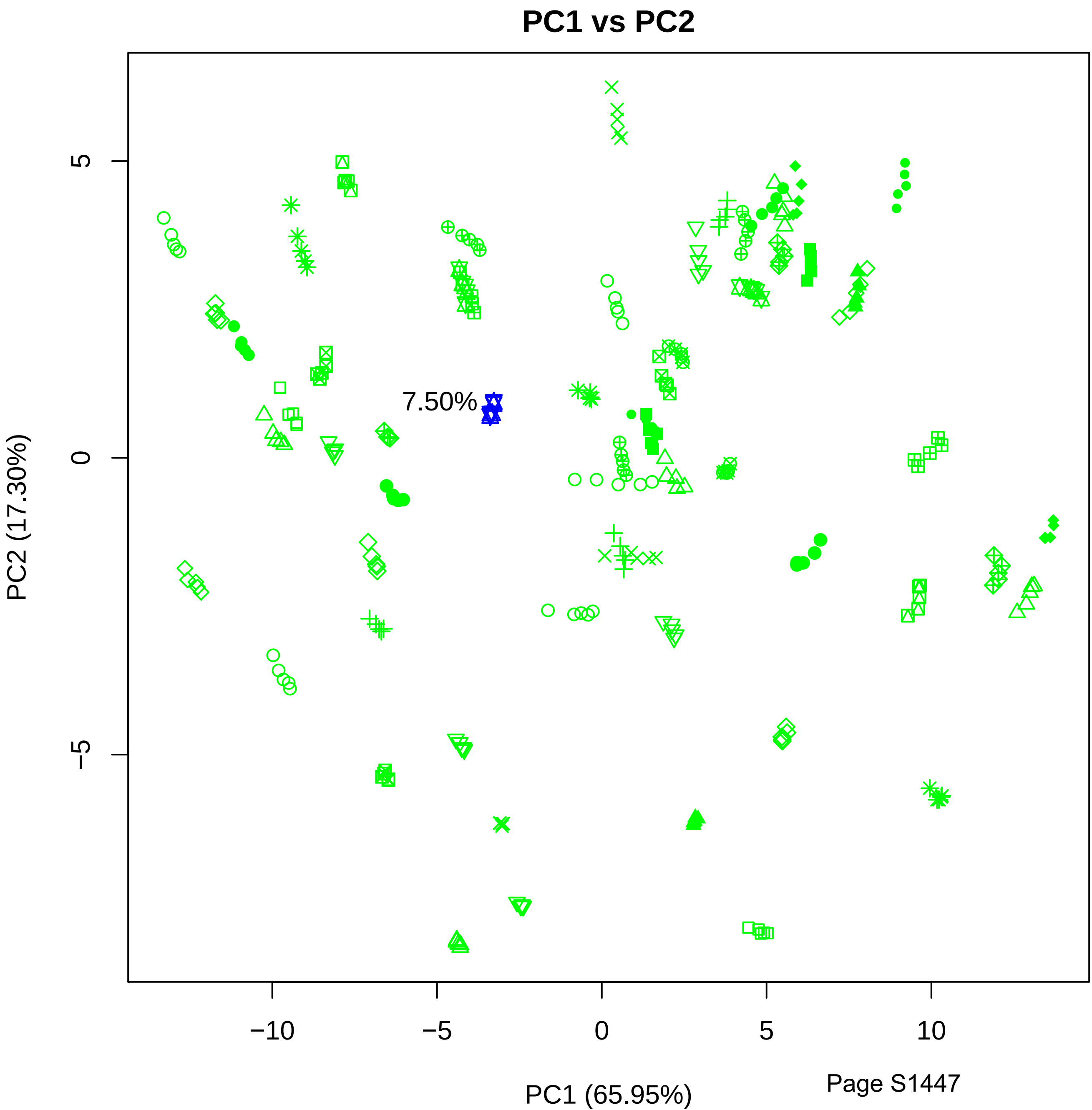

PC1 vs PC2
PC2 (17.30%)
PC1 (65.95%)
7.50%
5
0
−5
−10
−5
0
5
10

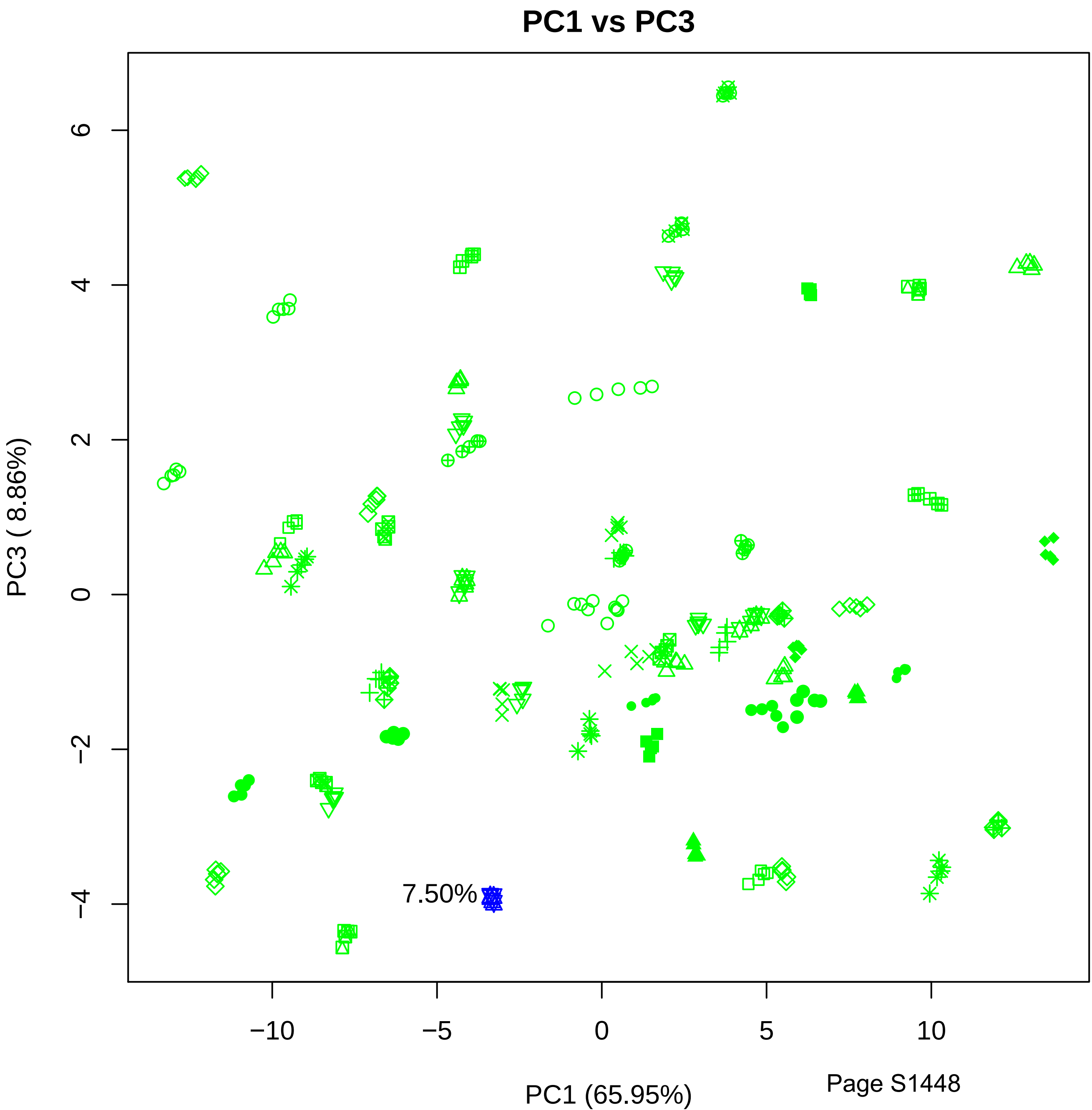

PC1 vs PC3
PC3 (8.86%)
6
4
2
0
−2
−4
7.50%
−10
−5
0
5
10
PC1 (65.95%)

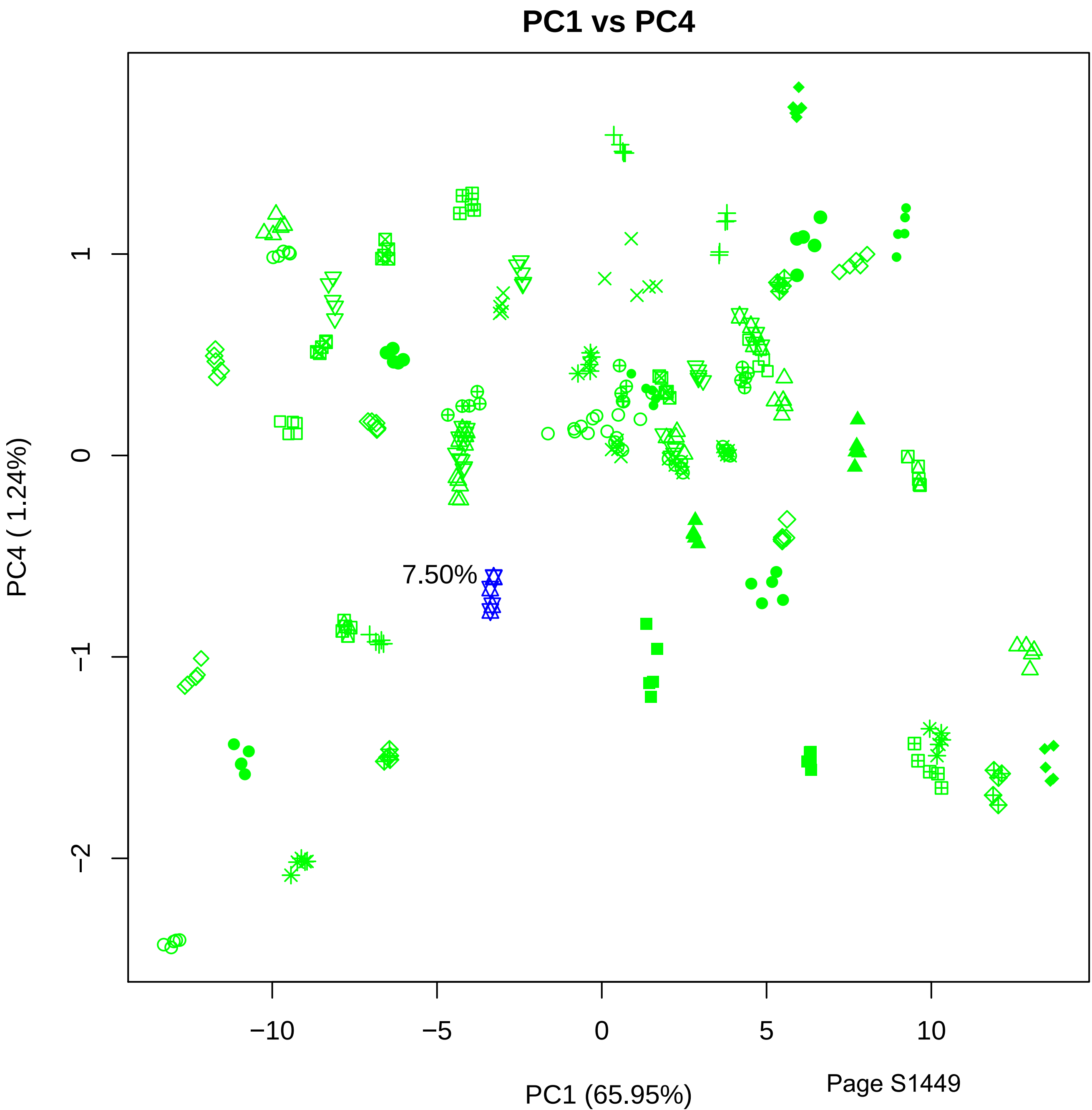

PC1 vs PC4
PC4 (1.24%)
PC1 (65.95%)
7.50%
−10
−5
0
5
10
−2
−1
0
1

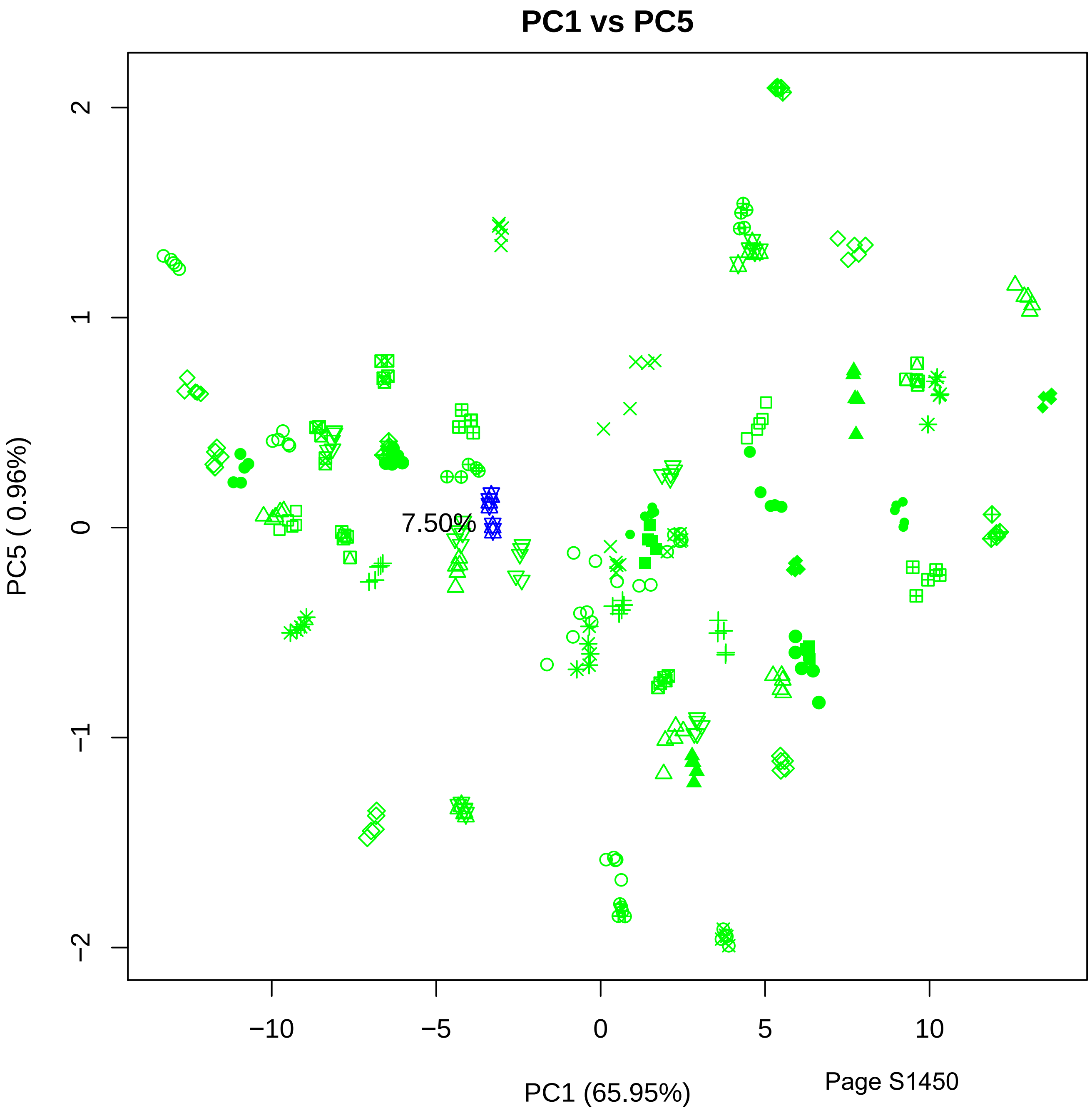

PC1 vs PC5
PC5 (0.96%)
PC1 (65.95%)
2
1
0
−1
−2
−10
−5
0
5
10
7.50%

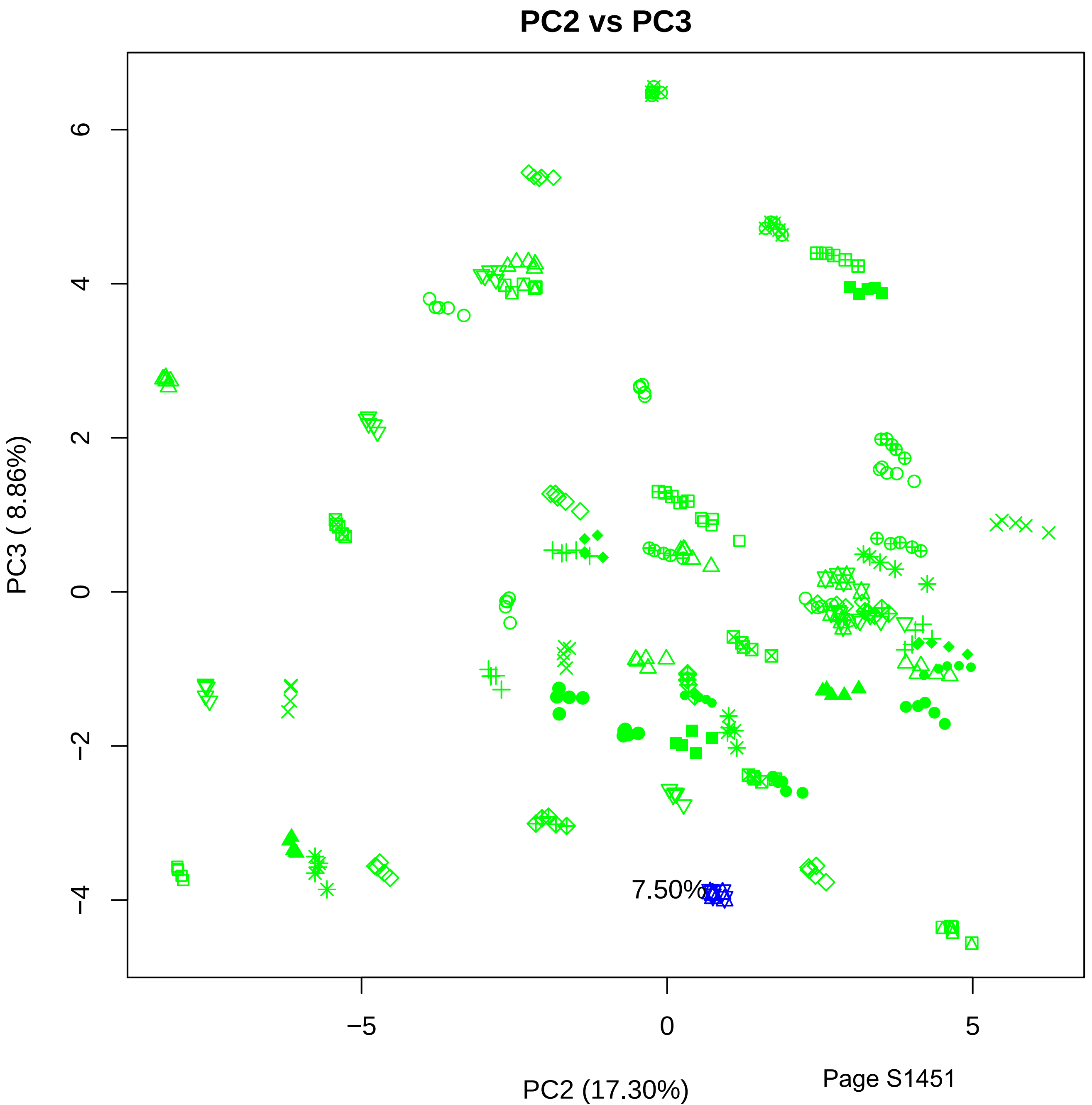
PC2 vs PC3
PC3 (8.86%)
PC2 (17.30%)
6
4
2
0
−2
−4
−5
0
5
7.50%

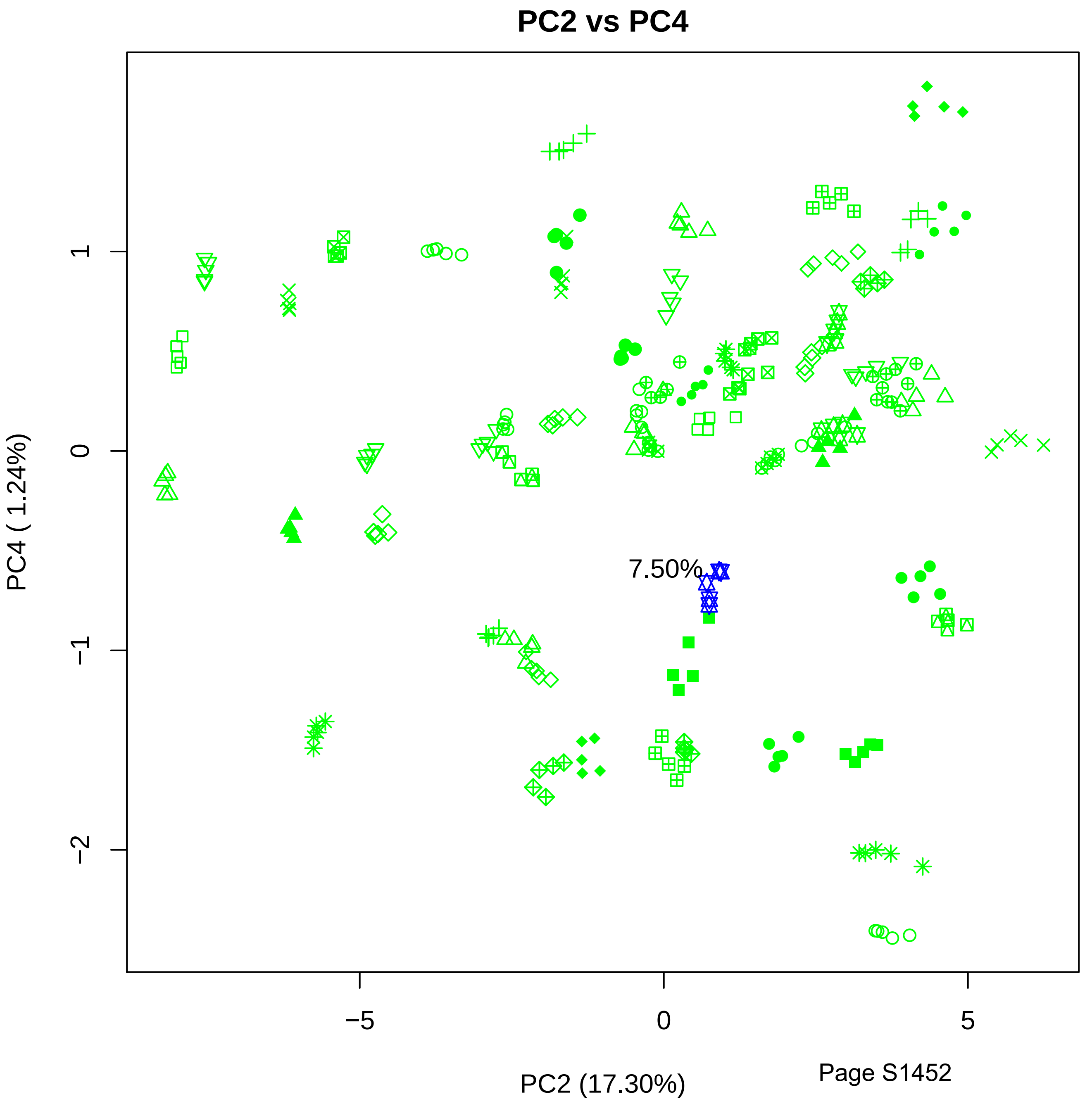

PC2 vs PC4
PC4 (1.24%)
PC2 (17.30%)
7.50%
1
0
−1
−2
−5
0
5

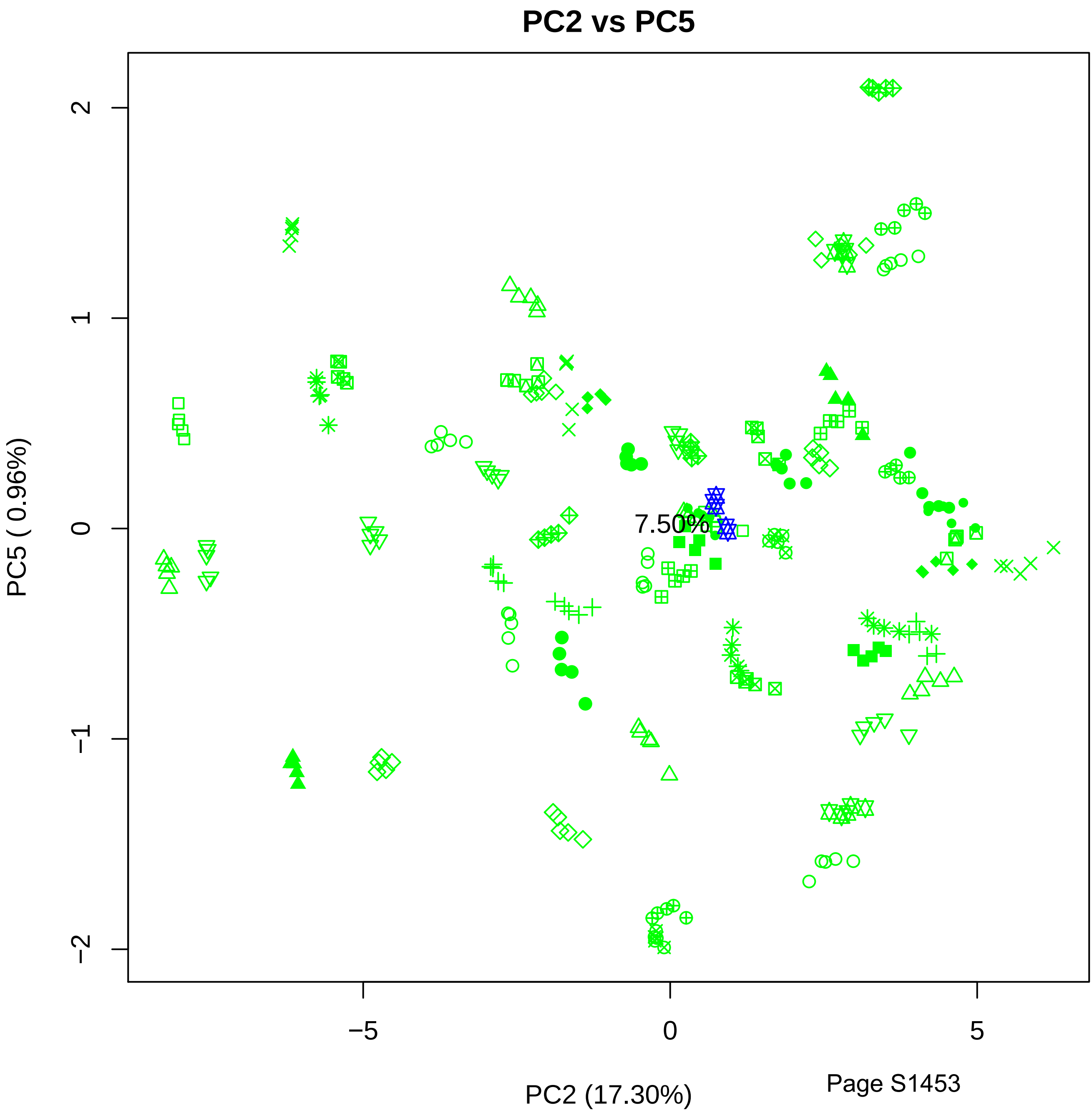
PC2 vs PC5
PC5 (0.96%)
PC2 (17.30%)
2
1
0
−1
−2
−5
0
5
7.50%

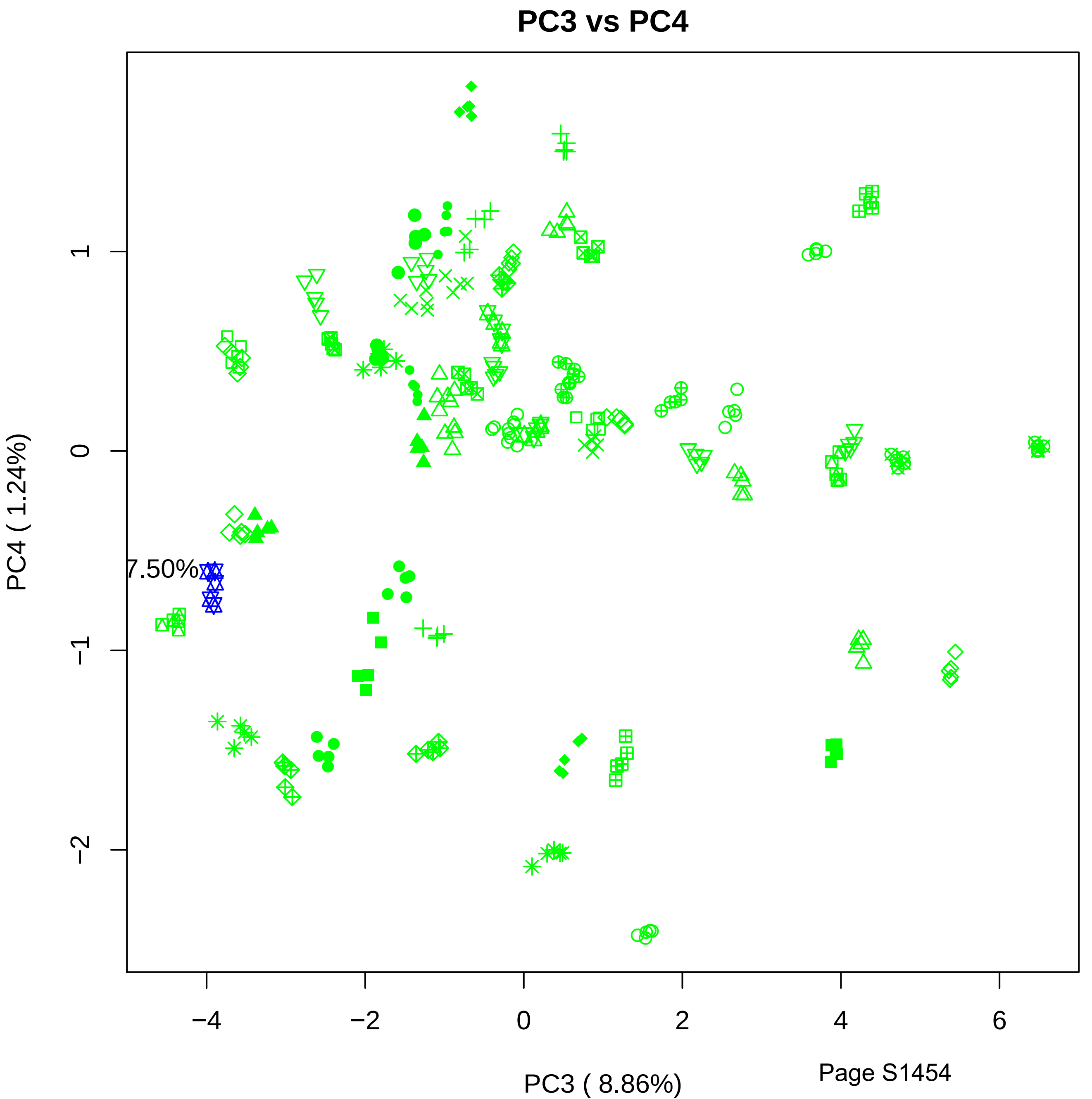
PC3 vs PC4
PC4 (1.24%)
PC3 (8.86%)
7.50%
1
0
−1
−2
−4
−2
0
2
4
6

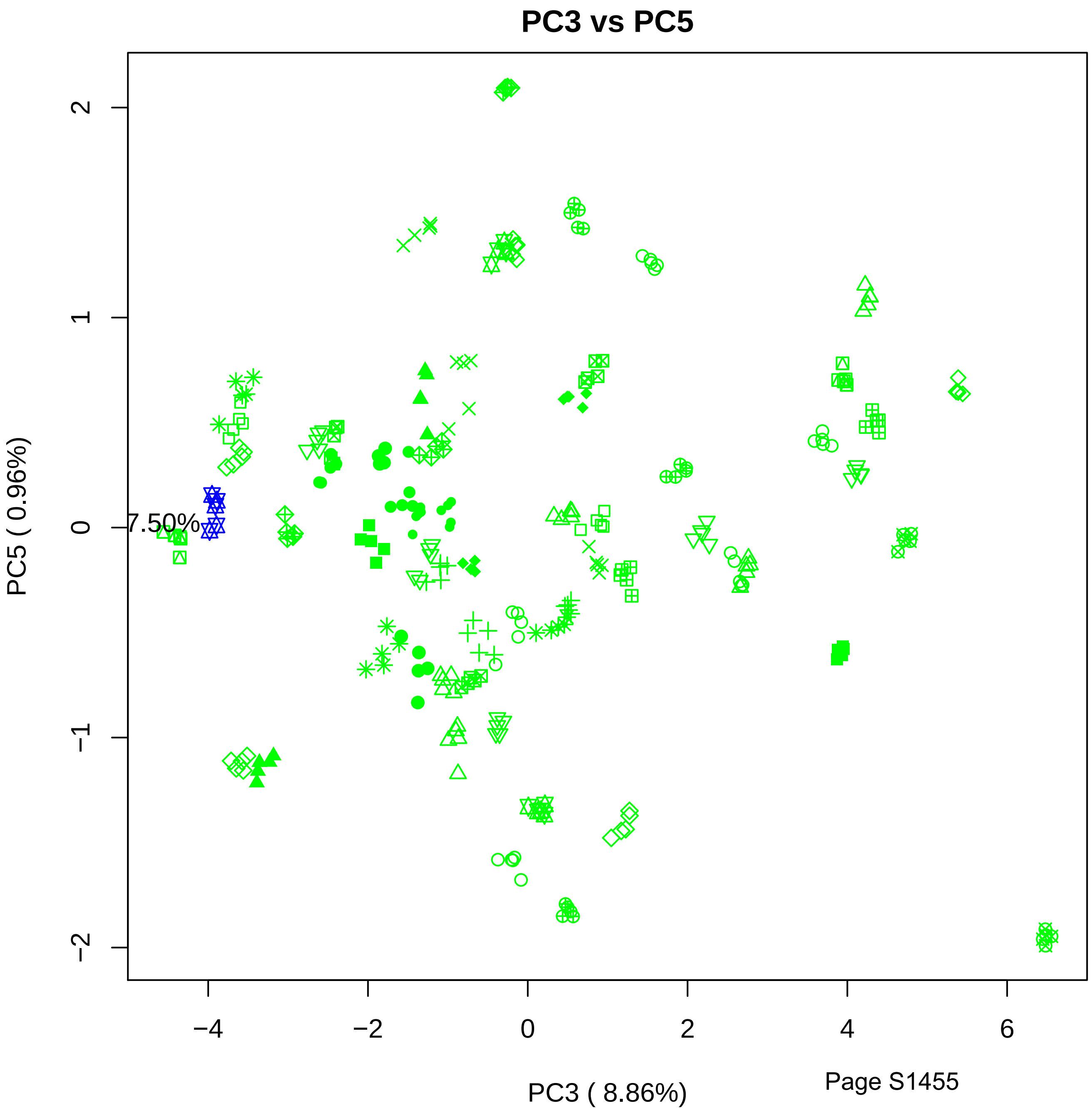
PC3 vs PC5
PC5 (0.96%)
PC3 (8.86%)
7.50%
2
1
0
−1
−2
−4
−2
0
2
4
6

PC4 vs PC5

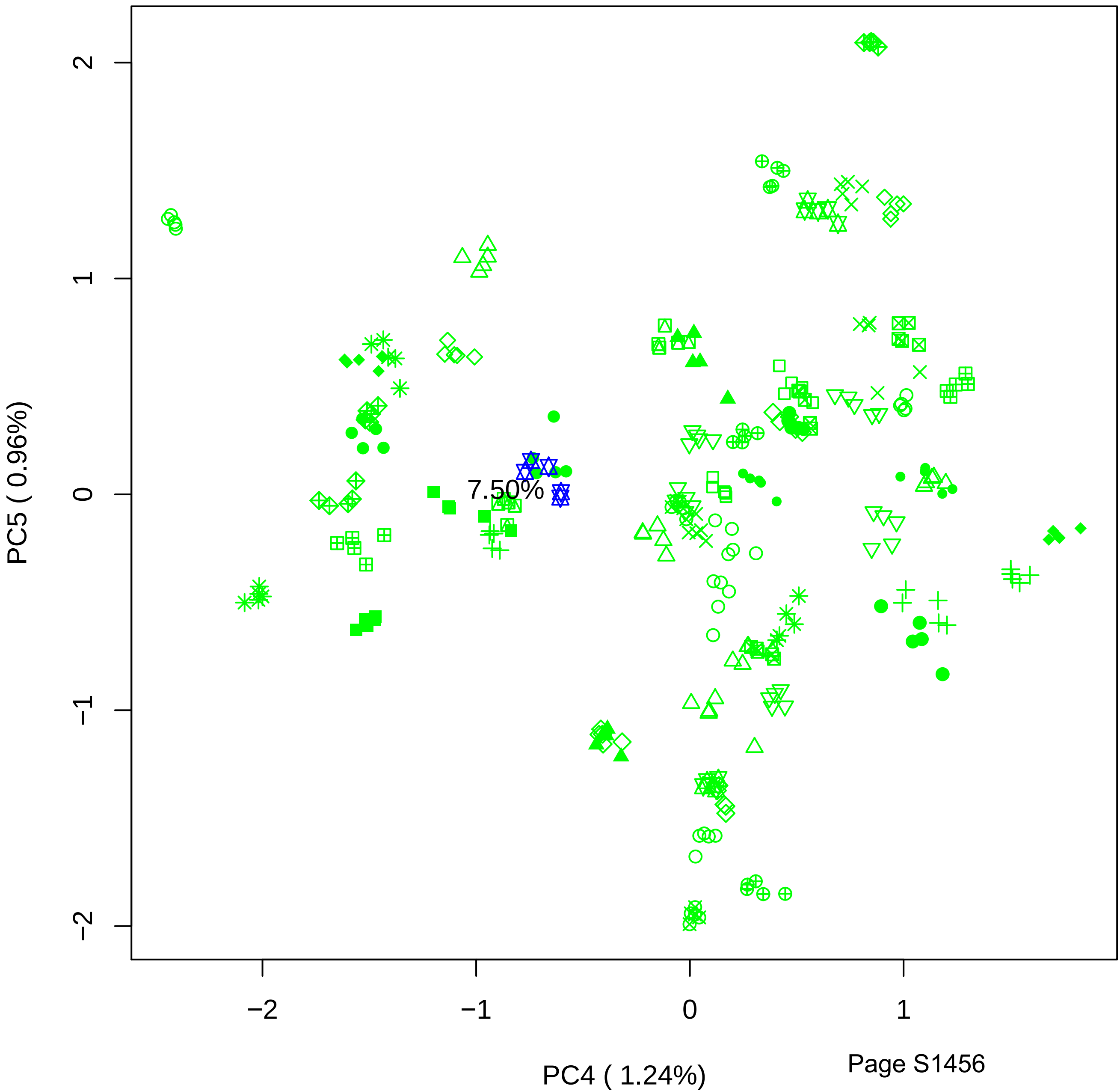

PCA of 10% BMH(d) vs PMH(d)

Scaled Matrix PCAed

7th order polynomial baseline correction performed

Raw spectra smoothed to 21 Neighbours, 2nd order PN, Savtisky Golay

Normalisation between 0 and 100

D2 spectra smoothed to 41 Neighbours, 2nd order PN, Savtisky Golay

Regions Analysed: 3601−2501 and 2001−700cm−1

Samples Analysed: 1 bContam's 0 BLH's 0 BMH's 0 OMH's 0 pContam's 0 PLH's 60 PMH's

Keele University AD

Fri Dec 14 14:22:58 2018

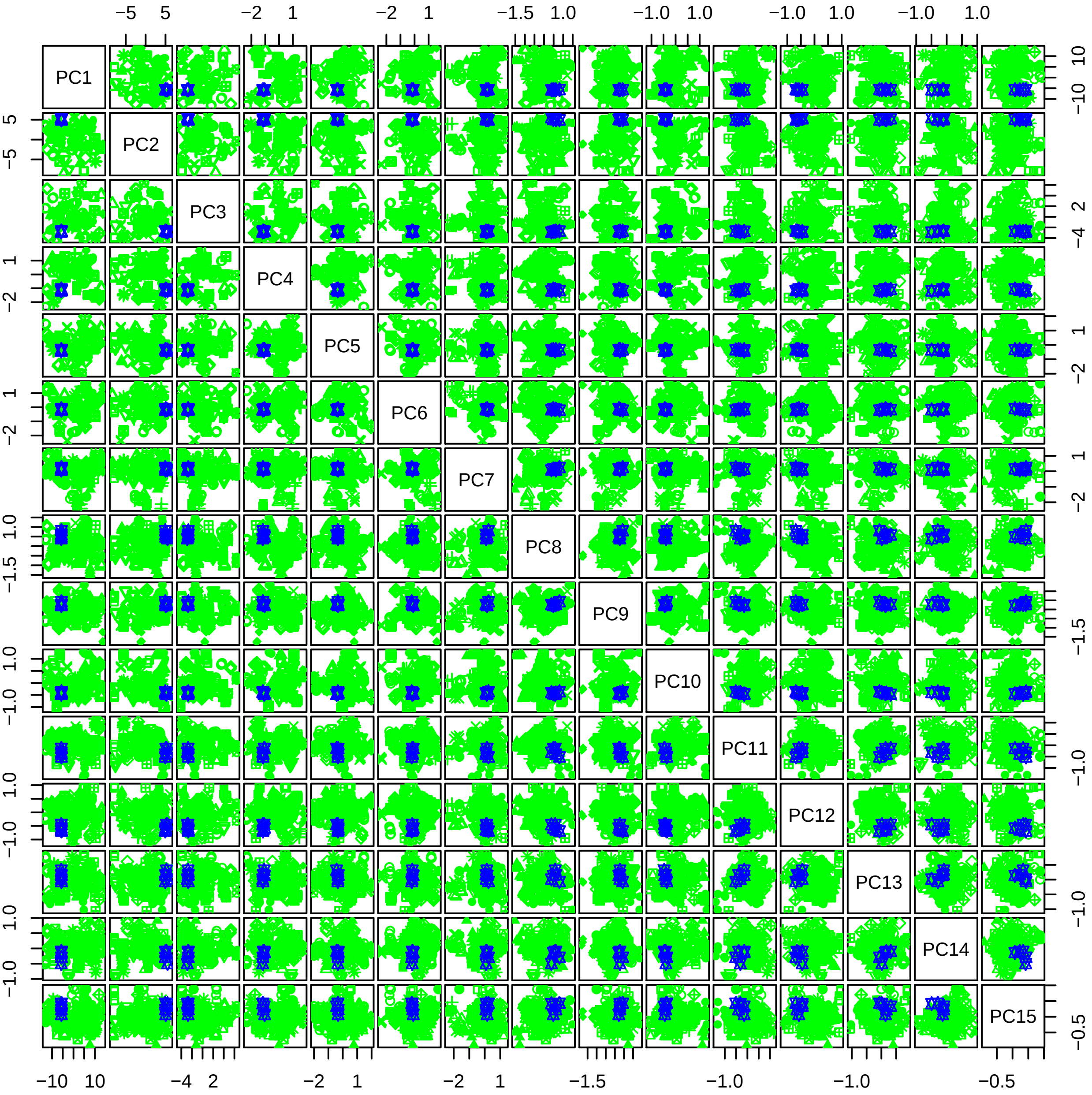
PC1
PC2
PC3
PC4
PC5
PC6
PC7
PC8
PC9
PC10
PC11
PC12
PC13
PC14
PC15

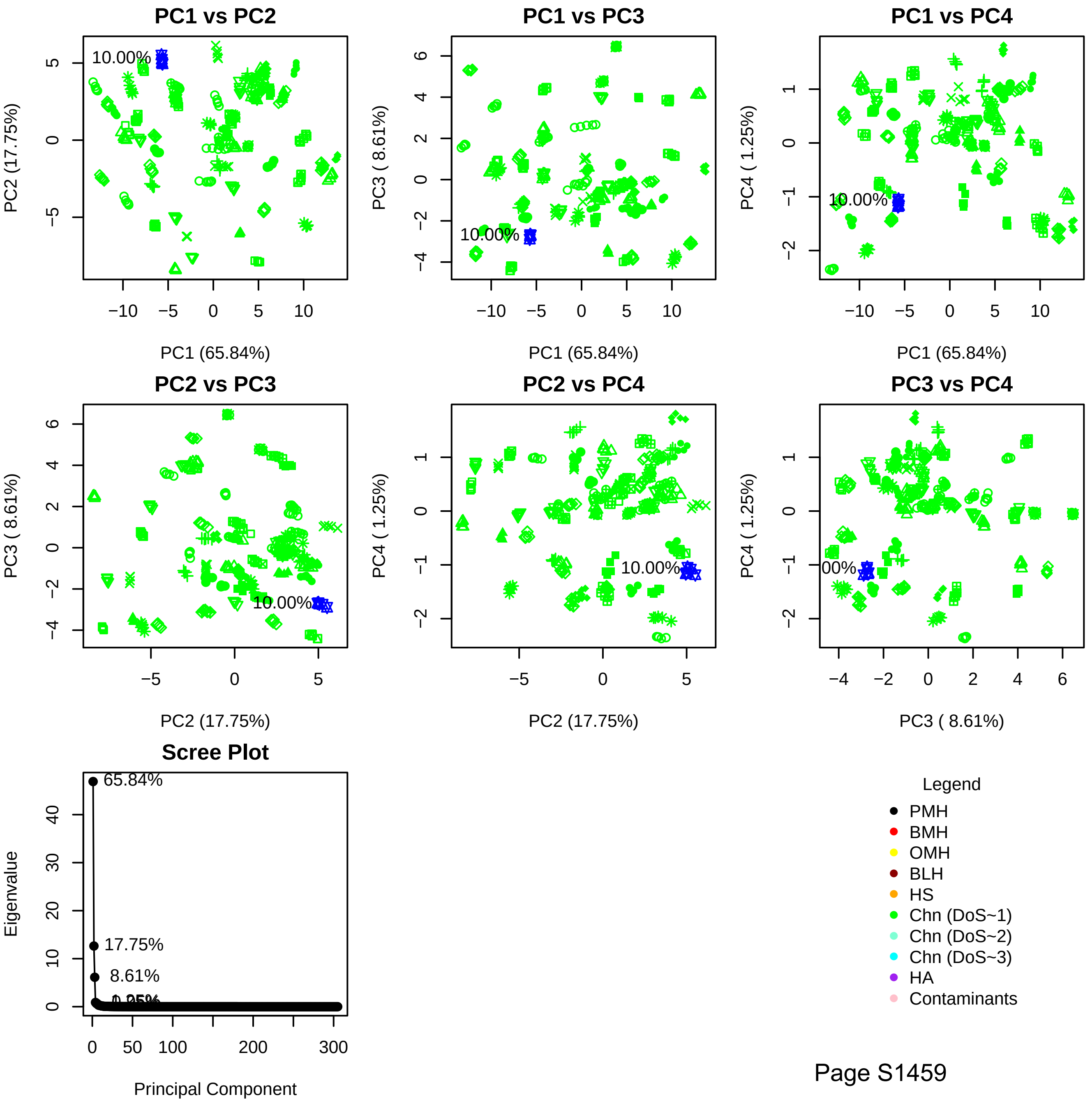
PC1 vs PC2
PC1 vs PC3
PC1 vs PC4
PC2 vs PC3
PC2 vs PC4
PC3 vs PC4
Scree Plot
PC1 (65.84%)
PC2 (17.75%)
PC3 (8.61%)
PC4 (1.25%)
10.00%
Eigenvalue
Principal Component
65.84%
17.75%
8.61%
Legend
PMH
BMH
OMH
BLH
HS
Chn (DoS~1)
Chn (DoS~2)
Chn (DoS~3)
HA
Contaminants

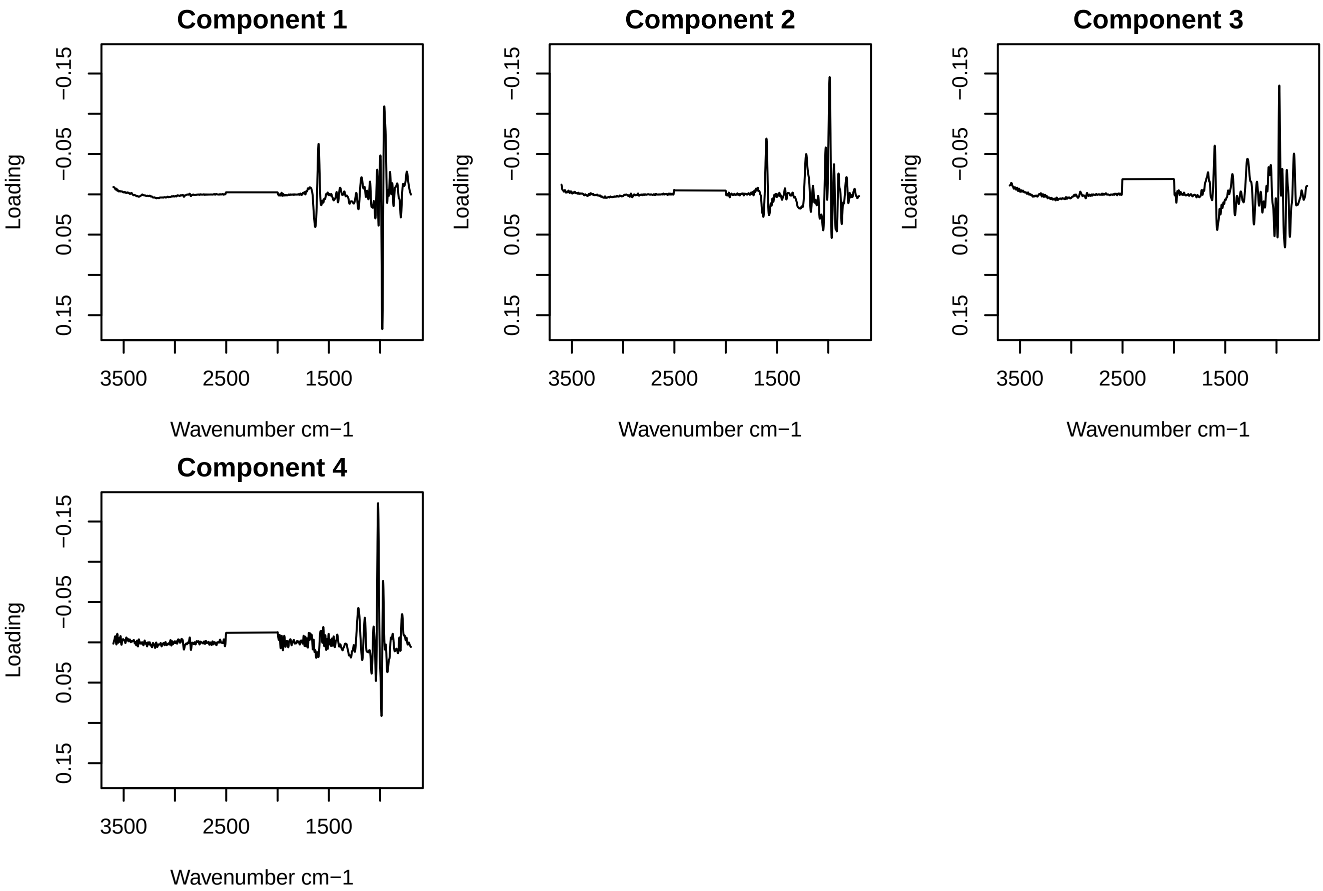
Component 1
Component 2
Component 3
Component 4
Loading
−0.15
−0.05
0.05
0.15
3500
2500
1500
Wavenumber cm−1

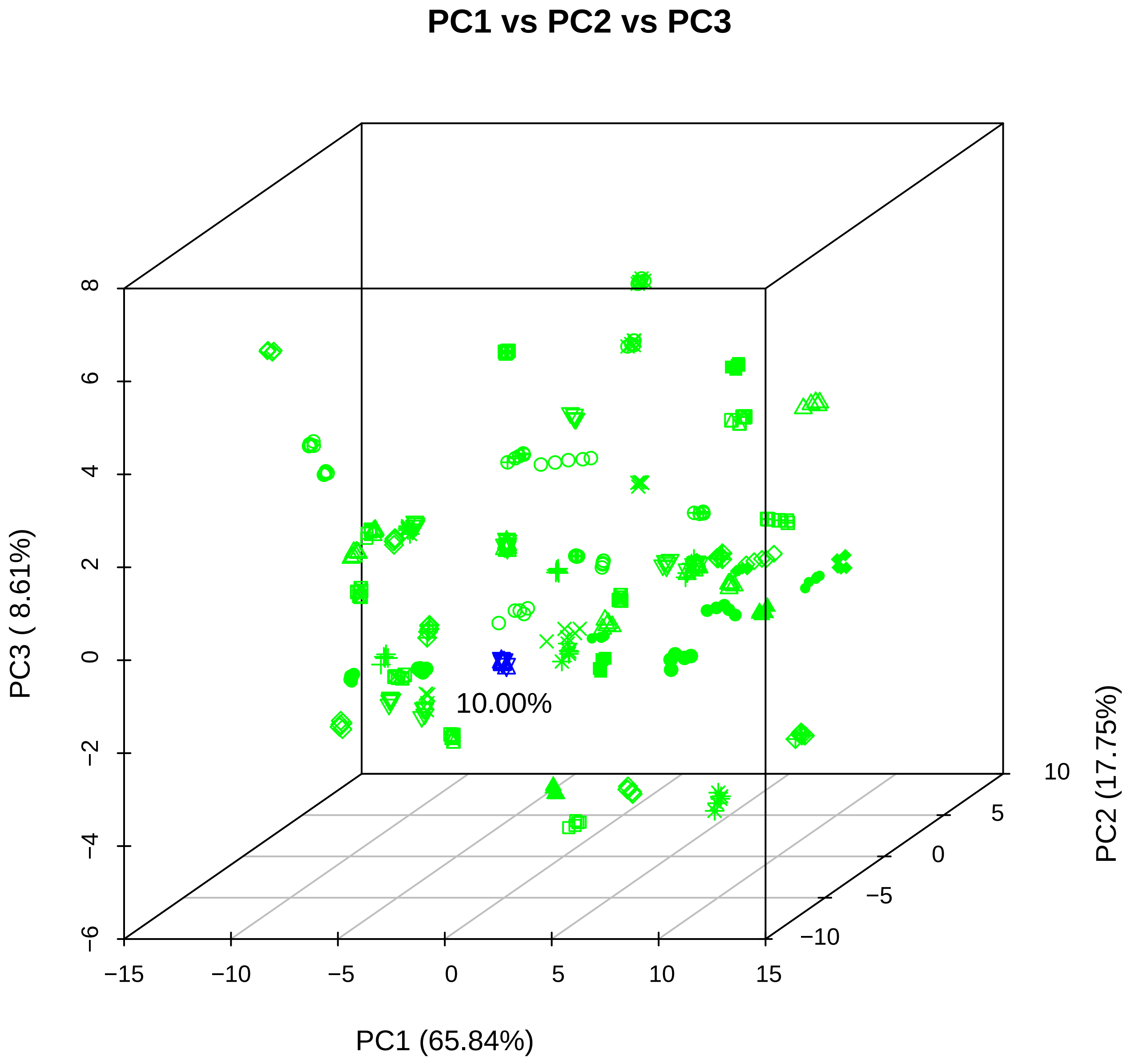

PC1 vs PC2 vs PC3
8
6
4
2
0
−2
−4
−6
PC3 (8.61%)
10.00%
−15
−10
−5
0
5
10
15
PC1 (65.84%)
10
5
0
−5
−10
PC2 (17.75%)

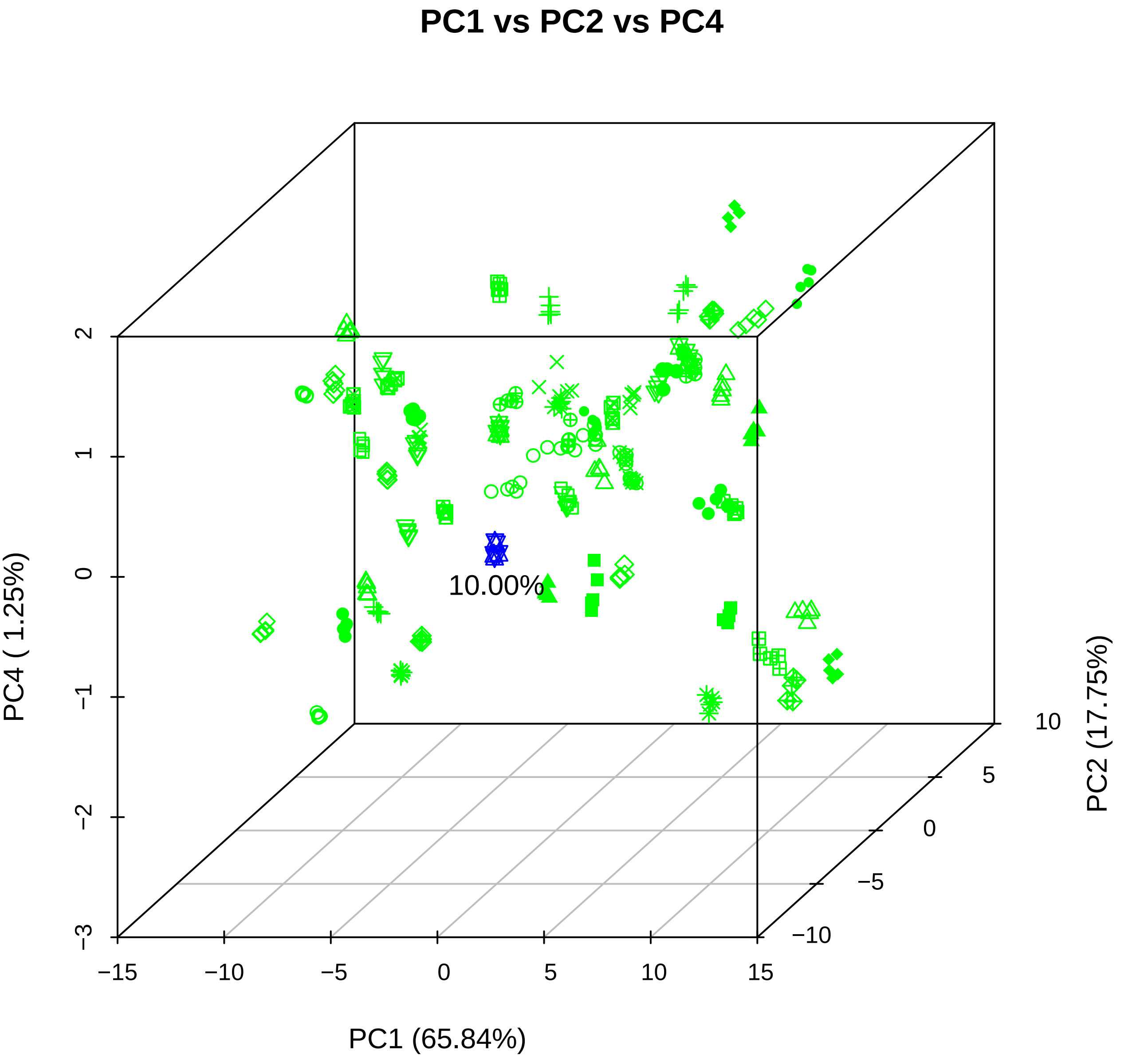
PC1 vs PC2 vs PC4
10.00%
PC1 (65.84%)
PC2 (17.75%)
PC4 (1.25%)
−15
−10
−5
0
5
10
15
−10
−5
0
5
10
−3
−2
−1
0
1
2

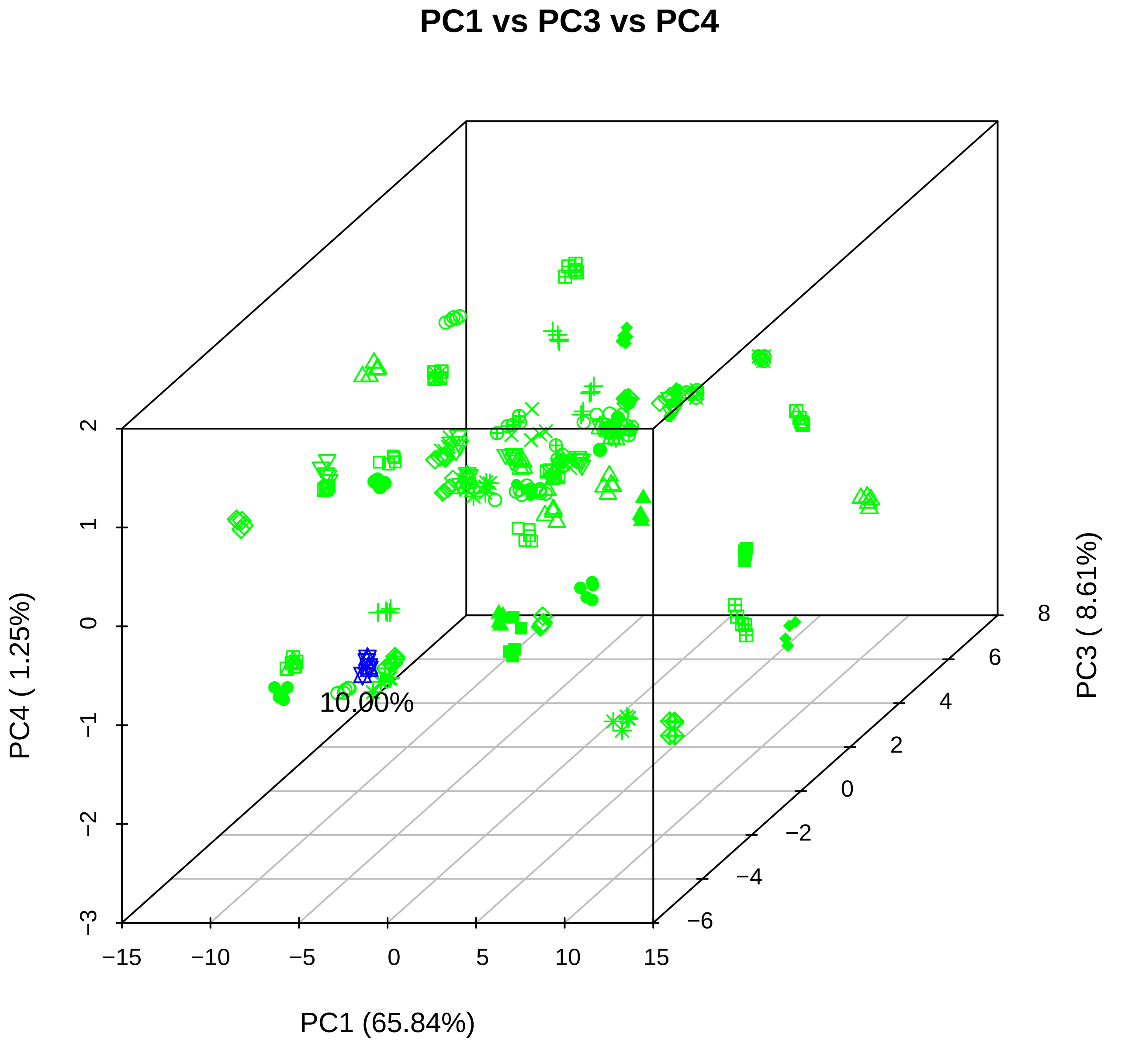

PC1 vs PC3 vs PC4
PC1 (65.84%)
PC4 (1.25%)
PC3 (8.61%)
10.00%
−15
−10
−5
0
5
10
15
−3
−2
−1
0
1
2
−6
−4
−2
0
2
4
6
8

PC2 vs PC3 vs PC5

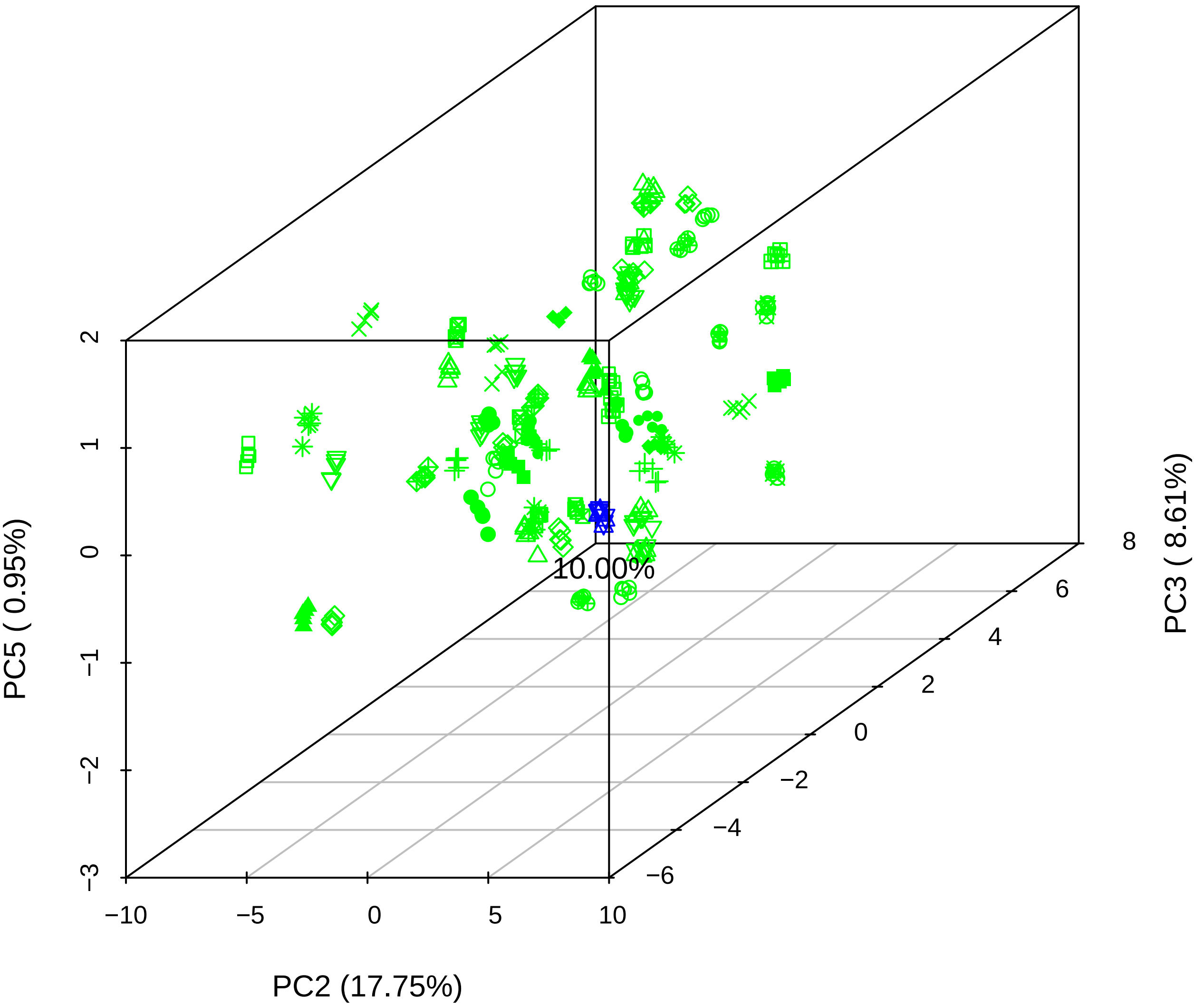

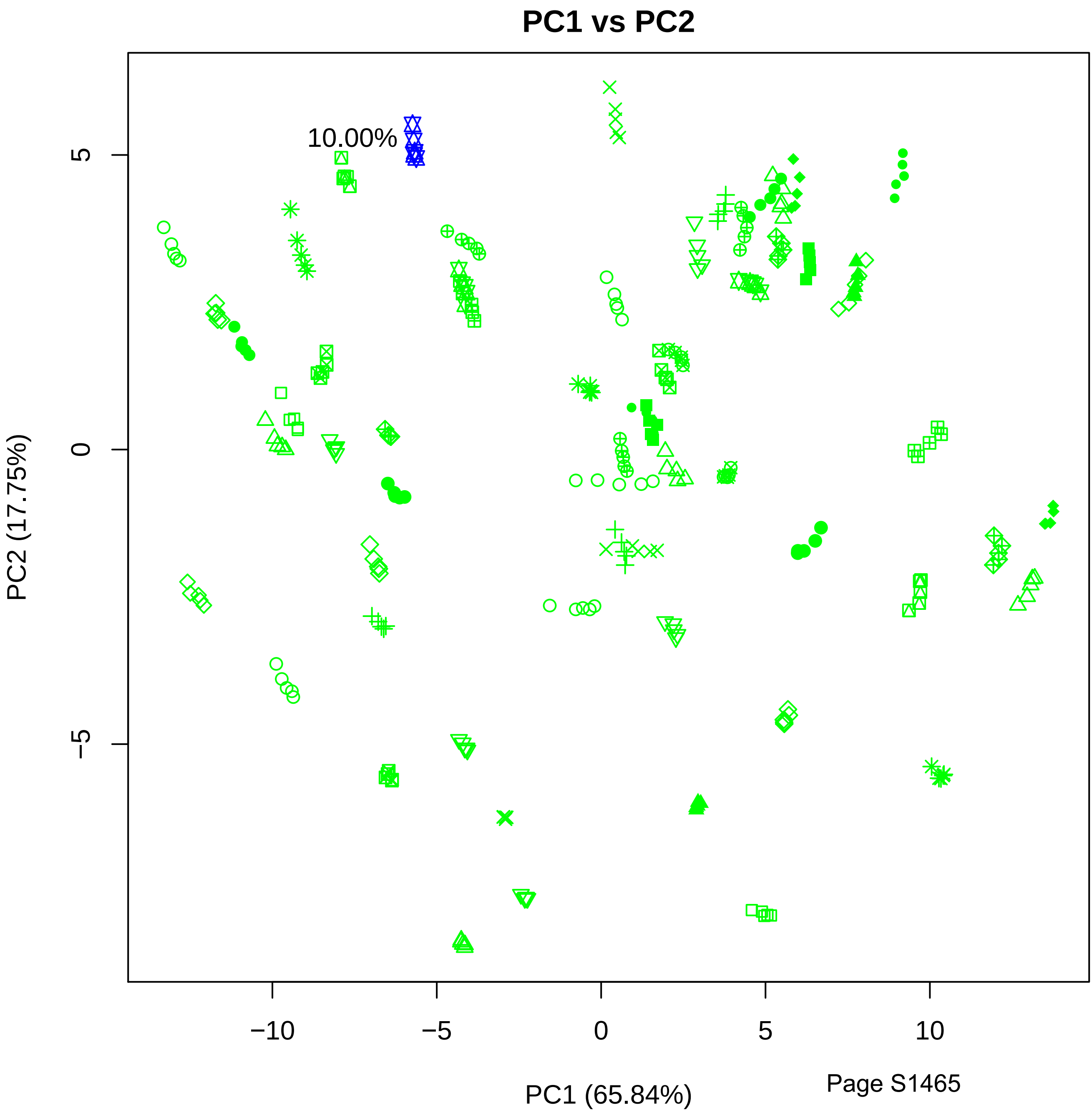

PC1 vs PC2
10.00%
5
0
−5
PC2 (17.75%)
−10
−5
0
5
10
PC1 (65.84%)

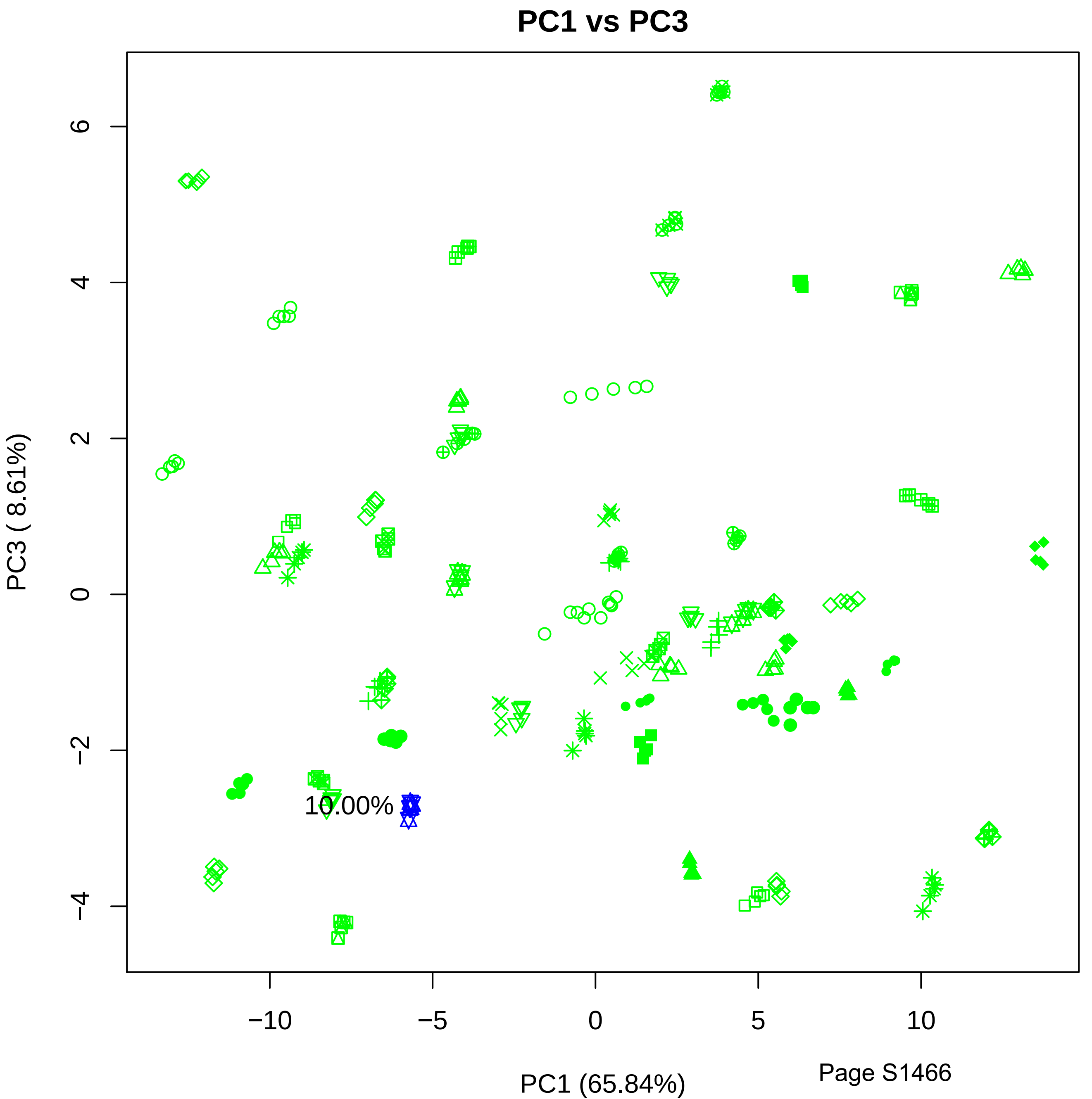

PC1 vs PC3
PC3 (8.61%)
PC1 (65.84%)
10.00%
6
4
2
0
−2
−4
−10
−5
0
5
10

PC1 vs PC4

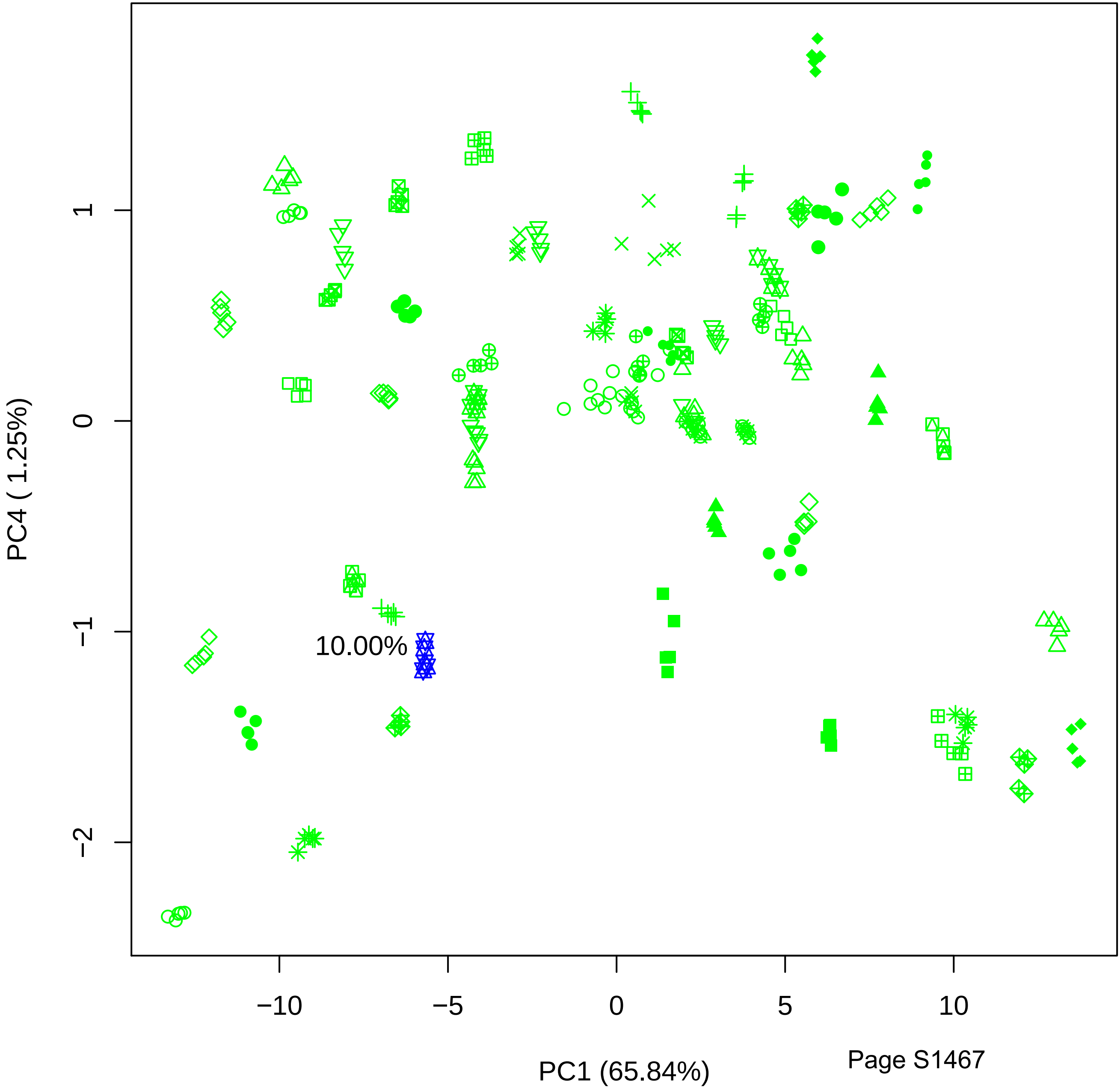

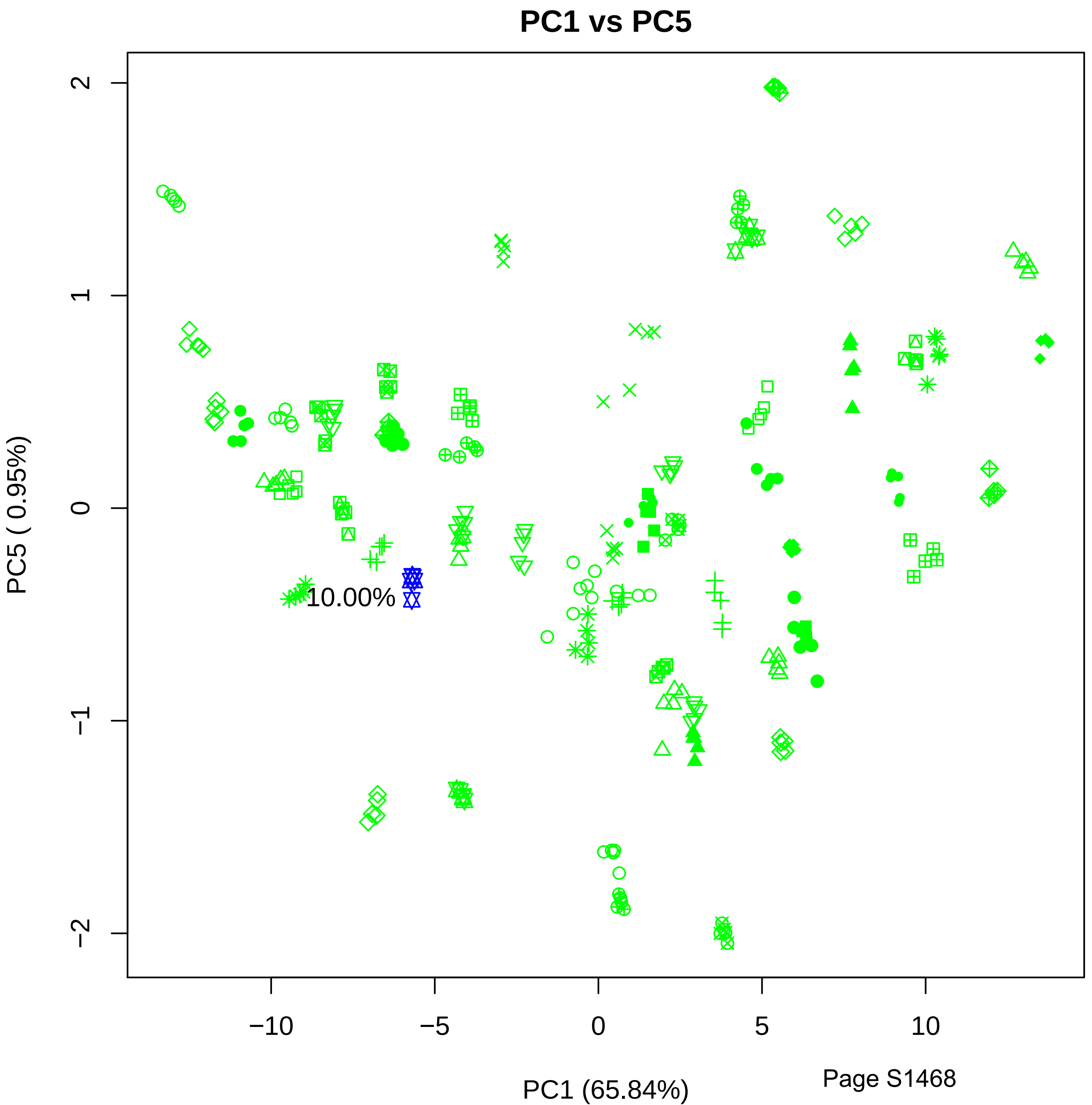
PC1 vs PC5
PC5 (0.95%)
PC1 (65.84%)
10.00%
2
1
0
−1
−2
−10
−5
0
5
10

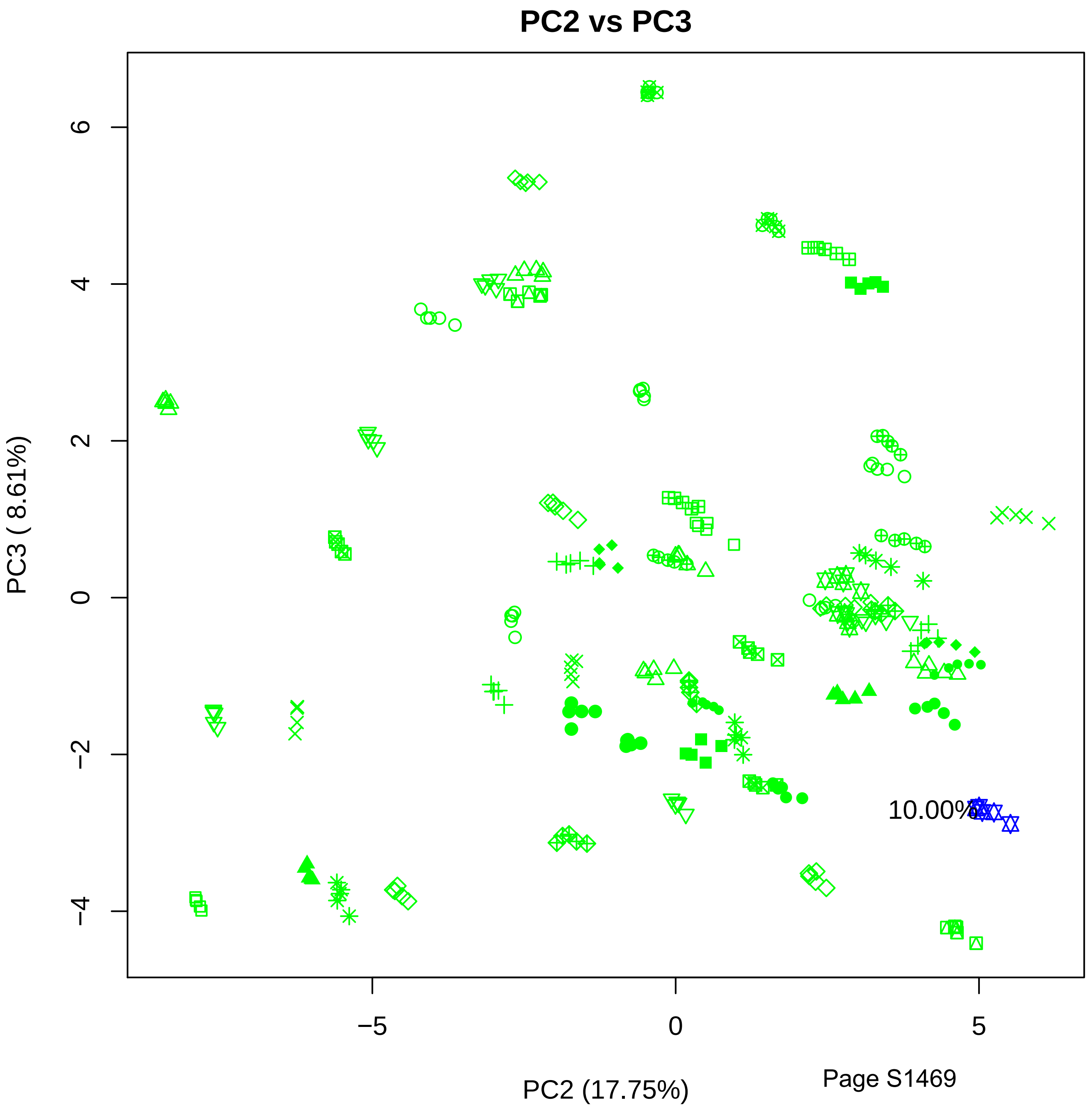
PC2 vs PC3
PC3 (8.61%)
PC2 (17.75%)
6
4
2
0
−2
−4
−5
0
5
10.00%

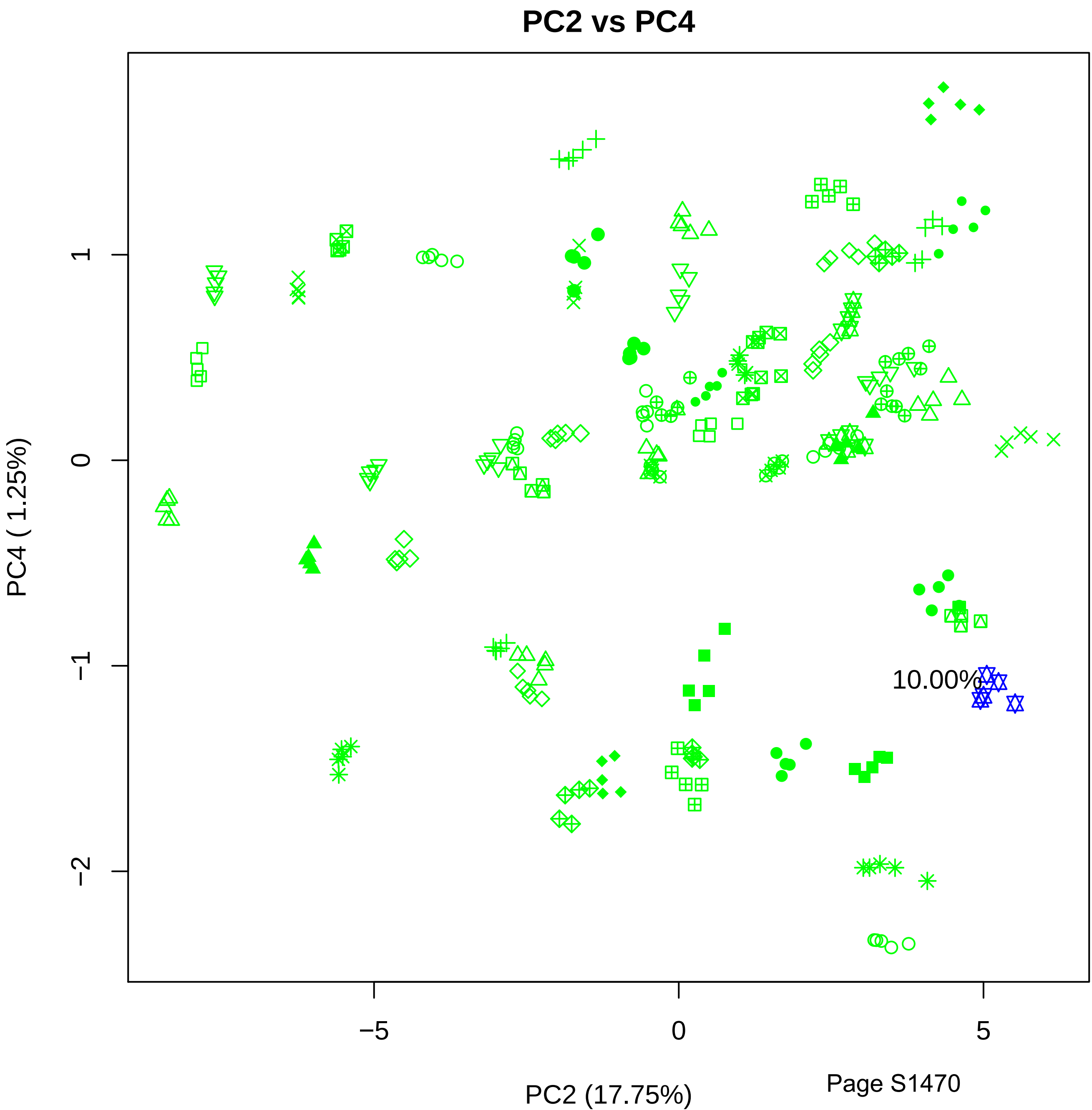

PC2 vs PC4
PC4 (1.25%)
PC2 (17.75%)
1
0
−1
−2
−5
0
5
10.00%

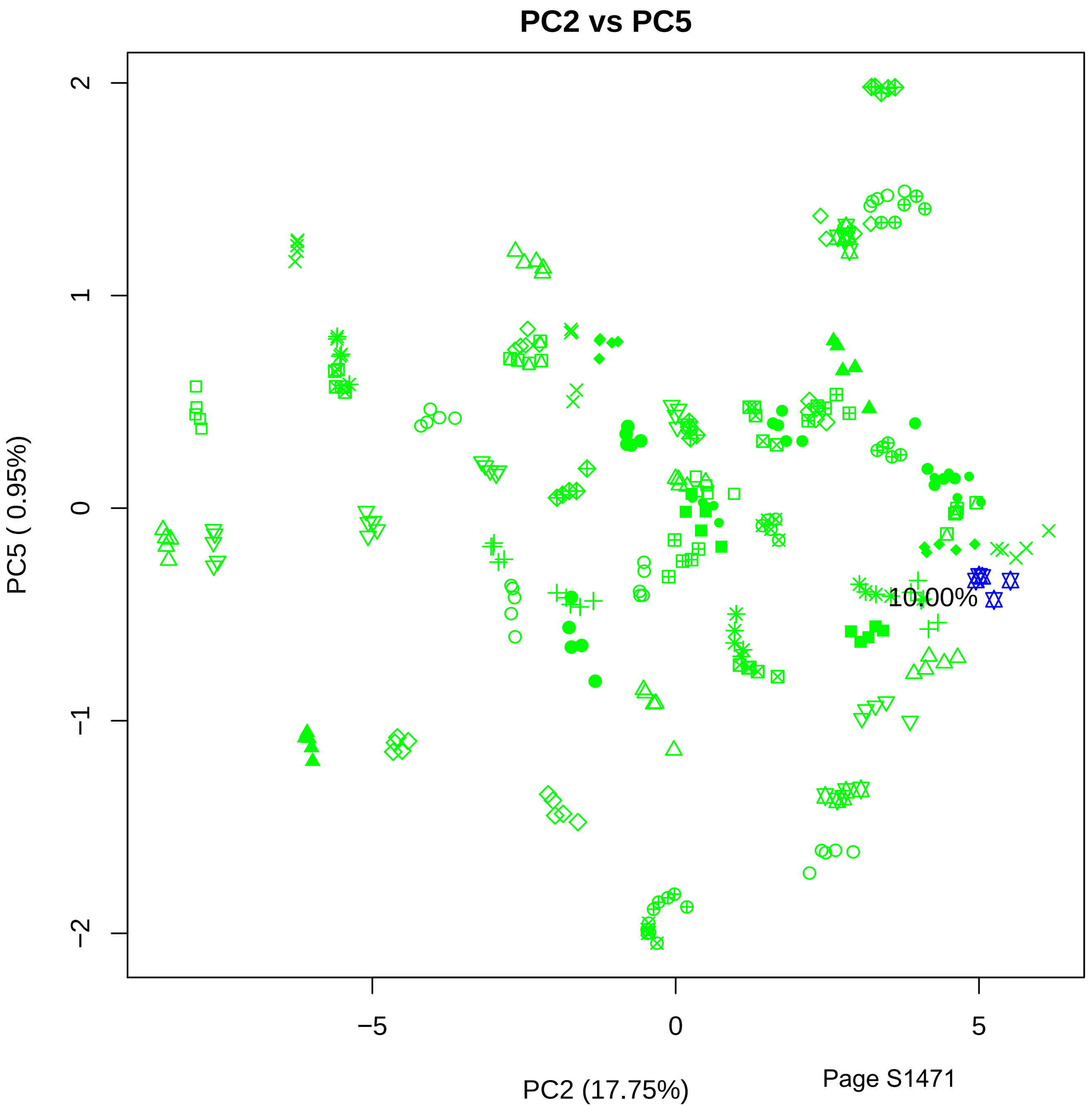
PC2 vs PC5
PC5 (0.95%)
PC2 (17.75%)
2
1
0
−1
−2
−5
0
5
10.00%

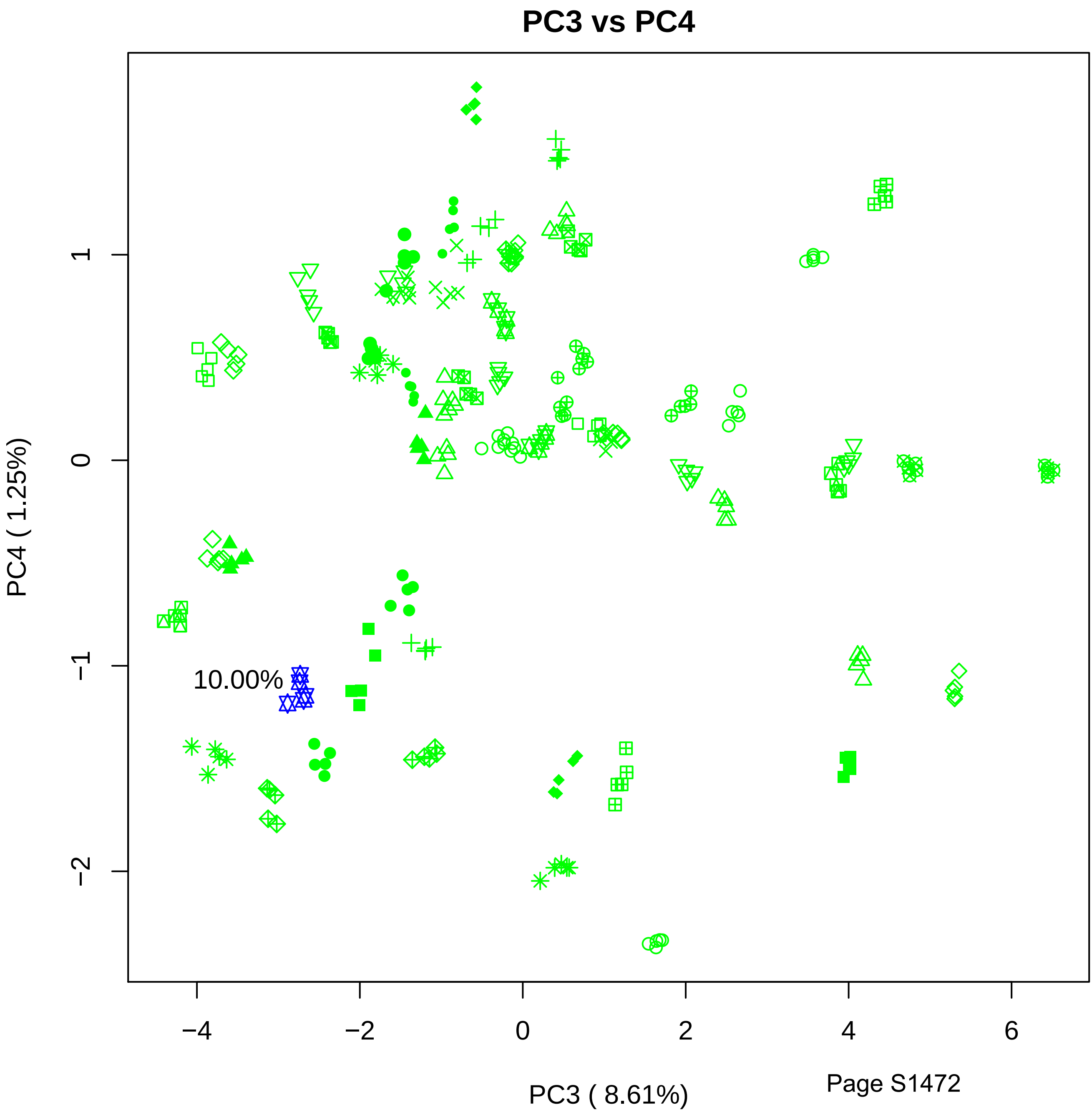

PC3 vs PC4
PC4 (1.25%)
PC3 (8.61%)
10.00%
1
0
−1
−2
−4
−2
0
2
4
6

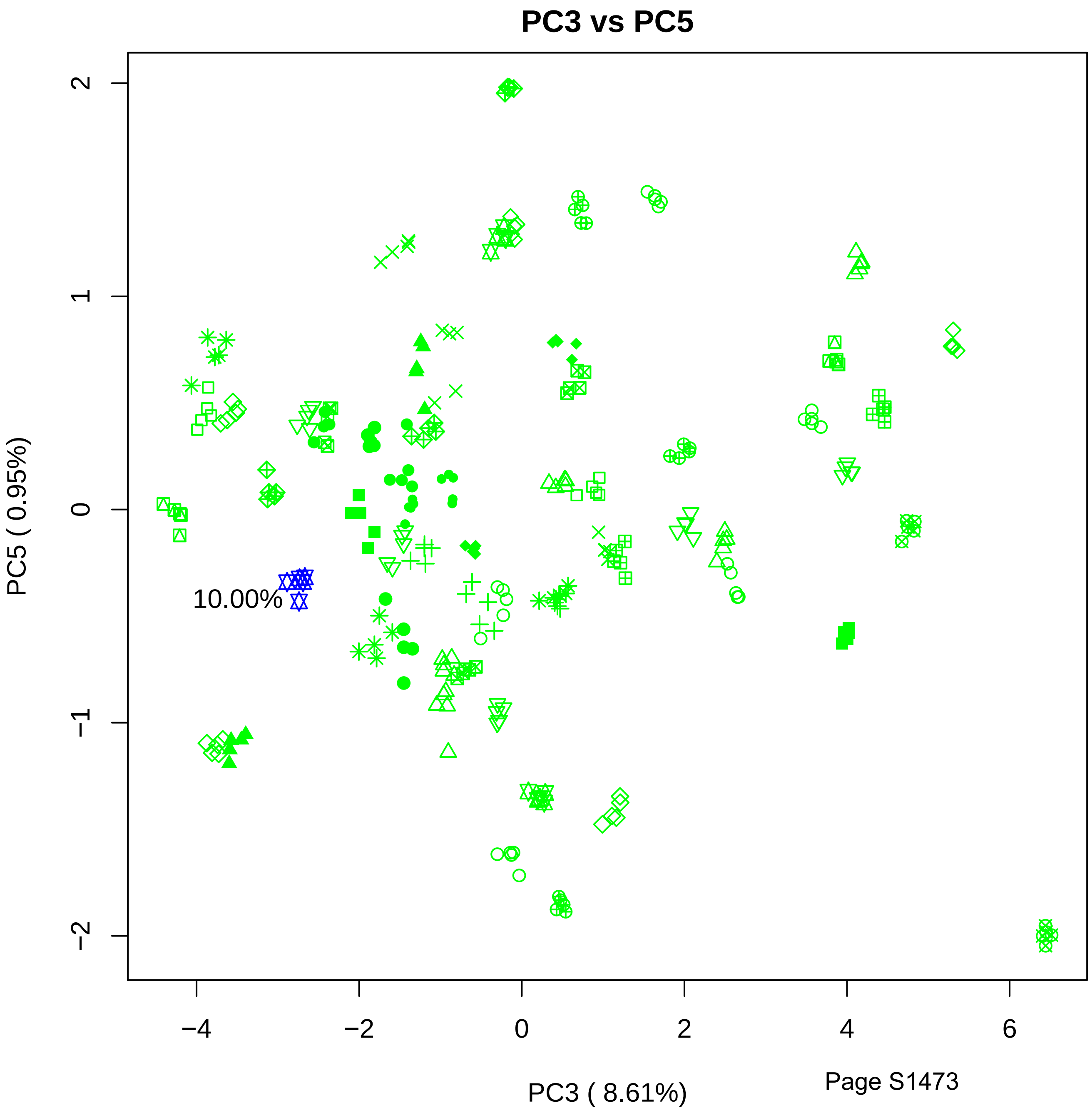
PC3 vs PC5
PC5 (0.95%)
PC3 (8.61%)
10.00%
2
1
0
−1
−2
−4
−2
0
2
4
6

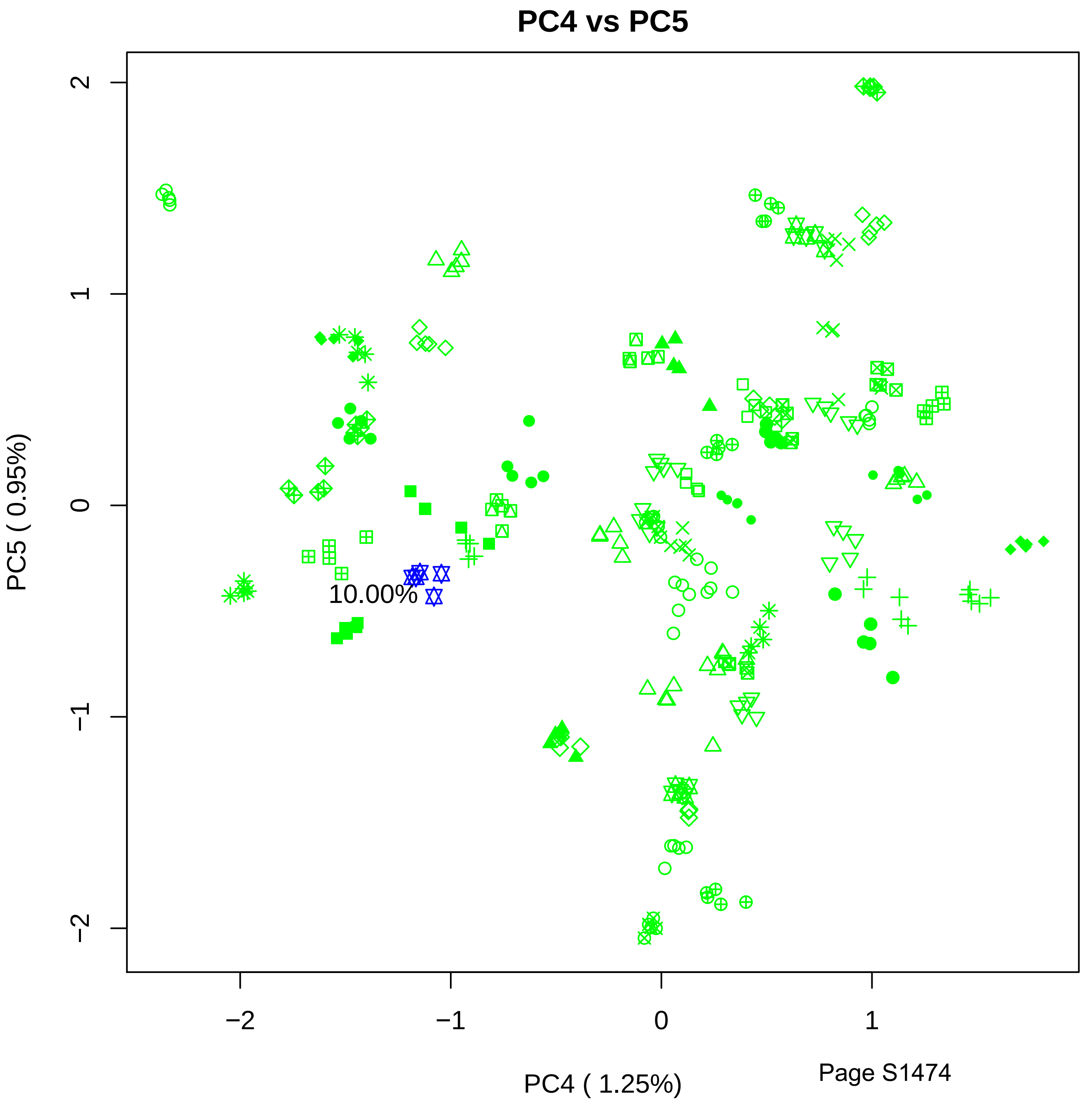

PC4 vs PC5
PC5 (0.95%)
PC4 (1.25%)
10.00%
2
1
0
−1
−2
−2
−1
0
1

PCA of 15% BMH(d) vs PMH(d)

Scaled Matrix PCAed

7th order polynomial baseline correction performed

Raw spectra smoothed to 21 Neighbours, 2nd order PN, Savtisky Golay

Normalisation between 0 and 100

D2 spectra smoothed to 41 Neighbours, 2nd order PN, Savtisky Golay

Regions Analysed: 3601−2501 and 2001−700cm−1

Samples Analysed: 1 bContam's 0 BLH's 0 BMH's 0 OMH's 0 pContam's 0 PLH's 60 PMH's

Keele University AD

Fri Dec 14 14:23:07 2018

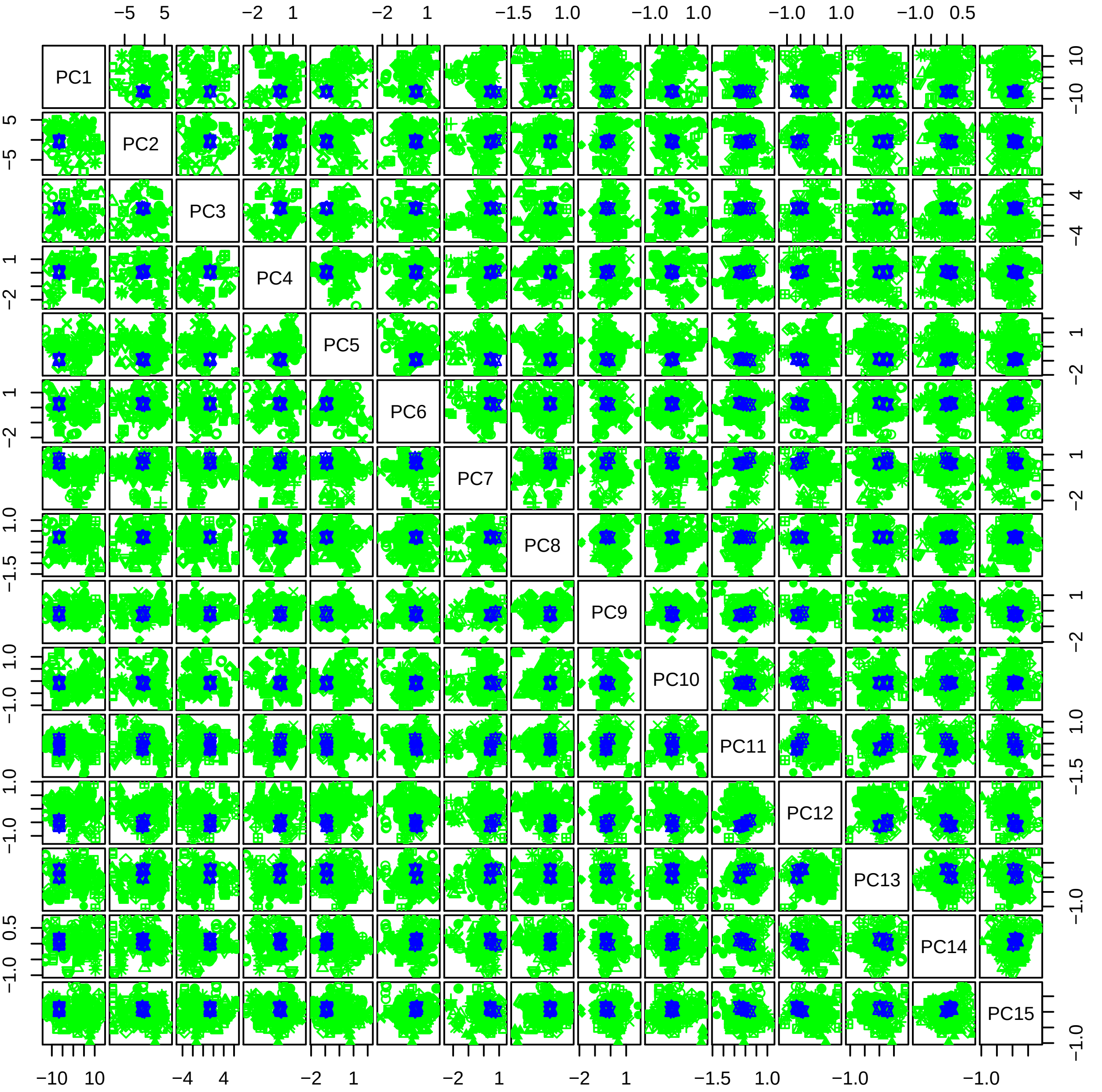
PC1
PC2
PC3
PC4
PC5
PC6
PC7
PC8
PC9
PC10
PC11
PC12
PC13
PC14
PC15

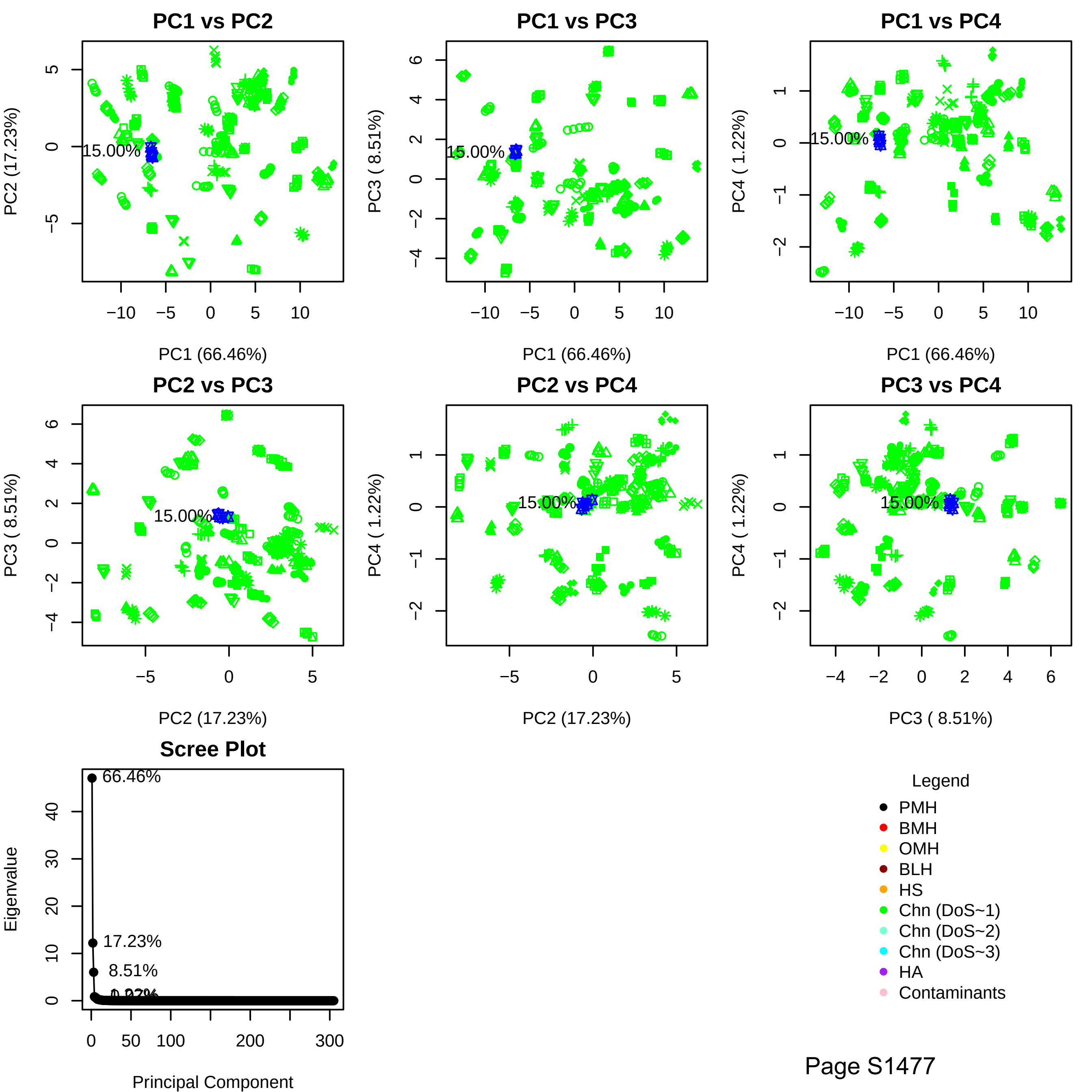
PC1 vs PC2
PC1 vs PC3
PC1 vs PC4
PC2 vs PC3
PC2 vs PC4
PC3 vs PC4
Scree Plot
PC1 (66.46%)
PC2 (17.23%)
PC3 (8.51%)
PC4 (1.22%)
15.00%
Eigenvalue
Principal Component
66.46%
17.23%
8.51%
Legend
PMH
BMH
OMH
BLH
HS
Chn (DoS~1)
Chn (DoS~2)
Chn (DoS~3)
HA
Contaminants

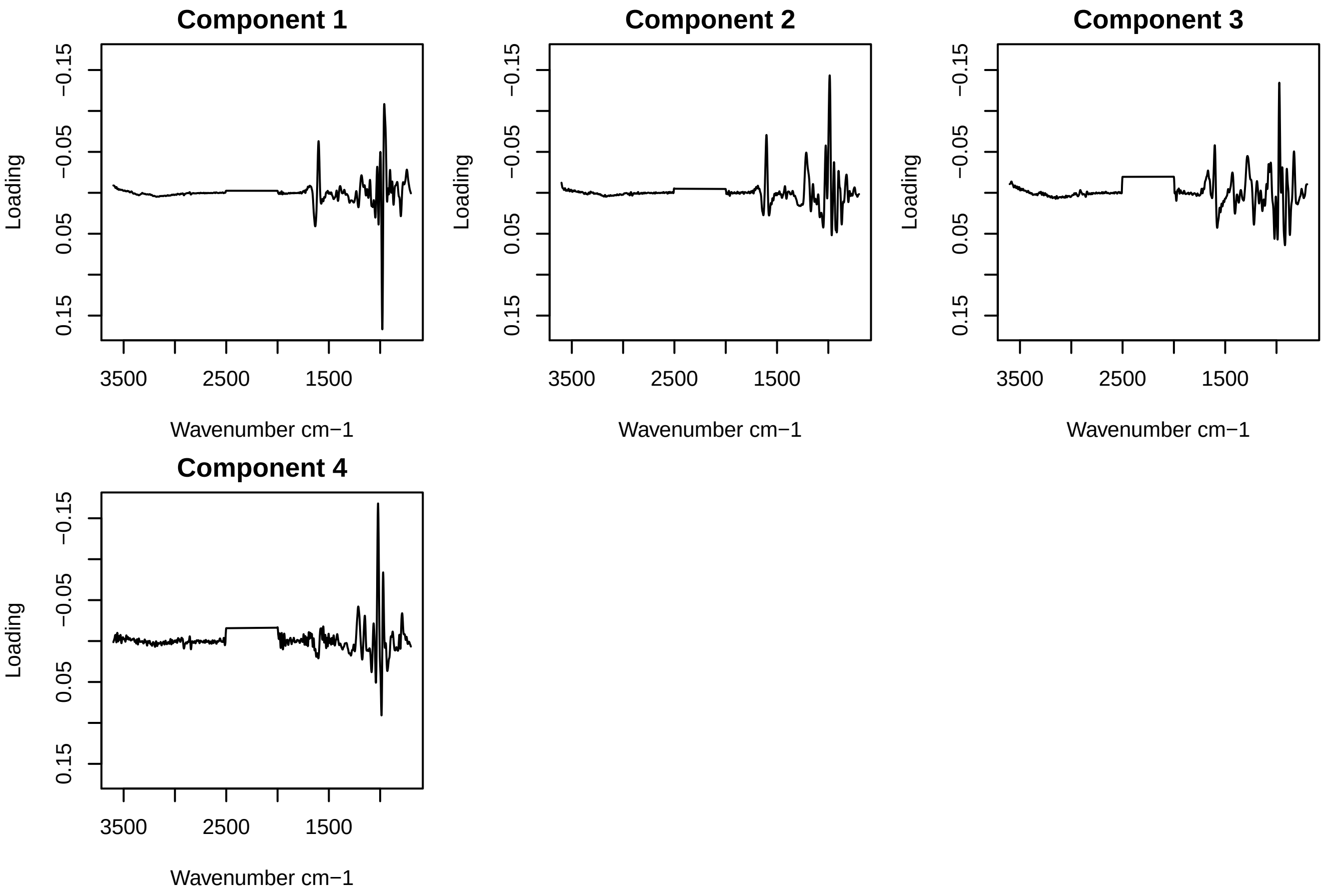
Component 1
Component 2
Component 3
Component 4
Loading
−0.15
−0.05
0.05
0.15
3500
2500
1500
Wavenumber cm−1

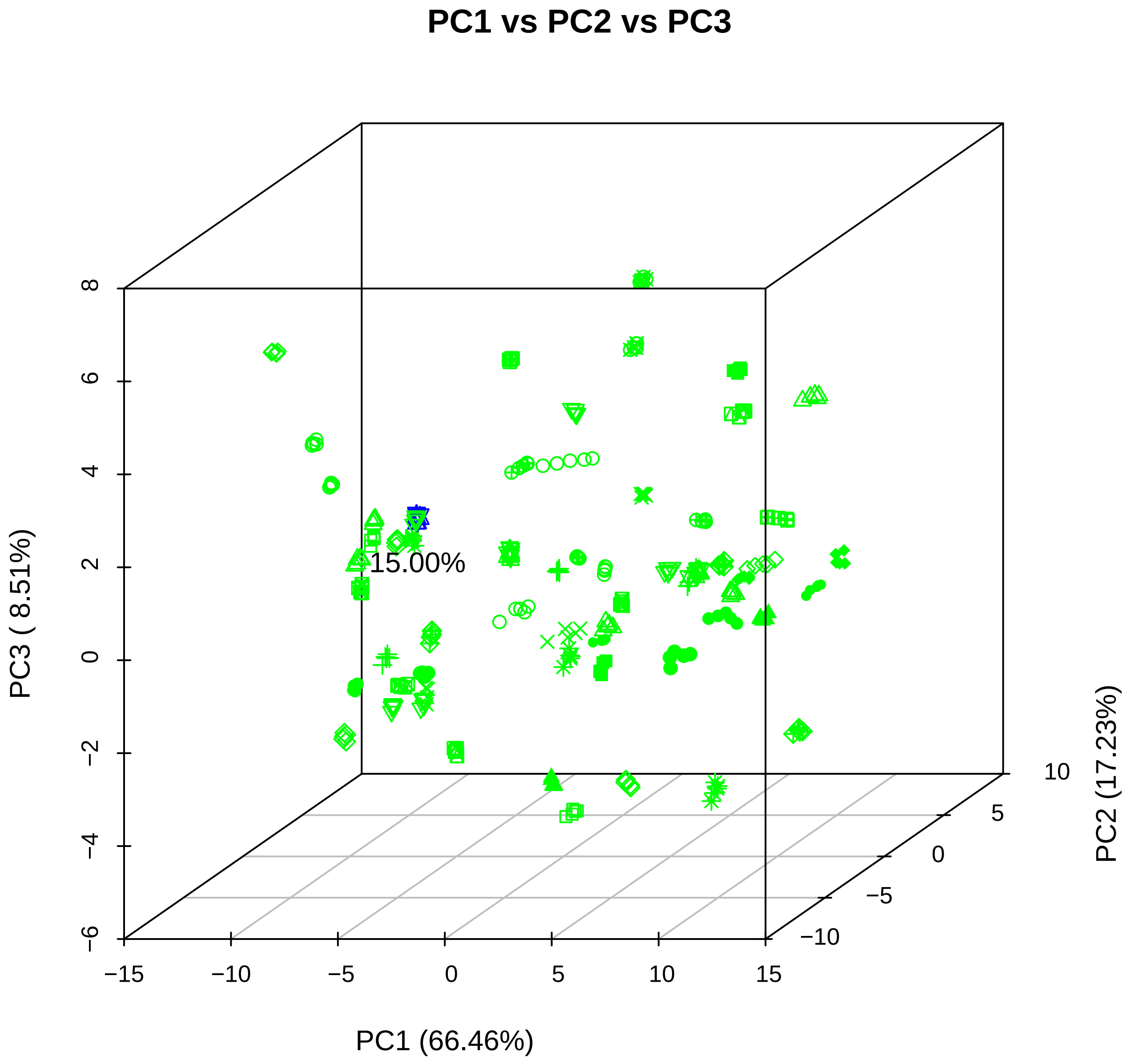

PC1 vs PC2 vs PC3
PC3 (8.51%)
PC1 (66.46%)
PC2 (17.23%)
15.00%
8
6
4
2
0
−2
−4
−6
−15
−10
−5
0
5
10
15
10
5
0
−5
−10

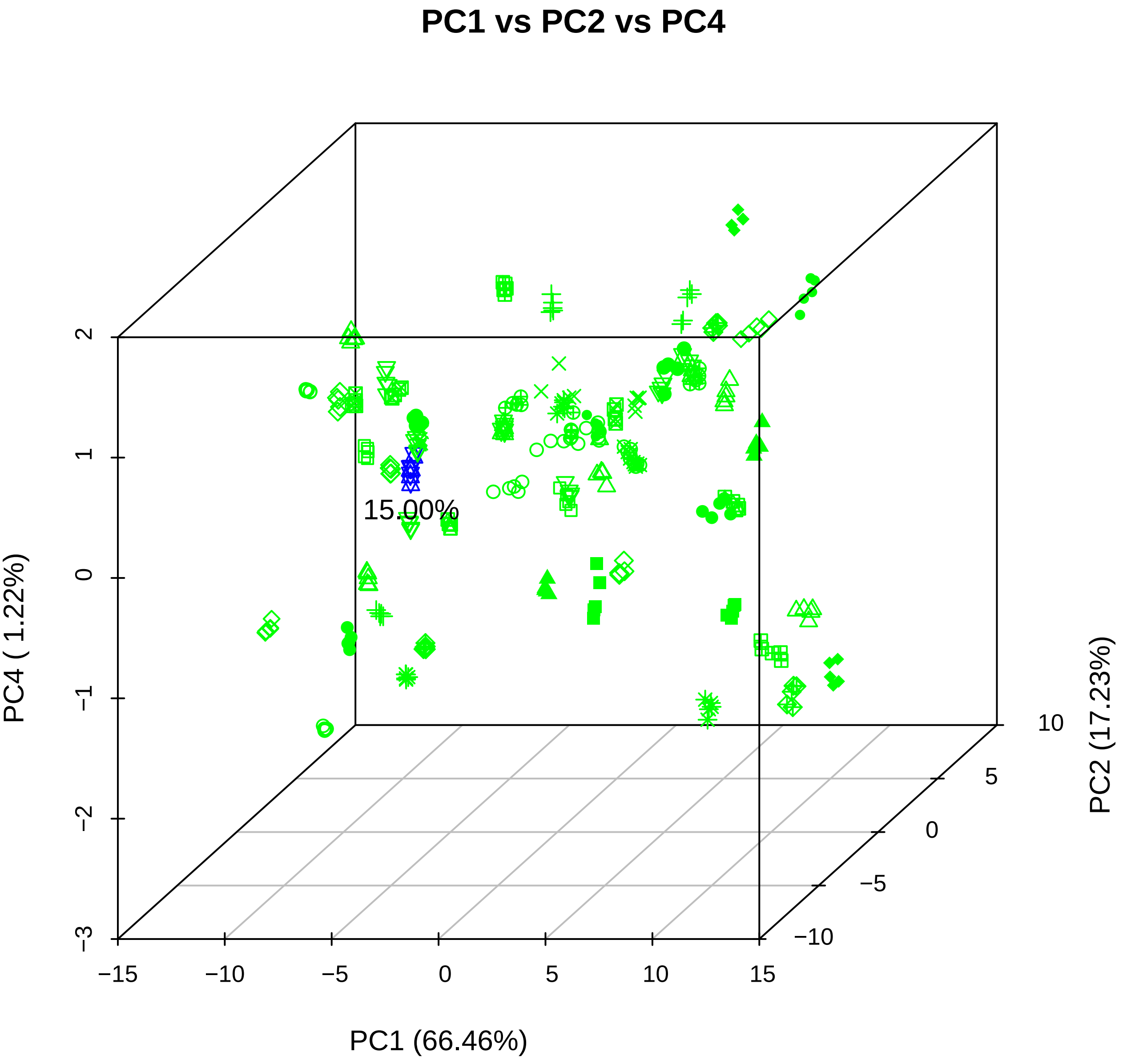
PC1 vs PC2 vs PC4
15.00%
2
1
0
−1
−2
−3
PC4 (1.22%)
−15
−10
−5
0
5
10
15
PC1 (66.46%)
10
5
0
−5
−10
PC2 (17.23%)

PC1 vs PC3 vs PC4

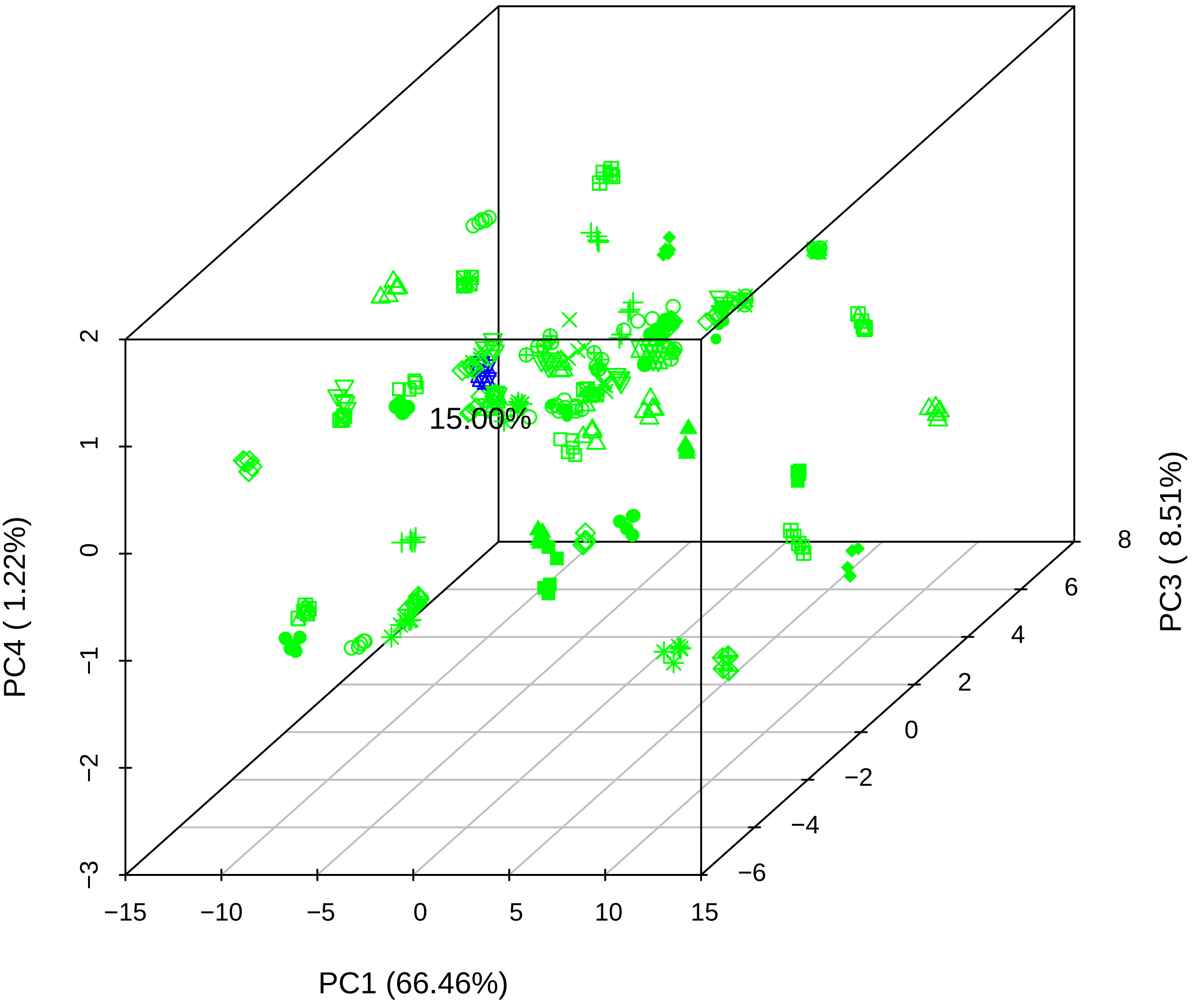

PC2 vs PC3 vs PC5

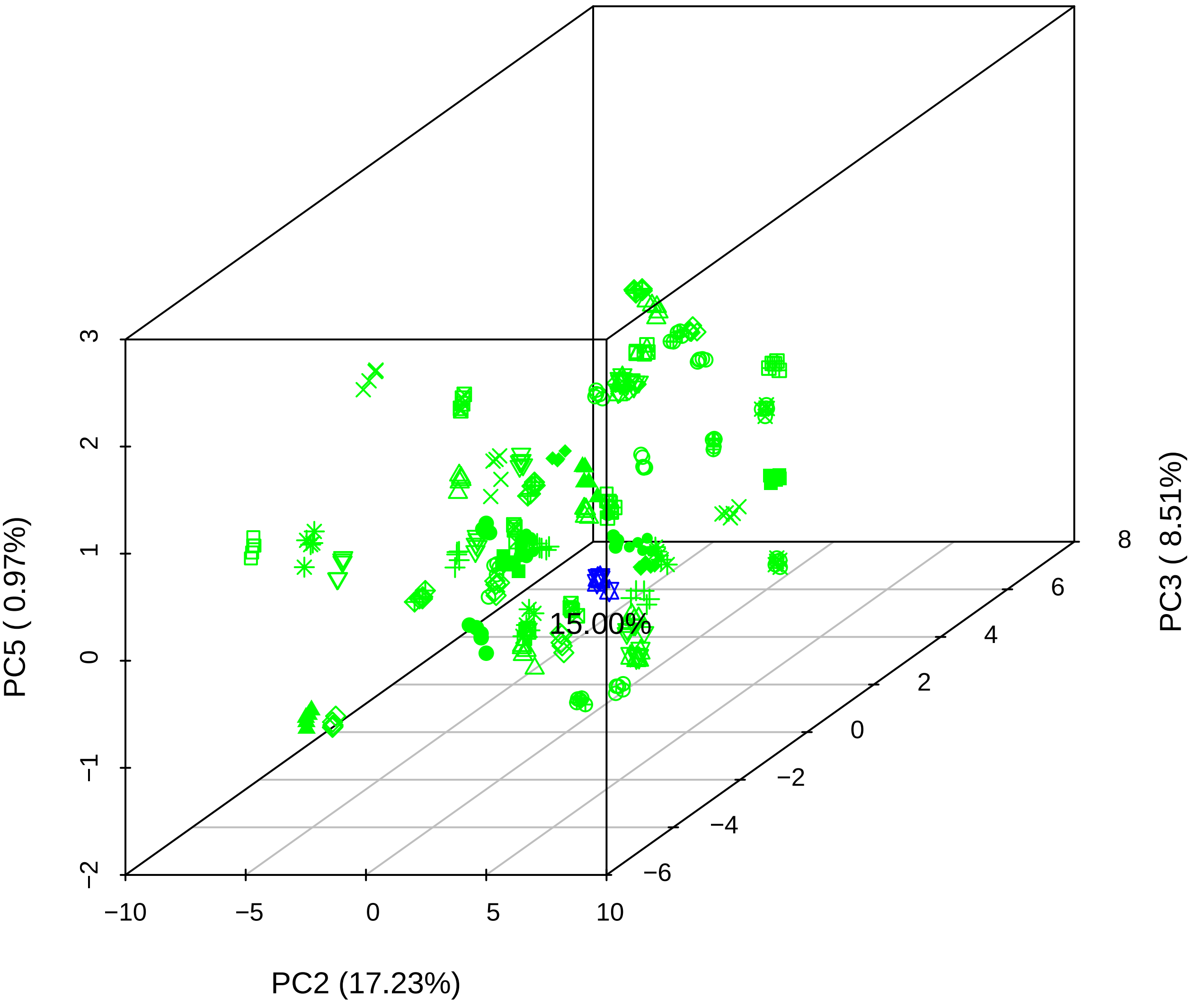

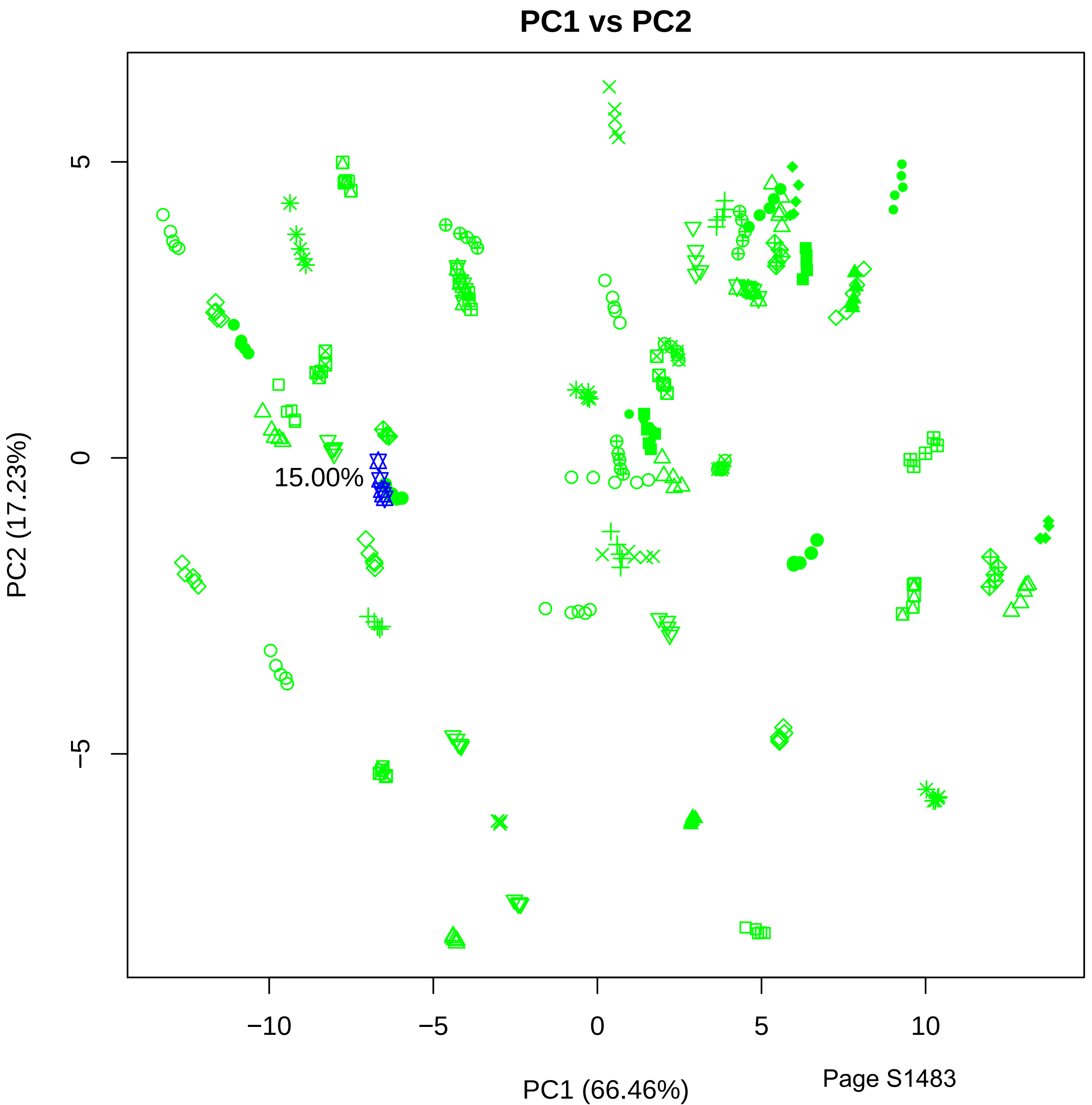
PC1 vs PC2
PC2 (17.23%)
PC1 (66.46%)
15.00%
5
0
−5
−10
−5
0
5
10

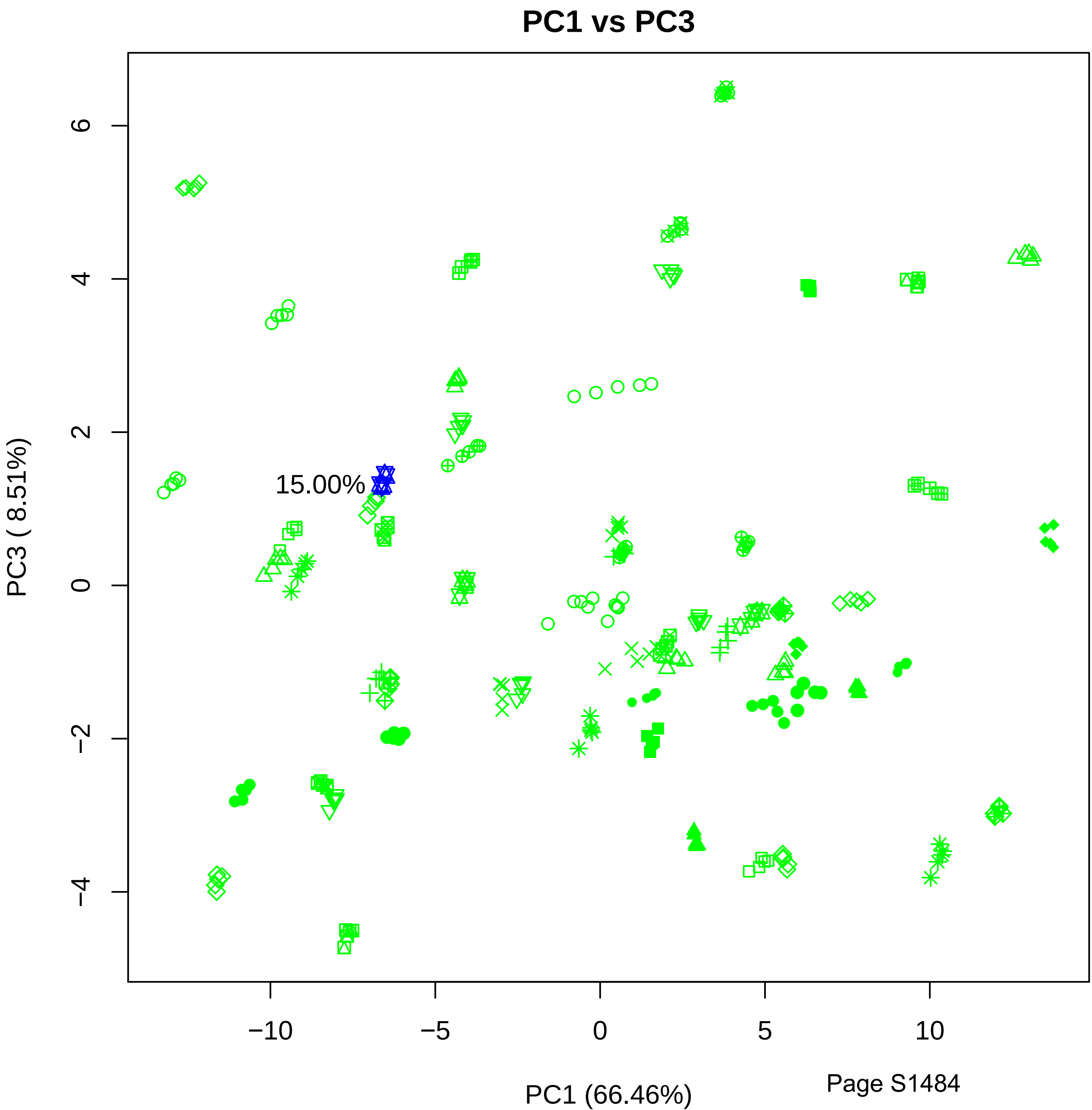
PC1 vs PC3
15.00%
PC3 (8.51%)
PC1 (66.46%)
6
4
2
0
−2
−4
−10
−5
0
5
10

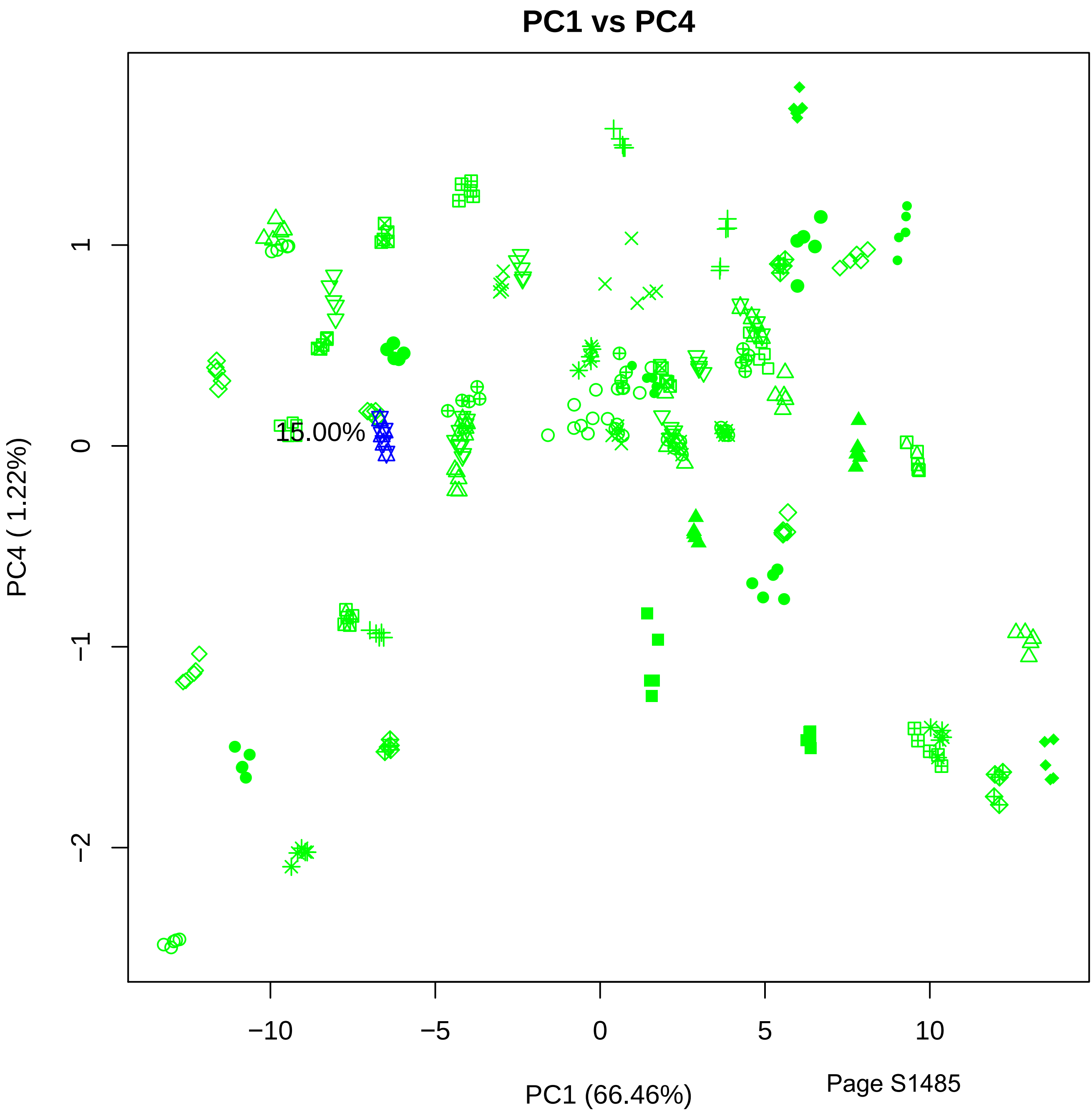

PC1 vs PC4
15.00%
PC4 (1.22%)
PC1 (66.46%)
−10
−5
0
5
10
−2
−1
0
1

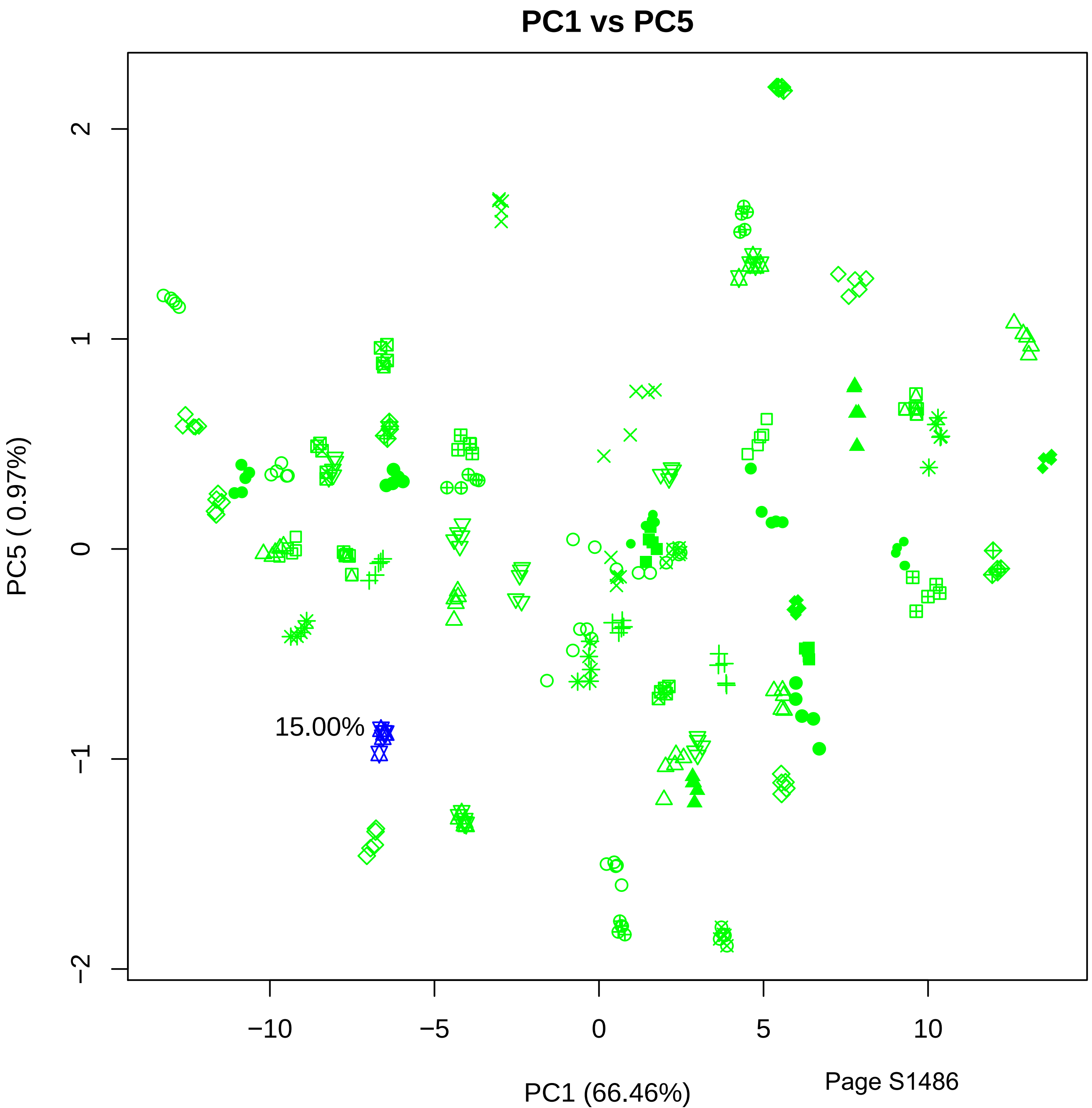
PC1 vs PC5
PC5 (0.97%)
PC1 (66.46%)
15.00%
2
1
0
−1
−2
−10
−5
0
5
10

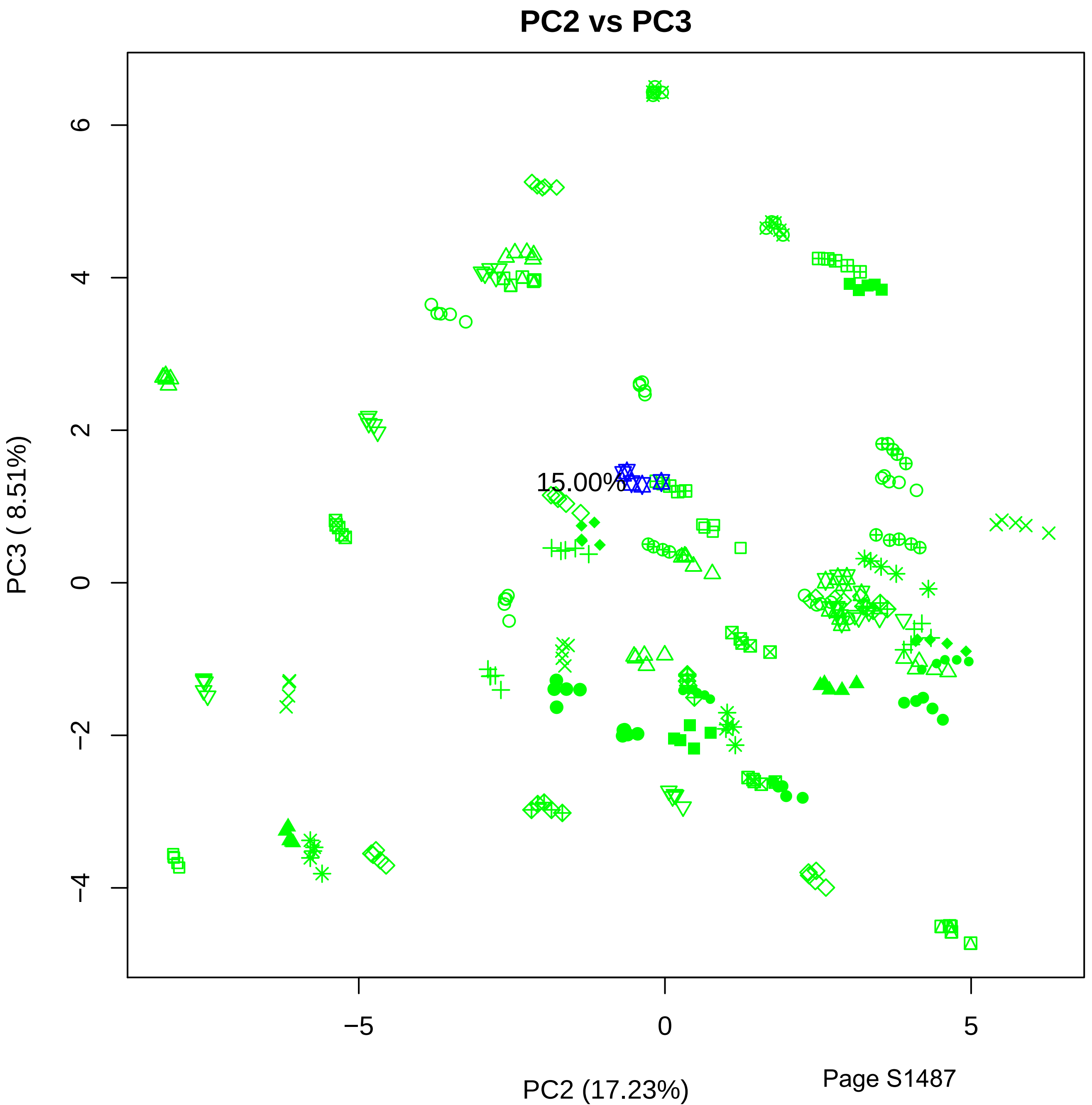

PC2 vs PC3
15.00%
PC3 (8.51%)
PC2 (17.23%)
−4
−2
0
2
4
6
−5
0
5

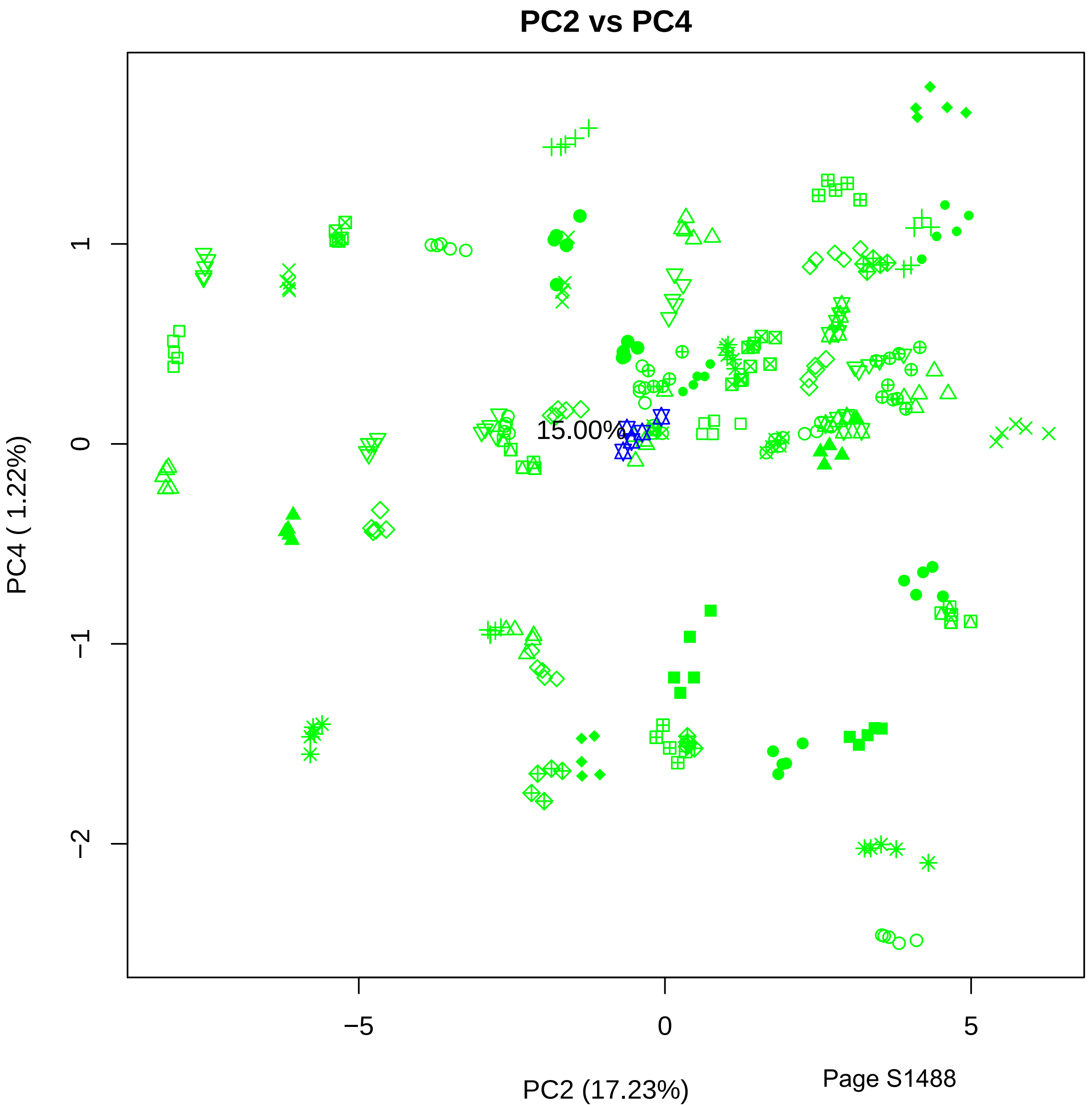
PC2 vs PC4
15.00%
1
0
−1
−2
−5
0
5
PC4 (1.22%)
PC2 (17.23%)

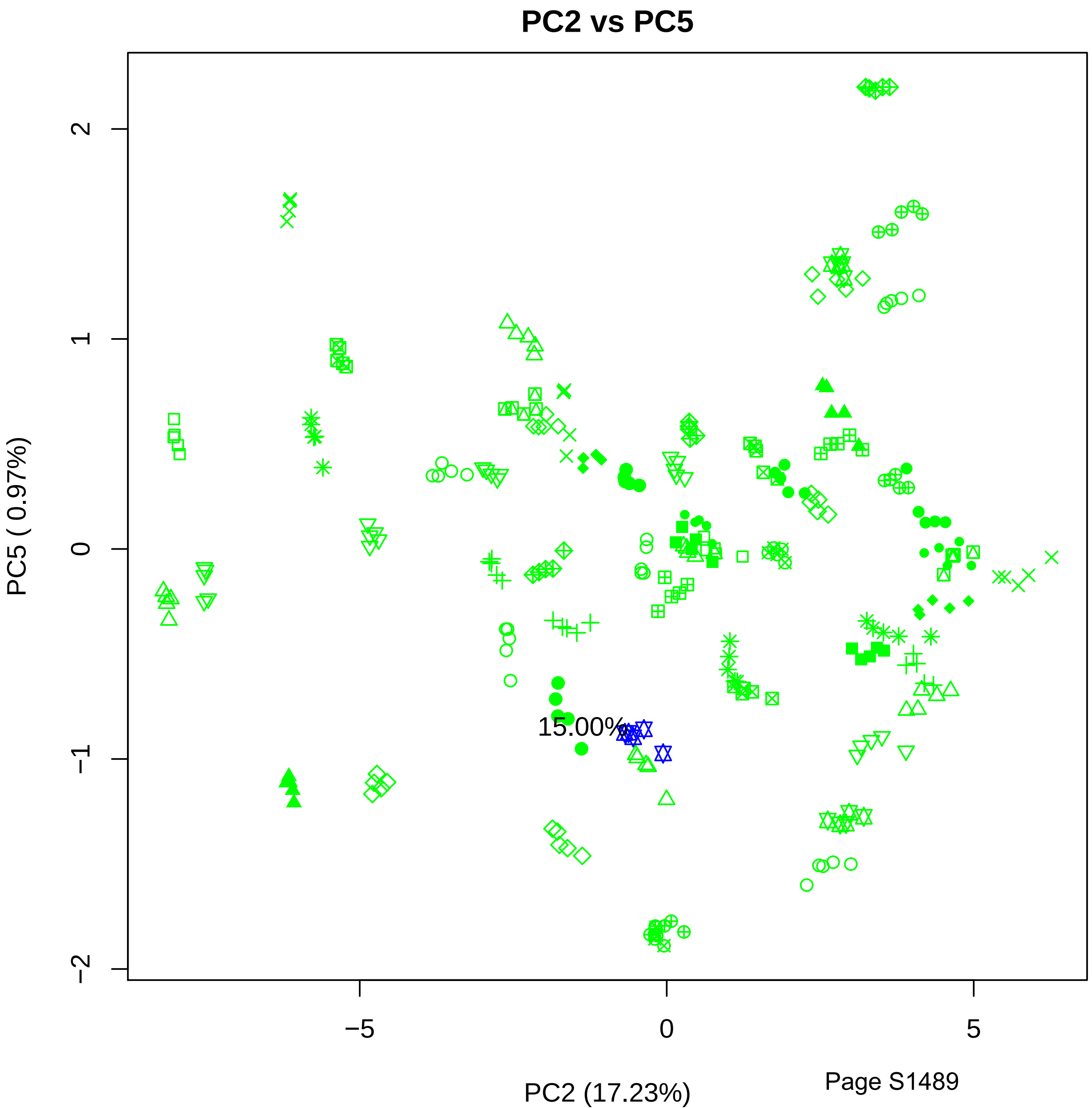
PC2 vs PC5
2
1
0
−1
−2
PC5 (0.97%)
15.00%
−5
0
5
PC2 (17.23%)

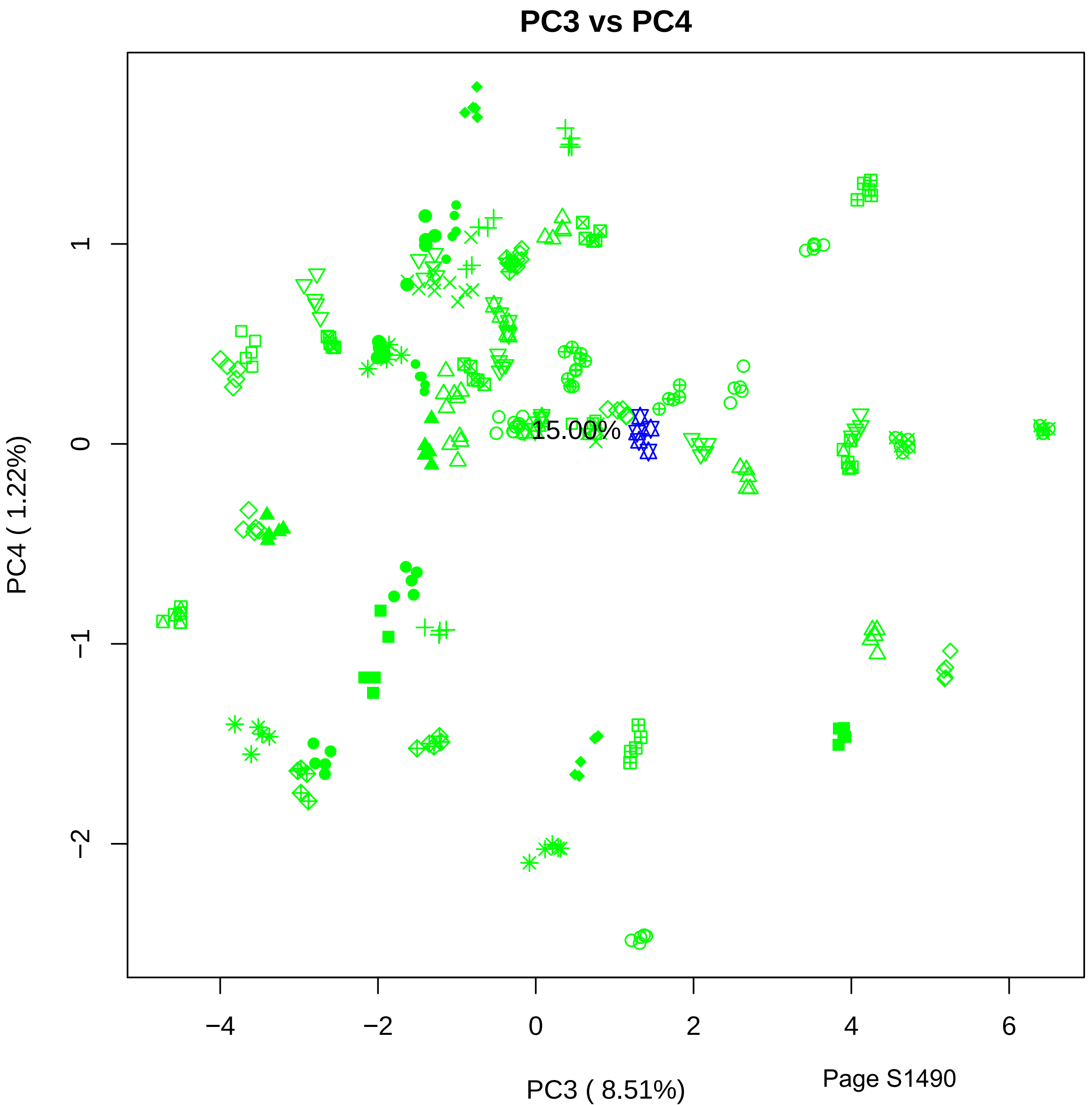
PC3 vs PC4
15.00%
PC4 (1.22%)
PC3 (8.51%)
1
0
−1
−2
−4
−2
0
2
4
6

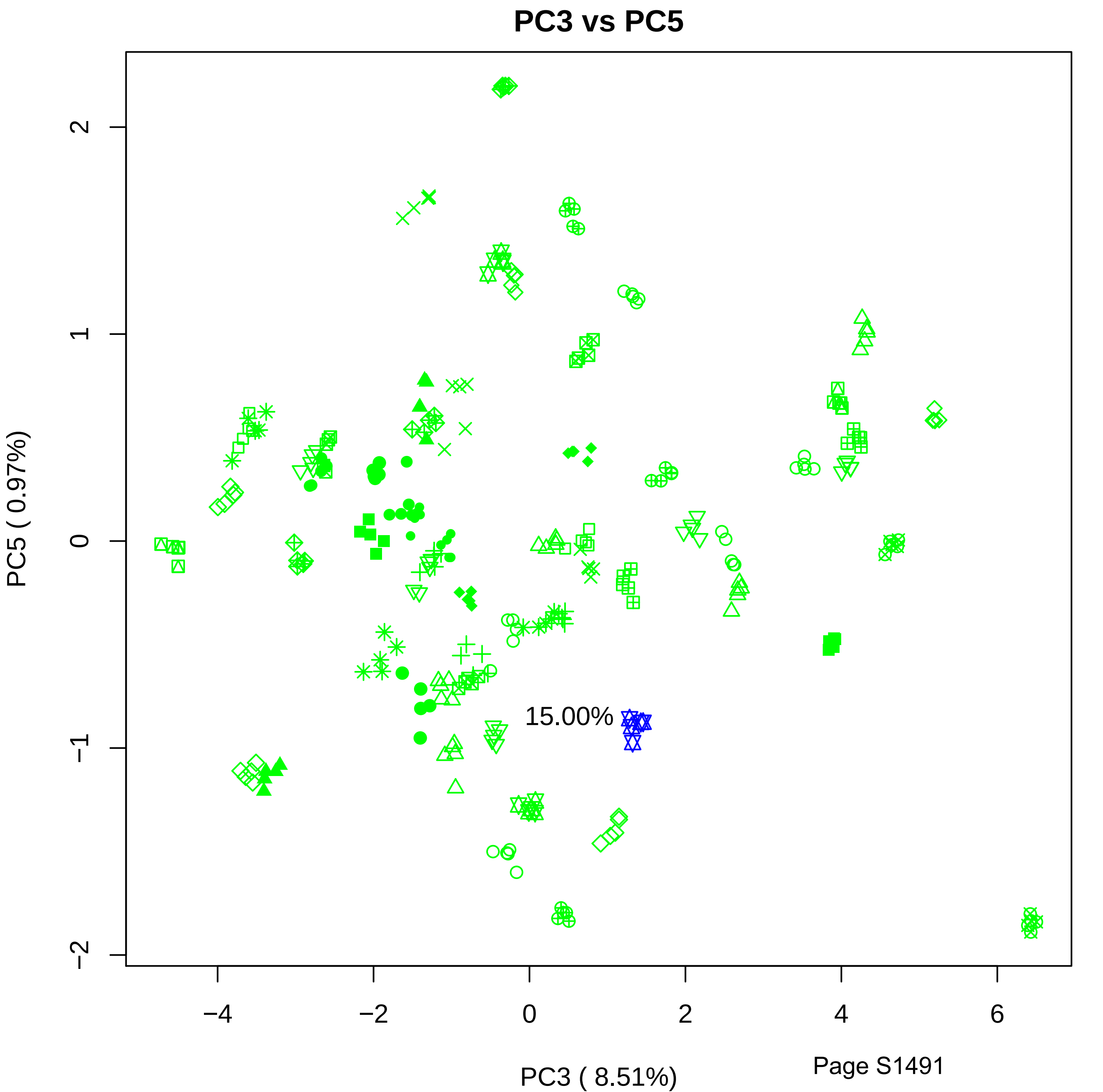
PC3 vs PC5
15.00%
PC5 (0.97%)
PC3 (8.51%)
2
1
0
−1
−2
−4
−2
0
2
4
6

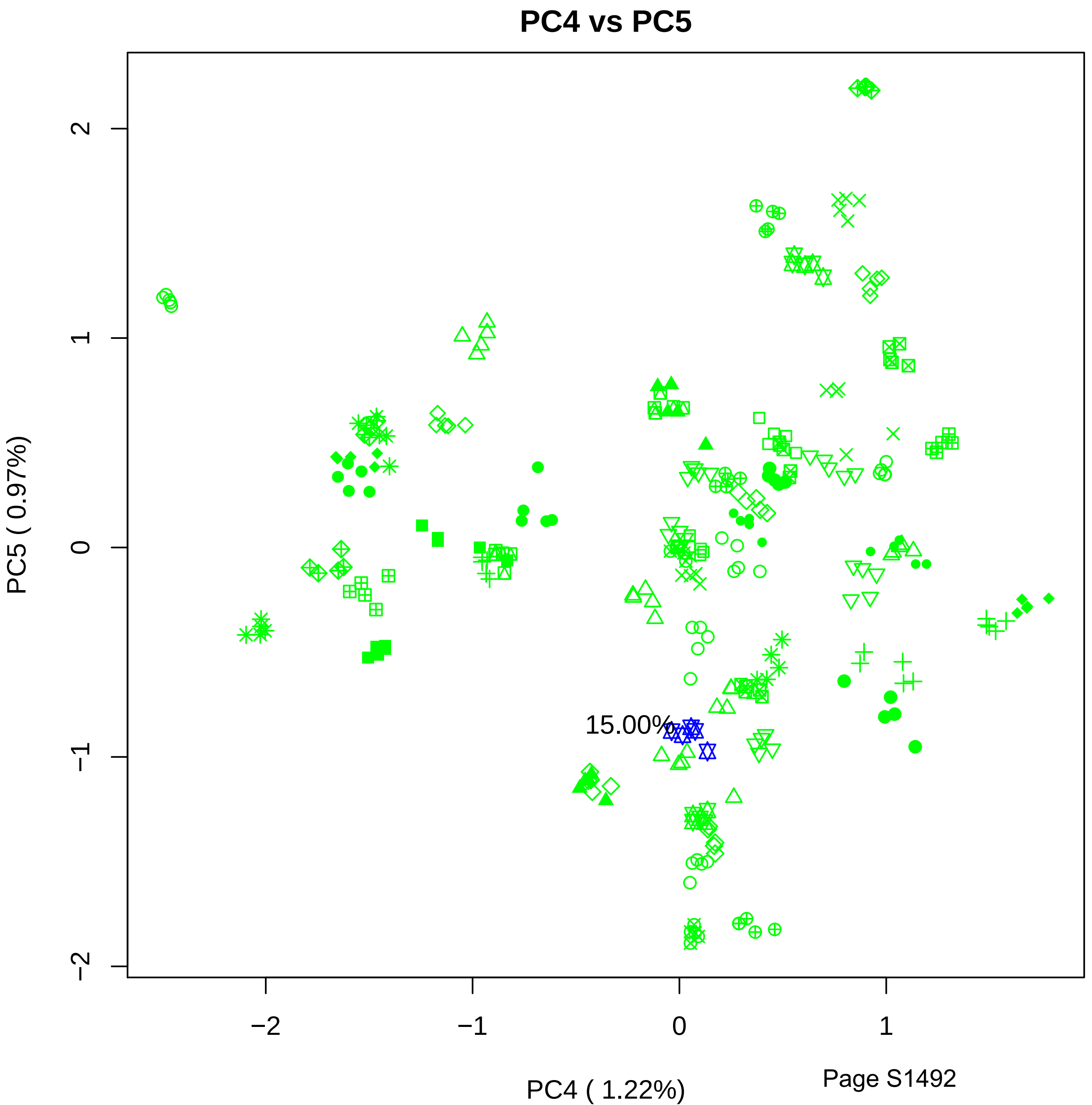
PC4 vs PC5
PC5 (0.97%)
PC4 (1.22%)
15.00%
2
1
0
−1
−2
−2
−1
0
1

PCA of 20% BMH(d) vs PMH(d)

Scaled Matrix PCAed

7th order polynomial baseline correction performed

Raw spectra smoothed to 21 Neighbours, 2nd order PN, Savtisky Golay

Normalisation between 0 and 100

D2 spectra smoothed to 41 Neighbours, 2nd order PN, Savtisky Golay

Regions Analysed: 3601−2501 and 2001−700cm−1

Samples Analysed: 1 bContam's 0 BLH's 0 BMH's 0 OMH's 0 pContam's 0 PLH's 60 PMH's

Keele University AD

Fri Dec 14 14:23:14 2018

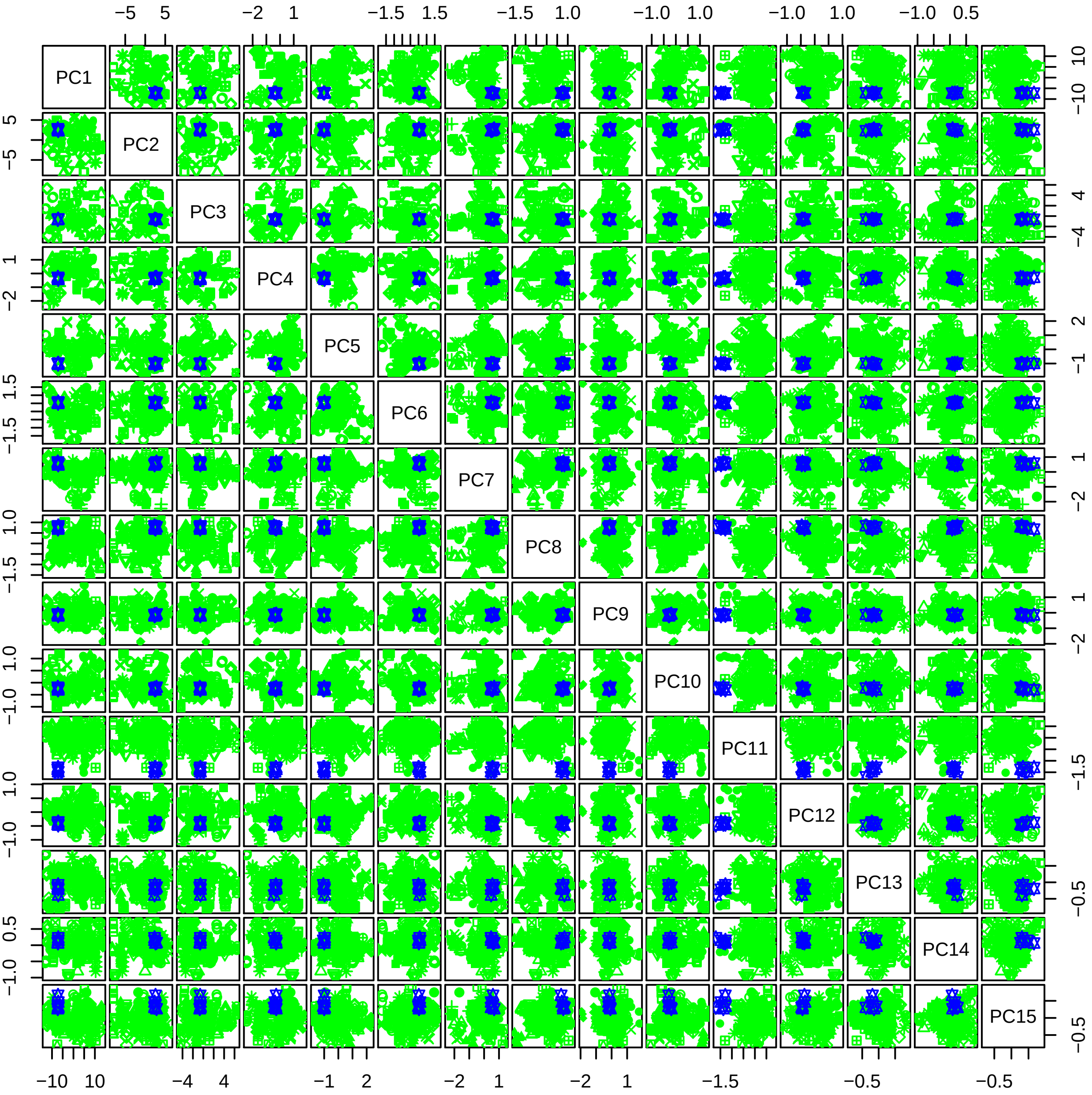
PC1
PC2
PC3
PC4
PC5
PC6
PC7
PC8
PC9
PC10
PC11
PC12
PC13
PC14
PC15

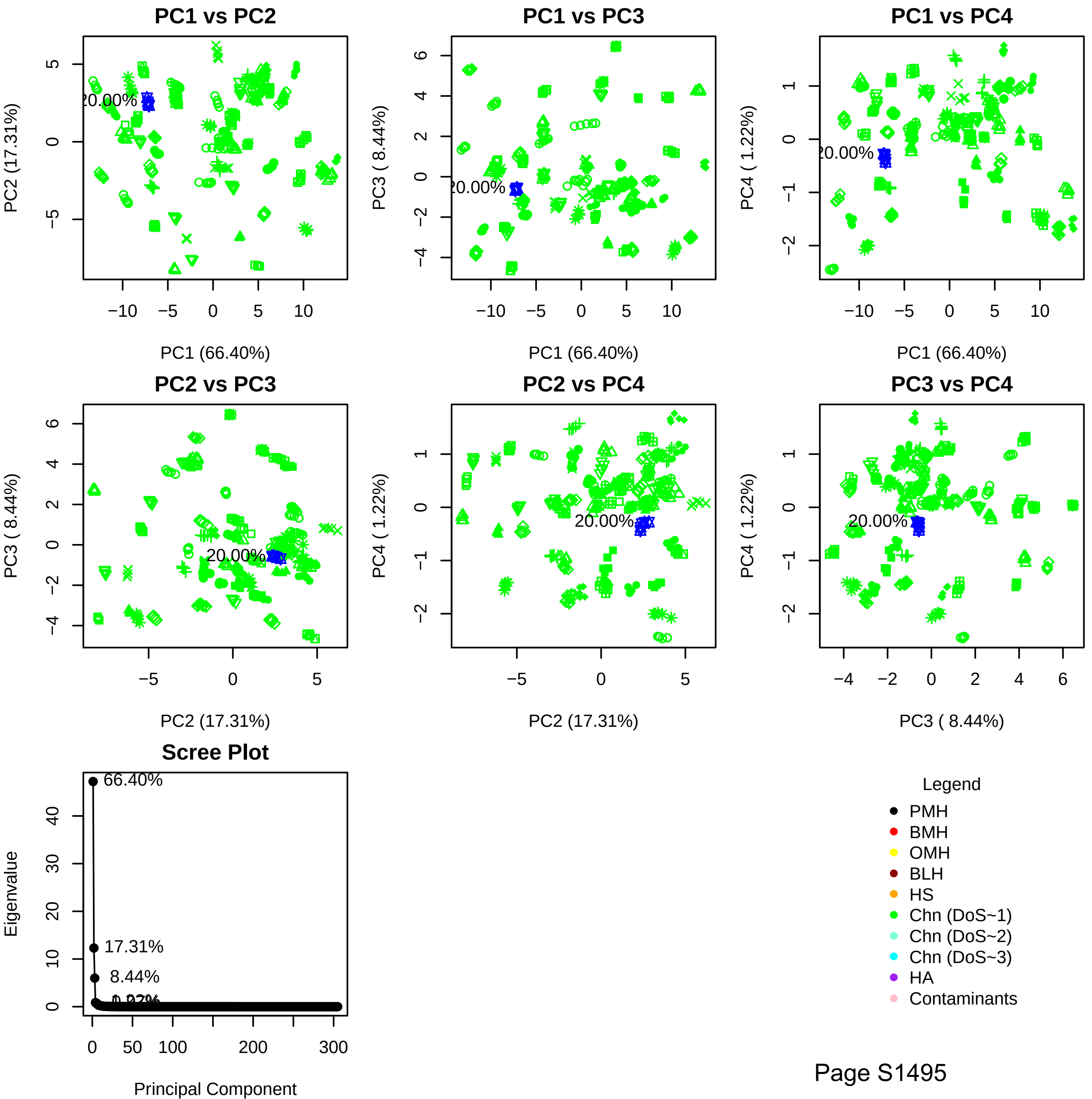

PC1 vs PC2
PC1 vs PC3
PC1 vs PC4
PC2 vs PC3
PC2 vs PC4
PC3 vs PC4
Scree Plot
PC1 (66.40%)
PC2 (17.31%)
PC3 (8.44%)
PC4 (1.22%)
20.00%
Eigenvalue
Principal Component
66.40%
17.31%
8.44%
Legend
PMH
BMH
OMH
BLH
HS
Chn (DoS~1)
Chn (DoS~2)
Chn (DoS~3)
HA
Contaminants

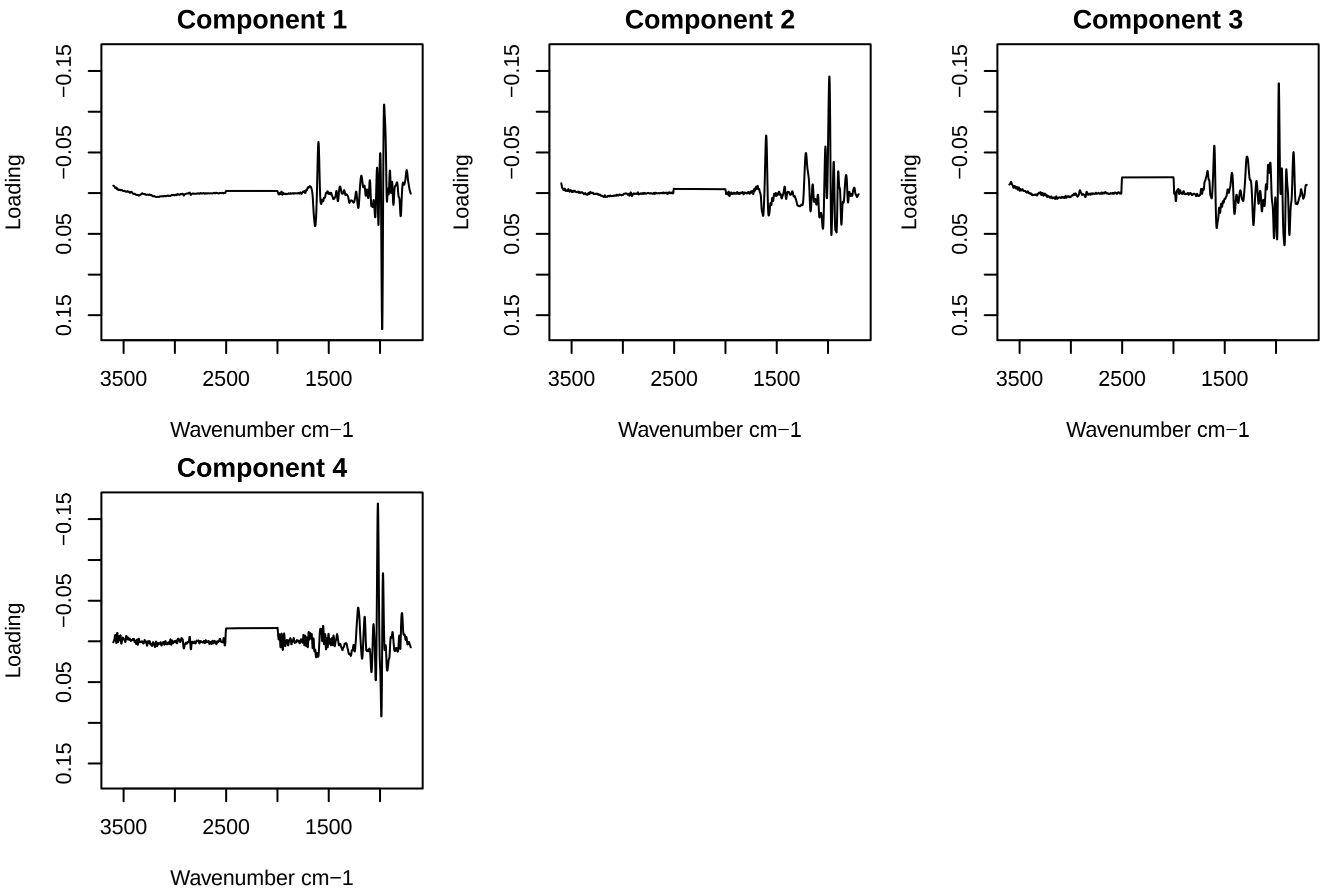
Component 1
Component 2
Component 3
Component 4
Loading
−0.15
−0.05
0.05
0.15
3500
2500
1500
Wavenumber cm−1

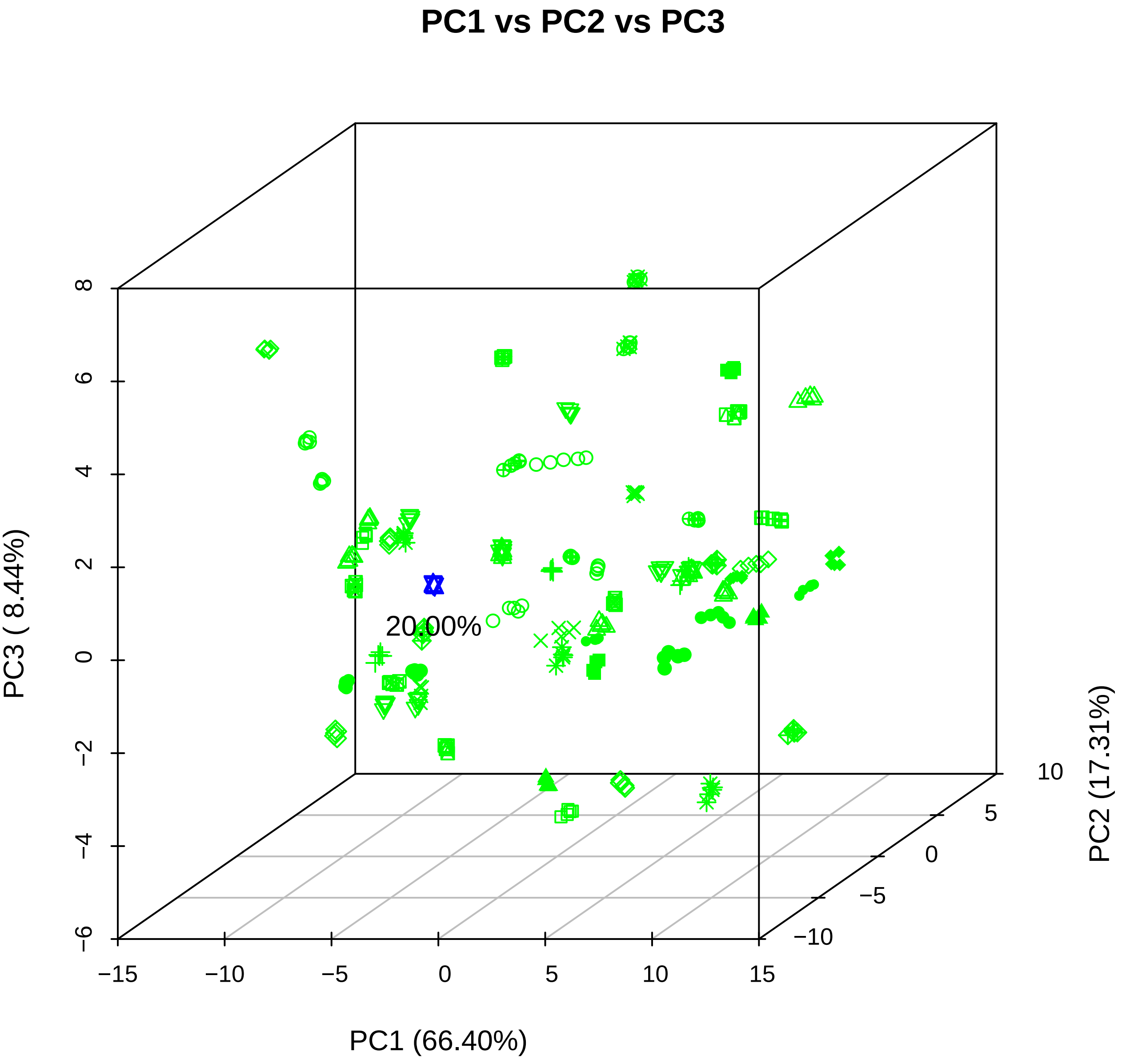

PC1 vs PC2 vs PC3
20.00%
PC1 (66.40%)
PC2 (17.31%)
PC3 (8.44%)
−15
−10
−5
0
5
10
15
−6
−4
−2
0
2
4
6
8

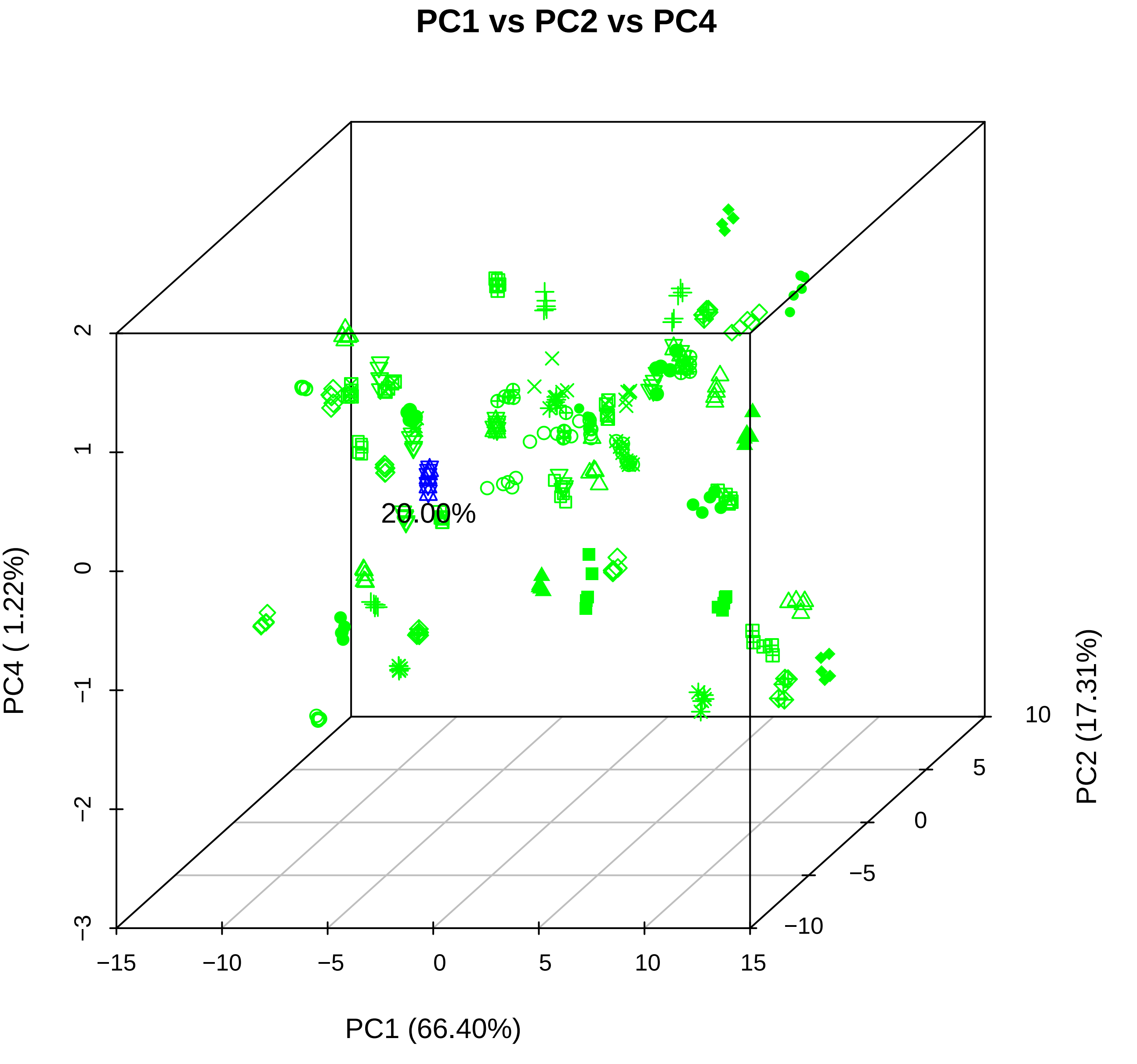

PC1 vs PC2 vs PC4
20.00%
PC1 (66.40%)
PC2 (17.31%)
PC4 (1.22%)
−15
−10
−5
0
5
10
15
−10
−5
0
5
10
−3
−2
−1
0
1
2

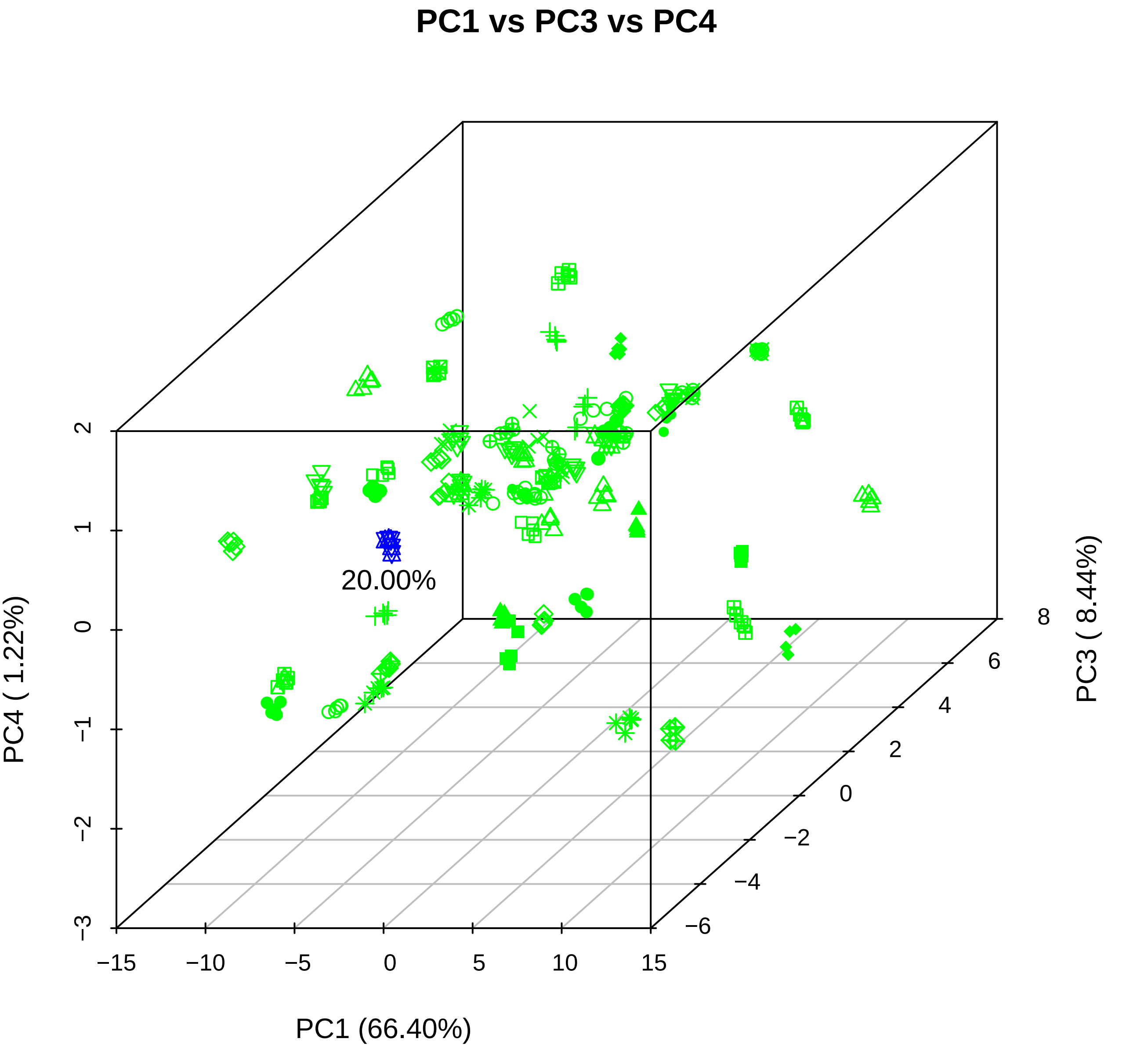

PC1 vs PC3 vs PC4
20.00%
PC1 (66.40%)
PC4 (1.22%)
PC3 (8.44%)
−15
−10
−5
0
5
10
15
−3
−2
−1
0
1
2
−6
−4
−2
0
2
4
6
8

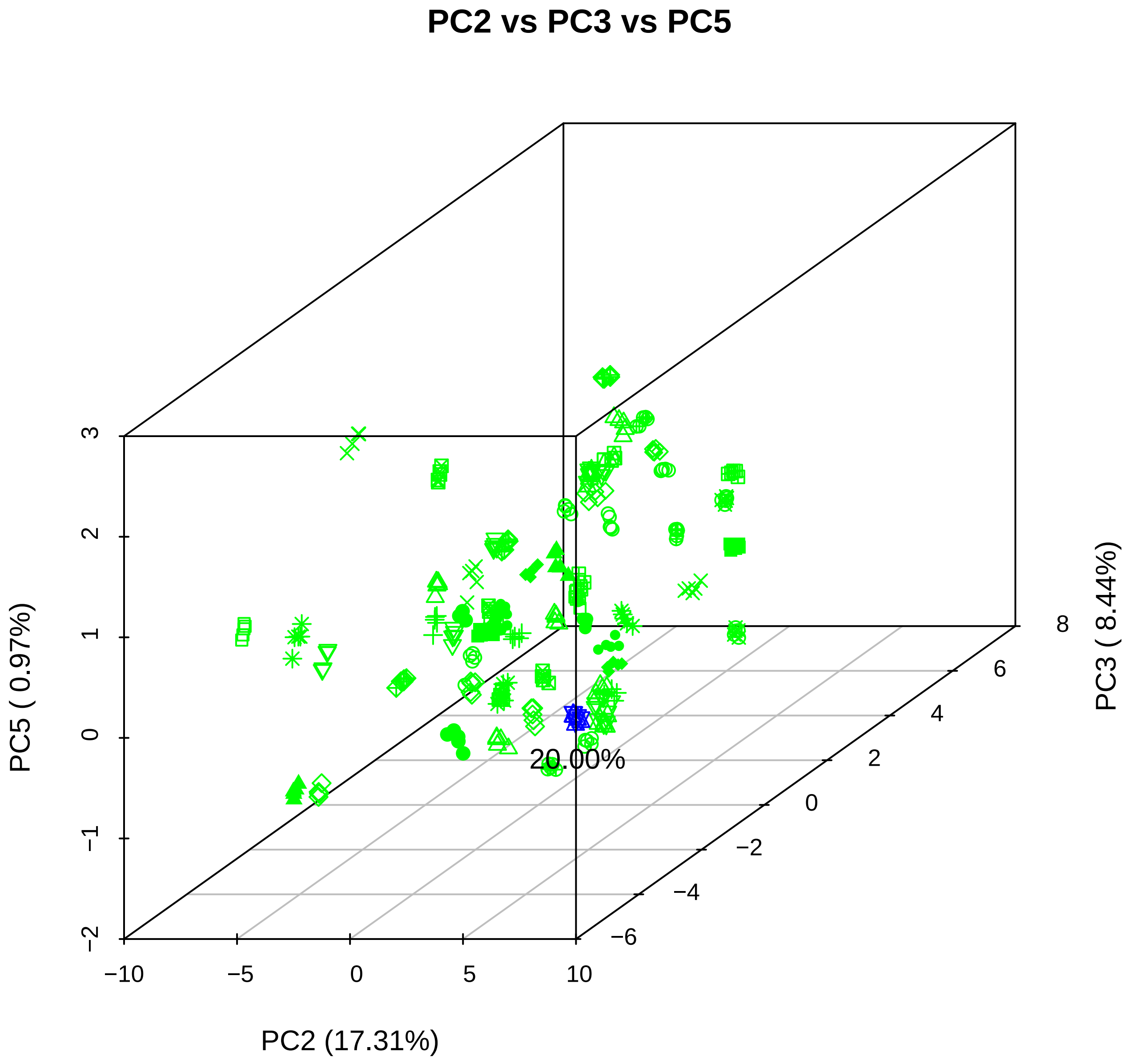
PC2 vs PC3 vs PC5
PC5 (0.97%)
PC2 (17.31%)
PC3 (8.44%)
20.00%
3
2
1
0
−1
−2
−10
−5
0
5
10
−6
−4
−2
0
2
4
6
8

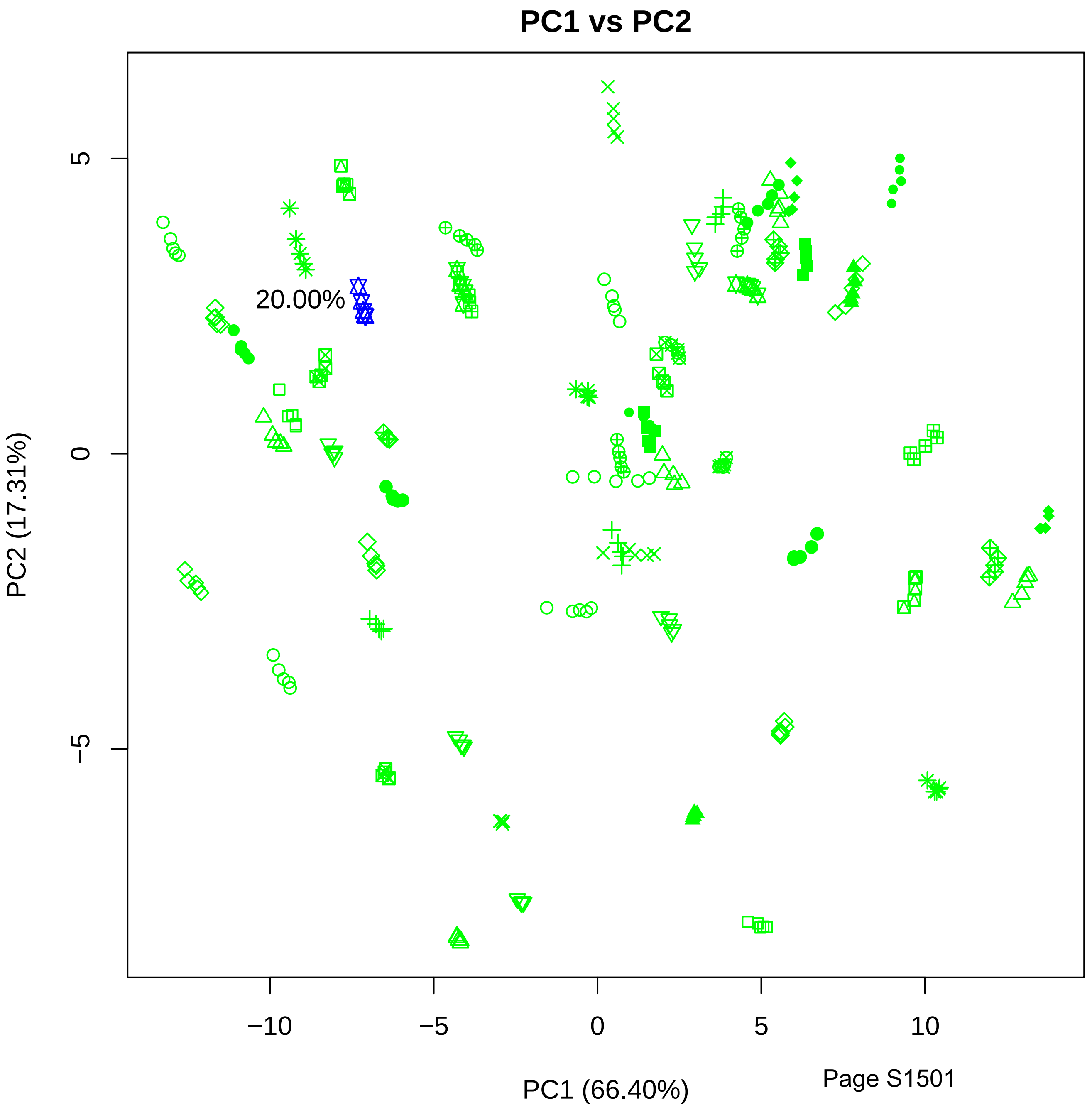
PC1 vs PC2
20.00%
PC2 (17.31%)
PC1 (66.40%)
5
0
−5
−10
−5
0
5
10

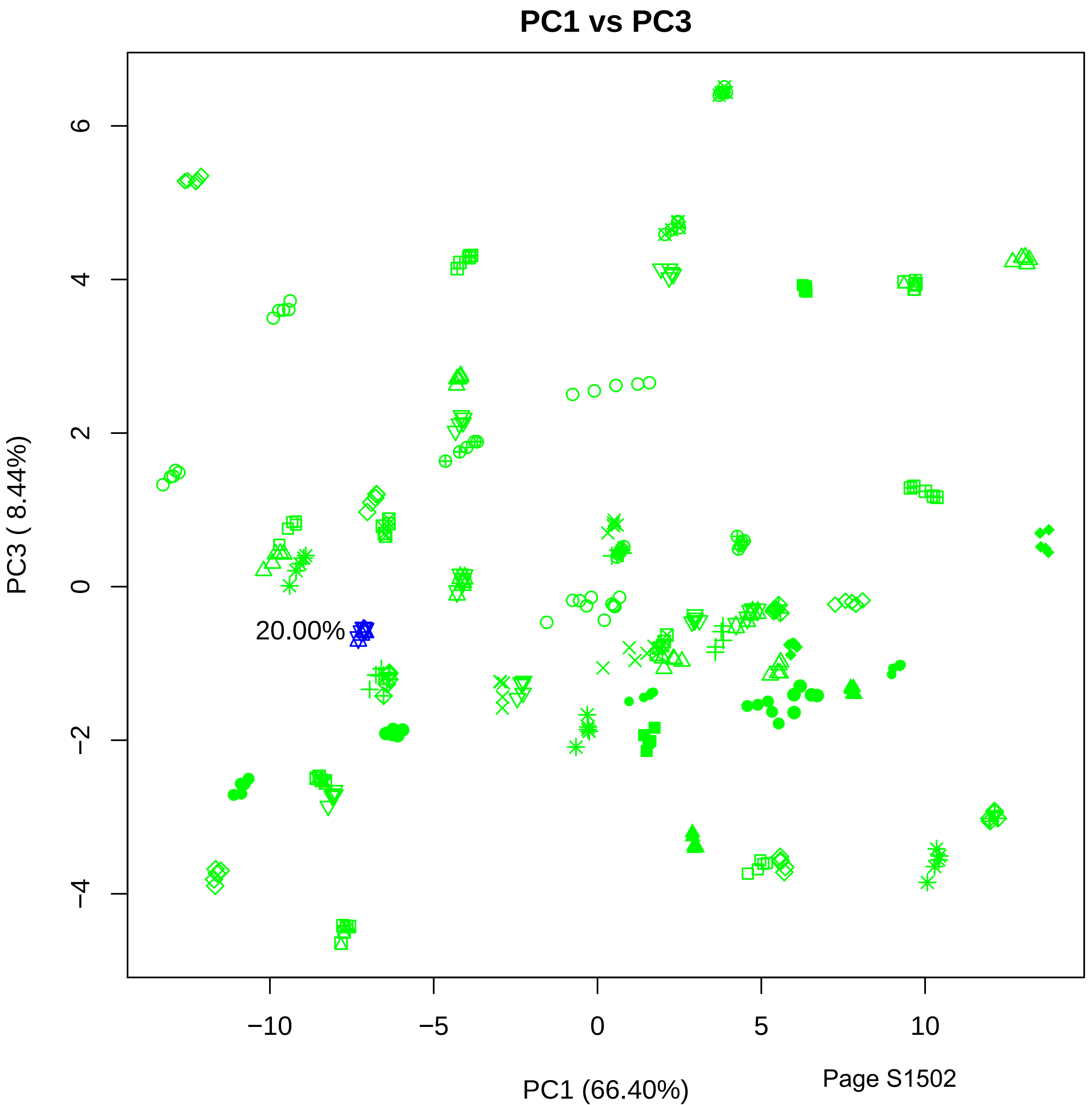
PC1 vs PC3
PC3 (8.44%)
PC1 (66.40%)
20.00%
6
4
2
0
−2
−4
−10
−5
0
5
10

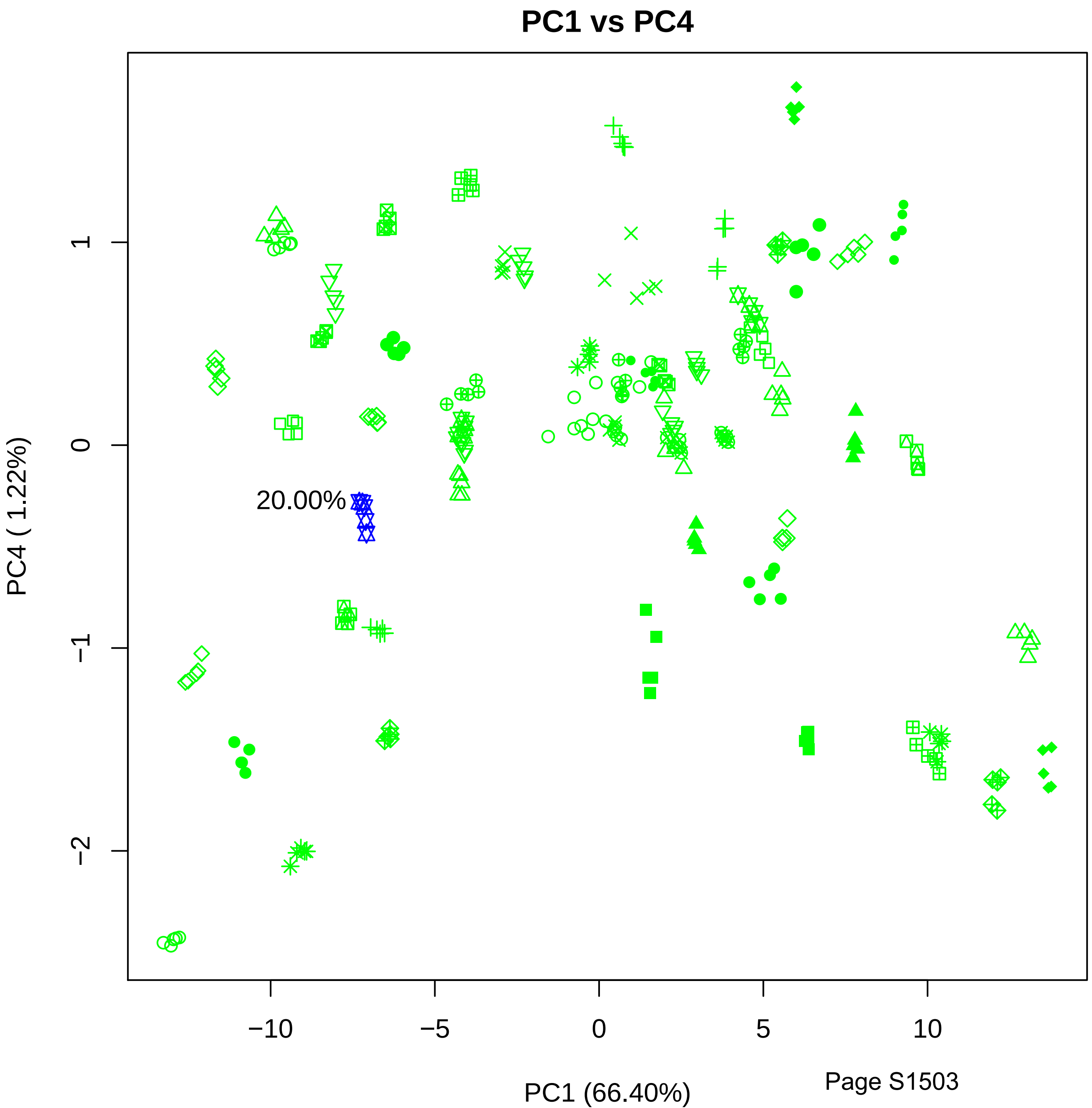
PC1 vs PC4
PC4 (1.22%)
PC1 (66.40%)
20.00%
1
0
−1
−2
−10
−5
0
5
10

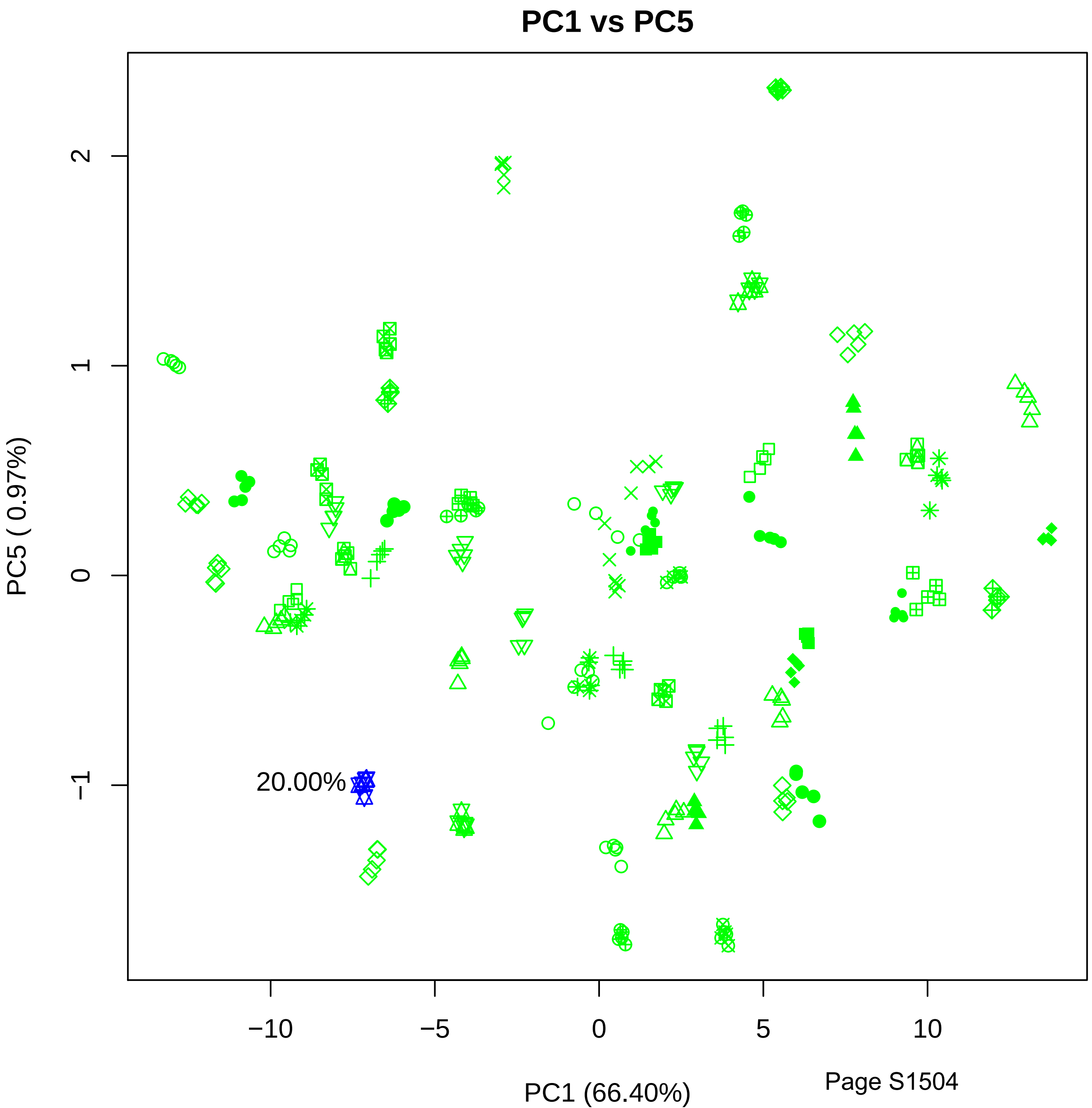
PC1 vs PC5
PC5 (0.97%)
PC1 (66.40%)
20.00%
2
1
0
−1
−10
−5
0
5
10

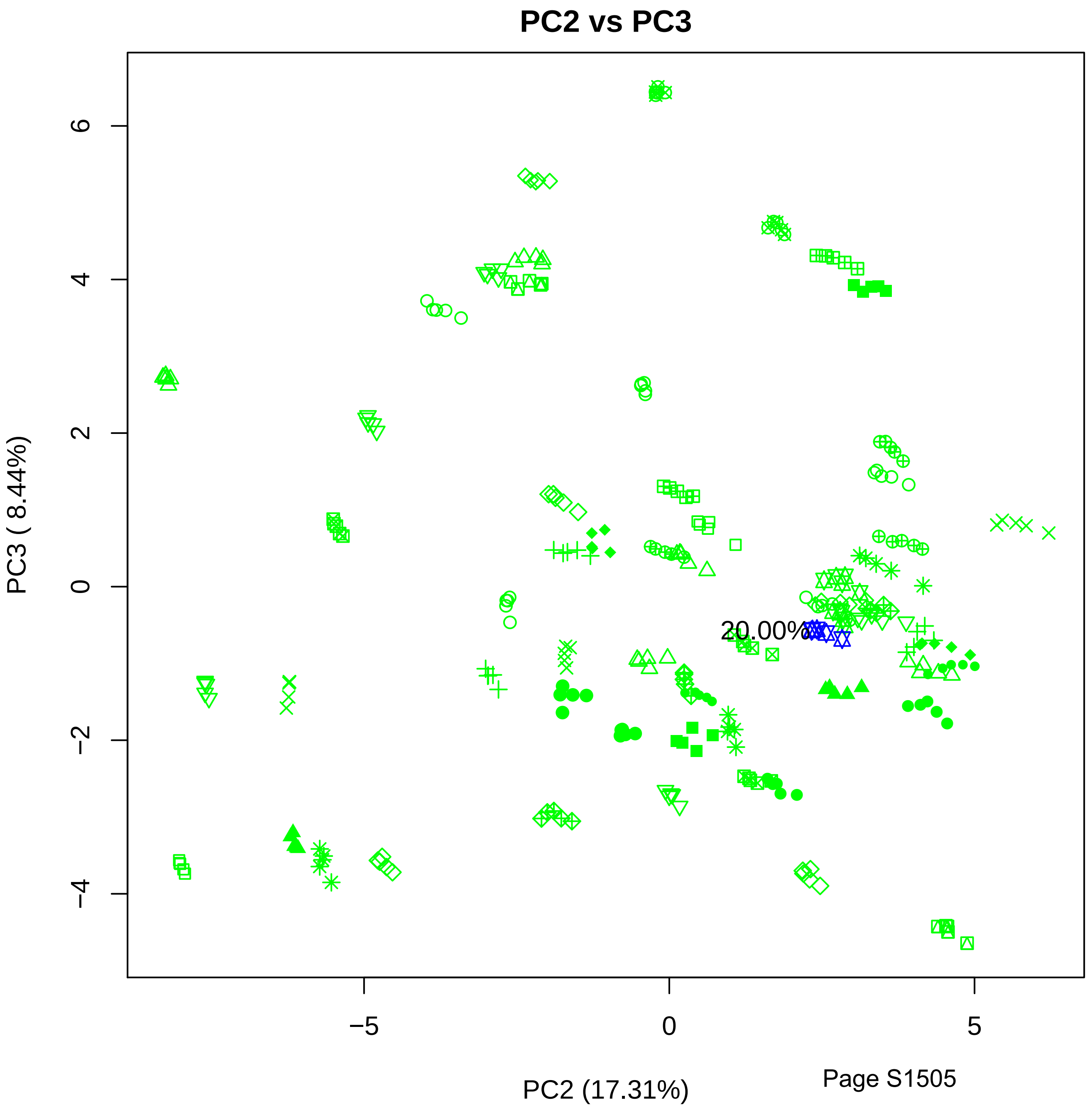

PC2 vs PC3
PC3 (8.44%)
PC2 (17.31%)
6
4
2
0
−2
−4
−5
0
5
20.00%

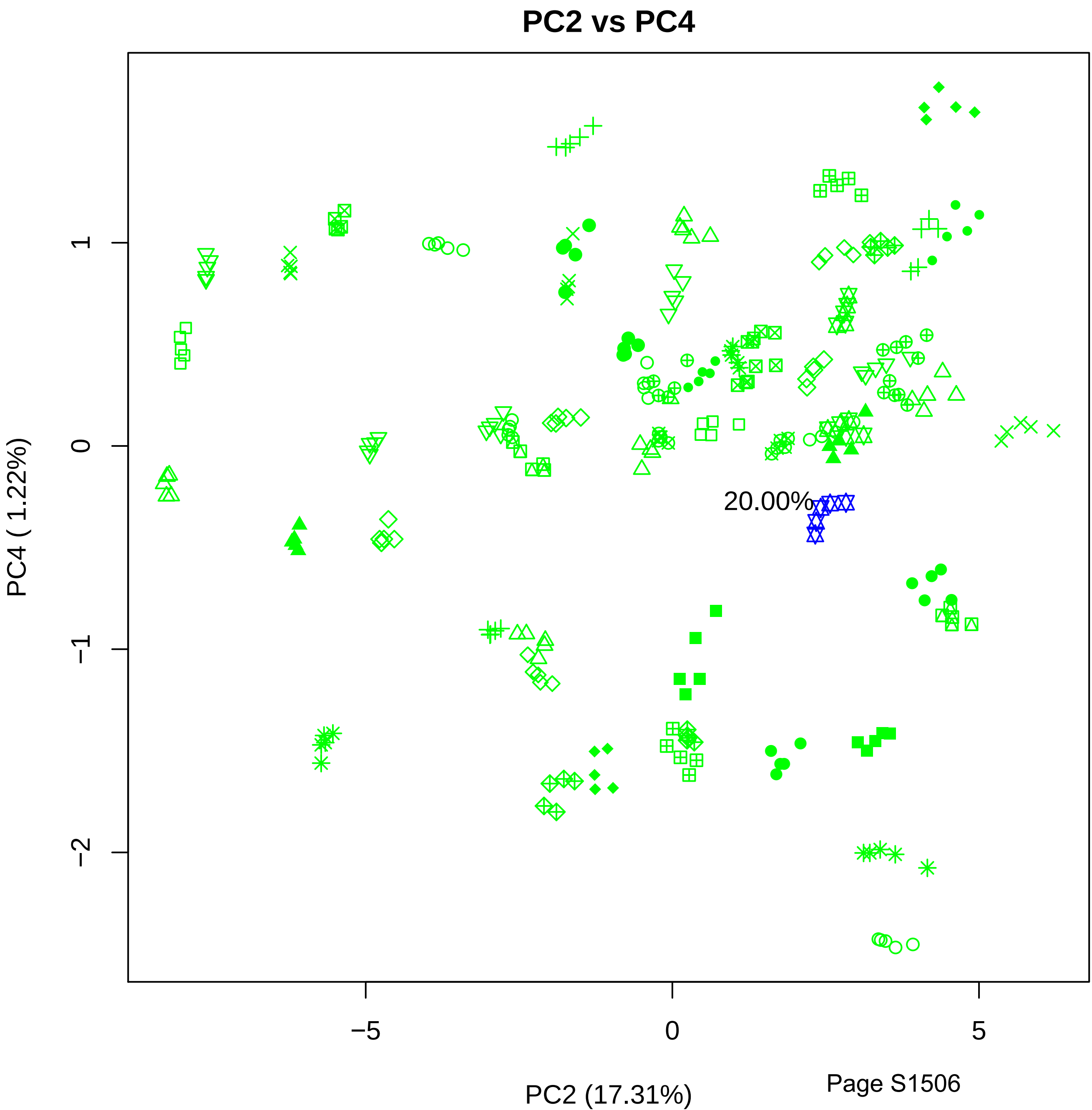

PC2 vs PC4
PC4 (1.22%)
PC2 (17.31%)
1
0
−1
−2
−5
0
5
20.00%

PC2 vs PC5

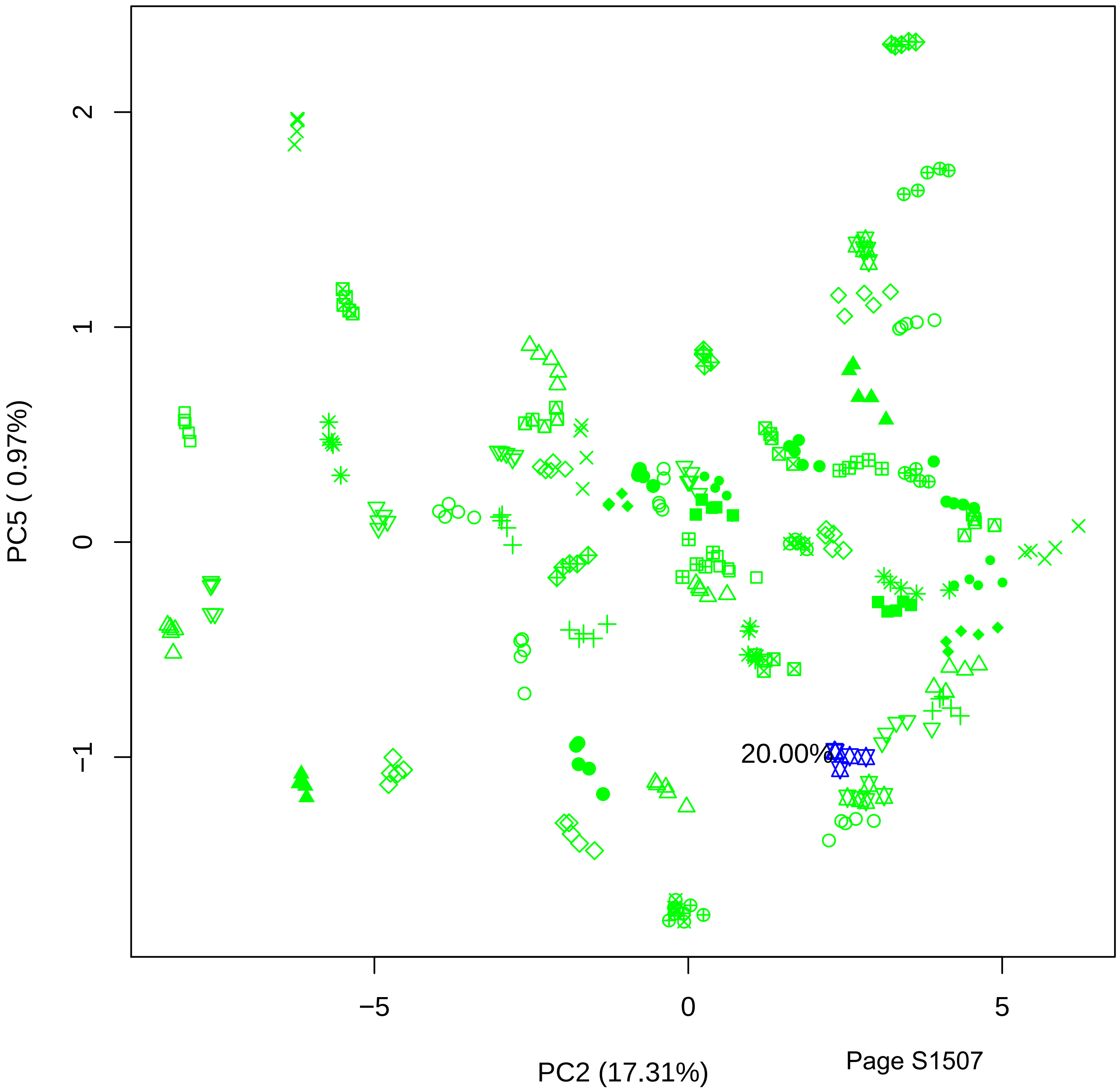

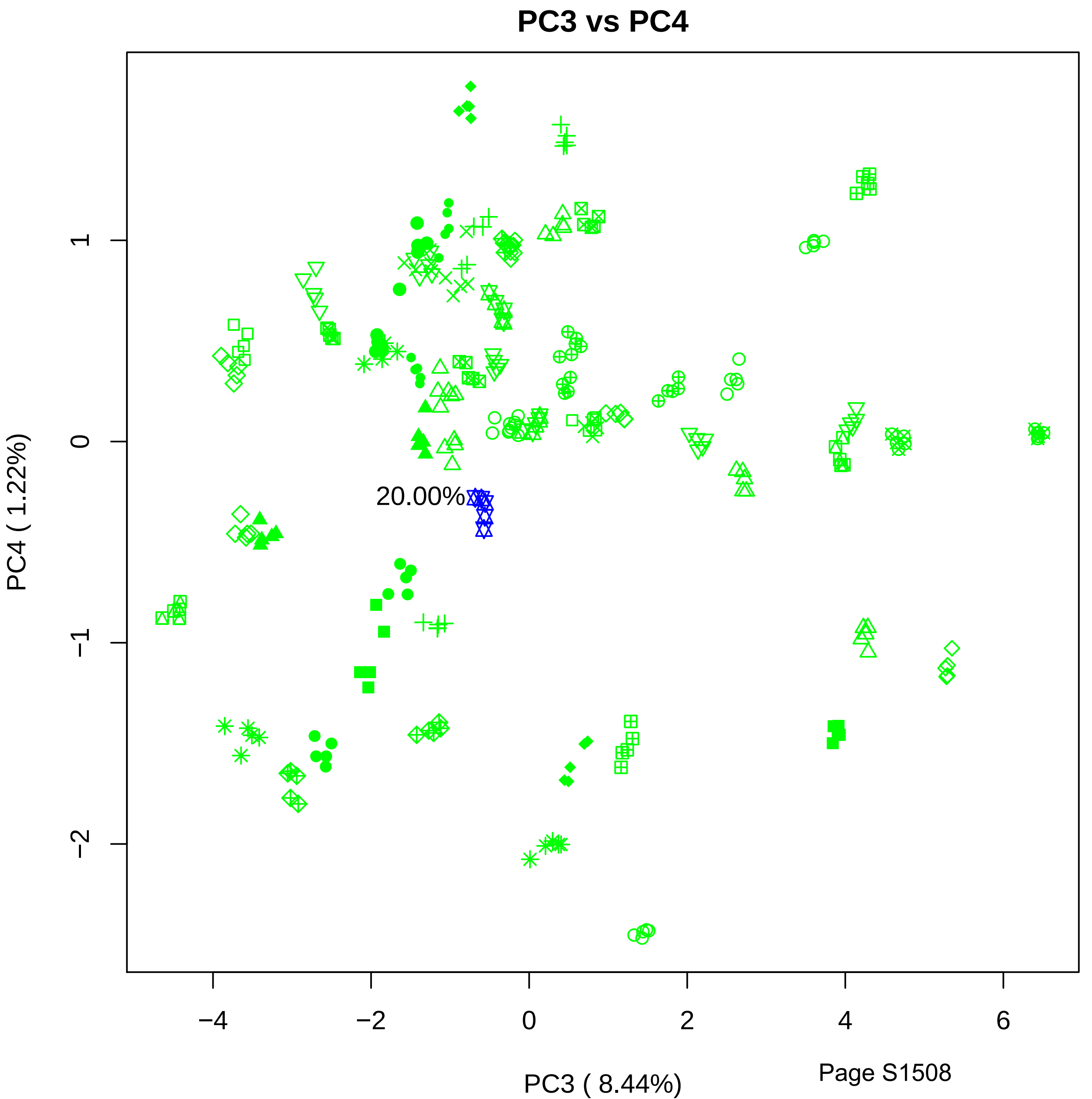
PC3 vs PC4
PC4 (1.22%)
PC3 (8.44%)
20.00%
1
0
−1
−2
−4
−2
0
2
4
6

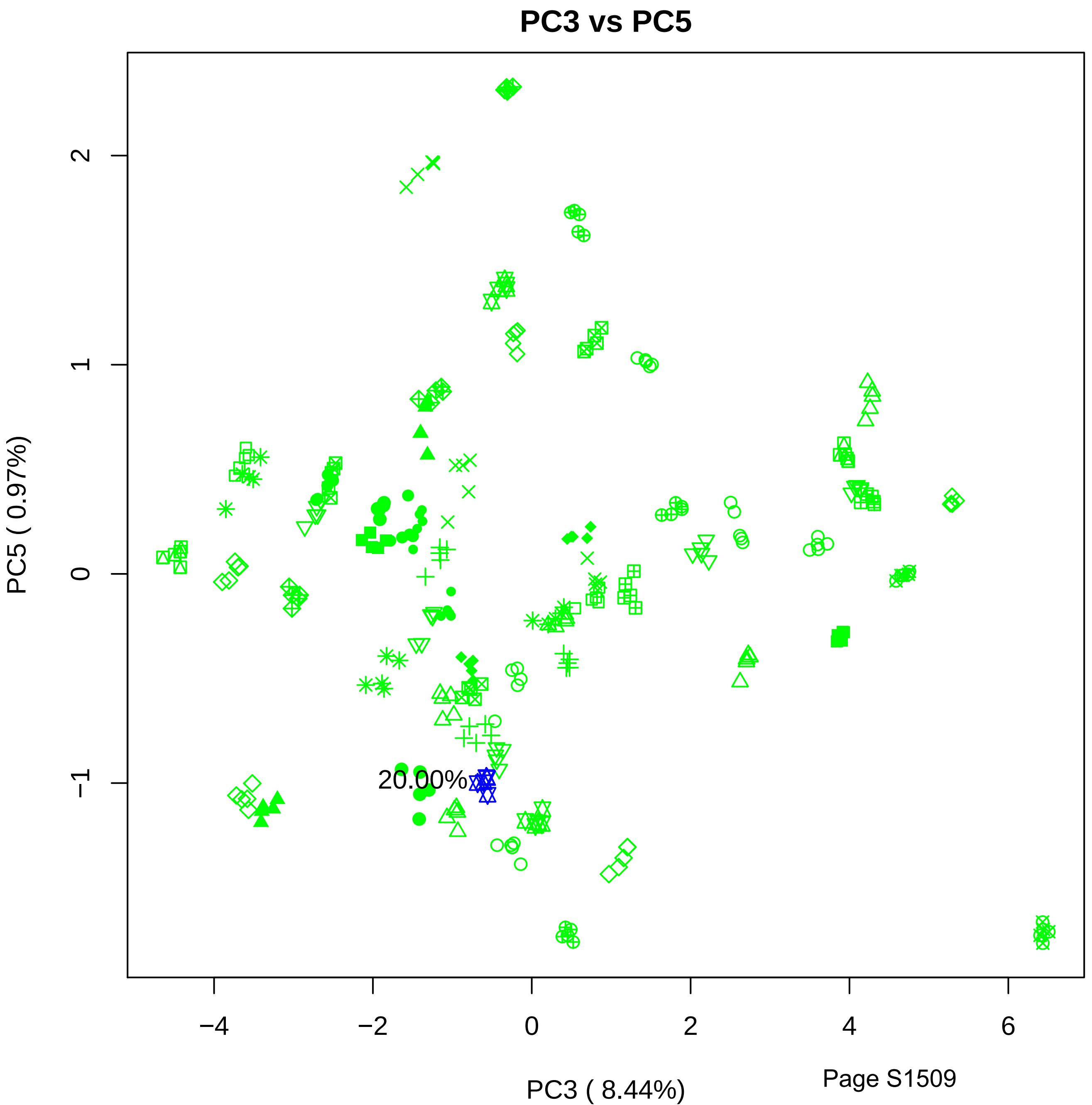

PC3 vs PC5
PC5 (0.97%)
PC3 (8.44%)
20.00%
2
1
0
−1
−4
−2
0
2
4
6

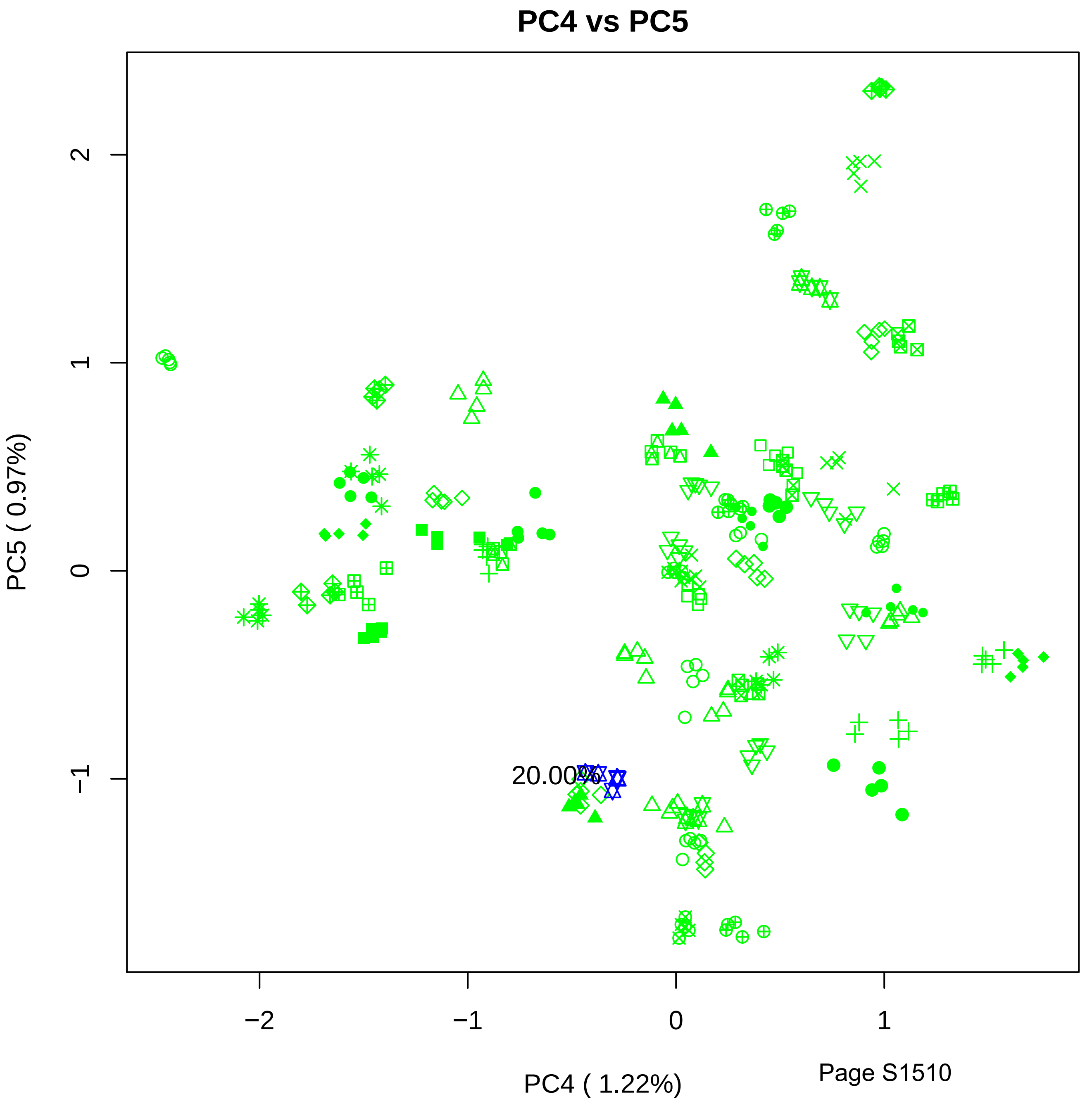

PC4 vs PC5
PC5 (0.97%)
PC4 (1.22%)
20.00%
2
1
0
−1
−2
−1
0
1

PCA of 40% BMH(d) vs PMH(d)

Scaled Matrix PCAed

7th order polynomial baseline correction performed

Raw spectra smoothed to 21 Neighbours, 2nd order PN, Savtisky Golay

Normalisation between 0 and 100

D2 spectra smoothed to 41 Neighbours, 2nd order PN, Savtisky Golay

Regions Analysed: 3601−2501 and 2001−700cm−1

Samples Analysed: 1 bContam's 0 BLH's 0 BMH's 0 OMH's 0 pContam's 0 PLH's 60 PMH's

Keele University AD

Fri Dec 14 14:23:23 2018

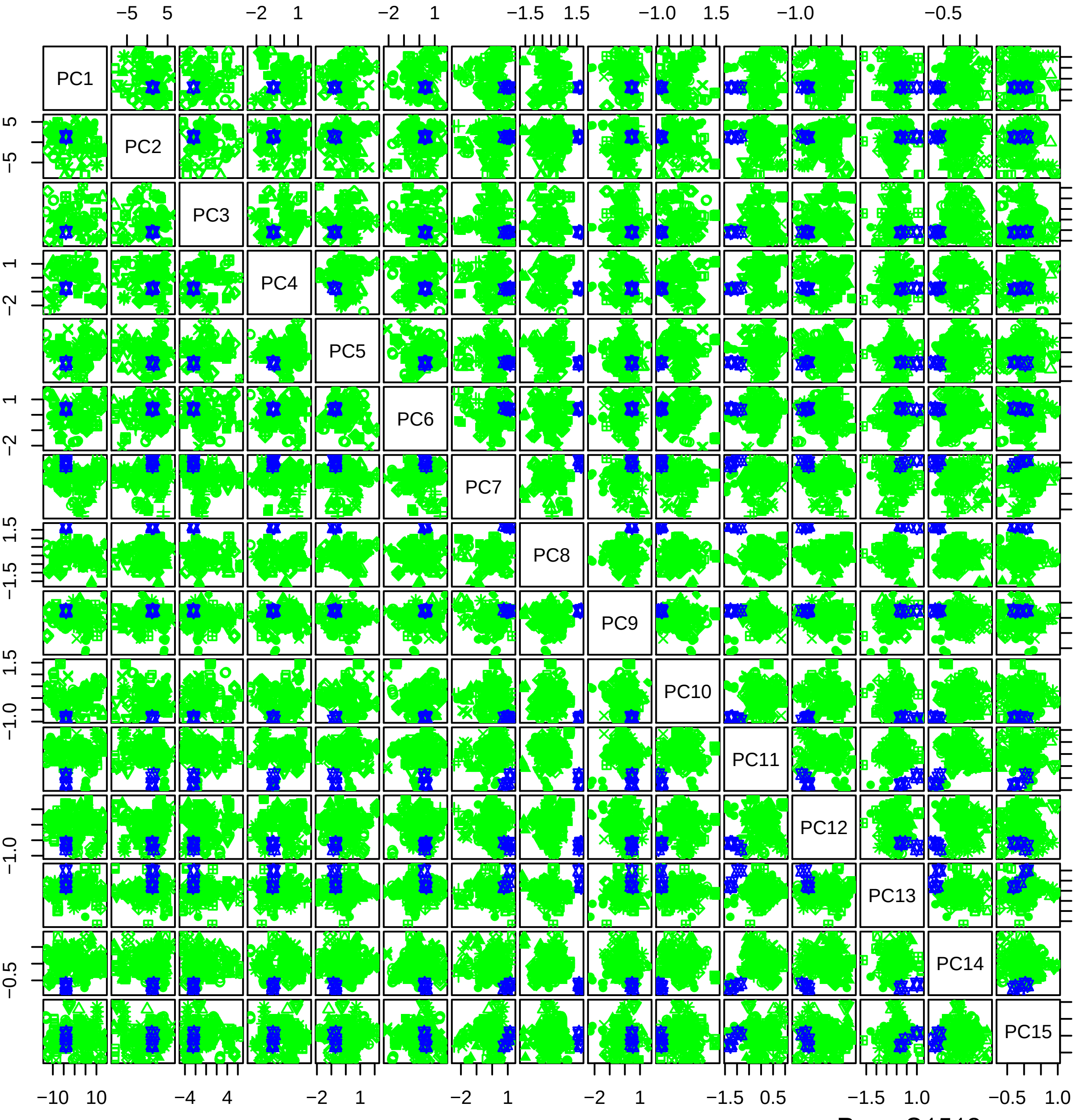
PC1
PC2
PC3
PC4
PC5
PC6
PC7
PC8
PC9
PC10
PC11
PC12
PC13
PC14
PC15

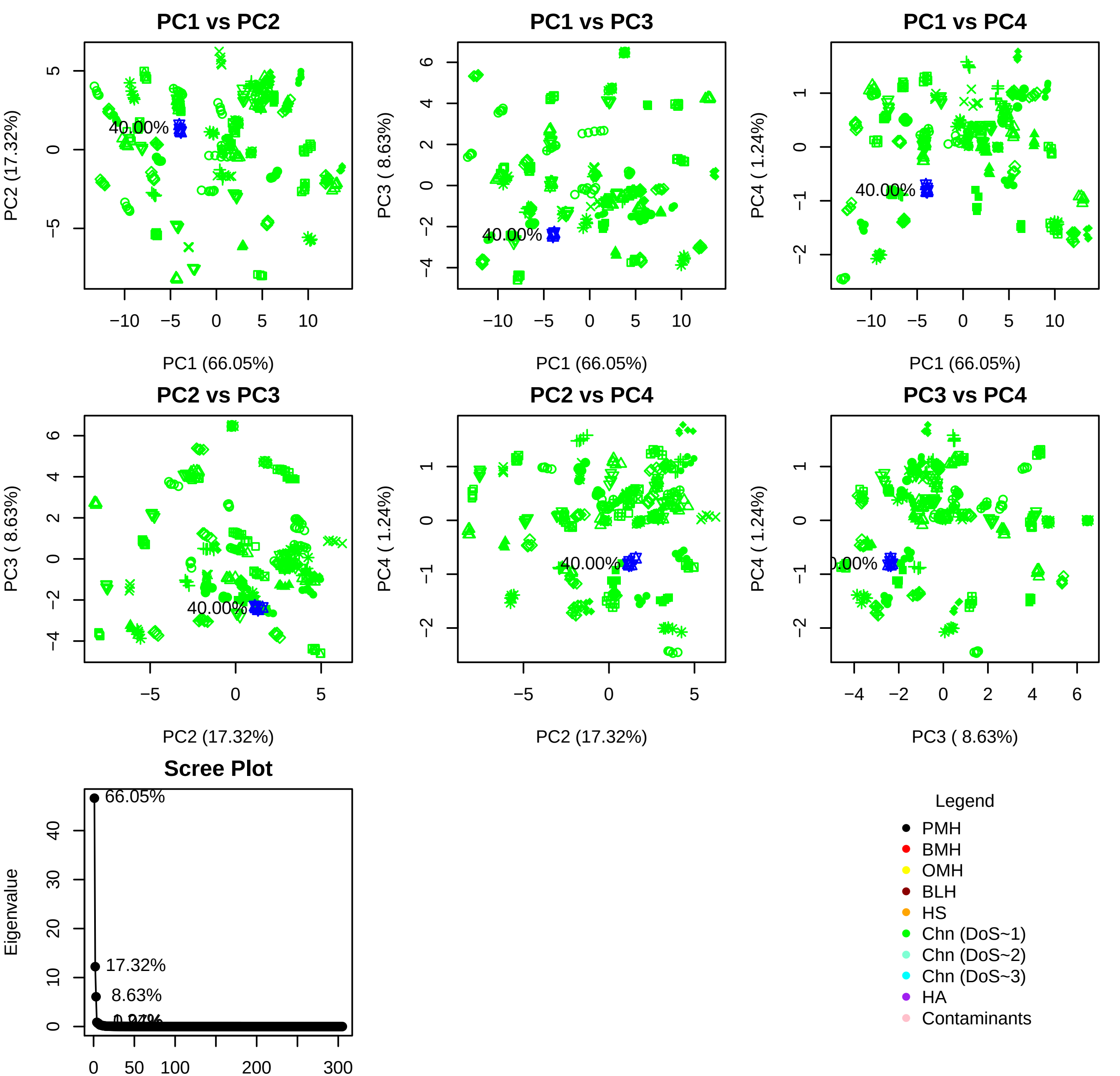
PC1 vs PC2
PC1 vs PC3
PC1 vs PC4
PC2 vs PC3
PC2 vs PC4
PC3 vs PC4
Scree Plot
PC1 (66.05%)
PC2 (17.32%)
PC3 (8.63%)
PC4 (1.24%)
40.00%
Eigenvalue
Principal Component
66.05%
17.32%
8.63%
Legend
PMH
BMH
OMH
BLH
HS
Chn (DoS~1)
Chn (DoS~2)
Chn (DoS~3)
HA
Contaminants

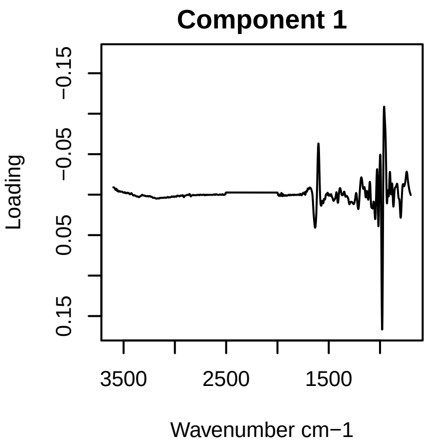
Component 1
Loading
−0.15
−0.05
0.05
0.15
3500
2500
1500
Wavenumber cm−1

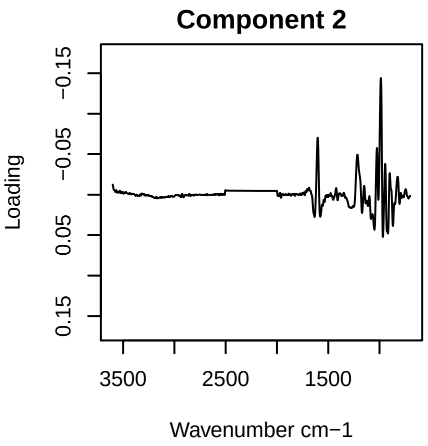
Component 2
Loading
−0.15
−0.05
0.05
0.15
3500
2500
1500
Wavenumber cm−1

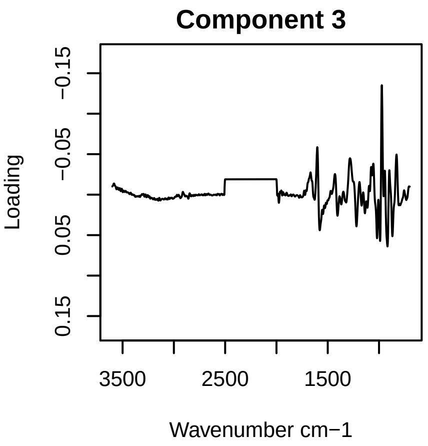
Component 3
Loading
−0.15
−0.05
0.05
0.15
3500
2500
1500
Wavenumber cm−1

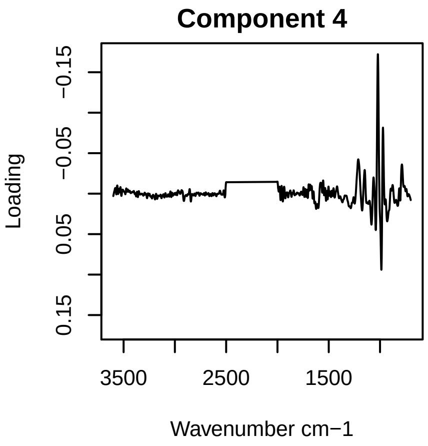
Component 4
Loading
−0.15
−0.05
0.05
0.15
3500
2500
1500
Wavenumber cm−1

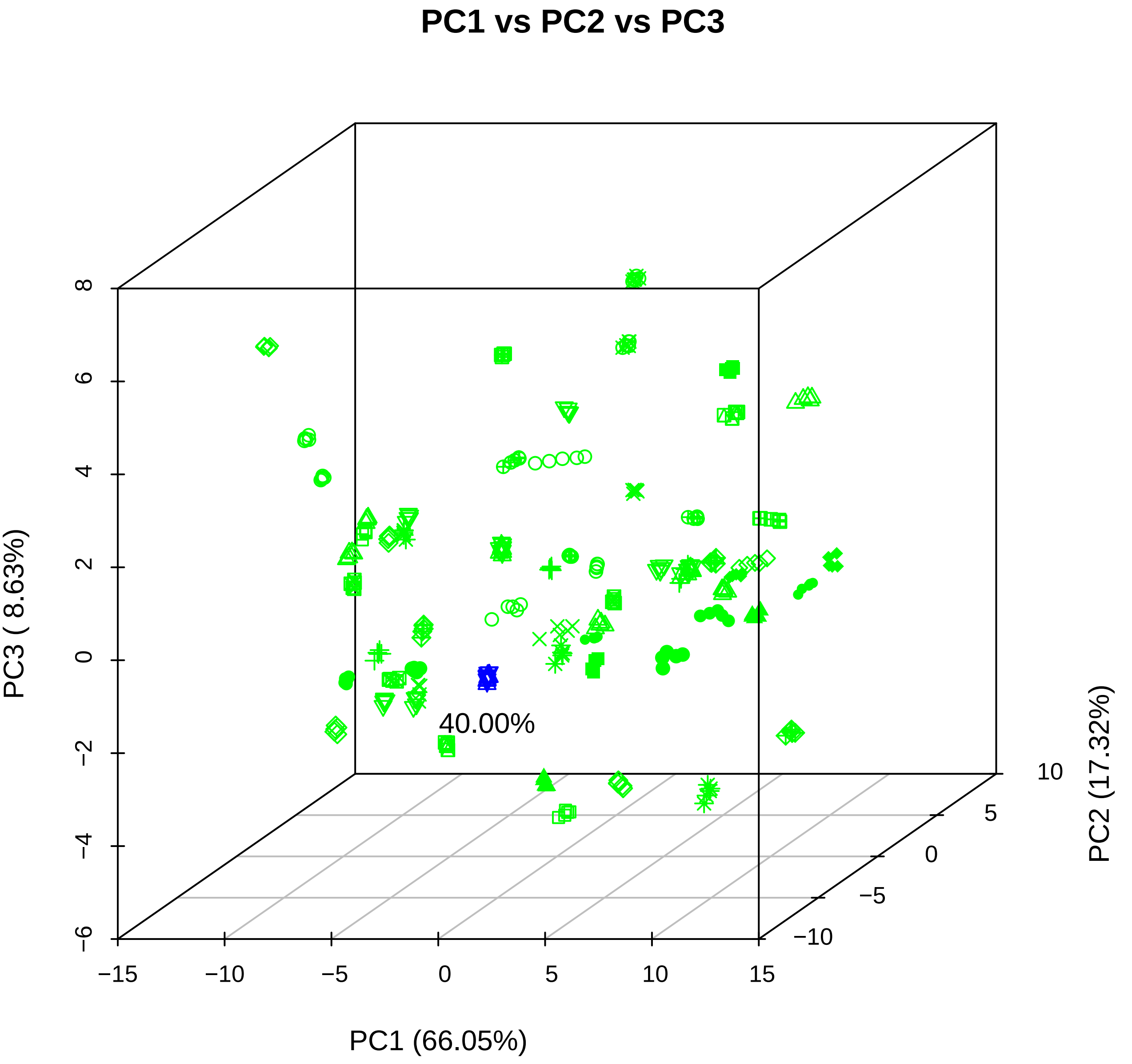
PC1 vs PC2 vs PC3
40.00%
PC1 (66.05%)
PC2 (17.32%)
PC3 (8.63%)
−15
−10
−5
0
5
10
15
−6
−4
−2
0
2
4
6
8
−10
−5
0
5
10

PC1 vs PC2 vs PC4

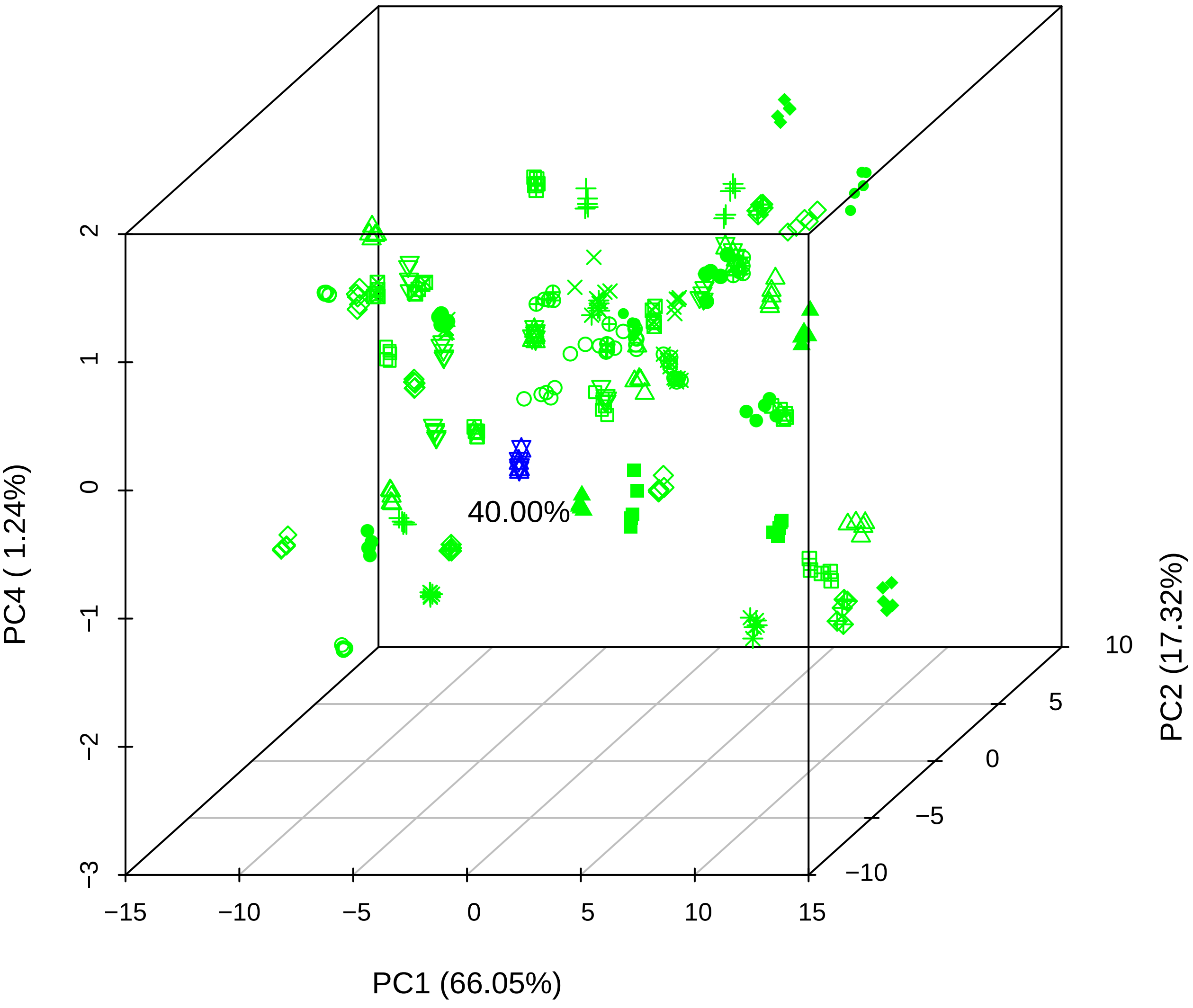

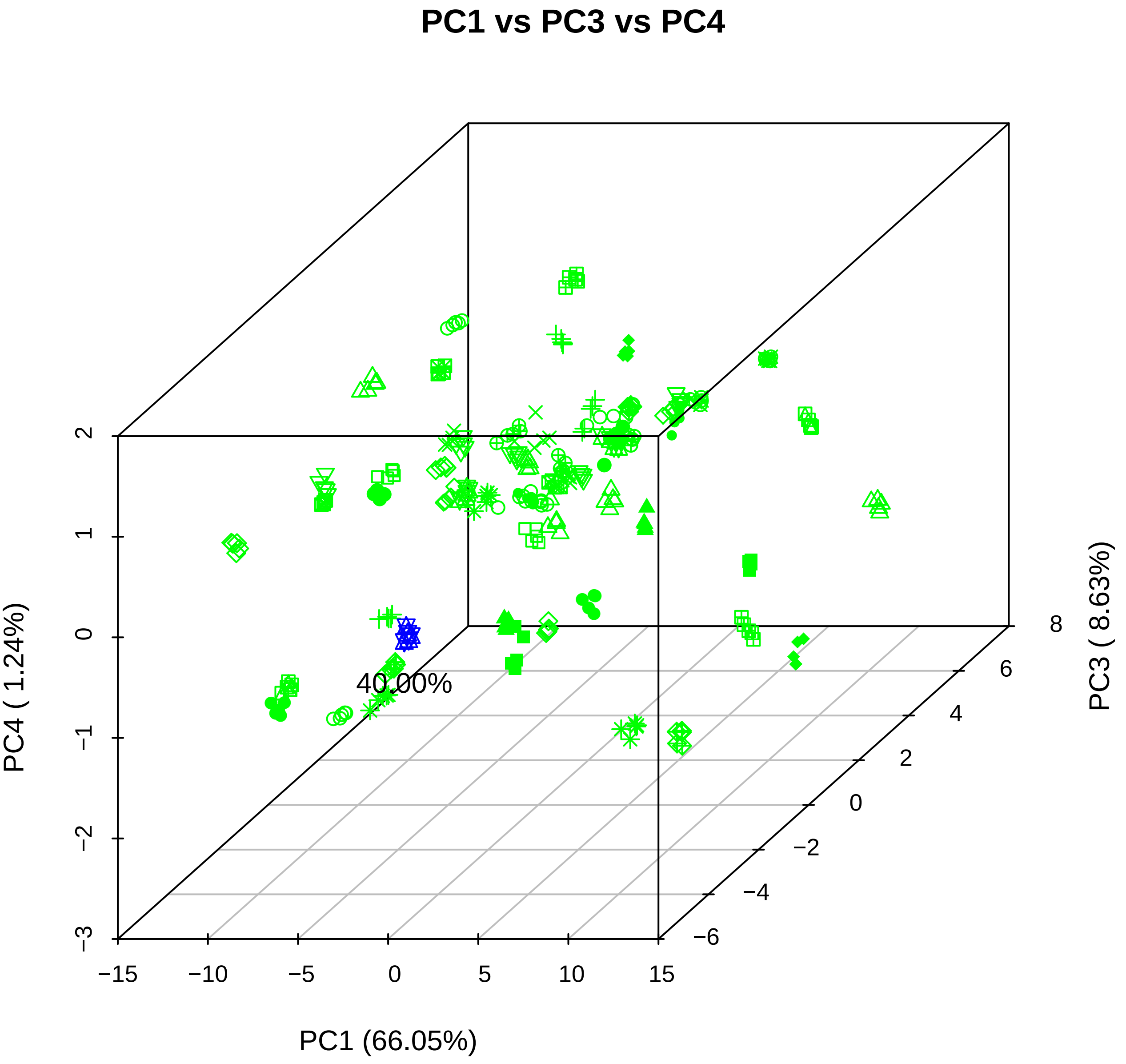
PC1 vs PC3 vs PC4
PC4 (1.24%)
PC3 (8.63%)
PC1 (66.05%)
40.00%
−15
−10
−5
0
5
10
15
−3
−2
−1
0
1
2
−6
−4
−2
0
2
4
6
8

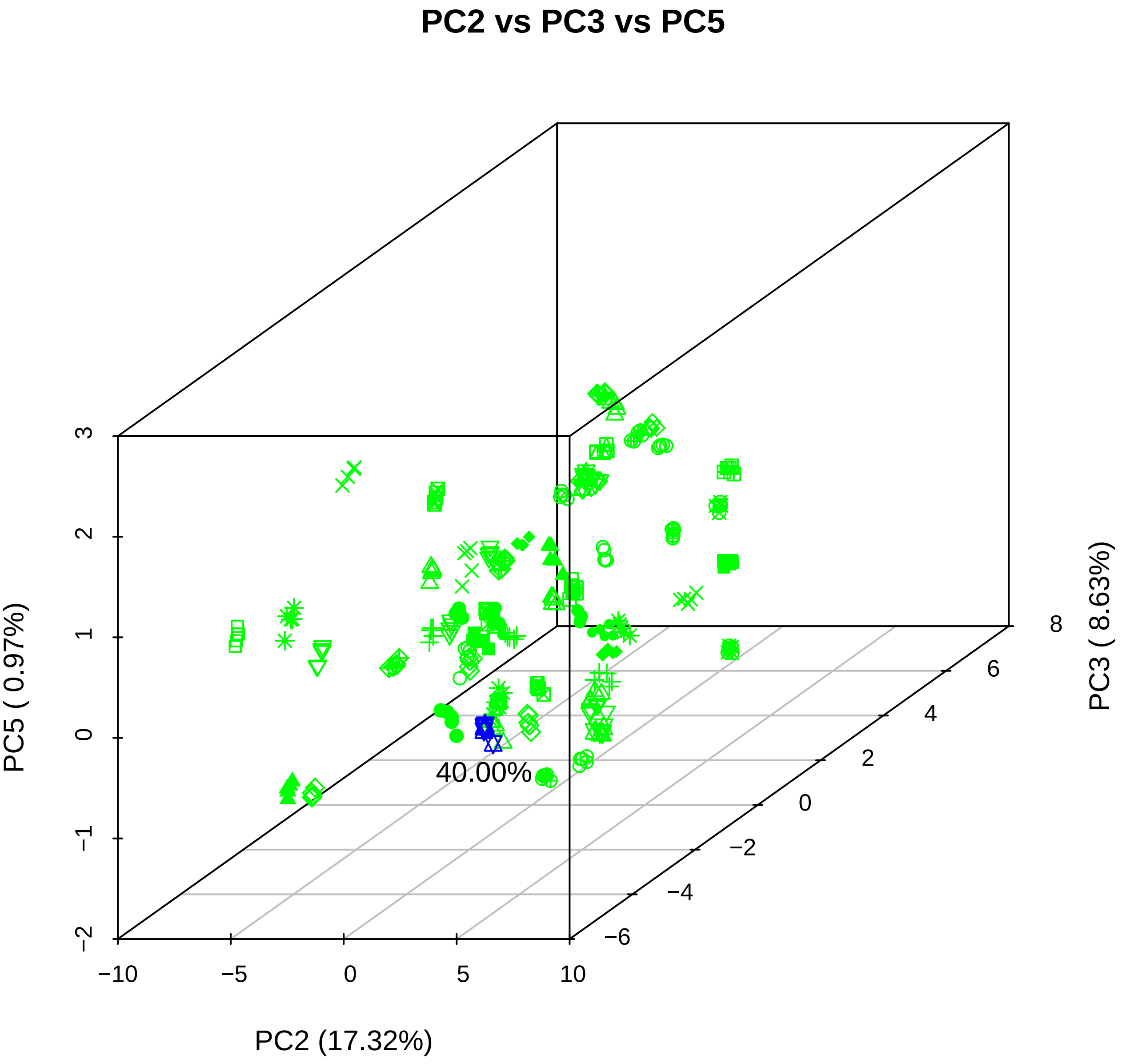
PC2 vs PC3 vs PC5
PC5 (0.97%)
3
2
1
0
−1
−2
−10
−5
0
5
10
PC2 (17.32%)
PC3 (8.63%)
8
6
4
2
0
−2
−4
−6
40.00%

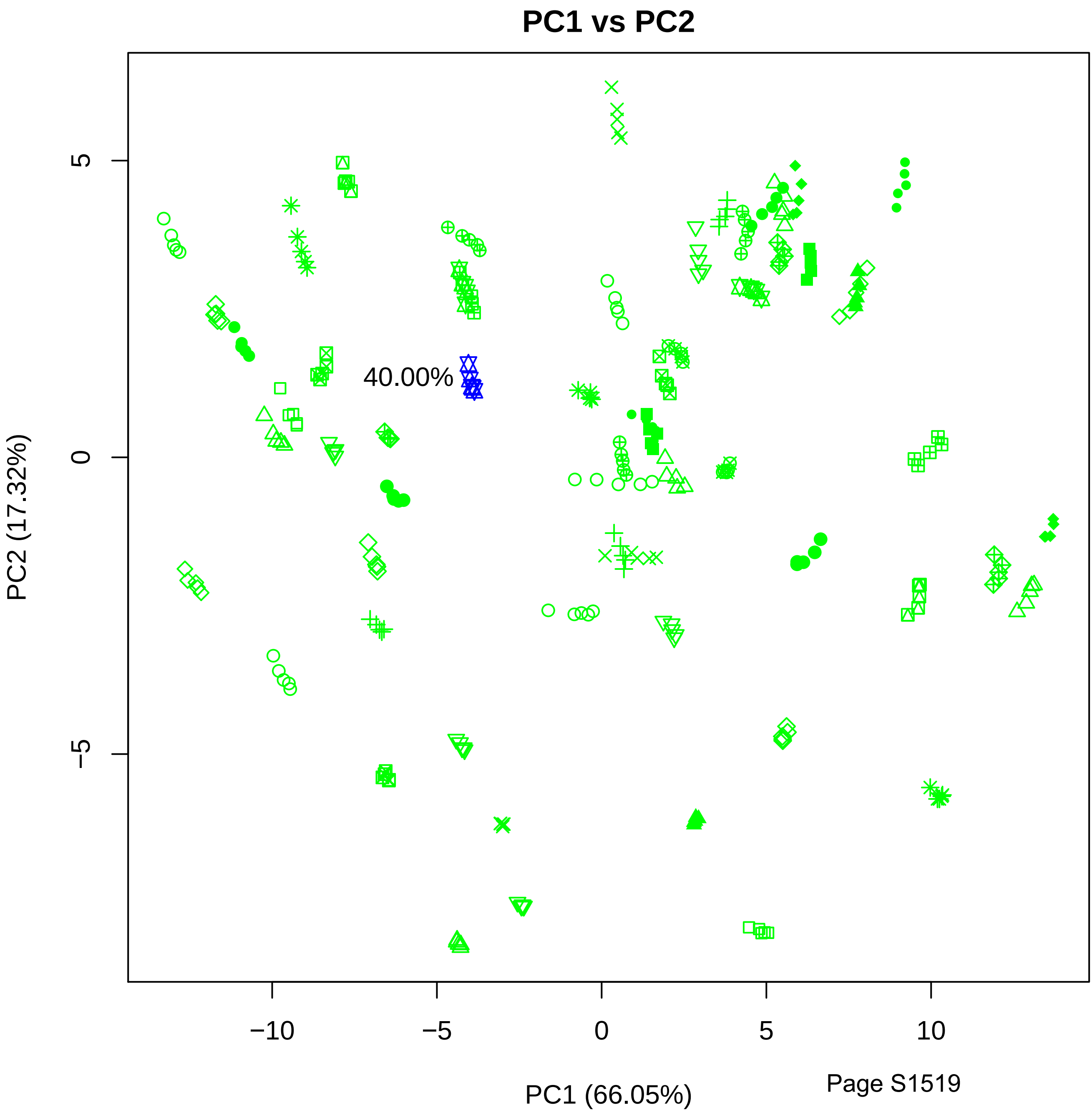

PC1 vs PC2
40.00%
5
0
−5
PC2 (17.32%)
−10
−5
0
5
10
PC1 (66.05%)

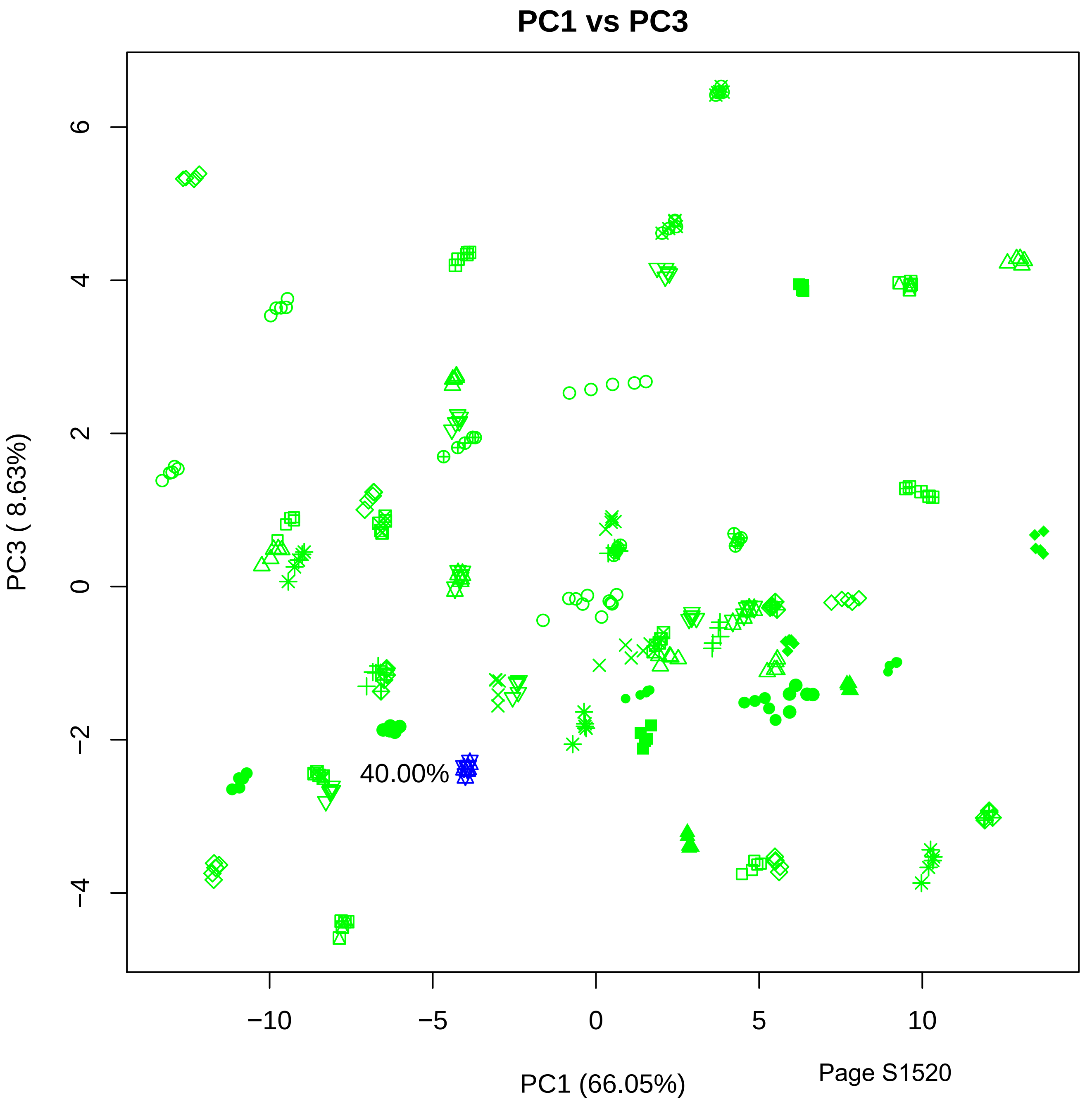

PC1 vs PC3
PC3 (8.63%)
PC1 (66.05%)
40.00%
6
4
2
0
−2
−4
−10
−5
0
5
10

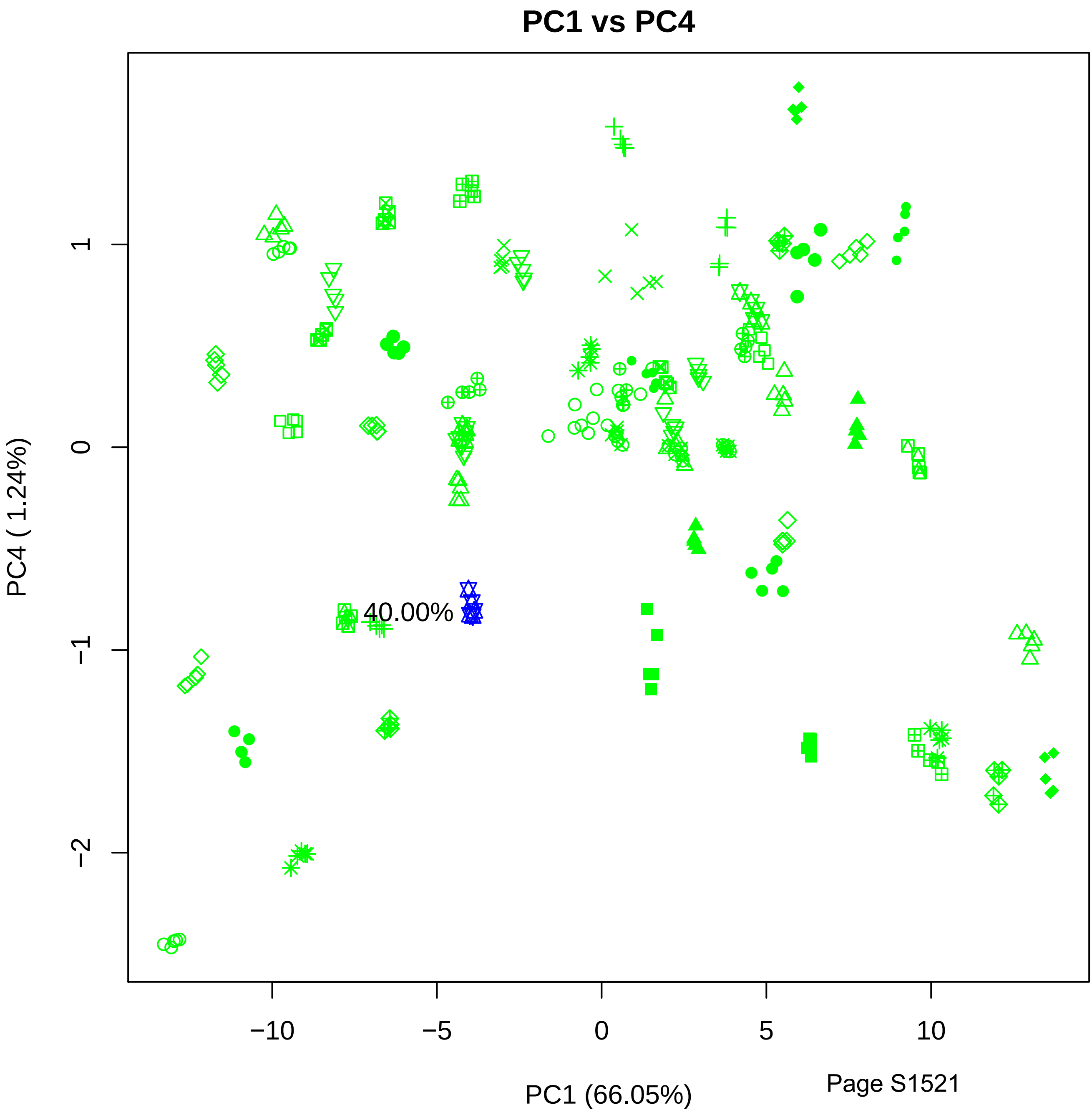

PC1 vs PC4
PC4 (1.24%)
PC1 (66.05%)
40.00%
1
0
−1
−2
−10
−5
0
5
10

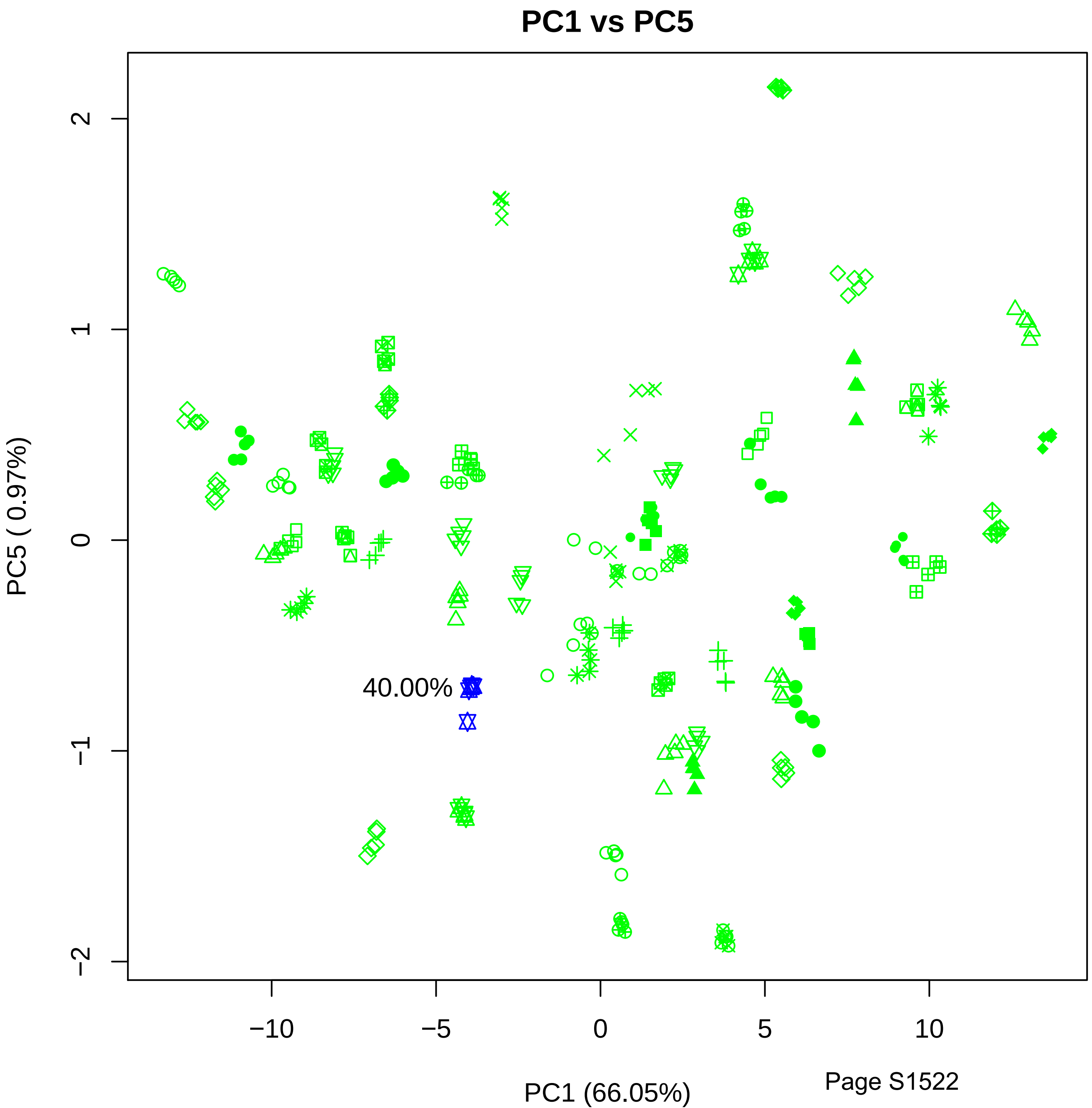
PC1 vs PC5
PC5 (0.97%)
PC1 (66.05%)
40.00%
2
1
0
−1
−2
−10
−5
0
5
10

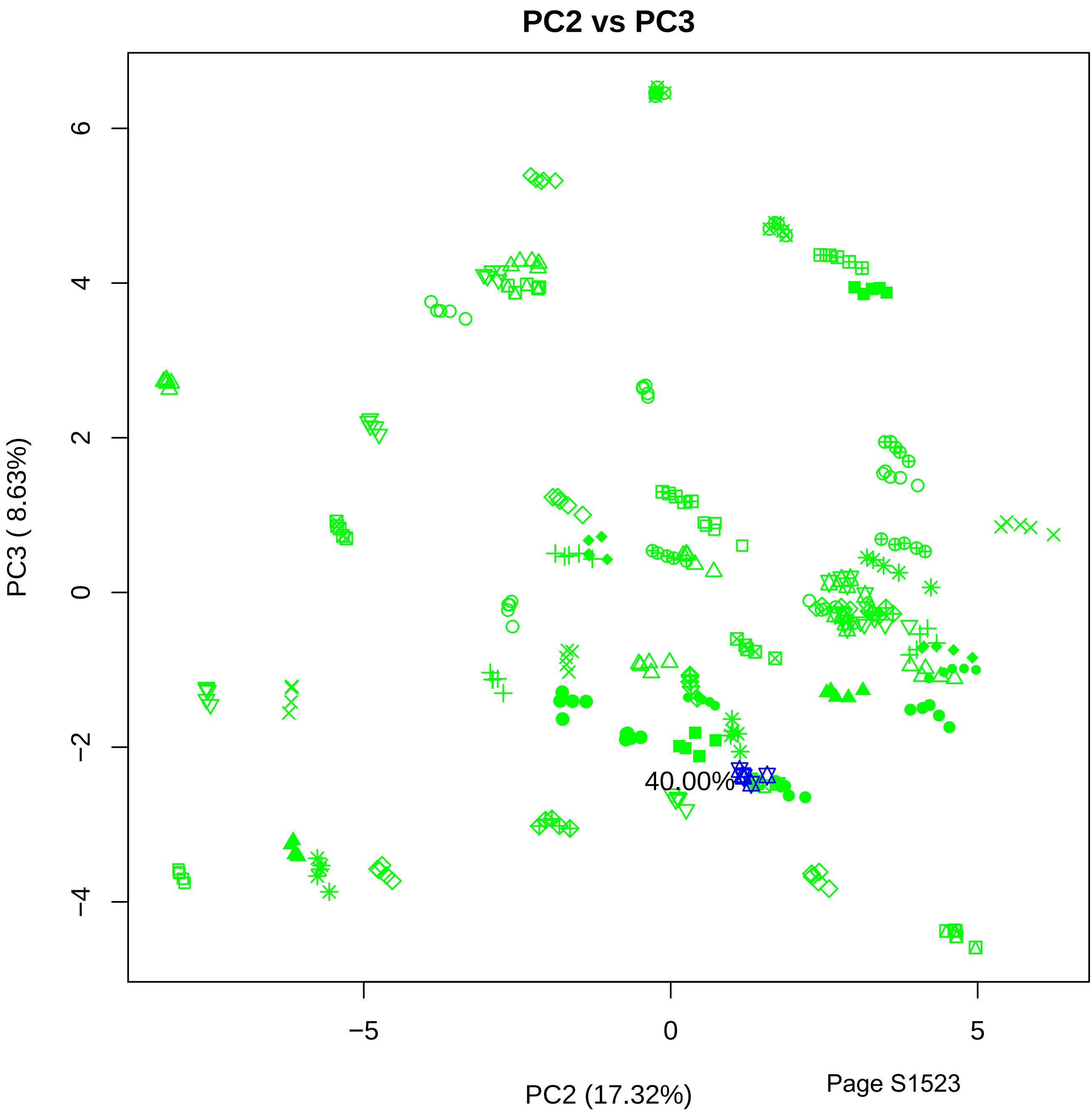
PC2 vs PC3
PC3 (8.63%)
6
4
2
0
−2
−4
−5
0
5
40.00%
PC2 (17.32%)

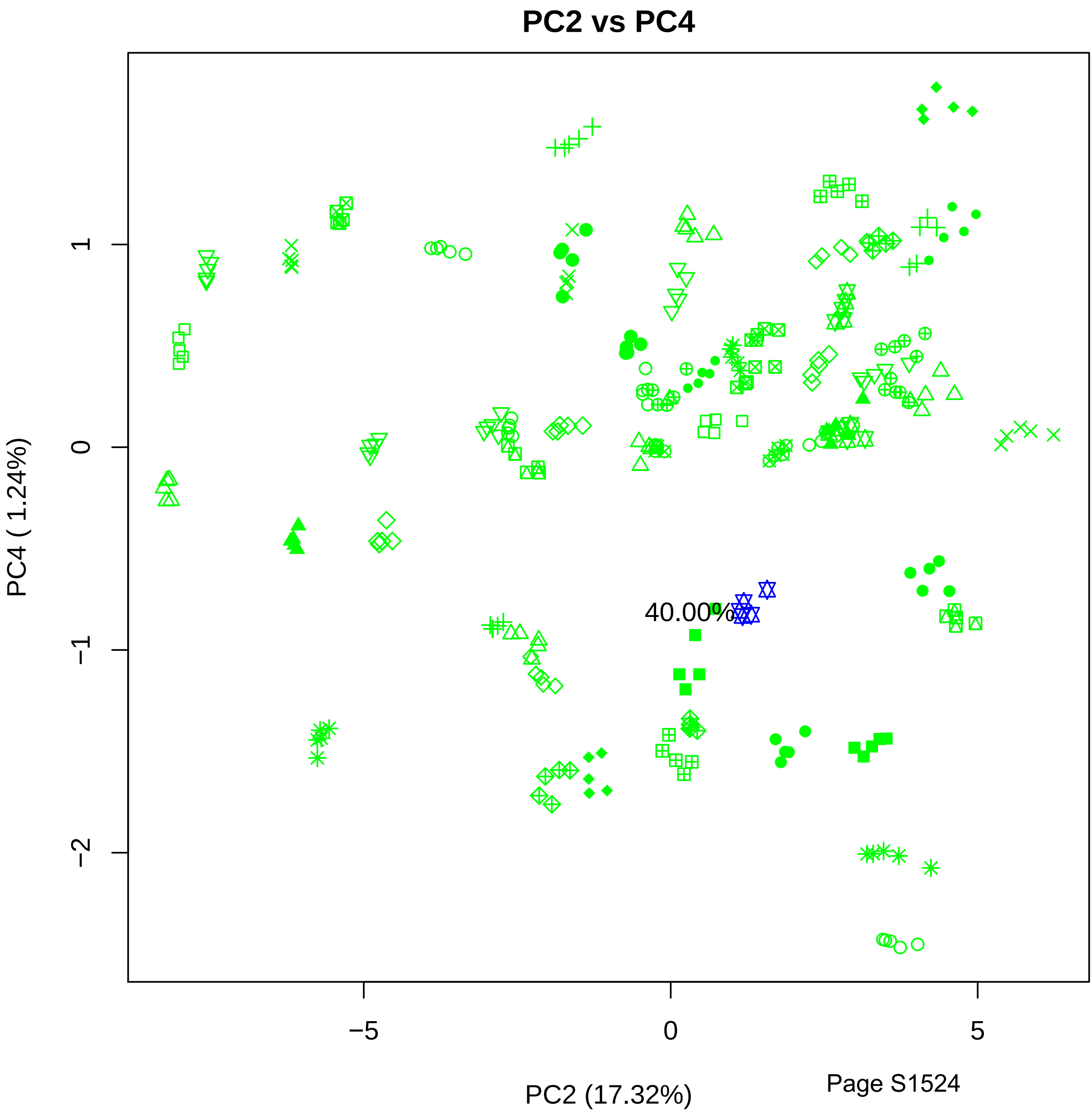
PC2 vs PC4
PC4 (1.24%)
PC2 (17.32%)
1
0
−1
−2
−5
0
5
40.00%

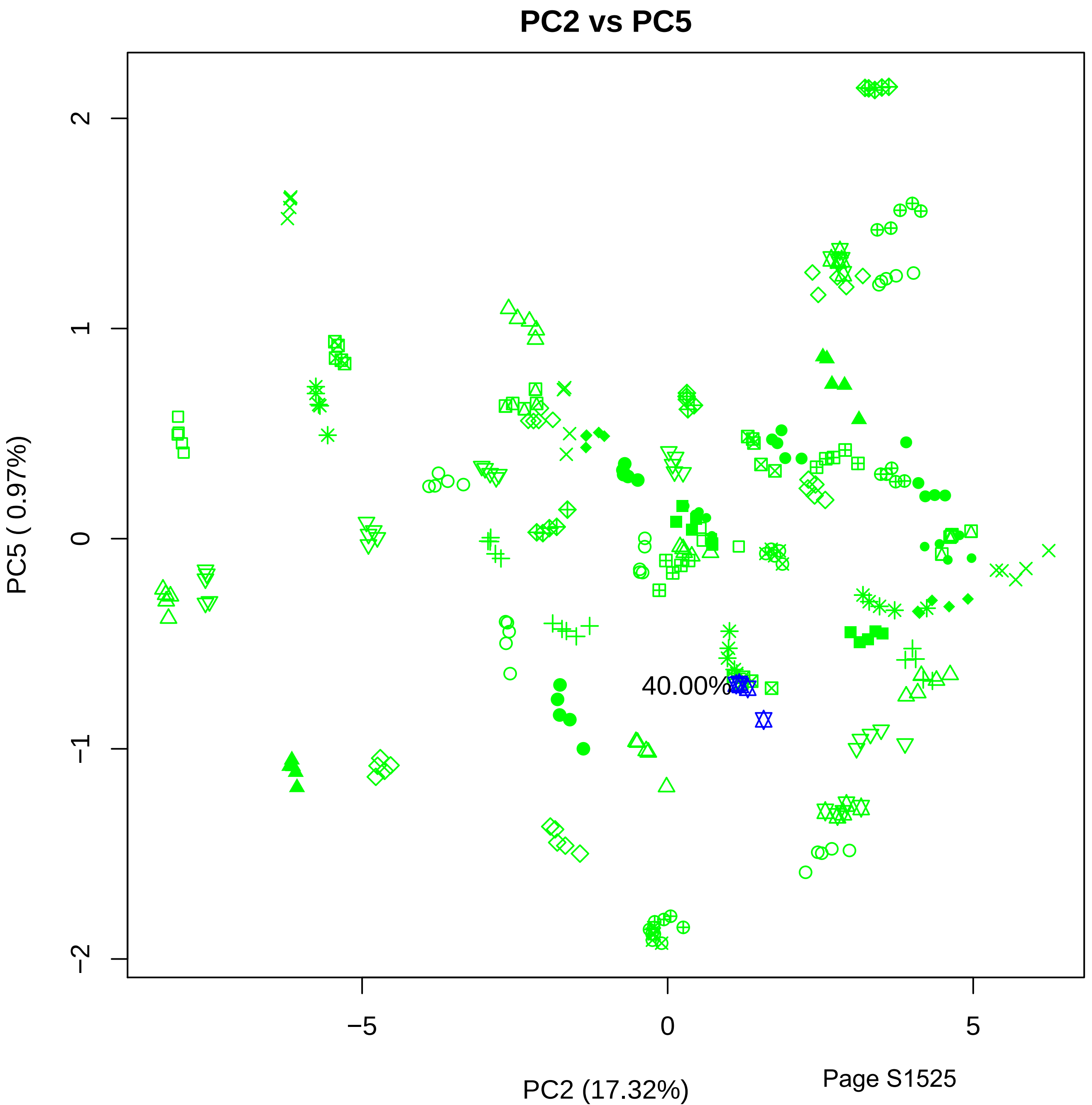
PC2 vs PC5
2
1
0
−1
−2
PC5 (0.97%)
−5
0
5
PC2 (17.32%)
40.00%

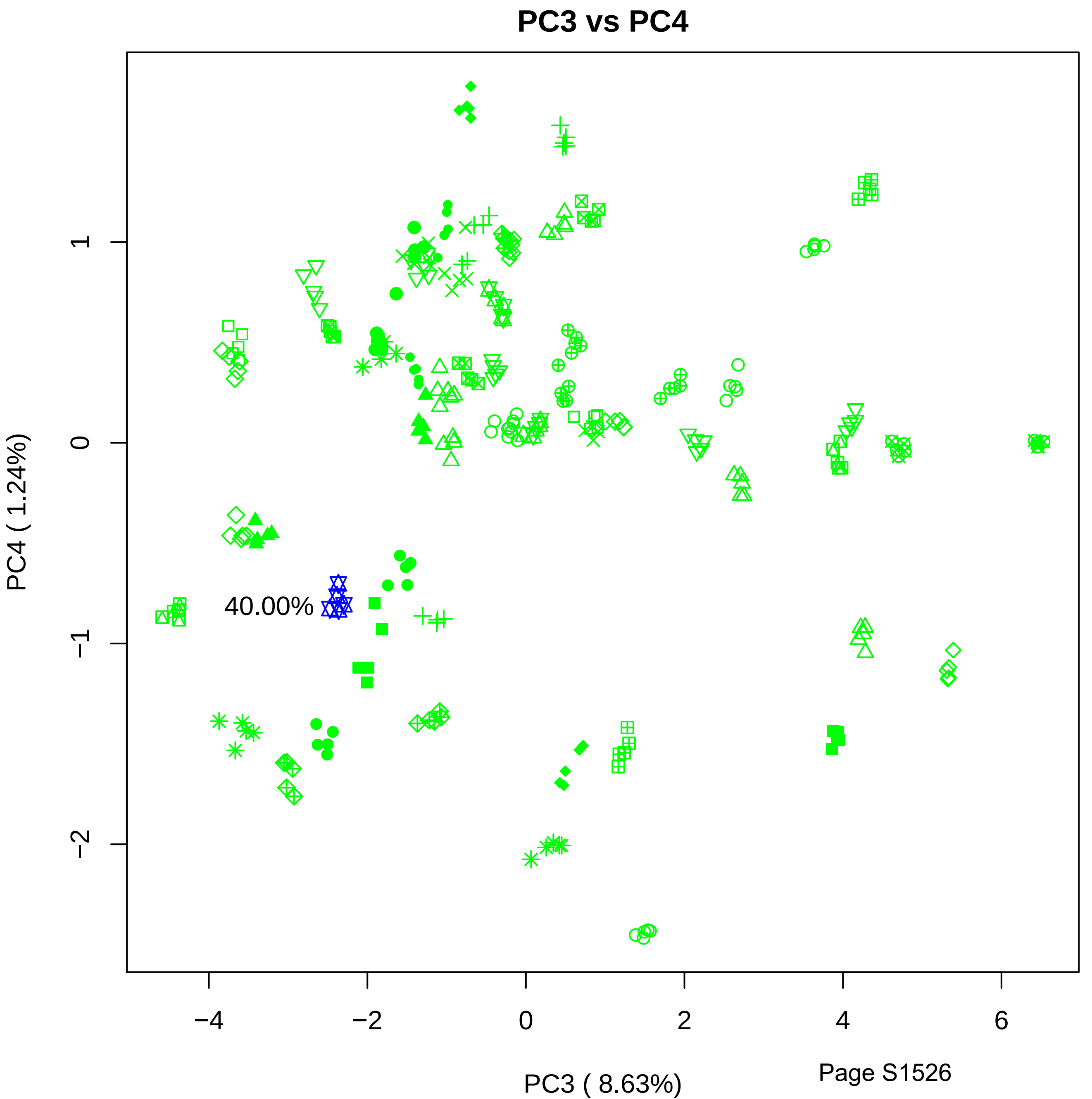

PC3 vs PC4
PC4 (1.24%)
PC3 (8.63%)
40.00%
1
0
−1
−2
−4
−2
0
2
4
6

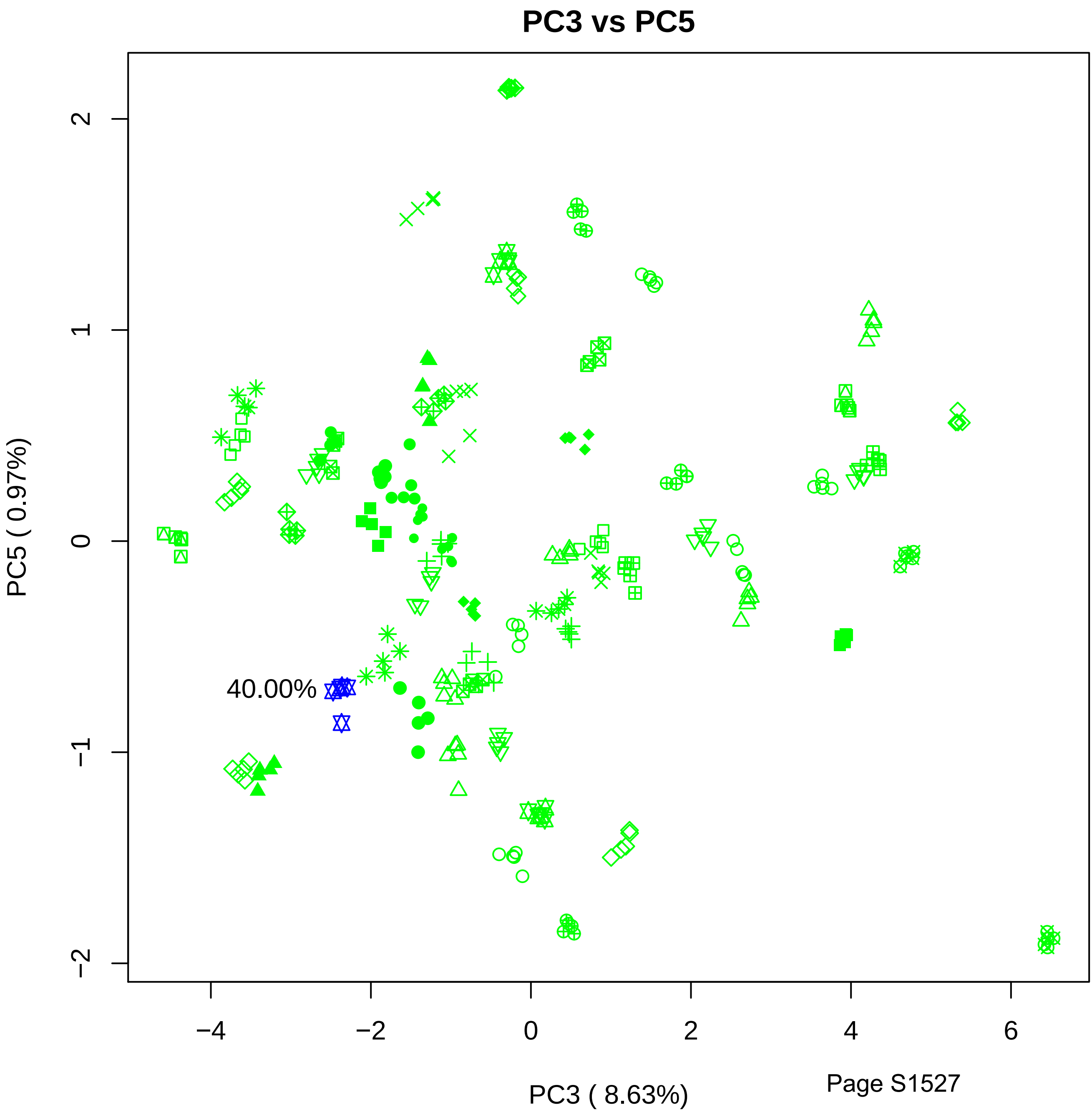

PC3 vs PC5
40.00%
PC5 (0.97%)
PC3 (8.63%)
−4
−2
0
2
4
6
−2
−1
0
1
2

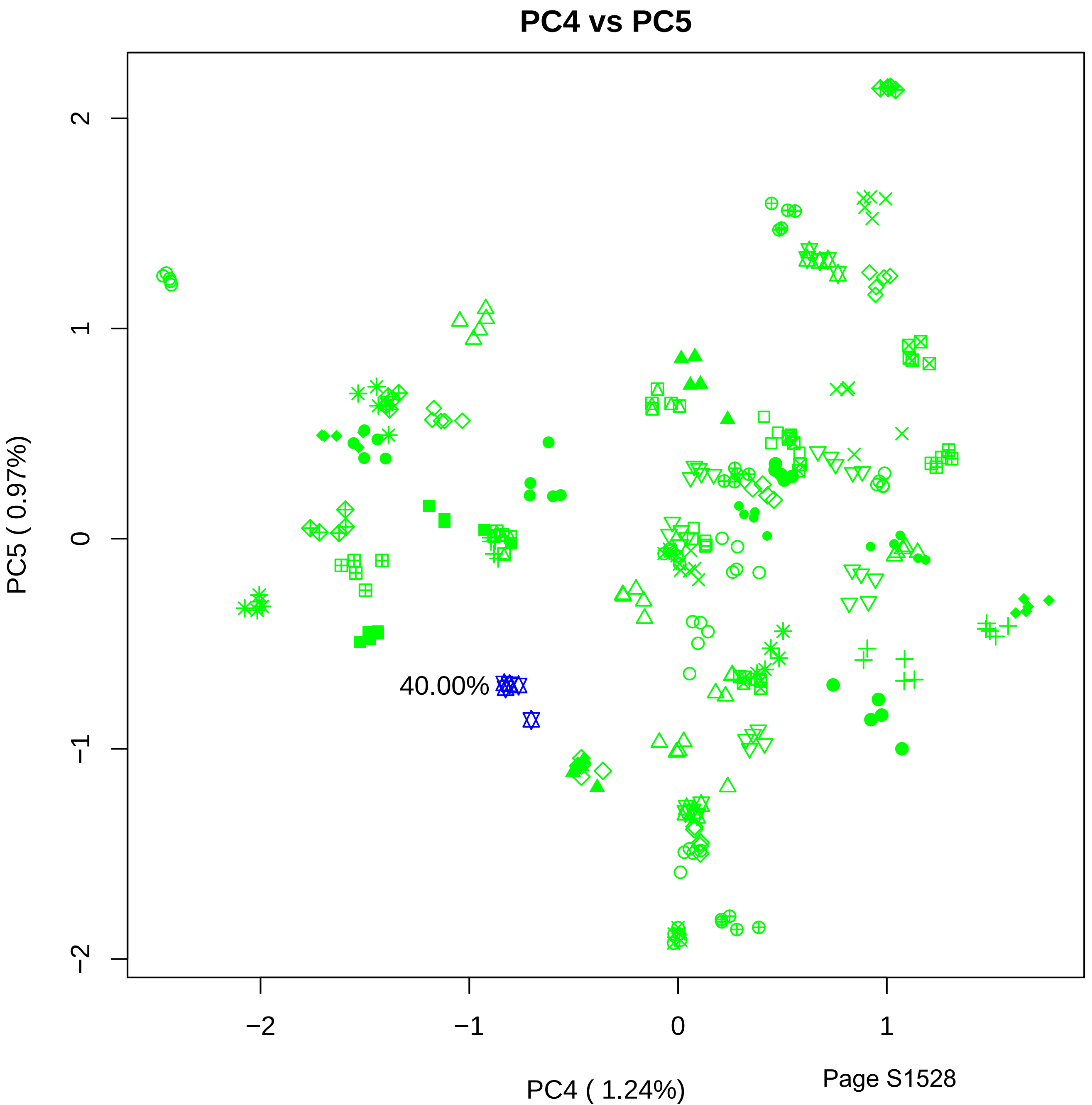
PC4 vs PC5
2
1
0
−1
−2
40.00%
−2
−1
0
1
PC5 (0.97%)
PC4 (1.24%)
